# iNuc-PhysChem: A Sequence-Based Predictor for Identifying Nucleosomes via Physicochemical Properties

**DOI:** 10.1371/journal.pone.0047843

**Published:** 2012-10-29

**Authors:** Wei Chen, Hao Lin, Peng-Mian Feng, Chen Ding, Yong-Chun Zuo, Kuo-Chen Chou

**Affiliations:** 1 Department of Physics, School of Sciences, Center for Genomics and Computational Biology, Hebei United University, Tangshan, China; 2 Key Laboratory for Neuro-Information of Ministry of Education, Center of Bioinformatics, School of Life Science and Technology, University of Electronic Science and Technology of China, Chengdu, China; 3 School of Public Health, Hebei United University, Tangshan, China; 4 The National Research Center for Animal Transgenic Biotechnology, Inner Mongolia University, Hohhot, China; 5 Gordon Life Science Institute, San Diego, California, United States of America; Semmelweis University, Hungary

## Abstract

Nucleosome positioning has important roles in key cellular processes. Although intensive efforts have been made in this area, the rules defining nucleosome positioning is still elusive and debated. In this study, we carried out a systematic comparison among the profiles of twelve DNA physicochemical features between the nucleosomal and linker sequences in the *Saccharomyces cerevisiae* genome. We found that nucleosomal sequences have some position-specific physicochemical features, which can be used for in-depth studying nucleosomes. Meanwhile, a new predictor, called **iNuc-PhysChem**, was developed for identification of nucleosomal sequences by incorporating these physicochemical properties into a 1788-D (dimensional) feature vector, which was further reduced to a 884-D vector via the IFS (incremental feature selection) procedure to optimize the feature set. It was observed by a cross-validation test on a benchmark dataset that the overall success rate achieved by **iNuc-PhysChem** was over 96% in identifying nucleosomal or linker sequences. As a web-server, **iNuc-PhysChem** is freely accessible to the public at http://lin.uestc.edu.cn/server/iNuc-PhysChem. For the convenience of the vast majority of experimental scientists, a step-by-step guide is provided on how to use the web-server to get the desired results without the need to follow the complicated mathematics that were presented just for the integrity in developing the predictor. Meanwhile, for those who prefer to run predictions in their own computers, the predictor's code can be easily downloaded from the web-server. It is anticipated that **iNuc-PhysChem** may become a useful high throughput tool for both basic research and drug design.

## Introduction

**Figure 1:**
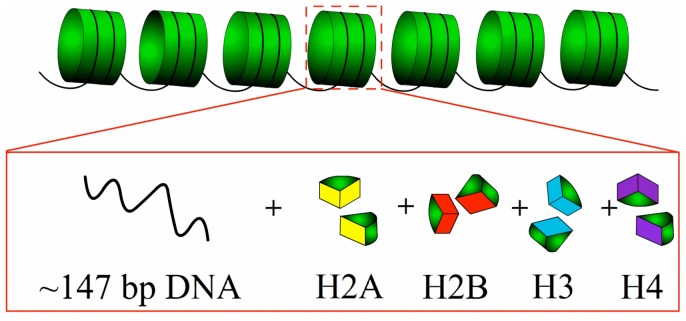
A schematic illustration to show the basic architecture of nucleosome. Nucleosomes form the fundamental repeating units of eukaryotic chromatin (upper panel), each of them consists of approximately 147 base pairs of DNA wrapped in 1.67 left-handed superhelical turns around a histone octamer consisting of 2 copies each of the core histones H2A, H2B, H3, and H4 (lower panel).

In eukaryotic cells, genomic DNA is highly compacted into several levels of chromatin structures that ultimately make up the chromosomes. At the lowest level of compaction, a ∼147 bp DNA sequence is tightly wrapped around the histone-octamer core (**Fig. 1**) into the elementary structural unit of chromatin, known as nucleosome [1]. The packaging of DNA around the histone-octamer modulates the accessibility of genomic regions to regulatory proteins. There are close relationships between nucleosome positioning and key cellular processes, as demonstrated in mRNA splicing, DNA replication, and DNA repair [2], [3], [4]. Consequently, revealing the mechanism involved in controlling nucleosome positioning is fundamentally important for in-depth understanding the subsequent steps of gene expression.

High-resolution genome-wide nucleosome maps are now available for several model organisms, such as *Saccharomyces cerevisiae*, *Caenorhabditis elegans*, *Drosophila melanogaster* and *Homo sapiens* [5], [6], [7], [8], [9]. These high-resolution data provide unprecedented opportunities for further investigating the roles of nucleosome positioning in gene regulation. However, experimental approach is expensive to perform genome-wide analysis of nucleosome distribution. In this regard, computational methods can be applied to the entire genome without this kind of disadvantage. Since the report of the nucleosome positioning code (∼10 bp repeating pattern of dinucleotides AA-TT-TA/GC) in yeast [8], lots of theoretical works have been done attempting to elucidate nucleosome occupancy signals that determine the preference of a particular region in binding to histones and forming a nucleosome [10], [11], [12]. Although of great interest and value, sequence-based predictions of nucleosome positioning have been limited in their accuracy and resolution, and to which extent nucleosome positioning in vivo is really dictated by the DNA sequence [10] is still an issue of controversy [13].

It was reported by Miele et al. [7] that DNA physical-chemical properties may determine nucleosome occupancy. Moreover, the recent study by Nozaki et al. [14] also suggested the existence of a highly bendable, fragile structure for nucleosomal DNA, implying that nucleosomal sequences indeed have distinct structural properties when compared with linker sequences.

In view of this, the present study was initiated in an attempt to develop a new method for predicting nucleosomal sequences based on the physicochemical properties of DNA.

According to a recent review [15], to establish a really useful statistical predictor for a biological system, we need to consider the following procedures: (1) construct or select a valid benchmark dataset to train and test the predictor; (2) formulate the biological samples with an effective mathematical expression that can truly reflect their intrinsic correlation with the target to be predicted; (3) introduce or develop a powerful algorithm (or engine) to operate the prediction; (4) properly perform cross-validation tests to objectively evaluate the anticipated accuracy of the predictor; (5) establish a user-friendly web-server for the predictor that is accessible to the public. Below, let us describe how to deal with these steps.

## Materials and Methods

### 1. Benchmark Dataset: Nucleosomal and Linker Sequences

The reference genome sequence of *Saccharomyces cerevisiae* was obtained from the Saccharomyces Genome Database (http://www.yeastgenome.org/). The nucleosome positions of *Saccharomyces cerevisiae* were derived from the published data obtained by Lee et al. [16], where each of the 1,206,683 DNA fragments in the dataset constructed by these authors was assigned a nucleosome formation score using a lasso model, with the high or low score to reflect its high or low propensity in forming nucleosome, respectively. The low score can also be interpreted as the propensity to inhibit the formation of nucleosome. To prepare a high quality benchmark dataset, 5,000 fragments of 150 bp with the highest scores were selected as the nucleosome-forming sequence samples to construct the positive set 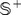, and 5,000 fragments of 150 bp with the lowest scores were selected as the nucleosome-inhibiting (or linker) sequence samples to construct the negative set 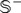; i.e., the benchmark dataset 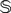 in this study can be formulated as
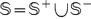
(1)where 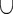 represents the symbol for “union” in the set theory, and
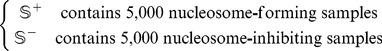
(2)For the convenience of readers, the 5,000 sequences in 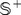 and 5,000 sequences in 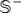 are given in the Information S1.

### 2. Feature Vectors based on DNA Physicochemical Properties

Owing to their important roles in various different biological processes, the intrinsic physicochemical properties of DNA sequences have been intensively studied by many investigators [17], [18], [19], [20], [21]. In the present study, the following twelve DNA physicochemical properties are to be considered: (1) A-philicity [22], (2) base stacking [23], (3) B-DNA twist [24], (4) bendability [25], (5) DNA bending stiffness [26], (6) DNA denaturation [27], (7) duplex disrupt energy [28], (8) duplex free energy [29], (9) propeller twist [30], (10) protein deformation [31], (11) protein-DNA twist [31], and (12) Z-DNA [32].

In order to quantitatively analyze the physical and chemical properties of the DNA sequence samples, we firstly converted the retrieved nucleosomal and linker sequences into numerical profiles according to the following schemes as validated by Florquin et al. [18]. The detailed procedures are as following steps.

#### Step 1

For any 2 base pair (bp) piece of DNA, there is a corresponding numerical value associated with any one of the aforementioned 12 physicochemical properties. Since the values of the 12 properties were at different levels, to make them easier to be handled, we normalized them into the range 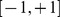 by means of the following equation
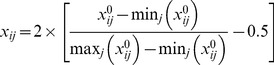
(3)where 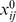 is the original value of the *i*-th DNA physicochemical property (*i* = 1, 2, …, 12) for the *j*-th (*j* = AA, AC, AG, AT, CA, CC, CG, CT, GA, GC, GG, GT, TA, TC, TG, TT) dinucleotide (see Information S2); while 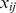 the corresponding normalized value (see **Table 1**).

**Table 1:** The normalized values for the 12 physicochemical properties of dinucleotide.

Dinuc-leotide	Physicochemical properties^a^											
	P(1)	P(2)	P(3)	P(4)	P(5)	P(6)	P(7)	P(8)	P(9)	P(10)	P(11)	P(12)
AA	0.85	0.85	−0.22	−0.96	−0.73	−0.62	−0.09	0.68	−1.00	1.00	0.36	0.23
AC	−1.00	−1.00	−0.60	−0.45	−0.27	0.37	−0.64	0.37	0.05	0.56	−0.48	0.50
AG	−0.56	−0.56	−1.00	0.45	−0.27	−0.18	−0.36	0.37	−0.12	−0.10	−0.39	0.04
AT	−0.01	−0.01	1.00	−1.00	−1.00	−0.48	−1.00	1.00	−0.31	−0.90	−1.00	1.00
CA	1.00	1.00	0.13	1.00	−0.27	−0.65	−0.09	0.16	0.75	−0.14	0.88	−0.77
CC	−0.87	−0.87	−0.25	0.81	1.00	0.15	1.00	−0.47	1.00	−0.75	−0.15	−0.35
CG	−0.14	−0.14	−0.89	0.80	0.18	−0.10	1.45	−1.00	0.64	−0.45	0.60	−1.00
CT	−0.56	−0.56	−1.00	−0.29	−0.27	−0.18	−0.36	0.37	−0.12	−1.00	−0.39	0.04
GA	0.87	0.87	0.43	1.24	−0.27	−0.30	−0.36	0.37	−0.02	−0.87	0.65	0.04
GC	0.32	0.32	0.24	1.17	0.18	1.00	1.00	−0.47	0.44	−0.54	0.01	0.27
GG	−0.87	−0.87	−0.25	0.63	1.00	0.15	1.00	−0.47	1.00	−0.14	−0.15	−0.35
GT	−1.00	−1.00	−0.60	−0.29	−0.27	0.37	−0.64	0.37	0.05	−0.90	−0.48	0.50
AA	0.32	0.32	−0.84	2.37	−1.00	−1.00	−0.45	1.00	0.29	−0.87	1.00	−0.31
AC	0.87	0.87	0.43	0.24	−0.27	−0.30	−0.36	0.37	−0.02	−0.45	0.65	0.04
AG	1.00	1.00	0.13	2.02	−0.27	−0.65	−0.09	0.16	0.75	0.56	0.88	−0.77
AT	0.85	0.85	−0.22	−1.00	−0.73	−0.62	−0.09	0.68	−1.00	−0.77	0.36	0.23

a In this table, the following symbols were used to represent the 12 physicochemical properties of DNA: P(1) for “A-philicity” [22], P(2) for “base stacking” [23], P(3) for “B-DNA twist” [24], P(4) for “bendability” [25], P(5) for “DNA bending stiffness” [26], P(6) for “DNA denaturation” [27], P(7) for “duplex disrupt energy” [28], P(8) for “duplex free energy” [29], P(9) for “propeller twist” [30], P(10) for “protein deformation” [31], P(11) for “protein-DNA twist” [31], and P(12) for “Z-DNA” [32].

#### Step 2

By means of a sliding window [33], [34] approach with a window size of 2 bp and a step size of 1 bp, a DNA sequence was replaced by the corresponding normalized physicochemical values. Thus, each of the sequences in 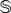 was translated into a numerical vector consisting of 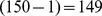 components, i.e., a 149-D (dimensional) numerical vector.

#### Step 3

After going through the above step with all the 12 physicochemical properties, each of the sequences in 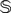 was translated to 12 different 149-D vectors corresponding to the 12 physicochemical features. By combining the 12 vectors, we obtain an integrated vector containing 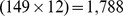 components; i.e., each of the nucleosomal sequences in 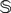 can be formulated as a 1788-D vector
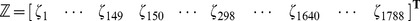
(4)where the first 149 components were derived from the property “A-philicity” or P(1), the second 149 components from the property “base stacking” or P(2), the last 149 components from the property “Z-DNA” or P(12) (cf. **Table 1**), and **T** the transposing operator.

### 3. Covariant or Quadratic Discriminant Function

The covariant discriminant (CD) or quadratic discriminant (QD) function has been widely used in the realm of bioinformatics, such as protein structural class prediction [35], [36], [37], protein coding region identification [38], protein subcellular location prediction [39], [40], splice site prediction [41], membrane protein type and location prediction [42], out membrane protein prediction [43], enzyme family class prediction [44], antimicrobial peptide classification [45], and prediction of protein cellular attributes [46].

Its formulation can be briefly described as follows. Suppose the standard feature vectors for the DNA sequences in 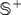 and 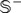 are, respectively, expressed by
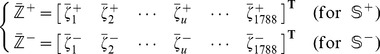
(5)where
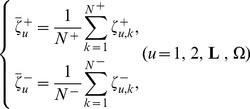
(6)where 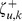 is the *u*-th component of the feature vector for the *k*-th sequence in the positive dataset 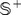, 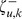 that for the *k*-th sequence in the negative dataset 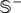, 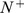 the total number of DNA sequences in 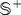, 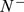 that in 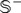, and 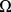 the total number of components in a feature vector. For the current case, we have 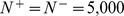 (cf. Information S1) and 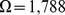 (cf. **Eq.4**).

Thus, whether a query DNA sequence belongs to the nucleosome-forming subset 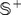 or nucleosome-inhibiting subset 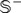 will be judged by
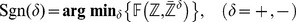
(7)where 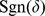 is the argument of 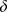 that minimizes 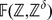, which is defined by
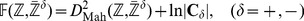
(8)where
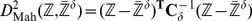
(9)is the squared Mahalanobis distance [47], [48], [49] between 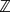 and 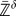,
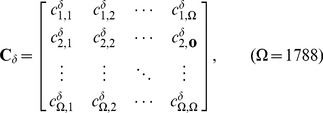
(10)is the covariance matrix [50] for the subset 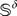 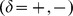, the 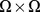 elements in 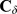 are given by
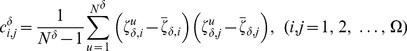
(11)

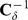
 is the inverse matrix of 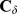, and 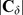 is the determinant of the matrix 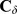. Therefore, the covariance discriminant function is also called the modified Mahalanobis discriminant function [51], [52]. More description about the covariance discriminant function and its application in biology can be found in a review [50].

### 4. Performance Evaluation

In statistical prediction, the following three cross-validation methods are often used to examine a predictor for its effectiveness in practical application: independent dataset test, subsampling (K-fold cross-validation) test, and jackknife test. However, as elaborated in ref. [53] and demonstrated by Eqs.28–32 of [15], among the three cross-validation methods, the jackknife test is deemed the least arbitrary that can always yield a unique result for a given benchmark dataset, and hence has been increasingly used and widely recognized by investigators to examine the accuracy of various predictors (see, e.g., [54], [55], [56], [57], [58], [59], [60]). However, since the current study would involve feature selection as described below, to reduce the computational time, the 5-fold cross-validation test would be adopted as done by many investigators using SVM (Support Vector Machine) as the prediction engine.

Also, to use a more intuitive and easier-to-understand method to measure the prediction quality, according to the definition [33], [61], the rates of correct predictions for the nucleosome-forming dataset 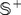 and the nucleosome-inhibiting dataset 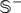 are respectively defined by
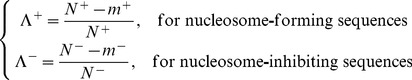
(12)where 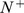 is the total number of nucleosome-forming sequences concerned and 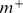 the number of nucleosome-forming sequences missed in prediction; 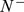 the total number of nucleosome-inhibiting sequences concerned and 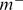 the number of nucleosome-inhibiting sequences missed in prediction. The overall success prediction rate is given by
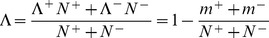
(13)It is clear from **Eqs.12**–**13** that, if and only if none of nucleosome-forming sequences and nucleosome-inhibiting sequences are mispredicted, i.e., 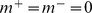 and 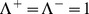, we have the overall success rate 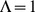. Otherwise, the overall success rate would be smaller than 1.

### 5. Feature Selection

Inclusion of redundant and noisy information would cause poor prediction results and increase computational time. To improve the prediction quality and gain deeper insights into the physicochemical properties of nucleosomal sequences, we performed feature selection using the wrapper-type feature selection algorithm called “fselect.py”, which can be downloaded at http://www.csie.ntu.edu.tw/~cjlin/libsvmtools. The basic idea of this algorithm is to rank each of the features involved according to a score as elaborated by Chen and Lin [62]. The ranked feature with a higher score indicates that it is a more highly relevant one for the target to be predicted. Based on the ranked features, we used the Incremental Feature Selection (IFS) [63] to determine the optimal number of features. During the IFS procedure, features in the ranked feature set were added one by one from higher to lower rank. A new feature set was composed when one feature had been added. Thus, the *N* feature sets thus formed would be composed of *N* ranked features. The 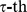 feature set can be formulated as
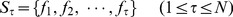
(14)For each of the *N* feature sets, a CD prediction model (cf. **Eq.7**) was constructed and examined with the 5-fold cross-validation on the benchmark dataset. By doing so, we obtained an IFS curve in a 2D Cartesian coordinate system with index 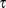 as its abscissa (or *X*-coordinate) and the overall success rate 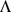 as its ordinate (or Y-coordinate). The optimal feature set is defined by
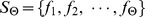
(15)with which the IFS curve reaches its peak. In other words, in the 2D coordinate system, when 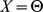 the value of 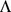 is the maximum. Thus, we can use the 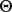 features in **Eq.15** to build the final predictor.

The predictor established by going through all the above procedures is called **iNuc-PhysChem**. Meanwhile, a user-friendly web-server for the predictor was also established as will be describe at the end of the paper.

## Results and Discussion

### 1. Graphic Profiles of Nucleosome and Non-nucleosome Sequences

**Figure 2:**
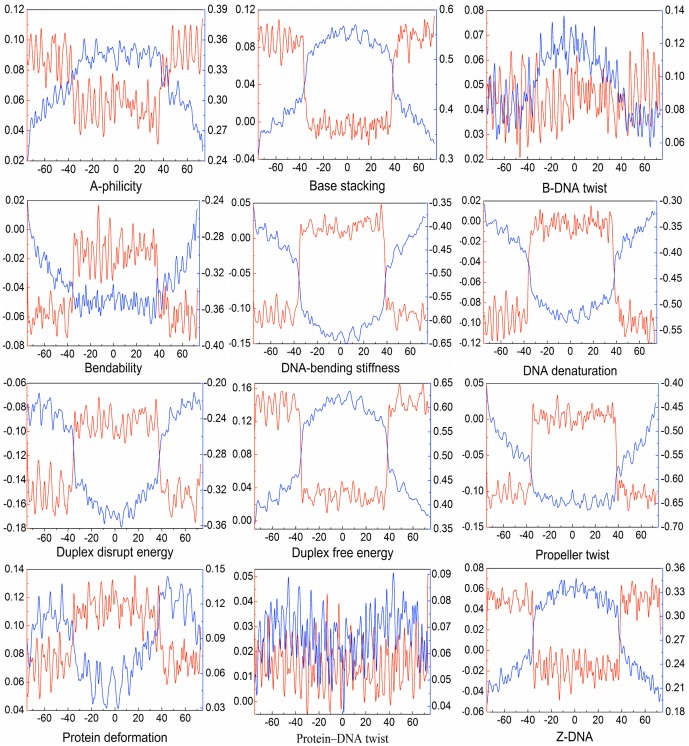
Graphic profiles to show the difference between nucleosomal (red) and linker (blue) sequences. It contains 12 panels drawn according to their standard feature vectors (cf. Eq.5), with each to reflect one of the 12 physicochemical features as marked at the bottom of each panel.

Different from the previous methods [10], [11], [12] that were mostly based on the sequence compositional features, we carried out a graphic profile comparison between nucleosomal and linker (non-nucleosomal) sequences in order to explore the specific features possessed by nucleosomal sequences. Using graphic approaches to study biological problems can provide an intuitive picture or useful insights for revealing complicated relations in these systems, as demonstrated by many previous studies on a series of important biological topics, such as enzyme-catalyzed reactions [64], [65], [66], [67], inhibition of HIV-1 reverse transcriptase [68], [69], protein folding kinetics [70], drug metabolism systems [71], and using wenxiang diagram or graph [72] to study protein-protein interactions [73], [74], [75]. To introduce graphic approach for the current study, let us use the conversion scheme [18] to transform the nucleosome and non-nucleosome sequences into the numerical vectors (cf. **Eq.4**). To intuitively show the difference between these two different types of sequences, a graphic expression of the standard feature vector (cf. **Eq.5**) for the nucleosomal sequences and that for the non-nucleosomal sequences are given in **Fig. 2**, which consists of 12 panels corresponding to 12 physicochemical properties of DNA sequences (cf. Section 2 of Materials and Method). The curves in the “A-philicity” panel reflect the first 149 components in the two standard feature vectors, those in the “base stacking” panel reflect the second 149 components, and so forth. It is interesting to note that, except for the “B-DNA twist” panel and “Protein-DNA twist” panel, the differences between the nucleosomal and non-nucleosomal sequences are quite remarkable in all the other 10 panels. These findings suggest that the two physicochemical properties might play a less role in distinguishing nucleosomal and non-nucleosomal sequences than the other 10 properties.

### 2. Comparison of the 12 Properties in Classification Performance

**Figure 3:**
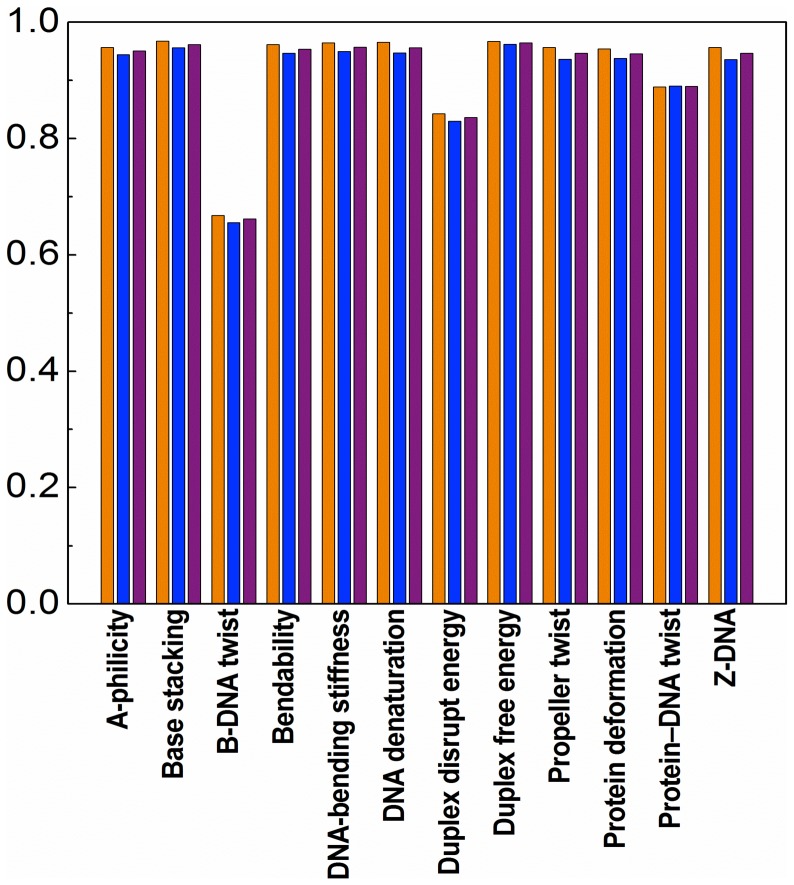
Comparison of success rates based on different physicochemical properties. The orange column shows 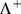, the rate of correct prediction for the nucleosome-forming dataset (cf. Eq.10); the blue column shows 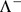, the rate of correct predictions for the nucleosome-inhibiting dataset; the purple column shows 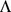, the overall success rate (cf. Eq.11).

In order to compare the 12 physicochemical properties for the classification performance, the feature vector **Eq.4**, standard vector **Eq.5**, and classifier **Eq.7** were reduced from the original 1788-D working space to twelve 149-D sub-working spaces. Each of the sub-working spaces corresponds to one of the 12 physicochemical properties. Shown in **Fig. 3** are their success rates in the classification performance when examined by the 5-fold cross-validation on the benchmark dataset 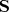. As can be seen from **Fig. 3**, the success rates obtained by using the “B-DNA twist” and “protein-DNA twist” properties are indeed remarkably lower than those by most of the other properties, quite consistent with the graphic profile analysis of last section.

### 3. Selection of Position Specific DNA Features

To identify the key features for nucleosomal sequence prediction, we used the wrapper-type feature selection algorithm and IFS approach as described in Section 5 of Materials and Method.

**Figure 4:**
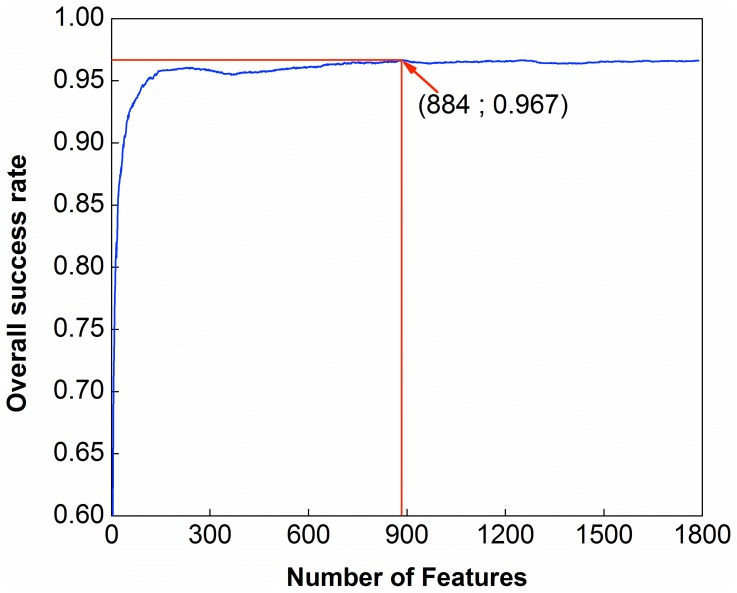
A plot to show the IFS procedure. When the top 884 of the 1,788 features were used to perform prediction, the overall success rate 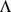 reached its peak of 0.967.

By adding the ranked features one by one according to the scores calculated by fselect.py, we built 1,788 individual CD predictors for the 1,788 sub-feature sets. We then tested the prediction performance for each of the 1,788 predictors and plotted the IFS curve as shown in **Fig. 4**, from which we can see that, when the top ranked 884 features were used, the overall success rate reached its peak, i.e., 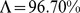 (cf. **Eq.13**), with 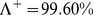 for the nucleosome-forming sequences and 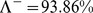 for the nucleosome-inhibiting sequences (cf. **Eq.12**).

**Figure 5:**
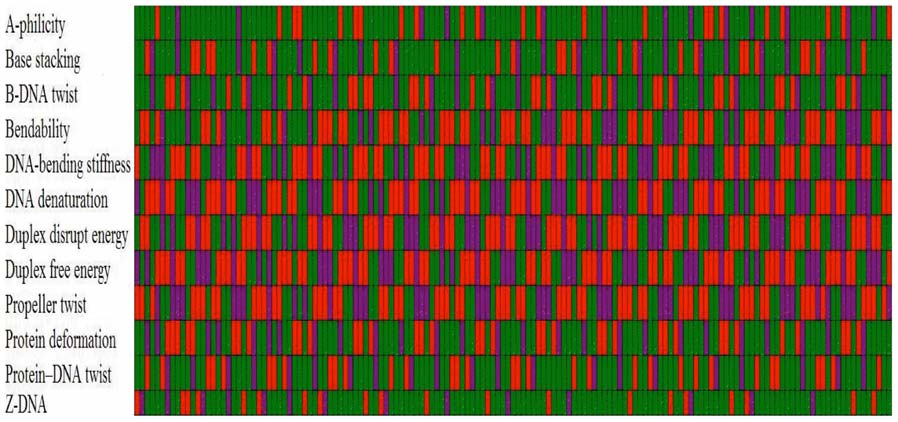
A distribution overall view for the 12 physicochemical features. The features that were included in the optimal feature set *S*_884_ are shown in the red and purple boxes: the former was positively correlated with nucleosomal sequences, while the latter negatively correlated with nucleosomal sequences. Those features that were not in the optimal feature set *S*_884_ are shown in the green boxes.

In other words, we have 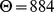 (cf. **Eq.15**) and the optimal feature set for the current biological system should be
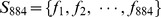
(16)To provide an overall view, a distribution of the 12 physicochemical features and their roles for the prediction model is given in **Fig. 5**, where the green boxes indicate the features that were not contained in the optimal feature set *S*_884_. The red and purple boxes indicate the features that were included in the optimal feature set *S*_884_: features in red boxes were positively correlated with nucleosomal sequences, while those in purple boxes were negatively correlated with nucleosomal sequences.

### 4. Comparison with Existing Methods

Based on the 2-mer absolute frequency of nucleotides, Zhang et al. [76] proposed a model to distinguish nucleosomal and linker sequences. When tested by the 5-fold cross-validation on the benchmark dataset, their method achieved an overall success rate of 95.70%, which is lower than that by the present method.

Furthermore, our model trained on the yeast data was also applied to the human genome. According to the human reference genome (hg 18), we randomly extracted 1000 nucleosomal and 1000 linker sequences from the high-resolution experimental data of human CD 4^+^ T cell [9]. Our model achieved an overall success rate of 98.5% for classifying the experimentally confirmed nucleosomal and linker sequences in the human genome. This result is higher than 93.8% obtained by using the model proposed by Peckham et al. [10], which has also been applied to predict human nucleosomal sequences by Gupta et al. [12]. All these results indicate that it is a quite promising approach by incorporating the DNA physicochemical features for predicting the nucleosomal sequences, and also suggest a conserved mechanism of nucleosome positioning across genomes.

Different with most current nucleosome positioning prediction methods that were solely relied on local sequence compositional information, in this study we developed a new method by incorporating the physicochemical features of DNA sequences. Our rationale to do so is that, different from the other nucleotide information, the physicochemical properties might affect DNA binding of regulatory proteins, either directly by hampering or favoring complex formation, or indirectly through the modulation of the chromatin structure and hence the DNA accessibility [77]. Therefore, the current method may become a useful vehicle for in-depth studying nucleosomes.

### 5. Web-Server Guide

For the convenience of the vast majority of experimental scientists, below let us give a step-by-step guide on how to use the **iNuc-PhysChem** web-server to get their desired results without the need to follow the complicated mathematic equations that were presented just for the integrity in developing the predictor.

#### Step 1

**Figure 6:**
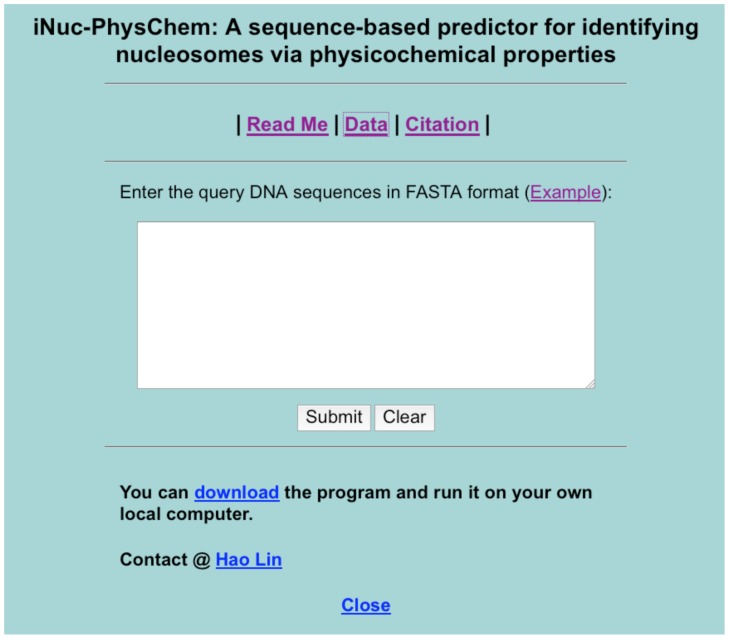
A screenshot to show the top page of the iNuc-PhysChem web-server. Its website address is at http://lin.uestc.edu.cn/server/iNuc-PhysChem.

Open the web server at http://lin.uestc.edu.cn/server/iNuc-PhysChem and you will see the top page of **iNuc-PhysChem** on your computer screen, as shown in **Fig. 6**. Click on the Read Me button to see a brief introduction about the predictor and the caveat when using it.

#### Step 2

Either type or copy and paste the query DNA sequence into the input box at the center of **Fig. 6**. The input sequence should be in the FASTA format. A sequence in FASTA format consists of a single initial line beginning with a greater-than symbol (“>”) in the first column, followed by lines of sequence data. The words right after the “>” symbol in the single initial line are optional and only used for the purpose of identification and description. All lines should be no longer than 120 characters and usually do not exceed 80 characters. The sequence ends if another line starting with a “>” appears; this indicates the start of another sequence. Example sequences in FASTA format can be seen by clicking on the Example button right above the input box.

#### Step 3

Click on the Submit button to see the predicted result. For example, if you use the three query DNA sequences in the Example window as the input, after clicking the Submit button, you will see the following shown on the screen of your computer: the outcome for the 1^st^ query sample (with 150 bp long) is “**nucleosome**”; the outcome for the 2^nd^ query sample (with 150 bp long) is “**linker**”; the outcome for the 3^rd^ query sample (with 502 bp long) contains 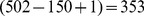 sub-results, in which the outcomes for the segments from #1 to #61 are of “**linker**”, those for the segments from #62 to #198 are of “nucleosome”, and those from #199 to #353 are of “**linker**”. All these results are fully consistent with the experimental observations as summarized in the Information S1. It takes about few seconds for the above computation before the predicted result appears on your computer screen; the more number of query sequences and longer of each sequence, the more time it is usually needed.

#### Step 4

Click on the Citation button to find the relevant papers that document the detailed development and algorithm of **iNuc-PhysChem**.

#### Step 5

Click on the Data button to download the benchmark datasets used to train and test the **iNuc-PhysChem** predictor.

#### Step 6

The program is also available by clicking the button download on the lower panel of **Fig. 6**.

### 6. Some Remarks

In this study although **iNuc-PhysChem** was trained by the dataset derived from *Saccharomyces cerevisiae*, it can be successfully used to identify nucleosome positioning for an independent DNA segment extracted from the *Saccharomyces cerevisiae* genome, as demonstrated by the 3^rd^ sequence in the Example window of the **iNuc-PhysChem** web-server. Particularly, it can be also successfully used to classify nucleosomal and linker sequences in the human genome, as elaborated in Section 4 of Results and Discussion. Therefore, it is anticipated that **iNuc-PhysChem** can be successfully used to identify nucleosome in the whole genome as well.

The current study was focused on the demonstration that the physicochemical properties of DNA are important for nucleosome positioning prediction. Since the physicochemical properties of DNA can be used to describe the interaction between DNA and chromatin remodeling complexes in vivo, here we just used the in vivo data for the current study. However, it is instructive to point out that although in vivo and in vitro nucleosome maps are similar, promoters and DNA replication regions, where nucleosomal sequences are depleted in vivo, are strongly affected by nucleosome remodeling [78], [79]. In view of this, we shall consider in our future work to use in vitro nucleosome maps [78] and the raw data from [80] to train the prediction model. Also, it is intriguing to analyze the impacts of different conformations (such as B- and Z-form) of DNA to nucleosome positioning, and will be investigated in our future studies as well.

Based on the results as reported in Section 4 of the Results and Discussion, we believe that the user-friendly web-server **iNuc-PhysChem** as proposed in this paper may serve as a useful tool for studying nucleosome positioning. Or at the very least, it can play a complimentary role to the existing methods in this area. Meanwhile, we also sincerely hope to hear any feedbacks (either positive or negative) from the users in using **iNuc-PhysChem** to generate their desired data. Their feedbacks will be very useful for us to improve the performance of **iNuc-PhysChem**.

## Supporting Information

Information S1
**The benchmark dataset** 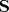 **consists of a positive dataset** 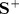 **and a negative dataset** 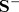 **.** The positive dataset contains 5,000 nucleosome-forming DNA segments, while the negative dataset contains 5,000 nucleosome-inhibiting DNA segments. Each of these segments is 150-bp long.(PDF)

Information S2 **The original numerical values for the 12 physicochemical properties of dinucleotide, where the physicochemical property “A-philicity”** [**22**] **is denoted by P(1); “base stacking”** [**23**] **by P(2); “B-DNA twist”** [**24**] **by P(3); “bendability”** [**25**] **by P(4); “DNA bending stiffness”** [**26**] **by P(5); “DNA denaturation”** [**27**] **by P(6); “duplex disrupt energy”** [**28**] **by P(7); “duplex free energy”** [**29**] **by P(8); “propeller twist”** [**30**] **by P(9); “protein deformation”** [**31**] **by P(10); “protein-DNA twist”** [**31**] **by P(11); and “Z-DNA”** [**32**] **by P(12).** Their values were taken from the papers cited above, respectively.
(PDF)

## References

[1] Luger K, Mader AW, Richmond RK, Sargent DF, Richmond TJ (1997) Crystal structure of the nucleosome core particle at 2.8 A resolution. Nature 389: 251–260. 9305837 10.1038/38444

[2] Berbenetz NM, Nislow C, Brown GW (2010) Diversity of eukaryotic DNA replication origins revealed by genome-wide analysis of chromatin structure. PLoS Genet 6. 10.1371/journal.pgen.1001092 PMC2932696 20824081

[3] Tilgner H, Nikolaou C, Althammer S, Sammeth M, Beato M, et al (2009) Nucleosome positioning as a determinant of exon recognition. Nat Struct Mol Biol 16: 996–1001. 19684599 10.1038/nsmb.1658

[4] Yasuda T, Sugasawa K, Shimizu Y, Iwai S, Shiomi T, et al (2005) Nucleosomal structure of undamaged DNA regions suppresses the non-specific DNA binding of the XPC complex. DNA Repair (Amst) 4: 389–395. 15661662 10.1016/j.dnarep.2004.10.008

[5] Yuan GC, Liu JS (2008) Genomic sequence is highly predictive of local nucleosome depletion. PLoS Comput Biol 4: e13. 18225943 10.1371/journal.pcbi.0040013 PMC2211532

[6] Valouev A, Ichikawa J, Tonthat T, Stuart J, Ranade S, et al (2008) A high-resolution, nucleosome position map of C. elegans reveals a lack of universal sequence-dictated positioning. Genome Res 18: 1051–1063. 18477713 10.1101/gr.076463.108 PMC2493394

[7] Miele V, Vaillant C, d'Aubenton-Carafa Y, Thermes C, Grange T (2008) DNA physical properties determine nucleosome occupancy from yeast to fly. Nucleic Acids Res 36: 3746–3756. 18487627 10.1093/nar/gkn262 PMC2441789

[8] Segal E, Fondufe-Mittendorf Y, Chen L, Thastrom A, Field Y, et al (2006) A genomic code for nucleosome positioning. Nature 442: 772–778. 16862119 10.1038/nature04979 PMC2623244

[9] Schones DE, Cui K, Cuddapah S, Roh TY, Barski A, et al (2008) Dynamic regulation of nucleosome positioning in the human genome. Cell 132: 887–898. 18329373 10.1016/j.cell.2008.02.022 PMC10894452

[10] Peckham HE, Thurman RE, Fu Y, Stamatoyannopoulos JA, Noble WS, et al (2007) Nucleosome positioning signals in genomic DNA. Genome Res 17: 1170–1177. 17620451 10.1101/gr.6101007 PMC1933512

[11] Ioshikhes IP, Albert I, Zanton SJ, Pugh BF (2006) Nucleosome positions predicted through comparative genomics. Nat Genet 38: 1210–1215. 16964265 10.1038/ng1878

[12] Gupta S, Dennis J, Thurman RE, Kingston R, Stamatoyannopoulos JA, et al (2008) Predicting human nucleosome occupancy from primary sequence. PLoS Comput Biol 4: e1000134. 18725940 10.1371/journal.pcbi.1000134 PMC2515632

[13] Jiang C, Pugh BF (2009) Nucleosome positioning and gene regulation: advances through genomics. Nat Rev Genet 10: 161–172. 19204718 10.1038/nrg2522 PMC4860946

[14] Nozaki T, Yachie N, Ogawa R, Saito R, Tomita M (2011) Computational analysis suggests a highly bendable, fragile structure for nucleosomal DNA. Gene 476: 10–14. 21338662 10.1016/j.gene.2011.02.004

[15] Chou KC (2011) Some remarks on protein attribute prediction and pseudo amino acid composition (50th Anniversary Year Review). Journal of Theoretical Biology 273: 236–247. 21168420 10.1016/j.jtbi.2010.12.024 PMC7125570

[16] Lee W, Tillo D, Bray N, Morse RH, Davis RW, et al (2007) A high-resolution atlas of nucleosome occupancy in yeast. Nat Genet 39: 1235–1244. 17873876 10.1038/ng2117

[17] Bajic VB, Tan SL, Suzuki Y, Sugano S (2004) Promoter prediction analysis on the whole human genome. Nat Biotechnol 22: 1467–1473. 15529174 10.1038/nbt1032

[18] Florquin K, Saeys Y, Degroeve S, Rouze P, Van de Peer Y (2005) Large-scale structural analysis of the core promoter in mammalian and plant genomes. Nucleic Acids Res 33: 4255–4264. 16049029 10.1093/nar/gki737 PMC1181242

[19] Heddi B, Abi-Ghanem J, Lavigne M, Hartmann B (2010) Sequence-dependent DNA flexibility mediates DNase I cleavage. J Mol Biol 395: 123–133. 19850052 10.1016/j.jmb.2009.10.023

[20] Milani P, Chevereau G, Vaillant C, Audit B, Haftek-Terreau Z, et al (2009) Nucleosome positioning by genomic excluding-energy barriers. Proc Natl Acad Sci U S A 106: 22257–22262. 20018700 10.1073/pnas.0909511106 PMC2799728

[21] Fujii S, Kono H, Takenaka S, Go N, Sarai A (2007) Sequence-dependent DNA deformability studied using molecular dynamics simulations. Nucleic Acids Res 35: 6063–6074. 17766249 10.1093/nar/gkm627 PMC2094071

[22] Ivanov VI, Minchenkova LE (1994) [The A-form of DNA: in search of the biological role]. Mol Biol (Mosk) 28: 1258–1271. 7885327

[23] Ornstein RL, Rein R, Breen DL, Macelroy RD (2004) An optimized potential function for the calculation of nucleic acid interaction energies I. Base stacking. Biopolymers 17: 2341–2360. 10.1002/bip.1978.360171005 24624489

[24] Gorin AA, Zhurkin VB, Olson WK (1995) B-DNA twisting correlates with base-pair morphology. J Mol Biol 247: 34–48. 7897660 10.1006/jmbi.1994.0120

[25] Brukner I, Sanchez R, Suck D, Pongor S (1995) Trinucleotide models for DNA bending propensity: comparison of models based on DNaseI digestion and nucleosome packaging data. J Biomol Struct Dyn 13: 309–317. 8579790 10.1080/07391102.1995.10508842

[26] Sivolob AV, Khrapunov SN (1995) Translational positioning of nucleosomes on DNA: the role of sequence-dependent isotropic DNA bending stiffness. J Mol Biol 247: 918–931. 7723041 10.1006/jmbi.1994.0190

[27] Blake RD, Delcourt SG (1998) Thermal stability of DNA. Nucleic Acids Res 26: 3323–3332. 9649614 10.1093/nar/26.14.3323 PMC147704

[28] Breslauer KJ, Frank R, Blocker H, Marky LA (1986) Predicting DNA duplex stability from the base sequence. Proc Natl Acad Sci U S A 83: 3746–3750. 3459152 10.1073/pnas.83.11.3746 PMC323600

[29] Sugimoto N, Nakano S, Yoneyama M, Honda K (1996) Improved thermodynamic parameters and helix initiation factor to predict stability of DNA duplexes. Nucleic Acids Res 24: 4501–4505. 8948641 10.1093/nar/24.22.4501 PMC146261

[30] el Hassan MA, Calladine CR (1996) Propeller-twisting of base-pairs and the conformational mobility of dinucleotide steps in DNA. J Mol Biol 259: 95–103. 8648652 10.1006/jmbi.1996.0304

[31] Olson WK, Gorin AA, Lu XJ, Hock LM, Zhurkin VB (1998) DNA sequence-dependent deformability deduced from protein-DNA crystal complexes. Proc Natl Acad Sci U S A 95: 11163–11168. 9736707 10.1073/pnas.95.19.11163 PMC21613

[32] Ho PS, Zhou GW, Clark LB (1990) Polarized electronic spectra of Z-DNA single crystals. Biopolymers 30: 151–163. 2224047 10.1002/bip.360300115

[33] Chou KC (2001) Prediction of protein signal sequences and their cleavage sites. PROTEINS: Structure, Function, and Genetics 42: 136–139. 10.1002/1097-0134(20010101)42:1<136::aid-prot130>3.0.co;2-f 11093267

[34] Chou KC (2001) Using subsite coupling to predict signal peptides. Protein Engineering 14: 75–79. 11297664 10.1093/protein/14.2.75

[35] Chou KC, Liu W, Maggiora GM, Zhang CT (1998) Prediction and classification of domain structural classes. PROTEINS: Structure, Function, and Genetics 31: 97–103. 9552161

[36] Zhou GP (1998) An intriguing controversy over protein structural class prediction. Journal of Protein Chemistry 17: 729–738. 9988519 10.1023/a:1020713915365

[37] Zhou GP, Assa-Munt N (2001) Some insights into protein structural class prediction. PROTEINS: Structure, Function, and Genetics 44: 57–59. 10.1002/prot.1071 11354006

[38] Zhang MQ (1997) Identification of protein coding regions in the human genome by quadratic discriminant analysis. Proc Natl Acad Sci U S A 94: 565–568. 9012824 10.1073/pnas.94.2.565 PMC19553

[39] Chou KC, Elrod DW (1998) Using discriminant function for prediction of subcellular location of prokaryotic proteins. Biochem Biophys Res Commun 252: 63–68. 9813147 10.1006/bbrc.1998.9498

[40] Zhou GP, Doctor K (2003) Subcellular location prediction of apoptosis proteins. PROTEINS: Structure, Function, and Genetics 50: 44–48. 10.1002/prot.10251 12471598

[41] Zhang L, Luo L (2003) Splice site prediction with quadratic discriminant analysis using diversity measure. Nucleic Acids Res 31: 6214–6220. 14576308 10.1093/nar/gkg805 PMC275452

[42] Chou KC, Elrod DW (1999) Prediction of membrane protein types and subcellular locations. PROTEINS: Structure, Function, and Genetics 34: 137–153. 10336379

[43] Lin H (2008) The modified Mahalanobis Discriminant for predicting outer membrane proteins by using Chou's pseudo amino acid composition. J Theor Biol 252: 350–356. 18355838 10.1016/j.jtbi.2008.02.004

[44] Chou KC (2005) Using amphiphilic pseudo amino acid composition to predict enzyme subfamily classes. Bioinformatics 21: 10–19. 15308540 10.1093/bioinformatics/bth466

[45] Chen W, Luo L (2009) Classification of antimicrobial peptide using diversity measure with quadratic discriminant analysis. J Microbiol Methods 78: 94–96. 19348863 10.1016/j.mimet.2009.03.013

[46] Chou KC (2001) Prediction of protein cellular attributes using pseudo amino acid composition. PROTEINS: Structure, Function, and Genetics (Erratum: ibid, 2001, Vol 44, 60) 43: 246–255. 10.1002/prot.1035 11288174

[47] Mahalanobis PC (1936) On the generalized distance in statistics. Proc Natl Inst Sci India 2: 49–55.

[48] Chou KC (1995) A novel approach to predicting protein structural classes in a (20-1)-D amino acid composition space. Proteins: Structure, Function & Genetics 21: 319–344. 10.1002/prot.340210406 7567954

[49] Pillai KCS (1985) Mahalanobis D2. In: Kotz S, Johnson NL, editors. Encyclopedia of Statistical Sciences. New York: John Wiley & Sons. This reference also presents a brief biography of Mahalanobis who was a man of great originality and who made considerable contributions to statistics. pp. 176–181.

[50] Chou KC, Shen HB (2007) Review: Recent progresses in protein subcellular location prediction. Analytical Biochemistry 370: 1–16. 17698024 10.1016/j.ab.2007.07.006

[51] Liu W, Chou KC (1998) Prediction of protein structural classes by modified Mahalanobis discriminant algorithm. Journal of Protein Chemistry 17: 209–217. 9588944 10.1023/a:1022576400291

[52] Chou KC (1999) A key driving force in determination of protein structural classes. Biochemical and Biophysical Research Communications 264: 216–224. 10527868 10.1006/bbrc.1999.1325

[53] Chou KC, Shen HB (2008) Cell-PLoc: A package of Web servers for predicting subcellular localization of proteins in various organisms (updated version: Cell-PLoc 2.0: An improved package of web-servers for predicting subcellular localization of proteins in various organisms, Natural Science, 2010, 2, 1090–1103; doi:10.4236/ns.2010.210136). Nature Protocols 3: 153–162. 18274516 10.1038/nprot.2007.494

[54] Mohabatkar H (2010) Prediction of cyclin proteins using Chou's pseudo amino acid composition. Protein & Peptide Letters 17: 1207–1214. 20450487 10.2174/092986610792231564

[55] Sahu SS, Panda G (2010) A novel feature representation method based on Chou's pseudo amino acid composition for protein structural class prediction. Computational Biology and Chemistry 34: 320–327. 21106461 10.1016/j.compbiolchem.2010.09.002

[56] Chou KC, Wu ZC, Xiao X (2012) iLoc-Hum: Using accumulation-label scale to predict subcellular locations of human proteins with both single and multiple sites. Molecular Biosystems 8: 629–641. 22134333 10.1039/c1mb05420a

[57] Esmaeili M, Mohabatkar H, Mohsenzadeh S (2010) Using the concept of Chou's pseudo amino acid composition for risk type prediction of human papillomaviruses. Journal of Theoretical Biology 263: 203–209. 19961864 10.1016/j.jtbi.2009.11.016

[58] Qin YF, Wang CH, Yu XQ, Zhu J, Liu TG, et al (2012) Predicting Protein Structural Class by Incorporating Patterns of Over- Represented k-mers into the General form of Chou's PseAAC. Protein & Peptide Letters 19: 388–397. 22316305 10.2174/092986612799789350

[59] Chou KC, Wu ZC, Xiao X (2011) iLoc-Euk: A Multi-Label Classifier for Predicting the Subcellular Localization of Singleplex and Multiplex Eukaryotic Proteins. PLoS One 6: e18258. 21483473 10.1371/journal.pone.0018258 PMC3068162

[60] Zhao XW, Ma ZQ, Yin MH (2012) Predicting protein-protein interactions by combing various sequence- derived features into the general form of Chou's Pseudo amino acid composition. Protein & Peptide Letters 19: 492–500. 22486644 10.2174/092986612800191080

[61] Chou KC (2001) Prediction of signal peptides using scaled window. Peptides 22: 1973–1979. 11786179 10.1016/s0196-9781(01)00540-x

[62] Chen YW, Lin C.J. (2006) Combining SVMs with Various Feature Selection Strategies. ; Guyon I, Elisseeff A, editors: Springer Physica Verlag Pub.

[63] Huang T, Niu S, Xu Z, Huang Y, Kong X, et al (2011) Predicting Transcriptional Activity of Multiple Site p53 Mutants Based on Hybrid Properties. PLoS ONE 6: e22940. 21857971 10.1371/journal.pone.0022940 PMC3152557

[64] Chou KC, Forsen S (1980) Graphical rules for enzyme-catalyzed rate laws. Biochemical Journal 187: 829–835. 7188428 10.1042/bj1870829 PMC1162468

[65] Zhou GP, Deng MH (1984) An extension of Chou's graphic rules for deriving enzyme kinetic equations to systems involving parallel reaction pathways. Biochemical Journal 222: 169–176. 6477507 10.1042/bj2220169 PMC1144157

[66] Chou KC (1989) Graphic rules in steady and non-steady enzyme kinetics. Journal of Biological Chemistry 264: 12074–12079. 2745429

[67] Andraos J (2008) Kinetic plasticity and the determination of product ratios for kinetic schemes leading to multiple products without rate laws: new methods based on directed graphs. Canadian Journal of Chemistry 86: 342–357.

[68] Althaus IW, Chou JJ, Gonzales AJ, Diebel MR, Chou KC, et al (1993) Steady-state kinetic studies with the non-nucleoside HIV-1 reverse transcriptase inhibitor U-87201E. Journal of Biological Chemistry 268: 6119–6124. 7681060

[69] Althaus IW, Gonzales AJ, Chou JJ, Diebel MR, Chou KC, et al (1993) The quinoline U-78036 is a potent inhibitor of HIV-1 reverse transcriptase. Journal of Biological Chemistry 268: 14875–14880. 7686907

[70] Chou KC (1990) Review: Applications of graph theory to enzyme kinetics and protein folding kinetics. Steady and non-steady state systems. Biophysical Chemistry 35: 1–24. 2183882 10.1016/0301-4622(90)80056-d

[71] Chou KC (2010) Graphic rule for drug metabolism systems. Current Drug Metabolism 11: 369–378. 20446902 10.2174/138920010791514261

[72] Chou KC, Lin WZ, Xiao X (2011) Wenxiang: a web-server for drawing wenxiang diagrams. Natural Science 3: 862–865 doi:810.4236/ns.2011.310111 (openly accessible at http://www.scirp.org/journal/NS/

[73] Zhou GP (2011) The disposition of the LZCC protein residues in wenxiang diagram provides new insights into the protein-protein interaction mechanism. Journal of Theoretical Biology 284: 142–148. 21718705 10.1016/j.jtbi.2011.06.006 PMC7094099

[74] Kurochkina N, Choekyi T (2011) Helix-helix interfaces and ligand binding. Journal of Theoretical Biology 283: 92–102. 21620863 10.1016/j.jtbi.2011.05.014

[75] Zhou GP (2011) The Structural Determinations of the Leucine Zipper Coiled-Coil Domains of the cGMP-Dependent Protein Kinase I alpha and its Interaction with the Myosin Binding Subunit of the Myosin Light Chains Phosphase. Proteins & Peptide Letters 18: 966–978. 10.2174/0929866511107010966 21592084

[76] Zhang Z, Zhang Y, Gutman I (2012) Predicting nucleosome positions in yeast: using the absolute frequency. J Biomol Struct Dyn 29: 1081–1088. 22292961 10.1080/073911012010525032

[77] Goni JR, Perez A, Torrents D, Orozco M (2007) Determining promoter location based on DNA structure first-principles calculations. Genome Biol 8: R263. 18072969 10.1186/gb-2007-8-12-r263 PMC2246265

[78] Zhang Y, Moqtaderi Z, Rattner BP, Euskirchen G, Snyder M, et al (2009) Intrinsic histone-DNA interactions are not the major determinant of nucleosome positions in vivo. Nat Struct Mol Biol 16: 847–852. 19620965 10.1038/nsmb.1636 PMC2823114

[79] Tanaka Y, Yoshimura I, Nakai K (2010) Positional variations among heterogeneous nucleosome maps give dynamical information on chromatin. Chromosoma 119: 391–404. 20224866 10.1007/s00412-010-0264-y PMC2926881

[80] Kaplan DL, Bruck I (2010) Methods to study how replication fork helicases unwind DNA. Methods Mol Biol 587: 127–135. 20225146 10.1007/978-1-60327-355-8_9

