# Supplementary material for: iNuc-PhysChem: A Sequence-Based Predictor for Identifying Nucleosomes via Physicochemical Properties
Source: PLoS One. 2012 Oct 29;7(10):e47843. doi: 10.1371/journal.pone.0047843 (PMC3483203; doi:10.1371/journal.pone.0047843)
Supplement: Information S1 — The benchmark dataset consists of a positive dataset and a negative dataset . The positive dataset contains 5,000 nucleosome-forming DNA segments, while the negative dataset contains 5,000 nucleosome-inhibiting DNA segments. Each of these segments is 150-bp long. (PDF) [file pone.0047843.s001.pdf]

Supporting Information S1. The benchmark dataset $\mathbb{S}$ consists of a positive dataset $\mathbb{S}^{+}$ and a negative dataset $\mathbb{S}^{-}$. The positive dataset contains 5,000 nucleosome-forming DNA segments, while the negative dataset contains 5,000 nucleosome-inhibiting DNA segments. Each of these segments is 150-bp long.

I. The positive dataset $\mathbb{S}^{+}$ contains the following 5,000 nucleosome-forming DNA sequences of 150-bp long

GCCCAGAACGAAGAAATCAGTGCCACGCCAACTCCAAATCCAGAAAGCAGCGCAGGTGCAGATGACACTTC
CAGAGAAGCAAGTGCAAGTGCTGAAGGTGCTGAGGCCATTGAAGGCGACTTCATGTCTACTTTGAAGCAAT
CGAAGAAG

AAGAAATCAGTGCCACGCCAACTCCAAATCCAGAAAGCAGCGCAGGTGCAGATGACACTTCCAGAGAAGCA
AGTGCAAGTGCTGAAGGTGCTGAGGCCATTGAAGGCGACTTCATGTCTACTTTGAAGCAATCGAAGAAGAA
GCAAGAAA

CCATGAGATGTCAGGAATTGTTTCCAAGGTTGGTCCTAAAGTGACAAAGGTGAAGGTTGGCGACCACGTGG
TCGTTGATGCTGCCAGCAGTTGTGCGGACCTGCATTGCTGGCCACACTCCAAATTTTACAATTCCAAACCA
TGTGATGC

TCAGGAATTGTTTCCAAGGTTGGTCCTAAAGTGACAAAGGTGAAGGTTGGCGACCACGTGGTCGTTGATGC
TGCCAGCAGTTGTGCGGACCTGCATTGCTGGCCACACTCCAAATTTTACAATTCCAAACCATGTGATGCTT
GTCAGAGG

GCCATGGAACGACACTTTTACCTCTACTTCTACCGAATTGACCACAGTCACCGGTACCAATGGTTTGCCAA
CTGATGAGACCATCATTGTCATCAGAACACCAACAACAGCCACTACTGCCATGACTACAACTCAGCCATGG
AACGACAC

CGTCACCGGTACCAACGGCGTTCCAACTGACGAAACCGTCATTGTCATCAGAACTCCAACCAGTGAAGGTC
TAATCAGCACCACCACTGAACCATGGACTGGCACTTTCACTTCGACTTCCACTGAGGTTACCACCATCACT
GGAACCAA

GTCCTGAAGATGACGAAGATGAATTGATGGACGACGTTATGGATGATTTGACTGGTTTGTTGGACTCCGTT
GACACAACTGGTAAAGGTGTTGTGGTCCAAGCATCCACCTTGGGTTCTTTGGAAGCTTTGTTGGATTTCTT
GAAAGACA

GTTTGGAAGAACCATTCTTCATTGAGCCATTCAATGATCAGACTGACACGTTGCTCGAAATCCTGGATGAA
GAAGCCAAGCAGTTCTTCACGAATCAGGTCACTGGCCTCTTGTGCTTCGATTCCTCTCGTAACCAATCTGA
TTAAGACG

AAATGACGCACGTCACCGGTACCAACGGCGTTCCAACTGACGAAACCGTCATTGTCATCAGAACTCCAACC
AGTGAAGGTCTAATCAGCACCACCACTGAACCATGGACTGGCACTTTCACTTCGACTTCCACTGAGGTTAC
CACCATCA

TAACAATCACGGTTTCGTCAGTTGGTTGACCGTTAGTACCGGTGACGGTGGTCATCTCAGTGGATGTAGAG
GTGAAAGTACCAGTCCATGGTTCAGTGGTGGTGCTGATTAGACCTTCACTAGTTGGAGTTCTGATGACAAT
GACGGTTT

GCCATGGAACGACACTTTTACCTCTACATCCACTGAAATCACCACCGTCACCGGTACCAATGGTTTGCCAA
CTGATGAGACCATCATTGTCATCAGAACACCAACAACAGCCACTACTGCCATGACTACACCTCAGCCATGG
AACGACAC

ATCACTTTATCGTGCATCTTGACCACGTTATTTCTGCTGGTGAACGAGTGGGGACAGTTCAATTCTGTGGT
AACAAGGCCACAATTGGTGGTGGACCGTGACCGACACGCAAAGCTGGAGCTTAATATGGATGTGACATTTC
CATCGATG

CCTCTACATCCACTGAAATCACCACCGTCACCGGTACCAATGGTTTGCCAACTGATGAGACCATCATTGTC
ATCAGAACACCAACAACAGCCACTACTGCCATGACTACACCTCAGCCATGGAACGACACTTTTACCTCTAC
ATCCACTG

TGACGAAGATGAATTGATGGACGACGTTATGGATGATTTGACTGGTTTGTTGGACTCCGTTGACACAACTG
GTAAAGGTGTTGTGGTCCAAGCATCCACCTTGGGTTCTTTGGAAGCTTTGTTGGATTTCTTGAAAGACATG
AAAATCCC

ATTGTCATCAGAACTCCAACCAGTGAAGGTCTAATCAGCACCACCACTGAACCATGGACTGGCACTTTCAC
TTCGACTTCCACTGAGGTTACCACCATCACTGGAACCAACGGTCAACCAACTGACGAAACTGTGATTGTTA
TCAGAACT

GAATTGATGGACGACGTTATGGATGATTTGACTGGTTTGTTGGACTCCGTTGACACAACTGGTAAAGGTGT
TGTGGTCCAAGCATCCACCTTGGGTTCTTTGGAAGCTTTGTTGGATTTCTTGAAAGACATGAAAATCCCTG
TGATGTCT

CACTGAAATCACCACCGTCACCGGTACCAATGGTTTGCCAACTGATGAGACCATCATTGTCATCAGAACAC
CAACAACAGCCACTACTGCCATGACTACACCTCAGCCATGGAACGACACTTTTACCTCTACATCCACTGAA
ATGACCAC

ACTTCTACCGAATTGACCACAGTCACCGGTACCAATGGTTTGCCAACTGATGAGACCATCATTGTCATCAG
AACACCAACAACAGCCACTACTGCCATGACTACAACTCAGCCATGGAACGACACTTTTACCTCTACTTCTA
CCGAATTG

CGCCACCTGGAGCACCACCAGCTTGGTACAACTTAGACATGATTGGGTTGGCAATGTCTTGCAACTCCTTC
AACTTGTCATCGAATTCTTCCTTGCTGGCAGTGGTGTTGCTGTCTAACCAAGAAATAGTCTCTTCAGCCTT
CTTGGTGA

AATTGACCACAGTCACCGGTACCAATGGTTTGCCAACTGATGAGACCATCATTGTCATCAGAACACCAACA
ACAGCCACTACTGCCATGACTACAACTCAGCCATGGAACGACACTTTTACCTCTACTTCTACCGAATTGAC
CACAGTCA

ACTTCTACCGAATTGACCACAGTCACCGGTACCAATGGTTTGCCAACTGATGAGACCATCATTGTCATCAG
AACACCAACAACAGCCACTACTGCCATGACTACAACTCAGCCATGGAACGACACTTTTACCTCTACATCCA
CTGAAATC

CTTAAAGAATCTCAAGAGTTCCCAGAACCAATCTTCACCCCATCGACCAAGGCTGAACAAGGTGAACATGA
CGAAAACATCTCTCCTGCCCAGGCCGCTGAGCTGGTGGGTGAAGATTTGTCACGTAGAGTGGCAGAACTGG
CTGTAAAA

ACCTCAAGGACTTAAAGAATCTCAAGAGTTCCCAGAACCAATCTTCACCCCATCGACCAAGGCTGAACAAG
GTGAACATGACGAAAACATCTCTCCTGCCCAGGCCGCTGAGCTGGTGGGTGAAGATTTGTCACGTAGAGTG
GCAGAACT

CCTCTACTTCTACCGAATTGACCACAGTCACCGGTACCAATGGTTTGCCAACTGATGAGACCATCATTGTC
ATCAGAACACCAACAACAGCCACTACTGCCATGACTACAACTCAGCCATGGAACGACACTTTTACCTCTAC
TTCTACCG

TAGAATCCAGATACAAATCACAAGGTAACCTTAGAGTTTGAGCGAGAACTAGTTCAGCAGGGTGTAGCTCG
TTCCTCAGCGGGTCTGTGGACAGGTCCATTGGAGACCCCTTCCACTCGATCTTGAGTGACTTGTTACTGTC
TGTAGGAA

TACCGAATTGACCACAGTCACCGGTACCAATGGTTTGCCAACTGATGAGACCATCATTGTCATCAGAACAC
CAACAACAGCCACTACTGCCATGACTACAACTCAGCCATGGAACGACACTTTTACCTCTACTTCTACCGAA
TTGACCAC

AATTGACCACAGTCACCGGTACCAATGGTTTGCCAACTGATGAGACCATCATTGTCATCAGAACACCAACA
ACAGCCACTACTGCCATGACTACAACTCAGCCATGGAACGACACTTTTACCTCTACATCCACTGAAATCAC
CACCGTCA

ACACTGGCCACACCAGTTCTAGCCACATCAAAACCAATACCAGCACCACCACCGCTGCCGGATGAAGCACT
GGTCAACAATTCTCTTCTCAGTTTGGCCAGCTTGGCCTTCAGTTGACCCAAATGGAAAGATGTGGCCTTGT
TCTTTTGG

ACTGAGCCATGGAACAGCACTTTTACCTCTACTTCTACCGAATTGACCACAGTCACCGGTACCAATGGTTT
GCCAACTGATGAGACCATCATTGTCATCAGAACACCAACAACAGCCACTACTGCCATGACTACAACTCAGC
CATGGAAC

ACTCAGCCATGGAACGACACTTTTACCTCTACTTCTACCGAATTGACCACAGTCACCGGTACCAATGGTTT
GCCAACTGATGAGACCATCATTGTCATCAGAACACCAACAACAGCCACTACTGCCATGACTACAACTCAGC
CATGGAAC

ACATCTACTGAAATGACGCACGTCACCGGTACCAACGGCGTTCCAACTGACGAAACCGTCATTGTCATCAG
AACTCCAACCAGTGAAGGTCTAATCAGCACCACCACTGAACCATGGACTGGCACTTTCACTTCGACTTCCA
CTGAGGTT

ATCACAATGTCTAGATTAGAAAGATTGACCTCATTAAACGTTGTTGCTGGTTCTGACTTGAGAAGAACCTC
CATCATTGGTACCATCGGTCCAAAGACCAACAACCCAGAAACCTTGGTTGCTTTGAGAAAGGCTGGTTTGA
ACATTGTC

GAACTCCAACCAGTGAAGGTCTAATCAGCACCACCACTGAACCATGGACTGGCACTTTCACTTCGACTTCC
ACTGAGGTTACCACCATCACTGGAACCAACGGTCAACCAACTGACGAAACTGTGATTGTTATCAGAACTCC
AACCAGTG

ATGGGATCGTTATGAGATGACCTCGTGGTCATGGAGTCCCTGAGTGATGGTATGGACATCCCAGAATCCAT
CGAGTTTGTGAACTCGCTGTCGTCATCATCACCAGTGTTGTCATTATTGCGAAGATGCCTGCCACTAGGAA
TCCATCGA

GGTGACGGTGGTCATCTCAGTGGATGTAGAGGTGAAAGTACCAGTCCATGGTTCAGTGGTGGTGCTGATTA
GACCTTCACTAGTTGGAGTTCTGATGACAATGACGGTTTCGTCAGTTGGAACGCCGTTGGTACCGGTGACG
GTGGTCAT

GCAACCGTTTCCTCCAAGTCATACACCACTGTTACTGTTACTCACTGTGACAACAATGGCTGTAACACCAA
GACTGTCACTTCTGAATGTTCTAAAGAAACTGCAGCAACCACCATTTCTCCAAAATCATACACTACTGTTA
CCGTTACT

ACCAACGGCGTTCCAACTGACGAAACCGTCATTGTCATCAGAACTCCAACCAGTGAAGGTCTAATCAGCAC
CACCACTGAACCATGGACTGGCACTTTCACTTCGACTTCCACTGAGGTTACCACCATCACTGGAACCAACG
GTCAACCA

GTTTGAAACAACCTCAAGGACTTAAAGAATCTCAAGAGTTCCCAGAACCAATCTTCACCCCATCGACCAAG
GCTGAACAAGGTGAACATGACGAAAACATCTCTCCTGCCCAGGCCGCTGAGCTGGTGGGTGAAGATTTGTC
ACGTAGAG

GGAGTTCTGATAACAATCACGGTTTCGTCAGTTGGTTGACCGTTAGTACCGGTGACGGTGGTCATCTCAGT
GGATGTAGAGGTGAAAGTACCAGTCCATGGTTCAGTGGTGGTGCTGATTAGACCTTCACTAGTTGGAGTTC
TGATGACA

GCCATGGAACAGCACTTTTACCTCTACTTCTACCGAATTGACCACAGTCACTGGCACCAATGGTGTACGAA
CTGACGAAACCATCATTGTAATCAGAACACCAACAACAGCCACTACTGCCATAACTACAACTGAGCCATGG
AACAGCAC

CTACAACTCAGCCATGGAACGACACTTTTACCTCTACATCCACTGAAATCACCACCGTCACCGGTACCAAT
GGTTTGCCAACTGATGAGACCATCATTGTCATCAGAACACCAACAACAGCCACTACTGCCATGACTACACC
TCAGCCAT

ATCTTCACCCCATCGACCAAGGCTGAACAAGGTGAACATGACGAAAACATCTCTCCTGCCCAGGCCGCTGA
GCTGGTGGGTGAAGATTTGTCACGTAGAGTGGCAGAACTGGCTGTAAAACTGTACTCCAAGTGCAAAGATT
ATGCTAAG

CGAAACCGTCATTGTCATCAGAACTCCAACCAGTGAAGGTCTAATCAGCACCACCACTGAACCATGGACTG
GCACTTTCACTTCGACTTCCACTGAGGTTACCACCATCACTGGAACCAACGGTCAACCAACTGACGAAACT
GTGATTGT

CTCAAGAGTTCCCAGAACCAATCTTCACCCCATCGACCAAGGCTGAACAAGGTGAACATGACGAAAACATC
TCTCCTGCCCAGGCCGCTGAGCTGGTGGGTGAAGATTTGTCACGTAGAGTGGCAGAACTGGCTGTAAAACT
GTACTCCA

CGTCACCGGTACCAACGGCGTTCCAACTGACGAAACCGTCATTGTCATCAGAACTCCAACTAGTGAAGGTC
TAATCAGCACCACCACTGAACCATGGACTGGTACTTTCACCTCTACATCCACTGAGATGACCACCGTCACC
GGTACTAA

GGAAGCACAGAAGCAAGAGGAGGCGCATCGATCGTGGCAGATGAGTCAGCAAACACCACAGGAAAGTGAAC
AGACCACAGCGAAAGAACAGGACCTTGATCAAGAGAGCGTGTTGAGCAACATTGACTTCAATACGGATTTG
AATCACAA

GAGGGTAGTTGACATGGAGTTAGAATTGGGTCAGTGTTAGTGTTAGTGTTAGTATTAGGGTGTGGTGTGTG
GGTGTGGTGTGGGTGTGGGTGTGGGTGTGGGTGTGGGTGTGGGTGTGGTGTGGTGTGTGGGTGTGGTGTGG
GTGTGGTG

TGGCTTAATCAACTTCTTCAACGGTTGGACCTTCAGCCTCTGGAGCTGGAGGAGCACCACCTGGGAAACCG
CCTGGAGCACCACCTGCAGCGCCACCTGGAGCACCACCAGCTTGGTACAACTTAGACATGATTGGGTTGGC
AATGTCTT

TTTCACTTCTACATCTACTGAAATGACGCACGTCACCGGTACCAACGGCGTTCCAACTGACGAAACCGTCA
TTGTCATCAGAACTCCAACCAGTGAAGGTCTAATCAGCACCACCACTGAACCATGGACTGGCACTTTCACT
TCGACTTC

ATTGAGCCATTCAATGATCAGACTGACACGTTGCTCGAAATCCTGGATGAAGAAGCCAAGCAGTTCTTCAC
GAATCAGGTCACTGGCCTCTTGTGCTTCGATTCCTCTCGTAACCAATCTGATTAAGACGTGTAACCATGAC
GAGGAGTC

TTCCAACTGACGAAACCGTCATTGTCATCAGAACTCCAACCAGTGAAGGTCTAATCAGCACCACCACTGAA
CCATGGACTGGCACTTTCACTTCGACTTCCACTGAGGTTACCACCATCACTGGAACCAACGGTCAACCAAC
TGACGAAA

TCATTAAACGTTGTTGCTGGTTCTGACTTGAGAAGAACCTCCATCATTGGTACCATCGGTCCAAAGACCAA
CAACCCAGAAACCTTGGTTGCTTTGAGAAAGGCTGGTTTGAACATTGTCCGTATGAACTTCTCTCACGGTT
CTTACGAA

TGCCACGCCAACTCCAAATCCAGAAAGCAGCGCAGGTGCAGATGACACTTCCAGAGAAGCAAGTGCAAGTG
CTGAAGGTGCTGAGGCCATTGAAGGCGACTTCATGTCTACTTTGAAGCAATCGAAGAAGAAGCAAGAAAAG
AAGGTTAT

AGCACCACCAGCTTGGTACAACTTAGACATGATTGGGTTGGCAATGTCTTGCAACTCCTTCAACTTGTCAT
CGAATTCTTCCTTGCTGGCAGTGGTGTTGCTGTCTAACCAAGAAATAGTCTCTTCAGCCTTCTTGGTGACG
GTGTCCTT

CAGTGAAGGTCTAATCAGCACCACCACTGAACCATGGACTGGCACTTTCACTTCGACTTCCACTGAGGTTA
CCACCATCACTGGAACCAACGGTCAACCAACTGACGAAACTGTGATTGTTATCAGAACTCCAACCAGTGAA
GGTCTAAT

CGTGCATCTTGACCACGTTATTTCTGCTGGTGAACGAGTGGGGACAGTTCAATTCTGTGGTAACAAGGCCA
CAATTGGTGGTGGACCGTGACCGACACGCAAAGCTGGAGCTTAATATGGATGTGACATTTCCATCGATGCC
ATGTGACC

ACATCTACTGAAATGACCACCATTACTGGAACCAACGGCGTTCCAACTGACGAAACCGTCATTGTCATCAG
AACTCCAACCAGTGAAGGTCTAATCAGCACCACCACTGAACCATGGACTGGTACTTTTACTTCTACATCTA
CTGAAATG

CCTTGCTGGCAGTGGTGTTGCTGTCTAACCAAGAAATAGTCTCTTCAGCCTTCTTGGTGACGGTGTCCTTG
TCAGCTTGTTCCAATTTGTCACCAGCTTCAGAAATGGTGTTCTTCAAAGAGTAAGCAATGGATTCCAATTG
GTTCTTGG

ACCATTCTTCATTGAGCCATTCAATGATCAGACTGACACGTTGCTCGAAATCCTGGATGAAGAAGCCAAGC
AGTTCTTCACGAATCAGGTCACTGGCCTCTTGTGCTTCGATTCCTCTCGTAACCAATCTGATTAAGACGTG
TAACCATG

CTAGATTAGAAAGATTGACCTCATTAAACGTTGTTGCTGGTTCTGACTTGAGAAGAACCTCCATCATTGGT
ACCATCGGTCCAAAGACCAACAACCCAGAAACCTTGGTTGCTTTGAGAAAGGCTGGTTTGAACATTGTCCG
TATGAACT

CGTTAGTACCGGTGACGGTGGTCATCTCAGTGGATGTAGAGGTGAAAGTACCAGTCCATGGTTCAGTGGTG
GTGCTGATTAGACCTTCACTAGTTGGAGTTCTGATGACAATGACGGTTTCGTCAGTTGGAACGCCGTTGGT
ACCGGTGA

AAGATTGACCTCATTAAACGTTGTTGCTGGTTCTGACTTGAGAAGAACCTCCATCATTGGTACCATCGGTC
CAAAGACCAACAACCCAGAAACCTTGGTTGCTTTGAGAAAGGCTGGTTTGAACATTGTCCGTATGAACTTC
TCTCACGG

GACAAACCCCACACTGGCCACACCAGTTCTAGCCACATCAAAACCAATACCAGCACCACCACCGCTGCCGG
ATGAAGCACTGGTCAACAATTCTCTTCTCAGTTTGGCCAGCTTGGCCTTCAGTTGACCCAAATGGAAAGAT
GTGGCCTT

GCAATGTCTTGCAACTCCTTCAACTTGTCATCGAATTCTTCCTTGCTGGCAGTGGTGTTGCTGTCTAACCA
AGAAATAGTCTCTTCAGCCTTCTTGGTGACGGTGTCCTTGTCAGCTTGTTCCAATTTGTCACCAGCTTCAG
AAATGGTG

GGTTTCGTCAGTTGGTTGACCGTTAGTACCGGTGACGGTGGTCATCTCAGTGGATGTAGAGGTGAAAGTAC
CAGTCCATGGTTCAGTGGTGGTGCTGATTAGACCTTCACTAGTTGGAGTTCTGATGACAATGACGGTTTCG
TCAGTTGG

GTTGGTTGACCGTTAGTACCGGTGACGGTGGTCATCTCAGTGGATGTAGAGGTGAAAGTACCAGTCCATGG
TTCAGTGGTGGTGCTGATTAGACCTTCACTAGTTGGAGTTCTGATGACAATGACGGTTTCGTCAGTTGGAA
CGCCGTTG

TTGTAGAAGAAATGACAGGGGAAGAAATGAATGAAGAAGAAATGACTGGAGAAGAAGTGACTAGAGAAGAA
GTGACTGAGGAAGAAATGACTAGAGAAGAAGTGTCTGAGGAAGAAATTACTGAGGAGGAAATCACAGAAGT
TCCATTGC

AAATGACCACCATTACTGGAACCAACGGCGTTCCAACTGACGAAACCGTCATTGTCATCAGAACTCCAACC
AGTGAAGGTCTAATCAGCACCACCACTGAACCATGGACTGGTACTTTTACTTCTACATCTACTGAAATGAC
CACCATTA

CTCTCGGTGACTCTTACACCAATAGCACCTCCTCCGCAGACTTGAGTTCTATCACTTCCGTCTCGTCAGCT
AGTGCAAGTGCCACCGCTTCCGACTCACTTTCTTCCAGTGACGGTACCGTTTATTTGCCATCCACAACAAT
TAGCGGTG

TACAAGGGAAGGAAGCACAGAAGCAAGAGGAGGCGCATCGATCGTGGCAGATGAGTCAGCAAACACCACAG
GAAAGTGAACAGACCACAGCGAAAGAACAGGACCTTGATCAAGAGAGCGTGTTGAGCAACATTGACTTCAA
TACGGATT

ATACAAATCACAAGGTAACCTTAGAGTTTGAGCGAGAACTAGTTCAGCAGGGTGTAGCTCGTTCCTCAGCG
GGTCTGTGGACAGGTCCATTGGAGACCCCTTCCACTCGATCTTGAGTGACTTGTTACTGTCTGTAGGAAGC
GTAGATAA

GAGATGGCAGTAGTTGGAGTTTTGACAATAATGACAGTTTCATCAGTTGGTTGACCGTTGGTTCCAGTGAT
GGTGGTCATCTCAGTAGATGTAGAGGTGAAAGTACCGGTCCATGGCTCGGTTGTAGTTGTAACCAAACCTT
CACTGGTT

AAAACCAACAAGGTGTTAACAACTTCGACGAAATCTTGAAGGTCACTGACGGTGTTATGGTTGCCAGAGGT
GACTTGGGTATTGAAATCCCAGCCCCAGAAGTCTTGGCTGTCCAAAAGAAATTGATTGCTAAGTCTAACTT
GGCTGGTA

GCCATGGAACGACACTTTTACCTCTACATCCACTGAAATCACCACCGTCACCGGTACCACCGGTTTGCCAA
CTGATGAGACCATCATTGTCATCAGAACACCAACAACAGCCACTACTGCCATGACTACAACTCAGCCATGG
AACGACAC

TTTTACCTCTACTTCTACCGAATTGACCACAGTCACCGGTACCAATGGTTTGCCAACTGATGAGACCATCA
TTGTCATCAGAACACCAACAACAGCCACTACTGCCATGACTACAACTCAGCCATGGAACGACACTTTTACC
TCTACTTC

TTTTACCTCTACTTCTACCGAATTGACCACAGTCACCGGTACCAATGGTTTGCCAACTGATGAGACCATCA
TTGTCATCAGAACACCAACAACAGCCACTACTGCCATGACTACAACTCAGCCATGGAACGACACTTTTACC
TCTACATC

ACCATGGACTGGCACTTTCACTTCGACTTCCACTGAGGTTACCACCATCACTGGAACCAACGGTCAACCAA
CTGACGAAACTGTGATTGTTATCAGAACTCCAACCAGTGAAGGTCTAATCAGCACCACCACTGAACCATGG
ACTGGTAC

ACTGTGCATGGTTTGAAACAACCTCAAGGACTTAAAGAATCTCAAGAGTTCCCAGAACCAATCTTCACCCC
ATCGACCAAGGCTGAACAAGGTGAACATGACGAAAACATCTCTCCTGCCCAGGCCGCTGAGCTGGTGGGTG
AAGATTTG

ACGGAACCATGGACCGGTACTTTCACTTCTACATCTACTGAAATGACCACCGTCACCGGTACCAACGGCGT
TCCAACTGACGAAACCGTCATTGTCATCAGAACTCCAACAACTGCTAGCACCATCATAACTACAACTGAGC
CATGGAAC

CCCAGAACCAATCTTCACCCCATCGACCAAGGCTGAACAAGGTGAACATGACGAAAACATCTCTCCTGCCC
AGGCCGCTGAGCTGGTGGGTGAAGATTTGTCACGTAGAGTGGCAGAACTGGCTGTAAAACTGTACTCCAAG
TGCAAAGA

ACATCCACTGAAATGACCACCGTCACCGGTACCAACGGCGTTCCAACTGACGAAACCGTCATTGTCATCAG
AACTCCAACTAGTGAAGGTCTAATCAGCACCACCACTGAACCATGGACTGGTACTTTCACCTCTACATCCA
CTGAGATG

TTCTGACTTGAGAAGAACCTCCATCATTGGTACCATCGGTCCAAAGACCAACAACCCAGAAACCTTGGTTG
CTTTGAGAAAGGCTGGTTTGAACATTGTCCGTATGAACTTCTCTCACGGTTCTTACGAATACCACAAGTCT
GTCATTGA

CTGTTCCATGGCTCAGTTGTAGTTATGATGGTGCTAGCAGTTGTTGGAGTTCTGATGACAATGACGGTTTC
GTCAGTTGGAACGCCGTTGGTACCGGTGACGGTGGTCATTTCAGTAGATGTAGAAGTGAAAGTACCGGTCC
ATGGTTCC

ACGCTGTGGTTCCCACCGCACGCTATCTTCCTGACTATTGCTCCATCATCTCCTGGCACAGACCTCTGTGG
GGTATCCATATCCTCATCGTGCCCCAGTCCCAGTTGCCTTTGCCCATTAGACCCAAACGCATACACACAAC
TCATCGAT

ACGGAACCATGGACCGGTACTTTCACTTCTACATCTACTGAAATGACGCACGTCACCGGTACCAACGGCGT
TCCAACTGACGAAACCGTCATTGTCATCAGAACTCCAACCAGTGAAGGTCTAATCAGCACCACCACTGAAC
CATGGACT

GGAACAGCACTTTTACCTCTACTTCTACCGAATTGACCACAGTCACCGGTACCAATGGTTTGCCAACTGAT
GAGACCATCATTGTCATCAGAACACCAACAACAGCCACTACTGCCATGACTACAACTCAGCCATGGAACGA
CACTTTTA

GGAACGACACTTTTACCTCTACTTCTACCGAATTGACCACAGTCACCGGTACCAATGGTTTGCCAACTGAT
GAGACCATCATTGTCATCAGAACACCAACAACAGCCACTACTGCCATGACTACAACTCAGCCATGGAACGA
CACTTTTA

AAGCAAGAGGAGGCGCATCGATCGTGGCAGATGAGTCAGCAAACACCACAGGAAAGTGAACAGACCACAGC
GAAAGAACAGGACCTTGATCAAGAGAGCGTGTTGAGCAACATTGACTTCAATACGGATTTGAATCACAATT
TGAATTTA

CTAGTTGTCGGTCCTGAAGATGACGAAGATGAATTGATGGACGACGTTATGGATGATTTGACTGGTTTGTT
GGACTCCGTTGACACAACTGGTAAAGGTGTTGTGGTCCAAGCATCCACCTTGGGTTCTTTGGAAGCTTTGT
TGGATTTC

TTAGAGTTTGAGCGAGAACTAGTTCAGCAGGGTGTAGCTCGTTCCTCAGCGGGTCTGTGGACAGGTCCATT
GGAGACCCCTTCCACTCGATCTTGAGTGACTTGTTACTGTCTGTAGGAAGCGTAGATAAGGGCGGAGAGTA
ATCTGGTA

TTCACTGGTTGGAGTTCTGATAACAATCACGGTTTCGTCAGTTGGTTGACCGTTAGTACCGGTGACGGTGG
TCATCTCAGTGGATGTAGAGGTGAAAGTACCAGTCCATGGTTCAGTGGTGGTGCTGATTAGACCTTCACTA
GTTGGAGT

TTGGTTACAACCACCACTGAACCATGGACTGGTACTTTTACTTCGACTTCCACTGAAATGTCTACTGTCAC
TGGAACCAATGGCTTGCCAACTGATGAAACTGTCATTGTTGTCAAAACTCCAACTACTGCCATCTCATCCA
GTTTGTCA

CGAAACCGTCATTGTCATCAGAACTCCAACTAGTGAAGGTCTAATCAGCACCACCACTGAACCATGGACTG
GTACTTTCACCTCTACATCCACTGAGATGACCACCGTCACCGGTACTAACGGTCAACCAACTGACGAAACC
GTGATTGT

ACTGCCATGACTACAACTCAGCCATGGAACGACACTTTTACCTCTACATCCACTGAAATCACCACCGTCAC
CGGTACCAATGGTTTGCCAACTGATGAGACCATCATTGTCATCAGAACACCAACAACAGCCACTACTGCCA
TGACTACA

AACTCCACTACAGCATCTGGTTCAATCGCTTCTACTGCTTCCACCGCTTCCACTACTTCTACTGCATCCGC
TGCATCCGCCACCAGCTTCACCTCAGGTTCCGCTTCTGTCTACACTACTACATTAACTTACTTGAATGCCA
CAAGTACA

GGTTGTTGTAGAAGAAATGACAGGGGAAGAAATGAATGAAGAAGAAATGACTGGAGAAGAAGTGACTAGAG
AAGAAGTGACTGAGGAAGAAATGACTAGAGAAGAAGTGTCTGAGGAAGAAATTACTGAGGAGGAAATCACA
GAAGTTCC

CCACCTGCAGCGCCACCTGGAGCACCACCAGCTTGGTACAACTTAGACATGATTGGGTTGGCAATGTCTTG
CAACTCCTTCAACTTGTCATCGAATTCTTCCTTGCTGGCAGTGGTGTTGCTGTCTAACCAAGAAATAGTCT
CTTCAGCC

AAATGACCACCGTCACCGGTACCAACGGCGTTCCAACTGACGAAACCGTCATTGTCATCAGAACTCCAACT
AGTGAAGGTCTAATCAGCACCACCACTGAACCATGGACTGGTACTTTCACCTCTACATCCACTGAGATGAC
CACCGTCA

GACACTTTTACCTCTACTTCTACCGAATTGACCACAGTCACCGGTACCAATGGTTTGCCAACTGATGAGAC
CATCATTGTCATCAGAACACCAACAACAGCCACTACTGCCATGACTACAACTCAGCCATGGAACGACACTT
TTACCTCT

ATCTCCACATCCTACCAGATTCCCGTCATTTGTCAGCATCACGCTGTGGTTCCCACCGCACGCTATCTTCC
TGACTATTGCTCCATCATCTCCTGGCACAGACCTCTGTGGGGTATCCATATCCTCATCGTGCCCCAGTCCC
AGTTGCCT

ACCACCGTCACCGGTACCAATGGTTTGCCAACTGATGAGACCATCATTGTCATCAGAACACCAACAACAGC
CACTACTGCCATGACTACACCTCAGCCATGGAACGACACTTTTACCTCTACATCCACTGAAATGACCACCG
TCACCGGT

GCACTGCTGTGGGTACGGCCCATTCTGTGGTGAATGTGACTGAGCAGTTTGAGGAGAGGCATGATGGGGGT
TCTCTGGAACAGCTGATGAAGCAGGTGTTGTTGTCTGTTGAGAGTTAGCCTTAGTGGAAGCCTTCTCACAT
TCTTCTGT

CTTCGACTTCCACTGAAATGTCTACTGTCACTGGAACCAATGGCTTGCCAACTGATGAAACTGTCATTGTT
GTCAAAACTCCAACTACTGCCATCTCATCCAGTTTGTCATCATCATCTTCAGGACAAATCACCAGCTCTAT
CACGTCTT

AGTCACCGGTACCAATGGTTTGCCAACTGATGAGACCATCATTGTCATCAGAACACCAACAACAGCCACTA
CTGCCATGACTACAACTCAGCCATGGAACGACACTTTTACCTCTACTTCTACCGAATTGACCACAGTCACC
GGTACCAA

GACACTTTTACCTCTACATCCACTGAAATCACCACCGTCACCGGTACCAATGGTTTGCCAACTGATGAGAC
CATCATTGTCATCAGAACACCAACAACAGCCACTACTGCCATGACTACACCTCAGCCATGGAACGACACTT
TTACCTCT

CAAGGTAACCTTAGAGTTTGAGCGAGAACTAGTTCAGCAGGGTGTAGCTCGTTCCTCAGCGGGTCTGTGGA
CAGGTCCATTGGAGACCCCTTCCACTCGATCTTGAGTGACTTGTTACTGTCTGTAGGAAGCGTAGATAAGG
GCGGAGAG

GGGACAGTTCAATTCTGTGGTAACAAGGCCACAATTGGTGGTGGACCGTGACCGACACGCAAAGCTGGAGC
TTAATATGGATGTGACATTTCCATCGATGCCATGTGACCTGGTGAATCTCGATATTATGGACGACTCTGGA
GAGATGCA

ATTGAATCTTGAAATTGAAGAGGTGACTACTGTTTTCGTCTCAGCAGCTCCAGTACTGGTAGTTGTCTCAG
CAGCTCCAGTATTGGTTGTTGTCTCACTGGTAGCACTGTTCATTTTAGAGCTGACAGACTGTTCATTCGTA
GTCTGTGG

AGCAGCTCCAGTACTGGTAGTTGTCTCAGCAGCTCCAGTATTGGTTGTTGTCTCACTGGTAGCACTGTTCA
TTTTAGAGCTGACAGACTCTTCATTCGTAGTCTGTGGCCTCCATGTTGGATAGACCGTAACAACATCATTC
ACAGTAGC

CCACCACTGAACCATGGACTGGTACTTTTACTTCGACTTCCACTGAAATGTCTACTGTCACTGGAACCAAT
GGCTTGCCAACTGATGAAACTGTCATTGTTGTCAAAACTCCAACTACTGCCATCTCATCCAGTTTGTCATC
ATCATCTT

AGTCACCGGTACCAATGGTTTGCCAACTGATGAGACCATCATTGTCATCAGAACACCAACAACAGCCACTA
CTGCCATGACTACAACTCAGCCATGGAACGACACTTTTACCTCTACATCCACTGAAATCACCACCGTCACC
GGTACCAA

TAGTTGGAGTTTTGACAATAATGACAGTTTCATCAGTTGGTTGACCGTTGGTTCCAGTGATGGTGGTCATC
TCAGTAGATGTAGAGGTGAAAGTACCGGTCCATGGCTCGGTTGTAGTTGTAACCAAACCTTCACTGGTTGG
AGTTCTGA

TTTCCAAGGTTGGTCCTAAAGTGACAAAGGTGAAGGTTGGCGACCACGTGGTCGTTGATGCTGCCAGCAGT
TGTGCGGACCTGCATTGCTGGCCACACTCCAAATTTTACAATTCCAAACCATGTGATGCTTGTCAGAGGGG
CAGTGAAA

GTCATCTCAGTGGATGTAGAGGTGAAAGTACCAGTCCATGGTTCAGTGGTGGTGCTGATTAGACCTTCACT
AGTTGGAGTTCTGATGACAATGACGGTTTCGTCAGTTGGAACGCCGTTGGTACCGGTGACGGTGGTCATTT
CAGTGGAT

GACAGAAGTACGAGCTTTCATGCATCAACACAGAAACAATCGCTACCAGGACATTCATTCACCAGGACTTC
CACAAGAAGGTCACCGACCTGCGAGCCAGGCTGCTGAACAGAACCACGCAGACCTGGTACGATATCAACAA
GGAGCGCC

CCTCGTGGTCATGGAGTCCCTGAGTGATGGTATGGACATCCCAGAATCCATCGAGTTTGTGAACTCGCTGT
CGTCATCATCACCAGTGTTGTCATTATTGCGAAGATGCCTGCCACTAGGAATCCATCGACGTACCATGGCT
ATAACTTT

ACTCCAAATCCAGAAAGCAGCGCAGGTGCAGATGACACTTCCAGAGAAGCAAGTGCAAGTGCTGAAGGTGC
TGAGGCCATTGAAGGCGACTTCATGTCTACTTTGAAGCAATCGAAGAAGAAGCAAGAAAAGAAGGTTATTG
AAGAGAAG

TGAGACCATCATTGTCATCAGAACACCAACAACAGCCACTACTGCCATGACTACAACTCAGCCATGGAACG
ACACTTTTACCTCTACATCCACTGAAATCACCACCGTCACCGGTACCAATGGTTTGCCAACTGATGAGACC
ATCATTGT

AAATAAGTGTCTAGATACCTATCATGATTTTTCCAAATAGTTCTTGCAAATGATGGCCATGCAGCTTTGAC
GGCAAGGACACCCTCTGCGTGGCTGGTGTTAAGTTCTTCACCAGTGTTAGGGTCAAGAACAACTGCATCAA
TACCGAAG

AACGGCAAAGTACAAGGGAAGGAAGCACAGAAGCAAGAGGAGGCGCATCGATCGTGGCAGATGAGTCAGCA
AACACCACAGGAAAGTGAACAGACCACAGCGAAAGAACAGGACCTTGATCAAGAGAGCGTGTTGAGCAACA
TTGACTTC

TAACCAAACCTTCACTGGTTGGAGTTCTGATAACAATCACGGTTTCGTCAGTTGGTTGACCGTTAGTACCG
GTGACGGTGGTCATCTCAGTGGATGTAGAGGTGAAAGTACCAGTCCATGGTTCAGTGGTGGTGCTGATTAG
ACCTTCAC

AACCTTCTCTTAGAATCCAGATACAAATCACAAGGTAACCTTAGAGTTTGAGCGAGAACTAGTTCAGCAGG
GTGTAGCTCGTTCCTCAGCGGGTCTGTGGACAGGTCCATTGGAGACCCCTTCCACTCGATCTTGAGTGACT
TGTTACTG

ACTACCTCATGAATTCTAGTGGATGAAAGAAGCAGCACGAACACCATTTCTACAGACAACGACACATGGAA
AGGTTCACCATTCCCAAAGAAAACAACGATGGCCACAAGGGTGTGGTCCTCCATTCTCCTACTGTTGGAAG
GAGATATT

TTGTCTCTAGTTTGCGATAGTGTAGATACCGTCCTTGGATAGAGCACTGGAGATGGCTGGCTTTAATCTGC
TGGAGTACCATGGAACACCGGTGATCATTCTGGTCACTTGGTCTGGAGCAATACCGGTCAACATGGTGGTG
AAGTCACC

CTACAACTCAGCCATGGAACGACACTTTTACCTCTACTTCTACCGAATTGACCACAGTCACCGGTACCAAT
GGTTTGCCAACTGATGAGACCATCATTGTCATCAGAACACCAACAACAGCCACTACTGCCATGACTACAAC
TCAGCCAT

TGGTGTCTCAAACTCCACTACAGCATCTGGTTCAATCGCTTCTACTGCTTCCACCGCTTCCACTACTTCTA
CTGCATCCGCTGCATCCGCCACCAGCTTCACCTCAGGTTCCGCTTCTGTCTACACTACTACATTAACTTAC
TTGAATGC

ACCACAGTCACCGGTACCAATGGTTTGCCAACTGATGAGACCATCATTGTCATCAGAACACCAACAACAGC
CACTACTGCCATGACTACAACTCAGCCATGGAACGACACTTTTACCTCTACTTCTACCGAATTGACCACAG
TCACCGGT

CATCGACCAAGGCTGAACAAGGTGAACATGACGAAAACATCTCTCCTGCCCAGGCCGCTGAGCTGGTGGGT
GAAGATTTGTCACGTAGAGTGGCAGAACTGGCTGTAAAACTGTACTCCAAGTGCAAAGATTATGCTAAGGA
GAAGGGCA

CATAACTACAACTGAGCCATGGAACAGCACTTTTACCTCTACTTCTACCGAATTGACCACAGTCACCGGTA
CCAATGGTTTGCCAACTGATGAGACCATCATTGTCATCAGAACACCAACAACAGCCACTACTGCCATGACT
ACAACTCA

CATGACTACAACTCAGCCATGGAACGACACTTTTACCTCTACTTCTACCGAATTGACCACAGTCACCGGTA
CCAATGGTTTGCCAACTGATGAGACCATCATTGTCATCAGAACACCAACAACAGCCACTACTGCCATGACT
ACAACTCA

GGTACTTTTACTTCGACTTCCACTGAAATGTCTACTGTCACTGGAACCAATGGCTTGCCAACTGATGAAAC
TGTCATTGTTGTCAAAACTCCAACTACTGCCATCTCATCCAGTTTGTCATCATCATCTTCAGGACAAATCA
CCAGCTCT

AGGTGACTACTGTTTTCGTCTCAGCAGCTCCAGTACTGGTAGTTGTCTCAGCAGCTCCAGTATTGGTTGTT
GTCTCACTGGTAGCACTGTTCATTTTAGAGCTGACAGACTGTTCATTCGTAGTCTGTGGCCTCCATGTAGA
ATAGACCG

TAATAAACTCATAACAGTGCAACGGCAAAGTACAAGGGAAGGAAGCACAGAAGCAAGAGGAGGCGCATCGA
TCGTGGCAGATGAGTCAGCAAACACCACAGGAAAGTGAACAGACCACAGCGAAAGAACAGGACCTTGATCA
AGAGAGCG

ACCAATGGTTTGCCAACTGATGAGACCATCATTGTCATCAGAACACCAACAACAGCCACTACTGCCATGAC
TACAACTCAGCCATGGAACGACACTTTTACCTCTACATCCACTGAAATCACCACCGTCACCGGTACCAATG
GTTTGCCA

CATGACTACAACTCAGCCATGGAACGACACTTTTACCTCTACATCCACTGAAATGACCACCGTCACCGGTA
CCAACGGCGTTCCAACTGACGAAACCGTCATTGTCATCAGAACTCCAACTAGTGAAGGTCTAATCAGCACC
ACCACTGA

TGGCAATGGGCCATGAGATGTCAGGAATTGTTTCCAAGGTTGGTCCTAAAGTGACAAAGGTGAAGGTTGGC
GACCACGTGGTCGTTGATGCTGCCAGCAGTTGTGCGGACCTGCATTGCTGGCCACACTCCAAATTTTACAA
TTCCAAAC

GTGTGGTGATGGATAGTGAGTGGATAGTGAGTGGATGGATGGTGGAGTGGGGGAATGAGACAGGGCATGGG
GTGGTGAGGTAAGTGCCGTGGATTGTGATGATGGAGAGGGAGGGTAGTTGACATGGAGTTAGAATTGGGTC
AGTGTTAG

TCATATCAGAGTCCGCTGAGGATGAATCAGTAAATGTATTACCTGACTCAGGTGATGGAGTGCTCAGAGGC
GTTCCAACTGATGATGGATACTGCGGAAACTGTGATTGTGGCCCAGGTGGAAAGTACATAGGCGACATTTG
ATAAGGTG

CTGAAAAGGACAAGGAAGATTTGAGATTCGGTGTCAAGAACGGTGTCCACATGGTCTTCGCTTCTTTCATC
AGAACCGCCAACGATGTTTTGACCATCAGAGAAGTCTTGGGTGAACAAGGTAAGGACGTCAAGATCATTGT
CAAGATTG

CAAACTGGATGAGATGGCAGTAGTTGGAGTTTTGACAATAATGACAGTTTCATCAGTTGGTTGACCGTTGG
TTCCAGTGATGGTGGTCATCTCAGTAGATGTAGAGGTGAAAGTACCGGTCCATGGCTCGGTTGTAGTTGTA
ACCAAACC

TGTCAGCATCACGCTGTGGTTCCCACCGCACGCTATCTTCCTGACTATTGCTCCATCATCTCCTGGCACAG
ACCTCTGTGGGGTATCCATATCCTCATCGTGCCCCAGTCCCAGTTGCCTTTGCCCATTAGACCCAAACGCA
TACACACA

ACTGAACCATGGACTGGTACTTTTACTTCTACATCTACTGAAATGACCACCATTACTGGAACCAACGGCGT
TCCAACTGACGAAACCGTCATTGTCATCAGAACTCCAACCAGTGAAGGTCTAATCAGCACCACCACTGAAC
CATGGACT

CACTGAGGTTACCACCATCACTGGAACCAACGGTCAACCAACTGACGAAACTGTGATTGTTATCAGAACTC
CAACCAGTGAAGGTCTAATCAGCACCACCACTGAACCATGGACTGGTACTTTCACTTCTACATCTACTGAA
ATGACCAC

ATTGTCATCAGAACTCCAACAACTGCTAGCACCATCATAACTACAACTGAGCCATGGAACAGCACTTTTAC
CTCTACTTCTACCGAATTGACCACAGTCACTGGCACCAATGGTGTACGAACTGACGAAACCATCATTGTAA
TCAGAACA

ATTTGTCCTGAAGAAGATGACAAACTGGATGAGATGGCAGTAGTTGGAGTTTTGACAATAATGACAGTTTC
ATCAGTTGGTTGACCGTTGGTTCCAGTGATGGTGGTCATCTCAGTAGATGTAGAGGTGAAAGTACCGGTCC
ATGGCTCG

CACCTTCTTGTCCCCATATATCTCCAATCCATTGAACTTCGGTGCAGACATCGTTGTCCACTCCGCTACAA
AGTACATCAACGGTCACTCAGACGTTGTGCTCGGTGTCCTGGCCACTAATAACAAGCCATTGTACGAGCGT
CTGCAGTT

CAGGGGAAGAAATGAATGAAGAAGAAATGACTGGAGAAGAAGTGACTAGAGAAGAAGTGACTGAGGAAGAA
ATGACTAGAGAAGAAGTGTCTGAGGAAGAAATTACTGAGGAGGAAATCACAGAAGTTCCATTGCTAGGATA
GAATGGGG

GCAACTCCTTCAACTTGTCATCGAATTCTTCCTTGCTGGCAGTGGTGTTGCTGTCTAACCAAGAAATAGTC
TCTTCAGCCTTCTTGGTGACGGTGTCCTTGTCAGCTTGTTCCAATTTGTCACCAGCTTCAGAAATGGTGTT
CTTCAAAG

CCGGTACCAATGGTTTGCCAACTGATGAGACCATCATTGTCATCAGAACACCAACAACAGCCACTACTGCC
ATGACTACACCTCAGCCATGGAACGACACTTTTACCTCTACATCCACTGAAATGACCACCGTCACCGGTAC
CAACGGTT

CTTCGACTTCCACTGAGGTTACCACCATCACTGGAACCAACGGTCAACCAACTGACGAAACTGTGATTGTT
ATCAGAACTCCAACCAGTGAAGGTCTAATCAGCACCACCACTGAACCATGGACTGGTACTTTCACTTCTAC
ATCTACTG

CGTTCATCAGAGCAGGCTTGATGGGATCGTTATGAGATGACCTCGTGGTCATGGAGTCCCTGAGTGATGGT
ATGGACATCCCAGAATCCATCGAGTTTGTGAACTCGCTGTCGTCATCATCACCAGTGTTGTCATTATTGCG
AAGATGCC

CTACAACTCAGCCATGGAACGACACTTTTACCTCTACATCCACTGAAATCACCACCGTCACCGGTACCACC
GGTTTGCCAACTGATGAGACCATCATTGTCATCAGAACACCAACAACAGCCACTACTGCCATGACTACAAC
TCAGCCAT

CCCGTCTGTTATCTCCACATCCTACCAGATTCCCGTCATTTGTCAGCATCACGCTGTGGTTCCCACCGCAC
GCTATCTTCCTGACTATTGCTCCATCATCTCCTGGCACAGACCTCTGTGGGGTATCCATATCCTCATCGTG
CCCCAGTC

GCTTGGTACAACTTAGACATGATTGGGTTGGCAATGTCTTGCAACTCCTTCAACTTGTCATCGAATTCTTC
CTTGCTGGCAGTGGTGTTGCTGTCTAACCAAGAAATAGTCTCTTCAGCCTTCTTGGTGACGGTGTCCTTGT
CAGCTTGT

GGTGAAAGTACCAGTCCATGGTTCAGTGGTGGTGCTGATTAGACCTTCACTAGTTGGAGTTCTGATGACAA
TGACGGTTTCGTCAGTTGGAACGCCGTTGGTACCGGTGACGGTGGTCATTTCAGTGGATGTAGAGGTAAAA
GTGTCGTT

CAGCTTCTCCCCTATCATTGCTGCCATCCATATGGAGAACAGGCACGACTTCGTCGTTTTCTGTGGGTGCT
ACAACCTTTCTCGCCTTCTTCCCAACGAACCTTGGCAACAATGGATCCATTTGCTTGGACACTACCTCAAT
ATGGTGGC

ATAACAGTGCAACGGCAAAGTACAAGGGAAGGAAGCACAGAAGCAAGAGGAGGCGCATCGATCGTGGCAGA
TGAGTCAGCAAACACCACAGGAAAGTGAACAGACCACAGCGAAAGAACAGGACCTTGATCAAGAGAGCGTG
TTGAGCAA

GCGTACCAGCGACAGAAGTACGAGCTTTCATGCATCAACACAGAAACAATCGCTACCAGGACATTCATTCA
CCAGGACTTCCACAAGAAGGTCACCGACCTGCGAGCCAGGCTGCTGAACAGAACCACGCAGACCTGGTACG
ATATCAAC

GAAGCAGTCTTAGAGCATACGTCAGATTCACAAGAAGAAATTGTAACTACTGTGGTTTGTTTTGTTGTTTC
TGTGGTTTGCTCTGTTGTCCCCTTGGTTTGCTTTGTTGTCTCCGTAGTTTGCTTTGTTATCTCTGTGGTAG
AAATAGGG

TTGCTCTAACAACTGCTGTTGCTGGTTAATAAATAGTTCTTGGTTGGAAACCATGGGTTGTGGTGGCTGTG
GCTGTGACTGTGACTGTAACTGCGATTGCAATGGTTCATCCTTGTATGGAGTATGCGTCGAAGGCAGCGGC
GACTCCTC

CATTACTGGAACCAACGGCGTTCCAACTGACGAAACCGTCATTGTCATCAGAACTCCAACCAGTGAAGGTC
TAATCAGCACCACCACTGAACCATGGACTGGTACTTTTACTTCTACATCTACTGAAATGACCACCATTACT
GGAACCAA

TTTTACTTCTACATCTACTGAAATGACCACCATTACTGGAACCAACGGCGTTCCAACTGACGAAACCGTCA
TTGTCATCAGAACTCCAACCAGTGAAGGTCTAATCAGCACCACCACTGAACCATGGACTGGTACTTTTACT
TCTACATC

GGCTGAACAAGGTGAACATGACGAAAACATCTCTCCTGCCCAGGCCGCTGAGCTGGTGGGTGAAGATTTGT
CACGTAGAGTGGCAGAACTGGCTGTAAAACTGTACTCCAAGTGCAAAGATTATGCTAAGGAGAAGGGCATC
ATCATCGC

TTCTAGGCTGCTAGTTGTCGGTCCTGAAGATGACGAAGATGAATTGATGGACGACGTTATGGATGATTTGA
CTGGTTTGTTGGACTCCGTTGACACAACTGGTAAAGGTGTTGTGGTCCAAGCATCCACCTTGGGTTCTTTG
GAAGCTTT

CGAATATGTAGACTTTGGTGATTCAGAAGAGATAGAAGAGGAAGAAGAGACAGAACTAGCTGAACTAGTTT
CGCTCTCAGAAGAACCAGAGGTGGAACTACTGGTTGGAATGACGGATGATTTAAATGATTCAGAGAATATA
GAAGTGGA

AAAGCTGGTGATTTGTCCTGAAGAAGATGACAAACTGGATGAGATGGCAGTAGTTGGAGTTTTGACAATAA
TGACAGTTTCATCAGTTGGTTGACCGTTGGTTCCAGTGATGGTGGTCATCTCAGTAGATGTAGAGGTGAAA
GTACCGGT

GTGACTACTGTTTTCGTCTCAGCAGCTCCAGTACTGGTAGTTGTCTCAGCAGCTCCAGTATTGGTTGTTGT
CTCACTGGTAGCACTGTTCATTTTAGAGCTGACAGACTCTTCATTCGTAGTCTGTGGCCTCCATGTTGGAT
AGACCGTA

CACTGAAATCACCACCGTCACCGGTACCACCGGTTTGCCAACTGATGAGACCATCATTGTCATCAGAACAC
CAACAACAGCCACTACTGCCATGACTACAACTCAGCCATGGAACGACACTTTTACCTCTACATCCACTGAA
ATGACCAC

GGTAAAAGTGCTGTTCCATGGCTCAGTTGTAGTTATGATGGTGCTAGCAGTTGTTGGAGTTCTGATGACAA
TGACGGTTTCGTCAGTTGGAACGCCGTTGGTACCGGTGACGGTGGTCATTTCAGTAGATGTAGAAGTGAAA
GTACCGGT

AGAAGACGCCGCCCAGAACGAAGAAATCAGTGCCACGCCAACTCCAAATCCAGAAAGCAGCGCAGGTGCAG
ATGACACTTCCAGAGAAGCAAGTGCAAGTGCTGAAGGTGCTGAGGCCATTGAAGGCGACTTCATGTCTACT
TTGAAGCA

ATTGTTATCAGAACTCCAACCAGTGAAGGTTTGGTTACAACCACCACTGAACCATGGACTGGTACTTTTAC
TTCGACTTCCACTGAAATGTCTACTGTCACTGGAACCAATGGCTTGCCAACTGATGAAACTGTCATTGTTG
TCAAAACT

ATAGGTGGTAATGATGAAGACGAATATGTAGACTTTGGTGATTCAGAAGAGATAGAAGAGGAAGAAGAGAC
AGAACTAGCTGAACTAGTTTCGCTCTCAGAAGAACCAGAGGTGGAACTACTGGTTGGAATGACGGATGATT
TAAATGAT

ATGATTCAGAGAGTATAGAAGCGGAGGTTGTTGTAGAAGAAATGACAGGGGAAGAAATGAATGAAGAAGAA
ATGACTGGAGAAGAAGTGACTAGAGAAGAAGTGACTGAGGAAGAAATGACTAGAGAAGAAGTGTCTGAGGA
AGAAATTA

TCAGCATGATTGAATCTTGAAATTGAAGAGGTGACTACTGTTTTCGTCTCAGCAGCTCCAGTACTGGTAGT
TGTCTCAGCAGCTCCAGTATTGGTTGTTGTCTCACTGGTAGCACTGTTCATTTTAGAGCTGACAGACTCTT
CATTCGTA

GCCATGGAACAGCACTTTTACCTCTACATCCACTGAAATGACCACCGTCACCGGTACCAACGGTTTGCCAA
CTGATGAAACCATCATTGTCATCAGAACACCAACAACAGCCACTACTGCCATAACTACAACTCAGCCATGG
AACGACAC

GGACCGGTACTTTCACTTCTACATCTACTGAAATGACGCACGTCACCGGTACCAACGGCGTTCCAACTGAC
GAAACCGTCATTGTCATCAGAACTCCAACCAGTGAAGGTCTAATCAGCACCACCACTGAACCATGGACTGG
CACTTTCA

CCTCCAAGTCATACACCACTGTTACTGTTACTCACTGTGACAACAATGGCTGTAACACCAAGACTGTCACT
TCTGAATGTTCTAAAGAAACTGCAGCAACCACCATTTCTCCAAAATCATACACTACTGTTACCGTTACTCA
CTGTGACG

CCGGTACCAATGGTTTGCCAACTGATGAGACCATCATTGTCATCAGAACACCAACAACAGCCACTACTGCC
ATGACTACAACTCAGCCATGGAACGACACTTTTACCTCTACTTCTACCGAATTGACCACAGTCACCGGTAC
CAATGGTT

TGAATCTTGAAATTGAAGAGGTGACTACTGTTTTCGTCTCAGCAGCTCCAGTACTGGTAGTTGTCTCAGCA
GCTCCAGTATTGGTTGTTGTCTCACTGGTAGCACTGTTCATTTTAGAGCTGACAGACTCTTCATTCGTAGT
CTGTGGCC

TCAGAGAATATAGAAGTGGAGGTTGTTGTAGAAGAAATGACAGGGGAAGAAATGAATGAAGAAGAAATGAC
TGGAGAAGAAGTGACTAGAGAAGAAGTGACTGAGGAAGAAATGACTAGAGAAGAAGTGTCTGAGGAAGAAA
TTACTGAG

CGTCTGTGTTTCAGCATGATTGAATCTTGAAATTGAAGAGGTGACTACTGTTTTCGTCTCAGCAGCTCCAG
TACTGGTAGTTGTCTCAGCAGCTCCAGTATTGGTTGTTGTCTCACTGGTAGCACTGTTCATTTTAGAGCTG
ACAGACTC

CCACCACTGAACCATGGACTGGCACTTTCACTTCGACTTCCACTGAGGTTACCACCATCACTGGAACCAAC
GGTCAACCAACTGACGAAACTGTGATTGTTATCAGAACTCCAACCAGTGAAGGTCTAATCAGCACCACCAC
TGAACCAT

AAGTCACTACTGGACCAGGATGCCTCGGACGCCACCAAAATTCAGCTCTTGCACGACGCCTGTACGGCGCA
CTCGCAAATCACAAGGGAATGCTCCCAGGGGCTCGGCCAGGACCGTCACTTGTATGCGCTCTACTGCCTCT
GGAACCAA

TTTAAATGATTCAGAGAATATAGAAGTGGAGGTTGTTGTAGAAGAAATGACAGGGGAAGAAATGAATGAAG
AAGAAATGACTGGAGAAGAAGTGACTAGAGAAGAAGTGACTGAGGAAGAAATGACTAGAGAAGAAGTGTCT
GAGGAAGA

TACAACTACAACGGAACCATGGACCGGTACTTTCACTTCTACATCTACTGAAATGACCACCGTCACCGGTA
CCAACGGCGTTCCAACTGACGAAACCGTCATTGTCATCAGAACTCCAACAACTGCTAGCACCATCATAACT
ACAACTGA

TGGTGGTCATCTCAGTAGATGTAGAGGTGAAAGTACCGGTCCATGGCTCGGTTGTAGTTGTAACCAAACCT
TCACTGGTTGGAGTTCTGATAACAATCACGGTTTCGTCAGTTGGTTGACCGTTAGTACCGGTGACGGTGGT
CATCTCAG

AAGTACCGGTCCATGGCTCGGTTGTAGTTGTAACCAAACCTTCACTGGTTGGAGTTCTGATAACAATCACG
GTTTCGTCAGTTGGTTGACCGTTAGTACCGGTGACGGTGGTCATCTCAGTGGATGTAGAGGTGAAAGTACC
AGTCCATG

GATGGAGAGGGAGGGTAGTTGACATGGAGTTAGAATTGGGTCAGTGTTAGTGTTAGTGTTAGTATTAGGGT
GTGGTGTGTGGGTGTGGTGTGGGTGTGGGTGTGGGTGTGGGTGTGGGTGTGGGTGTGGTGTGGTGTGTGGG
TGTGGTGT

TTTCAGCATGATTGAATCTTGAAATTGAAGAGGTGACTACTGTTTTCGTCTCAGCAGCTCCAGTACTGGTA
GTTGTCTCAGCAGCTCCAGTATTGGTTGTTGTCTCACTGGTAGCACTGTTCATTTTAGAGCTGACAGACTG
TTCATTCG

GCGGAGGTTGTTGTAGAAGAAATGACAGGGGAAGAAATGAATGAAGAAGAAATGACTGGAGAAGAAGTGAC
TAGAGAAGAAGTGACTGAGGAAGAAATGACTAGAGAAGAAGTGTCTGAGGAAGAAATTACTGAGGAGGAAA
TCACAGAA

ACCATGGACTGGTACTTTTACTTCGACTTCCACTGAAATGTCTACTGTCACTGGAACCAATGGCTTGCCAA
CTGATGAAACTGTCATTGTTGTCAAAACTCCAACTACTGCCATCTCATCCAGTTTGTCATCATCATCTTCA
GGACAAAT

TCCCACCGCACGCTATCTTCCTGACTATTGCTCCATCATCTCCTGGCACAGACCTCTGTGGGGTATCCATA
TCCTCATCGTGCCCCAGTCCCAGTTGCCTTTGCCCATTAGACCCAAACGCATACACACAACTCATCGATAC
AAGCCTGT

CCTCTACATCCACTGAAATCACCACCGTCACCGGTACCACCGGTTTGCCAACTGATGAGACCATCATTGTC
ATCAGAACACCAACAACAGCCACTACTGCCATGACTACAACTCAGCCATGGAACGACACTTTTACCTCTAC
ATCCACTG

CCAGCTTTGTCTGAAAAGGACAAGGAAGATTTGAGATTCGGTGTCAAGAACGGTGTCCACATGGTCTTCGC
TTCTTTCATCAGAACCGCCAACGATGTTTTGACCATCAGAGAAGTCTTGGGTGAACAAGGTAAGGACGTCA
AGATCATT

CCACACCACACCCACACACCCACACACCACACCACACACCACACCACACCCACACACACACATCCTAACAC
TACCCTAACACAGCCCTAATCTAACCCTGGCCAACCTGTCTCTCAACTTACCCTCCATTACCCTGCCTCCA
CTCGTTAC

GCCGTCTGTGTTTCAGCATGATTGAATCTTGAAATTGAAGAGGTGACTACTGTTTTCGTCTCAGCAGCTCC
AGTACTGGTAGTTGTCTCAGCAGCTCCAGTATTGGTTGTTGTCTCACTGGTAGCACTGTTCATTTTAGAGC
TGACAGAC

TTTGGTTGGTAGACAAGTCCTTCTTGTTCTTTCTCTTGAATTCTTGGATGAAGTGGTTGACCAATCTGTTG
TCAAAATCTTCACCACCCAAATGGGTGTCACCAGCGGTGGCCTTAACTTCAAAGATACCGTCTTCAATGGA
CAACAAAG

TATCTTCCACTCATTTGGATCAGCTTCTCCCCTATCATTGCTGCCATCCATATGGAGAACAGGCACGACTT
CGTCGTTTTCTGTGGGTGCTACAACCTTTCTCGCCTTCTTCCCAACGAACCTTGGCAACAATGGATCCATT
TGCTTGGA

TCAATGATCAGACTGACACGTTGCTCGAAATCCTGGATGAAGAAGCCAAGCAGTTCTTCACGAATCAGGTC
ACTGGCCTCTTGTGCTTCGATTCCTCTCGTAACCAATCTGATTAAGACGTGTAACCATGACGAGGAGTCGT
CATCGTCC

ATTGTCATCAGAACTCCAACTAGTGAAGGTCTAATCAGCACCACCACTGAACCATGGACTGGTACTTTCAC
CTCTACATCCACTGAGATGACCACCGTCACCGGTACTAACGGTCAACCAACTGACGAAACCGTGATTGTTA
TCAGAACT

ACTGCCATGACTACAACTCAGCCATGGAACGACACTTTTACCTCTACTTCTACCGAATTGACCACAGTCAC
CGGTACCAATGGTTTGCCAACTGATGAGACCATCATTGTCATCAGAACACCAACAACAGCCACTACTGCCA
TGACTACA

AAGGATAATGGAGCGACATCCAACAACAATAGATCTTGAGTCTTGGAAGATTCGTCACCAGTCAAAATAGC
AGCTTGAACAGCAGCACCGTAAGCAACAGCTTCATCTGGGTTGATAGATCTGTTTGGTTCCTTACCGTTGA
AGTAGTCA

ACCACCATCACTGGAACCAACGGTCAACCAACTGACGAAACTGTGATTGTTATCAGAACTCCAACCAGTGA
AGGTCTAATCAGCACCACCACTGAACCATGGACTGGTACTTTCACTTCTACATCTACTGAAATGACCACCG
TCACCGGT

GGTTGTTGGGGTGTGGTGATGGATAGTGAGTGGATAGTGAGTGGATGGATGGTGGAGTGGGGGAATGAGAC
AGGGCATGGGGTGGTGAGGTAAGTGCCGTGGATTGTGATGATGGAGAGGGAGGGTAGTTGACATGGAGTTA
GAATTGGG

GTTGTAGTTGTAACCAAACCTTCACTGGTTGGAGTTCTGATAACAATCACGGTTTCGTCAGTTGGTTGACC
GTTAGTACCGGTGACGGTGGTCATCTCAGTGGATGTAGAGGTGAAAGTACCAGTCCATGGTTCAGTGGTGG
TGCTGATT

AGCAGGCTTGATGGGATCGTTATGAGATGACCTCGTGGTCATGGAGTCCCTGAGTGATGGTATGGACATCC
CAGAATCCATCGAGTTTGTGAACTCGCTGTCGTCATCATCACCAGTGTTGTCATTATTGCGAAGATGCCTG
CCACTAGG

TAGACACAGAAGAGTTCTCGATGAAAGCTAGGGCAAAAAGCACTAGGGAGGGCAGGACACCAGCAACGGCT
TGCCCCACCATGACACCTTGACTGTACTCGGAACCGAAGACGTTGGCTATGGCCATGATACCATTCTGTGT
CATGGCTG

CCATGGCTCGGTTGTAGTTGTAACCAAACCTTCACTGGTTGGAGTTCTGATAACAATCACGGTTTCGTCAG
TTGGTTGACCGTTAGTACCGGTGACGGTGGTCATCTCAGTGGATGTAGAGGTGAAAGTACCAGTCCATGGT
TCAGTGGT

GACCACGTTATTTCTGCTGGTGAACGAGTGGGGACAGTTCAATTCTGTGGTAACAAGGCCACAATTGGTGG
TGGACCGTGACCGACACGCAAAGCTGGAGCTTAATATGGATGTGACATTTCCATCGATGCCATGTGACCTG
GTGAATCT

CTGCCATCCATATGGAGAACAGGCACGACTTCGTCGTTTTCTGTGGGTGCTACAACCTTTCTCGCCTTCTT
CCCAACGAACCTTGGCAACAATGGATCCATTTGCTTGGACACTACCTCAATATGGTGGCTGTTGTTCAATA
ATAGATTC

CATCAGTTGGTTGACCGTTGGTTCCAGTGATGGTGGTCATCTCAGTAGATGTAGAGGTGAAAGTACCGGTC
CATGGCTCGGTTGTAGTTGTAACCAAACCTTCACTGGTTGGAGTTCTGATAACAATCACGGTTTCGTCAGT
TGGTTGAC

TTTCGAATCTTCAGTATGAGTTGACGGAGGTGTGGAATCGGTTGGACTCACAGCTTTTGAAAGGACATTTC
TCGGTTGCTCAGGATGTAGTTCAATGTCGGATTGGAAGTCATGGTCAGATTCTATGTTAAGATCATTGGAT
TCTTGGAT

TGAACGAGTGGGGACAGTTCAATTCTGTGGTAACAAGGCCACAATTGGTGGTGGACCGTGACCGACACGCA
AAGCTGGAGCTTAATATGGATGTGACATTTCCATCGATGCCATGTGACCTGGTGAATCTCGATATTATGGA
CGACTCTG

GGACCGGTACTTTCACTTCTACATCTACTGAAATGACCACCGTCACCGGTACCAACGGCGTTCCAACTGAC
GAAACCGTCATTGTCATCAGAACTCCAACAACTGCTAGCACCATCATAACTACAACTGAGCCATGGAACAG
CACTTTTA

GTGACAAAGGTGAAGGTTGGCGACCACGTGGTCGTTGATGCTGCCAGCAGTTGTGCGGACCTGCATTGCTG
GCCACACTCCAAATTTTACAATTCCAAACCATGTGATGCTTGTCAGAGGGGCAGTGAAAATCTATGTACCC
ACGCCGGT

TTCCAACTGACGAAACCGTCATTGTCATCAGAACTCCAACTAGTGAAGGTCTAATCAGCACCACCACTGAA
CCATGGACTGGTACTTTCACCTCTACATCCACTGAGATGACCACCGTCACCGGTACTAACGGTCAACCAAC
TGACGAAA

TTGTTGCTGGTTCTGACTTGAGAAGAACCTCCATCATTGGTACCATCGGTCCAAAGACCAACAACCCAGAA
ACCTTGGTTGCTTTGAGAAAGGCTGGTTTGAACATTGTCCGTATGAACTTCTCTCACGGTTCTTACGAATA
CCACAAGT

GCTTTACCTCTGGCAATGGGCCATGAGATGTCAGGAATTGTTTCCAAGGTTGGTCCTAAAGTGACAAAGGT
GAAGGTTGGCGACCACGTGGTCGTTGATGCTGCCAGCAGTTGTGCGGACCTGCATTGCTGGCCACACTCCA
AATTTTAC

CGAAACCGTGATTGTTATCAGAACTCCAACCAGTGAAGGTTTGGTTACAACCACCACTGAACCATGGACTG
GTACTTTTACTTCGACTTCCACTGAAATGTCTACTGTCACTGGAACCAATGGCTTGCCAACTGATGAAACT
GTCATTGT

TGCCAACTGATGAGACCATCATTGTCATCAGAACACCAACAACAGCCACTACTGCCATGACTACAACTCAG
CCATGGAACGACACTTTTACCTCTACATCCACTGAAATCACCACCGTCACCGGTACCAATGGTTTGCCAAC
TGATGAGA

TTTTACCTCTACATCCACTGAAATGACCACCGTCACCGGTACCAACGGCGTTCCAACTGACGAAACCGTCA
TTGTCATCAGAACTCCAACTAGTGAAGGTCTAATCAGCACCACCACTGAACCATGGACTGGTACTTTCACC
TCTACATC

CAGAAAGCAGCGCAGGTGCAGATGACACTTCCAGAGAAGCAAGTGCAAGTGCTGAAGGTGCTGAGGCCATT
GAAGGCGACTTCATGTCTACTTTGAAGCAATCGAAGAAGAAGCAAGAAAAGAAGGTTATTGAAGAGAAGAA
GGATGGTA

TTCAATACCCAAGGATAATGGAGCGACATCCAACAACAATAGATCTTGAGTCTTGGAAGATTCGTCACCAG
TCAAAATAGCAGCTTGAACAGCAGCACCGTAAGCAACAGCTTCATCTGGGTTGATAGATCTGTTTGGTTCC
TTACCGTT

ATGATGAAGACGAATATGTAGACTTTGGTGATTCAGAAGAGATAGAAGAGGAAGAAGAGACAGAACTAGCT
GAACTAGTTTCGCTCTCAGAAGAACCAGAGGTGGAACTACTGGTTGGAATGACGGATGATTTAAATGATTC
AGAGAATA

ATGGTCTTCGCTTCTTTCATCAGAACCGCCAACGATGTTTTGACCATCAGAGAAGTCTTGGGTGAACAAGG
TAAGGACGTCAAGATCATTGTCAAGATTGAAAACCAACAAGGTGTTAACAACTTCGACGAAATCTTGAAGG
TCACTGAC

CTCGAAGCAACGATAGACAATCCGGTGATCAGTTTCGTGCTGTCCATTTGCAAGATGGCCTCTGTGGACAG
CTTGATAAGTATGTCATTGGGTAGCTGGGTCACATCCTGGTTGGAGTTCGAACTGTTCATCAATGAGTACA
CAGATGAG

GTCCGCTGAGGATGAATCAGTAAATGTATTACCTGACTCAGGTGATGGAGTGCTCAGAGGCGTTCCAACTG
ATGATGGATACTGCGGAAACTGTGATTGTGGCCCAGGTGGAAAGTACATAGGCGACATTTGATAAGGTGTA
TACGGAAT

CAACTTGTCATCGAATTCTTCCTTGCTGGCAGTGGTGTTGCTGTCTAACCAAGAAATAGTCTCTTCAGCCT
TCTTGGTGACGGTGTCCTTGTCAGCTTGTTCCAATTTGTCACCAGCTTCAGAAATGGTGTTCTTCAAAGAG
TAAGCAAT

CGGCGGGCTGATCACTTTATCGTGCATCTTGACCACGTTATTTCTGCTGGTGAACGAGTGGGGACAGTTCA
ATTCTGTGGTAACAAGGCCACAATTGGTGGTGGACCGTGACCGACACGCAAAGCTGGAGCTTAATATGGAT
GTGACATT

ACTCAGCCATGGAACGACACTTTTACCTCTACATCCACTGAAATGACCACCGTCACCGGTACCAACGGCGT
TCCAACTGACGAAACCGTCATTGTCATCAGAACTCCAACTAGTGAAGGTCTAATCAGCACCACCACTGAAC
CATGGACT

CTAATCAGCACCACCACTGAACCATGGACTGGCACTTTCACTTCGACTTCCACTGAGGTTACCACCATCAC
TGGAACCAACGGTCAACCAACTGACGAAACTGTGATTGTTATCAGAACTCCAACCAGTGAAGGTCTAATCA
GCACCACC

ACTGATGAGACCATCATTGTCATCAGAACACCAACAACAGCCACTACTGCCATGACTACAACTCAGCCATG
GAACGACACTTTTACCTCTACATCCACTGAAATGACCACCGTCACCGGTACCAACGGCGTTCCAACTGACG
AAACCGTC

GTTCAGTGGTGGTGCTGATTAGACCTTCACTAGTTGGAGTTCTGATGACAATGACGGTTTCGTCAGTTGGA
ACGCCGTTGGTACCGGTGACGGTGGTCATTTCAGTGGATGTAGAGGTAAAAGTGTCGTTCCATGGCTGAGT
TGTAGTCA

TGCTACGGATCGTATCTCTGCATATGACGTTATTATGGAAAACAGCATTCCTGAAAAGGGGATCCTATTGA
CCAAACTGTCAGAGTTCTGGTTCAAGTTCCTGTCCAACGATGTTCGTAATCATTTGGTCGACATCGCCCCA
GGTAAGAC

GCCATAAATTATCCAACGCTGCTTTACCTCTGGCAATGGGCCATGAGATGTCAGGAATTGTTTCCAAGGTT
GGTCCTAAAGTGACAAAGGTGAAGGTTGGCGACCACGTGGTCGTTGATGCTGCCAGCAGTTGTGCGGACCT
GCATTGCT

GAAGAAATGACAGGGGAAGAAATGAATGAAGAAGAAATGACTGGAGAAGAAGTGACTAGAGAAGAAGTGAC
TGAGGAAGAAATGACTAGAGAAGAAGTGTCTGAGGAAGAAATTACTGAGGAGGAAATCACAGAAGTTCCAT
TGCTAGGA

GTCAAGATTGAAAACCAACAAGGTGTTAACAACTTCGACGAAATCTTGAAGGTCACTGACGGTGTTATGGT
TGCCAGAGGTGACTTGGGTATTGAAATCCCAGCCCCAGAAGTCTTGGCTGTCCAAAAGAAATTGATTGCTA
AGTCTAAC

TGGATAAGATATATTGGGCAGGGGATAGATGGTTGTTGGGGTGTGGTGATGGATAGTGAGTGGATAGTGAG
TGGATGGATGGTGGAGTGGGGGAATGAGACAGGGCATGGGGTGGTGAGGTAAGTGCCGTGGATTGTGATGA
TGGAGAGG

AGGAATTCGAAGAAGACGCCGCCCAGAACGAAGAAATCAGTGCCACGCCAACTCCAAATCCAGAAAGCAGC
GCAGGTGCAGATGACACTTCCAGAGAAGCAAGTGCAAGTGCTGAAGGTGCTGAGGCCATTGAAGGCGACTT
CATGTCTA

AATGACAGGGGAAGAAATGAATGAAGAAGAAATGACTGGAGAAGAAGTGACTAGAGAAGAAGTGACTGAGG
AAGAAATGACTAGAGAAGAAGTGTCTGAGGAAGAAATTACTGAGGAGGAAATCACAGAAGTTCCATTGCTA
GGATAGAA

AAATGACCACCGTCACCGGTACCAACGGTTTGCCAACTGATGAAACCATCATTGTCATCAGAACACCAACA
ACAGCCACTACTGCCATGACTACAACTCAGCCATGGAACGACACTTTTACCTCTACATCCACTGAAATCAC
CACCGTCA

TGAGACCATCATTGTCATCAGAACACCAACAACAGCCACTACTGCCATGACTACAACTCAGCCATGGAACG
ACACTTTTACCTCTACTTCTACCGAATTGACCACAGTCACCGGTACCAATGGTTTGCCAACTGATGAGACC
ATCATTGT

AGTGGTGTTGCTGTCTAACCAAGAAATAGTCTCTTCAGCCTTCTTGGTGACGGTGTCCTTGTCAGCTTGTT
CCAATTTGTCACCAGCTTCAGAAATGGTGTTCTTCAAAGAGTAAGCAATGGATTCCAATTGGTTCTTGGAA
GCAATTCT

TCGAATTCTTCCTTGCTGGCAGTGGTGTTGCTGTCTAACCAAGAAATAGTCTCTTCAGCCTTCTTGGTGAC
GGTGTCCTTGTCAGCTTGTTCCAATTTGTCACCAGCTTCAGAAATGGTGTTCTTCAAAGAGTAAGCAATGG
ATTCCAAT

CCAACCTACAAGATCTCCTAAAGAAACAATTCCAGCCGCTAGACGACCCAAACGTCCAACAAGTGCTCCAT
CTCATGCTCCACTATGCCGTGCAAGTCGCCCCCATGGCTGTCATAAAGGAAATCGTCCATCATTGGGTCTC
AACTACAA

CGGTACAGAATTGATGTACGTATTCATTAAATCCGTGTACCTCGAAGCAACGATAGACAATCCGGTGATCA
GTTTCGTGCTGTCCATTTGCAAGATGGCCTCTGTGGACAGCTTGATAAGTATGTCATTGGGTAGCTGGGTC
ACATCCTG

TGGTCCTAAAGTGACAAAGGTGAAGGTTGGCGACCACGTGGTCGTTGATGCTGCCAGCAGTTGTGCGGACC
TGCATTGCTGGCCACACTCCAAATTTTACAATTCCAAACCATGTGATGCTTGTCAGAGGGGCAGTGAAAAT
CTATGTAC

ACATCCACTGAAATGACCACCGTCACCGGTACCAACGGTTTGCCAACTGATGAAACCATCATTGTCATCAG
AACACCAACAACAGCCACTACTGCCATGACTACAACTCAGCCATGGAACGACACTTTTACCTCTACATCCA
CTGAAATC

GCCCTCTTGCCACTTGCAAACAGACGCCACGTCGCATCACGTGGTGGCGATGTATCGTGGCCAGTTCTACT
GGTTCGACGTGCTGGACACACGCAACGAGCCCATCTTCGCCACCCCAGAACAACTGGAGTGGAACCTCTAC
TCGATCAT

CGATAGACAATCCGGTGATCAGTTTCGTGCTGTCCATTTGCAAGATGGCCTCTGTGGACAGCTTGATAAGT
ATGTCATTGGGTAGCTGGGTCACATCCTGGTTGGAGTTCGAACTGTTCATCAATGAGTACACAGATGAGTA
GGTGTTGC

CCAGTCCATGGTTCAGTGGTGGTGCTGATTAGACCTTCACTAGTTGGAGTTCTGATGACAATGACGGTTTC
GTCAGTTGGAACGCCGTTGGTACCGGTGACGGTGGTCATTTCAGTGGATGTAGAGGTAAAAGTGTCGTTCC
ATGGCTGA

GAACTCCAACTAGTGAAGGTCTAATCAGCACCACCACTGAACCATGGACTGGTACTTTCACCTCTACATCC
ACTGAGATGACCACCGTCACCGGTACTAACGGTCAACCAACTGACGAAACCGTGATTGTTATCAGAACTCC
AACCAGTG

TCAGCAGCTCCAGTACTGGTAGTTGTCTCAGCAGCTCCAGTATTGGTTGTTGTCTCACTGGTAGCACTGTT
CATTTTAGAGCTGACAGACTGTTCATTCGTAGTCTGTGGCCTCCATGTAGAATAGACCGTAACAACATCAT
TCACAGTA

CAGCACTACAACGGAACCATGGACCGGTACTTTCACTTCTACATCTACTGAAATGACGCACGTCACCGGTA
CCAACGGCGTTCCAACTGACGAAACCGTCATTGTCATCAGAACTCCAACCAGTGAAGGTCTAATCAGCACC
ACCACTGA

CTGAAGAAACTGGCGCTGTCTCCGCATCTATCTATTCATTCACACCTAGCTCGTTCAAGAGCAGCGGTGAC
ATTTCTTTGAGTTTGTCAAAGGCCAAGAAGGGTGAAGTCACCTTTTCTCCATACTCTAACGCTGGTACCTT
TTCTTTGT

TATGAGATGACCTCGTGGTCATGGAGTCCCTGAGTGATGGTATGGACATCCCAGAATCCATCGAGTTTGTG
AACTCGCTGTCGTCATCATCACCAGTGTTGTCATTATTGCGAAGATGCCTGCCACTAGGAATCCATCGACG
TACCATGG

GGTGAACAAGGTAAGGACGTCAAGATCATTGTCAAGATTGAAAACCAACAAGGTGTTAACAACTTCGACGA
AATCTTGAAGGTCACTGACGGTGTTATGGTTGCCAGAGGTGACTTGGGTATTGAAATCCCAGCCCCAGAAG
TCTTGGCT

AACAGCCACTACTGCCATGACTACAACTCAGCCATGGAACGACACTTTTACCTCTACATCCACTGAAATCA
CCACCGTCACCGGTACCAATGGTTTGCCAACTGATGAGACCATCATTGTCATCAGAACACCAACAACAGCC
ACTACTGC

TAGAAACACGTTTCGAATCTTCAGTATGAGTTGACGGAGGTGTGGAATCGGTTGGACTCACAGCTTTTGAA
AGGACATTTCTCGGTTGCTCAGGATGTAGTTCAATGTCGGATTGGAAGTCATGGTCAGATTCTATGTTAAG
ATCATTGG

AGTGACTGTGGTCAATTCGGTAGAAGTAGAGGTAAAAGTGCTGTTCCATGGCTCAGTTGTAGTTATGATGG
TGCTAGCAGTTGTTGGAGTTCTGATGACAATGACGGTTTCGTCAGTTGGAACGCCGTTGGTACCGGTGACG
GTGGTCAT

ATGGAGTCCCTGAGTGATGGTATGGACATCCCAGAATCCATCGAGTTTGTGAACTCGCTGTCGTCATCATC
ACCAGTGTTGTCATTATTGCGAAGATGCCTGCCACTAGGAATCCATCGACGTACCATGGCTATAACTTTCC
TTATGTTG

ACCAACGGCGTTCCAACTGACGAAACCGTCATTGTCATCAGAACTCCAACTAGTGAAGGTCTAATCAGCAC
CACCACTGAACCATGGACTGGTACTTTCACCTCTACATCCACTGAGATGACCACCGTCACCGGTACTAACG
GTCAACCA

TTGTTGCACTGGTAACAGGTGGTAATGATGAAGTAATTTCCTGACTTGTTGTTGTACTGGTAACAGGTGGT
AATGATGAAGCAGTTTCCTGGCTTGTTGTTGCAGTGGTAATAGGTGGTAATGATGAAGACGAATATGTAGA
CTTTGGTG

CAGACGTGATAAAGCTGGTGATTTGTCCTGAAGAAGATGACAAACTGGATGAGATGGCAGTAGTTGGAGTT
TTGACAATAATGACAGTTTCATCAGTTGGTTGACCGTTGGTTCCAGTGATGGTGGTCATCTCAGTAGATGT
AGAGGTGA

GAACTCCAACCAGTGAAGGTTTGGTTACAACCACCACTGAACCATGGACTGGTACTTTTACTTCGACTTCC
ACTGAAATGTCTACTGTCACTGGAACCAATGGCTTGCCAACTGATGAAACTGTCATTGTTGTCAAAACTCC
AACTACTG

GAACACCAACAACAGCCACTACTGCCATGACTACAACTCAGCCATGGAACGACACTTTTACCTCTACATCC
ACTGAAATCACCACCGTCACCGGTACCAATGGTTTGCCAACTGATGAGACCATCATTGTCATCAGAACACC
AACAACAG

CATCAGAACACCAACAACAGCCACTACTGCCATGACTACAACTCAGCCATGGAACGACACTTTTACCTCTA
CATCCACTGAAATGACCACCGTCACCGGTACCAACGGCGTTCCAACTGACGAAACCGTCATTGTCATCAGA
ACTCCAAC

AAGGGCTACTATAACATTTGTTCATATTTGGGGCTTTGTGGTGCGGGTGTGCAGAGCGGTGGATTTGCAGA
ACGTGTTGTGATGAACGAATCTCACTGCTACAAAGTACCGGACTTCGTGCCCTTAGACGTTGCAGCTTTGA
TTCAACCG

TCATTTGGATCAGCTTCTCCCCTATCATTGCTGCCATCCATATGGAGAACAGGCACGACTTCGTCGTTTTC
TGTGGGTGCTACAACCTTTCTCGCCTTCTTCCCAACGAACCTTGGCAACAATGGATCCATTTGCTTGGACA
CTACCTCA

TAGTGAGAGTTGGATAAGATATATTGGGCAGGGGATAGATGGTTGTTGGGGTGTGGTGATGGATAGTGAGT
GGATAGTGAGTGGATGGATGGTGGAGTGGGGGAATGAGACAGGGCATGGGGTGGTGAGGTAAGTGCCGTGG
ATTGTGAT

ACTGCCATGACTACAACTCAGCCATGGAACGACACTTTTACCTCTACATCCACTGAAATCACCACCGTCAC
CGGTACCACCGGTTTGCCAACTGATGAGACCATCATTGTCATCAGAACACCAACAACAGCCACTACTGCCA
TGACTACA

GATTGGGTTGGCAATGTCTTGCAACTCCTTCAACTTGTCATCGAATTCTTCCTTGCTGGCAGTGGTGTTGC
TGTCTAACCAAGAAATAGTCTCTTCAGCCTTCTTGGTGACGGTGTCCTTGTCAGCTTGTTCCAATTTGTCA
CCAGCTTC

GGCACTTTCACTTCGACTTCCACTGAGGTTACCACCATCACTGGAACCAACGGTCAACCAACTGACGAAAC
TGTGATTGTTATCAGAACTCCAACCAGTGAAGGTCTAATCAGCACCACCACTGAACCATGGACTGGTACTT
TCACTTCT

TATAAGAACTACTACCTCATGAATTCTAGTGGATGAAAGAAGCAGCACGAACACCATTTCTACAGACAACG
ACACATGGAAAGGTTCACCATTCCCAAAGAAAACAACGATGGCCACAAGGGTGTGGTCCTCCATTCTCCTA
CTGTTGGA

GAACTCCAACAACTGCTAGCACCATCATAACTACAACTGAGCCATGGAACAGCACTTTTACCTCTACTTCT
ACCGAATTGACCACAGTCACTGGCACCAATGGTGTACGAACTGACGAAACCATCATTGTAATCAGAACACC
AACAACAG

ATATTGGTTTTAGAAACACGTTTCGAATCTTCAGTATGAGTTGACGGAGGTGTGGAATCGGTTGGACTCAC
AGCTTTTGAAAGGACATTTCTCGGTTGCTCAGGATGTAGTTCAATGTCGGATTGGAAGTCATGGTCAGATT
CTATGTTA

TTTGACAATAATGACAGTTTCATCAGTTGGTTGACCGTTGGTTCCAGTGATGGTGGTCATCTCAGTAGATG
TAGAGGTGAAAGTACCGGTCCATGGCTCGGTTGTAGTTGTAACCAAACCTTCACTGGTTGGAGTTCTGATA
ACAATCAC

ACGTCCTCTCCCACCAAGGTAGCATCGTCGTCTCCCTTAAAGCCTACTTCGCCAACAGTTCCGGATGCAAG
TGTGGCGTCTTTGAGAAGCAGGTTTACTTTCAAGCCTTCAGATCCCAGCGAAGGAGCTCATACTTCGAAGC
CGCTCCCA

CTGTGGTCAATTCGGTAGAAGTAGAGGTAAAAGTGTCGTTCCATGGCTGAGTTGTAGTCATGGCAGTAGTG
GCTGTTGTTGGTGTTCTGATGACAATGATGGTCTCATCAGTTGGCAAACCATTGGTACCGGTGACTGTGGT
CAATTCGG

TTGTAGATACAATGGCTGGAGAAGCAGTCTTAGAGCATACGTCAGATTCACAAGAAGAAATTGTAACTACT
GTGGTTTGTTTTGTTGTTTCTGTGGTTTGCTCTGTTGTCCCCTTGGTTTGCTTTGTTGTCTCCGTAGTTTG
CTTTGTTA

AAATGACCACCGTCACCGGTACCAACGGCGTTCCAACTGACGAAACCGTCATTGTCATCAGAACTCCAACA
ACTGCTAGCACCATCATAACTACAACTGAGCCATGGAACAGCACTTTTACCTCTACTTCTACCGAATTGAC
CACAGTCA

CATTCTGTGGTGAATGTGACTGAGCAGTTTGAGGAGAGGCATGATGGGGGTTCTCTGGAACAGCTGATGAA
GCAGGTGTTGTTGTCTGTTGAGAGTTAGCCTTAGTGGAAGCCTTCTCACATTCTTCTGTTTTGGAAGCTGA
AACGTCTA

ATGACAGTTTCATCAGTTGGTTGACCGTTGGTTCCAGTGATGGTGGTCATCTCAGTAGATGTAGAGGTGAA
AGTACCGGTCCATGGCTCGGTTGTAGTTGTAACCAAACCTTCACTGGTTGGAGTTCTGATAACAATCACGG
TTTCGTCA

AATCAGAACACCAACAACAGCCACTACTGCCATAACTACAACTGAGCCATGGAACAGCACTTTTACCTCTA
CTTCTACCGAATTGACCACAGTCACCGGTACCAATGGTTTGCCAACTGATGAGACCATCATTGTCATCAGA
ACACCAAC

TTGTTGGAATCGTTCATCAGAGCAGGCTTGATGGGATCGTTATGAGATGACCTCGTGGTCATGGAGTCCCT
GAGTGATGGTATGGACATCCCAGAATCCATCGAGTTTGTGAACTCGCTGTCGTCATCATCACCAGTGTTGT
CATTATTG

TCCTGGATGAAGAAGCCAAGCAGTTCTTCACGAATCAGGTCACTGGCCTCTTGTGCTTCGATTCCTCTCGT
AACCAATCTGATTAAGACGTGTAACCATGACGAGGAGTCGTCATCGTCCAGTAATATGGAGGAGGAAGACG
AAGATTTG

AAGAAGATGACAAACTGGATGAGATGGCAGTAGTTGGAGTTTTGACAATAATGACAGTTTCATCAGTTGGT
TGACCGTTGGTTCCAGTGATGGTGGTCATCTCAGTAGATGTAGAGGTGAAAGTACCGGTCCATGGCTCGGT
TGTAGTTG

TAGAAGTGGAGGTTGTTGTAGAAGAAATGACAGGGGAAGAAATGAATGAAGAAGAAATGACTGGAGAAGAA
GTGACTAGAGAAGAAGTGACTGAGGAAGAAATGACTAGAGAAGAAGTGTCTGAGGAAGAAATTACTGAGGA
GGAAATCA

TCTTGCAATCGCTTCCTCAGGGCTCCCATGCGGTCTCTATCGGTGATGTGTACGGTGGTACCCACAGATAC
TTCACCAAAGTCGCCAACGCTCACGGTGTGGAAACCTCCTTCACTAACGATTTGTTGAACGATCTACCTCA
ATTGATAA

CAACTGCTACCGACGCAACTGCAACCGTTTCCTCCAAGTCATACACCACTGTTACTGTTACTCACTGTGAC
AACAATGGCTGTAACACCAAGACTGTCACTTCTGAATGTTCTAAAGAAACTGCAGCAACCACCATTTCTCC
AAAATCAT

AATAGCACCTCCTCCGCAGACTTGAGTTCTATCACTTCCGTCTCGTCAGCTAGTGCAAGTGCCACCGCTTC
CGACTCACTTTCTTCCAGTGACGGTACCGTTTATTTGCCATCCACAACAATTAGCGGTGATCTCACAGTTA
CTGGTAAA

CCGTTTCTGGTTCTAGGCTGCTAGTTGTCGGTCCTGAAGATGACGAAGATGAATTGATGGACGACGTTATG
GATGATTTGACTGGTTTGTTGGACTCCGTTGACACAACTGGTAAAGGTGTTGTGGTCCAAGCATCCACCTT
GGGTTCTT

CTCTTACACCAATAGCACCTCCTCCGCAGACTTGAGTTCTATCACTTCCGTCTCGTCAGCTAGTGCAAGTG
CCACCGCTTCCGACTCACTTTCTTCCAGTGACGGTACCGTTTATTTGCCATCCACAACAATTAGCGGTGAT
CTCACAGT

CGACGCAACTGCAACCGTTTCCTCCAAGTCATACACCACTGTTACTGTTACTCACTGTGACAACAATGGCT
GTAACACCAAGACTGTCACTTCTGAATGTTCTAAAGAAACTGCAGCAACCACCATTTCTCCAAAATCATAC
ACTACTGT

CTCAAAGCTCTTTGGTTGGTAGACAAGTCCTTCTTGTTCTTTCTCTTGAATTCTTGGATGAAGTGGTTGAC
CAATCTGTTGTCAAAATCTTCACCACCCAAATGGGTGTCACCAGCGGTGGCCTTAACTTCAAAGATACCGT
CTTCAATG

GTGTCAAGAACGGTGTCCACATGGTCTTCGCTTCTTTCATCAGAACCGCCAACGATGTTTTGACCATCAGA
GAAGTCTTGGGTGAACAAGGTAAGGACGTCAAGATCATTGTCAAGATTGAAAACCAACAAGGTGTTAACAA
CTTCGACG

GACACTTTTACCTCTACATCCACTGAAATCACCACCGTCACCGGTACCACCGGTTTGCCAACTGATGAGAC
CATCATTGTCATCAGAACACCAACAACAGCCACTACTGCCATGACTACAACTCAGCCATGGAACGACACTT
TTACCTCT

GGTCATGTGTAAGACGGCTAGGTAAATTTCAGTGCATATTGAACTCCAGGGACTTCACAAGTAGTGGTGGT
AGCGATGCAGCAGTCGTTCCTGAAGATATAGGCAGGTTTGTCAAATTTGTTGTTGATAGCGATGTAGAGGA
TGTGCTGA

ATCCAACGCTGCTTTACCTCTGGCAATGGGCCATGAGATGTCAGGAATTGTTTCCAAGGTTGGTCCTAAAG
TGACAAAGGTGAAGGTTGGCGACCACGTGGTCGTTGATGCTGCCAGCAGTTGTGCGGACCTGCATTGCTGG
CCACACTC

CACGAAGATGAGAAAGAGGCGATAGATGAGGCCAAGAAGATGAAAGTGCCGGGAGAGAACGAGGACGAAAG
CAAGGAAGAGGAAAAGAGTCAAGAACTGGAAGAGGCAATTGACAGCAAGGAGAAGAGCACCGACGCCAGGG
ACGAGCAA

CTACAACTGAGCCATGGAACAGCACTTTTACCTCTACTTCTACCGAATTGACCACAGTCACTGGCACCAAT
GGTGTACGAACTGACGAAACCATCATTGTAATCAGAACACCAACAACAGCCACTACTGCCATAACTACAAC
TGAGCCAT

TGAATGTGACTGAGCAGTTTGAGGAGAGGCATGATGGGGGTTCTCTGGAACAGCTGATGAAGCAGGTGTTG
TTGTCTGTTGAGAGTTAGCCTTAGTGGAAGCCTTCTCACATTCTTCTGTTTTGGAAGCTGAAACGTCTAAC
GGATCTTG

CTACAACTGAGCCATGGAACAGCACTTTTACCTCTACATCCACTGAAATGACCACCGTCACCGGTACCAAC
GGTTTGCCAACTGATGAAACCATCATTGTCATCAGAACACCAACAACAGCCACTACTGCCATAACTACAAC
TCAGCCAT

TGGATGTAGAGGTGAAAGTACCAGTCCATGGTTCAGTGGTGGTGCTGATTAGACCTTCACTAGTTGGAGTT
CTGATGACAATGACGGTTTCGTCAGTTGGAACGCCGTTGGTACCGGTGACGGTGGTCATTTCAGTGGATGT
AGAGGTAA

TTTTATTGAAATCGAATCCGAGTAGGTTCTTAGCAATGCTCAATGAAGTGTTTGAAGCGTCCTTGTTTAAC
GATGACAATGACATGGTTGCATCAGTTGGAGAAGCAGAATTGGTAAGTAGGCAATATGTTATTGATCTACT
ATTGGATG

TCCTGACTTGTTGTTGCACTGGTAACAGGTGGTAATGATGAAGTAATTTCCTGACTTGTTGTTGTACTGGT
AACAGGTGGTAATGATGAAGCAGTTTCCTGGCTTGTTGTTGCAGTGGTAATAGGTGGTAATGATGAAGACG
AATATGTA

CAGAATCGAGGCAACAAACAACGCTAGTTACTGTTACTTCCTGCGAATCTGGTGTGTGTTCCGAAACTGCT
TCACCTGCCATTGTTTCGACGGCCACGGCTACTGTGAATGATGTTGTTACGGTCTATCCTACATGGAGGCC
ACAGACTG

CGGTGGTCATTTCAGTGGATGTAGAGGTAAAAGTGTCGTTCCATGGCTGAGTTGTAGTCATGGCAGTAGTG
GCTGTTGTTGGTGTTCTGATGACAATGATGGTCTCATCAGTTGGCAAACCGTTGGTACCGGTGACGGTGGT
GATTTCAG

CGGTGGTCATTTCAGTGGATGTAGAGGTAAAAGTGTCGTTCCATGGCTGAGTTGTAGTCATGGCAGTAGTG
GCTGTTGTTGGTGTTCTGATGACAATGATGGTCTCATCAGTTGGCAAACCATTGGTACCGGTGACGGTGGT
GATTTCAG

CCGCCAACGGCTTCGGCATCGGCTACATCATCAGAGACCACTCCGTCTCTGTGGTGGTGTCCTCAAGGCAT
CGCCAGACTGCTCGGTTTGCGTCGCTCATGGAAAAGTCGCTGCTGGAGATCGACCGCATCTTCAAACGGCA
GCAAGCTC

TGGAAAGTGTCTAGAAGATTACCAAGAACCACGTTATTTGAGAGAGGATGGCAAGGTGACGACAATCACAC
CAAGACCACATTTTGGGTGCGCCTGGAAGCAAGACCTGAGAAACTGGGCCAAAATATTCAAACCAAGCATA
AGATAGTT

CCTCTATCTCTGGTGTCTCAAACTCCACTACAGCATCTGGTTCAATCGCTTCTACTGCTTCCACCGCTTCC
ACTACTTCTACTGCATCCGCTGCATCCGCCACCAGCTTCACCTCAGGTTCCGCTTCTGTCTACACTACTAC
ATTAACTT

CTGGTTGGCTTCGTCCTTGCCATTAGCAAACAGCTCTATCTTGGGAGTCAATGGTGTAGAAACTGCACTGT
TAGTTGCCGTGCCTGAGGGAGTGCTATCTCCCGTAGGTTGTTGTTTCTTTGCTGTCTTGCTGTCGGTGAGA
GTGTCGGC

GCTCAGTTGTAGTTATGATGGTGCTAGCAGTTGTTGGAGTTCTGATGACAATGACGGTTTCGTCAGTTGGA
ACGCCGTTGGTACCGGTGACGGTGGTCATTTCAGTAGATGTAGAAGTGAAAGTACCGGTCCATGGTTCCGT
TGTAGTTA

GAAATTGAAGAGGTGACTACTGTTTTCGTCTCAGCAGCTCCAGTACTGGTAGTTGTCTCAGCAGCTCCAGT
ATTGGTTGTTGTCTCACTGGTAGCACTGTTCATTTTAGAGCTGACAGACTGTTCATTCGTAGTCTGTGGCC
TCCATGTA

TGAAGGTTGGCGACCACGTGGTCGTTGATGCTGCCAGCAGTTGTGCGGACCTGCATTGCTGGCCACACTCC
AAATTTTACAATTCCAAACCATGTGATGCTTGTCAGAGGGGCAGTGAAAATCTATGTACCCACGCCGGTTT
TGTAGGAC

TTTCTGCTGGTGAACGAGTGGGGACAGTTCAATTCTGTGGTAACAAGGCCACAATTGGTGGTGGACCGTGA
CCGACACGCAAAGCTGGAGCTTAATATGGATGTGACATTTCCATCGATGCCATGTGACCTGGTGAATCTCG
ATATTATG

TCATCTAGCGCCTCTATCTCTGGTGTCTCAAACTCCACTACAGCATCTGGTTCAATCGCTTCTACTGCTTC
CACCGCTTCCACTACTTCTACTGCATCCGCTGCATCCGCCACCAGCTTCACCTCAGGTTCCGCTTCTGTCT
ACACTACT

CAGGTACCGATGTCGATTTGCCAGCTTTGTCTGAAAAGGACAAGGAAGATTTGAGATTCGGTGTCAAGAAC
GGTGTCCACATGGTCTTCGCTTCTTTCATCAGAACCGCCAACGATGTTTTGACCATCAGAGAAGTCTTGGG
TGAACAAG

CCTTCGCCAGGGAAGTTTCCCCAACCTACAAGATCTCCTAAAGAAACAATTCCAGCCGCTAGACGACCCAA
ACGTCCAACAAGTGCTCCATCTCATGCTCCACTATGCCGTGCAAGTCGCCCCCATGGCTGTCATAAAGGAA
ATCGTCCA

CCCCAGACTCAATAATTCCTTCATCTAGCGCCTCTATCTCTGGTGTCTCAAACTCCACTACAGCATCTGGT
TCAATCGCTTCTACTGCTTCCACCGCTTCCACTACTTCTACTGCATCCGCTGCATCCGCCACCAGCTTCAC
CTCAGGTT

GTTCCAGCACCAACTGCTACCGACGCAACTGCAACCGTTTCCTCCAAGTCATACACCACTGTTACTGTTAC
TCACTGTGACAACAATGGCTGTAACACCAAGACTGTCACTTCTGAATGTTCTAAAGAAACTGCAGCAACCA
CCATTTCT

GAGTCTGTGATCAAGAAATGTTCTTCCTACGGTGATTTGGTCACTTCGCAAATGAAGAAATGGGGTGTGCA
AGTGGAAGGCGATAACTCTGAGTTGGACCTGATGAACCTTGGGGAAGACGATGACGACGACAATGATGATG
GCAATAAC

GGGGATAGATGGTTGTTGGGGTGTGGTGATGGATAGTGAGTGGATAGTGAGTGGATGGATGGTGGAGTGGG
GGAATGAGACAGGGCATGGGGTGGTGAGGTAAGTGCCGTGGATTGTGATGATGGAGAGGGAGGGTAGTTGA
CATGGAGT

AATGGCTGGAGAAGCAGTCTTAGAGCATACGTCAGATTCACAAGAAGAAATTGTAACTACTGTGGTTTGTT
TTGTTGTTTCTGTGGTTTGCTCTGTTGTCCCCTTGGTTTGCTTTGTTGTCTCCGTAGTTTGCTTTGTTATC
TCTGTGGT

ATTGTCATCAGAACACCAACAACAGCCACTACTGCCATGACTACAACTCAGCCATGGAACGACACTTTTAC
CTCTACATCCACTGAAATCACCACCGTCACCGGTACCAATGGTTTGCCAACTGATGAGACCATCATTGTCA
TCAGAACA

CAGTTTGACACAGCTGCAGGTATCTTCCACTCATTTGGATCAGCTTCTCCCCTATCATTGCTGCCATCCAT
ATGGAGAACAGGCACGACTTCGTCGTTTTCTGTGGGTGCTACAACCTTTCTCGCCTTCTTCCCAACGAACC
TTGGCAAC

ATCCGTGTACCTCGAAGCAACGATAGACAATCCGGTGATCAGTTTCGTGCTGTCCATTTGCAAGATGGCCT
CTGTGGACAGCTTGATAAGTATGTCATTGGGTAGCTGGGTCACATCCTGGTTGGAGTTCGAACTGTTCATC
AATGAGTA

AACCAACTGACGAAACCGTGATTGTTATCAGAACTCCAACCAGTGAAGGTTTGGTTACAACCACCACTGAA
CCATGGACTGGTACTTTTACTTCGACTTCCACTGAAATGTCTACTGTCACTGGAACCAATGGCTTGCCAAC
TGATGAAA

TTGAGATTCGGTGTCAAGAACGGTGTCCACATGGTCTTCGCTTCTTTCATCAGAACCGCCAACGATGTTTT
GACCATCAGAGAAGTCTTGGGTGAACAAGGTAAGGACGTCAAGATCATTGTCAAGATTGAAAACCAACAAG
GTGTTAAC

CCTCAGCCATGGAACGACACTTTTACCTCTACATCCACTGAAATGACCACCGTCACCGGTACCAACGGTTT
GCCAACTGATGAAACCATCATTGTCATCAGAACACCAACAACAGCCACTACTGCCATAACTACAACTGAGC
CATGGAAC

ACTCAGCCATGGAACGACACTTTTACCTCTACATCCACTGAAATGACCACCGTCACCGGTACCAACGGTTT
GCCAACTGATGAAACCATCATTGTCATCAGAACACCAACAACAGCCACTACTGCCATGACTACAACTCAGC
CATGGAAC

AAGTGTCGTTCCATGGCTGAGTTGTAGTCATGGCAGTAGTGGCTGTTGTTGGTGTTCTGATGACAATGATG
GTCTCATCAGTTGGCAAACCATTGGTACCGGTGACTGTGGTCAATTCGGTAGAAGTAGAGGTAAAAGTGTC
GTTCCATG

TGGCATCTAGTTCTTCATATACGGCACCTCCCCTGAACGAAGATGGTCCTAAAGGGGTAGCTTCTGCAGTG
TCACAAGGCTCCGAATCCGTAGTCTCATGGACAACTTTAACACACGTATATTCCATCCTGGGTGCTTATGG
AGGGCCCA

GATGGAGAGTGCCATAAATTATCCAACGCTGCTTTACCTCTGGCAATGGGCCATGAGATGTCAGGAATTGT
TTCCAAGGTTGGTCCTAAAGTGACAAAGGTGAAGGTTGGCGACCACGTGGTCGTTGATGCTGCCAGCAGTT
GTGCGGAC

GTAGAAGAGAACGAACCTGAATGTGAATGGTGTGATGCAGAGTCTGGGGTCGTCATTGAACTTGAAGTCTT
GTAAGGGGAATTGAATGGAGATGGAGAGGATGAAGATGAGGTTGGAGTGAAGGCAAATGGTGGAGAAATGC
TATCTTTG

ACCAATGGTTTGCCAACTGATGAGACCATCATTGTCATCAGAACACCAACAACAGCCACTACTGCCATGAC
TACAACTCAGCCATGGAACGACACTTTTACCTCTACTTCTACCGAATTGACCACAGTCACCGGTACCAATG
GTTTGCCA

ATAAAACATCATCACAATGTCTAGATTAGAAAGATTGACCTCATTAAACGTTGTTGCTGGTTCTGACTTGA
GAAGAACCTCCATCATTGGTACCATCGGTCCAAAGACCAACAACCCAGAAACCTTGGTTGCTTTGAGAAAG
GCTGGTTT

GTTGTACTGATAACAGGTGGTAATGATGAAGTAATTTCCTGACTTGCTGTCGCACTGGTAACAGGTGGTAA
TGAAGAAGTAATTTCCTGACTTGTTGTTGTACTGGTAACAGGTGGTAATGATGAAGTAATTTCCTGACTTG
TTGTTGTA

ACGGCACCTCCCCTGAACGAAGATGGTCCTAAAGGGGTAGCTTCTGCAGTGTCACAAGGCTCCGAATCCGT
AGTCTCATGGACAACTTTAACACACGTATATTCCATCCTGGGTGCTTATGGAGGGCCCACGTGCTTGTATC
CGACAGCC

ACTGATGAGACCATCATTGTCATCAGAACACCAACAACAGCCACTACTGCCATGACTACAACTCAGCCATG
GAACGACACTTTTACCTCTACTTCTACCGAATTGACCACAGTCACCGGTACCAATGGTTTGCCAACTGATG
AGACCATC

TGAGCAGTTTGAGGAGAGGCATGATGGGGGTTCTCTGGAACAGCTGATGAAGCAGGTGTTGTTGTCTGTTG
AGAGTTAGCCTTAGTGGAAGCCTTCTCACATTCTTCTGTTTTGGAAGCTGAAACGTCTAACGGATCTTGAT
TTGTGTGG

TGACCATCAGAGAAGTCTTGGGTGAACAAGGTAAGGACGTCAAGATCATTGTCAAGATTGAAAACCAACAA
GGTGTTAACAACTTCGACGAAATCTTGAAGGTCACTGACGGTGTTATGGTTGCCAGAGGTGACTTGGGTAT
TGAAATCC

ATGTGAATGGTGTGATGCAGAGTCTGGGGTCGTCATTGAACTTGAAGTCTTGTAAGGGGAATTGAATGGAG
ATGGAGAGGATGAAGATGAGGTTGGAGTGAAGGCAAATGGTGGAGAAATGCTATCTTTGGTCAACCTTCTT
AAATGAGT

CCATCATTGTCATCAGAACACCAACAACAGCCACTACTGCCATGACTACAACTCAGCCATGGAACGACACT
TTTACCTCTACATCCACTGAAATGACCACCGTCACCGGTACCAACGGCGTTCCAACTGACGAAACCGTCAT
TGTCATCA

TGTCGATTTGCCAGCTTTGTCTGAAAAGGACAAGGAAGATTTGAGATTCGGTGTCAAGAACGGTGTCCACA
TGGTCTTCGCTTCTTTCATCAGAACCGCCAACGATGTTTTGACCATCAGAGAAGTCTTGGGTGAACAAGGT
AAGGACGT

GTAAGAGCGCCTGGTTGGCTTCGTCCTTGCCATTAGCAAACAGCTCTATCTTGGGAGTCAATGGTGTAGAA
ACTGCACTGTTAGTTGCCGTGCCTGAGGGAGTGCTATCTCCCGTAGGTTGTTGTTTCTTTGCTGTCTTGCT
GTCGGTGA

GTACTGGTAGTTGTCTCAGCAGCTCCAGTATTGGTTGTTGTCTCACTGGTAGCACTGTTCATTTTAGAGCT
GACAGACTCTTCATTCGTAGTCTGTGGCCTCCATGTTGGATAGACCGTAACAACATCATTCACAGTAGCCG
TGGCCGTC

CATGACTACACCTCAGCCATGGAACGACACTTTTACCTCTACATCCACTGAAATGACCACCGTCACCGGTA
CCAACGGTTTGCCAACTGATGAAACCATCATTGTCATCAGAACACCAACAACAGCCACTACTGCCATAACT
ACAACTGA

CATAACTACAACTCAGCCATGGAACGACACTTTTACCTCTACATCCACTGAAATGACCACCGTCACCGGTA
CCAACGGTTTGCCAACTGATGAAACCATCATTGTCATCAGAACACCAACAACAGCCACTACTGCCATGACT
ACAACTCA

CGGTCAACCAACTGACGAAACTGTGATTGTTATCAGAACTCCAACCAGTGAAGGTCTAATCAGCACCACCA
CTGAACCATGGACTGGTACTTTCACTTCTACATCTACTGAAATGACCACCGTCACCGGTACTAACGGTCAA
CCAACTGA

CACTGAGATGACCACCGTCACCGGTACTAACGGTCAACCAACTGACGAAACCGTGATTGTTATCAGAACTC
CAACCAGTGAAGGTTTGGTTACAACCACCACTGAACCATGGACTGGTACTTTTACTTCTACATCTACTGAA
ATGACCAC

CCTATCATTGCTGCCATCCATATGGAGAACAGGCACGACTTCGTCGTTTTCTGTGGGTGCTACAACCTTTC
TCGCCTTCTTCCCAACGAACCTTGGCAACAATGGATCCATTTGCTTGGACACTACCTCAATATGGTGGCTG
TTGTTCAA

TTGGGCATGACCATTCTGTTTTCAATGTCGTCATCTTCGTCGATGCTGTACTGAGACAGTTTCTTGTGTTG
TAAGAGCGCCTGGTTGGCTTCGTCCTTGCCATTAGCAAACAGCTCTATCTTGGGAGTCAATGGTGTAGAAA
CTGCACTG

TTGCTCGAAATCCTGGATGAAGAAGCCAAGCAGTTCTTCACGAATCAGGTCACTGGCCTCTTGTGCTTCGA
TTCCTCTCGTAACCAATCTGATTAAGACGTGTAACCATGACGAGGAGTCGTCATCGTCCAGTAATATGGAG
GAGGAAGA

CAGCATCTGGTTCAATCGCTTCTACTGCTTCCACCGCTTCCACTACTTCTACTGCATCCGCTGCATCCGCC
ACCAGCTTCACCTCAGGTTCCGCTTCTGTCTACACTACTACATTAACTTACTTGAATGCCACAAGTACAGT
CGTGGTTT

AATTGAAGAGGTGACTACTGTTTTCGTCTCAGCAGCTCCAGTACTGGTAGTTGTCTCAGCAGCTCCAGTAT
TGGTTGTTGTCTCACTGGTAGCACTGTTCATTTTAGAGCTGACAGACTCTTCATTCGTAGTCTGTGGCCTC
CATGTTGG

AAGAGTACGTAAAAACAGGTACTGTGCATGGTTTGAAACAACCTCAAGGACTTAAAGAATCTCAAGAGTTC
CCAGAACCAATCTTCACCCCATCGACCAAGGCTGAACAAGGTGAACATGACGAAAACATCTCTCCTGCCCA
GGCCGCTG

TAGTGTTGACATAAGGTGTTGTAGGATAAGACGGTGAACGTCCTCTTGGCTAAATCGGTGGCCAGATGCTC
AGTGGATTGCTGCAAAGAATCGTACTCCCTCTGTGGTTCCCCATCGGTATTGATTTCGATGAACCTACGTT
CATGCGGT

TACAACCACCACTGAACCATGGACTGGTACTTTTACTTCTACATCTACTGAAATGACCACCATTACTGGAA
CCAACGGCGTTCCAACTGACGAAACCGTCATTGTCATCAGAACTCCAACCAGTGAAGGTCTAATCAGCACC
ACCACTGA

TGCAGTGGTAATAGGTGGTAATGATGAAGACGAATATGTAGACTTTGGTGATTCAGAAGAGATAGAAGAGG
AAGAAGAGACAGAACTAGCTGAACTAGTTTCGCTCTCAGAAGAACCAGAGGTGGAACTACTGGTTGGAATG
ACGGATGA

CATCAGAACACCAACAACAGCCACTACTGCCATGACTACAACTCAGCCATGGAACGACACTTTTACCTCTA
CTTCTACCGAATTGACCACAGTCACCGGTACCAATGGTTTGCCAACTGATGAGACCATCATTGTCATCAGA
ACACCAAC

ATTTCCTCTACGGTACCATCGGCAGGCTGGTAGATCTCGGAGGCTGACTTGACGGACTCAATGGACCCTAG
CGACTCACCTTGGGCAATCTCAGTGCCCACTTCTGGCAACTCAACATAGGTAGCGTCCCCTAAGGCATCAG
TGGCGTAT

CCTCTACATCCACTGAGATGACCACCGTCACCGGTACTAACGGTCAACCAACTGACGAAACCGTGATTGTT
ATCAGAACTCCAACCAGTGAAGGTTTGGTTACAACCACCACTGAACCATGGACTGGTACTTTTACTTCTAC
ATCTACTG

TGGGGTGGCATAGTGTTGACATAAGGTGTTGTAGGATAAGACGGTGAACGTCCTCTTGGCTAAATCGGTGG
CCAGATGCTCAGTGGATTGCTGCAAAGAATCGTACTCCCTCTGTGGTTCCCCATCGGTATTGATTTCGATG
AACCTACG

CTGGTTGGAATGACGGATGATTTAAATGATTCAGAGAATATAGAAGTGGAGGTTGTTGTAGAAGAAATGAC
AGGGGAAGAAATGAATGAAGAAGAAATGACTGGAGAAGAAGTGACTAGAGAAGAAGTGACTGAGGAAGAAA
TGACTAGA

TGTTGTTGTACTGGTAACAGGTGGTAATGAAGAAGTAATTTCCTGACTTGTTGTTGCACTGGTAACAGGTG
GTAATGATGAAGTAATTTCCTGACTTGTTGTTGTACTGGTAACAGGTGGTAATGATGAAGCAGTTTCCTGG
CTTGTTGT

ACTTGAGATTTGTCCAATTTAGCATCTCTCAAGACCTTTTCAACTGGGTCCAAAGTAGATCTGAACAAGTC
AGCACACAATTCTTCGAATCTGGCTCTGGTGATGGAAGTGTAGAAATCGATACCTTCGAACAAAGAGTCAA
TTTCAACG

TGATCTTCCTAAACACCTTCGCAAGGTGCCTTTTAACGTGTTTCGTACTGTGCAGCGGTACAGCACGTTCC
TCTGACACAAACGACACTACTCCGGCGTCTGCAAAGCATTTGCAGACCACTTCTTTATTGACGTGTATGGA
CAATTCGC

GAGGTGCTGGAGGTGCTGGAGGTGTTGGAGGTACTGGAGGTATTGAAGGTGCTGAGAGTGCTGGAGGTGCT
GAAGATACTGGAGGTACTGGGGGTACTGGGGGTACTGAAGGTGCAGAGAGTGCTGGAGGTGCTGGAGGTAC
TGGAGGTG

AGGTGCTGGAGGTGTTGGAGGTACTGGAGGTATTGAAGGTGCTGAGAGTGCTGGAGGTGCTGAAGATACTG
GAGGTACTGGGGGTACTGGGGGTACTGAAGGTGCAGAGAGTGCTGGAGGTGCTGGAGGTACTGGAGGTGCA
GAGGGTAC

GCTGAGAGTGCTGGAGGTGCTGAAGATACTGGAGGTACTGGGGGTACTGGGGGTACTGAAGGTGCAGAGAG
TGCTGGAGGTGCTGGAGGTACTGGAGGTGCAGAGGGTACTGAAGGTGCAGAGGGTACTGGAGGTGCAGAGG
GTGCAGAA

AGAGGCGCTGGAGGTGCTGGAGGTGCTGGAGGTGTTGGAGGTACTGGAGGTATTGAAGGTGCTGAGAGTGC
TGGAGGTGCTGAAGATACTGGAGGTACTGGGGGTACTGGGGGTACTGAAGGTGCAGAGAGTGCTGGAGGTG
CTGGAGGT

GTACTGGAGGTATTGAAGGTGCTGAGAGTGCTGGAGGTGCTGAAGATACTGGAGGTACTGGGGGTACTGGG
GGTACTGAAGGTGCAGAGAGTGCTGGAGGTGCTGGAGGTACTGGAGGTGCAGAGGGTACTGAAGGTGCAGA
GGGTACTG

TATTGAAGGTGCTGAGAGTGCTGGAGGTGCTGAAGATACTGGAGGTACTGGGGGTACTGGGGGTACTGAAG
GTGCAGAGAGTGCTGGAGGTGCTGGAGGTACTGGAGGTGCAGAGGGTACTGAAGGTGCAGAGGGTACTGGA
GGTGCAGA

TGCGAGCTTGCTAATGGATAATCAGCGTGCTAACGCTGGGTCTACATCCGTTCCCACAAACATTCCTCCCC
CTAGGGGTAGGTCGAAGTCAGTGGTGGAGACTAACCTGTCTAACGTTGAGGCTGACTCCGGACATCATCAC
CACCACCG

ACTCCGTAGCTGCGAGCTTGCTAATGGATAATCAGCGTGCTAACGCTGGGTCTACATCCGTTCCCACAAAC
ATTCCTCCCCCTAGGGGTAGGTCGAAGTCAGTGGTGGAGACTAACCTGTCTAACGTTGAGGCTGACTCCGG
ACATCATC

AAACGATAAGGGACGCCGTCACTCCGTAGCTGCGAGCTTGCTAATGGATAATCAGCGTGCTAACGCTGGGT
CTACATCCGTTCCCACAAACATTCCTCCCCCTAGGGGTAGGTCGAAGTCAGTGGTGGAGACTAACCTGTCT
AACGTTGA

CTGGAGGTGCTGAAGATACTGGAGGTACTGGGGGTACTGGGGGTACTGAAGGTGCAGAGAGTGCTGGAGGT
GCTGGAGGTACTGGAGGTGCAGAGGGTACTGAAGGTGCAGAGGGTACTGGAGGTGCAGAGGGTGCAGAAGG
CACTGGGT

GGTGTTGGAGGTACTGGAGGTATTGAAGGTGCTGAGAGTGCTGGAGGTGCTGAAGATACTGGAGGTACTGG
GGGTACTGGGGGTACTGAAGGTGCAGAGAGTGCTGGAGGTGCTGGAGGTACTGGAGGTGCAGAGGGTACTG
AAGGTGCA

TAACGTCAATAACACGAGATGCTGACGATGTGACAGAAGTAGCTGTAGATTGAGCTTTGGAGGAGACTGGC
TTAGTCACAGTTGAACAGACGTTATTTGAACAACTAGTTATGGTAGTTTCAACAGTGGAAGCTTGAGTCTT
TGTGACGG

TGAAGATACTGGAGGTACTGGGGGTACTGGGGGTACTGAAGGTGCAGAGAGTGCTGGAGGTGCTGGAGGTA
CTGGAGGTGCAGAGGGTACTGAAGGTGCAGAGGGTACTGGAGGTGCAGAGGGTGCAGAAGGCACTGGGTGA
GGTGGCAC

AACACGAGATGCTGACGATGTGACAGAAGTAGCTGTAGATTGAGCTTTGGAGGAGACTGGCTTAGTCACAG
TTGAACAGACGTTATTTGAACAACTAGTTATGGTAGTTTCAACAGTGGAAGCTTGAGTCTTTGTGACGGTG
CTGTCAGT

TGAAGTTGCCTTTGAAATTATTGACGGCACCGGTAAGTATGTCACTCCAGGTCTGATCGACAGTCATGTCC
ACATTGCGTCGGTTGCAGGAGAGGCTGATCTGACCAAGTTAATGCTGATGCCAAAGTCAGTTGCATTACTC
AGAATAAG

GGAGGTACTGGGGGTACTGGGGGTACTGAAGGTGCAGAGAGTGCTGGAGGTGCTGGAGGTACTGGAGGTGC
AGAGGGTACTGAAGGTGCAGAGGGTACTGGAGGTGCAGAGGGTGCAGAAGGCACTGGGTGAGGTGGCACCT
TGTCCTCA

GTTAACCACACTAAATACACTCCAGACAAGAAGATTGTCTCCAACGCTTCTTGTACCACCAACTGTTTGGC
TCCATTGGCCAAGGTTATCAACGATGCTTTCGGTATTGAAGAAGGTTTGATGACCACTGTTCACTCCATGA
CCGCCACT

AGTCGACAACTCTGGTAGAGTAACCGTATTCGTTGTCGTACCAGGAAACCAACTTGACGAACTTTGGAGAC
AATTGGATACCAGCGGCAGCATCGAAGATGGAAGAGTTAGAGTCACCCAAGAAGTCAGAGGAGACAACAGC
GTCTTCAG

GGACGCCGTCACTCCGTAGCTGCGAGCTTGCTAATGGATAATCAGCGTGCTAACGCTGGGTCTACATCCGT
TCCCACAAACATTCCTCCCCCTAGGGGTAGGTCGAAGTCAGTGGTGGAGACTAACCTGTCTAACGTTGAGG
CTGACTCC

CATAATCGCACTTAAATACGCAAAATATACAAGATAGCCCTACAACTGCTCCATGGACTCCAAGGACACGC
AAAAGTTGCTTAAGGAGCACCGAATCCCCTGCATCGACGTTGGATGGTTGGTCAGGCCCAGTGCCTCCACC
AGCAAGAG

TAACCGTTGATGAAGTTGCCTTTGAAATTATTGACGGCACCGGTAAGTATGTCACTCCAGGTCTGATCGAC
AGTCATGTCCACATTGCGTCGGTTGCAGGAGAGGCTGATCTGACCAAGTTAATGCTGATGCCAAAGTCAGT
TGCATTAC

CTTGGCAACGTGTTCAACCAAGTCGACAACTCTGGTAGAGTAACCGTATTCGTTGTCGTACCAGGAAACCA
ACTTGACGAACTTTGGAGACAATTGGATACCAGCGGCAGCATCGAAGATGGAAGAGTTAGAGTCACCCAAG
AAGTCAGA

TCTGGTAGAGTAACCGTATTCGTTGTCGTACCAGGAAACCAACTTGACGAACTTTGGAGACAATTGGATAC
CAGCGGCAGCATCGAAGATGGAAGAGTTAGAGTCACCCAAGAAGTCAGAGGAGACAACAGCGTCTTCAGTG
TAACCCAA

TTGACGGCACCGGTAAGTATGTCACTCCAGGTCTGATCGACAGTCATGTCCACATTGCGTCGGTTGCAGGA
GAGGCTGATCTGACCAAGTTAATGCTGATGCCAAAGTCAGTTGCATTACTCAGAATAAGATACACTTTGGA
AGCCGCTC

TTCTTTCTTCAAACGATAAGGGACGCCGTCACTCCGTAGCTGCGAGCTTGCTAATGGATAATCAGCGTGCT
AACGCTGGGTCTACATCCGTTCCCACAAACATTCCTCCCCCTAGGGGTAGGTCGAAGTCAGTGGTGGAGAC
TAACCTGT

TTTGAAATTATTGACGGCACCGGTAAGTATGTCACTCCAGGTCTGATCGACAGTCATGTCCACATTGCGTC
GGTTGCAGGAGAGGCTGATCTGACCAAGTTAATGCTGATGCCAAAGTCAGTTGCATTACTCAGAATAAGAT
ACACTTTG

CTGACCAAGTTAATGCTGATGCCAAAGTCAGTTGCATTACTCAGAATAAGATACACTTTGGAAGCCGCTCT
GGCAAGAGGTTTCACAACAGTGAGAGACTGTGGGGGTGCAGAAGGCTTTCTGAAAGCAGAGATTCGTCAGG
GATCCTTG

TGTTGTTGGTGTTAACCACACTAAATACACTCCAGACAAGAAGATTGTCTCCAACGCTTCTTGTACCACCA
ACTGTTTGGCTCCATTGGCCAAGGTTATCAACGATGCTTTCGGTATTGAAGAAGGTTTGATGACCACTGTT
CACTCCAT

TTGACGCTATCACCAACACTGGTCCAAGAGAAGACACCACCAGAGTCGGTGGTGGTGGTGCTGCTAGACGT
CAAGCTGTCGATGTTTCTCCATTGAGAAGAGTTAACCAAGCTATTGCTTTGTTGACCATTGGTGCCAGAGA
AGCTGCTT

AAATTTAAGCCTTGGCAACGTGTTCAACCAAGTCGACAACTCTGGTAGAGTAACCGTATTCGTTGTCGTAC
CAGGAAACCAACTTGACGAACTTTGGAGACAATTGGATACCAGCGGCAGCATCGAAGATGGAAGAGTTAGA
GTCACCCA

CAGGCACTCGCATAATCGCACTTAAATACGCAAAATATACAAGATAGCCCTACAACTGCTCCATGGACTCC
AAGGACACGCAAAAGTTGCTTAAGGAGCACCGAATCCCCTGCATCGACGTTGGATGGTTGGTCAGGCCCAG
TGCCTCCA

GACAGAGAAATAATCTTTGTCAATGGTGACACTTTGAATTCCATCATAACCGTCTGGGATTTTATGTATTA
AGATTCTTTGCCTTTTAGATTTGATAAATATGCGCCGATATTAGACGTGTGTGTGTGTGTGTGTGTGTGTG
TGTGTGTG

TGACAGAAGTAGCTGTAGATTGAGCTTTGGAGGAGACTGGCTTAGTCACAGTTGAACAGACGTTATTTGAA
CAACTAGTTATGGTAGTTTCAACAGTGGAAGCTTGAGTCTTTGTGACGGTGCTGTCAGTACCATTTTGGAC
CACTGGAG

TGAAAGTCGTTGGGAGTATGATTTGAATCGCTATGTAAAGGAAATTCTTTCGAATGAGTTGCAATATATCG
ATAAGGATTATTTCATCCAAGAAATGAACAGAAGGTTGCAATCTAACAAACAGGAGATTTGGGAAGAGATC
ACAAATAG

TTCAATGGAAGAAGGAATCTCAGAAGAGGAAGTAATTACACTAGAAACTATTAGTTCACTGGAAATGGTGA
TTTGATTAGAGGAGATAGATTCTGCGGAAGTGGTTGCTTTACTGAAAGAGAGAGGTTCAGCAGAAGAGGTG
ATTTCACT

TTCATTTCTTGATGAAACTCATGGATACGTGGTCCGTTCATTGTGAAAGGATCACTGAGTTGCCACTTTGA
GTTTTCGTCAATCACAGGAGCGTCTGGTAGACCAGCAACCTTGGAGTACAAGCTTCCCACATCAATTCTAA
AGCCGTCT

GCTGACGATGTGACAGAAGTAGCTGTAGATTGAGCTTTGGAGGAGACTGGCTTAGTCACAGTTGAACAGAC
GTTATTTGAACAACTAGTTATGGTAGTTTCAACAGTGGAAGCTTGAGTCTTTGTGACGGTGCTGTCAGTAC
CATTTTGG

TGTTCAACCAAGTCGACAACTCTGGTAGAGTAACCGTATTCGTTGTCGTACCAGGAAACCAACTTGACGAA
CTTTGGAGACAATTGGATACCAGCGGCAGCATCGAAGATGGAAGAGTTAGAGTCACCCAAGAAGTCAGAGG
AGACAACA

TCATTGACTTGCTGCTGAATGTAGCCCAGGACATAGATTCCAACGATCTTTCGGAGCACGATTCCTGGCTG
CAGCAGTTCATCCAACATAATAACACGATTCGTTTCATGGGCGATGACACCTGGCTGAAACTGTTCCCACA
GCAATGGT

TGTGGGACTATGCTTTCTGTGGGCTCTAATTATCATCGTTGGCATGTTATTAGTTCCAGAGTCCCCAAGAT
ATCTGATTGAATGTGAGAGACATGAAGAGGCCTGTGTCTCCATCGCCAAGATCGACAAGGTTTCACCAGAG
GATCCATG

TCCAGCAGCATTCTTTCTTCAAACGATAAGGGACGCCGTCACTCCGTAGCTGCGAGCTTGCTAATGGATAA
TCAGCGTGCTAACGCTGGGTCTACATCCGTTCCCACAAACATTCCTCCCCCTAGGGGTAGGTCGAAGTCAG
TGGTGGAG

GTTGCATTACTCAGAATAAGATACACTTTGGAAGCCGCTCTGGCAAGAGGTTTCACAACAGTGAGAGACTG
TGGGGGTGCAGAAGGCTTTCTGAAAGCAGAGATTCGTCAGGGATCCTTGAATGGTCCCAGATTAATTACGT
GTGGACAT

GTACATACATATACACCACTCAGGCACTCGCATAATCGCACTTAAATACGCAAAATATACAAGATAGCCCT
ACAACTGCTCCATGGACTCCAAGGACACGCAAAAGTTGCTTAAGGAGCACCGAATCCCCTGCATCGACGTT
GGATGGTT

CCTTATTATCATCGGCACCTTCTTCCTCACTTCGGGTCTCCACCCAGCACCACCTCATGAGGCAAAGCGTC
CACACCATGGAAAAGGTCCCATGCACTCACCCAAATGTGAGAAGATTGAACCATTAAGTCCATCATTCAAA
CATTCCGT

ATACACCACTCAGGCACTCGCATAATCGCACTTAAATACGCAAAATATACAAGATAGCCCTACAACTGCTC
CATGGACTCCAAGGACACGCAAAAGTTGCTTAAGGAGCACCGAATCCCCTGCATCGACGTTGGATGGTTGG
TCAGGCCC

GGAGGATTCCTGTGGGACTATGCTTTCTGTGGGCTCTAATTATCATCGTTGGCATGTTATTAGTTCCAGAG
TCCCCAAGATATCTGATTGAATGTGAGAGACATGAAGAGGCCTGTGTCTCCATCGCCAAGATCGACAAGGT
TTCACCAG

GGTGTTAGAAATATTACTGAAGTGGACAAGACATTTGAGGATGTGCACGTTTCTGATAAGTCAATGATCTG
GAATTCTGATCTCGTAGAAACTCTGGAATTGCAAAATTTACTTACTTGTGCCACACAAACGGCTGTTTCTG
CTTCCAAA

TGGATGGTGGTTGGGAGTGGTATAATGAAATGGGACAGGGTAACGAGTGGGGAGGTAGGGTAATGGAGGGT
ACGTTAAGAGACAGGTTTATCAGGGTTGGATTAGAATAGGGTTAGGGTAGTGTTAGGGTAGTGTTAGGGTA
GTGTGGTG

CTAAATACACTCCAGACAAGAAGATTGTCTCCAACGCTTCTTGTACCACCAACTGTTTGGCTCCATTGGCC
AAGGTTATCAACGATGCTTTCGGTATTGAAGAAGGTTTGATGACCACTGTTCACTCCATGACCGCCACTCA
AAAGACTG

CAGTGGAGAAGATTTGAGATTTCTTTGTTGGAATAGTAGTGTTTCTTGGAATCAATCTTGTGAAAACACCA
CCTAAAGTTTCGATACCTAGAGACAATGGGGTAACATCTAATAATAAGACGTCAGTAACCTCACCGGACAA
GACAGCAC

ATGGATACGTGGTCCGTTCATTGTGAAAGGATCACTGAGTTGCCACTTTGAGTTTTCGTCAATCACAGGAG
CGTCTGGTAGACCAGCAACCTTGGAGTACAAGCTTCCCACATCAATTCTAAAGCCGTCTACACCATGGTCT
AACCAGTA

GGTCAAGGTACCAACCATGACCAAACGAGTAGGCAACCCAGCAAAGGAACCGAAAAATCCTAACTGTTTGG
CCAACTGAGCCAACAAACCGACTGTGGATTGGCCAGGAGCCTTCTTAGTCTTGTTGACCTTGGACAACAGA
GTGTCTGC

CACATACTTGCTCATCGAGGTTGCGTCTTGGAACCTGTCGGACAATGAGGCATACCTGCTCAATTTCTGTG
GGTCCAGAACTTCTTTCTGGAAAAGCGAAGTGGCCTCTTCATAACGATGAGGGTCTGGCTGCTTACCTTGC
AGGCCTGG

GCCAAAGTCAGTTGCATTACTCAGAATAAGATACACTTTGGAAGCCGCTCTGGCAAGAGGTTTCACAACAG
TGAGAGACTGTGGGGGTGCAGAAGGCTTTCTGAAAGCAGAGATTCGTCAGGGATCCTTGAATGGTCCCAGA
TTAATTAC

TAACCGTATTCGTTGTCGTACCAGGAAACCAACTTGACGAACTTTGGAGACAATTGGATACCAGCGGCAGC
ATCGAAGATGGAAGAGTTAGAGTCACCCAAGAAGTCAGAGGAGACAACAGCGTCTTCAGTGTAACCCAAGA
CACCCTTC

GATGAAACTCATGGATACGTGGTCCGTTCATTGTGAAAGGATCACTGAGTTGCCACTTTGAGTTTTCGTCA
ATCACAGGAGCGTCTGGTAGACCAGCAACCTTGGAGTACAAGCTTCCCACATCAATTCTAAAGCCGTCTAC
ACCATGGT

GATGAACTTGTTCATTTCTTGATGAAACTCATGGATACGTGGTCCGTTCATTGTGAAAGGATCACTGAGTT
GCCACTTTGAGTTTTCGTCAATCACAGGAGCGTCTGGTAGACCAGCAACCTTGGAGTACAAGCTTCCCACA
TCAATTCT

GAAGGAATCTCAGAAGAGGAAGTAATTACACTAGAAACTATTAGTTCACTGGAAATGGTGATTTGATTAGA
GGAGATAGATTCTGCGGAAGTGGTTGCTTTACTGAAAGAGAGAGGTTCAGCAGAAGAGGTGATTTCACTGA
AAGTAGAC

CAAGTTGTTGTTGACGCTATCACCAACACTGGTCCAAGAGAAGACACCACCAGAGTCGGTGGTGGTGGTGC
TGCTAGACGTCAAGCTGTCGATGTTTCTCCATTGAGAAGAGTTAACCAAGCTATTGCTTTGTTGACCATTG
GTGCCAGA

CAGAAACAAAAGGCCTGACGCTGGAGGAGGTGAATACTATGTGGCTGGAAGGTGTGCCAGCATGGAAATCA
GCCTCATGGGTGCCACCAGAAAGAAGAACCGCAGATTACGATGCTGACGCCATAGACCATGACGATAGACC
AATCTACA

TTCTTTGTCCCAGAAACAAAAGGCCTGACGCTGGAGGAGGTGAATACTATGTGGCTGGAAGGTGTGCCAGC
ATGGAAATCAGCCTCATGGGTGCCACCAGAAAGAAGAACCGCAGATTACGATGCTGACGCCATAGACCATG
ACGATAGA

ACTAAGACTACCGCTGCTGCTGTCTCTCAAATTGGTGATGGTCAAGTTCAAGCTACCACCAAGACTACTGC
TGCCGCCGTTTCTCAAATCGGTGATGGTCAAGTTCAAGCTACTACCAAGACTACCGCTGCTGCTGTCTCTC
AAATCGGT

TAGTGCTTGGTCAATTCATCGATTTGGATCTTACCGAATTCACCAACGTAACCGATCTTCTTACCCTTTAG
GGACTGGAAGTCTTCAGTGATACCACTGCCCTTTAAGTACAAGACACCGGTGAATGGTTCGTCCAACAAAG
AGGCAACA

GGAAGAGTTAGAGTCACCCAAGAAGTCAGAGGAGACAACAGCGTCTTCAGTGTAACCCAAGACACCCTTCA
ACTTACCTTCAGCGGCAGCCTTGACAACCTTCTTGATTTCATCGTAGGTGGTTTCCTTGTTCAACTTGACA
GTCAAGTC

TCGAAACGTCTGTAGACTCTTCCAGCAGCATTCTTTCTTCAAACGATAAGGGACGCCGTCACTCCGTAGCT
GCGAGCTTGCTAATGGATAATCAGCGTGCTAACGCTGGGTCTACATCCGTTCCCACAAACATTCCTCCCCC
TAGGGGTA

TCTTCGCCGGCCTGTTTGCCAAACATCTTCTTCCCCATAAACTGGAGGTTCTCCGTGTCCAGCATTTCGAA
GTCCTCTTCATCCTCGTCCAAAGAACTCATCTGTTCTCGTGTGCGCCTGTGTGTGTTTACTTTTCCCCAGC
ACTACGCA

TGTAGACTCTTCCAGCAGCATTCTTTCTTCAAACGATAAGGGACGCCGTCACTCCGTAGCTGCGAGCTTGC
TAATGGATAATCAGCGTGCTAACGCTGGGTCTACATCCGTTCCCACAAACATTCCTCCCCCTAGGGGTAGG
TCGAAGTC

TATCGATGATTCCATCTCTTTCCAAGGTAGCTCTAATTTGTTCTGAACCAGGGGTGACAAAGAAGGGGATA
CGTGGTTTCAAGCCGGCTTTGGAAGCTTGCTTGACCAAGTCGACAACACGACTCATGTCTTGATAAGATGA
ATTGGTAC

CCAGCCGTAAGTAAGTATAGTTATGACTTCATCTCTAGATGATATAGAACCAACAGCATATAATAATATGG
AAGCGGACGAGGAATATTGCAGACGCAACGATATACATGACCTTTCCAGCGTGGTCGGTGATGCTGTCTCA
CAAGGTGT

CGGTAAGTATGTCACTCCAGGTCTGATCGACAGTCATGTCCACATTGCGTCGGTTGCAGGAGAGGCTGATC
TGACCAAGTTAATGCTGATGCCAAAGTCAGTTGCATTACTCAGAATAAGATACACTTTGGAAGCCGCTCTG
GCAAGAGG

ATACCCCTAAAGACAGGTAATTTAGCAGCTTTAGAGATGGAGAGATCGCTTCTGTAGTGTTTATCACCGTG
GTCAGTTCACCAATTCTATATTTGCTAACATCTGCCAATGAATTAAGTTGAGTGTTAATTTCAGTCTTGTC
CACACCCT

TCCATCTCTTTCCAAGGTAGCTCTAATTTGTTCTGAACCAGGGGTGACAAAGAAGGGGATACGTGGTTTCA
AGCCGGCTTTGGAAGCTTGCTTGACCAAGTCGACAACACGACTCATGTCTTGATAAGATGAATTGGTACAT
GATCCAAT

CATCGAAGATGGAAGAGTTAGAGTCACCCAAGAAGTCAGAGGAGACAACAGCGTCTTCAGTGTAACCCAAG
ACACCCTTCAACTTACCTTCAGCGGCAGCCTTGACAACCTTCTTGATTTCATCGTAGGTGGTTTCCTTGTT
CAACTTGA

ACACCTAGCTTCATTGACTTGCTGCTGAATGTAGCCCAGGACATAGATTCCAACGATCTTTCGGAGCACGA
TTCCTGGCTGCAGCAGTTCATCCAACATAATAACACGATTCGTTTCATGGGCGATGACACCTGGCTGAAAC
TGTTCCCA

AGAGGATTGCTCGGATGTAATGGCGTATTCGGCAGCGTATGTTACACCGTGTCCTGACACAGAAGCACTGT
TGGAGTAAATGCTACTCGATGTGGCGGCACTGTTACTAATATTCTTCTCTGTTGAACTTGAAGCCTGAGCA
GTTTCCGA

ACTTTGGAGACAATTGGATACCAGCGGCAGCATCGAAGATGGAAGAGTTAGAGTCACCCAAGAAGTCAGAG
GAGACAACAGCGTCTTCAGTGTAACCCAAGACACCCTTCAACTTACCTTCAGCGGCAGCCTTGACAACCTT
CTTGATTT

TCAATTCATCGATTTGGATCTTACCGAATTCACCAACGTAACCGATCTTCTTACCCTTTAGGGACTGGAAG
TCTTCAGTGATACCACTGCCCTTTAAGTACAAGACACCGGTGAATGGTTCGTCCAACAAAGAGGCAACAGA
GGTCACTG

CCGTTAGTGGTAACGTCAATAACACGAGATGCTGACGATGTGACAGAAGTAGCTGTAGATTGAGCTTTGGA
GGAGACTGGCTTAGTCACAGTTGAACAGACGTTATTTGAACAACTAGTTATGGTAGTTTCAACAGTGGAAG
CTTGAGTC

CACCAACACTGGTCCAAGAGAAGACACCACCAGAGTCGGTGGTGGTGGTGCTGCTAGACGTCAAGCTGTCG
ATGTTTCTCCATTGAGAAGAGTTAACCAAGCTATTGCTTTGTTGACCATTGGTGCCAGAGAAGCTGCTTTC
AGAAACAT

GATTTGAGATTTCTTTGTTGGAATAGTAGTGTTTCTTGGAATCAATCTTGTGAAAACACCACCTAAAGTTT
CGATACCTAGAGACAATGGGGTAACATCTAATAATAAGACGTCAGTAACCTCACCGGACAAGACAGCACCT
TGCACAGC

TGTCTCTAGTTTGCGATAGTGTAGATACCGTCCTTGGATAGAGCACTGGAGATGGCTGGCTTTAATCTGCT
GGAGTACCATGGGACACCGGTGATCATTCTGGTCACTTGGTCTGGAGAAATACCAGTCAACATGGTGGTGA
AGTCACCG

TTGGGACTCTGAAAGCCATACCGGTCAACTTACCTTGCAATTCTGGCAAGACCTTACCGACAGCCTTAGCA
GCACCGGTAGAGGATGGGATGATGTTACCGGAAGCGGTTCTACCACCTCTCCAGTCCTTGTGGGATGGACC
GTCAACAG

TCCAAGGTAGCTCTAATTTGTTCTGAACCAGGGGTGACAAAGAAGGGGATACGTGGTTTCAAGCCGGCTTT
GGAAGCTTGCTTGACCAAGTCGACAACACGACTCATGTCTTGATAAGATGAATTGGTACATGATCCAATCA
AACCAGCG

GATGAGTCATGACTCAATAATCAAACCATGTTGAAAAGGCTGAAATTTCTAACAATTTCCGGACCATATTC
CTCATAATCTTTCTTAGTGTGACAGTAGCCTTTGAATTCGGCTGTTTGTGCCAGCAGCGAGCCACCAAACC
AGACAGCG

CTATAGAAATCTAAAATATCTAATCAGATCTTGGAGCTTCCTTGAAGGAGATGGCAGCTTCTTCACCCATG
GCAGAAATGATAGTGACCATCAAATCCTTACCTTCATCGAAAGCAGCTTGCATGCTGTCACCCAATTCACC
TTCTGGTG

ACAACTCAATCCAGCCGTAAGTAAGTATAGTTATGACTTCATCTCTAGATGATATAGAACCAACAGCATAT
AATAATATGGAAGCGGACGAGGAATATTGCAGACGCAACGATATACATGACCTTTCCAGCGTGGTCGGTGA
TGCTGTCT

GGGGTTGTTGGAAACGTTGAAGGGGAAGCCGGTGACTCTCTCATCCATCAGGATGCCGAAATAGTCACCCA
AGTACGTCCCTGTGATACCCAGCTTGTACATGGAACTCAAAACAAGCACTTGCCCCAAACCAAAGAGAGCC
ACACCCAG

CCGCTGCTGCTGTCTCTCAAATTGGTGATGGTCAAGTTCAAGCTACCACCAAGACTACTGCTGCCGCCGTT
TCTCAAATCGGTGATGGTCAAGTTCAAGCTACTACCAAGACTACCGCTGCTGCTGTCTCTCAAATCGGTGA
TGGTCAAG

TAATGCTGATGCCAAAGTCAGTTGCATTACTCAGAATAAGATACACTTTGGAAGCCGCTCTGGCAAGAGGT
TTCACAACAGTGAGAGACTGTGGGGGTGCAGAAGGCTTTCTGAAAGCAGAGATTCGTCAGGGATCCTTGAA
TGGTCCCA

GTAAGTATAGTTATGACTTCATCTCTAGATGATATAGAACCAACAGCATATAATAATATGGAAGCGGACGA
GGAATATTGCAGACGCAACGATATACATGACCTTTCCAGCGTGGTCGGTGATGCTGTCTCACAAGGTGTGC
CAGACATG

TTAGTTCACTGGAAATGGTGATTTGATTAGAGGAGATAGATTCTGCGGAAGTGGTTGCTTTACTGAAAGAG
AGAGGTTCAGCAGAAGAGGTGATTTCACTGAAAGTAGACCCGAAGGAAGAAACAGGTTCTGTCGTGGTCAC
TTGACTAG

CTCATCGAGGTTGCGTCTTGGAACCTGTCGGACAATGAGGCATACCTGCTCAATTTCTGTGGGTCCAGAAC
TTCTTTCTGGAAAAGCGAAGTGGCCTCTTCATAACGATGAGGGTCTGGCTGCTTACCTTGCAGGCCTGGAA
TCGTCTCC

TAAAGAACCATAAATACCAAATTGCAAGGAGGTCAAGGTACCAACCATGACCAAACGAGTAGGCAACCCAG
CAAAGGAACCGAAAAATCCTAACTGTTTGGCCAACTGAGCCAACAAACCGACTGTGGATTGGCCAGGAGCC
TTCTTAGT

TTGCAGCAGAGCTAGATTCAGATGAAACAGTAGCTTGGGTGGAAGCAGCGTTTGAGCTGGTCTCTGCTGAA
CTTTCAGAGGAGCTAGAAGACTCAGTGGCGGCACTAGAAGCTTCTGAAGTGGAGGTGGAAGAAGCCTCTGT
GGTAGAAG

TTGTACTCTGCACTTTGAGTATCATTCCAATTTGATCTTTCTTCTACCGGTACTTAGGGATAGCAAATGTC
GTCGAACAATGATCTTTGGTTGCATTTAGTATCCCAGTTAAATACTCAACAGAGAGCAGCGGCCCTCTTTG
ATTACACT

TTATGTCTTTTTCTTTGTCCCAGAAACAAAAGGCCTGACGCTGGAGGAGGTGAATACTATGTGGCTGGAAG
GTGTGCCAGCATGGAAATCAGCCTCATGGGTGCCACCAGAAAGAAGAACCGCAGATTACGATGCTGACGCC
ATAGACCA

TTAAGGAGTTAAATTTAAGCCTTGGCAACGTGTTCAACCAAGTCGACAACTCTGGTAGAGTAACCGTATTC
GTTGTCGTACCAGGAAACCAACTTGACGAACTTTGGAGACAATTGGATACCAGCGGCAGCATCGAAGATGG
AAGAGTTA

GCTGCTGAATGTAGCCCAGGACATAGATTCCAACGATCTTTCGGAGCACGATTCCTGGCTGCAGCAGTTCA
TCCAACATAATAACACGATTCGTTTCATGGGCGATGACACCTGGCTGAAACTGTTCCCACAGCAATGGTTT
GACTTCGC

AGGCCTGACGCTGGAGGAGGTGAATACTATGTGGCTGGAAGGTGTGCCAGCATGGAAATCAGCCTCATGGG
TGCCACCAGAAAGAAGAACCGCAGATTACGATGCTGACGCCATAGACCATGACGATAGACCAATCTACAAG
AGGTTCTT

CGTTGTCGTACCAGGAAACCAACTTGACGAACTTTGGAGACAATTGGATACCAGCGGCAGCATCGAAGATG
GAAGAGTTAGAGTCACCCAAGAAGTCAGAGGAGACAACAGCGTCTTCAGTGTAACCCAAGACACCCTTCAA
CTTACCTT

AGACTTTCGTTACTGTTTAAGTCATCGTATTGAGGGGGATACACCAGGTTTTGTTGAGAATTAGTTACTTT
TTCCTCATTCCAAGGGTTAACAAAGCAAAATTGAGAATTCTGTTGTTGTTGTTGTTGCTGCTGCTGCTGCT
GCTGGTGA

CTCACTGGTTTGGGTTGAGGCGCCGTTCAAAGTGCTACAAGATGTCGAGATTGTTCTAGTGAAGGCCGTCT
CATCTGTAGAAGAAGCAGCGGAGGTAGGGAAATTACTATCCGTACTACAAGAAGCTGTGATGGTGTTTGTG
ATCAGATT

GTGTGGGTGTGGTGTGGGTGTGGTGTGGGTGTGGTGTGGGTGTGTGGGTGTGGTGTGTGGGTGTGTGGTGT
GTGGGTGTGTGTGGGTGTGGTGTGTGTGGGTGTGGTGTGTGTGGGTGTGGTGTGTGTGTGTGTGGGTGTGG
TGTGTGTG

GGTGTGGGTGTGGTGTGGGTGTGGTGTGGGTGTGTGGGTGTGGTGTGTGGGTGTGTGGTGTGTGGGTGTGT
GTGGGTGTGGTGTGTGTGGGTGTGGTGTGTGTGGGTGTGGTGTGTGTGTGTGTGGGTGTGGTGTGTGTGTG
GGTGTGGG

GTGGTGTGGGTGTGGTGTGGGTGTGGGTGTGGTGTGGGTGTGGTGTGGGTGTGGTGTGGGTGTGTGGGTGT
GGTGTGTGGGTGTGTGGTGTGTGGGTGTGTGTGGGTGTGGTGTGTGTGGGTGTGGTGTGTGTGGGTGTGGT
GTGTGTGT

GGGTAGTGTTAGGGTTGTGGGTGTGTGGGTGTGGTGTGGGTGTGGTGTGGGTGTGGGTGTGGTGTGGGTGT
GGTGTGGGTGTGGTGTGGGTGTGTGGGTGTGGTGTGTGGGTGTGTGGTGTGTGGGTGTGTGTGGGTGTGGT
GTGTGTGG

TGTGGTGTGGGTGTGGGTGTGGTGTGGGTGTGGTGTGGGTGTGGTGTGGGTGTGTGGGTGTGGTGTGTGGG
TGTGTGGTGTGTGGGTGTGTGTGGGTGTGGTGTGTGTGGGTGTGGTGTGTGTGGGTGTGGTGTGTGTGTGT
GTGGGTGT

AGGGTTGTGGGTGTGTGGGTGTGGTGTGGGTGTGGTGTGGGTGTGGGTGTGGTGTGGGTGTGGTGTGGGTG
TGGTGTGGGTGTGTGGGTGTGGTGTGTGGGTGTGTGGTGTGTGGGTGTGTGTGGGTGTGGTGTGTGTGGGT
GTGGTGTG

GTGTGTGGGTGTGGTGTGGGTGTGGTGTGGGTGTGGGTGTGGTGTGGGTGTGGTGTGGGTGTGGTGTGGGT
GTGTGGGTGTGGTGTGTGGGTGTGTGGTGTGTGGGTGTGTGTGGGTGTGGTGTGTGTGGGTGTGGTGTGTG
TGGGTGTG

GGTTGGAGTAGGGTAGTGTTAGGGTTGTGGGTGTGTGGGTGTGGTGTGGGTGTGGTGTGGGTGTGGGTGTG
GTGTGGGTGTGGTGTGGGTGTGGTGTGGGTGTGTGGGTGTGGTGTGTGGGTGTGTGGTGTGTGGGTGTGTG
TGGGTGTG

GGTTCATCAGGGTTGGAGTAGGGTAGTGTTAGGGTTGTGGGTGTGTGGGTGTGGTGTGGGTGTGGTGTGGG
TGTGGGTGTGGTGTGGGTGTGGTGTGGGTGTGGTGTGGGTGTGTGGGTGTGGTGTGTGGGTGTGTGGTGTG
TGGGTGTG

TTGTGAGACAGGTTCATCAGGGTTGGAGTAGGGTAGTGTTAGGGTTGTGGGTGTGTGGGTGTGGTGTGGGT
GTGGTGTGGGTGTGGGTGTGGTGTGGGTGTGGTGTGGGTGTGGTGTGGGTGTGTGGGTGTGGTGTGTGGGT
GTGTGGTG

GAGGTGTAAGTTGTGAGACAGGTTCATCAGGGTTGGAGTAGGGTAGTGTTAGGGTTGTGGGTGTGTGGGTG
TGGTGTGGGTGTGGTGTGGGTGTGGGTGTGGTGTGGGTGTGGTGTGGGTGTGGTGTGGGTGTGTGGGTGTG
GTGTGTGG

TTGTTGCTGTTGCTGTTGCTGTTGTAGTTGTTGTTGTGGTTGTTGTGGTTGCTGTGGTTGTTGTTGTTGCT
GTGGTTGCTGTGGTTGCTGTGGTTGCTGTGGTTGCTGTGGTTGCTGTGGTTGTTGGGATTGTTGTTGTGGT
TGAGGTAC

TGCTGTTGCTGTTGTAGTTGTTGTTGTGGTTGTTGTGGTTGCTGTGGTTGTTGTTGTTGCTGTGGTTGCTG
TGGTTGCTGTGGTTGCTGTGGTTGCTGTGGTTGCTGTGGTTGTTGGGATTGTTGTTGTGGTTGAGGTACAG
TATTAGCT

GTACTGGTTGTTGTTGCTGTTGCTGTTGCTGTTGTAGTTGTTGTTGTGGTTGTTGTGGTTGCTGTGGTTGT
TGTTGTTGCTGTGGTTGCTGTGGTTGCTGTGGTTGCTGTGGTTGCTGTGGTTGCTGTGGTTGTTGGGATTG
TTGTTGTG

TTGTTGTGGTTGTTGTGGTTGCTGTGGTTGTTGTTGTTGCTGTGGTTGCTGTGGTTGCTGTGGTTGCTGTG
GTTGCTGTGGTTGCTGTGGTTGTTGGGATTGTTGTTGTGGTTGAGGTACAGTATTAGCTTGATCTTCTGGA
GGCTCTGA

GTTGTAGTTGTTGTTGTGGTTGTTGTGGTTGCTGTGGTTGTTGTTGTTGCTGTGGTTGCTGTGGTTGCTGT
GGTTGCTGTGGTTGCTGTGGTTGCTGTGGTTGTTGGGATTGTTGTTGTGGTTGAGGTACAGTATTAGCTTG
ATCTTCTG

CAGAGTCACTGGAACTTGAGCTTGAGCTAGAAGAAGAAGATGATGATGAGCTAGATTCAGAGTCACTGCTG
TCACTGCTGTCACTGCTGCTGCTGCTGCTGCTGCTGCTGCTGCTGCTTGAAGATTCGCCGCTAGAACTGGA
CGAGGAAG

TTGGGCTTGAGCTTGAGCTTGTACTGGTTGTTGTTGCTGTTGCTGTTGCTGTTGTAGTTGTTGTTGTGGTT
GTTGTGGTTGCTGTGGTTGTTGTTGTTGCTGTGGTTGCTGTGGTTGCTGTGGTTGCTGTGGTTGCTGTGGT
TGCTGTGG

GGAACTTGAGCTTGAGCTAGAAGAAGAAGATGATGATGAGCTAGATTCAGAGTCACTGCTGTCACTGCTGT
CACTGCTGCTGCTGCTGCTGCTGCTGCTGCTGCTGCTTGAAGATTCGCCGCTAGAACTGGACGAGGAAGAT
GAAGAAGA

TTGGGTAATGGAGGTGTAAGTTGTGAGACAGGTTCATCAGGGTTGGAGTAGGGTAGTGTTAGGGTTGTGGG
TGTGTGGGTGTGGTGTGGGTGTGGTGTGGGTGTGGGTGTGGTGTGGGTGTGGTGTGGGTGTGGTGTGGGTG
TGTGGGTG

GCTTGAGCTTGTACTGGTTGTTGTTGCTGTTGCTGTTGCTGTTGTAGTTGTTGTTGTGGTTGTTGTGGTTG
CTGTGGTTGTTGTTGTTGCTGTGGTTGCTGTGGTTGCTGTGGTTGCTGTGGTTGCTGTGGTTGCTGTGGTT
GTTGGGAT

CTTGAGCTAGAAGAAGAAGATGATGATGAGCTAGATTCAGAGTCACTGCTGTCACTGCTGTCACTGCTGCT
GCTGCTGCTGCTGCTGCTGCTGCTTGAAGATTCGCCGCTAGAACTGGACGAGGAAGATGAAGAAGAAG
AGGAGGAG

ATGCTGTTGTTGCTGCTGTTGCTGTTGTAGATGATGATGTTGTTGTTGAAGTTGTTGTTGTTGTTGCAACT
GGTGTTGCTGTTGTAGCTGCTGCTGTTGCTGCAACTGCTGTTGTTGTTGTAATTGCAGCTGTTGCTGCATC
TGCTGCTG

GTTGTTGTTGTTGTTGCAACTGGTGTTGCTGTTGTAGCTGCTGCTGTTGCTGCAACTGCTGTTGTTGTTGT
AATTGCAGCTGTTGCTGCATCTGCTGCTGGTGGTGAGTCAATTGAGGTTGCAGATTGGGTGGTAACAAACC
AGGATGAG

GAATGATGAGTTGGGTAATGGAGGTGTAAGTTGTGAGACAGGTTCATCAGGGTTGGAGTAGGGTAGTGTTA
GGGTTGTGGGTGTGTGGGTGTGGTGTGGGTGTGGTGTGGGTGTGGGTGTGGTGTGGGTGTGGTGTGGGTGT
GGTGTGGG

TGTTGTGGTTGCTGTGGTTGTTGTTGTTGCTGTGGTTGCTGTGGTTGCTGTGGTTGCTGTGGTTGCTGTGG
TTGCTGTGGTTGTTGGGATTGTTGTTGTGGTTGAGGTACAGTATTAGCTTGATCTTCTGGAGGCTCTGAAA
TAACAGCA

AAGAAGAAGATGATGATGAGCTAGATTCAGAGTCACTGCTGTCACTGCTGTCACTGCTGCTGCTGCTGCTG
CTGCTGCTGCTGCTGCTTGAAGATTCGCCGCTAGAACTGGACGAGGAAGATGAAGAAGAAGAGGAGGAGGA
GGAGGAGG

CTAGAAGATTCAGAGTCACTGGAACTTGAGCTTGAGCTAGAAGAAGAAGATGATGATGAGCTAGATTCAGA
GTCACTGCTGTCACTGCTGTCACTGCTGCTGCTGCTGCTGCTGCTGCTGCTGCTGCTTGAAGATTCGCCGC
TAGAACTG

ACAGGGTAACGAATGATGAGTTGGGTAATGGAGGTGTAAGTTGTGAGACAGGTTCATCAGGGTTGGAGTAG
GGTAGTGTTAGGGTTGTGGGTGTGTGGGTGTGGTGTGGGTGTGGTGTGGGTGTGGGTGTGGTGTGGGTGTG
GTGTGGGT

TTGTTGCAACTGGTGTTGCTGTTGTAGCTGCTGCTGTTGCTGCAACTGCTGTTGTTGTTGTAATTGCAGCT
GTTGCTGCATCTGCTGCTGGTGGTGAGTCAATTGAGGTTGCAGATTGGGTGGTAACAAACCAGGATGAGTA
GCGGTGTT

TGTTGTTGAAGTTGTTGTTGTTGTTGCAACTGGTGTTGCTGTTGTAGCTGCTGCTGTTGCTGCAACTGCTG
TTGTTGTTGTAATTGCAGCTGTTGCTGCATCTGCTGCTGGTGGTGAGTCAATTGAGGTTGCAGATTGGGTG
GTAACAAA

GAGTCTGAGTCCGAGCTTGAGTCGGAATCCGAATCTGAATCCGAGGAAGAGCTGCTAGAGCTGGAGCTAGA
GTCGGAGTCAGATGAAGAGTCGCTGGAGCTGGAGTCGGAGTCAGACGAAGAGTCGCTAGAGCTGGAGCTAG
AACTTGAT

GTCGGATTCACTAGAAGATTCAGAGTCACTGGAACTTGAGCTTGAGCTAGAAGAAGAAGATGATGATGAGC
TAGATTCAGAGTCACTGCTGTCACTGCTGTCACTGCTGCTGCTGCTGCTGCTGCTGCTGCTGCTGCTTGAA
GATTCGCC

TTCGACTTGACCTTGGACTGGATCTCCTGCGACATACCTCTGGTCGTTGAAGACAACTCTGCCACGTGGGG
CAAAGAAGAAGCATCAGTGACCTTGTTCTCCTCACCGGAAGAGCCGATCTCCTTCGATGCGGTAGAACCTT
GCAAGTGC

GCTGTTGTAGATGATGATGTTGTTGTTGAAGTTGTTGTTGTTGTTGCAACTGGTGTTGCTGTTGTAGCTGC
TGCTGTTGCTGCAACTGCTGTTGTTGTTGTAATTGCAGCTGTTGCTGCATCTGCTGCTGGTGGTGAGTCAA
TTGAGGTT

TTGAGTGGAAGATAATTTCTTCACCACTGGATGCTGTTGTTGCTGCTGTTGCTGTTGTAGATGATGATGTT
GTTGTTGAAGTTGTTGTTGTTGTTGCAACTGGTGTTGCTGTTGTAGCTGCTGCTGTTGCTGCAACTGCTGT
TGTTGTTG

ATGATGATGTTGTTGTTGAAGTTGTTGTTGTTGTTGCAACTGGTGTTGCTGTTGTAGCTGCTGCTGTTGCT
GCAACTGCTGTTGTTGTTGTAATTGCAGCTGTTGCTGCATCTGCTGCTGGTGGTGAGTCAATTGAGGTTGC
AGATTGGG

TGGTGCTTAGTGATTCGTTGAAGCTGCCCTCGCCTACACTTTCAGCTGCTGCTGGAGTGGATGATTGTGAC
GGAGAGGACCACCCCACGTGCCAGAATTGTTTCACTGTCAAAACGCCCCTATGGAGAAGAGATGAACACGG
TACTGTTC

CACAATACCGTGCTTCCAGATGGCAACAAATGGAGTGGTACCATCTTCACGGTAGTTCAACATGACAACCA
TGGCATCTGGGTCCATGGATTCACCAGTGAAGAACTCCCAGTCCTTGAAAGAACCAATAACCTTCTTGACG
TAAGTTTG

CCTTGGACTGGATCTCCTGCGACATACCTCTGGTCGTTGAAGACAACTCTGCCACGTGGGGCAAAGAAGAA
GCATCAGTGACCTTGTTCTCCTCACCGGAAGAGCCGATCTCCTTCGATGCGGTAGAACCTTGCAAGTGCAA
TGGCTTGA

GTCGGAATCCGAATCTGAATCCGAGGAAGAGCTGCTAGAGCTGGAGCTAGAGTCGGAGTCAGATGAAGAGT
CGCTGGAGCTGGAGTCGGAGTCAGACGAAGAGTCGCTAGAGCTGGAGCTAGAACTTGATTGAGAATCTGAT
TCAGAATC

TCACCACTGGATGCTGTTGTTGCTGCTGTTGCTGTTGTAGATGATGATGTTGTTGTTGAAGTTGTTGTTGT
TGTTGCAACTGGTGTTGCTGTTGTAGCTGCTGCTGTTGCTGCAACTGCTGTTGTTGTTGTAATTGCAGCTG
TTGCTGCA

AGCTAGGGACGAGGAAACTGGTGATAAAGAGCAAGAACAAGAAGAACAAGAGGGTGCTGACAATGAGGACG
ACGATGATGAGGATGACGAAGAGGATGAGGAGGACGAGGAGGAGGAAGAAGCTCTAAATGAAACAGCATCC
GATGAGAG

GAGGAAACTGGTGATAAAGAGCAAGAACAAGAAGAACAAGAGGGTGCTGACAATGAGGACGACGATGATGA
GGATGACGAAGAGGATGAGGAGGACGAGGAGGAGGAAGAAGCTCTAAATGAAACAGCATCCGATGAGAGCA
ATGACGAG

CAATTCTGCTTTGAGTGGAAGATAATTTCTTCACCACTGGATGCTGTTGTTGCTGCTGTTGCTGTTGTAGA
TGATGATGTTGTTGTTGAAGTTGTTGTTGTTGTTGCAACTGGTGTTGCTGTTGTAGCTGCTGCTGTTGCTG
CAACTGCT

TTTCTTCTCTCAATTCTGCTTTGAGTGGAAGATAATTTCTTCACCACTGGATGCTGTTGTTGCTGCTGTTG
CTGTTGTAGATGATGATGTTGTTGTTGAAGTTGTTGTTGTTGTTGCAACTGGTGTTGCTGTTGTAGCTGCT
GCTGTTGC

AAAGGAGCAATAAACGACAAACTACCGACAGTCTTTATGAAGAGGAAGTTGACAAACTCAGCGTTGGGTCA
CTGTTTGGGACTTGATTTCCTGACTGATCCGAGCGAGTCAGAACATGAATACAGGTGCATGTTTCAGACAG
TTCAGGAC

CCGAGCTTGAGTCGGAATCCGAATCTGAATCCGAGGAAGAGCTGCTAGAGCTGGAGCTAGAGTCGGAGTCA
GATGAAGAGTCGCTGGAGCTGGAGTCGGAGTCAGACGAAGAGTCGCTAGAGCTGGAGCTAGAACTTGATTG
AGAATCTG

ATTGTTCTTCTTGGGCTTGAGCTTGAGCTTGTACTGGTTGTTGTTGCTGTTGCTGTTGCTGTTGTAGTTGT
TGTTGTGGTTGTTGTGGTTGCTGTGGTTGTTGTTGTTGCTGTGGTTGCTGTGGTTGCTGTGGTTGCTGTGG
TTGCTGTG

TGCTTCCAGATGGCAACAAATGGAGTGGTACCATCTTCACGGTAGTTCAACATGACAACCATGGCATCTGG
GTCCATGGATTCACCAGTGAAGAACTCCCAGTCCTTGAAAGAACCAATAACCTTCTTGACGTAAGTTTGAG
CACCCTTT

TGATGATGAGCTAGATTCAGAGTCACTGCTGTCACTGCTGTCACTGCTGCTGCTGCTGCTGCTGCTGCTGC
TGCTGCTTGAAGATTCGCCGCTAGAACTGGACGAGGAAGATGAAGAAGAAGAGGAGGAGGAGGAGGAGGAG
GAGGAGGA

TGCTGCTGTTGCTGTTGTAGATGATGATGTTGTTGTTGAAGTTGTTGTTGTTGTTGCAACTGGTGTTGCTG
TTGTAGCTGCTGCTGTTGCTGCAACTGCTGTTGTTGTTGTAATTGCAGCTGTTGCTGCATCTGCTGCTGGT
GGTGAGTC

GTCTAGATTAATTGGTCTACATAGCGGCAAGATGTTTGTGTCACCTTCACATGTCAAAGAGGCGTCCAGGT
GGTACTTCCCCATGCACTTGGAATTGGTTCAGCGTTCCTCCATGGATAGTTCCTTATTGTACGGTAGCGAC
CCCAACTT

GTCGATATAGTTCGACTTGACCTTGGACTGGATCTCCTGCGACATACCTCTGGTCGTTGAAGACAACTCTG
CCACGTGGGGCAAAGAAGAAGCATCAGTGACCTTGTTCTCCTCACCGGAAGAGCCGATCTCCTTCGATGCG
GTAGAACC

TTAACTAACTCGTCCAACTTGAAGGCCCCTTCCTTCCCAGCAGCAACAACTTGCTTCACCAGACGAGGAGA
GCTGGACGATTCGGAAACCCACTGGTTCACAGATCCCAAAACAACGAGGTTGAAGGAAATCAATCTCTCCA
CAGGTGGC

TTAGCCAGGTCCTTGGTTGGGTCGATATAGTTCGACTTGACCTTGGACTGGATCTCCTGCGACATACCTCT
GGTCGTTGAAGACAACTCTGCCACGTGGGGCAAAGAAGAAGCATCAGTGACCTTGTTCTCCTCACCGGAAG
AGCCGATC

TATAAGAGAATGGTGCTTAGTGATTCGTTGAAGCTGCCCTCGCCTACACTTTCAGCTGCTGCTGGAGTGGA
TGATTGTGACGGAGAGGACCACCCCACGTGCCAGAATTGTTTCACTGTCAAAACGCCCCTATGGAGAAGAG
ATGAACAC

GAAGATCCACTGGAGCTAGAGTCGGATTCACTAGAAGATTCAGAGTCACTGGAACTTGAGCTTGAGCTAGA
AGAAGAAGATGATGATGAGCTAGATTCAGAGTCACTGCTGTCACTGCTGTCACTGCTGCTGCTGCTGCTGC
TGCTGCTG

GAATCTGAATCCGAGGAAGAGCTGCTAGAGCTGGAGCTAGAGTCGGAGTCAGATGAAGAGTCGCTGGAGCT
GGAGTCGGAGTCAGACGAAGAGTCGCTAGAGCTGGAGCTAGAACTTGATTGAGAATCTGATTCAGAATCTG
ATTCGGAA

TGGTGTTATTGTCTAGATTAATTGGTCTACATAGCGGCAAGATGTTTGTGTCACCTTCACATGTCAAAGAG
GCGTCCAGGTGGTACTTCCCCATGCACTTGGAATTGGTTCAGCGTTCCTCCATGGATAGTTCCTTATTGTA
CGGTAGCG

TGGAGCTAGAGTCGGATTCACTAGAAGATTCAGAGTCACTGGAACTTGAGCTTGAGCTAGAAGAAGAAGAT
GATGATGAGCTAGATTCAGAGTCACTGCTGTCACTGCTGTCACTGCTGCTGCTGCTGCTGCTGCTGCTGCT
GCTGCTTG

TGGGTTAACAGGTTGGCCATTTTGCATTTGAACTTCAGGTTGGGGTTGACCTTGAGGTTGCTGTTGCTGTT
GCTGTTGTGATTGCTTCTGTTGAGAGTCTTCAGAATGCTGGGCAACACCTTGATTTGGACTTTGCTGTTCC
TGACCGTT

AAATACGGAAAGCTAGGGACGAGGAAACTGGTGATAAAGAGCAAGAACAAGAAGAACAAGAGGGTGCTGAC
AATGAGGACGACGATGATGAGGATGACGAAGAGGATGAGGAGGACGAGGAGGAGGAAGAAGCTCTAAATGA
AACAGCAT

GAGTTTCAAGGACAACCTGTTCCGTGGGAAGTTTGTTTCCAGAGAAGACCCACGACAATCCGCTTCAGATG
TGCCGATGGACTCTCCTGACACGTCCGCATTGTCCTCAATCTCACCGTTGAATGTTTCCTCCTCTAAGGAG
GAATCCAG

GATAATTTCTTCACCACTGGATGCTGTTGTTGCTGCTGTTGCTGTTGTAGATGATGATGTTGTTGTTGAAG
TTGTTGTTGTTGTTGCAACTGGTGTTGCTGTTGTAGCTGCTGCTGTTGCTGCAACTGCTGTTGTTGTTGTA
ATTGCAGC

TTGTTGTTGAGGCGACTGCTGGAAGTGACCTTGATAAGGCGCTTGTTGTTGTTGCTGTTGTTGCTGCTGCT
GCTGCTGTTGTTGTTGTTGAGGCTGGGCTTGAAACTGGTTCAAGTATTGGTGAGGTGTCTGGTGAGAAGCA
TCTCTGTA

CCTTGGTTGGGTCGATATAGTTCGACTTGACCTTGGACTGGATCTCCTGCGACATACCTCTGGTCGTTGAA
GACAACTCTGCCACGTGGGGCAAAGAAGAAGCATCAGTGACCTTGTTCTCCTCACCGGAAGAGCCGATCTC
CTTCGATG

AGGAGAATAATGGTTCACCGTGTTCCTTGAAGTAAGCTTCATCAGCTTCCAACATACCATCGAACCATGGC
AACAACTTCTTGGCACAGTGGTCAGAGTGTAAGACAACTGGGATACCGTAAGCTGGAGCAATGGATCTGAT
GTAGTGGG

TTTGTTGTTGTGGGTTAACAGGTTGGCCATTTTGCATTTGAACTTCAGGTTGGGGTTGACCTTGAGGTTGC
TGTTGCTGTTGCTGTTGTGATTGCTTCTGTTGAGAGTCTTCAGAATGCTGGGCAACACCTTGATTTGGACT
TTGCTGTT

TGATTCGTTGAAGCTGCCCTCGCCTACACTTTCAGCTGCTGCTGGAGTGGATGATTGTGACGGAGAGGACC
ACCCCACGTGCCAGAATTGTTTCACTGTCAAAACGCCCCTATGGAGAAGAGATGAACACGGTACTGTTCTC
TGTAATGC

AGACCCAGGCTGTAACGCTGAAGGATTCGTATGTTAGCGACGACGTCGCCAACTCCACGGAACGCTACAAC
TTGTCCCCTTCTCCGGAGGACGAAGACTTCGAAGGCCCCACTGAAGAAGAAATGCAGACTTTAAGGCACGT
TGGTGGTA

CTAGATTCAGAGTCACTGCTGTCACTGCTGTCACTGCTGCTGCTGCTGCTGCTGCTGCTGCTGCTGCTTGA
AGATTCGCCGCTAGAACTGGACGAGGAAGATGAAGAAGAAGAGGAGGAGGAGGAGGAGGAGGAGGAGGAAG
AAGAAGAA

GTACTTTTCTTTAGCCAGGTCCTTGGTTGGGTCGATATAGTTCGACTTGACCTTGGACTGGATCTCCTGCG
ACATACCTCTGGTCGTTGAAGACAACTCTGCCACGTGGGGCAAAGAAGAAGCATCAGTGACCTTGTTCTCC
TCACCGGA

ACGTAGTCTCTGATACCAGTCAAGTAAGCGTATTGACAGTCAGTGTCCAAGTTGACCTTGACAACACCGTT
GTCAATACCAGTGTGGAATTCTTGGACAGTAGAACCGGAACCACCGTGGAAGACCAAGAACAATGGCTTTT
CTTCCTTG

CGAGCCTGAAGAGTCTGAGTCCGAGCTTGAGTCGGAATCCGAATCTGAATCCGAGGAAGAGCTGCTAGAGC
TGGAGCTAGAGTCGGAGTCAGATGAAGAGTCGCTGGAGCTGGAGTCGGAGTCAGACGAAGAGTCGCTAGAG
CTGGAGCT

CGTCCAACTTGAAGGCCCCTTCCTTCCCAGCAGCAACAACTTGCTTCACCAGACGAGGAGAGCTGGACGAT
TCGGAAACCCACTGGTTCACAGATCCCAAAACAACGAGGTTGAAGGAAATCAATCTCTCCACAGGTGGCAG
AGATTCCG

GACAACCTGTTCCGTGGGAAGTTTGTTTCCAGAGAAGACCCACGACAATCCGCTTCAGATGTGCCGATGGA
CTCTCCTGACACGTCCGCATTGTCCTCAATCTCACCGTTGAATGTTTCCTCCTCTAAGGAGGAATCCAGTG
ACACGCAA

GTCACTGCTGTCACTGCTGCTGCTGCTGCTGCTGCTGCTGCTGCTGCTTGAAGATTCGCCGCTAGAACTGG
ACGAGGAAGATGAAGAAGAAGAGGAGGAGGAGGAGGAGGAGGAGGAGGAAGAAGAAGAAGAAGATTTCTCT
TCAATCTC

GGCAATGCCGCTCGAAGTAGGAGTTTCAAGGACAACCTGTTCCGTGGGAAGTTTGTTTCCAGAGAAGACCC
ACGACAATCCGCTTCAGATGTGCCGATGGACTCTCCTGACACGTCCGCATTGTCCTCAATCTCACCGTTGA
ATGTTTCC

CCGAGGAAGAGCTGCTAGAGCTGGAGCTAGAGTCGGAGTCAGATGAAGAGTCGCTGGAGCTGGAGTCGGAG
TCAGACGAAGAGTCGCTAGAGCTGGAGCTAGAACTTGATTGAGAATCTGATTCAGAATCTGATTCGGAATC
AGAAGAAC

GCATTATGATGACGGCAACTACAACGCAGAATACTCAGGACACCAAGAAGTCGCAGGACCTGGTCATGAAG
CTGTCAAACTCGATGAATGAAGTAGTTCAAATCACCAGCGTGCTGACAATGATCAAGCCATTCAAGATCTT
CGAGCACG

CTGCAAGACAATTGGTGATGAAACTCGACAACGCTAGTTACAGTTCTGTCTCTGCATCTTCATCTCCATCT
CCCTCCACACCGACGAAAAGTGGCAAGATGAGATCAAGATCATCCTCACCTGTGCGTCCCAAGGCATACAC
TCCAAGCC

GCTGTGGTTGTTGTTGTTGCTGTGGTTGCTGTGGTTGCTGTGGTTGCTGTGGTTGCTGTGGTTGCTGTGGT
TGTTGGGATTGTTGTTGTGGTTGAGGTACAGTATTAGCTTGATCTTCTGGAGGCTCTGAAATAACAGCACT
AATTTCAA

TCCGTGGGAAGTTTGTTTCCAGAGAAGACCCACGACAATCCGCTTCAGATGTGCCGATGGACTCTCCTGAC
ACGTCCGCATTGTCCTCAATCTCACCGTTGAATGTTTCCTCCTCTAAGGAGGAATCCAGTGACACGCAAAG
CTCGCCTC

TGCTGCTGCTGCTGCTGCTGCTGCTGCTTGAAGATTCGCCGCTAGAACTGGACGAGGAAGATGAAGAAGAA
GAGGAGGAGGAGGAGGAGGAGGAGGAGGAAGAAGAAGAAGAAGATTTCTCTTCAATCTCCTTCTCCTTAAC
ACTTAATT

CTGATTCGGATTCCGAAGAAGATTCGTCAGAACTAGAGCTAGAGGAAGAAGAAGATCCACTGGAGCTAGAG
TCGGATTCACTAGAAGATTCAGAGTCACTGGAACTTGAGCTTGAGCTAGAAGAAGAAGATGATGATGAGCT
AGATTCAG

CATCAAATTCAAGCATAACGAAAAAGGGAAGTAGAAGGTCCAGTGGCTCATCACCCACTCGTCACACCACA
AGAGTGTGCGTGTCGTGTCATTCGAGTGATTCGCCCTGTTGGAGGCCATCGTGGTCGCCAAGAAAGCAAGA
TCAGCTAT

TGCATTTGCTGCGAAGAAGGAATTTCTAGTTAAGGATCTCACTGAGGAAAGGCACCTAGCCAAGGCCAAGC
AGCAGGATGGTTCGCAAGCTGGAGAGGTTCCTAATCCCTTCAACGATCCAAGTATGTCTAATGCTATGATG
AACATGGC

TCTTTTCTTCCACAATACCGTGCTTCCAGATGGCAACAAATGGAGTGGTACCATCTTCACGGTAGTTCAAC
ATGACAACCATGGCATCTGGGTCCATGGATTCACCAGTGAAGAACTCCCAGTCCTTGAAAGAACCAATAAC
CTTCTTGA

CTCGAAGTAGGAGTTTCAAGGACAACCTGTTCCGTGGGAAGTTTGTTTCCAGAGAAGACCCACGACAATCC
GCTTCAGATGTGCCGATGGACTCTCCTGACACGTCCGCATTGTCCTCAATCTCACCGTTGAATGTTTCCTC
CTCTAAGG

CTTGTTCAAGACGTAGTCTCTGATACCAGTCAAGTAAGCGTATTGACAGTCAGTGTCCAAGTTGACCTTGA
CAACACCGTTGTCAATACCAGTGTGGAATTCTTGGACAGTAGAACCGGAACCACCGTGGAAGACCAAGAAC
AATGGCTT

GAATTAGCCCAAACCGTATGCAAATCGACCGGTTTCTTAGTGAATGCAGAAGAACTGCGTTCCACCACTGA
CTCCAACAAGCCACCTTTTGCGCCTTGTCTTGTCAACGATCCGATTTCTTCATTATCATACAAAGTGACAG
TAGAGAAT

GGGCAGCAGTGGCAATGCCGCTCGAAGTAGGAGTTTCAAGGACAACCTGTTCCGTGGGAAGTTTGTTTCCA
GAGAAGACCCACGACAATCCGCTTCAGATGTGCCGATGGACTCTCCTGACACGTCCGCATTGTCCTCAATC
TCACCGTT

TGGTCGTTGAAGACAACTCTGCCACGTGGGGCAAAGAAGAAGCATCAGTGACCTTGTTCTCCTCACCGGAA
GAGCCGATCTCCTTCGATGCGGTAGAACCTTGCAAGTGCAATGGCTTGATCAGGAACTCCCTCAACAGAGT
ATTAGAAA

AGTCACTGCTGTCACTGCTGTCACTGCTGCTGCTGCTGCTGCTGCTGCTGCTGCTGCTTGAAGATTCGCCG
CTAGAACTGGACGAGGAAGATGAAGAAGAAGAGGAGGAGGAGGAGGAGGAGGAGGAGGAAGAAGAAGAAGA
AGATTTCT

AGACAAATCCAACATGTGGGAGGAGAATAATGGTTCACCGTGTTCCTTGAAGTAAGCTTCATCAGCTTCCA
ACATACCATCGAACCATGGCAACAACTTCTTGGCACAGTGGTCAGAGTGTAAGACAACTGGGATACCGTAA
GCTGGAGC

AAGCTGCCCTCGCCTACACTTTCAGCTGCTGCTGGAGTGGATGATTGTGACGGAGAGGACCACCCCACGTG
CCAGAATTGTTTCACTGTCAAAACGCCCCTATGGAGAAGAGATGAACACGGTACTGTTCTCTGTAATGCAT
GTGGCCTC

AGGAAGCCTCGTTAGTGCCTTCTTCAATATCCTTTATCAACTGTTTGGTGTCCTTGACAGGCTCTTGGTGC
TCTTCTTGCTTCTTCCTCTTGAACCATGACATAAAGTCCCCGTTTGCAGCGGAACTGTGAATACATCTGGC
TTTCTGCT

CACCAAGAGGTTAACTAACTCGTCCAACTTGAAGGCCCCTTCCTTCCCAGCAGCAACAACTTGCTTCACCA
GACGAGGAGAGCTGGACGATTCGGAAACCCACTGGTTCACAGATCCCAAAACAACGAGGTTGAAGGAAATC
AATCTCTC

TTTGCATTTGAACTTCAGGTTGGGGTTGACCTTGAGGTTGCTGTTGCTGTTGCTGTTGTGATTGCTTCTGT
TGAGAGTCTTCAGAATGCTGGGCAACACCTTGATTTGGACTTTGCTGTTCCTGACCGTTTTGAGTTTGGTA
ATAGTTGT

TCACTGCTGCTGCTGCTGCTGCTGCTGCTGCTGCTGCTTGAAGATTCGCCGCTAGAACTGGACGAGGAAGA
TGAAGAAGAAGAGGAGGAGGAGGAGGAGGAGGAGGAGGAAGAAGAAGAAGAAGATTTCTCTTCAATCTCCT
TCTCCTTA

ACAGCAATCTCAAAACACCAATAATGCGTTTGGTAATCAAAATCAGCTGGGGGGCAGTTCCTTCGGATCAA
AACCAGTTGGTTCAGGGTCGCTGTTTGGCCAGAGCAACAACACTTTAGGTAATACAACCAATAATAGAAAT
GGATTGTT

GTCAAATCCAAGCTACTACCACTGCTACCACCGAAGCTACCACCACTGCTGCCCCATCTTCCACCGTTGAA
ACTGTTTCTCCATCCAGCACCGAAACTATCTCTCAACAAACTGAAAATGGTGCTGCTAAGGCCGCTGTCGG
TATGGGTG

CCGTCGACACCCTCCTTGAACACCAAGAGGTTAACTAACTCGTCCAACTTGAAGGCCCCTTCCTTCCCAGC
AGCAACAACTTGCTTCACCAGACGAGGAGAGCTGGACGATTCGGAAACCCACTGGTTCACAGATCCCAAAA
CAACGAGG

AGAGAAGACCCACGACAATCCGCTTCAGATGTGCCGATGGACTCTCCTGACACGTCCGCATTGTCCTCAAT
CTCACCGTTGAATGTTTCCTCCTCTAAGGAGGAATCCAGTGACACGCAAAGCTCGCCTCCAAACTTCTCAA
GTAAGCGC

TGTAGTCCTTCTTGTTCAAGACGTAGTCTCTGATACCAGTCAAGTAAGCGTATTGACAGTCAGTGTCCAAG
TTGACCTTGACAACACCGTTGTCAATACCAGTGTGGAATTCTTGGACAGTAGAACCGGAACCACCGTGGAA
GACCAAGA

CCTCCTTGAACACCAAGAGGTTAACTAACTCGTCCAACTTGAAGGCCCCTTCCTTCCCAGCAGCAACAACT
TGCTTCACCAGACGAGGAGAGCTGGACGATTCGGAAACCCACTGGTTCACAGATCCCAAAACAACGAGGTT
GAAGGAAA

TTCAAGCTGCTAAGTCTACTGCCGCTGCCGTTTCTCAAATAACTGATGGTCAAGTTCAAGCTGCCAAGTCT
ACTGCTGCCGCTGCCTCTCAGATTTCTGACGGCCAAGTTCAGGCCACTACCTCTACTAAGGCTGCTGCATC
CCAAATTA

GCCACGTGGGGCAAAGAAGAAGCATCAGTGACCTTGTTCTCCTCACCGGAAGAGCCGATCTCCTTCGATGC
GGTAGAACCTTGCAAGTGCAATGGCTTGATCAGGAACTCCCTCAACAGAGTATTAGAAATGTTGGCGACGT
AGTCGACG

TGGTCAAGCAGATGCCTACATCACTCATTAGCATTATGATGACGGCAACTACAACGCAGAATACTCAGGAC
ACCAAGAAGTCGCAGGACCTGGTCATGAAGCTGTCAAACTCGATGAATGAAGTAGTTCAAATCACCAGCGT
GCTGACAA

AGTCAAGGAAACGCAAATGACGAATACGATGCCGAACAAATGCGGTTGAAGGAACACGAACACGAACAGAA
ACTGCTGGCCAGGGAACAAGAGTTAAGAGACATTGTTGCAAACACCAACGATAAACTAATAGACATATCGA
TGATAAAC

TTGGACGACGTGGTGTTATTGTCTAGATTAATTGGTCTACATAGCGGCAAGATGTTTGTGTCACCTTCACA
TGTCAAAGAGGCGTCCAGGTGGTACTTCCCCATGCACTTGGAATTGGTTCAGCGTTCCTCCATGGATAGTT
CCTTATTG

CTCATTTCCCACCAACTTGTTTTCCCAGTCGACCCAAAGCGCCTCCTTTGGATTCCAAAGTCCCCTCTGGA
CACACTTCTGCAGGTCCATCCCGAACGTCGGGTCTGTGTGCTTGTATTTGAGCACTGAATCCAGCTTTGGA
ATCTGCCT

GCTGCTAGAGCTGGAGCTAGAGTCGGAGTCAGATGAAGAGTCGCTGGAGCTGGAGTCGGAGTCAGACGAAG
AGTCGCTAGAGCTGGAGCTAGAACTTGATTGAGAATCTGATTCAGAATCTGATTCGGAATCAGAAGAACTA
CTTGAGCT

AGTTTCCTACTATGGCATTGGAGAATTGGATTCTCAGATTCTCAAACTTCAACCTCTTGGTCGTCGTCATT
TGAAAGAGCAGTTCCTCAGGTGTTTCTTGTTACTGCCGTCCCTTCCAGAAGAAATTCGACGATGACAATAG
TCGGAGAG

TGGAACTGCATTTGTTGTTGTGGGTTAACAGGTTGGCCATTTTGCATTTGAACTTCAGGTTGGGGTTGACC
TTGAGGTTGCTGTTGCTGTTGCTGTTGTGATTGCTTCTGTTGAGAGTCTTCAGAATGCTGGGCAACACCTT
GATTTGGA

AAGCATAACGAAAAAGGGAAGTAGAAGGTCCAGTGGCTCATCACCCACTCGTCACACCACAAGAGTGTGCG
TGTCGTGTCATTCGAGTGATTCGCCCTGTTGGAGGCCATCGTGGTCGCCAAGAAAGCAAGATCAGCTATGT
AACTCTTG

GATCTCCTGCGACATACCTCTGGTCGTTGAAGACAACTCTGCCACGTGGGGCAAAGAAGAAGCATCAGTGA
CCTTGTTCTCCTCACCGGAAGAGCCGATCTCCTTCGATGCGGTAGAACCTTGCAAGTGCAATGGCTTGATC
AGGAACTC

GTTACCGACTGGGGACATTATGTAGTCCTTCTTGTTCAAGACGTAGTCTCTGATACCAGTCAAGTAAGCGT
ATTGACAGTCAGTGTCCAAGTTGACCTTGACAACACCGTTGTCAATACCAGTGTGGAATTCTTGGACAGTA
GAACCGGA

TGATACCAGTCAAGTAAGCGTATTGACAGTCAGTGTCCAAGTTGACCTTGACAACACCGTTGTCAATACCA
GTGTGGAATTCTTGGACAGTAGAACCGGAACCACCGTGGAAGACCAAGAACAATGGCTTTTCTTCCTTGCA
ACCAACTT

AAACTCGACAACGCTAGTTACAGTTCTGTCTCTGCATCTTCATCTCCATCTCCCTCCACACCGACGAAAAG
TGGCAAGATGAGATCAAGATCATCCTCACCTGTGCGTCCCAAGGCATACACTCCAAGCCCAAGGTCACCCA
ATTATCAT

GGGCATATCTAAAGGAGCAATAAACGACAAACTACCGACAGTCTTTATGAAGAGGAAGTTGACAAACTCAG
CGTTGGGTCACTGTTTGGGACTTGATTTCCTGACTGATCCGAGCGAGTCAGAACATGAATACAGGTGCATG
TTTCAGAC

AACTTCAGGTTGGGGTTGACCTTGAGGTTGCTGTTGCTGTTGCTGTTGTGATTGCTTCTGTTGAGAGTCTT
CAGAATGCTGGGCAACACCTTGATTTGGACTTTGCTGTTCCTGACCGTTTTGAGTTTGGTAATAGTTGTAA
TTGTTCTT

AACCCTGATCAACCTGTCTCCAAACCTACCCTCACATTACCCTACCTCTCCACTCGTTACCCTGCCCCACT
CAACCATACCACTCCCACCCACCATCCATCTCTCTACTGTCACCAGCCCACCGTCCACCATAACCGTTACC
CTCCCATT

TCATAACAGAGGGCATATCTAAAGGAGCAATAAACGACAAACTACCGACAGTCTTTATGAAGAGGAAGTTG
ACAAACTCAGCGTTGGGTCACTGTTTGGGACTTGATTTCCTGACTGATCCGAGCGAGTCAGAACATGAATA
CAGGTGCA

CGATGAGTTCACCAAATGGAAAATGATCAAAGCTCAACCAAACATCAAGACTGGGTCACAGTCAGCTGAAC
AGAAAACTTCAAACAATCCTCACTCTCGGTTGTTCAAGGTCGTGCTGACCATATGGCATTATAGCGAGATT
TTGCTATT

TTAAGTGCAGGCGTTGGTTATGCTTCGTCTACAATTGGTTGGTGTGCACCGATGGGTCATGTTGGACCTTG
ATCATAGGTCTGTACACAGGCTCCCACATTTGGGCTTTGACCCACTGTAAACATTCGTCAAAGTCACGAGG
AACCTTGA

GGTTGGCCATTTTGCATTTGAACTTCAGGTTGGGGTTGACCTTGAGGTTGCTGTTGCTGTTGCTGTTGTGA
TTGCTTCTGTTGAGAGTCTTCAGAATGCTGGGCAACACCTTGATTTGGACTTTGCTGTTCCTGACCGTTTT
GAGTTTGG

CTGAAGGTAATGATGCCGCTCAAGGAATGGATTTCAGCCAAATGATGGGAGGTGCTGGAGGTGCTGGAGGT
GCTGGAGGCATGGACTTCAGCCAAATGATGGGAGGTGCTGGTGGCGCTGGTTCTCCAGATATGGCTCAATT
GCAGCAAT

CTAATTTCGCTAGACTTAGATCTTTTCTTCCACAATACCGTGCTTCCAGATGGCAACAAATGGAGTGGTAC
CATCTTCACGGTAGTTCAACATGACAACCATGGCATCTGGGTCCATGGATTCACCAGTGAAGAACTCCCAG
TCCTTGAA

GGGATTTCAATATCAAATTCTTCTTCAATAGCTACGAGCAGCTCGACAGTGTCCAAGGAGTCCAACCCCAA
ATCCTTGTGAAATTGGGTATCGCTGGAGATTTGCTTGTTGGCAATGTTGGGAGAGTTCTTATCAAACGCCT
TGATAACA

AACATGTGGGAGGAGAATAATGGTTCACCGTGTTCCTTGAAGTAAGCTTCATCAGCTTCCAACATACCATC
GAACCATGGCAACAACTTCTTGGCACAGTGGTCAGAGTGTAAGACAACTGGGATACCGTAAGCTGGAGCAA
TGGATCTG

AGACAACTCTGCCACGTGGGGCAAAGAAGAAGCATCAGTGACCTTGTTCTCCTCACCGGAAGAGCCGATCT
CCTTCGATGCGGTAGAACCTTGCAAGTGCAATGGCTTGATCAGGAACTCCCTCAACAGAGTATTAGAAATG
TTGGCGAC

GGGGACATTATGTAGTCCTTCTTGTTCAAGACGTAGTCTCTGATACCAGTCAAGTAAGCGTATTGACAGTC
AGTGTCCAAGTTGACCTTGACAACACCGTTGTCAATACCAGTGTGGAATTCTTGGACAGTAGAACCGGAAC
CACCGTGG

GATGCCTACATCACTCATTAGCATTATGATGACGGCAACTACAACGCAGAATACTCAGGACACCAAGAAGT
CGCAGGACCTGGTCATGAAGCTGTCAAACTCGATGAATGAAGTAGTTCAAATCACCAGCGTGCTGACAATG
ATCAAGCC

TGATGCCGCTCAAGGAATGGATTTCAGCCAAATGATGGGAGGTGCTGGAGGTGCTGGAGGTGCTGGAGGCA
TGGACTTCAGCCAAATGATGGGAGGTGCTGGTGGCGCTGGTTCTCCAGATATGGCTCAATTGCAGCAATTA
TTGGCTCA

GTCACACCAAAGGGATCCAATGTGGTGAGACCATACACTCCTGTGAGTGATCTTTCCCAGAAGGGTCACTT
CCAGCTGGTCGTCAAGCATTATGAAGGTGGTAAAATGACCTCACATTTATTTGGTCTTAAACCAAATGACA
CCGTTTCT

TGTCTTCCGATGCATTTGCTGCGAAGAAGGAATTTCTAGTTAAGGATCTCACTGAGGAAAGGCACCTAGCC
AAGGCCAAGCAGCAGGATGGTTCGCAAGCTGGAGAGGTTCCTAATCCCTTCAACGATCCAAGTATGTCTAA
TGCTATGA

AGAATCAATGAAATACGGAAAGCTAGGGACGAGGAAACTGGTGATAAAGAGCAAGAACAAGAAGAACAAGA
GGGTGCTGACAATGAGGACGACGATGATGAGGATGACGAAGAGGATGAGGAGGACGAGGAGGAGGAAGAAG
CTCTAAAT

TCTACAAGTACCTCTTCTTTCAGCTTTCACGAAACTTCTACAGCCTCCACCTCCGTTCAAATATCTTCTCA
GTTTGTGACTCCATCCTCCCCTATTTCCACAGTTGCCCCTCGTTCTACAGGGCTCAATAGTCAAACTGAAA
GTACAAAT

GTTTGTTTCCAGAGAAGACCCACGACAATCCGCTTCAGATGTGCCGATGGACTCTCCTGACACGTCCGCAT
TGTCCTCAATCTCACCGTTGAATGTTTCCTCCTCTAAGGAGGAATCCAGTGACACGCAAAGCTCGCCTCCA
AACTTCTC

GAAGAACAAGAGGGTGCTGACAATGAGGACGACGATGATGAGGATGACGAAGAGGATGAGGAGGACGAGGA
GGAGGAAGAAGCTCTAAATGAAACAGCATCCGATGAGAGCAATGACGAGGAAGACGAAGAGGATGAGGAAG
ACGTGAAA

CGTTAGGAGGAGAATCAATGAAATACGGAAAGCTAGGGACGAGGAAACTGGTGATAAAGAGCAAGAACAAG
AAGAACAAGAGGGTGCTGACAATGAGGACGACGATGATGAGGATGACGAAGAGGATGAGGAGGACGAGGAG
GAGGAAGA

TCATATTATATATAAGAGAATGGTGCTTAGTGATTCGTTGAAGCTGCCCTCGCCTACACTTTCAGCTGCTG
CTGGAGTGGATGATTGTGACGGAGAGGACCACCCCACGTGCCAGAATTGTTTCACTGTCAAAACGCCCCTA
TGGAGAAG

AGAAGTAAAAGCTGAAGATGATACTGGTGAAGAAGAAGAGGATGACCCAGTGATCGAAGAGTTTCCATTGA
AGATCTCCGGAGAAGAGGAGTCACTGCACGTGTTTCAGTATGCTAATAGACCAAGGCTAGTAGGACGCAAA
CCTGCTGA

GGCAGCATGCGGATTTTGGAATCCCCATCGTGAAGGTAGAAGCCCAGATCAGGGTCTTTCTTCATGGAGGT
AGAGACGGACGAGAGTGACGTGCTTGGGGTTGAGACGCCGTATGGATTGTACACTAATCGTTCGCTACTGA
ACTTGGAG

TTGTAGAGATCGTTAGGAGGAGAATCAATGAAATACGGAAAGCTAGGGACGAGGAAACTGGTGATAAAGAG
CAAGAACAAGAAGAACAAGAGGGTGCTGACAATGAGGACGACGATGATGAGGATGACGAAGAGGATGAGGA
GGACGAGG

ATTGGTCTACATAGCGGCAAGATGTTTGTGTCACCTTCACATGTCAAAGAGGCGTCCAGGTGGTACTTCCC
CATGCACTTGGAATTGGTTCAGCGTTCCTCCATGGATAGTTCCTTATTGTACGGTAGCGACCCCAACTTGG
TGGACGAA

TATGGCATTGGAGAATTGGATTCTCAGATTCTCAAACTTCAACCTCTTGGTCGTCGTCATTTGAAAGAGCA
GTTCCTCAGGTGTTTCTTGTTACTGCCGTCCCTTCCAGAAGAAATTCGACGATGACAATAGTCGGAGAGAT
ATTCCGGA

TAGACTTAGATCTTTTCTTCCACAATACCGTGCTTCCAGATGGCAACAAATGGAGTGGTACCATCTTCACG
GTAGTTCAACATGACAACCATGGCATCTGGGTCCATGGATTCACCAGTGAAGAACTCCCAGTCCTTGAAAG
AACCAATA

GCTGCTGCTGCTGCTGCTTGAAGATTCGCCGCTAGAACTGGACGAGGAAGATGAAGAAGAAGAGGAGGAGG
AGGAGGAGGAGGAGGAGGAAGAAGAAGAAGAAGATTTCTCTTCAATCTCCTTCTCCTTAACACTTAATTTT
GGCACTTC

GACAAACTCAGCGTTGGGTCACTGTTTGGGACTTGATTTCCTGACTGATCCGAGCGAGTCAGAACATGAAT
ACAGGTGCATGTTTCAGACAGTTCAGGACATCCCATCTCTCTCGCAACTGATCTTATTCAACAGCATGCCA
AACGTTCC

CCCTTAATTTGACCAACTCACTTGTTGAAATACTAGGTTTTGTCTCTTGACAGGCTTCCAATAAATCGTTT
ATTGTGACCACAGCTGTCAGCTCAGAAGCAGCTGAAGTGCTTGTCTTTGTCTCGTGCACCACATCTTGCTG
CAATAGAG

TATCAAATTCTTCTTCAATAGCTACGAGCAGCTCGACAGTGTCCAAGGAGTCCAACCCCAAATCCTTGTGA
AATTGGGTATCGCTGGAGATTTGCTTGTTGGCAATGTTGGGAGAGTTCTTATCAAACGCCTTGATAACATC
AATGACCC

CAAGATGATGGAATTAGCCCAAACCGTATGCAAATCGACCGGTTTCTTAGTGAATGCAGAAGAACTGCGTT
CCACCACTGACTCCAACAAGCCACCTTTTGCGCCTTGTCTTGTCAACGATCCGATTTCTTCATTATCATAC
AAAGTGAC

GACTATTCAAGACCGAACACGAAAGCACCACTAACATTTACAATTCCTGAGGGCATTGTCTACTCACGTGG
TGGTCAAATTCCAGCTGAGGAAGATGTTCCAATGATTCATTCTTTAGAGCCAACTATGAGAAACAAATTTG
AAATTCCA

TACAAATTCATTGGACGACGTGGTGTTATTGTCTAGATTAATTGGTCTACATAGCGGCAAGATGTTTGTGT
CACCTTCACATGTCAAAGAGGCGTCCAGGTGGTACTTCCCCATGCACTTGGAATTGGTTCAGCGTTCCTCC
ATGGATAG

TTTTGAGATAATTGTTCTTCTTGGGCTTGAGCTTGAGCTTGTACTGGTTGTTGTTGCTGTTGCTGTTGCTG
TTGTAGTTGTTGTTGTGGTTGTTGTGGTTGCTGTGGTTGTTGTTGTTGCTGTGGTTGCTGTGGTTGCTGTG
GTTGCTGT

AAACGACAAATTTACCGTTTCTGCCATCTACTCTGATTTACCACAACAAGAAAGAGACACCATCATGAAGG
AATTCAGAAGTGGTTCTTCCAGAATTTTGATCTCCACTGATTTGTTGGCTAGAGGTATCGATGTCCAACAA
GTTTCTTT

CGCCTACACTTTCAGCTGCTGCTGGAGTGGATGATTGTGACGGAGAGGACCACCCCACGTGCCAGAATTGT
TTCACTGTCAAAACGCCCCTATGGAGAAGAGATGAACACGGTACTGTTCTCTGTAATGCATGTGGCCTCTT
CCTGAAGT

CATTGGAAGCTTTAGGAAAGTCCACATTTGTCTTGGATGACTGGAAGAGAAAGTACTCCAACGACGACACC
AGACCAGTTGCTTTGCCATGGTTCTGGGAACACTACAACCCTGAAGAATACTCCATCTGGAAGGTTGGTTA
CAAATACA

GACATACCTCTGGTCGTTGAAGACAACTCTGCCACGTGGGGCAAAGAAGAAGCATCAGTGACCTTGTTCTC
CTCACCGGAAGAGCCGATCTCCTTCGATGCGGTAGAACCTTGCAAGTGCAATGGCTTGATCAGGAACTCCC
TCAACAGA

AGACCGACAATAGAACCTCTTGACAATAACGATTTATCCAACACCTGATCATAATTGAAATGCTCGGCCTC
CTCGTTCTCTTGACTGTCATCTTCATGAACAATGGTGTGCAAGTTATCAAACAAACCATTCGGATCGAAAT
GAACGATA

TGTAACGCTGAAGGATTCGTATGTTAGCGACGACGTCGCCAACTCCACGGAACGCTACAACTTGTCCCCTT
CTCCGGAGGACGAAGACTTCGAAGGCCCCACTGAAGAAGAAATGCAGACTTTAAGGCACGTTGGTGGTAAA
ATTCCTAT

CAAGTAAGCGTATTGACAGTCAGTGTCCAAGTTGACCTTGACAACACCGTTGTCAATACCAGTGTGGAATT
CTTGGACAGTAGAACCGGAACCACCGTGGAAGACCAAGAACAATGGCTTTTCTTCCTTGCAACCAACTTGT
TCTCTGGT

TTCTAAACAATAATTTAGAGCCAGCATGGTATCCGCAGTTTCACCACTTTGTGAAACAAACACGCATACAT
CGTCTCTGAAGACAGGGCATTTTCTGTCCAGAAAGTCAGACGCTAATTCCACACTAACTGGGATATCTGAT
AATTCTTC

ATTGGTGATGAAACTCGACAACGCTAGTTACAGTTCTGTCTCTGCATCTTCATCTCCATCTCCCTCCACAC
CGACGAAAAGTGGCAAGATGAGATCAAGATCATCCTCACCTGTGCGTCCCAAGGCATACACTCCAAGCCCA
AGGTCACC

CTGCTGCTTGAAGATTCGCCGCTAGAACTGGACGAGGAAGATGAAGAAGAAGAGGAGGAGGAGGAGGAGGA
GGAGGAGGAAGAAGAAGAAGAAGATTTCTCTTCAATCTCCTTCTCCTTAACACTTAATTTTGGCACTTCGT
CAACTTTA

CCTAATTGACTTATTAACCAGAAGGAAATCAGAAGGCGAGTGTTGTGACCTAAACAAGTGCACTCCGCTCT
TGCAATCAGAGCAGCCAGAATATATTGCATGTGTCCCACCAGTGATAAAGTCTTCTCAAAGTGAAAGAAAC
GTGCCACA

TCACTCATTAGCATTATGATGACGGCAACTACAACGCAGAATACTCAGGACACCAAGAAGTCGCAGGACCT
GGTCATGAAGCTGTCAAACTCGATGAATGAAGTAGTTCAAATCACCAGCGTGCTGACAATGATCAAGCCAT
TCAAGATC

GTCCTAGCATCTGCTCTGTTTGCTAAATTTGTCACACCAAAGGGATCCAATGTGGTGAGACCATACACTCC
TGTGAGTGATCTTTCCCAGAAGGGTCACTTCCAGCTGGTCGTCAAGCATTATGAAGGTGGTAAAATGACCT
CACATTTA

GTGATAAAGAGCAAGAACAAGAAGAACAAGAGGGTGCTGACAATGAGGACGACGATGATGAGGATGACGAA
GAGGATGAGGAGGACGAGGAGGAGGAAGAAGCTCTAAATGAAACAGCATCCGATGAGAGCAATGACGAGGA
AGACGAAG

ACTTCTCATGGCTACCCCAACGAAGCCAGACAAGATCTAGGAGATGTCTCCACCTTCCGAGCTACACTCTC
ACATCTCTTACATAGACACTGTGAAGTAAGTGATTGCACACAATCCAAAGAAGCACATCAAATAATATGTA
AATATTAC

GTATTAGAAATGTTGGCGACGTAGTCGACGAAGAACCACACCAGCCAGATCCCTGGCGTGTTGGAATGTGA
TTTCCAATTGTACAGCAAGCCGTCGACACCCTCCTTGAACACCAAGAGGTTAACTAACTCGTCCAACTTGA
AGGCCCCT

ACACCCACACACCACACCCACACACACACACCCACACCCACACCCACACACACACACCCACACACACCACA
CCCACACACCACACCCACACCCACACCACACCCACACACCCACACCCACACACCCACACCACACCCACACC
CACACACA

ACCACACCCACACACACACACCCACACCCACACCCACACACACACACCCACACACACCACACCCACACACC
ACACCCACACCCACACCACACCCACACACCCACACCCACACACCCACACCACACCCACACCCACACACACC
CACACAGT

CACACACACACCCACACCCACACCCACACACACACACCCACACACACCACACCCACACACCACACCCACAC
CCACACCACACCCACACACCCACACCCACACACCCACACCACACCCACACCCACACACACCCACACAGTAC
CTCAACAC

CCCACACCCACACCCACACACACACACCCACACACACCACACCCACACACCACACCCACACCCACACCACA
CCCACACACCCACACCCACACACCCACACCACACCCACACCCACACACACCCACACAGTACCTCAACACAA
CCCCTAAT

GGGTTAGGTGTGTGTGTGTGTGTGGGTGTGGTGTGTGGGTGTGTGGGTGTGGTGTGGTGTGTGTGTGTGTG
GTGTGTGGGTGTGGTGGGTGTGGTGTGGGTGTGGGTGTGGGTGTGGGTGTGGGTGTGGTGTGTATATCTAT
GTCACCTT

TGTGTGTGTGTGTGGGTGTGGTGTGTGGGTGTGTGGGTGTGGTGTGGTGTGTGTGTGTGTGGTGTGTGGGT
GTGGTGGGTGTGGTGTGGGTGTGGGTGTGGGTGTGGGTGTGGGTGTGGTGTGTATATCTATGTCACCTTAT
TGCATGCT

TATATACACCACACCCACACACCACACCCACACACACACACCCACACCCACACCCACACACACACACCCAC
ACACACCACACCCACACACCACACCCACACCCACACCACACCCACACACCCACACCCACACACCCACACCA
CACCCACA

GTTGATCAGGGTTAGAGTAGGGTAGTGTTAGGGTTAGGTGTGTGTGTGTGTGTGGGTGTGGTGTGTGGGTG
TGTGGGTGTGGTGTGGTGTGTGTGTGTGTGGTGTGTGGGTGTGGTGGGTGTGGTGTGGGTGTGGGTGTGGG
TGTGGGTG

TGTGGGTGTGGTGTGTGGGTGTGTGGGTGTGGTGTGGTGTGTGTGTGTGTGGTGTGTGGGTGTGGTGGGTG
TGGTGTGGGTGTGGGTGTGGGTGTGGGTGTGGGTGTGGTGTGTATATCTATGTCACCTTATTGCATGCTGG
ATGGTGTT

CAGTGACATATATATACACCACACCCACACACCACACCCACACACACACACCCACACCCACACCCACACAC
ACACACCCACACACACCACACCCACACACCACACCCACACCCACACCACACCCACACACCCACACCCACAC
ACCCACAC

ACACCATCCAGCATGCAATACAGTGACATATATATACACCACACCCACACACCACACCCACACACACACAC
CCACACCCACACCCACACACACACACCCACACACACCACACCCACACACCACACCCACACCCACACCACAC
CCACACAC

GTTAGAGTAGGGTAGTGTTAGGGTTAGGTGTGTGTGTGTGTGGGTGTGGTGTGTGGGTGTGTGGGTGTG
GTGTGGTGTGTGTGTGTGTGGTGTGTGGGTGTGGTGGGTGTGGTGTGGGTGTGGGTGTGGGTGTGGGTGTG
GGTGTGGT

GCATGCAATACAGTGACATATATATACACCACACCCACACACCACACCCACACACACACACCCACACCCAC
ACCCACACACACACACCCACACACACCACACCCACACACCACACCCACACCCACACCACACCCACACACCC
ACACCCAC

GGTAGTGTTAGGGTTAGGTGTGTGTGTGTGTGGGTGTGGTGTGTGGGTGTGTGGGTGTGGTGTGGTGTG
TGTGTGTGTGGTGTGTGGGTGTGGTGGGTGTGGTGTGGGTGTGGGTGTGGGTGTGGGTGTGGGTGTGGTGT
GTATATCT

CACCCACACACACACACCCACACACACCACACCCACACACCACACCCACACCCACACCACACCCACACACC
CACACCCACACACCCACACCACACCCACACCCACACACACCCACACAGTACCTCAACACAACCCCTAATCC
AACCCTGA

TGAGAGACAGGTTGATCAGGGTTAGAGTAGGGTAGTGTTAGGGTTAGGTGTGTGTGTGTGTGGGTGTGG
TGTGTGGGTGTGTGGGTGTGGTGTGGTGTGTGTGTGTGTGGTGTGTGGGTGTGGTGGGTGTGGTGTGGGTG
TGGGTGTG

CACACACCCACACACACCACACCCACACACCACACCCACACCCACACCACACCCACACACCCACACCCACA
CACCCACACCACACCCACACCCACACACACCCACACAGTACCTCAACACAACCCCTAATCCAACCCTGATG
AACCTGCC

CACACACCACACCCACACACCACACCCACACCCACACCACACCCACACACCCACACCCACACACCCACACC
ACACCCACACCCACACACACCCACACAGTACCTCAACACAACCCCTAATCCAACCCTGATGAACCTGCCTC
CAAACCTA

GTGTGTGGGTGTGTGGGTGTGGTGTGGTGTGTGTGTGTGTGGTGTGTGGGTGTGGTGGGTGTGGTGTGGGT
GTGGGTGTGGGTGTGGGTGTGGGTGTGGTGTGTATATCTATGTCACCTTATTGCATGCTGGATGGTGTTAG
ACAAGGCC

GAGGGTAAGTTGAGAGACAGGTTGATCAGGGTTAGAGTAGGGTAGTGTTAGGGTTAGGTGTGTGTGTGT
GTGGGTGTGGTGTGTGGGTGTGTGGGTGTGGTGTGGTGTGTGTGTGTGTGGTGTGTGGGTGTGGTGGGTGT
GGTGTGGG

GCCTTGTCTAACACCATCCAGCATGCAATACAGTGACATATATATACACCACACCCACACACCACACCCAC
ACACACACACCCACACCCACACCCACACACACACACCCACACACACCACACCCACACACCACACCCACACC
CACACCAC

TCTTCAAAGCCGGTTGATGGTCTCCGCTGCCGTTCATGATACGTGTCTTCATGACGTCTGCTGGAGAGCAC
ACAGTGGTGGCCACCAGGCCAGCCAACAGGGACGCTGTCAAGTGTGTATAGTTCTTGGATGCGTCGAAATC
TAATTTTG

ACCCACACACCACACCCACACCCACACCACACCCACACACCCACACCCACACACCCACACCACACCCACAC
CCACACACACCCACACAGTACCTCAACACAACCCCTAATCCAACCCTGATGAACCTGCCTCCAAACCTACC
CTCCATTA

ACTAAGTTTACAACAACAAAGCAGCGGCACCAGCCAACAAAGCACCAGCAGCTGGCAAAGCCTTAGCAGCA
GCACCAGTGTAAGAGGTGACAGAGTGAGTTGGAGCAGCAGAGGTGGTGTTCTTTGGAGCTTCAGTAGAGGT
AACTGGAG

GCAGCGGCACCAGCCAACAAAGCACCAGCAGCTGGCAAAGCCTTAGCAGCAGCACCAGTGTAAGAGGTGAC
AGAGTGAGTTGGAGCAGCAGAGGTGGTGTTCTTTGGAGCTTCAGTAGAGGTAACTGGAGCAGCAGTAGAAG
TACCGTTC

GGTCATCATGCCGTGCTGTTGGTACTGTCCATTCTGTGGAGGTGGTACTGAAGCAGGTTGAGGAGAGACAT
GATGATGGTTCTCTGGAACAGCTGATGTCCCAGGTGTTGTCTCTTCTTGAGAATTAACCTTAGTGGAATCT
CTATCAAA

CTTTGTTTGGGGTCATCATGCCGTGCTGTTGGTACTGTCCATTCTGTGGAGGTGGTACTGAAGCAGGTTGA
GGAGAGACATGATGATGGTTCTCTGGAACAGCTGATGTCCCAGGTGTTGTCTCTTCTTGAGAATTAACCTT
AGTGGAAT

CCGTGCTGTTGGTACTGTCCATTCTGTGGAGGTGGTACTGAAGCAGGTTGAGGAGAGACATGATGATGGTT
CTCTGGAACAGCTGATGTCCCAGGTGTTGTCTCTTCTTGAGAATTAACCTTAGTGGAATCTCTATCAAATT
CCGGTAAA

TATACTTCACGCTGTTTCATTCTATGTTCTGACAATTCTTTGTTCCTCTGTTCGACTTGGGGGGCAACTGG
TTGTTCCACATCTGGGTCTTGTTTCTTCAGCTGTGGCGTTGACTCAACTTCTTCTTCTGGCTTCATGGAAT
CTCTACAT

GTCTAACACCATCCAGCATGCAATACAGTGACATATATATACACCACACCCACACACCACACCCACACCAC
ACCCACACCCACACACACCACACACCCACACACTCTCTTACATCTACCTTTACTCTCGCTGTCACACCTTA
CCCGGCTT

GGCGATCGCTAAGCTTAATCAACTTCTTCGACAGTTGGACCTTCAGCTTCTGGAGCTGGAGGAGCACCACC
TGGGAAACCACCTGGAGCTGCGCCTTCTGGAGCACCACCAGCTTGGTACAATTTAGACATGATTGGGTTGG
CAACCTCT

CACACCCACACCCACACCACACCCACACACCCACACCCACACACCCACACCACACCCACACCCACACACAC
CCACACAGTACCTCAACACAACCCCTAATCCAACCCTGATGAACCTGCCTCCAAACCTACCCTCCATTACC
CTACCTCT

GTGTGGGTGTGGTGTGGTGTGTGTGTGTGTGGTGTGTGGGTGTGGTGGGTGTGGTGTGGGTGTGGGTGTGG
GTGTGGGTGTGGGTGTGGTGTGTATATCTATGTCACCTTATTGCATGCTGGATGGTGTTAGACAAGGCCGT
AGGGACAT

GTGTTATTAACGGACCCACGCACAGTGGTGCTATCCATAATTTGCTGCTGCAGGGCCTTCAGGCCCTGCAC
GCTAGCACACACCTTGGCGTCTGCAGTAGTGGTCTCCTTCTCAGTTTTGCTGCACCATTTGAAAGTGTTTG
TCCACATT

TGTCCCTACGGCCTTGTCTAACACCATCCAGCATGCAATACAGTGACATATATATACACCACACCCACACA
CCACACCCACACACACACACCCACACCCACACCCACACACACACACCCACACACACCACACCCACACACCA
CACCCACA

CTACGGCCTTGTCTAACACCATCCAGCATGCAATACAGTGACATATATATACACCACACCCACACACCACA
CCCACACCACACCCACACCCACACACACCACACACCCACACACTCTCTTACATCTACCTTTACTCTCGCTG
TCACACCT

GTTGGTTGGTGGGAACTGGAAGTGAAAGTTCTGTTCTTTAGGGTGCAGCGGTTGCTGTTGCTGTTGCTGTT
GCTGTTGCTGCTGTGGTTGGATTGATGCTTGTGATGTTTGTGGTGCCTGTGGTGCTTGTGGTACAGTTTTG
CTGCCAGT

TCGGCAAGGATCTTCAAAGCCGGTTGATGGTCTCCGCTGCCGTTCATGATACGTGTCTTCATGACGTCTGC
TGGAGAGCACACAGTGGTGGCCACCAGGCCAGCCAACAGGGACGCTGTCAAGTGTGTATAGTTCTTGGATG
CGTCGAAA

CAACAACAAAGCAGCGGCACCAGCCAACAAAGCACCAGCAGCTGGCAAAGCCTTAGCAGCAGCACCAGTGT
AAGAGGTGACAGAGTGAGTTGGAGCAGCAGAGGTGGTGTTCTTTGGAGCTTCAGTAGAGGTAACTGGAGCA
GCAGTAGA

CAGCCAACAAAGCACCAGCAGCTGGCAAAGCCTTAGCAGCAGCACCAGTGTAAGAGGTGACAGAGTGAGTT
GGAGCAGCAGAGGTGGTGTTCTTTGGAGCTTCAGTAGAGGTAACTGGAGCAGCAGTAGAAGTACCGTTCTT
TGGGGCTT

GAAGCCATAGCTTTGTTTGGGGTCATCATGCCGTGCTGTTGGTACTGTCCATTCTGTGGAGGTGGTACTGA
AGCAGGTTGAGGAGAGACATGATGATGGTTCTCTGGAACAGCTGATGTCCCAGGTGTTGTCTCTTCTTGAG
AATTAACC

CGGACCCACGCACAGTGGTGCTATCCATAATTTGCTGCTGCAGGGCCTTCAGGCCCTGCACGCTAGCACAC
ACCTTGGCGTCTGCAGTAGTGGTCTCCTTCTCAGTTTTGCTGCACCATTTGAAAGTGTTTGTCCACATTCT
ACTGAAGA

CCCACACCACACCCACACACCCACACCCACACACCCACACCACACCCACACCCACACACACCCACACAGTA
CCTCAACACAACCCCTAATCCAACCCTGATGAACCTGCCTCCAAACCTACCCTCCATTACCCTACCTCTCC
ACTCGTTA

TGCCCAGTTAGAAGCCATAGCTTTGTTTGGGGTCATCATGCCGTGCTGTTGGTACTGTCCATTCTGTGGAG
GTGGTACTGAAGCAGGTTGAGGAGAGACATGATGATGGTTCTCTGGAACAGCTGATGTCCCAGGTGTTGTC
TCTTCTTG

AGCACCAGCAGCTGGCAAAGCCTTAGCAGCAGCACCAGTGTAAGAGGTGACAGAGTGAGTTGGAGCAGCAG
AGGTGGTGTTCTTTGGAGCTTCAGTAGAGGTAACTGGAGCAGCAGTAGAAGTACCGTTCTTTGGGGCTTCA
GTGGTCAA

TCTATGTTCTGACAATTCTTTGTTCCTCTGTTCGACTTGGGGGGCAACTGGTTGTTCCACATCTGGGTCTT
GTTTCTTCAGCTGTGGCGTTGACTCAACTTCTTCTTCTGGCTTCATGGAATCTCTACATTCTTGTTGGAAA
GAGTCGAT

TATGAGGCTAGTTGGTTGGTGGGAACTGGAAGTGAAAGTTCTGTTCTTTAGGGTGCAGCGGTTGCTGTTGC
TGTTGCTGTTGCTGTTGCTGCTGTGGTTGGATTGATGCTTGTGATGTTTGTGGTGCCTGTGGTGCTTGTGG
TACAGTTT

ATCATCGACGTCTTCTACGCAAACGTCAACATCATCTTCATCCTCACTGTCGTCAACGCCGTCCTCTTCAT
CATCCCCATCAACAATCACTTCTGCACCTTCAACCTCCTCCACACCATCCACTACTGCCTATAATCAAGGA
AGCACTAT

GCTGTTTCATTCTATGTTCTGACAATTCTTTGTTCCTCTGTTCGACTTGGGGGGCAACTGGTTGTTCCACA
TCTGGGTCTTGTTTCTTCAGCTGTGGCGTTGACTCAACTTCTTCTTCTGGCTTCATGGAATCTCTACATTC
TTGTTGGA

TGGAACATCCTAGAATTAGGAATTTCTGTTTGACCGTTCTCGTTCCCCTCTTCTCCATTGCTCACATCAGA
CTCAGGCTCAGACTCAGACTCAGACCCAGATCCATCGTCACCTGCCAGAAGAGCGTCCCCAAACAGATTAC
CAGAATTC

CATCCCAGCAATGATCTGAGGAAACCACATCACTACACATCTGGCTGGTGAGATGGCTCTGGCCAAACACA
AAGAGAGAGAAACGGCTGGGTTCAAAGCCCCACCAGAAACACCAGATAGAAAACATCACAGAGAAACCGAA
ACCAAGGG

TCTCCGCTGCCGTTCATGATACGTGTCTTCATGACGTCTGCTGGAGAGCACACAGTGGTGGCCACCAGGCC
AGCCAACAGGGACGCTGTCAAGTGTGTATAGTTCTTGGATGCGTCGAAATCTAATTTTGTGACCAAGTAGT
TCTTAAAC

CGGTTGATGGTCTCCGCTGCCGTTCATGATACGTGTCTTCATGACGTCTGCTGGAGAGCACACAGTGGTGG
CCACCAGGCCAGCCAACAGGGACGCTGTCAAGTGTGTATAGTTCTTGGATGCGTCGAAATCTAATTTTGTG
ACCAAGTA

AATTTCTGTTTGACCGTTCTCGTTCCCCTCTTCTCCATTGCTCACATCAGACTCAGGCTCAGACTCAGACT
CAGACCCAGATCCATCGTCACCTGCCAGAAGAGCGTCCCCAAACAGATTACCAGAATTCTGTACTTCCATG
TTGTATAC

TATTTTACAGGGCGATCGCTAAGCTTAATCAACTTCTTCGACAGTTGGACCTTCAGCTTCTGGAGCTGGAG
GAGCACCACCTGGGAAACCACCTGGAGCTGCGCCTTCTGGAGCACCACCAGCTTGGTACAATTTAGACATG
ATTGGGTT

TAGAATTAGGAATTTCTGTTTGACCGTTCTCGTTCCCCTCTTCTCCATTGCTCACATCAGACTCAGGCTCA
GACTCAGACTCAGACCCAGATCCATCGTCACCTGCCAGAAGAGCGTCCCCAAACAGATTACCAGAATTCTG
TACTTCCA

TAGGGTAATGGAGGGTAAGTTGAGAGACAGGTTGATCAGGGTTAGAGTAGGGTAGTGTTAGGGTTAGGTGT
GTGTGTGTGTGTGGGTGTGGTGTGTGGGTGTGTGGGTGTGGTGTGGTGTGTGTGTGTGTGGTGTGTGGGTG
TGGTGGGT

ATAATAATAAACTAAGTTTACAACAACAAAGCAGCGGCACCAGCCAACAAAGCACCAGCAGCTGGCAAAGC
CTTAGCAGCAGCACCAGTGTAAGAGGTGACAGAGTGAGTTGGAGCAGCAGAGGTGGTGTTCTTTGGAGCTT
CAGTAGAG

GCGGACAGCGTCGGCAAGGATCTTCAAAGCCGGTTGATGGTCTCCGCTGCCGTTCATGATACGTGTCTTCA
TGACGTCTGCTGGAGAGCACACAGTGGTGGCCACCAGGCCAGCCAACAGGGACGCTGTCAAGTGTGTATAG
TTCTTGGA

GTACTTCAAGTATACTTCACGCTGTTTCATTCTATGTTCTGACAATTCTTTGTTCCTCTGTTCGACTTGGG
GGGCAACTGGTTGTTCCACATCTGGGTCTTGTTTCTTCAGCTGTGGCGTTGACTCAACTTCTTCTTCTGGC
TTCATGGA

CATCGGACAACAAGTTCATAACGTAACTACCTCTCTTGATAGCATCTTCAACAGTGAACAAGTTCTTGCCT
GGAACCCAACCGTCTTCGATGGCAGCCTTCCAAGAAGCACCATCTTTACGGACACCAATGATAACGTTCAA
ACCGTTGT

TGTATTTCTATATGAGGCTAGTTGGTTGGTGGGAACTGGAAGTGAAAGTTCTGTTCTTTAGGGTGCAGCGG
TTGCTGTTGCTGTTGCTGTTGCTGTTGCTGCTGTGGTTGGATTGATGCTTGTGATGTTTGTGGTGCCTGTG
GTGCTTGT

TATAGGATTTGGAGTAGGGTAGTAAATATAAGGATTCTCCTGTTGCTGTTGCTGTTGTTGGTCTTCTTGTT
GTTGTTGTTGTTGCTGTTGCTGTTGTTGCTGGGGAGCAGAGAGTGGTGGAGGTGGCATAAACATCATTGGG
TTGGGGTA

TCTACTCTACACTTGGTCTTGAGATTGAGAGGTGGTGGTAAGAAGAGAAAGAAGAAGGTCTACACCACCCC
AAAGAAGATCAAGCACAAGCACAAGAAGGTCAAGTTGGCTGTCTTGTCCTACTACAAGGTCGATGCTGAAG
GTAAGGTT

TACCCACAGTCATCAACTGGTCCTTGTGGAAATCCTGTTGCTGCTGCTGCTGCTGCTCCTGTTGTTGTTGT
TGTTGTTGAAGCTGTCTCTTTGGGACGAGGCTGCTTGCCCATTCCAGTTGTGCGTCCATTAAGTCTTGTTT
GTTCCTGT

AGAATCCTCTTCCACTTATGTCTCTTCGAGCAAGCAAGCTTCCTCCACTAGCGAGGCTCACTCTTCCAGTG
CTGCCTCTTCGACCGTGTCCCAAGAAACAGTCTCCTCTGCTCTACCAACTTCTACCGCCGTTATTTCTACT
TTCTCTGA

GGAGTAGGGTAGTAAATATAAGGATTCTCCTGTTGCTGTTGCTGTTGTTGGTCTTCTTGTTGTTGTTGTTG
TTGCTGTTGCTGTTGTTGCTGGGGAGCAGAGAGTGGTGGAGGTGGCATAAACATCATTGGGTTGGGGTAGG
CAAACGGG

AAGCTTAATCAACTTCTTCGACAGTTGGACCTTCAGCTTCTGGAGCTGGAGGAGCACCACCTGGGAAACCA
CCTGGAGCTGCGCCTTCTGGAGCACCACCAGCTTGGTACAATTTAGACATGATTGGGTTGGCAACCTCTTG
CAATTCCT

GGTGTGGTGTGTGTGTGTGTGGTGTGTGGGTGTGGTGGGTGTGGTGTGGGTGTGGGTGTGGGTGTGGGTGT
GGGTGTGGTGTGTATATCTATGTCACCTTATTGCATGCTGGATGGTGTTAGACAAGGCCGTAGGGACATAT
AGCATCTA

GGGAACTGGAAGTGAAAGTTCTGTTCTTTAGGGTGCAGCGGTTGCTGTTGCTGTTGCTGTTGCTGTTGCTG
CTGTGGTTGGATTGATGCTTGTGATGTTTGTGGTGCCTGTGGTGCTTGTGGTACAGTTTTGCTGCCAGTTT
GCTGCGAG

GGCCCTCTTTGCGGACAGCGTCGGCAAGGATCTTCAAAGCCGGTTGATGGTCTCCGCTGCCGTTCATGATA
CGTGTCTTCATGACGTCTGCTGGAGAGCACACAGTGGTGGCCACCAGGCCAGCCAACAGGGACGCTGTCAA
GTGTGTAT

CTGATTCGCATACCCACAGTCATCAACTGGTCCTTGTGGAAATCCTGTTGCTGCTGCTGCTGCTGCTCCTG
TTGTTGTTGTTGTTGTTGAAGCTGTCTCTTTGGGACGAGGCTGCTTGCCCATTCCAGTTGTGCGTCCATTA
AGTCTTGT

CACCAGCAGCCATCCCAGCAATGATCTGAGGAAACCACATCACTACACATCTGGCTGGTGAGATGGCTCTG
GCCAAACACAAAGAGAGAGAAACGGCTGGGTTCAAAGCCCCACCAGAAACACCAGATAGAAAACATCACAG
AGAAACCG

GACAATTCTTTGTTCCTCTGTTCGACTTGGGGGGCAACTGGTTGTTCCACATCTGGGTCTTGTTTCTTCAG
CTGTGGCGTTGACTCAACTTCTTCTTCTGGCTTCATGGAATCTCTACATTCTTGTTGGAAAGAGTCGATGA
TTTCCAAT

CTGGTAACGAGGGTGCTATCAGAGACAAGACCGCTGGTAGAAGAAACAACAGATCAAAGGATGTCACTGAC
TCTGCCACCACCAAGAAGTCCAACACCAGAAGGGCCACTGACCGCCACTCTAGAACTGGTAAGACTGACAC
CAAGAAGA

CATCAACTGGTCCTTGTGGAAATCCTGTTGCTGCTGCTGCTGCTGCTCCTGTTGTTGTTGTTGTTGTTGAA
GCTGTCTCTTTGGGACGAGGCTGCTTGCCCATTCCAGTTGTGCGTCCATTAAGTCTTGTTTGTTCCTGTAT
TAGTGTGT

AGATATTTTGGATTGCTTACTGGACGCTGAGGGTTTGTTTGTCGTGAGTGTTGGAGGAGGAGGAGGAGCCG
ATGGTACTGACGTTGCAACCGAAGGAGGAGGAGGAGGTGGTAGTGGTGCTAAAGGAATAGATGATGCTGAC
AAGGCAGA

AACTGGATGAAGATATTTTGGATTGCTTACTGGACGCTGAGGGTTTGTTTGTCGTGAGTGTTGGAGGAGGA
GGAGGAGCCGATGGTACTGACGTTGCAACCGAAGGAGGAGGAGGAGGTGGTAGTGGTGCTAAAGGAATAGA
TGATGCTG

TCTTCTACGCAAACGTCAACATCATCTTCATCCTCACTGTCGTCAACGCCGTCCTCTTCATCATCCCCATC
AACAATCACTTCTGCACCTTCAACCTCCTCCACACCATCCACTACTGCCTATAATCAAGGAAGCACTATCA
CCAGTATT

CTTGGATAAGATGGTCAGACGATACTTGTACAGTAGGCCCTCGCTGCTGCGACTGCGACTGCGACTGTGAC
TGTGACTGTGACTGGGGTTGTGGTTGTGGTTGTGACTGTTGTTGTTGTTGCTGCTGTTGTTTTTTCTGCCT
GGAAGTAT

TGACCGTTCTCGTTCCCCTCTTCTCCATTGCTCACATCAGACTCAGGCTCAGACTCAGACTCAGACCCAGA
TCCATCGTCACCTGCCAGAAGAGCGTCCCCAAACAGATTACCAGAATTCTGTACTTCCATGTTGTATACAG
CTTGTGCG

CAAGTTCATAACGTAACTACCTCTCTTGATAGCATCTTCAACAGTGAACAAGTTCTTGCCTGGAACCCAAC
CGTCTTCGATGGCAGCCTTCCAAGAAGCACCATCTTTACGGACACCAATGATAACGTTCAAACCGTTGTCT
CTCAAGTT

GAGTGGGGAGTAGGGTAATGGAGGGTAAGTTGAGAGACAGGTTGATCAGGGTTAGAGTAGGGTAGTGTTAG
GGTTAGGTGTGTGTGTGTGTGTGGGTGTGGTGTGTGGGTGTGTGGGTGTGGTGTGGTGTGTGTGTGTGTGG
TGTGTGGG

GACTTGGTCTTGTCGAACACGCCATACTGGTCACAGTCCATCACAGAACCGCAGTAGGGGTTTCCCTTGCC
AGGAACCCATAAGTCAGCTGAGCCTGTGTCTAACAGCACAGTGAGGTTTTGTGAAGGTGTACCAATGGCTA
GCTCAACA

GATTGCTTACTGGACGCTGAGGGTTTGTTTGTCGTGAGTGTTGGAGGAGGAGGAGGAGCCGATGGTACTGA
CGTTGCAACCGAAGGAGGAGGAGGAGGTGGTAGTGGTGCTAAAGGAATAGATGATGCTGACAAGGCAGAGG
ATGTTGAA

ATGGTCAGACGATACTTGTACAGTAGGCCCTCGCTGCTGCGACTGCGACTGCGACTGTGACTGTGACTGTG
ACTGGGGTTGTGGTTGTGGTTGTGACTGTTGTTGTTGTTGCTGCTGTTGTTTTTTCTGCCTGGAAGTATCT
TTCACAGC

ACTTTTGCTGTGTATTTCTATATGAGGCTAGTTGGTTGGTGGGAACTGGAAGTGAAAGTTCTGTTCTTTAG
GGTGCAGCGGTTGCTGTTGCTGTTGCTGTTGCTGTTGCTGCTGTGGTTGGATTGATGCTTGTGATGTTTGT
GGTGCCTG

GCCGAGGAAGAACTGGATGAAGATATTTTGGATTGCTTACTGGACGCTGAGGGTTTGTTTGTCGTGAGTGT
TGGAGGAGGAGGAGGAGCCGATGGTACTGACGTTGCAACCGAAGGAGGAGGAGGAGGTGGTAGTGGTGCTA
AAGGAATA

TACCACTGAAAGTACCAACTCCAGCACTAATGCTACTACCACTGCCAGCACCAACGTCAGGACTAGTGCTA
CTACCACTGCCAGCATCAACGTCAGGACTAGTGCGACTACCACTGAAAGTACCAACTCCAACACTAGTGCT
ACTACCAC

TCCACTTATGTCTCTTCGAGCAAGCAAGCTTCCTCCACTAGCGAGGCTCACTCTTCCAGTGCTGCCTCTTC
GACCGTGTCCCAAGAAACAGTCTCCTCTGCTCTACCAACTTCTACCGCCGTTATTTCTACTTTCTCTGAAG
GTTCTGGT

AGTGAAAGTTCTGTTCTTTAGGGTGCAGCGGTTGCTGTTGCTGTTGCTGTTGCTGTTGCTGCTGTGGTTGG
ATTGATGCTTGTGATGTTTGTGGTGCCTGTGGTGCTTGTGGTACAGTTTTGCTGCCAGTTTGCTGCGAGTA
TGTAATTA

GAAATTGAAGATTTGGTTGACAGAATTGAAAACCCAGAAAAGTACGCTGCTGCTGCTCCAGCTGCTACCTC
CGCTGCTTCCGGTGACGCTGCTCCAGCTGAAGAAGCTGCTGCTGAAGAAGAAGAAGAATCTGATGACGACA
TGGGTTTC

ACAGGGTAACGAGTGGGGAGTAGGGTAATGGAGGGTAAGTTGAGAGACAGGTTGATCAGGGTTAGAGTAGG
GTAGTGTTAGGGTTAGGTGTGTGTGTGTGTGTGGGTGTGGTGTGTGGGTGTGTGGGTGTGGTGTGGTGTGT
GTGTGTGT

AAATTCATATCTTGGATAAGATGGTCAGACGATACTTGTACAGTAGGCCCTCGCTGCTGCGACTGCGACTG
CGACTGTGACTGTGACTGTGACTGGGGTTGTGGTTGTGGTTGTGACTGTTGTTGTTGTTGCTGCTGTTGTT
TTTTCTGC

GATTACCACTGAAAGTACCAACTCCAGCACTAATGCTACTACCACTGCCAGCACCAACGTCAGGACTAGTG
CTACTACCACTGCCAGCATCAACGTCAGGACTAGTGCGACTACCACTGAAAGTACCAACTCCAACACTAGT
GCTACTAC

TTGAGCCGTCTGGAAACATCGCTGCTATATCACCAATCAATCCGACTGCTGCTCTTGAGGTAGCATCCTCA
CTGTACAGCTGAGGATCTTCTGCAACTTGTGCAATGAACTGGAATATAGTACCAACATAAGGGAACAGAGC
TTCAGGTT

TCACATCCTCGGATCCAATCTTAGTAACAGCATCGCCACTATCGCCTGCACCGCCAGCACCAGCAGCAGCA
GCCTTTTGCTCATCATGCTGGTGCTCATTCGTCGGTGTAGCAGTCTTATCCATTCTTGCGGCGTCGTCCTC
ACCATCGT

CCATTGCGACAGTGTTCCAAGGGACTCGTGCTCTTTCGATTGCCATCACTGCACCGAACACGATGATGTGT
ACACATGTTCCAAACTTTCCCAGACATTTGACGATGGTCCTTCTGCTTCCACTACTAAATTATTGGACCGG
TTGAAGCA

CTGTTCTTTAGGGTGCAGCGGTTGCTGTTGCTGTTGCTGTTGCTGTTGCTGCTGTGGTTGGATTGATGCTT
GTGATGTTTGTGGTGCCTGTGGTGCTTGTGGTACAGTTTTGCTGCCAGTTTGCTGCGAGTATGTAATTATG
GGGTGAGC

GAATGTATAAATAATAATAAACTAAGTTTACAACAACAAAGCAGCGGCACCAGCCAACAAAGCACCAGCAG
CTGGCAAAGCCTTAGCAGCAGCACCAGTGTAAGAGGTGACAGAGTGAGTTGGAGCAGCAGAGGTGGTGTTC
TTTGGAGC

AATTGTTTGATTGAGCCGTCTGGAAACATCGCTGCTATATCACCAATCAATCCGACTGCTGCTCTTGAGGT
AGCATCCTCACTGTACAGCTGAGGATCTTCTGCAACTTGTGCAATGAACTGGAATATAGTACCAACATAAG
GGAACAGA

GAGTGTCCTTGTACTTCAAGTATACTTCACGCTGTTTCATTCTATGTTCTGACAATTCTTTGTTCCTCTGT
TCGACTTGGGGGGCAACTGGTTGTTCCACATCTGGGTCTTGTTTCTTCAGCTGTGGCGTTGACTCAACTTC
TTCTTCTG

GTCATCGACGGTGACGGTAGCGGTGGAAACCAAAGCTGGGGAGACAGTTTCAGAACAGACGTGGTCTTCAC
AAGAAGTGATGGTGACCAAAGTGGTAGATTCTTGGCTGACAGTAGCAGTGGTAACGTTAGCAGCGGCAGAA
GCGACAGC

ACTCTTGCCCTGAAGAGGACTTCTTTGTCGCTATCCTTAGCCTCATCCAAGTCAACAAACTTGACACGCTT
CTGACCAGTTCTGTCAGAGTCACGAGACTGGATCAATGGCAACTTACCGTAGTTGTCCTTTGCGGTGTCTT
CGGCAGCG

TGATGTACACCCTCTCATCGAGATTGAGTTTCAATAAATCCAAAGGTGTCTCCATTTTCTTGAGTGTGCGT
GATGTTGTGTGTGTGTGTGTATTTATCTGCGTGTCTCTCCCCTGTTGTATTGGTTGCTAGAATGGATGGAA
ACATCCTG

GATTGAGCGGCATCGGACAACAAGTTCATAACGTAACTACCTCTCTTGATAGCATCTTCAACAGTGAACAA
GTTCTTGCCTGGAACCCAACCGTCTTCGATGGCAGCCTTCCAAGAAGCACCATCTTTACGGACACCAATGA
TAACGTTC

CTATATGTCCCTACGGCCTTGTCTAACACCATCCAGCATGCAATACAGTGACATATATATACACCACACCC
ACACACCACACCCACACCACACCCACACCCACACACACCACACACCCACACACTCTCTTACATCTACCTTT
ACTCTCGC

CAACTTCTGAATCATCGACGTCTTCTACGCAAACGTCAACATCATCTTCATCCTCACTGTCGTCAACGCCG
TCCTCTTCATCATCCCCATCAACAATCACTTCTGCACCTTCAACCTCCTCCACACCATCCACTACTGCCTA
TAATCAAG

ACCCACACACCCACACCCACACACCCACACCACACCCACACCCACACACACCCACACAGTACCTCAACACA
ACCCCTAATCCAACCCTGATGAACCTGCCTCCAAACCTACCCTCCATTACCCTACCTCTCCACTCGTTACC
CTGTCTCA

TCTATAGAAGATGCCAATGAACCTGCGGAGGATTCCAATGAACCTGTGGAGGATTCCAACAAACCTGTGAA
GGATTCCAACAAACCTGTGGAGGATTCCAACAAACCTGTGGAGGATTCCAACAAACCTGTGGAGGATTCCA
ACAAACCT

GAATGCCAGAATTGATTCCAGTCTTGTCTGAAACCATGTGGGACACCAAGAAGGAAGTCAAGGCTGCTGCT
ACTGCCGCCATGACCAAGGCTACCGAAACTGTTGACAACAAGGATATTGAACGTTTCATTCCAAGTTTGAT
TCAATGTA

ACTAATAGTAATCTTGGTCTGAACATTGTGTTCCGTCTGATTGAAACTGAGGACACCAATGTCAGATTGAC
CATTGTGGTGACTTCTAGAACGCTTCCTCGAGTGCAGGAGGTGATTAACCAGATTAAAGATTTTTACAACA
AATCAGGC

GTAATGGCTGTGCATTCAGCGATTAAAGTCCCGTCATCAAGTTCAAGCACTGGCACTGTGCCTGAATAGTT
CTTGGCAAGAAATTCAGGCTTCTTGTGCTCTCCCTTCCAGAGGTTGATCCTCACAAATTGCACACTTGATA
GCATGTTC

AATTCTCAACAAGAGACAACACCTGGGACATCAGCTGTTCCAGAGAACCATCATCATGTCTCTCCTCAACC
TGCTTCAGTACCACCTCCACAGAATGGACAGTACCAACAGCACGGCATGATGACCCCAAACAAAGCTATGG
CCTCTAAC

CTAGCGGCACCACCAGCAGCCATCCCAGCAATGATCTGAGGAAACCACATCACTACACATCTGGCTGGTGA
GATGGCTCTGGCCAAACACAAAGAGAGAGAAACGGCTGGGTTCAAAGCCCCACCAGAAACACCAGATAGAA
AACATCAC

ATGAAATCGAGGCTAGTTTCAAGATAACTTGTCTCATCTCATGGCTGAATCCAGTGAGTTCCATGTAGCCA
CCCCTTTGACCACATTCACCAGAAACACCCTTAGAAGTCGAATGCAAAGAAGCTAGCTGAACATTATCGAA
TTTACCTG

TTTTGACAGGTATAGGATTTGGAGTAGGGTAGTAAATATAAGGATTCTCCTGTTGCTGTTGCTGTTGTTGG
TCTTCTTGTTGTTGTTGTTGTTGCTGTTGCTGTTGTTGCTGGGGAGCAGAGAGTGGTGGAGGTGGCATAAA
CATCATTG

GACCCGCTGTCTGATTCGCATACCCACAGTCATCAACTGGTCCTTGTGGAAATCCTGTTGCTGCTGCTGCT
GCTGCTCCTGTTGTTGTTGTTGTTGTTGAAGCTGTCTCTTTGGGACGAGGCTGCTTGCCCATTCCAGTTGT
GCGTCCAT

GAACGTGGACGACTTGGTCTTGTCGAACACGCCATACTGGTCACAGTCCATCACAGAACCGCAGTAGGGGT
TTCCCTTGCCAGGAACCCATAAGTCAGCTGAGCCTGTGTCTAACAGCACAGTGAGGTTTTGTGAAGGTGTA
CCAATGGC

ACTAACGAACCATTGACTGAAATCAAGAACGTTATTGTCTCTAACAGAGTTGCTGCTCCAGCCAGAGCTGG
TGCCGTTGCTCCAGAAGACATCTGGGTTAGAGCCGTCAACACTGGTATGGAACCAGGTAAGACTTCTTTCT
TCCAAGCT

ACTTGGTCTTGAGATTGAGAGGTGGTGGTAAGAAGAGAAAGAAGAAGGTCTACACCACCCCAAAGAAGATC
AAGCACAAGCACAAGAAGGTCAAGTTGGCTGTCTTGTCCTACTACAAGGTCGATGCTGAAGGTAAGGTTAC
CAAATTGA

TCGACATCCGCAACTTCTGAATCATCGACGTCTTCTACGCAAACGTCAACATCATCTTCATCCTCACTGTC
GTCAACGCCGTCCTCTTCATCATCCCCATCAACAATCACTTCTGCACCTTCAACCTCCTCCACACCATCCA
CTACTGCC

GGCGAGGACTTGTTGGCTTTGAACGTGGACGACTTGGTCTTGTCGAACACGCCATACTGGTCACAGTCCAT
CACAGAACCGCAGTAGGGGTTTCCCTTGCCAGGAACCCATAAGTCAGCTGAGCCTGTGTCTAACAGCACAG
TGAGGTTT

TGCATTCAGCGATTAAAGTCCCGTCATCAAGTTCAAGCACTGGCACTGTGCCTGAATAGTTCTTGGCAAGA
AATTCAGGCTTCTTGTGCTCTCCCTTCCAGAGGTTGATCCTCACAAATTGCACACTTGATAGCATGTTCTT
CTCAGCCA

CCAAGACCTTCTGGTAACGAGGGTGCTATCAGAGACAAGACCGCTGGTAGAAGAAACAACAGATCAAAGGA
TGTCACTGACTCTGCCACCACCAAGAAGTCCAACACCAGAAGGGCCACTGACCGCCACTCTAGAACTGGTA
AGACTGAC

ACATACGAGTTCGTCTACCAGCATTGCCTCCATATCGTTCACATCATTCAGTTTCTCATCAGATTCAAGCA
CCAGCAGCTCTTCCTCTGCTTCTTCAGATTCTTCATCCTCCTCATCCTTTTCCATCTCTTCGACATCCGCA
ACTTCTGA

CACAGTGGTGCTATCCATAATTTGCTGCTGCAGGGCCTTCAGGCCCTGCACGCTAGCACACACCTTGGCGT
CTGCAGTAGTGGTCTCCTTCTCAGTTTTGCTGCACCATTTGAAAGTGTTTGTCCACATTCTACTGAAGAAA
ACTACGCT

GAGATTGAGAGGTGGTGGTAAGAAGAGAAAGAAGAAGGTCTACACCACCCCAAAGAAGATCAAGCACAAGC
ACAAGAAGGTCAAGTTGGCTGTCTTGTCCTACTACAAGGTCGATGCTGAAGGTAAGGTTACCAAATTGAGA
AGAGAATG

ATCCAGCATGCAATACAGTGACATATATATACACCACACCCACACACCACACCCACACCACACCCACACCC
ACACACACCACACACCCACACACTCTCTTACATCTACCTTTACTCTCGCTGTCACACCTTACCCGGCTTTC
TGACCGAA

AAACTTCCAACAACAACCACAATACACTCAAAATTATCAACAACAACCACAATACATTCAACCTCATCAAC
AACAACAGCAGCAGCAGCAGCAGCAACAGCAGCAACAGGGATATACTCCTGACCAAGGTGTAAGCTTAATT
GATCTTTG

ACGGGACACAATGAAATCGAGGCTAGTTTCAAGATAACTTGTCTCATCTCATGGCTGAATCCAGTGAGTTC
CATGTAGCCACCCCTTTGACCACATTCACCAGAAACACCCTTAGAAGTCGAATGCAAAGAAGCTAGCTGAA
CATTATCG

TTAAACGAAAGATAAATTTTGAATTAAGGCGGTCGTTACATCATACCTGGCATTCCTGGCATACCACCTGG
CATACCACCAGCGCCAGCAGCTGCTGGTGGTTCTGGGGCATCAACAATAGCAACTTCGGTAGTAGCTAATA
GTGAGGCA

GGTGCTCATTCGTCGGTGTAGCAGTCTTATCCATTCTTGCGGCGTCGTCCTCACCATCGTCGTCCTCCTGT
TCATCGTCGTCCTCTCGTTCGTCCTCGTCATCATCATCGTCCTCATCATCATCATCGTCATCCTCTTCATC
TTCACCAC

CTATTTTCTTCTTCTTCGATTTTGATTTAGATTCACTGCTTGCCGATGAAGATGGAGGCTGGGATGATGTT
GACTGAGGACTTGTCACAGTAATGTTCACATTGGCAAGTGGTGACTGAGAGTTGAATTCAGTTGAAACGTC
TCTATTAA

GTTGGTAATGTGCCCAGTTAGAAGCCATAGCTTTGTTTGGGGTCATCATGCCGTGCTGTTGGTACTGTCCA
TTCTGTGGAGGTGGTACTGAAGCAGGTTGAGGAGAGACATGATGATGGTTCTCTGGAACAGCTGATGTCCC
AGGTGTTG

TACCCTACTCAAATTCATATCTTGGATAAGATGGTCAGACGATACTTGTACAGTAGGCCCTCGCTGCTGCG
ACTGCGACTGCGACTGTGACTGTGACTGTGACTGGGGTTGTGGTTGTGGTTGTGACTGTTGTTGTTGTTGC
TGCTGTTG

CGTCGGTGTAGCAGTCTTATCCATTCTTGCGGCGTCGTCCTCACCATCGTCGTCCTCCTGTTCATCGTCGT
CCTCTCGTTCGTCCTCGTCATCATCATCGTCCTCATCATCATCATCGTCATCCTCTTCATCTTCACCACCG
TCATCATT

TGTTGGCTTTGAACGTGGACGACTTGGTCTTGTCGAACACGCCATACTGGTCACAGTCCATCACAGAACCG
CAGTAGGGGTTTCCCTTGCCAGGAACCCATAAGTCAGCTGAGCCTGTGTCTAACAGCACAGTGAGGTTTTG
TGAAGGTG

TCATCATGCTGGTGCTCATTCGTCGGTGTAGCAGTCTTATCCATTCTTGCGGCGTCGTCCTCACCATCGTC
GTCCTCCTGTTCATCGTCGTCCTCTCGTTCGTCCTCGTCATCATCATCGTCCTCATCATCATCATCGTCAT
CCTCTTCA

TTGGTCGAATTGGAACATCCTAGAATTAGGAATTTCTGTTTGACCGTTCTCGTTCCCCTCTTCTCCATTGC
TCACATCAGACTCAGGCTCAGACTCAGACTCAGACCCAGATCCATCGTCACCTGCCAGAAGAGCGTCCCCA
AACAGATT

ATGCACTGCTGTGGGTACGGCCCATTCTGTGGAGGTGGTACTGAAGCAGGTTGAGGAGAGGCATGATGGGG
GTTCTCTGGAACAGCTGATGAAGCAGGTGTTGTTGTCTGTTGAGAGTTAGCCTTAGTGGAAGCCTTATCAT
ATTCTTGA

CCAAAAGGAATCTACTCTACACTTGGTCTTGAGATTGAGAGGTGGTGGTAAGAAGAGAAAGAAGAAGGTCT
ACACCACCCCAAAGAAGATCAAGCACAAGCACAAGAAGGTCAAGTTGGCTGTCTTGTCCTACTACAAGGTC
GATGCTGA

ATAGATGGTTGTTGGTAATGTGCCCAGTTAGAAGCCATAGCTTTGTTTGGGGTCATCATGCCGTGCTGTTG
GTACTGTCCATTCTGTGGAGGTGGTACTGAAGCAGGTTGAGGAGAGACATGATGATGGTTCTCTGGAACAG
CTGATGTC

ACAGGGATTACTACGTCATCTTCTATATGCTTAAACCTGAGAGACTTGATCTTCTTGGTTTTGTTCACTTG
CAATGCTGGCGCCTCTGAGGCACCATTGGCTGGCAAGTCGATGGCATATAGATCTTTAATGTTGTCTGACA
GGTTCTCA

TGTTCCTCTGTTCGACTTGGGGGGCAACTGGTTGTTCCACATCTGGGTCTTGTTTCTTCAGCTGTGGCGTT
GACTCAACTTCTTCTTCTGGCTTCATGGAATCTCTACATTCTTGTTGGAAAGAGTCGATGATTTCCAATAT
TTCATTAC

ACTTTTCGGATATTTTACAGGGCGATCGCTAAGCTTAATCAACTTCTTCGACAGTTGGACCTTCAGCTTCT
GGAGCTGGAGGAGCACCACCTGGGAAACCACCTGGAGCTGCGCCTTCTGGAGCACCACCAGCTTGGTACAA
TTTAGACA

GGCATAAAAAGGCGAGGACTTGTTGGCTTTGAACGTGGACGACTTGGTCTTGTCGAACACGCCATACTGGT
CACAGTCCATCACAGAACCGCAGTAGGGGTTTCCCTTGCCAGGAACCCATAAGTCAGCTGAGCCTGTGTCT
AACAGCAC

TACCACCGAAAGTACCGACTCCAACACTAGTGCTACTACCACTGCTAGCACCAACTCCAGCACTAATGCCA
CTACCACTGCTAGCACCAACTCCAGCACTAATGCCACTACCACTGAAAGTACCAACGCTAGTGCCAAGGAG
GACGCCAA

GCTGGAGTTGGTAGTCGCATTGGTACTGGCATTAGCACTACCATGAATGCACGTGTTGCTGTCCTCATCAC
TGCTGCAATACTTTCTGCACCTGTCACTGCTATTGCTCTCCTGGAAGCTAGACGGTAACGCAACGATCGAC
ATGGAAGC

AAACCCAAGGTAGAGCGTATACTGGAAGCGGAGTTGGTAGCAGCCCTCTGGAGTGCCCTCTTGCTTTTATG
AGTCGACTTTGCAGTAGTGGTAGAGTTTGTCTTCTTGGTGGTGGTTGACCTCGTAGTGGTGGCAGTACTGG
TGCCATTG

CAGTCATGTGGAAGATAGCCAAAATCACGTGAATGCCATCCCAAGAAGGAATTCTCTGTTGAGCAGACCCA
CTTTAAGAAGCAACAATGGTTCTTCCTCTGCCAACCCCTTTGCTATCAATGTTTCTCCTGCATCTCAAATC
AGGGATGG

GTGTGTGTGTGGTGTGTGGGTGTGGTGGGTGTGGTGTGGGTGTGGGTGTGGGTGTGGGTGTGGGTGTGGTG
TGTATATCTATGTCACCTTATTGCATGCTGGATGGTGTTAGACAAGGCCGTAGGGACATATAGCATCTAGG
AAGTAACC

CTAGTGCGATTACCACTGAAAGTACCAACTCCAGCACTAATGCTACTACCACTGCCAGCACCAACGTCAGG
ACTAGTGCTACTACCACTGCCAGCATCAACGTCAGGACTAGTGCGACTACCACTGAAAGTACCAACTCCAA
CACTAGTG

ACTACGGTGACTTCACCACCATGTTGACTGGTATTGCCCCAGACCAAGTGACCAGAATGATCACCGGTGTT
CCATGGTACTCCAGCAGATTAAAGCCAGCTATCTCCAAGGCTCTATCCAAAGATGGTATCTACACTATCGC
AAACTAGG

TTTCGATGGTGGCCTGCATAAAATCAGGCCTTTGAAACGGAGCTTCCCCATCTCTTAGGATCGACTAACCC
ACGTCCAACTGCTGTTGACGTGGAACCTTTCCCCACTTCAGTCTTCAAAGTTCTCATTTGAATATTTGCTA
CTACCACC

GTGGTGTATTGAGTGATAACGTCATCGACGGTGACGGTAGCGGTGGAAACCAAAGCTGGGGAGACAGTTTC
AGAACAGACGTGGTCTTCACAAGAAGTGATGGTGACCAAAGTGGTAGATTCTTGGCTGACAGTAGCAGTGG
TAACGTTA

AAACTCCAGAAGTAAATGAGTCTATAGAAGATGCCAATGAACCTGCGGAGGATTCCAATGAACCTGTGGAG
GATTCCAACAAACCTGTGAAGGATTCCAACAAACCTGTGGAGGATTCCAACAAACCTGTGGAGGATTCCAA
CAAACCTG

TACTAAATTCATTAGATGTTCTAAAATCGTAAATACGATTCATCTGTGCAGACTCTTCACTGCTAGTGTTC
GATAGGACATCTGCATCGAAGTCCTCGTCGTCCTCTCCTGCAAAGGAGCTTTCATGCAAACTATCCAAAGA
TATTTCTG

CATCGTCGATGCTGAGCTATCAGTTGCTTCCGCACTCCAATGATGTGTCCCGGTCGAACTCCAGCTCGAGC
TTCCTTCCCTCCGTGCAGCAGCCTACGGAAGGTTCCGCTTCTGCATCGGAAACTTCTTCATCTGCATCACC
TTCACGTT

TCGTATCTGTGCCAACAAGTACATGACTACTGTCTCCGGTAGAGATGCTTTCCACTTAAGAGTCAGAGTCC
ATCCTTTCCATGTCTTGAGAATCAACAAGATGTTGTCTTGTGCCGGTGCGGATAGATTGCAACAAGGTATG
AGAGGTGC

TACTACCACCGAAAGTACCGACTCCAACACTAGTGCTACTACCACTGCTAGCACCAACTCCAGCACTAATG
CCACTACCACTGCTAGCACCAACTCCAGCACTAATGCCACTACCACTGAAAGTACCAACGCTAGTGCCAAG
GAGGACGC

CATTGACTGAAATCAAGAACGTTATTGTCTCTAACAGAGTTGCTGCTCCAGCCAGAGCTGGTGCCGTTGCT
CCAGAAGACATCTGGGTTAGAGCCGTCAACACTGGTATGGAACCAGGTAAGACTTCTTTCTTCCAAGCTTT
GGGTGTCC

CCCTTTCGATGGTGGCCTGCATAAAATCAGGCCTTTGAAACGGAGCTTCCCCATCTCTTAGGATCGACTAA
CCCACGTCCAACTGCTGTTGACGTGGAACCTTTCCCCACTTCAGTCTTCAAAGTTCTCATTTGAATATTTG
CTACTACC

GGTGGTGGTAAGAAGAGAAAGAAGAAGGTCTACACCACCCCAAAGAAGATCAAGCACAAGCACAAGAAGGT
CAAGTTGGCTGTCTTGTCCTACTACAAGGTCGATGCTGAAGGTAAGGTTACCAAATTGAGAAGAGAATGTA
GCAACCCA

GCCATACTGGTCACAGTCCATCACAGAACCGCAGTAGGGGTTTCCCTTGCCAGGAACCCATAAGTCAGCTG
AGCCTGTGTCTAACAGCACAGTGAGGTTTTGTGAAGGTGTACCAATGGCTAGCTCAACAGAATAAAAGCTT
TGCTCGTT

TGTCGAACACGCCATACTGGTCACAGTCCATCACAGAACCGCAGTAGGGGTTTCCCTTGCCAGGAACCCAT
AAGTCAGCTGAGCCTGTGTCTAACAGCACAGTGAGGTTTTGTGAAGGTGTACCAATGGCTAGCTCAACAGA
ATAAAAGC

TACCATCCCAAGGTTTGAATGGTTTCAACAGTTGTAGACGGTCTGAAGTTGGAGAAACTTTAACTTCAACG
GTGCTACGGTCTGCAGGTGGAGCTTGGTAAGTGTTCTCACCAGCATCATAACCTCTTTGAGGCAAACCATC
ACCATGTG

AGCTGCTACTTACCCTACTCAAATTCATATCTTGGATAAGATGGTCAGACGATACTTGTACAGTAGGCCCT
CGCTGCTGCGACTGCGACTGCGACTGTGACTGTGACTGTGACTGGGGTTGTGGTTGTGGTTGTGACTGTTG
TTGTTGTT

ACATGACTACTGTCTCCGGTAGAGATGCTTTCCACTTAAGAGTCAGAGTCCATCCTTTCCATGTCTTGAGA
ATCAACAAGATGTTGTCTTGTGCCGGTGCGGATAGATTGCAACAAGGTATGAGAGGTGCTTGGGGTAAGCC
ACACGGTT

TAGCTGGCCGTTTCAGAGGTAAGAGAGTTGTCTACTTGAAACATCTAGAAGACAACACTCTATTGGTCACT
GGTCCATTCAAGGTCAATGGTGTTCCATTGAGAAGAGTCAACGCTCGTTATGTCATTGCTACCTCTACCAA
AGTCTCCG

TGGCCGCTCGTGTCGACATTGGTCAAATTATCTTCTCTGTCAGAACCAAGGACAGCAACAAGGATGTTGTC
GTTGAAGGTTTGAGAAGAGCCAGATACAAGTTCCCAGGTCAACAAAAGATTATTTTGTCTAAGAAGTGGGG
TTTCACCA

GTGACGGTAGCGGTGGAAACCAAAGCTGGGGAGACAGTTTCAGAACAGACGTGGTCTTCACAAGAAGTGAT
GGTGACCAAAGTGGTAGATTCTTGGCTGACAGTAGCAGTGGTAACGTTAGCAGCGGCAGAAGCGACAGCGG
CGACAGCG

CATATCGTTCACATCATTCAGTTTCTCATCAGATTCAAGCACCAGCAGCTCTTCCTCTGCTTCTTCAGATT
CTTCATCCTCCTCATCCTTTTCCATCTCTTCGACATCCGCAACTTCTGAATCATCGACGTCTTCTACGCAA
ACGTCAAC

CTCGATGCATAACCCTGGTCGACCCGCTGTCTGATTCGCATACCCACAGTCATCAACTGGTCCTTGTGGAA
ATCCTGTTGCTGCTGCTGCTGCTGCTCCTGTTGTTGTTGTTGTTGTTGAAGCTGTCTCTTTGGGACGAGGC
TGCTTGCC

GTCTGAAGTTGGAGAAACTTTAACTTCAACGGTGCTACGGTCTGCAGGTGGAGCTTGGTAAGTGTTCTCAC
CAGCATCATAACCTCTTTGAGGCAAACCATCACCATGTGGTGGTTTCAACATGAACTCATTACCATCCTTG
TCCTTTAA

CGTTCATGATACGTGTCTTCATGACGTCTGCTGGAGAGCACACAGTGGTGGCCACCAGGCCAGCCAACAGG
GACGCTGTCAAGTGTGTATAGTTCTTGGATGCGTCGAAATCTAATTTTGTGACCAAGTAGTTCTTAAACAC
GTCATACG

TTCTCTATGTGAATAATAAATAGCCTTGAATCTTTCTAGTAGTGGAGTAGGGTCAAGATTAGGATGTGTCC
TGCCCCATGTTCTGCAGTCTGGTGGTTGAGAAGGTATGCCAGGTGGTTTATTAACTATGAAGTAATGCGTG
TTTTCAAA

ATTTCAGTCAAATTCCAGATTATAAGGAATATGATCAAAACCATTGCGACAGTGTTCCAAGGGACTCGTGC
TCTTTCGATTGCCATCACTGCACCGAACACGATGATGTGTACACATGTTCCAAACTTTCCCAGACATTTGA
CGATGGTC

TCGTCTACCAGCATTGCCTCCATATCGTTCACATCATTCAGTTTCTCATCAGATTCAAGCACCAGCAGCTC
TTCCTCTGCTTCTTCAGATTCTTCATCCTCCTCATCCTTTTCCATCTCTTCGACATCCGCAACTTCTGAAT
CATCGACG

ATGCGACTACCAACTCCAGCACTAATGCTACTACCACTGCCAGCACCAACGTCAGGACTAGTGCTACTACC
ACTGCCAGCATCAACGTCAGGACTAGTGCGATTACCACTGAAAGTACCAACTCCAGCACTAATGCTACTAC
CACTGCCA

GATAAATTTTGAATTAAGGCGGTCGTTACATCATACCTGGCATTCCTGGCATACCACCTGGCATACCACCA
GCGCCAGCAGCTGCTGGTGGTTCTGGGGCATCAACAATAGCAACTTCGGTAGTAGCTAATAGTGAGGCAAC
ACCAGAAG

TCAGAGTCCGCTGAGGATGAATCAGTAAATGTATTACCTGACTCAGGTGATGGAGTGCTCAGAGGCGTTCC
AACTGATGATGGATACTGCGGAAACTGTGATTGTGGCCCAGGTGGAAAGTACATAGGCGACATTTGATAAG
GTGTATAC

TACCTAAAATGATATCCTTCAAAGATATGTTTAATGAATATGCAGCCACAGCTTTGAATTCAGATTGACCT
TGTGTTGCATTAAGTCAGACACCTCTAGATTTGTGTGGCTGCTCTCAAGAAAGGATGTTGAAGAAGATTTA
TTTACGGA

AAAGAACAGACCAAGACCTTCTGGTAACGAGGGTGCTATCAGAGACAAGACCGCTGGTAGAAGAAACAACA
GATCAAAGGATGTCACTGACTCTGCCACCACCAAGAAGTCCAACACCAGAAGGGCCACTGACCGCCACTCT
AGAACTGG

ACTACAACATCCAAAAGGAATCTACTCTACACTTGGTCTTGAGATTGAGAGGTGGTGGTAAGAAGAGAAAG
AAGAAGGTCTACACCACCCCAAAGAAGATCAAGCACAAGCACAAGAAGGTCAAGTTGGCTGTCTTGTCCTA
CTACAAGG

GGTGGCCTGCATAAAATCAGGCCTTTGAAACGGAGCTTCCCCATCTCTTAGGATCGACTAACCCACGTCCA
ACTGCTGTTGACGTGGAACCTTTCCCCACTTCAGTCTTCAAAGTTCTCATTTGAATATTTGCTACTACCAC
CAAGATCT

ATTACACCAGAAGAGGCAAGAAGAAGCCCAATTACAACAGCAGCAAGCCCAATTGCTACAACAGCAAGCCC
AGTTCCAGCAACAACAACCCTTGAAGCAAACAAGGACTGGGAACCAGTCTATATCGGATAAATACAGCGAC
TTGAATAC

AACAGATGTGGTCGTCGTTCTTTCCATGTTCAAAAGAAGACCTGTTCCTCCTGTGGTTATCCAGCTGCTAA
GACCAGATCTTACAACTGGGGTGCCAAGGCTAAGAGAAGACACACTACTGGTACTGGTAGAATGAGATACT
TGAAACAC

AAGCTTCATGCTTTGGATACTCATCGATACATTCAGACTCCCCCACACCCAAGTGGAGATCCGCCAACTGA
ACAATTTTGAACTTTCCTTCATCAGTTTCTTGCAGCCGTTGAGCTCCTGTGTCTACAGGACGAGTACCAAC
GAATTTGT

CTGACGTTGTCGTTTTGCCACCAAAGGAAATCGTCAAGAGCAACACTTCCTCCAAGAAGGCTGACGTCCCA
CCTCCATCCGCTGACCCATCCAAGGCTAGAAAGAACAGACCAAGACCTTCTGGTAACGAGGGTGCTATCAG
AGACAAGA

TCTGCCTTGCAAGCTTCATGCTTTGGATACTCATCGATACATTCAGACTCCCCCACACCCAAGTGGAGATC
CGCCAACTGAACAATTTTGAACTTTCCTTCATCAGTTTCTTGCAGCCGTTGAGCTCCTGTGTCTACAGGAC
GAGTACCA

AGTACCGACTCCAACACTAGTGCTACTACCACTGCTAGCACCAACTCCAGCACTAATGCCACTACCACTGC
TAGCACCAACTCCAGCACTAATGCCACTACCACTGAAAGTACCAACGCTAGTGCCAAGGAGGACGCCAATA
AAGATGGC

GACTCCAACACTAGTGCTACTACCACCGAAAGTACCGACTCCAACACTAGTGCTACTACCACTGCTAGCAC
CAACTCCAGCACTAATGCCACTACCACTGCTAGCACCAACTCCAGCACTAATGCCACTACCACTGAAAGTA
CCAACGCT

GGGTTGTCCCGCACCATTCAACGGCGGCTATCTGGCGATGTTGAAAGAGGGGGGGAGTGGAGAGGGCTCTG
AGGGTCAAGTGGGAGGCTGTGACAGTTCTCATAAGCTGGGCACTGAGATACATGGAAAGAAATAGGCTGTT
CACAGTGT

CCTTAGCATTCTTCAAGACTTGAGTTTGGTATTCCTTGAATTCTGGAGTGGCAGCTTGCTTCAAAGCAGTG
GCCAAAGCAGCAATGGTATGGTTGTGTGGACCACCTTGGTGACCTGGGAAAACAGAGAAGTTAATTGGGTT
TTCCAAGT

ATCGAATTCTTCCTTGGTAGCAGTGGTGTTGCTGTCTAACCAAGCAATAGTTTCTTCAGCCTTCTTAGTGA
CAGCGTCCTTGTCAGCTTGCTCTAGCTTGTCACCAGCTTCAGAAATGGTGTTCTTCAAAGAGTAAGCAATG
GATTCCAA

TTTCAGAGGTAAGAGAGTTGTCTACTTGAAACATCTAGAAGACAACACTCTATTGGTCACTGGTCCATTCA
AGGTCAATGGTGTTCCATTGAGAAGAGTCAACGCTCGTTATGTCATTGCTACCTCTACCAAAGTCTCCGTG
GAAGGTGT

AACGTCAGGACTAGTGCGATTACCACTGAAAGTACCAACTCCAGCACTAATGCTACTACCACTGCCAGCAC
CAACGTCAGGACTAGTGCTACTACCACTGCCAGCATCAACGTCAGGACTAGTGCGACTACCACTGAAAGTA
CCAACTCC

AATTCCAGATTATAAGGAATATGATCAAAACCATTGCGACAGTGTTCCAAGGGACTCGTGCTCTTTCGATT
GCCATCACTGCACCGAACACGATGATGTGTACACATGTTCCAAACTTTCCCAGACATTTGACGATGGTCCT
TCTGCTTC

CCAAGAAGGAATTCTCTGTTGAGCAGACCCACTTTAAGAAGCAACAATGGTTCTTCCTCTGCCAACCCCTT
TGCTATCAATGTTTCTCCTGCATCTCAAATCAGGGATGGTTTGTACTGGGATAGGTCGGTCCATGATTTAC
GTGAGCTA

CCAAAGTAACGCAAACACTTGTCTTTCTCTTTCTCTGTTTTGTTCCGCCAGCTGCAAAGCTGCCTTTGAAG
TGTTCTGTGAGTCCATGCTCAGGACTTTGATTTCTGCATGCTTCACGATCTGCGCATACTCCTCGATGGTT
TTAGATTT

AGCAGCATCAACATATCTATGTTCAAAAGTCAAAGAAGATATCAACTCTTCTGCGACTGACTCAACTTCCT
CTGGGAATTTCTGAACAGCGATGGACATGGCCTCACGCCATCTTTTCGCTGATTGGTATGCCCCCATTGCT
TCTTTGAG

ACTTTGTCCGACTACAACATCCAAAAGGAATCTACTCTACACTTGGTCTTGAGATTGAGAGGTGGTGGTAA
GAAGAGAAAGAAGAAGGTCTACACCACCCCAAAGAAGATCAAGCACAAGCACAAGAAGGTCAAGTTGGCTG
TCTTGTCC

TGTCTTGTCCTACTACAAGGTCGATGCTGAAGGTAAGGTTACCAAATTGAGAAGAGAATGTAGCAACCCAA
CTTGTGGTGCTGGTGTTTTCTTGGCTAACCACAAGGACAGATTGTACTGTGGTAAGTGTCATTCCGTCTAC
AAGGTTAA

ATATTCTGTCACAATTATCAAGCAGCAAGTTATATAAAGTTGTTTCTCACAGCTTCTTGCACTCCCTTGCG
GTGCCTTTCCTGCACCACTAGTGTAACTGCTGATGTCTTCCATTTGATGTCAGAATCGATTTCTTGTTACG
CCACGAAC

ATCAACGTCAGGACTAGTGCGATTACCACTGAAAGTACCAACTCCAGCACTAATGCTACTACCACTGCCAG
CACCAACGTCAGGACTAGTGCTACTACCACTGCCAGCATCAACGTCAGGACTAGTGCGACTACCACTGAAA
GTACCAAC

GTGGTGGGTGGAGAATCATCCATAATGAGGACGACAACAACAGTCATGTGGAAGATAGCCAAAATCACGTG
AATGCCATCCCAAGAAGGAATTCTCTGTTGAGCAGACCCACTTTAAGAAGCAACAATGGTTCTTCCTCTGC
CAACCCCT

AGTTGGTAGTCGCATTGGTACTGGCATTAGCACTACCATGAATGCACGTGTTGCTGTCCTCATCACTGCTG
CAATACTTTCTGCACCTGTCACTGCTATTGCTCTCCTGGAAGCTAGACGGTAACGCAACGATCGACATGGA
AGCTGTCG

AGTGTTCCAAGGGACTCGTGCTCTTTCGATTGCCATCACTGCACCGAACACGATGATGTGTACACATGTTC
CAAACTTTCCCAGACATTTGACGATGGTCCTTCTGCTTCCACTACTAAATTATTGGACCGGTTGAAGCATA
ATTCCACC

TTTACTATATACTTTTGCTGTGTATTTCTATATGAGGCTAGTTGGTTGGTGGGAACTGGAAGTGAAAGTTC
TGTTCTTTAGGGTGCAGCGGTTGCTGTTGCTGTTGCTGTTGCTGTTGCTGCTGTGGTTGGATTGATGCTTG
TGATGTTT

GCGCCTTCTGGAGCACCACCAGCTTGGTACAATTTAGACATGATTGGGTTGGCAACCTCTTGCAATTCCTT
CAATTGGTCATCGAATTCTTCCTTGGTAGCAGTGGTGTTGCTGTCTAACCAAGCAATAGTTTCTTCAGCCT
TCTTAGTG

GCATTGCCTCCATATCGTTCACATCATTCAGTTTCTCATCAGATTCAAGCACCAGCAGCTCTTCCTCTGCT
TCTTCAGATTCTTCATCCTCCTCATCCTTTTCCATCTCTTCGACATCCGCAACTTCTGAATCATCGACGTC
TTCTACGC

GTAAAGAGAACCTTGCCTGGAGTCATAGCACTAGCGGCACCACCAGCAGCCATCCCAGCAATGATCTGAGG
AAACCACATCACTACACATCTGGCTGGTGAGATGGCTCTGGCCAAACACAAAGAGAGAGAAACGGCTGGGT
TCAAAGCC

CGTTCTCTAGGCTGATGGTAGGCAGGGCGATGCCATACTGGTGAAGTGTGGTCCGTCGGTCTGTCTCCTCC
AAACGTTGATGTTCTCCTGGTAGCTTTCTCATCACCGTCAATATTACAAGTGTAAGAATGAGAAATGCTCT
ATATGAGG

TGAATTTGTGACCCTTGTTGATACCTCTGGATTTCTTACCAGTGGCAGTCAAACCTCTAGCTTCACGGTGC
TTGTGAACTGGGTCACAGATCCAGTTGTAACGAGCATCTCTTCTGATAGCCTTGTGTTGAGGGTCGACCAA
GATAACTT

AAACCACGACCTATTTTCTTCTTCTTCGATTTTGATTTAGATTCACTGCTTGCCGATGAAGATGGAGGCTG
GGATGATGTTGACTGAGGACTTGTCACAGTAATGTTCACATTGGCAAGTGGTGACTGAGAGTTGAATTCAG
TTGAAACG

AGGTAACGACGTCGAAGACGCTGACGTTGTCGTTTTGCCACCAAAGGAAATCGTCAAGAGCAACACTTCCT
CCAAGAAGGCTGACGTCCCACCTCCATCCGCTGACCCATCCAAGGCTAGAAAGAACAGACCAAGACCTTCT
GGTAACGA

AGAAGAGAAAGAAGAAGGTCTACACCACCCCAAAGAAGATCAAGCACAAGCACAAGAAGGTCAAGTTGGCT
GTCTTGTCCTACTACAAGGTCGATGCTGAAGGTAAGGTTACCAAATTGAGAAGAGAATGTAGCAACCCAAC
TTGTGGTG

ACCGACTCCAACACTAGTGCTACTACCACCGAAAGTACCGACTCCAACACTAGTGCTACTACCACTGCTAG
CACCAACTCCAGCACTAATGCCACTACCACTGCTAGCACCAACTCCAGCACTAATGCCACTACCACTGAAA
GTACCAAC

CCAAAGGAAATCGTCAAGAGCAACACTTCCTCCAAGAAGGCTGACGTCCCACCTCCATCCGCTGACCCATC
CAAGGCTAGAAAGAACAGACCAAGACCTTCTGGTAACGAGGGTGCTATCAGAGACAAGACCGCTGGTAGAA
GAAACAAC

GATCCTGACTTCCAAGCTGCCTTACAACTAAGTAAAGAAGAAGAGGAGTTGAAGCAATTGCAGGAACTACA
GAGATTACAGAAGCAACAACAGTCTCTGTCTCAATTTCAAGCTCCTTTACAACAACAACAACCACAACAAC
AACCAGCG

ATAGAGATTCCACTAAGGTTAATTCTCAACAAGAGACAACACCTGGGACATCAGCTGTTCCAGAGAACCAT
CATCATGTCTCTCCTCAACCTGCTTCAGTACCACCTCCACAGAATGGACAGTACCAACAGCACGGCATGAT
GACCCCAA

AAACGTCAACATCATCTTCATCCTCACTGTCGTCAACGCCGTCCTCTTCATCATCCCCATCAACAATCACT
TCTGCACCTTCAACCTCCTCCACACCATCCACTACTGCCTATAATCAAGGAAGCACTATCACCAGTATTAT
TAACGGTA

AGATGCTATATGTCCCTACGGCCTTGTCTAACACCATCCAGCATGCAATACAGTGACATATATATACACCA
CACCCACACACCACACCCACACACACACACCCACACCCACACCCACACACACACACCCACACACACCACAC
CCACACAC

GGGTGCAGCGGTTGCTGTTGCTGTTGCTGTTGCTGTTGCTGCTGTGGTTGGATTGATGCTTGTGATGTTTG
TGGTGCCTGTGGTGCTTGTGGTACAGTTTTGCTGCCAGTTTGCTGCGAGTATGTAATTATGGGGTGAGCGG
CATTTACA

ATACCTCTGGATTTCTTACCAGTGGCAGTCAAACCTCTAGCTTCACGGTGCTTGTGAACTGGGTCACAGAT
CCAGTTGTAACGAGCATCTCTTCTGATAGCCTTGTGTTGAGGGTCGACCAAGATAACTTCGAAGTACTTGT
AAGTAGAA

TGAATCAACAAGGTTCTTCGACCCCATCTACAGCTACCGATGCAAATGCCGCCTCAACTGCTTCCACTCAC
ACAAACACCACGACATTCAAAAGACACATTGTTGCCGTGGATGACATATCGAAAATGAACTATGAAATGAT
AAAGAATT

GTGTCTATTGATGAAGGATCACGGCCTCTTGGCTAAGCCCACCCACGACCACATCATCAGATTGGCTCCTC
CTTTGGTCATCTCCGAAGAGGACTTGCAAACCGGTGTCGAAACCATTGCCAAGTGTATCGATCTGTTATAA
TATTAATA

CTTTGGATACTCATCGATACATTCAGACTCCCCCACACCCAAGTGGAGATCCGCCAACTGAACAATTTTGA
ACTTTCCTTCATCAGTTTCTTGCAGCCGTTGAGCTCCTGTGTCTACAGGACGAGTACCAACGAATTTGTAA
GTCAAATA

TGGAGTCATCGTGTTATTAACGGACCCACGCACAGTGGTGCTATCCATAATTTGCTGCTGCAGGGCCTTCA
GGCCCTGCACGCTAGCACACACCTTGGCGTCTGCAGTAGTGGTCTCCTTCTCAGTTTTGCTGCACCATTTG
AAAGTGTT

TTCCATCTCTTCGACATCCGCAACTTCTGAATCATCGACGTCTTCTACGCAAACGTCAACATCATCTTCAT
CCTCACTGTCGTCAACGCCGTCCTCTTCATCATCCCCATCAACAATCACTTCTGCACCTTCAACCTCCTCC
ACACCATC

ATGAGATCCAAGAATCCAATGATCTTAACATAGAATCTGACCATGACTTCCAATCTGACATTGAACTACAT
CCTGAGCAACCGAGAAATGTCCTTTCAAAAGCTGTGAGTCCAACCGATTCCACACCTCCGTCAACTCATAC
TGAAGATT

GTTGCCAGGTTTGAAGAGTAACTTTGCTACCATTGCTGACCCAGAAGCCAGAGAAGTTACTTTGAGAGCTT
TGAAGACTTTGAGAAGAGTTGGTAACGTTGGTGAAGACGATGCTATTCCAGAAGTTTCTCACGCTGGTGAC
GTTTCTAC

GGGTGCTATCAGAGACAAGACCGCTGGTAGAAGAAACAACAGATCAAAGGATGTCACTGACTCTGCCACCA
CCAAGAAGTCCAACACCAGAAGGGCCACTGACCGCCACTCTAGAACTGGTAAGACTGACACCAAGAAGAAG
GTTAACCA

AGTACCAACTCCAGCACTAATGCTACTACCACTGCCAGCACCAACGTCAGGACTAGTGCTACTACCACTGC
CAGCATCAACGTCAGGACTAGTGCGACTACCACTGAAAGTACCAACTCCAACACTAGTGCTACTACCACCG
AAAGTACC

GATTAAAGTCCCGTCATCAAGTTCAAGCACTGGCACTGTGCCTGAATAGTTCTTGGCAAGAAATTCAGGCT
TCTTGTGCTCTCCCTTCCAGAGGTTGATCCTCACAAATTGCACACTTGATAGCATGTTCTTCTCAGCCAAG
GCAATGCG

TTATCTGTAGGTTTAATGTTTTTTGAGTCCAGGCCAATTGTTGGAGTTGGGGGAGGGGGTGGAGCTTGTGG
AGGGTTGCTAACATGTGTTGGCAGAGACGCTGAAGGCAAAGGGGGTGCTGACACACTAGGAAGAGAAATAT
TAGAGCTT

ACTCCAGCACTAATGCTACTACCACTGCCAGCACCAACGTCAGGACTAGTGCTACTACCACTGCCAGCATC
AACGTCAGGACTAGTGCGACTACCACTGAAAGTACCAACTCCAACACTAGTGCTACTACCACCGAAAGTAC
CGACTCCA

TGCTACTACCACTGCCAGCACCAACGTCAGGACTAGTGCTACTACCACTGCCAGCATCAACGTCAGGACTA
GTGCGACTACCACTGAAAGTACCAACTCCAACACTAGTGCTACTACCACCGAAAGTACCGACTCCAACACT
AGTGCTAC

ATATAGATGCAGCAGAAGAAGAAGAAGAAGGAGAAGTTCTTCTTCCCCCCAGCAGACCTACATCTGCCAGG
CAGTTACATTTATCACTTGAAAGAGATGAGTTTGATCAGACACATAGAAAGAAGATTATTAAAGATGTACC
TGGTACGC

TAATCCCGCCTGGAGTCATCGTGTTATTAACGGACCCACGCACAGTGGTGCTATCCATAATTTGCTGCTGC
AGGGCCTTCAGGCCCTGCACGCTAGCACACACCTTGGCGTCTGCAGTAGTGGTCTCCTTCTCAGTTTTGCT
GCACCATT

GCAAAATAACGAGTGTCCTTGTACTTCAAGTATACTTCACGCTGTTTCATTCTATGTTCTGACAATTCTTT
GTTCCTCTGTTCGACTTGGGGGGCAACTGGTTGTTCCACATCTGGGTCTTGTTTCTTCAGCTGTGGCGTTG
ACTCAACT

TGCAATTCCTTCAATTGGTCATCGAATTCTTCCTTGGTAGCAGTGGTGTTGCTGTCTAACCAAGCAATAGT
TTCTTCAGCCTTCTTAGTGACAGCGTCCTTGTCAGCTTGCTCTAGCTTGTCACCAGCTTCAGAAATGGTGT
TCTTCAAA

CGTTTTGCCACCAAAGGAAATCGTCAAGAGCAACACTTCCTCCAAGAAGGCTGACGTCCCACCTCCATCCG
CTGACCCATCCAAGGCTAGAAAGAACAGACCAAGACCTTCTGGTAACGAGGGTGCTATCAGAGACAAGACC
GCTGGTAG

CTTGGTCTTGTAAGAGTAAGAGAAAGAACCGGTGTTGAAATCAGACAACAAAGCACCGACAGACAAAATCA
AGTCAGCAGATTCAACGGCTTCCTTAACTTCTGGCTTGGACAAGGTACCGACGTAAACACCACCGTATCTT
GGGTGTTG

AACTTCTTCGACAGTTGGACCTTCAGCTTCTGGAGCTGGAGGAGCACCACCTGGGAAACCACCTGGAGCTG
CGCCTTCTGGAGCACCACCAGCTTGGTACAATTTAGACATGATTGGGTTGGCAACCTCTTGCAATTCCTTC
AATTGGTC

GAGTCCGCTGAGGATGAATCAGTAAATGTATTACCTGACTCAGGTGATGGAGTGCTCAGAGGCGTTCCAAC
TGATGATGGATACTGCGGAAACTGTGATTGTGGCCCAGGTGGAAAGTACATAGGCGACATTTGATAAGGTG
TATACGGA

GGCAACCTCTTGCAATTCCTTCAATTGGTCATCGAATTCTTCCTTGGTAGCAGTGGTGTTGCTGTCTAACC
AAGCAATAGTTTCTTCAGCCTTCTTAGTGACAGCGTCCTTGTCAGCTTGCTCTAGCTTGTCACCAGCTTCA
GAAATGGT

TGATTGCCCCATAGAGAGCTATAAGCCGACGTGAAAGCTGCTGGTTCCAGCTTGGCTCATGTCGTCACCAG
TCACTAGTCACTTGGTCGCATTCATTGCTACTCATCTGCGAGTGAGCATATTTGAGATCTGACTTGCCAAG
GGATTAGA

GTAGGGTTAGGGTAGGGTTAGGGTAGTGTTAGGGTGTGGGTGTGGTGTGTGTGTGTGGGTGTGGTGTGGGT
GTGGGTGTGGTGTGTGGGTGTGGTGTGTGGGTGTGTGTGGGTGTGGTGTGGTGTGTGGGTGTGGTGTGGGT
GTGGTGTG

TAAGAATAGGGTAGGGTTAGGGTAGGGTTAGGGTAGTGTTAGGGTGTGGGTGTGGTGTGTGTGTGTGGGTG
TGGTGTGGGTGTGGGTGTGGTGTGTGGGTGTGGTGTGTGGGTGTGTGTGGGTGTGGTGTGGTGTGTGGGTG
TGGTGTGG

TAAATCAGGGTAAGAATAGGGTAGGGTTAGGGTAGGGTTAGGGTAGTGTTAGGGTGTGGGTGTGGTGTGTG
TGTGTGGGTGTGGTGTGGGTGTGGGTGTGGTGTGTGGGTGTGGTGTGTGGGTGTGTGTGGGTGTGGTGTGG
TGTGTGGG

AGAGACATGCTAAATCAGGGTAAGAATAGGGTAGGGTTAGGGTAGGGTTAGGGTAGTGTTAGGGTGTGGGT
GTGGTGTGTGTGTGTGGGTGTGGTGTGGGTGTGGGTGTGGTGTGTGGGTGTGGTGTGTGGGTGTGTGTGGG
TGTGGTGT

GCTCCAGTTCATCCACAACAACAACAACAACCAAATGCCTATTCACACCCTCCTGCTGCAGGTGCCCAAAC
GAGACAGCCAGGTATGTTTGCGCAGATGGCCTCCACTGCTGCAGGTGTTGCCGTTGGTAGCACCATTGGAC
ACACCCTA

ATCCACAACAACAACAACAACCAAATGCCTATTCACACCCTCCTGCTGCAGGTGCCCAAACGAGACAGCCA
GGTATGTTTGCGCAGATGGCCTCCACTGCTGCAGGTGTTGCCGTTGGTAGCACCATTGGACACACCCTAGG
TGCAGGTA

AAAGAGCTATTCCTTGACTTATCCAAACTAATCCATTACGACGCCACCAGCGTCACGCACACACCATCAGA
CACCACACTCATAGACGCTACTACCTGGTACAGTGTCAAGACAGAGAGGACTACAAAGGACTATAAAGAAT
CATTGCAA

GTTGTGCAGCAGAAACAGGATAAACAGTTGCAGCATCAAACACAAGAGCAGCAGCAGATACGAGAGGACCA
GCAGGAAGTCCCACCCCAACGTCCACGACAACAAAACAGATGGAAACCCTGGTGGAATTCCACTGCCGACG
ATGAGCCA

TTTACCTTACATGTCTCGTTCCCCAGTAGTGAAGTGTTTAGGTGTCTGGCTCAGCGTGAGAAGATCTTAGA
AGGCTTGACCGGTGCAGACTTTGTCGGCTTCCAGACGAGGGAGTATGCAAGACATTTCTTACAGACGTCTA
ACCGTCTG

TATAATGGATGTTGTGCAGCAGAAACAGGATAAACAGTTGCAGCATCAAACACAAGAGCAGCAGCAGATAC
GAGAGGACCAGCAGGAAGTCCCACCCCAACGTCCACGACAACAAAACAGATGGAAACCCTGGTGGAATTCC
ACTGCCGA

TCTACAATACTGGTTCCAGAAGTGGAACAGCAAGAACCTTCTCAGAGTGAAAGAACACCGTCTTTGTTTTC
GTCCGAAGGCTCTGGCTCAGAGTCGGAAGCACCCTTGCTTCCAGAGATCACCACGCCAGGTCCTCATCAGC
CTATGGGT

ATCATAAAGTTCTAAATGCGTCTCAGTAGCTACGCACAATTGCGACTGCTCACGCTTGGACCCTGCTTCCA
GATCGACGAAGTGTCCGATGCAGGAGTGCACGAAATTCGTCTGTTTCTTTAAAGTAAGATGGTACAAGTAT
AGCTCATC

CAAAGTCAGCTTCCACATCACAATCATCTTCTCGCTCCTTAACTTCAAGCACCAGTCCATCTTCAAGCACT
GGCTCATCTTCAAGCACCGGTTCATCTTCAGCTTCAAGCAGCTCCAAAAGTAAAGGCGTCGGAAATATTGT
TAATGTTT

AAATTAAACCACAGACGGTTATAAAGAAACTATCCAAGGTTGCCAACGACACAGGAAGACATGTCATTGTT
ACAACGGGTGTGGGGCAACATCAAATGTGGGCTGCTCAACACTGGACATGGAGAAATCCACATACTTTCAT
CACATCAG

ACAGACGGTTATAAAGAAACTATCCAAGGTTGCCAACGACACAGGAAGACATGTCATTGTTACAACGGGTG
TGGGGCAACATCAAATGTGGGCTGCTCAACACTGGACATGGAGAAATCCACATACTTTCATCACATCAGGT
GGTTTAGG

AATATGAGGGACCATTTGTGGGTTGACAATAGGATACGGTGTCTGTTGCTGCTGTTGATGTTGTTGCTGTT
GCTGTTGCTGTTGCTGCTGTTGCTGTTGCTGTTGCTGTTGCTGTTGTTGTTGTTGTTGTTGCTGTTGCTGT
TGCTGTTG

AACTCACTGAACGAACTAGACGTCACAGCCACAACGGTCGCAAAGTCAGCTTCCACATCACAATCATCTTC
TCGCTCCTTAACTTCAAGCACCAGTCCATCTTCAAGCACTGGCTCATCTTCAAGCACCGGTTCATCTTCAG
CTTCAAGC

TCCTTGACTTATCCAAACTAATCCATTACGACGCCACCAGCGTCACGCACACACCATCAGACACCACACTC
ATAGACGCTACTACCTGGTACAGTGTCAAGACAGAGAGGACTACAAAGGACTATAAAGAATCATTGCAACG
AACGGAGT

CTATTTCATACGACAAAGCTAACTCACTGAACGAACTAGACGTCACAGCCACAACGGTCGCAAAGTCAGCT
TCCACATCACAATCATCTTCTCGCTCCTTAACTTCAAGCACCAGTCCATCTTCAAGCACTGGCTCATCTTC
AAGCACCG

AGCATTTACATGAAGACGAAGAAATAAGATACTGTCTCGAGGGTGCTGGATACTTTGACGTCAGGGATGCT
TCCACACCAGAGAACTGGATTAGGTGTTTGGTAGAGTCAGGTGATTTACTGATTCTTCCACCAGGCATCTA
TCATCGTT

GGGTAAGTTAAGAGACATGCTAAATCAGGGTAAGAATAGGGTAGGGTTAGGGTAGGGTTAGGGTAGTGTTA
GGGTGTGGGTGTGGTGTGTGTGTGTGGGTGTGGTGTGGGTGTGGGTGTGGTGTGTGGGTGTGGTGTGTGGG
TGTGTGTG

ATGGAGGCTCAGGTGGTGGCGGTGTCTCCTCATGGGGTGGTGCTTCCACTTGGGGTGGCCAAGGTAATGGA
GGTGCATCCGCTTGGGGCGGTGCTGGCGGCGGTGCCTCAGCTTGGGGCGGCCAAGGTACTGGTGCTACTTC
TACTTGGG

TTCTACCAAGAGCATTTACATGAAGACGAAGAAATAAGATACTGTCTCGAGGGTGCTGGATACTTTGACGT
CAGGGATGCTTCCACACCAGAGAACTGGATTAGGTGTTTGGTAGAGTCAGGTGATTTACTGATTCTTCCAC
CAGGCATC

GAGCATGCAATTCGCAAGAATGATGGACCCTAATGCCGGTATGGGCTCTGCAGGTGGGGCTGCCTCTGCCT
TCCCCGCTCCTGGTGGCGATGCTCCAGAGGAAGGCTCCAACACGAACACTACTTCCTCATCCAACACAGGG
AACAACGC

ATCGAGACCTACTCAGACAAGGTCCGCTTCTACCATGGCGGCTCCAGTTCATCCACAACAACAACAACAAC
CAAATGCCTATTCACACCCTCCTGCTGCAGGTGCCCAAACGAGACAGCCAGGTATGTTTGCGCAGATGGCC
TCCACTGC

GATGAGGTTAGAAGAACAAGATTTGGTGTCGTAGTTATCAATGTCATCATCTTCTTCTTCGTCTTCTTCAT
CTCTATCCTGGTCGTCAAGACCATCCGTGTCACTGTGGTCAGTTTCATCGCCATGGTCCTTATCTGAATGA
TCTACTTC

GGAGGTACCATCTACAATACTGGTTCCAGAAGTGGAACAGCAAGAACCTTCTCAGAGTGAAAGAACACCGT
CTTTGTTTTCGTCCGAAGGCTCTGGCTCAGAGTCGGAAGCACCCTTGCTTCCAGAGATCACCACGCCAGGT
CCTCATCA

GGGCGACAAGTTTAGGCATTGAAGAGGGGAACGTTGGAAAGCTGTTGGTGGAGTTTGTCATGGAATCTGTG
TTGGAGAAGTTCTGCTCTGATGAGCAGAACATATTGTTGACCGACACCGTTGTGTCTATAGGTGACGATAA
CTCAGAGG

TGACGTCTGCAAATGTAAGCTTTCTTGGGGGCAATATGGAGGTATGTTTCAATGGTGGTGCCGCTGGAGCC
TGAGTTTGAGCTGAAGCTTCAACTGGGGCTGAAGTTTGGACTGGGGCTGAATTTTGGACTGGGGCTGAAGC
TTGAACTG

TACCCAGTTGAGATTGCTGAAGCTGTTTTCAACTATGGTGACTTCACCACCATGTTGACTGGTATTCCAGC
TGAACAAGTCACCAGAGTCATCACTGGTGTCCCATGGTACTCCACTAGATTGAGACCAGCTATTTCCAGTG
CTCTATCT

CTATGAACATTAATTCTTTCCCAAAGGAACTGAAGTAAGTGGGGTCCTGCCATGGTGAGTCATTGGTCACT
TGAGTTGATTTGGTTGAAGAAACAATTGTTGTCACCTCTTCATCTTCTGTGGAAACCACTACTTTATCTTT
TGCTTTCA

CATCTGCAGTATCATAAAGTTCTAAATGCGTCTCAGTAGCTACGCACAATTGCGACTGCTCACGCTTGGAC
CCTGCTTCCAGATCGACGAAGTGTCCGATGCAGGAGTGCACGAAATTCGTCTGTTTCTTTAAAGTAAGATG
GTACAAGT

GGATGGACTCTCTTGAAGCTAGATTACAATTCATTCAGGTCCTGAAGAACCTGCAAAAGACGCTGCACAAG
ACCAGAGACTCTATCACATCATCGTCGACCACCACACCACCGTCATCGCAACAAAAGCTGAACAATGACCC
TATACAGT

GGTTGACAATAGGATACGGTGTCTGTTGCTGCTGTTGATGTTGTTGCTGTTGCTGTTGCTGTTGCTGCTGT
TGCTGTTGCTGTTGCTGTTGCTGTTGTTGTTGTTGTTGTTGCTGTTGCTGTTGCTGTTGCGGTTGATGCTG
GGGTTGAT

ATCGTGGCTCCTTTAATGTTTGATTATCTGGTACAGTATTCCCACTGTCTGCTTCCTTAGACCATTTGGAA
TGTGATGCCGATTCAGATTCCACTCTTGGAAGGGTGATTCCTGTATGGATACTTCCCACATTTGATTCAGA
TGATTGAT

AGGTGGTGGCGGTGTCTCCTCATGGGGTGGTGCTTCCACTTGGGGTGGCCAAGGTAATGGAGGTGCATCCG
CTTGGGGCGGTGCTGGCGGCGGTGCCTCAGCTTGGGGCGGCCAAGGTACTGGTGCTACTTCTACTTGGGGT
GGTGCTTC

AGGATACGGTGTCTGTTGCTGCTGTTGATGTTGTTGCTGTTGCTGTTGCTGTTGCTGCTGTTGCTGTTGCT
GTTGCTGTTGCTGTTGTTGTTGTTGTTGTTGCTGTTGCTGTTGCTGTTGCGGTTGATGCTGGGGTTGATGC
TGAGGCTG

AGCGGCCCACAATTTAACTACTTTCAGTGCTCCTTCAACATCCAGTGCACAACTTGTAAGCAAATCAACTA
CCTCTAGCTCCATCCTGGTCACACCCAGAATCGACAGGTCTGGAAATTCCTCAACTGCCTCTAGAATTGCA
ACATCACT

ACTCAGACAAGGTCCGCTTCTACCATGGCGGCTCCAGTTCATCCACAACAACAACAACAACCAAATGCCTA
TTCACACCCTCCTGCTGCAGGTGCCCAAACGAGACAGCCAGGTATGTTTGCGCAGATGGCCTCCACTGCTG
CAGGTGTT

TGTTATTTCATTTTCTGCTAAATTTCTTTGGTTCCATTGAACTCGCTCTTCTGGTGTCATCTTGGCCAACA
TGTCTTGCTCATGTTTCAGTGACAGTGTGTCCTTTGGAAGGAAGGTACTCGATGTTCCAATTATCTCCCCC
TCCTCTTT

AGAAACAGGATAAACAGTTGCAGCATCAAACACAAGAGCAGCAGCAGATACGAGAGGACCAGCAGGAAGTC
CCACCCCAACGTCCACGACAACAAAACAGATGGAAACCCTGGTGGAATTCCACTGCCGACGATGAGCCAAA
CACGGGAA

ATAATTGTGTAAATAGTCGATTGTCGTCTGCGGTATGAAATAGCCACCGGTGGAAGGGTCATTCTCTAGCC
ACGCAAGCACACCGGTATTCTCACCATCCCCAGGTGCATCAGCAATTACCTCACCCAAGAAATCAGGAACA
TCAGCTGG

GAAATAAGATACTGTCTCGAGGGTGCTGGATACTTTGACGTCAGGGATGCTTCCACACCAGAGAACTGGAT
TAGGTGTTTGGTAGAGTCAGGTGATTTACTGATTCTTCCACCAGGCATCTATCATCGTTTCACCTTGACAA
CTAGCAAC

AGATGATACCATTTCAGCTACTATTTCATACGACAAAGCTAACTCACTGAACGAACTAGACGTCACAGCCA
CAACGGTCGCAAAGTCAGCTTCCACATCACAATCATCTTCTCGCTCCTTAACTTCAAGCACCAGTCCATCT
TCAAGCAC

TTTAGTTCCCCATCTGCAGTATCATAAAGTTCTAAATGCGTCTCAGTAGCTACGCACAATTGCGACTGCTC
ACGCTTGGACCCTGCTTCCAGATCGACGAAGTGTCCGATGCAGGAGTGCACGAAATTCGTCTGTTTCTTTA
AAGTAAGA

TGCCTCAAAAGGAGGTACCATCTACAATACTGGTTCCAGAAGTGGAACAGCAAGAACCTTCTCAGAGTGAA
AGAACACCGTCTTTGTTTTCGTCCGAAGGCTCTGGCTCAGAGTCGGAAGCACCCTTGCTTCCAGAGATCAC
CACGCCAG

CCAAGTGGAGAAGGTTGTTGTTTCTTACAAATTGCTAGATGCTCCAGCTGCCATCAGAACTGGTCAATTCG
GCTGGTCTGCTAACATGGAAAGAATCATGAAGGCTCAAGCCTTGAGAGACTCTTCCATGTCCTCCTACATG
TCTTCCAA

GAAGAACAAGATTTGGTGTCGTAGTTATCAATGTCATCATCTTCTTCTTCGTCTTCTTCATCTCTATCCTG
GTCGTCAAGACCATCCGTGTCACTGTGGTCAGTTTCATCGCCATGGTCCTTATCTGAATGATCTACTTCAT
GATATAAC

TAGCCACCGGTGGAAGGGTCATTCTCTAGCCACGCAAGCACACCGGTATTCTCACCATCCCCAGGTGCATC
AGCAATTACCTCACCCAAGAAATCAGGAACATCAGCTGGGGGAAATTCATTGGGTGCTGTTGGATAGTAGA
GTTTATAT

CGCTGGCGCAAATAACGCCACCGCCACCGGTGCAGCAGCTGGCACTGGCGCCACCCCGAACATGTCGTCAG
GTCAAAGTGCAGGCTTCAATCCGCTGGCCGACTTGACCAGTGCCAGATACGCTGGATATTTGAATATGCCA
TCTGCAGA

GATAAGTGATGTAGGTATATCCTGTGTAAGACTACCTCGTACGCCAAGATCATCTGTGGCAACGACCGCTT
CAACAGAGAGCTCTGAGCAAGGTCCCAAAATGAAGAGAATGGCAAGACGTAAGAGTGCAAAGTCTTTGGTA
AACTACAC

CGACAAAGCTAACTCACTGAACGAACTAGACGTCACAGCCACAACGGTCGCAAAGTCAGCTTCCACATCAC
AATCATCTTCTCGCTCCTTAACTTCAAGCACCAGTCCATCTTCAAGCACTGGCTCATCTTCAAGCACCGGT
TCATCTTC

CGGTATGAAATAGCCACCGGTGGAAGGGTCATTCTCTAGCCACGCAAGCACACCGGTATTCTCACCATCCC
CAGGTGCATCAGCAATTACCTCACCCAAGAAATCAGGAACATCAGCTGGGGGAAATTCATTGGGTGCTGTT
GGATAGTA

TCCTTCAACATCCAGTGCACAACTTGTAAGCAAATCAACTACCTCTAGCTCCATCCTGGTCACACCCAGAA
TCGACAGGTCTGGAAATTCCTCAACTGCCTCTAGAATTGCAACATCACTCCCTAACAAAACTACATTTGTT
TCTTCCCT

GCAAGGAATACCACATCCGCAACAATCGCAGCCACAGCAACAGCAACAACAACAACAACAACTGCAACAGC
AGCAACAGCAGCAACAACAACAACCCCTCACCGGCATTCATCAGCCTCACCAACAGGCTTTTGCCAACGCT
GCCTCCCC

ACCTCTGGCAAAGAAATCTAAGGCTTCTCTGGTATCAGCTCTGTTCCCCACGTAAGAGCCGACAATGGAGA
TAGACTTGACAACGTGGTTGAAGACATCAGAGGAGCACTTTGCACCGGCTGGCAAACCAACCAAGACAACA
GTACCGTT

ATGAAGAAATTTGCCGTATGAATTATTCCTGTATGTACACCAAATGTGTCATGATCTTCTTGTTTCTTCAA
TGTGGCGCTGGCATTCGAGAGTGATGATGCTTGGTGTGAGCACTTGAAATGTCTCCTCTTTAAGAAAGACA
ATCCTATC

TCTTGAAGCTAGATTACAATTCATTCAGGTCCTGAAGAACCTGCAAAAGACGCTGCACAAGACCAGAGACT
CTATCACATCATCGTCGACCACCACACCACCGTCATCGCAACAAAAGCTGAACAATGACCCTATACAGTTC
TACTTGAG

GCTGTTGATGTTGTTGCTGTTGCTGTTGCTGTTGCTGCTGTTGCTGTTGCTGTTGCTGTTGCTGTTGTTGT
TGTTGTTGTTGCTGTTGCTGTTGCTGTTGCGGTTGATGCTGGGGTTGATGCTGAGGCTGCTGGATCTGCTG
TTGAGATG

AACAATCGCAGCCACAGCAACAGCAACAACAACAACAACAACTGCAACAGCAGCAACAGCAGCAACAACAA
CAACCCCTCACCGGCATTCATCAGCCTCACCAACAGGCTTTTGCCAACGCTGCCTCCCCCTATCTGAATGC
TGAACAGA

CAGTAAGTAGCCATCGAGTAGACGGCAGAGTTGATACCACCAGCTGGAGCACCGACATTAACAATGGCAAT
CTTCAGTCTCTTGTCCTTTGGTAGCTTTGGTTCGTTGTGGTCAGCAGAGTTGATAGCCATGAAATTGTTTA
AATGTTCA

ATCAAAGAATACGAACCATTGACCAAGGCCTTGAAGGATATCTTGGGTGACCAAGTGGAGAAGGTTGTTGT
TTCTTACAAATTGCTAGATGCTCCAGCTGCCATCAGAACTGGTCAATTCGGCTGGTCTGCTAACATGGAAA
GAATCATG

ACCATTTGTGGGTTGACAATAGGATACGGTGTCTGTTGCTGCTGTTGATGTTGTTGCTGTTGCTGTTGCTG
TTGCTGCTGTTGCTGTTGCTGTTGCTGTTGCTGTTGTTGTTGTTGTTGTTGCTGTTGCTGTTGCTGTTGCG
GTTGATGC

TCCAGTGCACAACTTGTAAGCAAATCAACTACCTCTAGCTCCATCCTGGTCACACCCAGAATCGACAGGTC
TGGAAATTCCTCAACTGCCTCTAGAATTGCAACATCACTCCCTAACAAAACTACATTTGTTTCTTCCCTAA
GTTCAACA

GTTGCTGCTGTTGCTGTTGCTGTTGCTGTTGCTGTTGTTGTTGTTGTTGTTGCTGTTGCTGTTGCTGTTGC
GGTTGATGCTGGGGTTGATGCTGAGGCTGCTGGATCTGCTGTTGAGATGATTGCTGGGGCTGTGCTGGTGA
TTGCAAAT

TGATAAACTTGCTCTCGGTACGCTAGACAAAGAATTTCTCAATGAGGTCCTGGCCATAGCCTCTTCATCTA
CCATTCTTTCCAGAGACTTTCTTTGCTGGAGAACCAGCTCTGCTTCTGCGTCTTTACGTTCTTTGGTACTG
GTGCATAT

GGTGTCTCCTCATGGGGTGGTGCTTCCACTTGGGGTGGCCAAGGTAATGGAGGTGCATCCGCTTGGGGCGG
TGCTGGCGGCGGTGCCTCAGCTTGGGGCGGCCAAGGTACTGGTGCTACTTCTACTTGGGGTGGTGCTTCAG
CCTGGGGT

AGATTACAATTCATTCAGGTCCTGAAGAACCTGCAAAAGACGCTGCACAAGACCAGAGACTCTATCACATC
ATCGTCGACCACCACACCACCGTCATCGCAACAAAAGCTGAACAATGACCCTATACAGTTCTACTTGAGAA
ACTACAGA

ATAGTGAATTTCACCTTTCGCACACAGAAGAGGACAAGTTAAATGAGTTCCAGGTCATCACGAATTTCCCC
CCAGAAGACTTGCCAGATGTGGTGAGACTGTTGAGGAACCATGGTTGGCAGTTAGAACCAGCATTGAGCCG
ATATTTCG

ATTCAACAGCAGCAACATCACACGCAGAATTCACAACCACAACAGCAACAGCAACAACAACCACAGCAGCA
AATGTCACAGCAACAAATGTCACAGCATCCTCGACCACAGCAAGGAATACCACATCCGCAACAATCGCAGC
CACAGCAA

TGGGGTGGCCAAGGTAATGGAGGTGCATCCGCTTGGGGCGGTGCTGGCGGCGGTGCCTCAGCTTGGGGCGG
CCAAGGTACTGGTGCTACTTCTACTTGGGGTGGTGCTTCAGCCTGGGGTAACAAATCAAGTTGGGGCGGTG
CATCCACT

TGTTGCTGTTGCTGTTGTTGTTGTTGTTGTTGCTGTTGCTGTTGCTGTTGCGGTTGATGCTGGGGTTGATG
CTGAGGCTGCTGGATCTGCTGTTGAGATGATTGCTGGGGCTGTGCTGGTGATTGCAAATCATTGAAGTGTT
TATGTAGA

AAGGTTGTTGTTTCTTACAAATTGCTAGATGCTCCAGCTGCCATCAGAACTGGTCAATTCGGCTGGTCTGC
TAACATGGAAAGAATCATGAAGGCTCAAGCCTTGAGAGACTCTTCCATGTCCTCCTACATGTCTTCCAAGA
AGACTTTC

TGGAAGGGTCATTCTCTAGCCACGCAAGCACACCGGTATTCTCACCATCCCCAGGTGCATCAGCAATTACC
TCACCCAAGAAATCAGGAACATCAGCTGGGGGAAATTCATTGGGTGCTGTTGGATAGTAGAGTTTATATCC
TAACTTTT

TGCTGTTGCTGTTGCTGCTGTTGCTGTTGCTGTTGCTGTTGCTGTTGTTGTTGTTGTTGTTGCTGTTGCTG
TTGCTGTTGCGGTTGATGCTGGGGTTGATGCTGAGGCTGCTGGATCTGCTGTTGAGATGATTGCTGGGGCT
GTGCTGGT

AAACTCAATGATTCAACAGCAGCAACATCACACGCAGAATTCACAACCACAACAGCAACAGCAACAACAAC
CACAGCAGCAAATGTCACAGCAACAAATGTCACAGCATCCTCGACCACAGCAAGGAATACCACATCCGCAA
CAATCGCA

GCTGTTGTTGTTGTTGTTGTTGCTGTTGCTGTTGCTGTTGCGGTTGATGCTGGGGTTGATGCTGAGGCTGC
TGGATCTGCTGTTGAGATGATTGCTGGGGCTGTGCTGGTGATTGCAAATCATTGAAGTGTTTATGTAGATT
TATAGTTG

TACCATGGCGGCTCCAGTTCATCCACAACAACAACAACAACCAAATGCCTATTCACACCCTCCTGCTGCAG
GTGCCCAAACGAGACAGCCAGGTATGTTTGCGCAGATGGCCTCCACTGCTGCAGGTGTTGCCGTTGGTAGC
ACCATTGG

TATACAGCAAGGATGGACTCTCTTGAAGCTAGATTACAATTCATTCAGGTCCTGAAGAACCTGCAAAAGAC
GCTGCACAAGACCAGAGACTCTATCACATCATCGTCGACCACCACACCACCGTCATCGCAACAAAAGCTGA
ACAATGAC

ACAACGGTCGCAAAGTCAGCTTCCACATCACAATCATCTTCTCGCTCCTTAACTTCAAGCACCAGTCCATC
TTCAAGCACTGGCTCATCTTCAAGCACCGGTTCATCTTCAGCTTCAAGCAGCTCCAAAAGTAAAGGCGTCG
GAAATATT

AATAACGCCACCGCCACCGGTGCAGCAGCTGGCACTGGCGCCACCCCGAACATGTCGTCAGGTCAAAGTGC
AGGCTTCAATCCGCTGGCCGACTTGACCAGTGCCAGATACGCTGGATATTTGAATATGCCATCTGCAGACA
TGTTTGGC

GCGGATAAATTGCCTCAAAAGGAGGTACCATCTACAATACTGGTTCCAGAAGTGGAACAGCAAGAACCTTC
TCAGAGTGAAAGAACACCGTCTTTGTTTTCGTCCGAAGGCTCTGGCTCAGAGTCGGAAGCACCCTTGCTTC
CAGAGATC

TCACCTTTCGCACACAGAAGAGGACAAGTTAAATGAGTTCCAGGTCATCACGAATTTCCCCCCAGAAGACT
TGCCAGATGTGGTGAGACTGTTGAGGAACCATGGTTGGCAGTTAGAACCAGCATTGAGCCGATATTTCGAT
GGAGAGTG

CAAGAACCTTCTCAGAGTGAAAGAACACCGTCTTTGTTTTCGTCCGAAGGCTCTGGCTCAGAGTCGGAAGC
ACCCTTGCTTCCAGAGATCACCACGCCAGGTCCTCATCAGCCTATGGGTAATACCAGCAATAATGTGGTCG
AAATGATA

GTCTGTTGCTGCTGTTGATGTTGTTGCTGTTGCTGTTGCTGTTGCTGCTGTTGCTGTTGCTGTTGCTGTTG
CTGTTGTTGTTGTTGTTGTTGCTGTTGCTGTTGCTGTTGCGGTTGATGCTGGGGTTGATGCTGAGGCTGCT
GGATCTGC

CCACATCCGCAACAATCGCAGCCACAGCAACAGCAACAACAACAACAACAACTGCAACAGCAGCAACAGCA
GCAACAACAACAACCCCTCACCGGCATTCATCAGCCTCACCAACAGGCTTTTGCCAACGCTGCCTCCCCCT
ATCTGAAT

GCCACAGCAACAGCAACAACAACAACAACAACTGCAACAGCAGCAACAGCAGCAACAACAACAACCCCTCA
CCGGCATTCATCAGCCTCACCAACAGGCTTTTGCCAACGCTGCCTCCCCCTATCTGAATGCTGAACAGAAT
GCTGCCTA

CTATATCGAGATCGAGACCTACTCAGACAAGGTCCGCTTCTACCATGGCGGCTCCAGTTCATCCACAACAA
CAACAACAACCAAATGCCTATTCACACCCTCCTGCTGCAGGTGCCCAAACGAGACAGCCAGGTATGTTTGC
GCAGATGG

TATCAGAGTCCGCTGAGGATGAATCAGTAAATGTATTACCTGACTCAGGTGATGGAGTGCTCAGAGGCGTT
CCAACTGATGATGGATACTGCGGAAACTGTGATTGTGGCCCAGGTGGAAAGTACATAGGCGACATTTGATA
AGGTGTAT

TTTCCGATGGAATATGAGGGACCATTTGTGGGTTGACAATAGGATACGGTGTCTGTTGCTGCTGTTGATGT
TGTTGCTGTTGCTGTTGCTGTTGCTGCTGTTGCTGTTGCTGTTGCTGTTGCTGTTGTTGTTGTTGTTGTTG
CTGTTGCT

ACGAACTAGACGTCACAGCCACAACGGTCGCAAAGTCAGCTTCCACATCACAATCATCTTCTCGCTCCTTA
ACTTCAAGCACCAGTCCATCTTCAAGCACTGGCTCATCTTCAAGCACCGGTTCATCTTCAGCTTCAAGCAG
CTCCAAAA

TTGTTGCTGTTGCTGTTGCTGTTGCTGCTGTTGCTGTTGCTGTTGCTGTTGCTGTTGTTGTTGTTGTTGTT
GCTGTTGCTGTTGCTGTTGCGGTTGATGCTGGGGTTGATGCTGAGGCTGCTGGATCTGCTGTTGAGATGAT
TGCTGGGG

ATGTCTCGTTCCCCAGTAGTGAAGTGTTTAGGTGTCTGGCTCAGCGTGAGAAGATCTTAGAAGGCTTGACC
GGTGCAGACTTTGTCGGCTTCCAGACGAGGGAGTATGCAAGACATTTCTTACAGACGTCTAACCGTCTGCT
AATGGCGG

GGCGACGTCACTTCCTCATTAGCGGCCCACAATTTAACTACTTTCAGTGCTCCTTCAACATCCAGTGCACA
ACTTGTAAGCAAATCAACTACCTCTAGCTCCATCCTGGTCACACCCAGAATCGACAGGTCTGGAAATTCCT
CAACTGCC

GTAGTTATCAATGTCATCATCTTCTTCTTCGTCTTCTTCATCTCTATCCTGGTCGTCAAGACCATCCGTGT
CACTGTGGTCAGTTTCATCGCCATGGTCCTTATCTGAATGATCTACTTCATGATATAACTGCAGGGTGCCT
TCAACGAC

CGTCACAGCCACAACGGTCGCAAAGTCAGCTTCCACATCACAATCATCTTCTCGCTCCTTAACTTCAAGCA
CCAGTCCATCTTCAAGCACTGGCTCATCTTCAAGCACCGGTTCATCTTCAGCTTCAAGCAGCTCCAAAAGT
AAAGGCGT

TAATTCTTTCCCAAAGGAACTGAAGTAAGTGGGGTCCTGCCATGGTGAGTCATTGGTCACTTGAGTTGATT
TGGTTGAAGAAACAATTGTTGTCACCTCTTCATCTTCTGTGGAAACCACTACTTTATCTTTTGCTTTCACT
GTAATTTT

TTTCTTACAAATTGCTAGATGCTCCAGCTGCCATCAGAACTGGTCAATTCGGCTGGTCTGCTAACATGGAA
AGAATCATGAAGGCTCAAGCCTTGAGAGACTCTTCCATGTCCTCCTACATGTCTTCCAAGAAGACTTTCGA
AATTTCTC

TAGTAGAGCTTCCAAACAATCCTGTAGAGTTGGTCGCTCCAGAGCCAAAGCCACCACCACTTCCAGTTCCG
GTTCCAAACATGCCTGTTGCTGGCTTTTGACCAAATAATCCTCCTGTTGTAGCAGAAGTAGTGGAGTTATT
ATTACCCA

TTCGCAAGAATGATGGACCCTAATGCCGGTATGGGCTCTGCAGGTGGGGCTGCCTCTGCCTTCCCCGCTCC
TGGTGGCGATGCTCCAGAGGAAGGCTCCAACACGAACACTACTTCCTCATCCAACACAGGGAACAACGCAG
GGACTAAT

AGAGTTGTCGAAGTTTGTTTGGCTGACTTGCAAGGTTCTGAAGACCATTCTTTCAGAAAGGTCAAGTTGAG
AGTCGATGAAGTTCAAGGTAAGAACTTGTTGACCAACTTCCACGGTATGGACTTCACTACCGACAAATTGA
GATCAATG

GCCAGTAGTCAATCATGAGGATAGTGAATTTCACCTTTCGCACACAGAAGAGGACAAGTTAAATGAGTTCC
AGGTCATCACGAATTTCCCCCCAGAAGACTTGCCAGATGTGGTGAGACTGTTGAGGAACCATGGTTGGCAG
TTAGAACC

AGTGGAACAGCAAGAACCTTCTCAGAGTGAAAGAACACCGTCTTTGTTTTCGTCCGAAGGCTCTGGCTCAG
AGTCGGAAGCACCCTTGCTTCCAGAGATCACCACGCCAGGTCCTCATCAGCCTATGGGTAATACCAGCAAT
AATGTGGT

TGCTTCCACTTGGGGTGGCCAAGGTAATGGAGGTGCATCCGCTTGGGGCGGTGCTGGCGGCGGTGCCTCAG
CTTGGGGCGGCCAAGGTACTGGTGCTACTTCTACTTGGGGTGGTGCTTCAGCCTGGGGTAACAAATCAAGT
TGGGGCGG

TTTCGAATCTTCAGTATGAGTTGACGGAGGTGTGGAATCGGTTGGACTCACAGCTTTTGAAAGGACATTTC
TCAGTTGCTCAGGATGTAGTTCAATGTCAGATTGGAAGTCATGGTCAGATTCTATGTTAAGATCATTGGAT
TGTTGGAT

TTATCGACACTGAAATAAACAGAATCAGAAAACTGGTCGGTCCAACTGCTGAAGTCATTGGTGCTGTTTCC
GGTGGTGTTGATTCTACTGTTGCATCCAAATTGATGACAGAAGCCATCGGTGATAGATTCCATGCCATCTT
GGTCGATA

TCATCAAGACCTATATCGAGATCGAGACCTACTCAGACAAGGTCCGCTTCTACCATGGCGGCTCCAGTTCA
TCCACAACAACAACAACAACCAAATGCCTATTCACACCCTCCTGCTGCAGGTGCCCAAACGAGACAGCCAG
GTATGTTT

GGTCCGCTTCTACCATGGCGGCTCCAGTTCATCCACAACAACAACAACAACCAAATGCCTATTCACACCCT
CCTGCTGCAGGTGCCCAAACGAGACAGCCAGGTATGTTTGCGCAGATGGCCTCCACTGCTGCAGGTGTTGC
CGTTGGTA

GCTCTCGGTACGCTAGACAAAGAATTTCTCAATGAGGTCCTGGCCATAGCCTCTTCATCTACCATTCTTTC
CAGAGACTTTCTTTGCTGGAGAACCAGCTCTGCTTCTGCGTCTTTACGTTCTTTGGTACTGGTGCATATGT
CTGCCATT

AAACAAAGACACCGACACAGAACAAGAAACATGCAAATAGCTGGTTCATGTTCTTCAAAGCAACACCCATG
TGGTACACAACTACAGGCAACCAAGCAACTGGAACAACCCACCAAGCTGTTTTAGTTAATGGTTCCAAGAA
ATTACCAA

AAAACGCTATTATAATGGATGTTGTGCAGCAGAAACAGGATAAACAGTTGCAGCATCAAACACAAGAGCAG
CAGCAGATACGAGAGGACCAGCAGGAAGTCCCACCCCAACGTCCACGACAACAAAACAGATGGAAACCCTG
GTGGAATT

AACACCATCCAGCATGCAATACAGTGACATATATATACACCCACACCCACACACCACACCCACACCCACAC
ACCACACCCACACACCCTAACACAATCCTAACAGTACCCTATTCTAACCCTGATGAACCTGTCTCCAAACC
TACCCTCC

TTTCTTGGGGGCAATATGGAGGTATGTTTCAATGGTGGTGCCGCTGGAGCCTGAGTTTGAGCTGAAGCTTC
AACTGGGGCTGAAGTTTGGACTGGGGCTGAATTTTGGACTGGGGCTGAAGCTTGAACTGGGACTGAAGTTT
GGACTTGT

TTCCACATCACAATCATCTTCTCGCTCCTTAACTTCAAGCACCAGTCCATCTTCAAGCACTGGCTCATCTT
CAAGCACCGGTTCATCTTCAGCTTCAAGCAGCTCCAAAAGTAAAGGCGTCGGAAATATTGTTAATGTTTCC
TTTAGTCA

ACAAATAGCACCTATGAACAAGGTGAAAAAGGAAGAAGTAGTGGAGAGGATTGTCTGCGATGCCACCGATG
TGAATGCCAGCGAGGCATTGGTCACAAGGTTAGCTGTGAACCAAAGTATGCAGAACTCAGCACTTAACTTG
ATTGTTTC

AAGGAACAGCTATCTTTCGTTATGAATTTGTACTACGAGAAGAGTGGTGGTAGCAAATCTGACTGTAGCTT
CAGCGGTTCTGCCACTCTACAAACTGCCACCACGCAAGCTAGTTGCTCCTCCGCTTTGAAAGAGATTGGTA
GTATGGGT

ATTTGGTGTCGTAGTTATCAATGTCATCATCTTCTTCTTCGTCTTCTTCATCTCTATCCTGGTCGTCAAGA
CCATCCGTGTCACTGTGGTCAGTTTCATCGCCATGGTCCTTATCTGAATGATCTACTTCATGATATAACTG
CAGGGTGC

CAAAATAGGATTTACCTTACATGTCTCGTTCCCCAGTAGTGAAGTGTTTAGGTGTCTGGCTCAGCGTGAGA
AGATCTTAGAAGGCTTGACCGGTGCAGACTTTGTCGGCTTCCAGACGAGGGAGTATGCAAGACATTTCTTA
CAGACGTC

AAACTGGACTATGGAAAACTTTATCGACACTGAAATAAACAGAATCAGAAAACTGGTCGGTCCAACTGCTG
AAGTCATTGGTGCTGTTTCCGGTGGTGTTGATTCTACTGTTGCATCCAAATTGATGACAGAAGCCATCGGT
GATAGATT

ATCCAAACTAATCCATTACGACGCCACCAGCGTCACGCACACACCATCAGACACCACACTCATAGACGCTA
CTACCTGGTACAGTGTCAAGACAGAGAGGACTACAAAGGACTATAAAGAATCATTGCAACGAACGGAGTCT
CTGCTTAA

ATTCTCTAGCCACGCAAGCACACCGGTATTCTCACCATCCCCAGGTGCATCAGCAATTACCTCACCCAAGA
AATCAGGAACATCAGCTGGGGGAAATTCATTGGGTGCTGTTGGATAGTAGAGTTTATATCCTAACTTTTCC
ATTTCGGC

CACACAGAAGAGGACAAGTTAAATGAGTTCCAGGTCATCACGAATTTCCCCCCAGAAGACTTGCCAGATGT
GGTGAGACTGTTGAGGAACCATGGTTGGCAGTTAGAACCAGCATTGAGCCGATATTTCGATGGAGAGTGGA
AAGGCGAA

GTAGGTATATCCTGTGTAAGACTACCTCGTACGCCAAGATCATCTGTGGCAACGACCGCTTCAACAGAGAG
CTCTGAGCAAGGTCCCAAAATGAAGAGAATGGCAAGACGTAAGAGTGCAAAGTCTTTGGTAAACTACACTG
CCACCATT

ATATGGCACGTTCAAGAGGATCATCAAGACCTATATCGAGATCGAGACCTACTCAGACAAGGTCCGCTTCT
ACCATGGCGGCTCCAGTTCATCCACAACAACAACAACAACCAAATGCCTATTCACACCCTCCTGCTGCAGG
TGCCCAAA

CTTTCAGTGCTCCTTCAACATCCAGTGCACAACTTGTAAGCAAATCAACTACCTCTAGCTCCATCCTGGTC
ACACCCAGAATCGACAGGTCTGGAAATTCCTCAACTGCCTCTAGAATTGCAACATCACTCCCTAACAAAAC
TACATTTG

AGCCACCTGTAAAGAGCTATTCCTTGACTTATCCAAACTAATCCATTACGACGCCACCAGCGTCACGCACA
CACCATCAGACACCACACTCATAGACGCTACTACCTGGTACAGTGTCAAGACAGAGAGGACTACAAAGGAC
TATAAAGA

TTGCTGTTGCTGTTGCTGTTGCTGTTGTTGTTGTTGTTGTTGCTGTTGCTGTTGCTGTTGCGGTTGATGCT
GGGGTTGATGCTGAGGCTGCTGGATCTGCTGTTGAGATGATTGCTGGGGCTGTGCTGGTGATTGCAAATCA
TTGAAGTG

AAAGTAATGTCGACGATGATGGCTATCAAGAGGATGATGATGATGACGGTGATGACGAAGGTGATGGAAGG
GATAATGAAGAAGACAGCACTGCTGAGGAAGATGAAGTAGATGATGAAATCGAAACAGATATGAAGAACGC
TTCGATTA

TGATTGACGATTCCAACTCTCTATTCAAAGGTATGAACGACTCTACTGTCTGGATGTCTCACGGTGATAAA
TTGCACGGCTTGCCAACTGGTTACAAGACCATTGCCACCTCCGATAACTCCCCATACTGTGGTATTGTCCA
CGAAACTA

AAATAGTCGATTGTCGTCTGCGGTATGAAATAGCCACCGGTGGAAGGGTCATTCTCTAGCCACGCAAGCAC
ACCGGTATTCTCACCATCCCCAGGTGCATCAGCAATTACCTCACCCAAGAAATCAGGAACATCAGCTGGGG
GAAATTCA

TTGCCGTATGAATTATTCCTGTATGTACACCAAATGTGTCATGATCTTCTTGTTTCTTCAATGTGGCGCTG
GCATTCGAGAGTGATGATGCTTGGTGTGAGCACTTGAAATGTCTCCTCTTTAAGAAAGACAATCCTATCCT
TTCATGAT

GCAATATGGAGGTATGTTTCAATGGTGGTGCCGCTGGAGCCTGAGTTTGAGCTGAAGCTTCAACTGGGGCT
GAAGTTTGGACTGGGGCTGAATTTTGGACTGGGGCTGAAGCTTGAACTGGGACTGAAGTTTGGACTTGTTC
TTGAGCTG

CTTAGCAGCCAAGTTCCACAACATAATTTCACCGTCCTTACCAGCGGAAGCAATCAAAGTTCCGTCTGGGG
AAGCAGTCAAAGTGTTGATGTTGGAGTTGTGACCGATGAAGTCAGCTTCAATTTGGAATTGGTTTAAGTTC
CAAGCCTG

TCTCTAGCGGAGTTGGATCGCTATATCGTGTGTTATTAACGGTGGTTGTGTCACTTGGCATTGACGCAGAA
CTGGATGTCGTGCCAGACCATGATCGTCTCCCACTAGATTCCCCACCGAATTTTATATCTAATTCTGGATC
AATAAGTA

AGTAGAGATACGACAGCTGGACTAGAAGAGGATATAGGAGCAGAGAGAGAAGACAACACTTCACCTACTGC
CCCTCAAATCTCAACTTTGCCTCCAAGAAAATTGACATTTGAAGATGTCGTTAAACCAGATTACTCAAACG
CTCCAATA

ACGAACCATTGACCAAGGCCTTGAAGGATATCTTGGGTGACCAAGTGGAGAAGGTTGTTGTTTCTTACAAA
TTGCTAGATGCTCCAGCTGCCATCAGAACTGGTCAATTCGGCTGGTCTGCTAACATGGAAAGAATCATGAA
GGCTCAAG

GCAGAGATGGATAGGTGTCGGTGGTTTTCATACGGTAAGCCAAATCAGTGTAAGAGTGAACTTTAGAGGAA
AGACCAGACTCAGCAGAAGCTTCTGGTGCTGGTGGTGGAGTTGGCTTGGTTGAAACTGCGTTCTTAGTAGA
AGTAACAC

TATTTGTTTCGGCCAGGAAGCGTTTCAAGTAGGCTTGCGTGCATGCAGGGGATTGATCTTTGGTCTTCCAA
CTTCTCTTCGTGTCGATCTTTGTTGCGGAACGGCTGGCACTGCACCATTTCCATTAGGATGTACTACCTTA
GAAGGCGG

TGGAAACACGTTTCGAATCTTCAGTATGAGTTGACGGAGGTGTGGAATCGGTTGGACTCACAGCTTTTGAA
AGGACATTTCTCAGTTGCTCAGGATGTAGTTCAATGTCAGATTGGAAGTCATGGTCAGATTCTATGTTAAG
ATCATTGG

ATGACAGTGAAAAGTAATGTCGACGATGATGGCTATCAAGAGGATGATGATGATGACGGTGATGACGAAGG
TGATGGAAGGGATAATGAAGAAGACAGCACTGCTGAGGAAGATGAAGTAGATGATGAAATCGAAACAGATA
TGAAGAAC

CCTTCAGCAGACCAACCACGGACGTCTTCCCAGTGGAATTCCTTGATGTATTCTGGACCACCACGAACCAA
ACCTTCATAACCTTCCATGACAACAAAGGCACGACAACCTTTGAAGATAGCGGAACGCACGATGGCTCTAA
CGTTAGAG

TGCTGGACCAAACACTAACGGTTCTCAATTCTTCATTACCACTGTGCCTTGTCCATGGTTGGATGGAAAAC
ACGTTGTCTTTGGTGAGGTAACCAAAGGTATGGACATTGTCAAAGCAATCGAATCATACGGTACTGCTTCT
GGTAAACC

TCATCATGCACTGCTGTGGGTACGGCCCATTCTGTGGAGGTGGTACTGAAGCAGGTTGAGGAGAGGCATGA
TGGGGGTTCTCTGGAACAGCTGATGAAGCAGGTGTTGTTGTCTGTTGAGAGTTAGCCTTAGTGGAAGCCTT
ATCATATT

GGTATGTTTCAATGGTGGTGCCGCTGGAGCCTGAGTTTGAGCTGAAGCTTCAACTGGGGCTGAAGTTTGGA
CTGGGGCTGAATTTTGGACTGGGGCTGAAGCTTGAACTGGGACTGAAGTTTGGACTTGTTCTTGAGCTGGA
GCTTGTAG

CTTCCTCATTAGCGGCCCACAATTTAACTACTTTCAGTGCTCCTTCAACATCCAGTGCACAACTTGTAAGC
AAATCAACTACCTCTAGCTCCATCCTGGTCACACCCAGAATCGACAGGTCTGGAAATTCCTCAACTGCCTC
TAGAATTG

GGTGATTTACTGATTCTTCCACCAGGCATCTATCATCGTTTCACCTTGACAACTAGCAACCACATCAAGGC
CTTGAGACTGTTTAAGGACGAGCCCAAATGGCAAGCTATCAACAGGTCAAATCAGGCTGATTCATTGCCTG
TACGCAAG

AATTATTCCTGTATGTACACCAAATGTGTCATGATCTTCTTGTTTCTTCAATGTGGCGCTGGCATTCGAGA
GTGATGATGCTTGGTGTGAGCACTTGAAATGTCTCCTCTTTAAGAAAGACAATCCTATCCTTTCATGATCA
AGGGCTTC

TCGAATCTTCAGTATGAGTTGACGGAGGTGTGGAATCGGTTGGACTCACAGCTTTTGAAAGGACATTTCTC
GGTTGCTCAGGATGTAGTTCGATGTCAGATTGGAAGTCATGGTCAGATTCTATGTTAAGATCATCGGATTG
TTGGATCT

CCAGCTAATAAAGCTAACCAACCACAGTTTCTACCCATAACTTCAACAACGAAAGCTCTTGAGTGAGAGTT
GGCAGTGGCTTCAACGTAATCGATGGCCTTACAGATTCTGTCCAAGGCAGAGTAAGCACCAATAGTAGCAT
CCGTGGTG

CTATAAGCAATACTACGAATATTGACATCTTGAAACAAACCAAGGCAGGCGAACATCAACGTGATGGTCAC
CAACAGCACCCACACGGTGGTCATGGACCCATGAACAGATCACGTTTTTCTAATGCAGGGCCTTTTGGTGG
TGGTAGCA

ATATTATGTGCTGGTGTTACTGTATATAAAGCACTAAAAGAGGCAGACTTGAAAGCTGGTGACTGGGTTGC
CATCTCTGGTGCTGCAGGTGGCTTGGGTTCCTTGGCCGTTCAATATGCAACTGCGATGGGTTACAGAGTTC
TAGGTATT

AGATTGCTGAAGCTGTTTTCAACTATGGTGACTTCACCACCATGTTGACTGGTATTCCAGCTGAACAAGTC
ACCAGAGTCATCACTGGTGTCCCATGGTACTCCACTAGATTGAGACCAGCTATTTCCAGTGCTCTATCTAA
GGACGGTA

TTCGCCCTTGTAAGCAGCAACCAAGATAAGAGCGATGATACCACCGGAGATTTGACCCCAAGCTTGGTTAG
CAAAGACAGCACCCATGATGGCACCTCTCCATTTGGTAGTGGCAAATTCAGAAGTAATAATAGAAGATAGT
GGGTAGTC

GTGGTGTGGGTGTGGTGTGGTGTGTGGGTGTGGGTGTGGGTGTGGTGTGTGTGGGTGTGGTGTGGGTGTGT
GGGTGTGGGTGTGGTGTGTGGTGTGTGTGTGGGTGTGGGTGTGGGTGTGGTGTGTGGGTGTGGGTGTGGTG
TGTGGGTG

GGGTGTGGGTGTGGTGTGTGGGTGTGGTGTGTGGGTGTGGTGTGGGTGTGGTGTGTGGGTGTGTGTGGGTG
TGGTGTGGGTGTGGTGTGGTGTGTGGGTGTGGGTGTGGGTGTGGTGTGTGTGGGTGTGGTGTGGGTGTGTG
GGTGTGGG

TGTGGGTGTGGTGTGTGGGTGTGTGTGGGTGTGGTGTGGGTGTGGTGTGGTGTGTGGGTGTGGGTGTGGGT
GTGGTGTGTGTGGGTGTGGTGTGGGTGTGTGGGTGTGGGTGTGGTGTGTGGTGTGTGTGTGGGTGTGGGTG
TGGGTGTG

TGTGTGGGTGTGGGTGTGGGTGTGGTGTGTGTGGGTGTGGTGTGGGTGTGTGGGTGTGGGTGTGGTGTGTG
GTGTGTGTGTGGGTGTGGGTGTGGGTGTGGTGTGTGGGTGTGGGTGTGGTGTGTGGGTGTGGTGTGTGGGT
GTGTGTGG

GTGTGTGTGGGTGTGTGGGTGTGGGTGTGTGGGTGTGGGTGTGGTGTGTGGGTGTGGTGTGTGGGTGTGGT
GTGGGTGTGGTGTGTGGGTGTGTGTGGGTGTGGTGTGGGTGTGGTGTGGTGTGTGGGTGTGGGTGTGGGTG
TGGTGTGT

GTGGTGTGTGGGTGTGGTGTGTGGGTGTGGTGTGGGTGTGGTGTGTGGGTGTGTGTGGGTGTGGTGTGGGT
GTGGTGTGGTGTGTGGGTGTGGGTGTGGGTGTGGTGTGTGTGGGTGTGGTGTGGGTGTGTGGGTGTGGGTG
TGGTGTGT

GGTGTGGTGTGTGGGTGTGGTGTGGGTGTGGTGTGTGGGTGTGTGTGGGTGTGGTGTGGGTGTGGTGTGGT
GTGTGGGTGTGGGTGTGGGTGTGGTGTGTGTGGGTGTGGTGTGGGTGTGTGGGTGTGGGTGTGGTGTGTGG
TGTGTGTG

GTGTGTGGGTGTGTGTGGGTGTGGTGTGGGTGTGGTGTGGTGTGTGGGTGTGGGTGTGGGTGTGGTGTGTG
TGGGTGTGGTGTGGGTGTGTGGGTGTGGGTGTGGTGTGTGGTGTGTGTGTGGGTGTGGGTGTGGGTGTGGT
GTGTGGGT

GTGTGTGGGTGTGGTGTGGGTGTGGTGTGGTGTGTGGGTGTGGGTGTGGGTGTGGTGTGTGTGGGTGTGGT
GTGGGTGTGTGGGTGTGGGTGTGGTGTGTGGTGTGTGTGTGGGTGTGGGTGTGGGTGTGGTGTGTGGGTGT
GGGTGTGG

TGTGGTGTGGTGTGTGGGTGTGGGTGTGGGTGTGGTGTGTGTGGGTGTGGTGTGGGTGTGTGGGTGTGGGT
GTGGTGTGTGGTGTGTGTGTGGGTGTGGGTGTGGGTGTGGTGTGTGGGTGTGGGTGTGGTGTGTGGGTGTG
GTGTGTGG

GTGTGTGGGTGTGGGTGTGTGGGTGTGGGTGTGGTGTGTGGGTGTGGTGTGTGGGTGTGGTGTGGGTGTGG
TGTGTGGGTGTGTGTGGGTGTGGTGTGGGTGTGGTGTGGTGTGTGGGTGTGGGTGTGGGTGTGGTGTGTGT
GGGTGTGG

TGTGGGTGTGGTGTGTGTGGGTGTGTGGGTGTGGGTGTGTGGGTGTGGGTGTGGTGTGTGGGTGTGGTGTG
TGGGTGTGGTGTGGGTGTGGTGTGTGGGTGTGTGTGGGTGTGGTGTGGGTGTGGTGTGGTGTGTGGGTGTG
GGTGTGGG

GTGGGTGTGTGGGTGTGGGTGTGGTGTGTGGGTGTGGTGTGTGGGTGTGGTGTGGGTGTGGTGTGTGGGTG
TGTGTGGGTGTGGTGTGGGTGTGGTGTGGTGTGTGGGTGTGGGTGTGGGTGTGGTGTGTGTGGGTGTGGTG
TGGGTGTG

GTGGGTGTGGTGTGGGTGTGGTGTGTGGGTGTGTGTGGGTGTGGTGTGGGTGTGGTGTGGTGTGTGGGTGT
GGGTGTGGGTGTGGTGTGTGTGGGTGTGGTGTGGGTGTGTGGGTGTGGGTGTGGTGTGTGGTGTGTGTGTG
GGTGTGGG

AATAGGGTTAGGGTAGTGTTAGGGTAGTGTTAGGGTAGTGTGTGGGTGTGGTGTGTGTGGGTGTGTGGGTG
TGGGTGTGTGGGTGTGGGTGTGGTGTGTGGGTGTGGTGTGTGGGTGTGGTGTGGGTGTGGTGTGTGGGTGT
GTGTGGGT

GGGTAGTGTTAGGGTAGTGTTAGGGTAGTGTGTGGGTGTGGTGTGTGTGGGTGTGTGGGTGTGGGTGTGTG
GGTGTGGGTGTGGTGTGTGGGTGTGGTGTGTGGGTGTGGTGTGGGTGTGGTGTGTGGGTGTGTGTGGGTGT
GGTGTGGG

AGGGTAGTGTTAGGGTAGTGTGTGGGTGTGGTGTGTGTGGGTGTGTGGGTGTGGGTGTGTGGGTGTGGGTG
TGGTGTGTGGGTGTGGTGTGTGGGTGTGGTGTGGGTGTGGTGTGTGGGTGTGTGTGGGTGTGGTGTGGGTG
TGGTGTGG

TAGGGTAGTGTGTGGGTGTGGTGTGTGTGGGTGTGTGGGTGTGGGTGTGTGGGTGTGGGTGTGGTGTGTGG
GTGTGGTGTGTGGGTGTGGTGTGGGTGTGGTGTGTGGGTGTGTGTGGGTGTGGTGTGGGTGTGGTGTGGTG
TGTGGGTG

GTTGGATTAGAATAGGGTTAGGGTAGTGTTAGGGTAGTGTTAGGGTAGTGTGTGGGTGTGGTGTGTGTGGG
TGTGTGGGTGTGGGTGTGTGGGTGTGGGTGTGGTGTGTGGGTGTGGTGTGTGGGTGTGGTGTGGGTGTGGT
GTGTGGGT

GTTTATCAGGGTTGGATTAGAATAGGGTTAGGGTAGTGTTAGGGTAGTGTTAGGGTAGTGTGTGGGTGTGG
TGTGTGTGGGTGTGTGGGTGTGGGTGTGTGGGTGTGGGTGTGGTGTGTGGGTGTGGTGTGTGGGTGTGGTG
TGGGTGTG

AACACCATCCAGCATGCAATACAGTGACATATATATACCCACACCCACACCCACACACCACACCCACACAC
CCACACCACACCCACACCCACACACCCACACCCACACCCATCACAACCCTAACCCTACCCTATTCTAACCC
TGATGAAC

CTTGGCTCCATATTTCGACCTCGAAGAAATCCTGTTTACCACACCACTCAGGTCACCAGCACCTCTGATAA
CATGGTACTTCACACCAGGCAAGTCCTGACAACGCCCACCTCTGACATATACAATCGAATGCTCTTGAGCA
TCGTGGCC

TTTGGGGTCATCATGCCGTGCTGTTGGTACTGTCCATTCTGTGGAGGTGGTACTGAAGCAGGTTGAGGAGA
GACATGATGATGGTTCTCTGGAACAGCTGATGTCCCAGGTGTTGTCTCTTGTTGAGAATTAACCTTAGTGG
AATCTCTA

GGAGGTTGTACCTTCAGTGATGGTAATGTATGAGTCAATAGATAGACTAGTGACAGCAGACACAGGAGGTT
CTGTGAAGTCTGTGGAGGGGTCTGGCCTGGTGCCTGTTTCAGCTACTGCGCTCCCTGTCGATGTGGTCGTC
TTTACACT

GCCATCAGTAGATGAAGATAATACAGATGGTAGCGGCGGCGGTGGTGGTGTTTCTCCCTTGGGTTTGCCGA
ACAATGCCATGGGTGGAGGAGGAGGTGGTGGTGGTGGAGGGGGAGGAGCAGGAGAATCCCCAGTAGTTTCT
TGCTTGAC

TCATGCCGTGCTGTTGGTACTGTCCATTCTGTGGAGGTGGTACTGAAGCAGGTTGAGGAGAGACATGATGA
TGGTTCTCTGGAACAGCTGATGTCCCAGGTGTTGTCTCTTGTTGAGAATTAACCTTAGTGGAATCTCTATC
AAATTCCG

GGTGGTGGTGTTTCTCCCTTGGGTTTGCCGAACAATGCCATGGGTGGAGGAGGAGGTGGTGGTGGTGGAGG
GGGAGGAGCAGGAGAATCCCCAGTAGTTTCTTGCTTGACAGTGTCGTCTTCCGACTTTTTCTCTTTCTCCA
ACGATTCA

GATGAAGATAATACAGATGGTAGCGGCGGCGGTGGTGGTGTTTCTCCCTTGGGTTTGCCGAACAATGCCAT
GGGTGGAGGAGGAGGTGGTGGTGGTGGAGGGGGAGGAGCAGGAGAATCCCCAGTAGTTTCTTGCTTGACAG
TGTCGTCT

AGGTTTTGCATTGAGTTACCATTATTAGTCGTGTTCGAGACCTGCGTGTGGATGACGGGTGCAGAGTCAAT
CGACATGTCTGTCAGAGAGGGGTTGGAGTCCGTGGAGGAGACACTACTAAACCTTCTTTGGCGTTGTTGTG
ACTTAGAC

CATAGCTTTGTTTGGGGTCATCATGCCGTGCTGTTGGTACTGTCCATTCTGTGGAGGTGGTACTGAAGCAG
GTTGAGGAGAGACATGATGATGGTTCTCTGGAACAGCTGATGTCCCAGGTGTTGTCTCTTGTTGAGAATTA
ACCTTAGT

TTGTGTAAGTGGAGGTTGTACCTTCAGTGATGGTAATGTATGAGTCAATAGATAGACTAGTGACAGCAGAC
ACAGGAGGTTCTGTGAAGTCTGTGGAGGGGTCTGGCCTGGTGCCTGTTTCAGCTACTGCGCTCCCTGTCGA
TGTGGTCG

TAAGAGACAGGTTTATCAGGGTTGGATTAGAATAGGGTTAGGGTAGTGTTAGGGTAGTGTTAGGGTAGTGT
GTGGGTGTGGTGTGTGTGGGTGTGTGGGTGTGGGTGTGTGGGTGTGGGTGTGGTGTGTGGGTGTGGTGTGT
GGGTGTGG

TACTTGGCTTCTTGGCTCCATATTTCGACCTCGAAGAAATCCTGTTTACCACACCACTCAGGTCACCAGCA
CCTCTGATAACATGGTACTTCACACCAGGCAAGTCCTGACAACGCCCACCTCTGACATATACAATCGAATG
CTCTTGAG

AGCATGCAATACAGTGACATATATATACCCACACCCACACCCACACACCACACCCACACACCCACACCACA
CCCACACCCACACACCCACACCCACACCCATCACAACCCTAACCCTACCCTATTCTAACCCTGATGAACGT
GTCTCTCA

GGCACAATCAACGAAGAATACAGGTGCTACTTCATCTCCTTCGCCAGCAAGTGCTACAGCGGCTCCAGCAC
CACCACCTCCTCCACCAGCCCCACCAGCTTCCGTCTTTGAAATCTCTAATGATACACCAGCAACGAGTAGT
GATGCTAA

CCTTCAGTGATGGTAATGTATGAGTCAATAGATAGACTAGTGACAGCAGACACAGGAGGTTCTGTGAAGTC
TGTGGAGGGGTCTGGCCTGGTGCCTGTTTCAGCTACTGCGCTCCCTGTCGATGTGGTCGTCTTTACACTGG
AGGATGAA

AGATAAGGATGAAGGTATTAGTATGCAGCAATTCTACAATTGGGTGACTTCATACTCCAACAGGCTGGTCT
TCCACCAGTCTCAGGCCAAGTTTCAGCAAAGAGAGGACCATCCGGCCCCTGCAACGGAATCTTCGTCTTTG
ATGCCGCC

AGTTAGAGGCCATAGCTTTGTTTGGGGTCATCATGCCGTGCTGTTGGTACTGTCCATTCTGTGGAGGTGGT
ACTGAAGCAGGTTGAGGAGAGACATGATGATGGTTCTCTGGAACAGCTGATGTCCCAGGTGTTGTCTCTTG
TTGAGAAT

ATACAGATGGTAGCGGCGGCGGTGGTGGTGTTTCTCCCTTGGGTTTGCCGAACAATGCCATGGGTGGAGGA
GGAGGTGGTGGTGGTGGAGGGGGAGGAGCAGGAGAATCCCCAGTAGTTTCTTGCTTGACAGTGTCGTCTTC
CGACTTTT

AGCGGAGGCCAGGTTTTGCATTGAGTTACCATTATTAGTCGTGTTCGAGACCTGCGTGTGGATGACGGGTG
CAGAGTCAATCGACATGTCTGTCAGAGAGGGGTTGGAGTCCGTGGAGGAGACACTACTAAACCTTCTTTGG
CGTTGTTG

TGGTAATGTATGAGTCAATAGATAGACTAGTGACAGCAGACACAGGAGGTTCTGTGAAGTCTGTGGAGGGG
TCTGGCCTGGTGCCTGTTTCAGCTACTGCGCTCCCTGTCGATGTGGTCGTCTTTACACTGGAGGATGAACC
AGACGAAG

CGCAATCACCAATCGTACATAGCAGCAACAACACGCTGCACCACCACGAGCACCAACAACACTTGCCGCCC
ACACTAGAGTCGCTATCCTCCAAGTCGCACTCTGTGCCAGACTTAAATACAGCTACTCCCAGCTCTCCGAA
ACGAATGC

TCTAGGTGTTGGTATGCAAGGTGACATTTTCGGTATTGTTGTCCCAAGAAACACAACTGTTCCAACCATCA
AGAGAAGAACCTTCACAACTGTCAGTGACAACCAAACCACCGTTCAATTCCCAGTCTACCAAGGTGAACGT
GTCAACTG

AGTCCATGGAAGCTTTATGTTCTGTAGTATCATTTTGAATAGAAGAAGCAGAGAAAGCAGAGGAAGATGGT
GCCGACAACGAGGCGCCTGCAGTCGTGGTGTGTGTGACTGGGATGTTTAACTTTGACTGGACTGACTCATT
GTGCTGTA

AATATTCCTTCTACAATGACATCCATACTAAGTTTCAATAGAGACCAACAGCAGCCGCTATCTCAACCACT
GCCTCCTCCTCCACAACAGCAACAGGATCTCCACACGCACAATTTACATACTATACCAAGGAAACCTGGAA
TGGTTCAA

TTTCTCCCTTGGGTTTGCCGAACAATGCCATGGGTGGAGGAGGAGGTGGTGGTGGTGGAGGGGGAGGAGCA
GGAGAATCCCCAGTAGTTTCTTGCTTGACAGTGTCGTCTTCCGACTTTTTCTCTTTCTCCAACGATTCACC
GAACAATT

ACATAATTAGATGCTGTGACGAGCTTTATTGCGTATGTCCCACCCAGTTCCCTGTACAAGTGATTTGCCAC
AGACACACCAAACACACCAGCACCAACAACAACTATGTTCTTTGTGTTAATTGTCATATATTTTGCAATTT
CTTTCTTC

GGTATGCAAGGTGACATTTTCGGTATTGTTGTCCCAAGAAACACAACTGTTCCAACCATCAAGAGAAGAAC
CTTCACAACTGTCAGTGACAACCAAACCACCGTTCAATTCCCAGTCTACCAAGGTGAACGTGTCAACTGTA
AAGAAAAC

CATTTTGAATAGAAGAAGCAGAGAAAGCAGAGGAAGATGGTGCCGACAACGAGGCGCCTGCAGTCGTGGTG
TGTGTGACTGGGATGTTTAACTTTGACTGGACTGACTCATTGTGCTGTAGCCTAATGGCATCGGCCACGTC
AAATCTAG

AATACGCTGGTTACTATCAGCAGTGAAAGAGCATTCGAAGAGCAGATGATGGCGGCACAGGTGAGCCTGGC
GAAACAGAGAGAAGAGCTCGAGACTACGGCCCTCAGCTTGCCACGAGATATTGAATTGAGGGGTGAAGAAG
ATGATATT

CACCTCTACTCATACCACTCACAAGTATGGTAAGTTCAACAAGACTTCCAAGTCCAAGACCCCAAACCACA
CTGGTACTCACAAGTACGGTAAGTTCAACAAGACCTCCAAGTCTAAGACCCCAAACCATACCGGTACTCAC
AAGTATGG

AGCTGGAGGATTCAGGTTCTGATTCAAAGACCAGTACGAAGGCTCAGCCTGCCGAACCACAGGCAGAAAAG
AAACAAGAAGCGCCAGCTGAAGAGACCAAGACTTCTGCACCTGAAGCTAAGAAATCTGACGTTGCTGCTCC
TCAAGGTA

CTCCATTATCTCTAGGTGTTGGTATGCAAGGTGACATTTTCGGTATTGTTGTCCCAAGAAACACAACTGTT
CCAACCATCAAGAGAAGAACCTTCACAACTGTCAGTGACAACCAAACCACCGTTCAATTCCCAGTCTACCA
AGGTGAAC

TCTGTAGTATCATTTTGAATAGAAGAAGCAGAGAAAGCAGAGGAAGATGGTGCCGACAACGAGGCGCCTGC
AGTCGTGGTGTGTGTGACTGGGATGTTTAACTTTGACTGGACTGACTCATTGTGCTGTAGCCTAATGGCAT
CGGCCACG

CAAGGTTTCGGAAGACAAGGCCCACAAGGATTTGGAGGTCCTGGTCCACAAGAGTTTGGTGGTCCAGGTGG
CCAAGGATTCGGTGGTCCAAATCCTCAAGAATTCGGCGGGCCAGGTGGCCAAGGATTCGGTGGTCCAAACC
CTCAGGAA

TGAGTCAATAGATAGACTAGTGACAGCAGACACAGGAGGTTCTGTGAAGTCTGTGGAGGGGTCTGGCCTGG
TGCCTGTTTCAGCTACTGCGCTCCCTGTCGATGTGGTCGTCTTTACACTGGAGGATGAACCAGACGAAGAT
ACAGACGA

CGGTATTGTTGTCCCAAGAAACACAACTGTTCCAACCATCAAGAGAAGAACCTTCACAACTGTCAGTGACA
ACCAAACCACCGTTCAATTCCCAGTCTACCAAGGTGAACGTGTCAACTGTAAAGAAAACACTTTGTTGGGT
GAATTCGA

CTCTTTGTTCGATTCCAATGTCTGAAAATAACGAAGAACAACATCAACAACAACAACAACAGCAACCTGTT
GCCGTCGAAACTCCCTCGGCAGTTGAAGCTCCAGCATCAGCAGATCCTTCTTCTGAACAGAGCGTCGCTGT
CGAAGGCA

GCGGAATGACGCCATCAGTAGATGAAGATAATACAGATGGTAGCGGCGGCGGTGGTGGTGTTTCTCCCTTG
GGTTTGCCGAACAATGCCATGGGTGGAGGAGGAGGTGGTGGTGGTGGAGGGGGAGGAGCAGGAGAATCCCC
AGTAGTTT

ACGAAGAACAACATCAACAACAACAACAACAGCAACCTGTTGCCGTCGAAACTCCCTCGGCAGTTGAAGCT
CCAGCATCAGCAGATCCTTCTTCTGAACAGAGCGTCGCTGTCGAAGGCAATTCTGAACAAGCTGAAGACAA
TCAGGGTG

GAAGGTATTAGTATGCAGCAATTCTACAATTGGGTGACTTCATACTCCAACAGGCTGGTCTTCCACCAGTC
TCAGGCCAAGTTTCAGCAAAGAGAGGACCATCCGGCCCCTGCAACGGAATCTTCGTCTTTGATGCCGCCCG
CTAACACA

TTGAGTTACCATTATTAGTCGTGTTCGAGACCTGCGTGTGGATGACGGGTGCAGAGTCAATCGACATGTCT
GTCAGAGAGGGGTTGGAGTCCGTGGAGGAGACACTACTAAACCTTCTTTGGCGTTGTTGTGACTTAGACTT
CCTGTTTC

TAGAAAATACACATAATTAGATGCTGTGACGAGCTTTATTGCGTATGTCCCACCCAGTTCCCTGTACAAGT
GATTTGCCACAGACACACCAAACACACCAGCACCAACAACAACTATGTTCTTTGTGTTAATTGTCATATAT
TTTGCAAT

GTATGCAGCAATTCTACAATTGGGTGACTTCATACTCCAACAGGCTGGTCTTCCACCAGTCTCAGGCCAAG
TTTCAGCAAAGAGAGGACCATCCGGCCCCTGCAACGGAATCTTCGTCTTTGATGCCGCCCGCTAACACAAC
AGCAACAC

GTCCCAAGAAACACAACTGTTCCAACCATCAAGAGAAGAACCTTCACAACTGTCAGTGACAACCAAACCAC
CGTTCAATTCCCAGTCTACCAAGGTGAACGTGTCAACTGTAAAGAAAACACTTTGTTGGGTGAATTCGACT
TGAAGAAC

CCACTTTAACTTTGTCATCGGACGGTAGTTTAACTACTACCACCTCTACTCATACCACTCACAAGTATGGT
AAGTTCAACAAGACTTCCAAGTCCAAGACCCCAAACCACACTGGTACTCACAAGTACGGTAAGTTCAACAA
GACCTCCA

GATAGACTAGTGACAGCAGACACAGGAGGTTCTGTGAAGTCTGTGGAGGGGTCTGGCCTGGTGCCTGTTTC
AGCTACTGCGCTCCCTGTCGATGTGGTCGTCTTTACACTGGAGGATGAACCAGACGAAGATACAGACGAGG
ACACAGAC

CAGAGTTTAGGTTTCGAGCCAAACATCAATAACATAATATCAGGTCCTGGTGGAATGCATGTCGACACCGC
TAGGCTGCATCCTTTGGCTGGTCTAGACAAGGGTGTGGAGTATTTAGATCTGGAAGAAGAACAACTATCCT
CGTTAGAA

GTGTTCGAGACCTGCGTGTGGATGACGGGTGCAGAGTCAATCGACATGTCTGTCAGAGAGGGGTTGGAGTC
CGTGGAGGAGACACTACTAAACCTTCTTTGGCGTTGTTGTGACTTAGACTTCCTGTTTCTTGCAACACTTT
GCAGTTGA

TGAAAGCATCCTCATTACTGAACGCATGTCCTCCGGAGAATCCGCCCGCACCGCCAGGGAAGCCTCCAGCA
CCTCCAGCACCGCCAGGACCACCAGGACCAAAGCTTGGACCACCAGATCTAGCAGCCTCGAGACCGTATTG
ATCATATA

CATCACGATTTACTTGGCTTCTTGGCTCCATATTTCGACCTCGAAGAAATCCTGTTTACCACACCACTCAG
GTCACCAGCACCTCTGATAACATGGTACTTCACACCAGGCAAGTCCTGACAACGCCCACCTCTGACATATA
CAATCGAA

GATTCCAATGTCTGAAAATAACGAAGAACAACATCAACAACAACAACAACAGCAACCTGTTGCCGTCGAAA
CTCCCTCGGCAGTTGAAGCTCCAGCATCAGCAGATCCTTCTTCTGAACAGAGCGTCGCTGTCGAAGGCAAT
TCTGAACA

TTTGTCATCGGACGGTAGTTTAACTACTACCACCTCTACTCATACCACTCACAAGTATGGTAAGTTCAACA
AGACTTCCAAGTCCAAGACCCCAAACCACACTGGTACTCACAAGTACGGTAAGTTCAACAAGACCTCCAAG
TCTAAGAC

ATTTACAAGAAGATAAGGATGAAGGTATTAGTATGCAGCAATTCTACAATTGGGTGACTTCATACTCCAAC
AGGCTGGTCTTCCACCAGTCTCAGGCCAAGTTTCAGCAAAGAGAGGACCATCCGGCCCCTGCAACGGAATC
TTCGTCTT

GGTTAAATGCTTGGGTAGCTGGGAAGCCAAGGGACGATTGAGTCCCACTCCCTGCTGCTGTTGCTGCTGTT
GTTGTTGCTGCTGTTGTTGTTGCTGCAGAACGTTCAGTCCGGGCGGCAAACTAGCCACCGAAACAGGCGTT
CCTATATT

TCCAAACCATACCGGTACTCACAAGTACGGTAAGTTCAACAAGACCTCCAAATCCAAGACTCCAAACCACA
CTGGTACTCACAAGTACGGTAAGTTCAACAAGACCTCCAAGTCTAAGACCCCAAACCATACCGGTACTCAC
AAGTATGG

CACCAATCCTAATATTCCTTCTACAATGACATCCATACTAAGTTTCAATAGAGACCAACAGCAGCCGCTAT
CTCAACCACTGCCTCCTCCTCCACAACAGCAACAGGATCTCCACACGCACAATTTACATACTATACCAAGG
AAACCTGG

CCCCACGTAATGAGTTGTTTGATTCTCCCTTATGTGAGAGCTGCATGGCTTGGGGACAGTCAGAGATACAA
GAACAGCAGGATTAATTGAGGGCTGAGAGATCGGACCTTTGTCAACGTCCCAACCTGTGGGGACGTCCACC
GAAACAAT

ATTGATAACGATTGGCCTGTTGCTGGGCCTGGGCTTGTTGCAATTGTTGTTGCTGCACTTTGGCTTGTGCT
TGAGCCTGTGCTTGAGCCTGAGCCTGCTGTTGTTGTTGCTGTTGATTGTAATGATGGTGATGATCGGGTGA
TGAATATT

ACCACCAGCGTCCTATCAGAAGAAACTGGAGCAACGATTTCCTGGTGTCAAGTTTACGGTCGCCAAGAAGG
CAGACTCGCTCTACTGCATGGTCAGTGTGGCGAGTGTCGTTGCAAAAGTGACCAGAGATATACTAGTCGAA
TCCTTGAA

TCTGAAAATAACGAAGAACAACATCAACAACAACAACAACAGCAACCTGTTGCCGTCGAAACTCCCTCGGC
AGTTGAAGCTCCAGCATCAGCAGATCCTTCTTCTGAACAGAGCGTCGCTGTCGAAGGCAATTCTGAACAAG
CTGAAGAC

ATCGGTGGCATACTTTGGTTGGTTAAATGCTTGGGTAGCTGGGAAGCCAAGGGACGATTGAGTCCCACTCC
CTGCTGCTGTTGCTGCTGTTGTTGTTGCTGCTGTTGTTGTTGCTGCAGAACGTTCAGTCCGGGCGGCAAAC
TAGCCACC

CATACCACTCACAAGTATGGTAAGTTCAACAAGACTTCCAAGTCCAAGACCCCAAACCACACTGGTACTCA
CAAGTACGGTAAGTTCAACAAGACCTCCAAGTCTAAGACCCCAAACCATACCGGTACTCACAAGTATGGTA
AGTTCAAC

AGAAGAAGCAGAGAAAGCAGAGGAAGATGGTGCCGACAACGAGGCGCCTGCAGTCGTGGTGTGTGTGACTG
GGATGTTTAACTTTGACTGGACTGACTCATTGTGCTGTAGCCTAATGGCATCGGCCACGTCAAATCTAGAT
TCTAATAT

TTGCCAACGAACAAGGTAACAGAGTTACTCCATCTTTCGTTGCCTTCACCCCACAGGAAAGATTGATCGGT
GATGCTGCCAAGAACCAAGCTGCTTTGAACCCAAGAAACACTGTTTTTGATGCTAAGCGTTTGATTGGTAG
AAGATTCG

AATTTGTTTGCCCCAACTGTAGATCAAGTTGTGATTTGGAGGCCTCCTTTGAAAGTAGTGACGAGGAAGAT
GAAAGTGATGTGGAAAGTGAAGGTGATCAACTGGTGGATCAACTTAGCGTCTTAATGGAAACTTCAAAGGA
TGTTGATA

ACAATAACAACAATAACGGTGGATTTGGCGGTCCAGGCGGCCCTGGCGGTCAAGGTTTCGGAAGACAAGGC
CCACAAGGATTTGGAGGTCCTGGTCCACAAGAGTTTGGTGGTCCAGGTGGCCAAGGATTCGGTGGTCCAAA
TCCTCAAG

ATACAAGGCCCAGTTACTACCTGAAGTGACTGTAAGGGCACTCTGCTTTAAGCTGAAGGAAATGCTAGTGA
AGGAGTCAAACGTGATTCACATTCAGACCCCTGTCACAGTCGTGGGGGATATGCATGGACAGTTTCACGAT
ATGCTGGA

GTGACATTTTCGGTATTGTTGTCCCAAGAAACACAACTGTTCCAACCATCAAGAGAAGAACCTTCACAACT
GTCAGTGACAACCAAACCACCGTTCAATTCCCAGTCTACCAAGGTGAACGTGTCAACTGTAAAGAAAACAC
TTTGTTGG

CAACAACAACAGCAACCTGTTGCCGTCGAAACTCCCTCGGCAGTTGAAGCTCCAGCATCAGCAGATCCTTC
TTCTGAACAGAGCGTCGCTGTCGAAGGCAATTCTGAACAAGCTGAAGACAATCAGGGTGAAAATGATCCTT
CCGTAGTT

ACCACATCTACTCTTTGTTCGATTCCAATGTCTGAAAATAACGAAGAACAACATCAACAACAACAACAACA
GCAACCTGTTGCCGTCGAAACTCCCTCGGCAGTTGAAGCTCCAGCATCAGCAGATCCTTCTTCTGAACAGA
GCGTCGCT

TAGCGGCGGCGGTGGTGGTGTTTCTCCCTTGGGTTTGCCGAACAATGCCATGGGTGGAGGAGGAGGTGGTG
GTGGTGGAGGGGGAGGAGCAGGAGAATCCCCAGTAGTTTCTTGCTTGACAGTGTCGTCTTCCGACTTTTTC
TCTTTCTC

CAATAACGGTGGATTTGGCGGTCCAGGCGGCCCTGGCGGTCAAGGTTTCGGAAGACAAGGCCCACAAGGAT
TTGGAGGTCCTGGTCCACAAGAGTTTGGTGGTCCAGGTGGCCAAGGATTCGGTGGTCCAAATCCTCAAGAA
TTCGGCGG

CACCGTCAGGACAATAACAACAATAACGGTGGATTTGGCGGTCCAGGCGGCCCTGGCGGTCAAGGTTTCGG
AAGACAAGGCCCACAAGGATTTGGAGGTCCTGGTCCACAAGAGTTTGGTGGTCCAGGTGGCCAAGGATTCG
GTGGTCCA

TGGTTGTTGGTAATGTGCCCAGTTAGAGGCCATAGCTTTGTTTGGGGTCATCATGCCGTGCTGTTGGTACT
GTCCATTCTGTGGAGGTGGTACTGAAGCAGGTTGAGGAGAGACATGATGATGGTTCTCTGGAACAGCTGAT
GTCCCAGG

GGCCTTGTCTAACACCATCCAGCATGCAATACAGTGACATATATATACCCACACCCACACCCACACACCAC
ACCCACACACCCACACCACACCCACACCCACACACCCACACCCACACCCATCACAACCCTAACCCTACCCT
ATTCTAAC

GGCAGTTGGGTTAGCCACATTTTGGCCTTTAATATCTAAACCAACATGTCTGGAACCTGGTTCGAAGACAG
CATAGTCCCTGCCAAAGTTGGCACCTGCCACCAATCCTGGACCACCGATCAAAGCAGCGCCAATGTTGCCT
AAGATGGT

TCTGTAAGCTCGCAATCACCAATCGTACATAGCAGCAACAACACGCTGCACCACCACGAGCACCAACAACA
CTTGCCGCCCACACTAGAGTCGCTATCCTCCAAGTCGCACTCTGTGCCAGACTTAAATACAGCTACTCCCA
GCTCTCCG

TGTCAGAGAGGGGTTGGAGTCCGTGGAGGAGACACTACTAAACCTTCTTTGGCGTTGTTGTGACTTAGACT
TCCTGTTTCTTGCAACACTTTGCAGTTGATGCTGTTGCTGCTGATTAAACAATTGGGTGGCCTTGTTCGTG
TTTTCCAG

TACTTTGGTTGGTTAAATGCTTGGGTAGCTGGGAAGCCAAGGGACGATTGAGTCCCACTCCCTGCTGCTGT
TGCTGCTGTTGTTGTTGCTGCTGTTGTTGTTGCTGCAGAACGTTCAGTCCGGGCGGCAAACTAGCCACCGA
AACAGGCG

ATTATTAGTCGTGTTCGAGACCTGCGTGTGGATGACGGGTGCAGAGTCAATCGACATGTCTGTCAGAGAGG
GGTTGGAGTCCGTGGAGGAGACACTACTAAACCTTCTTTGGCGTTGTTGTGACTTAGACTTCCTGTTTCTT
GCAACACT

CAGTTACTACCTGAAGTGACTGTAAGGGCACTCTGCTTTAAGCTGAAGGAAATGCTAGTGAAGGAGTCAAA
CGTGATTCACATTCAGACCCCTGTCACAGTCGTGGGGGATATGCATGGACAGTTTCACGATATGCTGGAGA
TCTTCCAA

ACATCAACAACAACAACAACAGCAACCTGTTGCCGTCGAAACTCCCTCGGCAGTTGAAGCTCCAGCATCAG
CAGATCCTTCTTCTGAACAGAGCGTCGCTGTCGAAGGCAATTCTGAACAAGCTGAAGACAATCAGGGTGAA
AATGATCC

TAATGTGCCCAGTTAGAGGCCATAGCTTTGTTTGGGGTCATCATGCCGTGCTGTTGGTACTGTCCATTCTG
TGGAGGTGGTACTGAAGCAGGTTGAGGAGAGACATGATGATGGTTCTCTGGAACAGCTGATGTCCCAGGTG
TTGTCTCT

AGCTTTATGTTCTGTAGTATCATTTTGAATAGAAGAAGCAGAGAAAGCAGAGGAAGATGGTGCCGACAACG
AGGCGCCTGCAGTCGTGGTGTGTGTGACTGGGATGTTTAACTTTGACTGGACTGACTCATTGTGCTGTAGC
CTAATGGC

TACGTACTTATTTAATCTTTCTAGTAGACAATCTTTCTACCGGTGAAGGTTCTTCTTGCCTGTTGTCTGCC
TTCTGCACTCAATTCATCATTTTCATCATCTTCGTCCACCTTGGCTCTATTTAAGACTTTGATGAACGAGT
CCACTCTG

TCATATCCGCAATTTGTTTGCCCCAACTGTAGATCAAGTTGTGATTTGGAGGCCTCCTTTGAAAGTAGTGA
CGAGGAAGATGAAAGTGATGTGGAAAGTGAAGGTGATCAACTGGTGGATCAACTTAGCGTCTTAATGGAAA
CTTCAAAG

TAAGTTCAACAAGACTTCCAAGTCCAAGACTCCAAACCATACCGGTACTCACAAGTACGGTAAGTTCAACA
AGACCTCCAAATCCAAGACTCCAAACCACACTGGTACTCACAAGTACGGTAAGTTCAACAAGACCTCCAAG
TCTAAGAC

ATCCAAGCGATCTGTAAGCTCGCAATCACCAATCGTACATAGCAGCAACAACACGCTGCACCACCACGAGC
ACCAACAACACTTGCCGCCCACACTAGAGTCGCTATCCTCCAAGTCGCACTCTGTGCCAGACTTAAATACA
GCTACTCC

AGCTGAAGAAGCTGAAGAAGCCGAAATGGAGTTAGTGGAAGCGGAGAACATGCAACCAAGAGCAGACATAT
TGCCCACTGAGGAGCAGGAAGAAATGATGGCACAAGAAACTCCTAATTTAACTTCCACAAGAACAAGAATG
ATTGAAAT

GAAGACAAGGCCCACAAGGATTTGGAGGTCCTGGTCCACAAGAGTTTGGTGGTCCAGGTGGCCAAGGATTC
GGTGGTCCAAATCCTCAAGAATTCGGCGGGCCAGGTGGCCAAGGATTCGGTGGTCCAAACCCTCAGGAATT
CGGGGGCC

CTGTTGGTACTGTCCATTCTGTGGAGGTGGTACTGAAGCAGGTTGAGGAGAGACATGATGATGGTTCTCTG
GAACAGCTGATGTCCCAGGTGTTGTCTCTTGTTGAGAATTAACCTTAGTGGAATCTCTATCAAATTCCGGT
AAATTGGA

AAGGACTTTAAGCTGGAGGATTCAGGTTCTGATTCAAAGACCAGTACGAAGGCTCAGCCTGCCGAACCACA
GGCAGAAAAGAAACAAGAAGCGCCAGCTGAAGAGACCAAGACTTCTGCACCTGAAGCTAAGAAATCTGACG
TTGCTGCT

TGAATCTTCCCCCCACGTAATGAGTTGTTTGATTCTCCCTTATGTGAGAGCTGCATGGCTTGGGGACAGTC
AGAGATACAAGAACAGCAGGATTAATTGAGGGCTGAGAGATCGGACCTTTGTCAACGTCCCAACCTGTGGG
GACGTCCA

GCACGTGTGGTTGTGTAAGTGGAGGTTGTACCTTCAGTGATGGTAATGTATGAGTCAATAGATAGACTAGT
GACAGCAGACACAGGAGGTTCTGTGAAGTCTGTGGAGGGGTCTGGCCTGGTGCCTGTTTCAGCTACTGCGC
TCCCTGTC

CCTGCGTGTGGATGACGGGTGCAGAGTCAATCGACATGTCTGTCAGAGAGGGGTTGGAGTCCGTGGAGGAG
ACACTACTAAACCTTCTTTGGCGTTGTTGTGACTTAGACTTCCTGTTTCTTGCAACACTTTGCAGTTGATG
CTGTTGCT

GTGTTTTGACCCTTAGGAGCTCTGACAGCTAATTGATCTAAAGTGATACATTCACCGCCAGCCTTAACGAT
CTTGGCTCTGGCACCAGCAGTGAATCTCAAAGCAGCAACAGTGGTCTTTGGGAATTCGAAGATCCTGGCAT
CGTCAGTA

GAAGCTGTTTCAATTCCGGTGATGGCAAAGGTCCGTATTGGACACTTCGTGGAGGCACAGATCCTGGAAGA
GCTGCAAGTAGACTACATTGACGAAAGCGAGGTTTTGACTCCAGCTGATTGGACACATCACATTGAGAAGC
ATAACTTC

TCCTATCAGAAGAAACTGGAGCAACGATTTCCTGGTGTCAAGTTTACGGTCGCCAAGAAGGCAGACTCGCT
CTACTGCATGGTCAGTGTGGCGAGTGTCGTTGCAAAAGTGACCAGAGATATACTAGTCGAATCCTTGAAGA
GGGATCCC

GGTCATGCTCTCATATCCGCAATTTGTTTGCCCCAACTGTAGATCAAGTTGTGATTTGGAGGCCTCCTTTG
AAAGTAGTGACGAGGAAGATGAAAGTGATGTGGAAAGTGAAGGTGATCAACTGGTGGATCAACTTAGCGTC
TTAATGGA

ATCATGCACTGCTGTTGGTACGGCCCATTCTGTGGTGAATGTGACTGAGCAGTTTGAGGAGAGGCATGATG
GGGGTTCTCTGGAACAGCTGATGAAGCAGGTGTTGTTGTCTGTTGAGAGTTAGCCTTAGTGGAAGCCTTAT
CATATTCT

TCAGAGTCCGCTGAGGATGAATCAGTAAATGTATTACCTGACTCAGGTGATGGAGTGCTCAGAGGCGTTCC
AACTGATGATGGATACTGCGGAAACTGTGAATGTGGCCCAGGTGGAAAGTACATAGGCGACATTTGATAAG
GTGTATAC

ACGAAGAATACAGGTGCTACTTCATCTCCTTCGCCAGCAAGTGCTACAGCGGCTCCAGCACCACCACCTCC
TCCACCAGCCCCACCAGCTTCCGTCTTTGAAATCTCTAATGATACACCAGCAACGAGTAGTGATGCTAACA
AAGGCGGT

ACCGGTACTCACAAGTACGGTAAGTTCAACAAGACCTCCAAATCCAAGACTCCAAACCACACTGGTACTCA
CAAGTACGGTAAGTTCAACAAGACCTCCAAGTCTAAGACCCCAAACCATACCGGTACTCACAAGTATGGTA
AGTTCAAC

TCGGAGAGACTGTCGTTGGAAGGAACATCTACTGTACTTTCGTCGGCTACCACAACATGTGAGTCTTCATC
AGCGTCATCTTCACCATCCTCATAGTCTTCTGTTTCTTCTGGAACATTAGTATCCTCTTGTACTTCGCCTT
CCTGGTTC

TAAGAAGATTGGTCATGCTCTCATATCCGCAATTTGTTTGCCCCAACTGTAGATCAAGTTGTGATTTGGAG
GCCTCCTTTGAAAGTAGTGACGAGGAAGATGAAAGTGATGTGGAAAGTGAAGGTGATCAACTGGTGGATCA
ACTTAGCG

GCCTCGAACAATTTCGACGCTACAATGTACGGATCCGGTTCCTCCTCGGAAGACGCACTAGATGAGCACAG
AAGATGGAAGAGTTTGAGTACAAAGGACAAGTTTGTCGAAGGTCTATCTAATAACAAGTATAAGATCATCA
CCGGTGCC

CTCATTACTGAACGCATGTCCTCCGGAGAATCCGCCCGCACCGCCAGGGAAGCCTCCAGCACCTCCAGCAC
CGCCAGGACCACCAGGACCAAAGCTTGGACCACCAGATCTAGCAGCCTCGAGACCGTATTGATCATATATT
TCCCTTTT

CGAAGCTGCTTTGTCCGATGCTTTGGCTGCTTTGCAAATCGAAGACCCATCCGCTGATGAGTTGAGAAAGG
CAGAAGTTGGTTTGAAGAGAGTTGTCACCAAGGCCATGTCTTCTCGTTAAGATGTCATTCTGGTTAAGGTT
ATACACAT

GAAGTAAGATTCACGGCTTGGACATGTTCGATGAAATTGGTTTCGCTCCAGATGCTGAAGGTGAGTACAAG
TCCTCATACTGCTCTCACATGGATTGTTTCCGTTGGGTGAAGCGTGATTCTTATTTACCACAAGGTTCCCA
GGGTTTAA

TTAGATGTTGCTCCATTATCTCTAGGTGTTGGTATGCAAGGTGACATTTTCGGTATTGTTGTCCCAAGAAA
CACAACTGTTCCAACCATCAAGAGAAGAACCTTCACAACTGTCAGTGACAACCAAACCACCGTTCAATTCC
CAGTCTAC

GCAGAGTCAATCGACATGTCTGTCAGAGAGGGGTTGGAGTCCGTGGAGGAGACACTACTAAACCTTCTTTG
GCGTTGTTGTGACTTAGACTTCCTGTTTCTTGCAACACTTTGCAGTTGATGCTGTTGCTGCTGATTAAACA
ATTGGGTG

TGCCAAGGATCTAGGTGAGGCTTTGAGAAGAATAAACGAAGGTGCTGCAATGATCCGTACCAAAGGTGAAG
CAGGTACCGGTGACGTTTCCGAGGCCGTCAAGCACATCACCAAGATTAAGGCGGAGATCCAGCAGTATAAA
GAGAATTT

ATGATTCCGTATACACCTTATCAAATGTCGCCTATGTACTTTCCACCTGGGCCACATTCACAGTTTCCGCA
GTATCCATCATCAGTTGGAACGCCTCTGAGCACTCCATCACCTGAGTCAGGTAATACATTTACTGATTCAT
CCTCAGCG

GCCTTTAGTTCTGGAGAGGAGCTTTCTAGATCGATGAAGGTCTTGGCGACTTGTAATTCCAACTCACTTGG
AGCTTGGGATAAGATCTTGGATTGGACAGAGGACATTGGTCAGTTATGTGTCTAGTCTTTAGTGTGTTGGT
TTCTCTTC

GAATCTTCAGTATGAGTTGACGGAGGTGTGGAATCGGTTGGACTCACAGCTTTTGAAAGGACATTTCTCGG
TTGCTCAGGATGTAGTTCGATGTCAGATTGGAAGTCATGGTCAGATTCTATGTTAAGATCATCGGATTGTT
GGATCTCA

TGACATTGAGTCATATCTAGAAAAGTCGTCTAAGCAGTCTTCTCAAACCAGTGGTGCTGCCGCCGCCACTC
CTGCCGCCGCTACCTCAAGCACTACTGCTGGCTCTGCTCCATCGCCTTCTTCTACAGCATCATATGAGGAT
GTTCCAAT

TGTTACGTATAATACGCTGGTTACTATCAGCAGTGAAAGAGCATTCGAAGAGCAGATGATGGCGGCACAGG
TGAGCCTGGCGAAACAGAGAGAAGAGCTCGAGACTACGGCCCTCAGCTTGCCACGAGATATTGAATTGAGG
GGTGAAGA

ACAGTGACATATATATACCCACACCCACACCCACACACCACACCCACACACCCACACCACACCCACACCCA
CACACCCACACCCACACCCATCACAACCCTAACCCTACCCTATTCTAACCCTGATGAACGTGTCTCTCAAC
TTACCCTC

AACACACCCTAGCAGCAACAACGGTGCTAATAATAATGGCATGGGGAGCACGAACTCGTTGGACCAGTTTG
TGGCAACAGCCTCATCGTCATCTTCTCTGGTGACCAGCAGCGAGAATAGGCGCCCCCTAATAGGTGACGTT
ACCAATAG

TAACTACTACCACCTCTACTCATACCACTCACAAGTATGGTAAGTTCAACAAGACTTCCAAGTCCAAGACC
CCAAACCACACTGGTACTCACAAGTACGGTAAGTTCAACAAGACCTCCAAGTCTAAGACCCCAAACCATAC
CGGTACTC

AGAACGTGAAGCTCAGTCACGTGTATGTGGATACTGTTGGACCACCAGCGTCCTATCAGAAGAAACTGGAG
CAACGATTTCCTGGTGTCAAGTTTACGGTCGCCAAGAAGGCAGACTCGCTCTACTGCATGGTCAGTGTGGC
GAGTGTCG

ATGAGTTTGGAGTCCATGGAAGCTTTATGTTCTGTAGTATCATTTTGAATAGAAGAAGCAGAGAAAGCAGA
GGAAGATGGTGCCGACAACGAGGCGCCTGCAGTCGTGGTGTGTGTGACTGGGATGTTTAACTTTGACTGGA
CTGACTCA

ATGAAGAAGAAGCTGAAGAAGCTGAAGAAGCCGAAATGGAGTTAGTGGAAGCGGAGAACATGCAACCAAGA
GCAGACATATTGCCCACTGAGGAGCAGGAAGAAATGATGGCACAAGAAACTCCTAATTTAACTTCCACAAG
AACAAGAA

TGCTGTTGGTTAAGCATATTCATCAAAGAATTCAATTGAGCAGTGGGGTCAAATTGTTGCTGAGGTACAGG
GGGAGCCGCTGCGGCAGGTCCCTGAGAAGGTAATGGTTGGTTGGGGGCATAACCATACGGTTGCTGCATCA
TAGGTTGC

CCGTTGCTGTCGAAGTTGAGGATGAGGTCGATGTGGTGGATGAAGAAGTTGCCTGCGTAGAGGACGTTTCT
GAACTGGAAGAGGCAGAAGTGCTAGAGGAAGTGGTGGAAGGAGAAGAAGAAGTAGAAGTAGTACTAGAGGT
AGTAGAAG

GGGACCTATGCATCCAAACAATTCTCAACGATCTTTGCAGCAGCAGCAGCAGCAGCAGCAGCAACAAAAAC
AACAGCATCAACAATATCCATACCACCATCAAGGACCATCTCCATCTCCATCCCCATCTCCGTCTCCATTA
AACCCTTA

CTCTAGGAATTGAAAGCATCTTAGATACGTATGCACTAACTGGTCCTTCTTTATCACATGTCTTCATGGCT
TTCAGAAGTCTTGGATCCATGGCTGCTGTGTCGGATTCGGAGACCAAAATAGTGTTCAAACGATCGGTTTG
TGATTCCA

AAGAGGTACTTTGACCAAGAACTTGAAGCATATTGATGTTACCTTCACCAAGGTCAACAACCAATTGATCA
AGGTTGCTGTTCACAACGGTGACAGAAAGCACGTTGCCGCTTTGAGAACCGTTAAATCTTTGGTTGACAAC
ATGATCAC

TCTCAGTGGTTTCCTTTAGCTTGGTGTCGCTTTCCTCCAAGTCTTCTTCCAATTGCTGGTTCTTCTTGGAG
AAATTGTCGTTGTTGGACTGCAAGTGGTGAGAGTCCTCGGACAATTGCTTGGATTCAGCCAATTCTGCTTC
CAACTTCT

ACAGTGGACGACGAGGGCGAACTATATGGGTTAGACACCTCCGGCAACTCACCAGCCAATGAACACACAGC
TACCACAATTACACAGAATCACAGCGTGGTGGCCTCAAACGGAGACGTCGCATTCATCCCAGGAACTGCTA
CCGAAGGC

GGTAATTCTTCTCTAGGAATTGAAAGCATCTTAGATACGTATGCACTAACTGGTCCTTCTTTATCACATGT
CTTCATGGCTTTCAGAAGTCTTGGATCCATGGCTGCTGTGTCGGATTCGGAGACCAAAATAGTGTTCAAAC
GATCGGTT

TTAGCCACATTTTGGCCTTTAATATCTAAACCAACATGTCTGGAACCTGGTTCGAAGACAGCATAGTCCCT
GCCAAAGTTGGCACCTGCCACCAATCCTGGACCACCGATCAAAGCAGCGCCAATGTTGCCTAAGATGGTAC
CGTACATT

TTACATTGCCATCACACGTAAACTGGATACGCACGATTTGGCAAGAGACAGAGTAGATGACATCACTGAGG
TCCTTTCTACCATCCAACAACCATCCCTGATCATCGGTATCCAATCTGATGGACTGTTCACATATTCAGAA
CAAGAATT

GGATTTGGCGGTCCAGGCGGCCCTGGCGGTCAAGGTTTCGGAAGACAAGGCCCACAAGGATTTGGAGGTCC
TGGTCCACAAGAGTTTGGTGGTCCAGGTGGCCAAGGATTCGGTGGTCCAAATCCTCAAGAATTCGGCGGGC
CAGGTGGC

CCGAGAGCAATCATCCCTATATGCCGCTGCTTTAACTTCCGCTTCACCTCTCTTCTTTCTTGTGGTGAGCT
CTCTGTTTCCTTAGCAGCATCATCCTCGACTTTTCTCTCTGTGGTGATGGCCTTCGTAACGTTCTCCCCAG
AATATTGT

TCAATGTCGCTTGCATTGTCGTAGTGGAGGAAAACAATCTCTGCGCCTGTCTCAAGGGCGTACACCATGTG
TTGGACATAAACCTCGACAACATGGCTCTTGTTGGCCTGCTTCAATTCTGGTGTACCTGTAAGTTCTCCGT
CTTTTCCC

GACGAGCAATATTTACAAGAAGATAAGGATGAAGGTATTAGTATGCAGCAATTCTACAATTGGGTGACTTC
ATACTCCAACAGGCTGGTCTTCCACCAGTCTCAGGCCAAGTTTCAGCAAAGAGAGGACCATCCGGCCCCTG
CAACGGAA

CAATTCCGGTGATGGCAAAGGTCCGTATTGGACACTTCGTGGAGGCACAGATCCTGGAAGAGCTGCAAGTA
GACTACATTGACGAAAGCGAGGTTTTGACTCCAGCTGATTGGACACATCACATTGAGAAGCATAACTTCAA
GGTGCCAT

ACACCACACCCACACCACACCCACACCCACACACCACACCCACACACACACACACACCACACCCACACACA
CCCACACACCACACCACACCCACACACCACCCACACACACACAACACTACCCTAATCTAACCCTGTCCAAC
CTGTCTCC

CACACCACACCCACACCCACACACCACACCCACACACACACACACACCACACCCACACACACCCACACACC
ACACCACACCCACACACCACCCACACACACACAACACTACCCTAATCTAACCCTGTCCAACCTGTCTCCAA
ACTTACCC

TGTTTGGGGTCATCATGCCGTGCTGTTGGTACTGTCCATTCTGTGGAGGTGGTACTGAAGCAGGTTGAGGA
GAGACATGATGATGGTTCTCTGGAACAGCTGATGTCCCAGGTGTTGTCTCTTGTTGAGAATTAACCTTAGT
GGAATCTC

TTCAGCGCTGGTGTTCTCTAAGTTTATATCGTTGACAATGGTGCCTGTCTGCTTGGCCAACTGAGTGGCCT
TCACCATCAGTTCCACCTCCTCTCTGTTCTGTGTGTCCACTTGATTTTGTAGATGTGATATGTACTCTACC
GCCTGTGT

ACAGGGCGACTTCAGCGCTGGTGTTCTCTAAGTTTATATCGTTGACAATGGTGCCTGTCTGCTTGGCCAAC
TGAGTGGCCTTCACCATCAGTTCCACCTCCTCTCTGTTCTGTGTGTCCACTTGATTTTGTAGATGTGATAT
GTACTCTA

CCACACCCACACACCACACCCACACACACACACACACCACACCCACACACACCCACACACCACACCACACC
CACACACCACCCACACACACACAACACTACCCTAATCTAACCCTGTCCAACCTGTCTCCAAACTTACCCTC
CATTACCT

GCCATAGCTTTGTTTGGGGTCATCATGCCGTGCTGTTGGTACTGTCCATTCTGTGGAGGTGGTACTGAAGC
AGGTTGAGGAGAGACATGATGATGGTTCTCTGGAACAGCTGATGTCCCAGGTGTTGTCTCTTGTTGAGAAT
TAACCTTA

AAGTTGCTAGCTCTTCTATTGCTTCATCCACTAGCTCTTCTGTTGCACCATCCTCAAGTGAAGTTGTCAGC
TCTTCCGTTGCACCATCCTCAAGTGAAGTTGTCAGCTCTTCCGTTGCACCATCCTCAAGTGAAGTTGTCAG
CTCTTCCG

ACACCACACCCACACACACACACACACACCACACCCACACACACCCACACACCACACCACACCCACACACCAC
CCACACACACACAACACTACCCTAATCTAACCCTGTCCAACCTGTCTCCAAACTTACCCTCCATTACCTTA
CCTCCCCA

CTAGCTCTTCTGTTGCACCATCCTCAAGTGAAGTTGTCAGCTCTTCCGTTGCACCATCCTCAAGTGAAGTT
GTCAGCTCTTCCGTTGCACCATCCTCAAGTGAAGTTGTCAGCTCTTCCGTTGCTTCATCCTCAAGTGAAGT
TGCCAGCT

CATCATGCCGTGCTGTTGGTACTGTCCATTCTGTGGAGGTGGTACTGAAGCAGGTTGAGGAGAGACATGAT
GATGGTTCTCTGGAACAGCTGATGTCCCAGGTGTTGTCTCTTGTTGAGAATTAACCTTAGTGGAATCTCTA
TCAAATTC

CTGTTGACGAATTAATTACTGAAGGTAACGAAAAGTTGGCTGCTGTTCCAGCTGCTGGTCCAGCTTCTGCT
GGCGGTGCTGCTGCTGCCTCTGGTGATGCTGCTGCTGAAGAAGAAAAGGAAGAAGAAGCCGCTGAAGAATC
TGACGACG

GCTTCATCCACTAGCTCTTCTGTTGCACCATCCTCAAGTGAAGTTGTCAGCTCTTCCGTTGCACCATCCTC
AAGTGAAGTTGTCAGCTCTTCCGTTGCACCATCCTCAAGTGAAGTTGTCAGCTCTTCCGTTGCTTCATCCT
CAAGTGAA

AAGCACAGGCACAGGCACAGGCACAAGCCCAGGCGCAGGCCCAAGCCCAAGCCCAAGCACAAGCACAAGCA
CACGCTCAGCACCAGCCCTCCCAACAACCACAACAAGCTCAGCAACAACCTAACCCACTACATGGGTTGAC
ACCTACTG

TGTTGCACCATCCTCAAGTGAAGTTGTCAGCTCTTCCGTTGCACCATCCTCAAGTGAAGTTGTCAGCTCTT
CCGTTGCACCATCCTCAAGTGAAGTTGTCAGCTCTTCCGTTGCTTCATCCTCAAGTGAAGTTGCCAGCTCC
TCTGTTGC

CTCTTCTATTGCTTCATCCACTAGCTCTTCTGTTGCACCATCCTCAAGTGAAGTTGTCAGCTCTTCCGTTG
CACCATCCTCAAGTGAAGTTGTCAGCTCTTCCGTTGCACCATCCTCAAGTGAAGTTGTCAGCTCTTCCGTT
GCTTCATC

TCAGCTCTTCCGTTGCTTCATCCTCAAGTGAAGTTGCCAGCTCCTCTGTTGCGCCATCCTCAAGTGAAGTT
GTCAGCTCTTCCGTTGCACCATCCTCAAGTGAAGTTGTCAGCTCTTCCGTTGCTTCATCCTCAAGTGAAGT
TGCCAGCT

AAGCGCAAGCACAAGCACAAGCACAAGCACAAGCACAGGCACAGGCACAGGCACAAGCCCAGGCGCAGGCC
CAAGCCCAAGCCCAAGCACAAGCACAAGCACACGCTCAGCACCAGCCCTCCCAACAACCACAACAAGCTCA
GCAACAAC

GAAGGTAAGTCTGTTGACGAATTAATTACTGAAGGTAACGAAAAGTTGGCTGCTGTTCCAGCTGCTGGTCC
AGCTTCTGCTGGCGGTGCTGCTGCTGCCTCTGGTGATGCTGCTGCTGAAGAAGAAAAGGAAGAAGAAGCCG
CTGAAGAA

CCTCAAACTTGTAAGCGGTACCATACATGACGTAATCATAATCATCTGCAAGGGATCTGTCACCAGCCTGT
GGAGGTCTCCAGCTTCTTGTCGCAGAAGAGTCGTTAGCTGGGGTGTCTTCGAGGTTTAAAGAGGATGCAAT
AGTCACTG

AGTGAAGTTGTCAGCTCTTCCGTTGCTTCATCCTCAAGTGAAGTTGCCAGCTCCTCTGTTGCGCCATCCTC
AAGTGAAGTTGTCAGCTCTTCCGTTGCACCATCCTCAAGTGAAGTTGTCAGCTCTTCCGTTGCTTCATCCT
CAAGTGAA

GCCATCCTCAAGTGAAGTTGTCAGCTCTTCCGTTGCTTCATCCTCAAGTGAAGTTGCCAGCTCCTCTGTTG
CGCCATCCTCAAGTGAAGTTGTCAGCTCTTCCGTTGCACCATCCTCAAGTGAAGTTGTCAGCTCTTCCGTT
GCTTCATC

CCAGTTAGAAGCCATAGCTTTGTTTGGGGTCATCATGCCGTGCTGTTGGTACTGTCCATTCTGTGGAGGTG
GTACTGAAGCAGGTTGAGGAGAGACATGATGATGGTTCTCTGGAACAGCTGATGTCCCAGGTGTTGTCTCT
TGTTGAGA

TCTTCAAGTGAAGTTGCTAGCTCTTCTATTGCTTCATCCACTAGCTCTTCTGTTGCACCATCCTCAAGTGA
AGTTGTCAGCTCTTCCGTTGCACCATCCTCAAGTGAAGTTGTCAGCTCTTCCGTTGCACCATCCTCAAGTG
AAGTTGTC

GAACCGAAGAAGCGGAGCCTGATTCTGTGGCAGAAGATGAACCGGAGACTGATGAGCCTGATTCTGTGGCA
GAAGATGAACCGGAGACTGATGAGCCTGATTCTGTGGCAGAAGATGAACCGGAGGCGGATGAGCCTGATTC
AGTGGCAG

ACGACGAGTGCTGCACCACCTCCGCCACCAGCATTCCTAACTCAACAACCTCAATCTGGAGGAGCTCCAGC
TCCACCCCCACCTCCTCAAATGCCAGCTACATCAACATCCGGAGGCGGTTCATTCGCTGAAACTACTGGAG
ATGCAGGT

ACAAGCACAAGCACAAGCACAAGCACAGGCACAGGCACAGGCACAAGCCCAGGCGCAGGCCCAAGCCCAAG
CCCAAGCACAAGCACAAGCACACGCTCAGCACCAGCCCTCCCAACAACCACAACAAGCTCAGCAACAACCT
AACCCACT

GCACAAGCACAAGCACAGGCACAGGCACAGGCACAAGCCCAGGCGCAGGCCCAAGCCCAAGCCCAAGCACA
AGCACAAGCACACGCTCAGCACCAGCCCTCCCAACAACCACAACAAGCTCAGCAACAACCTAACCCACTAC
ATGGGTTG

CTCTTCCGTTGCACCATCCTCAAGTGAAGTTGTCAGCTCTTCCGTTGCACCATCCTCAAGTGAAGTTGTCA
GCTCTTCCGTTGCTTCATCCTCAAGTGAAGTTGCCAGCTCCTCTGTTGCGCCATCCTCAAGTGAAGTTGTC
AGCTCTTC

GGTTTAGTCGGTATCGATCAATTCTTGTTGAAGACTGGTACTTTGACCACCAGTGAAACTGCTCACAACAT
GAAGGTCATGAAATTCTCTGTCTCTCCAGTTGTGCAAGTCGCTGTCGAAGTCAAGAACGCTAACGACTTAC
CAAAATTG

CAAGTGAAGTTGTCAGCTCTTCCGTTGCACCATCCTCAAGTGAAGTTGTCAGCTCTTCCGTTGCTTCATCC
TCAAGTGAAGTTGCCAGCTCCTCTGTTGCGCCATCCTCAAGTGAAGTTGTCAGCTCTTCCGTTGCTTCATC
CTCAAGTG

CAAGTGAAGTTGTCAGCTCTTCCGTTGCACCATCCTCAAGTGAAGTTGTCAGCTCTTCCGTTGCTTCATCC
TCAAGTGAAGTTGCCAGCTCCTCTGTTGCGCCATCCTCAAGTGAAGTTGTCAGCTCTTCCGTTGCTTCTTC
TACAAGCG

TGGAACCACGAGGAACAACTAAGTCGATGTACTCATCTTGATCCAACAAGTCGGAAACATCCTGTCTGGTT
TCGATCAATTGCACAGAGCCCACAGGAACACCAGTCTCACTTTGGAATTGTGCAATGGTGTCGTTAACGAT
CTTTGCCA

TCCGTTGCACCATCCTCAAGTGAAGTTGTCAGCTCTTCCGTTGCTTCATCCTCAAGTGAAGTTGCCAGCTC
CTCTGTTGCGCCATCCTCAAGTGAAGTTGTCAGCTCTTCCGTTGCTTCATCCTCAAGTGAAGTTGCCAGCT
CCTCTGTT

CACACACACACACACACACCACACCCACACACACCCACACACCACACCACACCCACACACCACCCACACACAC
ACAACACTACCCTAATCTAACCCTGTCCAACCTGTCTCCAAACTTACCCTCCATTACCTTACCTCCCCACT
CGTTACCC

GCACCATCCTCAAGTGAAGTTGTCAGCTCTTCCGTTGCACCATCCTCAAGTGAAGTTGTCAGCTCTTCCGT
TGCTTCATCCTCAAGTGAAGTTGCCAGCTCCTCTGTTGCGCCATCCTCAAGTGAAGTTGTCAGCTCTTCCG
TTGCTTCA

TCCGTTGCACCATCCTCAAGTGAAGTTGTCAGCTCTTCCGTTGCTTCATCCTCAAGTGAAGTTGCCAGCTC
CTCTGTTGCGCCATCCTCAAGTGAAGTTGTCAGCTCTTCCGTTGCTTCTTCTACAAGCGAAGCTACTAGTT
CTTCTGCT

GATGAGCTAGGAACCGAAGAAGCGGAGCCTGATTCTGTGGCAGAAGATGAACCGGAGACTGATGAGCCTGA
TTCTGTGGCAGAAGATGAACCGGAGACTGATGAGCCTGATTCTGTGGCAGAAGATGAACCGGAGGCGGATG
AGCCTGAT

ACTAAGGCATTGGAACCACGAGGAACAACTAAGTCGATGTACTCATCTTGATCCAACAAGTCGGAAACATC
CTGTCTGGTTTCGATCAATTGCACAGAGCCCACAGGAACACCAGTCTCACTTTGGAATTGTGCAATGGTGT
CGTTAACG

TGTCAGCTCTTCCGTTGCACCATCCTCAAGTGAAGTTGTCAGCTCTTCCGTTGCTTCATCCTCAAGTGAAG
TTGCCAGCTCCTCTGTTGCGCCATCCTCAAGTGAAGTTGTCAGCTCTTCCGTTGCTTCATCCTCAAGTGAA
GTTGCCAG

TCCTCAAGTGAAGTTGTCAGCTCTTCCGTTGCACCATCCTCAAGTGAAGTTGTCAGCTCTTCCGTTGCACC
ATCCTCAAGTGAAGTTGTCAGCTCTTCCGTTGCTTCATCCTCAAGTGAAGTTGCCAGCTCCTCTGTTGCGC
CATCCTCA

AGCTCTTCCGTTGCTTCATCCTCAAGTGAAGTTGCCAGCTCCTCTGTTGCGCCATCCTCAAGTGAAGTTGT
CAGCTCTTCCGTTGCTTCATCCTCAAGTGAAGTTGCCAGCTCCTCTGTTGCGCCATCCTCAAGTGAAGTTG
TCAGCTCT

TGCTGCCACATCTTCAAGTGAAGTTGCTAGCTCTTCTATTGCTTCATCCACTAGCTCTTCTGTTGCACCAT
CCTCAAGTGAAGTTGTCAGCTCTTCCGTTGCACCATCCTCAAGTGAAGTTGTCAGCTCTTCCGTTGCACCA
TCCTCAAG

TGTCAGCTCTTCCGTTGCACCATCCTCAAGTGAAGTTGTCAGCTCTTCCGTTGCTTCATCCTCAAGTGAAG
TTGCCAGCTCCTCTGTTGCGCCATCCTCAAGTGAAGTTGTCAGCTCTTCCGTTGCTTCTTCTACAAGCGAA
GCTACTAG

CTGATTCTGTGACAGAACCGGATGAGCTAGGAACCGAAGAAGCGGAGCCTGATTCTGTGGCAGAAGATGAA
CCGGAGACTGATGAGCCTGATTCTGTGGCAGAAGATGAACCGGAGACTGATGAGCCTGATTCTGTGGCAGA
AGATGAAC

AAGTTGTCAGCTCTTCCGTTGCACCATCCTCAAGTGAAGTTGTCAGCTCTTCCGTTGCACCATCCTCAAGT
GAAGTTGTCAGCTCTTCCGTTGCTTCATCCTCAAGTGAAGTTGCCAGCTCCTCTGTTGCGCCATCCTCAAG
TGAAGTTG

GCCGGACTCTTCAGCTTCTGCCAATCAGCAGGTGCAACATGCTCAGCAACATGCTCAACAACAACATGAGG
CCCAAATGCATGCACAGGCACAAGCTCAGGCTCAGGCACAGGCACAGGTGGAACAACAGAAGCAGCAACAG
CAATTCTT

CTCCTCTGTTGCGCCATCCTCAAGTGAAGTTGTCAGCTCTTCCGTTGCACCATCCTCAAGTGAAGTTGTCA
GCTCTTCCGTTGCTTCATCCTCAAGTGAAGTTGCCAGCTCCTCTGTTGCGCCATCCTCAAGTGAAGTTGTC
AGCTCTTC

CTCAAGTGAAGTTGCCAGCTCCTCTGTTGCGCCATCCTCAAGTGAAGTTGTCAGCTCTTCCGTTGCTTCAT
CCTCAAGTGAAGTTGCCAGCTCCTCTGTTGCGCCATCCTCAAGTGAAGTTGTCAGCTCTTCCGTTGCACCA
TCCTCAAG

CGTTGCTTCATCCTCAAGTGAAGTTGCCAGCTCCTCTGTTGCGCCATCCTCAAGTGAAGTTGTCAGCTCTT
CCGTTGCACCATCCTCAAGTGAAGTTGTCAGCTCTTCCGTTGCTTCATCCTCAAGTGAAGTTGCCAGCTCC
TCTGTTGC

TGCAACAGTCACTACAGCAAATGCAGCATTTACAGCAATTGAAAATGCAGCAGCAACAACAACAGCAGCAA
CAACAACAGCAGCAACAACAACAGCAGCAACAACAACAGCAACAGCACATATATCCCTCCTCGACTCCTGG
TGTGGCTA

GTTGCCAGCTCCTCTGTTGCGCCATCCTCAAGTGAAGTTGTCAGCTCTTCCGTTGCTTCATCCTCAAGTGA
AGTTGCCAGCTCCTCTGTTGCGCCATCCTCAAGTGAAGTTGTCAGCTCTTCCGTTGCACCATCCTCAAGTG
AAGTTGTC

TGGGCCTCCTGTTCAGGATCCACTCTAGAAGGCTCCTCTTCATGATGTTCTTCCGTTTCCTGTGGTTGTTC
TGGTTGTTGTTCGTGTGACTGTTGGGTGTGATTGTCGCCGGACGTTTCCCCCAATCCTGCTAATAAAGGGT
CCTGTTCT

GACAGAACCGGATGAGCTAGGAACCGAAGAAGCGGAGCCTGATTCTGTGGCAGAAGATGAACCGGAGACTG
ATGAGCCTGATTCTGTGGCAGAAGATGAACCGGAGACTGATGAGCCTGATTCTGTGGCAGAAGATGAACCG
GAGGCGGA

ACGTAACCAGTAATACCCTGAATTCTGCCGGTGGAAATAGCCTCCTTCCACAGGCCACTGGAAGAAGAGGG
CCAGCACCACCACCTCCTCCAAGAGCATCTCGCCCCACACCAAACGTTACGATGCAACAAAATCCACAACA
GTACAATA

ACCGGAGACTGATGAGCCTGATTCTGTGGCAGAAGATGAACCGGAGACTGATGAGCCTGATTCTGTGGCAG
AAGATGAACCGGAGGCGGATGAGCCTGATTCAGTGGCAGAAGATGAACCGGAGGCGGATGAGCCTGATTCA
GTGGCAGA

CATCCTCAAGTGAAGTTGTCAGCTCTTCCGTTGCTTCATCCTCAAGTGAAGTTGCCAGCTCCTCTGTTGCG
CCATCCTCAAGTGAAGTTGTCAGCTCTTCCGTTGCTTCATCCTCAAGTGAAGTTGCCAGCTCCTCTGTTGC
GCCATCCT

GAGACTGAAGAGCCTGATGAAGTAATAGAGCTCGAGACTTCAGAGGACGAGGTTGATGGAGCAATGGAGCT
CGAGACTTCGGTGGAGGATGACGTTAACTCAGTGAGAGAGCTCGAGACATCAGAAGTAGATGAGGTCAACT
CAATAGAA

AGCGGAGCCTGATTCTGTGGCAGAAGATGAACCGGAGACTGATGAGCCTGATTCTGTGGCAGAAGATGAAC
CGGAGACTGATGAGCCTGATTCTGTGGCAGAAGATGAACCGGAGGCGGATGAGCCTGATTCAGTGGCAGAA
GATGAACC

CAAGCGGCGCAAGCGCAAGCACAAGCACAAGCACAAGCACAAGCACAGGCACAGGCACAGGCACAAGCCCA
GGCGCAGGCCCAAGCCCAAGCCCAAGCACAAGCACAAGCACACGCTCAGCACCAGCCCTCCCAACAACCAC
AACAAGCT

CCTCTGTTGCGCCATCCTCAAGTGAAGTTGTCAGCTCTTCCGTTGCTTCATCCTCAAGTGAAGTTGCCAGC
TCCTCTGTTGCGCCATCCTCAAGTGAAGTTGTCAGCTCTTCCGTTGCACCATCCTCAAGTGAAGTTGTCAG
CTCTTCCG

GCGCCATCCTCAAGTGAAGTTGTCAGCTCTTCCGTTGCACCATCCTCAAGTGAAGTTGTCAGCTCTTCCGT
TGCTTCATCCTCAAGTGAAGTTGCCAGCTCCTCTGTTGCGCCATCCTCAAGTGAAGTTGTCAGCTCTTCCG
TTGCTTCT

GTTCAGGATCCACTCTAGAAGGCTCCTCTTCATGATGTTCTTCCGTTTCCTGTGGTTGTTCTGGTTGTTGT
TCGTGTGACTGTTGGGTGTGATTGTCGCCGGACGTTTCCCCCAATCCTGCTAATAAAGGGTCCTGTTCTTC
ACTCATTC

GAGGATGAGCCTGATTCTGTGACAGAACCGGATGAGCTAGGAACCGAAGAAGCGGAGCCTGATTCTGTGGC
AGAAGATGAACCGGAGACTGATGAGCCTGATTCTGTGGCAGAAGATGAACCGGAGACTGATGAGCCTGATT
CTGTGGCA

GGAGCTCTGACAGCTAATTGATCCAAAGTGATACATTCACCACCAGCCTTAACAATCTTGGCTCTGGCACC
AGCAGTGAATCTCAAAGCAGCAACAGTGGTCTTTGGGAATTCAAAGATTCTGGCATCGTCAGTAACAGTAC
CAACAACG

GTATCGATCAATTCTTGTTGAAGACTGGTACTTTGACCACCAGTGAAACTGCTCACAACATGAAGGTCATG
AAATTCTCTGTCTCTCCAGTTGTGCAAGTCGCTGTCGAAGTCAAGAACGCTAACGACTTACCAAAATTGGT
CGAAGGTT

CAGAAGAGGTAGCAGCTTCTGAGCTGGTAGAAGTACTACTTGGAGACCAGGTGTTGCTGCTGCCTTCACCA
GTCCAGACAAAAGTAGCATCTTGGGTGACAGTCTTAGTGTAGACGTGACCGTTCTTGGTGGCAGTGATAGT
GGTAGTGA

GTGTTCTCTAAGTTTATATCGTTGACAATGGTGCCTGTCTGCTTGGCCAACTGAGTGGCCTTCACCATCAG
TTCCACCTCCTCTCTGTTCTGTGTGTCCACTTGATTTTGTAGATGTGATATGTACTCTACCGCCTGTGTGA
GAATTTGG

TCCTCAAGTGAAGTTGCCAGCTCCTCTGTTGCGCCATCCTCAAGTGAAGTTGTCAGCTCTTCCGTTGCACC
ATCCTCAAGTGAAGTTGTCAGCTCTTCCGTTGCTTCATCCTCAAGTGAAGTTGCCAGCTCCTCTGTTGCGC
CATCCTCA

GTTGCTCCTTACAACTCAACCATTGCTAGCTCTTCTTCCACTGCCCAGACTTCTATCTCGACCATTGCTCC
TTACAACTCCACAACCACCACCACCCCAGCTAGTTCTGCTTCCAGCGTTATTATCTCAACCAGAAACGGTA
CCACTGTT

GATGAGCCTGATTCTGTGGCAGAAGATGAACCGGAGACTGATGAGCCTGATTCTGTGGCAGAAGATGAACC
GGAGGCGGATGAGCCTGATTCAGTGGCAGAAGATGAACCGGAGGCGGATGAGCCTGATTCAGTGGCAGAAG
TTGAACCG

AAGTTGCCAGCTCCTCTGTTGCGCCATCCTCAAGTGAAGTTGTCAGCTCTTCCGTTGCACCATCCTCAAGT
GAAGTTGTCAGCTCTTCCGTTGCTTCATCCTCAAGTGAAGTTGCCAGCTCCTCTGTTGCGCCATCCTCAAG
TGAAGTTG

CCATCATTTGACTTCTGCTTCCAAGACTTATTATTCAAATCTTGCTGTGGGTGCTCGGATGGGTGATTAGA
ATTGGATGGAGACAAGAGTGGAGACGATTCATCAGCTTGGTCAGCAGCTTTGTGCTCCTTGTGAGAGCTAG
TGTACAAA

TTGCTTCATCCTCAAGTGAAGTTGCCAGCTCCTCTGTTGCGCCATCCTCAAGTGAAGTTGTCAGCTCTTCC
GTTGCTTCATCCTCAAGTGAAGTTGCCAGCTCCTCTGTTGCGCCATCCTCAAGTGAAGTTGTCAGCTCTTC
CGTTGCAC

GTGGTTGAAGCAGAAGAGGTAGCAGCTTCTGAGCTGGTAGAAGTACTACTTGGAGACCAGGTGTTGCTGCT
GCCTTCACCAGTCCAGACAAAAGTAGCATCTTGGGTGACAGTCTTAGTGTAGACGTGACCGTTCTTGGTGG
CAGTGATA

AAGCTCAAGCACAGGCGGCACAAGCGGCGCAAGCGCAAGCACAAGCACAAGCACAAGCACAAGCACAGGCA
CAGGCACAGGCACAAGCCCAGGCGCAGGCCCAAGCCCAAGCCCAAGCACAAGCACAAGCACACGCTCAGCA
CCAGCCCT

TGAAGTTGTCAGCTCTTCCGTTGCTTCATCCTCAAGTGAAGTTGCCAGCTCCTCTGTTGCGCCATCCTCAA
GTGAAGTTGTCAGCTCTTCCGTTGCTTCATCCTCAAGTGAAGTTGCCAGCTCCTCTGTTGCGCCATCCTCA
AGTGAAGT

TAGCTCAAGCGTTGCTCCTTACAACTCAACCATTGCTAGCTCTTCTTCCACTGCCCAGACTTCTATCTCGA
CCATTGCTCCTTACAACTCCACAACCACCACCACCCCAGCTAGTTCTGCTTCCAGCGTTATTATCTCAACC
AGAAACGG

GATACCACCCCACCAAAGGACATGTGGAAATTGATCGTCGATCCAATGGATGCTCCGGACCAACCACAAGA
TTTGACCATTGACTTTGAACGTGGTCTTCCAGTCAAGTTGACCTACACCGACAACAAGACTTCCAAGGAAG
TTTCCGTT

CACCAAAGGACATGTGGAAATTGATCGTCGATCCAATGGATGCTCCGGACCAACCACAAGATTTGACCATT
GACTTTGAACGTGGTCTTCCAGTCAAGTTGACCTACACCGACAACAAGACTTCCAAGGAAGTTTCCGTTAC
CAAGCCTT

CAGCTAATTGATCCAAAGTGATACATTCACCACCAGCCTTAACAATCTTGGCTCTGGCACCAGCAGTGAAT
CTCAAAGCAGCAACAGTGGTCTTTGGGAATTCAAAGATTCTGGCATCGTCAGTAACAGTACCAACAACGAC
AACAGTCT

AGATGAGCCGGAGACTGAAGAGCCTGATGAAGTAATAGAGCTCGAGACTTCAGAGGACGAGGTTGATGGAG
CAATGGAGCTCGAGACTTCGGTGGAGGATGACGTTAACTCAGTGAGAGAGCTCGAGACATCAGAAGTAGAT
GAGGTCAA

CAGAAGATGAACCGGAGACTGATGAGCCTGATTCTGTGGCAGAAGATGAACCGGAGACTGATGAGCCTGAT
TCTGTGGCAGAAGATGAACCGGAGGCGGATGAGCCTGATTCAGTGGCAGAAGATGAACCGGAGGCGGATGA
GCCTGATT

AGAAATTCTTGCCGGACTCTTCAGCTTCTGCCAATCAGCAGGTGCAACATGCTCAGCAACATGCTCAACAA
CAACATGAGGCCCAAATGCATGCACAGGCACAAGCTCAGGCTCAGGCACAGGCACAGGTGGAACAACAGAA
GCAGCAAC

ATTAATTACTGAAGGTAACGAAAAGTTGGCTGCTGTTCCAGCTGCTGGTCCAGCTTCTGCTGGCGGTGCTG
CTGCTGCCTCTGGTGATGCTGCTGCTGAAGAAGAAAAGGAAGAAGAAGCCGCTGAAGAATCTGACGACGAC
ATGGGTTT

CCTCCGCTGTTTCCTCTTCGACCGAGTCTGTTAGCTCTTCCTCTGTCAGTTCTTCCTCAGCCGTTTCCTCT
TCTGAAGCTGTCAGTTCCTCTCCAGTTTCCTCAGTTGTTTCATCTTCGGCCGGACCTGCTAGCTCAAGCGT
TGCTCCTT

CCCCTTTGGATATCAGACAAATAGCAACATGTCATCTCCACCCCCTCCTCCAGTGACAACTTTCAATACCC
TGACACCACAAATGACTGCAGCAACTGGACAACCTGCAGTTCCCCTTCCTCAGAATACTCAAGCACCTTCG
CAAGCCAC

AGCCTGATGAAGTAATAGAGCTCGAGACTTCAGAGGACGAGGTTGATGGAGCAATGGAGCTCGAGACTTCG
GTGGAGGATGACGTTAACTCAGTGAGAGAGCTCGAGACATCAGAAGTAGATGAGGTCAACTCAATAGAACT
ACTGATGG

CATCCTCAAGTGAAGTTGTCAGCTCTTCCGTTGCTTCATCCTCAAGTGAAGTTGCCAGCTCCTCTGTTGCG
CCATCCTCAAGTGAAGTTGTCAGCTCTTCCGTTGCTTCTTCTACAAGCGAAGCTACTAGTTCTTCTGCTGT
CACATCTT

CTGCACCACCTCCGCCACCAGCATTCCTAACTCAACAACCTCAATCTGGAGGAGCTCCAGCTCCACCCCCA
CCTCCTCAAATGCCAGCTACATCAACATCCGGAGGCGGTTCATTCGCTGAAACTACTGGAGATGCAGGTCG
TGATGCAC

GATTCTGTGGCAGAAGATGAACCGGAGACTGATGAGCCTGATTCTGTGGCAGAAGATGAACCGGAGACTGA
TGAGCCTGATTCTGTGGCAGAAGATGAACCGGAGGCGGATGAGCCTGATTCAGTGGCAGAAGATGAACCGG
AGGCGGAT

GACAAATCGCAGCTAGATAAGCCCTCAGTTTTACTGCAGGAAGCACGTGGAGAATCTGCTTCACCACCAGC
AGCGGCTGGAAATGGAGGCACACCTGGTGGACCTCCGGCTTCTTTAGCAGATGCGTTGGCAGCAGCTTTAA
ACAAAAGA

ACAGGCACAGGCACAAGCCCAGGCGCAGGCCCAAGCCCAAGCCCAAGCACAAGCACAAGCACACGCTCAGC
ACCAGCCCTCCCAACAACCACAACAAGCTCAGCAACAACCTAACCCACTACATGGGTTGACACCTACTGCA
AAGGATGT

GAAGATTTCAAGAAATTCTTGCCGGACTCTTCAGCTTCTGCCAATCAGCAGGTGCAACATGCTCAGCAACA
TGCTCAACAACAACATGAGGCCCAAATGCATGCACAGGCACAAGCTCAGGCTCAGGCACAGGCACAGGTGG
AACAACAG

ACAGGCGGCACAAGCGGCGCAAGCGCAAGCACAAGCACAAGCACAAGCACAAGCACAGGCACAGGCACAGG
CACAAGCCCAGGCGCAGGCCCAAGCCCAAGCCCAAGCACAAGCACAAGCACACGCTCAGCACCAGCCCTCC
CAACAACC

ATTCTGTGGCAGAAGATGAACCGGAGACTGATGAGCCTGATTCTGTGGCAGAAGATGAACCGGAGGCGGAT
GAGCCTGATTCAGTGGCAGAAGATGAACCGGAGGCGGATGAGCCTGATTCAGTGGCAGAAGTTGAACCGGA
GACTGATG

TTCTTCTGCTGTCACATCTTCCTCCGCTGTTTCCTCTTCGACCGAGTCTGTTAGCTCTTCCTCTGTCAGTT
CTTCCTCAGCCGTTTCCTCTTCTGAAGCTGTCAGTTCCTCTCCAGTTTCCTCAGTTGTTTCATCTTCGGCC
GGACCTGC

GTCAAGTAATTCCTCAAGTATTCTGCATCTTTACCTATTTCCATGACACACACATCCACATTAGAACCAGA
CCCCAAATCATTCCAGATACCAGCCTGGATTGCATCAGAGGCCAACTTTATGGCTTCCTCCTTCGTAAGGT
CTTGCTTC

TATCAGACAAATAGCAACATGTCATCTCCACCCCCTCCTCCAGTGACAACTTTCAATACCCTGACACCACA
AATGACTGCAGCAACTGGACAACCTGCAGTTCCCCTTCCTCAGAATACTCAAGCACCTTCGCAAGCCACAA
ATGTGCCA

TGCTTTAGGAAGGTACTATACACTCGCTTCTGTCATGCTCGAGTCCGCTTCATCTGTGAAAGATTCAGCCA
CGCTTGAAGTAACAACAGGAGGCAAGACGAGGGGTCTGGTGCCATACTGTTCATCAAATTCAGTAAAGTCG
AGTTGTGA

CCAAGACTTATTATTCAAATCTTGCTGTGGGTGCTCGGATGGGTGATTAGAATTGGATGGAGACAAGAGTG
GAGACGATTCATCAGCTTGGTCAGCAGCTTTGTGCTCCTTGTGAGAGCTAGTGTACAAATGATGCAAATAG
GAAGTAAT

GTCCGCTTTGGAAGGTAAGTCTGTTGACGAATTAATTACTGAAGGTAACGAAAAGTTGGCTGCTGTTCCAG
CTGCTGGTCCAGCTTCTGCTGGCGGTGCTGCTGCTGCCTCTGGTGATGCTGCTGCTGAAGAAGAAAAGGAA
GAAGAAGC

GAGCTGGTAGAAGTACTACTTGGAGACCAGGTGTTGCTGCTGCCTTCACCAGTCCAGACAAAAGTAGCATC
TTGGGTGACAGTCTTAGTGTAGACGTGACCGTTCTTGGTGGCAGTGATAGTGGTAGTGATACTAGAACCAG
AACCTTCA

GAGAAAAGTTGACAAATCGCAGCTAGATAAGCCCTCAGTTTTACTGCAGGAAGCACGTGGAGAATCTGCTT
CACCACCAGCAGCGGCTGGAAATGGAGGCACACCTGGTGGACCTCCGGCTTCTTTAGCAGATGCGTTGGCA
GCAGCTTT

ATCCAAAGTGATACATTCACCACCAGCCTTAACAATCTTGGCTCTGGCACCAGCAGTGAATCTCAAAGCAG
CAACAGTGGTCTTTGGGAATTCAAAGATTCTGGCATCGTCAGTAACAGTACCAACAACGACAACAGTCTTG
TTAGCAGC

ACCACCCCAGCTAGTTCTGCTTCCAGCGTTATTATCTCAACCAGAAACGGTACCACTGTTACTGAAACTGA
CAACACTCTTGTCACCAAAGAAACCACTGTCTGTGACTACTCTTCAACATCTGCCGTTCCAGCTTCCACCA
CCGGTTAC

AGGCGCAGGCCCAAGCCCAAGCCCAAGCACAAGCACAAGCACACGCTCAGCACCAGCCCTCCCAACAACCA
CAACAAGCTCAGCAACAACCTAACCCACTACATGGGTTGACACCTACTGCAAAGGATGTCGAAGTAATTAA
GCAATTGT

AAGCTACTAGTTCTTCTGCTGTCACATCTTCCTCCGCTGTTTCCTCTTCGACCGAGTCTGTTAGCTCTTCC
TCTGTCAGTTCTTCCTCAGCCGTTTCCTCTTCTGAAGCTGTCAGTTCCTCTCCAGTTTCCTCAGTTGTTTC
ATCTTCGG

AAGTCGATGTACTCATCTTGATCCAACAAGTCGGAAACATCCTGTCTGGTTTCGATCAATTGCACAGAGCC
CACAGGAACACCAGTCTCACTTTGGAATTGTGCAATGGTGTCGTTAACGATCTTTGCCATTTCTCTGAACG
TGTTCACA

GTCACATCTTCCTCCGCTGTTTCCTCTTCGACCGAGTCTGTTAGCTCTTCCTCTGTCAGTTCTTCCTCAGC
CGTTTCCTCTTCTGAAGCTGTCAGTTCCTCTCCAGTTTCCTCAGTTGTTTCATCTTCGGCCGGACCTGCTA
GCTCAAGC

CGTTCTTGGTGGCAGTGATAGTGGTAGTGATACTAGAACCAGAACCTTCATCAGCAGAAGAAGTCTCAGCA
GCAGAAGAAGTCTCAGCAGCAGAAGTGGTAGCGGCAGCAGAAGTGGTAGCGGCAGCAGAGGTTTCGGCGGC
AGAAGTTT

GGTATGACCACCATTGTCAGAGATTTGGACAGACCAGGTTCTAAGTTCCACAAGCGTGAAGTTGTCGAAGC
TGTCACCGTTGTTGACACTCCACCAGTTGTCGTTGTTGGTGTTGTCGGTTACGTCGAAACCCCAAGAGGTT
TGAGATCT

CCACTGAAAGTACCAACTCCAGCACTAATGCTACTACCACTGCCAGCACCAACGTCAGGACTAGTGCTACT
ACCACTGCCAGCATCAACGTCAGGACTAGTGCGACTACCACTGAAAGTACCAACTCCAACACTAGTGCTAC
TACCACCG

GCTCAAGCACAAGCTCAAGCACAGGCGGCACAAGCGGCGCAAGCGCAAGCACAAGCACAAGCACAAGCACA
AGCACAGGCACAGGCACAGGCACAAGCCCAGGCGCAGGCCCAAGCCCAAGCCCAAGCACAAGCACAAGCAC
ACGCTCAG

TCTACAAGCGAAGCTACTAGTTCTTCTGCTGTCACATCTTCCTCCGCTGTTTCCTCTTCGACCGAGTCTGT
TAGCTCTTCCTCTGTCAGTTCTTCCTCAGCCGTTTCCTCTTCTGAAGCTGTCAGTTCCTCTCCAGTTTCCT
CAGTTGTT

ACTTCTGCTTCCAAGACTTATTATTCAAATCTTGCTGTGGGTGCTCGGATGGGTGATTAGAATTGGATGGA
GACAAGAGTGGAGACGATTCATCAGCTTGGTCAGCAGCTTTGTGCTCCTTGTGAGAGCTAGTGTACAAATG
ATGCAAAT

CACTCTAGAAGGCTCCTCTTCATGATGTTCTTCCGTTTCCTGTGGTTGTTCTGGTTGTTGTTCGTGTGACT
GTTGGGTGTGATTGTCGCCGGACGTTTCCCCCAATCCTGCTAATAAAGGGTCCTGTTCTTCACTCATTCTT
AGTTATTA

GCACAAGCCCAGGCGCAGGCCCAAGCCCAAGCCCAAGCACAAGCACAAGCACACGCTCAGCACCAGCCCTC
CCAACAACCACAACAAGCTCAGCAACAACCTAACCCACTACATGGGTTGACACCTACTGCAAAGGATGTCG
AAGTAATT

GATTGTGTGATAGATGATTCAGATGCTGATGAGGATGAGCCTGATTCTGTGACAGAACCGGATGAGCTAGG
AACCGAAGAAGCGGAGCCTGATTCTGTGGCAGAAGATGAACCGGAGACTGATGAGCCTGATTCTGTGGCAG
AAGATGAA

ATTCTAACCGCCCCTTTGGATATCAGACAAATAGCAACATGTCATCTCCACCCCCTCCTCCAGTGACAACT
TTCAATACCCTGACACCACAAATGACTGCAGCAACTGGACAACCTGCAGTTCCCCTTCCTCAGAATACTCA
AGCACCTT

CCTCAAGTTCTGCTGCCACATCTTCAAGTGAAGTTGCTAGCTCTTCTATTGCTTCATCCACTAGCTCTTCT
GTTGCACCATCCTCAAGTGAAGTTGTCAGCTCTTCCGTTGCACCATCCTCAAGTGAAGTTGTCAGCTCTTC
CGTTGCAC

CCGGACCTGCTAGCTCAAGCGTTGCTCCTTACAACTCAACCATTGCTAGCTCTTCTTCCACTGCCCAGACT
TCTATCTCGACCATTGCTCCTTACAACTCCACAACCACCACCACCCCAGCTAGTTCTGCTTCCAGCGTTAT
TATCTCAA

GTTCTTGTATGATCTGTAAGCACCGAACAGGTAACCAATTTCACGACCACCAACACCGATATCACCAGCTG
GCACGTCAGTGTCTTGACCAATGTGTCTGCTCAATTCTCTCATGAAAGCATAACAGATTCTTCTGATTTCG
TTATTAGA

TAACATTATCGGTTTAGTCGGTATCGATCAATTCTTGTTGAAGACTGGTACTTTGACCACCAGTGAAACTG
CTCACAACATGAAGGTCATGAAATTCTCTGTCTCTCCAGTTGTGCAAGTCGCTGTCGAAGTCAAGAACGCT
AACGACTT

CCAAGCCCAAGCCCAAGCACAAGCACAAGCACACGCTCAGCACCAGCCCTCCCAACAACCACAACAAGCTC
AGCAACAACCTAACCCACTACATGGGTTGACACCTACTGCAAAGGATGTCGAAGTAATTAAGCAATTGTCC
TTGGATGC

CTTTACGTGTCACTGACGGTGCTTTGGTTGTCGTCGACACCATTGAAGGTGTCTGTGTCCAAACCGAAACT
GTTTTGAGACAAGCTTTGGGTGAAAGAATCAAGCCTGTTGTTGTTATCAACAAGGTCGACAGAGCTTTGTT
GGAATTGC

TTCCTCTTCGACCGAGTCTGTTAGCTCTTCCTCTGTCAGTTCTTCCTCAGCCGTTTCCTCTTCTGAAGCTG
TCAGTTCCTCTCCAGTTTCCTCAGTTGTTTCATCTTCGGCCGGACCTGCTAGCTCAAGCGTTGCTCCTTAC
AACTCAAC

ATTTCTTGATGAAACTCATGGATACGTGGTCCGTTCATTGTGAAAGGATCACTGGGTTGCCACTTTGAGTT
TTCGTCAATCACAGGAGCGTCTGGTAGACCAGCAACCTTGGAGTACAAGCTTCCCACATCAATTCTAAAGC
CGTCTACA

AGAAGAATCTTGCCAAAGCCATCTAGAACATCTAGACAAGTCCAAAAGAGACCAAGATCCAGAACCTTGAC
TGCTGTTCATGACAAGATCTTGGAAGACTTAGTCTTCCCAACTGAAATCGTTGGTAAGAGAGTTAGATATT
TGGTTGGT

CTCGAGACTTCAGAGGACGAGGTTGATGGAGCAATGGAGCTCGAGACTTCGGTGGAGGATGACGTTAACTC
AGTGAGAGAGCTCGAGACATCAGAAGTAGATGAGGTCAACTCAATAGAACTACTGATGGAAACGGAACCTG
ATGAAGTT

TGCTGGCGTAAGAGTCTTCACCACCCCAAACAAAAGTAGCATCTTGGGTGACAGTCTTAGTGTAGACATGA
CCGTTCTTGGTGGCAGTGATGGTGGTGGTGATACTCTCAGCAAGAGCAGTAGCGGCAACAGCAGATAGAAC
CAAAGCGG

AGCGGTACCAGATTGTGTGATAGATGATTCAGATGCTGATGAGGATGAGCCTGATTCTGTGACAGAACCGG
ATGAGCTAGGAACCGAAGAAGCGGAGCCTGATTCTGTGGCAGAAGATGAACCGGAGACTGATGAGCCTGAT
TCTGTGGC

TTCCAGCGTTATTATCTCAACCAGAAACGGTACCACTGTTACTGAAACTGACAACACTCTTGTCACCAAAG
AAACCACTGTCTGTGACTACTCTTCAACATCTGCCGTTCCAGCTTCCACCACCGGTTACAACAATTCTACT
AAGGTTTC

CATGTGGAAATTGATCGTCGATCCAATGGATGCTCCGGACCAACCACAAGATTTGACCATTGACTTTGAAC
GTGGTCTTCCAGTCAAGTTGACCTACACCGACAACAAGACTTCCAAGGAAGTTTCCGTTACCAAGCCTTTG
GATGTTTT

TTACAAGGCTGGTATGACCACCATTGTCAGAGATTTGGACAGACCAGGTTCTAAGTTCCACAAGCGTGAAG
TTGTCGAAGCTGTCACCGTTGTTGACACTCCACCAGTTGTCGTTGTTGGTGTTGTCGGTTACGTCGAAACC
CCAAGAGG

GATTGAGGAATTGAACTCGAGATACCTGGAGCAGAAGAAGCTGATGAACCGGAAACGGAGGAACCAGATTG
TGTAACAGAACCGGACGTGCTGGAGGCTGATGAACCGGAAGCGGTACCAGATTGTGTGATAGATGATTCAG
ATGCTGAT

TGATTTACTAGAAGATTTCAAGAAATTCTTGCCGGACTCTTCAGCTTCTGCCAATCAGCAGGTGCAACATG
CTCAGCAACATGCTCAACAACAACATGAGGCCCAAATGCATGCACAGGCACAAGCTCAGGCTCAGGCACAG
GCACAGGT

AGCAGCTTCTGAGCTGGTAGAAGTACTACTTGGAGACCAGGTGTTGCTGCTGCCTTCACCAGTCCAGACAA
AAGTAGCATCTTGGGTGACAGTCTTAGTGTAGACGTGACCGTTCTTGGTGGCAGTGATAGTGGTAGTGATA
CTAGAACC

TCAGCTCTTCCGTTGCTTCTTCTACAAGCGAAGCTACTAGTTCTTCTGCTGTCACATCTTCCTCCGCTGTT
TCCTCTTCGACCGAGTCTGTTAGCTCTTCCTCTGTCAGTTCTTCCTCAGCCGTTTCCTCTTCTGAAGCTGT
CAGTTCCT

CGTTGCTTCTTCTACAAGCGAAGCTACTAGTTCTTCTGCTGTCACATCTTCCTCCGCTGTTTCCTCTTCGA
CCGAGTCTGTTAGCTCTTCCTCTGTCAGTTCTTCCTCAGCCGTTTCCTCTTCTGAAGCTGTCAGTTCCTCT
CCAGTTTC

TAGATGATTCAGATGCTGATGAGGATGAGCCTGATTCTGTGACAGAACCGGATGAGCTAGGAACCGAAGAA
GCGGAGCCTGATTCTGTGGCAGAAGATGAACCGGAGACTGATGAGCCTGATTCTGTGGCAGAAGATGAACC
GGAGACTG

CAATACCCAAGACTCTGTAACCCATAGCCTTGGCGTATTGAACAGCCAAAGAACCTAGACCACCAGCAGCA
CCGGAGATAGCAACCCAGTGACCGGCCATCAAGTTAGCAGACTTCAAAGCCTTGTAGACGGTGATACCAGC
ACACAAGA

AGACCAGGTTCTAAGTTCCACAAGCGTGAAGTTGTCGAAGCTGTCACCGTTGTTGACACTCCACCAGTTGT
CGTTGTTGGTGTTGTCGGTTACGTCGAAACCCCAAGAGGTTTGAGATCTTTGACCACCGTCTGGGCTGAAC
ATTTGTCT

GCAGCCTTGGTAGAGGAAGCGACGGTGGAAGAGGTAGCCTCACTGGAAGAAGCGACAGCAGAAGAAGTAGC
AGCAGAAGAAGAAGCTTCACTGGAAGAAGCGACGGCAGAAGAAGTAGCTTCACTGGAGGAAGCGACGGCAG
AAGAAGTA

TCAAGGAAAGTATTGAATAATATTGTCGAAGAAACAAACGCACTTCAAAGGCCAGTTGTTGAAGTTGTCAA
GGAAGACAGAAGTGTTCCTGACCTAGCAGGTGTTCAGCAAGAGCAAGCAGAGAAATATTCCTATTCAAACA
ATAGTGGA

TCTTACGAGGCAGGTATCTTGGAAGACCCAGATACCACCCCACCAAAGGACATGTGGAAATTGATCGTCGA
TCCAATGGATGCTCCGGACCAACCACAAGATTTGACCATTGACTTTGAACGTGGTCTTCCAGTCAAGTTGA
CCTACACC

TTGCTTACTTCCTCAAACTTGTAAGCGGTACCATACATGACGTAATCATAATCATCTGCAAGGGATCTGTC
ACCAGCCTGTGGAGGTCTCCAGCTTCTTGTCGCAGAAGAGTCGTTAGCTGGGGTGTCTTCGAGGTTTAAAG
AGGATGCA

ATCATCTGTATTATAGATTTCGTTACCGATAGTCTTTCCTTCAGAATTCTTTCGGTCCTCTTCAATGTCAC
TGACAACTTCAGAAACCTCTTCACTCAGGTTGTCGTCATCCTCTAAATTGGTTGTAATAAAAGAACTAGGT
ATTTGTGT

CTAAGAAATTGCCAAGAAAGACTCACAGAGGTCTAAGAAAGGTTGCTTGTATTGGTGCTTGGCATCCAGCC
CACGTTATGTGGAGTGTTGCCAGAGCTGGTCAAAGAGGTTACCATTCCAGAACCTCCATTAACCACAAGAT
TTACAGAG

CCATTGTCAGAGATTTGGACAGACCAGGTTCTAAGTTCCACAAGCGTGAAGTTGTCGAAGCTGTCACCGTT
GTTGACACTCCACCAGTTGTCGTTGTTGGTGTTGTCGGTTACGTCGAAACCCCAAGAGGTTTGAGATCTTT
GACCACCG

TGCTGTTGGTACTGTCCATTCTGTGGAGGTGGTACTGAAGCAGGTTGAGGAGAGACATGATGATGGTTCTC
TGGAACAGCTGATGTCCCAGGTGTTGTCTCTTGTTGAGAATTAACCTTAGTGGAATCTCTATCAAATTCCG
GTAAATTG

AAGCTCAAGCTCAGGCACAGGCTCAAGCACAAGCTCAAGCACAGGCGGCACAAGCGGCGCAAGCGCAAGCA
CAAGCACAAGCACAAGCACAAGCACAGGCACAGGCACAGGCACAAGCCCAGGCGCAGGCCCAAGCCCAAGC
CCAAGCAC

TGAAGTTGTCAGCTCTTCCGTTGCTTCATCCTCAAGTGAAGTTGCCAGCTCCTCTGTTGCGCCATCCTCAA
GTGAAGTTGTCAGCTCTTCCGTTGCTTCTTCTACAAGCGAAGCTACTAGTTCTTCTGCTGTCACATCTTCC
TCCGCTGT

GATGGTTGTTGGTAATGTGCCCAGTTAGAAGCCATAGCTTTGTTTGGGGTCATCATGCCGTGCTGTTGGTA
CTGTCCATTCTGTGGAGGTGGTACTGAAGCAGGTTGAGGAGAGACATGATGATGGTTCTCTGGAACAGCTG
ATGTCCCA

CGATGTCTAACATCACTATTAGTGCATCTTCATTATTATCTCAAACTCCAACTCCAACAACACAATTGCAA
CAGCGGTTGAACTCAGCAGCTGCAGCCGCCGCCGCAGCTGCTTCACCATCGAATTCCACCCCAACTGGATA
CACAGCAG

GAACTTGTTCATTTCTTGATGAAACTCATGGATACGTGGTCCGTTCATTGTGAAAGGATCACTGGGTTGCC
ACTTTGAGTTTTCGTCAATCACAGGAGCGTCTGGTAGACCAGCAACCTTGGAGTACAAGCTTCCCACATCA
ATTCTAAA

GGAAGACCCAGATACCACCCCACCAAAGGACATGTGGAAATTGATCGTCGATCCAATGGATGCTCCGGACC
AACCACAAGATTTGACCATTGACTTTGAACGTGGTCTTCCAGTCAAGTTGACCTACACCGACAACAAGACT
TCCAAGGA

CGATGAGGTTGATTGAGGAATTGAACTCGAGATACCTGGAGCAGAAGAAGCTGATGAACCGGAAACGGAGG
AACCAGATTGTGTAACAGAACCGGACGTGCTGGAGGCTGATGAACCGGAAGCGGTACCAGATTGTGTGATA
GATGATTC

ACCGAGTCTGTTAGCTCTTCCTCTGTCAGTTCTTCCTCAGCCGTTTCCTCTTCTGAAGCTGTCAGTTCCTC
TCCAGTTTCCTCAGTTGTTTCATCTTCGGCCGGACCTGCTAGCTCAAGCGTTGCTCCTTACAACTCAACCA
TTGCTAGC

AGTAATAGAGCTCGAGACTTCAGAGGACGAGGTTGATGGAGCAATGGAGCTCGAGACTTCGGTGGAGGATG
ACGTTAACTCAGTGAGAGAGCTCGAGACATCAGAAGTAGATGAGGTCAACTCAATAGAACTACTGATGGAA
ACGGAACC

TTCAAAGATTCTGGCATCGTCAGTAACAGTACCAACAACGACAACAGTCTTGTTAGCAGCACCTTCTTGCT
TCAAAGCTCTAGCAATTCTAGAGACAGAAACAGGTGGTCTGTTGATCTTAGACAAGAACAAAGCCTTCAAG
ACAACCTT

CTAGAAAAAGTACTAATGAACATCACACCGTTTATTGTTGGGAGAAGTGTTCCATGATCATGGACAATTCA
TCAGTTTCAGCACCCCAGTTCTTGACAACAACAACGGAGGCACCGACAACCTTTCTGGCGTTACCTTCACG
GTCGATCT

GGTGGTTGCAGTGGTTGAAGCAGAAGAGGTAGCAGCTTCTGAGCTGGTAGAAGTACTACTTGGAGACCAGG
TGTTGCTGCTGCCTTCACCAGTCCAGACAAAAGTAGCATCTTGGGTGACAGTCTTAGTGTAGACGTGACCG
TTCTTGGT

ACTACAGCAAATGCAGCATTTACAGCAATTGAAAATGCAGCAGCAACAACAACAGCAGCAACAACAACAGC
AGCAACAACAACAGCAGCAACAACAACAGCAACAGCACATATATCCCTCCTCGACTCCTGGTGTGGCTAAT
TATTCGGC

CGCATACAAGGAATACAAAAGTTGGTAACGACATCGTCAGAGGTGTTTCCGGTGGTGAAAGGAAGCGTGTC
TCCATTGCTGAAGTCTCCATCTGTGGATCCAAATTTCAATGCTGGGATAATGCTACAAGGGGTTTGGATTC
CGCTACCG

TGTCAGCTCTGTTACCGACGTAAGAACCAACAATAGAGATGGACTTGACGACTTGGTTGAAGACATCAGAA
CAACACTTGGCACCAGCTGGCATACCGACCAAAACGGTGGTACCGTTAGCTCTAACGTATCTGGTAGAAGC
TTCAATAG

AGATGCTGATGAGGATGAGCCTGATTCTGTGACAGAACCGGATGAGCTAGGAACCGAAGAAGCGGAGCCTG
ATTCTGTGGCAGAAGATGAACCGGAGACTGATGAGCCTGATTCTGTGGCAGAAGATGAACCGGAGACTGAT
GAGCCTGA

AAAATGCTCCTGATTTACTAGAAGATTTCAAGAAATTCTTGCCGGACTCTTCAGCTTCTGCCAATCAGCAG
GTGCAACATGCTCAGCAACATGCTCAACAACAACATGAGGCCCAAATGCATGCACAGGCACAAGCTCAGGC
TCAGGCAC

TCAGGCACAGGCTCAAGCACAAGCTCAAGCACAGGCGGCACAAGCGGCGCAAGCGCAAGCACAAGCACAAG
CACAAGCACAAGCACAGGCACAGGCACAGGCACAAGCCCAGGCGCAGGCCCAAGCCCAAGCCCAAGCACAA
GCACAAGC

GGTTAATTCTCAAGAAGAGACAACACCTGGGACATCAGCTGTTCCAGAGAACCATCATCATGTCTCTCCTC
AACCTGCTTCAGTACCACCTCCACAGAATGGACAGTACCAACAGCACGGCATGATGACCCCAAACAAAGCT
ATGGCTTC

AGGTACTATACACTCGCTTCTGTCATGCTCGAGTCCGCTTCATCTGTGAAAGATTCAGCCACGCTTGAAGT
AACAACAGGAGGCAAGACGAGGGGTCTGGTGCCATACTGTTCATCAAATTCAGTAAAGTCGAGTTGTGAGT
TATCGCCC

CCTTCTTGGGTTACAAGGCTGGTATGACCACCATTGTCAGAGATTTGGACAGACCAGGTTCTAAGTTCCAC
AAGCGTGAAGTTGTCGAAGCTGTCACCGTTGTTGACACTCCACCAGTTGTCGTTGTTGGTGTTGTCGGTTA
CGTCGAAA

CAAGAAGAGACAACACCTGGGACATCAGCTGTTCCAGAGAACCATCATCATGTCTCTCCTCAACCTGCTTC
AGTACCACCTCCACAGAATGGACAGTACCAACAGCACGGCATGATGACCCCAAACAAAGCTATGGCTTCTA
ACTGGGCA

ATGCTTACATATGTATCTTGCGTTGCTTAGATGATATCATATCCTGGGGGTTCAAGACTCCACCGATCTCA
ACAGTGTCCATCGAGATTGTTCCAGATGAATGGAGGAAGCAAGTTATTAACGAAGTCGTGATAGCCCACGG
TAACCAAT

TGGATGGCACCGTTGCTTGGATGCAACCCCTGATTATATTCTTCTCGCACCAGGTGATTAGCTGAACCTGT
GGCGGTTGGCTTCAAAGGTTCTAACGGAAGTTGCTGTGGAACAGTGGTGTTATTCACATTGCTAGTTGGTA
GAGTATTG

GTACTTATATTGCTTTAGGAAGGTACTATACACTCGCTTCTGTCATGCTCGAGTCCGCTTCATCTGTGAAA
GATTCAGCCACGCTTGAAGTAACAACAGGAGGCAAGACGAGGGGTCTGGTGCCATACTGTTCATCAAATTC
AGTAAAGT

TTCTTCCTTGATTTGCACCCTCCGCTGCGATGTGTACAGACGTTGCTTTCTTTTCGCTGGACTGTCTGGCC
ACCTGGTTAGGTGGAGTTTGCTCCACATCCGACCTGAGACTACCCAACTTTGGATCGCTGAATGTAACAGT
TGGTATCC

AGGAACAACTAAGTCGATGTACTCATCTTGATCCAACAAGTCGGAAACATCCTGTCTGGTTTCGATCAATT
GCACAGAGCCCACAGGAACACCAGTCTCACTTTGGAATTGTGCAATGGTGTCGTTAACGATCTTTGCCATT
TCTCTGAA

TTTCCCAGAATCCAAAGTCGAGACCTACTATCAAGAATCCGCTGGTGTTGCAGATCTGATCACCACCTGCT
CAGGCGGTAGAAACGTCAAGGTTGCCACATACATGGCCAAGACCGGTAAGTCAGCCTTGGAAGCAGAAAAG
GAATTGCT

GGTAATGTGCCCAGTTAGAAGCCATAGCTTTGTTTGGGGTCATCATGCCGTGCTGTTGGTACTGTCCATTC
TGTGGAGGTGGTACTGAAGCAGGTTGAGGAGAGACATGATGATGGTTCTCTGGAACAGCTGATGTCCCAGG
TGTTGTCT

GAGGTAGCCTCACTGGAAGAAGCGACAGCAGAAGAAGTAGCAGCAGAAGAAGAAGCTTCACTGGAAGAAGC
GACGGCAGAAGAAGTAGCTTCACTGGAGGAAGCGACGGCAGAAGAAGTAGTTTCACTGGAGGAAGCAACAG
CGGAAGAA

TCTTTCAAGTTTCTTCCTTGATTTGCACCCTCCGCTGCGATGTGTACAGACGTTGCTTTCTTTTCGCTGGA
CTGTCTGGCCACCTGGTTAGGTGGAGTTTGCTCCACATCCGACCTGAGACTACCCAACTTTGGATCGCTGA
ATGTAACA

ATCTTCTCCCCTGTTGCAGTCTCGCTTGTGCTTGTTGTGCCTGTTGTGCCTGTTGCGCTTGTTGCGCCTGT
TGTGCTTGGAATTGTGCCTGCTGCATCTGCGCCTGCTGGGGCATAGGAGAATTTATCTGCGAACCTGGCAT
CACATTTG

TCCGCCACCAGCATTCCTAACTCAACAACCTCAATCTGGAGGAGCTCCAGCTCCACCCCCACCTCCTCAAA
TGCCAGCTACATCAACATCCGGAGGCGGTTCATTCGCTGAAACTACTGGAGATGCAGGTCGTGATGCACTT
TTAGCTTC

AAACGAATAAGTACTTATATTGCTTTAGGAAGGTACTATACACTCGCTTCTGTCATGCTCGAGTCCGCTTC
ATCTGTGAAAGATTCAGCCACGCTTGAAGTAACAACAGGAGGCAAGACGAGGGGTCTGGTGCCATACTGTT
CATCAAAT

TCACCACCGTCAATACCCAAGACTCTGTAACCCATAGCCTTGGCGTATTGAACAGCCAAAGAACCTAGACC
ACCAGCAGCACCGGAGATAGCAACCCAGTGACCGGCCATCAAGTTAGCAGACTTCAAAGCCTTGTAGACGG
TGATACCA

ACGCCATTGATGATGTAGTTGATGATGTAGTCGATGATGTAGTTGATGTTGTGGAGGACGTTGTTGAAGAA
GTTGTGGAAGACGACGCAGAAGTAGACTCCGTACTTGAATCTGAGCTGCTTATGCTATTGCTATTTGTGTC
AGAGTCAA

CACACACCACACCCACACACACCCACACACCACACCACACCCACACACCACCCACACACACACAACACTAC
CCTAATCTAACCCTGTCCAACCTGTCTCCAAACTTACCCTCCATTACCTTACCTCCCCACTCGTTACCCTG
CCCCATTT

AGATTTGGACAGACCAGGTTCTAAGTTCCACAAGCGTGAAGTTGTCGAAGCTGTCACCGTTGTTGACACTC
CACCAGTTGTCGTTGTTGGTGTTGTCGGTTACGTCGAAACCCCAAGAGGTTTGAGATCTTTGACCACCGTC
TGGGCTGA

CATCACCGTCATCATCTGTATTATAGATTTCGTTACCGATAGTCTTTCCTTCAGAATTCTTTCGGTCCTCT
TCAATGTCACTGACAACTTCAGAAACCTCTTCACTCAGGTTGTCGTCATCCTCTAAATTGGTTGTAATAAA
AGAACTAG

GAAGGTAAGGCCTTGTTGAAGGTTGTTATGAGAAAGTTCTTGCCAGCTGCCGATGCCTTATTGGAAATGAT
TGTCTTGCACTTGCCATCTCCAGTCACTGCTCAAGCCTACAGAGCTGAACAATTATACGAAGGTCCAGCTG
ACGATGCC

GCTAAGCGTAACTGGTGCAAGCCATAGGTAGCCTCACTACGGTACAGAGGTTACTTTGAAAGCAAGAACCC
TTCACATGTCACACCTGCACGCAGAGGCATGGAGAGGCCAGCACAAACCACCATGGATGGGTAAAGTCATA
GAAGGAAA

CATCACTATTAGTGCATCTTCATTATTATCTCAAACTCCAACTCCAACAACACAATTGCAACAGCGGTTGA
ACTCAGCAGCTGCAGCCGCCGCCGCAGCTGCTTCACCATCGAATTCCACCCCAACTGGATACACAGCAGAG
CAACAAAG

TATGTCACTCAATGACATGCTTGATCAGCAATCCTTTATGCTAGACACTGCGGGAACCAGGGCTCAGCCGC
TCCAGCAACAACAGCAGCAGCAACAACAGCAACAGCAGGCGTCGTTGCCTTCCCTTAATATTCAAACAGTT
TCGTCTAC

TAATACCCTGAATTCTGCCGGTGGAAATAGCCTCCTTCCACAGGCCACTGGAAGAAGAGGGCCAGCACCAC
CACCTCCTCCAAGAGCATCTCGCCCCACACCAAACGTTACGATGCAACAAAATCCACAACAGTACAATAAT
TCTAACCG

ACTGGAAGAAGAAGCTCCAGAAGCATATCCATTCCTTGCAAAACGTTCGTTTCTGCGTGCCTGTCTTCTCC
ACTTTTGTCTCGGTCAGCCAACAGGCACGTGGTGGGTGTGCCTGGAGATTAGAAATTGTAGTTCCTCAGAT
TATGTAAA

TGGTTGTTGCATATGTTAATGTTGTGGTATAAACAGATGGGGAGCCGGAAATCAAGGTGGAAGCCGTTGAA
CCAGTCACGGAAGCACCGAATGAAGTGGAAGCGGTTGAACCAGTTACGGAAGCACCGAATGAAGTGGAACC
GGAAGTAG

AGTAAACATTGTCATGGGTAATCTTCTCCCCTGTTGCAGTCTCGCTTGTGCTTGTTGTGCCTGTTGTGCCT
GTTGCGCTTGTTGCGCCTGTTGTGCTTGGAATTGTGCCTGCTGCATCTGCGCCTGCTGGGGCATAGGAGAA
TTTATCTG

AACAACAGCAACAAGCTCAAGCTCAAGCTCAAGCTCAAGCTCAGGCACAGGCTCAAGCACAAGCTCAAGCA
CAGGCGGCACAAGCGGCGCAAGCGCAAGCACAAGCACAAGCACAAGCACAAGCACAGGCACAGGCACAGGC
ACAAGCCC

AATGACATGCTTGATCAGCAATCCTTTATGCTAGACACTGCGGGAACCAGGGCTCAGCCGCTCCAGCAACA
ACAGCAGCAGCAACAACAGCAACAGCAGGCGTCGTTGCCTTCCCTTAATATTCAAACAGTTTCGTCTACAG
CAGCTGGT

TTCAGTGGCGGAGGATGAACCGGAGGCGGATGAGCCTGATTCAGTGGCAGAAGATGAACTGGAGACTGATG
AGCCTGATGAAGTAATAGAAGATGAGCCGGAGACTGAAGAGCCTGATGAAGTAATAGAGCTCGAGACTTCA
GAGGACGA

ACCGCCTCCACCACATAGGCACGTAACCAGTAATACCCTGAATTCTGCCGGTGGAAATAGCCTCCTTCCAC
AGGCCACTGGAAGAAGAGGGCCAGCACCACCACCTCCTCCAAGAGCATCTCGCCCCACACCAAACGTTACG
ATGCAACA

GTCATGGGTAATCTTCTCCCCTGTTGCAGTCTCGCTTGTGCTTGTTGTGCCTGTTGTGCCTGTTGCGCTTG
TTGCGCCTGTTGTGCTTGGAATTGTGCCTGCTGCATCTGCGCCTGCTGGGGCATAGGAGAATTTATCTGCG
AACCTGGC

GCTCTAACTTCCTTCTTGGGTTACAAGGCTGGTATGACCACCATTGTCAGAGATTTGGACAGACCAGGTTC
TAAGTTCCACAAGCGTGAAGTTGTCGAAGCTGTCACCGTTGTTGACACTCCACCAGTTGTCGTTGTTGGTG
TTGTCGGT

ATGAACCGGAAGCGGTACCAGATTGTGTGATAGATGATTCAGATGCTGATGAGGATGAGCCTGATTCTGTG
ACAGAACCGGATGAGCTAGGAACCGAAGAAGCGGAGCCTGATTCTGTGGCAGAAGATGAACCGGAGACTGA
TGAGCCTG

GTAAGCGGTACCATACATGACGTAATCATAATCATCTGCAAGGGATCTGTCACCAGCCTGTGGAGGTCTCC
AGCTTCTTGTCGCAGAAGAGTCGTTAGCTGGGGTGTCTTCGAGGTTTAAAGAGGATGCAATAGTCACTGTC
AAAGAATC

GGCCTTGTTATGGGCCTCCTGTTCAGGATCCACTCTAGAAGGCTCCTCTTCATGATGTTCTTCCGTTTCCT
GTGGTTGTTCTGGTTGTTGTTCGTGTGACTGTTGGGTGTGATTGTCGCCGGACGTTTCCCCCAATCCTGCT
AATAAAGG

TGCCAAAGCCATCTAGAACATCTAGACAAGTCCAAAAGAGACCAAGATCCAGAACCTTGACTGCTGTTCAT
GACAAGATCTTGGAAGACTTAGTCTTCCCAACTGAAATCGTTGGTAAGAGAGTTAGATATTTGGTTGGTGG
TAACAAGA

CCACCGAAAGTACCGACTCCAACACTAGTGCTACTACCACTGCTAGCACCAACTCCAGCACTAATGCCACT
ACCACTGCTAGCACCAACTCCAGCACTAATGCCACTACCACTGAAAGTACCAACGCTAGTGCCAAGGAGGA
CGCCAATA

TTAGCTCTTCCTCTGTCAGTTCTTCCTCAGCCGTTTCCTCTTCTGAAGCTGTCAGTTCCTCTCCAGTTTCC
TCAGTTGTTTCATCTTCGGCCGGACCTGCTAGCTCAAGCGTTGCTCCTTACAACTCAACCATTGCTAGCTC
TTCTTCCA

ATACATTCACCACCAGCCTTAACAATCTTGGCTCTGGCACCAGCAGTGAATCTCAAAGCAGCAACAGTGGT
CTTTGGGAATTCAAAGATTCTGGCATCGTCAGTAACAGTACCAACAACGACAACAGTCTTGTTAGCAGCAC
CTTCTTGC

CACATCCCGCACTTGACAGTATTCCAAACTTTATACACAGTGGCAAGACTGAAGACACCAAGGAACCGAAT
CAAGGGTGTCGATAGGGACACATTTGCCAAACACATGACTGAAGTGGCAATGGCAACTTACGGACTGAGCC
ACACTGCA

GAAACTCATGGATACGTGGTCCGTTCATTGTGAAAGGATCACTGGGTTGCCACTTTGAGTTTTCGTCAATC
ACAGGAGCGTCTGGTAGACCAGCAACCTTGGAGTACAAGCTTCCCACATCAATTCTAAAGCCGTCTACACC
ATGGTCTA

AGTGCGATTACCACTGAAAGTACCAACTCCAGCACTAATGCTACTACCACTGCCAGCACCAACGTCAGGAC
TAGTGCTACTACCACTGCCAGCATCAACGTCAGGACTAGTGCGACTACCACTGAAAGTACCAACTCCAACA
CTAGTGCT

CTTCTGATGTTAAAGTGTGTGGTGGTAAGACATTATTTCTCACCTTTGTCTTAGCTTGAGGTACTTCAGAG
TGTTGATCCTGTGAACCGATTGGATCAGGTGAGGAAGTACTCAGCGGTGGGATATACTGTGGCAGCGGATA
GTGTGGGT

AAAACTATGATCGATTATTTTGAAAGCCATGGTGCTCATAAATGCCCTGCTGACGCCAACCCAGCTGAATG
GATGCTAGAAGTTGTTGGTGCAGCTCCAGGCTCTCATGCAAATCAAGATTATTACGAAGTTTGGAGGAATT
CTGAAGAG

GATAACTCTTTCAGAGGCGGTAGTGGCTGGGGTAGCGATTCCAAGTCTTCTGGCTGGGGTAACAGCGGTGG
TTCAAACAACTCTTCTTGGTGGTGATTTCAGACAAACTAGGGTGAGGATTCTTCGTTTTCTGCTCTAGCAT
GTCTTATA

ATGCAGCATTTACAGCAATTGAAAATGCAGCAGCAACAACAACAGCAGCAACAACAACAGCAGCAACAACA
ACAGCAGCAACAACAACAGCAACAGCACATATATCCCTCCTCGACTCCTGGTGTGGCTAATTATTCGGCAA
TGGCTAAT

ATATCAAAGAAGTATTTTAATTCGACGAAACTCATATTCTCCACACAGTGCAGGAAAGGTTTGGACTTGAC
AAGACTCTTAAATCCTCGTCCGGAAGCGAACCTGTCTCCACTCAAACTATCCAGATAGTGATTGACGTTAT
CAATTGTA

CAAGCGTGAAGTTGTCGAAGCTGTCACCGTTGTTGACACTCCACCAGTTGTCGTTGTTGGTGTTGTCGGTT
ACGTCGAAACCCCAAGAGGTTTGAGATCTTTGACCACCGTCTGGGCTGAACATTTGTCTGACGAAGTCAAG
AGAAGATT

CCACCCCAAACAAAAGTAGCATCTTGGGTGACAGTCTTAGTGTAGACATGACCGTTCTTGGTGGCAGTGAT
GGTGGTGGTGATACTCTCAGCAAGAGCAGTAGCGGCAACAGCAGATAGAACCAAAGCGGAAGAGAATTTCA
TTTTAGGG

AAGTACTACTTGGAGACCAGGTGTTGCTGCTGCCTTCACCAGTCCAGACAAAAGTAGCATCTTGGGTGACA
GTCTTAGTGTAGACGTGACCGTTCTTGGTGGCAGTGATAGTGGTAGTGATACTAGAACCAGAACCTTCATC
AGCAGAAG

CCAGTTGAGATTGCTGAAGCTGTTTTCAACTATGGTGACTTCACCACCATGTTGACTGGTATTCCAGCTGA
ACAAGTCACCAGAGTCATCACTGGTGTCCCATGGTACTCTACCAGATTGAGACCGGCTATCTCCAGTGCTC
TATCTAAG

AGAAGAACAGCATAATATTGCTACCAAAGAAGCAGAATTGCTTGTTAAGAAAGAAGAGGAAAGGTCGAAGA
AGCTTGCAGCCACTAGAGTTTCTGGTGGCCATCTGGAGAGAGACAATGTGGTTAGGGAAGAAGATAAATTG
TGGACAGT

CAGGTATCTTGGAAGACCCAGATACCACCCCACCAAAGGACATGTGGAAATTGATCGTCGATCCAATGGAT
GCTCCGGACCAACCACAAGATTTGACCATTGACTTTGAACGTGGTCTTCCAGTCAAGTTGACCTACACCGA
CAACAAGA

GAGCCTGATGAAGTAATAGAAGATGAGCCGGAGACTGAAGAGCCTGATGAAGTAATAGAGCTCGAGACTTC
AGAGGACGAGGTTGATGGAGCAATGGAGCTCGAGACTTCGGTGGAGGATGACGTTAACTCAGTGAGAGAGC
TCGAGACA

GGAGACTGATGAGCCTGATGAAGTAATAGAAGATGAGCCGGAGACTGAAGAGCCTGATGAAGTAATAGAGC
TCGAGACTTCAGAGGACGAGGTTGATGGAGCAATGGAGCTCGAGACTTCGGTGGAGGATGACGTTAACTCA
GTGAGAGA

CCAATCCTGGACAGGGCGACTTCAGCGCTGGTGTTCTCTAAGTTTATATCGTTGACAATGGTGCCTGTCTG
CTTGGCCAACTGAGTGGCCTTCACCATCAGTTCCACCTCCTCTCTGTTCTGTGTGTCCACTTGATTTTGTA
GATGTGAT

GTCACTCAAGCCGTCGAATCATCTACATCCTCCTCATCCTCCTCATCCTCCTCCTCCTCTTCTGCCTCTTC
TTCTGGTGCTGCTCCTGCTGCATTCCAAGGAGCAAGTGTCGGTGCATTGGCCCTTGGTTTGATTTCTTACC
TATTATAA

TTCTAGCAGCTTTCTCTTGAATTGATTCAAGGCCTTGTTATGGGCCTCCTGTTCAGGATCCACTCTAGAAG
GCTCCTCTTCATGATGTTCTTCCGTTTCCTGTGGTTGTTCTGGTTGTTGTTCGTGTGACTGTTGGGTGTGA
TTGTCGCC

CTGATGAACCGGAAACGGAGGAACCAGATTGTGTAACAGAACCGGACGTGCTGGAGGCTGATGAACCGGAA
GCGGTACCAGATTGTGTGATAGATGATTCAGATGCTGATGAGGATGAGCCTGATTCTGTGACAGAACCGGA
TGAGCTAG

AGCGGTTGAACCAGTTACGGAAGCACCGAATGAAGTGGAACCGGAAGTAGAGTTGGTAGCCCCTGATGTGG
TAGATGTGGATGAGGGTGTAGCGCTGGAAGCAACAACACCTCCGTAATTTAGATAATAAGCATAGGCACCA
GCAAATGT

AGTCTTAGTGTAGACGTGACCGTTCTTGGTGGCAGTGATAGTGGTAGTGATACTAGAACCAGAACCTTCAT
CAGCAGAAGAAGTCTCAGCAGCAGAAGAAGTCTCAGCAGCAGAAGTGGTAGCGGCAGCAGAAGTGGTAGCG
GCAGCAGA

ATTGTCGTCTTCGTCTTCATCTTCGTCTTCCCCTTCTTCATCTTCATCTTCTTCGTCTGCAACGTCTCTTG
GGTCAACAGAAAGACCTGTTACACTTCTGGGTGTCTCAGATCCAACTACGTTATCGACACTATCGTCTGAC
TCATTCTC

AAAGTAGCATCTTGGGTGACAGTCTTAGTGTAGACGTGACCGTTCTTGGTGGCAGTGATAGTGGTAGTGAT
ACTAGAACCAGAACCTTCATCAGCAGAAGAAGTCTCAGCAGCAGAAGAAGTCTCAGCAGCAGAAGTGGTAG
CGGCAGCA

AGATTCAGCGGCAGAAGTGCTGCTGGCGTAAGAGTCTTCACCACCCCAAACAAAAGTAGCATCTTGGGTGA
CAGTCTTAGTGTAGACATGACCGTTCTTGGTGGCAGTGATGGTGGTGGTGATACTCTCAGCAAGAGCAGTA
GCGGCAAC

AGAGTCTTCACCACCCCAAACAAAAGTAGCATCTTGGGTGACAGTCTTAGTGTAGACATGACCGTTCTTGG
TGGCAGTGATGGTGGTGGTGATACTCTCAGCAAGAGCAGTAGCGGCAACAGCAGATAGAACCAAAGCGGAA
GAGAATTT

TTTCGTAAATTTCTGGCAAGGTAGACAAGCCGACAACCTTGATTGGAGACTTGACCAAACCTCTGGCGAAG
AAGTCCAAAGCTTCTCTGGTGTCAGCTCTGTTACCGACGTAAGAACCAACAATAGAGATGGACTTGACGAC
TTGGTTGA

ACTGGTGCAAGCCATAGGTAGCCTCACTACGGTACAGAGGTTACTTTGAAAGCAAGAACCCTTCACATGTC
ACACCTGCACGCAGAGGCATGGAGAGGCCAGCACAAACCACCATGGATGGGTAAAGTCATAGAAGGAAAAG
CACATGGT

CCGTTGTAGATACCGACGACGGAACCGATCATTTCTGGAACAATGATCATGTTTCTCATGTGGGTTCTGAC
TGGAGCTGGCTTTTCATTTTCTGGGGCAGCCAACTTGGCAGCTCTCAACTTCTTCATGAAACCGGCTGGCT
TGGAAGTC

AGTGGAAGAGGCAGCCTTGGTAGAGGAAGCGACGGTGGAAGAGGTAGCCTCACTGGAAGAAGCGACAGCAG
AAGAAGTAGCAGCAGAAGAAGAAGCTTCACTGGAAGAAGCGACGGCAGAAGAAGTAGCTTCACTGGAGGAA
GCGACGGC

GGAAACGGAGGAACCAGATTGTGTAACAGAACCGGACGTGCTGGAGGCTGATGAACCGGAAGCGGTACCAG
ATTGTGTGATAGATGATTCAGATGCTGATGAGGATGAGCCTGATTCTGTGACAGAACCGGATGAGCTAGGA
ACCGAAGA

AAGTAATAGAAGATGAGCCGGAGACTGAAGAGCCTGATGAAGTAATAGAGCTCGAGACTTCAGAGGACGAG
GTTGATGGAGCAATGGAGCTCGAGACTTCGGTGGAGGATGACGTTAACTCAGTGAGAGAGCTCGAGACATC
AGAAGTAG

TGCCAGCTGCCGATGCCTTATTGGAAATGATTGTCTTGCACTTGCCATCTCCAGTCACTGCTCAAGCCTAC
AGAGCTGAACAATTATACGAAGGTCCAGCTGACGATGCCAACTGTATTGCTATCAAGAACTGTGATCCAAA
GGCTGATT

TTTGATAGAGATTCCACTAAGGTTAATTCTCAAGAAGAGACAACACCTGGGACATCAGCTGTTCCAGAGAA
CCATCATCATGTCTCTCCTCAACCTGCTTCAGTACCACCTCCACAGAATGGACAGTACCAACAGCACGGCA
TGATGACC

TATACTCCCTAGATCCACGTCAAAATGCTCCAATCTTATTATTGGAGAACACTCAAAGCCAGACTCTCTTG
GTATCACGACCGTTCCTGATTTGACTCAGGAATTTGCCTGTCAGGGAGCTTTAATCCATTATCTGCTGCAT
ATAGAACG

TGATCAATCATCGATTAACCATTAGTGATAAGAAACAATGTCTTTGGAAAGAGAGGAACCACAACATTTCG
GAGCAGGGCCAGCTCAAATGCCTACACCAGTTTTGCAACAAGCTGCTAAAGACTTAATCAATTTCAATGAC
ATAGGTTT

CAAGACCGTCATGTCATCTTCTTGGCTGAAAGAAGAATCTTGCCAAAGCCATCTAGAACATCTAGACAAGT
CCAAAAGAGACCAAGATCCAGAACCTTGACTGCTGTTCATGACAAGATCTTGGAAGACTTAGTCTTCCCAA
CTGAAATC

GGCAGTGATAGTGGTAGTGATACTAGAACCAGAACCTTCATCAGCAGAAGAAGTCTCAGCAGCAGAAGAAG
TCTCAGCAGCAGAAGTGGTAGCGGCAGCAGAAGTGGTAGCGGCAGCAGAGGTTTCGGCGGCAGAAGTTTCG
GCGGCAGA

CGAAGGTTGTAAAACTATGATCGATTATTTTGAAAGCCATGGTGCTCATAAATGCCCTGCTGACGCCAACC
CAGCTGAATGGATGCTAGAAGTTGTTGGTGCAGCTCCAGGCTCTCATGCAAATCAAGATTATTACGAAGTT
TGGAGGAA

GCTTCTCTGGTGTCAGCTCTGTTACCGACGTAAGAACCAACAATAGAGATGGACTTGACGACTTGGTTGAA
GACATCAGAACAACACTTGGCACCAGCTGGCATACCGACCAAAACGGTGGTACCGTTAGCTCTAACGTATC
TGGTAGAA

AGCCGAAGAGGTAATAGAACTTAAGGTACCGGAGGTGATAGAACCAGAGGCAGTGGAAGCCGATGAGGTTG
ATTGAGGAATTGAACTCGAGATACCTGGAGCAGAAGAAGCTGATGAACCGGAAACGGAGGAACCAGATTGT
GTAACAGA

CACAACCACCACCACCCCAGCTAGTTCTGCTTCCAGCGTTATTATCTCAACCAGAAACGGTACCACTGTTA
CTGAAACTGACAACACTCTTGTCACCAAAGAAACCACTGTCTGTGACTACTCTTCAACATCTGCCGTTCCA
GCTTCCAC

CCACATAGGCACGTAACCAGTAATACCCTGAATTCTGCCGGTGGAAATAGCCTCCTTCCACAGGCCACTGG
AAGAAGAGGGCCAGCACCACCACCTCCTCCAAGAGCATCTCGCCCCACACCAAACGTTACGATGCAACAAA
ATCCACAA

TTGAACTCGAGATACCTGGAGCAGAAGAAGCTGATGAACCGGAAACGGAGGAACCAGATTGTGTAACAGAA
CCGGACGTGCTGGAGGCTGATGAACCGGAAGCGGTACCAGATTGTGTGATAGATGATTCAGATGCTGATGA
GGATGAGC

ATCGAGAAGGCTAGAGTGGAGGCCCAAAGTCACAGACTAACGTTGGAGGATCCCGTGACGGTGGAGTATCT
CACACGTTATGTCGCTGGTGTGCAACAAAGGTACACGCAGTCAGGAGGTGTTAGACCATTTGGTGTGTCGA
CGCTGATT

CTTAGAGAACTGATAGAAAAGCAGATCATGGGCAGTAGATCGGAGAATGTGGGAACAGTGACTAGAGAAGG
CTCCAGAGTGGAACAAGATGATGTCCTCATGGATGATGACTCTGACTCATCGGAATACGTGGATATGTGGA
TCGATTTA

TAGACGTGACCGTTCTTGGTGGCAGTGATAGTGGTAGTGATACTAGAACCAGAACCTTCATCAGCAGAAGA
AGTCTCAGCAGCAGAAGAAGTCTCAGCAGCAGAAGTGGTAGCGGCAGCAGAAGTGGTAGCGGCAGCAGAGG
TTTCGGCG

CAGCAAAATATGCAACAGTCACTACAGCAAATGCAGCATTTACAGCAATTGAAAATGCAGCAGCAACAACA
ACAGCAGCAACAACAACAGCAGCAACAACAACAGCAGCAACAACAACAGCAACAGCACATATATCCCTCCT
CGACTCCT

CGTCAGGACTAGTGCGATTACCACTGAAAGTACCAACTCCAGCACTAATGCTACTACCACTGCCAGCACCA
ACGTCAGGACTAGTGCTACTACCACTGCCAGCATCAACGTCAGGACTAGTGCGACTACCACTGAAAGTACC
AACTCCAA

CCGTCGAATCATCTACATCCTCCTCATCCTCCTCATCCTCCTCCTCCTCTTCTGCCTCTTCTTCTGGTGCT
GCTCCTGCTGCATTCCAAGGAGCAAGTGTCGGTGCATTGGCCCTTGGTTTGATTTCTTACCTATTATAAGC
TCGGTTTC

GCATTCCTAACTCAACAACCTCAATCTGGAGGAGCTCCAGCTCCACCCCCACCTCCTCAAATGCCAGCTAC
ATCAACATCCGGAGGCGGTTCATTCGCTGAAACTACTGGAGATGCAGGTCGTGATGCACTTTTAGCTTCAA
TTAGAGGG

TCTCTAACTACCTCAAGTTCTGCTGCCACATCTTCAAGTGAAGTTGCTAGCTCTTCTATTGCTTCATCCAC
TAGCTCTTCTGTTGCACCATCCTCAAGTGAAGTTGTCAGCTCTTCCGTTGCACCATCCTCAAGTGAAGTTG
TCAGCTCT

TTTTCCATCTTTTCGTAAATTTCTGGCAAGGTAGACAAGCCGACAACCTTGATTGGAGACTTGACCAAACC
TCTGGCGAAGAAGTCCAAAGCTTCTCTGGTGTCAGCTCTGTTACCGACGTAAGAACCAACAATAGAGATGG
ACTTGACG

ACTTGACAGTATTCCAAACTTTATACACAGTGGCAAGACTGAAGACACCAAGGAACCGAATCAAGGGTGTC
GATAGGGACACATTTGCCAAACACATGACTGAAGTGGCAATGGCAACTTACGGACTGAGCCACACTGCAGA
TACAAAAG

TTCAGTAAGATAACTTAGCGCACACTTTCCTACTTTAAGCTCACCAAATGGCATCCCCAGGATCGACAGCA
TTACCACACAAGCGACAAAGAGTCCGAAAGGCTTGCGTGCCCTGTAGGGAACGTAAAAGGAAATGTAATGG
CAAATCTC

CGACTAGGTTTGATAGCACGTTGGAAGACATACGGAAGGGCAAAAGAACTGTCTTCCCTGTGTCCGGATGC
ACGGTGGAGTCCAGTTGTTTCTTGGCTCGCCAGAACTCCGGAGTAGTCTCTTTTAGTTCACCGTGACGATA
CGCACTGA

GCTCAAGCTCAAGCTCAAGCTCAGGCACAGGCTCAAGCACAAGCTCAAGCACAGGCGGCACAAGCGGCGCA
AGCGCAAGCACAAGCACAAGCACAAGCACAAGCACAGGCACAGGCACAGGCACAAGCCCAGGCGCAGGCCC
AAGCCCAA

TCGTAAAGAATATCATCATAAAGTATGGTATACAAAATTTACTGTGCAACAACAGCACAATGCAGTCCATG
ATCTGCTCATCTGAGCACGAAAACCTAACCTGCAAGTACTGGCCTGTTAGCTTCCTCGCTTCTTGGTGTGA
GAATGGGT

ATTCTTGTTGAAGACTGGTACTTTGACCACCAGTGAAACTGCTCACAACATGAAGGTCATGAAATTCTCTG
TCTCTCCAGTTGTGCAAGTCGCTGTCGAAGTCAAGAACGCTAACGACTTACCAAAATTGGTCGAAGGTTTG
AAGAGATT

AGAAAGTTCTTGCCAGCTGCCGATGCCTTATTGGAAATGATTGTCTTGCACTTGCCATCTCCAGTCACTGC
TCAAGCCTACAGAGCTGAACAATTATACGAAGGTCCAGCTGACGATGCCAACTGTATTGCTATCAAGAACT
GTGATCCA

GTGTAACAGAACCGGACGTGCTGGAGGCTGATGAACCGGAAGCGGTACCAGATTGTGTGATAGATGATTCA
GATGCTGATGAGGATGAGCCTGATTCTGTGACAGAACCGGATGAGCTAGGAACCGAAGAAGCGGAGCCTGA
TTCTGTGG

AAAGGACTTGGAAGGTAAGGCCTTGTTGAAGGTTGTTATGAGAAAGTTCTTGCCAGCTGCCGATGCCTTAT
TGGAAATGATTGTCTTGCACTTGCCATCTCCAGTCACTGCTCAAGCCTACAGAGCTGAACAATTATACGAA
GGTCCAGC

AATTAGATCCTTGCTTACTTCCTCAAACTTGTAAGCGGTACCATACATGACGTAATCATAATCATCTGCAA
GGGATCTGTCACCAGCCTGTGGAGGTCTCCAGCTTCTTGTCGCAGAAGAGTCGTTAGCTGGGGTGTCTTCG
AGGTTTAA

AAACAGATGGGGAGCCGGAAATCAAGGTGGAAGCCGTTGAACCAGTCACGGAAGCACCGAATGAAGTGGAA
GCGGTTGAACCAGTTACGGAAGCACCGAATGAAGTGGAACCGGAAGTAGAGTTGGTAGCCCCTGATGTGGT
AGATGTGG

TCTTTGGGAATTCAAAGATTCTGGCATCGTCAGTAACAGTACCAACAACGACAACAGTCTTGTTAGCAGCA
CCTTCTTGCTTCAAAGCTCTAGCAATTCTAGAGACAGAAACAGGTGGTCTGTTGATCTTAGACAAGAACAA
AGCCTTCA

AGATGGGGTACTAAGAAATTGCCAAGAAAGACTCACAGAGGTCTAAGAAAGGTTGCTTGTATTGGTGCTTG
GCATCCAGCCCACGTTATGTGGAGTGTTGCCAGAGCTGGTCAAAGAGGTTACCATTCCAGAACCTCCATTA
ACCACAAG

TTATAGATTTCGTTACCGATAGTCTTTCCTTCAGAATTCTTTCGGTCCTCTTCAATGTCACTGACAACTTC
AGAAACCTCTTCACTCAGGTTGTCGTCATCCTCTAAATTGGTTGTAATAAAAGAACTAGGTATTTGTGTAG
ATAGTTGA

TCTGGTAGAAGCTTCAATAGCGGCTTCGGAAACGGAAACGTTGATGACACCGTGAGCACCACCGTCAGTGG
CCTTTAGAACAGCACCGACAATGTCCTTTTCCTTAGTGAAGTCAATGAAGACTTCACCACCGATGGATCTG
AATAATTC

GGGGCTCTTCAAGAAGCAGAGATAACTCTTTCAGAGGCGGTAGTGGCTGGGGTAGCGATTCCAAGTCTTCT
GGCTGGGGTAACAGCGGTGGTTCAAACAACTCTTCTTGGTGGTGATTTCAGACAAACTAGGGTGAGGATTC
TTCGTTTT

CACTGACTATTCCTTGTCAAAGTACAATCCTGACGATACCATTGCTCCTCCTCAAGATGCCACTGAAGAAT
CACAAACAAAATCTCTGAGATTCTTGCACAAGGGTGGAAGCAGAAGATCTCCAAAACAGATTGGAAGAAGA
AACACCGC

CACCAGCCTTAACAATCTTGGCTCTGGCACCAGCAGTGAATCTCAAAGCAGCAACAGTGGTCTTTGGGAAT
TCAAAGATTCTGGCATCGTCAGTAACAGTACCAACAACGACAACAGTCTTGTTAGCAGCACCTTCTTGCTT
CAAAGCTC

TATCAGATGCAACAACAGCAACAAGCTCAAGCTCAAGCTCAAGCTCAAGCTCAGGCACAGGCTCAAGCACA
AGCTCAAGCACAGGCGGCACAAGCGGCGCAAGCGCAAGCACAAGCACAAGCACAAGCACAAGCACAGGCAC
AGGCACAG

AAGATGAACTGGAGACTGATGAGCCTGATGAAGTAATAGAAGATGAGCCGGAGACTGAAGAGCCTGATGAA
GTAATAGAGCTCGAGACTTCAGAGGACGAGGTTGATGGAGCAATGGAGCTCGAGACTTCGGTGGAGGATGA
CGTTAACT

GAAGAAGTAGCAGCAGAAGAAGAAGCTTCACTGGAAGAAGCGACGGCAGAAGAAGTAGCTTCACTGGAGGA
AGCGACGGCAGAAGAAGTAGTTTCACTGGAGGAAGCAACAGCGGAAGAAGTAGCAGCAGAAGAAGCTTCAG
TAGAAGAG

GTGGTAGTGATACTAGAACCAGAACCTTCATCAGCAGAAGAAGTCTCAGCAGCAGAAGAAGTCTCAGCAGC
AGAAGTGGTAGCGGCAGCAGAAGTGGTAGCGGCAGCAGAGGTTTCGGCGGCAGAAGTTTCGGCGGCAGAAG
ATTCAGCG

GAGCTAAGAAAGTGTATAATTTGACTAACAATTTCAAGTAGACATTGTCAGACTTTGGAGCAGTTCTGTGA
CCAGATCTCTTGTGTTGCTTGGAAGTGTGATCGATACCCATTGTGAATCCGTAAGCTTGATGATCTCTTCT
TGTGATGG

GCAACAGTTCCAGGGAATCATCCTCATCAAAAGAACCTCCATCAGAGCTCAAAGTTCCATCATCCAGACAG
GACAAGCAGCTCAACAAGGATAAAGAATCTCCGCTTCTGAGTTGACCATGCTTATTGAACATATGGTGTGA
TTGCGATT

GATTGGAGACTTGACCAAACCTCTGGCGAAGAAGTCCAAAGCTTCTCTGGTGTCAGCTCTGTTACCGACGT
AAGAACCAACAATAGAGATGGACTTGACGACTTGGTTGAAGACATCAGAACAACACTTGGCACCAGCTGGC
ATACCGAC

AGCCCACATTTCTTACGAGGCAGGTATCTTGGAAGACCCAGATACCACCCCACCAAAGGACATGTGGAAAT
TGATCGTCGATCCAATGGATGCTCCGGACCAACCACAAGATTTGACCATTGACTTTGAACGTGGTCTTCCA
GTCAAGTT

GCCCAAGCACAAGCACAAGCACACGCTCAGCACCAGCCCTCCCAACAACCACAACAAGCTCAGCAACAACC
TAACCCACTACATGGGTTGACACCTACTGCAAAGGATGTCGAAGTAATTAAGCAATTGTCCTTGGATGCTT
CTAAGACC

TTTGGTTCATCATCGTCATAGTACTCGCCCTCCTCCTCGTCATCTTCTTCAGATCCGCTTGAGCCGTCTTC
TTCATCATCTGAGAATTCCTCTGTGCCAGAACTACATTCGTGGTCAGCGTTATCAAATTCAAATTGCTCAG
AAGCAGCA

TTGTGCACCGTAGAATTGTAGAATACAAATACATAAATAAGTGTGTTCCCGAAGGACTAAGGAATGACGGC
AGAGGAGTCTTGTGACCGCCCTGCTGCAAAACTTGGCAAGGACAGAAATTAAGTAATAGATACGCACAACC
TTGGAGCA

GAACCAGATTGTGTAACAGAACCGGACGTGCTGGAGGCTGATGAACCGGAAGCGGTACCAGATTGTGTGAT
AGATGATTCAGATGCTGATGAGGATGAGCCTGATTCTGTGACAGAACCGGATGAGCTAGGAACCGAAGAAG
CGGAGCCT

CATTGCTAGCTCTTCTTCCACTGCCCAGACTTCTATCTCGACCATTGCTCCTTACAACTCCACAACCACCA
CCACCCCAGCTAGTTCTGCTTCCAGCGTTATTATCTCAACCAGAAACGGTACCACTGTTACTGAAACTGAC
AACACTCT

TTGCACTAGAAGCCGAAGAGGTAATAGAACTTAAGGTACCGGAGGTGATAGAACCAGAGGCAGTGGAAGCC
GATGAGGTTGATTGAGGAATTGAACTCGAGATACCTGGAGCAGAAGAAGCTGATGAACCGGAAACGGAGGA
ACCAGATT

CTAAGTTCCACAAGCGTGAAGTTGTCGAAGCTGTCACCGTTGTTGACACTCCACCAGTTGTCGTTGTTGGT
GTTGTCGGTTACGTCGAAACCCCAAGAGGTTTGAGATCTTTGACCACCGTCTGGGCTGAACATTTGTCTGA
CGAAGTCA

ATTGATTCAAGGCCTTGTTATGGGCCTCCTGTTCAGGATCCACTCTAGAAGGCTCCTCTTCATGATGTTCT
TCCGTTTCCTGTGGTTGTTCTGGTTGTTGTTCGTGTGACTGTTGGGTGTGATTGTCGCCGGACGTTTCCCC
CAATCCTG

AAACTCTCATATAGGTCTTGGATTTTGGTCTCCCCTTCATCTCCGTCATGTCCGTCGCCTTCTTCATCTTC
TTCTGACCCCAGTCTAGACTGTGAACGTCTTTTGTCTCTTTCTGATATCCTTGATGATCTCCTTCTAATAC
CATCCCTT

CTTGGCTGAAAGAAGAATCTTGCCAAAGCCATCTAGAACATCTAGACAAGTCCAAAAGAGACCAAGATCCA
GAACCTTGACTGCTGTTCATGACAAGATCTTGGAAGACTTAGTCTTCCCAACTGAAATCGTTGGTAAGAGA
GTTAGATA

AGGCTACGATCCGTGGGATTTACTGAGCAAGAAATCGAACTGCTGAGGCAACAGTTTCGGGCCACGTATGG
AGATTTGGAGGAGGAGGAGGAGAGACTTGCTCAAAATGGCAACAGAGATGATGAGGGCCATGACATTAGAC
AGTTGGAG

ATGCAGATTCCAGAATTCTTATCGAGAAGGCTAGAGTGGAGGCCCAAAGTCACAGACTAACGTTGGAGGAT
CCCGTGACGGTGGAGTATCTCACACGTTATGTCGCTGGTGTGCAACAAAGGTACACGCAGTCAGGAGGTGT
TAGACCAT

TCTCAAAGCAGCAACAGTGGTCTTTGGGAATTCAAAGATTCTGGCATCGTCAGTAACAGTACCAACAACGA
CAACAGTCTTGTTAGCAGCACCTTCTTGCTTCAAAGCTCTAGCAATTCTAGAGACAGAAACAGGTGGTCTG
TTGATCTT

TGTATTTATGTCTTTCAAGTTTCTTCCTTGATTTGCACCCTCCGCTGCGATGTGTACAGACGTTGCTTTCT
TTTCGCTGGACTGTCTGGCCACCTGGTTAGGTGGAGTTTGCTCCACATCCGACCTGAGACTACCCAACTTT
GGATCGCT

CAGTGGAAGCCGATGAGGTTGATTGAGGAATTGAACTCGAGATACCTGGAGCAGAAGAAGCTGATGAACCG
GAAACGGAGGAACCAGATTGTGTAACAGAACCGGACGTGCTGGAGGCTGATGAACCGGAAGCGGTACCAGA
TTGTGTGA

ACAAAGTGATCAAGACATAGGGAAACGCCAACCACAATTTCAACAGCAGCAGCAGCCCCAACAGCAGCAGC
AGCAGCAGCAACAGCAACAGAGACAACACCAGGTCCAGACACAACAACAAAGACAGATACCTGATAGGAGA
TCTCTTTC

GGAAACGCCAACCACAATTTCAACAGCAGCAGCAGCCCCAACAGCAGCAGCAGCAGCAGCAACAGCAACAG
AGACAACACCAGGTCCAGACACAACAACAAAGACAGATACCTGATAGGAGATCTCTTTCACTTTCTCCTTG
TACAAGAG

CAAGACATAGGGAAACGCCAACCACAATTTCAACAGCAGCAGCAGCCCCAACAGCAGCAGCAGCAGCAGCA
ACAGCAACAGAGACAACACCAGGTCCAGACACAACAACAAAGACAGATACCTGATAGGAGATCTCTTTCAC
TTTCTCCT

AACAAGAAGGACAAAGTGATCAAGACATAGGGAAACGCCAACCACAATTTCAACAGCAGCAGCAGCCCCAA
CAGCAGCAGCAGCAGCAGCAACAGCAACAGAGACAACACCAGGTCCAGACACAACAACAAAGACAGATACC
TGATAGGA

CCGGCAGGGCATCGTAGTAGTCTGGATCAACGATGACGTGACAGGTGGAGCAGGCACAAGAACCGCCGCAT
GCGCCCTCCATGTCCAGGTTGTGACCTTGAGCGATGTCCAGGATGGTTTCGCCCTCACAGACTTCGTACGT
CTTCTGGG

CTGTTGGTAAGCAAATTGTCAACATCCCCTCTTTCATGGTCAGATTGGACTCTGAGAAGCACATTGACTTT
GCTCCAACATCTCCATTCGGTGGTGCTAGACCAGGCAGAGTTGCTAGAAGAAATGCTGCAAGGAAGGCGGA
AGCTTCTG

TCTTCAGGTTCCGGCAGGGCATCGTAGTAGTCTGGATCAACGATGACGTGACAGGTGGAGCAGGCACAAGA
ACCGCCGCATGCGCCCTCCATGTCCAGGTTGTGACCTTGAGCGATGTCCAGGATGGTTTCGCCCTCACAGA
CTTCGTAC

AGGCACATTGCTGTTGGTAAGCAAATTGTCAACATCCCCTCTTTCATGGTCAGATTGGACTCTGAGAAGCA
CATTGACTTTGCTCCAACATCTCCATTCGGTGGTGCTAGACCAGGCAGAGTTGCTAGAAGAAATGCTGCAA
GGAAGGCG

AATCACTCAAAGGCACATTGCTGTTGGTAAGCAAATTGTCAACATCCCCTCTTTCATGGTCAGATTGGACT
CTGAGAAGCACATTGACTTTGCTCCAACATCTCCATTCGGTGGTGCTAGACCAGGCAGAGTTGCTAGAAGA
AATGCTGC

AATCAGCCAGAACAAGAAGGACAAAGTGATCAAGACATAGGGAAACGCCAACCACAATTTCAACAGCAGCA
GCAGCCCCAACAGCAGCAGCAGCAGCAGCAACAGCAACAGAGACAACACCAGGTCCAGACACAACAACAAA
GACAGATA

TTGCTCGGGGATAGTAATAATGCGGGGGGGAAGACGAAACAGACCGTGTGTGGTGTGGGTGGGATTTCGTG
GGAGCACGTGGCGAGCAGAGGAAGAGCCTCACACAATGCTGCGTTCTTCATAAAGGGGATCAAAGCAAATA
GATAATCG

TCTCTTTCTCTTCTTGGTGGTCTTGAGGAGCTTTGGAAGTTGGAACCTCTGGCTGCACTCCAGTCAATATC
TGGCTCTTCTCTTTCTCTTCTTGGTGGCCTCGAGGAGCTTTGAAAGTTAGAGCCTCTAGCTGCACTCCAGT
CAATATCT

TTTAGGATCTGATGCTTATGTTGTTTCAAGATCAATGACCCAGAAGGTCTCGGATGGGGTCAAAGCCCTAA
TTTGTGGGGTTGTTGGCGTGGGGATGATGTGTTCTTTGTCACCTCAACTATCAATATTGTTACTATTCTTC
ACTCCACC

CAAACTGAGCACCTCTGGCAGCACCCCAATCCAAAGCTGGTTCTTCCTTTTCTCTTTCTCTTCTTGGTGGT
CTTGAGGAGCTTTGGAAGTTGGAACCTCTGGCTGCACTCCAGTCAATATCTGGCTCTTCTCTTTCTCTTCT
TGGTGGCC

ACCACTGAAAGTACCAACTCCAGCACTAATGCTACTACCACTGCCAGCACCAACGTCAGGACTAGTGCTAC
TACCACTGCCAGCATCAACGTCAGGACTAGTGCGACTACCACTGAAAGTACCAACTCCAACACTAGTGCTA
CTACCACC

AAGTGTGAGTTTGCTCGGGGATAGTAATAATGCGGGGGGGAAGACGAAACAGACCGTGTGTGGTGTGGGTG
GGATTTCGTGGGAGCACGTGGCGAGCAGAGGAAGAGCCTCACACAATGCTGCGTTCTTCATAAAGGGGATC
AAAGCAAA

TGAGGCTTACCAAACTGAGCACCTCTGGCAGCACCCCAATCCAAAGCTGGTTCTTCCTTTTCTCTTTCTCT
TCTTGGTGGTCTTGAGGAGCTTTGGAAGTTGGAACCTCTGGCTGCACTCCAGTCAATATCTGGCTCTTCTC
TTTCTCTT

ATCGTAGTAGTCTGGATCAACGATGACGTGACAGGTGGAGCAGGCACAAGAACCGCCGCATGCGCCCTCCA
TGTCCAGGTTGTGACCTTGAGCGATGTCCAGGATGGTTTCGCCCTCACAGACTTCGTACGTCTTCTGGGAG
CCATCCTT

GCTGTACGTTCAGTGACAATAAAGGATTGTCATGCATATGTGTGTGGCTGTGACTATGAGTATGTTGCAAC
AACGACTTCACAGAGGAAGCCTTGCCTTCCTTCTTTCCTTCACTTTCATTCTGTCTGGAATTCGATCCATT
CTGTGAAG

ATGATTTCTTCGGATTCTTCAATGGTTGGGCTTTCCTCTTCTTCGCAGTGGCTGTCTCAGAATCCTCATGA
TCTGCTTCCTTGTCACCAGAGTCATCCTCCTCAGGGACATGCTCTAGCTCCGACTCCTGACCAACGCTGTT
TCCGCCTG

AGCGTTGGAGATACCTTGACCTAATGGACCGGTAGTAACTTCAACACCTGGCAACTCAAATTCAGGATGAC
CTGGTGTTCTGGAACCCAACTGTCTGAACTGTTTCAAGTCTTCAATAGACAGATCGTAACCAGTCAAATGT
AGCATAGA

AACACTCTCAAGATAGAGTTGAGTCTTCTGATATCCAGGAAGCCTTGTCAAGATTGCAACAAGAGGACAAG
GTCATTGTCCTTGGCGAGGGTGTAAGGAGATCAGTTCGCCTGAATAACCGTGTCTGATGCTGTCTACTACA
ATCATTCC

AGGACCCCAGAATCAGCCAGAACAAGAAGGACAAAGTGATCAAGACATAGGGAAACGCCAACCACAATTTC
AACAGCAGCAGCAGCCCCAACAGCAGCAGCAGCAGCAGCAACAGCAACAGAGACAACACCAGGTCCAGACA
CAACAACA

CCCCTCTTCAATGATTTCTTCGGATTCTTCAATGGTTGGGCTTTCCTCTTCTTCGCAGTGGCTGTCTCAGA
ATCCTCATGATCTGCTTCCTTGTCACCAGAGTCATCCTCCTCAGGGACATGCTCTAGCTCCGACTCCTGAC
CAACGCTG

ACCACAATTTCAACAGCAGCAGCAGCCCCAACAGCAGCAGCAGCAGCAGCAACAGCAACAGAGACAACACC
AGGTCCAGACACAACAACAAAGACAGATACCTGATAGGAGATCTCTTTCACTTTCTCCTTGTACAAGAGCC
AATTCTTT

ACCTCTGGCAGCACCCCAATCCAAAGCTGGTTCTTCCTTTTCTCTTTCTCTTCTTGGTGGTCTTGAGGAGC
TTTGGAAGTTGGAACCTCTGGCTGCACTCCAGTCAATATCTGGCTCTTCTCTTTCTCTTCTTGGTGGCCTC
GAGGAGCT

GTTTTCATCATCTTCAGGTTCCGGCAGGGCATCGTAGTAGTCTGGATCAACGATGACGTGACAGGTGGAGC
AGGCACAAGAACCGCCGCATGCGCCCTCCATGTCCAGGTTGTGACCTTGAGCGATGTCCAGGATGGTTTCG
CCCTCACA

CTTTGGAAGTTGGAACCTCTGGCTGCACTCCAGTCAATATCTGGCTCTTCTCTTTCTCTTCTTGGTGGCCT
CGAGGAGCTTTGAAAGTTAGAGCCTCTAGCTGCACTCCAGTCAATATCTGGCTCTTCTCTTTCTCTTCTTG
GTGGTCTG

TTAATATGTTGTTGATTTCCTCATCTGGTCCAATCAAGACACCTGTTGGTGCCTGTGCCACATCCGTGGAA
TCTGTTGACATTGGTGTAGAAACCATCTTGTCTGGTAAGGCTAGAATCTGTATTGTCGGTGGTTACGATGA
TTTCCAAG

TTCTTCCTTTTCTCTTTCTCTTCTTGGTGGTCTTGAGGAGCTTTGGAAGTTGGAACCTCTGGCTGCACTCC
AGTCAATATCTGGCTCTTCTCTTTCTCTTCTTGGTGGCCTCGAGGAGCTTTGAAAGTTAGAGCCTCTAGCT
GCACTCCA

TCCTTAAACTGTGAAAGAAAGAATAGCGATACAAATGTCTAGGCAGTCCCTAGGACAGTGCCGGAGTTGTC
ATTGTGGAGAGGCAATGGTGTTTCTGTGGACATTAACGGTTCGTAATCTTTAGTTCTGTCGATATCCTCAT
CTCTGTAA

GCACCCCAATCCAAAGCTGGTTCTTCCTTTTCTCTTTCTCTTCTTGGTGGTCTTGAGGAGCTTTGGAAGTT
GGAACCTCTGGCTGCACTCCAGTCAATATCTGGCTCTTCTCTTTCTCTTCTTGGTGGCCTCGAGGAGCTTT
GAAAGTTA

TCTGAGAAGCACATTGACTTTGCTCCAACATCTCCATTCGGTGGTGCTAGACCAGGCAGAGTTGCTAGAAG
AAATGCTGCAAGGAAGGCGGAAGCTTCTGGTGAAGCTGCTGATGAAGCTGATGAGGCCGATGAAGAATAAT
TGTGCTGA

AATCATTTATCAACACCATGTCCGCTTGGGTTAATATGTTGTTGATTTCCTCATCTGGTCCAATCAAGACA
CCTGTTGGTGCCTGTGCCACATCCGTGGAATCTGTTGACATTGGTGTAGAAACCATCTTGTCTGGTAAGGC
TAGAATCT

CCAAAGCTGGTTCTTCCTTTTCTCTTTCTCTTCTTGGTGGTCTTGAGGAGCTTTGGAAGTTGGAACCTCTG
GCTGCACTCCAGTCAATATCTGGCTCTTCTCTTTCTCTTCTTGGTGGCCTCGAGGAGCTTTGAAAGTTAGA
GCCTCTAG

TTTAACATACACAAACACATACTATCAGAATACAATGACTAAGCTACACTTTGACACTGCTGAACCAGTCA
AGATCACACTTCCAAATGGTTTGACATACGAGCAACCAACCGGTCTATTCATTAACAACAAGTTTATGAAA
GCTCAAGA

TCCGCTTGGGTTAATATGTTGTTGATTTCCTCATCTGGTCCAATCAAGACACCTGTTGGTGCCTGTGCCAC
ATCCGTGGAATCTGTTGACATTGGTGTAGAAACCATCTTGTCTGGTAAGGCTAGAATCTGTATTGTCGGTG
GTTACGAT

CCGGTGTCATCAAGCCAGGTATGGTTGTTACTTTTGCCCCAGCTGGTGTTACCACTGAAGTCAAGTCCGTT
GAAATGCATCACGAACAATTGGAACAAGGTGTTCCAGGTGACAACGTTGGTTTCAACGTCAAGAACGTTTC
CGTTAAGG

TCTGGATCAACGATGACGTGACAGGTGGAGCAGGCACAAGAACCGCCGCATGCGCCCTCCATGTCCAGGTT
GTGACCTTGAGCGATGTCCAGGATGGTTTCGCCCTCACAGACTTCGTACGTCTTCTGGGAGCCATCCTTCA
GAATAAAA

CAAGAGTTTTAATCACTCAAAGGCACATTGCTGTTGGTAAGCAAATTGTCAACATCCCCTCTTTCATGGTC
AGATTGGACTCTGAGAAGCACATTGACTTTGCTCCAACATCTCCATTCGGTGGTGCTAGACCAGGCAGAGT
TGCTAGAA

TAATTTCTCGTTTAGGATCTGATGCTTATGTTGTTTCAAGATCAATGACCCAGAAGGTCTCGGATGGGGTC
AAAGCCCTAATTTGTGGGGTTGTTGGCGTGGGGATGATGTGTTCTTTGTCACCTCAACTATCAATATTGTT
ACTATTCT

GACCTATCCTTGTAGGTATTTTTGGTTTGTTGAGGCTTACCAAACTGAGCACCTCTGGCAGCACCCCAATC
CAAAGCTGGTTCTTCCTTTTCTCTTTCTCTTCTTGGTGGTCTTGAGGAGCTTTGGAAGTTGGAACCTCTGG
CTGCACTC

CTTTCCTCTTCTTCGCAGTGGCTGTCTCAGAATCCTCATGATCTGCTTCCTTGTCACCAGAGTCATCCTCC
TCAGGGACATGCTCTAGCTCCGACTCCTGACCAACGCTGTTTCCGCCTGGCGAATTCTTAATGTTGCTATC
GCTGCTGC

TTTATTCTCTTGACTCGTCCATCATAGAGCTTTATAGAAATGTCAGAGAAGGCTTCTGAAGAGAGACCCAT
ACGGCTGGCCGTTCTGGGGGGCACTTCTACCGGAAAGACCTCCCTGGTATCTAGGCTTACTGTTAATATCG
TCCACGAG

AGACGTACTCAAAATGTCTCAGAACGTTTACATTGTATCGACTGCCAGAACCCCAATTGGTTCATTCCAGG
GTTCTCTATCCTCCAAGACAGCAGTGGAATTGGGTGCTGTTGCTTTAAAAGGCGCCTTGGCTAAGGTTCCA
GAATTGGA

GCCTCTTGCAGCGCTCCAGTCGATATCAACTTCTTCTCTTTCTCTTCTTGGTGGTCTGGAAGAGCCTTGGA
AATTGGAACCTCTTGCAGCAGTCCAGTCAATATCAACTTCTTCTCTTTCTCTTCTTGGACCTCTGAAGTTA
GAACCTCT

CTTCGCAGTGGCTGTCTCAGAATCCTCATGATCTGCTTCCTTGTCACCAGAGTCATCCTCCTCAGGGACAT
GCTCTAGCTCCGACTCCTGACCAACGCTGTTTCCGCCTGGCGAATTCTTAATGTTGCTATCGCTGCTGCCC
TTAGCATT

GTTGGGGATTTAATTTCTCGTTTAGGATCTGATGCTTATGTTGTTTCAAGATCAATGACCCAGAAGGTCTC
GGATGGGGTCAAAGCCCTAATTTGTGGGGTTGTTGGCGTGGGGATGATGTGTTCTTTGTCACCTCAACTAT
CAATATTG

CCAAGATGCTTTGACAGCTTCCACTGAAAACGGTGTCAAGTTGAGACAAACCGATAGACTGTTGGATGGTG
TTGCTGATGGCTCCATGAGATCCAAGGCCGACAGGATGGCCAAGATGGAAAGAAGAGAAAGAAATAGACAT
GCCAAGCA

CAAGCCAGGTATGGTTGTTACTTTTGCCCCAGCTGGTGTTACCACTGAAGTCAAGTCCGTTGAAATGCATC
ACGAACAATTGGAACAAGGTGTTCCAGGTGACAACGTTGGTTTCAACGTCAAGAACGTTTCCGTTAAGGAA
ATCAGAAG

TTCTTGATCTGCTGTACGTTCAGTGACAATAAAGGATTGTCATGCATATGTGTGTGGCTGTGACTATGAGT
ATGTTGCAACAACGACTTCACAGAGGAAGCCTTGCCTTCCTTCTTTCCTTCACTTTCATTCTGTCTGGAAT
TCGATCCA

CAACACCATGTCCGCTTGGGTTAATATGTTGTTGATTTCCTCATCTGGTCCAATCAAGACACCTGTTGGTG
CCTGTGCCACATCCGTGGAATCTGTTGACATTGGTGTAGAAACCATCTTGTCTGGTAAGGCTAGAATCTGT
ATTGTCGG

GAAGATGCAACTTTCATGGAAGGATATCCCTACTGTCGCTCCAGCAAATGATTTGCTGGATATTGTCTTGA
ACAGAACCCAGAGAAAGACACCAACTGTGATCAGACCTGGTTTCAAGATTACAAGAATCAGAGCGTTCTAT
ATGCGTAA

ACAGGTGGAGCAGGCACAAGAACCGCCGCATGCGCCCTCCATGTCCAGGTTGTGACCTTGAGCGATGTCCA
GGATGGTTTCGCCCTCACAGACTTCGTACGTCTTCTGGGAGCCATCCTTCAGAATAAAAGTTATCTTCAGT
TCTTCGCC

CAAATCAATGAACACTCTCAAGATAGAGTTGAGTCTTCTGATATCCAGGAAGCCTTGTCAAGATTGCAACA
AGAGGACAAGGTCATTGTCCTTGGCGAGGGTGTAAGGAGATCAGTTCGCCTGAATAACCGTGTCTGATGCT
GTCTACTA

AACATCCCCTCTTTCATGGTCAGATTGGACTCTGAGAAGCACATTGACTTTGCTCCAACATCTCCATTCGG
TGGTGCTAGACCAGGCAGAGTTGCTAGAAGAAATGCTGCAAGGAAGGCGGAAGCTTCTGGTGAAGCTGCTG
ATGAAGCT

TTTGGTTTGTTGAGGCTTACCAAACTGAGCACCTCTGGCAGCACCCCAATCCAAAGCTGGTTCTTCCTTTT
CTCTTTCTCTTCTTGGTGGTCTTGAGGAGCTTTGGAAGTTGGAACCTCTGGCTGCACTCCAGTCAATATCT
GGCTCTTC

ATATCAGAGTCCGCTGAGGATGAATCAGTAAATGTATTACCTGACTCAGGTGATGGAGTGCTCAGAGGCGT
TCCAACTGATGATGGATACTGCGGAAACTGTGATTGTGGCCCAGGTGGAAAGTACATAGGCGACATTTGAT
AAGGTGTA

AGAATCAGAATGAGTATGAGTATGATTTGTTGATTTAGTGTCATGAGAGTGCTCCAAATCACCTTTAGAGT
CAGAGGTTACATCAACAAACTCGACGTCCACCTTCCCACGTTTGGCACCTTCATACGTAAATCCTCTCTTA
TAGTCGAA

TTACCTTCTTTTGGAAGGTTTAGTAGTCGTATCTCTTTCAATGATTCCGCCTGTATCCTCCAGACGCTCTT
CCTCAAGGGTCACCTGATCTCTTCTTTCCCACCAACGGTCTTCACATAACTCAAATGACTTCGTCTTTAAC
TGTTTACC

CTAAGCAGGTTATCAGACAGAGAGTTTGCCACAGGTAAATTCAAGTTTGTTCCTCCTGGGGTGAAGGTCGA
AAAACTGACACCAAAGCAAGAGGAAGAGTTGAAGGCCAAGACCATTGCAAAATATGAAGCTCTAGACAAGG
ATCCTAAA

ATAGTAATAATGCGGGGGGGAAGACGAAACAGACCGTGTGTGGTGTGGGTGGGATTTCGTGGGAGCACGTG
GCGAGCAGAGGAAGAGCCTCACACAATGCTGCGTTCTTCATAAAGGGGATCAAAGCAAATAGATAATCGAT
TCCAAAGA

ATTGGCTTCTAGAATTGAGAATAAGTTGAAATCTCAGTCTAGAATCAACAATGTTCTGAACAAGATTCATG
TTGCTCAACCTCAAGCAAGAGATGATGTCAAGAGAACACCATTTATCCCAGAATCTGTCAAGAATTTGAAG
AAATACGA

ATTATCAACAACAGCAGTCAAACACCGGTAACTACAACTACCACATCCACAACAACTGCGACATCTTCTCC
TGGGAAATTCTCCTCTTCTCCGAACTCCTCTGTACTGGAGAACAACAGATTAAACAGTATCAACAATTCAA
ATCAATAT

GCAAATTGTCAACATCCCCTCTTTCATGGTCAGATTGGACTCTGAGAAGCACATTGACTTTGCTCCAACAT
CTCCATTCGGTGGTGCTAGACCAGGCAGAGTTGCTAGAAGAAATGCTGCAAGGAAGGCGGAAGCTTCTGGT
GAAGCTGC

ATAACTCACAAGGACCCCAGAATCAGCCAGAACAAGAAGGACAAAGTGATCAAGACATAGGGAAACGCCAA
CCACAATTTCAACAGCAGCAGCAGCCCCAACAGCAGCAGCAGCAGCAGCAACAGCAACAGAGACAACACCA
GGTCCAGA

TTCTTTCAGACAATCTCTTAGCCAACAATTGAGCGTATTCGGCAGCAGCTTCTCTTTGAGCTTGAGCGTTT
CTGACCTTCAAAGCTCTTTGGTGTCTCTTTCTTTGCAATCTTTGAGGAGTAACCAATCTTTGGATCTTTGG
AGCCTTGG

TGTAGGTATTTTTGGTTTGTTGAGGCTTACCAAACTGAGCACCTCTGGCAGCACCCCAATCCAAAGCTGGT
TCTTCCTTTTCTCTTTCTCTTCTTGGTGGTCTTGAGGAGCTTTGGAAGTTGGAACCTCTGGCTGCACTCCA
GTCAATAT

GTAATTTGTCATTCGTGCCATTATTCTCTCCGAAAGTTTGTGAATGTGAGGATAATGGTGAGTTCATTTGT
GGCTGAGAGCTTACAACAGGTGGTGGAGATCTCACTGGAGAGTCTCCGTAGTTTACACTATATAGTGCCAT
ATCTTCGA

AGCCAAGTCTGTCCACCATGCAAGAGTTTTAATCACTCAAAGGCACATTGCTGTTGGTAAGCAAATTGTCA
ACATCCCCTCTTTCATGGTCAGATTGGACTCTGAGAAGCACATTGACTTTGCTCCAACATCTCCATTCGGT
GGTGCTAG

AGGTCGATTCTTTGCATAAGAGGCTACTGACCGAAGTTAATGAGGAGCAAGCAAGTCACTTAAGGCACTCC
CTGGACAACTTCGTCGCACAAGCCACGGACTTGCAGTTCAAACTGAAAAATGAGATTAAAAGTGCCCAAAG
GGATGGGA

GTCCACCATGCAAGAGTTTTAATCACTCAAAGGCACATTGCTGTTGGTAAGCAAATTGTCAACATCCCCTC
TTTCATGGTCAGATTGGACTCTGAGAAGCACATTGACTTTGCTCCAACATCTCCATTCGGTGGTGCTAGAC
CAGGCAGA

CAGATTGGACTCTGAGAAGCACATTGACTTTGCTCCAACATCTCCATTCGGTGGTGCTAGACCAGGCAGAG
TTGCTAGAAGAAATGCTGCAAGGAAGGCGGAAGCTTCTGGTGAAGCTGCTGATGAAGCTGATGAGGCCGAT
GAAGAATA

ACGTCAGGACTAGTGCGACTACCACTGAAAGTACCAACTCCAGCACTAATGCTACTACCACTGCCAGCACC
AACGTCAGGACTAGTGCTACTACCACTGCCAGCATCAACGTCAGGACTAGTGCGACTACCACTGAAAGTAC
CAACTCCA

CGATGACGTGACAGGTGGAGCAGGCACAAGAACCGCCGCATGCGCCCTCCATGTCCAGGTTGTGACCTTGA
GCGATGTCCAGGATGGTTTCGCCCTCACAGACTTCGTACGTCTTCTGGGAGCCATCCTTCAGAATAAAAGT
TATCTTCA

TAGTGCGACTACCACTGAAAGTACCAACTCCAGCACTAATGCTACTACCACTGCCAGCACCAACGTCAGGA
CTAGTGCTACTACCACTGCCAGCATCAACGTCAGGACTAGTGCGACTACCACTGAAAGTACCAACTCCAAC
ACTAGTGC

CCATCAGTAGAATTCGTTATGCTATCCTCTTCATCTGAAGATGGCTCGAAGACTCCTTCATTGTGTTTGGC
GGAGCTACTTGCTGCAGTTGTGTTGCTTGTATGACTCCCAGTAGAATGATGCTCTGTATACGCAAGTACCA
AACTTGCG

GATGTCTTGGACCAAGAACCGGACGCAGGTTTAGGTAATGGTGGTCTAGGTCGTCTTGCAGCTTGCTTCGT
CGACTCAATGGCAACGGAAGGCATCCCTGCCTGGGGTTATGGTCTACGTTATGAGTATGGTATCTTTGCTC
AAAAGATT

CCCAATCAAAGCATCTCTTTCCATTTCACCGACACGAATACCACCATGTCTCTTTCTACCTTTGACAGGCT
GCATAGTCAAACTATTCACAGGACCAGTGGAACGAACTTGGAACTTGTCATTGACCATATGACGTAATCTT
TGGTAGTA

GTCTTCTTGGAAGACATGTAGGAGGACATGGAAGAGTCTCTCAAGGCTTGAGCCTTCATGATTCTTTCCAT
GTTAGCAGACCAACCAAATTGACCAGTTCTGATAGCAGCTGGGGCATCCAACAATTTGTAAGAAACAACAA
CTTTCTCC

CGATATCAACTTCTTCTCTTTCTCTTCTTGGTGGTCTGGAAGAGCCTTGGAAATTGGAACCTCTTGCAGCA
GTCCAGTCAATATCAACTTCTTCTCTTTCTCTTCTTGGACCTCTGAAGTTAGAACCTCTAGCGGCACCCCA
ATCAAGAT

GGTGTTCATAAACGTGGTCAATTGCGTTGGCCTTTTAAGCATGAGCTCCAACGGTCCAGAGCCCATCTGCA
GTTGACAGGGCCTGAAGAAAAGATTCATGACAGTCATGTTCCTGGTGAGGGTGCCTTGGGTGTAACACTTA
TCCTTACA

TTTATATACATCCTTAAACTGTGAAAGAAAGAATAGCGATACAAATGTCTAGGCAGTCCCTAGGACAGTGC
CGGAGTTGTCATTGTGGAGAGGCAATGGTGTTTCTGTGGACATTAACGGTTCGTAATCTTTAGTTCTGTCG
ATATCCTC

GAGAGTAAATCAAACACACGTAGAAACAAGTAGCAACTAGAACAGGTTCAGTGACAGAGAAGTACAAAGGT
CTCAACAGACAAGCCCTCATCATTTCACCCATACTGACACCTTGCGCTTCCTGCTCAGTCATAATCTTGGG
ATTACCAG

GCCAGGATTCTTCTTGATCTGCTGTACGTTCAGTGACAATAAAGGATTGTCATGCATATGTGTGTGGCTGT
GACTATGAGTATGTTGCAACAACGACTTCACAGAGGAAGCCTTGCCTTCCTTCTTTCCTTCACTTTCATTC
TGTCTGGA

CTTTCATGGTCAGATTGGACTCTGAGAAGCACATTGACTTTGCTCCAACATCTCCATTCGGTGGTGCTAGA
CCAGGCAGAGTTGCTAGAAGAAATGCTGCAAGGAAGGCGGAAGCTTCTGGTGAAGCTGCTGATGAAGCTGA
TGAGGCCG

ACTGCTCGTACCATCAGTAGAATTCGTTATGCTATCCTCTTCATCTGAAGATGGCTCGAAGACTCCTTCAT
TGTGTTTGGCGGAGCTACTTGCTGCAGTTGTGTTGCTTGTATGACTCCCAGTAGAATGATGCTCTGTATAC
GCAAGTAC

TTCTTGGTGGTCTTGAGGAGCTTTGGAAGTTGGAACCTCTGGCTGCACTCCAGTCAATATCTGGCTCTTCT
CTTTCTCTTCTTGGTGGCCTCGAGGAGCTTTGAAAGTTAGAGCCTCTAGCTGCACTCCAGTCAATATCTGG
CTCTTCTC

TTGACAGCTTCCACTGAAAACGGTGTCAAGTTGAGACAAACCGATAGACTGTTGGATGGTGTTGCTGATGG
CTCCATGAGATCCAAGGCCGACAGGATGGCCAAGATGGAAAGAAGAGAAAGAAATAGACATGCCAAGCAAG
GTGAATCT

AGCTAGGTTTAGCCAAGTCTGTCCACCATGCAAGAGTTTTAATCACTCAAAGGCACATTGCTGTTGGTAAG
CAAATTGTCAACATCCCCTCTTTCATGGTCAGATTGGACTCTGAGAAGCACATTGACTTTGCTCCAACATC
TCCATTCG

AGAGTTGAAACCGGTGTCATCAAGCCAGGTATGGTTGTTACTTTTGCCCCAGCTGGTGTTACCACTGAAGT
CAAGTCCGTTGAAATGCATCACGAACAATTGGAACAAGGTGTTCCAGGTGACAACGTTGGTTTCAACGTCA
AGAACGTT

GAAGCCACCAAGGGAATGATCACCAACTTGCAAAGAAATGTGGATCGCTTGTACAAGCTGAACGTGAGAAG
CAACGATCCTCAATCCAGCTTGTCTAAGGTGGACATTCCCTTGGAAGTTGTTCAATACATTGAGGACGGTA
GAAATCCA

ATAAGTTGAAATCTCAGTCTAGAATCAACAATGTTCTGAACAAGATTCATGTTGCTCAACCTCAAGCAAGA
GATGATGTCAAGAGAACACCATTTATCCCAGAATCTGTCAAGAATTTGAAGAAATACGACCCTGAAGATCC
AAACAGGA

CCACTGAAAACGGTGTCAAGTTGAGACAAACCGATAGACTGTTGGATGGTGTTGCTGATGGCTCCATGAGA
TCCAAGGCCGACAGGATGGCCAAGATGGAAAGAAGAGAAAGAAATAGACATGCCAAGCAAGGTGAATCTGA
TAGACACA

GTTCCGATGAAATCTTCGGTGGTTATCTATATTTCGCACAAGCTCCTTCTGCGGCAGAATTTCACACTGAA
TCCGTGCAACGTGTCAAGAACTTGCATTTGGCAGATTGTTTGAGAGCTAACAAGTCTACGATGGCTTGGGG
TCTAGAAG

CAAGTTAGAGGATGTCTTGGACCAAGAACCGGACGCAGGTTTAGGTAATGGTGGTCTAGGTCGTCTTGCAG
CTTGCTTCGTCGACTCAATGGCAACGGAAGGCATCCCTGCCTGGGGTTATGGTCTACGTTATGAGTATGGT
ATCTTTGC

AGAAGGTAAATGAAGATTCTCCATCATCTTCTTCAAAACTAGCTGAACGACCTCGTCTTCCAAACAACGAC
TCCACTACTAGCATGCCTGAAAGTCCCACCGAGGTAGCTGGTGATGATGTTGATAGGGAGAAACCGCCAGA
GTCAAGTA

ATGGTTGTTACTTTTGCCCCAGCTGGTGTTACCACTGAAGTCAAGTCCGTTGAAATGCATCACGAACAATT
GGAACAAGGTGTTCCAGGTGACAACGTTGGTTTCAACGTCAAGAACGTTTCCGTTAAGGAAATCAGAAGAG
GTAACGTC

TGAATCTTCAGTATGAGTTGACGGAGGTGTGGAATCGGTTGGACTCACAGCTTTTGAAAGGACATTTCTCG
GTTGCTCAGGATGTAGTTCAATGTCAGATTGGAAGTCATGGTCAGATTCTATGTTAAGATCATTGGATTCT
TGGATCTC

ATCAGAGTCTGCTGAGGATGAATCAGTAAATGTATTACCTGACTCAGGTGATGGAGTGCTCAGAGGCGTTC
CAACTGATGATGGATACTGCGGAAACTGTGATTGTGGCCCAGGTGGAAAGTACATAGGCGACATTTGATAA
GGTGTATA

ACTGCTGTGGGTACGGCCCATTCTGTGGTGAATGTGACTGAGCAGTTTGAGGAGAGGCATGATGGGGGTTC
TCTGGAACAGCTGATGAAGCAGGTGTTGTTGTCTGTTGAGAGTTAGCCTTAGTGGAAGCCTTCTCACATTC
TTCTGTTT

TGCTCACGGGACATATCTTTACATGCGTTGTAAGTATTGGAAGCAAAGTGACACCACACCTCTTCCACGTG
TTATTCGTGGTGGATGTTACAGTTTCAGGGTCAGCCGTGAAGCTTTCTAAATTGAACGTTTCCTCGAGAAA
GCTGTTCT

GTCATCATGCACTGCTGTGGGTACGGCCCATTCTGTGGTGAATGTGACTGAGCAGTTTGAGGAGAGGCATG
ATGGGGGTTCTCTGGAACAGCTGATGAAGCAGGTGTTGTTGTCTGTTGAGAGTTAGCCTTAGTGGAAGCCT
TCTCACAT

TTAACTAGCTTTGGGGGAGAGCCATGGAAAATAGCACTCGGTCTTGTGGCGGAGTTGGATTTGCTTGACTG
TGGCCCCCTCACGCTGTTTAATGACGACGAGAGGCAGCAATGATGTTGTTCATTTCTTCGACCTTAGCCTT
GGCTCTGG

AGGGAATGATCACCAACTTGCAAAGAAATGTGGATCGCTTGTACAAGCTGAACGTGAGAAGCAACGATCCT
CAATCCAGCTTGTCTAAGGTGGACATTCCCTTGGAAGTTGTTCAATACATTGAGGACGGTAGAAATCCAGA
TATTTACA

CTCCTATACCGAAGATGCAACTTTCATGGAAGGATATCCCTACTGTCGCTCCAGCAAATGATTTGCTGGAT
ATTGTCTTGAACAGAACCCAGAGAAAGACACCAACTGTGATCAGACCTGGTTTCAAGATTACAAGAATCAG
AGCGTTCT

TGGCAGATAATAGTTGTTGCTGGTCCATTGGAGTTTGAGAAGGTGGTTGAGGAGGCGGACCAGCCTGATAA
CGACCATACCCTTGAGGAATTTGTTGTTGTGGAGGTGGTGGTGGAATAGAAGTCTGGTAGCGACCGTAGCT
CTGATTCA

GTTGAACGAATAATGGAATATCCGACTTAAGACTTTGGGCCACAGACTTCACGGTGCTCACGCTACTTGCA
TGAGGTCTTCTAAGTGTCTTCAACGAACCTTGGGGTGTTGAGCTAAATGTATTCAAGGTATTGGTTGAATG
GATGGATG

CGTTACTCACACTGCTCGTACCATCAGTAGAATTCGTTATGCTATCCTCTTCATCTGAAGATGGCTCGAAG
ACTCCTTCATTGTGTTTGGCGGAGCTACTTGCTGCAGTTGTGTTGCTTGTATGACTCCCAGTAGAATGATG
CTCTGTAT

TCCAGCACTAATGCTACTACCACTGCCAGCACCAACGTCAGGACTAGTGCTACTACCACTGCCAGCATCAA
CGTCAGGACTAGTGCGACTACCACTGAAAGTACCAACTCCAGCACTAATGCTACTACCACTGCCAGCACCA
ACGTCAGG

CAGCACTAATGCTACTACCACTGCCAGCACCAACGTCAGGACTAGTGCTACTACCACTGCCAGCATCAACG
TCAGGACTAGTGCGACTACCACTGAAAGTACCAACTCCAACACTAGTGCTACTACCACCGAAAGTACCGAC
TCCAACAC

GTTTGGTAGGGATCTGAAGTGTGCTAAAGTTTCAATAGGTTGATGTGTTGGACCATCTTCACCGACACCGA
TAGAGTCATGTGTAGCAACCCAAATAACTGGGTGGCCAGACAAAGCGGACAATCTAACGGCACCAGCAGCA
TAAGAAAC

GTTAAACGTGGAACAAGCTATATCACATCCGTTCCTAAACGAGGTGCGGAAGCCAGACGACGAGCCTGTTT
GCCTTCAGGGTCCTTTCGACTTCACCTATGAATCCGAGTTGAATTCAATGTCCAAATTAAGAGACTACTTG
GTTGAAGA

GCCTTTGAGAAATCTAACTGAAACGCACAACTTTAGTAGCACAAACCTGGACACAGATGGTACAGGCGACG
ATCATGATGGTGCTCCTCTTTCCTCATCTCCTTCTTTTGGACAACAAAATGACAATAGCACCAACGATAAT
GCTGGTCT

TTCGTGGTGGTGGGTTGTCCATTAGTGATATTGCTCAAGGTAAGTCTCAGAATGCACCTTCAGATGGCACC
GGCTCATCCACTCCACAACATCATGACGAAGACGAAGATGAATTATCTCGTCAAATCAAGGCGGCTGCCTC
TACCTTAG

CATGGCCTACAGATGAAATATGTGCTCAACTAATGACACAATTCCCACCAGGAACGCCGACCAGTGTCCTG
CTGCAGACTATTTCAGATGAGCTAGAGAAAAGTTCTGACAACCTGTTCACGTTATCTGATTTAAAGAGCAA
ACTGAAAG

AGCATCAGCAACAACAACAACAACAACAGCAACAGCAGCAACAGCAACAGCAACAGCAGCAGCAACAACAA
CAGCAACAACAACAACAACAGCAGCAGCAGCAGCAGCAGCAGCAAGGACAAATACCGCAATCTCAGCAAGT
TCCTCAAG

GCAACAACAACAGCAGCAGCAGCATCAGCAACAACAACAACAACAACAGCAACAGCAGCAACAGCAACAGC
AACAGCAGCAGCAACAACAACAGCAACAACAACAACAACAGCAGCAGCAGCAGCAGCAGCAGCAAGGACAA
ATACCGCA

CAGCAGCAGCAGCATCAGCAACAACAACAACAACAACAGCAACAGCAGCAACAGCAACAGCAACAGCAGCA
GCAACAACAACAGCAACAACAACAACAACAGCAGCAGCAGCAGCAGCAGCAGCAAGGACAAATACCGCAAT
CTCAGCAA

TCATGTGCAAATACAACAGCAGCAACAAAAGCAACAACAACAGCAGCAGCAGCATCAGCAACAACAACAAC
AACAACAGCAACAGCAGCAACAGCAACAGCAACAGCAGCAGCAACAACAACAGCAACAACAACAACAACAG
CAGCAGCA

AGCAACAAAAGCAACAACAACAGCAGCAGCAGCATCAGCAACAACAACAACAACAACAGCAACAGCAGCAA
CAGCAACAGCAACAGCAGCAGCAACAACAACAGCAACAACAACAACAACAGCAGCAGCAGCAGCAGCAGCA
GCAAGGAC

GTCATCATTGGCATCGTAGTCCCAAGCAGGACATGAGTTACAAGCACTCAAAGTGTTGGCACCAGCGGATT
CAGCTTCACTGACAGTCTGCAAGGTGATGTTGAATTGGTCACCTAAACCATCAATGAAATATTGAGCAGTG
TCATGACA

AGTTTAGGCATCATGTGCAAATACAACAGCAGCAACAAAAGCAACAACAACAGCAGCAGCAGCATCAGCAA
CAACAACAACAACAACAGCAACAGCAGCAACAGCAACAGCAACAGCAGCAGCAACAACAACAGCAACAACA
ACAACAAC

ATACAACAGCAGCAACAAAAGCAACAACAACAGCAGCAGCAGCATCAGCAACAACAACAACAACAACAGCA
ACAGCAGCAACAGCAACAGCAACAGCAGCAGCAACAACAACAGCAACAACAACAACAACAGCAGCAGCAGC
AGCAGCAG

GTGTCGTATTCATTTACAATGTCATCATTGGCATCGTAGTCCCAAGCAGGACATGAGTTACAAGCACTCAA
AGTGTTGGCACCAGCGGATTCAGCTTCACTGACAGTCTGCAAGGTGATGTTGAATTGGTCACCTAAACCAT
CAATGAAA

ACAACAACAACAACAACAGCAACAGCAGCAACAGCAACAGCAACAGCAGCAGCAACAACAACAGCAACAAC
AACAACAACAGCAGCAGCAGCAGCAGCAGCAGCAAGGACAAATACCGCAATCTCAGCAAGTTCCTCAAGTT
AGATCCAT

CCGACCAACTCATTGACCACAGATCTTAAATATTGGTACTCTTCCACATCTCCACGACTTGGCACTGCAAC
CTGTACCAGAACAACCTTGCCCCTCCATTCTGGATGCTCGTTCAGAAACACTTCCATGGCGTGCAACTTCT
GAGGCACA

TAATTGCTGCTGCTGTTGTTGTTGTTGTTGTTGTTGTTGTTGTTGTTGTTGTTGTTGTTGTTGTTGTTGTT
GTTGCTGTTGTTGTTGTTGTTGTTGTGCCTGCGCCTGTGCTTGTGCTTGTGCCTGTGCTTGTGCTTGTGCT
TGCGCTTG

CAGCAACAACAGTTTAGGCATCATGTGCAAATACAACAGCAGCAACAAAAGCAACAACAACAGCAGCAGCA
GCATCAGCAACAACAACAACAACAACAGCAACAGCAGCAACAGCAACAGCAACAGCAGCAGCAACAACAAC
AGCAACAA

CTTCCACATCTCCACGACTTGGCACTGCAACCTGTACCAGAACAACCTTGCCCCTCCATTCTGGATGCTCG
TTCAGAAACACTTCCATGGCGTGCAACTTCTGAGGCACACCTTTGATGTAATCCAGCCTGTCGACACCAAC
TATGATCT

CATTTACAATGTCATCATTGGCATCGTAGTCCCAAGCAGGACATGAGTTACAAGCACTCAAAGTGTTGGCA
CCAGCGGATTCAGCTTCACTGACAGTCTGCAAGGTGATGTTGAATTGGTCACCTAAACCATCAATGAAATA
TTGAGCAG

TATTGGTACTCTTCCACATCTCCACGACTTGGCACTGCAACCTGTACCAGAACAACCTTGCCCCTCCATTC
TGGATGCTCGTTCAGAAACACTTCCATGGCGTGCAACTTCTGAGGCACACCTTTGATGTAATCCAGCCTGT
CGACACCA

CATTGACCACAGATCTTAAATATTGGTACTCTTCCACATCTCCACGACTTGGCACTGCAACCTGTACCAGA
ACAACCTTGCCCCTCCATTCTGGATGCTCGTTCAGAAACACTTCCATGGCGTGCAACTTCTGAGGCACACC
TTTGATGT

AACAGCAGCAACAGCAACAGCAACAGCAGCAGCAACAACAACAGCAACAACAACAACAACAGCAGCAGCAG
CAGCAGCAGCAGCAAGGACAAATACCGCAATCTCAGCAAGTTCCTCAAGTTAGATCCATGAGTGGACAACC
TCCCACCA

CTCCCTAGTTTGCGATAGTGTAGATACCGTCCTTGGATAGAGCACTGGAGATGGCTGGCTTTAATCTGCTG
GAGTACCATGGAACACCGGTGATCATTCTGGTCACTTGGTCTGGGGCAATACCAGTCAACATGGTGGTGAA
GTCACCGT

CAGCAGGGATATAACCAGCAGGGATATAACCAACAAGGCCATCAACAACCAGTCTACGTCCAACAACAACC
ACCCCAGAGGGGTAACGAAGGTTGTCTGGCTGCATGTCTGGCTGCATTATGTATATGCTGCACCATGGATA
TGCTATTC

AGATCTTAAATATTGGTACTCTTCCACATCTCCACGACTTGGCACTGCAACCTGTACCAGAACAACCTTGC
CCCTCCATTCTGGATGCTCGTTCAGAAACACTTCCATGGCGTGCAACTTCTGAGGCACACCTTTGATGTAA
TCCAGCCT

CAACAACAGCAACAGCAGCAACAGCAACAGCAACAGCAGCAGCAACAACAACAGCAACAACAACAACAACA
GCAGCAGCAGCAGCAGCAGCAGCAAGGACAAATACCGCAATCTCAGCAAGTTCCTCAAGTTAGATCCATGA
GTGGACAA

TTGTTGTTGTTGTTGTTGTTGTTGTTGTTGTTGTTGTTGTTGTTGCTGTTGTTGTTGTTGTTGTTGTGCCT
GCGCCTGTGCTTGTGCTTGTGCCTGTGCTTGTGCTTGTGCTTGCGCTTGTGCATGTGCTTGTGCTTGTGCT
TGTGCCTG

GGGCTACAATCAGCAGGGATATAACCAGCAGGGATATAACCAACAAGGCCATCAACAACCAGTCTACGTCC
AACAACAACCACCCCAGAGGGGTAACGAAGGTTGTCTGGCTGCATGTCTGGCTGCATTATGTATATGCTGC
ACCATGGA

CAAACAACTCAAGATCCGTTAGACATTTCAGCTTCCAAAACAGAAGAATGTGAGAAGGTTTCCACTCAGGC
TAATTCTCAACAGCCAACAACACCTCCCTCATCTGCTGTTCCAGAGAACCATCATCATGCCTCTCCTCAAG
CTGCTCAA

AAGATCCGTTAGACATTTCAGCTTCCAAAACAGAAGAATGTGAGAAGGTTTCCACTCAGGCTAATTCTCAA
CAGCCAACAACACCTCCCTCATCTGCTGTTCCAGAGAACCATCATCATGCCTCTCCTCAAGCTGCTCAAGT
ACCATTGC

GTCATTTGTGGCCTTCGGAATATTTAGCTTTAAATATGGCTTCCCTGAGAGCTTTCCTCTATTGCTGTTCA
GTTGGTGCAGGTGATGGACCATCGCCTGCACTGGAGCTGGACCTTCCAAAGTATTTGATATAGCAACTGTC
GGGTGTAT

TTGAGATAACACCAGCACCTGCGACACCTCTTAAGATGTTAGCACCACAACCCTTGAATAGAGAACCAACA
CCTTCAGCAGCAACAATCTTCCTCAAACAGTCAAAGGCACCGTCGTACTTAACAGCTTGACCGGAGGTCAT
CATCATTC

ATCTGGTTATGTGTCTTCATTTGGTAATCACTGGGATGTTACTGGGGCAGCAGCAACTCCGTGTGTACCCC
TAACTCCGTGTGTACCCCTAAAGAACCTTGCCTGTCAAGGTGCATTGTTGGATCGGAATAGTAACCGTCTT
TACATGAA

AAACCGGTGTCATCAAGCCAGGTATGGTTGTTACTTTCGCCCCAGCTGGTGTTACCACTGAAGTCAAGTCC
GTTGAAATGCATCACGAACAATTGGAACAAGGTGTTCCAGGTGACAACGTTGGTTTCAACGTCAAGAACGT
TTCCGTTA

GCATCGTAGTCCCAAGCAGGACATGAGTTACAAGCACTCAAAGTGTTGGCACCAGCGGATTCAGCTTCACT
GACAGTCTGCAAGGTGATGTTGAATTGGTCACCTAAACCATCAATGAAATATTGAGCAGTGTCATGACATC
TCTTAGAA

GTTGTTGTTGTTGTTGTTGTTGTTGTTGTTGTTGTTGTTGTTGTTGTTGTTGTTGCTGTTGTTGTTGTTGT
TGTTGTGCCTGCGCCTGTGCTTGTGCTTGTGCCTGTGCTTGTGCTTGTGCTTGCGCTTGTGCATGTGCTTG
TGCTTGTG

AGTACTACAAACATGCAAGATTCGAACCCCAGCTATGGAGCTTCACCCATGCAAGGTCAGCCTCCAGTCGG
TGGACAACCGCCTGTGCCTGTAAGAATGCAGCCACAGCCACCGCAACCAATGCAACAGGGAAACATCTATC
CGATTGAG

TAACGTTTCAGCTTCCGCTAGCTCTTCTTCCTCTTCTTCTAAGAAGTCTAAGGGTGCTGCTCCAGAACTTG
TTCCAGCCACTTCATTCATGGGTGTCGTTGCTGCTGTTGGCGTTGCTTACTATAAGATTAAAGCAACAATT
TGTGTTTC

TTGTTGTTGTTGTTGCTGTTGTTGTTGTTGTTGTTGTGCCTGCGCCTGTGCTTGTGCTTGTGCCTGTGCTT
GTGCTTGTGCTTGCGCTTGTGCATGTGCTTGTGCTTGTGCTTGTGCCTGTGCTTGCGCCTGTGCTTGTGCT
TGTGCTTG

CTCAACTGTACTACAATAGCAACGTCAATGCTCACAACAACAACAACAACAGCAACAACAACAACAACAAC
AACAACAACAGCAACAACAACAACAATCTTTACAACCAGACGCAGTTCTCCACGAGGTACTTCAACTCGAA
CTCCTCTC

CTACAATAGCAACGTCAATGCTCACAACAACAACAACAACAGCAACAACAACAACAACAACAACAACAACA
GCAACAACAACAACAATCTTTACAACCAGACGCAGTTCTCCACGAGGTACTTCAACTCGAACTCCTCTCCC
TCGTTGAC

GTTTCTTATCTGTTCATTCCAAATATCTTTTATGCTCTCCCCCATGGTCCTGCTCCATGCAGTGCCATCAT
CCGTCACAACTAGCTTGCGGTTCCTGGTCTCCCCAGGTACCACTTCTTTTCTATCCACCAGGTCCTGTACC
TGATGATC

TGCCTGTGCTTGTGCTTGTGCTTGCGCTTGTGCATGTGCTTGTGCTTGTGCTTGTGCCTGTGCTTGCGCCT
GTGCTTGTGCTTGTGCTTGCGCTTGTGCTTGTGCTTGTGCTTGAGCTTGTGCTTGTGCTTGAGCTTGTGCT
TGAGCTTG

TCTGCAAGACCAACTAAAGTAGACCCTACCGGTATCTCTACCATTGTTCCTATGGCCTCTCATGTAGATCA
AACTGAGCATGACCTGGACATCTTGGTCACTGACCAAGGTTTGGCGGATCTAAGAGGTCTATCGCCTAAGG
AAAGAGCC

TGCATGTGCTTGTGCTTGTGCTTGTGCCTGTGCTTGCGCCTGTGCTTGTGCTTGTGCTTGCGCTTGTGCTT
GTGCTTGTGCTTGAGCTTGTGCTTGTGCTTGAGCTTGTGCTTGAGCTTGAGCTTGAGCTTGAGCTTGTAAT
TGAGGGGA

CAAGTAGGTTGTGTCGTATTCATTTACAATGTCATCATTGGCATCGTAGTCCCAAGCAGGACATGAGTTAC
AAGCACTCAAAGTGTTGGCACCAGCGGATTCAGCTTCACTGACAGTCTGCAAGGTGATGTTGAATTGGTCA
CCTAAACC

GTTGTTGTTGTTGTTGTTGTTGTTGCTGTTGTTGTTGTTGTTGTTGTGCCTGCGCCTGTGCTTGTGCTTGT
GCCTGTGCTTGTGCTTGTGCTTGCGCTTGTGCATGTGCTTGTGCTTGTGCTTGTGCCTGTGCTTGCGCCTG
TGCTTGTG

TGTTGCTGTTGTTGTTGTTGTTGTTGTGCCTGCGCCTGTGCTTGTGCTTGTGCCTGTGCTTGTGCTTGTGC
TTGCGCTTGTGCATGTGCTTGTGCTTGTGCTTGTGCCTGTGCTTGCGCCTGTGCTTGTGCTTGTGCTTGCG
CTTGTGCT

TTTCAGGAAACCATACCCATATCTGAAAGTGGACGACAAGTGTGGTGTTGACTCCATCCCGGTTGATCCTG
AAAAGGTTGTTGCGATTGTGGAGTCCACCATGAGGGACCAGGTCCCACCAAATACGCCCTCTGACGACATG
TCCAGGGC

CGGAGATGAAGAACCACTTGTCAATAGACTTGTCAATTGGAGCATCTGCCTTTGGAGAAGCTGGTGGGATG
GCATCAGAGGCAGCCTTGGAGTAGGTTGGGGTGTCAGGCAAAGTAACATTGGTCAAATCAATACCCTTGCT
GTCAGAAG

ATCGAACAGCCGCAGCGGTGGCATCAGTAGTTTCGGTAGTAGCCGTTGGTCTTGCAGGCATGGCAGGAGGA
GGGCCCTCTTCATCCTCATCTGGAGCTTGGTCTTCTTTACTTTGCATCATACGAGCTCTATCATTGTGTTG
AGGAGGTG

CTTGTGCTTGTGCCTGTGCTTGTGCTTGTGCTTGCGCTTGTGCATGTGCTTGTGCTTGTGCTTGTGCCTGT
GCTTGCGCCTGTGCTTGTGCTTGTGCTTGCGCTTGTGCTTGTGCTTGTGCTTGAGCTTGTGCTTGTGCTTG
AGCTTGTG

ACAGCAACAGCAACAGCAGCAGCAACAACAACAGCAACAACAACAACAACAGCAGCAGCAGCAGCAGCAGC
AGCAAGGACAAATACCGCAATCTCAGCAAGTTCCTCAAGTTAGATCCATGAGTGGACAACCTCCCACCAAT
GTTCAGCC

AGACATTTCAGCTTCCAAAACAGAAGAATGTGAGAAGGTTTCCACTCAGGCTAATTCTCAACAGCCAACAA
CACCTCCCTCATCTGCTGTTCCAGAGAACCATCATCATGCCTCTCCTCAAGCTGCTCAAGTACCATTGCCA
CAAAATGG

GTTGATTCTACCGACCAACTCATTGACCACAGATCTTAAATATTGGTACTCTTCCACATCTCCACGACTTG
GCACTGCAACCTGTACCAGAACAACCTTGCCCCTCCATTCTGGATGCTCGTTCAGAAACACTTCCATGGCG
TGCAACTT

GTTGTTGTTGTTGTTGTGCCTGCGCCTGTGCTTGTGCTTGTGCCTGTGCTTGTGCTTGTGCTTGCGCTTGT
GCATGTGCTTGTGCTTGTGCTTGTGCCTGTGCTTGCGCCTGTGCTTGTGCTTGTGCTTGCGCTTGTGCTTG
TGCTTGTG

TCCACGACTTGGCACTGCAACCTGTACCAGAACAACCTTGCCCCTCCATTCTGGATGCTCGTTCAGAAACA
CTTCCATGGCGTGCAACTTCTGAGGCACACCTTTGATGTAATCCAGCCTGTCGACACCAACTATGATCTTG
CAGCCCTT

TGTGCTTGTGCTTGCGCTTGTGCATGTGCTTGTGCTTGTGCTTGTGCCTGTGCTTGCGCCTGTGCTTGTGC
TTGTGCTTGCGCTTGTGCTTGTGCTTGTGCTTGAGCTTGTGCTTGTGCTTGAGCTTGTGCTTGAGCTTGAG
CTTGAGCT

TGCTGTTGTTGTTGTTGTTGTTGTTGTTGTTGTTGTTGTTGTTGTTGTTGTTGTTGTTGTTGTTGCTGTTG
TTGTTGTTGTTGTTGTGCCTGCGCCTGTGCTTGTGCTTGTGCCTGTGCTTGTGCTTGTGCTTGCGCTTGTG
CATGTGCT

ACATGCAAGATTCGAACCCCAGCTATGGAGCTTCACCCATGCAAGGTCAGCCTCCAGTCGGTGGACAACCG
CCTGTGCCTGTAAGAATGCAGCCACAGCCACCGCAACCAATGCAACAGGGAAACATCTATCCGATTGAGCC
TTCTCTTG

TTGTTGTGCCTGCGCCTGTGCTTGTGCTTGTGCCTGTGCTTGTGCTTGTGCTTGCGCTTGTGCATGTGCTT
GTGCTTGTGCTTGTGCCTGTGCTTGCGCCTGTGCTTGTGCTTGTGCTTGCGCTTGTGCTTGTGCTTGTGCT
TGAGCTTG

CAACAGCAGCAGCAACAACAACAGCAACAACAACAACAACAGCAGCAGCAGCAGCAGCAGCAGCAAGGACA
AATACCGCAATCTCAGCAAGTTCCTCAAGTTAGATCCATGAGTGGACAACCTCCCACCAATGTTCAGCCCA
CTATTGGC

TGGATCAGGCATGTACCTGTAATCGATCGTGGTTTCTTTGCTTCTCAACTTCACCGTTGATGAGCCTGTCC
AGCCCCTTGTTTCTGGCTCCATCAGTGAGCTAGTGTCACCCACAGAAATCAACTCAACTTGCCGCTGATAT
TCGTACTT

GAAGCACTAAATTCCTATTATGCTTCTCAAACGGATGACCAAAAGGATAGAAGAGAGGAAGCACATTGGAA
CAGACAGCAGGAGAAGGCCCTCAAGCAAGAAGCCTTCTCCACCAACTCTTCGAATAAAGCCATAAATACGG
AGCACGTT

TAAACAGAACCGGAGATGAAGAACCACTTGTCAATAGACTTGTCAATTGGAGCATCTGCCTTTGGAGAAGC
TGGTGGGATGGCATCAGAGGCAGCCTTGGAGTAGGTTGGGGTGTCAGGCAAAGTAACATTGGTCAAATCAA
TACCCTTG

CTGAATTTCTTGCTTCGCTTGTTGGTACGTTCCTGGACGCAACTTGAACAGAGCAGTTTCAGCTTCCGAGA
CGAATCCGTGGCTCACCAGCCACCTTGGAGATTCGTCCACTTTGAGCCAAGCTAAAATATTCGCTACCGCA
ATCACGGA

GTTCTTCAATGTTGATTCTAATGGATGTCTGTCATTCAATTGTGTGGAAAGAGGTACTGCACCGCCTTCTT
GCTGGCGTAATGTGTCTGGCAAGGAGGGCACTGCAGATGAAAGAACACTTTTGTCTTGATCTGTAGAGACT
TGAGATTT

CCAACACGTGCGAGAACGTATCGACATCTTCGTTTGTCACAGACCCGCTGTTGTTGTTGTCAAACTGAGAA
TTCCTGTTCGAGTCTGAGTCAGAGACAAACATCGGTCTCATCTTCTCCCAAAGTCGAGATGCAGGCACCAA
GAGCAGCC

TGTTGTTGTTGTTGTTGTTGTTGTTGTTGTTGTTGCTGTTGTTGTTGTTGTTGTTGTGCCTGCGCCTGTGC
TTGTGCTTGTGCCTGTGCTTGTGCTTGTGCTTGCGCTTGTGCATGTGCTTGTGCTTGTGCTTGTGCCTGTG
CTTGCGCC

TGTGCTTGTGCTTGTGCCTGTGCTTGCGCCTGTGCTTGTGCTTGTGCTTGCGCTTGTGCTTGTGCTTGTGC
TTGAGCTTGTGCTTGTGCTTGAGCTTGTGCTTGAGCTTGAGCTTGAGCTTGAGCTTGTAATTGAGGGGAAG
CTCCATTA

AAACACCATCTGAAGCAGCAAAAGAGATGGAGTTACCGAAACAACTCGACCAGACACCAGAACCGGCAGTC
CCCCCTTGGTTGTTCATGAATGTACAGTTGCTAGTAGAGCCTGGGACAAAGTAGGAACCTCTGGACCAGTC
ACCGTTAG

CTTGTGCCTGTGCTTGCGCCTGTGCTTGTGCTTGTGCTTGCGCTTGTGCTTGTGCTTGTGCTTGAGCTTGT
GCTTGTGCTTGAGCTTGTGCTTGAGCTTGAGCTTGAGCTTGAGCTTGTAATTGAGGGGAAGCTCCATTACT
GTACATTG

AACGTCAATGCTCACAACAACAACAACAACAGCAACAACAACAACAACAACAACAACAACAGCAACAACAA
CAACAATCTTTACAACCAGACGCAGTTCTCCACGAGGTACTTCAACTCGAACTCCTCTCCCTCGTTGACTT
CTTCCACT

TGAACCCACCTTTCAGGAAACCATACCCATATCTGAAAGTGGACGACAAGTGTGGTGTTGACTCCATCCCG
GTTGATCCTGAAAAGGTTGTTGCGATTGTGGAGTCCACCATGAGGGACCAGGTCCCACCAAATACGCCCTC
TGACGACA

CCAAGACATGCTCGTAGGCTTCCTTCGCACCTTGCCACTCTCCCATACTCTCCAAAACACTACCCAACTGA
AACCATATGTCCCACTCCTGCAAGGGAGCAGGAGGTTGAGGGAGAATGTATCTGAAGCATTCCAAAGCTTG
AGACCATT

TAATTCTCAACAAGAGACAACACCTGGGACATCAGCTGTTCCAGAGAACCATCATCATGTCTCTCCTCAAC
CTGCTTCAGTACCACCTCCACAGAATGGACAGTACCAACAGCACGGCATGATGACTCCAAACAAAGCTATG
GCTTCTAA

TTTCTGGTGAAATTCCATCTACCTTGAATGAAGAGAGTAAAGACAAGGTCAAGGTCAAGGAGAGCAGCTCT
GGTAAGTTCAAGAGAGTCATCACTTTGCCAGACTACCCAGGTGTGGATGCAGACAACATTAAAGCAGACTA
CGCAAATG

AGTTACCGAAACAACTCGACCAGACACCAGAACCGGCAGTCCCCCCTTGGTTGTTCATGAATGTACAGTTG
CTAGTAGAGCCTGGGACAAAGTAGGAACCTCTGGACCAGTCACCGTTAGTGTTAGAAGATGATGACGAAGA
TGATGAAG

CAAACAAGATCATTTGCAGTTGGTCGTACATTGAGATAACACCAGCACCTGCGACACCTCTTAAGATGTTA
GCACCACAACCCTTGAATAGAGAACCAACACCTTCAGCAGCAACAATCTTCCTCAAACAGTCAAAGGCACC
GTCGTACT

TTCGAACCCCAGCTATGGAGCTTCACCCATGCAAGGTCAGCCTCCAGTCGGTGGACAACCGCCTGTGCCTG
TAAGAATGCAGCCACAGCCACCGCAACCAATGCAACAGGGAAACATCTATCCGATTGAGCCTTCTCTTGAT
TCTACTGG

CTCTAGGACAATTCTATTCAAGTCTGACGCCGAAAACGGACCATTGGACCTCCCCTGCTGTTGAGACTGGC
CTTGACTCTGTTGCTGTGGCTGCTGGCCGGAATTGGCGCCAGCAGCATTATTAGTTCGTTGATTCTTTACT
GGTTGTGG

CGAGAACGTATCGACATCTTCGTTTGTCACAGACCCGCTGTTGTTGTTGTCAAACTGAGAATTCCTGTTCG
AGTCTGAGTCAGAGACAAACATCGGTCTCATCTTCTCCCAAAGTCGAGATGCAGGCACCAAGAGCAGCCAC
GAAGCAAA

CAACAACAACAGCAACAACAACAACAACAACAACAACAACAGCAACAACAACAACAATCTTTACAACCAGA
CGCAGTTCTCCACGAGGTACTTCAACTCGAACTCCTCTCCCTCGTTGACTTCTTCCACTTCTAACTCATCC
TCTCCATA

CTCATAAGCTGGGTATGAAATTCATCACCGATTTGGTTATCAACCACTGTTCTACAGAACACGAATGGTTC
AAAGAGAGCAGATCCTCGAAGACCAATCCGAAGCGTGACTGGTTCTTCTGGAGACCTCCTAAAGGTTATGA
CGCCGAAG

AATCGATCGTGGTTTCTTTGCTTCTCAACTTCACCGTTGATGAGCCTGTCCAGCCCCTTGTTTCTGGCTCC
ATCAGTGAGCTAGTGTCACCCACAGAAATCAACTCAACTTGCCGCTGATATTCGTACTTGATTGCATTGAT
GATGGAAC

ATGCTCAAGCTAACGTTTCAGCTTCCGCTAGCTCTTCTTCCTCTTCTTCTAAGAAGTCTAAGGGTGCTGCT
CCAGAACTTGTTCCAGCCACTTCATTCATGGGTGTCGTTGCTGCTGTTGGCGTTGCTTACTATAAGATTAA
AGCAACAA

CTGTCATCATGGTGAGATATTCGTTGTTTCTTACTTGGGAACTGGTGCTGTTGCAACTGTTGCTGCTCCGG
TTGTCTTTTCATGTGAGCAGAACTAAAATCTTGGTCTTGGTCAGAATCTCGATTATCCTTCACCATGACGA
GAATGCGT

GCAACATTGGAACTCCATCTTTTAACATGGCGCAAATTCCGCAACAGCTGTATCAGAGCCTCACACCACAA
CAATTGCAGATGATTCAGCAACGACACCAACAGTTACTGAGGAGTCGTCTACAACAACAACAACAACAACA
ACAACAAA

CTTGCGCTTGTGCATGTGCTTGTGCTTGTGCTTGTGCCTGTGCTTGCGCCTGTGCTTGTGCTTGTGCTTGC
GCTTGTGCTTGTGCTTGTGCTTGAGCTTGTGCTTGTGCTTGAGCTTGTGCTTGAGCTTGAGCTTGAGCTTG
AGCTTGTA

CGTTTGTCACAGACCCGCTGTTGTTGTTGTCAAACTGAGAATTCCTGTTCGAGTCTGAGTCAGAGACAAAC
ATCGGTCTCATCTTCTCCCAAAGTCGAGATGCAGGCACCAAGAGCAGCCACGAAGCAAAATACATAAAGGT
CAGATAGA

CACCAAGAGCTTGAGCGGAGGCAGCACCAGAAACACCATCTGAAGCAGCAAAAGAGATGGAGTTACCGAAA
CAACTCGACCAGACACCAGAACCGGCAGTCCCCCCTTGGTTGTTCATGAATGTACAGTTGCTAGTAGAGCC
TGGGACAA

CTTCAAGGTTTCAGCAATGTGAGTACCGTCAATGTTGGAAACGAAGTGGACATCCAAGACACCAGCGTAGT
GCTTCAAAGCCTCAGTGACCATGACTGGACCCAAATCGGAACCACCAATACCGATGTTAACAACATCGGTG
ATCTTCTT

TTGCCGCCGATGAAGAGGAAGAGGATTCGGTTGTCTTAGAATTGTTCGTGACATTCTTCACTCCAGCATCA
CGATGGCGGTGGTGGTGATGATGATGGTGGGGCTGAGTAGCATCTGTAGTAGTAGTAGTAGTAGTATTGTT
ATTAGATG

GCGACACCTCTTAAGATGTTAGCACCACAACCCTTGAATAGAGAACCAACACCTTCAGCAGCAACAATCTT
CCTCAAACAGTCAAAGGCACCGTCGTACTTAACAGCTTGACCGGAGGTCATCATCATTCTTCTTCTAACGG
TATCCAAT

GTCAAGTTGAACGTGATCGGATACTGATTTCTAGAAACAGGGGGGGCTGGTGTTGGTTCTGGCGAAGCCGC
TTGCTCCGACTGCTTTGATGTCACAGCTTTGGGTTTCTCCGCTACACTGTCTACCTTAGGATTATTGATCA
TGACTCAA

AGATTTAAATGAAGCACTAAATTCCTATTATGCTTCTCAAACGGATGACCAAAAGGATAGAAGAGAGGAAG
CACATTGGAACAGACAGCAGGAGAAGGCCCTCAAGCAAGAAGCCTTCTCCACCAACTCTTCGAATAAAGCC
ATAAATAC

GGTATGGTTGTTACTTTCGCCCCAGCTGGTGTTACCACTGAAGTCAAGTCCGTTGAAATGCATCACGAACA
ATTGGAACAAGGTGTTCCAGGTGACAACGTTGGTTTCAACGTCAAGAACGTTTCCGTTAAGGAAATCAGAA
GAGGTAAC

TCACCGTTAGTGTTAGAAGATGATGACGAAGATGATGAAGCAGCAGCAGCAGAAGAGGTGGTTTTAGAGGA
AGAGATGGAAGACTGGCTTTCACTCGTTTCAACTGCTTGAGCACTGCTGGACAAAACAGTAGAGTCTTTAG
TGTAAGAA

ATGTACCTGTAATCGATCGTGGTTTCTTTGCTTCTCAACTTCACCGTTGATGAGCCTGTCCAGCCCCTTGT
TTCTGGCTCCATCAGTGAGCTAGTGTCACCCACAGAAATCAACTCAACTTGCCGCTGATATTCGTACTTGA
TTGCATTG

GTTCAGCCTGTCTATGGCCAAGCCATGTCAAGTACTACAAACATGCAAGATTCGAACCCCAGCTATGGAGC
TTCACCCATGCAAGGTCAGCCTCCAGTCGGTGGACAACCGCCTGTGCCTGTAAGAATGCAGCCACAGCCAC
CGCAACCA

CATCAAGCCAGGTATGGTTGTTACTTTCGCCCCAGCTGGTGTTACCACTGAAGTCAAGTCCGTTGAAATGC
ATCACGAACAATTGGAACAAGGTGTTCCAGGTGACAACGTTGGTTTCAACGTCAAGAACGTTTCCGTTAAG
GAAATCAG

AAAGGCATCACTCCCTAGTTTGCGATAGTGTAGATACCGTCCTTGGATAGAGCACTGGAGATGGCTGGCTT
TAATCTGCTGGAGTACCATGGAACACCGGTGATCATTCTGGTCACTTGGTCTGGGGCAATACCAGTCAACA
TGGTGGTG

GATGCCTCCAATGCTCAAGCTAACGTTTCAGCTTCCGCTAGCTCTTCTTCCTCTTCTTCTAAGAAGTCTAA
GGGTGCTGCTCCAGAACTTGTTCCAGCCACTTCATTCATGGGTGTCGTTGCTGCTGTTGGCGTTGCTTACT
ATAAGATT

CTGAATTTGGAGATTTAAATGAAGCACTAAATTCCTATTATGCTTCTCAAACGGATGACCAAAAGGATAGA
AGAGAGGAAGCACATTGGAACAGACAGCAGGAGAAGGCCCTCAAGCAAGAAGCCTTCTCCACCAACTCTTC
GAATAAAG

GAACTAAATCTTTGTGGTTAGATAAAGGAACTAAAAGAACCTTTGTTGGAGCAACCAAAGGTGGGAAGGAA
AGAACACTTCTGGCATTGTCTTCTGGTCTGTTCCAGAAAGAGTGCTCAAAAACGGAGTAGATGATACGGCC
AATACCGA

CAATGTCATCCAAGTAGGTTGTGTCGTATTCATTTACAATGTCATCATTGGCATCGTAGTCCCAAGCAGGA
CATGAGTTACAAGCACTCAAAGTGTTGGCACCAGCGGATTCAGCTTCACTGACAGTCTGCAAGGTGATGTT
GAATTGGT

AGCAACAACAACAGCAACAACAACAACAACAGCAGCAGCAGCAGCAGCAGCAGCAAGGACAAATACCGCAA
TCTCAGCAAGTTCCTCAAGTTAGATCCATGAGTGGACAACCTCCCACCAATGTTCAGCCCACTATTGGCCA
ACTTCCTC

CTCACAACAACAACAACAACAGCAACAACAACAACAACAACAACAACAACAGCAACAACAACAACAATCTT
TACAACCAGACGCAGTTCTCCACGAGGTACTTCAACTCGAACTCCTCTCCCTCGTTGACTTCTTCCACTTC
TAACTCAT

CCCTACAATCGAAGATCACGTAGCGCACACCGTCCTTGAGATCCTGCCACAGATCCAGCGGAGGTCTGACA
AATTGCTTCCTTTGCAGAGACTTTTCACCTTGCGGAGACTGTTCACCTTCCATACTACTAGTATCTAACTC
TTTCTCTC

ACCAGATTTAATGGTGACATCACCAAGAGCTTGAGCGGAGGCAGCACCAGAAACACCATCTGAAGCAGCAA
AAGAGATGGAGTTACCGAAACAACTCGACCAGACACCAGAACCGGCAGTCCCCCCTTGGTTGTTCATGAAT
GTACAGTT

CTTGTACGAGATCGAACAGCCGCAGCGGTGGCATCAGTAGTTTCGGTAGTAGCCGTTGGTCTTGCAGGCAT
GGCAGGAGGAGGGCCCTCTTCATCCTCATCTGGAGCTTGGTCTTCTTTACTTTGCATCATACGAGCTCTAT
CATTGTGT

AGCAACAACAACAACAACAACAACAACAACAGCAACAACAACAACAATCTTTACAACCAGACGCAGTTCTC
CACGAGGTACTTCAACTCGAACTCCTCTCCCTCGTTGACTTCTTCCACTTCTAACTCATCCTCTCCATACA
ACCAAAGC

GGTCAACAACCTTCAAGGTTTCAGCAATGTGAGTACCGTCAATGTTGGAAACGAAGTGGACATCCAAGACA
CCAGCGTAGTGCTTCAAAGCCTCAGTGACCATGACTGGACCCAAATCGGAACCACCAATACCGATGTTAAC
AACATCGG

AATCGACAGAGTTTCTTATCTGTTCATTCCAAATATCTTTTATGCTCTCCCCCATGGTCCTGCTCCATGCA
GTGCCATCATCCGTCACAACTAGCTTGCGGTTCCTGGTCTCCCCAGGTACCACTTCTTTTCTATCCACCAG
GTCCTGTA

GGGTATGAAATTCATCACCGATTTGGTTATCAACCACTGTTCTACAGAACACGAATGGTTCAAAGAGAGCA
GATCCTCGAAGACCAATCCGAAGCGTGACTGGTTCTTCTGGAGACCTCCTAAAGGTTATGACGCCGAAGGC
AAGCCAAT

GAGCAACCAAATGGGTTTGAGCACCAAAGAAGTTGTTGTACCAGACAGACAACAAACCACCCAACAATGGA
ATGTTGTCTTCCAATGGGGTTTGGGTGAAGTGGTTGTCGACGGCTTCAGCACCCTTCAAGAAAGCCTCAAA
GTTGTCAT

GATAGAGATTCCACTAAGGTTAATTCTCAACAAGAGACAACACCTGGGACATCAGCTGTTCCAGAGAACCA
TCATCATGTCTCTCCTCAACCTGCTTCAGTACCACCTCCACAGAATGGACAGTACCAACAGCACGGCATGA
TGACTCCA

TGATTTTGAGCCAAGACATGCTCGTAGGCTTCCTTCGCACCTTGCCACTCTCCCATACTCTCCAAAACACT
ACCCAACTGAAACCATATGTCCCACTCCTGCAAGGGAGCAGGAGGTTGAGGGAGAATGTATCTGAAGCATT
CCAAAGCT

TGCGCCTGTGCTTGTGCTTGTGCCTGTGCTTGTGCTTGTGCTTGCGCTTGTGCATGTGCTTGTGCTTGTGC
TTGTGCCTGTGCTTGCGCCTGTGCTTGTGCTTGTGCTTGCGCTTGTGCTTGTGCTTGTGCTTGAGCTTGTG
CTTGTGCT

TCTATGGCCAAGCCATGTCAAGTACTACAAACATGCAAGATTCGAACCCCAGCTATGGAGCTTCACCCATG
CAAGGTCAGCCTCCAGTCGGTGGACAACCGCCTGTGCCTGTAAGAATGCAGCCACAGCCACCGCAACCAAT
GCAACAGG

GTGTCTTCATTTGGTAATCACTGGGATGTTACTGGGGCAGCAGCAACTCCGTGTGTACCCCTAACTCCGTG
TGTACCCCTAAAGAACCTTGCCTGTCAAGGTGCATTGTTGGATCGGAATAGTAACCGTCTTTACATGAACA
TCCACAAC

AGCCATGTCAAGTACTACAAACATGCAAGATTCGAACCCCAGCTATGGAGCTTCACCCATGCAAGGTCAGC
CTCCAGTCGGTGGACAACCGCCTGTGCCTGTAAGAATGCAGCCACAGCCACCGCAACCAATGCAACAGGGA
AACATCTA

TAAAGAAGTCCAAACAACTCAAGATCCGTTAGACATTTCAGCTTCCAAAACAGAAGAATGTGAGAAGGTTT
CCACTCAGGCTAATTCTCAACAGCCAACAACACCTCCCTCATCTGCTGTTCCAGAGAACCATCATCATGCC
TCTCCTCA

TGTTAGAAGATGATGACGAAGATGATGAAGCAGCAGCAGCAGAAGAGGTGGTTTTAGAGGAAGAGATGGAA
GACTGGCTTTCACTCGTTTCAACTGCTTGAGCACTGCTGGACAAAACAGTAGAGTCTTTAGTGTAAGAAGA
AGGAACAG

ACAGCAACAACAACAACAACAGCAGCAGCAGCAGCAGCAGCAGCAAGGACAAATACCGCAATCTCAGCAAG
TTCCTCAAGTTAGATCCATGAGTGGACAACCTCCCACCAATGTTCAGCCCACTATTGGCCAACTTCCTCAA
CTTCCAAA

ATTCCTATTATGCTTCTCAAACGGATGACCAAAAGGATAGAAGAGAGGAAGCACATTGGAACAGACAGCAG
GAGAAGGCCCTCAAGCAAGAAGCCTTCTCCACCAACTCTTCGAATAAAGCCATAAATACGGAGCACGTTGG
TGGGTTAT

ATGGTGACATCACCAAGAGCTTGAGCGGAGGCAGCACCAGAAACACCATCTGAAGCAGCAAAAGAGATGGA
GTTACCGAAACAACTCGACCAGACACCAGAACCGGCAGTCCCCCCTTGGTTGTTCATGAATGTACAGTTGC
TAGTAGAG

TTTAACATGGCGCAAATTCCGCAACAGCTGTATCAGAGCCTCACACCACAACAATTGCAGATGATTCAGCA
ACGACACCAACAGTTACTGAGGAGTCGTCTACAACAACAACAACAACAACAACAACAAACTTCACCGCCAC
CGCAAACG

CTACTCAAATGCAGATACCGAAATATGAGAACAAGCCATTCAAGCCTCCAAGAAGGGTTGGATCAAATAAG
TACACACAACTCAAACCAACCGCCACTGCAGTCACAACAGCCCCTATATCTAAAGCCAAAGTTACTGTCAA
CTTGAAAA

GTTGATTCTAATGGATGTCTGTCATTCAATTGTGTGGAAAGAGGTACTGCACCGCCTTCTTGCTGGCGTAA
TGTGTCTGGCAAGGAGGGCACTGCAGATGAAAGAACACTTTTGTCTTGATCTGTAGAGACTTGAGATTTAA
AGGTATCT

CAACAACAACAGCAACAACAACAACAATCTTTACAACCAGACGCAGTTCTCCACGAGGTACTTCAACTCGA
ACTCCTCTCCCTCGTTGACTTCTTCCACTTCTAACTCATCCTCTCCATACAACCAAAGCACCTTCGAATAC
ATTTTGCC

GTTTGGCACACAGTGAACAGATTGGTCTATTCTCGTCGCATCTGCGTTTCTTGAATCTGCATGCCCAGCAC
CCAGTAAATGTCCTCCCCTTGGTGGGGGGCACTTTGGAGGATGACGCTACTTTCTTGCTTCTCTGCTGCCT
CTTACTAT

AAAGAGATGGAGTTACCGAAACAACTCGACCAGACACCAGAACCGGCAGTCCCCCCTTGGTTGTTCATGAA
TGTACAGTTGCTAGTAGAGCCTGGGACAAAGTAGGAACCTCTGGACCAGTCACCGTTAGTGTTAGAAGATG
ATGACGAA

CATTTTGAATTTGCATTTGCTGTTGGACTTGCAGTTGTTGCTGAACTTGCATCTGCTGCTTGGCTTGCATC
TGTTGCTTTGCCTGCATCTGTTGTGCCTGCAATTGCTGGGCTTGTATCTGTTGCGCTTGCTGCTGATTCAT
GGAAATAA

ACATCTGAAGCAAACGTACCCTCAACTGTACTACAATAGCAACGTCAATGCTCACAACAACAACAACAACA
GCAACAACAACAACAACAACAACAACAACAGCAACAACAACAACAATCTTTACAACCAGACGCAGTTCTCC
ACGAGGTA

GTCTATGGTTGATTTGATGACTGTCATCATGGTGAGATATTCGTTGTTTCTTACTTGGGAACTGGTGCTGT
TGCAACTGTTGCTGCTCCGGTTGTCTTTTCATGTGAGCAGAACTAAAATCTTGGTCTTGGTCAGAATCTCG
ATTATCCT

GATTCCGGTACTACTCAAATGCAGATACCGAAATATGAGAACAAGCCATTCAAGCCTCCAAGAAGGGTTGG
ATCAAATAAGTACACACAACTCAAACCAACCGCCACTGCAGTCACAACAGCCCCTATATCTAAAGCCAAAG
TTACTGTC

TATAACAATAAAGCAGCTGCACCTGCAACAACAGCACCGAAAGAGGCAGCGCCTGCATTGACTCTTTGACC
GGCATTGGAAGCAGTTTCGACTGTAGAAGTGCTGACAGAAGAGGTAGTTGGAGCGACAGAAGAAGAAACGG
CCTTAGAA

ATCTAGTACTGTGCTATCCTCCTCAATCTGTGAATAGCGTCCCCCAGAGATTCTGGGCTCTTGGTCTACCG
ACAATCTTCCCGAAGTGCGACGGTTGGTGTCGGAAGTCGAGGATCTCGGAGTCGTAGTAGCACTATCGCTG
ATAGAGCC

TGAAGCAGCAAAAGAGATGGAGTTACCGAAACAACTCGACCAGACACCAGAACCGGCAGTCCCCCCTTGGT
TGTTCATGAATGTACAGTTGCTAGTAGAGCCTGGGACAAAGTAGGAACCTCTGGACCAGTCACCGTTAGTG
TTAGAAGA

TCGACATCTTCGTTTGTCACAGACCCGCTGTTGTTGTTGTCAAACTGAGAATTCCTGTTCGAGTCTGAGTC
AGAGACAAACATCGGTCTCATCTTCTCCCAAAGTCGAGATGCAGGCACCAAGAGCAGCCACGAAGCAAAAT
ACATAAAG

CCACTGTATCTTGTCTGAAAACAGCTCCGTACAATGGAGAATTAGGATTTGTGAACATGGTCTGGAAGTGA
CCCACCATGTGGGATAGATCCCTGTAGGTCACCTGAGTGTCCGTTTCAGGAACAATAACGGCGACATTATC
GGATACGC

AGATTCGTTATCTGGACCGAAGCTGCTTTCACCAAGTTGGACCAAGTCTGGGGTTCCGAAACCGTTGCCTC
CTCCAAGGTCGGCTACACTTTGCCATCCCATATCATCTCCACTTCTGATGTCACCAGAATTATCAACTCTT
CCGAAATC

TGGTCGAATGGAGCAACCAAATGGGTTTGAGCACCAAAGAAGTTGTTGTACCAGACAGACAACAAACCACC
CAACAATGGAATGTTGTCTTCCAATGGGGTTTGGGTGAAGTGGTTGTCGACGGCTTCAGCACCCTTCAAGA
AAGCCTCA

TCTTTCAGACAATCTCTTAGCCAACAATTGAGCGTATTCGGCAGCAGCTTCTCTTTGAGCTTGAGCGTTTC
TGACCTTCAAAGCTCTTTGGTGTCTCTTTCTTTGCAATCTTTGAGGAGTAACCAATCTTTGGATCTTTGGA
GCCTTGGT

CTGTCGACGTAAACAGTGGAAGTAACTTGAACATATTCAACAGCAACATCCCTCTTGTGCTGATGCTTATG
TCTGGAGACAACAGCTTCACCTTCCTTACAGGACTCGATTGATTGTTTCTCTGATCTTTTCTTCAAAGCAT
TAGATTCA

AATTCCATCTACCTTGAATGAAGAGAGTAAAGACAAGGTCAAGGTCAAGGAGAGCAGCTCTGGTAAGTTCA
AGAGAGTCATCACTTTGCCAGACTACCCAGGTGTGGATGCAGACAACATTAAAGCAGACTACGCAAATGGT
GTTTTGAC

GCCCTGTCGCCATCACCAAGGGTTTCTGCCAAAGAAGCAATGGAGAGCCAAGTTTCCGCAGTTGATTGTGT
TAATGGGTCGAGTGGCTGCTGAGGAACTGCTGCCTGCTGTTGCTGTTGCTGCTGTTGTTGTTGCTGTTGTT
GTTGCTGT

GGACATGTTAGTGCCAACTCACATGGTTTCTCAATACTAAGCAAACACCCTCACCCAAATAATCTTGTCCA
TTCCCACTCACTTTCTCACACAAATGCGAAGAGCCACCTGCCTATCAGTAGCACTAGCACTAAAGAGAACA
GCACGAAC

GGGGACGAATTCCACAGTACCGAACTGACCGTTGATTCTACCGACCAACTCATTGACCACAGATCTTAAAT
ATTGGTACTCTTCCACATCTCCACGACTTGGCACTGCAACCTGTACCAGAACAACCTTGCCCCTCCATTCT
GGATGCTC

AAACAGTGGAAGTAACTTGAACATATTCAACAGCAACATCCCTCTTGTGCTGATGCTTATGTCTGGAGACA
ACAGCTTCACCTTCCTTACAGGACTCGATTGATTGTTTCTCTGATCTTTTCTTCAAAGCATTAGATTCACC
ATTTGGGT

CGTTCACTTCAAATGCACACCACGAGAATAAAGTACTAGCGTCAGTTGAGGTCAAGTTCAAACCCTTGTTT
TCCTTGTTCAATCTCTTGGCAATGTCATCCAAGTAGGTTGTGTCGTATTCATTTACAATGTCATCATTGGC
ATCGTAGT

ATAACTATAAACACTTATAGAACAATTACCGTTTCTTCCTTCACACAACATGGCTTCTACTTCGAACACGT
TCCCTCCAAGCCAAAGCAATTCTTCCAACAACCTTCCAACTTCTAGACATGCATCCATTGTGGAGATGCTG
TCTACTCC

TGGTTTCCCAGTTCTTCAATGTTGATTCTAATGGATGTCTGTCATTCAATTGTGTGGAAAGAGGTACTGCA
CCGCCTTCTTGCTGGCGTAATGTGTCTGGCAAGGAGGGCACTGCAGATGAAAGAACACTTTTGTCTTGATC
TGTAGAGA

ATGTACAGTTGCTAGTAGAGCCTGGGACAAAGTAGGAACCTCTGGACCAGTCACCGTTAGTGTTAGAAGAT
GATGACGAAGATGATGAAGCAGCAGCAGCAGAAGAGGTGGTTTTAGAGGAAGAGATGGAAGACTGGCTTTC
ACTCGTTT

ATCTGAAAGTGGACGACAAGTGTGGTGTTGACTCCATCCCGGTTGATCCTGAAAAGGTTGTTGCGATTGTG
GAGTCCACCATGAGGGACCAGGTCCCACCAAATACGCCCTCTGACGACATGTCCAGGGCTATTGCAGGTCA
TTTGGTCG

TCTTGCGTTTACCATAGAGTTTTTGAGCATTTGGCCCATAACAACTGCATTGGGATCTTTCCTGAAGGTGG
GTCCCACGACAGAACAAACTTGTTGCCCCTGAAAGCAGGTGTGGCGATTATGGCTCTTGGTTGCATGGATA
AGCATCCT

ACAACAACAACAACAACAACAGCAACAACAACAACAATCTTTACAACCAGACGCAGTTCTCCACGAGGTAC
TTCAACTCGAACTCCTCTCCCTCGTTGACTTCTTCCACTTCTAACTCATCCTCTCCATACAACCAAAGCAC
CTTCGAAT

TGAAGAGGAAGAGGATTCGGTTGTCTTAGAATTGTTCGTGACATTCTTCACTCCAGCATCACGATGGCGGT
GGTGGTGATGATGATGGTGGGGCTGAGTAGCATCTGTAGTAGTAGTAGTAGTAGTATTGTTATTAGATGGT
CTCGCAGA

CTGGAGTTGGTAGTCGCATTGGTACTGGCATTAGCACTACCATGAATGCACGTGTCGCTGTCCTCATCACT
GCTGCAATACTTTCTGCACCTGTCACTGCTATTGCTCTCCTGGAAGCTAGACGGTAACGCAACGATCGACA
TGGAAGCT

GCACAAAATAATCTGGTTATGTGTCTTCATTTGGTAATCACTGGGATGTTACTGGGGCAGCAGCAACTCCG
TGTGTACCCCTAACTCCGTGTGTACCCCTAAAGAACCTTGCCTGTCAAGGTGCATTGTTGGATCGGAATAG
TAACCGTC

TGCTTTAGCGCATAGCGAAACTCCTACTAGTAGTAATAATAAGGAGGCAGTATCACAACCAAGTGAAGGGA
AGCACAAGCACAAGCACAAGCACAAGAGCAAGCACAAACACAAGAACAGTAGCTCCAAAGATGGCTCTTCC
GAAGAAAA

CCGTAAACTCATTGCCATACATATCCTCCAGATGGAAGGGTCCCCCAAGTGAAGGTTTACCGTATCCTCTG
TTTGCTTCAGCCTCCTTCTGTGTTTCCAATCTGCGTTTCTCCCTGTTGAAGAAATAAGAAAGTGCCCCACC
GACTGCTA

CATTTACCGCAGAAGGAGGATAGTTCCCAGTCTTTGCCTACTACAGCTGTTGCTCCACCCTCTTCCCACGC
CAATGTAGAGGCTTCAGCAGATGTACAGCATCTGGACACTGCGATTAAGCTAGATAATCAATATTACTTCA
AACTGATG

TTCTTAACGGTGGCCATAACCATATCACCTAGAGAGGCGGCTGGCAATCTGTTCAATCTGGAACCAGAGCC
TTTGACGGCGATAATGTACAAGTTTCTGGCACCACTGTTGTCAGCACAGTTCATGATGGCACCGACTGGTA
GACCTAAC

GCTTCCGCTAGCTCTTCTTCCTCTTCTTCTAAGAAGTCTAAGGGTGCTGCTCCAGAACTTGTTCCAGCCAC
TTCATTCATGGGTGTCGTTGCTGCTGTTGGCGTTGCTTACTATAAGATTAAAGCAACAATTTGTGTTTCTA
TTATTACT

TATGCTCTCCCCCATGGTCCTGCTCCATGCAGTGCCATCATCCGTCACAACTAGCTTGCGGTTCCTGGTCT
CCCCAGGTACCACTTCTTTTCTATCCACCAGGTCCTGTACCTGATGATCTTGTTTCAACCATGTAGCATCG
TGATCGTA

CTCCAGGTGCTCACTTGGGTAGATTCGTTATCTGGACCGAAGCTGCTTTCACCAAGTTGGACCAAGTCTGG
GGTTCCGAAACCGTTGCCTCCTCCAAGGTCGGCTACACTTTGCCATCCCATATCATCTCCACTTCTGATGT
CACCAGAA

CCATACCCATATCTGAAAGTGGACGACAAGTGTGGTGTTGACTCCATCCCGGTTGATCCTGAAAAGGTTGT
TGCGATTGTGGAGTCCACCATGAGGGACCAGGTCCCACCAAATACGCCCTCTGACGACATGTCCAGGGCTA
TTGCAGGT

CCAAAGTAGCTGGAGTAATCTTTTGAGCCAAGATAGAGGTAGTTGGTCTGTTACCTGAGAAGACCTTGTGT
GGGACCAAACCACCAGTGGCACCTTCAGCCTTAACTTGTTCTTCATCCTTACCAACCATTAAAGCTTCAGC
TTGAGCAA

TTGTTCATGAATGTACAGTTGCTAGTAGAGCCTGGGACAAAGTAGGAACCTCTGGACCAGTCACCGTTAGT
GTTAGAAGATGATGACGAAGATGATGAAGCAGCAGCAGCAGAAGAGGTGGTTTTAGAGGAAGAGATGGAAG
ACTGGCTT

CAACAACAACAGATATCTAAACTCTTTCCAATCTTCCTTGGAAAGTGTCTCTGACTCTTTACAATTCTCTT
CCAACGGTGACAACACTACTTTGGCCTTCGACAATTTGGTCTGGGCTAACAACATCACTTTGAGAGATGTT
AACTCTAT

AATCTCTTGGCAATGTCATCCAAGTAGGTTGTGTCGTATTCATTTACAATGTCATCATTGGCATCGTAGTC
CCAAGCAGGACATGAGTTACAAGCACTCAAAGTGTTGGCACCAGCGGATTCAGCTTCACTGACAGTCTGCA
AGGTGATG

GAACCACTTGTCAATAGACTTGTCAATTGGAGCATCTGCCTTTGGAGAAGCTGGTGGGATGGCATCAGAGG
CAGCCTTGGAGTAGGTTGGGGTGTCAGGCAAAGTAACATTGGTCAAATCAATACCCTTGCTGTCAGAAGAC
TTACCAGC

AGTAGGAACCTCTGGACCAGTCACCGTTAGTGTTAGAAGATGATGACGAAGATGATGAAGCAGCAGCAGCA
GAAGAGGTGGTTTTAGAGGAAGAGATGGAAGACTGGCTTTCACTCGTTTCAACTGCTTGAGCACTGCTGGA
CAAAACAG

AATCTCTTAGCCAACAATTGAGCGTATTCGGCAGCAGCTTCTCTTTGAGCTTGAGCGTTTCTGACCTTCAA
AGCTCTTTGGTGTCTCTTTCTTTGCAATCTTTGAGGAGTAACCAATCTTTGGATCTTTGGAGCCTTGGTGT
AAGTCTTT

TTGGTTTGTCTAATGAATAATTCCATTGTGCTCTATGATGTTGCACATGCACGTGTTTTGTTGTACCTCTA
CTCTGGGTCGAAGTGGTTTGATGCTCACCGTCCAGAGCCACTTTGTGTCCCGACGTACCTGTTCCATCCTT
CAAACGCC

TTATCTCCATGTTTGGCACACAGTGAACAGATTGGTCTATTCTCGTCGCATCTGCGTTTCTTGAATCTGCA
TGCCCAGCACCCAGTAAATGTCCTCCCCTTGGTGGGGGGCACTTTGGAGGATGACGCTACTTTCTTGCTTC
TCTGCTGC

CAGAAGAATGTGAGAAGGTTTCCACTCAGGCTAATTCTCAACAGCCAACAACACCTCCCTCATCTGCTGTT
CCAGAGAACCATCATCATGCCTCTCCTCAAGCTGCTCAAGTACCATTGCCACAAAATGGGCCGTACCCACA
GCAGCGCA

GTTTGTACAAATCCTCCAATGAGGCGATTGCTGTAATGCATCTTTGCCACACATCTGTCTATGCAGCATTG
CTCACCTTTCGTTAGATCAGGCTCACCAAAGCCCTCATGCGGAATACATTTCTCAAGACACGTACTGAGAA
TATTGTTG

CCCCCCTTGGTTGTTCATGAATGTACAGTTGCTAGTAGAGCCTGGGACAAAGTAGGAACCTCTGGACCAGT
CACCGTTAGTGTTAGAAGATGATGACGAAGATGATGAAGCAGCAGCAGCAGAAGAGGTGGTTTTAGAGGAA
GAGATGGA

GGCACTGCAACCTGTACCAGAACAACCTTGCCCCTCCATTCTGGATGCTCGTTCAGAAACACTTCCATGGC
GTGCAACTTCTGAGGCACACCTTTGATGTAATCCAGCCTGTCGACACCAACTATGATCTTGCAGCCCTTGA
AAGTTTCC

TGTGCTTGTGCTTGTGCTTGCGCTTGTGCTTGTGCTTGTGCTTGAGCTTGTGCTTGTGCTTGAGCTTGTGC
TTGAGCTTGAGCTTGAGCTTGAGCTTGTAATTGAGGGGAAGCTCCATTACTGTACATTGTTGCAGAAGAGT
TAATAGGC

ACAAGAAGAAGTTGGATTATGTTTTGGCTTTGAAGGTTGAAGATTTCTTGGAAAGAAGATTGCAAACTCAA
GTCTACAAGTTGGGTTTGGCCAAGTCTGTCCACCACGCCAGAGTTTTAATCACTCAAAGACACATTGCTGT
TGGTAAGC

ACCGGAATTTGATAGAGATTCCACTAAGGTTAATTCTCAACAAGAGACAACACCTGGGACATCAGCTGTTC
CAGAGAACCATCATCATGTCTCTCCTCAACCTGCTTCAGTACCACCTCCACAGAATGGACAGTACCAACAG
CACGGCAT

GTGCTATCCTCCTCAATCTGTGAATAGCGTCCCCCAGAGATTCTGGGCTCTTGGTCTACCGACAATCTTCC
CGAAGTGCGACGGTTGGTGTCGGAAGTCGAGGATCTCGGAGTCGTAGTAGCACTATCGCTGATAGAGCCCT
GTATGGCA

GAAAAGATTAAGCATTGCCAAGGCATCGCTCGATCTCAAACATCATACTTCTGAACTCAACTCTAGCACAT
CAGAGCCAGACAGTCAAAGACGGTCCAAAGATAGTTCTGTGCCCCTAATAATTGATAGCAGTGGTTCAGCA
AATTGAGA

TCTGGCAAGCGTCATTTGTGGCCTTCGGAATATTTAGCTTTAAATATGGCTTCCCTGAGAGCTTTCCTCTA
TTGCTGTTCAGTTGGTGCAGGTGATGGACCATCGCCTGCACTGGAGCTGGACCTTCCAAAGTATTTGATAT
AGCAACTG

TTCTAGTGGTGATGCCTCCAATGCTCAAGCTAACGTTTCAGCTTCCGCTAGCTCTTCTTCCTCTTCTTCTA
AGAAGTCTAAGGGTGCTGCTCCAGAACTTGTTCCAGCCACTTCATTCATGGGTGTCGTTGCTGCTGTTGGC
GTTGCTTA

TGCTTGCGCCTGTGCTTGTGCTTGTGCTTGCGCTTGTGCTTGTGCTTGTGCTTGAGCTTGTGCTTGTGCTT
GAGCTTGTGCTTGAGCTTGAGCTTGAGCTTGAGCTTGTAATTGAGGGGAAGCTCCATTACTGTACATTGTT
GCAGAAGA

ACGTGATCGGATACTGATTTCTAGAAACAGGGGGGGCTGGTGTTGGTTCTGGCGAAGCCGCTTGCTCCGAC
TGCTTTGATGTCACAGCTTTGGGTTTCTCCGCTACACTGTCTACCTTAGGATTATTGATCATGACTCAAGC
TTGCGATA

TCTGGACCGAAGCTGCTTTCACCAAGTTGGACCAAGTCTGGGGTTCCGAAACCGTTGCCTCCTCCAAGGTC
GGCTACACTTTGCCATCCCATATCATCTCCACTTCTGATGTCACCAGAATTATCAACTCTTCCGAAATCCA
ATCTGCTA

GGAAGAAGATGCAGCCTTGGAAGAAGATGCAGCCTCGGAAGAAGATGCAGCCTCGGAAGAAGCTGGGGAAA
CGGAGGCAAGAGCAGCAGCGATTTCAGAACTCAATCTTGTGGTGTACCATGGCAATTTAACAATTGTCTTA
GTGATAGC

ATTCAGATAGGTGTAATGGAAATTGTGTACCAGGCAAATTCACTCCTTGGAGTGATCTAGACAACCAAAAT
TGCCAGGCAACCTGGATAAGACACTCTATGTCTGAGGAAGAGTTAGAAATGTATTGAAGACATTAGCTTTG
AGAATTTG

TGACAATTGTTGCAAGTAGGCTGGGAATCTGTGCAAGTATTGGTCGAATGGAGCAACCAAATGGGTTTGAG
CACCAAAGAAGTTGTTGTACCAGACAGACAACAAACCACCCAACAATGGAATGTTGTCTTCCAATGGGGTT
TGGGTGAA

GCAGCACCAGAAACACCATCTGAAGCAGCAAAAGAGATGGAGTTACCGAAACAACTCGACCAGACACCAGA
ACCGGCAGTCCCCCCTTGGTTGTTCATGAATGTACAGTTGCTAGTAGAGCCTGGGACAAAGTAGGAACCTC
TGGACCAG

ATAACCAGCAGGGATATAACCAACAAGGCCATCAACAACCAGTCTACGTCCAACAACAACCACCCCAGAGG
GGTAACGAAGGTTGTCTGGCTGCATGTCTGGCTGCATTATGTATATGCTGCACCATGGATATGCTATTCTA
AGAGAACC

CCAGAAATCCCATTGGTTGTCTCCACTGACTTGGAATCTATTCAAAAGACCAAGGAAGCTGTTGCTGCTTT
GAAGGCTGTTGGTGCTCACTCCGACTTGTTGAAGGTCTTGAAGTCCAAGAAATTGAGAGCCGGTAAGGGTA
AGTACAGA

ACTCTTTCCAATCTTCCTTGGAAAGTGTCTCTGACTCTTTACAATTCTCTTCCAACGGTGACAACACTACT
TTGGCCTTCGACAATTTGGTCTGGGCTAACAACATCACTTTGAGAGATGTTAACTCTATTTCTTTCGGTAG
TTTGCAAA

CAAACGTACCCTCAACTGTACTACAATAGCAACGTCAATGCTCACAACAACAACAACAACAGCAACAACAA
CAACAACAACAACAACAACAGCAACAACAACAACAATCTTTACAACCAGACGCAGTTCTCCACGAGGTACT
TCAACTCG

TGCGATAGTGTAGATACCGTCCTTGGATAGAGCACTGGAGATGGCTGGCTTTAATCTGCTGGAGTACCATG
GAACACCGGTGATCATTCTGGTCACTTGGTCTGGGGCAATACCAGTCAACATGGTGGTGAAGTCACCGTAG
TTGAAAAC

GCTGCCAATCCTGAATTTCTTGCTTCGCTTGTTGGTACGTTCCTGGACGCAACTTGAACAGAGCAGTTTCA
GCTTCCGAGACGAATCCGTGGCTCACCAGCCACCTTGGAGATTCGTCCACTTTGAGCCAAGCTAAAATATT
CGCTACCG

TGTTGGACTTGCAGTTGTTGCTGAACTTGCATCTGCTGCTTGGCTTGCATCTGTTGCTTTGCCTGCATCTG
TTGTGCCTGCAATTGCTGGGCTTGTATCTGTTGCGCTTGCTGCTGATTCATGGAAATAACAGGTCTCACCA
TTGGAATT

GATGATGAAGCAGCAGCAGCAGAAGAGGTGGTTTTAGAGGAAGAGATGGAAGACTGGCTTTCACTCGTTTC
AACTGCTTGAGCACTGCTGGACAAAACAGTAGAGTCTTTAGTGTAAGAAGAAGGAACAGCAGGACCGACAA
CGGTATTG

ATTGGTCTATTCTCGTCGCATCTGCGTTTCTTGAATCTGCATGCCCAGCACCCAGTAAATGTCCTCCCCTT
GGTGGGGGGCACTTTGGAGGATGACGCTACTTTCTTGCTTCTCTGCTGCCTCTTACTATTGACCATTTGGT
TCTAGTGC

GGTAGAGTTGAAACCGGTGTCATCAAGCCAGGTATGGTTGTTACTTTCGCCCCAGCTGGTGTTACCACTGA
AGTCAAGTCCGTTGAAATGCATCACGAACAATTGGAACAAGGTGTTCCAGGTGACAACGTTGGTTTCAACG
TCAAGAAC

TTATTATTTGCATTTTGAATTTGCATTTGCTGTTGGACTTGCAGTTGTTGCTGAACTTGCATCTGCTGCTT
GGCTTGCATCTGTTGCTTTGCCTGCATCTGTTGTGCCTGCAATTGCTGGGCTTGTATCTGTTGCGCTTGCT
GCTGATTC

GGCATGATGTTGATTTTGAGCCAAGACATGCTCGTAGGCTTCCTTCGCACCTTGCCACTCTCCCATACTCT
CCAAAACACTACCCAACTGAAACCATATGTCCCACTCCTGCAAGGGAGCAGGAGGTTGAGGGAGAATGTAT
CTGAAGCA

CAGAGGCTAGACTGCTGGACACATGCAACGAAATTAGGATTGAAGCTCACCTGAGAAGGGAAACCACTGAC
GAGGGCCAAGTGCAACATAAGTTGGCTGCGCCCTTGGACCTTGAGCAACGGTTATTTTACTACCCATGCGA
CTTGTCCT

AAGCAGCTGCACCTGCAACAACAGCACCGAAAGAGGCAGCGCCTGCATTGACTCTTTGACCGGCATTGGAA
GCAGTTTCGACTGTAGAAGTGCTGACAGAAGAGGTAGTTGGAGCGACAGAAGAAGAAACGGCCTTAGAAGA
TGATTCGG

TCTCTTGCGAGACCTTCACTCTTTAGTTCAGAGTAAATATTTGTATCCATGATGATTAACACGTCTTGGTC
GGTTCTGGTTTCTTGTCCAGCTTGGACAGCAGATTCTGGCAAGCCTCTAATAGCATTCAAATCTCCTTTAA
CCAGTTCA

TTACTTTCGCCCCAGCTGGTGTTACCACTGAAGTCAAGTCCGTTGAAATGCATCACGAACAATTGGAACAA
GGTGTTCCAGGTGACAACGTTGGTTTCAACGTCAAGAACGTTTCCGTTAAGGAAATCAGAAGAGGTAACGT
CTGTGGTG

TCTGGACCAGTCACCGTTAGTGTTAGAAGATGATGACGAAGATGATGAAGCAGCAGCAGCAGAAGAGGTGG
TTTTAGAGGAAGAGATGGAAGACTGGCTTTCACTCGTTTCAACTGCTTGAGCACTGCTGGACAAAACAGTA
GAGTCTTT

TATTCAGGTACTCTAGGACAATTCTATTCAAGTCTGACGCCGAAAACGGACCATTGGACCTCCCCTGCTGT
TGAGACTGGCCTTGACTCTGTTGCTGTGGCTGCTGGCCGGAATTGGCGCCAGCAGCATTATTAGTTCGTTG
ATTCTTTA

GCAGCCTTGGAAGAAGATGCAGCCTCGGAAGAAGATGCAGCCTCGGAAGAAGCTGGGGAAACGGAGGCAAG
AGCAGCAGCGATTTCAGAACTCAATCTTGTGGTGTACCATGGCAATTTAACAATTGTCTTAGTGATAGCAT
CAAAGTCC

CCGGACCCACACTATCCGCTGCCACAGTATATCCCACCGCTGAGTACTTCCTCACCTGATCCAATCGATTC
ACAGGATCAACACTCTGAAGTACCTCAAGCTAAGACAAAGGTGAGAAATAATGTCTTACCACCACACACTT
TAACATCA

TTGCTGCAGCTGTTGTCTTGGTAGGGGCTGTAATTGCTGCTGCTGTTGTTGTTGTTGTTGTTGTTGTTGTT
GTTGTTGTTGTTGTTGTTGTTGTTGTTGTTGTTGCTGTTGTTGTTGTTGTTGTTGTGCCTGCGCCTGTGCT
TGTGCTTG

TGGCGCTGTGTGGTTTCCCAGTTCTTCAATGTTGATTCTAATGGATGTCTGTCATTCAATTGTGTGGAAAG
AGGTACTGCACCGCCTTCTTGCTGGCGTAATGTGTCTGGCAAGGAGGGCACTGCAGATGAAAGAACACTTT
TGTCTTGA

TTGCATTTGCTGTTGGACTTGCAGTTGTTGCTGAACTTGCATCTGCTGCTTGGCTTGCATCTGTTGCTTTG
CCTGCATCTGTTGTGCCTGCAATTGCTGGGCTTGTATCTGTTGCGCTTGCTGCTGATTCATGGAAATAACA
GGTCTCAC

ACCAGCACCTGCGACACCTCTTAAGATGTTAGCACCACAACCCTTGAATAGAGAACCAACACCTTCAGCAG
CAACAATCTTCCTCAAACAGTCAAAGGCACCGTCGTACTTAACAGCTTGACCGGAGGTCATCATCATTCTT
CTTCTAAC

ACCCCCCTCATTTACGCTAAGCACATTTATGAAAACATAGACGGCTACAAGTGCGTTCCGTCAAAGAGGCC
TCTTGAACAACTTTCCCCCACGGAACTCCACCAGGGAGATCGCCCCAATAAGGCTAGCTTTTCCAACAAGA
AGGCAATC

TAGCGGATATTTCACTATGTTTGATCCTCTCGATTTGTACACCCCTGATGACATCCAGGTTGAGGCTTTAC
AGTTCAATCTAGCAGAGAGAGAGCCAAAAGATCCTTGCTCACCGCAACGTGACGAAATATTAACGGCTGTA
GATGAAGA

GCAGTTGTTGCTGAACTTGCATCTGCTGCTTGGCTTGCATCTGTTGCTTTGCCTGCATCTGTTGTGCCTGC
AATTGCTGGGCTTGTATCTGTTGCGCTTGCTGCTGATTCATGGAAATAACAGGTCTCACCATTGGAATTGC
TGGGGAAT

GTTACGCCACTGCTTCTGCTATTGCTGCTACTGCTGTTGCCTCTTTGGTCTTGGCCAGAGGTCACAGAGTC
GAAAAGATTCCAGAAATCCCATTGGTTGTCTCCACTGACTTGGAATCTATTCAAAAGACCAAGGAAGCTGT
TGCTGCTT

TGCAAGTAGGCTGGGAATCTGTGCAAGTATTGGTCGAATGGAGCAACCAAATGGGTTTGAGCACCAAAGAA
GTTGTTGTACCAGACAGACAACAAACCACCCAACAATGGAATGTTGTCTTCCAATGGGGTTTGGGTGAAGT
GGTTGTCG

AATTCTACCCAACATGTCTTCAGACACAGGAATTCTCAAACTCTCACCAGTGAATTCCACGGTAGTCTTCT
TGACATCAATACCAGATGTACCTTCAAACACTTGCACAATGGCTCTATCTCCTCTAATTTCCAAAACTTGA
CCTTGTCT

TGAAACCGGTGGTGATCACACCAATGAATCCCGTGTAGATGAATGTTGTGCTGAGAAAGTGAACGACACTG
AGACTGGCTTGGATGTGGACAGCTGTTGCGGCGATGCTCAAACAGGTGGTGACCACACCAATGAATCCTGT
GTTGATGG

GTAGCCATCTGTCCTGCTCTCTTAGCGTGGAAATTTGCTGCAAACTGCCGCTGGAGCTAGCGTTTCCACCG
TTCTCCTGAGTATCCTCTGGGCTTGTGCTAACATGTTCGGACTCGTTGGTATTCATATTCTCGAGAGGTTT
GTGCCACT

TGCTTCGCTTGTTGGTACGTTCCTGGACGCAACTTGAACAGAGCAGTTTCAGCTTCCGAGACGAATCCGTG
GCTCACCAGCCACCTTGGAGATTCGTCCACTTTGAGCCAAGCTAAAATATTCGCTACCGCAATCACGGATC
CGGAAAAG

CATTTGCAGTTGGTCGTACATTGAGATAACACCAGCACCTGCGACACCTCTTAAGATGTTAGCACCACAAC
CCTTGAATAGAGAACCAACACCTTCAGCAGCAACAATCTTCCTCAAACAGTCAAAGGCACCGTCGTACTTA
ACAGCTTG

ACAACAATCTTTACAACCAGACGCAGTTCTCCACGAGGTACTTCAACTCGAACTCCTCTCCCTCGTTGACT
TCTTCCACTTCTAACTCATCCTCTCCATACAACCAAAGCACCTTCGAATACATTTTGCCGTCAACTTCGGC
AGCTTCCA

AACTTCTTACCAAACAAGATCATTTGCAGTTGGTCGTACATTGAGATAACACCAGCACCTGCGACACCTCT
TAAGATGTTAGCACCACAACCCTTGAATAGAGAACCAACACCTTCAGCAGCAACAATCTTCCTCAAACAGT
CAAAGGCA

CATTGGTTGTCTCCACTGACTTGGAATCTATTCAAAAGACCAAGGAAGCTGTTGCTGCTTTGAAGGCTGTT
GGTGCTCACTCCGACTTGTTGAAGGTCTTGAAGTCCAAGAAATTGAGAGCCGGTAAGGGTAAGTACAGAAA
CAGAAGAT

CCGTACCAAGGAGAAAAGTCCTGTGAATGTTGAGCACCAACACCCACTGCAGCACCATTAGGACCTCTGAA
GACCATTTGACATTTTTGAGTACCACCAGACATGTAGTGAGTCTTTGCAGCGGAATTGACAACATGATCGA
TAGCTTGC

GTGCAAGTATTGGTCGAATGGAGCAACCAAATGGGTTTGAGCACCAAAGAAGTTGTTGTACCAGACAGACA
ACAAACCACCCAACAATGGAATGTTGTCTTCCAATGGGGTTTGGGTGAAGTGGTTGTCGACGGCTTCAGCA
CCCTTCAA

GAAGATCACGTAGCGCACACCGTCCTTGAGATCCTGCCACAGATCCAGCGGAGGTCTGACAAATTGCTTCC
TTTGCAGAGACTTTTCACCTTGCGGAGACTGTTCACCTTCCATACTACTAGTATCTAACTCTTTCTCTCTC
CCATACAC

ATGTGTACATGAGGTAACTGGTCTATGGTTGATTTGATGACTGTCATCATGGTGAGATATTCGTTGTTTCT
TACTTGGGAACTGGTGCTGTTGCAACTGTTGCTGCTCCGGTTGTCTTTTCATGTGAGCAGAACTAAAATCT
TGGTCTTG

TTGTCTGAAAACAGCTCCGTACAATGGAGAATTAGGATTTGTGAACATGGTCTGGAAGTGACCCACCATGT
GGGATAGATCCCTGTAGGTCACCTGAGTGTCCGTTTCAGGAACAATAACGGCGACATTATCGGATACGCTA
AAAGTATC

TTTTGAGCATTTGGCCCATAACAACTGCATTGGGATCTTTCCTGAAGGTGGGTCCCACGACAGAACAAACT
TGTTGCCCCTGAAAGCAGGTGTGGCGATTATGGCTCTTGGTTGCATGGATAAGCATCCTGACGTCAATGTT
AAGATTGT

AGAGGATGGAGCGGCAGAGGATGTAGCTTCGGAAGAAGATGCAGCCTTGGAAGAAGATGCAGCCTCGGAAG
AAGATGCAGCCTCGGAAGAAGCTGGGGAAACGGAGGCAAGAGCAGCAGCGATTTCAGAACTCAATCTTGTG
GTGTACCA

ATGTAGCTTCGGAAGAAGATGCAGCCTTGGAAGAAGATGCAGCCTCGGAAGAAGATGCAGCCTCGGAAGAA
GCTGGGGAAACGGAGGCAAGAGCAGCAGCGATTTCAGAACTCAATCTTGTGGTGTACCATGGCAATTTAAC
AATTGTCT

TTGAGCGGAGGCAGCACCAGAAACACCATCTGAAGCAGCAAAAGAGATGGAGTTACCGAAACAACTCGACC
AGACACCAGAACCGGCAGTCCCCCCTTGGTTGTTCATGAATGTACAGTTGCTAGTAGAGCCTGGGACAAAG
TAGGAACC

ATGGATATCGCCACTGTATCTTGTCTGAAAACAGCTCCGTACAATGGAGAATTAGGATTTGTGAACATGGT
CTGGAAGTGACCCACCATGTGGGATAGATCCCTGTAGGTCACCTGAGTGTCCGTTTCAGGAACAATAACGG
CGACATTA

GCATGCCCCCTCTGCAAGACCAACTAAAGTAGACCCTACCGGTATCTCTACCATTGTTCCTATGGCCTCTC
ATGTAGATCAAACTGAGCATGACCTGGACATCTTGGTCACTGACCAAGGTTTGGCGGATCTAAGAGGTCTA
TCGCCTAA

TGGTCGTACATTGAGATAACACCAGCACCTGCGACACCTCTTAAGATGTTAGCACCACAACCCTTGAATAG
AGAACCAACACCTTCAGCAGCAACAATCTTCCTCAAACAGTCAAAGGCACCGTCGTACTTAACAGCTTGAC
CGGAGGTC

TTTGTGGTTAGATAAAGGAACTAAAAGAACCTTTGTTGGAGCAACCAAAGGTGGGAAGGAAAGAACACTTC
TGGCATTGTCTTCTGGTCTGTTCCAGAAAGAGTGCTCAAAAACGGAGTAGATGATACGGCCAATACCGAAA
GATGGTTC

CAATTCGTCTGTTGAATAATTGTTCCTCTTACTGGAACCTTTGCGTCCAGAAAGCTTAGCTTGTTGAATTG
ATGCAGACGATGAAGACGATGAAAAGGAGGAGACAGCAGCACCAGTATCATCTCTTGATAAGTCCATCTCA
TTCAAACT

AACCACATAAGTCTGATATCGTAACTACAATTATCTCCATGTTTGGCACACAGTGAACAGATTGGTCTATT
CTCGTCGCATCTGCGTTTCTTGAATCTGCATGCCCAGCACCCAGTAAATGTCCTCCCCTTGGTGGGGGGCA
CTTTGGAG

TAGTCGCATTGGTACTGGCATTAGCACTACCATGAATGCACGTGTCGCTGTCCTCATCACTGCTGCAATAC
TTTCTGCACCTGTCACTGCTATTGCTCTCCTGGAAGCTAGACGGTAACGCAACGATCGACATGGAAGCTGT
CGCCTGAT

CTCCACTGACTTGGAATCTATTCAAAAGACCAAGGAAGCTGTTGCTGCTTTGAAGGCTGTTGGTGCTCACT
CCGACTTGTTGAAGGTCTTGAAGTCCAAGAAATTGAGAGCCGGTAAGGGTAAGTACAGAAACAGAAGATGG
ACTCAAAG

ACCTGCAACAACAGCACCGAAAGAGGCAGCGCCTGCATTGACTCTTTGACCGGCATTGGAAGCAGTTTCGA
CTGTAGAAGTGCTGACAGAAGAGGTAGTTGGAGCGACAGAAGAAGAAACGGCCTTAGAAGATGATTCGGCA
GATGATGA

CTGGGAATCTGTGCAAGTATTGGTCGAATGGAGCAACCAAATGGGTTTGAGCACCAAAGAAGTTGTTGTAC
CAGACAGACAACAAACCACCCAACAATGGAATGTTGTCTTCCAATGGGGTTTGGGTGAAGTGGTTGTCGAC
GGCTTCAG

CTTGGCTCTCAACTTTTCAGCCATAGTCATTTTCTTTGGACCAGAAGATGGAGTTGGGGTTGCTGGGGTTG
CTGGAGTAGCTGGAGTAGCTGGGGAGGACCCACCAGCTGTTCTACCGGCTAGTCTCTGTTCGATGGCTTCT
TCCATTTC

AACAACCTTGCCCCTCCATTCTGGATGCTCGTTCAGAAACACTTCCATGGCGTGCAACTTCTGAGGCACAC
CTTTGATGTAATCCAGCCTGTCGACACCAACTATGATCTTGCAGCCCTTGAAAGTTTCCTTCAATTGTTGG
ATTCTCTT

CCCATGGTCCTGCTCCATGCAGTGCCATCATCCGTCACAACTAGCTTGCGGTTCCTGGTCTCCCCAGGTAC
CACTTCTTTTCTATCCACCAGGTCCTGTACCTGATGATCTTGTTTCAACCATGTAGCATCGTGATCGTAGA
AGTAGCCA

GTTCTTACATTGATAAGAAATGTCCATTCACTGGTTTAGTTTCCATCCGTGGTAAGATCTTGACCGGTACC
GTCGTCTCCACCAAGATGCACCGTACCATTGTCATCAGAAGAGCTTACTTGCATTACATTCCAAAGTACAA
CAGATACG

GCGAACAATAAATTACTATATTATCAACCATTTCTATCATCTTGGCACATTGCAGGTCGAACCTTCGGAAT
AGACCTCTCCTTCCCCAGACGTCCTGCCCACTCCGTTTTAGCGTGGTATTAGACCCAACCTTGTTTCTAGA
ATACCTTT

TCCGCAACAATCTCAACCCATTGCTAATCAATCAGCGACTTCTACCCCTCCTCCTCCTCCAGCACCACACA
ACTTACATCCCCAAATTGGTCAAGTGCCCTTAGCTCCAGCGCCTATTAATTTGCCTCCACAAATTGCTCAG
TTACCTTT

GTAGTAGGCAATCAAAGCACCCAAAGTAGCTGGAGTAATCTTTTGAGCCAAGATAGAGGTAGTTGGTCTGT
TACCTGAGAAGACCTTGTGTGGGACCAAACCACCAGTGGCACCTTCAGCCTTAACTTGTTCTTCATCCTTA
CCAACCAT

GTGCCAACTCACATGGTTTCTCAATACTAAGCAAACACCCTCACCCAAATAATCTTGTCCATTCCCACTCA
CTTTCTCACACAAATGCGAAGAGCCACCTGCCTATCAGTAGCACTAGCACTAAAGAGAACAGCACGAACAA
GGAGGAGG

CCCAAGCAGGACATGAGTTACAAGCACTCAAAGTGTTGGCACCAGCGGATTCAGCTTCACTGACAGTCTGC
AAGGTGATGTTGAATTGGTCACCTAAACCATCAATGAAATATTGAGCAGTGTCATGACATCTCTTAGAATT
AGAGGTAA

TACTGCAGCCAATGCAGTTTCTGCCACCGCTCAATCGTCTGCTAATGGTGCGACTCCAATGTCAAAGTCAT
CCTCTTCAACATCCCTCAACTCCCACTCTCCGTTGATGACAGCAATGGAAGACCCACCATCCCCACGTTCT
TCGGCCAT

AGACCCGCTGTTGTTGTTGTCAAACTGAGAATTCCTGTTCGAGTCTGAGTCAGAGACAAACATCGGTCTCA
TCTTCTCCCAAAGTCGAGATGCAGGCACCAAGAGCAGCCACGAAGCAAAATACATAAAGGTCAGATAGAAG
GTTAATGA

CCCAACTATAATAACTATAAACACTTATAGAACAATTACCGTTTCTTCCTTCACACAACATGGCTTCTACT
TCGAACACGTTCCCTCCAAGCCAAAGCAATTCTTCCAACAACCTTCCAACTTCTAGACATGCATCCATTGT
GGAGATGC

CCCAGCTGGTGTTACCACTGAAGTCAAGTCCGTTGAAATGCATCACGAACAATTGGAACAAGGTGTTCCAG
GTGACAACGTTGGTTTCAACGTCAAGAACGTTTCCGTTAAGGAAATCAGAAGAGGTAACGTCTGTGGTGAC
GCTAAGAA

TTTCAGCCTTTCTTTCAGACAATCTCTTAGCCAACAATTGAGCGTATTCGGCAGCAGCTTCTCTTTGAGCT
TGAGCGTTTCTGACCTTCAAAGCTCTTTGGTGTCTCTTTCTTTGCAATCTTTGAGGAGTAACCAATCTTTG
GATCTTTG

CACTGATAACGTCTGGTGCGAGGTTGATATACGGCAATTCTGGATCAGGCATGTACCTGTAATCGATCGTG
GTTTCTTTGCTTCTCAACTTCACCGTTGATGAGCCTGTCCAGCCCCTTGTTTCTGGCTCCATCAGTGAGCT
AGTGTCAC

TTCGAACACGTTCCCTCCAAGCCAAAGCAATTCTTCCAACAACCTTCCAACTTCTAGACATGCATCCATTG
TGGAGATGCTGTCTACTCCGCCATTGCTGCCCCACGTTCAAGTGAACGATACAGACGACAAGGAACAACCA
GAAGAGTC

CCTCTGTCAAATGGCTTTGGGCAACAGCAACAGCAACAGCAACAGCAACAGCAGCCTCTGAACCAAAACAA
CGCTTTGTTGCCACAAATGAACGTGGAAGGAGTTTCTGGGATGGCTGCGGTTCAGCCTGTCTATGGCCAAG
CCATGTCA

GGGGTTAATGTCCTCACCTTCTTGAATATCGTCTAATTCTTCAGACTCTTCTTCAGTGGCTTCTACTCTCT
TATGATGTTGCAATTCTGAGTCTGGTATCCTCTCCACCACAATATCCTCCAACTTAGGCGTAGCAGGGCTG
GTGCTTTC

AGCATTGCCAAGGCATCGCTCGATCTCAAACATCATACTTCTGAACTCAACTCTAGCACATCAGAGCCAGA
CAGTCAAAGACGGTCCAAAGATAGTTCTGTGCCCCTAATAATTGATAGCAGTGGTTCAGCAAATTGAGAAC
ATAACAAA

CATCACCAAGGGTTTCTGCCAAAGAAGCAATGGAGAGCCAAGTTTCCGCAGTTGATTGTGTTAATGGGTCG
AGTGGCTGCTGAGGAACTGCTGCCTGCTGTTGCTGTTGCTGCTGTTGTTGTTGCTGTTGTTGTTGCTGTTG
AGCGGGTT

CCCAAAGACCGCTATTGAAGGTTCTTACATTGATAAGAAATGTCCATTCACTGGTTTAGTTTCCATCCGTG
GTAAGATCTTGACCGGTACCGTCGTCTCCACCAAGATGCACCGTACCATTGTCATCAGAAGAGCTTACTTG
CATTACAT

CTTTAGTTCAGAGTAAATATTTGTATCCATGATGATTAACACGTCTTGGTCGGTTCTGGTTTCTTGTCCAG
CTTGGACAGCAGATTCTGGCAAGCCTCTAATAGCATTCAAATCTCCTTTAACCAGTTCAATACCGGCAACC
TCCAACTT

TGTCCAGCAGATTCAGATAGGTGTAATGGAAATTGTGTACCAGGCAAATTCACTCCTTGGAGTGATCTAGA
CAACCAAAATTGCCAGGCAACCTGGATAAGACACTCTATGTCTGAGGAAGAGTTAGAAATGTATTGAAGAC
ATTAGCTT

ACAAATTTAACAGCTGCTAATACTGCAGCCAATGCAGTTTCTGCCACCGCTCAATCGTCTGCTAATGGTGC
GACTCCAATGTCAAAGTCATCCTCTTCAACATCCCTCAACTCCCACTCTCCGTTGATGACAGCAATGGAAG
ACCCACCA

AAATATCTTTTATGCTCTCCCCCATGGTCCTGCTCCATGCAGTGCCATCATCCGTCACAACTAGCTTGCGG
TTCCTGGTCTCCCCAGGTACCACTTCTTTTCTATCCACCAGGTCCTGTACCTGATGATCTTGTTTCAACCA
TGTAGCAT

TTCACTATGTTTGATCCTCTCGATTTGTACACCCCTGATGACATCCAGGTTGAGGCTTTACAGTTCAATCT
AGCAGAGAGAGAGAGCCAAAAGATCCTTGCTCACCGCAACGTGACGAAATATTAACGGCTGTAGATGAAGAAG
AGAGTGAC

AGCAACAACAACAACAATCTTTACAACCAGACGCAGTTCTCCACGAGGTACTTCAACTCGAACTCCTCTCC
CTCGTTGACTTCTTCCACTTCTAACTCATCCTCTCCATACAACCAAAGCACCTTCGAATACATTTTGCCGT
CAACTTCG

CGAACTGACCGTTGATTCTACCGACCAACTCATTGACCACAGATCTTAAATATTGGTACTCTTCCACATCT
CCACGACTTGGCACTGCAACCTGTACCAGAACAACCTTGCCCCTCCATTCTGGATGCTCGTTCAGAAACAC
TTCCATGG

GAAATGACAGAAATCAAAATAACAGCATTTATAAAGTGAGGCACAACTTTGACACCATGTGATGCAACAGC
AATGACAAATGGGGAGGCATGGGATCCAGAGGAGTCACTAGACCCTAATAATTCGTCACTGTTGTATGGAA
CAAGAAAG

CAGTGAACAGATTGGTCTATTCTCGTCGCATCTGCGTTTCTTGAATCTGCATGCCCAGCACCCAGTAAATG
TCCTCCCCTTGGTGGGGGGCACTTTGGAGGATGACGCTACTTTCTTGCTTCTCTGCTGCCTCTTACTATTG
ACCATTTG

GCTGATCTCGCAAGTGCATTCCTAGACTTAATTCATATCTGCTCCTCAACTGTCGATGATGCCTGCTAAAC
TGCAGCTTGACGTACTGCGGACCCTGCAGTCCAGCGCTCGTCATGGAACGCAAACGCTGAAAAACTCCAAC
TTTCTCGA

ACGGCATTAAACTTTTCCAGCAAGTCCTTCCTCACCTGATCCTTCTCATCGTCAGGAATCTCTAGCCCAGG
CCATCCGAACCACTTGAAAGTGTACGTCTTCTTCAACCCTTCCAACGCCGTGACCAGCCCTCCGGACGACA
TTGCGTAC

AGGTACCATTGAGTTCTGGGAATTATCATCGAGCTGTATACCTAATGAATCGTGGGGTTTGTCTTCGTCTT
TGTTGTCTCCCAGGGATGAAGAAGCAGCAGCGGCAGCAGCGGCAATAGCATCGTCATCATCTTGCTGCTTT
TGTTGAAG

GAGTTCTGGGAATTATCATCGAGCTGTATACCTAATGAATCGTGGGGTTTGTCTTCGTCTTTGTTGTCTCC
CAGGGATGAAGAAGCAGCAGCGGCAGCAGCGGCAATAGCATCGTCATCATCTTGCTGCTTTTGTTGAAGAA
GTTGTTGT

TCCTCACCTTCTTGAATATCGTCTAATTCTTCAGACTCTTCTTCAGTGGCTTCTACTCTCTTATGATGTTG
CAATTCTGAGTCTGGTATCCTCTCCACCACAATATCCTCCAACTTAGGCGTAGCAGGGCTGGTGCTTTCTT
GGTCTTCT

GGTTCAGGGATATAGGTTATCACCATACTCCGTACACGAACTGTCCAACTTGTCCAGCAGATTCAGATAGG
TGTAATGGAAATTGTGTACCAGGCAAATTCACTCCTTGGAGTGATCTAGACAACCAAAATTGCCAGGCAAC
CTGGATAA

TGATGACGAAGATGATGAAGCAGCAGCAGCAGAAGAGGTGGTTTTAGAGGAAGAGATGGAAGACTGGCTTT
CACTCGTTTCAACTGCTTGAGCACTGCTGGACAAAACAGTAGAGTCTTTAGTGTAAGAAGAAGGAACAGCA
GGACCGAC

AAGACTTGTTATTGTCTGATTTGTCTAGATTCTTCGCAAATTTCCAGCCTTCAGAGGCTTCGCTGAACAAT
ACCGGATGACAAGAGTATCCTCCACAAACAATCTCGTGGTCGTTTATCCATACTAAGGACCTGTAAGGTAA
ACCTTCCG

CTTGTTCCTCTGGTTCTTCCTTGATTATACTTTGACCGAAGTTTGAGGTGGGAGTAAATTTCTTCAATCCA
GAGGAGGAGTGAGTTAAAGGTGGAGGCTGCTGTGGCTGTTGTTGTTGTAATGTTGCTTGGCCATTGTTATT
ATCATATG

GTTTCAAAGCTTTGAAAGCTGGCTTACTGACAGAAACTTATTTAGAGGCACAGTTTGTCCGTCAACACAAG
AAGAAATTTGCGTCTTTCAGTCTGACTTCTGATGTAGAAGAGAGAGTTATGGAATTAATCACCTCTGGTGA
TGTTTATA

TCCACAGTACCGAACTGACCGTTGATTCTACCGACCAACTCATTGACCACAGATCTTAAATATTGGTACTC
TTCCACATCTCCACGACTTGGCACTGCAACCTGTACCAGAACAACCTTGCCCCTCCATTCTGGATGCTCGT
TCAGAAAC

GACCTTAAGTTTGTTTTCAGCTTATTTGATAGTTTACTGGCTATGACACTTCCACCAGCTGTGACTGCAGG
AGCGTTGAGTAGGGAGGTGGAGGAAGATTGAGTCGGTTGGGCATAACTTGGATAGTTCCTTTTCTGCTCCA
GTAAACTG

GCCTTCGGAATATTTAGCTTTAAATATGGCTTCCCTGAGAGCTTTCCTCTATTGCTGTTCAGTTGGTGCAG
GTGATGGACCATCGCCTGCACTGGAGCTGGACCTTCCAAAGTATTTGATATAGCAACTGTCGGGTGTATCT
TTTGCTTT

CCTGGGACAAAGTAGGAACCTCTGGACCAGTCACCGTTAGTGTTAGAAGATGATGACGAAGATGATGAAGC
AGCAGCAGCAGAAGAGGTGGTTTTAGAGGAAGAGATGGAAGACTGGCTTTCACTCGTTTCAACTGCTTGAG
CACTGCTG

AACTCCATCTTTTAACATGGCGCAAATTCCGCAACAGCTGTATCAGAGCCTCACACCACAACAATTGCAGA
TGATTCAGCAACGACACCAACAGTTACTGAGGAGTCGTCTACAACAACAACAACAACAACAACAACAAACT
TCACCGCC

TCCAGATTCCAAGAAATTGTCAAGGAAACCTCCAACTTTATCAAGAAGGTTGGTTACAACCCAAAGACTGT
TCCATTCGTCCCAATCTCTGGTTGGAACGGTGACAACATGATTGAAGCTACCACCAACGCTCCATGGTACA
AGGGTTGG

TGAATTCCACGGTAGTCTTCTTGACATCAATACCAGATGTACCTTCAAACACTTGCACAATGGCTCTATCT
CCTCTAATTTCCAAAACTTGACCTTGTCTCACGGTTCCATCTGGCAATGTCAAATTAACAATTTCGTTGTA
ACGTGGGA

GTAGTTTCTGGGTCAACAACCTTCAAGGTTTCAGCAATGTGAGTACCGTCAATGTTGGAAACGAAGTGGAC
ATCCAAGACACCAGCGTAGTGCTTCAAAGCCTCAGTGACCATGACTGGACCCAAATCGGAACCACCAATAC
CGATGTTA

CGCAGTGGCACTCGAGGACAAGGAGTATCCAAATTGGTTATGGAGCGTATTGGATAGTGATCACGTTGTCG
AACATGCGGCTGAGGACCCAGAGGGACAGGCTCTCTTAAAGAGAAGAAAGAACATAAGGAAGGCGAACAGG
CAGCGAAT

TATTGCTCTCCTGGAAGCTAGACGGTAACGCAACGATCGACATGGAAGCTGTCGCCTGATTTTCAGCCAAT
CTGTCCATTCTTTCTATCAGTTCCACTGTGTCAGCAGACAGGTCTGTCCTGGAGCCACAGCATCCAACATG
CTGGCCCT

TAGACGATGCTGAAACCGGTGGTGATCACACCAATGAATCCCGTGTAGATGAATGTTGTGCTGAGAAAGTG
AACGACACTGAGACTGGCTTGGATGTGGACAGCTGTTGCGGCGATGCTCAAACAGGTGGTGACCACACCAA
TGAATCCT

GTAAGAGATGAGATCCGTAACTTATTCACACAAATGGGGGATCTGGCGCTGGAGGACGCTGTCGTCAAACT
AGGTGAGCTGAGAAGAGGAATTAGGCTGCTGGCACCAGACGACAAGGATGTCAAGTACCGCATGGATTGGG
CCAGACGC

GAAATCCTTAGAAATTGTTGTCATTGTTTCTATCCTGTTGACGATCGTCAAACAAGATCTGTCTGTTGAGG
ACGACAGCCCGTTTGAAGGAAGTTCCTCTTCTGCTGGTCTTCCAAGTCCAAATACGAACACAAACGCAGAT
TCGACCAC

GATTTGATGACTGTCATCATGGTGAGATATTCGTTGTTTCTTACTTGGGAACTGGTGCTGTTGCAACTGTT
GCTGCTCCGGTTGTCTTTTCATGTGAGCAGAACTAAAATCTTGGTCTTGGTCAGAATCTCGATTATCCTTC
ACCATGAC

TATCAAGGGATGAGCCTGTTAAAGATTTTAGATCACTTAAGTTTCGGAGCGGTAGTGATTTCAAATGCTGG
GGTGACGAGAAGACAAGTTCTCATGTTCATTCATCCAGTGTGAACTCAGTTAATTCCTTTACTTCTACCAC
CTCTTCTT

CCAATAAGGAAGACATCATCATCACGGTCCAATTCTTCCGCCATGGCACTATTCAAGGCCTCTCTGACGGT
CATCGTCTTTGTTGATGAGAATCTCAATGCTGCTGCTGCTGCAGAGGGTCTTACAAAAGAAGTTGGGGCAC
GACGTGCA

ATTCAGACTTAGAAGCCGGTTTAACAGTCCAAGCATCATCTGTCTCAGCTGATGTGGAAGGAGCTGTTTGA
GCAGGACTTGTTGTTTGTGGCAACAATTGCTGAGGCAGTGAGGTTTCCGGACTTGTAGCAACGTTCAATGA
ACCATCAG

GTCGTCAAACTAGGTGAGCTGAGAAGAGGAATTAGGCTGCTGGCACCAGACGACAAGGATGTCAAGTACCG
CATGGATTGGGCCAGACGCTGCACAGACCTCTTCGGCATTCAGCACTGCCACAACATCGACGTAAAACGTC
TGCTGGAT

CCATGGCTACGCTGCCAATCCTGAATTTCTTGCTTCGCTTGTTGGTACGTTCCTGGACGCAACTTGAACAG
AGCAGTTTCAGCTTCCGAGACGAATCCGTGGCTCACCAGCCACCTTGGAGATTCGTCCACTTTGAGCCAAG
CTAAAATA

TGGGAATAAATTTCCAAAGTGTCAGGGTCATTGAAGTCCAGTTGTTGGGGTGGGAGAGGCAAAGTGGAAGT
TGATGGTAAAGGCGCATAAAATCTCTCAGTGGACATCTGGGCTTGATTATTAACGTTTGTTTGGTACAACG
ATGAAGAA

GGACGTGTAAGTGGTTTTCATACAAATGAAGGATGCAAAGACGAGGAGGAGGAGGATGACATTGACCCACT
GCACAAGGAGAATGGCATTAACACGCCATCGCAGCAATCGCAAAATTACGGTATGTTAGAGGCTAAACATG
ATAATCCA

AAAACACAAAGATTATTATCGTCAGTAGGTCAGAGGCTAGACTGCTGGACACATGCAACGAAATTAGGATT
GAAGCTCACCTGAGAAGGGAAACCACTGACGAGGGCCAAGTGCAACATAAGTTGGCTGCGCCCTTGGACCT
TGAGCAAC

CTCTATGATGTTGCACATGCACGTGTTTTGTTGTACCTCTACTCTGGGTCGAAGTGGTTTGATGCTCACCG
TCCAGAGCCACTTTGTGTCCCGACGTACCTGTTCCATCCTTCAAACGCCATTTCGTGTAGCAATAGCCAGA
AGATTGAG

GTAGGGGCTGTAATTGCTGCTGCTGTTGTTGTTGTTGTTGTTGTTGTTGTTGTTGTTGTTGTTGTTGTTGT
TGTTGTTGTTGTTGCTGTTGTTGTTGTTGTTGTTGTGCCTGCGCCTGTGCTTGTGCTTGTGCCTGTGCTTG
TGCTTGTG

AGATATCTAAACTCTTTCCAATCTTCCTTGGAAAGTGTCTCTGACTCTTTACAATTCTCTTCCAACGGTGA
CAACACTACTTTGGCCTTCGACAATTTGGTCTGGGCTAACAACATCACTTTGAGAGATGTTAACTCTATTT
CTTTCGGT

ACGGCAATTCTGGATCAGGCATGTACCTGTAATCGATCGTGGTTTCTTTGCTTCTCAACTTCACCGTTGAT
GAGCCTGTCCAGCCCCTTGTTTCTGGCTCCATCAGTGAGCTAGTGTCACCCACAGAAATCAACTCAACTTG
CCGCTGAT

GAAACCTCGTGTTTGTACAAATCCTCCAATGAGGCGATTGCTGTAATGCATCTTTGCCACACATCTGTCTA
TGCAGCATTGCTCACCTTTCGTTAGATCAGGCTCACCAAAGCCCTCATGCGGAATACATTTCTCAAGACAC
GTACTGAG

TGGCTTCTACTTCGAACACGTTCCCTCCAAGCCAAAGCAATTCTTCCAACAACCTTCCAACTTCTAGACAT
GCATCCATTGTGGAGATGCTGTCTACTCCGCCATTGCTGCCCCACGTTCAAGTGAACGATACAGACGACAA
GGAACAAC

CCTAATTGTTGTAATACTTTGGCATGATGTTGATTTTGAGCCAAGACATGCTCGTAGGCTTCCTTCGCACC
TTGCCACTCTCCCATACTCTCCAAAACACTACCCAACTGAAACCATATGTCCCACTCCTGCAAGGGAGCAG
GAGGTTGA

TTGTCCGAAGACAAGAAGAAGTTGGATTATGTTTTGGCTTTGAAGGTTGAAGATTTCTTGGAAAGAAGATT
GCAAACTCAAGTCTACAAGTTGGGTTTGGCCAAGTCTGTCCACCACGCCAGAGTTTTAATCACTCAAAGAC
ACATTGCT

ATTCGTTCTCTATCGAGAACTCTAATTAACTCTACCAAGGCCGCAAGACCTGCCGCTGCTGCTTTGGCTTC
CACCAGAAGATTGGCTTCCACCAAGGCACAACCCACAGAAGTTTCCTCCATCTTAGAGGAAAGAATTAAGG
GTGTGTCC

AAAGTCGATAGTATCTTTAACTAATTCGTACTCCACTAAAACATCGTCGGTGGCTTCCAAACCACACTTCT
TTCTCAATTTTTGGATTCTGTTGACCAGCTCTCTTGCGAGACCTTCACTCTTTAGTTCAGAGTAAATATTT
GTATCCAT

CGCAGCGGTGGCATCAGTAGTTTCGGTAGTAGCCGTTGGTCTTGCAGGCATGGCAGGAGGAGGGCCCTCTT
CATCCTCATCTGGAGCTTGGTCTTCTTTACTTTGCATCATACGAGCTCTATCATTGTGTTGAGGAGGTGGT
AAAAAGTT

AAAGAGAAAAGGACGTGTAAGTGGTTTTCATACAAATGAAGGATGCAAAGACGAGGAGGAGGAGGATGACA
TTGACCCACTGCACAAGGAGAATGGCATTAACACGCCATCGCAGCAATCGCAAAATTACGGTATGTTAGAG
GCTAAACA

ACTCAAGCTTTGAACTTCGCCTTCAAAGATAAGATTAAGTTGATGTTTGGTTTCAAGAAAGAGGAAGGCTA
TGGTAAATGGTTTGCAGGTAATCTGGCTTCTGGTGGTGCAGCTGGTGCTCTTTCGTTATTATTTGTTTATT
CTTTAGAT

GCTTCTGCAGGAACAGTAGCAGTAGAAAGTTCTATGGTCTCAGCAGTGCTTTGGTCATTTGCTTTTGTTGT
TGAACTTTCTGATGATACAGTTGGTTGGACACTAGATGACTCTGGTTCCGTATTAAGCGGTTTTGTAGAAG
GAGAAACA

CAAATTTTGGTTTCTGGTGAAATTCCATCTACCTTGAATGAAGAGAGTAAAGACAAGGTCAAGGTCAAGGA
GAGCAGCTCTGGTAAGTTCAAGAGAGTCATCACTTTGCCAGACTACCCAGGTGTGGATGCAGACAACATTA
AAGCAGAC

GAGCATGTCAAAGATGTTGAAGTTGCTGTTAAGTTGTCCTGAATGCAATCCATGAATAATTGTAGACCAAC
CCTCCCCAGCTTTCGAGGCCTGTTTGATTGAAGTCGTGTGTTGGTAGCTTCATCTACTTGTAGGGAATCTA
GTAACTCA

CAGCTGCTAATACTGCAGCCAATGCAGTTTCTGCCACCGCTCAATCGTCTGCTAATGGTGCGACTCCAATG
TCAAAGTCATCCTCTTCAACATCCCTCAACTCCCACTCTCCGTTGATGACAGCAATGGAAGACCCACCATC
CCCACGTT

ACCAAGTTGGACCAAGTCTGGGGTTCCGAAACCGTTGCCTCCTCCAAGGTCGGCTACACTTTGCCATCCCA
TATCATCTCCACTTCTGATGTCACCAGAATTATCAACTCTTCCGAAATCCAATCTGCTATCAGACCAGCTG
GCCAAGCT

CTGTGGTTGTTGCATAATTCCATCGGTGTTGAATGTGTTCATCATCTGTGGTTGTTGCATAGCACCACCAG
TATTGAAAGTAGTCATCATTTGGAGCTCTTGCATAACACCACCAGTATTGAAGGTATTCATCGTTTGTGGT
TGCTGTTG

CTGTGAATGTTGAGCACCAACACCCACTGCAGCACCATTAGGACCTCTGAAGACCATTTGACATTTTTGAG
TACCACCAGACATGTAGTGAGTCTTTGCAGCGGAATTGACAACATGATCGATAGCTTGCATAGAGAAATTG
AACGACAT

CTTACCTTCTTGGCAATGTGGCACAAAATAATCTGGTTATGTGTCTTCATTTGGTAATCACTGGGATGTTA
CTGGGGCAGCAGCAACTCCGTGTGTACCCCTAACTCCGTGTGTACCCCTAAAGAACCTTGCCTGTCAAGGT
GCATTGTT

TGATGAACAGGAACTAAATCTTTGTGGTTAGATAAAGGAACTAAAAGAACCTTTGTTGGAGCAACCAAAGG
TGGGAAGGAAAGAACACTTCTGGCATTGTCTTCTGGTCTGTTCCAGAAAGAGTGCTCAAAAACGGAGTAGA
TGATACGG

TGAAGGTTGAAGATTTCTTGGAAAGAAGATTGCAAACTCAAGTCTACAAGTTGGGTTTGGCCAAGTCTGTC
CACCACGCCAGAGTTTTAATCACTCAAAGACACATTGCTGTTGGTAAGCAAATCGTCAACATCCCATCTTT
CATGGTCA

GCTTCCAAAACAGAAGAATGTGAGAAGGTTTCCACTCAGGCTAATTCTCAACAGCCAACAACACCTCCCTC
ATCTGCTGTTCCAGAGAACCATCATCATGCCTCTCCTCAAGCTGCTCAAGTACCATTGCCACAAAATGGGC
CGTACCCA

TAACCTTTAGCGTTCACTTCAAATGCACACCACGAGAATAAAGTACTAGCGTCAGTTGAGGTCAAGTTCAA
ACCCTTGTTTTCCTTGTTCAATCTCTTGGCAATGTCATCCAAGTAGGTTGTGTCGTATTCATTTACAATGT
CATCATTG

TGTTCATTCCAAATATCTTTTATGCTCTCCCCCATGGTCCTGCTCCATGCAGTGCCATCATCCGTCACAAC
TAGCTTGCGGTTCCTGGTCTCCCCAGGTACCACTTCTTTTCTATCCACCAGGTCCTGTACCTGATGATCTT
GTTTCAAC

CAGCAGCAGCAGAAGAGGTGGTTTTAGAGGAAGAGATGGAAGACTGGCTTTCACTCGTTTCAACTGCTTGA
GCACTGCTGGACAAAACAGTAGAGTCTTTAGTGTAAGAAGAAGGAACAGCAGGACCGACAACGGTATTGGT
GGAATCGG

ATTTAGACAGTGACCAGATCTTTAGCTTCACTAAAGAGTACGAAGACAAGACGCTGTTTGCTGCTTTGAAT
TTCAGTGGCGAAGAAATTGAATTCAGCCTCCCAAGAGAAGGTGCTTCTTTATCTTTTATTCTTGGAAATTA
TGATGATA

CTCCAGTAAAGAGCATGTCAAAGATGTTGAAGTTGCTGTTAAGTTGTCCTGAATGCAATCCATGAATAATT
GTAGACCAACCCTCCCCAGCTTTCGAGGCCTGTTTGATTGAAGTCGTGTGTTGGTAGCTTCATCTACTTGT
AGGGAATC

GTTTCTTCCTTCACACAACATGGCTTCTACTTCGAACACGTTCCCTCCAAGCCAAAGCAATTCTTCCAACA
ACCTTCCAACTTCTAGACATGCATCCATTGTGGAGATGCTGTCTACTCCGCCATTGCTGCCCCACGTTCAA
GTGAACGA

TGGCAAAAGAAACGTATTATGAAAAGATTAAGCATTGCCAAGGCATCGCTCGATCTCAAACATCATACTTC
TGAACTCAACTCTAGCACATCAGAGCCAGACAGTCAAAGACGGTCCAAAGATAGTTCTGTGCCCCTAATAA
TTGATAGC

GGTCAAGTGATCTTGCCAGCGCCCGGAGAGCCTCTGTCAAATGGCTTTGGGCAACAGCAACAGCAACAGCA
ACAGCAACAGCAGCCTCTGAACCAAAACAACGCTTTGTTGCCACAAATGAACGTGGAAGGAGTTTCTGGGA
TGGCTGCG

AAAATTGATATTACAGCATCAATTGACATCAATCATTAAATCATCATTAGCTTCCTGTGGATCAACAAACT
CCCTGCTGGCTCCTGCCAATAGTTCTCTTCTAATCTCAGGACCGATATCTTTATGGCCTCTTTGACGAACA
GTTTCCAA

TTTACGCTAAGCACATTTATGAAAACATAGACGGCTACAAGTGCGTTCCGTCAAAGAGGCCTCTTGAACAA
CTTTCCCCCACGGAACTCCACCAGGGAGATCGCCCCAATAAGGCTAGCTTTTCCAACAAGAAGGCAATCCT
GGAGAGTG

CCTGTACCAGAACAACCTTGCCCCTCCATTCTGGATGCTCGTTCAGAAACACTTCCATGGCGTGCAACTTC
TGAGGCACACCTTTGATGTAATCCAGCCTGTCGACACCAACTATGATCTTGCAGCCCTTGAAAGTTTCCTT
CAATTGTT

TGTTCATCATCTGTGGTTGTTGCATAATTCCATCGGTGTTGAATGTGTTCATCATCTGTGGTTGTTGCATA
GCACCACCAGTATTGAAAGTAGTCATCATTTGGAGCTCTTGCATAACACCACCAGTATTGAAGGTATTCAT
CGTTTGTG

AGATGATTGAACTGGTTCCCGAACATTGTTGTATTGGAAACAGCTTGACGAAGCAGCTCCAGATAAAGGTG
TTGAAGACCTCACTTCGTGACGATTCGGCTTCGATGTTTCCTCACGTTGTGATGTTACTTCAGGATCTTTA
TCGTTGTG

TATAACGAAATCAGCTCTTCAAATGGTATAGACTTGTGCATGAAATGGATGGGGACGAATTCCACAGTACC
GAACTGACCGTTGATTCTACCGACCAACTCATTGACCACAGATCTTAAATATTGGTACTCTTCCACATCTC
CACGACTT

TGTAATTTAGCAATATCCCAAGAACAATCATCGAAATGTCCCGTCCACAAGTTACTGTTCACTCTTTGACT
GGTGAAGCTACTGCCAATGCCTTGCCATTGCCAGCTGTCTTCTCCGCTCCTATCCGTCCAGACATTGTCCA
CACTGTTT

AGCTATGGAGCTTCACCCATGCAAGGTCAGCCTCCAGTCGGTGGACAACCGCCTGTGCCTGTAAGAATGCA
GCCACAGCCACCGCAACCAATGCAACAGGGAAACATCTATCCGATTGAGCCTTCTCTTGATTCTACTGGCT
CAACCCCA

TCACATGATTTTTCTACAAATAATCTTAAAACGTACGATATCGTGGAGAGTCTGTTCTCACTAAGTGATAG
CTTGTTTGTGGTTGACACTGTCAAGTTTCGAAGAAGGTCACAGTATTTTGTGTGTTCAGATGCAAGTAACT
CTACCAAA

CAACAACAACAGCAGCAGCAGCAGCAGCAGCAGCAAGGACAAATACCGCAATCTCAGCAAGTTCCTCAAGT
TAGATCCATGAGTGGACAACCTCCCACCAATGTTCAGCCCACTATTGGCCAACTTCCTCAACTTCCAAAAT
TAAACTTA

AACGTATTATGAAAAGATTAAGCATTGCCAAGGCATCGCTCGATCTCAAACATCATACTTCTGAACTCAAC
TCTAGCACATCAGAGCCAGACAGTCAAAGACGGTCCAAAGATAGTTCTGTGCCCCTAATAATTGATAGCAG
TGGTTCAG

CAACAACTTCTAGGTAGTAGTCTAAGTTGTATTTCTTACCTGGCTGAAGCATGTCACCAGAGTAACAAACA
GTAGCTTCGACAACACCACCGGCCTTCTTGACAGCATTCACACCAACTTTTAATTGTTCTAAATCATTCAA
GGCATCAA

GAGGGTTCGTCGGGCTTTGAACAGGATCTAAGGACGATGAAGTGCCTGCAACCGAAGAAGGTAGGTCATGA
TAAGTGGCATCTGAGTCGAAACTTTGAAGACTTCCACAAGCCATAAATTCGGCTATGTTTCTCTCCTCTTC
ATTCCAAT

ATCTTCCTTGGAAAGTGTCTCTGACTCTTTACAATTCTCTTCCAACGGTGACAACACTACTTTGGCCTTCG
ACAATTTGGTCTGGGCTAACAACATCACTTTGAGAGATGTTAACTCTATTTCTTTCGGTAGTTTGCAAACT
GTTAACGC

GGTTTTTCCTTTGCCGCCGATGAAGAGGAAGAGGATTCGGTTGTCTTAGAATTGTTCGTGACATTCTTCAC
TCCAGCATCACGATGGCGGTGGTGGTGATGATGATGGTGGGGCTGAGTAGCATCTGTAGTAGTAGTAGTAG
TAGTATTG

ACCTATTAGCTGGCAAAAGAAACGTATTATGAAAAGATTAAGCATTGCCAAGGCATCGCTCGATCTCAAAC
ATCATACTTCTGAACTCAACTCTAGCACATCAGAGCCAGACAGTCAAAGACGGTCCAAAGATAGTTCTGTG
CCCCTAAT

AGATCCGTAACTTATTCACACAAATGGGGGATCTGGCGCTGGAGGACGCTGTCGTCAAACTAGGTGAGCTG
AGAAGAGGAATTAGGCTGCTGGCACCAGACGACAAGGATGTCAAGTACCGCATGGATTGGGCCAGACGCTG
CACAGACC

ACAATCAGCAGGGCTACAATCAGCAGGGATATAACCAGCAGGGATATAACCAACAAGGCCATCAACAACCA
GTCTACGTCCAACAACAACCACCCCAGAGGGGTAACGAAGGTTGTCTGGCTGCATGTCTGGCTGCATTATG
TATATGCT

CCTGCAAGGGCATGTGCGGTTGTTGGAGCGATTGTAGCCAAATCGTGGGTTCTGACTCTTGTTGCAAGTAG
AGATTGATGAGCGTCTCTCCAGGTGGTGTCCATCAGTAGAGTACCATTGAACTGTCTGACTTGCTTGGCAA
ATTCAGAT

TACTGCTCAAGCTGATTTGGACAAAATCTCCGGTTGTAGTACCATTGTTGGTAACTTGACCATCACCGGTG
ACTTGGGTTCCGCTGCTTTGGCTAGTATCCAAGAGATTGATGGTTCCTTGACTATCTTCAACTCCAGTTCT
TTATCTTC

TGTTGAAGCTTTCAGTGAATACCCACCATTAGGTAGATTCGCTGTCAGAGACATGAGACAAACTGTCGCTG
TCGGTGTTATCAAGTCTGTTGACAAGACTGAAAAGGCCGCTAAGGTTACCAAGGCTGCTCAAAAGGCTGCT
AAGAAATA

CATGTGCGGTTGTTGGAGCGATTGTAGCCAAATCGTGGGTTCTGACTCTTGTTGCAAGTAGAGATTGATGA
GCGTCTCTCCAGGTGGTGTCCATCAGTAGAGTACCATTGAACTGTCTGACTTGCTTGGCAAATTCAGATGG
TCCCTTTT

CACCTGGGACATCAGCTGTTCCAGAGAACCATCATCATGTCTCTCCTCAACCTGCTTCAGTACCACCTCCA
CAGAATGGACAGTACCAACAGCACGGCATGATGACTCCAAACAAAGCTATGGCTTCTAACTGGGCACATTA
CCAACAAC

GGGTTAGATGTAGACGATGCTGAAACCGGTGGTGATCACACCAATGAATCCCGTGTAGATGAATGTTGTGC
TGAGAAAGTGAACGACACTGAGACTGGCTTGGATGTGGACAGCTGTTGCGGCGATGCTCAAACAGGTGGTG
ACCACACC

GCTCACAGCATATAACGAAATCAGCTCTTCAAATGGTATAGACTTGTGCATGAAATGGATGGGGACGAATT
CCACAGTACCGAACTGACCGTTGATTCTACCGACCAACTCATTGACCACAGATCTTAAATATTGGTACTCT
TCCACATC

GCTAGGTGTCAGCATTGGTGATGCATGAGCAGATGATTGAACTGGTTCCCGAACATTGTTGTATTGGAAAC
AGCTTGACGAAGCAGCTCCAGATAAAGGTGTTGAAGACCTCACTTCGTGACGATTCGGCTTCGATGTTTCC
TCACGTTG

TGTTGTCTTGGTAGGGGCTGTAATTGCTGCTGCTGTTGTTGTTGTTGTTGTTGTTGTTGTTGTTGTTGTTG
TTGTTGTTGTTGTTGTTGTTGTTGCTGTTGTTGTTGTTGTTGTTGTGCCTGCGCCTGTGCTTGTGCTTGTG
CCTGTGCT

TGGACCGATCCCTCCCAGTGTAGAAATTTCTTTGGCTATAATTAGCAGGTCCTTTGGGTTGGTCCTGTTGT
TGCTGCTGTTGTTCAACTCTAGATTGATACTGAGATTCATCTGGAAAGGTATTCCCTTGTGTACCTGTTTG
ATTTTGAG

ACACATTGCTGTTGGTAAGCAAATCGTCAACATCCCATCTTTCATGGTCAGATTGGACTCTGAAAAGCACA
TTGACTTCGCTCCAACTTCTCCATTCGGTGGTGCTAGACCAGGTAGAGTTGCTAGAAGAAACGCTGCTAGA
AAGGCTGA

ACTGGTTCCCGAACATTGTTGTATTGGAAACAGCTTGACGAAGCAGCTCCAGATAAAGGTGTTGAAGACCT
CACTTCGTGACGATTCGGCTTCGATGTTTCCTCACGTTGTGATGTTACTTCAGGATCTTTATCGTTGTGCC
ACCACGTA

AATATATTTAGCAACATTGGAACTCCATCTTTTAACATGGCGCAAATTCCGCAACAGCTGTATCAGAGCCT
CACACCACAACAATTGCAGATGATTCAGCAACGACACCAACAGTTACTGAGGAGTCGTCTACAACAACAAC
AACAACAA

TAAATGTGGTCTCCATGTTGCTCACTCTTTTCTAAAGAAACTTGCACCGGAAAGGTTTGCCAGTGCTCCTC
TGGCCGGGCTGCAAGTCTTCTGTGAGGGTGATGTACCAACTGGCAGTGGATTGTCTTCTTCGGCCGCATTC
ATTTGTGC

ATTGCCATACATATCCTCCAGATGGAAGGGTCCCCCAAGTGAAGGTTTACCGTATCCTCTGTTTGCTTCAG
CCTCCTTCTGTGTTTCCAATCTGCGTTTCTCCCTGTTGAAGAAATAAGAAAGTGCCCCACCGACTGCTAGG
AATAGAGC

GTTGTTGCATAGCACCCCCAGTGTTAAAAGTGTTCATCATCTGTGGTTGTTGCATAATTCCATCGGTGTTG
AATGTGTTCATCATCTGTGGTTGTTGCATAGCACCACCAGTATTGAAAGTAGTCATCATTTGGAGCTCTTG
CATAACAC

AGCTGCTTTCACCAAGTTGGACCAAGTCTGGGGTTCCGAAACCGTTGCCTCCTCCAAGGTCGGCTACACTT
TGCCATCCCATATCATCTCCACTTCTGATGTCACCAGAATTATCAACTCTTCCGAAATCCAATCTGCTATC
AGACCAGC

GTTGTTGCATAGCACCACCAGTATTGAAAGTAGTCATCATTTGGAGCTCTTGCATAACACCACCAGTATTG
AAGGTATTCATCGTTTGTGGTTGCTGTTGTTGAAGAACGCCCCCTGTACTTAAAGTATTCATGGCTCCACC
AGTGAACT

GAGCTGTATACCTAATGAATCGTGGGGTTTGTCTTCGTCTTTGTTGTCTCCCAGGGATGAAGAAGCAGCAG
CGGCAGCAGCGGCAATAGCATCGTCATCATCTTGCTGCTTTTGTTGAAGAAGTTGTTGTTGTTCTTGAATA
GCAGCAGC

CCCTACACCGGTTTCAAAGCTTTGAAAGCTGGCTTACTGACAGAAACTTATTTAGAGGCACAGTTTGTCCG
TCAACACAAGAAGAAATTTGCGTCTTTCAGTCTGACTTCTGATGTAGAAGAGAGAGTTATGGAATTAATCA
CCTCTGGT

TTGCAATTGGCTCCAGGTGCTCACTTGGGTAGATTCGTTATCTGGACCGAAGCTGCTTTCACCAAGTTGGA
CCAAGTCTGGGGTTCCGAAACCGTTGCCTCCTCCAAGGTCGGCTACACTTTGCCATCCCATATCATCTCCA
CTTCTGAT

GTTTGCAACAGAAATGACAGAAATCAAAATAACAGCATTTATAAAGTGAGGCACAACTTTGACACCATGTG
ATGCAACAGCAATGACAAATGGGGAGGCATGGGATCCAGAGGAGTCACTAGACCCTAATAATTCGTCACTG
TTGTATGG

GGTCTACCAGAACCGTCAAAAATTCTACCCAACATGTCTTCAGACACAGGAATTCTCAAACTCTCACCAGT
GAATTCCACGGTAGTCTTCTTGACATCAATACCAGATGTACCTTCAAACACTTGCACAATGGCTCTATCTC
CTCTAATT

AATTCACTAAACAAGGTAGTGTAAGCGTCATCGGTGTAAGTCATGACTTGTTGATACACACTCAAGACATC
AGATGGAATTTGGAATCCACTGTTGCCAGTTTCTAGACCCAAGTAGTCAGAAAGATGAGAGTTGATGTCAC
CGATAATA

GCCCGGAGAGCCTCTGTCAAATGGCTTTGGGCAACAGCAACAGCAACAGCAACAGCAACAGCAGCCTCTGA
ACCAAAACAACGCTTTGTTGCCACAAATGAACGTGGAAGGAGTTTCTGGGATGGCTGCGGTTCAGCCTGTC
TATGGCCA

CTCAATAGATCGTCATCACTATTCTCTAAGTCCTCATACTGCCTTCTCCTTCTTCGTCTTTGCACAGGAAG
GCCCATTTCATCAAGTTGCGCTGCACCTTCTTGTTCTTCGTCCTCGTCATCTATGTAGGCAACATTTCTCA
ATAGCCTA

CAGCAACAGCAACAGCAACAGCAGCCTCTGAACCAAAACAACGCTTTGTTGCCACAAATGAACGTGGAAGG
AGTTTCTGGGATGGCTGCGGTTCAGCCTGTCTATGGCCAAGCCATGTCAAGTACTACAAACATGCAAGATT
CGAACCCC

TCTGCGATTTCATCGCTCAGGAAGATGGGTACGGCATTAAACTTTTCCAGCAAGTCCTTCCTCACCTGATC
CTTCTCATCGTCAGGAATCTCTAGCCCAGGCCATCCGAACCACTTGAAAGTGTACGTCTTCTTCAACCCTT
CCAACGCC

GCTATTGAAGGTTCTTACATTGATAAGAAATGTCCATTCACTGGTTTAGTTTCCATCCGTGGTAAGATCTT
GACCGGTACCGTCGTCTCCACCAAGATGCACCGTACCATTGTCATCAGAAGAGCTTACTTGCATTACATTC
CAAAGTAC

ATGGCTTTGGGCAACAGCAACAGCAACAGCAACAGCAACAGCAGCCTCTGAACCAAAACAACGCTTTGTTG
CCACAAATGAACGTGGAAGGAGTTTCTGGGATGGCTGCGGTTCAGCCTGTCTATGGCCAAGCCATGTCAAG
TACTACAA

GGTATACTTCTGGTACATCCTTTGGAGGCTTTTGTTTCGAACTTCCATCTCCAGAATCCGATCGACTGGAT
GATGACTGTCCACCTCCAGAAGCGGAGCTTCTGGAGTTTTTCGAAGCTTCACCTTCTTGGTCCTTATTAAC
AATTTCAT

TATCGAGAACTCTAATTAACTCTACCAAGGCCGCAAGACCTGCCGCTGCTGCTTTGGCTTCCACCAGAAGA
TTGGCTTCCACCAAGGCACAACCCACAGAAGTTTCCTCCATCTTAGAGGAAAGAATTAAGGGTGTGTCCGA
CGAGGCCA

GAACATTGTTGTATTGGAAACAGCTTGACGAAGCAGCTCCAGATAAAGGTGTTGAAGACCTCACTTCGTGA
CGATTCGGCTTCGATGTTTCCTCACGTTGTGATGTTACTTCAGGATCTTTATCGTTGTGCCACCACGTAGA
GAATCCCT

TTGTTGTTGTCAAACTGAGAATTCCTGTTCGAGTCTGAGTCAGAGACAAACATCGGTCTCATCTTCTCCCA
AAGTCGAGATGCAGGCACCAAGAGCAGCCACGAAGCAAAATACATAAAGGTCAGATAGAAGGTTAATGAAC
TTGAGTTT

TCATTGCAACGTCGAATGTAGCACCACCCCAACATTCTAAAGCGAAAGCACCTGCAAGGGCATGTGCGGTT
GTTGGAGCGATTGTAGCCAAATCGTGGGTTCTGACTCTTGTTGCAAGTAGAGATTGATGAGCGTCTCTCCA
GGTGGTGT

GGTGATGTGGTGGGTGGAGCGCTCGATAATCCGTTTGAAGATGACATGGACGGTGTTGACTTCAACCAAGC
TTTCGAAGGAACAGATGATAACGAGGAGGCCAGTGTGAAACTTGATTTACAGGATGACGAAGATCATAAGT
TCCCCATT

TACCCTGATCCATGAAACTGGATAAGTCATTGGCGTCTTCATCGTGGCCAGCAAGCAGGTCATCTGCTTCA
TCCAAAAATTGATCATGGATCAGGGATGAGTAGCTTGGACCTGTTCTGTTTCCAAACCTTCCTCTAATTTT
GGAGATAG

AATTATCATCGAGCTGTATACCTAATGAATCGTGGGGTTTGTCTTCGTCTTTGTTGTCTCCCAGGGATGAA
GAAGCAGCAGCGGCAGCAGCGGCAATAGCATCGTCATCATCTTGCTGCTTTTGTTGAAGAAGTTGTTGTTG
TTCTTGAA

GGGCAGCAGAAGAGGATGGAGCGGCAGAGGATGTAGCTTCGGAAGAAGATGCAGCCTTGGAAGAAGATGCA
GCCTCGGAAGAAGATGCAGCCTCGGAAGAAGCTGGGGAAACGGAGGCAAGAGCAGCAGCGATTTCAGAACT
CAATCTTG

CTGAAATTGAGAAACGTGAAAACTACAAGAGAAAATTGGAAGCTGCCGAAGGAGATGCCACTGTTGTTACT
GAACGCTCTGATTCTGCTTCTTTCTTGGAAGAGAAGGAAGAACCTCAAGAGAATCATGATAACAAAGAGGA
GCAGTCGT

GTCCATGTCACTCAATAGATCGTCATCACTATTCTCTAAGTCCTCATACTGCCTTCTCCTTCTTCGTCTTT
GCACAGGAAGGCCCATTTCATCAAGTTGCGCTGCACCTTCTTGTTCTTCGTCCTCGTCATCTATGTAGGCA
ACATTTCT

CAACGATCGACATGGAAGCTGTCGCCTGATTTTCAGCCAATCTGTCCATTCTTTCTATCAGTTCCACTGTG
TCAGCAGACAGGTCTGTCCTGGAGCCACAGCATCCAACATGCTGGCCCTTTTTTTCCTTTCTTTGATTCAA
GTCCATAG

TCACTCAAAGACACATTGCTGTTGGTAAGCAAATCGTCAACATCCCATCTTTCATGGTCAGATTGGACTCT
GAAAAGCACATTGACTTCGCTCCAACTTCTCCATTCGGTGGTGCTAGACCAGGTAGAGTTGCTAGAAGAAA
CGCTGCTA

GATACTGTTCCATTTGTCTTTGGCGCTGTGTGGTTTCCCAGTTCTTCAATGTTGATTCTAATGGATGTCTG
TCATTCAATTGTGTGGAAAGAGGTACTGCACCGCCTTCTTGCTGGCGTAATGTGTCTGGCAAGGAGGGCAC
TGCAGATG

CAGACACCAGAACCGGCAGTCCCCCCTTGGTTGTTCATGAATGTACAGTTGCTAGTAGAGCCTGGGACAAA
GTAGGAACCTCTGGACCAGTCACCGTTAGTGTTAGAAGATGATGACGAAGATGATGAAGCAGCAGCAGCAG
AAGAGGTG

AACGAAAAGCGTTACGCCACTGCTTCTGCTATTGCTGCTACTGCTGTTGCCTCTTTGGTCTTGGCCAGAGG
TCACAGAGTCGAAAAGATTCCAGAAATCCCATTGGTTGTCTCCACTGACTTGGAATCTATTCAAAAGACCA
AGGAAGCT

TGCTATCCTTATCCTTCGAATGCCGGTATCTTTCCAAATCGTGCCTTTGCTTGGAACCAGTCCTGTGTGTT
TCATGCTTGCCATTTGACCGGGTTGACAGTTCGTGACCAGTACTGTGGCGCTTATTCTCAGTTATTGCGGT
GGAAGCAT

CGGTGACTCCTTGTGGCTGGCCCACAGTACCAGGCTTTCTCTTTCCTGGAGGCAGATTGTTTGAGGTCATT
TGATGTGATGCTTCGGTCATCGCATAGGCCTCCAAGACAGGTGCATTGAATTCCTTCTCCAGCTTATGGAA
CGTTGCTG

GGAGCGACAGAAGAAGAAACGGCCTTAGAAGATGATTCGGCAGATGATGATGGGGCAGCAGAAGAAGATGG
GGCAGCAGAAGAGGATGGAGCGGCAGAGGATGTAGCTTCGGAAGAAGATGCAGCCTTGGAAGAAGATGCAG
CCTCGGAA

TTCAAATATTTGTTGTAAAAGGTTCCCTTTTATAACAATAAAGCAGCTGCACCTGCAACAACAGCACCGAA
AGAGGCAGCGCCTGCATTGACTCTTTGACCGGCATTGGAAGCAGTTTCGACTGTAGAAGTGCTGACAGAAG
AGGTAGTT

TGCCGTATCCACCTTATCAAATGTCACCTATGTACGCTCCACCTGGGGCACAATCACAGTTTACACAATAT
CCACAATATGTTGGAACACATTTGAACACCCCGTCACCTGAGTCAGGTAATTCATTTCCTGATTCATCCTC
AGCAAAGT

CTTCGTTCCAGCATTTCTATCTGGCTGTAAATATTCTCATGCGGCCCCATTGAAGGCATTGTAGTTCTAGG
GACACCACTATGGCTCCCTCCGGGAGAGGCTGCAGGCGCACCTCCGCTATCTATGGTAATATGCTGAGCTG
TAGTTGCA

TGAGCCTGTTAAAGATTTTAGATCACTTAAGTTTCGGAGCGGTAGTGATTTCAAATGCTGGGGTGACGAGA
AGACAAGTTCTCATGTTCATTCATCCAGTGTGAACTCAGTTAATTCCTTTACTTCTACCACCTCTTCTTCA
AAGTGGAA

TGGTTACTGCACATCTGAAGCAAACGTACCCTCAACTGTACTACAATAGCAACGTCAATGCTCACAACAAC
AACAACAACAGCAACAACAACAACAACAACAACAACAACAGCAACAACAACAACAATCTTTACAACCAGAC
GCAGTTCT

TGGAGTAATCTTTTGAGCCAAGATAGAGGTAGTTGGTCTGTTACCTGAGAAGACCTTGTGTGGGACCAAAC
CACCAGTGGCACCTTCAGCCTTAACTTGTTCTTCATCCTTACCAACCATTAAAGCTTCAGCTTGAGCAAAG
AAGTTTGA

CTCCATGTTGCTCACTCTTTTCTAAAGAAACTTGCACCGGAAAGGTTTGCCAGTGCTCCTCTGGCCGGGCT
GCAAGTCTTCTGTGAGGGTGATGTACCAACTGGCAGTGGATTGTCTTCTTCGGCCGCATTCATTTGTGCCG
TTGCTTTA

GATAAAGGAACTAAAAGAACCTTTGTTGGAGCAACCAAAGGTGGGAAGGAAAGAACACTTCTGGCATTGTC
TTCTGGTCTGTTCCAGAAAGAGTGCTCAAAAACGGAGTAGATGATACGGCCAATACCGAAAGATGGTTCAA
TGACACTA

TACTCATACCGTAAAGACAACCTAATTGTTGTAATACTTTGGCATGATGTTGATTTTGAGCCAAGACATGC
TCGTAGGCTTCCTTCGCACCTTGCCACTCTCCCATACTCTCCAAAACACTACCCAACTGAAACCATATGTC
CCACTCCT

GTAAGCGTCATCGGTGTAAGTCATGACTTGTTGATACACACTCAAGACATCAGATGGAATTTGGAATCCAC
TGTTGCCAGTTTCTAGACCCAAGTAGTCAGAAAGATGAGAGTTGATGTCACCGATAATAGCTTGCAATTCA
GCAGTTTC

CTCACTCTTTTCTAAAGAAACTTGCACCGGAAAGGTTTGCCAGTGCTCCTCTGGCCGGGCTGCAAGTCTTC
TGTGAGGGTGATGTACCAACTGGCAGTGGATTGTCTTCTTCGGCCGCATTCATTTGTGCCGTTGCTTTAGC
TGTTGTTA

GAACGAGGACATGGATAAATACTTGTTGGAGTACCGCAGTTTGAAGCTCCTTCATCAGTCCAGTAATTCCT
TCCAGTCTCACAATGCGCCCTCCCACCAGTCGAACTACCACCCCCATTACAATCACATGAAATACAACAAC
ACTGGTAG

CTCTTTAGAATGCTGTCTTACTTCGTCTTCTACAGGGCTTGCTTCTGCAGGAACAGTAGCAGTAGAAAGTT
CTATGGTCTCAGCAGTGCTTTGGTCATTTGCTTTTGTTGTTGAACTTTCTGATGATACAGTTGGTTGGACA
CTAGATGA

GCAGCATCCTCGACTTCAGTTTACGCAGGAAGTTATTGGATCCCAGGACATCCATTGTGGGTACTACTGTA
CCTTCTCCGTATCCACCTAGAACACCATCCACAGTGGCGTCCACATCCGTCCAATAGTCGATGGCATCTTC
GTATTTGA

AAACAAGAACCAAATTTTGGTTTCTGGTGAAATTCCATCTACCTTGAATGAAGAGAGTAAAGACAAGGTCA
AGGTCAAGGAGAGCAGCTCTGGTAAGTTCAAGAGAGTCATCACTTTGCCAGACTACCCAGGTGTGGATGCA
GACAACAT

GATAACTGGTTCAACTCTATCAATAGATTGGTCTCTTTATTCACCAAAGGTGACGGTCATGCAAGAGAAAT
AGTTTGTCATGGCTTCATTAACTTAGAAGATGGCAAACTGGTGGAGCATTTGCTGAACAGTGATAGCAAGA
CAAAGGAA

CTTCCAATTTACCGGAATTTGATAGAGATTCCACTAAGGTTAATTCTCAACAAGAGACAACACCTGGGACA
TCAGCTGTTCCAGAGAACCATCATCATGTCTCTCCTCAACCTGCTTCAGTACCACCTCCACAGAATGGACA
GTACCAAC

TGAAATAGTGCTCCAGTAAAGAGCATGTCAAAGATGTTGAAGTTGCTGTTAAGTTGTCCTGAATGCAATCC
ATGAATAATTGTAGACCAACCCTCCCCAGCTTTCGAGGCCTGTTTGATTGAAGTCGTGTGTTGGTAGCTTC
ATCTACTT

ATTGACAAGACTCATAAGCTGGGTATGAAATTCATCACCGATTTGGTTATCAACCACTGTTCTACAGAACA
CGAATGGTTCAAAGAGAGCAGATCCTCGAAGACCAATCCGAAGCGTGACTGGTTCTTCTGGAGACCTCCTA
AAGGTTAT

GGACGACAAGTGTGGTGTTGACTCCATCCCGGTTGATCCTGAAAAGGTTGTTGCGATTGTGGAGTCCACCA
TGAGGGACCAGGTCCCACCAAATACGCCCTCTGACGACATGTCCAGGGCTATTGCAGGTCATTTGGTCGAG
TTTTTCAG

ACCTTGAATGAAGAGAGTAAAGACAAGGTCAAGGTCAAGGAGAGCAGCTCTGGTAAGTTCAAGAGAGTCAT
CACTTTGCCAGACTACCCAGGTGTGGATGCAGACAACATTAAAGCAGACTACGCAAATGGTGTTTTGACAT
TAACAGTT

TCCTTTAAGGATCTCGAGCTTTCGTAAGAAAGTGGCACAAACTGAATGGAATCACCGGGTTTAACCTGTCC
AACCTTCCACAGTTCTGCTTCTGGGACAACAGCTTGACACACAAAACCACCTAAGGAAGGACCATCGCAAG
TAATAATA

ATCAAAGCACCCAAAGTAGCTGGAGTAATCTTTTGAGCCAAGATAGAGGTAGTTGGTCTGTTACCTGAGAA
GACCTTGTGTGGGACCAAACCACCAGTGGCACCTTCAGCCTTAACTTGTTCTTCATCCTTACCAACCATTA
AAGCTTCA

TAACAACACACAATTGAAGGTCATTGACGGTTTTAACAAGGTTCAAACTGTTGGTGGTGCCATTGAAGTTA
CTGGTAACTTCTCAACCTTGGACTTATCTTCTTTGAAGTCTGTTAGAGGTGGTGCTAACTTCGACTCTTCT
TCCAGTAA

GCTAGTAGAGCCTGGGACAAAGTAGGAACCTCTGGACCAGTCACCGTTAGTGTTAGAAGATGATGACGAAG
ATGATGAAGCAGCAGCAGCAGAAGAGGTGGTTTTAGAGGAAGAGATGGAAGACTGGCTTTCACTCGTTTCA
ACTGCTTG

GAGTTATGGTCATCATCAAAATCTAGTACTGTGCTATCCTCCTCAATCTGTGAATAGCGTCCCCCAGAGAT
TCTGGGCTCTTGGTCTACCGACAATCTTCCCGAAGTGCGACGGTTGGTGTCGGAAGTCGAGGATCTCGGAG
TCGTAGTA

CACACACACCACACCCACACACACCCACACCCACACACCACACCCACACACACACCACACCCACACACACC
ACACCACACCCACACCACACCCACACCCACACACCACACCCACACCCACACCCCACACCCACACACCACAC
CCACACAC

CCCACACCACACCCACACACCCACACCCACACACCACACCCACACACACCACACCCACACACACCCACACC
CACACACCACACCCACACACACACCACACCCACACACACCACACCACACCCACACCACACCCACACCCACA
CACCACAC

CACCACACACACCACACCCACACACCCACACCACACCACACCCACACCACACCCACACACCCACACCCACA
CACCACACCCACACACACCACACCCACACACACCCACACCCACACACCACACCCACACACACACCACACCC
ACACACAC

CCACACACACCACACCACACCCACACCACACCCACACCCACACACCACACCCACACCCACACCCCACACCC
ACACACCACACCCACACACACCACACCCACACACACCCACACCACACCCACACACCACACCCACACACCCA
CACCCACA

CACACCACACCCACACCACACCCACACCCACACACCACACCCACACCCACACCCCACACCCACACACCACA
CCCACACACACCACACCCACACACACCCACACCACACCCACACACCACACCCACACACCCACACCCACACA
CACCACAC

CACACCCACACCACACCACACCCACACCACACCCACACACCCACACCCACACACCACACCCACACACACCA
CACCCACACACACCCACACCCACACACCACACCCACACACACACCACACCCACACACACCACACCACACCC
ACACCACA

CCACACCACACCCACACCACACCCACACACCCACACCCACACACCACACCCACACACACCACACCCACACA
CACCCACACCCACACACCACACCCACACACACACCACACCCACACACACCACACCACACCCACACCACACC
CACACCCA

ACCCACACACCCACACCCACACACCACACCCACACACACCACACCCACACACACCCACACCCACACACCAC
ACCCACACACACACCACACCCACACACACCACACCACACCCACACCACACCCACACCCACACACCACACCC
ACACCCAC

ACACCACACCCACACACACCACACCCACACACACCCACACCCACACACCACACCCACACACACACCACACC
CACACACACCACACCACACCCACACCACACCCACACCCACACACCACACCCACACCCACACCCCACACCCA
CACACCAC

CCCACACCCACACACCACACCCACACCCACACCCCACACCCACACACCACACCCACACACACCACACCCAC
ACACACCCACACCACACCCACACACCACACCCACACACCCACACCCACACACACCACACCCACACCACACC
CACACCCA

CCACACACCACACCCACACACACACCACACCCACACACACCACACCACACCCACACCACACCCACACCCAC
ACACCACACCCACACCCACACCCCACACCCACACACCACACCCACACACACCACACCCACACACACCCACA
CCACACCC

ACACCCACACCACACCCACACACCACACACACCACACCCACACACCCACACCACACCACACCCACACCACA
CCCACACACCCACACCCACACACCACACCCACACACACCACACCCACACACACCCACACCCACACACCACA
CCCACACA

CACACCCACACACCACACACACCACACCCACACACCCACACCACACCACACCCACACCACACCCACACACC
CACACCCACACACCACACCCACACACACCACACCCACACACACCCACACCCACACACCACACCCACACACA
CACCACAC

CCACACCCACACCCCACACCCACACACCACACCCACACACACCACACCCACACACACCCACACCACACCCA
CACACCACACCCACACACCCACACCCACACACACCACACCCACACCACACCCACACCCACACACCCACACC
CTAACACT

ACACCCACACACACCCACACCCACACACCACACCCACACACACACCACACCCACACACACCACACCACACC
CACACCACACCCACACCCACACACCACACCCACACCCACACCCCACACCCACACACCACACCCACACACAC
CACACCCA

ACACCCACACCCACACACCACACCCACACACACACCACACCCACACACACCACACCACACCCACACCACAC
CCACACCCACACACCACACCCACACCCACACCCCACACCCACACACCACACCCACACACACCACACCCACA
CACACCCA

CACCCACACACACACCACACCCACACACACCACACCACACCCACACCACACCCACACCCACACACCACACC
CACACCCACACCCCACACCCACACACCACACCCACACACACCACACCCACACACACCCACACCACACCCAC
ACACCACA

CACACCACACCCACACACACCACACCACACCCACACCACACCCACACCCACACACCACACCCACACCCACA
CCCCACACCCACACACCACACCCACACACACCACACCCACACACACCCACACCACACCCACACACCACACC
CACACACC

CCACACCCACACACCACACCCACACACACCACACCCACACACACCCACACCCACACACCACACCCACACAC
ACACCACACCCACACACACCACACCACACCCACACCACACCCACACCCACACACCACACCCACACCCACAC
CCCACACC

CACACCACACCCACACCCACACCCCACACCCACACACCACACCCACACACACCACACCCACACACACCCAC
ACCACACCCACACACCACACCCACACACCCACACCCACACACACCACACCCACACCACACCCACACCCACA
CACCCACA

CCCACACACCACACCCACACCACACCCACACACCACACACACCACACCCACACACCCACACCACACCACAC
CCACACCACACCCACACACCCACACCCACACACCACACCCACACACACCACACCCACACACACCCACACCC
ACACACCA

ACCACACCCACACACCCACACCACACCACACCCACACCACACCCACACACCCACACCCACACACCACACCC
ACACACACCACACCCACACACACCCACACCCACACACCACACCCACACACACACCACACCCACACACACCA
CACCACAC

CCACACCACACCCACACCCACACACCACACCCACACCCACACCCCACACCCACACACCACACCCACACACA
CCACACCCACACACACCCACACCACACCCACACACCACACCCACACACCCACACCCACACACACCACACCC
ACACCACA

ACCCCACACCCACACACCACACCCACACACACCACACCCACACACACCCACACCACACCCACACACCACAC
CCACACACCCACACCCACACACACCACACCCACACCACACCCACACCCACACACCCACACCCTAACACTAC
CCTAACAC

CACACACCACACCCACACACACCACACCCACACACACCCACACCACACCCACACACCACACCCACACACCC
ACACCCACACACACCACACCCACACCACACCCACACCCACACACCCACACCCTAACACTACCCTAACACTA
CCCTATTC

ACCCACACACACCACACCCACACACACCCACACCACACCCACACACCACACCCACACACCCACACCCACAC
ACACCACACCCACACCACACCCACACCCACACACCCACACCCTAACACTACCCTAACACTACCCTATTCTA
ACCCTGAT

ACCACACCCACACACACCCACACCACACCCACACACCACACCCACACACCCACACCCACACACACCACACC
CACACCACACCCACACCCACACACCCACACCCTAACACTACCCTAACACTACCCTATTCTAACCCTGATTT
TACCTGTC

TGAGGGTAGGTTTGGAGACAGGTAAAATCAGGGTTAGAATAGGGTAGTGTTAGGGTAGTGTGTGGGTGTGG
GTGTGTGGGTGTGGTGTGTGGGTGTGGTGTGTGTGGGTGTGGTGTGTGGGTGTGGGTGTGTGGGTGTGGTG
GGTGTGGT

TGAAGCTCGATACCGTTCGAGGTCTTTTCCACTTTCTCTGCCTTCTTGATCACAGGCTTCTCCACTTTCTT
TGCAGGAGTTGCCACTGGCTGGTGCTGGTGCTGCTTCTGCTGCTGCTTCTGCTGCTGCTTCTGCTTCTGCT
TCTTGTTG

CACACACCCACACCACACCCACACACCACACCCACACACCCACACCCACACACACCACACCCACACCACAC
CCACACCCACACACCCACACCCTAACACTACCCTAACACTACCCTATTCTAACCCTGATTTTACCTGTCTC
CCAACTTA

TTCAAGCGCTGGCGCAGTTGTTTCTTCAAGTTGACGTTCTCCTCGTCCATGGTCACACCGTCCAAATTCAC
CTGCTCCTCCCCCTGCGGTGGGGCCCCACTGCTCAACACAAGGTAGTACTTCTTATTCTTTTTAGACTCTG
GGTCGTCC

TGCTTCCGGCCTGGCTGCCTTGGCTTCTCAATTCTTTAAGTCAGGTAACAATTCCCAAGGTCAGGGACAAG
GTCAAGGTCAAGGTCAAGGTCAAGGACAAGGTCAAGGTCAAGGTTCTTTTACTGCTTTGGCGTCTTTGGCT
TCATCTTT

TACGAACCTGAGTCTACCGGTTCCTCCTTTCACGATTTCTTGCAATTGTTTGATGAAACCAAGGTCCAGTA
CGGACTGGCACGTGTGTCCCCACCAGGGTCAGACGTTGAGAAGATTATTATCATTGGTTGGTGTCCTGATT
CTGCGCCA

TAGTACACAAGGGAACTCGAACTATGTTCCTGAATCAACTGCAAGTGGAAGTTCACAATACCAGGACTGGT
CAAGCTCTTCTCTTCCGCTGTCACAAACCACTTGGGTTGTCATCAACACAACTAATACACAAGGGTCTGTA
ACGTCAAC

CTGGCTGCCTTGGCTTCTCAATTCTTTAAGTCAGGTAACAATTCCCAAGGTCAGGGACAAGGTCAAGGTCA
AGGTCAAGGTCAAGGACAAGGTCAAGGTCAAGGTTCTTTTACTGCTTTGGCGTCTTTGGCTTCATCTTTCA
TGAATTCC

CCAATTCCAGCACTCTTGCTGCCTCGAACAACGTTCCTGAATCAACTGCAAGCGGAAGTTCACAATACCAG
GACTGGTCAAGCTCTTCTCTTCCGCTGTCACAAACCACTTGGGTTGTCATCAACACAACTAATACACAAGG
GTCTGTAA

ACTCCTTGTCTTTGCCTCCCTTCAAGCGCTGGCGCAGTTGTTTCTTCAAGTTGACGTTCTCCTCGTCCATG
GTCACACCGTCCAAATTCACCTGCTCCTCCCCCTGCGGTGGGGCCCCACTGCTCAACACAAGGTAGTACTT
CTTATTCT

TTTGCCTCCCTTCAAGCGCTGGCGCAGTTGTTTCTTCAAGTTGACGTTCTCCTCGTCCATGGTCACACCGT
CCAAATTCACCTGCTCCTCCCCCTGCGGTGGGGCCCCACTGCTCAACACAAGGTAGTACTTCTTATTCTTT
TTAGACTC

CACTCTTGCTGCCTCGAACAACGTTCCTGAATCAACTGCAAGCGGAAGTTCACAATACCAGGACTGGTCAA
GCTCTTCTCTTCCGCTGTCACAAACCACTTGGGTTGTCATCAACACAACTAATACACAAGGGTCTGTAACG
TCAACCAC

CTCTTGGCAGACTCCTTGTCTTTGCCTCCCTTCAAGCGCTGGCGCAGTTGTTTCTTCAAGTTGACGTTCTC
CTCGTCCATGGTCACACCGTCCAAATTCACCTGCTCCTCCCCCTGCGGTGGGGCCCCACTGCTCAACACAA
GGTAGTAC

TGGCTTCTCAATTCTTTAAGTCAGGTAACAATTCCCAAGGTCAGGGACAAGGTCAAGGTCAAGGTCAAGGT
CAAGGACAAGGTCAAGGTCAAGGTTCTTTTACTGCTTTGGCGTCTTTGGCTTCATCTTTCATGAATTCCAA
CAACAATA

TTAGAAGTAGGAGATCCGTTGATGGCTGTGTTACTCAATGGTGCCACGGAAACCTGGGGAGGTGGTTGCTG
TTGTTGAAGTTGCTGCTGTTGCAGTTGCTGTTGTGGTAGTTGTTGTTGGGCGTTTGAGGACACCACAGGCA
AAGTGGTG

GCTCAACAACAACAGCAGCAATTACAGAAAAGCCGTTTGAATCAGCCATCCCAGTCGGCTCAACCTCCAGG
AGTGAATGTCCCAAATCCTCAAGGTGGGATTGCTGCAGTTCAATCAGATTTGGAACAGAATCAACGTGTTC
TCGTTCAC

GTTTTCGAATTCGAAAAGTTCGCTGCTGGTACTAAGGCTTTGTTAGACGAAGTTGTCAAGAGCTCTGCTGC
TGGTAACACCGTCATCATTGGTGGTGGTGACACTGCCACTGTCGCTAAGAAGTACGGTGTCACTGACAAGA
TCTCCCAT

ACTATGGTGACTTCACCACTATGTTGACTGGTATTCCAGCTGAACAAGTCACCAGAGTCATCACTGGTGTC
CCATGGTACTCCACTAGATTGAGACCAGCCATCTCCAGTGCTCTATCTAAGGACGGTATCTACACTGCTAT
TCCAAAAT

AACCTGGGGAGGTGGTTGCTGTTGTTGAAGTTGCTGCTGTTGCAGTTGCTGTTGTGGTAGTTGTTGTTGGG
CGTTTGAGGACACCACAGGCAAAGTGGTGGTAGGCAACTGTGAGCCAACCAGATTAGGACGGCTAGCTTGT
ACTGGGAA

AGTTGTCAAGAGCTCTGCTGCTGGTAACACCGTCATCATTGGTGGTGGTGACACTGCCACTGTCGCTAAGA
AGTACGGTGTCACTGACAAGATCTCCCATGTCTCTACTGGTGGTGGTGCTTCTTTGGAATTATTGGAAGGT
AAGGAATT

GAGATCCGTTGATGGCTGTGTTACTCAATGGTGCCACGGAAACCTGGGGAGGTGGTTGCTGTTGTTGAAGT
TGCTGCTGTTGCAGTTGCTGTTGTGGTAGTTGTTGTTGGGCGTTTGAGGACACCACAGGCAAAGTGGTGGT
AGGCAACT

AACAGCAGCAATTACAGAAAAGCCGTTTGAATCAGCCATCCCAGTCGGCTCAACCTCCAGGAGTGAATGTC
CCAAATCCTCAAGGTGGGATTGCTGCAGTTCAATCAGATTTGGAACAGAATCAACGTGTTCTCGTTCACCT
CATGGACA

ACTTTCTCTGCCTTCTTGATCACAGGCTTCTCCACTTTCTTTGCAGGAGTTGCCACTGGCTGGTGCTGGTG
CTGCTTCTGCTGCTGCTTCTGCTGCTGCTTCTGCTTCTGCTTCTTGTTGCCCTTGTACAACGCTTTCTGGT
ACTTCTCG

TCGACCAACTTCAAGAAGGCAGAGATACGTGTGGGTTTTGCAGACAACTCCACAATACAGCTTGTTTCGCT
GATGTCGACGACACGACCTCCAAAGTTGTTGGTCAAGTTGGTGATATCGTTCAAATGCTCGTGCTTCAACC
TTAATACC

TAGGGTAATGTGAGGGTAGGTTTGGAGACAGGTAAAATCAGGGTTAGAATAGGGTAGTGTTAGGGTAGTGT
GTGGGTGTGGGTGTGTGGGTGTGGTGTGTGGGTGTGGTGTGTGTGGGTGTGGTGTGTGGGTGTGGGTGTGT
GGGTGTGG

ACGGTGAAGATGGTGAGCTAATCGAGTACATGAGGATTAAGTCCGGCGTGGCCGATGTTCACGGAGTGGAC
GGCGTGCAGGGTGAGGGTGTCATCGACAGCAAATTGCTGGAAGAGTTCAAGGACAACGTGAGATACACCTT
GGAAAATG

AGTGGTCTCTTTAGAAGTAGGAGATCCGTTGATGGCTGTGTTACTCAATGGTGCCACGGAAACCTGGGGAG
GTGGTTGCTGTTGTTGAAGTTGCTGCTGTTGCAGTTGCTGTTGTGGTAGTTGTTGTTGGGCGTTTGAGGAC
ACCACAGG

GATGGCTGTGTTACTCAATGGTGCCACGGAAACCTGGGGAGGTGGTTGCTGTTGTTGAAGTTGCTGCTGTT
GCAGTTGCTGTTGTGGTAGTTGTTGTTGGGCGTTTGAGGACACCACAGGCAAAGTGGTGGTAGGCAACTGT
GAGCCAAC

CCTCCATTCTGTCTTCTCTGTTATCGTAACCAAATTCTTGCTGTTGATGGTGATCCGATGCCTCCTGGTCC
ATCGACTGTTGATTACCGCTGTGCCGACTGGTGATCCGGAAACTTCTCATGGGTGTGGGGGATTTAGGATC
ATCCATGG

ACAAATAAGAGCTCAACAACAACAGCAGCAATTACAGAAAAGCCGTTTGAATCAGCCATCCCAGTCGGCTC
AACCTCCAGGAGTGAATGTCCCAAATCCTCAAGGTGGGATTGCTGCAGTTCAATCAGATTTGGAACAGAAT
CAACGTGT

CCTTCTTGATCACAGGCTTCTCCACTTTCTTTGCAGGAGTTGCCACTGGCTGGTGCTGGTGCTGCTTCTGC
TGCTGCTTCTGCTGCTGCTTCTGCTTCTGCTTCTTGTTGCCCTTGTACAACGCTTTCTGGTACTTCTCGTC
CTCGCTGA

TTACTCAATGGTGCCACGGAAACCTGGGGAGGTGGTTGCTGTTGTTGAAGTTGCTGCTGTTGCAGTTGCTG
TTGTGGTAGTTGTTGTTGGGCGTTTGAGGACACCACAGGCAAAGTGGTGGTAGGCAACTGTGAGCCAACCA
GATTAGGA

GAAGAGAATGATGATGACTGGGATGATGATGAAGACGAGGCTGCTCAACCTCCTTTGCCTTCGAGGAATGT
TGCGTCAGGAGCACCAGTGCAAAAAGAAGAGCCTGAACAAGAAGAGATCGCCCCAAGCTTACCTTCTAGAA
ACTCGATC

AAACTCGATCCCAGCTCCAAAACAAGAAGAAGCACCTGAACAAGCACCTGAAGAAGAAATTGAAGAAGAAG
CTGAGGAAGCCGCTCCACAGCTGCCATCAAGAAGCTCTGCAGCTCCTCCTCCGCCTCCAAGACGAGCAACT
CCAGAGAA

TTCATGAGCTCCTTCTTTCTTAGAATGAAACTCTTGGCAGACTCCTTGTCTTTGCCTCCCTTCAAGCGCTG
GCGCAGTTGTTTCTTCAAGTTGACGTTCTCCTCGTCCATGGTCACACCGTCCAAATTCACCTGCTCCTCCC
CCTGCGGT

TAATTCTCAACAAGAGACAACACCTGGGACATCAGCTGTTCCAGAGAACCATCATCATGTCTCTCCTCAAC
CTGCTTCAGTACCACCTCCACAGAATGGACAGTACCAACAGCACGGCATGATGACCCCAAACAAAGCTATG
GCCTCTAA

CCGGACCCACACTATCCGTTGCCACAGTATATCCCACCACTGAGTACTTCCTCACCTGATCCAATCGATTC
ACAGAATCAACACTCTGAAGTACCTCAAGCTGAGACAAAGGTGAGAAATAACGTCTTACCACCACACACTT
TAACATCA

GTGCCACGGAAACCTGGGGAGGTGGTTGCTGTTGTTGAAGTTGCTGCTGTTGCAGTTGCTGTTGTGGTAGT
TGTTGTTGGGCGTTTGAGGACACCACAGGCAAAGTGGTGGTAGGCAACTGTGAGCCAACCAGATTAGGACG
GCTAGCTT

GGCGCAGTTGTTTCTTCAAGTTGACGTTCTCCTCGTCCATGGTCACACCGTCCAAATTCACCTGCTCCTCC
CCCTGCGGTGGGGCCCCACTGCTCAACACAAGGTAGTACTTCTTATTCTTTTTAGACTCTGGGTCGTCCAC
CACAAGCC

TGTTAGACGAAGTTGTCAAGAGCTCTGCTGCTGGTAACACCGTCATCATTGGTGGTGGTGACACTGCCACT
GTCGCTAAGAAGTACGGTGTCACTGACAAGATCTCCCATGTCTCTACTGGTGGTGGTGCTTCTTTGGAATT
ATTGGAAG

TACCGTTCGAGGTCTTTTCCACTTTCTCTGCCTTCTTGATCACAGGCTTCTCCACTTTCTTTGCAGGAGTT
GCCACTGGCTGGTGCTGGTGCTGCTTCTGCTGCTGCTTCTGCTGCTGCTTCTGCTTCTGCTTCTTGTTGCC
CTTGTACA

TCAGGAACAAATTCTCTCATTAGTAGAACTGTGTTTGAATGTTTGCGCTGCTTCAGCACCCAAGTCTTGTC
GTGCGAACACAGTACAACTTCAGACTTGTCTTTGTCCAATGACTTAAACCTTAACTGGTGATTTTGAACCG
GATCCTGT

CGCTGCTGGTACTAAGGCTTTGTTAGACGAAGTTGTCAAGAGCTCTGCTGCTGGTAACACCGTCATCATTG
GTGGTGGTGACACTGCCACTGTCGCTAAGAAGTACGGTGTCACTGACAAGATCTCCCATGTCTCTACTGGT
GGTGGTGC

TACCTTCTAGAAACTCGATCCCAGCTCCAAAACAAGAAGAAGCACCTGAACAAGCACCTGAAGAAGAAATT
GAAGAAGAAGCTGAGGAAGCCGCTCCACAGCTGCCATCAAGAAGCTCTGCAGCTCCTCCTCCGCCTCCAAG
ACGAGCAA

ATGGCTCCCACTTCAGGTTCAAGCCCAAGTTATAGGTCAACACCAAGCCAGCTGTGGTCAGTGACACTGGG
ATAGTGGCTGCCCCTTCCAAAGACCTGACAGGGCCGGCAGGTAGAACATCTTCACCCAAAAATTTGAGTTC
ATTGGGTG

ATTCTTTAAGTCAGGTAACAATTCCCAAGGTCAGGGACAAGGTCAAGGTCAAGGTCAAGGTCAAGGACAAG
GTCAAGGTCAAGGTTCTTTTACTGCTTTGGCGTCTTTGGCTTCATCTTTCATGAATTCCAACAACAATAAT
CAGCAAGG

ATAGAGTCGACCTCCATTCTGTCTTCTCTGTTATCGTAACCAAATTCTTGCTGTTGATGGTGATCCGATGC
CTCCTGGTCCATCGACTGTTGATTACCGCTGTGCCGACTGGTGATCCGGAAACTTCTCATGGGTGTGGGGG
ATTTAGGA

CCTTCTTTCTTAGAATGAAACTCTTGGCAGACTCCTTGTCTTTGCCTCCCTTCAAGCGCTGGCGCAGTTGT
TTCTTCAAGTTGACGTTCTCCTCGTCCATGGTCACACCGTCCAAATTCACCTGCTCCTCCCCCTGCGGTGG
GGCCCCAC

CTCCGTTATCCCAGGTTCCTTGGGTTTGTTGCCATCTGCGTCCTTGGCCTCTTTGCCAGACAAGAACACCG
CATTTGGTTTGTACGAACCATGCCACGGTTCTGCTCCAGATTTGCCAAAGAATAAGGTCAACCCTATCGCC
ACTATCTT

GCAAAGTTCGTTCGATCGTACTGTTACTCTCTCTCTTTCAAACAGAATTGTCCGAATCGTGTGACAACAAC
AGCCTGTTCTCACACACTCTTTTCTTCTAACCAAGGGGGTGGTTTAGTTTAGTAGAACCTCGTGAAACTTA
CATTTACA

TTGGAAGACCGAAGAAAATAGATAGACTTTCGAATAAGTTCAGCAACAACATGTGGTTGAAATCCTTGCTG
CTCTGCCTGTACTCCTTAGTACTCTGCCAAGTCCACGCTGCACCTTCATCAGGGAAGCAGATTACCTCCAA
GGATGTTG

TTCGTCGAGAACGGTGAAGATGGTGAGCTAATCGAGTACATGAGGATTAAGTCCGGCGTGGCCGATGTTCA
CGGAGTGGACGGCGTGCAGGGTGAGGGTGTCATCGACAGCAAATTGCTGGAAGAGTTCAAGGACAACGTGA
GATACACC

GGAAGAACCAGAAGAGAATGATGATGACTGGGATGATGATGAAGACGAGGCTGCTCAACCTCCTTTGCCTT
CGAGGAATGTTGCGTCAGGAGCACCAGTGCAAAAAGAAGAGCCTGAACAAGAAGAGATCGCCCCAAGCTTA
CCTTCTAG

ACGAATCTGCAAACGTCTTTGCTCTTCAGGCGACAAATCTCTCTTCGATCTGCGTCCACTTTGCAGAGGCA
TTTTAGTTGCGCCAACCACAGGGCCTGGAGAAGGAGTGTTGGACATACCGATGGGTGAAGGCGAAGCTTGA
ACTGGAAC

GGCAAGTTTAGCAACTGTTTCCAGCCTTCACCTTCAAACACATGTCCCAAGTGTCCACCACACCTTGCACA
ACATATCTCCACCCTCGCAGGCATTAAAGAATTGTCACGATGATATGTGATGGCTCCAGGGGATACCTCTT
CGTAGAAT

AAAGTGTCGTTGTAGTTCAGATGAGATAGCGACTCAATTTCCACGCCTGGGGCAAAGATGTTTACACAGGG
CCCCCAATTGCTGAACTTGGCAATCGTGTCAATGTGGTCATCAAAGGCCCCTACGGTGATAACGTTTTCTG
CAGACGCA

AGCTCTGCTGCTGGTAACACCGTCATCATTGGTGGTGGTGACACTGCCACTGTCGCTAAGAAGTACGGTGT
CACTGACAAGATCTCCCATGTCTCTACTGGTGGTGGTGCTTCTTTGGAATTATTGGAAGGTAAGGAATTGC
CAGGTGTT

CCGATGAAGCCTCCGTTATCCCAGGTTCCTTGGGTTTGTTGCCATCTGCGTCCTTGGCCTCTTTGCCAGAC
AAGAACACCGCATTTGGTTTGTACGAACCATGCCACGGTTCTGCTCCAGATTTGCCAAAGAATAAGGTCAA
CCCTATCG

GAAGAAAATAGATAGACTTTCGAATAAGTTCAGCAACAACATGTGGTTGAAATCCTTGCTGCTCTGCCTGT
ACTCCTTAGTACTCTGCCAAGTCCACGCTGCACCTTCATCAGGGAAGCAGATTACCTCCAAGGATGTTGAT
CTTCAGAA

GTAAATGTTAACCACCATTGCTATTTCCTTGCTAGGTTTGCCAAAGCCGTTGGCGAAGGCAGCACTAGACG
GCGAGTCACTTCCGTCTCTTTGTCCACTTCCTCTTCACCATCAAATCGAAGCCTGCAAGTGCCGTCCCTCT
TGGTTCCG

GGGAACTCGAACTATGTTCCTGAATCAACTGCAAGTGGAAGTTCACAATACCAGGACTGGTCAAGCTCTTC
TCTTCCGCTGTCACAAACCACTTGGGTTGTCATCAACACAACTAATACACAAGGGTCTGTAACGTCAACCA
CATCCCCG

CAGATTAATGAAAAGTATGGCCACTTCTTAGAAAAATCGAAGGTTGTTATTGATCTGTGTGCTGCTCCTGG
TTCATGGTGTCAAGTTGCATCCAAACTCTGTCCTGTCAACTCCTTAATTATTGGTGTTGATATTGTTCCAA
TGAAGCCG

CCCACGTCTTTTCATGAGCTCCTTCTTTCTTAGAATGAAACTCTTGGCAGACTCCTTGTCTTTGCCTCCCT
TCAAGCGCTGGCGCAGTTGTTTCTTCAAGTTGACGTTCTCCTCGTCCATGGTCACACCGTCCAAATTCACC
TGCTCCTC

GCCTTGACAGAGGGTAAAGTAGTGGTCTCTTTAGAAGTAGGAGATCCGTTGATGGCTGTGTTACTCAATGG
TGCCACGGAAACCTGGGGAGGTGGTTGCTGTTGTTGAAGTTGCTGCTGTTGCAGTTGCTGTTGTGGTAGTT
GTTGTTGG

TGCCCACTGTGATCTCCAGAGCAAAGTTCGTTCGATCGTACTGTTACTCTCTCTCTTTCAAACAGAATTGT
CCGAATCGTGTGACAACAACAGCCTGTTCTCACACACTCTTTTCTTCTAACCAAGGGGGTGGTTTAGTTTA
GTAGAACC

CATCATCATTGGCATCGTTGCCATCTCTTTCTATATCACCAACTGTTGACACTGTTTCTGTTCTACAACCA
ACTACTTCCATCGCAACACTAACTTGCACAGACTCACAATGCCAACAGGAGGTATCCACTATCTGTAATGG
ATCCAACT

TTGGTTCTGAGGCATACCGGCGTTATTACTGTTGTGGAACTTTCTCCTTTGAGGACCATTGCTGTTATGGT
GATGACTGTGGTGATGATTACGGTGGTGAGGTGGTTGTTGCTGTTGTGTAGATTGACCATTTTCTTGAGAG
GTAGCGTC

GATAGAGATTCCACTAAGGTTAATTCTCAACAAGAGACAACACCTGGGACATCAGCTGTTCCAGAGAACCA
TCATCATGTCTCTCCTCAACCTGCTTCAGTACCACCTCCACAGAATGGACAGTACCAACAGCACGGCATGA
TGACCCCA

ACTAAGGCTTTGTTAGACGAAGTTGTCAAGAGCTCTGCTGCTGGTAACACCGTCATCATTGGTGGTGGTGA
CACTGCCACTGTCGCTAAGAAGTACGGTGTCACTGACAAGATCTCCCATGTCTCTACTGGTGGTGGTGCTT
CTTTGGAA

AATAGGTTGTTCAGGAACAAATTCTCTCATTAGTAGAACTGTGTTTGAATGTTTGCGCTGCTTCAGCACCC
AAGTCTTGTCGTGCGAACACAGTACAACTTCAGACTTGTCTTTGTCCAATGACTTAAACCTTAACTGGTGA
TTTTGAAC

GTGGGAAGCCTTGGTTCTGAGGCATACCGGCGTTATTACTGTTGTGGAACTTTCTCCTTTGAGGACCATTG
CTGTTATGGTGATGACTGTGGTGATGATTACGGTGGTGAGGTGGTTGTTGCTGTTGTGTAGATTGACCATT
TTCTTGAG

CGTGGCTTGAGGATTTAGTTCTCTTAGCTCGCCAGTAATCTGAGAGGAGAGTGTGGAAACTGGACGTTCCT
GGGGAATAGAGGACAAGGAAGAAGAAGTAGGGGTAGCGCCTGAGCCGTGATTAGGCAAAGAGTCATAGAGT
TGAGAATC

TAGAATGAAACTCTTGGCAGACTCCTTGTCTTTGCCTCCCTTCAAGCGCTGGCGCAGTTGTTTCTTCAAGT
TGACGTTCTCCTCGTCCATGGTCACACCGTCCAAATTCACCTGCTCCTCCCCCTGCGGTGGGGCCCCACTG
CTCAACAC

ACCGAATGGCTCGACCAACTTCAAGAAGGCAGAGATACGTGTGGGTTTTGCAGACAACTCCACAATACAGC
TTGTTTCGCTGATGTCGACGACACGACCTCCAAAGTTGTTGGTCAAGTTGGTGATATCGTTCAAATGCTCG
TGCTTCAA

GGTGGTTGCTGTTGTTGAAGTTGCTGCTGTTGCAGTTGCTGTTGTGGTAGTTGTTGTTGGGCGTTTGAGGA
CACCACAGGCAAAGTGGTGGTAGGCAACTGTGAGCCAACCAGATTAGGACGGCTAGCTTGTACTGGGAAGG
CCGATGGC

CGGATAATGAGGAAGAACCAGAAGAGAATGATGATGACTGGGATGATGATGAAGACGAGGCTGCTCAACCT
CCTTTGCCTTCGAGGAATGTTGCGTCAGGAGCACCAGTGCAAAAAGAAGAGCCTGAACAAGAAGAGATCGC
CCCAAGCT

CACATTGAAGCGGATAATGAGGAAGAACCAGAAGAGAATGATGATGACTGGGATGATGATGAAGACGAGGC
TGCTCAACCTCCTTTGCCTTCGAGGAATGTTGCGTCAGGAGCACCAGTGCAAAAAGAAGAGCCTGAACAAG
AAGAGATC

CAGTGGAGTCGACTGAAGGAGTGGAGTCGACTGAGGCGGAACGTGTGGCAGGGAAGCAGGAGCAGGAGGAG
GAGTACCCTGTGGACGCCCACATGCAAAAGTACCTTTCACACCTGAAGAGCAAGTCTCGGTCGAGGTTCCA
CCGAAAGG

TTTATGACCAGGGAGGTCAACAGAAAGTTTTCTAGTTCTTACATCCCACACTTTCAACGTGGTATCTTTGG
AACATGACACCAGTAGTCGGCAGTCCGATGACCACGCAACCTGGTATACGCTGGCTACATGCCCTCTAAAT
GTGGAGAT

GCCCCAAGCTTACCTTCTAGAAACTCGATCCCAGCTCCAAAACAAGAAGAAGCACCTGAACAAGCACCTGA
AGAAGAAATTGAAGAAGAAGCTGAGGAAGCCGCTCCACAGCTGCCATCAAGAAGCTCTGCAGCTCCTCCTC
CGCCTCCA

AAAGTATGGGTGCTTCCGGCCTGGCTGCCTTGGCTTCTCAATTCTTTAAGTCAGGTAACAATTCCCAAGGT
CAGGGACAAGGTCAAGGTCAAGGTCAAGGTCAAGGACAAGGTCAAGGTCAAGGTTCTTTTACTGCTTTGGC
GTCTTTGG

TGTCACCGACGGCATCACCAATAAACTTGCAGACCAAGAGGTTGTTGACCTCATCACCTCTACGGTGAACT
CCTGGGGACTGAAAAAGGCGACTCCTCAATTTGTTGCAGAGGAGACAATCAAATTTATTCAGGCAATAGCC
ACTAAACA

ACCCAGTTGAGATTGCTGAAGCTGTTTTCAACTATGGTGACTTCACCACTATGTTGACTGGTATTCCAGCT
GAACAAGTCACCAGAGTCATCACTGGTGTCCCATGGTACTCCACTAGATTGAGACCAGCCATCTCCAGTGC
TCTATCTA

AAAGGAGTTCTTGGCAATGCCATCATACCGCTTCTTGCACACTCTAGGACACCGAATGGCTCGACCAACTT
CAAGAAGGCAGAGATACGTGTGGGTTTTGCAGACAACTCCACAATACAGCTTGTTTCGCTGATGTCGACGA
CACGACCT

CGTGCCACACTGGTAGAGTCTGTGTCTACGATGTGGTCAACGACAAGGGCTGGAGTGTAAACATGTTCAAC
CACAGAATCCACAAAGTCATTGACGAAGTTCTCCTTGGATACGAGCAGGCTGCCAAGTGCGTTGAACCAGA
GCCCTGCG

TGGTGGAGTCAGATGGAACTGGGGTAACATCTTCGATACGGCCAATTCTCAAACCAGATCTGGCCAAAGCT
CTCAAAGCAGCTTGACCACCTGGACCTGGAGTCTTGGTTCTAGTACCACCGGTAGCTCTGATCTTAACGTG
AACGGCAG

GGTCTTTTCCACTTTCTCTGCCTTCTTGATCACAGGCTTCTCCACTTTCTTTGCAGGAGTTGCCACTGGCT
GGTGCTGGTGCTGCTTCTGCTGCTGCTTCTGCTGCTGCTTCTGCTTCTGCTTCTTGTTGCCCTTGTACAAC
GCTTTCTG

AAACGTCTTTGCTCTTCAGGCGACAAATCTCTCTTCGATCTGCGTCCACTTTGCAGAGGCATTTTAGTTGC
GCCAACCACAGGGCCTGGAGAAGGAGTGTTGGACATACCGATGGGTGAAGGCGAAGCTTGAACTGGAACTT
GTACAGGA

ACCACCATTGCTATTTCCTTGCTAGGTTTGCCAAAGCCGTTGGCGAAGGCAGCACTAGACGGCGAGTCACT
TCCGTCTCTTTGTCCACTTCCTCTTCACCATCAAATCGAAGCCTGCAAGTGCCGTCCCTCTTGGTTCCGAT
GACAATTG

AGAAGAGATCGCCCCAAGCTTACCTTCTAGAAACTCGATCCCAGCTCCAAAACAAGAAGAAGCACCTGAAC
AAGCACCTGAAGAAGAAATTGAAGAAGAAGCTGAGGAAGCCGCTCCACAGCTGCCATCAAGAAGCTCTGCA
GCTCCTCC

TGATATCTTCGATGAAGAAACAAGTTCATTAATATCAACCTCAGCTGCCTCATCGGAGAAAGCCTCGTCCA
CCCTTTCTTCAACTGCACAACCTCATAGGACATCTCACTCTTCCTCTTCATTCGAGCTACCAGTCACTGCT
CCATCATC

GCTTGTCTTCAAAGGAGTTCTTGGCAATGCCATCATACCGCTTCTTGCACACTCTAGGACACCGAATGGCT
CGACCAACTTCAAGAAGGCAGAGATACGTGTGGGTTTTGCAGACAACTCCACAATACAGCTTGTTTCGCTG
ATGTCGAC

GCAAATGCCTACCCAAATCCTAACCTCCGAACAAATAAGAGCTCAACAACAACAGCAGCAATTACAGAAAA
GCCGTTTGAATCAGCCATCCCAGTCGGCTCAACCTCCAGGAGTGAATGTCCCAAATCCTCAAGGTGGGATT
GCTGCAGT

GCTAGGTTTGCCAAAGCCGTTGGCGAAGGCAGCACTAGACGGCGAGTCACTTCCGTCTCTTTGTCCACTTC
CTCTTCACCATCAAATCGAAGCCTGCAAGTGCCGTCCCTCTTGGTTCCGATGACAATTGCAGGATAAAAAG
TGGTCGTT

GATCTCCAGAGCAAAGTTCGTTCGATCGTACTGTTACTCTCTCTCTTTCAAACAGAATTGTCCGAATCGTG
TGACAACAACAGCCTGTTCTCACACACTCTTTTCTTCTAACCAAGGGGGTGGTTTAGTTTAGTAGAACCTC
GTGAAACT

GAAAAAAGAATACGAACCTGAGTCTACCGGTTCCTCCTTTCACGATTTCTTGCAATTGTTTGATGAAACCA
AGGTCCAGTACGGACTGGCACGTGTGTCCCCACCAGGGTCAGACGTTGAGAAGATTATTATCATTGGTTGG
TGTCCTGA

ATGATGACTGGGATGATGATGAAGACGAGGCTGCTCAACCTCCTTTGCCTTCGAGGAATGTTGCGTCAGGA
GCACCAGTGCAAAAAGAAGAGCCTGAACAAGAAGAGATCGCCCCAAGCTTACCTTCTAGAAACTCGATCCC
AGCTCCAA

ATGTGGTCAACGACAAGGGCTGGAGTGTAAACATGTTCAACCACAGAATCCACAAAGTCATTGACGAAGTT
CTCCTTGGATACGAGCAGGCTGCCAAGTGCGTTGAACCAGAGCCCTGCGTAGATTGCTACAACTGGAAGTT
TATTCCAA

AAGGAATTGAAGTACTTCGGTAAGGCTTTGGAGAACCCAACCAGACCATTCTTGGCCATCTTAGGTGGTGC
CAAGGTTGCTGACAAGATTCAATTGATTGACAACTTGTTGGACAAGGTCGACTCTATCATCATTGGTGGTG
GTATGGCT

TCCTCTTCATCCTCTTCTTGTCCACTATGGAATACAAAGACGTCAACGAGAGTGTCATTGTACGTTTGAAG
TGTGGCATGAATGGCTGGCGCAAGTTCCCCAACTGGAGAGGGCAATAAATGATGCGTAGAATTTACGATAG
GGAATTTA

ATACGACTCGTTCGTCGAGAACGGTGAAGATGGTGAGCTAATCGAGTACATGAGGATTAAGTCCGGCGTGG
CCGATGTTCACGGAGTGGACGGCGTGCAGGGTGAGGGTGTCATCGACAGCAAATTGCTGGAAGAGTTCAAG
GACAACGT

CGTATAGCAAAGATCTGAAATGGATACGGATAAGTTAATCTCAGAGGCTGAGTCTCATTTTTCTCAAGGAA
ACCATGCAGAAGCTGTTGCGAAGTTGACATCCGCAGCTCAGTCGAACCCCAATGACGAGCAAATGTCAACT
ATTGAATC

ACCGGAATTTGATAGAGATTCCACTAAGGTTAATTCTCAACAAGAGACAACACCTGGGACATCAGCTGTTC
CAGAGAACCATCATCATGTCTCTCCTCAACCTGCTTCAGTACCACCTCCACAGAATGGACAGTACCAACAG
CACGGCAT

AATACCCAAGTTTCTGCTACTTCCGAAGCTACTAGTACTAGCACTCAAGTGTCTGCTACTTCTGCGACGGC
CACTGCTAGCGAGAGTTCAACCACATCCCAGGTTTCTACTGCTTCCGAAACTATTAGCACTCTCGGTACTC
AAAACTTT

TACCACGTCGCTGGTGGAGCCAGTGGAGTCGACTGAAGGAGTGGAGTCGACTGAGGCGGAACGTGTGGCAG
GGAAGCAGGAGCAGGAGGAGGAGTACCCTGTGGACGCCCACATGCAAAAGTACCTTTCACACCTGAAGAGC
AAGTCTCG

CACAGTGTCTGGTGTCCTTGGGCAAGTTTAGCAACTGTTTCCAGCCTTCACCTTCAAACACATGTCCCAAG
TGTCCACCACACCTTGCACAACATATCTCCACCCTCGCAGGCATTAAAGAATTGTCACGATGATATGTGAT
GGCTCCAG

GGGGTAACATCTTCGATACGGCCAATTCTCAAACCAGATCTGGCCAAAGCTCTCAAAGCAGCTTGACCACC
TGGACCTGGAGTCTTGGTTCTAGTACCACCGGTAGCTCTGATCTTAACGTGAACGGCAGTGATACCGACTT
CCTTACAC

CCCTTCTTTCTGGTGGAGTCAGATGGAACTGGGGTAACATCTTCGATACGGCCAATTCTCAAACCAGATCT
GGCCAAAGCTCTCAAAGCAGCTTGACCACCTGGACCTGGAGTCTTGGTTCTAGTACCACCGGTAGCTCTGA
TCTTAACG

CTATTCAAGCTACTACCAATGACCAAACTTCAAAAACTATTCCAACTCTTGTTGACGCCACATCGTCATTA
CCACCAACATTGAGGTCATCCAGTATGGCACCAACAAGTGGTTCTGATTCAATCTCACACAACTTTACGAG
CCCCCCCT

CTATTTCCTTGCTAGGTTTGCCAAAGCCGTTGGCGAAGGCAGCACTAGACGGCGAGTCACTTCCGTCTCTT
TGTCCACTTCCTCTTCACCATCAAATCGAAGCCTGCAAGTGCCGTCCCTCTTGGTTCCGATGACAATTGCA
GGATAAAA

CTTTGGCATGACGAATCTGCAAACGTCTTTGCTCTTCAGGCGACAAATCTCTCTTCGATCTGCGTCCACTT
TGCAGAGGCATTTTAGTTGCGCCAACCACAGGGCCTGGAGAAGGAGTGTTGGACATACCGATGGGTGAAGG
CGAAGCTT

GTTGTTGAAGTTGCTGCTGTTGCAGTTGCTGTTGTGGTAGTTGTTGTTGGGCGTTTGAGGACACCACAGGC
AAAGTGGTGGTAGGCAACTGTGAGCCAACCAGATTAGGACGGCTAGCTTGTACTGGGAAGGCCGATGGCGA
ACCAGTAG

GCAACACCTGCATATGCGTTGTACCAAGCAATGAAAGCAACAACAACTCCCAGGACACCACCAGCTCTTGT
GACACCAAGTCTATTAGCAAAGTGACCAATAGACAACAGTAGGAAAGTTAATGCTAGTAAGAAGAACAACA
AAAAGAAC

GGCATCGTTGCCATCTCTTTCTATATCACCAACTGTTGACACTGTTTCTGTTCTACAACCAACTACTTCCA
TCGCAACACTAACTTGCACAGACTCACAATGCCAACAGGAGGTATCCACTATCTGTAATGGATCCAACTGT
GACGATGT

GACTGAAGGAGTGGAGTCGACTGAGGCGGAACGTGTGGCAGGGAAGCAGGAGCAGGAGGAGGAGTACCCTG
TGGACGCCCACATGCAAAAGTACCTTTCACACCTGAAGAGCAAGTCTCGGTCGAGGTTCCACCGAAAGGAT
GCTAGCAA

ATTACAGAAAAGCCGTTTGAATCAGCCATCCCAGTCGGCTCAACCTCCAGGAGTGAATGTCCCAAATCCTC
AAGGTGGGATTGCTGCAGTTCAATCAGATTTGGAACAGAATCAACGTGTTCTCGTTCACCTCATGGACATT
TTAGTTTC

AGTCTACCGGTTCCTCCTTTCACGATTTCTTGCAATTGTTTGATGAAACCAAGGTCCAGTACGGACTGGCA
CGTGTGTCCCCACCAGGGTCAGACGTTGAGAAGATTATTATCATTGGTTGGTGTCCTGATTCTGCGCCATT
GAAGACAA

TGTGTCTACGATGTGGTCAACGACAAGGGCTGGAGTGTAAACATGTTCAACCACAGAATCCACAAAGTCAT
TGACGAAGTTCTCCTTGGATACGAGCAGGCTGCCAAGTGCGTTGAACCAGAGCCCTGCGTAGATTGCTACA
ACTGGAAG

CGTCGAAAGTAATAGGTTGTTCAGGAACAAATTCTCTCATTAGTAGAACTGTGTTTGAATGTTTGCGCTGC
TTCAGCACCCAAGTCTTGTCGTGCGAACACAGTACAACTTCAGACTTGTCTTTGTCCAATGACTTAAACCT
TAACTGGT

TGGCGAAGGCAGCACTAGACGGCGAGTCACTTCCGTCTCTTTGTCCACTTCCTCTTCACCATCAAATCGAA
GCCTGCAAGTGCCGTCCCTCTTGGTTCCGATGACAATTGCAGGATAAAAAGTGGTCGTTTCAGGATATCTT
GCCAGAAC

TACATTTAATCCTTCTTGAGGTTTAAAGACGCACTGTTCACACAGTGTCTGGTGTCCTTGGGCAAGTTTAG
CAACTGTTTCCAGCCTTCACCTTCAAACACATGTCCCAAGTGTCCACCACACCTTGCACAACATATCTCCA
CCCTCGCA

CCGCCAATTTTAGTACACAAGGGAACTCGAACTATGTTCCTGAATCAACTGCAAGTGGAAGTTCACAATAC
CAGGACTGGTCAAGCTCTTCTCTTCCGCTGTCACAAACCACTTGGGTTGTCATCAACACAACTAATACACA
AGGGTCTG

ACCCAAATCCTAACCTCCGAACAAATAAGAGCTCAACAACAACAGCAGCAATTACAGAAAAGCCGTTTGAA
TCAGCCATCCCAGTCGGCTCAACCTCCAGGAGTGAATGTCCCAAATCCTCAAGGTGGGATTGCTGCAGTTC
AATCAGAT

TTGGCAATGCCATCATACCGCTTCTTGCACACTCTAGGACACCGAATGGCTCGACCAACTTCAAGAAGGCA
GAGATACGTGTGGGTTTTGCAGACAACTCCACAATACAGCTTGTTTCGCTGATGTCGACGACACGACCTCC
AAAGTTGT

AGCCGTTTGAATCAGCCATCCCAGTCGGCTCAACCTCCAGGAGTGAATGTCCCAAATCCTCAAGGTGGGAT
TGCTGCAGTTCAATCAGATTTGGAACAGAATCAACGTGTTCTCGTTCACCTCATGGACATTTTAGTTTCTC
AAATTAAA

ATCAAATAAAGGTTCTTCTAACAGAGGGTTTGACGTAGGGACTGTCATGTCAATGCTAAGTGGTTCTGGCG
GCGGGAGCCAAAGTATGGGTGCTTCCGGCCTGGCTGCCTTGGCTTCTCAATTCTTTAAGTCAGGTAACAAT
TCCCAAGG

TTCGTTCCAATGTCAAGTTCGATTTCGAAAATCATTTAATTGGTGGTGCTGCTATCGATGCTACAGGTGTT
CCACTTCCAGATGAGGCGCTGGAAGCCTCCAAGAAGGCTGATGCCGTTTTGTTAGGTGCTGTGGGTGGTCC
TAAATGGG

TCCACCAGGTGTTTTCGAATTCGAAAAGTTCGCTGCTGGTACTAAGGCTTTGTTAGACGAAGTTGTCAAGA
GCTCTGCTGCTGGTAACACCGTCATCATTGGTGGTGGTGACACTGCCACTGTCGCTAAGAAGTACGGTGTC
ACTGACAA

TCGAAAAGTTCGCTGCTGGTACTAAGGCTTTGTTAGACGAAGTTGTCAAGAGCTCTGCTGCTGGTAACACC
GTCATCATTGGTGGTGGTGACACTGCCACTGTCGCTAAGAAGTACGGTGTCACTGACAAGATCTCCCATGT
CTCTACTG

CTTCTTGCACACTCTAGGACACCGAATGGCTCGACCAACTTCAAGAAGGCAGAGATACGTGTGGGTTTTGC
AGACAACTCCACAATACAGCTTGTTTCGCTGATGTCGACGACACGACCTCCAAAGTTGTTGGTCAAGTTGG
TGATATCG

ATGATATGTAGTAAATGTTAACCACCATTGCTATTTCCTTGCTAGGTTTGCCAAAGCCGTTGGCGAAGGCA
GCACTAGACGGCGAGTCACTTCCGTCTCTTTGTCCACTTCCTCTTCACCATCAAATCGAAGCCTGCAAGTG
CCGTCCCT

AGTACTTCGGTAAGGCTTTGGAGAACCCAACCAGACCATTCTTGGCCATCTTAGGTGGTGCCAAGGTTGCT
GACAAGATTCAATTGATTGACAACTTGTTGGACAAGGTCGACTCTATCATCATTGGTGGTGGTATGGCTTT
CACCTTCA

GCTGTTTTCAACTATGGTGACTTCACCACTATGTTGACTGGTATTCCAGCTGAACAAGTCACCAGAGTCAT
CACTGGTGTCCCATGGTACTCCACTAGATTGAGACCAGCCATCTCCAGTGCTCTATCTAAGGACGGTATCT
ACACTGCT

GATTGCTGAAGCTGTTTTCAACTATGGTGACTTCACCACTATGTTGACTGGTATTCCAGCTGAACAAGTCA
CCAGAGTCATCACTGGTGTCCCATGGTACTCCACTAGATTGAGACCAGCCATCTCCAGTGCTCTATCTAAG
GACGGTAT

TTGGCACAATGGTAGACACCGGACTCGTTGGTGTGTAAATACGCACCGGTGTTGGGCCTTTCAGTGGCCTT
ATCTCTCAGCACCATCAGCTGCAATGGTGTCAGGGCATCGTTCCACTTCACGTCATTCGATTCGTCACTCA
TTTTCTTG

CCTTCTTGAGGTTTAAAGACGCACTGTTCACACAGTGTCTGGTGTCCTTGGGCAAGTTTAGCAACTGTTTC
CAGCCTTCACCTTCAAACACATGTCCCAAGTGTCCACCACACCTTGCACAACATATCTCCACCCTCGCAGG
CATTAAAG

AAGGAGATCTGCAGTGTCATGTATTCATGCTGCGGCACTTTCACAACGATGATGCTTCCGTCTTCCTGCTT
CTTGGAGAGAAGATCCGGATAGATGGCCTCGACGGCCTCCAGTTCTTCGACCAACTGTTCGTGATCGTCAT
CCATTGAG

CTTCAGGTTCAAGCCCAAGTTATAGGTCAACACCAAGCCAGCTGTGGTCAGTGACACTGGGATAGTGGCTG
CCCCTTCCAAAGACCTGACAGGGCCGGCAGGTAGAACATCTTCACCCAAAAATTTGAGTTCATTGGGTGAT
TTGTAGGC

TTTGGCTCCAGTTGCTAAGGAATTGCAATCATTGTTGGGTAAGGATGTCACCTTCTTGAACGACTGTGTCG
GTCCAGAAGTTGAAGCCGCTGTCAAGGCTTCTGCCCCAGGTTCCGTTATTTTGTTGGAAAACTTGCGTTAC
CACATCGA

TTATCGTAACCAAATTCTTGCTGTTGATGGTGATCCGATGCCTCCTGGTCCATCGACTGTTGATTACCGCT
GTGCCGACTGGTGATCCGGAAACTTCTCATGGGTGTGGGGGATTTAGGATCATCCATGGGAGAGAACTGGT
TAGTGAGC

CACAGGCTTCTCCACTTTCTTTGCAGGAGTTGCCACTGGCTGGTGCTGGTGCTGCTTCTGCTGCTGCTTCT
GCTGCTGCTTCTGCTTCTGCTTCTTGTTGCCCTTGTACAACGCTTTCTGGTACTTCTCGTCCTCGCTGATG
CAAGACGT

AGGGTAAAGTAGTGGTCTCTTTAGAAGTAGGAGATCCGTTGATGGCTGTGTTACTCAATGGTGCCACGGAA
ACCTGGGGAGGTGGTTGCTGTTGTTGAAGTTGCTGCTGTTGCAGTTGCTGTTGTGGTAGTTGTTGTTGGGC
GTTTGAGG

TTTGGTATTTAAGTCAATCTCAGTAGCACCTCGCCTTCCATCTGTTCCCTCTACATCTTCTTCATCTCTGA
GTAAAGATCTGATCTCATCTCTATGAGCTGCAAGATGAGCCCACTCTCTCTGTGTTGTACCATCACTGCTA
GTAAATGT

AACTGTTGACACTGTTTCTGTTCTACAACCAACTACTTCCATCGCAACACTAACTTGCACAGACTCACAAT
GCCAACAGGAGGTATCCACTATCTGTAATGGATCCAACTGTGACGATGTGACTTCAACTGCCACTACTCCT
CCATCTAC

CACCACACCCACACACCACACCCACACACCCACACCCACACACACCACACCCACACCACACCCACACCCAC
ACACCCACACCCTAACACTACCCTAACACTACCCTATTCTAACCCTGATTTTACCTGTCTCCCAACTTACT
CTCCATTA

GATTCGGACGTGATACCGCCTCTTTGGATAATCTTGTAGACCTGGAAGTAGAAATCCTCATTGTAAGGGTC
CTCAGTGACAATTTGAGACAACTGATATCTGGTGATGAAGTCCTTATCACGAGGAGTCATTAAACCTGAGT
ATTTCAAG

ACTCTAGGACACCGAATGGCTCGACCAACTTCAAGAAGGCAGAGATACGTGTGGGTTTTGCAGACAACTCC
ACAATACAGCTTGTTTCGCTGATGTCGACGACACGACCTCCAAAGTTGTTGGTCAAGTTGGTGATATCGTT
CAAATGCT

AGTAACTATTAATACCCAAGTTTCTGCTACTTCCGAAGCTACTAGTACTAGCACTCAAGTGTCTGCTACTT
CTGCGACGGCCACTGCTAGCGAGAGTTCAACCACATCCCAGGTTTCTACTGCTTCCGAAACTATTAGCACT
CTCGGTAC

TTTGAGCACTTCAACAGAATCAGAATCTTCATCTGGATATTTGTCGAAAGGAGTATGCTCAGGTACTGAAT
GTACGCAAGATGTGCCAACACAATCATCCTCACCTGCTTCAACGTTAGCATATTCCCCCTCTGTTTCTACA
TCATCATC

CCGGAGCCATTGTACTTTGGGGTGAAGGCGCCATTGCTGGAGGTGGTGCAGTAGACCACAAAGATTCCATT
GGCTTCAGGTCTAGAGGTCCTGCTGCTGCCGTTCCATCGGTCCTAGGGCCGCTAATTCCTTCTGCAGTAGC
TGCAACAT

CTTGCCCTTGTGAAGCTCGATACCGTTCGAGGTCTTTTCCACTTTCTCTGCCTTCTTGATCACAGGCTTCT
CCACTTTCTTTGCAGGAGTTGCCACTGGCTGGTGCTGGTGCTGCTTCTGCTGCTGCTTCTGCTGCTGCTTC
TGCTTCTG

AATATCCGAACCAATGTCGTCATCCATAGTATCGTTAACATTGTCCACAACATTCTCTCTCAACTTGTCTT
CCTCTACTTCGTCCTCGGCATCAACACTTGGAGGTGATATCAAAGGTAAAGACCATGAATTAAAGGCGCCT
ATAGCCCA

AACTGTCTTTGCATCCAAGAGAAACGCTGACTCACTACTTATGGTCTTCCAAGCAAAACGAGCTTGTGCTG
CAGGGCGACCAATACTTCAATGAAGATGCTGCAAGAACGAGTGACATATACAGTAACAACAACAATGACAG
GTCACTAA

CATCATACCGCTTCTTGCACACTCTAGGACACCGAATGGCTCGACCAACTTCAAGAAGGCAGAGATACGTG
TGGGTTTTGCAGACAACTCCACAATACAGCTTGTTTCGCTGATGTCGACGACACGACCTCCAAAGTTGTTG
GTCAAGTT

AGATGGAACTGGGGTAACATCTTCGATACGGCCAATTCTCAAACCAGATCTGGCCAAAGCTCTCAAAGCAG
CTTGACCACCTGGACCTGGAGTCTTGGTTCTAGTACCACCGGTAGCTCTGATCTTAACGTGAACGGCAGTG
ATACCGAC

ATCAGCCATCCCAGTCGGCTCAACCTCCAGGAGTGAATGTCCCAAATCCTCAAGGTGGGATTGCTGCAGTT
CAATCAGATTTGGAACAGAATCAACGTGTTCTCGTTCACCTCATGGACATTTTAGTTTCTCAAATTAAAGA
AAATGCTA

AGATCTGAAATGGATACGGATAAGTTAATCTCAGAGGCTGAGTCTCATTTTTCTCAAGGAAACCATGCAGA
AGCTGTTGCGAAGTTGACATCCGCAGCTCAGTCGAACCCCAATGACGAGCAAATGTCAACTATTGAATCAT
TAATTCAA

CACCATCCATGCAGCACAACCTCCGATTACAGAAAGTTTCAGTTTCCTTGTCTTTGCCACCAACAAACTGT
AGAATGGAGATGCAGTAGCAGCAACAAAGTCAGGAAGGTTTACTATCCGAGCAGCCGAAGTGGACGATTCT
TTCATCGA

GTGGAGTCGACTGAGGCGGAACGTGTGGCAGGGAAGCAGGAGCAGGAGGAGGAGTACCCTGTGGACGCCCA
CATGCAAAAGTACCTTTCACACCTGAAGAGCAAGTCTCGGTCGAGGTTCCACCGAAAGGATGCTAGCAAGT
ATGTGTCG

AGCCTGAACAAGAAGAGATCGCCCCAAGCTTACCTTCTAGAAACTCGATCCCAGCTCCAAAACAAGAAGAA
GCACCTGAACAAGCACCTGAAGAAGAAATTGAAGAAGAAGCTGAGGAAGCCGCTCCACAGCTGCCATCAAG
AAGCTCTG

GGTAGACACCGGACTCGTTGGTGTGTAAATACGCACCGGTGTTGGGCCTTTCAGTGGCCTTATCTCTCAGC
ACCATCAGCTGCAATGGTGTCAGGGCATCGTTCCACTTCACGTCATTCGATTCGTCACTCATTTTCTTGCT
CTTATTCC

ATGTCGACGATTTCGCTGATCTTTTCGTCTTCGTCGGCAGCTTCCTCGGTGCTTGTCTTCAAAGGAGTTCT
TGGCAATGCCATCATACCGCTTCTTGCACACTCTAGGACACCGAATGGCTCGACCAACTTCAAGAAGGCAG
AGATACGT

CGAAATTAATTCAGGCTTATCCAGATGTAAGATTTGTCAAGTGCGACGTGGACGAATCACCAGATATTGCC
AAAGAGTGTGAAGTGACGGCTATGCCCACCTTTGTTCTTGGCAAGGATGGCCAACTCATCGGCAAGATCAT
TGGAGCTA

TAACCTCCGAACAAATAAGAGCTCAACAACAACAGCAGCAATTACAGAAAAGCCGTTTGAATCAGCCATCC
CAGTCGGCTCAACCTCCAGGAGTGAATGTCCCAAATCCTCAAGGTGGGATTGCTGCAGTTCAATCAGATTT
GGAACAGA

CAATCGAATCTCACCAAGAGGTAGTGTGACATCGTTATTCCAACCCCACAGTTTTGAACCAGATTGAAGAT
TCTCAGACATAGGAGTTTGTAAAGTGGCATTGTACTCCTCCAGTTCCTTAGCAATTGCCTCTATGTTCAAT
CGAGGGAA

GCACTGTTCACACAGTGTCTGGTGTCCTTGGGCAAGTTTAGCAACTGTTTCCAGCCTTCACCTTCAAACAC
ATGTCCCAAGTGTCCACCACACCTTGCACAACATATCTCCACCCTCGCAGGCATTAAAGAATTGTCACGAT
GATATGTG

GTATTATCAACCGGACCCACACTATCCGTTGCCACAGTATATCCCACCACTGAGTACTTCCTCACCTGATC
CAATCGATTCACAGAATCAACACTCTGAAGTACCTCAAGCTGAGACAAAGGTGAGAAATAACGTCTTACCA
CCACACAC

GGCATCACCAATAAACTTGCAGACCAAGAGGTTGTTGACCTCATCACCTCTACGGTGAACTCCTGGGGACT
GAAAAAGGCGACTCCTCAATTTGTTGCAGAGGAGACAATCAAATTTATTCAGGCAATAGCCACTAAACACT
CAGACAAC

GACAACGAGCAATACTGTACCAATGACCACGTTTTGACTATGTGGACTCCTTACAAGGACGGCTACATTTC
ACAAGGAGGCTTTGCCTCCCACGTGAGGCTTCATGAACACTTTGCTATTCAAATACCAGAAAATATTCCAA
GTCCGCTA

TTGCATCGACAAGATTGGCTGTAGGGGGGCCATTTGCTGCGGAGCAGGAGCCGGAGCCATTGTACTTTGGG
GTGAAGGCGCCATTGCTGGAGGTGGTGCAGTAGACCACAAAGATTCCATTGGCTTCAGGTCTAGAGGTCCT
GCTGCTGC

CTTTCAACGTAGATTCAGGTGCCTTGACAGAGGGTAAAGTAGTGGTCTCTTTAGAAGTAGGAGATCCGTTG
ATGGCTGTGTTACTCAATGGTGCCACGGAAACCTGGGGAGGTGGTTGCTGTTGTTGAAGTTGCTGCTGTTG
CAGTTGCT

GATGAAGAAACAAGTTCATTAATATCAACCTCAGCTGCCTCATCGGAGAAAGCCTCGTCCACCCTTTCTTC
AACTGCACAACCTCATAGGACATCTCACTCTTCCTCTTCATTCGAGCTACCAGTCACTGCTCCATCATCCT
CTAGTTTA

TCGTCGGCAGCTTCCTCGGTGCTTGTCTTCAAAGGAGTTCTTGGCAATGCCATCATACCGCTTCTTGCACA
CTCTAGGACACCGAATGGCTCGACCAACTTCAAGAAGGCAGAGATACGTGTGGGTTTTGCAGACAACTCCA
CAATACAG

CTTCAACTAATAAAATGTCACACACTAATAAGATCGCATACGTGTTGAACAATGACACGGAGGAAACAGCC
TCGCCCTCTTCCGTTGGTTGTTTTGACAAGAAACAGCTCACTAAATTACTGATACATACTCTAAAGGAGCT
GGGCTACG

CTTCCTCGGTGCTTGTCTTCAAAGGAGTTCTTGGCAATGCCATCATACCGCTTCTTGCACACTCTAGGACA
CCGAATGGCTCGACCAACTTCAAGAAGGCAGAGATACGTGTGGGTTTTGCAGACAACTCCACAATACAGCT
TGTTTCGC

TGTAGTTCAGATGAGATAGCGACTCAATTTCCACGCCTGGGGCAAAGATGTTTACACAGGGCCCCCAATTG
CTGAACTTGGCAATCGTGTCAATGTGGTCATCAAAGGCCCCTACGGTGATAACGTTTTCTGCAGACGCAGG
TGAGGCCC

TACAGGTCCAAGGAACAACTTCCTCGCCACTTGACTACACCTTTCCAGTTGTCATCCGAAGTGCTGTCGCA
TATTGAAATTGACGACTCGACAGGTCTACGTGAAACAGAGTTGTCATTATTACAGAGTCTCAGACTGCCCA
CCACTCCA

GGAGCAGGAGCCGGAGCCATTGTACTTTGGGGTGAAGGCGCCATTGCTGGAGGTGGTGCAGTAGACCACAA
AGATTCCATTGGCTTCAGGTCTAGAGGTCCTGCTGCTGCCGTTCCATCGGTCCTAGGGCCGCTAATTCCTT
CTGCAGTA

GCCGTTTCTTTGTGGGGTTGATGGCTCCCACTTCAGGTTCAAGCCCAAGTTATAGGTCAACACCAAGCCAG
CTGTGGTCAGTGACACTGGGATAGTGGCTGCCCCTTCCAAAGACCTGACAGGGCCGGCAGGTAGAACATCT
TCACCCAA

CGCTTGAAGAATAATACTGAGGTGAACACACCCACGCCAAAACCCATGGCAGTCTTGACCAGCATGTTGGA
CAGGACAATATCCCACTTAGTGTCGAGGATCGTGGACACGGAGCTGCCATTCTTGTTAGAATCCTTGGAGG
GAGTGCTT

GTATTCATGCTGCGGCACTTTCACAACGATGATGCTTCCGTCTTCCTGCTTCTTGGAGAGAAGATCCGGAT
AGATGGCCTCGACGGCCTCCAGTTCTTCGACCAACTGTTCGTGATCGTCATCCATTGAGCTTTTCTTTCCT
CTCTCTTT

CCAAAGCCGTTGGCGAAGGCAGCACTAGACGGCGAGTCACTTCCGTCTCTTTGTCCACTTCCTCTTCACCA
TCAAATCGAAGCCTGCAAGTGCCGTCCCTCTTGGTTCCGATGACAATTGCAGGATAAAAAGTGGTCGTTTC
AGGATATC

TGGTTCTGGCGGCGGGAGCCAAAGTATGGGTGCTTCCGGCCTGGCTGCCTTGGCTTCTCAATTCTTTAAGT
CAGGTAACAATTCCCAAGGTCAGGGACAAGGTCAAGGTCAAGGTCAAGGTCAAGGACAAGGTCAAGGTCAA
GGTTCTTT

TTGTGATTAGCCTTTCTGTCTGATGACAGGATAGAGTCGACCTCCATTCTGTCTTCTCTGTTATCGTAACC
AAATTCTTGCTGTTGATGGTGATCCGATGCCTCCTGGTCCATCGACTGTTGATTACCGCTGTGCCGACTGG
TGATCCGG

TGTGGGGTTGATGGCTCCCACTTCAGGTTCAAGCCCAAGTTATAGGTCAACACCAAGCCAGCTGTGGTCAG
TGACACTGGGATAGTGGCTGCCCCTTCCAAAGACCTGACAGGGCCGGCAGGTAGAACATCTTCACCCAAAA
ATTTGAGT

GGCATACCGGCGTTATTACTGTTGTGGAACTTTCTCCTTTGAGGACCATTGCTGTTATGGTGATGACTGTG
GTGATGATTACGGTGGTGAGGTGGTTGTTGCTGTTGTGTAGATTGACCATTTTCTTGAGAGGTAGCGTCTT
TTGAATCT

CTGCTGTGTCGTTACAGAACTCGCTGATCGGGCTGCCCAAGGGCAAGTTCTCCACAGACGATGAAGAGGCC
ACGTCCAAGTTCATCCAGGCAAGGGCGATCAGAAGGAAAGTTCTTAGATATCTGCAATTGGTCACGGAGGG
TGAGTTTC

CCACACTTAACGAAATTAATTCAGGCTTATCCAGATGTAAGATTTGTCAAGTGCGACGTGGACGAATCACC
AGATATTGCCAAAGAGTGTGAAGTGACGGCTATGCCCACCTTTGTTCTTGGCAAGGATGGCCAACTCATCG
GCAAGATC

TTCTACAACCAACTACTTCCATCGCAACACTAACTTGCACAGACTCACAATGCCAACAGGAGGTATCCACT
ATCTGTAATGGATCCAACTGTGACGATGTGACTTCAACTGCCACTACTCCTCCATCTACGGTTACTGATAC
TATGACAT

ATCGAGTACATGAGGATTAAGTCCGGCGTGGCCGATGTTCACGGAGTGGACGGCGTGCAGGGTGAGGGTGT
CATCGACAGCAAATTGCTGGAAGAGTTCAAGGACAACGTGAGATACACCTTGGAAAATGACCCTGAGGAAG
GAGCCGAT

TTATTACGAGCGTTGCGTCCTTGGTAAGGTTTAAACTGTGGTGGGAAGCCTTGGTTCTGAGGCATACCGGC
GTTATTACTGTTGTGGAACTTTCTCCTTTGAGGACCATTGCTGTTATGGTGATGACTGTGGTGATGATTAC
GGTGGTGA

TCAGGTAACAATTCCCAAGGTCAGGGACAAGGTCAAGGTCAAGGTCAAGGTCAAGGACAAGGTCAAGGTCA
AGGTTCTTTTACTGCTTTGGCGTCTTTGGCTTCATCTTTCATGAATTCCAACAACAATAATCAGCAAGGTC
AAAATCAA

CTCTGGAGCGTCCTGTAGCCTTTGTTTTAGTAAAGATTCTAGCCCCACCAAACCATGCTTGAATTCACCAA
AGCAAGACATGGTCTCCAAGTGGCAAAATCCAACGTTTTCTTGTTCAACGATAAACTTTAAGGCATCCGAA
TCACAGTC

CGTGTAGTAGTTTGGTATTTAAGTCAATCTCAGTAGCACCTCGCCTTCCATCTGTTCCCTCTACATCTTCT
TCATCTCTGAGTAAAGATCTGATCTCATCTCTATGAGCTGCAAGATGAGCCCACTCTCTCTGTGTTGTACC
ATCACTGC

AGATTCAGGTGCCTTGACAGAGGGTAAAGTAGTGGTCTCTTTAGAAGTAGGAGATCCGTTGATGGCTGTGT
TACTCAATGGTGCCACGGAAACCTGGGGAGGTGGTTGCTGTTGTTGAAGTTGCTGCTGTTGCAGTTGCTGT
TGTGGTAG

CAGTCCAAACAAGAGCGTTTCGTCGAAAGTAATAGGTTGTTCAGGAACAAATTCTCTCATTAGTAGAACTG
TGTTTGAATGTTTGCGCTGCTTCAGCACCCAAGTCTTGTCGTGCGAACACAGTACAACTTCAGACTTGTCT
TTGTCCAA

GAGTGCGTGCATTTCATTCAATCACTAATAAACTATTACTTGGTCCCGGTGACCGAAGAAGACAAGACATG
GTCAAGGGCATTTGTTGGTCATCACGTGAGCGGGGATCTTAAGTGGCTGGAGACTATTGGTGTCAAATTCC
CTGGCAGA

CTTCACCACTATGTTGACTGGTATTCCAGCTGAACAAGTCACCAGAGTCATCACTGGTGTCCCATGGTACT
CCACTAGATTGAGACCAGCCATCTCCAGTGCTCTATCTAAGGACGGTATCTACACTGCTATTCCAAAATAG
AGACATGC

GACCAAACTTCAAAAACTATTCCAACTCTTGTTGACGCCACATCGTCATTACCACCAACATTGAGGTCATC
CAGTATGGCACCAACAAGTGGTTCTGATTCAATCTCACACAACTTTACGAGCCCCCCCTCTAAAACAAGTG
GTAACTAC

TGACAAAATTAAAGTGTCGTTGTAGTTCAGATGAGATAGCGACTCAATTTCCACGCCTGGGGCAAAGATGT
TTACACAGGGCCCCCAATTGCTGAACTTGGCAATCGTGTCAATGTGGTCATCAAAGGCCCCTACGGTGATA
ACGTTTTC

TTTCCAAGTCTTGCATCGACAAGATTGGCTGTAGGGGGGCCATTTGCTGCGGAGCAGGAGCCGGAGCCATT
GTACTTTGGGGTGAAGGCGCCATTGCTGGAGGTGGTGCAGTAGACCACAAAGATTCCATTGGCTTCAGGTC
TAGAGGTC

ACTATCCGTTGCCACAGTATATCCCACCACTGAGTACTTCCTCACCTGATCCAATCGATTCACAGAATCAA
CACTCTGAAGTACCTCAAGCTGAGACAAAGGTGAGAAATAACGTCTTACCACCACACACTTTAACATCAGA
AGAAAACT

GTGATTGTACAGCATCAGTGAAAACACTAGTGGGTTCCGAACTGTCAGCCAAAACACTAGTGGGTTCCGAA
TTGTCAGCCAAAACACTAGTGGGTTCCAAATTGTCAGCCAACACACTGGTGTATTCCGAATTATCAATCAA
GGCACTGG

CATGCTATGATGCCCACTGTGATCTCCAGAGCAAAGTTCGTTCGATCGTACTGTTACTCTCTCTCTTTCAA
ACAGAATTGTCCGAATCGTGTGACAACAACAGCCTGTTCTCACACACTCTTTTCTTCTAACCAAGGGGGTG
GTTTAGTT

TAAGTCTTTGGAAGTCGTAAGGATAAATCTGGAATCTTGTGACCACGTCAAAGACGTTATGTCTTGAAAGT
GTCCCGCATGCACCCTATGGCGGACGAAGGGAGCAAACTGTCTGTCTTTATTAACATCTGGAGTCTTCCAA
ATCTGTAA

AAGGCTTGCTTTTGCAACAGTACAGGTCCAAGGAACAACTTCCTCGCCACTTGACTACACCTTTCCAGTTG
TCATCCGAAGTGCTGTCGCATATTGAAATTGACGACTCGACAGGTCTACGTGAAACAGAGTTGTCATTATT
ACAGAGTC

CGACAAGGGCTGGAGTGTAAACATGTTCAACCACAGAATCCACAAAGTCATTGACGAAGTTCTCCTTGGAT
ACGAGCAGGCTGCCAAGTGCGTTGAACCAGAGCCCTGCGTAGATTGCTACAACTGGAAGTTTATTCCAAGC
AGAGACTG

CCGTGTTGTCCATGTCCATAAGGTCGAGTGCGTTAGATGCCGGTTGAGGTGGTGGAGGTGGAGGCAATGGC
GGTTTTAGGTGTGGAGACTTGGGCTTTGGGGGCAGTGGGGGAGTATTCTGCTCCTGCTGGAGCTGCGTATT
GGGCTTAG

CCATTGCTGGAGGTGGTGCAGTAGACCACAAAGATTCCATTGGCTTCAGGTCTAGAGGTCCTGCTGCTGCC
GTTCCATCGGTCCTAGGGCCGCTAATTCCTTCTGCAGTAGCTGCAACATATGATCTAGCCGTGGCACCGAC
GCCATTAC

TGGTGAGCTAATCGAGTACATGAGGATTAAGTCCGGCGTGGCCGATGTTCACGGAGTGGACGGCGTGCAGG
GTGAGGGTGTCATCGACAGCAAATTGCTGGAAGAGTTCAAGGACAACGTGAGATACACCTTGGAAAATGAC
CCTGAGGA

CCAGCTCCAAAACAAGAAGAAGCACCTGAACAAGCACCTGAAGAAGAAATTGAAGAAGAAGCTGAGGAAGC
CGCTCCACAGCTGCCATCAAGAAGCTCTGCAGCTCCTCCTCCGCCTCCAAGACGAGCAACTCCAGAGAAAA
AGCCAAAG

ATGGTTCTCCTGCAGCTATGTTGCTTTCTATATCAGCGATTGGACCTGGAGTCTGAGAGTCAGCGTCATCG
TTTAAGTCACCGGTTGGCGTTTCTTGTTTCAATTCATCTGTCTGTAATGTGTAGATCAAATTTTTCAATTC
GTTATAAT

CTGAATGTACTCATTGAAGGAGTCGGTGGCCAACTTAACGACAGCGGAGTCTTCAGGGGCCACAGCCTCTT
GTTGGGCGAAAACAGAGGAGGCGAGCAGCAGGGAGGACCATGACAGGACGGCACCAGCAGAAAACTTCATA
ACGGGATA

CACCTGGGACATCAGCTGTTCCAGAGAACCATCATCATGTCTCTCCTCAACCTGCTTCAGTACCACCTCCA
CAGAATGGACAGTACCAACAGCACGGCATGATGACCCCAAACAAAGCTATGGCCTCTAACTGGGCACATTA
CCAACAAC

ATGTTGAATCTCATGTTGTATGTGTACGACCAGTCAACATAGAGTCTCAAGATGTGCAGGGCAAAGATTGC
CGCAACTGACGCAGTGAACGCTTGTGCTATCTTGGGGTACAGGAACATGGAAGTCATTCTTGCAAATATTG
CATGAAAC

GCAATGTTGGACTTGGCAATGTTCCTGTGCACTTTCTTCGTGGTTTTCGTGCACTGGCTGGTGAAAAAGCG
GATCATCAACTGGAAGTGGACTGGGTTCGTTGCAGTGAGCATCTTCGAGTTGGCTTTCATCCCCGTGACGT
TCCCCATT

TGCGTCCACTTTGCAGAGGCATTTTAGTTGCGCCAACCACAGGGCCTGGAGAAGGAGTGTTGGACATACCG
ATGGGTGAAGGCGAAGCTTGAACTGGAACTTGTACAGGACCCATTGGCATTTGATTCATATTCACATTAAC
GCCAGGAG

GTTTAAAGACGCACTGTTCACACAGTGTCTGGTGTCCTTGGGCAAGTTTAGCAACTGTTTCCAGCCTTCAC
CTTCAAACACATGTCCCAAGTGTCCACCACACCTTGCACAACATATCTCCACCCTCGCAGGCATTAAAGAA
TTGTCACG

TCTCCGTTGTATATTGGTACTGAATTCAAGTTCAATTCGCCATGTGTCTCTCTGGTCTCAAATTCTGATTC
AGTCTTGGCGAATCCCACGACGTCCATGTACGGCTTGGACAGTCCAAACAAGAGCGTTTCGTCGAAAGTAA
TAGGTTGT

TTCGTCAAACGCCTCTTCAGAGAGTTGAGGCAAACCGTACTTCAAGTCTTCAGTCATGTCGTGGATGGCAA
ATAGAGGGAATTGTTCCTTCATGTTCAAGTTGCCGGCGTGTCTGCCGAATTTTCTGGCATCGATGCTAACA
AAGTTCAT

GTTGTGGAACTTTCTCCTTTGAGGACCATTGCTGTTATGGTGATGACTGTGGTGATGATTACGGTGGTGAG
GTGGTTGTTGCTGTTGTGTAGATTGACCATTTTCTTGAGAGGTAGCGTCTTTTGAATCTTCCTCACCTACT
GCCGATTC

ACCAGCAACAAGGCCAAAACTATCAGCATCAACAACAGGGTCAGCAGCAGCAACAAGGCCACTCCAGTTCA
TTCTCAGCTTTGGCTTCCATGGCAAGTTCCTACCTGGGCAATAACTCCAATTCAAATTCGAGTTATGGGGG
CCAGCAAC

CCCTTTGTATTAGAACACATTTGAATAACCAACCAAATTGTCCTCTCTGCCTTTTCGAGTTCAGAGAGTCC
TTGCTGAGAAGTGAGTTCCTGGTCAGTGAAATAATTCAAAGTTATACATCCCTACGATCTTCCTTACTAGA
TGCACTAA

ATTTCATTCAATCACTAATAAACTATTACTTGGTCCCGGTGACCGAAGAAGACAAGACATGGTCAAGGGCA
TTTGTTGGTCATCACGTGAGCGGGGATCTTAAGTGGCTGGAGACTATTGGTGTCAAATTCCCTGGCAGAGG
GTATGAAG

AACTACTTCCATCGCAACACTAACTTGCACAGACTCACAATGCCAACAGGAGGTATCCACTATCTGTAATG
GATCCAACTGTGACGATGTGACTTCAACTGCCACTACTCCTCCATCTACGGTTACTGATACTATGACATGT
ACTGGATC

AACGGCAGAGCATGTAGCTATCAGATCCAAATTGCAATATCGTCTAGAGCTGGCACAGGGAGCGGTAGGTT
CAGTACAGATCCCTGTTGTAGAGGTTGATGAACTACCGGAGGGATACGACCGGTCCTGAGAAAAGAGAAAG
GGAACACT

TAAGGCTTTGGAGAACCCAACCAGACCATTCTTGGCCATCTTAGGTGGTGCCAAGGTTGCTGACAAGATTC
AATTGATTGACAACTTGTTGGACAAGGTCGACTCTATCATCATTGGTGGTGGTATGGCTTTCACCTTCAAG
AAGGTTTT

CCAGGTTCCTTGGGTTTGTTGCCATCTGCGTCCTTGGCCTCTTTGCCAGACAAGAACACCGCATTTGGTTT
GTACGAACCATGCCACGGTTCTGCTCCAGATTTGCCAAAGAATAAGGTCAACCCTATCGCCACTATCTTGT
CTGCTGCA

GCCATACAGGAAATGCACGGCGCTTGAAGAATAATACTGAGGTGAACACACCCACGCCAAAACCCATGGCA
GTCTTGACCAGCATGTTGGACAGGACAATATCCCACTTAGTGTCGAGGATCGTGGACACGGAGCTGCCATT
CTTGTTAG

ATTCTAATGATGGAGATGGTGTTGAAAAAGTTCAAAGTGACCAGGGAACCGAGGATCAGCAAATGGAGAAG
GATCAGGACACTGTTATCAACGAAGATAGAGTTGCTGGTGAAAGAAATTTGCCTAACGAAGATTCAACTGA
TGCTGACT

CAACTTTCCTCCCACGTCTTTTCATGAGCTCCTTCTTTCTTAGAATGAAACTCTTGGCAGACTCCTTGTCT
TTGCCTCCCTTCAAGCGCTGGCGCAGTTGTTTCTTCAAGTTGACGTTCTCCTCGTCCATGGTCACACCGTC
CAAATTCA

AACAAGAAGAAGCACCTGAACAAGCACCTGAAGAAGAAATTGAAGAAGAAGCTGAGGAAGCCGCTCCACAG
CTGCCATCAAGAAGCTCTGCAGCTCCTCCTCCGCCTCCAAGACGAGCAACTCCAGAGAAAAAGCCAAAGGA
AAATCCTT

GGATGATGATGAAGACGAGGCTGCTCAACCTCCTTTGCCTTCGAGGAATGTTGCGTCAGGAGCACCAGTGC
AAAAAGAAGAGCCTGAACAAGAAGAGATCGCCCCAAGCTTACCTTCTAGAAACTCGATCCCAGCTCCAAAA
CAAGAAGA

CGTTGTTGTCTTTCCAAGTCTTGCATCGACAAGATTGGCTGTAGGGGGGCCATTTGCTGCGGAGCAGGAGC
CGGAGCCATTGTACTTTGGGGTGAAGGCGCCATTGCTGGAGGTGGTGCAGTAGACCACAAAGATTCCATTG
GCTTCAGG

CTCTTCGATCTGCGTCCACTTTGCAGAGGCATTTTAGTTGCGCCAACCACAGGGCCTGGAGAAGGAGTGTT
GGACATACCGATGGGTGAAGGCGAAGCTTGAACTGGAACTTGTACAGGACCCATTGGCATTTGATTCATAT
TCACATTA

TCTGTCGCAGTTGGCACAATGGTAGACACCGGACTCGTTGGTGTGTAAATACGCACCGGTGTTGGGCCTTT
CAGTGGCCTTATCTCTCAGCACCATCAGCTGCAATGGTGTCAGGGCATCGTTCCACTTCACGTCATTCGAT
TCGTCACT

TGGGTTTGTTGCCATCTGCGTCCTTGGCCTCTTTGCCAGACAAGAACACCGCATTTGGTTTGTACGAACCA
TGCCACGGTTCTGCTCCAGATTTGCCAAAGAATAAGGTCAACCCTATCGCCACTATCTTGTCTGCTGCAAT
GATGTTGA

TCGACGGCCGTCTTGGCTATCTGGTTCCGCAACAGCAACGAGGCGTCTTGCACCAGTTGTTTGCGGTCTAT
GGAAAGGATGTAGCCAACAAAGGTGTCGTACGAGCACTTGGACGAGTCGTACACAATCCGGACCACGTCGC
CTGCCTTC

ACGTACGGATTTCATTTCGTCGCACCAGGGGTCGTATTTAAGAAAGTTCAGGCGAGTAAGCTCCAACAGTG
ACTGAGAAAGTAGCAAAGAGCCAAACAACCCCCATAAGAGCTTTCTGACGCTGACGAATCGAGATTGGCGA
GGTATGAT

TGATGGTTCCGTTGTGGAATACTACCCCGGTTTCAGATCTATGCTGAGACACGCCTTAGCCTTGTCACCCT
TGGGTGCCGAGGGTGAGAGGAAGGTGCACTTGAAGATTGCCAAGGATGGTTCCGGAGTGGGTGCCGCCTTG
TGTGCGCT

CATTTGCTGCGGAGCAGGAGCCGGAGCCATTGTACTTTGGGGTGAAGGCGCCATTGCTGGAGGTGGTGCAG
TAGACCACAAAGATTCCATTGGCTTCAGGTCTAGAGGTCCTGCTGCTGCCGTTCCATCGGTCCTAGGGCCG
CTAATTCC

ACCATTACTAACTCTACCGTTACATTTGGATCGACCAGCAAGTATCCATCTACTAGTGTATCTAACCCAAC
AGAAGCCAGCCAACATGTGAGCTCTAGTGTGAACTCACTCACTGATTTTACTTCAAATTCAACCGAAACCA
TCGCAGTT

ACTGGTAGGATATTCTGCACCAACACCAATACCTACCAAACCTCTCATAACTGTTAACATCCAAAACATGC
CAGGTACAGTAGTACCGTGAGAGGCAGCACACAGAGCACTACCAATAACCAAGATAGCAGTGGCCACAAGG
ATACAAGA

CCAACTAAGGAAGCCCATGATCGATGCATATGTGGCGGAGATGAGGCAGAAGTTTGATGCCTCCTATGGAC
AATCCAGGGCGCAATTGGAAGCCAAACTGGCGCAGGTGGACAGTGAATGGCATATGGTACATGGTGATGTG
CATGCAAA

GAGAACCCAACCAGACCATTCTTGGCCATCTTAGGTGGTGCCAAGGTTGCTGACAAGATTCAATTGATTGA
CAACTTGTTGGACAAGGTCGACTCTATCATCATTGGTGGTGGTATGGCTTTCACCTTCAAGAAGGTTTTGG
AAAACACT

CGGAAGGATCCGATGGAGAATTTCTTCTCAATTTTGTTGTCGATGAACTGTCTCCATGACCTGTTGGAATT
CGAAGTGAATTGTTGGAATGCTGTGGGATTTTTGAACCTCACCAAATACTCCTCTGCGTATGCGAGGTTGA
CCAGCAAA

TGTCTTGAAGCCAATCCCCCACTGTGCTAGCAGTAGCGCTAGCTTGTATGCTGCTGCTGCTGCTGCTTCTT
GATGATGGTGATGTTTCGCCCAGTAGAGTCTCAGTTGCTGATGGTATTGAAGTTGTCATTTCTAGTTTTAT
CGTTAATG

TGGTAGAGTCTGTGTCTACGATGTGGTCAACGACAAGGGCTGGAGTGTAAACATGTTCAACCACAGAATCC
ACAAAGTCATTGACGAAGTTCTCCTTGGATACGAGCAGGCTGCCAAGTGCGTTGAACCAGAGCCCTGCGTA
GATTGCTA

ATATTGGTACTGAATTCAAGTTCAATTCGCCATGTGTCTCTCTGGTCTCAAATTCTGATTCAGTCTTGGCG
AATCCCACGACGTCCATGTACGGCTTGGACAGTCCAAACAAGAGCGTTTCGTCGAAAGTAATAGGTTGTTC
AGGAACAA

CAATGCTAAGTGGTTCTGGCGGCGGGAGCCAAAGTATGGGTGCTTCCGGCCTGGCTGCCTTGGCTTCTCAA
TTCTTTAAGTCAGGTAACAATTCCCAAGGTCAGGGACAAGGTCAAGGTCAAGGTCAAGGTCAAGGACAAGG
TCAAGGTC

ACCTTTGTTGGATTCGGACGTGATACCGCCTCTTTGGATAATCTTGTAGACCTGGAAGTAGAAATCCTCAT
TGTAAGGGTCCTCAGTGACAATTTGAGACAACTGATATCTGGTGATGAAGTCCTTATCACGAGGAGTCATT
AAACCTGA

ACTTGGCAATGTTCCTGTGCACTTTCTTCGTGGTTTTCGTGCACTGGCTGGTGAAAAAGCGGATCATCAAC
TGGAAGTGGACTGGGTTCGTTGCAGTGAGCATCTTCGAGTTGGCTTTCATCCCCGTGACGTTCCCCATTTA
CGTCTACT

GCAAGCACCAAGTCGTGCGACTGAATGTACTCATTGAAGGAGTCGGTGGCCAACTTAACGACAGCGGAGTC
TTCAGGGGCCACAGCCTCTTGTTGGGCGAAAACAGAGGAGGCGAGCAGCAGGGAGGACCATGACAGGACGG
CACCAGCA

GGCGGGAGCCAAAGTATGGGTGCTTCCGGCCTGGCTGCCTTGGCTTCTCAATTCTTTAAGTCAGGTAACAA
TTCCCAAGGTCAGGGACAAGGTCAAGGTCAAGGTCAAGGTCAAGGACAAGGTCAAGGTCAAGGTTCTTTTA
CTGCTTTG

AATCACAAGACATTTTTAATATCATCGATCCGGATGATGACGCCTCTTTCTCCTCTCCACCCCACCGGATC
AACCTTTTGCAGGATAACTTCTCACACGATCTGACTGTGACCTTTCAAGAATGGAAGACTATCCAAGACAC
CACAGACG

TCCAACTCTTGTTGACGCCACATCGTCATTACCACCAACATTGAGGTCATCCAGTATGGCACCAACAAGTG
GTTCTGATTCAATCTCACACAACTTTACGAGCCCCCCCTCTAAAACAAGTGGTAACTACGATGTTTTGACT
TCAAACTC

GAAATCGGTGACTCCATCTTCGACAAGGCTGGTGCTGAAATCGTTCCAAAGTTGATGGAAAAGGCCAAGGC
CAAGGGTGTCGAAGTCGTCTTGCCAGTCGACTTCATCATTGCTGATGCTTTCTCTGCTGATGCCAACACCA
AGACTGTC

ACTATGTTCCTGAATCAACTGCAAGTGGAAGTTCACAATACCAGGACTGGTCAAGCTCTTCTCTTCCGCTG
TCACAAACCACTTGGGTTGTCATCAACACAACTAATACACAAGGGTCTGTAACGTCAACCACATCCCCGGC
TTATGTTT

GTCTTCTCTGTTATCGTAACCAAATTCTTGCTGTTGATGGTGATCCGATGCCTCCTGGTCCATCGACTGTT
GATTACCGCTGTGCCGACTGGTGATCCGGAAACTTCTCATGGGTGTGGGGGATTTAGGATCATCCATGGGA
GAGAACTG

GATAACGTGATCCTGCCACAGGAGTCCATGGAGGTTTCGCCACGGTCGTCTACCACGTCGCTGGTGGAGCC
AGTGGAGTCGACTGAAGGAGTGGAGTCGACTGAGGCGGAACGTGTGGCAGGGAAGCAGGAGCAGGAGGAGG
AGTACCCT

GGAACCGGCTGCAGCAGTCCAGGACTCTGGACCTCTTGGATCTGTTCATCTCACAAGGCGTGAAGTTTACA
GTCATGTACAATGATGACAAGCTGCTGCAAAGGCTGAGAGGGATGGCTACGAACTCTGAAAACAGCGGGTC
CGGTGAAA

CGTAATTTCAGAGGTAGGTGAAGACACAATTGGTTCATTAGTGACACTCTCTTCATAGAAAGAAGAAGAAG
AAGAAGAAGAAGAAGAAGAAGCTGATGTTGGGATATCTGCAGAAGTGAATTGTGAATCAGCTGATGACGAT
ATCCAAAC

CCTGATTAAGTCCCACGCTCTCACCGTACCATCTAACGATGATGAGAACATGACCTGACCCCTTTTCGCAA
ACTGTACAGCAGTAACTGAAGAGGTGTGTTCTTCAAAAGTGGCCAAACAAAACCCTGATGTAATGTCCCAA
ACTTTGAT

CAATTGATTGACAACTTGTTGGACAAGGTCGACTCTATCATCATTGGTGGTGGTATGGCTTTCACCTTCAA
GAAGGTTTTGGAAAACACTGAAATCGGTGACTCCATCTTCGACAAGGCTGGTGCTGAAATCGTTCCAAAGT
TGATGGAA

AAGAGCGTTTCGTCGAAAGTAATAGGTTGTTCAGGAACAAATTCTCTCATTAGTAGAACTGTGTTTGAATG
TTTGCGCTGCTTCAGCACCCAAGTCTTGTCGTGCGAACACAGTACAACTTCAGACTTGTCTTTGTCCAATG
ACTTAAAC

CTTCCAATTTACCGGAATTTGATAGAGATTCCACTAAGGTTAATTCTCAACAAGAGACAACACCTGGGACA
TCAGCTGTTCCAGAGAACCATCATCATGTCTCTCCTCAACCTGCTTCAGTACCACCTCCACAGAATGGACA
GTACCAAC

TCATCATCATCATCATCATTGGCATCGTTGCCATCTCTTTCTATATCACCAACTGTTGACACTGTTTCTGT
TCTACAACCAACTACTTCCATCGCAACACTAACTTGCACAGACTCACAATGCCAACAGGAGGTATCCACTA
TCTGTAAT

AGTGGGGAGGTAGGGTAATGTGAGGGTAGGTTTGGAGACAGGTAAAATCAGGGTTAGAATAGGGTAGTGTT
AGGGTAGTGTGTGGGTGTGGGTGTGTGGGTGTGGTGTGTGGGTGTGGTGTGTGTGGGTGTGGTGTGTGGGT
GTGGGTGT

TGCAAAGGCCATGAAAGTCGAGCCTCTATCAGTAATGGGGTCCGACGCGGTCCAGCCCTCGAAGGGGTCTG
TGGGAATGTCACTCTGCTGGACCGGTTCTGTCTCCTCCTCTGGTTCAACGTACAAGACACCGTCGAGTTCT
GTGAGGAA

TGTACAACGACTTGCCCTTGTGAAGCTCGATACCGTTCGAGGTCTTTTCCACTTTCTCTGCCTTCTTGATC
ACAGGCTTCTCCACTTTCTTTGCAGGAGTTGCCACTGGCTGGTGCTGGTGCTGCTTCTGCTGCTGCTTCTG
CTGCTGCT

GAAGCCAACAAACGGCATTCGGTCACGTACGATAACGTGATCCTGCCACAGGAGTCCATGGAGGTTTCGCC
ACGGTCGTCTACCACGTCGCTGGTGGAGCCAGTGGAGTCGACTGAAGGAGTGGAGTCGACTGAGGCGGAAC
GTGTGGCA

ACACCACACCCACACCACACCCACACACACCACACCCACACACCACACCCACACCCACACACCCACACCCA
CACACCACACACCACACCACACCACACCCACACCCACACCACACCCACACCCACACACCACACCCACACCC
ACACACCA

CACACCACACCCACACACACCACACCCACACACCACACCCACACCCACACACCCACACCCACACACCACAC
ACCACACCACACCACACCCACACCCACACCACACCCACACCCACACACCACACCCACACCCACACACCACA
CACTACCC

CCACACACACCACACCCACACACCACACCCACACCCACACACCCACACCCACACACCACACACCACACCAC
ACCACACCCACACCCACACCACACCCACACCCACACACCACACCCACACCCACACACCACACACTACCCCT
AACACTAC

CTTGGCTTGTCTTAGTCTTAGGCTTAGGCTTCTCTCTGGTGTGGGTGTGTGGGTGTGTGGGTGTGGTGTGG
GTGTGGGTGTGGTGTGGGTGTGGTGGGTGTGGTGTGGTGTGTGTGGGTGTGGTGTGGTGTGTATATATATG
TCACTGTA

GTTGGTCAGGCTTGGCTTGTCTTAGTCTTAGGCTTAGGCTTCTCTCTGGTGTGGGTGTGTGGGTGTGTGGG
TGTGGTGTGGGTGTGGGTGTGGTGTGGGTGTGGTGGGTGTGGTGTGGTGTGTGTGGGTGTGGTGTGGTGTG
TATATATA

CACACCCACACACCACACCCACACCCACACACCCACACCCACACACCACACACCACACCACACCACACCCA
CACCCACACCACACCCACACCCACACACCACACCCACACCCACACACCACACACTACCCCTAACACTACCC
TATTCTAA

TGAGAGACAGGTTGGTCAGGCTTGGCTTGTCTTAGTCTTAGGCTTAGGCTTCTCTCTGGTGTGGGTGTGTG
GGTGTGTGGGTGTGGTGTGGGTGTGGGTGTGGTGTGGGTGTGGTGGGTGTGGTGTGGTGTGTGTGGGTGTG
GTGTGGTG

CACCACACCCACACCCACACACCCACACCCACACACCACACACCACACCACACCACACCCACACCCACACC
ACACCCACACCCACACACCACACCCACACCCACACACCACACACTACCCCTAACACTACCCTATTCTAACC
CTGATTTT

CTTAGTCTTAGGCTTAGGCTTCTCTCTGGTGTGGGTGTGTGGGTGTGTGGGTGTGGTGTGGGTGTGGGTGT
GGTGTGGGTGTGGTGGGTGTGGTGTGGTGTGTGTGGGTGTGGTGTGGTGTGTATATATATGTCACTGTATT
GCATGCTG

CTCAGCAGCAGAGGAAGTCTCAGCAGCAGAAGAAGTCTCAACAGCAGAGGAAGTCTCAACAGCAGAGGAAG
TCTCAACAGCAGAGGAAGTCTCAGCAGCAGAGGAAGTCTCAACAGCAGAAGAAGCCTCCACAATAGAGGCC
TCAGTAGT

ACACCCACACACCCACACCCACACACCACACACCACACCACACCACACCCACACCCACACCACACCCACAC
CCACACACCACACCCACACCCACACACCACACACTACCCCTAACACTACCCTATTCTAACCCTGATTTTAC
CTGTCTCC

TAGAGCAACTACCAGAATCTGTTGTCGATGCCCCAGTCGAAGAACAGCACCAAGAACCACCACAGGCTCCA
GATGCTCCACAAGAACCACAAGTTCCACAGGAATCTGCTCCACAGGAATCTGCTCCACAAGAACCACCAGC
TCCACAAG

AAATCTCAACAGCAGAGGAAGTCTCAACGGCAGAGGAAGTCTCAGCAGCAGAGGAAGTCTCAGCAGCAGAA
GAAGTCTCAACAGCAGAGGAAGTCTCAACAGCAGAGGAAGTCTCAACAGCAGAGGAAGTCTCAGCAGCAGA
GGAAGTCT

AGCAGAGGAAGTCTCAACGGCAGAGGAAGTCTCAGCAGCAGAGGAAGTCTCAGCAGCAGAAGAAGTCTCAA
CAGCAGAGGAAGTCTCAACAGCAGAGGAAGTCTCAACAGCAGAGGAAGTCTCAGCAGCAGAGGAAGTCTCA
ACAGCAGA

ACAGCAGAAGAAATCTCAACAGCAGAGGAAGTCTCAACGGCAGAGGAAGTCTCAGCAGCAGAGGAAGTCTC
AGCAGCAGAAGAAGTCTCAACAGCAGAGGAAGTCTCAACAGCAGAGGAAGTCTCAACAGCAGAGGAAGTCT
CAGCAGCA

CAGAGGAAGTCTCAGCAGCAGAGGAAGTCTCAGCAGCAGAAGAAGTCTCAACAGCAGAGGAAGTCTCAACA
GCAGAGGAAGTCTCAACAGCAGAGGAAGTCTCAGCAGCAGAGGAAGTCTCAACAGCAGAAGAAGCCTCCAC
AATAGAGG

GAGGAAGTCTCAGCAGCAGAAGAAGTCTCAACAGCAGAGGAAGTCTCAACAGCAGAGGAAGTCTCAACAGC
AGAGGAAGTCTCAGCAGCAGAGGAAGTCTCAACAGCAGAAGAAGCCTCCACAATAGAGGCCTCAGTAGTGG
AGGGAATT

GTCTCAACGGCAGAGGAAGTCTCAGCAGCAGAGGAAGTCTCAGCAGCAGAAGAAGTCTCAACAGCAGAGGA
AGTCTCAACAGCAGAGGAAGTCTCAACAGCAGAGGAAGTCTCAGCAGCAGAGGAAGTCTCAACAGCAGAAG
AAGCCTCC

TTGTTTGGGGTCATCATGCCGTGCTGTTGGTACTGTCCATTCTGTGGAGGTGGTACTGAAGCAGGTTGAGG
AGAGACATGATGATGGTTCTCTGGAACAGCTGATGTCCCAGGTGTTGTCTCTTGTTGAGAATTAACCTTAG
TGGAATCT

GAAACTCACGTAGAGCAACTACCAGAATCTGTTGTCGATGCCCCAGTCGAAGAACAGCACCAAGAACCACC
ACAGGCTCCAGATGCTCCACAAGAACCACAAGTTCCACAGGAATCTGCTCCACAGGAATCTGCTCCACAAG
AACCACCA

AGAAGTCTCAACAGCAGAAGAAATCTCAACAGCAGAGGAAGTCTCAACGGCAGAGGAAGTCTCAGCAGCAG
AGGAAGTCTCAGCAGCAGAAGAAGTCTCAACAGCAGAGGAAGTCTCAACAGCAGAGGAAGTCTCAACAGCA
GAGGAAGT

TCTCAACAGCAGAAGTCTCAACAGCAGAAGAAATCTCAACAGCAGAGGAAGTCTCAACGGCAGAGGAAGTC
TCAGCAGCAGAGGAAGTCTCAGCAGCAGAAGAAGTCTCAACAGCAGAGGAAGTCTCAACAGCAGAGGAAGT
CTCAACAG

TTAGAATAAGAAAGCGACACCGGCAGCGACGGCAGCACCGAAGACACCAGCACCCAAAGCGTTGGAAGCAG
CAGCACCAGTGGAGATCTTGGTGCTGGTGGTGTTGGAACCGTTGGTACGGTTGGAACCATTGGTAGAAACG
TTAGCAGC

ATTAAAGAACTTAGAATAAGAAAGCGACACCGGCAGCGACGGCAGCACCGAAGACACCAGCACCCAAAGCG
TTGGAAGCAGCAGCACCAGTGGAGATCTTGGTGCTGGTGGTGTTGGAACCGTTGGTACGGTTGGAACCATT
GGTAGAAA

AGTAGAGGAAGAAGAAGAAGTCTCAACAGCAGAAGTCTCAACAGCAGAAGAAATCTCAACAGCAGAGGAAG
TCTCAACGGCAGAGGAAGTCTCAGCAGCAGAGGAAGTCTCAGCAGCAGAAGAAGTCTCAACAGCAGAGGAA
GTCTCAAC

GGATAATGTCTGAAGCTCAAGAAACTCACGTAGAGCAACTACCAGAATCTGTTGTCGATGCCCCAGTCGAA
GAACAGCACCAAGAACCACCACAGGCTCCAGATGCTCCACAAGAACCACAAGTTCCACAGGAATCTGCTCC
ACAGGAAT

TGAAGCTCAAGAAACTCACGTAGAGCAACTACCAGAATCTGTTGTCGATGCCCCAGTCGAAGAACAGCACC
AAGAACCACCACAGGCTCCAGATGCTCCACAAGAACCACAAGTTCCACAGGAATCTGCTCCACAGGAATCT
GCTCCACA

GAAGAAGAAGTCTCAACAGCAGAAGTCTCAACAGCAGAAGAAATCTCAACAGCAGAGGAAGTCTCAACGGC
AGAGGAAGTCTCAGCAGCAGAGGAAGTCTCAGCAGCAGAAGAAGTCTCAACAGCAGAGGAAGTCTCAACAG
CAGAGGAA

AGAAGTCTCAACAGCAGAGGAAGTCTCAACAGCAGAGGAAGTCTCAACAGCAGAGGAAGTCTCAGCAGCAG
AGGAAGTCTCAACAGCAGAAGAAGCCTCCACAATAGAGGCCTCAGTAGTGGAGGGAATTTCAATAGCAGAA
ATACTAGA

CCCCAGTCGAAGAACAGCACCAAGAACCACCACAGGCTCCAGATGCTCCACAAGAACCACAAGTTCCACAG
GAATCTGCTCCACAGGAATCTGCTCCACAAGAACCACCAGCTCCACAAGAACAAAATGACGTTCCTCCACC
ATCTAATG

GGCCATAGCTTTGTTTGGGGTCATCATGCCGTGCTGTTGGTACTGTCCATTCTGTGGAGGTGGTACTGAAG
CAGGTTGAGGAGAGACATGATGATGGTTCTCTGGAACAGCTGATGTCCCAGGTGTTGTCTCTTGTTGAGAA
TTAACCTT

ACCAGAATCTGTTGTCGATGCCCCAGTCGAAGAACAGCACCAAGAACCACCACAGGCTCCAGATGCTCCAC
AAGAACCACAAGTTCCACAGGAATCTGCTCCACAGGAATCTGCTCCACAAGAACCACCAGCTCCACAAGAA
CAAAATGA

GTTGTCGATGCCCCAGTCGAAGAACAGCACCAAGAACCACCACAGGCTCCAGATGCTCCACAAGAACCACA
AGTTCCACAGGAATCTGCTCCACAGGAATCTGCTCCACAAGAACCACCAGCTCCACAAGAACAAAATGACG
TTCCTCCA

GAGGGTAATTTGAGAGACAGGTTGGTCAGGCTTGGCTTGTCTTAGTCTTAGGCTTAGGCTTCTCTCTGGTG
TGGGTGTGTGGGTGTGTGGGTGTGGTGTGGGTGTGGGTGTGGTGTGGGTGTGGTGGGTGTGGTGTGGTGTG
TGTGGGTG

GGCTTAGGCTTCTCTCTGGTGTGGGTGTGTGGGTGTGTGGGTGTGGTGTGGGTGTGGGTGTGGTGTGGGTG
TGGTGGGTGTGGTGTGGTGTGTGTGGGTGTGGTGTGGTGTGTATATATATGTCACTGTATTGCATGCTGGA
TGGTGTTA

GCGATGGCTCCGCTTGTTCGGTAGGCTCTGGCTTAGATTCTCCAGAACCCTCAGCAGGTGCTTCACCAGGC
TCGACCTGAGCTAACTCCTCACCAACAGTGACAGTGTCCTCTGGTTTGAAATTTAGCTTCGTAACAGTACC
TGATACTG

TAAGGGACAACTTGACCTTATGGACCTCTGATATTTCTGAATCTGGTCAAGAAGATCAACAACAACAACAA
CAACAGCAACAGCAACAGCAACAACAGCAACAACAAGCTCCAGCTGAACAAACTCAAGGTGAACCAACCAA
ATAAGAGC

TCATCATGCCGTGCTGTTGGTACTGTCCATTCTGTGGAGGTGGTACTGAAGCAGGTTGAGGAGAGACATGA
TGATGGTTCTCTGGAACAGCTGATGTCCCAGGTGTTGTCTCTTGTTGAGAATTAACCTTAGTGGAATCTCT
ATCAAATT

GGAAAGAGAAGATTGAGATGGAGATGATTTCGGTGGCGGAGCTTGAGACCCTGGTTGTAGTTGCTGCAGTT
GCTGCACATGACTGGACTCCTCCTGTAACTGCTGCCTTTGCTGTTGAATCTGGTGCTGCTTTAGCTGTTGC
TGTTGTTC

TTAACAGAAGTGGTCTCGATAGTAGAGGAAGAAGAAGAAGTCTCAACAGCAGAAGTCTCAACAGCAGAAGA
AATCTCAACAGCAGAGGAAGTCTCAACGGCAGAGGAAGTCTCAGCAGCAGAGGAAGTCTCAGCAGCAGAAG
AAGTCTCA

CAGCAGCAGAAGAAGTCTCAACAGCAGAGGAAGTCTCAACAGCAGAGGAAGTCTCAACAGCAGAGGAAGTC
TCAGCAGCAGAGGAAGTCTCAACAGCAGAAGAAGCCTCCACAATAGAGGCCTCAGTAGTGGAGGGAATTTC
AATAGCAG

TAGAAATATTGGAAAGAGAAGATTGAGATGGAGATGATTTCGGTGGCGGAGCTTGAGACCCTGGTTGTAGT
TGCTGCAGTTGCTGCACATGACTGGACTCCTCCTGTAACTGCTGCCTTTGCTGTTGAATCTGGTGCTGCTT
TAGCTGTT

GATTGAGATGGAGATGATTTCGGTGGCGGAGCTTGAGACCCTGGTTGTAGTTGCTGCAGTTGCTGCACATG
ACTGGACTCCTCCTGTAACTGCTGCCTTTGCTGTTGAATCTGGTGCTGCTTTAGCTGTTGCTGTTGTTCTC
TCAAGGTT

AGTATTGGTAGGAGATGTGGTTTTCACACTGGCATGACCATGTGTGTGAGTGACAGGGGAAGTGGTAGCAC
TGGAATGACCATGCGTGTGAGTAACAGGAGAAGTGGCAGCTTTGGAAGTGCTGTGGTTATGAGTGGTTGCA
ATACTTGG

CCCAGTTAGAGGCCATAGCTTTGTTTGGGGTCATCATGCCGTGCTGTTGGTACTGTCCATTCTGTGGAGGT
GGTACTGAAGCAGGTTGAGGAGAGACATGATGATGGTTCTCTGGAACAGCTGATGTCCCAGGTGTTGTCTC
TTGTTGAG

GAATTAATGTTAGAAATATTGGAAAGAGAAGATTGAGATGGAGATGATTTCGGTGGCGGAGCTTGAGACCC
TGGTTGTAGTTGCTGCAGTTGCTGCACATGACTGGACTCCTCCTGTAACTGCTGCCTTTGCTGTTGAATCT
GGTGCTGC

TGGTCTCGATAGTAGAGGAAGAAGAAGAAGTCTCAACAGCAGAAGTCTCAACAGCAGAAGAAATCTCAACA
GCAGAGGAAGTCTCAACGGCAGAGGAAGTCTCAGCAGCAGAGGAAGTCTCAGCAGCAGAAGAAGTCTCAAC
AGCAGAGG

GAAGGTAAGGACTTGAAGGAAATCCTATCTGGTTTCCATAACGCTGGCCCTGTTGCTGGTGCTGGTGCTGC
TTCTGGCGCTGCCGCTGCTGGTGGTGACGCTGCTGCTGAAGAAGAAAAAGAAGAAGAAGCTGCTGAAGAAT
CTGACGAC

AAAACCATAAGGATAATGTCTGAAGCTCAAGAAACTCACGTAGAGCAACTACCAGAATCTGTTGTCGATGC
CCCAGTCGAAGAACAGCACCAAGAACCACCACAGGCTCCAGATGCTCCACAAGAACCACAAGTTCCACAGG
AATCTGCT

ACCCACACCCACACACCACACACCACACCACACCACACCCACACCCACACCACACCCACACCCACACACCA
CACCCACACCCACACACCACACACTACCCCTAACACTACCCTATTCTAACCCTGATTTTACCTGTCTCCAA
ACCTACCC

AATGCTCATGAGTATTGGTAGGAGATGTGGTTTTCACACTGGCATGACCATGTGTGTGAGTGACAGGGGAA
GTGGTAGCACTGGAATGACCATGCGTGTGAGTAACAGGAGAAGTGGCAGCTTTGGAAGTGCTGTGGTTATG
AGTGGTTG

TAAACTCAAAATTAAAGAACTTAGAATAAGAAAGCGACACCGGCAGCGACGGCAGCACCGAAGACACCAGC
ACCCAAAGCGTTGGAAGCAGCAGCACCAGTGGAGATCTTGGTGCTGGTGGTGTTGGAACCGTTGGTACGGT
TGGAACCA

TAGGGTAATGGAGGGTAATTTGAGAGACAGGTTGGTCAGGCTTGGCTTGTCTTAGTCTTAGGCTTAGGCTT
CTCTCTGGTGTGGGTGTGTGGGTGTGTGGGTGTGGTGTGGGTGTGGGTGTGGTGTGGGTGTGGTGGGTGTG
GTGTGGTG

ATCACGTTCTCTAGAGTCTCTGTAAGCATCGGATGAAGGATCTCTTGCACCAGCACGACGAGAAGGTGGAA
CATACTGCCCTGGGATGGAACCAGCTCCGCCAACTTGGCCTGCCTTCTCTTCACTGGCGGCCTCGACACCG
CCTTCATT

GTCGGTATCGATCAATTCTTGTTGAAGACTGGTACTTTGACCACCAGTGAAACTGCTCACAACATGAAGGT
CATGAAATTCTCTGTCTCTCCAGTTGTGCAAGTCGCTGTCGAAGTCAAGAACGCTAACGACTTACCAAAAT
TGGTCGAA

ATAATGTCTCTAAACTCAAAATTAAAGAACTTAGAATAAGAAAGCGACACCGGCAGCGACGGCAGCACCGA
AGACACCAGCACCCAAAGCGTTGGAAGCAGCAGCACCAGTGGAGATCTTGGTGCTGGTGGTGTTGGAACCG
TTGGTACG

GAAGAAATCATCGCTGAAGGTCAAAAGAAGTTCGCTACTGTTCCAACTGGTGGTGCTTCTTCTGCTGCTGC
CGGTGCTGCCGGTGCTGCTGCCGGTGGTGATGCTGCTGAAGAAGAAAAGGAAGAAGAAGCTAAGGAAGAAT
CTGATGAT

AAAGCGACACCGGCAGCGACGGCAGCACCGAAGACACCAGCACCCAAAGCGTTGGAAGCAGCAGCACCAGT
GGAGATCTTGGTGCTGGTGGTGTTGGAACCGTTGGTACGGTTGGAACCATTGGTAGAAACGTTAGCAGCTT
GGACAGCG

GGAGATGTGGTTTTCACACTGGCATGACCATGTGTGTGAGTGACAGGGGAAGTGGTAGCACTGGAATGACC
ATGCGTGTGAGTAACAGGAGAAGTGGCAGCTTTGGAAGTGCTGTGGTTATGAGTGGTTGCAATACTTGGAT
GCCCTGCA

CCAGTTGAAGTTGCTGAAGCCGTTTTCAACTACGGTGACTTCACCACCATGTTGACTGGTATTGCCCCAGA
CCAAGTGACCAGAATGATCACCGGTGTTCCATGGTACTCCAGCAGATTAAAGCCAGCCATCTCCAGTGCTC
TATCCAAG

GCTACACTCCCTTAATAAAGTCAACATGAGCAGTAACAACAACACAAACACAGCACCTGCCAATGCAAATT
CTAGCCACCACCACCACCATCACCACCATCACCACCACCATCACGGTCATGGCGGAAGCAACTCGACGCTA
AACAATCC

GTAATAACAATGAAAGTGATGATGGCCTGACAATAGTTGAAGAGAGAACAACACGACCCAGAGTGACACTG
AACCTGCCAGGCGGGGAGAGGCTTGAGGTAACTGCGACGACAACAGACATACCGATAAGAAGGTCATTTGA
ATTCCAAG

CTTGACCTTATGGACCTCTGATATTTCTGAATCTGGTCAAGAAGATCAACAACAACAACAACAACAGCAAC
AGCAACAGCAACAACAGCAACAACAAGCTCCAGCTGAACAAACTCAAGGTGAACCAACCAAATAAGAGCGC
TGAGAAAT

CGGGTACTTGAATGATAATGGTAATAACAATGAAAGTGATGATGGCCTGACAATAGTTGAAGAGAGAACAA
CACGACCCAGAGTGACACTGAACCTGCCAGGCGGGGAGAGGCTTGAGGTAACTGCGACGACAACAGACATA
CCGATAAG

AGATAAGCTTTTAACAGAAGTGGTCTCGATAGTAGAGGAAGAAGAAGAAGTCTCAACAGCAGAAGTCTCAA
CAGCAGAAGAAATCTCAACAGCAGAGGAAGTCTCAACGGCAGAGGAAGTCTCAGCAGCAGAGGAAGTCTCA
GCAGCAGA

AGATTCTGAAATTACAGCCATAAGTACGAAGGAAGGACTACTGAGTGTACAAGGTATGACCTGTGGGTCTT
GTGTTTCTACAGTCACCAAACAAGTGGAAGGCATTGAGGGTGTTGAATCGGTAGTCGTTTCCTTGGTAACG
GAAGAGTG

GAGATGATTTCGGTGGCGGAGCTTGAGACCCTGGTTGTAGTTGCTGCAGTTGCTGCACATGACTGGACTCC
TCCTGTAACTGCTGCCTTTGCTGTTGAATCTGGTGCTGCTTTAGCTGTTGCTGTTGTTCTCTCAAGGTTCT
ATAATAGT

CACTACCACCAAAGAAACAGGTGTTACTACCAAACAAACCACAGCCAACCCAAGTCTAACCGTCTCCACAG
TCGTCCCAGTTTCATCCTCTGCTTCTTCTCATTCCGTTGTCATCAACAGTAACGGTGCTAACGTCGTCGTT
CCAGGTGC

CTAGAGTCTCTGTAAGCATCGGATGAAGGATCTCTTGCACCAGCACGACGAGAAGGTGGAACATACTGCCC
TGGGATGGAACCAGCTCCGCCAACTTGGCCTGCCTTCTCTTCACTGGCGGCCTCGACACCGCCTTCATTAG
TGGCTGGG

TTGGAAGACACGCCCACGGTGATCAATATAAAGCTACGGACACACTGATCCCAGGCCCAGGATCTTTGGAA
CTGGTCTACAAGCCATCCGACCCTACGACTGCTCAACCACAAACTTTGAAAGTGTATGACTACAAGGGCAG
TGGTGTGG

AGTGGGGAGGTAGGGTAATGGAGGGTAATTTGAGAGACAGGTTGGTCAGGCTTGGCTTGTCTTAGTCTTAG
GCTTAGGCTTCTCTCTGGTGTGGGTGTGTGGGTGTGTGGGTGTGGTGTGGGTGTGGGTGTGGTGTGGGTGT
GGTGGGTG

GACGTTGAATATGTTTGCTCTTTCAAGTAAGGTCAATAAACGCTGTCCAGCACCAGCTTCGCCACAGTCAG
CACATCCTTGCTCAATGTTCTTTGGCTGCACCGTTATGTTCCTTGACTTGTTAACATGACTATTATTACTC
TTATTTGC

GGATGAAGGATCTCTTGCACCAGCACGACGAGAAGGTGGAACATACTGCCCTGGGATGGAACCAGCTCCGC
CAACTTGGCCTGCCTTCTCTTCACTGGCGGCCTCGACACCGCCTTCATTAGTGGCTGGGTCTTCTAATGCT
GAAAGTTC

CCTTGGAGCAGAATTAATGTTAGAAATATTGGAAAGAGAAGATTGAGATGGAGATGATTTCGGTGGCGGAG
CTTGAGACCCTGGTTGTAGTTGCTGCAGTTGCTGCACATGACTGGACTCCTCCTGTAACTGCTGCCTTTGC
TGTTGAAT

TGTAAGCATCGGATGAAGGATCTCTTGCACCAGCACGACGAGAAGGTGGAACATACTGCCCTGGGATGGAA
CCAGCTCCGCCAACTTGGCCTGCCTTCTCTTCACTGGCGGCCTCGACACCGCCTTCATTAGTGGCTGGGTC
TTCTAATG

CTTAATAAAGTCAACATGAGCAGTAACAACAACACAAACACAGCACCTGCCAATGCAAATTCTAGCCACCA
CCACCACCATCACCACCATCACCACCACCATCACGGTCATGGCGGAAGCAACTCGACGCTAAACAATCCCA
AGTCGTCC

GGGCTCTTTGGAAGAAATCATCGCTGAAGGTCAAAAGAAGTTCGCTACTGTTCCAACTGGTGGTGCTTCTT
CTGCTGCTGCCGGTGCTGCCGGTGCTGCTGCCGGTGGTGATGCTGCTGAAGAAGAAAAGGAAGAAGAAGCT
AAGGAAGA

AGTCAAGTCCAGGCAGCTCAACTCCATAATATTGCCATTATCAGCTGCCACAAGTACTCTCTCTTGATCTT
GCACGCTGCTTGTTTCAATTACAGCCACCACTGGGTGCTGAAAGTAGTGTCTTGCCACCATATTTCGCTGC
ACGACATA

TGGCTTGTCATCGTGAGTCATTTCTTCAGCGTCGTAGTTGGCACCTCTTCTGGATGGTGGAACCCATGAGG
CAGACTTCCATGGTAGAACACCTTCTTCCCACATGGTGTTGACTTCTTCCAAAGTCAAACCCTTAGTTTCT
GGAACAAC

ATCAATTCTTGTTGAAGACTGGTACTTTGACCACCAGTGAAACTGCTCACAACATGAAGGTCATGAAATTC
TCTGTCTCTCCAGTTGTGCAAGTCGCTGTCGAAGTCAAGAACGCTAACGACTTACCAAAATTGGTCGAAGG
TTTGAAGA

TGTAAGAACCGACCATATCCTTAGTTTCGTGATACTGCTGCTGCTGCTGCTGTTGTTGTTGTTGCTGTTGC
TGCTGTTGTTGCTGTTGCTGCTGAAGTTCGTGATACCTCATTCTTTCCTGCAAAACTTGTTGGTCCTGTGA
GAAGACCT

CTATTACCAAGACGTTGAATATGTTTGCTCTTTCAAGTAAGGTCAATAAACGCTGTCCAGCACCAGCTTCG
CCACAGTCAGCACATCCTTGCTCAATGTTCTTTGGCTGCACCGTTATGTTCCTTGACTTGTTAACATGACT
ATTATTAC

AATCTAAGGGCACTACCACCAAAGAAACAGGTGTTACTACCAAACAAACCACAGCCAACCCAAGTCTAACC
GTCTCCACAGTCGTCCCAGTTTCATCCTCTGCTTCTTCTCATTCCGTTGTCATCAACAGTAACGGTGCTAA
CGTCGTCG

TGAAAGTGATGATGGCCTGACAATAGTTGAAGAGAGAACAACACGACCCAGAGTGACACTGAACCTGCCAG
GCGGGGAGAGGCTTGAGGTAACTGCGACGACAACAGACATACCGATAAGAAGGTCATTTGAATTCCAAGAA
GATCTAGG

TTTGCCAAGAAGCACGAAGCTAGACAAAGATTGGAATCCTACGTTGCCTCCATCGAACAAACTGTCACTGA
CCCAGTCTTGTCTTCTAAATTGAAGAGAGGTTCCAAGTCCAAGATTGAAGCTGCTTTGTCCGATGCTTTGG
CTGCTTTG

TACACATATCATCACGTTCTCTAGAGTCTCTGTAAGCATCGGATGAAGGATCTCTTGCACCAGCACGACGA
GAAGGTGGAACATACTGCCCTGGGATGGAACCAGCTCCGCCAACTTGGCCTGCCTTCTCTTCACTGGCGGC
CTCGACAC

CGGCAGCGACGGCAGCACCGAAGACACCAGCACCCAAAGCGTTGGAAGCAGCAGCACCAGTGGAGATCTTG
GTGCTGGTGGTGTTGGAACCGTTGGTACGGTTGGAACCATTGGTAGAAACGTTAGCAGCTTGGACAGCGGC
AACAGTAG

CAAGGCCAGAACAAGATACAGGCGCCCCTATCAAAATGTCTACTGGTGTCACAAGCTCTCCATTAAGTCCA
TCAGGCTCCACCCCAGAACATTCCACCAAGGTCTTGAACAACGGCGAAGAGGAGTTCATTTGTCACTACTG
TGACGCTA

TCAAAATGTCTACTGGTGTCACAAGCTCTCCATTAAGTCCATCAGGCTCCACCCCAGAACATTCCACCAAG
GTCTTGAACAACGGCGAAGAGGAGTTCATTTGTCACTACTGTGACGCTACTTTCAGGATTAGAGGATATCT
AACGAGAC

TGGACCAGTATTCGCTTTAGAATGCTCATGAGTATTGGTAGGAGATGTGGTTTTCACACTGGCATGACCAT
GTGTGTGAGTGACAGGGGAAGTGGTAGCACTGGAATGACCATGCGTGTGAGTAACAGGAGAAGTGGCAGCT
TTGGAAGT

CCGATCCTCAGCAAGTCCTGGAGACTTTAGGTTTACTTGTTGGTGATGTCTCTCTCTTCCAAAGTGCTGGT
ACGGTCGACCTGGATTCCAGAGGTCATGTCAAGAATAGTGAGAGCAGTTTGAAATCAAAGCTAGCATCTAA
AGCTTATG

CTTCAGAGGCGGCTTCAGAGGTGGCTTCAGAGGCGGTTTCTCCAGAGGCGGCTTCGGTGGCCCCAGAGGTG
GATTTGGTGGTCCAAGAGGTGGTTACGGTGGCTATTCCAGAGGTGGCTACGGTGGCTACTCCAGAGGCGGA
TATGGTGG

CGCCCACGGTGATCAATATAAAGCTACGGACACACTGATCCCAGGCCCAGGATCTTTGGAACTGGTCTACA
AGCCATCCGACCCTACGACTGCTCAACCACAAACTTTGAAAGTGTATGACTACAAGGGCAGTGGTGTGGCC
ATGGCCAT

AGAACAGCACCAAGAACCACCACAGGCTCCAGATGCTCCACAAGAACCACAAGTTCCACAGGAATCTGCTC
CACAGGAATCTGCTCCACAAGAACCACCAGCTCCACAAGAACAAAATGACGTTCCTCCACCATCTAATGCT
CCAATTTA

GATCAAATCAGAGTCTTGTCAAGAGCTCAAGAAGTGCCACACGAGGGTGGGTTCTCGGACCTGCTTTGGTC
AGACCCTGACAATGTAGAGGCTTGGCAAGTTTCCCCTCGTGGTGCAGGATGGCTCTTTGGCAGTAAAGTTG
CTAGAGAG

ACTTGAAGGAAATCCTATCTGGTTTCCATAACGCTGGCCCTGTTGCTGGTGCTGGTGCTGCTTCTGGCGCT
GCCGCTGCTGGTGGTGACGCTGCTGCTGAAGAAGAAAAAGAAGAAGAAGCTGCTGAAGAATCTGACGACGA
CATGGGTT

GGACCCAGTTCAGTTCTAGTTTTACAAATAAATACACGAGCGATGTCGGACTCAGAAGTCAATCAAGAAGC
TAAGCCAGAGGTCAAGCCAGAAGTCAAGCCTGAGACTCACATCAATTTAAAGGTGTCCGATGGATCTTCAG
AGATCTTC

CAACGAACAAGGTAACAGAGTCACCCCATCTTTCGTTGCTTTCACTCCAGAAGAAAGATTGATTGGTGATG
CTGCCAAGAACCAAGCTGCTTTGAACCCAAGAAACACTGTCTTCGATGCTAAGCGTTTGATTGGTAGAAGA
TTCGACGA

CCAGGATCAGGTCTTCCGTAGCGGTATCTAATGCTAACAACAGTCAGGCGAGAGCTAGGCAGCTATTGCAA
GGACAGCAACAGCCTGGCGTGCAGCAGATTTCACAACAACAACATCAACAGAATGAGAAGACTACAGCAAG
CAAAGTTG

ATCTTGGACCCTCACGATTTCGGTATGAAGAGGTATATCCACTTCGCCAACAGACTAACTGGCTGGAACGC
CATCAAAGCCAGAGTCGACCAGTTGAACTTGAACTTGACGGATGACCAAATCAAGGAAGTTACTGCTAAGA
TTAAGAAG

CAGGGTAACGAGTGGGGAGGTAGGGTAATGGAGGGTAATTTGAGAGACAGGTTGGTCAGGCTTGGCTTGTC
TTAGTCTTAGGCTTAGGCTTCTCTCTGGTGTGGGTGTGTGGGTGTGTGGGTGTGGTGTGGGTGTGGGTGTG
GTGTGGGT

ATGCTAACAACAGTCAGGCGAGAGCTAGGCAGCTATTGCAAGGACAGCAACAGCCTGGCGTGCAGCAGATT
TCACAACAACAACATCAACAGAATGAGAAGACTACAGCAAGCAAAGTTGTTCTGACGGTGAACGATCTCAG
TAGCGCTG

CGATTACCACTGAAAGTACCAACTCCAGCACTAATGCTACTACCACTGCCAGCACCAACGTCAGGACTAGT
GCTACTACCACTGCCAGCATCAACGTCAGGACTAGTGCGACTACCACTGAAAGTACCAACTCCAACACTAG
TGCTACTA

ACCCCTTTTCCATACATTCAATGAAGTACGCTGTTAGTTCGTCCAACTTTGCTGCATCAACCTGCAATGAG
GAGCAGATCTCAACCACTGCATCTTCCAATGCCTCAGCATTGACTTTATGTAAATTTTCAAATGACATTAC
TGTGTATC

ACACACCACACACCACACCACACCACACCCACACCCACACCACACCCACACCCACACACCACACCCACACC
CACACACCACACACTACCCCTAACACTACCCTATTCTAACCCTGATTTTACCTGTCTCCAAACCTACCCTC
ACATTACC

AAGAGCTCAAGAAGTGCCACACGAGGGTGGGTTCTCGGACCTGCTTTGGTCAGACCCTGACAATGTAGAGG
CTTGGCAAGTTTCCCCTCGTGGTGCAGGATGGCTCTTTGGCAGTAAAGTTGCTAGAGAGTTTAACCACGTT
AATGGACT

CAGCACGACGAGAAGGTGGAACATACTGCCCTGGGATGGAACCAGCTCCGCCAACTTGGCCTGCCTTCTCT
TCACTGGCGGCCTCGACACCGCCTTCATTAGTGGCTGGGTCTTCTAATGCTGAAAGTTCACTTAAAATTGT
CTTGAATG

CATCTGCTTCTTCCACCGATGTCACTTCCTCCTCTTCCATCTCCACTTCCTCTGGCTCAGTAACTATCACA
TCTTCTGAAGCTCCAGAATCCGACAACGGTACCAGCACAGCTGCACCAACTGAAACCTCAACAGAGGCTCC
AACCACTG

TAAGTACGAAGGAAGGACTACTGAGTGTACAAGGTATGACCTGTGGGTCTTGTGTTTCTACAGTCACCAAA
CAAGTGGAAGGCATTGAGGGTGTTGAATCGGTAGTCGTTTCCTTGGTAACGGAAGAGTGCCATGTTATTTA
TGAACCGT

ATGCAATTATTAAGGGACAACTTGACCTTATGGACCTCTGATATTTCTGAATCTGGTCAAGAAGATCAACA
ACAACAACAACAACAGCAACAGCAACAGCAACAACAGCAACAACAAGCTCCAGCTGAACAAACTCAAGGTG
AACCAACC

GCGACACCTTGCGATGGCTCCGCTTGTTCGGTAGGCTCTGGCTTAGATTCTCCAGAACCCTCAGCAGGTGC
TTCACCAGGCTCGACCTGAGCTAACTCCTCACCAACAGTGACAGTGTCCTCTGGTTTGAAATTTAGCTTCG
TAACAGTA

TTTTCACACTGGCATGACCATGTGTGTGAGTGACAGGGGAAGTGGTAGCACTGGAATGACCATGCGTGTGA
GTAACAGGAGAAGTGGCAGCTTTGGAAGTGCTGTGGTTATGAGTGGTTGCAATACTTGGATGCCCTGCATC
ACCTTTGG

GGCTTCAGAGGTGGCTTCAGAGGCGGTTTCTCCAGAGGCGGCTTCGGTGGCCCCAGAGGTGGATTTGGTGG
TCCAAGAGGTGGTTACGGTGGCTATTCCAGAGGTGGCTACGGTGGCTACTCCAGAGGCGGATATGGTGGCT
CCAGAGGT

CACACTGATCCCAGGCCCAGGATCTTTGGAACTGGTCTACAAGCCATCCGACCCTACGACTGCTCAACCAC
AAACTTTGAAAGTGTATGACTACAAGGGCAGTGGTGTGGCCATGGCCATGTACAATACTGACGAATCCATC
GAAGGGTT

AGCACGAAGCTAGACAAAGATTGGAATCCTACGTTGCCTCCATCGAACAAACTGTCACTGACCCAGTCTTG
TCTTCTAAATTGAAGAGAGGTTCCAAGTCCAAGATTGAAGCTGCTTTGTCCGATGCTTTGGCTGCTTTGCA
AATCGAAG

GATACTGCTGCTGCTGCTGCTGTTGTTGTTGTTGCTGTTGCTGCTGTTGTTGCTGTTGCTGCTGAAGTTCG
TGATACCTCATTCTTTCCTGCAAAACTTGTTGGTCCTGTGAGAAGACCTGCCTTAGTTGCATTTGAACTTG
TTTCAGAG

GGACAAGCCTATTACTATAGTCCTGAATACGGTTATGATGACGAGGATGGTGAAGAAGAGGACCAAGACGA
AGATATGGTGGGTGACAGCGGCACTACAAGACAGGAAGATGGTGGCGAGGACAGCAACTCGAGAAGATATC
CATCATAT

TCTGAGTAGGAGATAAGCTTTTAACAGAAGTGGTCTCGATAGTAGAGGAAGAAGAAGAAGTCTCAACAGCA
GAAGTCTCAACAGCAGAAGAAATCTCAACAGCAGAGGAAGTCTCAACGGCAGAGGAAGTCTCAGCAGCAGA
GGAAGTCT

GGCTCGAGATTCACGAACAAAATGCAAAGGTTTTCACTAGTCACTCACAGATCATTCTCTCACTCCTGTGT
GAAGCCCAAATCTGCATGCTCTTTGGTCAAACCAGTTCATCACTTGGTGAAAATTGATAAGTCAAAGTTAT
CCCCTAGA

ACGTCGAATCCAAATTGAGAGATGACTTGGAAAGATTAAAGAAAATCAGAGCTCATCGTGGTATCAGACAC
TTCTGGGGTTTGCGTGTTAGAGGTCAACACACCAAGACCACTGGTAGAAGAAGAGCTTAAACTGATAAGAT
TATTCAAG

ATTACAGCCATAAGTACGAAGGAAGGACTACTGAGTGTACAAGGTATGACCTGTGGGTCTTGTGTTTCTAC
AGTCACCAAACAAGTGGAAGGCATTGAGGGTGTTGAATCGGTAGTCGTTTCCTTGGTAACGGAAGAGTGCC
ATGTTATT

TTATAAACTACTTCGAGGATTTGCCACAGAGGTCAAGGATAAAGTCTGGTTTCTCGAAGGAGTCACGCAGG
TGCTGCCTCAAGATGTTGACGAATACAGTGGAGGTGGTGGTATGCATATGATGCTAGATTTCCTCGGTGGC
GGATTACC

TGAAGGCGAAGAATCCCACAGTGTCCAAGACTACCAAGAGGCCCACCAGCACCACCAACCACCTGAACCCC
AACCATATTATCCTCCTCCTCCTCCAGGTGAACACATGCACGGTCGCCCACCAATGCACCACCGTCAAGAA
GGAGAACT

TAAGGCTTTGGAAGGTAAGGACTTGAAGGAAATCCTATCTGGTTTCCATAACGCTGGCCCTGTTGCTGGTG
CTGGTGCTGCTTCTGGCGCTGCCGCTGCTGGTGGTGACGCTGCTGCTGAAGAAGAAAAAGAAGAAGAAGCT
GCTGAAGA

CAGTCAGGCGAGAGCTAGGCAGCTATTGCAAGGACAGCAACAGCCTGGCGTGCAGCAGATTTCACAACAAC
AACATCAACAGAATGAGAAGACTACAGCAAGCAAAGTTGTTCTGACGGTGAACGATCTCAGTAGCGCTGTT
GCTGAATA

TCCTGAATACGGTTATGATGACGAGGATGGTGAAGAAGAGGACCAAGACGAAGATATGGTGGGTGACAGCG
GCACTACAAGACAGGAAGATGGTGGCGAGGACAGCAACTCGAGAAGATATCCATCATATTACCATTGTAAT
ACTGCCAG

GGCATGACCATGTGTGTGAGTGACAGGGGAAGTGGTAGCACTGGAATGACCATGCGTGTGAGTAACAGGAG
AAGTGGCAGCTTTGGAAGTGCTGTGGTTATGAGTGGTTGCAATACTTGGATGCCCTGCATCACCTTTGGAA
TGTCTATG

TAGCAATCTATGATCATAAGTCAAAGCCAAGTACATCATTGGTCTTGAGACAATTTGTCCATTAACAGTGA
CAGGTCTCTCTTTGACACCATGCAAGCCTAGGACGGCTGTTTGTGGTGAATTGATGATAGGAGTACCGTAT
AATGAACC

GCGGATCCAAATGATGAGGAAGTTATAACAGAAGTTGCTATGCTTCACGATGCTGTTAACAGGACCAAACA
CGAGAAACATTCACTGTCAAGTTTGTTCTCCAGAGGCAGGTCCCAAAATCTTCAGAGGGCTTTGATTGCAG
CTTCAACG

CCAGGCCCAGGATCTTTGGAACTGGTCTACAAGCCATCCGACCCTACGACTGCTCAACCACAAACTTTGAA
AGTGTATGACTACAAGGGCAGTGGTGTGGCCATGGCCATGTACAATACTGACGAATCCATCGAAGGGTTTG
CTCATTCG

CTCACGATTTCGGTATGAAGAGGTATATCCACTTCGCCAACAGACTAACTGGCTGGAACGCCATCAAAGCC
AGAGTCGACCAGTTGAACTTGAACTTGACGGATGACCAAATCAAGGAAGTTACTGCTAAGATTAAGAAGCT
GGGTGATG

GAAAGAACTCTGATATGGATGTTGAAGATCGTCTCCAGGCCGTTGTCTTGACAGACTCTTATGAAACTAGG
TTTATGCCACTGACAGCTGTCAAGCCAAGGTGTTTGCTGCCACTGGCTAACGTACCTCTCATTGAATACAC
CTTAGAAT

CGTTTTCAACTACGGTGACTTCACCACCATGTTGACTGGTATTGCCCCAGACCAAGTGACCAGAATGATCA
CCGGTGTTCCATGGTACTCCAGCAGATTAAAGCCAGCCATCTCCAGTGCTCTATCCAAGGTCGGTATCTAC
ACTATCGC

TTCCACCGATGTCACTTCCTCCTCTTCCATCTCCACTTCCTCTGGCTCAGTAACTATCACATCTTCTGAAG
CTCCAGAATCCGACAACGGTACCAGCACAGCTGCACCAACTGAAACCTCAACAGAGGCTCCAACCACTGCT
ATCCCAAC

AATGATAATGGTAATAACAATGAAAGTGATGATGGCCTGACAATAGTTGAAGAGAGAACAACACGACCCAG
AGTGACACTGAACCTGCCAGGCGGGGAGAGGCTTGAGGTAACTGCGACGACAACAGACATACCGATAAGAA
GGTCATTT

AACACGACCTTCACCTTAGGCCAGGAACTTACTGAATCTACGGTGAATCACACTAATCATTCTGATGATGA
ACTCCCTGGACACCTCCTTCTCGATTCAGGAGCATCACGAACCCTTATAAGATCTGCTCATCACATACACT
CAGCATCA

CAAACAACTCAAGATCCGTTAGACATTTCAGCTTCCAAAACAGAAGAATGTGAGAAGGTTTTCACTCAGGC
TAATTCTCAACAGCCAACAACACCTCCCTCAGCTGCTGTTCCAGAGAACCATCATCATGCCTCTCCTCAAG
CTGCTCAA

CCCGTGGTGGTTCCAGAGGCGGCTTCGGTGGTAGAGGCGGTTCTCGTGGTGGTGCCCGTGGTGGCTCCAGA
GGTGGTAGAGGTGGCGCTGCTGGTGGTGCCCGTGGTGGTGCCAAGGTCGTTATTGAACCACATAGACATGC
CGGTGTTT

TGGACCTCTGATATTTCTGAATCTGGTCAAGAAGATCAACAACAACAACAACAACAGCAACAGCAACAGCA
ACAACAGCAACAACAAGCTCCAGCTGAACAAACTCAAGGTGAACCAACCAAATAAGAGCGCTGAGAAATAC
AAGGGGAA

TAGAATGCTCGATCAAATCAGAGTCTTGTCAAGAGCTCAAGAAGTGCCACACGAGGGTGGGTTCTCGGACC
TGCTTTGGTCAGACCCTGACAATGTAGAGGCTTGGCAAGTTTCCCCTCGTGGTGCAGGATGGCTCTTTGGC
AGTAAAGT

GGAGCTGGCGAGCTGGCTAGCTGTGGAGACTATTCCACCATGGTACACACTAGGATCCTCGATCTTGGTGA
GCCATGAGTAGAACTTGTTTGGTTCCACCCCTCCGGTTGCCATGTTTATATCTTCCTCAGCAAACTGTTTA
TCTTGCAT

TCGCTGAAGGTCAAAAGAAGTTCGCTACTGTTCCAACTGGTGGTGCTTCTTCTGCTGCTGCCGGTGCTGCC
GGTGCTGCTGCCGGTGGTGATGCTGCTGAAGAAGAAAAGGAAGAAGAAGCTAAGGAAGAATCTGATGATGA
CATGGGTT

CATCCATCTTCACGTTCTGTGCAATTTCTTTAATTGTATCTTGAGGAGGACCAGTCGCAGTGTTGCCAGAG
TACTTTCTACGATCTGCAGACTTCGAACTTGTGTCCGTGTAGACACTAGGGTCGACAGCGTTCATACCGGA
ACTAACAT

GTGCTGTTGGTACTGTCCATTCTGTGGAGGTGGTACTGAAGCAGGTTGAGGAGAGACATGATGATGGTTCT
CTGGAACAGCTGATGTCCCAGGTGTTGTCTCTTGTTGAGAATTAACCTTAGTGGAATCTCTATCAAATTCC
GGTAAATT

CACTTTGATCATGCAATTATTAAGGGACAACTTGACCTTATGGACCTCTGATATTTCTGAATCTGGTCAAG
AAGATCAACAACAACAACAACAACAGCAACAGCAACAGCAACAACAGCAACAACAAGCTCCAGCTGAACAA
ACTCAAGG

AGACATTTCAGCTTCCAAAACAGAAGAATGTGAGAAGGTTTTCACTCAGGCTAATTCTCAACAGCCAACAA
CACCTCCCTCAGCTGCTGTTCCAGAGAACCATCATCATGCCTCTCCTCAAGCTGCTCAAGTACCATTGCCA
CAAAATGG

AGATGGTTGTTGGTAATGTGCCCAGTTAGAGGCCATAGCTTTGTTTGGGGTCATCATGCCGTGCTGTTGGT
ACTGTCCATTCTGTGGAGGTGGTACTGAAGCAGGTTGAGGAGAGACATGATGATGGTTCTCTGGAACAGCT
GATGTCCC

AAGGGTTCATGCCAATCTTCAATGCATATCTCAGATAAACCAAGGAGTGCCCTCAGCGAAACCACTGATCC
TCACGCCTCCTCAGCTAGCCAACCAGCAGCAACCTCCACAGGATATTCTTTCTAAACTCTATCTTCTCTTG
GCAAGAGT

CGTCCACTCTCTCATTGTAATTGTTTAGCACAATTTCGTTCTCCCTGACAGAGTCCTTGTACAGCGCCAGG
TCTCCCGAATCAATGTTCTCCAAGTCGTCGCTGTCGTCAGCTTCAAGACGGTCGTCAGCACCCTCCAACTT
AGCTATGT

TACGAATATTCCAGGATCAGGTCTTCCGTAGCGGTATCTAATGCTAACAACAGTCAGGCGAGAGCTAGGCA
GCTATTGCAAGGACAGCAACAGCCTGGCGTGCAGCAGATTTCACAACAACAACATCAACAGAATGAGAAGA
CTACAGCA

GAGTCTTGTCAAGAGCTCAAGAAGTGCCACACGAGGGTGGGTTCTCGGACCTGCTTTGGTCAGACCCTGAC
AATGTAGAGGCTTGGCAAGTTTCCCCTCGTGGTGCAGGATGGCTCTTTGGCAGTAAAGTTGCTAGAGAGTT
TAACCACG

GTCTTCCGTAGCGGTATCTAATGCTAACAACAGTCAGGCGAGAGCTAGGCAGCTATTGCAAGGACAGCAAC
AGCCTGGCGTGCAGCAGATTTCACAACAACAACATCAACAGAATGAGAAGACTACAGCAAGCAAAGTTGTT
CTGACGGT

CCAATCATTATTGGAAGACACGCCCACGGTGATCAATATAAAGCTACGGACACACTGATCCCAGGCCCAGG
ATCTTTGGAACTGGTCTACAAGCCATCCGACCCTACGACTGCTCAACCACAAACTTTGAAAGTGTATGACT
ACAAGGGC

CCAGTTACCGAATCTAAGGGCACTACCACCAAAGAAACAGGTGTTACTACCAAACAAACCACAGCCAACCC
AAGTCTAACCGTCTCCACAGTCGTCCCAGTTTCATCCTCTGCTTCTTCTCATTCCGTTGTCATCAACAGTA
ACGGTGCT

CGCTGAATTAGAGTTTTGTGTGTAAGAACCGACCATATCCTTAGTTTCGTGATACTGCTGCTGCTGCTGCT
GTTGTTGTTGTTGCTGTTGCTGCTGTTGTTGCTGTTGCTGCTGAAGTTCGTGATACCTCATTCTTTCCTGC
AAAACTTG

TCCTCCGTTGAAATTATTGCCAACGAACAAGGTAACAGAGTCACCCCATCTTTCGTTGCTTTCACTCCAGA
AGAAAGATTGATTGGTGATGCTGCCAAGAACCAAGCTGCTTTGAACCCAAGAAACACTGTCTTCGATGCTA
AGCGTTTG

AAGATCCGTTAGACATTTCAGCTTCCAAAACAGAAGAATGTGAGAAGGTTTTCACTCAGGCTAATTCTCAA
CAGCCAACAACACCTCCCTCAGCTGCTGTTCCAGAGAACCATCATCATGCCTCTCCTCAAGCTGCTCAAGT
ACCATTGC

GAGTTTTGTGTGTAAGAACCGACCATATCCTTAGTTTCGTGATACTGCTGCTGCTGCTGCTGTTGTTGTTG
TTGCTGTTGCTGCTGTTGTTGCTGTTGCTGCTGAAGTTCGTGATACCTCATTCTTTCCTGCAAAACTTGTT
GGTCCTGT

ATTACTATAGTCCTGAATACGGTTATGATGACGAGGATGGTGAAGAAGAGGACCAAGACGAAGATATGGTG
GGTGACAGCGGCACTACAAGACAGGAAGATGGTGGCGAGGACAGCAACTCGAGAAGATATCCATCATATTA
CCATTGTA

CCGCTAATGTTATCTTAGGTTTGTGTCTGTCATTTATGCTTTCTTTCTGTGGTGTTACACAACCTGTCTCA
TTGATGCCTGGCTTCTGGACATTCATGTGGAAGGCTTCCCCATACACATATTTTGTTCAGAATCTGGTCGG
AATTATGC

TTCTCAACAAGAGACAACACCTGGGACATCAGCTGTTCCAGAGAACCATCATCATGTCTCTCCTCAACCTG
CTTCAGTACCACCTCCACAGAATGGACAGTACCAACAGCACGGCATGATGACCCCAAACAAAGCTATGGCC
TCTAACTG

TCCAACAGTACATCTGCTTCTTCCACCGATGTCACTTCCTCCTCTTCCATCTCCACTTCCTCTGGCTCAGT
AACTATCACATCTTCTGAAGCTCCAGAATCCGACAACGGTACCAGCACAGCTGCACCAACTGAAACCTCAA
CAGAGGCT

AATCCCATTGGTTGTCTCCACTGACTTGGAATCTATTCAAAAGACCAAGGAAGCTGTTGCTGCTTTGAAGG
CTGTTGGTGCTCACTCCGACTTGTTGAAGGTCTTGAAGTCCAAGAAATTGAGAGCCGGTAAGGGTAAGTAC
AGAAACAG

ACAAGATACAGGCGCCCCTATCAAAATGTCTACTGGTGTCACAAGCTCTCCATTAAGTCCATCAGGCTCCA
CCCCAGAACATTCCACCAAGGTCTTGAACAACGGCGAAGAGGAGTTCATTTGTCACTACTGTGACGCTACT
TTCAGGAT

AGATACTAAGAGATTCTGAAATTACAGCCATAAGTACGAAGGAAGGACTACTGAGTGTACAAGGTATGACC
TGTGGGTCTTGTGTTTCTACAGTCACCAAACAAGTGGAAGGCATTGAGGGTGTTGAATCGGTAGTCGTTTC
CTTGGTAA

CAAGAACCACCACAGGCTCCAGATGCTCCACAAGAACCACAAGTTCCACAGGAATCTGCTCCACAGGAATC
TGCTCCACAAGAACCACCAGCTCCACAAGAACAAAATGACGTTCCTCCACCATCTAATGCTCCAATTTATG
AAGGCGAA

TTCGCTTTAGAATGCTCATGAGTATTGGTAGGAGATGTGGTTTTCACACTGGCATGACCATGTGTGTGAGT
GACAGGGGAAGTGGTAGCACTGGAATGACCATGCGTGTGAGTAACAGGAGAAGTGGCAGCTTTGGAAGTGC
TGTGGTTA

ACACGAGTAGTGAACATCCTCTCTTCCTCATAAACATTATATTCATGTTCTGTGGAGGTGCATGCAATCTC
ATCACATGTCGCCGTGGCTGTCGTGACTGGAATATTGTGACCGATAGTATTAGAAGAATTGTTGTTATGCT
TGTTATCA

GTCTTCTGTCCCAGTTACCGAATCTAAGGGCACTACCACCAAAGAAACAGGTGTTACTACCAAACAAACCA
CAGCCAACCCAAGTCTAACCGTCTCCACAGTCGTCCCAGTTTCATCCTCTGCTTCTTCTCATTCCGTTGTC
ATCAACAG

GCCACCATACCATCTGGTCACTATTACCAAGACGTTGAATATGTTTGCTCTTTCAAGTAAGGTCAATAAAC
GCTGTCCAGCACCAGCTTCGCCACAGTCAGCACATCCTTGCTCAATGTTCTTTGGCTGCACCGTTATGTTC
CTTGACTT

ATGTTTGCTCTTTCAAGTAAGGTCAATAAACGCTGTCCAGCACCAGCTTCGCCACAGTCAGCACATCCTTG
CTCAATGTTCTTTGGCTGCACCGTTATGTTCCTTGACTTGTTAACATGACTATTATTACTCTTATTTGCTT
TATTGCCC

TCTTGTACAATGGCTTGTCATCGTGAGTCATTTCTTCAGCGTCGTAGTTGGCACCTCTTCTGGATGGTGGA
ACCCATGAGGCAGACTTCCATGGTAGAACACCTTCTTCCCACATGGTGTTGACTTCTTCCAAAGTCAAACC
CTTAGTTT

GTGGCTTCAGAGGCGGTTTCTCCAGAGGCGGCTTCGGTGGCCCCAGAGGTGGATTTGGTGGTCCAAGAGGT
GGTTACGGTGGCTATTCCAGAGGTGGCTACGGTGGCTACTCCAGAGGCGGATATGGTGGCTCCAGAGGTGG
TTACGATA

TTCTTATATTTCCAGCTAGGGAGCCATCACGCCTTGTTTGAGCAACAAGAAGAAGAGACCGATGAAGTTAT
GAGCACCATTTCCAGGAATCCACATCACTCTTTGAGTGTCAAGTCATCATTGGTGATACTTCTAGGTACAA
CTGTGATC

TCTCTTGCACCAGCACGACGAGAAGGTGGAACATACTGCCCTGGGATGGAACCAGCTCCGCCAACTTGGCC
TGCCTTCTCTTCACTGGCGGCCTCGACACCGCCTTCATTAGTGGCTGGGTCTTCTAATGCTGAAAGTTCAC
TTAAAATT

TGGTAATGTGCCCAGTTAGAGGCCATAGCTTTGTTTGGGGTCATCATGCCGTGCTGTTGGTACTGTCCATT
CTGTGGAGGTGGTACTGAAGCAGGTTGAGGAGAGACATGATGATGGTTCTCTGGAACAGCTGATGTCCCAG
GTGTTGTC

GCAACTAGGACAGCAGGCAGTTTAGAATTCTTGATCCAAATGGCTGTTTCAACTTCCTGTGCTGGAGAGGT
AATTTCGCCTTGCTCAACTTCTTCAGGTAACTCTTCTCCCACATCCAAATCTTCGTCACCCAAATCCCAGG
CACCTTCG

GGGTGATATTTAAGGAAGAAATCCACCCTTCTTTAAGGTTTCCACTGCACATGTTGAAATGTGTCTGAATC
TTGATGAGCCCAAAGGCGTGCCACCATACCATCTGGTCACTATTACCAAGACGTTGAATATGTTTGCTCTT
TCAAGTAA

TACGGTGACTTCACCACCATGTTGACTGGTATTGCCCCAGACCAAGTGACCAGAATGATCACCGGTGTTCC
ATGGTACTCCAGCAGATTAAAGCCAGCCATCTCCAGTGCTCTATCCAAGGTCGGTATCTACACTATCGCAA
ACTAGAGA

TAACGACAAGGTCTTAAACCATTTCTATCCAATATAGCACCACAGTAACGTCCATCAGTGAAAGTTAACAA
AGCTGGACCATCCCAAGGTTCCATCAGACATGCAGCCCAGTCGTACCATGCTTTTAGGTCAGAATCCATAT
CCTTATGA

TGGAAGGTAAGGGCTCTTTGGAAGAAATCATCGCTGAAGGTCAAAAGAAGTTCGCTACTGTTCCAACTGGT
GGTGCTTCTTCTGCTGCTGCCGGTGCTGCCGGTGCTGCTGCCGGTGGTGATGCTGCTGAAGAAGAAAAGGA
AGAAGAAG

CATATCAGAGTCCGCTGAGGATGAATCAGTAAATGTATTACCTGACTCAGGTGATGGAGTGCTCAGAGGCG
TTCCAACTGATGATGGATACTGCGGAAACTGTGATTGTGGCCCAGGTGGAAAGTACATAGGCGACATTTGA
TAAGGTGT

CCGGTAACATTATCGGTTTAGTCGGTATCGATCAATTCTTGTTGAAGACTGGTACTTTGACCACCAGTGAA
ACTGCTCACAACATGAAGGTCATGAAATTCTCTGTCTCTCCAGTTGTGCAAGTCGCTGTCGAAGTCAAGAA
CGCTAACG

AATGGCTTATCATCGTGAGCCATTTCTTCAGCGTCGTAGTTGGCACCTCTTCTAGATGGTGGAACCCATGA
GGCAGACTTCCATGGTAGAACACCTTCTTCCCACATGGTGTTGACTTCTTCCAAAGTCAAACCCTTAGTTT
CTGGAACA

CTCCCGAGATTAGAATGCTCGATCAAATCAGAGTCTTGTCAAGAGCTCAAGAAGTGCCACACGAGGGTGGG
TTCTCGGACCTGCTTTGGTCAGACCCTGACAATGTAGAGGCTTGGCAAGTTTCCCCTCGTGGTGCAGGATG
GCTCTTTG

TTAGTTTCGTGATACTGCTGCTGCTGCTGCTGTTGTTGTTGTTGCTGTTGCTGCTGTTGTTGCTGTTGCTG
CTGAAGTTCGTGATACCTCATTCTTTCCTGCAAAACTTGTTGGTCCTGTGAGAAGACCTGCCTTAGTTGCA
TTTGAACT

CTGCTCGGATCAAGGCCAGAACAAGATACAGGCGCCCCTATCAAAATGTCTACTGGTGTCACAAGCTCTCC
ATTAAGTCCATCAGGCTCCACCCCAGAACATTCCACCAAGGTCTTGAACAACGGCGAAGAGGAGTTCATTT
GTCACTAC

CCAATTTACCGGAATTTGATAGAGATTCCACTAAGGTTAATTCTCAACAAGAGACAACACCTGGGACATCA
GCTGTTCCAGAGAACCATCATCATGTCTCTCCTCAACCTGCTTCAGTACCACCTCCACAGAATGGACAGTA
CCAACAGC

TCTCCTTCATGGTACCGTAACGGTAGGCGTTGGTTCCTGAACAAGAATTAGCAATGCAACCACCAATCTGT
GCACCTGGACCAGGGTCACAGCCAAACATCAAACCGTGGTCGCTCAAATAGTCATTCAAATCCTCCCAGGG
TAGACCGG

AAAGAAACAGGTGTTACTACCAAACAAACCACAGCCAACCCAAGTCTAACCGTCTCCACAGTCGTCCCAGT
TTCATCCTCTGCTTCTTCTCATTCCGTTGTCATCAACAGTAACGGTGCTAACGTCGTCGTTCCAGGTGCTT
TAGGTTTG

AAGCTACGGACACACTGATCCCAGGCCCAGGATCTTTGGAACTGGTCTACAAGCCATCCGACCCTACGACT
GCTCAACCACAAACTTTGAAAGTGTATGACTACAAGGGCAGTGGTGTGGCCATGGCCATGTACAATACTGA
CGAATCCA

AACTTGCATAATCTTCAAAGTACACATATCATCACGTTCTCTAGAGTCTCTGTAAGCATCGGATGAAGGAT
CTCTTGCACCAGCACGACGAGAAGGTGGAACATACTGCCCTGGGATGGAACCAGCTCCGCCAACTTGGCCT
GCCTTCTC

TACTGGTGTCACAAGCTCTCCATTAAGTCCATCAGGCTCCACCCCAGAACATTCCACCAAGGTCTTGAACA
ACGGCGAAGAGGAGTTCATTTGTCACTACTGTGACGCTACTTTCAGGATTAGAGGATATCTAACGAGACAT
ATTAAGAA

CACGTTCTGTGCAATTTCTTTAATTGTATCTTGAGGAGGACCAGTCGCAGTGTTGCCAGAGTACTTTCTAC
GATCTGCAGACTTCGAACTTGTGTCCGTGTAGACACTAGGGTCGACAGCGTTCATACCGGAACTAACATTA
CTCTTTGG

GTAACAGCTTCTCTACCATCTAGCAATCTATGATCATAAGTCAAAGCCAAGTACATCATTGGTCTTGAGAC
AATTTGTCCATTAACAGTGACAGGTCTCTCTTTGACACCATGCAAGCCTAGGACGGCTGTTTGTGGTGAAT
TGATGATA

CTACTACCACCGAAAGTACCGACTCCAACACTAGTGCTACTACCACTGCTAGCACCAACTCCAGCACTAAT
GCCACTACCACTGCTAGCACCAACTCCAGCACTAATGCCACTACCACTGAAAGTACCAACGCTAGTGCCAA
GGAGGACG

GCGGTATCTAATGCTAACAACAGTCAGGCGAGAGCTAGGCAGCTATTGCAAGGACAGCAACAGCCTGGCGT
GCAGCAGATTTCACAACAACAACATCAACAGAATGAGAAGACTACAGCAAGCAAAGTTGTTCTGACGGTGA
ACGATCTC

CTTTGAAGAGGGCTCGAGATTCACGAACAAAATGCAAAGGTTTTCACTAGTCACTCACAGATCATTCTCTC
ACTCCTGTGTGAAGCCCAAATCTGCATGCTCTTTGGTCAAACCAGTTCATCACTTGGTGAAAATTGATAAG
TCAAAGTT

CACAGGCTCCAGATGCTCCACAAGAACCACAAGTTCCACAGGAATCTGCTCCACAGGAATCTGCTCCACAA
GAACCACCAGCTCCACAAGAACAAAATGACGTTCCTCCACCATCTAATGCTCCAATTTATGAAGGCGAAGA
ATCCCACA

GTGACTTTATCCTTGGAGCAGAATTAATGTTAGAAATATTGGAAAGAGAAGATTGAGATGGAGATGATTTC
GGTGGCGGAGCTTGAGACCCTGGTTGTAGTTGCTGCAGTTGCTGCACATGACTGGACTCCTCCTGTAACTG
CTGCCTTT

GTGTTACTACCAAACAAACCACAGCCAACCCAAGTCTAACCGTCTCCACAGTCGTCCCAGTTTCATCCTCT
GCTTCTTCTCATTCCGTTGTCATCAACAGTAACGGTGCTAACGTCGTCGTTCCAGGTGCTTTAGGTTTGGC
TGGTGTTG

TGTGTGTGAGTGACAGGGGAAGTGGTAGCACTGGAATGACCATGCGTGTGAGTAACAGGAGAAGTGGCAGC
TTTGGAAGTGCTGTGGTTATGAGTGGTTGCAATACTTGGATGCCCTGCATCACCTTTGGAATGTCTATGCG
CAGTGGGG

TAAAGAAGTCCAAACAACTCAAGATCCGTTAGACATTTCAGCTTCCAAAACAGAAGAATGTGAGAAGGTTT
TCACTCAGGCTAATTCTCAACAGCCAACAACACCTCCCTCAGCTGCTGTTCCAGAGAACCATCATCATGCC
TCTCCTCA

ATTAACTAATTCATCACCATTCCAAATACTTTCATTTATTTCATCCTTTTCTAGTAGCAAAAGTGACTTGC
CAGCGTCCTTCTGAGGTGGCATTCCTGCTGGAACTGGTGTGGTGGTTACGTTTGATAAATCATGTTGTGAG
ATGCTATT

CTTCTAGAAGACTTGGATGCTAGTGTAATTCTTTTAGGTTGACCATTACTTTGAGTATCACGCTCCCCAGC
AGCAGCAGCTTCTTCATCCTGCTCAGACATTTGTCCTTCATCAGTGCTATTATCTGAATCACGTACGTTAT
TCATCATA

ATATCAGAGTCCGCTGAGGATGAATCAGTAAATGTATTACCTGACTCAGGTGATGGAGTGCTCAGAGGCGT
TCCAACTGATGATGGATACTGCGGAAACTGTGATTGTGGCCCAGGTGGAAAGTACATAGGCGACATTTGAT
AAGGTGTA

GCCTCCAAAGTTTGATCCAAATGAAGTTAAGTACTTGTACTTGAGAGCTGTCGGTGGTGAAGTCGGTGCTT
CCGCCGCCTTGGCTCCAAAGATCGGTCCATTGGGTTTATCCCCAAAGAAGGTTGGTGAAGATATCGCCAAG
GCCACCAA

CCAGGAACTTACTGAATCTACGGTGAATCACACTAATCATTCTGATGATGAACTCCCTGGACACCTCCTTC
TCGATTCAGGAGCATCACGAACCCTTATAAGATCTGCTCATCACATACACTCAGCATCATCTAATCCTGAC
ATAAACGT

AGTTCCTAAGGGAACTTCTGACTATCAAGCGAAGTGGTACTTGGATGACGTCATTGACGCAAATGAGGAAG
AAGAGGCAGAACAGACCAATGGAAAGGATGAGACAATGATGGAAATTGATGATGAAATGATGGTGGAGCAA
GACAATGA

CTAGAATAGAGGTTCTTAGCGAACAAATAACCGAATAGAGTAGCTGAAGAGGCGATGACAGAGTATTTTAA
CCACTGGCTGCTGCTGTCGGTGCTGCCTGTGCCTGTTGAGGCGTATCTGTAGCATGATCTTCTCACCAGCC
TTCCTCTA

CAAATTGAGAGATGACTTGGAAAGATTAAAGAAAATCAGAGCTCATCGTGGTATCAGACACTTCTGGGGTT
TGCGTGTTAGAGGTCAACACACCAAGACCACTGGTAGAAGAAGAGCTTAAACTGATAAGATTATTCAAGCT
TAATCTTT

ATGGCGCCCACCTCTGTAGATTCAGATGCCGATGCACAATTAGCGGAGAGGTTGCAAAGGGAAGCTTACCA
ACAACAACAGCCTGACCAAGATTATGTCAGGCCACCTGATGAGGCAAGACATGAAGTACTTACTGAAACTT
CAGGATTC

ATCTTCAAAGTACACATATCATCACGTTCTCTAGAGTCTCTGTAAGCATCGGATGAAGGATCTCTTGCACC
AGCACGACGAGAAGGTGGAACATACTGCCCTGGGATGGAACCAGCTCCGCCAACTTGGCCTGCCTTCTCTT
CACTGGCG

TATTGTTATCATATGAATTATGATAATATCCGGAGTTGTTCCTGGAGTGAGTTCTACTTCTGCTGCCACCT
GCACTCATACCAGGTGAATTACCGTTTGAGTTGTAATGAGGGTGAGGCGGTTGAGGCGTGAAATGATTGTT
AGAGCCAG

CACTGCTGTGGGTACGGCCCATTCTGTGGAGGTGGTACTGAAGCAGGTTGAGGAGAGGCATGATGGGGGTT
CTCTGGAACAGCTGATGAAGCAGGTGTTGTTGTCTGTTGAGAGTTAGCCTTAGTGGAAGCCTTCTCACATT
CTTCTGTT

AAAATTGGTGGTCTCGACAACCTAACAACCATTGGCGGTACTTTGGAAGTTGTTGGTAACTTCACCTCCTT
GAACCTAGACTCTTTGAAGTCTGTCAAGGGTGGCGCAGATGTCGAATCAAAGTCAAGCAATTTCTCCTGTA
ATGCTTTG

GGTACCGTAACGGTAGGCGTTGGTTCCTGAACAAGAATTAGCAATGCAACCACCAATCTGTGCACCTGGAC
CAGGGTCACAGCCAAACATCAAACCGTGGTCGCTCAAATAGTCATTCAAATCCTCCCAGGGTAGACCGGCC
TGCACGGT

GACCATATCCTTAGTTTCGTGATACTGCTGCTGCTGCTGCTGTTGTTGTTGTTGCTGTTGCTGCTGTTGTT
GCTGTTGCTGCTGAAGTTCGTGATACCTCATTCTTTCCTGCAAAACTTGTTGGTCCTGTGAGAAGACCTGC
CTTAGTTG

AATGCGACTACCAACTCCAGCACTAATGCTACTACCACTGCCAGCACCAACGTCAGGACTAGTGCTACTAC
CACTGCCAGCATCAACGTCAGGACTAGTGCGATTACCACTGAAAGTACCAACTCCAGCACTAATGCTACTA
CCACTGCC

CTGAGTGTACAAGGTATGACCTGTGGGTCTTGTGTTTCTACAGTCACCAAACAAGTGGAAGGCATTGAGGG
TGTTGAATCGGTAGTCGTTTCCTTGGTAACGGAAGAGTGCCATGTTATTTATGAACCGTCCAAGACAACGC
TAGAAACC

AAGCCCCTGAGTCTTCTGTCCCAGTTACCGAATCTAAGGGCACTACCACCAAAGAAACAGGTGTTACTACC
AAACAAACCACAGCCAACCCAAGTCTAACCGTCTCCACAGTCGTCCCAGTTTCATCCTCTGCTTCTTCTCA
TTCCGTTG

TCTTTGGGCAAGGACACCGGCGTTGATTTCATCAGCCTGTTTGAGTACCCATGGATCCTCTGGTGAAACCT
TGTTGATCTTGGCGATGGAGACACAGGCCTCTTCATGTCTCTCACATTCAATCAGATATCTTGGGGACTCT
GGAACTAA

GGCAACCATGGAAAGAACTCTGATATGGATGTTGAAGATCGTCTCCAGGCCGTTGTCTTGACAGACTCTTA
TGAAACTAGGTTTATGCCACTGACAGCTGTCAAGCCAAGGTGTTTGCTGCCACTGGCTAACGTACCTCTCA
TTGAATAC

CTTTGGAAGCCTGTCATAAAGGTTGGGGTCAATCTATTATCATTGGTGTGGCTGCCGCTGGTGAAGAAATT
TCTACAAGGCCGTTCCAGCTGGTCACTGGTAGAGTGTGGAAAGGCTCTGCTTTTGGTGGCATCAAAGGTAG
ATCTGAAA

CGGTATGAAGAGGTATATCCACTTCGCCAACAGACTAACTGGCTGGAACGCCATCAAAGCCAGAGTCGACC
AGTTGAACTTGAACTTGACGGATGACCAAATCAAGGAAGTTACTGCTAAGATTAAGAAGCTGGGTGATGTC
AGATCGCT

GGTCACGTTGACTTCTCCTCTGAAGTTACTGCCGCTTTACGTGTCACTGACGGTGCTTTGGTTGTCGTCGA
CACCATTGAAGGTGTCTGTGTCCAAACCGAAACTGTTTTGAGACAAGCTTTGGGTGAGAGAATCAAGCCTG
TTGTTGTT

CAGGACGATAGAGGTAGCACAATGGCGGTAGCGCCACGCCTACCAGGATCAACAAGTCTACTAGAGTGCAG
GGGTACTCAACAGTGTCGATCTGGATGAGCGACAGCTTCCTCTTCTCCGCAGCGATGCGGAACATCTTGTT
GAAGATTT

ATGGAACAGGATCTACTTCTGGTTCTTGTTCAGCCTTTGCTCCCTCATCTGTCATTTCCACGTCTTCACCT
TCTACTTTCACTTCCTTTTCAGCTTCGTGTTGTCTGTGAGCTTCGATAGAATCCTTGATAGCGTATTTAGC
AGCCCTCT

ATTCTCAAGAAGAGACAACACCTGGGACATCAGCTGTTCCAGAGAACCATCATCATGTCTCTCCTCAACCT
GCTTCAGTACCACCTCCACAGAATGGACAGTACCAACAGCACGGCATGATGACCCCAAACAAAGCTATGGC
CTCTAACT

CAGTGGCTGATGGACCAGTATTCGCTTTAGAATGCTCATGAGTATTGGTAGGAGATGTGGTTTTCACACTG
GCATGACCATGTGTGTGAGTGACAGGGGAAGTGGTAGCACTGGAATGACCATGCGTGTGAGTAACAGGAGA
AGTGGCAG

GTGTCACTGACGGTGCTTTGGTTGTCGTCGACACCATTGAAGGTGTCTGTGTCCAAACCGAAACTGTTTTG
AGACAAGCTTTGGGTGAGAGAATCAAGCCTGTTGTTGTTATCAACAAGGTCGACAGAGCTTTGTTGGAATT
GCAAGTTT

TAGTCAATATGCCTCCAAAGTTTGATCCAAATGAAGTTAAGTACTTGTACTTGAGAGCTGTCGGTGGTGAA
GTCGGTGCTTCCGCCGCCTTGGCTCCAAAGATCGGTCCATTGGGTTTATCCCCAAAGAAGGTTGGTGAAGA
TATCGCCA

CAGAAGAATGTGAGAAGGTTTTCACTCAGGCTAATTCTCAACAGCCAACAACACCTCCCTCAGCTGCTGTT
CCAGAGAACCATCATCATGCCTCTCCTCAAGCTGCTCAAGTACCATTGCCACAAAATGGGCCGTACCCACA
GCAGCGAA

GACCCAAAGGAACCGGATTAGCGAACTGGTGCGGAGTATACTTTGCACAGTTTCCTTCCTCCCCATCCCCT
CTGTCTAGTGCATTGAGCAAATCTCTGCGCCTGTAGGTGGAGGATCCTAGAGTAATGAAGTCTCTGTCGGA
GTATATCT

GAATGTCTAGCGAAGATAAGAAACCTGTCGTCGACAAGAAGGAAGAGGCTGCTCCAAAGCCACCATCCTCT
GCTGTCTTCTCCATGTTTGGTGGTAAGAAGGCCGAAAAGCCAGAAACCAAGAAAGACGAAGAAGATACCAA
GGAGGAAA

ACCGGATCAGATACACTATTGTGGTGTAATCTGGACCTTGACTGTCTAAACAACTCCTCTTGTTGTCGTAG
CAGCTCTTCTTCCGAGAGACCTGACTTCTTGAACTTGGAATCCCTAGTTTCTTTCACCTTCTGGGAACCCT
TAAAATTC

TACTTACGAATCCTCCGTTGAAATTATTGCCAACGAACAAGGTAACAGAGTCACCCCATCTTTCGTTGCTT
TCACTCCAGAAGAAAGATTGATTGGTGATGCTGCCAAGAACCAAGCTGCTTTGAACCCAAGAAACACTGTC
TTCGATGC

TGTGTAGTTGCAGTGGCTGATGGACCAGTATTCGCTTTAGAATGCTCATGAGTATTGGTAGGAGATGTGGT
TTTCACACTGGCATGACCATGTGTGTGAGTGACAGGGGAAGTGGTAGCACTGGAATGACCATGCGTGTGAG
TAACAGGA

TGGAGAGGTAGGAGAGTATGTTGGGGAAGTTGGAGAAAAGCCTGGTGAGGAGACTCCAAATCCGGGAGATG
TAGGTGCTTCACCATAAGCACCAAATGGAGACGTGGCTTCACCATAATCAGCACCACCGTACGCTGTAAAT
CCTCCAGC

CATCTGGTCACTATTACCAAGACGTTGAATATGTTTGCTCTTTCAAGTAAGGTCAATAAACGCTGTCCAGC
ACCAGCTTCGCCACAGTCAGCACATCCTTGCTCAATGTTCTTTGGCTGCACCGTTATGTTCCTTGACTTGT
TAACATGA

CATCAACGTCAGGACTAGTGCGATTACCACTGAAAGTACCAACTCCAGCACTAATGCTACTACCACTGCCA
GCACCAACGTCAGGACTAGTGCTACTACCACTGCCAGCATCAACGTCAGGACTAGTGCGACTACCACTGAA
AGTACCAA

GAGGTAGCACAATGGCGGTAGCGCCACGCCTACCAGGATCAACAAGTCTACTAGAGTGCAGGGGTACTCAA
CAGTGTCGATCTGGATGAGCGACAGCTTCCTCTTCTCCGCAGCGATGCGGAACATCTTGTTGAAGATTTGC
TTGATTCC

TTTAGGTGCAATGAGAATTATGAGATCATATTATAAACTACTTCGAGGATTTGCCACAGAGGTCAAGGATA
AAGTCTGGTTTCTCGAAGGAGTCACGCAGGTGCTGCCTCAAGATGTTGACGAATACAGTGGAGGTGGTGGT
ATGCATAT

GAATCCCACAGTGTCCAAGACTACCAAGAGGCCCACCAGCACCACCAACCACCTGAACCCCAACCATATTA
TCCTCCTCCTCCTCCAGGTGAACACATGCACGGTCGCCCACCAATGCACCACCGTCAAGAAGGAGAACTCT
CGAACACC

AACAAGTTTTGCTACACTCCCTTAATAAAGTCAACATGAGCAGTAACAACAACACAAACACAGCACCTGCC
AATGCAAATTCTAGCCACCACCACCACCATCACCACCATCACCACCACCATCACGGTCATGGCGGAAGCAA
CTCGACGC

TGAACCAGCAGCTCTAGCTTCTTCAACACTAGCTTCAATAACTTCCAACTCTTGTTGAATGAATGGATGGT
CTGGGGCAACCTTGTTAACTTTGGAAAGAGATGCTCTTGCTTCGTCAATTTGACCAGCTTCAACCAAATAA
CGTGGGGA

GCCGCTTTACGTGTCACTGACGGTGCTTTGGTTGTCGTCGACACCATTGAAGGTGTCTGTGTCCAAACCGA
AACTGTTTTGAGACAAGCTTTGGGTGAGAGAATCAAGCCTGTTGTTGTTATCAACAAGGTCGACAGAGCTT
TGTTGGAA

AGGCAGCTCAACTCCATAATATTGCCATTATCAGCTGCCACAAGTACTCTCTCTTGATCTTGCACGCTGCT
TGTTTCAATTACAGCCACCACTGGGTGCTGAAAGTAGTGTCTTGCCACCATATTTCGCTGCACGACATACA
CTACCGGT

TTGGATCTGCTGAAGTAGCCACGTCACTTGAGCTAGTTGGATCTGTTGATGTGGTTACCTCGTCAGAGCTT
CTTGTACGATACATACCAGCAACACTTGATGAGGCATGTGGACCAACTGTTGTAGTAGACTCAATTAAAGA
TGTAGAGT

AAGGCCTTGTTGAAGGTTGTTATGAGAAAGTTCTTGCCAGCTGCCGATGCCTTATTGGAAATGATTGTCTT
GCACTTGCCATCTCCAGTCACTGCTCAAGCCTACAGAGCTGAACAATTATACGAAGGTCCAGCTGACGATG
CCAACTGT

CCGAATAGAGTAGCTGAAGAGGCGATGACAGAGTATTTTAACCACTGGCTGCTGCTGTCGGTGCTGCCTGT
GCCTGTTGAGGCGTATCTGTAGCATGATCTTCTCACCAGCCTTCCTCTAGGTTGTGCAATAGGCTTTATTA
GCCTTGTG

CGACACCATTGACCAACGGTAGAGTTCCAGCCTACGATGGTAGATTTGACCAAGGAGTGGTTCTTGATGGT
GGAGTTGCACAAAACAACAGATCTGGTGATTCTAACACCATCACCGATGGTGACATTAGGACCGATAACCA
CGTCTGGG

TACCGACTCCAACACTAGTGCTACTACCACCGAAAGTACCGACTCCAACACTAGTGCTACTACCACTGCTA
GCACCAACTCCAGCACTAATGCCACTACCACTGCTAGCACCAACTCCAGCACTAATGCCACTACCACTGAA
AGTACCAA

TCGTGAGTCATTTCTTCAGCGTCGTAGTTGGCACCTCTTCTGGATGGTGGAACCCATGAGGCAGACTTCCA
TGGTAGAACACCTTCTTCCCACATGGTGTTGACTTCTTCCAAAGTCAAACCCTTAGTTTCTGGAACAACTA
ACAAAACA

TGTTTCATCACAAGTAAGTGATTTTCTAAAGAGTCAATTAAACGTCCTGGAGTGGCCACCAGGATGTCACA
ACCCTCCGATAGAGAAAATGAAATTTCCTCCAAGGAGTGTCCACCAACAATAGAAATTACTTTGCAGTCAT
AGTTGCTC

TCACCACCATGTTGACTGGTATTGCCCCAGACCAAGTGACCAGAATGATCACCGGTGTTCCATGGTACTCC
AGCAGATTAAAGCCAGCCATCTCCAGTGCTCTATCCAAGGTCGGTATCTACACTATCGCAAACTAGAGACA
AACGCCAA

ATAAAGAGTATTTCGAAACAAACACGACAATTACACTTGCAAGTCAGTAGAGAAACCCAGTACAGAGCAAG
CAAAAGATGATCTCTGATGAACAGCTGAACTCCTTGGCCATCACCTTCGGTATTGTGATGATGACTTTAAT
TGTCATTT

AGGACACCGGCGTTGATTTCATCAGCCTGTTTGAGTACCCATGGATCCTCTGGTGAAACCTTGTTGATCTT
GGCGATGGAGACACAGGCCTCTTCATGTCTCTCACATTCAATCAGATATCTTGGGGACTCTGGAACTAATA
ACATGCCA

GATCTTTGGAACTGGTCTACAAGCCATCCGACCCTACGACTGCTCAACCACAAACTTTGAAAGTGTATGAC
TACAAGGGCAGTGGTGTGGCCATGGCCATGTACAATACTGACGAATCCATCGAAGGGTTTGCTCATTCGTC
TTTCAAGC

TATGTCACCGAACCTTCCATCCGCTAATGTTATCTTAGGTTTGTGTCTGTCATTTATGCTTTCTTTCTGTG
GTGTTACACAACCTGTCTCATTGATGCCTGGCTTCTGGACATTCATGTGGAAGGCTTCCCCATACACATAT
TTTGTTCA

AAATCGTCAATAAAGTCCTTGTTGAACTTTGCATCATTACTCCTTGCCTTCGGAACGTGAGGTGGAAGACT
CCACACTGCAACAGCATCGAAAGTATTAACTTCGGAGATCTCACCATAAGGTGAATCCAGGAATGGCGATA
TGATATCG

ATAAAAGACCAGATACCTTTCACAAGTGACATCATCGTCCTTGTCTCCTTGGAGAGAACAGAGCACATTAT
AAGCAGTCATCAAGTCACTTGGCGTCAAAGCAGATCTCTGGTCTTTAAACTCGCTTGTCACTAGGAGACTA
TGTTCAGG

ACAATGGAATCGCTGAATTAGAGTTTTGTGTGTAAGAACCGACCATATCCTTAGTTTCGTGATACTGCTGC
TGCTGCTGCTGTTGTTGTTGTTGCTGTTGCTGCTGTTGTTGCTGTTGCTGCTGAAGTTCGTGATACCTCAT
TCTTTCCT

CTTGGAAGGTAAGGCCTTGTTGAAGGTTGTTATGAGAAAGTTCTTGCCAGCTGCCGATGCCTTATTGGAAA
TGATTGTCTTGCACTTGCCATCTCCAGTCACTGCTCAAGCCTACAGAGCTGAACAATTATACGAAGGTCCA
GCTGACGA

CGAAGATAAGAAACCTGTCGTCGACAAGAAGGAAGAGGCTGCTCCAAAGCCACCATCCTCTGCTGTCTTCT
CCATGTTTGGTGGTAAGAAGGCCGAAAAGCCAGAAACCAAGAAAGACGAAGAAGATACCAAGGAGGAAACC
AAGAAGGA

TGCTCCAACTGCTTCTGCAAAGTGAATTGTTCAATCATCAAATTTGTCAACTCACTTTGGGAACGTCTGTG
TGCAACATGAATCTGCTTTGGGGCGTTTTTACCACCTCCTGTGGTTTGCACAAACATGTTATTTGGCTCTT
GGGATCTA

AAATGTTCCTGCTGTGGAGGATACCCCTGCTGCGATGGCATTTGCGGTGGAGGATACGCCTGTTGTTGCTG
ATGCGAAGGAGGAACCATGAATTGTGGATGATGTTGCTGCGGAGGAATAGCAAATTGTTGCTGAGATGGTA
ATTGTTGC

CTACTACTGAAGCTCCAACCACCGGTCTTCCAACCAACGGTACCACTTCAGCTTTCCCACCAACTACATCT
TTGCCACCAAGCAACACTACCACCACTCCTCCTTACAACCCATCTACTGACTACACCACTGACTACACTGT
AGTCACTG

GATGGAGGACCATTGCTTTATAGTCAATTTTGGTATCATCACCCAATGTGTTCTCTCCCACTGCTTTCAGT
CTGCGGAGGGACTCATCTTGAGTAAGACCTTCAGTCAGACTTGTGCCTATAAATTCAGCGGCTTCTTCTGT
AGTCAATG

TATGAGAAAGTTCTTGCCAGCTGCCGATGCCTTATTGGAAATGATTGTCTTGCACTTGCCATCTCCAGTCA
CTGCTCAAGCCTACAGAGCTGAACAATTATACGAAGGTCCAGCTGACGATGCCAACTGTATTGCTATCAAG
AACTGTGA

AACTATCAAGAGCCATCATTGACTGTGGTTTTGAACATCCTTCTGAGGTCCAGCAACATACCATTCCTCAG
TCAATTCATGGTACCGATGTCTTGTGTCAAGCAAAGTCTGGTTTAGGTAAGACAGCTGTCTTTGTCTTATC
CACTCTGC

TTAAAGACCTTCAATCTTTATATGATAAGATGAACGGCGGACCAGTGTGGTCCTCATTGAGTGAGTGCTTA
TCTCAATTTGAAAAGAGTCAAGCCATCAACACTTCAGCCACAATCTTACTTCCGTTGATCGAATCACTTAT
GGTTGTAT

ACCAGTACCAAAGATACAACCAATGACAGGCTCGCTAATCTCACCAGCACTCGATGGACGAGAGCCTGAAG
TGTAGCAGTGGGATAGGAAAGTGCCAACCGTGTCGTTGGTCAAAGCTACCACATTAATCATAGAGAGCCCC
TGAATGTC

ATTGGCTCCAGGTGCTCACTTGGGTAGATTCGTTATCTGGACCGAAGCTGCTTTCACCAAGTTGGACCAAG
TCTGGGGTTCCGAAACCGTTGCCTCCTCCAAGGTCGGTTACACTTTGCCATCCCACATCATCTCCACTTCT
GATGTCAC

TTGCTATTGTTATTGTTATCATATGAATTATGATAATATCCGGAGTTGTTCCTGGAGTGAGTTCTACTTCT
GCTGCCACCTGCACTCATACCAGGTGAATTACCGTTTGAGTTGTAATGAGGGTGAGGCGGTTGAGGCGTGA
AATGATTG

GTGTCCAAGACTACCAAGAGGCCCACCAGCACCACCAACCACCTGAACCCCAACCATATTATCCTCCTCCT
CCTCCAGGTGAACACATGCACGGTCGCCCACCAATGCACCACCGTCAAGAAGGAGAACTCTCGAACACCAG
ATTGTTTG

TGTTCGCAAATTATTTGGTGCTGAACATTCTCTTGTACAATGGCTTGTCATCGTGAGTCATTTCTTCAGCG
TCGTAGTTGGCACCTCTTCTGGATGGTGGAACCCATGAGGCAGACTTCCATGGTAGAACACCTTCTTCCCA
CATGGTGT

GGTGCTCACTTGGGTAGATTCGTTATCTGGACCGAAGCTGCTTTCACCAAGTTGGACCAAGTCTGGGGTTC
CGAAACCGTTGCCTCCTCCAAGGTCGGTTACACTTTGCCATCCCACATCATCTCCACTTCTGATGTCACCA
GAATTATC

TGCTGTTCCATAAACGTACATATATGACATCTACAAAACTACAGAGAAGGATATCATTCTGGGTCTGAATC
AGGGTTTGCCACACGCAAGCCATGCCCCAGAGAATAGCTGAGGTCTACCACATCAGAGTTCGTTGTGGAGA
CAGAGTCC

ACAGCAGAGGAAGTCTCAACAGCAGAGGAAGTCTCAACAGCAGAGGAAGTCTCAGCAGCAGAGGAAGTCTC
AACAGCAGAAGAAGCCTCCACAATAGAGGCCTCAGTAGTGGAGGGAATTTCAATAGCAGAAATACTAGAAG
TTGCAACG

GTTCTAAAGGCCTTGTTCCACAGACCTTACTTCCACGTTAGTGTCATCGAAGATGTTGCTGGTATCTCCAT
CTGTGGTGCTTTGAAGAACGTTGTTGCCTTAGGTTGTGGTTTCGTCGAAGGTCTAGGCTGGGGTAACAACG
CTTCTGCT

TAGGACAGAAGGACCCAGTTCAGTTCTAGTTTTACAAATAAATACACGAGCGATGTCGGACTCAGAAGTCA
ATCAAGAAGCTAAGCCAGAGGTCAAGCCAGAAGTCAAGCCTGAGACTCACATCAATTTAAAGGTGTCCGAT
GGATCTTC

GGCCACCTGATTTTCAGGAGTGGTGAGGAAATCCCAAAACATGTCAGCATCCCTTAGGTTGGTTTGTGGGT
TTCTCTTCTGTGTGTGGATAAAGTGAGGGAACTTGGAAGGGTCTCTGATAAAGAATACCGGTGTATTATTG
TAGACCCA

TTTGACTGTGAGATACTAAGAGATTCTGAAATTACAGCCATAAGTACGAAGGAAGGACTACTGAGTGTACA
AGGTATGACCTGTGGGTCTTGTGTTTCTACAGTCACCAAACAAGTGGAAGGCATTGAGGGTGTTGAATCGG
TAGTCGTT

ACAGAGCTACTTAGATCAGCTGAACTTGAACTGACTACGGAAGTAGTTGGATCAGCTGAAGTAGTTTGAAC
GGTCGAGGTAGTTGGGTCAGCTGAAGTAGTTGGGTCAACTGAAGTAGTTGGATCAACTGAAGTAGAACTGA
CTATAGAA

AACTCCAGCACTAATGCTACTACCACTGCCAGCACCAACGTCAGGACTAGTGCTACTACCACTGCCAGCAT
CAACGTCAGGACTAGTGCGACTACCACTGAAAGTACCAACTCCAACACTAGTGCTACTACCACCGAAAGTA
CCGACTCC

GGTAAGCGACTAAACCCTTAGCAATAGCTTGTCTGATGGCGTAGACTTGAGAAACATGACCACCACCAGTG
ACTCTAACTCTGATATCGATGTTGGAGAACTTGTCCAAACCAACCAACAATAATGGTTCGTAGACCTTGAA
TCTTAGGA

CCATAGTCACGTATAACTGTCCTAATTCCAATATGCTTGTCTCCTGTTCATTTCTACGCTTGTCATCCACA
CTGGCACCGGCAGCGGCGCTGCTCTGTGAGCTGTCTTTATCCAGCAGGCTCAAATAGACTTGTTCTGCCTC
ATTATACT

GCTTCTGCAAAGTGAATTGTTCAATCATCAAATTTGTCAACTCACTTTGGGAACGTCTGTGTGCAACATGA
ATCTGCTTTGGGGCGTTTTTACCACCTCCTGTGGTTTGCACAAACATGTTATTTGGCTCTTGGGATCTATT
GTTGTTAA

AAATTATTGCCAACGAACAAGGTAACAGAGTCACCCCATCTTTCGTTGCTTTCACTCCAGAAGAAAGATTG
ATTGGTGATGCTGCCAAGAACCAAGCTGCTTTGAACCCAAGAAACACTGTCTTCGATGCTAAGCGTTTGAT
TGGTAGAA

CGTTATCTGGACCGAAGCTGCTTTCACCAAGTTGGACCAAGTCTGGGGTTCCGAAACCGTTGCCTCCTCCA
AGGTCGGTTACACTTTGCCATCCCACATCATCTCCACTTCTGATGTCACCAGAATTATCAACTCTTCTGAA
ATCCAATC

ACCACCAACCACCTGAACCCCAACCATATTATCCTCCTCCTCCTCCAGGTGAACACATGCACGGTCGCCCA
CCAATGCACCACCGTCAAGAAGGAGAACTCTCGAACACCAGATTGTTTGTTAGACCTTTCCCATTGGACGT
TCAAGAAT

GTCTCGACAACCTAACAACCATTGGCGGTACTTTGGAAGTTGTTGGTAACTTCACCTCCTTGAACCTAGAC
TCTTTGAAGTCTGTCAAGGGTGGCGCAGATGTCGAATCAAAGTCAAGCAATTTCTCCTGTAATGCTTTGAA
AGCTTTGC

GACAGGTATTGAGGCGTTCGCAGAAGCATTGCTGGTAATTCCAAAGACTTTGGTGAAGAACTCAGGATTCG
ACCCATTGGACGTGCTCGCAATGGTGGAGGACGAGTTGGATGACGCTCAGGATTCTGACGAAACGAGATAT
GTTGGTGT

GAAGTGGTACTTGGATGACGTCATTGACGCAAATGAGGAAGAAGAGGCAGAACAGACCAATGGAAAGGATG
AGACAATGATGGAAATTGATGATGAAATGATGGTGGAGCAAGACAATGAAGAGGTAGCAGGCGACGAAGAA
TATGACAT

GCACCTGCTGCAATATCCTCAACGTACACGTCTTCACCATCGGCACCTGTTGCAGTATCCTCGACGTACAC
GTCTTCACCATCGGCTCTTGTTGTCCTGTCATCTACTTCCACATCCTCTCCATATGATATTGTTTACTCTC
CATCAACT

TTGTCCTCTTTGGAAGGTAAGGGCTCTTTGGAAGAAATCATCGCTGAAGGTCAAAAGAAGTTCGCTACTGT
TCCAACTGGTGGTGCTTCTTCTGCTGCTGCCGGTGCTGCCGGTGCTGCTGCCGGTGGTGATGCTGCTGAAG
AAGAAAAG

GCCCACCAGCACCACCAACCACCTGAACCCCAACCATATTATCCTCCTCCTCCTCCAGGTGAACACATGCA
CGGTCGCCCACCAATGCACCACCGTCAAGAAGGAGAACTCTCGAACACCAGATTGTTTGTTAGACCTTTCC
CATTGGAC

TAGCTTCTCGAACAGATGAGAGTACAATAACAGGAAGTGCGACGGATGCAGAAACAGGTGATGATGATGAT
GATGATGATGATGACGATGATGAAGATGAGGATGACGAAGATGAGCCCCCCTTATTGAAGTACACACGAAT
TAGTCAAC

CTCTACCATCTAGCAATCTATGATCATAAGTCAAAGCCAAGTACATCATTGGTCTTGAGACAATTTGTCCA
TTAACAGTGACAGGTCTCTCTTTGACACCATGCAAGCCTAGGACGGCTGTTTGTGGTGAATTGATGATAGG
AGTACCGT

GATACATTTCCTTCATTGCTGTTCTCGTTCTGAAGGTTATGTGAGTTTGTAGAAGAAGGGACAGAACTGCT
TCTTTGAAGTTGAACAAGCTGAACATCATCAACACGTTCGCCATTGAAAGAAGTAGGTACATTAACGTTTC
TTGGTTGT

CTAGTGCTACTACCACTGCTAGCACCAACTCCAGCACTAATGCCACTACCACTGCTAGCACCAACTCCAGC
ACTAATGCCACTACCACTGAAAGTACCAACGCTAGTGCCAAGGAGGACGCCAATAAAGATGGCAATGCTGA
GGATAATA

TCCTTTGCACCCATAGTCACGTATAACTGTCCTAATTCCAATATGCTTGTCTCCTGTTCATTTCTACGCTT
GTCATCCACACTGGCACCGGCAGCGGCGCTGCTCTGTGAGCTGTCTTTATCCAGCAGGCTCAAATAGACTT
GTTCTGCC

TTAGATCAGCTGAACTTGAACTGACTACGGAAGTAGTTGGATCAGCTGAAGTAGTTTGAACGGTCGAGGTA
GTTGGGTCAGCTGAAGTAGTTGGGTCAACTGAAGTAGTTGGATCAACTGAAGTAGAACTGACTATAGAACT
AGTTGGGT

AAATGGCTATGCCACCGCTACTGGTGCTGGTGCCGCTGCTGCCACTGCCACAGCGTCATCAACACATGCAG
CAGCAGCAGCAGCCGCTGCTGCCAACCATTCCACCCAGGAGTCGGGTTTCGATTACGAAGGCCTGATAGAT
TCCGAACT

AGATTTCTTGCTAAAGCCAGAACTATCAAGAGCCATCATTGACTGTGGTTTTGAACATCCTTCTGAGGTCC
AGCAACATACCATTCCTCAGTCAATTCATGGTACCGATGTCTTGTGTCAAGCAAAGTCTGGTTTAGGTAAG
ACAGCTGT

AATGGCGGTAGCGCCACGCCTACCAGGATCAACAAGTCTACTAGAGTGCAGGGGTACTCAACAGTGTCGAT
CTGGATGAGCGACAGCTTCCTCTTCTCCGCAGCGATGCGGAACATCTTGTTGAAGATTTGCTTGATTCCGG
TAGCAGTG

CTAAAGCCAGAACTATCAAGAGCCATCATTGACTGTGGTTTTGAACATCCTTCTGAGGTCCAGCAACATAC
CATTCCTCAGTCAATTCATGGTACCGATGTCTTGTGTCAAGCAAAGTCTGGTTTAGGTAAGACAGCTGTCT
TTGTCTTA

TAGAGATTCCACTAAGGTTAATTCTCAAGAAGAGACAACACCTGGGACATCAGCTGTTCCAGAGAACCATC
ATCATGTCTCTCCTCAACCTGCTTCAGTACCACCTCCACAGAATGGACAGTACCAACAGCACGGCATGATG
ACCCCAAA

CAGAACTCACACTATATGACTTTGAAGGTGCCAAATGGGGAAGGCCTGGAACGGACAACTGATCTGTGTCA
CGATGATGGTGATGATGATGTTGTTGTTGCTGGTCCCCTGGGCTGTCTAGCGAGTGCCTAGATTTAATAGC
ACCCAGTG

TTTGATCCAAATGAAGTTAAGTACTTGTACTTGAGAGCTGTCGGTGGTGAAGTCGGTGCTTCCGCCGCCTT
GGCTCCAAAGATCGGTCCATTGGGTTTATCCCCAAAGAAGGTTGGTGAAGATATCGCCAAGGCCACCAAGG
AATTCAAA

TTGGATGACGTCATTGACGCAAATGAGGAAGAAGAGGCAGAACAGACCAATGGAAAGGATGAGACAATGAT
GGAAATTGATGATGAAATGATGGTGGAGCAAGACAATGAAGAGGTAGCAGGCGACGAAGAATATGACATAG
AGGATAAT

GTCATCATGCACTGCTGTGGGTACGGCCCATTCTGTGGAGGTGGTACTGAAGCAGGTTGAGGAGAGGCATG
ATGGGGGTTCTCTGGAACAGCTGATGAAGCAGGTGTTGTTGTCTGTTGAGAGTTAGCCTTAGTGGAAGCCT
TATCATAT

TACCACTGCCAGCACCAACGTCAGGACTAGTGCTACTACCACTGCCAGCATCAACGTCAGGACTAGTGCGA
CTACCACTGAAAGTACCAACTCCAACACTAGTGCTACTACCACCGAAAGTACCGACTCCAACACTAGTGCT
ACTACCAC

TGTAAAATTTGCCTGCACTATCAGTAGGAGGCTCGATATCAAGAGGGTTCTTGTAGTCCAGAGCCACGTTC
TCAAGACCTACAGGTGAGTCCTCTAAGTTTCGTGTGGAAAGCGCTACCTCCTTCATCAACAAGACACGTTC
ATCCATAT

GCAAGTCCTGGAGACTTTAGGTTTACTTGTTGGTGATGTCTCTCTCTTCCAAAGTGCTGGTACGGTCGACC
TGGATTCCAGAGGTCATGTCAAGAATAGTGAGAGCAGTTTGAAATCAAAGCTAGCATCTAAAGCTTATGTT
ATGAAAAG

CTGCTGCTGCTGTTGTTGTTGTTGCTGTTGCTGCTGTTGTTGCTGTTGCTGCTGAAGTTCGTGATACCTCA
TTCTTTCCTGCAAAACTTGTTGGTCCTGTGAGAAGACCTGCCTTAGTTGCATTTGAACTTGTTTCAGAGCA
TTGAGAGT

TGATGTTGGCGAGTACGATGGTGATGTTGGTGAGTACGATGGTGATGTTGGTGAGTACGCTGGAGAGGTAG
GAGAGTATGTTGGGGAAGTTGGAGAAAAGCCTGGTGAGGAGACTCCAAATCCGGGAGATGTAGGTGCTTCA
CCATAAGC

CGGAATTTGATAGAGATTCCACTAAGGTTAATTCTCAAGAAGAGACAACACCTGGGACATCAGCTGTTCCA
GAGAACCATCATCATGTCTCTCCTCAACCTGCTTCAGTACCACCTCCACAGAATGGACAGTACCAACAGCA
CGGCATGA

GGAATTTGATAGAGATTCCACTAAGGTTAATTCTCAACAAGAGACAACACCTGGGACATCAGCTGTTCCAG
AGAACCATCATCATGTCTCTCCTCAACCTGCTTCAGTACCACCTCCACAGAATGGACAGTACCAACAGCAC
GGCATGAT

TGGGTAGATTCGTTATCTGGACCGAAGCTGCTTTCACCAAGTTGGACCAAGTCTGGGGTTCCGAAACCGTT
GCCTCCTCCAAGGTCGGTTACACTTTGCCATCCCACATCATCTCCACTTCTGATGTCACCAGAATTATCAA
CTCTTCTG

CAATCGCTCCGTATATGTGCAAGTATCATTCTCCAAAGATAAAAGCTGTGATAGCGGTACAGGACGGAGCT
GCTGTGGCTTCCGCAAAACAGTCAAGTCCAGGCAGCTCAACTCCATAATATTGCCATTATCAGCTGCCACA
AGTACTCT

AAACAGTGACCGTATGTCCGGCACGGTTCAACATATCAGCACACGCTAAACCTGCTGGACCAGAACCAATG
ACACCCACTGTAAAGCCAGTGCGTGTACTTGGTGGACAAGGCTTAATCCATCCTTCCTTGAAAGCATTGTC
AATGATAA

GCGTGCAGTGCAGGACGATAGAGGTAGCACAATGGCGGTAGCGCCACGCCTACCAGGATCAACAAGTCTAC
TAGAGTGCAGGGGTACTCAACAGTGTCGATCTGGATGAGCGACAGCTTCCTCTTCTCCGCAGCGATGCGGA
ACATCTTG

TATCGGTTTAGTCGGTATCGATCAATTCTTGTTGAAGACTGGTACTTTGACCACCAGTGAAACTGCTCACA
ACATGAAGGTCATGAAATTCTCTGTCTCTCCAGTTGTGCAAGTCGCTGTCGAAGTCAAGAACGCTAACGAC
TTACCAAA

ACTGAATCTACGGTGAATCACACTAATCATTCTGATGATGAACTCCCTGGACACCTCCTTCTCGATTCAGG
AGCATCACGAACCCTTATAAGATCTGCTCATCACATACACTCAGCATCATCTAATCCTGACATAAACGTAG
TTGATGCT

GAGGAATGCTTGCTAATCCAGGAGCTGGCGAGCTGGCTAGCTGTGGAGACTATTCCACCATGGTACACACT
AGGATCCTCGATCTTGGTGAGCCATGAGTAGAACTTGTTTGGTTCCACCCCTCCGGTTGCCATGTTTATAT
CTTCCTCA

TTAATACTGGGGTTGTGACTGACCAGGTGGCATCGCCATGAAATGTTCCTGCTGTGGAGGATACCCCTGCT
GCGATGGCATTTGCGGTGGAGGATACGCCTGTTGTTGCTGATGCGAAGGAGGAACCATGAATTGTGGATGA
TGTTGCTG

AACACCTACGGTGGTCTGGGGTCGCTGGGCACTCCAATAGTGAGAACAGACGCTGCAGCCATATGGGCCAC
GGGACAGACGTGGTGGCAGATCCCACCAGTGGCTCAGGTTGAGTTGAAAGGTCAATTGCCTCAGGGTGTTT
CCGGAAAA

CAGTTCTAGTTTTACAAATAAATACACGAGCGATGTCGGACTCAGAAGTCAATCAAGAAGCTAAGCCAGAG
GTCAAGCCAGAAGTCAAGCCTGAGACTCACATCAATTTAAAGGTGTCCGATGGATCTTCAGAGATCTTCTT
CAAGATCA

CACCATCATAAATAATATTACCGAAAATGACACCTGTGTCAGCAGAGGAAACTTTGACAGTAACAGTGAAT
TTGTGGAAGCCATGAGGAATCACGTTGGTCTTCTGTGGTGTGTCAATAATCTTCAAATCACCAAGAGTTGC
AAATTGCA

AAACTCCCTACACAATACGTTATACTCACATTAAATATCCAGCCTGCAGAAAGTGCCACCAAGATTGAATC
AACCACAATAGCATTTGTCAAGGAACTGCCTTGGGATCTACACACCATTGTACCTGTTTCGAATGGTGCTA
TCATTGTG

CCCCTAGTTCTCTTTGGGCAAGGACACCGGCGTTGATTTCATCAGCCTGTTTGAGTACCCATGGATCCTCT
GGTGAAACCTTGTTGATCTTGGCGATGGAGACACAGGCCTCTTCATGTCTCTCACATTCAATCAGATATCT
TGGGGACT

TAGATATTCTTTAATACTGGGGTTGTGACTGACCAGGTGGCATCGCCATGAAATGTTCCTGCTGTGGAGGA
TACCCCTGCTGCGATGGCATTTGCGGTGGAGGATACGCCTGTTGTTGCTGATGCGAAGGAGGAACCATGAA
TTGTGGAT

TTGTCTCCTTGGAGAGAACAGAGCACATTATAAGCAGTCATCAAGTCACTTGGCGTCAAAGCAGATCTCTG
GTCTTTAAACTCGCTTGTCACTAGGAGACTATGTTCAGGGACCACAGGAAATTTGTTGAGTAGCAGCTTAT
ATTCTCCG

CTTCCAACTAACGGTACTTCTACTGAAGCTCCAACTGATACTACTACTGAAGCTCCAACCACCGGTCTTCC
AACCAACGGTACCACTTCAGCTTTCCCACCAACTACATCTTTGCCACCAAGCAACACTACCACCACTCCTC
CTTACAAC

GTGTTATTCAACCCCTTTTCCATACATTCAATGAAGTACGCTGTTAGTTCGTCCAACTTTGCTGCATCAAC
CTGCAATGAGGAGCAGATCTCAACCACTGCATCTTCCAATGCCTCAGCATTGACTTTATGTAAATTTTCAA
ATGACATT

AGCAATGCTGAACACATTATTCTTGTCGTCTCTATCAATATGCAAATGCTCGGCTCCTGTCTGGGAGTGCA
CCAAATCTACCGCAGTGAGTCTCAGCTCCGGAACCGGTAGAATTCTTCTCACTTCATAACCATGGAATATA
CCTCCAAC

GTCATCTCAAAGGAAGATAACCTCTTAGAAGCAGTAGTAACTGAGCCTGCCTTCGATGAACTTGAGACGGA
AGATTGTACCGCTGACATGGGTGTGCTGGATGACGAAGATGTGTAACTGTTCTGTCTTATTAGAGTTGTTT
TCAATACC

CGGTAGGCGTTGGTTCCTGAACAAGAATTAGCAATGCAACCACCAATCTGTGCACCTGGACCAGGGTCACA
GCCAAACATCAAACCGTGGTCGCTCAAATAGTCATTCAAATCCTCCCAGGGTAGACCGGCCTGCACGGTGA
TGTCCAGG

CTGAACATTCTCTTGTACAATGGCTTGTCATCGTGAGTCATTTCTTCAGCGTCGTAGTTGGCACCTCTTCT
GGATGGTGGAACCCATGAGGCAGACTTCCATGGTAGAACACCTTCTTCCCACATGGTGTTGACTTCTTCCA
AAGTCAAA

ACTTTGTCAACTCGGATGTTCAAATGCTTAGCGACACGGTTCACATCTCTCCAATCCCTCTCGTAGCATGG
CTCCTTGCCAGGGTCGTCCAAAGATTGTGATTCAGACCAGTTCTGCATATATACTCCTCGGGTATTTGGAA
ATTCTCCG

CGTACAAACTCTAACAAGTCTGGGCTTCCTTTGATAAACAATGAAAACAGTGTCCCCAATCCTCCCAACAC
TGCAACCATACCGTTGCAGAAGTCCAGACTCATTGTAAATCCATTCATTCCAAGGAGGCCTTACTCTAATG
TACTGCCC

TATCAGAGTCCGCTGAGGATGAATCAGTAAATGTATTACCTGACTCAGGTGATGGAGTGCTCAGAGGCGTT
CCAACTGATGATGGATACTGCGGAAACTGTGATTGTGGCCCAGGTGGAAAGTACATAGGCGACATTTGATA
AGGTGTAT

TCCAATTTACCGGAATTTGATAGAGATTCCACTAAGGTTAATTCTCAAGAAGAGACAACACCTGGGACATC
AGCTGTTCCAGAGAACCATCATCATGTCTCTCCTCAACCTGCTTCAGTACCACCTCCACAGAATGGACAGT
ACCAACAG

TTTGTAATATATGGAACAGGATCTACTTCTGGTTCTTGTTCAGCCTTTGCTCCCTCATCTGTCATTTCCAC
GTCTTCACCTTCTACTTTCACTTCCTTTTCAGCTTCGTGTTGTCTGTGAGCTTCGATAGAATCCTTGATAG
CGTATTTA

GTCTTAAACCATTTCTATCCAATATAGCACCACAGTAACGTCCATCAGTGAAAGTTAACAAAGCTGGACCA
TCCCAAGGTTCCATCAGACATGCAGCCCAGTCGTACCATGCTTTTAGGTCAGAATCCATATCCTTATGATA
CGCTTCAG

CATTTCTTCAGCGTCGTAGTTGGCACCTCTTCTAGATGGTGGAACCCATGAGGCAGACTTCCATGGTAGAA
CACCTTCTTCCCACATGGTGTTGACTTCTTCCAAAGTCAAACCCTTAGTTTCTGGAACAACTAACAAAACA
TAGAAGAA

TGCAGGTGTAGGTGTATGAAGAGGTGTATCATATGCCATTGACGCCATGAACAGGCCTAAAACACCACCCA
GTGCAAACCCTGTTACACCACTCACAACTGATTTTCCAGGACAGGAAGTCATGAAGTTCATGATCATTTCA
GCACCCCG

GTTCTCAAAGTCTATCCATTTGCTTGGAATACCTAGCTTGTTAAGGTGATCAATGATCGATTGACCGTGTG
GACCTTGAACCTGCACTTCAGCACATTTGAATGTTTGTGATTCACTTATTGTTGTCGATCCGCTACAAATC
TTTCTTAA

CCTCTTCCATCTCCACTTCCTCTGGCTCAGTAACTATCACATCTTCTGAAGCTCCAGAATCCGACAACGGT
ACCAGCACAGCTGCACCAACTGAAACCTCAACAGAGGCTCCAACCACTGCTATCCCAACTAACGGTACCTC
TACTGAAG

CGAAAGTACCGACTCCAACACTAGTGCTACTACCACTGCTAGCACCAACTCCAGCACTAATGCCACTACCA
CTGCTAGCACCAACTCCAGCACTAATGCCACTACCACTGAAAGTACCAACGCTAGTGCCAAGGAGGACGCC
AATAAAGA

AACACATTATTCTTGTCGTCTCTATCAATATGCAAATGCTCGGCTCCTGTCTGGGAGTGCACCAAATCTAC
CGCAGTGAGTCTCAGCTCCGGAACCGGTAGAATTCTTCTCACTTCATAACCATGGAATATACCTCCAACTG
GATATTTT

TAAGTTCAAGACCCAAATATTCGATGATGTGGAAGTTGAGAAAGAAGTTGACATCAGGAAGAGAGTGTTCA
ATGTGTTCAACAAAACTATCGATGACTTCAATGGTGACCTTGTGGAATATAACAAATATTTGGAAGAGGTG
GAAGACAT

AGAGATTCCACTAAGGTTAATTCTCAACAAGAGACAACACCTGGGACATCAGCTGTTCCAGAGAACCATCA
TCATGTCTCTCCTCAACCTGCTTCAGTACCACCTCCACAGAATGGACAGTACCAACAGCACGGCATGATGA
CCCCAAAC

TGCTCCAATGCGTCCACTCTCTCATTGTAATTGTTTAGCACAATTTCGTTCTCCCTGACAGAGTCCTTGTA
CAGCGCCAGGTCTCCCGAATCAATGTTCTCCAAGTCGTCGCTGTCGTCAGCTTCAAGACGGTCGTCAGCAC
CCTCCAAC

GTGGTCACAATATGAAGACTATCCCAGTCATGAAATTGTCTCCACATATGACTCAAGATTGAGCGTCTCTG
CAGTTTCTTACTCACCGACAGATGGCACCTTAGTGTGCAATGGCTATGACGATACCATCCGCTTATTCGAT
GTCAAGAG

ACATCCTCTGGTGATAAGCTACTATCGTGATCTAAACAATCTTTGACAGGATCGAGTGAATTTCTGTTCAC
AAACCTGATGATTCCGTTGAGAACTCCAACAGATGTTTTCTCTCTGATTCTCAGCTCTCCTAATTTAGGGG
TATCTACA

TTGAAGAATAAACACGACCTTCACCTTAGGCCAGGAACTTACTGAATCTACGGTGAATCACACTAATCATT
CTGATGATGAACTCCCTGGACACCTCCTTCTCGATTCAGGAGCATCACGAACCCTTATAAGATCTGCTCAT
CACATACA

TCATCACCATTCCAAATACTTTCATTTATTTCATCCTTTTCTAGTAGCAAAAGTGACTTGCCAGCGTCCTT
CTGAGGTGGCATTCCTGCTGGAACTGGTGTGGTGGTTACGTTTGATAAATCATGTTGTGAGATGCTATTTC
GTAGAAAG

CTGAAGCTCCAACCACCGCTCTTCCAACTAACGGTACTTCTACTGAAGCTCCAACTGATACTACTACTGAA
GCTCCAACCACCGGTCTTCCAACCAACGGTACCACTTCAGCTTTCCCACCAACTACATCTTTGCCACCAAG
CAACACTA

TGAGTACGATGGTGATGTTGGTGAGTACGCTGGAGAGGTAGGAGAGTATGTTGGGGAAGTTGGAGAAAAGC
CTGGTGAGGAGACTCCAAATCCGGGAGATGTAGGTGCTTCACCATAAGCACCAAATGGAGACGTGGCTTCA
CCATAATC

AAAGTAATAACAGAGATATTTGACGCTACTGAGGATGCCAAGGAGGCAGACGAAAGTGAAAGAGGAATGCC
ACTTGCGACAGCATTGAATACATATCCCAAGGCAGCAGCTTGGTCACTATTGGTCTCTACAACTTTAATCA
TGGAAGGG

AAACTAAACCATTGTCCAAAGATACATTTCCTTCATTGCTGTTCTCGTTCTGAAGGTTATGTGAGTTTGTA
GAAGAAGGGACAGAACTGCTTCTTTGAAGTTGAACAAGCTGAACATCATCAACACGTTCGCCATTGAAAGA
AGTAGGTA

AGATTTGGAACACGACCACGCTGGTGTTCCATTGAAGATCTCCCCACCAGTTGTCGCTTACAGAGAAACTG
TTGAAAGTGAATCTTCTCAAACTGCTTTGTCCAAGTCTCCAAACAAGCATAACAGAATCTACTTGAAGGCT
GAACCAAT

CGGTGGGTTTGCCTCGCAATACATTAGGGGGACTTACTTCATGGATGTGCATCCTCCTCTTGCAAAGATGT
TGTATGCTGGTGTGGCATCGCTTGGTGGGTTCCAGGGTGATTTTGACTTCGAAAATATTGGTGACAGCTTT
CCATCTAC

GTTGTCGTCGACACCATTGAAGGTGTCTGTGTCCAAACCGAAACTGTTTTGAGACAAGCTTTGGGTGAGAG
AATCAAGCCTGTTGTTGTTATCAACAAGGTCGACAGAGCTTTGTTGGAATTGCAAGTTTCTAAGGAAGATT
TATACCAA

CAAACAAACCACAGCCAACCCAAGTCTAACCGTCTCCACAGTCGTCCCAGTTTCATCCTCTGCTTCTTCTC
ATTCCGTTGTCATCAACAGTAACGGTGCTAACGTCGTCGTTCCAGGTGCTTTAGGTTTGGCTGGTGTTGCT
ATGTTATT

GGTTGTGACTGACCAGGTGGCATCGCCATGAAATGTTCCTGCTGTGGAGGATACCCCTGCTGCGATGGCAT
TTGCGGTGGAGGATACGCCTGTTGTTGCTGATGCGAAGGAGGAACCATGAATTGTGGATGATGTTGCTGCG
GAGGAATA

CTTCAACACTAGCTTCAATAACTTCCAACTCTTGTTGAATGAATGGATGGTCTGGGGCAACCTTGTTAACT
TTGGAAAGAGATGCTCTTGCTTCGTCAATTTGACCAGCTTCAACCAAATAACGTGGGGATTCTGGAACGAA
AGTCATAC

GGAACTTCTGACTATCAAGCGAAGTGGTACTTGGATGACGTCATTGACGCAAATGAGGAAGAAGAGGCAGA
ACAGACCAATGGAAAGGATGAGACAATGATGGAAATTGATGATGAAATGATGGTGGAGCAAGACAATGAAG
AGGTAGCA

GTGATAAGCTACTATCGTGATCTAAACAATCTTTGACAGGATCGAGTGAATTTCTGTTCACAAACCTGATG
ATTCCGTTGAGAACTCCAACAGATGTTTTCTCTCTGATTCTCAGCTCTCCTAATTTAGGGGTATCTACAAA
GGTACCAA

TTATTTGGTGCTGAACATTCTCTTGTACAATGGCTTGTCATCGTGAGTCATTTCTTCAGCGTCGTAGTTGG
CACCTCTTCTGGATGGTGGAACCCATGAGGCAGACTTCCATGGTAGAACACCTTCTTCCCACATGGTGTTG
ACTTCTTC

CGTGGTCAACAAATTAAAGTTGTTTCTCAACTATTTCGAAAGTGCCTGGAGATTGATACTGTGATACCTAA
CATGCAATCTCAGGCCTCTGATGACCAATATGAGGGTGCCACTGTTATTGAGCCTATTCGTGGTTATTACG
ATGTACCG

TCGGAACTGGTTTCGGAACTACTGCTATCCACGTCCTCATCATCCTCGTCCCCATTGTCTTCATCTTCATC
TTCATCTTCATCTTCATCTTCATCTCCATCTTCACCTTCATCGTCATGGCGTTTGTCATTTGTATCTTCCG
TTTCTTCG

TACTGAAGCTCCAACTGATACTACTACTGAAGCTCCAACCACCGGTCTTCCAACCAACGGTACCACTTCAG
CTTTCCCACCAACTACATCTTTGCCACCAAGCAACACTACCACCACTCCTCCTTACAACCCATCTACTGAC
TACACCAC

CGTAAGACACGAAGACGTTGAAACTGTCAAAAGAGAAATACAAGAAGCTCTTTCAGAAAAGCCAACTCGTG
AACCAACCCCCAGTGTGAAGACAGAGCCTGTCGGAACCGGATTACAATCTTATTTGGAAGAAAGAGAAAGG
CAAGTCAA

AGTTCCTTAAAAGGGTTCATGCCAATCTTCAATGCATATCTCAGATAAACCAAGGAGTGCCCTCAGCGAAA
CCACTGATCCTCACGCCTCCTCAGCTAGCCAACCAGCAGCAACCTCCACAGGATATTCTTTCTAAACTCTA
TCTTCTCT

CTAATGCTACTACCACTGCCAGCACCAACGTCAGGACTAGTGCTACTACCACTGCCAGCATCAACGTCAGG
ACTAGTGCGACTACCACTGAAAGTACCAACTCCAACACTAGTGCTACTACCACCGAAAGTACCGACTCCAA
CACTAGTG

GAAGAATCATACAAGGATAGCACTTTGATCATGCAATTATTAAGGGACAACTTGACCTTATGGACCTCTGA
TATTTCTGAATCTGGTCAAGAAGATCAACAACAACAACAACAACAGCAACAGCAACAGCAACAACAGCAAC
AACAAGCT

TTCTTGCCAGCTGCCGATGCCTTATTGGAAATGATTGTCTTGCACTTGCCATCTCCAGTCACTGCTCAAGC
CTACAGAGCTGAACAATTATACGAAGGTCCAGCTGACGATGCCAACTGTATTGCTATCAAGAACTGTGATC
CAAAGGCT

TGGCTGTTTCAACTTCCTGTGCTGGAGAGGTAATTTCGCCTTGCTCAACTTCTTCAGGTAACTCTTCTCCC
ACATCCAAATCTTCGTCACCCAAATCCCAGGCACCTTCGTCTTCACCAATATCCTCATCGTTAAAGTTTTC
TTCGCCTA

ATCCATTCCACCGGTTTCAAAGATTTCTTGCTAAAGCCAGAACTATCAAGAGCCATCATTGACTGTGGTTT
TGAACATCCTTCTGAGGTCCAGCAACATACCATTCCTCAGTCAATTCATGGTACCGATGTCTTGTGTCAAG
CAAAGTCT

GTAGAAGCAGTAGCTGACAACAAATCTAAAGGGGAGACGAAAGTTGCTGTGTCTCAAGAGGGATTGAAAGA
TGTTAGTGACCATGTTGGCCTCGCCAACAAGGATGAGAGCAAGGACGACGACGACGATGATGACTGGGAAT
GAGTGATT

ACGGTACTTCTACTGAAGCTCCAACTGATACTACTACTGAAGCTCCAACCACCGGTCTTCCAACCAACGGT
ACCACTTCAGCTTTCCCACCAACTACATCTTTGCCACCAAGCAACACTACCACCACTCCTCCTTACAACCC
ATCTACTG

GGTGTTGGTATGCAAGGTGACATGTTCGGTATCGTTGTTCCAAGAAACACTACTGTTCCAACCATCAAGAG
AAGAACCTTTACTACATGTGCTGACAACCAAACCACCGTTCAATTCCCAGTCTACCAAGGTGAACGTGTTA
ACTGTAAA

GTCATTTACGGTGACGGTAGCAGTGGAAACCAAAGCTGGGGAAACTGTTTCAGAACAAACGTGGTCCTCAC
AAGAAGTGATAGTGACCAAAGTGGTAGATTCTTCTGTGACAGTAGCAGTGGTAATGTTAGAAGCGGCGGAG
GCAACAGC

GAAGGGCCAAGAGACGTAACATGGAAAGATTGCAATTGGTCACTGGCGGTGAAGCTCAGAACTCTGTGGAA
GACTTGTCGCCTCAGATTCTTGGGTTTTCTGGCTTGGTCTACCAAGAAACCATAGGCGAGGAAAAATTCAC
ATACGTTA

TACTGTCCATTCTGTGGAGGTGGTACTGAAGCAGGTTGAGGAGAGACATGATGATGGTTCTCTGGAACAGC
TGATGTCCCAGGTGTTGTCTCTTGTTGAGAATTAACCTTAGTGGAATCTCTATCAAATTCCGGTAAATTGG
AAGCTGAA

TCGAATCTTCAGTATGAGTTGACGGAGGTGTGGAATCGGTTGGACTCACAGCTTTTGAAAGGACATTTCTC
GGTTGCTCAGGATGTAGTTCAATGTCGGATTGGAAGTCATGGTCAGATTCTATGTTAAGATCATTGGATTC
TTGGATCT

AGTTGTACCACTTTGAAGAGGGCTCGAGATTCACGAACAAAATGCAAAGGTTTTCACTAGTCACTCACAGA
TCATTCTCTCACTCCTGTGTGAAGCCCAAATCTGCATGCTCTTTGGTCAAACCAGTTCATCACTTGGTGAA
AATTGATA

GTTGTCTCCACTGACTTGGAATCTATTCAAAAGACCAAGGAAGCTGTTGCTGCTTTGAAGGCTGTTGGTGC
TCACTCCGACTTGTTGAAGGTCTTGAAGTCCAAGAAATTGAGAGCCGGTAAGGGTAAGTACAGAAACAGAA
GATGGACT

TAAACACAACTATAGAAAAAGAATGTCTAGCGAAGATAAGAAACCTGTCGTCGACAAGAAGGAAGAGGCTG
CTCCAAAGCCACCATCCTCTGCTGTCTTCTCCATGTTTGGTGGTAAGAAGGCCGAAAAGCCAGAAACCAAG
AAAGACGA

CGTATGTCCGGCACGGTTCAACATATCAGCACACGCTAAACCTGCTGGACCAGAACCAATGACACCCACTG
TAAAGCCAGTGCGTGTACTTGGTGGACAAGGCTTAATCCATCCTTCCTTGAAAGCATTGTCAATGATAATT
CTTTCAAC

CGGTGCTTTGGTTGTCGTCGACACCATTGAAGGTGTCTGTGTCCAAACCGAAACTGTTTTGAGACAAGCTT
TGGGTGAGAGAATCAAGCCTGTTGTTGTTATCAACAAGGTCGACAGAGCTTTGTTGGAATTGCAAGTTTCT
AAGGAAGA

GAGGCGTTCGCAGAAGCATTGCTGGTAATTCCAAAGACTTTGGTGAAGAACTCAGGATTCGACCCATTGGA
CGTGCTCGCAATGGTGGAGGACGAGTTGGATGACGCTCAGGATTCTGACGAAACGAGATATGTTGGTGTGG
ACTTGAAC

TCTCTTGTACAATGGCTTATCATCGTGAGCCATTTCTTCAGCGTCGTAGTTGGCACCTCTTCTAGATGGTG
GAACCCATGAGGCAGACTTCCATGGTAGAACACCTTCTTCCCACATGGTGTTGACTTCTTCCAAAGTCAAA
CCCTTAGT

TCACCATGCGCACCATCATAAATAATATTACCGAAAATGACACCTGTGTCAGCAGAGGAAACTTTGACAGT
AACAGTGAATTTGTGGAAGCCATGAGGAATCACGTTGGTCTTCTGTGGTGTGTCAATAATCTTCAAATCAC
CAAGAGTT

ACTTCCCAAGCTTTCGTAGCACTCATTCTCCATAATTTAACTTGACGGTCGTCACTGCCGGAGACAATCAA
TGGTAAAGTTGGATGGAAAGAGGCCCAGTTGACACCTCTTGTGTGACCCTCTAGAATAAACTTGACAACAC
AGTCACCA

ATGTTCTACAACCCTCATAAATGTTGGTAAAATATTTCTTCAATGGCTCCAGCTTGACTACACCCACTTGC
TTGCTCAAGGCTTGAACAGCGGCATCAAAGGCACCAGCGGCAACTAGGACAGCAGGCAGTTTAGAATTCTT
GATCCAAA

ACCGAAGCTGCTTTCACCAAGTTGGACCAAGTCTGGGGTTCCGAAACCGTTGCCTCCTCCAAGGTCGGTTA
CACTTTGCCATCCCACATCATCTCCACTTCTGATGTCACCAGAATTATCAACTCTTCTGAAATCCAATCTG
CTATCAGA

GGCGCCCCTATCAAAATGTCTACTGGTGTCACAAGCTCTCCATTAAGTCCATCAGGCTCCACCCCAGAACA
TTCCACCAAGGTCTTGAACAACGGCGAAGAGGAGTTCATTTGTCACTACTGTGACGCTACTTTCAGGATTA
GAGGATAT

GAAAGAGAAGTCTGAGTAGGAGATAAGCTTTTAACAGAAGTGGTCTCGATAGTAGAGGAAGAAGAAGAAGT
CTCAACAGCAGAAGTCTCAACAGCAGAAGAAATCTCAACAGCAGAGGAAGTCTCAACGGCAGAGGAAGTCT
CAGCAGCA

TTGCTGAAGCCGTTTTCAACTACGGTGACTTCACCACCATGTTGACTGGTATTGCCCCAGACCAAGTGACC
AGAATGATCACCGGTGTTCCATGGTACTCCAGCAGATTAAAGCCAGCCATCTCCAGTGCTCTATCCAAGGT
CGGTATCT

TTACCGTCCAACCATGGGCATGGAACGGTGGTGATGAAGAATTGAGAACCGTTGGTGTTTGGACCGGCGTT
GGCCATGGACAACAAACCTGGTCTGTCGTGGTGCTTCTTGAAGTTTTCATCTGGGAATTTGCCACCGTAGA
TAGACTTA

TAGACAAAGATTGGAATCCTACGTTGCCTCCATCGAACAAACTGTCACTGACCCAGTCTTGTCTTCTAAAT
TGAAGAGAGGTTCCAAGTCCAAGATTGAAGCTGCTTTGTCCGATGCTTTGGCTGCTTTGCAAATCGAAGAC
CCATCTGC

GTGGTCTGGGGTCGCTGGGCACTCCAATAGTGAGAACAGACGCTGCAGCCATATGGGCCACGGGACAGACG
TGGTGGCAGATCCCACCAGTGGCTCAGGTTGAGTTGAAAGGTCAATTGCCTCAGGGTGTTTCCGGAAAAGA
TATCATTG

TGATGAATTGTTGGTTGCCAGAATTACCGGTAGATTAATTCACCCAGCCTCTGGCAGATCCTACCACAAGA
TCTTTAACCCACCAAAGGAAGACATGAAGGATGACGTCACCGGTGAAGCTTTAGTTCAAAGATCTGATGAC
AATGCAGA

GGTCATCATGCACTGCTGTGGGTACGGCCCATTCTGTGGAGGTGGTACTGAAGCAGGTTGAGGAGAGGCAT
GATGGGGGTTCTCTGGAACAGCTGATGAAGCAGGTGTTGTTGTCTGTTGAGAGTTAGCCTTAGTGGAAGCC
TTCTCACA

ACAAGGATAGCACTTTGATCATGCAATTATTAAGGGACAACTTGACCTTATGGACCTCTGATATTTCTGAA
TCTGGTCAAGAAGATCAACAACAACAACAACAACAGCAACAGCAACAGCAACAACAGCAACAACAAGCTCC
AGCTGAAC

CCGGTTTCAAAGATTTCTTGCTAAAGCCAGAACTATCAAGAGCCATCATTGACTGTGGTTTTGAACATCCT
TCTGAGGTCCAGCAACATACCATTCCTCAGTCAATTCATGGTACCGATGTCTTGTGTCAAGCAAAGTCTGG
TTTAGGTA

ATCAGCCTGTTTGAGTACCCATGGATCCTCTGGTGAAACCTTGTTGATCTTGGCGATGGAGACACAGGCCT
CTTCATGTCTCTCACATTCAATCAGATATCTTGGGGACTCTGGAACTAATAACATGCCAACGATGATAATT
AGAGCCCA

AAGATAGTTAGAGTAACTGGTATAAGAAACAATAATGCAAGACCAACCGTCTCTGTTGTTATTCGTGGTGC
AAACAACATGATCATTGATGAAACAGAGCGTTCCCTCCATGATGCACTATGTGTTATTCGTTGTCTAGTGA
AAGAGAGA

TACCTGATGGAGGACCATTGCTTTATAGTCAATTTTGGTATCATCACCCAATGTGTTCTCTCCCACTGCTT
TCAGTCTGCGGAGGGACTCATCTTGAGTAAGACCTTCAGTCAGACTTGTGCCTATAAATTCAGCGGCTTCT
TCTGTAGT

TGTGGACATCTATTTTGTTGGAGTTGTCTTATGAGCTGGTGTAAAGAGAGGCCAGAATGCCCCTTGTGCAG
ACAACACTGTCAACCACAGGAAATTCTGGTCCTGCGGCAATAGAGGAACTAATCAATAATTTGACTACTCT
TTTAGCAT

TGATAATATCCGGAGTTGTTCCTGGAGTGAGTTCTACTTCTGCTGCCACCTGCACTCATACCAGGTGAATT
ACCGTTTGAGTTGTAATGAGGGTGAGGCGGTTGAGGCGTGAAATGATTGTTAGAGCCAGAATTTCCTGACA
GGTATTGT

GGAGGGTTATCTCCCGAGATTAGAATGCTCGATCAAATCAGAGTCTTGTCAAGAGCTCAAGAAGTGCCACA
CGAGGGTGGGTTCTCGGACCTGCTTTGGTCAGACCCTGACAATGTAGAGGCTTGGCAAGTTTCCCCTCGTG
GTGCAGGA

AGATGCTCCACAAGAACCACAAGTTCCACAGGAATCTGCTCCACAGGAATCTGCTCCACAAGAACCACCAG
CTCCACAAGAACAAAATGACGTTCCTCCACCATCTAATGCTCCAATTTATGAAGGCGAAGAATCCCACAGT
GTCCAAGA

GGTGCTCTATCTGGTGCTAACATTGCCACCGAAGTCGCTCAAGAACACTGGTCTGAAACAACAGTTGCTTA
CCACATTCCAAAGGATTTCAGAGGCGAGGGCAAGGACGTCGACCATAAGGTTCTAAAGGCCTTGTTCCACA
GACCTTAC

CACCCACTTGCTTGCTCAAGGCTTGAACAGCGGCATCAAAGGCACCAGCGGCAACTAGGACAGCAGGCAGT
TTAGAATTCTTGATCCAAATGGCTGTTTCAACTTCCTGTGCTGGAGAGGTAATTTCGCCTTGCTCAACTTC
TTCAGGTA

AAAAGAATACGTCTGCGATGCCTACAAATTAGGCTGTCATTCTGCTCCAAAGCAAACTATTCATGTTTTGG
AACAACCAGACAGACCTCAACCAAGGTTGGACAGGAACAGAGACAGCGGTTACGGTGTTTCCGTTGGTAGA
ATCAGAGA

AACAAAATGACGTTCCTCCACCATCTAATGCTCCAATTTATGAAGGCGAAGAATCCCACAGTGTCCAAGAC
TACCAAGAGGCCCACCAGCACCACCAACCACCTGAACCCCAACCATATTATCCTCCTCCTCCTCCAGGTGA
ACACATGC

TCATCATGCACTGCTGTGGGTACGGCCCATTCTGTGGAGGTGGTACTGAAGCAGGTTGAGGAGAGGCATGA
TGGGGGTTCTCTGGAACAGCTGATGAAGCAGGTGTTGTTGTCTGTTGAGAGTTAGCCTTAGTGGAAGCCTT
ATCATATT

AAATGAGATCCAAGAATCCAATGATCTTAACATAGAATCTGACCATGACTTCCAATCCGACATTGAACTAC
ATCCTGAGCAACCGAGAAATGTCCTTTCAAAAGCTGTGAGTCCAACCGATTCCACACCTCCGTCAACTCAT
ACTGAAGA

ATGAGATCCAAGAATCCAATGATCTTAACATAGAATCTGACCATGACTTCCAATCCGACATTGAACTACAT
CCTGAGCAACCGAGAAATGTCCTTTCAAAAGCTGTGAGTCCAACCGATTCCACACCTCCGTCAACTCATAC
TGAAGATT

ATGTTTACGCTAAGGCTTTGGAAGGTAAGGACTTGAAGGAAATCCTATCTGGTTTCCATAACGCTGGCCCT
GTTGCTGGTGCTGGTGCTGCTTCTGGCGCTGCCGCTGCTGGTGGTGACGCTGCTGCTGAAGAAGAAAAAGA
AGAAGAAG

CTGTTGCTGAGCCATCAACTGTTGCTGCTGTGCTTGAACTTGCTCCAACTGCTTCTGCAAAGTGAATTGTT
CAATCATCAAATTTGTCAACTCACTTTGGGAACGTCTGTGTGCAACATGAATCTGCTTTGGGGCGTTTTTA
CCACCTCC

TGGTGACCCCCAGCTAGAATTTAAAGTTTTACAGGGATATAGGGACCTGGAAAGTGAAATGCACAAAGGCA
GAGCTCAGGTGACCAGAACAGGAGATATAGGTGTTGCTATGGACAATCTGAACGCTGTGGATTCCCTATTC
AATAAGGT

TTTCGAATCTTCAGTATGAGTTGACGGAGGTGTGGAATCGGTTGGACTCACAGCTTTTGAAAGGACATTTC
TCGGTTGCTCAGGATGTAGTTCAATGTCGGATTGGAAGTCATGGTCAGATTCTATGTTAAGATCATTGGAT
TCTTGGAT

AACGTTGATGACAGATAATGGCAACTTCAAACCCAAAGATTTAACCAAGTCAGAGGTTTGGTTGTCAACGT
TCAAAACACCGTTAGAACGAGACAAGTCCAAAGCAGAGACTTCTGGGACGTATTGATCCTTTCTTTCTCTT
TCTTCTTC

TATATGGTAAGGGTGATATTTAAGGAAGAAATCCACCCTTCTTTAAGGTTTCCACTGCACATGTTGAAATG
TGTCTGAATCTTGATGAGCCCAAAGGCGTGCCACCATACCATCTGGTCACTATTACCAAGACGTTGAATAT
GTTTGCTC

GGTACCGATGTCTTGTGTCAAGCAAAGTCTGGTTTAGGTAAGACAGCTGTCTTTGTCTTATCCACTCTGCA
ACAATTGGACCCTGTTCCAGGTGAAGTTGCCGTTGTTGTCATTTGTAATGCTAGAGAACTGGCCTATCAAA
TTCGTAAC

TCAAAAGAAGTTCGCTACTGTTCCAACTGGTGGTGCTTCTTCTGCTGCTGCCGGTGCTGCCGGTGCTGCTG
CCGGTGGTGATGCTGCTGAAGAAGAAAAGGAAGAAGAAGCTAAGGAAGAATCTGATGATGACATGGGTTTT
GGTTTATT

TCACGAACAAAATGCAAAGGTTTTCACTAGTCACTCACAGATCATTCTCTCACTCCTGTGTGAAGCCCAAA
TCTGCATGCTCTTTGGTCAAACCAGTTCATCACTTGGTGAAAATTGATAAGTCAAAGTTATCCCCTAGATT
TCCAGAAT

GCCAGTATTGCCTGTATTGGTACCAAACTGGGGCATTCCAAATGCTGAAGTGGTGGTTCCTCCTGTCATTG
TGTTTGCTGTTCCAAAACTAGGTCTTCCAAATGCACTTCCTCCCATGTTATTGGTACTAGCAGCATTATTT
GTGAAAGG

GAAATGCTCCTTTGTAATATATGGAACAGGATCTACTTCTGGTTCTTGTTCAGCCTTTGCTCCCTCATCTG
TCATTTCCACGTCTTCACCTTCTACTTTCACTTCCTTTTCAGCTTCGTGTTGTCTGTGAGCTTCGATAGAA
TCCTTGAT

ACCATAGGCGATCAAACAACGCCCCAATCACTCCTCCTAATAACCACTACCTAACCTCACATCCCAGTTCG
TCAAGAAGACTCTCTTCCTCTTCGAGAAGGTCGAGCATGGGGAATAATAACAATTCAGAATTGCCCCCGTC
AATGATCC

GCCTTAAATGATAGTGAGAGTGATGCTAACGACTCCGATAGTGAAGTTGAAGCTGATTATGGTCCCAACGA
TGTCCAGGATGTGATCGAGTACAGCTCAGATGAAGAAGAAGGAGTGAACAATAAGAAGAAGGCTGAAAACA
AGGACATC

ACACCATTGAAGGTGTCTGTGTCCAAACCGAAACTGTTTTGAGACAAGCTTTGGGTGAGAGAATCAAGCCT
GTTGTTGTTATCAACAAGGTCGACAGAGCTTTGTTGGAATTGCAAGTTTCTAAGGAAGATTTATACCAAAC
CTTTGCCA

TATCGGCACAACGGACCTGGGTTGACAAGAGCTTGAAGGCTCCAAGAGACAGCGACCGTCGGCGCCGAATG
ACGAAACTATGCTGTGTGTGACCAGGCGAACAACATTGAGAGAGTTGAGGAGCCGTACGGACAGCTGTTTG
TAGTGATA

TCTCTCTGGTGTGGGTGTGTGGGTGTGTGGGTGTGGTGTGGGTGTGGGTGTGGTGTGGGTGTGGTGGGTGT
GGTGTGGTGTGTGTGGGTGTGGTGTGGTGTGTATATATATGTCACTGTATTGCATGCTGGATGGTGTTAGA
CAAGGCCG

CCTACAAATTAGGCTGTCATTCTGCTCCAAAGCAAACTATTCATGTTTTGGAACAACCAGACAGACCTCAA
CCAAGGTTGGACAGGAACAGAGACAGCGGTTACGGTGTTTCCGTTGGTAGAATCAGAGAAGACCCATTGTT
AGATTTCA

ATTGTCCAAAGATACATTTCCTTCATTGCTGTTCTCGTTCTGAAGGTTATGTGAGTTTGTAGAAGAAGGGA
CAGAACTGCTTCTTTGAAGTTGAACAAGCTGAACATCATCAACACGTTCGCCATTGAAAGAAGTAGGTACA
TTAACGTT

AGCTCAATTAGTCGATGAAACCTTCTGTAATGCTCTAGAATACGGTTTACCACCAACTGGTGGTTGGGGTT
GTGGTATTGATAGACTGGCCATGTTCTTGACCGACTCCAACACCATTAGAGAAGTCTTATTGTTCCCAACT
TTGAAGCC

AAGAGAAGCTGTTAGTGGCCTGTCTTGCTGTCTTTACAGCGGTCATTAGATTGCATGGCTTGGCATGGCCT
GACAGCGTGGTGTTTGATGAAGTACATTTCGGTGGGTTTGCCTCGCAATACATTAGGGGGACTTACTTCAT
GGATGTGC

AGGTTCCTACATTGACAAGAAATGTCCATTCACTGGTTTAGTTTCCATCCGTGGTAAGATCTTGACCGGTA
CCGTCGTCTCCACCAAGATGCACCGTACCATTGTCATCAGAAGGGCTTACTTGCATTACATTCCAAAGTAC
AACAGATA

ATGAGAATTATGAGATCATATTATAAACTACTTCGAGGATTTGCCACAGAGGTCAAGGATAAAGTCTGGTT
TCTCGAAGGAGTCACGCAGGTGCTGCCTCAAGATGTTGACGAATACAGTGGAGGTGGTGGTATGCATATGA
TGCTAGAT

TGAAAGTACCAACTCCAGCACTAATGCTACTACCACTGCCAGCACCAACGTCAGGACTAGTGCTACTACCA
CTGCCAGCATCAACGTCAGGACTAGTGCGACTACCACTGAAAGTACCAACTCCAACACTAGTGCTACTACC
ACCGAAAG

GGTTATGATGACGAGGATGGTGAAGAAGAGGACCAAGACGAAGATATGGTGGGTGACAGCGGCACTACAAG
ACAGGAAGATGGTGGCGAGGACAGCAACTCGAGAAGATATCCATCATATTACCATTGTAATACTGCCAGGA
ATAATAGG

GCTGAAGAGTTCAAGGCTGCCGATGAAGCTTTTGCCAAGAAGCACGAAGCTAGACAAAGATTGGAATCCTA
CGTTGCCTCCATCGAACAAACTGTCACTGACCCAGTCTTGTCTTCTAAATTGAAGAGAGGTTCCAAGTCCA
AGATTGAA

ATATGAATTATGATAATATCCGGAGTTGTTCCTGGAGTGAGTTCTACTTCTGCTGCCACCTGCACTCATAC
CAGGTGAATTACCGTTTGAGTTGTAATGAGGGTGAGGCGGTTGAGGCGTGAAATGATTGTTAGAGCCAGAA
TTTCCTGA

CAGCAGGCAGTTTAGAATTCTTGATCCAAATGGCTGTTTCAACTTCCTGTGCTGGAGAGGTAATTTCGCCT
TGCTCAACTTCTTCAGGTAACTCTTCTCCCACATCCAAATCTTCGTCACCCAAATCCCAGGCACCTTCGTC
TTCACCAA

TATAGAAAAAGAATGTCTAGCGAAGATAAGAAACCTGTCGTCGACAAGAAGGAAGAGGCTGCTCCAAAGCC
ACCATCCTCTGCTGTCTTCTCCATGTTTGGTGGTAAGAAGGCCGAAAAGCCAGAAACCAAGAAAGACGAAG
AAGATACC

TGCTAATCCAGGAGCTGGCGAGCTGGCTAGCTGTGGAGACTATTCCACCATGGTACACACTAGGATCCTCG
ATCTTGGTGAGCCATGAGTAGAACTTGTTTGGTTCCACCCCTCCGGTTGCCATGTTTATATCTTCCTCAGC
AAACTGTT

CACTTGTATGTCCATGAGTAACTGAAGGGTTAGTTTTAGTACTGGAATGATCCTGTGCAGCAGCGGTGGAT
TTAGGACCAGTGCTTGAACCAGAGTGACCATGGCTAACAGGGTGGGTAGTTTTGACGTTGATATGTCCATG
TGTAGTTG

CAGAAGCATTGCTGGTAATTCCAAAGACTTTGGTGAAGAACTCAGGATTCGACCCATTGGACGTGCTCGCA
ATGGTGGAGGACGAGTTGGATGACGCTCAGGATTCTGACGAAACGAGATATGTTGGTGTGGACTTGAACAT
AGGTGATT

TCTTCAACTTGTTCTCAAAGTCTATCCATTTGCTTGGAATACCTAGCTTGTTAAGGTGATCAATGATCGAT
TGACCGTGTGGACCTTGAACCTGCACTTCAGCACATTTGAATGTTTGTGATTCACTTATTGTTGTCGATCC
GCTACAAA

CCAGTTCCCTGGACAAGCCTATTACTATAGTCCTGAATACGGTTATGATGACGAGGATGGTGAAGAAGAGG
ACCAAGACGAAGATATGGTGGGTGACAGCGGCACTACAAGACAGGAAGATGGTGGCGAGGACAGCAACTCG
AGAAGATA

AGTAAGAGAACAAGATCAATACCAAGGAGGTCCCTCGAAAGAGCTTGACAGGCTACAACCACCACCTTCAA
TGAAGAAAAGCCCTCCAAGGAAGAAGAAGAGCCTAAAGGACTTGATATATGAAACGAACAAGACATTCTAT
CAGGTGGA

TCCAGCTAGGGAGCCATCACGCCTTGTTTGAGCAACAAGAAGAAGAGACCGATGAAGTTATGAGCACCATT
TCCAGGAATCCACATCACTCTTTGAGTGTCAAGTCATCATTGGTGATACTTCTAGGTACAACTGTGATCAT
CTCTTTTT

TCCAGAGGCGGCTTCGGTGGCCCCAGAGGTGGATTTGGTGGTCCAAGAGGTGGTTACGGTGGCTATTCCAG
AGGTGGCTACGGTGGCTACTCCAGAGGCGGATATGGTGGCTCCAGAGGTGGTTACGATAGTCCTAGAGGTG
GTTACGAT

TACCATTTCATGGGTTGCTCTACTTTTTCCGAATATACTGTGGTGGCAGATGTCTCTGTGGTTGCCATCGA
TCCAAAAGCTCCCTTGGATGCTGCCTGTTTACTGGGTTGTGGTGTTACTACTGGTTTTGGGGCGGCTCTTA
AGACAGCT

GAAAATGGAGTAGCTTCTCGAACAGATGAGAGTACAATAACAGGAAGTGCGACGGATGCAGAAACAGGTGA
TGATGATGATGATGATGATGATGACGATGATGAAGATGAGGATGACGAAGATGAGCCCCCCTTATTGAAGT
ACACACGA

CTTTCGTAGCACTCATTCTCCATAATTTAACTTGACGGTCGTCACTGCCGGAGACAATCAATGGTAAAGTT
GGATGGAAAGAGGCCCAGTTGACACCTCTTGTGTGACCCTCTAGAATAAACTTGACAACACAGTCACCAAG
AGAGCCAT

TGAAGTAGCCACGTCACTTGAGCTAGTTGGATCTGTTGATGTGGTTACCTCGTCAGAGCTTCTTGTACGAT
ACATACCAGCAACACTTGATGAGGCATGTGGACCAACTGTTGTAGTAGACTCAATTAAAGATGTAGAGTCC
GTGGAAGT

CGTGATTGGCAATTGTGCTCTAATAGTTACTCTATTATTGCTGTTAATTGACAATGTTTAGTCACGTGCAA
CACAATTCAAGTCACGTGGAAAGGCCTTCACATGGTGATCCATCTTCTACATCTTCATCGGTCCTGCATAA
AGTCATAA

AGATGTTTATTCCAATCCTATTCCTGTCAGCTACCTAGATACCTTAAACTTGAACCACTCCATGTCTGTAC
GGCAAACACAACAGCAACAACAACAGCGGCAACAGCAGCAGTATGTAGACTCTTCATCGGCATTTCAATGC
TGTACAGA

TTGGGTTGATATTGTTAATAATAGCACCCACATTCTCAGAAGACCTTTGTGAAATCTGAGATGCAGTGCAA
GATGAAATTGAACCTAAAGGTGCCTTCTCTGAAAGAGTTGGACTTATAGATGCAGCATTGTTCCTTAAGGC
ATTCATTT

CAAGGACTTGTTGTTGTTAGATGTTGCTCCATTATCTCTAGGTGTTGGTATGCAAGGTGACATGTTCGGTA
TCGTTGTTCCAAGAAACACTACTGTTCCAACCATCAAGAGAAGAACCTTTACTACATGTGCTGACAACCAA
ACCACCGT

TACCTGACTCGGTGATGGACATTAACATAGACGATATTCTTGCAGAACTGGATAAGGAAACTACTGCTGTC
GACTCTACCAAGATCACGCAAGGTTCTTCTTCCACTACCCACAGAGATGCAAATACAATAGTGGGTTCGTC
CTTAGACT

AAGTGGACTAGGCAACCATGGAAAGAACTCTGATATGGATGTTGAAGATCGTCTCCAGGCCGTTGTCTTGA
CAGACTCTTATGAAACTAGGTTTATGCCACTGACAGCTGTCAAGCCAAGGTGTTTGCTGCCACTGGCTAAC
GTACCTCT

CAATTACGCTAAAACCATAAGGATAATGTCTGAAGCTCAAGAAACTCACGTAGAGCAACTACCAGAATCTG
TTGTCGATGCCCCAGTCGAAGAACAGCACCAAGAACCACCACAGGCTCCAGATGCTCCACAAGAACCACAA
GTTCCACA

TACTTTTGGTGGTAAGCGACTAAACCCTTAGCAATAGCTTGTCTGATGGCGTAGACTTGAGAAACATGACC
ACCACCAGTGACTCTAACTCTGATATCGATGTTGGAGAACTTGTCCAAACCAACCAACAATAATGGTTCGT
AGACCTTG

GGTAACAGAGTCACCCCATCTTTCGTTGCTTTCACTCCAGAAGAAAGATTGATTGGTGATGCTGCCAAGAA
CCAAGCTGCTTTGAACCCAAGAAACACTGTCTTCGATGCTAAGCGTTTGATTGGTAGAAGATTCGACGACG
AATCTGTT

AATAATATTACCGAAAATGACACCTGTGTCAGCAGAGGAAACTTTGACAGTAACAGTGAATTTGTGGAAGC
CATGAGGAATCACGTTGGTCTTCTGTGGTGTGTCAATAATCTTCAAATCACCAAGAGTTGCAAATTGCACA
TGTAGGTT

GACAACATGCTTACCGTCCAACCATGGGCATGGAACGGTGGTGATGAAGAATTGAGAACCGTTGGTGTTTG
GACCGGCGTTGGCCATGGACAACAAACCTGGTCTGTCGTGGTGCTTCTTGAAGTTTTCATCTGGGAATTTG
CCACCGTA

TATTTTGTTGGAGTTGTCTTATGAGCTGGTGTAAAGAGAGGCCAGAATGCCCCTTGTGCAGACAACACTGT
CAACCACAGGAAATTCTGGTCCTGCGGCAATAGAGGAACTAATCAATAATTTGACTACTCTTTTAGCATTG
TCCACAGG

AGCAGCAGCAGTAGCGTAAGACCAAGTTAGTATGGCTACGTTCAACCCACAAAACGAGATGGAGAACCAGG
CACGTGTACAAGAGTACAAGGTCTCCACTGGCAGAGGCGGAGCTGGCAACATCCATAAATCTATGTCCAAG
CCGTCTCC

CTCCGACTTAACCGGATCAGATACACTATTGTGGTGTAATCTGGACCTTGACTGTCTAAACAACTCCTCTT
GTTGTCGTAGCAGCTCTTCTTCCGAGAGACCTGACTTCTTGAACTTGGAATCCCTAGTTTCTTTCACCTTC
TGGGAACC

GCTTGAGACCCTGGTTGTAGTTGCTGCAGTTGCTGCACATGACTGGACTCCTCCTGTAACTGCTGCCTTTG
CTGTTGAATCTGGTGCTGCTTTAGCTGTTGCTGTTGTTCTCTCAAGGTTCTATAATAGTTTTTTGCCGCCT
TAAGGGCC

CGACTACTTTGGCCCAATTTTCCAACAGTACATCTGCTTCTTCCACCGATGTCACTTCCTCCTCTTCCATC
TCCACTTCCTCTGGCTCAGTAACTATCACATCTTCTGAAGCTCCAGAATCCGACAACGGTACCAGCACAGC
TGCACCAA

TTCTTAGCATTCATACCATCCATTTCTGTTAATAGTTGGTTGACGACTCTATCCGAAGCACCACCAGCATC
ACCCAAGGAACCACCTCTTGCCTTGGCAATGGAGTCTAATTCATCTAGAAAAACGACGGTTGGTGCAGCTG
CTCTTGCC

CTCCACTTCCTCTGGCTCAGTAACTATCACATCTTCTGAAGCTCCAGAATCCGACAACGGTACCAGCACAG
CTGCACCAACTGAAACCTCAACAGAGGCTCCAACCACTGCTATCCCAACTAACGGTACCTCTACTGAAGCT
CCAACCAC

TGGTTCCTGAACAAGAATTAGCAATGCAACCACCAATCTGTGCACCTGGACCAGGGTCACAGCCAAACATC
AAACCGTGGTCGCTCAAATAGTCATTCAAATCCTCCCAGGGTAGACCGGCCTGCACGGTGATGTCCAGGTC
CAGCTTGT

CACAATACGTTATACTCACATTAAATATCCAGCCTGCAGAAAGTGCCACCAAGATTGAATCAACCACAATA
GCATTTGTCAAGGAACTGCCTTGGGATCTACACACCATTGTACCTGTTTCGAATGGTGCTATCATTGTGGG
AACCAATG

CATAGAATCTGACCATGACTTCCAATCCGACATTGAACTACATCCTGAGCAACCGAGAAATGTCCTTTCAA
AAGCTGTGAGTCCAACCGATTCCACACCTCCGTCAACTCATACTGAAGATTCGAAACGTGTTTCTAAAACC
AATATTCG

TGAGATCATATTATAAACTACTTCGAGGATTTGCCACAGAGGTCAAGGATAAAGTCTGGTTTCTCGAAGGA
GTCACGCAGGTGCTGCCTCAAGATGTTGACGAATACAGTGGAGGTGGTGGTATGCATATGATGCTAGATTT
CCTCGGTG

TTCTTGTCTTCGTATTTCCTTTTCCTCTGCATCTTCATCATTCTCGTTGCTGGAGTCCTCAGATTCGCTAC
TGCTGAACATTAAGTCATCGGCTGCTTGCTCACTTGAAGCCTGAGAGGGCTCTTTCCTTATACTTTGAGTC
TCATCTTC

GCCATCAACTGTTGCTGCTGTGCTTGAACTTGCTCCAACTGCTTCTGCAAAGTGAATTGTTCAATCATCAA
ATTTGTCAACTCACTTTGGGAACGTCTGTGTGCAACATGAATCTGCTTTGGGGCGTTTTTACCACCTCCTG
TGGTTTGC

AGCGGGCCCTCAGGGCTGGTCTACTGCCTTATACTCTAGGATGTACATCCTACCACACACAACAAGCCTGT
CACACCATGACTACAGATCCTTCTGTCAAATTGAAGTCCGCCAAAGACTCCCTCGTCTCCTCCCTCTTCGA
GTTATCAA

AGAGGGCACTATTGCTAAGTTTGGGTTGATATTGTTAATAATAGCACCCACATTCTCAGAAGACCTTTGTG
AAATCTGAGATGCAGTGCAAGATGAAATTGAACCTAAAGGTGCCTTCTCTGAAAGAGTTGGACTTATAGAT
GCAGCATT

TGAAAGCAGTACGAATCGTCTCTTGGACGACTTTATCTGTCTCCACGTCGACTGCGGCCGTGGCTTCATCA
AGCACCAAAATCTTTGATGGAACCAACATTGCTCTTGCAAGACATAATAATTGTCTTTGTCCAACACTTAA
GTTGCCAC

ATTGAATCATAATCCGTATTCTCTCTTCGTTCTTTAAAGTTTGTTTGGTGAGAATCCTCAAGTGGTAATTC
CACTGTCTCCCTTGCATTTATTGGCTGGTTTGGAGAGTTAGGCACATCCTCATCAAAGATATTTGTCAAAG
TCATCGCT

GCTTCCTTCACCTCAGGTTGGACTTCCAAATTGATATTCTCCATCACCTCACTTTCATTTCTTGTTTCTGC
TCCAGTTGAAGGACCAACAGGCTCTTCTGTTTCGATTCTGATAGACGATAAGTCCGTGGATTTTATAGAAT
GATCATAA

ATCGAAGCACTACGTAGTTCTGAAATTTTGGAAGTATCTGCTGATGGAGAGAACGTCAAGAGACGTGTTCC
TTTGGACCTAACTGCTGCCAGAAATGCCAGAATTGAGCAAAACCAACGGACTTTGGCTGTAATGAATTTCC
CACATGAG

ACTTGGATGCTAGTGTAATTCTTTTAGGTTGACCATTACTTTGAGTATCACGCTCCCCAGCAGCAGCAGCT
TCTTCATCCTGCTCAGACATTTGTCCTTCATCAGTGCTATTATCTGAATCACGTACGTTATTCATCATAGA
GAAGATGT

CGGTGGCGGAGCTTGAGACCCTGGTTGTAGTTGCTGCAGTTGCTGCACATGACTGGACTCCTCCTGTAACT
GCTGCCTTTGCTGTTGAATCTGGTGCTGCTTTAGCTGTTGCTGTTGTTCTCTCAAGGTTCTATAATAGTTT
TTTGCCGC

CTTCATTGCTGTTCTCGTTCTGAAGGTTATGTGAGTTTGTAGAAGAAGGGACAGAACTGCTTCTTTGAAGT
TGAACAAGCTGAACATCATCAACACGTTCGCCATTGAAAGAAGTAGGTACATTAACGTTTCTTGGTTGTTG
TGGTTGTT

ACTTCTCCTCTGAAGTTACTGCCGCTTTACGTGTCACTGACGGTGCTTTGGTTGTCGTCGACACCATTGAA
GGTGTCTGTGTCCAAACCGAAACTGTTTTGAGACAAGCTTTGGGTGAGAGAATCAAGCCTGTTGTTGTTAT
CAACAAGG

CTACCAAGAGGCCCACCAGCACCACCAACCACCTGAACCCCAACCATATTATCCTCCTCCTCCTCCAGGTG
AACACATGCACGGTCGCCCACCAATGCACCACCGTCAAGAAGGAGAACTCTCGAACACCAGATTGTTTGTT
AGACCTTT

GATCTTAACATAGAATCTGACCATGACTTCCAATCCGACATTGAACTACATCCTGAGCAACCGAGAAATGT
CCTTTCAAAAGCTGTGAGTCCAACCGATTCCACACCTCCGTCAACTCATACTGAAGATTCGAAACGTGTTT
CTAAAACC

AACCACCGCTCTTCCAACTAACGGTACTTCTACTGAAGCTCCAACTGATACTACTACTGAAGCTCCAACCA
CCGGTCTTCCAACCAACGGTACCACTTCAGCTTTCCCACCAACTACATCTTTGCCACCAAGCAACACTACC
ACCACTCC

TATTACTGCACCAGTTGGGGCCAACTACGCTGGATCGACGTGGGATTGGCAGGGAAGAACTTCGAGGGAGG
TGGTCGGAGAAGAGAGTGGGATGCGGGCGTGGGGGTCTGCTGTTACTTTTATCGGCACAACGGACCTGGGT
TGACAAGA

GTAATCAATCATAACAGGAAGCGATAAGAAAGTTTACATTGCACAGCCACCCTCATTGCCTCTGTGATTGA
AGATCTGGGAGTGGGGCTAAAGGTGCAACTTCCGTACAAGAGTGACGAGATAAAGGATTTTCCACGGAATG
GCTGACAA

CCGCCCACGGTTTATCCACAACAAGTGCCATTTCCAGGTCAACCACAGGGGGGTCAATTTCCACAGCCTTC
ATCAGAACAGCAGGTGTTTAACCAGCTTCCACAAGTGACTCAAACTTTTCACAACTCTGCGCAGAATACGA
ATGCAACT

TAAACACCCTGGTACAGTCATCCAGGAACTAACGTATTTGGAACTCAGGAAAAGGAACTGGAGATACCACA
AGACGTTGAAGGCCTGGCTCACCAAAGATCCTATGATGGAACCTATTGTATCCGCTGATGGTTTAAGTGAA
AGGGGATC

CATCGTGAGCCATTTCTTCAGCGTCGTAGTTGGCACCTCTTCTAGATGGTGGAACCCATGAGGCAGACTTC
CATGGTAGAACACCTTCTTCCCACATGGTGTTGACTTCTTCCAAAGTCAAACCCTTAGTTTCTGGAACAAC
TAACAAAA

TGATAAGACTCCATACGATGACATGCGTTCATTGAGTTTGTATGTGGTGGCCACAAGCTCAACCTGGAGGA
GTGCCTTTTCTAATCTGGTCTCATCCATCTTTGATGTGTGCTTCTTTAATAATTGCGACACCACCCTACCA
ATATCGTA

GCAAACCAAACGTCAAACACCGCTCAGGTAGCTTCATCGAACCCCTATGCTCCACCACCACAACAAAGAGT
AGCAACCCCATTATCTGGAGGCGTGCCTCCAGCTCCGTTGCCAAAGGCCTCTAATCCATATGCTCCAACTG
CAACCACT

TATTCAATGTGGTGCTCTATCTGGTGCTAACATTGCCACCGAAGTCGCTCAAGAACACTGGTCTGAAACAA
CAGTTGCTTACCACATTCCAAAGGATTTCAGAGGCGAGGGCAAGGACGTCGACCATAAGGTTCTAAAGGCC
TTGTTCCA

GAACTTGCATCGGATGATCCATTGAATTGCAAGTGTTGCTGCTGTTGTTGTGGTTGCGGTTGCGGTTGTGG
CTGCAACTGTGATTGCGATTGAAACATCTGGGTTGTCTGTGGTGCTTGCGACGTGAGGTTAGTAAATCCAC
CGTTATTA

TGGCTTTGAGATCACTGGTCTTTGAACGAAGTTGGAGGCATCCATCTGGTCTGGTAAAGTAACATCTTGTT
CATCAACTTCAGCTTGCTCCAAGAAAGCAGAAGCAGCCGCTTCATCTCCGTTAGCCTTTGCTACGGCATAA
GCTAATGG

GGTTCTTAGCGAACAAATAACCGAATAGAGTAGCTGAAGAGGCGATGACAGAGTATTTTAACCACTGGCTG
CTGCTGTCGGTGCTGCCTGTGCCTGTTGAGGCGTATCTGTAGCATGATCTTCTCACCAGCCTTCCTCTAGG
TTGTGCAA

CATCGCCATGAAATGTTCCTGCTGTGGAGGATACCCCTGCTGCGATGGCATTTGCGGTGGAGGATACGCCT
GTTGTTGCTGATGCGAAGGAGGAACCATGAATTGTGGATGATGTTGCTGCGGAGGAATAGCAAATTGTTGC
TGAGATGG

ATGAATCATCGTTGAATGACAATAATGATAATGATAACAGCAAGGACAGGGAGGAAACATCTTCTCACGCA
AGGACGGTTTCTATTGACGACATTCTGAACTCTACTTTGGAACACGATAGTAATAGCATTGAAGAGCAGAG
TTTGGTGG

CCTAACAACCATTGGCGGTACTTTGGAAGTTGTTGGTAACTTCACCTCCTTGAACCTAGACTCTTTGAAGT
CTGTCAAGGGTGGCGCAGATGTCGAATCAAAGTCAAGCAATTTCTCCTGTAATGCTTTGAAAGCTTTGCAA
AAGAAAGG

GTGTTCCTGAGTCTGACCAAGGCACTAGTACAGAAGAAGAGGATGAAGTAGATGAAGAGCAAGTCCAGGCG
TATGCTCCACAGATTAGTGATGGATTGGATGGAGACCACCAGCTAAATTCTGTAACGAGCAAAGAAAATGT
ACTTGAAA

GCGCCACGCCTACCAGGATCAACAAGTCTACTAGAGTGCAGGGGTACTCAACAGTGTCGATCTGGATGAGC
GACAGCTTCCTCTTCTCCGCAGCGATGCGGAACATCTTGTTGAAGATTTGCTTGATTCCGGTAGCAGTGCG
GGTATCGT

CATCAGGAATACTCTGGAATAAATACCATGTGTAACTAGGACAACAACATCTCTGGGTCTTCTCTCTTGCC
TATCATGGAAGTGCCTGAATAAAGTCTCTTGGAAACTGGCGACTCTGTCATATACATCTGCCGCACTTTCT
CCATGAGG

GTTCTTTGCAGCGGCCTGATATAAAGCTGTTGCGTAAGTGCCCTCAACACCGAACAATCTCACCGGTGGGG
GAGCAGCAGCTTTGGAAGCAGCAGCTCTTAAGCTTGATGCAAATGACCTGGTAAAGACTCTATTAAACATG
ATTGTCCA

CTTGACGGTCGTCACTGCCGGAGACAATCAATGGTAAAGTTGGATGGAAAGAGGCCCAGTTGACACCTCTT
GTGTGACCCTCTAGAATAAACTTGACAACACAGTCACCAAGAGAGCCATCCAATAGATTTTGTTGTGCACT
CATTTGCT

CCCTAGCAGGCCCAGAATAAACGAAATACGTCCCCTTACAGATTTCAAAGAGCCCATTTCACTGGACACTC
ATTTTGATGGTGTCAGCCACTCACAAGAGACCATAGTCACAGAGGAAATACCAATGAGTATACTCAATGGT
GAGCAAGG

GACTGTCCAGCCGGTAACATTATCGGTTTAGTCGGTATCGATCAATTCTTGTTGAAGACTGGTACTTTGAC
CACCAGTGAAACTGCTCACAACATGAAGGTCATGAAATTCTCTGTCTCTCCAGTTGTGCAAGTCGCTGTCG
AAGTCAAG

AAACCTGTCGTCGACAAGAAGGAAGAGGCTGCTCCAAAGCCACCATCCTCTGCTGTCTTCTCCATGTTTGG
TGGTAAGAAGGCCGAAAAGCCAGAAACCAAGAAAGACGAAGAAGATACCAAGGAGGAAACCAAGAAGGAAG
GTGATGAT

GTGGCCTTGATTGTCATAATTGTTACAACCTGTCGATAAATCGAAGGTGGTTGTCCCCCATTGCCAAGTTT
GCCAGTACTGAGCAGCATCACCTTGTAGATATTCGAACTGGATTTGGAAATTTGGCATCCAAACTGTGCAA
CCGTCAGA

ATTGCTAAGTTTGGGTTGATATTGTTAATAATAGCACCCACATTCTCAGAAGACCTTTGTGAAATCTGAGA
TGCAGTGCAAGATGAAATTGAACCTAAAGGTGCCTTCTCTGAAAGAGTTGGACTTATAGATGCAGCATTGT
TCCTTAAG

ACATCTATCCAATAATCTTGCTCAAAATTAACTTTGTCAACTCGGATGTTCAAATGCTTAGCGACACGGTT
CACATCTCTCCAATCCCTCTCGTAGCATGGCTCCTTGCCAGGGTCGTCCAAAGATTGTGATTCAGACCAGT
TCTGCATA

GGTGATGTTGGTGAGTACGCTGGAGAGGTAGGAGAGTATGTTGGGGAAGTTGGAGAAAAGCCTGGTGAGGA
GACTCCAAATCCGGGAGATGTAGGTGCTTCACCATAAGCACCAAATGGAGACGTGGCTTCACCATAATCAG
CACCACCG

ATATTTCTGAATCTGGTCAAGAAGATCAACAACAACAACAACAACAGCAACAGCAACAGCAACAACAGCAA
CAACAAGCTCCAGCTGAACAAACTCAAGGTGAACCAACCAAATAAGAGCGCTGAGAAATACAAGGGGAAAT
GAAGATTT

GCAAGGACGTCGACCATAAGGTTCTAAAGGCCTTGTTCCACAGACCTTACTTCCACGTTAGTGTCATCGAA
GATGTTGCTGGTATCTCCATCTGTGGTGCTTTGAAGAACGTTGTTGCCTTAGGTTGTGGTTTCGTCGAAGG
TCTAGGCT

CGATGAAGCTTTTGCCAAGAAGCACGAAGCTAGACAAAGATTGGAATCCTACGTTGCCTCCATCGAACAAA
CTGTCACTGACCCAGTCTTGTCTTCTAAATTGAAGAGAGGTTCCAAGTCCAAGATTGAAGCTGCTTTGTCC
GATGCTTT

CGACCATAAGGTTCTAAAGGCCTTGTTCCACAGACCTTACTTCCACGTTAGTGTCATCGAAGATGTTGCTG
GTATCTCCATCTGTGGTGCTTTGAAGAACGTTGTTGCCTTAGGTTGTGGTTTCGTCGAAGGTCTAGGCTGG
GGTAACAA

ACTCCATAATATTGCCATTATCAGCTGCCACAAGTACTCTCTCTTGATCTTGCACGCTGCTTGTTTCAATT
ACAGCCACCACTGGGTGCTGAAAGTAGTGTCTTGCCACCATATTTCGCTGCACGACATACACTACCGGTGT
TTCTGGCT

GAACATATCGTTGCTGGTACCGGTGAATTGCATTTGGAAATTTGTTTGCAAGATTTGGAACACGACCACGC
TGGTGTTCCATTGAAGATCTCCCCACCAGTTGTCGCTTACAGAGAAACTGTTGAAAGTGAATCTTCTCAAA
CTGCTTTG

AGACGTATCTGAATGTAGGTTTGACTTTAATGGCGACCTTGTTCCACCCACAAGACAAATAGACTCTACCA
TTCACTCAGGGTTGCACCATCACAGCGACTCGCCTGAACTCGCCGGTTACACCATAGTGGAGTTAGAACAT
TTAGCAAG

TGATTGAGTCCAACATAAATGAAGGTAGACAAGATATTCCCAAAGAAGGTGGCAGAGAGGGATTGAAGTAT
GGTTGTTCTGTTACGGATGCTTGTATTGGCTGGGAGTCCACCGAACAGGTATTGGAGCTATTGGCAGAAGG
TGTTAGAA

ATCATCGTCCTTGTCTCCTTGGAGAGAACAGAGCACATTATAAGCAGTCATCAAGTCACTTGGCGTCAAAG
CAGATCTCTGGTCTTTAAACTCGCTTGTCACTAGGAGACTATGTTCAGGGACCACAGGAAATTTGTTGAGT
AGCAGCTT

ATGTTGGTAAAATATTTCTTCAATGGCTCCAGCTTGACTACACCCACTTGCTTGCTCAAGGCTTGAACAGC
GGCATCAAAGGCACCAGCGGCAACTAGGACAGCAGGCAGTTTAGAATTCTTGATCCAAATGGCTGTTTCAA
CTTCCTGT

ATCTGGTCAAGAAGATCAACAACAACAACAACAACAGCAACAGCAACAGCAACAACAGCAACAACAAGCTC
CAGCTGAACAAACTCAAGGTGAACCAACCAAATAAGAGCGCTGAGAAATACAAGGGGAAATGAAGATTTAC
CACTCCAG

TTCCCCGTCTTCGGAACTGGTTTCGGAACTACTGCTATCCACGTCCTCATCATCCTCGTCCCCATTGTCTT
CATCTTCATCTTCATCTTCATCTTCATCTTCATCTCCATCTTCACCTTCATCGTCATGGCGTTTGTCATTT
GTATCTTC

AGTATTGAGATATTACGTAGAGGTGCTGTAATAGGTACTGTTAGTGTGAGCACGCACCTCGAAAGTAGACT
GCAACTGTGGAGAATGTGTATTTCCAAGGCCGAGTCTACTTTGGGACCATCAGTAACCAGAGAGTTGTACC
AAGAATGC

CCTCGCTGGTGACATCCTTGTCATTTAGATATTCTTCCGGAATCTTAGACTTCAATTGCTGCATATCAAAG
GTTCCGTCACCATTCAAAGTCACAGAACACACTCTGAAGTGAGTCCCACCTAAATCTGCAGCCAGCAAAAC
ACCACGTT

CAACAACAACAACTACAGGAACAACAGTACTTAGCTTCTATGCAACAGCAGCAACAGGCAATGTCCAACAA
TCCATTTGCCAAATCAGAACAGAGCTCAAGTTCACCAAAACGGAACCAACTAGTAGCAGCTTCTTCTCCAC
AGCAACTG

CGTTGATTTCATCAGCCTGTTTGAGTACCCATGGATCCTCTGGTGAAACCTTGTTGATCTTGGCGATGGAG
ACACAGGCCTCTTCATGTCTCTCACATTCAATCAGATATCTTGGGGACTCTGGAACTAATAACATGCCAAC
GATGATAA

TAGCTCCCTTCTTTGGTCTCATCAGAGTAACGGCTAGGAACACCAGGTTCAATAGCAACACGGTCACCAAC
TTTGACCCTTGTGACGGCATCACCAACTTCCACAACCTGTCCGCTTGATTCATGACCTAAAACCATTGGCG
CCTTCAAT

ACCCTCATAAATGTTGGTAAAATATTTCTTCAATGGCTCCAGCTTGACTACACCCACTTGCTTGCTCAAGG
CTTGAACAGCGGCATCAAAGGCACCAGCGGCAACTAGGACAGCAGGCAGTTTAGAATTCTTGATCCAAATG
GCTGTTTC

ACAAGCTCTCCATTAAGTCCATCAGGCTCCACCCCAGAACATTCCACCAAGGTCTTGAACAACGGCGAAGA
GGAGTTCATTTGTCACTACTGTGACGCTACTTTCAGGATTAGAGGATATCTAACGAGACATATTAAGAAGC
ACGCCATC

GTTAGTGGCCTGTCTTGCTGTCTTTACAGCGGTCATTAGATTGCATGGCTTGGCATGGCCTGACAGCGTGG
TGTTTGATGAAGTACATTTCGGTGGGTTTGCCTCGCAATACATTAGGGGGACTTACTTCATGGATGTGCAT
CCTCCTCT

CCACAGTAGTTATTTTTTCTTAGATATTCTTTAATACTGGGGTTGTGACTGACCAGGTGGCATCGCCATGA
AATGTTCCTGCTGTGGAGGATACCCCTGCTGCGATGGCATTTGCGGTGGAGGATACGCCTGTTGTTGCTGA
TGCGAAGG

ACCACGATCTGATTGACGAGTTTGAATCTCAAGGTTTTGAAAAGGACAAGATTGTGGAAGTGTTGAGACGA
TTAGGCGTCAAGTCCTTAGACCCCAATGACAACAACACAGCCAACCGTATCATCGAGGAATTGTTGAAGTG
AATAGATA

ACCTTCCAAACGACACCATTGACCAACGGTAGAGTTCCAGCCTACGATGGTAGATTTGACCAAGGAGTGGT
TCTTGATGGTGGAGTTGCACAAAACAACAGATCTGGTGATTCTAACACCATCACCGATGGTGACATTAGGA
CCGATAAC

CCAGAGAATTAGCCATGCAGACTTTCAACGTTTTCAAGGATTTTGCCAGAGGAACAGAACTAAGAAGCGTC
CTCTTGACTGGTGGTGATTCCTTGGAAGAACAATTTGGCATGATGATGACCAACCCAGACGTTATTATTGC
AACACCTG

ATTATCTCTAGGTGTTGGTATGCAAGGTGACATGTTCGGTATCGTTGTTCCAAGAAACACTACTGTTCCAA
CCATCAAGAGAAGAACCTTTACTACATGTGCTGACAACCAAACCACCGTTCAATTCCCAGTCTACCAAGGT
GAACGTGT

AGGAAGATAACCTCTTAGAAGCAGTAGTAACTGAGCCTGCCTTCGATGAACTTGAGACGGAAGATTGTACC
GCTGACATGGGTGTGCTGGATGACGAAGATGTGTAACTGTTCTGTCTTATTAGAGTTGTTTTCAATACCGC
ATTCCCAC

CTAGATCGTACAAAGAATTGGTAGATCCTGATTTCCCAAGTGACAATGTTCTCCTCAGTGAAAGTCCTTCA
TCGTGGTTATCGTCTACTTCTTGGTCATCGTCATCTTCTTCCTCTTCGTCGTCGCTACTATTGTATTGTGA
GTCTTCAA

CATGTTCGGTATCGTTGTTCCAAGAAACACTACTGTTCCAACCATCAAGAGAAGAACCTTTACTACATGTG
CTGACAACCAAACCACCGTTCAATTCCCAGTCTACCAAGGTGAACGTGTTAACTGTAAAGAAAACACTTTG
TTGGGTGA

ATAAGCTCAGAACTTCGCTGGATCGCACAAGAGAAGAGATGCTTGCCAACCACCAACTGGACACAAGATAC
TCCGTGGAAAGAGCACGTGCCTCGCTGGATCTGCCGGGTATCAATCATGCAGAGACGCTGCTCAGCCAGCG
CAGCAGGG

CGCTTATTCGATGTCAAGAGTAGGGATCACCTTTCTGCGAAGTTGGAGCCCAAACTAACGATTCAGCACAA
TTGCCAAACAGGAAGATGGACAAGCATCCTCAAGGCCAGGTTCAAGCCGAACAAGAATGTTTTTGCAATAG
CTAACATG

CGGTGAATTGCATTTGGAAATTTGTTTGCAAGATTTGGAACACGACCACGCTGGTGTTCCATTGAAGATCT
CCCCACCAGTTGTCGCTTACAGAGAAACTGTTGAAAGTGAATCTTCTCAAACTGCTTTGTCCAAGTCTCCA
AACAAGCA

GTGATGTTGGTGAGTACGATGGTGATGTTGGTGAGTACGCTGGAGAGGTAGGAGAGTATGTTGGGGAAGTT
GGAGAAAAGCCTGGTGAGGAGACTCCAAATCCGGGAGATGTAGGTGCTTCACCATAAGCACCAAATGGAGA
CGTGGCTT

AAGTACATTTCGGTGGGTTTGCCTCGCAATACATTAGGGGGACTTACTTCATGGATGTGCATCCTCCTCTT
GCAAAGATGTTGTATGCTGGTGTGGCATCGCTTGGTGGGTTCCAGGGTGATTTTGACTTCGAAAATATTGG
TGACAGCT

TCATTTTCTTTGCCGCCGATGGAATAGCCTTTCTTGGATTGGATTGTTCACTGGCAGTCATAGCCAGCTGT
TCACTCATACCAAAGGCGAACGCTGCTGTGACAAATGTGGCAACGACACCTTTGAACCTCTGGATGGGTGT
ATCACCAC

GAGCATATTTCACAACACCTGTTTGTAAGTTGTAGGGAACGGTGAAACTCTTAGAATCAATCGTGGCACCC
ACATCTCCTGTGGTAATTCTTGTTTGTGCCTCTGGTGCAATGGTTATCATACTCGGATTAGCGGCTTTTGT
ACCAATCA

CGGTGGTTCCAGAGGTGGCTTCGGTGGTAGAGGCGGTTCTCGTGGTGGTGCCCGTGGTGGTTCCAGAGGCG
GCTTCGGTGGTAGAGGCGGTTCTCGTGGTGGTGCCCGTGGTGGCTCCAGAGGTGGTAGAGGTGGCGCTGCT
GGTGGTGC

GCCTGCACTATCAGTAGGAGGCTCGATATCAAGAGGGTTCTTGTAGTCCAGAGCCACGTTCTCAAGACCTA
CAGGTGAGTCCTCTAAGTTTCGTGTGGAAAGCGCTACCTCCTTCATCAACAAGACACGTTCATCCATATTT
TGCAAGTA

GTAAGATCTTATGTGTCCATGGAGGGTTATCTCCCGAGATTAGAATGCTCGATCAAATCAGAGTCTTGTCA
AGAGCTCAAGAAGTGCCACACGAGGGTGGGTTCTCGGACCTGCTTTGGTCAGACCCTGACAATGTAGAGGC
TTGGCAAG

AAACCTCGGCGGCTATGTCTCGTACTAACATGGATACAAGACACGCACATTCTGCTTTACTGGCAGCACCA
CAGAGTGCTACTGCAAATAGCAGGAGCAGCAACAGCAGCAGCGAGAGTAGTAGTAACAAAAACAATATCAA
TGTCGGCG

TTGTGCGAGACGGATCGATTGACGCCGACGATTGCGCAGGCACCTGCACCTGCGCCTGCGCCTGCGTGTCG
GTGCTAATGTTCTTCAAACTCCATACTGCCATACCTGGGCTGGCGGTCTTGTCATTGTCCCACAGGATCCT
TTCAACTT

CGATGTCGGACTCAGAAGTCAATCAAGAAGCTAAGCCAGAGGTCAAGCCAGAAGTCAAGCCTGAGACTCAC
ATCAATTTAAAGGTGTCCGATGGATCTTCAGAGATCTTCTTCAAGATCAAAAAGACCACTCCTTTAAGAAG
GCTGATGG

ACGAAAAGGACTTGGAAGGTAAGGCCTTGTTGAAGGTTGTTATGAGAAAGTTCTTGCCAGCTGCCGATGCC
TTATTGGAAATGATTGTCTTGCACTTGCCATCTCCAGTCACTGCTCAAGCCTACAGAGCTGAACAATTATA
CGAAGGTC

CGTCAGAGCTTCTTGTACGATACATACCAGCAACACTTGATGAGGCATGTGGACCAACTGTTGTAGTAGAC
TCAATTAAAGATGTAGAGTCCGTGGAAGTGGAAATCACGACAGAGATATTTAGACTTGTTGAAGTAGTAAT
ATCGTTTG

TACCTACGAAATCTTGGACCCTCACGATTTCGGTATGAAGAGGTATATCCACTTCGCCAACAGACTAACTG
GCTGGAACGCCATCAAAGCCAGAGTCGACCAGTTGAACTTGAACTTGACGGATGACCAAATCAAGGAAGTT
ACTGCTAA

GGAAGGACTACTGAGTGTACAAGGTATGACCTGTGGGTCTTGTGTTTCTACAGTCACCAAACAAGTGGAAG
GCATTGAGGGTGTTGAATCGGTAGTCGTTTCCTTGGTAACGGAAGAGTGCCATGTTATTTATGAACCGTCC
AAGACAAC

ACCATGGGCATGGAACGGTGGTGATGAAGAATTGAGAACCGTTGGTGTTTGGACCGGCGTTGGCCATGGAC
AACAAACCTGGTCTGTCGTGGTGCTTCTTGAAGTTTTCATCTGGGAATTTGCCACCGTAGATAGACTTACC
GCCGGTAC

GATGGTTGTGCTAGATCGTACAAAGAATTGGTAGATCCTGATTTCCCAAGTGACAATGTTCTCCTCAGTGA
AAGTCCTTCATCGTGGTTATCGTCTACTTCTTGGTCATCGTCATCTTCTTCCTCTTCGTCGTCGCTACTAT
TGTATTGT

TAACTATATTTAACGCTACAAATCTAACCTCATTCGCTGCTGATTCCTTGGAGTCCATCACAGATTCTTTG
AACCTACAGAGTTTGACAATCTTGACTTCTGCTTCATTTGGGTCTTTACAGAGCGTTGATAGTATAAAACT
GATTACTC

ACACAACATCTTGGCTTTGAGAAGGGCCAAGAGACGTAACATGGAAAGATTGCAATTGGTCACTGGCGGTG
AAGCTCAGAACTCTGTGGAAGACTTGTCGCCTCAGATTCTTGGGTTTTCTGGCTTGGTCTACCAAGAAACC
ATAGGCGA

GAGTACGATGGTGATGTTGGTGAGTACGATGGTGATGTTGGTGAGTACGCTGGAGAGGTAGGAGAGTATGT
TGGGGAAGTTGGAGAAAAGCCTGGTGAGGAGACTCCAAATCCGGGAGATGTAGGTGCTTCACCATAAGCAC
CAAATGGA

GAAATGATTGTTAGAGCCAGAATTTCCTGACAGGTATTGTCCTGTCTGTTGTGCCAATTGCTGTTGCTGAG
CCATCAACTGTTGCTGCTGTGCTTGAACTTGCTCCAACTGCTTCTGCAAAGTGAATTGTTCAATCATCAAA
TTTGTCAA

GAGTAACTGGTATAAGAAACAATAATGCAAGACCAACCGTCTCTGTTGTTATTCGTGGTGCAAACAACATG
ATCATTGATGAAACAGAGCGTTCCCTCCATGATGCACTATGTGTTATTCGTTGTCTAGTGAAAGAGAGAGG
TTTAATCG

GGATTCTTTTATTTACGACGAAAATTCAGTCATACAGTTACACCAACCAGTAATGTCATCGCTTGAAGAAA
TTCTCTTCTTCCTTCAAGCTCCAACTCAAACAACACTCTCTCTGGCTTTGATAAACTCGATCTATGATATG
CTTGATTA

AAACTTAAGTGACTGGAATAATGGTTGGCAATTTTGTCTTGCTGGTTTCATGCCTGCTGTCTGGACTATTG
GTTCCTTTGATTCATGTGTCCATCAATCTGAAGAAGCCAAAGATGCCAAGAAATCAGTCCCCATTGGTATA
ATCTCATC

ACTATCAAGCGAAGTGGTACTTGGATGACGTCATTGACGCAAATGAGGAAGAAGAGGCAGAACAGACCAAT
GGAAAGGATGAGACAATGATGGAAATTGATGATGAAATGATGGTGGAGCAAGACAATGAAGAGGTAGCAGG
CGACGAAG

CCGGATACAAGAGCGCCAAGCGATAGCAATAAAGTAATAACAGAGATATTTGACGCTACTGAGGATGCCAA
GGAGGCAGACGAAAGTGAAAGAGGAATGCCACTTGCGACAGCATTGAATACATATCCCAAGGCAGCAGCTT
GGTCACTA

CTGCTGAACTTGAACTGATAACAGAGCTACTTAGATCAGCTGAACTTGAACTGACTACGGAAGTAGTTGGA
TCAGCTGAAGTAGTTTGAACGGTCGAGGTAGTTGGGTCAGCTGAAGTAGTTGGGTCAACTGAAGTAGTTGG
ATCAACTG

CATACATTCAATGAAGTACGCTGTTAGTTCGTCCAACTTTGCTGCATCAACCTGCAATGAGGAGCAGATCT
CAACCACTGCATCTTCCAATGCCTCAGCATTGACTTTATGTAAATTTTCAAATGACATTACTGTGTATCTA
TGATTCGA

ATTGGTTTTAGAAACACGTTTCGAATCTTCAGTATGAGTTGACGGAGGTGTGGAATCGGTTGGACTCACAG
CTTTTGAAAGGACATTTCTCGGTTGCTCAGGATGTAGTTCAATGTCGGATTGGAAGTCATGGTCAGATTCT
ATGTTAAG

GTGAGTACGCTGGAGAGGTAGGAGAGTATGTTGGGGAAGTTGGAGAAAAGCCTGGTGAGGAGACTCCAAAT
CCGGGAGATGTAGGTGCTTCACCATAAGCACCAAATGGAGACGTGGCTTCACCATAATCAGCACCACCGTA
CGCTGTAA

GCCTGCACCTCTGGGACTGATTCCCCATCCGCCTCTATCGTCAGGGTCTGACCATAGAAGGTCACACATAG
GACCTTCATGAGGCACTTCCTGTATTCTGTTCAACTCTCTCACCTGATCTATGGTTTCTATCATGGGGGAA
AGTCCTCC

TGAACTGATAACAGAGCTACTTAGATCAGCTGAACTTGAACTGACTACGGAAGTAGTTGGATCAGCTGAAG
TAGTTTGAACGGTCGAGGTAGTTGGGTCAGCTGAAGTAGTTGGGTCAACTGAAGTAGTTGGATCAACTGAA
GTAGAACT

TGAAGTTACTGCCGCTTTACGTGTCACTGACGGTGCTTTGGTTGTCGTCGACACCATTGAAGGTGTCTGTG
TCCAAACCGAAACTGTTTTGAGACAAGCTTTGGGTGAGAGAATCAAGCCTGTTGTTGTTATCAACAAGGTC
GACAGAGC

CTTCACCAAAGACAACATGCTTACCGTCCAACCATGGGCATGGAACGGTGGTGATGAAGAATTGAGAACCG
TTGGTGTTTGGACCGGCGTTGGCCATGGACAACAAACCTGGTCTGTCGTGGTGCTTCTTGAAGTTTTCATC
TGGGAATT

TCATTGACGCAAATGAGGAAGAAGAGGCAGAACAGACCAATGGAAAGGATGAGACAATGATGGAAATTGAT
GATGAAATGATGGTGGAGCAAGACAATGAAGAGGTAGCAGGCGACGAAGAATATGACATAGAGGATAATGA
AGGATTTG

TGGAGTGAAGTCGTTGACGATATTAAACGTTCTTCTAATTTCCTCGGGAGACCTTCCCCTGATCATTTCAG
CAACCACCTTGCAGCCAGCGTCAAGCAAGGGCTTGATGTTGAGGTAGTTTGCCGCCAGAATAATTTCGTAT
AGCATCTC

CGACTCTCCAGGTCACGTTGACTTCTCCTCTGAAGTTACTGCCGCTTTACGTGTCACTGACGGTGCTTTGG
TTGTCGTCGACACCATTGAAGGTGTCTGTGTCCAAACCGAAACTGTTTTGAGACAAGCTTTGGGTGAGAGA
ATCAAGCC

GCTCTAGCTTCTTCAACACTAGCTTCAATAACTTCCAACTCTTGTTGAATGAATGGATGGTCTGGGGCAAC
CTTGTTAACTTTGGAAAGAGATGCTCTTGCTTCGTCAATTTGACCAGCTTCAACCAAATAACGTGGGGATT
CTGGAACG

CCTTGTTCCACAGACCTTACTTCCACGTTAGTGTCATCGAAGATGTTGCTGGTATCTCCATCTGTGGTGCT
TTGAAGAACGTTGTTGCCTTAGGTTGTGGTTTCGTCGAAGGTCTAGGCTGGGGTAACAACGCTTCTGCTGC
CATCCAAA

TTGCTGGTACCGGTGAATTGCATTTGGAAATTTGTTTGCAAGATTTGGAACACGACCACGCTGGTGTTCCA
TTGAAGATCTCCCCACCAGTTGTCGCTTACAGAGAAACTGTTGAAAGTGAATCTTCTCAAACTGCTTTGTC
CAAGTCTC

AGGTCATGGATGGTAAAGCAAACATTGAAGTTCGTCCAAGATTCGTCAACAAAGGTGAAATAGTCAAGAGA
CTAGTCTGGCATCAACATGGCAAACCACAGGACATGTTGAAGGGAATCAGTGAAAAACTACCTAAGGATGA
AATGCCTG

TTTTTGCTGTATTGTTCCAGGCAGAGCGGTTATAATTCACCGGACTTGGCAACAACAATTCTAGCCTTGGT
GGCACCGGAAGGAGAACCCAAGGACTCAACCTTCTTAACGATGTCGTAACCGTCAACAACTTCACCAAAGA
CAACATGC

CCACCGAAAGTACCGACTCCAACACTAGTGCTACTACCACCGAAAGTACCGACTCCAACACTAGTGCTACT
ACCACTGCTAGCACCAACTCCAGCACTAATGCCACTACCACTGCTAGCACCAACTCCAGCACTAATGCCAC
TACCACTG

ATTCTGTGGAGGTGGTACTGAAGCAGGTTGAGGAGAGGCATGATGGGGGTTCTCTGGAACAGCTGATGAAG
CAGGTGTTGTTGTCTGTTGAGAGTTAGCCTTAGTGGAAGCCTTCTCACATTCTTCTGTTTTGGAAGCTGAA
ACGTCTAA

CTGACTACGGAAGTAGTTGGATCAGCTGAAGTAGTTTGAACGGTCGAGGTAGTTGGGTCAGCTGAAGTAGT
TGGGTCAACTGAAGTAGTTGGATCAACTGAAGTAGAACTGACTATAGAACTAGTTGGGTCAACTGAAGTAG
TTGGATCA

AGGACTAGTGCGATTACCACTGAAAGTACCAACTCCAGCACTAATGCTACTACCACTGCCAGCACCAACGT
CAGGACTAGTGCTACTACCACTGCCAGCATCAACGTCAGGACTAGTGCGACTACCACTGAAAGTACCAACT
CCAACACT

GCCTACAGAGGTGTTATTCAACCCCTTTTCCATACATTCAATGAAGTACGCTGTTAGTTCGTCCAACTTTG
CTGCATCAACCTGCAATGAGGAGCAGATCTCAACCACTGCATCTTCCAATGCCTCAGCATTGACTTTATGT
AAATTTTC

TTACGTTTTGTTCTTATATTTCCAGCTAGGGAGCCATCACGCCTTGTTTGAGCAACAAGAAGAAGAGACCG
ATGAAGTTATGAGCACCATTTCCAGGAATCCACATCACTCTTTGAGTGTCAAGTCATCATTGGTGATACTT
CTAGGTAC

ACAGATAATGGCAACTTCAAACCCAAAGATTTAACCAAGTCAGAGGTTTGGTTGTCAACGTTCAAAACACC
GTTAGAACGAGACAAGTCCAAAGCAGAGACTTCTGGGACGTATTGATCCTTTCTTTCTCTTTCTTCTTCTT
GCAATTTG

TTTAGAATTCTTGATCCAAATGGCTGTTTCAACTTCCTGTGCTGGAGAGGTAATTTCGCCTTGCTCAACTT
CTTCAGGTAACTCTTCTCCCACATCCAAATCTTCGTCACCCAAATCCCAGGCACCTTCGTCTTCACCAATA
TCCTCATC

AATTATTTGGTGCTGAACATTCTCTTGTACAATGGCTTATCATCGTGAGCCATTTCTTCAGCGTCGTAGTT
GGCACCTCTTCTAGATGGTGGAACCCATGAGGCAGACTTCCATGGTAGAACACCTTCTTCCCACATGGTGT
TGACTTCT

AAGTGCCACCAAGATTGAATCAACCACAATAGCATTTGTCAAGGAACTGCCTTGGGATCTACACACCATTG
TACCTGTTTCGAATGGTGCTATCATTGTGGGAACCAATGAACTGGCATTTCTAGATAATACTGGCGTTTTA
CAATCGAC

CAAGAATCCAATGATCTTAACATAGAATCTGACCATGACTTCCAATCCGACATTGAACTACATCCTGAGCA
ACCGAGAAATGTCCTTTCAAAAGCTGTGAGTCCAACCGATTCCACACCTCCGTCAACTCATACTGAAGATT
CGAAACGT

ACTAAGGTTAATTCTCAAGAAGAGACAACACCTGGGACATCAGCTGTTCCAGAGAACCATCATCATGTCTC
TCCTCAACCTGCTTCAGTACCACCTCCACAGAATGGACAGTACCAACAGCACGGCATGATGACCCCAAACA
AAGCTATG

CCAACTCCAGCACTAATGCTACTACCACTGCCAGCACCAACGTCAGGACTAGTGCTACTACCACTGCCAGC
ATCAACGTCAGGACTAGTGCGATTACCACTGAAAGTACCAACTCCAGCACTAATGCTACTACCACTGCCAG
CACCAACG

AAACGAGAACCGCAATACTGGCGCTGGCGTAGATGTAAATACAAATGCAAATGCAAATGCAAATGCAACTG
CAAATGCAACTGCAAATGCAACTGCAAATGCAACTGCAGAGCTGAACCTCCCCACGGTCGATGAGCAAAGA
CAGTATAA

ATAGGACATTTATGGCTATCTGATAAGACTCCATACGATGACATGCGTTCATTGAGTTTGTATGTGGTGGC
CACAAGCTCAACCTGGAGGAGTGCCTTTTCTAATCTGGTCTCATCCATCTTTGATGTGTGCTTCTTTAATA
ATTGCGAC

TTTCTTCAGCGTCGTAGTTGGCACCTCTTCTGGATGGTGGAACCCATGAGGCAGACTTCCATGGTAGAACA
CCTTCTTCCCACATGGTGTTGACTTCTTCCAAAGTCAAACCCTTAGTTTCTGGAACAACTAACAAAACATA
GAAGAACA

TGATGACAAAGTAGAAGCAGTAGCTGACAACAAATCTAAAGGGGAGACGAAAGTTGCTGTGTCTCAAGAGG
GATTGAAAGATGTTAGTGACCATGTTGGCCTCGCCAACAAGGATGAGAGCAAGGACGACGACGACGATGAT
GACTGGGA

GAGTATGCTACAAGAATGACCGGCTGAAACGTCGGTGCCTAGACCAACCTTTATGCCCTGGTCCAGCAACC
ATCGAACCCTACACTCTCCAGAAGTCAGAGAGGAGTTGGAAATGGGACAATGAGATATACCACAGCGACGC
TGTTTAAT

ATGATCTTAACATAGAATCTGACCATGACTTCCAATCCGACATTGAACTACATCCTGAGCAACCGAGAAAT
GTCCTTTCAAAAGCTGTGAGTCCAACCGATTCCACACCTCCGTCAACTCATACTGAAGATTCGAAACGTGT
TTCTAAAA

AACTTCGCTGGATCGCACAAGAGAAGAGATGCTTGCCAACCACCAACTGGACACAAGATACTCCGTGGAAA
GAGCACGTGCCTCGCTGGATCTGCCGGGTATCAATCATGCAGAGACGCTGCTCAGCCAGCGCAGCAGGGAC
CGTTAATG

GTCACTGCCGGAGACAATCAATGGTAAAGTTGGATGGAAAGAGGCCCAGTTGACACCTCTTGTGTGACCCT
CTAGAATAAACTTGACAACACAGTCACCAAGAGAGCCATCCAATAGATTTTGTTGTGCACTCATTTGCTCC
TCAAATGA

AAAGCGCTAATTGACGTATTGCCAGTATTGCCTGTATTGGTACCAAACTGGGGCATTCCAAATGCTGAAGT
GGTGGTTCCTCCTGTCATTGTGTTTGCTGTTCCAAAACTAGGTCTTCCAAATGCACTTCCTCCCATGTTAT
TGGTACTA

GATAAATCAATAGTCAATATGCCTCCAAAGTTTGATCCAAATGAAGTTAAGTACTTGTACTTGAGAGCTGT
CGGTGGTGAAGTCGGTGCTTCCGCCGCCTTGGCTCCAAAGATCGGTCCATTGGGTTTATCCCCAAAGAAGG
TTGGTGAA

AATATTTCTTCAATGGCTCCAGCTTGACTACACCCACTTGCTTGCTCAAGGCTTGAACAGCGGCATCAAAG
GCACCAGCGGCAACTAGGACAGCAGGCAGTTTAGAATTCTTGATCCAAATGGCTGTTTCAACTTCCTGTGC
TGGAGAGG

CTTAGAATGATTGTGTCAGAGAAAGTAGCTAGATCTGTTGAAGTAGTTGGACCACTTGGTCTGGTTGAATA
AACTGAGGTTGAACTGACAATAAAACTGGTTGGTTCAGATGAAGTTGAACTAACTACAGAACTAGCTGGAT
CAATTGAG

GAGACAACACCTGGGACATCAGCTGTTCCAGAGAACCATCATCATGTCTCTCCTCAACCTGCTTCAGTACC
ACCTCCACAGAATGGACAGTACCAACAGCACGGCATGATGACCCCAAACAAAGCTATGGCCTCTAACTGGG
CACATTAC

AAAAGGCGGCAGTTCCTAAGGGAACTTCTGACTATCAAGCGAAGTGGTACTTGGATGACGTCATTGACGCA
AATGAGGAAGAAGAGGCAGAACAGACCAATGGAAAGGATGAGACAATGATGGAAATTGATGATGAAATGAT
GGTGGAGC

ATCTGTTGATGTGGTTACCTCGTCAGAGCTTCTTGTACGATACATACCAGCAACACTTGATGAGGCATGTG
GACCAACTGTTGTAGTAGACTCAATTAAAGATGTAGAGTCCGTGGAAGTGGAAATCACGACAGAGATATTT
AGACTTGT

TTTGTTTGCAAGATTTGGAACACGACCACGCTGGTGTTCCATTGAAGATCTCCCCACCAGTTGTCGCTTAC
AGAGAAACTGTTGAAAGTGAATCTTCTCAAACTGCTTTGTCCAAGTCTCCAAACAAGCATAACAGAATCTA
CTTGAAGG

CAACGAGTGCAGCAACAAAGGGTACAACAACAACAACAACAACAGCAGCAGCAGCAGCAACAGCAGCAACA
GCAACAGCAACAGCAACAGCAACGCCAGGGTCAAAACCAAAGAAAGATTTCTAGTTCTAATTCTACTGAAA
TACCCTCT

GGACCCACACTATCCGCTGCCACAGTATATCCCACCGCTGAGTACTTCCTCACCTGATCCAATCGATTCAC
AGGATCAACACTCTGAAGTACCTCAAGCTAAGACAAAGGTGAGAAATAATGTCTTACCACCACACACTTTA
ACATCAGA

CGATAGCAATAAAGTAATAACAGAGATATTTGACGCTACTGAGGATGCCAAGGAGGCAGACGAAAGTGAAA
GAGGAATGCCACTTGCGACAGCATTGAATACATATCCCAAGGCAGCAGCTTGGTCACTATTGGTCTCTACA
ACTTTAAT

TTAGATCTTGCAATTCCTGTTCCTGGATAGCGAACAAATCCTCTGGATTGTTTTCCTCAAAGGGGTCCACC
AAAGTCACATTGGTGGGGATTTGTACTTGATTTTGTGTTTCTGTTTCTATTTGCCCCTTTGTTTCTGATTG
TTGCGATA

TCAGTAGGAGGCTCGATATCAAGAGGGTTCTTGTAGTCCAGAGCCACGTTCTCAAGACCTACAGGTGAGTC
CTCTAAGTTTCGTGTGGAAAGCGCTACCTCCTTCATCAACAAGACACGTTCATCCATATTTTGCAAGTAGC
GTGACCAG

TCACCCCATCTTTCGTTGCTTTCACTCCAGAAGAAAGATTGATTGGTGATGCTGCCAAGAACCAAGCTGCT
TTGAACCCAAGAAACACTGTCTTCGATGCTAAGCGTTTGATTGGTAGAAGATTCGACGACGAATCTGTTCA
AAAGGACA

GGCTACTATTGTGGCTGTTGGATGATGAGAAATCTAATTTGGCTGCATCTTCATGATTCATTTCATCAAGC
ATGCCACCATCACGGCCCCACTTTCTCCCACTACCAACATTTCCTGTAGATTGCTTCTTACCTTTTGTATC
CCTCAATT

TAAAGTCCTTGTTGAACTTTGCATCATTACTCCTTGCCTTCGGAACGTGAGGTGGAAGACTCCACACTGCA
ACAGCATCGAAAGTATTAACTTCGGAGATCTCACCATAAGGTGAATCCAGGAATGGCGATATGATATCGGT
GGTTATTG

CCATCTGCCGCACTATGGAGGGTGATTCAACGGAAAAGGCGCAGGAAGTGCAGTAGTGACCGGCAAGCAGC
ATGTTGAAAGACATTGTTGGGAAATCCTGTTTGAGTCCTGGTGTGTCTTCATTCTCCTACCATTTGTAGCT
GGCGTAGA

CTGATGGAGAGAACGTCAAGAGACGTGTTCCTTTGGACCTAACTGCTGCCAGAAATGCCAGAATTGAGCAA
AACCAACGGACTTTGGCTGTAATGAATTTCCCACATGAGGACGTTGAAGCTTCCCAAATTCCTGAATTGCA
GGAGAACT

TGGTTCAAATACATCTATCCAATAATCTTGCTCAAAATTAACTTTGTCAACTCGGATGTTCAAATGCTTAG
CGACACGGTTCACATCTCTCCAATCCCTCTCGTAGCATGGCTCCTTGCCAGGGTCGTCCAAAGATTGTGAT
TCAGACCA

AGAAATTACGGCCTAGATTTGATTAGAGGTAAAATTGTGGACGAGATTCACGCTGGTGTTCTTGAGCCAAC
CATAAGCAAAGTCAAATCCTTGAAATCAGCATTGGAGGCTTGTGTAGCAATTCTAAGAATTGATACAATGA
TTACAGTG

CACACTACCCCTAACACTACCCTATTCTAACCCTGATTTTACCTGTCTCCAAACCTACCCTCACATTACCC
TACCTCCCCACTCGTTACCCTGCCCCACTCAACCATCCACTCCCAACCACCATCCATCTCTCTACTTACCA
CTAACCAC

AATGACTTACGTGTTCCTGAGTCTGACCAAGGCACTAGTACAGAAGAAGAGGATGAAGTAGATGAAGAGCA
AGTCCAGGCGTATGCTCCACAGATTAGTGATGGATTGGATGGAGACCACCAGCTAAATTCTGTAACGAGCA
AAGAAAAT

ATTGCGTCAAATGAGATCCAAGAATCCAATGATCTTAACATAGAATCTGACCATGACTTCCAATCCGACAT
TGAACTACATCCTGAGCAACCGAGAAATGTCCTTTCAAAAGCTGTGAGTCCAACCGATTCCACACCTCCGT
CAACTCAT

AACCTTCCATCCGCTAATGTTATCTTAGGTTTGTGTCTGTCATTTATGCTTTCTTTCTGTGGTGTTACACA
ACCTGTCTCATTGATGCCTGGCTTCTGGACATTCATGTGGAAGGCTTCCCCATACACATATTTTGTTCAGA
ATCTGGTC

CATTTGGAAATTTGTTTGCAAGATTTGGAACACGACCACGCTGGTGTTCCATTGAAGATCTCCCCACCAGT
TGTCGCTTACAGAGAAACTGTTGAAAGTGAATCTTCTCAAACTGCTTTGTCCAAGTCTCCAAACAAGCATA
ACAGAATC

TATTCTAGGATCCTTCATGTTGAGTTAATCGGTTATACTTCTGTCGCTGTGGCAAACTGATGTATGCGCCA
ACTTGAGACCATTGTCTCCACATTACAGAGGTGCTTCTAAGTCATCTGTTTCTAAACACTTTAGCTTTCTA
TAATATTC

AATCCGTATTCTCTCTTCGTTCTTTAAAGTTTGTTTGGTGAGAATCCTCAAGTGGTAATTCCACTGTCTCC
CTTGCATTTATTGGCTGGTTTGGAGAGTTAGGCACATCCTCATCAAAGATATTTGTCAAAGTCATCGCTCT
CTTTGCAG

CAACTACTGAACCGATTCAATTGAAGAATAAACACGACCTTCACCTTAGGCCAGGAACTTACTGAATCTAC
GGTGAATCACACTAATCATTCTGATGATGAACTCCCTGGACACCTCCTTCTCGATTCAGGAGCATCACGAA
CCCTTATA

TTAATGCTCTCTTATGCTTGTTCTTCTTTGCAATTTCAATTTGTCTCTTGAATGAGGATTTGGTGGTGTCC
AGGAATGCAACCTCCAGAAGTTTAACTTCTTCCTTCTTTTCCTCAGGATTAACTTCATCCAATAAAATAGA
AATAGATG

AAAGCGTTACGCCACTGCTTCTGCTATTGCTGCTACTGCTGTTGCTTCTTTGGTCTTGGCCAGAGGTCACA
GAGTCGAAAAGATTCCAGAAATCCCATTGGTTGTCTCCACTGACTTGGAATCTATTCAAAAGACCAAGGAA
GCTGTTGC

GCAGAATATACCGCCCACGGTTTATCCACAACAAGTGCCATTTCCAGGTCAACCACAGGGGGGTCAATTTC
CACAGCCTTCATCAGAACAGCAGGTGTTTAACCAGCTTCCACAAGTGACTCAAACTTTTCACAACTCTGCG
CAGAATAC

GTTGCTGCTGTGCTTGAACTTGCTCCAACTGCTTCTGCAAAGTGAATTGTTCAATCATCAAATTTGTCAAC
TCACTTTGGGAACGTCTGTGTGCAACATGAATCTGCTTTGGGGCGTTTTTACCACCTCCTGTGGTTTGCAC
AAACATGT

CATGGAGGATGGGTGACAAGGACAAACATTGGAATTACGATGTGTTCAATGCTCTGGGGCTGGAACACAAG
CTTATTCAGCGTGTGGGGAACATTGCGAGGGAAGAAAGCGTTATTCTGAAGGAACTAGCTAAGCTCGAATC
ACATCCTA

CCAAAGACTTTGGTGAAGAACTCAGGATTCGACCCATTGGACGTGCTCGCAATGGTGGAGGACGAGTTGGA
TGACGCTCAGGATTCTGACGAAACGAGATATGTTGGTGTGGACTTGAACATAGGTGATTCTTGCGACCCTA
CCATCGAG

ATATTGGTTTTAGAAACACGTTTCGAATCTTCAGTATGAGTTGACGGAGGTGTGGAATCGGTTGGACTCAC
AGCTTTTGAAAGGACATTTCTCGGTTGCTCAGGATGTAGTTCAATGTCGGATTGGAAGTCATGGTCAGATT
CTATGTTA

GTATCTTGGCTGGTTGAAGTGGTTGAAACCCTTAGAATGATTGTGTCAGAGAAAGTAGCTAGATCTGTTGA
AGTAGTTGGACCACTTGGTCTGGTTGAATAAACTGAGGTTGAACTGACAATAAAACTGGTTGGTTCAGATG
AAGTTGAA

TCCGCAAAACAGTCAAGTCCAGGCAGCTCAACTCCATAATATTGCCATTATCAGCTGCCACAAGTACTCTC
TCTTGATCTTGCACGCTGCTTGTTTCAATTACAGCCACCACTGGGTGCTGAAAGTAGTGTCTTGCCACCAT
ATTTCGCT

GACACCGTCTTCAATCTTGTATGTAATGATAGATCTAGATCCATCTGCATTCTCTATGATCTCCTCTGGTG
CAACTTCACTCATTATTCCTGGTGGTCTTTGGTCTTTTGATCCTTTCAATACAGCGAGTAACTAGGGCTTC
TCTTCCCT

TGATCTTTTCCGCCCCAGTTTCTAATTAATCTTTTCAGTCTCTGAATCTGTTTCAGCTGCTGAGGGGAGAC
AGGTGTGGATGTCATCGAATCAATATCTTCAAGAGACTCTGGAGATATATCGTCAAATAAAAGTTTGAATT
CCGTATGG

CTTAGTCTCCCTAGAATAGAGGTTCTTAGCGAACAAATAACCGAATAGAGTAGCTGAAGAGGCGATGACAG
AGTATTTTAACCACTGGCTGCTGCTGTCGGTGCTGCCTGTGCCTGTTGAGGCGTATCTGTAGCATGATCTT
CTCACCAG

TAGAAACACGTTTCGAATCTTCAGTATGAGTTGACGGAGGTGTGGAATCGGTTGGACTCACAGCTTTTGAA
AGGACATTTCTCGGTTGCTCAGGATGTAGTTCAATGTCGGATTGGAAGTCATGGTCAGATTCTATGTTAAG
ATCATTGG

ACCGATTCAATTGAAGAATAAACACGACCTTCACCTTAGGCCAGGAACTTACTGAATCTACGGTGAATCAC
ACTAATCATTCTGATGATGAACTCCCTGGACACCTCCTTCTCGATTCAGGAGCATCACGAACCCTTATAAG
ATCTGCTC

AGAATCCAATGATCTTAACATAGAATCTGACCATGACTTCCAATCCGACATTGAACTACATCCTGAGCAAC
CGAGAAATGTCCTTTCAAAAGCTGTGAGTCCAACCGATTCCACACCTCCGTCAACTCATACTGAAGATTCG
AAACGTGT

GCCTCGCAATACATTAGGGGGACTTACTTCATGGATGTGCATCCTCCTCTTGCAAAGATGTTGTATGCTGG
TGTGGCATCGCTTGGTGGGTTCCAGGGTGATTTTGACTTCGAAAATATTGGTGACAGCTTTCCATCTACGA
CGCCATAC

GTCTGAGCAACTTCTGAATTCATTGAAATTGATGAGCTTTGACTCCACACTGCGAGAAGTTGAAGTAGAGA
AGACATCTGATAATGATAGAAACAAGGAGTCTGGAGATCTCCAAATAGCAAGAAAGAAGGTCACATCAAAT
GTTATGCG

CGTCAGGACTAGTGCTACTACCACTGCCAGCATCAACGTCAGGACTAGTGCGATTACCACTGAAAGTACCA
ACTCCAGCACTAATGCTACTACCACTGCCAGCACCAACGTCAGGACTAGTGCTACTACCACTGCCAGCATC
AACGTCAG

GACGCCGACGATTGCGCAGGCACCTGCACCTGCGCCTGCGCCTGCGTGTCGGTGCTAATGTTCTTCAAACT
CCATACTGCCATACCTGGGCTGGCGGTCTTGTCATTGTCCCACAGGATCCTTTCAACTTCGACGGAAATTC
GATCAGGC

TGTCTTGCTGTCTTTACAGCGGTCATTAGATTGCATGGCTTGGCATGGCCTGACAGCGTGGTGTTTGATGA
AGTACATTTCGGTGGGTTTGCCTCGCAATACATTAGGGGGACTTACTTCATGGATGTGCATCCTCCTCTTG
CAAAGATG

TTCCCTTGCGGCGACACCTTGCGATGGCTCCGCTTGTTCGGTAGGCTCTGGCTTAGATTCTCCAGAACCCT
CAGCAGGTGCTTCACCAGGCTCGACCTGAGCTAACTCCTCACCAACAGTGACAGTGTCCTCTGGTTTGAAA
TTTAGCTT

CTGCTGTGGGTACGGCCCATTCTGTGGAGGTGGTACTGAAGCAGGTTGAGGAGAGGCATGATGGGGGTTCT
CTGGAACAGCTGATGAAGCAGGTGTTGTTGTCTGTTGAGAGTTAGCCTTAGTGGAAGCCTTATCATATTCT
TGAATTTT

TTGGAATCCTACGTTGCCTCCATCGAACAAACTGTCACTGACCCAGTCTTGTCTTCTAAATTGAAGAGAGG
TTCCAAGTCCAAGATTGAAGCTGCTTTGTCCGATGCTTTGGCTGCTTTGCAAATCGAAGACCCATCTGCTG
ATGAATTG

GACTCCAACACTAGTGCTACTACCACTGCTAGCACCAACTCCAGCACTAATGCCACTACCACTGCTAGCAC
CAACTCCAGCACTAATGCCACTACCACTGAAAGTACCAACGCTAGTGCCAAGGAGGACGCCAATAAAGATG
GCAATGCT

TGTACGCTCCACCTGGAGCACAATCACAGTTTACACAATATCCACAATATGTTGGAACACATTTGAACACC
CCGTCACCTGAGTCAGGTAATTCATTTCCTGATTCATCCTCAGCAAAGTCTAATATGACATCCACTAATCA
ACATGTCA

GCTCATTTAACCGATCCTCAGCAAGTCCTGGAGACTTTAGGTTTACTTGTTGGTGATGTCTCTCTCTTCCA
AAGTGCTGGTACGGTCGACCTGGATTCCAGAGGTCATGTCAAGAATAGTGAGAGCAGTTTGAAATCAAAGC
TAGCATCT

GTTCCAAGTCCAAGATTGAAGCTGCTTTGTCCGATGCTTTGGCTGCTTTGCAAATCGAAGACCCATCTGCT
GATGAATTGAGAAAGGCTGAAGTTGGTTTGAAGAGAGTTGTCACCAAGGCCATGTCTTCTCGTTAAGACTG
CATTCATC

CCTCCCACGGTGCAGCAGCAATCACCTTCCGTTATTAGGCAGTCTCCCACTCAACGGCGGAAAACGTCTAC
CACATCTTCCACATCTCGTGCACCACCACCTACAAATCCAGATGCCTCTTCTTCTTCATCATCTTTTGCTG
TACCGACA

AATGCAAAGGTTTTCACTAGTCACTCACAGATCATTCTCTCACTCCTGTGTGAAGCCCAAATCTGCATGCT
CTTTGGTCAAACCAGTTCATCACTTGGTGAAAATTGATAAGTCAAAGTTATCCCCTAGATTTCCAGAATTG
AAATATGA

AGGGTGCAAAACTATGATCGATTATTTTGAAAGTAAAGGAGCTCACAAATGTCCACCTGATGCAAACCCTG
CCGAATGGATGTTAGAGGTTGTAGGTGCCGCTCCTGGTTCTCACGCTACGCAAGATTATAATGAAGTCTGG
AGAAACTC

GGCACCAGCGGCAACTAGGACAGCAGGCAGTTTAGAATTCTTGATCCAAATGGCTGTTTCAACTTCCTGTG
CTGGAGAGGTAATTTCGCCTTGCTCAACTTCTTCAGGTAACTCTTCTCCCACATCCAAATCTTCGTCACCC
AAATCCCA

ACGTCACTTGAGCTAGTTGGATCTGTTGATGTGGTTACCTCGTCAGAGCTTCTTGTACGATACATACCAGC
AACACTTGATGAGGCATGTGGACCAACTGTTGTAGTAGACTCAATTAAAGATGTAGAGTCCGTGGAAGTGG
AAATCACG

TCTATCCATTTGCTTGGAATACCTAGCTTGTTAAGGTGATCAATGATCGATTGACCGTGTGGACCTTGAAC
CTGCACTTCAGCACATTTGAATGTTTGTGATTCACTTATTGTTGTCGATCCGCTACAAATCTTTCTTAAGT
CAGCCGCA

GGCTATGTCTCGTACTAACATGGATACAAGACACGCACATTCTGCTTTACTGGCAGCACCACAGAGTGCTA
CTGCAAATAGCAGGAGCAGCAACAGCAGCAGCGAGAGTAGTAGTAACAAAAACAATATCAATGTCGGCGTC
GGTGACGA

TTGTGTATATTATATGGTAAGGGTGATATTTAAGGAAGAAATCCACCCTTCTTTAAGGTTTCCACTGCACA
TGTTGAAATGTGTCTGAATCTTGATGAGCCCAAAGGCGTGCCACCATACCATCTGGTCACTATTACCAAGA
CGTTGAAT

CGGTTACTTCTAAAGAAGTCCAAACAACTCAAGATCCGTTAGACATTTCAGCTTCCAAAACAGAAGAATGT
GAGAAGGTTTTCACTCAGGCTAATTCTCAACAGCCAACAACACCTCCCTCAGCTGCTGTTCCAGAGAACCA
TCATCATG

TCGCAATAATCACACTAGCTACCATTCTTCATCGAATTCGGAGTCAAACATGGAAAGTCCTCGCTTGTCAG
ATGGTGAGTCTTCCACTCCGACCTCTATTGAAGAGTTAAACCCAACAATAAATAATTCGAGGCTGGTGAAG
AGAAACTA

TCTTTGTTCTTCCATATGAGAACGCCTGCGAGTATTCGAATTTGCTTCTGTTTCCACAGGAACATTGAAGT
TGGTCACATTATTAGCATCCATTTCACTGTCAAAGCCATCTGCATCTGCATCAAATTCATCGTCGTCGATG
TTATCGTA

TAAATTCACGTCTTTCTTCTTCATTGATGGTATGTGTAGTACCTGTTTGGGAGCCAGCAACAATAATCTTG
GCTTGAGTACCCTTACCCTTGTGCTGTAGTCCTGTGGCAGCAGTGGAAACAATAGGGGTTGAGTTAGGTGC
TACGTTGA

TTTATAATATTCTCCTTCATGGTACCGTAACGGTAGGCGTTGGTTCCTGAACAAGAATTAGCAATGCAACC
ACCAATCTGTGCACCTGGACCAGGGTCACAGCCAAACATCAAACCGTGGTCGCTCAAATAGTCATTCAAAT
CCTCCCAG

TCAATTCCCAGTCTACCAAGGTGAACGTGTTAACTGTAAAGAAAACACTTTGTTGGGTGAATTCGACTTGA
AGAACATCCCAATGATGCCAGCTGGTGAACCAGTCTTGGAAGCTATCTTCGAAGTTGATGCTAACGGTATC
TTGAAGGT

TTCGCTACTGTTCCAACTGGTGGTGCTTCTTCTGCTGCTGCCGGTGCTGCCGGTGCTGCTGCCGGTGGTGA
TGCTGCTGAAGAAGAAAAGGAAGAAGAAGCTAAGGAAGAATCTGATGATGACATGGGTTTTGGTTTATTCG
ATTAAGGA

AGAAAGGTTCCTATGTTGGTATCCATTCCACCGGTTTCAAAGATTTCTTGCTAAAGCCAGAACTATCAAGA
GCCATCATTGACTGTGGTTTTGAACATCCTTCTGAGGTCCAGCAACATACCATTCCTCAGTCAATTCATGG
TACCGATG

TATGGCTATCTGATAAGACTCCATACGATGACATGCGTTCATTGAGTTTGTATGTGGTGGCCACAAGCTCA
ACCTGGAGGAGTGCCTTTTCTAATCTGGTCTCATCCATCTTTGATGTGTGCTTCTTTAATAATTGCGACAC
CACCCTAC

TATTTTTAACCAGCAGGAGATCATCTGGAAGGTGTTGATACTGGACATCAAGAGCACTGCTACCATATCTT
CTGTTCTCAGAGTCAATGACCTGCTGAAAGCTGGTATCACCGTTCATTCCTTGATTAAACAAGACAGATCT
CCCTTGCC

ACTTCGCCAACAGACTAACTGGCTGGAACGCCATCAAAGCCAGAGTCGACCAGTTGAACTTGAACTTGACG
GATGACCAAATCAAGGAAGTTACTGCTAAGATTAAGAAGCTGGGTGATGTCAGATCGCTGAATATCGATGA
TGTTGACT

GTTGAAGACTGGTACTTTGACCACCAGTGAAACTGCTCACAACATGAAGGTCATGAAATTCTCTGTCTCTC
CAGTTGTGCAAGTCGCTGTCGAAGTCAAGAACGCTAACGACTTACCAAAATTGGTCGAAGGTTTGAAGAGA
TTGTCCAA

TGGGGGTCTGCTGTTACTTTTATCGGCACAACGGACCTGGGTTGACAAGAGCTTGAAGGCTCCAAGAGACA
GCGACCGTCGGCGCCGAATGACGAAACTATGCTGTGTGTGACCAGGCGAACAACATTGAGAGAGTTGAGGA
GCCGTACG

AGGACCATTGCTTTATAGTCAATTTTGGTATCATCACCCAATGTGTTCTCTCCCACTGCTTTCAGTCTGCG
GAGGGACTCATCTTGAGTAAGACCTTCAGTCAGACTTGTGCCTATAAATTCAGCGGCTTCTTCTGTAGTCA
ATGTGTGA

CCACATAACGGACGTACGAATCGTTTGAGCATTGAAACTTTTCTGTGTAGACTCTAGCTCCCTGAGGAATG
TACCACGACCTGTGGTAGGAATGATCACGAAAGGGAACATGATTGGTTGTTAAATTTCTAGTGTCATCAAT
CAATCCAG

GACTGGAATAATGGTTGGCAATTTTGTCTTGCTGGTTTCATGCCTGCTGTCTGGACTATTGGTTCCTTTGA
TTCATGTGTCCATCAATCTGAAGAAGCCAAAGATGCCAAGAAATCAGTCCCCATTGGTATAATCTCATCTA
TTGCTGTT

CAACCAACGGTACCACTTCAGCTTTCCCACCAACTACATCTTTGCCACCAAGCAACACTACCACCACTCCT
CCTTACAACCCATCTACTGACTACACCACTGACTACACTGTAGTCACTGAATATACTACTTACTGTCCAGA
ACCAACCA

CGTCAAACACCGCTCAGGTAGCTTCATCGAACCCCTATGCTCCACCACCACAACAAAGAGTAGCAACCCCA
TTATCTGGAGGCGTGCCTCCAGCTCCGTTGCCAAAGGCCTCTAATCCATATGCTCCAACTGCAACCACTCA
ACCCAACG

AACACTAGTGCTACTACCACCGAAAGTACCGACTCCAACACTAGTGCTACTACCACTGCTAGCACCAACTC
CAGCACTAATGCCACTACCACTGCTAGCACCAACTCCAGCACTAATGCCACTACCACTGAAAGTACCAACG
CTAGTGCC

CCCGTCCCATACTTCTCATCCAGCCACTCTCTCAGCTTGCCGAACTTGACGAACTTGTTGCAACCGATATC
TGGATTGGGTGTCGACCCTTCACTGTAGCCTCTCAACATTGGTTCAAATACATCTATCCAATAATCTTGCT
CAAAATTA

TTTGGATTTCCTAGAACAAATCGATAAAAATTCCACGTTAACTTTGCAGGACCTCTTCGATTCATTCACCT
TCGAAAAGATTCACTCTCATGTGGGTGTCAGGACTGATCTGTTTGATAGGAATCCGTCAGAAGTATTGATT
ACTGATTT

CCACTGCCAGCATCAACGTCAGGACTAGTGCGATTACCACTGAAAGTACCAACTCCAGCACTAATGCTACT
ACCACTGCCAGCACCAACGTCAGGACTAGTGCTACTACCACTGCCAGCATCAACGTCAGGACTAGTGCGAC
TACCACTG

GGTGGTACTGAAGCAGGTTGAGGAGAGGCATGATGGGGGTTCTCTGGAACAGCTGATGAAGCAGGTGTTGT
TGTCTGTTGAGAGTTAGCCTTAGTGGAAGCCTTCTCACATTCTTCTGTTTTGGAAGCTGAAACGTCTAACG
GATCTTGA

TGCCGTATCCACCTTATCAAATGTCACCTATGTACGCTCCACCTGGAGCACAATCACAGTTTACACAATAT
CCACAATATGTTGGAACACATTTGAACACCCCGTCACCTGAGTCAGGTAATTCATTTCCTGATTCATCCTC
AGCAAAGT

CTGCCGATGCCTTATTGGAAATGATTGTCTTGCACTTGCCATCTCCAGTCACTGCTCAAGCCTACAGAGCT
GAACAATTATACGAAGGTCCAGCTGACGATGCCAACTGTATTGCTATCAAGAACTGTGATCCAAAGGCTGA
TTTGATGT

CATTATGCTTTTGTTCGTCTTGCTTTGGAGAATATGCAGGAGATCCTGGGCTGTAGCCTGGAGATGTTGGG
GAGTAAGAAGGTGAAGTAGGGCTATAGTTTGGAGAGGTGGGAGAGTAAGAAGGTGATGTTGGGGAATAGGA
TGGTGATG

CAGACTAACTGGCTGGAACGCCATCAAAGCCAGAGTCGACCAGTTGAACTTGAACTTGACGGATGACCAAA
TCAAGGAAGTTACTGCTAAGATTAAGAAGCTGGGTGATGTCAGATCGCTGAATATCGATGATGTTGACTCT
ATCATCAA

AAGTTCTTTCCATGAGGCTTCCCCTAGTTCTCTTTGGGCAAGGACACCGGCGTTGATTTCATCAGCCTGTT
TGAGTACCCATGGATCCTCTGGTGAAACCTTGTTGATCTTGGCGATGGAGACACAGGCCTCTTCATGTCTC
TCACATTC

TGCTTGGAATACCTAGCTTGTTAAGGTGATCAATGATCGATTGACCGTGTGGACCTTGAACCTGCACTTCA
GCACATTTGAATGTTTGTGATTCACTTATTGTTGTCGATCCGCTACAAATCTTTCTTAAGTCAGCCGCAAG
TGATTCTG

TGAACTTGAACTGACTACGGAAGTAGTTGGATCAGCTGAAGTAGTTTGAACGGTCGAGGTAGTTGGGTCAG
CTGAAGTAGTTGGGTCAACTGAAGTAGTTGGATCAACTGAAGTAGAACTGACTATAGAACTAGTTGGGTCA
ACTGAAGT

CGCTTGTTCGGTAGGCTCTGGCTTAGATTCTCCAGAACCCTCAGCAGGTGCTTCACCAGGCTCGACCTGAG
CTAACTCCTCACCAACAGTGACAGTGTCCTCTGGTTTGAAATTTAGCTTCGTAACAGTACCTGATACTGGC
GAATTGAC

AAAGAAGAGAGGTTATTGGAATGCTAGCACGTTCCGTAATTATTGGTTTCTTGGCTACTGTTCAAGATGAT
AGGACGACCAAGACTGACGTCAAGCTTGCTGATCCTCACATGAACTTTATTAGAAAATTTGCCATTGAAGC
GATTATAA

ATCCTCCTCCTCCTCCAGGTGAACACATGCACGGTCGCCCACCAATGCACCACCGTCAAGAAGGAGAACTC
TCGAACACCAGATTGTTTGTTAGACCTTTCCCATTGGACGTTCAAGAATCCGAGTTGAATGAAATCTTTGG
TCCATTTG

GATCTTTCATAAACAGTGACCGTATGTCCGGCACGGTTCAACATATCAGCACACGCTAAACCTGCTGGACC
AGAACCAATGACACCCACTGTAAAGCCAGTGCGTGTACTTGGTGGACAAGGCTTAATCCATCCTTCCTTGA
AAGCATTG

CCAAAGGCGTGCCACCATACCATCTGGTCACTATTACCAAGACGTTGAATATGTTTGCTCTTTCAAGTAAG
GTCAATAAACGCTGTCCAGCACCAGCTTCGCCACAGTCAGCACATCCTTGCTCAATGTTCTTTGGCTGCAC
CGTTATGT

ATGGCTATGACGATACCATCCGCTTATTCGATGTCAAGAGTAGGGATCACCTTTCTGCGAAGTTGGAGCCC
AAACTAACGATTCAGCACAATTGCCAAACAGGAAGATGGACAAGCATCCTCAAGGCCAGGTTCAAGCCGAA
CAAGAATG

TACATCACTGAGGAACTAGGTATTCAATGTGGTGCTCTATCTGGTGCTAACATTGCCACCGAAGTCGCTCA
AGAACACTGGTCTGAAACAACAGTTGCTTACCACATTCCAAAGGATTTCAGAGGCGAGGGCAAGGACGTCG
ACCATAAG

GAAGTGCCACACGAGGGTGGGTTCTCGGACCTGCTTTGGTCAGACCCTGACAATGTAGAGGCTTGGCAAGT
TTCCCCTCGTGGTGCAGGATGGCTCTTTGGCAGTAAAGTTGCTAGAGAGTTTAACCACGTTAATGGACTGA
ACCTTATT

TGCAGTCATCTTATCGCCTTCCAGAGAATTAGCCATGCAGACTTTCAACGTTTTCAAGGATTTTGCCAGAG
GAACAGAACTAAGAAGCGTCCTCTTGACTGGTGGTGATTCCTTGGAAGAACAATTTGGCATGATGATGACC
AACCCAGA

CATCGAACAAACTGTCACTGACCCAGTCTTGTCTTCTAAATTGAAGAGAGGTTCCAAGTCCAAGATTGAAG
CTGCTTTGTCCGATGCTTTGGCTGCTTTGCAAATCGAAGACCCATCTGCTGATGAATTGAGAAAGGCTGAA
GTTGGTTT

TAAGGAAGAAATCCACCCTTCTTTAAGGTTTCCACTGCACATGTTGAAATGTGTCTGAATCTTGATGAGCC
CAAAGGCGTGCCACCATACCATCTGGTCACTATTACCAAGACGTTGAATATGTTTGCTCTTTCAAGTAAGG
TCAATAAA

TGGCTAAGTATGGATAAACACCATCACAACCATAACCTAGTAGGACACAGAAATGGTGAATTTCTCTGGCT
TCACCTGTTTCCAAAATCAAAGCAACTTGGGAACGCTGCTTGTTTCTGATTAGGTGATGATGAATACATGA
AATTGCAA

GTAGTCAAGAATCGTTCCCACTCAATTGACGAGGCAATTCGCAAAGACAGTTTTTCTCTTCTCGCCTCAGC
TTCTTGAGAAAGCTCCTTCAAAGGTGGGTTTGAACCGCATTGGTTCAAGTCTTCATGAAGACCATCTAGAA
GGAATTGG

TAGCTGAAGAGGCGATGACAGAGTATTTTAACCACTGGCTGCTGCTGTCGGTGCTGCCTGTGCCTGTTGAG
GCGTATCTGTAGCATGATCTTCTCACCAGCCTTCCTCTAGGTTGTGCAATAGGCTTTATTAGCCTTGTGCA
AGTACGCT

AATACACGAGCGATGTCGGACTCAGAAGTCAATCAAGAAGCTAAGCCAGAGGTCAAGCCAGAAGTCAAGCC
TGAGACTCACATCAATTTAAAGGTGTCCGATGGATCTTCAGAGATCTTCTTCAAGATCAAAAAGACCACTC
CTTTAAGA

CCGTCAACAACTTCACCAAAGACAACATGCTTACCGTCCAACCATGGGCATGGAACGGTGGTGATGAAGAA
TTGAGAACCGTTGGTGTTTGGACCGGCGTTGGCCATGGACAACAAACCTGGTCTGTCGTGGTGCTTCTTGA
AGTTTTCA

CAGACCTTACTTCCACGTTAGTGTCATCGAAGATGTTGCTGGTATCTCCATCTGTGGTGCTTTGAAGAACG
TTGTTGCCTTAGGTTGTGGTTTCGTCGAAGGTCTAGGCTGGGGTAACAACGCTTCTGCTGCCATCCAAAGA
GTCGGTTT

AGGAGAACCCAAGGACTCAACCTTCTTAACGATGTCGTAACCGTCAACAACTTCACCAAAGACAACATGCT
TACCGTCCAACCATGGGCATGGAACGGTGGTGATGAAGAATTGAGAACCGTTGGTGTTTGGACCGGCGTTG
GCCATGGA

GGCCCAATTTTCCAACAGTACATCTGCTTCTTCCACCGATGTCACTTCCTCCTCTTCCATCTCCACTTCCT
CTGGCTCAGTAACTATCACATCTTCTGAAGCTCCAGAATCCGACAACGGTACCAGCACAGCTGCACCAACT
GAAACCTC

TACCATCTAAATGAACATCTTGTTCTCTTAACATTTGTTCCTGAAACGGATTGGACATACCACCATCATTG
TTCTCAGCCATGCTGGTGTTAAGTGTTGAATTTTCCACCGTCTCTTCTAGAGGAATAGTTGTTTGAGTAGA
TTCCTGTA

AGCAAAAGACCGACGGTAACTCCTTCTTGATCAACTTGATCGACTCTCCAGGTCACGTTGACTTCTCCTCT
GAAGTTACTGCCGCTTTACGTGTCACTGACGGTGCTTTGGTTGTCGTCGACACCATTGAAGGTGTCTGTGT
CCAAACCG

CTTTCACCAAGTTGGACCAAGTCTGGGGTTCCGAAACCGTTGCCTCCTCCAAGGTCGGTTACACTTTGCCA
TCCCACATCATCTCCACTTCTGATGTCACCAGAATTATCAACTCTTCTGAAATCCAATCTGCTATCAGACC
AGCTGGCC

TGGTTACGATAGTCCAAGAGGTGGTTATTCCAGAGGTGGCTATGGTGGTCCAAGAAATGATTACGGTCCTC
CAAGAGGTAGCTACGGTGGTTCAAGAGGTGGTTATGATGGTCCAAGAGGCGATTATGGTCCTCCAAGAGAT
GCATACAG

AGATGTAAATACAAATGCAAATGCAAATGCAAATGCAACTGCAAATGCAACTGCAAATGCAACTGCAAATG
CAACTGCAGAGCTGAACCTCCCCACGGTCGATGAGCAAAGACAGTATAAGGTACAACTGCTATTGCATATC
AACAGCAT

GCGAGCATTTAAATCGTCAATAAAGTCCTTGTTGAACTTTGCATCATTACTCCTTGCCTTCGGAACGTGAG
GTGGAAGACTCCACACTGCAACAGCATCGAAAGTATTAACTTCGGAGATCTCACCATAAGGTGAATCCAGG
AATGGCGA

TTGATCCAAATGGCTGTTTCAACTTCCTGTGCTGGAGAGGTAATTTCGCCTTGCTCAACTTCTTCAGGTAA
CTCTTCTCCCACATCCAAATCTTCGTCACCCAAATCCCAGGCACCTTCGTCTTCACCAATATCCTCATCGT
TAAAGTTT

AATATAGCACCACAGTAACGTCCATCAGTGAAAGTTAACAAAGCTGGACCATCCCAAGGTTCCATCAGACA
TGCAGCCCAGTCGTACCATGCTTTTAGGTCAGAATCCATATCCTTATGATACGCTTCAGGAACCATCATCA
TAACAGCT

TAAACCCTTAGCAATAGCTTGTCTGATGGCGTAGACTTGAGAAACATGACCACCACCAGTGACTCTAACTC
TGATATCGATGTTGGAGAACTTGTCCAAACCAACCAACAATAATGGTTCGTAGACCTTGAATCTTAGGATT
TCTGGTTC

TGTTCGCTCTCATCCATCTTCACGTTCTGTGCAATTTCTTTAATTGTATCTTGAGGAGGACCAGTCGCAGT
GTTGCCAGAGTACTTTCTACGATCTGCAGACTTCGAACTTGTGTCCGTGTAGACACTAGGGTCGACAGCGT
TCATACCG

GGTCAAGATAGTTCTGTCCTTGAAAGCAGTACGAATCGTCTCTTGGACGACTTTATCTGTCTCCACGTCGA
CTGCGGCCGTGGCTTCATCAAGCACCAAAATCTTTGATGGAACCAACATTGCTCTTGCAAGACATAATAAT
TGTCTTTG

CCAACTGATACTACTACTGAAGCTCCAACCACCGGTCTTCCAACCAACGGTACCACTTCAGCTTTCCCACC
AACTACATCTTTGCCACCAAGCAACACTACCACCACTCCTCCTTACAACCCATCTACTGACTACACCACTG
ACTACACT

CAGAGGTGGCTATGGTGGTCCAAGAAATGATTACGGTCCTCCAAGAGGTAGCTACGGTGGTTCAAGAGGTG
GTTATGATGGTCCAAGAGGCGATTATGGTCCTCCAAGAGATGCATACAGAACCAGAGATGCTCCACGTGAA
AGATCACC

ATTCTATAAATCATTTTCTTTGCCGCCGATGGAATAGCCTTTCTTGGATTGGATTGTTCACTGGCAGTCAT
AGCCAGCTGTTCACTCATACCAAAGGCGAACGCTGCTGTGACAAATGTGGCAACGACACCTTTGAACCTCT
GGATGGGT

TTGAATGAGTCAGGGTAACGAGTGGGGAGGTAGGGTAATGGAGGGTAATTTGAGAGACAGGTTGGTCAGGC
TTGGCTTGTCTTAGTCTTAGGCTTAGGCTTCTCTCTGGTGTGGGTGTGTGGGTGTGTGGGTGTGGTGTGGG
TGTGGGTG

CATTTTCATTAACTTGCATAATCTTCAAAGTACACATATCATCACGTTCTCTAGAGTCTCTGTAAGCATCG
GATGAAGGATCTCTTGCACCAGCACGACGAGAAGGTGGAACATACTGCCCTGGGATGGAACCAGCTCCGCC
AACTTGGC

TTCAAGCCATAAGATTCTTTTGCCACCAAGATCAAGAGGTACAATCACTTGGATTGCTCCAGCTGGTGAGT
ACACTTTGGATGAGAAGATTTTGGAAGTTGAATTTGATGGCAAGAAGTCTGATTTCACTCTTTACCATACT
TGGCCTGT

GGTGTTTCCAACTTTGCTGTGGAAGACTTGCAAAGAATTCTGAAAGTTGCGGAAGTCAAGCCCCAAGTTAA
TCAAATTGAGTTCAGTCCCTTCTTGCAGAATCAAACACCAGGGATCTACAAATTTTGCCAAGAACATGATA
TATTGGTA

AAGCGCTCGAGAAAGTTGGAGTTTTTCAGCGTTTGCGTTCCATGACGAGCGCTGGACTGCAGGGTCCGCAG
TACGTCAAGCTGCAGTTTAGCAGGCATCATCGACAGTTGAGGAGCAGATATGAATTAAGTCTAGGAATGCA
CTTGCGAG

TGCTTTGGAGAATATGCAGGAGATCCTGGGCTGTAGCCTGGAGATGTTGGGGAGTAAGAAGGTGAAGTAGG
GCTATAGTTTGGAGAGGTGGGAGAGTAAGAAGGTGATGTTGGGGAATAGGATGGTGATGTTGGAGAGTATG
AAGGCGAT

TACCACTGCTAGCACCAACTCCAGCACTAATGCCACTACCACTGCTAGCACCAACTCCAGCACTAATGCCA
CTACCACTGAAAGTACCAACGCTAGTGCCAAGGAGGACGCCAATAAAGATGGCAATGCTGAGGATAATAGA
TTCCATCC

ATTGAATTAGGTCTGGGCTGATGTTTACGCTAAGGCTTTGGAAGGTAAGGACTTGAAGGAAATCCTATCTG
GTTTCCATAACGCTGGCCCTGTTGCTGGTGCTGGTGCTGCTTCTGGCGCTGCCGCTGCTGGTGGTGACGCT
GCTGCTGA

GACCAGGTGGCATCGCCATGAAATGTTCCTGCTGTGGAGGATACCCCTGCTGCGATGGCATTTGCGGTGGA
GGATACGCCTGTTGTTGCTGATGCGAAGGAGGAACCATGAATTGTGGATGATGTTGCTGCGGAGGAATAGC
AAATTGTT

GATGTCGTAACCGTCAACAACTTCACCAAAGACAACATGCTTACCGTCCAACCATGGGCATGGAACGGTGG
TGATGAAGAATTGAGAACCGTTGGTGTTTGGACCGGCGTTGGCCATGGACAACAAACCTGGTCTGTCGTGG
TGCTTCTT

GCCAGGAACACGGTAGTACTTGAGCCTAGGGACGACGAAAATGAGCGTCTCGAGGGCGTGAGTCAGGAACT
CGGCAACAATGATGAGTCGCAGCACAAGATCACTATCCAGCCACGCCTCTAAGGTGGAGCCCACAGGATTA
GGGACCAG

ACTGTCACTGACCCAGTCTTGTCTTCTAAATTGAAGAGAGGTTCCAAGTCCAAGATTGAAGCTGCTTTGTC
CGATGCTTTGGCTGCTTTGCAAATCGAAGACCCATCTGCTGATGAATTGAGAAAGGCTGAAGTTGGTTTGA
AGAGAGTT

CCACCAGTGAAACTGCTCACAACATGAAGGTCATGAAATTCTCTGTCTCTCCAGTTGTGCAAGTCGCTGTC
GAAGTCAAGAACGCTAACGACTTACCAAAATTGGTCGAAGGTTTGAAGAGATTGTCCAAGTCTGATCCATG
TGTCTTGA

CAAAGTAGACAAAGAGTTCATAATTGTGGCTTCATTACCACCACCAGTTCCGGTGGAGACGAAACAACCAG
CGACCTTACCATGCAAAGCACCCTTAGCCCACAACCCACCGGTACGGTCCCAGAAAGCCTTCCATTGAGCA
GGGAAGTT

ATGGTTGGCAATTTTGTCTTGCTGGTTTCATGCCTGCTGTCTGGACTATTGGTTCCTTTGATTCATGTGTC
CATCAATCTGAAGAAGCCAAAGATGCCAAGAAATCAGTCCCCATTGGTATAATCTCATCTATTGCTGTTTG
TTGGATTT

AGATTCCAGAAATCCCATTGGTTGTCTCCACTGACTTGGAATCTATTCAAAAGACCAAGGAAGCTGTTGCT
GCTTTGAAGGCTGTTGGTGCTCACTCCGACTTGTTGAAGGTCTTGAAGTCCAAGAAATTGAGAGCCGGTAA
GGGTAAGT

GGTTAACCAAGCTGAAGAGTTCAAGGCTGCCGATGAAGCTTTTGCCAAGAAGCACGAAGCTAGACAAAGAT
TGGAATCCTACGTTGCCTCCATCGAACAAACTGTCACTGACCCAGTCTTGTCTTCTAAATTGAAGAGAGGT
TCCAAGTC

GCAGCATATGGTTTTGTAAATCCGTGAGGTTTAAGACAAATTCTTGGCTCAGTTCAGGGACTGGTTTAGGA
ACCTCAGGTCTTCGCAAAGGTGGGCCTTGTGCTGGTGTTTGATTATTATCTAGATAGCTTGTAGGGTTTGG
CTTATGAA

ATATCAGTTAGACTAGATGATACTGTTTGGGTATCTTGGCTGGTTGAAGTGGTTGAAACCCTTAGAATGAT
TGTGTCAGAGAAAGTAGCTAGATCTGTTGAAGTAGTTGGACCACTTGGTCTGGTTGAATAAACTGAGGTTG
AACTGACA

CCTAATTCCAATATGCTTGTCTCCTGTTCATTTCTACGCTTGTCATCCACACTGGCACCGGCAGCGGCGCT
GCTCTGTGAGCTGTCTTTATCCAGCAGGCTCAAATAGACTTGTTCTGCCTCATTATACTGTTTTTCATTAA
CCAGTCTC

TGCCAAGTCACTGCTCGGATCAAGGCCAGAACAAGATACAGGCGCCCCTATCAAAATGTCTACTGGTGTCA
CAAGCTCTCCATTAAGTCCATCAGGCTCCACCCCAGAACATTCCACCAAGGTCTTGAACAACGGCGAAGAG
GAGTTCAT

TAATAGTTACTCTATTATTGCTGTTAATTGACAATGTTTAGTCACGTGCAACACAATTCAAGTCACGTGGA
AAGGCCTTCACATGGTGATCCATCTTCTACATCTTCATCGGTCCTGCATAAAGTCATAATATGGGGCTACT
GGAATGTA

ATGCAAATGGTGATCAGACGAGTTATTAAATCAGCCTTCTTCTCATCATCGTTCAGATACAACTGAAACAT
AATGTCAAGGAGAACTGCAGCAGCACTCGTTTCTTGTTTCAACAGGTTTGGTATTTTATCAACTACACGAG
CCTCTACT

CACTAATGCTACTACCACTGCCAGCACCAACGTCAGGACTAGTGCTACTACCACTGCCAGCATCAACGTCA
GGACTAGTGCGATTACCACTGAAAGTACCAACTCCAGCACTAATGCTACTACCACTGCCAGCACCAACGTC
AGGACTAG

ACTACCACTGCCAGCACCAACGTCAGGACTAGTGCTACTACCACTGCCAGCATCAACGTCAGGACTAGTGC
GATTACCACTGAAAGTACCAACTCCAGCACTAATGCTACTACCACTGCCAGCACCAACGTCAGGACTAGTG
CTACTACC

GCGTCGTAGTTGGCACCTCTTCTAGATGGTGGAACCCATGAGGCAGACTTCCATGGTAGAACACCTTCTTC
CCACATGGTGTTGACTTCTTCCAAAGTCAAACCCTTAGTTTCTGGAACAACTAACAAAACATAGAAGAACA
TGAAGACC

CTTCTAAGGATGGTGACCCCCAGCTAGAATTTAAAGTTTTACAGGGATATAGGGACCTGGAAAGTGAAATG
CACAAAGGCAGAGCTCAGGTGACCAGAACAGGAGATATAGGTGTTGCTATGGACAATCTGAACGCTGTGGA
TTCCCTAT

GTGGTGTATTAAGTGATAACGTCATTTACGGTGACGGTAGCAGTGGAAACCAAAGCTGGGGAAACTGTTTC
AGAACAAACGTGGTCCTCACAAGAAGTGATAGTGACCAAAGTGGTAGATTCTTCTGTGACAGTAGCAGTGG
TAATGTTA

GCGAACTGGTGCGGAGTATACTTTGCACAGTTTCCTTCCTCCCCATCCCCTCTGTCTAGTGCATTGAGCAA
ATCTCTGCGCCTGTAGGTGGAGGATCCTAGAGTAATGAAGTCTCTGTCGGAGTATATCTCTTGAATATTAG
GGAAGCCC

AGTTGTTCCCCCAGCAACATTACAGTCGTATGTAAATTGACATTGGACTTTTCTTCCTTCAATGATTTCCT
CCCTAGCTGACCTGGTCGTCACAGTAGCTGACATACCAAGAGATCTTGAAATATGGACAATTCCGTCCATA
ATGGATGA

CCTATTACTACCAGTTCCCTGGACAAGCCTATTACTATAGTCCTGAATACGGTTATGATGACGAGGATGGT
GAAGAAGAGGACCAAGACGAAGATATGGTGGGTGACAGCGGCACTACAAGACAGGAAGATGGTGGCGAGGA
CAGCAACT

CATGAGGCTTCCCCTAGTTCTCTTTGGGCAAGGACACCGGCGTTGATTTCATCAGCCTGTTTGAGTACCCA
TGGATCCTCTGGTGAAACCTTGTTGATCTTGGCGATGGAGACACAGGCCTCTTCATGTCTCTCACATTCAA
TCAGATAT

CGAAGAGAGTGATGGTTGTGCTAGATCGTACAAAGAATTGGTAGATCCTGATTTCCCAAGTGACAATGTTC
TCCTCAGTGAAAGTCCTTCATCGTGGTTATCGTCTACTTCTTGGTCATCGTCATCTTCTTCCTCTTCGTCG
TCGCTACT

CTCCAACACTAGTGCTACTACCACCGAAAGTACCGACTCCAACACTAGTGCTACTACCACCGAAAGTACCG
ACTCCAACACTAGTGCTACTACCACTGCTAGCACCAACTCCAGCACTAATGCCACTACCACTGCTAGCACC
AACTCCAG

GTGGTTACCTCGTCAGAGCTTCTTGTACGATACATACCAGCAACACTTGATGAGGCATGTGGACCAACTGT
TGTAGTAGACTCAATTAAAGATGTAGAGTCCGTGGAAGTGGAAATCACGACAGAGATATTTAGACTTGTTG
AAGTAGTA

CCTTCTTAACGATGTCGTAACCGTCAACAACTTCACCAAAGACAACATGCTTACCGTCCAACCATGGGCAT
GGAACGGTGGTGATGAAGAATTGAGAACCGTTGGTGTTTGGACCGGCGTTGGCCATGGACAACAAACCTGG
TCTGTCGT

TATTTTTTCTTAGATATTCTTTAATACTGGGGTTGTGACTGACCAGGTGGCATCGCCATGAAATGTTCCTG
CTGTGGAGGATACCCCTGCTGCGATGGCATTTGCGGTGGAGGATACGCCTGTTGTTGCTGATGCGAAGGAG
GAACCATG

TTAGTGTGCAATGGCTATGACGATACCATCCGCTTATTCGATGTCAAGAGTAGGGATCACCTTTCTGCGAA
GTTGGAGCCCAAACTAACGATTCAGCACAATTGCCAAACAGGAAGATGGACAAGCATCCTCAAGGCCAGGT
TCAAGCCG

CAGGGGCCAACGACAGTTCGTCGTTGAACGATTGTGGTAATAAATTGACAAGTTTTTGTGGCATGAACACC
GCAGAGGACCTCTTTCTCGCACGCCTATCAACAGAGGAGCCTTCTTCAATATGGCTGGTCACATCAACAAC
ATGACAAC

ACAACGGTCGGATCTTTCATAAACAGTGACCGTATGTCCGGCACGGTTCAACATATCAGCACACGCTAAAC
CTGCTGGACCAGAACCAATGACACCCACTGTAAAGCCAGTGCGTGTACTTGGTGGACAAGGCTTAATCCAT
CCTTCCTT

TCCATGAGTAACTGAAGGGTTAGTTTTAGTACTGGAATGATCCTGTGCAGCAGCGGTGGATTTAGGACCAG
TGCTTGAACCAGAGTGACCATGGCTAACAGGGTGGGTAGTTTTGACGTTGATATGTCCATGTGTAGTTGCA
GTGGCTGA

AAGGCAGCATCAATAGAGGAGTTCTTTGCAGCGGCCTGATATAAAGCTGTTGCGTAAGTGCCCTCAACACC
GAACAATCTCACCGGTGGGGGAGCAGCAGCTTTGGAAGCAGCAGCTCTTAAGCTTGATGCAAATGACCTGG
TAAAGACT

GTACTGGATGGTCTAGCACTTGTGCTTGCTCTTGAAGTATCATAGAATGACCGGCCCGCACCTGTAATCAC
ACCGTTTGAGTATGCTGCTGTCAGTTGACCTGGGACAGTGAACATATAGCCTGACCTATAGGATGGATAAA
GAGTCAAG

AGCTTCAATAACTTCCAACTCTTGTTGAATGAATGGATGGTCTGGGGCAACCTTGTTAACTTTGGAAAGAG
ATGCTCTTGCTTCGTCAATTTGACCAGCTTCAACCAAATAACGTGGGGATTCTGGAACGAAAGTCATACCA
CCGATCAT

CTGCTGTATTTGCAGGTGTAGGTGTATGAAGAGGTGTATCATATGCCATTGACGCCATGAACAGGCCTAAA
ACACCACCCAGTGCAAACCCTGTTACACCACTCACAACTGATTTTCCAGGACAGGAAGTCATGAAGTTCAT
GATCATTT

AGGTGTCTGTGTCCAAACCGAAACTGTTTTGAGACAAGCTTTGGGTGAGAGAATCAAGCCTGTTGTTGTTA
TCAACAAGGTCGACAGAGCTTTGTTGGAATTGCAAGTTTCTAAGGAAGATTTATACCAAACCTTTGCCAGA
ACTGTTGA

ATGAACAATATCGTTGCTGATAAAATGACAGAATTATTTAGTGCTGGCAATGAAGAAGATCCAAGAGATTT
TGATGTGTCTGTCATTCCAAGTCTTGGAAACAATGATGTCTTTCCACATAACATGTTTGCACTAGGACCAA
CTCTACAA

TTCCAGAGGCGGCTTCGGTGGTAGAGGCGGTTCTCGTGGTGGTGCCCGTGGTGGCTCCAGAGGTGGTAGAG
GTGGCGCTGCTGGTGGTGCCCGTGGTGGTGCCAAGGTCGTTATTGAACCACATAGACATGCCGGTGTTTAC
ATTGCTAG

AACCTTGTTAAAAAGCCAGAGTGGTCACAATATGAAGACTATCCCAGTCATGAAATTGTCTCCACATATGA
CTCAAGATTGAGCGTCTCTGCAGTTTCTTACTCACCGACAGATGGCACCTTAGTGTGCAATGGCTATGACG
ATACCATC

AACTTCCTGTGCTGGAGAGGTAATTTCGCCTTGCTCAACTTCTTCAGGTAACTCTTCTCCCACATCCAAAT
CTTCGTCACCCAAATCCCAGGCACCTTCGTCTTCACCAATATCCTCATCGTTAAAGTTTTCTTCGCCTAGT
GGTTCTTC

GTTGAACTTTGCATCATTACTCCTTGCCTTCGGAACGTGAGGTGGAAGACTCCACACTGCAACAGCATCGA
AAGTATTAACTTCGGAGATCTCACCATAAGGTGAATCCAGGAATGGCGATATGATATCGGTGGTTATTGTC
CTTGTAGG

GCCAAGCGCCTTAACAACTTCTGGAGACAACGTTTCCTCGACTTGATAAATGTCTGCAGAGCCTCCAGCGG
CTTCAATTCCCTTCTTTTCTGCCTCTGCGGTGGCAGCAACGTGACCATATAGTGTGTAAATGATGATAGCT
ACTCTTGG

TCCACCACCACAACAAAGAGTAGCAACCCCATTATCTGGAGGCGTGCCTCCAGCTCCGTTGCCAAAGGCCT
CTAATCCATATGCTCCAACTGCAACCACTCAACCCAACGGTTCCTCCTATCCTCCAACCGGTCCGTATACT
AATAACCA

TCTGGCTCAGTAACTATCACATCTTCTGAAGCTCCAGAATCCGACAACGGTACCAGCACAGCTGCACCAAC
TGAAACCTCAACAGAGGCTCCAACCACTGCTATCCCAACTAACGGTACCTCTACTGAAGCTCCAACCACTG
CTATCCCA

AAAGACCCTCAGAGACCACGGAGTAAAGTTTACGTTCTTGGAAATCCTGGCTGCCCAGCATGCGTTCATCC
GTGACGAGTTCAGCAAGGGCAGGTTCGACTCCGCTATCACTCAAAGTTGTCTCGGCTTCTTGTTCGAGCAG
TTCAACAG

TACTAGGGCACCTCCAAACGCAGTCGCTAGAGTTGCCAAGCTCTTGGACACCACTAGAGAGGTCAAGGTCA
ACGTCGAAGGTTTGCCAAGGGACATTAAGCAGGATGCTGTAAGAGTATGTTAATACGTGAAATGAGAGCTA
TTTGTTTA

GCTGCTTTGTCCGATGCTTTGGCTGCTTTGCAAATCGAAGACCCATCTGCTGATGAATTGAGAAAGGCTGA
AGTTGGTTTGAAGAGAGTTGTCACCAAGGCCATGTCTTCTCGTTAAGACTGCATTCATCACATATATATGA
ATATTACT

GCTCGATATCAAGAGGGTTCTTGTAGTCCAGAGCCACGTTCTCAAGACCTACAGGTGAGTCCTCTAAGTTT
CGTGTGGAAAGCGCTACCTCCTTCATCAACAAGACACGTTCATCCATATTTTGCAAGTAGCGTGACCAGAA
GGCGTCAG

CCGCTATGAGGTCCACAATTATAAAAGACCAGATACCTTTCACAAGTGACATCATCGTCCTTGTCTCCTTG
GAGAGAACAGAGCACATTATAAGCAGTCATCAAGTCACTTGGCGTCAAAGCAGATCTCTGGTCTTTAAACT
CGCTTGTC

TGGCCAGCTAACCACCACTACAGAAATCGAAAACTTCCCAGGTTTCCCAGATGGTCTAACAGGTAGCGAAC
TGATGGACAGAATGAGAGAACAATCCACGAAGTTTGGCACTGAAATTATCACGGAAACAGTTTCCAAAGTT
GATCTGTC

CACTTCGACCAAGCTGGTAAGAAGGTTTCTAGACGTAATGCTAGAGCTACCAGAGCCGCTAAGATTGCTCC
AAGACCTCTAGATCTCTTGAGACCTGTTGTTAGAGCCCCAACTGTTAAGTATAACAGAAAGGTCAGAGCTG
GCAGAGGT

CAGAGCCGCTAAGATTGCTCCAAGACCTCTAGATCTCTTGAGACCTGTTGTTAGAGCCCCAACTGTTAAGT
ATAACAGAAAGGTCAGAGCTGGCAGAGGTTTCACCTTGGCTGAAGTTAAGGCCGCTGGTTTGACTGCTGCT
TATGCCAG

AAGTTTCACCGATACACAAGATGACACCGACACCTTGACCTAAAGCGAACTTGGTCTTGTCAGCAATGAAC
TTGTCATCTTCGTGGAAGTAAGATCTTCTTTCGGAGTGACCCAAAATAACCCACTTAGCACCAACATCCTT
GATTTGGT

ACCGGCATTCGTTGCCAAATCTGCAGCAACCAATTTAGTTTCCATACCACCGGTCCCAACGTCAGAACCTG
AACCACCAGCAGTATTCACACCGGGCAAACCCTTTGAGAGATCTGGGACAACTAAGATCGGCATGGCATCT
GGGTTTGT

TTGCCAGGGGAATGAAGCGATTAGTACTAGTCAATGCCTTTTCTCCAAACTGACCTTGTGGAGAAGCCTGT
AGTTCAGACCCGCTCTGAGAATTGCTGCCATCTACGAAAGGACGAGGATACTCTTGCGTTTGTGTATGTTC
CGCAGATG

TTCCGGTGGTGAGTATGCTACAAGAATGACCGGCTGAAACGTCGGTGCCTAGACCAACCTTTATGCCCTGG
TCCAGCAACCATCGAACCCTACACTCTCCAGAAGTCAGAGAGGAGTTGGAAATGGGACAATGAGATATACC
ACAGCGAC

ATGAACATCTTGTTCTCTTAACATTTGTTCCTGAAACGGATTGGACATACCACCATCATTGTTCTCAGCCA
TGCTGGTGTTAAGTGTTGAATTTTCCACCGTCTCTTCTAGAGGAATAGTTGTTTGAGTAGATTCCTGTATT
CTTCGATC

ATGGTCTAACAGGTAGCGAACTGATGGACAGAATGAGAGAACAATCCACGAAGTTTGGCACTGAAATTATC
ACGGAAACAGTTTCCAAAGTTGATCTGTCTTCCAAACCATTCAAGCTATGGACCGAATTTAACGAAGACGC
AGAACCTG

GTGCTAATGCTTTACTCCAAGAAATTGGTGGTCCTCTAGATCCAAGTGAGTTAACACAAATCAAGAAGGGT
GATTTAGTGAATGAGAATGAAGACTTGGAAGAGTTGGACATAGAGGAAGAATATTCTGACTAATAGTCTTC
TAATTTAA

TCTATTCCTAGTATGCAGGCACCATTTTATGGTATGACACCAGGCGCCTCTGCAAATGCTCTACCTCCAAA
GCCGTACGTTCCAGCAACCACCACTAGTGCTCCTGTTCATACAGAAGGTAAATATGCGCCACCAAGCCAAC
CTTCGATG

AGGAACTAGGTATTCAATGTGGTGCTCTATCTGGTGCTAACATTGCCACCGAAGTCGCTCAAGAACACTGG
TCTGAAACAACAGTTGCTTACCACATTCCAAAGGATTTCAGAGGCGAGGGCAAGGACGTCGACCATAAGGT
TCTAAAGG

AAGAAGAATCCTCCGACTTAACCGGATCAGATACACTATTGTGGTGTAATCTGGACCTTGACTGTCTAAAC
AACTCCTCTTGTTGTCGTAGCAGCTCTTCTTCCGAGAGACCTGACTTCTTGAACTTGGAATCCCTAGTTTC
TTTCACCT

CAGGGCTGGTCTACTGCCTTATACTCTAGGATGTACATCCTACCACACACAACAAGCCTGTCACACCATGA
CTACAGATCCTTCTGTCAAATTGAAGTCCGCCAAAGACTCCCTCGTCTCCTCCCTCTTCGAGTTATCAAAA
GCTGCTAA

TTTGATTTAGAGGTCATGGATGGTAAAGCAAACATTGAAGTTCGTCCAAGATTCGTCAACAAAGGTGAAAT
AGTCAAGAGACTAGTCTGGCATCAACATGGCAAACCACAGGACATGTTGAAGGGAATCAGTGAAAAACTAC
CTAAGGAT

AGTGTCCATATAAAGGGTGTGACAAGATTCTTAGAAAGAATAAGTTCAAGACCCAAATATTCGATGATGTG
GAAGTTGAGAAAGAAGTTGACATCAGGAAGAGAGTGTTCAATGTGTTCAACAAAACTATCGATGACTTCAA
TGGTGACC

TTGTTCGTCTTGCTTTGGAGAATATGCAGGAGATCCTGGGCTGTAGCCTGGAGATGTTGGGGAGTAAGAAG
GTGAAGTAGGGCTATAGTTTGGAGAGGTGGGAGAGTAAGAAGGTGATGTTGGGGAATAGGATGGTGATGTT
GGAGAGTA

GTTAGATCAAGAGCAAACTACGATCTGGTCCTTTATGTCTACAACAAAGTTTCTGACTGTGTGAGACCAAT
CACAGTTCATATAGGTCCCGACGGCAGGTTGGGTTGCAACGTTGGATACGGGTTTCTTCACAGGATTCCAA
CAGTAAAG

ATGGATCCTCTGGTGAAACCTTGTTGATCTTGGCGATGGAGACACAGGCCTCTTCATGTCTCTCACATTCA
ATCAGATATCTTGGGGACTCTGGAACTAATAACATGCCAACGATGATAATTAGAGCCCACAGAAAGCATAG
TCCCACAG

AGAGACAACACCTGGGACATCAGCTGTTCCAGAGAACCATCATCATGTCTCTCCTCAACCTGCTTCAGTAC
CACCTCCACAGAATGGACAGTACCAACAGCACGGCATGATGACCCCAAACAAAGCTATGGCCTCTAACTGG
GCACATTA

ACAAGTTTTCGGTATTGCGTTCTCCATTATGGGTCTATTGCCCTCCATTGCATCTGTGATGGGTGGTGGGC
TCGGTGGTGGTCCAGCAACATTAGTGTGGGGTTGGTTCGTTGCTGCGTTTTTCATTTTACTGGTGGGTATT
ACCATGGC

CACTCAACAGCAACGAGTGCAGCAACAAAGGGTACAACAACAACAACAACAACAGCAGCAGCAGCAGCAAC
AGCAGCAACAGCAACAGCAACAGCAACAGCAACGCCAGGGTCAAAACCAAAGAAAGATTTCTAGTTCTAAT
TCTACTGA

CATGGTTTAAACTCGATAAAAGATTTGAAGGTTCCTTCCGACTCTTCTGGACCTGTCGGTGTATGCACATA
TGATGATCATCGTGTGGCCATGAGTTTCTCGCTTCTTGCAGGAATGGTAAATTCTCAAAATGAACGTGACG
AAGTTGCT

CATTAGTTGAAATTAGAGAGGCCAGTTCTAAACTTTTAATGTCTATTTCTTGTGTGGCTGACCTTGAAGTC
CGAACCGGAGCCGGTTGTGACACAGGATTGGATGGAGATGATGGTATTTGATGAAATTTGGAAGATGGAAG
CAAGGAAT

AATCCTATCTGGTTTCCATAACGCTGGCCCTGTTGCTGGTGCTGGTGCTGCTTCTGGCGCTGCCGCTGCTG
GTGGTGACGCTGCTGCTGAAGAAGAAAAAGAAGAAGAAGCTGCTGAAGAATCTGACGACGACATGGGTTTC
GGTTTATT

CTGGGACATCAGCTGTTCCAGAGAACCATCATCATGTCTCTCCTCAACCTGCTTCAGTACCACCTCCACAG
AATGGACAGTACCAACAGCACGGCATGATGACCCCAAACAAAGCTATGGCCTCTAACTGGGCACATTACCA
ACAACCAT

GGAGAGAACAGAGCACATTATAAGCAGTCATCAAGTCACTTGGCGTCAAAGCAGATCTCTGGTCTTTAAAC
TCGCTTGTCACTAGGAGACTATGTTCAGGGACCACAGGAAATTTGTTGAGTAGCAGCTTATATTCTCCGTC
ACCATTTA

AACCTCCTTACGTCGAAGATATGCAGATTGGGGCTCTTGAATCTTTGAGCCTTCCTCCATGTCGGTCTGCA
CTGAAACGCTGACCATGGTTGCCTTAGCGTGCACCTCTGTGGGATGGTGGCCCATCGTCTCGCTCCCAGGG
AACAAGCT

CGCTGAGGATGAATCAGTAAATGTATTACCTGACTCAGGTGATGGAGTGCTCAGAGGCGTTCCAACTGATG
ATGGATACTGCGGAAACTGTGATTGTGGCCCAGGTGGAAAGTACATAGGCGACATTTGATAAGGTGTATAC
GGAATCAT

TCCAGTACCGGATCTAGCAACAAAGAGAATGCAATAACATCAAGCTCTGAAACCACCACAATGGCTGGCCA
ATATGGTGAAAGTGGAAGCACAACAATAATGGATGAACAAGAAACTGGTACGTCCAGCCAGTATATTAGTG
TGACGACG

GTGTCCAATTGCTATCCTCTTACATCACTGAGGAACTAGGTATTCAATGTGGTGCTCTATCTGGTGCTAAC
ATTGCCACCGAAGTCGCTCAAGAACACTGGTCTGAAACAACAGTTGCTTACCACATTCCAAAGGATTTCAG
AGGCGAGG

AATGTACATATGGCTAATAGACCTTAATAGTGATACTCTGTTGGTTTTTGGCCTGCTGTGTTGGGGAAACC
GCATTGCAGTAACGACTTGTCCACAAGATGAGGATCTTCAGTGAGGTTCTCTAGTTTTTCTCTGTCTTTAA
CGAGGATC

GAGCACATTATAAGCAGTCATCAAGTCACTTGGCGTCAAAGCAGATCTCTGGTCTTTAAACTCGCTTGTCA
CTAGGAGACTATGTTCAGGGACCACAGGAAATTTGTTGAGTAGCAGCTTATATTCTCCGTCACCATTTAGA
TCTTCAGT

TGCTCTATCTGTGTTACGCCCACAAACCGGTATGCTCTTAAACAGTTCACCATTGAAGACTCCGTCCTTCA
CACCGTTGGGATTTGGCTTAATAGGTCAAAGAAGATGGAAATCAAGGGGTAACACCTATCAACCTAGTACA
TTGAAACG

GATACACAAGATGACACCGACACCTTGACCTAAAGCGAACTTGGTCTTGTCAGCAATGAACTTGTCATCTT
CGTGGAAGTAAGATCTTCTTTCGGAGTGACCCAAAATAACCCACTTAGCACCAACATCCTTGATTTGGTCA
ACGGAGTT

ATGTTCCGTACGCCGCCTTTGGAATCAGGGCCATTATTTAAGAGGCCATCTCTGTCATCTGAGTCCGCACA
CCACAGATCTTCTAGCTTACAGACGTCTAGATCCACTAATGCATTGTTGGAAGACGATTCCACGAAAGTGG
ATGCTACT

CGGATGATCCATTGAATTGCAAGTGTTGCTGCTGTTGTTGTGGTTGCGGTTGCGGTTGTGGCTGCAACTGT
GATTGCGATTGAAACATCTGGGTTGTCTGTGGTGCTTGCGACGTGAGGTTAGTAAATCCACCGTTATTATT
GCCTGAAC

CCTATCAATAGTTTCTAACTCATGTCAGTCTTGCGATCACAACCTCCTTCAGTTGTACCGCTACATCTGAC
AACATCCACCAGTCGCAAAACAGAACAGGAACCATCGCTATTGCACTCTGCAATAATTGAGCGACATCAAG
ACCGTTCG

AATGCTCCAAGTTTAAGCAACTTTGCCTCTTTGATAAGCACTGGTCAATTCAATTCTTCTCAAACTCTTGC
AAACAATTTGAGAGCGGACACACCAAGAAACCAAGTAAGTGGAAACTTTAAGGAAAACGAATACGAAGACA
ATGGCGAA

AAATCGTAAAGAACTTGCATCGGATGATCCATTGAATTGCAAGTGTTGCTGCTGTTGTTGTGGTTGCGGTT
GCGGTTGTGGCTGCAACTGTGATTGCGATTGAAACATCTGGGTTGTCTGTGGTGCTTGCGACGTGAGGTTA
GTAAATCC

TACGACCTGGATGCAAATGGTGATCAGACGAGTTATTAAATCAGCCTTCTTCTCATCATCGTTCAGATACA
ACTGAAACATAATGTCAAGGAGAACTGCAGCAGCACTCGTTTCTTGTTTCAACAGGTTTGGTATTTTATCA
ACTACACG

CGAACAATCAAGAAAGGACTGTGAAGCTATATGTAAACTAGGTTTAAAGGCCAAGATCCTTACACACATTC
GTTGTCACATGGACGATGCCAGAGTCGCCGTAGAGACTGGTGTCGACGGTGTCGATGTTGTTATCGGCACC
TCCAAATT

TTGAATGGTGATGCTTCTTCTTCTGCTGCCCCAAGCTCTTCTGCTGCTCCAACTTCTTCTGCTGCCCCAAG
CTCATCTGCTGCCCCAACTTCTTCTGCTGCCTCAAGCTCTTCTGAAGCTAAGTCTTCTTCTGCTGCCCCAA
GCTCTTCT

TTTGAAGTCTTTGAATGGTGATGCTTCTTCTTCTGCTGCCCCAAGCTCTTCTGCTGCTCCAACTTCTTCTG
CTGCCCCAAGCTCATCTGCTGCCCCAACTTCTTCTGCTGCCTCAAGCTCTTCTGAAGCTAAGTCTTCTTCT
GCTGCCCC

TGGAACCAGCTTTGAAGTCTTTGAATGGTGATGCTTCTTCTTCTGCTGCCCCAAGCTCTTCTGCTGCTCCA
ACTTCTTCTGCTGCCCCAAGCTCATCTGCTGCCCCAACTTCTTCTGCTGCCTCAAGCTCTTCTGAAGCTAA
GTCTTCTT

TCCTCTAGATTGGAACCAGCTTTGAAGTCTTTGAATGGTGATGCTTCTTCTTCTGCTGCCCCAAGCTCTTC
TGCTGCTCCAACTTCTTCTGCTGCCCCAAGCTCATCTGCTGCCCCAACTTCTTCTGCTGCCTCAAGCTCTT
CTGAAGCT

TCCATGGTACTCCTCTAGATTGGAACCAGCTTTGAAGTCTTTGAATGGTGATGCTTCTTCTTCTGCTGCCC
CAAGCTCTTCTGCTGCTCCAACTTCTTCTGCTGCCCCAAGCTCATCTGCTGCCCCAACTTCTTCTGCTGCC
TCAAGCTC

CACAATCAATGCTCGCCTTCAAGGTACTAAGATGTTGCCTCCAAACTTGACTGCTCCAAGGTTAGAAAGAG
AACACTCCTCTGTCCTTGACCAACTTGTCACAGACGCACAAGATACAGTCGACCGGTTTGTAGCCTGTGAC
AGTGATAG

GCTCAAGCATCACAATCAATGCTCGCCTTCAAGGTACTAAGATGTTGCCTCCAAACTTGACTGCTCCAAGG
TTAGAAAGAGAACACTCCTCTGTCCTTGACCAACTTGTCACAGACGCACAAGATACAGTCGACCGGTTTGT
AGCCTGTG

TTCTGCTGCCCCAAGCTCTTCTGCTGCTCCAACTTCTTCTGCTGCCCCAAGCTCATCTGCTGCCCCAACTT
CTTCTGCTGCCTCAAGCTCTTCTGAAGCTAAGTCTTCTTCTGCTGCCCCAAGCTCTTCTGAAGCTAAGTCT
TCTTCTGC

CCAAGCTCTTCTGCTGCTCCAACTTCTTCTGCTGCCCCAAGCTCATCTGCTGCCCCAACTTCTTCTGCTGC
CTCAAGCTCTTCTGAAGCTAAGTCTTCTTCTGCTGCCCCAAGCTCTTCTGAAGCTAAGTCTTCTTCTGCTG
CCCCAAGC

AGCAAGATGTTGACCATGGTTCCATGGTACTCCTCTAGATTGGAACCAGCTTTGAAGTCTTTGAATGGTGA
TGCTTCTTCTTCTGCTGCCCCAAGCTCTTCTGCTGCTCCAACTTCTTCTGCTGCCCCAAGCTCATCTGCTG
CCCCAACT

ATGCTTCTTCTTCTGCTGCCCCAAGCTCTTCTGCTGCTCCAACTTCTTCTGCTGCCCCAAGCTCATCTGCT
GCCCCAACTTCTTCTGCTGCCTCAAGCTCTTCTGAAGCTAAGTCTTCTTCTGCTGCCCCAAGCTCTTCTGA
AGCTAAGT

TTCTGCTGCCCCAAGCTCTTCTGAAGCTAAGTCTTCTTCTGCTGCTCCAAGCTCCACTGAAGCTAAGATAA
CTTCTGCTGCTCCAAGCTCCACTGGTGCCAAGACCTCTGCCATCTCTCAAATTACCGATGGTCAAATCCAA
GCTACCAA

CTGCTGCTCCAACTTCTTCTGCTGCCCCAAGCTCATCTGCTGCCCCAACTTCTTCTGCTGCCTCAAGCTCT
TCTGAAGCTAAGTCTTCTTCTGCTGCCCCAAGCTCTTCTGAAGCTAAGTCTTCTTCTGCTGCCCCAAGCTC
TTCTGAAG

GCTCGCCTTCAAGGTACTAAGATGTTGCCTCCAAACTTGACTGCTCCAAGGTTAGAAAGAGAACACTCCTC
TGTCCTTGACCAACTTGTCACAGACGCACAAGATACAGTCGACCGGTTTGTAGCCTGTGACAGTGATAGCA
GTAGCACT

GTAGTGACAACGTTGGCAAAGCGAAGGTACCACCAAAGCAGGAGCCACAGAAGACTGTGAGAACAGTCAAC
ACAGCAAATCAACAAGAAAAGCAACAACAGAGGCAGCAGCAGCCGTCTCCGCATAATGTTAAGGACCGCAA
GGAGCAAA

CTAAGTCTTCTTCTGCTGCCCCAAGCTCTTCTGAAGCTAAGTCTTCTTCTGCTGCTCCAAGCTCCACTGAA
GCTAAGATAACTTCTGCTGCTCCAAGCTCCACTGGTGCCAAGACCTCTGCCATCTCTCAAATTACCGATGG
TCAAATCC

TGACCATGGTTCCATGGTACTCCTCTAGATTGGAACCAGCTTTGAAGTCTTTGAATGGTGATGCTTCTTCT
TCTGCTGCCCCAAGCTCTTCTGCTGCTCCAACTTCTTCTGCTGCCCCAAGCTCATCTGCTGCCCCAACTTC
TTCTGCTG

TCTTCTGCTGCCTCAAGCTCTTCTGAAGCTAAGTCTTCTTCTGCTGCCCCAAGCTCTTCTGAAGCTAAGTC
TTCTTCTGCTGCCCCAAGCTCTTCTGAAGCTAAGTCTTCTTCTGCTGCCCCAAGCTCTTCTGAAGCTAAGT
CTTCTTCT

GCTTCCTGCGGATGAGGAAGCTGAATTACAAAGAAGGCTTCAACTGGGGTTGAGAGTGTCTCCAAGACGTA
GAGAACATCTTCACGCACCTGAGGAAGGTTCGTCGGGACCTCTTACCGAGGTCGGTACTCCAAGATTACCT
AACGTATC

GCCAGCGCTGGTAGTGACAACGTTGGCAAAGCGAAGGTACCACCAAAGCAGGAGCCACAGAAGACTGTGAG
AACAGTCAACACAGCAAATCAACAAGAAAAGCAACAACAGAGGCAGCAGCAGCCGTCTCCGCATAATGTTA
AGGACCGC

CTAAGGGACAATTTAACCTTATGGACTTCAGACATGTCCGAGTCCGGTCAAGCTGAAGACCAACAACAACA
ACAACAACATCAGCAACAGCAGCCACCTGCTGCCGCCGAAGGTGAAGCACCAAAGTAAGTATTCTGATAAA
TCTAAAGA

CTTAGCCATTAATTCTTGGACACCTTTACCTTTAGCAGACTTGATGGTGTCATCTTCAAATAAGCGTGGGG
AGTCCTCGATGTGGAAGTGACGTTGAGTCTCGGTGCAAGCACCGTTAATACCACTGGAGGCCTTGATAGAG
TTCCCACC

TACCACTGAAAGTACCAACTCCAGCACTAATGCTACTACCACTGCCAGCACCAACGTCAGGACTAGTGCTA
CTACCACTGCCAGCATCAACGTCAGGACTAGTGCGACTACCACTGAAAGTACCAACTCCAACACTAGTGCT
ACTACCAC

CATGCAACTGCTAAGGGACAATTTAACCTTATGGACTTCAGACATGTCCGAGTCCGGTCAAGCTGAAGACC
AACAACAACAACAACAACATCAGCAACAGCAGCCACCTGCTGCCGCCGAAGGTGAAGCACCAAAGTAAGTA
TTCTGATA

GCTGCCCCAAGCTCATCTGCTGCCCCAACTTCTTCTGCTGCCTCAAGCTCTTCTGAAGCTAAGTCTTCTTC
TGCTGCCCCAAGCTCTTCTGAAGCTAAGTCTTCTTCTGCTGCCCCAAGCTCTTCTGAAGCTAAGTCTTCTT
CTGCTGCC

GTCTTCTTCTGCTGCTCCAAGCTCCACTGAAGCTAAGATAACTTCTGCTGCTCCAAGCTCCACTGGTGCCA
AGACCTCTGCCATCTCTCAAATTACCGATGGTCAAATCCAAGCTACCAAGGCTGTTTCTGAGCAAACTGAA
AACGGTGC

AACTTCTTCTGCTGCCCCAAGCTCATCTGCTGCCCCAACTTCTTCTGCTGCCTCAAGCTCTTCTGAAGCTA
AGTCTTCTTCTGCTGCCCCAAGCTCTTCTGAAGCTAAGTCTTCTTCTGCTGCCCCAAGCTCTTCTGAAGCT
AAGTCTTC

TCTTCTGAAGCTAAGTCTTCTTCTGCTGCCCCAAGCTCTTCTGAAGCTAAGTCTTCTTCTGCTGCTCCAAG
CTCCACTGAAGCTAAGATAACTTCTGCTGCTCCAAGCTCCACTGGTGCCAAGACCTCTGCCATCTCTCAAA
TTACCGAT

TGCCCCAACTTCTTCTGCTGCCTCAAGCTCTTCTGAAGCTAAGTCTTCTTCTGCTGCCCCAAGCTCTTCTG
AAGCTAAGTCTTCTTCTGCTGCCCCAAGCTCTTCTGAAGCTAAGTCTTCTTCTGCTGCCCCAAGCTCTTCT
GAAGCTAA

CCTCAAGCTCTTCTGAAGCTAAGTCTTCTTCTGCTGCCCCAAGCTCTTCTGAAGCTAAGTCTTCTTCTGCT
GCCCCAAGCTCTTCTGAAGCTAAGTCTTCTTCTGCTGCCCCAAGCTCTTCTGAAGCTAAGTCTTCTTCTGC
TGCTCCAA

AAGTCTTCTTCTGCTGCCCCAAGCTCTTCTGAAGCTAAGTCTTCTTCTGCTGCCCCAAGCTCTTCTGAAGC
TAAGTCTTCTTCTGCTGCCCCAAGCTCTTCTGAAGCTAAGTCTTCTTCTGCTGCTCCAAGCTCCACTGAAG
CTAAGATA

CTGCTGCCCCAAGCTCTTCTGAAGCTAAGTCTTCTTCTGCTGCCCCAAGCTCTTCTGAAGCTAAGTCTTCT
TCTGCTGCCCCAAGCTCTTCTGAAGCTAAGTCTTCTTCTGCTGCTCCAAGCTCCACTGAAGCTAAGATAAC
TTCTGCTG

TGCTGGTGTTAGCAAGATGTTGACCATGGTTCCATGGTACTCCTCTAGATTGGAACCAGCTTTGAAGTCTT
TGAATGGTGATGCTTCTTCTTCTGCTGCCCCAAGCTCTTCTGCTGCTCCAACTTCTTCTGCTGCCCCAAGC
TCATCTGC

TGGTGAAGGAGTGAGAGTTTGACCTTGGCTTTAGAGTCTTATGATGGGGATGGTGGTGTAGGTGTTGATGA
GGATGATGAGGGAGTGCGGCCGGCAGCGTGGGAGGAGCGAACGGCATGGAAGAAGCAGCGTTTGAGACAAT
CGTAGTGG

AAGCTCTTCTGAAGCTAAGTCTTCTTCTGCTGCCCCAAGCTCTTCTGAAGCTAAGTCTTCTTCTGCTGCCC
CAAGCTCTTCTGAAGCTAAGTCTTCTTCTGCTGCTCCAAGCTCCACTGAAGCTAAGATAACTTCTGCTGCT
CCAAGCTC

AAGAACGGCAGCACTCCTGACACGCAGACCGCCAGCGCTGGTAGTGACAACGTTGGCAAAGCGAAGGTACC
ACCAAAGCAGGAGCCACAGAAGACTGTGAGAACAGTCAACACAGCAAATCAACAAGAAAAGCAACAACAGA
GGCAGCAG

AACTAGAAGGTGTTGCAAACCCCATTATGAGTAAATTTTACGGAGCTGCAGGTGGTGCCCCAGGAGCAGGC
CCAGTTCCGGGTGCTGGAGCAGGCCCCACTGGAGCACCAGACAACGGCCCAACGGTTGAAGAGGTTGATTA
GATAAATA

GCACTCCTGACACGCAGACCGCCAGCGCTGGTAGTGACAACGTTGGCAAAGCGAAGGTACCACCAAAGCAG
GAGCCACAGAAGACTGTGAGAACAGTCAACACAGCAAATCAACAAGAAAAGCAACAACAGAGGCAGCAGCA
GCCGTCTC

ATTTAACCTTATGGACTTCAGACATGTCCGAGTCCGGTCAAGCTGAAGACCAACAACAACAACAACAACAT
CAGCAACAGCAGCCACCTGCTGCCGCCGAAGGTGAAGCACCAAAGTAAGTATTCTGATAAATCTAAAGAGA
AATTACTA

GAAGCTAAGTCTTCTTCTGCTGCCCCAAGCTCTTCTGAAGCTAAGTCTTCTTCTGCTGCCCCAAGCTCTTC
TGAAGCTAAGTCTTCTTCTGCTGCTCCAAGCTCCACTGAAGCTAAGATAACTTCTGCTGCTCCAAGCTCCA
CTGGTGCC

CGATCAAGAAAGCTCTAATAATTCTTTCCTTAACACAGTTGGCACATCTGGAACCACCGTAAGCTCTGGAA
ACAGTCTTGTGGGTCTTGGAAACAGTGGCGTATTGTCTTGGTCTCAAAGTAGAGATACCTTGCAAAGCGCT
ACCACAGT

GCTCATCTGCTGCCCCAACTTCTTCTGCTGCCTCAAGCTCTTCTGAAGCTAAGTCTTCTTCTGCTGCCCCA
AGCTCTTCTGAAGCTAAGTCTTCTTCTGCTGCCCCAAGCTCTTCTGAAGCTAAGTCTTCTTCTGCTGCCCC
AAGCTCTT

TTCTGAAGCTAAGTCTTCTTCTGCTGCCCCAAGCTCTTCTGAAGCTAAGTCTTCTTCTGCTGCCCCAAGCT
CTTCTGAAGCTAAGTCTTCTTCTGCTGCCCCAAGCTCTTCTGAAGCTAAGTCTTCTTCTGCTGCTCCAAGC
TCCACTGA

GCACACTTATCATGCAACTGCTAAGGGACAATTTAACCTTATGGACTTCAGACATGTCCGAGTCCGGTCAA
GCTGAAGACCAACAACAACAACAACAACATCAGCAACAGCAGCCACCTGCTGCCGCCGAAGGTGAAGCACC
AAAGTAAG

CGTCTTCTGCATGCTTCCTGTCTTCTTCCTCACCACTAAGCCATGAGAAGAAGAGGAAGATTGTACTGAAG
GTGATGGTGCAGAAGGAGAAGGAGAAGAGGATGGTTCTGATATGGCAGCCAATAAGTCCTTTCTCGAATCA
TTGCTGAA

GCTGCTCCAAGCTCCACTGAAGCTAAGATAACTTCTGCTGCTCCAAGCTCCACTGGTGCCAAGACCTCTGC
CATCTCTCAAATTACCGATGGTCAAATCCAAGCTACCAAGGCTGTTTCTGAGCAAACTGAAAACGGTGCTG
CTAAGGCC

TAATGTCACCGCTTCCTGCGGATGAGGAAGCTGAATTACAAAGAAGGCTTCAACTGGGGTTGAGAGTGTCT
CCAAGACGTAGAGAACATCTTCACGCACCTGAGGAAGGTTCGTCGGGACCTCTTACCGAGGTCGGTACTCC
AAGATTAC

AGGTTGACTTTGCTGGTGTTAGCAAGATGTTGACCATGGTTCCATGGTACTCCTCTAGATTGGAACCAGCT
TTGAAGTCTTTGAATGGTGATGCTTCTTCTTCTGCTGCCCCAAGCTCTTCTGCTGCTCCAACTTCTTCTGC
TGCCCCAA

GATGAGGCATGCAGGTTGGATTATGCTCTTATCGACCCTGATTTCTTGCAAACAGTAGACGCAGGTGTCAG
GTTCACTGAACGGATCCCTCACCTGGACTGCAGTGTCTCTGACCATTTTGCATACTCATGCACCCTTAACA
TCGTCCCA

TGCCCCAAGCTCTTCTGAAGCTAAGTCTTCTTCTGCTGCCCCAAGCTCTTCTGAAGCTAAGTCTTCTTCTG
CTGCTCCAAGCTCCACTGAAGCTAAGATAACTTCTGCTGCTCCAAGCTCCACTGGTGCCAAGACCTCTGCC
ATCTCTCA

GATGAGGAAGCTGAATTACAAAGAAGGCTTCAACTGGGGTTGAGAGTGTCTCCAAGACGTAGAGAACATCT
TCACGCACCTGAGGAAGGTTCGTCGGGACCTCTTACCGAGGTCGGTACTCCAAGATTACCTAACGTATCTT
CTGCAGGT

ATGCACTGCTGTGGGTACGGCCCATTCTGTGGAGGTGGTACTGAAGCAGGTTGAGGAGAGGCATGATGGGG
GTTCTCTGGAACAGCTGATGAAGCAGGTGTTGTTGTCTGTTGAGAGTTAGCCTTAGTGGAAGCCTTATCAT
ATTCTTGA

TACCACCGAAAGTACCGACTCCAACACTAGTGCTACTACCACTGCTAGCACCAACTCCAGCACTAATGCCA
CTACCACTGCTAGCACCAACTCCAGCACTAATGCCACTACCACTGAAAGTACCAACGCTAGTGCCAAGGAG
GACGCCAA

CTAGTGCGACTACCACTGAAAGTACCAACTCCAGCACTAATGCTACTACCACTGCCAGCACCAACGTCAGG
ACTAGTGCTACTACCACTGCCAGCATCAACGTCAGGACTAGTGCGACTACCACTGAAAGTACCAACTCCAA
CACTAGTG

TTGTACTCTGAGGTTGACTTTGCTGGTGTTAGCAAGATGTTGACCATGGTTCCATGGTACTCCTCTAGATT
GGAACCAGCTTTGAAGTCTTTGAATGGTGATGCTTCTTCTTCTGCTGCCCCAAGCTCTTCTGCTGCTCCAA
CTTCTTCT

CTGGTTTAGTCTCAGCTAATTTCTTCAAATTGTTAGATAAGGCAGAAACAATTTCGAAACCTGGAGGCAAC
TTCCCAGACGATCTGTGAGTTCTTGCCACAGAGTGGCCACCCAATTGAGCCAATAGGTCCAATTTCAAATC
AAATTCGT

CCAAGCTCTTCTGAAGCTAAGTCTTCTTCTGCTGCTCCAAGCTCCACTGAAGCTAAGATAACTTCTGCTGC
TCCAAGCTCCACTGGTGCCAAGACCTCTGCCATCTCTCAAATTACCGATGGTCAAATCCAAGCTACCAAGG
CTGTTTCT

CTACACTACTTTGTACTCTGAGGTTGACTTTGCTGGTGTTAGCAAGATGTTGACCATGGTTCCATGGTACT
CCTCTAGATTGGAACCAGCTTTGAAGTCTTTGAATGGTGATGCTTCTTCTTCTGCTGCCCCAAGCTCTTCT
GCTGCTCC

TGTTGCAAACCCCATTATGAGTAAATTTTACGGAGCTGCAGGTGGTGCCCCAGGAGCAGGCCCAGTTCCGG
GTGCTGGAGCAGGCCCCACTGGAGCACCAGACAACGGCCCAACGGTTGAAGAGGTTGATTAGATAAATACA
AAGATGCG

TGCTGCTGCTGCTGTTGTTGTTGTTGTTGCTGTTGTTGTTGTTGTTGGTTGTTTGGTGAGACAAAGTCACC
CTTTGAGTCCACCAGCTCCGAGCCATCTCCCTCTTCAGACCAGTCTACAGTGACTTTATTGTTCAAAGTGG
AGTCAATG

GCCTGTGTTCATTAGACACAGAACTCCTCATGAAATTCAGGAAGCCAACAGTGTGGATATGTCCGCTTTGA
AGGACCCACAGACCGATGCTGACAGAGTCAAAGACCCTCAATGGTTAATTATGCTGGGTATTTGTACTCAC
TTAGGTTG

AAGGTACTAAGATGTTGCCTCCAAACTTGACTGCTCCAAGGTTAGAAAGAGAACACTCCTCTGTCCTTGAC
CAACTTGTCACAGACGCACAAGATACAGTCGACCGGTTTGTAGCCTGTGACAGTGATAGCAGTAGCACTAT
TGAATGAG

ACCACAGCCCCAAAGTTTTACTGCGTGAGACCAAACGCCGCAGTTGTTGCTCCAGGTGAAACAATCCAGGT
TCAAGTTATCTTCCTGGGTTTGACTGAAGAGCCTGCCGCAGACTTTAAGTGCCGTGACAAGTTCCTGGTTA
TCACGTTG

CATGCACTGCTGTGGGTACGGCCCATTCTGTGGAGGTGGTACTGAAGCAGGTTGAGGAGAGGCATGATGGG
GGTTCTCTGGAACAGCTGATGAAGCAGGTGTTGTTGTCTGTTGAGAGTTAGCCTTAGTGGAAGCCTTCTCA
CATTCTTC

AGCTGGATTACCATCTCTAATGCCAACTTCCATGCATATAGCTCAGGCCCCACCGTGTGCAGCTCCGTGCT
TCGCAGCTCCTGCAGAGCATCCTCGGGGATTGGGAACCTCTCATTTAGCAAGTAATTCACATAACACAGAT
TTAGAAAC

GCTCCACTGAAGCTAAGATAACTTCTGCTGCTCCAAGCTCCACTGGTGCCAAGACCTCTGCCATCTCTCAA
ATTACCGATGGTCAAATCCAAGCTACCAAGGCTGTTTCTGAGCAAACTGAAAACGGTGCTGCTAAGGCCTT
TGTTGGTA

GAATGAATCCATACTTCTCCTCATCATTAGACCGAACGATGGGGTCTTGGTTCAAAGAATTGGTGAAGGAG
TGAGAGTTTGACCTTGGCTTTAGAGTCTTATGATGGGGATGGTGGTGTAGGTGTTGATGAGGATGATGAGG
GAGTGCGG

TGGATACGGTGATGTTCAGTACTGGTATACTGGCGGTTCTCAGGTCGCTGAAGCTTCAGGGTCAGTATGTG
GGGGTGATGATCACGGCGTCGCACAACCCATACCAGGACAACGGGGTCAAGATCGTGGAACCAGACGGATC
GATGCTTT

TGAGTGTAAACGTGAGCCCTGTAATCACATAGAATAGAGTATGGAGGTTGCATCAAGTGAGTTCTGATGGC
AATCTGGTTGGACAAAGTCCCAGAGACACAGAACAAACCTGCTTCTTTGCCAGCCATGCGGGCAACGGTCT
GTTCGAGC

CTGAAGCTAAGTCTTCTTCTGCTGCTCCAAGCTCCACTGAAGCTAAGATAACTTCTGCTGCTCCAAGCTCC
ACTGGTGCCAAGACCTCTGCCATCTCTCAAATTACCGATGGTCAAATCCAAGCTACCAAGGCTGTTTCTGA
GCAAACTG

TGGCATCTGCAGTTAAATCAACAGTTTGTTCCTTACTATTTGTTCCAGGTTTCGTTGTCGGCGGTGCTGCG
TCAAGCATCGGGGAAGCACTCGGAGTGTTTGCTGTTTGTGAAAGAACGTCCATTGTGTGCAATTCTTCAAC
ATTATTCG

AGTGATGTCAACTGGAAACATCATAGCTCTTCCTTTCTTGCCTTCCACAGTGCCCTGTTGCTGTTCTTGTT
GCTGTTGAAAGCCTTGATTTTGAAGTGAGTCAGGCTGCTGAGATGGTTGATAATCCATTTTTTGCAAGGTA
TTTCCTGT

TAAAGGAATCCATTAAATTACGTCCTAGTGAAGATAATTTACAACTGGACGGTGTTGACAAGATTGCTGTG
TTACATGGTGGTACAAAGGCATTACAAGTAACACCTCCTGAACATAAATCGGATATCCCTCCTCCACCTGA
TGGAGGTG

CGTGAGCCCTGTAATCACATAGAATAGAGTATGGAGGTTGCATCAAGTGAGTTCTGATGGCAATCTGGTTG
GACAAAGTCCCAGAGACACAGAACAAACCTGCTTCTTTGCCAGCCATGCGGGCAACGGTCTGTTCGAGCCT
AACGGTGT

GACTCCAACACTAGTGCTACTACCACCGAAAGTACCGACTCCAACACTAGTGCTACTACCACTGCTAGCAC
CAACTCCAGCACTAATGCCACTACCACTGCTAGCACCAACTCCAGCACTAATGCCACTACCACTGAAAGTA
CCAACGCT

AGAATAGAGTATGGAGGTTGCATCAAGTGAGTTCTGATGGCAATCTGGTTGGACAAAGTCCCAGAGACACA
GAACAAACCTGCTTCTTTGCCAGCCATGCGGGCAACGGTCTGTTCGAGCCTAACGGTGTCAACATCTTCAC
CGTAGACA

AGTACCGACTCCAACACTAGTGCTACTACCACTGCTAGCACCAACTCCAGCACTAATGCCACTACCACTGC
TAGCACCAACTCCAGCACTAATGCCACTACCACTGAAAGTACCAACGCTAGTGCCAAGGAGGACGCCAATA
AAGATGGC

GCTTCCGCCACTGACGACTCCTACACTACTTTGTACTCTGAGGTTGACTTTGCTGGTGTTAGCAAGATGTT
GACCATGGTTCCATGGTACTCCTCTAGATTGGAACCAGCTTTGAAGTCTTTGAATGGTGATGCTTCTTCTT
CTGCTGCC

CTGTTGCTGTTGCTGCTGCTGCTGTTGTTGTTGTTGTTGCTGTTGTTGTTGTTGTTGGTTGTTTGGTGAGA
CAAAGTCACCCTTTGAGTCCACCAGCTCCGAGCCATCTCCCTCTTCAGACCAGTCTACAGTGACTTTATTG
TTCAAAGT

AACGTCAGGACTAGTGCGACTACCACTGAAAGTACCAACTCCAGCACTAATGCTACTACCACTGCCAGCAC
CAACGTCAGGACTAGTGCTACTACCACTGCCAGCATCAACGTCAGGACTAGTGCGACTACCACTGAAAGTA
CCAACTCC

ACGTTTCGAATCTTCAGTATGAGTTGACGGAGGTGTGGAATCGGTTGGACTCACAGCTTTTGAAAGGACAT
TTCTCAGTTGCTCAGGATGTAGTTCAATGTCAGATTGGAAGTCATGGTCAGATTCTATGTTAAGATCATTG
GATTGTTG

GTACTGCTTGGAGGGTGGAAATACGGAGCTTTTTCTCTTATCTCTGTTGTTGTCTTCCCCTCTGTGGTGAC
TGTGTCCCTGGCCATGGTGATGGTGGGAATGCATGTATGGAGAAGCACCGCTTGTCGAGTTTCGAATAGTT
TGAGAGGA

CAGTTGTTGCTCCAGGTGAAACAATCCAGGTTCAAGTTATCTTCCTGGGTTTGACTGAAGAGCCTGCCGCA
GACTTTAAGTGCCGTGACAAGTTCCTGGTTATCACGTTGCCTTCTCCTTACGACCTCAATGGCAAGGCTGT
TGCAGATG

CTTCCTGGGTTTGACTGAAGAGCCTGCCGCAGACTTTAAGTGCCGTGACAAGTTCCTGGTTATCACGTTGC
CTTCTCCTTACGACCTCAATGGCAAGGCTGTTGCAGATGTTTGGTCCGACTTGGAGGCTGAGTTCAAGCAA
CAGGCTAT

CACGCAGACCGCCAGCGCTGGTAGTGACAACGTTGGCAAAGCGAAGGTACCACCAAAGCAGGAGCCACAGA
AGACTGTGAGAACAGTCAACACAGCAAATCAACAAGAAAAGCAACAACAGAGGCAGCAGCAGCCGTCTCCG
CATAATGT

AGACTTTAAGTGCCGTGACAAGTTCCTGGTTATCACGTTGCCTTCTCCTTACGACCTCAATGGCAAGGCTG
TTGCAGATGTTTGGTCCGACTTGGAGGCTGAGTTCAAGCAACAGGCTATCTCTAAAAAGATAAAAGTCAAA
TATTTGAT

GTAATCACATAGAATAGAGTATGGAGGTTGCATCAAGTGAGTTCTGATGGCAATCTGGTTGGACAAAGTCC
CAGAGACACAGAACAAACCTGCTTCTTTGCCAGCCATGCGGGCAACGGTCTGTTCGAGCCTAACGGTGTCA
ACATCTTC

CTGACGACTCCTACACTACTTTGTACTCTGAGGTTGACTTTGCTGGTGTTAGCAAGATGTTGACCATGGTT
CCATGGTACTCCTCTAGATTGGAACCAGCTTTGAAGTCTTTGAATGGTGATGCTTCTTCTTCTGCTGCCCC
AAGCTCTT

ATGAGGGATCTGGCTTGGACCCACATAAGGGAGCTACCACATCTTCCACCCAAAAGAGTTCTTCCTCAACT
GCAACCTCTTCCTCCAAAACGTCATCCGATCACAGCAGTTCAACTAAGAAGAGTAGTAAGACTAGTTCCAC
CGCTTCAT

AGTACCAACTCCAGCACTAATGCTACTACCACTGCCAGCACCAACGTCAGGACTAGTGCTACTACCACTGC
CAGCATCAACGTCAGGACTAGTGCGACTACCACTGAAAGTACCAACTCCAACACTAGTGCTACTACCACCG
AAAGTACC

GGTACCGTTCTTTCCTCCACGAGGGCTGTTTGGTATGGTAAGGTCAGTGCTAGAATAAAGACGTCACATCT
TGCTGGTGTGGTCACTGGGTTCATTCTGTATTCCGGCGCAGGTGATGAACTTGATTACGAATTCGTCGGTG
CTGATCTA

TGCTACTACCACTGCCAGCACCAACGTCAGGACTAGTGCTACTACCACTGCCAGCATCAACGTCAGGACTA
GTGCGACTACCACTGAAAGTACCAACTCCAACACTAGTGCTACTACCACCGAAAGTACCGACTCCAACACT
AGTGCTAC

AGAGTCCGCTGAGGATGAATCAGTAAATGTATTACCTGACTCAGGTGATGGAGTGCTCAGAGGCGTTCCAA
CTGATGATGGATACTGCGGAAACTGTGATTGTGGCCCAGGTGGAAAGTACATAGGCGACATTTGATAAGGT
GTATACGG

CTTCTTCTGCTGCCCCAAGCTCTTCTGAAGCTAAGTCTTCTTCTGCTGCCCCAAGCTCTTCTGAAGCTAAG
TCTTCTTCTGCTGCTCCAAGCTCCACTGAAGCTAAGATAACTTCTGCTGCTCCAAGCTCCACTGGTGCCAA
GACCTCTG

CGACTACCAACTCCAGCACTAATGCTACTACCACTGCCAGCACCAACGTCAGGACTAGTGCTACTACCACT
GCCAGCATCAACGTCAGGACTAGTGCGACTACCACTGAAAGTACCAACTCCAGCACTAATGCTACTACCAC
TGCCAGCA

TTCCAACAATTCAGACCAAACCATTGCATTTAAGGTCAAGACCACAGCCCCAAAGTTTTACTGCGTGAGAC
CAAACGCCGCAGTTGTTGCTCCAGGTGAAACAATCCAGGTTCAAGTTATCTTCCTGGGTTTGACTGAAGAG
CCTGCCGC

CTCCAGCACTAATGCTACTACCACTGCCAGCACCAACGTCAGGACTAGTGCTACTACCACTGCCAGCATCA
ACGTCAGGACTAGTGCGACTACCACTGAAAGTACCAACTCCAGCACTAATGCTACTACCACTGCCAGCACC
AACGTCAG

TTGTCTGTTGCTTATAAGAACGTTATTGGTGCTCGTCGTGCCTCTTGGAGAATTGTTTCTTCTATTGAGCA
AAAGGAGGAGTCCAAGGAGAAGTCCGAACACCAGGTCGAGTTGATTTGTTCGTACCGTTCGAAGATTGAGA
CCGAACTA

TGCAATAATGAACAGTCACATGCACGCCCCATACGCCAAGCAGGGTGATGCCGCCTACTTGTGCCACAGAT
CTTGTCAGGCCTGGGATTTCAGCAGGCTCATTAAGCTTTACAGGCAGGCCGGTTATGCGGTGATTGTGGTG
GGTGACTT

TCATCATTAGACCGAACGATGGGGTCTTGGTTCAAAGAATTGGTGAAGGAGTGAGAGTTTGACCTTGGCTT
TAGAGTCTTATGATGGGGATGGTGGTGTAGGTGTTGATGAGGATGATGAGGGAGTGCGGCCGGCAGCGTGG
GAGGAGCG

CCTGTTGCCAAGTGATGTCAACTGGAAACATCATAGCTCTTCCTTTCTTGCCTTCCACAGTGCCCTGTTGC
TGTTCTTGTTGCTGTTGAAAGCCTTGATTTTGAAGTGAGTCAGGCTGCTGAGATGGTTGATAATCCATTTT
TTGCAAGG

CCCATTATGAGTAAATTTTACGGAGCTGCAGGTGGTGCCCCAGGAGCAGGCCCAGTTCCGGGTGCTGGAGC
AGGCCCCACTGGAGCACCAGACAACGGCCCAACGGTTGAAGAGGTTGATTAGATAAATACAAAGATGCGAT
GAAGTAGC

GAGGGTGGAAATACGGAGCTTTTTCTCTTATCTCTGTTGTTGTCTTCCCCTCTGTGGTGACTGTGTCCCTG
GCCATGGTGATGGTGGGAATGCATGTATGGAGAAGCACCGCTTGTCGAGTTTCGAATAGTTTGAGAGGATG
ATGACGGC

GAGTCCGCTGAGGATGAATCAGTAAATGTATTACCTGACTCAGGTGATGGAGTGCTCAGAGGCGTTCCAAC
TGATGATGGATACTGCGGAAACTGTGATTGTGGCCCAGGTGGAAAGTACATAGGCGACATTTGATAAGGTG
TATACGGA

TACCCAATTGAAGTTGCTGAGGCCGTTTTCAACTACGGTGACTTCACCACCATGTTGACTGGTATTGCCCC
AGACCAAGTGACCAGAATGATCACCGGTGTTCCATGGTACTCCAGCAGATTAAAGCCAGCCATTTCCAGTG
CTCTATCC

ATGGACAGTGATGAGGGATCTGGCTTGGACCCACATAAGGGAGCTACCACATCTTCCACCCAAAAGAGTTC
TTCCTCAACTGCAACCTCTTCCTCCAAAACGTCATCCGATCACAGCAGTTCAACTAAGAAGAGTAGTAAGA
CTAGTTCC

AGCTCTAATAATTCTTTCCTTAACACAGTTGGCACATCTGGAACCACCGTAAGCTCTGGAAACAGTCTTGT
GGGTCTTGGAAACAGTGGCGTATTGTCTTGGTCTCAAAGTAGAGATACCTTGCAAAGCGCTACCACAGTCA
CCACACTT

TCTTCAGTATGAGTTGACGGAGGTGTGGAATCGGTTGGACTCACAGCTTTTGAAAGGACATTTCTCAGTTG
CTCAGGATGTAGTTCAATGTCAGATTGGAAGTCATGGTCAGATTCTATGTTAAGATCATTGGATTGTTGGA
TCTCATTT

TTTTGGAAACACGTTTCGAATCTTCAGTATGAGTTGACGGAGGTGTGGAATCGGTTGGACTCACAGCTTTT
GAAAGGACATTTCTCAGTTGCTCAGGATGTAGTTCAATGTCAGATTGGAAGTCATGGTCAGATTCTATGTT
AAGATCAT

CGTAAGCAATCACGTACCTCAACATATTTCTTTCGCAGCACGCCACGCCTTTCGGTGCCAACTTGGAATGA
AGTCTGACCTGTTCAATGGTTTTGAGATTTACTGGGTCAACAGTTATCCATTTCTTAGAGAATTTGTCCCA
CACTTCAC

GCTGTTGTTGCTGTTGCTGTTGCTGCTGCTGCTGTTGTTGTTGTTGTTGCTGTTGTTGTTGTTGTTGGTTG
TTTGGTGAGACAAAGTCACCCTTTGAGTCCACCAGCTCCGAGCCATCTCCCTCTTCAGACCAGTCTACAGT
GACTTTAT

GGTACAAAGGCATTACAAGTAACACCTCCTGAACATAAATCGGATATCCCTCCTCCACCTGATGGAGGTGC
CTTAGCCATCGTCACCAAAACTGGCTTTGAAACATCTCAGGGTTCTTTAGTTCGAGTTATGATTTACTCCG
CTGAACGT

ATCTTCAGAAAAGAACGGCAGCACTCCTGACACGCAGACCGCCAGCGCTGGTAGTGACAACGTTGGCAAAG
CGAAGGTACCACCAAAGCAGGAGCCACAGAAGACTGTGAGAACAGTCAACACAGCAAATCAACAAGAAAAG
CAACAACA

CGTACTGCGCTGCGGAAGTGTGGATGAGTGGCATATACACTCTTCTGCAGAGGATTTTCGTACACAACGGT
GTGTGTGTGCTGTGAAGCTGTCTGCTTCCTTACTTGGCTGTTTGCTTGCTTGTGCGTCATGGTCACTTTTG
TTAGAAGT

ATCTTCAGTATGAGTTGACGGAGGTGTGGAATCGGTTGGACTCACAGCTTTTGAAAGGACATTTCTCGGTT
GCTCAGGATGTAGTTCGATGTCAGATTGGAAGTCATGGTCAGATTCTATGTTAAGATCATCGGATTGTTGG
ATCTCATT

TGCTACTACCACTGCTAGCACCAACTCCAGCACTAATGCCACTACCACTGCTAGCACCAACTCCAGCACTA
ATGCCACTACCACTGAAAGTACCAACGCTAGTGCCAAGGAGGACGCCAATAAAGATGGCAATGCTGAGGAT
AATAGATT

GCTTTCACGATACCGCATTACGTACTGCGCTGCGGAAGTGTGGATGAGTGGCATATACACTCTTCTGCAGA
GGATTTTCGTACACAACGGTGTGTGTGTGCTGTGAAGCTGTCTGCTTCCTTACTTGGCTGTTTGCTTGCTT
GTGCGTCA

GCTCGAGAAAGTTGGAGTTTTTCAGCGTTTGCGTTCCATGACGAGCGCTGGACTGCAGGGTCCGCAGTACG
TCAAGCTGCAGTTTAGCAGGCATCATCGACAGTTGAGGAGCAGATATGAATTAAGTCTAGGAATGCACTTG
CGAGATCA

GATAACGATATAATGTCACCGCTTCCTGCGGATGAGGAAGCTGAATTACAAAGAAGGCTTCAACTGGGGTT
GAGAGTGTCTCCAAGACGTAGAGAACATCTTCACGCACCTGAGGAAGGTTCGTCGGGACCTCTTACCGAGG
TCGGTACT

GTTGGTGACTTAGAACCTTCTGTTTCCGAAGCCCACTTATATGATATCTTCTCTCCAATCGGTTCAGTCTC
CTCCATTCGTGTCTGTCGTGATGCCATCACTAAGACCTCTTTGGGCTATGCTTATGTTAACTTTAACGACC
ATGAAGCC

TTTTGTTCTTCGATCAAGAAAGCTCTAATAATTCTTTCCTTAACACAGTTGGCACATCTGGAACCACCGTA
AGCTCTGGAAACAGTCTTGTGGGTCTTGGAAACAGTGGCGTATTGTCTTGGTCTCAAAGTAGAGATACCTT
GCAAAGCG

ATTCTTTCCTTAACACAGTTGGCACATCTGGAACCACCGTAAGCTCTGGAAACAGTCTTGTGGGTCTTGGA
AACAGTGGCGTATTGTCTTGGTCTCAAAGTAGAGATACCTTGCAAAGCGCTACCACAGTCACCACACTTTG
GTCTGGTA

TTGCCGTCGAGCTGTTGTTGCTGTTGCTGTTGCTGCTGCTGCTGTTGTTGTTGTTGTTGCTGTTGTTGTTG
TTGTTGGTTGTTTGGTGAGACAAAGTCACCCTTTGAGTCCACCAGCTCCGAGCCATCTCCCTCTTCAGACC
AGTCTACA

ATTGAAGACCCCCTTCTGATTCCTTCCATGAATTTAAATCGTAGGGTTGAATGTTTCCCAGCCAGTCTGGT
AAAGGTCTCCAGACGTTGGGGACAATGACGTCCTTGTCGTGATGCATTAGATCCTCCATGATGGTAGTGGG
GATGGTTT

CACCTTTACCTTTAGCAGACTTGATGGTGTCATCTTCAAATAAGCGTGGGGAGTCCTCGATGTGGAAGTGA
CGTTGAGTCTCGGTGCAAGCACCGTTAATACCACTGGAGGCCTTGATAGAGTTCCCACCGATCGAGGAAGC
CTTTTCGA

TGCCAGCATCAACGTCAGGACTAGTGCGACTACCACTGAAAGTACCAACTCCAGCACTAATGCTACTACCA
CTGCCAGCACCAACGTCAGGACTAGTGCTACTACCACTGCCAGCATCAACGTCAGGACTAGTGCGACTACC
ACTGAAAG

GCGAATATTGGTTTTAGAAACACGTTTCGAATCTTCAGTATGAGTTGACGGAGGTGTGGAATCGGTTGGAC
TCACAGCTTTTGAAAGGACATTTCTCGGTTGCTCAGGATGTAGTTCGATGTCAGATTGGAAGTCATGGTCA
GATTCTAT

GCCAAGAATTTGGATACGGTGATGTTCAGTACTGGTATACTGGCGGTTCTCAGGTCGCTGAAGCTTCAGGG
TCAGTATGTGGGGGTGATGATCACGGCGTCGCACAACCCATACCAGGACAACGGGGTCAAGATCGTGGAAC
CAGACGGA

GGCAAGGTAAGCCTGTGTTCATTAGACACAGAACTCCTCATGAAATTCAGGAAGCCAACAGTGTGGATATG
TCCGCTTTGAAGGACCCACAGACCGATGCTGACAGAGTCAAAGACCCTCAATGGTTAATTATGCTGGGTAT
TTGTACTC

GATGTTGCCTCCAAACTTGACTGCTCCAAGGTTAGAAAGAGAACACTCCTCTGTCCTTGACCAACTTGTCA
CAGACGCACAAGATACAGTCGACCGGTTTGTAGCCTGTGACAGTGATAGCAGTAGCACTATTGAATGAGTT
AACGGGCA

GCGTAATTTGTTGTCTGTTGCTTATAAGAACGTTATTGGTGCTCGTCGTGCCTCTTGGAGAATTGTTTCTT
CTATTGAGCAAAAGGAGGAGTCCAAGGAGAAGTCCGAACACCAGGTCGAGTTGATTTGTTCGTACCGTTCG
AAGATTGA

GCTGGTGTTTTGGATATCGGTATGGCTTTAGCTTCCGCCACTGACGACTCCTACACTACTTTGTACTCTGA
GGTTGACTTTGCTGGTGTTAGCAAGATGTTGACCATGGTTCCATGGTACTCCTCTAGATTGGAACCAGCTT
TGAAGTCT

TATGGCTTTAGCTTCCGCCACTGACGACTCCTACACTACTTTGTACTCTGAGGTTGACTTTGCTGGTGTTA
GCAAGATGTTGACCATGGTTCCATGGTACTCCTCTAGATTGGAACCAGCTTTGAAGTCTTTGAATGGTGAT
GCTTCTTC

ACGAACCCAAGGAGGAAAATAAGGACGTTCAAAAGCCCTCTGATGGACCTTCTGCTACTGCTTCAGAGAAC
GAACAAGCTGCTGCTTCCACTGCTGCTCCCGCTTTGTCACCAGAAGAAATTAAGGCCAAAGCATTGGATCT
TTTAAATA

TTCAAAGAATTGGTGAAGGAGTGAGAGTTTGACCTTGGCTTTAGAGTCTTATGATGGGGATGGTGGTGTAG
GTGTTGATGAGGATGATGAGGGAGTGCGGCCGGCAGCGTGGGAGGAGCGAACGGCATGGAAGAAGCAGCGT
TTGAGACA

CGAATATTGGTTTTGGAAACACGTTTCGAATCTTCAGTATGAGTTGACGGAGGTGTGGAATCGGTTGGACT
CACAGCTTTTGAAAGGACATTTCTCAGTTGCTCAGGATGTAGTTCAATGTCAGATTGGAAGTCATGGTCAG
ATTCTATG

CCAGCACTAATGCTACTACCACTGCCAGCACCAACGTCAGGACTAGTGCTACTACCACTGCCAGCATCAAC
GTCAGGACTAGTGCGACTACCACTGAAAGTACCAACTCCAACACTAGTGCTACTACCACCGAAAGTACCGA
CTCCAACA

ACAGTTTGTTCCTTACTATTTGTTCCAGGTTTCGTTGTCGGCGGTGCTGCGTCAAGCATCGGGGAAGCACT
CGGAGTGTTTGCTGTTTGTGAAAGAACGTCCATTGTGTGCAATTCTTCAACATTATTCGTTAATGTGATAT
TGTTACCA

CCCACCAGGATAGGTCATGTTCGTACCATGGCAACTTTAAAAACAACTGATAAGAAGGCCCCTGAGGACAT
CGAGGGCTCGGACACAGTGCAAATTGAGTTGCCTGAATCTTCCTTCGAGTCGTATATGCTAGAGCCTCCAG
ACTTGTCT

TGGATATCGGTATGGCTTTAGCTTCCGCCACTGACGACTCCTACACTACTTTGTACTCTGAGGTTGACTTT
GCTGGTGTTAGCAAGATGTTGACCATGGTTCCATGGTACTCCTCTAGATTGGAACCAGCTTTGAAGTCTTT
GAATGGTG

AACTTCTTCTGGATCTAACGAATACTTTAGGAATGAATCCATACTTCTCCTCATCATTAGACCGAACGATG
GGGTCTTGGTTCAAAGAATTGGTGAAGGAGTGAGAGTTTGACCTTGGCTTTAGAGTCTTATGATGGGGATG
GTGGTGTA

AACTACGGTGACTTCACCACCATGTTGACTGGTATTGCCCCAGACCAAGTGACCAGAATGATCACCGGTGT
TCCATGGTACTCCAGCAGATTAAAGCCAGCCATTTCCAGTGCTCTATCCAAGGACGGTATCTACACTATCG
CAAACTAG

CAAGATGACGAACAGAACGCTGGTGAAGACGATAACGATATAATGTCACCGCTTCCTGCGGATGAGGAAGC
TGAATTACAAAGAAGGCTTCAACTGGGGTTGAGAGTGTCTCCAAGACGTAGAGAACATCTTCACGCACCTG
AGGAAGGT

GTCCTTGTCGTGATGCATTAGATCCTCCATGATGGTAGTGGGGATGGTTTCGACATCCACGTCTCTCCAGT
AAACCCAGGAGTGGTATGGTTTCAGGGCCACGGACCCAAGCCAATTACGAGCCCTGGCCATCAGCTTTCTT
CTGGGGCC

CATTACAAGTAACACCTCCTGAACATAAATCGGATATCCCTCCTCCACCTGATGGAGGTGCCTTAGCCATC
GTCACCAAAACTGGCTTTGAAACATCTCAGGGTTCTTTAGTTCGAGTTATGATTTACTCCGCTGAACGTGT
TTCCGTCG

TACGCCTTGCCAAAGACTTACAACAAGTTGCACTACTGTGTCTCCTGTGCTATTCACGCCAGAATTGTTAG
AGTTAGATCCAGAGAAGACAGAAAGAACAGAGCTCCTCCACAAAGACCAAGATTTAACAGGGATAACAAAG
TTTCTCCA

GCAATCTGATCTCGCAAGTGCATTCCTAGGCTTAATTCATATCTGCTCCTCAACTGTCGATGATGCCTGCT
AAACTGCAGCTTGACGTACTGCGGACCCTGCAGTCCAGCGCTCGTCATGGAACGCAAACGCTGAAAAACTC
CAACTTTC

CACCAACGTCAGGACTAGTGCTACTACCACTGCCAGCATCAACGTCAGGACTAGTGCGACTACCACTGAAA
GTACCAACTCCAGCACTAATGCTACTACCACTGCCAGCACCAACGTCAGGACTAGTGCTACTACCACTGCC
AGCATCAA

TATTGAAGTGGCTACCGCATTGTCCAAATACATCAATGAAGGTAACTCTGTCGGTTCAGTCAACTTCCCAG
AAGTGGCATTGAAATCATTGTCTTACGACCAAGAGAACACTGTGCGTGTGTTATACATTCACCAAAATGTA
CCAGGTGT

CATCAAGTGAGTTCTGATGGCAATCTGGTTGGACAAAGTCCCAGAGACACAGAACAAACCTGCTTCTTTGC
CAGCCATGCGGGCAACGGTCTGTTCGAGCCTAACGGTGTCAACATCTTCACCGTAGACAGCGTCACCGATA
GAGGCCTC

ATCGAATAATAACAAAAGTATTAACAACAGTAATGCTGATTCTGCTCCTCCGTTGAGGTTGCACAATCCAG
TTTCGTACTCTCCTTCGAATGAACCCATACAACCTTCAAGCAGTCTATTGAGTCAATTGACACAGGACACC
GATAATCG

TCTCCTGTGCTATTCACGCCAGAATTGTTAGAGTTAGATCCAGAGAAGACAGAAAGAACAGAGCTCCTCCA
CAAAGACCAAGATTTAACAGGGATAACAAAGTTTCTCCAGCAGCTGCCGCAAAGAAAGCTTTGTAAATATG
ATGAGAGA

AAGGAGAAGGTTGAAATTTCTAAGGAGATCAGATCCATTTTCCAGATTGGAGGAAGGTGAACTTGGAACAG
ACGTAGATGGCTTCAAAGATGTGTACAGGATGAAGAGTAGCAGTATGTTTGATCTTGGTAAGAGTTCAGCC
ACAATGCA

CGATGAGGTAGGAAAAGTTGGTGTTCATTTGGCGTTGAATACTTCTGGGAAAGACAAGGTTCAGTTTGTCT
CCAGCAAGGACATTCTTGACATCTCAGCTTCTCTGGAGAAGATTGCTACCTAAATCAACCAACGGCTTCTT
CTCGCAGC

AACTGAAACCTACCCAATTGAAGTTGCTGAGGCCGTTTTCAACTACGGTGACTTCACCACCATGTTGACTG
GTATTGCCCCAGACCAAGTGACCAGAATGATCACCGGTGTTCCATGGTACTCCAGCAGATTAAAGCCAGCC
ATTTCCAG

TTGATGGTGTCATCTTCAAATAAGCGTGGGGAGTCCTCGATGTGGAAGTGACGTTGAGTCTCGGTGCAAGC
ACCGTTAATACCACTGGAGGCCTTGATAGAGTTCCCACCGATCGAGGAAGCCTTTTCGAGGATGGTTACAG
GGATGTTA

GTTGTCAAATGGCAAGGTAAGCCTGTGTTCATTAGACACAGAACTCCTCATGAAATTCAGGAAGCCAACAG
TGTGGATATGTCCGCTTTGAAGGACCCACAGACCGATGCTGACAGAGTCAAAGACCCTCAATGGTTAATTA
TGCTGGGT

CGCATTAGTACTGGCGTTAGTACTGGCATTAGCACTACCATGAATGCACGTGTTGCTGTCCTCATCACTGC
TGCAATACTTTCTGTACCTGTCACTGCTATTGCTCTCCTGGAAGTTAGACGGTAACGCAACGATCGACATG
GAAGCTGT

AGTACCAATGCGACTACCAACTCCAGCACTAATGCTACTACCACTGCCAGCACCAACGTCAGGACTAGTGC
TACTACCACTGCCAGCATCAACGTCAGGACTAGTGCGACTACCACTGAAAGTACCAACTCCAGCACTAATG
CTACTACC

TAAGGTCAAGACCACAGCCCCAAAGTTTTACTGCGTGAGACCAAACGCCGCAGTTGTTGCTCCAGGTGAAA
CAATCCAGGTTCAAGTTATCTTCCTGGGTTTGACTGAAGAGCCTGCCGCAGACTTTAAGTGCCGTGACAAG
TTCCTGGT

GGTTTGGATCCAAGTCAGAGTAACAGAAATGAGAGTGTATTTGAGTCTTGTTAGCAACACCAGAAGTAGCA
ACTCTGAAAGCTTCGGCAGCCCAGGTGTAGTAAGCAGATCTCTCAGTACCTTCTCTCAATGGTAAACCTTC
TCTTAAAG

AAGACCCAGTAACGTACACTTTATTACCCCCCTGTTGCCAAGTGATGTCAACTGGAAACATCATAGCTCTT
CCTTTCTTGCCTTCCACAGTGCCCTGTTGCTGTTCTTGTTGCTGTTGAAAGCCTTGATTTTGAAGTGAGTC
AGGCTGCT

CTGAATTACAAAGAAGGCTTCAACTGGGGTTGAGAGTGTCTCCAAGACGTAGAGAACATCTTCACGCACCT
GAGGAAGGTTCGTCGGGACCTCTTACCGAGGTCGGTACTCCAAGATTACCTAACGTATCTTCTGCAGGTCA
GGATGATG

TGCCGATGTGCTTGACCTCGTCACTCGAGGCTCTTCTTTACCGGGAGTGCTTGCATCTTTCGTTGAAGAAT
GTGCACTCAAGACCTCAGATGTGGAGTGTGGCCTCATTGGATTGGACTGTTTATTACTACAAACCAAGGGA
TATTGATG

AACAGTCACATGCACGCCCCATACGCCAAGCAGGGTGATGCCGCCTACTTGTGCCACAGATCTTGTCAGGC
CTGGGATTTCAGCAGGCTCATTAAGCTTTACAGGCAGGCCGGTTATGCGGTGATTGTGGTGGGTGACTTAA
ACTCCAGA

AAAGAAACAAACGAACCCAAGGAGGAAAATAAGGACGTTCAAAAGCCCTCTGATGGACCTTCTGCTACTGC
TTCAGAGAACGAACAAGCTGCTGCTTCCACTGCTGCTCCCGCTTTGTCACCAGAAGAAATTAAGGCCAAAG
CATTGGAT

CCATTGCATTTAAGGTCAAGACCACAGCCCCAAAGTTTTACTGCGTGAGACCAAACGCCGCAGTTGTTGCT
CCAGGTGAAACAATCCAGGTTCAAGTTATCTTCCTGGGTTTGACTGAAGAGCCTGCCGCAGACTTTAAGTG
CCGTGACA

AATACTTTAGGAATGAATCCATACTTCTCCTCATCATTAGACCGAACGATGGGGTCTTGGTTCAAAGAATT
GGTGAAGGAGTGAGAGTTTGACCTTGGCTTTAGAGTCTTATGATGGGGATGGTGGTGTAGGTGTTGATGAG
GATGATGA

ACCGAACGATGGGGTCTTGGTTCAAAGAATTGGTGAAGGAGTGAGAGTTTGACCTTGGCTTTAGAGTCTTA
TGATGGGGATGGTGGTGTAGGTGTTGATGAGGATGATGAGGGAGTGCGGCCGGCAGCGTGGGAGGAGCGAA
CGGCATGG

AAGAGAAACGAATACCAATTGTTGGACATTGATGACGGTTTCTTGTCTTTGATGAACATGGACGGTGACAC
TAAGGATGATGTCAAGGCTCCAGAAGGTGAATTGGGTGACAGTTTGCAAACTGCTTTTGATGAAGGTAAGG
ACTTGATG

CAGTATGCCAGCTGGTGTTTTGGATATCGGTATGGCTTTAGCTTCCGCCACTGACGACTCCTACACTACTT
TGTACTCTGAGGTTGACTTTGCTGGTGTTAGCAAGATGTTGACCATGGTTCCATGGTACTCCTCTAGATTG
GAACCAGC

CCACCAAGACCTCTTAAGGATTCGGCAGCCAAGAATTTCCAGCTTGAGCTACGGTCATTTGGATCAATGAA
CCCAGTTGGATGAACTTGAATTTGGTCCATGTCAATCAGATCAGCGCCTAACTTCTGCAGAAGCCTTTGAC
CATCACCA

GGATCTAACGAATACTTTAGGAATGAATCCATACTTCTCCTCATCATTAGACCGAACGATGGGGTCTTGGT
TCAAAGAATTGGTGAAGGAGTGAGAGTTTGACCTTGGCTTTAGAGTCTTATGATGGGGATGGTGGTGTAGG
TGTTGATG

TACATAAACATAGGAGAAGCAGGAGCGTTCATAATATTGATGACATTTCCTTAAATTTCGAGGCAACCTCC
ACACCACCAGCTCCGACCTCTGCTCCCTCAATTCCTGTTGAACATTCTAACCCATGTACTTCCATTGAAAT
ACCTAAGA

GTTTTAGAAACACGTTTCGAATCTTCAGTATGAGTTGACGGAGGTGTGGAATCGGTTGGACTCACAGCTTT
TGAAAGGACATTTCTCGGTTGCTCAGGATGTAGTTCGATGTCAGATTGGAAGTCATGGTCAGATTCTATGT
TAAGATCA

CTAGTGCTACTACCACCGAAAGTACCGACTCCAACACTAGTGCTACTACCACTGCTAGCACCAACTCCAGC
ACTAATGCCACTACCACTGCTAGCACCAACTCCAGCACTAATGCCACTACCACTGAAAGTACCAACGCTAG
TGCCAAGG

TGGCTTGGACCCACATAAGGGAGCTACCACATCTTCCACCCAAAAGAGTTCTTCCTCAACTGCAACCTCTT
CCTCCAAAACGTCATCCGATCACAGCAGTTCAACTAAGAAGAGTAGTAAGACTAGTTCCACCGCTTCATCA
TCATCATC

AGTTAAATCAACAGTTTGTTCCTTACTATTTGTTCCAGGTTTCGTTGTCGGCGGTGCTGCGTCAAGCATCG
GGGAAGCACTCGGAGTGTTTGCTGTTTGTGAAAGAACGTCCATTGTGTGCAATTCTTCAACATTATTCGTT
AATGTGAT

ACGCTAGTATTTCCAACAATTCAGACCAAACCATTGCATTTAAGGTCAAGACCACAGCCCCAAAGTTTTAC
TGCGTGAGACCAAACGCCGCAGTTGTTGCTCCAGGTGAAACAATCCAGGTTCAAGTTATCTTCCTGGGTTT
GACTGAAG

TGCTTGGTACCTGGCCATTACTAGAAGAAGAGAAACAATTAGTGTATTGGATTCGACAAGAGGCAAGCAAG
GGAGCCAAGTTTTCCGCATGTCTGGAAGGCAGATCAAAGAGTTGTATTATAAAGTATGGAGCAACTTGCGT
GAATCGAA

CCACTGCCAGCACCAACGTCAGGACTAGTGCTACTACCACTGCCAGCATCAACGTCAGGACTAGTGCGACT
ACCACTGAAAGTACCAACTCCAGCACTAATGCTACTACCACTGCCAGCACCAACGTCAGGACTAGTGCTAC
TACCACTG

TTGCTCTCCTGGAAGTTAGACGGTAACGCAACGATCGACATGGAAGCTGTCGCCTGTTTTTCAGCCAATCT
GTCCATTCTTTCTATCAGTTCCACTGTGTCAGCAGACAGGTCTGTCCTGGAGCCACAGCATCCAACATGCT
GGCCCTTT

GGCACATCTGGAACCACCGTAAGCTCTGGAAACAGTCTTGTGGGTCTTGGAAACAGTGGCGTATTGTCTTG
GTCTCAAAGTAGAGATACCTTGCAAAGCGCTACCACAGTCACCACACTTTGGTCTGGTAGCTAACTTCTTG
ACATGTTG

TTCTTCGTCAAATGCGTTCTTTAGTGCATTTATACTGCCGATGGAACCAGGAATTCTGTACAATCCCACTT
CATCTAGCCCACGCAGCTCAATCTCTTCCAGCAATTTCACAACAATTGTAGGTATCAAGGTATTCTCTCTT
TCACAAAC

TGGAGGTGGTACTGAAGCAGGTTGAGGAGAGGCATGATGGGGGTTCTCTGGAACAGCTGATGAAGCAGGTG
TTGTTGTCTGTTGAGAGTTAGCCTTAGTGGAAGCCTTCTCACATTCTTCTGTTTTGGAAGCTGAAACGTCT
AACGGATC

CAGTCCACCTCTCAAATCTGTGGGGGCTATTTCAGACAAAAGCATTGGACACAACACCGAACAGCCACCAG
CACCAAGACCGTAGATGATCTTACCGACAAAGTACTGGTACCATTTGTGATTTGAACTGATCTGAATAATT
GCACCAAC

AGAAAATACCGAACGTCATGTTCAGTTGGTACAATGAGACCAGTCCACCTCTCAAATCTGTGGGGGCTATT
TCAGACAAAAGCATTGGACACAACACCGAACAGCCACCAGCACCAAGACCGTAGATGATCTTACCGACAAA
GTACTGGT

TTTAGCAGACTTGATGGTGTCATCTTCAAATAAGCGTGGGGAGTCCTCGATGTGGAAGTGACGTTGAGTCT
CGGTGCAAGCACCGTTAATACCACTGGAGGCCTTGATAGAGTTCCCACCGATCGAGGAAGCCTTTTCGAGG
ATGGTTAC

AAAGAACCAAGCCCTAAGCCTTCTCACAAGAGGAACCAAAGTAACAGCAGCATTTCTTCTGCATGGTCCAA
GTTTAAGCACAAGTCTGCATCATCACCTGCTAATGCGGACACGGATATTCAAGATTCATCAACTCCATCAA
CTAGTCCA

TCTTTCCAAGCTGCTCACCCAACTGAAACCTACCCAATTGAAGTTGCTGAGGCCGTTTTCAACTACGGTGA
CTTCACCACCATGTTGACTGGTATTGCCCCAGACCAAGTGACCAGAATGATCACCGGTGTTCCATGGTACT
CCAGCAGA

AAGCGAGCCTAAAGAAACAAACGAACCCAAGGAGGAAAATAAGGACGTTCAAAAGCCCTCTGATGGACCTT
CTGCTACTGCTTCAGAGAACGAACAAGCTGCTGCTTCCACTGCTGCTCCCGCTTTGTCACCAGAAGAAATT
AAGGCCAA

GTCAAATTAACTTCAATTGCCGCTGGTGTTGCCGCCATCGCTGCTACTGCTTCCGCAACCACCACTCTAGC
TCAATCTGACGAAAGAGTCAACTTGGTTGAATTGGGTGTCTACGTATCTGATATCAGAGCTCATTTGGCTC
AATACTAC

AAACCTGCTGCATGAATGAAGATACATCCTGGCACACCGCTCTTGGTGCTCAGCTCTTCGTCTCTGAGGCC
TCTTAATGGCTCCGGCAAACCTCTCCTAAACTGGAAGGAGGTGGAGTTGATTGGTACAGTAGATACCCTCC
AGGCACCA

CTATTCAAGGGATAGTGTTGTCACAGAGGAGATTCCAATGACTTTCATGCACGGTGAAGATGGAAGTCCTA
GCGGATATCAGAAGCAAGAAACCTGGATGACTTCACCCAAGGAAACACAAGATCTTCTTCAATCGCCCCAA
TTTCAGCA

ATGACTTCTTCTTATGCTCAGATGATGCTCTGTGAACTAAGTGCGCAGTCCTTCAAACCTTTCCACTCAGG
TAATCTTGATAAAGTTTCCAAGATGGTCCATTCTCTGGGTCTCTTACATAAACAAACTTCACTCCGTATTT
CTCCACTT

GAGAGTGTATTTGAGTCTTGTTAGCAACACCAGAAGTAGCAACTCTGAAAGCTTCGGCAGCCCAGGTGTAG
TAAGCAGATCTCTCAGTACCTTCTCTCAATGGTAAACCTTCTCTTAAAGCTGGTTCATCAACTTGGATAAC
CTTGATAC

TAAACTCACTTGTCATTATTGAAAGTGAACGTGATCCAGAACCGCTTGTGGGGCTTCCTACAGAGGAAGGT
GAACTTGGATCCCAAGTCACCGGCGAACTCGCTGGTGATGACATGCCGAAATTATGTCTGCTTGAATTCAG
CTTGTTGT

AGGACTAGTGCTACTACCACTGCCAGCATCAACGTCAGGACTAGTGCGACTACCACTGAAAGTACCAACTC
CAGCACTAATGCTACTACCACTGCCAGCACCAACGTCAGGACTAGTGCTACTACCACTGCCAGCATCAACG
TCAGGACT

TCGACAGAATACGCTAGTATTTCCAACAATTCAGACCAAACCATTGCATTTAAGGTCAAGACCACAGCCCC
AAAGTTTTACTGCGTGAGACCAAACGCCGCAGTTGTTGCTCCAGGTGAAACAATCCAGGTTCAAGTTATCT
TCCTGGGT

AATTCTTGGACACCTTTACCTTTAGCAGACTTGATGGTGTCATCTTCAAATAAGCGTGGGGAGTCCTCGAT
GTGGAAGTGACGTTGAGTCTCGGTGCAAGCACCGTTAATACCACTGGAGGCCTTGATAGAGTTCCCACCGA
TCGAGGAA

AGATTGCTGTGTTACATGGTGGTACAAAGGCATTACAAGTAACACCTCCTGAACATAAATCGGATATCCCT
CCTCCACCTGATGGAGGTGCCTTAGCCATCGTCACCAAAACTGGCTTTGAAACATCTCAGGGTTCTTTAGT
TCGAGTTA

TCCTTCCATGAATTTAAATCGTAGGGTTGAATGTTTCCCAGCCAGTCTGGTAAAGGTCTCCAGACGTTGGG
GACAATGACGTCCTTGTCGTGATGCATTAGATCCTCCATGATGGTAGTGGGGATGGTTTCGACATCCACGT
CTCTCCAG

ACTTCACCCAAGGAAACACAAGATCTTCTTCAATCGCCCCAATTTCAGCAGGCCCAAACGTTCGGAAGAGG
CCCATCAACCAACGTCAGGTCATCCTTGGACCGCACAAGAGAACAGATGATCGCCACTAACCAACTAGATA
ACCGCTAC

CTGCGTGAGACCAAACGCCGCAGTTGTTGCTCCAGGTGAAACAATCCAGGTTCAAGTTATCTTCCTGGGTT
TGACTGAAGAGCCTGCCGCAGACTTTAAGTGCCGTGACAAGTTCCTGGTTATCACGTTGCCTTCTCCTTAC
GACCTCAA

CACGTTTCGAATCTTCAGTATGAGTTGACGGAGGTGTGGAATCGGTTGGACTCACAGCTTTTGAAAGGACA
TTTCTCGGTTGCTCAGGATGTAGTTCGATGTCAGATTGGAAGTCATGGTCAGATTCTATGTTAAGATCATC
GGATTGTT

ACTGCCAGCACCAACGTCAGGACTAGTGCTACTACCACTGCCAGCATCAACGTCAGGACTAGTGCGACTAC
CACTGAAAGTACCAACTCCAACACTAGTGCTACTACCACCGAAAGTACCGACTCCAACACTAGTGCTACTA
CCACCGAA

GATAGTGTTGTCACAGAGGAGATTCCAATGACTTTCATGCACGGTGAAGATGGAAGTCCTAGCGGATATCA
GAAGCAAGAAACCTGGATGACTTCACCCAAGGAAACACAAGATCTTCTTCAATCGCCCCAATTTCAGCAGG
CCCAAACG

GACAACTTAGGAGGATCGAACTATTCAAGGGATAGTGTTGTCACAGAGGAGATTCCAATGACTTTCATGCA
CGGTGAAGATGGAAGTCCTAGCGGATATCAGAAGCAAGAAACCTGGATGACTTCACCCAAGGAAACACAAG
ATCTTCTT

ACAATCCAGGTTCAAGTTATCTTCCTGGGTTTGACTGAAGAGCCTGCCGCAGACTTTAAGTGCCGTGACAA
GTTCCTGGTTATCACGTTGCCTTCTCCTTACGACCTCAATGGCAAGGCTGTTGCAGATGTTTGGTCCGACT
TGGAGGCT

GCAAATTGCTCACCTGACATCACATTGATGGCAATATCCAAGTCGTGAGAACCTTGTCCCAGAAGCTTGTC
ACGCACCCATCCGCCCGTGATCCGAAGAGTCAATGGCTCAGGCTTATTGTGGTACTTTTGATTGTACAAGT
CTGTATAA

TAGAAGGCATAGCACTTTCCGACAACTTAGGAGGATCGAACTATTCAAGGGATAGTGTTGTCACAGAGGAG
ATTCCAATGACTTTCATGCACGGTGAAGATGGAAGTCCTAGCGGATATCAGAAGCAAGAAACCTGGATGAC
TTCACCCA

ATATCTCCCACGGCTACGGCAAATACAAGCGGATCGCAACAGATCAATATCGACTCTGCCCTGAGAGACAG
GTCGAGTAACGTTGCAGCACAACCATCATTGTCGGACGCTTCAAGTGGCAGCAACGACAAAGAACTGAAAG
TGCTACTG

AGACACAAATCAGCGAGGCAGTTGATCTCGGACGAAAACAAGCGGATCAACGCCTTGTTGACCAAGGCTAA
CAAAGCTGCAGAGAGTTCTACTGCTGCTAGGCGACTTGTGCCCAAAGCGACGTACTTTAGCGTGGAAGCGC
CACCGTCT

TAGCTGGACTTCTGAATTAGTTTCATTACCAAATATCATCTTGACACCACACATTGGTGGCTCTACCGAAG
AAGCCCAAAGCTCAATCGGTATTGAAGTGGCTACCGCATTGTCCAAATACATCAATGAAGGTAACTCTGTC
GGTTCAGT

TTCTTCAAATTGTTAGATAAGGCAGAAACAATTTCGAAACCTGGAGGCAACTTCCCAGACGATCTGTGAGT
TCTTGCCACAGAGTGGCCACCCAATTGAGCCAATAGGTCCAATTTCAAATCAAATTCGTTTTTCAACCATT
CAATAGCC

ATACTTCTCCTCATCATTAGACCGAACGATGGGGTCTTGGTTCAAAGAATTGGTGAAGGAGTGAGAGTTTG
ACCTTGGCTTTAGAGTCTTATGATGGGGATGGTGGTGTAGGTGTTGATGAGGATGATGAGGGAGTGCGGCC
GGCAGCGT

GCTGCTATGAAGGATGGCGCTTATGTTATTAATGCTTCAAGAGGTACTGTCGTGGACATTCCATCTTTGAT
CCAAGCCGTGAAAGCCAACAAAATTGCAGGTGCTGCTTTGGATGTTTATCCACATGAACCAGCTAAGAACG
GTGAAGGT

TAGTGGATGTGTACTGCTTGGAGGGTGGAAATACGGAGCTTTTTCTCTTATCTCTGTTGTTGTCTTCCCCT
CTGTGGTGACTGTGTCCCTGGCCATGGTGATGGTGGGAATGCATGTATGGAGAAGCACCGCTTGTCGAGTT
TCGAATAG

AAGACAACCCGATGAGGCATGCAGGTTGGATTATGCTCTTATCGACCCTGATTTCTTGCAAACAGTAGACG
CAGGTGTCAGGTTCACTGAACGGATCCCTCACCTGGACTGCAGTGTCTCTGACCATTTTGCATACTCATGC
ACCCTTAA

CAGAACGCTGGCCAAGAATTTGGATACGGTGATGTTCAGTACTGGTATACTGGCGGTTCTCAGGTCGCTGA
AGCTTCAGGGTCAGTATGTGGGGGTGATGATCACGGCGTCGCACAACCCATACCAGGACAACGGGGTCAAG
ATCGTGGA

TCTTGACTACAGTAGTATAACTTTGTAGTTCGTTTATCAGACAGTGTAGGTCTGTTTGGTGACCTAGGGGG
TGGAAGAACTGGTATCCTGTTATTATCTCCGAGTGCTGTCAATTCTTGTACTATGCCAGAGTCCTTAATTG
AAGGTACA

CCCATTCTGTGGAGGTGGTACTGAAGCAGGTTGAGGAGAGGCATGATGGGGGTTCTCTGGAACAGCTGATG
AAGCAGGTGTTGTTGTCTGTTGAGAGTTAGCCTTAGTGGAAGCCTTATCATATTCTTGAATTTTGGAAGCT
GAAACGTC

AACCTGGATGACTTCACCCAAGGAAACACAAGATCTTCTTCAATCGCCCCAATTTCAGCAGGCCCAAACGT
TCGGAAGAGGCCCATCAACCAACGTCAGGTCATCCTTGGACCGCACAAGAGAACAGATGATCGCCACTAAC
CAACTAGA

AGGAAACACAAGATCTTCTTCAATCGCCCCAATTTCAGCAGGCCCAAACGTTCGGAAGAGGCCCATCAACC
AACGTCAGGTCATCCTTGGACCGCACAAGAGAACAGATGATCGCCACTAACCAACTAGATAACCGCTACTC
TGTGGAAA

CAAGTCAGAGTAACAGAAATGAGAGTGTATTTGAGTCTTGTTAGCAACACCAGAAGTAGCAACTCTGAAAG
CTTCGGCAGCCCAGGTGTAGTAAGCAGATCTCTCAGTACCTTCTCTCAATGGTAAACCTTCTCTTAAAGCT
GGTTCATC

TAACAGAAATGAGAGTGTATTTGAGTCTTGTTAGCAACACCAGAAGTAGCAACTCTGAAAGCTTCGGCAGC
CCAGGTGTAGTAAGCAGATCTCTCAGTACCTTCTCTCAATGGTAAACCTTCTCTTAAAGCTGGTTCATCAA
CTTGGATA

TGCCGTGACAAGTTCCTGGTTATCACGTTGCCTTCTCCTTACGACCTCAATGGCAAGGCTGTTGCAGATGT
TTGGTCCGACTTGGAGGCTGAGTTCAAGCAACAGGCTATCTCTAAAAAGATAAAAGTCAAATATTTGATAA
GTCCAGAT

GTGAGAGTTTGACCTTGGCTTTAGAGTCTTATGATGGGGATGGTGGTGTAGGTGTTGATGAGGATGATGAG
GGAGTGCGGCCGGCAGCGTGGGAGGAGCGAACGGCATGGAAGAAGCAGCGTTTGAGACAATCGTAGTGGGA
GTCACATT

TTATGTTATTAATGCTTCAAGAGGTACTGTCGTGGACATTCCATCTTTGATCCAAGCCGTGAAAGCCAACA
AAATTGCAGGTGCTGCTTTGGATGTTTATCCACATGAACCAGCTAAGAACGGTGAAGGTTCATTTAACGAT
GAGCTAAA

ATGGAGGTTGCATCAAGTGAGTTCTGATGGCAATCTGGTTGGACAAAGTCCCAGAGACACAGAACAAACCT
GCTTCTTTGCCAGCCATGCGGGCAACGGTCTGTTCGAGCCTAACGGTGTCAACATCTTCACCGTAGACAGC
GTCACCGA

TACTTCCGATCGTAAGCAATCACGTACCTCAACATATTTCTTTCGCAGCACGCCACGCCTTTCGGTGCCAA
CTTGGAATGAAGTCTGACCTGTTCAATGGTTTTGAGATTTACTGGGTCAACAGTTATCCATTTCTTAGAGA
ATTTGTCC

TCTTCTTCCTCACCACTAAGCCATGAGAAGAAGAGGAAGATTGTACTGAAGGTGATGGTGCAGAAGGAGAA
GGAGAAGAGGATGGTTCTGATATGGCAGCCAATAAGTCCTTTCTCGAATCATTGCTGAATTTTGAAACTGG
CTCAATCA

CTCGCAAGTGCATTCCTAGGCTTAATTCATATCTGCTCCTCAACTGTCGATGATGCCTGCTAAACTGCAGC
TTGACGTACTGCGGACCCTGCAGTCCAGCGCTCGTCATGGAACGCAAACGCTGAAAAACTCCAACTTTCTC
GAGCGCTT

CAATGGATACAATTCTTTACTTTTCTCATCTTTCAATGGTATTGACCCACGTCTGTGGTGTGTTTGTGAAG
CTTCAACGTCGTGAAAGAGGGTTGTGACCGGCTCATTGTACATATGCTTCTCCTCTATGTCGGCGTCTTCT
TTTGAATT

GTTGACAAATCTTCTACCGGTAATAGGGTTTAATAGGATACCACCAAGACCTCTTAAGGATTCGGCAGCCA
AGAATTTCCAGCTTGAGCTACGGTCATTTGGATCAATGAACCCAGTTGGATGAACTTGAATTTGGTCCATG
TCAATCAG

AACGTACACTTTATTACCCCCCTGTTGCCAAGTGATGTCAACTGGAAACATCATAGCTCTTCCTTTCTTGC
CTTCCACAGTGCCCTGTTGCTGTTCTTGTTGCTGTTGAAAGCCTTGATTTTGAAGTGAGTCAGGCTGCTGA
GATGGTTG

GGACAATGACGTCCTTGTCGTGATGCATTAGATCCTCCATGATGGTAGTGGGGATGGTTTCGACATCCACG
TCTCTCCAGTAAACCCAGGAGTGGTATGGTTTCAGGGCCACGGACCCAAGCCAATTACGAGCCCTGGCCAT
CAGCTTTC

AACAGAACGCTGGTGAAGACGATAACGATATAATGTCACCGCTTCCTGCGGATGAGGAAGCTGAATTACAA
AGAAGGCTTCAACTGGGGTTGAGAGTGTCTCCAAGACGTAGAGAACATCTTCACGCACCTGAGGAAGGTTC
GTCGGGAC

CCGTTTGCTAAATATCCAAATCTTTGAAACTATTTATCATGCCTCCATGGAAGCTTCTTGTGAACTAGCTC
AGAAGGACGGTCCATACGAAACTTTCCAAGGATCTCCTGCTTCTCAAGGTATACTACAGTTTGATATGTGG
GACCAAAA

TCTCTGAGGCCTCTTAATGGCTCCGGCAAACCTCTCCTAAACTGGAAGGAGGTGGAGTTGATTGGTACAGT
AGATACCCTCCAGGCACCAGAAGAGTCTGTGAAGAGAACAAACTCGATTTGCTTTTCAATGTTCTTCTCTC
TTTCCAGC

ATACGGAGCTTTTTCTCTTATCTCTGTTGTTGTCTTCCCCTCTGTGGTGACTGTGTCCCTGGCCATGGTGA
TGGTGGGAATGCATGTATGGAGAAGCACCGCTTGTCGAGTTTCGAATAGTTTGAGAGGATGATGACGGCGA
CGACGACG

CTGCTTCCCCAAATGGCGATGTCTTAGTCATGATCACTGGTGATCTGTTGATTGACGAAGAACAAAATCCA
CAGCGTTTCTCTCAAGTGTTCCATTTGATTCCTGATGGAAACTCTTACTACGTGTTTAACGATATCTTCCG
TTTGAATT

ACCTGGATCTTCTTCAAGTGCTCTAAAGAGAGATAAATCATCATCACCATCTTCCTCAGAAGTATTCTCAA
GAGCTCGCTCTCCAATGATTTCGACTACGGCCTCTGGGCTGTCATGGCGGTTATCTGTGACGATATTTTGT
GTTCTCGA

TCCAGGTGAAACAATCCAGGTTCAAGTTATCTTCCTGGGTTTGACTGAAGAGCCTGCCGCAGACTTTAAGT
GCCGTGACAAGTTCCTGGTTATCACGTTGCCTTCTCCTTACGACCTCAATGGCAAGGCTGTTGCAGATGTT
TGGTCCGA

AAGACCTCTGCCATCTCTCAAATTACCGATGGTCAAATCCAAGCTACCAAGGCTGTTTCTGAGCAAACTGA
AAACGGTGCTGCTAAGGCCTTTGTTGGTATGGGTGCTGGTGTTGTCGCAGCTGCCGCTATGTTGTTATAAG
TTATTGGT

AACGATGACGAGGACGAAGAAGAAGGAGCCGGCGATGATATTGAAGAGTTTATGAACGAAGAGTTCGCAGC
ATCAGTAGTGGTAGAGAGAGAAGATGGCACAGAAGCAGTCAAAGGCAATTTGTTTAAATACACGGTAGACC
ATTCATCC

TGATATACTACTGGGTGAAGATTTGGCTATAATGTAAGATTGAAGAGGGTGTGAGGCTCTCCGAATGGATG
AAAAGCCGTCATCTACCGAACTATTCGTGGTGGTTCCCACTGTGGAAGCTCGCCTATAAACTAAAGGTGTA
TTGGAAAG

GGGGTCTTGGTTCAAAGAATTGGTGAAGGAGTGAGAGTTTGACCTTGGCTTTAGAGTCTTATGATGGGGAT
GGTGGTGTAGGTGTTGATGAGGATGATGAGGGAGTGCGGCCGGCAGCGTGGGAGGAGCGAACGGCATGGAA
GAAGCAGC

GATGTTCAGTACTGGTATACTGGCGGTTCTCAGGTCGCTGAAGCTTCAGGGTCAGTATGTGGGGGTGATGA
TCACGGCGTCGCACAACCCATACCAGGACAACGGGGTCAAGATCGTGGAACCAGACGGATCGATGCTTTTG
GCCACATG

TAGTCAACATGACGTGTTCCGTTCTTATGTTCGAGCTAATGCCTCGTCTGCTCTTCCACGTTCAGATGTTC
CCTTGATTGCTCAAAACACTCTCAGCAGGCCGAGATTTCGATTTTAGCCAGCAGTACGGAAGTAATCCGAG
CCATGAAA

CGCCTTGTTGACCAAGGCTAACAAAGCTGCAGAGAGTTCTACTGCTGCTAGGCGACTTGTGCCCAAAGCGA
CGTACTTTAGCGTGGAAGCGCCACCGTCTATCAGGCCTGCCAAGAAGTACTGCGATGTTACTGGGTTGAAG
GGCTTCTA

ATTGGTTGTGCCTCTGCAATTATCTTCACCTCATTAGGTGCTGCTTACGGTACTGCTAAGTCTGGTGTTGG
TATCTGTGCCACTTGTGTGTTGAGACCAGACCTATTATTCAAGAACATTGTTCCTGTTATTATGGCTGGTA
TCATTGCC

AATTCTTTACTTTTCTCATCTTTCAATGGTATTGACCCACGTCTGTGGTGTGTTTGTGAAGCTTCAACGTC
GTGAAAGAGGGTTGTGACCGGCTCATTGTACATATGCTTCTCCTCTATGTCGGCGTCTTCTTTTGAATTTG
TCATTGCT

TCAAGAAGGCAGTTTGCCTTATAATATCCTCATACGGGATTAGTGTGGCCACTCTTCCTGGGTTTGCAGGT
GTACCCCTATGATCAGTGGAACTTTGCCAGAACCGACGAGCAAAACCGTGAATTATTGCCGGTATTCTATG
CGTGTAAT

CACCGTATTGAACACAGGAACCCGCCCCATTGCAATAATGAACAGTCACATGCACGCCCCATACGCCAAGC
AGGGTGATGCCGCCTACTTGTGCCACAGATCTTGTCAGGCCTGGGATTTCAGCAGGCTCATTAAGCTTTAC
AGGCAGGC

CTTTTCGTGATAGTCAACATGACGTGTTCCGTTCTTATGTTCGAGCTAATGCCTCGTCTGCTCTTCCACGT
TCAGATGTTCCCTTGATTGCTCAAAACACTCTCAGCAGGCCGAGATTTCGATTTTAGCCAGCAGTACGGAA
GTAATCCG

TTGGCCTTACAGCCACGGTCATTACATAAAGCAGTCATGAACAAACAACTCAACTTCTCACCACATGACAT
CACCAGATCTACTGTACGTGAACTCACTTCACCCAAAACTTTTGAAGCATTTAAATATTTCTTGACCAGTT
CAAGTTCT

CCCTTCTGATTCCTTCCATGAATTTAAATCGTAGGGTTGAATGTTTCCCAGCCAGTCTGGTAAAGGTCTCC
AGACGTTGGGGACAATGACGTCCTTGTCGTGATGCATTAGATCCTCCATGATGGTAGTGGGGATGGTTTCG
ACATCCAC

ATGTCATATCAGAGTCCGCTGAGGATGAATCAGTAAATGTATTACCTGACTCAGGTGATGGAGTGCTCAGA
GGCGTTCCAACTGATGATGGATACTGCGGAAACTGTGATTGTGGCCCAGGTGGAAAGTACATAGGCGACAT
TTGATAAG

TTCAAGTTATCTTCCTGGGTTTGACTGAAGAGCCTGCCGCAGACTTTAAGTGCCGTGACAAGTTCCTGGTT
ATCACGTTGCCTTCTCCTTACGACCTCAATGGCAAGGCTGTTGCAGATGTTTGGTCCGACTTGGAGGCTGA
GTTCAAGC

TCTTTCAAGCTTCGTGGTGCCTATAACATGATTGCCAAGTTGGACGATTCTCAAAGAAACCAGGGTGTTAT
TGCCTGTTCAGCTGGGAATCATGCCCAAGGTGTGGCCTTTGCTGCTAAACACTTGAAAATACCTGCTACTA
TCGTTATG

TGTCATATCAGAGTCCGCTGAGGATGAATCAGTAAATGTATTACCTGACTCAGGTGATGGAGTGCTCAGAG
GCGTTCCAACTGATGATGGATACTGCGGAAACTGTGATTGTGGCCCAGGTGGAAAGTACATAGGCGACATT
TGATAAGG

CCAACACTAGTGCTACTACCACTGCTAGCACCAACTCCAGCACTAATGCCACTACCACTGCTAGCACCAAC
TCCAGCACTAATGCCACTACCACTGAAAGTACCAACGCTAGTGCCAAGGAGGACGCCAATAAAGATGGCAA
TGCTGAGG

ATGCTTCCTGTCTTCTTCCTCACCACTAAGCCATGAGAAGAAGAGGAAGATTGTACTGAAGGTGATGGTGC
AGAAGGAGAAGGAGAAGAGGATGGTTCTGATATGGCAGCCAATAAGTCCTTTCTCGAATCATTGCTGAATT
TTGAAACT

AGATCTTCTTCAATCGCCCCAATTTCAGCAGGCCCAAACGTTCGGAAGAGGCCCATCAACCAACGTCAGGT
CATCCTTGGACCGCACAAGAGAACAGATGATCGCCACTAACCAACTAGATAACCGCTACTCTGTGGAAAGA
GCGAGAAC

GCACAATCCAGTTTCGTACTCTCCTTCGAATGAACCCATACAACCTTCAAGCAGTCTATTGAGTCAATTGA
CACAGGACACCGATAATCGATCAATGCTCTCCAATCACATCTCATCGAACAACGAAAACAAGCAACAGCCT
TCTTCATA

GATGAACTGCTCGAAACGGATACTGTCCTTGAAATTTCCATGTCTCCTGTGTTTACAGAAGGCATGATTCT
TCGCAGAGGACTTAAGTCTGTTGGCGGTCTTGAAAAGCTCTTTGATGGATGTGAAGTTTGAGAAAGAAGCG
AAGCACTG

CTCAGCTAATTTCTTCAAATTGTTAGATAAGGCAGAAACAATTTCGAAACCTGGAGGCAACTTCCCAGACG
ATCTGTGAGTTCTTGCCACAGAGTGGCCACCCAATTGAGCCAATAGGTCCAATTTCAAATCAAATTCGTTT
TTCAACCA

TAGTAGAAATGAAGAAAGCGTCTTCGGCAGATAAGTTCTCTACGGATTGAAGTTGTGCCTGCTGGACGTTT
TCCTTGTGGAAATCGATCTCTTCTTGAGGGGCACCTCTCTTGAAGACTATAATGTAGCGGTTGGGGATGAT
CTTGGAGA

AGACAGGCATAAGGAGAAGGTTGAAATTTCTAAGGAGATCAGATCCATTTTCCAGATTGGAGGAAGGTGAA
CTTGGAACAGACGTAGATGGCTTCAAAGATGTGTACAGGATGAAGAGTAGCAGTATGTTTGATCTTGGTAA
GAGTTCAG

CATGAATGAAGATACATCCTGGCACACCGCTCTTGGTGCTCAGCTCTTCGTCTCTGAGGCCTCTTAATGGC
TCCGGCAAACCTCTCCTAAACTGGAAGGAGGTGGAGTTGATTGGTACAGTAGATACCCTCCAGGCACCAGA
AGAGTCTG

TAGGCAAACGAACTTCTTCTGGATCTAACGAATACTTTAGGAATGAATCCATACTTCTCCTCATCATTAGA
CCGAACGATGGGGTCTTGGTTCAAAGAATTGGTGAAGGAGTGAGAGTTTGACCTTGGCTTTAGAGTCTTAT
GATGGGGA

TAGGTCATGTTCGTACCATGGCAACTTTAAAAACAACTGATAAGAAGGCCCCTGAGGACATCGAGGGCTCG
GACACAGTGCAAATTGAGTTGCCTGAATCTTCCTTCGAGTCGTATATGCTAGAGCCTCCAGACTTGTCTTA
TGAGACTT

GAAGCCCAAAGCTCAATCGGTATTGAAGTGGCTACCGCATTGTCCAAATACATCAATGAAGGTAACTCTGT
CGGTTCAGTCAACTTCCCAGAAGTGGCATTGAAATCATTGTCTTACGACCAAGAGAACACTGTGCGTGTGT
TATACATT

CGAAAGTACCGACTCCAACACTAGTGCTACTACCACCGAAAGTACCGACTCCAACACTAGTGCTACTACCA
CTGCTAGCACCAACTCCAGCACTAATGCCACTACCACTGCTAGCACCAACTCCAGCACTAATGCCACTACC
ACTGAAAG

GTAGGGTTGAATGTTTCCCAGCCAGTCTGGTAAAGGTCTCCAGACGTTGGGGACAATGACGTCCTTGTCGT
GATGCATTAGATCCTCCATGATGGTAGTGGGGATGGTTTCGACATCCACGTCTCTCCAGTAAACCCAGGAG
TGGTATGG

TCGGCAATGATATCGATGAAGAGGAAAGTGAATACGAAGAAGCTTTGTCGAAGAGGTCTCCACAGAAATCA
CCGAAAAAAAGACAAAGAGTCAGACAACCAGCAAGCAACTCTGGATCCCCAATCAAATCTACTCCAAGAAG
GTCAACGG

GAACGTCATGTTCAGTTGGTACAATGAGACCAGTCCACCTCTCAAATCTGTGGGGGCTATTTCAGACAAAA
GCATTGGACACAACACCGAACAGCCACCAGCACCAAGACCGTAGATGATCTTACCGACAAAGTACTGGTAC
CATTTGTG

TGTTGTTGCAAATACTCGTTCAAACCAGTAGCAAATTGCTCACCTGACATCACATTGATGGCAATATCCAA
GTCGTGAGAACCTTGTCCCAGAAGCTTGTCACGCACCCATCCGCCCGTGATCCGAAGAGTCAATGGCTCAG
GCTTATTG

TGAGGAGTTCCTAGTATTTCTGGGAGTGGGGATAAAATTCTGTTGTGACGGAAGGTCCTCTAACGACTGTG
AAGTCTTTCTAACATGGCCTCTGTAGGAAGCTCTCTGGTCAACGTTTGCGGGAGGCATCAATTGGTTATAA
ATGTTAGC

TTTCTTAACCTTTCTTGTTTGAGTCAGTTTAGCATCTTCTAGAACAGGAGTGGGTAAGAATGAAGTTGTCG
AGACTTCCAGATTGGATGGTACCAGAGAAACTTCTTCAGTGGATTCAATTGCCTTATCAGCCAATGAAACA
TCGTCAGT

TAGTGCTATCTGCCGATGTGCTTGACCTCGTCACTCGAGGCTCTTCTTTACCGGGAGTGCTTGCATCTTTC
GTTGAAGAATGTGCACTCAAGACCTCAGATGTGGAGTGTGGCCTCATTGGATTGGACTGTTTATTACTACA
AACCAAGG

AAATTAAGCATATCTGACTGTTTATCTGTTTATCTGTTTATACACGGTGAACTGTCACGAGAACCCTTACT
TGCACGATTAGCAGATTGTTCAGAGAGCCTCGCTCGTTGAAGTTCTTCTATATGCCAGCGTGTTTCATGTA
GCAGGAAA

ACTTTCATGCACGGTGAAGATGGAAGTCCTAGCGGATATCAGAAGCAAGAAACCTGGATGACTTCACCCAA
GGAAACACAAGATCTTCTTCAATCGCCCCAATTTCAGCAGGCCCAAACGTTCGGAAGAGGCCCATCAACCA
ACGTCAGG

CACCCACACACACCACACCCACACACCCACACCCACACACCACACCCACACCACACCCACACACCACACCC
ACACCACACCACACCCACACCACACCCACCACACCCACACCCACACACCACACCCACACCCTAATTCTACC
TCAACCCT

TATACCCACACACCCACACACACCACACCCACACACCCACACCCACACACCACACCCACACCACACCCACA
CACCACACCCACACCACACCACACCCACACCACACCCACCACACCCACACCCACACACCACACCCACACCC
TAATTCTA

GTGACATATATATACCCACACACCCACACACACCACACCCACACACCCACACCCACACACCACACCCACAC
CACACCCACACACCACACCCACACCACACCACACCCACACCACACCCACCACACCCACACCCACACACCAC
ACCCACAC

ACCATCCAGCATGCAATACAGTGACATATATATACCCACACACCCACACACACCACACCCACACACCCACA
CCCACACACCACACCCACACCACACCCACACACCACACCCACACCACACCACACCCACACCACACCCACCA
CACCCACA

ATGCAATACAGTGACATATATATACCCACACACCCACACACACCACACCCACACACCCACACCCACACACC
ACACCCACACCACACCCACACACCACACCCACACCACACCACACCCACACCACACCCACCACACCCACACC
CACACACC

CACCACACCCACACACCCACACCCACACACCACACCCACACCACACCCACACACCACACCCACACCACACC
ACACCCACACCACACCCACCACACCCACACCCACACACCACACCCACACCCTAATTCTACCTCAACCCTAC
CCTAATCC

ACACACCCACACCCACACACCACACCCACACCACACCCACACACCACACCCACACCACACCACACCCACAC
CACACCCACCACACCCACACCCACACACCACACCCACACCCTAATTCTACCTCAACCCTACCCTAATCCAA
CCCTTCCA

AACGAATGACTCACGTTATCAGGCTCATAGCTTGTGTGTGTGTGTGTGTGTGTGTGTGTGTGTGTGTGTGT
GTGTGTGTGTGTGTGTGTGTGATTGTTGTTCTAGTCGCTTGCTTTATAAAGTAACGACACTTTCTGGTG
CCAATATG

TCGGCTAAAAAACGAATGACTCACGTTATCAGGCTCATAGCTTGTGTGTGTGTGTGTGTGTGTGTGTGT
GTGTGTGTGTGTGTGTGTGTGTGTGTGTGTGATTGTTGTTCTAGTCGCTTGCTTTATAAAGTAACGACA
CTTTCTGG

CTTGTCTAACACCATCCAGCATGCAATACAGTGACATATATATACCCACACACCCACACACACCACACCCA
CACACCCACACCCACACACCACACCCACACCACACCCACACACCACACCCACACCACACCACACCCACACC
ACACCCAC

ATCTCTACTATCGGCTAAAAAACGAATGACTCACGTTATCAGGCTCATAGCTTGTGTGTGTGTGTGTGT
GTGTGTGTGTGTGTGTGTGTGTGTGTGTGTGTGTGTGTGTGTGATTGTTGTTCTAGTCGCTTGCTTTATAA
AGTAACGA

ACCCACACACCACACCCACACCACACCCACACACCACACCCACACCACACCACACCCACACCACACCCACC
ACACCCACACCCACACACCACACCCACACCCTAATTCTACCTCAACCCTACCCTAATCCAACCCTTCCATC
CTGTCTCT

TCAAATGCATATCTCTACTATCGGCTAAAAAACGAATGACTCACGTTATCAGGCTCATAGCTTGTGTGTGT
GTGTGTGTGTGTGTGTGTGTGTGTGTGTGTGTGTGTGTGTGTGTGTGTGTGTGATTGTTGTTCTAGTCGCT
TGCTTTAT

CACACCCACACCACACCCACACACCACACCCACACCACACCACACCCACACCACACCCACCACACCCACAC
CCACACACCACACCCACACCCTAATTCTACCTCAACCCTACCCTAATCCAACCCTTCCATCCTGTCTCTCA
ACTTACCC

AACCTATTGCATCCAAACCAGAAGTAATACAACCAGAATGTGGAGATGAGGAGCCAACAGGTGTGTATCCA
GAAGGTTCAGCCACAGGTTGGTCATCAATACCACTGGGGGAGCATGCAATATAATCAGATGGTTGCGAGGA
ATAGCTAG

GTAGAAACACTAGGTGTTTCCTGTGCACAGGGCGGTTCGTCGATGTTGAGGAAGTCCTGTGATCTTTCAAA
TGTGGGTGACGTGCTCACTTGGGGCGTGTTCAAGTGGGGATCTGTCATGAGGGTGAGTGTATGCGTGTGTG
TGTGTGTT

TCCCTACGGCCTTGTCTAACACCATCCAGCATGCAATACAGTGACATATATATACCCACACACCCACACAC
ACCACACCCACACACCCACACCCACACACCACACCCACACCACACCCACACACCACACCCACACCACACCA
CACCCACA

GGCGGTTCGTCGATGTTGAGGAAGTCCTGTGATCTTTCAAATGTGGGTGACGTGCTCACTTGGGGCGTGTT
CAAGTGGGGATCTGTCATGAGGGTGAGTGTATGCGTGTGTGTGTGTGTTGCGTAGATGTGTGATTTACTAT
GTGTTATG

GCCAAGAGTTTCTCTTCAATCTGTACTTGTGGAAAGCACGACCAGCCTTCAACAATGGTTTGTCAACTCTA
CCACCACCGGCAATGACACCGATGACACCTCTGGCATCAGAAGAGATAACCTTCTTGGCACCGGATGGTAA
TCTGACTC

TCACGTTATCAGGCTCATAGCTTGTGTGTGTGTGTGTGTGTGTGTGTGTGTGTGTGTGTGTGTGTGTGTGT
GTGTGTGTGTGTGATTGTTGTTCTAGTCGCTTGCTTTATAAAGTAACGACACTTTCTGGTGCCAATATGTG
AAAACGCA

GAAGATTGGTAACCTATTGCATCCAAACCAGAAGTAATACAACCAGAATGTGGAGATGAGGAGCCAACAGG
TGTGTATCCAGAAGGTTCAGCCACAGGTTGGTCATCAATACCACTGGGGGAGCATGCAATATAATCAGATG
GTTGCGAG

AGCCTGGTCTGCCTGTTGACGAGAACTCTTCTCATTGTTAACACCTGGTGGAGGACCTTTGGGTCTTGGCC
ATGTTGTTCCCCTTGGTGGTTCCCACTGAGAGCTGTTCGTAGATAAATCTACATAATACCAAGTCTGGTAT
TCATCATC

GAGGTTGAATGTAGAAACACTAGGTGTTTCCTGTGCACAGGGCGGTTCGTCGATGTTGAGGAAGTCCTGTG
ATCTTTCAAATGTGGGTGACGTGCTCACTTGGGGCGTGTTCAAGTGGGGATCTGTCATGAGGGTGAGTGTA
TGCGTGTG

AAGGTGGAGGAGCCTGGTCTGCCTGTTGACGAGAACTCTTCTCATTGTTAACACCTGGTGGAGGACCTTTG
GGTCTTGGCCATGTTGTTCCCCTTGGTGGTTCCCACTGAGAGCTGTTCGTAGATAAATCTACATAATACCA
AGTCTGGT

CGATGTTGAGGAAGTCCTGTGATCTTTCAAATGTGGGTGACGTGCTCACTTGGGGCGTGTTCAAGTGGGGA
TCTGTCATGAGGGTGAGTGTATGCGTGTGTGTGTGTGTTGCGTAGATGTGTGATTTACTATGTGTTATGTA
GCTGCTCT

TAGGTGTTTCCTGTGCACAGGGCGGTTCGTCGATGTTGAGGAAGTCCTGTGATCTTTCAAATGTGGGTGAC
GTGCTCACTTGGGGCGTGTTCAAGTGGGGATCTGTCATGAGGGTGAGTGTATGCGTGTGTGTGTGTGTTGC
GTAGATGT

GAAGTCCTGTGATCTTTCAAATGTGGGTGACGTGCTCACTTGGGGCGTGTTCAAGTGGGGATCTGTCATGA
GGGTGAGTGTATGCGTGTGTGTGTGTGTTGCGTAGATGTGTGATTTACTATGTGTTATGTAGCTGCTCTCA
TCGCTGCT

CCACACCCACACACCACACCCACACCACACCACACCCACACCACACCCACCACACCCACACCCACACACCA
CACCCACACCCTAATTCTACCTCAACCCTACCCTAATCCAACCCTTCCATCCTGTCTCTCAACTTACCCTC
CATTACCC

TGGGCCCCTGCTTGCACTTGCGGTGTTGATTGAGAAGAATAAGGTGGAGGAGCCTGGTCTGCCTGTTGACG
AGAACTCTTCTCATTGTTAACACCTGGTGGAGGACCTTTGGGTCTTGGCCATGTTGTTCCCCTTGGTGGTT
CCCACTGA

TGCTCCAACTGCACAACCTCCACCACTCCGCTGTGGAGGAAGGACCCCAAGGGTCTTCCCCTGTGCAATGC
TTGCGGCCTCTTCCTCAAGCTCCACGGCGTCACAAGGCCTCTGTCGTTGAAGACTGACATCATTAAGAAGA
GACAGAGG

CGGGTCTTTGGCCAAGAGTTTCTCTTCAATCTGTACTTGTGGAAAGCACGACCAGCCTTCAACAATGGTTT
GTCAACTCTACCACCACCGGCAATGACACCGATGACACCTCTGGCATCAGAAGAGATAACCTTCTTGGCAC
CGGATGGT

ACAGCACCTCTAGAGATGGTAGAAGCCTTACCAATATGTTGATGGTTACCACCACCGTGAGGGTGATCAAC
TGGATTCATGGCAACACCACGGGTCTTTGGCCAAGAGTTTCTCTTCAATCTGTACTTGTGGAAAGCACGAC
CAGCCTTC

CTTGCACTTGCGGTGTTGATTGAGAAGAATAAGGTGGAGGAGCCTGGTCTGCCTGTTGACGAGAACTCTTC
TCATTGTTAACACCTGGTGGAGGACCTTTGGGTCTTGGCCATGTTGTTCCCCTTGGTGGTTCCCACTGAGA
GCTGTTCG

ACACGAGTTGTCGTCGAGAACAGGATGTACTGATAGGACGTTTTCTCCACTGCCACCAATTGACATCTCAG
TGCTCTTCAGCACCCTCTTTGGTTTGGGTTGTGGTGGTGGCAAACTGAAGCTTTCGTTACTATCAGAGGAC
TCTATTGT

ATATCGATGAAGATGACGACATGTCATCTTACAACGACAAAGCAGCCTCGGTAGCGCACACCAGAGTCCTC
AATTCCTTGCATCTGTCCACCGACAGCAATACCGCCCACGAGACGTCCAATGCAAACGACAACCACAACCC
CTTCTACA

TTCATTAAGTGAAGATTGGTAACCTATTGCATCCAAACCAGAAGTAATACAACCAGAATGTGGAGATGAGG
AGCCAACAGGTGTGTATCCAGAAGGTTCAGCCACAGGTTGGTCATCAATACCACTGGGGGAGCATGCAATA
TAATCAGA

TACTCCGCCTTCGAACACCTCATCCAATCCGGATATAAAATGCTCCAACTGCACAACCTCCACCACTCCGC
TGTGGAGGAAGGACCCCAAGGGTCTTCCCCTGTGCAATGCTTGCGGCCTCTTCCTCAAGCTCCACGGCGTC
ACAAGGCC

CTGTACTTGTGGAAAGCACGACCAGCCTTCAACAATGGTTTGTCAACTCTACCACCACCGGCAATGACACC
GATGACACCTCTGGCATCAGAAGAGATAACCTTCTTGGCACCGGATGGTAATCTGACTCTAGTCTTGTTTT
CGTCTGGG

CTGTTTCAATTCCGGTGATGGCAAAGGTCCGTATTGGACACTTCGTGGAGGCACAGATCCTGGAAGAGCTG
CAAGTAGACTACATTGACGAAAGTGAGGTTTTGACTCCAGCTGATTGGACACATCACATTGAGAAGCATAA
CTTCAAGG

TAGAGATGGTAGAAGCCTTACCAATATGTTGATGGTTACCACCACCGTGAGGGTGATCAACTGGATTCATG
GCAACACCACGGGTCTTTGGCCAAGAGTTTCTCTTCAATCTGTACTTGTGGAAAGCACGACCAGCCTTCAA
CAATGGTT

CCAATATGTTGATGGTTACCACCACCGTGAGGGTGATCAACTGGATTCATGGCAACACCACGGGTCTTTGG
CCAAGAGTTTCTCTTCAATCTGTACTTGTGGAAAGCACGACCAGCCTTCAACAATGGTTTGTCAACTCTAC
CACCACCG

CATCCAATCCGGATATAAAATGCTCCAACTGCACAACCTCCACCACTCCGCTGTGGAGGAAGGACCCCAAG
GGTCTTCCCCTGTGCAATGCTTGCGGCCTCTTCCTCAAGCTCCACGGCGTCACAAGGCCTCTGTCGTTGAA
GACTGACA

AACGATAACGATATCGATGAAGATGACGACATGTCATCTTACAACGACAAAGCAGCCTCGGTAGCGCACAC
CAGAGTCCTCAATTCCTTGCATCTGTCCACCGACAGCAATACCGCCCACGAGACGTCCAATGCAAACGACA
ACCACAAC

GATATGAATATGACTATGAACATGAACCTTCACAACGCCTCAACCTCCTCCTTCAACAATGAAGCCTTCTG
GAAGCCTTTGGACTCCGCAATAGATCATCATTCTGGAGACACAAATCCAAACTCAAACATGAACACCACTC
CAAATGGC

TCTCTTCAATCTGTACTTGTGGAAAGCACGACCAGCCTTCAACAATGGTTTGTCAACTCTACCACCACCGG
CAATGACACCGATGACACCTCTGGCATCAGAAGAGATAACCTTCTTGGCACCGGATGGTAATCTGACTCTA
GTCTTGTT

GAGGCGGAAACGTTCTCCAGCTTTCGGCCTGATATGAATATGACTATGAACATGAACCTTCACAACGCCTC
AACCTCCTCCTTCAACAATGAAGCCTTCTGGAAGCCTTTGGACTCCGCAATAGATCATCATTCTGGAGACA
CAAATCCA

TGAGAAGAATAAGGTGGAGGAGCCTGGTCTGCCTGTTGACGAGAACTCTTCTCATTGTTAACACCTGGTGG
AGGACCTTTGGGTCTTGGCCATGTTGTTCCCCTTGGTGGTTCCCACTGAGAGCTGTTCGTAGATAAATCTA
CATAATAC

ACACCACACCCACACCACACCACACCCACACCACACCCACCACACCCACACCCACACACCACACCCACACC
CTAATTCTACCTCAACCCTACCCTAATCCAACCCTTCCATCCTGTCTCTCAACTTACCCTCCATTACCCTG
CCTCCCCA

GACCCACACTATCCGTTGCCACAGTATATCCCACCACTGAGTACTTCCTCACCTGATCCAATCGATTCACA
GAATCAACACTCTGAAGTACCTCAAGCTGAGACAAAGGTGAGAAATAACGTCTTACCACCACACACTTTAA
CATCAGAA

TCGAACACCTCATCCAATCCGGATATAAAATGCTCCAACTGCACAACCTCCACCACTCCGCTGTGGAGGAA
GGACCCCAAGGGTCTTCCCCTGTGCAATGCTTGCGGCCTCTTCCTCAAGCTCCACGGCGTCACAAGGCCTC
TGTCGTTG

GGATATAAAATGCTCCAACTGCACAACCTCCACCACTCCGCTGTGGAGGAAGGACCCCAAGGGTCTTCCCC
TGTGCAATGCTTGCGGCCTCTTCCTCAAGCTCCACGGCGTCACAAGGCCTCTGTCGTTGAAGACTGACATC
ATTAAGAA

TTCACTATTATCGATTTGCCCCTCAAAGTAGCTGTCTTGGCCACACTGTCACAAATGTCCCCAATTGGGGT
TTCAGATTGCAAAGTGACCAAAGCGGCAGTAATTTGGTGTGGAGCAAAAGGAATACACTTTCTCCTGTACA
CCTTGGAC

GCAATGGCCTTCAAATGCATATCTCTACTATCGGCTAAAAAACGAATGACTCACGTTATCAGGCTCATAGC
TTGTGTGTGTGTGTGTGTGTGTGTGTGTGTGTGTGTGTGTGTGTGTGTGTGTGTGTGTGTGATTGTTGT
TCTAGTCG

CGTTCTCCAGCTTTCGGCCTGATATGAATATGACTATGAACATGAACCTTCACAACGCCTCAACCTCCTCC
TTCAACAATGAAGCCTTCTGGAAGCCTTTGGACTCCGCAATAGATCATCATTCTGGAGACACAAATCCAAA
CTCAAACA

CTGTGCACAGGGCGGTTCGTCGATGTTGAGGAAGTCCTGTGATCTTTCAAATGTGGGTGACGTGCTCACTT
GGGGCGTGTTCAAGTGGGGATCTGTCATGAGGGTGAGTGTATGCGTGTGTGTGTGTGTTGCGTAGATGTGT
GATTTACT

GGCAACACCACGGGTCTTTGGCCAAGAGTTTCTCTTCAATCTGTACTTGTGGAAAGCACGACCAGCCTTCA
ACAATGGTTTGTCAACTCTACCACCACCGGCAATGACACCGATGACACCTCTGGCATCAGAAGAGATAACC
TTCTTGGC

CTGAGGCTGTTGAGGCTGATAGTAACGAGGTTGCTGTGCTTGGGCCCCTGCTTGCACTTGCGGTGTTGATT
GAGAAGAATAAGGTGGAGGAGCCTGGTCTGCCTGTTGACGAGAACTCTTCTCATTGTTAACACCTGGTGGA
GGACCTTT

ATGCTATATGTCCCTACGGCCTTGTCTAACACCATCCAGCATGCAATACAGTGACATATATATACCCACAC
ACCCACACACACCACACCCACACACCCACACCCACACACCACACCCACACCACACCCACACACCACACCCA
CACCACAC

TTCTCAACAAGAGACAACACCTGGGACATCAGCTGTTCCAGAGAACCATCATCATGTCTCTCCTCAACCTG
CTTCAGTACCACCTCCACAGAATGGACAGTACCAACAGCACGGCATGATGACCCCAAACAAAGCTATGGCC
TCTAACTG

TCGTCGAGAACAGGATGTACTGATAGGACGTTTTCTCCACTGCCACCAATTGACATCTCAGTGCTCTTCAG
CACCCTCTTTGGTTTGGGTTGTGGTGGTGGCAAACTGAAGCTTTCGTTACTATCAGAGGACTCTATTGTAA
TCTTTTCA

CGGTGTTGATTGAGAAGAATAAGGTGGAGGAGCCTGGTCTGCCTGTTGACGAGAACTCTTCTCATTGTTAA
CACCTGGTGGAGGACCTTTGGGTCTTGGCCATGTTGTTCCCCTTGGTGGTTCCCACTGAGAGCTGTTCGTA
GATAAATC

GATGGTTACCACCACCGTGAGGGTGATCAACTGGATTCATGGCAACACCACGGGTCTTTGGCCAAGAGTTT
CTCTTCAATCTGTACTTGTGGAAAGCACGACCAGCCTTCAACAATGGTTTGTCAACTCTACCACCACCGGC
AATGACAC

CGAATAGCAAGGTAGCTTCCATCCTGTACATGCAAGACCGTCACACAGCATGTCATCGGAAGAACCCCATG
CATCTATATCCTTTCCAGATGGCTCACATGTCCGGTCGTCCTCCACTGGTACTTCATCTGTGAACACAATC
GATGCTAC

CAATAACGATAACGATAACGATATCGATGAAGATGACGACATGTCATCTTACAACGACAAAGCAGCCTCGG
TAGCGCACACCAGAGTCCTCAATTCCTTGCATCTGTCCACCGACAGCAATACCGCCCACGAGACGTCCAAT
GCAAACGA

ACACCCACACCCTAATTCTACCTCAACCCTACCCTAATCCAACCCTTCCATCCTGTCTCTCAACTTACCCT
CCATTACCCTGCCTCCCCACTCGTTACCCTGCCTCCCCACCACCAACCCACAAATCCACCATAACAGTTAC
CCTCCAAT

CTGTTCTTCTTCTGATAGCTTCTTGGATAGTTGACTGGTATCCGTTGCTTTCAATGGAGGAACTGAACCGT
TTGCTTGACTTCCTTCGTCAAAATCTGAATCACTCTCTGTCTGTTCAAGATACAGATCTCTTAATTTCTGG
ATCTTCAT

CGGTATGGACAATCTGAAGGCAGTGCAGGAAGATGAAGATGAAGGGGTGACAGTTTCGAGGGCCCTGAGGG
GAATTCTACAGGAAATGTCTCAGGAAGGGACGCATCACTTGACAATAGCGTCGAACATAGAAAGTTTGGTG
CTGCAGCC

GCACAACCTCCACCACTCCGCTGTGGAGGAAGGACCCCAAGGGTCTTCCCCTGTGCAATGCTTGCGGCCTC
TTCCTCAAGCTCCACGGCGTCACAAGGCCTCTGTCGTTGAAGACTGACATCATTAAGAAGAGACAGAGGTC
GTCTACCA

AGTTTATGTTTATATACACCGGTGTAGGCTGTGCGTTGGTGTGAACACTTCCTGTTGCTGTTGCTGTTGAA
GCCGTCTCCGCATGCAACTGTAGTCATACAGCCTCTTGTCTGCCCTTATTCAATTGACCAGCTAACAATAG
CTTCGCTT

AGAAGCCTTACCAATATGTTGATGGTTACCACCACCGTGAGGGTGATCAACTGGATTCATGGCAACACCAC
GGGTCTTTGGCCAAGAGTTTCTCTTCAATCTGTACTTGTGGAAAGCACGACCAGCCTTCAACAATGGTTTG
TCAACTCT

CACAACGCCTCAACCTCCTCCTTCAACAATGAAGCCTTCTGGAAGCCTTTGGACTCCGCAATAGATCATCA
TTCTGGAGACACAAATCCAAACTCAAACATGAACACCACTCCAAATGGCAATCTGAGCCTGGATTGGTTGA
ATCTGAAT

CAGTTGTTTGGTATAGAAGCCGGTATGGACAATCTGAAGGCAGTGCAGGAAGATGAAGATGAAGGGGTGAC
AGTTTCGAGGGCCCTGAGGGGAATTCTACAGGAAATGTCTCAGGAAGGGACGCATCACTTGACAATAGCGT
CGAACATA

CATGGAACAGACACGAGTTGTCGTCGAGAACAGGATGTACTGATAGGACGTTTTCTCCACTGCCACCAATT
GACATCTCAGTGCTCTTCAGCACCCTCTTTGGTTTGGGTTGTGGTGGTGGCAAACTGAAGCTTTCGTTACT
ATCAGAGG

CCAATTTACCGGAATTTGATAGAGATTCCACTAAGGTTAATTCTCAACAAGAGACAACACCTGGGACATCA
GCTGTTCCAGAGAACCATCATCATGTCTCTCCTCAACCTGCTTCAGTACCACCTCCACAGAATGGACAGTA
CCAACAGC

GGAAAGCACGACCAGCCTTCAACAATGGTTTGTCAACTCTACCACCACCGGCAATGACACCGATGACACCT
CTGGCATCAGAAGAGATAACCTTCTTGGCACCGGATGGTAATCTGACTCTAGTCTTGTTTTCGTCTGGGTT
GTGACCGA

GAAATCGGTATGGGTATTCATAACGAGTCTGGTACCTATAAGTCTTCTCCGCTGCCATCGATTTCTGAGCT
CGTTTCCCAGATGCTTCCTCTTCTTCTCGATGAGGATGAAGACCGTTCTTATGTGAAGTTTGAGCCCAAAG
AGGACGTA

TTGACCAGAAACAGCACCTCTAGAGATGGTAGAAGCCTTACCAATATGTTGATGGTTACCACCACCGTGAG
GGTGATCAACTGGATTCATGGCAACACCACGGGTCTTTGGCCAAGAGTTTCTCTTCAATCTGTACTTGTGG
AAAGCACG

AGAGATTCCACTAAGGTTAATTCTCAACAAGAGACAACACCTGGGACATCAGCTGTTCCAGAGAACCATCA
TCATGTCTCTCCTCAACCTGCTTCAGTACCACCTCCACAGAATGGACAGTACCAACAGCACGGCATGATGA
CCCCAAAC

GGAATTTGATAGAGATTCCACTAAGGTTAATTCTCAACAAGAGACAACACCTGGGACATCAGCTGTTCCAG
AGAACCATCATCATGTCTCTCCTCAACCTGCTTCAGTACCACCTCCACAGAATGGACAGTACCAACAGCAC
GGCATGAT

CACCACTCCGCTGTGGAGGAAGGACCCCAAGGGTCTTCCCCTGTGCAATGCTTGCGGCCTCTTCCTCAAGC
TCCACGGCGTCACAAGGCCTCTGTCGTTGAAGACTGACATCATTAAGAAGAGACAGAGGTCGTCTACCAAG
ATAAACAA

ATTGTTTTAAATGAACCTTTCACTAAAGCAGTTCTTACGAACGGTTCGCTGAGAAACATTATCTGTTGTGA
TTCTGATCTCTCACTAGCTTCTTGCTGTTGAGACTGTGGTGTGGTAGATGTCAATTGCTGTGATTGCGGCG
TAGACTTT

GCTTTGTATCAAGAAACCCAAGATTACGTATAGGCTACTTCACTCAACATCATGTGGATTCTATGGATTTG
ACCACGTCTGCAGTGGACTGGATGTCCAAATCCTTCCCAGGTAAAACTGATGAAGAGTATAGACGTCATCT
AGGTTCAT

CTTTCGGCCTGATATGAATATGACTATGAACATGAACCTTCACAACGCCTCAACCTCCTCCTTCAACAATG
AAGCCTTCTGGAAGCCTTTGGACTCCGCAATAGATCATCATTCTGGAGACACAAATCCAAACTCAAACATG
AACACCAC

TTATCAACCGGACCCACACTATCCGTTGCCACAGTATATCCCACCACTGAGTACTTCCTCACCTGATCCAA
TCGATTCACAGAATCAACACTCTGAAGTACCTCAAGCTGAGACAAAGGTGAGAAATAACGTCTTACCACCA
CACACTTT

ACCCCTGCAAATAGACCTTTACTCTTAGAATAGGCAAAAACTGCAGCCACACCACCGGCGGAGGCTGATGC
AGCTGCTTCTGCACTTCTACCAAGAGGCCCAGCAGAAACGGAGACGTTACCACCCAACGTGATGGTCCCAA
ATTCAGAA

TCGAAGACAACAATAACGATAACGATAACGATATCGATGAAGATGACGACATGTCATCTTACAACGACAAA
GCAGCCTCGGTAGCGCACACCAGAGTCCTCAATTCCTTGCATCTGTCCACCGACAGCAATACCGCCCACGA
GACGTCCA

CTGTAATCCATGTGGCAAGTCCACCGTAATTGTCTTTGCCCTGTTGACCTGAACACCCTCACCATGGCACT
TGGTACAATTGTCCTGAGGCCGTTTCATGGTACCTTCACCGTTGCAAGTAGGACAAGTCGACATCATCTGA
AATCCGCC

GTCGACACGAATTCTCACCAAAATATCACCTCTTGATAACTTGACTGAATCTTTCAAGTCCGCCTCTACAG
CGATGTCAGGGTATGAGCCTTGGCCAGGGATCCTGACCACGTCGCCGTCCTGTAATCCATGTGGCAAGTCC
ACCGTAAT

ACCACCGTGAGGGTGATCAACTGGATTCATGGCAACACCACGGGTCTTTGGCCAAGAGTTTCTCTTCAATC
TGTACTTGTGGAAAGCACGACCAGCCTTCAACAATGGTTTGTCAACTCTACCACCACCGGCAATGACACCG
ATGACACC

AGCACCATCATGGATCAATTCTGTTCTGCACTTGTTTGGAGAGTTCAAGGACTTGAATTTGGCATCACAAT
GCTTCTGGACCAAAGAGGTCAGTTTCTCAGAATCCATGTCGGGGACGGTTCTAATGGAAAACTTACCGAAG
ACCTTAGC

TGTCATCGGAAGAACCCCATGCATCTATATCCTTTCCAGATGGCTCACATGTCCGGTCGTCCTCCACTGGT
ACTTCATCTGTGAACACAATCGATGCTACCTTATCCAGACCGAACTATATTAAGAAACCGTCCCTGCACAT
TATGTCGA

GATCTCGCAAGTGCATTCCTAGACTTAATTCATATCTGCTCCTCAACTGTCGATGATGCCTGCTAAACTGC
AGCTTGACGTACTGCGGACCCTGCAGTCCAGCGCTCGTCATGGAACGCAAACGCTGAAAAACTCCAACTTT
CTCGAGCG

TTGCTGTGCTTGGGCCCCTGCTTGCACTTGCGGTGTTGATTGAGAAGAATAAGGTGGAGGAGCCTGGTCTG
CCTGTTGACGAGAACTCTTCTCATTGTTAACACCTGGTGGAGGACCTTTGGGTCTTGGCCATGTTGTTCCC
CTTGGTGG

GTGAGAATACATGACCGAAGGAATGTTGTTATAGTTGACATGACCGTGACCAGTTTTCAACATTTCGACAG
CTGCAATACCTTCCTCTTCGGCTTTGTGAGCCAGCATTGGACCAAATGTAACATCTCCTACCACTTTAATG
TGTGGGAA

TGAGGCTGATAGTAACGAGGTTGCTGTGCTTGGGCCCCTGCTTGCACTTGCGGTGTTGATTGAGAAGAATA
AGGTGGAGGAGCCTGGTCTGCCTGTTGACGAGAACTCTTCTCATTGTTAACACCTGGTGGAGGACCTTTGG
GTCTTGGC

TACTTCCTAGATGCTATATGTCCCTACGGCCTTGTCTAACACCATCCAGCATGCAATACAGTGACATATAT
ATACCCACACACCCACACACACCACACCCACACACCCACACCCACACACCACACCCACACCACACCCACAC
ACCACACC

ATCCTGTACATGCAAGACCGTCACACAGCATGTCATCGGAAGAACCCCATGCATCTATATCCTTTCCAGAT
GGCTCACATGTCCGGTCGTCCTCCACTGGTACTTCATCTGTGAACACAATCGATGCTACCTTATCCAGACC
GAACTATA

CACACCACACCACACCCACACCACACCCACCACACCCACACCCACACACCACACCCACACCCTAATTCTAC
CTCAACCCTACCCTAATCCAACCCTTCCATCCTGTCTCTCAACTTACCCTCCATTACCCTGCCTCCCCACT
CGTTACCC

TCACACAGCATGTCATCGGAAGAACCCCATGCATCTATATCCTTTCCAGATGGCTCACATGTCCGGTCGTC
CTCCACTGGTACTTCATCTGTGAACACAATCGATGCTACCTTATCCAGACCGAACTATATTAAGAAACCGT
CCCTGCAC

GGTATGAGCCTTGGCCAGGGATCCTGACCACGTCGCCGTCCTGTAATCCATGTGGCAAGTCCACCGTAATT
GTCTTTGCCCTGTTGACCTGAACACCCTCACCATGGCACTTGGTACAATTGTCCTGAGGCCGTTTCATGGT
ACCTTCAC

GTCTACCTATTAAATGACGATGACGTACAAAGTATAATGACCTCAGGTGAAGATTCTAAATTGTTCATTTC
TACACCCCCACCACCACCGAAGATGGCCATCCCAGCAACAAAGCAAGGCGGATCGCTGGAAATCTCATTCG
ATTCGGAA

ATTCTCACCAAAATATCACCTCTTGATAACTTGACTGAATCTTTCAAGTCCGCCTCTACAGCGATGTCAGG
GTATGAGCCTTGGCCAGGGATCCTGACCACGTCGCCGTCCTGTAATCCATGTGGCAAGTCCACCGTAATTG
TCTTTGCC

AATGGATCAGAAACCCAATAAGCACCATCATGGATCAATTCTGTTCTGCACTTGTTTGGAGAGTTCAAGGA
CTTGAATTTGGCATCACAATGCTTCTGGACCAAAGAGGTCAGTTTCTCAGAATCCATGTCGGGGACGGTTC
TAATGGAA

GGTAGCTTCCATCCTGTACATGCAAGACCGTCACACAGCATGTCATCGGAAGAACCCCATGCATCTATATC
CTTTCCAGATGGCTCACATGTCCGGTCGTCCTCCACTGGTACTTCATCTGTGAACACAATCGATGCTACCT
TATCCAGA

TGATCTAGAAACTGGGGATGGCACGTTTGACTTAAACGAAGAGATCATTCCACTGAAGAACGAGGACGTCG
AAGAAGTCCTTGATTGATCGTGCTTGATCTCTGTAGGGTGTTCATCGGCGGCACTTTGGTTTGTAATCACT
ACTAAATT

CGTCGCCGTCCTGTAATCCATGTGGCAAGTCCACCGTAATTGTCTTTGCCCTGTTGACCTGAACACCCTCA
CCATGGCACTTGGTACAATTGTCCTGAGGCCGTTTCATGGTACCTTCACCGTTGCAAGTAGGACAAGTCGA
CATCATCT

ATCCAAACCAGAAGTAATACAACCAGAATGTGGAGATGAGGAGCCAACAGGTGTGTATCCAGAAGGTTCAG
CCACAGGTTGGTCATCAATACCACTGGGGGAGCATGCAATATAATCAGATGGTTGCGAGGAATAGCTAGTA
GAGCTAAA

AAAACTATTTCGAATAGCAAGGTAGCTTCCATCCTGTACATGCAAGACCGTCACACAGCATGTCATCGGAA
GAACCCCATGCATCTATATCCTTTCCAGATGGCTCACATGTCCGGTCGTCCTCCACTGGTACTTCATCTGT
GAACACAA

TCTGATAGCTTCTTGGATAGTTGACTGGTATCCGTTGCTTTCAATGGAGGAACTGAACCGTTTGCTTGACT
TCCTTCGTCAAAATCTGAATCACTCTCTGTCTGTTCAAGATACAGATCTCTTAATTTCTGGATCTTCATGT
TTAATCTG

GAAAAGAAGGAGTTTATGTTTATATACACCGGTGTAGGCTGTGCGTTGGTGTGAACACTTCCTGTTGCTGT
TGCTGTTGAAGCCGTCTCCGCATGCAACTGTAGTCATACAGCCTCTTGTCTGCCCTTATTCAATTGACCAG
CTAACAAT

TGACTATGAACATGAACCTTCACAACGCCTCAACCTCCTCCTTCAACAATGAAGCCTTCTGGAAGCCTTTG
GACTCCGCAATAGATCATCATTCTGGAGACACAAATCCAAACTCAAACATGAACACCACTCCAAATGGCAA
TCTGAGCC

TCTTAGAGACGTAAGCGGGCTTCACTATTATCGATTTGCCCCTCAAAGTAGCTGTCTTGGCCACACTGTCA
CAAATGTCCCCAATTGGGGTTTCAGATTGCAAAGTGACCAAAGCGGCAGTAATTTGGTGTGGAGCAAAAGG
AATACACT

CTCGTCTGCTGCCGCTTCCAGATCGCTATCTCCATTACTAAACGTTCCAGCACCAGAGGATGGCACGGAGA
GAATCTTACCTCAGAGTGCTCTTGGTCCCAATAGTGGCTCTGTGCCAGGAGTACATAGTAACGTATCACCT
GTTTTACT

CTGGATTCATGGCAACACCACGGGTCTTTGGCCAAGAGTTTCTCTTCAATCTGTACTTGTGGAAAGCACGA
CCAGCCTTCAACAATGGTTTGTCAACTCTACCACCACCGGCAATGACACCGATGACACCTCTGGCATCAGA
AGAGATAA

CACATTTGTTGGAAGGTAGTCAAAGAAGCCAAGATAGAACCACCAATCCAGACGGAGTACTTTCTTTCTGG
AGGAGCAATGATCTTGACCTTCATGGAAGATGGAGCCAAAGCGGTGATTTCCTTTTGCATTCTTTCGGCAA
TACCTGGG

GGTCTTTGGAATATCAGATTCTCGATCTACAACTTTCCTTAACAGTGGGTTTGCCATGTGTGCCCCAAAAC
CTGTGGCCAAGGTGGGAGACGAATACGTAACACCTAGAAGATTTACGTATCTTAGGAATTGATCACCATTA
GACTGTAC

TTGGCCAGGGATCCTGACCACGTCGCCGTCCTGTAATCCATGTGGCAAGTCCACCGTAATTGTCTTTGCCC
TGTTGACCTGAACACCCTCACCATGGCACTTGGTACAATTGTCCTGAGGCCGTTTCATGGTACCTTCACCG
TTGCAAGT

CAACCTCCTCCTTCAACAATGAAGCCTTCTGGAAGCCTTTGGACTCCGCAATAGATCATCATTCTGGAGAC
ACAAATCCAAACTCAAACATGAACACCACTCCAAATGGCAATCTGAGCCTGGATTGGTTGAATCTGAATTT
ATAGATCC

ACCTTTGAAATTATCCAACATGATAACGTCTGCACCAGCTTCAATGGCCTCTGTGGCTTCATCTTCACTCA
AACACTCCACTTCGATCTTCACAGCAAACCCGCACACGGCCCTGGCGTTCTTAACTGCGTTTGTTATAGAA
CCAGTAGC

CCACAAACAGCTAATCTAGCAGCTCTGGTACCGATCAATTCACAAAGTCTTCTAATCAACTTACGTTCTGG
CAGAGTGGTCTTGACACCAAAGTCCTTTTGGAAGATGTCATCAGTATCTTCCAAGTTTTCAAATGGATCAT
CCTCGATT

AACGAATCTGCGCCATTACAACCGTTCCCTGAGAGGCCGATGGTGAGAGATACAGTGGTTCTTGAACCTAG
TGGACATGTTGTCCTGAGATTTAGAGCTGACAACCCTGGTGTCTGGTATTTCCATTGTCATGTAGATTGGC
ACTTACAA

TCAGATTCAGATTTGGATTCAGAGGACAATAAACATGGCAAAGGCGACAATGACACTGCCCCCATATGGTT
ACAAGATGATGTGCATTCAGACGAAGATATTCAGCTGGACTCAGAGGACGATTCCGATACAGAAGCTGTTC
AAGCCCAA

ATTAGTACTGGCGTTAGTACTGGCATTAGCACTACCATGAATGCACGTGTCGCTGTCCTCATCACTGCTGC
AATACTTTCTGCACCTGTCACTGCTATTGCTCTCCTGGAAGCTAGACGGTAACGCAACGATCGACATGGAA
GCTGTCGC

CATGAACCTTCACAACGCCTCAACCTCCTCCTTCAACAATGAAGCCTTCTGGAAGCCTTTGGACTCCGCAA
TAGATCATCATTCTGGAGACACAAATCCAAACTCAAACATGAACACCACTCCAAATGGCAATCTGAGCCTG
GATTGGTT

ATCCTGACCACGTCGCCGTCCTGTAATCCATGTGGCAAGTCCACCGTAATTGTCTTTGCCCTGTTGACCTG
AACACCCTCACCATGGCACTTGGTACAATTGTCCTGAGGCCGTTTCATGGTACCTTCACCGTTGCAAGTAG
GACAAGTC

GATCTTTCAAATGTGGGTGACGTGCTCACTTGGGGCGTGTTCAAGTGGGGATCTGTCATGAGGGTGAGTGT
ATGCGTGTGTGTGTGTGTTGCGTAGATGTGTGATTTACTATGTGTTATGTAGCTGCTCTCATCGCTGCTGA
TAGTAGAT

GCCGCTTCCAGATCGCTATCTCCATTACTAAACGTTCCAGCACCAGAGGATGGCACGGAGAGAATCTTACC
TCAGAGTGCTCTTGGTCCCAATAGTGGCTCTGTGCCAGGAGTACATAGTAACGTATCACCTGTTTTACTTT
CAAGATCC

ATTGAGGGTACTGAGGCTGTTGAGGCTGATAGTAACGAGGTTGCTGTGCTTGGGCCCCTGCTTGCACTTGC
GGTGTTGATTGAGAAGAATAAGGTGGAGGAGCCTGGTCTGCCTGTTGACGAGAACTCTTCTCATTGTTAAC
ACCTGGTG

ATTATGGAAGCTGTTTCAATTCCGGTGATGGCAAAGGTCCGTATTGGACACTTCGTGGAGGCACAGATCCT
GGAAGAGCTGCAAGTAGACTACATTGACGAAAGTGAGGTTTTGACTCCAGCTGATTGGACACATCACATTG
AGAAGCAT

AGTATAATGACCTCAGGTGAAGATTCTAAATTGTTCATTTCTACACCCCCACCACCACCGAAGATGGCCAT
CCCAGCAACAAAGCAAGGCGGATCGCTGGAAATCTCATTCGATTCGGAAAACGATAGGGCTTTGCATTATC
AAGATGAC

TGTTGAAGGAGAACCGGATATGGTAGGCTTATGTATTTTCAACTTGCCACCAGATGTAGTGTTTGAAGCGT
TACTCCCTGGAGTTGACACTGATCCGTCTGAGGATGACCCCATTAGTGACGCAGAAGAGGTTCTTCTGTGA
ACCCCAGG

CACTAAAGCAGTTCTTACGAACGGTTCGCTGAGAAACATTATCTGTTGTGATTCTGATCTCTCACTAGCTT
CTTGCTGTTGAGACTGTGGTGTGGTAGATGTCAATTGCTGTGATTGCGGCGTAGACTTTTGTGCGGGGAAA
CTTGTTTG

AGAATCCACAAGGGAACCGAGAACTTGCATTAAATCGATCATTGGTTCAGCAACAACACCACCAAAGATAC
CAGAGTGTAAATCTGCACTTGGACCCTCAATGATGGTTTGATAGTAGTTGCAACCTCTTAGACCATAAGTC
AAAACAGG

AAGAAACCCAAGATTACGTATAGGCTACTTCACTCAACATCATGTGGATTCTATGGATTTGACCACGTCTG
CAGTGGACTGGATGTCCAAATCCTTCCCAGGTAAAACTGATGAAGAGTATAGACGTCATCTAGGTTCATTT
GGTATCAC

GCTCGTTTGAGGCGTTTTTGGCGATCCAGCCGTTCTTGAAAGCTCTAGCCTGACCGACCTTACCAACTTGA
GGACCCAGTTTGGACATCACATCTTTGATTTGAAGTTGACCCAACTCTTGGATGAGCTTGACTAGTTTAAT
TTCGTACG

CTAAGGTTAATTCTCAACAAGAGACAACACCTGGGACATCAGCTGTTCCAGAGAACCATCATCATGTCTCT
CCTCAACCTGCTTCAGTACCACCTCCACAGAATGGACAGTACCAACAGCACGGCATGATGACCCCAAACAA
AGCTATGG

GTATAGAAGCCGGTATGGACAATCTGAAGGCAGTGCAGGAAGATGAAGATGAAGGGGTGACAGTTTCGAGG
GCCCTGAGGGGAATTCTACAGGAAATGTCTCAGGAAGGGACGCATCACTTGACAATAGCGTCGAACATAGA
AAGTTTGG

TAGGCAATAGCATGGAACAGACACGAGTTGTCGTCGAGAACAGGATGTACTGATAGGACGTTTTCTCCACT
GCCACCAATTGACATCTCAGTGCTCTTCAGCACCCTCTTTGGTTTGGGTTGTGGTGGTGGCAAACTGAAGC
TTTCGTTA

TGACGTACAAAGTATAATGACCTCAGGTGAAGATTCTAAATTGTTCATTTCTACACCCCCACCACCACCGA
AGATGGCCATCCCAGCAACAAAGCAAGGCGGATCGCTGGAAATCTCATTCGATTCGGAAAACGATAGGGCT
TTGCATTA

TCAAACCGTCACCTTTGAAATTATCCAACATGATAACGTCTGCACCAGCTTCAATGGCCTCTGTGGCTTCA
TCTTCACTCAAACACTCCACTTCGATCTTCACAGCAAACCCGCACACGGCCCTGGCGTTCTTAACTGCGTT
TGTTATAG

GGGTGATCAACTGGATTCATGGCAACACCACGGGTCTTTGGCCAAGAGTTTCTCTTCAATCTGTACTTGTG
GAAAGCACGACCAGCCTTCAACAATGGTTTGTCAACTCTACCACCACCGGCAATGACACCGATGACACCTC
TGGCATCA

TTGGTATCGAATGTATGCAACAAGTCGAATTGGAAGAGTACTTGGCCAAGCAAGGCAGACCAGCTTCTGAT
GCTAAAATGTTGAGAATTGACAAGTTGGCTTGCTTGGGTTGCTGTTGCTTCTGTACCGTTCTTTACATCTG
CAACGATG

TTGGACCCGGGAGGTTGAATGTAGAAACACTAGGTGTTTCCTGTGCACAGGGCGGTTCGTCGATGTTGAGG
AAGTCCTGTGATCTTTCAAATGTGGGTGACGTGCTCACTTGGGGCGTGTTCAAGTGGGGATCTGTCATGAG
GGTGAGTG

TTTGACGACGATGACGATGATGATGACGTGGAGACGCACTCCATTGTGCACTCAGACCTGCTCAACGACAT
GGACAGCGCTTCCCAGCGTGCTTCACATAATGCTTCTGGTTTCCCTAATTTTCTGGACACTTCCTGCTCGT
CCTCCTTC

TTTCACAAGTTTGGAATAAAGGTGAAGGTTTCCTAGATATTCCAGGTGCACAAGACGAAATGGTTTTACCA
CCAGCTCCTCCGAAGAACTCCGACCCTTCCATTTTGAGAGCCTGGAAACTTCAAGTCAAAACAATTGCTAA
TAAATTCT

TAAATCTCCGGACTCTTCTCCTGCAAAGCTATATAATGCCACGGCCTTTGGAGAAGACGTGGATGGGGATG
TTGGAGCAGTGAATCTGCCCTGACTAGTTTGCGGTGTTGAAGCGGACGAGATTCTAGATTTAGAAAATCTG
TTCGACAA

TTGCTGTATCTTGACTCTCTCAGCACACTCCTCGTAGTGGTGCACAAGGGCCTTACCCTCCTCCGTGTTCT
TGAAATGTTCTCTCAAATCTTCCAACTGATCAGTGACTTCTTCCTCTTCTTCCTCATCTTCGTCGTCATCA
TCATCATC

GATCAAAGAAATTATGGAAGCTGTTTCAATTCCGGTGATGGCAAAGGTCCGTATTGGACACTTCGTGGAGG
CACAGATCCTGGAAGAGCTGCAAGTAGACTACATTGACGAAAGTGAGGTTTTGACTCCAGCTGATTGGACA
CATCACAT

TTGCGGTGCCAAGGATCTAGGTGAGGCTTTGAGAAGAATAAACGAAGGTGCTGCAATGATCCGTACCAAAG
GTGAAGCAGGTACCGGTGACGTTTCCGAGGCCGTCAAGCACATCACCAAGATTAAGGCGGAGATCCAGCAG
TATAAAGA

GACACAATGTCCCATCGTTCCGGGACAAACATACCTGTACAACTTCACTGTCCCAGAGCAGGTAGGAACGT
TCTGGTACCATGCACACATGGGTGCCCAGTACGGTGACGGTATGAGGGGTGCATTTATCATCCACGATCCG
GAGGAGCC

AGGACCCCAAGGGTCTTCCCCTGTGCAATGCTTGCGGCCTCTTCCTCAAGCTCCACGGCGTCACAAGGCCT
CTGTCGTTGAAGACTGACATCATTAAGAAGAGACAGAGGTCGTCTACCAAGATAAACAACAATATAACGCC
CCCTCCAT

ATGGATGGTCCTTCTATGGTGACACAATGTCCCATCGTTCCGGGACAAACATACCTGTACAACTTCACTGT
CCCAGAGCAGGTAGGAACGTTCTGGTACCATGCACACATGGGTGCCCAGTACGGTGACGGTATGAGGGGTG
CATTTATC

TGGTAATCGCATTAGTACTGGCGTTAGTACTGGCATTAGCACTACCATGAATGCACGTGTCGCTGTCCTCA
TCACTGCTGCAATACTTTCTGCACCTGTCACTGCTATTGCTCTCCTGGAAGCTAGACGGTAACGCAACGAT
CGACATGG

AAGGCTTATGTACGTCATGTCGCCGAAAGCTTTAAAGCTCAATTTACGTGAGAATGAACTGGAAGTCCTCA
AGACAGCTGGTCATCTATTGACAAGAGAATTCCTGAGAGATGTTACAATGAATTTAGTCCAAGATAATGAA
ACTAGGGG

ATGGCGCCTTTCCAATCTTCTTTGTCTACGTTCGTTAAAGTCAAGATCCTTCTCATTCTCAGATTCTGAAG
AAGAGGATCCGGAAGAAGAGGATCCATCATCATCTGAATGATGGTTGCACTCTTCTTCATCCTCATTTTGT
GGATGAAA

GGGCACCTCTACCGGAGTCGTGGACAATTTGCTTAACGATACCACGAATGTAACCGTGACGTTCAGCATAA
TCCAAAGTTCTCAACTTGGCAGCACCTTGTCTCAATCTGGTGTGGGAGGTAAAGATAGAACCAGCACCCTT
TCTTTGGT

TACTTGAGCATGTAAATCAGAGATTCTATGTAACAGCTGCCCAGAAACCAGAAAACGTCTCTTAACCACTT
CCATTGTTGGCATGTTGAAACCTCCTGCCGCCTTGATCATCCTTCGACTTAGGGAAGAATCTTAACAAATG
AAATTTAA

CAACAGCTATTTAGTGTATCCATCACCACCAAAAGTTATTAACTCCGAAATTAAAGCTCATGCCACCACAA
ACAATATCACATTGTCAGTTGGTGGCAACACAGAGACCAGTTTCAAGAGAGATCAGCAAGATGCTGGCCAT
AGTGACAT

CGTATGTCAGATCCTCGCATGATCAAAGAAATTATGGAAGCTGTTTCAATTCCGGTGATGGCAAAGGTCCG
TATTGGACACTTCGTGGAGGCACAGATCCTGGAAGAGCTGCAAGTAGACTACATTGACGAAAGTGAGGTTT
TGACTCCA

TCTTGATAACTTGACTGAATCTTTCAAGTCCGCCTCTACAGCGATGTCAGGGTATGAGCCTTGGCCAGGGA
TCCTGACCACGTCGCCGTCCTGTAATCCATGTGGCAAGTCCACCGTAATTGTCTTTGCCCTGTTGACCTGA
ACACCCTC

CCCATCGTTCCGGGACAAACATACCTGTACAACTTCACTGTCCCAGAGCAGGTAGGAACGTTCTGGTACCA
TGCACACATGGGTGCCCAGTACGGTGACGGTATGAGGGGTGCATTTATCATCCACGATCCGGAGGAGCCCT
TCGAATAT

GGGATTAACTCTGGAGGAAGTTAATGAAATGTACGAAGAAAGAATAAAGCCATGGAAGTCCGGAGGTTGGA
TTCCCAGTTCTAGAAGAACACCACAACCAACAAGCAGTACACCATTAGTTATTGTTGATAGTAAATAATTT
CTAAATAT

CCTAATTCTACCTCAACCCTACCCTAATCCAACCCTTCCATCCTGTCTCTCAACTTACCCTCCATTACCCT
GCCTCCCCACTCGTTACCCTGCCTCCCCACCACCAACCCACAAATCCACCATAACAGTTACCCTCCAATTA
CCCATATC

CTTTGTTAGGTTTGTCCAATATGGCGTCATTGAACTTGACGGGGTTGTTGAGGACCTCCTTAGAGACCATC
TCCCTGAGGTCACGAACGCTATCTTGTTTGAAGATGCCATAGGCAATAGCATGGAACAGACACGAGTTGTC
GTCGAGAA

TAGGCAAAAACTGCAGCCACACCACCGGCGGAGGCTGATGCAGCTGCTTCTGCACTTCTACCAAGAGGCCC
AGCAGAAACGGAGACGTTACCACCCAACGTGATGGTCCCAAATTCAGAAAACGACTTAACAGCATCCTGAG
TATTTAAA

TCCGGTGATGGCAAAGGTCCGTATTGGACACTTCGTGGAGGCACAGATCCTGGAAGAGCTGCAAGTAGACT
ACATTGACGAAAGTGAGGTTTTGACTCCAGCTGATTGGACACATCACATTGAGAAGCATAACTTCAAGGTG
CCATTTGT

ACCAGCCTTCAACAATGGTTTGTCAACTCTACCACCACCGGCAATGACACCGATGACACCTCTGGCATCAG
AAGAGATAACCTTCTTGGCACCGGATGGTAATCTGACTCTAGTCTTGTTTTCGTCTGGGTTGTGACCGATG
ATAATAAC

GAGACAACACCTGGGACATCAGCTGTTCCAGAGAACCATCATCATGTCTCTCCTCAACCTGCTTCAGTACC
ACCTCCACAGAATGGACAGTACCAACAGCACGGCATGATGACCCCAAACAAAGCTATGGCCTCTAACTGGG
CACATTAC

CGATGAGTTGCAAGGTTTAGTTAACAGTACTGTTACTCAGGCCATTATGTTTGGTGTCAGATGTGGTGCAG
CTGCTTTGACTTTGATTGTCATGTGGATGACATCGAGAAGCAGAAAAACGCCGATTTTCATTATCAACCAA
GTTTCATT

TATCTTGTTTGAAGATGCCATAGGCAATAGCATGGAACAGACACGAGTTGTCGTCGAGAACAGGATGTACT
GATAGGACGTTTTCTCCACTGCCACCAATTGACATCTCAGTGCTCTTCAGCACCCTCTTTGGTTTGGGTTG
TGGTGGTG

ATAAGGTCTGCAATGTACGTCGGAGAAGAGCCACTATCATCGGCGACTCCTGTGATCGTGTTCCACTGTGG
AGAGCCATATCGCACCTTGCCGTCCAGTTCCAGCTCCAAGATCATACACGGATTCTTCTCTGTGGCCAGCT
GCAGATAT

CACACCCACACCCACACACCACACCCACACCCTAATTCTACCTCAACCCTACCCTAATCCAACCCTTCCAT
CCTGTCTCTCAACTTACCCTCCATTACCCTGCCTCCCCACTCGTTACCCTGCCTCCCCACCACCAACCCAC
AAATCCAC

GAACCGGATATGGTAGGCTTATGTATTTTCAACTTGCCACCAGATGTAGTGTTTGAAGCGTTACTCCCTGG
AGTTGACACTGATCCGTCTGAGGATGACCCCATTAGTGACGCAGAAGAGGTTCTTCTGTGAACCCCAGGTT
GTTCAGAA

CAACAGCAACAGCTTGCTGTCGCACAGGCTCAGGCTCAGGCACAGGCACAGGCACAGGCTCAAGTTCAGGC
TCAGGCTCAGGCTCAGGCCCAGGCTCAGGCTCAAGCACAGCAGATCCAGATGCAACAGCTTCAGATGCAGC
AACAGCAA

GCAGCAACAGCAACAGCAACAGCTTGCTGTCGCACAGGCTCAGGCTCAGGCACAGGCACAGGCACAGGCTC
AAGTTCAGGCTCAGGCTCAGGCTCAGGCCCAGGCTCAGGCTCAAGCACAGCAGATCCAGATGCAACAGCTT
CAGATGCA

CTATGCAACAGCAGCAACAGCAACAGCAACAGCTTGCTGTCGCACAGGCTCAGGCTCAGGCACAGGCACAG
GCACAGGCTCAAGTTCAGGCTCAGGCTCAGGCTCAGGCCCAGGCTCAGGCTCAAGCACAGCAGATCCAGAT
GCAACAGC

TCAGCAGCAATACGCCATGGCTATGCAACAGCAGCAACAGCAACAGCAACAGCTTGCTGTCGCACAGGCTC
AGGCTCAGGCACAGGCACAGGCACAGGCTCAAGTTCAGGCTCAGGCTCAGGCTCAGGCCCAGGCTCAGGCT
CAAGCACA

TACGCCATGGCTATGCAACAGCAGCAACAGCAACAGCAACAGCTTGCTGTCGCACAGGCTCAGGCTCAGGC
ACAGGCACAGGCACAGGCTCAAGTTCAGGCTCAGGCTCAGGCTCAGGCCCAGGCTCAGGCTCAAGCACAGC
AGATCCAG

TATATGTAGATCAGCAGCAATACGCCATGGCTATGCAACAGCAGCAACAGCAACAGCAACAGCTTGCTGTC
GCACAGGCTCAGGCTCAGGCACAGGCACAGGCACAGGCTCAAGTTCAGGCTCAGGCTCAGGCTCAGGCCCA
GGCTCAGG

GCATTCTGGGTCCCTAGTTGTGGCTGAAGTGGTTGAAGTTGAGGTTGCTGTTGAGGTTGCTGTTGAGGTTG
CTGTTGAGGTTGCTGTTGAGGTTGCTGTTGAGGTTGCTGTTGAGGTTGCTGTTGAGGTTGCTGTTGAGGTT
GCTGTTGC

CAGGAGCATCTGTCTGCATTGCATTCTGGGTCCCTAGTTGTGGCTGAAGTGGTTGAAGTTGAGGTTGCTGT
TGAGGTTGCTGTTGAGGTTGCTGTTGAGGTTGCTGTTGAGGTTGCTGTTGAGGTTGCTGTTGAGGTTGCTG
TTGAGGTT

ACCGGAATGATATATGTAGATCAGCAGCAATACGCCATGGCTATGCAACAGCAGCAACAGCAACAGCAACA
GCTTGCTGTCGCACAGGCTCAGGCTCAGGCACAGGCACAGGCACAGGCTCAAGTTCAGGCTCAGGCTCAGG
CTCAGGCC

TGTTTGGGGTCATCATGCCGTGCTGTTGGTACTGTCCATTCTGTGGAGGTGGTACTGAAGCAGGTTGAGGA
GAGACATGATGATGGTTCTCTGGAACAGCTGATGTCCCAGGTGTTGTCTCTTGTTGAGAATTAACCTTAGT
GGAATCTC

TGTCTGCATTGCATTCTGGGTCCCTAGTTGTGGCTGAAGTGGTTGAAGTTGAGGTTGCTGTTGAGGTTGCT
GTTGAGGTTGCTGTTGAGGTTGCTGTTGAGGTTGCTGTTGAGGTTGCTGTTGAGGTTGCTGTTGAGGTTGC
TGTTGAGG

GATGGAGTTGCAGGAGCATCTGTCTGCATTGCATTCTGGGTCCCTAGTTGTGGCTGAAGTGGTTGAAGTTG
AGGTTGCTGTTGAGGTTGCTGTTGAGGTTGCTGTTGAGGTTGCTGTTGAGGTTGCTGTTGAGGTTGCTGTT
GAGGTTGC

CGAAACAAATACAAAGACTACAACAACAGAGAGCTCTACAGGCTCAGCTTCTTTCTCAACAACAGCAACAG
CAGCAACAGCAGCAGCAACATCACTCACCCCAAGCGCAGGCCCAGGCCAGTACCCAACAACCTACTCAAGG
TATGGTGC

GCCATAGCTTTGTTTGGGGTCATCATGCCGTGCTGTTGGTACTGTCCATTCTGTGGAGGTGGTACTGAAGC
AGGTTGAGGAGAGACATGATGATGGTTCTCTGGAACAGCTGATGTCCCAGGTGTTGTCTCTTGTTGAGAAT
TAACCTTA

TGATATGGGGGATGGAGTTGCAGGAGCATCTGTCTGCATTGCATTCTGGGTCCCTAGTTGTGGCTGAAGTG
GTTGAAGTTGAGGTTGCTGTTGAGGTTGCTGTTGAGGTTGCTGTTGAGGTTGCTGTTGAGGTTGCTGTTGA
GGTTGCTG

CATCATGCCGTGCTGTTGGTACTGTCCATTCTGTGGAGGTGGTACTGAAGCAGGTTGAGGAGAGACATGAT
GATGGTTCTCTGGAACAGCTGATGTCCCAGGTGTTGTCTCTTGTTGAGAATTAACCTTAGTGGAATCTCTA
TCAAATTC

AACCTCAATCCGAAACAAATACAAAGACTACAACAACAGAGAGCTCTACAGGCTCAGCTTCTTTCTCAACA
ACAGCAACAGCAGCAACAGCAGCAGCAACATCACTCACCCCAAGCGCAGGCCCAGGCCAGTACCCAACAAC
CTACTCAA

CCAGTTAGAGGCCATAGCTTTGTTTGGGGTCATCATGCCGTGCTGTTGGTACTGTCCATTCTGTGGAGGTG
GTACTGAAGCAGGTTGAGGAGAGACATGATGATGGTTCTCTGGAACAGCTGATGTCCCAGGTGTTGTCTCT
TGTTGAGA

CGATCCTGCTACCGGAATGATATATGTAGATCAGCAGCAATACGCCATGGCTATGCAACAGCAGCAACAGC
AACAGCAACAGCTTGCTGTCGCACAGGCTCAGGCTCAGGCACAGGCACAGGCACAGGCTCAAGTTCAGGCT
CAGGCTCA

CGGAAAAGGCTGATATGGGGGATGGAGTTGCAGGAGCATCTGTCTGCATTGCATTCTGGGTCCCTAGTTGT
GGCTGAAGTGGTTGAAGTTGAGGTTGCTGTTGAGGTTGCTGTTGAGGTTGCTGTTGAGGTTGCTGTTGAGG
TTGCTGTT

TTAGAACCATCAACGACTAACAGTTCCAGTACGTTTAGTCTGGTCACTTCAAGTGACAACAATTGGTGGAT
TCCAACTGAGTTAATCACGCAGGCACCAGAAGCTGCATCCACTGCATCTTCTACCGTTGGAGGAACACAAA
CTATGACT

TTAACAACGCCGGAAAAGGCTGATATGGGGGATGGAGTTGCAGGAGCATCTGTCTGCATTGCATTCTGGGT
CCCTAGTTGTGGCTGAAGTGGTTGAAGTTGAGGTTGCTGTTGAGGTTGCTGTTGAGGTTGCTGTTGAGGTT
GCTGTTGA

CCAGGCAAGCACAGCAGCATCATCAAGATCATGCGCCAGGCAATGCAGAGGACAAGACAACTGTAGGTGAG
TCCGTGAATGGTGTTCAACAGCCGGCCTCCTCCCAGTCAGATGCCATGAAACAAGACAAGTACGTCAGCAC
TAAACCAA

CTCTATTGAGGTCCTTGCTCAAGGAATGCGATGACATTGGTACAGCAAACATAGCTCAGGACCGTGGACGA
CTTCTGGGGGTTGCCACCAGTGACAACTCTTCAACCACGGAAGTTCAAGGAAGAACGAATAACGATTTGCA
ACAGGGGC

CACAAACCAAAACCTCAATCCGAAACAAATACAAAGACTACAACAACAGAGAGCTCTACAGGCTCAGCTTC
TTTCTCAACAACAGCAACAGCAGCAACAGCAGCAGCAACATCACTCACCCCAAGCGCAGGCCCAGGCCAGT
ACCCAACA

AGGAAGAAGCTTGGGAGGAAGAGGAAGATTGAGTAGATGTAGCAGCAGAGCTGGAGGCTGGAGATGCGGCA
GCAGAAACTTGTGAGTTGTTCTCAGAAGAGGAAGCAGCAGCGGTAGCTACAGAAGCAGCAGACGATGTTGT
ACTGGTAG

TTGTTTCTAATAGATTGTTGATTTATTTTGATTAAGCTGTTGTTTGTTGGGGAAGAGACTCACTTTGCTGG
TGTCCGACTTGTTCATGTTCTTCAACAGCACCTTCTTCTTCTTGTTCAGGTTCTTCAATTTGTCCATCTTC
TTGTTCGT

TAGATTGTTGATTTATTTTGATTAAGCTGTTGTTTGTTGGGGAAGAGACTCACTTTGCTGGTGTCCGACTT
GTTCATGTTCTTCAACAGCACCTTCTTCTTCTTGTTCAGGTTCTTCAATTTGTCCATCTTCTTGTTCGTCT
TCGGACCA

AGCTTGCTGTCGCACAGGCTCAGGCTCAGGCACAGGCACAGGCACAGGCTCAAGTTCAGGCTCAGGCTCAG
GCTCAGGCCCAGGCTCAGGCTCAAGCACAGCAGATCCAGATGCAACAGCTTCAGATGCAGCAACAGCAACA
GGCGCCAT

CTTCAACAGCACCTTCTTCTTCTTGTTCAGGTTCTTCAATTTGTCCATCTTCTTGTTCGTCTTCGGACCAC
CTGCCTTGACCGTCTTCCTCTTCCTCTTCTTCGCCTTCTTCAGGTTCGAAGTCTTCGTAATCCTGCTTGAA
ACTTAAAG

GGGAAATACTCACGAATGAATTTAATACCTTGAATGACTGGCCCTGCAGGATCGTCAGCCGCTGTACCCAC
TGGATCCTTGGTACCAGGGATGAGAGGCACACCAAACAAGATCACAGAACGCAAACCCTTGGCCACTAATG
GCTTTAAG

AATAAGCGTTCCAGGCAAGCACAGCAGCATCATCAAGATCATGCGCCAGGCAATGCAGAGGACAAGACAAC
TGTAGGTGAGTCCGTGAATGGTGTTCAACAGCCGGCCTCCTCCCAGTCAGATGCCATGAAACAAGACAAGT
ACGTCAGC

ACAAAGACTACAACAACAGAGAGCTCTACAGGCTCAGCTTCTTTCTCAACAACAGCAACAGCAGCAACAGC
AGCAGCAACATCACTCACCCCAAGCGCAGGCCCAGGCCAGTACCCAACAACCTACTCAAGGTATGGTGCCC
AATCATTT

CCTCAGCAGAGTTCTTCCTCAATATGGACACCATCGCCGGAGATTGGATCTGCTGCTTTCTGTCAACAAAC
CCAGTGCCTGCCCCTGTTGAGCTCTCTTGCTGCAACTGCTGCTGACGATGTTGATTTACCATCGATTTCAA
TGAGATGC

TTTTCAACTACGGTGACTTCACCACCATGTTGACTGGTATTGCCCCAGACCAAGTGACCAGAATGATCACC
GGTGTTCCATGGTACTCCAGCAGATTAAAGCCAGCCATCTCCAGTGCTCTATCCAAGGACGGTATCTACAC
TATCGCAA

AACAGTATTACGATCCTGCTACCGGAATGATATATGTAGATCAGCAGCAATACGCCATGGCTATGCAACAG
CAGCAACAGCAACAGCAACAGCTTGCTGTCGCACAGGCTCAGGCTCAGGCACAGGCACAGGCACAGGCTCA
AGTTCAGG

TGGTGGCTCTGTCACCTCCATGGAATTGTCATCATTTTTGTTGATGGTCCTTTCTTCTGTCGTTGCAACTT
GGGGTGACCAGCAAGCTGTGGCTGCCAAGGCTGCTTCATTGGCTGAAGGAGCAGCCGGTGCTGTTGCCTCC
TTTAACCC

TCAAAGACTTCAAGAAAGCCTTATCAATAGCATCCAAACCCTTCTTCTTTCTGGAAGCAGCCAAACAAGCA
GTCAACATCAAGTCGTCTGGAGAAACACCTTCAACAGTGTATGGTTCGTGCAAGGACAACTTGTTCTTGGT
CAAAGTAC

ACAGCAGCATCATCAAGATCATGCGCCAGGCAATGCAGAGGACAAGACAACTGTAGGTGAGTCCGTGAATG
GTGTTCAACAGCCGGCCTCCTCCCAGTCAGATGCCATGAAACAAGACAAGTACGTCAGCACTAAACCAAGA
AATAGTAA

ACCTTCTTCTTCTTGTTCAGGTTCTTCAATTTGTCCATCTTCTTGTTCGTCTTCGGACCACCTGCCTTGAC
CGTCTTCCTCTTCCTCTTCTTCGCCTTCTTCAGGTTCGAAGTCTTCGTAATCCTGCTTGAAACTTAAAGTA
GGCTTGAC

GGATATTGCTTCAAAGACTTCAAGAAAGCCTTATCAATAGCATCCAAACCCTTCTTCTTTCTGGAAGCAGC
CAAACAAGCAGTCAACATCAAGTCGTCTGGAGAAACACCTTCAACAGTGTATGGTTCGTGCAAGGACAACT
TGTTCTTG

TAGTTTGCGATAGTGTAGATACCGTCCTTGGATAGAGCACTGGAGATGGCTGGCTTTAATCTGCTGGAGTA
CCATGGAACACCGGTGATCATTCTGGTCACTTGGTCTGGGGCAATACCAGTCAACATGGTGGTGAAGTCAC
CGTAGTTG

AGGGAAGACCATCGACCACATGTGGCCATTGTAAAGAGCTGAGAAGAACCAAGAACTTCAACCCATCCGGT
GGGTGCATGTGTGCCTCTGCACGACGGCCAGCTGTTGGCAGCAAGGAAGATGAAACACGATGTCGTTGTGA
TGAGGGTG

TGATGCCTTAAATGACTTGGAACAATTGAAGGTCGGTGTAGATGCTGTGAAGAAGGCAGGTGGTGTTGTAG
AAGCCACTGTTTGTTTCTCTGGGGATATGCTTCAGCCAGGCAAGAAATACAATTTGGATTACTACTTGGAA
ATTGCTGA

TGTGGCCATTGTAAAGAGCTGAGAAGAACCAAGAACTTCAACCCATCCGGTGGGTGCATGTGTGCCTCTGC
ACGACGGCCAGCTGTTGGCAGCAAGGAAGATGAAACACGATGTCGTTGTGATGAGGGTGAACCTTGTAAAT
GTCATACC

CAAGAAAGCCTTATCAATAGCATCCAAACCCTTCTTCTTTCTGGAAGCAGCCAAACAAGCAGTCAACATCA
AGTCGTCTGGAGAAACACCTTCAACAGTGTATGGTTCGTGCAAGGACAACTTGTTCTTGGTCAAAGTACCG
GTTTTGTC

GGAGACGACGACGTCGAAAGTCTGCGAGGATCACGTGCTACAGCTGGTCTCATGGCTCTTGTAACTGAAGT
TTGTCTCTCTCTGCATGGTTTGGCAACAGCCTTCTTGTGTTTTGATTCTTTATTCATGTTTACGGCTCTGG
AGGTAGCG

TGGACCGGATGTACGAGGCTTCTTTGCCCCAATTTATTGACATCACCTGGAATGCAGGCGGTGGACGGTTG
TCACATCTGTCCACGGACTTGGTTGCGACAGCGCAGTCTGTGCTTGGTTTGGAAACGTGCATGCACCTTAC
CTGCACCA

TTATCAATAGCATCCAAACCCTTCTTCTTTCTGGAAGCAGCCAAACAAGCAGTCAACATCAAGTCGTCTGG
AGAAACACCTTCAACAGTGTATGGTTCGTGCAAGGACAACTTGTTCTTGGTCAAAGTACCGGTTTTGTCGG
AACACAAG

TACATTTCAACACCTGTTCTTGAATTTGCTGCGGATAATTAGACAGCTGCTGTTGTTGAGCAATTGCTTGC
GTTTGTTGCAACGGTACTTGAATTTGCTGCTGCTGCTGCTGCTGTAAATTAGATCCAGTTGAAGGCTTAGA
CCAAGCAA

ATTTACAAGCACCGCTGGACTCGTATGGGGTATAAGTGATACCTCTGACACCAGAGGCAAAGCTGCTGACG
TCTTCGCCGGAAGAACTAGAGGAAGAAGCTTGGGAGGAAGAGGAAGATTGAGTAGATGTAGCAGCAGAGCT
GGAGGCTG

ATCGACCACATGTGGCCATTGTAAAGAGCTGAGAAGAACCAAGAACTTCAACCCATCCGGTGGGTGCATGT
GTGCCTCTGCACGACGGCCAGCTGTTGGCAGCAAGGAAGATGAAACACGATGTCGTTGTGATGAGGGTGAA
CCTTGTAA

ATTGATGAATCTGCAACAACTCCGGCTATCCCTACTAAATCTGATGAAGCTGAAGCTGAAGTTGAGGCTGA
AGCAGGTGATGCTGGAACAAAGATTGGACTTGAAGCTGAAATCGAAACTACAACTGATGAAACTGATGATG
GTACAAAC

GTGTCCGACTTGTTCATGTTCTTCAACAGCACCTTCTTCTTCTTGTTCAGGTTCTTCAATTTGTCCATCTT
CTTGTTCGTCTTCGGACCACCTGCCTTGACCGTCTTCCTCTTCCTCTTCTTCGCCTTCTTCAGGTTCGAAG
TCTTCGTA

GGAAGAGACTCACTTTGCTGGTGTCCGACTTGTTCATGTTCTTCAACAGCACCTTCTTCTTCTTGTTCAGG
TTCTTCAATTTGTCCATCTTCTTGTTCGTCTTCGGACCACCTGCCTTGACCGTCTTCCTCTTCCTCTTCTT
CGCCTTCT

TCTTGTCTCTTGCGGCCTTGTTCTTTCTTTCAATGTAACGCTTGTGGTCTCTGTTAGAAGAGTCATCTGGG
ACATCTTCGTCCAAGAACTCAAAGGTTCTCCAGGAGTCAAATCTGTGCCAGAAAGCATAGAATTGTTCAAC
TTCCTTCT

GTGCTGGTAGTAGAAGTACTGGTGGTAGTGGTTGTCGTAGTTGCAGTGTCAGCAGTTGCTTCAGTGGCCTC
AGATGTGGTAGTAGAAGTAACGTCCCCTTCATCTGATGAAGACGATGACGATGACGATGACGATGACGATG
ACGATGAT

TTAATCACCTCACAAACCAAAACCTCAATCCGAAACAAATACAAAGACTACAACAACAGAGAGCTCTACAG
GCTCAGCTTCTTTCTCAACAACAGCAACAGCAGCAACAGCAGCAGCAACATCACTCACCCCAAGCGCAGGC
CCAGGCCA

GGAATTCATCAATATTTTGACTGACGATGGTTTATTAAACGAGGAGTGTGGTCCAGAGTGGCAAGGCATGA
AGAGGTTTGATGCCAGAAAGAAGGTCATTGAGCAGCTGAAGGAAAAGAACCTATACGTTGGCCAAGAAGAT
AATGAAAT

GTTGATATATTTAGAGTCTTTGATGCCTTAAATGACTTGGAACAATTGAAGGTCGGTGTAGATGCTGTGAA
GAAGGCAGGTGGTGTTGTAGAAGCCACTGTTTGTTTCTCTGGGGATATGCTTCAGCCAGGCAAGAAATACA
ATTTGGAT

ACCGGACCCACACTATCCGTTGCCACAGTATATCCCACCACTGAGTACTTCCTCACCTGATCCAATCGATT
CACAGAATCAACACTCTGAAGTACCTCAAGCTGAGACAAAGGTGAGAAATAACGTCTTACCACCACACACT
TTAACATC

ATTAAGCTGTTGTTTGTTGGGGAAGAGACTCACTTTGCTGGTGTCCGACTTGTTCATGTTCTTCAACAGCA
CCTTCTTCTTCTTGTTCAGGTTCTTCAATTTGTCCATCTTCTTGTTCGTCTTCGGACCACCTGCCTTGACC
GTCTTCCT

GAAGAACTAGAGGAAGAAGCTTGGGAGGAAGAGGAAGATTGAGTAGATGTAGCAGCAGAGCTGGAGGCTGG
AGATGCGGCAGCAGAAACTTGTGAGTTGTTCTCAGAAGAGGAAGCAGCAGCGGTAGCTACAGAAGCAGCAG
ACGATGTT

GGGAAGTGGATACTGACACGGACGATAACAACGAAGGAGATGGCTCTGACAATCACGAGGAAGGTGGAGAA
GAAGGAAGTAGAGGAGCTGATGCTGATGTGTCCAGTGCACAGCAGCGTGCCGAAAGGGTTGCGGACCCATG
GATATATC

GAACTTTGGAGACAATTGGATACCAGCGGAAGCATCGAAGATGGAAGAGTGAGAGTCACCCAAGAAGTCAG
AGGAGACAACAGCGTCTTCGGTGTAACCCAAAACACCCTTCAACTTACCTTCAGCGGCAGCCTTAACAACC
TTCTTGAT

CATCAAGATCATGCGCCAGGCAATGCAGAGGACAAGACAACTGTAGGTGAGTCCGTGAATGGTGTTCAACA
GCCGGCCTCCTCCCAGTCAGATGCCATGAAACAAGACAAGTACGTCAGCACTAAACCAAGAAATAGTAAGA
ACGATGGT

CATCCAAACCCTTCTTCTTTCTGGAAGCAGCCAAACAAGCAGTCAACATCAAGTCGTCTGGAGAAACACCT
TCAACAGTGTATGGTTCGTGCAAGGACAACTTGTTCTTGGTCAAAGTACCGGTTTTGTCGGAACACAAGAT
TTCGACAC

AACCTCTGATGAAAATCAATTTGTTCTGTTCCACCAAATTTGACAGAGGAGTGTGTTGCCACTCCTGCCTA
TGAACAGATAACAGATTCTGTGAATTCAAGGTGGACAAGGAGTTGCCATTATTGTTTGAGTATTCCGTGTT
ATCCAGCG

CACTTTGCTGGTGTCCGACTTGTTCATGTTCTTCAACAGCACCTTCTTCTTCTTGTTCAGGTTCTTCAATT
TGTCCATCTTCTTGTTCGTCTTCGGACCACCTGCCTTGACCGTCTTCCTCTTCCTCTTCTTCGCCTTCTTC
AGGTTCGA

GTGGCTGCTGCGAACATTCTCCTCAGCAGAGTTCTTCCTCAATATGGACACCATCGCCGGAGATTGGATCT
GCTGCTTTCTGTCAACAAACCCAGTGCCTGCCCCTGTTGAGCTCTCTTGCTGCAACTGCTGCTGACGATGT
TGATTTAC

CCTTCACCGAGCTTGCTACTGTGGCTGCTGCGAACATTCTCCTCAGCAGAGTTCTTCCTCAATATGGACAC
CATCGCCGGAGATTGGATCTGCTGCTTTCTGTCAACAAACCCAGTGCCTGCCCCTGTTGAGCTCTCTTGCT
GCAACTGC

CGCACAGGCTCAGGCTCAGGCACAGGCACAGGCACAGGCTCAAGTTCAGGCTCAGGCTCAGGCTCAGGCCC
AGGCTCAGGCTCAAGCACAGCAGATCCAGATGCAACAGCTTCAGATGCAGCAACAGCAACAGGCGCCATTA
TCTTTTCA

GTTCTTCCTCAATATGGACACCATCGCCGGAGATTGGATCTGCTGCTTTCTGTCAACAAACCCAGTGCCTG
CCCCTGTTGAGCTCTCTTGCTGCAACTGCTGCTGACGATGTTGATTTACCATCGATTTCAATGAGATGCTG
CTCAAAAT

AGCTGCTGACGTCTTCGCCGGAAGAACTAGAGGAAGAAGCTTGGGAGGAAGAGGAAGATTGAGTAGATGTA
GCAGCAGAGCTGGAGGCTGGAGATGCGGCAGCAGAAACTTGTGAGTTGTTCTCAGAAGAGGAAGCAGCAGC
GGTAGCTA

CGCTGACAGAGAATCCACTCAAACCACACTTAGTCTTGGAACCTCTGCCTGTAGCACCCTCGTCTCTGATT
TCACCACCAGAACCTGTAGCAGCACCTGGGAAAGGAGACACGGCTGTTGGATGGTTGTGAGTTTCGACTTT
GATAAGTA

ATTTATTTTGATTAAGCTGTTGTTTGTTGGGGAAGAGACTCACTTTGCTGGTGTCCGACTTGTTCATGTTC
TTCAACAGCACCTTCTTCTTCTTGTTCAGGTTCTTCAATTTGTCCATCTTCTTGTTCGTCTTCGGACCACC
TGCCTTGA

GCCACTTCGACAATTTCATCTTCTTCGTCGTCATCTTCTTCTTCTTCACTCTCCTCCTCTTCTTCCTCTTC
CGCATTCTTTGATGGCATCTCTTCCACTGCTCCCGCACCTGGCTCGCCTCTCATTAACATATTAACAATTC
TGTAACAT

CAAGTTATAAAGCCTTCTTGGCGGCATCAGCAGGGGAAACCTTGTTTTCTCTGTTGAATCTTGGTCTTTGA
GGTGGAGCTCTGTTCTTTCTGTCTTCTCTGGATCTGACTCTGACAATTCTGGCGTGAATAGCACAAGAAAC
ACAGTAGT

TACCAGGAGACCAACTTGACGAACTTTGGAGACAATTGGATACCAGCGGAAGCATCGAAGATGGAAGAGTG
AGAGTCACCCAAGAAGTCAGAGGAGACAACAGCGTCTTCGGTGTAACCCAAAACACCCTTCAACTTACCTT
CAGCGGCA

ATACAGTTCAGGGAAATACTCACGAATGAATTTAATACCTTGAATGACTGGCCCTGCAGGATCGTCAGCCG
CTGTACCCACTGGATCCTTGGTACCAGGGATGAGAGGCACACCAAACAAGATCACAGAACGCAAACCCTTG
GCCACTAA

TGCGGCCTTGTTCTTTCTTTCAATGTAACGCTTGTGGTCTCTGTTAGAAGAGTCATCTGGGACATCTTCGT
CCAAGAACTCAAAGGTTCTCCAGGAGTCAAATCTGTGCCAGAAAGCATAGAATTGTTCAACTTCCTTCTTG
GAAGAATC

GCTCGGACGAAATATTCGGTGGCTATCTATATTTCGCACAAGCACCTTCTGCTGCAGAATTTCACACCGAA
TCTGTGCAACGTGTCAAGAACTTGCATTTGGCAGATTGTTTGAGAGCTAATAAGTCCACGATGGCTTGGGG
TCTAGAAG

CTGGATCCTACGAGCATACCATTCACTCCAATCATCTCTGTCCGGATCTGCCTCAATGCCGATGACCAAAC
CTGGCTGGTGTGATATCTCATCACTTGAGAGAAGTGAGGGCTTTGAGTTCTTATAAGGAATAGGTTTATTC
AAATCTAT

AGGCGCCTTTACCATATCCTCCACCTTTCACTAATTACTATCAACAGCCTCAGCAACAGTACGCACCACCT
TCACAGCAAGCACCGGTGGAAGCACAACCGCAACAGAGCAGTGGGGCTAGTAGTGCCTTCAAGAGTTTTGG
TAGCAAAT

CCGTGGACGAATTCCCCAACGGTAGATTCGGTGATTCGTCTTCTCCTGCGTTCGGTGACTTGGATCTGTGT
GGTTCAGACTTGATCAGGCAATCAGCGAACAAATGTCTCGAATTATGGTCCACCCCTACTTCCATCAACGA
CGTCGCCT

TAGAAGTAGTTTAGGGTCATTTTTCAGTACATTGGTTGGCCTGGCTTGAGTTGCAACCAGTGCCAAGCATC
AGCGTTGGCCTCTCTCTTGTACATTGGTTGGCCTGGTCTCAAATTTAACCAGTGCCAAGCGTCGGCAACAG
CCTCTCTT

GTCTTCGCCGGAAGAACTAGAGGAAGAAGCTTGGGAGGAAGAGGAAGATTGAGTAGATGTAGCAGCAGAGC
TGGAGGCTGGAGATGCGGCAGCAGAAACTTGTGAGTTGTTCTCAGAAGAGGAAGCAGCAGCGGTAGCTACA
GAAGCAGC

GTGAATTTCTGCACTGAGAAACGTACCTGTAGTGATTATGACCTGATCTGCCCCAACCTGGGTACCGTCAT
CCAGAACCACACCTTTGATGACCTTGTGGCCACATCCGGGGTCATACAAGATCAAGTCAGCAACTTTGTTC
TGCAGCAG

AAACCACACTTAGTCTTGGAACCTCTGCCTGTAGCACCCTCGTCTCTGATTTCACCACCAGAACCTGTAGC
AGCACCTGGGAAAGGAGACACGGCTGTTGGATGGTTGTGAGTTTCGACTTTGATAAGTAATGGAATTCTTT
CCTTTGTA

TCAGTACCTTCTACAAGTTCCAGATCGTCCGTTTCTCAAGTCTCAGACACTCCGGTGCCTTCTACAAGTTC
AAGGTCGTCCGTTTCTCAAACATCTAGCTCACTACAGCCCACCACTACATCCTCCCAACGTTTCACCATTT
CCACTCAT

TTTAATACCTTGAATGACTGGCCCTGCAGGATCGTCAGCCGCTGTACCCACTGGATCCTTGGTACCAGGGA
TGAGAGGCACACCAAACAAGATCACAGAACGCAAACCCTTGGCCACTAATGGCTTTAAGTAGTCTTTTAGC
CTATTTAC

CGAGAGGGTTTAGAAGTAGTTTAGGGTCATTTTTCAGTACATTGGTTGGCCTGGCTTGAGTTGCAACCAGT
GCCAAGCATCAGCGTTGGCCTCTCTCTTGTACATTGGTTGGCCTGGTCTCAAATTTAACCAGTGCCAAGCG
TCGGCAAC

CGAACATTCTCCTCAGCAGAGTTCTTCCTCAATATGGACACCATCGCCGGAGATTGGATCTGCTGCTTTCT
GTCAACAAACCCAGTGCCTGCCCCTGTTGAGCTCTCTTGCTGCAACTGCTGCTGACGATGTTGATTTACCA
TCGATTTC

TTCAAATACGCCAGACAATTGATTTCTAACGAAGGTGTCTTGGTGGGTGGTTCTTCCGGTTCTGCCTTCAC
TGCGGTTGTGAAATACTGTGAAGACCACCCTGAACTGACTGAAGATGATGTCATTGTTGCCATATTCCCAG
ATTCCATC

TTAATTCTCAACAAGAGACAACACCTGGGACATCAGCTGTTCCAGAGAACCATCATCATGTCTCTCCTCAA
CCTGCTTCAGTACCACCTCCACAGAATGGACAGTACCAACAGCACGGCATGATGACCCCAAACAAAGCTAT
GGCCTCTA

GGATAATGGTGTTGATATATTTAGAGTCTTTGATGCCTTAAATGACTTGGAACAATTGAAGGTCGGTGTAG
ATGCTGTGAAGAAGGCAGGTGGTGTTGTAGAAGCCACTGTTTGTTTCTCTGGGGATATGCTTCAGCCAGGC
AAGAAATA

TTGGGAGGAAGAGGAAGATTGAGTAGATGTAGCAGCAGAGCTGGAGGCTGGAGATGCGGCAGCAGAAACTT
GTGAGTTGTTCTCAGAAGAGGAAGCAGCAGCGGTAGCTACAGAAGCAGCAGACGATGTTGTACTGGTAGCG
GCAGCTGG

TGACGAAGAAGGAGACGACGACGTCGAAAGTCTGCGAGGATCACGTGCTACAGCTGGTCTCATGGCTCTTG
TAACTGAAGTTTGTCTCTCTCTGCATGGTTTGGCAACAGCCTTCTTGTGTTTTGATTCTTTATTCATGTTT
ACGGCTCT

CAATTTCATCTTCTTCGTCGTCATCTTCTTCTTCTTCACTCTCCTCCTCTTCTTCCTCTTCCGCATTCTTT
GATGGCATCTCTTCCACTGCTCCCGCACCTGGCTCGCCTCTCATTAACATATTAACAATTCTGTAACATAA
TTCACCAA

TTATTGCCAAGTTGTCCGTTATCCCAATCAGAAGAGGTTACTGGGGTACCAACTTGGGTCAACCACATTCT
TTGGCCACCAAGACCACTGGTAAGTGTGGTTCCGTCACTGTTAGATTGATCCCAGCCCCAAGAGGTTCTGG
TATCGTCG

TGCTGTTGGTACTGTCCATTCTGTGGAGGTGGTACTGAAGCAGGTTGAGGAGAGACATGATGATGGTTCTC
TGGAACAGCTGATGTCCCAGGTGTTGTCTCTTGTTGAGAATTAACCTTAGTGGAATCTCTATCAAATTCCG
GTAAATTG

CCACTGAAAGTACCAACTCCAGCACTAATGCTACTACCACTGCCAGCACCAACGTCAGGACTAGTGCTACT
ACCACTGCCAGCATCAACGTCAGGACTAGTGCGACTACCACTGAAAGTACCAACTCCAACACTAGTGCTAC
TACCACCG

TTGGTTACGATCCAACTGCTCCTGTCGAAGAAGGCGAAGAAGAAGAAAGTGAAAGTGAAAGTGAAAGTGAA
GATCAAGTTGAGGAGGAAGATCAAGAAGTTGTTGCTGGAGAAGAAGATGATGACGATGACGAAGAGCTACA
AGCTCAAA

CCCAATTGTCTCCATCGAAGATCCATTTGCTGAAGATGACTGGGAAGCTTGGTCTCACTTCTTCAAGACCG
CTGGTATTCAAATTGTTGCTGATGACTTGACTGTCACCAACCCAAAGAGAATTGCTACCGCTATCGAAAAG
AAGGCTGC

GAGAATAGAGCCTCACAGGATATGTATCTCTATGAAGCGATGCCACCAACGCTGCCCCACAGGGATTGGAA
GGACTATTTTGTGATGGCTACTGCCACAGCTGGGCTGTTGTATGGTGCATATGAAGTAACTAGAAGGTATG
TGATACCA

GATGGTTGTTGGTAATGTGCCCAGTTAGAGGCCATAGCTTTGTTTGGGGTCATCATGCCGTGCTGTTGGTA
CTGTCCATTCTGTGGAGGTGGTACTGAAGCAGGTTGAGGAGAGACATGATGATGGTTCTCTGGAACAGCTG
ATGTCCCA

CGTTCGAAGAATTTGTCGAAGATAAACGATTCATTGCTCTAGAGGTATCTGATAATGATGACGATTGTGAC
ACTGATTTGACTGCTGACACAGCTGACGAGCTGGAAAGCTCTGCTATCTTAAAGATGAGGGAGTCTGATGC
ATCTTTAA

GACCCTAAATTCATTCCAAACAAACCATACAGACAATTCAACACATGGTGTGGTGAACCTGCAAGAATGAT
CATTGCAGGTGCCATTGGACAGGAAATCTCCGACAAGAAGTTGACTGAACAATGTTCAAGAGTAGGTGATT
ATTTGTTC

CAACGACTAACAGTTCCAGTACGTTTAGTCTGGTCACTTCAAGTGACAACAATTGGTGGATTCCAACTGAG
TTAATCACGCAGGCACCAGAAGCTGCATCCACTGCATCTTCTACCGTTGGAGGAACACAAACTATGACTTT
GCCCCATG

GTACTCTCACTTCAATCTCCCCGTCGCACAAGATCACAAGAGGATCTTTGATGGCGACAAGGGCTTGAACA
CCGGTGGGATGGGTGCCTATGCCCCCGCTCCTGTGGCCACACCATCTTTGTTGAAGACCATAGATTCACAG
ATTGTGAA

CAGGCTCAGGCACAGGCACAGGCACAGGCTCAAGTTCAGGCTCAGGCTCAGGCTCAGGCCCAGGCTCAGGC
TCAAGCACAGCAGATCCAGATGCAACAGCTTCAGATGCAGCAACAGCAACAGGCGCCATTATCTTTTCAGC
AAATGTCG

ATCAGACGCAAGGGAAGACCATCGACCACATGTGGCCATTGTAAAGAGCTGAGAAGAACCAAGAACTTCAA
CCCATCCGGTGGGTGCATGTGTGCCTCTGCACGACGGCCAGCTGTTGGCAGCAAGGAAGATGAAACACGAT
GTCGTTGT

CCTTTGATCTCCATATTGGTTATTTGTTGCGTTAAAGTGTTGCTGTTGCTGTTGCTGCTCATCCATGAACC
ACTGTTGCTGCTGCTGCTGTCTTGAACTATGATCAACTTCGGACGCATTTAAATCTATTGAATCTTCATTC
GTCATTAC

TTCGTTGTCGTACCAGGAGACCAACTTGACGAACTTTGGAGACAATTGGATACCAGCGGAAGCATCGAAGA
TGGAAGAGTGAGAGTCACCCAAGAAGTCAGAGGAGACAACAGCGTCTTCGGTGTAACCCAAAACACCCTTC
AACTTACC

TCATTGCTCTAGAGGTATCTGATAATGATGACGATTGTGACACTGATTTGACTGCTGACACAGCTGACGAG
CTGGAAAGCTCTGCTATCTTAAAGATGAGGGAGTCTGATGCATCTTTAAATGTTACCACCGGCAATAATAC
ATCAAGGA

GATAAACGATTCATTGCTCTAGAGGTATCTGATAATGATGACGATTGTGACACTGATTTGACTGCTGACAC
AGCTGACGAGCTGGAAAGCTCTGCTATCTTAAAGATGAGGGAGTCTGATGCATCTTTAAATGTTACCACCG
GCAATAAT

ACTCTGGTAGAGTAACCGTATTCGTTGTCGTACCAGGAGACCAACTTGACGAACTTTGGAGACAATTGGAT
ACCAGCGGAAGCATCGAAGATGGAAGAGTGAGAGTCACCCAAGAAGTCAGAGGAGACAACAGCGTCTTCGG
TGTAACCC

TTAGAGTCTTTGATGCCTTAAATGACTTGGAACAATTGAAGGTCGGTGTAGATGCTGTGAAGAAGGCAGGT
GGTGTTGTAGAAGCCACTGTTTGTTTCTCTGGGGATATGCTTCAGCCAGGCAAGAAATACAATTTGGATTA
CTACTTGG

CTAGCGCTGTTGCTAATCATGCGCACGACGATGAGGCTTCCACAGACGTTGAAGGCTCCACAGACGTCAAT
GTCAATGAACAGGCTCTGTTGCAAGAGGATTTTGACATGTGGAGTGAGACTATATTGCAGAAAACACAAGA
CGTCATAC

ATCATTTTTGTTGATGGTCCTTTCTTCTGTCGTTGCAACTTGGGGTGACCAGCAAGCTGTGGCTGCCAAGG
CTGCTTCATTGGCTGAAGGAGCAGCCGGTGCTGTTGCCTCCTTTAACCCAGGTTATTTCTGGATGTTCACC
AACTGTAT

CCAGAGGCAAAGCTGCTGACGTCTTCGCCGGAAGAACTAGAGGAAGAAGCTTGGGAGGAAGAGGAAGATTG
AGTAGATGTAGCAGCAGAGCTGGAGGCTGGAGATGCGGCAGCAGAAACTTGTGAGTTGTTCTCAGAAGAGG
AAGCAGCA

TGGAATTGTCATCATTTTTGTTGATGGTCCTTTCTTCTGTCGTTGCAACTTGGGGTGACCAGCAAGCTGTG
GCTGCCAAGGCTGCTTCATTGGCTGAAGGAGCAGCCGGTGCTGTTGCCTCCTTTAACCCAGGTTATTTCTG
GATGTTCA

AGTAGTAGAAGTGCTGGTAGTAGAAGTACTGGTGGTAGTGGTTGTCGTAGTTGCAGTGTCAGCAGTTGCTT
CAGTGGCCTCAGATGTGGTAGTAGAAGTAACGTCCCCTTCATCTGATGAAGACGATGACGATGACGATGAC
GATGACGA

GAATCCACTCAAACCACACTTAGTCTTGGAACCTCTGCCTGTAGCACCCTCGTCTCTGATTTCACCACCAG
AACCTGTAGCAGCACCTGGGAAAGGAGACACGGCTGTTGGATGGTTGTGAGTTTCGACTTTGATAAGTAAT
GGAATTCT

CAGTTCCAGTACGTTTAGTCTGGTCACTTCAAGTGACAACAATTGGTGGATTCCAACTGAGTTAATCACGC
AGGCACCAGAAGCTGCATCCACTGCATCTTCTACCGTTGGAGGAACACAAACTATGACTTTGCCCCATGCA
ATTGCAGC

GCTTGCTACTGTGGCTGCTGCGAACATTCTCCTCAGCAGAGTTCTTCCTCAATATGGACACCATCGCCGGA
GATTGGATCTGCTGCTTTCTGTCAACAAACCCAGTGCCTGCCCCTGTTGAGCTCTCTTGCTGCAACTGCTG
CTGACGAT

TACTTTGGACCAGGAAGAGGCTAGCGCTGTTGCTAATCATGCGCACGACGATGAGGCTTCCACAGACGTTG
AAGGCTCCACAGACGTCAATGTCAATGAACAGGCTCTGTTGCAAGAGGATTTTGACATGTGGAGTGAGACT
ATATTGCA

GTAAAGAGCTGAGAAGAACCAAGAACTTCAACCCATCCGGTGGGTGCATGTGTGCCTCTGCACGACGGCCA
GCTGTTGGCAGCAAGGAAGATGAAACACGATGTCGTTGTGATGAGGGTGAACCTTGTAAATGTCATACCAA
GAGGAAAA

CACGAATGAATTTAATACCTTGAATGACTGGCCCTGCAGGATCGTCAGCCGCTGTACCCACTGGATCCTTG
GTACCAGGGATGAGAGGCACACCAAACAAGATCACAGAACGCAAACCCTTGGCCACTAATGGCTTTAAGTA
GTCTTTTA

TTTCTCAAGTTTCAGACACGTCAGTACCTTCTACAAGTTCCAGATCGTCCGTTTCTCAAGTCTCAGACACT
CCGGTGCCTTCTACAAGTTCAAGGTCGTCCGTTTCTCAAACATCTAGCTCACTACAGCCCACCACTACATC
CTCCCAAC

TCTGGTGAAGGCTCGGACGAAATATTCGGTGGCTATCTATATTTCGCACAAGCACCTTCTGCTGCAGAATT
TCACACCGAATCTGTGCAACGTGTCAAGAACTTGCATTTGGCAGATTGTTTGAGAGCTAATAAGTCCACGA
TGGCTTGG

TCTTCTTCTTCTTCTTCGTCGGAAGAAGAGTCAGAGGAAGAGGAAGAGGAATCCTTGGATTCTTCCTTCTT
GGTTTCAGCTTCACTTTCGCTGTCAGAAGACGATGAAGAAGAGGATTCAGAATCAGAGGATGAAGATGAAG
ATGAAGAT

GCGTAGACGTATCTCATCTTGTACTTGTAACCCTTGGTGACACCAGTGATCATGTTGTCAACCAAAGACTT
GACGGTTCTCAAAGCAGCAACGTGCTTTCTGCCACCGTTGTGAACAGCAACCTTGATCAATTGGTTGTTGA
CCTTGGTG

TGTTTGTTGGGGAAGAGACTCACTTTGCTGGTGTCCGACTTGTTCATGTTCTTCAACAGCACCTTCTTCTT
CTTGTTCAGGTTCTTCAATTTGTCCATCTTCTTGTTCGTCTTCGGACCACCTGCCTTGACCGTCTTCCTCT
TCCTCTTC

TGATAGAGATTCCACTAAGGTTAATTCTCAACAAGAGACAACACCTGGGACATCAGCTGTTCCAGAGAACC
ATCATCATGTCTCTCCTCAACCTGCTTCAGTACCACCTCCACAGAATGGACAGTACCAACAGCACGGCATG
ATGACCCC

TGAATGACTGGCCCTGCAGGATCGTCAGCCGCTGTACCCACTGGATCCTTGGTACCAGGGATGAGAGGCAC
ACCAAACAAGATCACAGAACGCAAACCCTTGGCCACTAATGGCTTTAAGTAGTCTTTTAGCCTATTTACAC
CGATTCTA

TGTTCATGTTCTTCAACAGCACCTTCTTCTTCTTGTTCAGGTTCTTCAATTTGTCCATCTTCTTGTTCGTC
TTCGGACCACCTGCCTTGACCGTCTTCCTCTTCCTCTTCTTCGCCTTCTTCAGGTTCGAAGTCTTCGTAAT
CCTGCTTG

TTCAATCTCCCCGTCGCACAAGATCACAAGAGGATCTTTGATGGCGACAAGGGCTTGAACACCGGTGGGAT
GGGTGCCTATGCCCCCGCTCCTGTGGCCACACCATCTTTGTTGAAGACCATAGATTCACAGATTGTGAAGC
CTACGATT

GTATCAATTTCGTTAGAACCTTGTTGTCTAAGAGAAAGTTACAATGGATGGTTGACAAGGACTTGGTCGGA
AATTGGGACGATCCAAGGTTCCCAACTGTCAGGGGTGTGAGAAGAAGAGGTATGACTGTCGAAGGTTTGAG
GAACTTCG

TTCTTTCTTTCAATGTAACGCTTGTGGTCTCTGTTAGAAGAGTCATCTGGGACATCTTCGTCCAAGAACTC
AAAGGTTCTCCAGGAGTCAAATCTGTGCCAGAAAGCATAGAATTGTTCAACTTCCTTCTTGGAAGAATCTT
TGTTACCT

GCTACAGATGATCAGACGCAAGGGAAGACCATCGACCACATGTGGCCATTGTAAAGAGCTGAGAAGAACCA
AGAACTTCAACCCATCCGGTGGGTGCATGTGTGCCTCTGCACGACGGCCAGCTGTTGGCAGCAAGGAAGAT
GAAACACG

CGACAGCATTAGTAGTAGAAGTGCTGGTAGTAGAAGTACTGGTGGTAGTGGTTGTCGTAGTTGCAGTGTCA
GCAGTTGCTTCAGTGGCCTCAGATGTGGTAGTAGAAGTAACGTCCCCTTCATCTGATGAAGACGATGACGA
TGACGATG

GGTAATGTGCCCAGTTAGAGGCCATAGCTTTGTTTGGGGTCATCATGCCGTGCTGTTGGTACTGTCCATTC
TGTGGAGGTGGTACTGAAGCAGGTTGAGGAGAGACATGATGATGGTTCTCTGGAACAGCTGATGTCCCAGG
TGTTGTCT

TGCTTCATCAGCACTATTTTCGACAGCATTAGTAGTAGAAGTGCTGGTAGTAGAAGTACTGGTGGTAGTGG
TTGTCGTAGTTGCAGTGTCAGCAGTTGCTTCAGTGGCCTCAGATGTGGTAGTAGAAGTAACGTCCCCTTCA
TCTGATGA

TGGTTTCAGCTTCACTTTCGCTGTCAGAAGACGATGAAGAAGAGGATTCAGAATCAGAGGATGAAGATGAA
GATGAAGATTCAGATTCAGACTCAGACTCAGATTCAGATTCAGATGAAGATGAAGATGAGGATGAAGATTC
GGAGGAAG

TGGTTTGGCTTGGATAGTCCATTGACAAAACTGGATCCTACGAGCATACCATTCACTCCAATCATCTCTGT
CCGGATCTGCCTCAATGCCGATGACCAAACCTGGCTGGTGTGATATCTCATCACTTGAGAGAAGTGAGGGC
TTTGAGTT

CGGCTACTCAATCCACAACTCTATCTTTTACCGACGCAAACAGCAGTTCTGCTTCCGCTCCATTGGAAGTG
GCAACGTCTACGCCAACCCCATCTTCAAAGGCATCCTCTCTGTTGCTTACACCATCAACATCCTCTTTAAG
TCAGGTTG

CGGTGACTTCACCACCATGTTGACTGGTATTGCCCCAGACCAAGTGACCAGAATGATCACCGGTGTTCCAT
GGTACTCCAGCAGATTAAAGCCAGCCATCTCCAGTGCTCTATCCAAGGACGGTATCTACACTATCGCAAAC
TAGAGACA

ATCCTTGGATTCTTCCTTCTTGGTTTCAGCTTCACTTTCGCTGTCAGAAGACGATGAAGAAGAGGATTCAG
AATCAGAGGATGAAGATGAAGATGAAGATTCAGATTCAGACTCAGACTCAGATTCAGATTCAGATGAAGAT
GAAGATGA

CGTTTGTCTCTAGTTTGCGATAGTGTAGATACCGTCCTTGGATAGAGCACTGGAGATGGCTGGCTTTAATC
TGCTGGAGTACCATGGAACACCGGTGATCATTCTGGTCACTTGGTCTGGGGCAATACCAGTCAACATGGTG
GTGAAGTC

ACGGAAATAGAACCTCTGATGAAAATCAATTTGTTCTGTTCCACCAAATTTGACAGAGGAGTGTGTTGCCA
CTCCTGCCTATGAACAGATAACAGATTCTGTGAATTCAAGGTGGACAAGGAGTTGCCATTATTGTTTGAGT
ATTCCGTG

GTACGAGGCTTCTTTGCCCCAATTTATTGACATCACCTGGAATGCAGGCGGTGGACGGTTGTCACATCTGT
CCACGGACTTGGTTGCGACAGCGCAGTCTGTGCTTGGTTTGGAAACGTGCATGCACCTTACCTGCACCAAT
ATGCCCAT

TAGTCTTGGAACCTCTGCCTGTAGCACCCTCGTCTCTGATTTCACCACCAGAACCTGTAGCAGCACCTGGG
AAAGGAGACACGGCTGTTGGATGGTTGTGAGTTTCGACTTTGATAAGTAATGGAATTCTTTCCTTTGTAGA
GGTCCATT

GTGAGCATATGTTTGGGATGAGGACGTTGCTGGCATTGTCGAGGGCCTTTGGTGGGGTCTTGATTGGTGCC
CTTGCGCCAAGTTAGCTGCTGGTCTTGGTGGTCGTGGCGGTTGAGATTGCAATCTCTCCTCTTCCTTGATT
ACGTCCTC

ATCCAAGATTTCCTTCTTTGGCATACGAATAGATTTAATATTGCTCTTGGCCTTCTCACGCCCCACTTTCA
AGAAATTCGAGTTGTCTGACCTCATGGACATACCTGTGTGGCTCATCGTAACACCAACGGTTTCATCCAAA
GTTGTGAT

CCAACTTGACGAACTTTGGAGACAATTGGATACCAGCGGAAGCATCGAAGATGGAAGAGTGAGAGTCACCC
AAGAAGTCAGAGGAGACAACAGCGTCTTCGGTGTAACCCAAAACACCCTTCAACTTACCTTCAGCGGCAGC
CTTAACAA

TCAGAGGAAGAGGAAGAGGAATCCTTGGATTCTTCCTTCTTGGTTTCAGCTTCACTTTCGCTGTCAGAAGA
CGATGAAGAAGAGGATTCAGAATCAGAGGATGAAGATGAAGATGAAGATTCAGATTCAGACTCAGACTCAG
ATTCAGAT

TTGATGGTCCTTTCTTCTGTCGTTGCAACTTGGGGTGACCAGCAAGCTGTGGCTGCCAAGGCTGCTTCATT
GGCTGAAGGAGCAGCCGGTGCTGTTGCCTCCTTTAACCCAGGTTATTTCTGGATGTTCACCAACTGTATCA
CTTCTGCA

GACGATAACAACGAAGGAGATGGCTCTGACAATCACGAGGAAGGTGGAGAAGAAGGAAGTAGAGGAGCTGA
TGCTGATGTGTCCAGTGCACAGCAGCGTGCCGAAAGGGTTGCGGACCCATGGATATATCAAAGATCTAGAT
CAGCTATT

CACCAGTGATCATGTTGTCAACCAAAGACTTGACGGTTCTCAAAGCAGCAACGTGCTTTCTGCCACCGTTG
TGAACAGCAACCTTGATCAATTGGTTGTTGACCTTGGTGAAGGTAACATCAATGTGCTTCAAGTTCTTGGT
CAAAGTAC

CATTGGCCTTGATCTTATCGGCGGTGGACACTAACTCTTCGTCATTCAAACTGTCAACGATCAAACCAACT
TGTTCGTCAATCGTGGTGGCCTTGTCAGCATCACCAAAGTAGTCAGCTTCCTTAGCAGAGTTACGGATGGC
TCTCTTGT

GACAATTGGATACCAGCGGAAGCATCGAAGATGGAAGAGTGAGAGTCACCCAAGAAGTCAGAGGAGACAAC
AGCGTCTTCGGTGTAACCCAAAACACCCTTCAACTTACCTTCAGCGGCAGCCTTAACAACCTTCTTGATTT
CATCGTAG

ATCTCATCTTGTACTTGTAACCCTTGGTGACACCAGTGATCATGTTGTCAACCAAAGACTTGACGGTTCTC
AAAGCAGCAACGTGCTTTCTGCCACCGTTGTGAACAGCAACCTTGATCAATTGGTTGTTGACCTTGGTGAA
GGTAACAT

AAGAGTGTGAGTGAGCATATGTTTGGGATGAGGACGTTGCTGGCATTGTCGAGGGCCTTTGGTGGGGTCTT
GATTGGTGCCCTTGCGCCAAGTTAGCTGCTGGTCTTGGTGGTCGTGGCGGTTGAGATTGCAATCTCTCCTC
TTCCTTGA

ATTCCCCAACGGTAGATTCGGTGATTCGTCTTCTCCTGCGTTCGGTGACTTGGATCTGTGTGGTTCAGACT
TGATCAGGCAATCAGCGAACAAATGTCTCGAATTATGGTCCACCCCTACTTCCATCAACGACGTCGCCTTC
TTGGTCAT

TCTTCGTCTACTAGCGATGATGAAGAGGGAGATATTATTGCCCAGACGACAGAGCCCAAGCAAGATGCCAG
TCCTGATGATGATCGCTCAGGCCATTCTTCGCCCAGAGAGGAGGGACAACAGCAAATACGTGCCAAAGAAG
CTTCGGGA

TACAACTTCAAGTTCGTCCGTTTCTCAAGTTTCAGACACGTCAGTACCTTCTACAAGTTCCAGATCGTCCG
TTTCTCAAGTCTCAGACACTCCGGTGCCTTCTACAAGTTCAAGGTCGTCCGTTTCTCAAACATCTAGCTCA
CTACAGCC

ATTGACAAAACTGGATCCTACGAGCATACCATTCACTCCAATCATCTCTGTCCGGATCTGCCTCAATGCCG
ATGACCAAACCTGGCTGGTGTGATATCTCATCACTTGAGAGAAGTGAGGGCTTTGAGTTCTTATAAGGAAT
AGGTTTAT

GATCAGGGCCTTTCGGAAGACTTTCACATATTTCTTTATTGGCGCAGCCACCACAAGCATCGGACTTACCT
GCCATGTCTGATTCTGGACCAGGACAGTGTTCTGGTTCCGGCTGATTAAGTTCATACTCTGCTGGTAGCAC
TTCGTCGT

GGAAGAAGAGTCAGAGGAAGAGGAAGAGGAATCCTTGGATTCTTCCTTCTTGGTTTCAGCTTCACTTTCGC
TGTCAGAAGACGATGAAGAAGAGGATTCAGAATCAGAGGATGAAGATGAAGATGAAGATTCAGATTCAGAC
TCAGACTC

AGTTCGTCCGTTTCTCAAGTTTCAGACACGTCAGTACCTTCTACAAGTTCCAGATCGTCCGTTTCTCAAGT
CTCAGACACTCCGGTGCCTTCTACAAGTTCAAGGTCGTCCGTTTCTCAAACATCTAGCTCACTACAGCCCA
CCACTACA

GAGAAGAACCAAGAACTTCAACCCATCCGGTGGGTGCATGTGTGCCTCTGCACGACGGCCAGCTGTTGGCA
GCAAGGAAGATGAAACACGATGTCGTTGTGATGAGGGTGAACCTTGTAAATGTCATACCAAGAGGAAAAGC
AGCCGGAA

ATGCGCCAGGCAATGCAGAGGACAAGACAACTGTAGGTGAGTCCGTGAATGGTGTTCAACAGCCGGCCTCC
TCCCAGTCAGATGCCATGAAACAAGACAAGTACGTCAGCACTAAACCAAGAAATAGTAAGAACGATGGTGT
GGATACAG

CATGCACTGCTGTGGGTACGGCCCATTCTGTGGAGGTGGTACTGAAGCAGGTTGAGGAGAGGCATGATGGG
GGTTCTCTGGAACAGCTGATGAAGCAGGTGTTGTTGTCTGTTGAGAGTTAGCCTTAGTGGAAGCCTTATCA
TATTCTTG

TCAGACACACCGGTTTCTTATACAACTTCAAGTTCGTCCGTTTCTCAAGTTTCAGACACGTCAGTACCTTC
TACAAGTTCCAGATCGTCCGTTTCTCAAGTCTCAGACACTCCGGTGCCTTCTACAAGTTCAAGGTCGTCCG
TTTCTCAA

CAGAGTCCGCTGAGGATGAATCAGTAAATGTATTACCTGACTCAGGTGATGGAGTGCTCAGAGGCGTTCCA
ACTGATGATGGATACTGCGGAAACTGTGATTGTGGCCCAGGTGGAAAGTACATAGGCGACATTTGATAAGG
TGTATACG

CTCCAACACTAGTGCTACTACCACCGAAAGTACCGACTCCAACACTAGTGCTACTACCACTGCTAGCACCA
ACTCCAGCACTAATGCCACTACCACTGCTAGCACCAACTCCAGCACTAATGCCACTACCACTGAAAGTACC
AACGCTAG

CTACTACCACTGCTAGCACCAACTCCAGCACTAATGCCACTACCACTGCTAGCACCAACTCCAGCACTAAT
GCCACTACCACTGAAAGTACCAACGCTAGTGCCAAGGAGGACGCCAATAAAGATGGCAATGCTGAGGATAA
TAGATTCC

TTCGCTGCCGGTTGGGGTGTTATGGTTTCCCACAGATCTGGTGAAACTGAAGACACTTTCATTGCTGACTT
GGTCGTCGGTTTGAGAACTGGTCAAATCAAGACTGGTGCTCCAGCTAGATCCGAAAGATTGGCTAAATTGA
ACCAATTG

TACTCAATAACAAACGTAAAATACCTGGATCCGACTGAATTGCATCGTTGGATGCAAGAAGGACACACTAC
TACGCTGAGGGAGCCTTTCCAGGTAGTGGATGTGCGAGGCTCAGATTATATGGGGGGCCATATCAAGGACG
GATGGCAC

CAAGTTCAAGTTGATCGACATTAAAGAATTAAGGCACGACAAGAACTGGAACAGTGATAAGAAGGACAACG
GTCCTAAGACCATTCAACAGATTCATGAGGAAGAGGAGAGACAACGTCAATTGAAGAATAATTCAAGATCT
AATTCAAG

GCAGCACCGGTGGAGGATGGGATGATGTTACCGGAAGCGGTTCTACCACCTCTCCAGTCCTTGTGGGATGG
ACCGTCAACAGTCTTTTGAGTAGCAGTCAAAGAGTGGACAGTGGTCATCAAACCTTCTTCAATACCGAAAG
CATCGTTG

TGCTTCCTCTACGGAAATAGAACCTCTGATGAAAATCAATTTGTTCTGTTCCACCAAATTTGACAGAGGAG
TGTGTTGCCACTCCTGCCTATGAACAGATAACAGATTCTGTGAATTCAAGGTGGACAAGGAGTTGCCATTA
TTGTTTGA

CTATATTAATCAAGTTATAAAGCCTTCTTGGCGGCATCAGCAGGGGAAACCTTGTTTTCTCTGTTGAATCT
TGGTCTTTGAGGTGGAGCTCTGTTCTTTCTGTCTTCTCTGGATCTGACTCTGACAATTCTGGCGTGAATAG
CACAAGAA

AGCTGCAGCGTTAACAACGCCGGAAAAGGCTGATATGGGGGATGGAGTTGCAGGAGCATCTGTCTGCATTG
CATTCTGGGTCCCTAGTTGTGGCTGAAGTGGTTGAAGTTGAGGTTGCTGTTGAGGTTGCTGTTGAGGTTGC
TGTTGAGG

CCAGCACTAATGCTACTACCACTGCCAGCACCAACGTCAGGACTAGTGCTACTACCACTGCCAGCATCAAC
GTCAGGACTAGTGCGACTACCACTGAAAGTACCAACTCCAGCACTAATGCTACTACCACTGCCAGCACCAA
CGTCAGGA

GGTAGATTCGGTGATTCGTCTTCTCCTGCGTTCGGTGACTTGGATCTGTGTGGTTCAGACTTGATCAGGCA
ATCAGCGAACAAATGTCTCGAATTATGGTCCACCCCTACTTCCATCAACGACGTCGCCTTCTTGGTCATCA
ACTACTTG

GACGACCGTTTTAGAACCATCAACGACTAACAGTTCCAGTACGTTTAGTCTGGTCACTTCAAGTGACAACA
ATTGGTGGATTCCAACTGAGTTAATCACGCAGGCACCAGAAGCTGCATCCACTGCATCTTCTACCGTTGGA
GGAACACA

TGGAGGATGGGATGATGTTACCGGAAGCGGTTCTACCACCTCTCCAGTCCTTGTGGGATGGACCGTCAACA
GTCTTTTGAGTAGCAGTCAAAGAGTGGACAGTGGTCATCAAACCTTCTTCAATACCGAAAGCATCGTTGAT
AACCTTGG

TTTCTTCCACGACCCTAAATTCATTCCAAACAAACCATACAGACAATTCAACACATGGTGTGGTGAACCTG
CAAGAATGATCATTGCAGGTGCCATTGGACAGGAAATCTCCGACAAGAAGTTGACTGAACAATGTTCAAGA
GTAGGTGA

GAAGTATCATGCTCGAATGGTGAAAACATACCTGTAGAGTTGGAACCACCCCCAACACATGCAACAACTGC
GTCAGGTAATTTACCATTGTTCATGGCAGCAAACTGTTCCTTGGTTTCTTTACCAATGACACTTTGGAAAG
TTCTAACC

GTGCAGGATATTTCTTCCACGACCCTAAATTCATTCCAAACAAACCATACAGACAATTCAACACATGGTGT
GGTGAACCTGCAAGAATGATCATTGCAGGTGCCATTGGACAGGAAATCTCCGACAAGAAGTTGACTGAACA
ATGTTCAA

CACAGGCACAGGCACAGGCTCAAGTTCAGGCTCAGGCTCAGGCTCAGGCCCAGGCTCAGGCTCAAGCACAG
CAGATCCAGATGCAACAGCTTCAGATGCAGCAACAGCAACAGGCGCCATTATCTTTTCAGCAAATGTCGCA
AGGAGGAA

CCAGCATCAACGTCAGGACTAGTGCGACTACCACTGAAAGTACCAACTCCAGCACTAATGCTACTACCACT
GCCAGCACCAACGTCAGGACTAGTGCTACTACCACTGCCAGCATCAACGTCAGGACTAGTGCGACTACCAC
TGAAAGTA

AGTGCGACTACCACTGAAAGTACCAACTCCAGCACTAATGCTACTACCACTGCCAGCACCAACGTCAGGAC
TAGTGCTACTACCACTGCCAGCATCAACGTCAGGACTAGTGCGACTACCACTGAAAGTACCAACTCCAACA
CTAGTGCT

AGTAACCGTATTCGTTGTCGTACCAGGAGACCAACTTGACGAACTTTGGAGACAATTGGATACCAGCGGAA
GCATCGAAGATGGAAGAGTGAGAGTCACCCAAGAAGTCAGAGGAGACAACAGCGTCTTCGGTGTAACCCAA
AACACCCT

AAGACTTGACGGTCTCAGTCTTAGTTAACTTAAGTTGATTTGCACGAGAGTTGGTTTTTGAGGGGACATAC
TGTTGAGGTTGTTGGCCCTCAACGTCCACCATGGAAGTGTTTTGTGAGTTGAATGAAACAACAGATTCTTG
ATCACTCA

TAGAAGTACTGGTGGTAGTGGTTGTCGTAGTTGCAGTGTCAGCAGTTGCTTCAGTGGCCTCAGATGTGGTA
GTAGAAGTAACGTCCCCTTCATCTGATGAAGACGATGACGATGACGATGACGATGACGATGACGATGATGA
TGTAGAAG

CCCCAACCTGGGTACCGTCATCCAGAACCACACCTTTGATGACCTTGTGGCCACATCCGGGGTCATACAAG
ATCAAGTCAGCAACTTTGTTCTGCAGCAGAGACAAGTTGGGGTGTGCTTTCTTGTCGGAAAGTTCCCTTTG
CATGTATT

CAAGCCTTCTTTCAAATACGCCAGACAATTGATTTCTAACGAAGGTGTCTTGGTGGGTGGTTCTTCCGGTT
CTGCCTTCACTGCGGTTGTGAAATACTGTGAAGACCACCCTGAACTGACTGAAGATGATGTCATTGTTGCC
ATATTCCC

AATCTGATAGAATAACCAACTTCTTCGCCCAACTTGACATCCATTTCCTCAGCAACTCTCTGTGCAACAGA
CATTGCTGCGACACGACGAGGTTGAGTACATGCAACTTGAGTGTTCTCTAAGTGTGGCATCTCATCGAATA
AAACGAAT

CTAGCGATGATGAAGAGGGAGATATTATTGCCCAGACGACAGAGCCCAAGCAAGATGCCAGTCCTGATGAT
GATCGCTCAGGCCATTCTTCGCCCAGAGAGGAGGGACAACAGCAAATACGTGCCAAAGAAGCTTCGGGAGG
TCCAAGTG

GTTGTCCGTTATCCCAATCAGAAGAGGTTACTGGGGTACCAACTTGGGTCAACCACATTCTTTGGCCACCA
AGACCACTGGTAAGTGTGGTTCCGTCACTGTTAGATTGATCCCAGCCCCAAGAGGTTCTGGTATCGTCGCT
TCTCCAGC

TACTGACACGGACGATAACAACGAAGGAGATGGCTCTGACAATCACGAGGAAGGTGGAGAAGAAGGAAGTA
GAGGAGCTGATGCTGATGTGTCCAGTGCACAGCAGCGTGCCGAAAGGGTTGCGGACCCATGGATATATCAA
AGATCTAG

TAACATCTTAACTCTTAGACCCAAGTGTCTAGCTTCGCTAACAGTTTGAGCAGTATCGTCTCTTGGTGGAT
CCATACATGGCATAACACCCAAGATTTCCCAGTGACCTTCACCTCTCTTTCTAGCAACACCTAAAGCACGG
AACCCTCT

CGCCACAATGCCTTGAATAAGTGAACCGTAAAGGGTTATACAGCCTGAGCTTGGCGTTGCAGCCACCAGCC
CAGAAATGATACCGGAGCACAGACCAACTGTCGACCATTTCTTCTCCGATCTGTAATCCAGAAGACACCAC
GTCATCCC

GAGGAAGATTGAGTAGATGTAGCAGCAGAGCTGGAGGCTGGAGATGCGGCAGCAGAAACTTGTGAGTTGTT
CTCAGAAGAGGAAGCAGCAGCGGTAGCTACAGAAGCAGCAGACGATGTTGTACTGGTAGCGGCAGCTGGAG
CGGAGTTG

CCTCCTCAGTTGGAGAATGTTATCTTGAATAAATACTATGCCACGCAAGACCAGTTCAACGAGAACAACTC
AGGCGCTCTGCCCATTCCGAACCATGTGGTGCTTAACCACTTGGTTACTAGTAGCATTAAGCACAATACAC
TTTGTGTA

AATATCTGTTAACGATGGTGTAATGAGAGTAGTATGCGCACCAGTCCTAGATGACGCAGCTGCAGCTTCGC
AGCCTGCATGTCCAGCACCGATGACCACGACCTGTGTCTTGGTTGTGGGTTGGAAGCTTGTCAAAGAAGAT
ATGGTCAA

TTCGTAAAGGCGTTTGTCTCTAGTTTGCGATAGTGTAGATACCGTCCTTGGATAGAGCACTGGAGATGGCT
GGCTTTAATCTGCTGGAGTACCATGGAACACCGGTGATCATTCTGGTCACTTGGTCTGGGGCAATACCAGT
CAACATGG

TTTGAAACGTCGTTCGAAGAATTTGTCGAAGATAAACGATTCATTGCTCTAGAGGTATCTGATAATGATGA
CGATTGTGACACTGATTTGACTGCTGACACAGCTGACGAGCTGGAAAGCTCTGCTATCTTAAAGATGAGGG
AGTCTGAT

CAATGTAACGCTTGTGGTCTCTGTTAGAAGAGTCATCTGGGACATCTTCGTCCAAGAACTCAAAGGTTCTC
CAGGAGTCAAATCTGTGCCAGAAAGCATAGAATTGTTCAACTTCCTTCTTGGAAGAATCTTTGTTACCTAG
AGAAGGAA

CTAGTAGAATCAAGTTCAAGTTGATCGACATTAAAGAATTAAGGCACGACAAGAACTGGAACAGTGATAAG
AAGGACAACGGTCCTAAGACCATTCAACAGATTCATGAGGAAGAGGAGAGACAACGTCAATTGAAGAATAA
TTCAAGAT

CCCTTGGTGACACCAGTGATCATGTTGTCAACCAAAGACTTGACGGTTCTCAAAGCAGCAACGTGCTTTCT
GCCACCGTTGTGAACAGCAACCTTGATCAATTGGTTGTTGACCTTGGTGAAGGTAACATCAATGTGCTTCA
AGTTCTTG

GACTAGTGCTACTACCACTGCCAGCATCAACGTCAGGACTAGTGCGACTACCACTGAAAGTACCAACTCCA
GCACTAATGCTACTACCACTGCCAGCACCAACGTCAGGACTAGTGCTACTACCACTGCCAGCATCAACGTC
AGGACTAG

GGTAAACATTACAAAATTCTCCAAATTCGAAAGAAGTAAATCAAGCCACAAGAATGAAAGGCGTGTTCTTG
AGATACAAACCTCCCTGGTGAGGATGTTTGAGAAGAATGTCATGCTGAATATCTACCCCAGAACAGTTATC
GATATCGA

GCTGGTATCATTATTGCCAAGTTGTCCGTTATCCCAATCAGAAGAGGTTACTGGGGTACCAACTTGGGTCA
ACCACATTCTTTGGCCACCAAGACCACTGGTAAGTGTGGTTCCGTCACTGTTAGATTGATCCCAGCCCCAA
GAGGTTCT

TATGTTTCACCTATATTAATCAAGTTATAAAGCCTTCTTGGCGGCATCAGCAGGGGAAACCTTGTTTTCTC
TGTTGAATCTTGGTCTTTGAGGTGGAGCTCTGTTCTTTCTGTCTTCTCTGGATCTGACTCTGACAATTCTG
GCGTGAAT

CTGTCAGAAGACGATGAAGAAGAGGATTCAGAATCAGAGGATGAAGATGAAGATGAAGATTCAGATTCAGA
CTCAGACTCAGATTCAGATTCAGATGAAGATGAAGATGAGGATGAAGATTCGGAGGAAGAGGAAGAGACGG
CCTTAGCT

CGGTTTCTTATACAACTTCAAGTTCGTCCGTTTCTCAAGTTTCAGACACGTCAGTACCTTCTACAAGTTCC
AGATCGTCCGTTTCTCAAGTCTCAGACACTCCGGTGCCTTCTACAAGTTCAAGGTCGTCCGTTTCTCAAAC
ATCTAGCT

AACAAGTACTCAAACAGTGGTTGAAACAATTACTTTGGACCAGGAAGAGGCTAGCGCTGTTGCTAATCATG
CGCACGACGATGAGGCTTCCACAGACGTTGAAGGCTCCACAGACGTCAATGTCAATGAACAGGCTCTGTTG
CAAGAGGA

CAAGTCGACAACTCTGGTAGAGTAACCGTATTCGTTGTCGTACCAGGAGACCAACTTGACGAACTTTGGAG
ACAATTGGATACCAGCGGAAGCATCGAAGATGGAAGAGTGAGAGTCACCCAAGAAGTCAGAGGAGACAACA
GCGTCTTC

TCATTCCAAACAAACCATACAGACAATTCAACACATGGTGTGGTGAACCTGCAAGAATGATCATTGCAGGT
GCCATTGGACAGGAAATCTCCGACAAGAAGTTGACTGAACAATGTTCAAGAGTAGGTGATTATTTGTTCAA
GAAATTGG

CGAGCATACCATTCACTCCAATCATCTCTGTCCGGATCTGCCTCAATGCCGATGACCAAACCTGGCTGGTG
TGATATCTCATCACTTGAGAGAAGTGAGGGCTTTGAGTTCTTATAAGGAATAGGTTTATTCAAATCTATTA
TGTTGTTT

CAGGAAGAGGCTAGCGCTGTTGCTAATCATGCGCACGACGATGAGGCTTCCACAGACGTTGAAGGCTCCAC
AGACGTCAATGTCAATGAACAGGCTCTGTTGCAAGAGGATTTTGACATGTGGAGTGAGACTATATTGCAGA
AAACACAA

TTCATTACTATTATACATGTGATATCAGAACGGAAGGTTTTACTCAACTTGACTGCCATCCCTCGGTTTGT
AGTGAGGCAACAGCTCCACAACCTCAGCTGGTAGTCTACCATCGACCTTGATGGCGTACATACCAGCTATA
CTATGATC

GAGATCTATTTTTACTCAGCGGTAATATTAGATCATGGCTAGGGGGATCTGGAAGTACAATGATGTGCTCT
CCCCCTCTCAAACACAACACCAGGATGAACTAAGGGCTCATCTCGAAAGTCGAAGGTGCCTCATTCAGGTT
ATTAGTGG

TCAAGACTCTTTCGCTGCCGGTTGGGGTGTTATGGTTTCCCACAGATCTGGTGAAACTGAAGACACTTTCA
TTGCTGACTTGGTCGTCGGTTTGAGAACTGGTCAAATCAAGACTGGTGCTCCAGCTAGATCCGAAAGATTG
GCTAAATT

GGTGATGCTATTGGTTACGATCCAACTGCTCCTGTCGAAGAAGGCGAAGAAGAAGAAAGTGAAAGTGAAAG
TGAAAGTGAAGATCAAGTTGAGGAGGAAGATCAAGAAGTTGTTGCTGGAGAAGAAGATGATGACGATGACG
AAGAGCTA

CTTCTTCTTTCTGGAAGCAGCCAAACAAGCAGTCAACATCAAGTCGTCTGGAGAAACACCTTCAACAGTGT
ATGGTTCGTGCAAGGACAACTTGTTCTTGGTCAAAGTACCGGTTTTGTCGGAACACAAGATTTCGACACCA
GCCAAGGA

TACTGGATTGACGTATCCAGCTTGGGGATTTAAACAAGCTGAAATCGTCTGCAAAGACTCCTGGAGTCGTA
GGGACAAATCGACCTGGTGGAATGTAGAAGATCACTGGAACCACAATTTAGAAACCGCAGAAGATTACGTT
CCACCAAA

AGAGTCCGCTGAGGATGAATCAGTAAATGTATTACCTGACTCAGGTGATGGAGTGCTCAGAGGCGTTCCAA
CTGATGATGGATACTGCGGAAACTGTGATTGTGGCCCAGGTGGAAAGTACATAGGCGACATTTGATAAGGT
GTATACGG

CGCTATCTTCTTCTTTTCAAACGTGATGGGAGATGTCACGGTGTCAGCACCGCTTTCCTCATCACCTTTCC
TGCCACGGTAGTCATGGTTCCTTGGTCCCAGGGGAACTCCTCTGCCGTCTCGCTCATCACCGTCCTCCTCC
TCTTCCTT

CCACCGAAAGTACCGACTCCAACACTAGTGCTACTACCACTGCTAGCACCAACTCCAGCACTAATGCCACT
ACCACTGCTAGCACCAACTCCAGCACTAATGCCACTACCACTGAAAGTACCAACGCTAGTGCCAAGGAGGA
CGCCAATA

GTAGACTCTTTGGAATGAAAGGTTGGACATCCTTAATCATCCAACTCTTGATATCAGCTGGGACCTTTGGA
GAGTTGTAAGCACAAAGGATTCTTGTTTCTTCTGGACTGATTTGGTAAACCAAGATTGGCATATGATCACT
ACCAAGAA

AGTTCGGAAAGTTCAAGCTCTTTGATTTCCTCTTCAATTTCAACATCCGTTTCGACTTCTTCAGTGTACGT
TCCCTCCTCTTCAACTTCATCTCCACCTTCGTCCTCATCCGAATTGACATCATCCTCGTACTCATCATCCT
CATCCTCA

GTCACCTCCATGGAATTGTCATCATTTTTGTTGATGGTCCTTTCTTCTGTCGTTGCAACTTGGGGTGACCA
GCAAGCTGTGGCTGCCAAGGCTGCTTCATTGGCTGAAGGAGCAGCCGGTGCTGTTGCCTCCTTTAACCCAG
GTTATTTC

TTGATCGACATTAAAGAATTAAGGCACGACAAGAACTGGAACAGTGATAAGAAGGACAACGGTCCTAAGAC
CATTCAACAGATTCATGAGGAAGAGGAGAGACAACGTCAATTGAAGAATAATTCAAGATCTAATTCAAGAC
GTACGAAC

GCAGTCTTCTTCTTGTCTCTTGCGGCCTTGTTCTTTCTTTCAATGTAACGCTTGTGGTCTCTGTTAGAAGA
GTCATCTGGGACATCTTCGTCCAAGAACTCAAAGGTTCTCCAGGAGTCAAATCTGTGCCAGAAAGCATAGA
ATTGTTCA

TCTTCCTTCTTGGTTTCAGCTTCACTTTCGCTGTCAGAAGACGATGAAGAAGAGGATTCAGAATCAGAGGA
TGAAGATGAAGATGAAGATTCAGATTCAGACTCAGACTCAGATTCAGATTCAGATGAAGATGAAGATGAGG
ATGAAGAT

CAACCATGGTCCATCATGCCAGATTTGTACTTCTACAGAGACCCTGAAGAAGTTGAACAACAAGTTGCTGA
AGAAGCTACCACCGAAGAAGCTGGTGAAGAAGAAGCCAAGGAAGAGGTTACCGAAGAGCAAGCTGAAGCTA
CTGAATGG

GCACTATTTTCGACAGCATTAGTAGTAGAAGTGCTGGTAGTAGAAGTACTGGTGGTAGTGGTTGTCGTAGT
TGCAGTGTCAGCAGTTGCTTCAGTGGCCTCAGATGTGGTAGTAGAAGTAACGTCCCCTTCATCTGATGAAG
ACGATGAC

GAAGAGGTTACTGGGGTACCAACTTGGGTCAACCACATTCTTTGGCCACCAAGACCACTGGTAAGTGTGGT
TCCGTCACTGTTAGATTGATCCCAGCCCCAAGAGGTTCTGGTATCGTCGCTTCTCCAGCTGTCAAAAAGTT
GTTGCAAT

TGATAGTCACTCACTTGGCTTCATCATCCAATGTATTACGCAACATTGCTGCTCAGACCTTAGGTGATCTT
GTCCGTCGTGTAGGTGGTAATGCTTTGTCCCAACTGTTACCAAGTTTGGAGGAATCTTTGATAGAAACATC
AAACTCAG

AGTGACCTAGGCCACTTCGACAATTTCATCTTCTTCGTCGTCATCTTCTTCTTCTTCACTCTCCTCCTCTT
CTTCCTCTTCCGCATTCTTTGATGGCATCTCTTCCACTGCTCCCGCACCTGGCTCGCCTCTCATTAACATA
TTAACAAT

ACCTGATCTGCCCCAACCTGGGTACCGTCATCCAGAACCACACCTTTGATGACCTTGTGGCCACATCCGGG
GTCATACAAGATCAAGTCAGCAACTTTGTTCTGCAGCAGAGACAAGTTGGGGTGTGCTTTCTTGTCGGAAA
GTTCCCTT

TTATGTTCATGCCCTTGCTCATCAACTTGGTGGTTTCTACCACTTGCCTCATGGTGTTTGTAACGCTGTCT
TGTTGCCTCATGTTCAAGAGGCCAACATGCAATGTCCAAAGGCCAAGAAGAGATTAGGTGAAATTGCTTTG
CATTTCGG

TACCGACTCCAACACTAGTGCTACTACCACTGCTAGCACCAACTCCAGCACTAATGCCACTACCACTGCTA
GCACCAACTCCAGCACTAATGCCACTACCACTGAAAGTACCAACGCTAGTGCCAAGGAGGACGCCAATAAA
GATGGCAA

TTTCTTGCTTCTTAACAAATCACCATTCTTGTTATTAAAAACACTGAAGCCCAGTGATTGCCTCCCACGCT
CGTGACTGGTTCCTCCGAACCTGCTGGAGTCTGTTTCTTCTTGCAACACGTCCATTACATGGATTTCAAAA
ACAACCTC

TGGATAGTCCATTGACAAAACTGGATCCTACGAGCATACCATTCACTCCAATCATCTCTGTCCGGATCTGC
CTCAATGCCGATGACCAAACCTGGCTGGTGTGATATCTCATCACTTGAGAGAAGTGAGGGCTTTGAGTTCT
TATAAGGA

ATATCTGTTTATATGACTGAAAATATCTACATAGATTCAGAAGCGTCCAAGGATGTTGACAACAAGTGCTG
TCCAGCCTGTGAAATGCTCAGCACCAGTACCATGACCATCTATCGGACTGTAATTTTCATAACAATAACCT
TGTTCTTC

GTTCAAGCTCTTTGATTTCCTCTTCAATTTCAACATCCGTTTCGACTTCTTCAGTGTACGTTCCCTCCTCT
TCAACTTCATCTCCACCTTCGTCCTCATCCGAATTGACATCATCCTCGTACTCATCATCCTCATCCTCATC
CACCCTCT

ACCTCTGACACCAGAGGCAAAGCTGCTGACGTCTTCGCCGGAAGAACTAGAGGAAGAAGCTTGGGAGGAAG
AGGAAGATTGAGTAGATGTAGCAGCAGAGCTGGAGGCTGGAGATGCGGCAGCAGAAACTTGTGAGTTGTTC
TCAGAAGA

CTGCAACAACTCCGGCTATCCCTACTAAATCTGATGAAGCTGAAGCTGAAGTTGAGGCTGAAGCAGGTGAT
GCTGGAACAAAGATTGGACTTGAAGCTGAAATCGAAACTACAACTGATGAAACTGATGATGGTACAAACAC
CGTTTCTC

GGAAGAGGCACATTCATTGCCTGGGATTTGCCTACTGGTGAGAAGAGAGACTTACTATTGAAGAAATTGAA
GTTGAATGGTTGCAACGTTGGTGGATGTGCAGTCCATGCAGTGAGATTAAGACCTTCATTAACATTCGAGG
AGAAGCAT

TACCAACTCCAGCACTAATGCTACTACCACTGCCAGCACCAACGTCAGGACTAGTGCTACTACCACTGCCA
GCATCAACGTCAGGACTAGTGCGACTACCACTGAAAGTACCAACTCCAACACTAGTGCTACTACCACCGAA
AGTACCGA

AGCTTAGAAGGGGAAGTGGATACTGACACGGACGATAACAACGAAGGAGATGGCTCTGACAATCACGAGGA
AGGTGGAGAAGAAGGAAGTAGAGGAGCTGATGCTGATGTGTCCAGTGCACAGCAGCGTGCCGAAAGGGTTG
CGGACCCA

CTACTACCACTGCCAGCACCAACGTCAGGACTAGTGCTACTACCACTGCCAGCATCAACGTCAGGACTAGT
GCGACTACCACTGAAAGTACCAACTCCAACACTAGTGCTACTACCACCGAAAGTACCGACTCCAACACTAG
TGCTACTA

TGCTTGACCAGCGTGTTGAATGCTTCCTCTACGGAAATAGAACCTCTGATGAAAATCAATTTGTTCTGTTC
CACCAAATTTGACAGAGGAGTGTGTTGCCACTCCTGCCTATGAACAGATAACAGATTCTGTGAATTCAAGG
TGGACAAG

GTACTTGTAACCCTTGGTGACACCAGTGATCATGTTGTCAACCAAAGACTTGACGGTTCTCAAAGCAGCAA
CGTGCTTTCTGCCACCGTTGTGAACAGCAACCTTGATCAATTGGTTGTTGACCTTGGTGAAGGTAACATCA
ATGTGCTT

ATCTGGTTACATATCTGTTTATATGACTGAAAATATCTACATAGATTCAGAAGCGTCCAAGGATGTTGACA
ACAAGTGCTGTCCAGCCTGTGAAATGCTCAGCACCAGTACCATGACCATCTATCGGACTGTAATTTTCATA
ACAATAAC

AATCTTCAGTATGAGTTGACGGAGGTGTGGAATCGGTTGGACTCACAGCTTTTGAAAGGACATTTCTCGGT
TGCTCAGGATGTAGTTCAATGTCGGATTGGAAGTCATGGTCAGATTCTATGTTAAGATCATTGGATTCTTG
GATCTCAT

AAAGTACCGACTCCAACACTAGTGCTACTACCACCGAAAGTACCGACTCCAACACTAGTGCTACTACCACT
GCTAGCACCAACTCCAGCACTAATGCCACTACCACTGCTAGCACCAACTCCAGCACTAATGCCACTACCAC
TGAAAGTA

TTCACTTTCGCTGTCAGAAGACGATGAAGAAGAGGATTCAGAATCAGAGGATGAAGATGAAGATGAAGATT
CAGATTCAGACTCAGACTCAGATTCAGATTCAGATGAAGATGAAGATGAGGATGAAGATTCGGAGGAAGAG
GAAGAGAC

TGTATGATATAAGCAAATCAAGTTCATACGAAAATTGCAACCACTGGCTTACCGAACTGAGAGAGAACGCA
GATGACAACGTGGCTGTTGGGTTGATAGGGAACAAATCAGATCTGGCTCATTTAAGAGCAGTACCAACCGA
TGAAGCGA

ACTGCCAGCACCAACGTCAGGACTAGTGCTACTACCACTGCCAGCATCAACGTCAGGACTAGTGCGACTAC
CACTGAAAGTACCAACTCCAGCACTAATGCTACTACCACTGCCAGCACCAACGTCAGGACTAGTGCTACTA
CCACTGCC

TCCACTAAGGTTAATTCTCAACAAGAGACAACACCTGGGACATCAGCTGTTCCAGAGAACCATCATCATGT
CTCTCCTCAACCTGCTTCAGTACCACCTCCACAGAATGGACAGTACCAACAGCACGGCATGATGACCCCAA
ACAAAGCT

CGTCAGGACTAGTGCGACTACCACTGAAAGTACCAACTCCAGCACTAATGCTACTACCACTGCCAGCACCA
ACGTCAGGACTAGTGCTACTACCACTGCCAGCATCAACGTCAGGACTAGTGCGACTACCACTGAAAGTACC
AACTCCAA

CTATCTTTTACCGACGCAAACAGCAGTTCTGCTTCCGCTCCATTGGAAGTGGCAACGTCTACGCCAACCCC
ATCTTCAAAGGCATCCTCTCTGTTGCTTACACCATCAACATCCTCTTTAAGTCAGGTTGCTACAAATACTA
ATGTACAG

TGGTAGTACTGAGAATAGAGCCTCACAGGATATGTATCTCTATGAAGCGATGCCACCAACGCTGCCCCACA
GGGATTGGAAGGACTATTTTGTGATGGCTACTGCCACAGCTGGGCTGTTGTATGGTGCATATGAAGTAACT
AGAAGGTA

ACAGTCAAACATTGATGAATCTGCAACAACTCCGGCTATCCCTACTAAATCTGATGAAGCTGAAGCTGAAG
TTGAGGCTGAAGCAGGTGATGCTGGAACAAAGATTGGACTTGAAGCTGAAATCGAAACTACAACTGATGAA
ACTGATGA

TTGTTGAAGGTCAACCAAATCGGTACCTTGTCTGAATCCATCAAGGCTGCTCAAGACTCTTTCGCTGCCGG
TTGGGGTGTTATGGTTTCCCACAGATCTGGTGAAACTGAAGACACTTTCATTGCTGACTTGGTCGTCGGTT
TGAGAACT

TTGTTGTCTAAGAGAAAGTTACAATGGATGGTTGACAAGGACTTGGTCGGAAATTGGGACGATCCAAGGTT
CCCAACTGTCAGGGGTGTGAGAAGAAGAGGTATGACTGTCGAAGGTTTGAGGAACTTCGTCTTATCCCAAG
GTCCATCC

CCAGAGCCGGTCAAAGAACCAGATTTAAGGCTGTTGTCGTTGTTGGTGACTCTAACGGTCACGTTGGTTTG
GGTATCAAGACCGCCAAGGAAGTTGCTGGTGCCATCAGAGCTGGTATCATTATTGCCAAGTTGTCCGTTAT
CCCAATCA

CGTTAGAACCTTGTTGTCTAAGAGAAAGTTACAATGGATGGTTGACAAGGACTTGGTCGGAAATTGGGACG
ATCCAAGGTTCCCAACTGTCAGGGGTGTGAGAAGAAGAGGTATGACTGTCGAAGGTTTGAGGAACTTCGTC
TTATCCCA

TGAAAGAAATCGAGAGGGTTTAGAAGTAGTTTAGGGTCATTTTTCAGTACATTGGTTGGCCTGGCTTGAGT
TGCAACCAGTGCCAAGCATCAGCGTTGGCCTCTCTCTTGTACATTGGTTGGCCTGGTCTCAAATTTAACCA
GTGCCAAG

GGTTATTTCGGTAAGGTTGGTATGAGATACTTCCACAAGCAACAAGCTCATTTCTGGAAGCCAGTCTTGAA
CTTGGACAAATTGTGGACATTGATCCCAGAAGACAAGAGAGACCAATACTTGAAATCTGCTTCTAAGGAAA
CTGCTCCA

TTGGTATTTCTTTGGCCAACCAACAAGTCGTTCAAACTTAAAACTGAACTTGGTCTCTTCAACTGTACGGA
ACTCTGAGTTTGTGCTCTGAGACTTCCATTGTTGTTTGGATTTGCATTGTTCAATCCTTGAAAATCCGTTA
AAGATCGC

ACTACCAACTCCAGCACTAATGCTACTACCACTGCCAGCACCAACGTCAGGACTAGTGCTACTACCACTGC
CAGCATCAACGTCAGGACTAGTGCGACTACCACTGAAAGTACCAACTCCAGCACTAATGCTACTACCACTG
CCAGCACC

GTCCTTGCTCAAGGAATGCGATGACATTGGTACAGCAAACATAGCTCAGGACCGTGGACGACTTCTGGGG
TTGCCACCAGTGACAACTCTTCAACCACGGAAGTTCAAGGAAGAACGAATAACGATTTGCAACAGGGGCAG
ATGCAAAT

TTCAGACACGTCAGTACCTTCTACAAGTTCCAGATCGTCCGTTTCTCAAGTCTCAGACACTCCGGTGCCTT
CTACAAGTTCAAGGTCGTCCGTTTCTCAAACATCTAGCTCACTACAGCCCACCACTACATCCTCCCAACGT
TTCACCAT

ATTCTTCAACATCAAGTGCCGTTCCTCTGGATTGACACCTAATGCTGTGGTCTTGGTTGCTACTGTTAGGG
CATTGAAGTCACACGGTGGTGCTCCAGATGTCAAACCTGGCCAACCTTTACCTTCCGCATACACTGAAGAG
AATATCGA

TGTCTATATCCTCGCTGAATCCATCTAATGCATTATCAAGAGCCCTCGGTATTGCATCAACGAGATTGTTT
GGTGGTGCAAATCAACAGCAGCAACAGCAACAAATCACATCTTCCCCACCGTACAGTCAAACTTTGTTGAA
TTCCCAAC

CCTACTGGTGAGAAGAGAGACTTACTATTGAAGAAATTGAAGTTGAATGGTTGCAACGTTGGTGGATGTGC
AGTCCATGCAGTGAGATTAAGACCTTCATTAACATTCGAGGAGAAGCATGCTGATATCTTTATTGAAGCAT
TAGCCAAA

AGGAAGAGGAATCCTTGGATTCTTCCTTCTTGGTTTCAGCTTCACTTTCGCTGTCAGAAGACGATGAAGAA
GAGGATTCAGAATCAGAGGATGAAGATGAAGATGAAGATTCAGATTCAGACTCAGACTCAGATTCAGATTC
AGATGAAG

TCTTATTATATCTTTAAATATGTACATACGCTTGTTTATACGTGGAGGCGACAAAAAGGGTTGGTTGAAGG
GAGAGAGAAAGAGACGCACAGAGATTGGAGGAGGGGCTCAGTTCGCTGATTGTTTCTTCTTCTGTGTTAGG
TTTTCCAA

TTCGTGTCTTGAAGCACTCGTATAAAGTCTCTTAGTTTCGTCGGAGGCATGTTGCATTTCACCAACTGTCA
TAATCTCCCTGCCATCCTTCACTCTGTTTCTGATGAATTGATTCATTTCTTGATGGAACTCGTGAATACGT
GGTCCATT

GTAGGCGTATAAGAGTGTGAGTGAGCATATGTTTGGGATGAGGACGTTGCTGGCATTGTCGAGGGCCTTTG
GTGGGGTCTTGATTGGTGCCCTTGCGCCAAGTTAGCTGCTGGTCTTGGTGGTCGTGGCGGTTGAGATTGCA
ATCTCTCC

ATATATGTCAGAAATTGGTTGAGCCTTACCTAAAGTTTCAAGTAGTTGAGATACAGTTGGCATCTCAATAT
CGGAAGTGGAAGGTTCTGTCTCCTTCACTGTGTTCTCCTCTGTGGTGATTACGGAATTTATTGTTTCGTCG
TCACTTTT

TGTACTCACACTGATGGTCCGCTACAGATGATCAGACGCAAGGGAAGACCATCGACCACATGTGGCCATTG
TAAAGAGCTGAGAAGAACCAAGAACTTCAACCCATCCGGTGGGTGCATGTGTGCCTCTGCACGACGGCCAG
CTGTTGGC

CCATCTAATGCATTATCAAGAGCCCTCGGTATTGCATCAACGAGATTGTTTGGTGGTGCAAATCAACAGCA
GCAACAGCAACAAATCACATCTTCCCCACCGTACAGTCAAACTTTGTTGAATTCCCAACTTTTTCATGAAC
TTACTGAG

CAGCAGTTCTGCTTCCGCTCCATTGGAAGTGGCAACGTCTACGCCAACCCCATCTTCAAAGGCATCCTCTC
TGTTGCTTACACCATCAACATCCTCTTTAAGTCAGGTTGCTACAAATACTAATGTACAGACGAGTTTAACA
ACGGAATC

TACGACGGTTGACATTTGGAAGGCATTGGTTCCAAATACTCCAAGAGCTGTGAAAGAGATCCTTCCAACAT
TGACTGGTATGATAGTCACTCACTTGGCTTCATCATCCAATGTATTACGCAACATTGCTGCTCAGACCTTA
GGTGATCT

ACTGTCCATTCTGTGGAGGTGGTACTGAAGCAGGTTGAGGAGAGACATGATGATGGTTCTCTGGAACAGCT
GATGTCCCAGGTGTTGTCTCTTGTTGAGAATTAACCTTAGTGGAATCTCTATCAAATTCCGGTAAATTGGA
AGCTGAAA

TACCAGCGGAAGCATCGAAGATGGAAGAGTGAGAGTCACCCAAGAAGTCAGAGGAGACAACAGCGTCTTCG
GTGTAACCCAAAACACCCTTCAACTTACCTTCAGCGGCAGCCTTAACAACCTTCTTGATTTCATCGTAGGT
GGTTTCCT

GCGAATATTGGTTTTAGAAACACGTTTCGAATCTTCAGTATGAGTTGACGGAGGTGTGGAATCGGTTGGAC
TCACAGCTTTTGAAAGGACATTTCTCGGTTGCTCAGGATGTAGTTCAATGTCGGATTGGAAGTCATGGTCA
GATTCTAT

ATACCATCCAGGTTATTTCGGTAAGGTTGGTATGAGATACTTCCACAAGCAACAAGCTCATTTCTGGAAGC
CAGTCTTGAACTTGGACAAATTGTGGACATTGATCCCAGAAGACAAGAGAGACCAATACTTGAAATCTGCT
TCTAAGGA

CTGATGGTCCGCTACAGATGATCAGACGCAAGGGAAGACCATCGACCACATGTGGCCATTGTAAAGAGCTG
AGAAGAACCAAGAACTTCAACCCATCCGGTGGGTGCATGTGTGCCTCTGCACGACGGCCAGCTGTTGGCAG
CAAGGAAG

GGGTGCAAGGATCACTCAAAGTTGCATTTAAATCTGGATTATGTTCACTGGTCCAAAAATCGATGTGTCTC
CAAGCTAAGAGGGTTTCAGAGACACCATCGTTTGACTCCGTGGACGTAAATTCATCAATATTTATGTCTTC
ATTAGATG

TTTAAACACGACTTGAAGATTGTTAAATTAAGCTCCGCTAATGCCGACAATTTCGACTTCCAATCTCCGTT
TGCTGTCTTGTCTGATGATGAAACGTCCCAACATTTCACCACCTACCTAATCTCTCCAGCTGAACATCCAA
CAATTTGT

CCAACGTCAGGACTAGTGCTACTACCACTGCCAGCATCAACGTCAGGACTAGTGCGACTACCACTGAAAGT
ACCAACTCCAGCACTAATGCTACTACCACTGCCAGCACCAACGTCAGGACTAGTGCTACTACCACTGCCAG
CATCAACG

AGCCTTGTATGACATGAAGGACCATCCAATAATCGAGGAACACACAAGAAACGAGGCTTCACAAAACAATT
CCACCTCTAGCCTGTGGAAGTTCTGGAAACGAGAACCTACGACTGCTGTAAACGGAACGCAAGCTGTTGAT
AATAATAC

TATGGACTCGTACTCAATAACAAACGTAAAATACCTGGATCCGACTGAATTGCATCGTTGGATGCAAGAAG
GACACACTACTACGCTGAGGGAGCCTTTCCAGGTAGTGGATGTGCGAGGCTCAGATTATATGGGGGGCCAT
ATCAAGGA

CTTAAACAATATCAAACCGATTGATCCAATGACTATTCCTGGCTTGAAAGAAGCCGGCTGGACTCCCGAGA
TGGATGCGTTGGCACAACGTCCCAAGCGTGGTCCACACGATGCAGCAATACAGAATATACTCACAGAGCTA
CAAAATCA

TTCATACTCAGTTGCCATCCATGGCTTCTTTAGGTTACTTCAATCAGCCAAGTTCTACTTACTATGCTCCT
CCTGCACCACTTCAACAGCACCAGCAACCACCTATCCTTCCCCCTCCGGGCCTAATGTACACTAGTAACAA
TAACAGCA

ACTTCTGGTATCGTACGTGCTACGACGGTTGACATTTGGAAGGCATTGGTTCCAAATACTCCAAGAGCTGT
GAAAGAGATCCTTCCAACATTGACTGGTATGATAGTCACTCACTTGGCTTCATCATCCAATGTATTACGCA
ACATTGCT

TACCAATGCGACTACCAACTCCAGCACTAATGCTACTACCACTGCCAGCACCAACGTCAGGACTAGTGCTA
CTACCACTGCCAGCATCAACGTCAGGACTAGTGCGACTACCACTGAAAGTACCAACTCCAGCACTAATGCT
ACTACCAC

TAATTCTACAGCTATCACAGCAGATCTAGGTTCATCCGAAGCAGAGAACCTACTGCAGTTGAAGACAGGCT
TAGCTGCCATAGTCTCTACTGTCATTGAGGAATTCACACTGTTTATGGATATCGCTGAGAGAATCGCCGTG
TTACATCA

ATCCACAACTCTATCTTTTACCGACGCAAACAGCAGTTCTGCTTCCGCTCCATTGGAAGTGGCAACGTCTA
CGCCAACCCCATCTTCAAAGGCATCCTCTCTGTTGCTTACACCATCAACATCCTCTTTAAGTCAGGTTGCT
ACAAATAC

AATGAGATCCAAGAATCCAATGATCTTAACATAGAATCTGACCATGACTTCCAATCCGACATTGAACTACA
TCCTGAGCAACCGAGAAATGTCCTTTCAAAAGCTGTGAGTCCAACCGATTCCACACCTCCGTCAACTCATA
CTGAAGAT

GCACTGAGAAACGTACCTGTAGTGATTATGACCTGATCTGCCCCAACCTGGGTACCGTCATCCAGAACCAC
ACCTTTGATGACCTTGTGGCCACATCCGGGGTCATACAAGATCAAGTCAGCAACTTTGTTCTGCAGCAGAG
ACAAGTTG

TTGGGTGACCTGGTTTGGCTTGGATAGTCCATTGACAAAACTGGATCCTACGAGCATACCATTCACTCCAA
TCATCTCTGTCCGGATCTGCCTCAATGCCGATGACCAAACCTGGCTGGTGTGATATCTCATCACTTGAGAG
AAGTGAGG

GAATCCAACTCAGAGGCAGTTGAAGCCGAAAAAAAAGAAGAGGAAGCTCCCCAGCAACAACAATCAGAACA
ACAGCCGGAGCAAGGAGAGGCAGTGCCGGAGCCTGTGGAAGAAGAGAGTTAACCACCTCATAGAAAGACAA
ATAAAGAA

TGCCATCAGAGCTGGTATCATTATTGCCAAGTTGTCCGTTATCCCAATCAGAAGAGGTTACTGGGGTACCA
ACTTGGGTCAACCACATTCTTTGGCCACCAAGACCACTGGTAAGTGTGGTTCCGTCACTGTTAGATTGATC
CCAGCCCC

TCATGCACTGCTGTGGGTACGGCCCATTCTGTGGAGGTGGTACTGAAGCAGGTTGAGGAGAGGCATGATGG
GGGTTCTCTGGAACAGCTGATGAAGCAGGTGTTGTTGTCTGTTGAGAGTTAGCCTTAGTGGAAGCCTTATC
ATATTCTT

GTTGGGGTGTTATGGTTTCCCACAGATCTGGTGAAACTGAAGACACTTTCATTGCTGACTTGGTCGTCGGT
TTGAGAACTGGTCAAATCAAGACTGGTGCTCCAGCTAGATCCGAAAGATTGGCTAAATTGAACCAATTGTT
GAGAATCG

TCCGGCTATCCCTACTAAATCTGATGAAGCTGAAGCTGAAGTTGAGGCTGAAGCAGGTGATGCTGGAACAA
AGATTGGACTTGAAGCTGAAATCGAAACTACAACTGATGAAACTGATGATGGTACAAACACCGTTTCTCAC
ATATTGAA

TACCGGAATTTGATAGAGATTCCACTAAGGTTAATTCTCAACAAGAGACAACACCTGGGACATCAGCTGTT
CCAGAGAACCATCATCATGTCTCTCCTCAACCTGCTTCAGTACCACCTCCACAGAATGGACAGTACCAACA
GCACGGCA

TTCAACAATGCTTCTCTAGGTTATGTTCATGCCCTTGCTCATCAACTTGGTGGTTTCTACCACTTGCCTCA
TGGTGTTTGTAACGCTGTCTTGTTGCCTCATGTTCAAGAGGCCAACATGCAATGTCCAAAGGCCAAGAAGA
GATTAGGT

GAGTAGATGTAGCAGCAGAGCTGGAGGCTGGAGATGCGGCAGCAGAAACTTGTGAGTTGTTCTCAGAAGAG
GAAGCAGCAGCGGTAGCTACAGAAGCAGCAGACGATGTTGTACTGGTAGCGGCAGCTGGAGCGGAGTTGAC
AACAGCAT

ATCTTCAGTATGAGTTGACGGAGGTGTGGAATCGGTTGGACTCACAGCTTTTGAAAGGACATTTCTCGGTT
GCTCAGGATGTAGTTCAATGTCGGATTGGAAGTCATGGTCAGATTCTATGTTAAGATCATTGGATTCTTGG
ATCTCATT

GACGATATTCCAACCACCAAAGAAGCTTACTGGTACAGGCTGAAGTTTGATGCTTGGTTTCCTCAAAAGAC
TGCGGCAGACACTGTCATGAGATGGATTCCAAAGGCCGATTGGGGTTGTGCCGAAGATCCTTCAGGTAGAT
ACGCCAAA

CTGTATTATACAATTCTATGCTATGTGAACTGTCCTTGAAAGTGACCTAGGCCACTTCGACAATTTCATCT
TCTTCGTCGTCATCTTCTTCTTCTTCACTCTCCTCCTCTTCTTCCTCTTCCGCATTCTTTGATGGCATCTC
TTCCACTG

TTCAATTTCAAGCCTTGTATGACATGAAGGACCATCCAATAATCGAGGAACACACAAGAAACGAGGCTTCA
CAAAACAATTCCACCTCTAGCCTGTGGAAGTTCTGGAAACGAGAACCTACGACTGCTGTAAACGGAACGCA
AGCTGTTG

CTTCTTCGTCGGAAGAAGAGTCAGAGGAAGAGGAAGAGGAATCCTTGGATTCTTCCTTCTTGGTTTCAGCT
TCACTTTCGCTGTCAGAAGACGATGAAGAAGAGGATTCAGAATCAGAGGATGAAGATGAAGATGAAGATTC
AGATTCAG

AAAATTTACTTATGTTTCACCTATATTAATCAAGTTATAAAGCCTTCTTGGCGGCATCAGCAGGGGAAACC
TTGTTTTCTCTGTTGAATCTTGGTCTTTGAGGTGGAGCTCTGTTCTTTCTGTCTTCTCTGGATCTGACTCT
GACAATTC

GCTGAAGCCGTTTTCAACTACGGTGACTTCACCACCATGTTGACTGGTATTGCCCCAGACCAAGTGACCAG
AATGATCACCGGTGTTCCATGGTACTCCAGCAGATTAAAGCCAGCCATCTCCAGTGCTCTATCCAAGGACG
GTATCTAC

AATAACCAACTTCTTCGCCCAACTTGACATCCATTTCCTCAGCAACTCTCTGTGCAACAGACATTGCTGCG
ACACGACGAGGTTGAGTACATGCAACTTGAGTGTTCTCTAAGTGTGGCATCTCATCGAATAAAACGAATTG
AGGAATTT

TTCTCAAGTTTCAGACACACCGGTTTCTTATACAACTTCAAGTTCGTCCGTTTCTCAAGTTTCAGACACGT
CAGTACCTTCTACAAGTTCCAGATCGTCCGTTTCTCAAGTCTCAGACACTCCGGTGCCTTCTACAAGTTCA
AGGTCGTC

ATTGTTATTTCTGTATTATACAATTCTATGCTATGTGAACTGTCCTTGAAAGTGACCTAGGCCACTTCGAC
AATTTCATCTTCTTCGTCGTCATCTTCTTCTTCTTCACTCTCCTCCTCTTCTTCCTCTTCCGCATTCTTTG
ATGGCATC

TTAGTTAACTTAAGTTGATTTGCACGAGAGTTGGTTTTTGAGGGGACATACTGTTGAGGTTGTTGGCCCTC
AACGTCCACCATGGAAGTGTTTTGTGAGTTGAATGAAACAACAGATTCTTGATCACTCATATTTGTTTTGT
GTATTATT

TATAAAGTCTCTTAGTTTCGTCGGAGGCATGTTGCATTTCACCAACTGTCATAATCTCCCTGCCATCCTTC
ACTCTGTTTCTGATGAATTGATTCATTTCTTGATGGAACTCGTGAATACGTGGTCCATTCAATGTGTATGG
ATCACTGG

TCCTTCTTTGGCATACGAATAGATTTAATATTGCTCTTGGCCTTCTCACGCCCCACTTTCAAGAAATTCGA
GTTGTCTGACCTCATGGACATACCTGTGTGGCTCATCGTAACACCAACGGTTTCATCCAAAGTTGTGATCC
TTTCCTTA

TAGCATGCGACCACCCAGTAATCATACTGCTGACGCTATTGGTCACGTGGTTATGGCAGCTGCTGTTGACT
GCGGTGGCGTCCCGTTTCCACACCGTACGTGAGCACATGTCTGGATTGCTAGCTGCGTACATAGTGACAGG
CCTTGTCT

ACTGAGGGGGAGCTGCAGCGTTAACAACGCCGGAAAAGGCTGATATGGGGGATGGAGTTGCAGGAGCATCT
GTCTGCATTGCATTCTGGGTCCCTAGTTGTGGCTGAAGTGGTTGAAGTTGAGGTTGCTGTTGAGGTTGCTG
TTGAGGTT

ACTGAGGTCGCCGAAACCGAACAGTCAAACATTGATGAATCTGCAACAACTCCGGCTATCCCTACTAAATC
TGATGAAGCTGAAGCTGAAGTTGAGGCTGAAGCAGGTGATGCTGGAACAAAGATTGGACTTGAAGCTGAAA
TCGAAACT

ATATGGAACCAGTTGGTGCAGGCCACTATCAGTTCGTAGTGAAGTCAAAGCTGCTTGTTTCATATGCTGTG
CAGCTTCATCAGCTTGACTCTTCGCTGTCAAGGTTGAAATGACCTTATTGAAATAAATCTGTAATTCTTTG
GATAAAAC

TAACTTAACGGAAGTATCATGCTCGAATGGTGAAAACATACCTGTAGAGTTGGAACCACCCCCAACACATG
CAACAACTGCGTCAGGTAATTTACCATTGTTCATGGCAGCAAACTGTTCCTTGGTTTCTTTACCAATGACA
CTTTGGAA

AGTGATTATGACCTGATCTGCCCCAACCTGGGTACCGTCATCCAGAACCACACCTTTGATGACCTTGTGGC
CACATCCGGGGTCATACAAGATCAAGTCAGCAACTTTGTTCTGCAGCAGAGACAAGTTGGGGTGTGCTTTC
TTGTCGGA

AGCTGCCCCACTGCAAACAAGTGAATCAAGCAGTTTTACCACTGCATCAGCAGCTCTACCAGTAAGTTCAA
CAGACGTTGATGGCTCAAGCGCCTCACCTGTAGTGAGCATGAGCGCCGCAGGACAAATAGCTAGCTCAAGC
AGCACAGA

AGTGCTACTACCACCGAAAGTACCGACTCCAACACTAGTGCTACTACCACTGCTAGCACCAACTCCAGCAC
TAATGCCACTACCACTGCTAGCACCAACTCCAGCACTAATGCCACTACCACTGAAAGTACCAACGCTAGTG
CCAAGGAG

GGATTGGGTGATCTTCTTCGACAGTCTTCAAGACGAATAATGGAGCACCCCTTAACACAAACAATTCTTTCA
CCTTCTGGAGATTCGACAACGGCAGTAACCTTCTTGGAGACAGGGTCAAATGGATGGAATTCCAAAACCTT
GTACTTGG

CATCATTTAATGCCAATGTGTCGAGCAAGCAAATTCAGCAGCAGCAGCAGCAGCAGCAGCACAAGTCTCAA
GACACAGGTCTCACGCCGCTGGAGATACAATCACAACAACAAAAACTACGACAACAACAACTACAACAACA
GAAGTTTG

TTGCTCTTGGCCTTCTCACGCCCCACTTTCAAGAAATTCGAGTTGTCTGACCTCATGGACATACCTGTGTG
GCTCATCGTAACACCAACGGTTTCATCCAAAGTTGTGATCCTTTCCTTATTATTAAGCTTGACAATATTTC
TTCTCTGT

TGGTTTCTACCACTTGCCTCATGGTGTTTGTAACGCTGTCTTGTTGCCTCATGTTCAAGAGGCCAACATGC
AATGTCCAAAGGCCAAGAAGAGATTAGGTGAAATTGCTTTGCATTTCGGTGCTTCTCAAGAAGATCCAGAA
GAAACCAT

AAATTCCAAAGTGCAGGATATTTCTTCCACGACCCTAAATTCATTCCAAACAAACCATACAGACAATTCAA
CACATGGTGTGGTGAACCTGCAAGAATGATCATTGCAGGTGCCATTGGACAGGAAATCTCCGACAAGAAGT
TGACTGAA

ATAGAATCTGACCATGACTTCCAATCCGACATTGAACTACATCCTGAGCAACCGAGAAATGTCCTTTCAAA
AGCTGTGAGTCCAACCGATTCCACACCTCCGTCAACTCATACTGAAGATTCGAAACGTGTTTCTAAAACCA
ATATTCGC

CCTTGAATAAGTGAACCGTAAAGGGTTATACAGCCTGAGCTTGGCGTTGCAGCCACCAGCCCAGAAATGAT
ACCGGAGCACAGACCAACTGTCGACCATTTCTTCTCCGATCTGTAATCCAGAAGACACCACGTCATCCCAC
CAGTAATG

GTTTACGTTTAGTTCGGAAAGTTCAAGCTCTTTGATTTCCTCTTCAATTTCAACATCCGTTTCGACTTCTT
CAGTGTACGTTCCCTCCTCTTCAACTTCATCTCCACCTTCGTCCTCATCCGAATTGACATCATCCTCGTAC
TCATCATC

ATCAAAAGGTCGCTGACAGAGAATCCACTCAAACCACACTTAGTCTTGGAACCTCTGCCTGTAGCACCCTC
GTCTCTGATTTCACCACCAGAACCTGTAGCAGCACCTGGGAAAGGAGACACGGCTGTTGGATGGTTGTGAG
TTTCGACT

TTGAAACAATTACTTTGGACCAGGAAGAGGCTAGCGCTGTTGCTAATCATGCGCACGACGATGAGGCTTCC
ACAGACGTTGAAGGCTCCACAGACGTCAATGTCAATGAACAGGCTCTGTTGCAAGAGGATTTTGACATGTG
GAGTGAGA

GAATCAGAGGATGAAGATGAAGATGAAGATTCAGATTCAGACTCAGACTCAGATTCAGATTCAGATGAAGA
TGAAGATGAGGATGAAGATTCGGAGGAAGAGGAAGAGACGGCCTTAGCTTTTTCTTCTTTGGCTTGTTTGG
AAGCCTTA

TTTATTTTCGAATCTGATAGAATAACCAACTTCTTCGCCCAACTTGACATCCATTTCCTCAGCAACTCTCT
GTGCAACAGACATTGCTGCGACACGACGAGGTTGAGTACATGCAACTTGAGTGTTCTCTAAGTGTGGCATC
TCATCGAA

ACTACCACTGCCAGCATCAACGTCAGGACTAGTGCGACTACCACTGAAAGTACCAACTCCAGCACTAATGC
TACTACCACTGCCAGCACCAACGTCAGGACTAGTGCTACTACCACTGCCAGCATCAACGTCAGGACTAGTG
CGACTACC

GCAACGAATTCATCATTTAATGCCAATGTGTCGAGCAAGCAAATTCAGCAGCAGCAGCAGCAGCAGCAGCA
CAAGTCTCAAGACACAGGTCTCACGCCGCTGGAGATACAATCACAACAACAAAAACTACGACAACAACAAC
TACAACAA

TCAGAGCCAAAGCGAGAGTCAAGGTCAAGTTTCTTTCACCAATGAAGCTTCTCAGGATAGTTCCACCACCT
CTTTGGTAACAGCCTATTCTCAAGGTGTTCATTCGCACCAGTCTGCAACAATAGTGAGTGCCACAATCTCT
TCCCTCCC

ACCAAAGACTTGACGGTTCTCAAAGCAGCAACGTGCTTTCTGCCACCGTTGTGAACAGCAACCTTGATCAA
TTGGTTGTTGACCTTGGTGAAGGTAACATCAATGTGCTTCAAGTTCTTGGTCAAAGTACCTCTTGGACCAA
CAACCTTG

ATCATCAATATCTCCTTCCTCTTCATCATCAACAATAATATCATCGTCATCATCACTGCCGACATTCACTG
TGGCATCAACATCTTCGACAGTTGCCTCCTCCACACTTTCCACTAGCTCATCGTTGGTTATCTCTACGTCT
TCGTCAAC

CAACGGAATCGACGACCGTTTTAGAACCATCAACGACTAACAGTTCCAGTACGTTTAGTCTGGTCACTTCA
AGTGACAACAATTGGTGGATTCCAACTGAGTTAATCACGCAGGCACCAGAAGCTGCATCCACTGCATCTTC
TACCGTTG

TTTGATTTCCTCTTCAATTTCAACATCCGTTTCGACTTCTTCAGTGTACGTTCCCTCCTCTTCAACTTCAT
CTCCACCTTCGTCCTCATCCGAATTGACATCATCCTCGTACTCATCATCCTCATCCTCATCCACCCTCTTT
TCCTACTC

TCAACAAGTTTGACCAATCTAGCGTTATCAGCAGTCTTCTTCTTGTCTCTTGCGGCCTTGTTCTTTCTTTC
AATGTAACGCTTGTGGTCTCTGTTAGAAGAGTCATCTGGGACATCTTCGTCCAAGAACTCAAAGGTTCTCC
AGGAGTCA

TCACTTGGCTTCATCATCCAATGTATTACGCAACATTGCTGCTCAGACCTTAGGTGATCTTGTCCGTCGTG
TAGGTGGTAATGCTTTGTCCCAACTGTTACCAAGTTTGGAGGAATCTTTGATAGAAACATCAAACTCAGAT
TCGAGACA

TTAAGGTTCTCTTCGCATAGTCGGCAGCTTTCTTTCGGACGTTGAACACTCAACAAACCTTATCTAGTGCC
CAACCAGGTGTGCTTCTACGAGTCTTGCTCACTCAGACACACCTATCCCTATTGTTACGGCTATGGGGATG
GCACACAA

AATATGGACACCATCGCCGGAGATTGGATCTGCTGCTTTCTGTCAACAAACCCAGTGCCTGCCCCTGTTGA
GCTCTCTTGCTGCAACTGCTGCTGACGATGTTGATTTACCATCGATTTCAATGAGATGCTGCTCAAAATAC
TCTGTTCG

TTAACATGAGAGGTATTAAATCCACTGCTATGGTATTGTGTGGTTCTAACGACGACAAAGTTGAATTTGTC
GAGCCACCAAAGGACTCTAAGGCCGGTGACAAGGTGTTCTTTGAAGGTTTCGGTGACGAAGCTCCAATGAA
GCAATTGA

ACCGCTGGACTCGTATGGGGTATAAGTGATACCTCTGACACCAGAGGCAAAGCTGCTGACGTCTTCGCCGG
AAGAACTAGAGGAAGAAGCTTGGGAGGAAGAGGAAGATTGAGTAGATGTAGCAGCAGAGCTGGAGGCTGGA
GATGCGGC

CAACCACCAAAGAAGCTTACTGGTACAGGCTGAAGTTTGATGCTTGGTTTCCTCAAAAGACTGCGGCAGAC
ACTGTCATGAGATGGATTCCAAAGGCCGATTGGGGTTGTGCCGAAGATCCTTCAGGTAGATACGCCAAAAT
ACACGAAA

TCTTCAATTTCAACATCCGTTTCGACTTCTTCAGTGTACGTTCCCTCCTCTTCAACTTCATCTCCACCTTC
GTCCTCATCCGAATTGACATCATCCTCGTACTCATCATCCTCATCCTCATCCACCCTCTTTTCCTACTCCT
CCTCATTT

CCAGACAATTGATTTCTAACGAAGGTGTCTTGGTGGGTGGTTCTTCCGGTTCTGCCTTCACTGCGGTTGTG
AAATACTGTGAAGACCACCCTGAACTGACTGAAGATGATGTCATTGTTGCCATATTCCCAGATTCCATCAG
GTCGTACC

CACAGTCCAAGGAGTATTTCAAATTGTCACTTGAAGTGAAAACACCCCAGTGCTTCTCGACATCAGAGGTA
CCAGAGGTGTTTGGCTTCCAATCTTCATCAAAGGCTTCAAAAACAATAACGTTAACACCCCAAGCTCTCAT
GGAACAGA

TAAAATCAGAGTCAGATTCGTTGGTATTTCTTTGGCCAACCAACAAGTCGTTCAAACTTAAAACTGAACTT
GGTCTCTTCAACTGTACGGAACTCTGAGTTTGTGCTCTGAGACTTCCATTGTTGTTTGGATTTGCATTGTT
CAATCCTT

AGACTATTCACGTACAGGGTCCTCAGACGACGAGGATAGTGGAGCTTATGACGAGTGGATTCCATCGTTCT
GTTCCAGATTTGGTCACGAGTACTTCTGCCAAGTCCCGACAGAGTTTATCGAGGATGATTTCAATATGACC
TCGTTATC

GGTGGTAGTGGTTGTCGTAGTTGCAGTGTCAGCAGTTGCTTCAGTGGCCTCAGATGTGGTAGTAGAAGTAA
CGTCCCCTTCATCTGATGAAGACGATGACGATGACGATGACGATGACGATGACGATGATGATGTAGAAGTG
GACGAAGA

CAAGACCAAACATGTCCATCTGTATCTTCGGTGATGCTTTCGATGTTGACAGAGCTAAGTCTTGCGGTGTT
GACGCTATGTCCGTCGATGACTTGAAGAAGTTGAACAAGAACAAGAAGTTAATCAAGAAGTTGTCTAAGAA
GTACAACG

CAGCCTGAGCTTGGCGTTGCAGCCACCAGCCCAGAAATGATACCGGAGCACAGACCAACTGTCGACCATTT
CTTCTCCGATCTGTAATCCAGAAGACACCACGTCATCCCACCAGTAATGGCACTGAGACATGTATTCATGA
ATGCATAA

CGCTCAGCAGTTTAGAAGCAATATTGCTAATGAAATCAAAAGCATTCAAGGTCACGTGCCTGGGTTTGCAC
CTAACCTTGCCATCATTCAAGTAGGCAACAGACCAGACTCAGCCACATATGTACGCATGAAGCGTAAGGCA
GCTGAAGA

TTAGGTTGTTACTTGGCTTCTACTGGATTGACGTATCCAGCTTGGGGATTTAAACAAGCTGAAATCGTCTG
CAAAGACTCCTGGAGTCGTAGGGACAAATCGACCTGGTGGAATGTAGAAGATCACTGGAACCACAATTTAG
AAACCGCA

AGGTATGGCTTTCAACAATGCTTCTCTAGGTTATGTTCATGCCCTTGCTCATCAACTTGGTGGTTTCTACC
ACTTGCCTCATGGTGTTTGTAACGCTGTCTTGTTGCCTCATGTTCAAGAGGCCAACATGCAATGTCCAAAG
GCCAAGAA

TGACCACCTTGGACTTCACAGACGAAGACTCTTCTTCTTGTCGCAGAAGCACTCTGTTTGATGTCATCAGT
GTAATTGACTAATGCGTTCAAACAGGTATCAACACCAAGTGAGTATTCAGTACCTGGAACGTTGTTAGAAA
CAGTGGCT

ATCATCTCAGTACACTATATCATCCACTGGTATACTTTCTCAGGTTTCAGACACATCGGTGTCTTATACAA
CTTCAAGTTCGTCTGTTTCTCAAGTTTCAGACACACCAGTTTCTTATACAACTTCAAGTTCGTCTGTTTCT
CAAGTTTC

ATGGCTTCTTTAGGTTACTTCAATCAGCCAAGTTCTACTTACTATGCTCCTCCTGCACCACTTCAACAGCA
CCAGCAACCACCTATCCTTCCCCCTCCGGGCCTAATGTACACTAGTAACAATAACAGCAACGTTATTCCTC
CTCCTGTT

TGTCGCCGATGTGAATTTCTGCACTGAGAAACGTACCTGTAGTGATTATGACCTGATCTGCCCCAACCTGG
GTACCGTCATCCAGAACCACACCTTTGATGACCTTGTGGCCACATCCGGGGTCATACAAGATCAAGTCAGC
AACTTTGT

TCGTACGTGCTACGACGGTTGACATTTGGAAGGCATTGGTTCCAAATACTCCAAGAGCTGTGAAAGAGATC
CTTCCAACATTGACTGGTATGATAGTCACTCACTTGGCTTCATCATCCAATGTATTACGCAACATTGCTGC
TCAGACCT

AAGCCTTTGCAGATTCCTCCCTTGCCATTGAAGTTGCTAACACCTGTTCCTTCCGATATAGACATCTCCAG
AGCACAACAGCCAAAGCTTATCAACCAGCTTGCTCAAGAATTGGGTATTTACTCTCATGAGTTGGAGCTGT
ACGGACAT

GAGAGGTAAAGGAAGAGGCACATTCATTGCCTGGGATTTGCCTACTGGTGAGAAGAGAGACTTACTATTGA
AGAAATTGAAGTTGAATGGTTGCAACGTTGGTGGATGTGCAGTCCATGCAGTGAGATTAAGACCTTCATTA
ACATTCGA

CTTCTCTAGGTTATGTTCATGCCCTTGCTCATCAACTTGGTGGTTTCTACCACTTGCCTCATGGTGTTTGT
AACGCTGTCTTGTTGCCTCATGTTCAAGAGGCCAACATGCAATGTCCAAAGGCCAAGAAGAGATTAGGTGA
AATTGCTT

TTCTTCTTTTTCTTCTTCTTCTTCTTCGTCGGAAGAAGAGTCAGAGGAAGAGGAAGAGGAATCCTTGGATT
CTTCCTTCTTGGTTTCAGCTTCACTTTCGCTGTCAGAAGACGATGAAGAAGAGGATTCAGAATCAGAGGAT
GAAGATGA

GAAATTGGTTGAGCCTTACCTAAAGTTTCAAGTAGTTGAGATACAGTTGGCATCTCAATATCGGAAGTGGA
AGGTTCTGTCTCCTTCACTGTGTTCTCCTCTGTGGTGATTACGGAATTTATTGTTTCGTCGTCACTTTTAT
TTACAGTT

CTGAACACCACGATTTGCATCATCACTACTTTATTGGTAACTACGCTTCCTCTTTCAGATGGTGGGATTAC
TGTCTAGACACTGAATCTGGTCCAGAAGCTAAGGCCTCCAGAGAAGAAAGAATGAAGAAGAGAGCTGAAAA
CAATGCTC

TCAAGGCTGCTCAAGACTCTTTCGCTGCCGGTTGGGGTGTTATGGTTTCCCACAGATCTGGTGAAACTGAA
GACACTTTCATTGCTGACTTGGTCGTCGGTTTGAGAACTGGTCAAATCAAGACTGGTGCTCCAGCTAGATC
CGAAAGAT

AGAACGCATATAGTAGAGAGTAAATCAAACACACGTAGAAACAAGTGGCAACTAGAACAGGTTCAGTGACA
GCGAAGTACAAAGGTCTCAACAGACAAGCCCTCATCATTTCACTCATACTGACACCTTGCGCTTCCTGCTC
AGTCATAA

CCTTCTTGGTTTCTTCTTTTTCTTCTTCTTCTTCTTCGTCGGAAGAAGAGTCAGAGGAAGAGGAAGAGGAA
TCCTTGGATTCTTCCTTCTTGGTTTCAGCTTCACTTTCGCTGTCAGAAGACGATGAAGAAGAGGATTCAGA
ATCAGAGG

CACCTTTGGCTGCTTCCGGGGGTGTTGCTGACAACATAGGCGGAACTATGCAGAATTCAGGCAGCAGAGGG
ACGCTCGACGAGACTGTGCTGCAAACACTAAAGCGAGATGTGGTGGAGATTAATTCCAGACTGAAACAAGT
GGTATACC

TCTCCACCTTCGTCCTCATCCGAATTGACATCATCCTCGTACTCATCATCCTCATCCTCATCCACCCTCTT
TTCCTACTCCTCCTCATTTTCATCATCCTCATCCTCATCATCCTCATCATCATCCTCATCCTCATCATCAT
CATCATCA

GTTTGGGATGAGGACGTTGCTGGCATTGTCGAGGGCCTTTGGTGGGGTCTTGATTGGTGCCCTTGCGCCAA
GTTAGCTGCTGGTCTTGGTGGTCGTGGCGGTTGAGATTGCAATCTCTCCTCTTCCTTGATTACGTCCTCGT
AGCTCGGC

AAGCTGTTGAGCGAGCTATTTCGGGTATCCCAGCCTTCTCTGCAGACCGCCCCAGTTGGCTTGGCTCTGGT
GCTGTTCGTTAGCATCACATCGCCTGTGACAGGCAGAGGTAATAACGGCTTAAGGTTCTCTTCGCATAGTC
GGCAGCTT

ATCAAACCGATTGATCCAATGACTATTCCTGGCTTGAAAGAAGCCGGCTGGACTCCCGAGATGGATGCGTT
GGCACAACGTCCCAAGCGTGGTCCACACGATGCAGCAATACAGAATATACTCACAGAGCTACAAAATCATG
CAGCAGCT

AAGGAATTTGGAACGTGCCCCCGTTACTATTGTAATGGCATGCAGTTGCTGCCTTGTGGGTTGAGCGATAC
AGTTGGGAAACACACCGTAAGGTTGTACTGTCCCAGTTGTCAGGACTTGTATCTTCCCCAATCATCTAGAT
TCCTGTGC

CCCTTGGTTGAAAAGTTCATTAATTTCACTGAAGGTTCTACCAGTTGTCTCAGGCAGATCGATGATGACCC
AAGCTAAAGTGACTGCTGTGAAACCACCCCAGTATAGACCAGTTTTGGCACCCCAGTTCCAATCGCTCACG
TTTAGCAT

GATAGAGCACTGGAGATGGCTGGCTTTAATCTGCTGGAGTACCATGGAACACCGGTGATCATTCTGGTCAC
TTGGTCTGGGGCAATACCAGTCAACATGGTGGTGAAGTCACCGTAGTTGAAAACGGCTTCAGCAACTTCAA
CTGGGTAG

GGACGCCAACACCAAGAACAGGAGAAGAAGAGCCAATAAAAAGTCAAGTGAAACGTCACCTGATTCTACTC
CGGCCCCATCTGCTCCTGCCTCCACAAATGCCCCCACAAACAATAAAGAAACTTCTCCAGAGGAGAAGAAA
ATAAGATC

TCGATATCGTCATCGTCAGAAGATTCAGATGCAGCGTCATCGTAAGTCTTAGCAGGCTTTTCTTGAGTTGG
CTGATGAGCTGAAACAGAAGATGCTGAAGAGGATGATGAAGAGGATGATGTATCAGTCATATTGATATTGT
TTGATAAT

CTGCAAACAAGTGAATCAAGCAGTTTTACCACTGCATCAGCAGCTCTACCAGTAAGTTCAACAGACGTTGA
TGGCTCAAGCGCCTCACCTGTAGTGAGCATGAGCGCCGCAGGACAAATAGCTAGCTCAAGCAGCACAGATA
ATCCAACT

TGTATGAACGTGAAAGAAATCGAGAGGGTTTAGAAGTAGTTTAGGGTCATTTTTCAGTACATTGGTTGGCC
TGGCTTGAGTTGCAACCAGTGCCAAGCATCAGCGTTGGCCTCTCTCTTGTACATTGGTTGGCCTGGTCTCA
AATTTAAC

ACTCACTAAGTTCGTGTCTTGAAGCACTCGTATAAAGTCTCTTAGTTTCGTCGGAGGCATGTTGCATTTCA
CCAACTGTCATAATCTCCCTGCCATCCTTCACTCTGTTTCTGATGAATTGATTCATTTCTTGATGGAACTC
GTGAATAC

ATATGACTGAAAATATCTACATAGATTCAGAAGCGTCCAAGGATGTTGACAACAAGTGCTGTCCAGCCTGT
GAAATGCTCAGCACCAGTACCATGACCATCTATCGGACTGTAATTTTCATAACAATAACCTTGTTCTTCCC
AAACTTTG

ATCAACTTGGTGGTTTCTACCACTTGCCTCATGGTGTTTGTAACGCTGTCTTGTTGCCTCATGTTCAAGAG
GCCAACATGCAATGTCCAAAGGCCAAGAAGAGATTAGGTGAAATTGCTTTGCATTTCGGTGCTTCTCAAGA
AGATCCAG

ACTCTTAGACCCAAGTGTCTAGCTTCGCTAACAGTTTGAGCAGTATCGTCTCTTGGTGGATCCATACATGG
CATAACACCCAAGATTTCCCAGTGACCTTCACCTCTCTTTCTAGCAACACCTAAAGCACGGAACCCTCTAG
AAGCCAAT

CGGTACCTTGTCTGAATCCATCAAGGCTGCTCAAGACTCTTTCGCTGCCGGTTGGGGTGTTATGGTTTCCC
ACAGATCTGGTGAAACTGAAGACACTTTCATTGCTGACTTGGTCGTCGGTTTGAGAACTGGTCAAATCAAG
ACTGGTGC

AGTGCATTATTAATGAACAGTGCACCACCTCCGAGTAAAAGCAAACCTCCCAAGCCTGCAAATGCCCCCCT
TGGTGAAGGCACTTGACCTCTGCCTCCGGTTTGCTGTACCTTGGACAATTGTTTTTGGAACGCCTTTGCGT
ACCTTTGG

AGAGAAAGTTACAATGGATGGTTGACAAGGACTTGGTCGGAAATTGGGACGATCCAAGGTTCCCAACTGTC
AGGGGTGTGAGAAGAAGAGGTATGACTGTCGAAGGTTTGAGGAACTTCGTCTTATCCCAAGGTCCATCCAG
AAATGTCA

GACAGCCTTAGCAGCACCGGTGGAGGATGGGATGATGTTACCGGAAGCGGTTCTACCACCTCTCCAGTCCT
TGTGGGATGGACCGTCAACAGTCTTTTGAGTAGCAGTCAAAGAGTGGACAGTGGTCATCAAACCTTCTTCA
ATACCGAA

GAGGCCGTGGTTGGGAACTTGTAGGCGTATAAGAGTGTGAGTGAGCATATGTTTGGGATGAGGACGTTGCT
GGCATTGTCGAGGGCCTTTGGTGGGGTCTTGATTGGTGCCCTTGCGCCAAGTTAGCTGCTGGTCTTGGTGG
TCGTGGCG

GACGTAGAGGACGACGTAGGTGTATTGGTTGATTCGGTGGACTCCTGAGAAGCAATTGAGGTACTCGTTTG
TTGATTCAAAGTTGAAGTCGCAGCTGCTTGGTAAGAATGATGCTGACTTTGAGTGTTGCCTTCAACATTGG
CTTCACCG

TTTGATTCTTCCTTCTTGGTTTCTTCTTTTTCTTCTTCTTCTTCTTCGTCGGAAGAAGAGTCAGAGGAAGA
GGAAGAGGAATCCTTGGATTCTTCCTTCTTGGTTTCAGCTTCACTTTCGCTGTCAGAAGACGATGAAGAAG
AGGATTCA

CGACGCTTTGTTGTTGAAGGTCAACCAAATCGGTACCTTGTCTGAATCCATCAAGGCTGCTCAAGACTCTT
TCGCTGCCGGTTGGGGTGTTATGGTTTCCCACAGATCTGGTGAAACTGAAGACACTTTCATTGCTGACTTG
GTCGTCGG

AGCAAGCCAAGGATAATGGTGTTGATATATTTAGAGTCTTTGATGCCTTAAATGACTTGGAACAATTGAAG
GTCGGTGTAGATGCTGTGAAGAAGGCAGGTGGTGTTGTAGAAGCCACTGTTTGTTTCTCTGGGGATATGCT
TCAGCCAG

TAATGTCTGCTCCAGAAGCTCAACAACAAAAGAGAGGTGGTTTCGGTGGCCGTAACAGAGGCCGTCCAAAC
AGAAGAGGACCAAGAAACACTGAAGAAAAGGGATGGGTTCCAGTTACCAAACTAGGTAGATTAGTCAAGGC
TGGTAAGA

TCTAGCTTGACGCTGGACCAGATTACAGGCCTAATTGAAGTCAACGAAAAGGAATTGGATGCAACTACCAA
GGCAAAGACAGAAGACTTCGTCAAGGCATTCCAGGTCTTCGACAAGGAAAGTACAGGCAAGGTATCCGTTG
GTGACTTA

GTTGCCATCCATGGCTTCTTTAGGTTACTTCAATCAGCCAAGTTCTACTTACTATGCTCCTCCTGCACCAC
TTCAACAGCACCAGCAACCACCTATCCTTCCCCCTCCGGGCCTAATGTACACTAGTAACAATAACAGCAAC
GTTATTCC

ACCGTCCTTGGATAGAGCACTGGAGATGGCTGGCTTTAATCTGCTGGAGTACCATGGAACACCGGTGATCA
TTCTGGTCACTTGGTCTGGGGCAATACCAGTCAACATGGTGGTGAAGTCACCGTAGTTGAAAACGGCTTCA
GCAACTTC

ACCAGTGATTAAACCAGTGAAAGAAGCGATGTAACCAGAGTGACCACCTTGGACTTCACAGACGAAGACTC
TTCTTCTTGTCGCAGAAGCACTCTGTTTGATGTCATCAGTGTAATTGACTAATGCGTTCAAACAGGTATCA
ACACCAAG

CAAGGGCTTCTGAGACCATGAGGAACTCGCTACTTTACAAGATGTCCTACAAAGATTTCCCACAATTATTC
AATGGTGGCCAAGCCACTGACAGAGTGCGTCAACAAATGATCACACCATTAGACGTCCCACCATTAGACTA
CTTCGACG

CATGTTGTCAACCAAAGACTTGACGGTTCTCAAAGCAGCAACGTGCTTTCTGCCACCGTTGTGAACAGCAA
CCTTGATCAATTGGTTGTTGACCTTGGTGAAGGTAACATCAATGTGCTTCAAGTTCTTGGTCAAAGTACCT
CTTGGACC

AATTCTGGCAAGACCTTACCGACAGCCTTAGCAGCACCGGTGGAGGATGGGATGATGTTACCGGAAGCGGT
TCTACCACCTCTCCAGTCCTTGTGGGATGGACCGTCAACAGTCTTTTGAGTAGCAGTCAAAGAGTGGACAG
TGGTCATC

CACTATCCGTTGCCACAGTATATCCCACCACTGAGTACTTCCTCACCTGATCCAATCGATTCACAGAATCA
ACACTCTGAAGTACCTCAAGCTGAGACAAAGGTGAGAAATAACGTCTTACCACCACACACTTTAACATCAG
AAGAAAAC

CCTGTCGAAGAAGGCGAAGAAGAAGAAAGTGAAAGTGAAAGTGAAAGTGAAGATCAAGTTGAGGAGGAAGA
TCAAGAAGTTGTTGCTGGAGAAGAAGATGATGACGATGACGAAGAGCTACAAGCTCAAAAAGAGCTTGAAT
TGGAAGCA

ATTGCCTAAGGCTTTGGGTATCAAACCACAAATTTATGCTAAGCTGGAACTATACAATCCAGGTGGTTCCA
TCAAAGACAGAATTGCCAAGTCTATGGTGGAAGAAGCTGAAGCTTCCGGTAGAATTCATCCTTCCAGATCT
ACTCTGAT

TGAAGAGATACCCAATTGTCTCCATCGAAGATCCATTTGCTGAAGATGACTGGGAAGCTTGGTCTCACTTC
TTCAAGACCGCTGGTATTCAAATTGTTGCTGATGACTTGACTGTCACCAACCCAAAGAGAATTGCTACCGC
TATCGAAA

TTGACTTTGAGCCTTGTTAGTAGCTTCTTGTTGTGGTATACGGAACCCAGAAGCATCAACCTCACTCAACT
TGAAAGATTCTAACCAAACTTCTCTCAAGGAATCTTGGGAAGTCCCTGAAGATAGTAAGCTATTTTCACCA
TGTTTGAA

TTGGTCCATTCTTTCCTTCGTTCTCTTTATCTTTAATGTTTGGGTCAAGGTCTTTGACCACTTTGCCACAT
AACTGAGCATACATTGAAGACCAATGAGGTTCATCACAAGCCTTGTGGAAAATTTGTTCAATAACTATTTT
CAATGTTT

AAGAATCCAATGATCTTAACATAGAATCTGACCATGACTTCCAATCCGACATTGAACTACATCCTGAGCAA
CCGAGAAATGTCCTTTCAAAAGCTGTGAGTCCAACCGATTCCACACCTCCGTCAACTCATACTGAAGATTC
GAAACGTG

GGCCTCGGTGGAGGCCGTGGTTGGGAACTTGTAGGCGTATAAGAGTGTGAGTGAGCATATGTTTGGGATGA
GGACGTTGCTGGCATTGTCGAGGGCCTTTGGTGGGGTCTTGATTGGTGCCCTTGCGCCAAGTTAGCTGCTG
GTCTTGGT

GCCCTTGCAACTAGAGGCTTTCCAACAAGGATTCTAAACTCCCCTCCACAGACCCCATCAAGTCGAACTGG
TTCAAAAGTTATGACCAAAGGTGGCTCCAACGACGCTTCGAGCACTAAGGTGGAAGAGGAATTCAATGAAT
TTCAATCC

GCACTTATAGTGTATGATATAAGCAAATCAAGTTCATACGAAAATTGCAACCACTGGCTTACCGAACTGAG
AGAGAACGCAGATGACAACGTGGCTGTTGGGTTGATAGGGAACAAATCAGATCTGGCTCATTTAAGAGCAG
TACCAACC

GACAGCTCTTTCAACAAGTTTGACCAATCTAGCGTTATCAGCAGTCTTCTTCTTGTCTCTTGCGGCCTTGT
TCTTTCTTTCAATGTAACGCTTGTGGTCTCTGTTAGAAGAGTCATCTGGGACATCTTCGTCCAAGAACTCA
AAGGTTCT

GGCGGACCTGCTGGGAACGGTCGTCATAGAAATATCCCAAGCAAATGAAGTCGACGAGTCTGTCGTTGGCT
GCTCCATTCAGTTCATCCAGCAAAGCTGTTTCCAACAAGATCCTATCAATAAATGACTGGTTAAGGTCACT
CCTATCCC

ACGTTGCAACAGGATTTGATTCAGGATATGAGCAAACTTGTGGGCAGTTTGAAGCAAGGAGCTGTGGCATT
TCAATCAGCACTTGATGAAGATAAGCAAGTTCTTGGAGCTGCAGAGATAGGTATTCAAGTTGCTTCTCAAG
GTTTAATG

ACTCATAAGCTGGGTATGAAATTCATCACCGATTTGGTTATCAACCACTGTTCTACAGAACACGAATGGTT
CAAAGAGAGCAGATCCTCGAAGACCAATCCGAAGCGTGACTGGTTCTTCTGGAGACCTCCTAAGGGTTATG
ACGCCGAA

GAGCTTATCAAACCTGTTAAGAGTTGCCATAATAAACCTTCAGTGTTGGTGTTGGATGACAGGATTGTGGA
TGCGGCAACCAAAGATCTCTACGTGAATGGGTTCCAAGAAGAGATTCAGTATCAGAATCCTACACCGGAGA
ACTTGCAA

CTGAAACAGCACCAGTGATTAAACCAGTGAAAGAAGCGATGTAACCAGAGTGACCACCTTGGACTTCACAG
ACGAAGACTCTTCTTCTTGTCGCAGAAGCACTCTGTTTGATGTCATCAGTGTAATTGACTAATGCGTTCAA
ACAGGTAT

GTGATTCGTCTTCTCCTGCGTTCGGTGACTTGGATCTGTGTGGTTCAGACTTGATCAGGCAATCAGCGAAC
AAATGTCTCGAATTATGGTCCACCCCTACTTCCATCAACGACGTCGCCTTCTTGGTCATCAACTACTTGAA
TGGAAACT

TGATCTTAACATAGAATCTGACCATGACTTCCAATCCGACATTGAACTACATCCTGAGCAACCGAGAAATG
TCCTTTCAAAAGCTGTGAGTCCAACCGATTCCACACCTCCGTCAACTCATACTGAAGATTCGAAACGTGTT
TCTAAAAC

TAGGATATGAAACTACGAAATCAGTCGATAGGCAAATTGAGGACCAGGACACGTTGCAACAGGATTTGATT
CAGGATATGAGCAAACTTGTGGGCAGTTTGAAGCAAGGAGCTGTGGCATTTCAATCAGCACTTGATGAAGA
TAAGCAAG

AGGTGTATGTTGAATAGGGTTCTTTATCTTTATGGTTCTTTGCGAACGAAATATGTCTGGAAGGGTGCCAA
TCTCTGGTGACCACAATCCTGTGCCAGTCTCTATCAGCATCTTGCATCAAATCCGAGATAGGATTGATTAA
TTCCTCAC

AGCGTTATCAGCAGTCTTCTTCTTGTCTCTTGCGGCCTTGTTCTTTCTTTCAATGTAACGCTTGTGGTCTC
TGTTAGAAGAGTCATCTGGGACATCTTCGTCCAAGAACTCAAAGGTTCTCCAGGAGTCAAATCTGTGCCAG
AAAGCATA

AGCACTAATGCTACTACCACTGCCAGCACCAACGTCAGGACTAGTGCTACTACCACTGCCAGCATCAACGT
CAGGACTAGTGCGACTACCACTGAAAGTACCAACTCCAACACTAGTGCTACTACCACCGAAAGTACCGACT
CCAACACT

TGACAGAACTCAACCATGGTCCATCATGCCAGATTTGTACTTCTACAGAGACCCTGAAGAAGTTGAACAAC
AAGTTGCTGAAGAAGCTACCACCGAAGAAGCTGGTGAAGAAGAAGCCAAGGAAGAGGTTACCGAAGAGCAA
GCTGAAGC

GATCTCCTTACTAGCAGAAGCCTTCCTACCATCTATCTGCCCTTCACCGAGCTTGCTACTGTGGCTGCTGC
GAACATTCTCCTCAGCAGAGTTCTTCCTCAATATGGACACCATCGCCGGAGATTGGATCTGCTGCTTTCTG
TCAACAAA

AGTAGAGGACGACGTAGAGGACGACGTAGGTGTATTGGTTGATTCGGTGGACTCCTGAGAAGCAATTGAGG
TACTCGTTTGTTGATTCAAAGTTGAAGTCGCAGCTGCTTGGTAAGAATGATGCTGACTTTGAGTGTTGCCT
TCAACATT

CTGGAAGCAGCCAAACAAGCAGTCAACATCAAGTCGTCTGGAGAAACACCTTCAACAGTGTATGGTTCGTG
CAAGGACAACTTGTTCTTGGTCAAAGTACCGGTTTTGTCGGAACACAAGATTTCGACACCAGCCAAGGATT
CAATAGCA

CTAATTGAAGTCAACGAAAAGGAATTGGATGCAACTACCAAGGCAAAGACAGAAGACTTCGTCAAGGCATT
CCAGGTCTTCGACAAGGAAAGTACAGGCAAGGTATCCGTTGGTGACTTAAGGTACATGCTAACTGGCTTGG
GTGAAAAG

GTTGACAAGGACTTGGTCGGAAATTGGGACGATCCAAGGTTCCCAACTGTCAGGGGTGTGAGAAGAAGAGG
TATGACTGTCGAAGGTTTGAGGAACTTCGTCTTATCCCAAGGTCCATCCAGAAATGTCATTAACTTGGAAT
GGAACTTG

ACTACCACCGAAAGTACCGACTCCAACACTAGTGCTACTACCACCGAAAGTACCGACTCCAACACTAGTGC
TACTACCACTGCTAGCACCAACTCCAGCACTAATGCCACTACCACTGCTAGCACCAACTCCAGCACTAATG
CCACTACC

CCAAACTGTCCAAGACCAAACATGTCCATCTGTATCTTCGGTGATGCTTTCGATGTTGACAGAGCTAAGTC
TTGCGGTGTTGACGCTATGTCCGTCGATGACTTGAAGAAGTTGAACAAGAACAAGAAGTTAATCAAGAAGT
TGTCTAAG

CCGACGCAAACAGCAGTTCTGCTTCCGCTCCATTGGAAGTGGCAACGTCTACGCCAACCCCATCTTCAAAG
GCATCCTCTCTGTTGCTTACACCATCAACATCCTCTTTAAGTCAGGTTGCTACAAATACTAATGTACAGAC
GAGTTTAA

TATAAGTGATACCTCTGACACCAGAGGCAAAGCTGCTGACGTCTTCGCCGGAAGAACTAGAGGAAGAAGCT
TGGGAGGAAGAGGAAGATTGAGTAGATGTAGCAGCAGAGCTGGAGGCTGGAGATGCGGCAGCAGAAACTTG
TGAGTTGT

TTGGCGTTGCAGCCACCAGCCCAGAAATGATACCGGAGCACAGACCAACTGTCGACCATTTCTTCTCCGAT
CTGTAATCCAGAAGACACCACGTCATCCCACCAGTAATGGCACTGAGACATGTATTCATGAATGCATAAAC
TGACCTCA

GGTTTTAGAAACACGTTTCGAATCTTCAGTATGAGTTGACGGAGGTGTGGAATCGGTTGGACTCACAGCTT
TTGAAAGGACATTTCTCGGTTGCTCAGGATGTAGTTCAATGTCGGATTGGAAGTCATGGTCAGATTCTATG
TTAAGATC

GTTTTAGAAACACGTTTCGAATCTTCAGTATGAGTTGACGGAGGTGTGGAATCGGTTGGACTCACAGCTTT
TGAAAGGACATTTCTCGGTTGCTCAGGATGTAGTTCAATGTCGGATTGGAAGTCATGGTCAGATTCTATGT
TAAGATCA

TCACTCATTGTCCGGTGTAGACTATAAATCACTGGATGCAACTTGTCAATAACTTTCAACAACTTCTCAGC
ATTTGCAAAGCTGATTTCTTTGTTCTTTGGCAGTGTCAAAGCTGCGACATCATCAGGCCTGAGCTTGTTAT
CATGTCTT

CCTTCCTACCATCTATCTGCCCTTCACCGAGCTTGCTACTGTGGCTGCTGCGAACATTCTCCTCAGCAGAG
TTCTTCCTCAATATGGACACCATCGCCGGAGATTGGATCTGCTGCTTTCTGTCAACAAACCCAGTGCCTGC
CCCTGTTG

AAATCTTGGCTTTCCTTTTCATTGACAACAATACGACTTCCTTGATCTGAAACATTTGTCATAGGAGGTGG
AGGTGGGAAGGAAATCGGTTGTCCAAACCCATGTGGTGCTGGTGGCGGAATAGACATTCCATTCATATTAG
GGTAAGGC

TCTGAATCCATCAAGGCTGCTCAAGACTCTTTCGCTGCCGGTTGGGGTGTTATGGTTTCCCACAGATCTGG
TGAAACTGAAGACACTTTCATTGCTGACTTGGTCGTCGGTTTGAGAACTGGTCAAATCAAGACTGGTGCTC
CAGCTAGA

TCAAACCACAAATTTATGCTAAGCTGGAACTATACAATCCAGGTGGTTCCATCAAAGACAGAATTGCCAAG
TCTATGGTGGAAGAAGCTGAAGCTTCCGGTAGAATTCATCCTTCCAGATCTACTCTGATCGAACCTACTTC
TGGTAACA

ACACCTGGGACATCAGCTGTTCCAGAGAACCATCATCATGTCTCTCCTCAACCTGCTTCAGTACCACCTCC
ACAGAATGGACAGTACCAACAGCACGGCATGATGACCCCAAACAAAGCTATGGCCTCTAACTGGGCACATT
ACCAACAA

CTTACCTTGCAATTCTGGCAAGACCTTACCGACAGCCTTAGCAGCACCGGTGGAGGATGGGATGATGTTAC
CGGAAGCGGTTCTACCACCTCTCCAGTCCTTGTGGGATGGACCGTCAACAGTCTTTTGAGTAGCAGTCAAA
GAGTGGAC

CGAATTGACATCATCCTCGTACTCATCATCCTCATCCTCATCCACCCTCTTTTCCTACTCCTCCTCATTTT
CATCATCCTCATCCTCATCATCCTCATCATCATCCTCATCCTCATCATCATCATCATCATCATCATCATAT
TTCACCCT

TTATTGGTAACTACGCTTCCTCTTTCAGATGGTGGGATTACTGTCTAGACACTGAATCTGGTCCAGAAGCT
AAGGCCTCCAGAGAAGAAAGAATGAAGAAGAGAGCTGAAAACAATGCTCAAAAGAAGACTAACTAAGAGAA
GAAACATA

GTAGGCGTCCTTAGGAAAGATAGTTTGATTGATACCGAAGGCAGACGTGCCGGTCTCGGAAATGATAACAT
CACCTTCTTGCAAGAATTTGGACAATTCGTTCCACAACCACTCTTGTTTCAAGGGCGTGCTAGCAGGTACA
CCTTTGTT

TGGCAGGAACCCCTTGGTTGAAAAGTTCATTAATTTCACTGAAGGTTCTACCAGTTGTCTCAGGCAGATCG
ATGATGACCCAAGCTAAAGTGACTGCTGTGAAACCACCCCAGTATAGACCAGTTTTGGCACCCCAGTTCCA
ATCGCTCA

TTAATGGCAGACTGTGTCTTTGTACATATCCCTTGATTGGCACAGTGAAAGATCTTCTCAAAGTGTACTGG
ATATGACTCAGTCTTGTCGGATCCTTGGCCAACCTTTGAGCACTAAGAGCATCCTTCCGTCGTTTAGTCAA
CTGAGAAA

AAGAAGCGATGTAACCAGAGTGACCACCTTGGACTTCACAGACGAAGACTCTTCTTCTTGTCGCAGAAGCA
CTCTGTTTGATGTCATCAGTGTAATTGACTAATGCGTTCAAACAGGTATCAACACCAAGTGAGTATTCAGT
ACCTGGAA

AATTCAAAGACTTAAACAATATCAAACCGATTGATCCAATGACTATTCCTGGCTTGAAAGAAGCCGGCTGG
ACTCCCGAGATGGATGCGTTGGCACAACGTCCCAAGCGTGGTCCACACGATGCAGCAATACAGAATATACT
CACAGAGC

ACAGTCTTCAAGACGAATAATGGAGCACCCTTAACACAAACAATTCTTTCACCTTCTGGAGATTCGACAAC
GGCAGTAACCTTCTTGGAGACAGGGTCAAATGGATGGAATTCCAAAACCTTGTACTTGGTCAAAGCGTCCT
TAGCCTTT

AGGACGTTGCTGGCATTGTCGAGGGCCTTTGGTGGGGTCTTGATTGGTGCCCTTGCGCCAAGTTAGCTGCT
GGTCTTGGTGGTCGTGGCGGTTGAGATTGCAATCTCTCCTCTTCCTTGATTACGTCCTCGTAGCTCGGCAG
TTCATCAT

ATCCCAATCAGAAGAGGTTACTGGGGTACCAACTTGGGTCAACCACATTCTTTGGCCACCAAGACCACTGG
TAAGTGTGGTTCCGTCACTGTTAGATTGATCCCAGCCCCAAGAGGTTCTGGTATCGTCGCTTCTCCAGCTG
TCAAAAAG

AGAGGATTCAGAATCAGAGGATGAAGATGAAGATGAAGATTCAGATTCAGACTCAGACTCAGATTCAGATT
CAGATGAAGATGAAGATGAGGATGAAGATTCGGAGGAAGAGGAAGAGACGGCCTTAGCTTTTTCTTCTTTG
GCTTGTTT

AGCTCCGCTAATGCCGACAATTTCGACTTCCAATCTCCGTTTGCTGTCTTGTCTGATGATGAAACGTCCCA
ACATTTCACCACCTACCTAATCTCTCCAGCTGAACATCCAACAATTTGTACTTTCACACCAGAAAAGGGTG
GTAAACCG

GGGTCAAATATGCCTGTGAAACGTGTATCAGGGGTCACAGGGCGGCGCAGTGTACTCACACTGATGGTCCG
CTACAGATGATCAGACGCAAGGGAAGACCATCGACCACATGTGGCCATTGTAAAGAGCTGAGAAGAACCAA
GAACTTCA

TGAAGAACAATAGCTAGTTTCACAAATTTCATCATCTTAACCCTCTCTGGATCCTGGAAGAACACGTTTAC
TTCCTGGGCTGTTTCCACAACACTCAGGCCGTCTTCAACACTTTGATATTTTGCGATTACTAAAGAAAGTT
CCATAAAT

CGTGTTCAACCAAGTCGACAACTCTGGTAGAGTAACCGTATTCGTTGTCGTACCAGGAGACCAACTTGACG
AACTTTGGAGACAATTGGATACCAGCGGAAGCATCGAAGATGGAAGAGTGAGAGTCACCCAAGAAGTCAGA
GGAGACAA

TCCAACTGCTCCTGTCGAAGAAGGCGAAGAAGAAGAAAGTGAAAGTGAAAGTGAAAGTGAAGATCAAGTTG
AGGAGGAAGATCAAGAAGTTGTTGCTGGAGAAGAAGATGATGACGATGACGAAGAGCTACAAGCTCAAAAA
GAGCTTGA

GTGGAAGCGCTCGAGAAAGTTGGAGTTTTTCAGCGTTTGCGTTCCATGACGAGCGCTGGACTGCAGGGTCC
GCAGTACGTCAAGCTGCAGTTTAGCAGGCATCATCGACAGTTGAGGAGCAGATATGAATTAAGTCTAGGAA
TGCACTTG

CTACAAGTTCCAGATCGTCCGTTTCTCAAGTCTCAGACACTCCGGTGCCTTCTACAAGTTCAAGGTCGTCC
GTTTCTCAAACATCTAGCTCACTACAGCCCACCACTACATCCTCCCAACGTTTCACCATTTCCACTCATGG
AGCGCTTT

CAAACAGTGGTTGAAACAATTACTTTGGACCAGGAAGAGGCTAGCGCTGTTGCTAATCATGCGCACGACGA
TGAGGCTTCCACAGACGTTGAAGGCTCCACAGACGTCAATGTCAATGAACAGGCTCTGTTGCAAGAGGATT
TTGACATG

ACGACGTAGGTGTATTGGTTGATTCGGTGGACTCCTGAGAAGCAATTGAGGTACTCGTTTGTTGATTCAAA
GTTGAAGTCGCAGCTGCTTGGTAAGAATGATGCTGACTTTGAGTGTTGCCTTCAACATTGGCTTCACCGAC
ACCACTCA

GCCCTTGCTCATCAACTTGGTGGTTTCTACCACTTGCCTCATGGTGTTTGTAACGCTGTCTTGTTGCCTCA
TGTTCAAGAGGCCAACATGCAATGTCCAAAGGCCAAGAAGAGATTAGGTGAAATTGCTTTGCATTTCGGTG
CTTCTCAA

CTGGGTCAACGACGTAGACAATACCATCAATTGTTAAAGAAGTTTCAGCGATGTTCGTGGAGATAACAACT
TTCCTGCCTGGTCTACCATTATGTGACTCTGGAGCAGGCTCGAATATTCTTTGTTGTTGATGCGGTGGTAA
TGAACCAT

TTACCACAACTTGAAGTCTTTGACCAAGAAGAGATACGGTGCTTCTGCCGGTAACGTCGGTGACGAAGGTG
GTGTTGCTCCAAACATTCAAACTGCTGAAGAAGCTTTGGACTTGATTGTTGACGCTATCAAGGCTGCTGGT
CACGACGG

CATTTCGTCAAGCAAGCCAAGGATAATGGTGTTGATATATTTAGAGTCTTTGATGCCTTAAATGACTTGGA
ACAATTGAAGGTCGGTGTAGATGCTGTGAAGAAGGCAGGTGGTGTTGTAGAAGCCACTGTTTGTTTCTCTG
GGGATATG

GCATACGAATAGATTTAATATTGCTCTTGGCCTTCTCACGCCCCACTTTCAAGAAATTCGAGTTGTCTGAC
CTCATGGACATACCTGTGTGGCTCATCGTAACACCAACGGTTTCATCCAAAGTTGTGATCCTTTCCTTATT
ATTAAGCT

ATGAAGCCAGTAAATATCCCACACTTTAAAGAAGTCATTATCATGTCGACGGTCTGCGATCATTGTGGTTA
TAAGTCTAATGAGGTGAAGACCGGTGGTGCCATCCCTGACAAAGGAAGAAGGATTACTTTATACTGTGACG
ATGCAGCT

GATGTAGAAATACCGAACCAGGAATTAGTCAAACCGAATCCAACACCAAGAATTGACCACAACGAAAATGA
CTTTCTTAGATGAACTTCACCATCTGCTGCTACAGCACCCTTGCTATCCATATCATCCAATGGCTTTATTT
CCTCCTCA

CGTTGCAGTTGAGGCACCTTTTGAGGACATTTCTCCTAACCGTAGACTGCACACCCAAATGCCTGAAGTCT
CATCAGGTTCCCATACCATCAATGAAGTATCAGCAGTAGCAGCTAGTAGTTGTAAGCGACTTTCGTGCCAC
TGAAGGGA

AGTTAGCTGGTGCTTGACCAGCGTGTTGAATGCTTCCTCTACGGAAATAGAACCTCTGATGAAAATCAATT
TGTTCTGTTCCACCAAATTTGACAGAGGAGTGTGTTGCCACTCCTGCCTATGAACAGATAACAGATTCTGT
GAATTCAA

ATTTGTCGAAGATAAACGATTCATTGCTCTAGAGGTATCTGATAATGATGACGATTGTGACACTGATTTGA
CTGCTGACACAGCTGACGAGCTGGAAAGCTCTGCTATCTTAAAGATGAGGGAGTCTGATGCATCTTTAAAT
GTTACCAC

CGTTTCTCGAAGACTGCGCACAATACTTACAGTCCTCAGAACAACCACCAGATTTGATGTTCATCAATGTG
CACAATTGCACTTTGGTTGGATCGTGCCACTTTCTGTGCTGCAATTGTGCTGCATGAGTAAGTTCGAGCAG
TGGGGTAT

AGACACACCGGTTTCTTATACAACTTCAAGTTCGTCTGTTTCTCAAGTTTCAGACACACCAGTTTCTTATA
CAACTTCAAGTTCATCTGTTTCTCAAGTTTCAGACACACCGGTTTCTTATACAACTTCAAGTTCGTCCGTT
TCTCAAGT

TATTAGAAGTTTTATTTTCGAATCTGATAGAATAACCAACTTCTTCGCCCAACTTGACATCCATTTCCTCA
GCAACTCTCTGTGCAACAGACATTGCTGCGACACGACGAGGTTGAGTACATGCAACTTGAGTGTTCTCTAA
GTGTGGCA

TGACCAATCTAGCGTTATCAGCAGTCTTCTTCTTGTCTCTTGCGGCCTTGTTCTTTCTTTCAATGTAACGC
TTGTGGTCTCTGTTAGAAGAGTCATCTGGGACATCTTCGTCCAAGAACTCAAAGGTTCTCCAGGAGTCAAA
TCTGTGCC

GGATAAATTCGAGCTCCACTTCGAACCTCGTGGGGAGAGGATTTTGATCCGAGGAAGGTGTCGCTGGTGCG
TTTCCATTAGTGCTTGACATCCTGGGTGGAGCTTGGGGGAACTTTGCCGTCTTATGAGTGCTATAAGTTTT
GGCTTACT

AAATGCAAGATTTAAAGTAAATTCACTTAAGCCTTGGCAACGTGTTCAACCAAGTCGACAACTCTGGTAGA
GTAACCGTATTCGTTGTCGTACCAGGAGACCAACTTGACGAACTTTGGAGACAATTGGATACCAGCGGAAG
CATCGAAG

GCTACAGTCTACTACTTTGGATAGCTCAAGTTTAGCTAGCTCCTCTGCGTCGAGTTCAGACCTTACAGATT
ATGGCGTCTCCAGTACAGCAAGCATACCGCTGTTGTCAGCCTCAGAACAAGCAAGTACTTCCAGCAGTTTT
AGCGTTGT

ATTCATCACCGATTTGGTTATCAACCACTGTTCTACAGAACACGAATGGTTCAAAGAGAGCAGATCCTCGA
AGACCAATCCGAAGCGTGACTGGTTCTTCTGGAGACCTCCTAAGGGTTATGACGCCGAAGGCAAGCCAATT
CCTCCAAA

TCTCCTTCCTCTTCATCATCAACAATAATATCATCGTCATCATCACTGCCGACATTCACTGTGGCATCAAC
ATCTTCGACAGTTGCCTCCTCCACACTTTCCACTAGCTCATCGTTGGTTATCTCTACGTCTTCGTCAACGT
TTACGTTT

AATATTTTGACTGACGATGGTTTATTAAACGAGGAGTGTGGTCCAGAGTGGCAAGGCATGAAGAGGTTTGA
TGCCAGAAAGAAGGTCATTGAGCAGCTGAAGGAAAAGAACCTATACGTTGGCCAAGAAGATAATGAAATGA
CCATTCCA

TTATACATGTGATATCAGAACGGAAGGTTTTACTCAACTTGACTGCCATCCCTCGGTTTGTAGTGAGGCAA
CAGCTCCACAACCTCAGCTGGTAGTCTACCATCGACCTTGATGGCGTACATACCAGCTATACTATGATCTA
CGCTCAGC

CTGGGATTTGCCTACTGGTGAGAAGAGAGACTTACTATTGAAGAAATTGAAGTTGAATGGTTGCAACGTTG
GTGGATGTGCAGTCCATGCAGTGAGATTAAGACCTTCATTAACATTCGAGGAGAAGCATGCTGATATCTTT
ATTGAAGC

TGCTGTAGGTGCACTTATAGTGTATGATATAAGCAAATCAAGTTCATACGAAAATTGCAACCACTGGCTTA
CCGAACTGAGAGAGAACGCAGATGACAACGTGGCTGTTGGGTTGATAGGGAACAAATCAGATCTGGCTCAT
TTAAGAGC

TGGCTGAAGTGGTTGAAGTTGAGGTTGCTGTTGAGGTTGCTGTTGAGGTTGCTGTTGAGGTTGCTGTTGAG
GTTGCTGTTGAGGTTGCTGTTGAGGTTGCTGTTGAGGTTGCTGTTGAGGTTGCTGTTGCGCGATGTCTGGT
TGCTGTTG

TGCTCTACGTTACTAAAGTAACGGATTACAAATTAAACAAAAGATGCTTCAAACCAGTTTCGGCATTCCAT
GGAGGGCTCAGTGTTATCATTGCCCCACATTGGATGGAGATGTTTAACTCTATAGAACTACAAATGTTAAT
ATCAGGTG

TGTCTTGATAGCGCCTGAAATGCGTTGAATTCGTTCCTTTTCCTCCTCTTCAGTTTCCTCTGCACGTTGTC
TTGTACCCACACTTGGCCAAGAAAACCCATCTCTCTCGACAGGAGGGTTTAAAGTGTATGGAGAGGTAGGT
CTAATGTT

GCGTGTTGAATGCTTCCTCTACGGAAATAGAACCTCTGATGAAAATCAATTTGTTCTGTTCCACCAAATTT
GACAGAGGAGTGTGTTGCCACTCCTGCCTATGAACAGATAACAGATTCTGTGAATTCAAGGTGGACAAGGA
GTTGCCAT

GAGCTCCACTTCGAACCTCGTGGGGAGAGGATTTTGATCCGAGGAAGGTGTCGCTGGTGCGTTTCCATTAG
TGCTTGACATCCTGGGTGGAGCTTGGGGGAACTTTGCCGTCTTATGAGTGCTATAAGTTTTGGCTTACTTT
GCCTCATT

TCCGAAGCGGATTTACAAGCACCGCTGGACTCGTATGGGGTATAAGTGATACCTCTGACACCAGAGGCAAA
GCTGCTGACGTCTTCGCCGGAAGAACTAGAGGAAGAAGCTTGGGAGGAAGAGGAAGATTGAGTAGATGTAG
CAGCAGAG

GATTACAGGCCTAATTGAAGTCAACGAAAAGGAATTGGATGCAACTACCAAGGCAAAGACAGAAGACTTCG
TCAAGGCATTCCAGGTCTTCGACAAGGAAAGTACAGGCAAGGTATCCGTTGGTGACTTAAGGTACATGCTA
ACTGGCTT

CGTACAGGGTCCTCAGACGACGAGGATAGTGGAGCTTATGACGAGTGGATTCCATCGTTCTGTTCCAGATT
TGGTCACGAGTACTTCTGCCAAGTCCCGACAGAGTTTATCGAGGATGATTTCAATATGACCTCGTTATCCC
AAGAGGTA

AGACCGGTATCGTTTCTCGAAGACTGCGCACAATACTTACAGTCCTCAGAACAACCACCAGATTTGATGTT
CATCAATGTGCACAATTGCACTTTGGTTGGATCGTGCCACTTTCTGTGCTGCAATTGTGCTGCATGAGTAA
GTTCGAGC

CACCTCACTTAAGCCCATGGGGTGATGCTATTGGTTACGATCCAACTGCTCCTGTCGAAGAAGGCGAAGAA
GAAGAAAGTGAAAGTGAAAGTGAAAGTGAAGATCAAGTTGAGGAGGAAGATCAAGAAGTTGTTGCTGGAGA
AGAAGATG

CTTAGCCTTTGGATATTGCTTCAAAGACTTCAAGAAAGCCTTATCAATAGCATCCAAACCCTTCTTCTTTC
TGGAAGCAGCCAAACAAGCAGTCAACATCAAGTCGTCTGGAGAAACACCTTCAACAGTGTATGGTTCGTGC
AAGGACAA

GAAAATCAATTTGTTCTGTTCCACCAAATTTGACAGAGGAGTGTGTTGCCACTCCTGCCTATGAACAGATA
ACAGATTCTGTGAATTCAAGGTGGACAAGGAGTTGCCATTATTGTTTGAGTATTCCGTGTTATCCAGCGTG
GAGAGAGA

GAGATACGGTGCTTCTGCCGGTAACGTCGGTGACGAAGGTGGTGTTGCTCCAAACATTCAAACTGCTGAAG
AAGCTTTGGACTTGATTGTTGACGCTATCAAGGCTGCTGGTCACGACGGTAAGATCAAGATCGGTTTGGAC
TGTGCTTC

CCACGGAAGTTGCTTCATCAGCACTATTTTCGACAGCATTAGTAGTAGAAGTGCTGGTAGTAGAAGTACTG
GTGGTAGTGGTTGTCGTAGTTGCAGTGTCAGCAGTTGCTTCAGTGGCCTCAGATGTGGTAGTAGAAGTAAC
GTCCCCTT

GACACACCAGTTTCTTATACAACTTCAAGTTCGTCTGTTTCTCAAGTTTCAGACACACCGGTTTCTTATAC
AACTTCAAGTTCGTCTGTTTCTCAAGTTTCAGACACACCAGTTTCTTATACAACTTCAAGTTCATCTGTTT
CTCAAGTT

TGAGCGCACCTCAAGTGAGAAACGCAGGCCCAGAGATTCTCCAGGCAGGAAGGAATTGGGACAGATCCTGC
CTGGAGGGATGGAGGAATTTACTTCGTAGGCTGGAACAGGCTCATCTCAGCCAACGATTGTAAATGTGCAG
GGCCATCG

TTCGACTTCTTCAGTGTACGTTCCCTCCTCTTCAACTTCATCTCCACCTTCGTCCTCATCCGAATTGACAT
CATCCTCGTACTCATCATCCTCATCCTCATCCACCCTCTTTTCCTACTCCTCCTCATTTTCATCATCCTCA
TCCTCATC

TTGAAACCAGTTAACATGAGAGGTATTAAATCCACTGCTATGGTATTGTGTGGTTCTAACGACGACAAAGT
TGAATTTGTCGAGCCACCAAAGGACTCTAAGGCCGGTGACAAGGTGTTCTTTGAAGGTTTCGGTGACGAAG
CTCCAATG

TTCAGGTAAGCCATCAACATGTCCACTAGGTAGTCCATGTGGCGGACCTGCTGGGAACGGTCGTCATAGAA
ATATCCCAAGCAAATGAAGTCGACGAGTCTGTCGTTGGCTGCTCCATTCAGTTCATCCAGCAAAGCTGTTT
CCAACAAG

TCTCAATGGGCCGTGGACGAATTCCCCAACGGTAGATTCGGTGATTCGTCTTCTCCTGCGTTCGGTGACTT
GGATCTGTGTGGTTCAGACTTGATCAGGCAATCAGCGAACAAATGTCTCGAATTATGGTCCACCCCTACTT
CCATCAAC

AGAAGAAAAAAAGCTGTTGAGCGAGCTATTTCGGGTATCCCAGCCTTCTCTGCAGACCGCCCCAGTTGGCT
TGGCTCTGGTGCTGTTCGTTAGCATCACATCGCCTGTGACAGGCAGAGGTAATAACGGCTTAAGGTTCTCT
TCGCATAG

CATTTCTCCACTTTCGCATACCTTGAAGTGATGAGACCCAACCAGCTCACCTTTCACCCCATCACTCCACT
CCCCATCAAAGAACCTGTACATTTACCCATACAACTGACACATGTATCGTGGACGGCCTTAATAACGTGCA
CCATGGTA

CTTTTGAATAATCTTGAAAAAGCCATCTTGGTCCAAACTACCACCAGCAGCAGATTGCTTGTCTGGATGGT
ACTTGACAACTTGTTTTCTGTGAGCCTTGATGATTTGACTTTCAGTAGCTCTGAAACGCAACTTAGACAAA
CCCATAGC

GTAGGCGACTTGTTACCAACTTGGTTGTAAACTTGCAACAAAGCAGAGGCACCTAGTGACTTAGTCTCTTG
TTTAGCTGATAGATCAACCAAGACAAGGACAGAATCATCTGTGTTTCTATTTAGCAATGGAGTCCACGTTT
TACTAGTG

CTTGGGATGGGGTAGCTTGGTTACCGTTAACCAATTCGTTACCAATGGAAACAGTGGTGACATCGTCCCAA
GAACCGTAAGATTCAACAGCAGACTTGATGGTGTTGACACCGTCTTGGATTTGGTCAACGTAGTAAATACC
TAAAAAGA

AACACTTACCAATATCTGTTAACGATGGTGTAATGAGAGTAGTATGCGCACCAGTCCTAGATGACGCAGCT
GCAGCTTCGCAGCCTGCATGTCCAGCACCGATGACCACGACCTGTGTCTTGGTTGTGGGTTGGAAGCTTGT
CAAAGAAG

TAGAAGAAGAAGTAGAGGACGACGTAGAGGACGACGTAGGTGTATTGGTTGATTCGGTGGACTCCTGAGAA
GCAATTGAGGTACTCGTTTGTTGATTCAAAGTTGAAGTCGCAGCTGCTTGGTAAGAATGATGCTGACTTTG
AGTGTTGC

TCGTAACCCATATCATCTTGTGGCGAGTCGTAGAATGGTGAGATCCAAATGGCATCGGCACCAAGCTCTTT
GATGTACTCCAGCTTGGAGGCAATCCCTTTCATGTCACCCCAGCCATCGTCATTAGAGTCTTTGAAACTTG
CTGGGTAA

GTGGTAGTTGGCTTGTTTCCAAATAACCCACCTCCAATGGTGGATTGTTGAGGTTGTGCTGTTTGTTGCCC
AAATAAACCTGTTCCAGTAGCTGTAGTTCCTGAAGCGGTAGTGCTTGAACTTCCTAGGCTGCCAAGAGCAG
GCTTATTA

GTACATGCATTCACTCATTGTCCGGTGTAGACTATAAATCACTGGATGCAACTTGTCAATAACTTTCAACA
ACTTCTCAGCATTTGCAAAGCTGATTTCTTTGTTCTTTGGCAGTGTCAAAGCTGCGACATCATCAGGCCTG
AGCTTGTT

ATTTCGCACAAGCACCTTCTGCTGCAGAATTTCACACCGAATCTGTGCAACGTGTCAAGAACTTGCATTTG
GCAGATTGTTTGAGAGCTAATAAGTCCACGATGGCTTGGGGTCTAGAAGCTCGTGTTCCCTTCTTAGACAA
AGACTTTT

TTTTTATGACACCAATCTATCATTTCTCCACTTTCGCATACCTTGAAGTGATGAGACCCAACCAGCTCACC
TTTCACCCCATCACTCCACTCCCCATCAAAGAACCTGTACATTTACCCATACAACTGACACATGTATCGTG
GACGGCCT

GTCAGATTCGTTGGTATTTCTTTGGCCAACCAACAAGTCGTTCAAACTTAAAACTGAACTTGGTCTCTTCA
ACTGTACGGAACTCTGAGTTTGTGCTCTGAGACTTCCATTGTTGTTTGGATTTGCATTGTTCAATCCTTGA
AAATCCGT

TTTAAAGTAAATTCACTTAAGCCTTGGCAACGTGTTCAACCAAGTCGACAACTCTGGTAGAGTAACCGTAT
TCGTTGTCGTACCAGGAGACCAACTTGACGAACTTTGGAGACAATTGGATACCAGCGGAAGCATCGAAGAT
GGAAGAGT

ATTCAGGATATGATTTCTAGGGGCAAACTCTTTGTCATAGGTGAAGAAGAGACGAAAGTTACTAAGGAGCT
CTACCTCGTTGACTCTGAAACCGGAAGAGGGGAGGGATTTCCCACTGATAATGTTGTCAAGTACAAGAATA
TTGCTGAG

TATGAGATACTTCCACAAGCAACAAGCTCATTTCTGGAAGCCAGTCTTGAACTTGGACAAATTGTGGACAT
TGATCCCAGAAGACAAGAGAGACCAATACTTGAAATCTGCTTCTAAGGAAACTGCTCCAGTTATTGACACT
TTGGCAGC

ACGAAGGAGATGGCTCTGACAATCACGAGGAAGGTGGAGAAGAAGGAAGTAGAGGAGCTGATGCTGATGTG
TCCAGTGCACAGCAGCGTGCCGAAAGGGTTGCGGACCCATGGATATATCAAAGATCTAGATCAGCTATTAA
TATAGAAA

TATTTGTATGCAGGAACGACACTTCTGGTATCGTACGTGCTACGACGGTTGACATTTGGAAGGCATTGGTT
CCAAATACTCCAAGAGCTGTGAAAGAGATCCTTCCAACATTGACTGGTATGATAGTCACTCACTTGGCTTC
ATCATCCA

GAGAAATCTTACGAAATTCTAAAAGAGCATGATGTCGATTATGTCTTGGTCATCTTTGGTGGTCTAATTGG
GTTTGGTGGTGATGACATCAACAAATTCTTGTGGATGATCAGAATTAGCGAGGGAATCTGGCCAGAAGAGA
TAAAAGAG

TGGTATTGTGTGGTTCTAACGACGACAAAGTTGAATTTGTCGAGCCACCAAAGGACTCTAAGGCCGGTGAC
AAGGTGTTCTTTGAAGGTTTCGGTGACGAAGCTCCAATGAAGCAATTGAATCCAAAAAAGAAGATCTGGGA
ACACTTAC

CTCATGACGGAGAGATACTATGCCATATATCTTCATTGTGCGACGAGCCTGGATGCCACAATAGCACCACC
GCAACTTGAACTTGTAACTGATCCTCAGCTTTCTATGGACAGTTTCCTTGAAATGATTAGGGTATTTACTG
TACCAGGA

CACGTTTCGAATCTTCAGTATGAGTTGACGGAGGTGTGGAATCGGTTGGACTCACAGCTTTTGAAAGGACA
TTTCTCGGTTGCTCAGGATGTAGTTCAATGTCGGATTGGAAGTCATGGTCAGATTCTATGTTAAGATCATT
GGATTCTT

GGCTATCTATATTTCGCACAAGCACCTTCTGCTGCAGAATTTCACACCGAATCTGTGCAACGTGTCAAGAA
CTTGCATTTGGCAGATTGTTTGAGAGCTAATAAGTCCACGATGGCTTGGGGTCTAGAAGCTCGTGTTCCCT
TCTTAGAC

CTTACCAATATTCAACTCCCAAGGTTGTTCATTACCTGGTATCAAAAGGTCGCTGACAGAGAATCCACTCA
AACCACACTTAGTCTTGGAACCTCTGCCTGTAGCACCCTCGTCTCTGATTTCACCACCAGAACCTGTAGCA
GCACCTGG

TCTTCAAATGTTTTGTAACCCCTTTGATCTCCATATTGGTTATTTGTTGCGTTAAAGTGTTGCTGTTGCTG
TTGCTGCTCATCCATGAACCACTGTTGCTGCTGCTGCTGTCTTGAACTATGATCAACTTCGGACGCATTTA
AATCTATT

CCAAATTCGAAAGAAGTAAATCAAGCCACAAGAATGAAAGGCGTGTTCTTGAGATACAAACCTCCCTGGTG
AGGATGTTTGAGAAGAATGTCATGCTGAATATCTACCCCAGAACAGTTATCGATATCGAGATCCATGTCCT
TGAGCAAG

CGTCAATGGCGTCGTAATTAATCTTCTTGGAGAAACTTGCCTTCTGCAACATGTTCTTGACACTATCAGCA
GTTGTGAAATCACCACTTTCCTCAGCTGCCTTCAATGCAGCGTGTAAACCAGACTTATCTTGTAAGTCTGA
CATCAATC

TTCTTGCACTCTTTGCCAGTCAAGGAATTCCAAATCATTGACACTTTGTTGCCAGGTTTGCAAGACGAAGT
CATGAACATCAAGCCAGTTCAAAAGCAAACCAGAGCCGGTCAAAGAACCAGATTTAAGGCTGTTGTCGTTG
TTGGTGAC

TATTGATGGACACCACCTGGAGAGACGCTCATCAATCTCTACTTGCAACAAGAGTCAGAACCCACGATTTG
GCTACAATCGCTCCAACAACCGCACATGCCCTTGCAGGTCGTTTCGCCTTAGAATGTTGGGGTGGTGCCAC
ATTCGATG

AAGTTCAAAGCAAGATTAGGCTTTCTGATGAATTGGTAGCGCAGAGTCTGGCAAAGTCTATTTCAAGTTCA
CAGCGTCCGTTTACCACAATTGTTTGAGAACTCTTCTCCTTCCTGTGCCTATAGCCACACTTGCAAACATT
ACATGGTG

GGTAATATTAGATCATGGCTAGGGGGATCTGGAAGTACAATGATGTGCTCTCCCCCTCTCAAACACAACAC
CAGGATGAACTAAGGGCTCATCTCGAAAGTCGAAGGTGCCTCATTCAGGTTATTAGTGGTGCCTGTTGTGT
CTTCCATG

ACTTTTGATGAGAATCCTTTGCTCTTGGGAGATATGGGTGAGTAGTCGTTGTGAAAACAATGGCTTCTGCA
CCTTGAGAAATAGGTGCAATGTCCACATCAGGAGTCAGGTAGGAAGATACTTTATCAATGAATTCTTGCGT
CATAATGA

GGAGTGTACACTGAAACAGCACCAGTGATTAAACCAGTGAAAGAAGCGATGTAACCAGAGTGACCACCTTG
GACTTCACAGACGAAGACTCTTCTTCTTGTCGCAGAAGCACTCTGTTTGATGTCATCAGTGTAATTGACTA
ATGCGTTC

CAGGTTTCAGACACATCGGTGTCTTATACAACTTCAAGTTCGTCTGTTTCTCAAGTTTCAGACACACCAGT
TTCTTATACAACTTCAAGTTCGTCTGTTTCTCAAGTTTCAGACACACCGGTTTCTTATACAACTTCAAGTT
CGTCTGTT

TTTTGTAACCCCTTTGATCTCCATATTGGTTATTTGTTGCGTTAAAGTGTTGCTGTTGCTGTTGCTGCTCA
TCCATGAACCACTGTTGCTGCTGCTGCTGTCTTGAACTATGATCAACTTCGGACGCATTTAAATCTATTGA
ATCTTCAT

ACAAGAGACAACACCTGGGACATCAGCTGTTCCAGAGAACCATCATCATGTCTCTCCTCAACCTGCTTCAG
TACCACCTCCACAGAATGGACAGTACCAACAGCACGGCATGATGACCCCAAACAAAGCTATGGCCTCTAAC
TGGGCACA

GATTTCTAACGAAGGTGTCTTGGTGGGTGGTTCTTCCGGTTCTGCCTTCACTGCGGTTGTGAAATACTGTG
AAGACCACCCTGAACTGACTGAAGATGATGTCATTGTTGCCATATTCCCAGATTCCATCAGGTCGTACCTA
ACCAAATT

ACCACCATGTTGACTGGTATTGCCCCAGACCAAGTGACCAGAATGATCACCGGTGTTCCATGGTACTCCAG
CAGATTAAAGCCAGCCATCTCCAGTGCTCTATCCAAGGACGGTATCTACACTATCGCAAACTAGAGACAAA
CGCCTTTA

TTGGGAACTTGTAGGCGTATAAGAGTGTGAGTGAGCATATGTTTGGGATGAGGACGTTGCTGGCATTGTCG
AGGGCCTTTGGTGGGGTCTTGATTGGTGCCCTTGCGCCAAGTTAGCTGCTGGTCTTGGTGGTCGTGGCGGT
TGAGATTG

TGACGGTTCTCAAAGCAGCAACGTGCTTTCTGCCACCGTTGTGAACAGCAACCTTGATCAATTGGTTGTTG
ACCTTGGTGAAGGTAACATCAATGTGCTTCAAGTTCTTGGTCAAAGTACCTCTTGGACCAACAACCTTGAC
GATTCTGG

CGTCCTCATCCGAATTGACATCATCCTCGTACTCATCATCCTCATCCTCATCCACCCTCTTTTCCTACTCC
TCCTCATTTTCATCATCCTCATCCTCATCATCCTCATCATCATCCTCATCCTCATCATCATCATCATCATC
ATCATCAT

AAGGCAAAGAAGAACGCATATAGTAGAGAGTAAATCAAACACACGTAGAAACAAGTGGCAACTAGAACAGG
TTCAGTGACAGCGAAGTACAAAGGTCTCAACAGACAAGCCCTCATCATTTCACTCATACTGACACCTTGCG
CTTCCTGC

CTCGCTGAATCCATCTAATGCATTATCAAGAGCCCTCGGTATTGCATCAACGAGATTGTTTGGTGGTGCAA
ATCAACAGCAGCAACAGCAACAAATCACATCTTCCCCACCGTACAGTCAAACTTTGTTGAATTCCCAACTT
TTTCATGA

AGAAGGCTTCCACTAAGGCTAACTCTCAACAGACAACAACACCTGCTTCATCAGCTGTTCCAGAGAACCCC
CATCATGCCTCTCCTCAAACTGCTCAGTCACATTCACCACAGAATGGGCCGTACCCACAGCAGTGCATGAT
GACCCAAA

TGGGTATGAAATTCATCACCGATTTGGTTATCAACCACTGTTCTACAGAACACGAATGGTTCAAAGAGAGC
AGATCCTCGAAGACCAATCCGAAGCGTGACTGGTTCTTCTGGAGACCTCCTAAGGGTTATGACGCCGAAGG
CAAGCCAA

AACGTAAAGGAATATGGCCAGCATGCATCTGTGGAATATTTGAAGGGTCTGGCACTTCAGATAACCCTGGC
AACAGCTCATATGCCACTTTGTACTCCTCAGATGACGGAAGGGATACTGAGATCCTTATTAAAGCAGACAT
GGCTAGCC

AATCTGGTATACTGGTAGCACTAGAACTAGCACTATTTATTGGAGAGCCCACAACCTTCTTCTTTCTGCCT
CTTTTACCTCGAGGGTGGGAGTTTGTATGAATTCTTCTGTGTCTCGTCAGTTCATCGCTTCTACTGAACCT
TTTCACAC

AATATTGACGAAAATTGCATCTACGTTCTTATTGGATAAGATCAATTCGAAACCTTGTTTGATGGTCTCAG
GGGTGGCACCACCACCACAATCCAAAAAGTTCGCAGGATCGCCTCCATTTAATTTGATGACATCCATAGTA
GCCATAGC

ACGTACCTGTAGTGATTATGACCTGATCTGCCCCAACCTGGGTACCGTCATCCAGAACCACACCTTTGATG
ACCTTGTGGCCACATCCGGGGTCATACAAGATCAAGTCAGCAACTTTGTTCTGCAGCAGAGACAAGTTGGG
GTGTGCTT

GTAAGGTTGGTATGAGATACTTCCACAAGCAACAAGCTCATTTCTGGAAGCCAGTCTTGAACTTGGACAAA
TTGTGGACATTGATCCCAGAAGACAAGAGAGACCAATACTTGAAATCTGCTTCTAAGGAAACTGCTCCAGT
TATTGACA

CACTTGCCTCATGGTGTTTGTAACGCTGTCTTGTTGCCTCATGTTCAAGAGGCCAACATGCAATGTCCAAA
GGCCAAGAAGAGATTAGGTGAAATTGCTTTGCATTTCGGTGCTTCTCAAGAAGATCCAGAAGAAACCATCA
AGGCTTTG

CAAAAGCAAACCAGAGCCGGTCAAAGAACCAGATTTAAGGCTGTTGTCGTTGTTGGTGACTCTAACGGTCA
CGTTGGTTTGGGTATCAAGACCGCCAAGGAAGTTGCTGGTGCCATCAGAGCTGGTATCATTATTGCCAAGT
TGTCCGTT

TTCCAAGAAATGTACGACTACTGTGTTCAAATGATCAAGGATGGTAAAGCTTACTGTGACGACACTCCAAC
TGAAAAGATGAGAGAAGAACGTATGGATGGTGTTGCTTCTGCCAGAAGAGATCGTTCTGTTGAAGAGAACT
TAAGAATT

ACAATGGATGGTTGACAAGGACTTGGTCGGAAATTGGGACGATCCAAGGTTCCCAACTGTCAGGGGTGTGA
GAAGAAGAGGTATGACTGTCGAAGGTTTGAGGAACTTCGTCTTATCCCAAGGTCCATCCAGAAATGTCATT
AACTTGGA

TAGAAGATTATACAGATAATTACTATCTTGGATACATAGATGCACCAGTCACAAGAAATCCATGTTCAGAC
CACCTAATTCCAGATTGTTCAGGTCTAGAGAATCCAGTTCTTTCCCCCCGTTATTCATGTTATTCATGCCC
ATATTCAT

CAATGCAGAGGACAAGACAACTGTAGGTGAGTCCGTGAATGGTGTTCAACAGCCGGCCTCCTCCCAGTCAG
ATGCCATGAAACAAGACAAGTACGTCAGCACTAAACCAAGAAATAGTAAGAACGATGGTGTGGATACAGCT
ATATACCA

TTCTTCGCCCAACTTGACATCCATTTCCTCAGCAACTCTCTGTGCAACAGACATTGCTGCGACACGACGAG
GTTGAGTACATGCAACTTGAGTGTTCTCTAAGTGTGGCATCTCATCGAATAAAACGAATTGAGGAATTTGT
GTCGTTTT

ACCAGACAACTGCACTAACTGAAAGATTCTTCCCAGGATTCGAACTTGATGCTGATGGAGACACTATCATC
GGTGCCACAACCCACTTACAAGAAGAATACGACTCTGACTATGATTCAGAAGATAATCTGACCCAGAATGG
ATACGTCC

AAAGGTTTGACTTCAGTATTCTGTCCTGGTTTAACATTAGACAGGTGTTGAGAGGCGCCTGTATCCGTCAC
AGAAGCACTTGCTGTCGTCGACGTTACAGGTGTTTGGAGGCTGTTATCATTCAAAGCAGTGACAATAGCAC
CTCTAATA

TCCCTAGTTGTGGCTGAAGTGGTTGAAGTTGAGGTTGCTGTTGAGGTTGCTGTTGAGGTTGCTGTTGAGGT
TGCTGTTGAGGTTGCTGTTGAGGTTGCTGTTGAGGTTGCTGTTGAGGTTGCTGTTGAGGTTGCTGTTGCGC
GATGTCTG

ATGTCATATCAGAGTCCGCTGAGGATGAATCAGTAAATGTATTACCTGACTCAGGTGATGGAGTGCTCAGA
GGCGTTCCAACTGATGATGGATACTGCGGAAACTGTGATTGTGGCCCAGGTGGAAAGTACATAGGCGACAT
TTGATAAG

TTGGAGAAGAAGTTATAGCAGCGCTGCTACTAGAGGAACCTTCAGAGGTGGTAGCGGCTGTTTCCTGTGTG
GATGAAGTAGAGGACGAAGTAGAGGACGAAGTAGAGGAAGAAGTAGAAGAAGAAGTAGAGGAAGAAGTAGA
AGAAGAAG

TGCTAATCATGCGCACGACGATGAGGCTTCCACAGACGTTGAAGGCTCCACAGACGTCAATGTCAATGAAC
AGGCTCTGTTGCAAGAGGATTTTGACATGTGGAGTGAGACTATATTGCAGAAAACACAAGACGTCATACAG
CTATTTGA

GAAGATGAACCTTATTATTTCTTACAAATGAATCCCTACATCAAAGACTTGAAGGAAAGAGAGAAGGCACA
AAGTTCTCCAAAGCAGGACCATGACGAATTTTTGAAGTCCTTGGCAAACACTTTCCTTTCCTTGGATACAG
AAGGCCGT

ACACGTTTCGAATCTTCAGTATGAGTTGACGGAGGTGTGGAATCGGTTGGACTCACAGCTTTTGAAAGGAC
ATTTCTCGGTTGCTCAGGATGTAGTTCAATGTCGGATTGGAAGTCATGGTCAGATTCTATGTTAAGATCAT
TGGATTCT

GATAGACGCTTGTCTATATCCTCGCTGAATCCATCTAATGCATTATCAAGAGCCCTCGGTATTGCATCAAC
GAGATTGTTTGGTGGTGCAAATCAACAGCAGCAACAGCAACAAATCACATCTTCCCCACCGTACAGTCAAA
CTTTGTTG

AAATCTTGTAAAGACTTGACGGTCTCAGTCTTAGTTAACTTAAGTTGATTTGCACGAGAGTTGGTTTTTGA
GGGGACATACTGTTGAGGTTGTTGGCCCTCAACGTCCACCATGGAAGTGTTTTGTGAGTTGAATGAAACAA
CAGATTCT

ATTGTATCAATAAAGGCGAATTGGTACCTGCCAACAAATATCTTCCAGGGGAGGCTCTTCCACCTCACTTA
AGCCCATGGGGTGATGCTATTGGTTACGATCCAACTGCTCCTGTCGAAGAAGGCGAAGAAGAAGAAAGTGA
AAGTGAAA

GCTTCCAATTTACCGGAATTTGATAGAGATTCCACTAAGGTTAATTCTCAACAAGAGACAACACCTGGGAC
ATCAGCTGTTCCAGAGAACCATCATCATGTCTCTCCTCAACCTGCTTCAGTACCACCTCCACAGAATGGAC
AGTACCAA

TATGGTTTCCCACAGATCTGGTGAAACTGAAGACACTTTCATTGCTGACTTGGTCGTCGGTTTGAGAACTG
GTCAAATCAAGACTGGTGCTCCAGCTAGATCCGAAAGATTGGCTAAATTGAACCAATTGTTGAGAATCGAA
GAAGAATT

TTAAAGCGTTCATGAAAAGTCACAGTCCAAGGAGTATTTCAAATTGTCACTTGAAGTGAAAACACCCCAGT
GCTTCTCGACATCAGAGGTACCAGAGGTGTTTGGCTTCCAATCTTCATCAAAGGCTTCAAAAACAATAACG
TTAACACC

GGTCTCAGTCTTAGTTAACTTAAGTTGATTTGCACGAGAGTTGGTTTTTGAGGGGACATACTGTTGAGGTT
GTTGGCCCTCAACGTCCACCATGGAAGTGTTTTGTGAGTTGAATGAAACAACAGATTCTTGATCACTCATA
TTTGTTTT

TCAACCAAATCGGTACCTTGTCTGAATCCATCAAGGCTGCTCAAGACTCTTTCGCTGCCGGTTGGGGTGTT
ATGGTTTCCCACAGATCTGGTGAAACTGAAGACACTTTCATTGCTGACTTGGTCGTCGGTTTGAGAACTGG
TCAAATCA

TCAAGTGAGAAACGCAGGCCCAGAGATTCTCCAGGCAGGAAGGAATTGGGACAGATCCTGCCTGGAGGGAT
GGAGGAATTTACTTCGTAGGCTGGAACAGGCTCATCTCAGCCAACGATTGTAAATGTGCAGGGCCATCGTT
ACAATCCT

ACTAACTTCAATCGCTGCTGGTGTTGCCGCCATCGCTGCTACTGCTTCCGCAACCACCACTCTAGCTCAAT
CTGACGAAAGAGTCAACTTGGTTGAATTGGGTGTCTACGTCTCTGATATCAGAGCTCACTTGGCCCAATAC
TACTCTTT

GGTGTTGCTCCAAACATTCAAACTGCTGAAGAAGCTTTGGACTTGATTGTTGACGCTATCAAGGCTGCTGG
TCACGACGGTAAGATCAAGATCGGTTTGGACTGTGCTTCCTCTGAATTCTTCAAGGACGGTAAGTACGACT
TGGACTTC

TTAGGGTCATTTTTCAGTACATTGGTTGGCCTGGCTTGAGTTGCAACCAGTGCCAAGCATCAGCGTTGGCC
TCTCTCTTGTACATTGGTTGGCCTGGTCTCAAATTTAACCAGTGCCAAGCGTCGGCAACAGCCTCTCTTTT
CGCCAAAG

GCGATGTATGGTAGCTTTGTTTCTACCTTGTGGTACTTTGACAACTCTTCGTCGTAGTGGAAGTCACCACT
GTTGACCCAAACGATCTTTGTGTAACCTTCGGACACCTCATTCAAAGTGCTTGCCATTCGAACATGATAGC
CGAGTGGT

ACATAAACAGGACTACGTCACCGACGGAAGCAAACGAAGTATCTTCACGATCTCATGCAGTGCTGCAAATA
CACATCATGCAGACAAATAAGCTTGTAGACTTGACCTCGCAACATACATTTGCTACACTTTCAATAATCGA
TCTAGCTG

TAGTAGAGAGTAAATCAAACACACGTAGAAACAAGTGGCAACTAGAACAGGTTCAGTGACAGCGAAGTACA
AAGGTCTCAACAGACAAGCCCTCATCATTTCACTCATACTGACACCTTGCGCTTCCTGCTCAGTCATAATC
TTGGGATT

ACTTTAATTGAACCATTAGAAGAACATAGCAAGAATTTCATGTTCGTCACCGAGTTTGTCACTAGCTCATT
GGAGACCGTTTTCAGAGAAACCGATGATGAAGAGCAGAATTTCTTACAAGGTCATGTTAAAGATAATATCG
TGGTACAG

AATGACAGGGAAGGCAAAGAAGAACGCATATAGTAGAGAGTAAATCAAACACACGTAGAAACAAGTGGCAA
CTAGAACAGGTTCAGTGACAGCGAAGTACAAAGGTCTCAACAGACAAGCCCTCATCATTTCACTCATACTG
ACACCTTG

GTAACCAGAGTGACCACCTTGGACTTCACAGACGAAGACTCTTCTTCTTGTCGCAGAAGCACTCTGTTTGA
TGTCATCAGTGTAATTGACTAATGCGTTCAAACAGGTATCAACACCAAGTGAGTATTCAGTACCTGGAACG
TTGTTAGA

GTGGTGAAAGATTCTTCAACATCAAGTGCCGTTCCTCTGGATTGACACCTAATGCTGTGGTCTTGGTTGCT
ACTGTTAGGGCATTGAAGTCACACGGTGGTGCTCCAGATGTCAAACCTGGCCAACCTTTACCTTCCGCATA
CACTGAAG

TCCGGTGTAGACTATAAATCACTGGATGCAACTTGTCAATAACTTTCAACAACTTCTCAGCATTTGCAAAG
CTGATTTCTTTGTTCTTTGGCAGTGTCAAAGCTGCGACATCATCAGGCCTGAGCTTGTTATCATGTCTTGC
CAATAAAA

CAATTCTATGCTATGTGAACTGTCCTTGAAAGTGACCTAGGCCACTTCGACAATTTCATCTTCTTCGTCGT
CATCTTCTTCTTCTTCACTCTCCTCCTCTTCTTCCTCTTCCGCATTCTTTGATGGCATCTCTTCCACTGCT
CCCGCACC

CCGTCGCACAAGATCACAAGAGGATCTTTGATGGCGACAAGGGCTTGAACACCGGTGGGATGGGTGCCTAT
GCCCCCGCTCCTGTGGCCACACCATCTTTGTTGAAGACCATAGATTCACAGATTGTGAAGCCTACGATTGA
TGGGATGA

TTCAATATGACCTCGTTATCCCAAGAGGTACCCCACTACCGGAAAGCTCTGGATTTGATATTGGATTTGGA
GGCTATGAGTGATGAAGAAGAGGACGAAGACGACGTGGTGGAAGAAGACGAAGTGGATCAAGAGATGCAGA
GTAATGAC

GAAGCACTCGTATAAAGTCTCTTAGTTTCGTCGGAGGCATGTTGCATTTCACCAACTGTCATAATCTCCCT
GCCATCCTTCACTCTGTTTCTGATGAATTGATTCATTTCTTGATGGAACTCGTGAATACGTGGTCCATTCA
ATGTGTAT

AAACCACCTCACGCAAGAAGAACAGGCGGTGGACGTATTGTCCCCGGAGTTCCTCCTGGTGCAGCAAAGAA
GACCATCCCAGGGCTAGTTCCGGGCATGAGTGCCAACAAGGACGCCAACACCAAGAACAGGAGAAGAAGAG
CCAATAAA

GATTTCGCTCGTATCAATTTCGTTAGAACCTTGTTGTCTAAGAGAAAGTTACAATGGATGGTTGACAAGGA
CTTGGTCGGAAATTGGGACGATCCAAGGTTCCCAACTGTCAGGGGTGTGAGAAGAAGAGGTATGACTGTCG
AAGGTTTG

GATTAAAGCATCGATATCGTCATCGTCAGAAGATTCAGATGCAGCGTCATCGTAAGTCTTAGCAGGCTTTT
CTTGAGTTGGCTGATGAGCTGAAACAGAAGATGCTGAAGAGGATGATGAAGAGGATGATGTATCAGTCATA
TTGATATT

AGCTAACTACAACAAGTACTCAAACAGTGGTTGAAACAATTACTTTGGACCAGGAAGAGGCTAGCGCTGTT
GCTAATCATGCGCACGACGATGAGGCTTCCACAGACGTTGAAGGCTCCACAGACGTCAATGTCAATGAACA
GGCTCTGT

TGCTAGCACCAACTCCAGCACTAATGCCACTACCACTGCTAGCACCAACTCCAGCACTAATGCCACTACCA
CTGAAAGTACCAACGCTAGTGCCAAGGAGGACGCCAATAAAGATGGCAATGCTGAGGATAATAGATTCCAT
CCAGTCAC

CAAAAGCAGGTATTACAACTTAATAGAGATTCGTACCCCTTCGAGGCGGGTTTGGAGCTACCTTTCGAAAT
GGTGACAGAAGTCCCCATTCCTAGGCGACCACCGCCACCACAGGCTGCAAATAACACAAACTCTGTATCAA
ATAACACA

CATCACTACTTTATTGGTAACTACGCTTCCTCTTTCAGATGGTGGGATTACTGTCTAGACACTGAATCTGG
TCCAGAAGCTAAGGCCTCCAGAGAAGAAAGAATGAAGAAGAGAGCTGAAAACAATGCTCAAAAGAAGACTA
ACTAAGAG

CATGAAAAGTCACAGTCCAAGGAGTATTTCAAATTGTCACTTGAAGTGAAAACACCCCAGTGCTTCTCGAC
ATCAGAGGTACCAGAGGTGTTTGGCTTCCAATCTTCATCAAAGGCTTCAAAAACAATAACGTTAACACCCC
AAGCTCTC

GTGGATGAAGAGGAAGATACAGAAGACGAAGAAGATACAGTGGAAGAAACCGAGGAAGACACAGTGGAAGA
AGCCGAGGAGGACACAGTGGAAGAAACCGAGGAAGATACAGTGGAAGACGCCGAGGAAGATACAGTAGAAG
AGGATGTA

CCCATTTGGTGTGCAATAGTAAAGATGATGTCAACAAAATCTTGACCTCCTGTGTTGCTGCCATCCAATCG
ATAAGGTCACACCAAACAGCAAAGAAAGGACGTAAGGGACAGGTATCCCATGCTGTTGCAGCTCCTTTGGT
ATCTTTCA

CCATCAACATGTCCACTAGGTAGTCCATGTGGCGGACCTGCTGGGAACGGTCGTCATAGAAATATCCCAAG
CAAATGAAGTCGACGAGTCTGTCGTTGGCTGCTCCATTCAGTTCATCCAGCAAAGCTGTTTCCAACAAGAT
CCTATCAA

GATCTTATCGGCGGTGGACACTAACTCTTCGTCATTCAAACTGTCAACGATCAAACCAACTTGTTCGTCAA
TCGTGGTGGCCTTGTCAGCATCACCAAAGTAGTCAGCTTCCTTAGCAGAGTTACGGATGGCTCTCTTGTTC
TTTTTCTT

GTGGTGTGTGGTGTGTGGGTGTGGTGTGGTGTGGGTGTGGGTGTGGTGTGTGGGTGTGGTGTGTGTGGGTG
TGGGTGTGGTGTGGGTGTGGGTGTGTGTGGGTGTGTGTGTGTGGGTGTGTGTGGGTGTGGGTGTGGGTGTG
GGTGTGGT

GTGTGTGTGTGGGTGTGGTGTGTGGGTGTGGGTGTGGGTGTGGGTGTGGTGTGGGTGTGGTGTGTGGGTGT
GGTGTGTGGGTGTGGTGTGGGTGTGGTGTGGTGTGTGGGTGTGTGGGTGTGGTGTGGTGTGTGGGTGTGGT
GTGGGTGT

GTGTGTGGGTGTGGTGTGGTGTGGGTGTGGGTGTGGTGTGTGGGTGTGGTGTGTGTGGGTGTGGGTGTGGT
GTGGGTGTGGGTGTGTGTGGGTGTGTGTGTGTGGGTGTGTGTGGGTGTGGGTGTGGGTGTGGGTGTGGTGT
GTGTATAT

GGTGTGGTGTGTGGGTGTGGGTGTGTGTGTGGGTGTGGTGTGTGGGTGTGGGTGTGGGTGTGGGTGTGGTG
TGGGTGTGGTGTGTGGGTGTGGTGTGTGGGTGTGGTGTGGGTGTGGTGTGGTGTGTGGGTGTGTGGGTGTG
GTGTGGTG

TAAGAGACAGGTTAGATAGGGTGGTGTGTGGTGTGTGGGTGTGGTGTGGTGTGGGTGTGGGTGTGGTGTGT
GGGTGTGGTGTGTGTGGGTGTGGGTGTGGTGTGGGTGTGGGTGTGTGTGGGTGTGTGTGTGTGGGTGTGTG
TGGGTGTG

GTTAGATAGGGTGGTGTGTGGTGTGTGGGTGTGGTGTGGTGTGGGTGTGGGTGTGGTGTGTGGGTGTGGTG
TGTGTGGGTGTGGGTGTGGTGTGGGTGTGGGTGTGTGTGGGTGTGTGTGTGTGGGTGTGTGTGGGTGTGGG
TGTGGGTG

GTGGGTGTGGGTGTGTGTGTGGGTGTGGTGTGTGGGTGTGGGTGTGGGTGTGGGTGTGGTGTGGGTGTGGT
GTGTGGGTGTGGTGTGTGGGTGTGGTGTGGGTGTGGTGTGGTGTGTGGGTGTGTGGGTGTGGTGTGGTGTG
TGGGTGTG

AGATAGATGTGAAAAGTGTGGGTGTGGTGTGTGGGTGTGGGTGTGTGTGTGGGTGTGGTGTGTGGGTGTGG
GTGTGGGTGTGGGTGTGGTGTGGGTGTGGTGTGTGGGTGTGGTGTGTGGGTGTGGTGTGGGTGTGGTGTGG
TGTGTGGG

GAAAAGTGTGGGTGTGGTGTGTGGGTGTGGGTGTGTGTGTGGGTGTGGTGTGTGGGTGTGGGTGTGGGTGT
GGGTGTGGTGTGGGTGTGGTGTGTGGGTGTGGTGTGTGGGTGTGGTGTGGGTGTGGTGTGGTGTGTGGGTG
TGTGGGTG

AGGAGTGACAGCGAGAGTAAAGATAGATGTGAAAAGTGTGGGTGTGGTGTGTGGGTGTGGGTGTGTGTGTG
GGTGTGGTGTGTGGGTGTGGGTGTGGGTGTGGGTGTGGTGTGGGTGTGGTGTGTGGGTGTGGTGTGTGGGT
GTGGTGTG

GCGAGAGTAAAGATAGATGTGAAAAGTGTGGGTGTGGTGTGTGGGTGTGGGTGTGTGTGTGGGTGTGGTGT
GTGGGTGTGGGTGTGGGTGTGGGTGTGGTGTGGGTGTGGTGTGTGGGTGTGGTGTGTGGGTGTGGTGTGGG
TGTGGTGT

GTGGTGTGGTGTGGGTGTGGGTGTGGTGTGTGGGTGTGGTGTGTGTGGGTGTGGGTGTGGTGTGGGTGTGG
GTGTGTGTGGGTGTGTGTGTGTGGGTGTGTGTGGGTGTGGGTGTGGGTGTGGGTGTGGTGTGTGTATATAT
ATGTCACT

AGGGTAATGTGAGGGTAGGTTAAGAGACAGGTTAGATAGGGTGGTGTGTGGTGTGTGGGTGTGGTGTGGTG
TGGGTGTGGGTGTGGTGTGTGGGTGTGGTGTGTGTGGGTGTGGGTGTGGTGTGGGTGTGGGTGTGTGTGGG
TGTGTGTG

GAGGGTAGGTTAAGAGACAGGTTAGATAGGGTGGTGTGTGGTGTGTGGGTGTGGTGTGGTGTGGGTGTGGG
TGTGGTGTGTGGGTGTGGTGTGTGTGGGTGTGGGTGTGGTGTGGGTGTGGGTGTGTGTGGGTGTGTGTGTG
TGGGTGTG

GTGGGTGTGGGTGTGGTGTGTGGGTGTGGTGTGTGTGGGTGTGGGTGTGGTGTGGGTGTGGGTGTGTGTGG
GTGTGTGTGTGTGGGTGTGTGTGGGTGTGGGTGTGGGTGTGGGTGTGGTGTGTGTATATATATGTCACTGT
ATTGCATG

GTGTGGTGTGTGGGTGTGGTGTGTGTGGGTGTGGGTGTGGTGTGGGTGTGGGTGTGTGTGGGTGTGTGTGT
GTGGGTGTGTGTGGGTGTGGGTGTGGGTGTGGGTGTGGTGTGTGTATATATATGTCACTGTATTGCATGCT
GGATGGTG

GTGGGGAGGTAGGGTAATGTGAGGGTAGGTTAAGAGACAGGTTAGATAGGGTGGTGTGTGGTGTGTGGGTG
TGGTGTGGTGTGGGTGTGGGTGTGGTGTGTGGGTGTGGTGTGTGTGGGTGTGGGTGTGGTGTGGGTGTGGG
TGTGTGTG

AAGCCGGGTAAGGAGTGACAGCGAGAGTAAAGATAGATGTGAAAAGTGTGGGTGTGGTGTGTGGGTGTGGG
TGTGTGTGTGGGTGTGGTGTGTGGGTGTGGGTGTGGGTGTGGGTGTGGTGTGGGTGTGGTGTGTGGGTGTG
GTGTGTGG

AGGGTAACGAGTGGGGAGGTAGGGTAATGTGAGGGTAGGTTAAGAGACAGGTTAGATAGGGTGGTGTGTGG
TGTGTGGGTGTGGTGTGGTGTGGGTGTGGGTGTGGTGTGTGGGTGTGGTGTGTGTGGGTGTGGGTGTGGTG
TGGGTGTG

CGGCCCCTTTGAACCTCTTCATCTTTACCTCGTATTCCGGCACGTCCTCCTGCTGCTCGTGCGGTGCAGGT
GCAGGTGCAGGTGCCTGTTTCAGGTTTCTGTGAGCAATCTCGTTCTTATCAATCTCCTCTATCTTGGCTCC
GGAGATTT

TTCGGTCAGAAAGCCGGGTAAGGAGTGACAGCGAGAGTAAAGATAGATGTGAAAAGTGTGGGTGTGGTGTG
TGGGTGTGGGTGTGTGTGTGGGTGTGGTGTGTGGGTGTGGGTGTGGGTGTGGGTGTGGTGTGGGTGTGGTG
TGTGGGTG

AGCTTGTAGGCGGCCCCTTTGAACCTCTTCATCTTTACCTCGTATTCCGGCACGTCCTCCTGCTGCTCGTG
CGGTGCAGGTGCAGGTGCAGGTGCCTGTTTCAGGTTTCTGTGAGCAATCTCGTTCTTATCAATCTCCTCTA
TCTTGGCT

TGAACCATCATGACCACTGGATCCATCACTTGCATAAACGACAGGGAAGTCGACCGATGTCCATGATGCCA
CGGTACTGTTGTGACTTGTACCAGAGCTGACAACAACCAAACCTTGGATACTAGAACTTAGTGATTGAATT
AAGCTTTG

CACTGCTGTGGGTACGGCCCATTCTGTGGAGGTGGTACTGAAGCAGGTTGAGGAGAGGCATGATGGAGGTT
CTCTGGAACAGCTGATGAAGCAGGTGTTGTTGTCTGTTGAGAGTTAGCCTTAGTGGAAGCCTTATCATATT
CTTGAATT

CTAGTTTGTGATAGTGTAGATACCGTCCTTGGATAGAGCACTGGAGATGGCTGGCTTTAATCTGCTGGAGT
ACCATGGAACACCGGTGATCATTCTGGTCACTTGGTCTGGGGCAATACCAGTCAACATGGTGGTGAAGTCA
CCGTAGTT

CCCACTTGAGCATACGAGTACACTGAACAAGTTTATTGACAATGCAAGGGAGGAGGAAGACGCTGAAGAGG
ACTCTCAACCGACAGAAGAACCTGTACAGAAGGAAACACAGGACGCCAGCGACAGCGACAGCGACAGCGAC
GATGACTA

ACACATAGGTACTTGCTGAACGAAACAACCAAAAGAGTTGCTTGGTGCCACGATTCTCCCAAGCCTCCAAG
TGACAGAATCACCTTCACTCTGCCTGTGTTGAAAGACGCCAAAGCCCTGTGTTTTGTGGCTGAGGGCAGTT
CCAAACAA

GGAAGACCTAAATTCTTGATTAGACTTGTTACTCCTAATATGTTGCGGCACTCAAGTGGTGACATGGCACC
ACTGCCGATAACAATGCCTCTGGACCTCGAACTCCTGATGACGCTTCTTACATTGGATACGAACTGCCTCC
GAGCTTGT

GACCACGTCCGTAACCATCATCATCATAATCATCATCATAACGACTGCGTGACCCCAGCGACATTCCTCTT
CTTCTTGATGATCTTGTGTTTGGACGTGTTGAGGATTCATCAGTAGAATCAAAGGAATCTGGAATATCGTC
GTAATAAT

GCCTGTTTCGGAAGCGTTTGCCAAGTTGTGTGTGAATGAGAAGCCTCCTGCTGAATCTGCCGTTGCGGTGA
AGTCTTTGGTCTTCAAACCAAAGACTCCAAAGTCCGCCACCCCTGTTCCTATCGTTGTGGTGGCTTTGCAA
TCTACTAC

CTTGGTGTAATGCAATTCGTTGATTTGACCGGAGATATAAGGTTCCAATGCACCGAAGTCCCACTTCAAGT
CTGGCAAGGTGACTTTGGTTCTCCTTGCTGTGGTGGAGAGCAATGACAAACCACCCTTCTTGGTTAAATTA
GCAGCTGC

CATACGAGTACACTGAACAAGTTTATTGACAATGCAAGGGAGGAGGAAGACGCTGAAGAGGACTCTCAACC
GACAGAAGAACCTGTACAGAAGGAAACACAGGACGCCAGCGACAGCGACAGCGACAGCGACGATGACTACA
ATCCGGGC

GGACACACTTGTTCCTTATTCCCTGGAGAAACACATAGGTACTTGCTGAACGAAACAACCAAAAGAGTTGC
TTGGTGCCACGATTCTCCCAAGCCTCCAAGTGACAGAATCACCTTCACTCTGCCTGTGTTGAAAGACGCCA
AAGCCCTG

TCATTCCTAAGGGACTTGACCCCACAAGAGGTGGAAAGACGTGAGATTCCATTGGCTGAGGTGCTTCCAGA
ACCTTCTGGTGGTGAAGATACCGGTCTGGTTCCCCCATTACCTCCCACCGATATCGGTAGACATGTCAAAT
ACCAATGG

GATTATCTCGCTTATCAGCAGAGTACTTTAATAAGAAGTCTTCGACCTCAGTTCCTATCTCGGTAGGTGAC
ACACCTACTACCACGCCAGGGTCCATTGAAGCTGCAGGTGTGGTTGCATGGAATGAAATTCTATCTCAGAC
TGATATGA

CCCTGGAGAAACACATAGGTACTTGCTGAACGAAACAACCAAAAGAGTTGCTTGGTGCCACGATTCTCCCA
AGCCTCCAAGTGACAGAATCACCTTCACTCTGCCTGTGTTGAAAGACGCCAAAGCCCTGTGTTTTGTGGCT
GAGGGCAG

GCCTTCGGAAGTATCTTCGTTGGGGTCAGATCACCAGAATGATGGTGAGGATTCAGACACTGACAGTGACA
ACTTTTTGCAAGACCCTGAAGACGATGTGGATGAAGAAAGCACTGGTAGAGGTACAGTCACTACCACTTCC
ACATCCAC

CACTGAACAAGTTTATTGACAATGCAAGGGAGGAGGAAGACGCTGAAGAGGACTCTCAACCGACAGAAGAA
CCTGTACAGAAGGAAACACAGGACGCCAGCGACAGCGACAGCGACAGCGACGATGACTACAATCCGGGCTT
ATCCATGG

TGCAACCGTTTCCTCCAAATCATACACCACTGTTACTGTTACTCACTGTGACAACAATGGCTGTAACACCA
AGACTGTCACTTCTGAATGTTCTAAAGAAACTGCAGCAACCACCATTTCTCCAAAATCATACACTACTGTT
ACCGTTAC

CTTTTGCGGTTCATTCCTAAGGGACTTGACCCCACAAGAGGTGGAAAGACGTGAGATTCCATTGGCTGAGG
TGCTTCCAGAACCTTCTGGTGGTGAAGATACCGGTCTGGTTCCCCCATTACCTCCCACCGATATCGGTAGA
CATGTCAA

GTGTCACATCATCAACATCTACCACCACAAGTACTACGTCGTCAACCCTCATTTCCACGAGCACATCATCA
TCATCATCATCAACTCCAACCACAACATCGTCAGCCCCCATTTCTACAAGCACGACGTCGTCAACTTCCAC
TTCAACAA

CCTTCGCCTCTTGAAATAACAAATCCAACTTTGCCATCAACTTCGACAGCTGGGGCACCGTTAGCGCACTT
GCTGACATCACACTTGTCAAATGGCAAACCAATGAAGTCACGGAAGTTAATGATAGTATTCTTAGGATTTC
TGATTAAT

GACCACTGACGGTGTACGAAATCAATTTCAGAGTAATGGTTGGTGTTCAAATAACTGTGCTGGTCATCAGT
TTGCCATCGTACAGGGGTTCATGTGCTGGTGCAGTGATTCGGAACCGAGCACTCAGACATCGGTGGGAGAC
TGCAGTGG

ACCGCTGCTATGATGGGAGGCTTTTGCGGTTCATTCCTAAGGGACTTGACCCCACAAGAGGTGGAAAGACG
TGAGATTCCATTGGCTGAGGTGCTTCCAGAACCTTCTGGTGGTGAAGATACCGGTCTGGTTCCCCCATTAC
CTCCCACC

ATAAGAAGTCTTCGACCTCAGTTCCTATCTCGGTAGGTGACACACCTACTACCACGCCAGGGTCCATTGAA
GCTGCAGGTGTGGTTGCATGGAATGAAATTCTATCTCAGACTGATATGATTTCCAAGGATCATGATCAGTT
ATCAACCG

AAGAATTCGCAGCTTGTAGGCGGCCCCTTTGAACCTCTTCATCTTTACCTCGTATTCCGGCACGTCCTCCT
GCTGCTCGTGCGGTGCAGGTGCAGGTGCAGGTGCCTGTTTCAGGTTTCTGTGAGCAATCTCGTTCTTATCA
ATCTCCTC

TTCTTCCTTACCTTCGCCTCTTGAAATAACAAATCCAACTTTGCCATCAACTTCGACAGCTGGGGCACCGT
TAGCGCACTTGCTGACATCACACTTGTCAAATGGCAAACCAATGAAGTCACGGAAGTTAATGATAGTATTC
TTAGGATT

GTCGACTTCTACGACCGTAAGTGTCACATCATCAACATCTACCACCACAAGTACTACGTCGTCAACCCTCA
TTTCCACGAGCACATCATCATCATCATCATCAACTCCAACCACAACATCGTCAGCCCCCATTTCTACAAGC
ACGACGTC

CGAAAGTACCGACTCCAACACTAGTGCTACTACCACTGCTAGCACCAACTCCAGCACTAATGCCACTACCA
CTGCTAGCACCAACTCCAGCACTAATGCCACTACCACTGAAAGTACCAACGCTAGTGCCAAGGAGGACGCC
AATAAAGA

AGAAGAACAAATCAAAATATGCCTGTTTCGGAAGCGTTTGCCAAGTTGTGTGTGAATGAGAAGCCTCCTGC
TGAATCTGCCGTTGCGGTGAAGTCTTTGGTCTTCAAACCAAAGACTCCAAAGTCCGCCACCCCTGTTCCTA
TCGTTGTG

CGTCAGAAACGAACGCCTTTTGTTTAGTTAATTGGTAAATCAGCCATGTGTCCACAGTGCCGAACATCAGG
TCGTTCTCCTCATACGCCTTGGTACACAGAGGCTCATTGTCGAGGAACCAGCGCAGCTTGGAACAGGAGAA
ATACGTGG

GTTCCTTATTCCCTGGAGAAACACATAGGTACTTGCTGAACGAAACAACCAAAAGAGTTGCTTGGTGCCAC
GATTCTCCCAAGCCTCCAAGTGACAGAATCACCTTCACTCTGCCTGTGTTGAAAGACGCCAAAGCCCTGTG
TTTTGTGG

TCCGTCAATCTGTAACGACCCAAATCGTATTAAAGTTTCGCCGTCCTGTTCACTGAACCTTCCCTCATTTG
GAGAATCTCTCCTCGCCAGCGACGCAAAGTCCTTAGGCAACTCTAGTTCACCTTGAATCTCCAGCATCATC
ATCCCAAG

CCAAGTTGTGTGTGAATGAGAAGCCTCCTGCTGAATCTGCCGTTGCGGTGAAGTCTTTGGTCTTCAAACCA
AAGACTCCAAAGTCCGCCACCCCTGTTCCTATCGTTGTGGTGGCTTTGCAATCTACTACTACTCCTTCTGC
GTTGATTG

GTCAACCCTCATTTCCACGAGCACATCATCATCATCATCATCAACTCCAACCACAACATCGTCAGCCCCCA
TTTCTACAAGCACGACGTCGTCAACTTCCACTTCAACAAGTACAACATCGCCAACTTCTTCTTCAGCACCT
ACAAGCTC

GAACCTCTTCATCTTTACCTCGTATTCCGGCACGTCCTCCTGCTGCTCGTGCGGTGCAGGTGCAGGTGCAG
GTGCCTGTTTCAGGTTTCTGTGAGCAATCTCGTTCTTATCAATCTCCTCTATCTTGGCTCCGGAGATTTTG
TTCTCGAT

CGCTATTCTCCATCTATGATCAGTCCACGTCTCCATATCTCTTGCACTGTTTCGAACTGTTGAACTTGACC
TCCAGATCGTTTGCTGCTGTGATCAGAGAGCTGCATCCAGAATTGAGAAACTGTGTTACTCTCTTTTATTT
GATTTTAA

TTTTTCCAATTATGGAACGCCTGTTCCTGATCCATGGCCTGCACTTGCGACCACAATTCCACACCTGAGGC
ACCTGCCTCTTTTCCAGCATGTGGCAACTGTCCCCACGACAGGGCATCCCAGAATCCTCTGGTAAATCTTA
AATGAAAC

GCTCTCGGTGACTCTTACACCAATAGCACCTCCTCCGCAGACTTGAGTTCTATCACTTCCGTCTCGTCAGC
TAGTGCAAGTGCCACCGCTTCCGACTCACTTTCTTCCAGTGACGGTACCGTTTATTTGCCATCCACAACAA
TTAGCGGT

TGCAGAACACCGCTATTCTCCATCTATGATCAGTCCACGTCTCCATATCTCTTGCACTGTTTCGAACTGTT
GAACTTGACCTCCAGATCGTTTGCTGCTGTGATCAGAGAGCTGCATCCAGAATTGAGAAACTGTGTTACTC
TCTTTTAT

GATGATGGTGCAGGAACTACAGACGAAGATTACATGGCCTGGAAGGACTCCATCCTGGAGGTTTTGAAAGA
CGAACTGCATTTGGACGAACAGGAAGCCAAGTTCACCTCTCAATTCCAGTACACTGTGTTGAACGAAATCA
CTGACTCC

AGAATATATCGTATGATAACGTAAGAGTATTCAGTATCAACGGCAACAACAACAACAACAACAACAACAAC
AGCACAACAACAGCACAACAACATCGAACGGTTGCTACTGCGACGTTACGGATTATATCCACAGTATATGG
CGTGCAGC

GCGTTTGTCTCTAGTTTGTGATAGTGTAGATACCGTCCTTGGATAGAGCACTGGAGATGGCTGGCTTTAAT
CTGCTGGAGTACCATGGAACACCGGTGATCATTCTGGTCACTTGGTCTGGGGCAATACCAGTCAACATGGT
GGTGAAGT

GCTGAAGTTTTGCAGAACACCGCTATTCTCCATCTATGATCAGTCCACGTCTCCATATCTCTTGCACTGTT
TCGAACTGTTGAACTTGACCTCCAGATCGTTTGCTGCTGTGATCAGAGAGCTGCATCCAGAATTGAGAAAC
TGTGTTAC

TGTAACAGGGTTGGCTCCTGCAGTTGCAGGCACATAATAACCCTGTTGTGGTTGCTGTTGTTGCTGTTGCT
GTTGTTGCTGTTGCTGTTGTTGCTGTTGTTGGAGATGATTCTGAGTGTAGTGAGCCTGAAATTCCATTTTA
TCAAAACG

GAATCTTCAGTATGAGTTGACGGAGGTGTGGAATCGGTTGGACTCACAGCTTTTGAAAGGACATTTCTCAG
TTGCTCAGGATGTAGTTCAATGTCAGATTGGAAGTCATGGTCAGATTCTATGTTAAGATCATTGGATTGTT
GGATCTCA

TGAGTGGGGCAGGGTAACGAGTGGGGAGGTAGGGTAATGTGAGGGTAGGTTAAGAGACAGGTTAGATAGGG
TGGTGTGTGGTGTGTGGGTGTGGTGTGGTGTGGGTGTGGGTGTGGTGTGTGGGTGTGGTGTGTGTGGGTGT
GGGTGTGG

GTTTGTTTCCTCACGTGTGTTGGAAAAGGAAAAGGACCATGTTGAAGGCTTTGCTCCAGAAGTTGCCTGGG
TCACCAGAGCTGGCTCCTCTGAATTGGAAGAACCAATCGCCATTAGACCAACTTCTGAAACTGTCATGTAT
CCTTACTA

GTGCTACACCAGGAACCCCGCCCACTTGAGCATACGAGTACACTGAACAAGTTTATTGACAATGCAAGGGA
GGAGGAAGACGCTGAAGAGGACTCTCAACCGACAGAAGAACCTGTACAGAAGGAAACACAGGACGCCAGCG
ACAGCGAC

CAAATGATTGAGAATATATCGTATGATAACGTAAGAGTATTCAGTATCAACGGCAACAACAACAACAACAA
CAACAACAACAGCACAACAACAGCACAACAACATCGAACGGTTGCTACTGCGACGTTACGGATTATATCCA
CAGTATAT

AAAAGAGTTGCTTGGTGCCACGATTCTCCCAAGCCTCCAAGTGACAGAATCACCTTCACTCTGCCTGTGTT
GAAAGACGCCAAAGCCCTGTGTTTTGTGGCTGAGGGCAGTTCCAAACAAAATATAATGCATGAGATCTTTG
ACTTGAAA

CATCTATGATCAGTCCACGTCTCCATATCTCTTGCACTGTTTCGAACTGTTGAACTTGACCTCCAGATCGT
TTGCTGCTGTGATCAGAGAGCTGCATCCAGAATTGAGAAACTGTGTTACTCTCTTTTATTTGATTTTAAGG
GCTTTGGA

CTTATCAGCAGAGTACTTTAATAAGAAGTCTTCGACCTCAGTTCCTATCTCGGTAGGTGACACACCTACTA
CCACGCCAGGGTCCATTGAAGCTGCAGGTGTGGTTGCATGGAATGAAATTCTATCTCAGACTGATATGATT
TCCAAGGA

CAGTCCACGTCTCCATATCTCTTGCACTGTTTCGAACTGTTGAACTTGACCTCCAGATCGTTTGCTGCTGT
GATCAGAGAGCTGCATCCAGAATTGAGAAACTGTGTTACTCTCTTTTATTTGATTTTAAGGGCTTTGGATA
CCATCGAA

GTTTATTGACAATGCAAGGGAGGAGGAAGACGCTGAAGAGGACTCTCAACCGACAGAAGAACCTGTACAGA
AGGAAACACAGGACGCCAGCGACAGCGACAGCGACAGCGACGATGACTACAATCCGGGCTTATCCATGGAT
TTCCTCAC

CGAAACAACCAAAAGAGTTGCTTGGTGCCACGATTCTCCCAAGCCTCCAAGTGACAGAATCACCTTCACTC
TGCCTGTGTTGAAAGACGCCAAAGCCCTGTGTTTTGTGGCTGAGGGCAGTTCCAAACAAAATATAATGCAT
GAGATCTT

GATTATCTTCTACCTCCTCTGAAACCACCACGAGATCCGCCACGAGATCCTCCTCTAAATGATCCGCCACG
AGATCCACCTCTGAAAGAACTGCCACCACGGCCTCCTCTGAAAGAACTACCACCACGGCCTCCTCTGAAAC
CTCCTCTG

GAAGCGTTTGCCAAGTTGTGTGTGAATGAGAAGCCTCCTGCTGAATCTGCCGTTGCGGTGAAGTCTTTGGT
CTTCAAACCAAAGACTCCAAAGTCCGCCACCCCTGTTCCTATCGTTGTGGTGGCTTTGCAATCTACTACTA
CTCCTTCT

GTACTACGTCGTCAACCCTCATTTCCACGAGCACATCATCATCATCATCATCAACTCCAACCACAACATCG
TCAGCCCCCATTTCTACAAGCACGACGTCGTCAACTTCCACTTCAACAAGTACAACATCGCCAACTTCTTC
TTCAGCAC

TACATGGCCTGGAAGGACTCCATCCTGGAGGTTTTGAAAGACGAACTGCATTTGGACGAACAGGAAGCCAA
GTTCACCTCTCAATTCCAGTACACTGTGTTGAACGAAATCACTGACTCCATGTCGCTTGGTGAACCCTCTG
CTCACTAT

GATGGTGAGGATTCAGACACTGACAGTGACAACTTTTTGCAAGACCCTGAAGACGATGTGGATGAAGAAAG
CACTGGTAGAGGTACAGTCACTACCACTTCCACATCCACTGAGTCAAGAGGCCGTCCATCTTCTTGTATCT
TCGTGGCA

ATTCAGACACTGACAGTGACAACTTTTTGCAAGACCCTGAAGACGATGTGGATGAAGAAAGCACTGGTAGA
GGTACAGTCACTACCACTTCCACATCCACTGAGTCAAGAGGCCGTCCATCTTCTTGTATCTTCGTGGCAAG
CTTAGCAG

CCTTGTCTGAATCCATCAAGGCTGCTCAAGACTCTTTCGCTGCCAACTGGGGTGTTATGGTTTCCCACAGA
TCTGGTGAAACTGAAGACACTTTCATTGCTGACTTGGTTGTCGGTTTGAGAACTGGTCAAATCAAGACTGG
TGCTCCAG

AATTCTTGATTAGACTTGTTACTCCTAATATGTTGCGGCACTCAAGTGGTGACATGGCACCACTGCCGATA
ACAATGCCTCTGGACCTCGAACTCCTGATGACGCTTCTTACATTGGATACGAACTGCCTCCGAGCTTGTAC
GTCACGCA

TGTGAATGAGAAGCCTCCTGCTGAATCTGCCGTTGCGGTGAAGTCTTTGGTCTTCAAACCAAAGACTCCAA
AGTCCGCCACCCCTGTTCCTATCGTTGTGGTGGCTTTGCAATCTACTACTACTCCTTCTGCGTTGATTGCC
AACGCTAC

ATTCTGTGGAGGTGGTACTGAAGCAGGTTGAGGAGAGGCATGATGGAGGTTCTCTGGAACAGCTGATGAAG
CAGGTGTTGTTGTCTGTTGAGAGTTAGCCTTAGTGGAAGCCTTATCATATTCTTGAATTTTGGAAGCTGAA
ACGTCTAA

GCAAGTATGGCGGTGTTGTGTGGTGTATGCGGAATAAAAGAGTTCAAATACAAGTGTCCAAGATGTTTGGT
GCAAACTTGCTCTTTGGAGTGTTCCAAGAAGCACAAGACAAGGGACAATTGCTCAGGTCAAACACATGATC
CTAAGGAG

TGAGAACTGATGAGCTAGGCCGAGAAAGCTGGGAATATTTAACCCCTCAGCAAGCCGCAAACGACCCACCA
TCCACTTTCACGCAGTGGCTTCTTCAAGATCCCAAATTTCCTCAACCTCATCCAGAAAGAAATAAGCATTC
ACCAGATT

CTTCTTGCAGGCGGTCATATTCTTCCTGGTTGAAGAGGGGGAACTCTGCCACCATGGATGCACACGTATTT
GGATCATCTTCGTCGCTGTTGTACTGTGAGCTCTTGTTTGTGCTATGTTCTCGGTGCCCCTTCATGGTTTG
TATCTCCC

AATGGTGAGAGAGATGGATATCGGTAGTCTGATGGGATTGTTGAACTTGGTGTGATCGTGAGAGGTAGCGA
TGCCAGAAGCAGCAGGTGGCGTCTGGTTTGGGGGAGATGAGGGAACAGTATTAGTAGACATCCAGGAATAA
CCACTATA

TGACCACTGGATCCATCACTTGCATAAACGACAGGGAAGTCGACCGATGTCCATGATGCCACGGTACTGTT
GTGACTTGTACCAGAGCTGACAACAACCAAACCTTGGATACTAGAACTTAGTGATTGAATTAAGCTTTGAG
AATATCCT

CATATCAGAGTCCGCTGAGGATGAATCAGTAAATGTATTACCTGACTCAGGTGATGGAGTGCTCAGAGGCG
TTCCAACTGATGATGGATACTGCGGAAACTGTGATTGTGGCCCAGGTGGAAAGTACATAGGCGACATTTGA
TAAGGTGT

GTTTCAAGTAGCTCTATCACGAGTAGTAGTAGTACGAGTATTGTGGACACAACCACAATCTCGCCGACTCT
GACGTCAACGAGTACGACCCCATTGACAACCGCCTCAACCAGTACAACACCATCAACTGATATAACATCGG
CACTGCCC

GATCTGTCACCATCTGTGTACTGGAGGGCTCCATTCAACGCCCTGGCTGATGTGACCCAGTTGGTGGAGTT
CATTGTTCTGGACGTGGATTCTACAGGTATAAGCAGGGGAAATCGTGTCTTAGCTGACATTACTGTTGCTA
GAACGTCT

TGTAAAGTTTGCAACACCTTGTTGTTTGCCGTGTTCAGCACGTTGCTCTGCGAGTGGAACTGCGACCGAGT
CTCCCAGGCTTGGGAGATGAGACGGTCGACTACGTTGTTGGACTGATCGATTCGCCTTGTTTCGTTCTGAA
TGTACTCG

ATCAAAATATGCCTGTTTCGGAAGCGTTTGCCAAGTTGTGTGTGAATGAGAAGCCTCCTGCTGAATCTGCC
GTTGCGGTGAAGTCTTTGGTCTTCAAACCAAAGACTCCAAAGTCCGCCACCCCTGTTCCTATCGTTGTGGT
GGCTTTGC

AAAGAATCAGATGAAACTACTGTATGCCTACTAGAAGAGTGTTCCGTTTCGACTTGGCACTGCAAATTGTT
TGATTCTTCAAGCAGCTCTGGGGCCCTCCAACCACTTGTGCCAGAAGGGTTATTCAAATTTGTTCTAAATG
AAGACTGA

TTACCGATGATGGCACCATCGGATTATCCAGTTCTTCGATTTCGTATCCAGGCGGTGGTGGTGGTGGTGGT
GGAAGTGCTAAGTCGCTGTCCTCTTCAAAACCAGGAGGTGGGGGCGGTAGTCCACTCATCTTTCTTTGACG
CTATTTTT

ATCAACATCTACCACCACAAGTACTACGTCGTCAACCCTCATTTCCACGAGCACATCATCATCATCATCAT
CAACTCCAACCACAACATCGTCAGCCCCCATTTCTACAAGCACGACGTCGTCAACTTCCACTTCAACAAGT
ACAACATC

ACCACCACAAGTACTACGTCGTCAACCCTCATTTCCACGAGCACATCATCATCATCATCATCAACTCCAAC
CACAACATCGTCAGCCCCCATTTCTACAAGCACGACGTCGTCAACTTCCACTTCAACAAGTACAACATCGC
CAACTTCT

TGTTCTCAATAAGAATTCGCAGCTTGTAGGCGGCCCCTTTGAACCTCTTCATCTTTACCTCGTATTCCGGC
ACGTCCTCCTGCTGCTCGTGCGGTGCAGGTGCAGGTGCAGGTGCCTGTTTCAGGTTTCTGTGAGCAATCTC
GTTCTTAT

GAATTAGACGTGGCCATCTGTTTTGGATAATTTTCTTCACTCGCTGCGGCGTTGCCATTATCCACATCTGA
AGGCTGCTGTTGCTGTTGCTGTTGCTGTTGCTGCTGCTGTTGCTGCTGCTGTAATTGTAATTGCCTTTGCT
CTTCTAAT

TGCCAACTGGGGTGTTATGGTTTCCCACAGATCTGGTGAAACTGAAGACACTTTCATTGCTGACTTGGTTG
TCGGTTTGAGAACTGGTCAAATCAAGACTGGTGCTCCAGCTAGATCCGAAAGATTGGCTAAGTTGAACCAA
TTGTTGAG

ATTACAGCTTTCCATTTATAGAGCCCTCCGTTAGACTGGAAGTGCTAGGTGAGGGTGTTACTGGCGACAAG
CACTCGTCTGTCAATCTGCACAATTTGTGGGAGCACATGATCTATGTAAAAGATCCTACGCTGTCCTTTAT
CTTGACCC

TATGGAACGCCTGTTCCTGATCCATGGCCTGCACTTGCGACCACAATTCCACACCTGAGGCACCTGCCTCT
TTTCCAGCATGTGGCAACTGTCCCCACGACAGGGCATCCCAGAATCCTCTGGTAAATCTTAAATGAAACTG
ACGCGTGG

TGGGTGTGGTGTGTGTGGGTGTGGGTGTGGTGTGGGTGTGGGTGTGTGTGGGTGTGTGTGTGTGGGTGTGT
GTGGGTGTGGGTGTGGGTGTGGGTGTGGTGTGTGTATATATATGTCACTGTATTGCATGCTGGATGGTGTT
AGACAAGG

CGGTGTTGTGTGGTGTATGCGGAATAAAAGAGTTCAAATACAAGTGTCCAAGATGTTTGGTGCAAACTTGC
TCTTTGGAGTGTTCCAAGAAGCACAAGACAAGGGACAATTGCTCAGGTCAAACACATGATCCTAAGGAGTA
TATATCGA

TGGTGTATGCGGAATAAAAGAGTTCAAATACAAGTGTCCAAGATGTTTGGTGCAAACTTGCTCTTTGGAGT
GTTCCAAGAAGCACAAGACAAGGGACAATTGCTCAGGTCAAACACATGATCCTAAGGAGTATATATCGAGT
GAGGCGTT

CGAACATCAGGTCGTTCTCCTCATACGCCTTGGTACACAGAGGCTCATTGTCGAGGAACCAGCGCAGCTTG
GAACAGGAGAAATACGTGGAGAGCAATGGCAATCCAGTCTTCTGTCTAAGCTGCAGTTGCCTATCGACGCT
AGTGTTTT

CCAACTCCAGCACTAATGCTACTACCACTGCCAGCATCAACGTCAGGACTAGTGCGACTACCACTGCCAGC
ATCAACGTCAGGACTAGTGCGACTACCACTGAAAGTACCAACTCCAACACTAATGCTACTACCACTGAAAG
TACCAACT

GTAAGAGAGTAGAAGAACAAATCAAAATATGCCTGTTTCGGAAGCGTTTGCCAAGTTGTGTGTGAATGAGA
AGCCTCCTGCTGAATCTGCCGTTGCGGTGAAGTCTTTGGTCTTCAAACCAAAGACTCCAAAGTCCGCCACC
CCTGTTCC

TGTTGAAGAACACCACAGTGATCGAGTTTCCCACGATATTCGTTGCCATGACAGAGGCTGACCTACCAGAG
GGCTACGAGGTGCTACACCAGGAACCCCGCCCACTTGAGCATACGAGTACACTGAACAAGTTTATTGACAA
TGCAAGGG

GCTGCTGACGCTTTGTTGTTGAAGGTTAACCAAATCGGTACCTTGTCTGAATCCATCAAGGCTGCTCAAGA
CTCTTTCGCTGCCAACTGGGGTGTTATGGTTTCCCACAGATCTGGTGAAACTGAAGACACTTTCATTGCTG
ACTTGGTT

GCGTATCGTTAATGGTGAGAGAGATGGATATCGGTAGTCTGATGGGATTGTTGAACTTGGTGTGATCGTGA
GAGGTAGCGATGCCAGAAGCAGCAGGTGGCGTCTGGTTTGGGGGAGATGAGGGAACAGTATTAGTAGACAT
CCAGGAAT

CACCACAGTGATCGAGTTTCCCACGATATTCGTTGCCATGACAGAGGCTGACCTACCAGAGGGCTACGAGG
TGCTACACCAGGAACCCCGCCCACTTGAGCATACGAGTACACTGAACAAGTTTATTGACAATGCAAGGGAG
GAGGAAGA

TAACCATAAAGTAAGAGAGTAGAAGAACAAATCAAAATATGCCTGTTTCGGAAGCGTTTGCCAAGTTGTGT
GTGAATGAGAAGCCTCCTGCTGAATCTGCCGTTGCGGTGAAGTCTTTGGTCTTCAAACCAAAGACTCCAAA
GTCCGCCA

GGTACAGATCTGAAAGATTGCCAGCCAACTTACTACAAGCTCAACGTGACTACTTTGGTGCTCACACTTTC
AGAGTGTTGCCAGAATGTGCTTCTGACAACTTGCCAGTAGACAAGGATATCCATATCAACTGGACTGGCCA
CGGTGGTA

AAACTCCTCATCGTCGTCGTCTTCGTCTGACGATGTCTCGTCCTCGCCAGCTCCAGTGGTCGTCCTAGCAT
TTCCATCTTGCTTGTGCAAAGGATGCACCTGCTCCTTTTCAACAAGGTCCTGTAAACACGAGATTTTATCG
GAAAGCTG

CTCTTCTAGCGCCCCAGACTCAATAATTCCTTCATCTAGCGCCTCCATCTCTGGTGTCTCAAACTCCACTA
CAGCATCTGGTTCAATCGCTTCTACTGCTTCCACCGCTTCCACTACTTCTACTGCATCCGCTGCATCCGCC
ACCAGCTT

AAGAGCCAAGAGCTACTATGGCAATGGCATCAAAACATATCCCTTGCCATCTTCACCGAAATCGCCGACAT
CAGAAGAAACACACTCATCTATGACAGCATCTGGAGAGGACTCACACCTACTTAAGCAATAGATTGTGCTA
TCTGGGAC

ATTTCCACGAGCACATCATCATCATCATCATCAACTCCAACCACAACATCGTCAGCCCCCATTTCTACAAG
CACGACGTCGTCAACTTCCACTTCAACAAGTACAACATCGCCAACTTCTTCTTCAGCACCTACAAGCTCGT
CTAATACA

GCTGCTGTCAAATTTGTTGCTTATGAACAGATTAGGAATACCTTGATTCCCTCAAAAGAATTTGAGTCACA
TTGGAGAAGGTTGGTGAGTGGTTCACTGGCAGGATTATGCAGTGTTTTCATAACATATCCGTTAGATCTCG
TGAGGGTT

ACTCTTTCGCTGCCAACTGGGGTGTTATGGTTTCCCACAGATCTGGTGAAACTGAAGACACTTTCATTGCT
GACTTGGTTGTCGGTTTGAGAACTGGTCAAATCAAGACTGGTGCTCCAGCTAGATCCGAAAGATTGGCTAA
GTTGAACC

GTTTTCATTTCTTCTTGCAGGCGGTCATATTCTTCCTGGTTGAAGAGGGGGAACTCTGCCACCATGGATGC
ACACGTATTTGGATCATCTTCGTCGCTGTTGTACTGTGAGCTCTTGTTTGTGCTATGTTCTCGGTGCCCCT
TCATGGTT

AAACTCCACTACAGCATCTGGTTCAATCGCTTCTACTGCTTCCACCGCTTCCACTACTTCTACTGCATCCG
CTGCATCCGCCACCAGCTTCACCTCAGGTTCCGCTTCTGTCTACACTACTACATTAACTTACTTGAATGCC
ACAAGTAC

CCTCATGAATTCTAGTGGATGAAAGAAGCAGCACGAACACCATTTCTACAGACAACGACACATGGAAAGGT
TCACCATTCCCAAAGAAAACAACGATGGCCACAAGGGTGTGGTCCTCCATTCTCCTACTGTTGGAAGGAGA
TATTATCC

ACAGGCTATTATTACAGCTTTCCATTTATAGAGCCCTCCGTTAGACTGGAAGTGCTAGGTGAGGGTGTTAC
TGGCGACAAGCACTCGTCTGTCAATCTGCACAATTTGTGGGAGCACATGATCTATGTAAAAGATCCTACGC
TGTCCTTT

CCAAATCCATCCAAGCCAAGACCAAATGAAGAATTAGTGTTCGGCCAGACATTCACCGATCATATGTTGAC
CATTCCTTGGTCAGCCAAAGAAGGGTGGGGCACTCCACACATCAAGCCTTACGGTAATCTTTCTCTTGACC
CATCTGCT

CGGTGAAGCTGATGATGGTGCAGGAACTACAGACGAAGATTACATGGCCTGGAAGGACTCCATCCTGGAGG
TTTTGAAAGACGAACTGCATTTGGACGAACAGGAAGCCAAGTTCACCTCTCAATTCCAGTACACTGTGTTG
AACGAAAT

GACATCTCTTTGGGACCTAAAGGCCACCGTCATTTGAGGGGACATCGTCGGCCAGACAGATGCTGAAACGG
TCTCATTCAGATCATCGCCAACTGTCGTAGTAGAGTCAACAGCAGTAACCCACGTTCGCAGTGAGGACCAT
ATGATAAA

TTACAACAGTGGATTCAAGATGAAGAGGGCGGCAAGATCACGATCCCTCTGCCTTTGAAGAAACGACACAG
ATCCCAGCAACACAATGACCAACAACCGCCACAACCACGCACCAAAGAGCTGATCATTCCGTCACATATTA
GCCACTAT

TGTTTAGTTAATTGGTAAATCAGCCATGTGTCCACAGTGCCGAACATCAGGTCGTTCTCCTCATACGCCTT
GGTACACAGAGGCTCATTGTCGAGGAACCAGCGCAGCTTGGAACAGGAGAAATACGTGGAGAGCAATGGCA
ATCCAGTC

CAGGAACTACAGACGAAGATTACATGGCCTGGAAGGACTCCATCCTGGAGGTTTTGAAAGACGAACTGCAT
TTGGACGAACAGGAAGCCAAGTTCACCTCTCAATTCCAGTACACTGTGTTGAACGAAATCACTGACTCCAT
GTCGCTTG

GTAACCATCATCATCATAATCATCATCATAACGACTGCGTGACCCCAGCGACATTCCTCTTCTTCTTGATG
ATCTTGTGTTTGGACGTGTTGAGGATTCATCAGTAGAATCAAAGGAATCTGGAATATCGTCGTAATAATGG
CTTGTGTT

TGAACTGTCCCAGTTATATTTGTAACAGGGTTGGCTCCTGCAGTTGCAGGCACATAATAACCCTGTTGTGG
TTGCTGTTGTTGCTGTTGCTGTTGTTGCTGTTGCTGTTGTTGCTGTTGTTGGAGATGATTCTGAGTGTAGT
GAGCCTGA

ACGACCGTAAGTGTCACATCATCAACATCTACCACCACAAGTACTACGTCGTCAACCCTCATTTCCACGAG
CACATCATCATCATCATCATCAACTCCAACCACAACATCGTCAGCCCCCATTTCTACAAGCACGACGTCGT
CAACTTCC

CGCAATACCACGAGGCCAAGGAGCGTATCAGGCAGAAGGAAGTAACTGCAGGTGAAGCACAGGACGAAGCC
AGCTTGCAGCAGCAACAGCAGCAAGATTTGCAGCAACAGCAACAAGTAGTGACTACAGTTGCCTCGCAAAG
TCCTCATG

CAAATCGGTACCTTGTCTGAATCCATCAAGGCTGCTCAAGACTCTTTCGCTGCCAACTGGGGTGTTATGGT
TTCCCACAGATCTGGTGAAACTGAAGACACTTTCATTGCTGACTTGGTTGTCGGTTTGAGAACTGGTCAAA
TCAAGACT

CTAGTGCTACTACCACTGCTAGCACCAACTCCAGCACTAATGCCACTACCACTGCTAGCACCAACTCCAGC
ACTAATGCCACTACCACTGAAAGTACCAACGCTAGTGCCAAGGAGGACGCCAATAAAGATGGCAATGCTGA
GGATAATA

TTCATTTCAATATCTCTGTCTGCGTCAAGATCACCACCATACTCCAAATCAGAGTCGCTGTCTTCTTCTTC
TTCATCATCATCATCCTCATCATCATCATCTTCATCATCATCCTCATCGCCTTCTTCTCCATCATCATCAT
CATCGTCA

TCTCTGGACACGTGCTGTATGTAGTTCACCACATCCATGCCGTCCACAACCTGTCCAAATACCACATGCTT
TCCATCGAGCCACGACGCTTCTTCCGTAGTGGTGATGAAGAACTGAGATCCATTGGTATCTTTACCACGAT
TAGCCATC

GGCATCAACTTCACCTTCTTCGTCATCTTCTTCTGCTGCGGCGTTGACACTGCCTTCTGCGGCGGCTTGCA
TGTCAGCTTGGATGTCTTCTGGGGACTTGGTAGCGACGTCTTCGTTAGATGGCATAATACCGCTAGCTTGA
GCTTGTTG

GTATCTTCGTTGGGGTCAGATCACCAGAATGATGGTGAGGATTCAGACACTGACAGTGACAACTTTTTGCA
AGACCCTGAAGACGATGTGGATGAAGAAAGCACTGGTAGAGGTACAGTCACTACCACTTCCACATCCACTG
AGTCAAGA

CTTTGTTGTTGAAGGTTAACCAAATCGGTACCTTGTCTGAATCCATCAAGGCTGCTCAAGACTCTTTCGCT
GCCAACTGGGGTGTTATGGTTTCCCACAGATCTGGTGAAACTGAAGACACTTTCATTGCTGACTTGGTTGT
CGGTTTGA

TCCTCCAAATCATACACCACTGTTACTGTTACTCACTGTGACAACAATGGCTGTAACACCAAGACTGTCAC
TTCTGAATGTTCTAAAGAAACTGCAGCAACCACCATTTCTCCAAAATCATACACTACTGTTACCGTTACTC
ACTGTGAC

GAACGCCTTTTGTTTAGTTAATTGGTAAATCAGCCATGTGTCCACAGTGCCGAACATCAGGTCGTTCTCCT
CATACGCCTTGGTACACAGAGGCTCATTGTCGAGGAACCAGCGCAGCTTGGAACAGGAGAAATACGTGGAG
AGCAATGG

ATACATTCGTTTCATTTCAATATCTCTGTCTGCGTCAAGATCACCACCATACTCCAAATCAGAGTCGCTGT
CTTCTTCTTCTTCATCATCATCATCCTCATCATCATCATCTTCATCATCATCCTCATCGCCTTCTTCTCCA
TCATCATC

GCTGCTCAAGACTCTTTCGCTGCCAACTGGGGTGTTATGGTTTCCCACAGATCTGGTGAAACTGAAGACAC
TTTCATTGCTGACTTGGTTGTCGGTTTGAGAACTGGTCAAATCAAGACTGGTGCTCCAGCTAGATCCGAAA
GATTGGCT

CAACGGTCAACCAACTGACGAAACTGTTATTGTTATCAGAACTCCAACCAGTGAAGGTCTAATCAGCACCA
CCACTGAACCATGGACTGGTACTTTCACCTCTACATCTACTGAGGTTACCACCATCACTGGTACCAACGGT
CAACCAAC

CAGTTATATTTGTAACAGGGTTGGCTCCTGCAGTTGCAGGCACATAATAACCCTGTTGTGGTTGCTGTTGT
TGCTGTTGCTGTTGTTGCTGTTGCTGTTGTTGCTGTTGTTGGAGATGATTCTGAGTGTAGTGAGCCTGAAA
TTCCATTT

CGAGGCCAAGGAGCGTATCAGGCAGAAGGAAGTAACTGCAGGTGAAGCACAGGACGAAGCCAGCTTGCAGC
AGCAACAGCAGCAAGATTTGCAGCAACAGCAACAAGTAGTGACTACAGTTGCCTCGCAAAGTCCTCATGCA
ACTGCAAC

GAACTCTGCCACCATGGATGCACACGTATTTGGATCATCTTCGTCGCTGTTGTACTGTGAGCTCTTGTTTG
TGCTATGTTCTCGGTGCCCCTTCATGGTTTGTATCTCCCAGAGGAGAAAATCGTCACTCAAGTTAAACACG
ACCTTGCC

GAGTACTTTAATAAGAAGTCTTCGACCTCAGTTCCTATCTCGGTAGGTGACACACCTACTACCACGCCAGG
GTCCATTGAAGCTGCAGGTGTGGTTGCATGGAATGAAATTCTATCTCAGACTGATATGATTTCCAAGGATC
ATGATCAG

TGGCCATCTGTTTTGGATAATTTTCTTCACTCGCTGCGGCGTTGCCATTATCCACATCTGAAGGCTGCTGT
TGCTGTTGCTGTTGCTGTTGCTGCTGCTGTTGCTGCTGCTGTAATTGTAATTGCCTTTGCTCTTCTAATAG
CGGTTGTC

TCCACAGTGCCGAACATCAGGTCGTTCTCCTCATACGCCTTGGTACACAGAGGCTCATTGTCGAGGAACCA
GCGCAGCTTGGAACAGGAGAAATACGTGGAGAGCAATGGCAATCCAGTCTTCTGTCTAAGCTGCAGTTGCC
TATCGACG

CTACGAAATGAGAATCCACAAGAGATACATCGACTTGGAAGCTCCTGTTCAAATCGTTAAGAGAATCACTC
AAATCACCATTGAACCTGGTGTGGATGTCGAAGTTGTTGTTGCTTCCAACTAAGCTGGTTCTAACTGGAAA
TAATTTCC

AAATTCGAGTATTGAGTTCTGATTGTGGAGTTGCTGGCAATCAGAGTTGGCTCACAGGCATCAAAGAACCA
TTGAACATTCTTCTGACCAGACAAAACGCCCACAGGAACAGTGTAAGGAGGGAAAACACCAGAATACACAA
CTTTATCT

AACAATATATGCGTACTCTTCGTCGCTGACGGTCTTGTATCTGACCCAGGGGAAAGACTGGTACTGCAATT
GTGGCTCTTCGACCTTCAAGTACTCAACTTTCAGGCCACTGCATGTACAGTACGGAATCTCGAAATCAATG
TTGACCAA

AAAACACAGCGACCACTGACGGTGTACGAAATCAATTTCAGAGTAATGGTTGGTGTTCAAATAACTGTGCT
GGTCATCAGTTTGCCATCGTACAGGGGTTCATGTGCTGGTGCAGTGATTCGGAACCGAGCACTCAGACATC
GGTGGGAG

GAGGCATTAGAAGATATTTGTGCGCTCGTAAAATTCGAGTATTGAGTTCTGATTGTGGAGTTGCTGGCAAT
CAGAGTTGGCTCACAGGCATCAAAGAACCATTGAACATTCTTCTGACCAGACAAAACGCCCACAGGAACAG
TGTAAGGA

TTCGGAGCTTTGTTCTCAATAAGAATTCGCAGCTTGTAGGCGGCCCCTTTGAACCTCTTCATCTTTACCTC
GTATTCCGGCACGTCCTCCTGCTGCTCGTGCGGTGCAGGTGCAGGTGCAGGTGCCTGTTTCAGGTTTCTGT
GAGCAATC

GAAGGTTAACCAAATCGGTACCTTGTCTGAATCCATCAAGGCTGCTCAAGACTCTTTCGCTGCCAACTGGG
GTGTTATGGTTTCCCACAGATCTGGTGAAACTGAAGACACTTTCATTGCTGACTTGGTTGTCGGTTTGAGA
ACTGGTCA

CGTACTCTTCATACATTCGTTTCATTTCAATATCTCTGTCTGCGTCAAGATCACCACCATACTCCAAATCA
GAGTCGCTGTCTTCTTCTTCTTCATCATCATCATCCTCATCATCATCATCTTCATCATCATCCTCATCGCC
TTCTTCTC

TAGGCAAGCTCGGTGAAGCTGATGATGGTGCAGGAACTACAGACGAAGATTACATGGCCTGGAAGGACTCC
ATCCTGGAGGTTTTGAAAGACGAACTGCATTTGGACGAACAGGAAGCCAAGTTCACCTCTCAATTCCAGTA
CACTGTGT

TGGTCCTGATGGACACACTTGTTCCTTATTCCCTGGAGAAACACATAGGTACTTGCTGAACGAAACAACCA
AAAGAGTTGCTTGGTGCCACGATTCTCCCAAGCCTCCAAGTGACAGAATCACCTTCACTCTGCCTGTGTTG
AAAGACGC

ATTGAAATTCCTGAGCATGAGAGAATAGAATTAATTAGATACATCGTCAATACAGCACATCCGGTTGATGG
TGGCTGGGGTCTACATTCTGTTGACAAATCCACCGTGTTTGGTACAGTATTGAACTATGTAATCTTACGTT
TATTGGGT

CTTGATCAGTTCCGGTGTACGTGAAACTTTACGTTATTTGGTGCAACACAAAATGGTTGATGCTGTCGTTA
CTTCTGCTGGTGGTGTGGAAGAAGATTTGATCAAATGTCTTGCTCCAACTTACTTGGGTGAATTTGCTTTG
AAAGGTAA

ATTGGACAGTTTTTTCCAATTATGGAACGCCTGTTCCTGATCCATGGCCTGCACTTGCGACCACAATTCCA
CACCTGAGGCACCTGCCTCTTTTCCAGCATGTGGCAACTGTCCCCACGACAGGGCATCCCAGAATCCTCTG
GTAAATCT

CTGGTGGAGTTGGCTATACATGAGAAATGCATCGGAGAATTGTTGAAGAACACCACAGTGATCGAGTTTCC
CACGATATTCGTTGCCATGACAGAGGCTGACCTACCAGAGGGCTACGAGGTGCTACACCAGGAACCCCGCC
CACTTGAG

CACTTCTATATTCTCTGAATCATCTACATCATCCGTCATTCCAACCAGTAGTTCCACCTCTGGTTCTTCTG
AGAGCAAAACGAGTTCGGCTAGTTCTTCCTCTTCTTCCTCTTCTATCTCTTCTGAATCACCAAAGTCTCCT
ACAAATTC

TCAAGAGGATTTTGGACTTTGGATGCCTTATACTTCTTGAGATGGAAGGAGCTGATGCTACTGTGGTTGGT
GTTTCCACAATTGTTCACTGTCAACTGATGAGGTCCATCTTCCATACTGAACATGCCCTTGTAAATACGAT
TTGCATAA

TACCACTGAAAGTACCAACTCCAGCACTAATGCTACTACCACTGCCAGCATCAACGTCAGGACTAGTGCGA
CTACCACTGAAAGTACCAACTCCAACACTAGTGCTACTACCACCGAAAGTACCGACTCCAACACTAGTGCT
ACTACCAC

TTCACATAAGTTTGATGGTGCTTGGTGTAATGCAATTCGTTGATTTGACCGGAGATATAAGGTTCCAATGC
ACCGAAGTCCCACTTCAAGTCTGGCAAGGTGACTTTGGTTCTCCTTGCTGTGGTGGAGAGCAATGACAAAC
CACCCTTC

CACTAATGCTACTACCACTGCCAGCATCAACGTCAGGACTAGTGCGACTACCACTGCCAGCATCAACGTCA
GGACTAGTGCGACTACCACTGAAAGTACCAACTCCAACACTAATGCTACTACCACTGAAAGTACCAACTCC
AGCACTAA

TGATGGGAGGCTTTTGCGGTTCATTCCTAAGGGACTTGACCCCACAAGAGGTGGAAAGACGTGAGATTCCA
TTGGCTGAGGTGCTTCCAGAACCTTCTGGTGGTGAAGATACCGGTCTGGTTCCCCCATTACCTCCCACCGA
TATCGGTA

TACCGACTCCAACACTAGTGCTACTACCACCGAAAGTACCGACTCCAACACTAGTGCTACTACCACTGCTA
GCACCAACTCCAGCACTAATGCCACTACCACTGCTAGCACCAACTCCAGCACTAATGCCACTACCACTGAA
AGTACCAA

TGGTGTTCAAATAACTGTGCTGGTCATCAGTTTGCCATCGTACAGGGGTTCATGTGCTGGTGCAGTGATTC
GGAACCGAGCACTCAGACATCGGTGGGAGACTGCAGTGGCACTTGTCCCGGTTATGGCTACGAAGATTGTG
GTAATGCG

TTTCTAGCTAGATTATCTTCTACCTCCTCTGAAACCACCACGAGATCCGCCACGAGATCCTCCTCTAAATG
ATCCGCCACGAGATCCACCTCTGAAAGAACTGCCACCACGGCCTCCTCTGAAAGAACTACCACCACGGCCT
CCTCTGAA

TACCACTGCTAGCACCAACTCCAGCACTAATGCCACTACCACTGCTAGCACCAACTCCAGCACTAATGCCA
CTACCACTGAAAGTACCAACGCTAGTGCCAAGGAGGACGCCAATAAAGATGGCAATGCTGAGGATAATAGA
TTCCATCC

CTGGTGTCTCAAACTCCACTACAGCATCTGGTTCAATCGCTTCTACTGCTTCCACCGCTTCCACTACTTCT
ACTGCATCCGCTGCATCCGCCACCAGCTTCACCTCAGGTTCCGCTTCTGTCTACACTACTACATTAACTTA
CTTGAATG

ACCAACCTTTAAATACGGTGAATATTCACAATGAAAGGAATGAGTACACACCTGAACTTTGTCAAAGAGAA
GAATCCTCGAATAAAGAACCTTCAGACTCAGTTCCTCAAGAAGTTTCATCCTCTAGAGATAATAGGGCATC
AAATAGAA

GTATCCTCATTTACCGATGATGGCACCATCGGATTATCCAGTTCTTCGATTTCGTATCCAGGCGGTGGTGG
TGGTGGTGGTGGAAGTGCTAAGTCGCTGTCCTCTTCAAAACCAGGAGGTGGGGGCGGTAGTCCACTCATCT
TTCTTTGA

AGACGAAGATTACATGGCCTGGAAGGACTCCATCCTGGAGGTTTTGAAAGACGAACTGCATTTGGACGAAC
AGGAAGCCAAGTTCACCTCTCAATTCCAGTACACTGTGTTGAACGAAATCACTGACTCCATGTCGCTTGGT
GAACCCTC

TATCTCTGTCTGCGTCAAGATCACCACCATACTCCAAATCAGAGTCGCTGTCTTCTTCTTCTTCATCATCA
TCATCCTCATCATCATCATCTTCATCATCATCCTCATCGCCTTCTTCTCCATCATCATCATCATCGTCATC
GTCATCGT

AACGATCGACATGGAAGCTGTCGCCTGATTTTCAGCCAATCTGTCCATTCTTTCTATCAGTTCCACTGTGT
CAGCAGACAGGTCTGTCCTGGAGCCACAGCATCCAACATGCTGGCCCTTTTTTCCTTTCTTTGATTCAAGT
CCATAGAA

TTTTCTTCACTCGCTGCGGCGTTGCCATTATCCACATCTGAAGGCTGCTGTTGCTGTTGCTGTTGCTGTTG
CTGCTGCTGTTGCTGCTGCTGTAATTGTAATTGCCTTTGCTCTTCTAATAGCGGTTGTCTCACAAACAGCC
TGAAATCT

CTAATGCTACTACCACTGAAAGTACCAACTCCAGCACTAATGCTACTACCACTGCCAGCATCAACGTCAGG
ACTAGTGCGACTACCACTGAAAGTACCAACTCCAACACTAGTGCTACTACCACCGAAAGTACCGACTCCAA
CACTAGTG

TTGGCTCCTGCAGTTGCAGGCACATAATAACCCTGTTGTGGTTGCTGTTGTTGCTGTTGCTGTTGTTGCTG
TTGCTGTTGTTGCTGTTGTTGGAGATGATTCTGAGTGTAGTGAGCCTGAAATTCCATTTTATCAAAACGCT
GAGGAATA

GGTACGGCCCATTCTGTGGAGGTGGTACTGAAGCAGGTTGAGGAGAGGCATGATGGAGGTTCTCTGGAACA
GCTGATGAAGCAGGTGTTGTTGTCTGTTGAGAGTTAGCCTTAGTGGAAGCCTTATCATATTCTTGAATTTT
GGAAGCTG

ATCTTTACCTCGTATTCCGGCACGTCCTCCTGCTGCTCGTGCGGTGCAGGTGCAGGTGCAGGTGCCTGTTT
CAGGTTTCTGTGAGCAATCTCGTTCTTATCAATCTCCTCTATCTTGGCTCCGGAGATTTTGTTCTCGATAG
GGAACAAT

TCGTCGTCGTCTTCGTCTGACGATGTCTCGTCCTCGCCAGCTCCAGTGGTCGTCCTAGCATTTCCATCTTG
CTTGTGCAAAGGATGCACCTGCTCCTTTTCAACAAGGTCCTGTAAACACGAGATTTTATCGGAAAGCTGCT
TATTGGCC

GCTATCAGACTAGGCAAGCTCGGTGAAGCTGATGATGGTGCAGGAACTACAGACGAAGATTACATGGCCTG
GAAGGACTCCATCCTGGAGGTTTTGAAAGACGAACTGCATTTGGACGAACAGGAAGCCAAGTTCACCTCTC
AATTCCAG

TGAAGAGGGCGGCAAGATCACGATCCCTCTGCCTTTGAAGAAACGACACAGATCCCAGCAACACAATGACC
AACAACCGCCACAACCACGCACCAAAGAGCTGATCATTCCGTCACATATTAGCCACTATGTCATGAATCTA
CCAGACAG

TGGGACCTAAAGGCCACCGTCATTTGAGGGGACATCGTCGGCCAGACAGATGCTGAAACGGTCTCATTCAG
ATCATCGCCAACTGTCGTAGTAGAGTCAACAGCAGTAACCCACGTTCGCAGTGAGGACCATATGATAAATA
GGTATATG

CACCTTGTGGAATACTTGTGTTACTTATGAACCCAATTCCTGAACCATCATGACCACTGGATCCATCACTT
GCATAAACGACAGGGAAGTCGACCGATGTCCATGATGCCACGGTACTGTTGTGACTTGTACCAGAGCTGAC
AACAACCA

GCGTACTCTTCGTCGCTGACGGTCTTGTATCTGACCCAGGGGAAAGACTGGTACTGCAATTGTGGCTCTTC
GACCTTCAAGTACTCAACTTTCAGGCCACTGCATGTACAGTACGGAATCTCGAAATCAATGTTGACCAACT
TATCTCGA

CCAAAGGACGAACTGACAAGTGTACAGAACAAGAAACTAAAGACTGCAGATGGCAATAATACCCCAGTCAC
GAAAGGTGTGCTGCTACACGAATCCATCCACAGCGTGGAGGATGCGTATGGAAAATTGCCTGAGGACGCCT
TGGCGTTT

CTGTTCCTGATCCATGGCCTGCACTTGCGACCACAATTCCACACCTGAGGCACCTGCCTCTTTTCCAGCAT
GTGGCAACTGTCCCCACGACAGGGCATCCCAGAATCCTCTGGTAAATCTTAAATGAAACTGACGCGTGGCA
GTAGATTC

ATTTCCCAATGTTTGTTTCCTCACGTGTGTTGGAAAAGGAAAAGGACCATGTTGAAGGCTTTGCTCCAGAA
GTTGCCTGGGTCACCAGAGCTGGCTCCTCTGAATTGGAAGAACCAATCGCCATTAGACCAACTTCTGAAAC
TGTCATGT

TACTAGAAACCCAAATCCATCCAAGCCAAGACCAAATGAAGAATTAGTGTTCGGCCAGACATTCACCGATC
ATATGTTGACCATTCCTTGGTCAGCCAAAGAAGGGTGGGGCACTCCACACATCAAGCCTTACGGTAATCTT
TCTCTTGA

TCACGTGTGTTGGAAAAGGAAAAGGACCATGTTGAAGGCTTTGCTCCAGAAGTTGCCTGGGTCACCAGAGC
TGGCTCCTCTGAATTGGAAGAACCAATCGCCATTAGACCAACTTCTGAAACTGTCATGTATCCTTACTACG
CAAAATGG

TCCCACGCTGGTGGTGCTTTGGCTTTGCAAGAATTCATGATTGCTCCAACTGGTGCTAAGACCTTCGCTGA
AGCCATGAGAATTGGTTCCGAAGTTTACCACAACTTGAAGTCTTTGACCAAGAAGAGATACGGTGCTTCTG
CCGGTAAC

TAACGGTCAATTAACTGATGAAACTGTCATTGTCATCAGAACTCCAACAACAGCTAGCACCATCACAACTA
CCACCGAGCCATGGACCGGTACTTTCACCTCTACATCCACTGAGATGACTACTGTCACCGGTACCAACGGT
CAACCAAC

TTTTGGATAATTTTCTTCACTCGCTGCGGCGTTGCCATTATCCACATCTGAAGGCTGCTGTTGCTGTTGCT
GTTGCTGTTGCTGCTGCTGTTGCTGCTGCTGTAATTGTAATTGCCTTTGCTCTTCTAATAGCGGTTGTCTC
ACAAACAG

GCACATCATCATCATCATCATCATCAACTCCAACCACAACATCGTCAGCCCCCATTTCTACAAGCACGACGTCG
TCAACTTCCACTTCAACAAGTACAACATCGCCAACTTCTTCTTCAGCACCTACAAGCTCGTCTAATACAAC
ACCAACGA

AAATTTAGAGCACGTTGTAAATTGTCAGTAACATATTGCTCCCAAACTTTCCATTCTGGTTGAAACTTGTT
GCAAGAGTACCAAGACATCCAAGTGGCAAAACCTTCATTCAACCATAAGCCTTCCCACCAATCCATGGTGA
CCAAGTTG

TCTATAGTTAAGTTTCCCATGACATCTCTTTGGGACCTAAAGGCCACCGTCATTTGAGGGGACATCGTCGG
CCAGACAGATGCTGAAACGGTCTCATTCAGATCATCGCCAACTGTCGTAGTAGAGTCAACAGCAGTAACCC
ACGTTCGC

ATGAAACTACTGTATGCCTACTAGAAGAGTGTTCCGTTTCGACTTGGCACTGCAAATTGTTTGATTCTTCA
AGCAGCTCTGGGGCCCTCCAACCACTTGTGCCAGAAGGGTTATTCAAATTTGTTCTAAATGAAGACTGACC
AGAGTCTA

ACTTGCTGAACGAAACAACCAAAAGAGTTGCTTGGTGCCACGATTCTCCCAAGCCTCCAAGTGACAGAATC
ACCTTCACTCTGCCTGTGTTGAAAGACGCCAAAGCCCTGTGTTTTGTGGCTGAGGGCAGTTCCAAACAAAA
TATAATGC

CGATCCAAAATGTTTAGCGGCGTCGTAGACGACTCACTTGCCGGCATTTTGTCAGCTTGTGAGACGTCCTC
TGAAGGCTGCGACTTCTGTCTGCATGATTGTTTCAACACCCCAAGTACTTCAGCCGCTTCTACCTCTTCCT
CTGATAAT

GGAATAAAAGAGTTCAAATACAAGTGTCCAAGATGTTTGGTGCAAACTTGCTCTTTGGAGTGTTCCAAGAA
GCACAAGACAAGGGACAATTGCTCAGGTCAAACACATGATCCTAAGGAGTATATATCGAGTGAGGCGTTGA
AACAGGCG

AATACTTGTGTTACTTATGAACCCAATTCCTGAACCATCATGACCACTGGATCCATCACTTGCATAAACGA
CAGGGAAGTCGACCGATGTCCATGATGCCACGGTACTGTTGTGACTTGTACCAGAGCTGACAACAACCAAA
CCTTGGAT

CCAACTGCTACCGACGCAACTGCAACCGTTTCCTCCAAATCATACACCACTGTTACTGTTACTCACTGTGA
CAACAATGGCTGTAACACCAAGACTGTCACTTCTGAATGTTCTAAAGAAACTGCAGCAACCACCATTTCTC
CAAAATCA

AAAGAGCTAGGTGGTGTTACACTCGGTTCTATGACTGCTAACATCACGGGTCTTGCTGCTATTCAAGGTCT
GTGTACATGTCTGGACACACTGTGTGCGCAGGCATACGGTGCCAAAAACTACCACTTGGTGGGTGTGCTAG
TGCAGAGA

TGGCTATACATGAGAAATGCATCGGAGAATTGTTGAAGAACACCACAGTGATCGAGTTTCCCACGATATTC
GTTGCCATGACAGAGGCTGACCTACCAGAGGGCTACGAGGTGCTACACCAGGAACCCCGCCCACTTGAGCA
TACGAGTA

AACTCCAACACTAATGCTACTACCACTGAAAGTACCAACTCCAGCACTAATGCTACTACCACTGCCAGCAT
CAACGTCAGGACTAGTGCGACTACCACTGAAAGTACCAACTCCAACACTAGTGCTACTACCACCGAAAGTA
CCGACTCC

CCAAGGAGCTATTCGACACCCACAGAGTTATCGTTAATGAATGGAGTGGTTTTGTTCCAGCATTGAACAAG
AAGAACGTTATCTTAGCACCATGGTGTGGTGTGATGGAATGTGAAGAAGATATCAAGGAATCTTCTGCAAA
GAAAGACG

ACCTGAGAAACTTAACTGTTTGCAGATTCCACACCTTCCCCCTTCAGTGCAGTGGTACAGTTCTTGGAAAT
ATCAAGAAGTGAATCTGAACAAGGACTGGCTGGCAAAAAGACAGAGAGGGCTCGAGTACTTCCTCAATCAC
ATCATCCT

CGAAGGGCGTTTCCCCACTATACTCTTCGCTGCAAGCGTGGGTGTGTGCCTTTTCCCACTGGAAACCAAAG
GAATGATCAATGTCGATACCAAGACATTGAGGAACATGAGTCCTTTGACGATTCTTACGCCATAGTCTGTC
ATGCGACC

ATCCATCACTTGCATAAACGACAGGGAAGTCGACCGATGTCCATGATGCCACGGTACTGTTGTGACTTGTA
CCAGAGCTGACAACAACCAAACCTTGGATACTAGAACTTAGTGATTGAATTAAGCTTTGAGAATATCCTTC
TTCGTATA

CTGCTGGTGGCCAATTGACAACAACCACCGATATCGAAAATTTCCCAGGGTTTCCTGAATCGTTGAGTGGC
AGTGAACTGATGGAGAGGATGAGGAAACAATCTGCCAAGTTTGGCACTAACATAATTACCGAGACTGTCTC
TAAAGTCG

CCTATCGCTCGGAAGACCTAAATTCTTGATTAGACTTGTTACTCCTAATATGTTGCGGCACTCAAGTGGTG
ACATGGCACCACTGCCGATAACAATGCCTCTGGACCTCGAACTCCTGATGACGCTTCTTACATTGGATACG
AACTGCCT

CCGGTCTGGAATTCGAACGAATGAATCTCTGCGAAAAAGACCCCATCGAACTCTCACTTCTATGAGTGTGT
CTTCTTCTCCTCCTTCTCCTCCTTCTCCTTCTCCTTCTTCTGGGAACAATTCGATAATCTGGATACTCCCT
GACTTCCA

ATCGGAGAATTGTTGAAGAACACCACAGTGATCGAGTTTCCCACGATATTCGTTGCCATGACAGAGGCTGA
CCTACCAGAGGGCTACGAGGTGCTACACCAGGAACCCCGCCCACTTGAGCATACGAGTACACTGAACAAGT
TTATTGAC

TGGTTTTGGAAACACGTTTCGAATCTTCAGTATGAGTTGACGGAGGTGTGGAATCGGTTGGACTCACAGCT
TTTGAAAGGACATTTCTCAGTTGCTCAGGATGTAGTTCAATGTCAGATTGGAAGTCATGGTCAGATTCTAT
GTTAAGAT

GATTCCGTTCCTTATATCGAAGAGGCCTTGCAAGTGTATGCTGCAGGATTTGACAAAGAGTGGAAGCTGTT
TAATACTGAGAAGGAAGAGAGTCCTTTCGACCTGGAAGACATTCAGCTCCCCAAAGAAGCTTACCGATTTA
AGCTTACC

TCTTGGGTCAAATCACAAAGGCCTACAGAGAAGAACCAGATTTGGAAAACTTGTTGTTCAACAAGTTCTTC
GCTGATGCCGTCACCAAGGCTCAATCTGGTTGGAGAAAGTCAATTGCGTTGGCTACCACCTACGGTATCCC
AACACCAG

GCCACACGTCGATCTCAGGCCGATCTGACAATCTTTAAAGGCATCAACAAGAGCAGCAGCTTGCCGACGTG
GCAAAAGTCAAAGAAGAACAGAGGCAACTCGTCCTACGTGAAGCCACATACAACAAGTTAGGAACTATTAG
GCGGAACC

CTACTACCACCGAAAGTACCGACTCCAACACTAGTGCTACTACCACTGCTAGCACCAACTCCAGCACTAAT
GCCACTACCACTGCTAGCACCAACTCCAGCACTAATGCCACTACCACTGAAAGTACCAACGCTAGTGCCAA
GGAGGACG

TGCGTCAAGATCACCACCATACTCCAAATCAGAGTCGCTGTCTTCTTCTTCTTCATCATCATCATCCTCAT
CATCATCATCTTCATCATCATCCTCATCGCCTTCTTCTCCATCATCATCATCATCGTCATCGTCATCGTCA
TCATCCTC

GGATTCAAGATGAAGAGGGCGGCAAGATCACGATCCCTCTGCCTTTGAAGAAACGACACAGATCCCAGCAA
CACAATGACCAACAACCGCCACAACCACGCACCAAAGAGCTGATCATTCCGTCACATATTAGCCACTATGT
CATGAATC

ACTCCAACTAGTGAGGGTTTGATTACTACAACTACCGAACCATGGACCGGTACTTTCACCTCTACATCCAC
TGAGGTTACCACCATCACTGGTACCAACGGTCAACCAACTGACGAAACCGTGATTGTCATTAGAACTCCAA
CTAGTGAG

AATGCAAGGGAGGAGGAAGACGCTGAAGAGGACTCTCAACCGACAGAAGAACCTGTACAGAAGGAAACACA
GGACGCCAGCGACAGCGACAGCGACAGCGACGATGACTACAATCCGGGCTTATCCATGGATTTCCTCACTG
CATGAGCC

GCGTCAAGTCTTAGCAAATGCAGAACGCTCATATAATCAACTAATGGATCGTGCAGCTAACGCACATATCT
CACCACCAGTTCCAGGCCCTGCACTCTACGCAGGAATGACTCATGCTAATAATACTCCAGTAATGCCACCT
CAGAGGCA

AAGCGCTCGAGAAAGTTGGAGTTTTTCAGCGTTTGCGTTCCATGACGAGCGCTGGACTGCAGGGTCCGCAG
TACGTCAAGCTGCAGTTTAGCAGGCATCATCGACAGTTGAGGAGCAGATATGAATTAAGTCTAGGAATGCA
CTTGCGAG

AACACTAGTGCTACTACCACCGAAAGTACCGACTCCAACACTAGTGCTACTACCACTGCTAGCACCAACTC
CAGCACTAATGCCACTACCACTGCTAGCACCAACTCCAGCACTAATGCCACTACCACTGAAAGTACCAACG
CTAGTGCC

TACCTCCTCTGAAACCACCACGAGATCCGCCACGAGATCCTCCTCTAAATGATCCGCCACGAGATCCACCT
CTGAAAGAACTGCCACCACGGCCTCCTCTGAAAGAACTACCACCACGGCCTCCTCTGAAACCTCCTCTGCT
ACCACCGC

GCCCCAGACTCAATAATTCCTTCATCTAGCGCCTCCATCTCTGGTGTCTCAAACTCCACTACAGCATCTGG
TTCAATCGCTTCTACTGCTTCCACCGCTTCCACTACTTCTACTGCATCCGCTGCATCCGCCACCAGCTTCA
CCTCAGGT

CTTCCAACATTGTCAAGAACGCTGAACAACACAACTTGGTCAAGAAGGGTCCAGTCAGACTACCAACCAAG
GTTTTGAAGATCTCCACCAGAAAGACTCCAAATGGTGAAGGTTCTAAGACTTGGGAAACCTACGAAATGAG
AATCCACA

TTTGTTCCAGCATTGAACAAGAAGAACGTTATCTTAGCACCATGGTGTGGTGTGATGGAATGTGAAGAAGA
TATCAAGGAATCTTCTGCAAAGAAAGACGATGGTGAAGAATTCGAGGAAGATGACAAGGCACCAAGTATGG
GTGCCAAA

AACACGTTTCGAATCTTCAGTATGAGTTGACGGAGGTGTGGAATCGGTTGGACTCACAGCTTTTGAAAGGA
CATTTCTCAGTTGCTCAGGATGTAGTTCAATGTCAGATTGGAAGTCATGGTCAGATTCTATGTTAAGATCA
TTGGATTG

TCCCAAGCTTCGAAGGGCGTTTCCCCACTATACTCTTCGCTGCAAGCGTGGGTGTGTGCCTTTTCCCACTG
GAAACCAAAGGAATGATCAATGTCGATACCAAGACATTGAGGAACATGAGTCCTTTGACGATTCTTACGCC
ATAGTCTG

GCTTCCTCTGAATTCTTCAAGGACGGTAAGTACGACTTGGACTTCAAGAACCCAGAATCTGACAAATCCAA
GTGGTTGACTGGTGTCGAATTAGCTGACATGTACCACTCCTTGATGAAGAGATACCCAATTGTCTCCATCG
AAGATCCA

TGGGGTCAGATCACCAGAATGATGGTGAGGATTCAGACACTGACAGTGACAACTTTTTGCAAGACCCTGAA
GACGATGTGGATGAAGAAAGCACTGGTAGAGGTACAGTCACTACCACTTCCACATCCACTGAGTCAAGAGG
CCGTCCAT

GCAGAAATTGCACCTTGTGGAATACTTGTGTTACTTATGAACCCAATTCCTGAACCATCATGACCACTGGA
TCCATCACTTGCATAAACGACAGGGAAGTCGACCGATGTCCATGATGCCACGGTACTGTTGTGACTTGTAC
CAGAGCTG

ATAACTGTGCTGGTCATCAGTTTGCCATCGTACAGGGGTTCATGTGCTGGTGCAGTGATTCGGAACCGAGC
ACTCAGACATCGGTGGGAGACTGCAGTGGCACTTGTCCCGGTTATGGCTACGAAGATTGTGGTAATGCGGA
TAAGGATC

ATCCATCAAGGCTGCTCAAGACTCTTTCGCTGCCAACTGGGGTGTTATGGTTTCCCACAGATCTGGTGAAA
CTGAAGACACTTTCATTGCTGACTTGGTTGTCGGTTTGAGAACTGGTCAAATCAAGACTGGTGCTCCAGCT
AGATCCGA

ATTCATTGAGGTTTTCATTTCTTCTTGCAGGCGGTCATATTCTTCCTGGTTGAAGAGGGGGAACTCTGCCA
CCATGGATGCACACGTATTTGGATCATCTTCGTCGCTGTTGTACTGTGAGCTCTTGTTTGTGCTATGTTCT
CGGTGCCC

TGGCCCTCTCTCCGTCAATCTGTAACGACCCAAATCGTATTAAAGTTTCGCCGTCCTGTTCACTGAACCTT
CCCTCATTTGGAGAATCTCTCCTCGCCAGCGACGCAAAGTCCTTAGGCAACTCTAGTTCACCTTGAATCTC
CAGCATCA

AGGCCACCGTCATTTGAGGGGACATCGTCGGCCAGACAGATGCTGAAACGGTCTCATTCAGATCATCGCCA
ACTGTCGTAGTAGAGTCAACAGCAGTAACCCACGTTCGCAGTGAGGACCATATGATAAATAGGTATATGAG
TATCCACA

AAATACGGTGAATATTCACAATGAAAGGAATGAGTACACACCTGAACTTTGTCAAAGAGAAGAATCCTCGA
ATAAAGAACCTTCAGACTCAGTTCCTCAAGAAGTTTCATCCTCTAGAGATAATAGGGCATCAAATAGAAGA
TTTCAGCA

ATATAATCAACTAATGGATCGTGCAGCTAACGCACATATCTCACCACCAGTTCCAGGCCCTGCACTCTACG
CAGGAATGACTCATGCTAATAATACTCCAGTAATGCCACCTCAGAGGCAGAGTTACCAAAGCAATGAGTAC
TCACCTTA

TCGGCCAGACATTCACCGATCATATGTTGACCATTCCTTGGTCAGCCAAAGAAGGGTGGGGCACTCCACAC
ATCAAGCCTTACGGTAATCTTTCTCTTGACCCATCTGCTTGTGTATTCCATTATGCATTTGAATTATTTGA
AGGTTTGA

GTCATTTAAGGATTCCGTTCCTTATATCGAAGAGGCCTTGCAAGTGTATGCTGCAGGATTTGACAAAGAGT
GGAAGCTGTTTAATACTGAGAAGGAAGAGAGTCCTTTCGACCTGGAAGACATTCAGCTCCCCAAAGAAGCT
TACCGATT

TTCACCACAATAGACTGCTCGCTGGCGTTGACCTGCTCTGAGGCATCAAAAAACTCCTCATCGTCGTCGTC
TTCGTCTGACGATGTCTCGTCCTCGCCAGCTCCAGTGGTCGTCCTAGCATTTCCATCTTGCTTGTGCAAAG
GATGCACC

TGCCAGTACCAATGCGACTACCAACTCCAGCACTAATGCTACTACCACTGCCAGCATCAACGTCAGGACTA
GTGCGACTACCACTGCCAGCATCAACGTCAGGACTAGTGCGACTACCACTGAAAGTACCAACTCCAACACT
AATGCTAC

AGGTTCTGATCATCCAAAGATCTTTGCGTACCAAAATTCATGGGGTTTATCCACTCGTGTCATTGGTGTCA
TGGTCATGATCCACTCTGACAACAAGGGTTTGGTTATCCCACCAAGAGTGTCCCAATTCCAATCCGTTGTC
ATTCCAGT

TAAGCAATTGGAAAACGTCTCTTCCAACATTGTCAAGAACGCTGAACAACACAACTTGGTCAAGAAGGGTC
CAGTCAGACTACCAACCAAGGTTTTGAAGATCTCCACCAGAAAGACTCCAAATGGTGAAGGTTCTAAGACT
TGGGAAAC

TATTTTCGCCACAGGCTATTATTACAGCTTTCCATTTATAGAGCCCTCCGTTAGACTGGAAGTGCTAGGTG
AGGGTGTTACTGGCGACAAGCACTCGTCTGTCAATCTGCACAATTTGTGGGAGCACATGATCTATGTAAAA
GATCCTAC

GCTGAAGAAACTGGCGCTGTCTCCGCATCTATCTATTCATTCACACCTAGCTCGTTCAAGAGCAGCGGTGA
CATTTCTTTGAGTTTGTCAAAGGCCAAGAAGGGTGAAGTCACCTTTTCTCCATACTCTAACGCTGGTACCT
TTTCTTTG

TTTAGGGACGAAAGAGCTAGGTGGTGTTACACTCGGTTCTATGACTGCTAACATCACGGGTCTTGCTGCTA
TTCAAGGTCTGTGTACATGTCTGGACACACTGTGTGCGCAGGCATACGGTGCCAAAAACTACCACTTGGTG
GGTGTGCT

ACTACCACTGCCAGCATCAACGTCAGGACTAGTGCGACTACCACTGCCAGCATCAACGTCAGGACTAGTGC
GACTACCACTGAAAGTACCAACTCCAACACTAATGCTACTACCACTGAAAGTACCAACTCCAGCACTAATG
CTACTACC

ACTCGGTTCTATGACTGCTAACATCACGGGTCTTGCTGCTATTCAAGGTCTGTGTACATGTCTGGACACAC
TGTGTGCGCAGGCATACGGTGCCAAAAACTACCACTTGGTGGGTGTGCTAGTGCAGAGATGTGCTGTGATC
ACCATCTT

TTATTGTTAGGCGTATCGTTAATGGTGAGAGAGATGGATATCGGTAGTCTGATGGGATTGTTGAACTTGGT
GTGATCGTGAGAGGTAGCGATGCCAGAAGCAGCAGGTGGCGTCTGGTTTGGGGGAGATGAGGGAACAGTAT
TAGTAGAC

ATGACTGCTAACATCACGGGTCTTGCTGCTATTCAAGGTCTGTGTACATGTCTGGACACACTGTGTGCGCA
GGCATACGGTGCCAAAAACTACCACTTGGTGGGTGTGCTAGTGCAGAGATGTGCTGTGATCACCATCTTGG
CGTTCTTG

CGTATTCCGGCACGTCCTCCTGCTGCTCGTGCGGTGCAGGTGCAGGTGCAGGTGCCTGTTTCAGGTTTCTG
TGAGCAATCTCGTTCTTATCAATCTCCTCTATCTTGGCTCCGGAGATTTTGTTCTCGATAGGGAACAATGG
TGGTAGTG

GTATTGAAGTGCCAATATTTGGCGACAATGGAAACCGTAACCCAACTGGTAATACCAACCCAGCAACAACA
ACAGCTATCCAAAGCAACAACAACACCAACAATGCTTCTCCGGCAACATCTACAGTTTCCTTACAACTACC
TAATTTAC

GGATGCCTTATACTTCTTGAGATGGAAGGAGCTGATGCTACTGTGGTTGGTGTTTCCACAATTGTTCACTG
TCAACTGATGAGGTCCATCTTCCATACTGAACATGCCCTTGTAAATACGATTTGCATAACGGCCAACAGTA
GCACCAAT

CGTGCTGTATGTAGTTCACCACATCCATGCCGTCCACAACCTGTCCAAATACCACATGCTTTCCATCGAGC
CACGACGCTTCTTCCGTAGTGGTGATGAAGAACTGAGATCCATTGGTATCTTTACCACGATTAGCCATCGA
TAGTCTGC

CATCTCCCTTCACTCTTCCAGGTTTGAGTACGCTTCCTCTAAATAGGGGACGACTTGCTTTCTGGTGGCCT
CGACGTTCAGTTGCTTAAACATCGCCACACCTCCATCAAGATTGCCCCCAAATAATTGAAGTTGGAGCTTG
TCGCTAAC

TGAAAGTACCAACTCCAACACTAATGCTACTACCACTGAAAGTACCAACTCCAGCACTAATGCTACTACCA
CTGCCAGCATCAACGTCAGGACTAGTGCGACTACCACTGAAAGTACCAACTCCAACACTAGTGCTACTACC
ACCGAAAG

GCAACACCTTGTTGTTTGCCGTGTTCAGCACGTTGCTCTGCGAGTGGAACTGCGACCGAGTCTCCCAGGCT
TGGGAGATGAGACGGTCGACTACGTTGTTGGACTGATCGATTCGCCTTGTTTCGTTCTGAATGTACTCGTC
AGCATCCC

GGTGATGACACACCTCACAATTCACAAAGAGAATACGATATCCCCAATGTAGTGCAATGTAAAGAGGTGAA
GAAAGTGCAAGAAGAGGAAGCTGATCCGTCGAAGATGGTTCATGTTATCTTCTATTCCTTTATGGAACTCG
ATATATTA

CTCATTAATTGTTATTGGAACAGCGCCTATTTTACATTCCTGGACGAGAGAAGTCGCAGTGGTGTTTTTGG
TGAACTCAAGGAAAGCCTTACATGAACCAATGGTTGGGTGACCAGCAAATGGCAATTCACTTCTTGGAGTA
AAGATCCT

TCCGCTGAGGATGAATCAGTAAATGTATTACCTGACTCAGGTGATGGAGTGCTCAGAGGCGTTCCAACTGA
TGATGGATACTGCGGAAACTGTGATTGTGGCCCAGGTGGAAAGTACATAGGCGACATTTGATAAGGTGTAT
ACGGAATC

ACGTTATTTGGTGCAACACAAAATGGTTGATGCTGTCGTTACTTCTGCTGGTGGTGTGGAAGAAGATTTGA
TCAAATGTCTTGCTCCAACTTACTTGGGTGAATTTGCTTTGAAAGGTAAATCTTTGCGTGACCAAGGTATG
AATCGTAT

AATCACAAAGGCCTACAGAGAAGAACCAGATTTGGAAAACTTGTTGTTCAACAAGTTCTTCGCTGATGCCG
TCACCAAGGCTCAATCTGGTTGGAGAAAGTCAATTGCGTTGGCTACCACCTACGGTATCCCAACACCAGCC
TTTTCCAC

ACTTGATGTTTTGTTGGATAGCAATCATTTTTCTTGCGTTTGCGGGAGAGGGCTCCTTGGCCAGAAGATCT
GAGAGTTCTTGGAATTGGTCAACAGCAGTGTTGAATCCGTTCACATAAGTTTGATGGTGCTTGGTGTAATG
CAATTCGT

TCATCATAATCATCATCATAACGACTGCGTGACCCCAGCGACATTCCTCTTCTTCTTGATGATCTTGTGTT
TGGACGTGTTGAGGATTCATCAGTAGAATCAAAGGAATCTGGAATATCGTCGTAATAATGGCTTGTGTTGT
CGCTGTAT

TTTATGGATCCGACTACTTTGCAAACCGCGGATCTGTCACCATCTGTGTACTGGAGGGCTCCATTCAACGC
CCTGGCTGATGTGACCCAGTTGGTGGAGTTCATTGTTCTGGACGTGGATTCTACAGGTATAAGCAGGGGAA
ATCGTGTC

AGAACTACTACCTCATGAATTCTAGTGGATGAAAGAAGCAGCACGAACACCATTTCTACAGACAACGACAC
ATGGAAAGGTTCACCATTCCCAAAGAAAACAACGATGGCCACAAGGGTGTGGTCCTCCATTCTCCTACTGT
TGGAAGGA

TCACCAGAATGATGGTGAGGATTCAGACACTGACAGTGACAACTTTTTGCAAGACCCTGAAGACGATGTGG
ATGAAGAAAGCACTGGTAGAGGTACAGTCACTACCACTTCCACATCCACTGAGTCAAGAGGCCGTCCATCT
TCTTGTAT

ATTGCTCTCCTGGAAGCTAGACGGTAACGCAACGATCGACATGGAAGCTGTCGCCTGATTTTCAGCCAATC
TGTCCATTCTTTCTATCAGTTCCACTGTGTCAGCAGACAGGTCTGTCCTGGAGCCACAGCATCCAACATGC
TGGCCCTT

CAGCCATGTGTCCACAGTGCCGAACATCAGGTCGTTCTCCTCATACGCCTTGGTACACAGAGGCTCATTGT
CGAGGAACCAGCGCAGCTTGGAACAGGAGAAATACGTGGAGAGCAATGGCAATCCAGTCTTCTGTCTAAGC
TGCAGTTG

GTTGAATCCGTTCACATAAGTTTGATGGTGCTTGGTGTAATGCAATTCGTTGATTTGACCGGAGATATAAG
GTTCCAATGCACCGAAGTCCCACTTCAAGTCTGGCAAGGTGACTTTGGTTCTCCTTGCTGTGGTGGAGAGC
AATGACAA

AGGAACCCCGCCCACTTGAGCATACGAGTACACTGAACAAGTTTATTGACAATGCAAGGGAGGAGGAAGAC
GCTGAAGAGGACTCTCAACCGACAGAAGAACCTGTACAGAAGGAAACACAGGACGCCAGCGACAGCGACAG
CGACAGCG

TTTGATGGTGCTTGGTGTAATGCAATTCGTTGATTTGACCGGAGATATAAGGTTCCAATGCACCGAAGTCC
CACTTCAAGTCTGGCAAGGTGACTTTGGTTCTCCTTGCTGTGGTGGAGAGCAATGACAAACCACCCTTCTT
GGTTAAAT

TTCTCTGAATCATCTACATCATCCGTCATTCCAACCAGTAGTTCCACCTCTGGTTCTTCTGAGAGCAAAAC
GAGTTCGGCTAGTTCTTCCTCTTCTTCCTCTTCTATCTCTTCTGAATCACCAAAGTCTCCTACAAATTCTT
CTTCATCA

CTCTCACTTCTATGAGTGTGTCTTCTTCTCCTCCTTCTCCTCCTTCTCCTTCTCCTTCTTCTGGGAACAAT
TCGATAATCTGGATACTCCCTGACTTCCAAGTCCAAAGACACAGATATCCCGATTGATAGGTACATAGACC
AGTCAATC

GTGAAACTTTACGTTATTTGGTGCAACACAAAATGGTTGATGCTGTCGTTACTTCTGCTGGTGGTGTGGAA
GAAGATTTGATCAAATGTCTTGCTCCAACTTACTTGGGTGAATTTGCTTTGAAAGGTAAATCTTTGCGTGA
CCAAGGTA

ACTCCAACAACAGCTAGCACCATCACAACTACCACCGAGCCATGGACCGGTACTTTCACCTCTACATCCAC
TGAGATGACTACTGTCACCGGTACCAACGGTCAACCAACTGACGAAACTGTTATTGTCATTAGAACTCCAA
CTAGTGAG

AAGACCCTGAAGACGATGTGGATGAAGAAAGCACTGGTAGAGGTACAGTCACTACCACTTCCACATCCACT
GAGTCAAGAGGCCGTCCATCTTCTTGTATCTTCGTGGCAAGCTTAGCAGCAGCCCTATCCGATGACGAATT
ATGTCTGT

ACTTTGACCTCCACCAAGGTTAAGCAATTGGAAAACGTCTCTTCCAACATTGTCAAGAACGCTGAACAACA
CAACTTGGTCAAGAAGGGTCCAGTCAGACTACCAACCAAGGTTTTGAAGATCTCCACCAGAAAGACTCCAA
ATGGTGAA

GCGTTGGTTACGTCAGAAACGAACGCCTTTTGTTTAGTTAATTGGTAAATCAGCCATGTGTCCACAGTGCC
GAACATCAGGTCGTTCTCCTCATACGCCTTGGTACACAGAGGCTCATTGTCGAGGAACCAGCGCAGCTTGG
AACAGGAG

GGTCTTGTATCTGACCCAGGGGAAAGACTGGTACTGCAATTGTGGCTCTTCGACCTTCAAGTACTCAACTT
TCAGGCCACTGCATGTACAGTACGGAATCTCGAAATCAATGTTGACCAACTTATCTCGAGGAGTAACGTGG
GAAGTTTG

GACTGCGATGTTCTACGTGGAGTATAGTAACTAGCAGGATTGAGCCTCCTGCACTCTTTGAGCCTCAATAG
AGAAGCGATCTCCTCGGAGAGTTTTCTTCCCTTATGCTGGTGACCATCTTTAAGTTGTTTGTGTTGTCCTT
GTGAAACT

TTGAAATAACAAATCCAACTTTGCCATCAACTTCGACAGCTGGGGCACCGTTAGCGCACTTGCTGACATCA
CACTTGTCAAATGGCAAACCAATGAAGTCACGGAAGTTAATGATAGTATTCTTAGGATTTCTGATTAATTG
TTGCAAAG

GTTACGAAGAATTGAAGAAGAAGGGTATTCTTTTCGTTGGTTCTGGTGTCTCCGGTGGTGAGGAAGGTGCC
CGTTACGGTCCATCTTTGATGCCAGGTGGTTCTGAAGAAGCTTGGCCACATATTAAGAACATCTTCCAATC
CATCTCTG

AACTAGTGAGGGTTTGATTACTACAACTACCGAACCATGGACCGGAACTTTCACCTCTACATCCACTGAGA
TGACTACTGTGACCGGTACCAACGGTCAACCAACTGACGAAACTGTTATTGTCATTAGAACTCCAACTAGT
GAGGGTTT

GCCACAGTTATTACAACAGTGGATTCAAGATGAAGAGGGCGGCAAGATCACGATCCCTCTGCCTTTGAAGA
AACGACACAGATCCCAGCAACACAATGACCAACAACCGCCACAACCACGCACCAAAGAGCTGATCATTCCG
TCACATAT

CAGTAAGTTTTTCTTCCTTACCTTCGCCTCTTGAAATAACAAATCCAACTTTGCCATCAACTTCGACAGCT
GGGGCACCGTTAGCGCACTTGCTGACATCACACTTGTCAAATGGCAAACCAATGAAGTCACGGAAGTTAAT
GATAGTAT

TAACGCTTCTACCACCGTCTCTTCTACGATCTCTTCTAGCGCCCCAGACTCAATAATTCCTTCATCTAGCG
CCTCCATCTCTGGTGTCTCAAACTCCACTACAGCATCTGGTTCAATCGCTTCTACTGCTTCCACCGCTTCC
ACTACTTC

TACAGCGAGAGATTATCTCGCTTATCAGCAGAGTACTTTAATAAGAAGTCTTCGACCTCAGTTCCTATCTC
GGTAGGTGACACACCTACTACCACGCCAGGGTCCATTGAAGCTGCAGGTGTGGTTGCATGGAATGAAATTC
TATCTCAG

GTCTGAATCACCATCCCCACGTCAATCTACGTATTCCTTGACTGCAGGCAGTCCACCAAATGACCCAAGCA
CGTTGGCAAGCCCATTTCACGATCCCATTCTTCCCAGAAGAACTTCTACTTTTCTTCATTCGCCCATTCAA
AAGCAACA

AGACAACATAACCGCTGCTATGATGGGAGGCTTTTGCGGTTCATTCCTAAGGGACTTGACCCCACAAGAGG
TGGAAAGACGTGAGATTCCATTGGCTGAGGTGCTTCCAGAACCTTCTGGTGGTGAAGATACCGGTCTGGTT
CCCCCATT

ATGACCACCATCACCGATACTAACGGTCAATTAACTGATGAAACTGTCATTGTCATCAGAACTCCAACAAC
AGCTAGCACCATCACAACTACCACCGAGCCATGGACCGGTACTTTCACCTCTACATCCACTGAGATGACTA
CTGTCACC

CCAATTGACAACAACCACCGATATCGAAAATTTCCCAGGGTTTCCTGAATCGTTGAGTGGCAGTGAACTGA
TGGAGAGGATGAGGAAACAATCTGCCAAGTTTGGCACTAACATAATTACCGAGACTGTCTCTAAAGTCGAT
TTATCTTC

AGAACCCACTAGGTTCTGATCATCCAAAGATCTTTGCGTACCAAAATTCATGGGGTTTATCCACTCGTGTC
ATTGGTGTCATGGTCATGATCCACTCTGACAACAAGGGTTTGGTTATCCCACCAAGAGTGTCCCAATTCCA
ATCCGTTG

GACACACCGGCAAATAAACCTTTGCTTTTGGAATAGGCAAAAACTGCCGAGACACCACCTGTGGATGCAGA
AGCTGCAGCTTCTGCACTTCTTCCAAGAGGACCTGCGGAAACTGACACATTACCACCTAAAGTAATTGTAC
CAAATTCA

GGCCATGCTACAGATGCTTTTATCCAACACCTCCGCCACCAAATGCCGTGACCTCTTGCCAAGAAGGCGGT
GTGATAGGACCTTGCATTGGACTAGTTGGAACAATGATGGCTGTAGAAACTTTGAAGCTTATCCTAGGAAT
CTACACCA

TTAGAACTCCAACTAGTGAGGGTTTGATTACTACAACTACCGAACCATGGACCGGAACTTTCACCTCTACA
TCCACTGAGATGACTACTGTGACCGGTACCAACGGTCAACCAACTGACGAAACTGTTATTGTCATTAGAAC
TCCAACTA

TTTGGACTTTGGATGCCTTATACTTCTTGAGATGGAAGGAGCTGATGCTACTGTGGTTGGTGTTTCCACAA
TTGTTCACTGTCAACTGATGAGGTCCATCTTCCATACTGAACATGCCCTTGTAAATACGATTTGCATAACG
GCCAACAG

CACTGTCCAGTTTATGGATCCGACTACTTTGCAAACCGCGGATCTGTCACCATCTGTGTACTGGAGGGCTC
CATTCAACGCCCTGGCTGATGTGACCCAGTTGGTGGAGTTCATTGTTCTGGACGTGGATTCTACAGGTATA
AGCAGGGG

CAATTGTCTTTGATGTTGAGGATAGCGATGAAGCTGTGACATCAGAGGTGGACAAGCCGACTAAGGAGAAT
CAGTCGGAGGATGACGATGCCAAGGGTGGATCAACAGGAAAGGAACAACCTGGGTCTTACACGCCTAAAGA
AGGCACTG

GTAAAGGTCATCAAGAGGATTTTGGACTTTGGATGCCTTATACTTCTTGAGATGGAAGGAGCTGATGCTAC
TGTGGTTGGTGTTTCCACAATTGTTCACTGTCAACTGATGAGGTCCATCTTCCATACTGAACATGCCCTTG
TAAATACG

ATTTTGAGGTTGAACTGTCCCAGTTATATTTGTAACAGGGTTGGCTCCTGCAGTTGCAGGCACATAATAAC
CCTGTTGTGGTTGCTGTTGTTGCTGTTGCTGTTGTTGCTGTTGCTGTTGTTGCTGTTGTTGGAGATGATTC
TGAGTGTA

GTAACTTCTGTCAAATGTTCTTCATCATAAACACGTCTAACGCTTTTCAGTAGAGCAGCACGGACTTTCAT
GACTGCAGACAAGGCATCTCCTCTCAAGGCCAAATGACGTTGGTCCAACAGTAAAGAAGGGTCTGAGCCCT
CTGCGATT

ACAGCATCTGGTTCAATCGCTTCTACTGCTTCCACCGCTTCCACTACTTCTACTGCATCCGCTGCATCCGC
CACCAGCTTCACCTCAGGTTCCGCTTCTGTCTACACTACTACATTAACTTACTTGAATGCCACAAGTACAG
TCGTGGTT

CACCCATTTGGTCACGCTTTGGTTGCCGGTATTGAAAGATACCCATTGAAGGTCACCAAGAAGCACGGTGC
CAAGAAGGTTGCTAAGAGAACCAAGATCAAGCCTTTCATCAAGGTCGTCAACTACAACCATTTATTGCCAA
CCAGATAC

TACTACAAGCTCAACGTGACTACTTTGGTGCTCACACTTTCAGAGTGTTGCCAGAATGTGCTTCTGACAAC
TTGCCAGTAGACAAGGATATCCATATCAACTGGACTGGCCACGGTGGTAATGTTTCTTCCTCTACATACCA
AGCTTAAA

CAGTGTGTACGTTTCAAGTAGCTCTATCACGAGTAGTAGTAGTACGAGTATTGTGGACACAACCACAATCT
CGCCGACTCTGACGTCAACGAGTACGACCCCATTGACAACCGCCTCAACCAGTACAACACCATCAACTGAT
ATAACATC

ATTCGTAAAGGCGTTTGTCTCTAGTTTGTGATAGTGTAGATACCGTCCTTGGATAGAGCACTGGAGATGGC
TGGCTTTAATCTGCTGGAGTACCATGGAACACCGGTGATCATTCTGGTCACTTGGTCTGGGGCAATACCAG
TCAACATG

TGTCATCAGAACTCCAACAACAGCTAGCACCATCACAACTACCACCGAGCCATGGACCGGTACTTTCACCT
CTACATCCACTGAGATGACTACTGTCACCGGTACCAACGGTCAACCAACTGACGAAACTGTTATTGTCATT
AGAACTCC

CATTGGTGTACAAAATGCCTATTTCCCAATGTTTGTTTCCTCACGTGTGTTGGAAAAGGAAAAGGACCATG
TTGAAGGCTTTGCTCCAGAAGTTGCCTGGGTCACCAGAGCTGGCTCCTCTGAATTGGAAGAACCAATCGCC
ATTAGACC

GCCTCCATCTCTGGTGTCTCAAACTCCACTACAGCATCTGGTTCAATCGCTTCTACTGCTTCCACCGCTTC
CACTACTTCTACTGCATCCGCTGCATCCGCCACCAGCTTCACCTCAGGTTCCGCTTCTGTCTACACTACTA
CATTAACT

CCCCATCGAACTCTCACTTCTATGAGTGTGTCTTCTTCTCCTCCTTCTCCTCCTTCTCCTTCTCCTTCTTC
TGGGAACAATTCGATAATCTGGATACTCCCTGACTTCCAAGTCCAAAGACACAGATATCCCGATTGATAGG
TACATAGA

CTGCACGCCTGCTAGCTTTGTGTTGGTCAACTTAATCAGCTCGTCCAGACTGCCAAACTGCTCGTCGATTG
CCTTTGCCAAAGCGCCGGTGGGTGGTTCACCACCGCCCTGCGACTCTGGAGCCAGGTTTTCCCAGAATAGA
CAGTGGTT

AGATCTGTTTTCTTGGGTCAAATCACAAAGGCCTACAGAGAAGAACCAGATTTGGAAAACTTGTTGTTCAA
CAAGTTCTTCGCTGATGCCGTCACCAAGGCTCAATCTGGTTGGAGAAAGTCAATTGCGTTGGCTACCACCT
ACGGTATC

TGATGTTGAGGATAGCGATGAAGCTGTGACATCAGAGGTGGACAAGCCGACTAAGGAGAATCAGTCGGAGG
ATGACGATGCCAAGGGTGGATCAACAGGAAAGGAACAACCTGGGTCTTACACGCCTAAAGAAGGCACTGCT
GGGGAACG

ATCATCATCATCAACTCCAACCACAACATCGTCAGCCCCCATTTCTACAAGCACGACGTCGTCAACTTCCA
CTTCAACAAGTACAACATCGCCAACTTCTTCTTCAGCACCTACAAGCTCGTCTAATACAACACCAACGAGC
ACAACGTT

GTATGATAACGTAAGAGTATTCAGTATCAACGGCAACAACAACAACAACAACAACAACAACAGCACAACAA
CAGCACAACAACATCGAACGGTTGCTACTGCGACGTTACGGATTATATCCACAGTATATGGCGTGCAGCGA
TAAGGCTC

CCGACGCAACTGCAACCGTTTCCTCCAAATCATACACCACTGTTACTGTTACTCACTGTGACAACAATGGC
TGTAACACCAAGACTGTCACTTCTGAATGTTCTAAAGAAACTGCAGCAACCACCATTTCTCCAAAATCATA
CACTACTG

TCACCGGTACCAACGGTCAACCAACTGACGAAACTGTTATTGTTATCAGAACTCCAACCAGTGAAGGTCTA
ATCAGCACCACCACTGAACCATGGACTGGTACTTTCACCTCTACATCTACTGAGGTTACCACCATCACTGG
TACCAACG

CGTAGATTCAAACCAACATACGGCAGCTGGTCTATAGTTAAGTTTCCCATGACATCTCTTTGGGACCTAAA
GGCCACCGTCATTTGAGGGGACATCGTCGGCCAGACAGATGCTGAAACGGTCTCATTCAGATCATCGCCAA
CTGTCGTA

GAGGATATAGACCAATTTTTGCCTTCGGAAGTATCTTCGTTGGGGTCAGATCACCAGAATGATGGTGAGGA
TTCAGACACTGACAGTGACAACTTTTTGCAAGACCCTGAAGACGATGTGGATGAAGAAAGCACTGGTAGAG
GTACAGTC

CCACCGAAAGTACCGACTCCAACACTAGTGCTACTACCACCGAAAGTACCGACTCCAACACTAGTGCTACT
ACCACTGCTAGCACCAACTCCAGCACTAATGCCACTACCACTGCTAGCACCAACTCCAGCACTAATGCCAC
TACCACTG

ACCCAATTCCTGAACCATCATGACCACTGGATCCATCACTTGCATAAACGACAGGGAAGTCGACCGATGTC
CATGATGCCACGGTACTGTTGTGACTTGTACCAGAGCTGACAACAACCAAACCTTGGATACTAGAACTTAG
TGATTGAA

GAAAACGTCTCTTCCAACATTGTCAAGAACGCTGAACAACACAACTTGGTCAAGAAGGGTCCAGTCAGACT
ACCAACCAAGGTTTTGAAGATCTCCACCAGAAAGACTCCAAATGGTGAAGGTTCTAAGACTTGGGAAACCT
ACGAAATG

CATCTGTGTACTGGAGGGCTCCATTCAACGCCCTGGCTGATGTGACCCAGTTGGTGGAGTTCATTGTTCTG
GACGTGGATTCTACAGGTATAAGCAGGGGAAATCGTGTCTTAGCTGACATTACTGTTGCTAGAACGTCTGA
CTTGGGTG

CTAGTGCTTGCCGTGCTACTGTACGTAAAGAGAAACTCCATCAAGGAACTGCTGATGTCCGATGACGGAGA
TATCACAGCTGTCAGCTCGGGCAACAGAGACATTGCTCAGGTGGTGACCGAAAACAACAAGAACTACTTGG
TGTTGTAT

GCGGTCATATTCTTCCTGGTTGAAGAGGGGGAACTCTGCCACCATGGATGCACACGTATTTGGATCATCTT
CGTCGCTGTTGTACTGTGAGCTCTTGTTTGTGCTATGTTCTCGGTGCCCCTTCATGGTTTGTATCTCCCAG
AGGAGAAA

CACCTCCATTTTCACCACAATAGACTGCTCGCTGGCGTTGACCTGCTCTGAGGCATCAAAAAACTCCTCAT
CGTCGTCGTCTTCGTCTGACGATGTCTCGTCCTCGCCAGCTCCAGTGGTCGTCCTAGCATTTCCATCTTGC
TTGTGCAA

GAATTAGTGTTCGGCCAGACATTCACCGATCATATGTTGACCATTCCTTGGTCAGCCAAAGAAGGGTGGGG
CACTCCACACATCAAGCCTTACGGTAATCTTTCTCTTGACCCATCTGCTTGTGTATTCCATTATGCATTTG
AATTATTT

TCTTCCTGGTTGAAGAGGGGGAACTCTGCCACCATGGATGCACACGTATTTGGATCATCTTCGTCGCTGTT
GTACTGTGAGCTCTTGTTTGTGCTATGTTCTCGGTGCCCCTTCATGGTTTGTATCTCCCAGAGGAGAAAAT
CGTCACTC

GTTCAAAGCATACTTTAATCTGTTTCTTAGAAAGACAATCAATGGCAAGGATTCACGCAATTTGTGTGGAC
CAGCAGATGGTCTTGGGGCGTAACAACCGGACAACTTGTCCAATAACCAATGGTGTGGAGCTGCTAATCTC
TTTAGATG

TCTTGCTGCTATTCAAGGTCTGTGTACATGTCTGGACACACTGTGTGCGCAGGCATACGGTGCCAAAAACT
ACCACTTGGTGGGTGTGCTAGTGCAGAGATGTGCTGTGATCACCATCTTGGCGTTCTTGCCAATGATGTAT
GTTTGGTT

TCTAGAAGATTACCAAGAACCACGTTATTTGAGAGAGGATGGCAAGGTGACGACAATCACACCAAGACCAC
ATTTTGGGTGCGCCTGGAAGCAAGACCTGAGAAACTGGGCCAAAATATTCAAACCAAGCATAAGATAGTTG
GAGGTAGG

GTGATCGTTCCAGCGAGATGCTTCCTAAACAAAGAAGTATCATAGGATCCCACGTGCAACGTCCACCGTCT
CAAACAACTTTAGGAAGGTCAAGGGCTGGAAGCAATACAATGAATAAAGTTTCAGGCTTAGATATTGCTCG
AAGACCAA

TCAATCACGGTATTCAAATAGCAATGTTCACAATACATTAGCGTCTTCGAGTGGATCTCTTCCCACAGAAT
CTGCTCTGCTTTTGCAACAAAGACCACCTTCAGTTTTGAGATACAACACAGATAATTTGAAGTCTAAGTTT
CATTATTT

TATGTGTTGCGTTGCAACAATATTAGAAACTTCCCCTCCGGAACCAAAGTTCACGTCTCCAACCCAATCCC
ACTTGGCCGTGGATTGGGTTCCTCTGGTGCAGCAGTTGTGGCAGGTGTTATTTTAGGTAACGAAGTGGCCC
AATTGGGT

TACGTACCAAAGAAGTGTTATAAAAGTGATATAACTATGTCAGAGGATCTTTCACCTACAAGCAGCAGGGT
GGATTTGAGCAATCCTCATGGGTTTACCAAAGAGGGAGTGGATTTATCGAAGCTGTCACCACAAGAACTAA
AATTGTAT

CATCCAAAGATCTTTGCGTACCAAAATTCATGGGGTTTATCCACTCGTGTCATTGGTGTCATGGTCATGAT
CCACTCTGACAACAAGGGTTTGGTTATCCCACCAAGAGTGTCCCAATTCCAATCCGTTGTCATTCCAGTGG
GCATCACA

CTTCTACGATCTCTTCTAGCGCCCCAGACTCAATAATTCCTTCATCTAGCGCCTCCATCTCTGGTGTCTCA
AACTCCACTACAGCATCTGGTTCAATCGCTTCTACTGCTTCCACCGCTTCCACTACTTCTACTGCATCCGC
TGCATCCG

TCATCCTTATTCAAGTCACCGGCATCAACTTCACCTTCTTCGTCATCTTCTTCTGCTGCGGCGTTGACACT
GCCTTCTGCGGCGGCTTGCATGTCAGCTTGGATGTCTTCTGGGGACTTGGTAGCGACGTCTTCGTTAGATG
GCATAATA

CCACCAAGGTTAAGCAATTGGAAAACGTCTCTTCCAACATTGTCAAGAACGCTGAACAACACAACTTGGTC
AAGAAGGGTCCAGTCAGACTACCAACCAAGGTTTTGAAGATCTCCACCAGAAAGACTCCAAATGGTGAAGG
TTCTAAGA

TGTACAGAACAAGAAACTAAAGACTGCAGATGGCAATAATACCCCAGTCACGAAAGGTGTGCTGCTACACG
AATCCATCCACAGCGTGGAGGATGCGTATGGAAAATTGCCTGAGGACGCCTTGGCGTTTCTTAAGGAAAAC
AGTGCGGA

GTGGTATGGTTGAGTGGGGCAGGGTAACGAGTGGGGAGGTAGGGTAATGTGAGGGTAGGTTAAGAGACAGG
TTAGATAGGGTGGTGTGTGGTGTGTGGGTGTGGTGTGGTGTGGGTGTGGGTGTGGTGTGTGGGTGTGGTGT
GTGTGGGT

TTGCGCTAACGAAACCACATGGATCCTCCACACAACCACTTAGTTTGGCAAATCCCAAGACCAAGAGGAAC
TTTCCTCACCATTCGGGGCAGGTGACGAAGCAAGAGCGAAGCTGGATTCTACGCTGGATTAGTGAAGCGCC
AGCGAGGA

ATTCAACAATGTAAAGGTCATCAAGAGGATTTTGGACTTTGGATGCCTTATACTTCTTGAGATGGAAGGAG
CTGATGCTACTGTGGTTGGTGTTTCCACAATTGTTCACTGTCAACTGATGAGGTCCATCTTCCATACTGAA
CATGCCCT

ACTTGTACCAAGCAGATGCTTCCCAAGCTTCGAAGGGCGTTTCCCCACTATACTCTTCGCTGCAAGCGTGG
GTGTGTGCCTTTTCCCACTGGAAACCAAAGGAATGATCAATGTCGATACCAAGACATTGAGGAACATGAGT
CCTTTGAC

AGATTTCTAACACTGTCCAGTTTATGGATCCGACTACTTTGCAAACCGCGGATCTGTCACCATCTGTGTAC
TGGAGGGCTCCATTCAACGCCCTGGCTGATGTGACCCAGTTGGTGGAGTTCATTGTTCTGGACGTGGATTC
TACAGGTA

TCACCGATACTAACGGTCAATTAACTGATGAAACTGTCATTGTCATCAGAACTCCAACAACAGCTAGCACC
ATCACAACTACCACCGAGCCATGGACCGGTACTTTCACCTCTACATCCACTGAGATGACTACTGTCACCGG
TACCAACG

GACTCCAACACTAGTGCTACTACCACTGCTAGCACCAACTCCAGCACTAATGCCACTACCACTGCTAGCAC
CAACTCCAGCACTAATGCCACTACCACTGAAAGTACCAACGCTAGTGCCAAGGAGGACGCCAATAAAGATG
GCAATGCT

TCACCTTCTTCGTCATCTTCTTCTGCTGCGGCGTTGACACTGCCTTCTGCGGCGGCTTGCATGTCAGCTTG
GATGTCTTCTGGGGACTTGGTAGCGACGTCTTCGTTAGATGGCATAATACCGCTAGCTTGAGCTTGTTGTT
GAGCAGCG

CAGCTTTGAAGCTGAAGTTTTGCAGAACACCGCTATTCTCCATCTATGATCAGTCCACGTCTCCATATCTC
TTGCACTGTTTCGAACTGTTGAACTTGACCTCCAGATCGTTTGCTGCTGTGATCAGAGAGCTGCATCCAGA
ATTGAGAA

CCGCCATGGAACTTGATGTTTTGTTGGATAGCAATCATTTTTCTTGCGTTTGCGGGAGAGGGCTCCTTGGC
CAGAAGATCTGAGAGTTCTTGGAATTGGTCAACAGCAGTGTTGAATCCGTTCACATAAGTTTGATGGTGCT
TGGTGTAA

CTCTGTCATTTCTTCTTCTACAACAACCTCCACTTCTATATTCTCTGAATCATCTACATCATCCGTCATTC
CAACCAGTAGTTCCACCTCTGGTTCTTCTGAGAGCAAAACGAGTTCGGCTAGTTCTTCCTCTTCTTCCTCT
TCTATCTC

ATTGGTAAATCAGCCATGTGTCCACAGTGCCGAACATCAGGTCGTTCTCCTCATACGCCTTGGTACACAGA
GGCTCATTGTCGAGGAACCAGCGCAGCTTGGAACAGGAGAAATACGTGGAGAGCAATGGCAATCCAGTCTT
CTGTCTAA

TTTTGTCCATTGAAACCACCTCTGGCGCCGCCTCTACCTCTGAAGTTCCCTCTGCCTCTGAAACCTCCTCT
GTAGCCGCCTCTGAAACCTCCTCTAAAACCTCCTCTGCCTCTGAAACCGCCTCTGCCTCTGAAACCTCCTC
TTTGCATT

TTCGACCTCAGTTCCTATCTCGGTAGGTGACACACCTACTACCACGCCAGGGTCCATTGAAGCTGCAGGTG
TGGTTGCATGGAATGAAATTCTATCTCAGACTGATATGATTTCCAAGGATCATGATCAGTTATCAACCGAC
TTTGAAAA

AAACTGTTATTGTTATCAGAACTCCAACCAGTGAAGGTCTAATCAGCACCACCACTGAACCATGGACTGGT
ACTTTCACCTCTACATCTACTGAGGTTACCACCATCACTGGTACCAACGGTCAACCAACTGACGAAACCGT
GATTGTCA

TGTTATCAGAACTCCAACCAGTGAAGGTCTAATCAGCACCACCACTGAACCATGGACTGGTACTTTCACCT
CTACATCTACTGAGGTTACCACCATCACTGGTACCAACGGTCAACCAACTGACGAAACCGTGATTGTCATT
AGAACTCC

ACACGTCTAACGCTTTTCAGTAGAGCAGCACGGACTTTCATGACTGCAGACAAGGCATCTCCTCTCAAGGC
CAAATGACGTTGGTCCAACAGTAAAGAAGGGTCTGAGCCCTCTGCGATTTTGTTTGTAAAGGAGTCTTCAC
CACCGGGG

CGGTGAATGTACCACCTTCTTCATTAACAATTTTATAACGCTGGTTGGGAAGATTGGATATCCCTACAGGA
GGAGGAACAGGGGCAGCAGTCGCAGTGGCAGCACTCATTATTTGATACAATATGTGATGTAGTTCGTTATC
AATACTCT

AATGCGACTACCAACTCCAGCACTAATGCTACTACCACTGCCAGCATCAACGTCAGGACTAGTGCGACTAC
CACTGCCAGCATCAACGTCAGGACTAGTGCGACTACCACTGAAAGTACCAACTCCAACACTAATGCTACTA
CCACTGAA

GGTGGTGCCAAGACTGGTTCTGGTAAAACTATTGCATTTGCAGGGCCTATGCTGACTAAATGGTCCGAAGA
TCCGTCAGGGATGTTTGGAGTGGTCTTGACCCCAACCAGAGAGTTAGCCATGCAAATTGCCGAACAATTTA
CTGCACTT

ATCCACTGAGATGACCACCATCACCGATACTAACGGTCAATTAACTGATGAAACTGTCATTGTCATCAGAA
CTCCAACAACAGCTAGCACCATCACAACTACCACCGAGCCATGGACCGGTACTTTCACCTCTACATCCACT
GAGATGAC

ATCTACTGAGATGACTACTGTCACCGGTACCAACGGTCAACCAACTGACGAAACTGTTATTGTTATCAGAA
CTCCAACCAGTGAAGGTCTAATCAGCACCACCACTGAACCATGGACTGGTACTTTCACCTCTACATCTACT
GAGGTTAC

TCCGGTGTACGTGAAACTTTACGTTATTTGGTGCAACACAAAATGGTTGATGCTGTCGTTACTTCTGCTGG
TGGTGTGGAAGAAGATTTGATCAAATGTCTTGCTCCAACTTACTTGGGTGAATTTGCTTTGAAAGGTAAAT
CTTTGCGT

ACAGAGGCTGACCTACCAGAGGGCTACGAGGTGCTACACCAGGAACCCCGCCCACTTGAGCATACGAGTAC
ACTGAACAAGTTTATTGACAATGCAAGGGAGGAGGAAGACGCTGAAGAGGACTCTCAACCGACAGAAGAAC
CTGTACAG

TAATGAATCTAAATTTAGAGCACGTTGTAAATTGTCAGTAACATATTGCTCCCAAACTTTCCATTCTGGTT
GAAACTTGTTGCAAGAGTACCAAGACATCCAAGTGGCAAAACCTTCATTCAACCATAAGCCTTCCCACCAA
TCCATGGT

AGCTACTATGGCAATGGCATCAAAACATATCCCTTGCCATCTTCACCGAAATCGCCGACATCAGAAGAAAC
ACACTCATCTATGACAGCATCTGGAGAGGACTCACACCTACTTAAGCAATAGATTGTGCTATCTGGGACAG
TAACAATG

TGTCAAGAACGCTGAACAACACAACTTGGTCAAGAAGGGTCCAGTCAGACTACCAACCAAGGTTTTGAAGA
TCTCCACCAGAAAGACTCCAAATGGTGAAGGTTCTAAGACTTGGGAAACCTACGAAATGAGAATCCACAAG
AGATACAT

AGCACCAACTCCAGCACTAATGCCACTACCACTGCTAGCACCAACTCCAGCACTAATGCCACTACCACTGA
AAGTACCAACGCTAGTGCCAAGGAGGACGCCAATAAAGATGGCAATGCTGAGGATAATAGATTCCATCCAG
TCACCGAC

ATGGAAAGTGTCTAGAAGATTACCAAGAACCACGTTATTTGAGAGAGGATGGCAAGGTGACGACAATCACA
CCAAGACCACATTTTGGGTGCGCCTGGAAGCAAGACCTGAGAAACTGGGCCAAAATATTCAAACCAAGCAT
AAGATAGT

TTTGGGTGCTGTTCAGGAGTTCTTAAAGCAGCAGAAGGTTGTTGTTGTGGTTGATAACGAGCGCCAAACTG
CTGAGATTGAGTTTGCGCTTGAGGCTGAGATGGAGGTTGGCCTGTTGCACGTAGCTGTTGCTGTTGCTGTT
GCTGCTGT

TTAGCTGACATGTACCACTCCTTGATGAAGAGATACCCAATTGTCTCCATCGAAGATCCATTTGCTGAAGA
TGACTGGGAAGCTTGGTCTCACTTCTTCAAGACCGCTGGTATCCAAATTGTTGCTGATGACTTGACTGTCA
CCAACCCA

ACTCCAACCAGTGAAGGTCTAATCAGCACCACCACTGAACCATGGACTGGTACTTTCACCTCTACATCTAC
TGAGGTTACCACCATCACTGGTACCAACGGTCAACCAACTGACGAAACCGTGATTGTCATTAGAACTCCAA
CTAGTGAG

TCGTCGCTGTTGTACTGTGAGCTCTTGTTTGTGCTATGTTCTCGGTGCCCCTTCATGGTTTGTATCTCCCA
GAGGAGAAAATCGTCACTCAAGTTAAACACGACCTTGCCGATGTTTGCCTTGAATCTAATTTGCTTGCTGA
GGTCTATT

TCTAGTGGATGAAAGAAGCAGCACGAACACCATTTCTACAGACAACGACACATGGAAAGGTTCACCATTCC
CAAAGAAAACAACGATGGCCACAAGGGTGTGGTCCTCCATTCTCCTACTGTTGGAAGGAGATATTATCCGA
CCGACTGT

CAACAACCTCCACTTCTATATTCTCTGAATCATCTACATCATCCGTCATTCCAACCAGTAGTTCCACCTCT
GGTTCTTCTGAGAGCAAAACGAGTTCGGCTAGTTCTTCCTCTTCTTCCTCTTCTATCTCTTCTGAATCACC
AAAGTCTC

CATTCGAATTCCACCAGAACTGACTTCCGATGAGGATAGTTTGCCAGCAGAAAGCGAGGATGAATCCGTAG
CGGGTGGAGGAAAGGAGGAGGAAGAGCCTGATCTCATTGATGCTCAAGAAATATATGATTTGATAGCTCAT
ATTTCCGA

TTAACTGATGAAACTGTCATTGTCATCAGAACTCCAACAACAGCTAGCACCATCACAACTACCACCGAGCC
ATGGACCGGTACTTTCACCTCTACATCCACTGAGATGACTACTGTCACCGGTACCAACGGTCAACCAACTG
ACGAAACT

ACCTACCAGAGGGCTACGAGGTGCTACACCAGGAACCCCGCCCACTTGAGCATACGAGTACACTGAACAAG
TTTATTGACAATGCAAGGGAGGAGGAAGACGCTGAAGAGGACTCTCAACCGACAGAAGAACCTGTACAGAA
GGAAACAC

CACACGTATTTGGATCATCTTCGTCGCTGTTGTACTGTGAGCTCTTGTTTGTGCTATGTTCTCGGTGCCCC
TTCATGGTTTGTATCTCCCAGAGGAGAAAATCGTCACTCAAGTTAAACACGACCTTGCCGATGTTTGCCTT
GAATCTAA

TACCGTCCTTGGATAGAGCACTGGAGATGGCTGGCTTTAATCTGCTGGAGTACCATGGAACACCGGTGATC
ATTCTGGTCACTTGGTCTGGGGCAATACCAGTCAACATGGTGGTGAAGTCACCGTAGTTGAAAACGGCTTC
AGCAACTT

AGAATCCACAAGAGATACATCGACTTGGAAGCTCCTGTTCAAATCGTTAAGAGAATCACTCAAATCACCAT
TGAACCTGGTGTGGATGTCGAAGTTGTTGTTGCTTCCAACTAAGCTGGTTCTAACTGGAAATAATTTCCAT
TAGATTCC

TGAGCTAGGCCGAGAAAGCTGGGAATATTTAACCCCTCAGCAAGCCGCAAACGACCCACCATCCACTTTCA
CGCAGTGGCTTCTTCAAGATCCCAAATTTCCTCAACCTCATCCAGAAAGAAATAAGCATTCACCAGATTTT
TCAGCCTT

TTGTCTTCTGTCTTAAACACGAAAAGCTTAGGTTCCGTGGCATCGCTCTTCTGTTGAGATGCGAACAAAGC
ATATGCTGGGGTTTTGAATGGATGGTCTTGTGAACCGGCTGTGGTCAATTCGTCTACACCGGTAGCCTCCT
TAATGTAC

CAAAATGCCTATTTCCCAATGTTTGTTTCCTCACGTGTGTTGGAAAAGGAAAAGGACCATGTTGAAGGCTT
TGCTCCAGAAGTTGCCTGGGTCACCAGAGCTGGCTCCTCTGAATTGGAAGAACCAATCGCCATTAGACCAA
CTTCTGAA

GTTATTGGAACAGCGCCTATTTTACATTCCTGGACGAGAGAAGTCGCAGTGGTGTTTTTGGTGAACTCAAG
GAAAGCCTTACATGAACCAATGGTTGGGTGACCAGCAAATGGCAATTCACTTCTTGGAGTAAAGATCCTCA
ACTTGTAA

TCTCGCAAGTGCATTCCTAGACTTAATTCATATCTGCTCCTCAACTGTCGATGATGCCTGCTAAACTGCAG
CTTGACGTACTGCGGACCCTGCAGTCCAGCGCTCGTCATGGAACGCAAACGCTGAAAAACTCCAACTTTCT
CGAGCGCT

GATCTCAGGCCGATCTGACAATCTTTAAAGGCATCAACAAGAGCAGCAGCTTGCCGACGTGGCAAAAGTCA
AAGAAGAACAGAGGCAACTCGTCCTACGTGAAGCCACATACAACAAGTTAGGAACTATTAGGCGGAACCGC
TTTCTAAG

GGTGTTATGGTTTCCCACAGATCTGGTGAAACTGAAGACACTTTCATTGCTGACTTGGTTGTCGGTTTGAG
AACTGGTCAAATCAAGACTGGTGCTCCAGCTAGATCCGAAAGATTGGCTAAGTTGAACCAATTGTTGAGAA
TCGAAGAA

ATCAGAGGTGGACAAGCCGACTAAGGAGAATCAGTCGGAGGATGACGATGCCAAGGGTGGATCAACAGGAA
AGGAACAACCTGGGTCTTACACGCCTAAAGAAGGCACTGCTGGGGAACGTGAGAATGAGAATGAAAGCAAC
GTTAAGCC

GTCAAATTGTACTATCCCTGATCCTTCAATACATACTACTAGCACTATCACAACTACCACCGAGCCATGGA
CCGGTACTTTCACTTCTACATCCACTGAGATGACCACCATCACCGATACTAACGGTCAATTAACTGATGAA
ACTGTCAT

AGACGATGTGGATGAAGAAAGCACTGGTAGAGGTACAGTCACTACCACTTCCACATCCACTGAGTCAAGAG
GCCGTCCATCTTCTTGTATCTTCGTGGCAAGCTTAGCAGCAGCCCTATCCGATGACGAATTATGTCTGTCG
GTGACTGA

GGCTCCTTGGCCAGAAGATCTGAGAGTTCTTGGAATTGGTCAACAGCAGTGTTGAATCCGTTCACATAAGT
TTGATGGTGCTTGGTGTAATGCAATTCGTTGATTTGACCGGAGATATAAGGTTCCAATGCACCGAAGTCCC
ACTTCAAG

ACCATGGATGCACACGTATTTGGATCATCTTCGTCGCTGTTGTACTGTGAGCTCTTGTTTGTGCTATGTTC
TCGGTGCCCCTTCATGGTTTGTATCTCCCAGAGGAGAAAATCGTCACTCAAGTTAAACACGACCTTGCCGA
TGTTTGCC

AGTTTTCTCTCGTACTCTTCATACATTCGTTTCATTTCAATATCTCTGTCTGCGTCAAGATCACCACCATA
CTCCAAATCAGAGTCGCTGTCTTCTTCTTCTTCATCATCATCATCCTCATCATCATCATCTTCATCATCAT
CCTCATCG

AAATTATCCTGATGTGAGTTTTCCGGTGATGGGGGTGCCGCAGTGTCGATGAACGCCTGAGACAGGAACTT
TGAGTCCAGTACTTCAGAGACCAGATGTTTGTCTTCAGCGGCCACATATATGCCCAGACCATATGCTTTGA
AAGTGATC

CAATAGCACCTCCTCCGCAGACTTGAGTTCTATCACTTCCGTCTCGTCAGCTAGTGCAAGTGCCACCGCTT
CCGACTCACTTTCTTCCAGTGACGGTACCGTTTATTTGCCATCCACAACAATTAGCGGTGATCTCACAGTT
ACTGGTAA

TGAAAGTTCGAATAACAACGCAGAGGGTGAAGACATAATCAGAAATGAAGAAGTCGAAGATGAGATCAAAT
CATCACTTGGCAACCACAAGTCAAGCCAGTACGCAAATGCGTTCGATTCGGAAATAATCAAGAGGGAATTA
AGATCAAG

CGCAAGCTGATCTCGCAAGTGCATTCCTAGACTTAATTCATATCTGCTCCTCAACTGTCGATGATGCCTGC
TAAACTGCAGCTTGACGTACTGCGGACCCTGCAGTCCAGCGCTCGTCATGGAACGCAAACGCTGAAAAACT
CCAACTTT

CGGAAGGAATGTATCCTCATTTACCGATGATGGCACCATCGGATTATCCAGTTCTTCGATTTCGTATCCAG
GCGGTGGTGGTGGTGGTGGTGGAAGTGCTAAGTCGCTGTCCTCTTCAAAACCAGGAGGTGGGGGCGGTAGT
CCACTCAT

TGAAGAGGGGGAACTCTGCCACCATGGATGCACACGTATTTGGATCATCTTCGTCGCTGTTGTACTGTGAG
CTCTTGTTTGTGCTATGTTCTCGGTGCCCCTTCATGGTTTGTATCTCCCAGAGGAGAAAATCGTCACTCAA
GTTAAACA

AAATGTTAGGCTCAGCAATCAAGTGAGATTCAGAATTTACTGGGTGGCTCTGATTTTGGTGTTCTGTTGAC
ACATTTCGATTGTGTACCTCCCTGTTGAGGCGATCAGCATCTGAAAAATCTTTTGGTGCAGGTGATGGAGC
GGAAGCTA

CAGGCCAAGTGCTGGTGACGAAGATTCTGCTCATCCTGATAAGAACAAAGAAATTTCGATGCCTACTCCGG
ATTCCAATACTTTGGTGGTCCAGTCAGAAGAAGGTGGAGCTCATTCACTTGAGGTAGATACCAATCGAAGG
TCCGATAA

CATTTGAGGGGACATCGTCGGCCAGACAGATGCTGAAACGGTCTCATTCAGATCATCGCCAACTGTCGTAG
TAGAGTCAACAGCAGTAACCCACGTTCGCAGTGAGGACCATATGATAAATAGGTATATGAGTATCCACATA
CCGTGTCT

TCTAATAGACGTTACGAAGAATTGAAGAAGAAGGGTATTCTTTTCGTTGGTTCTGGTGTCTCCGGTGGTGA
GGAAGGTGCCCGTTACGGTCCATCTTTGATGCCAGGTGGTTCTGAAGAAGCTTGGCCACATATTAAGAACA
TCTTCCAA

TCGCATTGGTACTGGCGTTAGTACTGGCATTAGCACTACCATGAATGCACGTGTCGCTGTCCTCATCACTG
CTGCAATACTTTCTGTACCTGTCACTGCTATTGCTCTCCTGGAAGCTAGACGGTAACGCAACGATCGACAT
GGAAGCTG

TCGATCGAATTTGAAACTATGTCCTGTTGCTTTAAAGGTGGTAGAACAACATCGTGGGTCAGAGAGGATGG
CTCGTTCAAGTCAATTGATAGATCCTTACTGGACAGGTTCATTGCCGCATACTTCAAACACAATCACCGTC
TATTTCCC

ATACATTCTGAACAGGTTGAGTTGGTTGAACAGGTTGAGTTGGCTGAACAGGTTGAGTTGGCTGAACAGGT
TGAGTTGGCTGAACAGGTTGAGTTGGTTGAACAGGTTGTGTTGATTGAACAGGTTGAGTTGGTTGAGCGAC
TTCCACAT

AGATTGACTGACAGAGCTTGATACATCAGAAACGGATGAAGAGGATTGGCTGACAGAGCTTGAGACAACAG
AGGAAGAGGATGGACCGACAGAGCTCGAGACACCAGAAGTGGATGAAGCTGATTGTGTGATAGAACTTAAG
ATATCGGT

AGAGCTTGAGACATCAGAAGTAGAGGAAGCTGATTGACTGACAGAGCTTGAGACATCAGAAGCTGAGGAAG
CTGATTGACTGACAGAGCTTGAGACATCAGAAGCTGAGGAAGAAGATTGACTGACAGAGCTTGATACATCA
GAAGCTGA

TAGAGGAAGCTGATTGACTGACAGAGCTTGAGACATCAGAAGCTGAGGAAGCTGATTGACTGACAGAGCTT
GAGACATCAGAAGCTGAGGAAGAAGATTGACTGACAGAGCTTGATACATCAGAAGCTGAAGAAGAAGATTG
ACTGACAG

ACAGAGCTTGATACATCAGAAACGGATGAAGAGGATTGGCTGACAGAGCTTGAGACAACAGAGGAAGAGGA
TGGACCGACAGAGCTCGAGACACCAGAAGTGGATGAAGCTGATTGTGTGATAGAACTTAAGATATCGGTAG
CGGACGAG

ATTGACTGACAGAGCTTGAGACATCAGAAGTAGAGGAAGCTGATTGACTGACAGAGCTTGAGACATCAGAA
GCTGAGGAAGCTGATTGACTGACAGAGCTTGAGACATCAGAAGCTGAGGAAGAAGATTGACTGACAGAGCT
TGATACAT

TTTGCATTATATACATTCTGAACAGGTTGAGTTGGTTGAACAGGTTGAGTTGGCTGAACAGGTTGAGTTGG
CTGAACAGGTTGAGTTGGCTGAACAGGTTGAGTTGGTTGAACAGGTTGTGTTGATTGAACAGGTTGAGTTG
GTTGAGCG

GTAGAGCTGGATGGAGTTGGTACTGGAGCAGAAGAGCTTTCAGTGGTAGAGCTGGTTACTGGAGCAGAAGA
GCTTTCAGTGGTGGAGCTTGATGGGGTTGGAGCTGGAGCAGAAGAGCTTTCAGTAGTAGAGCTGGATGGAG
TTGGTACT

ACATCAGAAGTAGAGGAAGCTGATTGACTGACAGAGCTTGAGACATCAGAAGCTGAGGAAGCTGATTGACT
GACAGAGCTTGAGACATCAGAAGCTGAGGAAGAAGATTGACTGACAGAGCTTGATACATCAGAAGCTGAAG
AAGAAGAT

CCAGAGCTTGAGACACCAGAAGTAGAGGAAGCTGATTGACTGACAGAGCTTGAGACATCAGAAGTAGAGGA
AGCTGATTGACTGACAGAGCTTGAGACATCAGAAGCTGAGGAAGAAGATTGACTGACAGAGCTTGATACAT
CAGAAGCT

CTGAAGAAGAAGATTGACTGACAGAGCTTGATACATCAGAAACGGATGAAGAGGATTGGCTGACAGAGCTT
GAGACAACAGAGGAAGAGGATGGACCGACAGAGCTCGAGACACCAGAAGTGGATGAAGCTGATTGTGTGAT
AGAACTTA

ACTGACAGAGCTTGATACATCAGAAGCTGAAGAAGAAGATTGACTGACAGAGCTTGAGACATCAGAAGTAG
AGGAAGCTGATTGACTGACAGAGCTTGAGACATCAGAAGCTGAAGAAGAAGATTGACTGACAGAGCTTGAT
ACATCAGA

ATGGAGTTGGTACTGGAGCAGAAGAGCTTTCAGTGGTAGAGCTGGTTACTGGAGCAGAAGAGCTTTCAGTG
GTGGAGCTTGATGGGGTTGGAGCTGGAGCAGAAGAGCTTTCAGTAGTAGAGCTGGATGGAGTTGGTACTGG
AGCAGAAG

TGACTGACAGAGCTTGATACATCAGAAGCTGAAGAAGAAGATTGACTGACAGAGCTTGAGACATCAGAAGT
AGAGGAAGCTGATTGACTGACAGAGCTTGAGACATCAGAAGCTGAGGAAGCTGATTGACTGACAGAGCTTG
AGACATCA

GAAGAAGAAGATTGACTGACAGAGCTTGAGACATCAGAAGTAGAGGAAGCTGATTGACTGACAGAGCTTGA
GACATCAGAAGCTGAGGAAGCTGATTGACTGACAGAGCTTGAGACATCAGAAGCTGAGGAAGAAGATTGAC
TGACAGAG

TCTTCTTCTGCTTCTGAATCCTCTTCAGCTGCCTCCTCTTCTGCTTCTGAATCCTCTTCAGCTGCTTCTTC
CTCTGCTTCAGAATCTTCTTCTGCTGCCTCCTCTTCTGCTTCTGAAGCTGCTAAGTCTTCTAGCTCTGCCA
AGTCTTCT

AGCTTGATACATCAGAAGCTGAAGAAGAAGATTGACTGACAGAGCTTGAGACATCAGAAGTAGAGGAAGCT
GATTGACTGACAGAGCTTGAGACATCAGAAGCTGAGGAAGCTGATTGACTGACAGAGCTTGAGACATCAGA
AGCTGAGG

ATCAGAAGCTGAAGAAGAAGATTGACTGACAGAGCTTGAGACATCAGAAGTAGAGGAAGCTGATTGACTGA
CAGAGCTTGAGACATCAGAAGCTGAGGAAGCTGATTGACTGACAGAGCTTGAGACATCAGAAGCTGAGGAA
GAAGATTG

TCTGCTGCCTCCTCTTCTGCTTCTGAAGCTGCTAAGTCTTCTAGCTCTGCCAAGTCTTCTGGCTCTTCTGC
TGCTTCATCTGCTGCTTCATCTGCTTCTTCCAAGGCCTCTTCTGCAGCTTCCTCTTCTGCAAAGGCCTCCT
CTTCTGCA

CTTCATCATCGGATTCTTGCTTTGCATTATATACATTCTGAACAGGTTGAGTTGGTTGAACAGGTTGAGTT
GGCTGAACAGGTTGAGTTGGCTGAACAGGTTGAGTTGGCTGAACAGGTTGAGTTGGTTGAACAGGTTGTGT
TGATTGAA

GGATTCTTGCTTTGCATTATATACATTCTGAACAGGTTGAGTTGGTTGAACAGGTTGAGTTGGCTGAACAG
GTTGAGTTGGCTGAACAGGTTGAGTTGGCTGAACAGGTTGAGTTGGTTGAACAGGTTGTGTTGATTGAACA
GGTTGAGT

ATACATCAGAAACGGATGAAGAGGATTGGCTGACAGAGCTTGAGACAACAGAGGAAGAGGATGGACCGACA
GAGCTCGAGACACCAGAAGTGGATGAAGCTGATTGTGTGATAGAACTTAAGATATCGGTAGCGGACGAGGA
TGTCTCAG

TGATTGGCTGCCAGAGCTTGAGACACCAGAAGTAGAGGAAGCTGATTGACTGACAGAGCTTGAGACATCAG
AAGTAGAGGAAGCTGATTGACTGACAGAGCTTGAGACATCAGAAGCTGAGGAAGAAGATTGACTGACAGAG
CTTGATAC

TTCAGCTGCTTCTTCTTCTGCTTCTGAATCCTCTTCAGCTGCCTCCTCTTCTGCTTCTGAATCCTCTTCAG
CTGCTTCTTCCTCTGCTTCAGAATCTTCTTCTGCTGCCTCCTCTTCTGCTTCTGAAGCTGCTAAGTCTTCT
AGCTCTGC

TGAGCTTGAGCTACCAGAAGCGGATGAGACTGATTGGCTGCCAGAGCTTGAGACACCAGAAGTAGAGGAAG
CTGATTGACTGACAGAGCTTGAGACATCAGAAGTAGAGGAAGCTGATTGACTGACAGAGCTTGAGACATCA
GAAGCTGA

CGAATCTGGTTCCGAATCTGCCACTGCTTCCTCAGACGCTTCTTCTGCTTCTGAATCCTCTTCAGCTGCTT
CTTCTTCTGCTTCTGAATCCTCTTCAGCTGCCTCCTCTTCTGCTTCTGAATCCTCTTCAGCTGCTTCTTCC
TCTGCTTC

CTGAATCCTCTTCAGCTGCTTCTTCTTCTGCTTCTGAATCCTCTTCAGCTGCCTCCTCTTCTGCTTCTGAA
TCCTCTTCAGCTGCTTCTTCCTCTGCTTCAGAATCTTCTTCTGCTGCCTCCTCTTCTGCTTCTGAAGCTGC
TAAGTCTT

CTCAGACGCTTCTTCTGCTTCTGAATCCTCTTCAGCTGCTTCTTCTTCTGCTTCTGAATCCTCTTCAGCTG
CCTCCTCTTCTGCTTCTGAATCCTCTTCAGCTGCTTCTTCCTCTGCTTCAGAATCTTCTTCTGCTGCCTCC
TCTTCTGC

AAGAGCTTTCAGTAGTAGAGCTTGATGGAGTTGGTACTGGAGCAGAAGAGCTTTCAGTAGTAGAGCTTGAT
GGAGTTGGCACTGGAGCAGAAGAGCTTTCAGTGGTGGAGCTGGTTACTGGAGTAGAAGAGCTTTCAGTAGT
AGAGCTGG

ATCCTCTTCAGCTGCTTCTTCCTCTGCTTCAGAATCTTCTTCTGCTGCCTCCTCTTCTGCTTCTGAAGCTG
CTAAGTCTTCTAGCTCTGCCAAGTCTTCTGGCTCTTCTGCTGCTTCATCTGCTGCTTCATCTGCTTCTTCC
AAGGCCTC

GAAGCTGAGGAAGAAGATTGACTGACAGAGCTTGATACATCAGAAGCTGAAGAAGAAGATTGACTGACAGA
GCTTGAGACATCAGAAGTAGAGGAAGCTGATTGACTGACAGAGCTTGAGACATCAGAAGCTGAAGAAGAAG
ATTGACTG

AACAGGTTGAGTTGGTTGAACAGGTTGAGTTGGCTGAACAGGTTGAGTTGGCTGAACAGGTTGAGTTGGCT
GAACAGGTTGAGTTGGTTGAACAGGTTGTGTTGATTGAACAGGTTGAGTTGGTTGAGCGACTTCCACATAT
TTTTCTTC

TGACAGAGCTTGAGACATCAGAAGTAGAGGAAGCTGATTGACTGACAGAGCTTGAGACATCAGAAGCTGAG
GAAGAAGATTGACTGACAGAGCTTGATACATCAGAAGCTGAAGAAGAAGATTGACTGACAGAGCTTGAGAC
ATCAGAAG

CCACTGCTTCCTCAGACGCTTCTTCTGCTTCTGAATCCTCTTCAGCTGCTTCTTCTTCTGCTTCTGAATCC
TCTTCAGCTGCCTCCTCTTCTGCTTCTGAATCCTCTTCAGCTGCTTCTTCCTCTGCTTCAGAATCTTCTTC
TGCTGCCT

AGACACCAGAAGTAGAGGAAGCTGATTGACTGACAGAGCTTGAGACATCAGAAGTAGAGGAAGCTGATTGA
CTGACAGAGCTTGAGACATCAGAAGCTGAGGAAGAAGATTGACTGACAGAGCTTGATACATCAGAAGCTGA
AGAAGAAG

TCCGAATCTGCCACTGCTTCCTCAGACGCTTCTTCTGCTTCTGAATCCTCTTCAGCTGCTTCTTCTTCTGC
TTCTGAATCCTCTTCAGCTGCCTCCTCTTCTGCTTCTGAATCCTCTTCAGCTGCTTCTTCCTCTGCTTCAG
AATCTTCT

TCTTCTGCTTCTGAATCCTCTTCAGCTGCTTCTTCTTCTGCTTCTGAATCCTCTTCAGCTGCCTCCTCTTC
TGCTTCTGAATCCTCTTCAGCTGCTTCTTCCTCTGCTTCAGAATCTTCTTCTGCTGCCTCCTCTTCTGCTT
CTGAAGCT

CATTGTCTGTTTAAAGCGACAAATGGCGTCGAGTAGGGAATCCCACTTGCCAAGCATGCAGCCCTGTGTTC
TGTAGCACCGGCTCCAACATTGAGAACATTTCCACAGGCAACTTCTTCGATTAAGTTCAAATCAGCCCTCA
AAGGTTCC

GGAAGAAGATTGACTGACAGAGCTTGATACATCAGAAGCTGAAGAAGAAGATTGACTGACAGAGCTTGAGA
CATCAGAAGTAGAGGAAGCTGATTGACTGACAGAGCTTGAGACATCAGAAGCTGAGGAAGCTGATTGACTG
ACAGAGCT

GAAGAGCTTTCAGTGGTAGAGCTGGTTACTGGAGCAGAAGAGCTTTCAGTGGTGGAGCTTGATGGGGTTGG
AGCTGGAGCAGAAGAGCTTTCAGTAGTAGAGCTGGATGGAGTTGGTACTGGAGCAGAAGAGCTTTCAGTGG
TAGAGCTG

GCTGATTGACTGACAGAGCTTGAGACATCAGAAGTAGAGGAAGCTGATTGACTGACAGAGCTTGAGACATC
AGAAGCTGAGGAAGAAGATTGACTGACAGAGCTTGATACATCAGAAGCTGAAGAAGAAGATTGACTGACAG
AGCTTGAG

CACAGGTGAGGGGCAGTGTTGATAAGATGTTTACGCTCGTTGAGTGCCTCGATGACCAGATCCAGTTGTGC
CTTGGAGAACTCCCAGAAGGCCTTCTCTAAGTACCGCACCCCACCGTGAATCATCTTGGTAGATTTGGACG
ACGTTCCC

TACTGGAGCAGAAGAGCTTTCAGTGGTAGAGCTGGTTACTGGAGCAGAAGAGCTTTCAGTGGTGGAGCTTG
ATGGGGTTGGAGCTGGAGCAGAAGAGCTTTCAGTAGTAGAGCTGGATGGAGTTGGTACTGGAGCAGAAGAG
CTTTCAGT

AAGAAGATTGACTGACAGAGCTTGATACATCAGAAGCTGAAGAAGAAGATTGACTGACAGAGCTTGAGACA
TCAGAAGTAGAGGAAGCTGATTGACTGACAGAGCTTGAGACATCAGAAGCTGAAGAAGAAGATTGACTGAC
AGAGCTTG

TCCACCAACAACTGCTGTAATTGGTTCTTGTTCATTAGTTCCATGCCTCGTTGCAAGGGCAGAGGCTGCCC
TCTGATGAGCGATCCTGGCTGAGGCCTCCTCTTGGAGACGCCCCCATATTTCATCACATTCTTGTTACGAG
GTAGTTCT

AGTAGAGGAAGCTGATTGACTGACAGAGCTTGAGACATCAGAAGTAGAGGAAGCTGATTGACTGACAGAGC
TTGAGACATCAGAAGCTGAGGAAGAAGATTGACTGACAGAGCTTGATACATCAGAAGCTGAAGAAGAAGAT
TGACTGAC

GCTGCTTCTTCCTCTGCTTCAGAATCTTCTTCTGCTGCCTCCTCTTCTGCTTCTGAAGCTGCTAAGTCTTC
TAGCTCTGCCAAGTCTTCTGGCTCTTCTGCTGCTTCATCTGCTGCTTCATCTGCTTCTTCCAAGGCCTCTT
CTGCAGCT

ACAGAGCTTGAGACATCAGAAGCTGAGGAAGCTGATTGACTGACAGAGCTTGAGACATCAGAAGCTGAGGA
AGAAGATTGACTGACAGAGCTTGATACATCAGAAGCTGAAGAAGAAGATTGACTGACAGAGCTTGAGACAT
CAGAAGTA

CGGATGAGACTGATTGGCTGCCAGAGCTTGAGACACCAGAAGTAGAGGAAGCTGATTGACTGACAGAGCTT
GAGACATCAGAAGTAGAGGAAGCTGATTGACTGACAGAGCTTGAGACATCAGAAGCTGAGGAAGAAGATTG
ACTGACAG

TCCTTAGGTCAAACCGTTTCCGAATCTGGTTCCGAATCTGCCACTGCTTCCTCAGACGCTTCTTCTGCTTC
TGAATCCTCTTCAGCTGCTTCTTCTTCTGCTTCTGAATCCTCTTCAGCTGCCTCCTCTTCTGCTTCTGAAT
CCTCTTCA

AAACATGAAATGTGAAGACGTTGCCCCTCGTCATAGGTAAGCGGTGGTCATCACAACCATCAGCGGTGACA
ATAACACAGCAAACAGTCTTGTTTGACTCTCCTGCCTGCATGGTGTTTGAAAGTGAATCGTCAGGATTTGC
AACAACCA

AAACCGTTTCCGAATCTGGTTCCGAATCTGCCACTGCTTCCTCAGACGCTTCTTCTGCTTCTGAATCCTCT
TCAGCTGCTTCTTCTTCTGCTTCTGAATCCTCTTCAGCTGCCTCCTCTTCTGCTTCTGAATCCTCTTCAGC
TGCTTCTT

CTACCAGAAGCGGATGAGACTGATTGGCTGCCAGAGCTTGAGACACCAGAAGTAGAGGAAGCTGATTGACT
GACAGAGCTTGAGACATCAGAAGTAGAGGAAGCTGATTGACTGACAGAGCTTGAGACATCAGAAGCTGAGG
AAGAAGAT

GTTGGTTGAACAGGTTGAGTTGGCTGAACAGGTTGAGTTGGCTGAACAGGTTGAGTTGGCTGAACAGGTTG
AGTTGGTTGAACAGGTTGTGTTGATTGAACAGGTTGAGTTGGTTGAGCGACTTCCACATATTTTTCTTCTT
GGAATGGA

ATCTTTTGTTCTTCAATCAAGAAAGCTCTGACGATTCTTTCCTTGACACAGTTGGCACATCTGGAACCACC
GTAAGCTCTGGAAACAGTCTTGTGGGTCTTGGAGACAGTAGCGTATTGTCTTGGTCTCAAAGTGGAAATAC
CTTGTAGA

GCTTTCAGTAGTAGAGCTGGATGGAGTTGGTACTGGAGCAGAAGAGCTTTCAGTGGTAGAGCTGGTTACTG
GAGCAGAAGAGCTTTCAGTGGTGGAGCTTGATGGGGTTGGAGCTGGAGCAGAAGAGCTTTCAGTAGTAGAG
CTGGATGG

CAGAAGCTGAGGAAGAAGATTGACTGACAGAGCTTGATACATCAGAAGCTGAAGAAGAAGATTGACTGACA
GAGCTTGAGACATCAGAAGTAGAGGAAGCTGATTGACTGACAGAGCTTGAGACATCAGAAGCTGAGGAAGC
TGATTGAC

CTGGTTACTGGAGTAGAAGAGCTTTCAGTAGTAGAGCTGGATGGAGTTGGTACTGGAGCAGAAGAGCTTTC
AGTGGTAGAGCTGGTTACTGGAGCAGAAGAGCTTTCAGTGGTGGAGCTTGATGGGGTTGGAGCTGGAGCAG
AAGAGCTT

CAGAAGCTGAAGAAGAAGATTGACTGACAGAGCTTGAGACATCAGAAGTAGAGGAAGCTGATTGACTGACA
GAGCTTGAGACATCAGAAGCTGAAGAAGAAGATTGACTGACAGAGCTTGATACATCAGAAACGGATGAAGA
GGATTGGC

TGATTGACTGACAGAGCTTGAGACATCAGAAGCTGAGGAAGCTGATTGACTGACAGAGCTTGAGACATCAG
AAGCTGAGGAAGAAGATTGACTGACAGAGCTTGATACATCAGAAGCTGAAGAAGAAGATTGACTGACAGAG
CTTGAGAC

CAGTATTACCTCCTTAGGTCAAACCGTTTCCGAATCTGGTTCCGAATCTGCCACTGCTTCCTCAGACGCTT
CTTCTGCTTCTGAATCCTCTTCAGCTGCTTCTTCTTCTGCTTCTGAATCCTCTTCAGCTGCCTCCTCTTCT
GCTTCTGA

TGTGAAGACGTTGCCCCTCGTCATAGGTAAGCGGTGGTCATCACAACCATCAGCGGTGACAATAACACAGC
AAACAGTCTTGTTTGACTCTCCTGCCTGCATGGTGTTTGAAAGTGAATCGTCAGGATTTGCAACAACCAAT
AGTGTACT

AGAAGAAGATTGACTGACAGAGCTTGAGACATCAGAAGTAGAGGAAGCTGATTGACTGACAGAGCTTGAGA
CATCAGAAGCTGAAGAAGAAGATTGACTGACAGAGCTTGATACATCAGAAACGGATGAAGAGGATTGGCTG
ACAGAGCT

ATTGACTGACAGAGCTTGAGACATCAGAAGCTGAAGAAGAAGATTGACTGACAGAGCTTGATACATCAGAA
ACGGATGAAGAGGATTGGCTGACAGAGCTTGAGACAACAGAGGAAGAGGATGGACCGACAGAGCTCGAGAC
ACCAGAAG

GATGGGGTTGGAGCTGGAGCAGAAGAGCTTTCAGTAGTAGAGCTGGATGGAGTTGGTACTGGAGCAGAAGA
GCTTTCAGTGGTAGAGCTGGTTACTGGAGCAGAAGAGCTTTCAGTAGTAGAGCTGGATGGAGTTGGTACTG
GAGCAGAA

CGTTGAACTACGCCCTCAGTAAAGTGGCTTTGAACTCCAGTGAATGTTTGAACAAGATGTTCCCCACCGAG
GAACAACCCTTGGCTTCGGCACTCTTGCAATTCAGTGATGTGCAGGCTAAGATTGCTCAAGCTAGAATTCA
ACAAGATA

AGAGCTTGAGACATCAGAAGCTGAAGAAGAAGATTGACTGACAGAGCTTGATACATCAGAAACGGATGAAG
AGGATTGGCTGACAGAGCTTGAGACAACAGAGGAAGAGGATGGACCGACAGAGCTCGAGACACCAGAAGTG
GATGAAGC

GAACCCAAGACGTTGAACTACGCCCTCAGTAAAGTGGCTTTGAACTCCAGTGAATGTTTGAACAAGATGTT
CCCCACCGAGGAACAACCCTTGGCTTCGGCACTCTTGCAATTCAGTGATGTGCAGGCTAAGATTGCTCAAG
CTAGAATT

GAGTAGAAGAGCTTTCAGTAGTAGAGCTGGATGGAGTTGGTACTGGAGCAGAAGAGCTTTCAGTGGTAGAG
CTGGTTACTGGAGCAGAAGAGCTTTCAGTGGTGGAGCTTGATGGGGTTGGAGCTGGAGCAGAAGAGCTTTC
AGTAGTAG

CCAGGTGCTGTAGATGGGGATCAGAATTGGTAGCACCGTGCACAGGTGAGGGGCAGTGTTGATAAGATGTT
TACGCTCGTTGAGTGCCTCGATGACCAGATCCAGTTGTGCCTTGGAGAACTCCCAGAAGGCCTTCTCTAAG
TACCGCAC

ATGGGGTTGGTACTGGAGCAGAAGAGCTTTCGGTAGTAGAGCTGGATGGAGTTGGCACTGGAGCAGAAGAG
CTTTCAGTAGTAGAGCTGGATGGAGTTGGTACTGGAGCAGAAGAGCTTTCAGTGGTAGAGCTGGTTACTGG
AGCAGAAG

ACTGACAGAGCTTGAGACATCAGAAGCTGAGGAAGAAGATTGACTGACAGAGCTTGATACATCAGAAGCTG
AAGAAGAAGATTGACTGACAGAGCTTGAGACATCAGAAGTAGAGGAAGCTGATTGACTGACAGAGCTTGAG
ACATCAGA

GAAGTAGAGGAAGCTGATTGACTGACAGAGCTTGAGACATCAGAAGCTGAGGAAGAAGATTGACTGACAGA
GCTTGATACATCAGAAGCTGAAGAAGAAGATTGACTGACAGAGCTTGAGACATCAGAAGTAGAGGAAGCTG
ATTGACTG

CCTCTTCTGCTTCTGAAGCTGCTAAGTCTTCTAGCTCTGCCAAGTCTTCTGGCTCTTCTGCTGCTTCATCT
GCTGCTTCATCTGCTTCTTCCAAGGCCTCTTCTGCAGCTTCCTCTTCTGCAAAGGCCTCCTCTTCTGCAGA
AAAATCTA

CCCCATCAAAGAACCCAAGACGTTGAACTACGCCCTCAGTAAAGTGGCTTTGAACTCCAGTGAATGTTTGA
ACAAGATGTTCCCCACCGAGGAACAACCCTTGGCTTCGGCACTCTTGCAATTCAGTGATGTGCAGGCTAAG
ATTGCTCA

GCCTCCTCTTCTGCTTCTGAATCCTCTTCAGCTGCTTCTTCCTCTGCTTCAGAATCTTCTTCTGCTGCCTC
CTCTTCTGCTTCTGAAGCTGCTAAGTCTTCTAGCTCTGCCAAGTCTTCTGGCTCTTCTGCTGCTTCATCTG
CTGCTTCA

AAGATCATACCGCTTACCATTAAGGATGTAGAGAATCTAGCCACCGACAACACTTCTGATGGCAGCTCTCC
GCAGGATGACCCAACAATGACTGATGGTGCAGACGAATCAGACACACCGTCGAACGAGCAAGAAACTGTCT
TAGATGAA

TCATCTTCATCTTCATCATCGGATTCTTGCTTTGCATTATATACATTCTGAACAGGTTGAGTTGGTTGAAC
AGGTTGAGTTGGCTGAACAGGTTGAGTTGGCTGAACAGGTTGAGTTGGCTGAACAGGTTGAGTTGGTTGAA
CAGGTTGT

GCTGATTGACTGACAGAGCTTGAGACATCAGAAGCTGAGGAAGAAGATTGACTGACAGAGCTTGATACATC
AGAAGCTGAAGAAGAAGATTGACTGACAGAGCTTGAGACATCAGAAGTAGAGGAAGCTGATTGACTGACAG
AGCTTGAG

TACTGGAGCAGAAGAGCTTTCGGTAGTAGAGCTGGATGGAGTTGGCACTGGAGCAGAAGAGCTTTCAGTAG
TAGAGCTGGATGGAGTTGGTACTGGAGCAGAAGAGCTTTCAGTGGTAGAGCTGGTTACTGGAGCAGAAGAG
CTTTCAGT

CTTCTTAACAATCTTTTGTTCTTCAATCAAGAAAGCTCTGACGATTCTTTCCTTGACACAGTTGGCACATC
TGGAACCACCGTAAGCTCTGGAAACAGTCTTGTGGGTCTTGGAGACAGTAGCGTATTGTCTTGGTCTCAAA
GTGGAAAT

TGGGGTTGGAGCTGGAGCAGAAGAGCTTTCAGTAGTAGAGCTTGATGGAGTTGGTACTGGAGCAGAAGAGC
TTTCAGTAGTAGAGCTTGATGGAGTTGGCACTGGAGCAGAAGAGCTTTCAGTGGTGGAGCTGGTTACTGGA
GTAGAAGA

CAGTGGTAGAGCTGGTTACTGGAGCAGAAGAGCTTTCAGTGGTGGAGCTTGATGGGGTTGGAGCTGGAGCA
GAAGAGCTTTCAGTAGTAGAGCTGGATGGAGTTGGTACTGGAGCAGAAGAGCTTTCAGTGGTAGAGCTGGT
TACTGGAG

CTTCTGAATCCTCTTCAGCTGCCTCCTCTTCTGCTTCTGAATCCTCTTCAGCTGCTTCTTCCTCTGCTTCA
GAATCTTCTTCTGCTGCCTCCTCTTCTGCTTCTGAAGCTGCTAAGTCTTCTAGCTCTGCCAAGTCTTCTGG
CTCTTCTG

TAAGGATGTAGAGAATCTAGCCACCGACAACACTTCTGATGGCAGCTCTCCGCAGGATGACCCAACAATGA
CTGATGGTGCAGACGAATCAGACACACCGTCGAACGAGCAAGAAACTGTCTTAGATGAAAACATACCTTAT
CCAACACA

ACATCAGAAGCTGAAGAAGAAGATTGACTGACAGAGCTTGATACATCAGAAACGGATGAAGAGGATTGGCT
GACAGAGCTTGAGACAACAGAGGAAGAGGATGGACCGACAGAGCTCGAGACACCAGAAGTGGATGAAGCTG
ATTGTGTG

CTTGATACATCAGAAGCTGAAGAAGAAGATTGACTGACAGAGCTTGAGACATCAGAAGTAGAGGAAGCTGA
TTGACTGACAGAGCTTGAGACATCAGAAGCTGAAGAAGAAGATTGACTGACAGAGCTTGATACATCAGAAA
CGGATGAA

TTGCCCCTCGTCATAGGTAAGCGGTGGTCATCACAACCATCAGCGGTGACAATAACACAGCAAACAGTCTT
GTTTGACTCTCCTGCCTGCATGGTGTTTGAAAGTGAATCGTCAGGATTTGCAACAACCAATAGTGTACTGC
TATTCGCA

CAGGTTGAGTTGGCTGAACAGGTTGAGTTGGCTGAACAGGTTGAGTTGGCTGAACAGGTTGAGTTGGTTGA
ACAGGTTGTGTTGATTGAACAGGTTGAGTTGGTTGAGCGACTTCCACATATTTTTCTTCTTGGAATGGAGT
TGCTCTAG

TGACAGAGCTTGAGACATCAGAAGCTGAGGAAGAAGATTGACTGACAGAGCTTGATACATCAGAAGCTGAA
GAAGAAGATTGACTGACAGAGCTTGAGACATCAGAAGTAGAGGAAGCTGATTGACTGACAGAGCTTGAGAC
ATCAGAAG

GATAAGAACGTGTCCGATGCCATGGACTCGTTATCTAAGGCGAAGGAGGACTTGAAACAGTACGGCAGCCA
CTGGTGGTCTGGATGGACTTCCAAGGTCGACAATGACAAGCAGGCTTTAAAAGATGAGGCCCAAAAGAAGT
ACGATGAA

GCTAAGTCTTCTAGCTCTGCCAAGTCTTCTGGCTCTTCTGCTGCTTCATCTGCTGCTTCATCTGCTTCTTC
CAAGGCCTCTTCTGCAGCTTCCTCTTCTGCAAAGGCCTCCTCTTCTGCAGAAAAATCTACTAATAGCTCCT
CCTCTGCT

GCTGGAGCAGAAGAGCTTTCAGTAGTAGAGCTTGATGGAGTTGGTACTGGAGCAGAAGAGCTTTCAGTAGT
AGAGCTTGATGGAGTTGGCACTGGAGCAGAAGAGCTTTCAGTGGTGGAGCTGGTTACTGGAGTAGAAGAGC
TTTCAGTA

GGTAGAGCTGGTTACTGGAGCAGAAGAGCTTTCAGTAGTAGAGCTGGATGGAGTTGGTACTGGAGCAGAAG
AGCTTTCAGTGGTAGAGCTGGTTACTGGAGCAGAAGAGCTTTCAGTAGTAGAGCTGGATGGAGTTGGTACT
GGAGCAGA

TGGATGGGGTTGGTACTGGAGCAGAAGAGCTTTCAGTAGTAGAGCTGGATGGAGTTGGTACTGGAGCAGAA
GAGCTTTCAGTGGTAGAGCTGGTTACTGGAGCAGAAGAGCTTTCAGTAGTAGAGCTTGATGGGGTTGGAGC
TGGAGCAG

TGGTAGAGCTGGTTACTGGAGCAGAAGAGCTTTCAGTAGTAGAGCTGGATGGAGTTGGTACTGGAGCAGAA
GAGCTTTCAGTGGTAGAGCTGGTTACTGGAGCAGAAGAGCTTTCAGTGGTAGAGCTGGTTACTGGAGCAGA
AGAGCTTT

CCTCTGCTTCAGAATCTTCTTCTGCTGCCTCCTCTTCTGCTTCTGAAGCTGCTAAGTCTTCTAGCTCTGCC
AAGTCTTCTGGCTCTTCTGCTGCTTCATCTGCTGCTTCATCTGCTTCTTCCAAGGCCTCTTCTGCAGCTTC
CTCTTCTG

TGGTAGAGCTGGTTACTGGAGCAGAAGAGCTTTCAGTAGTAGAGCTGGATGGAGTTGGTACTGGAGCAGAA
GAGCTTTCAGTAGTAGAGCTGGATGGGGTTGGTACTGGAGCAGAAGAGCTTTCAGTAGTAGAGCTGGATGG
AGTTGGTA

TGACTGACAGAGCTTGAGACATCAGAAGTAGAGGAAGCTGATTGACTGACAGAGCTTGAGACATCAGAAGC
TGAAGAAGAAGATTGACTGACAGAGCTTGATACATCAGAAACGGATGAAGAGGATTGGCTGACAGAGCTTG
AGACAACA

AGACATCAGAAGCTGAGGAAGCTGATTGACTGACAGAGCTTGAGACATCAGAAGCTGAGGAAGAAGATTGA
CTGACAGAGCTTGATACATCAGAAGCTGAAGAAGAAGATTGACTGACAGAGCTTGAGACATCAGAAGTAGA
GGAAGCTG

GCAGAAGAGCTTTCAGTAGTAGAGCTGGATGGAGTTGGTACTGGAGCAGAAGAGCTTTCAGTGGTAGAGCT
GGTTACTGGAGCAGAAGAGCTTTCAGTGGTAGAGCTGGTTACTGGAGCAGAAGAGCTTTCAGTGGTAGAGC
TGGTTACT

CTTTCAGTGGTAGAGCTGGTTACTGGAGCAGAAGAGCTTTCAGTGGTAGAGCTGGTTACTGGAGCAGAAGA
GCTTTCAGTGGTAGAGCTGGTTACTGGAGCAGAAGAGCTTTCAGTAGTAGAGCTGGATGGAGTTGGTACTG
GAGCAGAA

GGTAGAGCTGGTTACTGGAGCAGAAGAGCTTTCAGTAGTAGAGCTGGATGGAGTTGGTACTGGAGCAGAAG
AGCTTTCAGTGGTAGAGCTGGTTACTGGAGCAGAAGAGCTTTCAGTGGTAGAGCTGGTTACTGGAGCAGAA
GAGCTTTC

CTTGATGGAGTTGGTACTGGAGCAGAAGAGCTTTCAGTAGTAGAGCTTGATGGAGTTGGCACTGGAGCAGA
AGAGCTTTCAGTGGTGGAGCTGGTTACTGGAGTAGAAGAGCTTTCAGTAGTAGAGCTGGATGGAGTTGGTA
CTGGAGCA

TTCTGAAGCTGCTAAGTCTTCTAGCTCTGCCAAGTCTTCTGGCTCTTCTGCTGCTTCATCTGCTGCTTCAT
CTGCTTCTTCCAAGGCCTCTTCTGCAGCTTCCTCTTCTGCAAAGGCCTCCTCTTCTGCAGAAAAATCTACT
AATAGCTC

GCTGGTTACTGGAGCAGAAGAGCTTTCAGTGGTGGAGCTTGATGGGGTTGGAGCTGGAGCAGAAGAGCTTT
CAGTAGTAGAGCTGGATGGAGTTGGTACTGGAGCAGAAGAGCTTTCAGTGGTAGAGCTGGTTACTGGAGCA
GAAGAGCT

AGCTGGATGGAGTTGGTACTGGAGCAGAAGAGCTTTCAGTAGTAGAGCTGGATGGAGTTGGTACTGGAGCA
GAAGAGCTTTCAGTGGTAGAGCTGGTTACTGGAGCAGAAGAGCTTTCAGTGGTAGAGCTGGTTACTGGAGC
AGAAGAGC

CTGCTTCTGAATCCTCTTCAGCTGCTTCTTCCTCTGCTTCAGAATCTTCTTCTGCTGCCTCCTCTTCTGCT
TCTGAAGCTGCTAAGTCTTCTAGCTCTGCCAAGTCTTCTGGCTCTTCTGCTGCTTCATCTGCTGCTTCATC
TGCTTCTT

TTGCTCCAGGCCCCATCAAAGAACCCAAGACGTTGAACTACGCCCTCAGTAAAGTGGCTTTGAACTCCAGT
GAATGTTTGAACAAGATGTTCCCCACCGAGGAACAACCCTTGGCTTCGGCACTCTTGCAATTCAGTGATGT
GCAGGCTA

AGAATCTTCTTCTGCTGCCTCCTCTTCTGCTTCTGAAGCTGCTAAGTCTTCTAGCTCTGCCAAGTCTTCTG
GCTCTTCTGCTGCTTCATCTGCTGCTTCATCTGCTTCTTCCAAGGCCTCTTCTGCAGCTTCCTCTTCTGCA
AAGGCCTC

GGAGTTGGTACTGGAGCAGAAGAGCTTTCAGTGGTAGAGCTGGTTACTGGAGCAGAAGAGCTTTCAGTGGT
AGAGCTGGTTACTGGAGCAGAAGAGCTTTCAGTGGTAGAGCTGGTTACTGGAGCAGAAGAGCTTTCAGTGG
TAGAGCTG

CAGAAGAGCTTTCAGTAGTAGAGCTGGATGGAGTTGGTACTGGAGCAGAAGAGCTTTCAGTGGTAGAGCTG
GTTACTGGAGCAGAAGAGCTTTCAGTGGTAGAGCTGGTTACTGGAGCAGAAGAGCTTTCAGTAGTAGAGCT
TGATGGGG

AGCTGAGGAAGCTGATTGACTGACAGAGCTTGAGACATCAGAAGCTGAGGAAGAAGATTGACTGACAGAGC
TTGATACATCAGAAGCTGAAGAAGAAGATTGACTGACAGAGCTTGAGACATCAGAAGTAGAGGAAGCTGAT
TGACTGAC

TTGGTACTGGAGCAGAAGAGCTTTCAGTAGTAGAGCTTGATGGAGTTGGCACTGGAGCAGAAGAGCTTTCA
GTGGTGGAGCTGGTTACTGGAGTAGAAGAGCTTTCAGTAGTAGAGCTGGATGGAGTTGGTACTGGAGCAGA
AGAGCTTT

CGTCGTCATCTTCGTCGTTGCCCTCACCTTCCTCTTCAGAATCCACATCCACGACCCTCAACCGCATCGAG
GGGTCCTGCCTCAACACGGTCCACTTGAGGAACTCTTCCTCCCTTACTCTGCTGCGGCTACTGGCCCTGCT
TCTCGACC

GATGGAGTTGGTACTGGAGCAGAAGAGCTTTCAGTAGTAGAGCTGGATGGAGTTGGTACTGGAGCAGAAGA
GCTTTCAGTAGTAGAGCTGGATGGAGTTGGTACTGGAGCAGAAGAGCTTTCAGTGGTAGAGCTGGTTACTG
GAGCAGAA

GAAAGCTCTGACGATTCTTTCCTTGACACAGTTGGCACATCTGGAACCACCGTAAGCTCTGGAAACAGTCT
TGTGGGTCTTGGAGACAGTAGCGTATTGTCTTGGTCTCAAAGTGGAAATACCTTGTAGAGCACTACCACAG
TCACCACA

GAGGAAGCTGATTGACTGACAGAGCTTGAGACATCAGAAGCTGAAGAAGAAGATTGACTGACAGAGCTTGA
TACATCAGAAACGGATGAAGAGGATTGGCTGACAGAGCTTGAGACAACAGAGGAAGAGGATGGACCGACAG
AGCTCGAG

TGAGACATCAGAAGTAGAGGAAGCTGATTGACTGACAGAGCTTGAGACATCAGAAGCTGAGGAAGAAGATT
GACTGACAGAGCTTGATACATCAGAAGCTGAAGAAGAAGATTGACTGACAGAGCTTGAGACATCAGAAGTA
GAGGAAGC

GTCCTGTATCAGCAGCTCAATGTTCGACCTAATCTTGTAATGCTGGTCGAACGACAGTTGCGACAGCAGCT
CGTGTGAGGGTCGCTGGCGATGTGCCCAGCGCATTCCCCCACATTTCCATGAACGCATAATCTTCCACATA
CTACCGTG

AAGCTGATTGACTGACAGAGCTTGAGACATCAGAAGCTGAGGAAGAAGATTGACTGACAGAGCTTGATACA
TCAGAAGCTGAAGAAGAAGATTGACTGACAGAGCTTGAGACATCAGAAGTAGAGGAAGCTGATTGACTGAC
AGAGCTTG

CTAGCTCTGCCAAGTCTTCTGGCTCTTCTGCTGCTTCATCTGCTGCTTCATCTGCTTCTTCCAAGGCCTCT
TCTGCAGCTTCCTCTTCTGCAAAGGCCTCCTCTTCTGCAGAAAAATCTACTAATAGCTCCTCCTCTGCTAC
CTCCAAGA

AGAGCTGGATGGAGTTGGTACTGGAGCAGAAGAGCTTTCAGTGGTAGAGCTGGTTACTGGAGCAGAAGAGC
TTTCAGTGGTAGAGCTGGTTACTGGAGCAGAAGAGCTTTCAGTGGTAGAGCTGGTTACTGGAGCAGAAGAG
CTTTCAGT

CAGTTGAAATTGCTGAAGCTGTTTTCAACTATGGTGACTTCACCACCATGTTGACTGGTATTCCAGCTGAA
CAAGTCACCAGAGTTATCACTGGTGTTCCATGGTACTCTACCAGATTGAGACCAGCTATCTCCAGTGCTCT
ATCTAAGG

AGTAGTAGAGCTTGATGGAGTTGGTACTGGAGCAGAAGAGCTTTCAGTAGTAGAGCTTGATGGAGTTGGCA
CTGGAGCAGAAGAGCTTTCAGTGGTGGAGCTGGTTACTGGAGTAGAAGAGCTTTCAGTAGTAGAGCTGGAT
GGAGTTGG

ATTGAGGGAATGAGCTTGAGCTACCAGAAGCGGATGAGACTGATTGGCTGCCAGAGCTTGAGACACCAGAA
GTAGAGGAAGCTGATTGACTGACAGAGCTTGAGACATCAGAAGTAGAGGAAGCTGATTGACTGACAGAGCT
TGAGACAT

AGTAGAGCTGGATGGAGTTGGTACTGGAGCAGAAGAGCTTTCAGTGGTAGAGCTGGTTACTGGAGCAGAAG
AGCTTTCAGTGGTAGAGCTGGTTACTGGAGCAGAAGAGCTTTCAGTAGTAGAGCTGGATGGAGTTGGTACT
GGAGCAGA

GGTTACTGGAGCAGAAGAGCTTTCAGTAGTAGAGCTGGATGGAGTTGGTACTGGAGCAGAAGAGCTTTCAG
TGGTAGAGCTGGTTACTGGAGCAGAAGAGCTTTCAGTGGTAGAGCTGGTTACTGGAGCAGAAGAGCTTTCA
GTGGTAGA

GATGAGGTTGATTGAGGGAATGAGCTTGAGCTACCAGAAGCGGATGAGACTGATTGGCTGCCAGAGCTTGA
GACACCAGAAGTAGAGGAAGCTGATTGACTGACAGAGCTTGAGACATCAGAAGTAGAGGAAGCTGATTGAC
TGACAGAG

CTGGAGCAGAAGAGCTTTCAGTAGTAGAGCTGGATGGGGTTGGTACTGGAGCAGAAGAGCTTTCAGTAGTA
GAGCTGGATGGAGTTGGTACTGGAGCAGAAGAGCTTTCAGTGGTAGAGCTGGTTACTGGAGCAGAAGAGCT
TTCAGTAG

ATTTCTTGATGAAACTCATGGATACGTGGTCCGTTCATTGTGAAAGGATCACTGAGTTGCCACTTTGAGTT
TTCGTCAATCACAGGAGCGTCTGGTAGACCAGCAACCTTGGAGTACAAGCTTCCCACATCAATTCTAAAGC
CGTCTACA

CCTTAACGACCTTCTTAACAATCTTTTGTTCTTCAATCAAGAAAGCTCTGACGATTCTTTCCTTGACACAG
TTGGCACATCTGGAACCACCGTAAGCTCTGGAAACAGTCTTGTGGGTCTTGGAGACAGTAGCGTATTGTCT
TGGTCTCA

TGGTTACTGGAGCAGAAGAGCTTTCAGTAGTAGAGCTTGATGGGGTTGGAGCTGGAGCAGAAGAGCTTTCA
GTAGTAGAGCTTGATGGAGTTGGTACTGGAGCAGAAGAGCTTTCAGTAGTAGAGCTTGATGGAGTTGGCAC
TGGAGCAG

CTTTCAGTAGTAGAGCTGAATGGAATTGAAGATGGAGCGGAGGAAGTGATGTTGCTAGAGGAAGATGGGGT
TGGTACTGGTGCTACAGAGCTTTCAGTGGTGGAGCTGGATACTGGAGCAGAAGAGCTTTCAGTAGTAGAGC
TTGATGGG

TTCAGTAGTAGAGCTGGATGGAGTTGGTACTGGAGCAGAAGAGCTTTCAGTGGTAGAGCTGGTTACTGGAG
CAGAAGAGCTTTCAGTGGTAGAGCTGGTTACTGGAGCAGAAGAGCTTTCAGTAGTAGAGCTTGATGGGGTT
GGTACTGG

ACCTGAAGAACATTGTCTGTTTAAAGCGACAAATGGCGTCGAGTAGGGAATCCCACTTGCCAAGCATGCAG
CCCTGTGTTCTGTAGCACCGGCTCCAACATTGAGAACATTTCCACAGGCAACTTCTTCGATTAAGTTCAAA
TCAGCCCT

GTTGGTACTGGTGCTACAGAGCTTTCAGTAGTAGAGCTTGATGGGGTTGGTACTGGAGCAGAAGAGCTTTC
GGTAGTAGAGCTGGATGGAGTTGGCACTGGAGCAGAAGAGCTTTCAGTAGTAGAGCTGGATGGAGTTGGTA
CTGGAGCA

TAGAGCTGAATGGAATTGAAGATGGAGCGGAGGAAGTGATGTTGCTAGAGGAAGATGGGGTTGGTACTGGT
GCTACAGAGCTTTCAGTGGTGGAGCTGGATACTGGAGCAGAAGAGCTTTCAGTAGTAGAGCTTGATGGGGT
TGGTACTG

AGCTTTCAGTAGTAGAGCTGGATGGAGTTGGTACTGGAGCAGAAGAGCTTTCAGTGGTAGAGCTGGTTACT
GGAGCAGAAGAGCTTTCAGTGGTAGAGCTGGTTACTGGAGCAGAAGAGCTTTCAGTAGTAGAGCTGGATGG
AGTTGGTA

TTTCAGTAGTAGAGCTGGATGGAGTTGGTACTGGAGCAGAAGAGCTTTCAGTGGTAGAGCTGGTTACTGGA
GCAGAAGAGCTTTCAGTGGTAGAGCTGGTTACTGGAGCAGAAGAGCTTTCAGTGGTAGAGCTGGTTACTGG
AGCAGAAG

TAATGTAGGATTCCGTCTTGCCAGCATAGCGGATAAACACCGCAAGGAGGTGTTGTGGTCGGTAATGAGTG
GCACGAGGTGTTTGCTAGGCGTCGGACTACCAGTTGATGTGACTGCTACGGAAACTTTGACTCATGATGAA
CAAGGACC

AGAGCTGGATGGAGTTGGTACTGGAGCAGAAGAGCTTTCAGTGGTAGAGCTGGTTACTGGAGCAGAAGAGC
TTTCAGTAGTAGAGCTTGATGGGGTTGGAGCTGGAGCAGAAGAGCTTTCAGTAGTAGAGCTTGATGGAGTT
GGTACTGG

CGGATGGGTGGACAACATTTAAAGTAGGTGGCTCCGTCTGGGATCTGCTCTGCAGCAATACACAAATAGTG
GAAACAAGTCTCTGTTGTGACTGGTAGCCCTGACGCACGTGCCTTTCTGATCAAGGGAATTGCTTTCATTG
ATGCCAGA

CGCCCTCAGTAAAGTGGCTTTGAACTCCAGTGAATGTTTGAACAAGATGTTCCCCACCGAGGAACAACCCT
TGGCTTCGGCACTCTTGCAATTCAGTGATGTGCAGGCTAAGATTGCTCAAGCTAGAATTCAACAAGATACC
TTGATTCA

GGAGTTGGTACTGGAGCAGAAGAGCTTTCAGTAGTAGAGCTGGATGGGGTTGGTACTGGAGCAGAAGAGCT
TTCAGTAGTAGAGCTGGATGGAGTTGGTACTGGAGCAGAAGAGCTTTCAGTGGTAGAGCTGGTTACTGGAG
CAGAAGAG

AGCTTGAGACATCAGAAGTAGAGGAAGCTGATTGACTGACAGAGCTTGAGACATCAGAAGCTGAAGAAGAA
GATTGACTGACAGAGCTTGATACATCAGAAACGGATGAAGAGGATTGGCTGACAGAGCTTGAGACAACAGA
GGAAGAGG

AGCAGAAGAGCTTTCAGTAGTAGAGCTTGATGGAGTTGGCACTGGAGCAGAAGAGCTTTCAGTGGTGGAGC
TGGTTACTGGAGTAGAAGAGCTTTCAGTAGTAGAGCTGGATGGAGTTGGTACTGGAGCAGAAGAGCTTTCA
GTGGTAGA

GGTGCTGCTTCTATGCTGTGCTGCTTTGCTGTCTTTGCCTCCGTTGGTGTGACAAAGCTGTGGCCTCAAGG
AAGCAGTCACCAAGACATTACTTCTCAGGGTGCCGGTAACTGTATGATTGTGTTTACTATGTTCTTCATCT
TTTCGTTT

CTTCAATCAAGAAAGCTCTGACGATTCTTTCCTTGACACAGTTGGCACATCTGGAACCACCGTAAGCTCTG
GAAACAGTCTTGTGGGTCTTGGAGACAGTAGCGTATTGTCTTGGTCTCAAAGTGGAAATACCTTGTAGAGC
ACTACCAC

AGGAAGTGATGTTGCTAGAGGAAGATGGGGTTGGTACTGGTGCTACAGAGCTTTCAGTGGTGGAGCTGGAT
ACTGGAGCAGAAGAGCTTTCAGTAGTAGAGCTTGATGGGGTTGGTACTGGAACAGAAGAGCTTTCAGTGCT
AGAGCTGA

AGAGCTTTCAGTGGTAGAGCTGGTTACTGGAGCAGAAGAGCTTTCAGTGGTAGAGCTGGTTACTGGAGCAG
AAGAGCTTTCAGTGGTAGAGCTGGTTACTGGAGCAGAAGAGCTTTCAGTGGTAGAGCTGGTTACTGGAGCA
GAAGAGCT

GTAGAGCTGGATGGAGTTGGTACTGGAGCAGAAGAGCTTTCAGTGGTAGAGCTGGTTACTGGAGCAGAAGA
GCTTTCAGTAGTAGAGCTGGATGGAGTTGGTACTGGAGCAGAAGAGCTTTCAGTAGTAGAGCTGGATGGAG
TTGGTACT

GCTGGATGGAGTTGGCACTGGAGCAGAAGAGCTTTCAGTAGTAGAGCTGGATGGAGTTGGTACTGGAGCAG
AAGAGCTTTCAGTGGTAGAGCTGGTTACTGGAGCAGAAGAGCTTTCAGTAGTAGAGCTGGATGGAGTTGGT
ACTGGAGC

AGTGGTGGAGCTGGTTACTGGAGTAGAAGAGCTTTCAGTAGTAGAGCTGGATGGAGTTGGTACTGGAGCAG
AAGAGCTTTCAGTGGTAGAGCTGGTTACTGGAGCAGAAGAGCTTTCAGTGGTGGAGCTTGATGGGGTTGGA
GCTGGAGC

ATCAGAAGTAGAGGAAGCTGATTGACTGACAGAGCTTGAGACATCAGAAGCTGAAGAAGAAGATTGACTGA
CAGAGCTTGATACATCAGAAACGGATGAAGAGGATTGGCTGACAGAGCTTGAGACAACAGAGGAAGAGGAT
GGACCGAC

GGTTACTGGAGCAGAAGAGCTTTCAGTGGTAGAGCTGGTTACTGGAGCAGAAGAGCTTTCAGTAGTAGAGC
TTGATGGGGTTGGTACTGGAGCAGAAGAGCTTTCAGTGGTAGAGCTGGTTACTGGAGCAGAAGAGCTTTCA
GTAGTAGA

GAACTTGTTCATTTCTTGATGAAACTCATGGATACGTGGTCCGTTCATTGTGAAAGGATCACTGAGTTGCC
ACTTTGAGTTTTCGTCAATCACAGGAGCGTCTGGTAGACCAGCAACCTTGGAGTACAAGCTTCCCACATCA
ATTCTAAA

GCAGAAGAGCTTTCAGTAGTAGAGCTGGATGGAGTTGGTACTGGAGCAGAAGAGCTTTCAGTGGTAGAGCT
GGTTACTGGAGCAGAAGAGCTTTCAGTAGTAGAGCTTGATGGGGTTGGAGCTGGAGCAGAAGAGCTTTCAG
TAGTAGAG

CTGGAGCAGAAGAGCTTTCAGTGGTAGAGCTGGTTACTGGAGCAGAAGAGCTTTCAGTGGTAGAGCTGGTT
ACTGGAGCAGAAGAGCTTTCAGTGGTAGAGCTGGTTACTGGAGCAGAAGAGCTTTCAGTGGTAGAGCTGGT
TACTGGAG

GAAGAGCTTTCGGTAGTAGAGCTGGATGGAGTTGGCACTGGAGCAGAAGAGCTTTCAGTAGTAGAGCTGGA
TGGAGTTGGTACTGGAGCAGAAGAGCTTTCAGTGGTAGAGCTGGTTACTGGAGCAGAAGAGCTTTCAGTAG
TAGAGCTG

AGAAGAGCTTTCAGTAGTAGAGCTGGATGGAGTTGGTACTGGAGCAGAAGAGCTTTCAGTAGTAGAGCTGG
ATGGAGTTGGTACTGGAGCAGAAGAGCTTTCAGTGGTAGAGCTGGTTACTGGAGCAGAAGAGCTTTCAGTG
GTAGAGCT

GTACTGGAGCAGAAGAGCTTTCAGTGGTAGAGCTGGTTACTGGAGCAGAAGAGCTTTCAGTGGTAGAGCTG
GTTACTGGAGCAGAAGAGCTTTCAGTAGTAGAGCTGGATGGAGTTGGTACTGGAGCAGAAGAGCTTTCAGT
AGTAGAGC

GGAGCAGAAGAGCTTTCAGTGGTGGAGCTTGATGGGGTTGGAGCTGGAGCAGAAGAGCTTTCAGTAGTAGA
GCTGGATGGAGTTGGTACTGGAGCAGAAGAGCTTTCAGTGGTAGAGCTGGTTACTGGAGCAGAAGAGCTTT
CAGTAGTA

CAGAAGAGCTTTCAGTAGTAGAGCTGGATGGAGTTGGTACTGGAGCAGAAGAGCTTTCAGTGGTAGAGCTG
GTTACTGGAGCAGAAGAGCTTTCAGTAGTAGAGCTGGATGGAGTTGGTACTGGAGCAGAAGAGCTTTCAGT
GGTAGAGC

GAGAATCTAGCCACCGACAACACTTCTGATGGCAGCTCTCCGCAGGATGACCCAACAATGACTGATGGTGC
AGACGAATCAGACACACCGTCGAACGAGCAAGAAACTGTCTTAGATGAAAACATACCTTATCCAACACATC
TACTATCT

AGACTCAGAGCGGATGGGTGGACAACATTTAAAGTAGGTGGCTCCGTCTGGGATCTGCTCTGCAGCAATAC
ACAAATAGTGGAAACAAGTCTCTGTTGTGACTGGTAGCCCTGACGCACGTGCCTTTCTGATCAAGGGAATT
GCTTTCAT

AGCCTAGTTAATGGATTTATGCGGTATTCATTTCGTAAACTTGACATCGTGTGACCTTTCATTGACAGTCA
ACTGGAAGACATCTCCCCTAGCATAATGTCCGACAGGGTCAAGATCAAAGCGAGCTTCAGCAATCAAGTCA
GTGTTAAT

TTTCAGTAGTAGAGCTGGATGGAGTTGGTACTGGAGCAGAAGAGCTTTCAGTGGTAGAGCTGGTTACTGGA
GCAGAAGAGCTTTCAGTAGTAGAGCTTGATGGGGTTGGAGCTGGAGCAGAAGAGCTTTCAGTAGTAGAGCT
TGATGGAG

CTTGGCGATTAGACTCAGAGCGGATGGGTGGACAACATTTAAAGTAGGTGGCTCCGTCTGGGATCTGCTCT
GCAGCAATACACAAATAGTGGAAACAAGTCTCTGTTGTGACTGGTAGCCCTGACGCACGTGCCTTTCTGAT
CAAGGGAA

TCTCCGTGAGGGCGGTTCTTTCTGTAGCTGTTGGTTTTTTCCAGATCTTATGTGACACACCTGCACAACAA
TGTCATTCTCTGTCAGCTGCAAGACTCCCAAGACCACGAAGCTGCTGGTATCTTCTATTTCAGAGAGCGCT
GTTGCTCT

CCGTCGCCGTCGTCGTCATCTTCGTCGTTGCCCTCACCTTCCTCTTCAGAATCCACATCCACGACCCTCAA
CCGCATCGAGGGGTCCTGCCTCAACACGGTCCACTTGAGGAACTCTTCCTCCCTTACTCTGCTGCGGCTAC
TGGCCCTG

GTGGTAGAGCTGGTTACTGGAGCAGAAGAGCTTTCAGTAGTAGAGCTTGATGGGGTTGGAGCTGGAGCAGA
AGAGCTTTCAGTAGTAGAGCTTGATGGAGTTGGTACTGGAGCAGAAGAGCTTTCAGTAGTAGAGCTTGATG
GAGTTGGC

AGAGCTGGTTACTGGAGCAGAAGAGCTTTCAGTAGTAGAGCTTGATGGGGTTGGTACTGGAGCAGAAGAGC
TTTCAGTGGTAGAGCTGGTTACTGGAGCAGAAGAGCTTTCAGTAGTAGAGCTGGATGGAGTTGGTACTGGA
GCAGAAGA

GTACTGGAGCAGAAGAGCTTTCAGTAGTAGAGCTGGATGGAGTTGGTACTGGAGCAGAAGAGCTTTCAGTA
GTAGAGCTGGATGGAGTTGGTACTGGAGCAGAAGAGCTTTCAGTGGTAGAGCTGGTTACTGGAGCAGAAGA
GCTTTCAG

CTCTTCAGCTGCCTCCTCTTCTGCTTCTGAATCCTCTTCAGCTGCTTCTTCCTCTGCTTCAGAATCTTCTT
CTGCTGCCTCCTCTTCTGCTTCTGAAGCTGCTAAGTCTTCTAGCTCTGCCAAGTCTTCTGGCTCTTCTGCT
GCTTCATC

GTTACTGGAGCAGAAGAGCTTTCAGTAGTAGAGCTGGATGGAGTTGGTACTGGAGCAGAAGAGCTTTCAGT
GGTAGAGCTGGTTACTGGAGCAGAAGAGCTTTCAGTGGTAGAGCTGGTTACTGGAGCAGAAGAGCTTTCAG
TAGTAGAG

AAAGTGGCTTTGAACTCCAGTGAATGTTTGAACAAGATGTTCCCCACCGAGGAACAACCCTTGGCTTCGGC
ACTCTTGCAATTCAGTGATGTGCAGGCTAAGATTGCTCAAGCTAGAATTCAACAAGATACCTTGATTCAAA
CCAAATTC

AAGAGCTTTCAGTAGTAGAGCTTGATGGGGTTGGTACTGGAGCAGAAGAGCTTTCAGTGGTAGAGCTGGTT
ACTGGAGCAGAAGAGCTTTCAGTAGTAGAGCTGGATGGAGTTGGTACTGGAGCAGAAGAACTTTCAGTAGT
AGAGCTTG

CGCTTACCATTAAGGATGTAGAGAATCTAGCCACCGACAACACTTCTGATGGCAGCTCTCCGCAGGATGAC
CCAACAATGACTGATGGTGCAGACGAATCAGACACACCGTCGAACGAGCAAGAAACTGTCTTAGATGAAAA
CATACCTT

TGGAATTGAAGATGGAGCGGAGGAAGTGATGTTGCTAGAGGAAGATGGGGTTGGTACTGGTGCTACAGAGC
TTTCAGTGGTGGAGCTGGATACTGGAGCAGAAGAGCTTTCAGTAGTAGAGCTTGATGGGGTTGGTACTGGA
ACAGAAGA

TACATCAACGAATCCGTCAACGAGTTTTCAAGAAGCGTGGCTTCCAAGTTGACAGAGTTGACTCATGCTAC
ATCTGCGTCTGAGGCACAAAACATCTTAGTTGCTCCAGGCCCCATCAAAGAACCCAAGACGTTGAACTACG
CCCTCAGT

GGTTACTGGAGCAGAAGAGCTTTCAGTAGTAGAGCTGGATGGAGTTGGTACTGGAGCAGAAGAGCTTTCAG
TAGTAGAGCTGGATGGGGTTGGTACTGGAGCAGAAGAGCTTTCAGTAGTAGAGCTGGATGGAGTTGGTACT
GGAGCAGA

AGTTGGTACTGGAGCAGAAGAGCTTTCAGTAGTAGAGCTGGATGGAGTTGGTACTGGAGCAGAAGAGCTTT
CAGTGGTAGAGCTGGTTACTGGAGCAGAAGAGCTTTCAGTGGTAGAGCTGGTTACTGGAGCAGAAGAGCTT
TCAGTAGT

TGAGACATCAGAAGCTGAGGAAGAAGATTGACTGACAGAGCTTGATACATCAGAAGCTGAAGAAGAAGATT
GACTGACAGAGCTTGAGACATCAGAAGTAGAGGAAGCTGATTGACTGACAGAGCTTGAGACATCAGAAGCT
GAAGAAGA

CTCTAGTTTGCGATAGTGTAGATACCGTCCTTGGATAGAGCACTGGAGATGGCTGGCTTTAATCTGCTGGA
GTACCATGGGACACCGGTGATCATTCTGGTCACTTGGTCTGGAGAAATACCAGTCAACATGGTGGTGAAGT
CACCGTAG

TGTCATCAAATCCACCAACAACTGCTGTAATTGGTTCTTGTTCATTAGTTCCATGCCTCGTTGCAAGGGCA
GAGGCTGCCCTCTGATGAGCGATCCTGGCTGAGGCCTCCTCTTGGAGACGCCCCCATATTTCATCACATTC
TTGTTACG

AAGTGCAACAGATAATGCCGCAGTATTTATATCCACCAGGGATGGGACCACAAGCTCAGCTTCCTACAATG
AGCTCAAACTCGGAGTCCCAGACACCAGTGATGAGCTCACAGTTTCTTTCCTTGAACCAGCATGGCCTTTA
CCAACAAA

CATATCGTCGCCGTCGCCGTCGTCGTCATCTTCGTCGTTGCCCTCACCTTCCTCTTCAGAATCCACATCCA
CGACCCTCAACCGCATCGAGGGGTCCTGCCTCAACACGGTCCACTTGAGGAACTCTTCCTCCCTTACTCTG
CTGCGGCT

GGAAGATGGGGTTGGTACTGGTGCTACAGAGCTTTCAGTAGTAGAGCTTGATGGGGTTGGTACTGGAGCAG
AAGAGCTTTCGGTAGTAGAGCTGGATGGAGTTGGCACTGGAGCAGAAGAGCTTTCAGTAGTAGAGCTGGAT
GGAGTTGG

TAGAGCTTGATGGAGTTGGCACTGGAGCAGAAGAGCTTTCAGTGGTGGAGCTGGTTACTGGAGTAGAAGAG
CTTTCAGTAGTAGAGCTGGATGGAGTTGGTACTGGAGCAGAAGAGCTTTCAGTGGTAGAGCTGGTTACTGG
AGCAGAAG

CGTTCTCCCTAATGTTAACTTGCATATCTGTGTCCACAATACCTGGGGCGACGGCAATGGCTTTCACTTGC
CTTTCCTCGTTGGCCAGAGTCATGGCGAAGTGGTTCAGAGCGGCTTTTGAAGAACCGTAAGCTCCCCAACT
GCTGAAGT

GAGTTGGTACTGGAGCAGAAGAGCTTTCAGTGGTAGAGCTGGTTACTGGAGCAGAAGAGCTTTCAGTAGTA
GAGCTGGATGGAGTTGGTACTGGAGCAGAAGAGCTTTCAGTGGTAGAGCTGGTTACTGGAGCAGAAGAGCT
TTCAGTGG

AGTAGAAAAGCTTTCAGTAGTAGAGCTGAATGGAATTGAAGATGGAGCGGAGGAAGTGATGTTGCTAGAGG
AAGATGGGGTTGGTACTGGTGCTACAGAGCTTTCAGTGGTGGAGCTGGATACTGGAGCAGAAGAGCTTTCA
GTAGTAGA

ACGTACTGTGTTCTCTTGATCGTAGTCCAAAGACTTCAAACTGACTTCTGGGAAGTTCACAGAACCGACAG
AGTTACCTTCATTGATGTATTTGGACAATGCAGTAGCCACCTCAATACCGATTGAACTTTGAGCTTCTTCT
GTAGAGCC

TAGATGGGGATCAGAATTGGTAGCACCGTGCACAGGTGAGGGGCAGTGTTGATAAGATGTTTACGCTCGTT
GAGTGCCTCGATGACCAGATCCAGTTGTGCCTTGGAGAACTCCCAGAAGGCCTTCTCTAAGTACCGCACCC
CACCGTGA

AGTTGATGAGCACGGTGATGAATAATACACCTACAACAGTTGCCGCATTGGCTGCGGTTGCTGCAGCCTCT
GAAACAAATGGAAAGCTGGGGTCCGAGGAACAACCTGAAATCACTATTCCAAAGCCGAGGAGTAGTGCACA
GTTGGAGC

CCGCAACCACAAGTGCAACAGATAATGCCGCAGTATTTATATCCACCAGGGATGGGACCACAAGCTCAGCT
TCCTACAATGAGCTCAAACTCGGAGTCCCAGACACCAGTGATGAGCTCACAGTTTCTTTCCTTGAACCAGC
ATGGCCTT

CCGGCAAAGAAATCGTAGAATTTACAGCCCATATAGATGTACGGGACCTGCCAGGTGCTGTAGATGGGGAT
CAGAATTGGTAGCACCGTGCACAGGTGAGGGGCAGTGTTGATAAGATGTTTACGCTCGTTGAGTGCCTCGA
TGACCAGA

ATGGAGTTGGTACTGGAGCAGAAGAGCTTTCAGTGGTAGAGCTGGTTACTGGAGCAGAAGAGCTTTCAGTA
GTAGAGCTGGATGGAGTTGGTACTGGAGCAGAAGAGCTTTCAGTAGTAGAGCTGGATGGAGTTGGTACTGG
AGCAGAAG

AGCAGAAGAGCTTTCAGTGGTAGAGCTGGTTACTGGAGCAGAAGAGCTTTCAGTGGTAGAGCTGGTTACTG
GAGCAGAAGAGCTTTCAGTGGTAGAGCTGGTTACTGGAGCAGAAGAGCTTTCAGTAGTAGAGCTGGATGGA
GTTGGTAC

GGTGGAGCTTGATGGGGTTGGAGCTGGAGCAGAAGAGCTTTCAGTAGTAGAGCTGGATGGAGTTGGTACTG
GAGCAGAAGAGCTTTCAGTGGTAGAGCTGGTTACTGGAGCAGAAGAGCTTTCAGTAGTAGAGCTGGATGGA
GTTGGTAC

GCTGGTTACTGGAGCAGAAGAGCTTTCAGTAGTAGAGCTGGATGGAGTTGGTACTGGAGCAGAAGAGCTTT
CAGTAGTAGAGCTGGATGGAGTTGGTACTGGAGCAGAAGAGCTTTCAGTAGTAGAGCTGGATGGAGTTGGT
ACTGGAGC

GTAGAGCTTGATGGGGTTGGTACTGGAGCAGAAGAGCTTTCGGTAGTAGAGCTGGATGGAGTTGGCACTGG
AGCAGAAGAGCTTTCAGTAGTAGAGCTGGATGGAGTTGGTACTGGAGCAGAAGAGCTTTCAGTGGTAGAGC
TGGTTACT

TGAAAGTACAGGTAGATGTTAGTAAGGACGTAGAAGAAGGCAGCCTCAATGCTCTGCCTCCATCTGGAATC
ACAGAATCAGACGACAAAGCTGAAAAGTTCACTAAACATCCTGAATCTAGTTTAGAAGAGCTACAGAAGCA
TCAAGAAC

AGAGCTTTCAGTAGTAGAGCTGGATGGGGTTGGTACTGGAGCAGAAGAGCTTTCAGTAGTAGAGCTGGATG
GAGTTGGTACTGGAGCAGAAGAGCTTTCAGTGGTAGAGCTGGTTACTGGAGCAGAAGAGCTTTCAGTAGTA
GAGCTTGA

TGGAGCAGAAGAGCTTTCAGTGGTAGAGCTGGTTACTGGAGCAGAAGAGCTTTCAGTAGTAGAGCTGGATG
GAGTTGGTACTGGAGCAGAAGAGCTTTCAGTGGTAGAGCTGGTTACTGGAGCAGAAGAGCTTTCAGTGGTA
GAGCTGGT

GAAACTCATGGATACGTGGTCCGTTCATTGTGAAAGGATCACTGAGTTGCCACTTTGAGTTTTCGTCAATC
ACAGGAGCGTCTGGTAGACCAGCAACCTTGGAGTACAAGCTTCCCACATCAATTCTAAAGCCGTCTACACC
ATGGTCTA

GGAGTTGGTACTGGAGCAGAAGAGCTTTCAGTGGTAGAGCTGGTTACTGGAGCAGAAGAGCTTTCAGTAGT
AGAGCTTGATGGGGTTGGAGCTGGAGCAGAAGAGCTTTCAGTAGTAGAGCTTGATGGAGTTGGTACTGGAG
CAGAAGAG

AGCAGCTCAATGTTCGACCTAATCTTGTAATGCTGGTCGAACGACAGTTGCGACAGCAGCTCGTGTGAGGG
TCGCTGGCGATGTGCCCAGCGCATTCCCCCACATTTCCATGAACGCATAATCTTCCACATACTACCGTGAA
GGCGTCTT

CCGCCGTTAAACCTGAAGAACATTGTCTGTTTAAAGCGACAAATGGCGTCGAGTAGGGAATCCCACTTGCC
AAGCATGCAGCCCTGTGTTCTGTAGCACCGGCTCCAACATTGAGAACATTTCCACAGGCAACTTCTTCGAT
TAAGTTCA

AGCTTTCAGTGGTAGAGCTGGTTACTGGAGCAGAAGAGCTTTCAGTAGTAGAGCTGGATGGAGTTGGTACT
GGAGCAGAAGAGCTTTCAGTGGTAGAGCTGGTTACTGGAGCAGAAGAGCTTTCAGTAGTAGAGCTGGATGG
AGTTGGTA

AGCTTTCAGTGGTAGAGCTGGTTACTGGAGCAGAAGAGCTTTCAGTAGTAGAGCTGGATGGAGTTGGTACT
GGAGCAGAAGAGCTTTCAGTGGTAGAGCTGGTTACTGGAGCAGAAGAGCTTTCAGTGGTAGAGCTGGTTAC
TGGAGCAG

GACTTTCCTCAGATAGTCTTGATAATGGAATCCAACTAGTACCTGAAGTTGTTAAACTACCTCAACTTCCA
CCGCCCCCTCCTCCACCTCCCCCTCCTCCACTTCCACAGTCTCTTTTGACTGAAGCAGAAGCTAAACCGGA
TGGTGTTT

CTTGAGACATCAGAAGCTGAGGAAGAAGATTGACTGACAGAGCTTGATACATCAGAAGCTGAAGAAGAAGA
TTGACTGACAGAGCTTGAGACATCAGAAGTAGAGGAAGCTGATTGACTGACAGAGCTTGAGACATCAGAAG
CTGAGGAA

GTAGTAGAGCTGGATGGGGTTGGTACTGGAGCAGAAGAGCTTTCAGTAGTAGAGCTGGATGGAGTTGGTAC
TGGAGCAGAAGAGCTTTCAGTGGTAGAGCTGGTTACTGGAGCAGAAGAGCTTTCAGTAGTAGAGCTTGATG
GGGTTGGA

AGTAGTAGAGCTTGATGGGGTTGGTACTGGAGCAGAAGAGCTTTCAGTGGTAGAGCTGGTTACTGGAGCAG
AAGAGCTTTCAGTAGTAGAGCTGGATGGAGTTGGTACTGGAGCAGAAGAACTTTCAGTAGTAGAGCTTGAT
GGGGTTGG

TAGACACTTCGAGACCAGATCTTGAATGGCAAGAATTGACTTCATTCTCATCACAACCACTGGAACCGTTG
TCGGATACAGAAGAGCCAGACCTTGCGATTGCAGATGTTGAGACAAAGTTAGAAACAAAAGTCGATGAGCT
AAGGTACC

TTTAAACAGGTAGAGTTATTCGTAACCAAAGAAAGGGTGCTGGTTCTATCTTCACCTCTCACACCAGATTA
AGACAAGGTGCTGCCAAGTTGAGAACTTTGGACTATGCTGAACGTCATGGTTACATCCGTGGTATCGTTAA
GCAAATTG

TTTGTGGTTTGCTTATGTAAGTTAGAGATGGCGATTCTAGCGGCTAGAGTGGCATAATCAGGGTGCACAGT
GGTCATGTATGCACATGTTTCAGCTGCAAGATTGTCCAGCTCAACGGTAGTAACACCGGAGTACACACCAG
AAATAATA

TCAGTAGTAGAGCTGGATGGAGTTGGTACTGGAGCAGAAGAGCTTTCAGTAGTAGAGCTGGATGGAGTTGG
TACTGGAGCAGAAGAGCTTTCAGTGGTAGAGCTGGTTACTGGAGCAGAAGAGCTTTCAGTGGTAGAGCTGG
TTACTGGA

TCAGTAGTAGAGCTGGATGGAGTTGGTACTGGAGCAGAAGAGCTTTCAGTGGTAGAGCTGGTTACTGGAGC
AGAAGAGCTTTCAGTAGTAGAGCTGGATGGAGTTGGTACTGGAGCAGAAGAGCTTTCAGTGGTAGAGCTGG
TTACTGGA

GCTGTTTCATCTGCTGCCTTAGCCAAGAATGAGAAAATCTCTGATGCCGCTGCATCTGCCACTGCCTCAAC
ATCTCAAGGGGCATCCTCCTCCTCCTCCTCCTCCTCGGCAACTTCTACCCTAGAAAGCAGCTCTGTTTCTT
CATCTAGT

CCAACGAAGAAATCAAATTAGAACTGCTAGATTTCGAATCTGACGAAGGTAAGAAGGTCTTTTGGCATTCG
TCTGCCCACGTCTTGGGTGAATCTTGTGAGTGCCACCTAGGTGCCCATATTTGTTTAGGTCCTCCAACTGA
TGATGGGT

GAGCTTTCAGTGGTAGAGCTGGTTACTGGAGCAGAAGAGCTTTCAGTAGTAGAGCTGGATGGAGTTGGTAC
TGGAGCAGAAGAGCTTTCAGTGGTAGAGCTGGTTACTGGAGCAGAAGAGCTTTCAGTGGTAGAGCTGGTTA
CTGGAGCA

ACTGCTGTAATTGGTTCTTGTTCATTAGTTCCATGCCTCGTTGCAAGGGCAGAGGCTGCCCTCTGATGAGC
GATCCTGGCTGAGGCCTCCTCTTGGAGACGCCCCCATATTTCATCACATTCTTGTTACGAGGTAGTTCTTC
CTCTTCTT

TAGAGCAACAATTGGTGAGGAGTAACCTTCCACCTTAGTAGTTGGAGACTGAACTGCACCGAAAAGAGCAC
CTTGACATTGTTCCTTCAACACTTTAACAGTCTCATCAGGCAACGCCTTTCCTGTTTCTTGGAATGTTTGG
AAACCGGC

GAACAATCTGAGTTGATGAGCACGGTGATGAATAATACACCTACAACAGTTGCCGCATTGGCTGCGGTTGC
TGCAGCCTCTGAAACAAATGGAAAGCTGGGGTCCGAGGAACAACCTGAAATCACTATTCCAAAGCCGAGGA
GTAGTGCA

CTTACGCCATTGTCTCATCACTACAACTGTTTAGCTACTAACTTGCTTGACATCCAAAGAGCAGGCCAATC
ACTAGAGAATGTCTGGGATCACTCAGATGGGTACACACAAGGCAGTGGATGTGGTTGTTACCGCCACGACT
AGGGTCAC

TAGAGTTATTCGTAACCAAAGAAAGGGTGCTGGTTCTATCTTCACCTCTCACACCAGATTAAGACAAGGTG
CTGCCAAGTTGAGAACTTTGGACTATGCTGAACGTCATGGTTACATCCGTGGTATCGTTAAGCAAATTGTC
CACGACTC

GAGCTGGATGGAGTTGGTACTGGAGCAGAAGAGCTTTCAGTGGTAGAGCTGGTTACTGGAGCAGAAGAGCT
TTCAGTGGTAGAGCTGGTTACTGGAGCAGAAGAGCTTTCAGTAGTAGAGCTTGATGGGGTTGGTACTGGAG
CAGAAGAG

AGAAGAGCTTTCAGTAGTAGAGCTGGATGGAGTTGGTACTGGAGCAGAAGAGCTTTCAGTGGTAGAGCTGG
TTACTGGAGCAGAAGAGCTTTCAGTAGTAGAGCTGGATGGAGTTGGTACTGGAGCAGAAGAGCTTTCAGTG
GTAGAGCT

TACTGGAGCAGAAGAGCTTTCAGTGGTAGAGCTGGTTACTGGAGCAGAAGAGCTTTCAGTGGTAGAGCTGG
TTACTGGAGCAGAAGAGCTTTCAGTAGTAGAGCTGGATGGAGTTGGTACTGGAGCAGAAGAGCTTTCAGTG
GTAGAGCT

GAAGAGCTTTCAGTGGTAGAGCTGGTTACTGGAGCAGAAGAGCTTTCAGTGGTAGAGCTGGTTACTGGAGC
AGAAGAGCTTTCAGTAGTAGAGCTGGATGGAGTTGGTACTGGAGCAGAAGAGCTTTCAGTGGTAGAGCTGG
TTACTGGA

CAGTGGTAGAGCTGGTTACTGGAGCAGAAGAGCTTTCAGTAGTAGAGCTGGATGGAGTTGGTACTGGAGCA
GAAGAGCTTTCAGTAGTAGAGCTGGATGGAGTTGGTACTGGAGCAGAAGAGCTTTCAGTAGTAGAGCTGGA
TGGAGTTG

GATGGAGCGGAGGAAGTGATGTTGCTAGAGGAAGATGGGGTTGGTACTGGTGCTACAGAGCTTTCAGTGGT
GGAGCTGGATACTGGAGCAGAAGAGCTTTCAGTAGTAGAGCTTGATGGGGTTGGTACTGGAACAGAAGAGC
TTTCAGTG

TAGCACCGTGCACAGGTGAGGGGCAGTGTTGATAAGATGTTTACGCTCGTTGAGTGCCTCGATGACCAGAT
CCAGTTGTGCCTTGGAGAACTCCCAGAAGGCCTTCTCTAAGTACCGCACCCCACCGTGAATCATCTTGGTA
GATTTGGA

TGGAGCAGAAGAGCTTTCAGTGGTAGAGCTGGTTACTGGAGCAGAAGAGCTTTCAGTGGTAGAGCTGGTTA
CTGGAGCAGAAGAGCTTTCAGTAGTAGAGCTTGATGGGGTTGGTACTGGAGCAGAAGAGCTTTCAGTGGTA
GAGCTGGT

GTGGTAGAGCTGGTTACTGGAGCAGAAGAGCTTTCAGTGGTAGAGCTGGTTACTGGAGCAGAAGAGCTTTC
AGTGGTAGAGCTGGTTACTGGAGCAGAAGAGCTTTCAGTGGTAGAGCTGGTTACTGGAGCAGAAGAGCTTT
CAGTAGTA

ACTGGAGCAGAAGAGCTTTCAGTAGTAGAGCTTGATGGGGTTGGTACTGGAGCAGAAGAGCTTTCAGTGGT
AGAGCTGGTTACTGGAGCAGAAGAGCTTTCAGTAGTAGAGCTGGATGGAGTTGGTACTGGAGCAGAAGAAC
TTTCAGTA

AGTATCATACAGTTCTTCTATCCGATATATAATGAACGATAGGTGTGACACCACTGACTTGAGTAATTCTT
TCCAGCCACGCAAGCAAGACACCCCCTTGTTTCTGGCCCTGAGGTACGGACATTCCTTTGTAGAGATACTT
TACCAGCA

TATACAAGACACGTACTGTGTTCTCTTGATCGTAGTCCAAAGACTTCAAACTGACTTCTGGGAAGTTCACA
GAACCGACAGAGTTACCTTCATTGATGTATTTGGACAATGCAGTAGCCACCTCAATACCGATTGAACTTTG
AGCTTCTT

CTGTTGTAACAAGGGTGGTAGCTTCAGTAGTGTATTCGCCAGTACCAGTTGAACAAGGAACAGTGGTTGTG
ACAGTCTTTGGAGAGCATCCAGAGGTAGTTTCACCGGCAGAGTTTGTACCTGTAGAGCAAACCGTAGTAGT
AATAGTGG

TGCGTCCATTCTTCTGCACCAGCAAGCTTAATATCTCTTGTGTGACAAGGTTTCAGGTTGTGGAATCCGCC
AACTTCGACAACTTTGTCGACTGTTTCCTCAGCCATTTGTCTGTAAGTAGTCCATTTACCACCTGCAATAG
TAATTAGG

TCAGTGGTAGAGCTGGTTACTGGAGCAGAAGAGCTTTCAGTGGTAGAGCTGGTTACTGGAGCAGAAGAGCT
TTCAGTAGTAGAGCTGGATGGAGTTGGTACTGGAGCAGAAGAGCTTTCAGTAGTAGAGCTGGATGGGGTTG
GTACTGGA

GAGCTGGATGGAGTTGGTACTGGAGCAGAAGAGCTTTCAGTGGTAGAGCTGGTTACTGGAGCAGAAGAGCT
TTCAGTAGTAGAGCTGGATGGAGTTGGTACTGGAGCAGAAGAGCTTTCAGTGGTAGAGCTGGTTACTGGAG
CAGAAGAG

TATTGGTGGTAAACATGAAATGTGAAGACGTTGCCCCTCGTCATAGGTAAGCGGTGGTCATCACAACCATC
AGCGGTGACAATAACACAGCAAACAGTCTTGTTTGACTCTCCTGCCTGCATGGTGTTTGAAAGTGAATCGT
CAGGATTT

TGCTGAAGCTGTTTTCAACTATGGTGACTTCACCACCATGTTGACTGGTATTCCAGCTGAACAAGTCACCA
GAGTTATCACTGGTGTTCCATGGTACTCTACCAGATTGAGACCAGCTATCTCCAGTGCTCTATCTAAGGAC
GGTATTTA

TTCAGGAGCTTCAGAAGTGACAGTCTTGGTCTTACATCCATTGTCATCACAGTGGGTGACGGTGGCAGTGG
TGTACGATTGAGAAGAAACAGTAGTTGTGGTGGTAGCTTCAGGAGCTTCAGAGGTGACAGTCTTGGTGTTA
CAGCCATT

ACGGTAATGACTACTATCTTGGTCACATCTATGATATGATGCAGCAGAGACTGAAGCCTCAGGTGGTGACT
GTTATGGGTGATCTTTTCTCCAGTCAATGGATCGGTGATTCCGAATTCCATAATAGAACGAAAAGGTATAT
TAGCAGAA

TTGGTTCTTGTTCATTAGTTCCATGCCTCGTTGCAAGGGCAGAGGCTGCCCTCTGATGAGCGATCCTGGCT
GAGGCCTCCTCTTGGAGACGCCCCCATATTTCATCACATTCTTGTTACGAGGTAGTTCTTCCTCTTCTTGC
TCCTCGTT

GAATGCCCTGTCTTACTCAACAATTCCAATACTGATAGATGAGGGTCAAGTAAGCCTTTGTCTTCCCCAGT
TTGTGCTTCAGAGCATGTGTGAATAGTCGTTGACGAGGAATCAGGTGGCATGCTTTTAGATGAGGATCCTT
TACCTTCC

TCATCGACTTGAGAATACCACAAAGATCTGCAATAAATCACATTGTGGCTCCGAACTTAGTGAATGTGGAT
CCAAACTTGTTGTGGGACAAGCAGACGAACACACCTATCTACAAGGATGACATATTGGAACATTTACTGAA
GGAGAATG

TCAGAATTGGTAGCACCGTGCACAGGTGAGGGGCAGTGTTGATAAGATGTTTACGCTCGTTGAGTGCCTCG
ATGACCAGATCCAGTTGTGCCTTGGAGAACTCCCAGAAGGCCTTCTCTAAGTACCGCACCCCACCGTGAAT
CATCTTGG

GCGGTGGTCATCACAACCATCAGCGGTGACAATAACACAGCAAACAGTCTTGTTTGACTCTCCTGCCTGCA
TGGTGTTTGAAAGTGAATCGTCAGGATTTGCAACAACCAATAGTGTACTGCTATTCGCAGGAGCACAAGCT
CGCCGGTG

TTTCAGTGGTAGAGCTGGTTACTGGAGCAGAAGAGCTTTCAGTAGTAGAGCTTGATGGGGTTGGTACTGGA
GCAGAAGAGCTTTCAGTGGTAGAGCTGGTTACTGGAGCAGAAGAGCTTTCAGTAGTAGAGCTGGATGGAGT
TGGTACTG

TGGTAGAGCTGGTTACTGGAGCAGAAGAGCTTTCAGTGGTAGAGCTGGTTACTGGAGCAGAAGAGCTTTCA
GTAGTAGAGCTTGATGGGGTTGGTACTGGAGCAGAAGAGCTTTCAGTGGTAGAGCTGGTTACTGGAGCAGA
AGAGCTTT

AGCTGGATGGAGTTGGTACTGGAGCAGAAGAGCTTTCAGTGGTAGAGCTGGTTACTGGAGCAGAAGAGCTT
TCAGTAGTAGAGCTGGATGGAGTTGGTACTGGAGCAGAAGAGCTTTCAGTGGTAGAGCTGGTTACTGGAGC
AGAAGAGC

CAGTGGTAGAGCTGGTTACTGGAGCAGAAGAGCTTTCAGTGGTAGAGCTGGTTACTGGAGCAGAAGAGCTT
TCAGTAGTAGAGCTGGATGGAGTTGGTACTGGAGCAGAAGAGCTTTCAGTGGTAGAGCTGGTTACTGGAGC
AGAAGAGC

GAAGGCCCCACGTTCTCCCTAATGTTAACTTGCATATCTGTGTCCACAATACCTGGGGCGACGGCAATGGC
TTTCACTTGCCTTTCCTCGTTGGCCAGAGTCATGGCGAAGTGGTTCAGAGCGGCTTTTGAAGAACCGTAAG
CTCCCCAA

TGGAGTTGGCACTGGAGCAGAAGAGCTTTCAGTGGTGGAGCTGGTTACTGGAGTAGAAGAGCTTTCAGTAG
TAGAGCTGGATGGAGTTGGTACTGGAGCAGAAGAGCTTTCAGTGGTAGAGCTGGTTACTGGAGCAGAAGAG
CTTTCAGT

ATGTTCAAGTCTAGAGAACGTTCTTATAGAGAATTGCCATGGAGAGTTGCAGACTTCGGTGTTATCCACAG
AAATGAATTTTCTGGTGCCTTGTCTGGTTTGACTCGTGTCAGAAGATTCCAACAAGATGATGCTCATATCT
TCTGTACC

AGAGCTTTCAGTGGTAGAGCTGGTTACTGGAGCAGAAGAGCTTTCAGTAGTAGAGCTTGATGGGGTTGGAG
CTGGAGCAGAAGAGCTTTCAGTAGTAGAGCTTGATGGAGTTGGTACTGGAGCAGAAGAGCTTTCAGTAGTA
GAGCTTGA

TCATAGGTAAGCGGTGGTCATCACAACCATCAGCGGTGACAATAACACAGCAAACAGTCTTGTTTGACTCT
CCTGCCTGCATGGTGTTTGAAAGTGAATCGTCAGGATTTGCAACAACCAATAGTGTACTGCTATTCGCAGG
AGCACAAG

AGGTTTGGACGCCGTACGTGTCTATTGTGGGGTGCTGCTTCTATGCTGTGCTGCTTTGCTGTCTTTGCCTC
CGTTGGTGTGACAAAGCTGTGGCCTCAAGGAAGCAGTCACCAAGACATTACTTCTCAGGGTGCCGGTAACT
GTATGATT

AGTAGAAGCGGATGAGGTTGATTGAGGGAATGAGCTTGAGCTACCAGAAGCGGATGAGACTGATTGGCTGC
CAGAGCTTGAGACACCAGAAGTAGAGGAAGCTGATTGACTGACAGAGCTTGAGACATCAGAAGTAGAGGAA
GCTGATTG

ACCAGGAACGTTACGATGAATATACAAGACACGTACTGTGTTCTCTTGATCGTAGTCCAAAGACTTCAAAC
TGACTTCTGGGAAGTTCACAGAACCGACAGAGTTACCTTCATTGATGTATTTGGACAATGCAGTAGCCACC
TCAATACC

CTTTCAGTAGTAGAGCTTGATGGAGTTGGCACTGGAGCAGAAGAGCTTTCAGTGGTGGAGCTGGTTACTGG
AGTAGAAGAGCTTTCAGTAGTAGAGCTGGATGGAGTTGGTACTGGAGCAGAAGAGCTTTCAGTGGTAGAGC
TGGTTACT

GAGCTTTCAGTGGTAGAGCTGGTTACTGGAGCAGAAGAGCTTTCAGTGGTAGAGCTGGTTACTGGAGCAGA
AGAGCTTTCAGTAGTAGAGCTTGATGGGGTTGGTACTGGAGCAGAAGAGCTTTCAGTGGTAGAGCTGGTTA
CTGGAGCA

CCTTTAGCAACCAAAGGTAAAACTCGTTACACTTACTCAACTGGAACGAGATCAACCAGTCTTCAATGAAG
TCAGCAACGAACTCCCTCTGCAAAGCAGAATAGTCCTTCTTGAACGCAGGTCCGGAGCGATAGTCTCCGTC
GCTGCGAC

TGGTACTGGAGCAGAAGAGCTTTCAGTAGTAGAGCTGGATGGAGTTGGTACTGGAGCAGAAGAGCTTTCAG
TGGTAGAGCTGGTTACTGGAGCAGAAGAGCTTTCAGTAGTAGAGCTTGATGGGGTTGGAGCTGGAGCAGAA
GAGCTTTC

GAGCTGGAGCAGAAGAGCTTTCAGTAGTAGAGCTGGATGGAGTTGGTACTGGAGCAGAAGAGCTTTCAGTG
GTAGAGCTGGTTACTGGAGCAGAAGAGCTTTCAGTAGTAGAGCTGGATGGAGTTGGTACTGGAGCAGAAGA
GCTTTCAG

GTTGGCACTGGAGCAGAAGAGCTTTCAGTAGTAGAGCTGGATGGAGTTGGTACTGGAGCAGAAGAGCTTTC
AGTGGTAGAGCTGGTTACTGGAGCAGAAGAGCTTTCAGTAGTAGAGCTGGATGGAGTTGGTACTGGAGCAG
AAGAGCTT

GAGCAGAAGAGCTTTCAGTAGTAGAGCTGGATGGAGTTGGTACTGGAGCAGAAGAGCTTTCAGTGGTAGAG
CTGGTTACTGGAGCAGAAGAGCTTTCAGTAGTAGAGCTGGATGGAGTTGGTACTGGAGCAGAAGAGCTTTC
AGTAGTAG

TAGAGCTTGATGGGGTTGGAGCTGGAGCAGAAGAGCTTTCAGTAGTAGAGCTTGATGGAGTTGGTACTGGA
GCAGAAGAGCTTTCAGTAGTAGAGCTTGATGGAGTTGGCACTGGAGCAGAAGAGCTTTCAGTGGTGGAGCT
GGTTACTG

ATCCCACCAATTCCGGTAGCCTGAGGCTGGTTAAACGCCATTTGCTGTTGTGGTTGAGGCTGGCCTTGGGG
CTGGTTTGAAGACTGACCTTGCTGCTGCTGCGGGTAGAATCCATTTGGTTGTTGCTGCTGTTGCTGCTGTT
GCTGCTGG

ACTCATGCTACATCTGCGTCTGAGGCACAAAACATCTTAGTTGCTCCAGGCCCCATCAAAGAACCCAAGAC
GTTGAACTACGCCCTCAGTAAAGTGGCTTTGAACTCCAGTGAATGTTTGAACAAGATGTTCCCCACCGAGG
AACAACCC

TGGTTCTAAGGGAGCTTGGTTTTGCGGTTGAAGGTAAAATCCTGTTTGCTGCGGCTTCAAAGGTTGCAAGG
ACTGTGCTTGCTGAGGAGGTGGCACCTGTTGAGGTTGAGATTGATAATAACCAGTGCCCTGGGCCTGAAGG
GGCTGCTG

GAATTTGATACTGCTAAGAAGCGTTTCGAAGAGGCAGTGGATCGTAATGAGAAGGAGCTCTTGTCCACGGT
GATGAGAGAGAAGAAGGCCGCTCTGGACAGAGCATCCATTGAGTACGAAAGGTACGGAGAGCCAGAGACT
TTAATGAG

AACCGGAGGCAGTAGAAGCGGATGAGGTTGATTGAGGGAATGAGCTTGAGCTACCAGAAGCGGATGAGACT
GATTGGCTGCCAGAGCTTGAGACACCAGAAGTAGAGGAAGCTGATTGACTGACAGAGCTTGAGACATCAGA
AGTAGAGG

ACGGGACCTGCCAGGTGCTGTAGATGGGGATCAGAATTGGTAGCACCGTGCACAGGTGAGGGGCAGTGTTG
ATAAGATGTTTACGCTCGTTGAGTGCCTCGATGACCAGATCCAGTTGTGCCTTGGAGAACTCCCAGAAGGC
CTTCTCTA

TTTTTCCTCATTTAAACAGGTAGAGTTATTCGTAACCAAAGAAAGGGTGCTGGTTCTATCTTCACCTCTCA
CACCAGATTAAGACAAGGTGCTGCCAAGTTGAGAACTTTGGACTATGCTGAACGTCATGGTTACATCCGTG
GTATCGTT

AACATCTTAGTTGCTCCAGGCCCCATCAAAGAACCCAAGACGTTGAACTACGCCCTCAGTAAAGTGGCTTT
GAACTCCAGTGAATGTTTGAACAAGATGTTCCCCACCGAGGAACAACCCTTGGCTTCGGCACTCTTGCAAT
TCAGTGAT

II. The negative dataset $\mathbb{S}^-$ contains the following 5,000 nucleosome-inhibiting DNA sequences of 150-bp long

AAAAGATGGTTTAGCGAAGGCACCATTATGAAGATAGACACATTCTTCTTTTTTTTTTTTTTTTTTTTTTT
TTTTTTTTTTTTTCATTTACTTTTATTTCGCGCGGTCGGTAAATTTTTCGTGGGTTTCTTTGAATCTATTA
GCCGACAT

AAGATAGACACATTCTTCTTTTTTTTTTTTTTTTTTTTTTTTTTTTTTTTTTTTCATTTACTTTTATTTCG
CGCGGTCGGTAAATTTTTCGTGGGTTTCTTTGAATCTATTAGCCGACATAAGAATAATGCATAAATAATAT
TTTTAATG

TTAGCGAAGGCACCATTATGAAGATAGACACATTCTTCTTTTTTTTTTTTTTTTTTTTTTTTTTTTTTTTT
TTTCATTTACTTTTATTTCGCGCGGTCGGTAAATTTTTCGTGGGTTTCTTTGAATCTATTAGCCGACATAA
GAATAATG

CATTCTTCTTTTTTTTTTTTTTTTTTTTTTTTTTTTTTTTTTTTTCATTTACTTTTATTTCGCGCGGTCGGT
AAATTTTTCGTGGGTTTCTTTGAATCTATTAGCCGACATAAGAATAATGCATAAATAATATTTTTAATGTC
TTCCTATG

TTTTTTTTTTTTTTTTTTTTTTTTTTTTTTTTTTCATTTACTTTTATTTCGCGCGGTCGGTAAATTTTTCG
TGGGTTTCTTTGAATCTATTAGCCGACATAAGAATAATGCATAAATAATATTTTTAATGTCTTCCTATGCC
CAAAAGAA

CACCATTATGAAGATAGACACATTCTTCTTTTTTTTTTTTTTTTTTTTTTTTTTTTTTTTTTTTCATTTAC
TTTTATTTCGCGCGGTCGGTAAATTTTTCGTGGGTTTCTTTGAATCTATTAGCCGACATAAGAATAATGCA
TAAATAAT

AAAGCGTCAGAAAAGATGGTTTAGCGAAGGCACCATTATGAAGATAGACACATTCTTCTTTTTTTTTTTTT
TTTTTTTTTTTTTTTTTTTTTTTCATTTACTTTTATTTCGCGCGGTCGGTAAATTTTTCGTGGGTTTCTTT
GAATCTAT

TTCGAACTATAAAGCGTCAGAAAAGATGGTTTAGCGAAGGCACCATTATGAAGATAGACACATTCTTCTTT
TTTTTTTTTTTTTTTTTTTTTTTTTTTTTTTTTCATTTACTTTTATTTCGCGCGGTCGGTAAATTTTTCGT
GGGTTTCT

GAATGCTGTGTTCGAACTATAAAGCGTCAGAAAAGATGGTTTAGCGAAGGCACCATTATGAAGATAGACAC
ATTCTTCTTTTTTTTTTTTTTTTTTTTTTTTTTTTTTTTTTTTTCATTTACTTTTATTTCGCGCGGTCGGTA
AATTTTTC

CGCGGTTGAAGAATGCTGTGTTCGAACTATAAAGCGTCAGAAAAGATGGTTTAGCGAAGGCACCATTATGA
AGATAGACACATTCTTCTTTTTTTTTTTTTTTTTTTTTTTTTTTTTTTTTTTTTCATTTACTTTTATTTCGC
GCGGTCGG

TTTTTTTTTTTTTTTTTTTTTTTTCATTTACTTTTATTTCGCGCGGTCGGTAAATTTTTCGTGGGTTTCTT
TGAATCTATTAGCCGACATAAGAATAATGCATAAATAATATTTTTAATGTCTTCCTATGCCCAAAAGAAGA
AGTCTTGA

AATATCGATTTAAATTCGAAGTGTTGTTTGCAGGATATAAATCAAAAAAAAAAAAAAAAAAAAAAAATTAA
ATAAATAAATAAAATAAATACAATGATATCGATAACGGTGAAATTCTTTTCATGGATTTTTGTTGCCCAAG
AAAATAAC

TAACTTATGAAATATCGATTTAAATTCGAAGTGTTGTTTGCAGGATATAAATCAAAAAAAAAAAAAAAAAA
AAAAAATTAAATAAATAAATAAAATAAATACAATGATATCGATAACGGTGAAATTCTTTTCATGGATTTTT
GTTGCCCA

TAAATTCGAAGTGTTGTTTGCAGGATATAAATCAAAAAAAAAAAAAAAAAAAAAAAATTAAATAAATAAAT
AAAATAAATACAATGATATCGATAACGGTGAAATTCTTTTCATGGATTTTTGTTGCCCAAGAAAATAACAA
TAACGTTT

TTCAAGTGATTTTAACTTTACGCGGTTGAAGAATGCTGTGTTCGAACTATAAAGCGTCAGAAAAGATGGTT
TAGCGAAGGCACCATTATGAAGATAGACACATTCTTCTTTTTTTTTTTTTTTTTTTTTTTTTTTTTTTT
TTCATTTA

CGCGCCGCGCAGGTACCCCGCGCATCTCTTCTTCTCGAAGAAAGCGGAAAAAACAAAAAAAAAAGTATAAA
TAGTGGAGTCTTTTCCCATTTAACATTTAGAAAAAAATTCGAATGGAAATTTCTTGCCGAACATTTAACCG
GAGACCCT

TTTAACTTTACGCGGTTGAAGAATGCTGTGTTCGAACTATAAAGCGTCAGAAAAGATGGTTTAGCGAAGGC
ACCATTATGAAGATAGACACATTCTTCTTTTTTTTTTTTTTTTTTTTTTTTTTTTTTTTTTCATTTACT
TTTATTTC

GTGTTGTTTGCAGGATATAAATCAAAAAAAAAAAAAAAAAAAAAAAATTAAATAAATAAATAAAATAAATA
CAATGATATCGATAACGGTGAAATTCTTTTCATGGATTTTTGTTGCCCAAGAAAATAACAATAACGTTTTC
TTTATGAT

GTTTCTTTTTTTTTTTTTTTTTTCGCGACTACTCAGCCATCTTGCATTTTTAAAGAAAAAGATAATCATTA
ATGCCTTCACGGGAATACGTATAGAACATTATTAAAAGTATATGAATGGCATATATATATAGAACACCACC
CTTGGAAA

GTGTTAGTTGTAACTTATGAAATATCGATTTAAATTCGAAGTGTTGTTTGCAGGATATAAATCAAAAAAAA
AAAAAAAAAAAAAAAATTAAATAAATAAATAAAATAAATACAATGATATCGATAACGGTGAAATTCTTTTC
ATGGATTT

CTATACCAATCACTTTTTCATTTTTTTTCAAAAGCTCATCGGAAAATTTTTCAAAAAAAAAAAAAAAAAAA
AAAAAGGTTTATTACCCTACTGCATTTTGATAATCTGAACATAATGAGCTAATGAAAGCAATTCTCATTTA
AAAACAAG

ACCTTTTATGTAATGATTTAAGTCTTGTCACATGACATAATAATAAATAATTTTAAAAATATAAAATATTT
TTAATAGTTTTTAAATATTTTACAGTTTATTTTTTAAATTTATTTATATGTTTTTGTTTTCCGAAGCAGTC
AAAGTATT

TTTTTTTTTTTTTTTTCATTTACTTTTATTTCGCGGTCGGTAAATTTTTCGTGGGTTTCTTTGAATCTATT
AGCCGACATAAGAATAATGCATAAATAATATTTTTAATGTCTTCCTATGCCCAAAAGAAGAAGTCTTGAAG
TTGCCGCA

TCCTAACCGCCGCGCCGCGCAGGTACCCCGCGCATCTCTTCTTCTCGAAGAAAGCGGAAAAAACAAAAAAA
AAAGTATAAATAGTGGAGTCTTTTCCCATTTAACATTTAGAAAAAAATTCGAATGGAAATTTCTTGCCGAA
CATTTAAC

TTTTTAAGAATGTCGGGTAATAAACAGATTGTTTTTCTGGGAGGATAATCTTTTCTTTTTTCCTGTTGGTA
TTCTAAATTAACCTTGCTGTTTCTTTTTTTTTTTTTTTTTTTCGCGACTACTCAGCCATCTTGCATTTTTA
AAGAAAAA

ACATTGCTTTTTATTCAAGATTATTGGTTTTCCTAACCGCCGCGCCGCGCAGGTACCCCGCGCATCTCTTC
TTCTCGAAGAAAGCGGAAAAAACAAAAAAAAAAGTATAAATAGTGGAGTCTTTTCCCATTTAACATTTAGA
AAAAAATT

TAATGATTTAAGTCTTGTCACATGACATAATAATAAATAATTTTAAAAATATAAAATATTTTTAATAGTTT
TTAAATATTTTACAGTTTATTTTTTAAATTTATTTATATGTTTTTGTTTTCCGAAGCAGTCAAAGTATTTT
AATTTTCG

CACTTTTTCATTTTTTTTCAAAAGCTCATCGGAAAATTTTTCAAAAAAAAAAAAAAAAAAAAAAAAGGTTT
ATTACCCTACTGCATTTTGATAATCTGAACATAATGAGCTAATGAAAGCAATTCTCATTTAAAAACAAGTA
TTCTCTCT

TTATTGGTTTTCCTAACCGCCGCGCCGCGCAGGTACCCCGCGCATCTCTTCTTCTCGAAGAAAGCGGAAAA
AACAAAAAAAAAAGTATAAATAGTGGAGTCTTTTCCCATTTAACATTTAGAAAAAAATTCGAATGGAAATT
TCTTGCCG

GTTTCTATAAGTGTTAGTTGTAACTTATGAAATATCGATTTAAATTCGAAGTGTTGTTTGCAGGATATAAA
TCAAAAAAAAAAAAAAAAAAAAAAAATTAAATAAATAAATAAAATAAATACAATGATATCGATAACGGTGA
AATTCTTT

TGATCTTTTGCATATTTTTTTTTTTTTTTGGGCTATAAAGTATATATAGATACAAATATATGATGAATCATT
AAAGAGGAGGTTATTACTAAGTGAAAGAAAAAGAAAAAAAAAAAAGATCAAAACCAAACTTCGTATTCGAGC
CTAAAAAA

TTTTCTTTTTTCCTGTTGGTATTCTAAAATTAACCTTGCTGTTTCTTTTTTTTTTTTTTTTCGCGCGACTA
CTCAGCCATCTTGCATTTTTAAAGAAAAAGATAATCATTAATGCCTTCACGGGAATACGTATAGAACATTA
TTAAAAGT

ATCAAAAAAAAAAAAAAAAAAAAAAAAATTAAATAAATAAATAAAATAAATACAATGATATCGATAACGGTG
AAATTCTTTTCATGGATTTTTGTTGCCCAAGAAAATAACAATAACGTTTTCTTTATGATACATATATCTAC
TTTTTCAA

CATGAAACTCAAAAATCATCAAAAAAAGAAAAGCTAAATGTATACTTTTTTGTCTACATTAGTTACCTTTT
ATTACATGAGAAAGTTATTTTTCTTCTTTTTTTTTTTTTTTTTTGAAACTTTTTCCTCTCGGAAAATAAA
AGATATAT

TGCTTTTGTTTTTTTAAGAATGTCGGGTAATAAACAGATTGTTTTTCTGGGAGGATAATCTTTTCTTTTTT
CCTGTTGGTATTCTAAAATTAACCTTGCTGTTTCTTTTTTTTTTTTTTTTCGCGCGACTACTCAGCCATCT
TGCATTTT

TTTTTTTTTTTCGCGCGACTACTCAGCCATCTTGCATTTTTAAAGAAAAAGATAATCATTAATGCCTTCAC
GGGAATACGTATAGAACATTATTAAAAGTATATGAATGGCATATATATATAGAACACCACCCTTGGAAAAC
ATTTATAC

ATATCTGCCTCTATACCAATCACTTTTTCATTTTTTTTCAAAAGCTCATCGGAAAATTTTTCAAAAAAAAA
AAAAAAAAAAAAAAAAGGTTTATTACCCTACTGCATTTTGATAATCTGAACATAATGAGCTAATGAAAGCAA
TTCTCATT

CACTGTCAAAAGAAAGAACTAAGCAATGCAATATCTGCCTCTATACCAATCACTTTTTCATTTTTTTTCAA
AAGCTCATCGGAAAATTTTTCAAAAAAAAAAAAAAAAAAAAAAAAAGGTTTATTACCCTACTGCATTTTGAT
AATCTGAA

GAACTTCAAAACCTTTTATGTAATGATTTAAGTCTTGTCACATGACATAATAATAAATAATTTTAAAAATA
TAAAATATTTTTAATAGTTTTTAAATATTTTACAGTTTATTTTTTAAATTTATTTATATGTTTTTGTTTTC
CGAAGCAG

TCCTCAGAATTGCCATAGATGAGTATTTACTGATCTTTTGCATATTTTTTTTTTTTTTTGGGCTATAAAGTA
TATATAGATACAAATATATGATGAATCATTAAAGAGGAGGTTATTACTAAGTGAAAGAAAAAGAAAAAAAA
AAAGATCA

AGAAAGAACTAAGCAATGCAATATCTGCCTCTATACCAATCACTTTTTCATTTTTTTTCAAAAGCTCATCG
GAAAATTTTTCAAAAAAAAAAAAAAAAAAAAAAAAAGGTTTATTACCCTACTGCATTTTGATAATCTGAACA
TAATGAGC

ACGTATGTGCACATGTGATTTGCTTTTGTTTTTTTAAGAATGTCGGGTAATAAACAGATTGTTTTTCTGGG
AGGATAATCTTTTCTTTTTTCCTGTTGGTATTCTAAAATTAACCTTGCTGTTTCTTTTTTTTTTTTTTTTC
GCGCGACT

CAGGATATAAATCAAAAAAAAAAAAAAAAAAAAAAAATTAAATAAATAAATAAAATAAATACAATGATATC
GATAACGGTGAAATTCTTTTCATGGATTTTTGTTGCCCAAGAAAATAACAATAACGTTTTCTTTATGATAC
ATATATCT

GAGTATTTACTGATCTTTTGCATATTTTTTTTTTTTTTGGGCTATAAAGTATATATAGATACAAATATATG
ATGAATCATTAAAGAGGAGGTTATTACTAAGTGAAAGAAAAAGAAAAAAAAAAAGATCAAAACCAAACTTC
GTATTCGA

TGCCATAGATGAGTATTTACTGATCTTTTGCATATTTTTTTTTTTTTTGGGCTATAAAGTATATATAGATA
CAAATATATGATGAATCATTAAAGAGGAGGTTATTACTAAGTGAAAGAAAAAGAAAAAAAAAAAGATCAAA
ACCAAACT

TCATACTTCTGTTTCTATAAGTGTTAGTTGTAACTTATGAAATATCGATTTAAATTCGAAGTGTTGTTTGC
AGGATATAAATCAAAAAAAAAAAAAAAAAAAAAAAATTAAATAAATAAATAAAATAAATACAATGATATCG
ATAACGGT

TTATTCAAGATTATTGGTTTTCCTAACCGCCGCGCCGCGCAGGTACCCCGCGCATCTCTTCTTCTCGAAGA
AAGCGGAAAAAACAAAAAAAAAAGTATAAATAGTGGAGTCTTTTCCCATTTAACATTTAGAAAAAAATTCG
AATGGAAA

TAACCTTGCTGTTTCTTTTTTTTTTTTTTTTTTCGCGACTACTCAGCCATCTTGCATTTTTAAAGAAAAAG
ATAATCATTAATGCCTTCACGGGAATACGTATAGAACATTATTAAAAGTATATGAATGGCATATATATATA
GAACACCA

ACACATTTCTGCTGATGTGTTTTTTTTTTTCAACTTATTACGCGATTCGTTTTTTTTTTACGGTAACAGAA
TACAGAATAAATTCACGTACAAAAATAGAGAATATATAAAATAATAGGTTGACGATTATATTGGATCTTCC
CCTGGGGT

GTTTTTCTGGGAGGATAATCTTTTCTTTTTTCCTGTTGGTATTCTAAAATTAACCTTGCTGTTTCTTTTTT
TTTTTTTTTTTTCGCGACTACTCAGCCATCTTGCATTTTTAAAGAAAAAGATAATCATTAATGCCTTCACG
GGAATACG

TGTCGGGTAATAAACAGATTGTTTTTCTGGGAGGATAATCTTTTCTTTTTTCCTGTTGGTATTCTAAAATT
AACCTTGCTGTTTCTTTTTTTTTTTTTTTTTTCGCGACTACTCAGCCATCTTGCATTTTTAAAGAAAAAGA
TAATCATT

AAGCAATGCAATATCTGCCTCTATACCAATCACTTTTTCATTTTTTTTCAAAAGCTCATCGGAAAATTTTT
CAAAAAAAAAAAAAAAAAAAAAAAAGGTTTATTACCCTACTGCATTTTGATAATCTGAACATAATGAGCTA
ATGAAAGC

TTTTTTTTCAAAAGCTCATCGGAAAATTTTTCAAAAAAAAAAAAAAAAAAAAAAAAGGTTTATTACCCTAC
TGCATTTTGATAATCTGAACATAATGAGCTAATGAAAGCAATTCTCATTTAAAAACAAGTATTCTCTCTTA
TTGAAGTA

GCTGATGTGTTTTTTTTTTTCAACTTATTACGCGATTCGTTTTTTTTTTACGGTAACAGAATACAGAATAA
ATTCACGTACAAAAATAGAGAATATATAAAATAATAGGTTGACGATTATATTGGATCTTCCCCTGGGGTTC
AAGAGTCG

CGCGCGCAAGCCAGCGGTAAAGGGAAAAGAACGGAGGACGATTACATACAAGATGAACGAATAAATAAATT
AATAATAAATAATAATAAAAAGTACAGTAGCATTAAATATTATTAAGTTTAATGATTAAAAATTGGTTAAT
TGTCAAGA

CTGAAGAGAACACTGTCAAAAGAAAGAACTAAGCAATGCAATATCTGCCTCTATACCAATCACTTTTTCAT
TTTTTTTCAAAAGCTCATCGGAAAATTTTTCAAAAAAAAAAAAAAAAAAAAAAAAGGTTTATTACCCTACT
GCATTTTG

ATAGTGGCTCATGATCTGTAAATGATCGGTTGACCGCAGTATTATATAATAACATCCGTATAAGTACATAT
ACTACCATGTCTGTTCTCTACATTGCTTTTTATTCAAGATTATTGGTTTTCCTAACCGCCGCGCCGCGCAG
GTACCCCG

TCCGTAAAGGATGGTTTAATAATAAGAAATTTATAATATTAATAATACATATATACAAAAATTTATATTTA
TATACATGCGCCTAACTATTCATACTATTAATTTCATATTATTAAGCTTTTTTTTTTTCATTTATCATTTT
TTTTCGTA

ACATGTGATTTGCTTTTGTTTTTTTAAGAATGTCGGGTAATAAACAGATTGTTTTTCTGGGAGGATAATCT
TTTCTTTTTTCCTGTTGGTATTCTAAAATTAACCTTGCTGTTTCTTTTTTTTTTTTTTTTCGCGCGACTAC
TCAGCCAT

TATACTTTTTTGTCTACATTAGTTACCTTTTATTACATGAGAAAGTTATTTTTCTTCTTTTTTTTTTTTTT
TTTTTGAAACTTTTTCCTCTCGGAAAATAAAAGATATATTTACAAGTGAAAGCTTATTGTAATGTGTCATT
TTAAACAT

TAAGGGAATAGGCCGTTATTTCCGTAAAGGATGGTTTAATAATAAGAAATTTATAATATTAATAATACATA
TATACAAAAATTTATATTTATATACATGCGCCTAACTATTCATACTATTAATTTCATATTATTAAGCTTTT
TTTTTTTC

TTTTTTTTTTCAACTTATTACGCGATTCGTTTTTTTTTTACGGTAACAGAATACAGAATAAATTCACGTAC
AAAAATAGAGAATATATAAAATAATAGGTTGACGATTATATTGGATCTTCCCCTGGGGTTCAAGAGTCGAG
ACCGAGTC

TGATTAGTAACATGAAACTCAAAAATCATCAAAAAAAGAAAAGCTAAATGTATACTTTTTTGTCTACATTA
GTTACCTTTTATTACATGAGAAAGTTATTTTTCTTCTTTTTTTTTTTTTTTTTTTGAAACTTTTTCCTCTC
GGAAAATA

AGCCTTGCGTTTCGAGAAAGTGAAAACCAATTGAATACAAAATAAAAAAAAAGAAGAAAGAAATAGCAGGT
CTAAGATATATAAGAAAGTTAATATCATTTTTGAACATTTTATTTTAGACGCCTTCAGCCGCGCGACGCCC
GGAGTAAT

ATTCTAAAATTAACCTTGCTGTTTCTTTTTTTTTTTTTTTTCGCGCGACTACTCAGCCATCTTGCATTTTT
AAAGAAAAAGATAATCATTAATGCCTTCACGGGAATACGTATAGAACATTATTAAAAGTATATGAATGGCA
TATATATA

CTTTTTTTTTTTTTTTTTTTTTTGAGATTCTTCATATTCTGTTATTAAATTTTTAGACTTTAATTTTTACTT
TCTATGTAACGTTCACTCTTACCCTAAATATTAAACTATTTGATAAAATATTACACTGAAAGGTGTTACAA
CTTTTCCT

TGATAAGTACTCATACTTCTGTTTCTATAAGTGTTAGTTGTAACTTATGAAATATCGATTTAAATTCGAAG
TGTTGTTTGCAGGATATAAATCAAAAAAAAAAAAAAAAAAAAAAAATTAAATAAATAAATAAAATAAATAC
AATGATAT

AGTCTTGTCACATGACATAATAATAAATAATTTTAAAAATATAAAATATTTTTAATAGTTTTTAAATATTT
TACAGTTTATTTTTTAAATTTATTTATATGTTTTTGTTTTCCGAAGCAGTCAAAGTATTTTAATTTTCGGA
GCTTTCAT

GGAAAATTTTTCAAAAAAAAAAAAAAAAAAAAAAAAAAGGTTTATTACCCTACTGCATTTTGATAATCTGAAC
ATAATGAGCTAATGAAAGCAATTCTCATTTAAAAACAAGTATTCTCTCTTATTGAAGTATGCATTATCTAT
CATTATAA

AAAAATCATCAAAAAAAGAAAAGCTAAATGTATACTTTTTTGTCTACATTAGTTACCTTTTATTACATGAG
AAAGTTATTTTTCTTCTTTTTTTTTTTTTTTTTTTGAAACTTTTTCCTCTCGGAAAATAAAAGATATATTT
ACAAGTGA

TAATAAATAATTTTAAAAATATAAAATATTTTTAATAGTTTTTAAATATTTTACAGTTTATTTTTTAAATT
TATTTATATGTTTTTGTTTTCCGAAGCAGTCAAAGTATTTTAATTTTCGGAGCTTTCATTTCAAGCGCCTT
TTTTTTAC

AGTGTAAACATTCAAGTGATTTTAACTTTACGCGGTTGAAGAATGCTGTGTTCGAACTATAAAGCGTCAGA
AAAGATGGTTTAGCGAAGGCACCATTATGAAGATAGACACATTCTTCTTTTTTTTTTTTTTTTTTTTTTTT
TTTTTTTT

GAGGATAATCTTTTCTTTTTTCCTGTTGGTATTCTAAAATTAACCTTGCTGTTTCTTTTTTTTTTTTTTTT
CGCGCGACTACTCAGCCATCTTGCATTTTTAAAGAAAAAGATAATCATTAATGCCTTCACGGGAATACGTA
TAGAACAT

TGTGTCAATGCTACTATTTTGGAGATTAATCTCAGTACAAAACAATATTAAAAAGAGGTGAATTATTTTTC
CCCCCTTATTTTTTTTTTGTTAAAATTGATCCAAATGTAAATAAACAATCACAAGGAAAAAAAAAAAAAAA
AAAAAAAT

TTTTGTATGTACACATTTCTGCTGATGTGTTTTTTTTTTTCAACTTATTACGCGATTCGTTTTTTTTTTAC
GGTAACAGAATACAGAATAAATTCACGTACAAAAATAGAGAATATATAAAATAATAGGTTGACGATTATAT
TGGATCTT

TGTGACTAGAGGAAGTAAGGAGAAAAAACGATAGTAATCGTATTTTAGGTTGTGCGTTTTTATAATTTTTT
TTTTTTTGTAATTCTATGCAAATGTAATATAAGTATATTTAAAGAAATAATGAGTCCTGTGAAAACAAAAA
GAAAAAAA

AGCGAAAAGAAAAAGAAAAAATAGAAAATTTGGGTGGGGGGGGCGGAAGATCCCACGCCGCGCAAGAGATA
TTTCAATATTACTACTACATAGTATATGCGGCGCTACCATACGTACAACTTTTTTTTCTTTTTTTTTTTT
TTGCCTTC

AAAGCTCATCGGAAAATTTTTCAAAAAAAAAAAAAAAAAAAAAAAAGGTTTATTACCCTACTGCATTTTGA
TAATCTGAACATAATGAGCTAATGAAAGCAATTCTCATTTAAAAACAAGTATTCTCTCTTATTGAAGTATG
CATTATCT

TTAATTGTTCTGATTAGTAACATGAAACTCAAAAATCATCAAAAAAAGAAAAGCTAAATGTATACTTTTTT
GTCTACATTAGTTACCTTTTATTACATGAGAAAGTTATTTTTCTTCTTTTTTTTTTTTTTTTTTTGAAACT
TTTTCCTC

CGGTGGAAGAAAAACAGCGAAAAAAAATAACCGATACCCCCTTTTCGAATACAAATGCTTGTATATTCAAT
TATGAATTATTTTTTTTTTTTTTCATTTCTTATATTATTTTTTGTTCGAGAATCACTTTTTCAAGATGGTA
ACAACATC

GGAGATTAATCTCAGTACAAAACAATATTAAAAAGAGGTGAATTATTTTTCCCCCCTTATTTTTTTTTTGT
TAAAATTGATCCAAATGTAAATAAACAATCACAAGGAAAAAAAAAAAAAAAAAAAAAATAGCCGCCATGAC
CCCGGATC

AAAAGAAAAAATAGAAAATTTGGGTGGGGGGGGCGGAAGATCCCACGCCGCGCAAGAGATATTTCAATATT
ACTACTACATAGTATATGCGGCGCTACCATACGTACAACTTTTTTTTCTTTTTTTTTTTTTTGCCTTCTA
AATTTGTA

CGAACACCGTCATTGATCAAATAGGTCTATAATATTAATATACATTTATATAATCTACGGTATTTATATCA
TCAAAAAAAAGTAGTTTTTTTATTTTATTTTGTTCGTTAATTTTCAATTTCTATGGAAACCCGTTCGTAAA
ATTGGCGT

ATAGAGTAAGTTTTGTATGTACACATTTCTGCTGATGTGTTTTTTTTTTTCAACTTATTACGCGATTCGTT
TTTTTTTTACGGTAACAGAATACAGAATAAATTCACGTACAAAAATAGAGAATATATAAAATAATAGGTTG
ACGATTAT

AGCGGTCTTTTCCTCAGAATTGCCATAGATGAGTATTTACTGATCTTTTGCATATTTTTTTTTTTTTTGGG
CTATAAAGTATATATAGATACAAATATATGATGAATCATTAAAGAGGAGGTTATTACTAAGTGAAAGAAAA
AGAAAAAA

GGAAGTAAGGAGAAAAAACGATAGTAATCGTATTTTAGGTTGTGCGTTTTTATAATTTTTTTTTTTTTGTA
ATTCTATGCAAATGTAATATAAGTATATTTAAAGAAATAATGAGTCCTGTGAAAACAAAAAGAAAAAAAGA
TCATTAAT

GATCACTTTAGCTGGTCCCATTCGAAGAACCTTTTTTTTTTTTTTTTTTTTTGAGATTCTTCATATTCTGT
TATTAAATTTTTAGACTTTAATTTTTACTTTCTATGTAACGTTCACTCTTACCCTAAATATTAAACTATTT
GATAAAAT

AACAATATTAAAAAGAGGTGAATTATTTTTCCCCCCTTATTTTTTTTTTGTTAAAATTGATCCAAATGTAA
ATAAACAATCACAAGGAAAAAAAAAAAAAAAAAAAAAATAGCCGCCATGACCCCGGATCGTCGGTTGTGAT
ACGGTCAG

TCGCGCGACTACTCAGCCATCTTGCATTTTTAAAGAAAAAGATAATCATTAATGCCTTCACGGGAATACGT
ATAGAACATTATTAAAAGTATATGAATGGCATATATATATAGAACACCACCCTTGGAAAACATTTATACCC
CTTAAACT

CCTTATGTTGTTTGCTTAGTTTTTTGATATTAGTGTTGCTTATGTGAAATTTCGCGATTTCAATTAAAATA
ATAAATACATATATAAAGAATATACACAGAGGGAAGCAAAAGTAAACTAAAAGTGATACTTACACGAGCTT
TTTTGGTT

GGCCGTTATTTCCGTAAAGGATGGTTTAATAATAAGAAATTTATAATATTAATAATACATATATACAAAAA
TTTATATTTATATACATGCGCCTAACTATTCATACTATTAATTTCATATTATTAAGCTTTTTTTTTTTCAT
TTATCATT

TTCGAAGAACCTTTTTTTTTTTTTTTTTTTTTGAGATTCTTCATATTCTGTTATTAAATTTTTAGACTTTA
ATTTTTACTTTCTATGTAACGTTCACTCTTACCCTAAATATTAAACTATTTGATAAAATATTACACTGAAA
GGTGTTAC

TTTGCTTAGTTTTTTGATATTAGTGTTGCTTATGTGAAATTTCGCGATTTCAATTAAAATAATAAATACAT
ATATAAAGAATATACACAGAGGGAAGCAAAAGTAAACTAAAAGTGATACTTACACGAGCTTTTTTGGTTCC
AAACTGTT

AATAAGAAATTTATAATATTAATAATACATATATACAAAAATTTATATTTATATACATGCGCCTAACTATT
CATACTATTAATTTCATATTATTAAGCTTTTTTTTTTTCATTTATCATTTTTTTTCGTAACCTCTCATACC
TGTACAGG

AACATCCGTATAAGTACATATACTACCATGTCTGTTCTCTACATTGCTTTTTATTCAAGATTATTGGTTTT
CCTAACCGCCGCGCCGCGCAGGTACCCCGCGCATCTCTTCTTCTCGAAGAAAGCGGAAAAAACAAAAAAAA
AAGTATAA

TAAGTACATATACTACCATGTCTGTTCTCTACATTGCTTTTTATTCAAGATTATTGGTTTTCCTAACCGCC
GCGCCGCGCAGGTACCCCGCGCATCTCTTCTTCTCGAAGAAAGCGGAAAAAACAAAAAAAAAAGTATAAAT
AGTGGAGT

AGTAATTAACGCCCATTAAAAAGAAGGCATAGGAGGCATATACATATATATATATATATATATATATGGCT
GCTGACAGATATTCTGCACTTAAAAACTAAAAATATTATACCAACTTTTCTTTTTCTTCCCGTTCAGTTTG
CTTGATTG

TGGGTGGGGGGGGCGGAAGATCCCACGCCGCGCAAGAGATATTTCAATATTACTACTACATAGTATATGCG
GCGCTACCATACGTACAACTTTTTTTTCTTTTTTTTTTTTTTTGCCTTCTAAATTTGTAATTCGGTCACAC
TTTTGTCG

CTTTTACTCGCCGAGCGAACGTGCACCAAAAAGGGAAAGGAAAAAAAGAAAAAAAAAGGAAAAAGGAAACT
CAAAACTTGGATAAATAGAAGCACTCAAACTAAATTAAACTGCCAAAAAAAAAAAAAATAAAAAGGGAAAA
GTTTAAAC

CATGAAGTTTGATCACTTTAGCTGGTCCCATTCGAAGAACCTTTTTTTTTTTTTTTTTTTTTGAGATTCTT
CATATTCTGTTATTAAATTTTTAGACTTTAATTTTTACTTTCTATGTAACGTTCACTCTTACCCTAAATAT
TAAACTAT

CTCAGTACAAAACAATATTAAAAAGAGGTGAATTATTTTTCCCCCCTTATTTTTTTTTGTTAAAATTGAT
CCAAATGTAAATAAACAATCACAAGGAAAAAAAAAAAAAAAAAAAAAATAGCCGCCATGACCCCGGATCGT
CGGTTGTG

AAATGCTTCACGAACACCGTCATTGATCAAATAGGTCTATAATATTAATATACATTTATATAATCTACGGT
ATTTATATCATCAAAAAAAAGTAGTTTTTTTATTTTATTTTGTTCGTTAATTTTCAATTTCTATGGAAACC
CGTTCGTA

AAAAGAGGTGAATTATTTTTCCCCCCTTATTTTTTTTTGTTAAAATTGATCCAAATGTAAATAAACAATC
ACAAGGAAAAAAAAAAAAAAAAAAAAAATAGCCGCCATGACCCCGGATCGTCGGTTGTGATACGGTCAGGG
TAGCGCCC

TAAACAGATTGTTTTTCTGGGAGGATAATCTTTTCTTTTTTCCTGTTGGTATTCTAAAATTAACCTTGCTG
TTTCTTTTTTTTTTTTTTTTTTCGCGACTACTCAGCCATCTTGCATTTTTAAAGAAAAAGATAATCATTAA
TGCCTTCA

ATGGTTTAATAATAAGAAATTTATAATATTAATAATACATATATACAAAAATTTATATTTATATACATGCG
CCTAACTATTCATACTATTAATTTCATATTATTAAGCTTTTTTTTTTTCATTTATCATTTTTTTTCGTAAC
CTCTCATA

TTCGAGAAAGTGAAAACCAATTGAATACAAAATAAAAAAAAAGAAGAAAGAAATAGCAGGTCTAAGATATA
TAAGAAAGTTAATATCATTTTTGAACATTTTATTTTAGACGCCTTCAGCCGCGCGACGCCCGGAGTAATCA
TATGCCCA

TCCTCGTTAATGATAAGTACTCATACTTCTGTTTCTATAAGTGTTAGTTGTAACTTATGAAATATCGATTT
AAATTCGAAGTGTTGTTTGCAGGATATAAATCAAAAAAAAAAAAAAAAAAAAAAAATTAAATAAATAAATA
AAATAAAT

AGAAAAAACGATAGTAATCGTATTTTAGGTTGTGCGTTTTTATAATTTTTTTTTTTTGTAATTCTATGCA
AATGTAATATAAGTATATTTAAAGAAATAATGAGTCCTGTGAAAACAAAAAGAAAAAAAGATCATTAATGT
ATGTTAAC

TAGTTATGTACTGAAGAGAACACTGTCAAAAGAAAGAACTAAGCAATGCAATATCTGCCTCTATACCAATC
ACTTTTTCATTTTTTTTTCAAAAGCTCATCGGAAAATTTTCAAAAAAAAAAAAAAAAAAAAAAAAGGTTTA
TTACCCTA

GATTTATTTAAGCCTTGCGTTTCGAGAAAGTGAAAACCAATTGAATACAAAATAAAAAAAAAGAAGAAAGA
AATAGCAGGTCTAAGATATATAAGAAAGTTAATATCATTTTTGAACATTTTATTTTAGACGCCTTCAGCCG
CGCGACGC

CTTTGACATTGAACTTCAAAACCTTTTATGTAATGATTTAAGTCTTGTCACATGACATAATAATAAATAAT
TTTAAAAATATAAAATATTTTTAATAGTTTTTAAATATTTTACAGTTTATTTTTTAAATTTATTTATATGT
TTTTGTTT

AGGGCATTTTTCCTCGTTAATGATAAGTACTCATACTTCTGTTTCTATAAGTGTTAGTTGTAACTTATGAA
ATATCGATTTAAATTCGAAGTGTTGTTTGCAGGATATAAATCAAAAAAAAAAAAAAAAAAAAAAAATTAAA
TAAATAAA

TGAAAACCAATTGAATACAAAATAAAAAAAAAGAAGAAAGAAATAGCAGGTCTAAGATATATAAGAAAGTT
AATATCATTTTTGAACATTTTATTTTAGACGCCTTCAGCCGCGCGACGCCCGGAGTAATCATATGCCCATG
ACTTTACC

TTTTTGATATTAGTGTTGCTTATGTGAAATTTCGCGATTTCAATTAAAATAATAAATACATATATAAAGAA
TATACACAGAGGGAAGCAAAAGTAAACTAAAAGTGATACTTACACGAGCTTTTTTGGTTCCAAACTGTTCA
TGATGATG

CATATTTTTTTTTTTTTGGGCTATAAAGTATATATAGATACAAATATATGATGAATCATTAAAGAGGAGG
TTATTACTAAGTGAAAGAAAAAGAAAAAAAAAAGATCAAAACCAAACTTCGTATTCGAGCCTAAAAAACA
GAATATAA

TACTACCATGTCTGTTCTCTACATTGCTTTTTATTCAAGATTATTGGTTTTCCTAACCGCCGCGCCGCGCA
GGTACCCCGCGCATCTCTTCTTCTCGAAGAAAGCGGAAAAAACAAAAAAAAAAGTATAAATAGTGGAGTCT
TTTCCCAT

ATAGAAAATTTGGGTGGGGGGGGCGGAAGATCCCACGCCGCGCAAGAGATATTTCAATATTACTACTACAT
AGTATATGCGGCGCTACCATACGTACAACTTTTTTTTCTTTTTTTTTTTTTTGCCTTCTAAATTTGTAAT
TCGGTCAC

CAGAGGGAAGATAGAGTAAGTTTTGTATGTACACATTTCTGCTGATGTGTTTTTTTTTTCAACTTATTAC
GCGATTCGTTTTTTTTTACGGTAACAGAATACAGAATAAATTCACGTACAAAAATAGAGAATATATAAAA
TAATAGGT

TCTGTTCTCTACATTGCTTTTTATTCAAGATTATTGGTTTTCCTAACCGCCGCGCCGCGCAGGTACCCCGC
GCATCTCTTCTTCTCGAAGAAAGCGGAAAAAACAAAAAAAAAAGTATAAATAGTGGAGTCTTTTCCCATTT
AACATTTA

GGGCGGAAGATCCCACGCCGCGCAAGAGATATTTCAATATTACTACTACATAGTATATGCGGCGCTACCAT
ACGTACAACTTTTTTTTCTTTTTTTTTTTTTTGCCTTCTAAATTTGTAATTCGGTCACACTTTTGTCGCA
GTGTTGCA

ATTATATAATAACATCCGTATAAGTACATATACTACCATGTCTGTTCTCTACATTGCTTTTTATTCAAGAT
TATTGGTTTTCCTAACCGCCGCGCCGCGCAGGTACCCCGCGCATCTCTTCTTCTCGAAGAAAGCGGAAAAA
ACAAAAAA

AGTCTTTACCAGCGGTCTTTTCCTCAGAATTGCCATAGATGAGTATTTACTGATCTTTTGCATATTTTTT
TTTTTTGGGCTATAAAGTATATATAGATACAAATATATGATGAATCATTAAAGAGGAGGTTATTACTAAG
TGAAAGAA

TATACTCAAACGGTGGAAGAAAAACAGCGAAAAAAAATAACCGATACCCCCTTTTCGAATACAAATGCTTG
TATATTCAATTATGAATTATTTTTTTTTTTTTCATTTCTTATATTATTTTTTGTTCGAGAATCACTTTTT
CAAGATGG

ATGATCTGTAAATGATCGGTTGACCGCAGTATTATATAATAACATCCGTATAAGTACATATACTACCATGT
CTGTTCTCTACATTGCTTTTTATTCAAGATTATTGGTTTTCCTAACCGCCGCGCCGCGCAGGTACCCCGCG
CATCTCTT

CTACTATTTTGGAGATTAATCTCAGTACAAAACAATATTAAAAAGAGGTGAATTATTTTTCCCCCTTATT
TTTTTTTTGTTAAAATTGATCCAAATGTAAATAAACAATCACAAGGAAAAAAAAAAAAAAAAAAAAATAG
CCGCCATG

TCCTGTTGGTATTCTAAAATTAACCTTGCTGTTTCTTTTTTTTTTTTTTTCGCGCGACTACTCAGCCATC
TTGCATTTTTAAAGAAAAAGATAATCATTAATGCCTTCACGGGAATACGTATAGAACATTATTAAAAGTAT
ATGAATGG

ACCACAAAGCGAGGTCGCTTTTGAAGAGCCCTCGGTAGCATAACATTTTTAATTATTACGACTGTTTTTTT
TATTCATTATGTAGAGATAATTAAATGTTATAGATGCTCTATACTCAAACGGTGGAAGAAAAACAGCGAAA
AAAAATAA

AGTTACCTTTTATTACATGAGAAAGTTATTTTTCTTCTTTTTTTTTTTTTTTTTTTGAAACTTTTTCCTCT
CGGAAAATAAAAGATATATTTACAAGTGAAAGCTTATTGTAATGTGTCATTTTAAACATCAAATAACAGAC
CTTTACAT

AAAACAGCGAAAAAAAATAACCGATACCCCCTTTTCGAATACAAATGCTTGTATATTCAATTATGAATTAT
TTTTTTTTTTTTCATTTCTTATATTATTTTTTGTTCGAGAATCACTTTTTCAAGATGGTAACAACATCTT
CGTCTTCC

TTTCTCTATTTTTTTTTTAATTGAAAAATTTCCTTTCTCTATAGCGTATAGAATATATGTTACATGTATAT
ATATATATAAAGTAAAAACGTTCGGAAAATTCCTCATTATACCCAGATCATTAAAAGACATTTTCGTTATT
ATCAATTG

AGGTATTATTTTTTTTTTTTTTTGATAAGAAATTTAAGTGTTACAGAATGGGCCATCTTACAAAAATAATAG
TCTTTATGTATTTTTATATATGTAAAAGAATTGAAATATTTTATAACTGGTTGTTATTATGGTACAGTGCG
CTGCCCAA

CATTGATCAAATAGGTCTATAATATTAATATACATTTATATAATCTACGGTATTTATATCATCAAAAAAAA
GTAGTTTTTTTATTTTATTTTGTTCGTTAATTTTCAATTTCTATGGAAACCCGTTCGTAAAATTGGCGTTT
GTCTCTAG

TCCCACGCCGCGCAAGAGATATTTCAATATTACTACTACATAGTATATGCGGCGCTACCATACGTACAACT
TTTTTTTCTTTTTTTTTTTTTTTTGCCTTCTAAATTTGTAATTCGGTCACACTTTTGTCGCAGTGTTGCAAA
CGTCTTGA

AGTGTTAGCGATAATCAAGAAGTGAAACTCTTTCTCTATTTTTTTTTTAATTGAAAAATTTCCTTTCTCTA
TAGCGTATAGAATATATGTTACATGTATATATATATATAAAGTAAAAACGTTCGGAAAATTCCTCATTATA
CCCAGATC

GCCCATTAAAAAGAAGGCATAGGAGGCATATACATATATATATATATATATATATATGGCTGCTGACAGAT
ATTCTGCACTTAAAAACTAAAAATATTATACCAACTTTTCTTTTTCTTCCCGTTCAGTTTGCTTGATTGGC
CCAGCTCT

ATTATATTGACTTTGACATTGAACTTCAAAACCTTTTATGTAATGATTTAAGTCTTGTCACATGACATAAT
AATAAATAATTTTAAAAATATAAAATATTTTTAATAGTTTTTAAATATTTTACAGTTTATTTTTTAAATTT
ATTTATAT

ATTAGCCCAACAGCTACTAAAGGTATTATTTTTTTTTTTTTTTGATAAGAAATTTAAGTGTTACAGAATGGG
CCATCTTACAAAAATAATAGTCTTTATGTATTTTTATATATGTAAAAGAATTGAAATATTTTATAACTGGT
TGTTATTA

TTGAATACAAAATAAAAAAAAAGAAGAAAGAAATAGCAGGTCTAAGATATATAAGAAAGTTAATATCATTT
TTGAACATTTTATTTTAGACGCCTTCAGCCGCGCGACGCCCGGAGTAATCATATGCCCATGACTTTACCAA
AAGGCAAC

AAAAAAAGAAAAGCTAAATGTATACTTTTTTGTCTACATTAGTTACCTTTTATTACATGAGAAAGTTATTT
TTCTTCTTTTTTTTTTTTTTTTTTTTGAAACTTTTTCCTCTCGGAAAATAAAAGATATATTTACAAGTGAAA
GCTTATTG

AAAAAAATAACCGATACCCCCTTTTCGAATACAAATGCTTGTATATTCAATTATGAATTATTTTTTTTTTT
TTTCATTTCTTATATTATTTTTTGTTCGAGAATCACTTTTTCAAGATGGTAACAACATCTTCGTCTTCCAA
AATGTGAC

GTGCACCAAAAAGGGAAAGGAAAAAAAGAAAAAAAAAAGGAAAAAGGAAACTCAAAACTTGGATAAATAGAA
GCACTCAAACTAAATTAAACTGCCAAAAAAAAAAAAAAATAAAAAGGGAAAAGTTTAAACATCAAAGTACAC
CTTTCACC

GCTGGTCCCATTCGAAGAACCTTTTTTTTTTTTTTTTTTTTTTGAGATTCTTCATATTCTGTTATTAAATTT
TTAGACTTTAATTTTTACTTTCTATGTAACGTTCACTCTTACCCTAAATATTAAACTATTTGATAAAATAT
TACACTGA

AATGATCGGTTGACCGCAGTATTATATAATAACATCCGTATAAGTACATATACTACCATGTCTGTTCTCTA
CATTGCTTTTTATTCAAGATTATTGGTTTTCCTAACCGCCGCGCCGCGCAGGTACCCCGCGCATCTCTTCT
TCTCGAAG

CGTATTCATAAAATGCTTCACGAACACCGTCATTGATCAAATAGGTCTATAATATTAATATACATTTATAT
AATCTACGGTATTTATATCATCAAAAAAAAGTAGTTTTTTTATTTTATTTTGTTCGTTAATTTTCAATTTC
TATGGAAA

TTTTTCTGGGACTGGAATACCAAGCACTACAACGACACTTTATTAAAACAGTAAATAGATAATATGATTAT
GTAATTTTAGAAACTAATTATGAATACCGATTTATTTTTTTTTTTTTTTTTCACTTTTGCTGGCAAGAAAT
ACGAAATT

GAAGGTGTCGTTAATTGTTCTGATTAGTAACATGAAACTCAAAAATCATCAAAAAAAGAAAAGCTAAATGT
ATACTTTTTTGTCTACATTAGTTACCTTTTATTACATGAGAAAGTTATTTTTCTTCTTTTTTTTTTTTTTT
TTTTGAAA

TTTTTTTTTTTTGATAAGAAATTTAAGTGTTACAGAATGGGCCATCTTACAAAAATAATAGTCTTTATGTA
TTTTTATATATGTAAAAGAATTGAAATATTTTATAACTGGTTGTTATTATGGTACAGTGCGCTGCCCAATC
CACGTGGA

CATGACATAATAATAAATAATTTTAAAAATATAAAATATTTTTAATAGTTTTTAAATATTTTACAGTTTAT
TTTTTAAATTTATTTATATGTTTTTGTTTTCCGAAGCAGTCAAAGTATTTTAATTTTCGGAGCTTTCATTT
CAAGCGCC

GGATGAAAAAAAATGTATAATGTGACTAGAGGAAGTAAGGAGAAAAAACGATAGTAATCGTATTTTAGGTT
GTGCGTTTTTATAATTTTTTTTTTTTTTGTAATTCTATGCAAATGTAATATAAGTATATTTAAAGAAATAAT
GAGTCCTG

CAACATCCTGCATGAAGTTTGATCACTTTAGCTGGTCCCATTCGAAGAACCTTTTTTTTTTTTTTTTTTT
TGAGATTCTTCATATTCTGTTATTAAATTTTAGACTTTAATTTTTACTTTCTATGTAACGTTCACTCTTA
CCCTAAAT

AAAAAAAGAAAAAAAAAAGGAAAAAGGAAACTCAAAACTTGGATAAATAGAAGCACTCAAACTAAATTAAAC
TGCCAAAAAAAAAAAAAAATAAAAAGGGAAAAGTTTAAACATCAAAGTACACCTTTCACCCCTCCACACACC
ATGGAACA

GTAGAGGAAGAGACTGTCATAGGGAAGAGCCCTTTCTACATACTACTACATAATATATATATATATAGTATAG
AAATTGGTATATCACTACTTGTACAAATATCATATTGTACGATAATCGCGAAGAACGACGCACTGGTGGGA
AGAAGTGG

CGTCTGTGAAGGATGAAAAAAAATGTATAATGTGACTAGAGGAAGTAAGGAGAAAAAACGATAGTAATCGT
ATTTTAGGTTGTGCGTTTTTATAATTTTTTTTTTTTTTGTAATTCTATGCAAATGTAATATAAGTATATTTA
AAGAAATA

TCAAAAAAAAAAAAAAAAAAAAAAAAAGGTTTATTACCCTACTGCATTTTGATAATCTGAACATAATGAGCT
AATGAAAGCAATTCTCATTTAAAAACAAGTATTCTCTCTTATTGAAGTATGCATTATCTATCATTATAAAT
TCTTTTAT

AAATGTATAATGTGACTAGAGGAAGTAAGGAGAAAAAACGATAGTAATCGTATTTTAGGTTGTGCGTTTTT
ATAATTTTTTTTTTTTTGTAATTCTATGCAAATGTAATATAAGTATATTTAAAGAAATAATGAGTCCTGTG
AAAACAAA

ACTTTCAAAAAAAGAAAAAGTAAAGTATGATAAAACGGAGCACTTGCCAAAGTAATTAACGCCCATTAAAA
AGAAGGCATAGGAGGCATATACATATATATATATATATATATATATGGCTGCTGACAGATATTCTGCACTT
AAAAACTA

CTATAACTTTCCTTATGTTGTTTGCTTAGTTTTTTGATATTAGTGTTGCTTATGTGAAATTTCGCGATTTC
AATTAAAATAATAAATACATATATAAAGAATATACACAGAGGGAAGCAAAAGTAAACTAAAAGTGATACTT
ACACGAGC

TGGAAATCATCGCGCGCAAGCCAGCGGTAAAGGGAAAAGAACGGAGGACGATTACATACAAGATGAACGAA
TAAATAAATTAATAATAAATAATAATAAAAAGTACAGTAGCATTAAATATTATTAAGTTTAATGATTAAAA
ATTGGTTA

ATAGATGCTCTATACTCAAACGGTGGAAGAAAAACAGCGAAAAAAAATAACCGATACCCCCTTTTCGAATA
CAAATGCTTGTATATTCAATTATGAATTATTTTTTTTTTTTTTCATTTCTTATATTATTTTTTGTTCGAGA
ATCACTTT

TGGATTACCTCTTTTACTCGCCGAGCGAACGTGCACCAAAAAGGGAAAGGAAAAAAAGAAAAAAAAAGGAA
AAAGGAAACTCAAAACTTGGATAAATAGAAGCACTCAAACTAAATTAAACTGCCAAAAAAAAAAAAAAATAA
AAAGGGAA

AGACTGTCATAGGGAAGAGCCCTTTCTACATACTACTACATAATATATATATATAGTATAGAAATTGGTAT
ATCACTACTTGTACAAATATCATATTGTACGATAATCGCGAAGAACGACGCACTGGTGGGAAGAAGTGGAA
AACAGAAG

CACTTGCCAAAGTAATTAACGCCCATTAAAAAGAAGGCATAGGAGGCATATACATATATATATATATATAT
ATATATGGCTGCTGACAGATATTCTGCACTTAAAAACTAAAAATATTATACCAACTTTTCTTTTTCTTCCC
GTTCAGTT

AAAAAAAAAAAAAAAAAAATAGCCGCCATGACCCCGGATCGTCGGTTGTGATACGGTCAGGGTAGCGCCCT
GGTCAAACTTCAGAACTAAAAAAATAATAAGGAAGAAAAAAATAGCTAATTTTTCCGGCAGAAAGATTTTC
GCTACCCG

TGTCTACATTAGTTACCTTTTATTACATGAGAAAGTTATTTTTCTTCTTTTTTTTTTTTTTTTTTGAAAC
TTTTTCCTCTCGGAAAATAAAAGATATATTTACAAGTGAAAGCTTATTGTAATGTGTCATTTTAAACATCA
AATAACAG

AGTGAAACTCTTTCTCTATTTTTTTTTTTAATTGAAAAATTTCCTTTCTCTATAGCGTATAGAATATATGTT
ACATGTATATATATATATAAAGTAAAAACGTTCGGAAAATTCCTCATTATACCCAGATCATTAAAAGACAT
TTTCGTTA

ATAATCAAGAAGTGAAACTCTTTCTCTATTTTTTTTTTTAATTGAAAAATTTCCTTTCTCTATAGCGTATAG
AATATATGTTACATGTATATATATATATAAAGTAAAAACGTTCGGAAAATTCCTCATTATACCCAGATCAT
TAAAAGAC

TATTACATGAGAAAGTTATTTTTCTTCTTTTTTTTTTTTTTTTTTGAAACTTTTTCCTCTCGGAAAATAA
AAGATATATTTACAAGTGAAAGCTTATTGTAATGTGTCATTTTAAACATCAAATAACAGACCTTTACATCA
AATAAGCA

CACGAGCTACAACAAAATACTAAGGGAATAGGCCGTTATTTCCGTAAAGGATGGTTTAATAATAAGAAATT
TATAATATTAATAATACATATATACAAAAATTTATATTTATATACATGCGCCTAACTATTCATACTATTAA
TTTCATAT

TCATCGTCATATGCTATACCTTCAAGCATCTAGTATCGCATAAATAAAAAATAGTATTTGTATATCAAAAA
ATGATCCTGTGATTTTTTCATATGTAACGTATAAATGTAAAAATGTGCTTCTTCTGGTATTTTTAATCAAG
TGGAAAGA

AAAGATTTTTTTTTTTTTAATTTTTTTTTTTTTAATTTTTTTTTTTTTCATAGACTTTTATTTAAATAAATCAC
GTCTATATATGTATCAGTATATAACGTAAAAAAAAAAAACACCGTCAGTTAAACAAAACATAAATAAAAAAA
AAAAGAAG

AGCGCAATCATTGAATAGTCAAAGATTTTTTTTTTTTTAATTTTTTTTTTTTTAATTTTTTTTTTTTTCATAGA
CTTTTATTTAAATAAATCACGTCTATATATGTATCAGTATATAACGTAAAAAAAAAAAACACCGTCAGTTAA
ACAAAACA

AACGAAAAAAAAAAAAAAAAAAACACAGAAAAGAATGCAGAAAGATGTCAACTGAAAAAAAAAAAAGGTGAACA
CAGGAAAAAAAAATAAAAAAAAAAAAAAAAAAAGGAGGACGAAACAAAAAAGTGAAAAAAAATGAAAATTTT
TTTGGAAA

CAATAGGGCGAAAAAACAGGCAACGAACGAACAATGGAAAAACGAAAAAAAAAAAAAAAAAACACAGAAAAG
AATGCAGAAAGATGTCAACTGAAAAAAAAAAAAGGTGAACACAGGAAAAAAAAATAAAAAAAAAAAAAAAAA
AGGAGGAC

TTGAATAGTCAAAGATTTTTTTTTTTTTAATTTTTTTTTTTTTAATTTTTTTTTTTTTCATAGACTTTTATTTA
AATAAATCACGTCTATATATGTATCAGTATATAACGTAAAAAAAAAAAACACCGTCAGTTAAACAAAACATA
AATAAAAA

AATACGCTTAAGCGCAATCATTGAATAGTCAAAGATTTTTTTTTTTTTAATTTTTTTTTTTTTAATTTTTTTTT
TTTTCATAGACTTTTATTTAAATAAATCACGTCTATATATGTATCAGTATATAACGTAAAAAAAAAAAACAC
CGTCAGTT

ACAATGGAAAAACGAAAAAAAAAAAAAAAAAACACAGAAAAGAATGCAGAAAGATGTCAACTGAAAAAAAAA
AAGGTGAACACAGGAAAAAAAAATAAAAAAAAAAAAAAAAAAAGGAGGACGAAACAAAAAAGTGAAAAAAAA
TGAAAATT

AAAAAAAAAAAACACAGAAAAGAATGCAGAAAGATGTCAACTGAAAAAAAAAAAAGGTGAACACAGGAAAAAA
AATAAAAAAAAAAAAAAAAAAAGGAGGACGAAACAAAAAAGTGAAAAAAAATGAAAATTTTTTTGGAAAAC
CAAGAAAT

CAACGAACGAACAATGGAAAAACGAAAAAAAAAAAAAAAAAACACAGAAAAGAATGCAGAAAGATGTCAACT
GAAAAAAAAAAAAGGTGAACACAGGAAAAAAAAATAAAAAAAAAAAAAAAAAAAGGAGGACGAAACAAAAAAG
TGAAAAAA

AATGTTAACGAATACGCTTAAGCGCAATCATTGAATAGTCAAAGATTTTTTTTTTTTTAATTTTTTTTTTTTT
AATTTTTTTTTTTTCATAGACTTTTATTTAAATAAATCACGTCTATATATGTATCAGTATATAACGTAAAA
AAAAAAAC

TTTTTTTAATTTTTTTTTTTTTAATTTTTTTTTTTTTCATAGACTTTTATTTAAATAAATCACGTCTATATAT
GTATCAGTATATAACGTAAAAAAAAAAAACACCGTCAGTTAAACAAAACATAAATAAAAAAAAAAAGAAGTG
TTCAAATC

GGCTCGAGAACAATAGGGCGAAAAAACAGGCAACGAACGAACAATGGAAAAACGAAAAAAAAAAAAAAAAA
CACAGAAAAGAATGCAGAAAGATGTCAACTGAAAAAAAAAAAAGGTGAACACAGGAAAAAAAAATAAAAAAAA
AAAAAAAA

AAAAAACAGGCAACGAACGAACAATGGAAAAACGAAAAAAAAAAAAAAAAAACACAGAAAAGAATGCAGAAA
GATGTCAACTGAAAAAAAAAAAAGGTGAACACAGGAAAAAAAAATAAAAAAAAAAAAAAAAAAAGGAGGACGA
AACAAAAA

ACTTTTTTTTATATTGAATCTAATATATTATCAAACGGAAACTTCGGCTGAATTTCATACGTATATTGATT
AAAGTGGAAAGGGCATCGGAAAAGTAAGAAAAGCTTAAAAAAATTGAAAAAAAAAAAAAAAAAAATAAAAA
ATAAAAAA

ATATTGAATCTAATATATTATCAAACGGAAACTTCGGCTGAATTTCATACGTATATTGATTAAAGTGGAAA
GGGCATCGGAAAAGTAAGAAAAGCTTAAAAAAATTGAAAAAAAAAAAAAAAAAAATAAAAAATAAAAAATA
AAAAAACC

GTATATCGTTAATGTTAACGAATACGCTTAAGCGCAATCATTGAATAGTCAAAGATTTTTTTTTTTTAATT
TTTTTTTTTTAATTTTTTTTTTTTCATAGACTTTTATTTAAATAAATCACGTCTATATATGTATCAGTATA
TAACGTAA

TTTTTTTTTTTAATTTTTTTTTTTTCATAGACTTTTATTTAAATAAATCACGTCTATATATGTATCAGTAT
ATAACGTAAAAAAAAAAACACCGTCAGTTAAACAAAACATAAATAAAAAAAAAAAGAAGTGTTCAAATCAA
AGTGTCAA

ACACAGAAAAGAATGCAGAAAGATGTCAACTGAAAAAAAAAAAGGTGAACACAGGAAAAAAAATAAAAAAA
AAAAAAAAAAAAGGAGGACGAAACAAAAAAGTGAAAAAAAATGAAAATTTTTTTGGAAAACCAAGAAATGA
ATTATATT

AAAATTTTTAGGCTCGAGAACAATAGGGCGAAAAAACAGGCAACGAACGAACAATGGAAAAACGAAAAAAA
AAAAAAAAAACACAGAAAAGAATGCAGAAAGATGTCAACTGAAAAAAAAAAAGGTGAACACAGGAAAAAAA
ATAAAAAA

TAATATATTATCAAACGGAAACTTCGGCTGAATTTCATACGTATATTGATTAAAGTGGAAAGGGCATCGGA
AAAGTAAGAAAAGCTTAAAAAAATTGAAAAAAAAAAAAAAAAAAATAAAAAATAAAAAATAAAAAAACCTA
TACAATAC

AATCATATAAACTTTTTTTTATATTGAATCTAATATATTATCAAACGGAAACTTCGGCTGAATTTCATACG
TATATTGATTAAAGTGGAAAGGGCATCGGAAAAGTAAGAAAAGCTTAAAAAAATTGAAAAAAAAAAAAAAA
AAAATAAA

GCCATTGCCTTTGTTTACGAGTATATCGTTAATGTTAACGAATACGCTTAAGCGCAATCATTGAATAGTCA
AAGATTTTTTTTTTTTAATTTTTTTTTTTTAATTTTTTTTTTTTCATAGACTTTTATTTAAATAAATCACG
TCTATATA

TCAAACGGAAACTTCGGCTGAATTTCATACGTATATTGATTAAAGTGGAAAGGGCATCGGAAAAGTAAGAA
AAGCTTAAAAAAATTGAAAAAAAAAAAAAAAAAAATAAAAAATAAAAAATAAAAAAACCTATACAATACAT
ACATATGT

GTTGACGTCGAAAATACATCCGCGCAAATTTTGACAGGCTATTTTGGCAGCGCAATCAAAGAATAAACTTG
GTATAGTAAGGAAGCCAGTTACTGTACGCATGAATTACGGTAGCTTTTTTTATTTATTTATTTTTTTTTTT
TTTTGGAC

GTACATTTTTATTTTTCCCATTAAGTGAAATTTCCTTCAAATTATTCTCGAAGCTTTCATGCCATTGGTGA
CACGAAAAAAAAATCTTGTTGTATTTTATCTAACGCGTCGCGACGCGTTAGATAAATATAACAGTAAATGA
TGGTATAA

TTTTACTTCTCTGCATTTTTTTCGCGTCGCGTCGCAAATTGTGATGGTTAAAAGAAAAATGAAAAAATATT
TTTCGCGTCGTTATCACCCGGAGGAATAAAAAAAAGAAACATACACATCACGTGACCACAAGAATGTTAGA
AAGGCTTA

GCTTAGTGACGCGTTTTCGCGCGTCAGTTTCAAGTTTTTCTTGGCTTTTTTTTTTTCATTTTCGTAAAGGG
TCTTAAAAGGATTAAAAAATGCAGTATTGAAATAAAGAACAATTACGAACGGTGAAGCTGCTATTTTGGTT
ATTATACC

TTTTCCTTATGCTTAGTGACGCGTTTTCGCGCGTCAGTTTCAAGTTTTTCTTGGCTTTTTTTTTTTCATTT
TCGTAAAGGGTCTTAAAAGGATTAAAAAATGCAGTATTGAAATAAAGAACAATTACGAACGGTGAAGCTGC
TATTTTGG

AAAGAGTAATCTCGATAAGGGTACATTTTTATTTTTCCCATTAAGTGAAATTTCCTTCAAATTATTCTCGA
AGCTTTCATGCCATTGGTGACACGAAAAAAAAATCTTGTTGTATTTTATCTAACGCGTCGCGACGCGTTAG
ATAAATAT

CTTTTTTTTTTACAGTTCCACTTGCATCCCTCATTTTTCACATTCCTTTTTTTATAGAAAAGCATATCACT
ATATAAAAATTCTATTATAGTTGTAACAGCATAGCATATTATAGACTTTTTTTTTCTGGAAACGGTTAAAC
CGTTTGGC

ATGTCCCAGGTCATTTATCTGCCATTGCCTTTGTTTACGAGTATATCGTTAATGTTAACGAATACGCTTAA
GCGCAATCATTGAATAGTCAAAGATTTTTTTTTTTTAATTTTTTTTTTTTAATTTTTTTTTTTTCATAGAC
TTTTATTT

AAAATACATCCGCGCAAATTTTGACAGGCTATTTTGGCAGCGCAATCAAAGAATAAACTTGGTATAGTAAG
GAAGCCAGTTACTGTACGCATGAATTACGGTAGCTTTTTTTATTTTATTTATTTTTTTTTTTTTTGGACAA
ATAGAAAA

TCCAGCATTCTTTTACTTCTCTGCATTTTTTTCGCGTCGCGTCGCAAATTGTGATGGTTAAAAGAAAAATG
AAAAAATATTTTTCGCGTCGTTATCACCCGGAGGAATAAAAAAAAGAAACATACACATCACGTGACCACAA
GAATGTTA

AGATGTCAACTGAAAAAAAAAAAGGTGAACACAGGAAAAAAAATAAAAAAAAAAAAAAAAAAAGGAGGACG
AAACAAAAAAAGTGAAAAAAAAATGAAAATTTTTTGGAAAACCAAGAAATGAATTATATTTCCGTGTGAGAC
GACATCGT

TTGTTTACGAGTATATCGTTAATGTTAACGAATACGCTTAAGCGCAATCATTGAATAGTCAAAGATTTTTT
TTTTTTAATTTTTTTTTTTTAATTTTTTTTTTTTTCATAGACTTTATTTAAATAAATCACGTCTATATATG
TATCAGTA

TGTTAAAAATGGTGACTGTATCTACGTATCTATAAAAAAAGGTTAACTACCGGAAAATCATTTCTTCTCGT
AAAGTGTATATTTAGGTTCGGAAATCATAAACATTGTTTTTTTGTGTTTTTTATTTTTAAATAAAAAGAAT
AAATATTT

AATGGCACTGTATTGATGCATTTTCCTTATGCTTAGTGACGCGTTTTCGCGCGTCAGTTTCAAGTTTTTCT
TGGCTTTTTTTTTTTCATTTTCGTAAAGGGTCTTAAAAGGATTAAAAAATGCAGTATTGAAATAAAGAACA
ATTACGAA

AAAATTCATGATCGACCGCGCAAAATAAATAGATTTGCAAATAAGTTTTGTATGTACATTTATTAATATAT
ATAATATATCAAAAGAAAAAAATCAAAAAAAAAAAAAAAAAAAAATTGCACTCTTATTCAGTCATCAATTA
CAAAACCT

CAGAAAATGATGACAGTCTAGCTCCTATAGACGAAAAGAAGGTCCCAATGCTAAAGGACACCAAGAATAAA
ATCGAAAATGAGCATGTAATATGGGAAAAAGTGTACGAATCAAAGACAAAATAATATAGTTGCAATAATAG
TAATAATA

ACTTCGGCTGAATTTCATACGTATATTGATTAAAGTGGAAAGGGCATCGGAAAAGTAAGAAAAGCTTAAAA
AAATTGAAAAAAAAAAAAAAAAAAATAAAAAAATAAAAAATAAAAAACCTATACAATACATACATATGTAT
ATGAATAT

TAATGGCACCGTTGACGTCGAAAATACATCCGCGCAAATTTTGACAGGCTATTTTGGCAGCGCAATCAAAG
AATAAACTTGGTATAGTAAGGAAGCCAGTTACTGTACGCATGAATTACGGTAGCTTTTTTTATTTTATTTA
TTTTTTTT

ACTGTGTTGTTGATTTCTTATCCAGCATTCTTTTACTTCTCTGCATTTTTTTCGCGTCGCGTCGCAAATTG
TGATGGTTAAAAGAAAAATGAAAAAATATTTTTCGCGTCGTTATCACCCGGAGGAATAAAAAAAAGAAACA
TACACATC

TCATTTATCTGCCATTGCCTTTGTTTACGAGTATATCGTTAATGTTAACGAATACGCTTAAGCGCAATCAT
TGAATAGTCAAAGATTTTTTTTTTTTTAATTTTTTTTTTTTTAATTTTTTTTTTTTTCATAGACTTTTATTTAA
ATAAATCA

CCAATGTTCAACTGTGTTGTTGATTTCTTATCCAGCATTCTTTTACTTCTCTGCATTTTTTTCGCGTCGCG
TCGCAAATTGTGATGGTTAAAAGAAAAATGAAAAAATATTTTTCGCGTCGTTATCACCCGGAGGAATAAAA
AAAAGAAA

TATAAAAAAAGGTTAACTACCGGAAAATCATTTCTTCTCGTAAAGTGTATATTTAGGTTCGGAAATCATAA
ACATTGTTTTTTTGTGTTTTTTATTTTTAAATAAAAAGAATAAATATTTTATATTAAAAAATAAATTTTAA
AGTAAATT

GCCATTGGTGACACGAAAAAAAAAATCTTGTTGTATTTTATCTAACGCGTCGCGACGCGTTAGATAAATATA
ACAGTAAATGATGGTATAAATGCCACCGTCGATATTTAAATGAGATTGCTTATATATCGCATTATTACCTG
CGAAAATC

GAATGCAGAAAGATGTCAACTGAAAAAAAAAAAAGGTGAACACAGGAAAAAAAATAAAAAAAAAAAAAAAA
AAGGAGGACGAAACAAAAAAGTGAAAAAAAATGAAAATTTTTTTGGAAAACCAAGAAATGAATTATATTTC
CGTGTGAG

TGAATAAATACAGCACTTTCTCGCATCTACCTCAACTGAAAAAAAAAAAAAAAAAATAAAAAACGCCCCTTT
AAGACGAAGTGACAAAGAGGCAATAGTTCAAAAGAAAAAAAAAAAAATATAAAGACTGTTGATAGTTGAATT
TTTATAAA

CGCGCAAATTTTGACAGGCTATTTTGGCAGCGCAATCAAAGAATAAACTTGGTATAGTAAGGAAGCCAGTT
ACTGTACGCATGAATTACGGTAGCTTTTTTTATTTTATTTATTTTTTTTTTTTTTGGACAAATAGAAAAAT
TTTTATTA

AGCATCCACAACAACTAAACTGGTATTTTGTTATACACAACGCGTCTTGGTTAGGCATCTGTTTTGTTTTA
GCTATTGTTCATCGCGTTTTTTTTTTTTCTTATCTCTTTTACCGAGGGGCGAAAATAGCGATAGATCGAGAA
TATAAATA

ATATACTAGTGAGACCTTTTCCCCCGTTCCCAAAAAGAAAAAAAAAAAAAAAAAAAAGAAAACGAGCCTTCA
TCACGTGCATTAAAGAAATAGTAATCCCAACGCACTCATCCGCTATATACATAGCGAAAAACTATATGCCC
ACTTCACA

CAATTCGCGAAAATGAGCGAAAATTTTTCAAGCCCACCGCTAAAAAAAAAAAAAAAAAAGAAAAAAAGGGAC
ATAAATACGTATATCTCCAGAAAATGCCCAGTGGAGTGGATGAATACCTGATGGGCTATATTACTACATTA
TTGAAAAC

GCTCCTATAGACGAAAAGAAGGTCCCAATGCTAAAGGACACCAAGAATAAAATCGAAAATGAGCATGTAAT
ATGGGAAAAAGTGTACGAATCAAAGACAAAATAATATAGTTGCAATAATAGTAATAATAATAATAATAATA
ATAATAAT

ACAACTAAACTGGTATTTTGTTATACACAACGCGTCTTGGTTAGGCATCTGTTTTGTTTTAGCTATTGTTC
ATCGCGTTTTTTTTTTTCTTATCTCTTTTACCGAGGGGCGAAAATAGCGATAGATCGAGAATATAAATAAA
TAAAATAT

TATGTACATTTATTAATATATATAATATATCAAAAGAAAAAAATCAAAAAAAAAAAAAAAAAAAAAATTGCA
CTCTTATTCAGTCATCAATTACAAAACCTAGAGATAGCGATGGTGCATATTCAATAAAAAACTCCTTATAC
TGTCGAGA

GCGTTTTCGCGCGTCAGTTTCAAGTTTTTCTTGGCTTTTTTTTTTTCATTTTCGTAAAGGGTCTTAAAAGG
ATTAAAAAAATGCAGTATTGAAATAAAGAACAATTACGAACGGTGAAGCTGCTATTTTGGTTATTATACCCT
TCCAGGAC

AGATATAATTTTTAAAAAAAAAATTAGAAAAAGCAAATATAATTCAGGTCCCACTTGGAATAATGGCACTGT
ATTGATGCATTTTCCTTATGCTTAGTGACGCGTTTTCGCGCGTCAGTTTCAAGTTTTTCTTGGCTTTTTTT
TTTTCATT

CAAGTCTTTAAGAGCTACATTAAATTGAAATTGTCGAGTGTCGCCAACAACAATTCGCGAAAATGAGCGAA
AATTTTTCAAGCCCACCGCTAAAAAAAAAAAAAAAAAAGAAAAAAAGGGACATAAATACGTATATCTCCAGA
AAATGCCC

CAGCACTTTCTCGCATCTACCTCAACTGAAAAAAAAAAAAAAAAAATAAAAAACGCCCCTTTAAGACGAAGT
GACAAAGAGGCAATAGTTCAAAAGAAAAAAAAAAAAATATAAAGACTGTTGATAGTTGAATTTTTATAAACG
TTATGTTA

ATTTTTCCCATTAAGTGAAATTTCCTTCAAATTATTCTCGAAGCTTTCATGCCATTGGTGACACGAAAAAA
AAATCTTGTTGTATTTTATCTAACGCGTCGCGACGCGTTAGATAAATATAACAGTAAATGATGGTATAAAT
GCCACCGT

ATCGCGCGTCCTCAATATTTGAGTTTTTCAAATAGTGAGGTGTGGATGTATAGAGGAATTACACACTTTTA
AGTATGTGATGTATGGGCGCACAGTACCAATTTAACTTTTTTTTTTTTTCATTTTTTAGCTTGATTTTCAA
AAAACTTA

CTCTTTACTGAGGTGGCCCTTGAATAAATACAGCACTTTCTCGCATCTACCTCAACTGAAAAAAAAAAAAA
AAAATAAAAAACGCCCCTTTAAGACGAAGTGACAAAGAGGCAATAGTTCAAAAGAAAAAAAAAAAAATATAA
AGACTGTT

AATTTCATACGTATATTGATTAAAGTGGAAAGGGCATCGGAAAAGTAAGAAAAGCTTAAAAAAATTGAAAA
AAAAAAAAAAAAAAATAAAAAATAAAAAATAAAAAAACCTATACAATACATACATATGTATATGAATATAA
ACCTGGAA

TCTACGTATCTATAAAAAAAGGTTAACTACCGGAAAATCATTTCTTCTCGTAAAGTGTATATTTAGGTTCG
GAAATCATAAACATTGTTTTTTTGTGTTTTTTATTTTTAAATAAAAAGAATAAATATTTTATATTAAAAAA
TAAATTTT

TCTTTCCTTTATATACTAGTGAGACCTTTTCCCCCGTTCCCAAAAAGAAAAAAAAAAAAAAAAAAAAGAAAA
CGAGCCTTCATCACGTGCATTAAAGAAATAGTAATCCCAACGCACTCATCCGCTATATACATAGCGAAAAA
CTATATGC

ATCGACCGCGCAAAATAAATAGATTTGCAAATAAGTTTTGTATGTACATTTATTAATATATATAATATATC
AAAAGAAAAAAAATCAAAAAAAAAAAAAAAAAAAAAATTGCACTCTTATTCAGTCATCAATTACAAAACCTAG
AGATAGCG

TCGCCAACAACAATTCGCGAAAATGAGCGAAAATTTTTCAAGCCCACCGCTAAAAAAAAAAAAAAAAAAGAA
AAAAAGGGACATAAATACGTATATCTCCAGAAAATGCCCAGTGGAGTGGATGAATACCTGATGGGCTATAT
TACTACAT

TATTGACATAATCTACGATGTGAGAGCTGTATGATATTGTGGAACTTTGTATATATTGAAAAAATTTTCCA
GTGGAATTTTACTCGCGATGACTCAAGAGCGTGAAAAAAAAAAAAAAAAAAAAAATTAATTGCTAAAAAATC
AAATATAT

ATTCAGGTCCCACTTGGAATAATGGCACTGTATTGATGCATTTTCCTTATGCTTAGTGACGCGTTTTCGCG
CGTCAGTTTCAAGTTTTTCTTGGCTTTTTTTTTTTCATTTTCGTAAAGGGTCTTAAAAGGATTAAAAAATG
CAGTATTG

GGAACTTTGTATATATTGAAAAAATTTTCCAGTGGAATTTTACTCGCGATGACTCAAGAGCGTGAAAAAAA
AAAAAAAAAAAAAATTAATTGCTAAAAAATCAAATATATGCTTCCAATCGGATTTGAACCGATGATCTCCA
CATTACTA

CATAAAATGCTCCCATCTCAAAAGTAGGGCAAAATTCATGATCGACCGCGCAAAATAAATAGATTTGCAAA
TAAGTTTTGTATGTACATTTATTAATATATATAATATATCAAAAGAAAAAAATCAAAAAAAAAAAAAAAAA
AAAATTGC

CTGCATTTTTTTCGCGTCGCGTCGCAAATTGTGATGGTTAAAAGAAAAATGAAAAAATATTTTTCGCGTCG
TTATCACCCGGAGGAATAAAAAAAAGAAACATACACATCACGTGACCACAAGAATGTTAGAAAGGCTTACC
AGTACTAG

AGGAAAGGAAAAAATTTTTAGGCTCGAGAACAATAGGGCGAAAAAACAGGCAACGAACGAACAATGGAAAA
ACGAAAAAAAAAAAAAAAAAACACAGAAAAGAATGCAGAAAGATGTCAACTGAAAAAAAAAAGGTGAACAC
AGGAAAAA

GCTTCAAACTTTTTGTCATTTGTCTTCCATCCTTCCCTTATTCTCAATATATGTAATACGTCGTATTTG
ATATATATATATATATATATATAATATAATTTAACGGTTGTTATCTGCTACATCTGTAAAATAAAAATAAA
AATGCTTG

AAATAGAATAAGCATCCACAACAACTAAACTGGTATTTTGTTATACACAACGCGTCTTGGTTAGGCATCTG
TTTGTTTTAGCTATTGTTCATCGCGTTTTTTTTTTTTCTTATCTCTTTTACCGAGGGGCGAAAATAGCGAT
AGATCGAG

TAATTTTTTTTTTTTCATAGACTTTTATTTAAATAAATCACGTCTATATATGTATCAGTATATAACGTAAA
AAAAAAAACACCGTCAGTTAAACAAAACATAAATAAAAAAAAAAAGAAGTGTTCAAATCAAAGTGTCAAAT
CAAAATTT

ACTGAGGGTAAAAGAGTAATCTCGATAAGGGTACATTTTTATTTTTCCCATTAAGTGAAATTTCCTTCAAA
TTATTCTCGAAGCTTTCATGCCATTGGTGACACGAAAAAAAAATCTTGTTGTATTTTATCTAACGCGTCGC
GACGCGTT

CAAAAAGAAAAAAAAAAAAAAAAAAAGAAAACGAGCCTTCATCACGTGCATTAAAGAAATAGTAATCCCAA
CGCACTCATCCGCTATATACATAGCGAAAAACTATATGCCCACTTCACAAATACGAGTTTGCATGTATAAT
ATATAAGA

TGATTTCTTATCCAGCATTCTTTTACTTCTCTGCATTTTTTTCGCGTCGCGTCGCAAATTGTGATGGTTAA
AAGAAAAATGAAAAAATATTTTTCGCGTCGTTATCACCCGGAGGAATAAAAAAAAGAAACATACACATCAC
GTGACCAC

TCGCATCTACCTCAACTGAAAAAAAAAAAAAAAAATAAAAAACGCCCCTTTAAGACGAAGTGACAAAGAGG
CAATAGTTCAAAAGAAAAAAAAAAAATATAAAGACTGTTGATAGTTGAATTTTTATAAACGTTATGTTAAG
TATATGTA

TTTATTTTTTCTTTTTTTTTTACAGTTCCACTTGCATCCCTCATTTTTCACATTCCTTTTTTTATAGAAAA
GCATATCACTATATAAAAATTCTATTATAGTTGTAACAGCATAGCATATTATAGACTTTTTTTTTCTGGAA
ACGGTTAA

CACTTGGAATAATGGCACTGTATTGATGCATTTTCCTTATGCTTAGTGACGCGTTTTCGCGCGTCAGTTTC
AAGTTTTTCTTGGCTTTTTTTTTTTCATTTTCGTAAAGGGTCTTAAAAGGATTAAAAAATGCAGTATTGAA
ATAAAGAA

GGTGACTGTATCTACGTATCTATAAAAAAAGGTTAACTACCGGAAAATCATTTCTTCTCGTAAAGTGTATA
TTTAGGTTCGGAAATCATAAACATTGTTTTTTTGTGTTTTTTATTTTTAAATAAAAAGAATAAATATTTTA
TATTAAAA

AAATGAGCGAAAATTTTTCAAGCCCACCGCTAAAAAAAAAAAAAAAAAGAAAAAAAGGGACATAAATACGT
ATATCTCCAGAAAATGCCCAGTGGAGTGGATGAATACCTGATGGGCTATATTACTACATTATTGAAAACGT
TTACCAAA

AAAAAATAGCCAAATGTCAAACTCAAATAGTGTTTGTTGTACGCTTATGTAATGATAGTAATAGAATCCAA
AAAAAAAAAAAAATATACATGCTTTTTCATATCCTCTCTCACCCTATCTTTTTTTTTCTTCTAATTTTGGCT
CCGTTCAT

CTCGATAAGGGTACATTTTTATTTTTCCCATTAAGTGAAATTTCCTTCAAATTATTCTCGAAGCTTTCATG
CCATTGGTGACACGAAAAAAAAAATCTTGTTGTATTTTATCTAACGCGTCGCGACGCGTTAGATAAATATAA
CAGTAAAT

TGAAAAAAAAAAAAGGTGAACACAGGAAAAAAAAATAAAAAAAAAAAAAAAAAAAAGGAGGACGAAACAAAAAA
GTGAAAAAAAAATGAAAATTTTTTTGGAAAACCAAGAAATGAATTATATTTCCGTGTGAGACGACATCGTCG
AATATGAT

TGGTATTTTGTTATACACAACGCGTCTTGGTTAGGCATCTGTTTTGTTTTAGCTATTGTTCATCGCGTTTT
TTTTTTTCTTATCTCTTTTACCGAGGGGCGAAAATAGCGATAGATCGAGAATATAAATAAATAAAATATTG
TTGTGTTG

GAACACGTGGATGTCCCAGGTCATTTATCTGCCATTGCCTTTGTTTACGAGTATATCGTTAATGTTAACGA
ATACGCTTAAGCGCAATCATTGAATAGTCAAAGATTTTTTTTTTTTAATTTTTTTTTTTTAATTTTTTTTT
TTTCATAG

ACTATAAATTAGTAAATATACTATCCGTTGCAAGTCTTTAAGAGCTACATTAAATTGAAATTGTCGAGTGT
CGCCAACAACAATTCGCGAAAATGAGCGAAAATTTTTCAAGCCCACCGCTAAAAAAAAAAAAAAAAAGAAA
AAAAGGGA

TTGTGTGCAATGTTAAAAATGGTGACTGTATCTACGTATCTATAAAAAAAGGTTAACTACCGGAAAATCAT
TTCTTCTCGTAAAGTGTATATTTAGGTTCGGAAATCATAAACATTGTTTTTTTGTGTTTTTTATTTTTAAA
TAAAAAGA

GGTTAACTACCGGAAAATCATTTCTTCTCGTAAAGTGTATATTTAGGTTCGGAAATCATAAACATTGTTTT
TTTGTGTTTTTTATTTTTAAATAAAAAGAATAAATATTTTATATTAAAAAATAAATTTTAAAGTAAATTAA
TTATTTAA

AAAATTTTCCAGTGGAATTTTACTCGCGATGACTCAAGAGCGTGAAAAAAAAAAAAAAAAAAAAAATTAATT
GCTAAAAAATCAAATATATGCTTCCAATCGGATTTGAACCGATGATCTCCACATTACTAGTGTGGCGCCTT
ACCAACTT

CCTTGTATTTATATAAACAAAGATATAATTTTTAAAAAAAAAATTAGAAAAAGCAAATATAATTCAGGTCCC
ACTTGGAATAATGGCACTGTATTGATGCATTTTCCTTATGCTTAGTGACGCGTTTTCGCGCGTCAGTTTCA
AGTTTTTC

GTCAAACAGGAAATATTGCCTATTTTCGTACAAGGTTACTTCCTAGATGCTATATGTCCCTTTACATAATA
AATTAAAAAATTTTTTTTATAAATTATAATAATTTCTTTTTATTTCTAATAGTATCTTGGGATTAAATAAA
TCACTTAC

TATTGATGCATTTTCCTTATGCTTAGTGACGCGTTTTCGCGCGTCAGTTTCAAGTTTTTCTTGGCTTTTTT
TTTTTCATTTTCGTAAAGGGTCTTAAAAGGATTAAAAAATGCAGTATTGAAATAAAGAACAATTACGAACG
GTGAAGCT

ATAAGTTTTGTATGTACATTTATTAATATATATAATATATCAAAAGAAAAAAATCAAAAAAAAAAAAAAAA
AAAAATTGCACTCTTATTCAGTCATCAATTACAAAACCTAGAGATAGCGATGGTGCATATTCAATAAAAAA
CTCCTTAT

ATTATTCTCGAAGCTTTCATGCCATTGGTGACACGAAAAAAAAATCTTGTTGTATTTTATCTAACGCGTCG
CGACGCGTTAGATAAATATAACAGTAAATGATGGTATAAATGCCACCGTCGATATTTAAATGAGATTGCTT
ATATATCG

TGACAGTCTAGCTCCTATAGACGAAAAGAAGGTCCCAATGCTAAAGGACACCAAGAATAAAATCGAAAATG
AGCATGTAATATGGGAAAAAGTGTACGAATCAAAGACAAAATAATATAGTTGCAATAATAGTAATAATAAT
AATAATAA

ACACGAAAAAAAAATCTTGTTGTATTTTATCTAACGCGTCGCGACGCGTTAGATAAATATAACAGTAAATG
ATGGTATAAATGCCACCGTCGATATTTAAATGAGATTGCTTATATATCGCATTATTACCTGCGAAAATCTA
ATTTACGA

ATGCAAAATTCGTGGCTCAGTGCTCTCAAGACAATGTTGCAACCCTTTGCGTCAATTATATATATATATAT
ATATATATATATATCCCGTCCGCTTTCTTTTTTTTGTCAGTTGGGTCGCAACGCAGGGTCTCGAGACCTGA
AAAAAGCT

ATATATTGAAAAAATTTTCCAGTGGAATTTTACTCGCGATGACTCAAGAGCGTGAAAAAAAAAAAAAAAAA
AAAATTAATTGCTAAAAAATCAAATATATGCTTCCAATCGGATTTGAACCGATGATCTCCACATTACTAGT
GTGGCGCC

TGGCCATCCTAAATAGAATAAGCATCCACAACAACTAAACTGGTATTTTGTTATACACAACGCGTCTTGGT
TAGGCATCTGTTTTGTTTTAGCTATTGTTCATCGCGTTTTTTTTTTTCTTATCTCTTTTACCGAGGGGCGA
AAATAGCG

TATTTTTTTTTTATTTTTTTGTAGGTTCTTTGGCCATCCTAAATAGAATAAGCATCCACAACAACTAAACT
GGTATTTTGTTATACACAACGCGTCTTGGTTAGGCATCTGTTTTGTTTTAGCTATTGTTCATCGCGTTTTT
TTTTTTCT

GTAGGTTCTTTGGCCATCCTAAATAGAATAAGCATCCACAACAACTAAACTGGTATTTTGTTATACACAAC
GCGTCTTGGTTAGGCATCTGTTTTGTTTTAGCTATTGTTCATCGCGTTTTTTTTTTTCTTATCTCTTTTAC
CGAGGGGC

AAAGTAGGGCAAAATTCATGATCGACCGCGCAAAATAAATAGATTTGCAAATAAGTTTTGTATGTACATTT
ATTAATATATATAATATATCAAAAGAAAAAAATCAAAAAAAAAAAAAAAAAAAAAATTGCACTCTTATTCAG
TCATCAAT

AGGTGGCCCTTGAATAAATACAGCACTTTCTCGCATCTACCTCAACTGAAAAAAAAAAAAAAAAATAAAAA
ACGCCCCTTTAAGACGAAGTGACAAAGAGGCAATAGTTCAAAAGAAAAAAAAAAAATATAAAGACTGTTGA
TAGTTGAA

CTGGGCGCGACATTCCAAAAAAAAAATAAAAAAGAAGAGAAAATACTGGCTTATGAATACAAAAATTACAT
AAACGAAGCAAAATGATTATTTCGAATAAAACAGTCTATATATAAGTCAAATACAAGATAGGAAGAAAAAC
AGAAAAAA

TCGAGGGGTTTTTTATTCGAGATAGTAACTTCTGACTTTTCGCTTTTATACAGCACAGCAGAAAAAAAAAG
CCGCCGAGGCGCGCGCGTTCATGCAATGGCTCAGTAACCTCGTGATAGAAAAAGGGCAACAATATTGGGCT
ATTTTAGG

AGAGCTACATTAAATTGAAATTGTCGAGTGTCGCCAACAACAATTCGCGAAAATGAGCGAAAATTTTTCAA
GCCCACCGCTAAAAAAAAAAAAAAAAAGAAAAAAAGGGACATAAATACGTATATCTCCAGAAAATGCCCAG
TGGAGTGG

TTGTCGAGTGTCGCCAACAACAATTCGCGAAAATGAGCGAAAATTTTTCAAGCCCACCGCTAAAAAAAAAA
AAAAAAAGAAAAAAAGGGACATAAATACGTATATCTCCAGAAAATGCCCAGTGGAGTGGATGAATACCTGA
TGGGCTAT

CCATACCATCTATTGACATAATCTACGATGTGAGAGCTGTATGATATTGTGGAACTTTGTATATATTGAA
AAATTTTCCAGTGGAATTTTACTCGCGATGACTCAAGAGCGTGAAAAAAAAAAAAAAAAAAAAATTAATTG
CTAAAAAA

ACGAAAAGAAGGTCCCAATGCTAAAGGACACCAAGAATAAAATCGAAAATGAGCATGTAATATGGGAAAAA
GTGTACGAATCAAAGACAAAATAATATAGTTGCAATAATAGTAATAATAATAATAATAATAATAATAATAA
TAATAATA

TCCCATCTCAAAAGTAGGGCAAAATTCATGATCGACCGCGCAAAATAAATAGATTTGCAAATAAGTTTTGT
ATGTACATTTATTAATATATATATAATATATCAAAAGAAAAAAATCAAAAAAAAAAAAAAAAAAAAATTGCAC
TCTTATTC

CAAACAAGATCCTTGTATTTATATAAACAAAGATATAATTTTTAAAAAAAAATTAGAAAAAGCAAATATAA
TTCAGGTCCCACTTGGAATAATGGCACTGTATTGATGCATTTTCCTTATGCTTAGTGACGCGTTTTCGCGC
GTCAGTTT

TCTTTTGCTGTTACTATGTGTTTAAGAGAGAGCTGTTCTACAAAGAACTTTTCATTTACGCGTTTTTTCCT
TTTTTTTTTTTTCTCATCCTAAACGCGTAAATCTGTTCCCTTTTTCATGTAATGGGCGTACAACCGACGAT
GGAAAAAT

TTTTATTCGAGATAGTAACTTCTGACTTTTCGCTTTTATACAGCACAGCAGAAAAAAAAAAGCCGCCGAGGC
GCGCGCGTTCATGCAATGGCTCAGTAACCTCGTGATAGAAAAAGGGCAACAATATTGGGCTATTTTAGGCA
AAGAAACT

AAAATGCTCAAAAAAAATAGCCAAATGTCAAACTCAAATAGTGTTTGTTGTACGCTTATGTAATGATAGTAA
TAGAATCCAAAAAAAAAAAAAAATATACATGCTTTTTCATATCCTCTCTCACCCTATCTTTTTTTTTTCTTCT
AATTTTGG

AAAGGAAAGCAGGAAAGGAAAAAAATTTTTAGGCTCGAGAACAATAGGGCGAAAAAAACAGGCAACGAACGAA
CAATGGAAAAACGAAAAAAAAAAAAAAAAAACACAGAAAAGAATGCAGAAAGATGTCAACTGAAAAAAAAAA
AGGTGAAC

CGTGGCTCAGTGCTCTCAAGACAATGTTGCAACCCTTTGCGTCAATTATATATATATATATATATATATATAT
ATATCCCGTCCGCTTTCTTTTTTTTGTCAGTTGGGTCGCAACGCAGGGTCTCGAGACCTGAAAAAAGCTTC
ATAGCGAT

TTTAAAAAAAAATTAGAAAAAGCAAATATAATTCAGGTCCCACTTGGAATAATGGCACTGTATTGATGCAT
TTTCCTTATGCTTAGTGACGCGTTTTCGCGCGTCAGTTTCAAGTTTTTCTTGGCTTTTTTTTTTTCATTTT
CGTAAAGG

AGGATGATGGGAAAAAAAAAGATAATTTTTTTTTTTTTGTTTTTCCCTGCTTCCTTCTTGTTTATTGGTATTA
TTATGTTACGATATTCATTCATTATCCTATTGATATTTTCTTTATATTCACTAAAAAAAAAATTTATTCTAT
AAGACTGA

AAATATTGCCTATTTTCGTACAAGGTTACTTCCTAGATGCTATATGTCCCTTTACATAATAAATTAAAAAA
TTTTTTTTATAAATTATAATAATTTCTTTTTATTTCTAATAGTATCTTGGGATTAAATAAATCACTTACAA
TATTTATT

CCTTCCCTTATTCTCAATATATATGTAATACGTCGTATTTGATATATATATATATATATATATATAATATAAT
TTAACGGTTGTTATCTGCTACATCTGTAAAATAAAAATAAAAATGCTTGAAGCGGCTGTACTTAAAATAAA
CTCATCTA

TAGAACAATAAGGATGATGGGAAAAAAAAAGATAATTTTTTTTTTTTTGTTTTTCCCTGCTTCCTTCTTGTTT
ATTGGTATTATTATGTTACGATATTCATTCATTATCCTATTGATATTTTCTTTATATTCACTAAAAAAAAAA
TTTATTCT

GAGACCTTTTCCCCCGTTCCCAAAAAGAAAAAAAAAAAAAAAAAAAGAAAACGAGCCTTCATCACGTGCAT
TAAAGAAATAGTAATCCCAACGCACTCATCCGCTATATACATAGCGAAAAACTATATGCCCACTTCACAAA
TACGAGTT

ATATACACTGAATCATATAAACTTTTTTTTATATTGAATCTAATATATTATCAAACGGAAACTTCGGCTGA
ATTTCATACGTATATTGATTAAAGTGGAAAGGGCATCGGAAAAGTAAGAAAAGCTTAAAAAAAATTGAAAAA
AAAAAAAA

AGATGGCAGTTCGAGGGGTTTTTATTCGAGATAGTAACTTCTGACTTTTCGCTTTTATACAGCACAGCAG
AAAAAAAAAGCCGCCGAGGCGCGCGCGTTCATGCAATGGCTCAGTAACCTCGTGATAGAAAAAGGGCAACA
ATATTGGG

TATTCATATGTATCAAAGATACCTATGGGAACTGCTAAAAGCAACTATTTTGATCCCGTAGAACACGATTT
ATTTCATTAAATTTTTTTTTTTTTTTTTTTTTTTCACTTTCTTGCGATGAGATGCACATAGTAAAAGAAAA
GTATACCA

ACAGGAAAAAAAATAAAAAAAAAAAAAAAAAAAAAGGAGGACGAAACAAAAAAGTGAAAAAAAATGAAAATTT
TTTTGGAAAACCAAGAAATGAATTATATTTCCGTGTGAGACGACATCGTCGAATATGATTCAGGGTAACAG
TATTGATG

GACAGAAAAATAATGGCACCGTTGACGTCGAAAATACATCCGCGCAAATTTTGACAGGCTATTTTGGCAGC
GCAATCAAAGAATAAACTTGGTATAGTAAGGAAGCCAGTTACTGTACGCATGAATTACGGTAGCTTTTTTT
ATTTTATT

ACATATTTATGCTTCAAACTTTTTGTCATTTGTCTTCCATCCTTCCCTTATTCTCAATATATATGTAATAC
GTCGTATTTGATATATATATATATATATATATAATATAATTTAACGGTTGTTATCTGCTACATCTGTAAAA
TAAAAATA

TATTTTCGTACAAGGTTACTTCCTAGATGCTATATGTCCCTTTACATAATAAATTAAAAAATTTTTTTTAT
AAATTATAATAATTTCTTTTTATTTCTAATAGTATCTTGGGATTAAATAAATCACTTACAATATTTATTTT
ATTATATT

AAATTTTTCAAGCCCACCGCTAAAAAAAAAAAAAAAAAAGAAAAAAAGGGACATAAATACGTATATCTCCAG
AAAATGCCCAGTGGAGTGGATGAATACCTGATGGGCTATATTACTACATTATTGAAAACGTTTACCAAAAG
TTTCGTTA

TATTAATATATATAATATATCAAAAGAAAAAAATCAAAAAAAAAAAAAAAAAAAAAAATTGCACTCTTATTCA
GTCATCAATTACAAAACCTAGAGATAGCGATGGTGCATATTCAATAAAAAACTCCTTATACTGTCGAGAAA
GCTTATTA

TTTCCTTCAAATTATTCTCGAAGCTTTCATGCCATTGGTGACACGAAAAAAAAAATCTTGTTGTATTTTATC
TAACGCGTCGCGACGCGTTAGATAAATATAACAGTAAATGATGGTATAAATGCCACCGTCGATATTTAAAT
GAGATTGC

CTCTTTTCTTCTCTTTACTGAGGTGGCCCTTGAATAAATACAGCACTTTCTCGCATCTACCTCAACTGAAA
AAAAAAAAAAAAAATAAAAAACGCCCCTTTAAGACGAAGTGACAAAGAGGCAATAGTTCAAAAGAAAAAAA
AAAAATAT

TAAAATCTGCGTCAAACAGGAAATATTGCCTATTTTCGTACAAGGTTACTTCCTAGATGCTATATGTCCCT
TTACATAATAAATTAAAAAATTTTTTTTATAAATTATAATAATTTCTTTTTATTTCTAATAGTATCTTGGG
ATTAAATA

ATATAAACAAAGATATAATTTTTAAAAAAAAAATTAGAAAAAGCAAATATAATTCAGGTCCCACTTGGAATA
ATGGCACTGTATTGATGCATTTTCCTTATGCTTAGTGACGCGTTTTCGCGCGTCAGTTTCAAGTTTTTCTT
GGCTTTTT

ACTGCTAAAAGCAACTATTTTGATCCCGTAGAACACGATTTATTTCATTAAATTTTTTTTTTTTTTTTTTT
TTTTCACTTTCTTGCGATGAGATGCACATAGTAAAAGAAAAGTATACCAGTGTAGTGCGTAAATTAATACC
TATATAAT

AAAAAAAAAAAAAAAAGAAAACGAGCCTTCATCACGTGCATTAAAGAAATAGTAATCCCAACGCACTCATC
CGCTATATACATAGCGAAAAACTATATGCCCACTTCACAAATACGAGTTTGCATGTATAATATATAAGATT
TATAAATG

TTAAGTGAAATTTCCTTCAAATTATTCTCGAAGCTTTCATGCCATTGGTGACACGAAAAAAAAATCTTGTT
GTATTTTATCTAACGCGTCGCGACGCGTTAGATAAATATAACAGTAAATGATGGTATAAATGCCACCGTCG
ATATTTAA

ATCTACGATGTGAGAGCTGTATGATATTGTGGAACTTTGTATATATTGAAAAAATTTTCCAGTGGAATTTT
ACTCGCGATGACTCAAGAGCGTGAAAAAAAAAAAAAAAAAAAAATTAATTGCTAAAAAATCAAATATATGC
TTCCAATC

AAGCTTTCATGCCATTGGTGACACGAAAAAAAAATCTTGTTGTATTTTATCTAACGCGTCGCGACGCGTTA
GATAAATATAACAGTAAATGATGGTATAAATGCCACCGTCGATATTTAAATGAGATTGCTTATATATCGCA
TTATTACC

ATGATATTGTGGAACTTTGTATATATTGAAAAAATTTTCCAGTGGAATTTTACTCGCGATGACTCAAGAGC
GTGAAAAAAAAAAAAAAAAAAAAATTAATTGCTAAAAAATCAAATATATGCTTCCAATCGGATTTGAACCG
ATGATCTC

TATCAAAGATACCTATGGGAACTGCTAAAAGCAACTATTTTGATCCCGTAGAACACGATTTATTTCATTAA
ATTTTTTTTTTTTTTTTTTTTTTTCACTTTCTTGCGATGAGATGCACATAGTAAAAGAAAAGTATACCAGT
GTAGTGCG

AAATGTAAAAACTATAAATTAGTAAATATACTATCCGTTGCAAGTCTTTAAGAGCTACATTAAATTGAAAT
TGTCGAGTGTCGCCAACAACAATTCGCGAAAATGAGCGAAAATTTTTCAAGCCCACCGCTAAAAAAAAAAA
AAAAAAGA

CCGCATATGCATTGTGTAGATCCAAAAGTAAGGACAAGATATCATGGGATGAAGAAGAACAGGCGCGATTA
ATGGGCGTTGTAAAATTTAATTCAGAGCATTACAGGGACTAGGAAATAATACTAATTAAATAATTCTAATA
ATTCTAAT

CGGAAAATCATTTCTTCTCGTAAAGTGTATATTTAGGTTCGGAAATCATAAACATTGTTTTTTTGTGTTTT
TTATTTTTAAATAAAAAGAATAAATATTTTATATTAAAAAATAAATTTTAAAGTAAATTAATTATTTAACA
CGTGATTA

GATTTTAACCAAGAGCAATCCTATGGAACGTTCTGTCCCACTTCACTCAAGTTTTTGAAAGGGTAAAAATT
AAAAAAATTATAGAATTAACAGTAAAATGTGCTACGTTCCAAAAAAAAAAAATATGCACTATAGTATATTA
CTACATGT

TTTTGTCATTTGTCTTCCATCCTTCCCTTATTCTCAATATATATGTAATACGTCGTATTTGATATATATAT
ATATATATATATAATATAATTTAACGGTTGTTATCTGCTACATCTGTAAAATAAAAATAAAAATGCTTGAA
GCGGCTGT

TAAATTGAAATTGTCGAGTGTCGCCAACAACAATTCGCGAAAATGAGCGAAAATTTTTCAAGCCCACCGCT
AAAAAAAAAAAAAAAAAGAAAAAAAAGGACATAAATACGTATATCTCCAGAAAATGCCCAGTGGAGTGGAT
GAATACCT

GCCGCTGGTAATCGCGCGTCCTCAATATTTGAGTTTTTCAAATAGTGAGGTGTGGATGTATAGAGGAATTA
CACACTTTTAAGTATGTGATGTATGGGCGCACAGTACCAATTTAACTTTTTTTTTTTTTCATTTTTTAGCT
TGATTTTC

AGCTAATAGATTTATTTTTTCTTTTTTTTTTACAGTTCCACTTGCATCCCTCATTTTTCACATTCCTTTTT
TTATAGAAAAGCATATCACTATATAAAAATTCTATTATAGTTGTAACAGCATAGCATATTATAGACTTTTT
TTTTCTGG

AGGATATTGTCTTGGTGGCGTCTTTTGCTGTTACTATGTGTTTAAGAGAGAGCTGTTCTACAAAGAACTTT
TCATTTACGCGTTTTTTCCTTTTTTTTTTTTTCTCATCCTAAACGCGTAAATCTGTTCCCTTTTTCATGTA
ATGGGCGT

AAAGGTGAACACAGGAAAAAAAAATAAAAAAAAAAAAAAAAAAGGAGGACGAAACAAAAAAGTGAAAAAAA
ATGAAAATTTTTTTGGAAAACCAAGAAATGAATTATATTTCCGTGTGAGACGACATCGTCGAATATGATTC
AGGGTAAC

GACCATTTATTGGCTTAAAAGATTTTAACCAAGAGCAATCCTATGGAACGTTCTGTCCCACTTCACTCAAG
TTTTTGAAAGGGTAAAAATTAAAAAAATTATAGAATTAACAGTAAAATGTGCTACGTTCCAAAAAAAAAAA
ATATGCAC

TATATGTCCCTTTACATAATAAATTAAAAAATTTTTTTTATAAATTATAATAATTTCTTTTTATTTCTAAT
AGTATCTTGGGATTAAATAAATCACTTACAATATTTATTTTATTATATTGCTTTGTTCAATTAAATTTTAA
TACGAAAT

CTCAACTGAAAAAAAAAAAAAAAAATAAAAAACGCCCCTTTAAGACGAAGTGACAAAGAGGCAATAGTTCA
AAAGAAAAAAAAAAAATATAAAGACTGTTGATAGTTGAATTTTTATAAACGTTATGTTAAGTATATGTATT
GATGGAAG

TACAGTTCCACTTGCATCCCTCATTTTTCACATTCCTTTTTTTATAGAAAAGCATATCACTATATAAAAAT
TCTATTATAGTTGTAACAGCATAGCATATTATAGACTTTTTTTTTCTGGAAACGGTTAAACCGTTTGGCTG
AGATGCAA

TGAGAGCTGTATGATATTGTGGAACTTTGTATATATTGAAAAAATTTTCCAGTGGAATTTTACTCGCGATG
ACTCAAGAGCGTGAAAAAAAAAAAAAAAAAAAAATTAATTGCTAAAAAATCAAATATATGCTTCCAATCGG
ATTTGAAC

GTATATTGATTAAAGTGGAAAGGGCATCGGAAAAGTAAGAAAAGCTTAAAAAAATTGAAAAAAAAAAAAAA
AAAAATAAAAAATAAAAAATAAAAAAACCTATACAATACATACATATGTATATGAATATAAACCTGGAACG
ATGTGTCT

CACCGTCATTGATCAAATAGGTCTATAATATTAATATACATTTATATAATCTACGGTATTTATATCATCAA
AAAAAAGTAGTTTTTTATTTATTTTATTTTGTTCGTTAATTTTCAATGTCTATGGAAGCCCGTTCGTAAAA
TTGGCGTT

AGTAAATATACTATCCGTTGCAAGTCTTTAAGAGCTACATTAAATTGAAATTGTCGAGTGTCGCCAACAAC
AATTCGCGAAAATGAGCGAAAATTTTTCAAGCCCACCGCTAAAAAAAAAAAAAAAAAGAAAAAAAAGGACA
TAAATACG

CTATCCGTTGCAAGTCTTTAAGAGCTACATTAAATTGAAATTGTCGAGTGTCGCCAACAACAATTCGCGAA
AATGAGCGAAAATTTTTCAAGCCCACCGCTAAAAAAAAAAAAAAAAAGAAAAAAAAGGACATAAATACGTA
TATCTCCA

ACCTATGGGAACTGCTAAAAGCAACTATTTTGATCCCGTAGAACACGATTTATTTCATTAAATTTTTTTTTT
TTTTTTTTTTTTTTCACTTTCTTGCGATGAGATGCACATAGTAAAAGAAAAGTATACCAGTGTAGTGCGTA
AATTAATA

AGCAAATATAATTCAGGTCCCACTTGGAATAATGGCACTGTATTGATGCATTTTCCTTATGCTTAGTGACG
CGTTTTCGCGCGTCAGTTTCAAGTTTTTCTTGGCTTTTTTTTTTTCATTTTCGTAAAGGGTCTTAAAAGGA
TTAAAAAA

AGTGCTATAGCAAGTGGGCTTTTGCGTGCTTATCTTTCTCACTCCAACCGCTGCATCGTTTTCAGTAAGAA
GGAGCCCTTTCCCCATAGCAAATGATAGAAAAATAGTCAATAATTGAACGAAGAAAAAAAAAAAGAAAGTTT
GTTCATAA

AAGCTTAAACCAAGGGAAGCAAAATTTGAAATACCGAAGGTAGAACAATAAGGATGATGGGAAAAAAAAAGA
TAATTTTTTTTTTTTGTTTTTCCCTGCTTCCTTCTTGTTTATTGGTATTATTATGTTACGATATTCATTCA
TTATCCTA

ACGCTTTATAGATCACTTTTTTTTTTTTTTGAGGAACCGATTAATTAATACATCGTAGCCTCTGCTTATTG
CATAACACAACAAAAAAAATACAATAATACGCAACTTTTGTTTATAGAAAAATAAAAATGGAACATATGATA
TTCTCTGT

GCAACTATTTTGATCCCGTAGAACACGATTTATTTCATTAAATTTTTTTTTTTTTTTTTTTTTTTCACTTT
CTTGCGATGAGATGCACATAGTAAAAGAAAAGTATACCAGTGTAGTGCGTAAATTAATACCTATATAATAA
TTCATTTT

GAGGGTTTGAAAAATGCTCAAAAAAATAGCCAAATGTCAAACTCAAATAGTGTTTGTTGTACGCTTATGTA
ATGATAGTAATAGAATCCAAAAAAAAAAAAAATATACATGCTTTTTCATATCCTCTCTCACCCTATCTTTT
TTTTTCTT

ATCTCAAGGAATGCAAAATTCGTGGCTCAGTGCTCTCAAGACAATGTTGCAACCCTTTGCGTCAATTATAT
ATATATATATATATATATATATATATCCCGTCCGCTTTCTTTTTTTTGTCAGTTGGGTCGCAACGCAGGGTCT
CGAGACCT

GAAAAAAAAAAAAGCCGCCGAGGCGCGCGTTCATGCAATGGCTCAGTAACCTCGTGATAGAAAAAGGGCAAC
AATATTGGGCTATTTTAGGCAAAGAAACTCAGCTATTCGAAAAGGGCATCCATTTCATTTCCGGTTTTCTA
TCTAGCCA

GATAGTAACTTCTGACTTTTCGCTTTTATACAGCACAGCAGAAAAAAAAAAGCCGCCGAGGCGCGCGTTC
ATGCAATGGCTCAGTAACCTCGTGATAGAAAAAGGGCAACAATATTGGGCTATTTTAGGCAAAGAAACTCA
GCTATTCG

TAAATGTTATGTTCCATGTTGTGTTTTCCACCAAAAAACCGTGAAGGACAATTCAATGAGCAAAGGGTTTA
ACGGAGCTATAACCTGCTTTTGTAGATGAAAAAATAGAAAATTCAAATATATATATATATATATATTTATA
ATAGCATG

TAAAGTGGAAAGGGCATCGGAAAAGTAAGAAAAGCTTAAAAAAAATTGAAAAAAAAAAAAAAAAAATAAAA
AATAAAAAATAAAAAAACCTATACAATACATACATATGTATATGAATATAAACCTGGAACGATGTGTCTTT
AAAAATAG

CAAAATAAATAGATTTGCAAATAAGTTTTGTATGTACATTTATTAATATATATAATATATCAAAAGAAAAA
AATCAAAAAAAAAAAAAAAAAAAAATTGCACTCTTATTCAGTCATCAATTACAAAACCTAGAGATAGCGAT
GGTGCATA

ATGTTTGATGCCATACCATCTATTGACATAATCTACGATGTGAGAGCTGTATGATATTGTGGAACTTTGTA
TATATTGAAAAAATTTTCCAGTGGAATTTTACTCGCGATGACTCAAGAGCGTGAAAAAAAAAAAAAAAAAA
AAATTAAT

GCTGATTCCGCCGCATATGCATTGTGTAGATCCAAAAGTAAGGACAAGATATCATGGGATGAAGAAGAACA
GGCGCGATTAATGGGCGTTGTAAAATTTAATTCAGAGCATTACAGGGACTAGGAAATAATACTAATTAAAT
AATTCTAA

TAGATTTGTGAGCGCATTGGCGCGACCCATTTTTTTAACACAGAAAAAAAAGTAAAAAATAGAAATTACCCA
ATTAAGTGTTTTTATAGTAAACAATCCTTGATATCTTAGTTGAAAATAAACTCGAATTATGTACAAGTTTC
CTTCCACT

CTCATAGATGCAGAAAATGATGACAGTCTAGCTCCTATAGACGAAAAGAAGGTCCCAATGCTAAAGGACAC
CAAGAATAAAATCGAAAATGAGCATGTAATATGGGAAAAAGTGTACGAATCAAAGACAAAATAATATAGTT
GCAATAAT

TTAAAAGAAAGCGGCAATAGCCTTTCCTTTCTTAGCCGGAAAACTTACTACGGGAACAAGTTTTTTTTATT
AGTATAATGTAGATATAAAAGCTTAGAGTTTATATAAATATAATAAAAGGTTAGCACTGCTGTTGCAAAAA
TATCGAAT

CAGCACAGCAGAAAAAAAAAAGCCGCCGAGGCGCGCGCGTTCATGCAATGGCTCAGTAACCTCGTGATAGAA
AAAGGGCAACAATATTGGGCTATTTTAGGCAAAGAAACTCAGCTATTCGAAAAGGGCATCCATTTCATTTC
CGGTTTTC

TGGCTTAAAAGATTTTAACCAAGAGCAATCCTATGGAACGTTCTGTCCCACTTCACTCAAGTTTTTGAAAG
GGTAAAAATTAAAAAAATTATAGAATTAACAGTAAAATGTGCTACGTTCCAAAAAAAAAAAAATATGCACTA
TAGTATAT

CAAGTGGGCTTTTGCGTGCTTATCTTTCTCACTCCAACCGCTGCATCGTTTTCAGTAAGAAGGAGCCCTTT
CCCCATAGCAAATGATAGAAAAATAGTCAATAATTGAACGAAGAAAAAAAAAAAGAAAGTTTGTTCATAAAA
GAATATCA

AGCCCACCGCTAAAAAAAAAAAAAAAAAAGAAAAAAAGGGACATAAATACGTATATCTCCAGAAAATGCCCA
GTGGAGTGGATGAATACCTGATGGGCTATATTACTACATTATTGAAAACGTTTACCAAAAGTTTCGTTACT
GTATAAAA

TCCTAGATGCTATATGTCCCTTTACATAATAAATTAAAAAATTTTTTTTATAAATTATAATAATTTCTTTT
TATTTCTAATAGTATCTTGGGATTAAATAAATCACTTACAATATTTATTTTATTATATTGCTTTGTTCAAT
TAAATTTT

CAAGGTTACTTCCTAGATGCTATATGTCCCTTTACATAATAAATTAAAAAATTTTTTTTATAAATTATAAT
AATTTCTTTTTATTTCTAATAGTATCTTGGGATTAAATAAATCACTTACAATATTTATTTTATTATATTGC
TTTGTTCA

TTACTATGTGTTTAAGAGAGAGCTGTTCTACAAAGAACTTTTCATTTACGCGTTTTTTCCTTTTTTTTTTT
TTCTCATCCTAAACGCGTAAATCTGTTCCCTTTTTCATGTAATGGGCGTACAACCGACGATGGAAAAATGA
ATCTTGCA

TAAAAAAAAAAAAAAAAAAGAAAAAAAGGGACATAAATACGTATATCTCCAGAAAATGCCCAGTGGAGTGGA
TGAATACCTGATGGGCTATATTACTACATTATTGAAAACGTTTACCAAAAGTTTCGTTACTGTATAAAAAA
ATTGGGGT

TGTCTTCCATCCTTCCCTTATTCTCAATATATATGTAATACGTCGTATTTGATATATATATATATATATAT
ATAATATAATTTAACGGTTGTTATCTGCTACATCTGTAAAATAAAAATAAAAATGCTTGAAGCGGCTGTAC
TTAAAATA

GGAAATCATAAACATTGTTTTTTTGTGTTTTTTATTTTTAAATAAAAAGAATAAATATTTTATATTAAAAA
ATAAATTTTAAAGTAAATTAATTATTTAACACGTGATTAAGTTACTCTAATATAATCCTATTTAATAATTA
GTAGAGTT

GGTCCCAATGCTAAAGGACACCAAGAATAAAATCGAAAATGAGCATGTAATATGGGAAAAAGTGTACGAAT
CAAAGACAAAATAATATAGTTGCAATAATAGTAATAATAATAATAATAATAATAATAATAATAATAATAAT
AATAATAA

CGCGACCCATTTTTTTAACACAGAAAAAAAGTAAAAAATAGAAATTACCCAATTAAGTGTTTTTATAGTAA
ACAATCCTTGATATCTTAGTTGAAAATAAACTCGAATTATGTACAAGTTTCCTTCCACTTAATCTATGATC
CTTCTTTA

ATTTAGGTTCGGAAATCATAAACATTGTTTTTTTGTGTTTTTTATTTTTAAATAAAAAGAATAAATATTTT
ATATTAAAAAATAAATTTTAAAGTAAATTAATTATTTAACACGTGATTAAGTTACTCTAATATAATCCTAT
TTAATAAT

TGATCCCGTAGAACACGATTTATTTCATTAAATTTTTTTTTTTTTTTTTTTTTTTCACTTTCTTGCGATGA
GATGCACATAGTAAAAGAAAAGTATACCAGTGTAGTGCGTAAATTAATACCTATATAATAATTCATTTTCG
GTGACTCA

TGTATTTTATCTAACGCGTCGCGACGCGTTAGATAAATATAACAGTAAATGATGGTATAAATGCCACCGTC
GATATTTAAATGAGATTGCTTATATATCGCATTATTACCTGCGAAAATCTAATTTACGACTCCTTGAAAAA
ATTCCATC

AACGGCCCTTTTCGTGACTGCAAATGATACTAATTCCTGTATTTAAATATACTCATATTTAACGAACAATT
ATGATGAATGAAAAAACCTGATATAATAGACTTAACTAAGCGATCATAAAAAAATTCAAAAAGTTATTTTA
TAAAACAA

AGTGGAATTTTACTCGCGATGACTCAAGAGCGTGAAAAAAAAAAAAAAAAAAAAAATTAATTGCTAAAAAAT
CAAATATATGCTTCCAATCGGATTTGAACCGATGATCTCCACATTACTAGTGTGGCGCCTTACCAACTTGG
CCATAGAA

GAACTTTTTTTTGATTTGTTATATAATAACAGAAAATCCAGATTTGTACAGAAAGAAAATCCGTGATAGTT
TAATGGTCAGAATGGGCGCTTGTCGCGTGCCAGATCGGGGTTCAATTCCCCGTCGCGGAGATTTTTTTGGC
TACTGTTG

TATCTTTCTCACTCCAACCGCTGCATCGTTTTCAGTAAGAAGGAGCCCTTTCCCCATAGCAAATGATAGAA
AAATAGTCAATAATTGAACGAAGAAAAAAAAAAGAAAGTTTGTTCATAAAAGAATATCATATTATATATAT
ATATAGGG

ATCTTTGGGCTAAAATCTGCGTCAAACAGGAAATATTGCCTATTTTCGTACAAGGTTACTTCCTAGATGCT
ATATGTCCCTTTACATAATAAATTAAAAAATTTTTTTTATAAATTATAATAATTTCTTTTTATTTCTAATA
GTATCTTG

GAATTGAGGAAATTCCTTTTCTCCTTTGTTTTCTCTTTATCCCCGCGTAAATACGCGGAATGCCAATAATC
AAGTACACATTCTTTCTCCAAGTTGGCCAAAAAATAGTTCAAATTCTTTAAACTAAGCGCTATTAATTATT
ATTAATTA

GAAAAAAAAGATAATTTTTTTTTTTTTGTTTTTCCCTGCTTCCTTCTTGTTTATTGGTATTATTATGTTACG
ATATTCATTCATTATCCTATTGATATTTTCTTTATATTCACTAAAAAAAAATTTATTCTATAAGACTGACT
ATAATTTT

ATTCGTACAGTTGTGTGCAATGTTAAAAATGGTGACTGTATCTACGTATCTATAAAAAAAGGTTAACTACC
GGAAAATCATTTCTTCTCGTAAAGTGTATATTTAGGTTCGGAAATCATAAACATTGTTTTTTTGTGTTTTT
TATTTTTA

CGCTTTTATACAGCACAGCAGAAAAAAAAAAGCCGCCGAGGCGCGCGCGTTCATGCAATGGCTCAGTAACCT
CGTGATAGAAAAAGGGCAACAATATTGGGCTATTTTAGGCAAAGAAACTCAGCTATTCGAAAAGGGCATCC
ATTTCATT

TTGACAGGCTATTTTGGCAGCGCAATCAAAGAATAAACTTGGTATAGTAAGGAAGCCAGTTACTGTACGCA
TGAATTACGGTAGCTTTTTTTATTTTATTTATTTTTTTTTTTTTTTGGACAAATAGAAAAATTTTTATTATT
ATGTAAGA

TTTAAGAGAGAGCTGTTCTACAAAGAACTTTTCATTTACGCGTTTTTTCCTTTTTTTTTTTTTTCTCATCCT
AAACGCGTAAATCTGTTCCCTTTTTCATGTAATGGGCGTACAACCGACGATGGAAAAATGAATCTTGCAAT
TTTAATAC

GGGTGCGCGCGCTAGTATCTTTCCACATTAAGAAATATACCATAAAGGTTACTTAGACATCACTATGGCTA
TATATATATATATATATATATATATATATATATATGTAACTTAGCACCATCGCGCGTGCATCACTGCATGTGT
TAACCGAA

GCTTGTGTGTGGGTGCGCGCGCTAGTATCTTTCCACATTAAGAAATATACCATAAAGGTTACTTAGACATC
ACTATGGCTATATATATATATATATATATATATATATATATATATGTAACTTAGCACCATCGCGCGTGCATCA
CTGCATGT

AATAGGGTGAACGAAAAAAATTGGGAGATCTTGCCGACGGCTACGTATATATATATATATATATATAAATATA
TATATATATATTCATGTATACTGTATATGTACAATCATATATAAAGTTACTCTTCCCTTTTCTTTTTTTTT
TTACATTA

AAAAGACGAAGCTTGTGTGTGGGTGCGCGCGCTAGTATCTTTCCACATTAAGAAATATACCATAAAGGTTA
CTTAGACATCACTATGGCTATATATATATATATATATATATATATATATATATATGTAACTTAGCACCATCGC
GCGTGCAT

ACGAAAAAAAATTGGGAGATCTTGCCGACGGCTACGTATATATATATATATATATATAAATATATATATATATA
TTCATGTATACTGTATATGTACAATCATATATAAAGTTACTCTTCCCTTTTCTTTTTTTTTTTTACATTATT
ACGTATAT

GCGCGCTCCCTTAGCATGGGAGAGGTCTCCGGTTCGATTCCGGACTCGTCCATTTTAAAATTTCTTTTTTT
TTTTTTCTCTTTCCGTATGTAAGCTTTTTTCCTTTTTTTTTTTTTTTTTTTTTTTTTTTTTCTTCACTACTT
TTCAATGA

GTAGTCGGTAGCGCGCTCCCTTAGCATGGGAGAGGTCTCCGGTTCGATTCCGGACTCGTCCATTTTAAAAT
TTCTTTTTTTTTTTTTCTCTTTCCGTATGTAAGCTTTTTTCCTTTTTTTTTTTTTTTTTTTTTTTTTTTTTTC
TTCACTAC

ATGCATGGTAAATAGGGTGAACGAAAAAAAATTGGGAGATCTTGCCGACGGCTACGTATATATATATATATA
TATAAATATATATATATATATATTCATGTATACTGTATATGTACAATCATATATAAAGTTACTCTTCCCTTTT
CTTTTTTT

TGTATTATTATTATATTATTATTATTATTATTATTATTATTATTATTATTACGGTATGTCAAGTATTATTTTTT
TTTTAAAAATAAAGAAAAAAAATTGTACAAATATATATATTTATATATTACTCTATATGGTTTTTTTTTTTT
AAGTGCAG

TTAGCATGGGAGAGGTCTCCGGTTCGATTCCGGACTCGTCCATTTTAAAATTTCTTTTTTTTTTTTTCTCT
TTCCGTATGTAAGCTTTTTTCCTTTTTTTTTTTTTTTTTTTTTTTTTTTTTCTTCACTACTTTTCAATGAAA
CTCTCTTC

GCTAGTATCTTTCCACATTAAGAAATATACCATAAAGGTTACTTAGACATCACTATGGCTATATATATATA
TATATATATATATATATATATATGTAACTTAGCACCATCGCGCGTGCATCACTGCATGTGTTAACCGAAAA
GTTTGGCG

TCCTACTAGCTTTTTTTTTCTTCAATTCGATAGTTTAGATGGGTTTATATCTAAATATATATATATATAGTATT
TTATTTCATATTCAATCTCCTAGTATACATGAAATATTTAACGCTTTTTTTTGCCCTTGTTTTTATTTTTT
TTCTTTTT

GGCGTGTGGCGTAGTCGGTAGCGCGCTCCCTTAGCATGGGAGAGGTCTCCGGTTCGATTCCGGACTCGTCC
ATTTTAAAATTTCTTTTTTTTTTTTTCTCTTTCCGTATGTAAGCTTTTTTCCTTTTTTTTTTTTTTTTTTT
TTTTTTTT

TTATATTATTATTATTATTATTATTATTATTATTATTATTACGGTATGTCAAGTATTATTTTTTTTTTAAAAAT
AAAGAAAAAAAATTGTACAAATATATATATTTATATATTACTCTATATGGTTTTTTTTTTTTTAAGTGCAGGC
GTTGGTTA

AATTCCAAAAAAAAAAAGTAAAAAAAACAAAACTTTGATTGTTTTTTAATGATGTTAATGATTTTTTTTTTC
TTTCTTTATCATAAAAAAAAAAGTTAAAATGAAAAACAAATATGGGTCTGGAAGGCCATTATTTTTTTTTTA
TTTATATA

ATTATTATTATTATTATTATTATTATTACGGTATGTCAAGTATTATTTTTTTTTTTAAAAATAAAGAAAAAA
ATTGTACAAATATATATATTTATATATTACTCTATATGGTTTTTTTTTTTTTAAGTGCAGGCGTTGGTTATG
CTTCGTCT

TGATGCGATGCGATGTTAGCGCAAACAATAACGCGTACGATAATAAAACTTCAAATGCGCCAATTCTTTTTT
TTTTTTTTTTTTTTTTTTTTGATTTCTTTTCATATATCCGATTTAAACCGCCTAGGGAAGAGTAAAAACCCG
AATTGTTC

TTTTTTTTCTTCAATTCGATAGTTTAGATGGGTTTATATCTAAATATATATATATAGTATTTTATTTCATA
TTCAATCTCCTAGTATACATGAAATATTTAACGCTTTTTTTTGCCCTTGTTTTTATTTTTTTTCTTTTTTG
AAGCTGCA

ACGCGTACGATAATAAAACTTCAAATGCGCCAATTCTTTTTTTTTTTTTTTTTTTTTTTTTGATTTCTTTTC
ATATATCCGATTTAAACCGCCTAGGGAAGAGTAAAAACCCGAATTGTTCCGTCTATACTTGGTTGTATGGG
TGTTTTTT

TGCAAGGACCATCATTGTTTGAAAAAAGAGAGGATATTACTAGTAGGTAATAACAGCTATGTCGCGTTATA
TATATTATATATAAAAATTAAAAAATAGAAAATAAAATTTCAAATGCCCTCTGTGGGAATTGAACCCACGA
TCCCCGCA

TGCGAAGAAAGAAAAAGAAAATATTCACCTCTAGGAGCCAAAAACATAAAAAAAAAAAATAATTAAAAAAAA
CTTTTATTGAATGCTTAATTATTTAGTGACGCGATTACCTATATACCAGGTTATTTATTTTTTCTTTTTTC
AAAGGAAA

GTTGAAATACCCTATACTAATTGTTTGCTTTGTCTTTTTGTATATATCCGAACGTATCTATCTGAAATTTT
TCAAATTTATAAAAAATAATATATATAAAAGGAAAAAAAAAAAAAATCGATGCCCTACTAATAGAGATTGGA
GCTGAAAA

ATTGTATTATTGTATTATTATTATATTATTATTATTATTATTATTATTATTATTATTACGGTATGTCAAGT
ATTATTTTTTTTTTTAAAAATAAAGAAAAAAAATTGTACAAATATATATATTTATATATTACTCTATATGGTT
TTTTTTTT

GAAAAAGAAAATATTCACCTCTAGGAGCCAAAAACATAAAAAAAAAAAATAATTAAAAAAAACTTTTATTGA
ATGCTTAATTATTTAGTGACGCGATTACCTATATACCAGGTTATTTATTTTTTCTTTTTTCAAAGGAAAAC
AATACAAA

CTATCAAATTCGTTGGTTTTTTTTTCTGGGAAAACTCTATCAAAGTATAAGGTGCGCTTTTAACGTAATTTA
TAATGATTTTTTAGAAATAATAATGTATATAGTATAAAAAAATAAACAAACTTTAAAAAAAAGAGTGAAGA
ACTGTAAA

TTCCACATTAAGAAATATACCATAAAGGTTACTTAGACATCACTATGGCTATATATATATATATATATATA
TATATATATATATGTAACTTAGCACCATCGCGCGTGCATCACTGCATGTGTTAACCGAAAAGTTTGGCGAA
CACTTCAC

AGAAATATACCATAAAGGTTACTTAGACATCACTATGGCTATATATATATATATATATATATATATATATA
TATGTAACTTAGCACCATCGCGCGTGCATCACTGCATGTGTTAACCGAAAAGTTTGGCGAACACTTCACCG
ACACGGTC

AATACATCAAAACCTTCATTTGCGCGATGTTACGCGTCGCGTTTCCTATAAAAAATACATAAGCTAGTGAA
TAAAAGCTTTAAACGCGTTAGGGGGATCTGCAATTAAAGTAAATAAATGAAATAAATGAAATAGATGAAAA
ACTGGTTA

CCAAAATAAAATTTTTTTTTAAGTCCCAATTGAAATACTTCTCCTTATAAATATCATGAAAGGAAGAAAAA
AAAGTGAAAAAAAAAAAAAAAACCAATAAAAAAATAAAAAAATATCAATTCTACGAGAAATGCGTGCGAAA
TCGATGAG

AGAGGTCTCCGGTTCGATTCCGGACTCGTCCATTTTAAAATTTCTTTTTTTTTTTTTCTCTTTCCGTATGT
AAGCTTTTTTCCTTTTTTTTTTTTTTTTTTTTTTTTTTTTCTTCACTACTTTTCAATGAAACTCTCTTCAA
AAGACAGC

CGGACTCGTCCATTTTAAAATTTCTTTTTTTTTTTTTCTCTTTCCGTATGTAAGCTTTTTTCCTTTTTTTT
TTTTTTTTTTTTTTTTTTTTCTTCACTACTTTTCAATGAAACTCTCTTCAAAAGACAGCATGCAGTAAAAG
CGCCGATA

AACCTTCATTTGCGCGATGTTACGCGTCGCGTTTCCTATAAAAAATACATAAGCTAGTGAATAAAAGCTTT
AAACGCGTTAGGGGGATCTGCAATTAAAGTAAATAAATGAAATAAATGAAATAGATGAAAAACTGGTTACA
CTAATTAT

GTAATTTATTATTAATAATTATATTTTATCAATATTTATACGATATAAAAATTTAAAAAAATTATAATATA
GAAATAATAAATAATAATAATATGATAATAAAATAATATATAAATAAAAATGATAAGATTATATTAATTTA
TTTAATTT

GTAATATTTATAAAATATAAAAAGAAGAAATTTTTATTCTTTTTATATTTTATTATAAAATAGTTCAATTA
TTTATATTATTTAAATGTTGTAATTTATTATTAATAATTATATTTTATCAATATTTATACGATATAAAAAT
TTAAAAAA

CAACAACAAAAATTCCAAAAAAAAAAAGTAAAAAAACAAAACTTTGATTGTTTTTTAATGATGTTAATGAT
TTTTTTTTTCTTTCTTTATCATAAAAAAAAAGTTAAAATGAAAAACAAATATGGGTCTGGAAGGCCATTAT
TTTTTTTT

AGGAAGAAAAAAAAGTGAAAAAAAAAAAAAAAACCAATAAAAAAATAAAAAAATATCAATTCTACGAGAAA
TGCGTGCGAAATCGATGAGTTTTGTTTTGCCTCTGAAAAATTCTGGAAAATTTTTCTTAGCGGAAAAAAGA
AAAAAAAG

CGATGTTAGCGCAAACAATAACGCGTACGATAATAAAACTTCAAATGCGCCAATTCTTTTTTTTTTTTTTT
TTTTTTTTTGATTTCTTTTCATATATCCGATTTAAACCGCCTAGGGAAGAGTAAAAACCCGAATTGTTCCG
TCTATACT

GGTTCGATTCCGGACTCGTCCATTTTAAAATTTCTTTTTTTTTTTTTCTCTTTCCGTATGTAAGCTTTTTT
CCTTTTTTTTTTTTTTTTTTTTTTTTTTTTCTTCACTACTTTTCAATGAAACTCTCTTCAAAAGACAGCAT
GCAGTAAA

CGTTGGTTTTTTTTCTGGGAAAACTCTATCAAAGTATAAGGTGCGCTTTTAACGTAATTTATAATGATTTT
TTAGAAATAATAATGTATATAGTATAAAAAAATAAACAAACTTTAAAAAAAAGAGTGAAGAACTGTAAAGC
TCATTTCA

GCAAACAATAACGCGTACGATAATAAAACTTCAAATGCGCCAATTCTTTTTTTTTTTTTTTTTTTTTTTTG
ATTTCTTTTCATATATCCGATTTAAACCGCCTAGGGAAGAGTAAAAACCCGAATTGTTCCGTCTATACTTG
GTTGTATG

ATATTCACCTCTAGGAGCCAAAAACATAAAAAAAAAAATAATTAAAAAAAACTTTTATTGAATGCTTAATT
ATTTAGTGACGCGATTACCTATATACCAGGTTATTTATTTTTTCTTTTTTCAAAGGAAAACAATACAAAAT
ATTACTGT

TGCGCGATGTTACGCGTCGCGTTTCCTATAAAAAATACATAAGCTAGTGAATAAAAGCTTTAAACGCGTTA
GGGGGATCTGCAATTAAAGTAAATAAATGAAATAAATGAAATAGATGAAAAACTGGTTACACTAATTATAA
CGTTAATA

CGCTTTTCATCGATGTATATAAGGAAGAATACCTCATTATAATACATCAAAACCTTCATTTGCGCGATGTT
ACGCGTCGCGTTTCCTATAAAAAATACATAAGCTAGTGAATAAAAGCTTTAAACGCGTTAGGGGGATCTGC
AATTAAAG

TTATTATTATTATTATTACGGTATGTCAAGTATTATTTTTTTTTTAAAAATAAAGAAAAAAATTGTACAAA
TATATATATTTATATATTACTCTATATGGTTTTTTTTTTTTAAGTGCAGGCGTTGGTTATGCTTCGTCTAC
AATTGGTT

CTCAGTCAGCTGATGCGATGCGATGTTAGCGCAAACAATAACGCGTACGATAATAAAACTTCAAATGCGCC
AATTCTTTTTTTTTTTTTTTTTTTTTTTTGATTTCTTTTCATATATCCGATTTAAACCGCCTAGGGAAGAG
TAAAAACC

TTCCCCTCTGGTTAACAAAAACCAGTATATTAACAGAAATAAATAATAATAACAAAAAAATATGAATATAA
GAATAAATTGCAATAAATAGTAATATTTATAAAATATAAAAAGAAGAAATTTTTATTCTTTTTATATTTTA
TTATAAAA

TTTAAATGTTGTAATTTATTATTAATAATTATATTTTATCAATATTTATACGATATAAAAATTTAAAAAAA
TTATAATATAGAAATAATAAATAATAATAATATGATAATAAAATAATATATAAATAAAAATGATAAGATTA
TATTAATT

TAAAATATAAAAAGAAGAAATTTTTATTCTTTTTATATTTTATTATAAAATAGTTCAATTATTTATATTAT
TTAAATGTTGTAATTTATTATTAATAATTATATTTTATCAATATTTATACGATATAAAAATTTAAAAAAAT
TATAATAT

CTAGGAGCCAAAAACATAAAAAAAAAATAATTAAAAAAAAACTTTTATTGAATGCTTAATTATTTAGTGAC
GCGATTACCTATATACCAGGTTATTTATTTTTTCTTTTTTCAAAGGAAAACAATACAAAATATTACTGTTA
CCATTTTT

CATTTTAAAATTTCTTTTTTTTTTTTTTCTCTTTCCGTATGTAAGCTTTTTTCCTTTTTTTTTTTTTTTTT
TTTTTTTTTTCTTCACTACTTTTCAATGAAACTCTCTTCAAAAGACAGCATGCAGTAAAAGCGCCGATATG
AGTGACGA

ATTAATAATTATATTTTATCAATATTTATACGATATAAAAATTTAAAAAAATTATAATATAGAAATAATAA
ATAATAATAATATGATAATAAAATAATATATAAATAAAAATGATAAGATTATATTAATTTATTTAATTTTC
AAATTATT

TGTATAGACACCACATTGAAGGGGCGTCACAGCAATTTTTTTTATTTTTTTTTGATACTCAATAATTACGA
GCATATAATTTAGTACTTCCAAAAAAAAGTTAAAAAAAAATAGTGACAAAATGTGTCAGTAAAATATAT
ATGAGAAC

ATTTTTTTTTAAGTCCCAATTGAAATACTTCTCCTTATAAATATCATGAAAGGAAGAAAAAAAAGTGAAAA
AAAAAAAAAAAACCAATAAAAAAATAAAAAAATATCAATTCTACGAGAAATGCGTGCGAAATCGATGAGTT
TTGTTTTG

TTTTTATTCTTTTTATATTTTATTATAAAATAGTTCAATTATTTATATTATTTAAATGTTGTAATTTATTA
TTAATAATTATATTTTATCAATATTTATACGATATAAAAATTTAAAAAAATTATAATATAGAAATAATAAA
TAATAATA

CGATGTATATAAGGAAGAATACCTCATTATAATACATCAAAACCTTCATTTGCGCGATGTTACGCGTCGCG
TTTCCTATAAAAAATACATAAGCTAGTGAATAAAAGCTTTAAACGCGTTAGGGGGATCTGCAATTAAAGTA
AATAAATG

AAAACATTAACCAAAATAAAATTTTTTTTTAAGTCCCAATTGAAATACTTCTCCTTATAAATATCATGAAA
GGAAGAAAAAAAAGTGAAAAAAAAAAAAAAAACCAATAAAAAAATAAAAAAATATCAATTCTACGAGAAAT
GCGTGCGA

ATATTTTATCAATATTTATACGATATAAAAATTTAAAAAAATTATAATATAGAAATAATAAATAATAATAA
TATGATAATAAAATAATATATAAATAAAAATGATAAGATTATATTAATTTATTTAATTTTCAAATTATTAA
ATAAATAA

GCTTGGGTTAATGTTCTTGTGCTATTGTTGCTGTTGTTATTGTTTTTATTATTGTATTATTGTATTATTAT
TATATTATTATTATTATTATTATTATTATTATTACGGTATGTCAAGTATTATTTTTTTTTTAAAAATA
AAGAAAAA

CGGCACGAAGGTTGAAATACCCTATACTAATTGTTTGCTTTGTCTTTTTGTATATATCCGAACGTATCTAT
CTGAAATTTTTCAAATTTATAAAAAATAATATATATAAAAGGAAAAAAAAAAAAATCGATGCCCTACTAAT
AGAGATTG

TATTTGTCAGTGCAAGGACCATCATTGTTTGAAAAAAGAGAGGATATTACTAGTAGGTAATAACAGCTATG
TCGCGTTATATATATTATATATAAAAATTAAAAAATAGAAAATAAAATTTCAAATGCCCTCTGTGGGAATT
GAACCCAC

TTCCCTTGGCACCATGTATATTCACTTTTATTGTCTCTTGATCGAGCTATAACCAAATTTTTTTTTTTTTT
TTTTTTTTTCGTTTTTATAATTTTTTCTTTGGTATTCAATTGTATATAGTGCTCAAACAGCAACGTACATA
ATAGCTAG

CTGTTGTTATTGTTTTTATTATTGTATTATTGTATTATTATTATATTATTATTATTATTATTATTATTATT
ATTATTACGGTATGTCAAGTATTATTTTTTTTTTAAAAATAAAGAAAAAAATTGTACAAATATATATATTT
ATATATTA

GCTATTGTTGCTGTTGTTATTGTTTTTATTATTGTATTATTGTATTATTATTATATTATTATTATTATTAT
TATTATTATTATTATTACGGTATGTCAAGTATTATTTTTTTTTTAAAAATAAAGAAAAAAATTGTACAAAT
ATATATAT

ACCATGTATATTCACTTTTATTGTCTCTTGATCGAGCTATAACCAAATTTTTTTTTTTTTTTTTTTTTTC
GTTTTTATAATTTTTTCTTTGGTATTCAATTGTATATAGTGCTCAAACAGCAACGTACATAATAGCTAGCA
AACAATAG

AGTCTTGGTACTATCAAATTCGTTGGTTTTTTTTCTGGGAAAACTCTATCAAAGTATAAGGTGCGCTTTTA
ACGTAATTTATAATGATTTTTTAGAAATAATAATGTATATAGTATAAAAAAATAAACAAACTTTAAAAAAA
AGAGTGAA

CGATATAAAAATTTAAAAAAATTATAATATAGAAATAATAAATAATAATAATATGATAATAAAATAATATA
TAAATAAAAATGATAAGATTATATTAATTTATTTAATTTTCAAATTATTAAATAAATAATAACCTAATACT
TACTTTTC

ATCATTGTTTGAAAAAAGAGAGGATATTACTAGTAGGTAATAACAGCTATGTCGCGTTATATATATTATAT
ATAAAAATTAAAAAATAGAAAATAAAATTTCAAATGCCCTCTGTGGGAATTGAACCCACGATCCCCGCATT
ACGAGTGC

CTTTTTCCTATTCCCCTCTGGTTAACAAAAACCAGTATATTAACAGAAATAAATAATAATAACAAAAAAAT
ATGAATATAAGAATAAATTGCAATAAATAGTAATATTTATAAAATATAAAAAGAAGAAATTTTTATTCTTT
TTATATTT

AAGTAGTAAACCATTGAATGAAAACATTAACCAAAATAAAATTTTTTTTTAAGTCCCAATTGAAATACTTC
TCCTTATAAATATCATGAAAGGAAGAAAAAAAAGTGAAAAAAAAAAAAAAAAACCAATAAAAAAATAAAAAA
ATATCAAT

CATCTTTTCGCGCGAAAAAACAATATGATGCCTTTTTGAAACATGTTATTATTTGATTTTTTAATGGCTTC
TATTACGCGTCGTGGTTGGTGAACCTGGTACAAAATAAATAAAAAAATACCTGCTAAAAAATATCAAATAA
AGTAAGGA

GACTCAGTCACTCAGTCAGCTGATGCGATGCGATGTTAGCGCAAACAATAACGCGTACGATAATAAAACTT
CAAATGCGCCAATTCTTTTTTTTTTTTTTTTTTTTTTTTGATTTCTTTTCATATATCCGATTTAAACCGCC
TAGGGAAG

TAGTTCAATTATTTATATTATTTAAATGTTGTAATTTATTATTAATAATTATATTTTATCAATATTTATAC
GATATAAAAATTTAAAAAAATTATAATATAGAAATAATAAATAATAATAATATGATAATAAAATAATATAT
AAATAAAA

AGTATATAGCTGCGAAGAAAGAAAAAGAAAATATTCACCTCTAGGAGCCAAAAACATAAAAAAAAAAAATAA
TTAAAAAAAACTTTTATTGAATGCTTAATTATTTAGTGACGCGATTACCTATATACCAGGTTATTTATTTT
TTCTTTTT

AAGGAAGAATACCTCATTATAATACATCAAAACCTTCATTTGCGCGATGTTACGCGTCGCGTTTCCTATAA
AAAATACATAAGCTAGTGAATAAAAGCTTTAAACGCGTTAGGGGGATCTGCAATTAAAGTAAATAAATGAA
ATAAATGA

GCATTTTTCCTGTCCACCACTGTACGCCATTGTACACAAAAGTATTTTTTTATATTTTTTTTACAATTTTT
TATAATTTTTTTTTATGTTTAGTGTATATTTGCTAGAAAGAAACAAGATCTTTTTTGGTAGTGTAAATGCT
TAAACTTT

TTTTCTGGGAAAACTCTATCAAAGTATAAGGTGCGCTTTTAACGTAATTTATAATGATTTTTTAGAAATAA
TAATGTATATAGTATAAAAAAAATAAACAAACTTTAAAAAAAAGAGTGAAGAACTGTAAAGCTCATTTCATA
GGTTTCAA

ATGTTCTTGTGCTATTGTTGCTGTTGTTATTGTTTTATTATTGTATTATTGTATTATTATTATATTATTA
TTATTATTATTATTATTATTATTATTACGGTATGTCAAGTATTATTTTTTTTTTAAAAATAAAGAAAAAAA
TTGTACAA

ACCTCATTATAATACATCAAAACCTTCATTTGCGCGATGTTACGCGTCGCGTTTCCTATAAAAAATACATA
AGCTAGTGAATAAAAGCTTTAAACGCGTTAGGGGGATCTGCAATTAAAGTAAATAAATGAAATAAATGAAA
TAGATGAA

GAAAATTCTGGCATTTTTCCTGTCCACCACTGTACGCCATTGTACACAAAAGTATTTTTTTATATTTTTTT
TACAATTTTTTATAATTTTTTTTTATGTTTAGTGTATATTTGCTAGAAAGAAACAAGATCTTTTTTGGTAG
TGTAAATG

CCTATACTAATTGTTTGCTTTGTCTTTTTGTATATATCCGAACGTATCTATCTGAAATTTTTCAAATTTAT
AAAAAATAATATATATAAAAGGAAAAAAAAAAAAAATCGATGCCCTACTAATAGAGATTGGAGCTGAAAAGA
AAAAGTGT

ATTTATATTATTTAAATGTTGTAATTTATTATTAATAATTATATTTTATCAATATTTATACGATATAAAAA
TTTAAAAAAATTATAATATAGAAATAATAAATAATAATAATATGATAATAAAATAATATATAAATAAAAAT
GATAAGAT

TTGGGAGATCTTGCCGACGGCTACGTATATATATATATATATATATAAATATATATATATATATATTCATGTATA
CTGTATATGTACAATCATATATAAAGTTACTCTTCCCTTTTCTTTTTTTTTTTACATTATTACGTATATAC
TGCTTTAT

TTGTTTGCTTTGTCTTTTTGTATATATCCGAACGTATCTATCTGAAATTTTTCAAATTTATAAAAAATAAT
ATATATAAAAGGAAAAAAAAAAAAAATCGATGCCCTACTAATAGAGATTGGAGCTGAAAAGAAAAAGTGTAC
ATCAAACG

AAAACATAAAAAAAAAAAATAATTAAAAAAAACTTTTATTGAATGCTTAATTATTTAGTGACGCGATTACCT
ATATACCAGGTTATTTATTTTTTCTTTTTTCAAAGGAAAACAATACAAAATATTACTGTTACCATTTTTGT
TACTATTA

TCGTTTTTCCACAATACAAAAAAACACAGTCCTTTGTACTATCCCTTTTATTTCATTATTTTTTCTTTTTT
AAGATACCACTAGATATTATCATATATAGCATATTATATAACATAAAAAGTCAAGAAAAAAAAATGTTTTTA
TCACTTTC

GCTAAAATGAAAGTAGTAAACCATTGAATGAAAACATTAACCAAAATAAAATTTTTTTTTAAGTCCCAATT
GAAATACTTCTCCTTATAAATATCATGAAAGGAAGAAAAAAAAAGTGAAAAAAAAAAAAAAAAACCAATAAAA
AAATAAAA

GTATGTCAAGTATTATTTTTTTTTTTAAAAATAAAGAAAAAAAATTGTACAAATATATATATTTATATATTAC
TCTATATGGTTTTTTTTTTTTTAAGTGCAGGCGTTGGTTATGCTTCGTCTACAATTGGTTGGTGTGCACCGA
TGGGTCAT

ATAGGCGCGCGCGGTGTGATGGTGATGATAATGATGATGATGATGATAATGATGAATAAACGTTTCGGTTT
ACGAGTTTCTTTAAATATACATATATGATATAAAAAAAAAAAATACGTACCACTTACAATTTGCAAATATAG
ATGATCGA

GAAAAAAGAGAGGATATTACTAGTAGGTAATAACAGCTATGTCGCGTTATATATATTATATATAAAAATTA
AAAAATAGAAAATAAAATTTCAAATGCCCTCTGTGGGAATTGAACCCACGATCCCCGCATTACGAGTGCGA
TGCCTTAC

CTGTTTGACATGCTAGGACTTTCCCTTGGCACCATGTATATTCACTTTTATTGTCTCTTGATCGAGCTATA
ACCAAATTTTTTTTTTTTTTTTTTTTTTTCGTTTTTATAATTTTTTCTTTGGTATTCAATTGTATATAGTG
CTCAAACA

AAAAGACTCTCGGCACGAAGGTTGAAATACCCTATACTAATTGTTTGCTTTGTCTTTTTGTATATATCCGA
ACGTATCTATCTGAAATTTTTCAAATTTATAAAAAATAATATATATAAAAGGAAAAAAAAAAAAAATCGATG
CCCTACTA

AAGCCAAGGGGACTCAGTCACTCAGTCAGCTGATGCGATGCGATGTTAGCGCAAACAATAACGCGTACGAT
AATAAAACTTCAAATGCGCCAATTCTTTTTTTTTTTTTTTTTTTTTTTTGATTTCTTTTCATATATCCGAT
TTAAACCG

ATATCATGAAAGGAAGAAAAAAAAAGTGAAAAAAAAAAAAAAAAACCAATAAAAAAATAAAAAAATATCAATT
CTACGAGAAATGCGTGCGAAATCGATGAGTTTTGTTTTGCCTCTGAAAAATTCTGGAAAATTTTTCTTAGC
GGAAAAAA

TTGTCTCTTGATCGAGCTATAACCAAATTTTTTTTTTTTTTTTTTTTTTTCGTTTTTATAATTTTTTCTTT
GGTATTCAATTGTATATAGTGCTCAAACAGCAACGTACATAATAGCTAGCAAACAATAGATGAAATAATAG
AATAATAA

GCAGCTCTTCTTTTTTTTCATGCTCGAGGTTTTTAAGGGCGAACTTTAACTAGATGGATTATTCAGTTTTTT
TTAAAAGGATCAAAATCCTACATTATATATATATATATATATATATGTTTGTGTGTGTATATAAAAAAAAA
ATCATTTC

CTTTTTATGCTGCTATTGTGGAGGAACCGTCATCTTTTCGCGCGAAAAAACAATATGATGCCTTTTTGAAA
CATGTTATTATTTGATTTTTTAATGGCTTCTATTACGCGTCGTGGTTGGTGAACCTGGTACAAAATAAATA
AAAAAATA

GGAAAAACAAAAATGATTAAATAGGCGCGCGCGGTGTGATGGTGATGATAATGATGATGATGATGATAATG
ATGAATAAACGTTTCGGTTTACGAGTTTCTTTAAATATACATATATGATATAAAAAAAAAAAATACGTACCA
CTTACAAT

TATTATTTTTTTTTTTAAAAATAAAGAAAAAAAATTGTACAAATATATATATTTATATATTACTCTATATGGT
TTTTTTTTTTTAAGTGCAGGCGTTGGTTATGCTTCGTCTACAATTGGTTGGTGTGCACCGATGGGTCATGT
TGGACCTT

TTAAAAAATAAAAAAATAAAAAAATGAACGCCGGCCCCAGAGGGAAGAACAAGCTATTTATTTTCTTTTTT
ACTTTATCTTTCATGGTCATATATATATATATATATATATATATATATATCTTCTATAGGCTACTATACATGA
AGGCGTTC

TTCACTTTTATTGTCTCTTGATCGAGCTATAACCAAATTTTTTTTTTTTTTTTTTTTTTTCGTTTTTATAA
TTTTTTCTTTGGTATTCAATTGTATATAGTGCTCAAACAGCAACGTACATAATAGCTAGCAAACAATAGAT
GAAATAAT

TGCTAGGACTTTCCCTTGGCACCATGTATATTCACTTTTATTGTCTCTTGATCGAGCTATAACCAAATTTT
TTTTTTTTTTTTTTTTTTTCGTTTTTATAATTTTTTCTTTGGTATTCAATTGTATATAGTGCTCAAACAGC
AACGTACA

CCATTGAATGAAAACATTAACCAAAATAAAATTTTTTTTTAAGTCCCAATTGAAATACTTCTCCTTATAAA
TATCATGAAAGGAAGAAAAAAAAAGTGAAAAAAAAAAAAAAAAACCAATAAAAAAATAAAAAAATATCAATTC
TACGAGAA

AAATGATTAAATAGGCGCGCGCGGTGTGATGGTGATGATAATGATGATGATGATGATAATGATGAATAAAC
GTTTCGGTTTACGAGTTTCTTTAAATATACATATATGATATAAAAAAAAAAAATACGTACCACTTACAATTT
GCAAATAT

AAAAAATAAAAAAATGAACGCCGGCCCCAGAGGGAAGAACAAGCTATTTATTTTCTTTTTTACTTTATCTT
TCATGGTCATATATATATATATATATATATATATATATATATCTTCTATAGGCTACTATACATGAAGGCGTTCGC
CCTTACAA

AATATTTATACGATATAAAAATTTAAAAAAATTATAATATAGAAATAATAAATAATAATAATATGATAATA
AAATAATATATAAATAAAAATGATAAGATTATATTAATTTATTTAATTTTCAAATTATTAAATAAATAATA
ACCTAATA

TATTATTACGGTATGTCAAGTATTATTTTTTTTTTTAAAAATAAAGAAAAAAAATTGTACAAATATATATATT
TATATATTACTCTATATGGTTTTTTTTTTTTAAGTGCAGGCGTTGGTTATGCTTCGTCTACAATTGGTTGG
TGTGCACC

GAAAGGAACATATTTGTCAGTGCAAGGACCATCATTGTTTGAAAAAAGAGAGGATATTACTAGTAGGTAAT
AACAGCTATGTCGCGTTATATATATTATATATAAAAATTAAAAAATAGAAAATAAAATTTCAAATGCCCTC
TGTGGGAA

TGTCTTTTTGTATATATCCGAACGTATCTATCTGAAATTTTTCAAATTTATAAAAAATAATATATATAAAA
GGAAAAAAAAAAAAAATCGATGCCCTACTAATAGAGATTGGAGCTGAAAAGAAAAAGTGTACATCAAACGCT
ATATCTCG

CTCCTTATAAATATCATGAAAGGAAGAAAAAAAAAGTGAAAAAAAAAAAAAAAAACCAATAAAAAAATAAAAA
AATATCAATTCTACGAGAAATGCGTGCGAAATCGATGAGTTTTGTTTTGCCTCTGAAAAATTCTGGAAAAT
TTTTCTTA

CAAGAAAAAAATTGAGGGATGGGTCAACCTTCCATAGATTCTATATGGAATAATAAAATTTACTTCTTACTA
ACATTATATCAGGGTGAATATTACTGACAAAAATAATAACTTAAGTCTTCTTTATAATATGATGATCGACG
CGCGGGGT

GCTTAGGCGTCCTTTTCCTTCAACAACAAAAATTCCAAAAAAAAAAAAGTAAAAAAACAAAACTTTGATTGT
TTTTTAATGATGTTAATGATTTTTTTTTTTCTTTCTTTATCATAAAAAAAAAAGTTAAAATGAAAAACAAATA
TGGGTCTG

AAAGAAGAAATTTTTATTCTTTTTATATTTTATTATAAAATAGTTCAATTATTTATATTATTTAAATGTTG
TAATTTATTATTAATAATTATATTTTATCAATATTTATACGATATAAAAATTTAAAAAAATTATAATATAG
AAATAATA

AAAAAAAGTAAAAAAACAAAACTTTGATTGTTTTTTAATGATGTTAATGATTTTTTTTTTCTTTCTTTATC
ATAAAAAAAAAGTTAAAATGAAAAACAAATATGGGTCTGGAAGGCCATTATTTTTTTTTTATTTATATACC
GTTTCTGG

ATTGCTAGTTGCTTGGGTTAATGTTCTTGTGCTATTGTTGCTGTTGTTATTGTTTTTATTATTGTATTATT
GTATTATTATTATATTATTATTATTATTATTATTATTATTATTATTACGGTATGTCAAGTATTATTTTTTT
TTTAAAAA

TATAACATTGTCATAGTCTCATGCATGGTAAATAGGGTGAACGAAAAAAATTGGGAGATCTTGCCGACGGC
TACGTATATATATATATATATATAAATATATATATATATATTCATGTATACTGTATATGTACAATCATATA
TAAAGTTA

TCAAATGCGCCAATTCTTTTTTTTTTTTTTTTTTTTTTTTGATTTCTTTTCATATATCCGATTTAAACCGC
CTAGGGAAGAGTAAAAACCCGAATTGTTCCGTCTATACTTGGTTGTATGGGTGTTTTTTCTTCGATGCTTT
TCGTATAA

TTTCTTCAAGAAAAGACGAAGCTTGTGTGTGGGTGCGCGCGCTAGTATCTTTCCACATTAAGAAATATACC
ATAAAGGTTACTTAGACATCACTATGGCTATATATATATATATATATATATATATATATATATGTAACTTA
GCACCATC

TTGTTAAAAAGCTAAAATGAAAGTAGTAAACCATTGAATGAAAACATTAACCAAAATAAAATTTTTTTTA
AGTCCCAATTGAAATACTTCTCCTTATAAATATCATGAAAGGAAGAAAAAAAAGTGAAAAAAAAAAAAAAA
ACCAATAA

ATGCTGTCAACGCTTTTCATCGATGTATATAAGGAAGAATACCTCATTATAATACATCAAAACCTTCATTT
GCGCGATGTTACGCGTCGCGTTTCCTATAAAAAATACATAAGCTAGTGAATAAAAGCTTTAAACGCGTTAG
GGGGATCT

CACTAAAAAGAAAGTGGTTCACCAGTATGCGTCGGAAACGTCATATAAATCTATATATGTATATGTACATA
TATATATATATATATATATATTAGTGAGTATTTTAATGTATATCAAGTTTTGATAAAGATTGCAACTTGGC
TTGAAATA

ATATATACATTCCTACTAGCTTTTTTTTCTTCAATTCGATAGTTTAGATGGGTTTATATCTAAATATATAT
ATATAGTATTTTATTTCATATTCAATCTCCTAGTATACATGAAATATTTAACGCTTTTTTTTGCCCTTGTT
TTTATTTT

GATGATTCAAGAAAATTCTGGCATTTTTCCTGTCCACCACTGTACGCCATTGTACACAAAAGTATTTTTTT
ATATTTTTTTTACAATTTTTTATAATTTTTTTTTTATGTTTAGTGTATATTTGCTAGAAAGAAACAAGATCT
TTTTTGGT

CCACATTGAAGGGGCGTCACAGCAATTTTTTTTTATTTTTTTTTGATACTCAATAATTACGAGCATATAATT
TTAGTACTTCCAAAAAAAAGTTAAAAAAAAAATAGTGACAAAATGTGTCAGTAAAATATATATGAGAACAT
GGAATGTA

TAAGCTCTGCTGTGCTCTTCTCGTTAAGGAAAAAATGCGATGGTATATATATAGATATATATATATATATA
TGTATCTGTATATATATGTTCATACATATATATATATCTATTATTGTTGTTATTATTATCGTGATTATTAT
TTAGGTCC

ACAATACAAAAAAACACAGTCCTTTGTACTATCCCTTTTATTTCATTATTTTTTCTTTTTTAAGATACCAC
TAGATATTATCATATATAGCATATTATATAACATAAAAAGTCAAGAAAAAAAATGTTTTTATCACTTTCTA
TAACTGCA

AGTCACCAGGTGTATAGACACCACATTGAAGGGGCGTCACAGCAATTTTTTTTTATTTTTTTTTGATACTCA
ATAATTACGAGCATATAATTTAGTACTTCCAAAAAAAAGTTAAAAAAAAAATAGTGACAAAATGTGTCAG
TAAAATAT

TGTCCACCACTGTACGCCATTGTACACAAAAGTATTTTTTATATTTTTTTTACAATTTTTTATAATTTTT
TTTTATGTTTAGTGTATATTTGCTAGAAAGAAACAAGATCTTTTTTGGTAGTGTAAATGCTTAAACTTTGT
CTCTTTTT

ATCGAGCTATAACCAAATTTTTTTTTTTTTTTTTTTTTTCGTTTTATAATTTTTTTCTTTGGTATTCAAT
TGTATATAGTGCTCAAACAGCAACGTACATAATAGCTAGCAAACAATAGATGAAATAATAGAATAATAACA
AATAACTA

TTGAGGGATGGGTCAACCTTCCATAGATTCTATATGGAATAATAAAATTTACTTCTTACTAACATTATATC
AGGGTGAATATTACTGACAAAAATAATAACTTAAGTCTTCTTTATAATATGATGATCGACGCGCGGGGTAA
CGCGCTCT

ATTTAAAAAAATTATAATATAGAAATAATAAATAATAATAATATGATAATAAAATAATATATAAATAAAAA
TGATAAGATTATATTAATTTATTTAATTTTCAAATTATTAAATAAATAATAACCTAATACTTACTTTTCCT
AATATTAT

ATCCCTTTTATTTCATTATTTTTTCTTTTTTAAGATACCACTAGATATTATCATATATAGCATATTATATA
ACATAAAAAGTCAAGAAAAAAAAATGTTTTTATCACTTTCTATAACTGCATATCTTTTTTTGCATTTCGAAT
GATTGCTT

ATAAAATGCTTCACGAACACCGTCATTGATCAAATAGGTTTATAATATTAATATACATTTATATAATCTAC
GGTATTTATATCATCAAAAAAAAAGTAGTTTTTTTATTTTATTTTGTTCGTTAATTTTCAATGTCTATGGA
AACCCGTT

TCATAGTCTCATGCATGGTAAATAGGGTGAACGAAAAAAAATTGGGAGATCTTGCCGACGGCTACGTATATA
TATATATATATATAAATATATATATATATATTCATGTATACTGTATATGTACAATCATATATAAAGTTACT
CTTCCCTT

TTTTTTTCATGCTCGAGGTTTTTAAGGGCGAACTTTAACTAGATGGATTATTCAGTTTTTTTTTAAAAGGAT
CAAAATCCTACATTATATATATATATATATATATATGTTTGTGTGTATATAAAAAAAAAATCATTTCTT
GATCAAGA

CCTTTTCCTTCAACAACAAAAATTCCAAAAAAAAAAAGTAAAAAAACAAAACTTTGATTGTTTTTTAATGA
TGTTAATGATTTTTTTTTTTCTTTCTTTATCATAAAAAAAAAGTTAAAATGAAAAACAAATATGGGTCTGGA
AGGCCATT

TAATGCATTAGAACGTTACCTGGTCATTTGGATGGAGATCTAAGTAACACTTACTATCTCCTATGGTACTA
TCCTTTACCAAAAAAAAAAAAAAAAAAAAAAAAAAAAAAAATCAGCAAAGTGAAGTACCCTCTTGATGTATA
AATACATT

TAATAAAACTTCAAATGCGCCAATTCTTTTTTTTTTTTTTTTTTTTTTTGATTTCTTTTCATATATCCGA
TTTAAACCGCCTAGGGAAGAGTAAAAACCCGAATTGTTCCGTCTATACTTGGTTGTATGGGTGTTTTTTCT
TCGATGCT

CACTCCGGAAAAACTTTATAATAGATGTGGGGAAATCACCTTTGACGATGTGCAACGGAGAAACTATTTT
TTTTTTCTTTTTTTCTTTTTTTTTTTTTTTTGGTCCGAACAATGCGATGAGCCAGACGAAGAAAAAAATA
AAAAATGA

AAAGAAAAAGGAAAGGAACATATTTGTCAGTGCAAGGACCATCATTGTTTGAAAAAAGAGAGGATATTACT
AGTAGGTAATAACAGCTATGTCGCGTTATATATATTATATATAAAAATTAAAAAATAGAAAATAAAATTTC
AAATGCCC

TTTCTTTTTTTTTTTTCTCTTTCCGTATGTAAGCTTTTTTCCTTTTTTTTTTTTTTTTTTTTTTTTTTTT
CTTCACTACTTTTCAATGAAACTCTCTTCAAAAGACAGCATGCAGTAAAAGCGCCGATATGAGTGACGACG
ATTATATG

TTTCCAATGTATGCTGTCAACGCTTTTCATCGATGTATATAAGGAAGAATACCTCATTATAATACATCAAA
ACCTTCATTTGCGCGATGTTACGCGTCGCGTTTCCTATAAAAAATACATAAGCTAGTGAATAAAAGCTTTA
AACGCGTT

TATTATAAAATAGTTCAATTATTTATATTATTTAAATGTTGTAATTTATTATTAATAATTATATTTTATCA
ATATTTATACGATATAAAAATTTAAAAAAATTATAATATAGAAATAATAAATAATAATAATATGATAATAA
AATAATAT

AATGATAGTATTCTTCGCCTGCTTAGGCGTCCTTTTCCTTCAACAACAAAAATTCCAAAAAAAAAAAGTAA
AAAAACAAAACTTTGATTGTTTTTAATGATGTTAATGATTTTTTTTTCTTTCTTTATCATAAAAAAAAA
GTTAAAAT

AAAAGTGAAAAAAAAAAAAAAAAACCAATAAAAAAATAAAAAAATATCAATTCTACGAGAAATGCGTGCGAA
ATCGATGAGTTTTGTTTTGCCTCTGAAAAATTCTGGAAAATTTTTCTTAGCGGAAAAAAGAAAAAAAAGAA
ACGTGATG

TGTTTTTATTATTGTATTATTGTATTATTATTATATTATTATTATTATTATTATTATTATTATTATTACGG
TATGTCAAGTATTATTTTTTTTTTAAAAATAAAGAAAAAAATTGTACAAATATATATATTTATATATTACT
CTATATGG

TTGCCGACGGCTACGTATATATATATATATATATAAATATATATATATATATTCATGTATACTGTATATGT
ACAATCATATATAAAGTTACTCTTCCCTTTTCTTTTTTTTTTACATTATTACGTATATACTGCTTTATTA
TCGGAAAG

TGATGAGTCGAAGCCAAGGGGACTCAGTCACTCAGTCAGCTGATGCGATGCGATGTTAGCGCAAACAATAA
CGCGTACGATAATAAAACTTCAAATGCGCCAATTCTTTTTTTTTTTTTTTTTTTTTTGATTTCTTTTCA
TATATCCG

CCTTTGTACTATCCCTTTTATTTCATTATTTTTTCTTTTTTAAGATACCACTAGATATTATCATATATAGC
ATATTATATAACATAAAAAGTCAAGAAAAAAAAATGTTTTTATCACTTTCTATAACTGCATATCTTTTTTTG
CATTTCGA

AAAAAGAAAAAAACCCTGAAGTCTACTTCATGATTACCCCATACTTACGCGTGGTTTAATTCTATGTAACG
ATTAACGTCCATGTAATCATTATATATATATATATTGTAATAATCTCTCTAATTGAGTATCACAATCTGCT
GCGGTTAC

TTTTATATTTTATTATAAAATAGTTCAATTATTTATATTATTTAAATGTTGTAATTTATTATTAATAATTA
TATTTTATCAATATTTATACGATATAAAAATTTAAAAAAATTATAATATAGAAATAATAAATAATAATAAT
ATGATAAT

AAAACACAGTCCTTTGTACTATCCCTTTTATTTCATTATTTTTTCTTTTTTAAGATACCACTAGATATTAT
CATATATAGCATATTATATAACATAAAAAGTCAAGAAAAAAAAATGTTTTTATCACTTTCTATAACTGCATA
TCTTTTTT

CTTTTTTAAGCACTCCGGAAAAACTTTATAATAGATGTGGGGAAATCACCTTTGACGATGTGCAACGGAGA
AACTATTTTTTTTTTTCTTTTTTTCTTTTTTTTTTTTTTTTTGGTCCGAACAATGCGATGAGCCAGACGAA
GAAAAAAA

TACTTTGGCGAATCAACAAATTGAAGTTGCGCAATGTGCGATTCGCTTATGTATCAAAAAAAACTAAAAAA
TGAATAGAGAAAATCATATATATAAAAGAAAAAAATATCGTACACCTCTTAGGTAACTTGGAATATATTAA
ACATGTAA

GCAATAAATAGTAATATTTATAAAATATAAAAAGAAGAAATTTTTATTCTTTTTATATTTTATTATAAAAT
AGTTCAATTATTTATATTATTTAAATGTTGTAATTTATTATTAATAATTATATTTTATCAATATTTATACG
ATATAAAA

AAAAAAAATAATTAAAAAAAACTTTTATTGAATGCTTAATTATTTAGTGACGCGATTACCTATATACCAGG
TTATTTATTTTTTCTTTTTTCAAAGGAAAACAATACAAAATATTACTGTTACCATTTTTGTTACTATTATT
ATTGTCTT

TTCTTCGCCTGCTTAGGCGTCCTTTTCCTTCAACAACAAAAATTCCAAAAAAAAAAAGTAAAAAAACAAAA
CTTTGATTGTTTTTTAATGATGTTAATGATTTTTTTTTTCTTTCTTTATCATAAAAAAAAAGTTAAAATGA
AAAACAAA

AAAGGTCTGATATGTAGGGGAGGGCATATAATCATTTTAATATATATGTATATATTCTTACATATATATAT
AATATCGCCGTAACTTATAGCGTAAACTATATAAAATACGTCTTAGTCTATAAGCTATCACGGATAGCGTC
TTGGACGT

TGAAATACTTCTCCTTATAAATATCATGAAAGGAAGAAAAAAAAAGTGAAAAAAAAAAAAAAAAACCAATAAA
AAAATAAAAAAATATCAATTCTACGAGAAATGCGTGCGAAATCGATGAGTTTTGTTTTGCCTCTGAAAAAT
TCTGGAAA

GAACAAAATGTGCTTATCTGAGCTAATATTTTTTCATTTTTGTCCTATAAATAATAATGGAAAAAAAAAAC
TCATCGCGTGGTTGAACAGAAAAAAAACAATAAGAGTGAGTAAGCAGTGAAAAATGTAAGATTTACGCTTT
ATATAAGC

GAGGAACCGTCATCTTTTCGCGCGAAAAAACAATATGATGCCTTTTTGAAACATGTTATTATTTGATTTTT
TAATGGCTTCTATTACGCGTCGTGGTTGGTGAACCTGGTACAAAATAAATAAAAAAATACCTGCTAAAAAA
TATCAAAT

TTGGAAAAGTAGTAATGGTTATTCAAAAAGTTAGCAGCGAGATCTTTTTTCTATCGCGCGTTAAAACGTTT
GAACAGATATATATTATTCAGCCACAACTATCTATATATAATATTTCTTCGATTTTTCGTTTTCTCAAGTT
TCGTCTTA

GATTAGACATTTTTTCAATTTTGTCGTTGAGTTCCAATCCTTCCTTATTTATGGTTAACAAGGAAAGAGGTG
GAAGTTAAAAAAATGGAAATTAAAAAAAAAAAAAAATCAAAAAAAGAAAAATAAAAATAGAAAAATTGGCA
CGGAAATC

AACTTTAACTAGATGGATTATTCAGTTTTTTTTAAAAGGATCAAAATCCTACATTATATATATATATATATAT
ATATATGTTTGTGTGTGTATATAAAAAAAAAATCATTTCTTGATCAAGATCAGCCTCTTATATATGTCGCA
TCAATCTT

AGAAAGAAAATTGTTAAAAAGCTAAAATGAAAGTAGTAAACCATTGAATGAAAACATTAACCAAAATAAAA
TTTTTTTTTAAGTCCCAATTGAAATACTTCTCCTTATAAATATCATGAAAGGAAGAAAAAAAAAGTGAAAAA
AAAAAAAA

ACTAATATTTAGTATATAGCTGCGAAGAAAGAAAAAGAAAATATTCACCTCTAGGAGCCAAAAACATAAAA
AAAAAAATAATTAAAAAAAACTTTTATTGAATGCTTAATTATTTAGTGACGCGATTACCTATATACCAGGT
TATTTATT

TCGTTAAGGAAAAAATGCGATGGTATATATATAGATATATATATATATATATATGTATCTGTATATATATGTT
CATACATATATATATATCTATTATTGTTGTTATTATTATCGTGATTATTATTTAGGTCCTCCCTTCTTTTT
ACCTGTTT

CATAAAGGTTACTTAGACATCACTATGGCTATATATATATATATATATATATATATATATATATATGTAACTT
AGCACCATCGCGCGTGCATCACTGCATGTGTTAACCGAAAAGTTTGGCGAACACTTCACCGACACGGTCAT
TTAGATCT

AACATTCTTATTTTATACGTTCGGAAATCATGAACATTTATTTTTATGATTATGTAATTAAAAAAATATAA
TAAATTTATTTTGAAAATTAATTTTTTTAAAATTTTAAATATGTTTTTATCATGTGACTTATGAAAATGAT
ATTTACGG

GTTACCCTGTAAAGGTCTGATATGTAGGGGAGGGCATATAATCATTTTAATATATATGTATATATTCTTAC
ATATATATATAATATCGCCGTAACTTATAGCGTAAACTATATAAAATACGTCTTAGTCTATAAGCTATCAC
GGATAGCG

TAAAAAAAAAAAGTGATCTATAATGGGAAAGAAAAATCCAAACTTCTTCAAAGAGTATATATATGTACAGG
ATATTAAAATGTAATGATAGTAGTAATAATAGTATTAACATAAGCTAAGCGGATCTTGCTGTTTCCAAAGC
TTTTCTTG

TGCTATTGTGGAGGAACCGTCATCTTTTCGCGCGAAAAAACAATATGATGCCTTTTTGAAACATGTTATTA
TTTGATTTTTTAATGGCTTCTATTACGCGTCGTGGTTGGTGAACCTGGTACAAAATAAATAAAAAAATACC
TGCTAAAA

TCTCGAAGAAATGTCAATCCCTTTTTTAAGCACTCCGGAAAAACTTTATAATAGATGTGGGGAAATCACCT
TTGACGATGTGCAACGGAGAAACTATTTTTTTTTTTCTTTTTTTCTTTTTTTTTTTTTTTTGGTCCGAAC
AATGCGAT

TTTTTTTTTTTTTTTTTTTTCGTTTTTATAATTTTTTCTTTGGTATTCAATTGTATATAGTGCTCAAACAG
CAACGTACATAATAGCTAGCAAACAATAGATGAAATAATAGAATAATAACAAATAACTATATAAGAGTGGC
AGGAAAAA

AAAAAAAAAACTCATCGCGTGGTTGAACAGAAAAAAAACAATAAGAGTGAGTAAGCAGTGAAAAATGTAAG
ATTTACGCTTTATATAAGCAGATTTTGGTTTCCTTTATATATTTTTGCATTTGTCTATCCTTAACATTGGA
TAGCAATT

CCTTCTACTATAATGCATTAGAACGTTACCTGGTCATTTGGATGGAGATCTAAGTAACACTTACTATCTCC
TATGGTACTATCCTTTACCAAAAAAAAAAAAAAAAAAAAAAAAAAAAAAATCAGCAAAGTGAAGTACCCTC
TTGATGTA

TTTAAGGGCGAACTTTAACTAGATGGATTATTCAGTTTTTTTTAAAAGGATCAAAATCCTACATTATATAT
ATATATATATATATATGTTTGTGTGTGTATATAAAAAAAAAATCATTTCTTGATCAAGATCAGCCTCTTAT
ATATGTCG

TTCCAATCCTTCCTTATTTATGGTTAACAAGGAAAGAGGTGGAAGTTAAAAAAATGGAAATTAAAAAAAAA
AAAAAATCAAAAAAAGAAAAATAAAAATAGAAAAATTGGCACGGAAATCTACAGGATTTAGCACTAGTAGG
GGGTTTAT

AAAAAAATGAAAAATCAATGAATAAGAGGGCTTGAGTTCCAGGTTCTAGAAATTTCCCTCACTTATATATA
TATATATATCTATATACATATAGTTTTGAGTATATCATAACAGGATTCTCTTTCAATGAAACCTATGTTTT
CCGCGGCA

GGTCAACCTTCCATAGATTCTATATGGAATAATAAAATTTACTTCTTACTAACATTATATCAGGGTGAATA
TTACTGACAAAAATAATAACTTAAGTCTTCTTTATAATATGATGATCGACGCGCGGGGTAACGCGCTCTTC
CCATCTTT

ATATGATAATAAAATAATATATAAATAAAAATGATAAGATTATATTAATTTATTTAATTTTCAAATTATTA
AATAAATAATAACCTAATACTTACTTTTCCTAATATTATTAAAAATGGTTATTGTCAAACCCATTAACTGA
TTATATTA

GGAAATCACCTTTGACGATGTGCAACGGAGAAACTATTTTTTTTTTTCTTTTTTTCTTTTTTTTTTTTTTT
TTGGTCCGAACAATGCGATGAGCCAGACGAAGAAAAAAATAAAAAATGAGAATGAGTAATTATGTTAAGAC
CTTTAGAA

AAGATGTTTTAACTGATCTTAATTTATATGTAGAGGATATATGTAGTATATCTATTTTTTGCTGATTGACT
TATCAATTAGCTTCATAATACAACATTGTTTTTGTTTTTCGTTACTCGCGACTTTCTTCGCGGCAGAGCCG
CGCTACAG

AAAGTGGTTCACCAGTATGCGTCGGAAACGTCATATAAATCTATATATGTATATGTACATATATATATATA
TATATATATATTAGTGAGTATTTTAATGTATATCAAGTTTTGATAAAGATTGCAACTTGGCTTGAAATAAA
CGCATTTT

TTTTATACGTTCGGAAATCATGAACATTTATTTTTATGATTATGTAATTAAAAAAATATAATAAATTTATT
TTGAAAATTAATTTTTTTAAAATTTTAAATATGTTTTTATCATGTGACTTATGAAAATGATATTTACGGTT
CAAGAGAA

TGTACACAAAAGTATTTTTTTATATTTTTTTTACAATTTTTTATAATTTTTTTTTATGTTTAGTGTATATT
TGCTAGAAAGAAACAAGATCTTTTTTGGTAGTGTAAATGCTTAAACTTTGTCTCTTTTTCCTTTTTACTTT
GGAAAAAT

AACCAAATTTTTTTTTTTTTTTTTTTTTTTCGTTTTTATAATTTTTTCTTTGGTATTCAATTGTATATAGT
GCTCAAACAGCAACGTACATAATAGCTAGCAAACAATAGATGAAATAATAGAATAATAACAAATAACTATA
TAAGAGTG

TTCAATTAGTTCGTTTTTCCACAATACAAAAAAACACAGTCCTTTGTACTATCCCTTTTATTTCATTATTT
TTTCTTTTTTAAGATACCACTAGATATTATCATATATAGCATATTATATAACATAAAAAGTCAAGAAAAAA
AATGTTTT

CCGGATTATAAAGCCGATGTAGCAGAAAAACGGGTTCACGGAATACAATAGCCAAATAACAATAAATAAAT
AAATAAATAAATAAGAAAAAAAAAAAAGCAAGTAAATAAAACAAAACGGGATGCAACAAGCCAAGGAGGGAA
ACAATAGA

GCATTGTTGCTTGCAATTAAGTGTAGGTGTTTCCCTATCCACTTAGCTATGAACAAAATGTGCTTATCTGA
GCTAATATTTTTTCATTTTTGTCCTATAAATAATAATGGAAAAAAAAAACTCATCGCGTGGTTGAACAGAA
AAAAAACA

TTTCAAAAGAAAAAAGAAAAAAACCCTGAAGTCTACTTCATGATTACCCCATACTTACGCGTGGTTTAATT
CTATGTAACGATTAACGTCCATGTAATCATTATATATATATATATTGTAATAATCTCTCTAATTGAGTATC
ACAATCTG

GCTTGCAATCCCTTTTTTACTTTCAATTCTCCGTTAGGTTATTTATTCTACTACACGCAGTTTTTTTTTTT
CAATGGGTTATATATCTCTAATATATACACATTTATATGTTTAGACTTATACAGAAGCAAAAAAAAAATGCA
AAAGAAGC

GCTCGAGGTTTTTAAGGGCGAACTTTAACTAGATGGATTATTCAGTTTTTTTTAAAAGGATCAAAATCCTA
CATTATATATATATATATATATATATATGTTTGTGTGTGTATATAAAAAAAAAATCATTTCTTGATCAAGATC
AGCCTCTT

TTCCCTATCCACTTAGCTATGAACAAAATGTGCTTATCTGAGCTAATATTTTTTCATTTTTGTCCTATAAA
TAATAATGGAAAAAAAAAACTCATCGCGTGGTTGAACAGAAAAAAAACAATAAGAGTGAGTAAGCAGTGAA
AAATGTAA

GAACGTTACCTGGTCATTTGGATGGAGATCTAAGTAACACTTACTATCTCCTATGGTACTATCCTTTACCA
AAAAAAAAAAAAAAAAAAAAAAAAAAAAAAATCAGCAAAGTGAAGTACCCTCTTGATGTATAAATACATTGC
ACATCATT

AACAAAAAAATATGAATATAAGAATAAATTGCAATAAATAGTAATATTTATAAAATATAAAAAGAAGAAAT
TTTTATTCTTTTTATATTTTATTATAAAATAGTTCAATTATTTATATTATTTAAATGTTGTAATTTATTAT
TAATAATT

TCATACCTGTCTTTTTATGCTGCTATTGTGGAGGAACCGTCATCTTTTCGCGCGAAAAAACAATATGATGC
CTTTTTGAAACATGTTATTATTTGATTTTTTAATGGCTTCTATTACGCGTCGTGGTTGGTGAACCTGGTAC
AAAATAAA

TACAACTATTCTTTTTCCTATTCCCCTCTGGTTAACAAAAACCAGTATATTAACAGAAATAAATAATAATA
ACAAAAAAATATGAATATAAGAATAAATTGCAATAAATAGTAATATTTATAAAATATAAAAAGAAGAAATT
TTTATTCT

TGTACGCCATTGTACACAAAAGTATTTTTTTATATTTTTTTTACAATTTTTTATAATTTTTTTTTATGTTT
AGTGTATATTTGCTAGAAAGAAACAAGATCTTTTTTGGTAGTGTAAATGCTTAAACTTTGTCTCTTTTTCC
TTTTTACT

ATTCGTACAATCATTTTTACGGTCGAACTTCTGTAGTTACTCGATATATAAATATAAATAACGGACATAAA
AAAAAAAATTGTAACATTATAAATCATAAAATCATAATCATATCTATTTCTAACTTCTGACTATTGCGCAA
TTCGTTAG

AAGTCCCAATTGAAATACTTCTCCTTATAAATATCATGAAAGGAAGAAAAAAAAAGTGAAAAAAAAAAAAAA
AACCAATAAAAAAATAAAAAAATATCAATTCTACGAGAAATGCGTGCGAAATCGATGAGTTTTGTTTTGCC
TCTGAAAA

CCATAGATTCTATATGGAATAATAAAATTTACTTCTTACTAACATTATATCAGGGTGAATATTACTGACAA
AAATAATAACTTAAGTCTTCTTTATAATATGATGATCGACGCGCGGGGTAACGCGCTCTTCCCATCTTTGT
TTCCTTTC

TACGCGTCGCGTTTCCTATAAAAAATACATAAGCTAGTGAATAAAAGCTTTAAACGCGTTAGGGGGATCTG
CAATTAAAGTAAATAAATGAAATAAATGAAATAGATGAAAAACTGGTTACACTAATTATAACGTTAATAAA
ACTAGTTG

AAATAATAATAACAAAAAAATATGAATATAAGAATAAATTGCAATAAATAGTAATATTTATAAAATATAAA
AAGAAGAAATTTTTATTCTTTTTATATTTATTATAAAATAGTTCAATTATTTATATTATTTAAATGTTGT
AATTTATT

GATGGAGATCTAAGTAACACTTACTATCTCCTATGGTACTATCCTTTACCAAAAAAAAAAAAAAAAAAAAA
AAAAAAAAAATCAGCAAAGTGAAGTACCCTCTTGATGTATAAATACATTGCACATCATTGTTGAGAAATAG
TTTTGGAA

TGTCGTTGAGTTCCAATCCTTCCTTATTTATGGTTAACAAGGAAAGAGGTGGAAGTTAAAAAAATGGAAAT
TAAAAAAAAAAAAAAATCAAAAAAAGAAAAATAAAAATAGAAAAATTGGCACGGAAATCTACAGGATTTAG
CACTAGTA

TCACGAACACCGTCATTGATCAAATAGGTTTATAATATTAATATACATTTATATAATCTACGGTATTTATA
TCATCAAAAAAAAAGTAGTTTTTTTATTTTATTTTGTTCGTTAATTTTCAATGTCTATGGAAACCCGTTCG
TAAAATTG

AGCAATTTTTTTTATTTTTTTTTGATACTCAATAATTACGAGCATATAATTTTAGTACTTCCAAAAAAAAG
TTAAAAAAAAAATAGTGACAAAATGTGTCAGTAAAATATATATGAGAACATGGAATGTATTAAAGTAATTC
CATTTCTA

TTTTCGCCGCGCGGTAGGCGAAAAGCAAAGAAAACTACAGTAATATCCAGAAATAGATGCATTTTATGTGC
GAAATAAAACTCATGCTGAAAAAAATAGTAATAATTAATAGGGTAACGAATAGAATGCAAACCTTATCACG
TGAAAGAG

GTAAAAAAAAAAAAAAGAAAAAGCTGTAAAAGACGACAACGTTGGCGTCGCGAAAATATTAATAGAACCATA
TGTTGGATAATGTAGCCTCTATTATACACTTATCCCGATTACATTGTTTTAGCGACGGTAATCATCTTTTT
ATCCGGCA

ATCTACTATGGGCGTGTGGCGTAGTCGGTAGCGCGCTCCCTTAGCATGGGAGAGGTCTCCGGTTCGATTCC
GGACTCGTCCATTTTAAAATTTCTTTTTTTTTTTTTCTCTTTCCGTATGTAAGCTTTTTTCCTTTTTTTTT
TTTTTTTT

AAACGCTGCTATTTAAAAGGAAAAATTTTCCCAATTACGCGGGTAATACAAAAAAGATATAGCCCATTGTT
ATAAAAATATGTATGTGTTCAATATAGTGGTATATAGGTGTTATTTGCGCTGAATGATGTACTTGTCAATG
TCTGCGGG

AATCCCGTGAAGTCTTGGTACTATCAAATTCGTTGGTTTTTTTTCTGGGAAAACTCTATCAAAGTATAAGG
TGCGCTTTTAACGTAATTTATAATGATTTTTTAGAAATAATAATGTATATAGTATAAAAAAATAAACAAAC
TTTAAAAA

AAAAATGCGATGGTATATATATAGATATATATATATATATATGTATCTGTATATATATGTTCATACATATA
TATATATCTATTATTGTTGTTATTATTATCGTGATTATTATTTAGGTCCTCCCTTCTTTTTACCTGTTTTT
AATCTACC

TTATTTTAAGGTTACCCTGTAAAGGTCTGATATGTAGGGGAGGGCATATAATCATTTTAATATATATGTAT
ATATTCTTACATATATATATAATATCGCCGTAACTTATAGCGTAAACTATATAAAATACGTCTTAGTCTAT
AAGCTATC

CTTTCATATTTTTATTTTCGCGTTACACTAAATTCAAATATTAAAAAAGGATCGCTATTAATGTGTAGAAA
TTGAAAAAATTACATACAGAAGCGTATAAAATTCTTATCATCTATTTTCACCCTTCTACTGAGTTATTTCG
GATATTTT

ATTCAAAAAGTTAGCAGCGAGATCTTTTTTCTATCGCGCGTTAAAACGTTTGAACAGATATATATTATTCA
GCCACAACTATCTATATATAATATTTCTTCGATTTTTCGTTTTCTCAAGTTTCGTCTTATTGATTGATCAG
AAGTTTAC

AAGGAAGGAGAGGATGTTGAAGTTCCTTTCATTACCATTCATCTATGAGTAACTAATTAACCAAAATAAAA
AAATAAATTAAATATATACATTAAATATGTATATATATATGTATGACTTTTGACAACTTTTTCTTTGGTAA
AAACTTAT

AAACTTTATAATAGATGTGGGGAAATCACCTTTGACGATGTGCAACGGAGAAACTATTTTTTTTTTTCTTT
TTTTCTTTTTTTTTTTTTTTTTGGTCCGAACAATGCGATGAGCCAGACGAAGAAAAAAATAAAAAATGAGA
ATGAGTAA

AGTAATGGTTATTCAAAAAGTTAGCAGCGAGATCTTTTTTCTATCGCGCGTTAAAACGTTTGAACAGATAT
ATATTATTCAGCCACAACTATCTATATATAATATTTCTTCGATTTTTCGTTTTCTCAAGTTTCGTCTTATT
GATTGATC

TCCTTATTTATGGTTAACAAGGAAAGAGGTGGAAGTTAAAAAAATGGAAATTAAAAAAAAAAAAAAAATCAA
AAAAAGAAAAATAAAAATAGAAAAATTGGCACGGAAATCTACAGGATTTAGCACTAGTAGGGGGTTTATTA
AGCACTAC

TGGTTAACAAGGAAAGAGGTGGAAGTTAAAAAAATGGAAATTAAAAAAAAAAAAAAAATCAAAAAAAGAAAA
ATAAAAATAGAAAAATTGGCACGGAAATCTACAGGATTTAGCACTAGTAGGGGGTTTATTAAGCACTACAG
GTCGACTT

AGCATTTTTTTATATACTATATTATGAGAAATCTTTCTGAAAAATTATGAATTACTTTTTTCTGCTGATAG
TACAACCCGCTATATACTGTTACAAGTAGTGAATAAAGAAGTAAAAAAAAAAAAAACTTTCAAAGGAAAGAA
TATGAAGT

TTCAAAGTCCAAAAGACTCTCGGCACGAAGGTTGAAATACCCTATACTAATTGTTTGCTTTGTCTTTTTGT
ATATATCCGAACGTATCTATCTGAAATTTTTCAAATTTATAAAAAATAATATATATAAAAGGAAAAAAAAA
AAAATCGA

TATATATCCGAACGTATCTATCTGAAATTTTTCAAATTTATAAAAAATAATATATATAAAAGGAAAAAAAA
AAAAATCGATGCCCTACTAATAGAGATTGGAGCTGAAAAGAAAAAGTGTACATCAAACGCTATATCTCGAG
GTTTGGGA

ATAGATGTGGGGAAATCACCTTTGACGATGTGCAACGGAGAAACTATTTTTTTTTTTCTTTTTTTCTTTTT
TTTTTTTTTTTGGTCCGAACAATGCGATGAGCCAGACGAAGAAAAAAATAAAAAATGAGAATGAGTAATT
ATGTTAAG

TTTTCAATTTTGTCGTTGAGTTCCAATCCTTCCTTATTTATGGTTAACAAGGAAAGAGGTGGAAGTTAAAA
AAATGGAAATTAAAAAAAAAAAAAAATCAAAAAAAGAAAAATAAAAATAGAAAAATTGGCACGGAAATCTA
CAGGATTT

TATGAATATAAGAATAAATTGCAATAAATAGTAATATTTATAAAATATAAAAAGAAGAAATTTTTATTCTT
TTTATATTTTATTATAAAATAGTTCAATTATTTATATTATTTAAATGTTGTAATTTATTATTAATAATTAT
ATTTTATC

AATCAACAAATTGAAGTTGCGCAATGTGCGATTCGCTTATGTATCAAAAAAAACTAAAAAATGAATAGAGA
AAATCATATATATAAAAGAAAAAAATATCGTACACCTCTTAGGTAACTTGGAATATATTAAACATGTAAAG
AAGGAGAA

TTGAAGTTGCGCAATGTGCGATTCGCTTATGTATCAAAAAAAACTAAAAAATGAATAGAGAAAATCATATA
TATAAAAGAAAAAAATATCGTACACCTCTTAGGTAACTTGGAATATATTAAACATGTAAAGAAGGAGAAAA
CATTAACC

AAACTCTATCAAAGTATAAGGTGCGCTTTTAACGTAATTTATAATGATTTTTTAGAAATAATAATGTATAT
AGTATAAAAAAATAAACAAACTTTAAAAAAAAGAGTGAAGAACTGTAAAGCTCATTTCATAGGTTTCAAAA
GAAAGCTT

TAAGTAACACTTACTATCTCCTATGGTACTATCCTTTACCAAAAAAAAAAAAAAAAAAAAAAAAAAAAAAA
TCAGCAAAGTGAAGTACCCTCTTGATGTATAAATACATTGCACATCATTGTTGAGAAATAGTTTTGGAAGT
TGTCTAGT

TGCTTTTTCCTGCTCATTTATAGTTAACTTGATTTGAAATTACGCGTCGCGGGGAATCGCGTAAAACTCGA
TATTAAATCCTGTGATTTTGCAAAAGTAAATTTTTTACTATCTACATCTCTCCAATTTGCACAACTTTGAC
AGGACTGA

CATGCCATCTTAAGCTCTGCTGTGCTCTTCTCGTTAAGGAAAAAATGCGATGGTATATATATAGATATATA
TATATATATATGTATCTGTATATATATGTTCATACATATATATATATCTATTATTGTTGTTATTATTATCG
TGATTATT

CGCATACACAATATATACATTCCTACTAGCTTTTTTTTCTTCAATTCGATAGTTTAGATGGGTTTATATCT
AAATATATATATATAGTATTTTATTTCATATTCAATCTCCTAGTATACATGAAATATTTAACGCTTTTTTT
TGCCCTTG

AGATGGATTATTCAGTTTTTTTTTAAAAGGATCAAAATCCTACATTATATATATATATATATATATATGTTT
GTGTGTGTATATAAAAAAAAAATCATTTCTTGATCAAGATCAGCCTCTTATATATGTCGCATCAATCTTCT
GTAGGGAT

ATCTACCATCAAACTGCCATTTTTGATCCCAAACGCTGCTATTTAAAAGGAAAAATTTTCCCAATTACGCG
GGTAATACAAAAAAGATATAGCCCATTGTTATAAAAATATGTATGTGTTCAATATAGTGGTATATAGGTGT
TATTTGCG

TTTGTAAGTACTTTCATATTTTTATTTTCGCGTTACACTAAATTCAAATATTAAAAAAGGATCGCTATTAA
TGTGTAGAAATTGAAAAAATTACATACAGAAGCGTATAAAATTCTTATCATCTATTTTCACCCTTCTACTG
AGTTATTT

TAACAGAAATAAATAATAATAACAAAAAAATATGAATATAAGAATAAATTGCAATAAATAGTAATATTTAT
AAAATATAAAAAGAAGAAATTTTTATTCTTTTTATATTTTATTATAAAATAGTTCAATTATTTATATTATT
TAAATGTT

AAATATCATAATAAACAAACCCCTCCCGCGAAAACAAAAAAAGAGAGCTAAAAAAAGTACTTCTTCGCGTT
ATAGATGGTGCCGCACCGTACGTCATAACTAAAAATAGCGTTAGCCACAGCTCCCTGAACGAAAAGGGCCC
TTCGGCCC

TTTTTTTCTTCCGTAGACGAGGAAAAACAAAAATGATTAAATAGGCGCGCGCGGTGTGATGGTGATGATAA
TGATGATGATGATGATAATGATGAATAAACGTTTCGGTTTACGAGTTTCTTTAAATATACATATATGATAT
AAAAAAAA

TGTGCTCTTCTCGTTAAGGAAAAAATGCGATGGTATATATATAGATATATATATATATATATGTATCTGTA
TATATATGTTCATACATATATATATATCTATTATTGTTGTTATTATTATCGTGATTATTATTTAGGTCCTC
CCTTCTTT

AAATTCTCGTAACATTCTTATTTTATACGTTCGGAAATCATGAACATTTATTTTTATGATTATGTAATTAA
AAAAATATAATAAATTTATTTTGAAAATTAATTTTTTTAAAATTTTAAATATGTTTTTATCATGTGACTTA
TGAAAATG

CGTCATTGATCAAATAGGTTTATAATATTAATATACATTTATATAATCTACGGTATTTATATCATCAAAAA
AAAAGTAGTTTTTTTATTTTATTTTGTTCGTTAATTTTCAATGTCTATGGAAACCCGTTCGTAAAATTGGC
GTTTGTCT

ATTACTTTTTTCTGCTGATAGTACAACCCGCTATATACTGTTACAAGTAGTGAATAAAGAAGTAAAAAAAA
AAAAACTTTCAAAGGAAAGAATATGAAGTGAACATTTTTTTCTACGATTAAGATCCCCCTGTTTCTTTTTT
CTTTTTGA

AACCTTTTCAACCATAAAAATAGAAAGGGCACAGGGTTGGGGTATTTGAATTTTTTTTTTGGGTTTTTTCG
TATTACTTATTACTTACCTTCTTTTCTATATAATTTTGTTTTTCCCTGGTAGAGCGGAATCTTCCCACTAA
ATTTTTAG

TCTATATGTGATAAAATGATGATTATTGTAAATCCAAAACGTAAAACCGAAAATATGATAAAAACGAGAAA
AAAAGTAAGTTAAAAAATAAAAAAATAAAAAAATGAACGCCGGCCCCAGAGGGAAGAACAAGCTATTTATT
TTCTTTTT

GGAAGTTAAAAAAATGGAAATTAAAAAAAAAAAAAAATCAAAAAAAGAAAAATAAAAATAGAAAAATTGGC
ACGGAAATCTACAGGATTTAGCACTAGTAGGGGGTTTATTAAGCACTACAGGTCGACTTTAAACCAGAAAA
AATTATCA

TGTTTATATCACTAATATTTAGTATATAGCTGCGAAGAAAGAAAAAGAAAATATTCACCTCTAGGAGCCAA
AAACATAAAAAAAAAAATAATTAAAAAAAACTTTTATTGAATGCTTAATTATTTAGTGACGCGATTACCTA
TATACCAG

ATGTCAATCCCTTTTTTAAGCACTCCGGAAAAACTTTATAATAGATGTGGGGAAATCACCTTTGACGATGT
GCAACGGAGAAACTATTTTTTTTTTTCTTTTTTTCTTTTTTTTTTTTTTTTTGGTCCGAACAATGCGATGA
GCCAGACG

AAAAAACAAAACTTTGATTGTTTTTTAATGATGTTAATGATTTTTTTTTTCTTTCTTTATCATAAAAAAAA
AGTTAAAATGAAAAACAAATATGGGTCTGGAAGGCCATTATTTTTTTTTTATTTATATACCGTTTCTGGTA
CTTAGTTA

TATTATGGGTAATGATAGTATTCTTCGCCTGCTTAGGCGTCCTTTTCCTTCAACAACAAAAATTCCAAAAA
AAAAAAGTAAAAAAACAAAACTTTGATTGTTTTTTAATGATGTTAATGATTTTTTTTTTCTTTCTTTATCA
TAAAAAAA

AAACGCGAAGGAACGAAAGAAGATGAAAAAAAATGCAGCGATAAAATGATATTGTGGTTAATCTAAATTTAT
ATATATATATATATATACATATATATATAGAAATCTATTGTTATACACAAAAATACTTATTTTTTAATATA
GATGGCTG

ACGAGAAAAGAAACGCGAAGGAACGAAAGAAGATGAAAAAAATGCAGCGATAAAATGATATTGTGGTTAAT
CTAAATTTATATATATATATATATATACATATATATATAGAAATCTATTGTTATACACAAAAATACTTATT
TTTTAATA

GGAAGCCAAGAATTGAGAAAAAAGAAAAACCCGCGAGTAAGGAAATTAAATACAGGTGTACACATACACGC
ACACATATATATATATATATATATATGTATATGTGTATATAGGAAGCGCGCATGTTAGTATATACGATTCG
TTGGAAAG

AAAGAAAAACCCGCGAGTAAGGAAATTAAATACAGGTGTACACATACACGCACACATATATATATATATAT
ATATGTATATGTGTATATAGGAAGCGCGCATGTTAGTATATACGATTCGTTGGAAAGGGGCCGTCCACC
AAACGTGA

TGACCTTTTTTGATTATGTTACCACCCCATTTTTGCATTCTTCTTTTTTTTTTTTTTTTTGGGTCTGAAAA
TTTTTTTGCTTCTACTTAATTATAATTTTATATAATCATATATGGATCTGATTCTTAAAAAAAAATGAAAA
AAAGACAA

CTGGAAATGTAAGATGAATTCAAGTATCAAGTGCCGTTGGTATTATAGTTTATCAATTAATACGTAAATAT
ATATATATATATATATATATATATATATATGACTATAGTGGCATACTTCTCAATGGACTGTTGCTTCTATCAT
TTTATACA

TAATCACTTTCAACCGCGGTTTATCCGGCCCCACCCATGCATAACCCTAAATTATTAGATCACTTAGCACG
TGAAAAAGAAACGTTTTTAATGTTTTTTTTTTTTTTTTCTTTTTCTTTTTTTGCGTTGGTGAAAATTTTTT
CGCTTCCT

TTTATTGATTTTAAAAACAATATAAATGAAAGAAAAAAATGTGTTTTAGATTAGGCTTATAAGATATGACC
CATATCATAATTAAAAAAAGCGATTTAGTTGTTCTTTTAAAAAAAAAGAGAAAAAAAAAAAAAAAAAAAA
GGGAATGA

CCATTTTTTTCTATTTACCCTTCTTCTTTTTATTTTACGCGTCTCGCGTTTTATATATTAATAGTAATAAT
ATTTAATAGTGTATGTTTGAAATTTTTATATGTCTTTATTTCGCTAACGTAAAAATCCCTTCTTCTTTGTA
GGGACGGG

TTTTGCATTCTTCTTTTTTTTTTTTTTTTTGGGTCTGAAAATTTTTTTGCTTCTACTTAATTATAATTTTA
TATAATCATATATGGATCTGATTCTTAAAAAAAAATGAAAAAAAGACAAAACAAACCATATAGGTAACAAT
TTCTCTTA

TATCACTCGGCTCTCTAAAAGAAGTCATGTTCCCTTCGTTGGGTTAATTTTTTTTTTTTTTTTTTTTGTTT
ATATGTTTTTTTTTTTTTTTGCCAGGTATTGTTAGCGCCAGATAGCAAAAAATTTTATTCAGGGATACTGAA
CACCGGGG

TTCTTCATTTCCATTTTTTTTCTATTTACCCTTCTTCTTTTTATTTTACGCGTCTCGCGTTTTATATATTAA
TAGTAATAATATTTAATAGTGTATGTTTGAAATTTTTATATGTCTTTATTTCGCTAACGTAAAAATCCCTT
CTTCTTTG

AGATGAAAAAAATGCAGCGATAAAATGATATTGTGGTTAATCTAAATTTATATATATATATATATATACAT
ATATATATAGAAATCTATTGTTATACACAAAAATACTTATTTTTTAATATAGATGGCTGCAGATAAAGTAA
TAGTTTTA

AACTCTGTTTTTCTTCATTTCCATTTTTTTTCTATTTACCCTTCTTCTTTTTATTTTACGCGTCTCGCGTTT
TATATATTAATAGTAATAATATTTAATAGTGTATGTTTGAAATTTTTATATGTCTTTATTTCGCTAACGTA
AAAATCCC

TTAAAAACAATATAAATGAAAGAAAAAAATGTGTTTTAGATTAGGCTTATAAGATATGACCCATATCATAA
TTAAAAAAAGCGATTTAGTTGTTCTTTTAAAAAAAAAGAGAAAAAAAAAAAAAAAAAAAAAAGGAATGAGT
TTGTACAT

CAACCGCGGTTTATCCGGCCCCACCCATGCATAACCCTAAATTATTAGATCACTTAGCACGTGAAAAAGAA
ACGTTTTTAATGTTTTTTTTTTTTTTTTTCTTTTTCTTTTTTTGCGTTGGTGAAAATTTTTTCGCTTCCTCG
AGTATAAT

AATTGAGAAAAAAGAAAAACCCGCGAGTAAGGAAATTAAATACAGGTGTACACATACACGCACACATATAT
ATATATATATATATGTATATGTGTATATAGGAAGCGCGCGCATGTTAGTATATACGATTCGTTGGAAAGGG
GCCGTCCA

ATTCAATATCAAAAAAAGAAATCCATTGGGCCCTTAGTGCATTTTTATTTTTTTTTTTTTTTTTTTTTTTT
TTCTCTTTCTGAATTAGAGTACATCTCATCGCAGGGTCTCTTTTTGCACGTTTTTGATGTTGCTTATGAGA
CGCTTTGC

TTTTCCTAAGAATTTTCATTATTAGAAGGAGTAATGCTTACGGTACACATACATACACATATATATATATA
AATATATATATATATAAGGACATATACAGATTTTTTTTTTTCGCGTACTAAAGTACAGAACAAAGAAAATA
AGAAAAGA

GTTGAATTTACAAAAGGTAATTTATAGTACGTGAACATGTGTGCGTATATACATATATATATATATATATA
TATATATATATATATATATACTTTAGCATCGAAACAGCTAGTCCTTTTTAGAGTTCAATTTTTCCACAAAG
GCTTTTTT

ACCACCCCATTTTTGCATTCTTCTTTTTTTTTTTTTTTTTTGGGTCTGAAAATTTTTTGCTTCTACTTAAT
TATAATTTTATATAATCATATATGGATCTGATTCTTAAAAAAAAATGAAAAAAAGACAAAACAAACCATAT
AGGTAACA

TTAGTTGAATAACTCTGTTTTTCTTCATTTCCATTTTTTTCTATTTACCCTTCTTCTTTTTATTTTACGCG
TCTCGCGTTTTATATATTAATAGTAATAATATTTAATAGTGTATGTTTGAAATTTTTATATGTCTTTATTT
CGCTAACG

TGCGGCAATTACTTTTCCCCAAAAGCTCATAATCGATCAATTAACGCGTAAACGAAAAACAAAAAAAAAAA
AAATACGTAAATTAGGTCTTCATACGAGCGTTTTTTTTTTTTTGAGGATCGTTTGCATGTCTAGCGCGGCA
AGCGTGTT

AAATGTTTTTATTGTTCGGATAACAAATACAATAGTGTTATTAAAAAATAAAACTTATTTAAAAATAGTAA
TTTAAATTATTATTTTATTTTAATAAACTTTTTAATAATATTTATTACACGTGATTTAATATATCCTGTTT
TTTTTTCA

CTATTTACCCTTCTTCTTTTTATTTTACGCGTCTCGCGTTTTATATATTAATAGTAATAATATTTAATAGT
GTATGTTTGAAATTTTTATATGTCTTTATTTCGCTAACGTAAAAATCCCTTCTTCTTTGTAGGGACGGGGG
AGTGGTTA

AGTCACTATTATTTTTTTTTTTTATTCATAAGTACAATTGAATAATATTATTGATTTTTTTTATTATTGTTCT
TATTCTTTTTTTATCATTTCTATATAAAATATTATATGATTTAGTTTATCGTAAAAAAATTAAAAAATACA
GAGGAAAG

AATTTTCATTATTAGAAGGAGTAATGCTTACGGTACACATACATACACATATATATATATATAAATATATATA
TATATAAGGACATATACAGATTTTTTTTTTTCGCGTACTAAAGTACAGAACAAAGAAAATAAGAAAAGAAG
GCGATCAT

TCGAGAAAGAGGAAGCCAAGAATTGAGAAAAAAGAAAAACCCGCGAGTAAGGAAATTAAATACAGGTGTAC
ACATACACGCACACATATATATATATATATATATATGTATATGTGTATATAGGAAGCGCGCGCATGTTAGTAT
ATACGATT

TTCCTTTCTCTTTTCCTAAGAATTTTCATTATTAGAAGGAGTAATGCTTACGGTACACATACATACACATA
TATATATATAAATATATATATATATATAAGGACATATACAGATTTTTTTTTTTTCGCGTACTAAAGTACAGAAC
AAAGAAAA

CGACAGCCGCTGCGGCAATTACTTTTCCCCAAAAGCTCATAATCGATCAATTAACGCGTAAACGAAAAACA
AAAAAAAAAAAAAATACGTAAATTAGGTCTTCATACGAGCGTTTTTTTTTTTTTGAGGATCGTTTGCATGTC
TAGCGCGG

CAAAAGGTAATTTATAGTACGTGAACATGTGTGCGTATATACATATATATATATATATATATATATATATA
TATATATATACTTTAGCATCGAAACAGCTAGTCCTTTTTAGAGTTCAATTTTTCCACAAAGGCTTTTTTAG
CCAATTCA

AACAATCTTGCGGCACACCGCTTGAACGAAACGTTCGTAAAAATATTTATAATTCTTATTATTATTATTTA
TTATTATTATAATATATATAATTTAATAATCTTAATATTTATTATCATTGTTTCATATTTTTTTTTATAAA
TATTTATT

CGACAGTGAAAAATGCACAGAACATTCAAAATTATACTTATAATATGACTCTTTTCTTTTATTGCAGTTGG
TATTATATACATATATATATATATATATATATGTATATGTTCCCCGAGACAATGGTTTTATTTCCAAAAAA
AGTGTAAG

AATTATATACTCGAGAAAGAGGAAGCCAAGAATTGAGAAAAAAGAAAAACCCGCGAGTAAGGAAATTAAAT
ACAGGTGTACACATACACGCACACATATATATATATATATATATGTATATGTGTATATAGGAAGCGCGCGC
ATGTTAGT

AAGAAAGAGTTTTATTGATTTTAAAAACAATATAAATGAAAGAAAAAAATGTGTTTTAGATTAGGCTTATA
AGATATGACCCATATCATAATTAAAAAAAGCGATTTAGTTGTTCTTTTAAAAAAAAAGAGAAAAAAAAAAA
AAAAAAAA

AAAATCATCACTGGAAATGTAAGATGAATTCAAGTATCAAGTGCCGTTGGTATTATAGTTTATCAATTAAT
ACGTAAATATATATATATATATATATATATATATATATATGACTATAGTGGCATACTTCTCAATGGACTGTTG
CTTCTATC

AATGCAGCGATAAAATGATATTGTGGTTAATCTAAATTTATATATATATATATATATACATATATATATAG
AAATCTATTGTTATACACAAAAATACTTATTTTTTAATATAGATGGCTGCAGATAAAGTAATAGTTTTATA
TATAGGTA

AAAAAAAGAAATCCATTGGGCCCTTAGTGCATTTTTATTTTTTTTTTTTTTTTTTTTTTTTCTCTTTCT
GAATTAGAGTACATCTCATCGCAGGGTCTCTTTTTGCACGTTTTTGATGTTGCTTATGAGACGCTTTGCCT
TTTTAACT

TTCTTTTTTTTTTTTTTTTGGGTCTGAAAATTTTTTTGCTTCTACTTAATTATAATTTTATATAATCATA
TATGGATCTGATTCTTAAAAAAAAATGAAAAAAAGACAAAACAAACCATATAGGTAACAATTTCTCTTAGT
GACATAAA

TGATTATGTTACCACCCCATTTTTGCATTCTTCTTTTTTTTTTTTTTTTTTGGGTCTGAAAATTTTTTTGCT
TCTACTTAATTATAATTTTATATAATCATATATGGATCTGATTCTTAAAAAAAAATGAAAAAAAGACAAAA
CAAACCAT

CCGCGAGTAAGGAAATTAAATACAGGTGTACACATACACGCACACATATATATATATATATATATGTATAT
GTGTATATAGGAAGCGCGCGCATGTTAGTATATACGATTCGTTGGAAAGGGGCCGTCCACCAAACGTGACT
TGACGAGT

GCAAGCTATTCGACAGTGAAAAATGCACAGAACATTCAAAATTATACTTATAATATGACTCTTTTCTTTTA
TTGCAGTTGGTATTATATACATATATATATATATATATATATGTATATGTTCCCCGAGACAATGGTTTTAT
TTCCAAAA

AATTCTTATTATTATTATTTATTATTATTATAATATATATAATTTAATAATCTTAATATTTATTATCATTG
TTTCATATTTTTTTTTATAAATATTTATTTAAATAAAAAATATTAAAGAGTATTCAAAAAAGTCTTACTGT
GGGTAATG

ATAAATATGTTTAATGAATGATTCAATATCAAAAAAAGAAATCCATTGGGCCCTTAGTGCATTTTTATTTT
TTTTTTTTTTTTTTTTTTTTTCTCTTTCTGAATTAGAGTACATCTCATCGCAGGGTCTCTTTTTGCACGT
TTTTGATG

CAGTTCCAGAAAAAGAGGCTGTTGAATTTACAAAAGGTAATTTATAGTACGTGAACATGTGTGCGTATATA
CATATATATATATATATATATATATATATATATATATACTTTAGCATCGAAACAGCTAGTCCTTTTTAG
AGTTCAAT

GAGTAGTGTAGTGGGTGACCATACGCGAAACTCAGGTGCTGCAATCTTTATTTCTTTTTTTTTTTTTTTT
TTTTTTTTTTTTCTAGTTTCTTGGCTTCCTATGCTAAATCCCATAACTAACCTACCATTCGATTCAGAAAA
ATTCGCAC

GTTGTCAGAATTAGGTGCCTCCTGCTTTTTTTTTTTTTCCTGCTCTTATATCCGTTATATCCGAATGATTTT
TATCGCTTGTTTAAAAAATACTTTCCCGATATATATATATAGTCTCCCTTTAAATTTGTTTCCGGTAAGTT
TTTAACAC

CATCCCATTCCATTCGCTACTTCCTTTCTCTTTTCCTAAGAATTTTCATTATTAGAAGGAGTAATGCTTAC
GGTACACATACATACACATATATATATATAAATATATATATATATAAGGACATATACAGATTTTTTTTTT
CGCGTACT

CAACTGGCCTTATCACTCGGCTCTCTAAAAGAAGTCATGTTCCCTTCGTTGGGTTAATTTTTTTTTTTTTT
TTTTTTGTTTATATGTTTTTTTTTTTTTTGCCAGGTATTGTTAGCGCCAGATAGCAAAAAATTTTATTCAG
GGATACTG

GTTTGAAAAAAAAAAAAAAAAAATTTCCCTTATCAATGATATCCTTACGATTATATAAATTCCTTACCTAAA
CCTATTATTTGTGTACATATATCAGAGTATTATTACATATATAACCTTTTTCTCTAAAACAGGAAAAAAAA
AAGAAAAC

CTAGTCTTCAAGTCACTATTATTTTTTTTTTTTATTCATAAGTACAATTGAATAATATTATTGATTTTTTTA
TTATTGTTCTTATTCTTTTTTTATCATTTCTATATAAAATATTATATGATTTAGTTTATCGTAAAAAAATT
AAAAAATA

CATTCGCTACTTCCTTTCTCTTTTCCTAAGAATTTTCATTATTAGAAGGAGTAATGCTTACGGTACACATA
CATACACATATATATATATAAATATATATATATATAAGGACATATACAGATTTTTTTTTTTCGCGTACTAA
AGTACAGA

TATAAATGAAAGAAAAAAATGTGTTTTAGATTAGGCTTATAAGATATGACCCATATCATAATTAAAAAAAG
CGATTTAGTTGTTCTTTTAAAAAAAAAGAGAAAAAAAAAAAAAAAAAAAAAAGGGAATGAGTTTGTACATAC
TATTATAT

ATTTTTTTTTTTTATTCATAAGTACAATTGAATAATATTATTGATTTTTTTATTATTGTTCTTATTCTTTTT
TTATCATTTCTATATAAAATATTATATGATTTAGTTTATCGTAAAAAAATTAAAAAATACAGAGGAAAGCA
AAAGTAAA

TTAATGAATGATTCAATATCAAAAAAAGAAATCCATTGGGCCCTTAGTGCATTTTTATTTTTTTTTTTTTTT
TTTTTTTTTTTTCTCTTTCTGAATTAGAGTACATCTCATCGCAGGGTCTCTTTTTGCACGTTTTTGATGTT
GCTTATGA

ATTAGAAGGAGTAATGCTTACGGTACACATACATACACATATATATATATAAATATATATATATATAAGGA
CATATACAGATTTTTTTTTTTCGCGTACTAAAGTACAGAACAAAGAAAATAAGAAAAGAAGGCGATCATTG
AACTACTG

TAACAGATATTTAGTTGAATAACTCTGTTTTTCTTCATTTCCATTTTTTTCTATTTACCCTTCTTCTTTTT
ATTTTACGCGTCTCGCGTTTTATATATTAATAGTAATAATATTTAATAGTGTATGTTTGAAATTTTTATAT
GTCTTTAT

GAACGAAAGAAGATGAAAAAAATGCAGCGATAAAATGATATTGTGGTTAATCTAAATTTATATATATATAT
ATATATACATATATATATAGAAATCTATTGTTATACACAAAAATACTTATTTTTTAATATAGATGGCTGCA
GATAAAGT

CTCTCTAAAAGAAGTCATGTTCCCTTCGTTGGGTTAATTTTTTTTTTTTTTTTTTTTGTTTATATGTTTTT
TTTTTTTTTGCCAGGTATTGTTAGCGCCAGATAGCAAAAAATTTTATTCAGGGATACTGAACACCGGGGAA
GTTAACTG

AAAAGAGGCTGTTGAATTTACAAAAGGTAATTTATAGTACGTGAACATGTGTGCGTATATACATATATATA
TATATATATATATATATATATATATATATACTTTAGCATCGAAACAGCTAGTCCTTTTTAGAGTTCAATTT
TTCCACAA

TCCCTGGATTTTGAGATGGGAAAAGTTTCAATGCGTTCTATCGTATAGTATGATGTATTCAAAAAAAATGC
GATAATTCTTTAGTTGTCGGCACTTCGGAAGCCAAAAAAAAAAAAAAAAAAAAAAAAGAAAAAAAGAAAAG
GGTGTCTA

AGAAAAAAATGTGTTTTAGATTAGGCTTATAAGATATGACCCATATCATAATTAAAAAAAGCGATTTAGTT
GTTCTTTTAAAAAAAAAGAGAAAAAAAAAAAAAAAAAAAAAGGGAATGAGTTTGTACATACTATTATATTA
ATGTAGTA

CCCAGAAAAAAAAATCATCACTGGAAATGTAAGATGAATTCAAGTATCAAGTGCCGTTGGTATTATAGTTT
ATCAATTAATACGTAAATATATATATATATATATATATATATATATATGACTATAGTGGCATACTTCTCAA
TGGACTGT

GGAGTGAATCTTAAAAGCCCGAAAAGGAAATTCAAAATCTGTCTATTTATAGGCCGTCGCGCTCTACGAAA
ACGCGAAATTATTCAAACGGAAAACGGAAAAAAATCTAAAAAAAGAAATTAATTGAGAGATCTCACGGAAA
TGCCGCGA

AAAAAAGAAAAGACTCCGTTTAATCACTTTCAACCGCGGTTTATCCGGCCCCACCCATGCATAACCCTAAA
TTATTAGATCACTTAGCACGTGAAAAAGAAACGTTTTAATGTTTTTTTTTTTTTTTTTCTTTTTCTTTTTT
TGCGTTGG

ACTTTTACGACAACTGGCCTTATCACTCGGCTCTCTAAAAGAAGTCATGTTCCCTTCGTTGGGTTAATTTT
TTTTTTTTTTTTTTTTGTTTATATGTTTTTTTTTTTTTTGCCAGGTATTGTTAGCGCCAGATAGCAAAAAA
TTTTATTC

ACGTTCGTAAAAATATTTATAATTCTTATTATTATTATTTATTATTATTATAATATATATAATTTAATAAT
CTTAATATTTATTATCATTGTTTCATATTTTTTTTTATAAATATTTATTTAAATAAAAAATATTAAAGAGT
ATTCAAAA

ACTTTTCCCCAAAAGCTCATAATCGATCAATTAACGCGTAAACGAAAAACAAAAAAAAAAAAAATACGTAA
ATTAGGTCTTCATACGAGCGTTTTTTTTTTTTTTTGAGGATCGTTTGCATGTCTAGCGCGGCAAGCGTGTTTA
CAGGCATT

TAAAAAAATTTTCCGCAGGATATAGAAAAAAAAGAAATGAAATTATAGTAGCGGTTATTTCCGTGGGGTGC
TTTTTTACACCTGTACATCTTTTCCCTCCGTACATTTTTTTTTATTTTTTTTTTTGGTTTTTTTTTTCGAT
ATTTTTCC

CGGACCCTGAATTACTGACACAAAATGAAGGGATCAGTGGCCCTGAAAAAAAAAAAAAAAAAATAAACGGC
GCGCGGAGTAATTATGATTATGATTATGGTTATGATTATTATTACAACTCACTATTCTAAGTCTATATCGT
AAATAAGG

TAACAAATACAATAGTGTTATTAAAAAATAAAACTTATTTAAAAATAGTAATTTAAATTATTATTTTATTT
TAATAAACTTTTAATAATATTTATTACACGTGATTTAATATATCCTGTTTTTTTTTTCATCATTCTCTTTC
TTTCTTAT

GTAATGCTTACGGTACACATACATACACATATATATATATAAATATATATATATATAAGGACATATACAGA
TTTTTTTTTTCGCGTACTAAAGTACAGAACAAAGAAAATAAGAAAAGAAGGCGATCATTGAACTACTGTG
ACTTGCCA

AAAAAAAAAAAAAAAATAAAAAATAAAAAGACCAAATAGTAAATAGTAACTTACATACATTAGTAAATGGT
ACACTCTTACACACTATCATCCTCATCGTATATTATAATAGATATATACAATACATGTTTTTACCCGGATC
ATAGAATT

AACCATAGAGATGAACTTAAGAAAAAAGGGCCACGGGATTTTTTTTCGCGTTTTGGCGTTTTCATATCAA
AAAAAAAAAACCATATTAACATCTGTACATTATTATTAACTAAACATCCGTACACAAAAGGTTTTCCAAGA
CAAAACGT

GTGGGTGACCATACGCGAAACTCAGGTGCTGCAATCTTTATTTCTTTTTTTTTTTTTTTTTTTTTTTTT
TTCTAGTTTCTTGGCTTCCTATGCTAAATCCCATAACTAACCTACCATTCGATTCAGAAAAATTCGCACTA
TCCAGCTG

GAACATAAATGTTATGGACAAGAGTAGATATCAAAATAAAACAAAATAAAATAGAGTAAGTGAAAGACAAA
TGGAGGAAAAAAAAAAAAAAAAAAAAAAAAAAAAAAATAGTAAAAAGTGAAAGGAGAACGATGATAACACTA
ATCACACC

AAGATGAATTCAAGTATCAAGTGCCGTTGGTATTATAGTTTATCAATTAATACGTAAATATATATATATAT
ATATATATATATATATATGACTATAGTGGCATACTTCTCAATGGACTGTTGCTTCTATCATTTTATACATG
CGTTCAAC

CGGCACACCGCTTGAACGAAACGTTCGTAAAAATATTTATAATTCTTATTATTATTATTTATTATTATTAT
AATATATATAATTTAATAATCTTAATATTTATTATCATTGTTTCATATTTTTTTTTATAAATATTTATTTA
AATAAAAA

TGCAACGAAAAAAAAAAAAAAAAAATAAAAAATAAAAAGACCAAATAGTAAATAGTAACTTACATACATTAGT
AAATGGTACACTCTTACACACTATCATCCTCATCGTATATTATAATAGATATATACAATACATGTTTTTAC
CCGGATCA

ATTAAAAAAAGCGATTTAGTTGTTCTTTTAAAAAAAAAAGAGAAAAAAAAAAAAAAAAAAAAAAGGGAATGAG
TTTGTACATACTATTATATTAATGTAGTATCACGTTAAAAAGCCGTAGTCATCATTAATCGTCCAAAAGTC
TTCTACCG

AGACTCCGTTTAATCACTTTCAACCGCGGTTTATCCGGCCCCACCCATGCATAACCCTAAATTATTAGATC
ACTTAGCACGTGAAAAAGAAACGTTTTTAATGTTTTTTTTTTTTTTTTCTTTTTCTTTTTTTGCGTTGGTG
AAAATTTT

ATTACTGACACAAAATGAAGGGATCAGTGGCCCTGAAAAAAAAAAAAAAAAAAATAAACGGCGCGCGGAGTA
ATTATGATTATGATTATGGTTATGATTATTATTACAACTCACTATTCTAAGTCTATATCGTAAATAAGGGT
CGTGTCCG

CGGGAAGCTTTGTGAAAGCCCTTCTCTTTCAACCCATCTTTGCAACGAAAAAAAAAAAAAAAAAATAAAAAAT
AAAAAGACCAAATAGTAAATAGTAACTTACATACATTAGTAAATGGTACACTCTTACACACTATCATCCTC
ATCGTATA

ATTATTATTTATTATTATTATAATATATATAATTTAATAATCTTAATATTTATTATCATTGTTTCATATTT
TTTTTTATAAATATTTATTTAAATAAAAAATATTAAAGAGTATTCAAAAAAGTCTTACTGTGGGTAATGGA
TATTTCCT

GAATATAGGGGAACATAAATGTTATGGACAAGAGTAGATATCAAAATAAAACAAAATAAAATAGAGTAAGT
GAAAGACAAATGGAGGAAAAAAAAAAAAAAAAAAAAAAAAAAAAAAATAGTAAAAAGTGAAAGGAGAACGAT
GATAACAC

GGATCAGTGGCCCTGAAAAAAAAAAAAAAAAAATAAACGGCGCGCGGAGTAATTATGATTATGATTATGGT
TATGATTATTATTACAACTCACTATTCTAAGTCTATATCGTAAATAAGGGTCGTGTCCGTAGAATTCCCCT
GCCAGCCT

CTTGAACGAAACGTTCGTAAAAATATTTATAATTCTTATTATTATTATTTATTATTATTATAATATATATA
ATTTAATAATCTTAATATTTATTATCATTGTTTCATATTTTTTTTTATAAATATTTATTTAAATAAAAAAT
ATTAAAGA

CCCTGAAAAAAAAAAAAAAAAAATAAACGGCGCGCGGAGTAATTATGATTATGATTATGGTTATGATTATT
ATTACAACTCACTATTCTAAGTCTATATCGTAAATAAGGGTCGTGTCCGTAGAATTCCCCTGCCAGCCTCT
GTTGCCAC

GTATACTGGGAACAATCTTGCGGCACACCGCTTGAACGAAACGTTCGTAAAAATATTTATAATTCTTATTA
TTATTATTTATTATTATTATAATATATATAATTTAATAATCTTAATATTTATTATCATTGTTTCATATTTT
TTTTTATA

ATTATACTTATAATATGACTCTTTTCTTTTATTGCAGTTGGTATTATATACATATATATATATATATATAT
ATGTATATGTTCCCCGAGACAATGGTTTTATTTCCAAAAAAAGTGTAAGTAAGTTATTTTCTGTGTATAAA
AAAATAAA

AAACATGTGCGCGTTTGTGACAAAAACATAAATAAAACAAAAATAATAAAAAGTCAAGTGAAAAGCACTGA
AATCTAAAAAATAAATACATATACATATATATATATATATACCTATTTCGTTTCCGCATAGAAGAAATTGG
AGGCAACT

TCAAAATAAAACAAAATAAAATAGAGTAAGTGAAAGACAAATGGAGGAAAAAAAAAAAAAAAAAAAAAAAA
AAAAAATAGTAAAAAGTGAAAGGAGAACGATGATAACACTAATCACACCTCCGCATTTTCAACCAATGCTG
CAAGTTTC

CCATATCATAATTAAAAAAAGCGATTTAGTTGTTCTTTTAAAAAAAAAGAGAAAAAAAAAAAAAAAAAAAA
AGGGAATGAGTTTGTACATACTATTATATTAATGTAGTATCACGTTAAAAAGCCGTAGTCATCATTAATCG
TCCAAAAG

AGCAAATAATGAATATAGGGGAACATAAATGTTATGGACAAGAGTAGATATCAAAATAAAACAAAATAAAA
TAGAGTAAGTGAAAGACAAATGGAGGAAAAAAAAAAAAAAAAAAAAAAAAAAAAAATAGTAAAAAGTGAAA
GGAGAACG

TTAAAAGCCCGAAAAGGAAATTCAAAATCTGTCTATTTATAGGCCGTCGCGCTCTACGAAAACGCGAAATT
ATTCAAACGGAAAACGGAAAAAAATCTAAAAAAAGAAATTAATTGAGAGATCTCACGGAAATGCCGCGAGG
AATGTTTC

CTTTGTGAAAGCCCTTCTCTTTCAACCCATCTTTGCAACGAAAAAAAAAAAAAAAATAAAAAATAAAAAGA
CCAAATAGTAAATAGTAACTTACATACATTAGTAAATGGTACACTCTTACACACTATCATCCTCATCGTAT
ATTATAAT

AAGGATGTCCTGACCTTTTTTTGATTATGTTACCACCCCATTTTTGCATTCTTCTTTTTTTTTTTTTTTTG
GGTCTGAAAATTTTTTTGCTTCTACTTAATTATAATTTTATATAATCATATATGGATCTGATTCTTAAAAA
AAAATGAA

GAAGTCATGTTCCCTTCGTTGGGTTAATTTTTTTTTTTTTTTTTTTTGTTTATATGTTTTTTTTTTTTTTG
CCAGGTATTGTTAGCGCCAGATAGCAAAAAATTTATTCAGGATACTGAACACCGGGGAAGTTAACTGAG
TATATCGG

TTTTTTGATTTATTTATTTACTTTTACGACAACTGGCCTTATCACTCGGCTCTCTAAAAGAAGTCATGTT
CCCTTCGTTGGGTTAATTTTTTTTTTTTTTTTTTTTGTTTATATGTTTTTTTTTTTTTTGCCAGGTATTGT
TAGCGCCA

CGGATTCTTCCAGTTCCAGAAAAAGAGGCTGTTGAATTTACAAAAGGTAATTTATAGTACGTGAACATGTG
TGCGTATATACATATATATATATATATATATATATATATATATATATATACTTTAGCATCGAAACAGCTAG
TCCTTTTT

CATACTATTGTCCCTGGATTTTGAGATGGGAAAAGTTTCAATGCGTTCTATCGTATAGTATGATGTATTCA
AAAAAAATGCGATAATTCTTTAGTTGTCGGCACTTCGGAAGCCAAAAAAAAAAAAAAAAAAAAAAAAGAAA
AAAAGAAA

AAATATTTATAATTCTTATTATTATTATTTATTATTATTATAATATATATAATTTAATAATCTTAATATTT
ATTATCATTGTTTCATATTTTTTTTTATAAATATTTATTTAAATAAAAAATATTAAAGAGTATTCAAAAAA
GTCTTACT

ATACGCGAAACTCAGGTGCTGCAATCTTTATTTCTTTTTTTTTTTTTTTTTTTTTTTTTTTTTCTAGTTTC
TTGGCTTCCTATGCTAAATCCCATAACTAACCTACCATTCGATTCAGAAAAATTCGCACTATCCAGCTGCA
CTCTTCTT

ACTATAAATGTTTGAATATGAAGATATATATGTAGGAAGAATTTTTATTGAATATAGAAATTTAAAGAAAT
ATTATTAAAAACCGGAAAATGAAAAAGAAGAGTTAAAAAAAAAAAAAACGGAACTAGTAAAAGATGTGAAA
AAAAGAAG

CATTATACAATTTTTTTGCTTATTTTTTTCTTGCTAGGAATATACACAATAGCGTTCTTGGGATGAATAAT
AATAATATCAGTTTTTCTTCTTTATATGAAAAATGAAAAAAAAAAAAAATAAGTAAAAAATTTTCGCTTTC
TTTTCGGG

CTTATTATATACGAGAAAAGAAACGCGAAGGAACGAAAGAAGATGAAAAAAAATGCAGCGATAAAATGATAT
TGTGGTTAATCTAAATTTATATATATATATATATATACATATATATATAGAAATCTATTGTTATACACAAA
AATACTTA

GCTCGGGAAGCTTTGTGAAAGCCCTTCTCTTTCAACCCATCTTTGCAACGAAAAAAAAAAAAAAAATAAAA
AATAAAAAGACCAAATAGTAAATAGTAACTTACATACATTAGTAAATGGTACACTCTTACACACTATCATC
CTCATCGT

TTGAGATGGGAAAAGTTTCAATGCGTTCTATCGTATAGTATGATGTATTCAAAAAAAATGCGATAATTCTT
TAGTTGTCGGCACTTCGGAAGCCAAAAAAAAAAAAAAAAAAAAAAAAGAAAAAAAGAAAAGGGTGTCTAAG
ATGTACGG

ATGAACTTAAGAAAAAAAGGGCCACGGATTTTTTTTTCGCGTTTTGGCGTTTTCATATCAAAAAAAAAAAA
CCATATTAACATCTGTACATTATTATTAACTAAACATCCGTACACAAAAGGTTTTCCAAGACAAAACGTTG
TCGAGACT

ACAAAAAAAAAATTAAAAAAAAAAAAAAAACAATCAAAAAAGACACACAACTACAGAACAAAGGAAATCTT
TTTCTATGCACAAGCACAAGAGTACTTAGAGAGTAGTGATATCCCCTTTTTTTTCTACTATTTTTTTTTTT
CCCATTTT

GTACACAAATATGCATATATACCGTCATGAAGTAGCTTAACTTAAATAAAAGTTTTTTTTTTTTAGAGTTA
CCAATATCGCAAACATGAAAATGAGAATAAAAAAAAAAAAGCAGAAAAAAAAAAAGCATAACATGGAGAAA
AAAATAAT

TATTTTTTTTCTTGCTAGGAATATACACAATAGCGTTCTTGGGATGAATAATAATAATATCAGTTTTTCTTC
TTTATATGAAAAATGAAAAAAAAAAAAAATAAGTAAAAAATTTTCGCTTTCTTTTCGGGGAGCAGCCAAAT
AAAGATTG

CTTCTCTTTCAACCCATCTTTGCAACGAAAAAAAAAAAAAAAATAAAAAATAAAAAGACCAAATAGTAAAT
AGTAACTTACATACATTAGTAAATGGTACACTCTTACACACTATCATCCTCATCGTATATTATAATAGATA
TATACAAT

TCTTCCGGTACGACAGCCGCTGCGGCAATTACTTTTCCCCAAAAGCTCATAATCGATCAATTAACGCGTAA
ACGAAAAACAAAAAAAAAAAAAATACGTAAATTAGGTCTTCATACGAGCGTTTTTTTTTTTTTGAGGATCG
TTTGCATG

TTTATAGTACGTGAACATGTGTGCGTATATACATATATATATATATATATATATATATATATATATATATA
CTTTAGCATCGAAACAGCTAGTCCTTTTTAGAGTTCAATTTTTCCACAAAGGCTTTTTTAGCCAATTCAAT
TAGGATAT

GAAAAAAGGGCCACGGGATTTTTTTTTCGCGTTTTGGCGTTTTCATATCAAAAAAAAAAAACCATATTAAC
ATCTGTACATTATTATTAACTAAACATCCGTACACAAAAGGTTTTCCAAGACAAAACGTTGTCGAGACTAT
TCTCTTTC

GAATTTTCGCGTTTTGATGAAGCACAGGAAGAATTTCTTTTTTTTTTGGCTTCTTCTGGTTCCGTTTTTTA
CGCGCACAAATCTAAAAAAAGAAATAATTATAACCTAGTCTCGAAAATTTTCATCGATCCATTCGTTCCTT
TTTTTCGA

CAAAATGAAGGGATCAGTGGCCCTGAAAAAAAAAAAAAAAAAAAATAAACGGCGCGGAGTAATTATGATTA
TGATTATGGTTATGATTATTATTACAACTCACTATTCTAAGTCTATATCGTAAATAAGGGTCGTGTCCGTA
GAATTCCC

AAAACAACACTCTTCCGGTACGACAGCCGCTGCGGCAATTACTTTTCCCCAAAAGCTCATAATCGATCAAT
TAACGCGTAAACGAAAAACAAAAAAAAAAAAAATACGTAAATTAGGTCTTCATACGAGCGTTTTTTTTTTT
TTGAGGAT

AAAAGCTCATAATCGATCAATTAACGCGTAAACGAAAAACAAAAAAAAAAAAAATACGTAAATTAGGTCTT
CATACGAGCGTTTTTTTTTTTTTGAGGATCGTTTGCATGTCTAGCGCGGCAAGCGTGTTTACAGGCATTCG
TTGTGTGT

AAGATATGACCCATATCATAATTAAAAAAAGCGATTTAGTTGTTCTTTTAAAAAAAAAAGAGAAAAAAAAA
AAAAAAAAAAAGGGAATGAGTTTGTACATACTATTATATTAATGTAGTATCACGTTAAAAAGCCGTAGTCA
TCATTAAT

GTTATGGACAAGAGTAGATATCAAAATAAAACAAAATAAAATAGAGTAAGTGAAAGACAAATGGAGGAAAA
AAAAAAAAAAAAAAAAAAAAAAAAAATAGTAAAAAGTGAAAGGAGAACGATGATAACACTAATCACACCTC
CGCATTTT

GCCCTTCTCTTTCAACCCATCTTTGCAACGAAAAAAAAAAAAAAAATAAAAAATAAAAAGACCAAATAGTA
AATAGTAACTTACATACATTAGTAAATGGTACACTCTTACACACTATCATCCTCATCGTATATTATAATAG
ATATATAC

TCTGCCGCTTACGTAAAAACAGAACTCTGACCAGAAGAGATAAAAACAAAAAAAAAATAAAGGACAGTAAA
TATGAAAAATAAGATATTATTATATTAAATTAAATTAAATTAAATTAAACTACGATGGGAGCGTTATGCCA
AAAAAGAT

ATATTAAAACAACAAAAAATTAGTAAAAATACTATAAATGTTTGAATATGAAGATATATATGTAGGAAGAA
TTTTTATTGAATATAGAAATTTAAAGAAATATTATTAAAAACCGGAAAATGAAAAAGAAGAGTTAAAAAAA
AAAAAAAC

TCCCTTCGTTGGGTTAATTTTTTTTTTTTTTTTTTTTGTTTATATGTTTTTTTTTTTTTTGCCAGGTATTG
TTAGCGCCAGATAGCAAAAAATTTTATTCAGGGATACTGAACACCGGGGAAGTTAACTGAGTATATCGGTT
ATCATTAT

AAAAAAAAAAAAATAAAAAATAAAAAGACCAAATAGTAAATAGTAACTTACATACATTAGTAAATGGTACA
CTCTTACACACTATCATCCTCATCGTATATTATAATAGATATATACAATACATGTTTTTACCCGGATCATA
GAATTCTT

ATTGTTCGGATAACAAATACAATAGTGTTATTAAAAAATAAAACTTATTTAAAAATAGTAATTTAAATTAT
TATTTTATTTTAATAAACTTTTTAATAATATTTATTACACGTGATTTAATATATCCTGTTTTTTTTTCATC
ATTCTCTT

TTATCGCGCAGGTGAGAGAGTCCTGGAATTTCCAGCGGATGGTGACGCGAGGCATCCGTAGTGCACAATAA
AAAGTTAGTTTTTCATTAACCGAAAAAAAAAAAAAAATAAAATATATTAATGTTTGGGTTTCAACGTTTCC
GTGGGTCT

TTTGAATATGAAGATATATATGTAGGAAGAATTTTTATTGAATATAGAAATTTAAAGAAATATTATTAAAA
ACCGGAAAATGAAAAAGAAGAGTTAAAAAAAAAAAAACGGAACTAGTAAAAGATGTGAAAAAAAAGAAGAG
AAAAGTAG

TGTGAAAGCCCTTCTCTTTCAACCCATCTTTGCAACGAAAAAAAAAAAAAAAATAAAAAATAAAAAGACCA
AATAGTAAATAGTAACTTACATACATTAGTAAATGGTACACTCTTACACACTATCATCCTCATCGTATATT
ATAATAGA

ACATCCAATGGCGGGTAATAATTATAGTATTTTTCTTTTCTATTTTATTTATTTTTTTTTTTTTTTTTTGAT
TTTCTAGAATAATCAATCAATGGTAAATAAACAAAAGTTGCTTCTGTTGTAAAAAATACATCAAAGGGGCT
AAAATATC

ATTTATATTTCATCTTTTCTTTTTTTTTTTTTTACAATTCAGTAGTTTTATATAACTTCCCATAAATGTAT
TATTTTTTTAATACGACTATAATATACCTCTATACCCTTATTTATCTCTTTACCTTTGCTTTTAAGCTAAA
CAAATCTT

TTTTTTTGCTTATTTTTTTCTTGCTAGGAATATACACAATAGCGTTCTTGGGATGAATAATAATAATATCA
GTTTTCTTCTTTATATGAAAAATGAAAAAAAAAAAAAAATAAGTAAAAAATTTTCGCTTTCTTTTCGGGGA
GCAGCCAA

GAAAAGGAAGACAAAGACAATCAATAGAATGTATTATGTATGACATATAATAATAATAATTATAATAATAA
TAATAATAGTAGTAGCAGTAATAATAATATTATTATAGTATATTAAAACAACAAAAAATTAGTAAAAATAC
TATAAATG

CACCCGCGTAAAGAGTTTTTACCCCGAAAACAAATTTTTATGCTTGAAAAATAGCTAATAAAATGTTTTTA
TTGTTCGGATAACAAATACAATAGTGTTATTAAAAAATAAAACTTATTTAAAAATAGTAATTTAAATTATT
ATTTTATT

AAGCTGGTAAGAGCCTGACCGAGTAGTGTAGTGGGTGACCATACGCGAAACTCAGGTGCTGCAATCTTTAT
TTCTTTTTTTTTTTTTTTTTTTTTTTTTTTTTCTAGTTTCTTGGCTTCCTATGCTAAATCCCATAACTAAC
CTACCATT

AGGAATGAAAAAAAGTAAAGCATGGCAATGAATGACACAAGTGAAATAGAAAAGTGAAAAATTAAAAAAAA
AAAAAAAAAAAAAAAAACTGATTTATACTCATGAAAATCTTATTCGAGTTCATTCAAGACAAGCTTGACAT
TGATCTAC

ATTAAAAAAAAAAAAAAAAACAATCAAAAAAGACACACAACTACAGAACAAAGGAAATCTTTTTCTATGCA
CAAGCACAAGAGTACTTAGAGAGTAGTGATATCCCCTTTTTTTTCTACTATTTTTTTTTTTCCCATTTTTT
TCCTCCAA

AGCACAGGAAGAATTTCTTTTTTTTTTGGCTTCTTCTGGTTCCGTTTTTTACGCGCACAAATCTAAAAAAA
GAAATAATTATAACCTAGTCTCGAAAATTTTCATCGATCCATTCGTTCCTTTTTTTCGATTTTTTCAGATC
AAAATTCT

ATGCATATATACCGTCATGAAGTAGCTTAACTTAAATAAAAGTTTTTTTTTTTTAGAGTTACCAATATCGC
AAACATGAAAATGAGAATAAAAAAAAAAAAGCAGAAAAAAAAAAAGCATAACATGGAGAAAAAAATAATAA
ATAATTGA

TTCTTCTTTTTATTTTACGCGTCTCGCGTTTTATATATTAATAGTAATAATATTTAATAGTGTATGTTTGA
AATTTTTATATGTCTTTATTTCGCTAACGTAAAAATCCCTTCTTCTTTGTAGGGACGGGGGAGTGGTTAAC
ATTGGGAG

AAGCACGAAATGTCCGTTATTAAAAATTGGGGAGTGAATCTTAAAAGCCCGAAAAGGAAATTCAAAATCTG
TCTATTTATAGGCCGTCGCGCTCTACGAAAACGCGAAATTATTCAAACGGAAAACGGAAAAAAAATCTAAAA
AAAGAAAT

AATAGTGTTATTAAAAAATAAAACTTATTTAAAAATAGTAATTTAAATTATTATTTTATTTTAATAAACTT
TTTAATAATATTTATTACACGTGATTTAATATATCCTGTTTTTTTTTCATCATTCTCTTTCTTTCTTATGT
TAACCTCG

AGAGTAGATATCAAAATAAAACAAAATAAAATAGAGTAAGTGAAAGACAAATGGAGGAAAAAAAAAAAAAA
AAAAAAAAAAAAAAAATAGTAAAAAGTGAAAGGAGAACGATGATAACACTAATCACACCTCCGCATTTTCA
ACCAATGC

AAATATTAGCCTTCAAAGCCGCACACGTTTCTTTTTCCGAAAAATGAAAAATTTTTCACTGAAAAAAAAAA
AAAAAAAAAGAAGAAATAGAAAGGTATAAATAGTTGAATAATTTGCCCTTCCGAGATCCAACAAGATTTTT
GAGTGTTG

GAAGAAGCAAAAAAAAGAAAAGACTCCGTTTAATCACTTTCAACCGCGGTTTATCCGGCCCCACCCATGCA
TAACCCTAAATTATTAGATCACTTAGCACGTGAAAAAGAAACGTTTTTAATGTTTTTTTTTTTTTTTTCTT
TTTCTTTT

CAGCTGATTCATTTATATTTCATCTTTTCTTTTTTTTTTTTTTACAATTCAGTAGTTTTATATAACTTCCC
ATAAATGTATTATTTTTTTAATACGACTATAATATACCTCTATACCCTTATTTATCTCTTTACCTTTGCTT
TTAAGCTA

TTGAAGTACACACCCGCGTAAAGAGTTTTTACCCCGAAAACAAATTTTTATGCTTGAAAAATAGCTAATAA
AATGTTTTTATTGTTCGGATAACAAATACAATAGTGTTATTAAAAAATAAAACTTATTTAAAAATAGTAAT
TTAAATTA

AAAAAGAAACGAAGAAGCAAAAAAAAGAAAAGACTCCGTTTAATCACTTTCAACCGCGGTTTATCCGGCCC
CACCCATGCATAACCCTAAATTATTAGATCACTTAGCACGTGAAAAAGAAACGTTTTTAATGTTTTTTTTT
TTTTTTTC

ATATATTAGGGCATCCATAAAAATAGATTAGGGCAGCTCTGCCATTTTTTTCATAGCCGAAAGAAGAAAAA
CTGAAAAAAAAAAATGATACGAAAAAACCGCGAAACGACGCCTTTTGTTGCGATTGTCGGGCATCGCAAGTT
ACGACGCG

GTATTACGGGCCGTGCTCAAATTAGTTAGTTGGCTCTGCCTATACGCATATATGTATATATATATATATAT
ATATGTATGTATGTGTATATGTATATGTACGTATACCTTACATCATCATCTTTCTTTATATTCTCTCTAAT
GCGCATTT

GTACAATTGAATAATATTATTGATTTTTTTATTATTGTTCTTATTCTTTTTTTATCATTTCTATATAAAAT
ATTATATGATTTAGTTTATCGTAAAAAAATTAAAAAATACAGAGGAAAGCAAAAGTAAAACTTTTCGGATA
TTTTACAG

CTTTGCAACGAAAAAAAAAAAAAAAAATAAAAAATAAAAAGACCAAATAGTAAATAGTAACTTACATACATT
AGTAAATGGTACACTCTTACACACTATCATCCTCATCGTATATTATAATAGATATATACAATACATGTTTT
TACCCGGA

CTTTCACGCTCGGGAAGCTTTGTGAAAGCCCTTCTCTTTCAACCCATCTTTGCAACGAAAAAAAAAAAAAA
AATAAAAAATAAAAAGACCAAATAGTAAATAGTAACTTACATACATTAGTAAATGGTACACTCTTACACAC
TATCATCC

TAGAGCATAAGTTTTGTATAATTAAAATCTTCTAAACCTAATAAATATGTTTAATGAATGATTCAATATCA
AAAAAAGAAATCCATTGGGCCCTTAGTGCATTTTTATTTTTTTTTTTTTTTTTTTTTTTTTTCTCTTTCTG
AATTAGAG

GAAAAGGAAATTCAAAATCTGTCTATTTATAGGCCGTCGCGCTCTACGAAAACGCGAAATTATTCAAACGG
AAAACGGAAAAAAATCTAAAAAAAGAAATTAATTGAGAGATCTCACGGAAATGCCGCGAGGAATGTTTCTC
GAGGCTGA

CATCTTTTCTTTTTTTTTTTTTACAATTCAGTAGTTTTATATAACTTCCCATAAATGTATTATTTTTTTTA
ATACGACTATAATATACCTCTATACCCTTATTTATCTCTTTACCTTTGCTTTTAAGCTAAACAAATCTTCA
TTAACAGG

TGCTTGAAAAATAGCTAATAAAATGTTTTTATTGTTCGGATAACAAATACAATAGTGTTATTAAAAAATAA
AACTTATTTAAAAATAGTAATTTAAATTATTATTTTATTTTAATAAACTTTTTAATAATATTTATTACACG
TGATTTAA

TTTTGATAGTATAATATGCCTTTGTCTGCCCGGACCCTGAATTACTGACACAAAATGAAGGGATCAGTGGC
CCTGAAAAAAAAAAAAAAAAAATAAACGGCGCGCGGAGTAATTATGATTATGATTATGGTTATGATTATTA
TTACAACT

ACGTAAAAACAGAACTCTGACCAGAAGAGATAAAAACAAAAAAAAAATAAAGGACAGTAAATATGAAAAAT
AAGATATTATTATATTAAATTAAATTAAATTAAATTAAACTACGATGGGAGCGTTATGCCAAAAAAGATAA
AATTCTGA

TTATTTATTTACTTTTACGACAACTGGCCTTATCACTCGGCTCTCTAAAAGAAGTCATGTTCCCTTCGTTG
GGTTAATTTTTTTTTTTTTTTTTTTTTGTTTATATGTTTTTTTTTTTTTGCCAGGTATTGTTAGCGCCAGA
TAGCAAAA

GAGCCTGACCGAGTAGTGTAGTGGGTGACCATACGCGAAACTCAGGTGCTGCAATCTTTATTTCTTTTTTT
TTTTTTTTTTTTTTTTTTTTTTCTAGTTTCTTGGCTTCCTATGCTAAATCCCATAACTAACCTACCATTCG
ATTCAGAA

CCACCCATGCATAACCCTAAATTATTAGATCACTTAGCACGTGAAAAAGAAACGTTTTTAATGTTTTTTTT
TTTTTTTTCTTTTTCTTTTTTTGCGTTGGTGAAAATTTTTTCGCTTCCTCGAGTATAATTATCTCATCTCA
TCTTTCAT

AACCCATCTTTGCAACGAAAAAAAAAAAAAAAATAAAAAATAAAAAGACCAAATAGTAAATAGTAACTTAC
ATACATTAGTAAATGGTACACTCTTACACACTATCATCCTCATCGTATATTATAATAGATATATACAATAC
ATGTTTTT

TTATTCATAAGTACAATTGAATAATATTATTGATTTTTTTATTATTGTTCTTATTCTTTTTTTATCATTTC
TATATAAAATATTATATGATTTAGTTTATCGTAAAAAAATTAAAAAATACAGAGGAAAGCAAAAGTAAAAC
TTTTCGGA

GGCATGACAAGCAAGCTATTCGACAGTGAAAAATGCACAGAACATTCAAAATTATACTTATAATATGACTC
TTTTCTTTTATTGCAGTTGGTATTATATACATATATATATATATATATATATGTATATGTTCCCCGAGACA
ATGGTTTT

ACGGTAAGCTGTTCTTAATTGAACTTTGAAACATCCAATGGCGGGTAATAATTATAGTATTTTTCTTTTCT
ATTTTATTTATTTTTTTTTTTTTTTTTGATTTTCTAGAATAATCAATCAATGGTAAATAAACAAAAGTTGC
TTCTGTTG

ATTGACGTGTAAAAAAAAAAAAAAAAAAATGTTAATGGAACGACCGATCTAAAGAACCATATATAATGAAT
GACTAAAAAAAGCAAAATAGTTAAGAATCCAAGAAAGATACTAAGAAAAGGCGAAAAGAAAAAGAAAAAGT
ATGTCCGC

ACTGGGCGACGAATTTTCGCGTTTTGATGAAGCACAGGAAGAATTTCTTTTTTTTTTGGCTTCTTCTGGTT
CCGTTTTTTACGCGCACAAATCTAAAAAAAGAAATAATTATAACCTAGTCTCGAAAATTTTCATCGATCCA
TTCGTTCC

TTATTTTAAATAGAGCATAAGTTTTGTATAATTAAAATCTTCTAAACCTAATAAATATGTTTAATGAATGA
TTCAATATCAAAAAAAGAAATCCATTGGGCCCTTAGTGCATTTTTATTTTTTTTTTTTTTTTTTTTTTTTT
TCTCTTTC

AACAAAAAATTAGTAAAAATACTATAAATGTTTGAATATGAAGATATATATGTAGGAAGAATTTTTATTGA
ATATAGAAATTTAAAGAAATATTATTAAAAACCGGAAAATGAAAAAGAAGAGTTAAAAAAAAAAAAAAACGG
AACTAGTA

ATCCTTTGTAGAGGTTAATGCCGAAGTCTTCCTATAATGTAATGTTCTAACAAAAATTTTTACTGATTTAT
AAAACTTATATAGATAGATAGACACATATATATATCTATATATAGAAACACAACTAAAGTTTACCATGTTT
TATATAAT

ACCGATATTCCTAGTCTTCAAGTCACTATTATTTTTTTTTTTATTCATAAGTACAATTGAATAATATTATT
GATTTTTTTATTATTGTTCTTATTCTTTTTTTATCATTTCTATATAAAATATTATATGATTTAGTTTATCG
TAAAAAAA

CCTGTCAGTATAAAAAAATTTTCCGCAGGATATAGAAAAAAAAGAAATGAAATTATAGTAGCGGTTATTTC
CGTGGGGTGCTTTTTTACACCTGTACATCTTTTCCCTCCGTACATTTTTTTTATTTTTTTTTTTGGTTTTT
TTTTTTCG

ATGTTTTTTTTTTTTTTTTTCTTTTTCTTTTTTTGCGTTGGTGAAAATTTTTTCGCTTCCTCGAGTATAATT
ATCTCATCTCATCTTTCATATAAGATAAGAAGTTTTATAAAAACCTTTTGCATCAAAATTTTGTAGAATAT
CTCTTTTT

ATTCAAATCATAACAGATATTTAGTTGAATAACTCTGTTTTTCTTCATTTCCATTTTTTTCTATTTACCCT
TCTTCTTTTTATTTTACGCGTCTCGCGTTTTATATATTAATAGTAATAATATTTAATAGTGTATGTTTGAA
ATTTTTAT

GTTTTGATGAAGCACAGGAAGAATTTCTTTTTTTTTTGGCTTCTTCTGGTTCCGTTTTTTACGCGCACAAA
TCTAAAAAAAGAAATAATTATAACCTAGTCTCGAAAATTTTCATCGATCCATTCGTTCCTTTTTTTCGATT
TTTTCAGA

AATGGAAAAAAGCAAATAATGAATATAGGGGAACATAAATGTTATGGACAAGAGTAGATATCAAAATAAAA
CAAAATAAAATAGAGTAAGTGAAAGACAAATGGAGGAAAAAAAAAAAAAAAAAAAAAAAAAAAAAAATAGTA
AAAAGTGA

TTATACAGGAAAGAAAGAGTTTTATTGATTTTAAAAACAATATAAATGAAAGAAAAAAAATGTGTTTTAGAT
TAGGCTTATAAGATATGACCCATATCATAATTAAAAAAAGCGATTTAGTTGTTCTTTTAAAAAAAAAAGAGA
AAAAAAAA

GGGTTAATTTTTTTTTTTTTTTTTTTTTGTTTATATGTTTTTTTTTTTTTTTGCCAGGTATTGTTAGCGCCAG
ATAGCAAAAAATTTTATTCAGGGATACTGAACACCGGGGAAGTTAACTGAGTATATCGGTTATCATTATAA
ACACAAGA

ATTCGCATTTAGGAATGAAAAAAAGTAAAGCATGGCAATGAATGACACAAGTGAAATAGAAAAGTGAAAAA
TTAAAAAAAAAAAAAAAAAAAAAAAAAACTGATTTATACTCATGAAAATCTTATTCGAGTTCATTCAAGACA
AGCTTGAC

AAGAATACACGTACACAAATATGCATATATACCGTCATGAAGTAGCTTAACTTAAATAAAAGTTTTTTTTT
TTTAGAGTTACCAATATCGCAAACATGAAAATGAGAATAAAAAAAAAAAAAGCAGAAAAAAAAAAAAGCATAA
CATGGAGA

AATAAAGGTATTTTGAAAATTATAAAAAAAAGTTTTAATAATATAGATTTGGTAAAATAAATAGGCGTATA
TACACATTGTATACTGTTGATATTATCAAGTTTTTATTATTAAAGAGCTCTTTTTATTTTTTATTTATAGG
GGAAAATG

GATCTGTTATAATATTAATACGCATTTTTTTTTATGTTTTCTCGTTTCTTTTCTTTTTTTGTTTTATATCT
TACACATTTTTAATATACTAAAAGTAAAATAAAAAAATACGTACTGTTTCCCTTTGTTTTCTCCTCCTCTT
TGCTTATA

AGAGGAGAAAGAAAAGGAAGACAAAGACAATCAATAGAATGTATTATGTATGACATATAATAATAATAATT
ATAATAATAATAATAATAGTAGTAGCAGTAATAATAATATTATTATAGTATATTAAAACAACAAAAAATTA
GTAAAAAT

TTAGGCTTATAAGATATGACCCATATCATAATTAAAAAAAGCGATTTAGTTGTTCTTTTAAAAAAAAAGAG
AAAAAAAAAAAAAAAAAAAAAGGGAATGAGTTTGTACATACTATTATATTAATGTAGTATCACGTTAAAAA
GCCGTAGT

ATATCGATCCCATACTATTGTCCCTGGATTTTGAGATGGGAAAAGTTTCAATGCGTTCTATCGTATAGTAT
GATGTATTCAAAAAAAAATGCGATAATTCTTTAGTTGTCGGCACTTCGGAAGCCAAAAAAAAAAAAAAAAA
AAAAAAGA

ACAAAATAAAATAGAGTAAGTGAAAGACAAATGGAGGAAAAAAAAAAAAAAAAAAAAAAAAAAAAAATAGT
AAAAAGTGAAAGGAGAACGATGATAACACTAATCACACCTCCGCATTTTCAACCAATGCTGCAAGTTTCTC
AACACTGG

GTTCTTAATTGAACTTTGAAACATCCAATGGCGGGTAATAATTATAGTATTTTTCTTTTCTATTTTATTTA
TTTTTTTTTTTTTTTTTGATTTTCTAGAATAATCAATCAATGGTAAATAAACAAAAGTTGCTTCTGTTGTA
AAAAATAC

GAATTTCTTTTTTTTTTGGCTTCTTCTGGTTCCGTTTTTTACGCGCACAAATCTAAAAAAAGAAATAATTA
TAACCTAGTCTCGAAAATTTTCATCGATCCATTCGTTCCTTTTTTTCGATTTTTTCAGATCAAAATTCTTG
TTTCTTTC

TTCATTAATATTATTTTAAATAGAGCATAAGTTTTGTATAATTAAAATCTTCTAAACCTAATAAATATGTT
TAATGAATGATTCAATATCAAAAAAAAGAAATCCATTGGGCCCTTAGTGCATTTTTATTTTTTTTTTTTTT
TTTTTTTT

AAGATATATATGTAGGAAGAATTTTTATTGAATATAGAAATTTAAAGAAATATTATTAAAAACCGGAAAAT
GAAAAAGAAGAGTTAAAAAAAAAAAAAACGGAACTAGTAAAAGATGTGAAAAAAAGAAGAGAAAAGTAGAA
CGAGAACA

AGTTTTTTTTTTTTAGAGTTACCAATATCGCAAACATGAAAATGAGAATAAAAAAAAAAAAGCAGAAAAAA
AAAAAGCATAACATGGAGAAAAAAATAATAAATAATTGAGTTTAGTTGAGACGCCGGTCTTCGCCAGGCCA
GGAATTGT

AGTTTTGAATTCTTTCACCCATCATTCAAAATGCATATGCATACTATATTTGTCTAGTTTTTAATTATATA
ATAATAATAACAGAAACAAAAAATCGCATTCATCGTTTAATCTTATTTTTCTTTTTTCACCTTTTTCTTGT
GAAATTGC

CCGTGGGTGCTTTTTTACACCTGTACATCTTTTCCCTCCGTACATTTTTTTTTATTTTTTTTTTTGGTTTT
TTTTTTTCGATATTTTTCCCTCCGAAACTAGTTAGCACAATAATGCTGACTAAGGAAACTTTTCATCTCAG
AATTGATG

ATAGCTAATAAAATGTTTTTATTGTTCGGATAACAAATACAATAGTGTTATTAAAAAATAAAACTTATTTA
AAAATAGTAATTTAAATTATTATTTTATTTTAATAAACTTTTTAATAATATTTATTACACGTGATTTAATA
TATCCTGT

TTAGGTGCCTCCTGCTTTTTTTTTTTTCCTGCTCTTATATCCGTTATATCCGAATGATTTTTATCGCTTGT
TTAAAAAATACTTTCCCGATATATATATATAGTCTCCCTTTAAATTTGTTTCCGGTAAGTTTTTAACACCA
ATAAATGA

ACATATGCTTACTCTATATTTTCTCCAGTGCCTTCAAGCTCCGTATATATTATGTACTAGTTTTATTACAT
TAAAGATACATAACTTTTTTTTTTTTTTTCAGTTCACTGAAATAAGAAAAGGCGGGCGAGGTGAAAAACAA
AAAAAAAA

TGTCCGTTATTAAAAATTGGGGAGTGAATCTTAAAAGCCCGAAAAGGAAATTCAAAATCTGTCTATTTATA
GGCCGTCGCGCTCTACGAAAACGCGAAATTATTCAAACGGAAAACGGAAAAAAATCTAAAAAAAGAAATTA
ATTGAGAG

TTTTTTTTGCTGGATTTTATATTTTTCACAGCGAAAAGGACGGGAAGCTAAAAGATTAAAAAATAAATATT
GCAGATACCATATGTAATTTAGTAAAATATTTTTATAAAATATTATTATTTTCTTGTTCTTCAGTGATTTA
TTGTAACC

AAAAGTAGGTAATGGAAAAAAGCAAATAATGAATATAGGGGAACATAAATGTTATGGACAAGAGTAGATAT
CAAAATAAAACAAAATAAAATAGAGTAAGTGAAAGACAAATGGAGGAAAAAAAAAAAAAAAAAAAAAAAAA
AAAAATAG

CCTTTTTCGGGTAGTTGAAGAGAAAAGAGGAAGAGAATAGAAAAAAAAAAAAAGCGAAATTTTTACATCCT
CTATTGTAATGTAATTTCTTTTTTTTTATTAGGTATATATGTGAGATACAATTCTCGAAGTCATCGGGAAG
CTTTACCA

CATTGATGAACTAGTGCCGCGCTTAAAAGTATAATGTTGCTCTATTATTTTTCAAAAATTCATACAAAAGA
AAACGGGAATTAAGTAAGTATACAAAATATAATTATATTACGATGTTCGAAATTCTATATATGAATTTAAG
CGGAGTCG

GATATAAGAAACATAACTATTACATACGAAAATGTGCATGTTATCTATATCCTTCTTTATATAGATGCTGT
TAACTTCTTTTTTTTTTTTTGGGAAAATCAACTGTTAAACGCGACAGTAAAAGCAGCAAAACATTAATTTT
GCTTCCAA

TCGCATTTTTTTTTTTTTGCTGGATTTTATATTTTTCACAGCGAAAAGGACGGGAAGCTAAAAGATTAAAA
AATAAATATTGCAGATACCATATGTAATTTAGTAAAATATTTTTATAAAATATTATTATTTTCTTGTTCTT
CAGTGATT

AAGTTTGAGCCAAGCCACGCGACGCTCCATACTGTATAGACCTTTATTATAAAAACATCTATTTCTTTATA
TGTCCCTCTATCTTATTTTTCTTTTACTTTTATTTGTATAATACGCGAAAAATTATAGAATCATTTATTAT
CAAAAACA

ACACCTGTGAAGGTAAAGAAAGAGCATTTCTCTTTTTTTTATTTTTTTTTTTTTTTACTTTCTTTACATTA
AAAAAAATATGAAGAGTGTAATAGGTAAGTATAAGTATTATTTAATCATTACAATTTAGCTTCAATAGTAT
AGAAGCGC

ATCCATTGGGCCCTTAGTGCATTTTTATTTTTTTTTTTTTTTTTTTTTTTTTTCTCTTTCTGAATTAGAGT
ACATCTCATCGCAGGGTCTCTTTTTGCACGTTTTTGATGTTGCTTATGAGACGCTTTGCCTTTTTAACTTC
TTCAGTAT

GTTTTGTATAATTAAAATCTTCTAAACCTAATAAATATGTTTAATGAATGATTCAATATCAAAAAAAGAAA
TCCATTGGGCCCTTAGTGCATTTTTATTTTTTTTTTTTTTTTTTTTTTTTTTCTCTTTCTGAATTAGAGTA
CATCTCAT

CAAGTATCAAGTGCCGTTGGTATTATAGTTTATCAATTAATACGTAAATATATATATATATATATATATAT
ATATATATGACTATAGTGGCATACTTCTCAATGGACTGTTGCTTCTATCATTTTTATACATGCGTTCAACTT
TGAAGATT

AAAAAAAAAAAAAAAAAAATGTTAATGGAACGACCGATCTAAAGAACCATATATAATGAATGACTAAAAAA
AGCAAAATAGTTAAGAATCCAAGAAAGATACTAAGAAAAGGCGAAAAGAAAAAGAAAAAGTATGTCCGCTA
ACAAAGAT

CTTTTTATTTTTTATTTATAGGGGAAAATGACCGATATTCCTAGTCTTCAAGTCACTATTATTTTTTTTTT
TATTCATAAGTACAATTGAATAATATTATTGATTTTTTTATTATTGTTCTTATTCTTTTTTTATCATTTCT
ATATAAAA

TTCCGCAGGATATAGAAAAAAAAGAAATGAAATTATAGTAGCGGTTATTTCCGTGGGGTGCTTTTTTACAC
CTGTACATCTTTTCCCTCCGTACATTTTTTTTATTTTTTTTTTTGGTTTTTTTTTTTCGATATTTTTCCCT
CCGAAACT

TTCAACCCATCTTTGCAACGAAAAAAAAAAAAAAAATAAAAAATAAAAAGACCAAATAGTAAATAGTAACT
TACATACATTAGTAAATGGTACACTCTTACACACTATCATCCTCATCGTATATTATAATAGATATATACAA
TACATGTT

AAGTTATACTATTGACGTGTAAAAAAAAAAAAAAAAAAATGTTAATGGAACGACCGATCTAAAGAACCATA
TATAATGAATGACTAAAAAAAGCAAAATAGTTAAGAATCCAAGAAAGATACTAAGAAAAGGCGAAAAGAAA
AAGAAAAA

ATACAAAATTTTTCAATTTTTTTTCCAAGATTCTTGTACGATTAATTATTTTTTTTTTGCGTCCTACAGCG
TGATGAAAATTTCGCCTGCTGCAAGATGAGCGGGAACGGGCGAAATGTGCACGCGCACAACTTACGAAACG
CGGATGAG

AGGTATCGGTGTTGTCAGAATTAGGTGCCTCCTGCTTTTTTTTTTTTCCTGCTCTTATATCCGTTATATCC
GAATGATTTTTATCGCTTGTTTAAAAAATACTTTCCCGATATATATATATAGTCTCCCTTTAAATTTGTTT
CCGGTAAG

TTTGTCTGCCCGGACCCTGAATTACTGACACAAAATGAAGGGATCAGTGGCCCTGAAAAAAAAAAAAAAAA
AATAAACGGCGCGCGGAGTAATTATGATTATGATTATGGTTATGATTATTATTACAACTCACTATTCTAAG
TCTATATC

AACATTCAAAATTATACTTATAATATGACTCTTTTCTTTTATTGCAGTTGGTATTATATACATATATATAT
ATATATATATATGTATATGTTCCCCGAGACAATGGTTTTATTTCCAAAAAAAGTGTAAGTAAGTTATTTTC
TGTGTATA

TAATATGACTCTTTTCTTTTATTGCAGTTGGTATTATATACATATATATATATATATATATATGTATATGT
TCCCCGAGACAATGGTTTTATTTCCAAAAAAAGTGTAAGTAAGTTATTTTCTGTGTATAAAAAAATAAAAG
AAGCCAGA

TTTTTTTGGCTTCTTCTGGTTCCGTTTTTTACGCGCACAAATCTAAAAAAAGAAATAATTATAACCTAGTC
TCGAAAATTTTCATCGATCCATTCGTTCCTTTTTTTCGATTTTTTCAGATCAAAATTCTTGTTTCTTTCTT
TGTCTTAG

AACAGAAAATGCAAAGGACTAATAACTTGAATCATTTTTATATCAGGTTTATATAGGTATACGTTCAGTCT
AATTAATAATTTATAGTTTACAGTAGTTTTTTACAGTAGTTTTTTTTTTTTTTTTTCAGTTCCATGATTTT
TTTGTTCC

CGGGCAAATCCTTTCACGCTCGGGAAGCTTTGTGAAAGCCCTTCTCTTTCAACCCATCTTTGCAACGAAAA
AAAAAAAAAAAATAAAAAATAAAAAGACCAAATAGTAAATAGTAACTTACATACATTAGTAAATGGTACAC
TCTTACAC

GCATCCATAAAAATAGATTAGGGCAGCTCTGCCATTTTTTTCATAGCCGAAAGAAGAAAAACTGAAAAAAA
AAATGATACGAAAAAACCGCGAAACGACGCCTTTTGTTGCGATTGTCGGGCATCGCAAGTTACGACGCGTT
GTCCCTTG

AATCGATCAATTAACGCGTAAACGAAAAACAAAAAAAAAAAAAATACGTAAATTAGGTCTTCATACGAGCG
TTTTTTTTTTTTTGAGGATCGTTTGCATGTCTAGCGCGGCAAGCGTGTTTACAGGCATTCGTTGTGTGTTC
ATGTTTAG

ACAAAGACAATCAATAGAATGTATTATGTATGACATATAATAATAATAATTATAATAATAATAATAATAGT
AGTAGCAGTAATAATAATATTATTATAGTATATTAAAACAACAAAAAATTAGTAAAAATACTATAAATGTT
TGAATATG

TGATGTATTCAAAAAAAATGCGATAATTCTTTAGTTGTCGGCACTTCGGAAGCCAAAAAAAAAAAAAAAAA
AAAAAAAGAAAAAAAGAAAAGGGTGTCTAAGATGTACGGATCTTCATTGGGATGGAAAACAGGCAAGTAAA
AAGAATAG

AATCAATAAAAACATTATATGCGTTTCGAACAAAATTAAAGAAAAAGAATAAATATAGATTAAAAAAAAAA
AGAAGAAATTAAAAGAATTTCTACTAAATCCCAATTGTTATATATTTGTTAAATGCCAAAAAAGTTTATAA
AAAATTTA

TTTTTTTTTTGGGTCTGAAAATTTTTTTGCTTCTACTTAATTATAATTTTATATAATCATATATGGATCTG
ATTCTTAAAAAAAAATGAAAAAAAGACAAAACAAACCATATAGGTAACAATTTCTCTTAGTGACATAAAAG
AAGAAGGA

TCAATAGAATGTATTATGTATGACATATAATAATAATAATTATAATAATAATAATAATAGTAGTAGCAGTA
ATAATAATATTATTATAGTATATTAAAACAACAAAAAATTAGTAAAAATACTATAAATGTTTGAATATGAA
GATATATA

ACCGTCATGAAGTAGCTTAACTTAAATAAAAGTTTTTTTTTTTTAGAGTTACCAATATCGCAAACATGAAA
ATGAGAATAAAAAAAAAAAGCAGAAAAAAAAAGCATAACATGGAGAAAAAAAAATAATAAATAATTGAGT
TTAGTTGA

ACCCATATTCCAGCTGATTCATTTATATTTCATCTTTTCTTTTTTTTTTTTTTACAATTCAGTAGTTTTAT
ATAACTTCCCATAAATGTATTATTTTTTTAATACGACTATAATATACCTCTATACCCTTATTTATCTCTTT
ACCTTTGC

ATTATTATTATAATATATATATAATTTAATAATCTTAATATTTATTATCATTGTTTCATATTTTTTTATAA
ATATTTATTTAAATAAAAAATATTAAAGAGTATTCAAAAAAGTCTTACTGTGGGTAATGGATATTTCCTCA
TGTTTGCC

ATCCTTTCACGCTCGGGAAGCTTTGTGAAAGCCCTTCTCTTTCAACCCATCTTTGCAACGAAAAAAAAAAA
AAAAATAAAAAATAAAAAGACCAAATAGTAAATAGTAACTTACATACATTAGTAAATGGTACACTCTTACA
CACTATCA

AATAAACTTAAAAAAGAACGTATAGAACTCTCTTCTTCAATGAATAGTAATCCAATTTTTTTTATTAACTA
TACAACGTTATGACTTTAAAAATTAAAAAATATGTTTATGAGTAGTAAGAAAAACAAAAGGAAACAAAAAC
AAAAAAAG

GCTCAAAGGGCTCGATAACGACATAGCATTATAGACACTGTGCAGCGTAAAGCTGACATATAAAGAAATTA
CAAAAAAAAATTAAAAAAAAAAAAAAAAACAATCAAAAAAGACACACAACTACAGAACAAAGGAAATCTTT
TTCTATGC

AACTGTAGTTAAGCTGGTAAGAGCCTGACCGAGTAGTGTAGTGGGTGACCATACGCGAAACTCAGGTGCTG
CAATCTTTATTTCTTTTTTTTTTTTTTTTTTTTTTTTTTTTTCTAGTTTCTTGGCTTCCTATGCTAAATCC
CATAACTA

ATGCGTTCTATCGTATAGTATGATGTATTCAAAAAAAATGCGATAATTCTTTAGTTGTCGGCACTTCGGAA
GCCAAAAAAAAAAAAAAAAAAAAAAAAGAAAAAAAGAAAAGGGTGTCTAAGATGTACGGATCTTCATTGGG
ATGGAAAA

TATTTTTTTTCCAAAGTGACAGGTGCCCCGGGTAACCCAGTTCCTCACTATTTTTTACTGCGGAAGCGGAA
GCGGAAAATACGGAAACGCGCGGGAACATACAAAACATACAAAATATACCTTTCTCACACAAGAAATATAT
GCTACTTG

AAAAAAAAAAAATTTCCCTTATCAATGATATCCTTACGATTATATAAATTCCTTACCTAAACCTATTATTT
GTGTACATATATCAGAGTATTATTACATATATAACCTTTTTCTCTAAAACAGGAAAAAAAAAAGAAAACGA
TAACATGC

GCGGTTATTTCCGTGGGGTGCTTTTTTACACCTGTACATCTTTTCCCTCCGTACATTTTTTTTATTTTTTT
TTTTGGTTTTTTTTTTTCGATATTTTTCCCTCCGAAACTAGTTAGCACAATAATGCTGACTAAGGAAACTT
TTCATCTC

GTGCCGTTGGTATTATAGTTTATCAATTAATACGTAAATATATATATATATATATATATATATATATATGA
CTATAGTGGCATACTTCTCAATGGACTGTTGCTTCTATCATTTTATACATGCGTTCAACTTTGAAGATTAT
CACAAAAA

CCTGCTTTTTTTTTTTTCCTGCTCTTATATCCGTTATATCCGAATGATTTTTATCGCTTGTTTAAAAAATA
CTTTCCCGATATATATATATAGTCTCCCTTTAAATTTGTTTCCGGTAAGTTTTTAACACCAATAAATGAAA
AGAAATGA

GAACTTTGAAACATCCAATGGCGGGTAATAATTATAGTATTTTTCTTTTCTATTTTATTTATTTTTTTTTT
TTTTTTTGATTTTCTAGAATAATCAATCAATGGTAAATAAACAAAAGTTGCTTCTGTTGTAAAAAATACAT
CAAAGGGG

ATTTTATTTTTTTTTTTTTTTTTTTTTTTTTTTCTCTTTCTGAATTAGAGTACATCTCATCGCAGGGTCTC
TTTTTGCACGTTTTTGATGTTGCTTATGAGACGCTTTGCCTTTTTAACTTCTTCAGTATATTTTAAATATA
TACCAATT

CTTAAATAAAAGTTTTTTTTTTTTAGAGTTACCAATATCGCAAACATGAAAATGAGAATAAAAAAAAAAAA
GCAGAAAAAAAAAAAGCATAACATGGAGAAAAAAATAATAAATAATTGAGTTTAGTTGAGACGCCGGTCTT
CGCCAGGC

TCTACGAATGAAAATTCATAGACAGTAAAAAGTAATGAAAAATAAAATAAAAACTACTTTTTTTTTGATGAT
ATAAATACCGTAGATTATATAAATGTATATTAATATTATAAACCTATTTGATCAATGACAGTGTTCGTGAA
GCATTTTA

TTCAAAATCTGTCTATTTATAGGCCGTCGCGCTCTACGAAAACGCGAAATTATTCAAACGGAAAACGGAAA
AAAATCTAAAAAAAGAAATTAATTGAGAGATCTCACGGAAATGCCGCGAGGAATGTTTCTCGAGGCTGAGC
GGCGTGGT

TCATCTCTCTTAATTTTTCATGACTGACGTTTTTTCTTCATTTTAATTATCATAGTATTTGTTTGAAAAAA
AAAAAAAAAAATTTCCCTTATCAATGATATCCTTACGATTATATAAATTCCTTACCTAAACCTATTATTTG
TGTACATA

AGGTAAAGAAAGAGCATTTCTCTTTTTTTTATTTTTTTTTTTTTTACTTTCTTTACATTAAAAAAAAATAT
GAAGAGTGTAATAGGTAAGTATAAGTATTATTTAATCATTACAATTTAGCTTCAATAGTATAGAAGCGCAC
CACTTCAT

TCAGCATTACAGTTTTGAATTCTTTCACCCATCATTCAAAATGCATATGCATACTATATTTGTCTAGTTTT
TAATTATATAATAATAATAACAGAAACAAAAAATCGCATTCATCGTTTAATCTTATTTTTCTTTTTTCACC
TTTTTCTT

CTTTAGATGAGCTATTGGTGATTCGCATTTAGGAATGAAAAAAAGTAAAGCATGGCAATGAATGACACAAG
TGAAATAGAAAAGTGAAAAATTAAAAAAAAAAAAAAAAAAAAAAAAACTGATTTATACTCATGAAAATCTT
ATTCGAGT

CTCGCCGAGAAAAACTTCAATTTAAGCTATTCTCCAAAAATCTTAGCGTATATTTTTTTTCCAAAGTGACA
GGTGCCCCGGGTAACCCAGTTCCTCACTATTTTTTACTGCGGAAGCGGAAGCGGAAAATACGGAAACGCGC
GGGAACAT

CGCGCGTTTCTTTTTTTTTCTTGCTAACATTTTTCAACTTTAAAATGAAACGATGCATGAGTGATAGAGGC
ATTGAACTTTAGTAAACAATAGAAATAACCATCGGAGTTATTATTTTTTTCTGCTTATTCTTTGGTCGCAA
AAAAGTGT

AAAAAAAATGCGATAATTCTTTAGTTGTCGGCACTTCGGAAGCCAAAAAAAAAAAAAAAAAAAAAAAAGAA
AAAAAGAAAAGGGTGTCTAAGATGTACGGATCTTCATTGGGATGGAAAACAGGCAAGTAAAAAGAATAGTA
ATTATTAC

AGCCAAAAAAAAAAAAAAAAAAAAAAAAGAAAAAAAGAAAAGGGTGTCTAAGATGTACGGATCTTCATTGG
GATGGAAAACAGGCAAGTAAAAAGAATAGTAATTATTACAATGGAGAAGATAAGATAAAGTTAGCGGCAAC
AAATAAAG

TAATATATACCTTTAGATGAGCTATTGGTGATTCGCATTTAGGAATGAAAAAAAGTAAAGCATGGCAATGA
ATGACACAAGTGAAATAGAAAAGTGAAAAATTAAAAAAAAAAAAAAAAAAAAAAAAACTGATTTATACTCA
TGAAAATC

TGCTTTTTCCCCTGTCAGTATAAAAAAATTTTCCGCAGGATATAGAAAAAAAAGAAATGAAATTATAGTAG
CGGTTATTTCCGTGGGGTGCTTTTTTACACCTGTACATCTTTTCCCTCCGTACATTTTTTTTATTTTTTTT
TTTGGTTT

CACATTTGCTTTTTTTGATTTTATTTATTTACTTTTACGACAACTGGCCTTATCACTCGGCTCTCTAAAAG
AAGTCATGTTCCCTTCGTTGGGTTAATTTTTTTTTTTTTTTTTGTTTATATGTTTTTTTTTTTTTTTTTGC
CAGGTATT

ATAGAGTAAGTGAAAGACAAATGGAGGAAAAAAAAAAAAAAAAAAAAAAAAAAAAAATAGTAAAAAGTGAA
AGGAGAACGATGATAACACTAATCACACCTCCGCATTTTCAACCAATGCTGCAAGTTTCTCAACACTGGCT
AAATGTCT

GGAAATTAAATACAGGTGTACACATACACGCACACATATATATATATATATATATGTATATGTGTATATAG
GAAGCGCGCGCATGTTAGTATATACGATTCGTTGGAAAGGGGCCGTCCACCAAACGTGACTTGACGAGTTG
ACAAATTG

TCTTTTTTTTATTTTTTTTTTTTTTTACTTTCTTTACATTAAAAAAAATATGAAGAGTGTAATAGGTAAGT
ATAAGTATTATTTAATCATTACAATTTAGCTTCAATAGTATAGAAGCGCACCACTTCATCAATGGCTTTAG
CCACGTAT

ATAATATTATTGATTTTTTTATTATTGTTCTTATTCTTTTTTTATCATTTCTATATAAAATATTATATGAT
TTAGTTTATCGTAAAAAAATTAAAAAATACAGAGGAAAGCAAAAGTAAAACTTTTCGGATATTTTACAGGG
CGATCGCT

CACGAAAGCAAACCATAGAGATGAACTTAAGAAAAAAAGGGCCACGGATTTTTTTTTTCGCGTTTTGGCGTT
TTCATATCAAAAAAAAAAAAAAACCATATTAACATCTGTACATTATTATTAACTAAACATCCGTACACAAAAGG
TTTTCCAA

AATAAAAGTACAGCTCTAGGTCTATAAAAAAAAAAATTGGTTAAAGAAAAAATATACAGGTTTGTATATAG
AATCATTAATTAAAAAGATAATAGTATAAAACACATCAGATAAACAAAAGTTTGCCCAATAAAATTATATT
TTCCATCA

TCCTGGAATTTCCAGCGGATGGTGACGCGAGGCATCCGTAGTGCACAATAAAAAGTTAGTTTTTCATTAAC
CGAAAAAAAAAAAAAAATAAAATATATTAATGTTTGGGTTTCAACGTTTCCGTGGGTCTGGGTCCACAACA
GAAAACTG

GCAAAGGACTAATAACTTGAATCATTTTTATATCAGGTTTATATAGGTATACGTTCAGTCTAATTAATAAT
TTATAGTTTACAGTAGTTTTTTACAGTAGTTTTTTTTTTTTTTTTTCAGTTCCATGATTTTTTTGTTCCTT
TGATTCGT

GTCCGGGCAAATCCTTTCACGCTCGGGAAGCTTTGTGAAAGCCCTTCTCTTTCAACCCATCTTTGCAACGA
AAAAAAAAAAAAAAATAAAAAATAAAAAGACCAAATAGTAAATAGTAACTTACATACATTAGTAAATGGTA
CACTCTTA

ATTATTAGATCACTTAGCACGTGAAAAAGAAACGTTTTTAATGTTTTTTTTTTTTTTTTCTTTTTCTTTTT
TTGCGTTGGTGAAAATTTTTTCGCTTCCTCGAGTATAATTATCTCATCTCATCTTTCATATAAGATAAGAA
GTTTTATA

GCGATTTAGTTGTTCTTTTAAAAAAAAAAGAGAAAAAAAAAAAAAAAAAAAAGGGAATGAGTTTGTACATA
CTATTATATTAATGTAGTATCACGTTAAAAAGCCGTAGTCATCATTAATCGTCCAAAAGTCTTCTACCGGA
CCAGATGT

GCCGAGAAAAACTTCAATTTAAGCTATTCTCCAAAAATCTTAGCGTATATTTTTTTTCCAAAGTGACAGGT
GCCCCGGGTAACCCAGTTCCTCACTATTTTTTACTGCGGAAGCGGAAGCGGAAAATACGGAAACGCGCGGG
AACATACA

TTATCCGGCCCCACCCATGCATAACCCTAAATTATTAGATCACTTAGCACGTGAAAAAGAAACGTTTTTAA
TGTTTTTTTTTTTTTTTTCTTTTTCTTTTTTTGCGTTGGTGAAAATTTTTTCGCTTCCTCGAGTATAATTA
TCTCATCT

CAAAACCCGACATTGATGAACTAGTGCCGCGCTTAAAAGTATAATGTTGCTCTATTATTTTTCAAAAATTC
ATACAAAAGAAAACGGGAATTAAGTAAGTATACAAAATATAATTATATTACGATGTTCGAAATTCTATATA
TGAATTTA

CTACCTCCGTTAACAAGACATTAGATTAGTGATCACACCCAATTTTTAATTTAGTAACCCAAAACTAAATA
AGTATTTACTCAACTTTTTTTTAAAAAAAAAAAACTTAATTGAATTTTGCTCGCGATCTTTAGGTCCGGAG
TTTTCGTT

CGTATTTCTCGCCGAGAAAAACTTCAATTTAAGCTATTCTCCAAAAATCTTAGCGTATATTTTTTTTCCAA
AGTGACAGGTGCCCCGGGTAACCCAGTTCCTCACTATTTTTTACTGCGGAAGCGGAAGCGGAAAATACGGA
AACGCGCG

AACTCCGCGAGGTTCAGGAACCTGAAACAAAATCAATAAAAACATTATATGCGTTTCGAACAAAATTAAAG
AAAAAGAATAAATATAGATTAAAAAAAAAAAGAAGAAATTAAAAGAATTTCTACTAAATCCCAATTGTTAT
ATATTTGT

GTGTTTTAGATTAGGCTTATAAGATATGACCCATATCATAATTAAAAAAAGCGATTTAGTTGTTCTTTTAA
AAAAAAAAGAGAAAAAAAAAAAAAAAAAAAAGGGAATGAGTTTGTACATACTATTATATTAATGTAGTATC
ACGTTAAA

ACACTTGTCTTTATGAAAGAGTGGAAAAATGGTGGGAGTAAGTTATACTATTGACGTGTAAAAAAAAAAAA
AAAAAAATGTTAATGGAACGACCGATCTAAAGAACCATATATAATGAATGACTAAAAAAAGCAAAATAGT
TAAGAATC

AGCAATCTCATCGCGAAATTTTTCTCAAAATTTTTCAGTTAAAAAAAAAAAAGCACGTGCTCAAAAATGTT
TTTAGAAAATCATTTATTTAAGATAGGAAAAATGCAATTAATCATTGAATCAATATGTTCACCACTTAAAT
TACCCACT

TTTTTTTCCAAAGTGACAGGTGCCCCGGGTAACCCAGTTCCTCACTATTTTTTACTGCGGAAGCGGAAGCG
GAAAATACGGAAACGCGCGGGAACATACAAAACATACAAAATATACCTTTCTCACACAAGAAATATATGCT
ACTTGCAA

CTTCAAAGCCGCACACGTTTCTTTTTCCGAAAAATGAAAAATTTTTCACTGAAAAAAAAAAAAAAAAAAAAG
AAGAAATAGAAAGGTATAAATAGTTGAATAATTTGCCCTTCCGAGATCCAACAAGATTTTTGAGTGTTGGG
ACCCTTGG

CAAGTGTATCGATCTGTTATAATATTAATACGCATTTTTTTTATGTTTTCTCGTTTCTTTTCTTTTTTTTG
TTTTATATCTTACACATTTTTAATATACTAAAAGTAAAATAAAAAAAATACGTACTGTTTCCCTTTGTTTTC
TCCTCCTC

ACCCGCAGGTGAAAATTTTAGCAAAGTTACACTTTTTTTTTCATTTTTTTTTTTCTTTTCTCCTCCATCTA
ATTTCACCTGCGGTTACCGGCGGAATAACTCGCGCACCATTTCTCTGTCTCGGGCCTCGCGTTCCTCTTTT
TCGACTCG

TAAAATGATATTGTGGTTAATCTAAATTTATATATATATATATATATACATATATATATAGAAATCTATTG
TTATACACAAAAATACTTATTTTTTAATATAGATGGCTGCAGATAAAGTAATAGTTTTATATATAGGTATA
TTTACTGC

TCGGGCATTTAAAATCTTGGATCGATAACTTAACTGGGTGTAATAAAATATATAAAGATATAAAATAGATA
TATATATATAAAACGAACTTTATCAAGAAGAACTGCAAATGAAGCGTACTCTCAAATCCTCGAAAACGAAA
AACACTCC

GGTGAGAGAGTCCTGGAATTTCCAGCGGATGGTGACGCGAGGCATCCGTAGTGCACAATAAAAAGTTAGTT
TTTCATTAACCGAAAAAAAAAAAAAAAATAAAATATATTAATGTTTGGGTTTCAACGTTTCCGTGGGTCTGG
GTCCACAA

TAAGAAGGTAACATGAGAAAACAGACAAGAAAAAGAAACAAATAATATAGACTGATAGAAAAAAATACTGC
TTACTACCGCCGGTATAATATATATATATATATATATTTACATAGATGATTGCATAGTGTTTTAAAAAGCT
TTCCTAGG

CATTTGCGGCATCTTATAACCCACACACCGCATTTAGGACCCGTTTTTTTTCTTTTTTTTTTTGTTGAAAA
AATTCAACAGTTCCTATTTTTTTCTTTTCATTCTTTTTTTTTGCCTTGCCTTCGTTTCGAGAATTCTTTTT
CTTTTTCT

AAAAGTTTCAATGCGTTCTATCGTATAGTATGATGTATTCAAAAAAAAATGCGATAATTCTTTAGTTGTCGG
CACTTCGGAAGCCAAAAAAAAAAAAAAAAAAAAAAAAAGAAAAAAAGAAAAGGGTGTCTAAGATGTACGGAT
CTTCATTG

TAAAAATTGGGGAGTGAATCTTAAAAGCCCGAAAAGGAAATTCAAAATCTGTCTATTTATAGGCCGTCGCG
CTCTACGAAAACGCGAAATTATTCAAACGGAAAACGGAAAAAAAATCTAAAAAAAGAAATTAATTGAGAGAT
CTCACGGA

TCCCAATAGCACATCAACCAACCCATATTCCAGCTGATTCATTTATATTTCATCTTTTCTTTTTTTTTTTT
TTACAATTCAGTAGTTTTATATAACTTCCCATAAATGTATTATTTTTTTAATACGACTATAATATACCTCT
ATACCCTT

TGGATTTTATATTTTTCACAGCGAAAAGGACGGGAAGCTAAAAGATTAAAAAATAAATATTGCAGATACCA
TATGTAATTTAGTAAAATATTTTTATAAAATATTATTATTTTCTTGTTCTTCAGTGATTTATTGTAACCAT
GGAGAAAT

GACCGACGGTACCCGCAGGTGAAAATTTTAGCAAAGTTACACTTTTTTTTTCATTTTTTTTTTTCTTTTCT
CCTCCATCTAATTTCACCTGCGGTTACCGGCGGAATAACTCGCGCACCATTTCTCTGTCTCGGGCCTCGCG
TTCCTCTT

CGGTACACATACATACACATATATATATATAAATATATATATATATAAGGACATATACAGATTTTTTTTTT
TCGCGTACTAAAGTACAGAACAAAGAAAATAAGAAAAGAAGGCGATCATTGAACTACTGTGACTTGCCAAT
ATGGTCTA

CTGAAGCGTCATCATCTATCTGTATTTTTAATGCAATCATAGTATAAAAATTTTTAACCAAATTCAAAAAT
TATAATATTTTAAAAGTTTATGCTTTTGTCCTTTTATATTCAAGTCATTTGGCCATTATTACGCTTTTTTT
TTTATTAG

TAGGAAAAATCAACGAGCCAAAAAAAAAAAAAACGGAACCGTTCGGCATGTATATATTCACTATTTCATAT
ACGCGTTTGCACAATATACTCTATCTTATCCGTATCTATACGAGCCGGTTGCTGCACCTTACATGTCATAG
TTGAAATC

TACTCCCTACAAACATGTGCGCGTTTGTGACAAAAACATAAATAAAACAAAAATAATAAAAAGTCAAGTGA
AAAGCACTGAAATCTAAAAAATAAATACATATACATATATATATATATATACCTATTTCGTTTCCGCATAG
AAGAAATT

TCTTTGTTATATGGGTACAAGATATAAGAAACATAACTATTACATACGAAAATGTGCATGTTATCTATATC
CTTCTTTATATAGATGCTGTTAACTTCTTTTTTTTTTTTTGGGAAAATCAACTGTTAAACGCGACAGTAAA
AGCAGCAA

GCACTTCGGAAGCCAAAAAAAAAAAAAAAAAAAAAAAAGAAAAAAAGAAAAGGGTGTCTAAGATGTACGGA
TCTTCATTGGGATGGAAAACAGGCAAGTAAAAAGAATAGTAATTATTACAATGGAGAAGATAAGATAAAGT
TAGCGGCA

CCGTCCGATCAACTGTAGTTAAGCTGGTAAGAGCCTGACCGAGTAGTGTAGTGGGTGACCATACGCGAAAC
TCAGGTGCTGCAATCTTTATTTCTTTTTTTTTTTTTTTTTTTTTTTTTTTTTCTAGTTTCTTGGCTTCCTA
TGCTAAAT

TAATTTTTCATGACTGACGTTTTTTCTTCATTTTAATTATCATAGTATTTGTTTGAAAAAAAAAAAAAAAA
ATTTCCCTTATCAATGATATCCTTACGATTATATAAATTCCTTACCTAAACCTATTATTTGTGTACATATA
TCAGAGTA

TAGTAAAAATACTATAAATGTTTGAATATGAAGATATATATGTAGGAAGAATTTTTATTGAATATAGAAAT
TTAAAGAAATATTATTAAAAACCGGAAAATGAAAAAGAAGAGTTAAAAAAAAAAAAAACGGAACTAGTAAA
AGATGTGA

TTTTTTTTTTTTTACAATTCAGTAGTTTATATAACTTCCCATAAATGTATTATTTTTTTTAATACGACTAT
AATATACCTCTATACCCTTATTTATCTCTTTACCTTTGCTTTTAAGCTAAACAAATCTTCATTAACAGGTA
TTATATTA

AATAAAACAAAAATAATAAAAAGTCAAGTGAAAAGCACTGAAATCTAAAAAATAAATACATATACATATAT
ATATATATATACCTATTTCGTTTCCGCATAGAAGAAATTGGAGGCAACTAGTTGCAGGTTGTTTTCCTTAG
CCCATTTG

TATAGAAAAAAAAGAAATGAAATTATAGTAGCGGTTATTTCCGTGGGGTGCTTTTTTACACCTGTACATCT
TTTCCCTCCGTACATTTTTTTTTTATTTTTTTTGGTTTTTTTTTTTTTCGATATTTTCCCTCCGAAACTAG
TTAGCACA

ATTAAAATCTTCTAAACCTAATAAATATGTTTAATGAATGATTCAATATCAAAAAAAGAAATCCATTGGGC
CCTTAGTGCATTTTTATTTTTTTTTTTTTTTTTTTTTTTTTTCTCTTTCTGAATTAGAGTACATCTCATCG
CAGGGTCT

AGTTGAATATTTGAAGTACACACCCGCGTAAAGAGTTTTTACCCCGAAAACAAATTTTTATGCTTGAAAAA
TAGCTAATAAAATGTTTTTATTGTTCGGATAACAAATACAATAGTGTTATTAAAAAATAAAACTTATTTAA
AAATAGTA

GGTTTTTTTTTTTGTTTTGTCAAGAGCCGCGCACAAAATATGGTATTATTATAGAAAAGTTGGACTTAAAA
GCTTGAAAAAGCTTAGATTTTATATTCATATGCTGGTATATATGTACGACTAAATAATATAAATTTGATTA
TAACAATC

CAGCTTTCTTTTTTTCAAAAAACTTTTTCTATTATTAGATTAGATTTTTTTTAGTGTGTATATTAATAACG
TAAAATTTCTTTATCAACGACTTGTAGTATTCTTTTACTTTTATTTTTTTTACATATGCAACGTTATATTC
TTTTTTAA

TGATTTTTTTATTATTGTTCTTATTCTTTTTTTATCATTTCTATATAAAATATTATATGATTTAGTTTATC
GTAAAAAAATTAAAAAATACAGAGGAAAGCAAAAGTAAAACTTTTCGGATATTTTACAGGGCGATCGCTAA
GCTTAATC

GATGTCAGACAACGTGCAGCATCTTTTTTTCCTACCATTGAATATATATATAGTAATTACGGCAACCGTTT
ATATAAAAATTCAAAAGATTAAATATTTTAATATATTAGTGTCTGGTTTAAAAGTCATACTTCTCTAATCA
CTACCGTG

AGGCCGTCGCGCTCTACGAAAACGCGAAATTATTCAAACGGAAAACGGAAAAAAAATCTAAAAAAAGAAATT
AATTGAGAGATCTCACGGAAATGCCGCGAGGAATGTTTCTCGAGGCTGAGCGGCGTGGTCTGTGCAAAAAA
ATGGCAAT

TCAGCGTAGTCCCAGAAAAAAAAAATCATCACTGGAAATGTAAGATGAATTCAAGTATCAAGTGCCGTTGGT
ATTATAGTTTATCAATTAATACGTAAATATATATATATATATATATATATATATATATGACTATAGTGGCA
TACTTCTC

TAATTTTTATTAAGTTTTGATCAACTTAGGATTTTCCTATTTTATTTTTGTTTATTATATTAAGTGTTCAT
TATTTTAAGTAGTTATATATTATATAATACAACCCCGATTAGACAAACTTCATAAGTTTTTCGGGTGGGAC
GGTATCAA

AGCTATTTCTCACATTTGCTTTTTTTGATTTTATTTATTTACTTTTACGACAACTGGCCTTATCACTCGGC
TCTCTAAAAGAAGTCATGTTCCCTTCGTTGGGTTAATTTTTTTTTTTTTTTTTTTTGTTTATATGTTTTTT
TTTTTTTT

TCTTTCACCCATCATTCAAAATGCATATGCATACTATATTTGTCTAGTTTTTAATTATATAATAATAATAA
CAGAAACAAAAAAATCGCATTCATCGTTTAATCTTATTTTTCTTTTTTCACCTTTTTCTTGTGAAATTGCCA
AGCGCTTA

TTCAAAATTCTTAGAATATGCATCCACATTTATATACATATATATATATATATATATATTATAACTTCCAA
AAAGGAGATTGGACAATTTATATCTATAAGGCATTAATATTTAACTATTTACAAGCACTAGTGGTAAATTC
ATGGCGTC

AGAGCATTTCTCTTTTTTTTATTTTTTTTTTTTTTTACTTTCTTTACATTAAAAAAAATATGAAGAGTGTA
ATAGGTAAGTATAAGTATTATTTAATCATTACAATTTAGCTTCAATAGTATAGAAGCGCACCACTTCATCA
ATGGCTTT

GCTATTGGTGATTCGCATTTAGGAATGAAAAAAAGTAAAGCATGGCAATGAATGACACAAGTGAAATAGAA
AAGTGAAAAATTAAAAAAAAAAAAAAAAAAAAAAAAACTGATTTATACTCATGAAAATCTTATTCGAGTTC
ATTCAAGA

TCTTAGCGTATATTTTTTTTCCAAAGTGACAGGTGCCCCGGGTAACCCAGTTCCTCACTATTTTTTACTGC
GGAAGCGGAAGCGGAAAATACGGAAACGCGCGGGAACATACAAAACATACAAAATATACCTTTCTCACACA
AGAAATAT

TATTACTCACCCTGCCCCACGCTCAAAGGGCTCGATAACGACATAGCATTATAGACACTGTGCAGCGTAAA
GCTGACATATAAAGAAATTACAAAAAAAAATTAAAAAAAAAAAAAAAAACAATCAAAAAAGACACACAACT
ACAGAACA

CATAGTATTTGTTTGAAAAAAAAAAAAAAAAAATTTCCCTTATCAATGATATCCTTACGATTATATAAATTC
CTTACCTAAACCTATTATTTGTGTACATATATCAGAGTATTATTACATATATAACCTTTTTCTCTAAAACA
GGAAAAAA

AACATTATATGCGTTTCGAACAAAATTAAAGAAAAAGAATAAATATAGATTAAAAAAAAAAAGAAGAAATT
AAAAGAATTTCTACTAAATCCCAATTGTTATATATTTGTTAAATGCCAAAAAAGTTTATAAAAAATTTAGA
ATGTATAA

GCGGGTAATAATTATAGTATTTTTCTTTTCTATTTTATTTATTTTTTTTTTTTTTTTTGATTTTCTAGAAT
AATCAATCAATGGTAAATAAACAAAAGTTGCTTCTGTTGTAAAAAATACATCAAAGGGGCTAAAATATCAA
TATACGTT

AAGAGTTTTTACCCCGAAAACAAATTTTTATGCTTGAAAAATAGCTAATAAAATGTTTTTATTGTTCGGAT
AACAAATACAATAGTGTTATTAAAAAATAAAACTTATTTAAAAATAGTAATTTAAATTATTATTTTATTTT
AATAAACT

GGTGGGGAGTAAGTTATACTATTGACGTGTAAAAAAAAAAAAAAAAAAAAATGTTAATGGAACGACCGATCTA
AAGAACCATATATAATGAATGACTAAAAAAAGCAAAATAGTTAAGAATCCAAGAAAGATACTAAGAAAAGG
CGAAAAGA

AAGGATTGCTAACAGAAAATGCAAAGGACTAATAACTTGAATCATTTTTATATCAGGTTTATATAGGTATA
CGTTCAGTCTAATTAATAATTTATAGTTTACAGTAGTTTTTTACAGTAGTTTTTTTTTTTTTTTTCAGTT
CCATGATT

CTTTTCTTTTATTGCAGTTGGTATTATATACATATATATATATATATATATATGTATATGTTCCCCGAGAC
AATGGTTTTATTTCCAAAAAAAGTGTAAGTAAGTTATTTTCTGTGTATAAAAAAATAAAAGAAGCCAGATG
AAGTGCCA

AATATTAATACGCATTTTTTTTATGTTTTCTCGTTTCTTTTCTTTTTTTTGTTTTATATCTTACACATTTT
TAATATACTAAAAGTAAAATAAAAAAATACGTACTGTTTCCCTTTGTTTTCTCCTCCTCTTTGCTTATATC
TCCACTGC

GCAACAATATTTTTTGCTTATTTTGATATCCTGAAATCCTTTTTTTTTTTTTTTTTTTTTTTTTTTTTTTT
TGATTTCATTTCGTTTTTTTTTTTTTGAACATTATATATTGTATTGTTTTCTCCCTGTAGCGAGAACTAATT
GATTCTTT

AGAAATAGCAATAACACGCGTGCAGAAGATTGAGAAGACTACTACGTATTTTTTTTTATATGCATCCTTTT
TTCCACATATGTATGCGCGGTGCGCGCGCGCCATTAAAAGATTTTTTTCTTAGCTCTTTTTCTTTTTTTCTT
TTTCTTTC

AAAAGCGTAAGCAATGGTGTAGATACTTCTTATATAGACGTTATTATTACTATTATTATTATTATTATTAT
TATTATTATTATTATTATTATTATTATTATTATTATTATTATTATTATTATTATTATTATTATTATTATTA
TTATTATT

ATAACACGCGTGCAGAAGATTGAGAAGACTACTACGTATTTTTTTTTATATGCATCCTTTTTTCCACATAT
GTATGCGCGGTGCGCGCGCGCCATTAAAAGATTTTTTTCTTAGCTCTTTTTCTTTTTTCTTTTTCTTTCCA
CTGAGATG

GCAATGGTGTAGATACTTCTTATATAGACGTTATTATTACTATTATTATTATTATTATTATTATTATTATT
ATTATTATTATTATTATTATTATTATTATTATTATTATTATTATTATTATTATTATTATTATTATTATTAT
TATTATAT

TATATAGACGTTATTATTACTATTATTATTATTATTATTATTATTATTATTATTATTATTATTATTATTAT
TATTATTATTATTATTATTATTATTATTATTATTATTATTATTATTATTATTATTATTATATGACATTAATTAC
TTGTAATT

TTTTGCTTATTTTGATATCCTGAAATCCTTTTTTTTTTTTTTTTTTTTTTTTTTTTTTTTTTGATTTCATT
TCGTTTTTTTTTTTTGAACATTATATATTGTATTGTTTTCTCCCTGTAGCGAGAACTAATTGATTCTTTCT
CCTTTCCC

ATATATACGTTTGTAACAAATCGATAAATAGCAACAATATTTTTGCTTATTTTGATATCCTGAAATCCTTT
TTTTTTTTTTTTTTTTTTTTTTTTTTTTTTTGATTTCATTTCGTTTTTTTTTTTTGAACATTATATATTGT
ATTGTTTT

TCGATAAATAGCAACAATATTTTTGCTTATTTTGATATCCTGAAATCCTTTTTTTTTTTTTTTTTTTTTTT
TTTTTTTTTTTGATTTCATTTCGTTTTTTTTTTTTGAACATTATATATTGTATTGTTTTCTCCCTGTAGCG
AGAACTAA

AGATACTTCTTATATAGACGTTATTATTACTATTATTATTATTATTATTATTATTATTATTATTATTATTA
TTATTATTATTATTATTATTATTATTATTATTATTATTATTATTATTATTATTATTATTATTATTATATGA
CATTAATT

TTGTAACAAATCGATAAATAGCAACAATATTTTTGCTTATTTTGATATCCTGAAATCCTTTTTTTTTTTTT
TTTTTTTTTTTTTTTTTTTTTGATTTCATTTCGTTTTTTTTTTTTGAACATTATATATTGTATTGTTTTCT
CCCTGTAG

TTATTATTACTATTATTATTATTATTATTATTATTATTATTATTATTATTATTATTATTATTATTATTATT
ATTATTATTATTATTATTATTATTATTATTATTATTATTATTATTATATGACATTAATTACTTGTAATTAT
GTTATAAC

TACCTATAATTGTTCATATTATATATACGTTTGTAACAAATCGATAAATAGCAACAATATTTTTGCTTATT
TTGATATCCTGAAATCCTTTTTTTTTTTTTTTTTTTTTTTTTTTTTTTTTTGATTTCATTTCGTTTTTTTT
TTTTGAAC

CTCAAAATTAAGAAATAGCAATAACACGCGTGCAGAAGATTGAGAAGACTACTACGTATTTTTTTTTATAT
GCATCCTTTTTTCCACATATATGTATGCGCGGTGCGCGCGCCATTAAAAGATTTTTTTCTTAGCTCTTTTT
CTTTTTTC

AGCGATGCTATATCATCTGAGATGAGATGAGATGAGATGTTTATTATGATCCATTTCTTTTCTTTTTTTAT
TTTTTTTTTTTTTTTTTTTTTCATTTTTCAACGCGGGTAATAGAGAATACATCCACTGTCCGGGTAAATTA
TGTTACAG

TGTTCATATTATATATACGTTTGTAACAAATCGATAAATAGCAACAATATTTTTGCTTATTTTGATATCCT
GAAATCCTTTTTTTTTTTTTTTTTTTTTTTTTTTTTTTTTTGATTTCATTTCGTTTTTTTTTTTTGAACAT
TATATATT

TCACTTCTGAACATACCCATCTCAAAATTAAGAAATAGCAATAACACGCGTGCAGAAGATTGAGAAGACTA
CTACGTATTTTTTTTTATATGCATCCTTTTTTCCACATATATGTATGCGCGGTGCGCGCGCCATTAAAAGA
TTTTTTTC

GATCTCGGCGCGGAAAAATCAGCGCCCCACGCCAAAAGGTTCGTATTTTTTCTTTTTTTTTCTAATCTTCC
ATCTATTCGGTAGCGATGATTCATTTCTCTGAAAAAAAAAAAAAAAAAAAAAAATGAAAAAGAATATTTTT
TTGATGAA

TTTGATATCCTGAAATCCTTTTTTTTTTTTTTTTTTTTTTTTTTTTTTTTTTGATTTCATTTCGTTTTTTT
TTTTTGAACATTATATATTGTATTGTTTTCTCCCTGTAGCGAGAACTAATTGATTCTTTCTCCTTTCCCCC
TAAATACT

TATTATTATTATTATTATTATTATTATTATTATTATTATTATTATTATTATTATTATTATTATTATTATTA
TTATTATTATTATTATTATTATTATTATTATTATTATATGACATTAATTACTTGTAATTATGTTATAACAG
TGAAATAA

ATATAACTTTTTTAGATGTTAGCGATGCTATATCATCTGAGATGAGATGAGATGAGATGTTTATTATGATC
CATTTCTTTTCTTTTTTTATTTTTTTTTTTTTTTTTTTTTTCATTTTTCAACGCGGGTAATAGAGAATACA
TCCACTGT

TTTAGATGTTAGCGATGCTATATCATCTGAGATGAGATGAGATGAGATGTTTATTATGATCCATTTCTTTT
CTTTTTTTATTTTTTTTTTTTTTTTTTTTTTCATTTTTCAACGCGGGTAATAGAGAATACATCCACTGTCC
GGGTAAAT

GGCAATGAAAATATAACTTTTTTAGATGTTAGCGATGCTATATCATCTGAGATGAGATGAGATGAGATGTT
TATTATGATCCATTTCTTTTCTTTTTTTATTTTTTTTTTTTTTTTTTTTTTCATTTTTCAACGCGGGTAAT
AGAGAATA

TATCATCTGAGATGAGATGAGATGAGATGTTTATTATGATCCATTTCTTTTCTTTTTTTATTTTTTTTTTT
TTTTTTTTTTTCATTTTTCAACGCGGGTAATAGAGAATACATCCACTGTCCGGGTAAATTATGTTACAGTT
AGCGCTTA

TGAAATCCTTTTTTTTTTTTTTTTTTTTTTTTTTTTTTTTTTGATTTCATTTCGTTTTTTTTTTTTGAACA
TTATATATTGTATTGTTTTCTCCCTGTAGCGAGAACTAATTGATTCTTTCTCCTTTCCCCCTAAATACTCA
ACAGTACT

TGAGAAGACTACTACGTATTTTTTTTTATATGCATCCTTTTTTCCACATATGTATGCGCGGTGCGCGCGCG
CCATTAAAAGATTTTTTTCTTAGCTCTTTTTCTTTTTTCTTTTTCTTTCCACTGAGATGCGTTCTTCGGTA
TATAGTGA

ACATACCCATCTCAAAATTAAGAAATAGCAATAACACGCGTGCAGAAGATTGAGAAGACTACTACGTATTT
TTTTTTATATGCATCCTTTTTTCCACATATGTATGCGCGGTGCGCGCGCGCCATTAAAAGATTTTTTTCTT
AGCTCTTT

GTTTTTTGTTTTCGCTTTTTTTATTTTTTGTTCCTAATTTTTTTTTTTTTTACATTTTATTAGCTATACAC
TTGGCGCAAGGATATTTTATATACCGGATATAATAGATAATTAAAAGACTACTAATTAAAAATGATGTAAT
ATGGTAAT

CCATTTCTTTTCTTTTTTTATTTTTTTTTTTTTTTTTTTTTTCATTTTTCAACGCGGGTAATAGAGAATAC
ATCCACTGTCCGGGTAAATTATGTTACAGTTAGCGCTTAATGTTCACTTCACAGTATTATGAGCTGTTTTT
GTAATAAA

TTATTATGATCCATTTCTTTTCTTTTTTTATTTTTTTTTTTTTTTTTTTTTTCATTTTTCAACGCGGGTAA
TAGAGAATACATCCACTGTCCGGGTAAATTATGTTACAGTTAGCGCTTAATGTTCACTTCACAGTATTATG
AGCTGTTT

ACTACGTATTTTTTTTTATATGCATCCTTTTTTCCACATATGTATGCGCGGTGCGCGCGCGCCATTAAAAG
ATTTTTTTCTTAGCTCTTTTTCTTTTTTCTTTTTCTTTCCACTGAGATGCGTTCTTCGGTATATAGTGAGA
ACACTTAC

AAATATAATTGGCAATGAAAATATAACTTTTTTAGATGTTAGCGATGCTATATCATCTGAGATGAGATGAG
ATGAGATGTTTATTATGATCCATTTCTTTTCTTTTTTTATTTTTTTTTTTTTTTTTTTTTTCATTTTTCAA
CGCGGGTA

ATTATTATTATTATTATTATTATTATTATTATTATTATTATTATTATTATTATTATTATTATTATTATTAT
TATTATTATTATTATTATTATTATTATATGACATTAATTACTTGTAATTATGTTATAACAGTGAAATAAAT
AAAATATC

ATTTTCCTCATTGGTCTTTGTACCTATAATTGTTCATATTATATATACGTTTGTAACAAATCGATAAATAG
CAACAATATTTTTGCTTATTTTGATATCCTGAAATCCTTTTTTTTTTTTTTTTTTTTTTTTTTTTTTTTTT
GATTTCAT

TTGGTCTTTGTACCTATAATTGTTCATATTATATATACGTTTGTAACAAATCGATAAATAGCAACAATATT
TTTGCTTATTTTGATATCCTGAAATCCTTTTTTTTTTTTTTTTTTTTTTTTTTTTTTTTTTGATTTCATTT
CGTTTTTT

GAGATCTTTTGGTCAAACAATCTAGATTGTTCTGAAGATCAAAATAATATATATATATATATATATATATA
TATATATGTTTATACACTTAACTTACGTACAAAAATATAGATTTCGAAACGAATCAGTTGCTCTTAGCCTG
CACATCTA

GATTGGACTAGGCATCACCCAGACTTTACTTTTTCGGCATACATATCTATTTATATATAGTTTAATACGTA
AATACAAATATATATATACTGTAAATTTTAATCATTAAGCTTTTCTTTTTTTTTTGTTCACGCTTTCACCC
TGTGAGGC

GAGACGAGTGTCACTTCTGAACATACCCATCTCAAAATTAAGAAATAGCAATAACACGCGTGCAGAAGATT
GAGAAGACTACTACGTATTTTTTTTATATGCATCCTTTTTTTCCACATATGTATGCGCGGTGCGCGCGCGC
CATTAAAA

TTTTTTTTTTTTTTTTTTTTTTTTTTTTTTTTGATTTCATTTCGTTTTTTTTTTTTGAACATTATATATTG
TATTGTTTTCTCCCTGTAGCGAGAACTAATTGATTCTTTCTCCTTTCCCCCTAAATACTCAACAGTACTCT
CGAGCCAT

TGCAGAAGATTGAGAAGACTACTACGTATTTTTTTTTATATGCATCCTTTTTTCCACATATGTATGCGCGG
TGCGCGCGCGCCATTAAAAGATTTTTTTCTTAGCTCTTTTTCTTTTTTCTTTTTCTTTCCACTGAGATGCG
TTCTTCGG

ATTTTATTTTACGTTTGTTAATTCATTATACTGATAATATTTTTGAATTTTTTTTTTTTTTGATACATTTT
TTTTAATCGCTGTTTTGTCTGTTTTTTTCGATTCAGTTATAGGGAAAAAAACGGGAAAGGAAAGAGAAAAA
AAAATTAG

CTTTCCACACATTTTCCTCATTGGTCTTTGTACCTATAATTGTTCATATTATATATACGTTTGTAACAAAT
CGATAAATAGCAACAATATTTTTGCTTATTTTGATATCCTGAAATCCTTTTTTTTTTTTTTTTTTTTTTTT
TTTTTTTT

TAGTAAGAAAGATTGGACTAGGCATCACCCAGACTTTACTTTTTCGGCATACATATCTATTTATATATAGT
TTAATACGTAAATACAAATATATATATACTGTAAATTTTAATCATTAAGCTTTTCTTTTTTTTTTGTTCAC
GCTTTCAC

GTAATAAGTTTTCACCTTTTCCGGTGAATAATGAAAAAAAAAAAAACGAATACAAAAAAAAAGTAATTTCT
TTCAAGTTTATATATATAACAATATAATATTAAGTAATTTTAATCGAAAATTTTTTCCTCTAAAGAACCCA
AAAATAAG

CATTAACGGAGTTTTTTGTTTTCGCTTTTTTTATTTTTTGTTCCTAATTTTTTTTTTTTTTACATTTTATT
AGCTATACACTTGGCGCAAGGATATTTTATATACCGGATATAATAGATAATTAAAAGACTACTAATTAAAA
ATGATGTA

TTTTTTCTTTCTTTTTTTTTTTACCTGGGACGACATTCTAACTAATGAAATTTACGATTAATTATTCTTTT
TTTTTTTTTTCAAGAAATAAATATAACTGTAACATATATATATTAATTATGGGGGATCACAAAGAAAAATA
GTAACTTT

TGCATCCTTTTTTCCACATATGTATGCGCGGTGCGCGCGCGCCATTAAAAGATTTTTTTCTTAGCTCTTTT
TCTTTTTTCTTTTTCTTTCCACTGAGATGCGTTCTTCGGTATATAGTGAGAACACTTACAACTGTATACGA
ACATCGAA

ACGCGACATGGTGGGGGACGTGTAGTCGTGCCCTGCGATCCGCTTTATTACGTATATTGCCTTCACATGCC
GTGAAAGCCGACAGGGACAACGCGCGATAAAAAAAAAATTAAAAGTAAAACTACGTACGCGGCGGCGCGAC
TCGAATCC

GGCATCACCCAGACTTTACTTTTTCGGCATACATATCTATTTATATATAGTTTAATACGTAAATACAAATA
TATATATACTGTAAATTTTAATCATTAAGCTTTTCTTTTTTTTTTGTTCACGCTTTCACCCTGTGAGGCGA
ATGAGGTT

CACACGCGAAGGCAGTAGGCGATGAAAGGAAGTTTTTTAATGTCATTAAGAAAAAGATAGGGAACTTCTAT
TTCCTGTCTTTCATATATATATATATATATATATATATATATATGTGCATATGTACATCGTTGAGTTGTTC
ATGAAATA

TTTTGAATTTTTTTTTTTTTTGATACATTTTTTTAATCGCTGTTTTGTCTGTTTTTTTTCGATTCAGTTAT
AGGGAAAAAAACGGGAAAGGAAAGAGAAAAAAAAATTAGTGCAGAGCAATAAGAAGCGAAAATCAAAAAAA
AGTTTTGG

TTTTTTTTTTAATTTTTTTTTTTTTTGATCTCATTTCTTTTTTTATTGAATCTTTTTTTTTTGTAAGAAAA
TTAAGGTTTATTAGGCAGAGTATACCGAGTCGTTTGAAGTCATCTCCGGGTAGTGATTTTTATCACGTGAC
ACTTTTTT

TCTTTTTTTATTTTTTTTTTTTTTTTTTTTCATTTTTCAACGCGGGTAATAGAGAATACATCCACTGTC
CGGGTAAATTATGTTACAGTTAGCGCTTAATGTTCACTTCACAGTATTATGAGCTGTTTTTGTAATAAAGA
CGACTATA

TTTTTTTATATGCATCCTTTTTTCCACATATGTATGCGCGGTGCGCGCGCCATTAAAAGATTTTTTTTTCT
TAGCTCTTTTTCTTTTTTCTTTTTCTTTCCACTGAGATGCGTTCTTCGGTATATAGTGAGAACACTTACAA
CTGTATAC

TTCAGTTGCTGATCTCGGCGCGGAAAAATCAGCGCCCCACGCCAAAAGGTTCGTATTTTTTCTTTTTTTTT
CTAATCTTCCATCTATTCGGTAGCGATGATTCATTTCTCTGAAAAAAAAAAAAAAAAAAAAAAATGAAAAA
GAATATTT

ATTAGTTTCTTTTTTTTTTTAATTTTTTTTTTTTTTGATCTCATTTCTTTTTTTATTGAATCTTTTTTTTT
TGTAAGAAAATTAAGGTTTATTAGGCAGAGTATACCGAGTCGTTTGAAGTCATCTCCGGGTAGTGATTTTT
ATCACGTG

GCCAAAAGGTTCGTATTTTTTCTTTTTTTTTCTAATCTTCCATCTATTCGGTAGCGATGATTCATTTCTCT
GAAAAAAAAAAAAAAAAAAAAAAATGAAAAAGAATATTTTTTTGATGAACTTGTATTTCTCTTATCTGGTT
GATATATA

CGGCTGATAAATTAGTTTCTTTTTTTTTTTAATTTTTTTTTTTTTTGATCTCATTTCTTTTTTTATTGAAT
CTTTTTTTTTTGTAAGAAAATTAAGGTTTATTAGGCAGAGTATACCGAGTCGTTTGAAGTCATCTCCGGGT
AGTGATTT

TTCACCTTTTCCGGTGAATAATGAAAAAAAAAAAACGAATACAAAAAAAAAAGTAATTTCTTTCAAGTTTA
TATATATAACAATATAATATTAAGTAATTTTAATCGAAAATTTTTCCTCTAAAGAACCCAAAAATAAGCA
ACTATTGA

CGGAAAAATCAGCGCCCCACGCCAAAAGGTTCGTATTTTTTCTTTTTTTTTCTAATCTTCCATCTATTCGG
TAGCGATGATTCATTTCTCTGAAAAAAAAAAAAAAAAAAAAAAATGAAAAAGAATATTTTTTTGATGAACT
TGTATTTC

AATACATAGCCTAACAAGCAATTTTATTTTACGTTTGTTAATTCATTATACTGATAATATTTTTGAATTTT
TTTTTTTTTTGATACATTTTTTTAATCGCTGTTTTGTCTGTTTTTTTTCGATTCAGTTATAGGGAAAAAAA
CGGGAAAG

GTATATGTTTTCTTTTTTTTTTTTTTTTTTTTTTGAATGATATTAAGTTATACGAATGTTCGATAATATAT
ATGTATGAATAAATAAAAAGTTTTTAAAGTAGCCTTAAAGCTAGGCTATAATCATGCATCCTCAAATTCTG
TTAAAGTA

CTGATAATATTTTTGAATTTTTTTTTTTTTTGATACATTTTTTTAATCGCTGTTTTGTCTGTTTTTTTTCG
ATTCAGTTATAGGGAAAAAAACGGGAAAGGAAAGAGAAAAAAAAATTAGTGCAGAGCAATAAGAAGCGAAA
ATCAAAAA

ATATATATGTGTATATGTTTTCTTTTTTTTTTTTTTTTTTTTTGAATGATATTAAGTTATACGAATGTTC
GATAATATATATGTATGAATAAATAAAAAGTTTTAAAGTAGCCTTAAAGCTAGGCTATAATCATGCATCC
TCAAATTC

ACGTTTGTTAATTCATTATACTGATAATATTTTTGAATTTTTTTTTTTTTTTGATACATTTTTTTTTAATCGC
TGTTTTGTCTGTTTTTTTTCGATTCAGTTATAGGGAAAAAAACGGGAAAGGAAAGAGAAAAAAAAATTAGTG
CAGAGCAA

TTCGCTTTTTTTATTTTTTGTTCCTAATTTTTTTTTTTTTTTACATTTATTAGCTATACACTTGGCGCAAG
GATATTTTATATACCGGATATAATAGATAATTAAAAGACTACTAATTAAAAATGATGTAATATGGTAATAG
GTATGGAA

AGGAAAAAATAAAAGCGTAAGCAATGGTGTAGATACTTCTTATATAGACGTTATTATTACTATTATTATTA
TTATTATTATTATTATTATTATTATTATTATTATTATTATTATTATTATTATTATTATTATTATTATTATT
ATTATTAT

GATGAGATGTTTATTATGATCCATTTCTTTTCTTTTTTTATTTTTTTTTTTTTTTTTTTTTCATTTTTCA
ACGCGGGTAATAGAGAATACATCCACTGTCCGGGTAAATTATGTTACAGTTAGCGCTTAATGTTCACTTCA
CAGTATTA

ATGAAATATTCATTAACGGAGTTTTTTGTTTTCGCTTTTTTTATTTTTTGTTCCTAATTTTTTTTTTTTTTT
ACATTTTATTAGCTATACACTTGGCGCAAGGATATTTTATATACCGGATATAATAGATAATTAAAAGACTA
CTAATTAA

CAAAAGGTATATAATTCATTCAATAGATACAACTGCTCCATGAAAGGAAAAACCTTCATTTACGGTCGCGT
TTTTTTTCGTGATATAAAAAAGGTAAAAAAAACGCGTCGCGTTAGAAAAAAGGTGTGTGTGAAGAGAAAGA
CCCATTGA

CAAAAATTCAAATTTTCTCTCTCTCTCTCTATAAACATAAATATATATGTGTATATGTTTTCTTTTTTTTT
TTTTTTTTTTTTTGAATGATATTAAGTTATACGAATGTTCGATAATATATATGTATGAATAAATAAAAAGT
TTTTAAAG

CTTTTTTTTTTTTACCTGGGACGACATTCTAACTAATGAAATTTACGATTAATTATTCTTTTTTTTTTTTTT
CAAGAAATAAATATAACTGTAACATATATATATTAATTATGGGGGATCACAAAGAAAAATAGTAACTTTAA
GAATCAAA

TTTTTTATTCATAATTTGGTAATAACGGTTGATAAACGAGCGGCTGATAAATTAGTTTCTTTTTTTTTTTTA
ATTTTTTTTTTTTTTTGATCTCATTTCTTTTTTTTATTGAATCTTTTTTTTTTTGTAAGAAAATTAAGGTTTAT
TAGGCAGA

CCGGTGAATAATGAAAAAAAAAAAAAACGAATACAAAAAAAAAAAGTAATTTCTTTCAAGTTTATATATATAAC
AATATAATATTAAGTAATTTTAATCGAAAATTTTTTCCTCTAAAGAACCCAAAAATAAGCAACTATTGAAT
GAATATCT

GCAAATGATTCAAAAATTCAAATTTTCTCTCTCTCTCTCTATAAACATAAATATATATGTGTATATGTTTT
CTTTTTTTTTTTTTTTTTTTTTTGAATGATATTAAGTTATACGAATGTTCGATAATATATATGTATGAATA
AATAAAAA

AAATATTATTTTTTTTTTTTTTGAAAAAAATGTCGGACTTTATTCCTCCTAATTATTAATAAAATACGAATA
TATATCTAAATATAATTAATGCTTATTTACATGAAAAATCATCAATCGTAAACAGTTGATTAAAAAACAAA
AACTTTAT

GATGAGATGAGATGAGATGTTTATTATGATCCATTTCTTTTCTTTTTTTATTTTTTTTTTTTTTTTTTTTT
TCATTTTTCAACGCGGGTAATAGAGAATACATCCACTGTCCGGGTAAATTATGTTACAGTTAGCGCTTAAT
GTTCACTT

TTATTATTATTATTATTATTATTATTATTATTATTATTATTATTATTATTATTATTATTATTATTATTATT
ATTATTATTATTATTATATGACATTAATTACTTGTAATTATGTTATAACAGTGAAATAAATAAAATATCAA
ATTGTAAA

AGATTTTTAAGATCCTTATAACCTGTAAAAAAAAAAAAAAAAAAAAAAAGAAGGGTTTAAATAAAAAATCG
GACTTACTCAAAGGGTTGAAAAGCACTTTAATATAGGTTTTTAGTTTCGGGTAAGAAGATGTGTCAAAGGT
CTCGAAAA

TAAATAGGTTAAATTTTCTTTTTTTTTTTTCCATTTCATTTTCATTTTCATTTCCTGCAGTTATTTTTTTT
CTTATTAATCTATATCCATGTAATCGTTAGCGATATTGTTGTTAGTCAACGCGGCCATGGCAGTACCGGGT
GCTTGTGA

TAATTCGATAATTAGAGGTATCTTTATTTGGATAGAATATGCATATATATATATAAGCGTATATATATATA
TATATATATATGTATATACATATGGACATTAAGTAAATATAAATCTATAAGTGGGTATCGGGATCAGAGCC
ACTTTCTT

ATAATATGGTTTTTTTATTCATAATTTGGTAATAACGGTTGATAAACGAGCGGCTGATAAATTAGTTTCTT
TTTTTTTTTAATTTTTTTTTTTTTTGATCTCATTTCTTTTTTTATTGAATCTTTTTTTTTTGTAAGAAAAT
TAAGGTTT

TCACCACTTTTTTCTGGAAAAATACATAGCCTAACAAGCAATTTTATTTTACGTTTGTTAATTCATTATAC
TGATAATATTTTTGAATTTTTTTTTTTTTTGATACATTTTTTTAATCGCTGTTTTGTCTGTTTTTTTTCGA
TTCAGTTA

AATAACGGTTGATAAACGAGCGGCTGATAAATTAGTTTCTTTTTTTTTTTAATTTTTTTTTTTTTTGATCT
CATTTCTTTTTTTATTGAATCTTTTTTTTTTGTAAGAAAATTAAGGTTTATTAGGCAGAGTATACCGAGTC
GTTTGAAG

TTTTTTTTTTTGATACATTTTTTTAATCGCTGTTTTGTCTGTTTTTTTTCGATTCAGTTATAGGGAAAAAA
ACGGGAAAGGAAAGAGAAAAAAAAATTAGTGCAGAGCAATAAGAAGCGAAAATCAAAAAAAAGTTTTGGAT
CTGCAAGA

CAGTAATCGTTTTCTTTTTTTTTTTTTCATTAATTTATATGCTATCCTTTTAAAAATAGACATGTCATTTC
AAACGACATGTTAAAAATTATATATATGTTTATATATAGATTGATATATGTTGGAGCCGTTGGACAATTAT
GGGCAGCT

TCCGGTTGTGTCTTTTCATAATAATATGGTTTTTTTATTCATAATTTGGTAATAACGGTTGATAAACGAGC
GGCTGATAAATTAGTTTCTTTTTTTTTTTAATTTTTTTTTTTTTTGATCTCATTTCTTTTTTTATTGAATC
TTTTTTTT

ATAAACATAAATATATATGTGTATATGTTTTCTTTTTTTTTTTTTTTTTTTTTTGAATGATATTAAGTTAT
ACGAATGTTCGATAATATATATGTATGAATAAATAAAAAGTTTTTAAAGTAGCCTTAAAGCTAGGCTATAA
TCATGCAT

CTAACAAGCAATTTTATTTTACGTTTGTTAATTCATTATACTGATAATATTTTTGAATTTTTTTTTTTTTT
GATACATTTTTTTAATCGCTGTTTTGTCTGTTTTTTTTCGATTCAGTTATAGGGAAAAAAACGGGAAAGGA
AAGAGAAA

TTTCTTTTTTTTTTTTTCATTAATTTATATGCTATCCTTTTAAAAATAGACATGTCATTTCAAACGACATG
TTAAAAATTATATATATGTTTATATATAGATTGATATATGTTGGAGCCGTTGGACAATTATGGGCAGCTAT
CTGAACAT

AAATCGGTTGGGCTTAACTTTAAAGAAAAAAGTTGAGATTAGATTTATTGTGTTATAAATATAGATATACA
ATTCTTTATAAAAAAAATATATATATATATCATTGTTATTAAATAAAGAGTTTTCCTAGTATATAGATTAA
AAAACTAC

AATTTTCTCTCTCTCTCTCTATAAACATAAATATATATGTGTATATGTTTTCTTTTTTTTTTTTTTTTTT
TTTGAATGATATTAAGTTATACGAATGTTCGATAATATATATGTATGAATAAATAAAAAGTTTTTAAAGTA
GCCTTAAA

CAAGTTTCCCAGATTTTTAAGATCCTTATAACCTGTAAAAAAAAAAAAAAAAAAAAAAAGAAGGGTTTAA
ATAAAAATCGGACTTACTCAAAGGGTTGAAAAGCACTTTAATATAGGTTTTTAGTTTCGGGTAAGAAGATG
TGTCAAAG

AATTCACCTTTTTTTTCTTTCTTTTTTTTTTACCTGGGACGACATTCTAACTAATGAAATTTACGATTAA
TTATTCTTTTTTTTTTTTTTCAAGAAATAAATATAACTGTAACATATATATATTAATTATGGGGATCACA
AAGAAAAA

ATGAAAAAAAAAAAAACGAATACAAAAAAAAAAGTAATTTCTTTCAAGTTTATATATATAACAATATAATAT
TAAGTAATTTTAATCGAAAATTTTTTCCTCTAAAGAACCCAAAAATAAGCAACTATTGAATGAATATCTAA
AATGATCG

GTGCAATATAGTAATAAGTTTTCACCTTTTCCGGTGAATAATGAAAAAAAAAAAAACGAATACAAAAAAAA
AGTAATTTCTTTCAAGTTTATATATATAACAATATAATATTAAGTAATTTTAATCGAAAATTTTTTCCTCT
AAAGAACC

TTCAGACATCTTCGAGAATTTTTTTCGCGCGCTTTCTATCAAAAAAAAAATGAAAAATAAATAAATAAACAA
AGGAAAATAAACAACAACATATACTGTTCTTATATAACATAGAACATCTATTGTTTCCGTGTTTTGCTCTT
TCCTGCTT

TTTCCTATTCTCACCACTTTTTTCTGGAAAAATACATAGCCTAACAAGCAATTTTATTTTACGTTTGTTAA
TTCATTATACTGATAATATTTTTGAATTTTTTTTTTTTTTGATACATTTTTTTAATCGCTGTTTTGTCTG
TTTTTTTC

TCGTATTTTTTCTTTTTTTTTTCTAATCTTCCATCTATTCGGTAGCGATGATTCATTTCTCTGAAAAAAAAA
AAAAAAAAAAAAATGAAAAAGAATATTTTTTTGATGAACTTGTATTTCTCTTATCTGGTTGATATATATG
CTATCATT

ATAATTTGGTAATAACGGTTGATAAACGAGCGGCTGATAAATTAGTTTCTTTTTTTTTTTAATTTTTTTT
TTTTTGATCTCATTTCTTTTTTTATTGAATCTTTTTTTTTTGTAAGAAAATTAAGGTTTATTAGGCAGAGT
ATACCGAG

TTCGAGAATTTTTTTCGCGCGCTTTCTATCAAAAAAAAAATGAAAAATAAATAAATAAACAAAGGAAAATAA
ACAACAACATATACTGTTCTTATATAACATAGAACATCTATTGTTTCCGTGTTTTGCTCTTTCCTGCTTTT
GTTCGATC

AAGTGAAAAATAATTCGATAATTAGAGGTATCTTTATTTGGATAGAATATGCATATATATATATATAAGCGTA
TATATATATATATATATATATGTATATACATATGGACATTAAGTAAATATAAATCTATAAGTGGGTATCGG
GATCAGAG

CTCTCTCTCTATAAACATAAATATATATGTGTATATGTTTTCTTTTTTTTTTTTTTTTTTTTTGAATGAT
ATTAAGTTATACGAATGTTCGATAATATATATGTATGAATAAATAAAAAGTTTTTAAAGTAGCCTTAAAGC
TAGGCTAT

ATGAGAAAAAAAAATCGGTTGGGCTTAACTTTAAAGAAAAAAGTTGAGATTAGATTTATTGTGTTATAAATA
TAGATATACAATTCTTTATAAAAAAAAATATATATATATATATCATTGTTATTAAATAAAGAGTTTTCCTAGTA
TATAGATT

TCTTTTCATAATAATATGGTTTTTTTATTCATAATTTGGTAATAACGGTTGATAAACGAGCGGCTGATAAA
TTAGTTTCTTTTTTTTTTTTAATTTTTTTTTTTTTTGATCTCATTTCTTTTTTTTATTGAATCTTTTTTTTTT
GTAAGAAA

GATAAACGAGCGGCTGATAAATTAGTTTCTTTTTTTTTTAATTTTTTTTTTTTTTGATCTCATTTCTTTT
TTTATTGAATCTTTTTTTTTGTAAGAAAATTAAGGTTTATTAGGCAGAGTATACCGAGTCGTTTGAAGTC
ATCTCCGG

AAAAGCAAGAAAAAAGCGACACCAGTAAATTTCAGACATCTTCGAGAATTTTTTCGCGCGCTTTCTATCA
AAAAAAAATGAAAAATAAATAAATAAACAAAGGAAAATAAACAACAACATATACTGTTCTTATATAACATA
GAACATCT

TTTTGTACAAGTTGCGGATTACTTCTCTTTGTTTACCTTTTTGCTTTTATTTTTTTTTTAATACGTGCAT
ATATATATATATATTTAGTTTAGGTCAGAATACAAAGATAATTATGTATAAATGGGCGCATTTGGAAACTA
TTTTACAT

TTTTCTTTCCCATTTTGCTGGTAGTCAACAAAGTTGTCTTAAGGAAAAAAAAATAAAGAAAATATTGAAAA
CTAATAAGTTTTTTTCCTGTAAAAGTGTATAAAATATATAGGAGATCCCGTCAAATAATAGAAAAAAAATT
TTTTGCCA

AACTGGACAGTAGTAAGAAAGATTGGACTAGGCATCACCCAGACTTTACTTTTTCGGCATACATATCTATT
TATATATAGTTTAATACGTAAATACAAATATATATATACTGTAAATTTTAATCATTAAGCTTTTCTTTTTT
TTTTGTTC

TTTCTGGAAAAATACATAGCCTAACAAGCAATTTTATTTTACGTTTGTTAATTCATTATACTGATAATATT
TTTGAATTTTTTTTTTTTTTGATACATTTTTTTTAATCGCTGTTTTGTCTGTTTTTTTCGATTCAGTTATA
GGGAAAAA

CCGGCTTGGCTTCAGTTGCTGATCTCGGCGCGGAAAAATCAGCGCCCCACGCCAAAAGGTTCGTATTTTTT
CTTTTTTTTTCTAATCTTCCATCTATTCGGTAGCGATGATTCATTTCTCTGAAAAAAAAAAAAAAAAAAA
AAATGAAA

GCGAGCCGAGGAGACGAGTGTCACTTCTGAACATACCCATCTCAAAATTAAGAAATAGCAATAACACGCGT
GCAGAAGATTGAGAAGACTACTACGTATTTTTTTTTATATGCATCCTTTTTTCCACATATGTATGCGCGGT
GCGCGCGC

TTTCGTTGTGAAATATTATTTTTTTTTTTTTTGAAAAAAATGTCGGACTTTATTCCTCCTAATTATTAATAA
AATACGAATATATATCTAAATATAATTAATGCTTATTTACATGAAAAATCATCAATCGTAAACAGTTGATT
AAAAAACA

ATTCATTATACTGATAATATTTTTGAATTTTTTTTTTTTTTTGATACATTTTTTTTAATCGCTGTTTTGTCT
GTTTTTTTCGATTCAGTTATAGGGAAAAAAAACGGGAAAGGAAAGAGAAAAAAAAAATTAGTGCAGAGCAATA
AGAAGCGA

TCTTTTTTTTTCTAATCTTCCATCTATTCGGTAGCGATGATTCATTTCTCTGAAAAAAAAAAAAAAAAAAA
AAAATGAAAAAGAATATTTTTTTGATGAACTTGTATTTCTCTTATCTGGTTGATATATATGCTATCATTTA
TTTTCTTA

ACCCACTCAAAAATATAATTGGCAATGAAAATATAACTTTTTTAGATGTTAGCGATGCTATATCATCTGAG
ATGAGATGAGATGAGATGTTTATTATGATCCATTTCTTTTCTTTTTTTATTTTTTTTTTTTTTTTTTTTT
CATTTTTC

AGAAAAGTGTCAAGTTTCCCAGATTTTTAAGATCCTTATAACCTGTAAAAAAAAAAAAAAAAAAAAAAAAG
AAGGGTTTAAATAAAAATCGGACTTACTCAAAGGGTTGAAAAGCACTTTAATATAGGTTTTTAGTTTCGGG
TAAGAAGA

AGCGCCCCACGCCAAAAGGTTCGTATTTTTTCTTTTTTTTTCTAATCTTCCATCTATTCGGTAGCGATGAT
TCATTTCTCTGAAAAAAAAAAAAAAAAAAAAAAAAATGAAAAAGAATATTTTTTTGATGAACTTGTATTTCTC
TTATCTGG

TGCTACAGGACCCTAGGGTCTCGCTAAAAAAATGACTACGCGCGCTTATATAATTACTCGAATAAGACAAA
AGAAAAAAACCCTTGCGATATGTCGTAGACACGTAAAAATGGCCCATTTAGTGGTAGTCGCAAACTAGAGG
GGTAAAAA

TTTTTTTTTTTTTTTTTTTTTTCATTTTTCAACGCGGGTAATAGAGAATACATCCACTGTCCGGGTAAATT
ATGTTACAGTTAGCGCTTAATGTTCACTTCACAGTATTATGAGCTGTTTTTGTAATAAAGACGACTATAAC
ATAACAAA

GTTGCGGATTACTTCTCTTTGTTTACCTTTTTGCTTTTATTTTTTTTTTTAATACGTGCATATATATATAT
ATATTTAGTTTAGGTCAGAATACAAAGATAATTATGTATAAATGGGCGCATTTGGAAACTATTTTACATAC
AACTGAAC

AGACTTTACTTTTTCGGCATACATATCTATTTATATATAGTTTAATACGTAAATACAAATATATATATACT
GTAAATTTTAATCATTAAGCTTTTCTTTTTTTTTTGTTCACGCTTTCACCCTGTGAGGCGAATGAGGTTAG
CTTTTTCC

GGTCAAACAATCTAGATTGTTCTGAAGATCAAAATAATATATATATATATATATATATATATATATATGTT
TATACACTTAACTTACGTACAAAAATATAGATTTCGAAACGAATCAGTTGCTCTTAGCCTGCACATCTAGA
AATGTATT

AATTCGTCACACGCGACATGGTGGGGGACGTGTAGTCGTGCCCTGCGATCCGCTTTATTACGTATATTGCC
TTCACATGCCGTGAAAGCCGACAGGGACAACGCGCGATAAAAAAAAAATTAAAAGTAAAACTACGTACGCG
GCGGCGCG

ACTTCTTATTATCACGAAAAACGTCGTCTTTTCATGCAAGGAAGGACTACAGTATATACATATAAATATAT
ATTTATGTAATTATTTTTTTCTTTTTTTTTAAATTTTCTAGCGAGAATGAAGAAAAGCCGACCTCATCTCA
TCCTATTT

AATAATTTACTATTCAAAATATTTGCCTTTTCTTTTTTAATTTTTGTTTATTCTTTAATGTATAATTAAAT
AAAAAAATATTATTATATTTACTAATTAAGAGCGAAGCGTTTTATGTAGCTCCTTGGCCATACATACATTG
CGCGCAAT

CTAAGCTGCCCGACCTCACGAAATCAGTCATGCGGTAGTCCATTATAATATAACTATTATTATTATTACTA
TTACTATTATTATTATTATTATTATTATCTATTGTTTATATTCTCTTTAGCTTTTTATCACCTACGACGAC
GGATATAT

AAATAATGTTATAAACGATTGAAGGGATCCGCTGAGGAATGAAAAAAAAAAATAAGAAAGTAAGAGAAGGG
AAAAAAAAAAGAAAAAAAGAAAAAAAGAAAAAAAGAAAAAAAGAGGGAATGATCGAAGATAAAAATTCTCA
GAAATTTG

AGTCATGTACTGACCCATTAATTTTCTTTTCTCTTTTTTTTTTTTTTTTTTTTTTTTTCCTCTATTTCCTTT
TTTTGTTCTAGTCGGTTACGCAGTTCTGAGTAATGTAATATAGCGTTCCTTTTCCTCTCAGATTTTTTTGA
CCTTATTT

GAAAATGTTTTATTCTATATTGGTAGAGTTAATAAATAATTATATAATAAATTATTGTATGATAATATAAA
TAATAGTTTATGTAATATTTTAAGTTATTATTTATAATAAAAAAGTAACTATTGAACCTGTTAAATTAAAA
TATATTCA

AAAAAGCGACACCAGTAAATTTCAGACATCTTCGAGAATTTTTTTCGCGCGCTTTCTATCAAAAAAAAAATG
AAAAATAAATAAATAAACAAAGGAAAATAAACAACAACATATACTGTTCTTATATAACATAGAACATCTAT
TGTTTCCG

TTTCCACATATGTATGCGCGGTGCGCGCGCGCCATTAAAAGATTTTTTTCTTAGCTCTTTTTCTTTTTTTCT
TTTTCTTTCCACTGAGATGCGTTCTTCGGTATATAGTGAGAACACTTACAACTGTATACGAACATCGAAGG
AGCACCTA

ACCAAACCAGCAAAAGGTATATAATTCATTCAATAGATACAACTGCTCCATGAAAGGAAAAACCTTCATTT
ACGGTCGCGTTTTTTTTCGTGATATAAAAAAGGTAAAAAAAACGCGTCGCGTTAGAAAAAAGGTGTGTGTG
AAGAGAAA

GATCCTTATAACCTGTAAAAAAAAAAAAAAAAAAAAAAAAGAAGGGTTTAAATAAAAATCGGACTTACTCA
AAGGGTTGAAAAGCACTTTAATATAGGTTTTTAGTTTCGGGTAAGAAGATGTGTCAAAGGTCTCGAAAAGG
AAACATTA

CAGATATAGTGTTGAATGATAATTCACCTTTTTTTTCTTTCTTTTTTTTTTTACCTGGGACGACATTCTAA
CTAATGAAATTTACGATTAATTATTCTTTTTTTTTTTTTTCAAGAAATAAATATAACTGTAACATATATAT
ATTAATTA

GAAAAAGCATCAGTTACGAGACTCCTGAAGGAGCGCCGGAAAGTTTCTTTTTTTTTTTTTTTTTTTTAATT
TTTTTTGTGTGGGGAAAGACCGACTACCAGTAATACTTTAAAGATATTATTAAAAGAAATCCCGAAAAAAG
AAAGACCA

TCTTTTTTTTTTTTTTTTTTTTTTGAATGATATTAAGTTATACGAATGTTCGATAATATATATGTATGAAT
AAATAAAAAGTTTTTAAAGTAGCCTTAAAGCTAGGCTATAATCATGCATCCTCAAATTCTGTTAAAGTAGG
TCCCTCAT

AAAAATGCATATCCGAAAGGACCAAACCAGCAAAAGGTATATAATTCATTCAATAGATACAACTGCTCCAT
GAAAGGAAAAACCTTCATTTACGGTCGCGTTTTTTTTCGTGATATAAAAAAGGTAAAAAAAACGCGTCGCG
TTAGAAAA

CTTCTTTTCTTTTTGTACAAGTTGCGGATTACTTCTCTTTGTTTACCTTTTTGCTTTTATTTTTTTTTTTA
ATACGTGCATATATATATATATATTTAGTTTAGGTCAGAATACAAAGATAATTATGTATAAATGGGCGCAT
TTGGAAAC

GAAGGGATCCGCTGAGGAATGAAAAAAAAAATAAGAAAGTAAGAGAAGGGAAAAAAAAAAGAAAAAAAAGA
AAAAAAGAAAAAAAGAAAAAAAGAGGGAATGATCGAAGATAAAAATTCTCAGAAATTTGAGAAGAAGGAAA
AACTCTAC

GCCTTGTAAGTACTTGGCTCACGAATACATATCAAGATACTTATGATATATATATATAGAAAAAGCTTACT
TTTCTTGGAGTTATTGTTATTATCATCGCGAAGAACGATTGTATAACCCGGTTCAACGCGAAACGAATCGT
TAAACTGG

AGTGCTTTTTTTTTCTTTCCCATTTTGCTGGTAGTCAACAAAGTTGTCTTAAGGAAAAAAAAATAAAGAAA
ATATTGAAAACTAATAAGTTTTTTTCCTGTAAAAGTGTATAAAATATATAGGAGATCCCGTCAAATAATAG
AAAAAAAA

TAAACTTTCGAGAAAAGTGTCAAGTTTCCCAGATTTTTAAGATCCTTATAACCTGTAAAAAAAAAAAAAAA
AAAAAAAAAGAAGGGTTTAAATAAAAATCGGACTTACTCAAAGGGTTGAAAAGCACTTTAATATAGGTTTT
TAGTTTCG

ATAATTCATTCAATAGATACAACTGCTCCATGAAAGGAAAAACCTTCATTTACGGTCGCGTTTTTTTTCGT
GATATAAAAAAGGTAAAAAAAACGCGTCGCGTTAGAAAAAAGGTGTGTGTGAAGAGAAAGACCCATTGAAT
GGAGAGTT

AGAAGTACATTTTGATGATGGTTGTGATACTGTGGGTGTATTATTACTATTATTATTATTATTATTATTAT
TATTATTATTATTACTATTATTACTATTATTATTATTATTATTATTGTTGTTGTTGTTATTGTTATTGTTA
TTGTTAGG

ACCAGTAAATTTCAGACATCTTCGAGAATTTTTTTCGCGCGCTTTCTATCAAAAAAAAATGAAAAATAAAT
AAATAAACAAAGGAAAATAAACAACAACATATACTGTTCTTATATAACATAGAACATCTATTGTTTCCGTG
TTTTGCTC

GGTTCAAAGCGCCTTGTAAGTACTTGGCTCACGAATACATATCAAGATACTTATGATATATATATATAGAA
AAAGCTTACTTTTCTTGGAGTTATTGTTATTATCATCGCGAAGAACGATTGTATAACCCGGTTCAACGCGA
AACGAATC

CTCAAGTATAACCCACTCAAAAATATAATTGGCAATGAAAATATAACTTTTTTAGATGTTAGCGATGCTAT
ATCATCTGAGATGAGATGAGATGAGATGTTTATTATGATCCATTTCTTTTCTTTTTTTTATTTTTTTTTTT
TTTTTTTT

TCCTCGACACCAAAAAAAATGAATAAATAATGTATAAAACATATAACTTTGCAAATGATTCAAAAATTCAA
ATTTTCTCTCTCTCTCTCTATAAACATAAATATATATGTGTATATGTTTTCTTTTTTTTTTTTTTTTTTTT
TTGAATGA

TTTTTTTTTTTGAAAAAAATGTCGGACTTTATTCCTCCTAATTATTAATAAAATACGAATATATATCTAAA
TATAATTAATGCTTATTTACATGAAAAATCATCAATCGTAAACAGTTGATTAAAAAACAAAAACTTTATCG
GTTCCATT

GCTGAGGAATGAAAAAAAAAAAATAAGAAAGTAAGAGAAGGGAAAAAAAAAAGAAAAAAAAGAAAAAAAAGAAA
AAAAGAAAAAAAAGAGGGAATGATCGAAGATAAAAATTCTCAGAAATTTGAGAAGAAGGAAAAACTCTACTT
AAATAAAG

AAGAGAAGGAAAATAATGTTATAAACGATTGAAGGGATCCGCTGAGGAATGAAAAAAAAAAAATAAGAAAGT
AAGAGAAGGGAAAAAAAAAAGAAAAAAAAGAAAAAAAAGAAAAAAAAGAAAAAAAAGAGGGAATGATCGAAGATA
AAAATTCT

ACCCAATCGGTTTCCTATTCTCACCACTTTTTTCTGGAAAAATACATAGCCTAACAAGCAATTTTATTTTA
CGTTTGTTAATTCATTATACTGATAATATTTTTGAATTTTTTTTTTTTTTGATACATTTTTTTTAATCGCT
GTTTTGTC

CAGTTACGAGACTCCTGAAGGAGCGCCGGAAAGTTTCTTTTTTTTTTTTTTTTTTTTTAATTTTTTTTTGTGT
GGGGAAAGACCGACTACCAGTAATACTTTAAAGATATTATTAAAAGAAATCCCGAAAAAAGAAAGACCATG
AGAAAACC

TTTAACTTAATAAACTTTCGAGAAAAGTGTCAAGTTTCCCAGATTTTTAAGATCCTTATAACCTGTAAAAA
AAAAAAAAAAAAAAAAAAAGAAGGGTTTAAATAAAAATCGGACTTACTCAAAGGGTTGAAAAGCACTTTAA
TATAGGTT

ATCCGAAAGGACCAAACCAGCAAAAGGTATATAATTCATTCAATAGATACAACTGCTCCATGAAAGGAAAA
ACCTTCATTTACGGTCGCGTTTTTTTTCGTGATATAAAAAAGGTAAAAAAAACGCGTCGCGTTAGAAAAAA
GGTGTGTG

GATAAATTTCCCCGTTTTCTTTTACTTTTACTTCATTTAATATAATTCATATTTATTTCCCATGTACCAAT
TAATTACTCCTAAAAGAACAGAAATAGAAAAAAGTTGTATATAACAAGCCAAAAAAAAAAAAGAAAAAAGG
AAACACAG

TGACCCATTAATTTTCTTTTCTCTTTTTTTTTTTTTTTTTTTTTTTTTCCTCTATTTCCTTTTTTTGTTCTA
GTCGGTTACGCAGTTCTGAGTAATGTAATATAGCGTTCCTTTTCCTCTCAGATTTTTTTGACCTTATTTTG
TTTCCACT

AGCATATGTTAAATATTTTATTTATAGATTTTTCGTTGTGAAATATTATTTTTTTTTTTTTGAAAAAAATG
TCGGACTTTATTCCTCCTAATTATTAATAAAATACGAATATATATCTAAATATAATTAATGCTTATTTACA
TGAAAAAT

TGATACATTTTTTTTAATCGCTGTTTTGTCTGTTTTTTTCGATTCAGTTATAGGGAAAAAAACGGGAAAGG
AAAGAGAAAAAAAAAATTAGTGCAGAGCAATAAGAAGCGAAAATCAAAAAAAAAGTTTTGGATCTGCAAGACT
TGCTGTCA

TACTATCTAATACCTCTGTTCTTATACGGTTCCAAATTGAAAAAAAAATTAACTGCGAAAAGATGTCACAC
GTCAGTAAATCCGCGCAAAACCCCGTTATATTTCCGACGTCGTGCGGTAGTTTCTGCGCGGAACGGCCCCG
CGGAATAG

GTTCCGAAGAAAAAAATAAAAAAAAAGGGCCGACGGAGGGTTAGGTCAAATTAAATATTGAGCTAGGTTTA
GTTATCTCTTTTTTAGGTTTTTTATATACAAAAAAGATTATATTTAGGGTACATATGGCTGGAGCATAATG
TCTGGAAT

AGATTTATTGTGTTATAAATATAGATATACAATTCTTTATAAAAAAAATATATATATATATCATTGTTATT
AAATAAAGAGTTTTCCTAGTATATAGATTAAAAAACTACTCTATTAAATGAGAGCTAAAAAAAGCAGGCTG
CCAAAAAA

AAATATTTTATTTATAGATTTTTCGTTGTGAAATATTATTTTTTTTTTTTTGAAAAAAATGTCGGACTTTA
TTCCTCCTAATTATTAATAAAATACGAATATATATCTAAATATAATTAATGCTTATTTACATGAAAAATCA
TCAATCGT

ATTTTCTTTTCTCTTTTTTTTTTTTTTTTTTTTTTTTTCCTCTATTTCCTTTTTTTGTTCTAGTCGGTTACG
CAGTTCTGAGTAATGTAATATAGCGTTCCTTTTCCTCTCAGATTTTTTTGACCTTATTTTGTTTCCACTTT
TTACTACT

CGCTTTATTACGTATATTGCCTTCACATGCCGTGAAAGCCGACAGGGACAACGCGCGATAAAAAAAAAATT
AAAAGTAAAACTACGTACGCGGCGGCGCGACTCGAATCCTTGACTCCGACTGAGGAGACCCGAACAACGCT
CCCTGCTC

CTACTTTTTGAATAGGCATAGTTCACACTTTTATCTCCTTAGTTTGTACTACTCTTTTTTTATTTTTTTT
TTTTCTTCCACTCAGCTATTAGATTATTTATTATTTTATTATCTGTGGTATCATTTTCTACAGCCATACCT
TGATTTTA

TTTTTATATTTTCGGCATTTTTATGCTCGGCATTTCCCAACAGAAGCGGATATATTTTAAGGGCGCGGAGA
TGAGCCAAACGGCAAGCGATATCAAAATATCTGCATAATAACGGCAATCAACGCGCACGCGAATGGCCAAG
AGGGTACA

GCATTCAATACACACGCGAAGGCAGTAGGCGATGAAAGGAAGTTTTTTAATGTCATTAAGAAAAAGATAGG
GAACTTCTATTTCCTGTCTTTCATATATATATATATATATATATATATATATATGTGCATATGTACATCGT
TGAGTTGT

CCCTGCGATCCGCTTTATTACGTATATTGCCTTCACATGCCGTGAAAGCCGACAGGGACAACGCGCGATAA
AAAAAAAATTAAAAGTAAAACTACGTACGCGGCGGCGCGACTCGAATCCTTGACTCCGACTGAGGAGACCC
GAACAACG

TCATCTCCGGGTAGTGATTTTTATCACGTGACACTTTTTTATTCCTTTTCTCTTTTCTTTTTTAAATTTTT
TTTTTTTTTTTCATTGATTAACCGCCTCTTCTTTATCACTCTCGCATTCTGTCTACTAAACGGTAAAAAA
ACGAAGTG

TTATTTTTTGTTCCTAATTTTTTTTTTTTTTTACATTTTATTAGCTATACACTTGGCGCAAGGATATTTTAT
ATACCGGATATAATAGATAATTAAAAGACTACTAATTAAAAATGATGTAATATGGTAATAGGTATGGAAGA
ATCAGGGA

CGTATATTGCCTTCACATGCCGTGAAAGCCGACAGGGACAACGCGCGATAAAAAAAAAATTAAAAGTAAAA
CTACGTACGCGGCGGCGCGACTCGAATCCTTGACTCCGACTGAGGAGACCCGAACAACGCTCCCTGCTCTC
CGCCTTCG

TAACAAGATCATGAAATATTCATTAACGGAGTTTTTTGTTTTCGCTTTTTTTATTTTTTGTTCCTAATTTT
TTTTTTTTTACATTTTATTAGCTATACACTTGGCGCAAGGATATTTTATATACCGGATATAATAGATAAT
TAAAAGAC

CCCCTTCTCGCGCTGGCGAAGAAGTGAAAAAAAAAAAATAAAAAAATGGAAATTTCGGACGATCGTCATTTT
GAAAATACATAGTGAGCCAATATTATACTAGGAGTATATATATTTAGAGGCCAGTTCTGCTCTCTTGTCAA
TTGCCCGG

GATGCCGTATCTTTCCACACATTTTCCTCATTGGTCTTTGTACCTATAATTGTTCATATTATATATACGTT
TGTAACAAATCGATAAATAGCAACAATATTTTTGCTTATTTTGATATCCTGAAATCCTTTTTTTTTTTTTT
TTTTTTTT

TGAAACCATGCAGATATAGTGTTGAATGATAATTCACCTTTTTTTTCTTTCTTTTTTTTTTTACCTGGGAC
GACATTCTAACTAATGAAATTTACGATTAATTATTCTTTTTTTTTTTTTTTCAAGAAATAAATATAACTGTA
ACATATAT

TCCAAATTGAAAAAAAAAATTAACTGCGAAAAGATGTCACACGTCAGTAAATCCGCGCAAAACCCCGTTATA
TTTCCGACGTCGTGCGGTAGTTTCTGCGCGGAACGGCCCCGCGGAATAGGCACATTTTCTTCCGGCCGCAG
AAAGCTCT

TCACGTACATAGGTACACGCAGATGGGACAGGCGCATAAGTACGCGCTAATACATGTATTGATATAAGTTA
TGTTTTTATTTATCAAAATGATATTGTTGCATTTTTTTTTTTAAATCTCACGTTATTTTTCATTTATATAC
ATACACTT

GGCTTAACTTTAAAGAAAAAAGTTGAGATTAGATTTATTGTGTTATAAATATAGATATACAATTCTTTATA
AAAAAAATATATATATATATCATTGTTATTAAATAAAGAGTTTTCCTAGTATATAGATTAAAAAACTACTC
TATTAAAT

CCTGCCGGTACCGGCTTGGCTTCAGTTGCTGATCTCGGCGCGGAAAAATCAGCGCCCCACGCCAAAAGGTT
CGTATTTTTTCTTTTTTTTTCTAATCTTCCATCTATTCGGTAGCGATGATTCATTTCTCTGAAAAAAAAAA
AAAAAAAA

GTAGCGATGATTCATTTCTCTGAAAAAAAAAAAAAAAAAAAAAAAAATGAAAAAGAATATTTTTTTGATGAAC
TTGTATTTCTCTTATCTGGTTGATATATATGCTATCATTTATTTTCTTATCAAGTTTCCAAATTTCTAATC
CTTTCTCC

TTTATAGATTTTTCGTTGTGAAATATTATTTTTTTTTTTTTGAAAAAAATGTCGGACTTTATTCCTCCTAA
TTATTAATAAAATACGAATATATATCTAAATATAATTAATGCTTATTTACATGAAAAATCATCAATCGTAA
ACAGTTGA

TTATCGCTTTTCACGTCGAAAGGAAAAAATAAAAGCGTAAGCAATGGTGTAGATACTTCTTATATAGACGT
TATTATTACTATTATTATTATTATTATTATTATTATTATTATTATTATTATTATTATTATTATTATTATTA
TTATTATT

GTGGGGGACGTGTAGTCGTGCCCTGCGATCCGCTTTATTACGTATATTGCCTTCACATGCCGTGAAAGCCG
ACAGGGACAACGCGCGATAAAAAAAAAATTAAAAGTAAAACTACGTACGCGGCGGCGCGACTCGAATCCTT
GACTCCGA

AGAAGATCTGCAGTAATCGTTTTCTTTTTTTTTTTTTCATTAATTTATATGCTATCCTTTTAAAAATAGAC
ATGTCATTTCAAACGACATGTTAAAAATTATATATATGTTTATATATAGATTGATATATGTTGGAGCCGTT
GGACAATT

AAATTTTCTTTTTTTTTTTTTCCATTTCATTTTCATTTTCATTTCCTGCAGTTATTTTTTTTCTTATTAATC
TATATCCATGTAATCGTTAGCGATATTGTTGTTAGTCAACGCGGCCATGGCAGTACCGGGTGCTTGTGATG
CAATTATA

GAAAAAAAAAAATAAGAAAGTAAGAGAAGGGAAAAAAAAAAAGAAAAAAAGAAAAAAAGAAAAAAAGAAAAA
AAGAGGGAATGATCGAAGATAAAAATTCTCAGAAATTTGAGAAGAAGGAAAAACTCTACTTAAATAAAGAT
AAACTTTT

TCACGTCGAAAGGAAAAAATAAAAGCGTAAGCAATGGTGTAGATACTTCTTATATAGACGTTATTATTACT
ATTATTATTATTATTATTATTATTATTATTATTATTATTATTATTATTATTATTATTATTATTATTATTAT
TATTATTA

CTTATACGGTTCCAAATTGAAAAAAAAAATTAACTGCGAAAAGATGTCACACGTCAGTAAATCCGCGCAAAA
CCCCGTTATATTTCCGACGTCGTGCGGTAGTTTCTGCGCGGAACGGCCCCGCGGAATAGGCACATTTTCTT
CCGGCCGC

AAAAAATAAAAAAAAAGGGCCGACGGAGGGTTAGGTCAAATTAAATATTGAGCTAGGTTTAGTTATCTCTT
TTTTAGGTTTTTTATATACAAAAAAGATTATATTTAGGGTACATATGGCTGGAGCATAATGTCTGGAATGG
GTATTGCG

TGTATGCGCGGTGCGCGCGCGCCATTAAAAGATTTTTTTCTTAGCTCTTTTTCTTTTTTCTTTTTCTTTCC
ACTGAGATGCGTTCTTCGGTATATAGTGAGAACACTTACAACTGTATACGAACATCGAAGGAGCACCTAAC
AGAAAGAA

CCAAGTTCCTGAGATCTTTTGGTCAAACAATCTAGATTGTTCTGAAGATCAAAATAATATATATATATATA
TATATATATATATATATGTTTATACACTTAACTTACGTACAAAAATATAGATTTCGAAACGAATCAGTTGC
TCTTAGCC

AGTTGAGATTAGATTTATTGTGTTATAAATATAGATATACAATTCTTTATAAAAAAAATATATATATATAT
CATTGTTATTAAATAAAGAGTTTTCCTAGTATATAGATTAAAAAACTACTCTATTAAATGAGAGCTAAAAA
AAGCAGGC

GGGGTTATGAATGAGAAAAAAAAATCGGTTGGGCTTAACTTTAAAGAAAAAAGTTGAGATTAGATTTATTGT
GTTATAAATATAGATATACAATTCTTTATAAAAAAAATATATATATATATCATTGTTATTAAATAAAGAGT
TTTCCTAG

CATCTATTCGGTAGCGATGATTCATTTCTCTGAAAAAAAAAAAAAAAAAAAAAAAATGAAAAAGAATATTTT
TTTGATGAACTTGTATTTCTCTTATCTGGTTGATATATATGCTATCATTTATTTTCTTATCAAGTTTCCAA
ATTTCTAA

TTAGTGTCAAACTTCTTATTATCACGAAAAACGTCGTCTTTTCATGCAAGGAAGGACTACAGTATATACAT
ATAAATATATATTTATGTAATTATTTTTTTCTTTTTTTTTAAATTTTCTAGCGAGAATGAAGAAAAGCCGA
CCTCATCT

TACCTCTGTTCTTATACGGTTCCAAATTGAAAAAAAAAATTAACTGCGAAAAGATGTCACACGTCAGTAAAT
CCGCGCAAAACCCCGTTATATTTCCGACGTCGTGCGGTAGTTTCTGCGCGGAACGGCCCCGCGGAATAGGC
ACATTTTC

AAAATGTTTAAGATTATTAAATCACACAGGAATGAAATTGTTATTAGAAAAATTATAATATACATTCTATA
TAGGGAAAAATTTCTTCTGACTTTGAAATTTAGATGCTAATAGTTTTTTTTATTATTTCTATTTTTTTTT
TTTTAAGA

GTTGAATGATAATTCACCTTTTTTTTCTTTCTTTTTTTTTTTACCTGGGACGACATTCTAACTAATGAAAT
TTACGATTAATTATTCTTTTTTTTTTTTTTCAAGAAATAAATATAACTGTAACATATATATATTAATTATG
GGGGATCA

TTCATTTCTCTGAAAAAAAAAAAAAAAAAAAAAAAATGAAAAAGAATATTTTTTTGATGAACTTGTATTTCT
CTTATCTGGTTGATATATATGCTATCATTTATTTTCTTATCAAGTTTCCAAATTTCTAATCCTTTCTCCAC
CATCCCTA

TTTTGAAGGGGGGTTCAAAGCGCCTTGTAAGTACTTGGCTCACGAATACATATCAAGATACTTATGATATAT
ATATATAGAAAAAGCTTACTTTTCTTGGAGTTATTGTTATTATCATCGCGAAGAACGATTGTATAACCCGG
TTCAACGC

CTCAAATTACTGCTACAGGACCCTAGGGTCTCGCTAAAAAAATGACTACGCGCGCTTATATAATTACTCGA
ATAAGACAAAAGAAAAAAACCCTTGCGATATGTCGTAGACACGTAAAAATGGCCCATTTAGTGGTAGTCGC
AAACTAGA

TATTCAAAATATTTGCCTTTTCTTTTTTAATTTTTGTTTATTCTTTAATGTATAATTAAATAAAAAAATAT
TATTATATTTACTAATTAAGAGCGAAGCGTTTTATGTAGCTCCTTGGCCATACATACATTGCGCGCAATGT
TTATGCTT

GAGATGGAAGAAGTTGATTAATCTCTTTTTTCGCCTTCATGTTTTATATATTATATAAATTTGTTTACTTA
TTTTTACTATTTGTAATAATGATTCTGCTTTACGCGCCTTTAAAAAAGTTGGTCTTAATGTAAACATGAAC
CATAGCAC

AGATGGGACAGGCGCATAAGTACGCGCTAATACATGTATTGATATAAGTTATGTTTTTATTTATCAAAATG
ATATTGTTGCATTTTTTTTTTTTAAATCTCACGTTATTTTTCATTTATATACATACACTTAAGAGAATGTCA
GATTTTCT

TCAAAACCAACATACCAATTAATTAAAACTTGCTTTTAATCTGCATACATAAAACTTAGATCAAATAAATA
AAAAAAAAAGTAAAACAACCATATATACTATTTCATAAGCAAAAGTAATCAAATTAAACAAAAAAAAAGAAA
ATACAAAT

ACCTAAAGGTAAGTGAAAAATAATTCGATAATTAGAGGTATCTTTATTTGGATAGAATATGCATATATATA
TATAAGCGTATATATATATATATATATATATGTATATACATATGGACATTAAGTAAATATAAATCTATAAG
TGGGTATC

ATTAGAGGTATCTTTATTTGGATAGAATATGCATATATATATATAAGCGTATATATATATATATATATATA
TGTATATACATATGGACATTAAGTAAATATAAATCTATAAGTGGGTATCGGGATCAGAGCCACTTTCTTAG
AACTCCTC

GACCTAGGTTTAACAAGATCATGAAATATTCATTAACGGAGTTTTTTGTTTTCGCTTTTTTTATTTTTTGT
TCCTAATTTTTTTTTTTTTTACATTTTATTAGCTATACACTTGGCGCAAGGATATTTTATATACCGGATAT
AATAGATA

GTAGTGATTTTTATCACGTGACACTTTTTTATTCCTTTTCTCTTTTCTTTTTTAAATTTTTTTTTTTTTTT
TTCATTGATTAACCGCCTCTTCTTTATCACTCTCGCATTCTGTCTACTAAACGGTAAAAAAACGAAGTGAA
AAGTTCAA

ATATAACTTTGCAAATGATTCAAAAATTCAAATTTTCTCTCTCTCTCTCTATAAACATAAATATATATGTG
TATATGTTTTCTTTTTTTTTTTTTTTTTTTTTTGAATGATATTAAGTTATACGAATGTTCGATAATATATA
TGTATGAA

TGTTATAAATATAGATATACAATTCTTTATAAAAAAAATATATATATATATCATTGTTATTAAATAAAGAG
TTTTCCTAGTATATAGATTAAAAAACTACTCTATTAAATGAGAGCTAAAAAAAGCAGGCTGCCAAAAAAAT
AAAGCATT

TAATACTAATAAAAGCAAGAAAAAAGCGACACCAGTAAATTTCAGACATCTTCGAGAATTTTTTTCGCGCG
CTTTCTATCAAAAAAAAATGAAAAATAAATAAATAAACAAAGGAAAATAAACAACAACATATACTGTTCTT
ATATAACA

CCCTAGGGTCTCGCTAAAAAAATGACTACGCGCGCTTATATAATTACTCGAATAAGACAAAAGAAAAAAAC
CCTTGCGATATGTCGTAGACACGTAAAAATGGCCCATTTAGTGGTAGTCGCAAACTAGAGGGGTAAAAATA
ACCTGTCC

GTGGTGTTTCTTTAACTTAATAAACTTTCGAGAAAAGTGTCAAGTTTCCCAGATTTTTAAGATCCTTATAA
CCTGTAAAAAAAAAAAAAAAAAAAAAAAAAGAAGGGTTTAAATAAAAAATCGGACTTACTCAAAGGGTTGAAA
AGCACTTT

TATCCAATAGCTATGTACTCAAGTACCGTGTATACACTATAAAATCTCATCTCATCGCTAAAGCTAATATT
GAAAAAAAAAAAAAAAAAAAAAGGAAAAGGCAAATAAAAAATTTTCAATTCGGGTAACGAAGAATCGACTC
TCGCCGGG

GTTTACCTTTTTGCTTTTATTTTTTTTTTTAATACGTGCATATATATATATATATTTAGTTTAGGTCAGAA
TACAAAGATAATTATGTATAAATGGGCGCATTTGGAAACTATTTTACATACAACTGAACAAGTTCGGAACA
ATCTATCA

AAGTACCGTGTATACACTATAAAATCTCATCTCATCGCTAAAGCTAATATTGAAAAAAAAAAAAAAAAAAA
AAGGAAAAGGCAAATAAAAAATTTTCAATTCGGGTAACGAAGAATCGACTCTCGCCGGGTAAACAATTCTT
AGTCGTCA

AAAAAGAGATGAAAAAATGCGATGAGCCAAGCATGATTTGAATCGGATGGATTCTCAACTGATATTTATAT
GGCTTTTTCAGTTTTTTTTTTTTTTTTTTTTTTTTTCAGCATCTCGAAGAGCAAAAAAAAGGAACGAAAAA
GTGCCACG

ACAAAATTGGACTAATATTAAGGGCGACAGTTTTTCTTAAAAGTAAGATTTTTTACTTAAAAATTCTTGT
TCTTAACGAAATTACATACGTATTAAAATTATTTATTAGATAGTCGGTATTGTCTTTTTGTGAAAAAAAAA
AATTATAA

CAAAAAAAATGAATAAATAATGTATAAAACATATAACTTTGCAAATGATTCAAAAATTCAAATTTTCTCTC
TCTCTCTCTATAAACATAAATATATATGTGTATATGTTTTCTTTTTTTTTTTTTTTTTTTTTTGAATGATA
TTAAGTTA

GTATACCGAGTCGTTTGAAGTCATCTCCGGGTAGTGATTTTTATCACGTGACACTTTTTTATTCCTTTTCT
CTTTTCTTTTTTAAATTTTTTTTTTTTTTTTTCATTGATTAACCGCCTCTTCTTTATCACTCTCGCATTCT
GTCTACTA

AAGGAAAAAAAAATAAAGAAAATATTGAAAACTAATAAGTTTTTTTCCTGTAAAAGTGTATAAAATATATA
GGAGATCCCGTCAAATAATAGAAAAAAAATTTTTTGCCACATATACCTGTAGCCCCAGGAATACTGAAAAA
ATGGAGTA

TTTTTTTTTTTACATTTTATTAGCTATACACTTGGCGCAAGGATATTTTATATACCGGATATAATAGATAA
TTAAAAGACTACTAATTAAAAATGATGTAATATGGTAATAGGTATGGAAGAATCAGGGAGTTTTCAGAATG
TTCTTTGC

ATAAACGATTGAAGGGATCCGCTGAGGAATGAAAAAAAAAAATAAGAAAGTAAGAGAAGGGAAAAAAAAAA
GAAAAAAAGAAAAAAAGAAAAAAAGAAAAAAAGAGGGAATGATCGAAGATAAAAATTCTCAGAAATTTGAG
AAGAAGGA

TTCTCTCGGTGTGCAATATAGTAATAAGTTTTCACCTTTTCCGGTGAATAATGAAAAAAAAAAAACGAATA
CAAAAAAAAAAGTAATTTCTTTCAAGTTTATATATATAACAATATAATATTAAGTAATTTTAATCGAAAAT
TTTTTCCT

TAAAGAAAAAAGTTGAGATTAGATTTATTGTGTTATAAATATAGATATACAATTCTTTATAAAAAAAATAT
ATATATATATCATTGTTATTAAATAAAGAGTTTTCCTAGTATATAGATTAAAAAACTACTCTATTAAATGA
GAGCTAAA

GGCGCATAAGTACGCGCTAATACATGTATTGATATAAGTTATGTTTTTATTTATCAAAATGATATTGTTGC
ATTTTTTTTTTTAAATCTCACGTTATTTTTCATTTATATACATACACTTAAGAGAATGTCAGATTTTCTCT
TTTCGGCC

GGATGCCCTTTTAGTGTCAAACTTCTTATTATCACGAAAAACGTCGTCTTTTCATGCAAGGAAGGACTACA
GTATATACATATAAATATATATTTATGTAATTATTTTTTTCTTTTTTTTTTAAATTTCTAGCGAGAATGAA
GAAAAGCC

CAATAGATACAACTGCTCCATGAAAGGAAAAACCTTCATTTACGGTCGCGTTTTTTTTCGTGATATAAAAA
AGGTAAAAAAAAACGCGTCGCGTTAGAAAAAAGGTGTGTGTGAAGAGAAAGACCCATTGAATGGAGAGTTCG
GTTCTTGG

ACTCCTGAAGGAGCGCCGGAAAGTTTCTTTTTTTTTTTTTTTTTTTTTAATTTTTTTTGTGTGGGGAAAGAC
CGACTACCAGTAATACTTTAAAGATATTATTAAAAGAAATCCCGAAAAAAGAAAGACCATGAGAAAACCGA
GTGCGTTT

TGCCTTTATGAGTCATGTACTGACCCATTAATTTTCTTTTCTCTTTTTTTTTTTTTTTTTTTTTTTTCCTC
TATTTCCTTTTTTTGTTCTAGTCGGTTACGCAGTTCTGAGTAATGTAATATAGCGTTCCTTTTCCTCTCAG
ATTTTTTT

ACCTGTAAAAAAAAAAAAAAAAAAAAAAAAAAGAAGGGTTTAAATAAAAATCGGACTTACTCAAAGGGTTGAA
AAGCACTTTAATATAGGTTTTTAGTTTCGGGTAAGAAGATGTGTCAAAGGTCTCGAAAAGGAAACATTAGG
GCAAATAC

TTACTAAGAAAAGGAGAGGAAAAGGACGTGCATATATTCTTTTAATACATTTAAAATATGATTCTAATCAT
TTCTTATATAATAGTGTATAGTCTAATCATTCATCGTGTATAGTAACCCTTTTTGTTTTTTTTTATGCAAA
GTTTTTTT

GATCTCCCCTTCCGGTTGTGTCTTTTCATAATAATATGGTTTTTTTATTCATAATTTGGTAATAACGGTTG
ATAAACGAGCGGCTGATAAATTAGTTTCTTTTTTTTTTTTAATTTTTTTTTTTTTTGATCTCATTTCTTTTT
TTATTGAA

ATCAAGATACTTATGATATATATATATATAGAAAAAGCTTACTTTTCTTGGAGTTATTGTTATTATCATCGCG
AAGAACGATTGTATAACCCGGTTCAACGCGAAACGAATCGTTAAACTGGTGAAATGTTAACGCGAGTGTCA
GAGATATA

ATAATCATAATCAAAACCAACATACCAATTAATTAAAACTTGCTTTTAATCTGCATACATAAAACTTAGAT
CAAATAAATAAAAAAAAAAAGTAAAACAACCATATATACTATTTCATAAGCAAAAGTAATCAAATTAAACAA
AAAAAAGA

AAGAAGATACAATGAAAGAAAAGAGAAGGAAAATAATGTTATAAACGATTGAAGGGATCCGCTGAGGAATG
AAAAAAAAAAAATAAGAAAGTAAGAGAAGGGAAAAAAAAAAAGAAAAAAAGAAAAAAAGAAAAAAAGAAAAAA
AGAGGGAA

CATACCAATTAATTAAAACTTGCTTTTAATCTGCATACATAAAACTTAGATCAAATAAATAAAAAAAAAAAG
TAAAACAACCATATATACTATTTCATAAGCAAAAGTAATCAAATTAAACAAAAAAAAAGAAAATACAAATAT
TAAATTTA

ACTACGAAACGATAAATTTCCCCGTTTTCTTTTACTTTTACTTCATTTAATATAATTCATATTTATTTCCC
ATGTACCAATTAATTACTCCTAAAAGAACAGAAATAGAAAAAAGTTGTATATAACAAGCCAAAAAAAAAAA
AGAAAAAA

AAAAAAAATTAACTGCGAAAAGATGTCACACGTCAGTAAATCCGCGCAAAACCCCGTTATATTTCCGACGT
CGTGCGGTAGTTTCTGCGCGGAACGGCCCCGCGGAATAGGCACATTTTCTTCCGGCCGCAGAAAGCTCTCT
TTATCAAA

CTGATGGTGATGAAACCATGCAGATATAGTGTTGAATGATAATTCACCTTTTTTTTCTTTCTTTTTTTTTT
TACCTGGGACGACATTCTAACTAATGAAATTTACGATTAATTATTCTTTTTTTTTTTTTTTCAAGAAATAAA
TATAACTG

ATTCTTTTATCTTCTTTTCTTTTTGTACAAGTTGCGGATTACTTCTCTTTGTTTACCTTTTTGCTTTTATT
TTTTTTTTTAATACGTGCATATATATATATATATTTAGTTTAGGTCAGAATACAAAGATAATTATGTATAA
ATGGGCGC

GGATTTCGATGACCTAGGTTTAACAAGATCATGAAATATTCATTAACGGAGTTTTTTGTTTTCGCTTTTTT
TATTTTTTGTTCCTAATTTTTTTTTTTTTTTACATTTTATTAGCTATACACTTGGCGCAAGGATATTTTATA
TACCGGAT

CTATGTACTCAAGTACCGTGTATACACTATAAAATCTCATCTCATCGCTAAAGCTAATATTGAAAAAAAAA
AAAAAAAAAAAAAGGAAAAGGCAAATAAAAAATTTTCAATTCGGGTAACGAAGAATCGACTCTCGCCGGGTA
AACAATTC

TTCCTAATTTTTTTTTTTTTTTACATTTTATTAGCTATACACTTGGCGCAAGGATATTTTATATACCGGATA
TAATAGATAATTAAAAGACTACTAATTAAAAATGATGTAATATGGTAATAGGTATGGAAGAATCAGGGAGT
TTTCAGAA

AGTATACAGTTATCCAATAGCTATGTACTCAAGTACCGTGTATACACTATAAAATCTCATCTCATCGCTAA
AGCTAATATTGAAAAAAAAAAAAAAAAAAAAAAGGAAAAGGCAAATAAAAAATTTTCAATTCGGGTAACGAA
GAATCGAC

TCACTTGTTTATACAACTTTACGCGTCGCGTCGCGACATGTCTATAGATTAAGTCACTTTTAACGCCATCA
TTCCGGGTAGTGCATATTTTCCCTTGGCGGCTAAATAGATGAAAAGGTAAAAGTTTAATCTGAGAAAAAAA
CTGCATTG

ACTTCTCTTTGTTTACCTTTTTGCTTTTATTTTTTTTTTTAATACGTGCATATATATATATATATATTTAGTT
TAGGTCAGAATACAAAGATAATTATGTATAAATGGGCGCATTTGGAAACTATTTTACATACAACTGAACAA
GTTCGGAA

ATTAGGCAGAGTATACCGAGTCGTTTGAAGTCATCTCCGGGTAGTGATTTTTATCACGTGACACTTTTTA
TTCCTTTTCTCTTTTCTTTTTTAAATTTTTTTTTTTTTTTTCATTGATTAACCGCCTCTTCTTTATCACT
CTCGCATT

AGATTATTAAATCACACAGGAATGAAATTGTTATTAGAAAAATTATAATATACATTCTATATAGGGAAAAA
TTTCTTCTGACTTTGAAATTTAGATGCTAATAGTTTTTTTATTATTTCTATTTTTTTTTTTTTTAAGATC
AGTTGTTT

GCATTTTCAGTAAAAAATGCAGACTTTATTATTATTTAATCGTGCTTCTTATATATGACATTCTACCAAAT
CGGTAGTCATGTATATTTTTTTCGTATATACTTTATATATTTTTTTCTAAAAAACTAATGACGGCTAAAAT
TAAGTCAT

CCATGAAAACCTCAAATTACTGCTACAGGACCCTAGGGTCTCGCTAAAAAAATGACTACGCGCGCTTATAT
AATTACTCGAATAAGACAAAAGAAAAAAACCCTTGCGATATGTCGTAGACACGTAAAAATGGCCCATTTAG
TGGTAGTC

TTTTCTTTTCAACTTCTTTTTTTTTTTTTTCGTTTATGAAGCCGATGCTAATGCATCAATTTTTTTTTTTTG
GTGATGAGTTTTGAAAGAAAATGAAAAAAAAAAAAATTTCTATTATTGTCAGTGGAGTTTTTGAACAGATCA
CCTTGTGA

AATGAAAGAAAAGAGAAGGAAAATAATGTTATAAACGATTGAAGGGATCCGCTGAGGAATGAAAAAAAAAA
ATAAGAAAGTAAGAGAAGGGAAAAAAAAAAGAAAAAAAGAAAAAAAGAAAAAAAGAAAAAAAGAGGGAATG
ATCGAAGA

AGCGATTGTATCTGTATAGAGGTTGGTTTCTTTTTCTTACCTTTCTCTTTTTATTTTTTTCTCGCATTGGT
TCAAAAAAAAAAAAATGATGGTTTAAATTTCGTAAAATACGAAAAATGAAGGGAAGGTCCCACCCGGATTCG
AACTGGGG

CTTCACATGCCGTGAAAGCCGACAGGGACAACGCGCGATAAAAAAAAAATTAAAAGTAAAACTACGTACGC
GGCGGCGCGACTCGAATCCTTGACTCCGACTGAGGAGACCCGAACAACGCTCCCTGCTCTCCGCCTTCGGC
GCCTTCCG

CTGACATCTACCAAGTTCCTGAGATCTTTTGGTCAAACAATCTAGATTGTTCTGAAGATCAAAATAATATA
TATATATATATATATATATATATATATGTTTATACACTTAACTTACGTACAAAAATATAGATTTCGAAACG
AATCAGTT

TCGTTTGAAGTCATCTCCGGGTAGTGATTTTTATCACGTGACACTTTTTTATTCCTTTTCTCTTTTCTTTT
TTAAATTTTTTTTTTTTTTTTTCATTGATTAACCGCCTCTTCTTTATCACTCTCGCATTCTGTCTACTAAA
CGGTAAAA

TTATGATATATATATATATAGAAAAAGCTTACTTTTCTTGGAGTTATTGTTATTATCATCGCGAAGAACGATT
GTATAACCCGGTTCAACGCGAAACGAATCGTTAAACTGGTGAAATGTTAACGCGAGTGTCAGAGATATACA
TAGTATGA

TGTAGTCGTGCCCTGCGATCCGCTTTATTACGTATATTGCCTTCACATGCCGTGAAAGCCGACAGGGACAA
CGCGCGATAAAAAAAAAAATTAAAAGTAAAACTACGTACGCGGCGGCGCGACTCGAATCCTTGACTCCGACT
GAGGAGAC

TAGAAATATGACAAAAGAAGGAAGATAGGCATAGTGAATTTTATTTCTAATTTCACTTTTTATGTTTTTTT
TTTCGATATTTAATTTTTTCACGCATTACGAATATATTTCTATATACAAACATATGTAGTAGTACTAGTAG
CTGTCATC

GTGCAGGACCCTATGGCTCGAGAAGTTGCTACCGGTGTTTAATTTTTTTTTTATCTTAATATTAGGAAGAG
AAAAAGCATCAGTTACGAGACTCCTGAAGGAGCGCCGGAAAGTTTCTTTTTTTTTTTTTTTTTTTTAATTT
TTTTTGTG

GTTTCGTTTTTGCTTATACGACCAATCATTACCGCGTTCGGCTTTTCCAAAAAAAAAAAACTATAGTAAGG
ATAATGAAAAGAAAAAAGGGTCCCATGATTACTAAAAAGCTCTAATTCAAAAAAAAAGTCTTTGATAGGCA
GCTAGGAA

TGTATGCTTGTTTTTTCTTTTCTGTTTGTTGTAGTCTCTATAAATTTAAATGCACTTATCTTTTTATGCAA
AAAAAGCTGGGCGAGTTATTTGTTTCTATATTTGTATAATTTTTTTTTTGTGAAGATGAATATAAACTGA
CAAAAAAT

AAGCGAGTTTTTCAACCCTTCCACAATCTTCTCTTTATTAATAAATATGTTGGAGATATCTCCTGTATGGG
TAATATGTATATATGTAAAAAAAAAAAAAGAAAAACAAAAGAACCAAGATCGGGCTACGATGGATAAAAAA
AAAAGAAA

AAAACTTAGATCAAATAAATAAAAAAAAAAAGTAAAACAACCATATATACTATTTCATAAGCAAAAGTAATC
AAATTAAACAAAAAAAAGAAAATACAAATATTAAATTTAAATTAAGGCAATAAACATATATTATACACATA
AAAAGAAT

TTATCACGTGACACTTTTTTATTCCTTTTCTCTTTTCTTTTTTAAATTTTTTTTTTTTTTTTTCATTGATT
AACCGCCTCTTCTTTATCACTCTCGCATTCTGTCTACTAAACGGTAAAAAAACGAAGTGAAAAGTTCAATG
TGGGACCC

TCGAACAAAAGTGTAAGCAGACGTGAAATAGCAAACCTTTGAATGCATTTATATATTTTAATATACTTATA
ACAACGGATAAATGTTTAGATCCCTTTTGAAAAAAAAAAAAAAATTAGGATGAACCGAAAAAAATCATACTC
GTATTCCT

CATTGTGGGGTAGTTTTTACGAAAAAAATGAAAAGTTGTAAGTATAGTATATATTTTTTTTCTATGTAAGT
TTTATAAGATTCTATTCGCTATTACCACCGGTAAATTAAAAAGAACACTATTGTTACATTATATGTTTTTA
AATCATCA

TTCGGCATTTTTATGCTCGGCATTTCCCAACAGAAGCGGATATATTTTAAGGGCGCGGAGATGAGCCAAAC
GGCAAGCGATATCAAAATATCTGCATAATAACGGCAATCAACGCGCACGCGAATGGCCAAGAGGGTACAAT
AGCAGCAA

TCTGTATAGAGGTTGGTTTCTTTTTCTTACCTTTCTCTTTTTATTTTTTTCTCGCATTGGTTCAAAAAAAA
AAAATGATGGTTTAAATTTCGTAAAATACGAAAAATGAAGGGAAGGTCCCACCCGGATTCGAACTGGGGTT
GTTCGGAT

GATGAGCCAAGCATGATTTGAATCGGATGGATTCTCAACTGATATTTATATGGCTTTTTCAGTTTTTTTTT
TTTTTTTTTTTTTTTCAGCATCTCGAAGAGCAAAAAAAAAGGAACGAAAAAGTGCCACGGCAAAGAGTTTT
TCACATTA

ATTTATTATTGAAAATGTTTTATTCTATATTGGTAGAGTTAATAAATAATTATATAATAAATTATTGTATG
ATAATATAAATAATAGTTTATGTAATATTTTAAGTTATTATTTATAATAAAAAAGTAACTATTGAACCTGT
TAAATTAA

ATCATCGCATACCCAATCGGTTTCCTATTCTCACCACTTTTTTCTGGAAAAATACATAGCCTAACAAGCAA
TTTTATTTTACGTTTGTTAATTCATTATACTGATAATATTTTTGAATTTTTTTTTTTTTTGATACATTTTT
TTTAATCG

ACCGGTGTTTAATTTTTTTTTTATCTTAATATTAGGAAGAGAAAAAGCATCAGTTACGAGACTCCTGAAGG
AGCGCCGGAAAGTTTCTTTTTTTTTTTTTTTTTTTTAATTTTTTTTGTGTGGGGAAAGACCGACTACCAGT
AATACTTT

AGAGTTTGAGAAAAAGAGATGAAAAAATGCGATGAGCCAAGCATGATTTGAATCGGATGGATTCTCAACTG
ATATTTATATGGCTTTTTCAGTTTTTTTTTTTTTTTTTTTTTTTTCAGCATCTCGAAGAGCAAAAAAAAAG
GAACGAAA

ATATATAGATAGACAACTGGTCTTCTGCATCCGGTAATAGGCATTGGCTTTAATACTAATAAAAGCAAGAA
AAAAGCGACACCAGTAAATTTCAGACATCTTCGAGAATTTTTTTCGCGCGCTTTCTATCAAAAAAAAAATGA
AAAATAAA

ACCATTTTTTCGTAAAAAAAATTTAAGCCCAAGAGTTTGAGAAAAAGAGATGAAAAAATGCGATGAGCCAAG
CATGATTTGAATCGGATGGATTCTCAACTGATATTTATATGGCTTTTTCAGTTTTTTTTTTTTTTTTTTTT
TTTTTCAG

TATTCTATATTGGTAGAGTTAATAAATAATTATATAATAAATTATTGTATGATAATATAAATAATAGTTTA
TGTAATATTTTAAGTTATTATTTATAATAAAAAAGTAACTATTGAACCTGTTAAATTAAAATATATTCAAA
AAACATTA

AAGTTGTCTTAAGGAAAAAAAAAATAAAGAAAATATTGAAAACTAATAAGTTTTTTCCTGTAAAAGTGTAT
AAAATATATAGGAGATCCCGTCAAATAATAGAAAAAAAATTTTTTGCCACATATACCTGTAGCCCCAGGAA
TACTGAAA

GTAGTCAACAAAGTTGTCTTAAGGAAAAAAAAAATAAAGAAAATATTGAAAACTAATAAGTTTTTTCCTGT
AAAAGTGTATAAAATATATAGGAGATCCCGTCAAATAATAGAAAAAAAATTTTTTGCCACATATACCTGTA
GCCCCAGG

ATTAGGAAGAGAAAAAGCATCAGTTACGAGACTCCTGAAGGAGCGCCGGAAAGTTTCTTTTTTTTTTTTTT
TTTTTTAATTTTTTTTGTGTGGGGAAAGACCGACTACCAGTAATACTTTAAAGATATTATTAAAAGAAATC
CCGAAAAA

GGCAGTAGGCGATGAAAGGAAGTTTTTTAATGTCATTAAGAAAAAGATAGGGAACTTCTATTTCCTGTCTT
TCATATATATATATATATATATATATATATATGTGCATATGTACATCGTTGAGTTGTTCATGAAATAAT
GAATATAA

CCGCTTTTTAATTTTTAATCAATGTTACATACCTAGCGTAATTTTCCTATATATAAAATATCAGCTTTATA
TAAATATATTATTGAAACTGTACCTATAATAATAAATTTAAAATTAAGTAAATACAAATGCTTCAAAATTT
GTTAGTAT

CTTCTTGAGGTATTTCATTTTTATTCTTTTTTTGATATCTATATATAAGTATTTCGATATAGTAAAAAGAA
AACCCTTTCCTATGATGGACTAAAAAATATAATTATAAATAAATTATGAATAGTTGACGCATTAGAAGATA
TTGAGGAC

CATTTTGCTGGTAGTCAACAAAGTTGTCTTAAGGAAAAAAAAATAAAGAAAATATTGAAAACTAATAAGTT
TTTTTCCTGTAAAAGTGTATAAAATATATAGGAGATCCCGTCAAATAATAGAAAAAAAATTTTTTGCCACA
TATACCTG

CAAAAAAAAAAAAGAAAAAAGGAAACACAGCTTATTTCAAATTGCAATCTGCGTATTTCGTAAGTAACGAA
TGATCTCACAGCATTGCTTTTTTTTTTTTTTTTTTTATATATATGTACATTACCTATGTCACATATGACCA
TGATGAGT

AGTTGAAATCAAGAAGATACAATGAAAGAAAAGAGAAGGAAAATAATGTTATAAACGATTGAAGGGATCCG
CTGAGGAATGAAAAAAAAAAAAATAAGAAAGTAAGAGAAGGAAAAAAAAAGAAAAAAAGAAAAAAAGAAAA
AAAGAAAA

TAAACTGTACGGATATATATATGATATTACGGTGCCATATGATATTATACAATATTATACTGTACTGTACT
ATATTATATTATATTGTATTAATTATATTATACTATACCCTATCATACCAAATATTTTGGCTATATTAATT
TTTATCTA

ATTTTTAATCAATGTTACATACCTAGCGTAATTTTCCTATATATAAAATATCAGCTTTATATAAATATATT
ATTGAAACTGTACCTATAATAATAAATTTAAAATTAAGTAAATACAAATGCTTCAAAATTTGTTAGTATTG
TTTTCTTC

GATATACTTCCTTTTTGCCTTATCTTTCACTGTTTCCATCTCTTTTCTTTTTTTTTTTTTTTTTTTTTTTT
TTTTTTTTTTTTTTTTTTGAATCTTAAAATTCTCGAAACTGCACTGAAATGGCGCAGGAAACTGACCTCAT
CGCATGCC

TAAAAACCTTGATATACTTCCTTTTTGCCTTATCTTTCACTGTTTCCATCTCTTTTCTTTTTTTTTTTTTT
TTTTTTTTTTTTTTTTTTTTTTTTTTTTGAATCTTAAAATTCTCGAAACTGCACTGAAATGGCGCAGGAAA
CTGACCTC

GGAGCTATTAAATCTTTATAGTAAATTTTTTTTTTACTTTTTTTTTTTTTTTTTTTTTTTTTATTATTTAC
AAGCGTCTATATATTTTCTATTATAGAATATTGTCATTTATTACATTGGTTCATCTGTAAATCTCATCAAA
CGGTCTCG

TATTTTGATGTAAAAACCTTGATATACTTCCTTTTTGCCTTATCTTTCACTGTTTCCATCTCTTTTCTTTT
TTTTTTTTTTTTTTTTTTTTTTTTTTTTTTTTTTTTTTGAATCTTAAAATTCTCGAAACTGCACTGAAATG
GCGCAGGA

CTTTTTGCCTTATCTTTCACTGTTTCCATCTCTTTTCTTTTTTTTTTTTTTTTTTTTTTTTTTTTTTTTTT
TTTTTTTTGAATCTTAAAATTCTCGAAACTGCACTGAAATGGCGCAGGAAACTGACCTCATCGCATGCCAA
CGAAGATT

GCATCCTGCAACTGTTCTGTGGAGCTATTAAATCTTTATAGTAAATTTTTTTTTTACTTTTTTTTTTTTTT
TTTTTTTTTTTATTATTTACAAGCGTCTATATATTTTCTATTATAGAATATTGTCATTTATTACATTGGTT
CATCTGTA

TTCATCGCTTATAGAAGACATATTTTGATGTAAAAACCTTGATATACTTCCTTTTTGCCTTATCTTTCACT
GTTTCCATCTCTTTTCTTTTTTTTTTTTTTTTTTTTTTTTTTTTTTTTTTTTTTTTTTGAATCTTAAAATT
CTCGAAAC

GTTGCAAATTTTCATCGCTTATAGAAGACATATTTTGATGTAAAAACCTTGATATACTTCCTTTTTGCCTT
ATCTTTCACTGTTTCCATCTCTTTTCTTTTTTTTTTTTTTTTTTTTTTTTTTTTTTTTTTTTTTTTTTGAA
TCTTAAAA

ACTGTTCTGTGGAGCTATTAAATCTTTATAGTAAATTTTTTTTTACTTTTTTTTTTTTTTTTTTTTTTTTT
TATTATTTACAAGCGTCTATATATTTTCTATTATAGAATATTGTCATTTATTACATTGGTTCATCTGTAAA
TCTCATCA

ACATTCTAAGGCATCCTGCAACTGTTCTGTGGAGCTATTAAATCTTTATAGTAAATTTTTTTTTACTTTTT
TTTTTTTTTTTTTTTTTTTTTATTATTTACAAGCGTCTATATATTTTCTATTATAGAATATTGTCATTTAT
TACATTGG

ATAGAAGACATATTTTGATGTAAAAACCTTGATATACTTCCTTTTTGCCTTATCTTTCACTGTTTCCATCT
CTTTTCTTTTTTTTTTTTTTTTTTTTTTTTTTTTTTTTTTTTTTTTTTGAATCTTAAAATTCTCGAAACTG
CACTGAAA

TCGGGTAAGATATATGTGCTAGCGCCACGCGATGGCTGGTTTGTCTTTTTAAGTTCTTAATATTTATTTTT
ATATTTTGATTTTTTTTTTTTTTTTTTTTTTCCATTAGTGAATGACAGCAATGTATTAAGCTGCTGCCGCG
CATACAAA

TTTTGCTTTGGTTGCAAATTTTCATCGCTTATAGAAGACATATTTTGATGTAAAAACCTTGATATACTTCC
TTTTTGCCTTATCTTTCACTGTTTCCATCTCTTTTCTTTTTTTTTTTTTTTTTTTTTTTTTTTTTTTTTTT
TTTTTTTG

TATCTTTCACTGTTTCCATCTCTTTTCTTTTTTTTTTTTTTTTTTTTTTTTTTTTTTTTTTTTTTTTTTGA
ATCTTAAAATTCTCGAAACTGCACTGAAATGGCGCAGGAAACTGACCTCATCGCATGCCAACGAAGATTGA
AAAAAATT

CTATAAATAACAAACAATCGCGCGGAACGTCAAGCGTATATACTATATAAAAAGAAAACAAAAAAAATCAA
TAACCCATTAAAGATACATAATCAATAAAAAACGATATTAAAAAGCACGTAAAGTACTTAATATTTTTTTA
ATAATAGA

AATCTTTATAGTAAATTTTTTTTTACTTTTTTTTTTTTTTTTTTTTTTTTTTATTATTTACAAGCGTCTAT
ATATTTTCTATTATAGAATATTGTCATTTATTACATTGGTTCATCTGTAAATCTCATCAAACGGTCTCGTA
TCGGCGAA

TTGGGTTTTTTTCCCTTTGCGAATACCCGGGCTGCCAAAATAGAAAAGGAACAAATGGTAGGTCTTTAAGA
TAAGAAAGATAAGATAAGACATCTTTTTTTTTTTTTTTTTTTTTTTTTTTTTTTTCCGATAACAAAGAAGA
ATCCTTCG

AGCGCCACGCGATGGCTGGTTTGTCTTTTTAAGTTCTTAATATTTATTTTTATATTTTGATTTTTTTTTTT
TTTTTTTTTTTCCATTAGTGAATGACAGCAATGTATTAAGCTGCTGCCGCGCATACAAAATGTACTGCGTT
AAATTAGA

ATGACTTTATGACAACCATGATAGTACAGAAGAGAGACCTTTTTTCTTTTTTTTTTTTTTTTTCTTTTTTT
TTTTTTTTCTTTTTTTTCTTTTTTTTTTTTTGAGCCTTTTCGAATCTAGACTCTATGTGAGTCTATTCTCG
ATGGGGAG

TGAAAAAATGAAAGAGTAATGAACAAACATATTTTTTTTATAAAATGCATAAAAACATATAGATATATATA
TATTTACGTGGAAAAATGTTAAAACTATTGATAAGAAAAAACGCTCAAAACAAGTAGTAAGCAGGCTTTTT
TTTTTTTT

GTAAATTTTTTTTTACTTTTTTTTTTTTTTTTTTTTTTTTTTATTATTTACAAGCGTCTATATATTTTCTA
TTATAGAATATTGTCATTTATTACATTGGTTCATCTGTAAATCTCATCAAACGGTCTCGTATCGGCGAAGC
AACTGTCC

TGTTTCCATCTCTTTTCTTTTTTTTTTTTTTTTTTTTTTTTTTTTTTTTTTTTTTTTTTGAATCTTAAAAT
TCTCGAAACTGCACTGAAATGGCGCAGGAAACTGACCTCATCGCATGCCAACGAAGATTGAAAAAAATTAA
CATATAAG

ATATAACGCGAGGTTCAATGGCCTCTTTACCATGAAAAAAAAAAAAAAAAAAAAAAAAAGGTAAGGAAAAA
GAGTATTTTCAATTCGTTTCTGAACATATAAATATAAATAACCGAAAAATTAGCCCTTGAACATAATTAAC
ACTCTTCT

AATGGCTTTTAATGACATTAATGACTTTATGACAACCATGATAGTACAGAAGAGAGACCTTTTTTCTTTTT
TTTTTTTTTTTCTTTTTTTTTTTTTTTTCTTTTTTTCTTTTTTTTTTTTTTGAGCCTTTTCGAATCTAGAC
TCTATGTG

GGTTACCCGACTTCTTTACCCGGCTCTCCGTCGGGTAAGATATATGTGCTAGCGCCACGCGATGGCTGGTT
TGTCTTTTTAAGTTCTTAATATTTATTTTTATATTTGATTTTTTTTTTTTTTTTTTTTTTTCCATTAGTGA
ATGACAGC

AAGAAGGACACTATAAATAACAAACAATCGCGCGGAACGTCAAGCGTATATACTATATAAAAAGAAAACAA
AAAAAATCAATAACCCATTAAAGATACATAATCAATAAAAAACGATATTAAAAAGCACGTAAAGTACTTAA
TATTTTTT

GAATACCCGGGCTGCCAAAATAGAAAAGGAACAAATGGTAGGTCTTTAAGATAAGAAAGATAAGATAAGAC
ATCTTTTTTTTTTTTTTTTTTTTTTTTTTTTTTTTCCGATAACAAAGAAGAATCCTTCGGTCTCTTCTTAC
TGCTGTTA

AGTAAGCAGTATATAACGCGAGGTTCAATGGCCTCTTTACCATGAAAAAAAAAAAAAAAAAAAAAAAAAGG
TAAGGAAAAAGAGTATTTTCAATTCGTTTCTGAACATATAAATATAAATAACCGAAAAATTAGCCCTTGAA
CATAATTA

GACAACCATGATAGTACAGAAGAGAGACCTTTTTTCTTTTTTTTTTTTTTTTCTTTTTTTTTTTTTTTTCT
TTTTTTCTTTTTTTTTTTTTTGAGCCTTTTCGAATCTAGACTCTATGTGAGTCTATTCTCGATGGGGAGTA
TCGGAAAT

GAATGCTGTCTTGGGTTTTTTTCCCTTTGCGAATACCCGGGCTGCCAAAATAGAAAAGGAACAAATGGTAG
GTCTTTAAGATAAGAAAGATAAGATAAGACATCTTTTTTTTTTTTTTTTTTTTTTTTTTTTTTTTCCGATA
ACAAAGAA

ATGAAAAGGGTGTTCTTGGCTTTACAAGCGCATTAAACATTTAATATTATATAATATTTATAATATACATG
TTATTCCAATAGTGAAAAAAAAAAAAAAATGAAAATGAAAATGAAAAATTCGGCCCTTAAAAATGAACAAC
CTTTTACC

AAATTCCTCAATGAAAAGGGTGTTCTTGGCTTTACAAGCGCATTAAACATTTAATATTATATAATATTTAT
AATATACATGTTATTCCAATAGTGAAAAAAAAAAAAAAATGAAAATGAAAATGAAAAATTCGGCCCTTAAA
AATGAACA

TAAAAGTTGAAAATTCCTCAATGAAAAGGGTGTTCTTGGCTTTACAAGCGCATTAAACATTTAATATTATA
TAATATTTATAATATACATGTTATTCCAATAGTGAAAAAAAAAAAAAAATGAAAATGAAAATGAAAAATTC
GGCCCTTA

AGTCCAATCGGACTGATTCGTAAAAATTTTTGCAGACATTTTTGATATATATTCAATTCATATTTTATCTC
TTTTTTTTTTTTTTTTTTCAGACGTGGAATGGTGGGAAAGCCAAAATGTCACTGACCGCGGCTAAAAGTTA
AAAAATAC

TTATTTTAATGGTTACCCGACTTCTTTACCCGGCTCTCCGTCGGGTAAGATATATGTGCTAGCGCCACGCG
ATGGCTGGTTTGTCTTTTTAAGTTCTTAATATTTATTTTTATATTTGATTTTTTTTTTTTTTTTTTTTTTT
CCATTAGT

TTCCCTTTGCGAATACCCGGGCTGCCAAAATAGAAAAGGAACAAATGGTAGGTCTTTAAGATAAGAAAGAT
AAGATAAGACATCTTTTTTTTTTTTTTTTTTTTTTTTTTTTTTTTCCGATAACAAAGAAGAATCCTTCGGT
CTCTTCTT

AATGACATTAATGACTTTATGACAACCATGATAGTACAGAAGAGAGACCTTTTTTCTTTTTTTTTTTTTTTT
TCTTTTTTTTTTTTTTTTTCTTTTTTTCTTTTTTTTTTTTTTGAGCCTTTTCGAATCTAGACTCTATGTGAG
TCTATTCT

CAAACAATCGCGCGGAACGTCAAGCGTATATACTATATAAAAAGAAAACAAAAAAAAATCAATAACCCATTA
AAGATACATAATCAATAAAAAACGATATTAAAAAGCACGTAAAGTACTTAATATTTTTTTAATAATAGACA
TTTTCGCC

CTTCTTTACCCGGCTCTCCGTCGGGTAAGATATATGTGCTAGCGCCACGCGATGGCTGGTTTGTCTTTTTA
AGTTCTTAATATTTATTTTTATATTTTGATTTTTTTTTTTTTTTTTTTTTTCCATTAGTGAATGACAGCAA
TGTATTAA

TTTTTTTTTGTTACCCCTAACGATATTACAGCTATTTCCTAATGGCTTTTAATGACATTAATGACTTTATG
ACAACCATGATAGTACAGAAGAGAGACCTTTTTTCTTTTTTTTTTTTTTTTTCTTTTTTTTTTTTTTTTTCTT
TTTTTCTT

TATATGTGCTAGCGCCACGCGATGGCTGGTTTGTCTTTTTAAGTTCTTAATATTTATTTTTATATTTTGAT
TTTTTTTTTTTTTTTTTTTTTCCATTAGTGAATGACAGCAATGTATTAAGCTGCTGCCGCGCATACAAAAT
GTACTGCG

GTTGTACTTGACATTCTAAGGCATCCTGCAACTGTTCTGTGGAGCTATTAAATCTTTATAGTAAATTTTTT
TTTACTTTTTTTTTTTTTTTTTTTTTTTTTTTATTATTTACAAGCGTCTATATATTTTCTATTATAGAATAT
TGTCATTT

GCTATTTCCTAATGGCTTTTAATGACATTAATGACTTTATGACAACCATGATAGTACAGAAGAGAGACCTT
TTTTCTTTTTTTTTTTTTTTTTCTTTTTTTTTTTTTTTTTCTTTTTTTCTTTTTTTTTTTTTTTGAGCCTTTTC
GAATCTAG

ATAGTACAGAAGAGAGACCTTTTTTCTTTTTTTTTTTTTTTTTCTTTTTTTTTTTTTTTTTCTTTTTTTTCTTT
TTTTTTTTTTGAGCCTTTTCGAATCTAGACTCTATGTGAGTCTATTCTCGATGGGGAGTATCGGAAATTG
AATTTTAA

AATGAACAAGGAATGCTGTCTTGGGTTTTTTTCCCTTTGCGAATACCCGGGCTGCCAAAATAGAAAAGGAA
CAAATGGTAGGTCTTTAAGATAAGAAAGATAAGATAAGACATCTTTTTTTTTTTTTTTTTTTTTTTTTTTT
TTTTCCGA

TGTGAAATGGTTCTAGTTAGTCACGTGCAGCTTTTAAAAATATTTAAAACATTTAAAAAATATACATT
TTTTTATTATTTTTTTATATATTAATGTTAAAATTTATTTATGTATTTGTCTTCCGAAAAGTAAAATAACG
TTCAAATT

AGAGAGACCTTTTTTCTTTTTTTTTTTTTTTTTCTTTTTTTTTTTTTTTTTCTTTTTTTCTTTTTTTTTTTTTT
TGAGCCTTTTCGAATCTAGACTCTATGTGAGTCTATTCTCGATGGGGAGTATCGGAAATTGAATTTTAATT
CGAATGAC

TCTTTTCTTTTTTTTTTTTTTTTTTTTTTTTTTTTTTTTTTTTTTTTTTGAATCTTAAAATTCTCGAAACT
GCACTGAAATGGCGCAGGAAACTGACCTCATCGCATGCCAACGAAGATTGAAAAAAATTAACATATAAGGC
TTATGAAA

TTCAATTTTTTAGATTTTAATTAGCGGTTTTTTTTTGGTTTTTCTTAAGATTGGTTTTATGATTTTTTTTT
TTCTTTTTCAAATTTTTTTATTATTTTTTCAATTTTTCTTTTTAAAAGGCTTGTGGTTGACTAGTCCTTTC
TTTTTAGG

GTTGGTGGATCGGCCGTACGTGTTTAGTGAGGGTACTATTTAAACTTACTTTAAATAGCAAAATAGGAAAA
GCTACGAGAAACTGAAAAGTAAAAAAAAAAAAAAAAAAATAAAAAAAAAAGAAAAAAAAAAGGAGAAACATGA
TTGCCAGT

ATTGAGTTGTTGTGAAATGGTTCTAGTTAGTCACGTGCAGCTTTTTAAAAATATTTTAAAACATTTTAAAA
AATATACATTTTTTTATTATTTTTTTATATATTAATGTTAAAATTTATTTATGTATTTGTCTTCCGAAAAG
TAAAATAA

GCTTTTGTTCGTTGGTGGATCGGCCGTACGTGTTTAGTGAGGGTACTATTTAAACTTACTTTAAATAGCAA
AATAGGAAAAGCTACGAGAAACTGAAAAGTAAAAAAAAAAAAAAAAAATAAAAAAAAAAGAAAAAAAAAGG
AGAAACAT

CTTAAAAAAAAAAAAAAAAAAAAAGCGTCTTAGAGGGAATATAAGAGAAAAGAAGAGATAGCATAACACAT
GATTTATTTTTATTTTTGCGTTTTTTTTTTTTCTTAATTGTTTATCAATGTATCTGTGAGAGTATCTACAT
AAAAAAAA

CCGCGCCAGTATATTAGTTACTAATCATACAGTTTCTTGTTTCAATTTTTTAGATTTTAATTAGCGGTTTT
TTTTTGGTTTTTCTTAAGATTGGTTTTATGATTTTTTTTTTTCTTTTTCAAATTTTTTTATTATTTTTTCA
ATTTTTCT

TACTATATAAAAAGAAAACAAAAAAAATCAATAACCCATTAAAGATACATAATCAATAAAAAACGATATTA
AAAAGCACGTAAAGTACTTAATATTTTTTTAATAATAGACATTTTCGCCCTTACTTTTTAAATAAAAAACA
TAAAAAAA

ATAAAAATACACCTTCGTACAAGGTGCTAATAATGTTGAGAATTCGAAATTCCTTTTTAAAGGCGTATTCC
GTATTGAATGATTGAAAAATTTATTTCTTTTTTTATTTCTTTTTTTTTTTTTTTTTTTTTTTTTTACGCCG
ATGCTCAT

TTACCCCTAACGATATTACAGCTATTTCCTAATGGCTTTTAATGACATTAATGACTTTATGACAACCATGA
TAGTACAGAAGAGAGACCTTTTTTCTTTTTTTTTTTTTTTTCTTTTTTTTTTTTTTTTCTTTTTTTCTTTT
TTTTTTTT

CGGCCGTACGTGTTTAGTGAGGGTACTATTTAAACTTACTTTAAATAGCAAAATAGGAAAAGCTACGAGAA
ACTGAAAAGTAAAAAAAAAAAAAAAAAATAAAAAAAAAAGAAAAAAAAAGGAGAAACATGATTGCCAGTAC
TCGCAAGA

CGATATTACAGCTATTTCCTAATGGCTTTTAATGACATTAATGACTTTATGACAACCATGATAGTACAGAA
GAGAGACCTTTTTTCTTTTTTTTTTTTTTTTCTTTTTTTTTTTTTTTTCTTTTTTTCTTTTTTTTTTTTTT
GAGCCTTT

CATTTTTTAAAAGTTTTCAATGAAAAAATGAAAGAGTAATGAACAAACATATTTTTTTTATAAAATGCATA
AAAACATATAGATATATATATATTTACGTGGAAAAATGTTAAAACTATTGATAAGAAAAAACGCTCAAAAC
AAGTAGTA

ATTCGCCAGTAAATGCGTATCTTGGAGAAAATATCAATTTGTTATTTTGTATATATAAAAAATTAAAAAAA
AAAAAAAAAAAATACTCAAATACTCAAATACGTATACATTATACTGAAATACTTCGCTTGTCTTCGAATCT
AAATCCTC

ACCTTCGTACAAGGTGCTAATAATGTTGAGAATTCGAAATTCCTTTTTAAAGGCGTATTCCGTATTGAATG
ATTGAAAAATTTATTTCTTTTTTTATTTCTTTTTTTTTTTTTTTTTTTTTTTTTTACGCCGATGCTCATCG
CAGAAAAT

ATTGATTTTATTTTTTTTTGTTACCCCTAACGATATTACAGCTATTTCCTAATGGCTTTTAATGACATTAA
TGACTTTATGACAACCATGATAGTACAGAAGAGAGACCTTTTTTCTTTTTTTTTTTTTTTTCTTTTTTTTT
TTTTTTTC

CGGCTCTCCGTCGGGTAAGATATATGTGCTAGCGCCACGCGATGGCTGGTTTGTCTTTTTAAGTTCTTAAT
ATTTATTTTATATTTTGATTTTTTTTTTTTTTTTTTTTTTTCCATTAGTGAATGACAGCAATGTATTAAGC
TGCTGCCG

GATAAGTACAAGTCCAATCGGACTGATTCGTAAAAATTTTTGCAGACATTTTTGATATATATTCAATTCAT
ATTTTATCTCTTTTTTTTTTTTTTTTTTCAGACGTGGAATGGTGGGAAAGCCAAAATGTCACTGACCGCGG
CTAAAAGT

TACACAATAACGCCGACGAAAAATCTAATTAAGAAGGACACTATAAATAACAAACAATCGCGCGGAACGTC
AAGCGTATATACTATATAAAAAGAAAACAAAAAAAATCAATAACCCATTAAAGATACATAATCAATAAAAA
ACGATATT

AACAGTCCGTAAACAAACACATTCCCACAAAACACCGCACCGCGAAAAAAAAAAAAAACAGGAAAAAACGC
GAAATGTCCGTAACTTTACCCGGTAATAGTCACGTGATAAAGGTGCATTGGTCTAGCTGGCTGTTCTGGCC
AACGCGAC

TTTTTCTTTTTTTTTTTTTTTTCTTTTTTTTTTTTTTTTCTTTTTTCTTTTTTTTTTTTTTTGAGCCTTTT
CGAATCTAGACTCTATGTGAGTCTATTCTCGATGGGGAGTATCGGAAATTGAATTTTAATTCGAATGACTT
CTAATGCA

AGGTTCAATGGCCTCTTTACCATGAAAAAAAAAAAAAAAAAAAAAAAAAGGTAAGGAAAAAGAGTATTTTC
AATTCGTTTCTGAACATATAAATATAAATAACCGAAAAATTAGCCCTTGAACATAATTAACACTCTTCTTT
GATATTTA

TCCTTTTTAAAGGCGTATTCCGTATTGAATGATTGAAAAATTTATTTCTTTTTTTATTTCTTTTTTTTTTT
TTTTTTTTTTTTTTTACGCCGATGCTCATCGCAGAAAATTTTTCCTTCAGTTTATTTGTCTTATAAAAAGA
CTGTCCTA

CTTTCCCTCCGCAGGAAATGAAAGATGAACCACCCATTACATTCGATTTTTTTTTTAATTATATTTTTGAC
TATTATTTAATCATTAAAAACACATATATTTCATAAATTCGTAAGGTCGTTAGTTCTATCGTAAAAGTGAA
AAAGTTTT

GTCTACTATAATAATTCACCAAAAAACAGCAGAGCACGTCCTATATATATATATATATATATATATATATA
TACATATATATATACGAATATATTTATGACAGTCTAAATCGTTGCTCCTGTTCAATTTTAACGCTCTTTTA
TAACTGCT

AAGTTTTCAATGAAAAAATGAAAGAGTAATGAACAAACATATTTTTTTTATAAAATGCATAAAAACATATA
GATATATATATATTTACGTGGAAAAATGTTAAAACTATTGATAAGAAAAAACGCTCAAAACAAGTAGTAAG
CAGGCTTT

ATATAACCAGATAAAAATACACCTTCGTACAAGGTGCTAATAATGTTGAGAATTCGAAATTCCTTTTTAAA
GGCGTATTCCGTATTGAATGATTGAAAAATTTATTTCTTTTTTTATTTCTTTTTTTTTTTTTTTTTTTTTT
TTTTACGC

CTATACAAAAAGTGTGCTTTTTTCTTCTATTTTTTTTTCTTTAATGACTTTGTGTGTTTATTACCCTTTAA
TTAATTACCAAAAAAAAAATAAACTACGTTTTTAATTTCTAATTTATCAAAATTTCTTTTTTTCACATATA
ACTATATA

TTCTAGTTAGTCACGTGCAGCTTTTTAAAAATATTTTAAAACATTTTAAAAAATATACATTTTTTTATTAT
TTTTTTATATATTAATGTTAAAATTTATTTATGTATTTGTCTTCCGAAAAGTAAAATAACGTTCAAATTTG
GTATTTAA

AATTCGAAATTCCTTTTTAAAGGCGTATTCCGTATTGAATGATTGAAAAATTTATTTCTTTTTTTATTTCT
TTTTTTTTTTTTTTTTTTTTTTTTTACGCCGATGCTCATCGCAGAAAATTTTTCCTTCAGTTTATTTGTCT
TATAAAAA

CGCCGACGAAAAATCTAATTAAGAAGGACACTATAAATAACAAACAATCGCGCGGAACGTCAAGCGTATAT
ACTATATAAAAAGAAAACAAAAAAAATCAATAACCCATTAAAGATACATAATCAATAAAAAACGATATTAA
AAAGCACG

AAATCTAATTAAGAAGGACACTATAAATAACAAACAATCGCGCGGAACGTCAAGCGTATATACTATATAAA
AAGAAAACAAAAAAAATCAATAACCCATTAAAGATACATAATCAATAAAAAACGATATTAAAAAGCACGTA
AAGTACTT

TAATAGTCATAATGAACAAGGAATGCTGTCTTGGGTTTTTTTCCCTTTGCGAATACCCGGGCTGCCAAAAT
AGAAAAGGAACAAATGGTAGGTCTTTAAGATAAGAAAGATAAGATAAGACATCTTTTTTTTTTTTTTTTT
TTTTTTTT

CAAGCGTATATACTATATAAAAAGAAAACAAAAAAAATCAATAACCCATTAAAGATACATAATCAATAAAA
AACGATATTAAAAAGCACGTAAAGTACTTAATATTTTTTTAATAATAGACATTTTCGCCCTTACTTTTTAA
ATAAAAAA

CAAGGATCGTGCTTTTGTTCGTTGGTGGATCGGCCGTACGTGTTTAGTGAGGGTACTATTTAAACTTACTT
TAAATAGCAAAATAGGAAAAGCTACGAGAAACTGAAAAGTAAAAAAAAAAAAAAAAAAATAAAAAAAAAAGA
AAAAAAAA

AAGGTGCTAATAATGTTGAGAATTCGAAATTCCTTTTTAAAGGCGTATTCCGTATTGAATGATTGAAAAAT
TTATTTCTTTTTTTATTTCTTTTTTTTTTTTTTTTTTTTTTTTTTACGCCGATGCTCATCGCAGAAAATTT
TTCCTTCA

TGTTCTTGGCTTTACAAGCGCATTAAACATTTAATATTATATAATATTTATAATATACATGTTATTCCAAT
AGTGAAAAAAAAAAAAAAAATGAAAATGAAAATGAAAAATTCGGCCCTTAAAAATGAACAACCTTTTACCAG
CCGGCATT

TAATGTTGAGAATTCGAAATTCCTTTTTAAAGGCGTATTCCGTATTGAATGATTGAAAAATTTATTTCTTT
TTTTATTTCTTTTTTTTTTTTTTTTTTTTTTTTTTACGCCGATGCTCATCGCAGAAAATTTTTCCTTCAGT
TTATTTGT

GCTGCCAAAATAGAAAAGGAACAAATGGTAGGTCTTTAAGATAAGAAAGATAAGATAAGACATCTTTTTTT
TTTTTTTTTTTTTTTTTTTTTTTTTCCGATAACAAAGAAGAATCCTTCGGTCTCTTCTTACTGCTGTTACT
GCTTATCA

AAATGCGTATCTTGGAGAAAATATCAATTTGTTATTTTGTATATATAAAAAATTAAAAAAAAAAAAAAAAA
AATACTCAAATACTCAAATACGTATACATTATACTGAAATACTTCGCTTGTCTTCGAATCTAAATCCTCTC
TCTCTTCT

CGCGGAACGTCAAGCGTATATACTATATAAAAAGAAAACAAAAAAAATCAATAACCCATTAAAGATACATA
ATCAATAAAAAACGATATTAAAAAGCACGTAAAGTACTTAATATTTTTTTAATAATAGACATTTTCGCCCT
TACTTTTT

TGCTGATTTTTTATTTTAATGGTTACCCGACTTCTTTACCCGGCTCTCCGTCGGGTAAGATATATGTGCTA
GCGCCACGCGATGGCTGGTTTGTCTTTTTAAGTTCTTAATATTTATTTTTATATTTTGATTTTTTTTTTTT
TTTTTTTT

TATTTGGAATGGGCATCGATTTAAAATTTTCATTTAGTGGGATGTAAAAAAAAAATTTTAGTTCGCGCGTAC
AGTTTTTCAACATTTGCCATATGATGTAGAATGGCGCTATCAAATCATCACTCTTATTACAGGTGAATCGC
GCGCTAAC

CTAATCATACAGTTTCTTGTTTCAATTTTTTAGATTTTAATTAGCGGTTTTTTTTTTGGTTTTTCTTAAGAT
TGGTTTTATGATTTTTTTTTTTTCTTTTTCAAATTTTTTTATTATTTTTTCAATTTTTCTTTTTAAAAGGCT
TGTGGTTG

AGTTTCTTGTTTCAATTTTTTAGATTTTAATTAGCGGTTTTTTTTTTGGTTTTTCTTAAGATTGGTTTTATG
ATTTTTTTTTTTCTTTTTCAAATTTTTTTATTATTTTTTCAATTTTTCTTTTTAAAAGGCTTGTGGTTGAC
TAGTCCTT

CTAATCATGACATTTTTTAAAAGTTTTCAATGAAAAAATGAAAGAGTAATGAACAAACATATTTTTTTTAT
AAAATGCATAAAAACATATAGATATATATATATTTACGTGGAAAAATGTTAAAACTATTGATAAGAAAAAA
CGCTCAAA

CACCCATTACATTCGATTTTTTTTTTTAATTATATTTTTGACTATTATTTAATCATTAAAAACACATATATT
TCATAAATTCGTAAGGTCGTTAGTTCTATCGTAAAAGTGAAAAAGTTTTGAGCCAGTTTTCAAAAACCCGC
AGGATCCC

AGTGTGCTTTTTTCTTCTATTTTTTTTTTCTTTAATGACTTTGTGTGTTTATTACCCTTTAATTAATTACCA
AAAAAAAAATAAACTACGTTTTTAATTTCTAATTTATCAAAATTTCTTTTTTTCACATATAACTATATATT
TATATGTC

AAAGAGTAATGAACAAACATATTTTTTTTATAAAATGCATAAAAACATATAGATATATATATATTTACGTG
GAAAAATGTTAAAACTATTGATAAGAAAAAACGCTCAAAACAAGTAGTAAGCAGGCTTTTTTTTTTTTTGC
TGCTTGTT

CTTTTTTTTTTTTTTTTTTCAGACGTGGAATGGTGGGAAAGCCAAAATGTCACTGACCGCGGCTAAAAGTT
AAAAAATACATGAGACGACATTCTTTTATGTATATATATTTACACATGTATACAAACAAATACACACGCAA
GTAGAGAG

TCACGTGCAGCTTTTTAAAAATATTTTAAAACATTTTAAAAAATATACATTTTTTTATTATTTTTTTATAT
ATTAATGTTAAAATTTATTTATGTATTTGTCTTCCGAAAAGTAAAATAACGTTCAAATTTGGTATTTAAGG
ATATCTGC

TGACCATATGAGTAAGCAGTATATAACGCGAGGTTCAATGGCCTCTTTACCATGAAAAAAAAAAAAAAAAA
AAAAAAAAGGTAAGGAAAAAGAGTATTTTCAATTCGTTTCTGAACATATAAATATAAATAACCGAAAAATT
AGCCCTTG

TTTTTTTTTTTTTTTTTCCGTGCGTTCTCCCCGTGCCTTGATAAGCAATAATGGAAAAAATAAAAATAGCAG
CAATTGGTTATACAATGTATATATAAAACATGAATGATTGAATAATTTACAAGCGAAATAATCGCATTATA
TACGTGGC

AGGCGTATTCCGTATTGAATGATTGAAAAATTTATTTCTTTTTTTATTTCTTTTTTTTTTTTTTTTTTTTT
TTTTTACGCCGATGCTCATCGCAGAAAATTTTTCCTTCAGTTTATTTGTCTTATAAAAAGACTGTCCTACG
CTCAAATA

TAAGTAAAAAGAACGGATTATGAAAGAAAACGCGCATAATATATATATGTATGTATTTTCTAATTTATATT
AGCAACTAAATTAAATTACATCAATACTATTTTTTTTTTTTTACTTTACAAGGTTTATTTCAAAGTCTTCAA
CAATTTTT

ATATTAGTTACTAATCATACAGTTTCTTGTTTCAATTTTTTAGATTTTAATTAGCGGTTTTTTTTTTGGTTT
TTCTTAAGATTGGTTTTATGATTTTTTTTTTTCTTTTTCAAATTTTTTTATTATTTTTTCAATTTTTCTTT
TTAAAAGG

TTTTACTTTTTTTTTTTTTTTTTTTTTTTTTTATTATTTACAAGCGTCTATATATTTTCTATTATAGAATA
TTGTCATTTATTACATTGGTTCATCTGTAAATCTCATCAAACGGTCTCGTATCGGCGAAGCAACTGTCCCA
AACTTCAT

AGGTTAAAATAGAAGGCCGATGAGGCCTTATGTAAGTCTAATATGGTATTCTTAAAAAAAAAAAAAAAAAA
AAAGCGTCTTAGAGGGAATATAAGAGAAAAGAAGAGATAGCATAACACATGATTTATTTTTATTTTTGCGT
TTTTTTTT

GCCTCTTTACCATGAAAAAAAAAAAAAAAAAAAAAAAAAAAGGTAAGGAAAAAGAGTATTTTCAATTCGTTTC
TGAACATATAAATATAAATAACCGAAAAATTAGCCCTTGAACATAATTAACACTCTTCTTTGATATTTAAA
TCACAAGT

TCATGTGTACGTTGTACTTGACATTCTAAGGCATCCTGCAACTGTTCTGTGGAGCTATTAAATCTTTATAG
TAAATTTTTTTTTACTTTTTTTTTTTTTTTTTTTTTTTTTTATTATTTACAAGCGTCTATATATTTTCTAT
TATAGAAT

CTTTTTAAAAATATTTTAAAACATTTTAAAAAATATACATTTTTTTATTATTTTTTTATATATTAATGTTA
AAATTTATTTATGTATTTGTCTTCCGAAAAGTAAAATAACGTTCAAATTTGGTATTTAAGGATATCTGCGT
TTAGGCGC

AAACAAACACATTCCCACAAAACACCGCACCGCGAAAAAAAAAAAAACAGGAAAAAAACGCGAAATGTCCG
TAACTTTACCCGGTAATAGTCACGTGATAAAGGTGCATTGGTCTAGCTGGCTGTTCTGGCCAACGCGACAG
CAGGGTTC

TGTTTAGTGAGGGTACTATTTAAACTTACTTTAAATAGCAAAATAGGAAAAGCTACGAGAAACTGAAAAGT
AAAAAAAAAAAAAAAAAATAAAAAAAAAAGAAAAAAAAAGGAGAAACATGATTGCCAGTACTCGCAAGAAC
TAAGATAG

TCTTCCGCTTTAAAAGTTGAAAATTCCTCAATGAAAAGGGTGTTCTTGGCTTTACAAGCGCATTAAACATT
TAATATTATATAATATTTATAATATACATGTTATTCCAATAGTGAAAAAAAAAAAAAAATGAAAATGAAAA
TGAAAAAT

ACAAGGTTTATTTTGCTTTGGTTGCAAATTTTCATCGCTTATAGAAGACATATTTTGATGTAAAAACCTTG
ATATACTTCCTTTTTGCCTTATCTTTCACTGTTTCCATCTCTTTTCTTTTTTTTTTTTTTTTTTTTTTTTT
TTTTTTTT

TGAAAATAAAATTCGCCAGTAAATGCGTATCTTGGAGAAAATATCAATTTGTTATTTTGTATATATAAAAA
ATTAAAAAAAAAAAAAAAAAAATACTCAAATACTCAAATACGTATACATTATACTGAAATACTTCGCTTGT
CTTCGAAT

CGTATTGAATGATTGAAAAATTTATTTCTTTTTTTATTTCTTTTTTTTTTTTTTTTTTTTTTTTTTACGCC
GATGCTCATCGCAGAAAATTTTTCCTTCAGTTTATTTGTCTTATAAAAAGACTGTCCTACGCTCAAATAAC
TTATACTT

CCATTTTTTTATTGATTTTATTTTTTTTTGTTACCCCTAACGATATTACAGCTATTTCCTAATGGCTTTTA
ATGACATTAATGACTTTATGACAACCATGATAGTACAGAAGAGAGACCTTTTTTCTTTTTTTTTTTTTTTT
CTTTTTTT

ATTGCTGATTTGACCATATGAGTAAGCAGTATATAACGCGAGGTTCAATGGCCTCTTTACCATGAAAAAAA
AAAAAAAAAAAAAAAAAAGGTAAGGAAAAAGAGTATTTTCAATTCGTTTCTGAACATATAAATATAAATAA
CCGAAAAA

TCATTCCCTTCCGCGCCAGTATATTAGTTACTAATCATACAGTTTCTTGTTTCAATTTTTTAGATTTTAAT
TAGCGGTTTTTTTTTGGTTTTTCTTAAGATTGGTTTTATGATTTTTTTTTTTCTTTTTCAAATTTTTTTAT
TATTTTTT

TAATATATTCTAAGTAAAAAGAACGGATTATGAAAGAAAACGCGCATAATATATATATGTATGTATTTTCT
AATTTATATTAGCAACTAAATTAAATTACATCAATACTATTTTTTTTTTTTACTTTACAAGGTTTATTTCA
AAGTCTTC

GAACAAACATATTTTTTTTATAAAATGCATAAAAACATATAGATATATATATATTTACGTGGAAAAATGTT
AAAACTATTGATAAGAAAAAACGCTCAAAACAAGTAGTAAGCAGGCTTTTTTTTTTTTTGCTGCTTGTTTT
AGAGAACC

TAGATTTTAATTAGCGGTTTTTTTTTGGTTTTTCTTAAGATTGGTTTTATGATTTTTTTTTTTCTTTTTCA
AATTTTTTTATTATTTTTTCAATTTTTCTTTTTAAAAGGCTTGTGGTTGACTAGTCCTTTCTTTTTAGGGG
TTGCTTTC

TTTTTTTTTTTTTTTTTTTTTTTTTTTTTTTTTTTTTTTGAATCTTAAAATTCTCGAAACTGCACTGAAAT
GGCGCAGGAAACTGACCTCATCGCATGCCAACGAAGATTGAAAAAAATTAACATATAAGGCTTATGAAAGC
GACATCTT

ATAATTACCCATCTATGTTGTTTCCCCAAGAATGTTTGCTGCCATATAATAAGAACAAAAAAAAAAATAAC
AAAAAAAAAGAAAAAAAAAATCACTTGTAATCTTAAATTAATATTATACACCGGTTGTCCGTTTTTACCTC
TATGCCGT

GATTGAAAAATTTATTTCTTTTTTTATTTCTTTTTTTTTTTTTTTTTTTTTTTTTTACGCCGATGCTCATC
GCAGAAAATTTTTCCTTCAGTTTATTTGTCTTATAAAAAGACTGTCCTACGCTCAAATAACTTATACTTTT
CTGTATCT

ACGCGGTCGCGCGCCGTTTGACGTCCTATAGGAGAATGCCAAAAAGGGTGTTTTGCTGGAAAATATACATA
TATAAATATATATAAAGAATAGTGAAACAATAGAAATATTAGTAATACAGCCATAGCAGTAGTAGTGATAG
TACTAGCA

TTTCTTCTATTTTTTTTTCTTTAATGACTTTGTGTGTTTATTACCCTTTAATTAATTACCAAAAAAAAAAT
AAACTACGTTTTTAATTTCTAATTTATCAAAATTTCTTTTTTTCACATATAACTATATATTTATATGTCAC
CCTATTCA

TCTTGATACTCTGTATTTAGTACACAATAACGCCGACGAAAAATCTAATTAAGAAGGACACTATAAATAAC
AAACAATCGCGCGGAACGTCAAGCGTATATACTATATAAAAAGAAAACAAAAAAAATCAATAACCCATTAA
AGATACAT

TTGTTTTGTCCTTTTGCAGTTTGCTTTTATCCCTGTGATGAAAAAAAAAAAAACTAAAATTTTTTGCCGGC
GCATCGAAAAAATCTGTTACCCGACCCGTTATTGGTGATAATACCCATTTTTAACGCGATCACATATCATA
AACTGCAG

TTCAAAGAGCGAAAAGAAGGCGCGTCGCGTCGACGCGCCTTTTTAGGCTAGAAAATAAACAGAAAACAAAA
ACAAAAACAAAAAAAGGCGAAAAAACAAACGAAAAAACAAACGACAGTAGATAGAGGAGAAGGTTTTTGAC
AGGTTTGT

CATGAAAAAAAAAAAAAAAAAAAAAAAAAGGTAAGGAAAAAGAGTATTTTCAATTCGTTTCTGAACATATA
AATATAAATAACCGAAAAATTAGCCCTTGAACATAATTAACACTCTTCTTTGATATTTAAATCACAAGTAC
TTTTCTTT

GCCGCAATTAATTGAGTTGTTGTGAAATGGTTCTAGTTAGTCACGTGCAGCTTTTTAAAAATATTTTAAAA
CATTTTAAAAAATATACATTTTTTTATTATTTTTTTATATATTAATGTTAAAATTTATTTATGTATTTGTC
TTCCGAAA

ATTCCCACAAAACACCGCACCGCGAAAAAAAAAAAAAACAGGAAAAAACGCGAAATGTCCGTAACTTTACC
CGGTAATAGTCACGTGATAAAGGTGCATTGGTCTAGCTGGCTGTTCTGGCCAACGCGACAGCAGGGTTCAT
ACTTTCAG

GAAATCGCGTGGCTTGGTAACGCGATAATGACGCGACACAGCCATTAGTGTGAAATTTGATTTTCTTGGCC
AAACTAGGTATAATATTTGTTACAAATTATTGATTTTAATATATATCTCGGGTTCATTTTTTACGTCGGTA
CTCCAAAG

TAAAAATTTTTGCAGACATTTTTGATATATATTCAATTCATATTTTATCTCTTTTTTTTTTTTTTTTTTCA
GACGTGGAATGGTGGGAAAGCCAAAATGTCACTGACCGCGGCTAAAAGTTAAAAAATACATGAGACGACAT
TCTTTTAT

TTAAATAGCAAAATAGGAAAAGCTACGAGAAACTGAAAAGTAAAAAAAAAAAAAAAAAATAAAAAAAAAAG
AAAAAAAAAGGAGAAACATGATTGCCAGTACTCGCAAGAACTAAGATAGAGAGAAAAAGGACGATATATCA
AGAAATAA

TATTTCTACGCTATACAAAAAGTGTGCTTTTTTCTTCTATTTTTTTTTCTTTAATGACTTTGTGTGTTTAT
TACCCTTTAATTAATTACCAAAAAAAAAAATAAACTACGTTTTTAATTTCTAATTTATCAAAATTTCTTTTT
TTCACATA

CGTGTATTATTTCAAAGAGCGAAAAGAAGGCGCGTCGCGTCGACGCGCCTTTTTAGGCTAGAAAATAAACA
GAAAACAAAAACAAAAACAAAAAAAGGCGAAAAAACAAACGAAAAAACAAACGACAGTAGATAGAGGAGAA
GGTTTTTG

ATTGAGTTGTATTGCTGATTTGACCATATGAGTAAGCAGTATATAACGCGAGGTTCAATGGCCTCTTTACC
ATGAAAAAAAAAAAAAAAAAAAAAAAAAAGGTAAGGAAAAAGAGTATTTTCAATTCGTTTCTGAACATATAA
ATATAAAT

TCAAGTTTGTCATGGAATATGAAATAGAAAAGGAGTGATGGTTTTCTTTATTTTTAACCCTGTAAAATTTT
TTTATTTTTTTATTTTTACTCTTATATATGTATATGTTTGTGTTTATTTCTGAATAAAAATTTATAAGAAC
CAAATTAA

TCATTTTTTTTTTGATTGTCTTGCAGAAGTTGAAGAGGTCTTTACTGTAGCATATACAAGTGCTTAATTCT
TGATTTTTATTTTCTTTTTTTCATAATACACGTTATAGAAAAAGCCCCATTCTCAGTTTATGAAAAAACGC
GATATCAC

TTTATTTCTCGTCTACTATAATAATTCACCAAAAAACAGCAGAGCACGTCCTATATATATATATATATATA
TATATATATATACATATATATATACGAATATATTTATGACAGTCTAAATCGTTGCTCCTGTTCAATTTTAA
CGCTCTTT

AAAGAAAACAAAAAAAATCAATAACCCATTAAAGATACATAATCAATAAAAAACGATATTAAAAAGCACGT
AAAGTACTTAATATTTTTTTAATAATAGACATTTTCGCCCTTACTTTTTAAATAAAAAACATAAAAAAATA
TCAAATGT

AAAAAAATCAATAACCCATTAAAGATACATAATCAATAAAAAACGATATTAAAAAGCACGTAAAGTACTTA
ATATTTTTTTAATAATAGACATTTTCGCCCTTACTTTTTAAATAAAAAACATAAAAAAATATCAAATGTAC
ATCAAAAT

ATGCTATTGTGAGGTTAGAATGAAAATAAAATTCGCCAGTAAATGCGTATCTTGGAGAAAATATCAATTTG
TTATTTTGTATATATAAAAAATTAAAAAAAAAAAAAAAAAAATACTCAAATACTCAAATACGTATACATTA
TACTGAAA

AAAGATGAACCACCCATTACATTCGATTTTTTTTTTAATTATATTTTTGACTATTATTTAATCATTAAAAA
CACATATATTTCATAAATTCGTAAGGTCGTTAGTTCTATCGTAAAAGTGAAAAAGTTTTGAGCCAGTTTTC
AAAAACCC

CGATTGTAAATTTATTTCTCGTCTACTATAATAATTCACCAAAAAACAGCAGAGCACGTCCTATATATATA
TATATATATATATATATATATACATATATATATACGAATATATTTATGACAGTCTAAATCGTTGCTCCTGT
TCAATTTT

ACACTTAATTCGTATCATTCGAAACGGAAAAAGAAATGTGGATTTTCTTTTTCAATTTTTTTTTTTTTCAT
GATTTTTTTGGGCGACGCAGAGATGAGATAAAAAAATTTTTCAACTGCTATTCTCAATCGAATTTTTTTG
TTATCCTC

ATTTTCAATTTTCATCTTTTTTTTCACGATGCGAAGTTTCTTAGATAATTATCGCCATATACGAAACGCGT
TAGGAAACGCGTTCTTTCCTTTATAATAAGCAATATTATATCAACTTAAGGTGGAAGTAATGTGGCAAAAT
CGATAGCG

TGAGGCCTTATGTAAGTCTAATATGGTATTCTTAAAAAAAAAAAAAAAAAAAAAAGCGTCTTAGAGGGAATA
TAAGAGAAAAGAAGAGATAGCATAACACATGATTTATTTTTATTTTTGCGTTTTTTTTTTTCTTAATTGT
TTATCAAT

TTCAAAAGTACGTATATATGTATATACCATCTTTTTTTACGTTAAAATAGTGAAAAAGGAAAGGAGGAAAG
AAAAAACTAATATAATTAATTGTATTATATATGTACTTTTCATTTTGGAATGTGAAAAAATTCCAGGCGGA
AAAAATTG

GAAAAGAAGGCGCGTCGCGTCGACGCGCCTTTTTAGGCTAGAAAATAAACAGAAAACAAAAACAAAAACAA
AAAAAGGCGAAAAAACAAACGAAAAAACAAACGACAGTAGATAGAGGAGAAGGTTTTTGACAGGTTTGTGT
AATTGGTT

GGGCATCGATTTAAAATTTTCATTTAGTGGGATGTAAAAAAAAATTTTAGTTCGCGCGTACAGTTTTTCAA
CATTTGCCATATGATGTAGAATGGCGCTATCAAATCATCACTCTTATTACAGGTGAATCGCGCGCTAACAC
TTCAATTA

GACTGATTCGTAAAAATTTTTGCAGACATTTTTGATATATATTCAATTCATATTTTATCTCTTTTTTTTTT
TTTTTTTTCAGACGTGGAATGGTGGGAAAGCCAAAATGTCACTGACCGCGGCTAAAAGTTAAAAAATACAT
GAGACGAC

TATATATATATATATATATACGTGCATTTTTCTTAAAGAAATATAACTTTTTTCTTGTACTGCCTGCAATCTC
TATTCTTCATTCATCACACATCTATTCAAACGCGTTAAAATTTTTTTGTTAGCCTATAAGTAAAACGCGTC
GCGTGTAT

ATATGGTATTCTTAAAAAAAAAAAAAAAAAAAAAAGCGTCTTAGAGGGAATATAAGAGAAAAGAAGAGATAG
CATAACACATGATTTATTTTTATTTTTGCGTTTTTTTTTTTTCTTAATTGTTTATCAATGTATCTGTGAGA
GTATCTAC

AAAATTTTCTCCATTTTTTTATTGATTTTATTTTTTTTTGTTACCCCTAACGATATTACAGCTATTTCCTA
ATGGCTTTTAATGACATTAATGACTTTATGACAACCATGATAGTACAGAAGAGAGACCTTTTTTCTTTTTT
TTTTTTTT

TACCCCTTCCCACAGATATATAATATATTCTAAGTAAAAAGAACGGATTATGAAAGAAAACGCGCATAATA
TATATATGTATGTATTTTCTAATTTATATTAGCAACTAAATTAAATTACATCAATACTATTTTTTTTTTTT
ACTTTACA

TTTACAAGCGCATTAAACATTTAATATTATATAATATTTATAATATACATGTTATTCCAATAGTGAAAAAA
AAAAAAAAATGAAAATGAAAATGAAAAATTCGGCCCTTAAAAATGAACAACCTTTTACCAGCCGGCATTAA
ATGTCGTG

CTTTTGCAGTTTGCTTTTATCCCTGTGATGAAAAAAAAAAAAAACTAAAATTTTTTGCCGGCGCATCGAAAA
AATCTGTTACCCGACCCGTTATTGGTGATAATACCCATTTTTAACGCGATCACATATCATAAACTGCAGTG
AGAGGGTC

TTTTTTTTCTTTAATGACTTTGTGTGTTTATTACCCTTTAATTAATTACCAAAAAAAAAAATAAACTACGTT
TTTAATTTCTAATTTATCAAAATTTCTTTTTTTCACATATAACTATATATTTATATGTCACCCTATTCAAG
TTCGTTAG

ATGAAGATTTTTAATTTGAAGTCGAGTACATCTCTTTCAGTGAGTAACTTATATATTTTTGAAGTTAACAT
CCTTATTCTTTTTTTATATTTTTATTTTTTTTATGTTTATTTTCTATTACTGATTTTCACAAAGAAAGGTA
AAAAAAAA

TATATACCATCTTTTTTTACGTTAAAATAGTGAAAAAGGAAAGGAGGAAAGAAAAAACTAATATAATTAAT
TGTATTATATATGTACTTTTCATTTTGGAATGTGAAAAAATTCCAGGCGGAAAAAATTGAAAAAATCAAAC
ACTGCGCT

GGGTACTATTTAAACTTACTTTAAATAGCAAAATAGGAAAAGCTACGAGAAACTGAAAAGTAAAAAAAAAA
AAAAAAAATAAAAAAAAAAGAAAAAAAAAGGAGAAACATGATTGCCAGTACTCGCAAGAACTAAGATAGAG
AGAAAAAG

TAGAAAAGGAACAAATGGTAGGTCTTTAAGATAAGAAAGATAAGATAAGACATCTTTTTTTTTTTTTTTT
TTTTTTTTTTTTTTTCCGATAACAAAGAAGAATCCTTCGGTCTCTTCTTACTGCTGTTACTGCTTATCAAG
AGTGTCTG

GAAATAGAAAAGGAGTGATGGTTTTCTTTATTTTTAACCCTGTAAAATTTTTTTATTTTTTTTATTTTTACT
CTTATATATGTATATGTTTGTGTTTATTTCTGAATAAAAATTTATAAGAACCAAATTAAAACCGTTAATAG
CCCACCCA

TTAATGACTTTGTGTGTTTATTACCCTTTAATTAATTACCAAAAAAAAAATAAACTACGTTTTTAATTTCT
AATTTATCAAAATTTCTTTTTTTCACATATAACTATATATTTATATGTCACCCTATTCAAGTTCGTTAGTT
TTCTTTCA

GGTATTTTTTTTTTTTTTTATTGATTTTTTTTTCACTCTTATTTTTTCCCTCATTTATTCATCATACATTTA
ACGAACTCTATTTCTTTTCAAAATCCTTATCTACCTATTTTTTTTTCTTTATTTTCTTTCCTCTGTCAGTT
AGTCAGTA

AAAAAAAGCTGAAAGCTTAAAGAAGCAAATTTTTGAGAAGGTTCAGAAAGAATAAAATAACAGACTATACA
GTTTAATTATATATATATATATATATACGTGCATTTTTCTTAAAGAAATATAACTTTTTTCTTGTACTGCC
TGCAATCT

CGCGATAATGACGCGACACAGCCATTAGTGTGAAATTTGATTTTCTTGGCCAAACTAGGTATAATATTTGT
TACAAATTATTGATTTTAATATATATCTCGGGTTCATTTTTTACGTCGGTACTCCAAAGGATCAAAACACT
TACATTTT

GATGGCTGGTTTGTCTTTTTAAGTTCTTAATATTTATTTTTATATTTTGATTTTTTTTTTTTTTTTTTT
TCCATTAGTGAATGACAGCAATGTATTAAGCTGCTGCCGCGCATACAAAATGTACTGCGTTAAATTAGATT
AGCTTAAA

CACAGATATATAATATATTCTAAGTAAAAAGAACGGATTATGAAAGAAAACGCGCATAATATATATATGTA
TGTATTTTCTAATTTATATTAGCAACTAAATTAAATTACATCAATACTATTTTTTTTTTTTACTTTACAAG
GTTTATTT

GAAAGCTTAAAGAAGCAAATTTTTGAGAAGGTTCAGAAAGAATAAAATAACAGACTATACAGTTTAATTAT
ATATATATATATATATACGTGCATTTTTCTTAAAGAAATATAACTTTTTTCTTGTACTGCCTGCAATCTCT
ATTCTTCA

TGCTGATGCATCAAGTTTGTCATGGAATATGAAATAGAAAAGGAGTGATGGTTTTCTTTATTTTTAACCCT
GTAAAATTTTTTTATTTTTTTTATTTTTACTCTTATATATGTATATGTTTGTGTTTATTTCTGAATAAAAT
TTATAAGA

CGCGAAAAAAAAAAAAAACAGGAAAAAACGCGAAATGTCCGTAACTTTACCCGGTAATAGTCACGTGATAA
AGGTGCATTGGTCTAGCTGGCTGTTCTGGCCAACGCGACAGCAGGGTTCATACTTTCAGTAGAACAAAAAT
CGGGGGAA

ATAATTCACCAAAAAACAGCAGAGCACGTCCTATATATATATATATATATATATATATATATACATATATA
TATACGAATATATTTATGACAGTCTAAATCGTTGCTCCTGTTCAATTTTAACGCTCTTTTATAACTGCTGT
GGCGCCAT

TTGATATATTATTGAGTTGTATTGCTGATTTGACCATATGAGTAAGCAGTATATAACGCGAGGTTCAATGG
CCTCTTTACCATGAAAAAAAAAAAAAAAAAAAAAAAAAAGGTAAGGAAAAAGAGTATTTTCAATTCGTTTCT
GAACATAT

TAAACTTACTTTAAATAGCAAAATAGGAAAAGCTACGAGAAACTGAAAAGTAAAAAAAAAAAAAAAAAATA
AAAAAAAAAGAAAAAAAAAAGGAGAAACATGATTGCCAGTACTCGCAAGAACTAAGATAGAGAGAAAAAGGA
CGATATAT

ATCCATTGATACGGCCACGCCCGTTAAAACGCTAAGATATATATATATGTAGCTAAGATATATATATATGT
ATATATTCGGTATTTAGTAGATATTTCCTTAAGGCGATATTCGCCCGAGAGCTATTTCACAGTTTTTTTT
GCAGTCGT

TTAGCGGTTTTTTTTTGGTTTTTCTTAAGATTGGTTTTATGATTTTTTTTTTTCTTTTTCAAATTTTTTTA
TTATTTTTTCAATTTTTCTTTTTAAAAGGCTTGTGGTTGACTAGTCCTTTCTTTTTAGGGGTTGCTTTCTA
TGAGAACG

TATTACGGTTTTGATATATTATTGAGTTGTATTGCTGATTTGACCATATGAGTAAGCAGTATATAACGCGA
GGTTCAATGGCCTCTTTACCATGAAAAAAAAAAAAAAAAAAAAAAAAAAAGGTAAGGAAAAAGAGTATTTTCA
ATTCGTTT

TTAAAATTTTCATTTAGTGGGATGTAAAAAAAAAATTTTAGTTCGCGCGTACAGTTTTTCAACATTTGCCAT
ATGATGTAGAATGGCGCTATCAAATCATCACTCTTATTACAGGTGAATCGCGCGCTAACACTTCAATTACA
GACACATA

AGATAAAGGGGGCTTTGACGTATAAGTGTCGGTATTTTTTTTTTTTTTTATTGATTTTTTTTTTCACTCTTAT
TTTTTCCCTCATTTATTCATCATACATTTAACGAACTCTATTTCTTTTCAAAATCCTTATCTACCTATTTT
TTTTTCTT

GCAGGAAATGAAAGATGAACCACCCATTACATTCGATTTTTTTTTTTAATTATATTTTTGACTATTATTTAA
TCATTAAAAACACATATATTTCATAAATTCGTAAGGTCGTTAGTTCTATCGTAAAAGTGAAAAAGTTTTGA
GCCAGTTT

CGTATCATTCGAAACGGAAAAAGAAATGTGGATTTTCTTTTTCAATTTTTTTTTTTTTCATGATTTTTTTG
GGCGACGCAGAGATGAGATAAAAAAAATTTTTCAACTGCTATTCTCAATCGAATTTTTTTTGTTATCCTCAC
CTATTTGT

GAGGTTAGAATGAAAATAAAATTCGCCAGTAAATGCGTATCTTGGAGAAAATATCAATTTGTTATTTTGTA
TATATAAAAAATTAAAAAAAAAAAAAAAAAAAATACTCAAATACTCAAATACGTATACATTATACTGAAATA
CTTCGCTT

CTGTATTTAGTACACAATAACGCCGACGAAAAATCTAATTAAGAAGGACACTATAAATAACAAACAATCGC
GCGGAACGTCAAGCGTATATACTATATAAAAAGAAAACAAAAAAAATCAATAACCCATTAAAGATACATAA
TCAATAAA

TTTTTATTTCTTTTTTTTTTTTTTTTTTTTTTTTTTACGCCGATGCTCATCGCAGAAAATTTTTCCTTCAG
TTTATTTGTCTTATAAAAAGACTGTCCTACGCTCAAATAACTTATACTTTTCTGTATCTCATTCAAATTAT
TTTCTTGT

TCTTGTACTGCCTGCAATCTCTATTCTTCATTCATCACACATCTATTCAAACGCGTTAAAATTTTTTTGTT
AGCCTATAAGTAAAACGCGTCGCGTGTATCCAATTTTACTGAATTAAATTCTTTACTCATTGGATGATATA
TATCTTCT

GCAAATCAATGATAAGTACAAGTCCAATCGGACTGATTCGTAAAAATTTTTGCAGACATTTTTGATATATA
TTCAATTCATATTTTATCTCTTTTTTTTTTTTTTTTTTCAGACGTGGAATGGTGGGAAAGCCAAAATGTCA
CTGACCGC

AACACCGCACCGCGAAAAAAAAAAAAAAACAGGAAAAAACGCGAAATGTCCGTAACTTTACCCGGTAATAGT
CACGTGATAAAGGTGCATTGGTCTAGCTGGCTGTTCTGGCCAACGCGACAGCAGGGTTCATACTTTCAGTA
GAACAAAA

AACTGAAAAGTAAAAAAAAAAAAAAAAAATAAAAAAAAAAGAAAAAAAAAAGGAGAAACATGATTGCCAGTA
CTCGCAAGAACTAAGATAGAGAGAAAAAGGACGATATATCAAGAAATAAAAGGGAAGGTAATTCAAGTAAG
CACTGCTT

ACCCAAAAAAAAAAAAAAGACATCAAATCTCTTTTTTTTTTTTTTGTAGTTCTGTTGCCGTATGCTATCTA
CTCCTAAGGCTTTTTTCTACTACGGCAATCTATTCACAATGTGCAATTAGGCTAAATGCTAAAAAAAGAAA
AAAAAATC

AATGATGGGTCAAGGATCGTGCTTTTGTTCGTTGGTGGATCGGCCGTACGTGTTTAGTGAGGGTACTATTT
AAACTTACTTTAAATAGCAAAATAGGAAAAGCTACGAGAAACTGAAAAGTAAAAAAAAAAAAAAAAAATAA
AAAAAAAA

TATCTTGCAGATCCATTGATACGGCCACGCCCGTTAAAACGCTAAGATATATATATATGTAGCTAAGATAT
ATATATATGTATATATTCGGTATTTAGTAGATATTTCCTTAAGGCGATATTCGCCCGAGAGCTATTTCACA
GTTTTTTT

TGCATTTTTCTTAAAGAAATATAACTTTTTTCTTGTACTGCCTGCAATCTCTATTCTTCATTCATCACACA
TCTATTCAAACGCGTTAAAATTTTTTTGTTAGCCTATAAGTAAAACGCGTCGCGTGTATCCAATTTTACTG
AATTAAAT

TGTAAGTCTAATATGGTATTCTTAAAAAAAAAAAAAAAAAAAAAGCGTCTTAGAGGGAATATAAGAGAAAA
GAAGAGATAGCATAACACATGATTTATTTTTATTTTTGCGTTTTTTTTTTTTCTTAATTGTTTATCAATGT
ATCTGTGA

AATCAATCTTAACGCATATGCCCTTGTCAGAGCCGTCCTTCGAATAAGCATATTTGGAATGGGCATCGATT
TAAAATTTTTCATTTAGTGGGATGTAAAAAAAAATTTTAGTTCGCGTACAGTTTTTTCAACATTTGCCATA
TGATGTAG

AGAAGGCCGATGAGGCCTTATGTAAGTCTAATATGGTATTCTTAAAAAAAAAAAAAAAAAAAAAGCGTCTT
AGAGGGAATATAAGAGAAAAGAAGAGATAGCATAACACATGATTTATTTTTATTTTTGCGTTTTTTTTTTT
TCTTAATT

CGAAATGTAACTAATCATGACATTTTTTAAAAGTTTTCAATGAAAAAATGAAAGAGTAATGAACAAACATA
TTTTTTTTATAAAATGCATAAAAACATATAGATATATATATATTTACGTGGAAAAATGTTAAAACTATTGA
TAAGAAAA

CTTTTTTTACGTTAAAATAGTGAAAAAGGAAAGGAGGAAAGAAAAAACTAATATAATTAATTGTATTATAT
ATGTACTTTTCATTTTGGAATGTGAAAAAATTCCAGGCGGAAAAAATTGAAAAAATCAAACACTGCGCTTG
TGAGAAAA

ACGCGTCCCTTTGGAGAAGATCGATCCTTCTTTGGGTCTTTCAATTTGATTGGAAAAAAAATATTTAAAAA
AATAGAAGAAAAATATAGAATATATAGAATATATAGAATCAACTACTGCAGTAGATAAATAAAGAAGACTA
TTCATATT

ATTAGTGTTAATAGCGGTGACAGTAGTTTTATTGTATCTCACCTTTTTTTATGTATAATCATATAATGACT
TAATTATTTTTGAAATGTTTCTTTTTTTTTTTTTAGTTATAACCCTTTTTACGGATTCTTACGTACTTATT
TAATCTTT

TTATTGAAAATCCTTTGGAAATGCTATTGTGAGGTTAGAATGAAAATAAAATTCGCCAGTAAATGCGTATC
TTGGAGAAAATATCAATTTGTTATTTGTATATATAAAAAATTAAAAAAAAAAAAAAAAAAAATACTCAAAT
ACTCAAAT

TTAATTTGAAGTCGAGTACATCTCTTTCAGTGAGTAACTTATATATTTTTGAAGTTAACATCCTTATTCTT
TTTTTATATTTTTATTTTTTTTATGTTTATTTTCTATTACTGATTTTCACAAAGAAAGGTAAAAAAAAACA
TACTTCAG

TATAAGTGTCGGTATTTTTTTTTTTTTTTATTGATTTTTTTTCACTCTTATTTTTTCCCTCATTTATTCAT
CATACATTTAACGAACTCTATTTCTTTTCAAAATCCTTATCTACCTATTTTTTTTTTCTTTATTTCTTTCC
TCTGTCAG

ATGCAGACATTCTTCCGCTTTAAAAGTTGAAAATTCCTCAATGAAAAGGGTGTTCTTGGCTTTACAAGCGC
ATTAAACATTTAATATTATATAATATTTATAATATACATGTTATTCCAATAGTGAAAAAAAAAAAAAAATG
AAAATGAA

TTTTTTTTTTTTTTTTTTTTTTATTATTTACAAGCGTCTATATATTTTCTATTATAGAATATTGTCATTTA
TTACATTGGTTCATCTGTAAATCTCATCAAACGGTCTCGTATCGGCGAAGCAACTGTCCCAAACTTCATCT
ACAATTTT

TTTTTTTTTTTTTTTTCAGTTTTAAAATTAGTCCTGATCAAAAAGCCATCACGTGGATCCCCGAAAAAAAA
CGGATCCGGGTAACCAGAAGTATTTTTTTCAAGGGAATAAAAGTTACCCTACCAAAATAAAAAAATTCAAG
TAGGGTAA

CTTGGAGAAAATATCAATTTGTTATTTTGTATATATAAAAAATTAAAAAAAAAAAAAAAAAAATACTCAAA
TACTCAAATACGTATACATTATACTGAAATACTTCGCTTGTCTTCGAATCTAAATCCTCTCTCTCTTCTGT
CTCAAAAT

CGTATATATGTATATACCATCTTTTTTTACGTTAAAATAGTGAAAAAGGAAAGGAGGAAAGAAAAAACTAA
TATAATTAATTGTATTATATATGTACTTTTCATTTTGGAATGTGAAAAAATTCCAGGCGGAAAAAATTGAA
AAAATCAA

ACTATCCATCCGTGTATTATTTCAAAGAGCGAAAAGAAGGCGCGTCGCGTCGACGCGCCTTTTTAGGCTAG
AAAATAAACAGAAAACAAAAACAAAAACAAAAAAAGGCGAAAAAACAAACGAAAAAACAAACGACAGTAGA
TAGAGGAG

TTTCTCTCCCCGATTGTAAATTTATTTCTCGTCTACTATAATAATTCACCAAAAAACAGCAGAGCACGTCC
TATATATATATATATATATATATATATATATACATATATATATACGAATATATTTATGACAGTCTAAATCG
TTGCTCCT

GCGGCTGCTCGTATAAAATGACGCGGTCGCGCGCCGTTTGACGTCCTATAGGAGAATGCCAAAAAGGGTGT
TTTGCTGGAAAATATACATATATAAATATATATAAAGAATAGTGAAACAATAGAAATATTAGTAATACAGC
CATAGCAG

CGCATAACGAGCAAATCAATGATAAGTACAAGTCCAATCGGACTGATTCGTAAAAATTTTTGCAGACATTT
TTGATATATATTCAATTCATATTTATCTCTTTTTTTTTTTTTTTTTTTCAGACGTGGAATGGTGGGAAAGC
CAAAATGT

TTTTTTTTTTTTTTTTTTTTTTTTTTACGCCGATGCTCATCGCAGAAAATTTTTCCTTCAGTTTATTTGTC
TTATAAAAAGACTGTCCTACGCTCAAATAACTTATACTTTTCTGTATCTCATTCAAATTATTTTCTTGTCA
ACAACCTG

CAAAAGGTTGTACCCCTTCCCACAGATATATAATATATTCTAAGTAAAAAGAACGGATTATGAAAGAAAAC
GCGCATAATATATATATGTATGTATTTTCTAATTTATATTAGCAACTAAATTAAATTACATCAATACTATT
TTTTTTTT

TAAATGGCATTCATTCCCTTCCGCGCCAGTATATTAGTTACTAATCATACAGTTTCTTGTTTCAATTTTTT
AGATTTTAATTAGCGGTTTTTTTTTTGGTTTTTCTTAAGATTGGTTTTATGATTTTTTTTTTCTTTTTCAA
ATTTTTTT

TTGTCTTTTTAAGTTCTTAATATTTATTTTTATATTTTGATTTTTTTTTTTTTTTTTTTTTTCCATTAGTG
AATGACAGCAATGTATTAAGCTGCTGCCGCGCATACAAAATGTACTGCGTTAAATTAGATTAGCTTAAATT
AGTGCTTG

AACAACAAGTAGCTCTTCTCTTGTTTTGTCCTTTTGCAGTTTGCTTTTATCCCTGTGATGAAAAAAAAAAA
AACTAAAATTTTTTGCCGGCGCATCGAAAAAATCTGTTACCCGACCCGTTATTGGTGATAATACCCATTTT
TAACGCGA

AGGAGTGATGGTTTTCTTTATTTTTAACCCTGTAAAATTTTTTTATTTTTTTATTTTTACTCTTATATATG
TATATGTTTGTGTTTATTTCTGAATAAAAATTTATAAGAACCAAATTAAAACCGTTAATAGCCCACCCAAA
ATTGAAGT

AGGAAGTCAATAAGCATGGTCTATTCTTTTCTTTTATGTAGTTTATAATAGGCAACTTTAATGTTCCCAGA
AGACGCGTTTTCCATTACTTATTATACCCAAAAAAAAAAAATTAAAAAAAAACAACTTCGAACATGCAAACG
CAAAGCGA

TAAAAAAAAAAAAAAAAAAATAAAAAAAAAAGAAAAAAAAAGGAGAAACATGATTGCCAGTACTCGCAAGAA
CTAAGATAGAGAGAAAAAGGACGATATATCAAGAAATAAAAGGGAAGGTAATTCAAGTAAGCACTGCTTAG
CTGTCTGC

TATTTTATCTCTTTTTTTTTTTTTTTTTTCAGACGTGGAATGGTGGGAAAGCCAAAATGTCACTGACCGCG
GCTAAAAGTTAAAAAATACATGAGACGACATTCTTTTATGTATATATATTTACACATGTATACAAACAAAT
ACACACGC

CCCTGTGATGAAAAAAAAAAAAAACTAAAATTTTTTGCCGGCGCATCGAAAAAATCTGTTACCCGACCCGTT
ATTGGTGATAATACCCATTTTTAACGCGATCACATATCATAAACTGCAGTGAGAGGGTCGTTTACAGTAGT
TATAGTAC

TCTTTTTCCTTATTTCTACGCTATACAAAAAGTGTGCTTTTTTCTTCTATTTTTTTTTCTTTAATGACTTT
GTGTGTTTATTACCCTTTAATTAATTACCAAAAAAAAAAATAAACTACGTTTTTAATTTCTAATTTATCAAA
ATTTCTTT

GAAAATTCAGATCGCTTTATACGAGTAAGTATGCCAAATACACCAAATATACCAAAGCATTGATTGCGTAT
ACTGAGAATACATATATATATATATATATATATATATGTATGGTCATTGCTATTTACAAAAGGTTGTATGG
AGTGGGAA

ATTCGATTTTTTTTTTTAATTATATTTTTGACTATTATTTAATCATTAAAAACACATATATTTCATAAATTC
GTAAGGTCGTTAGTTCTATCGTAAAAGTGAAAAAGTTTTGAGCCAGTTTTCAAAAACCCGCAGGATCCCAC
TATGTATG

ACATTGACTCACTATCCATCCGTGTATTATTTCAAAGAGCGAAAAGAAGGCGCGTCGCGTCGACGCGCCTT
TTTAGGCTAGAAAATAAACAGAAAACAAAAACAAAAACAAAAAAAGGCGAAAAAACAAACGAAAAAACAAA
CGACAGTA

AGTTTAATTTTGGGTGGTAATGGCTGTGTGACTAGCTATAGAAAGAAAAAAATTAAAAAAAAAAAAAAAAA
TCAAGTAGTTCCTGCACTGCGACGTCCATTATAGCATTATGAATTGGTCCCTGATTTACGCATGCGATAAA
CTATTTTT

AAAAAAAAAAAAAAAGCGTCTTAGAGGGAATATAAGAGAAAAGAAGAGATAGCATAACACATGATTTATTTT
TATTTTTGCGTTTTTTTTTTTTCTTAATTGTTTATCAATGTATCTGTGAGAGTATCTACATAAAAAAAATA
ACCATATG

TCTTTTTTTAGAAATCGCGTGGCTTGGTAACGCGATAATGACGCGACACAGCCATTAGTGTGAAATTTGAT
TTTCTTGGCCAAACTAGGTATAATATTTGTTACAAATTATTGATTTTAATATATATCTCGGGTTCATTTTT
TACGTCGG

TTAAAGAAATATAACTTTTTTCTTGTACTGCCTGCAATCTCTATTCTTCATTCATCACACATCTATTCAAA
CGCGTTAAAATTTTTTTGTTAGCCTATAAGTAAAACGCGTCGCGTGTATCCAATTTTACTGAATTAAATTC
TTTACTCA

AAGATTCATAAGGACTGCCAATAAAAAAGACTATTTGTGAATTTTTTTATAGATTTGTAAAGGTTAAAATA
GAAGGCCGATGAGGCCTTATGTAAGTCTAATATGGTATTCTTAAAAAAAAAAAAAAAAAAAAAAGCGTCTTA
GAGGGAAT

TTTTTTTTTTTTCTTTTTTTTTTTTTTTTTCTTTTTTCTTTTTTTTTTTTTTGAGCCTTTTCGAATCTAGA
CTCTATGTGAGTCTATTCTCGATGGGGAGTATCGGAAATTGAATTTTAATTCGAATGACTTCTAATGCATC
ACTACAGA

GGCTTTGACGTATAAGTGTCGGTATTTTTTTTTTTTTTTATTGATTTTTTTTCACTCTTATTTTTTCCCTC
ATTTATTCATCATACATTTAACGAACTCTATTTCTTTTCAAAATCCTTATCTACCTATTTTTTTTTCTTTA
TTTTCTTT

TGCAGACATTTTTGATATATATTCAATTCATATTTTATCTCTTTTTTTTTTTTTTTTTTCAGACGTGGAAT
GGTGGGAAAGCCAAAATGTCACTGACCGCGGCTAAAAGTTAAAAAATACATGAGACGACATTCTTTTATGT
ATATATAT

TTAATGTCGAAACAGTCCGTAAACAAACACATTCCCACAAAACACCGCACCGCGAAAAAAAAAAAAAACAG
GAAAAAACGCGAAATGTCCGTAACTTTACCCGGTAATAGTCACGTGATAAAGGTGCATTGGTCTAGCTGGC
TGTTCTGG

GGACCAAGTGTCATGTGTACGTTGTACTTGACATTCTAAGGCATCCTGCAACTGTTCTGTGGAGCTATTAA
ATCTTTATAGTAAATTTTTTTTTACTTTTTTTTTTTTTTTTTTTTTTTTTTATTATTTACAAGCGTCTATA
TATTTTCT

GATGTAAAAAAAAAATTTTAGTTCGCGTACAGTTTTTTCAACATTTGCCATATGATGTAGAATGGCGCTAT
CAAATCATCACTCTTATTACAGGTGAATCGCGCTAACACTTCAATTACAGACACATACTCCTGAATGGG
TGCTTTCA

AAATAGGAAAAGCTACGAGAAACTGAAAAGTAAAAAAAAAAAAAAAAAAATAAAAAAAAAAGAAAAAAAAG
GAGAAACATGATTGCCAGTACTCGCAAGAACTAAGATAGAGAGAAAAAGGACGATATATCAAGAAATAAAA
GGGAAGGT

ATAGCGGTGACAGTAGTTTTATTGTATCTCACCTTTTTTTATGTATAATCATATAATGACTTAATTATTTT
TGAAATGTTTCTTTTTTTTTTTTTAGTTATAACCCTTTTTACGGATTCTTACGTACTTATTTAATCTTTCT
AGTAGACA

CCTAAAAACCACCCAAAAAAAAAAAAAAGACATCAAATCTCTTTTTTTTTTTTTTGTAGTTCTGTTGCCGT
ATGCTATCTACTCCTAAGGCTTTTTTCTACTACGGCAATCTATTCACAATGTGCAATTAGGCTAAATGCTA
AAAAAAGA

GTTCAGAAAGAATAAAATAACAGACTATACAGTTTAATTATATATATATATATATATACGTGCATTTTTCT
TAAAGAAATATAACTTTTTTCTTGTACTGCCTGCAATCTCTATTCTTCATTCATCACACATCTATTCAAAC
GCGTTAAA

GTATAAAATGACGCGGTCGCGCGCCGTTTGACGTCCTATAGGAGAATGCCAAAAAGGGTGTTTTGCTGGAA
AATATACATATATAAATATATATAAAGAATAGTGAAACAATAGAAATATTAGTAATACAGCCATAGCAGTA
GTAGTGAT

GTCATCGAACCTTTTTAAGAGATAAAGGGGGCTTTGACGTATAAGTGTCGGTATTTTTTTTTTTTTTTATT
GATTTTTTTTTCACTCTTATTTTTTCCCTCATTTATTCATCATACATTTAACGAACTCTATTTCTTTTCAA
AATCCTTA

TCCTTTGGAAATGCTATTGTGAGGTTAGAATGAAAATAAAATTCGCCAGTAAATGCGTATCTTGGAGAAAA
TATCAATTTGTTATTTTGTATATATAAAAAATTAAAAAAAAAAAAAAAAAAATACTCAAATACTCAAATAC
GTATACAT

TCTATGTATAATATAACCAGATAAAAATACACCTTCGTACAAGGTGCTAATAATGTTGAGAATTCGAAATT
CCTTTTTAAAGGCGTATTCCGTATTGAATGATTGAAAAATTTATTTCTTTTTTTATTTCTTTTTTTTTTTT
TTTTTTTT

CGACGACGCAGCGGCTGCTCGTATAAAATGACGCGGTCGCGCGCCGTTTGACGTCCTATAGGAGAATGCCA
AAAAGGGTGTTTTGCTGGAAAATATACATATATAAATATATATAAAGAATAGTGAAACAATAGAAATATTA
GTAATACA

ACACTTTTTTACTCGGAAATTTTCTCTCCCCGATTGTAAATTTATTTCTCGTCTACTATAATAATTCACCA
AAAAACAGCAGAGCACGTCCTATATATATATATATATATATATATATATATACATATATATATACGAATAT
ATTTATGA

TCTGATACGCCTTTCCCTCCGCAGGAAATGAAAGATGAACCACCCATTACATTCGATTTTTTTTTTAATTA
TATTTTTGACTATTATTTAATCATTAAAAACACATATATTTCATAAATTCGTAAGGTCGTTAGTTCTATCG
TAAAAGTG

ATTTTTTTTATAAAATGCATAAAAACATATAGATATATATATATTTACGTGGAAAAATGTTAAAACTATTG
ATAAGAAAAAACGCTCAAAACAAGTAGTAAGCAGGCTTTTTTTTTTTTTGCTGCTTGTTTTAGAGAACCTG
TATATCAA

TTTATTTCTTTTTTTATTTCTTTTTTTTTTTTTTTTTTTTTTTTTTACGCCGATGCTCATCGCAGAAAATT
TTTCCTTCAGTTTATTTGTCTTATAAAAAGACTGTCCTACGCTCAAATAACTTATACTTTTCTGTATCTCA
TTCAAATT

ATAAAAAAGACTATTTGTGAATTTTTTTATAGATTTGTAAAGGTTAAAATAGAAGGCCGATGAGGCCTTAT
GTAAGTCTAATATGGTATTCTTAAAAAAAAAAAAAAAAAAAAAGCGTCTTAGAGGGAATATAAGAGAAAAG
AAGAGATA

GAGGGCTATATCATTACCGAACTTAAGTTTTCTATTGGAATATTATACTTTATAAACTAAATAATTACCCA
TCTATGTTGTTTCCCCAAGAATGTTTGCTGCCATATAATAAGAACAAAAAAAAAAATAACAAAAAAAAAGA
AAAAAAAA

TACATAAAACAGTTTAATTTTGGGTGGTAATGGCTGTGTGACTAGCTATAGAAAGAAAAAAATTAAAAAAA
AAAAAAAAAATCAAGTAGTTCCTGCACTGCGACGTCCATTATAGCATTATGAATTGGTCCCTGATTTACGC
ATGCGATA

CTTTTTTAAGAGATAAAGGGGGCTTTGACGTATAAGTGTCGGTATTTTTTTTTTTTTTATTGATTTTTTTT
TCACTCTTATTTTTTCCCTCATTTATTCATCATACATTTAACGAACTCTATTTCTTTTCAAAATCCTTATC
TACCTATT

TTTTTTTTTTTGCACACCTTTAAAACCTATAGAAACTCCTTCATATAAAATGTACTAGTTATAAGATGACT
TTTTATATTCAAAATTAGAAGAAAACTTAACTAAAATTAAAAAAATAGCGGCTATTAAAAGGTATTAAAAA
GTGAGAAA

ATTTTTTCATGTTTTTCTTTTCTGATACGCCTTTCCCTCCGCAGGAAATGAAAGATGAACCACCCATTACA
TTCGATTTTTTTTTTAATTATATTTTTGACTATTATTTAATCATTAAAAACACATATATTTCATAAATTCG
TAAGGTCG

TCATCTCATTTAAATGGCATTCATTCCCTTCCGCGCCAGTATATTAGTTACTAATCATACAGTTTCTTGTT
TCAATTTTTTAGATTTTAATTAGCGGTTTTTTTTTTGGTTTTTCTTAAGATTGGTTTTATGATTTTTTTTT
TCTTTTTC

CTTTAATTAGTTTAGGTATACTTTCCTGCGAAGGTCTTGTTGGAATATGTAGTAATCTCTACCCGCGTTTT
TAGTTAATGCTGAATGGTTTTTGATGGCTCCTTTTTTCTTAGAGTGACAGGCGCGGCAGCCCCTTAACGCG
GGTACCTG

GTGCCGCGCGCCATCATGGTCCGGTAACGGTCGTAGTGAATGACTCATATTTTTCCATCTCTTTCGGCCTT
GCCAAAAAATGAAAAAAAAAAAAAAAAAAAAAAAAAAAATTAAAAACGATGTCCAGAATCACAACTACAAC
ATAGTTTT

CAGAACCGTCTTCCTTGTGCTGTTTATGATGTCTTTTGTCTCCTATTGTTTTCGAATTTTATTTTTTTTTT
TTTTTATTTTTTTATTTTTTTTTGTTTTATATAATTCCTCGCGCTGCCTTAATGATAATTCTTGATTTACT
AATTGTTT

CTGTTCTCGCGCGTTTTTTTTTTTTTTTTTGCTTTTTTTGGTTCCTTTCGCGGATATCGTATTATCGATATT
ACAACAAAAATGTTTGGAGATCTCCTTCGCTGTCTTTAAGTAAAAGGCTTTAACCGCTTATTTTTCTCTCT
TTTTTTCC

TTCCTTGTGCTGTTTATGATGTCTTTTGTCTCCTATTGTTTTCGAATTTTATTTTTTTTTTTTTTTTATTTT
TTTATTTTTTTTTGTTTTATATAATTCCTCGCGCTGCCTTAATGATAATTCTTGATTTACTAATTGTTTCA
TGACTTCT

GTCTTTTGTCTCCTATTGTTTTCGAATTTTATTTTTTTTTTTTTTTTATTTTTTTATTTTTTTTTGTTTTAT
ATAATTCCTCGCGCTGCCTTAATGATAATTCTTGATTTACTAATTGTTTCATGACTTCTGGTGCTAAAGGG
AAGGGGGA

GTCTATTTTGGTGCCGCGCGCCATCATGGTCCGGTAACGGTCGTAGTGAATGACTCATATTTTTCCATCTC
TTTCGGCCTTGCCAAAAAATGAAAAAAAAAAAAAAAAAAAAAAAAAAAAATTAAAAACGATGTCCAGAATCA
CAACTACA

AGCAACGAAACAGAACCGTCTTCCTTGTGCTGTTTATGATGTCTTTTGTCTCCTATTGTTTTCGAATTTTA
TTTTTTTTTTTTTTATTTTTTTATTTTTTTTTGTTTTATATAATTCCTCGCGCTGCCTTAATGATAATTC
TTGATTTA

TGTTTATGATGTCTTTTGTCTCCTATTGTTTTCGAATTTTATTTTTTTTTTTTTTTTATTTTTTTATTTTTT
TTTGTTTTATATAATTCCTCGCGCTGCCTTAATGATAATTCTTGATTTACTAATTGTTTCATGACTTCTGG
TGCTAAAG

GTGAGATGCCCAAGTGAGAATTTTTTTTAAATAATGATGTACTTTAATACAATATATATATATATATATATAT
ATATATATATATATATATATAAGGAATGATAACTCTATTTAAGTAGATTATGACATTGTAGTAGAAGAGGG
CGTGCTAC

TCTTTTGCTTGAGCACAGAGGTGAGATGCCCAAGTGAGAATTTTTTTTAAATAATGATGTACTTTAATACA
ATATATATATATATATATATATATATATATATATATATATATATAAGGAATGATAACTCTATTTAAGTAGATTAT
GACATTGT

CCAGAAAGTTAAAGGGCAAAAAAGCTCACATGACACGCTTGTTTTGGAAAACGTCATAGAAAAACCACGTG
AAAAAAAAAAAAAAAAAAAAAAAAAAAAGAAAAAGAAAAAGGGCAGCTGCCAACTAGTACACACTACACAG
ATATTTAT

TACTTCATTCGTCTATTTTGGTGCCGCGCGCCATCATGGTCCGGTAACGGTCGTAGTGAATGACTCATATT
TTTCCATCTCTTTCGGCCTTGCCAAAAAATGAAAAAAAAAAAAAAAAAAAAAAAAAAAAATTAAAAACGATG
TCCAGAAT

AAAGGGCAAAAAAGCTCACATGACACGCTTGTTTTGGAAAACGTCATAGAAAAACCACGTGAAAAAAAAAA
AAAAAAAAAAAAAAAAAAGAAAAAGAAAAAGGGCAGCTGCCAACTAGTACACACTACACAGATATTTATGA
AAAACACT

TTTTTTTCTTTATTCTTTTTTCTCCTCTTTATGAATACAGATATTCAATTTTTTGAATATCTGATTGTTTT
TATTTTCATTTCAATTTTTTTTTTTTTTTCAATTTTTTTTATCCGATCGTTGCGCCAAGCTTCTATCGCTTA
AAACAATG

TCCTATTGTTTTCGAATTTTATTTTTTTTTTTTTTTTATTTTTTTATTTTTTTTTGTTTTATATAATTCCTC
GCGCTGCCTTAATGATAATTCTTGATTTACTAATTGTTTCATGACTTCTGGTGCTAAAGGGAAGGGGGAAA
AGGGAAGC

GCTTGTTATATACTTCATTCGTCTATTTTGGTGCCGCGCGCCATCATGGTCCGGTAACGGTCGTAGTGAAT
GACTCATATTTTTCCATCTCTTTCGGCCTTGCCAAAAAATGAAAAAAAAAAAAAAAAAAAAAAAAAAATT
AAAAACGA

CAAGTGAGAATTTTTTTAAATAATGATGTACTTTAATACAATATATATATATATATATATATATATATAT
ATATATATATAAGGAATGATAACTCTATTTAAGTAGATTATGACATTGTAGTAGAAGAGGGCGTGCTACTT
GCGCTCTG

GACTCTCCAACCAGAAAGTTAAAGGGCAAAAAAGCTCACATGACACGCTTGTTTTGGAAAACGTCATAGAA
AAACCACGTGAAAAAAAAAAAAAAAAAAAAAAAAAAAGAAAAAGAAAAAGGGCAGCTGCCAACTAGTACA
CACTACAC

TACGGGACAGCAGAGATAGGCTGTTCTCGCGCGTTTTTTTTTTTTTTTGCTTTTTTTGGTTCCTTTCGCG
GATATCGTATTATCGATATTACAACAAAAATGTTTGGAGATCTCCTTCGCTGTCTTTAAGTAAAAGGCTTT
AACCGCTT

TATTAGACATAGCAACGAAACAGAACCGTCTTCCTTGTGCTGTTTATGATGTCTTTTGTCTCCTATTGTTT
TCGAATTTTATTTTTTTTTTTTTTTATTTTTTTATTTTTTTTGTTTTATATAATTCCTCGCGCTGCCTTA
ATGATAAT

TTCCTTATAACTATCGTGTTTATTTTCTTAGTGGTGGTTCTTTTATTATTACCCGTTTTCTTCGGTAATAT
TTTATAGTGAAAAATTTTGGTAAAAAAAAAAAAAAGAAAAAATTTTTTTTATTTCAAATCTCATCTCATC
TCTTAAAA

TCCCTGTGCATACGGGACAGCAGAGATAGGCTGTTCTCGCGCGTTTTTTTTTTTTTTTGCTTTTTTTGGT
TCCTTTCGCGGATATCGTATTATCGATATTACAACAAAAATGTTTGGAGATCTCCTTCGCTGTCTTTAAGT
AAAAGGCT

AAAAAAAAAAAAAAGGTGAAAGGAAGCGAGCATTAATTTTTTATTTTAATATGTAACTGTGATATGTATT
AACAAAGTATCTTTCATAATTTTTGACGAATATTTTTACTTCTTTTTTACTATTGAAGGTTGGGCTGTGAT
TCATCGCG

GAGCACAGAGGTGAGATGCCCAAGTGAGAATTTTTTTAAATAATGATGTACTTTAATACAATATATATAT
ATATATATATATATATATATATATATATATAAGGAATGATAACTCTATTTAAGTAGATTATGACATTGTAG
TAGAAGAG

CGGTCTTTTGGACTCTCCAACCAGAAAGTTAAAGGGCAAAAAAGCTCACATGACACGCTTGTTTTGGAAAA
CGTCATAGAAAAACCACGTGAAAAAAAAAAAAAAAAAAAAAAAAAAAGAAAAAGAAAAAGGGCAGCTGCC
AACTAGTA

GAGGCGAAATCGATCAATTAGTTGAACAATTAACTTAAAATCTAATATATATAAATATATATATATATATA
TATAGGAAATATACGGCACGTTATACTAGAGATATTAAACATCCCAACTCGGTTAATGGGTACAATAAAAA
AAAGAGGG

CCGCGCGGGAATTCACATTGCAGTATGGACGGACATCCGGCCATCGCGCGCGGGCCCGGTATTTGATCTCC
GTTTTAGAAGCACAGAAAAAAAATAATATGATGTTATGATGTTTAATAGATGGATTCATATCGTCCGGGTAA
AAACGCTT

TATTTTCTTAGTGGTGGTTCTTTTATTATTACCCGTTTTCTTCGGTAATATTTTATAGTGAAAAATTTTGG
TAAAAAAAAAAAAAAGAAAAAATTTTTTTTATTTCAAATCTCATCTCATCTCTTAAAAAGGTTTATTCTG
TTTACTCA

CAGAGATAGGCTGTTCTCGCGCGTTTTTTTTTTTTTTTGCTTTTTTTGGTTCCTTTCGCGGATATCGTAT
TATCGATATTACAACAAAAATGTTTGGAGATCTCCTTCGCTGTCTTTAAGTAAAAGGCTTTAACCGCTTAT
TTTTCTCT

AAAAAAAGAAATGACAAAAAAAGAGAAAAAAAAAAAATGAAACTACATAGTTAATTAATAATAGAAGTATTT
GTCAATAGTATGATAATGAAATCGATATTATGGAAGATATTAACCGCGCGCCGTATTAGTGTACACTATAT
TAAACTAC

AAAGCTCACATGACACGCTTGTTTTGGAAAACGTCATAGAAAAACCACGTGAAAAAAAAAAAAAAAAAAAA
AAAAAAAAGAAAAAGAAAAAGGGCAGCTGCCAACTAGTACACACTACACAGATATTTATGAAAAACACTAA
CTTCTCCT

CAATGAAATTTCAATATCGCATATAATAATTTTTGTTATCAAATATATATTTTTCGGAAGTCATATACATT
AAATAATTAAAACATTATTTTTAAAAATTAAAAAAATATATTTAGCAAATAGTTTTTACTTTTTTTTAAA
TGCAAAAT

CTATCGTGTTTATTTTCTTAGTGGTGGTTCTTTTATTATTACCCGTTTTCTTCGGTAATATTTTATAGTGA
AAAATTTTGGTAAAAAAAAAAAAAAAAGAAAAAATTTTTTTTATTTCAAATCTCATCTCATCTCTTAAAAAG
GTTTATTC

CTTACCTTTTCGGTCTTTTGGACTCTCCAACCAGAAAGTTAAAGGGCAAAAAAGCTCACATGACACGCTTG
TTTTGGAAAACGTCATAGAAAAACCACGTGAAAAAAAAAAAAAAAAAAAAAAAAAAAAAGAAAAAGAAAAAG
GGCAGCTG

TTTTACGATAAAATTTCTTCCAATTAACATCCCAGGCCTTGCTTCAATTTATCAATTGGTATATTAACTTT
CAACACATTTTGTTTTTGTTTTTATTTTTGTTTTTTTTATTTTTTTTTTTTTTTCGCGTTTCGTGCTTTAA
TGAAACAT

TCCTTCATGTACTGAGATACTGCTGCAAGCGAAATATCACGAATAATAATATAAAAAAAAAAAAAAAAAAA
AAAAAAGATGTTTAAATGTTAACAACCTAACAGCAATAAAAAATAAAAATAAAGGTAGAAGATTGCAAGAA
AGAAACTT

ATATTTGTCTTTCAAACGCGGTTTTAACATATCGCGAACAACGGTATTCGGGTAACAGCCTATCACTATTT
TTACCGGCGGCTATTTTTCCTAAACCTAGTATATATACGCGAAAATTGGCTACCTGCAAAATTCCAATAGT
GGCGAATG

AAAACGTTTTCTTACCTTTTCGGTCTTTTGGACTCTCCAACCAGAAAGTTAAAGGGCAAAAAAGCTCACAT
GACACGCTTGTTTTGGAAAACGTCATAGAAAAACCACGTGAAAAAAAAAAAAAAAAAAAAAAAAAAAAAAGAA
AAAGAAAA

CAGGAGAACTTCCCTGTGCATACGGGACAGCAGAGATAGGCTGTTCTCGCGCGTTTTTTTTTTTTTTTTGC
TTTTTTTGGTTCCTTTCGCGGATATCGTATTATCGATATTACAACAAAAATGTTTGGAGATCTCCTTCGCT
GTCTTTAA

TTCAAACGCGGTTTTAACATATCGCGAACAACGGTATTCGGGTAACAGCCTATCACTATTTTTACCGGCGG
CTATTTTTCCTAAACCTAGTATATATACGCGAAAATTGGCTACCTGCAAAATTCCAATAGTGGCGAATGTA
TTGGAATT

TTAACCGCGCGCCGTATTAGTGTACACTATATTAAACTACATTTTGCTTCTTACTGAATTTATAAATTATG
ATTATATTATTATTACTATTATGACTACTGTATATATTTTTTAGAATTAGCATCGGGAACCGATGAGCGTT
AGCTGAAA

ACTGGCAAGGTATTAGACATAGCAACGAAACAGAACCGTCTTCCTTGTGCTGTTTATGATGTCTTTTGTCT
CCTATTGTTTTCGAATTTTATTTTTTTTTTTTTTTATTTTTTTTATTTTTTTTTGTTTTATATAATTCCTCG
CGCTGCCT

GCCAAAAGATTCTTTTGCTTGAGCACAGAGGTGAGATGCCCAAGTGAGAATTTTTTTTAAATAATGATGTA
CTTTAATACAATATATATATATATATATATATATATATATATATATATATAAGGAATGATAACTCTATTTA
AGTAGATT

GTTTTGGAAAACGTCATAGAAAAACCACGTGAAAAAAAAAAAAAAAAAAAAAAAAAAAAGAAAAAGAAAAA
GGGCAGCTGCCAACTAGTACACACTACACAGATATTTATGAAAAACACTAACTTCTCCTTTTGGAACTGTT
GACGTACC

TGAACCTCAACAGCTACACATTCTTTTATAATCCTTAATATTCTATATATACATATATGAAAAAATAGAAA
ACGCGAAAACTTGTCATTTTTTTTTTAGGCGTTTTTATAATATACTGAAAATAAAAAGAGGCTCTTTAAAT
GTTGACAC

TTTTTCACTTTTTTGAGGTTTTATTTATCTTGTATTCTTTTTTTCTATTATTTTAAAGGTTCAATTTCTAT
CGACGGTTTTTTTAATTATATATATATATATATATATATATGTATATAATGAATGTGTAATATTCCAAGTA
TACGTACC

AAATATATATTTTTTCGGAAGTCATATACATTAAATAATTAAAACATTATTTTTAAAATTAAAAAAATATA
TTTTAGCAAATAGTTTTTACTTTTTTTTAAATGCAAAATAAGTTCACGTGATATTATTAATAATATTTTAC
CCAAAAAT

TCTGCATGGCGCTTGTTATATACTTCATTCGTCTATTTTGGTGCCGCGCCATCATGGTCCGGTAACGGT
CGTAGTGAATGACTCATATTTTTCCATCTCTTTCGGCCTTGCCAAAAAATGAAAAAAAAAAAAAAAAAAAA
AAAAAAAA

AAAAAAAGATCGGGGTGTAAAAATTCTATGCCAAGAAAAAAATTAAAAACAAAATAAAAAAAAAAAGAAAC
TTGCGGCTCTTCACTCTTAAAAGACAAAAAGTATTAGGATGATAAAAAAAATTCATAAAAAAAAAAAATTC
TATGGCCT

CAATAGTGTCTCCTTCATGTACTGAGATACTGCTGCAAGCGAAATATCACGAATAATAATATAAAAAAAAA
AAAAAAAAAAAAAAAAGATGTTTAAATGTTAACAACCTAACAGCAATAAAAAATAAAAATAAAGGTAGAAG
ATTGCAAG

ATATAATAATTTTGTTATCAAATATATATTTTTTCGGAAGTCATATACATTAAATAATTAAAACATTATTT
TTAAAAATTAAAAAAATATATTTTAGCAAATAGTTTTTACTTTTTTTTAAATGCAAAATAAGTTCACGTGA
TATTATTA

TTTTGTTATCAAATATATATTTTTTCGGAAGTCATATACATTAAATAATTAAAACATTATTTTTAAAATTA
AAAAAATATATTTTAGCAAATAGTTTTTACTTTTTTTTAAATGCAAAATAAGTTCACGTGATATTATTAAT
AATATTTT

TGACACGCTTGTTTTGGAAAACGTCATAGAAAAACCACGTGAAAAAAAAAAAAAAAAAAAAAAAAAAAAGA
AAAAGAAAAAGGGCAGCTGCCAACTAGTACACACTACACAGATATTTATGAAAAACACTAACTTCTCCTTT
TGGAACTG

AAATATTGGGAGGTTTTAACTAATTTTTCCAGGATATTTCAACTTTTCATAAATCAAATTTTTTTTACTTT
TTTTTTTGCTTTGCTATAGTATTTTAGAAAAGGACAAAAAAATACGAAAAAAGACAAAAAACCATATAAAA
AAATTGAT

AAATTTCTTCCAATTAACATCCCAGGCCTTGCTTCAATTTATCAATTGGTATATTAACTTTCAACACATTT
TGTTTTTGTTTTTATTTTTGTTTTTTTTATTTTTTTTTTTTTTTCGCGTTTCGTGCTTTAATGAAACATGT
GAAGTGTT

TGCCCCATTGATTCAAATGTCCATGCGCGTTGATTGATGTGTGATATATATATATATATATATGTATATAG
ATGTATATTATTATGTTCAGAGGTGATTTTATCTTTGTACGCGTACCATTTTTGTAAAGTGACCTTGTTTT
GACTTTTT

CGATTAATCTATTGTTCAGCAGCTCTTCTCTACCCTGTCATTCTAGTATTTTTTTTTTTTTTTTTTGGTTT
TACTTTTTTTTCTTCTTGCCTTTTTTTCTTGTTACTTTTTTTCTAGTTTTTTTTCCTTCCACTAAGCTTTT
TCCTTGAT

ACTGAGATACTGCTGCAAGCGAAATATCACGAATAATAATATAAAAAAAAAAAAAAAAAAAAAAAAAGATG
TTTAAATGTTAACAACCTAACAGCAATAAAAAATAAAAATAAAGGTAGAAGATTGCAAGAAAGAAACTTTA
CAATATTT

AGAATTATAAGAGGCGAAATCGATCAATTAGTTGAACAATTAACTTAAAATCTAATATATATAAATATATA
TATATATATATATAGGAAATATACGGCACGTTATACTAGAGATATTAAACATCCCAACTCGGTTAATGGGT
ACAATAAA

CATGAATGCTAAAAAAAGAAATGACAAAAAAAAGAGAAAAAAAAAATGAAACTACATAGTTAATTAATAAT
AGAAGTATTTGTCAATAGTATGATAATGAAATCGATATTATGGAAGATATTAACCGCGCGCCGTATTAGTG
TACACTAT

ATTGTTCAGCAGCTCTTCTCTACCCTGTCATTCTAGTATTTTTTTTTTTTTTTTTTGGTTTTACTTTTTTTT
TCTTCTTGCCTTTTTTTCTTGTTACTTTTTTTCTAGTTTTTTTTCCTTCCACTAAGCTTTTTCCTTGATTT
ATCCTTGG

AAGTTCCGCACTCCATCAAGTGAACCTCAACAGCTACACATTCTTTTATAATCCTTAATATTCTATATATA
CATATATGAAAAAATAGAAAACGCGAAAACTTGTCATTTTTTTTTTTAGGCGTTTTATAATATACTGAAAA
TAAAAAGA

GGCTTCTTGTATATGATTCTAAAATAATGTGTGAAAAAAAAAATAAAATAAAAAAAAGAGGAAAATAATAT
AGAATAACTATTAAGTTTCATTAAAAAAAAACCATTTGAATATACGACCAAAAACGTTACGCTTTCATAAA
GTGTGAAT

ATAGAAGTAAGGCGTTGCATTTCCTTATAACTATCGTGTTTATTTTCTTAGTGGTGGTTCTTTTATTATTA
CCCGTTTTCTTCGGTAATATTTTATAGTGAAAAATTTTGGTAAAAAAAAAAAAAAAAGAAAAAATTTTTTTT
ATTTCAAA

TTCGAATTTTATTTTTTTTTTTTTTTATTTTTTTTATTTTTTTTTGTTTATATAATTCCTCGCGCTGCCTT
AATGATAATTCTTGATTTACTAATTGTTTCATGACTTCTGGTGCTAAAGGGAAGGGGGAAAAGGGAAGCGA
AAACTTCA

TACGTATGTAAAAAAAAGATCGGGGTGTAAAAATTCTATGCCAAGAAAAAAATTAAAAACAAAATAAAAAA
AAAAAGAAACTTGCGGCTCTTCACTCTTAAAAGACAAAAAGTATTAGGATGATAAAAAAAATTCATAAAAA
AAAAAAAT

CAATTAACATCCCAGGCCTTGCTTCAATTTATCAATTGGTATATTAACTTTCAACACATTTTGTTTTTGTT
TTTATTTTGTTTTTTTTATTTTTTTTTTTTTTTTCGCGTTTCGTGCTTTAATGAAACATGTGAAGTGTTTC
GCAATGAC

TAACTCTTTTTCTAATAGGGGCAACTTACTTTTGTTTTCTCATTTGGCGAAAAAAAGTAAGTAGAAATAAA
TAAAAAAATAATATTATAATGTGCTGTATATATAAATACAAATGCGAAAGCTAATGCAGATTTTGCCTAGT
ACTCTCTA

ATAGAACCTTTTTTTATCTTCTTCACTTGTTCTGTTTTCTTTTTTTTTTTTTTTTTTTTTTTCAAGTTCCA
CGAAAAAATTTCTCGTTTTCCTTTAGTCACTCTTGTCACATAAGGATTATCCGAACCCCGCCCCGCGGAA
TTAATTAG

ATGGAAGATATTAACCGCGCGCCGTATTAGTGTACACTATATTAAACTACATTTTGCTTCTTACTGAATTT
ATAAATTATGATTATATTATTATTACTATTATGACTACTGTATATATTTTTTAGAATTAGCATCGGGAACC
GATGAGCG

ACGTAGCCACAAAAAAAAAAAAAAAAGGTGAAAGGAAGCGAGCATTAATTTTTTATTTAATATGTAACTGT
GATATGTATTAACAAAGTATCTTTCATAATTTTTGACGAATATTTTTACTTCTTTTTTACTATTGAAGGTT
GGCTGTG

TGTATTCTTTTTTTCTATTATTTTAAAGGTTCAATTTCTATCGACGGTTTTTTAATTATATATATATATA
TATATATATATGTATATAATGAATGTGTAATATTCCAAGTATACGTACCTTGGGTTTATATTTTTTAACTT
GTATATTC

ATAGTGCGATATAGAACCTTTTTTTATCTTCTTCACTTGTTCTGTTTTCTTTTTTTTTTTTTTTTTTTTT
TCAAGTTCCACGAAAAAATTTTCTCGTTTTCCTTTAGTCACTCTTGTCACATAAGGATTATCCGAACCCCG
CCCCGCGG

TCTAATAGGGGCAACTTACTTTTGTTTTCTCATTTGGCGAAAAAAAGTAAGTAGAAATAAATAAAAAAATA
ATATTATAATGTGCTGTATATATAAATACAAATGCGAAAGCTAATGCAGATTTTGCCTAGTACTCTCTAGA
AATAGTAA

GGCGTTGCATTTCCTTATAACTATCGTGTTTATTTTCTTAGTGGTGGTTCTTTTATTATTACCCGTTTTCT
TCGGTAATATTTTATAGTGAAAAATTTTGGTAAAAAAAAAAAAAAAGAAAAAATTTTTTTTATTTCAAATC
TCATCTCA

CGTCTCACTTCAATAGTGTCTCCTTCATGTACTGAGATACTGCTGCAAGCGAAATATCACGAATAATAATA
TAAAAAAAAAAAAAAAAAAAAAAAAAGATGTTTAAATGTTAACAACCTAACAGCAATAAAAAAATAAAAATA
AAGGTAGA

TTATTTATCTTGTATTCTTTTTTTCTATTATTTTAAAGGTTCAATTTCTATCGACGGTTTTTTAATTATA
TATATATATATATATATATATGTATATAATGAATGTGTAATATTCCAAGTATACGTACCTTGGGTTTATAT
TTTTTAAC

AACTATTTACTTAACTTTTAACTCGGGAAATATGTATCACTATACTAAAAAACATTAATTTAATCAAAGAA
AAATAAATTTAATGTATGTATCTATATATATATATATATATCACTTAAAACAGACAATAGCCATAATCAAC
TGGGTTAT

GGGGTCTAGTCGACATGTTTGCTTGGTGTCTTAGCAGCTCTTATAGTTCTAATGCTATAACTAAGAAAGTA
ATAATAATAATAATAAAAAGCTTTATATAATGTTTTATTATGGAAGTTTCGTTTTTGTGGCGCGACGCGAT
CAGCCAAA

TGCTGCAAGCGAAATATCACGAATAATAATATAAAAAAAAAAAAAAAAAAAAAAAAAAGATGTTTAAATGTT
AACAACCTAACAGCAATAAAAAAATAAAAATAAAGGTAGAAGATTGCAAGAAAGAAACTTTACAATATTTTG
TTTGGATG

AGGTTTCATGATGGAAACAAACTATAAAGGATTGTCTTGTTTCCTTTTTTTTTTTTTTTTTTTGTTTTTT
TATGACTAAATAAAGTAAATCATATGAGGTTTTATTTTTTATTTCGTTGATTTGGGTTACAGTATGGAAAT
GGGGCATT

TTTTTTTTTTTTTTTTGGTTTTACTTTTTTTTCTTCTTGCCTTTTTTTCTTGTTACTTTTTTTCTAGTTTT
TTTTCCTTCCACTAAGCTTTTTCCTTGATTTATCCTTGGGTTCTTCTTTCTACTCCTTTAGATTTTTTTTT
TATATATT

TTTTATTATTACCCGTTTTCTTCGGTAATATTTTATAGTGAAAAATTTTGGTAAAAAAAAAAAAAAAGAAA
AAATTTTTTTTATTTCAAATCTCATCTCATCTCTTAAAAAGGTTTATTCTGTTTACTCAAACTCTTCATCA
AAAACAAA

TTTTTTTTAAATAATGATGTACTTTAATACAATATATATATATATATATATATATATATATATATATATAT
AAGGAATGATAACTCTATTTAAGTAGATTATGACATTGTAGTAGAAGAGGGCGTGCTACTTGCGCTCTGTT
TCTTCTTA

ACGATAAATTTCCCCGTTTTCTTTTACTTTTACTTCATTTAATATAATTCATATTTATTTCCCATGTACCA
ATTAATTACTCATAAAAAGAACAGAAATAGAAAAAAGTTGTATATAACAAGCCAAAAAAAAGAAAAAAAAA
GGAAAAAA

CAAAATTGTACATGAATGCTAAAAAAAGAAATGACAAAAAAAGAGAAAAAAAAAAAATGAAACTACATAGTT
AATTAATAATAGAAGTATTTGTCAATAGTATGATAATGAAATCGATATTATGGAAGATATTAACCGCGCGC
CGTATTAG

CCCAAATTTTTTATGCATATACAAATGTTGAAAAGAAACGAATATTATTAGCAAGTAATATATAGTATATA
GTATATATATATATTTATATATGTAAACATATAATGAATATATAAATTGGTATGTAAAAGAGTTGCGAGCT
TCATGGCG

TATTCTTTTTTCTCCTCTTTATGAATACAGATATTCAATTTTTTGAATATCTGATTGTTTTTATTTTCATT
TCAATTTTTTTTTTTTTTCAATTTTTTTTATCCGATCGTTGCGCCAAGCTTCTATCGCTTAAAACAATGAC
ACTTCGCT

CTTTTTTTTATTTAGGAGAAACAATATATATATATATGTATATACGTATGTATGATTCATATTTTAATCGC
ATTTTTATTTCATCTAAAACAATTACTCTTTTTGATTATTTATAGAAAAAAAACAACAATGATACGTTTGC
TTCGCGTT

TGGAATGAATGGCAATATTCTTTTTTAGGTTAACCGGCCGGACAGTAATATAGTAATCGTTTTGTACGTTT
TTCAAGAAGCGACGCACAACTGTTTTCCATTTTTTTTTTTTTTTTTCAGTGATCATCGTCCATGAAAAAA
ATTTTTCA

TCAATATCGCATATAATAATTTTTGTTATCAAATATATATTTTTCGGAAGTCATATACATTAAATAATTAA
AACATTATTTTTAAAAATTAAAAAAATATATTTTAGCAAATAGTTTTTACTTTTTTTTAAATGCAAAATAA
GTTCACGT

TTTTAAAGGTTCAATTTCTATCGACGGTTTTTTTAATTATATATATATATATATATATATATGTATATAAT
GAATGTGTAATATTCCAAGTATACGTACCTTGGGTTTATATTTTTTAACTTGTATATTCTTTTACGATAAA
ATTTCTTC

CAATTTGTTGATATTTGTCTTTCAAACGCGGTTTTAACATATCGCGAACAACGGTATTCGGGTAACAGCCT
ATCACTATTTTTACCGGCGGCTATTTTTCCTAAACCTAGTATATATACGCGAAAATTGGCTACCTGCAAAA
TTCCAATA

AATGACGGGAAAAGAACACGGGAAACGCTACTATTGTTGTATAGTGCGATATAGAACCTTTTTTTATCTTC
TTCACTTGTTCTGTTTTCTTTTTTTTTTTTTTTTTTTTTTTCAAGTTCCACGAAAAAATTTTCTCGTTTTC
CTTTAGTC

TTCCTAATGTTAACTCTTTTTCTAATAGGGGCAACTTACTTTTGTTTTCTCATTTGGCGAAAAAAAGTAAG
TAGAAATAAATAAAAAAATAATATTATAATGTGCTGTATATATAAATACAAATGCGAAAGCTAATGCAGAT
TTTGCCTA

CATCGCATATCAGGAGAACTTCCCTGTGCATACGGGACAGCAGAGATAGGCTGTTCTCGCGCGTTTTTTTT
TTTTTTTTGCTTTTTTTTGGTTCCTTTCGCGGATATCGTATTATCGATATTACAACAAAAATGTTTGGAGAT
CTCCTTCG

AAACATTATTTTTAAAAATTAAAAAAATATATTTTAGCAAATAGTTTTTACTTTTTTTTAAATGCAAAATA
AGTTCACGTGATATTATTAATAATATTTTACCCAAAAATATGAGGGCATCGGAAAATTTTTCAGTAATGAA
TATTAATG

AAATACTGAACAATGAAATTTCAATATCGCATATAATAATTTTTGTTATCAAATATATATTTTTCGGAAGT
CATATACATTAAATAATTAAAACATTATTTTTAAAAATTAAAAAAATATATTTTAGCAAATAGTTTTTACT
TTTTTTTA

AAAAAAATACAGTTGTTCTTTACGTGCATCGATCATATAAGAAAAGGGAAAAAAGCCGTAAAGTAAAAAAA
TAAAGAGCTTTCCATGGCTGGTTTTTTTTCTTTTTTTTTTTTTTTTTGAAGACTACAGCAGGTATTACCC
GGAGTGAA

ATTATACGCGGGGATCCGGCGTCACAAGCCACGGCCCGTCCCGTCCGCGTGGTTTTTAATTCTGGCTCCGC
GTAGTACATTTTGTAGCTTGCCTTTTTTCCGGCTTCTAGAAAACTGGAAAAACCATAAGAAAAAAATGGGA
AGGGGAAA

GCAACTTACTTTTGTTTTCTCATTTGGCGAAAAAAAGTAAGTAGAAATAAATAAAAAAATAATATTATAAT
GTGCTGTATATATAAATACAAATGCGAAAGCTAATGCAGATTTTGCCTAGTACTCTCTAGAAATAGTAATG
TCCCTTTC

ATTCATCCTCGGCTTCTTGTATATGATTCTAAAATAATGTGTGAAAAAAAAAATAAAATAAAAAAAAGAGG
AAAATAATATAGAATAACTATTAAGTTTCATTAAAAAAAAACCATTTGAATATACGACCAAAAACGTTACG
CTTTCATA

TTTAATTCGGAAAAATAAAAAAAAAAAAAAAAAACATAAGGAAATTTTAATTAAATAGTCTATCTACACATT
TCCTAAAAGAAAATATTATTGCATTACTTTTTTGAAGATCTATAAAGGGCACTGTCTTACTTTTTAAAATC
TGACCCAC

CATCTACAAGCCCTATACCGAGGAAAGCGAATAGTCGCCATGCTAAACGCGCGGAACAAGGCCATATTTAT
ATATTTAATGCTTTTAACTATTATTAGTTTTTCTCACCCAGGGCTGGTTTCTTTACCCTGTGTGATGAAAG
TGCGCGCC

AAATCTGTAACTTTTAAAACTTCTGTTTCCGAACTCCTCATTTTTCATGCATAAATATATAAATATATATA
TATATATATATAAAGCATACATTTCAGTACCATGGGGTATATGATCCTCCCATTACGTATTCTAAAGTTCG
CGTTTTCT

CCATCATGGTCCGGTAACGGTCGTAGTGAATGACTCATATTTTTCCATCTCTTTCGGCCTTGCCAAAAAAT
GAAAAAAAAAAAAAAAAAAAAAAAAAAAATTAAAAACGATGTCCAGAATCACAACTACAACATAGTTTTGC
CTCTTACT

CTCCATCAAGTGAACCTCAACAGCTACACATTCTTTTATAATCCTTAATATTCTATATATACATATATGAA
AAAATAGAAAACGCGAAAACTTGTCATTTTTTTTTTAGGCGTTTTTATAATATACTGAAAATAAAAAGAGG
CTCTTTAA

TTTAGTGGAAAAATACTGAACAATGAAATTTCAATATCGCATATAATAATTTTTGTTATCAAATATATATT
TTTCGGAAGTCATATACATTAAATAATTAAAACATTATTTTTAAAAATTAAAAAAATATATTTTAGCAAAT
AGTTTTTA

ATGACAAAAAAAGAGAAAAAAAAAAAATGAAACTACATAGTTAATTAATAATAGAAGTATTTGTCAATAGTA
TGATAATGAAATCGATATTATGGAAGATATTAACCGCGCGCCGTATTAGTGTACACTATATTAAACTACAT
TTTGCTTC

TTTATTTTTTTTTCTAGGAATAAAAATGAAGAACGGGAAGAAAAGGAAAAATGAAAATGAAGAATATGAAG
AGTAAACCTACTTAAAAAAAAAAACCATACTTTAGTATTTAATGTAGCTACCACGCGTATTCGATACACTA
TTATTTTT

AAAAAGGTGAAAGGAAGCGAGCATTAATTTTTTATTTTAATATGTAACTGTGATATGTATTAACAAAGTAT
CTTTCATAATTTTTGACGAATATTTTTACTTCTTTTTTACTATTGAAGGTTGGGCTGTGATTCATCGCGCA
GAAAACAA

ACTGAGCAATAAAACGTTTTCTTACCTTTTCGGTCTTTTGGACTCTCCAACCAGAAAGTTAAAGGGCAAAA
AAGCTCACATGACACGCTTGTTTTGGAAAACGTCATAGAAAAACCACGTGAAAAAAAAAAAAAAAAAAAAA
AAAAAAAG

AAGATGACAAGGTAATGCATCATTCTATACGTGTCATTCTGAACGAGGCGCGCTTTCCTTTTTTTCTTTTTG
CTTTTTCTTTTTTTTTCTCTTGAACTCGAGAAAAAAAAATATAAAAGAGATGGAGGAACGGGAAAAAGTTAG
TTGTGGTG

TTCTAGTATTTTTTTTTTTTTTTTTTGGTTTTACTTTTTTTTCTTCTTGCCTTTTTTTCTTGTTACTTTTT
TTCTAGTTTTTTTTCCTTCCACTAAGCTTTTTCCTTGATTTATCCTTGGGTTCTTCTTTCTACTCCTTTAG
ATTTTTTT

ATGGAAACAAACTATAAAGGATTGTCTTGTTTCCTTTTTTTTTTTTTTTTTTTTGTTTTTTTATGACTAAA
TAAAGTAAATCATATGAGGTTTTATTTTTTATTTCGTTGATTTGGGTTACAGTATGGAAATGGGGCATTTG
ACACAAGT

TTGTAAACAACGTCTCACTTCAATAGTGTCTCCTTCATGTACTGAGATACTGCTGCAAGCGAAATATCACG
AATAATAATATAAAAAAAAAAAAAAAAAAAAAAAAAGATGTTTAAATGTTAACAACCTAACAGCAATAAAA
AATAAAAA

TTTCGTCAATCAATTTGTTGATATTTGTCTTTCAAACGCGGTTTTAACATATCGCGAACAACGGTATTCGG
GTAACAGCCTATCACTATTTTTACCGGCGGCTATTTTTCCTAAACCTAGTATATATACGCGAAAATTGGCT
ACCTGCAA

CCCAGGCCTTGCTTCAATTTATCAATTGGTATATTAACTTTCAACACATTTTGTTTTTGTTTTTATTTTTG
TTTTTTTATTTTTTTTTTTTTTTTCGCGTTTCGTGCTTTAATGAAACATGTGAAGTGTTTCGCAATGACTA
AGTAGCCT

GCTTCAATTTATCAATTGGTATATTAACTTTCAACACATTTTGTTTTTGTTTTTATTTTTGTTTTTTTTAT
TTTTTTTTTTTTTTCGCGTTTCGTGCTTTAATGAAACATGTGAAGTGTTTCGCAATGACTAAGTAGCCTCC
ACAGATGA

GTGGTGGTTCTTTTATTATTACCCGTTTTCTTCGGTAATATTTTATAGTGAAAAATTTTGGTAAAAAAAAA
AAAAAAGAAAAAATTTTTTTTATTTCAAATCTCATCTCATCTCTTAAAAAGGTTTATTCTGTTTACTCAAA
CTCTTCAT

AAAATAAAAAATAAAATTTTTTTCTCCCGATAATCAATTTTCTTAATTAATTAATTGCGTTACGATTCCGT
TTTTTTACTTCTTTTATCTCATTATCTATCTAAGTTATTTAAAAAAAAGAAAGAACTTTTTATGAACTTTC
CTCTTTTC

AGCTCTTCTCTACCCTGTCATTCTAGTATTTTTTTTTTTTTTTTTTGGTTTTACTTTTTTTTCTTCTTGCC
TTTTTTTCTTGTTACTTTTTTTCTAGTTTTTTTTCCTTCCACTAAGCTTTTTCCTTGATTTATCCTTGGGT
TCTTCTTT

GTTCTTTTCTTTTTTTTCTTTATTCTTTTTTCTCCTCTTTATGAATACAGATATTCAATTTTTTGAATATC
TGATTGTTTTTATTTTCATTTCAATTTTTTTTTTTTTTCAATTTTTTTTATCCGATCGTTGCGCCAAGCTT
CTATCGCT

TCATATACATTAAATAATTAAAACATTATTTTTAAAAATTAAAAAAATATATTTTAGCAAATAGTTTTTAC
TTTTTTTTAAATGCAAAATAAGTTCACGTGATATTATTAATAATATTTTACCCAAAAATATGAGGGCATCG
GAAAATTT

CACCAAAACCAAGAAAGTAGTTGACAAAAAAATGAAAACCACTATTATTTAAATTACGGAAAAAAAAAAAC
CAATAAACAAAAAACATAATAAAAGAAATGAAAAAAAAGAATATTAGTAGAGCATACTGAATTGTTTTCAG
AAAGAGAG

ATTGTTATTCCGATTAATCTATTGTTCAGCAGCTCTTCTCTACCCTGTCATTCTAGTATTTTTTTTTTTTT
TTTTTGGTTTTACTTTTTTTTCTTCTTGCCTTTTTTTCTTGTTACTTTTTTTCTAGTTTTTTTTCCTTCCA
CTAAGCTT

AAAGTGGCATTGAAATTTAGCTTTTTTTTATTTAGGAGAAACAATATATATATATATGTATATACGTATGT
ATGATTCATATTTTAATCGCATTTTTATTTCATCTAAAACAATTACTCTTTTTGATTATTTATAGAAAAAA
AACAACAA

ACTATAAAGGATTGTCTTGTTTCCTTTTTTTTTTTTTTTTTTTTGTTTTTTTATGACTAAATAAAGTAAAT
CATATGAGGTTTTATTTTTTATTTCGTTGATTTGGGTTACAGTATGGAAATGGGGCATTTGACACAAGTTA
TTTTATTC

CGACATGTTTGCTTGGTGTCTTAGCAGCTCTTATAGTTCTAATGCTATAACTAAGAAAGTAATAATAATAA
TAATAAAAAGCTTTATATAATGTTTTATTATGGAAGTTTCGTTTTTGTGGCGCGACGCGATCAGCCAAAAT
CAGCGGAA

GCGTTTTTTTTTTTTTTTTGCTTTTTTTGGTTCCTTTCGCGGATATCGTATTATCGATATTACAACAAAAA
TGTTTGGAGATCTCCTTCGCTGTCTTTAAGTAAAAGGCTTTAACCGCTTATTTTTCTCTCTTTTTTTTCCTT
TTACCCCT

GAAATACTTTTTGTTACCCGACTTGTAGCTTCATTTTTTCATTTTTTTTTTTTTTTTTTATTTTTATTTTG
TATTGTTCTTTGAAAGAATCATGCATGGAAGCAATAACTATTATACGGAAGTTATATACAAGAAATCATTA
TATAGTTA

AATCGATATTATGGAAGATATTAACCGCGCGCCGTATTAGTGTACACTATATTAAACTACATTTTGCTTCT
TACTGAATTTATAAATTATGATTATATTATTATTACTATTATGACTACTGTATATATTTTTTAGAATTAGC
ATCGGGAA

ATTCAAATGTCCATGCGCGTTGATTGATGTGTGATATATATATATATATATATGTATATAGATGTATATTA
TTATGTTCAGAGGTGATTTTATCTTTGTACGCGTACCATTTTTGTAAAGTGACCTTGTTTTGACTTTTTAA
CGAGTAAA

AATGGCATAAAACTATTTACTTAACTTTTAACTCGGGAAATATGTATCACTATACTAAAAAACATTAATTT
AATCAAAGAAAAATAAATTTAATGTATGTATCTATATATATATATATATATCACTTAAAACAGACAATAGC
CATAATCA

ACCTATTGCCATTGTTATTCCGATTAATCTATTGTTCAGCAGCTCTTCTCTACCCTGTCATTCTAGTATTT
TTTTTTTTTTTTTTTGGTTTTACTTTTTTTTCTTCTTGCCTTTTTTTCTTGTTACTTTTTTTCTAGTTTTT
TTTCCTTC

TACCACCTATGGGAAAGGCAGTAACTCACAAGCATTCACGTACATAATTCTGTTTTTTTTTTTTTTATTCA
TTTCTAGCACGCTATCGGGGTTATTTTTTTTTTTGTAATATATAGTGATGGTATAATGTTGTATAGATACT
GGTATCGC

CTTTTAAAACTTCTGTTTCCGAACTCCTCATTTTTCATGCATAAATATATAAATATATATATATATATATA
TAAAGCATACATTTCAGTACCATGGGGTATATGATCCTCCCATTACGTATTCTAAAGTTCGCGTTTTCTCA
CAATGCAA

CAAATAAGTCAGTTTTTTTTTTTGTTTTCACCATAACAAAGAGATGGAAAGTTATACTAGATAAAGATTTT
TAAAAAAATATAAAAGAAAATAAAACGAAATTTATATTTACACAGTGCATCTTGTCCCTCTCTTTCTCGCT
AAAACTTA

GCGCGAGAAACAAAATTGTACATGAATGCTAAAAAAAGAAATGACAAAAAAAGAGAAAAAAAAAAATGAAA
CTACATAGTTAATTAATAATAGAAGTATTTGTCAATAGTATGATAATGAAATCGATATTATGGAAGATATT
AACCGCGC

TTTTGAGGTTTTATTTATCTTGTATTCTTTTTTTCTATTATTTTAAAGGTTCAATTTCTATCGACGGTTTT
TTTAATTATATATATATATATATATATATATGTATATAATGAATGTGTAATATTCCAAGTATACGTACCTT
GGGTTTAT

GATGGCCCCGTTTCGTCAATCAATTTGTTGATATTTGTCTTTCAAACGCGGTTTTAACATATCGCGAACAA
CGGTATTCGGGTAACAGCCTATCACTATTTTTACCGGCGGCTATTTTTCCTAAACCTAGTATATATACGCG
AAAATTGG

AAAGAAATTTGGCCACTGCATCGCCTTTCCTGCCCCATTGATTCAAATGTCCATGCGCGTTGATTGATGTG
TGATATATATATATATATATATGTATATAGATGTATATTATTATGTTCAGAGGTGATTTTATCTTTGTACG
CGTACCAT

ACGTCATAGAAAAACCACGTGAAAAAAAAAAAAAAAAAAAAAAAAAAAAAAGAAAAAGAAAAAGGGCAGCTGC
CAACTAGTACACACTACACAGATATTTATGAAAAACACTAACTTCTCCTTTTGGAACTGTTGACGTACCTT
CCCTTACC

CCAAGAAAAAAATTAAAAACAAAATAAAAAAAAAAAAGAAACTTGCGGCTCTTCACTCTTAAAAGACAAAAA
GTATTAGGATGATAAAAAAAATTCATAAAAAAAAAAAAATTCTATGGCCTCAGTTCTCGAAGAGCCCATTTA
TAATTGTT

CAGCTACACATTCTTTTATAATCCTTAATATTCTATATATACATATATGAAAAAATAGAAAACGCGAAAAC
TTGTCATTTTTTTTTTAGGCGTTTTTATAATATACTGAAAATAAAAAGAGGCTCTTTAAATGTTGACACTC
TACTCCAA

ACCCGTTTTCTTCGGTAATATTTTATAGTGAAAAATTTTGGTAAAAAAAAAAAAAAAAGAAAAAATTTTTTT
TATTTCAAATCTCATCTCATCTCTTAAAAAGGTTTATTCTGTTTACTCAAACTCTTCATCAAAAACAAAAG
TACTGCGC

ATTATCGAGGATAGAAGTAAGGCGTTGCATTTCCTTATAACTATCGTGTTTATTTTCTTAGTGGTGGTTCT
TTTATTATTACCCGTTTTCTTCGGTAATATTTTATAGTGAAAAATTTTGGTAAAAAAAAAAAAAAAAGAAAA
AATTTTTT

CGGGGTGTAAAAATTCTATGCCAAGAAAAAAATTAAAAACAAAATAAAAAAAAAAAAGAAACTTGCGGCTCT
TCACTCTTAAAAGACAAAAAGTATTAGGATGATAAAAAAAATTCATAAAAAAAAAAAAATTCTATGGCCTCA
GTTCTCGA

TAAATAATTAAAACATTATTTTTAAAAATTAAAAAAATATATTTTAGCAAATAGTTTTTACTTTTTTTTAA
ATGCAAAATAAGTTCACGTGATATTATTAATAATATTTTACCCAAAAATATGAGGGCATCGGAAAATTTTT
CAGTAATG

TTTTCTATTATTTTAAAGGTTCAATTTCTATCGACGGTTTTTTTAATTATATATATATATATATATATATA
TGTATATAATGAATGTGTAATATTCCAAGTATACGTACCTTGGGTTTATATTTTTTAACTTGTATATTCTT
TTACGATA

CCATACTCTTTTTTTCCTAATCCTTTTCAAGTTTAAATCCATGATTTAATATCTATTTATACATATAAATA
ATATAAGTAATGGTTTAATGCATTGTTTCAAAAAAAAAATAAAAAAAAAAGGGAAGAAATTATATATAAAT
ATACTTCT

TAAAGAAAATAAAATAAAAAATAAAATTTTTTTCTCCCGATAATCAATTTTCTTAATTAATTAATTGCGTT
ACGATTCCGTTTTTTTACTTCTTTTATCTCATTATCTATCTAAGTTATTTAAAAAAAAGAAAGAACTTTTT
ATGAACTT

TTGTATATTCTTTTACGATAAAATTTCTTCCAATTAACATCCCAGGCCTTGCTTCAATTTATCAATTGGTA
TATTAACTTTCAACACATTTTGTTTTTGTTTTTATTTTTGTTTTTTTTATTTTTTTTTTTTTTTCGCGTTT
CGTGCTTT

TTTCTAGCTCTTTTTCACTTTTTTGAGGTTTTATTTATCTTGTATTCTTTTTTTCTATTATTTTAAAGGTT
CAATTTCTATCGACGGTTTTTTTAATTATATATATATATATATATATATATGTATATAATGAATGTGTAAT
ATTCCAAG

TTTTTATCTTCTTCACTTGTTCTGTTTTCTTTTTTTTTTTTTTTTTTTTTTTCAAGTTCCACGAAAAAATT
TTCTCGTTTTCCTTTAGTCACTCTTGTCACATAAGGATTATCCGAACCCCGCCCCGCGGAATTAATTAGTT
GGCTAGAT

CCGGTAACGGTCGTAGTGAATGACTCATATTTTTCCATCTCTTTCGGCCTTGCCAAAAAATGAAAAAAAAA
AAAAAAAAAAAAAAAAAAATTAAAAACGATGTCCAGAATCACAACTACAACATAGTTTTGCCTCTTACTGG
CTTGATTG

TTTTTAGTACGAGGTGTACGGACAAAAGATACTAACGGACAAAACGCGGTTGCAACTGAAATTTTTTTTTC
ACTATTTTCGTAGGGCACATTGGTGTAAATATATATATATATACTCAAGGTTCATCTCGTTTTTTTTAATA
TTCTTGTT

AGGTTTTAACTAATTTTTCCAGGATATTTCAACTTTTCATAAATCAAATTTTTTTTTACTTTTTTTTTGCT
TTGCTATAGTATTTTAGAAAAGGACAAAAAAATACGAAAAAAGACAAAAAACCATATAAAAAAATTGATTA
GAAGAACT

TTTCTAATTATTCCGTTAGCGCCGCTTACTTTTAATGTTCTTAAGATAAAAAAACATCATTAATATTATTA
TTAAAAAATTCATATATATACCTATATCTATATGCACTATTTATGCTTCCACTTTTCTTTTCTTTCTAAAT
ACATCATT

GAATAATAATATAAAAAAAAAAAAAAAAAAAAAAAAAGATGTTTAAATGTTAACAACCTAACAGCAATAAA
AAATAAAAATAAAGGTAGAAGATTGCAAGAAAGAAACTTTACAATATTTTGTTTGGATGCCCAACACCGGC
GTCTTGGT

GGTAATGCATCATTCTATACGTGTCATTCTGAACGAGGCGCGCTTTCCTTTTTTCTTTTTGCTTTTTCTTT
TTTTTTCTCTTGAACTCGAGAAAAAAAATATAAAAGAGATGGAGGAACGGGAAAAAGTTAGTTGTGGTGAT
AGGTGGCA

GAATATATTTAATGACGGGAAAAGAACACGGGAAACGCTACTATTGTTGTATAGTGCGATATAGAACCTTT
TTTTATCTTCTTCACTTGTTCTGTTTCTTTTTTTTTTTTTTTTTTTTTTTTCAAGTTCCACGAAAAAATTT
TCTCGTTT

AAATAAACCCTTTCCTCTTTTTTACGGCGACCATACTCTTTTTTTCCTAATCCTTTTCAAGTTTAAATCCA
TGATTTAATATCTATTTATACATATAAATAATATAAGTAATGGTTTAATGCATTGTTTCAAAAAAAAAATA
AAAAAAAA

ATCAATTGGTATATTAACTTTCAACACATTTTGTTTTTGTTTTTATTTTTGTTTTTTTTTATTTTTTTTTT
TTTTCGCGTTTCGTGCTTTAATGAAACATGTGAAGTGTTTCGCAATGACTAAGTAGCCTCCACAGATGATC
AGCAGGTA

AGTTGTTCTTTACGTGCATCGATCATATAAGAAAAGGGAAAAAAGCCGTAAAGTAAAAAAATAAAGAGCTT
TCCATGGCTGGTTTTTTTTCTTTTTTTTTTTTTTTTTTGAAGACTACAGCAGGTATTACCCGGAGTGAAAA
AGGTTTCT

GGTTGCCATGGGACACAAAAACGTAGCCACAAAAAAAAAAAAAAAGGTGAAAGGAAGCGAGCATTAATTTT
TTATTTTAATATGTAACTGTGATATGTATTAACAAAGTATCTTTCATAATTTTTGACGAATATTTTTACTT
CTTTTTTA

GGACATCCGGCCATCGCGCGCGGGCCCGGTATTTGATCTCCGTTTTAGAAGCACAGAAAAAAATAATATGA
TGTTATGATGTTTAATAGATGGATTCATATCGTCCGGGTAAAAACGCTTTTGGAGAAATCAAGGAAAATCG
CCGAGATC

CATTCTATACGTGTCATTCTGAACGAGGCGCGCTTTCCTTTTTTCTTTTTGCTTTTTCTTTTTTTTTCTCT
TGAACTCGAGAAAAAAAATATAAAAGAGATGGAGGAACGGGAAAAAGTTAGTTGTGGTGATAGGTGGCAAG
TGGTATTC

AGAAACCTGTATTCATCCTCGGCTTCTTGTATATGATTCTAAAATAATGTGTGAAAAAAAAAATAAAATAA
AAAAAAGAGGAAAATAATATAGAATAACTATTAAGTTTCATTAAAAAAAAACCATTTGAATATACGACCAA
AAACGTTA

AAATAGTGACTTTAATTCGGAAAAATAAAAAAAAAAAAAAAAACATAAGGAAATTTTAATTAAATAGTCTA
TCTACACATTTCCTAAAAGAAAATATTATTGCATTACTTTTTTGAAGATCTATAAAGGGCACTGTCTTACT
TTTTAAAA

TGGAAAATGAGCCAAAAGATTCTTTTGCTTGAGCACAGAGGTGAGATGCCCAAGTGAGAATTTTTTTTAAA
TAATGATGTACTTTAATACAATATATATATATATATATATATATATATATATATATATAAGGAATGATA
ACTCTATT

AACATTAATTTAATCAAAGAAAAATAAATTTAATGTATGTATCTATATATATATATATATATCACTTAAAA
CAGACAATAGCCATAATCAACTGGGTTATTATTATACAATATTAAAGGAAGAACGCATATTTGAAAAAAAA
ATAAAATG

TTGCAAAAACTAAACTCTACTCACGCACACTGGAATGAATGGCAATATTCTTTTTTAGGTTAACCGGCCGG
ACAGTAATATAGTAATCGTTTTGTACGTTTTTCAAGAAGCGACGCACAACTGTTTTCCATTTTTTTTTTTT
TTTTTTCA

CGATCAATTAGTTGAACAATTAACTTAAAATCTAATATATATAAATATATATATATATATATATAGGAAAT
ATACGGCACGTTATACTAGAGATATTAAACATCCCAACTCGGTTAATGGGTACAATAAAAAAAAGAGGGAT
TAATGTTT

AAAATAATGTGTGAAAAAAAAAATAAAATAAAAAAAAGAGGAAAATAATATAGAATAACTATTAAGTTTCA
TTAAAAAAAAACCATTTGAATATACGACCAAAAACGTTACGCTTTCATAAAGTGTGAATAAGCAAGGGAAC
TATACTTG

TTTTCGGAAGTCATATACATTAAATAATTAAAACATTATTTTTAAAAATTAAAAAAATATATTTTAGCAAA
TAGTTTTTACTTTTTTTTAAATGCAAAATAAGTTCACGTGATATTATTAATAATATTTTACCCAAAAATAT
GAGGGCAT

GTAGTGAGGCGATTAGATGTCGTTTTCATGTACAATAATGATATTATTTTTTTTTTTTCAATTTTTAGTAT
TTTACATGGCATTCTTACACTTTTATAAAGTATTATTTATATTACTAGTAGCAGTAAGTGATCGCCCTTTT
TTTTAGCA

GTAGTCTAATGTTCTTTTCTTTTTTTTCTTTATTCTTTTTTCTCCTCTTTATGAATACAGATATTCAATTT
TTTGAATATCTGATTGTTTTTATTTTCATTTCAATTTTTTTTTTTTTTCAATTTTTTTTATCCGATCGTTG
CGCCAAGC

AGTTTTTTTTTTTGTTTTCACCATAACAAAGAGATGGAAAGTTATACTAGATAAAGATTTTTAAAAAATAT
AAAAGAAAATAAAACGAAATTTATATTTTACACAGTGCATCTTGTCCCTCTCTTTCTCGCTAAAACTTAGT
TTCTTCTT

ATTGTCTTGTTTCCTTTTTTTTTTTTTTTTTTTTGTTTTTTTATGACTAAATAAAGTAAATCATATGAGGT
TTTATTTTTTATTTCGTTGATTTGGGTTACAGTATGGAAATGGGGCATTTGACACAAGTTATTTTATTCAT
ATGTTTTA

ATAGAGAGCTTTTAATTTTTATTTTTTTTCACTTGTATTTTTGACGGGCTATTAATTTTAAATGAAACTTT
TCTACATGATGGAACATAAACATAAATATAGGTATAGATAAATTGTTCTTCTTTTTTTTTTTTTTTCTTTA
AGAGAAAG

AGTTGTACAAAAATAGTGACTTTAATTCGGAAAAATAAAAAAAAAAAAAAAAACATAAGGAAATTTTAATT
AAATAGTCTATCTACACATTTCCTAAAAGAAAATATTATTGCATTACTTTTTTGAAGATCTATAAAGGGCA
CTGTCTTA

GGCAATATTCTTTTTTAGGTTAACCGGCCGGACAGTAATATAGTAATCGTTTTGTACGTTTTTCAAGAAGC
GACGCACAACTGTTTTCCATTTTTTTTTTTTTTTTTTCAGTGATCATCGTCCATGAAAAAAATTTTTCATT
TGTCTCTT

GGACACAAAAACGTAGCCACAAAAAAAAAAAAAAAAGGTGAAAGGAAGCGAGCATTAATTTTTTATTTTAAT
ATGTAACTGTGATATGTATTAACAAAGTATCTTTCATAATTTTTGACGAATATTTTTACTTCTTTTTTACT
ATTGAAGG

GTTTTAACATATCGCGAACAACGGTATTCGGGTAACAGCCTATCACTATTTTTACCGGCGGCTATTTTTCC
TAAACCTAGTATATATACGCGAAAATTGGCTACCTGCAAAATTCCAATAGTGGCGAATGTATTGGAATTAA
TCTGCAAG

GCTTGGTGTCTTAGCAGCTCTTATAGTTCTAATGCTATAACTAAGAAAGTAATAATAATAATAATAAAAAG
CTTTATATAATGTTTTATTATGGAAGTTTCGTTTTTGTGGCGCGACGCGATCAGCCAAAATCAGCGGAAAA
ATTCGTCG

TAAAAAGAAAAAAGCGAATATTTCAACACTGTGTTCTAAGAAAAGGCTCTATAGCTTTTTTTATTAGTTTT
ATTAATTTGAAAAATGCCTTGTTTGTTTTATATATATATATGTGTGTATTTAGGGCAGCCGCGGCCAACAG
GGGGTATG

ATTTTTTTTTTTTTTTATTTTTTTATTTTTTTTTTGTTTTATATAATTCCTCGCGCTGCCTTAATGATAATT
CTTGATTTACTAATTGTTTCATGACTTCTGGTGCTAAAGGGAAGGGGGAAAAGGGAAGCGAAAACTTCAAG
AGCAGAAC

TATACTTTTCCCTGCATAATCATCCCACTTTTTTTTTTTTTTTTTTTAATTCATTTTCAAGATTTTAGACG
TTGTATAATACATTTTTACCCACTACTGCGGTTTATTGTAATATTTTGTGAGATTTTATGAATGCGTTCCA
TCATCTAT

GAAATATCACGAATAATAATATAAAAAAAAAAAAAAAAAAAAAAAAAAGATGTTTAAATGTTAACAACCTAA
CAGCAATAAAAAAATAAAAATAAAGGTAGAAGATTGCAAGAAAGAAACTTTACAATATTTTGTTTGGATGCC
CAACACCG

TTAACTTTTAACTCGGGAAATATGTATCACTATACTAAAAAACATTAATTTAATCAAAGAAAAATAAATTT
AATGTATGTATCTATATATATATATATATATATCACTTAAAACAGACAATAGCCATAATCAACTGGGTTATTA
TTATACAA

GTGTCATTCTGAACGAGGCGCGCTTTCCTTTTTTCTTTTTGCTTTTTCTTTTTTTTTCTCTTGAACTCGAG
AAAAAAAATATAAAAGAGATGGAGGAACGGGAAAAAGTTAGTTGTGGTGATAGGTGGCAAGTGGTATTCCG
TAAGAACA

ATGAATACAGATATTCAATTTTTTGAATATCTGATTGTTTTTATTTTCATTTCAATTTTTTTTTTTTTTCA
ATTTTTTTTATCCGATCGTTGCGCCAAGCTTCTATCGCTTAAAACAATGACACTTCGCTGTGAAATGACAC
AATGGCTT

TTGAATATTTGGAATGAGAAAGAAATATAGTTACATAATGTACAAAAAAGAAGCAAGAAAGGAATATATAT
ATATATATATATATATATATATATGTATATTGTTATTTAGAATATCTCTCTCTCTTCGAGAATCTCTATAT
CCTCGAAA

GAAAAGAAATTCTTTATTTCACAAAAATTTCTCCCCACTTTTAATGTAATTTTTCTTTATATAATATATAT
ATATATATACATGAGTATCAATACAATATAACCTAATCTAGCTTTTTATCCAATGGACTTGACTATCATCA
GTACTAGA

TCGTAGTGAATGACTCATATTTTTCCATCTCTTTCGGCCTTGCCAAAAAATGAAAAAAAAAAAAAAAAAAA
AAAAAAAAATTAAAAACGATGTCCAGAATCACAACTACAACATAGTTTTGCCTCTTACTGGCTTGATTGGA
AGTTAGCT

AGAAATATAGTTACATAATGTACAAAAAAGAAGCAAGAAAGGAATATATATATATATATATATATATATAT
ATATGTATATTGTTATTTAGAATATCTCTCTCTCTTCGAGAATCTCTATATCCTCGAAAAGAACTTTTAGC
ATATTCAC

TGCTGCACGTGTAGTCTAATGTTCTTTTCTTTTTTTTCTTTATTCTTTTTTCTCCTCTTTATGAATACAGA
TATTCAATTTTTTGAATATCTGATTGTTTTTATTTTCATTTCAATTTTTTTTTTTTTTTCAATTTTTTTTTAT
CCGATCGT

TTAGCAGCTCTTATAGTTCTAATGCTATAACTAAGAAAGTAATAATAATAATAATAAAAAGCTTTATATAA
TGTTTTATTATGGAAGTTTCGTTTTTGTGGCGCGACGCGATCAGCCAAAATCAGCGGAAAAATTCGTCGGA
ACATTTTT

TCCCCGTTTTCTTTTACTTTTACTTCATTTAATATAATTCATATTTATTTCCCATGTACCAATTAATTACT
CATAAAAAGAACAGAAATAGAAAAAAGTTGTATATAACAAGCCAAAAAAAAGAAAAAAAAAGGAAAAAAAA
GAAAACAC

CAATCAGGTTGGGGTCTAGTCGACATGTTTGCTTGGTGTCTTAGCAGCTCTTATAGTTCTAATGCTATAAC
TAAGAAAGTAATAATAATAATAATAAAAAGCTTTATATAATGTTTTATTATGGAAGTTTCGTTTTTGTGGC
GCGACGCG

GTTTTCAATCCACCAAAACCAAGAAAGTAGTTGACAAAAAAATGAAAACCACTATTATTTAAATTACGGAA
AAAAAAAAACCAATAAACAAAAAACATAATAAAAGAAATGAAAAAAAAGAATATTAGTAGAGCATACTGAA
TTGTTTTC

AAAAGCAAGAGCAGCAAGAGCAGGACAAAAAGAAAAAATCTAACCATTAAAAACGGCGATATATATTGGGC
ATAAAACCTATATAAAATAACAGCAATAATAAAAATGCAAAATATAACATAGCACGGTATAGTTAGCTCAT
ATATGTTT

TCGCCTTTCCTGCCCCATTGATTCAAATGTCCATGCGCGTTGATTGATGTGTGATATATATATATATATAT
ATGTATATAGATGTATATTATTATGTTCAGAGGTGATTTTATCTTTGTACGCGTACCATTTTTGTAAAGTG
ACCTTGTT

GAAAATCCTACTCTGTAATAAAATAATCCCGGGGTAATGGATTGGATTTTTCTATCAGGATCGTTTACGTA
TATATAATACACTGTCGGAGTTTAGCGCGCTTACAGCCGCTACTTGTGGAGTCCTTTTTTTTTTTTTTTT
TTGGGGTT

TGAGTGTCGAAGAAACCTGTATTCATCCTCGGCTTCTTGTATATGATTCTAAAATAATGTGTGAAAAAAAA
AATAAAATAAAAAAAAGAGGAAAATAATATAGAATAACTATTAAGTTTCATTAAAAAAAAACCATTTGAAT
ATACGACC

ATAACTACAGAAATATTGGGAGGTTTTAACTAATTTTTCCAGGATATTTCAACTTTTCATAAATCAAATTT
TTTTTACTTTTTTTTTTGCTTTGCTATAGTATTTTAGAAAAGGACAAAAAAATACGAAAAAAGACAAAAAA
CCATATAA

AGTTTTTTTTTTAATGTTCTTCTCTCTCTTCTTCCTGTTTTTTCATGGCAATAATTTTGACTAGTTTTTCT
TTTTTTTTTTTTTTTTCATTCTTTTTCATTATTACTTAAGTTCTGTAAAGATATATGGAAGAGAAAATAA
AAAGAATA

CCAGTCTTTTGTCTCTTTTTTTTTTTTCTTCTTTTTTTTTTTTTTTTTTTTTTTTGCAGCATTATGATAGAA
CAATAGGGCTCAAGATCGCACCAAGGCTAACAGTAACGGTATACGCAATCGTAGTGAAAGTGATTTTCAAT
CAAGCATC

CAGGACAAAAAGAAAAAATCTAACCATTAAAAACGGCGATATATATTGGGCATAAAACCTATATAAAATAA
CAGCAATAATAAAAATGCAAAATATAACATAGCACGGTATAGTTAGCTCATATATGTTTTAACGAGCCTTA
CATTATAT

AAGATTTGAACCGCGCGGGAATTCACATTGCAGTATGGACGGACATCCGGCCATCGCGCGCGGGCCCGGTA
TTTGATCTCCGTTTTAGAAGCACAGAAAAAAATAATATGATGTTATGATGTTTAATAGATGGATTCATATC
GTCCGGGT

ACCATTGACGACACTGCCTCCCAAATTTTAAAATTTGAGGGCAGAATATAAACTTTAAATGAAAATAGATA
ATATTTATATATATTAACGTTATTACAATTATTTTTTATCATCTAGTACATCTCTGCGCATTTTTCTCTTC
TATATACA

CAGAAAATTCGTTTTCAATCCACCAAAACCAAGAAAGTAGTTGACAAAAAAATGAAAACCACTATTATTTA
AATTACGGAAAAAAAAAAACCAATAAACAAAAAACATAATAAAAGAAATGAAAAAAAAGAATATTAGTAGA
GCATACTG

CGGCAAAGCGAGACGATGCAGTAATCAGCGGCAATGAACTACGTATGTAAAAAAAAAGATCGGGGTGTAAA
AATTCTATGCCAAGAAAAAAATTAAAAACAAAATAAAAAAAAAAAGAAACTTGCGGCTCTTCACTCTTAAA
AGACAAAA

CTCTTTTTTTTTTTTTTTTTTTTTTTTCTCCTTCATTTTGACTTTTCCTTCTGTGTTAACATGATGAGTTT
ATACGTGCATCCTAACTAGTAGAATGTATATATATTATATTATGATTATATATATAATTTTGATTAAAATA
ACAATTAT

GGAATGAGAAAGAAATATAGTTACATAATGTACAAAAAAGAAGCAAGAAAGGAATATATATATATATATAT
ATATATATATATATGTATATTGTTATTTAGAATATCTCTCTCTCTTCGAGAATCTCTATATCCTCGAAAAG
AACTTTTA

ATATGATTCTAAAATAATGTGTGAAAAAAAAAATAAAATAAAAAAAAGAGGAAAATAATATAGAATAACTA
TTAAGTTTCATTAAAAAAAAACCATTTGAATATACGACCAAAAACGTTACGCTTTCATAAAGTGTGAATAA
GCAAGGGA

ATCTATAGGGCATATACAAAAAAAAAAGGTAGGTGTAAAAGATAATAAAATACAACCCTATTAGCTTTGAT
AAAAAATTAGTAGGGTTAGAATGTCATATGGTAGGGTGATATAAACGCGTGAACTTTACGCGTTTATATGT
ACTAAGTT

ATTTTTTAACTTGTATATTCTTTTACGATAAAATTTCTTCCAATTAACATCCCAGGCCTTGCTTCAATTTA
TCAATTGGTATATTAACTTTCAACACATTTTGTTTTTGTTTTTATTTTTGTTTTTTTTATTTTTTTTTTTT
TTTCGCGT

TACCACATTGTTTCTAATTATTCCGTTAGCGCCGCTTACTTTTAATGTTCTTAAGATAAAAAAACATCATT
AATATTATTATTAAAAAATTCATATATATACCTATATCTATATGCACTATTTATGCTTCCACTTTTCTTTT
CTTTCTAA

TTTACGGCGACCATACTCTTTTTTTCCTAATCCTTTTCAAGTTTAAATCCATGATTTAATATCTATTTATA
CATATAAATAATATAAGTAATGGTTTAATGCATTGTTTCAAAAAAAAAAATAAAAAAAAAAGGAAGAAATT
ATATATAA

AAATTTGAGGGCAGAATATAAACTTTAAATGAAAATAGATAATATTTATATATATTAACGTTATTACAATT
ATTTTTTATCATCTAGTACATCTCTGCGCATTTTTCTCTTCTATATACAGCTTAATATGTCGAAAACGCGA
AGCAAGAA

AACAACTTTTGAATATATTTAATGACGGGAAAAGAACACGGGAAACGCTACTATTGTTGTATAGTGCGATA
TAGAACCTTTTTTTATCTTCTTCACTTGTTCTGTTTCTTTTTTTTTTTTTTTTTTTTTTTTCAAGTTCCAC
GAAAAAAT

ATAAAAAAAAAAAAAAAAAAAAAAAAGATGTTTAAATGTTAACAACCTAACAGCAATAAAAAAATAAAAAT
AAAGGTAGAAGATTGCAAGAAAGAAACTTTACAATATTTTGTTTGGATGCCCAACACCGGCGTCTTGGTGT
TCTTCTAT

TATGTATAGCAGAATTATAAGAGGCGAAATCGATCAATTAGTTGAACAATTAACTTAAAATCTAATATATA
TAAATATATATATATATATATATAGGAAATATACGGCACGTTATACTAGAGATATTAAACATCCCAACTCG
GTTAATGG

TTATAGTTCTAATGCTATAACTAAGAAAGTAATAATAATAATAATAAAAAGCTTTATATAATGTTTTATTA
TGGAAGTTTCGTTTTTGTGGCGCGACGCGATCAGCCAAAATCAGCGGAAAAATTCGTCGGAACATTTTTTT
CTTTTTTG

TTTTTTTTTTTTTTTTTCTCCTTCATTTTGACTTTTCCTTCTGTGTTAACATGATGAGTTTATACGTGCAT
CCTAACTAGTAGAATGTATATATATTATATTATGATTATATATATAATTTTGATTAAAATAACAATTATCT
TAAGAAAA

AAATTTTTGTTGTGCACAAAACTAGTTATTGTACTTCCCGCCTTTGCTAAAGACGCGTAAGAAAAAAAAGT
ACAAATAATGCCCTATAAAGAAAAAAATTTAAATAAAACGCGAACTTAGTTTGGACGTAATACTTCTCCTT
CTCGGGCC

TTTCCTCTTTTTTACGGCGACCATACTCTTTTTTTCCTAATCCTTTTCAAGTTTAAATCCATGATTTAATA
TCTATTTATACATATAAATAATATAAGTAATGGTTTAATGCATTGTTTCAAAAAAAAAATAAAAAAAAAAG
GGAAGAAA

GCAGCAAGAGCAGGACAAAAAGAAAAAATCTAACCATTAAAAACGGCGATATATATTGGGCATAAAACCTA
TATAAAATAACAGCAATAATAAAAATGCAAAATATAACATAGCACGGTATAGTTAGCTCATATATGTTTTA
ACGAGCCT

CTTCTAAAAATAACGGAGTTTGAATCTCTAAATCTGTTTTAACTTCTTTTTACTATTATTTTTAGTCTTAG
TATCTCATCTCATCTCAATTTCTATATTCCACTATAAAATTTTTCACTCTTTCTGCGCGCGCCAATGTCCC
CGCAACTA

TTGTCCCCGTTTCCTAATGTTAACTCTTTTTCTAATAGGGGCAACTTACTTTTGTTTTCTCATTTGGCGAA
AAAAAGTAAGTAGAAATAAATAAAAAAATAATATTATAATGTGCTGTATATATAAATACAAATGCGAAAGC
TAATGCAG

TTACTACAAAACGATAAATTTCCCCGTTTTCTTTTACTTTTACTTCATTTAATATAATTCATATTTATTTC
CCATGTACCAATTAATTACTCATAAAAAGAACAGAAATAGAAAAAAGTTGTATATAACAAGCCAAAAAAAA
GAAAAAAA

CCCCTGATTTCCCAAATTTTTTATGCATATACAAATGTTGAAAAGAAACGAATATTATTAGCAAGTAATAT
ATAGTATATAGTATATATATATATATTTATATATGTAAACATATAATGAATATATAAATTGGTATGTAAAAGA
GTTGCGAG

CAGTATGGACGGACATCCGGCCATCGCGCGCGGGCCCGGTATTTGATCTCCGTTTTAGAAGCACAGAAAAA
AATAATATGATGTTATGATGTTTAATAGATGGATTCATATCGTCCGGGTAAAAACGCTTTTGGAGAAATCA
AGGAAAAT

TTCTGTCTCTGTTATATTTCCACATGTCATCATTTCAAATATATGTACTTTAAAGAAAATAAAATAAAAAA
TAAAATTTTTTTCTCCCGATAATCAATTTTCTTAATTAATTAATTGCGTTACGATTCCGTTTTTTTACTTC
TTTTATCT

TTTTTTTTTCCCTAATGCATACATGATTTTTACGTCTTTATATAATACTGTTCTATTCATATTCTAGCCTA
GTTTAATTATAATAATATCTATAATGTTTTTTTATATATCCGCATATCTGTGAGCTTGGGTATACAGAAAA
AAATGGCA

ATCTTTTCTTTATACTTTTCCCTGCATAATCATCCCACTTTTTTTTTTTTTTTTTTTAATTCATTTTCAAG
ATTTTAGACGTTGTATAATACATTTTTACCCACTACTGCGGTTTATTGTAATATTTTGTGAGATTTTATGA
ATGCGTTC

TGACTCATATTTTTCCATCTCTTTCGGCCTTGCCAAAAAAATGAAAAAAAAAAAAAAAAAAAAAAAAAAAT
TAAAAACGATGTCCAGAATCACAACTACAACATAGTTTTGCCTCTTACTGGCTTGATTGGAAGTTAGCTGT
TAAATTTC

GGCACCCACCCATCGCATATCAGGAGAACTTCCCTGTGCATACGGGACAGCAGAGATAGGCTGTTCTCGCG
CGTTTTTTTTTTTTTTTTGCTTTTTTTGGTTCCTTTCGCGGATATCGTATTATCGATATTACAACAAAAAT
GTTTGGAG

TCAATTTCTATCGACGGTTTTTTTAATTATATATATATATATATATATATATGTATATAATGAATGTGTAA
TATTCCAAGTATACGTACCTTGGGTTTATATTTTTTAACTTGTATATTCTTTTACGATAAAATTTCTTCCA
ATTAACAT

AACTTTTCATAAATCAAATTTTTTTTACTTTTTTTTTTGCTTTGCTATAGTATTTTAGAAAAGGACAAAAA
AATACGAAAAAAGACAAAAAACCATATAAAAAAATTGATTAGAAGAACTTATAATGGCATTGGCAAATTCC
AGACCTTT

AACTAATTTATCACCGCTAACGTTACGCGTTTGGCGTCCTAAAAAAAAGAAGGAAAAAAAAACCTATTTAT
TCACTCTATTTCGCCATAAAACGACATATAAATATTATACAGATGATGAAAGCCCACAAATTATGTTTAGT
AGCGTTCT

CCATCGCGCGCGGGCCCGGTATTTGATCTCCGTTTTAGAAGCACAGAAAAAAATAATATGATGTTATGATG
TTTAATAGATGGATTCATATCGTCCGGGTAAAAACGCTTTTGGAGAAATCAAGGAAAATCGCCGAGATCAC
TAGGAAAT

AGCCAAAGACAAGTTCCGCACTCCATCAAGTGAACCTCAACAGCTACACATTCTTTTATAATCCTTAATAT
TCTATATATACATATATGAAAAAAATAGAAAACGCGAAAACTTGTCATTTTTTTTTAGGCGTTTTTATAAT
ATACTGAA

ATCCTTAATATTCTATATATACATATATGAAAAAAATAGAAAACGCGAAAACTTGTCATTTTTTTTTAGGC
GTTTTTATAATATACTGAAAATAAAAAGAGGCTCTTTAAATGTTGACACTCTACTCCAATATCAACTGTAA
AAAATCTC

TACGTGCATCGATCATATAAGAAAAGGGAAAAAAGCCGTAAAGTAAAAAAATAAAGAGCTTTCCATGGCTG
GTTTTTTTTCTTTTTTTTTTTTTTTTTTGAAGACTACAGCAGGTATTACCCGGAGTGAAAAAGGTTTCTCA
CACTAAAA

AAAAAACACATTTCTTAACAGATGGCTGAAAAATTTTTTTTTTTTTTTTGGTTTTGCTTTGTATACACATG
TATCTATTTTTATAAAGATGAAATATATACGTCTAAGAGCTAAAATGAAAAACTATACTAATCACTTATAT
CTATTCTG

TTGTTACCCGACTTGTAGCTTCATTTTTTCATTTTTTTTTTTTTTTTTTATTTTTATTTTGTATTGTTCTT
TGAAAGAATCATGCATGGAAGCAATAACTATTATACGGAAGTTATATACAAGAAATCATTATATAGTTACA
GCATCATA

TACAATATTTTGTTTGGATGCCCAACACCGGCGTCTTGGTGTTCTTCTATAATATTTATTTTATTTATTTT
TTATTTATATATGTACATATATATTCCTATTATCTATTAAGAAAGTAAACTTAGTGTAAATCTACCACTGT
TCCAAGGC

ATGATAATGAAATCGATATTATGGAAGATATTAACCGCGCGCCGTATTAGTGTACACTATATTAAACTACA
TTTTGCTTCTTACTGAATTTATAAATTATGATTATATTATTATTACTATTATGACTACTGTATATATTTTT
TAGAATTA

GGGATCCGGCGTCACAAGCCACGGCCCGTCCCGTCCGCGTGGTTTTTAATTCTGGCTCCGCGTAGTACATT
TTGTAGCTTGCCTTTTTTTCCGGCTTCTAGAAAACTGGAAAAACCATAAGAAAAAAATGGAAGGGGAAAAC
AAAAACCA

TATCTTATTGAAAAAACACATTTCTTAACAGATGGCTGAAAAATTTTTTTTTTTTTTTTGGTTTTGCTTTG
TATACACATGTATCTATTTTTATAAAGATGAAATATATACGTCTAAGAGCTAAAATGAAAAACTATACTAA
TCACTTAT

GATGGCTGAAAAATTTTTTTTTTTTTTTTGGTTTTGCTTTGTATACACATGTATCTATTTTTATAAAGATG
AAATATATACGTCTAAGAGCTAAAATGAAAAACTATACTAATCACTTATATCTATTCTGGTGATTCATCAT
CTTCAGCT

GTGTTGAAACAAAAAAATACAGTTGTTCTTTACGTGCATCGATCATATAAGAAAAGGGAAAAAAGCCGTAA
AGTAAAAAAATAAAGAGCTTTCCATGGCTGGTTTTTTTTCTTTTTTTTTTTTTTTTTTGAAGACTACAGCA
GGTATTAC

TGGCATTAACTTTATTTGCCCACTTATATTTAGAGATCTTGAAATACTTTTTGTTACCCGACTTGTAGCTT
CATTTTTTCATTTTTTTTTTTTTTTTTTTATTTTTATTTGTATTGTTCTTTGAAAGAATCATGCATGGAAG
CAATAACT

AGAATATTGCGCTCGTGTTAGTGTTGAAACAAAAAAATACAGTTGTTCTTTACGTGCATCGATCATATAAG
AAAAGGGAAAAAAGCCGTAAAGTAAAAAAATAAAGAGCTTTCCATGGCTGGTTTTTTTTCTTTTTTTTTTT
TTTTTTTG

TATACTAAAAAACATTAATTTAATCAAAGAAAAATAAATTTAATGTATGTATCTATATATATATATATATA
TCACTTAAAACAGACAATAGCCATAATCAACTGGGTTATTATTATACAATATTAAAGGAAGAACGCATATT
TGAAAAAA

AAATTTTTTTTTTTTTTTTGGTTTTGCTTTGTATACACATGTATCTATTTTTATAAAGATGAAATATATAC
GTCTAAGAGCTAAAATGAAAAACTATACTAATCACTTATATCTATTCTGGTGATTCATCATCTTCAGCTTC
TGTATCCG

GAAGTGAAACGCGGGGACAACCATGATCTTTTTTGAGTAGGATAATTTTTTATGTCGGTTTGATATATTAT
AAAGCAAGTCATATATAATATCATACTGATTTTAACCAAACGATAATCAATCAGAAAAAGAAAATAAATAA
AAAATCTA

TTTGTTTTCTCATTTGGCGAAAAAAAGTAAGTAGAAATAAATAAAAAAATAATATTATAATGTGCTGTATA
TATAAATACAAATGCGAAAGCTAATGCAGATTTTGCCTAGTACTCTCTAGAAATAGTAATGTCCCTTTCCA
GCTCAACC

GATCATATAAGAAAAGGGAAAAAAGCCGTAAAGTAAAAAAATAAAGAGCTTTCCATGGCTGGTTTTTTTTC
TTTTTTTTTTTTTTTTTTGAAGACTACAGCAGGTATTACCCGGAGTGAAAAAGGTTTCTCACACTAAAATT
TACGTGTT

GTTCTTTCCTCTGTGGTGGTCCAGTCTTTTGTCTCTTTTTTTTTTTCTTCTTTTTTTTTTTTTTTTTTTTT
TTTGCAGCATTATGATAGAACAATAGGGCTCAAGATCGCACCAAGGCTAACAGTAACGGTATACGCAATCG
TAGTGAAA

TTTAATTTTTATTTTTTTTCACTTGTATTTTTGACGGGCTATTAATTTTAAATGAAACTTTTCTACATGAT
GGAACATAAACATAAATATAGGTATAGATAAATTGTTCTTCTTTTTTTTTTTTTTTCTTTAAGAGAAAGAG
TAAGTAAA

TTCTGTTTCCGAACTCCTCATTTTTCATGCATAAATATATAAATATATATATATATATATATAAAGCATAC
ATTTCAGTACCATGGGGTATATGATCCTCCCATTACGTATTCTAAAGTTCGCGTTTTCTCACAATGCAAGT
TTTTATAT

CTGTGGTGGTCCAGTCTTTTGTCTCTTTTTTTTTTTCTTCTTTTTTTTTTTTTTTTTTTTTTTTGCAGCAT
TATGATAGAACAATAGGGCTCAAGATCGCACCAAGGCTAACAGTAACGGTATACGCAATCGTAGTGAAAGT
GATTTTCA

TACCCTGTCATTCTAGTATTTTTTTTTTTTTTTTTTGGTTTACTTTTTTTTCTTCTTGCCTTTTTTTTCTT
GTTACTTTTTTTCTAGTTTTTTTTCCTTCCACTAAGCTTTTTCCTTGATTTATCCTTGGGTTCTTCTTTCT
ACTCCTTT

AAAAATATTAAAATTGAGACCAAATAAAGATCCTGAATCATAATATATACGTGTGTACTTCACTTTCTATA
GAGGAAAAAAAGCTAAAAAAAAAAAAAAAATGATAAAATAAACGAAAAAAACCATAAAACGATCATGATAGA
ATAATGTA

AAATCAAATTTTTTTTACTTTTTTTTTTGCTTTGCTATAGTATTTTAGAAAAGGACAAAAAAATACGAAAA
AAGACAAAAAACCATATAAAAAAATTGATTAGAAGAACTTATAATGGCATTGGCAAATTCCAGACCTTTGC
AAATACCA

CAATCAATCAATAACTACAGAAATATTGGGAGGTTTTAACTAATTTTTCCAGGATATTTCAACTTTTCATA
AATCAAATTTTTTTTACTTTTTTTTTTGCTTTGCTATAGTATTTTAGAAAAGGACAAAAAAATACGAAAAA
AGACAAAA

TTTATAAGAAATAGAGAGCTTTTAATTTTTATTTTTTTTCACTTGTATTTTTGACGGGCTATTAATTTTAA
ATGAAACTTTTCTACATGATGGAACATAAACATAAATATAGGTATAGATAAATTGTTCTTCTTTTTTTTTT
TTTTTCTT

ATCGAACAAGAAAAGCAAGAGCAGCAAGAGCAGGACAAAAAGAAAAAATCTAACCATTAAAAACGGCGATA
TATATTGGGCATAAAACCTATATAAAATAACAGCAATAATAAAAATGCAAAATATAACATAGCACGGTATA
GTTAGCTC

GACATATCATGGTTGCCATGGGACACAAAAACGTAGCCACAAAAAAAAAAAAAAAAGGTGAAAGGAAGCGAG
CATTAATTTTTTATTTTAATATGTAACTGTGATATGTATTAACAAAGTATCTTTCATAATTTTTGACGAAT
ATTTTTAC

AGGATATTTCAACTTTTCATAAATCAAATTTTTTTTACTTTTTTTTTTGCTTTGCTATAGTATTTTAGAAA
AGGACAAAAAAATACGAAAAAAGACAAAAAACCATATAAAAAAATTGATTAGAAGAACTTATAATGGCATT
GGCAAATT

GTTTAGTCAGCATCTACAAGCCCTATACCGAGGAAAGCGAATAGTCGCCATGCTAAACGCGCGGAACAAGG
CCATATTTATATATTTAATGCTTTTAACTATTATTAGTTTTTCTCACCCAGGGCTGGTTTCTTTACCCTGT
GTGATGAA

GACCAACCCGATTTGTTAGGATCTTTTCTTTATACTTTTCCCTGCATAATCATCCCACTTTTTTTTTTTTT
TTTTTTAATTCATTTTCAAGATTTTAGACGTTGTATAATACATTTTTACCCACTACTGCGGTTTATTGTAA
TATTTTGT

TGATGTGTTCATTTTTTTTATTGATAAAAATAGGGGAAAGTGTCTATATATTTTGCGTATAAAATTCTCTAC
ATTTTTTTTTCTTTGTTTAGTATGTGTATTGAAACATTTTATTTATAGGTTAGAGAAACAAAAAAAAAAAA
ACTGCAAT

TAATTTTTCCAGGATATTTCAACTTTTCATAAATCAAATTTTTTTTACTTTTTTTTTTGCTTTGCTATAGT
ATTTTAGAAAAGGACAAAAAAATACGAAAAAAGACAAAAAACCATATAAAAAAATTGATTAGAAGAACTTA
TAATGGCA

TCTGTTTTCTTTTTTTTTTTTTTTTTTTTTTTTCAAGTTCCACGAAAAAATTTTCTCGTTTTCCTTTAGTCA
CTCTTGTCACATAAGGATTATCCGAACCCCGCCCCGCGGAATTAATTAGTTGGCTAGATTCATGCTTGGTA
GAATGAAT

TAATCAAAGAAAAATAAATTTAATGTATGTATCTATATATATATATATATATCACTTAAAACAGACAATAG
CCATAATCAACTGGGTTATTATTATACAATATTAAAGGAAGAACGCATATTTGAAAAAAAAATAAAATGGG
TAATGAAT

TATTAAAGGAAGAACGCATATTTGAAAAAAAAATAAAATGGGTAATGAATTTTAGCACAAATACTTCACAA
AAGGAAATATACTAGTAGTAAATGTATATATTTATATGTTTATACAATGAATGGTACAAGAAATTTTGATA
TTCTTGCT

CTGAAGTGTGTGGTCGGTAAAACAACAGCGTAGATGCTTTTAAAAAAATTGTAGTATCACAGTTATATGTA
TAGCAAAGAAAAAGAGTCGCCTCTAAAAAAAAAAAAAAAACTCCATAAAAACAACAAAAAGAGACGGACGCG
GTTAACGA

ATATTAACTTTCAACACATTTTGTTTTTGTTTTTATTTTTGTTTTTTTTATTTTTTTTTTTTTTTCGCGTT
TCGTGCTTTAATGAAACATGTGAAGTGTTTCGCAATGACTAAGTAGCCTCCACAGATGATCAGCAGGTATT
GTACTTTA

AGTTGGTATATGCTGCACGTGTAGTCTAATGTTCTTTTCTTTTTTTTCTTTATTCTTTTTTCTCCTCTTTA
TGAATACAGATATTCAATTTTTTGAATATCTGATTGTTTTTATTTTCATTTCAATTTTTTTTTTTTTTCAA
TTTTTTTT

TTCTTTTATAATCCTTAATATTCTATATATACATATATGAAAAAATAGAAAACGCGAAAACTTGTCATTTT
TTTTTTAGGCGTTTTTATAATATACTGAAAATAAAAAGAGGCTCTTTAAATGTTGACACTCTACTCCAATA
TCAACTGT

TATAATTATATGAGTTTTGAAAAAAAGTACTGGCATCATTTAAATAAGCGGGCTAAACTTTGTTTATGTT
TAGGTTAACGATAGACATATTTATTTTTTTTTCTAGGAATAAAAATGAAGAACGGGAAGAAAAGGAAAAAT
GAAAATGA

GCAGAATATAAACTTTAAATGAAAATAGATAATATTTATATATATTAACGTTATTACAATTATTTTTTATC
ATCTAGTACATCTCTGCGCATTTTTCTCTTCTATATACAGCTTAATATGTCGAAAACGCGAAGCAAGAAAG
AAAAGAAA

CACTTATATTTAGAGATCTTGAAATACTTTTTGTTACCCGACTTGTAGCTTCATTTTTTCATTTTTTTTTT
TTTTTTTTATTTTTATTTTGTATTGTTCTTTGAAAGAATCATGCATGGAAGCAATAACTATTATACGGAAG
TTATATAC

TTGACAAAAAAAATGAAAACCACTATTATTTAAATTACGGAAAAAAAAAAAACCAATAAACAAAAAACATAAT
AAAAGAAATGAAAAAAAAGAATATTAGTAGAGCATACTGAATTGTTTTCAGAAAGAGAGTGAAAGGTAACA
AAAATTTA

ACACTGCCTCCCAAATTTTAAAATTTGAGGGCAGAATATAAACTTTAAATGAAAATAGATAATATTTATAT
ATATTAACGTTATTACAATTATTTTTTATCATCTAGTACATCTCTGCGCATTTTTCTCTTCTATATACAGC
TTAATATG

CCAATACCCGCGGGCAGTAACCGCATCTTTGGAAATACCGGGTAATAATAAATGCCCGGGAAAAAAGAGCA
TAAAACAGAGAGGCGATATAGGAATGAATATGTGCTATTTACGGAAGGGAAAAACACTTATTGTTTTTCTG
AAATGTGA

ACTAGTTATTGTACTTCCCGCCTTTGCTAAAGACGCGTAAGAAAAAAAAGTACAAATAATGCCCTATAAAG
AAAAAAATTTAAATAAAACGCGAACTTAGTTTGGACGTAATACTTCTCCTTCTCGGGCCGGATATTCGTAT
AGCTCGTA

CCATGCGCGTTGATTGATGTGTGATATATATATATATATATATGTATATAGATGTATATTATTATGTTCAG
AGGTGATTTTATCTTTGTACGCGTACCATTTTTGTAAAGTGACCTTGTTTTGACTTTTTAACGAGTAAAAA
AACGAGTT

AGAAAAAAATCTAACCATTAAAAACGGCGATATATATTGGGCATAAAACCTATATAAAATAACAGCAATAAT
AAAAATGCAAAATATAACATAGCACGGTATAGTTAGCTCATATATGTTTTAACGAGCCTTACATTATATCA
TTTAATAC

CAAACTCTTTTTTAACTCCAGAAAAGAAATTCTTTATTTCACAAAAATTTCTCCCCACTTTTAATGTAATT
TTTCTTTATATAATATATATATATATATACATGAGTATCAATACAATATAACCTAATCTAGCTTTTTATCC
AATGGACT

TAAAAAAAAAAAAGAGTAAGAAATTAACGTTACCACCTATGGGAAAGGCAGTAACTCACAAGCATTCACGT
ACATAATTCTGTTTTTTTTTTTTTTATTCATTTCTAGCACGCTATCGGGGTTATTTTTTTTTTTGTAATAT
ATAGTGAT

TTAAAATAAATATGTATAGCAGAATTATAAGAGGCGAAATCGATCAATTAGTTGAACAATTAACTTAAAAT
CTAATATATATAAATATATATATATATATATATATAGGAAATATACGGCACGTTATACTAGAGATATTAAACA
TCCCAACT

TTTCACATGTTTTAGTGGAAAAATACTGAACAATGAAATTTCAATATCGCATATAATAATTTTTGTTATCA
AATATATATTTTTCGGAAGTCATATACATTAAATAATTAAAACATTATTTTTAAAAATTAAAAAAATATAT
TTTAGCAA

TAAGCAACTAGTTTAGCACAACATCCAACCAAGAGGTTTCTCGCGTATTTCTCTCATTTTTTTACCCATTT
TACAAATTTTTTTTGCTATTTGAGCCATAGTACCCATTAATAGGTCTCGTCCATTCCCTTGTTTTTTTTTT
ATTGTTTC

ATCGCGAACAACGGTATTCGGGTAACAGCCTATCACTATTTTTACCGGCGGCTATTTTTCCTAAACCTAGT
ATATATACGCGAAAATTGGCTACCTGCAAAATTCCAATAGTGGCGAATGTATTGGAATTAATCTGCAAGGA
AAATAAGC

CCCTATACCGAGGAAAGCGAATAGTCGCCATGCTAAACGCGCGGAACAAGGCCATATTTATATATTTAATG
CTTTTAACTATTATTAGTTTTTCTCACCCAGGGCTGGTTTCTTTACCCTGTGTGATGAAAGTGCGCGCCTA
AAGTTATG

CATTTCAAATATATGTACTTTAAAGAAAATAAAATAAAAAATAAAATTTTTTTCTCCCGATAATCAATTTT
CTTAATTAATTAATTGCGTTACGATTCCGTTTTTTTACTTCTTTTATCTCATTATCTATCTAAGTTATTTA
AAAAAAAG

GTATAAACAAAGCCAAAGACAAGTTCCGCACTCCATCAAGTGAACCTCAACAGCTACACATTCTTTTATAA
TCCTTAATATTCTATATATACATATATGAAAAAATAGAAAACGCGAAAACTTGTCATTTTTTTTTTAGGCG
TTTTTATA

CCTCTAAAAAAAAAAAAAAACTCCATAAAAACAACAAAAAGAGACGGACGCGGTTAACGAAGTAGTAACTT
GATGAAAATGAATAAAAAAGAATAAAATTAACAAATAGAAAAGTTGAATCTTTTAAAACTCAAAGTCGCCA
TCGATCAA

CAGTTTTTCGTTTCATCATCATCATCGTATTATTATTATTGTTATTATTATTATTATCATTACTTTTATTA
ATATTAACTATTTTTTTAGTATGATTTCGGGTATATTTATTTTAATTAGATACTTTTAACCGTTTCAAACA
ATTAAAGG

GGTGAAAGAAGATGCGCGTTTATGGGGAGAAAACCCGTAGAAAACGCGAAAAGCCGCTGAAAATACAAAGG
GAAAAAGCCGCTCTCATTTCTTATTTAGGAAAAGAAATGAGCGGGGTAAACGGAGATCGCTGCGGACGGAA
GGCGCGAA

CATCCCACTTTTTTTTTTTTTTTTTTTAATTCATTTTCAAGATTTTAGACGTTGTATAATACATTTTTACC
CACTACTGCGGTTTATTGTAATATTTTGTGAGATTTTATGAATGCGTTCCATCATCTATTTACTACAAAAC
GATAAATT

AAAACCACGTGAAAAAAAAAAAAAAAAAAAAAAAAAAAAGAAAAAGAAAAAGGGCAGCTGCCAACTAGTAC
ACACTACACAGATATTTATGAAAAACACTAACTTCTCCTTTTGGAACTGTTGACGTACCTTCCCTTACCAA
TGCGGACG

TGAAATTTAGCTTTTTTTTATTTAGGAGAAACAATATATATATATATGTATATACGTATGTATGATTCATA
TTTTAATCGCATTTTTATTTCATCTAAAACAATTACTCTTTTTGATTATTTATAGAAAAAAAACAACAATG
ATACGTTT

ACGAAGTTAAAAAAAAAAACAAGAAAAAGAAACAAGATCAAAAGAAACGATAAAGGCTGGCTTATAAAATA
ATAGCATCGCGAACGATCAATAAGGGGCCCTGTTTATTTTTAACAAAGTTTACATATTTCGTTTTCCTCTT
TAAGCTGA

GTTATATTTCCACATGTCATCATTTCAAATATATGTACTTTAAAGAAAATAAAATAAAAAATAAAATTTTT
TTCTCCCGATAATCAATTTTCTTAATTAATTAATTGCGTTACGATTCCGTTTTTTTACTTCTTTTATCTCA
TTATCTAT

AGGAATAAAATTAGAATTTTTTTTGAATAATACGAAATAGAAAAGAAAAAGGTATGAAAAGTGCGCATCAC
GATTTATCATCCTTCTTTAGTGGGATTTTTTTTTTTTATCTTTTTGTATGTTTTTTCCAACTTCTTTTTTT
TTTTTTGT

ACTGCATGGTTTTCTAGCTCTTTTTCACTTTTTTGAGGTTTTATTTATCTTGTATTCTTTTTTTCTATTAT
TTTAAAGGTTCAATTTCTATCGACGGTTTTTTTAATTATATATATATATATATATATATATGTATATAATG
AATGTGTA

GGTATTAAATACCTGTACATTTGAATATTTGGAATGAGAAAGAAATATAGTTACATAATGTACAAAAAAGA
AGCAAGAAAGGAATATATATATATATATATATATATATATATGTATATTGTTATTTAGAATATCTCTCT
CTCTTCGA

GGCCACTGCATCGCCTTTCCTGCCCCATTGATTCAAATGTCCATGCGCGTTGATTGATGTGTGATATATAT
ATATATATATATGTATATAGATGTATATTATTATGTTCAGAGGTGATTTTATCTTTGTACGCGTACCATTT
TTGTAAAG

GAATACATCATATAATACGAATGACACAGGGGCACCCACCCATCGCATATCAGGAGAACTTCCCTGTGCAT
ACGGGACAGCAGAGATAGGCTGTTCTCGCGCTTTTTTTTTTTTTTTTTGCTTTTTTTGGTTCCTTTCGCGG
ATATCGTA

GCTCGTGTTAGTGTTGAAACAAAAAAATACAGTTGTTCTTTACGTGCATCGATCATATAAGAAAAGGGAAA
AAAGCCGTAAAGTAAAAAAATAAAGAGCTTTCCATGGCTGGTTTTTTTTCTTTTTTTTTTTTTTTTTTGAA
GACTACAG

TCTTTATTTCACAAAAATTTCTCCCCACTTTTAATGTAATTTTTCTTTATATAATATATATATATATATAC
ATGAGTATCAATACAATATAACCTAATCTAGCTTTTTATCCAATGGACTTGACTATCATCAGTACTAGAAA
GTGTCATT

TGGTCGGTAAAACAACAGCGTAGATGCTTTTAAAAAAATTGTAGTATCACAGTTATATGTATAGCAAAGAA
AAAGAGTCGCCTCTAAAAAAAAAAAAAAACTCCATAAAAACAACAAAAAGAGACGGACGCGGTTAACGAAG
TAGTAACT

ATTATAATTCATCTATAGGGCATATACAAAAAAAAAAGGTAGGTGTAAAAGATAATAAAATACAACCCTAT
TAGCTTTGATAAAAAATTAGTAGGGTTAGAATGTCATATGGTAGGGTGATATAAACGCGTGAACTTTACGC
GTTTATAT

GTCCTTGCAAAAAAATATTAAAATTGAGACCAAATAAAGATCCTGAATCATAATATATACGTGTGTACTTC
ACTTTCTATAGAGGAAAAAAAGCTAAAAAAAAAAAAAAATGATAAAATAAACGAAAAAAACCATAAAACGA
TCATGATA

CCAAATTTTAAAATTTGAGGGCAGAATATAAACTTTAAATGAAAATAGATAATATTTATATATATTAACGT
TATTACAATTATTTTTTATCATCTAGTACATCTCTGCGCATTTTTCTCTTCTATATACAGCTTAATATGTC
GAAAACGC

TCTCCTCTTTATGAATACAGATATTCAATTTTTTGAATATCTGATTGTTTTTATTTTCATTTCAATTTTTT
TTTTTTTTCAATTTTTTTTATCCGATCGTTGCGCCAAGCTTCTATCGCTTAAAACAATGACACTTCGCTGT
GAAATGAC

CTTCCTGTTTTTTCATGGCAATAATTTTGACTAGTTTTTCTTTTTTTTTTTTTTTTTTCATTCTTTTTCAT
TATTACTTAAGTTCTGTAAAGATATATGGAAGAGAAAATAAAAAGAATAAAACCAAAGATAGTGGTACAAC
TGACGTTA

CTGAAGTTGAAGTTGTACAAAAATAGTGACTTTAATTCGGAAAAATAAAAAAAAAAAAAAAAACATAAGGA
AATTTTAATTAAATAGTCTATCTACACATTTCCTAAAAGAAAATATTATTGCATTACTTTTTTGAAGATCT
ATAAAGGG

CTATCATTTTTTCTCGCCGTTTCTAGTAGAAAAGGGCAACTTTTACTTAAAAATCATACAATTGACTTACT
TTTTTTTTTTTACACAGATACCTTTTTATTTTGAACTACGCGCAGTTTGAAAGAGGAAGAGATAATCCTAT
GATTACTC

GTAAAAAAAATTTTTTTTTTGAGGTTTTTGCTTCCATTTCTTTAGACTGAACTAATTTATTTTTTTTATTAT
TATTCAGTAATTTTTATATATTTATTTATGGACTTATTGAGGTGCTTTGGGGGTGATGACGTCCCCAGTGA
GGCCCCCA

GTGTGTACTTCACTTTCTATAGAGGAAAAAAAAGCTAAAAAAAAAAAAAAAATGATAAAATAAACGAAAAAAA
CCATAAAACGATCATGATAGAATAATGTACATTATTAAGAATGCTTCATTGATGACATGCAGTGCGAAAAG
AAAGGAAC

GATGCGCGTTTATGGGGAGAAAACCCGTAGAAAACGCGAAAAGCCGCTGAAAATACAAAGGGAAAAAGCCG
CTCTCATTTCTTATTTAGGAAAAGAAATGAGCGGGGTAAACGGAGATCGCTGCGGACGGAAGGCGCGAAAT
ACCCACTG

CATAGTCTTCGTAAAAAAAATTTTTTTTTTGAGGTTTTTGCTTCCATTTCTTTAGACTGAACTAATTTATTT
TTTTTATTATTATTCAGTAATTTTTATATATTTATTTATGGACTTATTGAGGTGCTTTGGGGGTGATGACG
TCCCCAGT

TTGGTTTTATGAGTTTTCACCCCCGTTCTTTACCACATTGTTTCTAATTATTCCGTTAGCGCCGCTTACTT
TTAATGTTCTTAAGATAAAAAAACATCATTAATATTATTATTAAAAAATTCATATATATACCTATATCTAT
ATGCACTA

AACAGAAATAGAAAAAAGTTGTATATAACAAGCCAAAAAAAAGAAAAAAAAAAGGAAAAAAAAAGAAAACACA
GCTTATTTCAAATTGCAATCTGCGTATTTCGTAAGTAACGAATGATCTCACAGCATTGCTTTTTTTTTTTT
TTATATAT

ATTTGTTAGGATCTTTTCTTTATACTTTTCCCTGCATAATCATCCCACTTTTTTTTTTTTTTTTTTTAATT
CATTTTCAAGATTTTAGACGTTGTATAATACATTTTTACCCACTACTGCGGTTTATTGTAATATTTTGTGA
GATTTTAT

AAGAAAGTAGTTGACAAAAAAAATGAAAACCACTATTATTTAAATTACGGAAAAAAAAAAAACCAATAAACAA
AAAACATAATAAAAGAAATGAAAAAAAAAGAATATTAGTAGAGCATACTGAATTGTTTTCAGAAAGAGAGTG
AAAGGTAA

AAAATTTCTTATCATGGTAGTGATCACAAATAGATCACATGATATATTTTTTATTTTTAATTTTTTTTAAT
TATAAAAATAATTTTTTTCTTTAAATTAAACAAAAATAAAAAATTGTTTTTTGTTGGTTAAGATTTCCGAA
AATAGAAA

TCACGTGTGCAGCATAGTGTGGTATATTGTTTTTTTATATTTTATTTTTTATTTTTTTTTTTTTGCAGAAA
ACAGTGTTTGTATTATATGCTTTAGATATGCTAAAAAAAAATGAAAAAAAAATGGAACAAAATATGTGTTTTT
TTATTTAA

GTTCATCATTTCACGTGTGCAGCATAGTGTGGTATATTGTTTTTTTATATTTTATTTTTTATTTTTTTTTT
TTTGCAGAAAACAGTGTTTGTATTATATGCTTTAGATATGCTAAAAAAAAATGAAAAAAAAATGGAACAAAAT
ATGTGTTT

TGACGTACAAGTTCATCATTTCACGTGTGCAGCATAGTGTGGTATATTGTTTTTTTATATTTTATTTTTTA
TTTTTTTTTTTTTGCAGAAAACAGTGTTTGTATTATATGCTTTAGATATGCTAAAAAAAATGAAAAAAAAT
GGAACAAA

AGCATAGTGTGGTATATTGTTTTTTTATATTTTATTTTTTATTTTTTTTTTTTTGCAGAAAACAGTGTTTG
TATTATATGCTTTAGATATGCTAAAAAAAATGAAAAAAAATGGAACAAAATATGTGTTTTTTTATTTAACG
TTAAAATT

TTTTTTATATTTTATTTTTTATTTTTTTTTTTTTGCAGAAAACAGTGTTTGTATTATATGCTTTAGATATG
CTAAAAAAAATGAAAAAAAATGGAACAAAATATGTGTTTTTTTATTTAACGTTAAAATTAAAGATTAAGTT
ATTTATTC

GGTATATTGTTTTTTTATATTTTATTTTTTATTTTTTTTTTTTTGCAGAAAACAGTGTTTGTATTATATGC
TTTAGATATGCTAAAAAAAATGAAAAAAAATGGAACAAAATATGTGTTTTTTTATTTAACGTTAAAATTAA
AGATTAAG

TTTTTATGTTACCCGTCCAACGTTCTCACCCGGCTCCGCGCTATTTTTTTTTTTTTTTTTTTTTTTTTTCA
GACCCACACAAAATCCGCGTAGCCGAGATTGCTTATGTATGTTATCATATTGCCTGTTTGAGATATGCAGG
ATCAGCAT

TTTATTTTTTATTTTTTTTTTTTTGCAGAAAACAGTGTTTGTATTATATGCTTTAGATATGCTAAAAAAAA
TGAAAAAAAATGGAACAAAATATGTGTTTTTTTATTTAACGTTAAAATTAAAGATTAAGTTATTTATTCGA
CGTCAGCA

ATCATGGTAGTGATCACAAATAGATCACATGATATATTTTTATTTTTAATTTTTTTTTAATTATAAAAATA
ATTTTTTTCTTTAAATTAAACAAAAATAAAAAATTGTTTTTTGTTGGTTAAGATTTCCGAAAATAGAAATA
TTATTCAG

TTTTTTCAACGGCCGCTCCTTAAAGACCATCCGCATGCTAGAATTATATACTAATATGATTTTGTAAAGAT
AGGAATATTATAATTATAATTATATTTAATTTAATAAGAAAAGAAACGAAAAAAAAAAAAAAAATGGAATT
AAAAAGTT

TGCACGTGGCTGCAGACACATTTTGTATGGAGGGAATGCTTGATTATATAAAGAGCTCTGTTTTTATGTTA
CCCGTCCAACGTTCTCACCCGGCTCCGCGCTATTTTTTTTTTTTTTTTTTTTTTTTTTCAGACCCACACAA
AATCCGCG

TTTTGTATGGAGGGAATGCTTGATTATATAAAGAGCTCTGTTTTTATGTTACCCGTCCAACGTTCTCACCC
GGCTCCGCGCTATTTTTTTTTTTTTTTTTTTTTTTTTTCAGACCCACACAAAATCCGCGTAGCCGAGATTG
CTTATGTA

TAGATCACATGATATATTTTTATTTTTAATTTTTTTTTAATTATAAAAATAATTTTTTTCTTTAAATTAAA
CAAAAATAAAAAATTGTTTTTTGTTGGTTAAGATTTCCGAAAATAGAAATATTATTCAGTTGAAAGACAAA
AAAACATA

TCTTAAAAGAATTTTTTTTTTTTTTTCTGTCCTCCTAATATCTTTTATCTTTAATACTGTAGGGGCGCAAG
TTTCTTTTTTTTTTTTTTTTTTATGTTGCGTTTAGTTTTCTCTTGGCAAAAGTTTTTCGCACCCCGATCTT
TTTTTGCA

GATATATTTTTATTTTTAATTTTTTTTTAATTATAAAAATAATTTTTTTCTTTAAATTAAACAAAAATAAA
AAATTGTTTTTTGTTGGTTAAGATTTCCGAAAATAGAAATATTATTCAGTTGAAAGACAAAAAAACATAAA
TATTTCTA

ATTTCCAGAAGAAAGTGACATCGATGAATGCACTCAAGAGATTTTATGTGTTGTGAATATGTATATATTTA
TTTAGAAAAAAAAAAAAAAAAAAAAACCAAAATCATATACCTTAGGCATCCCGTTTCCCTCTTCATCATTT
TTGTCCCT

ACCCGTCCAACGTTCTCACCCGGCTCCGCGCTATTTTTTTTTTTTTTTTTTTTTTTTTCAGACCCACACA
AAATCCGCGTAGCCGAGATTGCTTATGTATGTTATCATATTGCCTGTTTGAGATATGCAGGATCAGCATAG
CATGTCTC

TGCAGACACATTTTGTATGGAGGGAATGCTTGATTATATAAAGAGCTCTGTTTTTATGTTACCCGTCCAAC
GTTCTCACCCGGCTCCGCGCTATTTTTTTTTTTTTTTTTTTTTTTTTCAGACCCACACAAAATCCGCGTA
GCCGAGAT

AAGAGCTCTGTTTTTATGTTACCCGTCCAACGTTCTCACCCGGCTCCGCGCTATTTTTTTTTTTTTTTTT
TTTTTTTTCAGACCCACACAAAATCCGCGTAGCCGAGATTGCTTATGTATGTTATCATATTGCCTGTTTGA
GATATGCA

TTTTGACATGTTTTTTCAACGGCCGCTCCTTAAAGACCATCCGCATGCTAGAATTATATACTAATATGATT
TTGTAAAGATAGGAATATTATAATTATAATTATATTTAATTTAATAAGAAAAGAAACGAAAAAAAAAAAAA
AAATGGAA

TGATCACAAATAGATCACATGATATATTTTTTATTTTTAATTTTTTTTAATTATAAAAATAATTTTTTTCT
TTAAATTAAACAAAAATAAAAAATTGTTTTTTGTTGGTTAAGATTTCCGAAAATAGAAATATTATTCAGTT
GAAAGACA

GTGTCTTGCCTAGACAATCTAAAAAAGGCTGCACACCCATGCATCATTCTAAAAAAATTATTTTTTTTCTT
TTCATTTACTTTTCGTTTTTTTTTTTTTTTTTTTCAGTTCGATTTCTTGGTCGGACGCGATGGCAAATTTT
TCATCGAG

ACCATAGATTTCTTAAAAGAATTTTTTTTTTTTTTCTGTCCTCCTAATATCTTTTATCTTTAATACTGTA
GGGGCGCAAGTTTCTTTTTTTTTTTTTTTTTATGTTGCGTTTAGTTTTTCTCTTGGCAAAAGTTTTTCGCA
CCCCGATC

CAATTTATATCTGAGAAAGATTCTACACACAGATATATATATATATATATATATTATTGTATATATACATA
CCTACATTATTTTTAAAGGCTTGCATGATGCTGTTATGTAAAAAAAAAAAAAAAAAAATCAGTGTAACATAA
TAGACGTA

AAAAAAGGCTGCACACCCATGCATCATTCTAAAAAAATTATTTTTTTTCTTTTCATTTACTTTTCGTTTTT
TTTTTTTTTTTTTTCAGTTCGATTTCTTGGTCGGACGCGATGGCAAATTTTTCATCGAGAGATTATCGTTA
TAAAGGCC

CCGCATGCTAGAATTATATACTAATATGATTTTGTAAAGATAGGAATATTATAATTATAATTATATTTAAT
TTAATAAGAAAAGAAACGAAAAAAAAAAAAAAAAATGGAATTAAAAAGTTTTGCCGTACATAAAATATTGAA
AAGACTTT

TGATTATATAAAGAGCTCTGTTTTTATGTTACCCGTCCAACGTTCTCACCCGGCTCCGCGCTATTTTTTTTT
TTTTTTTTTTTTTTTTTCAGACCCACACAAAATCCGCGTAGCCGAGATTGCTTATGTATGTTATCATATT
GCCTGTTT

GAAAGTGACATCGATGAATGCACTCAAGAGATTTTATGTGTTGTGAATATGTATATATTTATTTAGAAAAA
AAAAAAAAAAAAAAAACCAAAATCATATACCTTAGGCATCCCGTTTCCCTCTTCATCATTTTTGTCCCTTT
ATATCAAT

TGACTGATATAAAATTTCTTATCATGGTAGTGATCACAAATAGATCACATGATATATTTTTTATTTTTAAT
TTTTTTTAATTATAAAAATAATTTTTTTCTTTAAATTAAACAAAAATAAAAAATTGTTTTTTGTTGGTTAA
GATTTCCG

ATTTTTTTTTTTTTGCAGAAAACAGTGTTTGTATTATATGCTTTAGATATGCTAAAAAAAAATGAAAAAAAA
TGGAACAAAATATGTGTTTTTTTATTTAACGTTAAAATTAAAGATTAAGTTATTTATTCGACGTCAGCATC
AAAAGTTT

ATTTTTTTTTTTTTTTCTGTCCTCCTAATATCTTTTATCTTTAATACTGTAGGGGCGCAAGTTTCTTTTTT
TTTTTTTTTTTATGTTGCGTTTAGTTTTTCTCTTGGCAAAAGTTTTTCGCACCCCGATCTTTTTTTGCATA
CGTAGTTC

CGTACCTCAGTAGAATCAATCAATTTATATCTGAGAAAGATTCTACACACAGATATATATATATATATATA
TATTATTGTATATATACATACCTACATTATTTTTAAAGGCTTGCATGATGCTGTTATGTAAAAAAAAAAAA
AAAAAATC

ATTTTCAGCATTGGGGGCGGGTTGAGTATAGATACGAGAAGCGCCTATTTCGTATGAGGTTTTTTTTTTCT
ATTATTATGTATATTAGGCATATATATACATATATATATTTTGCATACATATTCATTTTTAAAAAAATGCA
ACTGCTAC

TAGACAATCTAAAAAAGGCTGCACACCCATGCATCATTCTAAAAAAATTATTTTTTTTCTTTTCATTTACT
TTTCGTTTTTTTTTTTTTTTTTTTCAGTTCGATTTCTTGGTCGGACGCGATGGCAAATTTTTCATCGAGAG
ATTATCGT

GGCCGCTCCTTAAAGACCATCCGCATGCTAGAATTATATACTAATATGATTTTGTAAAGATAGGAATATTA
TAATTATAATTATATTTAATTTAATAAGAAAAGAAACGAAAAAAAAAAAAAAAATGGAATTAAAAAGTTTT
GCCGTACA

TCTATCTAAACAGTGGTCAATGGATTCTTCGAGAGCTAAGACCATAGATTTCTTAAAAGAATTTTTTTTTT
TTTTTCTGTCCTCCTAATATCTTTTATCTTTAATACTGTAGGGGCGCAAGTTTCTTTTTTTTTTTTTTTTT
ATGTTGCG

TAGAATCAATCAATTTATATCTGAGAAAGATTCTACACACAGATATATATATATATATATATATTATTGTA
TATATACATACCTACATTATTTTTAAAGGCTTGCATGATGCTGTTATGTAAAAAAAAAAAAAAAAAATCAG
TGTAACAT

GAGAGCTAAGACCATAGATTTCTTAAAAGAATTTTTTTTTTTTTTTCTGTCCTCCTAATATCTTTTATCTT
TAATACTGTAGGGGCGCAAGTTTCTTTTTTTTTTTTTTTTTATGTTGCGTTTAGTTTTTCTCTTGGCAAAA
GTTTTTCG

TAAAGACCATCCGCATGCTAGAATTATATACTAATATGATTTTGTAAAGATAGGAATATTATAATTATAAT
TATATTTAATTTAATAAGAAAAGAAACGAAAAAAAAAAAAAAAATGGAATTAAAAAGTTTTGCCGTACATA
AAATATTG

CACAAAGTGGTGACGTACAAGTTCATCATTTCACGTGTGCAGCATAGTGTGGTATATTGTTTTTTTATATT
TTATTTTTTATTTTTTTTTTTTTTTGCAGAAAACAGTTTGTATTATATGCTTTAGATATGCTAAAAAAAAT
GAAAAAAA

TCGATGAATGCACTCAAGAGATTTTATGTGTTGTGAATATGTATATATTTATTTAGAAAAAAAAAAAAAAA
AAAAAACCAAAATCATATACCTTAGGCATCCCGTTTCCCTCTTCATCATTTTTGTCCCTTTATATCAATTT
TTTTAGAC

AGGGAATGCTTGATTATATAAAGAGCTCTGTTTTTATGTTACCCGTCCAACGTTCTCACCCGGCTCCGCGC
TATTTTTTTTTTTTTTTTTTTTTTTTTTCAGACCCACACAAAATCCGCGTAGCCGAGATTGCTTATGTATG
TTATCATA

GATACGAGAAGCGCCTATTTCGTATGAGGTTTTTTTTTTCTATTATTATGTATATTAGGCATATATATACA
TATATATATTTTGCATACATATTCATTTTTAAAAAAATGCAACTGCTACACGCTTTGAAAGATTCAATGAA
AAGTTTTT

GCACACCCATGCATCATTCTAAAAAAATTATTTTTTTTCTTTTCATTTACTTTTCGTTTTTTTTTTTTTTT
TTTTCAGTTCGATTTCTTGGTCGGACGCGATGGCAAATTTTTCATCGAGAGATTATCGTTATAAAGGCCTG
TTGATTTT

CTTCTCATTTTTCATTTCTTTTAAAATTATTTCAGTATAGTTATTATAATTAACATTGGAATAACATTAAT
AATAATAATTTTTTAATAGTAATAATGATAATAATAATAATAAAAATAAAAGAATAAATTTAAAAACAAAA
AAAATTCT

CGTTCTCACCCGGCTCCGCGCTATTTTTTTTTTTTTTTTTTTTTTTTTTCAGACCCACACAAAATCCGCGT
AGCCGAGATTGCTTATGTATGTTATCATATTGCCTGTTTGAGATATGCAGGATCAGCATAGCATGTCTCTA
ACCAAATT

CAGGTGAAATGTGTCTTGCCTAGACAATCTAAAAAAGGCTGCACACCCATGCATCATTCTAAAAAAATTAT
TTTTTTTCTTTTCATTTACTTTTCGTTTTTTTTTTTTTTTTTTTCAGTTCGATTTCTTGGTCGGACGCGAT
GGCAAATT

AAAAAAATTATTTTTTTTCTTTTCATTTACTTTTCGTTTTTTTTTTTTTTTTTTTCAGTTCGATTTCTTGG
TCGGACGCGATGGCAAATTTTTCATCGAGAGATTATCGTTATAAAGGCCTGTTGATTTTCAAAGAGATAGA
AATCTTCT

ACAGTGAAATTGATCTCAGGATCATTAGTAATTTTTCTCACTATAAGAAAAAAAAAAAAACATATATTTTG
CTATGAATTATAATAAGTAAATATATACATATATATGTACAGAGAAACTGACGAAGAAAATAAATCACCCG
TTAATGCT

ATACCTTGATGGCTGAACAGTAGTCAGAAAATTAAAAAAAAAAAAAAAGCGCTTATTACGGCGTATACACG
TAACTATATAGACGTAGATCATTGAATATTGATATGATATAATATTTTCCCCGGGATTGCAGAATACTGAC
ACGCCTTT

GAATTATATACTAATATGATTTTGTAAAGATAGGAATATTATAATTATAATTATATTTAATTTAATAAGAA
AAGAAACGAAAAAAAAAAAAAAAAATGGAATTAAAAAGTTTTGCCGTACATAAAATATTGAAAAGACTTTTA
CATTAATG

TTTTTTTATAGTATTACTTTTTTGTGACATCAAAATGAAGCACTAAATAATTATAGAAAATATTCAGCAAA
AAAAAAGAAAAACCTTCTTCTGAAAAAAAAAAAAAAAAAAATTCAAAAAAAGACACCTTTCTACCTTGGCAG
TAAATTTC

AATCGCATTCTCTATCTAAACAGTGGTCAATGGATTCTTCGAGAGCTAAGACCATAGATTTCTTAAAAGAA
TTTTTTTTTTTTTTTCTGTCCTCCTAATATCTTTTATCTTTAATACTGTAGGGGCGCAAGTTTCTTTTTTT
TTTTTTTT

GGCTGAACAGTAGTCAGAAAATTAAAAAAAAAAAAAAAGCGCTTATTACGGCGTATACACGTAACTATATA
GACGTAGATCATTGAATATTGATATGATATAATATTTTCCCCGGGATTGCAGAATACTGACACGCCTTTTA
TTTATCGT

TGAAAAAATAGAAAGAAAAATACCCATTTTTTCAAGGAAAATAAAATTTATATATTACATACATTTCTATA
AAACAGTTTAAATTCAAAAATATAAACAAAAACATAAAAATAAGAAAAGTTCAAGTCTTCGTTATTTTCTG
TTTGTGGA

GTTGAGTATAGATACGAGAAGCGCCTATTTCGTATGAGGTTTTTTTTTTCTATTATTATGTATATTAGGCA
TATATATACATATATATATATTTTGCATACATATTCATTTTTAAAAAAATGCAACTGCTACACGCTTTGAAAG
ATTCAATG

AAGTGTATTGCTCAGTAAGTATTATCATCTATTAAAGAAGAATAAAAATTATCATGTATTTATTTACAAGT
GCGTATCCTATATATATATATATATATACTTTCGCGATAAAATTTTTTACCAAATACCCTTCTTAACACCT
GGCAAATT

TCTATCATTTGAATTGAATATATAATTATATACAGTGAGATATACCAATTGCAGTAACAAAATGGCAAATA
TGTATATCAGTAATATAATTAGCATATAAAATAAAAAAAAAAAAATGCTTATTTGTCACCCGCTGACTGCGT
TTCCTTTT

ACATAAAATCTTTTTTTATAGTATTACTTTTTTGTGACATCAAAATGAAGCACTAAATAATTATAGAAAAT
ATTCAGCAAAAAAAAAGAAAAACCTTCTTCTGAAAAAAAAAAAAAAAAAATTCAAAAAAAGACACCTTTCT
ACCTTGGC

CCGAGTAACAATTTTCAGCATTGGGGGCGGGTTGAGTATAGATACGAGAAGCGCCTATTTCGTATGAGGTT
TTTTTTTTCTATTATTATGTATATTAGGCATATATATACATATATATATTTTGCATACATATTCATTTTTA
AAAAAATG

TTTCACAAATTATCCATTCTACATAAAATCTTTTTTTATAGTATTACTTTTTTGTGACATCAAAATGAAGC
ACTAAATAATTATAGAAAATATTCAGCAAAAAAAAAGAAAAACCTTCTTCTGAAAAAAAAAAAAAAAAAAT
TCAAAAAA

TCCCTTTTTGCCACCCGCTAATACTTTTATTTCTGTTCAACTATATATACATATATATATATTTACATATT
TTTACCTCCCTATCTCTATTGATAATAAAACAAATAAAAATCGTATATTTTTGTATATGTATTATCGTTTT
GAGGAGCG

GAATTGAATATATAATTATATACAGTGAGATATACCAATTGCAGTAACAAAATGGCAAATATGTATATCAG
TAATATAATTAGCATATAAAATAAAAAAAAAAAATGCTTATTTGTCACCCGCTGACTGCGTTTCCTTTTGA
ATTATAAA

GCAAGCACTACGTATCACGACAAACCAACAGCCGCGCCGTTTTTATATTCGGAAAGAGAATTCTTGTCGTT
ATTGAATAATAATAACAAAATTTCTCCACTTTTCACCATATTACCACTCCCTTTTTTTCAAGTAAAAAAAA
AAAAAAAA

TTGGGGGCGGGTTGAGTATAGATACGAGAAGCGCCTATTTCGTATGAGGTTTTTTTTTTCTATTATTATGT
ATATTAGGCATATATATACATATATATATTTTGCATACATATTCATTTTTAAAAAAATGCAACTGCTACAC
GCTTTGAA

TTATTTTTAATTTTTTTTTAATTATAAAAATAATTTTTTTCTTTAAATTAAACAAAAATAAAAAATTGTTTT
TTGTTGGTTAAGATTTCCGAAAATAGAAATATTATTCAGTTGAAAGACAAAAAAACATAAATATTTCTATG
AGCAAACA

TTCTATTTGAATATATAAAATTATATTCTCAATTTTCACGTATATATTCACCTCACAAATAATATCATTCC
TTTAAAAAAAAATATGTATTATTGTATAATGTTTCAGCATAGTCGTTTTTTAAGCCCTTTTTTTTAAACTT
AAAAAACA

GAGAACCAAAAAAAAAATTAAAAAACGTGAAAACGATGGTTTAACAACTTTTTTCGAATTTGGTATACGTGG
AAAAACGAATGTATAGATGCATTTTTAAAGAATATATATAAAATTTAGTAATTGTATTCCGCGAGCGGCGC
AATAGGTG

TAATTTAGACATACCTTGATGGCTGAACAGTAGTCAGAAAATTAAAAAAAAAAAAAAAAGCGCTTATTACGG
CGTATACACGTAACTATATAGACGTAGATCATTGAATATTGATATGATATAATATTTTCCCCGGGATTGCA
GAATACTG

AAAAGATACTTTTTTCTTTCTTCTTCTACGTCTCCTTTTTTTTTTTTAAGAAAATTTAACTTATACCACTA
TTTTGTTCGCAATTGATCAAGAAAAAAATACAATTGAAAAGGTTTTACATTTTTAATTTTTCTGCTCATCGC
GCTTTTTT

CTCTACATATAAGTGTATTGCTCAGTAAGTATTATCATCTATTAAAGAAGAATAAAAATTATCATGTATTT
ATTTACAAGTGCGTATCCTATATATATATATATATATACTTTCGCGATAAAATTTTTTACCAAATACCCTT
CTTAACAC

AAAAAAACACGTCCCAAAACAGTCCTTCTTTAACAGTCGGTACCAGTAATAAATACTGTGTTGTTTGTTTG
CCCACCGCTAAATAAACGAACGGCGAAAAAAATAGGCGGCAAAAGGCGGGTGATTCGAAAAGTATCGCGCG
CGACGAGG

ATATATATAAAAAAAAAAAAAAAGCAATGCTGTGAGATCATTCGTTACTTACGAAATACGCAGATTGCAATTT
GAAATAAGCTGTGTTTTCTTTTTTTTCCTTTTTTTTTCTTTTTTTTGGCTTGTTATATACAACTTTTTTCT
ATTTCTGT

TTTTTCTTTCTTCTTCTACGTCTCCTTTTTTTTTTTTAAGAAAATTTAACTTATACCACTATTTTGTTCGC
AATTGATCAAGAAAAAATACAATTGAAAAGGTTTTACATTTTTAATTTTTCTGCTCATCGCGCTTTTTTAA
AAGGATAA

TAACTTGTGTCAAATGCCCCATTTCCATACTGTAACCCAAATCAACGAAATAAAAAATAAAACCTCATATG
ATTTACTTTATTTAGTCATAAAAAAACAAAAAAAAAAAAAAAAAAAAAGGAAACAAGACAATCCTTTATAG
TTTGTTTC

CCAGGAATGCTTTCACAAATTATCCATTCTACATAAAATCTTTTTTTATAGTATTACTTTTTTGTGACATC
AAAATGAAGCACTAAATAATTATAGAAAATATTCAGCAAAAAAAAAGAAAAACCTTCTTCTGAAAAAAAAA
AAAAAAAA

CTAGGCTCTGATTTCCAGAAGAAAGTGACATCGATGAATGCACTCAAGAGATTTTATGTGTTGTGAATATG
TATATATTTATTTAGAAAAAAAAAAAAAAAAAAAAAACCAAAATCATATACCTTAGGCATCCCGTTTCCCTC
TTCATCAT

TGGATTCTTCGAGAGCTAAGACCATAGATTTCTTAAAAGAATTTTTTTTTTTTTTTCTGTCCTCCTAATAT
CTTTTATCTTTAATACTGTAGGGGCGCAAGTTTCTTTTTTTTTTTTTTTTTATGTTGCGTTTAGTTTTTCT
CTTGGCAA

AAAAAAATGAAAAAAAATACCAGAAAAAAAAATAACAAATCATATGCACTTCTTCATTAACGATTATGAAGAA
GTCTTATTTCTAACTAATATTATTATTATTATTATTATTATTATCATTCACCCCGCTTTCTTTTATTATAA
TTTGTTCT

CAGTGGTCAATGGATTCTTCGAGAGCTAAGACCATAGATTTCTTAAAAGAATTTTTTTTTTTTTTTCTGTC
CTCCTAATATCTTTTATCTTTAATACTGTAGGGGCGCAAGTTTCTTTTTTTTTTTTTTTTTATGTTGCGTT
TAGTTTTT

CTGAGAAAGATTCTACACACAGATATATATATATATATATATATTATTGTATATATACATACCTACATTAT
TTTTAAAGGCTTGCATGATGCTGTTATGTAAAAAAAAAAAAAAAAAAATCAGTGTAACATAATAGACGTAAC
ACGTTCAA

TTAAAATTATTTCAGTATAGTTATTATAATTAACATTGGAATAACATTAATAATAATAATTTTTTAATAGT
AATAATGATAATAATAATAATAAAAATAAAAGAATAAATTTAAAAACAAAAAAAATTCTAATATTAATGAT
GATAATAG

CTAATGCATGGCCGGCTGGCCACATAAAAGGAAAAACACTCGATGATGATAAAATATATATTAATAATACA
ATTTTTTTATTTATTTATTTAATCATATGTATAGATATAGAAAAAGGGCAGTAGAGAAAAGAGGAATAGAA
AGGAGAGA

TCTTCAATATCAGGTGAAATGTGTCTTGCCTAGACAATCTAAAAAAGGCTGCACACCCATGCATCATTCTA
AAAAAATTATTTTTTTTCTTTTCATTTACTTTTCGTTTTTTTTTTTTTTTTTTCAGTTCGATTTCTTGGT
CGGACGCG

GAGATTATCTTGACTGATATAAAATTTCTTATCATGGTAGTGATCACAAATAGATCACATGATATATTTTT
TATTTTTAATTTTTTTTTAATTATAAAAATAATTTTTTTCTTTAAATTAAACAAAAATAAAAAAATTGTTTTT
TGTTGGTT

ACTTTTGCCAATTTTTTGCGCACAAAGTGGTGACGTACAAGTTCATCATTTCACGTGTGCAGCATAGTGTG
GTATATTGTTTTTTTATATTTATTTTTTATTTTTTTTTTTTTGCAGAAAACAGTGTTTGTATTATATGCT
TTAGATAT

AGCCAAAAAAAAATAATAAAAAAAGCATAATTGGAGGCAGTAAAGCATTATAGTCATACACAATAATAATAA
TAATAATAATAATAATAATAATAATAATAGTAGTAGTAGTAATGGTAATAAAAAAGTTAGAAAAGAAATTA
GATACAGA

CCACCCGCTAATACTTTTATTTCTGTTCAACTATATATACATATATATATATTTACATATTTTTACCTCCC
TATCTCTATTGATAATAAAACAAATAAAAATCGTATATTTTTGTATATGTATTATCGTTTTGAGGAGCGAG
AGCGAAAA

GACCGTACTTTGCACGTGGCTGCAGACACATTTTGTATGGAGGGAATGCTTGATTATATATAAAGAGCTCTGT
TTTTATGTTACCCGTCCAACGTTCTCACCCGGCTCCGCGCTATTTTTTTTTTTTTTTTTTTTTTTTTTCAG
ACCCACAC

ATCATTAGTAATTTTTCTCACTATAAGAAAAAAAAAAAAAACATATATTTTGCTATGAATTATAATAAGTAA
ATATATACATATATATGTACAGAGAAACTGACGAAGAAAATAAATCACCCGTTAATGCTTCTTAGGTGCTC
TTGAAAGT

AATTTACAGGAAGATTTTCTTGAAAAAAATAGAAAGAAAAAATACCCATTTTTTCAAGGAAAATAAAATTTAT
ATATTACATACATTTCTATAAAACAGTTTAAATTCAAAAATATAAACAAAAACATAAAAATAAGAAAAGTT
CAAGTCTT

AAGATTTTCTTGAAAAAAATAGAAAGAAAAAATACCCATTTTTTCAAGGAAAATAAAATTTATATATTACATA
CATTTCTATAAAACAGTTTAAATTCAAAAATATAAACAAAAACATAAAAATAAGAAAAGTTCAAGTCTTCG
TTATTTTC

TAATGTACATATATATATATAAAAAAAAAAAAAAGCAATGCTGTGAGATCATTCGTTACTTACGAAATACGCAG
ATTGCAATTTGAAATAAGCTGTGTTTTCTTTTTTTTCCTTTTTTTTTCTTTTTTTTGGCTTGTTATATACA
ACTTTTTT

TCAATGATTAAATGAAATGATGCATATAAGTAGCGCTTTTTTTAATATTATAAGTTTGGATAAAAGTTACC
AATTTTTTCGTAGGATATATATTTTAAAGGCACTATATTGAAAATATCATGCATGCTTATATAAAAGCTTT
TTTTTTTC

GATAAAACATATGAATAAAATAACTTGTGTCAAATGCCCCATTTCCATACTGTAACCCAAATCAACGAAAT
AAAAAATAAAACCTCATATGATTTACTTTATTTAGTCATAAAAAAACAAAAAAAAAAAAAAAAAAAAAGGA
AACAAGAC

GCATCATTCTAAAAAAATTATTTTTTTTCTTTTCATTTACTTTTCGTTTTTTTTTTTTTTTTTTTCAGTTC
GATTTCTTGGTCGGACGCGATGGCAAATTTTTCATCGAGAGATTATCGTTATAAAGGCCTGTTGATTTTCA
AAGAGATA

ATTTTTCTCACTATAAGAAAAAAAAAAAAAACATATATTTTGCTATGAATTATAATAAGTAAATATATACAT
ATATATGTACAGAGAAACTGACGAAGAAAATAAATCACCCGTTAATGCTTCTTAGGTGCTCTTGAAAGTTT
TTATAAAC

GACAGCGATTTCAACAGTTACTACTGTGCTTAATGCCCTTATTTGATAGTTAGTTCTTCTTATAATAAATA
ATCATTGTATATTGATTATTCGTATAGTATGATATTTAATTGAAAAAAAAATTTTTTTTTTTTAAGAATA
TCATTCAA

GCCGCGCCGTTTTTATATTCGGAAAGAGAATTCTTGTCGTTATTGAATAATAATAACAAAATTTCTCCACT
TTTCACCATATTACCACTCCCTTTTTTTCAAGTAAAAAAAAAAAAAAAAAAGAATCTTATTGCTCCTTAAAA
AAGGATTC

TAACACACCTATCAAAAAATTATTCAGCAATTCCAATCTCGTTAGTAAAATATATTCTTATTTTTTTTTTT
TTTCTCTGATTGTATTATTTCTGGAGTTTTGACTTATTTTTTTACCACATCGCGCTTTTCGTCCCCAATCT
CTCTGATA

CGAAAAATGACATTTTTAGTTTGTGTAAAATTAATTTGATAAATAAATGAATGAAAATGATCAATAATAAG
CGAATAACTATATTTTTAATTACCTTATTTTTACAATATTTTTCACTTGCACTTTTATTTTATTTTCAGCG
CGCAGCTC

ATTTTTTGCGCACAAAGTGGTGACGTACAAGTTCATCATTTCACGTGTGCAGCATAGTGTGGTATATTGTT
TTTTTATATTTTATTTTTTATTTTTTTTTTTTTGCAGAAAACAGTGTTTGTATTATATGCTTTAGATATGC
TAAAAAAA

AAAAAATTAAAAAACGTGAAAACGATGGTTTAACAACTTTTTTCGAATTTGGTATACGTGGAAAAACGAAT
GTATAGATGCATTTTTAAAGAATATATATAAAATTTAGTAATTGTATTCCGCGAGCGGCGCAATAGGTGAT
TTCATTTA

ATTATCATCTATTAAAGAAGAATAAAAATTATCATGTATTTATTTACAAGTGCGTATCCTATATATATATA
TATATATACTTTCGCGATAAAATTTTTTACCAAATACCCTTCTTAACACCTGGCAAATTACCCTTCAGAGC
GTTTTCTC

CTCAGTAAGTATTATCATCTATTAAAGAAGAATAAAAATTATCATGTATTTATTTACAAGTGCGTATCCTA
TATATATATATATATATACTTTCGCGATAAAATTTTTTACCAAATACCCTTCTTAACACCTGGCAAATTAC
CCTTCAGA

ACTAATGCGTGAATACGCGCTGGCATGTCCCCATTCATGCATACATATATTCGTAAACATACACATATTCA
TAAAAATTTTCAGTTATATTATATTATAATCTTTTTTTAACTATACAGAGAAGATATTAAAAAAAAATGTAT
CCAAAGTG

AGCACACGACGACCGTACTTTGCACGTGGCTGCAGACACATTTTGTATGGAGGGAATGCTTGATTATATAA
AGAGCTCTGTTTTTATGTTACCCGTCCAACGTTCTCACCCGGCTCCGCGCTATTTTTTTTTTTTTTTTTTT
TTTTTTTC

CTACTGTGCTTAATGCCCTTATTTGATAGTTAGTTCTTCTTATAATAAATAATCATTGTATATTGATTATT
CGTATAGTATGATATTTAATTGAAAAAAAAATTTTTTTTTTTTAAGAATATCATTCAAAGGCATCAATCA
CAACCTTG

TAGTCAGAAAATTAAAAAAAAAAAAAAAAGCGCTTATTACGGCGTATACACGTAACTATATAGACGTAGATC
ATTGAATATTGATATGATATAATATTTTCCCCGGGATTGCAGAATACTGACACGCCTTTTATTTATCGTTC
ATCGTGTA

TATAATTATATACAGTGAGATATACCAATTGCAGTAACAAAATGGCAAATATGTATATCAGTAATATAATT
AGCATATAAAATAAAAAAAAAAAAATGCTTATTTGTCACCCGCTGACTGCGTTTCCTTTTGAATTATAAAAG
GTTCCTTA

CTGCACTCAGGACGGGCCAAGAAAGCATGTAGCGTGAAGAGAAACTAATTATTAAGAAAAAAGAAAAAAAA
AAAAAAAAAAAAAAAGAAAAGGAAAAAGGAAAAAGATAAACCAATCATACAATCTTTACAGAAAGGGAGTA
CTGAGAAG

TGATCTCAGGATCATTAGTAATTTTTCTCACTATAAGAAAAAAAAAAAAAACATATATTTTGCTATGAATTA
TAATAAGTAAATATATACATATATATGTACAGAGAAACTGACGAAGAAAATAAATCACCCGTTAATGCTTC
TTAGGTGC

CCTCCCATTTGACAGCGATTTCAACAGTTACTACTGTGCTTAATGCCCTTATTTGATAGTTAGTTCTTCTT
ATAATAAATAATCATTGTATATTGATTATTCGTATAGTATGATATTTAATTGAAAAAAAAATTTTTTTTT
TTTAAGAA

ATTTTGTTCGCAATTGATCAAGAAAAAATACAATTGAAAAGGTTTTACATTTTTAATTTTTCTGCTCATCG
CGCTTTTTTAAAAGGATAAATAAACATTTCTTTAAAAAACATCTTCAATAAGAAAAATCGGTTAAAAAAAC
TTTTCTTC

GGAAAACCCGAAAAAAATGAAAAAAATACCAGAAAAAAAAATAACAAATCATATGCACTTCTTCATTAACGA
TTATGAAGAAGTCTTATTTCTAACTAATATTATTATTATTATTATTATTATTATCATTCACCCCGCTTTCT
TTTATTAT

AAAAAGTTGATATTGAAAAAAAAAAAAAAAAGAGAACCAAAAAAAAAATTAAAAAACGTGAAAACGATGGTTT
AACAACTTTTTTCGAATTTGGTATACGTGGAAAAACGAATGTATAGATGCATTTTTAAAGAATATATATAA
AATTTAGT

AGCGATGACTAAAAGTGAAAAATTTCAAAGCCAAAAGAAAAAAAAAAAAAAGAAAATATTATTTCAGCGGCT
AATAGTTACCCGCATCAAAATAATATATCTCTGTACGGTAATGAAAATTTTTGAAATATGCGATGAGCTTA
GAACTAAA

AGTACCCAGAGACAAAAAAGAAAGAAAAGAAAAAAGAAACAGTCTACAAATTCTATTTGAATATATAAAAT
TATATTCTCAATTTTCACGTATATATTCACCTCACAAATAATATCATTCCTTTAAAAAAAAATATGTATTA
TTGTATAA

ATTTTATGTGTTGTGAATATGTATATATTTATTTAGAAAAAAAAAAAAAAAAAAAAAACCAAAATCATATAC
CTTAGGCATCCCGTTTCCCTCTTCATCATTTTTGTCCCTTTATATCAATTTTTTTAGACTTCATATAATTT
TGTATCAA

AGTCTACAAATTCTATTTGAATATATAAAATTATATTCTCAATTTTCACGTATATATTCACCTCACAAATA
ATATCATTCCTTTAAAAAAAAATATGTATTATTGTATAATGTTTCAGCATAGTCGTTTTTTAAGCCCTTTT
TTTTAAAC

TATCCATTCTACATAAAATCTTTTTTTATAGTATTACTTTTTTGTGACATCAAAATGAAGCACTAAATAAT
TATAGAAAATATTCAGCAAAAAAAAAGAAAAACCTTCTTCTGAAAAAAAAAAAAAAAAAAATTCAAAAAAAG
ACACCTTT

TGCATATAAGTAGCGCTTTTTTTAATATTATAAGTTTGGATAAAAGTTACCAATTTTTTCGTAGGATATAT
ATTTTAAAGGCACTATATTGAAAATATCATGCATGCTTATATAAAAGCTTTTTTTTTCTATAGGGTGACA
TTTAGGAG

ATGAATAAAATAACTTGTGTCAAATGCCCCATTTCCATACTGTAACCCAAATCAACGAAATAAAAAATAAA
ACCTCATATGATTTACTTTATTTAGTCATAAAAAAACAAAAAAAAAAAAAAAAAAAAAAGGAAACAAGACAA
TCCTTTAT

ATATATAAAATTATATTCTCAATTTTCACGTATATATTCACCTCACAAATAATATCATTCCTTTAAAAAAA
AATATGTATTATTGTATAATGTTTCAGCATAGTCGTTTTTTAAGCCCTTTTTTTTAAACTTAAAAAACAAA
TCAATGAG

AAAAGTGAAAAATTTCAAAGCCAAAAGAAAAAAAAAAAAAAGAAAATATTATTTCAGCGGCTAATAGTTACC
CGCATCAAAATAATATATCTCTGTACGGTAATGAAAATTTTTGAAATATGCGATGAGCTTAGAACTAAAGT
AAGCAATG

GCGCCTATTTCGTATGAGGTTTTTTTTTTCTATTATTATGTATATTAGGCATATATATACATATATATATT
TTGCATACATATTCATTTTTAAAAAAAATGCAACTGCTACACGCTTTGAAAGATTCAATGAAAAGTTTTTCC
GACGACCA

GCCGGCTGGCCACATAAAAGGAAAAACACTCGATGATGATAAAATATATATTAATAATACAATTTTTTTAT
TTATTTATTTAATCATATGTATAGATATAGAAAAAGGGCAGTAGAGAAAAGAGGAATAGAAAGGAGAGAGA
TAATAAAG

AAAAAAAAAAGAGAACCAAAAAAAAAATTAAAAAACGTGAAAACGATGGTTTAACAACTTTTTTCGAATTTG
GTATACGTGGAAAAACGAATGTATAGATGCATTTTTAAAGAATATATATAAAATTTAGTAATTGTATTCCG
CGAGCGGC

ATTACTTTTTTACATAAAAAAATTTCCCATCTCACGATCAAAAACAGGCATGAGAAAAAAATCAAAATTTATA
AAATTAATTTCTAATAAATTAACTGTAATGACATAAAATAAGAGGCTGCGACAGTCGAATTTTTTCTTTTT
TTTTTTTT

TTATCTTCCGTTTTGACATGTTTTTTCAACGGCCGCTCCTTAAAGACCATCCGCATGCTAGAATTATATAC
TAATATGATTTTGTAAAGATAGGAATATTATAATTATAATTATATTTAATTTAATAAGAAAAGAAACGAAA
AAAAAAAA

AAAAAAAAAAAAAAAAAATCAGTGTAACATAATAGACGTAACACGTTCAATGACATGATAATACTATATAT
AGTAACGATAAAATGTAAATGGTAATAGATATAATTATATGAATATTTAAGTAATGCATTGATAAGTGATC
AATTCATA

GACGGGCCAAGAAAGCATGTAGCGTGAAGAGAAACTAATTATTAAGAAAAAAGAAAAAAAAAAAAAAAAAA
AAAAAGAAAAGGAAAAAGGAAAAAGATAAACCAATCATACAATCTTTACAGAAAGGGAGTACTGAGAAGAA
GGCCAAAA

TTCATTTCTTTTAAAATTATTTCAGTATAGTTATTATAATTAACATTGGAATAACATTAATAATAATAATT
TTTTAATAGTAATAATGATAATAATAATAATAAAAATAAAAGAATAAATTTAAAAACAAAAAAAATTCTAA
TATTAATG

GATCCATGTGTCTATCATTTGAATTGAATATATAATTATATACAGTGAGATATACCAATTGCAGTAACAAA
ATGGCAAATATGTATATCAGTAATATAATTAGCATATAAAATAAAAAAAAAAAAATGCTTATTTGTCACCCG
CTGACTGC

TAAGAGGAAAGTATTTGTACAAAACGAAAAACTAAAGGCAAATATATATATAGATGTTGCCGCGCACCTTT
TTTTTAATGAATATTCACACAAATATTGAAAATAAAAACAAACAAAATTCAAATAACAACATGTAAGACCA
AAAAAAAA

TGTGTTTTCTTTTTTTTTCCTTTTTTTTTTCTTTTTTTTTGGCTTGTTATATACAACTTTTTTCTATTTCTGTT
CTTTTTAGAAGTAATTAATTGGTACATAGGAAATAAATATGAATTATATTAAATGAAGTAAAAGTAAAAGA
AAACGGGG

ACGGCAAAAAATACTGTAAGGAAGTTGTCTATATATGTACATATGGATATTGCGGTATAATAAAAAAGCTT
TAAAATTATAAAGAAACCCGACTTTTATCACATATAATTATATTATAAAAAAATAATGGCATCTGTATGAA
CTCGCGAT

GTTGCACTATAGTAAAAACTACAGTGAAATTGATCTCAGGATCATTAGTAATTTTTCTCACTATAAGAAAA
AAAAAAAAACATATATTTTGCTATGAATTATAATAAGTAAATATATACATATATATGTACAGAGAAACTGA
CGAAGAAA

CACTCAAGAGATTTTATGTGTTGTGAATATGTATATATTTATTTAGAAAAAAAAAAAAAAAAAAAAAACCAA
AATCATATACCTTAGGCATCCCGTTTCCCTCTTCATCATTTTTGTCCCTTTATATCAATTTTTTTAGACTT
CATATAAT

TTTCTTTTTCTATCTCTATTTTCTTTCTTTTCGTGTCTTATAATAATAATAATAATAATAATAATAAAAAT
AGTAATAATAAAAATAGTAATAAATAAAGATGGATTTCTTTAATTTGAATAATAATAATAATAATAATAAT
ACTACTAC

TCTTATTCTTCTCTACATATAAGTGTATTGCTCAGTAAGTATTATCATCTATTAAAGAAGAATAAAAATTA
TCATGTATTTATTTACAAGTGCGTATCCTATATATATATATATATATATACTTTCGCGATAAAATTTTTTACC
AAATACCC

AGTCTCTAAGATGCGCTTGAAAAATTTTTCATACGAAAAAAAAAAAAAAAAAAAAAAAAAAAAACACTCGCAA
GAGTAATCTCAATAATAATTTCCGTCATTAGCGATTATAGCATGAAGGGAGGCCGAGAGGCCGCTTCTGCG
GTATGATA

CAATTGATCAAGAAAAAAATACAATTGAAAAGGTTTTACATTTTTAATTTTTCTGCTCATCGCGCTTTTTTA
AAAGGATAAATAAACATTTCTTTAAAAAACATCTTCAATAAGAAAAATCGGTTAAAAAAACTTTTCTTCTC
AAAGCATA

TAGCGCTTTTTTTAATATTATAAGTTTGGATAAAAGTTACCAATTTTTTCGTAGGATATATATTTTAAAGG
CACTATATTGAAAATATCATGCATGCTTATATAAAAGCTTTTTTTTTTCTATAGGGTGACATTTAGGAGCA
ATAATTTT

AGCATAAATTAATTGAACGGTCCCTTTTTGCCACCCGCTAATACTTTTATTTCTGTTCAACTATATATACA
TATATATATATTTACATATTTTTACCTCCCTATCTCTATTGATAATAAAACAAATAAAAATCGTATATTTT
TGTATATG

GAAGTTTGAAAAAAAGTTGATATTGAAAAAAAAAAAAAAAGAGAACCAAAAAAAAATTAAAAAACGTGAAA
ACGATGGTTTAACAACTTTTTTCGAATTTGGTATACGTGGAAAAACGAATGTATAGATGCATTTTTAAAGA
ATATATAT

CGTATGAGGTTTTTTTTTTCTATTATTATGTATATTAGGCATATATATACATATATATATTTTGCATACAT
ATTCATTTTTAAAAAAATGCAACTGCTACACGCTTTGAAAGATTCAATGAAAAGTTTTTCCGACGACCAGA
ATTGGCTA

CGGCTCCGCGCTATTTTTTTTTTTTTTTTTTTTTTTTTTCAGACCCACACAAAATCCGCGTAGCCGAGATT
GCTTATGTATGTTATCATATTGCCTGTTTGAGATATGCAGGATCAGCATAGCATGTCTCTAACCAAATTGT
GGGTCAGT

ATACAAGAAATGATCTTAAAATCATGCTCTTACCCGTTCTGTAAATAGTGAAAAATTTTCATCGCGAAGTA
TGAACGAAAAAAAAAAAAGAAAATAGATGAACTTTTAGCATTTAATTAGTGGCAAACGCCTACCCCTCCTT
CCCACTTT

AGATGAGATGAGTCTCTAAGATGCGCTTGAAAAATTTTTCATACGAAAAAAAAAAAAAAAAAAAAAAAAAA
ACACTCGCAAGAGTAATCTCAATAATAATTTCCGTCATTAGCGATTATAGCATGAAGGGAGGCCGAGAGGC
CGCTTCTG

TAGGGGAAAATATGGTCGGTTAACACACCTATCAAAAAATTATTCAGCAATTCCAATCTCGTTAGTAAAAT
ATATTCTTATTTTTTTTTTTTTTCTCTGATTGTATTATTTCTGGAGTTTTGACTTATTTTTTTACCACATC
GCGCTTTT

CCTCCTAATATCTTTTATCTTTAATACTGTAGGGGCGCAAGTTTCTTTTTTTTTTTTTTTTTATGTTGCGT
TTAGTTTTTCTCTTGGCAAAAGTTTTTCGCACCCCGATCTTTTTTTGCATACGTAGTTCACTGCCGCTGCT
TACGGCAG

TTTTTTTCCTTTTTTTTTTCTTTTTTTTTGGCTTGTTATATACAACTTTTTTCTATTTCTGTTCTTTTTAGAA
GTAATTAATTGGTACATAGGAAATAAATATGAATTATATTAAATGAAGTAAAAGTAAAAGAAAACGGGGAA
ATTTATCG

TCAACAGTTACTACTGTGCTTAATGCCCTTATTTGATAGTTAGTTCTTCTTATAATAAATAATCATTGTAT
ATTGATTATTCGTATAGTATGATATTTAATTGAAAAAAAAATTTTTTTTTTTTAAGAATATCATTCAAAG
GCATCAAT

AATAATTAAGAGATGAGATGAGTCTCTAAGATGCGCTTGAAAAATTTTTCATACGAAAAAAAAAAAAAAAA
AAAAAAAAAAACACTCGCAAGAGTAATCTCAATAATAATTTCCGTCATTAGCGATTATAGCATGAAGGGAG
GCCGAGAG

TGAATATTATTAATTTAGACATACCTTGATGGCTGAACAGTAGTCAGAAAATTAAAAAAAAAAAAAAAGCG
CTTATTACGGCGTATACACGTAACTATATAGACGTAGATCATTGAATATTGATATGATATAATATTTTCCC
CGGGATTG

TTATATTCTCAATTTTCACGTATATATTCACCTCACAAATAATATCATTCCTTTAAAAAAAAATATGTATT
ATTGTATAATGTTTCAGCATAGTCGTTTTTAAGCCCTTTTTTTAAACTTAAAAAACAAATCAATGAGAT
ATTGTATA

TTATAGTCATGCTTCTTCTTTTGAATATTACCGATATTAGTAGTTTATATTTGATATTTCTATCAGTATTA
TTTAGATTTTTTTTTTTTTTTAATTGTTAATGCTTTTTCTTAGAACTTTCCAAAAACGAAAGGAAAATGG
GACATTCA

GCAATATGGCTAATTACACACCTGGAGAAAAAATCAGATATGTATATATAAGAATATTATAATACTGTATA
TTAAAAATGATTAAAATAAAGAAAAAAATGAATCGGGCGTTTAATTGCTTATTATCTTGAAGAAGCGAAAG
TACACTAT

CTCGAACTATAGCGATGACTAAAAGTGAAAAATTTCAAAGCCAAAAGAAAAAAAAAAAAAAGAAAATATTAT
TTCAGCGGCTAATAGTTACCCGCATCAAAATAATATATCTCTGTACGGTAATGAAAATTTTTGAAATATGC
GATGAGCT

TCTTTTATCTTTAATACTGTAGGGGCGCAAGTTTCTTTTTTTTTTTTTTTTTATGTTGCGTTTAGTTTTTC
TCTTGGCAAAAGTTTTTCGCACCCCGATCTTTTTTTGCATACGTAGTTCACTGCCGCTGCTTACGGCAGCG
TTTCACTT

GAATACGCGCTGGCATGTCCCCATTCATGCATACATATATTCGTAAACATACACATATTCATAAAAATTTT
CAGTTATATTATATTATAATCTTTTTTTAACTATACAGAGAAGATATTAAAAAAAATGTATCCAAAGTGAG
TTAAAAAA

CTAATATGATTTTGTAAAGATAGGAATATTATAATTATAATTATATTTAATTTAATAAGAAAAGAAACGAA
AAAAAAAAAAAAAATGGAATTAAAAAGTTTTGCCGTACATAAAATATTGAAAAGACTTTTACATTAATGCT
GGAACAAT

AATTGAACGGTCCCTTTTTGCCACCCGCTAATACTTTTATTTCTGTTCAACTATATATACATATATATATA
TTTACATATTTTTACCTCCCTATCTCTATTGATAATAAAACAAATAAAAATCGTATATTTTTGTATATGTA
TTATCGTT

GCTGTTATGTAAAAAAAAAAAAAAAAAAATCAGTGTAACATAATAGACGTAACACGTTCAATGACATGATAA
TACTATATATAGTAACGATAAAATGTAAATGGTAATAGATATAATTATATGAATATTTAAGTAATGCATTG
ATAAGTGA

TTCTTCTACGTCTCCTTTTTTTTTTTTAAGAAAATTTAACTTATACCACTATTTTGTTCGCAATTGATCAA
GAAAAAAATACAATTGAAAAGGTTTTACATTTTTAATTTTTCTGCTCATCGCGCTTTTTTAAAAGGATAAAT
AAACATTT

AGCCTGGTATCTAGGCTCTGATTTCCAGAAGAAAGTGACATCGATGAATGCACTCAAGAGATTTTATGTGT
TGTGAATATGTATATATTTATTTAGAAAAAAAAAAAAAAAAAAAAAACCAAAATCATATACCTTAGGCATCC
CGTTTCCC

TTTTTTTCAGAACTTCTTTTTCTGATTCAAAAATTCATTAAGCATAACTAAAACTGAAAATCAAAACTTAT
TAATATACAAAAATCTATATATTTCAAAATGTAAATCGTATCAAAACGTCACTCTATATGTAATAAAAACC
CAAAAAGT

AGTAAAAACTACAGTGAAATTGATCTCAGGATCATTAGTAATTTTTCTCACTATAAGAAAAAAAAAAAAAAC
ATATATTTTGCTATGAATTATAATAAGTAAATATATACATATATATGTACAGAGAAACTGACGAAGAAAAT
AAATCACC

TTCAGTATAGTTATTATAATTAACATTGGAATAACATTAATAATAATAATTTTTTAATAGTAATAATGATA
ATAATAATAATAAAAATAAAAGAATAAATTTAAAAACAAAAAAAATTCTAATATTAATGATGATAATAGTA
GTAATACT

GAAAAATACCGAGTAATAAAAGGTAAAAGGGCGTTAAATGCGCGCTTTTATGTTTGTATAAATAAAACACG
TTGATATTTTGTCTTATATAAACTTTATTCCAATAACCGCGACACCTTACCAAACACATATTATTACAACA
CATTTTTA

GAAGTTGTCTATATATGTACATATGGATATTGCGGTATAATAAAAAAGCTTTAAAATTATAAAGAAACCCG
ACTTTTATCACATATAATTATATTATAAAAAAATAATGGCATCTGTATGAACTCGCGATTAAGCTCACTTT
TTTCTAAT

TTGTGAATATGTATATATTTATTTAGAAAAAAAAAAAAAAAAAAAAACCAAAATCATATACCTTAGGCATC
CCGTTTCCCTCTTCATCATTTTTGTCCCTTTATATCAATTTTTTTAGACTTCATATAATTTTGTATCAACT
GTACCCTA

TTTTGCAGAAAACAGTGTTTGTATTATATGCTTTAGATATGCTAAAAAAAATGAAAAAAAATGGAACAAAA
TATGTGTTTTTTTTATTTAACGTTAAAATTAAAGATTAAGTTATTTATTCGACGTCAGCATCAAAGTTTGA
CCTTCAAC

TCTCCTTTTTTTTTTTTAAGAAAATTTAACTTATACCACTATTTTGTTCGCAATTGATCAAGAAAAAATAC
AATTGAAAAGGTTTTACATTTTTAATTTTTCTGCTCATCGCGCTTTTTTAAAAGGATAAATAAACATTTCT
TTAAAAAA

CGTATCACGACAAACCAACAGCCGCGCCGTTTTTATATTCGGAAAGAGAATTCTTGTCGTTATTGAATAAT
AATAACAAAATTTCTCCACTTTTCACCATATTACCACTCCCTTTTTTTCAAGTAAAAAAAAAAAAAAAAAG
AATCTTAT

GTATATATTTATTTAGAAAAAAAAAAAAAAAAAAAAACCAAAATCATATACCTTAGGCATCCCGTTTCCCT
CTTCATCATTTTTGTCCCTTTATATCAATTTTTTTAGACTTCATATAATTTTGTATCAACTGTACCCTACA
TCCCTTGG

ATTTAGAAAAAAAAAAAAAAAAAAAAACCAAAATCATATACCTTAGGCATCCCGTTTCCCTCTTCATCATT
TTTGTCCCTTTATATCAATTTTTTTAGACTTCATATAATTTTGTATCAACTGTACCCTACATCCCTTGGTA
TTCAAAAT

TTTCCTACAAGATAAAACATATGAATAAAATAACTTGTGTCAAATGCCCCATTTCCATACTGTAACCCAAA
TCAACGAAATAAAAAATAAAACCTCATATGATTTACTTTATTTAGTCATAAAAAAACAAAAAAAAAAAAAA
AAAAAAAG

TTCTTGAGGAAGACTTGCTTGAATATTACGAGACAGCTGAAGTAAGTTTGAATAATTAAGAGATGAGATGA
GTCTCTAAGATGCGCTTGAAAAATTTTTCATACGAAAAAAAAAAAAAAAAAAAAAAAAAAACACTCGCAAG
AGTAATCT

GAAAATATAACCTCTTTGGGGAAAAGAGAAATAGATCCTTTCTCAAAAATTTAGAAAGGAAAAAAAATCAA
AATATAATAAAATAGATTTTTTTTAGGTTAATTTTTTGCCAATTTAGCTAGACTTTTGAAAAGATCCAAAA
ATTTACTA

AGTGGGATAGAAATGAAATTCTTTTCTTTTAAATGGTATTAATAATTAATATATTAAATAATTATATTGAT
TCTATACATAAAACATTAGTGTGAAATTTTCGGAAAATAATAATAGAAAGTAAAATCAAATGTAAAATTAA
AAAAATGA

GTATAAGTACTCTATTAAACATCGTTATTTTCTCTTTTATTACTATTAATATTATTATATTTTGAGAATAG
AAAAGACTAATATATATATAGTATAATTTTTAAAATTAATTCCGTACAAAAGGCAAAAGAAATTGTCTTCT
TACTTAAA

CCTCTTTGGGGAAAAGAGAAATAGATCCTTTCTCAAAAATTTAGAAAGGAAAAAAAATCAAAATATAATAA
AATAGATTTTTTTTAGGTTAATTTTTTGCCAATTTAGCTAGACTTTTGAAAAGATCCAAAAATTTACTAAA
ATTGAATC

TTATACCACTATTTTGTTCGCAATTGATCAAGAAAAAATACAATTGAAAAGGTTTTACATTTTTAATTTTT
CTGCTCATCGCGCTTTTTTAAAAGGATAAATAAACATTTCTTTAAAAAACATCTTCAATAAGAAAAATCGG
TTAAAAAA

TTTCAAATTTCTCGAACTATAGCGATGACTAAAAGTGAAAAATTTCAAAGCCAAAAGAAAAAAAAAAAAAG
AAAATATTATTTCAGCGGCTAATAGTTACCCGCATCAAAATAATATATCTCTGTACGGTAATGAAAATTTT
TGAAATAT

GATACATTTTTTGATCGTGTGATGAAAGTATTAACATAGCTTTAGGGTTCTTCCATTGGATATCCAAAATA
GATATAAAGGTATATATATATATATATATATATATATGTTTATAAAGCAAACCGAAAGCAAAAATATCTGT
ATAACTTA

CAGAAGGAATCATTAGCTAAAAAGTTTATTATTAAAATCAGACATATATCTCATTATAATATTTTTTTTT
ATAATACCACCATTTGTTCTCATATAATACTGATAAATATCGCATCGAATGGAAAAGACAAATAGGAGGCC
GCTATTGT

TAGAAAAAAATTATAGAAGATTGAAACTGAGCAATATGGCTAATTACACACCTGGAGAAAAAATCAGATAT
GTATATATAAGAATATTATAATACTGTATATTAAAAATGATTAAAATAAAGAAAAAAATGAATCGGGCGTT
TAATTGCT

GTCTACGTTCCTAATGCATGGCCGGCTGGCCACATAAAAGGAAAAACACTCGATGATGATAAAATATATAT
TAATAATACAATTTTTTTATTTATTTATTTAATCATATGTATAGATATAGAAAAAGGGCAGTAGAGAAAAG
AGGAATAG

AGAAAAAATACAATTGAAAAGGTTTTACATTTTTAATTTTTCTGCTCATCGCGCTTTTTTAAAAGGATAAA
TAAACATTTCTTTAAAAAACATCTTCAATAAGAAAAATCGGTTAAAAAAACTTTTCTTCTCAAAGCATACC
TAATAACA

ATGTCAAACGCGTAGGAAAGCCAAGCTCAATTACGCGTCTCTTTTATTTTTAAACTATCAAATTGCCGCGC
ATGCTAGATGAATAAGGAAAAAAAAAAGGGGGACGGAAAACATTGCAACCAACACATTATTCTTTAGCGGCT
TTTTGTGA

AATTATGATACTGCACTCAGGACGGGCCAAGAAAGCATGTAGCGTGAAGAGAAACTAATTATTAAGAAAAA
AGAAAAAAAAAAAAAAAAAAAAAAAAGAAAAGGAAAAAGGAAAAAGATAAACCAATCATACAATCTTTACAG
AAAGGGAG

TTACTAAATTAATTATGATACTGCACTCAGGACGGGCCAAGAAAGCATGTAGCGTGAAGAGAAACTAATTA
TTAAGAAAAAAGAAAAAAAAAAAAAAAAAAAAAAAAAAGAAAAGGAAAAAGGAAAAAGATAAACCAATCATACA
ATCTTTAC

TAACATTGGAATAACATTAATAATAATAATTTTTTAATAGTAATAATGATAATAATAATAATAAAAATAAA
AGAATAAATTTAAAAACAAAAAAAATTCTAATATTAATGATGATAATAGTAGTAATACTGGTGGTATTATA
TATGTTGA

ATTAAAGAAGAATAAAAATTATCATGTATTTATTTACAAGTGCGTATCCTATATATATATATATATATATACT
TTCGCGATAAAATTTTTTACCAAATACCCTTCTTAACACCTGGCAAATTACCCTTCAGAGCGTTTTCTCTA
AATTGATA

ACTACGTTAAGAAAAATACCGAGTAATAAAAGGTAAAAGGGCGTTAAATGCGCGCTTTTATGTTTGTATAA
ATAAAACACGTTGATATTTTGTCTTATATAAACTTTATTCCAATAACCGCGACACCTTACCAAACACATAT
TATTACAA

ATTTCAACGCTTGTTTAAACATGTGCTAGGTAAGCGAGTTTTTTACTTTTACTTCCAATAAATATATATAT
ATATATACGTATACATGTATGGGCGGGCTACAACAAAATGAAATAAAAAGCGTCAGGATCATTGTTTTTTA
CCATACTT

GACAAAAAAGAAAGAAAAGAAAAAAGAAACAGTCTACAAATTCTATTTGAATATATAAAATTATATTCTCA
ATTTTCACGTATATATTCACCTCACAAATAATATCATTCCTTTAAAAAAAAATATGTATTATTGTATAATG
TTTCAGCA

TAATTACCCGTACTAAAGGTTTGGAAAAGAAAAAAGAGACCGCCTCGTTTCTTTTTCTTCGTCGAAAAAGG
CAATAAAAATTTTTATCACGTTTCTTTTTCTTGAAAATTTTTTTTTTTGATTTTTTTCTCTTTCGATGACC
TCCCATTG

TTTAGGGTTCTTCCATTGGATATCCAAAATAGATATAAAGGTATATATATATATATATATATATATATGTT
TATAAAGCAAACCGAAAGCAAAAATATCTGTATAACTTAGTTACAAGGGAAGGGAAGAGAATGCATTGGTT
AATAGGAA

AATGCAACACGAAGTTTGAAAAAAAGTTGATATTGAAAAAAAAAAAAAAAAGAGAACCAAAAAAAAAATTAAA
AAACGTGAAAACGATGGTTTAACAACTTTTTTCGAATTTGGTATACGTGGAAAAACGAATGTATAGATGCA
TTTTTAAA

AAGAAAAAAAAAAAATACAACGATAGTTCTTTGTTTTTCTTTAGGAAATATCTGAATAGAAAAGGCTTCAAC
GACCAGAATTGAAAAAATAAAATAGTATTAGCAAAGAAAAATATGATATTTTTTTTCAAAAAAAAAAAGAG
TGTCCTTG

GGTTACCATTACTTACGATTTTTTTTTTTTCGTTATGAACCCTGTGCCCGAGGGGTTTTCAGGCACATAGA
TCAAAGTTCTACTCGTTTACCTGTAATCTTGGCGCGTTTAGGTCTCTTTTTTTTTCTTTTTTTTTTCTTCT
TTTTTCAT

TATAGGTTTTTTAGATCCAATTTTTTTTTGTTGGCCATCTTAATATTACTACAAATTATATTAAAAAAGGT
TTTTGTTAAATTTAGTAAAATTCCGAAAACAAAATTCGATTTTGCGTGTGGATTAGTTTTTGTCTGTTTCA
TGTACGGG

CTGTAGTTTCAGCCAAAAAAAAATAATAAAAAAAAGCATAATTGGAGGCAGTAAAGCATTATAGTCATACACA
ATAATAATAATAATAATAATAATAATAATAATAATAATAGTAGTAGTAGTAATGGTAATAAAAAAGTTAGA
AAAGAAAT

CAATTGAAAAGGTTTTACATTTTTAATTTTTCTGCTCATCGCGCTTTTTTAAAAGGATAAATAAACATTTC
TTTAAAAAACATCTTCAATAAGAAAAATCGGTTAAAAAAACTTTTCTTCTCAAAGCATACCTAATAACAAT
ATAATCCC

TGAGTTTTCAAAAAATAAATGAGGAAACACACACATGTGTCTAGCGTAAATATTCTTCAGTTATATAGTAA
TTGTATTATATACTAAAATGAAAATCAGTAAGATTTTTTTTTACAGGCCTTCTTGGCGTTAGTTTTTCCG
GTTTTTCA

TTAAAAACAAAAAAAAATTCTAATATTAATGATGATAATAGTAGTAATACTGGTGGTATTATATATGTTGAA
GTGAACACGCATATCACTATAAACTATATATACATATATATAGATAGATAGATAGATGCAAAAAATTTTTT
CCTTTTAG

TTTTTAATAGTAATAATGATAATAATAATAATAAAAATAAAAGAATAAATTTAAAAACAAAAAAAATTCTA
ATATTAATGATGATAATAGTAGTAATACTGGTGGTATTATATATGTTGAAGTGAACACGCATATCACTATA
AACTATAT

CAAATGCCCCATTTCCATACTGTAACCCAAATCAACGAAATAAAAAATAAAACCTCATATGATTTACTTTA
TTTAGTCATAAAAAAACAAAAAAAAAAAAAAAAAAAAAGGAAACAAGACAATCCTTTATAGTTTGTTTCCA
TCATGAAA

TTGCATAATTAATTTACAGGAAGATTTTCTTGAAAAAATAGAAAGAAAAATACCCATTTTTTCAAGGAAAA
TAAAATTTATATATTACATACATTTCTATAAAACAGTTTAAATTCAAAAATATAAACAAAAACATAAAAAT
AAGAAAAG

GAAAAGAGAAATAGATCCTTTCTCAAAAATTTAGAAAGGAAAAAAAATCAAAATATAATAAAATAGATTTT
TTTTAGGTTAATTTTTTGCCAATTTAGCTAGACTTTTGAAAAGATCCAAAAATTTACTAAAATTGAATCTT
TCTGGTAT

AAAGCATAATTGGAGGCAGTAAAGCATTATAGTCATACACAATAATAATAATAATAATAATAATAATAATA
ATAATAATAGTAGTAGTAGTAATGGTAATAAAAAAGTTAGAAAAGAAATTAGATACAGATATTCGTAGTAA
AGGCCACG

GCAAAAAAACGAAAATGTAATGGAGTCATGCAAACATGACTAATAGTAACAAAAAAAAAAGATATAGTAGA
ATTGAGTAAGTAAGTTCTATGTATATTGTTGTTATTTTCTCTAAAAAAAAAAAAAAAACTATGTAAAATTAG
GAATTAAG

GAATTGGCTAGCAAAAAAACGAAAATGTAATGGAGTCATGCAAACATGACTAATAGTAACAAAAAAAAAAG
ATATAGTAGAATTGAGTAAGTAAGTTCTATGTATATTGTTGTTATTTTCTCTAAAAAAAAAAAAAAAACTAT
GTAAAATT

ATAACATTAATAATAATAATTTTTTAATAGTAATAATGATAATAATAATAATAAAAATAAAAGAATAAATT
TAAAAACAAAAAAAATTCTAATATTAATGATGATAATAGTAGTAATACTGGTGGTATTATATATGTTGAAG
TGAACACG

AAGACGCCTACTTAAACTGCAATGAATAGTCACCCATAGGTATAAGAAAAAAAAAAAAAAATAAAAGAGTAC
ATAATATACATACAACTAAAATTAAAATGCATAGAAACCTAAGAAAAAAATATTTGTTATACCAAAAAAAAT
ACCACAAA

ATTAAAACAAAGTACCCAGAGACAAAAAAGAAAGAAAAGAAAAAAGAAACAGTCTACAAATTCTATTTGAA
TATATAAAATTATATTCTCAATTTTCACGTATATATTCACCTCACAAATAATATCATTCCTTTAAAAAAAA
ATATGTAT

AAGACCTGGCAGTGGAGGCATGACAGAGGATATCGCGAAACGATTGTTTAAGAGTATATTATAAAGTTGAC
TTGTTTCCTTATAATTATAATTATAAATATATATATATAGATTATTCACAGGAGAAATTGGGGGCACAAAT
GAGGGGTA

CCTTTAAAACTATTAAAGAAGCTGTTCGCAACTCACAAAAAAAAATAAAAAAATAAAGCAGAACGAAAAAAA
AAAAAAACAAAAAAAACAGTTAACTATTCTAATTACTTAGAATCTTTAAAAGTCTTACTGTAATTCGATCA
GTGGAGAA

CTTAAACTGCAATGAATAGTCACCCATAGGTATAAGAAAAAAAAAAAAAAATAAAAGAGTACATAATATACA
TACAACTAAAATTAAAATGCATAGAAACCTAAGAAAAAAATATTTGTTATACCAAAAAAAATACCACAAATG
ATACGTAA

CCTGGAAAAATGGGGAAATTTCATGTACGTCTCCATCAATAGTCATTTTTAAATTTTAGGTTATTTTGTGT
TTGCATATATAAATGGTATATATATATATATATATATATTCATATAAATCTAGGGCTACATTTATAATATCAG
AGATACAT

ACCACCCGGGTGTGGATGTGGATGCAAAATAACAGTGAAATGAGTAATAACAATGGTCATAAACATGTGGT
GTGCAATGTGTCATCTTCGCGCGCGTGCTAAAAAAAACGAATAACGGTGGTGATCCCGCGGCTTTCCACAC
TGACGGGG

CGGTGATAGACGAGCTTCGCTTATAGTCATGCTTCTTCTTTTGAATATTACCGATATTAGTAGTTTATATT
TGATATTTCTATCAGTATTATTTAGATTTTTTTTTTTTTTTTAATTGTTAATGCTTTTTCTTAGAACTTTC
CAAAAACG

AAAGAAAAGAAAAAAGAAACAGTCTACAAATTCTATTTGAATATATAAAATTATATTCTCAATTTTCACGT
ATATATTCACCTCACAAATAATATCATTCCTTTAAAAAAAAATATGTATTATTGTATAATGTTTCAGCATA
GTCGTTTT

GTTAGTTTATAAATAAATTTTATATCACTATATGTGTGGTGAAAAGGAAGAGCAATCCTGCTAAAGCTTTT
ATATCTAAACGCCAAAAAAATAAAAAAAAAAGGAAATGGGAGAAAAGTTTTCAGCGCGCGAACTCGCGAAA
AAACTTCC

TCATTATTACTAACTAGTGCATCACCACTTGTCTAAATACCAAACGCAAAATATATATATATTCTTACTTCAT
AACGTTCAAAATAAAGTTTTTAGTTTAAAATAAAATAAAATAAAAAAAAAAAAAAAAAAAAAAAAAGAACCT
TTTGTTTT

TTTATTCTTGCCATCCGTGTACGCTAGGAGAGGATTATTAAAATAAGTGATATATACATATATATATATATAT
ATATATATATATAATACACTAATTATTTTATGTGATGTTGATCACGCGAAACGGTAAACGGCTCTGTTCGC
GCTTTCTT

TATATCACTATATGTGTGGTGAAAAGGAAGAGCAATCCTGCTAAAGCTTTTATATCTAAACGCCAAAAAAA
TAAAAAAAAAAGGAAATGGGAGAAAAGTTTTCAGCGCGCGAACTCGCGAAAAAACTTCCTCTTTAGCAATG
GTGACATA

AAAAAATTCGATTATAAAATAGTACAATGTCCAGAAGCTTGGTTTTTAATAGTATATAATAAAGATAAAGA
TAGATAGAGAACATAACTCTAAACTTTAAGAGTGAATTTTTTTTTTTTAGTTTAGTTTTTTTTTTTTTTTT
TTTTTACA

CAACTCCGTGATCGCGCGCCACGGGCCGTCGGCGGCTGTTAATTGAAGAAAAAAAAAAATGAAGAACCACAA
GGGGTGATCCATATAGGTGACTAGCATCATCCCCTGCGACGCGCGGCCCGCCGGGCCAAAGGCGGGCAATG
CGCGCTGC

AAATAAATTTTATATCACTATATGTGTGGTGAAAAGGAAGAGCAATCCTGCTAAAGCTTTTATATCTAAAC
GCCAAAAAAATAAAAAAAAAAGGAAATGGGAGAAAAGTTTTCAGCGCGCGAACTCGCGAAAAAACTTCCTC
TTTAGCAA

CCATCCGTGTACGCTAGGAGAGGATTATTAAAATAAGTGATATATACATATATATATATATATATATATATAT
ATAATACACTAATTATTTTATGTGATGTTGATCACGCGAAACGGTAAACGGCTCTGTTCGCGCTTTCTTTG
TTTACATT

TAACTAGTGCATCACCACTTGTCTAAATACCAAACGCAAAATATATATATATTCTTACTTCATAACGTTCAAA
ATAAAGTTTTTAGTTTAAAATAAAATAAAATAAAAAAAAAAAAAAAAAAAAAAAAAAGAACCTTTTGTTTTCG
CCCAGTAT

TTACTATATTAATATCACGTCACACGACGCACAGTGAGAAGTGAAAAATTTTTTTTCAATCTGAAAAAAAA
AAAAAAAAAAAAAAAAAATTTATATAAACGAATGGTATCTCCATCACATTTCTTTTAGCCTCGCAACTTGT
ACTTTTCA

TAACCCGTAGTATTAGTTCCCCATATAATTCCGACCGAGAAAGGTGCACCCACTTGTCATACAAATTGTAC
ATATATATATATATATATATATATATATATATATATATGTATATATTATACCTGCAAGCAATTGCACGCGAGT
ACTCTGCG

CTGAAGCCAGTTTATTCTTGCCATCCGTGTACGCTAGGAGAGGATTATTAAAATAAGTGATATATACATAT
ATATATATATATATATATATATATAATACACTAATTATTTTATGTGATGTTGATCACGCGAAACGGTAAACGG
CTCTGTTC

ATCGCGCGCCACGGGCCGTCGGCGGCTGTTAATTGAAGAAAAAAAAAAATGAAGAACCACAAGGGGTGATCC
ATATAGGTGACTAGCATCATCCCCTGCGACGCGCGGCCCGCCGGGCCAAAGGCGGGCAATGCGCGCTGCTG
ATTGGCCT

TAATTTAAAGCATATTCAATCATAATAAAAAAAAAAAAAAGAGAATTATTAGGAAAAATAAAAACAAAATAA
TATATAATATATGATGTAGAAAAAAATCTTGAAATTTTTATCGATTCGAATAAAATGGAATTTAAAAAAGT
GGGAAAGA

AATATCACGTCACACGACGCACAGTGAGAAGTGAAAAATTTTTTTTCAATCTGAAAAAAAAAAAAAAAAAA
AAAAAAAATTTATATAAACGAATGGTATCTCCATCACATTTCTTTTAGCCTCGCAACTTGTACTTTTCATC
ACTTTTCT

CATATTCAATCATAATAAAAAAAAAAAAAGAGAATTATTAGGAAAAATAAAAACAAAATAATATATAATAT
ATGATGTAGAAAAAAATCTTGAAATTTTTATCGATTCGAATAAAATGGAATTTAAAAAAGTGGGAAAGAAA
CAATGAGA

CACACGACGCACAGTGAGAAGTGAAAAATTTTTTTTCAATCTGAAAAAAAAAAAAAAAAAAAAAAAAAATT
TATATAAACGAATGGTATCTCCATCACATTTCTTTTAGCCTCGCAACTTGTACTTTTCATCACTTTTCTTG
TAATTTAG

ATTTACTTGTGTTAGTTTATAAATAAATTTTATATCACTATATGTGTGGTGAAAAGGAAGAGCAATCCTGC
TAAAGCTTTTATATCTAAACGCCAAAAAAATAAAAAAAAAAGGAAATGGGAGAAAAGTTTTCAGCGCGCGA
ACTCGCGA

ATCACCACTTGTCTAAATACCAAACGCAAAATATATATATTCTTACTTCATAACGTTCAAAATAAAGTTTT
TAGTTTAAAATAAAATAAAATAAAAAAAAAAAAAAAAAAAAAAAAGAACCTTTTGTTTTCGCCCAGTATAT
GGGTAAAT

CATAATAAAAAAAAAAAAAGAGAATTATTAGGAAAAATAAAAACAAAATAATATATAATATATGATGTAGA
AAAAAATCTTGAAATTTTTATCGATTCGAATAAAATGGAATTTAAAAAAGTGGGAAAGAAACAATGAGAAT
TCATAGGA

TGTTGTTGTATCATTATTACTAACTAGTGCATCACCACTTGTCTAAATACCAAACGCAAAATATATATATT
CTTACTTCATAACGTTCAAAATAAAGTTTTTAGTTTAAAATAAAATAAAATAAAAAAAAAAAAAAAAAAAA
AAAAGAAC

TCTTACTTCATAACGTTCAAAATAAAGTTTTTAGTTTAAAATAAAATAAAATAAAAAAAAAAAAAAAAAAA
AAAAAGAACCTTTTGTTTTCGCCCAGTATATGGGTAAATTAACGGTTCCTTTTATTAGTCATTTTCAACTC
TATACCAT

ATTATAAAATAGTACAATGTCCAGAAGCTTGGTTTTTAATAGTATATAATAAAGATAAAGATAGATAGAGA
ACATAACTCTAAACTTTAAGAGTGAATTTTTTTTTTTTTAGTTTAGTTTTTTTTTTTTTTTTTTTTACAAT
AATGTATT

GTCTAAATACCAAACGCAAAATATATATATTCTTACTTCATAACGTTCAAAATAAAGTTTTTAGTTTAAAA
TAAAATAAAATAAAAAAAAAAAAAAAAAAAAAAAAGAACCTTTTGTTTTCGCCCAGTATATGGGTAAATTA
ACGGTTCC

ACTTCATCATGATCATTAATTTCATATTCATATTCGGTTATTTTTTTTTTTTTTTATATTTTTTTTCAATG
GTGTTTTTGATGGTTTTTATTATATATATATATTTTTTTATCTTTTATCTTGTGTAAGCACCCCTCATTAC
TTGTATCG

TGCCTACTTCTACTTCTGGGAAAGGCATTTTTACTCGATCGCGTTAATATATGCATCAAGAAAATAAAAAA
TAAAACGCGAAGAGCTAAAAAAAAAAAAGAAAACCTACTATAAATAACCGATTAGAATCGAGTTTTTGTAT
TGAAATGG

GTAAAAATTAACTTCATCATGATCATTAATTTCATATTCATATTCGGTTATTTTTTTTTTTTTTTATATTT
TTTTTCAATGGTGTTTTTGATGGTTTTTATTATATATATATATTTTTTTATCTTTTATCTTGTGTAAGCAC
CCCTCATT

ACAGTGAGAAGTGAAAAATTTTTTTTCAATCTGAAAAAAAAAAAAAAAAAAAAAAAAAATTTATATAAACG
AATGGTATCTCCATCACATTTCTTTTAGCCTCGCAACTTGTACTTTTCATCACTTTTCTTGTAATTTAGCA
ATATCCCA

CCGACCGAGAAAGGTGCACCCACTTGTCATACAAATTGTACATATATATATATATATATATATATATATAT
ATATATGTATATATTATACCTGCAAGCAATTGCACGCGAGTACTCTGCGTGAGTCAAAAGACTGTGGTAAA
ATGGTACC

CAAACGCAAAATATATATATTCTTACTTCATAACGTTCAAAATAAAGTTTTTAGTTTAAAATAAAATAAAA
TAAAAAAAAAAAAAAAAAAAAAAAAGAACCTTTTGTTTTCGCCCAGTATATGGGTAAATTAACGGTTCCTT
TTATTAGT

ACGCTAGGAGAGGATTATTAAAATAAGTGATATATACATATATATATATATATATATATATATATAATACACT
AATTATTTTATGTGATGTTGATCACGCGAAACGGTAAACGGCTCTGTTCGCGCTTTCTTTGTTTACATTTT
AGTGAAGT

GCAAAAAAAAATAAAAGAAAAGGTAATAAATTGAATGAATGTAGATATATAAATATCAATAGAAATACGTAC
GCGGAATTATTTCTCTTTGTAACAAATTGATCAAAAGCTTTGTATTTTTTTTTTTTTTAGCTCGGCTGCGC
GCTATTAC

ATATGCACTTTTACTATATTAATATCACGTCACACGACGCACAGTGAGAAGTGAAAAATTTTTTTTCAATC
TGAAAAAAAAAAAAAAAAAAAAAAAAAAATTTATATAAACGAATGGTATCTCCATCACATTTCTTTTAGCCT
CGCAACTT

AAAGGCATTTTTACTCGATCGCGTTAATATATGCATCAAGAAAATAAAAAATAAAACGCGAAGAGCTAAAA
AAAAAAAAGAAAACCTACTATAAATAACCGATTAGAATCGAGTTTTTGTATTGAAATGGCGGTAATAAGCG
TTAAACCT

GATCATTAATTTCATATTCATATTCGGTTATTTTTTTTTTTTTTTTATATTTTTTTTCAATGGTGTTTTTGA
TGGTTTTTATTATATATATATATTTTTTTATCTTTTATCTTGTGTAAGCACCCCTCATTACTTGTATCGTG
CGATATAC

TATGTGTGGTGAAAAGGAAGAGCAATCCTGCTAAAGCTTTTATATCTAAACGCCAAAAAAATAAAAAAAAA
AGGAAATGGGAGAAAAGTTTTCAGCGCGCGAACTCGCGAAAAAACTTCCTCTTTAGCAATGGTGACATATA
GATCTCAA

TACTTCTGGGAAAGGCATTTTTACTCGATCGCGTTAATATATGCATCAAGAAAATAAAAAATAAAACGCGA
AGAGCTAAAAAAAAAAAAGAAAACCTACTATAAATAACCGATTAGAATCGAGTTTTTGTATTGAAATGGCG
GTAATAAG

TTTTAAAAAAAAAAAACGAAAAAAAACATAACTAAATTTAAAGTGCAGCCCAACAATAACCCTGAAAAATCT
AAATATCTTAGAATTTTTTTATTTTGATTATTATATATTATTATTATTCTTATGGTAAATAATGCCCCTAC
TTTTCTTC

GAGAACGTTCCTGAAGCCAGTTTATTCTTGCCATCCGTGTACGCTAGGAGAGGATTATTAAAATAAGTGAT
ATATACATATATATATATATATATATATATATAATACACTAATTATTTTATGTGATGTTGATCACGCGAAA
CGGTAAAC

TATGTCTACCACATAAAAATGACCTTATAACACAACTTCCTTAGTATGGAAGTAATACATATATACATATA
TACATATATACATACATATATATATATATATATATATATATACATCTTTTGAACCAATTCCCTATAGACTT
GGTTGTAA

ACCCTATCTCCCAGTGATAAACTTTAATTTTTTTTTCGTGCATATATATATATACATATATAATATAAAACA
AAATATCATGAATTTCTATCTAAAGTAGTGGGAAAAAATATGTCGTTAAAAATGGGTATAATATCCAAGCA
AAGCTTTT

CTATGCTTTTCCTCTCGATGAGATGAGCTGTGAAAAATTTTGAAAAAAAAAAAAAAAAAAAAAAAAAAAAA
AATTTGTTATCACCAATAACGAGAAACCTAACTCTAAAACTTAGGGTCAAAGAACAGAACCCTAGGCTTTG
TATCCAGT

TAACGTTCAAAATAAAGTTTTTAGTTTAAAATAAAATAAAATAAAAAAAAAAAAAAAAAAAAAAAAGAACC
TTTTGTTTTCGCCCAGTATATGGGTAAATTAACGGTTCCTTTTATTAGTCATTTTCAACTCTATACCATGC
TCCAAAAC

ATATATATATTCTTACTTCATAACGTTCAAAATAAAGTTTTTAGTTTAAAATAAAATAAAATAAAAAAAAA
AAAAAAAAAAAAAAAGAACCTTTTGTTTTCGCCCAGTATATGGGTAAATTAACGGTTCCTTTTATTAGTCA
TTTTCAAC

TTCACCCTGGCAGATAGGAAACCCTATCTCCCAGTGATAAACTTTAATTTTTTTTCGTGCATATATATATA
TACATATATAATATAAAACAAAATATCATGAATTTCTATCTAAAGTAGTGGGAAAAAATATGTCGTTAAAA
ATGGGTAT

ATGGATGTCTTGCCTACTTCTACTTCTGGGAAAGGCATTTTTACTCGATCGCGTTAATATATGCATCAAGA
AAATAAAAAATAAAACGCGAAGAGCTAAAAAAAAAAAAGAAAACCTACTATAAATAACCGATTAGAATCGA
GTTTTTGT

ACTTTAATTTTTTTTCGTGCATATATATATATACATATATAATATAAAACAAAATATCATGAATTTCTATC
TAAAGTAGTGGGAAAAAATATGTCGTTAAAAATGGGTATAATATCCAAGCAAAGCTTTTGGCTTTTTTTTT
CTTGAAAT

AGCAGTTACGCAACTCCGTGATCGCGCGCCACGGGCCGTCGGCGGCTGTTAATTGAAGAAAAAAAAAATGA
AGAACCACAAGGGGTGATCCATATAGGTGACTAGCATCATCCCCTGCGACGCGCGGCCCGCCGGGCCAAAG
GCGGGCAA

TATGTTTTTCATACGTGATTTGAAGTTTTTAAGTATCTGAAATACATACGCGCGCGTATGCATATGTATTA
GTTAAATTACTCGAATGTCCTTTATATAATATTACATTTTTACACACACATTCTTGAAGAAGAAATCCCGA
TCGTAAAA

ACATAAAAATGACCTTATAACACAACTTCCTTAGTATGGAAGTAATACATATATACATATATACATATATA
CATACATATATATATATATATATATATATACATCTTTTGAACCAATTCCCTATAGACTTGGTTGTAATT
CGTTAGAA

TATTAGTTCCCCATATAATTCCGACCGAGAAAGGTGCACCCACTTGTCATACAAATTGTACATATATATAT
ATATATATATATATATATATATATATGTATATATTATACCTGCAAGCAATTGCACGCGAGTACTCTGCGTG
AGTCAAAA

CCATATAATTCCGACCGAGAAAGGTGCACCCACTTGTCATACAAATTGTACATATATATATATATATATAT
ATATATATATATATATGTATATATTATACCTGCAAGCAATTGCACGCGAGTACTCTGCGTGAGTCAAAAGA
CTGTGGTA

CCAGTGATAAACTTTAATTTTTTTTCGTGCATATATATATATACATATATAATATAAAACAAAATATCATG
AATTTCTATCTAAAGTAGTGGGAAAAAATATGTCGTTAAAAATGGGTATAATATCCAAGCAAAGCTTTTGG
CTTTTTTT

TTCTGAAATTTTTAGGGTGGTAATTTAAAGCATATTCAATCATAATAAAAAAAAAAAAAGAGAATTATTAG
GAAAAATAAAAACAAAATAATATATAATATATGATGTAGAAAAAAATCTTGAAATTTTTATCGATTCGAAT
AAAATGGA

TTTTTCGACCTTAGAATGGCAGAAAAATGGCAAATACTGTAAAATACTTAACATTTAACTAAATTTTATTC
TATGCTTTTCCTCTCGATGAGATGAGCTGTGAAAAATTTTGAAAAAAAAAAAAAAAAAAAAAAAAAAAAAA
ATTTGTTA

AGTTATACGCGAAAGTAAAAGTGAGAGCTTTTCAGGGGTTAAAAGCTGGGCGTGTTCCATGACGTATTTAC
CGAGGTCGTATTATCAAAGAAAATGAAAAAAAAAAAAAAAAAAAAAAAAGCGAAGAGGAAAAAGACAAACG
AAAAACAA

AAAAACGAAAAAAAACATAACTAAATTTAAAGTGCAGCCCAACAATAACCCTGAAAAATCTAAATATCTTA
GAATTTTTTTATTTTGATTATTATATATTATTATTATTCTTATGGTAAATAATGCCCCTACTTTTCTTCTA
AGGAAGTG

AATAAAGTTTTTAGTTTAAAATAAAATAAAATAAAAAAAAAAAAAAAAAAAAAAAAAGAACCTTTTGTTTTC
GCCCAGTATATGGGTAAATTAACGGTTCCTTTTATTAGTCATTTTCAACTCTATACCATGCTCCAAAACGG
CGTTCGGC

ATACGTGATTTGAAGTTTTTAAGTATCTGAAATACATACGCGCGCGTATGCATATGTATTAGTTAAATTAC
TCGAATGTCCTTTATATAATATTACATTTTTACACACACATTCTTGAAGAAGAAATCCCGATCGTAAAAAA
AAACACCG

TCGTAGTTAGTTCTGAAATTTTTAGGGTGGTAATTTAAAGCATATTCAATCATAATAAAAAAAAAAAAAAGA
GAATTATTAGGAAAAATAAAAACAAAATAATATATAATATATGATGTAGAAAAAAATCTTGAAATTTTTAT
CGATTCGA

TTTCTTATTTTTAAAATCGATAGTTCTTCAGGATCTTGGTCACATATATATATATATATATATATACACTT
ATATAAAATAAGACGTTTATTTAATCTTGTTTTCTTACGAGTTTCTTTTTTTTTCTTTTTTTATTTTCTAC
TTTTGCAA

GTTATAGCTCTAACCCGTAGTATTAGTTCCCCATATAATTCCGACCGAGAAAGGTGCACCCACTTGTCATA
CAAATTGTACATATATATATATATATATATATATATATATATATATGTATATATTATACCTGCAAGCAATT
GCACGCGA

CTATATACTTTCGTAGTTAGTTCTGAAATTTTTAGGGTGGTAATTTAAAGCATATTCAATCATAATAAAAA
AAAAAAAAGAGAATTATTAGGAAAAATAAAAACAAAATAATATATAATATATGATGTAGAAAAAAATCTTG
AAATTTTT

AATATAGAAATTTTAAAAAAAAAAAACGAAAAAAAACATAACTAAATTTAAAGTGCAGCCCAACAATAACCC
TGAAAAATCTAAATATCTTAGAATTTTTTTATTTTGATTATTATATATTATTATTATTCTTATGGTAAATA
ATGCCCCT

AAATTTTATTCTATGCTTTTCCTCTCGATGAGATGAGCTGTGAAAAATTTTGAAAAAAAAAAAAAAAAAAA
AAAAAAAAAAAATTTGTTATCACCAATAACGAGAAACCTAACTCTAAAACTTAGGGTCAAAGAACAGAACC
CTAGGCTT

GGCTTGAAATATGGATGTCTTGCCTACTTCTACTTCTGGGAAAGGCATTTTTACTCGATCGCGTTAATATA
TGCATCAAGAAAATAAAAAATAAAACGCGAAGAGCTAAAAAAAAAAAAGAAAACCTACTATAAATAACCGA
TTAGAATC

CAGATAGGAAACCCTATCTCCCAGTGATAAACTTTAATTTTTTTTCGTGCATATATATATATACATATATA
ATATAAAACAAAATATCATGAATTTCTATCTAAAGTAGTGGGAAAAAATATGTCGTTAAAAATGGGTATAA
TATCCAAG

CATATGCTCATCGGGAAAACCTTTTAAGCAATAATAGATTTTAAGTATGTAATGATACAAATAATAATATA
TTTATATACGCATATATTTACATGTGCATATATGAAAAATAACCTAATTCACGTTACCCACCTTTTTTTTT
AGCCTTTT

ACGTTTCTCAGTAAAAATTAACTTCATCATGATCATTAATTTCATATTCATATTCGGTTATTTTTTTTTTT
TTTTATATTTTTTTTCAATGGTGTTTTTGATGGTTTTTATTATATATATATATTTTTTTATCTTTTATCTT
GTGTAAGC

CCTCTCGATGAGATGAGCTGTGAAAAATTTTGAAAAAAAAAAAAAAAAAAAAAAAAAAAAAAAAATTTGTTAT
CACCAATAACGAGAAACCTAACTCTAAAACTTAGGGTCAAAGAACAGAACCCTAGGCTTTGTATCCAGTTG
AATCACTA

TTACTCGATCGCGTTAATATATGCATCAAGAAAATAAAAAATAAAACGCGAAGAGCTAAAAAAAAAAAAAGA
AAACCTACTATAAATAACCGATTAGAATCGAGTTTTTGTATTGAAATGGCGGTAATAAGCGTTAAACCTCG
ACGAAGAG

TTTTTTTTTTTTTTTTATATTTTTTTTTCAATGGTGTTTTTGATGGTTTTTATTATATATATATATATTTTTTTTA
TCTTTTATCTTGTGTAAGCACCCCTCATTACTTGTATCGTGCGATATACAAATACCAAACAAACTCTATAA
TCATAGAA

AGAAAAATGGCAAATACTGTAAAATACTTAACATTTAACTAAATTTTATTCTATGCTTTTCCTCTCGATGA
GATGAGCTGTGAAAAATTTTGAAAAAAAAAAAAAAAAAAAAAAAAAAAAAAAATTTGTTATCACCAATAACG
AGAAACCT

TAAAAATCTCCGGGGCTAGCTTTTGCCGGGGAACCCATCCCGAAAAAATTGCAAAAAAAAAAAATAGCCGCC
GACCGTTGGTCGCTATTCACGGAATGATAGAAAAATAGCCGCGCTGCTCGTCCTGGGTGACCTTTTGTATA
TTGTATAA

CATAAAATGCTTCACGAACACCGTCATTGATCAAATAGGTTTATAATATTAATATACATTTATATAATCGG
CGGTATTTATATCATCAAAAAAAGTAGTTTTTTATTTTATTTTTTCATTACTTTTCACTGTCTATGGATTT
TCATTCGT

TTGTTGCCACTTTTTAATGTGAACTAAAAAAATAAAAATGAATATAAGGTACGTCTCCAAAAGAAATGTAA
ATATAGAAATTTTAAAAAAAAAAAACGAAAAAAAACATAACTAAATTTAAAGTGCAGCCCAACAATAACCCT
GAAAAATC

TTAGTTTAAAATAAAATAAAATAAAAAAAAAAAAAAAAAAAAAAAAAGAACCTTTTGTTTTCGCCCAGTATA
TGGGTAAATTAACGGTTCCTTTTATTAGTCATTTTCAACTCTATACCATGCTCCAAAACGGCGTTCGGCAC
AGGTTTTT

AAGTAAACAATATGTTTTTCATACGTGATTTGAAGTTTTTAAGTATCTGAAATACATACGCGCGCGTATGC
ATATGTATTAGTTAAATTACTCGAATGTCCTTTATATAATATTACATTTTTACACACACATTCTTGAAGAA
GAAATCCC

AGCATGTAGTCAACAAAAAGGGAGTAAAACATGCAGTGGCTCTATATATATAATTATCGTAGAATAATTCA
AAAAAAAATAAAAAACGAAAAAATTCATTCTTCATCACTCATCAAAGGCACTATTTCGTCATAACGCGGAG
GCTCGTTA

CGCCAAAAAAATAAAAAAAAAAGGAAATGGGAGAAAAGTTTTCAGCGCGCGAACTCGCGAAAAAACTTCCT
CTTTAGCAATGGTGACATATAGATCTCAAGGTTCTCAATTAAAAGACCTTCTTTGTAGCTTTTAGTGTGAT
TTTTCTGG

AAGGTGCACCCACTTGTCATACAAATTGTACATATATATATATATATATATATATATATATATATATGTAT
ATATTATACCTGCAAGCAATTGCACGCGAGTACTCTGCGTGAGTCAAAAGACTGTGGTAAAATGGTACCCA
TCTGATGC

TTCATATTCATATTCGGTTATTTTTTTTTTTTTTTTATATTTTTTTTTCAATGGTGTTTTTGATGGTTTTTAT
TATATATATATATTTTTTTTATCTTTTATCTTGTGTAAGCACCCCTCATTACTTGTATCGTGCGATATACAA
ATACCAAA

TTTAGGGTGGTAATTTAAAGCATATTCAATCATAATAAAAAAAAAAAAAAGAGAATTATTAGGAAAAATAAA
AACAAAATAATATATAATATATGATGTAGAAAAAAATCTTGAAATTTTTATCGATTCGAATAAAATGGAAT
TTAAAAAA

TGAAGTTTTTAAGTATCTGAAATACATACGCGCGCGTATGCATATGTATTAGTTAAATTACTCGAATGTCC
TTTATATAATATTACATTTTTACACACACATTCTTGAAGAAGAAATCCCGATCGTAAAAAAAAACACCGTG
CTCCAAAT

TAATCTTGAGCAAATTGATCCTACATAAATCATGTGACTTATTTATTTAATTATTATTAAGTAAAAAAGAT
TTTCTATTTAAATTTATTAATTAATTTTTTTTCTTAAATAATTATTTTATGTTTTTGTTTTCCGAAAAAGA
AAAATATT

TGATCAAAAAAAAAAAGGAAAATCAAGCGCGGGTAATAACGGCACCATTAAATACTAGTAGTACTATATAG
ATGGTATAAAGAAATGATTTAAAACAATACGATTATATGATATTTATACATCTGTTTCAGTTGAGCTTTTT
TTCCATAG

ATAAAATAAAATAAAAAAAAAAAAAAAAAAAAAAAAGAACCTTTTGTTTTCGCCCAGTATATGGGTAAATT
AACGGTTCCTTTTATTAGTCATTTTCAACTCTATACCATGCTCCAAAACGGCGTTCGGCACAGGTTTTTA
TTTTACCA

ACGGCGAAAGTGAAAGGTGCAAGTGGAAAGAAAGGAAGGCTAAAGCAAAAGTGTTTCTTATATATAATTTT
ATGTACCAGAGGAAGCAAAGTACGAAAACTATCAGTTGAAATCCAAAAAAAAAAAGTTACGCGTTTCGCGT
TTTGGTGC

TATTCGGTTATTTTTTTTTTTTTTTATATTTTTTTTCAATGGTGTTTTTGATGGTTTTTATTATATATATA
TATTTTTTTATCTTTTATCTTGTGTAAGCACCCCTCATTACTTGTATCGTGCGATATACAAATACCAAACA
AACTCTAT

GAAAAGGAAGAGCAATCCTGCTAAAGCTTTTATATCTAAACGCCAAAAAAATAAAAAAAAAAGGAAATGGG
AGAAAAGTTTTCAGCGCGCGAACTCGCGAAAAAACTTCCTCTTTAGCAATGGTGACATATAGATCTCAAGG
TTCTCAAT

TGCCTGAAACTTTTTCGACCTTAGAATGGCAGAAAAATGGCAAATACTGTAAAATACTTAACATTTAACTA
AATTTTATTCTATGCTTTTCCTCTCGATGAGATGAGCTGTGAAAAATTTTGAAAAAAAAAAAAAAAAAAA
AAAAAAAA

TTACCAAAAAAAAGTTCATCTTTCTTATTTTTAAAATCGATAGTTCTTCAGGATCTTGGTCACATATATAT
ATATATATATATATACACTTATATAAAATAAGACGTTTATTTAATCTTGTTTTCTTACGAGTTTCTTTTTT
TTTCTTTT

ATTAAAGAGAAAAAAATTCGATTATAAAATAGTACAATGTCCAGAAGCTTGGTTTTTAATAGTATATAATA
AAGATAAAGATAGATAGAGAACATAACTCTAAACTTTAAGAGTGAATTTTTTTTTTTTAGTTTAGTTTTTT
TTTTTTTT

AATATATTCATCGTCATATACGGAACATTCAGTTATACGCGAAAGTAAAAGTGAGAGCTTTTCAGGGGTTA
AAAGCTGGGCGTGTTCCATGACGTATTTACCGAGGTCGTATTATCAAAGAAAATGAAAAAAAAAAAAAAAA
AAAAAAAA

AAAAAAAAAGAGAATTATTAGGAAAAATAAAAACAAAATAATATATAATATATGATGTAGAAAAAAATCTT
GAAATTTTTATCGATTCGAATAAAATGGAATTTAAAAAAGTGGGAAAGAAACAATGAGAATTCATAGGATA
TAATGAAA

TATCAGAATTAGAGCCTTAAAAAAAGCCGCAAGACAGGTTAAAAAGGAATAGAATTATCGTTCTCGAGATA
GTTTTTATACAATACATATATATATATATATATATATATTTACAAGAAAAGCATGGCTTATGTATTATACT
TGCTTATG

ACATTTAACTAAATTTTATTCTATGCTTTTCCTCTCGATGAGATGAGCTGTGAAAAATTTTGAAAAAAAAA
AAAAAAAAAAAAAAAAAAAAAATTTGTTATCACCAATAACGAGAAACCTAACTCTAAAACTTAGGGTCAAA
GAACAGAA

AAAGTTCATCTTTCTTATTTTTAAAATCGATAGTTCTTCAGGATCTTGGTCACATATATATATATATATAT
ATATACACTTATATAAAATAAGACGTTTATTTAATCTTGTTTTCTTACGAGTTTCTTTTTTTTTCTTTTTT
TATTTTCT

GGTTTTTAATAGTATATAATAAAGATAAAGATAGATAGAGAACATAACTCTAAACTTTAAGAGTGAATTTT
TTTTTTTTAGTTTAGTTTTTTTTTTTTTTTTTTTTTACAATAATGTATTGTTCTTGGAAAATAACACAATC
TGTTCAAA

TTAGAATGGCAGAAAAATGGCAAATACTGTAAAATACTTAACATTTAACTAAATTTTATTCTATGCTTTTC
CTCTCGATGAGATGAGCTGTGAAAAATTTTGAAAAAAAAAAAAAAAAAAAAAAAAAAAAAAATTTGTTATC
ACCAATAA

TTTTTGACAAGAGAACGTTCCTGAAGCCAGTTTATTCTTGCCATCCGTGTACGCTAGGAGAGGATTATTAA
AATAAGTGATATATACATATATATATATATATATATATATATAATACACTAATTATTTTATGTGATGTTGA
TCACGCGA

GACCTTATAACACAACTTCCTTAGTATGGAAGTAATACATATATACATATATACATATATACATACATATA
TATATATATATATATATATATACATCTTTTGAACCAATTCCCTATAGACTTGGTTGTAATTCGTTAGAAAG
ATTTTCAT

CTTCGATGCATTTAGATAATTTTTGGAAACATTTTTTTTCTTGATGTATATTTTTTGTATTGTAGAAATCG
CGCGTACTGTACTTGTATATCGCTTTATAAGCGCTTTTAATTGATTGTTCATGACGAGGATAGGCGGATAG
GCGGAGGT

CTAATTAAATAGCATGTAGTCAACAAAAAGGGAGTAAAACATGCAGTGGCTCTATATATATAATTATCGTA
GAATAATTCAAAAAAAATAAAAAAACGAAAAAATTCATTCTTCATCACTCATCAAAGGCACTATTTCGTCA
TAACGCGG

GTGAGAGCTTTTCAGGGGTTAAAAGCTGGGCGTGTTCCATGACGTATTTACCGAGGTCGTATTATCAAAGA
AAATGAAAAAAAAAAAAAAAAAAAAAAAAGCGAAGAGGAAAAAGACAAACGAAAAACAAAACGAAATAACG
CATTGTAT

TAACTGTATAGAAACGTTCGTGGCGGAAAAAACTACGTAAAAAGGCGGTATTTATCTATTATTTGGCCAAA
AAAAAAAAAAAATACATACTACATATACATATACGCCATAAAAAATCTCTGCATCTATCTTATTTCCCATT
ATTTGGAC

CGTAGAAGATCAATCAATTGAAGTTAAAAAGGAGAAGTAATAATTATAGCATAATATATATTCATAATGTA
TAGGCATATTTATTTTTTTATTTTTTTATTTCATGTTCTATTTAATGACGAATCACGAAGAAAATATATCT
AAGAAAAG

TATTATCTTCCCATGCCGCTCCTGCGTGGGATTCCCTTTATTATAACTTTTAAGGTTTCTACCCACATATA
TGCAATTTTTATCATGTTTTTCTCTTTTTTTTTTTTTTTTTTTTTTTTCTCTCTTTGTTGTGTTTTCGAGA
AAAAGTTC

AAAAACATAACTAAATTTAAAGTGCAGCCCAACAATAACCCTGAAAAATCTAAATATCTTAGAATTTTTTT
ATTTTGATTATTATATATTATTATTATTCTTATGGTAAATAATGCCCCTACTTTTCTTCTAAGGAAGTGAG
TTACCACC

TCGGGAAAACCTTTTAAGCAATAATAGATTTTAAGTATGTAATGATACAAATAATAATATATTTATATACG
CATATATTTACATGTGCATATATGAAAAATAACCTAATTCACGTTACCCACCTTTTTTTTTAGCCTTTTCT
TTATTTAT

CAAATACTGTAAAATACTTAACATTTAACTAAATTTTATTCTATGCTTTTCCTCTCGATGAGATGAGCTGT
GAAAAATTTTGAAAAAAAAAAAAAAAAAAAAAAAAAAAAAAATTTGTTATCACCAATAACGAGAAACCTAA
CTCTAAAA

AGCTAAGGGGAGCAGTTACGCAACTCCGTGATCGCGCGCCACGGGCCGTCGGCGGCTGTTAATTGAAGAAA
AAAAAAATGAAGAACCACAAGGGGTGATCCATATAGGTGACTAGCATCATCCCCTGCGACGCGCGGCCCGC
CGGGCCAA

GCACCGTGCTAATAACGCGGGGATCAGCGGTTCGATCCCGCTAGAGACCATTTTTTTGCAATTCAAATAAC
GTCGTTTATTTTTTATATAAAAATAAAAATCAAAAAGAATTGCGCAAGCCCGGAATCGAACCGGGGGCCCA
ACGATGGC

CATTACCGTCTGTTTTATTTACTGTCACCTTGATGAGCGACTAAAAAGATAGAACGCGGGTTTTTCGCGCA
ATGTAACAAAGCTCCAGAAATTTTTTATAAATACTAATGTTATAGCAAAAAAAAATAAGGACCTTAAAGTCG
TAAACTAA

TTAAAATCGATAGTTCTTCAGGATCTTGGTCACATATATATATATATATATATATACACTTATATAAAATA
AGACGTTTATTTAATCTTGTTTTCTTACGAGTTTCTTTTTTTTTCTTTTTTTATTTTCTACTTTTGCAAGG
AACAGTGA

CTCCTAATGCCTTCGATGCATTTAGATAATTTTTGGAAACATTTTTTTTCTTGATGTATATTTTTTGTATT
GTAGAAATCGCGCGTACTGTACTTGTATATCGCTTTATAAGCGCTTTTAATTGATTGTTCATGACGAGGAT
AGGCGGAT

GTATAAAAAGAAATAGACCAATATCAACATCAATTTTATGTATTTTTTTTTTCTTTTGGTATAGCATTGAT
ATATTGAAATTTGTATATATTGCTGCGAACACTATTTAAAACAGGTTTTTTTTTTATTTTGGCAGTTTGAA
ACCCTTTC

GTTGGTTAAGGCACCGTGCTAATAACGCGGGGATCAGCGGTTCGATCCCGCTAGAGACCATTTTTTTGCAA
TTCAAATAACGTCGTTTATTTTTTATATAAAAATAAAAATCAAAAAGAATTGCGCAAGCCCGGAATCGAAC
CGGGGGCC

ATAATATTGAATATGCACTTTTACTATATTAATATCACGTCACACGACGCACAGTGAGAAGTGAAAAATTT
TTTTTCAATCTGAAAAAAAAAAAAAAAAAAAAAAAAAAATTTATATAAACGAATGGTATCTCCATCACATTT
CTTTTAGC

GTGAAAAATTTTTTTTCAATCTGAAAAAAAAAAAAAAAAAAAAAAAAAAATTTATATAAACGAATGGTATCT
CCATCACATTTCTTTTAGCCTCGCAACTTGTACTTTTCATCACTTTTCTTGTAATTTAGCAATATCCCAAG
AACAATCA

TTTATAAGAAACATCATGTGATGACATTTAAAAAGTGGGAAAGAATACTACTAATATAAAACTATTTACTG
GATATTTAGAATTTTTTTTTTTTCTTCTTTATACGTAAAAGTTGTTCAAAAAATTTCATCGTTTTCTTTTT
TTATGAAT

GAACTAAAAAAATAAAAATGAATATAAGGTACGTCTCCAAAAGAAATGTAAATATAGAAATTTTAAAAAAA
AAAACGAAAAAAAACATAACTAAATTTAAAGTGCAGCCCAACAATAACCCTGAAAAATCTAAATATCTTAG
AATTTTTT

AGCAATCCTGCTAAAGCTTTTATATCTAAACGCCAAAAAAATAAAAAAAAAAGGAAATGGGAGAAAAGTTT
TCAGCGCGCGAACTCGCGAAAAAACTTCCTCTTTAGCAATGGTGACATATAGATCTCAAGGTTCTCAATTA
AAAGACCT

TTTTTAATGTGAACTAAAAAAATAAAAATGAATATAAGGTACGTCTCCAAAAGAAATGTAAATATAGAAAT
TTTAAAAAAAAAAACGAAAAAAAACATAACTAAATTTAAAGTGCAGCCCAACAATAACCCTGAAAAATCTA
AATATCTT

AAGGGAAAAAAAATGATTCAATATGTATATATTTATTCATGTATATGTATTTTGAATTAAAAAAAAACGTTT
TATAAATACTCAATAAAGCCGTTGTATTTTCTTTATTACTATCGAAATTCTTGCTTTAAACATTTCGCGCA
TCGCCGGC

AATAACGCGGGGATCAGCGGTTCGATCCCGCTAGAGACCATTTTTTTGCAATTCAAATAACGTCGTTTATT
TTTTATATAAAAATAAAAATCAAAAAGAATTGCGCAAGCCCGGAATCGAACCGGGGGCCCAACGATGGCAA
CGTTGGAT

AGATGAGCTGTGAAAAATTTTGAAAAAAAAAAAAAAAAAAAAAAAAAAAAAAATTTGTTATCACCAATAAC
GAGAAACCTAACTCTAAAACTTAGGGTCAAAGAACAGAACCCTAGGCTTTGTATCCAGTTGAATCACTAAT
TACGAAAG

AAATACAGAAACTACAAAAGGCAAAAAAAATAAAAGAAAAGGTAATAAATTGAATGAATGTAGATATATAA
ATATCAATAGAAATACGTACGCGGAATTATTTCTCTTTGTAACAAATTGATCAAAAGCTTTGTATTTTTTT
TTTTTTTA

CTAAATTTAAAGTGCAGCCCAACAATAACCCTGAAAAATCTAAATATCTTAGAATTTTTTTATTTTGATTA
TTATATATTATTATTATTCTTATGGTAAATAATGCCCCTACTTTTCTTCTAAGGAAGTGAGTTACCACCAA
AATAAGGA

GCATAATATCTTAGATAGATGTCTCAAGAGACTATCCTAAATAATATTGAATATGCACTTTTACTATATTA
ATATCACGTCACACGACGCACAGTGAGAAGTGAAAAATTTTTTTTCAATCTGAAAAAAAAAAAAAAAAAAAA
AAAAAAAT

AAAGAACCACTCAGACTTTTTACCATCCTTTAGCTTTACCTAATATAATGAAATTTGTCTAAGTAACATAA
AAAAAAGGAGTAAAGAAAAAAAAAAAATAAGTAAATGAAATAAAATATAAAAAAATTCTTACTACGGTTTA
AAACAAAG

CTATGGATGTGGCTTGAAATATGGATGTCTTGCCTACTTCTACTTCTGGGAAAGGCATTTTTACTCGATCG
CGTTAATATATGCATCAAGAAAATAAAAAATAAAACGCGAAGAGCTAAAAAAAAAAAAGAAAACCTACTAT
AAATAACC

AAAATACTTAACATTTAACTAAATTTTATTCTATGCTTTTCCTCTCGATGAGATGAGCTGTGAAAAATTTT
GAAAAAAAAAAAAAAAAAAAAAAAAAAAAAAATTTGTTATCACCAATAACGAGAAACCTAACTCTAAAACT
TAGGGTCA

ACATTAGGTGACGTTTCTCAGTAAAAATTAACTTCATCATGATCATTAATTTCATATTCATATTCGGTTAT
TTTTTTTTTTTTTTATATTTTTTTTCAATGGTGTTTTTGATGGTTTTTATTATATATATATATTTTTTTAT
CTTTTATC

CAAATTGATCCTACATAAATCATGTGACTTATTTATTTAATTATTATTAAGTAAAAAAGATTTTCTATTTA
AATTTATTAATTAATTTTTTTTCTTAAATAATTATTTTATGTTTTTGTTTTCCGAAAAAGAAAAATATTTC
AACAAGAA

AAAAGCTCATCGCATTTTTTCGGTCCTGATGTTAAAAATTTATTTCGTTTATTAAAAAAAAAAAAAACATT
ACTGAAACGTATAAAAGAAAAGTCTTAATCTATAAATATGGTTTTATCATAATTAGACTGTTTTACTCTGT
CCATTGGT

TTTTGGATCAGTAACCGTTATTTGAGCATAACACAGGTTTTTAAATATATTATTATATATCATGGTATATG
TGTAAAATTTTTTTGCTGACTGGTTTTGTTTATTTATTTAGCTTTTTAAAAATTTACTTTCTTCTTGTTA
ATTTTTTC

TCAGACTTTTTACCATCCTTTAGCTTTACCTAATATAATGAAATTTGTCTAAGTAACATAAAAAAAAGGAG
TAAAGAAAAAAAAAAAATAAGTAAATGAAATAAAATATAAAAAAATTCTTACTACGGTTTAAAACAAAGTG
CTCTTCCG

AATATTATTACATATGCTCATCGGGAAAACCTTTTAAGCAATAATAGATTTTAAGTATGTAATGATACAAA
TAATAATATATTTATATACGCATATATTTACATGTGCATATATGAAAAATAACCTAATTCACGTTACCCAC
CTTTTTTT

GCCTACTGTGTGCAAAGATATGTATTCGCTCGTTCAGTGTTTTTTTAAAAATATGTATAGAATTTGTCATT
ATCTGCGTTAAAAAATAGTTATAAAGTATATACAATAACAATAAATGATAAAGAAATATGCAGTGAAAAGA
AAAAATTA

AGAGCCTTAAAAAAAGCCGCAAGACAGGTTAAAAAGGAATAGAATTATCGTTCTCGAGATAGTTTTTATAC
AATACATATATATATATATATATATATATTTACAAGAAAAGCATGGCTTATGTATTATACTTGCTTATGTA
CCAATATA

TGTTATTTTTTTTCACAAGGAGATAAAAAAAAAAACTTTAACATGTCACTTTAAGCATGTATGTAATACGT
CTATTAATTATATGTAACTTTTATAACTATTTTCATTTCTATGTGATCCATTTGCAGTTATGCACCTTTTT
TTTTTTTC

GGAGGTATCTCTATGGATGTGGCTTGAAATATGGATGTCTTGCCTACTTCTACTTCTGGGAAAGGCATTTT
TACTCGATCGCGTTAATATATGCATCAAGAAAATAAAAAATAAAACGCGAAGAGCTAAAAAAAAAAAAGAA
AACCTACT

CCAGAAGCTTGGTTTTTAATAGTATATAATAAAGATAAAGATAGATAGAGAACATAACTCTAAACTTTAAG
AGTGAATTTTTTTTTTTTAGTTTAGTTTTTTTTTTTTTTTTTTTTTACAATAATGTATTGTTCTTGGAAAA
TAACACAA

CGGAACATTCAGTTATACGCGAAAGTAAAAGTGAGAGCTTTTCAGGGGTTAAAAGCTGGGCGTGTTCCATG
ACGTATTTACCGAGGTCGTATTATCAAAGAAAATGAAAAAAAAAAAAAAAAAAAAAAAAGCGAAGAGGAAA
AAGACAAA

TTTTTTAAAAATATGTATAGAATTTGTCATTATCTGCGTTAAAAAATAGTTATAAAGTATATACAATAACA
ATAAATGATAAAGAAATATGCAGTGAAAAGAAAAAATTATGAAGCTTTTCCTTTCAGTGTTTTCTACCCTT
CTTCTTGC

GATTTTCTTTTTTCTTTTCTTTTTTTTTTTTGACGACTCTGTAATACTCTATTTATTATTTAGTTTAGTTT
TCTATTTAATATGATGGAATTCATAAAAAAAAAAAATACACATAGCCATGCCAGGTAATTGCAGATCAACT
GATAAATT

ACGCGCTGTTGGAGGTATCTCTATGGATGTGGCTTGAAATATGGATGTCTTGCCTACTTCTACTTCTGGGA
AAGGCATTTTACTCGATCGCGTTAATATATGCATCAAGAAAATAAAAAATAAAACGCGAAGAGCTAAAAA
AAAAAAAG

ACTTCTTTTTTACTTCATTTTTTTTTTTTTTTTTGGATAGTAAATAATGCACTTTTGACTAGATCTTGAGT
ATTTAGTTGAATTGAAATTAAAGATACAGTGTATAAAAATTATACAAAAAATAAAGGAAACTTGTATATTG
AAAATAGG

CTAAAGCTTTTATATCTAAACGCCAAAAAAATAAAAAAAAAAGGAAATGGGAGAAAAGTTTTCAGCGCGCG
AACTCGCGAAAAAACTTCCTCTTTAGCAATGGTGACATATAGATCTCAAGGTTCTCAATTAAAAGACCTTC
TTTGTAGC

GTCTCAAGAGACTATCCTAAATAATATTGAATATGCACTTTTACTATATTAATATCACGTCACACGACGCA
CAGTGAGAAGTGAAAAATTTTTTTCAATCTGAAAAAAAAAAAAAAAAAAAAAAAAAAATTTATATAAACGA
ATGGTATC

AGAACGCGGGTTTTTCGCGCAATGTAACAAAGCTCCAGAAATTTTTTATAAATACTAATGTTATAGCAAAA
AAAATAAGGACCTTAAAGTCGTAAACTAAATTGAAAAGCCCAAATCATTCAAATTCTATATCGTTGAACTT
TTAATAGG

TGAGGAATATAGGTTTCTTGGAGTTTTGAAAGTTGTTTTATTTATAATGTTATTATTATTATTATTATTAT
TATTATTATTATTATTATTATTATTATTATTATTATTATTATTGTTATTTTGGTGGTTCCTATCGTTATTT
AACAGTTT

CCCCTCCACTGTAAAGTAAATGTATATTATTAAAAGGCAAAAATAAGAATATAATACTTCAGTAATGAGCC
TTTTTTATTCGGTCTTGTTACTTCTTTTTTACTTCATTTTTTTTTTTTTTTTTGGATAGTAAATAATGCAC
TTTTGACT

TGCAAAGATATGTATTCGCTCGTTCAGTGTTTTTTTAAAAATATGTATAGAATTTGTCATTATCTGCGTTA
AAAAATAGTTATAAAGTATATACAATAACAATAAATGATAAAGAAATATGCAGTGAAAAGAAAAAATTATG
AAGCTTTT

CTAAAAAGATAGAACGCGGGTTTTTCGCGCAATGTAACAAAGCTCCAGAAATTTTTTATAAATACTAATGT
TATAGCAAAAAAAATAAGGACCTTAAAGTCGTAAACTAAATTGAAAAGCCCAAATCATTCAAATTCTATAT
CGTTGAAC

TAGATTGGTTGATGAAAAATCTTATCATAGGGCACCTTCCAGGTAAAAGATCTACTTACTATTAGTCAGTA
CGTCCTTTTTTCATTTGTTTATAATTTTTTTTTTTTTTTTTTTTTTCTCCTCTCAATATTTTAGAAGTAAC
ATGACAGA

TTTACGTAATTAAAAATCTCCGGGGCTAGCTTTTGCCGGGGAACCCATCCCGAAAAAATTGCAAAAAAAAA
AATAGCCGCCGACCGTTGGTCGCTATTCACGGAATGATAGAAAAATAGCCGCGCTGCTCGTCCTGGGTGAC
CTTTTGTA

TAAAAAATCATATTTTAGACTCACTTGCTAGTAAACCGGTTGATGCTATTGTTAATATATAACGACAAATG
TTCAAGCAATTATATAACCATTTTCTTCCATATTCACCTTAAACGAGGTCGCCTTTATTTTTTTTTTTTTT
TTTTTCAG

ATCTATGAAGTATGTCTACCACATAAAAATGACCTTATAACACAACTTCCTTAGTATGGAAGTAATACATA
TATACATATATACATATATACATACATATATATATATATATATATATATACATCTTTTGAACCAATTCC
CTATAGAC

TATATCTAAACGCCAAAAAAAATAAAAAAAAAAAGGAAATGGGAGAAAAGTTTTCAGCGCGAACTCGCGAA
AAAACTTCCTCTTTAGCAATGGTGACATATAGATCTCAAGGTTCTCAATTAAAAGACCTTCTTTGTAGCTT
TTAGTGTG

TCATCGTATTCATAAAATGCTTCACGAACACCGTCATTGATCAAATAGGTTTATAATATTAATATACATTT
ATATAATCGGCGGTATTTATATCATCAAAAAAAGTAGTTTTTTATTTTATTTTTTTCATTACTTTTCACTGT
CTATGGAT

AGTATATAATAAAGATAAAGATAGATAGAGAACATAACTCTAAACTTTAAGAGTGAATTTTTTTTTTTTAG
TTTAGTTTTTTTTTTTTTTTTTTTTTACAATAATGTATTGTTCTTGGAAAATAACACAATCTGTTCAAAGT
GGACAGAA

ATTCAATTTTTCTTCATAGCAATATTTTTTTTCTGTCCAAGTTTATTTTTTTTCCATAATTTCTGATTTAC
TACCATTGAAAATTATAAAAGGAAAAATATTACGCGCTACCATTTAATAAGTAGAAACAACTAAGTTCTCG
AATGAGAT

ACATCATGTGATGACATTTAAAAAGTGGGAAAGAATACTACTAATATAAAACTATTTACTGGATATTTAGA
ATTTTTTTTTTTTCTTCTTTATACGTAAAAGTTGTTCAAAAAATTTCATCGTTTTCTTTTTTTATGAATCT
TTGATTCT

TATCGCAAACTAGAGACTGATGCCTTTACGAATGAAATTCCATAAACCTTAAGAAATAACGGAAAAAAAAA
AAAAAGCTTTTTTTTTTTTTGATAGTATAAATAGTTGATTATATGTAAGTGTATATTAATATTGTTACTAC
TGGTCAAT

CTTGAACTATTTGGCCCATACGTAGAAGATCAATCAATTGAAGTTAAAAAGGAGAAGTAATAATTATAGCA
TAATATATATTCATAATGTATAGGCATATTTATTTTTTATTTTTTTTATTTCATGTTCTATTTAATGACGA
ATCACGAA

ATGCAATTTTTATCATGTTTTTCTCTTTTTTTTTTTTTTTTTTTTTTTTCTCTCTTTGTTGTGTTTTCGAG
AAAAAGTTCAATTGTTTAAAAGGAATTAAGCAAATTATATATATGCGACTGTTGGTTTCCAGTTCTAATCT
GTACTCTT

GAAACGTTCGTGGCGGAAAAAACTACGTAAAAAGGCGGTATTTATCTATTATTTGGCCAAAAAAAAAAAAA
AATACATACTACATATACATATACGCCATAAAAAATCTCTGCATCTATCTTATTTCCCATTATTTGGACAA
ATGCTTAC

ATCTGTCTCCGGTTATATATGTCTAAATAGAATTCGGGTAGCATTTTATTTTTATCGCTTATAGGAAAAAG
ATATTTGCATATATATATCTATATATCCTATATACATCTATATAGTTATTGTGTAAGGGGTTTTTATAAGG
TCCGATAC

CATAAAAAATATCTGTCTCCGGTTATATATGTCTAAATAGAATTCGGGTAGCATTTTATTTTTATCGCTTA
TAGGAAAAAGATATTTGCATATATATATCTATATATCCTATATACATCTATATAGTTATTGTGTAAGGGGT
TTTTATAA

TTTTTTCAATCTGAAAAAAAAAAAAAAAAAAAAAAAAAAAATTTATATAAACGAATGGTATCTCCATCACATT
TCTTTTAGCCTCGCAACTTGTACTTTTCATCACTTTTCTTGTAATTTAGCAATATCCCAAGAACAATCATC
GAAATGTC

TATCATGTTTTTCTCTTTTTTTTTTTTTTTTTTTTTTTTTCTCTCTTTGTTGTGTTTTCGAGAAAAAGTTCA
ATTGTTTAAAAGGAATTAAGCAAATTATATATATGCGACTGTTGGTTTCCAGTTCTAATCTGTACTCTTTT
TTTTGAAT

AATTACTGGATAACTGTATAGAAACGTTCGTGGCGGAAAAAACTACGTAAAAAGGCGGTATTTATCTATTA
TTTGGCCAAAAAAAAAAAAAAATACATACTACATATACATATACGCCATAAAAAATCTCTGCATCTATCTT
ATTTCCCA

AGTATATATCCAAAGAATACCTTAAATAGAAAAGGAAAGATAATAAATACTAAACACTACTATATATTCAG
GTAAAATACAAAAATTATAACATTTTTAAAACTTTTTTTTTTGAAAGTCCTTCTCGCTTTAGGATTTTTT
CCCATTAA

TGATGAGCGACTAAAAAGATAGAACGCGGGTTTTTCGCGCAATGTAACAAAGCTCCAGAAATTTTTTATAA
ATACTAATGTTATAGCAAAAAAAATAAGGACCTTAAAGTCGTAAACTAAATTGAAAAGCCCAAATCATTCA
AATTCTAT

AGGATTATTAAAATAAGTGATATATACATATATATATATATATATATATATATAATACACTAATTATTTTA
TGTGATGTTGATCACGCGAAACGGTAAACGGCTCTGTTCGCGCTTTCTTTGTTTACATTTTAGTGAAGTAT
TGTCAAGA

TAAAAGAAAAGGTAATAAATTGAATGAATGTAGATATATAAATATCAATAGAAATACGTACGCGGAATTAT
TTCTCTTTGTAACAAATTGATCAAAAGCTTTGTATTTTTTTTTTTTTTAGCTCGGCTGCGCGCTATTACAG
TAATTACA

TAAGAACATTTTTGCTTCTGGACAGACAGAACACGTAAAAAAAGAAACACGAAATTACGACTAACTTTCGC
GTAGGGGTAAAATTACCTAATGCGCGTAATTAAAAAAAAAAGCACAGCAGTGTAAATTGGAGCAATAGGAG
AAAATGAC

CGTTTCATTATATTATCTTCCCATGCCGCTCCTGCGTGGGATTCCCTTTATTATAACTTTTAAGGTTTCTA
CCCACATATATGCAATTTTTATCATGTTTTTCTCTTTTTTTTTTTTTTTTTTTTTTTTCTCTCTTTGTTGT
GTTTTCGA

GAAAGTAAAAGTGAGAGCTTTTCAGGGGTTAAAAGCTGGGCGTGTTCCATGACGTATTTACCGAGGTCGTA
TTATCAAAGAAAATGAAAAAAAAAAAAAAAAAAAAAAAAAAGCGAAGAGGAAAAAGACAAACGAAAAACAAAA
CGAAATAA

TAAAAGGCAAAAATAAGAATATAATACTTCAGTAATGAGCCTTTTTTATTCGGTCTTGTTACTTCTTTTTT
ACTTCATTTTTTTTTTTTTTTTGGATAGTAAATAATGCACTTTTGACTAGATCTTGAGTATTTAGTTGAA
TTGAAATT

TGAAAAATTTTGAAAAAAAAAAAAAAAAAAAAAAAAAAAAAAATTTGTTATCACCAATAACGAGAAACCTA
ACTCTAAAACTTAGGGTCAAAGAACAGAACCCTAGGCTTTGTATCCAGTTGAATCACTAATTACGAAAGAA
TGCTGGTG

TTTTTCTCATTGTTGTTGTATCATTATTACTAACTAGTGCATCACCACTTGTCTAAATACCAAACGCAAAA
TATATATATTCTTACTTCATAACGTTCAAAATAAAGTTTTTAGTTTAAAATAAAATAAAATAAAAAAAAAA
AAAAAAAA

AAGAAACATCGCGTAATGCAACAGTGAGACACTTGCCGTCATATATAAGGTTTTGGATCAGTAACCGTTAT
TTGAGCATAACACAGGTTTTTAAATATATTATTATATATCATGGTATATGTGTAAAATTTTTTTGCTGACT
GGTTTTGT

TCATTATCATCGTTTCATCGACATTAGGTGACGTTTCTCAGTAAAAATTAACTTCATCATGATCATTAATT
TCATATTCATATTCGGTTATTTTTTTTTTTTTTATATTTTTTTTTCAATGGTGTTTTTGATGGTTTTTATT
ATATATAT

ATTTTTATTAAAGGGAAAAAAATGATTCAATATGTATATATTTATTCATGTATATGTATTTTGAATTAAAA
AAAAACGTTTTATAAATACTCAATAAAGCCGTTGTATTTTCTTTATTACTATCGAAATTCTTGCTTTAAAC
ATTTCGCG

CAATCAATTGAAGTTAAAAAGGAGAAGTAATAATTATAGCATAATATATATTCATAATGTATAGGCATATT
TATTTTTTATTTTTTTTATTTCATGTTCTATTTAATGACGAATCACGAAGAAAATATATCTAAGAAAAGAT
CTTTTGAA

CCTTGATCTTTTTCTTTTTTTTTTTTCAATTACGGATTATGCATATATAGGTGGGTATACGTACGTAAAAA
AACAAAAACACACATGTAGACATCTATATACGTTTAAAAAAGCTTTAGATTCAGATCTTTGGGTTTTACAA
AAAAAAAA

AAGTGGAAAGAAAGGAAGGCTAAAGCAAAAGTGTTTCTTATATATAATTTTATGTACCAGAGGAAGCAAAG
TACGAAAACTATCAGTTGAAATCCAAAAAAAAAAAGTTACGCGTTTCGCGTTTTGGTGCGTCGGATGGAGA
AGAAAAAT

AAGTAATAAAAAAATTGCATTTTATAAGAAACATCATGTGATGACATTTAAAAAGTGGGAAAGAATACTAC
TAATATAAAACTATTTACTGGATATTTAGAATTTTTTTTTTTTCTTCTTTATACGTAAAAGTTGTTCAAAA
AATTTCAT

ATAATATCATTAAATATGTTTTACCAAAAAAAAGTTCATCTTTCTTATTTTTAAAATCGATAGTTCTTCAG
GATCTTGGTCACATATATATATATATATATATATACACTTATATAAAATAAGACGTTTATTTAATCTTGTT
TTCTTACG

AAAAAGCCGCAAGACAGGTTAAAAAGGAATAGAATTATCGTTCTCGAGATAGTTTTTATACAATACATATA
TATATATATATATATATATTTACAAGAAAAGCATGGCTTATGTATTATACTTGCTTATGTACCAATATATA
CATATATG

TTAGATAGATGTCTCAAGAGACTATCCTAAATAATATTGAATATGCACTTTTACTATATTAATATCACGTC
ACACGACGCACAGTGAGAAGTGAAAAATTTTTTTTCAATCTGAAAAAAAAAAAAAAAAAAAAAAAAAATTT
ATATAAAC

TTGGCCCATACGTAGAAGATCAATCAATTGAAGTTAAAAAGGAGAAGTAATAATTATAGCATAATATATAT
TCATAATGTATAGGCATATTTATTTTTTATTTTTTTTATTTCATGTTCTATTTAATGACGAATCACGAAGA
AAATATAT

ATAGATAGAGAACATAACTCTAAACTTTAAGAGTGAATTTTTTTTTTTTAGTTTAGTTTTTTTTTTTTTTT
TTTTTTACAATAATGTATTGTTCTTGGAAAATAACACAATCTGTTCAAAGTGGACAGAAACCCTAAAACAA
AAATAATA

CGTTTCATCGACATTAGGTGACGTTTCTCAGTAAAAATTAACTTCATCATGATCATTAATTTCATATTCAT
ATTCGGTTATTTTTTTTTTTTTTTATATTTTTTTTCAATGGTGTTTTTGATGGTTTTATTATATATATAT
ATTTTTTT

ATTAATGCATACATATCATAGTATAAAAAGAAATAGACCAATATCAACATCAATTTTATGTATTTTTTTTT
TCTTTTGGTATAGCATTGATATATTGAAATTTGTATATATTGCTGCGAACACTATTTAAAACAGGTTTTTT
TTTTATTT

TTTAGATAATTTTTGGAAACATTTTTTTTCTTGATGTATATTTTTTGTATTGTAGAAATCGCGCGTACTGT
ACTTGTATATCGCTTTATAAGCGCTTTTAATTGATTGTTCATGACGAGGATAGGCGGATAGGCGGAGGTAT
GCCTCTTA

TTTTTCGTGCATATATATATATATACATATATAATATAAAACAAAATATCATGAATTTCTATCTAAAGTAGTG
GGAAAAAATATGTCGTTAAAAATGGGTATAATATCCAAGCAAAGCTTTTGGCTTTTTTTTTCTTGAAATGA
GTGAAGGG

AAAGATAAAGATAGATAGAGAACATAACTCTAAACTTTAAGAGTGAATTTTTTTTTTTTTAGTTTAGTTTTT
TTTTTTTTTTTTTTTTACAATAATGTATTGTTCTTGGAAAATAACACAATCTGTTCAAAGTGGACAGAAAC
CCTAAAAC

TCGTCGATCTAAAAGCTCATCGCATTTTTTCGGTCCTGATGTTAAAAATTTATTTCGTTTATTAAAAAAAA
AAAAAACATTACTGAAACGTATAAAAGAAAAGTCTTAATCTATAAATATGGTTTTATCATAATTAGACTGT
TTTACTCT

TGTTGTATTCACAGCTACTTCTCCTAATGCCTTCGATGCATTTAGATAATTTTTGGAAACATTTTTTTTCT
TGATGTATATTTTTTGTATTGTAGAAATCGCGCGTACTGTACTTGTATATCGCTTTATAAGCGCTTTTAAT
TGATTGTT

CTGAAAAAAAAAAAAAAAAAAAAAAAAAAAATTTATATAAACGAATGGTATCTCCATCACATTTCTTTTAGCC
TCGCAACTTGTACTTTTCATCACTTTTCTTGTAATTTAGCAATATCCCAAGAACAATCATCGAAATGTCCC
GTCCACAA

AATAAAAATGAATATAAGGTACGTCTCCAAAAGAAATGTAAATATAGAAATTTTAAAAAAAAAAACGAAAA
AAAACATAACTAAATTTAAAGTGCAGCCCAACAATAACCCTGAAAAATCTAAATATCTTAGAATTTTTTTA
TTTTGATT

AGTACAATGTCCAGAAGCTTGGTTTTTAATAGTATATAATAAAGATAAAGATAGATAGAGAACATAACTCT
AAACTTTAAGAGTGAATTTTTTTTTTTTTAGTTTAGTTTTTTTTTTTTTTTTTTTTTACAATAATGTATTGT
TCTTGGAA

GGTGTTGTATATTTACTTGTGTTAGTTTATAAATAAATTTTATATCACTATATGTGTGGTGAAAAGGAAGA
GCAATCCTGCTAAAGCTTTTATATCTAAACGCCAAAAAAATAAAAAAAAAAGGAAATGGGAGAAAAGTTTT
CAGCGCGC

CGTTCAGTGTTTTTTTTAAAAATATGTATAGAATTTGTCATTATCTGCGTTAAAAAATAGTTATAAAGTATA
TACAATAACAATAAATGATAAAGAAATATGCAGTGAAAAGAAAAAATTATGAAGCTTTTCCTTTCAGTGTT
TTCTACCC

CGCGGAATTATTTCTCTTTGTAACAAATTGATCAAAAGCTTTGTATTTTTTTTTTTTTTAGCTCGGCTGCG
CGCTATTACAGTAATTACATGTAAATAAACTATTCCAAAGAATACAAAAGAAGAGCTTACAGTAGCCAGTC
AAAGTCGC

ATGGCTTCGCTAAGAACATTTTTGCTTCTGGACAGACAGAACACGTAAAAAAAGAAACACGAAATTACGAC
TAACTTTCGCGTAGGGGTAAAATTACCTAATGCGCGTAATTAAAAAAAAAAGCACAGCAGTGTAAATTGGA
GCAATAGG

CCTTTGAATCCTTTTTGATGAAAAAAATAGAAAAAAAAAAAAAAAAAAAAATGAAAGCAATATCGACTGTAT
GTATATGCGCACCATAATTTTCCTATTAATAGTTTAAGCATAATTAGTGTACTTACTAAAAATAATGTAAC
TTTGCTCT

CGGGGCTAGCTTTTGCCGGGGAACCCATCCCGAAAAAATTGCAAAAAAAAAAAATAGCCGCCGACCGTTGGT
CGCTATTCACGGAATGATAGAAAAATAGCCGCGCTGCTCGTCCTGGGTGACCTTTTGTATATTGTATAAAG
ATAAACAT

AAAAAAATAGAAAAAAAAAAAAAAAAAAAAATGAAAGCAATATCGACTGTATGTATATGCGCACCATAATTT
TCCTATTAATAGTTTAAGCATAATTAGTGTACTTACTAAAAATAATGTAACTTTGCTCTTTGCACCAATAG
ATGTTACT

GGAAATGGATTTATTACTGAAATATGTAGAAATATTTAACTCGATAGATTTGATCATACACATATAATGCA
AAATATATATATATATATATATATATATATATATATATATATGTGTGTGTGTGTGTGTGTGTGTGTGTAAA
CGTTCTTT

ACATATCATAGTATAAAAAGAAATAGACCAATATCAACATCAATTTTATGTATTTTTTTTTTCTTTTGGTA
TAGCATTGATATATTGAAATTTGTATATATTGCTGCGAACACTATTTAAAACAGGTTTTTTTTTTATTTTG
GCAGTTTG

AGATATTGTGTAATCGCCTCAAGTAAACAATATGTTTTTCATACGTGATTTGAAGTTTTTAAGTATCTGAA
ATACATACGCGCGCGTATGCATATGTATTAGTTAAATTACTCGAATGTCCTTTATATAATATTACATTTTT
ACACACAC

GGGAAATTTAAAAATGTAACGTTTAGCATTTGATAGACATAGAATAAAAATAAGTAACTATAAAAAAAGGA
AAAAAAAAATTGGGCAAAATAGGGATCATATTATAACTATTTATATACAGATTCGAAATATGAAGAAGAGA
CTATGAAA

TGTATATTATTAAAAGGCAAAAATAAGAATATAATACTTCAGTAATGAGCCTTTTTTATTCGGTCTTGTTA
CTTCTTTTTTACTTCATTTTTTTTTTTTTTTTTGGATAGTAAATAATGCACTTTTGACTAGATCTTGAGTA
TTTAGTTG

AAAAGCTGGGCGTGTTCCATGACGTATTTACCGAGGTCGTATTATCAAAGAAAATGAAAAAAAAAAAAAAA
AAAAAAAAAGCGAAGAGGAAAAAGACAAACGAAAAACAAAACGAAATAACGCATTGTATGCTGGGGCCTCA
ATTGAACT

AATACATACGCGCGCGTATGCATATGTATTAGTTAAATTACTCGAATGTCCTTTATATAATATTACATTTT
TACACACACATTCTTGAAGAAGAAATCCCGATCGTAAAAAAAAACACCGTGCTCCAAATGGCCTTCAATCT
TTTCGAAT

AGGAACCGCAGACATGATGTCCTCTTCTTTCAAATTACTGGATATATATATTATTATTATTGTTACCATTA
ACGTTATTATTATTAACTATTACTATTACATTAACTTAAAAATAAATATGAAACAACGAAGAACAAAAAGA
ATTATGAA

AGTAATGAGCCTTTTTTATTCGGTCTTGTTACTTCTTTTTTACTTCATTTTTTTTTTTTTTTTTGGATAGT
AAATAATGCACTTTTGACTAGATCTTGAGTATTTAGTTGAATTGAAATTAAAGATACAGTGTATAAAAATT
ATACAAAA

ACTGAATCATTTGTTGCCACTTTTTAATGTGAACTAAAAAAATAAAAATGAATATAAGGTACGTCTCCAAA
AGAAATGTAAATATAGAAATTTTAAAAAAAAAAACGAAAAAAAACATAACTAAATTTAAAGTGCAGCCCAA
CAATAACC

AAAAAAAAAGGTTAAGGATAAAGATAAGTAAACTAATGGAATATATAAACAAATAAGTAAGTAGATACTGT
AAATAGAACACACGTTTTTTAATTTATTTTTTTTTTACATATCTTAATTTAATATAAATCAAAAAGCCCA
AGCCTCAA

ATAAAAAAAAAAAAAAAAAAAAAAAAGAACCTTTTGTTTTCGCCCAGTATATGGGTAAATTAACGGTTCCT
TTTATTAGTCATTTTCAACTCTATACCATGCTCCAAAACGGCGTTCGGCACAGGTTTTTTATTTTACCACG
ATAACTCA

ACTACAAAAGGCAAAAAAAATAAAAGAAAAGGTAATAAATTGAATGAATGTAGATATATAAATATCAATAG
AAATACGTACGCGGAATTATTTCTCTTTGTAACAAATTGATCAAAAGCTTTGTATTTTTTTTTTTTTTTAGC
TCGGCTGC

TTTTTAAGTTCATTCTCTTTCATTTTCGGTAGTGAGATGGCAGTTCGAGGGGTTTTTTATTCGAGATAGTA
ACTTCTGGCTTTTCGCTTTTATACAGCACAGCAGAAAAAAAAAGCCGCCGAGGCGCGCGCGTTCATGCAAT
GGCTCAGT

TCTAATTTACCCGATGCTGGTTTTGTATCGAAAAAGATTTTCAGGTTTTAGGTTTTATTTTTTGTCTATTT
TTAAATATATAATATAATAAAAAATAAATTGTGTCTAATATCCATAATTTGTACATTTAGATTATTTCTTT
TGGTTCTT

CAAACAAACAAAAAAAAAAAAAAAAAAATGGTCGCAATCTCAATGATTTGGTTTTTTACCAAGCGTATGCC
CAGAATATTTGCATTAGCTTTTAATTTAATTTCAATATTTCTTTTGATTTTTCTTCTCATCGGCTGTTACA
ACCCGTCA

GAAGAAATAAATTCTTAGGGTCGACTCCACCATTTCAACTTTAGTATAATATGACAATAAAACATTATTGG
GACTGAGAACGATTATATTAAAATTAGTAAAATACATACATAACTACTTATAAAAAAAAAAAGAGAAATTT
GCCATTTT

CTTTTTTATTCGGTCTTGTTACTTCTTTTTTACTTCATTTTTTTTTTTTTTTTTGGATAGTAAATAATGCA
CTTTTGACTAGATCTTGAGTATTTAGTTGAATTGAAATTAAAGATACAGTGTATAAAAATTATACAAAAAA
TAAAGGAA

GTCAAAAACAAGATATTGTGTAATCGCCTCAAGTAAACAATATGTTTTTCATACGTGATTTGAAGTTTTTA
AGTATCTGAAATACATACGCGCGCGTATGCATATGTATTAGTTAAATTACTCGAATGTCCTTTATATAATA
TTACATTT

GGTTTTTTATTCGAGATAGTAACTTCTGGCTTTTCGCTTTTATACAGCACAGCAGAAAAAAAAAGCCGCCG
AGGCGCGCGCGTTCATGCAATGGCTCAGTAACCTCGTGATAGAAAAAGGGCAACAATATTGGGCTATTTTA
GGCAAAGA

AAAAAAAAAAAAAAAAAATGGTCGCAATCTCAATGATTTGGTTTTTTACCAAGCGTATGCCCAGAATATTT
GCATTAGCTTTTAATTTAATTTCAATATTTCTTTTGATTTTTCTTCTCATCGGCTGTTACAACCCGTCAAA
TCAGTCAA

AAGACAGGTTAAAAAGGAATAGAATTATCGTTCTCGAGATAGTTTTTATACAATACATATATATATATATA
TATATATATTTACAAGAAAAGCATGGCTTATGTATTATACTTGCTTATGTACCAATATATACATATATGGC
CTTATTAG

GTAAAGTAAATGTATATTATTAAAAGGCAAAAATAAGAATATAATACTTCAGTAATGAGCCTTTTTTATTC
GGTCTTGTTACTTCTTTTTTACTTCATTTTTTTTTTTTTTTTTGGATAGTAAATAATGCACTTTTGACTAG
ATCTTGAG

GATGAAAAATCTTATCATAGGGCACCTTCCAGGTAAAAGATCTACTTACTATTAGTCAGTACGTCCTTTTT
TCATTTGTTTATAATTTTTTTTTTTTTTTTTTTTTTCTCCTCTCAATATTTTAGAAGTAACATGACAGAGC
AACACGTT

AGGTTTCTTGGAGTTTTGAAAGTTGTTTTATTTATAATGTTATTATTATTATTATTATTATTATTATTATT
ATTATTATTATTATTATTATTATTATTATTATTGTTATTTTGGTGGTTCCTATCGTTATTTAACAGTTTCG
ATGGAGGA

CCATGCCGCTCCTGCGTGGGATTCCCTTTATTATAACTTTTAAGGTTTCTACCCACATATATGCAATTTTT
ATCATGTTTTTCTCTTTTTTTTTTTTTTTTTTTTTTTTCTCTCTTTGTTGTGTTTTCGAGAAAAAGTTCAA
TTGTTTAA

ACTTATATGGAGCTAAGGGGAGCAGTTACGCAACTCCGTGATCGCGCGCCACGGGCCGTCGGCGGCTGTTA
ATTGAAGAAAAAAAAAAATGAAGAACCACAAGGGGTGATCCATATAGGTGACTAGCATCATCCCCTGCGACG
CGCGGCCC

AAGAAATGTAAATATAGAAATTTTAAAAAAAAAAACGAAAAAAAACATAACTAAATTTAAAGTGCAGCCCA
ACAATAACCCTGAAAAATCTAAATATCTTAGAATTTTTTTATTTTGATTATTATATATTATTATTATTCTT
ATGGTAAA

TTCAGGGGTTAAAAGCTGGGCGTGTTCCATGACGTATTTACCGAGGTCGTATTATCAAAGAAAATGAAAAA
AAAAAAAAAAAAAAAAAAAGCGAAGAGGAAAAAGACAAACGAAAAACAAAACGAAATAACGCATTGTATGC
TGGGGCCT

ACATCTGTTTTTCCTTACTCTGTTTTTATTTTGTTGGTAACCTTTGAATCCTTTTTGATGAAAAAAATAGA
AAAAAAAAAAAAAAAAAAATGAAAGCAATATCGACTGTATGTATATGCGCACCATAATTTTCCTATTAATA
GTTTAAGC

CAACAAAAAGGGAGTAAAACATGCAGTGGCTCTATATATATAATTATCGTAGAATAATTCAAAAAAAAATA
AAAAACGAAAAAATTCATTCTTCATCACTCATCAAAGGCACTATTTCGTCATAACGCGGAGGCTCGTTACC
ATGAGAGA

TAATCGCCTCAAGTAAACAATATGTTTTTCATACGTGATTTGAAGTTTTTAAGTATCTGAAATACATACGC
GCGCGTATGCATATGTATTAGTTAAATTACTCGAATGTCCTTTATATAATATTACATTTTTACACACACAT
TCTTGAAG

TCTTCATAGCAATATTTTTTTTCTGTCCAAGTTTTATTTTTTTTCCATAATTTCTGATTTACTACCATTGAA
AATTATAAAAGGAAAAATATTACGCGCTACCATTTAATAAGTAGAAACAACTAAGTTCTCGAATGAGATGT
TTGCACTG

GAAATACGTACGCGGAATTATTTCTCTTTGTAACAAATTGATCAAAAGCTTTGTATTTTTTTTTTTTTTAG
CTCGGCTGCGCGCTATTACAGTAATTACATGTAAATAAACTATTCCAAAGAATACAAAAGAAGAGCTTACA
GTAGCCAG

ACTGTCACCTTGATGAGCGACTAAAAAGATAGAACGCGGGTTTTTCGCGCAATGTAACAAAGCTCCAGAAA
TTTTTTATAAATACTAATGTTATAGCAAAAAAAAATAAGGACCTTAAAGTCGTAAACTAAATTGAAAAGCCC
AAATCATT

AATATAAGGTACGTCTCCAAAAGAAATGTAAATATAGAAATTTTAAAAAAAAAACGAAAAAAAAACATAAC
TAAATTTAAAGTGCAGCCCAACAATAACCCTGAAAAATCTAAATATCTTAGAATTTTTTTATTTTGATTAT
TATATATT

TTGTTGGTAACCTTTGAATCCTTTTTGATGAAAAAAATAGAAAAAAAAAAAAAAAAAAAATGAAAGCAATA
TCGACTGTATGTATATGCGCACCATAATTTTCCTATTAATAGTTTAAGCATAATTAGTGTACTTACTAAAA
ATAATGTA

AAAATTGCATTTTATAAGAAACATCATGTGATGACATTTAAAAAGTGGGAAAGAATACTACTAATATAAAA
CTATTTACTGGATATTTAGAATTTTTTTTTTTTCTTCTTTATACGTAAAAGTTGTTCAAAAAATTTCATCG
TTTTCTTT

TTAGTATAATATGACAATAAAACATTATTGGGACTGAGAACGATTATATTAAAATTAGTAAAATACATACA
TAACTACTTATAAAAAAAAAAAGAGAAATTTGCCATTTTCACGAGTATAAGCACAGATTGTACGAAACTAT
TTCATATA

TCATCAGTTTTAGTAAAAAACGAACAAAAACACAATAAAATATAAATCAATATATTTAGGTTTACTGGGTT
CTTTAACAGTTGTATAATAGTTATTTTTTATTACAAAAATATAGGTTTTAATAAAAAAAAATAGGGTTCTA
TTTGTTTT

GTAAAAAAGATTTTCTATTTAAATTTATTAATTAATTTTTTTTTCTTAAATAATTATTTTATGTTTTTGTTT
TCCGAAAAAGAAAAATATTTCAACAAGAAGTATCAATATATTTCTTGCAAAAAATTGTTCACCGAGAATTG
ACAAAAAA

CTTATCATAGGGCACCTTCCAGGTAAAAGATCTACTTACTATTAGTCAGTACGTCCTTTTTTCATTTGTTT
ATAATTTTTTTTTTTTTTTTTTTTTTCTCCTCTCAATATTTTAGAAGTAACATGACAGAGCAACACGTTTA
TTTTCCTA

TGTATTCGCTCGTTCAGTGTTTTTTTAAAAATATGTATAGAATTTGTCATTATCTGCGTTAAAAAATAGTT
ATAAAGTATATACAATAACAATAAATGATAAAGAAATATGCAGTGAAAAGAAAAAATTATGAAGCTTTTCC
TTTCAGTG

AGTGCAGCCCAACAATAACCCTGAAAAATCTAAATATCTTAGAATTTTTTTATTTTGATTATTATATATTA
TTATTATTCTTATGGTAAATAATGCCCCTACTTTTCTTCTAAGGAAGTGAGTTACCACCAAAATAAGGAAA
TATCGCCT

CTCAAAAAAAAAAAAAAAAAATGACACATAGCTGAAGTGATAAACTTACTACACTGGTATAACGCTTATTA
GAGGAAATCTAATAATAAACTGTTAGCTATAAATAGTATACAATAACAATGAGCGCTAAAAGTATGACATA
TTAAACAT

TGGCGGAAAAAACTACGTAAAAAGGCGGTATTTATCTATTATTTGGCCAAAAAAAAAAAAAAATACATACT
ACATATACATATACGCCATAAAAAATCTCTGCATCTATCTTATTTCCCATTATTTGGACAAATGCTTACGT
GCTAATGT

TTCTCTTTTTTTTTTTTTTTTTTTTTTTTCTCTCTTTGTTGTGTTTTCGAGAAAAAGTTCAATTGTTTAAA
AGGAATTAAGCAAATTATATATATGCGACTGTTGGTTTCCAGTTCTAATCTGTACTCTTTTTTTTGAATTT
GCACTGTT

TACCGTAGAGATTCTTGCAACCTCGCTTAAACTCTCGCTTTTATATAATATTTCTCCTTATTGCGCGCTTC
GTTGAAAATTTCGCTAAACACGGGGTTTAAGTTTAAGTTTACAGGATTTATCCGGAAGTTTTCGCGGACCC
CACACAAT

ATTTTTTGCTCGTTTCATTATATTATCTTCCCATGCCGCTCCTGCGTGGGATTCCCTTTATTATAACTTTT
AAGGTTTCTACCCACATATATGCAATTTTTATCATGTTTTTCTCTTTTTTTTTTTTTTTTTTTTTTTTCTC
TCTTTGTT

ATAAAAAAAAAAAAGGAAATGGGAGAAAAGTTTTCAGCGCGAACTCGCGAAAAAACTTCCTCTTTAGCAAT
GGTGACATATAGATCTCAAGGTTCTCAATTAAAAGACCTTCTTTGTAGCTTTTAGTGTGATTTTTCTGGTT
TGAGAATA

CGCATTTTTTTCGGTCCTGATGTTAAAAATTTATTTCGTTTATTAAAAAAAAAAAAACATTACTGAAACGT
ATAAAAGAAAAGTCTTAATCTATAAATATGGTTTTATCATAATTAGACTGTTTTACTCTGTCCATTGGTCA
TTATAAAG

GAGTGAATTTTTTTTTTTTTAGTTTAGTTTTTTTTTTTTTTTTTTTTACAATAATGTATTGTTCTTGGAAA
ATAACACAATCTGTTCAAAGTGGACAGAAACCCTAAAACAAAAATAATAACGGGGAAGGAAAAAAATAGCA
AGTGAAAC

TTTTGTATCGAAAAAGATTTTCAGGTTTTAGGTTTTATTTTTTGTCTATTTTTAAATATATAATATAATAA
AAAATAAATTGTGTCTAATATCCATAATTTGTACATTTAGATTATTTCTTTTGGTTCTTTATTCTGACCTA
TATAGACT

AAATAAACGTTTCACCCTGGCAGATAGGAAACCCTATCTCCCAGTGATAAACTTTAATTTTTTTTCGTGCA
TATATATATATACATATATAATATAAAACAAAATATCATGAATTTCTATCTAAAGTAGTGGGAAAAAATAT
GTCGTTAA

TGAATCTTGGTGTTGTATTCACAGCTACTTCTCCTAATGCCTTCGATGCATTTAGATAATTTTTGGAAACA
TTTTTTTTCTTGATGTATATTTTTTGTATTGTAGAAATCGCGCGTACTGTACTTGTATATCGCTTTATAAG
CGCTTTTA

GTAACCGTTATTTGAGCATAACACAGGTTTTTAAATATATTATTATATATCATGGTATATGTGTAAAATTT
TTTTGCTGACTGGTTTTGTTTATTTATTTAGCTTTTTAAAAATTTTACTTTCTTCTTGTTAATTTTTTCTG
ATTGCTCT

TAAACTTTAAGAGTGAATTTTTTTTTTTTTAGTTTAGTTTTTTTTTTTTTTTTTTTTACAATAATGTATTG
TTCTTGGAAAATAACACAATCTGTTCAAAGTGGACAGAAACCCTAAAACAAAAATAATAACGGGGAAGGAA
AAAAATAG

CATTCTCTTTCATTTTCGGTAGTGAGATGGCAGTTCGAGGGGTTTTTTATTCGAGATAGTAACTTCTGGCT
TTTCGCTTTTATACAGCACAGCAGAAAAAAAAAAGCCGCCGAGGCGCGCGCGTTCATGCAATGGCTCAGTAA
CCTCGTGA

CAGTTCGAGGGGTTTTTTATTCGAGATAGTAACTTCTGGCTTTTCGCTTTTATACAGCACAGCAGAAAAAA
AAAGCCGCCGAGGCGCGCGCGTTCATGCAATGGCTCAGTAACCTCGTGATAGAAAAAGGGCAACAATATTG
GGCTATTT

TGTTTTATTTACTGTCACCTTGATGAGCGACTAAAAAGATAGAACGCGGGTTTTTCGCGCAATGTAACAAA
GCTCCAGAAATTTTTTATAAATACTAATGTTATAGCAAAAAAAATAAGGACCTTAAAGTCGTAAACTAAAT
TGAAAAGC

TATCGAGTAAGGTAGAAACATCTCTGTGATTAAACATCGTACATATAAATACCATATATAAAAGTATGTGG
GAAAAGAACGTTCAAAAAAAAAAAGTAAGTAGTAGGTTAAAAATAAAATCTCAAAAGAGGGAAAAAAAAAA
AAGAGGGA

TAAAAAAAGGAAAAAAAAAAATTGGGCAAAATAGGGATCATATTATAACTATTTATATACAGATTCGAAATA
TGAAGAAGAGACTATGAAAGGTGGGTGAATATCAATATTAAATAAAAATCAAACAATGATTGAAAAAACGG
TAAAAGAT

CTGAATCTTTTTTTCCGGCTGTCCTCTTACAGTTGAGTTACAATATTTTGCCAGAAATATTTACATTTTTT
TACCCAATCTCTATAATAATTATTTTAATTCCTAATCCGCTTCAGGCCCGCGGTCGTTGAAGTGCTATAGT
TTATATTC

CATTTTCGGTAGTGAGATGGCAGTTCGAGGGGTTTTTTATTCGAGATAGTAACTTCTGGCTTTTCGCTTTT
ATACAGCACAGCAGAAAAAAAAAAGCCGCCGAGGCGCGCGCGTTCATGCAATGGCTCAGTAACCTCGTGATA
GAAAAAGG

AAGTATCTGAAATACATACGCGCGCGTATGCATATGTATTAGTTAAATTACTCGAATGTCCTTTATATAAT
ATTACATTTTTACACACACATTCTTGAAGAAGAAATCCCGATCGTAAAAAAAAACACCGTGCTCCAAATGG
CCTTCAAT

TCCATAAATCTATCAGAATTAGAGCCTTAAAAAAAGCCGCAAGACAGGTTAAAAAGGAATAGAATTATCGT
TCTCGAGATAGTTTTTATACAATACATATATATATATATATATATATATTTACAAGAAAAGCATGGCTTAT
GTATTATA

CTTTTTGATGAAAAAAATAGAAAAAAAAAAAAAAAAAAAATGAAAGCAATATCGACTGTATGTATATGCGC
ACCATAATTTTCCTATTAATAGTTTAAGCATAATTAGTGTACTTACTAAAAATAATGTAACTTTGCTCTTT
GCACCAAT

ATAGTTATCATGTTTCTTATCCCCTCCACTGTAAAGTAAATGTATATTATTAAAAGGCAAAAATAAGAATA
TAATACTTCAGTAATGAGCCTTTTTATTCGGTCTTGTTACTTCTTTTTACTTCATTTTTTTTTTTTTTTTT
TTGGATAG

ATATCAACATCAATTTTATGTATTTTTTTTTTCTTTTGGTATAGCATTGATATATTGAAATTTGTATATAT
TGCTGCGAACACTATTTAAAACAGGTTTTTTTTTTATTTTGGCAGTTTGAAACCCTTTCCTCTGATGACTT
TAGTGTAG

ATTTATTTAATTATTATTAAGTAAAAAAGATTTTCTATTTAAATTTATTAATTAATTTTTTTTCTTAAATA
ATTATTTTATGTTTTTGTTTTCCGAAAAAGAAAAATATTTCAACAAGAAGTATCAATATATTTCTTGCAAA
AAATTGTT

ACAGCTACTTCTCCTAATGCCTTCGATGCATTTAGATAATTTTTGGAAACATTTTTTTTCTTGATGTATAT
TTTTTGTATTGTAGAAATCGCGCGTACTGTACTTGTATATCGCTTTATAAGCGCTTTTAATTGATTGTTCA
TGACGAGG

TTCACGAACACCGTCATTGATCAAATAGGTTTATAATATTAATATACATTTATATAATCGGCGGTATTTAT
ATCATCAAAAAAAGTAGTTTTTTATTTTATTTTTTCATTACTTTTCACTGTCTATGGATTTTCATTCGTAA
AGGCATCA

TGAAAGGTGCAAGTGGAAAGAAAGGAAGGCTAAAGCAAAAGTGTTTCTTATATATAATTTTATGTACCAGA
GGAAGCAAAGTACGAAAACTATCAGTTGAAATCCAAAAAAAAAAAGTTACGCGTTTCGCGTTTTGGTGCGT
CGGATGGA

GTGAAAAAGGACGGCGAAAGTGAAAGGTGCAAGTGGAAAGAAAGGAAGGCTAAAGCAAAAGTGTTTCTTAT
ATATAATTTTATGTACCAGAGGAAGCAAAGTACGAAAACTATCAGTTGAAATCCAAAAAAAAAAAGTTACG
CGTTTCGC

ACCTTAATTAAAAGAACCACTCAGACTTTTTACCATCCTTTAGCTTTACCTAATATAATGAAATTTGTCTA
AGTAACATAAAAAAAAGGAGTAAAGAAAAAAAAAAAATAAGTAAATGAAATAAAATATAAAAAAATTCTTA
CTACGGTT

GTATCTACACTATCGCAAACTAGAGACTGATGCCTTTACGAATGAAATTCCATAAACCTTAAGAAATAACG
GAAAAAAAAAAAAAAGCTTTTTTTTTTTTTGATAGTATAAATAGTTGATTATATGTAAGTGTATATTAATA
TTGTTACT

TCATATAGATACATCTGTTTTTCCTTACTCTGTTTTTATTTTGTTGGTAACCTTTGAATCCTTTTTGATGA
AAAAAATAGAAAAAAAAAAAAAAAAAAAATGAAAGCAATATCGACTGTATGTATATGCGCACCATAATTTT
CCTATTAA

CGGCTGAAATTATGAAAGAAGAAGAACATCACTTTACACGGATCGCACGCCCATAATTCTTTTTTTTTTTT
TTTTCATATCTTCGACGTTTGCCACTGCCTTCTCTTTTTCTTTCTTTTTTGGCGGCCGGTGGCCAAACGCG
CCAAAACC

AAAAAAGGAAAATCAAGCGCGGGTAATAACGGCACCATTAAATACTAGTAGTACTATATAGATGGTATAAA
GAAATGATTTAAAACAATACGATTATATGATATTTATACATCTGTTTCAGTTGAGCTTTTTTTCCATAGAG
CTGGTGGT

AGGTCATGAACTGAATCTTTTTTTCCGGCTGTCCTCTTACAGTTGAGTTACAATATTTTGCCAGAAATATT
TACATTTTTTTACCCAATCTCTATAATAATTATTTTAATTCCTAATCCGCTTCAGGCCCGCGGTCGTTGAA
GTGCTATA

TATATTTAGGTTTACTGGGTTCTTTAACAGTTGTATAATAGTTATTTTTTATTACAAAAATATAGGTTTTA
ATAAAAAAAAATAGGGTTCTATTTGTTTTACATTTATTGATTTGTTTTTCCTGGCGATACCCTCGAAAAAA
GCCAGGCC

TCCATCCTTTTGATCAAAAAAAAAAAAGGAAAATCAAGCGCGGGTAATAACGGCACCATTAAATACTAGTAG
TACTATATAGATGGTATAAAGAAATGATTTAAAACAATACGATTATATGATATTTATACATCTGTTTCAGT
TGAGCTTT

TTGAAAAAACGGTAAAAGATCATAAAAAGCCCTCCAGGTAATATGTCAATAAGTAAATATACTTTATCGAA
GGAAAGATCAAAAAAAAAAAAAAAAAAAAATGCAAACGAACATAGCCAAAGGACCACAATATTAATCTGAC
AATAAATT

TAACATGCATTCCTATAAAAATTAAAGAGAAAAAAATTCGATTATAAAATAGTACAATGTCCAGAAGCTTG
GTTTTTAATAGTATATAATAAAGATAAAGATAGATAGAGAACATAACTCTAAACTTTAAGAGTGAATTTTT
TTTTTTTA

CTTTTTTTTTTTTTTTTTTTTTTTTTTTGTGCTGCAAAGTTTCTTAAAGCCTTCGGGCTTACGAAATCCTT
TATCGCCGAAAGGGGACCGCTTCGAAAAGTGGATATAAAACAAGGTATTTATTTTTATAGACAATGACCAA
ATGACAGG

TACCATCCTTTAGCTTTACCTAATATAATGAAATTTGTCTAAGTAACATAAAAAAAAGGAGTAAAGAAAAA
AAAAAAATAAGTAAATGAAATAAAATATAAAAAAATTCTTACTACGGTTTAAAACAAAGTGCTCTTCCGCT
AACGTTTC

ACTATCCTAAATAATATTGAATATGCACTTTTACTATATTAATATCACGTCACACGACGCACAGTGAGAAG
TGAAAAATTTTTTTCAATCTGAAAAAAAAAAAAAAAAAAAAAAAAAATTTATATAAACGAATGGTATCTC
CATCACAT

TATAAATCAATATATTTAGGTTTACTGGGTTCTTTAACAGTTGTATAATAGTTATTTTTTATTACAAAAAT
ATAGGTTTTAATAAAAAAAAAATAGGGTTCTATTTGTTTTACATTTATTGATTTGTTTTTCCTGGCGATACC
CTCGAAAA

TCATTACTAGTTTAAACTGTATTTTTATTAAAGGGAAAAAAATGATTCAATATGTATATATTTATTCATGT
ATATGTATTTTGAATTAAAAAAAAACGTTTTATAAATACTCAATAAAGCCGTTGTATTTTCTTTATTACTA
TCGAAATT

ACGTCTCCAAAAGAAATGTAAATATAGAAATTTTAAAAAAAAAAAACGAAAAAAAACATAACTAAATTTAAA
GTGCAGCCCAACAATAACCCTGAAAAATCTAAATATCTTAGAATTTTTTTATTTTGATTATTATATATTAT
TATTATTC

CACACACACAAAAAAAAAAAGGTTAAGGATAAAGATAAGTAAACTAATGGAATATATAAACAAATAAGTAAG
TAGATACTGTAAATAGAACACACGTTTTTTAATTTATTTTTTTTTTTACATATCTTAATTTAATATAAATC
AAAAAGCC

CACAACTTCCTTAGTATGGAAGTAATACATATATACATATATACATATATACATACATATATATATATATA
TATATATATATACATCTTTTGAACCAATTCCCTATAGACTTGGTTGTAATTCGTTAGAAAGATTTTCATTA
ATTTTATT

TTTAAACTGTATTTTTATTAAAGGGAAAAAAATGATTCAATATGTATATATTTATTCATGTATATGTATTT
TGAATTAAAAAAAAACGTTTTATAAATACTCAATAAAGCCGTTGTATTTTCTTTATTACTATCGAAATTCT
TGCTTTAA

GTCGTATATTAGGCTCTTTTTCTTTTTCTTTTACCAGACTTGGTTTTTTTCTCTCTTTAGTAATTGTTTCT
ATATATGGCTTATCAATTCTATATATTTTTTAACTTGAATATATGAATGACATCTTTAATAATCACATCTC
GGCAAACG

TAAATCCTATTCATATAGATACATCTGTTTTTCCTTACTCTGTTTTTATTTTGTTGGTAACCTTTGAATCC
TTTTTGATGAAAAAAATAGAAAAAAAAAAAAAAAAAAAAAATGAAAGCAATATCGACTGTATGTATATGCGCA
CCATAATT

TGCCTTTACGAATGAAATTCCATAAACCTTAAGAAATAACGGAAAAAAAAAAAAAAGCTTTTTTTTTTTTT
GATAGTATAAATAGTTGATTATATGTAAGTGTATATTAATATTGTTACTACTGGTCAATCGTGATATTTAT
GAACAACT

AGATAAAAAAAAAAACTTTAACATGTCACTTTAAGCATGTATGTAATACGTCTATTAATTATATGTAACTT
TTATAACTATTTTCATTTCTATGTGATCCATTTGCAGTTATGCACCTTTTTTTTTTTCCGAATTGTGCTA
TGTATATA

GAAGAAAACCAAAATCCGGGATGAGTTGAAAGTCAAAAAGACTGTATATATAAATTTCAACTTTTGTAGAA
GATGCAGAAAAAGAAAATGATATGGTATGCAGAAAAAGAAATAAACCGCTATTATCCTCGCGGTTTGTCAT
TATAACAG

AGAATTATCGTTCTCGAGATAGTTTTTATACAATACATATATATATATATATATATATATATTTACAAGAAAA
GCATGGCTTATGTATTATACTTGCTTATGTACCAATATATACATATATGGCCTTATTAGGGCAACAAACGC
CAAAAATA

TGCTGTTTTCACCGAAACTTTGAAACACGATTAAACTTATTAGTACATTAAAAATATTCTTTTATTTTTTA
TATACATACATAAGCCTTTAATACCTTTAAAGCGGTAACGACTGCCTCTTGAACTTAAAATTATTCTTTTT
TTTGGATA

TCTTTTTCTTTTACCAGACTTGGTTTTTTTCTCTCTTTAGTAATTGTTTCTATATATGGCTTATCAATTCT
ATATATTTTTTAACTTGAATATATGAATGACATCTTTAATAATCACATCTCGGCAAACGACTGATGAGTTC
CACCTGCT

GGTTATATATGTCTAAATAGAATTCGGGTAGCATTTTATTTTTATCGCTTATAGGAAAAAGATATTTGCAT
ATATATATCTATATATCCTATATACATCTATATAGTTATTGTGTAAGGGGTTTTTATAAGGTCCGATACTT
ACAGAGAT

TATGAAAGAAGAACATCACTTTACACGGATCGCACGCCCATAATTCTTTTTTTTTTTTTTTTCATATC
TTCGACGTTTGCCACTGCCTTCTCTTTTTCTTTCTTTTTTGGCGGCCGGTGGCCAAACGCGCCAAAACCGA
AACGCTTA

AAAATCATTACGAGAGAATGATAAATTGTAACAGAATTTATAAATCCTATTCATATAGATACATCTGTTTT
TCCTTACTCTGTTTTTATTTTGTTGGTAACCTTTGAATCCTTTTTGATGAAAAAAATAGAAAAAAAAAAAA
AAAAAAAA

AATATTTTTTTTCTGTCCAAGTTTATTTTTTTTTCCATAATTTCTGATTTACTACCATTGAAAATTATAAAA
GGAAAAATATTACGCGCTACCATTTAATAAGTAGAAACAACTAAGTTCTCGAATGAGATGTTTGCACTGAT
ACAGCGAG

AAAATGTAACGTTTAGCATTTGATAGACATAGAATAAAAATAAGTAACTATAAAAAAAGGAAAAAAAAAAT
TGGGCAAAATAGGGATCATATTATAACTATTTATATACAGATTCGAAATATGAAGAAGAGACTATGAAAGG
TGGGTGAA

AAATAGACCAATATCAACATCAATTTTATGTATTTTTTTTTTCTTTTGGTATAGCATTGATATATTGAAAT
TTGTATATATTGCTGCGAACACTATTTAAAACAGGTTTTTTTTTTATTTTGGCAGTTTGAAACCCTTTCCT
CTGATGAC

AACATAACTCTAAACTTTAAGAGTGAATTTTTTTTTTTTTAGTTTAGTTTTTTTTTTTTTTTTTTTTACAA
TAATGTATTGTTCTTGGAAAATAACACAATCTGTTCAAAGTGGACAGAAACCCTAAAACAAAAATAATAAC
GGGGAAGG

TTTCTTTTTTTTTTTTTTTATGGAAAGGACCACTCTTACATAACTAGAATAGCATTAAGAATCAGATTTAC
AGATAATGATGTCATTATTAAATATATATATATATATATTGTCACTCCGTTCAAGTCGACAACCAATAAAA
AATTTAAA

TGATAAGCATATTCAATTTTTCTTCATAGCAATATTTTTTTCTGTCCAAGTTTTATTTTTTTTCCATAATT
TCTGATTTACTACCATTGAAAATTATAAAAGGAAAAATATTACGCGCTACCATTTAATAAGTAGAAACAAC
TAAGTTCT

ATAACCTAACCTAATTAAATAGCATGTAGTCAACAAAAAGGGAGTAAAACATGCAGTGGCTCTATATATAT
AATTATCGTAGAATAATTCAAAAAAAAATAAAAAACGAAAAAATTCATTCTTCATCACTCATCAAAGGCAC
TATTTCGT

AGTTCCATACGAAACTTTCATCTAATTTACCCGATGCTGGTTTTGTATCGAAAAAGATTTTCAGGTTTTAG
GTTTTATTTTTTGTCTATTTTTAAATATATAATATAATAAAAAATAAATTGTGTCTAATATCCATAATTTG
TACATTTA

TCCTTCCTTTTGGGTCATAAAATCGCTGGGATGGCCCTTGAAAAAATTTTTTCAGTGAAAAAAAAAAGAAT
TTTCGAGAAAAAAAAAAAAATTAGGAATTGTGCGTGGTCATTCAATACGGAGAATTAAGTAACACGCAACAA
CTGCATTC

GAAATCAGTAAAATAAACGTTTCACCCTGGCAGATAGGAAACCCTATCTCCCAGTGATAAACTTTAATTTT
TTTTCGTGCATATATATATATACATATATAATATAAAACAAAATATCATGAATTTCTATCTAAAGTAGTGG
GAAAAAAT

TTTTTTTTCCCGTAGTACCACTGGATCTGCCTAAATCGCGCTTCAGGAAAACCAGGGACCACCCTATTTTT
TTATATTTCATCATCTAAATTAAGTGTATACATATATGGATAAAGTCATTTTGAGTTTACATAATCGTATG
TTCGAGAG

AGTGAGATGGCAGTTCGAGGGGTTTTTTATTCGAGATAGTAACTTCTGGCTTTTCGCTTTTATACAGCACA
GCAGAAAAAAAAAAGCCGCCGAGGCGCGCGCGTTCATGCAATGGCTCAGTAACCTCGTGATAGAAAAAGGGC
AACAATAT

AAACTCTAATCATTACCGTCTGTTTTATTTACTGTCACCTTGATGAGCGACTAAAAAGATAGAACGCGGGT
TTTTCGCGCAATGTAACAAAGCTCCAGAAATTTTTTATAAATACTAATGTTATAGCAAAAAAAAATAAGGAC
CTTAAAGT

GAAACTTTCATCTAATTTACCCGATGCTGGTTTTGTATCGAAAAAGATTTTCAGGTTTTAGGTTTTATTTT
TTGTCTATTTTTAAATATATAATATAATAAAAAATAAATTGTGTCTAATATCCATAATTTGTACATTTAGA
TTATTTCT

ATTCTTAGGGTCGACTCCACCATTTCAACTTTAGTATAATATGACAATAAAACATTATTGGGACTGAGAAC
GATTATATTAAAATTAGTAAAATACATACATAACTACTTATAAAAAAAAAAAGAGAAATTTGCCATTTTCA
CGAGTATA

ATAATACTTCAGTAATGAGCCTTTTTTATTCGGTCTTGTTACTTCTTTTTTACTTCATTTTTTTTTTTTTT
TTTGGATAGTAAATAATGCACTTTTGACTAGATCTTGAGTATTTAGTTGAATTGAAATTAAAGATACAGTG
TATAAAAA

TCCCATTTGCATAATATCATTAAATATGTTTTACCAAAAAAAAGTTCATCTTTCTTATTTTTAAAATCGAT
AGTTCTTCAGGATCTTGGTCACATATATATATATATATATATATACACTTATATAAAATAAGACGTTTATT
TAATCTTG

TTTCACAAGGAGATAAAAAAAAAAAACTTTAACATGTCACTTTAAGCATGTATGTAATACGTCTATTAATTA
TATGTAACTTTTATAACTATTTTCATTTCTATGTGATCCATTTGCAGTTATGCACCTTTTTTTTTTTTTCCG
AATTGTGC

TCGACTCCACCATTTCAACTTTAGTATAATATGACAATAAAACATTATTGGGACTGAGAACGATTATATTA
AAATTAGTAAAATACATACATAACTACTTATAAAAAAAAAAAGAGAAATTTGCCATTTTCACGAGTATAAG
CACAGATT

TAGCTTTACCTAATATAATGAAATTTGTCTAAGTAACATAAAAAAAAGGAGTAAAGAAAAAAAAAAAAATAA
GTAAATGAAATAAAATATAAAAAAATTCTTACTACGGTTTAAAACAAAGTGCTCTTCCGCTAACGTTTCAA
GTTTCTAA

TAGTAAAAAACGAACAAAAACACAATAAAATATAAATCAATATATTTAGGTTTACTGGGTTCTTTAACAGT
TGTATAATAGTTATTTTTTATTACAAAAATATAGGTTTTAATAAAAAAAAATAGGGTTCTATTTGTTTTAC
ATTTATTG

ACCTTCCTGTTGAGGAATATAGGTTTCTTGGAGTTTTGAAAGTTGTTTTATTTATAATGTTATTATTATTA
TTATTATTATTATTATTATTATTATTATTATTATTATTATTATTATTATTATTGTTATTTTGGTGGTTCCT
ATCGTTAT

TCCTAATCCGCTTCAGGCCCGCGGTCGTTGAAGTGCTATAGTTTATATTCAGTACATTGTCTTTAATCTCT
CAGTATTATTTTATCAATAGTAAATACTTCAAGTCCATCGCACGGCGTGGTGGTGATAAAGGGAAAATGCG
CGCGGGAT

ATAATAACACTGACATAACGCTATAACGAAACAGCGTAACAAAATACTCTTTACATGGTTTATTTTTATAT
ATTCATTTAACATTATCAAAACTAACATTAAGTCCTTTATTTATCAAAACAAACGAAAAAAAAAAAATGCGA
GACAAATG

ATGACATTTAAAAAGTGGGAAAGAATACTACTAATATAAAACTATTTACTGGATATTTAGAATTTTTTTTT
TTTCTTCTTTATACGTAAAAGTTGTTCAAAAAATTTCATCGTTTTCTTTTTTTATGAATCTTTGATTCTTG
TTGAAAAG

TTTCTTCCTTTTTTCACGATTTTTTGCATGGGCATTTGTCACAATATATATTTTTATTCAGTTATATAAAT
ATACTCCTTTTCACAAAAGAAGGCCTAATATTATAGGAAATCAGCATTAAAAACATTATGTATTTCTTTTT
TCAGCGGC

TTTCACTGCATCCTTCCTTTTGGGTCATAAAATCGCTGGGATGGCCCTTGAAAAAATTTTTTCAGTGAAAA
AAAAAAGAATTTTCGAGAAAAAAAAAAAAATTAGGAATTGTGCGTGGTCATTCAATACGGAGAATTAAGTAA
CACGCAAC

AAAAGTGGGAAAGAATACTACTAATATAAAACTATTTACTGGATATTTAGAATTTTTTTTTTTTCTTCTTT
ATACGTAAAAGTTGTTCAAAAAATTTCATCGTTTTCTTTTTTTATGAATCTTTGATTCTTGTTGAAAAGAA
TTTCTAAA

TCAAATGAGCTGATAAGCATATTCAATTTTTCTTCATAGCAATATTTTTTTCTGTCCAAGTTTTATTTTTT
TTCCATAATTTCTGATTTACTACCATTGAAAATTATAAAAGGAAAAATATTACGCGCTACCATTTAATAAG
TAGAAACA

CCCTTGTTTCTTTACGTAATTAAAAATCTCCGGGGCTAGCTTTTGCCGGGGAACCCATCCCGAAAAAATTG
CAAAAAAAAAAATAGCCGCCGACCGTTGGTCGCTATTCACGGAATGATAGAAAAATAGCCGCGCTGCTCGT
CCTGGGTG

CCGATGCTGGTTTTGTATCGAAAAAGATTTTCAGGTTTTAGGTTTTATTTTTTGTCTATTTTTAAATATAT
AATATAATAAAAAATAAATTGTGTCTAATATCCATAATTTGTACATTTAGATTATTTCTTTTGGTTCTTTA
TTCTGACC

TACTTCATTTTTTTTTTTTTTTTTGGATAGTAAATAATGCACTTTTGACTAGATCTTGAGTATTTAGTTGA
ATTGAAATTAAAGATACAGTGTATAAAAATTATACAAAAAATAAAGGAAACTTGTATATTGAAAATAGGAT
TGCCAATA

TAAGTATAAAAACGAGCGAGAAATTTTGTTAAAGAATTGATATCATATTTTATAATTTACATAAACTAAAT
AGGGAAAGAGTAGTATAGCGTGCATAAGTTTGAAAAAAAAAAACTATTCATACACTCATATATACTTTTAT
ATGGTCTT

CAATCTTTTATTAACAAGAGGGCGGTTTCCAATTCTAAAGATGTATAAACAAAAATTCTTATATAACTTAG
GCGACACATAATATGCGCTTAAATAACATATAGTGAGTATATTATCAAAAAGCATTCATTTTTTTTTATTT
ATTAGTCC

TTTGCTTCTGGACAGACAGAACACGTAAAAAAAGAAACACGAAATTACGACTAACTTTCGCGTAGGGGTAA
AATTACCTAATGCGCGTAATTAAAAAAAAAAGCACAGCAGTGTAAATTGGAGCAATAGGAGAAAATGACCT
GTAAAGGA

TTTCTTATGTTCATCGTATTCATAAAATGCTTCACGAACACCGTCATTGATCAAATAGGTTTATAATATTA
ATATACATTTATATAATCGGCGGTATTTATATCATCAAAAAAAGTAGTTTTTTATTTTATTTTTTCATTAC
TTTTCACT

AATAGCTAATGGAAATGGATTTATTACTGAAATATGTAGAAATATTTAACTCGATAGATTTGATCATACAC
ATATAATGCAAAATATATATATATATATATATATATATATATATATATATATATATGTGTGTGTGTGTGTGTG
TGTGTGTA

ACTCGTGGTGCTATATACTTTCGTAGTTAGTTCTGAAATTTTTAGGGTGGTAATTTAAAGCATATTCAATC
ATAATAAAAAAAAAAAAAGAGAATTATTAGGAAAAATAAAAACAAAATAATATATAATATATGATGTAGAA
AAAAATCT

CACGGGAAGTAGGGCCTGACACAATTTTCGGTTCTTTTTTTTCTATAGATAATGGTTATATGTTATATTTA
CGGATATTTTTACGTTTACTTTGTAAGAAAGAGCTAAAAAGATGTAAAGATATCACTTCGTTTTTTTCCCT
CAAAAAAT

GAGTACTTCTTACTTAACACTCCCTGCGCCAATTCTGACTCTTATAATAAATAGCTCGAGACGTAACTGCC
AAAATTGTAAAAAAAAAATGAAAAACTATTAGAAAATTAATTGCGAAAAAAAAAAGGTTAGTCATATGTGC
CTTTGCGG

AAAGAGTGCTACTGAATCATTTGTTGCCACTTTTTAATGTGAACTAAAAAAATAAAAATGAATATAAGGTA
CGTCTCCAAAAGAAATGTAAATATAGAAATTTTAAAAAAAAAAACGAAAAAAAACATAACTAAATTTAAAG
TGCAGCCC

AATGAAATTCCATAAACCTTAAGAAATAACGGAAAAAAAAAAAAAAAGCTTTTTTTTTTTTGATAGTATAA
ATAGTTGATTATATGTAAGTGTATATTAATATTGTTACTACTGGTCAATCGTGATATTTATGAACAACTTG
GCGGAGCA

CTACATAAATCATGTGACTTATTTATTTAATTATTATTAAGTAAAAAAGATTTTCTATTTAAATTTATTAA
TTAATTTTTTTTCTTAAATAATTATTTTATGTTTTTGTTTTCCGAAAAAGAAAAATATTTCAACAAGAAGT
ATCAATAT

ACGGGCAGCCAAAAATACTGGGTGTTGTATATTTACTTGTGTTAGTTTATAAATAAATTTTATATCACTAT
ATGTGTGGTGAAAAGGAAGAGCAATCCTGCTAAAGCTTTTATATCTAAACGCCAAAAAAATAAAAAAAAAA
GGAAATGG

TCTTGGCCCAGTTGGTTAAGGCACCGTGCTAATAACGCGGGGATCAGCGGTTCGATCCCGCTAGAGACCAT
TTTTTTGCAATTCAAATAACGTCGTTTATTTTTTATATAAAAATAAAAATCAAAAAGAATTGCGCAAGCCC
GGAATCGA

TAGAAAATAAAAATACAGAAACTACAAAAGGCAAAAAAAATAAAAGAAAAGGTAATAAATTGAATGAATGT
AGATATATAAATATCAATAGAAATACGTACGCGGAATTATTTCTCTTTGTAACAAATTGATCAAAAGCTTT
GTATTTTT

TCGTCATATACGGAACATTCAGTTATACGCGAAAGTAAAAGTGAGAGCTTTTCAGGGGTTAAAAGCTGGGC
GTGTTCCATGACGTATTTACCGAGGTCGTATTATCAAAGAAAATGAAAAAAAAAAAAAAAAAAAAAAAAGC
GAAGAGGA

TCTTCAATTTTTCTATATGCATTTGAAACAGAAAAAAAGATATAAAAATACCAGCTATAACAAAAATCCAT
GTAAATAAAATTACTGTATATTTTTTTATGTAATCTAATCAAAAAAAATTCACGTCTAATACTAGTCATAA
ATGGAGTT

GTGTCGGCATTTTTTTTTTCCCGTAGTACCACTGGATCTGCCTAAATCGCGCTTCAGGAAAACCAGGGACCA
CCCTATTTTTTTATATTTCATCATCTAAATTAAGTGTATACATATATGGATAAAGTCATTTTGAGTTTACA
TAATCGTA

GTAAGTTGACTTTCACTGCATCCTTCCTTTTGGGTCATAAAATCGCTGGGATGGCCCTTGAAAAAATTTTT
TCAGTGAAAAAAAAAAAGAATTTTCGAGAAAAAAAAAAAAATTAGGAATTGTGCGTGGTCATTCAATACGGAG
AATTAAGT

TTTTTTAGCTACCTATATTCCACCATAACATCAATCATGCGGTTGCTGGTGTATTTACCAATAATGTTTAA
TGTATATATATATATATATATATATGGGGCCGTATACTTACATATAGTAGATGTCAAGCGTAGGCGCTTCCCC
TGCCGGCT

TGACTTGATAGTGAATTATGAGGATCTAGCGACAACACAGGCAGCGTCATAGTTGATGATCTTTTTTTTTT
TTTTTTTTTTTTTTTTGTGCTGCAAAGTTTCTTAAAGCCTTCGGGCTTACGAAATCCTTTATCGCCGAAA
GGGGACCG

AGGATCTAGCGACAACACAGGCAGCGTCATAGTTGATGATCTTTTTTTTTTTTTTTTTTTTTTTTTTGTG
CTGCAAAGTTTCTTAAAGCCTTCGGGCTTACGAAATCCTTTATCGCCGAAAGGGGACCGCTTCGAAAAGTG
GATATAAA

ATAGTAGAACTTTCTTCCTTTTTTCACGATTTTTTGCATGGGCATTTGTCACAATATATATTTTTATTCAG
TTATATAAATATACTCCTTTTCACAAAAGAAGGCCTAATATTATAGGAAATCAGCATTAAAAACATTATGT
ATTTCTTT

AAAATAAAAAATAAAACGCGAAGAGCTAAAAAAAAAAAAGAAAACCTACTATAAATAACCGATTAGAATCG
AGTTTTTGTATTGAAATGGCGGTAATAAGCGTTAAACCTCGACGAAGAGAGAAGATCCTACAGGAGGTAAA
AAACAGCT

CTGGATCTGCCTAAATCGCGCTTCAGGAAAACCAGGGACCACCCTATTTTTTTATATTTCATCATCTAAAT
TAAGTGTATACATATATGGATAAAGTCATTTTGAGTTTACATAATCGTATGTTCGAGAGAGGTCTTTCATA
AAAAAATC

TTCCTTACTCTGTTTTTATTTTGTTGGTAACCTTTGAATCCTTTTTGATGAAAAAAATAGAAAAAAAAAAA
AAAAAAAAATGAAAGCAATATCGACTGTATGTATATGCGCACCATAATTTTCCTATTAATAGTTTAAGCAT
AATTAGTG

CGTTGACTCCATTTTTTGCTCGTTTCATTATATTATCTTCCCATGCCGCTCCTGCGTGGGATTCCCTTTAT
TATAACTTTTAAGGTTTCTACCCACATATATGCAATTTTTATCATGTTTTTCTCTTTTTTTTTTTTTTTTT
TTTTTTTC

TAAATATGTTTTACCAAAAAAAAGTTCATCTTTCTTATTTTTAAAATCGATAGTTCTTCAGGATCTTGGTC
ACATATATATATATATATATATATATACACTTATATAAAATAAGACGTTTATTTAATCTTGTTTTCTTACGAG
TTTCTTTT

AATCGCTGGGATGGCCCTTGAAAAAATTTTTTCAGTGAAAAAAAAAAAGAATTTTCGAGAAAAAAAAAAAAAT
TAGGAATTGTGCGTGGTCATTCAATACGGAGAATTAAGTAACACGCAACAACTGCATTCTGCCTTAGTTGT
CTCAAGGC

CTAAATCGCGCTTCAGGAAAACCAGGGACCACCCTATTTTTTTATATTTCATCATCTAAATTAAGTGTATA
CATATATGGATAAAGTCATTTTGAGTTTACATAATCGTATGTTCGAGAGAGGTCTTTCATAAAAAAATCAA
GTTATCAA

AAAAAGGAATAGAATTATCGTTCTCGAGATAGTTTTTATACAATACATATATATATATATATATATATATT
TACAAGAAAAGCATGGCTTATGTATTATACTTGCTTATGTACCAATATATACATATATGGCCTTATTAGGG
CAACAAAC

CTATAACGAAACAGCGTAACAAAATACTCTTTACATGGTTTATTTTTATATATTCATTTAACATTATCAAA
ACTAACATTAAGTCCTTTATTTATCAAAACAAACGAAAAAAAAAAATGCGAGACAAATGATCCAGTCATTT
ATTGTCAA

ACATATGTACATATGACATACGTATTAGCCGCTGAGGACGCGGACGTATAAAAGGACAATACTTATATGGA
GCTAAGGGGAGCAGTTACGCAACTCCGTGATCGCGCGCCACGGGCCGTCGGCGGCTGTTAATTGAAGAAAA
AAAAAATG

CACTTAGCCAATGAGCATGGGTTGCAGATAACTCATACCGAAAACTATAATGACTTGATAGTGAATTATGA
GGATCTAGCGACAACACAGGCAGCGTCATAGTTGATGATCTTTTTTTTTTTTTTTTTTTTTTTTTTTGTGC
TGCAAAGT

CATTTCAACTTTAGTATAATATGACAATAAAACATTATTGGGACTGAGAACGATTATATTAAAATTAGTAA
AATACATACATAACTACTTATAAAAAAAAAAGAGAAATTTGCCATTTTCACGAGTATAAGCACAGATTGT
ACGAAACT

TTTTGGAAACATTTTTTTTCTTGATGTATATTTTTTGTATTGTAGAAATCGCGCGTACTGTACTTGTATAT
CGCTTTATAAGCGCTTTTAATTGATTGTTCATGACGAGGATAGGCGGATAGGCGGAGGTATGCCTCTTAAT
ATTTATAA

GGTGGGTGAATATCAATATTAAATAAAAATCAAACAATGATTGAAAAAACGGTAAAAGATCATAAAAAGCC
CTCCAGGTAATATGTCAATAAGTAAATATACTTTATCGAAGGAAAGATCAAAAAAAAAAAAAAAAAAAAAT
GCAAACGA

GCAAAAACAATATCGAGTAAGGTAGAAACATCTCTGTGATTAAACATCGTACATATAAATACCATATATAA
AAGTATGTGGGAAAAGAACGTTCAAAAAAAAAAAGTAAGTAGTAGGTTAAAAATAAAATCTCAAAAGAGGG
AAAAAAAA

ATAAACTTACTCTTCAATTTTTCTATATGCATTTGAAACAGAAAAAAAGATATAAAAATACCAGCTATAAC
AAAAATCCATGTAAATAAAATTACTGTATATTTTTTTATGTAATCTAATCAAAAAAAATTCACGTCTAATA
CTAGTCAT

AAGAAATAACGGAAAAAAAAAAAAAAAGCTTTTTTTTTTTTGATAGTATAAATAGTTGATTATATGTAAGT
GTATATTAATATTGTTACTACTGGTCAATCGTGATATTTATGAACAACTTGGCGGAGCAAGGACAAGAATC
CTCTCTTC

GATGAGACGAAGTATATATCCAAAGAATACCTTAAATAGAAAAGGAAAGATAATAAATACTAAACACTACT
ATATATTCAGGTAAAATACAAAAATTATAACATTTTAAAACTTTTTTTTTTTTGAAAGTCCTTCTCGCTTT
AGGATTTT

CATGTGACTTATTTATTTAATTATTATTAAGTAAAAAAGATTTTCTATTTAAATTTATTAATTAATTTTTT
TTCTTAAATAATTATTTTATGTTTTTGTTTTCCGAAAAAGAAAAATATTTCAACAAGAAGTATCAATATAT
TTCTTGCA

CTGCTAATTGATAGTTATCATGTTTCTTATCCCCTCCACTGTAAAGTAAATGTATATTATTAAAAGGCAAA
AATAAGAATATAATACTTCAGTAATGAGCCTTTTTTATTCGGTCTTGTTACTTCTTTTTTACTTCATTTTT
TTTTTTTT

TATCAATATTAAATAAAAATCAAACAATGATTGAAAAAACGGTAAAAGATCATAAAAAGCCCTCCAGGTAA
TATGTCAATAAGTAAATATACTTTATCGAAGGAAAGATCAAAAAAAAAAAAAAAAAAAAATGCAAACGAAC
ATAGCCAA

TTTGATTGTAGATTTTCTTTTTTCTTTTCTTTTTTTTTTTTGACGACTCTGTAATACTCTATTTATTATTT
AGTTTAGTTTTCTATTTAATATGATGGAATTCATAAAAAAAAAAAATACACATAGCCATGCCAGGTAATTG
CAGATCAA

TGATTTTTGGTAAGGGGACGTTCTAGAAACGTCTACTATTTTGGATGATTTACATAGTTTTAAAATAAAAA
AGGCTCAATTTTACCTTCCGCGCATTTCTATTTTTTTAATCTAAAACCTCAAATCTTACAGTCTCTCCTAG
CCGCTTTC

TATGCTCAGCTAGATTGGTTGATGAAAAATCTTATCATAGGGCACCTTCCAGGTAAAAGATCTACTTACTA
TTAGTCAGTACGTCCTTTTTTCATTTGTTTATAATTTTTTTTTTTTTTTTTTTTTTCTCCTCTCAATATTT
TAGAAGTA

AATATCAATAGAAATACGTACGCGGAATTATTTCTCTTTGTAACAAATTGATCAAAAGCTTTGTATTTTTT
TTTTTTTTAGCTCGGCTGCGCGCTATTACAGTAATTACATGTAAATAAACTATTCCAAAGAATACAAAAGA
AGAGCTTA

TAATGTTGCATCATTACTAGTTTAAACTGTATTTTTATTAAAGGGAAAAAAATGATTCAATATGTATATAT
TTATTCATGTATATGTATTTTGAATTAAAAAAAAACGTTTTATAAATACTCAATAAAGCCGTTGTATTTTC
TTTATTAC

GGTAATAAATTGAATGAATGTAGATATATAAATATCAATAGAAATACGTACGCGGAATTATTTCTCTTTGT
AACAAATTGATCAAAAGCTTTGTATTTTTTTTTTTTTTAGCTCGGCTGCGCGCTATTACAGTAATTACATG
TAAATAAA

TCTTAACGAATAATGTTGCATCATTACTAGTTTAAACTGTATTTTTATTAAAGGGAAAAAAATGATTCAAT
ATGTATATATTTATTCATGTATATGTATTTTGAATTAAAAAAAAACGTTTTATAAATACTCAATAAAGCCG
TTGTATTT

TTCAGCCTGTTCCATCCTTTTGATCAAAAAAAAAAAGGAAAATCAAGCGCGGGTAATAACGGCACCATTAA
ATACTAGTAGTACTATATAGATGGTATAAAGAAATGATTTAAAACAATACGATTATATGATATTTATACAT
CTGTTTCA

AACTACGTAAAAAGGCGGTATTTATCTATTATTTGGCCAAAAAAAAAAAAAAATACATACTACATATACAT
ATACGCCATAAAAAATCTCTGCATCTATCTTATTTCCCATTATTTGGACAAATGCTTACGTGCTAATGTCC
TTACCCTC

AACGAGCGAGAAATTTTGTTAAAGAATTGATATCATATTTTATAATTTACATAAACTAAATAGGGAAAGAG
TAGTATAGCGTGCATAAGTTTGAAAAAAAAAAACTATTCATACACTCATATATACTTTTATATGGTCTTTT
TTATATTC

AAGTTAAGGAGGACTTATGGAATGAATTCCTCTCTTCAATTAACGAAAAGTAAAATATATAGATAAAAATC
ACTGCATAGGGAAAAAAACTTTAAATTTTAGGTATACTGAAGACAACAAAAAATACATAACTTAAAATAAA
GGGGCATT

TTCCCCTCAGTGTTATTTTTTTTCACAAGGAGATAAAAAAAAAAACTTTAACATGTCACTTTAAGCATGTA
TGTAATACGTCTATTAATTATATGTAACTTTTATAACTATTTTCATTTCTATGTGATCCATTTGCAGTTAT
GCACCTTT

CTTTATATACCAATAGCAGCAACATCCGGCCCCTTGTTTCTTTACGTAATTAAAAATCTCCGGGGCTAGCT
TTTGCCGGGGAACCCATCCCGAAAAAATTGCAAAAAAAAAAATAGCCGCCGACCGTTGGTCGCTATTCACG
GAATGATA

AATTCGGGTAGCATTTTATTTTTATCGCTTATAGGAAAAAGATATTTGCATATATATATCTATATATCCTA
TATACATCTATATAGTTATTGTGTAAGGGGTTTTTATAAGGTCCGATACTTACAGAGATTAATTTGTAAAT
AAAAGCAG

GTAAACCGGTTGATGCTATTGTTAATATATAACGACAAATGTTCAAGCAATTATATAACCATTTTCTTCCA
TATTCACCTTAAACGAGGTCGCCTTTATTTTTTTTTTTTTTTTTTTCAGGGCATCGATCGGCCCGGAGTCG
TCGCCTTC

ACGGGCCGTCGGCGGCTGTTAATTGAAGAAAAAAAAAATGAAGAACCACAAGGGGTGATCCATATAGGTGA
CTAGCATCATCCCCTGCGACGCGCGGCCCGCCGGGCCAAAGGCGGGCAATGCGCGCTGCTGATTGGCCTCG
AGGACAAC

TCTACTTACTATTAGTCAGTACGTCCTTTTTTCATTTGTTTATAATTTTTTTTTTTTTTTTTTTTTTCTCC
TCTCAATATTTTAGAAGTAACATGACAGAGCAACACGTTTATTTTCCTACATATAGTCATAAGTATAAAAA
CGAGCGAG

GCTGGCTACTACAACTGCTAAAAACAATAGCATTAATAATAGTAATAGTAATAATAATAGTAATAATAATA
AAAATAATAATAATAATCATAATAATAATAATCTACTCGGTAATGACATCAGTCAGATGGCCTTTTTACTC
GATTACCC

AATCCAAAAAAAAAAAGTTACGCGTTTCGCGTTTTGGTGCGTCGGATGGAGAAGAAAAATGCTAAATAATT
AGATATTGAAGAATCAGCATAAGTTGTGCGAAAAAGGAAGAGAATACATAAAAGAAAAGAAGGATATATAA
GTAAAATA

CAATTTTATGTATTTTTTTTTTCTTTTGGTATAGCATTGATATATTGAAATTTGTATATATTGCTGCGAAC
ACTATTTAAAACAGGTTTTTTTTTTATTTTGGCAGTTTGAAACCCTTTCCTCTGATGACTTTAGTGTAGTA
AATGTAAA

AAATAAAAATCAAACAATGATTGAAAAAACGGTAAAAGATCATAAAAAGCCCTCCAGGTAATATGTCAATA
AGTAAATATACTTTATCGAAGGAAAGATCAAAAAAAAAAAAAAAAAAAAATGCAAACGAACATAGCCAAAG
GACCACAA

TATTGATGAGGAATATAAAAATGTAATTAGGCAAACCTACGCGCGAAAATATAAGAAGCAAAGATATAAAG
TGTAGTATATTTGAATATTTACATGAACGAAACGGCGACGTATAATGGTAATCGGCTGGGAACTTCATTAA
AAAGTCAA

CAAAGGGGGGGACCCTGAGAAAACTTCTCCCGAAAAAAAAAAAAAGAAATATACCCACGCGTTCTTTACCC
GGAAATAAATATCTTGATTTAGCCGCCGAGATTGTTATATATGCATCCAAGACCTCTGAATGGTGGCTAAT
TAAGACAC

ATAAAATGGAATTTAAAAAAGTGGGAAAGAAACAATGAGAATTCATAGGATATAATGAAAGGTAAATGCTT
TAAAAAAAAATTAAGAGGGGAAAATAAAAGTAGAAAAGCAAATAAAAACAAAACATGGAATAAAAGTAACT
TCAAAAAA

ATGCCTTCTACACTTAGCCAATGAGCATGGGTTGCAGATAACTCATACCGAAAACTATAATGACTTGATAG
TGAATTATGAGGATCTAGCGACAACACAGGCAGCGTCATAGTTGATGATCTTTTTTTTTTTTTTTTTTTTT
TTTTTTGT

GGTAGAAACATCTCTGTGATTAAACATCGTACATATAAATACCATATATAAAAGTATGTGGAAAAGAACG
TTCAAAAAAAAAAAGTAAGTAGTAGGTTAAAAATAAAATCTCAAAAGAGGGAAAAAAAAAAAAGAGGGATA
ATTTTGGC

TGGCATCTCTAAAGAGTGCTACTGAATCATTTGTTGCCACTTTTTAATGTGAACTAAAAAAATAAAAATGA
ATATAAGGTACGTCTCCAAAAGAAATGTAAATATAGAAATTTAAAAAAAAAAAACGAAAAAAAACATAACT
AAATTTAA

ATCCTTCGCTTAATATTTGATACGACTTTTTTGATTTCCATTATTATTATTTGTTACTATTATTATTTATC
ATTTGGGTTTCGGTTTTTTGTAATAATTTTCTTTTTTTTTTTTGGCTCTATTTCACTAAGACATCGTATAT
ATGCCAGG

ACAGAATTTATAAATCCTATTCATATAGATACATCTGTTTTTCCTTACTCTGTTTTTATTTTGTTGGTAAC
CTTTGAATCCTTTTTGATGAAAAAAATAGAAAAAAAAAAAAAAAAAAAAATGAAAGCAATATCGACTGTATG
TATATGCG

ACCCACATATATGCAATTTTTATCATGTTTTTCTCTTTTTTTTTTTTTTTTTTTTTTTTTCTCTCTTTGTTG
TGTTTTCGAGAAAAAGTTCAATTGTTTAAAAGGAATTAAGCAAATTATATATATGCGACTGTTGGTTTCCA
GTTCTAAT

TGGGAATCCAGGGAAATTTAAAAATGTAACGTTTAGCATTTGATAGACATAGAATAAAAATAAGTAACTAT
AAAAAAAGGAAAAAAAAAAATTGGGCAAAATAGGGATCATATTATAACTATTTATATACAGATTCGAAATAT
GAAGAAGA

TAAAAACAGGAAAAAAGATAGATCTAGAGCTTCACGAATCTATTTATTAGTTTCAACGCGCTATATTATTT
CGCGAGAACCAGTAATTCTCTATTTAAAAGCACTCTATTGCCTATCACAAAGTGCTCTGTTGTGTGTTGTT
TTCAATGT

ACCTATATTCCACCATAACATCAATCATGCGGTTGCTGGTGTATTTACCAATAATGTTTAATGTATATATA
TATATATATATATGGGGCCGTATACTTACATATAGTAGATGTCAAGCGTAGGCGCTTCCCCTGCCGGCTGT
GAGGGCGC

AACTTTTGCTACCTTAATTAAAAGAACCACTCAGACTTTTTACCATCCTTTAGCTTTACCTAATATAATGA
AATTTGTCTAAGTAACATAAAAAAAAGGAGTAAAGAAAAAAAAAAAAATAAGTAAATGAAATAAAATATAAA
AAAATTCT

AGTACATCGCAGGAACCGCAGACATGATGTCCTCTTCTTTCAAATTACTGGATATATATATTATTATTATT
GTTACCATTAACGTTATTATTATTAACTATTACTATTACATTAACTTAAAAATAAATATGAAACAACGAAG
AACAAAAA

GAAGAACATCACTTTACACGGATCGCACGCCCATAATTCTTTTTTTTTTTTTTTTTCATATCTTCGACGTTT
GCCACTGCCTTCTCTTTTTCTTTCTTTTTTGGCGGCCGGTGGCCAAACGCGCCAAAACCGAAACGCTTATA
AAATGTAG

AGGCTCTTTTTCTTTTTCTTTTACCAGACTTGGTTTTTTTCTCTCTTTAGTAATTGTTTCTATATATGGCT
TATCAATTCTATATATTTTTTAACTTGAATATATGAATGACATCTTTAATAATCACATCTCGGCAAACGAC
TGATGAGT

TAAAAACACATAGAAAATAAAAATACAGAAACTACAAAAGGCAAAAAAAATAAAAGAAAAGGTAATAAATT
GAATGAATGTAGATATATAAATATCAATAGAAATACGTACGCGGAATTATTTCTCTTTGTAACAAATTGAT
CAAAAGCT

AACATCCGGCCCCTTGTTTCTTTACGTAATTAAAAATCTCCGGGGCTAGCTTTTGCCGGGGAACCCATCCC
GAAAAAATTGCAAAAAAAAAAATAGCCGCCGACCGTTGGTCGCTATTCACGGAATGATAGAAAAATAGCCG
CGCTGCTC

GTTGCATCTGTACCGTAGAGATTCTTGCAACCTCGCTTAAACTCTCGCTTTTATATAATATTTCTCCTTAT
TGCGCGCTTCGTTGAAAATTTCGCTAAACACGGGGTTTAAGTTTAAGTTTACAGGATTTATCCGGAAGTTT
TCGCGGAC

CCTGCGTGGGATTCCCTTTATTATAACTTTTAAGGTTTCTACCCACATATATGCAATTTTTATCATGTTTT
TCTCTTTTTTTTTTTTTTTTTTTTTTTTTCTCTCTTTGTTGTGTTTTCGAGAAAAAGTTCAATTGTTTAAAA
GGAATTAA

GAACAAAGAAAAGAAACATCGCGTAATGCAACAGTGAGACACTTGCCGTCATATATAAGGTTTTGGATCAG
TAACCGTTATTTGAGCATAACACAGGTTTTTAAATATATTATTATATATCATGGTATATGTGTAAAATTTT
TTTGCTGA

GTGACGCTAATATTATGTACCGCCCTTCCCCACACACACAAAAAAAAAAGGTTAAGGATAAAGATAAGTAA
ACTAATGGAATATATAAACAAATAAGTAAGTAGATACTGTAAATAGAACACACGTTTTTAATTTATTTTT
TTTTTTAC

TTATAACTTTTAAGGTTTCTACCCACATATATGCAATTTTTATCATGTTTTTCTCTTTTTTTTTTTTTTTT
TTTTTTTTCTCTCTTTGTTGTGTTTTCGAGAAAAAGTTCAATTGTTTAAAAGGAATTAAGCAAATTATATA
TATGCGAC

TACACTTCAAACGGTTGGTGAAACAAAAAAAAAAAAAAGAACCCATCCTAAAATACTGTTATTATTACTAAA
TAGTGCTAAATAATACATATGCCTTGATTTTATTTCCGAAATTTTTTGTTTTGTCAATTTAGGCTCTATTA
GCTTCTTC

TAGTCCAGGTACTGCTGCTATTTGATTGTAGATTTTCTTTTTTCTTTTCTTTTTTTTTTTTGACGACTCTG
TAATACTCTATTTATTATTTAGTTTAGTTTTCTATTTAATATGATGGAATTCATAAAAAAAAAAAAATACAC
ATAGCCAT

CCAAGCCAGCTGCTGTTTTCACCGAAACTTTGAAACACGATTAAACTTATTAGTACATTAAAAATATTCTT
TTATTTTTTATATACATACATAAGCCTTTAATACCTTTAAAGCGGTAACGACTGCCTCTTGAACTTAAAAT
TATTCTTT

ATGCATCAAGAAAATAAAAAAATAAAACGCGAAGAGCTAAAAAAAAAAAAAGAAAACCTACTATAAATAACCG
ATTAGAATCGAGTTTTTGTATTGAAATGGCGGTAATAAGCGTTAAACCTCGACGAAGAGAGAAGATCCTAC
AGGAGGTA

ATAACGATAAGCTGGCTACTACAACTGCTAAAAACAATAGCATTAATAATAGTAATAGTAATAATAATAGT
AATAATAATAAAAATAATAATAATAATCATAATAATAATAATCTACTCGGTAATGACATCAGTCAGATGGC
CTTTTTAC

TTTCTACCCTGTGCTCTTTGTTGGCGGCTTTATTGATGAGGAATATAAAAATGTAATTAGGCAAACCTACG
CGCGAAAATATAAGAAGCAAAGATATAAAGTGTAGTATATTTGAATATTTACATGAACGAAACGGCGACGT
ATAATGGT

AAAATTTTTTTCATTCATTAACATGTGAAGGCAACATTCATCGATCTCCATCCCTACTGTCAAAAATATAA
GTATATATATATATATATTTATATAGTCTATATATATATAGTCCCACTCTCAAACTAATATAACCTTTACA
AACCTGTC

CTTTTAAGCAATAATAGATTTTAAGTATGTAATGATACAAATAATAATATATTTATATACGCATATATTTA
CATGTGCATATATGAAAAATAACCTAATTCACGTTACCCACCTTTTTTTTTAGCCTTTTCTTTATTTATAT
ATGTATCC

TGTTTTTATTTTGTTGGTAACCTTTGAATCCTTTTTGATGAAAAAAATAGAAAAAAAAAAAAAAAAAAAAT
GAAAGCAATATCGACTGTATGTATATGCGCACCATAATTTTCCTATTAATAGTTTAAGCATAATTAGTGTA
CTTACTAA

TCCTACAAGCGCTATAACATATGAAATATACATTAGTCGAATATTAACTTTGGGAGGTAATTACAATTCCT
ATCCGCTTTATATAATTTAGTGGAACTGGCACGCCGCTCGGAAAAATTGGACTTCAAATTACCCCGCGCGG
AGATTTGC

ACTCTTCTCATTTTTAAGTTCATTCTCTTTCATTTTCGGTAGTGAGATGGCAGTTCGAGGGGTTTTTTATT
CGAGATAGTAACTTCTGGCTTTTCGCTTTTATACAGCACAGCAGAAAAAAAAAAGCCGCCGAGGCGCGCGCG
TTCATGCA

TTTCTTTTTTTTTTTTCAATTACGGATTATGCATATATAGGTGGGTATACGTACGTAAAAAAACAAAAACA
CACATGTAGACATCTATATACGTTTAAAAAAGCTTTAGATTCAGATCTTTGGGTTTTACAAAAAAAAAAACG
GACAAAAT

CATATAGTCATAAGTATAAAAACGAGCGAGAAATTTTGTTAAAGAATTGATATCATATTTTATAATTTACA
TAAACTAAATAGGGAAAGAGTAGTATAGCGTGCATAAGTTTGAAAAAAAAAAACTATTCATACACTCATAT
ATACTTTT

TTGTTGATTGTCATCAGTTTTAGTAAAAAACGAACAAAAACACAATAAAATATAAATCAATATATTTAGGT
TTACTGGGTTCTTTAACAGTTGTATAATAGTTATTTTTTATTACAAAAATATAGGTTTTAATAAAAAAAAA
TAGGGTTC

AAAAAGATTTTCAGGTTTTAGGTTTTATTTTTTGTCTATTTTTAAATATATAATATAATAAAAAATAAATT
GTGTCTAATATCCATAATTTGTACATTTAGATTATTTCTTTTGGTTCTTTATTCTGACCTATATAGACTAC
TCAACACA

GGCTTTTCCGCGTATAAGAAATAATAACACTGACATAACGCTATAACGAAACAGCGTAACAAAATACTCTT
TACATGGTTTATTTTTATATATTCATTTAACATTATCAAAACTAACATTAAGTCCTTTATTTATCAAAACA
AACGAAAA

AGTCATTGATTACACTTCAAACGGTTGGTGAAACAAAAAAAAAAAAAAGAACCCATCCTAAAATACTGTTAT
TATTACTAAATAGTGCTAAATAATACATATGCCTTGATTTTATTTCCGAAATTTTTTGTTTTGTCAATTTA
GGCTCTAT

CCCGTAATTAAGGTGTTTATATAAACTTACTCTTCAATTTTTCTATATGCATTTGAAACAGAAAAAAAGAT
ATAAAAATACCAGCTATAACAAAAATCCATGTAAATAAAATTACTGTATATTTTTTATGTAATCTAATCA
AAAAAAAT

GTTGCCTTTCTTCCCCTCAGTGTTATTTTTTTTCACAAGGAGATAAAAAAAAAAACTTTAACATGTCACTT
TAAGCATGTATGTAATACGTCTATTAATTATATGTAACTTTTATAACTATTTTCATTTCTATGTGATCCAT
TTGCAGTT

ACAGACAAAACAAACTATCATCTTAACGAATAATGTTGCATCATTACTAGTTTAAACTGTATTTTTATTAA
AGGGAAAAAAAATGATTCAATATGTATATATTTATTCATGTATATGTATTTTGAATTAAAAAAAAACGTTTT
ATAAATAC

TAGTTCTTCAGGATCTTGGTCACATATATATATATATATATATATATACACTTATATAAAATAAGACGTTTAT
TTAATCTTGTTTTCTTACGAGTTTCTTTTTTTTTCTTTTTTTATTTTCTACTTTTGCAAGGAACAGTGACC
TTGCCAAT

ACGGTTGGTGAAACAAAAAAAAAAAAAAGAACCCATCCTAAAATACTGTTATTATTACTAAATAGTGCTAAA
TAATACATATGCCTTGATTTTATTTCCGAAATTTTTTGTTTTGTCAATTTAGGCTCTATTAGCTTCTTCAT
CAGGTTCA

ATTCCCTTTATTATAACTTTTAAGGTTTCTACCCACATATATGCAATTTTTATCATGTTTTTCTCTTTTTT
TTTTTTTTTTTTTTTTTCTCTCTTTGTTGTGTTTTCGAGAAAAAGTTCAATTGTTTAAAAGGAATTAAGC
AAATTATA

TAATATAATGAAATTTGTCTAAGTAACATAAAAAAAAGGAGTAAAGAAAAAAAAAAAAATAAGTAAATGAAA
TAAAATATAAAAAAATTCTTACTACGGTTTAAAACAAAGTGCTCTTCCGCTAACGTTTCAAGTTTCTAAAG
AATCGTTA

TTGGAACGCGAAAAAACAAAACAGACGCGAGAACGCGACGCGAAAAAATATATTTTTCCGTTTTTTTTTTT
TTTCGGAACAAAGATAACGCGTTAAAACCTGTGGATGTAATAAGGATCTGAAACACTGACTAAAACGCGTC
CTCATTTA

ATTTTGGTCATTGGAACGCGAAAAAACAAAACAGACGCGAGAACGCGACGCGAAAAAATATATTTTTCCGT
TTTTTTTTTTTTCGGAACAAAGATAACGCGTTAAAACCTGTGGATGTAATAAGGATCTGAAACACTGACT
AAAACGCG

GTCTCCTTCGTCAAAGACGACCACTTTCCCATTTTGGTCATTGGAACGCGAAAAAACAAAACAGACGCGAG
AACGCGACGCGAAAAAATATATTTTTCCGTTTTTTTTTTTTTTCGGAACAAAGATAACGCGTTAAAACCTG
TGGATGTA

AGAGGTTTTCGTCTCCTTCGTCAAAGACGACCACTTTCCCATTTTGGTCATTGGAACGCGAAAAAACAAAA
CAGACGCGAGAACGCGACGCGAAAAAATATATTTTTCCGTTTTTTTTTTTTTTCGGAACAAAGATAACGCG
TTAAAACC

AAAAAACAAAACAGACGCGAGAACGCGACGCGAAAAAATATATTTTTCCGTTTTTTTTTTTTTTCGGAACA
AAGATAACGCGTTAAAACCTGTGGATGTAATAAGGATCTGAAACACTGACTAAAACGCGTCCTCATTTAAA
ATCAAAAA

CCACTTTCCCATTTTGGTCATTGGAACGCGAAAAAACAAAACAGACGCGAGAACGCGACGCGAAAAAATAT
ATTTTTCCGTTTTTTTTTTTTTTCGGAACAAAGATAACGCGTTAAAACCTGTGGATGTAATAAGGATCTGA
AACACTGA

GAAAAAAATGGATAAAAAAACAAAAAAAAAAAAAAAAACAAAAAAAAAACAGCTTTGCTGGAGGATGATTA
ACTTTTAATTTTTTTAATTATTAGGTTTTTCAATTTTTTATGGTTTTTTGTGTAAAATGCAATGAACGAAT
CCATTTTT

TCAAAGACGACCACTTTCCCATTTTGGTCATTGGAACGCGAAAAAACAAAACAGACGCGAGAACGCGACGC
GAAAAAATATATTTTTCCGTTTTTTTTTTTTTTCGGAACAAAGATAACGCGTTAAAACCTGTGGATGTAAT
AAGGATCT

GATAAAAAAACAAAAAAAAAAAAAAAAACAAAAAAAAAACAGCTTTGCTGGAGGATGATTAACTTTTAATT
TTTTTAATTATTAGGTTTTTCAATTTTTTATGGTTTTTTGTGTAAAATGCAATGAACGAATCCATTTTTTA
TTTTGCAA

ACAGACGCGAGAACGCGACGCGAAAAAATATATTTTTCCGTTTTTTTTTTTTTTCGGAACAAAGATAACGC
GTTAAAACCTGTGGATGTAATAAGGATCTGAAACACTGACTAAAACGCGTCCTCATTTAAAATCAAAAAAT
TACTTCTT

TGGAACAAATGAAAAAAATGGATAAAAAAACAAAAAAAAAAAAAAAAACAAAAAAAAAACAGCTTTGCTGG
AGGATGATTAACTTTTAATTTTTTTAATTATTAGGTTTTTCAATTTTTTATGGTTTTTTGTGTAAAATGCA
ATGAACGA

TTACGCTAAAATAATAGTTTATTTTATTTTTTGAATATTTTTTATTTATATACGTATATATAGACTATTAT
TTATCTTTTAATGATTATTAAGATTTTTATTAAAAAAAAATTCGCTCCTCTTTTAATGCCTTTATGCAGTT
TTTTTTTC

CAAAAAAAAAAAAAAAAACAAAAAAAAAACAGCTTTGCTGGAGGATGATTAACTTTTAATTTTTTTAATTA
TTAGGTTTTTCAATTTTTTATGGTTTTTTGTGTAAAATGCAATGAACGAATCCATTTTTTATTTTGCAAGT
GTGAGAGG

ATCGATAGATCAATTTTTTTCTTTTCTCTTTCCCCATCCTTTACGCTAAAATAATAGTTTATTTTATTTTT
TGAATATTTTTTATTTATATACGTATATATAGACTATTATTTATCTTTTAATGATTATTAAGATTTTTATT
AAAAAAAA

ACGTTTTAGAAGAGGTTTTCGTCTCCTTCGTCAAAGACGACCACTTTCCCATTTTGGTCATTGGAACGCGA
AAAAACAAAACAGACGCGAGAACGCGACGCGAAAAAATATATTTTTCCGTTTTTTTTTTTTTTCGGAACAA
AGATAACG

CAATTTTTTTCTTTTCTCTTTCCCCATCCTTTACGCTAAAATAATAGTTTATTTTATTTTTTGAATATTTT
TTATTTATATACGTATATATAGACTATTATTTATCTTTTAATGATTATTAAGATTTTTATTAAAAAAAAAT
TCGCTCCT

ATTAAACTGATGGAACAAATGAAAAAAATGGATAAAAAAACAAAAAAAAAAAAAAAAACAAAAAAAAAACA
GCTTTGCTGGAGGATGATTAACTTTTAATTTTTTTAATTATTAGGTTTTTCAATTTTTTATGGTTTTTTGT
GTAAAATG

GGAATCAAAGATCTATAAATTTTATATTCATGAGCACATGTAGTTTTTTTTTTTTTAGTTTTAAATATAAG
AAAAAAGATAGTATATATATATATATATATATACATATACATATAAAAGGAAAGTAAGCTTATACAATACACA
AATAAATT

ATTGAAAAAAATAGTACAAATAAGTCACATGATGATATTTGATTTTATTATATTTTTAAAAAAAGTAAAAAA
TAAAAAGTAGTTTATTTTTAAAAAAATAAAATTTAAAATATTAGTGTATTTGATTTCCGAAAGTTAAAAAAG
AAATAGTA

GAAAAGAGAAAGGGAACACTTCTAATTTATCTTTAAAATTATATATATATATATATATATATGTGTGTTTG
TATACTCTGTGGGTATTTCCGTGTATATGGTTAATAATAGTAGTATCTTGTCAGTTTTTTTTATGTTTTTC
TTCGCGCG

AAATCCCATTAATTAAAAAAGAATTGTAACCTTATTTAAAAAAAAAAAATAGTACATAACAATAAAAAAAAA
AAAGATAATAATTTTGAATTTATTGCTAGACATTCTTACGTTTATTGTGTTCATGTTAACAAAAACACATG
CCCGCGGT

ATCTATAAATTTTATATTCATGAGCACATGTAGTTTTTTTTTTTTTAGTTTTAAATATAAGAAAAAAGATA
GTATATATATATATATATATACATATACATATAAAAGGAAAGTAAGCTTATACAATACACAAATAAATTCA
TATCAAAG

TTTTATTATACCCTTTTTTTCTCTCTTTACTGTACAGAAGATGCGTCACTAATTCAAAAAAAAAAAAAAAA
AAAAAAATATAAAAAAGAAAGATTTTGGAGAAGCTATATCCTTTTGTGACATACAATAGAGAAACTTGTTC
TGTTGGTA

CTTTTCTCTTTCCCCATCCTTTACGCTAAAATAATAGTTTATTTTATTTTTTGAATATTTTTTATTTATAT
ACGTATATATAGACTATTATTTATCTTTTAATGATTATTAAGATTTTTATTAAAAAAAAAATTCGCTCCTCT
TTTAATGC

TCCCCATCCTTTACGCTAAAATAATAGTTTATTTTATTTTTTGAATATTTTTTATTTATATACGTATATAT
AGACTATTATTTATCTTTTAATGATTATTAAGATTTTTATTAAAAAAAAAATTCGCTCCTCTTTTAATGCCT
TTATGCAG

TCTCTTTCGTATTAAACTGATGGAACAAATGAAAAAAATGGATAAAAAAACAAAAAAAAAAAAAAAAACAA
AAAAAAAACAGCTTTGCTGGAGGATGATTAACTTTTAATTTTTTTAATTATTAGGTTTTTCAATTTTTTAT
GGTTTTTT

ATAATAGTTTATTTTATTTTTTGAATATTTTTTATTTATATACGTATATATAGACTATTATTTATCTTTTA
ATGATTATTAAGATTTTTATTAAAAAAAAAATTCGCTCCTCTTTTAATGCCTTTATGCAGTTTTTTTTTCCC
ATTCGATA

CTCTCTCCCAACGTTTTAGAAGAGGTTTTCGTCTCCTTCGTCAAAGACGACCACTTTCCCATTTTGGTCAT
TGGAACGCGAAAAAACAAAACAGACGCGAGAACGCGACGCGAAAAAATATATTTTTCCGTTTTTTTTTTTT
TTCGGAAC

TTTATATTCATGAGCACATGTAGTTTTTTTTTTTTTAGTTTTAAATATAAGAAAAAAGATAGTATATATAT
ATATATATATACATATACATATAAAAGGAAAGTAAGCTTATACAATACACAAATAAATTCATATCAAAGAA
TAATGAAC

AAAGCTATTCATTGAAAAAAATAGTACAAATAAGTCACATGATGATATTTGATTTTATTATATTTTTAAAAA
AAGTAAAAAATAAAAAGTAGTTTATTTTTAAAAAAATAAAATTTAAAATATTAGTGTATTTGATTTCCGAAA
GTTAAAAA

CCAGGATAATTTTCAGGACACGTGTTTCGAAAGGTTTGTCGCTCCGAAAAAATCAAAAAAAACAAAAAACG
GGAAATAACAATAACGACAAAAATGGAAAAAAAAAAAATTTTAGACGCGGCGCTTGCACCCCGCATTATAA
GTGGTGTG

GAACGCGACGCGAAAAAATATATTTTTCCGTTTTTTTTTTTTTTCGGAACAAAGATAACGCGTTAAAACCT
GTGGATGTAATAAGGATCTGAAACACTGACTAAAACGCGTCCTCATTTAAAATCAAAAAATTACTTCTTCT
CCTCCCTT

CGTACGTCATCACTACGGGAAACCAAAAAAAAAAAAAAGAAAACAGTACAAAAAGGTTTACTTTACCCATA
GTTAGTATACGTAGATTTTATACACGCACTATTTTTCTTATATACAGGAGATGGGTGGCCACAGAACCCGC
GCCTAGCC

TAAATCGCATTTCTTTTCGTCCACATTTGCAAACAAAACTTTTCAATAATAATTTTATAAATAGTATCAAT
ATATATATATATATATATATTTATTTGTTTACTTTTTCTATCAGTGTTTTCAATTTTTTATTAAACAATGT
TTGATTTT

CACACCTTTATGAGAAGCGAATTTTTTTTTTTTTTTGGTTTAAATATATATATATACATATATATAGATAT
AGATATACACATGTATAGATGTATTCTAGTTATGCTTATACCTAGAACTGATCAGACCAGATCATACCTGG
TATAGAGC

GAATTGTAACCTTATTTAAAAAAAAAAAATAGTACATAACAATAAAAAAAAAAAAAGATAATAATTTTGAATT
TATTGCTAGACATTCTTACGTTTATTGTGTTCATGTTAACAAAAACACATGCCCGCGGTTATTATCGCTTT
TTAATTGC

TAGTTTTTTTTTTTTTAGTTTTAAATATAAGAAAAAAGATAGTATATATATATATATATATACATATACAT
ATAAAAGGAAAGTAAGCTTATACAATACACAAATAAATTCATATCAAAGAATAATGAACCGCAATATTCAG
AAACACAG

TTTCAGGACACGTGTTTCGAAAGGTTTGTCGCTCCGAAAAAATCAAAAAAAACAAAAAACGGGAAATAACA
ATAACGACAAAAATGGAAAAAAAAAAAATTTTAGACGCGGCGCTTGCACCCCGCATTATAAGTGGTGTGCC
GACGGACG

GGTGAAACTCTCGAGCATACATTACCTTACGTGTGTTAGTGTACTATATTATATATATATATATGTATATA
TATAAAGGGAGGAGTTTTTAATTATAATTGTAATTTCGTATTTTTTCTGCATTATACAGTTTTTTCCGATT
TTAAACGA

ATTTTTATTTTCTCTTTCGTATTAAACTGATGGAACAAATGAAAAAAATGGATAAAAAAACAAAAAAAAAA
AAAAAAACAAAAAAAAAACAGCTTTGCTGGAGGATGATTAACTTTTAATTTTTTTAATTATTAGGTTTTTC
AATTTTTT

AAATATAAGCTAAATCGCATTTCTTTTCGTCCACATTTGCAAACAAAACTTTTCAATAATAATTTTATAAA
TAGTATCAATATATATATATATATATATATATTTATTTGTTTACTTTTTCTATCAGTGTTTTCAATTTTTTAT
TAAACAAT

CCTCTGACGAAGGTGGTAGAGAACAAGAAAATGAATAACATTATATCATATTCCTATATATATATATATATAT
ATATATATATATCATATAACGGTGGAAAATCCCGGCGATATTACAGATAAACATTACACCCGCATGAATGT
GAGCCACT

CCCTTTTTTTCTCTCTTTACTGTACAGAAGATGCGTCACTAATTCAAAAAAAAAAAAAAAAAAAAAAAATAT
AAAAAAGAAAGATTTTGGAGAAGCTATATCCTTTTGTGACATACAATAGAGAAACTTGTTCTGTTGGTAAG
ATATCTCA

GCAAAAGATAATAGTGTCATCACACCTTTATGAGAAGCGAATTTTTTTTTTTTTTTGGTTTAAATATATAT
ATATACATATATATAGATATAGATATACACATGTATAGATGTATTCTAGTTATGCTTATACCTAGAACTGA
TCAGACCA

GAAAATTGTTCAAAGTTTTTATTTTTATTTTCTCTTTCGTATTAAACTGATGGAACAAATGAAAAAAATGG
ATAAAAAAACAAAAAAAAAAAAAAAAACAAAAAAAAAAACAGCTTTGCTGGAGGATGATTAACTTTTAATTT
TTTTAATT

AACATATTATCACGTGCAGCCCAGGATAATTTTCAGGACACGTGTTTCGAAAGGTTTGTCGCTCCGAAAAA
ATCAAAAAAAACAAAAAACGGGAAATAACAATAACGACAAAAATGGAAAAAAAAAAAATTTTAGACGCGGC
GCTTGCAC

CACTACGGGAAACCAAAAAAAAAAAAAAAGAAAACAGTACAAAAAGGTTTACTTTACCCATAGTTAGTATAC
GTAGATTTTATACACGCACTATTTTTCTTATATACAGGAGATGGGTGGCCACAGAACCCGCGCCTAGCCTA
TTTCTTTC

GACAATAAAAAGGAATTTGACAGCACAAAAGAGAAAAAAAAAACTATACATAAAATTTTTTACATATGAAT
TGCTTAGAGTAAAATAGGCATTTTTCATATCTTCTTCCCAATTATTAAATTAGCAAAAAATTGAATAAAAA
AAGCAGGT

ATTTTATTTTTTGAATATTTTTTATTTATATACGTATATATAGACTATTATTTATCTTTTAATGATTATTA
AGATTTTTATTAAAAAAAAATTCGCTCCTCTTTTAATGCCTTTATGCAGTTTTTTTTTCCCATTCGATATT
TCTATGTT

TGAGCACATGTAGTTTTTTTTTTTTTAGTTTTAAATATAAGAAAAAAGATAGTATATATATATATATATAT
ACATATACATATAAAAGGAAAGTAAGCTTATACAATACACAAATAAATTCATATCAAAGAATAATGAACCG
CAATATTC

AAGGTTTGTCGCTCCGAAAAAATCAAAAAAAACAAAAAACGGGAAATAACAATAACGACAAAAATGGAAAA
AAAAAAAATTTTAGACGCGGCGCTTGCACCCCGCATTATAAGTGGTGTGCCGACGGACGGTCAACCCCTTT
CAGGGGGC

ATTATTGATGCTAATACGGGTTTTTTATGAAAAAAAAAGGATAACAAACTTATATAAACTTATATAAAAAC
ATATACAATAAATGATCTACAAAGGGTAGGAAATAAAAAATAAGGGAGAAAAAAAAATACATGCGTAAGTA
CATTAAAA

AGGAATTTGACAGCACAAAAGAGAAAAAAAAAACTATACATAAAATTTTTTACATATGAATTGCTTAGAGT
AAAATAGGCATTTTTCATATCTTCTTCCCAATTATTAAATTAGCAAAAAATTGAATAAAAAAAGCAGGTGG
TTTAAGAA

GTACAATGAATTCAAAACATGAAAATTGTTCAAAGTTTTTATTTTTATTTTCTCTTTCGTATTAAACTGAT
GGAACAAATGAAAAAAATGGATAAAAAAACAAAAAAAAAAAAAAAAACAAAAAAAAAAACAGCTTTGCTGGA
GGATGATT

CCACATTTGCAAACAAAACTTTTCAATAATAATTTTATAAATAGTATCAATATATATATATATATATATAT
TTATTTGTTTACTTTTTCTATCAGTGTTTTCAATTTTTTATTAAACAATGTTTGATTTTTTAAATCGCAAT
TTAATACC

AATTAAAAAAGAATTGTAACCTTATTTAAAAAAAAAAAATAGTACATAACAATAAAAAAAAAAAAAGATAATA
ATTTTGAATTTATTGCTAGACATTCTTACGTTTATTGTGTTCATGTTAACAAAAACACATGCCCGCGGTTA
TTATCGCT

TGCTTTCATCCCTCTGACGAAGGTGGTAGAGAACAAGAAAATGAATAACATTATATCATATTCCTATATAT
ATATATATATATATATATATATCATATAACGGTGGAAAATCCCGGCGATATTACAGATAAACATTACACCC
GCATGAAT

TAGTACAAATAAGTCACATGATGATATTTGATTTTATTATATTTTTAAAAAAAGTAAAAAATAAAAAGTAG
TTTATTTTTAAAAAATAAAATTTAAAATATTAGTGTATTTGATTTCCGAAAGTTAAAAAAGAAATAGTAAG
AAATATAT

ATATTTAATACTAGATGTATATACGTTGTGTGTATGCGCTACTATAGTATAGCTTAACTGTTTTATTATAC
CCTTTTTTTTCTCTCTTTACTGTACAGAAGATGCGTCACTAATTCAAAAAAAAAAAAAAAAAAAAAAAATATA
AAAAAGAA

ATAGTGTCATCACACCTTTATGAGAAGCGAATTTTTTTTTTTTTTTTGGTTTAAATATATATATATACATAT
ATATAGATATAGATATACACATGTATAGATGTATTCTAGTTATGCTTATACCTAGAACTGATCAGACCAGA
TCATACCT

CGTGATCGTCATCCATTGAGCTTTTCTTTCCTCTCTCTTTTTTTTTTTCTTGTTACATATTCCTATATATAT
GTATATATATCTATATATATATATATCCCAGCGTATATGACTGCACAAGACGCAATTTTCTTGAACGGTTT
TTTCTTTT

CTAATACGGGTTTTTTATGAAAAAAAAAAGGATAACAAACTTATATAAACTTATATAAAAACATATACAATA
AATGATCTACAAAGGGTAGGAAATAAAAAATAAGGGAGAAAAAAAAAATACATGCGTAAGTACATTAAAATT
ACAGGAAA

TTTAGAGAAGGGAATCAAAGATCTATAAATTTTATATTCATGAGCACATGTAGTTTTTTTTTTTTTTAGTTT
TAAATATAAGAAAAAAGATAGTATATATATATATATATATACATATACATATAAAAGGAAAGTAAGCTTAT
ACAATACA

TGATGATAATGTACAATGAATTCAAAACATGAAAATTGTTCAAAGTTTTTATTTTTATTTTCTCTTTCGTA
TTAAACTGATGGAACAAATGAAAAAAATGGATAAAAAAACAAAAAAAAAAAAAAAAACAAAAAAAAAAACAG
CTTTGCTG

CACGTGCAGCCCAGGATAATTTTCAGGACACGTGTTTCGAAAGGTTTGTCGCTCCGAAAAAATCAAAAAAA
ACAAAAAAACGGGAAATAACAATAACGACAAAAATGGAAAAAAAAAAAAATTTTAGACGCGGCGCTTGCACCC
CGCATTAT

TTATTGCTTATGATGATAATGTACAATGAATTCAAAACATGAAAATTGTTCAAAGTTTTTATTTTTATTTT
CTCTTTCGTATTAAACTGATGGAACAAATGAAAAAAATGGATAAAAAAACAAAAAAAAAAAAAAAAACAAA
AAAAAAAC

CGTGTTTCGAAAGGTTTGTCGCTCCGAAAAAATCAAAAAAAACAAAAAAACGGGAAATAACAATAACGACAA
AAATGGAAAAAAAAAAAAATTTTAGACGCGGCGCTTGCACCCCGCATTATAAGTGGTGTGCCGACGGACGGT
CAACCCCT

TGTATGCGCTACTATAGTATAGCTTAACTGTTTTATTATACCCTTTTTTTTCTCTCTTTACTGTACAGAAGA
TGCGTCACTAATTCAAAAAAAAAAAAAAAAAAAAAAAATATAAAAAAGAAAGATTTTGGAGAAGCTATATCC
TTTTGTGA

TTCAAAACATGAAAATTGTTCAAAGTTTTTATTTTTATTTTCTCTTTCGTATTAAACTGATGGAACAAATG
AAAAAAATGGATAAAAAAACAAAAAAAAAAAAAAAAACAAAAAAAAAAACAGCTTTGCTGGAGGATGATTAA
CTTTTAAT

ATGATATTTGATTTTATTATATTTTTAAAAAAAGTAAAAAATAAAAAGTAGTTTATTTTTAAAAAAATAAAA
TTTAAAATATTAGTGTATTTGATTTCCGAAAGTTAAAAAAGAAATAGTAAGAAATATATATTTCATTGAAT
GGATATAT

TCACATCATGAAATATAAGCTAAATCGCATTTCTTTTCGTCCACATTTGCAAACAAAACTTTTCAATAATA
ATTTTATAAATAGTATCAATATATATATATATATATATATATTTATTTGTTTACTTTTTCTATCAGTGTTTTC
AATTTTTT

TATATATATATTTATTTGTTTACTTTTTCTATCAGTGTTTTCAATTTTTTATTAAACAATGTTTGATTTTT
TAAATCGCAATTTAATACCTAAATATAAAAAATGTTATTATATTGCAAAAACCCATCAACCTTGAAAAAAA
GTAGAAAC

AAAAAAAACAAAAAAAAAAACAGCTTTGCTGGAGGATGATTAACTTTTAATTTTTTTAATTATTAGGTTTTT
CAATTTTTTATGGTTTTTTGTGTAAAATGCAATGAACGAATCCATTTTTTATTTTGCAAGTGTGAGAGGCC
TTTATAAG

ATTTTATTATATTTTTAAAAAAAGTAAAAAATAAAAAGTAGTTTATTTTTAAAAAATAAAATTTAAAATAT
TAGTGTATTTGATTTCCGAAAGTTAAAAAAGAAATAGTAAGAAATATATATTTCATTGAATGGATATATGA
AACGTTTA

ATACGTTGTGTGTATGCGCTACTATAGTATAGCTTAACTGTTTTATTATACCCTTTTTTTCTCTCTTTACT
GTACAGAAGATGCGTCACTAATTCAAAAAAAAAAAAAAAAAAAAAAAATATAAAAAAGAAAGATTTTGGAGA
AGCTATAT

ACTATAGTATAGCTTAACTGTTTTATTATACCCTTTTTTTCTCTCTTTACTGTACAGAAGATGCGTCACTA
ATTCAAAAAAAAAAAAAAAAAAAAAAAATATAAAAAAGAAAGATTTTGGAGAAGCTATATCCTTTTGTGACA
TACAATAG

TTCTTTTCGTCCACATTTGCAAACAAAACTTTTCAATAATAATTTTATAAATAGTATCAATATATATATAT
ATATATATATTTATTTGTTTACTTTTTCTATCAGTGTTTTCAATTTTTTATTAAACAATGTTTGATTTTTT
AAATCGCA

CTTATTTAAAAAAAAAAAATAGTACATAACAATAAAAAAAAAAAAAGATAATAATTTTGAATTTATTGCTAGA
CATTCTTACGTTTATTGTGTTCATGTTAACAAAAACACATGCCCGCGGTTATTATCGCTTTTTAATTGCTT
TCTTAAAA

TCTTGCGTTACTATGGTGGTATTATTGATGCTAATACGGGTTTTTTATGAAAAAAAAAGGATAACAAACTT
ATATAAACTTATATAAAAACATATACAATAAATGATCTACAAAGGGTAGGAAATAAAAAATAAGGGAGAAA
AAAAAATA

AAGTCACATGATGATATTTGATTTTATTATATTTTTAAAAAAAGTAAAAAATAAAAAGTAGTTTATTTTTA
AAAAATAAAATTTAAAATATTAGTGTATTTGATTTCCGAAAGTTAAAAAAGAAATAGTAAGAAATATATAT
TTCATTGA

TTGAATTGAAATCGATAGATCAATTTTTTTCTTTTCTCTTTCCCCATCCTTTACGCTAAAATAATAGTTTA
TTTTATTTTTTGAATATTTTTTATTTATATACGTATATATAGACTATTATTTATCTTTTAATGATTATTAA
GATTTTTA

TCATATATACTTTTACTGACACCTTTCGTCACTATTTTTTGTTTAACTTTTTTTTTGGAAGTACTAAAATT
ATATGCTCGTAATTATTGAGTATCAAAAAAAAAAAAAAAAAAAAATTGCTGTGACACCCCTTCAATGTGGTG
TCTATACA

CAGCACAAAAGAGAAAAAAAAAAACTATACATAAAATTTTTTACATATGAATTGCTTAGAGTAAAATAGGCA
TTTTTCATATCTTCTTCCCAATTATTAAATTAGCAAAAAATTGAATAAAAAAAGCAGGTGGTTTAAGAATT
GCGCAGGG

ATGTAATTATCTCTCTCCCAACGTTTTAGAAGAGGTTTTCGTCTCCTTCGTCAAAGACGACCACTTTCCCA
TTTTGGTCATTGGAACGCGAAAAAACAAAACAGACGCGAGAACGCGACGCGAAAAAATATATTTTTCCGTT
TTTTTTTT

CCGACAGGGTAACATATTATCACGTGCAGCCCAGGATAATTTTCAGGACACGTGTTTCGAAAGGTTTGTCG
CTCCGAAAAAAATCAAAAAAAAACAAAAAACGGGAAATAACAATAACGACAAAAATGGAAAAAAAAAAAATTT
TAGACGCG

GCGCGTACATGATACGAGACGACAAGATATGCAAAAGATAATAGTGTCATCACACCTTTATGAGAAGCGAA
TTTTTTTTTTTTTTTGGTTTAAATATATATATATACATATATATAGATATAGATATACACATGTATAGATG
TATTCTAG

CTAGATGTATATACGTTGTGTGTATGCGCTACTATAGTATAGCTTAACTGTTTTATTATACCCTTTTTTTC
TCTCTTTACTGTACAGAAGATGCGTCACTAATTCAAAAAAAAAAAAAAAAAAAAAAAATATAAAAAAGAAAG
ATTTTGGA

AAGTCCTAGATAATCATCTTTTGTATTTTTTTTCTTCCTTTTATTAGTATGTATACATATATATTACTTTT
TATATTCTTTATAGCGCATTTTAAACACCTTTCTGAAAAAAATGCTGAGATTTTTGATGCTAATCGAAAGAT
TAAAAAAA

CAAAGTTTTTATTTTTATTTTCTCTTTCGTATTAAACTGATGGAACAAATGAAAAAAATGGATAAAAAAAC
AAAAAAAAAAAAAAAAACAAAAAAAAAACAGCTTTGCTGGAGGATGATTAACTTTTAATTTTTTTAATTAT
TAGGTTTT

AGGTGGTAGAGAACAAGAAAATGAATAACATTATATCATATTCCTATATATATATATATATATATATATAT
ATCATATAACGGTGGAAAATCCCGGCGATATTACAGATAAACATTACACCCGCATGAATGTGAGCCACTAC
TATATTAT

AGCCACTTAGACAGAGAGAAAACGAAGTGAGAAGAGGCTAGTATTTTTATATCTTAAAAAAAAAAAAAAAC
AAAAAAAAAAAAACATAAATATTTGTATCTTATTGCTTATGATGATAATGTACAATGAATTCAAAACATGA
AAATTGTT

TCGAGCATACATTACCTTACGTGTGTTAGTGTACTATATTATATATATATATATGTATATATATAAAGGGA
GGAGTTTTTAATTATAATTGTAATTTCGTATTTTTTCTGCATTATACAGTTTTTTCCGATTTTAAACGACT
TTATTTAA

TAAACATATAAAAGTACGATTAGTTGGTGTGGTTATATAAGTCATAAAATAATTATCATATTAAAAAATTG
AAAAATAAAAAACTACTCTTTTTTATTTCAATAGTTCTCGTTATTAGTAGGTCGTGCTCTTAAAAGATTAC
CCTTTCAG

AGCTTAACTGTTTTATTATACCCTTTTTTTCTCTCTTTACTGTACAGAAGATGCGTCACTAATTCAAAAAA
AAAAAAAAAAAAAAAAATATAAAAAAGAAAGATTTTGGAGAAGCTATATCCTTTTGTGACATACAATAGAG
AAACTTGT

AGAGTAGAAAATTTTCATAACGAATCTCTTCTATTCTTATGTACCGTTCCGCCAAATTTTTCATGAAAAAA
TTTGGGAAAAAGAAAAAAAAAAAAAAATAGTATATAAAGTAAAAAAGATGCGCAAGCCCGGAATCGAACCGG
GGGCCCAA

GCTCCGAAAAAATCAAAAAAAAACAAAAAACGGGAAATAACAATAACGACAAAAATGGAAAAAAAAAAAATT
TTAGACGCGGCGCTTGCACCCCGCATTATAAGTGGTGTGCCGACGGACGGTCAACCCCTTTCAGGGGGCCC
CCTATCTT

GAGAAAAAAAAAACTATACATAAAATTTTTTACATATGAATTGCTTAGAGTAAAATAGGCATTTTTCATAT
CTTCTTCCCAATTATTAAATTAGCAAAAAATTGAATAAAAAAAGCAGGTGGTTTAAGAATTGCGCAGGGAG
AAGAAAGA

ATAATTACTAATATTTAATACTAGATGTATATACGTTGTGTGTATGCGCTACTATAGTATAGCTTAACTGT
TTTATTATACCCTTTTTTTCTCTCTTTACTGTACAGAAGATGCGTCACTAATTCAAAAAAAAAAAAAAAAA
AAAAAATA

GGTCTTAGATGACAATAAAAAGGAATTTGACAGCACAAAAGAGAAAAAAAAAACTATACATAAAATTTTTT
ACATATGAATTGCTTAGAGTAAAATAGGCATTTTTCATATCTTCTTCCCAATTATTAAATTAGCAAAAAAT
TGAATAAA

AGGGAACACTTCTAATTTATCTTTAAAATTATATATATATATATATATATATGTGTGTTTGTATACTCTGT
GGGTATTTCCGTGTATATGGTTAATAATAGTAGTATCTTGTCAGTTTTTTTATGTTTTTCTTCGCGCGTC
AACTTTCT

GAAAATAAAAAAGCCAATGAGAACAATATTTTGTGTCGTTTTTTTTATTTTCGCTTCAATTCTCTATACAC
GTACGTCATCACTACGGGAAACCAAAAAAAAAAAAAAGAAAACAGTACAAAAAGGTTTACTTTACCCATAG
TTAGTATA

ATTTTTAAAAAAAGTAAAAAATAAAAAGTAGTTTATTTTTAAAAAATAAAATTTAAAATATTAGTGTATTT
GATTTCCGAAAGTTAAAAAAGAAATAGTAAGAAATATATATTTCATTGAATGGATATATGAAACGTTTACT
GGTGGAAG

TCCAATGGTTGGTGAAACTCTCGAGCATACATTACCTTACGTGTGTTAGTGTACTATATTATATATATATA
TATGTATATATATAAAGGGAGGAGTTTTAATTATAATTGTAATTTCGTATTTTTTCTGCATTATACAGTT
TTTTCCGA

AAACAAAACTTTTCAATAATAATTTTATAAATAGTATCAATATATATATATATATATATATATTTATTTGTTT
ACTTTTTCTATCAGTGTTTTCAATTTTTTATTAAACAATGTTTGATTTTTTAAATCGCAATTTAATACCTA
AATATAAA

TTCCATGTTCTCATATATACTTTTACTGACACCTTTCGTCACTATTTTTTGTTTAACTTTTTTTTTGGAAG
TACTAAAATTATATGCTCGTAATTATTGAGTATCAAAAAAAAAAAAAAAAAAAAATTGCTGTGACACCCCTT
CAATGTGG

ATCCATTGAGCTTTTCTTTCCTCTCTCTTTTTTTTTTCTTGTTACATATTCCTATATATATGTATATATAT
CTATATATATATATATCCCAGCGTATATGACTGCACAAGACGCAATTTTCTTGAACGGTTTTTTCTTTTCA
GCACTGCG

TTTTTTATGAAAAAAAAAGGATAACAAACTTATATAAACTTATATAAAAACATATACAATAAATGATCTAC
AAAGGGTAGGAAATAAAAAATAAGGGAGAAAAAAAAATACATGCGTAAGTACATTAAAATTACAGGAAAAA
TCTTACTT

CTTTAAAATTATATATATATATATATATATATATGTGTGTTTGTATACTCTGTGGGTATTTCCGTGTATATGG
TTAATAATAGTAGTATCTTGTCAGTTTTTTTTATGTTTTTCTTCGCGCGTCAACTTTCTACCAAGAGAAAA
ACAATATA

ACCCTACTGCTTTAGAGAAGGGAATCAAAGATCTATAAATTTTATATTCATGAGCACATGTAGTTTTTTTT
TTTTTAGTTTTAAATATAAGAAAAAAGATAGTATATATATATATATATATACATATACATATAAAAGGAAA
GTAAGCTT

GACAAGATATGCAAAAGATAATAGTGTCATCACACCTTTATGAGAAGCGAATTTTTTTTTTTTTTTGGTTT
AAATATATATATATACATATATATAGATATAGATATACACATGTATAGATGTATTCTAGTTATGCTTATAC
CTAGAACT

TTTTTTATTTTCGCTTCAATTCTCTATACACGTACGTCATCACTACGGGAAACCAAAAAAAAAAAAAAGAA
AACAGTACAAAAAGGTTTACTTTACCCATAGTTAGTATACGTAGATTTTATACACGCACTATTTTTCTTAT
ATACAGGA

GGAATGGGCGTCCGCTCCACCGTGGTCAAAGACAGGGGCAAAGAGCTCCTAGGTCTATATATATATCTATA
TACATATTTATATATATTATTAGAACTTTACAATATAGTATATACCATTCATTGTTTAAGTTTCGGGTAAT
ACTTTTTT

ACAGAGAGAAAACGAAGTGAGAAGAGGCTAGTATTTTTATATCTTAAAAAAAAAAAAAAACAAAAAAAAAA
AAACATAAATATTTGTATCTTATTGCTTATGATGATAATGTACAATGAATTCAAAACATGAAAATTGTTCA
AAGTTTTT

GTGTAAAATAGAAAATAAAAAAGCCAATGAGAACAATATTTTGTGTCGTTTTTTTTATTTTCGCTTCAATT
CTCTATACACGTACGTCATCACTACGGGAAACCAAAAAAAAAAAAAAGAAAACAGTACAAAAAGGTTTACT
TTACCCAT

TGAAAAAAAAAATGTGAAAGAGAGTAGAAAATTTTCATAACGAATCTCTTCTATTCTTATGTACCGTTCCG
CCAAATTTTTCATGAAAAAATTTGGGAAAAAGAAAAAAAAAAAAAAATAGTATATAAAGTAAAAAAGATGCG
CAAGCCCG

AATGATATCCGGCTTTTCCTTATAGGGAATAGTGGTGAAAGTTACGTAAATATATACATATAGAGCGGAAT
GGTACTGCATATTTTTATTATTTTTTTTTTTGAATATATATAAAGCATCGTCGCTTAAGACTAAACCTTCG
AGGATCTG

AAACTTACACGAATAGGATAACATGTATGCTAGCAGAATATATATGAAAGAAAAAAATTATTGATGCCTTT
AAACTTATACTATTATACTATATTATGTTATATTATATTATTAGTTTTATAGATATATTGAGATATGTTGA
ATATGATG

ATTACCAATTAGACATGCTGCTTGCAACAAGAAAATGCACGCGTAACAAAATATATATATATATATATATA
TATATGTATGTCCATACGGGTTTTTCGTTTTGTTGTGGTTGTAACAGCACAACAAAATGCTACACGGTGGC
AAATTGGA

ACAATATGGAAAATCCACAGAAAGCTATTCATTGAAAAAATAGTACAAATAAGTCACATGATGATATTTGA
TTTTATTATATTTTTAAAAAAAGTAAAAAATAAAAAGTAGTTTATTTTTAAAAAATAAAATTTAAAATATT
AGTGTATT

CTATGGTGGTATTATTGATGCTAATACGGGTTTTTTATGAAAAAAAAAGGATAACAAACTTATATAAACTT
ATATAAAAACATATACAATAAATGATCTACAAAGGGTAGGAAATAAAAAATAAGGGAGAAAAAAAAAATACA
TGCGTAAG

CTCTCTTTACTGTACAGAAGATGCGTCACTAATTCAAAAAAAAAAAAAAAAAAAAAAAATATAAAAAAGAAA
GATTTTGGAGAAGCTATATCCTTTTGTGACATACAATAGAGAAACTTGTTCTGTTGGTAAGATATCTCATC
TGAAATAC

TCGCTTCAATTCTCTATACACGTACGTCATCACTACGGGAAACCAAAAAAAAAAAAAAAGAAAACAGTACAA
AAAGGTTTACTTTACCCATAGTTAGTATACGTAGATTTTATACACGCACTATTTTTCTTATATACAGGAGA
TGGGTGGC

ACACCCCCCTGGGTGGAAACAAGTCCTAGATAATCATCTTTTGTATTTTTTTTCTTCCTTTTATTAGTATG
TATACATATATATTACTTTTTATATTCTTTATAGCGCATTTTAAACACCTTTCTGAAAAAATGCTGAGATT
TTTGATGC

GCTTATTTTTTTAATTCTTATGAAAAAAAAAATGTGAAAGAGAGTAGAAAATTTTCATAACGAATCTCTTC
TATTCTTATGTACCGTTCCGCCAAATTTTTCATGAAAAAATTTGGGAAAAAGAAAAAAAAAAAAAAATAGTA
TATAAAGT

TCAAATTGGTAAATCCCATTAATTAAAAAAGAATTGTAACCTTATTTAAAAAAAAAAAATAGTACATAACAA
TAAAAAAAAAAAAGATAATAATTTTGAATTTATTGCTAGACATTCTTACGTTTATTGTGTTCATGTTAACA
AAAACACA

TCAGCGCCAAACAATATGGAAAATCCACAGAAAGCTATTCATTGAAAAAATAGTACAAATAAGTCACATGA
TGATATTTGATTTTATTATATTTTTAAAAAAAGTAAAAAATAAAAAGTAGTTTATTTTTAAAAAATAAAAT
TTAAAATA

TCCGCTCCACCGTGGTCAAAGACAGGGGCAAAGAGCTCCTAGGTCTATATATATATCTATATACATATTTA
TATATATTATTAGAACTTTACAATATAGTATATACCATTCATTGTTTAAGTTTCGGGTAATACTTTTTTTT
TCCTTGTC

TGATTCACAACCATTTTAGATGTCTGCAGCTTTTTTTTTTTTTGATTTTTTTACTAATTTTAGTGCGATTTT
AGAATAACTCTTGGATTAAATAAATAAAAAAAAATACAATTATCACTCAGATTTTGAAGCAATCTGATAAAA
ATTGCAGC

TAGCAGAATATATATGAAAGAAAAAAATTATTGATGCCTTTAAACTTATACTATTATACTATATTATGTTA
TATTATATTATTAGTTTTATAGATATATTGAGATATGTTGAATATGATGACGAGGATGGTGGTTTAAGTGT
ATGGATTG

AACGAAGTGAGAAGAGGCTAGTATTTTTATATCTTAAAAAAAAAAAAAAACAAAAAAAAAAAAACATAAAT
ATTTGTATCTTATTGCTTATGATGATAATGTACAATGAATTCAAAACATGAAAATTGTTCAAAGTTTTTAT
TTTTATTT

TCATGATGGTAGCCACTTAGACAGAGAGAAAACGAAGTGAGAAGAGGCTAGTATTTTTATATCTTAAAAAA
AAAAAAAAACAAAAAAAAAAAAACATAAATATTTGTATCTTATTGCTTATGATGATAATGTACAATGAATT
CAAAACAT

ATTGGAGCTAACCCTACTGCTTTAGAGAAGGGAATCAAAGATCTATAAATTTTATATTCATGAGCACATGT
AGTTTTTTTTTTTTTAGTTTTAAATATAAGAAAAAAGATAGTATATATATATATATATATACATATACATA
TAAAAGGA

ATATCATATCTCATGATGGTAGCCACTTAGACAGAGAGAAAACGAAGTGAGAAGAGGCTAGTATTTTTATA
TCTTAAAAAAAAAAAAAAACAAAAAAAAAAAAACATAAATATTTGTATCTTATTGCTTATGATGATAATGT
ACAATGAA

TGGCTGATTACGGATCACGTTCAAATTGGTAAATCCCATTAATTAAAAAAGAATTGTAACCTTATTTAAAA
AAAAAAATAGTACATAACAATAAAAAAAAAAAAGATAATAATTTTGAATTTATTGCTAGACATTCTTACGT
TTATTGTG

AGGGAGGGATAATCGATCCCCAAATGGACAATACGATGTAACACCGTTATATAATTAATAAATGTATATAT
GTAACTATTTTTTGAAAAAAATAAAATGGTAATTTATTAAATATTTGGACCGGTCAACAATCTCAGATATA
GTTGAAAA

GAAGAGGCTAGTATTTTTATATCTTAAAAAAAAAAAAAAACAAAAAAAAAAAAACATAAATATTTGTATCT
TATTGCTTATGATGATAATGTACAATGAATTCAAAACATGAAAATTGTTCAAAGTTTTTATTTTTATTTTC
TCTTTCGT

CGGATCACGTTCAAATTGGTAAATCCCATTAATTAAAAAAGAATTGTAACCTTATTTAAAAAAAAAAATAG
TACATAACAATAAAAAAAAAAAAGATAATAATTTTGAATTTATTGCTAGACATTCTTACGTTTATTGTGTT
CATGTTAA

ATTTTCATAACGAATCTCTTCTATTCTTATGTACCGTTCCGCCAAATTTTTCATGAAAAAATTTGGAAAA
AGAAAAAAAAAAAAAATAGTATATAAAGTAAAAAAGATGCGCAAGCCCGGAATCGAACCGGGGGCCCAACG
ATGGCAAC

AAATCCACAGAAAGCTATTCATTGAAAAAATAGTACAAATAAGTCACATGATGATATTTGATTTTATTATA
TTTTTAAAAAAAGTAAAAAATAAAAAGTAGTTTATTTTTAAAAAATAAAATTTAAAATATTAGTGTATTTG
ATTTCCGA

AATTCAAAAAAAAAAAAAAAAAAAAAAATATAAAAAAGAAAGATTTTGGAGAAGCTATATCCTTTTGTGAC
ATACAATAGAGAAACTTGTTCTGTTGGTAAGATATCTCATCTGAAATACAACAAGGAAAACCAAAAGCCAT
TTTTTCAT

AAAAAAAAACAGCTTTGCTGGAGGATGATTAACTTTTAATTTTTTTAATTATTAGGTTTTTCAATTTTTTA
TGGTTTTTTGTGTAAAATGCAATGAACGAATCCATTTTTTATTTTGCAAGTGTGAGAGGCCTTTATAAGGA
AGACTCGG

AATAAAAAAAAGGGAGGGATAATCGATCCCCAAATGGACAATACGATGTAACACCGTTATATAATTAATAA
ATGTATATATGTAACTATTTTTTGAAAAAAATAAAATGGTAATTTATTAAATATTTGGACCGGTCAACAAT
CTCAGATA

TTGAATATTTTTTATTTATATACGTATATATAGACTATTATTTATCTTTTAATGATTATTAAGATTTTTAT
TAAAAAAAAATTCGCTCCTCTTTTAATGCCTTTATGCAGTTTTTTTTTCCCATTCGATATTTCTATGTTCG
GGTTCAGC

GATACGAGACGACAAGATATGCAAAAGATAATAGTGTCATCACACCTTTATGAGAAGCGAATTTTTTTTTT
TTTTTGGTTTAAATATATATATATACATATATATAGATATAGATATACACATGTATAGATGTATTCTAGTT
ATGCTTAT

CAGCTTTAAAATTACCAATTAGACATGCTGCTTGCAACAAGAAAATGCACGCGTAACAAAATATATATATA
TATATATATATATATGTATGTCCATACGGGTTTTTCGTTTTGTTGTGGTTGTAACAGCACAACAAAATGCT
ACACGGTG

TATATAAAAACATATACAATAAATGATCTACAAAGGGTAGGAAATAAAAAATAAGGGAGAAAAAAAAAATAC
ATGCGTAAGTACATTAAAATTACAGGAAAAATCTTACTTTAGTTCTGATATTTCACCATCGCGATACACCA
ACCCCATA

TTCTACTTTTATATCCCGTTTGGCTGATTACGGATCACGTTCAAATTGGTAAATCCCATTAATTAAAAAAG
AATTGTAACCTTATTTAAAAAAAAAAAATAGTACATAACAATAAAAAAAAAAAAAGATAATAATTTTGAATTT
ATTGCTAG

TTGTGTCGTTTTTTTTATTTTCGCTTCAATTCTCTATACACGTACGTCATCACTACGGGAAACCAAAAAAA
AAAAAAAGAAAACAGTACAAAAAGGTTTACTTTACCCATAGTTAGTATACGTAGATTTTATACACGCACTA
TTTTTCTT

GTCTTCCAGGGAAAAAAAAAAAAAAAAAAAAAAGAAAGCCAAAATAAGGAGCCTTGAAATGTGTATCTGATAT
ATATAATGTGTATGTAAATATCTATTTAATTAGCCATTGGGATTTCAACTTCTTGTTTGAAACAGAAGGAC
GGATAAAA

CTTTTCTTTCCTCTCTCTTTTTTTTTTTCTTGTTACATATTCCTATATATATGTATATATATCTATATATAT
ATATATCCCAGCGTATATGACTGCACAAGACGCAATTTTCTTGAACGGTTTTTTCTTTTCAGCACTGCGAA
AGAGAGAG

AATGTGAAAGAGAGTAGAAAATTTTCATAACGAATCTCTTCTATTCTTATGTACCGTTCCGCCAAATTTTT
CATGAAAAAATTTGGGAAAAAGAAAAAAAAAAAAAAATAGTATATAAAGTAAAAAAGATGCGCAAGCCCGGA
ATCGAACC

AGACATGCTGCTTGCAACAAGAAAATGCACGCGTAACAAAATATATATATATATATATATATATATGTATG
TCCATACGGGTTTTTCGTTTTGTTGTGGTTGTAACAGCACAACAAAATGCTACACGGTGGCAAATTGGAAA
AAGAGACT

TATTTAGAGATGCTTTCATCCCTCTGACGAAGGTGGTAGAGAACAAGAAAATGAATAACATTATATCATAT
TCCTATATATATATATATATATATATATATATATCATATAACGGTGGAAAATCCCGGCGATATTACAGATAAA
CATTACAC

AATCGATCCCCAAATGGACAATACGATGTAACACCGTTATATAATTAATAAATGTATATATGTAACTATTT
TTTGAAAAAAATAAAATGGTAATTTATTAAATATTTGGACCGGTCAACAATCTCAGATATAGTTGAAAAAA
TCTTTAAT

GAACTTCCATAGACATTGAAAAATAACGAAAGAATAAAAAAAAAATCTTTTTTTTTTTTGATATAAATAC
AGTTGACTATATAAACATATATCAATACTATAAGCCTATATTTGATCAAGACGACATTATTTAGGTGCATC
AACAACGG

CTTTACTAATGAACTTCCATAGACATTGAAAAATAACGAAAGAATAAAAAAAAAATCTTTTTTTTTTTTTG
ATATAAATACAGTTGACTATATAAACATATATCAATACTATAAGCCTATATTTGATCAAGACGACATTATT
TAGGTGCA

CGTGGTCAAAGACAGGGGCAAAGAGCTCCTAGGTCTATATATATATCTATATACATATTTATATATATTAT
TAGAACTTTACAATATAGTATATACCATTCATTGTTTAAGTTTCGGGTAATACTTTTTTTTTCCTTGTCAT
AACCCCAA

CTTTAATACATTCCATGTTCTCATATATACTTTTACTGACACCTTTCGTCACTATTTTTTGTTTAACTTTT
TTTTTGGAAGTACTAAAATTATATGCTCGTAATTATTGAGTATCAAAAAAAAAAAAAAAAAAAATTGCTGT
GACACCCC

GTCTAGCACTTGAGTATACCTCTATATGCTGTGTAAAATAGAAAATAAAAAAGCCAATGAGAACAATATTT
TGTGTCGTTTTTTTTATTTTCGCTTCAATTCTCTATACACGTACGTCATCACTACGGGAAACCAAAAAAAA
AAAAAAGA

TTAATTCTTATGAAAAAAAAAAATGTGAAAGAGAGTAGAAAATTTTCATAACGAATCTCTTCTATTCTTATG
TACCGTTCCGCCAAATTTTTCATGAAAAAATTTGGGAAAAAGAAAAAAAAAAAAAAATAGTATATAAAGTAA
AAAAGATG

GATGTATGTTTTTCTTTATGTAGAGCTTGGCAGCTTTAAAATTACCAATTAGACATGCTGCTTGCAACAAG
AAAATGCACGCGTAACAAAATATATATATATATATATATATATATGTATGTCCATACGGGTTTTTCGTTTT
GTTGTGGT

CCATTTTAGATGTCTGCAGCTTTTTTTTTTTTGATTTTTTTACTAATTTTAGTGCGATTTTAGAATAACTC
TTGGATTAAATAAATAAAAAAAAATACAATTATCACTCAGATTTTGAAGCAATCTGATAAAAATTGCAGCAT
ATTATTAT

CCACGGCCTAATCTCTGTAAGGATTGTATTTTATTTTTTATTTATTTATTATTTAGATTTTCTAAAAATAA
AAAAAAATCATACACTATTTTGATTACACTACTATGTAATTTATCTTTGAAAACAACCATGAAAAACTCAA
TTTTACAC

AAGTCAAAAAAATAGTTTTTGGGATATATCTTGTGATCAAATGCTTTGCAAAAGGGTAAAACAGCGTTTACA
ATAGGATAGCCAAAAGTAAAAAAAAAAAAAAAAAAAAAAAAAGAAGGAAGTTAGTATCGAGCTCAAACAATTT
TAGTTTTG

TTTTTCTTTGTTCTTTACGTTTGATTCTATTTCAACCATAATTTATTTTTTCGTTATTTAATAATTACTAA
TATTTAATACTAGATGTATATACGTTGTGTGTATGCGCTACTATAGTATAGCTTAACTGTTTTATTATACC
CTTTTTTT

TGAGAAGCGAATTTTTTTTTTTTTTTGGTTTAAATATATATATATACATATATATAGATATAGATATACAC
ATGTATAGATGTATTCTAGTTATGCTTATACCTAGAACTGATCAGACCAGATCATACCTGGTATAGAGCTT
AATCGATA

ACTCCACACTCCACGGCCTAATCTCTGTAAGGATTGTATTTTATTTTTTATTTATTTATTATTTAGATTTT
CTAAAAATAAAAAAAAATCATACACTATTTTGATTACACTACTATGTAATTTATCTTTGAAAACAACCATG
AAAAACTC

ATATCCCGTTTGGCTGATTACGGATCACGTTCAAATTGGTAAATCCCATTAATTAAAAAAGAATTGTAACC
TTATTTAAAAAAAAAAAATAGTACATAACAATAAAAAAAAAAAAAGATAATAATTTTGAATTTATTGCTAGAC
ATTCTTAC

TCCGCAAGAATGATTCACAACCATTTTAGATGTCTGCAGCTTTTTTTTTTTTGATTTTTTTACTAATTTTA
GTGCGATTTTAGAATAACTCTTGGATTAAATAAATAAAAAAAAATACAATTATCACTCAGATTTTGAAGCAA
TCTGATAA

TAATCATCTTTTGTATTTTTTTTTCTTCCTTTTATTAGTATGTATACATATATATTACTTTTTATATTCTTT
ATAGCGCATTTTAAACACCTTTCTGAAAAAATGCTGAGATTTTTGATGCTAATCGAAAGATTAAAAAAAGG
TCTATAGA

CCATTCTATTTAAACATATAAAAGTACGATTAGTTGGTGTGGTTATATAAGTCATAAAATAATTATCATAT
TAAAAAATTGAAAAATAAAAAACTACTCTTTTTTATTTCAATAGTTCTCGTTATTAGTAGGTCGTGCTCTT
AAAAGATT

CGAGTATGACGTCTTCCAGGGAAAAAAAAAAAAAAAAAAAAGAAAGCCAAAATAAGGAGCCTTGAAATGTG
TATCTGATATATATAATGTGTATGTAAATATCTATTTAATTAGCCATTGGGATTTCAACTTCTTGTTTGAA
ACAGAAGG

CGAAAAAATATATTTTTCCGTTTTTTTTTTTTTTCGGAACAAAGATAACGCGTTAAAACCTGTGGATGTAA
TAAGGATCTGAAACACTGACTAAAACGCGTCCTCATTTAAAATCAAAAAATTACTTCTTCTCCTCCCTTAC
GGTGCCCT

TTTTTTTGGTTTGTGTACGTATCCCACCGTACTTACCATCTTCTCTCCTTTATATATATATATATATATGT
ATATTTTCAGTGTATATACATACATTCTTATACAATACCGTATAAGAACGTATGTATGTATGTATGATTTT
CTCACATT

AAAAAAAATAGTACATAACAATAAAAAAAAAAAAGATAATAATTTTGAATTTATTGCTAGACATTCTTACG
TTTATTGTGTTCATGTTAACAAAAACACATGCCCGCGGTTATTATCGCTTTTTAATTGCTTTCTTAAAAAG
AGTCGGTT

CTCATATGCAGCGCGTACATGATACGAGACGACAAGATATGCAAAAGATAATAGTGTCATCACACCTTTAT
GAGAAGCGAATTTTTTTTTTTTTTTGGTTTAAATATATATATATACATATATATAGATATAGATATACACA
TGTATAGA

AGACATTGAAAAATAACGAAAGAATAAAAAAAAAATCTTTTTTTTTTTTTGATATAAATACAGTTGACTAT
ATAAACATATATCAATACTATAAGCCTATATTTGATCAAGACGACATTATTTAGGTGCATCAACAACGGAA
AAGAAACT

GAATAGGATAACATGTATGCTAGCAGAATATATATGAAAGAAAAAAATTATTGATGCCTTTAAACTTATAC
TATTATACTATATTATGTTATATTATATTATTAGTTTTATAGATATATTGAGATATGTTGAATATGATGAC
GAGGATGG

TCGTATTTGCAATTTTTGGAAATTAATTGTGTAGCTTTTCTTCTCTTAATTGGTGGCAGTTTTTATATATT
CAGTTTAGAATACCCTTTTTTTTTTTTTTTTTTTCTTCGCTTTTCGCATCTCATCGCTGGAAGTGGTGCTC
TTTTACAG

TATATATATATATATATATATTTATTTGTTTACTTTTTCTATCAGTGTTTTCAATTTTTTATTAAACAATG
TTTGATTTTTTAAATCGCAATTTAATACCTAAATATAAAAAATGTTATTATATTGCAAAAACCCATCAACC
TTGAAAAA

AAAGTACGATTAGTTGGTGTGGTTATATAAGTCATAAAATAATTATCATATTAAAAAATTGAAAAATAAAA
AACTACTCTTTTTTATTTCAATAGTTCTCGTTATTAGTAGGTCGTGCTCTTAAAAGATTACCCTTTCAGTA
GATGGTAA

TCTATATGCTGTGTAAAATAGAAAATAAAAAAGCCAATGAGAACAATATTTTGTGTCGTTTTTTTTATTTT
CGCTTCAATTCTCTATACACGTACGTCATCACTACGGGAAACCAAAAAAAAAAAAAAGAAAACAGTACAAA
AAGGTTTA

GAACAATATTTTGTGTCGTTTTTTTTATTTTCGCTTCAATTCTCTATACACGTACGTCATCACTACGGGAA
ACCAAAAAAAAAAAAAAGAAAACAGTACAAAAAGGTTTACTTTACCCATAGTTAGTATACGTAGATTTTAT
ACACGCAC

CGAATCTCTTCTATTCTTATGTACCGTTCCGCCAAATTTTTCATGAAAAAATTTGGGAAAAAGAAAAAAAA
AAAAAATAGTATATAAAGTAAAAAAGATGCGCAAGCCCGGAATCGAACCGGGGGCCCAACGATGGCAACGT
TGGATTTT

GTATTTTTATATCTTAAAAAAAAAAAAAAACAAAAAAAAAAAAACATAAATATTTGTATCTTATTGCTTAT
GATGATAATGTACAATGAATTCAAAACATGAAAATTGTTCAAAGTTTTATTTTTATTTTCTCTTTCGTAT
TAAACTGA

GTTTTGGACAAATAAAAAAAAGGGAGGGATAATCGATCCCCAAATGGACAATACGATGTAACACCGTTATA
TAATTAATAAATGTATATATGTAACTATTTTTTGAAAAAAATAAAATGGTAATTTATTAAATATTTGGACC
GGTCAACA

TTCTTTTCCATCCAATGGTTGGTGAAACTCTCGAGCATACATTACCTTACGTGTGTTAGTGTACTATATTA
TATATATATATATGTATATATATATAAAGGGAGGAGTTTTTAATTATAATTGTAATTTCGTATTTTTTTCTGCA
TTATACAG

GAACAAGAAAATGAATAACATTATATCATATTCCTATATATATATATATATATATATATATATCATATAAC
GGTGGAAAATCCCGGCGATATTACAGATAAACATTACACCCGCATGAATGTGAGCCACTACTATATTATAA
CTTAGTGA

GACAGGGGCAAAGAGCTCCTAGGTCTATATATATATCTATATACATATTTATATATATTATTAGAACTTTA
CAATATAGTATATACCATTCATTGTTTAAGTTTCGGGTAATACTTTTTTTTTCCTTGTCATAACCCCAAAA
ATTTTCGA

ATTAAACTAAACACCCCCCTGGGTGGAAACAAGTCCTAGATAATCATCTTTTGTATTTTTTTCTTCCTTT
TATTAGTATGTATACATATATATTACTTTTATATTCTTTATAGCGCATTTTAAACACCTTTCTGAAAAAA
TGCTGAGA

TTTATTTGTTTACTTTTTCTATCAGTGTTTTCAATTTTTTATTAAACAATGTTTGATTTTTTAAATCGCAA
TTTAATACCTAAATATAAAAAATGTTATTATATTGCAAAAACCCATCAACCTTGAAAAAAAGTAGAAACGT
TTTATTTA

GAAAAAAAAAAAAAAAAAAAAGAAAGCCAAAATAAGGAGCCTTGAAATGTGTATCTGATATATATAATGTG
TATGTAAATATCTATTTAATTAGCCATTGGGATTTCAACTTCTTGTTTGAAACAGAAGGACGGATAAAATA
CGTGATCA

TTTCAATAATAATTTTATAAATAGTATCAATATATATATATATATATATATTTATTTGTTTACTTTTTCTA
TCAGTGTTTTCAATTTTTTATTAAACAATGTTTGATTTTTTAAATCGCAATTTAATACCTAAATATAAAAA
ATGTTATT

CTCTTCATACTCGTATTTGCAATTTTTGGAAATTAATTGTGTAGCTTTTCTTCTCTTAATTGGTGGCAGTT
TTTATATATTCAGTTTAGAATACCCTTTTTTTTTTTTTTTTTTTCTTCGCTTTTCGCATCTCATCGCTGGA
AGTGGTGC

GGCTTTTCCTTATAGGGAATAGTGGTGAAAGTTACGTAAATATATACATATAGAGCGGAATGGTACTGCAT
ATTTTTATTATTTTTTTTTTTGAATATATATAAAGCATCGTCGCTTAAGACTAAACCTTCGAGGATCTGAG
GCCAGCGC

CCGGTCCTGAGAAAAGAGAAAGGAACACTTCTAATTTATCTTTAAAATTATATATATATATATATATATA
TGTGTGTTTGTATACTCTGTGGGTATTTCCGTGTATATGGTTAATAATAGTAGTATCTTGTCAGTTTTTTT
TATGTTTT

ATAGTTTTTGGGATATATCTTGTGATCAAATGCTTTGCAAAAGGGTAAAACAGCGTTTACAATAGGATAGC
CAAAAGTAAAAAAAAAAAAAAAAAAAAAAAGAAGGAAGTTAGTATCGAGCTCAAACAATTTTAGTTTTGCT
GGACATAT

AGCGCGCAACTGAAAAGATTTCTGATGGTAACAATACTACGGATAGCAATCAAAAACATCATCCTTATCAT
AATCACCCTTCCAACGATAGCGGTAATGAAAAGAATTGACTTTTTTTTTTTTTTTTTTTTCATTTCTTTTC
CTCACCGA

TCTGTTTGGAAGCGCGCAACTGAAAAGATTTCTGATGGTAACAATACTACGGATAGCAATCAAAAACATCA
TCCTTATCATAATCACCCTTCCAACGATAGCGGTAATGAAAAGAATTGACTTTTTTTTTTTTTTTTTTCA
TTTTCTTT

TATTTAAACCATGCATGTGTGTACATACATACATACACACATACATACATACATACATATATTTATGTATA
TTTATGTATATATATATATATATATATATGCGTAATTATGCAGATTTCTCCTTGTGTGCTACAGCGGGTAC
TCTGACGC

ACATGTATGCTAGCAGAATATATATGAAAGAAAAAAATTATTGATGCCTTTAAACTTATACTATTATACTA
TATTATGTTATATTATATTATTAGTTTTATAGATATATTGAGATATGTTGAATATGATGACGAGGATGGTG
GTTTAAGT

AGAGACATGCCTTTACTAATGAACTTCCATAGACATTGAAAAATAACGAAAGAATAAAAAAAAAATCTTTT
TTTTTTTTGATATAAATACAGTTGACTATATAAACATATATCAATACTATAAGCCTATATTTGATCAAGA
CGACATTA

TAGAGCTTGGCAGCTTTAAAATTACCAATTAGACATGCTGCTTGCAACAAGAAAATGCACGCGTAACAAAA
TATATATATATATATATATATATATGTATGTCCATACGGGTTTTTCGTTTTGTTGTGGTTGTAACAGCACA
ACAAAATG

TATATAACCACGAGTATGACGTCTTCCAGGGAAAAAAAAAAAAAAAAAAAAAGAAAGCCAAAATAAGGAGCC
TTGAAATGTGTATCTGATATATATAATGTGTATGTAAATATCTATTTAATTAGCCATTGGGATTTCAACTT
CTTGTTTG

AAATAACGAAAGAATAAAAAAAAAATCTTTTTTTTTTTTGATATAAATACAGTTGACTATATAAACATAT
ATCAATACTATAAGCCTATATTTGATCAAGACGACATTATTTAGGTGCATCAACAACGGAAAAGAAACTTT
CTTCTAGG

ATGCGTCACTAATTCAAAAAAAAAAAAAAAAAAAAAAAAATATAAAAAAGAAAGATTTTGGAGAAGCTATATC
CTTTTGTGACATACAATAGAGAAACTTGTTCTGTTGGTAAGATATCTCATCTGAAATACAACAAGGAAAAC
CAAAAGCC

GGGTGGAAACAAGTCCTAGATAATCATCTTTTGTATTTTTTTTCTTCCTTTTATTAGTATGTATACATATA
TATTACTTTTTATATTCTTTATAGCGCATTTTAAACACCTTTCTGAAAAAATGCTGAGATTTTTGATGCTA
ATCGAAAG

ATTGTTTAACTCTTGCGTTACTATGGTGGTATTATTGATGCTAATACGGGTTTTTTATGAAAAAAAAAGGA
TAACAAACTTATATAAACTTATATAAAAACATATACAATAAATGATCTACAAAGGGTAGGAAATAAAAAAT
AAGGGAGA

GCTAAAGCATGGTCTTAGATGACAATAAAAAGGAATTTGACAGCACAAAAGAGAAAAAAAAAACTATACAT
AAAATTTTTTACATATGAATTGCTTAGAGTAAAATAGGCATTTTTCATATCTTCTTCCCAATTATTAAATT
AGCAAAAA

TCATCATACTAAACTTACACGAATAGGATAACATGTATGCTAGCAGAATATATATGAAAGAAAAAAATTAT
TGATGCCTTTAAACTTATACTATTATACTATATTATGTTATATTATATTATTAGTTTTATAGATATATTGA
GATATGTT

ATAGAACAAACTCTTCATACTCGTATTTGCAATTTTTGGAAATTAATTGTGTAGCTTTTCTTCTCTTAATT
GGTGGCAGTTTTTATATATTCAGTTTAGAATACCCTTTTTTTTTTTTTTTTTTTCTTCGCTTTTCGCATCT
CATCGCTG

ATCTTAAAAAAAAAAAAAAAACAAAAAAAAAAAAACATAAATATTTGTATCTTATTGCTTATGATGATAATG
TACAATGAATTCAAAACATGAAAATTGTTCAAAGTTTTTATTTTTATTTTCTCTTTCGTATTAAACTGATG
GAACAAAT

TTTTACTGACACCTTTCGTCACTATTTTTTGTTTAACTTTTTTTTTGGAAGTACTAAAATTATATGCTCGT
AATTATTGAGTATCAAAAAAAAAAAAAAAAAAAATTGCTGTGACACCCCTTCAATGTGGTGTCTATACACC
TGGTGACT

ATATATATATATATATATATATGTGTGTTTGTATACTCTGTGGGTATTTCCGTGTATATGGTTAATAATAG
TAGTATCTTGTCAGTTTTTTTTTTATGTTTTTCTTCGCGTCAACTTTCTACCAAGAGAAAAACAATATAAG
GTCTCCTT

GCAATTTGAGATAGAACAAACTCTTCATACTCGTATTTGCAATTTTTGGAAATTAATTGTGTAGCTTTTCT
TCTCTTAATTGGTGGCAGTTTTATATATTCAGTTTAGAATACCCTTTTTTTTTTTTTTTTTTTTCTTCGCT
TTTCGCAT

TGTACAGAAGATGCGTCACTAATTCAAAAAAAAAAAAAAAAAAAAAAATATAAAAAAGAAAGATTTTGGAG
AAGCTATATCCTTTTGTGACATACAATAGAGAAACTTGTTCTGTTGGTAAGATATCTCATCTGAAATACAA
CAAGGAAA

TCTAATTTATCTTTAAAATTATATATATATATATATATATATGTGTGTTTGTATACTCTGTGGGTATTTCC
GTGTATATGGTTAATAATAGTAGTATCTTGTCAGTTTTTTTTTTATGTTTTTCTTCGCGTCAACTTTCTAC
CAAGAGAA

AATGGAATTACTTTAATACATTCCATGTTCTCATATATACTTTTACTGACACCTTTCGTCACTATTTTTTG
TTTAACTTTTTTTTTGGAAGTACTAAAATTATATGCTCGTAATTATTGAGTATCAAAAAAAAAAAAAAAAA
AAATTGCT

GTGGACATCTGTATATTGTGTTAGTGTGTGTTTATCGAGGTTACAAATATAAAAATCTTGTGATATATGTC
TTTTAAAGATACTCTTTTTGAAGCTCATCGCATTTTTTTTTTTTTTTTTCATTTTAAAATTTTTCACCGGT
TTCTCGGG

AAAAAGTGAAAAATAAACAGAATGTGATAACGGTAAAAGAAAATCAAAAATAAACAAAAAAAAATACATAT
ATAACTATTATTCATGATAGTAAACAGGTGAATTAAGATATTTAGTACAATTGTCTATTACCCCAATAGGC
ATAATCCT

TCTTTTTTTTTTTTTATTCAGAATATCTTCAAGTTTTTGTATCTTTTTGTATTATTATTAAACTTCTTTAT
AATATTTGTCTTATAAAATCCTATCATAACATGACTATGGCTTGGCCTAGACTCGGGTGCCATCTTTTTTA
ATGCGACT

AGGGCATTAAGCTTATTTTTTTAATTCTTATGAAAAAAAAAATGTGAAAGAGAGTAGAAAATTTTCATAAC
GAATCTCTTCTATTCTTATGTACCGTTCCGCCAAATTTTTCATGAAAAAATTTGGGAAAAAGAAAAAAAAA
AAAAATAG

AACCAAAAAAAAAAAAAAGAAAACAGTACAAAAAGGTTTACTTTACCCATAGTTAGTATACGTAGATTTTA
TACACGCACTATTTTTCTTATATACAGGAGATGGGTGGCCACAGAACCCGCGCCTAGCCTATTTCTTTCTT
CTTAGACC

GTAAAAAATAAAAAAAATAACCATGGAATATGTTTCTAAGAATTCGCAATCCTTGTTTATATAACTATAGC
TTTTTTTATGTTATAAGAAAACATTTATTCCTTTAACCAAAAGGAAAAAGGTAACAACCCCACTTACCCCT
CTGTCAAT

TGTGATCAAATGCTTTGCAAAAGGGTAAAACAGCGTTTACAATAGGATAGCCAAAAGTAAAAAAAAAAAAA
AAAAAAAAAAGAAGGAAGTTAGTATCGAGCTCAAACAATTTTAGTTTTGCTGGACATATTTTCAGTTTCAT
TACTGTAT

AAGAGCGAAAAAAAAGTGAAAAATAAACAGAATGTGATAACGGTAAAAGAAAATCAAAAATAAACAAAAAA
AAATACATATATAACTATTATTCATGATAGTAAACAGGTGAATTAAGATATTTAGTACAATTGTCTATTAC
CCCAATAG

TGAGTATACCTCTATATGCTGTGTAAAATAGAAAATAAAAAAGCCAATGAGAACAATATTTTGTGTCGTTT
TTTTTATTTTCGCTTCAATTCTCTATACACGTACGTCATCACTACGGGAAACCAAAAAAAAAAAAAAGAAA
ACAGTACA

GAGCCGCCCATGCGGAATCATGTCTCCGCGCAGAGGACTCATTAGTTTCCGTTCGCGGTGATTAGTACTCG
CGAAAAGAGAAAAAGAAAAATAGTAGGAACATTTCGCAATTGATCAAAATGTAAACAGTGCAGTAAAGAAT
GGGACCAT

CGGCAAGATCATTGGAGCTAACCCTACTGCTTTAGAGAAGGGAATCAAAGATCTATAAATTTTATATTCAT
GAGCACATGTAGTTTTTTTTTTTTTAGTTTTAAATATAAGAAAAAAGATAGTATATATATATATATATATA
CATATACA

ACCAACTGTTCGTGATCGTCATCCATTGAGCTTTTCTTTCCTCTCTCTTTTTTTTTTCTTGTTACATATTC
CTATATATATGTATATATATCTATATATATATATATCCCAGCGTATATGACTGCACAAGACGCAATTTTCT
TGAACGGT

TTAGTGTGTGTTTATCGAGGTTACAAATATAAAAATCTTGTGATATATGTCTTTTAAAGATACTCTTTTTG
AAGCTCATCGCATTTTTTTTTTTTTTTTTTCATTTTAAAATTTTCACCGGTTTCTCGGGTAACAGCTCTAA
ATGAAATC

TCAATTTTTTATTAAACAATGTTTGATTTTTTAAATCGCAATTTAATACCTAAATATAAAAAATGTTATTA
TATTGCAAAAACCCATCAACCTTGAAAAAAAGTAGAAACGTTTTATTTAATTCTATCAATACATCATAAAA
TACGAACG

CAAAAACATCATCCTTATCATAATCACCCTTCCAACGATAGCGGTAATGAAAAGAATTGACTTTTTTTTTT
TTTTTTTTTCATTTTCTTTTCCTCACCGAAAACTAATTATTCTTAATCTGATGGATTCATCGCAAACGGTT
GGATGAGT

CAGTTCTTCGACCAACTGTTCGTGATCGTCATCCATTGAGCTTTTCTTTCCTCTCTCTTTTTTTTTTCTTG
TTACATATTCCTATATATATGTATATATATCTATATATATATATATCCCAGCGTATATGACTGCACAAGAC
GCAATTTT

TTATCTCCTCTTAATGCCTTAAGTCAAAAAATAGTTTTTGGGATATATCTTGTGATCAAATGCTTTGCAAA
AGGGTAAAACAGCGTTTACAATAGGATAGCCAAAAGTAAAAAAAAAAAAAAAAAAAAAAAGAAGGAAGTTA
GTATCGAG

ACTGAAACAGCCATTCTATTTAAACATATAAAAGTACGATTAGTTGGTGTGGTTATATAAGTCATAAAATA
ATTATCATATTAAAAAATTGAAAAATAAAAAACTACTCTTTTTTATTTCAATAGTTCTCGTTATTAGTAGG
TCGTGCTC

TTCTTTACGTTTGATTCTATTTCAACCATAATTTATTTTTTCGTTATTTAATAATTACTAATATTTAATAC
TAGATGTATATACGTTGTGTGTATGCGCTACTATAGTATAGCTTAACTGTTTATTATACCCTTTTTTTTCT
CTCTTTAC

CTATTCTTATGTACCGTTCCGCCAAATTTTTCATGAAAAAAATTTGGGAAAAAGAAAAAAAAAAAAATAGT
ATATAAAGTAAAAAAGATGCGCAAGCCCGGAATCGAACCGGGGGCCCAACGATGGCAACGTTGGATTTTAC
CACTAAAC

TCTCTATACACGTACGTCATCACTACGGGAAACCAAAAAAAAAAAAAAGAAAACAGTACAAAAAGGTTTAC
TTTACCCATAGTTAGTATACGTAGATTTTATACACGCACTATTTTTCTTATATACAGGAGATGGGTGGCCA
CAGAACCC

TATATGACCCTGTATTTAATGTACGCGGTTAAAAAATTTTTATATTTTTAAATAAGTATCAACTTGGATAA
TATGATCTTGCCCGCAAGGTGGTTTTATATGAATAATTACTTTTTTTTTTTTTTTTTTTTTTTTTTTTT
TTTTAATT

TGTATTTAATGTACGCGGTTAAAAAATTTTTATATTTTTAAATAAGTATCAACTTGGATAATATGATCTTG
CCCGCAAGGTGGTTTTATATGAATAATTACTTTTTTTTTTTTTTTTTTTTTTTTTTTTTTTTTTTAATTGA
ACAATAGA

GTACGCGGTTAAAAAATTTTTATATTTTTAAATAAGTATCAACTTGGATAATATGATCTTGCCCGCAAGGT
GGTTTTATATGAATAATTACTTTTTTTTTTTTTTTTTTTTTTTTTTTTTTTTTTTAATTGAACAATAGATG
CAGAGGAA

GCTCTTCCTGTATATGACCCTGTATTTAATGTACGCGGTTAAAAAATTTTTATATTTTTAAATAAGTATCA
ACTTGGATAATATGATCTTGCCCGCAAGGTGGTTTTATATGAATAATTACTTTTTTTTTTTTTTTTTTTTT
TTTTTTTT

ATACATGTATCTTTTTGGTTGGTTAATTTATCTAAACAGTTTTATATATATATGTATATATATATATATAT
ATACACAGTATTAATATTCTTTCCTTGAGATTACTCTCTATAAAAAAAAAAAAAAAAAAGCTTTTAAAAAA
AAAAAAAC

CTTTTTGGTTGGTTAATTTATCTAAACAGTTTTATATATATATGTATATATATATATATATATACACAGTA
TTAATATTCTTTCCTTGAGATTACTCTCTATAAAAAAAAAAAAAAAAAAGCTTTTAAAAAAAAAAAAACTC
AAAAAAAG

ATGCCGCGCGCGCGAATTGCTCATGATATAATAATAGCTAATAATATGATAATATGATAATAATAGATAAG
ATTACTGATAACATTATTTTAGTATTCAAAAGCGTATTACATATATATAGTCGAGTAAATACTTTTTTTTC
AATGGGGA

GTATATAACAGGGCACTTTCGGCAGTGAATCAATAGGAGGCGTATAAGAAAAGCCAATTAGTACCTATATA
TATATATATATATATATATATATATATATATTCCTTTATTATAATTCCTATCAGCGATTTTTGCATCACAC
CCAAGAAA

GGGCACTTTCGGCAGTGAATCAATAGGAGGCGTATAAGAAAAGCCAATTAGTACCTATATATATATATATA
TATATATATATATATATATATTCCTTTATTATAATTCCTATCAGCGATTTTTGCATCACACCCAAGAAAAA
TCTATAAT

GGCAGTGAATCAATAGGAGGCGTATAAGAAAAGCCAATTAGTACCTATATATATATATATATATATATATA
TATATATATATTCCTTTATTATAATTCCTATCAGCGATTTTTGCATCACACCCAAGAAAAATCTATAATAG
AGACACAA

AATAGGTAAAAATAATAAAAATACATGTATCTTTTTGGTTGGTTAATTTATCTAAACAGTTTTATATATAT
ATGTATATATATATATATATATACACAGTATTAATATTCTTTCCTTGAGATTACTCTCTATAAAAAAAAAA
AAAAAAAA

AAAAAATTTTTATATTTTTAAATAAGTATCAACTTGGATAATATGATCTTGCCCGCAAGGTGGTTTTATAT
GAATAATTACTTTTTTTTTTTTTTTTTTTTTTTTTTTTTTTTTTTAATTGAACAATAGATGCAGAGGAATG
CTCCAAAA

GCTATCTCAAATGCCGCGCGCGCGAATTGCTCATGATATAATAATAGCTAATAATATGATAATATGATAAT
AATAGATAAGATTACTGATAACATTATTTTAGTATTCAAAAGCGTATTACATATATATAGTCGAGTAAATA
CTTTTTTT

TATATACCGTATATCGGTTCTTTCCAATTTTTTTTTTTTTTTTTTTTTTTTTTTTTTTTTTTGGTTGTAAT
ATTAAAAGAATAAACATTTATCTGATATGTAATTGCATTTATAAAATGTACAGTACCGCATTTAAAGTTTG
CTGATAAT

AACAAAAAAAAAATTGAAAAGTAACTACAATTGTCACTAGTTATTATTGGCCAAGTCATAAAGGGACCTTG
TCTCGATTTTATATACCGTATATCGGTTCTTTCCAATTTTTTTTTTTTTTTTTTTTTTTTTTTTTTTTTTT
GGTTGTAA

AATAAGTATCAACTTGGATAATATGATCTTGCCCGCAAGGTGGTTTTATATGAATAATTACTTTTTTTTTT
TTTTTTTTTTTTTTTTTTTTTTTTTAATTGAACAATAGATGCAGAGGAATGCTCCAAAATTTATTTCCTTG
AACAATAT

CAATAGGAGGCGTATAAGAAAAGCCAATTAGTACCTATATATATATATATATATATATATATATATATATA
TTCCTTTATTATAATTCCTATCAGCGATTTTTGCATCACACCCAAGAAAAATCTATAATAGAGACACAAAA
AAATGAAC

TTATTATTGGCCAAGTCATAAAGGGACCTTGTCTCGATTTTATATACCGTATATCGGTTCTTTCCAATTTT
TTTTTTTTTTTTTTTTTTTTTTTTTTTTTTTGGTTGTAATATTAAAAGAATAAACATTTATCTGATATGTA
ATTGCATT

TTTCCAATTTTTTTTTTTTTTTTTTTTTTTTTTTTTTTTTTTGGTTGTAATATTAAAAGAATAAACATTTA
TCTGATATGTAATTGCATTTATAAAATGTACAGTACCGCATTTAAAGTTTGCTGATAATTAAATCTAGAAT
TTTATACC

TATGATTGTCGTATATAACAGGGCACTTTCGGCAGTGAATCAATAGGAGGCGTATAAGAAAAGCCAATTAG
TACCTATATATATATATATATATATATATATATATATATATTCCTTTATTATAATTCCTATCAGCGATTTT
TGCATCAC

CCAAGTCATAAAGGGACCTTGTCTCGATTTTATATACCGTATATCGGTTCTTTCCAATTTTTTTTTTTTTT
TTTTTTTTTTTTTTTTTTTTTGGTTGTAATATTAAAAGAATAAACATTTATCTGATATGTAATTGCATTTA
TAAAATGT

ATATCGGTTCTTTCCAATTTTTTTTTTTTTTTTTTTTTTTTTTTTTTTTTTTGGTTGTAATATTAAAAGAA
TAAACATTTATCTGATATGTAATTGCATTTATAAAATGTACAGTACCGCATTTAAAGTTTGCTGATAATTA
AATCTAGA

ACTCCACTTTGCTTCTATAGTCCTATCTATTGTTCTTTTTATTCCTAGCATAACCTTATTGTATTGTTCTT
TTTCTTTTTCCTTTTTCTTTTTTTTTTTTTTTTTTTTTTTTTTTTCACATCTTTTTCCACGGCCGCTCAAA
GGGGTCTC

AATAATAAAAATACATGTATCTTTTTGGTTGGTTAATTTATCTAAACAGTTTTATATATATATGTATATAT
ATATATATATATACACAGTATTAATATTCTTTCCTTGAGATTACTCTCTATAAAAAAAAAAAAAAAAAAGC
TTTTAAAA

GATTAAGTTTGCTATCTCAAATGCCGCGCGCGAATTGCTCATGATATAATAATAGCTAATAATATGATA
ATATGATAATAATAGATAAGATTACTGATAACATTATTTTAGTATTCAAAAGCGTATTACATATATATAGT
CGAGTAAA

GGTTAATTTATCTAAACAGTTTTATATATATATGTATATATATATATATATATACACAGTATTAATATTCT
TTCCTTGAGATTACTCTCTATAAAAAAAAAAAAAAAAAAGCTTTTAAAAAAAAAAAAACTCAAAAAAAGAA
AGAATATT

GTCTCGATTTTATATACCGTATATCGGTTCTTTCCAATTTTTTTTTTTTTTTTTTTTTTTTTTTTTTTTTT
TGGTTGTAATATTAAAAGAATAAACATTTATCTGATATGTAATTGCATTTATAAAATGTACAGTACCGCAT
TTAAAGTT

ATATGATCTTGCCCGCAAGGTGGTTTTATATGAATAATTACTTTTTTTTTTTTTTTTTTTTTTTTTTTTTT
TTTTTAATTGAACAATAGATGCAGAGGAATGCTCCAAAATTTATTTCCTTGAACAATATTACAAGAGGCAG
ATATATAC

GCTTCTATAGTCCTATCTATTGTTCTTTTTATTCCTAGCATAACCTTATTGTATTGTTCTTTTTCTTTTTC
CTTTTTCTTTTTTTTTTTTTTTTTTTTTTTTTTTTCACATCTTTTTCCACGGCCGCTCAAAGGGGTCTCAC
ACTGGATT

TTGTCACTAGTTATTATTGGCCAAGTCATAAAGGGACCTTGTCTCGATTTTATATACCGTATATCGGTTCT
TTCCAATTTTTTTTTTTTTTTTTTTTTTTTTTTTTTTTTTTGGTTGTAATATTAAAAGAATAAACATTTAT
CTGATATG

ATTCAAAATGCCTACTTACCGCATTGTATGTGGGAGAATGAACATATCTGTAATTGTATTATATAAAAGTA
GTTAGTTTTTTTTTTTTTTTTTTTTTTTTTTTTTTTTTATGTGTAATGCAAATACAAAAAGAACTGCAGGC
ATTTTGAA

AATTGGATGTAACAAAAAAAAAATTGAAAAGTAACTACAATTGTCACTAGTTATTATTGGCCAAGTCATAA
AGGGACCTTGTCTCGATTTTATATACCGTATATCGGTTCTTTCCAATTTTTTTTTTTTTTTTTTTTTTTTT
TTTTTTTT

CACCGCAGTTATTTATGATCATTTCGAACGGGAAGTATGGATGAATCTTTTTTTTTTTTTTTTTATAGCAC
GCAACTGAAAAAAAAAAAAAGAAAAATTTTTCATCTTCGCTCGACGTTTCTTTTGTAGTACTCATCTCTTT
TTATATAA

CTGTTGGACGGTTGTCCGCGCGAGTTCGAACCTCGCATCCTTCAATAACTTATTTTTTTCCTCAATCTTCG
ATATTAGACATTTTTTTTTTCCTATGCCTCCCTTTTCCAAGTAATGTTTTTTTTTTTTTTTTTTTTTTTAG
CTCCTCTC

AACTTGGATAATATGATCTTGCCCGCAAGGTGGTTTTATATGAATAATTACTTTTTTTTTTTTTTTTTTTT
TTTTTTTTTTTTTTTAATTGAACAATAGATGCAGAGGAATGCTCCAAAATTTATTTCCTTGAACAATATTA
CAAGAGGC

TATATTTTTAAATAAGTATCAACTTGGATAATATGATCTTGCCCGCAAGGTGGTTTTATATGAATAATTAC
TTTTTTTTTTTTTTTTTTTTTTTTTTTTTTTTTTTAATTGAACAATAGATGCAGAGGAATGCTCCAAAATT
TATTTCCT

AAGGGACCTTGTCTCGATTTTATATACCGTATATCGGTTCTTTCCAATTTTTTTTTTTTTTTTTTTTTTTT
TTTTTTTTTTTGGTTGTAATATTAAAAGAATAAACATTTATCTGATATGTAATTGCATTTATAAAATGTAC
AGTACCGC

GTTGTCCGCGCGAGTTCGAACCTCGCATCCTTCAATAACTTATTTTTTTCCTCAATCTTCGATATTAGACA
TTTTTTTTTTCCTATGCCTCCCTTTTCCAAGTAATGTTTTTTTTTTTTTTTTTTTTTTTAGCTCCTCTCAC
AAAAGTAC

TCTTTTCCTTTTTGTCCTTGTCCTTTCTTTCCTATTTATATATATATATATATATATATATATAATACTACTA
TGTTATATTACATTATATTATTATCACAATAATTATTACGTTCATCGTGCCGAAACGTCACCTCCCACGGT
TTGGATCT

ATTTATGATCATTTCGAACGGGAAGTATGGATGAATCTTTTTTTTTTTTTTTTTATAGCACGCAACTGAAA
AAAAAAAAAAGAAAAATTTTTCATCTTCGCTCGACGTTTCTTTTGTAGTACTCATCTCTTTTTATATAAAG
ATTAATTA

CCTACTTACCGCATTGTATGTGGGAGAATGAACATATCTGTAATTGTATTATATAAAAGTAGTTAGTTTTT
TTTTTTTTTTTTTTTTTTTTTTTTTTTTATGTGTAATGCAAATACAAAAAGAACTGCAGGCATTTTGAAAG
TTCACTCG

GGTGTTGAAAGCTCTTCCTGTATATGACCCTGTATTTAATGTACGCGGTTAAAAAATTTTTATATTTTTAA
ATAAGTATCAACTTGGATAATATGATCTTGCCCGCAAGGTGGTTTTATATGAATAATTACTTTTTTTTTTT
TTTTTTTT

TCCTATCTATTGTTCTTTTTATTCCTAGCATAACCTTATTGTATTGTTCTTTTTCTTTTTCCTTTTTCTTT
TTTTTTTTTTTTTTTTTTTTTTTTTCACATCTTTTTCCACGGCCGCTCAAAGGGGTCTCACACTGGATTAA
AAGCTGAA

GCTATTGGTAACCATGAAGTGTAAGGCGACGCAGGAGAGATGAAAAGAATGAAATTAAAAGAAATCCAAAA
AAAAAAAAAAAAAAAAAAAAGAAAAAGAAAGAAAAAGGGGGAATAACACAGCCGCGGGTAACATGAAGCGG
CGCGAGCG

CCACGATTAGACTCCACTTTGCTTCTATAGTCCTATCTATTGTTCTTTTTATTCCTAGCATAACCTTATTG
TATTGTTCTTTTTCTTTTTCCTTTTTCTTTTTTTTTTTTTTTTTTTTTTTTTTTTCACATCTTTTTCCACG
GCCGCTCA

AACGGAATAAGAGGAAAAGACCTTTTATCTATTCAAATTGTTTCCCTTAGGTATATATATATATATATATA
TATATATATATTTTCCCTGTATATATCTATGTAAATGACGAAAACGCATGACATTTTAAAACCTACCCCGG
GTTTGACC

ACCATGAAGTGTAAGGCGACGCAGGAGAGATGAAAAGAATGAAATTAAAAGAAATCCAAAAAAAAAAAAAA
AAAAAAAAAAGAAAAAGAAAGAAAAAGGGGGAATAACACAGCCGCGGGTAACATGAAGCGGCGCGAGCGAT
ACTTCCGT

TGACCTGTACACTAATTAAAACACTTTTATCCATTTTTTTTCGTATAAGCATATTTTACATCTTTATATAT
ATTTATTTTTTTTTTTTTTATTTTGGTAGAATAGATTACATATACAACTTTTTCATAATGTCAAACTATAG
AGTTTTAT

AAAAAGAAAGAACGGAATAAGAGGAAAAGACCTTTTATCTATTCAAATTGTTTCCCTTAGGTATATATATA
TATATATATATATATATATATTTTCCCTGTATATATCTATGTAAATGACGAAAACGCATGACATTTTAAAA
CCTACCCC

GAGGAAAAGACCTTTTATCTATTCAAATTGTTTCCCTTAGGTATATATATATATATATATATATATATATA
TTTTCCCTGTATATATCTATGTAAATGACGAAAACGCATGACATTTTAAAACCTACCCCGGGTTTGACCAC
AACCCACC

CTGTGTGACGGATTAAGTTTGCTATCTCAAATGCCGCGCGCGCGAATTGCTCATGATATAATAATAGCTAA
TAATATGATAATATGATAATAATAGATAAGATTACTGATAACATTATTTTAGTATTCAAAAGCGTATTACA
TATATATA

GTAAATCAGTAAAAAAAAAAATTAACAGTTTTTTTTTTTCATTTTTTTTTTTTATTCTTATTTATGTATGA
TACTTTATTATTATTTCTCTTAATTATTTATTTATTTAACTAACACGATGAGCACTTTTAACTGCAATGGT
TAAACTGT

GACTTAAGATCTGTTGGACGGTTGTCCGCGCGAGTTCGAACCTCGCATCCTTCAATAACTTATTTTTTTCC
TCAATCTTCGATATTAGACATTTTTTTTTTCCTATGCCTCCCTTTTCCAAGTAATGTTTTTTTTTTTTTTT
TTTTTTTT

GTAACTACAATTGTCACTAGTTATTATTGGCCAAGTCATAAAGGGACCTTGTCTCGATTTTATATACCGTA
TATCGGTTCTTTCCAATTTTTTTTTTTTTTTTTTTTTTTTTTTTTTTTTTTGGTTGTAATATTAAAAGAAT
AAACATTT

TGTTCTTTTTATTCCTAGCATAACCTTATTGTATTGTTCTTTTTCTTTTTCCTTTTTCTTTTTTTTTTTTT
TTTTTTTTTTTTTTTCACATCTTTTTCCACGGCCGCTCAAAGGGGTCTCACACTGGATTAAAAGCTGAAAA
CAAGAAAT

TGTTCGAAAAAATAAGTATTTGAAAACGAAAAAATAGTTTGTATGTTTGTATATAAGTTGTTATTATCAAA
AATTAGAGGGAAAAAGTAACTAAATATATATTAAAAAAAAAAAAAAAATAAATGTATGTTGGAATGAAATA
GCGTAGTA

CACATTGTTATATATGTATCTATAAAGCGAAATTTTTTAAAATTAATTGATTAATTAATATTTTGTATCTA
AAGTGTTCATACTATATTATTTACTTAGATTATACATAATTAAAAAAAAAAAAAACTTTTTTTCCTTGTTT
TCATTTTG

ATAGTACTCTAAAAAGAAAGAACGGAATAAGAGGAAAAGACCTTTTATCTATTCAAATTGTTTCCCTTAGG
TATATATATATATATATATATATATATATATTTTCCCTGTATATATCTATGTAAATGACGAAAACGCATGA
CATTTTAA

AAATTGAAAAGTAACTACAATTGTCACTAGTTATTATTGGCCAAGTCATAAAGGGACCTTGTCTCGATTTT
ATATACCGTATATCGGTTCTTTCCAATTTTTTTTTTTTTTTTTTTTTTTTTTTTTTTTTTTGGTTGTAATA
TTAAAAGA

TATATGTATCTATAAAGCGAAATTTTTTAAAATTAATTGATTAATTAATATTTTGTATCTAAAGTGTTCAT
ACTATATTATTTACTTAGATTATACATAATTAAAAAAAAAAAAAACTTTTTTTCCTTGTTTTCATTTTGTT
TTTATTAT

TCTAAACAGTTTTATATATATATGTATATATATATATATATATACACAGTATTAATATTCTTTCCTTGAGA
TTACTCTCTATAAAAAAAAAAAAAAAAAAGCTTTTAAAAAAAAAAAAACTCAAAAAAAGAAAGAATATTCA
AAGAAAGT

CGTAGCCCAATCTACCACTTTTTTTTTTCATTTTTTAAAGTGTTATACTTAGTTATGCTCTAGGATAATGA
ACTACTTTTTTTTTTTTTTTTTTACTGTTATCATAAATATATATACCTTATTGTTGTTTGCAACCGTCGGT
TAATTCCT

AGGGGATAAGGGGCTTCGTCCTACACGATGCTCCTGTCGTTTCTTTTTTTCATTTCTTTTTTCGCCCAGCG
GGAAGTACGCGGAAAACAAGAAACAAAAAAAAAATAAAAAAATAAAAAATAAAACAGAAACTAAAATCTGA
TGATGAAA

GGTTAACCCTTTATTAAAATAAAAAAAAAAAGCATGCATAACACTAATAATATATTATTAAAAAAAAAGCC
AATCTATATTATTTAAATTATCCGAAATTACTTTTATTACATTATGACATTCTATAGTCTATGCAAGCGGA
TCAAAAAA

TTTCACTTTGTGTTCGAAAAAATAAGTATTTGAAAACGAAAAAATAGTTTGTATGTTTGTATATAAGTTGT
TATTATCAAAAATTAGAGGGAAAAAGTAACTAAATATATATTAAAAAAAAAAAAAAAATAAATGTATGTTG
GAATGAAA

CTTTTTTTTTTTTTTTTTTTTTTTTTTTTTTTTTTTAATTGAACAATAGATGCAGAGGAATGCTCCAAAAT
TTATTTCCTTGAACAATATTACAAGAGGCAGATATATACTTTTCCTTATTTCCCTTAGTAAAGAATGATGC
ATGTATAC

GTAAGGCGACGCAGGAGAGATGAAAAGAATGAAATTAAAAGAAATCCAAAAAAAAAAAAAAAAAAAAAAAA
GAAAAAGAAAGAAAAAGGGGGAATAACACAGCCGCGGGTAACATGAAGCGGCGCGAGCGATACTTCCGTGT
TAAGTGAT

TATTATTATTGTTAGTATTATTATTATTATTATTATTATTATTATTATTATTATTATTATTATTATTATTA
TTATTATTATTATTATATTATTATTAATACTAAATATTGACACTGCTTTACGCACTAGATTGTGCGGGTAA
CTGAATAT

ATGTTACCCGTTTATACCTCTATACGATTATTAAGGGAGGGTAATACTTTCTCAATTTTTTTTTTTTCAGT
TTTTAATATTATTTCCTTAAGATTTTTCGGTGGCGTTTCGCGACAACTTACGAGAAAAAAAAAAAAGAGGA
GACTTGAA

TTCTTTTTTTCATTTCTTTTTTCGCCCAGCGGGAAGTACGCGGAAAACAAGAAACAAAAAAAAAATAAAAA
AATAAAAAAATAAAACAGAAACTAAAATCTGATGATGAAATTGATATTCAATTGGAGAAAGAAGAAAGATCT
TGTTTAAT

GGGCTTCGTCCTACACGATGCTCCTGTCGTTTCTTTTTTTCATTTCTTTTTTCGCCCAGCGGGAAGTACGC
GGAAAACAAGAAACAAAAAAAAAATAAAAAAATAAAAAATAAAACAGAAACTAAAATCTGATGATGAAATT
GATATTCA

ATAGCCCATACACCGCAGTTATTTATGATCATTTCGAACGGGAAGTATGGATGAATCTTTTTTTTTTTTT
TTTATAGCACGCAACTGAAAAAAAAAAAAGAAAAATTTTTCATCTTCGCTCGACGTTTCTTTTGTAGTAC
TCATCTCT

ACGCTATATACTAAATAACAATAAGAAAAAGAAAAAGAAGCACAATATAGGAAAGCGTTCTAATTAACTAA
AGAAAAAAAAAAAATAAAAAAAAAATAAAAAAAGGTAACTAAAAAGGACAAAAAACATAACAGCTGTTTA
TAAAGTAC

CAAATTAGCTATTATTTCTCATTTGGTGATAATAATATATACATACATATATATATATATATAAATATA
TAGGTATATATATAAATAACCTAAGACAAGTTAAATGAGTATAAAAAACCTTGAACGTAAATGAAAAAAA
AAAAATAC

TGCTCGCATAATGTTACCCGTTTATACCTCTATACGATTATTAAGGGAGGGTAATACTTTCTCAATTTTT
TTTTTTCAGTTTTAATATTATTTCCTTAAGATTTTCGGTGGCGTTTCGCGACAACTTACGAGAAAAAAAA
AAAAAGAG

TGAATAATTACTTTTTTTTTTTTTTTTTTTTTTTTTTTTTTTTTTAATTGAACAATAGATGCAGAGGAAT
GCTCCAAAATTTATTTCCTTGAACAATATTACAAGAGGCAGATATATACTTTTCCTTATTTCCCTTAGTAA
AGAATGAT

AAAAAAAAAAAAAAAAAAATAAACAAATGAGCAACCCATCAAATACCAAGAACAAAGAATTTAATATAAT
ATATAGTTATTAAATTTAAATGTATATATGCAGTTCTGCTCTTATTTTGTGACCAATTTCTTTAAGTCATC
CAAACCGA

GTTAGTATTATTATTATTATTATTATTATTATTATTATTATTATTATTATTATTATTATTATTATTATTAT
TATTATATTATTATTAATACTAAATATTGACACTGCTTTACGCACTAGATTGTGCGGGTAACTGAATATTT
TTTGAAAA

ACCAAGATGGATTCAAAATGCCTACTTACCGCATTGTATGTGGGAGAATGAACATATCTGTAATTGTATTA
TATAAAAGTAGTTAGTTTTTTTTTTTTTTTTTTTTTTTTTTTTTTTTATGTGTAATGCAAATACAAAAAG
AACTGCAG

TTTTTTGCCACCACGATTAGACTCCACTTTGCTTCTATAGTCCTATCTATTGTTCTTTTTATTCCTAGCAT
AACCTTATTGTATTGTTCTTTTTCTTTTTCCTTTTTCTTTTTTTTTTTTTTTTTTTTTTTTTTTCACATC
TTTTTCCA

TGAAAAGAATGAAATTAAAAGAAATCCAAAAAAAAAAAAAAAAAAAAAAAGAAAAAGAAAGAAAAAGGGG
GAATAACACAGCCGCGGGTAACATGAAGCGGCGCGAGCGATACTTCCGTGTTAAGTGATATCCATCTTTCT
GCGGGTAA

GCCCGCAAGGTGGTTTTATATGAATAATTACTTTTTTTTTTTTTTTTTTTTTTTTTTTTTTTTTTAATTG
AACAATAGATGCAGAGGAATGCTCCAAAATTTATTTCCTTGAACAATATTACAAGAGGCAGATATATACTT
TTCCTTAT

ACTAATTAAAACACTTTTATCCATTTTTTTCGTATAAGCATATTTTACATCTTTATATATATTTATTTTT
TTTTTTTTATTTTGGTAGAATAGATTACATATACAACTTTTTCATAATGTCAAACTATAGAGTTTTATTT
CACATAAC

GTTTTTTTTTTTTTTTTGTACAGATTAGAAACCATACAGTGTAAACCATTATATAGGTCAATTAATATAT
TTATAGATCATCCTGTCAATTTTTTTTTATTTTTTTTGCAAAGCTCCCTTTTTCCTGAAACCAACAACAA
AATATAAA

CCCGGCCTCTTAAATAAAAACAATTACCACTGCAAAAATGTTCTTCAAACGCGTTTATTATATAATGAAAA
AGAAATTCAAATTTTTCGCGTTTGAAAATTTATGCTAAACAAATAACTCGAGGAGGGACGCGTCGAGAAAA
TCGCGTCT

CCAGCTGTAAAAAACCAGCAGATATCGCATAAAAAAATGGCCAGCTATAGAATAAAATTAGCCCAGCTATA
TTTTTATAGCGTCTTTTGATGACGCGAAAAGTAGACTTCGCGTAACACGAAAAATGCTGGTGCAGGTGGAT
TTTTTTGG

CTCCTGTCGTTTCTTTTTTTCATTTCTTTTTTCGCCCAGCGGGAAGTACGCGGAAAACAAGAAACAAAAAA
AAAATAAAAAAATAAAAAATAAAACAGAAACTAAAATCTGATGATGAAATTGATATTCAATTGGAGAAAGA
AGAAAGAT

TCTACCACTTTTTTTTTTCATTTTTTAAAGTGTTATACTTAGTTATGCTCTAGGATAATGAACTACTTTTT
TTTTTTTTTTTTTACTGTTATCATAAATATATATACCTTATTGTTGTTTGCAACCGTCGGTTAATTCCTTA
TCAAGGTT

GCAGGAGAGATGAAAAGAATGAAATTAAAAGAAATCCAAAAAAAAAAAAAAAAAAAAAAAAAGAAAAAGAAA
GAAAAAGGGGGAATAACACAGCCGCGGGTAACATGAAGCGGCGCGAGCGATACTTCCGTGTTAAGTGATAT
CCATCTTT

AGTAGAAGAGTTATGTTACAAATGTTGATATATATATATATATATATATATATATATATGTATGGACATTTGT
GATGAAAATATATCTTGAACAAGCGCGATTAAGGTCATAATAAAAAATCACAGAAAATTTTTCAGGTCGCG
GGCGTTCT

GCATTGTATGTGGGAGAATGAACATATCTGTAATTGTATTATATAAAAGTAGTTAGTTTTTTTTTTTTTTT
TTTTTTTTTTTTTTTTTTATGTGTAATGCAAATACAAAAAGAACTGCAGGCATTTTGAAAGTTCACTCGTA
TAGGAAAC

TCATGGGAACATAGCCCATACACCGCAGTTATTTATGATCATTTCGAACGGGAAGTATGGATGAATCTTTT
TTTTTTTTTTTTTATAGCACGCAACTGAAAAAAAAAAAAAGAAAAATTTTTCATCTTCGCTCGACGTTTCT
TTTGTAGT

AGTAAAAAATAATAGGTAAAAATAATAAAAATACATGTATCTTTTTGGTTGGTTAATTTATCTAAACAGTT
TTATATATATATGTATATATATATATATATATACACAGTATTAATATTCTTTCCTTGAGATTACTCTCTAT
AAAAAAAA

AATCAAAAATCAAATTAGCTATTATTTCTCATTTGGTGATAATAATATATACATACATATATATATATATA
TATAAATATATAGGTATATATATAAATAACCTAAGACAAGTTAAATGAGTATAAAAAACCTTGAACGTAAA
TGAAAAAA

AGTTATAGTTCACATTGTTATATATGTATCTATAAAGCGAAATTTTTTAAAATTAATTGATTAATTAATAT
TTTGTATCTAAAGTGTTCATACTATATTATTTACTTAGATTATACATAATTAAAAAAAAAAAAAACTTTTT
TTCCTTGT

TATTCAAAAAGGTGTTGAAAGCTCTTCCTGTATATGACCCTGTATTTAATGTACGCGGTTAAAAAATTTTT
ATATTTTTAAATAAGTATCAACTTGGATAATATGATCTTGCCCGCAAGGTGGTTTTATATGAATAATTACT
TTTTTTTT

AATAAGTATTTGAAAACGAAAAAATAGTTTGTATGTTTGTATATAAGTTGTTATTATCAAAAATTAGAGGG
AAAAAGTAACTAAATATATATTAAAAAAAAAAAAAAAATAAATGTATGTTGGAATGAAATAGCGTAGTAAG
GATAAAGG

ATTGCAGTTTTCTTTTCCTTTTTGTCCTTGTCCTTTCTTTCCTATTTATATATATATATATATATATATAT
AATACTACTATGTTATATTACATTATATTATTATCACAATAATTATTACGTTCATCGTGCCGAAACGTCAC
CTCCCACG

CACTCTGTACACGCTATATACTAAATAACAATAAGAAAAAGAAAAAGAAGCACAATATAGGAAAGCGTTCT
AATTAACTAAAGAAAAAAAAAAAAATAAAAAAAAAATAAAAAAAAGGTAACTAAAAAGGACAAAAAACATAA
CAGCTGTT

TGACAGAAAACCAAAAGCAAGCCGATTTCAGGTTAACCCTTTATTAAAATAAAAAAAAAAAGCATGCATAA
CACTAATAATATATTATTAAAAAAAAAAGCCAATCTATATTATTTAAATTATCCGAAATTACTTTTATTACA
TTATGACA

ATCATCTTTGAAGGCTTGTGCTGATCGAACGAAGCAAATCCTACGAGTAAATACATAAGCGTATACATATA
TATATATATATATATATATATATATATATGTATATATATATATGTGTGTGTGTAATTGTGTGTATTCAA
CTGAACTA

AATATATGAGAAAAAGTTGTTTTCACTTTGTGTTCGAAAAAATAAGTATTTGAAAACGAAAAAATAGTTTG
TATGTTTGTATATAAGTTGTTATTATCAAAAATTAGAGGGAAAAAGTAACTAAATATATATTAAAAAAAAA
AAAAAAAT

AAGCAACCTATGACCTGTACACTAATTAAAACACTTTTATCCATTTTTTTTCGTATAAGCATATTTTACAT
CTTTATATATATTTATTTTTTTTTTTTTTATTTTGGTAGAATAGATTACATATACAACTTTTTCATAATGT
CAAACTAT

GTAGTATTATCTGTGTGACGGATTAAGTTTGCTATCTCAAATGCCGCGCGCGAATTGCTCATGATATAA
TAATAGCTAATAATATGATAATATGATAATAATAGATAAGATTACTGATAACATTATTTTAGTATTCAAAA
GCGTATTA

GTGAACATATTGCTCGCATAATGTTACCCGTTTATACCTCTATACGATTATTAAGGGAGGGTAATACTTTC
TCAATTTTTTTTTTTTTCAGTTTTTAATATTATTTCCTTAAGATTTTTCGGTGGCGTTTCGCGACAACTTAC
GAGAAAAA

CAAGGAATTTGTAAATCAGTAAAAAAAAAAAATTAACAGTTTTTTTTTTTCATTTTTTTTTTTTATTCTTAT
TTATGTATGATACTTTATTATTATTTCTCTTAATTATTTATTTATTTAACTAACACGATGAGCACTTTTAA
CTGCAATG

AGCTTCGTTGTCATGGGAACATAGCCCATACACCGCAGTTATTTATGATCATTTCGAACGGGAAGTATGGA
TGAATCTTTTTTTTTTTTTTTTTTATAGCACGCAACTGAAAAAAAAAAAAAAGAAAAATTTTTCATCTTCGCT
CGACGTTT

TGGTTTTATATGAATAATTACTTTTTTTTTTTTTTTTTTTTTTTTTTTTTTTTTTTTTTAATTGAACAATAGAT
GCAGAGGAATGCTCCAAAATTTATTTCCTTGAACAATATTACAAGAGGCAGATATATACTTTTCCTTATTT
CCCTTAGT

GTTACATCTGTCACGGCATTCCAGCTGTAAAAAACCAGCAGATATCGCATAAAAAAATGGCCAGCTATAGA
ATAAAATTAGCCCAGCTATATTTTTATAGCGTCTTTTGATGACGCGAAAAGTAGACTTCGCGTAACACGAA
AAATGCTG

TAAATAAAAACAATTACCACTGCAAAAATGTTCTTCAAACGCGTTTATTATATAATGAAAAAGAAATTCAA
ATTTTTCGCGTTTGAAAATTTATGCTAAACAAATAACTCGAGGAGGGACGCGTCGAGAAAATCGCGTCTTT
CTACTCTC

TTTATATATATATGTATATATATATATATATATACACAGTATTAATATTCTTTCCTTGAGATTACTCTCTA
TAAAAAAAAAAAAAAAAAAGCTTTTAAAAAAAAAAAAAACTCAAAAAAAGAAAGAATATTCAAAGAAAGTTT
CATCTTGT

CTACACGATGCTCCTGTCGTTTCTTTTTTTCATTTCTTTTTTCGCCCAGCGGGAAGTACGCGGAAAACAAG
AAACAAAAAAAAAAATAAAAAAATAAAAAATAAAACAGAAACTAAAATCTGATGATGAAATTGATATTCAAT
TGGAGAAA

TTTTTTTTTTTTTATAGCACGCAACTGAAAAAAAAAAAAAAGAAAAATTTTTCATCTTCGCTCGACGTTTC
TTTTGTAGTACTCATCTCTTTTTATATAAAGATTAATTAGTTATTGTCGCTTTGCTTTTCCTTCTTTAAAA
AATGTTTC

ATTATATTCTAGCTTCGTTGTCATGGGAACATAGCCCATACACCGCAGTTATTTATGATCATTTCGAACGG
GAAGTATGGATGAATCTTTTTTTTTTTTTTTTTATAGCACGCAACTGAAAAAAAAAAAAAGAAAAATTTTT
CATCTTCG

CGATGCATAATTGGCATCTAGCCCCAATGACGATACACTAATATATATATATATATATATATCTATATATC
TGCAAGAAATAAGCGTGTGTAGTAAGTAAAAGGAAAAATAGAAATTTTTGCTCAAAAATAACGGTCCCATT
TTGATACA

TTTCTGTAGGGTTTTTTTTTTTTTTTTTGTACAGATTAGAAACCATACAGTGTAAACCATTATATAGGTCA
ATTAATATATTTATAGATCATCCTGTCAATTTTTTTTTATTTTTTTTTGCAAAGCTCCCTTTTTCCTGAAA
CCAACAAC

TATTTTAAATAATCAAAAATCAAATTAGCTATTATTTCTCATTTGGTGATAATAATATATACATACATATA
TATATATATATATAAATATATAGGTATATATATAAATAACCTAAGACAAGTTAAATGAGTATAAAAAACCT
TGAACGTA

CCAGTCAGCGACATCTGTCTTTTTTTAATTACTATTGCTGTGTACTTATAGAGTCGGTGGCATTTATTTTA
TTTTTAGTATTTTTTTATACGTGCGTATTCTATTGTTCATTTTTTTTACCAATTTTTTTTTATTTTTATAT
TTTGTTTC

CATTTCTTTTTTCGCCCAGCGGGAAGTACGCGGAAAACAAGAAACAAAAAAAAAATAAAAAAATAAAAAAT
AAAACAGAAACTAAAATCTGATGATGAAATTGATATTCAATTGGAGAAAGAAGAAAGATCTTGTTTAATTT
GAAATAAA

ATAAGAAAAAGAAAAAGAAGCACAATATAGGAAAGCGTTCTAATTAACTAAAGAAAAAAAAAAAATAAAAA
AAAAATAAAAAAAAGGTAACTAAAAAGGACAAAAAACATAACAGCTGTTTATAAAGTACTTTATACGTAAG
AAACTACT

TTATTAAAATAAAAAAAAAAAAGCATGCATAACACTAATAATATATTATTAAAAAAAAGCCAATCTATATT
ATTTAAATTATCCGAAATTACTTTTATTACATTATGACATTCTATAGTCTATGCAAGCGGATCAAAAAACC
ATTCCATC

CTCGCAACAAACCAAGATGGATTCAAAATGCCTACTTACCGCATTGTATGTGGGAGAATGAACATATCTGT
AATTGTATTATATAAAAGTAGTTAGTTTTTTTTTTTTTTTTTTTTTTTTTTTTTTTTTATGTGTAATGCAA
ATACAAAA

AAAAAGTTGTTTTCACTTTGTGTTCGAAAAAATAAGTATTTGAAAACGAAAAAATAGTTTGTATGTTTGTA
TATAAGTTGTTATTATCAAAAATTAGAGGGAAAAAGTAACTAAATATATATTAAAAAAAAAAAAAAAATAA
ATGTATGT

ATATTCAACAATTGCAGTTTTCTTTTCCTTTTTGTCCTTGTCCTTTCTTTCCTATTTATATATATATATAT
ATATATATATAATACTACTATGTTATATTACATTATATTATTATCACAATAATTATTACGTTCATCGTGCC
GAAACGTC

CAAAGCCGCGGCGTTCCCGGAAAAAAAAAAGGAAGGCCACGCTATTGGTAACCATGAAGTGTAAGGCGACG
CAGGAGAGATGAAAAGAATGAAATTAAAAGAAATCCAAAAAAAAAAAAAAAAAAAAAAAAGAAAAAGAAAG
AAAAAGGG

TTAATTAATATTTTGTATCTAAAGTGTTCATACTATATTATTTACTTAGATTATACATAATTAAAAAAAAA
AAAAACTTTTTTTCCTTGTTTTCATTTTGTTTTTATTATTAAATATTAAAAAAAGTGTCCATAACATCAAT
GTTTTTTT

TTAAGATCTTCACTCTGTACACGCTATATACTAAATAACAATAAGAAAAAGAAAAAGAAGCACAATATAGG
AAAGCGTTCTAATTAACTAAAGAAAAAAAAAAAATAAAAAAAAAATAAAAAAAAGGTAACTAAAAAGGACA
AAAAACAT

CGTATAAGAAAAGCCAATTAGTACCTATATATATATATATATATATATATATATATATATATTCCTTTATT
ATAATTCCTATCAGCGATTTTTGCATCACACCCAAGAAAAATCTATAATAGAGACACAAAAAAAATGAACGT
AACTGAAA

AACACTGCTAAAGCAACCTATGACCTGTACACTAATTAAAACACTTTTATCCATTTTTTTTCGTATAAGCA
TATTTTACATCTTTATATATATTTATTTTTTTTTTTTTTTATTTTGGTAGAATAGATTACATATACAACTTT
TTCATAAT

GAAATTAAAAGAAATCCAAAAAAAAAAAAAAAAAAAAAAAAAGAAAAAGAAAGAAAAAGGGGGAATAACACA
GCCGCGGGTAACATGAAGCGGCGCGAGCGATACTTCCGTGTTAAGTGATATCCATCTTTCTGCGGGTAATA
AGTCTAGT

AAAAAAAAAAGGAAGGCCACGCTATTGGTAACCATGAAGTGTAAGGCGACGCAGGAGAGATGAAAAGAATG
AAATTAAAAGAAATCCAAAAAAAAAAAAAAAAAAAAAAAAAGAAAAAGAAAGAAAAAGGGGGAATAACACAG
CCGCGGGT

TGAAAACGAAAAAATAGTTTGTATGTTTGTATATAAGTTGTTATTATCAAAAATTAGAGGGAAAAAGTAAC
TAAATATATATTAAAAAAAAAAAAAAAAATAAATGTATGTTGGAATGAAATAGCGTAGTAAGGATAAAGGTG
TAATAGAT

AAAAAAAAAAATTAACAGTTTTTTTTTTTCATTTTTTTTTTTTATTCTTATTTATGTATGATACTTTATTA
TTATTTCTCTTAATTATTTATTTATTTAACTAACACGATGAGCACTTTTAACTGCAATGGTTAAACTGTAG
CAATGTTG

TTTTGTATCTAAAGTGTTCATACTATATTATTTACTTAGATTATACATAATTAAAAAAAAAAAAAACTTTT
TTTCCTTGTTTTCATTTTGTTTTTATTATTAAATATTAAAAAAAGTGTCCATAACATCAATGTTTTTTTTT
ACCTTTGA

CTAAATAACAATAAGAAAAAGAAAAAGAAGCACAATATAGGAAAGCGTTCTAATTAACTAAAGAAAAAAAA
AAAATAAAAAAAAAAATAAAAAAAAGGTAACTAAAAAGGACAAAAAACATAACAGCTGTTTATAAAGTACTT
TATACGTA

AACAAACCAATATTTTAAATAATCAAAAATCAAATTAGCTATTATTTCTCATTTGGTGATAATAATATATA
CATACATATATATATATATATATAAATATATAGGTATATATATAAATAACCTAAGACAAGTTAAATGAGTA
TAAAAAAC

ATCTGAAAACTTCTTTTGCGATTCATAACCAACAGAAAATTTTGGAGGAAAACAATGTAGCTGTATAAATA
TATATATATATATATGTATGTATACTTATTTATTTATATATATATCACAAAAGGCTACCAAAAAGATTAGC
CGCCGAGG

TGATGCAGTAGTGAACATATTGCTCGCATAATGTTACCCGTTTATACCTCTATACGATTATTAAGGGAGGG
TAATACTTTCTCAATTTTTTTTTTTTTCAGTTTTTAATATTATTTCCTTAAGATTTTTCGGTGGCGTTTCGC
GACAACTT

ACACTTTTATCCATTTTTTTTCGTATAAGCATATTTTACATCTTTATATATATTTATTTTTTTTTTTTTTTA
TTTTGGTAGAATAGATTACATATACAACTTTTTCATAATGTCAAACTATAGAGTTTTATTTCACATAACGG
AAGAAGCC

TTTTTTTTTTTCTTCTTTTATTATTTTTTATGTTTATTCCATATATACACTTATAATTTTTTTTTTTGCGA
TTAATATAAGTTTGCCTGGATACACGAACATATATATGATTAAAATGGTTGTCTCGAACAAAAAAATTCAC
TTTTTAGA

AAAACCAGCAGATATCGCATAAAAAAAATGGCCAGCTATAGAATAAAATTAGCCCAGCTATATTTTTATAGC
GTCTTTTGATGACGCGAAAAGTAGACTTCGCGTAACACGAAAAATGCTGGTGCAGGTGGATTTTTTTGGCA
GATACTAC

GCGAGAATATTTTTTTGCCACCACGATTAGACTCCACTTTGCTTCTATAGTCCTATCTATTGTTCTTTTTA
TTCCTAGCATAACCTTATTGTATTGTTCTTTTTCTTTTTCCTTTTTCTTTTTTTTTTTTTTTTTTTTTTTT
TTTTCACA

TAACCTTATTGTATTGTTCTTTTTCTTTTTCCTTTTTCTTTTTTTTTTTTTTTTTTTTTTTTTTTTCACAT
CTTTTTCCACGGCCGCTCAAAGGGGTCTCACACTGGATTAAAAGCTGAAAACAAGAAATGGCTCTTTCGAT
AAGGTTGC

TAAAAAAAACTTTGGAAGCGTGCACACTCAGTCTTAAATCTGCCCTATACTACCGAAGATAAATACATGAA
CTACACGTATCATATAACATCATATCCTTTTTTTTTTTTTTTTTTTTTTTTTCTAGGCTTTACTAATACAA
GGGCCTTA

TTTTTTTTCATTTTTTAAAGTGTTATACTTAGTTATGCTCTAGGATAATGAACTACTTTTTTTTTTTTTTT
TTTACTGTTATCATAAATATATATACCTTATTGTTGTTTGCAACCGTCGGTTAATTCCTTATCAAGGTTCC
CCAAGTTC

TTTTTTAATTACTATTGCTGTGTACTTATAGAGTCGGTGGCATTTATTTTATTTTTAGTATTTTTTTATAC
GTGCGTATTCTATTGTTCATTTTTTACCAATTTTTTTTTTTATTTTTATATTTTGTTTCTTGCGTGGAGTT
TTTACAGG

ATTATTTCTCATTTGGTGATAATAATATATACATACATATATATATATATATATAAATATATAGGTATATA
TATAAATAACCTAAGACAAGTTAAATGAGTATAAAAAACCTTGAACGTAAATGAAAAAAAAAAAAATACTA
GTAAATTG

GCTATTATCCTTGTTCCCCCCAAGGACTGCAGTAGAAGAGTTATGTTACAAATGTTGATATATATATATAT
ATATATATATATATATGTATGGACATTTGTGATGAAAATATATCTTGAACAAGCGCGATTAAGGTCATAAT
AAAAAATC

ATCGAATTTACGTAGCCCAATCTACCACTTTTTTTTTTCATTTTTTAAAGTGTTATACTTAGTTATGCTCT
AGGATAATGAACTACTTTTTTTTTTTTTTTTTTACTGTTATCATAAATATATATACCTTATTGTTGTTTGC
AACCGTCG

CATATTTTTTCCAGTCACTATCATCATCATCATATTTTGTATATATACATTTATTATATAAAATATCATAC
CAATTTTTGCCAAAGAACACCGTTTCCCTCTTCGCGTTGACCACAGTAATTGCAGAAACGACAAAAAAAAA
ACAAAAAA

CCAGAGCCAGAATTGGATGTAACAAAAAAAAAATTGAAAAGTAACTACAATTGTCACTAGTTATTATTGGC
CAAGTCATAAAGGGACCTTGTCTCGATTTTATATACCGTATATCGGTTCTTTCCAATTTTTTTTTTTTTTT
TTTTTTTT

TTTTTTTTGTACAGATTAGAAACCATACAGTGTAAACCATTATATAGGTCAATTAATATATTTATAGATCA
TCCTGTCAATTTTTTTTTATTTTTTTTTGCAAAGCTCCCTTTTTCCTGAAACCAACAACAAAATATAAAAA
AAAAATGA

CATCCTTATAATTATATTCTAGCTTCGTTGTCATGGGAACATAGCCCATACACCGCAGTTATTTATGATCA
TTTCGAACGGGAAGTATGGATGAATCTTTTTTTTTTTTTTTTTATAGCACGCAACTGAAAAAAAAAAAAAG
AAAAATTT

CAAGGACTGCAGTAGAAGAGTTATGTTACAAATGTTGATATATATATATATATATATATATATATATATGTAT
GGACATTTGTGATGAAAATATATCTTGAACAAGCGCGATTAAGGTCATAATAAAAAATCACAGAAAATTTT
TCAGGTCG

ATTTCGAACGGGAAGTATGGATGAATCTTTTTTTTTTTTTTTTTATAGCACGCAACTGAAAAAAAAAAAAA
GAAAAATTTTCATCTTCGCTCGACGTTTCTTTTGTAGTACTCATCTCTTTTTATATAAAGATTAATTAGT
TATTGTCG

ATGAATCTTTTTTTTTTTTTTTTATAGCACGCAACTGAAAAAAAAAAAAAAGAAAAATTTTTCATCTTCGC
TCGACGTTTCTTTTGTAGTACTCATCTCTTTTTATATAAAGATTAATTAGTTATTGTCGCTTTGCTTTTCC
TTCTTTAA

TTTTTTTTTTTTTTTTTTTTTTTTTTTTTTTTGGTTGTAATATTAAAAGAATAAACATTTATCTGATATGT
AATTGCATTTATAAAATGTACAGTACCGCATTTAAAGTTTGCTGATAATTAAATCTAGAATTTTATACCTA
GGATCATC

TTATGTTACAAATGTTGATATATATATATATATATATATATATATATGTATGGACATTTGTGATGAAAATA
TATCTTGAACAAGCGCGATTAAGGTCATAATAAAAAATCACAGAAAATTTTTCAGGTCGCGGGCGTTCTCT
CACCCGGC

ATTTCCCTTCCGAGTCACTTATTTTTTTTTTCTTCTGAAAAAATTAATTAGATTAATTTCAATTAATATCA
TTTCCGCTTATCTGACTTCTTTCATTTTTTTTCTCTATATTTCGCGTTTACTAGGAAAGAAAAGGAAAAAA
AATTTTTC

TTAAAAAAAAAAAAAACTTTTTTTCCTTGTTTTCATTTTGTTTTTATTATTAAATATTAAAAAAAGTGTCC
ATAACATCAATGTTTTTTTTTACCTTTGATTAGTTTTCATTGGCGAAACCATAATCCCCGTTCCATTCCCA
TCATAACT

TCACGGCATTCCAGCTGTAAAAAACCAGCAGATATCGCATAAAAAAATGGCCAGCTATAGAATAAAATTAG
CCCAGCTATATTTTTATAGCGTCTTTTGATGACGCGAAAAGTAGACTTCGCGTAACACGAAAAATGCTGGT
GCAGGTGG

AGAAGAAAAAACAAAGAAGCCTCGCAACAAACCAAGATGGATTCAAAATGCCTACTTACCGCATTGTATGT
GGGAGAATGAACATATCTGTAATTGTATTATATAAAAGTAGTTAGTTTTTTTTTTTTTTTTTTTTTTTTTT
TTTTTTTA

AACACTTCAGAAAAATATTCTGCTACTATTCCTTACTTTACTATAAGAATTTTGTTTTCCAAAAAAAAAAA
ATATAAAAAAAATAATCATACTCTATTACTATGGCTAACGTAGAAAAACCAAACGATTGTTCAGGCTTTCC
CGTTGTTG

CCTCGCATCCTTCAATAACTTATTTTTTTCCTCAATCTTCGATATTAGACATTTTTTTTTTCCTATGCCTC
CCTTTTCCAAGTAATGTTTTTTTTTTTTTTTTTTTTTTTAGCTCCTCTCACAAAAGTACTGAGGACTGAAA
TGTAAAAA

AAAAAAAAAAAAAAAAAAAAAGAAAAAGAAAGAAAAAGGGGGAATAACACAGCCGCGGGTAACATGAAGCG
GCGCGAGCGATACTTCCGTGTTAAGTGATATCCATCTTTCTGCGGGTAATAAGTCTAGTCAAGTCCGCTGT
ATTTTTTC

GAAATCCAAAAAAAAAAAAAAAAAAAAAAAAGAAAAAGAAAGAAAAAGGGGGAATAACACAGCCGCGGGTA
ACATGAAGCGGCGCGAGCGATACTTCCGTGTTAAGTGATATCCATCTTTCTGCGGGTAATAAGTCTAGTCA
AGTCCGCT

GCTGAGAGGAAACACTGCTAAAGCAACCTATGACCTGTACACTAATTAAAACACTTTTATCCATTTTTTTT
CGTATAAGCATATTTTACATCTTTATATATATTTATTTTTTTTTTTTTTATTTTGGTAGAATAGATTACAT
ATACAACT

TATAAAGCGAAATTTTTTAAAATTAATTGATTAATTAATATTTTGTATCTAAAGTGTTCATACTATATTAT
TTACTTAGATTATACATAATTAAAAAAAAAAAAAACTTTTTTTCCTTGTTTTCATTTTGTTTTTATTATTA
AATATTAA

TTCTATGTAACATATTTTTTCCAGTCACTATCATCATCATCATATTTTGTATATATACATTTATTATATAA
AATATCATACCAATTTTTGCCAAAGAACACCGTTTCCCTCTTCGCGTTGACCACAGTAATTGCAGAAACGA
CAAAAAAA

ACTAGTAGTAATAGTAGTCAGATACAAAAATTCTATGTAACATATTTTTTCCAGTCACTATCATCATCATC
ATATTTTGTATATATACATTTATTATATAAAATATCATACCAATTTTTGCCAAAGAACACCGTTTCCCTCT
TCGCGTTG

CCTTTTATCTATTCAAATTGTTTCCCTTAGGTATATATATATATATATATATATATATATATTTTCCCTGT
ATATATCTATGTAAATGACGAAAACGCATGACATTTTAAAACCTACCCCGGGTTTGACCACAACCCACCGT
TCATCTAA

ATTCCTAGCATAACCTTATTGTATTGTTCTTTTTCTTTTTCCTTTTTCTTTTTTTTTTTTTTTTTTTTTT
TTTTTCACATCTTTTTCCACGGCCGCTCAAAGGGGTCTCACACTGGATTAAAAGCTGAAAACAAGAAATGG
CTCTTTCG

GTTGGAATAACGCGAAAAGAAGGGTACGGCGTAATCACCCCACACGGCTTGTATTTTTTTCGGAGAACTAA
GTCATAGACGTAATGCTAATTTTTCCGTCTAGCCGATGTTTATGCGGCTTTTATTTTTTTTTTTTTTTTT
TCTCATCT

GAAGAAAAAAAAAAACTGATATAGAACCCAAAAATGTTCGGCTTTTAAAATACAAATATGTACAGGTGTTAA
TGTCTAAATAATGATAATCCAAACGCGATAATTTTTTTTTCGCGTTTGTTTTTGTTTCAGAAGAATAAGTA
AACACTTC

TTATTATTATTATTATTATTATTATTATTATTATTATTATTATTATTATTATTATTATTATTATTATATTA
TTATTAATACTAAATATTGACACTGCTTTACGCACTAGATTGTGCGGGTAACTGAATATTTTTTGAAAATG
AAGAAAAA

ATATAAGTTGTTATTATCAAAAATTAGAGGGAAAAAGTAACTAAATATATATTAAAAAAAAAAAAAAAAATA
AATGTATGTTGGAATGAAATAGCGTAGTAAGGATAAAGGTGTAATAGATATATATTATAAGAAATAATCCG
GTGTCTTC

TGTACTTATAGAGTCGGTGGCATTTATTTTATTTTTAGTATTTTTTTATACGTGCGTATTCTATTGTTCAT
TTTTTTACCAATTTTTTTTTTATTTTTATATTTTGTTTCTTGCGTGGAGTTTTTACAGGAAGTTAATCACA
ACAGAGGG

TTCTTTTGCGATTCATAACCAACAGAAAATTTTGGAGGAAAACAATGTAGCTGTATAAATATATATATATA
TATATGTATGTATACTTATTTATTTATATATATATCACAAAAGGCTACCAAAAAGATTAGCCGCCGAGGTA
CATCGTGA

TGCAAAAAAAAAATTTTTTTGTCATTTCTTCGAATCAACTCAATGAAATTTGAAAAATAGCAGAAAATAAGT
TGATAAGATCGCAATGTAATATATATATATATATATATATATATTACAATTCGTGAACTGGCACAAGGAAAAG
CGTGTAAT

TTCGGTAACTAGGGGATAAGGGGCTTCGTCCTACACGATGCTCCTGTCGTTTCTTTTTTTCATTTCTTTTT
TCGCCCAGCGGGAAGTACGCGGAAAACAAGAAACAAAAAAAAAATAAAAAAATAAAAAATAAAACAGAAAC
TAAAATCT

TATATATATATATACACAGTATTAATATTCTTTCCTTGAGATTACTCTCTATAAAAAAAAAAAAAAAAAAG
CTTTTAAAAAAAAAAAAAACTCAAAAAAAGAAAGAATATTCAAAGAAAGTTTCATCTTGTCAGTTGAAATGA
ATAGTTTA

TTCTCAAAAATGTAGCGTCGCTAAAATGGTTTTCTCTACCTTTAGTTTTACGCCAGATTTTTACTAGACGC
GTTTTAGTTTACTTTTTCCTACCCTGTTCGTTTTTTTTTTTTTTTTCGCACCAAATTTTTCAACCGTTCCA
TGGAAGCC

AATAGGTATAAAAGGAGTTGAAATTAAAAATAATAATAAATAATGATATAAAAATTATAAGTTTCTCCTTT
CGGAACTAAAAAATAATATTAATAATAATATATAAATATTATATCTTTTTTATAATATAACATAGATACGA
GTTTTTAT

GTCATAATTTGTAGTATTATCTGTGTGACGGATTAAGTTTGCTATCTCAAATGCCGCGCGCGCGAATTGCT
CATGATATAATAATAGCTAATAATATGATAATATGATAATAATAGATAAGATTACTGATAACATTATTTTA
GTATTCAA

TTATTATCAAAAATTAGAGGGAAAAAGTAACTAAATATATATTAAAAAAAAAAAAAAAAATAAATGTATGTT
GGAATGAAATAGCGTAGTAAGGATAAAGGTGTAATAGATATATATTATAAGAAATAATCCGGTGTCTTCCT
GGTGACGG

GGCCCATGCCTTCGTTACCTTGGAATGGTATGTAAAGTGATAAAAATATACACGTATATATATATATATAT
ATATATATAAGTACGTGAGATCTATTTGTATAATATAATGCTAATAAAATAGTATCAACGATACTGTTGAT
AAAGGTAA

CTGAAAGAGTCTTTGACAAAATTCCAGCCATTTTTTCAACCTTTTTTTTATCCGTATATATTAAGGTATGC
TATATGTGTATTTTCCAATGTTTAGTTTTATAATAATACTTCTTCAGCATCAAATATGCTTGCTTATGTA
AAAATACA

GTATTGTTCTTTTTCTTTTTCCTTTTTCTTTTTTTTTTTTTTTTTTTTTTTTTTTTTCACATCTTTTTCCAC
GGCCGCTCAAAGGGGTCTCACACTGGATTAAAAGCTGAAAACAAGAAATGGCTCTTTCGATAAGGTTGCAA
CATTTTGA

TTATACATAATTAAAAAAAAAAAAAAACTTTTTTTCCTTGTTTTCATTTTGTTTTTATTATTAAATATTAAA
AAAAGTGTCCATAACATCAATGTTTTTTTTTACCTTTGATTAGTTTTCATTGGCGAAACCATAATCCCCGT
TCCATTCC

TCTTCGAAAACTGATGAGAATTCTTCATTATTTTTTTTTTTCTTCTTTTATTATTTTTTATGTTTATTCCA
TATATACACTTATAATTTTTTTTTTTGCGATTAATATAAGTTTGCCTGGATACACGAACATATATATGATT
AAAATGGT

AAATTAAAAATAATAATAAATAATGATATAAAAATTATAAGTTTCTCCTTTCGGAACTAAAAAATAATATT
AATAATAATATATAAATATTATATCTTTTTTATAATATAACATAGATACGAGTTTTTATGTTCCTTTGGCA
ATAGCTTT

GTTTAACATATTTCTGTAGGGTTTTTTTTTTTTTTTTGTACAGATTAGAAACCATACAGTGTAAACCATT
ATATAGGTCAATTAATATATTTATAGATCATCCTGTCAATTTTTTTTTATTTTTTTTTGCAAAGCTCCCTT
TTTCCTGA

GGTTACCTGGCTGATTTAGGGTTATATGTACTATTATTAGTATTATTATTGTTAGTATTATTATTATTATT
ATTATTATTATTATTATTATTATTATTATTATTATTATTATTATTATTATTATTATATTATTATTAATACT
AAATATTG

AGAAGGAAGCGACACATTTGTGATGCTTTGAGGTCAGTAATGTAATATATATATATATATATATATATATA
TATTATATAGTGTAACTGCTCTTATTTATCTTGTCTATTCGAACCCAACAAAAAAGGCTGCAGTGGGAATC
TGTTCATG

CTGATTTAGGGTTATATGTACTATTATTAGTATTATTATTGTTAGTATTATTATTATTATTATTATTATTA
TTATTATTATTATTATTATTATTATTATTATTATTATTATTATTATATTATTATTAATACTAAATATTGAC
ACTGCTTT

TATATATATATATATATATGTTATATTATTGTTAATTAAATATTATTATGCGTATTTTCCTTTTCTTTATTAG
TATAGTATTAATGACAGTAATAATAATAATAATAGTAACAATATCTCTTTTTTTTTTTTCAGTGAGCTTTTA
TTTTTTTT

ACACATAAGCAAAAAAAAAAAAAAAAAAAAATAAACAAATGAGCAACCCATCAAATACCAAGAACAAAGAAT
TTAATATAATATATAGTTATTAAATTTAAATGTATATATGCAGTTCTGCTCTTATTTTGTGACCAATTTCT
TTAAGTCA

CCAAAAGCAAGCCGATTTCAGGTTAACCCTTTATTAAAATAAAAAAAAAAAGCATGCATAACACTAATAAT
ATATTATTAAAAAAAAAGCCAATCTATATTATTTAAATTATCCGAAATTACTTTTATTACATTATGACATT
CTATAGTC

AGCTGACGATCCAGTCAGCGACATCTGTCTTTTTTTAATTACTATTGCTGTGTACTTATAGAGTCGGTGGC
ATTTATTTTATTTTTAGTATTTTTTTATACGTGCGTATTCTATTGTTCATTTTTTTACCAATTTTTTTTTT
ATTTTTAT

ATTCATAACCAACAGAAAATTTTGGAGGAAAACAATGTAGCTGTATAAATATATATATATATATATGTATG
TATACTTATTTATTTATATATATATCACAAAAGGCTACCAAAAAGATTAGCCGCCGAGGTACATCGTGAGG
AGTGATAT

TTCTTCATTATTTTTTTTTTTCTTCTTTTATTATTTTTTATGTTTATTCCATATATACACTTATAATTTTT
TTTTTTGCGATTAATATAAGTTTGCCTGGATACACGAACATATATATGATTAAAATGGTTGTCTCGAACAA
AAAAATTC

GGTCATCTAAAGAGGCGCATTTCTCAAAAATGTAGCGTCGCTAAAATGGTTTTCTCTACCTTTAGTTTTAC
GCCAGATTTTTACTAGACGCGTTTTAGTTTACTTTTTCCTACCCTGTTCGTTTTTTTTTTTTTTTTCGCAC
CAAATTTT

TAGTATTAAAAATTACCGTTACTGGTTAAAATGGAATACAGCTCATCCATTTAAAAAAAAAAAAAAATAGA
ATAACAATAATTAAGCAGAAAAAATGTTAACGAATATAATAAATCGATTTGTCCATTTTCATAAATTTTTT
TTTTGTAT

TAAAAGGCGAAGAAGGAAGCGACACATTTGTGATGCTTTGAGGTCAGTAATGTAATATATATATATATATA
TATATATATATATTATATAGTGTAACTGCTCTTATTTATCTTGTCTATTCGAACCCAACAAAAAAGGCTGC
AGTGGGAA

TGGGAGAATGAACATATCTGTAATTGTATTATATAAAAGTAGTTAGTTTTTTTTTTTTTTTTTTTTTTTTT
TTTTTTTTATGTGTAATGCAAATACAAAAAGAACTGCAGGCATTTTGAAAGTTCACTCGTATAGGAAACTT
TGATAGAG

CGAAGAAGTTGGTTACCTGGCTGATTTAGGGTTATATGTACTATTATTAGTATTATTATTGTTAGTATTAT
TATTATTATTATTATTATTATTATTATTATTATTATTATTATTATTATTATTATTATTATTATTATATTAT
TATTAATA

AATTACCGTTACTGGTTAAAATGGAATACAGCTCATCCATTTAAAAAAAAAAAAAAATAGAATAACAATAA
TTAAGCAGAAAAAATGTTAACGAATATAATAAATCGATTTGTCCATTTTCATAAATTTTTTTTTTGTATTA
TTTCATTA

GGAAGGCCACGCTATTGGTAACCATGAAGTGTAAGGCGACGCAGGAGAGATGAAAAGAATGAAATTAAAAG
AAATCCAAAAAAAAAAAAAAAAAAAAAAAAGAAAAAGAAAGAAAAAGGGGGAATAACACAGCCGCGGGTAA
CATGAAGC

TATGTATATATATATATATATACACAGTATTAATATTCTTTCCTTGAGATTACTCTCTATAAAAAAAAA
AAAAAAAAAGCTTTTAAAAAAAAAAAAACTCAAAAAAAGAAAGAATATTCAAAGAAAGTTTCATCTTGTCA
GTTGAAAT

ACTATTGCTGTGTACTTATAGAGTCGGTGGCATTTATTTTATTTTTAGTATTTTTTTATACGTGCGTATTC
TATTGTTCATTTTTTTACCAATTTTTTTTTTATTTTTATATTTTGTTTCTTGCGTGGAGTTTTTACAGGAA
GTTAATCA

ACAAAGAAGCCTCGCAACAAACCAAGATGGATTCAAAATGCCTACTTACCGCATTGTATGTGGGAGAATGA
ACATATCTGTAATTGTATTATATAAAAGTAGTTAGTTTTTTTTTTTTTTTTTTTTTTTTTTTTTTTTTATG
TGTAATGC

GATGCAGCAAAGTAAAAAATAATAGGTAAAAATAATAAAAATACATGTATCTTTTTGGTTGGTTAATTTAT
CTAAACAGTTTTATATATATATGTATATATATATATATATATACACAGTATTAATATTCTTTCCTTGAGAT
TACTCTCT

GCCGATTTCAGGTTAACCCTTTATTAAAATAAAAAAAAAAAAGCATGCATAACACTAATAATATATTATTAA
AAAAAAAGCCAATCTATATTATTTAAATTATCCGAAATTACTTTTATTACATTATGACATTCTATAGTCTA
TGCAAGCG

AACTCGCCTGCACTCCGAAACGAAATGCGATACAGTACGTGTCATGCGGTTAGTATATATACATATATATA
TATATATATATATTCAAATAAATTATAACGTATTAAATAATATGTGAAAAAAGAGGGAGAGTTAGATAGGA
TCAGTTGG

TTTTTTCAACCTTTTTTTTATCCGTATATATTAAGGTATGCTATATGTGTATTTTCCAATGTTTAGTTTTT
ATAATAATACTTCTTCAGCATCAAATATGCTTGCTTATGTAAAAATACAATCAAGTTTTGTCCCTTGCTTG
CGCGAAGA

CTTTTTCGGGACGTATTCTCGGGAAATAAGCAGGTCCAGATCCGCGTAGCATGAATGGGGCGCCAAAACAG
TCGCGGGTAACGAAAGAGCGCAACCACGCGTATATTCCGGGCCGGTGAGGGGCAACGCGCATTGTCCCGGG
GCGATATA

TTGTTCCCCCCAAGGACTGCAGTAGAAGAGTTATGTTACAAATGTTGATATATATATATATATATATATAT
ATATATGTATGGACATTTGTGATGAAAATATATCTTGAACAAGCGCGATTAAGGTCATAATAAAAAATCAC
AGAAAATT

TGATAAACGTGAAGGGAGTGTCATATCTACCGACATAAAGATAGAGATCTTTTTTTTTCCTTTATTTTTTT
TTTTGATTTTTTTTTTTGATTTTCTTGATATTAGTTTTTAGAAGTCCTTTGACGGGGCAGCCGGAGAATAT
ATACCAAC

AAAAACGCGTTAAAACTTGCATTCCAATTTATCAAAAAGTTTGCCAGGACATATAGGATATAAATGCATTG
CGAAGTAAAACACTTCTCAAAAAATTAAAAAAAAAAAAAAAAATAAAACTAACGTATTCACTTCTTTTACT
TTAGTGGA

CGGAGTGCTTGAAGAAAAAAAAAAACTGATATAGAACCCAAAAATGTTCGGCTTTTAAAATACAAATATGTA
CAGGTGTTAATGTCTAAATAATGATAATCCAAACGCGATAATTTTTTTTTCGCGTTTGTTTTTGTTTCAGA
AGAATAAG

GTAGAAATTAATAGTACTCTAAAAAGAAAGAACGGAATAAGAGGAAAAGACCTTTTATCTATTCAAATTGT
TTCCCTTAGGTATATATATATATATATATATATATATATATTTTCCCTGTATATATCTATGTAAATGACGA
AAACGCAT

TAAGGCGGCAGACTTAAGATCTGTTGGACGGTTGTCCGCGCGAGTTCGAACCTCGCATCCTTCAATAACTT
ATTTTTTTCCTCAATCTTCGATATTAGACATTTTTTTTTTCCTATGCCTCCCTTTTCCAAGTAATGTTTTT
TTTTTTTT

TTATTATAGTTCTATCTTCATGTTTTTAGAGGTTTTTTTTAAATGAGTTTATTAAAATTAAATTTTGAATG
TTATTTTTGAATAAAAATATAAAAAAAAATAATATTAATAATTTTTCATTAACTGCTCATGATATCAGTGCA
TTAGAAAA

CCAATATATGCATGGGCTGAGATAGAGGTACAAGGAATTTGTAAATCAGTAAAAAAAAAAAATTAACAGTTT
TTTTTTTTCATTTTTTTTTTTTTATTCTTATTTATGTATGATACTTTATTATTATTTCTCTTAATTATTTAT
TTATTTAA

CCAGTACGCCAATAAAGTGTGTCGCGCAGAATAGATCTTCATGTAATTCCTTTTTCCCTTTTATGCAATCT
ATAATGTTAATCAGCGTAGATCACTATATACGCTTTTTTTTTTTTTTTTCAGCTCCGATTAGAGAGCAAAA
GAGATGTA

ACTTTCTCTTTTTTTTTCACACGCGTTTGCAACATGCCCGTATTACATGAGCTTATTTGACTTTAGATGGT
TTTCAGTTCTTTTTTTTTTTTTTTTTTTTTCAGTAGATTAATGCGAAGAGTTAAATCACCAATTCAAAGA
TGGCAGAT

ATAAAAAGCATTACCGTCACGCATCGTGTTGCAAATAGCATATACATATATATATATATATATATACATAT
AACGTATACGTTAGAAAAATTTGAATACACACATTCATTTATCCTATTATTATCATTTTCATTCTTACCCG
CTCCCCGC

CTCCAATTGGGCGAGAATATTTTTTTGCCACCACGATTAGACTCCACTTTGCTTCTATAGTCCTATCTATT
GTTCTTTTTATTCCTAGCATAACCTTATTGTATTGTTCTTTTTCTTTTTCCTTTTTCTTTTTTTTTTTTTT
TTTTTTTT

GATAGAGGTACAAGGAATTTGTAAATCAGTAAAAAAAAAAAATTAACAGTTTTTTTTTTTCATTTTTTTTTT
TTATTCTTATTTATGTATGATACTTTATTATTATTTCTCTTAATTATTTATTTATTTAACTAACACGATGA
GCACTTTT

TCTATCTTCATGTTTTTAGAGGTTTTTTTAAATGAGTTTATTAAAATTAAATTTTGAATGTTATTTTTGA
ATAAAAATATAAAAAAAATAATATTAATAATTTTTCATTAACTGCTCATGATATCAGTGCATTAGAAAATC
TTTTGGTT

AATTTTTTAAAATTAATTGATTAATTAATATTTTGTATCTAAAGTGTTCATACTATATTATTTACTTAGAT
TATACATAATTAAAAAAAAAAAAAACTTTTTTTCCTTGTTTTCATTTTGTTTTTATTATTAAATATTAAAA
AAAGTGTC

AAAGTGTTCATACTATATTATTTACTTAGATTATACATAATTAAAAAAAAAAAAAACTTTTTTTCCTTGTT
TTCATTTTGTTTTTATTATTAAATATTAAAAAAAGTGTCCATAACATCAATGTTTTTTTTTACCTTTGATT
AGTTTTCA

AAAAAGGTGAGCGCTATATAGATTTAGTAATTTACATGTTACAGAAGCAAAAAGCAAATATATATATATTT
TTTATCATATTAGTTTTTATCCTCAGTAGTACTACTGAAATAATAATCTCAGATTTTTTTTTTCACTTGC
CGAGGGAC

TAATGATATAAAAATTATAAGTTTCTCCTTTCGGAACTAAAAAATAATATTAATAATAATATATAAATATT
ATATCTTTTTTATAATATAACATAGATACGAGTTTTTATGTTCCTTTGGCAATAGCTTTTCGTGACTTGTT
TTAAATAC

AAGGCTTGTGCTGATCGAACGAAGCAAATCCTACGAGTAAATACATAAGCGTATACATATATATATATATA
TATATATATATATATATGTATATATATATATGTGTGTGTGTGTAATTGTGTGTATTCAACTGAACTATG
AAGAGTCT

AAAGGAGTTGAAATTAAAAATAATAATAAATAATGATATAAAAATTATAAGTTTCTCCTTTCGGAACTAAA
AAATAATATTAATAATAATATATAAATATTATATCTTTTTTATAATATAACATAGATACGAGTTTTTATGT
TCCTTTGG

GCAGCAACAACGATGCATAATTGGCATCTAGCCCCAATGACGATACACTAATATATATATATATATATATA
TCTATATATCTGCAAGAAATAAGCGTGTGTAGTAAGTAAAAGGAAAAATAGAAATTTTTGCTCAAAAATAA
CGGTCCCA

TGCTACTTTCCCGGTTCTCCCTTTATTATAAACACTTCAGAAAAATATTCTGCTACTATTCCTTACTTTAC
TATAAGAATTTTGTTTTCCAAAAAAAAAAAAATATAAAAAAAATAATCATACTCTATTACTATGGCTAACGT
AGAAAAAC

TTTATACCTCTATACGATTATTAAGGGAGGGTAATACTTTCTCAATTTTTTTTTTTTTCAGTTTTTAATATT
ATTTCCTTAAGATTTTTCGGTGGCGTTTCGCGACAACTTACGAGAAAAAAAAAAAAAGAGGAGACTTGAAGA
AAGAGTGC

GAAAAAGAAGCACAATATAGGAAAGCGTTCTAATTAACTAAAGAAAAAAAAAAAAATAAAAAAAAAAATAAAA
AAAAGGTAACTAAAAAGGACAAAAAACATAACAGCTGTTTATAAAGTACTTTATACGTAAGAAACTACTGG
CACTATTC

TCCGCGTAGCATGAATGGGGCGCCAAAACAGTCGCGGGTAACGAAAGAGCGCAACCACGCGTATATTCCGG
GCCGGTGAGGGGCAACGCGCATTGTCCCGGGGCGATATAATCCATGTACCGGAACCCGCACTTTTTCGACT
TTCTTTCC

AGTTAAGAAAAAGTCTAAAAATGGTTTTTTTCATCCAAAATATTAAATTTTACTTTTATTACATACAACTT
TTTAAACTAATATACACATTTTAGCAGATGCGCGCACCTGCGTTGTTACCCACAACTCTTATGAGGCCCGCG
GACAGCAT

AAACTATTCTTTTTATGAATTGACAGAAAACCAAAAGCAAGCCGATTTCAGGTTAACCCTTTATTAAAATA
AAAAAAAAAAGCATGCATAACACTAATAATATATTATTAAAAAAAAAGCCAATCTATATTATTTAAATTAT
CCGAAATT

TTAATATGAGAAAAAGGTGAGCGCTATATAGATTTAGTAATTTACATGTTACAGAAGCAAAAAGCAAATAT
ATATATATTTTTTATCATATTAGTTTTTATCCTCAGTAGTACTACTGAAATAATAATCTCAGATTTTTTTT
TTTCACTT

AAATTCCTAAAGTTATAGTTCACATTGTTATATATGTATCTATAAAGCGAAATTTTTTAAAATTAATTGAT
TAATTAATATTTTGTATCTAAAGTGTTCATACTATATTATTTACTTAGATTATACATAATTAAAAAAAAAA
AAAACTTT

TTGGATTTCGTTTATTAGCTGTTTAACATATTTCTGTAGGGTTTTTTTTTTTTTTTTTGTACAGATTAGAA
ACCATACAGTGTAAACCATTATATAGGTCAATTAATATATTTATAGATCATCCTGTCAATTTTTTTTTATT
TTTTTTTG

CAAACAGGCGTATGATTGTCGTATATAACAGGGCACTTTCGGCAGTGAATCAATAGGAGGCGTATAAGAAA
AGCCAATTAGTACCTATATATATATATATATATATATATATATATATATATATTCCTTTATTATAATTCCTAT
CAGCGATT

CGAGTCACTTATTTTTTTTTTCTTCTGAAAAAATTAATTAGATTAATTTCAATTAATATCATTTCCGCTTA
TCTGACTTCTTTCATTTTTTTTTCTCTATATTTCGCGTTTACTAGGAAAGAAAAGGAAAAAAAAATTTTTCCC
CCTCCATC

CATGGGCTGAGATAGAGGTACAAGGAATTTGTAAATCAGTAAAAAAAAAAAATTAACAGTTTTTTTTTTTCA
TTTTTTTTTTTTATTCTTATTTATGTATGATACTTTATTATTATTTCTCTTAATTATTTATTTATTTAACT
AACACGAT

CAGGTAAGTCTAATTTATTTAAAAAAAATCCACGAGCATATAGATTTATAACAATAATAATAATTAATATTC
ATCAATTTTTTAGTAAATTCATATATTTTACTCCTACTCTATGTATTTTCAGTAAGTCTACTAGTCGTATT
ACCCGTCG

TTTCTTGAAATCTTCGAAAACTGATGAGAATTCTTCATTATTTTTTTTTTTCTTCTTTTATTATTTTTTAT
GTTTATTCCATATATACACTTATAATTTTTTTTTTTGCGATTAATATAAGTTTGCCTGGATACACGAACAT
ATATATGA

CGCGAAAAGAAGGGTACGGCGTAATCACCCCACACGGCTTGTATTTTTTTCGGAGAACTAAGTCATAGACG
TAATGCTAATTTTTCCGTCTAGCCGATGTTTATGCGGCTTTTATTTTTTTTTTTTTTTTTTCTCATCTTT
GTTCTTCC

CACTCCGAAACGAAATGCGATACAGTACGTGTCATGCGGTTAGTATATATACATATATATATATATATATA
TATTCAAATAAATTATAACGTATTAAATAATATGTGAAAAAAGAGGGAGAGTTAGATAGGATCAGTTGGTC
AACTCATG

ATTTTATGCGTCGGGTGACTTCAATTTGGCGCCCGTTGAGATTATCTAAAAAATGCTTTTATTTATTTAAG
CGCTTTTTGTTTTGACGAATGTATTTATGTTTTTGTATATATATGAGCGTTTTTTTTTTCCAGATTTAACT
AGTTTTCT

GTCGCGCAGAATAGATCTTCATGTAATTCCTTTTTCCCTTTTATGCAATCTATAATGTTAATCAGCGTAGA
TCACTATATACGCTTTTTTTTTTTTTTTTCAGCTCCGATTAGAGAGCAAAAGAGATGTAACATCGATCCTG
CTATAATT

TTTACTTAGATTATACATAATTAAAAAAAAAAAAAACTTTTTTTCCTTGTTTTCATTTTGTTTTTATTATT
AAATATTAAAAAAAGTGTCCATAACATCAATGTTTTTTTTTACCTTTGATTAGTTTTCATTGGCGAAACCA
TAATCCCC

GTTATATGTACTATTATTAGTATTATTATTGTTAGTATTATTATTATTATTATTATTATTATTATTATTAT
TATTATTATTATTATTATTATTATTATTATTATTATATTATTATTAATACTAAATATTGACACTGCTTTAC
GCACTAGA

GCATTTTTAAAGAATAAATATTACTTTTTTCGAAATCTGGTGTTTAAATATTTTTAAGAAAGTTAAAAAAT
AAGGAATATTTGATAAACAAAAAAAAATTAGCCTTTATATATCCATTTTTGTTTATGTATTCCTTTTTTTA
TTTTCTTA

AGCACGTTTGTTAAGATCTTCACTCTGTACACGCTATATACTAAATAACAATAAGAAAAAGAAAAAGAAGC
ACAATATAGGAAAGCGTTCTAATTAACTAAAGAAAAAAAAAAAATAAAAAAAAAATAAAAAAAAGGTAACT
AAAAAGGA

AGAGGCGCATTTCTCAAAAATGTAGCGTCGCTAAAATGGTTTTCTCTACCTTTAGTTTTACGCCAGATTTT
TACTAGACGCGTTTTAGTTTACTTTTTCCTACCCTGTTCGTTTTTTTTTTTTTTTCGCACCAAATTTTTC
AACCGTTC

AAATTAGAGGGAAAAAGTAACTAAATATATATTAAAAAAAAAAAAAAAAATAAATGTATGTTGGAATGAAAT
AGCGTAGTAAGGATAAAGGTGTAATAGATATATATTATAAGAAATAATCCGGTGTCTTCCTGGTGACGGTT
GGTTCACC

CACATGCAGTAATAGGTATAAAAGGAGTTGAAATTAAAAATAATAATAAATAATGATATAAAAATTATAAG
TTTCTCCTTTCGGAACTAAAAAATAATATTAATAATAATATATAAATATTATATCTTTTTTATAATATAAC
ATAGATAC

AAGCAACGCGACCGTCGTGGGTTCAAACCCCACCTCGAGCACTTTCTCTTTTTTTTTCACACGCGTTTGCA
ACATGCCCGTATTACATGAGCTTATTTGACTTTAGATGGTTTTCAGTTCTTTTTTTTTTTTTTTTTTTTT
CAGTAGAT

GCTCGTGTAAGTGTCGGCCCGGAACTTGATTCCCCCCGGACTAATTAGAAGTTCCGAGAACGCGCGCAAGC
CGTCCCGCGCGTAGAAGGCTAAAAAGCAGCCGGAGGAAAAAAAATAACGCAAATGTGGGCTCCCCTTCCCC
TGTGCGGC

AACCATAGATGGTCATCTAAAGAGGCGCATTTCTCAAAAATGTAGCGTCGCTAAAATGGTTTTCTCTACCT
TTAGTTTTACGCCAGATTTTTACTAGACGCGTTTTAGTTTACTTTTTCCTACCCTGTTCGTTTTTTTTTTT
TTTTTCGC

TTTTATGAATTGACAGAAAACCAAAAGCAAGCCGATTTCAGGTTAACCCTTTATTAAAATAAAAAAAAAAA
GCATGCATAACACTAATAATATATTATTAAAAAAAAAGCCAATCTATATTATTTAAATTATCCGAAATTAC
TTTTATTA

ACTGGTTAAAATGGAATACAGCTCATCCATTTAAAAAAAAAAAAAAATAGAATAACAATAATTAAGCAGAA
AAAATGTTAACGAATATAATAAATCGATTTGTCCATTTTCATAAATTTTTTTTTGTATTATTTCATTATC
TGTGCCTC

GTGTCGGCCCGGAACTTGATTCCCCCCGGACTAATTAGAAGTTCCGAGAACGCGCGCAAGCCGTCCCGCGC
GTAGAAGGCTAAAAAGCAGCCGGAGGAAAAAAAATAACGCAAATGTGGGCTCCCCTTCCCCTGTGCGGCCA
CGTCGTGC

CATACGTATGGTATACAGTATCAAAAGTAGCAGAATTTGTCTTCATTATGATATATATATATATATATGTACA
TATATATAGGGTGCTAAAAAGTATTCTTTTTGCTATTATTATTGCTTAAATCGCCGACCTTTTTCCCATTG
TCACGAGA

GTATACAGTATCAAAAGTAGCAGAATTTGTCTTCATTATGATATATATATATATATATGTACATATATATAGG
GTGCTAAAAAGTATTCTTTTTGCTATTATTATTGCTTAAATCGCCGACCTTTTTCCCATTGTCACGAGAAC
ATAATTGT

TAAAAAGGCTTTAATATGAGAAAAAGGTGAGCGCTATATAGATTTAGTAATTTACATGTTACAGAAGCAAA
AAGCAAATATATATATATTTTTTATCATATTAGTTTTTATCCTCAGTAGTACTACTGAAATAATAATCTCA
GATTTTTT

AAACGTGTAGTATTATTATTATTATTCTATTTCCTTTCCGTTTTTATTATTTATATATATATATATATATATA
TATATATTTGGGTGTTTATATTTAGGTAGGGCATAAGGATTTACTGTCGGCATGAACCTACTTGAACAAAA
AAATAAAA

TTTTTTTTATTTTTTTTTGCAAAGCTCCCTTTTTCCTGAAACCAACAACAAAATATAAAAAAAAAAATGAT
AAGCAGGATTTTTTCTTTTAATAATTCCTTAAAATTAACTAACAAGCTGAAGCCCTTACAACAAAGTAAGT
CAAACCCA

GATTTTATTTTCCTCAATGTGCTCGTGTAAGTGTCGGCCCGGAACTTGATTCCCCCCGGACTAATTAGAAG
TTCCGAGAACGCGCGCAAGCCGTCCCGCGCGTAGAAGGCTAAAAAGCAGCCGGAGGAAAAAAAATAACGCA
AATGTGGG

TAATAATAAATAATGATATAAAAATTATAAGTTTCTCCTTTCGGAACTAAAAAATAATATTAATAATAATA
TATAAATATTATATCTTTTTATAATATAACATAGATACGAGTTTTTATGTTCCTTTGGCAATAGCTTTTC
GTGACTTG

AATAAAGTGTGTCGCGCAGAATAGATCTTCATGTAATTCCTTTTTCCCTTTTATGCAATCTATAATGTTAA
TCAGCGTAGATCACTATATACGCTTTTTTTTTTTTTTTTCAGCTCCGATTAGAGAGCAAAAGAGATGTAAC
ATCGATCC

ACCTTGCTTAATCATCTTTGAAGGCTTGTGCTGATCGAACGAAGCAAATCCTACGAGTAAATACATAAGCG
TATACATATATATATATATATATATATATATATATATGTATATATATATATGTGTGTGTGTGTAATTGT
GTGTATTC

ACACCTTTGGAGCACGTTTGTTAAGATCTTCACTCTGTACACGCTATATACTAAATAACAATAAGAAAAAG
AAAAAGAAGCACAATATAGGAAAGCGTTCTAATTAACTAAAGAAAAAAAAAAAAATAAAAAAAAAAATAAAAA
AAAGGTAA

AAAATAGTTTGTATGTTTGTATATAAGTTGTTATTATCAAAAATTAGAGGGAAAAAGTAACTAAATATATA
TTAAAAAAAAAAAAAAAAATAAATGTATGTTGGAATGAAATAGCGTAGTAAGGATAAAGGTGTAATAGATAT
ATATTATA

GTATGTTTGTATATAAGTTGTTATTATCAAAAATTAGAGGGAAAAAGTAACTAAATATATATTAAAAAAAA
AAAAAAAATAAATGTATGTTGGAATGAAATAGCGTAGTAAGGATAAAGGTGTAATAGATATATATTATAAG
AAATAATC

TTCGCCCAGCGGGAAGTACGCGGAAAACAAGAAACAAAAAAAAAATAAAAAAATAAAAAATAAAACAGAAA
CTAAAATCTGATGATGAAATTGATATTCAATTGGAGAAAGAAGAAAGATCTTGTTTAATTTGAAATAAAAG
CAAGAAGA

AATATAATGGACACATAAGCAAAAAAAAAAAAAAAAAAAAAATAAACAAATGAGCAACCCATCAAATACCAAG
AACAAAGAATTTAATATAATATATAGTTATTAAATTTAAATGTATATATGCAGTTCTGCTCTTATTTTGTG
ACCAATTT

CTATTATTAGTATTATTATTGTTAGTATTATTATTATTATTATTATTATTATTATTATTATTATTATTATT
ATTATTATTATTATTATTATTATTATATTATTATTAATACTAAATATTGACACTGCTTTACGCACTAGATT
GTGCGGGT

ACTGAAGTGCAGCTGACGATCCAGTCAGCGACATCTGTCTTTTTTTAATTACTATTGCTGTGTACTTATAG
AGTCGGTGGCATTTATTTTATTTTTAGTATTTTTTTATACGTGCGTATTCTATTGTTCATTTTTTTACCAA
TTTTTTTT

AAGCCAATTAGTACCTATATATATATATATATATATATATATATATATATATATATTCCTTTATTATAATTCCTA
TCAGCGATTTTTGCATCACACCCAAGAAAAATCTATAATAGAGACACAAAAAAATGAACGTAACTGAAAAT
GCTCTATT

AATGTTGATATATATATATATATATATATATATATATATATGTATGGACATTTGTGATGAAAATATATCTTGAAC
AAGCGCGATTAAGGTCATAATAAAAAATCACAGAAAATTTTTCAGGTCGCGGGCGTTCTCTCACCCGGCCG
CCCAAAGT

GAAGGGAGTGTCATATCTACCGACATAAAGATAGAGATCTTTTTTTTTCCTTTATTTTTTTTTTTGATTTT
TTTTTTTGATTTTCTTGATATTAGTTTTTAGAAGTCCTTTGACGGGGCAGCCGGAGAATATATACCAACGG
CATTCTAT

CGATCGAGTAATCGAATTTACGTAGCCCAATCTACCACTTTTTTTTTTCATTTTTTAAAGTGTTATACTTA
GTTATGCTCTAGGATAATGAACTACTTTTTTTTTTTTTTTTTTACTGTTATCATAAATATATATACCTTAT
TGTTGTTT

AGTAAAACAACCAATATATGCATGGGCTGAGATAGAGGTACAAGGAATTTGTAAATCAGTAAAAAAAAAAA
TTAACAGTTTTTTTTTTTCATTTTTTTTTTTTATTCTTATTTATGTATGATACTTTATTATTATTTCTCTT
AATTATTT

AAGCAAATTGCAAAGCCGCGGCGTTCCCGGAAAAAAAAAAAGGAAGGCCACGCTATTGGTAACCATGAAGTG
TAAGGCGACGCAGGAGAGATGAAAAGAATGAAATTAAAAGAAATCCAAAAAAAAAAAAAAAAAAAAAAAAG
AAAAAGAA

GTAATCACCCCACACGGCTTGTATTTTTTTCGGAGAACTAAGTCATAGACGTAATGCTAATTTTTCCGTCT
AGCCGATGTTTATGCGGCTTTTATTTTTTTTTTTTTTTTTTCTCATCTTTGTTCTTCCGGTTTCCAAGAT
GTCTCACA

GATACAAAAATTCTATGTAACATATTTTTTCCAGTCACTATCATCATCATCATATTTTGTATATATACATT
TATTATATAAAATATCATACCAATTTTTGCCAAAGAACACCGTTTCCCTCTTCGCGTTGACCACAGTAATT
GCAGAAAC

CACAATATAGGAAAGCGTTCTAATTAACTAAAGAAAAAAAAAAAAATAAAAAAAAAAATAAAAAAAAAGGTAAC
TAAAAAGGACAAAAAACATAACAGCTGTTTATAAAGTACTTTATACGTAAGAAACTACTGGCACTATTCAT
TCCAATAA

TATATATATATATATATATATATATATATGTATGGACATTTGTGATGAAAATATATCTTGAACAAGCGCGATT
AAGGTCATAATAAAAAATCACAGAAAATTTTTCAGGTCGCGGGCGTTCTCTCACCCGGCCGCCCAAAGTAT
TATATGAA

GGCCGTTGATATTTCGCGATGGTATGCCGAAAAAGGAAATAACGCAGATTGCGCGTAGTAACGAATGGATT
TGACGCGTTTCCTTCGATGAAAAGTGGGAAATAATAATAAAAAATTCTTGTCAGACGAAACTTGGCGCAAA
ACAAATGA

TTTAATTGCTTGCAAAAGGTCACATGCTTATAATCAACTTTTTTAAAAATTTAAAATACTTTTTTATTTTT
TATTTTTAAACATAAATGAAATAATTTATTTATTGTTTATGATTACCGAAACATAAAACCTGCTCAAGAAA
AAGAAACT

TACGCTAGATGTTTCGAACCACCCAGGTCACACTGTCATTATTAGTAGTACATATATATATATATATATAT
AATTATCATTTAAAAGGAGATATATTACTGATAATCAAAATAGTAAAAACTACATCCGGAGTATGACAGTC
CAACGATT

TAATCAGCGAAAAAAAATGGCGAGGTGAAAAAAATCGATGAGATGAAAAAATTTTTTACTAGTAAAGGCGTA
TTGATGATATATTAGCAGCTAAAGTATAGCAAGGTTTGTCAATTTGAAGCAGTCCGCGTGTTTTACAATGA
GCGTCATT

GTGGGTAAATGATATATAAATTTTTATAAAAACTCTTGGCCAAGAAAAAAAAAAAAAGACTATGTCTAAACG
TAACGTTATCATTATTATTAAATCATTTTAATGTTTTATAAGTTTTGTTTTTCATTTCATACCCTTTAAGG
AGCATTTG

TGAGAACAGTAATATATGAGAAAAAGTTGTTTTCACTTTGTGTTCGAAAAAATAAGTATTTGAAAACGAAA
AAATAGTTTGTATGTTTGTATATAAGTTGTTATTATCAAAAATTAGAGGGAAAAAGTAACTAAATATATAT
TAAAAAAA

ATAGTAGTCAGATACAAAAATTCTATGTAACATATTTTTTCCAGTCACTATCATCATCATCATATTTTGTA
TATATACATTTATTATATAAAATATCATACCAATTTTTGCCAAAGAACACCGTTTCCCTCTTCGCGTTGAC
CACAGTAA

GCATATAAAGTATTCAAAAAGGTGTTGAAAGCTCTTCCTGTATATGACCCTGTATTTAATGTACGCGGTTA
AAAAATTTTTATATTTTTAAATAAGTATCAACTTGGATAATATGATCTTGCCCGCAAGGTGGTTTTATATG
AATAATTA

TGATTTCATTCCCGGCCTCTTAAATAAAAACAATTACCACTGCAAAAATGTTCTTCAAACGCGTTTATTAT
ATAATGAAAAAGAAATTCAAATTTTTCGCGTTTGAAAATTTATGCTAAACAAATAACTCGAGGAGGGACGC
GTCGAGAA

AGGGAAAGAGCGAAGAAGTTGGTTACCTGGCTGATTTAGGGTTATATGTACTATTATTAGTATTATTATTG
TTAGTATTATTATTATTATTATTATTATTATTATTATTATTATTATTATTATTATTATTATTATTATTATT
ATTATATT

CATTTATTTTATTTTTAGTATTTTTTTATACGTGCGTATTCTATTGTTCATTTTTTTACCAATTTTTTTTT
TATTTTTATATTTTGTTTCTTGCGTGGAGTTTTTACAGGAAGTTAATCACAACAGAGGGTTCAATCTCCAA
GGCATAAA

TTCTTCGGACTTTCTTGAAATCTTCGAAAACTGATGAGAATTCTTCATTATTTTTTTTTTTTCTTCTTTTAT
TATTTTTTATGTTTATTCCATATATACACTTATAATTTTTTTTTTTGCGATTAATATAAGTTTGCCTGGAT
ACACGAAC

ACAAAACTTACAAATAAATGATTAATTTTAGACAATATTTTTTTTTTTTTTTTTTATTATGTATATAGTTAA
CATGTATAAAGTAATAGCAGATTCTCTCTAAAAATATAACATAACATTTCATTTCAATTCGTACTAAATAA
TTTTTTAT

GAGTCGGTGGCATTTATTTTATTTTTAGTATTTTTTTATACGTGCGTATTCTATTGTTCATTTTTTTACCA
ATTTTTTTTTTATTTTTATATTTTGTTTCTTGCGTGGAGTTTTTACAGGAAGTTAATCACAACAGAGGGTT
CAATCTCC

AATTTTTTTGTCATTTCTTCGAATCAACTCAATGAAATTTGAAAAATAGCAGAAAATAAGTTGATAAGATC
GCAATGTAATATATATATATATATATATATATTACAATTCGTGAACTGGCACAAGGAAAAGCGTGTAATTA
AAAATGTT

GAAAAAGTAACTAAATATATATTAAAAAAAAAAAAAAAATAAATGTATGTTGGAATGAAATAGCGTAGTAA
GGATAAAGGTGTAATAGATATATATTATAAGAAATAATCCGGTGTCTTCCTGGTGACGGTTGGTTCACCGG
GTCTCACA

CGAGTTCGAACCTCGCATCCTTCAATAACTTATTTTTTTCCTCAATCTTCGATATTAGACATTTTTTTTTT
CCTATGCCTCCCTTTTCCAAGTAATGTTTTTTTTTTTTTTTTTTTTTTTAGCTCCTCTCACAAAAGTACTG
AGGACTGA

TGTGGTTCATGCTGAGAGGAAACACTGCTAAAGCAACCTATGACCTGTACACTAATTAAAACACTTTTATC
CATTTTTTTTCGTATAAGCATATTTTACATCTTTATATATATTTATTTTTTTTTTTTTTATTTTGGTAGAA
TAGATTAC

TATCAAATAGTAAAAAAAACTTTGGAAGCGTGCACACTCAGTCTTAAATCTGCCCTATACTACCGAAGATA
AATACATGAACTACACGTATCATATAACATCATATCCTTTTTTTTTTTTTTTTTTTTTTTTCTAGGCTTTT
ACTAATAC

AGTGCTACATAAAAACGCGTTAAAACTTGCATTCCAATTTATCAAAAAGTTTGCCAGGACATATAGGATAT
AAATGCATTGCGAAGTAAAACACTTCTCAAAAAATTAAAAAAAAAAAAAAAAATAAAACTAACGTATTCAC
TTCTTTTA

ATCTTTTCTCCACATGCAGTAATAGGTATAAAAGGAGTTGAAATTAAAAATAATAATAAATAATGATATAA
AAATTATAAGTTTCTCCTTTCGGAACTAAAAAATAATATTAATAATAATATATAAATATTATATCTTTTTT
ATAATATA

GAAAGCGTTCTAATTAACTAAAGAAAAAAAAAAAATAAAAAAAAATAAAAAAAAAGGTAACTAAAAAGGAC
AAAAAACATAACAGCTGTTTATAAAGTACTTTATACGTAAGAAACTACTGGCACTATTCATTCCAATAAAA
GGACAGGT

CATACATATATATATATATATATATATATGTTATATTATTGTTAATTAAATATTATTATGCGTATTTTCCTTT
TCTTTATTAGTATAGTATTAATGACAGTAATAATAATAATAATAGTAACAATATCTCTTTTTTTTTTTCAG
TGAGCTTT

GTTCAAGAGATGATAAACGTGAAGGGAGTGTCATATCTACCGACATAAAGATAGAGATCTTTTTTTTTCCT
TTATTTTTTTTTTTGATTTTTTTTTTTGATTTCTTGATATTAGTTTTTAGAAGTCCTTTGACGGGGCAGC
CGGAGAAT

AACATATCTGTAATTGTATTATATAAAAGTAGTTAGTTTTTTTTTTTTTTTTTTTTTTTTTTTTTTTTTAT
GTGTAATGCAAATACAAAAAGAACTGCAGGCATTTTGAAAGTTCACTCGTATAGGAAACTTTGATAGAGAG
GCAATTCC

AGTATTATGTGTCATAATTTGTAGTATTATCTGTGTGACGGATTAAGTTTGCTATCTCAAATGCCGCGCGC
GCGAATTGCTCATGATATAATAATAGCTAATAATATGATAATATGATAATAATAGATAAGATTACTGATAA
CATTATTT

TACCCTGTTTTTCTTTGTCGTTTTCTTCTATAATGAACAATTTACTTTACGGTCTTTAATCGATTTTAATG
ATTAGTTGGGCTTTTGGCTACATATGTAAAATTACATATAATATAATGGACACATAAGCAAAAAAAAAAAA
AAAAAAAA

TTTATTAGCTGTTTAACATATTTCTGTAGGGTTTTTTTTTTTTTTTTTGTACAGATTAGAAACCATACAGT
GTAAACCATTATATAGGTCAATTAATATATTTATAGATCATCCTGTCAATTTTTTTTTATTTTTTTTTGCA
AAGCTCCC

GTTTCGAACCACCCAGGTCACACTGTCATTATTAGTAGTACATATATATATATATATATATAATTATCATT
TAAAAGGAGATATATTACTGATAATCAAAATAGTAAAAACTACATCCGGAGTATGACAGTCCAACGATTAT
GTAGTATA

GGTGGCCTTCTTCGGTAACTAGGGGATAAGGGGCTTCGTCCTACACGATGCTCCTGTCGTTTCTTTTTTTC
ATTTCTTTTTTCGCCCAGCGGGAAGTACGCGGAAAACAAGAAACAAAAAAAAAATAAAAAAATAAAAAATA
AAACAGAA

AATTACTTTTTGAAAAGGAAAAATATTCAGGTTTGTTGTTTTTATGTAAGTTGTATGATTTGATATACATA
TATATATATATATAATATATATTGTACATGTGTTTTTCCGGGGAAGAATGGATTATCCGGAGGTGTGAATA
AAATGATG

TTTGTCCTTGTCCTTTCTTTCCTATTTATATATATATATATATATATATATATAATACTACTATGTTATATTA
CATTATATTATTATCACAATAATTATTACGTTCATCGTGCCGAAACGTCACCTCCCACGGTTTGGATCTAT
TGAAAGAG

CTTTGACAAAATTCCAGCCATTTTTTCAACCTTTTTTTTATCCGTATATATTAAGGTATGCTATATGTGTA
TTTTCCAATGTTTAGTTTTATAATAATACTTCTTCAGCATCAAATATGCTTGCTTATGTAAAAATACAAT
CAAGTTTT

TTCAATAACTTATTTTTTTCCTCAATCTTCGATATTAGACATTTTTTTTTTCCTATGCCTCCCTTTTCCAA
GTAATGTTTTTTTTTTTTTTTTTTTTTTTAGCTCCTCTCACAAAAGTACTGAGGACTGAAATGTAAAAACT
AATAACAC

TCGGGTGACTTCAATTTGGCGCCCGTTGAGATTATCTAAAAAATGCTTTTATTTATTTAAGCGCTTTTTGT
TTTGACGAATGTATTTATGTTTTTGTATATATATGAGCGTTTTTTTTTTCCAGATTTAACTAGTTTTCTTT
TTCACCTG

TTAAAGTTTTTTTTTTCCAGTTCTTTTTTCCCATCGTTTTTTTTTCTAGAAGCCGGGTTTTTCACCGTCAA
AAAATTTCCTAAAAAAGATCTGTCAATCATTAAGTTTTGCTTTTTCGGCTTTTAAGCCATGCTTTCAGGCA
AAAATTAA

ACTGTCCTCCGTTCTGTAAAATTCACGCTCTTAGTCCCTTTTCATAATTCCTTAACTTTTTGCGTACAAAA
TGATATGTTTATTATATTTTTCTTTTTTTTTTTTCAAATTTTTTCTTTTTCTTGAAAAATTTTTCAAATTG
GAAAGCTC

ACTAGAAAAGGTTCAAGAGATGATAAACGTGAAGGGAGTGTCATATCTACCGACATAAAGATAGAGATCTT
TTTTTTTCCTTTATTTTTTTTTTTGATTTTTTTTTTTGATTTTCTTGATATTAGTTTTTAGAAGTCCTTTG
ACGGGGCA

TGCCGCGCGTTTGAAAGGAAACTGATCTGAAAGTGATTTATGATGCCAAAAAAAAAAAAAAAATCTTTTGAA
GTACAATGTATGTATGCATGAGTTTATAAAAAATGATGTAAAGTGCGGAATAGTAGTACTGCTAATGAGAA
AAACACGG

AGGGTACGGCGTAATCACCCCACACGGCTTGTATTTTTTTCGGAGAACTAAGTCATAGACGTAATGCTAAT
TTTTCCGTCTAGCCGATGTTTATGCGGCTTTTATTTTTTTTTTTTTTTTTTCTCATCTTTGTTCTTCCGG
TTTCCAAG

CTAAAATGGTTTTCTCTACCTTTAGTTTTACGCCAGATTTTTACTAGACGCGTTTTAGTTTACTTTTTCCT
ACCCTGTTCGTTTTTTTTTTTTTTTTTCGCACCAAATTTTTCAACCGTTCCATGGAAGCCGGGATCGACCGT
CCTACACT

ACTGAAGCCGTTATTATAGTTCTATCTTCATGTTTTTAGAGGTTTTTTTTAAATGAGTTTATTAAAATTAA
ATTTTGAATGTTATTTTTGAATAAAAATATAAAAAAAATAATATTAATAATTTTTCATTAACTGCTCATGA
TATCAGTG

ACGAGGCCCTATAAAAAGCATTACCGTCACGCATCGTGTTGCAAATAGCATATACATATATATATATATAT
ATATACATATAACGTATACGTTAGAAAAATTTGAATACACACATTCATTTATCCTATTATTATCATTTTCA
TTCTTACC

TTCGTTACCTTGGAATGGTATGTAAAGTGATAAAAATATACACGTATATATATATATATATATATATATAA
GTACGTGAGATCTATTTGTATAATATAATGCTAATAAAATAGTATCAACGATACTGTTGATAAAGGTAAGT
CGATACCT

AACTGCATTACTTTTTCGGGACGTATTCTCGGGAAATAAGCAGGTCCAGATCCGCGTAGCATGAATGGGGC
GCCAAAACAGTCGCGGGTAACGAAAGAGCGCAACCACGCGTATATTCCGGGCCGGTGAGGGGCAACGCGCA
TTGTCCCG

TAATTTATTTAAAAAAATCCACGAGCATATAGATTTATAACAATAATAATAATTAATATTCATCAATTTTT
TAGTAAATTCATATATTTTACTCCTACTCTATGTATTTTCAGTAAGTCTACTAGTCGTATTACCCGTCGGT
TTCAAGGG

AACAGAAAATTTTGGAGGAAAACAATGTAGCTGTATAAATATATATATATATATATGTATGTATACTTATT
TATTTATATATATATCACAAAAGGCTACCAAAAAGATTAGCCGCCGAGGTACATCGTGAGGAGTGATATTC
TGGGGAAA

AGGAAAAAAAGAAAAGAAAAAAAAAATTGGGAAGAATGGGGATAAAAAAGATTTGTAACATTATAAACAAAA
ATAAGAAATAATTGTTTTTACGTACTTATTGTAAATAAAACAAAGGGATAACGGACAAAAAGGCATCGTTT
CTTTTGTA

GAAACATGAAGATGCAGCAAAGTAAAAAATAATAGGTAAAAATAATAAAAATACATGTATCTTTTTGGTTG
GTTAATTTATCTAAACAGTTTTATATATATATGTATATATATATATATATATACACAGTATTAATATTCTT
TCCTTGAG

GACATTCCCGGATTTTATTTTCCTCAATGTGCTCGTGTAAGTGTCGGCCCGGAACTTGATTCCCCCCGGAC
TAATTAGAAGTTCCGAGAACGCGCGCAAGCCGTCCCGCGCGTAGAAGGCTAAAAAGCAGCCGGAGGAAAAA
AAATAACG

TGTAGCGTCGCTAAAATGGTTTTCTCTACCTTTAGTTTTACGCCAGATTTTTACTAGACGCGTTTTAGTTT
ACTTTTTCCTACCCTGTTCGTTTTTTTTTTTTTTTTCGCACCAAATTTTTCAACCGTTCCATGGAAGCCGG
GATCGACC

ATATTTGATTATCATCGCCCGCTGGTCACCGCATTTTTAAAGAATAAATATTACTTTTTTCGAAATCTGGT
GTTTAAATATTTTTAAGAAAGTTAAAAAATAAGGAATATTTGATAAACAAAAAAAAATTAGCCTTTATATA
TCCATTTT

CTTACCGCGCCCATTTCGGAAACTGCATTACTTTTTCGGGACGTATTCTCGGGAAATAAGCAGGTCCAGAT
CCGCGTAGCATGAATGGGGCGCCAAAACAGTCGCGGGTAACGAAAGAGCGCAACCACGCGTATATTCCGGG
CCGGTGAG

TGCACACTCAGTCTTAAATCTGCCCTATACTACCGAAGATAAATACATGAACTACACGTATCATATAACAT
CATATCCTTTTTTTTTTTTTTTTTTTTTTTTCTAGGCTTTTACTAATACAAGGGCCTTAGCCATTCGCTAA
ACGTAATG

CCCATTCTTGCCAGTACGCCAATAAAGTGTGTCGCGCAGAATAGATCTTCATGTAATTCCTTTTTCCCTTT
TATGCAATCTATAATGTTAATCAGCGTAGATCACTATATACGCTTTTTTTTTTTTTTTTCAGCTCCGATTA
GAGAGCAA

CTGATCGAACGAAGCAAATCCTACGAGTAAATACATAAGCGTATACATATATATATATATATATATATATA
TATATATATGTATATATATATATGTGTGTGTGTGTAATTGTGTGTATTCAACTGAACTATGAAGAGTCTTT
GACCTCTT

TGTTTTTAGAGGTTTTTTTTAAATGAGTTTATTAAAATTAAATTTTGAATGTTATTTTTGAATAAAAATAT
AAAAAAAATAATATTAATAATTTTTCATTAACTGCTCATGATATCAGTGCATTAGAAAATCTTTTGGTTCC
ACTTTTCC

AGAGTAAGTATGGTCAGCAGAACAAAAAAGAGTTCCTACATTTATACGAAGGTAAAATTGTAAAAAACATG
AATGCGGCCTATCCGGCTCGAAAATTGTTCCTCATATATATTAAAAAAAAATCACGTGGTGCAAAAAAAAA
TTTTTTTG

AGGCCTTTAAAAGCAACGCGACCGTCGTGGGTTCAAACCCCACCTCGAGCACTTTCTCTTTTTTTTTCACA
CGCGTTTGCAACATGCCCGTATTACATGAGCTTATTTGACTTTAGATGGTTTTCAGTTCTTTTTTTTTTTT
TTTTTTTT

GGAAGTATGGATGAATCTTTTTTTTTTTTTTTTTATAGCACGCAACTGAAAAAAAAAAAAAGAAAAATTTT
TCATCTTCGCTCGACGTTTCTTTTGTAGTACTCATCTCTTTTTATATAAAGATTAATTAGTTATTGTCGCT
TTGCTTTT

ATAAAAAAAAAAAAAAAAAAAGCTTTTAAAAAAAAAAAAAACTCAAAAAAAGAAAGAATATTCAAAGAAAGTT
TCATCTTGTCAGTTGAAATGAATAGTTTATTGTGGAAATTATAACAATAAAAGTTAGCTAAAAGCGAAAGT
GTCGATGA

ACAACTGCGCATATTGCTAAGAACTGCACTGAAGGCATGAATTGTCTATTCCGTTAGAAATATAATTATTT
ACGAAAGCATCGCGCAGACGCAATTTTTTTAACATTTTTTTTTTTTCAAGTACTCTGGAAAAGTTTTCATG
AAGATCAT

GCAAGTAATAGCAGCAACAACGATGCATAATTGGCATCTAGCCCCAATGACGATACACTAATATATATATA
TATATATATATCTATATATCTGCAAGAAATAAGCGTGTGTAGTAAGTAAAAGGAAAAATAGAAATTTTTGC
TCAAAAAT

CTGATGAGAATTCTTCATTATTTTTTTTTTTTCTTCTTTTATTATTTTTTATGTTTATTCCATATATACACT
TATAATTTTTTTTTTTGCGATTAATATAAGTTTGCCTGGATACACGAACATATATATGATTAAAATGGTTG
TCTCGAAC

TGTACGACCTGTTGGAATAACGCGAAAAGAAGGGTACGGCGTAATCACCCCACACGGCTTGTATTTTTTTC
GGAGAACTAAGTCATAGACGTAATGCTAATTTTTCCGTCTAGCCGATGTTTATGCGGCTTTTATTTTTTTT
TTTTTTTT

GATATATAAATTTTTATAAAAACTCTTGGCCAAGAAAAAAAAAAAAAGACTATGTCTAAACGTAACGTTATC
ATTATTATTAAATCATTTTAATGTTTTATAAGTTTTGTTTTTCATTTCATACCCTTTAAGGAGCATTTGGC
ATTGTGTG

CCATTTTTTTTCGTATAAGCATATTTTACATCTTTATATATATTTATTTTTTTTTTTTTTTATTTTGGTAGA
ATAGATTACATATACAACTTTTTCATAATGTCAAACTATAGAGTTTTATTTCACATAACGGAAGAAGCCTA
TTGGAGCC

ACATCTGTCTTTTTTTTAATTACTATTGCTGTGTACTTATAGAGTCGGTGGCATTTATTTTATTTTTAGTAT
TTTTTTATACGTGCGTATTCTATTGTTCATTTTTTTACCAATTTTTTTTTTTATTTTTATATTTTGTTTCTT
GCGTGGAG

TGTATTACCATAAAAAGGCTTTAATATGAGAAAAAGGTGAGCGCTATATAGATTTAGTAATTTACATGTTA
CAGAAGCAAAAAGCAAATATATATATATATTTTTTATCATATTAGTTTTTATCCTCAGTAGTACTACTGAAAT
AATAATCT

GATATTAGACATTTTTTTTTTTCCTATGCCTCCCTTTTCCAAGTAATGTTTTTTTTTTTTTTTTTTTTTTTA
GCTCCTCTCACAAAAGTACTGAGGACTGAAATGTAAAAACTAATAACACAATAGAAATAATCATATGATGA
TATAGAAA

AAACGTATGTGGCCCATGCCTTCGTTACCTTGGAATGGTATGTAAAGTGATAAAAATATACACGTATATAT
ATATATATATATATATATAAGTACGTGAGATCTATTTGTATAATATAATGCTAATAAAATAGTATCAACGA
TACTGTTG

TGGTCTCCCGGAAACATGAAGATGCAGCAAAGTAAAAAATAATAGGTAAAAATAATAAAAATACATGTATC
TTTTTGGTTGGTTAATTTATCTAAACAGTTTTATATATATATGTATATATATATATATATATACACAGTAT
TAATATTC

ATTTCGCGATGGTATGCCGAAAAAGGAAATAACGCAGATTGCGCGTAGTAACGAATGGATTTGACGCGTTT
CCTTCGATGAAAAGTGGGAAATAATAATAAAAAATTCTTGTCAGACGAAACTTGGCGCAAAACAAATGATT
TCTTTACC

AATTACATATAATATAATGGACACATAAGCAAAAAAAAAAAAAAAAAAAATAAACAAATGAGCAACCCATC
AAATACCAAGAACAAAGAATTTAATATAATATATAGTTATTAAATTTAAATGTATATATGCAGTTCTGCTC
TTATTTTG

TGACCAAGGTGAAATTAGCAGGCAGTTTGAACAAAACTTACAAATAAATGATTAATTTTAGACAATATTTT
TTTTTTTTTTTTTATTATGTATATAGTTAACATGTATAAAGTAATAGCAGATTCTCTCTAAAAATATAACA
TAACATTT

AATTAATTGATTAATTAATATTTTGTATCTAAAGTGTTCATACTATATTATTTACTTAGATTATACATAAT
TAAAAAAAAAAAAAACTTTTTTTCCTTGTTTTCATTTTGTTTTTATTATTAAATATTAAAAAAAGTGTCCA
TAACATCA

TGCCCGTTCAAAACTATTCTTTTTATGAATTGACAGAAAACCAAAAGCAAGCCGATTTCAGGTTAACCCTT
TATTAAAATAAAAAAAAAAAGCATGCATAACACTAATAATATATTATTAAAAAAAAAGCCAATCTATATTA
TTTAAATT

TAATTGTATTATATAAAAGTAGTTAGTTTTTTTTTTTTTTTTTTTTTTTTTTTTTTTTTATGTGTAATGCA
AATACAAAAAGAACTGCAGGCATTTTGAAAGTTCACTCGTATAGGAAACTTTGATAGAGAGGCAATTCCTT
ACTTTCGT

GTCTTAAATCTGCCCTATACTACCGAAGATAAATACATGAACTACACGTATCATATAACATCATATCCTTT
TTTTTTTTTTTTTTTTTTTTTCTAGGCTTTTACTAATACAAGGGCCTTAGCCATTCGCTAAACGTAATGAA
CGATATTG

TGTTATACTTAGTTATGCTCTAGGATAATGAACTACTTTTTTTTTTTTTTTTTTACTGTTATCATAAATAT
ATATACCTTATTGTTGTTTGCAACCGTCGGTTAATTCCTTATCAAGGTTCCCCAAGTTCGGATCATTACCA
TCAATTTC

TTTTCTTTTTCCTTTTTCTTTTTTTTTTTTTTTTTTTTTTTTTTTTCACATCTTTTTCCACGGCCGCTCAA
AGGGGTCTCACACTGGATTAAAAGCTGAAAACAAGAAATGGCTCTTTCGATAAGGTTGCAACATTTTGATT
ACTACGTT

AACAGAGTGTTACGCCAACATACTAAACCAGAGAAGTAAAACGTTTTACTTATATATAATAGTATTCGTAA
ATTTATAAAAATCAAAAAACCAATATAAGCCTGGTTAAAAAAAAAAAAAGATCAGTATCGTAAAGTCGTAA
AGTTAGGT

TTTTTATAAAAACTCTTGGCCAAGAAAAAAAAAAAAGACTATGTCTAAACGTAACGTTATCATTATTATTA
AATCATTTTAATGTTTTATAAGTTTTGTTTTTCATTTCATACCCTTTAAGGAGCATTTGGCATTGTGTGCG
CCCTGAAG

TTACCGTCACGCATCGTGTTGCAAATAGCATATACATATATATATATATATATATACATATAACGTATACG
TTAGAAAAATTTGAATACACACATTCATTTATCCTATTATTATCATTTTCATTCTTACCCGCTCCCCGCTT
TCATCCGG

ATATAAAAGTAGTTAGTTTTTTTTTTTTTTTTTTTTTTTTTTTTTTTTTATGTGTAATGCAAATACAAAAA
GAACTGCAGGCATTTTGAAAGTTCACTCGTATAGGAAACTTTGATAGAGAGGCAATTCCTTACTTTCGTTA
TTCCTTTC

ATATAATCTTACTGTCCTCCGTTCTGTAAAATTCACGCTCTTAGTCCCTTTTCATAATTCCTTAACTTTTT
GCGTACAAAATGATATGTTTATTATATTTTTCTTTTTTTTTTTTCAAATTTTTTCTTTTTCTTGAAAAATT
TTTCAAAT

TGCAAAAGGTCACATGCTTATAATCAACTTTTTTAAAAATTTAAAATACTTTTTTATTTTTTATTTTTAAA
CATAAATGAAATAATTTATTTATTGTTTATGATTACCGAAACATAAAACCTGCTCAAGAAAAAGAAACTGT
TTTGTCCT

AAAAATATTCTGCTACTATTCCTTACTTTACTATAAGAATTTTGTTTTCCAAAAAAAAAAAATATAAAAAA
AATAATCATACTCTATTACTATGGCTAACGTAGAAAAACCAAACGATTGTTCAGGCTTTCCCGTTGTTGAC
TTGAATTC

TTTTTTAAAGTGTTATACTTAGTTATGCTCTAGGATAATGAACTACTTTTTTTTTTTTTTTTTTACTGTTA
TCATAAATATATATACCTTATTGTTGTTTGCAACCGTCGGTTAATTCCTTATCAAGGTTCCCCAAGTTCGG
ATCATTAC

CTAGGGGTAGAATTACTTTTTGAAAAGGAAAAATATTCAGGTTTGTTGTTTTTATGTAAGTTGTATGATTT
GATATACATATATATATATATATAATATATATTGTACATGTGTTTTTCCGGGGAAGAATGGATTATCCGGA
GGTGTGAA

CAGGTCCAGATCCGCGTAGCATGAATGGGGCGCCAAAACAGTCGCGGGTAACGAAAGAGCGCAACCACGCG
TATATTCCGGGCCGGTGAGGGGCAACGCGCATTGTCCCGGGGCGATATAATCCATGTACCGGAACCCGCAC
TTTTTCGA

ATCCTGTCAATTTTTTTTTATTTTTTTTTGCAAAGCTCCCTTTTTCCTGAAACCAACAACAAAATATAAAA
AAAAAATGATAAGCAGGATTTTTTCTTTTAATAATTCCTTAAAATTAACTAACAAGCTGAAGCCCTTACAA
CAAAGTAA

CACCTCGAGCACTTTCTCTTTTTTTTTCACACGCGTTTGCAACATGCCCGTATTACATGAGCTTATTTGAC
TTTAGATGGTTTTCAGTTCTTTTTTTTTTTTTTTTTTTTTCAGTAGATTAATGCGAAGAGTTAAATCACC
AATTCAAA

TTGCTGGTGTTGGCGCTACCATTGTTTTTTTGTTTTATGAGTTACGTTTACAAAAGTTCTTAACATTTACT
GTACGATAATATACTAAGGTGAAATACTAAAGTAAAATAAAATAAGAGAAAGAAAAAAAAAAAAAAAAGAA
AAAAGTAA

TTGAAAAAAGACTACAAAAAGAAATCAAGTCGATCGAGTAATCGAATTTACGTAGCCCAATCTACCACTTT
TTTTTTTCATTTTTTAAAGTGTTATACTTAGTTATGCTCTAGGATAATGAACTACTTTTTTTTTTTTTTT
TTACTGTT

TATTATTATTATTATTATTATTATTATTATTATTATTATTATTATTATTATTATTATATTATTATTAATAC
TAAATATTGACACTGCTTTACGCACTAGATTGTGCGGGTAACTGAATATTTTTTGAAAATGAAGAAAAAAA
AATTTCGA

TGTCATTTCTCATAACTACTTTATCACGTTAGAAATTACTTATTATTATTAAATTAATACAAAATTTAGTA
ACCAAATAAATATAAATAAATATGTATATTTAAATTTTAAAAAAAAAATCCTATAGAGCAAAAGGATTTTC
CATTATAA

ATGCACCTAAAACTCGCCTGCACTCCGAAACGAAATGCGATACAGTACGTGTCATGCGGTTAGTATATATA
CATATATATATATATATATATATTCAAATAAATTATAACGTATTAAATAATATGTGAAAAAAGAGGGAGAG
TTAGATAG

GGTTTTTTTTAAATGAGTTTATTAAAATTAAATTTTGAATGTTATTTTTGAATAAAAATATAAAAAAAATA
ATATTAATAATTTTTCATTAACTGCTCATGATATCAGTGCATTAGAAAATCTTTTGGTTCCACTTTTCCTA
TAAAAATA

GAAAAAAAAAAGCACCTATTGCATATAAAGTATTCAAAAAGGTGTTGAAAGCTCTTCCTGTATATGACCCT
GTATTTAATGTACGCGGTTAAAAAATTTTTATATTTTTAAATAAGTATCAACTTGGATAATATGATCTTGC
CCGCAAGG

TATGTTCCAGGATATGAAATGAAATACCTTTTGTTTCACCTTTTAAATAATTTAATGTTATATATACAACT
TTATCGTATCATATTCGCAATTACATTATACAAGAATGAGTTTTTTTTTTTCGCGGACAAAAGAAGAACAT
GTCAACGC

TCCTTATTTTTTTGAGAAAAAAATGCTTGAGAAAAAAATGCTTGAGAAAAGATGTATTTTTTTTTATTCGTTA
GCATTTTTCCAGATATCGTTGAACATTGCTAGAATACTATCAAACCCGACTTTCCCCTTCACCGCGTTATC
ACTAAAAC

TAAAACTTGCATTCCAATTTATCAAAAAGTTTGCCAGGACATATAGGATATAAATGCATTGCGAAGTAAAA
CACTTCTCAAAAAATTAAAAAAAAAAAAAAAAAATAAAACTAACGTATTCACTTCTTTTACTTTAGTGGAGA
GAATAACG

CCGTTTTTTTGACATTAAAAGTTTTCTTAATGCCGCGCGTTTGAAAGGAAACTGATCTGAAAGTGATTTAT
GATGCCAAAAAAAAAAAAAAAATCTTTTGAAGTACAATGTATGTATGCATGAGTTTATAAAAAATGATGTAA
AGTGCGGA

CGACATAAAGATAGAGATCTTTTTTTTTTCCTTTATTTTTTTTTTTTGATTTTTTTTTTTTGATTTTCTTGATA
TTAGTTTTTAGAAGTCCTTTGACGGGGCAGCCGGAGAATATATACCAACGGCATTCTATTGTCTTTTTTTC
TTCCTTCC

TTTTTTTCACACGCGTTTGCAACATGCCCGTATTACATGAGCTTATTTGACTTTAGATGGTTTTCAGTTCT
TTTTTTTTTTTTTTTTTTTTCAGTAGATTAATGCGAAGAGTTAAATCACCAATTCAAAGATGGCAGATGG
ACCACCTT

GTTTTTGCTTCCGTTTTTTTGACATTAAAAGTTTTCTTAATGCCGCGCGTTTGAAAGGAAACTGATCTGAA
AGTGATTTATGATGCCAAAAAAAAAAAAAAAATCTTTTGAAGTACAATGTATGTATGCATGAGTTTATAAAA
AATGATGT

TATATAGGTCAATTAATATATTTATAGATCATCCTGTCAATTTTTTTTTTATTTTTTTTTTGCAAAGCTCCCT
TTTTCCTGAAACCAACAACAAAATATAAAAAAAAAAATGATAAGCAGGATTTTTTCTTTTAATAATTCCTTA
AAATTAAC

GGTATCAAGCAGTAAAACAACCAATATATGCATGGGCTGAGATAGAGGTACAAGGAATTTGTAAATCAGTA
AAAAAAAAAATTAACAGTTTTTTTTTTTCATTTTTTTTTTTTATTCTTATTTATGTATGATACTTTATTAT
TATTTCTC

AGAATTGACGTATAACTTCTTTTGAAAGTCTTTGAAATTACATGGTAGTACATACATATATATATATATAT
ATATATGTTATATTATTGTTAATTAAATATTATTATGCGTATTTTCCTTTTCTTTATTAGTATAGTATTAA
TGACAGTA

ATTTTTTTTTTCTTCTGAAAAAATTAATTAGATTAATTTCAATTAATATCATTTCCGCTTATCTGACTTCT
TTCATTTTTTTTCTCTATATTTCGCGTTTACTAGGAAAGAAAAGGAAAAAAAATTTTTCCCCCTCCATCTG
TCCCAAAT

TACGCCAACATACTAAACCAGAGAAGTAAAACGTTTTACTTATATATAATAGTATTCGTAAATTTATAAAA
ATCAAAAAACCAATATAAGCCTGGTTAAAAAAAAAAAAAAGATCAGTATCGTAAAGTCGTAAAGTTAGGTCA
TTGTTTTT

CTTTTTTTTATCCGTATATATTAAGGTATGCTATATGTGTATTTTCCAATGTTTAGTTTTTATAATAATAC
TTCTTCAGCATCAAATATGCTTGCTTATGTAAAAATACAATCAAGTTTTGTCCCTTGCTTGCGCGAAGATG
AAAACTTC

ATTCACGCTCTTAGTCCCTTTTCATAATTCCTTAACTTTTTGCGTACAAAATGATATGTTTATTATATTTT
TCTTTTTTTTTTTTCAAATTTTTTCTTTTTCTTGAAAAATTTTTCAAATTGGAAAGCTCATCTCTCTTGAA
TGTATAAT

CATGGTAGTACATACATATATATATATATATATATATATGTTATATTATTGTTAATTAAATATTATTATGCGT
ATTTTCCTTTTCTTTATTAGTATAGTATTAATGACAGTAATAATAATAATAATAGTAACAATATCTCTTTT
TTTTTTTC

TTTGGAAGCGTGCACACTCAGTCTTAAATCTGCCCTATACTACCGAAGATAAATACATGAACTACACGTAT
CATATAACATCATATCCTTTTTTTTTTTTTTTTTTTTTTTTCTAGGCTTTTACTAATACAAGGGCCTTAGC
CATTCGCT

TGTACTGCTGAAAAAAAAAAAAAAAAAAGAAAAAAAAGAAAAAGATATCTTGGTTTGAATAAGTAAAATTAA
TAGAATCCAGTTTCCAGTGATCGGTACAGGCTAAATAATAAATATCTCTACTTTTATTTATTCTTTTCGTT
TTCTTGTG

TAAATGAGGGAAAAATGCAAATAATCTTGAATGATACTCAAAACTTTTGATTTATTGTAATTTCTTTCTGA
ACCATATTTATTACATTCAAACTTTTTTTTTTTTGCCTTTTATAAAAAAAAAATACTTATAAGAAAAGAGAA
AGAAAATG

GAGTACCGGGAGTATTATGTGTCATAATTTGTAGTATTATCTGTGTGACGGATTAAGTTTGCTATCTCAAA
TGCCGCGCGCGCGAATTGCTCATGATATAATAATAGCTAATAATATGATAATATGATAATAATAGATAAGA
TTACTGAT

ACTATTAAGTTACGCTAGATGTTTCGAACCACCCAGGTCACACTGTCATTATTAGTAGTACATATATATAT
ATATATATATAATTATCATTTAAAAGGAGATATATTACTGATAATCAAAATAGTAAAAACTACATCCGGAG
TATGACAG

TTACAAATCGACGAGGCCCTATAAAAAGCATTACCGTCACGCATCGTGTTGCAAATAGCATATACATATAT
ATATATATATATATACATATAACGTATACGTTAGAAAAATTTGAATACACACATTCATTTATCCTATTATT
ATCATTTT

TACTATATTATTTACTTAGATTATACATAATTAAAAAAAAAAAAAAACTTTTTTTCCTTGTTTTCATTTTGT
TTTTATTATTAAATATTAAAAAAAGTGTCCATAACATCAATGTTTTTTTTTACCTTTGATTAGTTTTCATT
GGCGAAAC

TTTTACGAACGGGTTTCCATAGACATTGAAAATGAACGAACAAAATAAAATAAATAAAAAACTACTTTTTT
TTGATGATATAAATACCGTAGATTATATAAATGTATATTAATATTATAAACCTATTTGATCAATGACAGTG
TTCGTGAA

TGGTCAGCAGAACAAAAAAGAGTTCCTACATTTATACGAAGGTAAAATTGTAAAAAACATGAATGCGGCCT
ATCCGGCTCGAAAATTGTTCCTCATATATATTAAAAAAAAAATCACGTGGTGCAAAAAAAAAATTTTTTTGTC
ATTTCTTC

GTTCTGTAAAATTCACGCTCTTAGTCCCTTTTCATAATTCCTTAACTTTTTGCGTACAAAATGATATGTTT
ATTATATTTTTCTTTTTTTTTTTTTCAAATTTTTTCTTTTTCTTGAAAAATTTTTCAAATTGGAAAGCTCAT
CTCTCTTG

CAATTACCACTGCAAAAATGTTCTTCAAACGCGTTTATTATATAATGAAAAAGAAATTCAAATTTTTCGCG
TTTGAAAATTTATGCTAAACAAATAACTCGAGGAGGGACGCGTCGAGAAAATCGCGTCTTTCTACTCTCAC
AGTGCTTG

GAACACGAATACTAGTAGTAATAGTAGTCAGATACAAAAATTCTATGTAACATATTTTTTCCAGTCACTAT
CATCATCATCATATTTTGTATATATACATTTATTATATAAAATATCATACCAATTTTTGCCAAAGAACACC
GTTTCCCT

GTTCAAACCCCACCTCGAGCACTTTCTCTTTTTTTTTCACACGCGTTTGCAACATGCCCGTATTACATGAG
CTTATTTGACTTTAGATGGTTTTCAGTTCTTTTTTTTTTTTTTTTTTTTTTCAGTAGATTAATGCGAAGAG
TTAAATCA

CCTGTACCTGCTTTTGTTATCGTTGTACATCCATGCACACGCTCATATATATATATATATATATATATATG
TATATGTACATATACCGCTTACCACCTACCGTGAAGTATATGTAAGGGTCTTCGCACCCGGATATCCCTTG
TGGGATCT

AATGCAAAATCTAGGGGTAGAATTACTTTTTGAAAAGGAAAAATATTCAGGTTTGTTGTTTTTATGTAAGT
TGTATGATTTGATATACATATATATATATATATAATATATATTGTACATGTGTTTTTCCGGGGAAGAATGG
ATTATCCG

AAAATTATAAGTTTCTCCTTTCGGAACTAAAAAATAATATTAATAATAATATATAAATATTATATCTTTTT
TATAATATAACATAGATACGAGTTTTTATGTTCCTTTGGCAATAGCTTTTCGTGACTTGTTTTAAATACAA
GGCATATA

GAAAATGCCCGCGAGATCTAATATTAACGAAAAAACTGAAATAAAAGCAACGCTTATACATATACATGCAT
GCATTAATATATGTATATATATCGGTACGTATAGATTGTAAAATTACACTTACTACTAAAGGCAGTATGAA
AGACGCCC

GCTCATCCATTTAAAAAAAAAAAAAATAGAATAACAATAATTAAGCAGAAAAAATGTTAACGAATATAAT
AAATCGATTTGTCCATTTTCATAAATTTTTTTTTTGTATTATTTCATTATCTGTGCCTCAAAGCATTAACT
TTTCTTGC

ATTAGCATTATGTCATTTCTCATAACTACTTTATCACGTTAGAAATTACTTATTATTATTAAATTAATACA
AAATTTAGTAACCAAATAAATATAAATAAATATGTATATTTAAATTTAAAAAAAAAAATCCTATAGAGCAA
AAGGATTT

TAGTGGTGTACGGAGTGCTTGAAGAAAAAAAAAACTGATATAGAACCCAAAAATGTTCGGCTTTTAAAATA
CAAATATGTACAGGTGTTAATGTCTAAATAATGATAATCCAAACGCGATAATTTTTTTTTCGCGTTTGTTT
TTGTTTCA

TGCTACTATTCCTTACTTTACTATAAGAATTTTGTTTTCCAAAAAAAAAAAATATAAAAAAAATAATCATA
CTCTATTACTATGGCTAACGTAGAAAAACCAAACGATTGTTCAGGCTTTCCCGTTGTTGACTTGAATTCGT
GCTTTTCT

GGATGCACTTCTGAAAGAGTCTTTGACAAAATTCCAGCCATTTTTTCAACCTTTTTTTTATCCGTATATAT
TAAGGTATGCTATATGTGTATTTTCCAATGTTTAGTTTTTATAATAATACTTCTTCAGCATCAAATATGCT
TGCTTATG

GCGTTCCCGGAAAAAAAAAAAGGAAGGCCACGCTATTGGTAACCATGAAGTGTAAGGCGACGCAGGAGAGAT
GAAAAGAATGAAATTAAAAGAAATCCAAAAAAAAAAAAAAAAAAGAAAAAGAAAGAAAAAGGGG
AATAACAC

TGTTTTTGCTTCTTATGGATTGGATGTTATAACAGTAGAAGTAATTTTAGGATAATTTTAAATAAAAATAA
TATTAAAAATAATAATAATACTAATAATAAATGAGAATAAAATGTTGATAAATGAGGGATAAGGTCTATTC
ATACGTCT

TGTAGTGTACTTTAAACATTTTTTTATCTTCATAGCAATAATATAAGCCTTTTACCACCCATAAACCATAA
AGTAGACCCAAACATTTTTAAAAAAATTTTACGTTATAATTTTTTCTTTGTCGTTTTTTCTGAGCGCGCAA
AGTAGCGG

ATGTACTCAGTCTAATCTACTTTGATTTAATTTAATTCAAGTTATATAAATCTATTCTTTTCCCTTTAATT
TTAATATTTTAATTTCAATCCTTTTCTTATTTTCTATTTTTGCTTTTTTTTTTTCTTTTTTCCATATTCTC
AGGCCACG

ATATTGCTAAGAACTGCACTGAAGGCATGAATTGTCTATTCCGTTAGAAATATAATTATTTACGAAAGCAT
CGCGCAGACGCAATTTTTTTAACATTTTTTTTTTTTCAAGTACTCTGGAAAAGTTTTCATGAAGATCATGA
ATACGTTA

TATACACAGTATTAATATTCTTTCCTTGAGATTACTCTCTATAAAAAAAAAAAAAAAAAAAGCTTTTAAAAA
AAAAAAAACTCAAAAAAAGAAAGAATATTCAAAGAAAGTTTCATCTTGTCAGTTGAAATGAATAGTTTATT
GTGGAAAT

TACAAAGAAGTTTCACTAAAAGTTGTCGCGATTGTATACATATATGCGTATATATACAATTCTACATGTTT
TATATTTATTCCATTATATTCTCGTTAAATTTTAACTTTAAATTAAGAGGGCAAAGAGGCCCAGCGGGAGT
TAATTTTT

AATTTTACAACCGAGTAAATATCTTATAATCATTAAAATTATTAAACTATTATACAATTTTTTTTACTTTA
CTTTAATTTTTATATACCCATATAAATAATAATATTAATTATAACCAAAGGAAGTGATTTCATTATGCTTG
AGTAGAGG

GCGCTATATAGATTTAGTAATTTACATGTTACAGAAGCAAAAAGCAAATATATATATATTTTTTTATCATAT
TAGTTTTTATCCTCAGTAGTACTACTGAAATAATAATCTCAGATTTTTTTTTTTCACTTGCCGAGGGACCT
TGTCCTAA

TGCTCACTTAGTGCTGCAGGTAAATTCCGTTTTTTTCCAAATTAATATTTATGAACGTTATGATGTCAAGT
TTTTTCAAGAAGTAATTATCCGCGAAAAAAAGAATATAAAAAATACAAATGTGCATAGATCCTCACATAGT
ATACAACT

TGCCCTATACTACCGAAGATAAATACATGAACTACACGTATCATATAACATCATATCCTTTTTTTTTTTTT
TTTTTTTTTTCTAGGCTTTTACTAATACAAGGGCCTTAGCCATTCGCTAAACGTAATGAACGATATTGAA
GCAAATGA

CACATGCTTATAATCAACTTTTTTAAAAATTTAAAATACTTTTTTATTTTTTATTTTTAAACATAAATGAA
ATAATTTATTTATTGTTTATGATTACCGAAACATAAAACCTGCTCAAGAAAAAGAAACTGTTTTGTCCTTG
GAAAAAAA

GAAAACTATGTTTAATTGAAATAACATTCATCAGCAAAATTAATAAACTAAATAAAATAAATAAATAAATA
AATAAATAAATAAATAAATAAATAAATAAATAAATAATATACGATCATAAAATAAATAATAACTCAAGCTT
TTTAGACT

ACATTTCTTTTAATCAGCGAAAAAAATGGCGAGGTGAAAAAAATCGATGAGATGAAAAAATTTTTTACTAG
TAAAGGCGTATTGATGATATATTAGCAGCTAAAGTATAGCAAGGTTTGTCAATTTGAAGCAGTCCGCGTGT
TTTACAAT

AGATATTATTGAGTACCGGGAGTATTATGTGTCATAATTTGTAGTATTATCTGTGTGACGGATTAAGTTTG
CTATCTCAAATGCCGCGCGCGCGAATTGCTCATGATATAATAATAGCTAATAATATGATAATATGATAATA
ATAGATAA

CCCGCATTAGTAATTGCCCGCTTTTCTTTTCTTCCGCGGGTGGGCCCCATAAATAGAAAAAAAAAAGAAAGA
AAGCGTTTAAATAAATAGAGTGAGCGGATTTCTATTATCTGAAAACCGGGTTATAATGCACGTGATATGCA
CGTGGGAG

CAAATAAATGATTAATTTTAGACAATATTTTTTTTTTTTTTTTTATTATGTATATAGTTAACATGTATAAA
GTAATAGCAGATTCTCTCTAAAAATATAACATAACATTTCATTTCAATTCGTACTAAATAATTTTTTATAT
AACAGATA

GCGTCGAGAAAATCGCGTCTTTCTACTCTCACAGTGCTTGCATACGTATGGTATACAGTATCAAAAGTAGC
AGAATTTGTCTTCATTATGATATATATATATATATGTACATATATATAGGGTGCTAAAAAGTATTCTTTTT
GCTATTAT

TGAAAAAAGTTCGCTTCTTAACCATATTAGAAATCTAGAAGAAATAAAAAAAAATCTGGCATTAGATATATA
TTTTGACTGTAGGCCTTCTGTATATTATATAATACTACTAATAACATCAAATGTTTGTGACTTTTCGGGTA
ACCGGGCT

ACATATGTAAAATTACATATAATATAATGGACACATAAGCAAAAAAAAAAAAAAAAAAAAATAAACAAATGA
GCAACCCATCAAATACCAAGAACAAAGAATTTAATATAATATATAGTTATTAAATTTAAATGTATATATGC
AGTTCTGC

GTAACTATTCTGTGGTTCATGCTGAGAGGAAACACTGCTAAAGCAACCTATGACCTGTACACTAATTAAAA
CACTTTTATCCATTTTTTTTCGTATAAGCATATTTTACATCTTTATATATATTTATTTTTTTTTTTTTTAT
TTTGGTAG

ACACATTCTTGTTACATCTGTCACGGCATTCCAGCTGTAAAAAACCAGCAGATATCGCATAAAAAAAATGGC
CAGCTATAGAATAAAATTAGCCCAGCTATATTTTTATAGCGTCTTTTGATGACGCGAAAAGTAGACTTCGC
GTAACACG

ACAGTGTATTAGTGCTACATAAAAACGCGTTAAAACTTGCATTCCAATTTATCAAAAAGTTTGCCAGGACA
TATAGGATATAAATGCATTGCGAAGTAAAACACTTCTCAAAAAATTAAAAAAAAAAAAAAAAAATAAAACTA
ACGTATTC

TTTGTACACGAGAATTGACGTATAACTTCTTTTGAAAGTCTTTGAAATTACATGGTAGTACATACATATAT
ATATATATATATATATGTTATATTATTGTTAATTAAATATTATTATGCGTATTTTCCTTTTCTTTATTAGT
ATAGTATT

AGTCTGAATAATAGAATGAGTTACATACGTAAACGTGTAGTATTATTATTATTATTCTATTTCCTTTCCGT
TTTTATTATTTATATATATATATATATATATATATATTTGGGTGTTTATATTTAGGTAGGGCATAAGGATT
TACTGTCG

TATACTAAGGTGAAATACTAAAGTAAAATAAAATAAGAGAAAGAAAAAAAAAAAAAAAAAAGAAAAAAGTAAT
GATGATGTTCCATAATTGCAGAATTAATAACTACTTAATAACTTCTCAAATTGGCGAAGGCGCGTACGGTC
TGGTTTAC

AATTCCTATTGTTGTTGGAGGTTATTTGGGCGCTTTGGTGGGCTATACTATCGGTGCCTATATATAATATC
GGTAATTTAAAAATTTGTATATAAAAAAATATAAGTATGATAGAGGTAACATAAACTGGTCCTATGCACAA
TTGAAAAA

GATATCGCATAAAAAAAATGGCCAGCTATAGAATAAAATTAGCCCAGCTATATTTTTATAGCGTCTTTTGAT
GACGCGAAAAGTAGACTTCGCGTAACACGAAAAATGCTGGTGCAGGTGGATTTTTTTGGCAGATACTACGG
TCAATTCT

ATTTGGTGATAATAATATATACATACATATATATATATATATATAAATATATAGGTATATATATAAATAAC
CTAAGACAAGTTAAATGAGTATAAAAAACCTTGAACGTAAATGAAAAAAAAAAAAAATACTAGTAAATTGAC
AACATAAA

TCCACAGAAAAAATTCCTAAAGTTATAGTTCACATTGTTATATATGTATCTATAAAGCGAAATTTTTTAAA
ATTAATTGATTAATTAATATTTTGTATCTAAAGTGTTCATACTATATTATTTACTTAGATTATACATAATT
AAAAAAAA

AAAATCGCTGAATTCCCAGAAGAAGGCATTGATCCGTCCACTTATTTGAATAATTAATAAAGGAAAAAAGA
TACGTTTTTATATAGATTATATAAAGATTTTGTATTATTCAAACGAACAAAACAAACAAAAAAGGGGAAGT
GGAAAAAA

ACTTAAGATTGGTGGCCTTCTTCGGTAACTAGGGGATAAGGGGCTTCGTCCTACACGATGCTCCTGTCGTT
TCTTTTTTTCATTTCTTTTTTCGCCCAGCGGGAAGTACGCGGAAAACAAGAAACAAAAAAAAAATAAAAAA
ATAAAAAA

TATAACTTCTTTTGAAAGTCTTTGAAATTACATGGTAGTACATACATATATATATATATATATATATGTTA
TATTATTGTTAATTAAATATTATTATGCGTATTTTCCTTTTCTTTATTAGTATAGTATTAATGACAGTAAT
AATAATAA

CCGCAGCAGCCCGCGGCTTCTGTCCTTATTCGCCCATTTTTTTTTGATTTTCGACTTTTTCACTCTGGCTA
GTTTTATTACGCATATTTGTATATATATATACGCATAAATTTCTGAAATGGATTACCTGGTTTATCAGAAA
CGAGAGCT

TGAGATGGCGTATTTCTACTCCAGCATTCTAGTTAAGAAAAAGTCTAAAAATGGTTTTTTTCATCCAAAAT
ATTAAATTTTACTTTTATTACATACAACTTTTTAAACTAATATACACATTTTAGCAGATGCGCGCACCTGC
GTTGTTAC

ATTTTTAGTATTTTTTTATACGTGCGTATTCTATTGTTCATTTTTTTACCAATTTTTTTTTTATTTTTATA
TTTTGTTTCTTGCGTGGAGTTTTTACAGGAAGTTAATCACAACAGAGGGTTCAATCTCCAAGGCATAAAAT
AAACAAAA

TCCTCAATGTGCTCGTGTAAGTGTCGGCCCGGAACTTGATTCCCCCCGGACTAATTAGAAGTTCCGAGAAC
GCGCGCAAGCCGTCCCGCGCGTAGAAGGCTAAAAAGCAGCCGGAGGAAAAAAAATAACGCAAATGTGGGCT
CCCCTTCC

GTCTTAAATGTTCTTCGGACTTTCTTGAAATCTTCGAAAACTGATGAGAATTCTTCATTATTTTTTTTTTT
CTTCTTTTATTATTTTTTATGTTTATTCCATATATACACTTATAATTTTTTTTTTTGCGATTAATATAAGT
TTGCCTGG

CCAGTCACTATCATCATCATCATATTTTGTATATATACATTTATTATATAAAATATCATACCAATTTTTGC
CAAAGAACACCGTTTCCCTCTTCGCGTTGACCACAGTAATTGCAGAAACGACAAAAAAAAAACAAAAAAAA
GAAGCACC

AAATACATGAACTACACGTATCATATAACATCATATCCTTTTTTTTTTTTTTTTTTTTTTTTCTAGGCTTT
TACTAATACAAGGGCCTTAGCCATTCGCTAAACGTAATGAACGATATTGAAGCAAATGAAGTAAAACAATG
TGGAAAGC

ATTAACAGTTTTTTTTTTTCATTTTTTTTTTTTATTCTTATTTATGTATGATACTTTATTATTATTTCTCT
TAATTATTTATTTATTTAACTAACACGATGAGCACTTTTAACTGCAATGGTTAAACTGTAGCAATGTTGGT
AAAAAAGC

GTTTTCTTAATGCCGCGCGTTTGAAAGGAAACTGATCTGAAAGTGATTTATGATGCCAAAAAAAAAAAAAA
ATCTTTTGAAGTACAATGTATGTATGCATGAGTTTATAAAAAATGATGTAAAGTGCGGAATAGTAGTACTG
CTAATGAG

CGATTTTAATGATTAGTTGGGCTTTTGGCTACATATGTAAAATTACATATAATATAATGGACACATAAGCA
AAAAAAAAAAAAAAAAAAATAAACAAATGAGCAACCCATCAAATACCAAGAACAAAGAATTTAATATAATA
TATAGTTA

CTGAATCATAGGTCAAGAAAATGATATACAAAATGGGTTCAAAAAAAATTTTTATCGAATATTTTCCTGTA
ATAATATTGTTTCTTTTAGAAAAATTTTGGTTTTCTATAATATATTTATTCTCCATGTTTTAATTTTCTCA
GCTATTAA

CCTATCGTCTATGTACTCAGTCTAATCTACTTTGATTTAATTTAATTCAAGTTATATAAATCTATTCTTTT
CCCTTTAATTTTAATATTTTAATTTCAATCCTTTTCTTATTTTCTATTTTTGCTTTTTTTTTTTCTTTTTT
CCATATTC

TCGTATGCTTACCTTGCTTAATCATCTTTGAAGGCTTGTGCTGATCGAACGAAGCAAATCCTACGAGTAAA
TACATAAGCGTATACATATATATATATATATATATATATATATATATATGTATATATATATATGTGTGTGT
GTGTAATT

TAAGCAAAGTTTAAAGTTTTTTTTTTCCAGTTCTTTTTTCCCATCGTTTTTTTTTCTAGAAGCCGGGTTTT
TCACCGTCAAAAAATTTCCTAAAAAAGATCTGTCAATCATTAAGTTTTGCTTTTTCGGCTTTTAAGCCATG
CTTTCAGG

CCTTTTTTTGACAAATTTGAAAATAAATATAAAAAGGGCCATAAAATATTGAGTTAAAATTGACGATTTTT
TTTATAGAAATGACTTGAATTTACGATTTAAAATAAAAATATACCTGGCATATAACTAACTATACAAATCA
TCATCTTC

TCATATCTACCGACATAAAGATAGAGATCTTTTTTTTTCCTTTATTTTTTTTTTTGATTTTTTTTTTTGAT
TTTCTTGATATTAGTTTTTAGAAGTCCTTTGACGGGGCAGCCGGAGAATATATACCAACGGCATTCTATTG
TCTTTTTT

GTGATAATATTAGTGGTGTACGGAGTGCTTGAAGAAAAAAAAAAACTGATATAGAACCCAAAAATGTTCGGC
TTTTAAAATACAAATATGTACAGGTGTTAATGTCTAAATAATGATAATCCAAACGCGATAATTTTTTTTTC
GCGTTTGT

TTTACTTTACGGTCTTTAATCGATTTTAATGATTAGTTGGGCTTTTGGCTACATATGTAAAATTACATATA
ATATAATGGACACATAAGCAAAAAAAAAAAAAAAAAAAATAAACAAATGAGCAACCCATCAAATACCAAGA
ACAAAGAA

TCGAGAACACAATTTTACAACCGAGTAAATATCTTATAATCATTAAAATTATTAAACTATTATACAATTTT
TTTTACTTTACTTTAATTTTTATATACCCATATAAATAATAATATTAATTATAACCAAAGGAAGTGATTTC
ATTATGCT

GGACATTTTTCACTATACGTTGATGCAGTAGTGAACATATTGCTCGCATAATGTTACCCGTTTATACCTCT
ATACGATTATTAAGGGAGGGTAATACTTTCTCAATTTTTTTTTTTTCAGTTTTTAATATTATTTCCTTAAG
ATTTTTCG

TTAAAAAAAAAAAAAAAATAGAATAACAATAATTAAGCAGAAAAAAATGTTAACGAATATAATAAATCGATTT
GTCCATTTTCATAAATTTTTTTTTTGTATTATTTCATTATCTGTGCCTCAAAGCATTAACTTTTCTTGCAG
TCAGAGAG

GAATTATAAAACAACTGCGCATATTGCTAAGAACTGCACTGAAGGCATGAATTGTCTATTCCGTTAGAAAT
ATAATTATTTACGAAAGCATCGCGCAGACGCAATTTTTTTAACATTTTTTTTTTTTCAAGTACTCTGGAAA
AGTTTTCA

TTAATATCAAAGTCTGAATAATAGAATGAGTTACATACGTAAACGTGTAGTATTATTATTATTATTCTATT
TCCTTTCCGTTTTTATTATTTATATATATATATATATATATATATATTTGGGTGTTTATATTTAGGTAGGG
CATAAGGA

ATTTTTTTTTTCCTATGCCTCCCTTTTCCAAGTAATGTTTTTTTTTTTTTTTTTTTTTTTAGCTCCTCTCA
CAAAAGTACTGAGGACTGAAATGTAAAAACTAATAACACAATAGAAATAATCATATGATGATATAGAAAAG
AAGAGAGG

CCATTTCGGAAACTGCATTACTTTTTCGGGACGTATTCTCGGGAAATAAGCAGGTCCAGATCCGCGTAGCA
TGAATGGGGCGCCAAAACAGTCGCGGGTAACGAAAGAGCGCAACCACGCGTATATTCCGGGCCGGTGAGGG
GCAACGCG

AAGAAAAAAAAATCTTCAATACAGTGTATTAGTGCTACATAAAAACGCGTTAAAACTTGCATTCCAATTTA
TCAAAAAGTTTGCCAGGACATATAGGATATAAATGCATTGCGAAGTAAAACACTTCTCAAAAAATTAAAAA
AAAAAAAA

GGGTTTCCATAGACATTGAAAATGAACGAACAAAATAAAATAAATAAAAAACTACTTTTTTTTGATGATAT
AAATACCGTAGATTATATAAATGTATATTAATATTATAAACCTATTTGATCAATGACAGTGTTCGTGAAGC
ATTTTATG

GTAGACATGACCACTTGGAGAAGTATTGGTTCTGGCCCAATAATGGAATTTTGAGAATGAATATTTTCGCG
TATATATTTTAATTAAGGGTTTATATACATAAAAGATATATCTAATGATCTGATCTGAGTCCCGACTTTTT
ATATGTGC

TGTCTTCAACTAAAAGGCGAAGAAGGAAGCGACACATTTGTGATGCTTTGAGGTCAGTAATGTAATATATA
TATATATATATATATATATATTATATAGTGTAACTGCTCTTATTTATCTTGTCTATTCGAACCCAACAA
AAAAGGCT

CTTGCCATGTAATAATAGATGTCCAAAAATTTCATCTGCCATTTTTTTATTTTCCCGTTCGTTCACTTGCT
CTAGTTTTTTTTTTTTTTTTTTTCAATACTCGGATTTGAAAATGTTAATGGGAGTGGCGCTAAAAACCTTC
ATCTGTAG

AGAATAAATATTACTTTTTTCGAAATCTGGTGTTTAAATATTTTTAAGAAAGTTAAAAAATAAGGAATATT
TGATAAACAAAAAAAAAATTAGCCTTTATATATCCATTTTTGTTTATGTATTCCTTTTTTTATTTTCTTATG
TGTTTGGG

AGGTAAATAACCTATCGTCTATGTACTCAGTCTAATCTACTTTGATTTAATTTAATTCAAGTTATATAAAT
CTATTCTTTTCCCTTTAATTTTAATATTTTAATTTCAATCCTTTTCTTATTTTCTATTTTTGCTTTTTTTT
TTTCTTTT

GGTCTTTAATCGATTTTAATGATTAGTTGGGCTTTTGGCTACATATGTAAAATTACATATAATATAATGGA
CACATAAGCAAAAAAAAAAAAAAAAAAAAATAAACAAATGAGCAACCCATCAAATACCAAGAACAAAGAATT
TAATATAA

ATTTCCTTTTCTTTTTTTCAAAGAGGTCATATTTATCACCAACTATATATTTTTTTGAAGAAAAGATATCA
TTACAACGATAAGTAGTTACAATATTTTTTTTATTTAGTTTAAAATAATGAGTAAATGACGTCGCATTTCT
TCGCTTTT

ATATTTGCCATTTGAATTTTTTTCGGCGCTTTTCTTTTTTTTTTTCTTTTTTTTTTAGCTTGAATTATCGA
TGAATGATGAAATGACTCTTAATGAACTTCGAAGTTATATATACAGCCACTTTTTTGTTTAGAAAGAAAAG
TATAATTA

TAATTTAACATCAAAAGGAATCTATGGTGTGGCCGTTGATATTTCGCGATGGTATGCCGAAAAAGGAAATA
ACGCAGATTGCGCGTAGTAACGAATGGATTTGACGCGTTTCCTTCGATGAAAAGTGGGAAATAATAATAAA
AAATTCTT

TACCGAAGATAAATACATGAACTACACGTATCATATAACATCATATCCTTTTTTTTTTTTTTTTTTTTTTT
TCTAGGCTTTTACTAATACAAGGGCCTTAGCCATTCGCTAAACGTAATGAACGATATTGAAGCAAATGAAG
TAAAACAA

AAAAAAAAAAAAAAAAAAGAAAAAAAAAGAAAAAGATATCTTGGTTTGAATAAGTAAAATTAATAGAATCCAG
TTTCCAGTGATCGGTACAGGCTAAATAATAAATATCTCTACTTTTATTTATTCTTTTCGTTTTCTTGTGTT
CAGGATAG

AATCTTCAATACAGTGTATTAGTGCTACATAAAAACGCGTTAAAACTTGCATTCCAATTTATCAAAAAGTT
TGCCAGGACATATAGGATATAAATGCATTGCGAAGTAAAACACTTCTCAAAAAATTAAAAAAAAAAAAAAA
AATAAAAC

GAAATCAAGTCGATCGAGTAATCGAATTTACGTAGCCCAATCTACCACTTTTTTTTTTCATTTTTTAAAGT
GTTATACTTAGTTATGCTCTAGGATAATGAACTACTTTTTTTTTTTTTTTTTTACTGTTATCATAAATATA
TATACCTT

GTGAGCAAAACGAAGAGAGAAAAAAAATTTTTTCTTAAAAGTTTTTTTTCATTTTGTGAGCTTATTCTTCTT
TTCTATATATTCTTGATATCTTAGATTATACATATTATTCTCTTACATTTCACGATTGCCCTTTTGGTGTT
TAGCATTC

ATGGAATACAGCTCATCCATTTAAAAAAAAAAAAAAATAGAATAACAATAATTAAGCAGAAAAAATGTTAA
CGAATATAATAAATCGATTTGTCCATTTTCATAAATTTTTTTTTTGTATTATTTCATTATCTGTGCCTCAA
AGCATTAA

TTAGTCCCTTTTCATAATTCCTTAACTTTTTGCGTACAAAATGATATGTTTATTATATTTTTCTTTTTTTT
TTTTCAAATTTTTTCTTTTTCTTGAAAAATTTTTCAAATTGGAAAGCTCATCTCTCTTGAATGTATAATAC
TTTCTTCC

AAAAAAAAAAAGCATGCATAACACTAATAATATATTATTAAAAAAAAAGCCAATCTATATTATTTAAATTA
TCCGAAATTACTTTTATTACATTATGACATTCTATAGTCTATGCAAGCGGATCAAAAAACCATTCCATCTT
GGTGCCAC

AAAAATAGATAAAACAATGAGATAATATTTATTAACTTCTACGTAAATAAAAAGTTACATAAGGATCAACC
TTTTTTTCTTTATTTTCTTTTTTTTTTTTATTTTTCATTTTCAATTACAAACTAAGACATAACAGTAAGCA
GTGCTTTT

GGAACTTGATTCCCCCCGGACTAATTAGAAGTTCCGAGAACGCGCGCAAGCCGTCCCGCGCGTAGAAGGCT
AAAAAGCAGCCGGAGGAAAAAAAATAACGCAAATGTGGGCTCCCCTTCCCCTGTGCGGCCACGTCGTGCCT
CCCCACCA

GTGCACGCAATATGTTCCAGGATATGAAATGAAATACCTTTTGTTTCACCTTTTAAATAATTTAATGTTAT
ATATACAACTTTATCGTATCATATTCGCAATTACATTATACAAGAATGAGTTTTTTTTTTTCGCGGACAAA
AGAAGAAC

TCAAAAGGAATCTATGGTGTGGCCGTTGATATTTCGCGATGGTATGCCGAAAAAGGAAATAACGCAGATTG
CGCGTAGTAACGAATGGATTTGACGCGTTTCCTTCGATGAAAAGTGGGAAATAATAATAAAAAATTCTTGT
CAGACGAA

CTCAACACTGAGGAGAACACAAAAAAAATAGTGGAATGTGATAGTTTGAAATAAGGATATTAGTTTATACT
CTAATATACAAATATAAAAGTTGTTTCTTTTTCGTTCTTTTTTTTTTTTGGACTTGTCTTCGTTTTTATTA
CATACATG

GAAAAAAGTACAAAAATGATAAAAGGCAAAAAAGATGAGAAGTTTTTTTTGTGATAAAATTAATTATTAAA
AAATACACACGTAGTTAAAATTAACAGCGGGGGAGGAAACCTTAGAGAAAGAGAAACAAAAAAAAAAGAAA
GGAAACAA

TGCCATATTATATTACAGAGAACAGAGTGTTACGCCAACATACTAAACCAGAGAAGTAAAACGTTTTACTT
ATATATAATAGTATTCGTAAATTTATAAAAATCAAAAAACCAATATAAGCCTGGTTAAAAAAAAAAAAAGA
TCAGTATC

GGGAAATAAGCAGGTCCAGATCCGCGTAGCATGAATGGGGCGCCAAAACAGTCGCGGGTAACGAAAGAGCG
CAACCACGCGTATATTCCGGGCCGGTGAGGGGCAACGCGCATTGTCCCGGGGCGATATAATCCATGTACCG
GAACCCGC

GAAGGGAGGGGTTTTTGCTTCCGTTTTTTTGACATTAAAAGTTTTCTTAATGCCGCGCGTTTGAAAGGAAA
CTGATCTGAAAGTGATTTATGATGCCAAAAAAAAAAAAAAATCTTTTGAAGTACAATGTATGTATGCATGA
GTTTATAA

TTACTTTTTTCGAAATCTGGTGTTTAAATATTTTTAAGAAAGTTAAAAAATAAGGAATATTTGATAAACAA
AAAAAAATTAGCCTTTATATATCCATTTTTGTTTATGTATTCCTTTTTTTATTTTCTTATGTGTTTGGGAT
TTACTGTC

TTGTTGCTATGTCCTTGTACAAACGTATGTGGCCCATGCCTTCGTTACCTTGGAATGGTATGTAAAGTGAT
AAAAATATACACGTATATATATATATATATATATATATAAGTACGTGAGATCTATTTGTATAATATAATGC
TAATAAAA

CGCCAAAACAGTCGCGGGTAACGAAAGAGCGCAACCACGCGTATATTCCGGGCCGGTGAGGGGCAACGCGC
ATTGTCCCGGGGCGATATAATCCATGTACCGGAACCCGCACTTTTTCGACTTTCTTTCCCCTTTTCCTGTC
TCCTATTG

TATACGATTATTAAGGGAGGGTAATACTTTCTCAATTTTTTTTTTTTCAGTTTTTAATATTATTTCCTTAA
GATTTTTTCGGTGGCGTTTCGCGACAACTTACGAGAAAAAAAAAAAGAGGAGACTTGAAGAAAGAGTGCAA
AGGGATAG

TCTATGGTGTGGCCGTTGATATTTCGCGATGGTATGCCGAAAAAGGAAATAACGCAGATTGCGCGTAGTAA
CGAATGGATTTGACGCGTTTCCTTCGATGAAAAGTGGGAAATAATAATAAAAAATTCTTGTCAGACGAAAC
TTGGCGCA

ATGAATGGGGCGCCAAAACAGTCGCGGGTAACGAAAGAGCGCAACCACGCGTATATTCCGGGCCGGTGAGG
GGCAACGCGCATTGTCCCGGGGCGATATAATCCATGTACCGGAACCCGCACTTTTTCGACTTTCTTTCCCC
TTTTCCTG

GGCTTTTGGAGTGAGCAAAACGAAGAGAGAAAAAAATTTTTTCTTAAAAGTTTTTTTTCATTTTGTGAGCT
TATTCTTCTTTTCTATATATTCTTGATATCTTAGATTATACATATTATTCTCTTACATTTCACGATTGCCC
TTTTGGTG

TATTTTTTTCCTCAATCTTCGATATTAGACATTTTTTTTTTCCTATGCCTCCCTTTTCCAAGTAATGTTTT
TTTTTTTTTTTTTTTTTTTAGCTCCTCTCACAAAAGTACTGAGGACTGAAATGTAAAAACTAATAACACAA
TAGAAATA

GTGGCAAATCAAAAGCGTAAGGAGATAAGAAAACAAAAAAGGGCTACATTTTAGAATAATTTGAATCATAT
GTAGAAAAGCTTGTAATAAGAAACCGTCATATTCTTTTTTTTTTTTTTGACCGCGTATGAATCAGTAATTT
ATTTCATT

TATTACAGAGAACAGAGTGTTACGCCAACATACTAAACCAGAGAAGTAAAACGTTTTACTTATATATAATA
GTATTCGTAAATTTATAAAAATCAAAAAACCAATATAAGCCTGGTTAAAAAAAAAAAAAGATCAGTATCGT
AAAGTCGT

CCCAACGTTGGGCCTAGGCCCTTACCGCGCCCATTTCGGAAACTGCATTACTTTTTCGGGACGTATTCTCG
GGAAATAAGCAGGTCCAGATCCGCGTAGCATGAATGGGGCGCCAAAACAGTCGCGGGTAACGAAAGAGCGC
AACCACGC

ACGCTATAGAATGCACCTAAAACTCGCCTGCACTCCGAAACGAAATGCGATACAGTACGTGTCATGCGGTT
AGTATATATACATATATATATATATATATATATTCAAATAAATTATAACGTATTAAATAATATGTGAAAAA
AGAGGGAG

TTGGCATCTAGCCCCAATGACGATACACTAATATATATATATATATATATATCTATATATCTGCAAGAAAT
AAGCGTGTGTAGTAAGTAAAAGGAAAAATAGAAATTTTTGCTCAAAAATAACGGTCCCATTTTGATACAAT
GTTTTTGG

GAAATTAGCAGGCAGTTTGAACAAAACTTACAAATAAATGATTAATTTTAGACAATATTTTTTTTTTTTTT
TTTATTATGTATATAGTTAACATGTATAAAGTAATAGCAGATTCTCTCTAAAAATATAACATAACATTTCA
TTTCAATT

TGCAGTGGTCATTTGAAAATGGTCATTAAATTAAACAAGTAATATGAGAGGAATGAGAGGGAAATAAATAA
CTGAAAGATAAAGGAGATAAGGAAAAAAAAAAAAAAAAAAAAAAAAAAAGGAACTTTAGTTAGTAATAATA
GAGTTTTT

TATTTCTACTCCAGCATTCTAGTTAAGAAAAAGTCTAAAAATGGTTTTTTTCATCCAAAATATTAAATTTT
ACTTTTATTACATACAACTTTTTAAACTAATATACACATTTTAGCAGATGCGCGCACCTGCGTTGTTACCA
CAACTCTT

CCAGCATTCTAGTTAAGAAAAAGTCTAAAAATGGTTTTTTTCATCCAAAATATTAAATTTTACTTTTATTA
CATACAACTTTTTAAACTAATATACACATTTTAGCAGATGCGCGCACCTGCGTTGTTACCACAACTCTTAT
GAGGCCCG

GGAGAACTGTCCGCAGCAGCCCGCGGCTTCTGTCCTTATTCGCCCATTTTTTTTTGATTTTCGACTTTTTC
ACTCTGGCTAGTTTTATTACGCATATTTGTATATATATATACGCATAAATTTCTGAAATGGATTACCTGGT
TTATCAGA

AAGATAATTCTGGTCTCCCGGAAACATGAAGATGCAGCAAAGTAAAAAATAATAGGTAAAAATAATAAAAA
TACATGTATCTTTTTGGTTGGTTAATTTATCTAAACAGTTTTATATATATATGTATATATATATATATATA
TACACAGT

ATTGAAATTTAAATTTGACATAAGCAAAGTTTAAAGTTTTTTTTTTCCAGTTCTTTTTTCCCATCGTTTTT
TTTTCTAGAAGCCGGGTTTTTCACCGTCAAAAAATTTCCTAAAAAAGATCTGTCAATCATTAAGTTTTGCT
TTTTCGGC

TTTTGGATAAAACTTTTCCTACTGTTCTTTTGAACTGGATTTGAATTTTATATCGATCATCTTGCCATGTA
ATAATAGATGTCCAAAAATTTCATCTGCCATTTTTTTATTTTCCCGTTCGTTCACTTGCTCTAGTTTTTTT
TTTTTTTT

TTGGACTGAACCCGCATTAGTAATTGCCCGCTTTTCTTTTCTTCCGCGGGTGGGCCCCATAAATAGAAAAA
AAAAGAAAGAAAGCGTTTAAATAAATAGAGTGAGCGGATTTCTATTATCTGAAAACCGGGTTATAATGCAC
GTGATATG

ATCTTAAGCGGAAGGGAGGGGTTTTTGCTTCCGTTTTTTTGACATTAAAAGTTTTCTTAATGCCGCGCGTT
TGAAAGGAAACTGATCTGAAAGTGATTTATGATGCCAAAAAAAAAAAAAAAATCTTTTGAAGTACAATGTAT
GTATGCAT

TGTACGATAATATACTAAGGTGAAATACTAAAGTAAAATAAAATAAGAGAAAGAAAAAAAAAAAAAAAAAGA
AAAAAGTAATGATGATGTTCCATAATTGCAGAATTAATAACTACTTAATAACTTCTCAAATTGGCGAAGGC
GCGTACGG

ATAGAATGAGTTACATACGTAAACGTGTAGTATTATTATTATTATTCTATTTCCTTTCCGTTTTTATTATT
TATATATATATATATATATATATATATATTTGGGTGTTTATATTTAGGTAGGGCATAAGGATTTACTGTCGGC
ATGAACCT

AACTATATATTTTTTTGAAGAAAAGATATCATTACAACGATAAGTAGTTACAATATTTTTTTTATTTAGTT
TAAAATAATGAGTAAATGACGTCGCATTTCTTCGCTTTTGTTATTTTTATTCTCCTCTCATTTTTGAGGAG
AATGTTAC

AGGAGAAATAGTAGAAATTAATAGTACTCTAAAAAGAAAGAACGGAATAAGAGGAAAAGACCTTTTATCTA
TTCAAATTGTTTCCCTTAGGTATATATATATATATATATATATATATATATATTTTCCCTGTATATATCTATG
TAAATGAC

GTTATTTGGGCGCTTTGGTGGGCTATACTATCGGTGCCTATATATAATATCGGTAATTTAAAAATTTGTAT
ATAAAAAAATATAAGTATGATAGAGGTAACATAAACTGGTCCTATGCACAATTGAAAAATCTATGACAAAA
ACTAAATG

TTGGAGGTCTCCATTGTGAATGTTCTCACTCGATTTTTTAAGTGTCTATTTAGTGTATTATGCCGTGAATG
CGCAAGATATATACGTATACTTAAGAATGTAGTTTATATTTGACAATTTTTTTCAGCTCATCGCATTTTTT
TTTCTAAA

TTTTTTTTTTTTTTTTTTTTTTTTTTAATTGAACAATAGATGCAGAGGAATGCTCCAAAATTTATTTCCTT
GAACAATATTACAAGAGGCAGATATATACTTTTCCTTATTTCCCTTAGTAAAGAATGATGCATGTATACTA
TTACAAAT

CCGGTTCTCCCTTTATTATAAACACTTCAGAAAAATATTCTGCTACTATTCCTTACTTTACTATAAGAATT
TTGTTTTCCAAAAAAAAAAAATATAAAAAAAATAATCATACTCTATTACTATGGCTAACGTAGAAAAACCA
AACGATTG

AGAAACCGTCATATTCTTTTTTTTTTTTTGACCGCGTATGAATCAGTAATTTATTTCATTTTGAATTACAT
CATTATAAAAAGTTCTTAGTAATATATGCATTGGGTATACAATCACGTGTACTATCAAATAAAGAAATGAA
AATACGGA

GGCCTAGGCCCTTACCGCGCCCATTTCGGAAACTGCATTACTTTTTCGGGACGTATTCTCGGGAAATAAGC
AGGTCCAGATCCGCGTAGCATGAATGGGGCGCCAAAACAGTCGCGGGTAACGAAAGAGCGCAACCACGCGT
ATATTCCG

TGCAAAAATGTTCTTCAAACGCGTTTATTATATAATGAAAAAGAAATTCAAATTTTTCGCGTTTGAAAATT
TATGCTAAACAAATAACTCGAGGAGGGACGCGTCGAGAAAATCGCGTCTTTCTACTCTCACAGTGCTTGCA
TACGTATG

TAGTAACTACATATTCAACAATTGCAGTTTTCTTTTCCTTTTTGTCCTTGTCCTTTCTTTCCTATTTATAT
ATATATATATATATATATATAATACTACTATGTTATATTACATTATATTATTATCACAATAATTATTACGT
TCATCGTG

GGCCTTGAAAAAATAGCCTGTAAATGAGGGAAAAATGCAAATAATCTTGAATGATACTCAAAACTTTTGAT
TTATTGTAATTTCTTTCTGAACCATATTTATTACATTCAAACTTTTTTTTTTTTGCCTTTTATAAAAAAAA
ATACTTAT

TTAAGGGAGGGTAATACTTTCTCAATTTTTTTTTTTTCAGTTTTTAATATTATTTCCTTAAGATTTTTCGG
TGGCGTTTCGCGACAACTTACGAGAAAAAAAAAAAAGAGGAGACTTGAAGAAAGAGTGCAAAGGGATAGCG
GGATTCTA

AAAAAGTAGATGCCATATTATATTACAGAGAACAGAGTGTTACGCCAACATACTAAACCAGAGAAGTAAAA
CGTTTTACTTATATATAATAGTATTCGTAAATTTATAAAAATCAAAAAACCAATATAAGCCTGGTTAAAAA
AAAAAAAA

GACAATATTTTTTTTTTTTTTTTTATTATGTATATAGTTAACATGTATAAAGTAATAGCAGATTCTCTCTA
AAAATATAACATAACATTTCATTTCAATTCGTACTAAATAATTTTTTATATAACAGATACATAAGATATTT
ATATAGAG

GTAACTATATTATATAAAATATTTTGATACCCTGTACCTGCTTTTGTTATCGTTGTACATCCATGCACACG
CTCATATATATATATATATATATATATATGTATATGTACATATACCGCTTACCACCTACCGTGAAGTATAT
GTAAGGGT

TTATCGTTATTTCTTTATTTTTTTCTTTTTCGCAAAGATCGACCTAAGACGCGTTTGCAAAAAAAAAACCA
TTTACTTACTGCGGGTAAAATATTAAAAGAAACTTAGGCGTTAGCCAAGAACCGATAAGAAAATAAAAATA
AAAAAGGT

ATATTCTTTTTTTTTTTTTGACCGCGTATGAATCAGTAATTTATTTCATTTTGAATTACATCATTATAAAA
AGTTCTTAGTAATATATGCATTGGGTATACAATCACGTGTACTATCAAATAAAGAAATGAAAATACGGATA
TACCTCTG

CCTTTTTCTTTTTTTTTTTTTTTTTTTTTTTTTTTTCACATCTTTTTCCACGGCCGCTCAAAGGGGTCTCA
CACTGGATTAAAAGCTGAAAACAAGAAATGGCTCTTTCGATAAGGTTGCAACATTTTGATTACTACGTTGA
TTAGAATA

TCGCGAATGGACGAAATTGTATGCAAAGAAGAAACCCGAGTGATCATTTCTGTATAAGTTTTCTCAACTGA
ATATATAAATATATATCATTACTATGCTGCTGCTGCAAAAAAAAAAAAAAAATTTACGATAAATGCAAAAA
CCATAGAT

ATTGTTTTTTTGTTTTATGAGTTACGTTTACAAAAGTTCTTAACATTTACTGTACGATAATATACTAAGGT
GAAATACTAAAGTAAAATAAAATAAGAGAAAGAAAAAAAAAAAAAAAAAGAAAAAAGTAATGATGATGTTCC
ATAATTGC

GAGGAGGGACGCGTCGAGAAAATCGCGTCTTTCTACTCTCACAGTGCTTGCATACGTATGGTATACAGTAT
CAAAAGTAGCAGAATTTGTCTTCATTATGATATATATATATATATATGTACATATATATAGGGTGCTAAAAAG
TATTCTTT

AGTAATGTTTTTTTTTTTTTTTTTTTTTTTAGCTCCTCTCACAAAAGTACTGAGGACTGAAATGTAAAAAC
TAATAACACAATAGAAATAATCATATGATGATATAGAAAAGAAGAGAGGAGACAATCATAATATTATACGA
ACTCATGG

ATTAATTTTAGACAATATTTTTTTTTTTTTTTTTATTATGTATATAGTTAACATGTATAAAGTAATAGCAG
ATTCTCTCTAAAAATATAACATAACATTTCATTTCAATTCGTACTAAATAATTTTTTATATAACAGATACA
TAAGATAT

CATAAATTAACCAATCATTTTGCTACTTTCCCGGTTCTCCCTTTATTATAAACACTTCAGAAAAATATTCT
GCTACTATTCCTTACTTTACTATAAGAATTTTGTTTTCCAAAAAAAAAAAATATAAAAAAAAATAATCATAC
TCTATTAC

AGCACCTATTGCATATAAAGTATTCAAAAAGGTGTTGAAAGCTCTTCCTGTATATGACCCTGTATTTAATG
TACGCGGTTAAAAAATTTTTATATTTTTAAATAAGTATCAACTTGGATAATATGATCTTGCCCGCAAGGTG
GTTTTATA

TCATTTTTAAATATTTGATTATCATCGCCCGCTGGTCACCGCATTTTTAAAGAATAAATATTACTTTTTTC
GAAATCTGGTGTTTAAATATTTTTAAGAAAGTTAAAAAATAAGGAATATTTGATAAACAAAAAAAAAATTAG
CCTTTATA

GTTGTTGGAGGTTATTTGGGCGCTTTGGTGGGCTATACTATCGGTGCCTATATATAATATCGGTAATTTAA
AAATTTGTATATAAAAAAATATAAGTATGATAGAGGTAACATAAACTGGTCCTATGCACAATTGAAAAATC
TATGACAA

AAATAACGCAGACGTCTGAGCAGGAGAAGTAAAACGCGAAGGGGAAATAATGCTTTTTAGTCTTGTAATTA
TATTAATTAATTAAATAAGTTTTTCTTTATCGGAATTTTATTTAAGACTTTCATTTCTCTAATGCTATTGT
CAGTTACA

CCGCGGCTTCTGTCCTTATTCGCCCATTTTTTTTTGATTTTCGACTTTTTCACTCTGGCTAGTTTTATTAC
GCATATTTGTATATATATATACGCATAAATTTCTGAAATGGATTACCTGGTTTATCAGAAACGAGAGCTTT
AATAAAGA

ACTACACGTATCATATAACATCATATCCTTTTTTTTTTTTTTTTTTTTTTTTTCTAGGCTTTTACTAATACA
AGGGCCTTAGCCATTCGCTAAACGTAATGAACGATATTGAAGCAAATGAAGTAAAACAATGTGGAAAGCAA
ATAAATAA

TATTATTATTATTATTCTATTTCCTTTCCGTTTTTATTATTTATATATATATATATATATATATATATATTTG
GGTGTTTATATTTAGGTAGGGCATAAGGATTTACTGTCGGCATGAACCTACTTGAACAAAAAAATAAAAAT
CAAAAGAA

ATAGAGATCTTTTTTTTTCCTTTATTTTTTTTTTTGATTTTTTTTTTTGATTTCTTGATATTAGTTTTTA
GAAGTCCTTTGACGGGGCAGCCGGAGAATATATACCAACGGCATTCTATTGTCTTTTTTTCTTCCTTCCTT
GCCAGGCA

ATATGATTCTGCTATTATCCTTGTTCCCCCCAAGGACTGCAGTAGAAGAGTTATGTTACAAATGTTGATAT
ATATATATATATATATATATATATATGTATGGACATTTGTGATGAAAATATATCTTGAACAAGCGCGATTA
AGGTCATA

GAAGTTAAAAGTGCACGCAATATGTTCCAGGATATGAAATGAAATACCTTTTGTTTCACCTTTTAAATAAT
TTAATGTTATATATACAACTTTATCGTATCATATTCGCAATTACATTATACAAGAATGAGTTTTTTTTTTT
CGCGGACA

ATTTGAAAATGGTCATTAAATTAAACAAGTAATATGAGAGGAATGAGAGGGAAATAAATAACTGAAAGATA
AAGGAGATAAGGAAAAAAAAAAAAAAAAAAAAAAAAAAGGGAACTTTAGTTAGTAATAATAGAGTTTTTGG
ACTGATCA

TTGCAGAAAGAACAAACCAATATTTTAAATAATCAAAAATCAAATTAGCTATTATTTCTCATTTGGTGATA
ATAATATATACATACATATATATATATATATATAAATATATAGGTATATATATAAATAACCTAAGACAAGT
TAAATGAG

AATCACGTGGTGCAAAAAAAATTTTTTTTGTCATTTCTTCGAATCAACTCAATGAAATTTGAAAAATAGCA
GAAAATAAGTTGATAAGATCGCAATGTAATATATATATATATATATATATATTACAATTCGTGAACTGGCA
CAAGGAAA

TGGCGCTACCATTGTTTTTTTGTTTTATGAGTTACGTTTACAAAAGTTCTTAACATTTACTGTACGATAAT
ATACTAAGGTGAAATACTAAAGTAAAATAAAATAAGAGAAAGAAAAAAAAAAAAAAAAGAAAAAAGTAATG
ATGATGTT

CTATGCAACTTGATTTTTGTTTTTCTTGGCCTGAGCCATTACCGTTATTATTATCGTTATTTCTTTATTTT
TTTCTTTTTCGCAAAGATCGACCTAAGACGCGTTTGCAAAAAAAAAACCATTTACTTACTGCGGGTAAAAT
ATTAAAAG

TTTGACTAACAAGCAAATTGCAAAGCCGCGGCGTTCCCGGAAAAAAAAAAGGAAGGCCACGCTATTGGTAA
CCATGAAGTGTAAGGCGACGCAGGAGAGATGAAAAGAATGAAATTAAAAGAAATCCAAAAAAAAAAAAAAA
AAAAAAAA

CATAACTACTTTATCACGTTAGAAATTACTTATTATTATTAAATTAATACAAAATTTAGTAACCAAATAAA
TATAAATAAATATGTATATTTAAATTTTAAAAAAAAAATCCTATAGAGCAAAAGGATTTTCCATTATAATA
TTAGCTGT

TAATTAAATATTATTATGCGTATTTTCCTTTTCTTTATTAGTATAGTATTAATGACAGTAATAATAATAAT
AATAGTAACAATATCTCTTTTTTTTTTTCAGTGAGCTTTATTTTTTTTTTCATTGCTCTTCTTTTGGCCTC
TTTTGTTT

TTGGATGCCGTTGAAAAAAGACTACAAAAAGAAATCAAGTCGATCGAGTAATCGAATTTACGTAGCCCAAT
CTACCACTTTTTTTTTTCATTTTTTAAAGTGTTATACTTAGTTATGCTCTAGGATAATGAACTACTTTTTT
TTTTTTTT

GGAAATATTGATTCTTATAGTGTTATAAGTGATAATATACGTTTATGTAAATAATATCTTTTTCACAACGT
TCAAAAGTGGCCTTTTTTCTTTTTTCGATATTCTGTATTTATTACGATTTTTTTTTTTTTTTCAGATATTT
ATTTTTGC

TATTATTATTAAATTAATACAAAATTTAGTAACCAAATAAATATAAATAAATATGTATATTTAAATTTTAA
AAAAAAAATCCTATAGAGCAAAAGGATTTTCCATTATAATATTAGCTGTACACCTCTTCCGCATTTTTTGA
GGGTGGTT

ATGAAAACTTTGTTTTTGCTTCTTATGGATTGGATGTTATAACAGTAGAAGTAATTTTAGGATAATTTTAA
ATAAAAATAATATTAAAAATAATAATAATACTAATAATAAATGAGAATAAAATGTTGATAAATGAGGGATA
AGGTCTAT

TCCAGACATGATCTTAAGCGGAAGGGAGGGGTTTTTGCTTCCGTTTTTTTGACATTAAAAGTTTTCTTAAT
GCCGCGCGTTTGAAAGGAAACTGATCTGAAAGTGATTTATGATGCCAAAAAAAAAAAAAAATCTTTTGAAG
TACAATGT

GGTCAAGAAAATGATATACAAAATGGGTTCAAAAAAAATTTTTATCGAATATTTTCCTGTAATAATATTGT
TTCTTTTAGAAAAATTTTGGTTTTCTATAATATATTTATTCTCCATGTTTTAATTTTCTCAGCTATTAATG
TTCAACTT

GATTTAGTAATTTACATGTTACAGAAGCAAAAAGCAAATATATATATATATTTTTTATCATATTAGTTTTTAT
CCTCAGTAGTACTACTGAAATAATAATCTCAGATTTTTTTTTTTCACTTGCCGAGGGACCTTGTCCTAAGT
GGCAAAGA

CGATTTAAATAGCGATGATAAAGTGCAAGTTCATAAGCAAACAGCGACCGTAAAATGGGATAAAAAAAGAA
AAAAATATGTTAATACTCAAGGTATTGATAACAAGAAATATATTATTGGTGAAAGTGGCCAAAAAAATCGCG
GCAAGCTT

TCACGATCCAAGCACTATTTGCCATTTTTGTGCCCTTTCAAATTATTCTTTTTTATCGCATTGTCATTCTG
GTACTATTAAAGTAAAATTAAAAAGATTGAGGAAAATTTTTGGTAAAAACGACAACTGCAGGACTCGAACC
TGCGCGGG

AATACTTATTCATCCTTATAATTATATTCTAGCTTCGTTGTCATGGGAACATAGCCCATACACCGCAGTTA
TTTATGATCATTTCGAACGGGAAGTATGGATGAATCTTTTTTTTTTTTTTTTATAGCACGCAACTGAAAA
AAAAAAAA

CTTCATTTTACAAAATCTGGTGTAAATCCTCGATTCCATTGTGATTTAAAAATAATTAATTACTGTATAAG
ATTCTATATAGACAATTAATTGAAAATAGTATTACTTCTTTAACTCGCTTAGGATTTTTTTTATTTTTTCT
CTCTTTTC

ATGGCTTAATATGAAAACTTTGTTTTTGCTTCTTATGGATTGGATGTTATAACAGTAGAAGTAATTTTAGG
ATAATTTTAAATAAAAATAATATTAAAAATAATAATAATACTAATAATAAATGAGAATAAAATGTTGATAA
ATGAGGGA

TATCAAAAAGGTCTTAAATGTTCTTCGGACTTTCTTGAAATCTTCGAAAACTGATGAGAATTCTTCATTAT
TTTTTTTTTCTTCTTTTATTATTTTTTATGTTTATTCCATATATACACTTATAATTTTTTTTTTTGCGAT
TAATATAA

TCAATTTGGCGCCCGTTGAGATTATCTAAAAAATGCTTTTATTTATTTAAGCGCTTTTTGTTTTGACGAAT
GTATTTATGTTTTTGTATATATATGAGCGTTTTTTTTTTCCAGATTTAACTAGTTTTCTTTTTCACCTGTA
TGCACACC

GTCCTTGTACAAACGTATGTGGCCCATGCCTTCGTTACCTTGGAATGGTATGTAAAGTGATAAAAATATAC
ACGTATATATATATATATATATATATATATAAGTACGTGAGATCTATTTGTATAATATAATGCTAATAAAATA
GTATCAAC

ATCATCGCCCGCTGGTCACCGCATTTTTAAAGAATAAATATTACTTTTTTCGAAATCTGGTGTTTAAATAT
TTTTAAGAAAGTTAAAAAATAAGGAATATTTGATAAACAAAAAAAAATTAGCCTTTATATATCCATTTTTG
TTTATGTA

TGTCATTTCGTACTAGATTAAAGGGAAAATTGTATCGTAAATAAGTAATAAAGTTAATGAAAATTCGGAAA
CAAAAATGGATAGATATTAAACAGTGAACAAATGAAAAATTTCAGAAAAAAAGAATTAAAAAAAAAAAGGG
AAAAAATA

AATTACAACTAATACTATATTATTTGTCTCCTTTTTTTTATTAGTATTAAAAATTACCGTTACTGGTTAAAA
TGGAATACAGCTCATCCATTTAAAAAAAAAAAAAAATAGAATAACAATAATTAAGCAGAAAAAATGTTAAC
GAATATAA

AAATTTGACATAAGCAAAGTTTAAAGTTTTTTTTTCCAGTTCTTTTTTCCCATCGTTTTTTTTTCTAGAA
GCCGGGTTTTTCACCGTCAAAAAATTTCCTAAAAAAGATCTGTCAATCATTAAGTTTTGCTTTTTCGGCTT
TTAAGCCA

TCTATTGGATAGTCAATGACACATTCGTGACCGAACAATGAGATATTATTGAGTACCGGGAGTATTATGTG
TCATAATTTGTAGTATTATCTGTGTGACGGATTAAGTTTGCTATCTCAAATGCCGCGCGCGCGAATTGCTC
ATGATATA

TTTAATTGAAATAACATTCATCAGCAAAATTAATAAACTAAATAAAATAAATAAATAAATAAATAAATAAA
TAAATAAATAAATAAATAAATAAATAATATACGATCATAAAATAAATAATAACTCAAGCTTTTTAGACTTG
ACTAGCTA

AGGCTATAAATCCTTATTTTTTTGAGAAAAAATGCTTGAGAAAAAATGCTTGAGAAAAGATGTATTTTTTT
TTATTCGTTAGCATTTTTCCAGATATCGTTGAACATTGCTAGAATACTATCAAACCCGACTTTCCCCTTCA
CCGCGTTA

CCTTACTTTACTATAAGAATTTTGTTTTCCAAAAAAAAAAAAATATAAAAAAAAATAATCATACTCTATTACT
ATGGCTAACGTAGAAAAACCAAACGATTGTTCAGGCTTTCCCGTTGTTGACTTGAATTCGTGCTTTTCTAA
CGGCTTCA

TTTAAACATTTTTTTATCTTCATAGCAATAATATAAGCCTTTTACCACCCATAAACCATAAAGTAGACCCA
AACATTTTTAAAAAAATTTTACGTTATAATTTTTTCTTTGTCGTTTTTTCTGAGCGCGCAAAGTAGCGGTG
AAATTTTG

GACACATTTGTGATGCTTTGAGGTCAGTAATGTAATATATATATATATATATATATATATATATTATATAG
TGTAACTGCTCTTATTTATCTTGTCTATTCGAACCCAACAAAAAAGGCTGCAGTGGGAATCTGTTCATGTG
GCTGTATC

ACGTATTCTCGGGAAATAAGCAGGTCCAGATCCGCGTAGCATGAATGGGGCGCCAAAACAGTCGCGGGTAA
CGAAAGAGCGCAACCACGCGTATATTCCGGGCCGGTGAGGGGCAACGCGCATTGTCCCGGGGCGATATAAT
CCATGTAC

AACCAGCCCGCGAGCAATAAATCCGAAGAGAAGTGCATAATATTTTAGTCTATTTAATACATTTGACGTTA
TTTGATCTTCACGTTGTTTATATATATATATATCAAGTATGCTATAACGCTAAAAAAAAATGCTCATAATA
CTATGCAA

AAAAATGTGACAAGAAAAAAGTGGTGACAAAAAAGACAAAATAATTATTCAGAGATGATAAACTTAAAGCA
AAACTTGGTAACAAACTTTCATTAAAACAAAATATGTATTCTTTTTTCATTTTCATTTTTTTTTTACTGTT
TTTTTGTT

AATAATATATACATACATATATATATATATATATAAATATATAGGTATATATATAAATAACCTAAGACAAG
TTAAATGAGTATAAAAAACCTTGAACGTAAATGAAAAAAAAAAAAAATACTAGTAAATTGACAACATAAAAG
ACAAAAAT

TGTCTTGAATACTGAAGCCGTTATTATAGTTCTATCTTCATGTTTTTAGAGGTTTTTTTTAAATGAGTTTA
TTAAAATTAAATTTTGAATGTTATTTTTGAATAAAAATATAAAAAAAAATAATATTAATAATTTTTCATTAA
CTGCTCAT

ACATACATATATATATATATATATATAAATATATAGGTATATATATAAATAACCTAAGACAAGTTAAATGAGT
ATAAAAAACCTTGAACGTAAATGAAAAAAAAAAAAAATACTAGTAAATTGACAACATAAAAGACAAAAATAA
ATGAAAGA

AGGAGAACACAAAAAAAAATAGTGGAATGTGATAGTTTGAAATAAGGATATTAGTTTATACTCTAATATACA
AATATAAAAGTTGTTTCTTTTTCGTTCTTTTTTTTTTTTGGACTTGTCTTCGTTTTTATTACATACATGCA
TGCGCTTA

CTTTTTTTCAAAGAGGTCATATTTATCACCAACTATATATTTTTTTGAAGAAAAGATATCATTACAACGAT
AAGTAGTTACAATATTTTTTTTATTTAGTTTAAAATAATGAGTAAATGACGTCGCATTTCTTCGCTTTTGT
TATTTTTA

AACTCTTGGCCAAGAAAAAAAAAAAAGACTATGTCTAAACGTAACGTTATCATTATTATTAAATCATTTTA
ATGTTTTATAAGTTTTGTTTTTCATTTCATACCCTTTAAGGAGCATTTGGCATTGTGTGCGCCCTGAAGGC
TAAATAAG

GACGTGGAACTAAATTAAAATATACGAACTCTAAGGAAAAATACGTAGCATAATAACGCATATAACTAACA
ATTTGGATAACTATCCATAAAAAAAAAAAATAGTCACATAATACGAACATTAATATCCTTTTTTTGTTACA
TTTTCTTC

TAATGAAAATGGGAAAAAGAGTCAGAGCTATGACCAAGGTGAAATTAGCAGGCAGTTTGAACAAAACTTAC
AAATAAATGATTAATTTTAGACAATATTTTTTTTTTTTTTTTTATTATGTATATAGTTAACATGTATAAAG
TAATAGCA

TCATATAACATCATATCCTTTTTTTTTTTTTTTTTTTTTTTTCTAGGCTTTTACTAATACAAGGGCCTTAG
CCATTCGCTAAACGTAATGAACGATATTGAAGCAAATGAAGTAAAACAATGTGGAAAGCAAATAAATAATG
GTTATAGT

AAAAAAATGGCCAGCTATAGAATAAAATTAGCCCAGCTATATTTTTATAGCGTCTTTTGATGACGCGAAAA
GTAGACTTCGCGTAACACGAAAAATGCTGGTGCAGGTGGATTTTTTTGGCAGATACTACGGTCAATTCTCC
GCTCGAAG

GGTAAAATTGTAAAAAACATGAATGCGGCCTATCCGGCTCGAAAATTGTTCCTCATATATATTAAAAAAAA
ATCACGTGGTGCAAAAAAAAATTTTTTTGTCATTTCTTCGAATCAACTCAATGAAATTTGAAAAATAGCAG
AAAATAAG

TACAGTTACATATAAAAAAAGGTGTTTTTGAGTTAGTTAGTATTATATATTAATTTTCTTGAGGGGGTGGT
ATTATTAAAGAATTAAATTCAATATTATTACTATTAATTACACTAAGGTGCCGGGTATTATTGACGTTTTT
TTTTTCTT

TGTAGAAAAGCTTGTAAATAAGAAACCGTCATATTCTTTTTTTTTTTTTGACCGCGTATGAATCAGTAATT
TATTTCATTTTGAATTACATCATTATAAAAAGTTCTTAGTAATATATGCATTGGGTATACAATCACGTGTA
CTATCAAA

TGAAAAGGAAAAATATTCAGGTTTGTTGTTTTTATGTAAGTTGTATGATTTGATATACATATATATATATA
TATAATATATATTGTACATGTGTTTTTCCGGGGAAGAATGGATTATCCGGAGGTGTGAATAAAATGATGAC
GATTATAG

CCATTGTGAATGTTCTCACTCGATTTTTTAAGTGTCTATTTAGTGTATTATGCCGTGAATGCGCAAGATAT
ATACGTATACTTAAGAATGTAGTTTATATTTGACAATTTTTTTCAGCTCATCGCATTTTTTTTTCTAAATT
CTTTACCT

AATCGCGTCTTTCTACTCTCACAGTGCTTGCATACGTATGGTATACAGTATCAAAAGTAGCAGAATTTGTC
TTCATTATGATATATATATATATATGTACATATATATAGGGTGCTAAAAAGTATTCTTTTTGCTATTATTA
TTGCTTAA

TGAAATACTAAAGTAAAATAAAATAAGAGAAAGAAAAAAAAAAAAAAAAGAAAAAAGTAATGATGATGTTC
CATAATTGCAGAATTAATAACTACTTAATAACTTCTCAAATTGGCGAAGGCGCGTACGGTCTGGTTTACCG
TGCCTTAG

ACCGTCGTGGGTTCAAACCCCACCTCGAGCACTTTCTCTTTTTTTTTCACACGCGTTTGCAACATGCCCGT
ATTACATGAGCTTATTTGACTTTAGATGGTTTTCAGTTCTTTTTTTTTTTTTTTTTTTTTTCAGTAGATTA
ATGCGAAG

AGTTATGCTCTAGGATAATGAACTACTTTTTTTTTTTTTTTTTTACTGTTATCATAAATATATATACCTTA
TTGTTGTTTGCAACCGTCGGTTAATTCCTTATCAAGGTTCCCCAAGTTCGGATCATTACCATCAATTTCCA
ACATTTTC

TTTGAAAGTCTTTGAAATTACATGGTAGTACATACATATATATATATATATATATATATGTTATATTATTGTT
AATTAAATATTATTATGCGTATTTTCCTTTTCTTTATTAGTATAGTATTAATGACAGTAATAATAATAATA
ATAGTAAC

ATCCATGATCATGATTTACGACGCGAGATGTTTCGCGCCTCACTTTGAAGAATGCCAAATATAAAAGTATA
AATATGGGAACTATTCTTATTTGTGAAGCTGCTGTAAAACCTTATATGTAGCTTCTACAATCGCGATGTGC
TCAGCATC

TTTTTTTTTTTTTTATTATGTATATAGTTAACATGTATAAAGTAATAGCAGATTCTCTCTAAAAATATAAC
ATAACATTTCATTTCAATTCGTACTAAATAATTTTTTATATAACAGATACATAAGATATTTATATAGAGGA
AAACGAAA

GATGTTAATAACGCGCCGGCAAGAGCGAAAGTGAGTAAGTGATTTTTCGTTTAATGAATTTATTATCGCAT
GACTTGCGGTATAGAAAATAGTTATAAGAGTGCTGAAAAAAAAAAGAAGTACAGAAGTTTTCGTTTCTTTG
AAAGTCGC

AATAATAGTATCAAAAATAATGATAGTCATAATAAAAATAATAATAATAATAATAATAACAATAATAATAA
TAATGATAATAATAATAGTATCGAAAATAATGATAGTAATAGTAATAATAAACATGATCATGGTAGTCGCA
GTAATACT

ACTGGCAAAGTTACAAATCGACGAGGCCCTATAAAAAGCATTACCGTCACGCATCGTGTTGCAAATAGCAT
ATACATATATATATATATATATATACATATAACGTATACGTTAGAAAAATTTGAATACACACATTCATTTA
TCCTATTA

AAAATAAAAATAAAAAAGGTAATGAGGAATTAGATGCAAAGAGATATAGATATAAACACACACACACAAAT
ATATATTTATATACATATGTATGGTGATATATATTCAAAAAAAAAAAGTACAGTAACTTAGAAACTATTAG
TGGGACGG

ACCAGAACTCTCGATTGAGGCGAAGAAGGCGGAGAAAAAAGCCGGTAAAAAAAAAAAAGCGGAATCAATCAG
CACAATCAGAGGCGTCAAGAAGCGGCGCGGGGATAATGCTTTTTTCCCGTGGCGTTCACATTTTTGTTCCG
CGGAAAGC

TAATGAACAATTTACTTTACGGTCTTTAATCGATTTTAATGATTAGTTGGGCTTTTGGCTACATATGTAAA
ATTACATATAATATAATGGACACATAAGCAAAAAAAAAAAAAAAAAAAAATAAACAAATGAGCAACCCATCA
AATACCAA

TAATTAACTAAAGAAAAAAAAAAAAATAAAAAAAAAAATAAAAAAAAAGGTAACTAAAAAGGACAAAAAACATA
ACAGCTGTTTATAAAGTACTTTATACGTAAGAAACTACTGGCACTATTCATTCCAATAAAAGGACAGGTAT
AAAGTGTC

TTACATGTATCCATATACTGAAAAATAGATAAAACAATGAGATAATATTTATTAACTTCTACGTAAATAAA
AAGTTACATAAGGATCAACCTTTTTTTCTTTATTTTCTTTTTTTTTTTTATTTTTCATTTTCAATTACAAA
CTAAGACA

CGATTTTTTAAGTGTCTATTTAGTGTATTATGCCGTGAATGCGCAAGATATATACGTATACTTAAGAATGT
AGTTTATATTTGACAATTTTTTTCAGCTCATCGCATTTTTTTTTCTAAATTCTTTACCTCTCGTGCTCCGT
GACTAGGA

TGGAAAAAGAAAAGGTAAAATTTTTTTGAAATTTGGTTAACAAGGATAGAAAGTAAAAAATGGTAAATTTT
TTTTTTTGGAGTTTCTAGTTGAGGTTTTTATAATTATATTATTATTAACAGAGTTTGATTTCTTAGTCAAA
TATTCTGG

TATCGTACCAAACGAGATGTTTGCAGAAAGAACAAACCAATATTTTAAATAATCAAAAATCAAATTAGCTA
TTATTTCTCATTTGGTGATAATAATATATACATACATATATATATATATATATAAATATATAGGTATATAT
ATAAATAA

ATGTAACGTCAACCAGCCCGCGAGCAATAAATCCGAAGAGAAGTGCATAATATTTTAGTCTATTTAATACA
TTTGACGTTATTTGATCTTCACGTTGTTTATATATATATATATCAAGTATGCTATAACGCTAAAAAAAAAT
GCTCATAA

TTTCACTGGATACAAAGAAGTTTCACTAAAAGTTGTCGCGATTGTATACATATATGCGTATATATACAATT
CTACATGTTTTATATTTATTCCATTATATTCTCGTTAAATTTTAACTTTAAATTAAGAGGGCAAAGAGGCC
CAGCGGGA

CAAACGCCAATTTTACGAACGGGTTTCCATAGACATTGAAAATGAACGAACAAAATAAAATAAATAAAAAA
CTACTTTTTTTTGATGATATAAATACCGTAGATTATATAAATGTATATTAATATTATAAACCTATTTGATC
AATGACAG

TTTTTAAGAAAGTTAAAAAATAAGGAATATTTGATAAACAAAAAAAAATTAGCCTTTATATATCCATTTTT
GTTTATGTATTCCTTTTTTTATTTTCTTATGTGTTTGGGATTTACTGTCGCAGTCTTTTCAAATTTTCCGG
GCTAAACC

GTCCAAAAATTTCATCTGCCATTTTTTTATTTTCCCGTTCGTTCACTTGCTCTAGTTTTTTTTTTTTTTTT
TTTCAATACTCGGATTTGAAAATGTTAATGGGAGTGGCGCTAAAAACCTTCATCTGTAGTTGAATGCTATG
ACATGAGC

TGCATAATCATGTATTACCATAAAAAGGCTTTAATATGAGAAAAAGGTGAGCGCTATATAGATTTAGTAAT
TTACATGTTACAGAAGCAAAAAGCAAATATATATATATTTTTTATCATATTAGTTTTTATCCTCAGTAGTA
CTACTGAA

TTTTTTTTTGCAAAGCTCCCTTTTTCCTGAAACCAACAACAAAATATAAAAAAAAAATGATAAGCAGGATT
TTTTCTTTTAATAATTCCTTAAAATTAACTAACAAGCTGAAGCCCTTACAACAAAGTAAGTCAAACCCAAT
TCACAAAA

CCCGGAAAATTGTAGTGGTGCAAGAAGGGAAAAGAATGATGGCGAAAAATGCACGCGCGTAAATAATGATT
TGTCGAGTCCTATTGACCACAAAACCCGCGGAAACCTAGTTAGCCCAAAAGGAAATGGATAGCATATGAAA
AACGAAAC

TTTGGCCCGAAAGATAATTCTGGTCTCCCGGAAACATGAAGATGCAGCAAAGTAAAAAATAATAGGTAAAA
ATAATAAAAATACATGTATCTTTTTGGTTGGTTAATTTATCTAAACAGTTTTATATATATATGTATATATA
TATATATA

ACTACAAAAAGAAATCAAGTCGATCGAGTAATCGAATTTACGTAGCCCAATCTACCACTTTTTTTTTTCAT
TTTTTAAAGTGTTATACTTAGTTATGCTCTAGGATAATGAACTACTTTTTTTTTTTTTTTTTTACTGTTAT
CATAAATA

GTGTAGATGAACTAGAAAAGGTTCAAGAGATGATAAACGTGAAGGGAGTGTCATATCTACCGACATAAAGA
TAGAGATCTTTTTTTTTCCTTTATTTTTTTTTTTGATTTTTTTTTTTGATTTCTTGATATTAGTTTTTAG
AAGTCCTT

AGTCTTTTTTTTTTTCAAATATTCATCATCAAAGGTTACGAAATCTTTTGAGCTATCTTAAACATTCGTTCT
TTTTATCAAATTTCAATTACTAACTTATTTTTTCAAAAAAAATTGCCTCTCCCGGTTTTTAATCATTATTT
TTTTCGAT

TTTTTTCCAAATTAATATTTATGAACGTTATGATGTCAAGTTTTTTCAAGAAGTAATTATCCGCGAAAAAA
AGAATATAAAAAATACAAATGTGCATAGATCCTCACATAGTATACAACTAAAAAGCAAACAAAAGAACATC
CTCAAATG

TTCCATTGCTTTTCCATTTTTGCCCGTTCAAAACTATTCTTTTTATGAATTGACAGAAAACCAAAAGCAAG
CCGATTTCAGGTTAACCCTTTATTAAAATAAAAAAAAAAAGCATGCATAACACTAATAATATATTATTAAA
AAAAAAGC

TTTCCTTGAGATTACTCTCTATAAAAAAAAAAAAAAAAAAGCTTTTAAAAAAAAAAAAACTCAAAAAAAGA
AAGAATATTCAAAGAAAGTTTCATCTTGTCAGTTGAAATGAATAGTTTATTGTGGAAATTATAACAATAAA
AGTTAGCT

ATCGTTTTGTGCGAATACATTTCATTTTGTTCCCTGCGTAAGAAGTATGCATAGTAAAATTAGCGTAGATA
TAGTTTTTTGAGGTATATACTTATTCATAGCTTATAAATATTTACAGTGTTGTAACTCTAGTAAAAACAAA
AGAGGTCA

ACGCTTGGAAGATTTGAAAGCCCATTCTTGCCAGTACGCCAATAAAGTGTGTCGCGCAGAATAGATCTTCA
TGTAATTCCTTTTTCCCTTTTATGCAATCTATAATGTTAATCAGCGTAGATCACTATATACGCTTTTTTTT
TTTTTTTT

CAAATCTCCTTTGGACTGAACCCGCATTAGTAATTGCCCGCTTTTCTTTTCTTCCGCGGGTGGGCCCCATA
AATAGAAAAAAAAAGAAAGAAAGCGTTTAAATAAATAGAGTGAGCGGATTTCTATTATCTGAAAACCGGGT
TATAATGC

AAAATATTTATGCAGTGGTCATTTGAAAATGGTCATTAAATTAAACAAGTAATATGAGAGGAATGAGAGGG
AAATAAATAACTGAAAGATAAAGGAGATAAGGAAAAAAAAAAAAAAAAAAAAAAAAAAGGGAACTTTAGTT
AGTAATAA

GAGAAAAAAAAAAAATTTCAAGGGCCCGCAATCTCAAAATGGAGTCAATTGTGTCAAGTCTTTTTTTTTTT
TTTCATCGTTATCTCACTTTTTATTGCTTATCTTAAGATAATTTAGCTGATATACTATATAGAATAGCCGA
AGTGTCAG

TATCGATCATCTTGCCATGTAATAATAGATGTCCAAAAATTTCATCTGCCATTTTTTTATTTTCCCGTTCG
TTCACTTGCTCTAGTTTTTTTTTTTTTTTTTTTCAATACTCGGATTTGAAAATGTTAATGGGAGTGGCGCT
AAAAACCT

TTTTGTATCTTGAGATGGCGTATTTCTACTCCAGCATTCTAGTTAAGAAAAAGTCTAAAAATGGTTTTTTT
CATCCAAAATATTAAATTTTACTTTTATTACATACAACTTTTTAAACTAATATACACATTTTAGCAGATGC
GCGCACCT

TAATACCGTAATGATAGCAGTTTATTGTAGAAAAACCATGTTATTACCCTTCCCTTTTTATTTCTTTTCGC
GTTGCAAATCACATATAACGAGGTGGCTTGTATTTGTCAAACCAAAAAAAAAAATGAAAATCGAAAAATGG
AAAAACAG

AACTAGTACCTGTCATTCACGACATGTGAACAAATAAAAACATTTATTTAAAAATTTTATGTATTCAAATA
TTTTCGGGAAAGAGATAAAAGTAACGACACTTAAAAATTTAAAAAATCACAATACTTTATTTACTCAGTCT
TTTGATCA

AAGAAAAAAAAAAATAAAAAAAAAATAAAAAAAAAGGTAACTAAAAAGGACAAAAAACATAACAGCTGTTT
ATAAAGTACTTTATACGTAAGAAACTACTGGCACTATTCATTCCAATAAAAGGACAGGTATAAAGTGTCTG
CTTATCTT

GATATGAAATGAAATACCTTTTGTTTCACCTTTTAAATAATTTAATGTTATATATACAACTTTATCGTATC
ATATTCGCAATTACATTATACAAGAATGAGTTTTTTTTTTTCGCGGACAAAAGAAGAACATGTCAACGCCA
ACCCCCTA

AACTAAAATGTCCAGACATGATCTTAAGCGGAAGGGAGGGGTTTTTGCTTCCGTTTTTTTGACATTAAAAG
TTTTCTTAATGCCGCGCGTTTGAAAGGAAACTGATCTGAAAGTGATTTATGATGCCAAAAAAAAAAAAAAA
TCTTTTGA

CGAAATGCGATACAGTACGTGTCATGCGGTTAGTATATATACATATATATATATATATATATATTCAAATA
AATTATAACGTATTAAATAATATGTGAAAAAAGAGGGAGAGTTAGATAGGATCAGTTGGTCAACTCATGGT
AGTATTCG

AAGATGGCCAGGCTAGTTAAATCTATTATACAATAATTATTGGTTATATATTTAAAAAACAGAAACATTTT
TTGCTTTAAAAGCACGCCATAGTATTGCTTTTAATATTGTGATCTGCGTCCTTTTTTTTCTCAGGAAAAAA
AAATTTTA

CATTTTTGATAACTAGTACCTGTCATTCACGACATGTGAACAAATAAAAACATTTATTTAAAAATTTTATG
TATTCAAATATTTTCGGGAAAGAGATAAAAGTAACGACACTTAAAAATTTAAAAAATCACAATACTTTATT
TACTCAGT

ATGATAGCAGTTTATTGTAGAAAAACCATGTTATTACCCTTCCCTTTTTATTTCTTTTCGCGTTGCAAATC
ACATATAACGAGGTGGCTTGTATTTGTCAAACCAAAAAAAAAAAATGAAAATCGAAAAATGGAAAAACAGAG
AGAGAAAC

TGATAAGAGATTCTAAGAAAGTGGCAAATCAAAAGCGTAAGGAGATAAGAAAACAAAAAAGGGCTACATTT
TAGAATAATTTGAATCATATGTAGAAAAGCTTGTAAATAAGAAACCGTCATATTCTTTTTTTTTTTTTGAC
CGCGTATG

AGCGATGATAAAGTGCAAGTTCATAAGCAAACAGCGACCGTAAAATGGGATAAAAAAAGAAAAAAAATATGT
TAATACTCAAGGTATTGATAACAAGAAATATATTATTGGTGAAAGTGGCCAAAAAAATCGCGGCAAGCTTCA
GGTCTGGT

CTTAAGCATGTATCAAATAGTAAAAAAAACTTTGGAAGCGTGCACACTCAGTCTTAAATCTGCCCTATACT
ACCGAAGATAAATACATGAACTACACGTATCATATAACATCATATCCTTTTTTTTTTTTTTTTTTTTTTT
CTAGGCTT

CCAATCATTTTGCTACTTTCCCGGTTCTCCCTTTATTATAAACACTTCAGAAAAATATTCTGCTACTATTC
CTTACTTTACTATAAGAATTTTGTTTTCCAAAAAAAAAAAATATAAAAAAAAATAATCATACTCTATTACTA
TGGCTAAC

GCTAGGTGGAATTCGCGCTGAAACAGATCACAATGAAGATCTAAGAATACAGAAACAATAAAGGTTAATGC
CCAGTTTTACATTTTTTTTTTTTCACTTTCACTTTGCTAGCGAGCGATGCGATTGAGAAAAAAAAATAGAAA
ATTTTTTC

TTTATAGATCATCCTGTCAATTTTTTTTTATTTTTTTTTGCAAAGCTCCCTTTTTCCTGAAACCAACAACA
AAATATAAAAAAAAAAATGATAAGCAGGATTTTTTCTTTTAATAATTCCTTAAAATTAACTAACAAGCTGAA
GCCCTTAC

CCAGAGTATCTTTACCCCCACATTCAATATGAGGTGTCAGCCATAATTATTTACATGATAATACTGATATT
TTTTCTTTTTCGCGATATATTGAAAAGAAAAAAAAATTGCGAGGTGATTCCTCATCGCATGTATTCATTTT
AACGACAT

ATTATAGAAAGTATTTGTCCTACCAAAAAAGGTTGGAGATAAAATTAATGCATTTTTCATTAGATATATAT
ATATATTCATATATATTGTTTAAGTTACCATTTTGTATGTTTTTCTTTGACCTTACTATGCAACCAATGCA
GGTGGTGG

AAAAGCGTAAGGAGATAAGAAAACAAAAAAGGGCTACATTTTAGAATAATTTGAATCATATGTAGAAAAGC
TTGTAAATAAGAAACCGTCATATTCTTTTTTTTTTTTTGACCGCGTATGAATCAGTAATTTATTTCATTTT
GAATTACA

CAGTCGCTTTTAATTTAACATCAAAAGGAATCTATGGTGTGGCCGTTGATATTTCGCGATGGTATGCCGAA
AAAGGAAATAACGCAGATTGCGCGTAGTAACGAATGGATTTGACGCGTTTCCTTCGATGAAAAGTGGGAAA
TAATAATA

ACGTATAGTTGATGATTAGAAACGAGCTTTTTTATGCCCATCGTCATCTATATATATATTTGTATCACTTT
AATAGTTATTTTAATATTTGATAACAACCTTTCTAATAGTATTGTTATGGAAGGTTTAGTGCTGGAGGAAA
AAAAAGAA

TTCTTACGCGTTGCGTGTAAAACATCCTCTCATTCAAGACAGGGTTTTCTAAAAGCAATAGGGGTAGTTTA
ATAATTCTTATATAATCATCATATACACTATTTTTAGTTCTTAATTCTTTAATACAAACTTATTAATGTGC
TCTCCATT

TTTTCCGCGATTAATTCGCCGAGAATACGACAAAAAAAAAGATAAACATCAGAAAATTCTATTATTAGATT
AGATTTTATATTAGATAATGAATTAATACAGGTAATAATTATAGAGAGGAAATAAAAATAAACAGATAAGT
TATTTACA

CAAATTTTCTACCTCTTCGTTAAAACCTTCTTCGCTTGTACTTATATTATTATGTTGGCTTTTCAAGTTTA
TATATTTTTAAAGGAAAAAAATAACTTTTTCATTTTTCGTTATAAAACAGAACCGTTATACATATTGAGAT
GGTTAAGG

GGCTTATCTGCCACAGTTTAATTTTTTTTTAATTTTATTTCTTTTTTTTTTATTTAACTAGAATCTGGCTA
AAAATAAGCAACGGTCCGTGTAGATTACTGTTTCGAACGAGGAACAACTGAAGGAAAAGTTTTTTATTTTC
CGCGAAGT

ATTACGCTTTCTACCAACATTTGCCACCCTTGGAACCAGAAGAGAATATACATCATTCGGGGTTGCCCTGC
CCATTTATATCGTATATTGTGATGATATACCTTTTTTTTTTTTTTCGCTGCGTTTTTTTTCTCGACACGTG
TCGAAGAA

TTTCTCCATATTTTGCGGCCCAACTTGTTGCAGAATTGAAAACTTTGTATAATACTAATAATAATTTATAA
TAATAACAATAATAATTTATAACAATAATAACAATGCCAAACAATTTATATGGGTAACCAGTTTTTGGTAC
TGTTTGCC

ATTCAAATTGTTTCCCTTAGGTATATATATATATATATATATATATATATATATTTTCCCTGTATATATCTAT
GTAAATGACGAAAACGCATGACATTTTAAAACCTACCCCGGGTTTGACCACAACCCACCGTTCATCTAATA
TTAACCCG

GATTTGAAAGCCCATTCTTGCCAGTACGCCAATAAAGTGTGTCGCGCAGAATAGATCTTCATGTAATTCCT
TTTTCCCTTTTATGCAATCTATAATGTTAATCAGCGTAGATCACTATATACGCTTTTTTTTTTTTTTTTCA
GCTCCGAT

TCTTGTTTTCCTTCATTTTACAAAATCTGGTGTAAATCCTCGATTCCATTGTGATTTAAAAATAATTAATT
ACTGTATAAGATTCTATATAGACAATTAATTGAAAATAGTATTACTTCTTTAACTCGCTTAGGATTTTTTT
TATTTTTT

GTTTTTTAATAAAAAAAGAAGCAGTTACAGGCGCATTACAAAGTTTGAGAAGAAAGGAAAAATGTCATTTG
GTTATATAAATTTTTTTTCTTAATGGAGATTGAGGCAGCAATAAAATGTGGGATGAAAAAAAAAAAAAAAG
GCACTTGA

AATTCCCAGAAGAAGGCATTGATCCGTCCACTTATTTGAATAATTAATAAAGGAAAAAAGATACGTTTTTA
TATAGATTATATAAAGATTTGTATTATTCAAACGAACAAAACAAACAAAAAAGGGGAAGTGGAAAAAAAT
TAGCAGTT

ACATTCGTGACCGAACAATGAGATATTATTGAGTACCGGGAGTATTATGTGTCATAATTTGTAGTATTATC
TGTGTGACGGATTAAGTTTGCTATCTCAAATGCCGCGCGCGCGAATTGCTCATGATATAATAATAGCTAAT
AATATGAT

TATATGTACTAACATATGTACTAAACAGAAACACAATATGAATTACAACTAATACTATATTATTTGTCTCC
TTTTTTTATTAGTATTAAAAATTACCGTTACTGGTTAAAATGGAATACAGCTCATCCATTTAAAAAAAAAA
AAAAATAG

GCACGCGCGTAAATAATGATTTGTCGAGTCCTATTGACCACAAAACCCGCGGAAACCTAGTTAGCCCAAAA
GGAAATGGATAGCATATGAAAAACGAAACGAAACGTCCTTTTGCACGGCAGAAAGAGCTTAGTAGGACCCG
GGACAAAT

TCCGCGTCCGCTAATTCGCGCGGCTTGCCACTTAACCCCGTCCAGTTAATTTGCCAAGCAGCGGCCGCACG
TTGGAACGTTTTCGCCGATTTATTTATTATTATTTATACATGTTGACGTTGGTGCCCTTTTGTTTATACTG
GAGCCTAA

TATATATGTTATATTATTGTTAATTAAATATTATTATGCGTATTTTCCTTTTCTTTATTAGTATAGTATTA
ATGACAGTAATAATAATAATAATAGTAACAATATCTCTTTTTTTTTTTCAGTGAGCTTTTATTTTTTTTTC
ATTGCTCT

TCTAATAGGGCGTGTTCACAATTTTATGCGTCGGGTGACTTCAATTTGGCGCCCGTTGAGATTATCTAAAA
AATGCTTTTATTTATTTAAGCGCTTTTTGTTTTGACGAATGTATTTATGTTTTTGTATATATATGAGCGTT
TTTTTTTT

GAAATTACTAAAAAAAAGAAAAAAAAAAGAACGGGGGTGTAATAATTTGTAGTTCATTATTGCAATTATAT
ATCTATATCTATATATGTATATAACATTAACATGTGCATGTACACACGTAATCGCGCGTGTACATGTCTAT
ATGTGTTA

AACTTTTTAAGTTGAGAACTAAAAAGCAGTATTGTTTTCGAATATGTATTATTTTTTTTTATTTTTTTTAT
TACTCGTTATCAATATATATATATACATGTATATTATTACTAAAAGAATGAGAAAATAAGATAGGATCCTG
GAAGGGGG

AATCTAAAGAGAAATTACTAAAAAAAAGAAAAAAAAAAGAACGGGGGTGTAATAATTTGTAGTTCATTATT
GCAATTATATATCTATATCTATATATGTATATAACATTAACATGTGCATGTACACACGTAATCGCGCGTGT
ACATGTCT

CTTCCGGCAGAACTTCGAAAGTGAAAAAAAAAAAAAAAAAAAAATAATAATAATATTAATACTACTTATAT
ACTATTAAAAAATCGTTTAGAAATGATTCATTTATAAAATTCGTTACTTGCGGTCATTGGCAATAGATTCA
GCTGCGGT

TCGATGAGCTCTTCCGGCAGAACTTCGAAAGTGAAAAAAAAAAAAAAAAAAAAATAATAATAATATTAATA
CTACTTATATACTATTAAAAAATCGTTTAGAAATGATTCATTTATAAAATTCGTTACTTGCGGTCATTGGC
AATAGATT

TATTCTGATAAATCTAAAGAGAAATTACTAAAAAAAAGAAAAAAAAAAGAACGGGGGTGTAATAATTTGTA
GTTCATTATTGCAATTATATATCTATATCTATATATGTATATAACATTAACATGTGCATGTACACACGTAA
TCGCGCGT

TTCTTGGCCGTCGATGAGCTCTTCCGGCAGAACTTCGAAAGTGAAAAAAAAAAAAAAAAAAAAATAATAAT
AATATTAATACTACTTATATACTATTAAAAAATCGTTTAGAAATGATTCATTTATAAAATTCGTTACTTGC
GGTCATTG

AAATAGCTTCCTCTTTGTGGCCGGGGCGCGGGGGGACGAGGCAAAAAGCAAAGAAAAGCAAAAAAAATAAA
AAAAAAAACAAAAAAACAGGGGTATGAGAAAAAGACACGCTTTTCCACGCGCAGCAAAAAGGAAAAAGGAA
AAGGAAAC

TGTAAAATGTTTCTTGGCCGTCGATGAGCTCTTCCGGCAGAACTTCGAAAGTGAAAAAAAAAAAAAAAAAA
AAATAATAATAATATTAATACTACTTATATACTATTAAAAAATCGTTTAGAAATGATTCATTTATAAAATT
CGTTACTT

CCTTTTCAAAAGGAGCATGATACCAATAATTTATATTGCCTTACGCCTCTTATTTTTTATATCCTCTTTTT
AGATTTTATTAATTTTTTTTTTTATTTATATACATATAATGTGCCCTGCTTTTTACAACCATACGTTTTTT
GTTTTTTA

TCGACGAGGAAACTTTTTAAGTTGAGAACTAAAAAGCAGTATTGTTTTCGAATATGTATTATTTTTTTTTA
TTTTTTTTATTACTCGTTATCAATATATATATATACATGTATATTATTACTAAAAGAATGAGAAAATAAGA
TAGGATCC

AAAAAAAGAAAAAAAAAAAGAACGGGGGTGTAATAATTTGTAGTTCATTATTGCAATTATATATCTATATCT
ATATATGTATATAACATTAACATGTGCATGTACACACGTAATCGCGCGTGTACATGTCTATATGTGTTACT
TGAACTAT

AAATTTCGGCATTGAACTTGATTCTAAATGCTACATACAATTACGCGCGCTGTTTTGATATACACACACAT
ATATATATATATACATTGTGTAAGAAAAATTCAAAGCTTAGTAAAACATACAGATCATGGAGAAAAAAAAA
AAACCGAA

CTACATACAATTACGCGCGCTGTTTTGATATACACACACATATATATATATATACATTGTGTAAGAAAAAT
TCAAAGCTTAGTAAAACATACAGATCATGGAGAAAAAAAAAAAAACCGAATACTTGTGTTACCAGAAGGATC
CACTGACT

AAAACTCATCGCTCTTCAGGGAAATCTTACGCGCTATTTTTACAGTCGCGCGGGTCTTAACCATAATTTTT
TACCGCTGCAAATATTTGAATGCTATAAGAGCAGATAAAAAATAGATTATAGAAATTAACCTGTTAAAACA
ATTATCCA

CTCTTTGTGGCCGGGGCGCGGGGGGACGAGGCAAAAAGCAAAGAAAAGCAAAAAAAATAAAAAAAAAAACA
AAAAAACAGGGGTATGAGAAAAAGACACGCTTTTCCACGCGCAGCAAAAAGGAAAAAGGAAAAGGAAACTC
TTTATTAT

ATTGAACTTGATTCTAAATGCTACATACAATTACGCGCGCTGTTTTGATATACACACACATATATATATAT
ATACATTGTGTAAGAAAAATTCAAAGCTTAGTAAAACATACAGATCATGGAGAAAAAAAAAAAAACCGAATA
CTTGTGTT

CCGGGGCGCGGGGGGACGAGGCAAAAAGCAAAGAAAAGCAAAAAAAATAAAAAAAAAAACAAAAAAACAGG
GGTATGAGAAAAAGACACGCTTTTCCACGCGCAGCAAAAAGGAAAAAGGAAAAGGAAACTCTTTATTATTG
GACCTTAA

ACAGTAACCTAATTGCTCAAATTTTGAGAGTTAAAGGAAAGAATAAAATATATAAATGATACGTTTATACC
CGCTAAAAAAAAAAAATATAAAACAAAAAAAGGATCTCATAAGACGATTTGTTGGGATATGTCCACATCTT
TCGGCTAT

CACGTGAACCACAGTAACCTAATTGCTCAAATTTTGAGAGTTAAAGGAAAGAATAAAATATATAAATGATA
CGTTTATACCCGCTAAAAAAAAAAAATATAAAACAAAAAAAGGATCTCATAAGACGATTTGTTGGGATATG
TCCACATC

ATAACCCTGACATTACATTTAGGGAATAATCGCCCCAATATAGGTTACAAAACATTAATATATATGTAAAT
ATATGTGCGCGTATATATATATATATATATATATAAAGGGAAAATTAATATTCTATCATTAAAGTGAAGAA
GAAAGGTG

AGGAGCATGATACCAATAATTTATATTGCCTTACGCCTCTTATTTTTTATATCCTCTTTTTAGATTTTATT
AATTTTTTTTTTTATTTATATACATATAATGTGCCCTGCTTTTTACAACCATACGTTTTTTGTTTTTTAAT
TTTCTTGC

TGAAGCAACTCAAGCGCAATGAGAAAAGTGACTATACAAATATGTAAAAAATTGCTACCAAAAAAAAAAAA
AAAAAATTTAAAAACTCTGATAATATAGTAAAAATTATTGGTACATTGTGAATTAAAATTTATGCGTTTGC
CCTCAAAT

AACTTCGAAAGTGAAAAAAAAAAAAAAAAAAAAAAATAATAATAATATTAATACTACTTATATACTATTAAAA
AATCGTTTAGAAATGATTCATTTATAAAATTCGTTACTTGCGGTCATTGGCAATAGATTCAGCTGCGGTTC
TCCCAAAG

TTGATACAGTATAACCCTGACATTACATTTAGGGAATAATCGCCCCAATATAGGTTACAAAACATTAATAT
ATATGTAAATATATGTGCGCGTATATATATATATATATATATATAAAGGGAAAATTAATATTCTATCATTA
AAGTGAAG

AGACATATCTTTTAAATGTTAAAACTCATCGCTCTTCAGGGAAATCTTACGCGCTATTTTTACAGTCGCGC
GGGTCTTAACCATAATTTTTTACCGCTGCAAATATTTGAATGCTATAAGAGCAGATAAAAAAATAGATTATA
GAAATTAA

AACTTGATCATCGACGAGGAAACTTTTTAAGTTGAGAACTAAAAAGCAGTATTGTTTTCGAATATGTATTA
TTTTTTTTTATTTTTTTTTATTACTCGTTATCAATATATATATATACATGTATATTATTACTAAAAGAATGA
GAAAATAA

ACACTTGAATCACGTGAACCACAGTAACCTAATTGCTCAAATTTTGAGAGTTAAAGGAAAGAATAAAATAT
ATAAATGATACGTTTATACCCGCTAAAAAAAAAAAAATATAAAACAAAAAAAGGATCTCATAAGACGATTTG
TTGGGATA

GCTCGGTGGCAAATAGCTTCCTCTTTGTGGCCGGGGCGCGGGGGGACGAGGCAAAAAGCAAAGAAAAGCAA
AAAAAATAAAAAAAAAAAACAAAAAAACAGGGGTATGAGAAAAAGACACGCTTTTCCACGCGCAGCAAAAAG
GAAAAAGG

TTGTGCCAGGCGGCAAAAAGACCCCTACGGAGGCGTTGTAAAAAGGCAAAAATTAACGCGAATTTGTATAG
TGCATCTAAAAAAAAATAATTAAGTATAAAAATAGATAAAGGAAATCAAGTGTTTCTTTTTTTTTTTTTGGT
TTATTGTT

TTTAAATGTTAAAACTCATCGCTCTTCAGGGAAATCTTACGCGCTATTTTTACAGTCGCGCGGGTCTTAAC
CATAATTTTTTACCGCTGCAAATATTTGAATGCTATAAGAGCAGATAAAAAAATAGATTATAGAAATTAACC
TGTTAAAA

GAAATCTTACGCGCTATTTTTACAGTCGCGCGGGTCTTAACCATAATTTTTTACCGCTGCAAATATTTGAA
TGCTATAAGAGCAGATAAAAAAATAGATTATAGAAATTAACCTGTTAAAACAATTATCCAAAATGTAGTAAG
TATTTATC

GTTGAGAACTAAAAAGCAGTATTGTTTTCGAATATGTATTATTTTTTTTTATTTTTTTTTATTACTCGTTAT
CAATATATATATATACATGTATATTATTACTAAAAGAATGAGAAAATAAGATAGGATCCTGGAAGGGGGAT
CAGGTAAA

AAAAAGCAGTATTGTTTTCGAATATGTATTATTTTTTTTTATTTTTTTTTATTACTCGTTATCAATATATAT
ATATACATGTATATTATTACTAAAAGAATGAGAAAATAAGATAGGATCCTGGAAGGGGGATCAGGTAAAGA
TATAAAGT

TCTATTTTTTTTTTTTTTTTTTTTTTTTTCGACCTCATCTCATCGCGCTTTCCATTCATCATATTGTACGA
AAACAAAGCACAATCGTAATAAAAGACATCCTGATAATTAGCAAAGGGCTAACCATAATAGTCTAAGTTTA
GTAGTTCA

CATTACATTTAGGGAATAATCGCCCCAATATAGGTTACAAAACATTAATATATATGTAAATATATGTGCGC
GTATATATATATATATATATATATAAAGGGAAAATTAATATTCTATCATTAAAGTGAAGAAGAAAGGTGGA
TTTAAATT

GCTCTTCAGGGAAATCTTACGCGCTATTTTTACAGTCGCGCGGGTCTTAACCATAATTTTTTACCGCTGCA
AATATTTGAATGCTATAAGAGCAGATAAAAAAATAGATTATAGAAATTAACCTGTTAAAACAATTATCCAAA
ATGTAGTA

ATAACTTTTATGTAAAATGTTTCTTGGCCGTCGATGAGCTCTTCCGGCAGAACTTCGAAAGTGAAAAAAAA
AAAAAAAAAAAAATAATAATAATATTAATACTACTTATATACTATTAAAAAATCGTTTAGAAATGATTCAT
TTATAAAA

ATTCTAAATGCTACATACAATTACGCGCGCTGTTTTGATATACACACACATATATATATATATACATTGTG
TAAGAAAAATTCAAAGCTTAGTAAAACATACAGATCATGGAGAAAAAAAAAAAAACCGAATACTTGTGTTAC
CAGAAGGA

ACAACAAATAAAATGAAGGATCTATGGATAAGTGGATGTTTCTATTTTTTTTTTTTTTTTTTTTTTTTCG
ACCTCATCTCATCGCGCTTTCCATTCATCATATTGTACGAAAACAAAGCACAATCGTAATAAAAGACATCC
TGATAATT

AGGGAATAATCGCCCCAATATAGGTTACAAAACATTAATATATATGTAAATATATGTGCGCGTATATATAT
ATATATATATATATAAAGGGAAAATTAATATTCTATCATTAAAGTGAAGAAGAAAGGTGGATTTAAATTCC
AAGTCTTT

ACTTTAGCAATTGATACAGTATAACCCTGACATTACATTTAGGGAATAATCGCCCCAATATAGGTTACAAA
ACATTAATATATATGTAAATATATGTGCGCGTATATATATATATATATATATATAAAGGGAAAATTAATAT
TCTATCAT

TCTATGGATAAGTGGATGTTTCTATTTTTTTTTTTTTTTTTTTTTTTTCGACCTCATCTCATCGCGCTTT
CCATTCATCATATTGTACGAAAACAAAGCACAATCGTAATAAAAGACATCCTGATAATTAGCAAAGGGCTA
ACCATAAT

AATGAAATAGGCCGTCTACGGCCGTCTACGGCCTATTCCATTGCTAAAAATTTGAATCGTATAAAGGGATA
TTACCCGGAAAAGAAACGCATTAAAAAAAAAAAAAAAAAAAAAACAGAAAAAGTGGTTAAGTGATTGACTG
ACCCTTGA

TTATATTGCCTTACGCCTCTTATTTTTTATATCCTCTTTTTAGATTTTATTAATTTTTTTTTTTATTTATA
TACATATAATGTGCCCTGCTTTTTACAACCATACGTTTTTGTTTTTAATTTTCTTGCATTTTAAAACTG
AATAGTGA

CGGCAAAAAGACCCCTACGGAGGCGTTGTAAAAAGGCAAAAATTAACGCGAATTTGTATAGTGCATCTAAA
AAAAATAATTAAGTATAAAAATAGATAAAGGAAATCAAGTGTTTCTTTTTTTTTTTTTGGTTTATTGTTTG
ACACTGGT

AGGAGGTTTTCCTTTTCAAAAGGAGCATGATACCAATAATTTATATTGCCTTACGCCTCTTATTTTTTATA
TCCTCTTTTTAGATTTTATTAATTTTTTTTTTTATTTATATACATATAATGTGCCCTGCTTTTTACAACCA
TACGTTTT

AAAATTTCTATGAAACATCAAATTAATCACGTGCTTTTTAAAAAATATAAATTTAATTTCATTTTCTATTT
CAATATTTATTAAATAAAAAATTTGAAAAATATATAAAAATTGTAGCAGTATTAGATTTCCGAAAAGAAAA
AAAGGTTT

ATTGCCATCCTTGTGCCAGGCGGCAAAAAGACCCCTACGGAGGCGTTGTAAAAAGGCAAAAATTAACGCGA
ATTTGTATAGTGCATCTAAAAAAAATAATTAAGTATAAAAATAGATAAAGGAAATCAAGTGTTTCTTTTTT
TTTTTTTG

TACCAATAATTTATATTGCCTTACGCCTCTTATTTTTTATATCCTCTTTTTAGATTTTATTAATTTTTTTT
TTTATTTATATACATATAATGTGCCCTGCTTTTTACAACCATACGTTTTTGTTTTTAATTTTCTTGCAT
TTTAAAAC

GAGAAAAGTGACTATACAAATATGTAAAAAATTGCTACCAAAAAAAAAAAAAAAAAAATTTAAAAACTCTGA
TAATATAGTAAAAATTATTGGTACATTGTGAATTAAAATTTATGCGTTTGCCCTCAAATCCTTTTCTATAT
CGTCCAAT

GATGTGCATATAATGTATATACATACATTTAGCATATGTAATACATTCGTACATGTATAGAAATATATGTT
TATATATATATATATATATATATGTATATACGAACTCGGTTTAACGCATAGTTGGTAAAAGATTTAATGTA
GATAAAAA

AATTAATCACGTGCTTTTTAAAAAATATAAATTTAATTTCATTTTCTATTTCAATATTTATTAAATAAAAA
ATTTGAAAAATATATAAAAATTGTAGCAGTATTAGATTTCCGAAAAGAAAAAAAGGTTTCAAGAAAAGGAA
AAGAAAAA

TTACGCCTCTTATTTTTTATATCCTCTTTTTAGATTTTATTAATTTTTTTTTTTATTTATATACATATAAT
GTGCCCTGCTTTTTACAACCATACGTTTTTTGTTTTTTAATTTTCTTGCATTTTAAAACTGAATAGTGACC
TGGGTATT

CAAGCGCAATGAGAAAAGTGACTATACAAATATGTAAAAAATTGCTACCAAAAAAAAAAAAAAAAAATTTA
AAAACTCTGATAATATAGTAAAAATTATTGGTACATTGTGAATTAAAATTTATGCGTTTGCCCTCAAATCC
TTTTCTAT

ACTATACAAATATGTAAAAAATTGCTACCAAAAAAAAAAAAAAAAAATTTAAAAACTCTGATAATATAGTA
AAAATTATTGGTACATTGTGAATTAAAATTTATGCGTTTGCCCTCAAATCCTTTTCTATATCGTCCAATTT
TGAGTCAA

GATTGATCTCAGACATATCTTTTAAATGTTAAAACTCATCGCTCTTCAGGGAAATCTTACGCGCTATTTTT
ACAGTCGCGCGGGTCTTAACCATAATTTTTTACCGCTGCAAATATTTGAATGCTATAAGAGCAGATAAAAA
ATAGATTA

CTTTTTATAACGTTTTCTTTCTTTTTTTTTCCTTCTTACATAGTATTCAACTGTATATTTAACATGTTTTA
CGTATTTTTAAGAAAAAATTACTAAACGCGATAATATTAAGCAAATATTTATCTCATAGTTCTCGAACTCA
TTTATTTC

ACATACATTTAGCATATGTAATACATTCGTACATGTATAGAAATATATGTTTATATATATATATATATATA
TATGTATATACGAACTCGGTTTAACGCATAGTTGGTAAAAGATTTAATGTAGATAAAAAACACATAAGTTT
TATACGCA

TAGGTAGCTGGTAAAAAAGCGATTGATCTCAGACATATCTTTTAAATGTTAAAACTCATCGCTCTTCAGGG
AAATCTTACGCGCTATTTTTACAGTCGCGCGGGTCTTAACCATAATTTTTTACCGCTGCAAATATTTGAAT
GCTATAAG

CTTTATCAGCAATGAAATAGGCCGTCTACGGCCGTCTACGGCCTATTCCATTGCTAAAAATTTGAATCGTA
TAAAGGGATATTACCCGGAAAAGAAACGCATTAAAAAAAAAAAAAAAAAAAAAACAGAAAAAGTGGTTAAG
TGATTGAC

ATTATTAGGAGATGTGCATATAATGTATATACATACATTTAGCATATGTAATACATTCGTACATGTATAGA
AATATATGTTTATATATATATATATATATATATGTATATACGAACTCGGTTTAACGCATAGTTGGTAAAAG
ATTTAATG

TGTTGACTAATAAAATCAATACCAAACCTGACAACAAATAAAATGAAGGATCTATGGATAAGTGGATGTTT
CTATTTTTTTTTTTTTTTTTTTTTTTTTCGACCTCATCTCATCGCGCTTTCCATTCATCATATTGTACGAA
AACAAAGC

CACGTTGACGTGCTTCTTAGTCCTTGTTCCTCTTTTTTTTTTGTTAGGTTAACCATTTATTTCCTAAACGG
GAAATAAGCAGGCGGGGAGCTGAATATAATTAACTTTCGCGTCATGGTTAGTGACGCGTCACTTCAAACGC
GGGTAACA

TAAAATCAATACCAAACCTGACAACAAATAAAATGAAGGATCTATGGATAAGTGGATGTTTCTATTTTTTT
TTTTTTTTTTTTTTTTTTCGACCTCATCTCATCGCGCTTTCCATTCATCATATTGTACGAAAACAAAGCAC
AATCGTAA

TGAAACATCAAATTAATCACGTGCTTTTTAAAAAATATAAATTTAATTTCATTTTCTATTTCAATATTTAT
TAAATAAAAAATTTGAAAAATATATAAAAATTGTAGCAGTATTAGATTTCCGAAAAGAAAAAAAGGTTTCA
AGAAAAGG

AACTAAAAAAGGAATGAAAAAAAAAAAAAAAAGTTCAAAAATAGTAACAGATATCTTTCGAAAAAGTTAAA
AACTGGTAAAAAATTTTGAATAGAGTATATAAAAACTAATAAATATGTTTAGTTATCATACACCATAGAAT
AACTACTA

CAACCATAACAAATTTCGGCATTGAACTTGATTCTAAATGCTACATACAATTACGCGCGCTGTTTTGATAT
ACACACACATATATATATATATACATTGTGTAAGAAAAATTCAAAGCTTAGTAAAACATACAGATCATGGA
GAAAAAAA

GACATGCACTTGAAGCAACTCAAGCGCAATGAGAAAAGTGACTATACAAATATGTAAAAAATTGCTACCAA
AAAAAAAAAAAAAAAATTTAAAAACTCTGATAATATAGTAAAAATTATTGGTACATTGTGAATTAAAATTT
ATGCGTTT

AAAAGAGGAAGCTCGGTGGCAAATAGCTTCCTCTTTGTGGCCGGGGCGCGGGGGGACGAGGCAAAAAGCAA
AGAAAAGCAAAAAAAATAAAAAAAAAAACAAAAAAACAGGGGTATGAGAAAAAGACACGCTTTTCCACGCG
CAGCAAAA

GCCGTCTACGGCCGTCTACGGCCTATTCCATTGCTAAAAATTTGAATCGTATAAAGGGATATTACCCGGAA
AAGAAACGCATTAAAAAAAAAAAAAAAAAAAAAACAGAAAAAGTGGTTAAGTGATTGACTGACCCTTGATA
GTTTTGTA

GTGCTTTTTAAAAAATATAAATTTAATTTCATTTTCTATTTCAATATTTATTAAATAAAAAATTTGAAAAA
TATATAAAAATTGTAGCAGTATTAGATTTCCGAAAAGAAAAAAAGGTTTCAAGAAAAGGAAAAGAAAAACT
AAACGTAG

AATTGCTCAAATTTTGAGAGTTAAAGGAAAGAATAAAATATATAAATGATACGTTTATACCCGCTAAAAAA
AAAAAATATAAAACAAAAAAAGGATCTCATAAGACGATTTGTTGGGATATGTCCACATCTTTCGGCTATAT
ACTACATT

CGTTTTCTTTCTTTTTTTTTCCTTCTTACATAGTATTCAACTGTATATTTAACATGTTTTACGTATTTTTA
AGAAAAAATTACTAAACGCGATAATATTAAGCAAATATTTATCTCATAGTTCTCGAACTCATTTATTTCCC
ATTGATGC

GTAAAAAAGCGATTGATCTCAGACATATCTTTTAAATGTTAAAACTCATCGCTCTTCAGGGAAATCTTACG
CGCTATTTTTACAGTCGCGCGGGTCTTAACCATAATTTTTTACCGCTGCAAATATTTGAATGCTATAAGAG
CAGATAAA

ACCAAACCTGACAACAAATAAAATGAAGGATCTATGGATAAGTGGATGTTTCTATTTTTTTTTTTTTTTTT
TTTTTTTTCGACCTCATCTCATCGCGCTTTCCATTCATCATATTGTACGAAAACAAAGCACAATCGTAATA
AAAGACAT

TATTCTTTTTTGTTGACTAATAAAATCAATACCAAACCTGACAACAAATAAAATGAAGGATCTATGGATAA
GTGGATGTTTCTATTTTTTTTTTTTTTTTTTTTTTTTTCGACCTCATCTCATCGCGCTTTCCATTCATCAT
ATTGTACG

TAATGTATATACATACATTTAGCATATGTAATACATTCGTACATGTATAGAAATATATGTTTATATATATA
TATATATATATATGTATATACGAACTCGGTTTAACGCATAGTTGGTAAAAGATTTAATGTAGATAAAAAAC
ACATAAGT

TGCTTCTTAGTCCTTGTTCCTCTTTTTTTTTTTGTTAGGTTAACCATTTATTTCCTAAACGGGAAATAAGCA
GGCGGGGAGCTGAATATAATTAACTTTCGCGTCATGGTTAGTGACGCGTCACTTCAAACGCGGGTAACAAA
ATCAAGTT

AGCATATGTAATACATTCGTACATGTATAGAAATATATGTTTATATATATATATATATATATATGTATATA
CGAACTCGGTTTAACGCATAGTTGGTAAAAGATTTAATGTAGATAAAAAACACATAAGTTTTATACGCACG
TAATCATG

CATAAGCCTTAACCGCAGCCCTCCTCTCCCCTAAACGTTTCTTTTTCTTTTTCCATTTTTTTTTTTTTTTT
AGTTTTACTTTCTTTTTATTCGGAAAAATCTTTTCTATATTTTTCAAGGGTAACCTCATCGCCATGCAATT
AATCACAT

AAAAAAAAGAACGGGGGTGTAATAATTTGTAGTTCATTATTGCAATTATATATCTATATCTATATATGTAT
ATAACATTAACATGTGCATGTACACACGTAATCGCGCGTGTACATGTCTATATGTGTTACTTGAACTATAC
TGTTTTGA

CAGATGGATACAGAATAGGAATCCTAAATATAAACACTATATTTATTTATTTTTTTCGAATAAAAGTCATG
TTAGCAATATTTATATAGTTGATTTTCCTGCATTTATTATATAATTGGCGGCATAGTAACATCAAAAAAAA
AAAAAAAA

ATCGGTTAAGGTTATAAAGTTAGGTAGCTGGTAAAAAAGCGATTGATCTCAGACATATCTTTTAAATGTTA
AAACTCATCGCTCTTCAGGGAAATCTTACGCGCTATTTTTACAGTCGCGCGGGTCTTAACCATAATTTTTT
ACCGCTGC

GTTATAAAGTTAGGTAGCTGGTAAAAAAGCGATTGATCTCAGACATATCTTTTAAATGTTAAAACTCATCG
CTCTTCAGGGAAATCTTACGCGCTATTTTTACAGTCGCGCGGGTCTTAACCATAATTTTTTACCGCTGCAA
ATATTTGA

GGACTCTCAAAAAAAAAATGTATTAATAAATAAAAGAACTTAAATATAGACTTTTTATTCGCATGCATATAA
AAATAATTAATTAATAAATAACTGTAAAAAACTAGAATAATAAAAGTAATAAATATCTCCTCCGACGACAT
AGCCTATT

TCGCCATAAGAGGAGGTTTTCCTTTTCAAAAGGAGCATGATACCAATAATTTATATTGCCTTACGCCTCTT
ATTTTTTATATCCTCTTTTTAGATTTTATTAATTTTTTTTTTTATTTATATACATATAATGTGCCCTGCTT
TTTACAAC

AGCAATGTGCACACTTGAATCACGTGAACCACAGTAACCTAATTGCTCAAATTTTGAGAGTTAAAGGAAAG
AATAAAATATATAAATGATACGTTTATACCCGCTAAAAAAAAAAAAATATAAAACAAAAAAAGGATCTCATA
AGACGATT

TTCTTCCATCTTTTTAATCTTTGTTTCCTTTTTTTACATCATATTACTTCTACCTACTCTACTCTATTCTA
ATTTCAATTGATTTTTTTTATTTTATTTTTTTTTTTGTTAATAAAAGAAGAAAAAACTTAAAAACGAATAA
GAGGCTGT

GGGTTTTTTTCTTTGCAATTTTTTTTTCGTTCTCCTAAAGCATACACAAATAAATCCTTTTTTTTATTTTC
TATTTATTTTGTTATTTATCATCTATATAGCAATAATATACTTTGTTTTTATTCGTATTTCACACTTTTCT
TTTTCCTT

TCCTTGTTCCTCTTTTTTTTTTGTTAGGTTAACCATTTATTTCCTAAACGGGAAATAAGCAGGCGGGGAGC
TGAATATAATTAACTTTCGCGTCATGGTTAGTGACGCGTCACTTCAAACGCGGGTAACAAAATCAAGTTGT
TTTTAGCC

AAAAAAATGTATTAATAAATAAAAGAACTTAAATATAGACTTTTTATTCGCATGCATATAAAAATAATTAA
TTAATAAATAACTGTAAAAAACTAGAATAATAAAAGTAATAAATATCTCCTCCGACGACATAGCCTATTTG
GCAGCCTT

TCTTTTTTTTTTGTTAGGTTAACCATTTATTTCCTAAACGGGAAATAAGCAGGCGGGGAGCTGAATATAAT
TAACTTTCGCGTCATGGTTAGTGACGCGTCACTTCAAACGCGGGTAACAAAATCAAGTTGTTTTTAGCCGC
CGAAGGTA

GCAACAAACCGCGAAAAAGCATAAGAAACATAAAAAAAAATATAAAAAAAAAGGAGATAATAATAAGATGTGA
AGGATTTGAATTAGTGGAAAAATGATAAGTCTACACTATTATTATTAACGAAATGCAATTTTCCTTTATTT
TCTTTTAT

TTGCCCTTAAACGTATACGAGGTATTCATTAATGTGTGTTGTAAGTATTTAGATTGACATCTATAAGAGAG
ATGAACATATATATATATATATATATATATATACATGCATATTTACTATAAACGCGGTTTATTCTGCCAGG
CAAAGGCA

ACCCCTACGGAGGCGTTGTAAAAAGGCAAAAATTAACGCGAATTTGTATAGTGCATCTAAAAAAAAATAATT
AAGTATAAAAATAGATAAAGGAAATCAAGTGTTTCTTTTTTTTTTTTTGGTTTATTGTTTGACACTGGTGA
TTTGCTTA

ATTAAAAAAAAAAAAAAAAAAAAAAAACAGAAAAAGTGGTTAAGTGATTGACTGACCCTTGATAGTTTTGTAC
AATTATACACTCGTTCTGATTAAAAACTTGTTTATAAAAATCTTTTTAAAAGAAAGAGAAGATCGTGTTTA
TTGCTTTT

TTACGCGCGCTGTTTTGATATACACACACATATATATATATATACATTGTGTAAGAAAAATTCAAAGCTTA
GTAAAACATACAGATCATGGAGAAAAAAAAAAAAACCGAATACTTGTGTTACCAGAAGGATCCACTGACTGG
ACTTGGGT

ACAGCTAAAACAGAGACAAAACTGCATGCCTCTTCTCCCCTTTATTATCACCTTTAAAAAAGATAAAAAAA
GAAACTGGAAAAAAGGTAAAAAAAAAAAAAAAATTAAATAAATACGAATACGTTTTCTCATGACCATTGTA
CTAGTAAT

GGTATTCATTAATGTGTGTTGTAAGTATTTAGATTGACATCTATAAGAGAGATGAACATATATATATATAT
ATATATATATATACATGCATATTTACTATAAACGCGGTTTATTCTGCCAGGCAAAGGCAATTTGCTTTTGT
TCGCCAGG

AGTGGATGTTTCTATTTTTTTTTTTTTTTTTTTTTTTTTCGACCTCATCTCATCGCGCTTTCCATTCATCA
TATTGTACGAAAACAAAGCACAATCGTAATAAAAGACATCCTGATAATTAGCAAAGGGCTAACCATAATAG
TCTAAGTT

GAACTGAATTCTTTATCAGCAATGAAATAGGCCGTCTACGGCCGTCTACGGCCTATTCCATTGCTAAAAAT
TTGAATCGTATAAAGGGATATTACCCGGAAAAGAAACGCATTAAAAAAAAAAAAAAAAAAAAAAACAGAAAA
AGTGGTTA

GATCCTTGCGGGGTCTTGCCTATTCTTTTTTGTTGACTAATAAAATCAATACCAAACCTGACAACAAATAA
AATGAAGGATCTATGGATAAGTGGATGTTTCTATTTTTTTTTTTTTTTTTTTTTTTTTCGACCTCATCTCA
TCGCGCTT

GCGCTATTTTTACAGTCGCGCGGGTCTTAACCATAATTTTTTACCGCTGCAAATATTTGAATGCTATAAGA
GCAGATAAAAAATAGATTATAGAAATTAACCTGTTAAAACAATTATCCAAAATGTAGTAAGTATTTATCTT
ACGTAAAA

AGGCGTTGTAAAAAGGCAAAAATTAACGCGAATTTGTATAGTGCATCTAAAAAAAAATAATTAAGTATAAAA
ATAGATAAAGGAAATCAAGTGTTTCTTTTTTTTTTTTTGGTTTATTGTTTGACACTGGTGATTTGCTTAAC
CAGCGCCT

TAAACACTATATTTATTTATTTTTTTTCGAATAAAAGTCATGTTAGCAATATTTATATAGTTGATTTTCCTG
CATTTATTATATAATTGGCGGCATAGTAACATCAAAAAAAAAAAAAAAAAACATTCAAATTGCACTACTTAT
AGCAGTAG

TATTTTTTATATCCTCTTTTTAGATTTTATTAATTTTTTTTTTTTATTTATATACATATAATGTGCCCTGCT
TTTTACAACCATACGTTTTTTGTTTTTTAATTTTCTTGCATTTTAAAACTGAATAGTGACCTGGGTATTAT
ACTACAGT

GGAATGAAAAAAAAAAAAAAAAAGTTCAAAAATAGTAACAGATATCTTTCGAAAAAGTTAAAAACTGGTAAA
AAATTTTGAATAGAGTATATAAAAACTAATAAATATGTTTAGTTATCATACACCATAGAATAACTACTAGA
GATATTAT

AAGAACAAATAGGCTACACAGGACTCTCAAAAAAAAAATGTATTAATAAATAAAAGAACTTAAATATAGACT
TTTTATTCGCATGCATATAAAAATAATTAATTAATAAATAACTGTAAAAAACTAGAATAATAAAAGTAATA
AATATCTC

ATCCTCTTTTTAGATTTTATTAATTTTTTTTTTTATTTATATACATATAATGTGCCCTGCTTTTTACAACC
ATACGTTTTTTGTTTTTTAATTTTCTTGCATTTTAAAACTGAATAGTGACCTGGGTATTATACTACAGTTG
AAATTGCT

TCAAAATGCAGCACCAATTGCAAATAACGACATTGATAATAATTTACAGTCTTTTTATTTTGATAATAGCA
ACTAAAAAAAGGAATGAAAAAAAAAAAAAAAGTTCAAAAATAGTAACAGATATCTTTCGAAAAAGTTAAAA
ACTGGTAA

GGGTCTTGCCTATTCTTTTTTGTTGACTAATAAAATCAATACCAAACCTGACAACAAATAAAATGAAGGAT
CTATGGATAAGTGGATGTTTCTATTTTTTTTTTTTTTTTTTTTTTTTTCGACCTCATCTCATCGCGCTTTC
CATTCATC

TCTTTATTCTCTTTTTTATAACGTTTTCTTTCTTTTTTTTCCTTCTTACATAGTATTCAACTGTATATTTA
ACATGTTTTACGTATTTTTAAGAAAAAATTACTAAACGCGATAATATTAAGCAAATATTTATCTCATAGTT
CTCGAACT

CGCCCCAATATAGGTTACAAAACATTAATATATATGTAAATATATGTGCGCGTATATATATATATATATAT
ATATAAAGGGAAAATTAATATTCTATCATTAAAGTGAAGAAGAAAGGTGGATTTAAATTCCAAGTCTTTTC
AATTGTTC

TTTTTACATCATATTACTTCTACCTACTCTACTCTATTCTAATTTCAATTGATTTTTTTTATTTTATTTTT
TTTTTTGTTAATAAAAGAAGAAAAAACTTAAAAACGAATAAGAGGCTGTCTTTGTTCCGTAGTATATGCCT
ATTTCCTT

GCCGTCTACGGCCTATTCCATTGCTAAAAATTTGAATCGTATAAAGGGATATTACCCGGAAAAGAAACGCA
TTAAAAAAAAAAAAAAAAAAAAAACAGAAAAAGTGGTTAAGTGATTGACTGACCCTTGATAGTTTTGTACA
ATTATACA

AAAACGTAGGCAACCATAACAAATTTCGGCATTGAACTTGATTCTAAATGCTACATACAATTACGCGCGCT
GTTTTGATATACACACACATATATATATATATACATTGTGTAAGAAAAATTCAAAGCTTAGTAAAACATAC
AGATCATG

ATATCTTTCGAAAAAGTTAAAAACTGGTAAAAAATTTTGAATAGAGTATATAAAAACTAATAAATATGTTT
AGTTATCATACACCATAGAATAACTACTAGAGATATTATTTCATTTTCGTTTTCATTTTTTTTATTTTTTT
CTTTTCAA

GAAATGGCTCATTATTAGGAGATGTGCATATAATGTATATACATACATTTAGCATATGTAATACATTCGTA
CATGTATAGAAATATATGTTTATATATATATATATATATATATGTATATACGAACTCGGTTTAACGCATAG
TTGGTAAA

GCACCAATTGCAAATAACGACATTGATAATAATTTACAGTCTTTTTATTTTGATAATAGCAACTAAAAAAG
GAATGAAAAAAAAAAAAAAAAGTTCAAAAATAGTAACAGATATCTTTCGAAAAAGTTAAAAACTGGTAAAA
AATTTTGA

CCTGACAAAAAAAAAAAAAATTAATTTTGGCCTGGAAAATTCTTCTCCTTAAATACAAAAAGATTCTTTTT
TATAGAATGTCATAATTCACGAACAGTTAAAAAAATATAAACACACAACGTAACACAGATGCCGGGTTACG
ATATCCGA

ATCTGCATCTAAGAACAAATAGGCTACACAGGACTCTCAAAAAAAAATGTATTAATAAATAAAAGAACTTA
AATATAGACTTTTTATTCGCATGCATATAAAAATAATTAATTAATAAATAACTGTAAAAAACTAGAATAAT
AAAAGTAA

CCGCAATACTGAAATGGCTCATTATTAGGAGATGTGCATATAATGTATATACATACATTTAGCATATGTAA
TACATTCGTACATGTATAGAAATATATGTTTATATATATATATATATATATATGTATATACGAACTCGGTT
TAACGCAT

TCTACACTATTATTATTAACGAAATGCAATTTTCCTTTATTTTCTTTTATTACTATTATCATTACTATTAT
TATTAGTATTATTATTTTTTTACTTATTTTTATATTAAACTATGTTAAATTACTATATTATTATTTTGTCT
AACTCTAT

ATTGCTACCAAAAAAAAAAAAAAAAAAAATTTAAAAACTCTGATAATATAGTAAAAATTATTGGTACATTGTG
AATTAAAATTTATGCGTTTGCCCTCAAATCCTTTTCTATATCGTCCAATTTTGAGTCAATGTTCTCAATTG
TGATACCA

AAATGAAGGATCTATGGATAAGTGGATGTTTCTATTTTTTTTTTTTTTTTTTTTTTTTTCGACCTCATCTC
ATCGCGCTTTCCATTCATCATATTGTACGAAAACAAAGCACAATCGTAATAAAAGACATCCTGATAATTAG
CAAAGGGC

ACTCAAATAATCAAAATGCAGCACCAATTGCAAATAACGACATTGATAATAATTTACAGTCTTTTTATTTT
GATAATAGCAACTAAAAAAGGAATGAAAAAAAAAAAAAAAAAGTTCAAAAATAGTAACAGATATCTTTCGAA
AAAGTTAA

CCATACACCCCATAAGCCTTAACCGCAGCCCTCCTCTCCCCTAAACGTTTCTTTTTCTTTTTCCATTTTTT
TTTTTTTTTTAGTTTTACTTTCTTTTTATTCGGAAAAATCTTTTCTATATTTTTCAAGGGTAACCTCATCG
CCATGCAA

TTCTCTCCATTTCTTCCATCTTTTTAATCTTTGTTTCCTTTTTTTACATCATATTACTTCTACCTACTCTA
CTCTATTCTAATTTCAATTGATTTTTTTTATTTTATTTTTTTTTTTTGTTAATAAAAGAAGAAAAAACTTAA
AAACGAAT

TATGTAAAAAATTGCTACCAAAAAAAAAAAAAAAAAAAATTTAAAAACTCTGATAATATAGTAAAAATTATTG
GTACATTGTGAATTAAAATTTATGCGTTTGCCCTCAAATCCTTTTCTATATCGTCCAATTTTGAGTCAATG
TTCTCAAT

CTTTTTATTTTGATAATAGCAACTAAAAAAGGAATGAAAAAAAAAAAAAAAAAGTTCAAAAATAGTAACAGA
TATCTTTCGAAAAAGTTAAAAACTGGTAAAAAATTTTGAATAGAGTATATAAAAACTAATAAATATGTTTA
GTTATCAT

TTTTTAATCTTTGTTTCCTTTTTTTACATCATATTACTTCTACCTACTCTACTCTATTCTAATTTCAATTG
ATTTTTTTTATTTTATTTTTTTTTTTTGTTAATAAAAGAAGAAAAAACTTAAAAACGAATAAGAGGCTGTCT
TTGTTCCG

TGATAATAGCAACTAAAAAAGGAATGAAAAAAAAAAAAAAAAAGTTCAAAAATAGTAACAGATATCTTTCGA
AAAAGTTAAAAACTGGTAAAAAATTTTGAATAGAGTATATAAAAACTAATAAATATGTTTAGTTATCATAC
ACCATAGA

ACATTGATGATCTTTATTCTCTTTTTATAACGTTTTCTTTCTTTTTTTTTTCCTTCTTACATAGTATTCAAC
TGTATATTTAACATGTTTTACGTATTTTTAAGAAAAAATTACTAAACGCGATAATATTAAGCAAATATTTA
TCTCATAG

TCCCTTTAGTGCAACAAACCGCGAAAAAGCATAAGAAACATAAAAAAAAATATAAAAAAAAAGGAGATAATAA
TAAGATGTGAAGGATTTGAATTAGTGGAAAAATGATAAGTCTACACTATTATTATTAACGAAATGCAATTT
TCCTTTAT

AGGCTACACAGGACTCTCAAAAAAAAAATGTATTAATAAATAAAAGAACTTAAATATAGACTTTTTATTCGC
ATGCATATAAAAATAATTAATTAATAAATAACTGTAAAAAACTAGAATAATAAAAGTAATAAATATCTCCT
CCGACGAC

AAATGATAAGTCTACACTATTATTATTAACGAAATGCAATTTTCCTTTATTTTCTTTTATTACTATTATCA
TTACTATTATTATTAGTATTATTATTTTTTTACTTATTTTTATATTAAACTATGTTAAATTACTATATTAT
TATTTTGT

TCCTATAAAATATTAAAAAGAACCAAAAAAAAAAATGGATAGAAGAATGCTTGAGGCAATAATAATGATTAT
CTATTTTATATATACATACATAATCATTTAAGTTTTTTTTTAAAGATATTTCGATTTAGCCGAATCTGCT
CAATGCCT

ATATTAAACTAAAATTTCTATGAAACATCAAATTAATCACGTGCTTTTTAAAAAATATAAATTTAATTTCA
TTTTCTATTTCAATATTTATTAAATAAAAAATTTGAAAAATATATAAAAATTGTAGCAGTATTAGATTTCC
GAAAAGAA

CTCTGCTATTTTGTGACGTTCAATTTTAATTGACGCGAAAAAGAAAAAATAAGAAGGGCAAAAAGAAAAAG
CGCAGCGGGTAGCAAATTTGGAATCGCATAAAAAGAAAAAAAAAAATATCAAAGAAAAAGAGTCATCTCAAA
CATATGTC

GAAATGCAATTTTCCTTTATTTTCTTTTATTACTATTATCATTACTATTATTATTAGTATTATTATTTTTT
TACTTATTTTTATATTAAACTATGTTAAATTACTATATTATTATTTTGTCTAACTCTATATTGAGGCTGTC
TTTCGTTG

TATTAAAAAGAACCAAAAAAAAAAATGGATAGAAGAATGCTTGAGGCAATAATAATGATTATCTATTTTATA
TATACATACATAATCATTTAAGTTTTTTTTTAAAGATATTTCGATTTAGCCGAATCTGCTCAATGCCTCT
TCGATGAC

GAACGATGAATGCATATACATATGTCGCGACTCCTTTTTTTCCATTATAGCGGCATCGCTTTATTTCGCGT
TTTAATTCATTTTCTTTTTTCTTCATTTCTTTCTGCATATGCAAACGCGGCAAGAAGTGCCCACGGATCCG
AAGAAAAC

AATGAGATCTACTTTAGCAATTGATACAGTATAACCCTGACATTACATTTAGGGAATAATCGCCCCAATAT
AGGTTACAAAACATTAATATATATGTAAATATATGTGCGCGTATATATATATATATATATATATAAAGGGA
AAATTAAT

CGTTTCTTTTTCTTATCGGTTTTATCCGGGTAATAAATTCAGCATATAGAAAAATTTTTCATTTCTATTTT
CTTTTTCTTTTCATTTTTCCTTTTTCTCAGAAAATTTTTTTTTCTAATGCTGGAATGAGATGAGTTAGAAA
ATTTTTCA

TCTGTGGAAAACTCAAATAATCAAAATGCAGCACCAATTGCAAATAACGACATTGATAATAATTTACAGTC
TTTTTATTTTGATAATAGCAACTAAAAAAGGAATGAAAAAAAAAAAAAAAAAGTTCAAAAATAGTAACAGAT
ATCTTTCG

CTTTTTTTTTCCTTCTTACATAGTATTCAACTGTATATTTAACATGTTTTACGTATTTTTAAGAAAAAATT
ACTAAACGCGATAATATTAAGCAAATATTTATCTCATAGTTCTCGAACTCATTTATTTCCCATTGATGCCA
TGAAAACC

AATTTACAGTCTTTTTATTTTGATAATAGCAACTAAAAAAGGAATGAAAAAAAAAAAAAAAAAGTTCAAAAA
TAGTAACAGATATCTTTCGAAAAAGTTAAAAACTGGTAAAAAATTTTGAATAGAGTATATAAAAACTAATA
AATATGTT

GTTTTAAGGGTAATAGGAAATATCACGCTACCTGACAAAAAAAAAAAAAAATTAATTTTGGCCTGGAAAATT
CTTCTCCTTAAATACAAAAAGATTCTTTTTTATAGAATGTCATAATTCACGAACAGTTAAAAAAATATAAA
CACACAAC

AACGCGTTTTAGACTCACCACTATTTTACTGTCTAAATATTTTTAATGGATATTTATATTAAAACCATTAT
CTTGGTTGGTTCCCTAACATCTTGATCATCGAGAAAAAAAAAAAAAAAAAGGACATGAAAAATTCTGACAAT
GAAAAAAA

GTGAAAAAAAAAAAAAAAAAAAAAATAATAATAATATTAATACTACTTATATACTATTAAAAAATCGTTTAG
AAATGATTCATTTATAAAATTCGTTACTTGCGGTCATTGGCAATAGATTCAGCTGCGGTTCTCCCAAAGAC
AACGCATT

CATTGATAATAATTTACAGTCTTTTTATTTTGATAATAGCAACTAAAAAAGGAATGAAAAAAAAAAAAAAA
AGTTCAAAAATAGTAACAGATATCTTTCGAAAAAGTTAAAAACTGGTAAAAAATTTTGAATAGAGTATATA
AAAACTAA

CTTTGCAATTTTTTTTTTCGTTCTCCTAAAGCATACACAAATAAATCCTTTTTTTTTATTTTCTATTTATTTT
GTTATTTATCATCTATATAGCAATAATATACTTTGTTTTTATTCGTATTTCACACTTTTCTTTTTCCTTAT
GCAGGCAG

AACTTCTATTGTTAAAGTAGACAATTGTATCGTTTCTTTTTCTTATCGGTTTTATCCGGGTAATAAATTCA
GCATATAGAAAAATTTTTCATTTCTATTTTCTTTTTCTTTTCATTTTTCCTTTTTCTCAGAAAATTTTTTT
TTCTAATG

ATTTATTTATTTTTTTCGAATAAAAGTCATGTTAGCAATATTTATATAGTTGATTTTCCTGCATTTATTAT
ATAATTGGCGGCATAGTAACATCAAAAAAAAAAAAAAAAACATTCAAATTGCACTACTTATAGCAGTAGCA
TCTATAAA

CACTGTAAAAAATTCTAATTATCGGTTAAGGTTATAAAGTTAGGTAGCTGGTAAAAAAGCGATTGATCTCA
GACATATCTTTTAAATGTTAAAACTCATCGCTCTTCAGGGAAATCTTACGCGCTATTTTTACAGTCGCGCG
GGTCTTAA

ATTTTGAGAGTTAAAGGAAAGAATAAAATATATAAATGATACGTTTATACCCGCTAAAAAAAAAAAATATA
AAACAAAAAAAGGATCTCATAAGACGATTTGTTGGGATATGTCCACATCTTTCGGCTATATACTACATTTA
GCTGTTTC

ATTGTTTTCGAATATGTATTATTTTTTTTTATTTTTTTTATTACTCGTTATCAATATATATATATACATGT
ATATTATTACTAAAAGAATGAGAAAATAAGATAGGATCCTGGAAGGGGGATCAGGTAAAGATATAAAGTGA
GATGAAGA

ACGTATACGAGGTATTCATTAATGTGTGTTGTAAGTATTTAGATTGACATCTATAAGAGAGATGAACATAT
ATATATATATATATATATATATACATGCATATTTACTATAAACGCGGTTTATTCTGCCAGGCAAAGGCAAT
TTGCTTTT

ATTAAAAGTTTCCTATAAAATATTAAAAAGAACCAAAAAAAAAAATGGATAGAAGAATGCTTGAGGCAATAA
TAATGATTATCTATTTTATATATACATACATAATCATTTAAGTTTTTTTTTTAAAGATATTTCGATTTAGC
CGAATCTG

ACTAATTATATATCACTACTAAAAAAAGCCAGAAAAAGAAAAAAAGTAAAATAGAAAATAGAAAATAGAAA
ATAGAAAAATAGAAATTAAAATTAAGAGCATTTTAAAATTAAGTCTACAAAGTAATACTTGGCATATTGCT
TAACTCCT

AAAAAAAAAAAAAAAACAGAAAAAGTGGTTAAGTGATTGACTGACCCTTGATAGTTTTGTACAATTATACAC
TCGTTCTGATTAAAAACTTGTTTATAAAAATCTTTTTAAAAGAAAGAGAAGATCGTGTTTATTGCTTTTCT
CAAAAAGA

GTTAAAGTAGACAATTGTATCGTTTCTTTTTCTTATCGGTTTTATCCGGGTAATAAATTCAGCATATAGAA
AAATTTTTCATTTCTATTTTCTTTTTCTTTTCATTTTTCCTTTTTCTCAGAAAATTTTTTTTTCTAATGCT
GGAATGAG

AAGAAAAAAGAATGAAGTGAAAAGCAAAAAAAAAAAAAAGATAAAAATGAAAAAACAAAAAAAACGGAGGGTA
TAATCACATGCAGATATAATTTTCGGAAATACATTATAGAAATATAATGTGTCACTTATCCTCTTCATTGT
GGTTATCC

ATTACCCGGAAAAGAAACGCATTAAAAAAAAAAAAAAAAAAAAAAAACAGAAAAAGTGGTTAAGTGATTGACT
GACCCTTGATAGTTTTGTACAATTATACACTCGTTCTGATTAAAAACTTGTTTATAAAAATCTTTTTAAAA
GAAAGAGA

AGAAGCCAAAAAAACGTAGGCAACCATAACAAATTTCGGCATTGAACTTGATTCTAAATGCTACATACAAT
TACGCGCGCTGTTTTGATATACACACACATATATATATATATACATTGTGTAAGAAAAATTCAAAGCTTAG
TAAAACAT

AACCGCAGCCCTCCTCTCCCCTAAACGTTTCTTTTTCTTTTTCCATTTTTTTTTTTTTTTTAGTTTTACTT
TCTTTTTATTCGGAAAAATCTTTTCTATATTTTTCAAGGGTAACCTCATCGCCATGCAATTAATCACATAG
ACTCCTTA

AAAAGGCAAAAATTAACGCGAATTTGTATAGTGCATCTAAAAAAAAATAATTAAGTATAAAAATAGATAAAG
GAAATCAAGTGTTTCTTTTTTTTTTTTTGGTTTATTGTTTGACACTGGTGATTTGCTTAACCAGCGCCTCC
AGCTTCGC

CGAAAACGAAAACATTTGATCAGATAAGTGATCTGCAAATAAGATATAATAAAAGTAACAGTTATTTATTT
AATTAAAAACGCAAATATGTAGTAATACGTGGACATATAAATTTCATGAATTAAAAAAAAAAAATGAAAT
TCAGATTG

TTGTTTCCTTTTTTTACATCATATTACTTCTACCTACTCTACTCTATTCTAATTTCAATTGATTTTTTTTA
TTTTATTTTTTTTTTTGTTAATAAAAGAAGAAAAAACTTAAAAACGAATAAGAGGCTGTCTTTGTTCCGTA
GTATATGC

ATTTTTTTTTTTCATATAAAGAGCTTGCGCGCGTGTTGTTGTTCGCTATCCATTTCCATTAGGAACATTTT
TGGATATTATTTTTCAGACCCGTAATATACTTAACACATATACCACTAAAGGCAAAAGAAAGAGAGATCTG
AAGTGAGA

CAGAGACAAAACTGCATGCCTCTTCTCCCCTTTATTATCACCTTTAAAAAAGATAAAAAAAGAAACTGGAA
AAAAGGTAAAAAAAAAAAAAAAATTAAATAAATACGAATACGTTTTCTCATGACCATTGTACTAGTAATCA
CAACCATG

TGGTTTTATTTTTTTTTTTTGAATTTTTATTTTCGTGACGTGCTTTTTTTTTCCGTTTTTGGTTTTAATGCCC
CAAAATAAAATAATCCCCTTTTGTATACGTTTTTCTTTGCCTGCCTTTTTATTTCTCAGTGCATTGCCCTT
TCTTTCGC

CAGAATAGGAATCCTAAATATAAACACTATATTTATTTATTTTTTTCGAATAAAAGTCATGTTAGCAATAT
TTATATAGTTGATTTTCCTGCATTTATTATATAATTGGCGGCATAGTAACATCAAAAAAAAAAAAAAAAAC
ATTCAAAT

TTTCAACTCAATAACTTTTATGTAAAATGTTTCTTGGCCGTCGATGAGCTCTTCCGGCAGAACTTCGAAAG
TGAAAAAAAAAAAAAAAAAAAAATAATAATAATATTAATACTACTTATATACTATTAAAAAATCGTTTAGA
AATGATTC

CAATATATGAGAACGATGAATGCATATACATATGTCGCGACTCCTTTTTTTCCATTATAGCGGCATCGCTT
TATTTCGCGTTTTAATTCATTTTCTTTTTTCTTCATTTCTTTCTGCATATGCAAACGCGGCAAGAAGTGCC
CACGGATC

GAGAGTACATTTGCCCTTAAACGTATACGAGGTATTCATTAATGTGTGTTGTAAGTATTTAGATTGACATC
TATAAGAGAGATGAACATATATATATATATATATATATATATACATGCATATTTACTATAAACGCGGTTTA
TTCTGCCA

AACCAAAAAAAAAAATGGATAGAAGAATGCTTGAGGCAATAATAATGATTATCTATTTTATATATACATACA
TAATCATTTAAGTTTTTTTTTTAAAGATATTTCGATTTAGCCGAATCTGCTCAATGCCTCTTCGATGACAT
CCTTGTGG

TTTCAAAGAAAAATGCCTCCAACGCGTTTTAGACTCACCACTATTTTACTGTCTAAATATTTTTAATGGAT
ATTTATATTAAAACCATTATCTTGGTTGGTTCCCTAACATCTTGATCATCGAGAAAAAAAAAAAAAAAAGG
ACATGAAA

TTTCCCCCGCCCATACACCCCATAAGCCTTAACCGCAGCCCTCCTCTCCCCTAAACGTTTCTTTTTCTTTT
TCCATTTTTTTTTTTTTTTTTAGTTTTACTTTCTTTTTATTCGGAAAAATCTTTTCTATATTTTTCAAGGGT
AACCTCAT

CGAGAAAAAAAAAAAAAAAAGGACATGAAAAATTCTGACAATGAAAAAAAAGAAATTAAAGACTCATAATT
ATTTATTGATAAGGCGAAGACGTAGCAACTATATGTACGTAGAAGTCAATGGCAATCGCGATAAAGCTGTG
CATTAATG

ATAAAGGATATTACCCGGAAAAGAAACGCATTAAAAAAAAAAAAAAAAAAAAAAACAGAAAAAGTGGTTAA
GTGATTGACTGACCCTTGATAGTTTTGTACAATTATACACTCGTTCTGATTAAAAACTTGTTTATAAAAAT
CTTTTTAA

ACAAAAGAAAATGGTGCCCTAAATATCTTTCAGATGAAAAACTAATTATATATCACTACTAAAAAAAGCCA
GAAAAAGAAAAAAAGTAAAATAGAAAATAGAAAATAGAAAATAGAAAAATAGAAATTAAAATTAAGAGCAT
TTTAAAAT

CATTAAGAATGTAGTGATGAGTACTGTAACTGGTTTTATTTTTTTTTTTGAATTTTTATTTTCGTGACGTG
CTTTTTTTTCCGTTTTTGGTTTTAATGCCCCAAAATAAAATAATCCCCTTTTGTATACGTTTTTCTTTGCC
TGCCTTTT

CACAAATAATTCCCTTTAGTGCAACAAACCGCGAAAAAGCATAAGAAACATAAAAAAAATATAAAAAAAAG
GAGATAATAATAAGATGTGAAGGATTTGAATTAGTGGAAAAATGATAAGTCTACACTATTATTATTAACGA
AATGCAAT

CTGGGTAAGACTACCTACGACCAGTCATGGATTAGATTAGTAATAGTTTACGCTAATATTAAATTGTTTAA
CAAACTAAGTAGTCAAAAAAAAAGAAAAAAAAAAAAAAAAAAAGAAAAGGTGACATTATGCAGAACATATT
TTATCGAT

GAAGAAGGGTCTGGGTAAGACTACCTACGACCAGTCATGGATTAGATTAGTAATAGTTTACGCTAATATTA
AATTGTTTAACAAACTAAGTAGTCAAAAAAAAAGAAAAAAAAAAAAAAAAAAAGAAAAGGTGACATTATGC
AGAACATA

TAATAATAGTAATAACAGTAGTAATAGTAATAATAGTAACAATAATAATAATAATAATAATAATAATAATA
ATAACAATAATAATAATATTAATAATATTAATAATGTCAATACTAATGCAGGGAACGGTAACAATCCAAAT
AGATTCCA

ATCCTAAATATAAACACTATATTTATTTATTTTTTTCGAATAAAAGTCATGTTAGCAATATTTATATAGTT
GATTTTCCTGCATTTATTATATAATTGGCGGCATAGTAACATCAAAAAAAAAAAAAAAAACATTCAAATTG
CACTACTT

AAGGATTTGAATTAGTGGAAAAATGATAAGTCTACACTATTATTATTAACGAAATGCAATTTTCCTTTATT
TTCTTTTATTACTATTATCATTACTATTATTATTAGTATTATTATTTTTTTACTTATTTTTATATTAAACT
ATGTTAAA

TACAGTCGCGCGGGTCTTAACCATAATTTTTTACCGCTGCAAATATTTGAATGCTATAAGAGCAGATAAAA
AATAGATTATAGAAATTAACCTGTTAAAACAATTATCCAAAATGTAGTAAGTATTTATCTTACGTAAAAAG
TTCGATGC

TTTTTTTTTTTTTTTTTTTCGACCTCATCTCATCGCGCTTTCCATTCATCATATTGTACGAAAACAAAGCA
CAATCGTAATAAAAGACATCCTGATAATTAGCAAAGGGCTAACCATAATAGTCTAAGTTTAGTAGTTCAAA
TGTTTGTT

CAATTTTAATTGACGCGAAAAAGAAAAAATAAGAAGGGCAAAAAGAAAAAGCGCAGCGGGTAGCAAATTTG
GAATCGCATAAAAAGAAAAAAAAAATATCAAAGAAAAAGAGTCATCTCAAACATATGTCTGCAGATACTTC
ATTATCAG

GCTGAAGAAGAGCCGATTTTACCCCAGTCGTATTGTTGGTAGCCTACATAGAGTCGTACGCGTTAGTTAGT
GTACCAAAAAAAAAAAAAAAAAAATACTCAAAGTATTCAAAATATTTGACGAAAAATGGCACATACCTGCAT
CTAACCTG

TTTAACAAAACTGTATATATGCGCTAAAAGCAGTATAATCATTATTTGTTGTTGTTATTATTATTATTATT
GTTATTATTATTATTATTATTATTATTGAGATTAATATTACCATCTCTCTGTTTTCTTTTCTTTCCCTTCT
AGCATATA

AATGTTTGTTGACATGCACTTGAAGCAACTCAAGCGCAATGAGAAAAGTGACTATACAAATATGTAAAAAA
TTGCTACCAAAAAAAAAAAAAAAAAAATTTAAAAACTCTGATAATATAGTAAAAATTATTGGTACATTGTGA
ATTAAAAT

AAAAAGTTAAAAACTGGTAAAAAATTTTGAATAGAGTATATAAAAACTAATAAATATGTTTAGTTATCATA
CACCATAGAATAACTACTAGAGATATTATTTCATTTTCGTTTTCATTTTTTTTATTTTTTTCTTTTCAATA
AGGTAACA

TTATAGGAGGATCTGCATCTAAGAACAAATAGGCTACACAGGACTCTCAAAAAAAAATGTATTAATAAATA
AAAGAACTTAAATATAGACTTTTTATTCGCATGCATATAAAAATAATTAATTAATAAATAACTGTAAAAAA
CTAGAATA

AATGTGTGTTGTAAGTATTTAGATTGACATCTATAAGAGAGATGAACATATATATATATATATATATATAT
ATACATGCATATTTACTATAAACGCGGTTTATTCTGCCAGGCAAAGGCAATTTGCTTTTGTTCGCCAGGCT
CAATGATA

AAAAAAAAAATTAATTTTGGCCTGGAAAATTCTTCTCCTTAAATACAAAAAGATTCTTTTTTATAGAATGT
CATAATTCACGAACAGTTAAAAAAATATAAACACACAACGTAACACAGATGCCGGGTTACGATATCCGAAA
AAGTTGAT

GAATAAAATATATATAAATGATACGTTTATACCCGCTAAAAAAAAAAAAATATAAAACAAAAAAAGGATCTCAT
AAGACGATTTGTTGGGATATGTCCACATCTTTCGGCTATATACTACATTTAGCTGTTTCCCGTTTCTTTGT
ATTATTCC

AAATTCATGTGTTTTAAGGGTAATAGGAAATATCACGCTACCTGACAAAAAAAAAAAAAATTAATTTTGGC
CTGGAAAATTCTTCTCCTTAAATACAAAAAGATTCTTTTTTATAGAATGTCATAATTCACGAACAGTTAAA
AAAATATA

AGCCGATTTTACCCCAGTCGTATTGTTGGTAGCCTACATAGAGTCGTACGCGTTAGTTAGTGTACCAAAAA
AAAAAAAAAAAAATACTCAAAGTATTCAAAATATTTGACGAAAAATGGCACATACCTGCATCTAACCTGAA
CAGCTTGT

CAGATAAGTGATCTGCAAATAAGATATAATAAAAGTAACAGTTATTTATTTAATTAAAAACGCAAATATGT
AGTAATACGTGGGACATATAAATTTCATGAATTAAAAAAAAAAAATGAAATTCAGATTGAAAATAATGGGG
ATATAAAA

TTGTGACGTTCAATTTTAATTGACGCGAAAAAGAAAAAATAAGAAGGGCAAAAAGAAAAAGCGCAGCGGGT
AGCAAATTTGGAATCGCATAAAAAGAAAAAAAAAAATATCAAAGAAAAAGAGTCATCTCAAACATATGTCTG
CAGATACT

CTGTCAACTTTTTGAACATGATTAAAAGTTTCCTATAAAATATTAAAAAGAACCAAAAAAAAAATGGATAG
AAGAATGCTTGAGGCAATAATAATGATTATCTATTTTATATATACATACATAATCATTTAAGTTTTTTTTT
TAAAGATA

TTAACTTTCGCGTCATGGTTAGTGACGCGTCACTTCAAACGCGGGTAACAAAATCAAGTTGTTTTTAGCCG
CCGAAGGTAATTGTATCCATAATGAAGGCTAGATTAAACCAATAATTTGTAGTGACAAAATTTATAGTCAG
TAGCAGGT

TTTGAATCGTATAAAGGGATATTACCCGGAAAAGAAACGCATTAAAAAAAAAAAAAAAAAAAAAAAACAGAAA
AAGTGGTTAAGTGATTGACTGACCCTTGATAGTTTTGTACAATTATACACTCGTTCTGATTAAAAACTTGT
TTATAAAA

GGCGAACTCATCTGTGGAAAACTCAAATAATCAAAATGCAGCACCAATTGCAAATAACGACATTGATAATA
ATTTACAGTCTTTTTATTTTGATAATAGCAACTAAAAAAGGAATGAAAAAAAAAAAAAAAAAGTTCAAAAAT
AGTAACAG

AATTCTAATTATCGGTTAAGGTTATAAAGTTAGGTAGCTGGTAAAAAAGCGATTGATCTCAGACATATCTT
TTAAATGTTAAAACTCATCGCTCTTCAGGGAAATCTTACGCGCTATTTTTACAGTCGCGCGGGTCTTAACC
ATAATTTT

TGACGCGAAAAAGAAAAAATAAGAAGGGCAAAAAGAAAAAGCGCAGCGGGTAGCAAATTTGGAATCGCATA
AAAAGAAAAAAAAAAATATCAAAGAAAAAGAGTCATCTCAAACATATGTCTGCAGATACTTCATTATCAGCT
TTGAAAAC

ACAATTGTATCGTTTCTTTTTCTTATCGGTTTTATCCGGGTAATAAATTCAGCATATAGAAAAATTTTTCA
TTTCTATTTTCTTTTTCTTTTCATTTTTCCTTTTTCTCAGAAAATTTTTTTTTCTAATGCTGGAATGAGAT
GAGTTAGA

GACACTTTATAAAAAATAAAGGAAAAGTAAAATATAATGCTGAAAATATAGTTGCCTTAAAATGCCATTGA
TATGATTTTTTTTTAACCTTAAATCTATTGGCCTATAATATTCGCGGCAAAATGAGCATTTGAAAATCAGT
AATAGAAT

CGTGAGCAACAACTTGATCATCGACGAGGAAACTTTTTAAGTTGAGAACTAAAAAGCAGTATTGTTTTCGA
ATATGTATTATTTTTTTTTATTTTTTTTTATTACTCGTTATCAATATATATATATACATGTATATTATTACT
AAAAGAAT

TTTCAACTTTTTCTATAATTTTCATGCATACATAAATCTGATATCGAAATAAAAATTACATTACACGGTAA
AAAAAATGTTTTCAAAGCTTTTGATCGTAAGCGATTGAAAAAATATAACGAATACTCCGATACGGGGAGTC
GAACCCCG

ATAATAACAATAATAATAGTAATAACAGTAGTAATAGTAATAATAGTAACAATAATAATAATAATAATAAT
AATAATAATAATAACAATAATAATAATATTAATAATATTAATAATGTCAATACTAATGCAGGGAACGGTAA
CAATCCAA

AAAAAAAAAAAAAGTTCAAAAATAGTAACAGATATCTTTCGAAAAAGTTAAAAACTGGTAAAAAATTTTGAA
TAGAGTATATAAAAACTAATAAATATGTTTAGTTATCATACACCATAGAATAACTACTAGAGATATTATTT
CATTTTCG

ATGTGTCACACCTCGATCGGCAACGTGAATTGGTTACCGTCTATTTAGTAATCAGTACAGAGATATTGAAG
ATGCCGCTATTTTTTTTTTTTTATATTTCCGCCGTATTTTATTCCTGTGTAGTCGGGTAATGGATGCTATT
TGTATGCG

TAGGTTACAAAACATTAATATATATGTAAATATATGTGCGCGTATATATATATATATATATATATAAAGGG
AAAATTAATATTCTATCATTAAAGTGAAGAAGAAAGGTGGATTTAAATTCCAAGTCTTTTCAATTGTTCTA
AACGTTCA

TATTGTTGGTAGCCTACATAGAGTCGTACGCGTTAGTTAGTGTACCAAAAAAAAAAAAAAAAAAATACTCAA
AGTATTCAAAATATTTGACGAAAAATGGCACATACCTGCATCTAACCTGAACAGCTTGTTGGACATGTTTT
AAAATTTT

GTAAAAAGAGAAACCCGAACAGGACAGAGCGTACATTTTTTATATACCTGTTCTTTTGTGCTTGTAGACGT
ATATCGTATTATTAAATTAAAAGTAAACCTAATTTGAACCATCGAGATAAAAAAAAAAAAAAACGGAGCACT
TTTTTGCT

TGAAAATGGAGGAAAAAAAAAAAAGACGAAATCCAAAAGGCTAAGGAAACGGGTAACCCGTGGTACCCGGGG
CAGAATCAAACTTATAATTACTAAAGATATACCATATATTACGATCCCTGCGCCCGGCGTGCTCCACATTA
AGTAAATG

AAAGATAGAGGAACTGAATTCTTTATCAGCAATGAAATAGGCCGTCTACGGCCGTCTACGGCCTATTCCAT
TGCTAAAAATTTGAATCGTATAAAGGGATATTACCCGGAAAAGAAACGCATTAAAAAAAAAAAAAAAAAAA
AAACAGAA

TTGAGCGACAGCTGAAGAAGAGCCGATTTTACCCCAGTCGTATTGTTGGTAGCCTACATAGAGTCGTACGC
GTTAGTTAGTGTACCAAAAAAAAAAAAAAAAAAATACTCAAAGTATTCAAAATATTTGACGAAAAATGGCAC
ATACCTGC

ATCTGCAAATAAGATATAATAAAAGTAACAGTTATTTATTTAATTAAAAACGCAAATATGTAGTAATACGT
GGGACATATAAATTTCATGAATTAAAAAAAAAAAATGAAATTCAGATTGAAAATAATGGGGATATAAAAAA
AGTGGACA

AAAAAATTTTTGTTCAAAAAATATAGGAAATTTTAGAAAACGAATGTTGAATGCGTTATAAATAATACAGA
ATGTACGTGTATATAAAAATATAATAAAAAAAAATGCAAGAGTTATGGCTTTGTAATAAAATTGATATCAAA
AAATTACC

ATGCGTAATGACATTGATGATCTTTATTCTCTTTTTATAACGTTTTCTTTCTTTTTTTTTCCTTCTTACAT
AGTATTCAACTGTATATTTAACATGTTTTACGTATTTTTAAGAAAAAATTACTAAACGCGATAATATTAAG
CAAATATT

TCGCTGAACCGGGTTTTTTTCTTTGCAATTTTTTTTCGTTCTCCTAAAGCATACACAAATAAATCCTTTT
TTTTATTTTCTATTTATTTTGTTATTTATCATCTATATAGCAATAATATACTTTGTTTTTATTCGTATTTC
ACACTTTT

TTGCTAAAAATTTGAATCGTATAAAGGGATATTACCCGGAAAAGAAACGCATTAAAAAAAAAAAAAAAAAAA
AAAACAGAAAAAGTGGTTAAGTGATTGACTGACCCTTGATAGTTTTGTACAATTATACACTCGTTCTGATT
AAAAACTT

GTAGTGATGAGTACTGTAACTGGTTTTATTTTTTTTTTTGAATTTTTATTTTCGTGACGTGCTTTTTTTTC
CGTTTTTGGTTTTAATGCCCCAAAATAAAATAATCCCCTTTTGTATACGTTTTTCTTTGCCTGCCTTTTTA
TTTCTCAG

TAATTTTTTTTTTTATTTATATACATATAATGTGCCCTGCTTTTTACAACCATACGTTTTTTGTTTTTTAA
TTTTCTTGCATTTTAAAACTGAATAGTGACCTGGGTATTATACTACAGTTGAAATTGCTCTCGTGAGGTAC
ATACTTGA

TTTATTATCACCTTTAAAAAAGATAAAAAAAGAAACTGGAAAAAAGGTAAAAAAAAAAAAAAAAATTAAATA
AATACGAATACGTTTTCTCATGACCATTGTACTAGTAATCACAACCATGATACTTGAATAACTTTTCTGTT
TTGTGCTG

ATATATTCAAAAAAAAAAAACAAAACAAAAAAATAATAACGTGATAAACATTAATGAACAATGTATTTACA
TTCTTAAGCATAGGTGAGAAATTACCTTCTTTACTTTTTTTTTTTTTTTTGGTGATATTGTATATTAAAAT
ATATAGTA

GATACCATAACGAAATAAACTTATGTGTAATTTCAACTTTTTCTATAATTTTCATGCATACATAAATCTGA
TATCGAAATAAAAATTACATTACACGGTAAAAAAAATGTTTTCAAAGCTTTTGATCGTAAGCGATTGAAAA
AATATAAC

TCATATAAACCAAAGAAACTTTTATACTTTTAAATATGCTAAACTATAGAAATTTAATGAACAAGCCCGCT
AAATTCACATTTTTCTTTTTGTTGTTGATATTTTTTTATTTTTATTTTTTTTTCATTTTAAGTTTTCCCCT
CACAGCAT

ATGACGAGAAGTAAAAAGAGAAACCCGAACAGGACAGAGCGTACATTTTTTATATACCTGTTCTTTTGTGC
TTGTAGACGTATATCGTATTATTAAATTAAAAGTAAACCTAATTTGAACCATCGAGATAAAAAAAAAAAAA
ACGGAGCA

ATATACATTGATGCGTAATGACATTGATGATCTTTATTCTCTTTTTATAACGTTTTCTTTCTTTTTTTTTC
CTTCTTACATAGTATTCAACTGTATATTTAACATGTTTTACGTATTTTTAAGAAAAAATTACTAAACGCGA
TAATATTA

CTTTTCAAGTTCGCCATAAGAGGAGGTTTTCCTTTTCAAAAGGAGCATGATACCAATAATTTATATTGCCT
TACGCCTCTTATTTTTTATATCCTCTTTTTAGATTTTATTAATTTTTTTTTTTATTTATATACATATAATG
TGCCCTGC

TTACCTTCCAGCATTTTTTCAAATGTGTTCACACGCCCTGTAACGTTTATATATAAATATTTTAATATTTA
ACTTAGCTTATGCATATATAGTTAAAAGGGATAATAACAATAAGAATAAAAAACACAAACTGCTGAGATAA
GAAAAGGA

AAGTGAAGGCAGGTGCCTAAGATTGGGTGGGAAAGTAAATTTACATATAGTTTTTTCTAGGAAAAAAAATAA
AAATAATGAAGGAAGGTATACTTATATAAGCGCTGTTTATCGAAACAGACGAGAATAAAAAATGGTTTTAA
AAAAATCA

GACTGATTCAATATTAAACTAAAATTTCTATGAAACATCAAATTAATCACGTGCTTTTTAAAAAATATAAA
TTTAATTTCATTTTCTATTTCAATATTTATTAAATAAAAAATTTGAAAAATATATAAAAATTGTAGCAGTA
TTAGATTT

AATTAACGCGAATTTGTATAGTGCATCTAAAAAAAATAATTAAGTATAAAAATAGATAAAGGAAATCAAGT
GTTTCTTTTTTTTTTTTGGTTTATTGTTTGACACTGGTGATTTGCTTAACCAGCGCCTCCAGCTTCGCGT
TCGTGAGA

TAGAACCTTAAAAAACAGTATTGGTAATTGCAATAGCACCAACCGTAAGTGAGCTAAACTAGTGAAAGATT
TTCTAAATCATAACGCGCGGAATTCCAGTTAATATATTACGTAGTCATCCCTCATCATAGAACTGTTTTTT
TTTTTTGC

TCTTGATCATCGAGAAAAAAAAAAAAAAAAAGGACATGAAAAATTCTGACAATGAAAAAAAAGAAATTAAAG
ACTCATAATTATTTATTGATAAGGCGAAGACGTAGCAACTATATGTACGTAGAAGTCAATGGCAATCGCGA
TAAAGCTG

TGCATATACATATGTCGCGACTCCTTTTTTTCCATTATAGCGGCATCGCTTTATTTCGCGTTTTAATTCAT
TTTCTTTTTTCTTCATTTCTTTCTGCATATGCAAACGCGGCAAGAAGTGCCCACGGATCCGAAGAAAACCT
AATACATT

TTTGAAACTCTTTAACAAAACTGTATATATGCGCTAAAAGCAGTATAATCATTATTTGTTGTTGTTATTAT
TATTATTATTGTTATTATTATTATTATTATTATTATTGAGATTAATATTACCATCTCTCTGTTTTCTTTTC
TTTCCCTT

CAAATAACGACATTGATAATAATTTACAGTCTTTTTATTTTGATAATAGCAACTAAAAAAGGAATGAAAAA
AAAAAAAAAAAGTTCAAAAATAGTAACAGATATCTTTCGAAAAAGTTAAAAACTGGTAAAAAAATTTTGAAT
AGAGTATA

TGTCCTTCAGCAAAAGAAAAAGCAGTTGGGCTCCTGTACGCCATATATACTTTATATGCGGTTTTTACCCC
GGAAATCGCGTTTATTAATGTCAATGCGCGATTTCTATTATGGGTTGAAAAAGTTGCACTTCTGTTCCTTT
CCAAACGA

TATTATTAACGAAATGCAATTTTCCTTTATTTTCTTTTATTACTATTATCATTACTATTATTATTAGTATT
ATTATTTTTTACTTATTTTTATATTAAACTATGTTAAATTACTATATTATTATTTTGTCTAACTCTATAT
TGAGGCTG

TAATGGTCCTCAAAGAAATAAAAGAAAAGGAAGAAGAAGTAATTGTAATATCAAACGGTTTTTTATAGTAT
ATTCTTCTTATTCTATATTTATATATCAATGTTTTATAATAAGATGTTTATTCATAGCATATCTGGTGGAT
CGTCTCTA

ACAGATAAAATCTAACAAGGATATATTCAAAAAAAAAAAAACAAAACAAAAAAATAATAACGTGATAAACAT
TAATGAACAATGTATTTACATTCTTAAGCATAGGTGAGAAATTACCTTCTTTACTTTTTTTTTTTTTTTG
GTGATATT

TGTATAATAGGTGCCATCTGGACACTTTATAAAAAATAAAGGAAAAGTAAAATATAATGCTGAAAATATAG
TTGCCTTAAAATGCCATTGATATGATTTTTTTTTAACCTTAAATCTATTGGCCTATAATATTCGCGGCAAA
ATGAGCAT

ATATTACTTCTACCTACTCTACTCTATTCTAATTTCAATTGATTTTTTTTATTTTATTTTTTTTTTTGTTA
ATAAAAGAAGAAAAAACTTAAAAACGAATAAGAGGCTGTCTTTGTTCCGTAGTATATGCCTATTTCCTTCT
AGAAGTTT

ACTTACAACCAAAAAATTTTTGTTCAAAAAATATAGGAAATTTTAGAAAACGAATGTTGAATGCGTTATAA
ATAATACAGAATGTACGTGTATATAAAAATATAATAAAAAAAATGCAAGAGTTATGGCTTTGTAATAAAAT
TGATATCA

AGGACAGAGCGTACATTTTTTATATACCTGTTCTTTTGTGCTTGTAGACGTATATCGTATTATTAAATTAA
AAGTAAACCTAATTTGAACCATCGAGATAAAAAAAAAAAAAAACGGAGCACTTTTTTGCTCATACAAGCAAA
ACCGCATT

GTGAGGTTGGCGTGAGCAACAACTTGATCATCGACGAGGAAACTTTTTAAGTTGAGAACTAAAAAGCAGTA
TTGTTTTCGAATATGTATTATTTTTTTTTATTTTTTTTATTACTCGTTATCAATATATATATATACATGTA
TATTATTA

AAATATTCCACACAAATCTATGCACATTACATTCTAGAATAAATTAATAAATAAAAATATATACATATATA
TTAATATGTATATATGTATGAATATAGTTTTCATTACAAAATAAGGCTTACTGTAGAGCATGTTGGAAATA
TTCAGGAT

TCACCACACACGAAAACGAAAACATTTGATCAGATAAGTGATCTGCAAATAAGATATAATAAAAGTAACAG
TTATTTATTTAATTAAAAACGCAAATATGTAGTAATACGTGGACATATAAATTTCATGAATTAAAAAAAA
AAAATGAA

ATTTTGCGCTTGTATAATAGGTGCCATCTGGACACTTTATAAAAAATAAAGGAAAAGTAAAATATAATGCT
GAAAATATAGTTGCCTTAAAATGCCATTGATATGATTTTTTTTTAACCTTAAATCTATTGGCCTATAATAT
TCGCGGCA

TATCACTACTAAAAAAAGCCAGAAAAAGAAAAAAAGTAAAATAGAAAATAGAAAATAGAAAATAGAAAAAT
AGAAATTAAAATTAAGAGCATTTTAAAATTAAGTCTACAAAGTAATACTTGGCATATTGCTTAACTCCTCA
CGCAGAGC

ATTAGTGGAAAAATGATAAGTCTACACTATTATTATTAACGAAATGCAATTTTCCTTTATTTTCTTTTATT
ACTATTATCATTACTATTATTATTAGTATTATTATTTTTTTACTTATTTTTATATTAAACTATGTTAAATT
ACTATATT

TTAAAGGAAAGAATAAAATATATAAATGATACGTTTATACCCGCTAAAAAAAAAAAAATATAAAACAAAAAA
AGGATCTCATAAGACGATTTGTTGGGATATGTCCACATCTTTCGGCTATATACTACATTTAGCTGTTTCCC
GTTTCTTT

CAAAGAAACTTTTATACTTTTAAATATGCTAAACTATAGAAATTTAATGAACAAGCCCGCTAAATTCACAT
TTTTCTTTTTGTTGTTGATATTTTTTTATTTTTATTTTTTTTTCATTTTAAGTTTTCCCCTCACAGCATAT
CCTTGAAG

ACATCAGAACCCGGTGTTAGAATGCAAGGTATAATCAGCAAAAAAAAAAAAAAATGTGTAAAAAAAAAAAGGT
CGGTTACCAGCTTTACTAAATGTTTTCTATTGAATAAAATCATAGAATTCCATAAGTGACTGTGACATTTT
CCACCCAG

ACCCCAGTCGTATTGTTGGTAGCCTACATAGAGTCGTACGCGTTAGTTAGTGTACCAAAAAAAAAAAAAAA
AAATACTCAAAGTATTCAAAATATTTGACGAAAAATGGCACATACCTGCATCTAACCTGAACAGCTTGTTG
GACATGTT

AAATGCCTCCAACGCGTTTTAGACTCACCACTATTTTACTGTCTAAATATTTTTAATGGATATTTATATTA
AAACCATTATCTTGGTTGGTTCCCTAACATCTTGATCATCGAGAAAAAAAAAAAAAAAAAGGACATGAAAAA
TTCTGACA

ACGGGGGTGTAATAATTTGTAGTTCATTATTGCAATTATATATCTATATCTATATATGTATATAACATTAA
CATGTGCATGTACACACGTAATCGCGCGTGTACATGTCTATATGTGTTACTTGAACTATACTGTTTTGACG
TGTATGTT

GTAAGTATTTAGATTGACATCTATAAGAGAGATGAACATATATATATATATATATATATATATACATGCAT
ATTTACTATAAACGCGGTTTATTCTGCCAGGCAAAGGCAATTTGCTTTTGTTCGCCAGGCTCAATGATAAC
GTTGATGA

ATCGAAAAAATGGCTTGGTGAGAAGAAGGGTCTGGGTAAGACTACCTACGACCAGTCATGGATTAGATTAGT
AATAGTTTACGCTAATATTAAATTGTTTAACAAACTAAGTAGTCAAAAAAAAGAAAAAAAAAAAAAAAAA
AAGAAAAG

AAATATCTTTCAGATGAAAAACTAATTATATATCACTACTAAAAAAAGCCAGAAAAAGAAAAAAAGTAAAA
TAGAAAATAGAAAATAGAAAATAGAAAAATAGAAATTAAAATTAAGAGCATTTTAAAATTAAGTCTACAAA
GTAATACT

ATACATTCGTACATGTATAGAAATATATGTTTATATATATATATATATATATATGTATATACGAACTCGGT
TTAACGCATAGTTGGTAAAAGATTTAATGTAGATAAAAAACACATAAGTTTTATACGCACGTAATCATGAC
TATCGCTA

TTTATTTTCTTTCTCTCCATTTCTTCCATCTTTTTAATCTTTGTTTCCTTTTTTTACATCATATTACTTCT
ACCTACTCTACTCTATTCTAATTTCAATTGATTTTTTTTATTTTATTTTTTTTTTTGTTAATAAAAGAAGA
AAAAACTT

AGGAAAAAAACAAAAAGAAAGCTGAAGAAAAGTATCTCAAGATATTTTCAGTAAATTTTTTTTTTTTTGAA
TTTTCCGTTTAATATTAAAAGACCTTAAATCGATAAGTTATGTATCTGTATATACAGGGTGTACTCAATTT
AGCAATAA

TTTTTTTCGTTCTCCTAAAGCATACACAAATAAATCCTTTTTTTTTATTTTCTATTTATTTTGTTATTTATC
ATCTATATAGCAATAATATACTTTGTTTTATTCGTATTTCACACTTTTCTTTTTCCTTATGCAGGCAGTG
TAATTCAT

TGACTCTTCTATTTTTTTTTTTCATATAAAGAGCTTGCGCGCGTGTTGTTGTTCGCTATCCATTTCCATTA
GGAACATTTTTGGATATTATTTTTCAGACCCGTAATATACTTAACACATATACCACTAAAGGCAAAAGAAA
GAGAGATC

CTCCTCTCCCCTAAACGTTTCTTTTTCTTTTTCCATTTTTTTTTTTTTTTTAGTTTTACTTTCTTTTTATT
CGGAAAAAATCTTTTCTATATTTTTCAAGGGTAACCTCATCGCCATGCAATTAATCACATAGACTCCTTAAG
TAACAATG

TCTAACAAGGATATATTCAAAAAAAAAAAAACAAAACAAAAAAAATAATAACGTGATAAACATTAATGAACAA
TGTATTTACATTCTTAAGCATAGGTGAGAAATTACCTTCTTTACTTTTTTTTTTTTTTTTGGTGATATTGT
ATATTAAA

GCTGTTGTGTGTAAATAGATTGAACGCGTTGGGAATGTAAAACGCGATTAAGAATTTCTGATTTAACGCAG
GTGCTAGGTTATTAATAGTTTCTTACGCGGATATGTAGGTTCAAGGGTCTATGTACGTATGGAGGAATAGC
AAAAAAAG

TGTTTACGCGTTTTATCCCATTATATGGCACCCAAATCAAATTTAAAAAGAAAAAACGCGTAAACAGTGTC
GGGTAAGTTCATCCTCTGTTACTTTAATTGCTTCTTTTTTTGAAATTCTAAGTAAACGCGTCATTTTGATC
CTCAGGAC

CTTATCTACATTTCAAAGAAAAATGCCTCCAACGCGTTTTAGACTCACCACTATTTTACTGTCTAAATATT
TTTAATGGATATTTATATTAAAACCATTATCTTGGTTGGTTCCCTAACATCTTGATCATCGAGAAAAAAAA
AAAAAAAA

CTAAACGTTTCTTTTTCTTTTTCCATTTTTTTTTTTTTTTTAGTTTTACTTTCTTTTTATTCGGAAAAATC
TTTTCTATATTTTTCAAGGGTAACCTCATCGCCATGCAATTAATCACATAGACTCCTTAAGTAACAATGGC
AAGGCGAT

CTCGTGACATACAGATAAAATCTAACAAGGATATATTCAAAAAAAAAAAACAAAACAAAAAAATAATAACG
TGATAAACATTAATGAACAATGTATTTACATTCTTAAGCATAGGTGAGAAATTACCTTCTTTACTTTTTTT
TTTTTTTT

GCCTATTCCATTGCTAAAAATTTGAATCGTATAAAGGGATATTACCCGGAAAAGAAACGCATTAAAAAAAA
AAAAAAAAAAAAAACAGAAAAAGTGGTTAAGTGATTGACTGACCCTTGATAGTTTTGTACAATTATACACT
CGTTCTGA

AGAGAAATATGAAAAGCAAGAATATAAAAATAGAACAGGAGCACTTAGTTCGGTTTTTTGCCATCATGTTT
AATATATATATATATATATATATATATATATATATATGTGCGTGCGTGTGTGTGTGTGTGTGTGTGTACAT
GTATGCGG

TCTTATCGGTTTTATCCGGGTAATAAATTCAGCATATAGAAAAATTTTTCATTTCTATTTTCTTTTTCTTT
TCATTTTTCCTTTTTCTCAGAAAATTTTTTTTTCTAATGCTGGAATGAGATGAGTTAGAAAATTTTTCACT
TTAAAACT

TTCTATAATTTTCATGCATACATAAATCTGATATCGAAATAAAAATTACATTACACGGTAAAAAAAATGTT
TTCAAAGCTTTTGATCGTAAGCGATTGAAAAAATATAACGAATACTCCGATACGGGGAGTCGAACCCCGGT
CTCCACGG

TGTTCAAAAAATATAGGAAATTTTAGAAAACGAATGTTGAATGCGTTATAAATAATACAGAATGTACGTGT
ATATAAAAATATAATAAAAAAAATGCAAGAGTTATGGCTTTGTAATAAAATTGATATCAAAAAATTACCCA
AATAAACT

AAACCCCAGGAAATATTCCACACAAATCTATGCACATTACATTCTAGAATAAATTAATAAATAAAAATATA
TACATATATATTAATATGTATATATGTATGAATATAGTTTTCATTACAAAATAAGGCTTACTGTAGAGCAT
GTTGGAAA

AAACAGATAGAAAAAGTATAACCCTAGAATCGAAATCAAAAATTAGAAAATAAACGTATTGGAATTTTGTG
CAATTATCATATTAAAATACAAAAACAATAAAAAAACTAAAAAGCTTTGAAACTTTCTTTAATTTTTTTCT
AATTTTCT

AACCATTTATTTCCTAAACGGGAAATAAGCAGGCGGGGAGCTGAATATAATTAACTTTCGCGTCATGGTTA
GTGACGCGTCACTTCAAACGCGGGTAACAAAATCAAGTTGTTTTTAGCCGCCGAAGGTAATTGTATCCATA
ATGAAGGC

AAAGCAAAGTAAGAAAAAAGAATGAAGTGAAAAGCAAAAAAAAAAAAAAGATAAAAATGAAAAAACAAAAAA
ACGGAGGGTATAATCACATGCAGATATAATTTTCGGAAATACATTATAGAAATATAATGTGTCACTTATCC
TCTTCATT

CAGATGAAAAACTAATTATATATCACTACTAAAAAAAAGCCAGAAAAAGAAAAAAAGTAAAATAGAAAATAG
AAAATAGAAAATAGAAAAATAGAAATTAAAATTAAGAGCATTTTAAAATTAAGTCTACAAAGTAATACTTG
GCATATTG

TTGTATAAAGTATACAAGATTGTAAAAATGATTGAAGTATTTTTATATATGTATATTACCTATAAAATTTT
TTATATTACGTACCGAAGGGGTTTTTTTAATGCTAAAGCGATTCATCTTGCTGTAGGTATATTTTTAAATC
ATCTGGAA

GTGCCATCTGGACACTTTATAAAAAATAAAGGAAAAGTAAAATATAATGCTGAAAATATAGTTGCCTTAAA
ATGCCATTGATATGATTTTTTTTTAACCTTAAATCTATTGGCCTATAATATTCGCGGCAAAATGAGCATTT
GAAAATCA

TAGGTTCGGGTGACGTGAAGATAACGAAAAATGAGTAAAAATTATCTTCTAATTATACATTAGTATTAGCG
TGAAAATGTACACATATACATATATATATATATATATATATACTTGAAGGTCTGAAGGAGTTTCAAATGCT
TCTACTCC

TCTACAAACTGGTCCTTAAGATGTTTTATTGAAGACATCATTTTTTATTTTTTTAAGCAGAACGTGCTGTG
CTCTCCCTTTTGGGCCTTTTTACGATTTTCAAAAAAATTGGTCTCTAGCGGGATCGAACCGCTGATCCCCG
CGTTATTA

ATAATCGTGGTCGGAACGTGCAATATATGAGAACGATGAATGCATATACATATGTCGCGACTCCTTTTTTT
CCATTATAGCGGCATCGCTTTATTTCGCGTTTTAATTCATTTTCTTTTTTCTTCATTTCTTTCTGCATATG
CAAACGCG

GGGGGACGAGGCAAAAAGCAAAGAAAAGCAAAAAAAATAAAAAAAAAAACAAAAAAACAGGGGTATGAGAA
AAAGACACGCTTTTCCACGCGCAGCAAAAAGGAAAAAGGAAAAGGAAACTCTTTATTATTGGACCTTAAAC
CTGAAAAC

GCATTTTTTCAAATGTGTTCACACGCCCTGTAACGTTTATATATAAATATTTTAATATTTAACTTAGCTTA
TGCATATATAGTTAAAAGGGATAATAACAATAAGAATAAAAAACACAAACTGCTGAGATAAGAAAAGGAAG
CGAATAGT

ACCTTGAAGACTATATTTCTTTTCATCACGTGCTATAAAAATAATTATAATTTAAATTTTTTAATATAAAT
ATATAAATTAAAAATAGAAAGTAAAAAAAGAAATTAAAGAAAAAATAGTTTTTGTTTTCCGAAGATGTAAA
ATAGGTTG

CTATATTTCTTTTCATCACGTGCTATAAAAATAATTATAATTTAAATTTTTTAATATAAATATATAAATTA
AAAATAGAAAGTAAAAAAAGAAATTAAAGAAAAAATAGTTTTTGTTTTCCGAAGATGTAAAATAGGTTGAA
AGTTAGAA

AATGCCTTCGCTTGTATTACGCAAAAAAAAAAAAAATAAATAAAAAAAAAAAAAAAAAATAAGGTATAAA
TCGTTGGTTCTTTTATGCACAATTATTTAACTATAGTTATCTATTTACGTAAAGGCTTCTATTTTTCCTTA
TCTACAAG

GCATGTAAACAATGCCTTCGCTTGTATTACGCAAAAAAAAAAAAAATAAATAAAAAAAAAAAAAAAAAAT
AAGGTATAAATCGTTGGTTCTTTTATGCACAATTATTTAACTATAGTTATCTATTTACGTAAAGGCTTCTA
TTTTTCCT

CTTGTATTACGCAAAAAAAAAAAAAATAAATAAAAAAAAAAAAAAAAAATAAGGTATAAATCGTTGGTTC
TTTTATGCACAATTATTTAACTATAGTTATCTATTTACGTAAAGGCTTCTATTTTTCCTTATCTACAAGAA
ATTGCATG

GCAAAAAAAAAAAAAATAAATAAAAAAAAAAAAAAAAAATAAGGTATAAATCGTTGGTTCTTTTATGCAC
AATTATTTAACTATAGTTATCTATTTACGTAAAGGCTTCTATTTTTCCTTATCTACAAGAAATTGCATGAA
GTTTAATT

CCTCACTTCAGCGGCTAAAAAAAAAAAAAACACCCCGAGTATTAGTGAATATAGATATATATATATATATA
TATATATATAGGGGGGCCGATAAAAAAGAACTTGCAATATTACGCAGATCAGGGATTTACTAAAAGAAAAG
TAATAAAG

GTATAATATAACCTTGAAGACTATATTTCTTTTCATCACGTGCTATAAAAATAATTATAATTTAAATTTTT
TAATATAAATATATAAATTAAAAATAGAAAGTAAAAAAAGAAATTAAAGAAAAAATAGTTTTTGTTTTCCG
AAGATGTA

CTATTCTCTCGAGCTCAGTTTTGGGTTTTGGCATGTAAACAATGCCTTCGCTTGTATTACGCAAAAAAAAA
AAAAATAAATAAAAAAAAAAAAAAAAAAATAAGGTATAAATCGTTGGTTCTTTTATGCACAATTATTTAAC
TATAGTTA

GAGCTCAGTTTTGGGTTTTGGCATGTAAACAATGCCTTCGCTTGTATTACGCAAAAAAAAAAAAAATAAAT
AAAAAAAAAAAAAAAAAAATAAGGTATAAATCGTTGGTTCTTTTATGCACAATTATTTAACTATAGTTATC
TATTTACG

GATCTGTCCTTACGCGTAATAATTACAAGAAGGTGAGAAAATTGCCTTTTTTTGCTAATTTTTTTTTTTTG
GTACCATATTCAATAATTGATGCTAAATTATAAATTGATTTTATTGTTTTCCTCTTTCCTACAATTAATCT
CTTTTTCT

CCCAAAAAAAAAAAGTACTCGCTTCTTTCCATGTCCGCTTCATATATATATACACATACTAATCAAACTCT
ATGTATACATAGAATAAAAAGAAGAACACTATATTTATTTCATAAAAAAAAAAAAAAAATAACAAAAAAAG
TGCAACAT

CTTTAACCTTCCGAGCTATTGATTTTTGATCCTCACTTCAGCGGCTAAAAAAAAAAAAAACACCCCGAGTA
TTAGTGAATATAGATATATATATATATATATATATATATATAGGGGGGCCGATAAAAAAGAACTTGCAATATT
ACGCAGAT

ACGCACACGCGCACGCATCCACATTCACTTTCACTTATAAAATTTCTTCTCTCTAGTTTTATATACGTTGT
ATTAGAAATATAAAATATGACAATAGTACACTTTGTAGGCTCTCTTTTTTTTTTTTTTTTTTTTTCTTATA
TATTTTTT

CCGAGCTATTGATTTTTGATCCTCACTTCAGCGGCTAAAAAAAAAAAAAACACCCCGAGTATTAGTGAATA
TAGATATATATATATATATATATATATATAGGGGGGCCGATAAAAAAGAACTTGCAATATTACGCAGATCA
GGGATTTA

ATATAAACCTGTATAATATAACCTTGAAGACTATATTTCTTTTCATCACGTGCTATAAAAATAATTATAAT
TTAAATTTTTTAATATAAATATATAAATTAAAAATAGAAAGTAAAAAAAGAAATTAAAGAAAAAATAGTTT
TTGTTTTC

GTAAAATAATATATAAACCTGTATAATATAACCTTGAAGACTATATTTCTTTTCATCACGTGCTATAAAAA
TAATTATAATTTAAATTTTTTAATATAAATATATAAATTAAAAATAGAAAGTAAAAAAAGAAATTAAAGAA
AAAATAGT

TTGGGTTTTGGCATGTAAACAATGCCTTCGCTTGTATTACGCAAAAAAAAAAAAAATAAATAAAAAAAAAA
AAAAAAAAATAAGGTATAAATCGTTGGTTCTTTTATGCACAATTATTTAACTATAGTTATCTATTTACGTA
AAGGCTTC

GCACACGCACACGCACACGCGCACGCATCCACATTCACTTTCACTTATAAAATTTCTTCTCTCTAGTTTTA
TATACGTTGTATTAGAAATATAAAATATGACAATAGTACACTTTGTAGGCTCTCTTTTTTTTTTTTTTTTT
TTTTCTTA

TTTCATCACGTGCTATAAAAATAATTATAATTTAAATTTTTTAATATAAATATATAAATTAAAAATAGAAA
GTAAAAAAAGAAATTAAAGAAAAAATAGTTTTTGTTTTCCGAAGATGTAAAATAGGTTGAAAGTTAGAAAT
TAGTATTA

GATTTTTGATCCTCACTTCAGCGGCTAAAAAAAAAAAAAAACACCCCGAGTATTAGTGAATATAGATATATA
TATATATATATATATATATAGGGGGGCCGATAAAAAAGAACTTGCAATATTACGCAGATCAGGGATTTACT
AAAAGAAA

ACTAGACAGAGCGCATAAAAAAAAGCCTAATCCCTTTTCCGTCTGTATACTTGTGCTCCCTTATAAAAAAAA
TTAAAAATTAAAAAATAAAAATAAGAATAGTGTATAAAATTATTTTTGCTTTGATTTTAGTTTATTTATTT
ACTTTCTA

CGTTCGTCAACTATTCTCTCGAGCTCAGTTTTGGGTTTTGGCATGTAAACAATGCCTTCGCTTGTATTACG
CAAAAAAAAAAAAAAATAAATAAAAAAAAAAAAAAAAAAAATAAGGTATAAATCGTTGGTTCTTTTATGCACA
ATTATTTA

AAAGCCTAATCCCTTTTCCGTCTGTATACTTGTGCTCCCTTATAAAAAAAATTAAAAATTAAAAAATAAAA
ATAAGAATAGTGTATAAAATTATTTTTGCTTTGATTTTAGTTTATTTATTTACTTTCTAAATACAGTAGGT
TAAATAAG

GCGGCTAAAAAAAAAAAAAAACACCCCGAGTATTAGTGAATATAGATATATATATATATATATATATATATA
GGGGGGCCGATAAAAAAGAACTTGCAATATTACGCAGATCAGGGATTTACTAAAAGAAAAGTAATAAAGGA
CTGCTGCT

TGCTATAAAAATAATTATAATTTAAATTTTTTAATATAAATATATAAATTAAAAATAGAAAGTAAAAAAAG
AAATTAAAGAAAAAATAGTTTTTGTTTTCCGAAGATGTAAAATAGGTTGAAAGTTAGAAATTAGTATTATA
ATAGCAAA

GCGCATAAAAAAAGCCTAATCCCTTTTCCGTCTGTATACTTGTGCTCCCTTATAAAAAAAATTAAAAATTA
AAAAATAAAAATAAGAATAGTGTATAAAATTATTTTTGCTTTGATTTTAGTTTATTTATTTACTTTCTAAA
TACAGTAG

CCCTTTTCCGTCTGTATACTTGTGCTCCCTTATAAAAAAAATTAAAAATTAAAAAATAAAAATAAGAATAG
TGTATAAAATTATTTTTGCTTTGATTTTAGTTTATTTATTTACTTTCTAAATACAGTAGGTTAAATAAGGA
TCGGTTTC

AGACTTTTTCCGATCTAAATAATGCATATAACCGTATCTTTTTTATATGAACCTAGTTTTGCTGTATAAGA
GCCGAGAAAAAAATATAGGAAAATATAGGAAAATATGCAAAATAGAAACACGAATTTTTTTTTTGTTAGAG
AAAAAAAG

TACGCGTAATAATTACAAGAAGGTGAGAAAATTGCCTTTTTTTGCTAATTTTTTTTTTTTGGTACCATATT
CAATAATTGATGCTAAATTATAAATTGATTTTATTGTTTTCCTCTTTCCTACAATTAATCTCTTTTTCTGG
CTTTATGA

GAGTAATAAAAAAATTCTAAAAAAAAAAACGCTTAAATCGAGGTGGAATATATAAACTTAAGTACGCATATA
CTCTAGTTCAGTTTAAAAATTAGCTGTTTTTTTAAAAAAAAAAATTCTCTTTCTTTACAAAATGTTCTTAA
ATGTATGT

TCACTTATAAAATTTCTTCTCTCTAGTTTTATATACGTTGTATTAGAAATATAAAATATGACAATAGTACA
CTTTGTAGGCTCTCTTTTTTTTTTTTTTTTTTTTTCTTATATATTTTTTTACAATGACCACGGTTTGTACA
CCTGGGGC

GATACACGGTCCAATGGATAAACATTTTTTATCAACACTATGATATATAAATATAATAGTTTTTCGTATAT
ATATTCCTTTTTTTGGTCAATTTTTGAAATTTTCGTAGAAAAGGGAGAGACAAAACACATTATATCAATGA
AAACGTAC

GCACGCATCCACATTCACTTTCACTTATAAAATTTCTTCTCTCTAGTTTTATATACGTTGTATTAGAAATA
TAAAATATGACAATAGTACACTTTGTAGGCTCTCTTTTTTTTTTTTTTTTTTTTTCTTATATATTTTTTTA
CAATGACC

ACACTTTTATGATCTGTCCTTACGCGTAATAATTACAAGAAGGTGAGAAAATTGCCTTTTTTTGCTAATTT
TTTTTTTTTGGTACCATATTCAATAATTGATGCTAAATTATAAATTGATTTTATTGTTTTCCTCTTTCCTA
CAATTAAT

CTTACGGCTTCAAAAAAGCACCGAAACTAATCGAGAAAGCTTATAATATCGGTATACATTTGAATGTATTT
TAACTATTTCTATATCAAAAAAAAAAAAAAAAAATTGTATATTTTTCGTTATTCTCTAATTCGTATCACATT
TTATCCCT

TTATAGATCCCCCAAAAAAAAAAAGTACTCGCTTCTTTCCATGTCCGCTTCATATATATATACACATACTA
ATCAAACTCTATGTATACATAGAATAAAAAGAAGAACACTATATTTATTTCATAAAAAAAAAAAAAAAAATA
ACAAAAAA

ATGAATAGCAGAGTAATAAAAAAAATTCTAAAAAAAAAAACGCTTAAATCGAGGTGGAATATATAAACTTAAG
TACGCATATACTCTAGTTCAGTTTAAAAATTAGCTGTTTTTTTAAAAAAAAAAATTCTCTTTCTTTACAAA
ATGTTCTT

AGTTTAATTTTGGGTGGTAATGGCTGTGTGACTAGCTATAGAAAGAAAAAAATTAAAAAAAAAAAAAAAAA
TCAAGTAGTTCCTGCACTGCGACGTCCATTATAGCATTATAAATTGGTCCCTGATTTACGCATGCGATAAA
CTATTTTT

TCGAAAAGACAGCGAGAAGGGACACTGGAAAAATGGCATAAAACTTTTCAAGGTTATTATTAGTAAGTTTT
TTTATTATATGATATATATATATATAAAAATGAAATAAATTATTTCTTGATGGAGCCACTTATTTTTCTTA
TACCACCG

ATCCAGCTTCCGTTCGTCAACTATTCTCTCGAGCTCAGTTTTGGGTTTTGGCATGTAAACAATGCCTTCGC
TTGTATTACGCAAAAAAAAAAAAAAATAAATAAAAAAAAAAAAAAAAAAAATAAGGTATAAATCGTTGGTTCT
TTTATGCA

AAAAAATAAATAAAAAAAAAAAAAAAAAAAAATAAGGTATAAATCGTTGGTTCTTTTATGCACAATTATTTAA
CTATAGTTATCTATTTACGTAAAGGCTTCTATTTTTCCTTATCTACAAGAAATTGCATGAAGTTTAATTTT
TTTTGTCA

AACGTACAGCACTTTAATTAATATAGACGTCTCTCTATGATTTTTTTAAATATTGTATATTTTTGTTTATA
TCTATATATCAGTATGCGCGCATACTTAGTAAAAGTATAAAAAGCCATTTGCTGCTTCATCGAATATTTTG
GCTTTCGT

CGGATGTAAAAGAAAACAGTACTAGACAGAGCGCATAAAAAAAGCCTAATCCCTTTTCCGTCTGTATACTT
GTGCTCCCTTATAAAAAAAATTAAAAATTAAAAAATAAAAATAAGAATAGTGTATAAAATTATTTTTGCTT
TGATTTTA

ATTATATTCAATGAATAGCAGAGTAATAAAAAAAATTCTAAAAAAAAAAACGCTTAAATCGAGGTGGAATATA
TAAACTTAAGTACGCATATACTCTAGTTCAGTTTAAAAATTAGCTGTTTTTTTAAAAAAAAAAATTCTCTT
TCTTTACA

GAGAAATGAAATTAGATCATAAATTAACTCTAACAAAGGAGAATATATATACGTACATATATATATATATA
TATATAAGTTATTGTAATTGAGAAAAAAATAAACATCATATAAAACTTCCGTTTTAATGAACGGGGGAAAA
TCCATGAT

TTGCCCTTTTTCTATCACGAGGTTACTGAGCCATTGCATGAACGCGCGCGCCTCGGCGGCTTTTTTTTCTA
CTCTATAAAAGCGAAAAGCCAGAAGTTACTATCTCGAATAAAAAACCCCTCGAACTGCCATCTCATTACCG
AAAATGAA

GACACTGGAAAAATGGCATAAAACTTTTCAAGGTTATTATTAGTAAGTTTTTTTATTATATGATATATATA
TATATAAAAATGAAATAAATTATTTCTTGATGGAGCCACTTATTTTTCTTATACCACCGGCGGCGCTTGTA
GTCCTTCT

TCTGTATACTTGTGCTCCCTTATAAAAAAAATTAAAAATTAAAAAATAAAAATAAGAATAGTGTATAAAAT
TATTTTTGCTTTGATTTTAGTTTATTTATTTACTTTCTAAATACAGTAGGTTAAATAAGGATCGGTTTCTT
GAAGAAAT

AGCGAGAAGGGACACTGGAAAAATGGCATAAAACTTTTCAAGGTTATTATTAGTAAGTTTTTTTATTATAT
GATATATATATATATAAAAATGAAATAAATTATTTCTTGATGGAGCCACTTATTTTTCTTATACCACCGGC
GGCGCTTG

TAAAGGATTTGGTCTGTAAGTCCATTTTTCATTTTTTCAAAATTAATAGTTTTTTCGGAATATACATATAC
ATTTTTTTACATACACATAAAAATATTTCTCTCCCTTTTACGTTCATCCGCCAATCGATAAAAATGCATTT
TATATACA

TCTTAACAACCTTTAACCTTCCGAGCTATTGATTTTTGATCCTCACTTCAGCGGCTAAAAAAAAAAAAAAC
ACCCCGAGTATTAGTGAATATAGATATATATATATATATATATATATATAGGGGGGCCGATAAAAAAGAAC
TTGCAATA

TCCATTTTTCATTTTTTCAAAATTAATAGTTTTTTCGGAATATACATATACATTTTTTTACATACACATAA
AAATATTTCTCTCCCTTTTACGTTCATCCGCCAATCGATAAAAATGCATTTTATATACATGAAATGGCGAA
TTCTTTTT

AAAAAGAACAACAAAAATAAGTAAAATAATATATAAACCTGTATAATATAACCTTGAAGACTATATTTCTT
TTCATCACGTGCTATAAAAATAATTATAATTTAAATTTTTTAATATAAATATATAAATTAAAAATAGAAAG
TAAAAAAA

TTTAGTTTAGTGCAGCCCACATACTACTTTTTTCCTTTTGTTTTTTTTTTTTATATTTCAAAGGTTAAACT
CGCTTAGACTATGTCTATAATATAAAAAAAAAAATAGCTCTATTCCGTTTCTTTTATTCTATTTGATATTTC
CATCACAC

AGAAAACAGTACTAGACAGAGCGCATAAAAAAAAGCCTAATCCCTTTTCCGTCTGTATACTTGTGCTCCCTT
ATAAAAAAAATTAAAAATTAAAAAATAAAAATAAGAATAGTGTATAAAATTATTTTTGCTTTGATTTTAGT
TTATTTAT

ACATTCACTTTCACTTATAAAATTTCTTCTCTCTAGTTTTATATACGTTGTATTAGAAATATAAAATATGA
CAATAGTACACTTTGTAGGCTCTCTTTTTTTTTTTTTTTTTTTTTCTTATATATTTTTTTACAATGACCAC
GGTTTGTA

GTTCCTTAATATTATATTCAATGAATAGCAGAGTAATAAAAAAATTCTAAAAAAAAAACGCTTAAATCGAG
GTGGAATATATAAACTTAAGTACGCATATACTCTAGTTCAGTTTAAAAATTAGCTGTTTTTTTAAAAAAAA
AAATTCTC

AATTTCTTCTCTCTAGTTTTATATACGTTGTATTAGAAATATAAAATATGACAATAGTACACTTTGTAGGC
TCTCTTTTTTTTTTTTTTTTTTTTTCTTATATATTTTTTTACAATGACCACGGTTTGTACACCTGGGGCTG
CAATTTTG

CTTCAAAAAATTCTAATTTAGCCTCGAAATTGAGAAATGAAATTAGATCATAAATTAACTCTAACAAAGGAG
AATATATATACGTACATATATATATATATATATATAAGTTATTGTAATTGAGAAAAAAATAAACATCATAT
AAAACTTC

TGGTATGATTCAATACAGAATGCCAAGGGAAACGTACAGCACTTTAATTAATATAGACGTCTCTCTATGAT
TTTTTTAAATATTGTATATTTTTGTTTATATCTATATATCAGTATGCGCGCATACTTAGTAAAAGTATAAA
AAGCCATT

TTGATGATTGAGGATGAAATACACTTTTATGATCTGTCCTTACGCGTAATAATTACAAGAAGGTGAGAAAA
TTGCCTTTTTTTGCTAATTTTTTTTTTTTTGGTACCATATTCAATAATTGATGCTAAATTATAAATTGATTT
TATTGTTT

AAATGGCATAAAACTTTTCAAGGTTATTATTAGTAAGTTTTTTATTATATGATATATATATATATATAAAAA
TGAAATAAATTATTTCTTGATGGAGCCACTTATTTTTCTTATACCACCGGCGGCGCTTGTAGTCCTTCTCC
CTGACGTT

AGTATTTGGCTTTTATTTTTTTTTTTTCATTTTCTTACTCGCGCTTTTCTTTATTAAGGGCTTTCTCAATT
AGATGTTAATAATGTCATAAGGCATCGTTATTCCGCTTTATATTGGTAACTCAGCAGTGGTCATTGCCTTC
GTTCACGA

AACTTTTTCTCTCCCGGAGAACAAAGTAAGTGGAAAAAATTATACTAATAACAATCTAAAAATGTTTATTA
GCTAGGATTATACACATAAATATATATATATGTGTGTGTCTATATTTACATATACTAGACCGCAAAAAAAA
CATAAGGG

AATGCATATAACCGTATCTTTTTTATATGAACCTAGTTTTGCTGTATAAGAGCCGAGAAAAAAAATATAGGA
AAATATAGGAAAATATGCAAAATAGAAACACGAATTTTTTTTTGTTAGAGAAAAAAAGAGATAAGCCCTG
TAGGGGCT

ACCTCACGGTTTTAGTTTAGTGCAGCCCACATACTACTTTTTTCCTTTTGTTTTTTTTTTTTATATTTCAA
AGGTTAAACTCGCTTAGACTATGTCTATAATATAAAAAAAAAATAGCTCTATTCCGTTTCTTTTATTCTAT
TTGATATT

GAAAGAAAAAAATTAAAAAAAAAAAAAAAAAATCAAGTAGTTCCTGCACTGCGACGTCCATTATAGCATTAT
AAATTGGTCCCTGATTTACGCATGCGATAAACTATTTTTAGCGCAGCCGCATATTATCCGAGAATAACTTC
CGACATAA

ACAAAAATAAGTAAAATAATATATAAACCTGTATAATATAACCTTGAAGACTATATTTCTTTTCATCACGT
GCTATAAAAATAATTATAATTTAAATTTTTTAATATAAATATATAAATTAAAAATAGAAAGTAAAAAAAGA
AATTAAAG

TACATAAAACAGTTTAATTTTGGGTGGTAATGGCTGTGTGACTAGCTATAGAAAGAAAAAAATTAAAAAAA
AAAAAAAAAATCAAGTAGTTCCTGCACTGCGACGTCCATTATAGCATTATAAATTGGTCCCTGATTTACGC
ATGCGATA

ATATCTAAATACCTCACGGTTTTAGTTTAGTGCAGCCCACATACTACTTTTTTCCTTTTGTTTTTTTTTTT
TATATTTCAAAGGTTAAACTCGCTTAGACTATGTCTATAATATAAAAAAAAAATAGCTCTATTCCGTTTCT
TTTATTCT

CCAATGGATAAACATTTTTTATCAACACTATGATATATAAATATAATAGTTTTTCGTATATATATTCCTTT
TTTTGGTCAATTTTTGAAATTTTCGTAGAAAAGGGAGAGACAAAACACATTATATCAATGAAAACGTACAA
AAAGTAGA

CTGGCTGGGGTTCCACCTCAAAGAGCCACGCTCTGCTTTTTTTCTATCTGTTTGTGTCATATCTATCTGTC
TATTTATCTATATATATATATTTTTTATATAAAACTATAAAGAATTCTTGATGTATGCCCTTAGGTTGGGCA
GCTTTTCA

TTCCACCTCAAAGAGCCACGCTCTGCTTTTTTTCTATCTGTTTGTGTCATATCTATCTGTCTATTTATCTA
TATATATATTTTTTATATAAAACTATAAAGAATTCTTGATGTATGCCCTTAGGTTGGGCAGCTTTTCAAC
CTTAGACT

TCTTAATGTATAAAGGATTTGGTCTGTAAGTCCATTTTTCATTTTTTCAAAATTAATAGTTTTTTCGGAAT
ATACATATACATTTTTTTACATACACATAAAAATATTTCTCTCCCTTTTACGTTCATCCGCCAATCGATAA
AAATGCAT

TGCAGCCCACATACTACTTTTTTCCTTTTGTTTTTTTTTTTTATATTTCAAAGGTTAAACTCGCTTAGACT
ATGTCTATAATATAAAAAAAAAATAGCTCTATTCCGTTTCTTTTATTCTATTTGATATTTCCATCACACTT
TCATCTTA

TAAAAAAAAAAAAAAAAAAATAAGGTATAAATCGTTGGTTCTTTTATGCACAATTATTTAACTATAGTTAT
CTATTTACGTAAAGGCTTCTATTTTTCCTTATCTACAAGAAATTGCATGAAGTTTAATTTTTTTTGTCACC
TTTGATCT

TGGGTGGTAATGGCTGTGTGACTAGCTATAGAAAGAAAAAAAATTAAAAAAAAAAAAAAAATCAAGTAGTT
CCTGCACTGCGACGTCCATTATAGCATTATAAATTGGTCCCTGATTTACGCATGCGATAAACTATTTTTAG
CGCAGCCG

CAAAAAAGCACCGAAACTAATCGAGAAAGCTTATAATATCGGTATACATTTGAATGTATTTTAACTATTTC
TATATCAAAAAAAAAAAAAAAAATTGTATATTTTTCGTTATTCTCTAATTCGTATCACATTTTATCCCTAA
GGGAATCT

CAAAATAAACAACTTTTTCTCTCCCGGAGAACAAAGTAAGTGGAAAAAATTATACTAATAACAATCTAAAA
ATGTTTATTAGCTAGGATTATACACATAAATATATATATATGTGTGTGTCTATATTTACATATACTAGACC
GCAAAAAA

AGGATGAAATACACTTTTATGATCTGTCCTTACGCGTAATAATTACAAGAAGGTGAGAAAATTGCCTTTTT
TTGCTAATTTTTTTTTTTTGGTACCATATTCAATAATTGATGCTAAATTATAAATTGATTTTATTGTTTTC
CTCTTTCC

AACTGAAAAGCGATGAAGAGATGATCATATGATACACGGTCCAATGGATAAACATTTTTTATCAACACTAT
GATATATAAATATAATAGTTTTTCGTATATATATTCCTTTTTTTGGTCAATTTTTGAAATTTTCGTAGAAA
AGGGAGAG

TGTGCTCCCTTATAAAAAAAATTAAAAATTAAAAAATAAAAATAAGAATAGTGTATAAAATTATTTTTGCT
TTGATTTTAGTTTATTTATTTACTTTCTAAATACAGTAGGTTAAATAAGGATCGGTTTCTTGAAGAAATTG
TAACAAGA

AGAGTATGTACGGATGTAAAAGAAAACAGTACTAGACAGAGCGCATAAAAAAAGCCTAATCCCTTTTCCGT
CTGTATACTTGTGCTCCCTTATAAAAAAAATTAAAAATTAAAAAATAAAAATAAGAATAGTGTATAAAATT
ATTTTTGC

TATGACTCTTTCGAAAAGACAGCGAGAAGGGACACTGGAAAAATGGCATAAAACTTTTCAAGGTTATTATT
AGTAAGTTTTTTTATTATATGATATATATATATATAAAAATGAAATAAATTATTTCTTGATGGAGCCACTT
ATTTTTCT

AAGAGCCACGCTCTGCTTTTTTTCTATCTGTTTGTGTCATATCTATCTGTCTATTTATCTATATATATATT
TTTTTATATAAAACTATAAAGAATTCTTGATGTATGCCCTTAGGTTGGGCAGCTTTTCAACCTTAGACTTG
ATGCTAAC

GTCAAAAGTTTTTTTGGCCATATGCAGTAAAAACTTACCCTTCAAATTGCAAAACCATTAGTTTTATTATA
TTTTTTTTTTTTTCTTTCATTGAAGTATACACGAAATCCCATACGCAAATAAACAGTCGTTGTATCATCAA
TTTCGGCT

CTCTTGTAATTACCTCATCTCATTATCAAGAAGCAAAATTTTAACTATGTATTTTGACCTTATTTTTGTAC
TCCACTTTTTTTTTTAGTTTTTTTTAGTTATTTTTTTAATTTTGAAGTAACGGCGATGAGGTGAAGTTTTT
TTTAAACT

CAATACAGAATGCCAAGGGAAACGTACAGCACTTTAATTAATATAGACGTCTCTCTATGATTTTTTTAAAT
ATTGTATATTTTTGTTTATATCTATATATCAGTATGCGCGCATACTTAGTAAAAGTATAAAAAGCCATTTG
CTGCTTCA

TTTTATTTTTTTTTTTTCATTTTCTTACTCGCGCTTTTCTTTATTAAGGGCTTTCTCAATTAGATGTTAAT
AATGTCATAAGGCATCGTTATTCCGCTTTATATTGGTAACTCAGCAGTGGTCATTGCCTTCGTTCACGACC
CTCCCTTA

AATTAAAAAAAAAAAAAAAAAATCAAGTAGTTCCTGCACTGCGACGTCCATTATAGCATTATAAATTGGTCC
CTGATTTACGCATGCGATAAACTATTTTTAGCGCAGCCGCATATTATCCGAGAATAACTTCCGACATAAGA
AAATTCGC

TACCTCATCTCATTATCAAGAAGCAAAATTTTAACTATGTATTTTGACCTTATTTTTGTACTCCACTTTTT
TTTTTTAGTTTTTTTTAGTTATTTTTTAATTTTGAAGTAACGGCGATGAGGTGAAGTTTTTTTTAAACTGA
AAAAGAAG

GATGTGTGATGTTCCTTAATATTATATTCAATGAATAGCAGAGTAATAAAAAAATTCTAAAAAAAAAACGC
TTAAATCGAGGTGGAATATATAAACTTAAGTACGCATATACTCTAGTTCAGTTTAAAAATTAGCTGTTTTT
TTAAAAAA

TGCCAAGGGAAACGTACAGCACTTTAATTAATATAGACGTCTCTCTATGATTTTTTTAAATATTGTATATT
TTTGTTTATATCTATATATCAGTATGCGCGCATACTTAGTAAAAGTATAAAAAGCCATTTGCTGCTTCATC
GAATATTT

TTATATCAATGAAAACGTACAAAAAGTAGATAAAGTCAGTGCTTAAACACGTCTTTTCCTTAAAAATACTT
TATTATTTTTATTTTATTGAGAGGGTGGTTTAAAAATAGAAATAGAGAGAGAGGTACATACATAAACATAC
GCGCACAA

ATACTACTTTTTTCCTTTTGTTTTTTTTTTTTATATTTCAAAGGTTAAACTCGCTTAGACTATGTCTATAA
TATAAAAAAAAAATAGCTCTATTCCGTTTCTTTTATTCTATTTGATATTTCCATCACACTTTCATCTTAAT
CACGGATG

ATCCGCATACGCACACGCACACGCACACGCGCACGCATCCACATTCACTTTCACTTATAAAATTTCTTCTC
TCTAGTTTTATATACGTTGTATTAGAAATATAAAATATGACAATAGTACACTTTGTAGGCTCTCTTTTTTT
TTTTTTTT

AAGATGAATGTCTCTTTGTTAATTTAGCTAGAGTTTTCCTTATTTATTTTTTTTTATTTACACTAATTCAAT
CTTTTTCGTTATATTTAGATATTATTAAAAATTATTATTATGGAAAAAATTCTTAAAAGGATAGACAGAGG
GACGCCGT

ATGATGAACCATGAAGAGCATGGTTAGATTGCTGCCAATTTTTTTTCCTATTTCGTCATTTTTTTATCAAG
ATTTTCCAGTTTTTTTTTTTTTTTTGGCATTGCTTACTATACGCGAGGGAGGTTCAATTGACACTTTCCGCT
ACGGCGAT

CATAAGGAGGAAAAAGAAAATTAAAAGAAAAAAAAAAAAGAAATAAAAAAAGAAAACGGTACTGGATATTGA
GATAAATTTTCCTTCAATTAAGATAATAAAACATGTTATATAAAATCAGACAAAATAATATGTAAATTTTT
AACGTATT

ATTAGAGGCATCTCCGACAATCAGAAGAACACTTCTTGTTATTATAATTACCCATATATTTTTTTTTTTTT
CAAATTGACTACTAGGAAAAAGTGACAATTTATAGTAAAACTGGCTAGAAAAACCTACAAGAAAAACAAAA
CCAGGTTA

GAACTGCTTCGACTTAATAAAACTAGCTCTTCAGAGGAAAAAGTGATTACCCTCGTACTTTTCATTTCATA
TATATATATATATATATATATGTACCTATATATTCTTCTTATTATGGCTTTTCTAATTCTTGCAAGATTCT
TTGACAGC

TCTAATTTAGCCTCGAAATTGAGAAATGAAATTAGATCATAAATTAACTCTAACAAAGGAGAATATATATA
CGTACATATATATATATATATATATATAAGTTATTGTAATTGAGAAAAAAAATAAACATCATATAAAACTTCCG
TTTTAATG

ACCGTATCTTTTTTATATGAACCTAGTTTTGCTGTATAAGAGCCGAGAAAAAAAATATAGGAAAATATAGGA
AAATATGCAAAATAGAAACACGAATTTTTTTTTTGTTAGAGAAAAAAAGAGATAAGCCCTGTAGGGGCTCG
AACCCCTA

ACTAGCTATAGAAAGAAAAAAATTAAAAAAAAAAAAAAAAATCAAGTAGTTCCTGCACTGCGACGTCCATT
ATAGCATTATAAATTGGTCCCTGATTTACGCATGCGATAAACTATTTTTAGCGCAGCCGCATATTATCCGA
GAATAACT

CCTCGAAATTGAGAAATGAAATTAGATCATAAATTAACTCTAACAAAGGAGAATATATATACGTACATATA
TATATATATATATATAAGTTATTGTAATTGAGAAAAAAATAAACATCATATAAAACTTCCGTTTTAATGAA
CGGGGGAA

TTTTATATGAACCTAGTTTTGCTGTATAAGAGCCGAGAAAAAAATATAGGAAAATATAGGAAAATATGCAA
AATAGAAACACGAATTTTTTTTTTGTTAGAGAAAAAAAGAGATAAGCCCTGTAGGGGCTCGAACCCCTAAC
CTTATGAT

TGATTCTTTTTTCCCTTTTTCTCAATATTCGCAGGTTATTTTTCCTTTATATATATACATTGTTTGTATCT
TTTTTTGATTACCATACTTACTATTTTTATATTAGTCATATTAGATGTCACTTTTATATTATCTAAACAAT
TCAAACTT

ATAATTATAATTTAAATTTTTTAATATAAATATATAAATTAAAAATAGAAAGTAAAAAAAGAAATTAAAGA
AAAAATAGTTTTTGTTTTCCGAAGATGTAAAATAGGTTGAAAGTTAGAAATTAGTATTATAATAGCAAAAA
AAATTTAA

GGTTACTGAGCCATTGCATGAACGCGCGCGCCTCGGCGGCTTTTTTTTCTACTCTATAAAAGCGAAAAGCC
AGAAGTTACTATCTCGAATAAAAAACCCCTCGAACTGCCATCTCATTACCGAAAATGAAAGAAAATGAACT
TAAAAATG

TTGCATATTACTTACGGCTTCAAAAAAGCACCGAAACTAATCGAGAAAGCTTATAATATCGGTATACATTT
GAATGTATTTTAACTATTTCTATATCAAAAAAAAAAAAAAAAATTGTATATTTTTCGTTATTCTCTAATTC
GTATCACA

CCATTGCATGAACGCGCGCGCCTCGGCGGCTTTTTTTTCTACTCTATAAAAGCGAAAAGCCAGAAGTTACT
ATCTCGAATAAAAAACCCCTCGAACTGCCATCTCATTACCGAAAATGAAAGAAAATGAACTTAAAAATGAG
AAAAGTGT

CGATGAAGAGATGATCATATGATACACGGTCCAATGGATAAACATTTTTTATCAACACTATGATATATAAA
TATAATAGTTTTTCGTATATATATTCCTTTTTTTGGTCAATTTTTGAAATTTTCGTAGAAAAGGGAGAGAC
AAAACACA

ACTTTAATTAATATAGACGTCTCTCTATGATTTTTTTAAATATTGTATATTTTTGTTTATATCTATATATC
AGTATGCGCGCATACTTAGTAAAAGTATAAAAAGCCATTTGCTGCTTCATCGAATATTTTGGCTTTCGTTG
TACAAAGC

TTTGGACGGCGCGGCCGACACATTTTGCTGTTTCCACTCCGCGGGGATTAAAGACGCCAGTTCGTGCAGCG
CGACCGCTAATCGATTACGCCGTGCTTGCTCTGCATGCTTGTGTGATTCGCGCTTGTCATCGTCCACGAGC
GCGCCCGA

TTATGTTGATATCCAGCTTCCGTTCGTCAACTATTCTCTCGAGCTCAGTTTTGGGTTTTGGCATGTAAACA
ATGCCTTCGCTTGTATTACGCAAAAAAAAAAAAATAAATAAAAAAAAAAAAAAAAAAAATAAGGTATAAAT
CGTTGGTT

TTTTTGGCCATATGCAGTAAAAACTTACCCTTCAAATTGCAAAACCATTAGTTTTATTATATTTTTTTTT
TTTCTTTCATTGAAGTATACACGAAATCCCATACGCAAATAAACAGTCGTTGTATCATCAATTTCGGCTGT
CTGGATGG

AAAAGTACTCGCTTCTTTCCATGTCCGCTTCATATATATATACACATACTAATCAAACTCTATGTATACAT
AGAATAAAAAGAAGAACACTATATTTATTTCATAAAAAAAAAAAAAAATAACAAAAAAAAGTGCAACATTT
ATCAAAAG

TTAAAAGAAAAAAAAAAAGAAATAAAAAAAGAAAACGGTACTGGATATTGAGATAAATTTTCCTTCAATTA
AGATAATAAAACATGTTATATAAAATCAGACAAAATAATATGTAAATTTTTAACGTATTATAATCTTAAAA
AGTTTATT

CATTAGACAGAGACTTTTTCCGATCTAAATAATGCATATAACCGTATCTTTTTTATATGAACCTAGTTTTG
CTGTATAAGAGCCGAGAAAAAAAATATAGGAAAATATAGGAAAATATGCAAAATAGAAACACGAATTTTTT
TTTGTTAG

AAAATTCTAAAAAAAAAACGCTTAAATCGAGGTGGAATATATAAACTTAAGTACGCATATACTCTAGTTCA
GTTTAAAAATTAGCTGTTTTTTTAAAAAAAAAAATTCTCTTTCTTTACAAAATGTTCTTAAATGTATGTAT
GTGTGTGT

TATAAAAAAATTAAAAATTAAAAAAATAAAAATAAGAATAGTGTATAAAATTATTTTTGCTTTGATTTTAG
TTTATTTATTTACTTTCTAAATACAGTAGGTTAAATAAGGATCGGTTTCTTGAAGAAATTGTAACAAGACT
CAGAATAA

TTTTTTTGTGACATATTAAAATTGCGAAATTGCAAACGAAAATATAAATATATATGAATGTTAATAAATAA
ATTATTTAATTGATGATTGAGGATGAAATACACTTTTATGATCTGTCCTTACGCGTAATAATTACAAGAAG
GTGAGAAA

TTGAACTTTAAGTATTTGGCTTTTATTTTTTTTTTTTCATTTTCTTACTCGCGCTTTTCTTTATTAAGGGC
TTTCTCAATTAGATGTTAATAATGTCATAAGGCATCGTTATTCCGCTTTATATTGGTAACTCAGCAGTGGT
CATTGCCT

CGATCTAAATAATGCATATAACCGTATCTTTTTTATATGAACCTAGTTTTGCTGTATAAGAGCCGAGAAAA
AAATATAGGAAAATATAGGAAAATATGCAAAATAGAAACACGAATTTTTTTTTGTTAGAGAAAAAAAAGAG
ATAAGCCC

ATGTCCGCTTCATATATATATACACATACTAATCAAACTCTATGTATACATAGAATAAAAAGAAGAACACT
ATATTTATTTCATAAAAAAAAAAAAAAAATAACAAAAAAAGTGCAACATTTATCAAAAGCTCAGTGTGCGT
TATGCTTC

GTGCATTGGCATTAGAGGCATCTCCGACAATCAGAAGAACACTTCTTGTTATTATAATTACCCATATATTT
TTTTTTTTTTCAAATTGACTACTAGGAAAAAGTGACAATTTATAGTAAAACTGGCTAGAAAAACCTACAAG
AAAAACAA

ATAAATCGCTTGATTCTTTTTTCCCTTTTTCTCAATATTCGCAGGTTATTTTTCCTTTATATATATACATT
GTTTGTATCTTTTTTTGATTACCATACTTACTATTTTTATATTAGTCATATTAGATGTCACTTTTATATTA
TCTAAACA

GGTCTGTAAGTCCATTTTTCATTTTTTCAAAATTAATAGTTTTTTCGGAATATACATATACATTTTTTTAC
ATACACATAAAAATATTTCTCTCCCTTTTACGTTCATCCGCCAATCGATAAAAATGCATTTTATATACATG
AAATGGCG

CTCTTCGACTACTTTCTGCAAGTAAATCATTCGGAAGTGCGACAATATAATGACAAAAGACGATAAGTTAC
TTCTATTAATTGAACTTTAAGTATTTGGCTTTTATTTTTTTTTTTTCATTTTCTTACTCGCGCTTTTCTTT
ATTAAGGG

TGGCTGTGTGACTAGCTATAGAAAGAAAAAAATTAAAAAAAAAAAAAAAAAATCAAGTAGTTCCTGCACTGC
GACGTCCATTATAGCATTATAAATTGGTCCCTGATTTACGCATGCGATAAACTATTTTTAGCGCAGCCGCA
TATTATCC

GACTTAATAAAACTAGCTCTTCAGAGGAAAAAGTGATTACCCTCGTACTTTTCATTTCATATATATATATA
TATATATATATGTACCTATATATTCTTCTTATTATGGCTTTTCTAATTCTTGCAAGATTCTTTGACAGCTC
CATCTTTC

CTCTGCTTTTTTTCTATCTGTTTGTGTCATATCTATCTGTCTATTTATCTATATATATATTTTTTTATATA
AAACTATAAAGAATTCTTGATGTATGCCCTTAGGTTGGGCAGCTTTTCAACCTTAGACTTGATGCTAACGC
CGCTCTGT

ATGATCATATGATACACGGTCCAATGGATAAACATTTTTTATCAACACTATGATATATAAATATAATAGTT
TTTCGTATATATATTCCTTTTTTTGGTCAATTTTTGAAATTTTCGTAGAAAAGGGAGAGACAAAACACATT
ATATCAAT

CATTATCAAGAAGCAAAATTTTAACTATGTATTTTGACCTTATTTTTGTACTCCACTTTTTTTTTTTAGTT
TTTTTTAGTTATTTTTTAATTTTGAAGTAACGGCGATGAGGTGAAGTTTTTTTAAACTGAAAAAGAAGAT
CGAATGCA

AACATTTTTTATCAACACTATGATATATAAATATAATAGTTTTTCGTATATATATTCCTTTTTTTGGTCAA
TTTTTGAAATTTTCGTAGAAAAGGGAGAGACAAAACACATTATATCAATGAAAACGTACAAAAAGTAGATA
AAGTCAGT

CTTGATAATTCTGTGGGCGCTTTTTTTGTGACATATTAAAATTGCGAAATTGCAAACGAAAATATAAATAT
ATATGAATGTTAATAAATAAATTATTTAATTGATGATTGAGGATGAAATACACTTTTATGATCTGTCCTTA
CGCGTAAT

AACTAGCTCTTCAGAGGAAAAAGTGATTACCCTCGTACTTTTCATTTCATATATATATATATATATATATA
TGTACCTATATATTCTTCTTATTATGGCTTTTCTAATTCTTGCAAGATTCTTTGACAGCTCCATCTTTCGT
TTGGTGAA

AAATTAGGGACTTCAAAAATTCTAATTTAGCCTCGAAATTGAGAAATGAAATTAGATCATAAATTAACTCT
AACAAAGGAGAATATATATACGTACATATATATATATATATATATAAGTTATTGTAATTGAGAAAAAAATA
AACATCAT

TTAATAAATAAATTATTTAATTGATGATTGAGGATGAAATACACTTTTATGATCTGTCCTTACGCGTAATA
ATTACAAGAAGGTGAGAAAATTGCCTTTTTTTGCTAATTTTTTTTTTTTGGTACCATATTCAATAATTGAT
GCTAAATT

TTTCCTTTTGTTTTTTTTTTTTTATATTTCAAAGGTTAAACTCGCTTAGACTATGTCTATAATATAAAAAAA
AAATAGCTCTATTCCGTTTCTTTTATTCTATTTGATATTTCCATCACACTTTCATCTTAATCACGGATGTA
TACTGATA

AGTTTCTGTGCCTAAAATAGCCCAACATTGTTGCCCTTTTTCTATCACGAGGTTACTGAGCCATTGCATGA
ACGCGCGCGCCTCGGCGGCTTTTTTTTCTACTCTATAAAAGCGAAAAGCCAGAAGTTACTATCTCGAATAA
AAAACCCC

GACACATAAGAAACAGATTTTTCACGAATTGAAAAGGCAAAATTCAAAAAATTAGGACTAGGCGATATAGA
TTTGTATATAATTTTATATAAAAGGAAATATGTACATCATTTTCTTTCACTGTAGGCAGCTGTTTCAAGTT
CTTTTTTT

TGAAAATATGGAAAGTACAAGTTTTTAGTTGAGAAGGGTTTAAGAAAGTTTTGAAAATGATCTAAAAAAAT
ATAAAAGCAATCAAAGAAATAAAAGCTGGAAAAATGCGTAATAACCGAAGTGACTAAAATTTCTTTACGCG
CCAAATAA

AGCTTACCCGCTTCCCCACACATTTTGGTAGTATCTGTCCTCTTGTTATTGTTACTGTAATTGTGTATATA
TGTTCTCGCGTGTGTCTTATTTACTTATTTAGTTATTATATTATATGGGTCTGCAAGGTAGAGGCGCGCTT
GTTCAACA

TAGCTCGGCTGCACCATTCTAAACGCGGGGGGAGTTTCTTTTTTTTTTTTTTTCAGCCTTTTCTTCGTAAG
CAAAGTATCCCACGTAAAAGGTTCCTTATACTAACACATATGTACTGTAAAGGTAAAATTACTTCTATTTT
TTCTACTG

GAATCTGAATTTATAGATCCCCCAAAAAAAAAAAGTACTCGCTTCTTTCCATGTCCGCTTCATATATATAT
ACACATACTAATCAAACTCTATGTATACATAGAATAAAAAGAAGAACACTATATTTATTTCATAAAAAAAA
AAAAAAAA

ATTTATACCATTCCCTACTGAAGTATGTAATTCAAAATATATATATGTATATGTATATGTACATATATATG
TATATACTATCTTAACAACCTTTAACCTTCCGAGCTATTGATTTTTGATCCTCACTTCAGCGGCTAAAAAA
AAAAAAAA

CCCAACATTGTTGCCCTTTTTCTATCACGAGGTTACTGAGCCATTGCATGAACGCGCGCGCCTCGGCGGCT
TTTTTTTCTACTCTATAAAAGCGAAAAGCCAGAAGTTACTATCTCGAATAAAAAACCCCTCGAACTGCCAT
CTCATTAC

CATAACAAATTTGCATATTACTTACGGCTTCAAAAAAGCACCGAAACTAATCGAGAAAGCTTATAATATCG
GTATACATTTGAATGTATTTTAACTATTTCTATATCAAAAAAAAAAAAAAAAAATTGTATATTTTTCGTTAT
TCTCTAAT

TGCAAACGAAAATATAAATATATATGAATGTTAATAAATAAATTATTTAATTGATGATTGAGGATGAAATA
CACTTTTATGATCTGTCCTTACGCGTAATAATTACAAGAAGGTGAGAAAATTGCCTTTTTTTGCTAATTTT
TTTTTTTT

TACGGTGTTTTTATGATCCCAGATGTTCAAAAAAAATCAATAATTATCAATTAATCGTGTACTTTAATAAT
CCATATATGAATCATTTAGGTAATTAGCTCTTTTTTGCTTGAAAAATTCATCCATTCGGCTCGCGTTGTCT
AAAAAAAA

ACTGTAAGCACACAGAGCTATGGGATGTGTGGCAATGACTGTATATATATGTATATATATATATATATATA
TATATATATTTATTTATTTATTTATTTAAAATATAGCTACGTATGCGCCAGTTGTTTTTATCAGCTTTGCT
TCTGGCCA

TTTTTAATGAACTGTAAGCACACAGAGCTATGGGATGTGTGGCAATGACTGTATATATATGTATATATATA
TATATATATATATATATATTTATTTATTTATTTATTTAAAATATAGCTACGTATGCGCCAGTTGTTTTTAT
CAGCTTTG

ATATTTTTTGTTTTTAATGAACTGTAAGCACACAGAGCTATGGGATGTGTGGCAATGACTGTATATATATG
TATATATATATATATATATATATATATATTTATTTATTTATTTATTTAAAATATAGCTACGTATGCGCCAG
TTGTTTTT

CACAGAGCTATGGGATGTGTGGCAATGACTGTATATATATGTATATATATATATATATATATATATATATT
TATTTATTTATTTATTTAAAATATAGCTACGTATGCGCCAGTTGTTTTTATCAGCTTTGCTTCTGGCCACA
TTTCAACC

AATACAAAGGAGAGTAAAGATGTGAAAATGAATTAAAAGGAAAAGTATTATGCATATATATATATATATAT
ATATATATATATTTATATATTTATACACATAGAATTATTTTACAGTAAAGGATGCTACACCACCCCGGGCT
AACTTTAT

ATACAATACATGTCATTTTTAACAATGATGCAAATAACTTTTAAGGCCTTAACACTCTACATAATATGCTA
AAAACATTAAAAAAAAAAAAAAGGAAAATAAAAAAAAAAAAAAAAAAAAAAAACGGGAGATTAACCGAATAGC
AAACTCTT

CCGAACTCTTATACAATACATGTCATTTTTAACAATGATGCAAATAACTTTTAAGGCCTTAACACTCTACA
TAATATGCTAAAAACATTAAAAAAAAAAAAAAGGAAAATAAAAAAAAAAAAAAAAAAAAAAAACGGGAGATTA
ACCGAATA

TGTCATTTTTAACAATGATGCAAATAACTTTTAAGGCCTTAACACTCTACATAATATGCTAAAAACATTAA
AAAAAAAAAAAGGAAAATAAAAAAAAAAAAAAAAAAAAAAAACGGGAGATTAACCGAATAGCAAACTCTTAA
ACTATGAA

AGAGTAAAGATGTGAAAATGAATTAAAAGGAAAAGTATTATGCATATATATATATATATATATATATATAT
ATTTATATATTTATACACATAGAATTATTTTACAGTAAAGGATGCTACACCACCCCGGGCTAACTTTATGG
CTTCTACT

TGTACTCCAGTATGGACCTCTTAGGTGAGTGATCTTATTAAAAAAAATAAAAAAAATAAAAAAATATGAAA
AAAAAAGAAAATTAAAAACTAAACAAATTTTATGACAGGAATAAAAACTATAAAAAATGAAAAGCAAAAAA
AAAAAGGC

TATGGACCTCTTAGGTGAGTGATCTTATTAAAAAAAATAAAAAAAATAAAAAAATATGAAAAAAAAAGAAA
ATTAAAAACTAAACAAATTTTATGACAGGAATAAAAACTATAAAAAATGAAAAGCAAAAAAAAAAAGGCTA
CTGGATTT

GATTATAGACTGTGAATATTGCATATGCAACTTTGACTCAAATTTTTCCAAAATTTGATATATATATATAT
ATATATATGTTTGTATGTATATATATATATACGTATATATATCATATATACGAAAAGTAGAAAAAAAAAAGG
TGATATTT

GATCTTATTAAAAAAAATAAAAAAAATAAAAAAATATGAAAAAAAAAGAAAATTAAAAACTAAACAAATTT
TATGACAGGAATAAAAACTATAAAAAATGAAAAGCAAAAAAAAAAAGGCTACTGGATTTTTAACATCTTGT
CCTTTTCT

GAACAATGATAATACAAAGGAGAGTAAAGATGTGAAAATGAATTAAAAGGAAAAGTATTATGCATATATAT
ATATATATATATATATATATATTTATATATTTATACACATAGAATTATTTTACAGTAAAGGATGCTACACC
ACCCCGGG

TTAGGTGAGTGATCTTATTAAAAAAAATAAAAAAAATAAAAAAATATGAAAAAAAAAGAAAATTAAAAACT
AAACAAATTTTATGACAGGAATAAAAACTATAAAAAATGAAAAGCAAAAAAAAAAAGGCTACTGGATTTTT
AACATCTT

TTCCTGAACTCGCATTTTTTTTAATGATTTTTTATGACCTCTTATATATTCTTTCATTATATAACCTCTATA
TTACATCAAAAGATGGAAAAATATAAAAAAATTAAAAAAAAAAAAAAGAAATTTTTTCCAAAAGAGTATAT
TTATATGT

TTCGTATAATTGGTACATAGGTATATATCAAATAAAAATATGCATTATTCTAAAAAAAAAAAAAAAAAAAA
AAAAAAAAAAATCAAAAATGTCTCTCTAAAGGGTGATTATAAAAAACTTGGTTTGGGCAAGTTTTTAGCCT
CCCTTGGA

AAAAAAATAAAAAAAATAAAAAAATATGAAAAAAAAAGAAAATTAAAAACTAAACAAATTTTATGACAGGA
ATAAAAACTATAAAAAATGAAAAGCAAAAAAAAAAAGGCTACTGGATTTTTAACATCTTGTCCTTTTCTTT
CCCTTTAT

TTTTTTTTTCTTTTTCACCGTATTTTTGTGCGTCCTTTTTCAATTATAGCTTTTTTTTTATTTTTTTTTTT
TCTCGTACTGTTTCACTGACAAAAGTTTTTTTTTCAAGAAAAATTTTCGATGCCGCGTTCTCTGTGTGCAA
CGGATGGA

CATCGAAGAGCCGAACTCTTATACAATACATGTCATTTTTAACAATGATGCAAATAACTTTTAAGGCCTTA
ACACTCTACATAATATGCTAAAAACATTAAAAAAAAAAAAAAGGAAAATAAAAAAAAAAAAAAAAAAAAAA
CGGGAGAT

TGTGAATATTGCATATGCAACTTTGACTCAAATTTTTCCAAAATTTGATATATATATATATATATATATGT
TTGTATGTATATATATATATACGTATATATATCATATATACGAAAAGTAGAAAAAAAAAAGGTGATATTTCG
CTCGTGGA

GCATATGCAACTTTGACTCAAATTTTTCCAAAATTTGATATATATATATATATATATATATGTTTGTATGTAT
ATATATATATACGTATATATATCATATATACGAAAAGTAGAAAAAAAAAAGGTGATATTTCGCTCGTGGAAA
AGCTAATG

CTGCGGGAGTGAACAATGATAATACAAAGGAGAGTAAAGATGTGAAAATGAATTAAAAGGAAAAGTATTAT
GCATATATATATATATATATATATATATATATATTTATATATTTATACACATAGAATTATTTTACAGTAAAGG
ATGCTACA

GGATGACCAGATCCAAAACAGACATAAGCATTCGTATAATTGGTACATAGGTATATATCAAATAAAAATAT
GCATTATTCTAAAAAAAAAAAAAAAAAAAAAAAAAAAAAAATCAAAAATGTCTCTCTAAAGGGTGATTATA
AAAAACTT

AATATAAAAAAAATTAAAAAAAAAAAAAGAAATTTTTTCCAAAAGAGTATATTTATATGTATGTATACATG
TAGGGAAAATAAGAAACTTTATTAATAGTAAAAAAGCATATATACTTTATTATTAACTCTTTTGTTTTTCT
CGAGAAGC

GAGAAACGATTTTTTTTTTCTTTTTCACCGTATTTTTGTGCGTCCTTTTTCAATTATAGCTTTTTTTTTAT
TTTTTTTTTTTCTCGTACTGTTTCACTGACAAAAGTTTTTTTTTCAAGAAAAATTTTCGATGCCGCGTTCT
CTGTGTGC

ATCCAAAACAGACATAAGCATTCGTATAATTGGTACATAGGTATATATCAAATAAAAATATGCATTATTCT
AAAAAAAAAAAAAAAAAAAAAAAAAAAAAAATCAAAAATGTCTCTCTAAAGGGTGATTATAAAAAACTTGG
TTTGGGCA

TCAGTTAGATCTTCTATTATTTCCTTTTTTTTCTTTTTGTTTGTAATTGTTTTTTATTTTTTTTTTTGCGC
GAAACTTTGCTATATTGGGTAACGCGTAAAATACTTTTTATTATTGCAGTAAGGCGGAAGGGTCTTCCCCT
TTGCATGT

AGAAAAAATGTGAACAAAAATAGTGTCATCTCCAGTAAGGCTTAAATGTATATTGATTTTCTATATATATA
TATATATATATATATTTATGAAATAAACCATATAATTATAAGTAGTAGCTTGGAAAAACGCCAAAGTTG
TACTTAGA

CACAGTGCCCTTATGATCAGGAACTGTTTAAAAACTTTTTTTTTTTTTTTTTTTTTTTTTTAAAATCTAAA
TATGTTGCTTGAATTATAAATACAATAACGGGAAAACATATGATGTGTCATGTTCAAAAAGCTTTGAATAG
GTGTCTTC

AACAATGATGCAAATAACTTTTAAGGCCTTAACACTCTACATAATATGCTAAAAACATTAAAAAAAAAAAA
AGGAAAATAAAAAAAAAAAAAAAAAAAAAAAACGGAGATTAACCGAATAGCAAACTCTTAAACTATGAAAA
GGCAATCA

AAGATGGAAAAATATAAAAAAAATTAAAAAAAAAAAAAGAAATTTTTTCCAAAAGAGTATATTTATATGTA
TGTATACATGTAGGGAAAATAAGAAACTTTATTAATAGTAAAAAAGCATATATACTTTATTATTAACTCTT
TTGTTTTT

AAAAAAAATTGTCATCTGAGAAATTGCTTTGGATGACCAGATCCAAAACAGACATAAGCATTCGTATAATT
GGTACATAGGTATATATCAAATAAAAATATGCATTATTCTAAAAAAAAAAAAAAAAAAAAAAAAAAAAAAA
TCAAAAAT

TTATTTACATCACAGTGCCCTTATGATCAGGAACTGTTTAAAAACTTTTTTTTTTTTTTTTTTTTTTTTTT
AAAATCTAAATATGTTGCTTGAATTATAAATACAATAACGGGAAAACATATGATGTGTCATGTTCAAAAAG
CTTTGAAT

TGGTACATAGGTATATATCAAATAAAAATATGCATTATTCTAAAAAAAAAAAAAAAAAAAAAAAAAAAAAA
ATCAAAAATGTCTCTCTAAAGGGTGATTATAAAAAACTTGGTTTGGGCAAGTTTTTAGCCTCCCTTGGACC
ATTAGTGG

GACATAAGCATTCGTATAATTGGTACATAGGTATATATCAAATAAAAATATGCATTATTCTAAAAAAAAAA
AAAAAAAAAAAAAAAAAAAAATCAAAAATGTCTCTCTAAAGGGTGATTATAAAAAACTTGGTTTGGGCAAG
TTTTTAGC

AAATTGCTTTGGATGACCAGATCCAAAACAGACATAAGCATTCGTATAATTGGTACATAGGTATATATCAA
ATAAAAATATGCATTATTCTAAAAAAAAAAAAAAAAAAAAAAAAAAAAAATCAAAAATGTCTCTCTAAAG
GGTGATTA

CTTTTTTTATTGTACTCCAGTATGGACCTCTTAGGTGAGTGATCTTATTAAAAAAAATAAAAAAAAATAAAA
AAATATGAAAAAAAAAGAAAATTAAAAACTAAACAAATTTTATGACAGGAATAAAAACTATAAAAAAATGAA
AAGCAAAA

TCGCGACTCGATAACGACGTGAGAAACGATTTTTTTTTTCTTTTTCACCGTATTTTTGTGCGTCCTTTTTC
AATTATAGCTTTTTTTTTATTTTTTTTTTTTCTCGTACTGTTTCACTGACAAAAGTTTTTTTTTCAAGAAA
AATTTTCG

CTTCTATTATTTCCTTTTTTTTTCTTTTTGTTTGTAATTGTTTTTTATTTTTTTTTTTGCGCGAAACTTTGC
TATATTGGGTAACGCGTAAAATACTTTTTATTATTGCAGTAAGGCGGAAGGGTCTTCCCCTTTGCATGTTA
AATAGCAT

TTTCATTATATAACCTCTATATTACATCAAAAGATGGAAAAATATAAAAAAATTAAAAAAAAAAAAAAAGAA
ATTTTTTCCAAAAGAGTATATTTATATGTATGTATACATGTAGGGAAAATAAGAAACTTTATTAATAGTAA
AAAAGCAT

CGATAATATGTTCCTGAACTCGCATTTTTTTAATGATTTTTTATGACCTCTTATATATTCTTTCATTATAT
AACCTCTATATTACATCAAAAGATGGAAAAATATAAAAAAATTAAAAAAAAAAAAAAAGAAATTTTTTCCAA
AAGAGTAT

AAAAAAATTGAAAAATCATAAATTAAAAAAAAAAAAAATCAATTGAATTTTTTTTTTTTCATGATTACGTTTT
GACATTTTTCCTTTTTTTTTCTCTTATTACGATTTACCTTTTTTATTTATTTTTTTCATTTTAGTATTTTA
TTCTTCGT

GCTGATATGTTTATTTACATCACAGTGCCCTTATGATCAGGAACTGTTTAAAAACTTTTTTTTTTTTTTT
TTTTTTTTTTAAAATCTAAATATGTTGCTTGAATTATAAATACAATAACGGGAAAACATATGATGTGTCAT
GTTCAAAA

ATAACGACGTGAGAAACGATTTTTTTTTTCTTTTTCACCGTATTTTTGTGCGTCCTTTTTCAATTATAGCT
TTTTTTTTATTTTTTTTTTTTTCTCGTACTGTTTCACTGACAAAAGTTTTTTTTTCAAGAAAAATTTTCGAT
GCCGCGTT

GTCATCTGAGAAATTGCTTTGGATGACCAGATCCAAAACAGACATAAGCATTCGTATAATTGGTACATAGG
TATATATCAAATAAAAATATGCATTATTCTAAAAAAAAAAAAAAAAAAAAAAAAAAAAAATCAAAAATGT
CTCTCTAA

AATACAAAAAACTATTTTCTTTTCGGAAGGCAAAAACAAAAATTATAATAAAAATAAAAATATGTATAATA
TAATTTTTATCTTCTAACTTTTTATTTATTGAATTTTTATATAGTAAATATAACACGTGATATATTTGAAA
TGTGATTA

ACTACCATAAAAAAAAAATTGAAAAATCATAAATTAAAAAAAAAAAAAATCAATTGAATTTTTTTTTTTTCATG
ATTACGTTTTGACATTTTTCCTTTTTTTTTCTCTTATTACGATTTACCTTTTTTATTTATTTTTTTCATTT
TAGTATTT

TTAAGGCCTTAACACTCTACATAATATGCTAAAAACATTAAAAAAAAAAAAAAGGAAAATAAAAAAAAAAAA
AAAAAAAAAAACGGGAGATTAACCGAATAGCAAACTCTTAAACTATGAAAAGGCAATCAATCCACTGTTTT
CTTTTTCC

CACTTATTGAAGTCCACATAGATACACACAGATAAACATATATATATTATGTATATATATATATATATATA
TATATATATATATATGTATATATCAACGCCCATACCATTATCATTATCAATAAGTTTGCGAGGCGGGTACC
CTCCCTCG

TGGGATGTGTGGCAATGACTGTATATATATGTATATATATATATATATATATATATATATATTTATTTATTTA
TTTATTTAAAATATAGCTACGTATGCGCCAGTTGTTTTTATCAGCTTTGCTTCTGGCCACATTTCAACCAG
GTCTCAGA

CAAAACTGCAACTACCATAAAAAAAAAATTGAAAAATCATAAATTAAAAAAAAAAAAAATCAATTGAATTTTT
TTTTTTCATGATTACGTTTTGACATTTTTCCTTTTTTTTTCTCTTATTACGATTTACCTTTTTTATTTATT
TTTTTCAT

AATTAAAAAAAAAAAAAATCAATTGAATTTTTTTTTTTCATGATTACGTTTTGACATTTTTCCTTTTTTTTT
CTCTTATTACGATTTACCTTTTTTATTTATTTTTTTCATTTTAGTATTTTATTCTTCGTTATTTATGTATA
GAAATTTT

CAAATAACTTTTAAGGCCTTAACACTCTACATAATATGCTAAAAACATTAAAAAAAAAAAAAAGGAAAATAA
AAAAAAAAAAAAAAAAAAAAACGGGAGATTAACCGAATAGCAAACTCTTAAACTATGAAAAGGCAATCAAT
CCACTGTT

AAAAATCATAAATTAAAAAAAAAAAAAATCAATTGAATTTTTTTTTTTCATGATTACGTTTTGACATTTTTC
CTTTTTTTTTCTCTTATTACGATTTACCTTTTTTATTTATTTTTTTCATTTTAGTATTTTATTCTTCGTTA
TTTATGTA

AAAAAATAAAAAAATATGAAAAAAAAAGAAAATTAAAAACTAAACAAATTTTATGACAGGAATAAAAACTA
TAAAAAATGAAAAGCAAAAAAAAAAAAGGCTACTGGATTTTTAACATCTTGTCCTTTTCTTTCCCTTTATCA
TTTTTTCT

TATGATGGGCGCTATCCTCATCGCGACTCGATAACGACGTGAGAAACGATTTTTTTTTTCTTTTTCACCGT
ATTTTTGTGCGTCCTTTTTCAATTATAGCTTTTTTTTTATTTTTTTTTTTTCTCGTACTGTTTCACTGACA
AAAGTTTT

TTATGATCAGGAACTGTTTAAAAACTTTTTTTTTTTTTTTTTTTTTTTTTTAAAATCTAAATATGTTGCTT
GAATTATAAATACAATAACGGGAAAACATATGATGTGTCATGTTCAAAAAGCTTTGAATAGGTGTCTTCCA
TCAAAAGG

AACACTCTACATAATATGCTAAAAACATTAAAAAAAAAAAAAAGGAAAATAAAAAAAAAAAAAAAAAAAAA
ACGGGAGATTAACCGAATAGCAAACTCTTAAACTATGAAAAGGCAATCAATCCACTGTTTTCTTTTTCCAT
TTCTTCTA

GCTATCCTCATCGCGACTCGATAACGACGTGAGAAACGATTTTTTTTTTCTTTTTCACCGTATTTTTGTGC
GTCCTTTTTCAATTATAGCTTTTTTTTTATTTTTTTTTTTTCTCGTACTGTTTCACTGACAAAAGTTTTTT
TTTCAAGA

TGTTTGCTTGATATTTTTTGTTTTTAATGAACTGTAAGCACACAGAGCTATGGGATGTGTGGCAATGACTG
TATATATATGTATATATATATATATATATATATATATATATTTATTTATTTATTTATTTAAAATATAGCTACG
TATGCGCC

TGTGAAAATGAATTAAAAGGAAAAGTATTATGCATATATATATATATATATATATATATATATATTTATATAT
TTATACACATAGAATTATTTTACAGTAAAGGATGCTACACCACCCCGGGCTAACTTTATGGCTTCTACTAT
TTCAGGTT

AATTAAAAAAAAAAAAAAGAAATTTTTTCCAAAAGAGTATATTTATATGTATGTATACATGTAGGGAAAAT
AAGAAACTTTATTAATAGTAAAAAAGCATATATACTTTATTATTAACTCTTTTGTTTTTCTCGAGAAGCTT
AATTTTGG

TAAAAAAAAAAAAAAAAAAAAAAAAAAAAAAAATCAAAAATGTCTCTCTAAAGGGTGATTATAAAAAACTTG
GTTTGGGCAAGTTTTTAGCCTCCCTTGGACCATTAGTGGCGAAAGTGGAGCCTGCATAAGAGTGACTGAGT
TTTTTGGC

TGAACAAAAATAGTGTCATCTCCAGTAAGGCTTAAATGTATATTGATTTTCTATATATATATATATATATA
TATATATTTATGAAATAAACCATATAATTATAAGTAGTAGCTTGGAAAAACGCCAAAGTTGTACTTAGAAT
GAGGTTTT

AATAAAAATATGCATTATTCTAAAAAAAAAAAAAAAAAAAAAAAAAAAAAATCAAAAATGTCTCTCTAAA
GGGTGATTATAAAAAACTTGGTTTGGGCAAGTTTTTAGCCTCCCTTGGACCATTAGTGGCGAAAGTGGAGC
CTGCATAA

TAACCTCTATATTACATCAAAAGATGGAAAAATATAAAAAAATTAAAAAAAAAAAAAAAGAAATTTTTTCCA
AAAGAGTATATTTATATGTATGTATACATGTAGGGAAAATAAGAAACTTTATTAATAGTAAAAAAGCATAT
ATACTTTA

CGCATTTTTTTAATGATTTTTTATGACCTCTTATATATTCTTTCATTATATAACCTCTATATTACATCAAA
AGATGGAAAAATATAAAAAAATTAAAAAAAAAAAAAAAGAAATTTTTTCCAAAAGAGTATATTTATATGTAT
GTATACAT

TAAGTTCTTTTAAGTATCACATTACAATAACAAAACTGCAACTACCATAAAAAAAAAATTGAAAAATCATAA
ATTAAAAAAAAAAAAAATCAATTGAATTTTTTTTTTTTCATGATTACGTTTTGACATTTTTCCTTTTTTTTTC
TCTTATTA

ATTACGTAAACGATAATATGTTCCTGAACTCGCATTTTTTTAATGATTTTTTATGACCTCTTATATATTCT
TTCATTATATAACCTCTATATTACATCAAAAGATGGAAAAATATAAAAAAATTAAAAAAAAAAAAAAAGAAA
TTTTTTCC

ATTACATCAAAAGATGGAAAAATATAAAAAAATTAAAAAAAAAAAAAAAGAAATTTTTTCCAAAAGAGTATA
TTTATATGTATGTATACATGTAGGGAAAATAAGAAACTTTATTAATAGTAAAAAAGCATATATACTTTATT
ATTAACTC

TTTCTGACGGCATCGAAGAGCCGAACTCTTATACAATACATGTCATTTTTAACAATGATGCAAATAACTTT
TAAGGCCTTAACACTCTACATAATATGCTAAAAACATTAAAAAAAAAAAAAAGGAAAATAAAAAAAAAAAAA
AAAAAAAA

CGGTTTCCATATTGGAACGGCACCTTCCTTTTTTGGAAGTCACGTTCCTGCAGCGCGTTTCTTTTTTTTTT
TTTTTTTTTTTTTATTTTTCGGTTTTTTTCGCACGTTTCTTTTTGGTGAGCGAGAAAGCTCGTTGCCTCC
TAATATAA

TTATATATTCTTTCATTATATAACCTCTATATTACATCAAAAGATGGAAAAATATAAAAAAATTAAAAAAA
AAAAAAAGAAATTTTTTCCAAAAGAGTATATTTATATGTATGTATACATGTAGGGAAAATAAGAAACTTTA
TTAATAGT

ATTGGAACGGCACCTTCCTTTTTTGGAAGTCACGTTCCTGCAGCGCGTTTCTTTTTTTTTTTTTTTTTTT
TTTTATTTTTCGGTTTTTTTCGCACGTTTCTTTTTGGTGAGCGAGAAAGCTCGTTGCCTCCTAATATAAAG
GATGAGAT

GTATATATCAAATAAAAATATGCATTATTCTAAAAAAAAAAAAAAAAAAAAAAAAAAAAAATCAAAAATG
TCTCTCTAAAGGGTGATTATAAAAAACTTGGTTTGGGCAAGTTTTTAGCCTCCCTTGGACCATTAGTGGCG
AAAGTGGA

ACTATTTTCTTTTCGGAAGGCAAAAACAAAAATTATAATAAAAATAAAAATATGTATAATATAATTTTTAT
CTTCTAACTTTTTATTTATTGAATTTTTATATAGTAAATATAACACGTGATATATTTGAAATGTGATTATA
TTATATAT

CTATCCCTCGGATTATAGACTGTGAATATTGCATATGCAACTTTGACTCAAATTTTTCCAAAATTTGATAT
ATATATATATATATATATGTTTGTATGTATATATATATATACGTATATATATCATATATACGAAAGTAGA
AAAAAAAA

TCTTTAACTGGAAAAAAAAAGCTTTCACGACTCAAAGAAAAAAAAAAAAAATTTTTTAGGGTAAAAAAAAA
CTAAGGGCTAATATGAAAAATACAAAGGTTATGTTACTTTAAAATGAGGTTTGGAAGAAGGAGAAAGACAA
AAAAGCAG

TGAAAAAAGTTCATGTTTTGTTATTTCTTTCTCACATACTTTATAAAAAAAGATAATTTTTTAAAAAAAA
AAAAAAATACAAGTGGAACATTATCAAATAAAAAGCATTATAGCGCCACATGCGATGAACCTGAGCTTTTA
TCTTCATT

TTAGAGCATAAGAAAAAATGTGAACAAAAATAGTGTCATCTCCAGTAAGGCTTAAATGTATATTGATTTTC
TATATATATATATATATATATATATATATTTATGAAATAAACCATATAATTATAAGTAGTAGCTTGGAAAAAC
GCCAAAGT

ATTACAATAACAAAACTGCAACTACCATAAAAAAAAAATTGAAAAATCATAAATTAAAAAAAAAAAAAATCAA
TTGAATTTTTTTTTTTCATGATTACGTTTTGACATTTTTCCTTTTTTTTTCTCTTATTACGATTTACCTTT
TTTATTTA

TAATGATTTTTTATGACCTCTTATATATTCTTTCATTATATAACCTCTATATTACATCAAAAGATGGAAAA
ATATAAAAAAATTAAAAAAAAAAAAAAGAAATTTTTTCCAAAAGAGTATATTTATATGTATGTATACATGT
AGGGAAAA

AGTCCACATAGATACACACAGATAAACATATATATATTATGTATATATATATATATATATATATATATATA
TATATGTATATATCAACGCCCATACCATTATCATTATCAATAAGTTTGCGAGGCGGGTACCCTCCCTCGTG
CTATTATA

TGAGTGCCTGCGGTTTCCATATTGGAACGGCACCTTCCTTTTTTGGAAGTCACGTTCCTGCAGCGCGTTTC
TTTTTTTTTTTTTTTTTTTTTTTATTTTTCGGTTTTTTTCGCACGTTTCTTTTTGGTGAGCGAGAAAGCT
CGTTGCCT

GAACTGTTTAAAAACTTTTTTTTTTTTTTTTTTTTTTTTTTTAAAATCTAAATATGTTGCTTGAATTATAAA
TACAATAACGGGAAAACATATGATGTGTCATGTTCAAAAAGCTTTGAATAGGTGTCTTCCATCAAAAGGGT
CACAGGAA

TTTTTTATTCTTCACAGCGTTTTTTCGACGGAGGAGGGAAAAAAAAAAAATTGTATTTAAGGCATCAAATGC
GCGACATCATATACCTTTGTCCGCTGTTATCATACCTTGAACCTCTACATTTCAGCCTAGCGCGCGCAAAA
AGAAAAAA

TTACCCGTCCTGCACAATCATGAGTGCCTGCGGTTTCCATATTGGAACGGCACCTTCCTTTTTTGGAAGTC
ACGTTCCTGCAGCGCGTTTCTTTTTTTTTTTTTTTTTTTTTTTATTTTTCGGTTTTTTTCGCACGTTTCT
TTTTGGTG

CACCTTCCTTTTTTGGAAGTCACGTTCCTGCAGCGCGTTTCTTTTTTTTTTTTTTTTTTTTTTTTATTTTT
CGGTTTTTTTCGCACGTTTCTTTTTGGTGAGCGAGAAAGCTCGTTGCCTCCTAATATAAAGGATGAGATGA
GATGAGCA

TTTTGGAAAGCTAAGGATAAAAACAATTCTTTTCATTAGATGTATTTTTTCATCGTATTAGGTGTAATATA
TATATATATGTATATATATATATATATATAATATATATGTAGTATACACACGTGGACGACCATTAACGAAT
GTTGTATA

AAAATCGATGGGCTGGGAAAACGCGTCATCAATTCAACCGAATAAGGAAAACTAAGCCACTTCACGCGGTT
CGCGATATTTGTCCAGCCTCTTTTTCCGAAAAAAAAAAAATTAAATAATAAAATGAAACGGACAGGAATTG
AACCTGCA

CGTCAAGTGAGAAGAGTTTTGGCCTTCGCTTCAGTTAGATCTTCTATTATTTCCTTTTTTTTTCTTTTTGTT
TGTAATTGTTTTTATTTTTTTTTTTGCGCGAAACTTTGCTATATTGGGTAACGCGTAAAATACTTTTTAT
TATTGCAG

TTCCTTTTTTTTCTTTTTGTTTGTAATTGTTTTTTATTTTTTTTTTTTGCGCGAAACTTTGCTATATTGGGT
AACGCGTAAAATACTTTTTATTATTGCAGTAAGGCGGAAGGGTCTTCCCCTTTGCATGTTAAATAGCATAC
ATGGCACC

AGTCGCCAAAAAAATCGATGGGCTGGGAAAACGCGTCATCAATTCAACCGAATAAGGAAAACTAAGCCACT
TCACGCGGTTCGCGATATTTGTCCAGCCTCTTTTTCCGAAAAAAAAAAAATTAAATAATAAAATGAAACGG
ACAGGAAT

ACCTTAAGTTTTAGTAGGCAGGACAATTTCAGTGTTTGTAAAAATATATTTATATTTATATATTTAAATAT
ACTAAATAAAAATAATACTATCATCGTTGTGATGCAAAAAAAAAAAAAAAATGAACAGAAAATAATAGTGAT
GTGAATGG

ATAATATGCTAAAAACATTAAAAAAAAAAAAAAGGAAAATAAAAAAAAAAAAAAAAAAAAAAAACGGGAGATT
AACCGAATAGCAAACTCTTAAACTATGAAAAGGCAATCAATCCACTGTTTTCTTTTTCCATTTCTTCTAGC
CTATTTGA

AGAAGGCGATCACTTATTGAAGTCCACATAGATACACACAGATAAACATATATATATTATGTATATATATA
TATATATATATATATATATATATATGTATATATCAACGCCCATACCATTATCATTATCAATAAGTTTGCGA
GGCGGGTA

TTATTAGTTACTTTTTTTATTGTACTCCAGTATGGACCTCTTAGGTGAGTGATCTTATTAAAAAAAAATAAA
AAAAATAAAAAAATATGAAAAAAAAAGAAAATTAAAAACTAAACAAATTTTATGACAGGAATAAAAACTAT
AAAAAATG

GCAGACATGACCCTTTTCTCGATATGTTTTTCTGTGATTTTTTTTTTTCAATTGTTCGAAATAACCTGTAT
TGTTCAATACTGGACAATTGTTCTGATATATATATATATATATAATTTTCAATGAAAAAAAAAAAAAAAAG
AAAGAAAA

GGCCTTCGCTTCAGTTAGATCTTCTATTATTTCCTTTTTTTTCTTTTTGTTTGTAATTGTTTTTTATTTTT
TTTTTTGCGCGAAACTTTGCTATATTGGGTAACGCGTAAAATACTTTTTATTATTGCAGTAAGGCGGAAGG
GTCTTCCC

TAGTGTCATCTCCAGTAAGGCTTAAATGTATATTGATTTTCTATATATATATATATATATATATATATTTA
TGAAATAAACCATATAATTATAAGTAGTAGCTTGGAAAAACGCCAAAGTTGTACTTAGAATGAGGTTTTAA
AAAAAAAT

ACCCACTTGTTTTACCGCAGAGGGCCCTTTTTTTGTTTACCGTCGCAAAAAAAAAACGCGAAGAATACGCG
TTGGTCGTTAAATTTTGTGGTACGCGTAAAACTGCTATTTGGTATCCTCTTTAGGGACGCGCCCATTGTCT
ATACGGTA

TATTGAATAGTGATGTTATGACCTTTTTTTTTACGCCTTTTTTTTTTTTTTTTTTTTTTTTAATTTATCTC
ACTTTTCCCTTCTATTTTATGTACTAACAGATAATTTTATAATGATTTCATGTATTTTTGGTTCAGAATTT
TGACAAGG

TGCATTATTCTAAAAAAAAAAAAAAAAAAAAAAAAAAAAAAAATCAAAAATGTCTCTCTAAAGGGTGATTAT
AAAAAACTTGGTTTGGGCAAGTTTTTAGCCTCCCTTGGACCATTAGTGGCGAAAGTGGAGCCTGCATAAGA
GTGACTGA

TAATTTCCGTAATACAAAAAACTATTTTCTTTTCGGAAGGCAAAAACAAAAATTATAATAAAAATAAAAAT
ATGTATAATATAATTTTTATCTTCTAACTTTTTATTTATTGAATTTTTATATAGTAAATATAACACGTGAT
ATATTTGA

CATCACTTTTCTATACTTTCTCTTCCCCGCGTGTTTTCCGTACACCAACAATATATGCCATAATACACGTA
ACATTTTTTTATAAAAAGAAAAGGTAAGTGATATATATAAAATAGCGCCATGAGTAGGAAACTTTTCTGTT
ACTGCAGA

AAAAAAATCAATTGAATTTTTTTTTTTCATGATTACGTTTTGACATTTTTCCTTTTTTTTTCTCTTATTAC
GATTTACCTTTTTTATTTATTTTTTTCATTTTAGTATTTTATTCTTCGTTATTTATGTATAGAAATTTTCA
TTTTCATT

TTATGACCTCTTATATATTCTTTCATTATATAACCTCTATATTACATCAAAAGATGGAAAAATATAAAAAA
ATTAAAAAAAAAAAAAAAGAAATTTTTTCCAAAAGAGTATATTTATATGTATGTATACATGTAGGAAAATA
AGAAACTT

AATACAATTTTTTTTTTATTCTTCACAGCGTTTTTTCGACGGAGGAGGGAAAAAAAAAAAATTGTATTTAAGG
CATCAAATGCGCGACATCATATACCTTTGTCCGCTGTTATCATACCTTGAACCTCTACATTTCAGCCTAGC
GCGCGCAA

TTTTTGGGCTACATTATTCTTGAAAAAAGTTCATGTTTTGTTATTTCTTTCTCACATACTTTATAAAAAAA
GATAATTTTTTTAAAAAAAAAAAAAAAATACAAGTGGAACATTATCAAATAAAAAGCATTATAGCGCCACAT
GCGATGAA

AATTAAAAGGAAAAGTATTATGCATATATATATATATATATATATATATATATTTATATATTTATACACAT
AGAATTATTTTACAGTAAAGGATGCTACACCACCCCGGGCTAACTTTATGGCTTCTACTATTTCAGGTTCC
CTTTCGCG

TTTCATTTTCGGTAGTGAGATGGCAGTTCGAGGGGTTTTTTATTCAAAATAATAACTTCTGGCTTTTCGCT
TTTATATAGCAGAAAAAAAAAAGCCGTCGAGGCGCGCGTTCATGCAATGGCTCAGTAACCTCGGGATAGAA
AAAGGGCA

TTTACCGCAGAGGGCCCTTTTTTTGTTTACCGTCGCAAAAAAAAAAACGCGAAGAATACGCGTTGGTCGTTA
AATTTGTGGTACGCGTAAAACTGCTATTTGGTATCCTCTTTAGGGACGCGCCCATTGTCTATACGGTAAT
ATAGGGCT

TCGGCTATTACGTCAAGTGAGAAGAGTTTTGGCCTTCGCTTCAGTTAGATCTTCTATTATTTCCTTTTTTT
TCTTTTTGTTTGTAATTGTTTTTTATTTTTTTTTTTGCGCGAAACTTTGCTATATTGGGTAACGCGTAAAA
TACTTTTT

AATGATTAATGCTGATATGTTTATTTACATCACAGTGCCCTTATGATCAGGAACTGTTTAAAAACTTTTTT
TTTTTTTTTTTTTTTTTTTTAAAATCTAAATATGTTGCTTGAATTATAAATACAATAACGGAAAACATAT
GATGTGTC

TTTTTCACCGTATTTTTGTGCGTCCTTTTTCAATTATAGCTTTTTTTTTATTTTTTTTTTTTCTCGTACTG
TTTCACTGACAAAAGTTTTTTTTTCAAGAAAAATTTTCGATGCCGCGTTCTCTGTGTGCAACGGATGGATG
GTAGATGG

CTTTGACTCAAATTTTTTCCAAAATTTGATATATATATATATATATATATATGTTTGTATGTATATATATATAT
ACGTATATATATCATATATACGAAAAGTAGAAAAAAAAAAGGTGATATTTCGCTCGTGGAAAAGCTAATGCC
ACAGCTTG

TAGTTTTTTTTTTTTTTTTTTTTTTATTTTGTATGGCATACTAACTATACAAATATTTATATGTACATTTAT
ACAGTAACCTATTATTTACCGATATTCACCGGTATTTTACTTTAATAGATGCTGGTAATAACGAAAAAAAT
TGATAACT

GAAAATAGGAAAAAAAAAATTGTCATCTGAGAAATTGCTTTGGATGACCAGATCCAAAACAGACATAAGCAT
TCGTATAATTGGTACATAGGTATATATCAAATAAAAATATGCATTATTCTAAAAAAAAAAAAAAAAAAAAA
AAAAAAAA

GGCTGGGAAAACGCGTCATCAATTCAACCGAATAAGGAAAACTAAGCCACTTCACGCGGTTCGCGATATTT
GTCCAGCCTCTTTTTCCGAAAAAAAAAAAAATTAAATAATAAAATGAAACGGACAGGAATTGAACCTGCAAC
CCTTCGAT

ATCAAATTTGACCTTAAGTTTTAGTAGGCAGGACAATTTCAGTGTTTGTAAAAATATATTTATATTTATAT
ATTTAAATATACTAAATAAAAATAATACTATCATCGTTGTGATGCAAAAAAAAAAAAAAATGAACAGAAAA
TAATAGTG

CATTTTTCTGTAATTTCCGTAATACAAAAAACTATTTTCTTTTCGGAAGGCAAAAACAAAAATTATAATAA
AAATAAAAATATGTATAATATAATTTTTATCTTCTAACTTTTTATTTATTGAATTTTTATATAGTAAATAT
AACACGTG

TTCACAGCGTTTTTTCGACGGAGGAGGGAAAAAAAAAAATTGTATTTAAGGCATCAAATGCGCGACATCAT
ATACCTTTGTCCGCTGTTATCATACCTTGAACCTCTACATTTCAGCCTAGCGCGCAAAAAGAAAAAAAAAA
ATCATAAG

AAATAAAAGAAAATCGACATAATGCACCTCAGTATAAATTGTAAAATATAACATAAATCAAAGAAAAACGA
GAAAACGATATGATTATCCGATACCGACAAATTCTCATGTTTATTTTTTTTTTTTTTTTTTTTTTTTTTTA
ATTTTTCT

CCTGGGTTATCTGCGGGAGTGAACAATGATAATACAAAGGAGAGTAAAGATGTGAAAATGAATTAAAAGGA
AAAGTATTATGCATATATATATATATATATATATATATATATATATTTATATATTTATACACATAGAATTATTTT
ACAGTAAA

TGATGTTATGACCTTTTTTTTTTTACGCCTTTTTTTTTTTTTTTTTTTTTTAATTTATCTCACTTTTCCCT
TCTATTTTATGTACTAACAGATAATTTTATAATGATTTCATGTATTTTTGGTTCAGAATTTTGACAAGGCA
ACTCTTGA

AAAAGAAGACTAACTAAGAGAAGAAACATACTTCAAAAAAAAAAAAAGAAAAACAACAAAAAAAACGTATA
AAATGAAATAAATTTCGAACCGCTTTTTTTCCTTTTTTTTTTTTTTACTTTGTTTGACCTCCCCTAACTCT
TTCTTTTT

CCATCGAAATAGTCGCCAAAAAAATCGATGGGCTGGGAAAACGCGTCATCAATTCAACCGAATAAGGAAAA
CTAAGCCACTTCACGCGGTTCGCGATATTTGTCCAGCCTCTTTTTCCGAAAAAAAAAAAATTAAATAATAA
AATGAAAC

TGACGAAAGAAGACACGTCGCCTAAACGCGTAATGAATCGCAATATACGAAAAGCTAAACGGGTTTTTCAG
AATCCTCTTACCCAGCCTTGGATAATAGTTTTGGCCGAAAAAAAAAAAAAGAAAGAAAAAAATTGAAATTT
CGCGATCC

CCTGTGGTAGACCCACTTGTTTTACCGCAGAGGGCCCTTTTTTTGTTTACCGTCGCAAAAAAAAAAACGCGA
AGAATACGCGTTGGTCGTTAAATTTTGTGGTACGCGTAAAACTGCTATTTGGTATCCTCTTTAGGGACGCG
CCCATTGT

TGAATTGGATATCAAATTTGACCTTAAGTTTTAGTAGGCAGGACAATTTCAGTGTTTGTAAAAATATATTT
ATATTTATATATTTAAATATACTAAATAAAAATAATACTATCATCGTTGTGATGCAAAAAAAAAAAAAAAT
GAACAGAA

ATACCAAGCTTTTTGGAAAGCTAAGGATAAAAACAATTCTTTTCATTAGATGTATTTTTTCATCGTATTAG
GTGTAATATATATATATATGTATATATATATATATATATAATATATATGTAGTATACACACGTGGACGACC
ATTAACGA

AGAAAAGAAAGGGCCTAAAAACCTCGCCCCAGTGCATAATAAAAGTAAAAAAAAAAAAATCAAACTTTATT
GGGATAAAATAAAAATCAGAAAAACCCTTATATATGTATATGTAGTTAAATGAAGCCAGTTTTTTTCGTCT
GGTTTGTT

GAAGAGTTTTGGCCTTCGCTTCAGTTAGATCTTCTATTATTTCCTTTTTTTTCTTTTTGTTTGTAATTGTT
TTTTATTTTTTTTTTTGCGCGAAACTTTGCTATATTGGGTAACGCGTAAAATACTTTTTATTATTGCAGTA
AGGCGGAA

TAAGTATCACATTACAATAACAAAACTGCAACTACCATAAAAAAAAAATTGAAAAATCATAAATTAAAAAAA
AAAAAATCAATTGAATTTTTTTTTTTCATGATTACGTTTTGACATTTTTCCTTTTTTTTTCTCTTATTACG
ATTTACCT

TGCACAATCATGAGTGCCTGCGGTTTCCATATTGGAACGGCACCTTCCTTTTTTGGAAGTCACGTTCCTGC
AGCGCGTTTCTTTTTTTTTTTTTTTTTTTTTTTTATTTTTCGGTTTTTTTCGCACGTTTCTTTTTGGTGAG
CGAGAAAG

GGGATGATCATATGATGGGCGCTATCCTCATCGCGACTCGATAACGACGTGAGAAACGATTTTTTTTTTCT
TTTTCACCGTATTTTTGTGCGTCCTTTTTCAATTATAGCTTTTTTTTTATTTTTTTTTTTTCTCGTACTGT
TTCACTGA

ACGAAACTGGTAAGTTCTTTTAAGTATCACATTACAATAACAAAACTGCAACTACCATAAAAAAAAAATTGA
AAAATCATAAATTAAAAAAAAAAAAAATCAATTGAATTTTTTTTTTTCATGATTACGTTTTGACATTTTTCC
TTTTTTTT

CAAAATTAAACATTTTTCTGTAATTTCCGTAATACAAAAAACTATTTTCTTTTCGGAAGGCAAAAACAAAA
ATTATAATAAAAATAAAAATATGTATAATATAATTTTTATCTTCTAACTTTTTATTTATTGAATTTTTATA
TAGTAAAT

CTGGAAAACGCCATCGAAATAGTCGCCAAAAAAATCGATGGGCTGGGAAAACGCGTCATCAATTCAACCGA
ATAAGGAAAACTAAGCCACTTCACGCGGTTCGCGATATTTGTCCAGCCTCTTTTTCCGAAAAAAAAAAAAT
TAAATAAT

GTCATGCTCACCCTTAAGTTCTCAACCTTTTAGTTTTTTTTTTTTTTTTTTTTTTATTTTGTATGGCATACT
AACTATACAAATATTTATATGTACATTTATACAGTAACCTATTATTTACCGATATTCACCGGTATTTTACT
TTAATAGA

TGAAAAAAAAAAAAAGCTCAGTCGCGCGTGGCCTTTCTCTATGGAGATTTCTCAAACGATATTAATCCTGTA
TATCTCTATTAATTTAGTTAATACATACACGATATATCGTCGCTTTTATTTTTTTTTTTCAAATACTTCTT
CTTCCTAA

TTGACACCGCGGCGCAAATGTTAGAAGTTCAAAATGAAGCCTAAGTATCACGCTAATTGAAGTTTTTTTTG
ATCACTCCAATAGGCAAATCTATAGATATATAAAAAATATAGACAAGACTTTTTTTTACATTGCCAGTTTT
CTTTTTTC

GAATGAAGAAGAGAGCTGAAAACAATGCTCAAAAGAAGACTAACTAAGAGAAGAAACATACTTCAAAAAAA
AAAAAAAGAAAAACAACAAAAAAACGTATAAAATGAAATAAATTTCGAACCGCTTTTTTTCCTTTTTTTTT
TTTTTACT

TTTTGGAAGTCACGTTCCTGCAGCGCGTTTCTTTTTTTTTTTTTTTTTTTTTTTTTATTTTTCGGTTTTTTT
CGCACGTTTCTTTTTGGTGAGCGAGAAAGCTCGTTGCCTCCTAATATAAAGGATGAGATGAGATGAGCAGT
GCCGCAAA

TTTCGGAAGGCAAAAACAAAAATTATAATAAAAATAAAAATATGTATAATATAATTTTTATCTTCTAACTT
TTTATTTATTGAATTTTTATATAGTAAATATAACACGTGATATATTTGAAATGTGATTATATTATATATAT
TGATGTTT

GAGAGCCAAGTCTTTCTTTATAATGCATAGCAATAAATAGTCATATATATATATATATATATATATATATG
TACATATATATGAACATATATATGAACAAATAAATGATAATGGTATGTGATAGTCATTATTAACGATTAAG
CGTTTTTC

CACGTTCCTGCAGCGCGTTTCTTTTTTTTTTTTTTTTTTTTTTTTTATTTTTCGGTTTTTTTCGCACGTTTC
TTTTTGGTGAGCGAGAAAGCTCGTTGCCTCCTAATATAAAGGATGAGATGAGATGAGCAGTGCCGCAAATG
AGGGATGT

TAACGCACTGATTACGTAAACGATAATATGTTCCTGAACTCGCATTTTTTTAATGATTTTTTATGACCTCT
TATATATTCTTTCATTATATAACCTCTATATTACATCAAAAGATGGAAAAATATAAAAAAATTAAAAAAAA
AAAAAAGA

GAAAAAAAAAGCTTTCACGACTCAAAGAAAAAAAAAAAAAATTTTTTTAGGGTAAAAAAAAACTAAGGGCTA
ATATGAAAAATACAAAGGTTATGTTACTTTAAAATGAGGTTTGGAAGAAGGAGAAAGACAAAAAAGCAGGG
CACATGTC

CGCGCATACATGCGTATTCATATTTCCCTTTTGCATTTGTCTTCTTCGGTTTTCCTGGTAGAATTTCCCTT
AATGGGAGAGTAGTAACAATGCAAAAAATGAAGAAAATTGAGAAAAAACAAAAAAGAAAAAAAAAAAAAAA
AAAAAAAA

ATAACCTGTATTGTTCAATACTGGACAATTGTTCTGATATATATATATATATATATAATTTTCAATGAAAAAA
AAAAAAAAAAGAAAGAAAATTAGAAGCTAGAAAGAAATAAGGAAAAGGAAAAGCAAACAACAATAGGAAAA
AAAAGCAT

CTATACTTTCTCTTCCCCGCGTGTTTTCCGTACACCAACAATATATGCCATAATACACGTAACATTTTTTT
ATAAAAAGAAAAGGTAAGTGATATATATATAAAATAGCGCCATGAGTAGGAAACTTTTCTGTTACTGCAGATA
TGTGCCAG

TTTTTTACTTTTGCCATCTTATACTCATCCTAATCATCTGTTTCATTTTCCTTCTATTTCTTTTTTTTTTT
TTTTTTTTTTTTTTTTTTTCAGTTTTCGAAGAAACTGCCTTTTTAGAATTGTAAAAGACGAAGTGAAGTAT
TCAGGAGT

ATCGGTTATAACTGCCGATACCCGTATCTTTTGTGTTTGATTCAAGGAATACTTATTTAGAGTAGAAAGAA
TATATTTACTTAGATAACTTAATGTATAATATATAAAAAAAATCAAAAAAACAGGAAAAAAAAAAAATCATG
AGTGAGTG

ACATTATTCTTGAAAAAAGTTCATGTTTTGTTATTTCTTTCTCACATACTTTATAAAAAAAGATAATTTTT
TTAAAAAAAAAAAAAAAATACAAGTGGAACATTATCAAATAAAAAGCATTATAGCGCCACATGCGATGAACC
TGAGCTTT

GTACAAAGATAAATAAAAGAAAATCGACATAATGCACCTCAGTATAAATTGTAAAATATAACATAAATCAA
AGAAAAACGAGAAAACGATATGATTATCCGATACCGACAAATTCTCATGTTTATTTTTTTTTTTTTTTTTT
TTTTTTTT

GATATGTTTTTCTGTGATTTTTTTTTTTCAATTGTTCGAAATAACCTGTATTGTTCAATACTGGACAATTG
TTCTGATATATATATATATATATATAATTTTCAATGAAAAAAAAAAAAAAAAAGAAAGAAAATTAGAAGCTAGA
AAGAAATA

TAACTAAGAGAAGAAACATACTTCAAAAAAAAAAAAAAAGAAAAACAACAAAAAAAACGTATAAAATGAAATA
AATTTCGAACCGCTTTTTTTCCTTTTTTTTTTTTTTACTTTGTTTGACCTCCCCTAACTCTTTCTTTTTAC
CTTCACAA

CCTAAACGCGTAATGAATCGCAATATACGAAAAGCTAAACGGGTTTTTCAGAATCCTCTTACCCAGCCTTG
GATAATAGTTTTGGCCGAAAAAAAAAAAAAAGAAAGAAAAAAAATTGAAATTTCGCGATCCGAACAAACAATG
AACAGAAA

TCCAGTAAGGCTTAAATGTATATTGATTTTCTATATATATATATATATATATATATATTTATGAAATAAAC
CATATAATTATAAGTAGTAGCTTGGAAAAACGCCAAAGTTGTACTTAGAATGAGGTTTTAAAAAAAAAATTA
TAAAAAAA

GCCGAGGTATTTAGAGCATAAGAAAAAATGTGAACAAAAATAGTGTCATCTCCAGTAAGGCTTAAATGTAT
ATTGATTTTCTATATATATATATATATATATATATATATTTATGAAATAAACCATATAATTATAAGTAGTAGC
TTGGAAAA

CTCAACCTTTTAGTTTTTTTTTTTTTTTTTTTTTATTTTGTATGGCATACTAACTATACAAATATTTATAT
GTACATTTATACAGTAACCTATTATTTACCGATATTCACCGGTATTTTACTTTAATAGATGCTGGTAATAA
CGAAAAAA

TACTGATTGTTGAAAAAAAAAAAAGCTCAGTCGCGCGTGGCCTTTCTCTATGGAGATTTCTCAAACGATAT
TAATCCTGTATATCTCTATTAATTTAGTTAATACATACACGATATATCGTCGCTTTTATTTTTTTTTTTCA
AATACTTC

AGGGCCCTTTTTTTGTTTACCGTCGCAAAAAAAAAACGCGAAGAATACGCGTTGGTCGTTAAATTTTGTGG
TACGCGTAAAACTGCTATTTGGTATCCTCTTTAGGGACGCGCCCATTGTCTATACGGTAATATAGGGCTTT
CTTCATTA

AGTATAAATTGTAAAATATAACATAAATCAAAGAAAAACGAGAAAACGATATGATTATCCGATACCGACAA
ATTCTCATGTTTATTTTTTTTTTTTTTTTTTTTTTTTTTTAATTTTTCTTGGGCCTTTACATTGAGTACAG
GGGCATAA

AAAAACATTAAAAAAAAAAAAAGGAAAATAAAAAAAAAAAAAAAAAAAAAAACGGGAGATTAACCGAATAG
CAAACTCTTAAACTATGAAAAGGCAATCAATCCACTGTTTTCTTTTTCCATTTCTTCTAGCCTATTTGAAC
TGTCTGCT

ATTTCCTCTAAGAAAAGAAAGGGCCTAAAAACCTCGCCCCAGTGCATAATAAAAGTAAAAAAAAAAAAATC
AAACTTTATTGGGATAAAATAAAAATCAGAAAAACCCTTATATATGTATATGTAGTTAAATGAAGCCAGTT
TTTTTCGT

ACGCGTCATCAATTCAACCGAATAAGGAAAACTAAGCCACTTCACGCGGTTCGCGATATTTGTCCAGCCTC
TTTTTCCGAAAAAAAAAAAATTAAATAATAAAATGAAACGGACAGGAATTGAACCTGCAACCCTTCGATTG
CAATCTTA

TGGTAATTCAGAGAGCCAAGTCTTTCTTTATAATGCATAGCAATAAATAGTCATATATATATATATATATA
TATATATATGTACATATATATGAACATATATATGAACAAATAAATGATAATGGTATGTGATAGTCATTATT
AACGATTA

TATGATATCAAGAAGGCGATCACTTATTGAAGTCCACATAGATACACACAGATAAACATATATATATTATG
TATATATATATATATATATATATATATATATATATGTATATATCAACGCCCATACCATTATCATTATCAAT
AAGTTTGC

GAAAACATGGTCTTGCTTCACCCTATTCTCGCCGAGAGCTGTACAAGATATTTTTTACTTTTGCCATCTTA
TACTCATCCTAATCATCTGTTTCATTTTCCTTCTATTTCTTTTTTTTTTTTTTTTTTTTTTTTTTTTTTCA
GTTTTCGA

ACCTTTTTTTTTTACGCCTTTTTTTTTTTTTTTTTTTTTTTAATTTATCTCACTTTTCCCTTCTATTTTAT
GTACTAACAGATAATTTTATAATGATTTCATGTATTTTTGGTTCAGAATTTTGACAAGGCAACTCTTGAAT
AATTGTCG

AACATTATACAAGTTTTTCATTTTGAGGGGTCTTTAACTGGAAAAAAAAAGCTTTCACGACTCAAAGAAAA
AAAAAAAAATTTTTTTAGGGTAAAAAAAAACTAAGGGCTAATATGAAAAATACAAAGGTTATGTTACTTTA
AAATGAGG

ATTGAATTTTTTTTTTTCATGATTACGTTTTGACATTTTTCCTTTTTTTTTCTCTTATTACGATTTACCTT
TTTTATTTATTTTTTTCATTTTAGTATTTTATTCTTCGTTATTTATGTATAGAAATTTTCATTTTCATTTA
GATTCAGA

AGACACGTCGCCTAAACGCGTAATGAATCGCAATATACGAAAAGCTAAACGGGTTTTTCAGAATCCTCTTA
CCCAGCCTTGGATAATAGTTTTGGCCGAAAAAAAAAAAAAGAAAGAAAAAAATTGAAATTTCGCGATCCGA
ACAAACAA

TAAATGTATATACTGATTGTTGAAAAAAAAAAAAAGCTCAGTCGCGCGTGGCCTTTCTCTATGGAGATTTCT
CAAACGATATTAATCCTGTATATCTCTATTAATTTAGTTAATACATACACGATATATCGTCGCTTTTATTT
TTTTTTTT

AAATTTCACAGTCATGCTCACCCTTAAGTTCTCAACCTTTTAGTTTTTTTTTTTTTTTTTTTTTATTTTGT
ATGGCATACTAACTATACAAATATTTATATGTACATTTATACAGTAACCTATTATTTACCGATATTCACCG
GTATTTTA

AAATCATGTGTTACCCGTCCTGCACAATCATGAGTGCCTGCGGTTTCCATATTGGAACGGCACCTTCCTTT
TTTGGAAGTCACGTTCCTGCAGCGCGTTTCTTTTTTTTTTTTTTTTTTTTTTTTATTTTTCGGTTTTTTTC
GCACGTTT

AGATTTTTGCCTATAAAGTTTAAAATACTTCCCCGCTATTTGTATATATGCATATCTTTATATTATGAAAG
ATATTTTTTTATTTACTTTTAGTCCGTATACTTTTATTTTGTTTTGTTAGACAATCTCGAAGAACAAAAAA
AAAAGATA

CATGATAACCAAAAATAGTCTTAACAAAATACATATAAGTAAATAATGCTAAATAAAAAAGCAAAAAAATA
AAATTTATATCACACTTGTAAAACCTTTAAATGATCTTCTTTTTTACCTGTTTTATTCACTTGTTTTTTTT
TTTCTGAG

GGGCCTAAAAACCTCGCCCCAGTGCATAATAAAAGTAAAAAAAAAAAAAATCAAACTTTATTGGGATAAAAT
AAAAATCAGAAAAACCCTTATATATGTATATGTAGTTAAATGAAGCCAGTTTTTTTCGTCTGGTTTGTTTC
AGCCTATA

ATTCACAATAGGAAAAAAAAAAATGGGTTTGGTGACTGCAGTAAAGACGTGCTAAAAAAAAAAAAGTTGTTT
TTATCTGAACCTTAAATATATCATTTATGAGTGTATATATTATACAAACGCAATAACAGTGGCTCCCGCCC
TCCACCTC

CGTCGCAAAAAAAAAAACGCGAAGAATACGCGTTGGTCGTTAAATTTTGTGGTACGCGTAAAACTGCTATTT
GGTATCCTCTTTAGGGACGCGCCCATTGTCTATACGGTAATATAGGGCTTTCTTCATTACCTGCTTGCGTC
AATTAGCT

CAAAGGATGCTCCCGGTTGTGTGATTGAAAACGGAACTAAGCTTTACTAATCAACAATATTAACTTTATAA
TAGATTCATATATGCGCGTACATATATTATATACAATTTTTCTACACAAGAATGCTTAATAAAGATACGAT
AATTTTTT

ATGGCGCAAGTACTTATTGTGACCTTTGGGGTACCGTTACCGTCAGTTTTCTTCAGCTAAGGCGCGCGCGC
CAGATAACTAAAAAAAAATATAGTTGCTGCTTAAAAAACAATACACCCGTACTCTCTTGCCTGTAAAAACC
TCGAAGGA

ACTGCCGATACCCGTATCTTTTGTGTTTGATTCAAGGAATACTTATTTAGAGTAGAAAGAATATATTTACT
TAGATAACTTAATGTATAATATATAAAAAAAATCAAAAAAACAGGAAAAAAAAAAAATCATGAGTGAGTGGC
ACTTTTAA

CCCTTTTCTCGATATGTTTTTCTGTGATTTTTTTTTTTTCAATTGTTCGAAATAACCTGTATTGTTCAATAC
TGGACAATTGTTCTGATATATATATATATATATATAATTTTCAATGAAAAAAAAAAAAAAAAAGAAAGAAAATT
AGAAGCTA

AAGTTTTTCATTTTGAGGGGTCTTTAACTGGAAAAAAAAAAGCTTTCACGACTCAAAGAAAAAAAAAAAAAT
TTTTTTAGGGTAAAAAAAAAACTAAGGGCTAATATGAAAAATACAAAGGTTATGTTACTTTAAAATGAGGTT
TGGAAGAA

CTAAGGATAAAAACAATTCTTTTCATTAGATGTATTTTTTTCATCGTATTAGGTGTAATATATATATATATG
TATATATATATATATATATAATATATATGTAGTATACACACGTGGACGACCATTAACGAATGTTGTATATG
CTTATGCG

ATAACTAATTGTGTGCGTTGCGCGGGGTTGTGTGGCTCAAAAAGATCTGTTTTTGTATTAGCAATTTTTTA
TTTCCCTTTTTAAATGAATTTTTTTTCTTTCTGTTTCGAGACAAGGCCTTTTGCAAAAAAAAAAAAAAGTAA
AAAAGTGA

TATTCTACGAAAACGAGATGTATTGAATAGTGATGTTATGACCTTTTTTTTTACGCCTTTTTTTTTTTTTT
TTTTTTTTTTAATTTATCTCACTTTTCCCTTCTATTTTATGTACTAACAGATAATTTTATAATGATTTCAT
GTATTTTT

ACATAAATCAAAGAAAAACGAGAAAACGATATGATTATCCGATACCGACAAATTCTCATGTTTATTTTTTT
TTTTTTTTTTTTTTTTTTTAATTTTTCTTGGGCCTTTACATTGAGTACAGGGGCATAAGTGCGAGGAGTT
ACCAGACG

CACTTATGCAAATGATTAATGCTGATATGTTTATTTACATCACAGTGCCCTTATGATCAGGAACTGTTTAA
AAACTTTTTTTTTTTTTTTTTTTTTTTTTTAAAATCTAAATATGTTGCTTGAATTATAAATACAATAACGG
GAAAACAT

AAAACTTTTTTTTTTTTTTTTTTTTTTTTTTAAAATCTAAATATGTTGCTTGAATTATAAATACAATAACG
GGAAAACATATGATGTGTCATGTTCAAAAAGCTTTGAATAGGTGTCTTCCATCAAAAGGGTCACAGGAACA
TGAATTTC

GTGTGCGTTGCGCGGGGTTGTGTGGCTCAAAAAGATCTGTTTTTGTATTAGCAATTTTTTATTTCCCTTTT
TAAATGAATTTTTTTTCTTTCTGTTTCGAGACAAGGCCTTTTGCAAAAAAAAAAAAAAGTAAAAAAGTGAAA
TTTGATAG

TCCACAGATAGTGCTACTTTAGTAAACGTTCACATCAAAAGGTAGTATTAATTAAAAAAAAAAAAAAAGCCA
ATAATTTCGCGCAAGCTTTGCTCTACATAATAAAAATACAATTAGGATAGAAATGAATATTTTTTTAGCGG
CAGTTTTA

GCACTAGAACATGGCGCAAGTACTTATTGTGACCTTTGGGGTACCGTTACCGTCAGTTTTCTTCAGCTAAG
GCGCGCGCGCCAGATAACTAAAAAAAAATATAGTTGCTGCTTAAAAAACAATACACCCGTACTCTCTTGCC
TGTAAAAA

AGATAGTTATAAATCTATGATTGTATGGTTTCTTTGACATATTTATAGTTAATATATATATATATATGTGT
TTAATTTTATAAAAATTCCATCGTTTATATTCGATTATTTTTATATGTCAACCTTTTTTTTAAGAAATGTT
CATATTTA

AAACGAGATGTATTGAATAGTGATGTTATGACCTTTTTTTTTACGCCTTTTTTTTTTTTTTTTTTTTTTT
AATTTATCTCACTTTTCCCTTCTATTTTATGTACTAACAGATAATTTTATAATGATTTCATGTATTTTTGG
TTCAGAAT

GGAAAAAAAAAAATGGGTTTGGTGACTGCAGTAAAGACGTGCTAAAAAAAAAAAAAGTTGTTTTTATCTGAAC
CTTAAATATATCATTTATGAGTGTATATATTATACAAACGCAATAACAGTGGCTCCCGCCCTCCACCTCCG
CCTGCTAC

AATATGAAATCAAAATTAAACATTTTTCTGTAATTTCCGTAATACAAAAAACTATTTTCTTTTCGGAAGGC
AAAAACAAAAATTATAATAAAAATAAAAATATGTATAATATAATTTTTATCTTCTAACTTTTTATTTATTG
AATTTTTA

TTTTTTTTCAATTGTTCGAAATAACCTGTATTGTTCAATACTGGACAATTGTTCTGATATATATATATATA
TATAATTTTCAATGAAAAAAAAAAAAAAAAAGAAAGAAAATTAGAAGCTAGAAAGAAATAAGGAAAAGGAAA
AGCAAACA

AATTCAGGTTAATTCTAAAAACTATATTGCAATCTTTTAAATATATGTATACACGTTCATTACCACGTATA
CATATCCATATATATATATATATATATACATACTATTTATGATAAATTTTTAACAAGCAAACCCATTATTA
AATGCTAT

TTAGTAGGCAGGACAATTTCAGTGTTTGTAAAAATATATTTATATTTATATATTTAAATATACTAAATAAA
AATAATACTATCATCGTTGTGATGCAAAAAAAAAAAAAAAATGAACAGAAAATAATAGTGATGTGAATGGTT
TCGTGCCA

GAGAGCTGAAAACAATGCTCAAAAGAAGACTAACTAAGAGAAGAAACATACTTCAAAAAAAAAAAAAAAGAA
AAACAACAAAAAAACGTATAAAATGAAATAAATTTCGAACCGCTTTTTTTCCTTTTTTTTTTTTTTTACTTT
GTTTGACC

TCTTGCTTCACCCTATTCTCGCCGAGAGCTGTACAAGATATTTTTTACTTTTGCCATCTTATACTCATCCT
AATCATCTGTTTCATTTTCCTTCTATTTCTTTTTTTTTTTTTTTTTTTTTTTTTTTTTTCAGTTTTCGAAG
AAACTGCC

TTTTTTTCATGATTACGTTTTGACATTTTTCCTTTTTTTTTCTCTTATTACGATTTACCTTTTTTATTTAT
TTTTTTCATTTTAGTATTTTATTCTTCGTTATTTATGTATAGAAATTTTCATTTTCATTTAGATTCAGATT
TGGTTATC

AAAGAAAAAAAAAAAAAAACTGAAATTTCCACCTTACCCGTTTTTTTAATGGAACAGTGATTGTTACGACAT
TGAGATTTTAGATTTATATAATCACATACATATTAAATAGTTAATGCACCTTTAATTCTTTTGCACTTCAT
GTTCTATA

TGTAATGTGACACAATTTTGCTATTGCGGAAACTGCAGCATAGTCAGTGTAATAATCGTACTGTTGCTTTT
TCATTTTTTTTTTTTTTTTTTTTTTTCTCATATAGAAAAACTGTTATTGAATGTTTTGACTAGTCTCATGA
AATAAACA

CCTTTTTTACTTTTTGGGCTACATTATTCTTGAAAAAAGTTCATGTTTTGTTATTTCTTTCTCACATACTT
TATAAAAAAAGATAATTTTTTTAAAAAAAAAAAAAAAATACAAGTGGAACATTATCAAATAAAAAGCATTAT
AGCGCCAC

CAAACTTATGCCATTTCTATCATGATAACCAAAAATAGTCTTAACAAAATACATATAAGTAAATAATGCTA
AATAAAAAAGCAAAAAAATAAAATTTATATCACACTTGTAAAACCTTTAAATGATCTTCTTTTTTACCTGT
TTTATTCA

AGATTTTGTATCGGCTATTACGTCAAGTGAGAAGAGTTTTGGCCTTCGCTTCAGTTAGATCTTCTATTATT
TCCTTTTTTTTCTTTTTGTTTGTAATTGTTTTTTATTTTTTTTTTTGCGCGAAACTTTGCTATATTGGGTA
ACGCGTAA

GTTCATTCTCTTTCATTTTCGGTAGTGAGATGGCAGTTCGAGGGGTTTTTTATTCAAAATAATAACTTCTG
GCTTTTCGCTTTTATATAGCAGAAAAAAAAAGCCGTCGAGGCGCGCGCGTTCATGCAATGGCTCAGTAACCT
CGGGATAG

CCCTTAAGTTCTCAACCTTTTAGTTTTTTTTTTTTTTTTTTTTTTTATTTTGTATGGCATACTAACTATACAA
ATATTTATATGTACATTTATACAGTAACCTATTATTTACCGATATTCACCGGTATTTTACTTTAATAGATG
CTGGTAAT

ATAAAAATCATTAGATCCACATTACTAATTATTGAATATGGACAATAATATCAAATATATTATTATCAAAT
TGATTATTTTGTGACGCGGCGTCAAATTTTAATCGCGTCTGACGAATGAGCGGGTAATAAACTGTCTTTTT
TTCATGAA

TCTGTGATTTTTTTTTTTTCAATTGTTCGAAATAACCTGTATTGTTCAATACTGGACAATTGTTCTGATATA
TATATATATATATAATTTTCAATGAAAAAAAAAAAAAAAAAGAAAGAAAATTAGAAGCTAGAAAGAAATAAG
GAAAAGGA

GTTGCTTTGTATTGAGCTGTATAAAAATCATTAGATCCACATTACTAATTATTGAATATGGACAATAATAT
CAAATATATTATTATCAAATTGATTATTTTGTGACGCGGCGTCAAATTTTAATCGCGTCTGACGAATGAGC
GGGTAATA

TTTTTTTTTTTTTTTTTTTTTTTTTTTTTTCAGTTTTCGAAGAAACTGCCTTTTTAGAATTGTAAAAGACG
AAGTGAAGTATTCAGGAGTATATTATTACATACATACAAAGCAAGACAAAGAAACATTTTTAGATCTAACA
TTTTACTT

GAAAAAAAAAAATTAAAATAAAGTCACAGAAATTCAATGAATATTATAAATTTTTCTTGTTTGTTTCCCTA
GTTGTTATTTTTATAAAAAAAATTCTTGTAGACAATAAAATAAGAAATGCCCATTTTGTAACTTAGCGAAA
GATGCCCA

AATTTTTCCAAAATTTGATATATATATATATATATATATGTTTGTATGTATATATATATATACGTATATAT
ATCATATATACGAAAAGTAGAAAAAAAAAGGTGATATTTCGCTCGTGGAAAAGCTAATGCCACAGCTTGTG
TTTCGTGT

AGGGGTTTTTTATTCAAAATAATAACTTCTGGCTTTTCGCTTTTATATAGCAGAAAAAAAAGCCGTCGAGG
CGCGCGCGTTCATGCAATGGCTCAGTAACCTCGGGATAGAAAAAGGGCAACAATGTTGAGCTATTTTAGGC
ACAGAAAC

TTTTGAGGGGTCTTTAACTGGAAAAAAAAAGCTTTCACGACTCAAAGAAAAAAAAAAAAATTTTTTTAGGG
TAAAAAAAAACTAAGGGCTAATATGAAAAATACAAAGGTTATGTTACTTTAAAATGAGGTTTGGAAGAAGG
AGAAAGAC

GAGGCGCGGGTTCAAACCCCGCGGGTATCAATATTTTTTTGAATCTACGCACTTCAATAAGAATTAGGCTG
TTAGGATAGCATTTTTTTTCTATGTACCAAAAGTATTCATACGTGGCTCCTTGTTACCCCACAAAACTTTT
TTAGACTT

ATATATGTATACACGTTCATTACCACGTATACATATCCATATATATATATATATATACATACTATTTAT
GATAAATTTTTAACAAGCAAACCCATTATTAAATGCTATAAATAAAAGACAGCTTCTTTCTAATTATTATT
CAAACTTA

TTTCCTGGTAGAATTTCCCTTAATGGGAGAGTAGTAACAATGCAAAAAATGAAGAAAATTGAGAAAAAACA
AAAAGAAAAAAAAAAAAAAAAAAAAAAAAGAGAGCTATGTAGGTTGATTGTGTTGTTCTATGTAGTGCCCT
TTATTTTT

TTTTACTTTCCTGGAAAACGCCATCGAAATAGTCGCCAAAAAAAATCGATGGGCTGGAAAACGCGTCATCA
ATTCAACCGAATAAGGAAAACTAAGCCACTTCACGCGGTTCGCGATATTTGTCCAGCCTCTTTTTCCGAAA
AAAAAAAA

TTTTTCGACGGAGGAGGGAAAAAAAAAAATTGTATTTAAGGCATCAAATGCGCGACATCATATACCTTTGT
CCGCTGTTATCATACCTTGAACCTCTACATTTCAGCCTAGCGCGCGCAAAAAGAAAAAAAAATCATAAGCA
AGAGTGGT

GTACAAGATATTTTTTACTTTTGCCATCTTATACTCATCCTAATCATCTGTTTCATTTTCCTTCTATTTCT
TTTTTTTTTTTTTTTTTTTTTTTTTTTTTCAGTTTTCGAAGAAACTGCCTTTTTAGAATTGTAAAAGACGA
AGTGAAGT

TGACATTTTTCCTTTTTTTTTCTCTTATTACGATTTACCTTTTTTATTTATTTTTTTCATTTTAGTATTTT
ATTCTTCGTTATTTATGTATAGAAATTTTCATTTTCATTTAGATTCAGATTTGGTTATCTTTTTTCATTAT
ATATCTTT

GCCGAGAGCTGTACAAGATATTTTTTACTTTTGCCATCTTATACTCATCCTAATCATCTGTTTCATTTTCC
TTCTATTTCTTTTTTTTTTTTTTTTTTTTTTTTTTTTTTCAGTTTTCGAAGAAACTGCCTTTTTAGAATTG
TAAAAGAC

AAGAAACATACTTCAAAAAAAAAAAAAAGAAAAACAACAAAAAAACGTATAAAATGAAATAAATTTCGAAC
CGCTTTTTTTCCTTTTTTTTTTTTTTACTTTGTTTGACCTCCCCTAACTCTTTCTTTTTACCTTCACAATT
GTTTATTT

GCATGGTTGGTGAATTGGATATCAAATTTGACCTTAAGTTTTAGTAGGCAGGACAATTTCAGTGTTTGTAA
AAATATATTTATATTTATATATTTAAATATACTAAATAAAAATAATACTATCATCGTTGTGATGCAAAAAA
AAAAAAAA

TATATAGAATTTAATAAAGACAAATGCAAAAGTTTTTTTAAAGTATGGATGTATAATAATAATAACGATAA
TACGAACACTATTGGGACTAATAATGATTTATAGTTTTTTTACACAGGGGATTTAACGTTCCGCGACCCT
TTTTCCTT

AAAAGTCTTCAGCAGTAGTTGCAAAAGTGTCCTTTTTTACTTTTTGGGCTACATTATTCTTGAAAAAAGTT
CATGTTTTGTTATTTCTTTCTCACATACTTTATAAAAAAAGATAATTTTTTTAAAAAAAAAAAAAAAATACA
AGTGGAAC

TCGCGCGTGGCCTTTCTCTATGGAGATTTCTCAAACGATATTAATCCTGTATATCTCTATTAATTTAGTTA
ATACATACACGATATATCGTCGCTTTTATTTTTTTTTTTCAAATACTTCTTCTTCCTAAAAGAGTTAAAAA
CTCTTCTC

GGCAATGACTGTATATATATGTATATATATATATATATATATATATATATATTTATTTATTTATTTATTTAAA
ATATAGCTACGTATGCGCCAGTTGTTTTTATCAGCTTTGCTTCTGGCCACATTTCAACCAGGTCTCAGAGG
ATATATGG

TAATGGGAGAGTAGTAACAATGCAAAAAATGAAGAAAATTGAGAAAAAAACAAAAAAGAAAAAAAAAAAAAA
AAAAAAAAAGAGAGCTATGTAGGTTGATTGTGTTGTTCTATGTAGTGCCCTTTATTTTTCTTTGGATTTAA
ATTTACAG

TTCTCCATCACCCATTTTTCATTTCATTTGCCGGGCGGAAAAAAAAAAAGGAAAAAAAAAAAAAAAAAAAAT
AAATGAACACATGGAAATAAGTCAAGGATTAGCGGATATGTAGTTCCAGTCCGGGTTATACCATCACGTGA
TAATAAAT

TTGCCATCTTATACTCATCCTAATCATCTGTTTCATTTTCCTTCTATTTCTTTTTTTTTTTTTTTTTTTTT
TTTTTTTTTCAGTTTTCGAAGAAACTGCCTTTTTAGAATTGTAAAAGACGAAGTGAAGTATTCAGGAGTAT
ATTATTAC

GTCTTTTATATATTATATTATTTGATACTTAACACAAAAATAAGTACGGATCACGTTTTTATTTTTTTATT
TCATTTTTCTTGTAATGATATATTATTAATAAAAAACTAACAAAAGTTAATTATCTTAACGGGAAGTGGCG
GCAACCTT

AAAAACATTCTTTTGATCTCGAAGTAGGAAAACTGACATTAGAAAAAGAAAAAAAAAAACAATCTGCGAATT
ATGCATTTCTTTTTTTCGCTTTTTTATTTTCTTTTTTCTTTTTTTCGACCTCCAATAGGGCTTTGGAAGCC
CTATATGT

CTTTCTTTCGTGTAATGTGACACAATTTTGCTATTGCGGAAACTGCAGCATAGTCAGTGTAATAATCGTAC
TGTTGCTTTTTCATTTTTTTTTTTTTTTTTTTTTTTCTCATATAGAAAAACTGTTATTGAATGTTTTGACT
AGTCTCAT

GTGTGGGGCCCGTTCAAGAATGGGAGGTATTGGGACGGGTGGACGTTTAAGCATACATTTATGTGCACTGT
AAGTAATTATATAGAATTTTTTTTTTTTTTTTTTTTTCATATCTGTCAATACTTGAAAATGTTCTAGTTA
GAGATTTA

TCATTTTTAAGTTCATTCTCTTTCATTTTCGGTAGTGAGATGGCAGTTCGAGGGGTTTTTTATTCAAAATA
ATAACTTCTGGCTTTTCGCTTTTATATAGCAGAAAAAAAAAGCCGTCGAGGCGCGCGCGTTCATGCAATGGC
TCAGTAAC

CATTTCAGTAAAAAGTCTTCAGCAGTAGTTGCAAAAGTGTCCTTTTTTACTTTTTGGGCTACATTATTCTT
GAAAAAAGTTCATGTTTTGTTATTTCTTTCTCACATACTTTATAAAAAAAGATAATTTTTTTAAAAAAAAA
AAAAAATA

AAATGATCATGTACCTTCTTCTCGCGAAAGTTTTAATTAAAATTTACTGATAGTTTTTTAATGATGAACTT
CTATAACTAAACCATAATAATGAAAATTACAGGTAATATTTATATCACAAGAAACGGCCCCTCTTTTCTGT
TTTTATAT

TATTCTTACTATCATAATTAAATATTTTTATCGTTATTAATTATTATTATTATTACTATTATTATTATTAT
TATTATTATTATTATTATTATTTATTATTTATTAATTATTAATTGTTAATATTATTAATTCTTGTCTATTG
TCTTTTGA

AATTCTAAAAACTATATTGCAATCTTTTAAATATATGTATACACGTTCATTACCACGTATACATATCCATA
TATATATATATATATATACATACTATTTATGATAAATTTTTAACAAGCAAACCCATTATTAAATGCTATAA
ATAAAAGA

GTAAAATATAACATAAATCAAAGAAAAACGAGAAAACGATATGATTATCCGATACCGACAAATTCTCATGT
TTATTTTTTTTTTTTTTTTTTTTTTTTTTAATTTTTCTTGGGCCTTTACATTGAGTACAGGGGCATAAGT
GCGAGGAG

AAAAATAGTCTTAACAAAATACATATAAGTAAATAATGCTAAATAAAAAAGCAAAAAAATAAAATTTATAT
CACACTTGTAAAACCTTTAAATGATCTTCTTTTTTACCTGTTTATTCACTTGTTTTTTTTTTTTCTGAGTT
GTTGCGCC

GCAAAAGTGTCCTTTTTTACTTTTTGGGCTACATTATTCTTGAAAAAAGTTCATGTTTTGTTATTTCTTTC
TCACATACTTTATAAAAAAAGATAATTTTTTTAAAAAAAAAAAAAAATACAAGTGGAACATTATCAAATAA
AAAGCATT

CCCTATTCTCGCCGAGAGCTGTACAAGATATTTTTTACTTTTGCCATCTTATACTCATCCTAATCATCTGT
TTCATTTTCCTTCTATTTCTTTTTTTTTTTTTTTTTTTTTTTTTTTTTTCAGTTTTCGAAGAAACTGCCTT
TTTAGAAT

GATTGGGAAGTGCAGACAGCAACAGCTACACTTAAGTACTTTTAATATTGGATTGAAAATTTTCGTTCTTG
CGATGAGCTGCCCTTGCAAATTTTTCACCAAAAAAAAAAAAAAAAAAAGAAATGGAGTGAAAAAAGAAACG
AAAAAAAA

TAGCGACGAAAAATGCGAGATCTCGACCAAAAAAAGGGGGTAGGGTAATAAAATTAACCCTATTATTTTTT
AACTTTAAAACCTATAATGTGCTAATATTTTATTATAAACCTCCTTTTTTTGCGTTCAAACCCTGACACAT
TTTAAGCC

AAGATTATTATTATTAGTTACTTTTTTTATTGTACTCCAGTATGGACCTCTTAGGTGAGTGATCTTATTAA
AAAAAATAAAAAAAATAAAAAAATATGAAAAAAAAAGAAAATTAAAAACTAAACAAATTTTATGACAGGAA
TAAAAACT

CGGTTTTTCATTTTTCATACTGGTAATTCAGAGAGCCAAGTCTTTCTTTATAATGCATAGCAATAAATAGT
CATATATATATATATATATATATATATATGTACATATATATGAACATATATATGAACAAATAAATGATAAT
GGTATGTG

GTACCTTCTTCTCGCGAAAGTTTTAATTAAAATTTACTGATAGTTTTTTAATGATGAACTTCTATAACTAA
ACCATAATAATGAAAATTACAGGTAATATTTATATCACAAGAAACGGCCCCTCTTTTCTGTTTTTATATAG
TAACCATT

TACTCGAAAAGGAAGAACACTAAAAGTACTTACATTTTCACATGTATGTATACCTATATATATATATATAT
ATACTCTTATAGATATATTTACAAATTAAAGGAAAAAATAATAAAATAACCTCCCTGTCACAAGTTAAAAC
ACGGCCCC

GAATTTCCCTTAATGGGAGAGTAGTAACAATGCAAAAAATGAAGAAAATTGAGAAAAAACAAAAAAGAAAA
AAAAAAAAAAAAAAAAAAAGAGAGCTATGTAGGTTGATTGTGTTGTTCTATGTAGTGCCCTTTATTTTTCT
TTGGATTT

TTTCTCAAAAACCTCATGGTTTCTCCATCACCCATTTTTCATTTCATTTGCCGGGCGGAAAAAAAAAAGGA
AAAAAAAAAAAAAAAAAAATAAATGAACACATGGAAATAAGTCAAGGATTAGCGGATATGTAGTTCCAGTC
CGGGTTAT

TCTTCCCCGCGTGTTTTCCGTACACCAACAATATATGCCATAATACACGTAACATTTTTTTATAAAAAGAA
AAGGTAAGTGATATATATAAAATAGCGCCATGAGTAGGAAACTTTTCTGTTACTGCAGATATGTGCCAGAC
TGGCTCAG

TTTTTCATACTGGTAATTCAGAGAGCCAAGTCTTTCTTTATAATGCATAGCAATAAATAGTCATATATATA
TATATATATATATATATATGTACATATATATGAACATATATATGAACAAATAAATGATAATGGTATGTGAT
AGTCATTA

TATTTCCCTTTTGCATTTGTCTTCTTCGGTTTTCCTGGTAGAATTTCCCTTAATGGGAGAGTAGTAACAAT
GCAAAAAATGAAGAAAATTGAGAAAAAACAAAAAAGAAAAAAAAAAAAAAAAAAAAAAAAAGAGCTATGTA
GGTTGATT

AACAATGCTCAAAAGAAGACTAACTAAGAGAAGAAACATACTTCAAAAAAAAAAAAAAGAAAAACAACAAA
AAAACGTATAAAATGAAATAAATTTCGAACCGCTTTTTTTCCTTTTTTTTTTTTTTACTTTGTTTGACCTC
CCCTAACT

TTCTTCCGTCCCGTGCGTGAGGGGGGCGCGGCCATTCGGTTTTTGCAATATGACCTGTGGGCCAAAAATCG
AAAAAAAAAAAAAATAAGAGGCGGCTGCGGAATTTTATAAGACAAGCGCAGGGCCAAAGAAAAAATAATAAT
TGACGTGG

GAACCGTACACGCTTTTAGCCAAAAAAAAAAGAAAGGTAGAATATAAAATAAAAACTCGTATTTCGTATTA
AGACAAATATACAGAAATACAAATGAGATCATAAACGAACTAAAATAGGAAAATGAAAAAAAAAAAAAAAG
ATTAGCGA

CAAAAACAAAAATTATAATAAAAATAAAAATATGTATAATATAATTTTTATCTTCTAACTTTTTATTTATT
GAATTTTTATATAGTAAATATAACACGTGATATATTTGAAATGTGATTATATTATATATATTGATGTTTAC
ATATTTTT

AAAAGCTCAGTCGCGCGTGGCCTTTCTCTATGGAGATTTCTCAAACGATATTAATCCTGTATATCTCTATT
AATTTAGTTAATACATACACGATATATCGTCGCTTTTATTTTTTTTTTTCAAATACTTCTTCTTCCTAAAA
GAGTTAAA

AGAATCATTCTTTTTTTTTTTTTTTTTTTTTTTTTGCCTCACATTATATTCTTGGCATCTGCCTAGCTTTC
GACTTTTTCGTTCAATTCCTAAAGCGCTAAAAAAATAGTCTCCCCCTGAAAGTGGAGTAGGATAGTAGTAT
ATGAAGTG

CGCGGGGTTGTGTGGCTCAAAAAGATCTGTTTTTGTATTAGCAATTTTTTATTTCCCTTTTTAAATGAATT
TTTTTTCTTTCTGTTTCGAGACAAGGCCTTTTGCAAAAAAAAAAAAAGTAAAAAAGTGAAATTTGATAGTG
TAAGGGGA

CGGCATCTTTTCTTTTTCTTTTCTTTTTTTTTTTTTCACTCTCCCTTTTAATGCCTCCACGGAGGTTCGAA
TGGGTTATACTAGAAAGAAAAAAAAAATAGTGATAATTAACAATGAAAAAAAAAAACAGCAACAAAATGTG
GTATTGGG

ATTGAGCTGTATAAAAATCATTAGATCCACATTACTAATTATTGAATATGGACAATAATATCAAATATATT
ATTATCAAATTGATTATTTTGTGACGCGGCGTCAAATTTTAATCGCGTCTGACGAATGAGCGGGTAATAAA
CTGTCTTT

AAGAAAAACGAGAAAACGATATGATTATCCGATACCGACAAATTCTCATGTTTATTTTTTTTTTTTTTTTT
TTTTTTTTTTAATTTTTCTTGGGCCTTTACATTGAGTACAGGGGCATAAGTGCGAGGAGTTACCAGACGAC
AACTAGCA

CCATCGTAATGGGATGATCATATGATGGGCGCTATCCTCATCGCGACTCGATAACGACGTGAGAAACGATT
TTTTTTTTCTTTTTCACCGTATTTTTGTGCGTCCTTTTTCAATTATAGCTTTTTTTTTATTTTTTTTTTTT
CTCGTACT

ATAATAAAAAAACTCCTTTATTAAGAGCGGAAGAATTTAATAATGAAGATGGGAATAAGCAAAACAAAAAC
AAAGAAGGGAAAAAAAATAAAAAATCGTATTTATTTATTTAAAAAATCATGTTGATGACGACAATGGAAAA
AAAAAACC

AAATTTGATATATATATATATATATATATATGTTTGTATGTATATATATATATACGTATATATATCATATATA
CGAAAAGTAGAAAAAAAAAGGTGATATTTCGCTCGTGGAAAAGCTAATGCCACAGCTTGTGTTTCGTGTAG
TTTGCCTT

TGGCAGTTCGAGGGGTTTTTTATTCAAAATAATAACTTCTGGCTTTTCGCTTTTATATAGCAGAAAAAAAA
GCCGTCGAGGCGCGCGCGTTCATGCAATGGCTCAGTAACCTCGGGATAGAAAAAGGGCAACAATGTTGAGC
TATTTTAG

CTGGACTGAAAAAAAAGGCAATTCGCGTACAATTTTCGTTGATCGTTCTTTATATAACCTTTGCATTAAATA
AATTTAACAAAAAAAAGTTCTTTCTAAAATAATATTATGGTGATACATGAATGTGCTTTAGTTTTTTCGTAG
GCTCATCC

TCATGTTTTGTTATTTCTTTCTCACATACTTTATAAAAAAAGATAATTTTTTTAAAAAAAAAAAAAAAATAC
AAGTGGAACATTATCAAATAAAAAGCATTATAGCGCCACATGCGATGAACCTGAGCTTTTATCTTCATTAA
TATATAGT

TCTTTTTCTTTTCTTTTTTTTTTTTTCACTCTCCCTTTTAATGCCTCCACGGAGGTTCGAATGGGTTATAC
TAGAAAGAAAAAAAAAATAGTGATAATTAACAATGAAAAAAAAAAAAACAGCAACAAAATGTGGTATTGGGGT
CACACCAC

TTTTGTTTACCGTCGCAAAAAAAAAAACGCGAAGAATACGCGTTGGTCGTTAAATTTTGTGGTACGCGTAAA
ACTGCTATTTGGTATCCTCTTTAGGGACGCGCCCATTGTCTATACGGTAATATAGGGCTTTCTTCATTACC
TGCTTGCG

CCATTTCTATCATGATAACCAAAAATAGTCTTAACAAAATACATATAAGTAAATAATGCTAAATAAAAAAG
CAAAAAAATAAAATTTATATCACACTTGTAAAACCTTTAAATGATCTTCTTTTTTACCTGTTTTATTCACT
TGTTTTTT

AATGCACCTCAGTATAAATTGTAAAATATAACATAAATCAAAGAAAAACGAGAAAACGATATGATTATCCG
ATACCGACAAATTCTCATGTTTATTTTTTTTTTTTTTTTTTTTTTTTTTAATTTTTCTTGGGCCTTTACA
TTGAGTAC

CAAAATTTTTGAAAAACGAAATAAAGAAGAAAGATTATTATTATTAGTTACTTTTTTTATTGTACTCCAGT
ATGGACCTCTTAGGTGAGTGATCTTATTAAAAAAAATAAAAAAAATAAAAAAATATGAAAAAAAAAGAAAA
TTAAAAAC

CACATCAAAAGGTAGTATTAATTAAAAAAAAAAAAAAAGCCAATAATTTCGCGCAAGCTTTGCTCTACATAA
TAAAAATACAATTAGGATAGAAATGAATATTTTTTTAGCGGCAGTTTTACCATAAGTAGCAAAACCCTAAC
AATACAAT

AGTAAACGTTCACATCAAAAGGTAGTATTAATTAAAAAAAAAAAAAAAGCCAATAATTTCGCGCAAGCTTTG
CTCTACATAATAAAAATACAATTAGGATAGAAATGAATATTTTTTTAGCGGCAGTTTTACCATAAGTAGCA
AAACCCTA

TACACTCTTCTCATTTTTAAGTTCATTCTCTTTCATTTTCGGTAGTGAGATGGCAGTTCGAGGGGTTTTTT
ATTCAAAATAATAACTTCTGGCTTTTCGCTTTTATATAGCAGAAAAAAAAGCCGTCGAGGCGCGCGCGTTC
ATGCAATG

GTTGCGGTGCAAAAACATTCTTTTGATCTCGAAGTAGGAAAACTGACATTAGAAAAAGAAAAAAAAAAACAA
TCTGCGAATTATGCATTTCTTTTTTTCGCTTTTTTATTTTCTTTTTTCTTTTTTTCGACCTCCAATAGGGC
TTTGGAAG

GATTACGTTTTGACATTTTTCCTTTTTTTTTCTCTTATTACGATTTACCTTTTTTATTTATTTTTTTCATT
TTAGTATTTTATTCTTCGTTATTTATGTATAGAAATTTTCATTTTCATTTAGATTCAGATTTGGTTATCTT
TTTTCATT

TCAAAATATGTATTCTTACTATCATAATTAAATATTTTTATCGTTATTAATTATTATTATTATTACTATTA
TTATTATTATTATTATTATTATTATTATTATTTATTATTTATTAATTATTAATTGTTAATATTATTAATTC
TTGTCTAT

TTTTTTCTTTCTTTTGAATGTGTGTATAAAAGAGAGAAAAAAATGGTTATTTTCCAATTAATTAATTATTAA
CGATTGTTAAAGAAAAAATTTATCAACGAGGTTGATAGAAAAAAAAAGTTTTTGTAGATATAGAAGAAAAA
AAGATTTT

CTTCAAAAAAAAAAAAAAGAAAAACAACAAAAAAAACGTATAAAATGAAATAAATTTCGAACCGCTTTTTTT
CCTTTTTTTTTTTTTTTACTTTGTTTGACCTCCCCTAACTCTTTCTTTTTACCTTCACAATTGTTTATTTTA
ATATATGA

CACAATTTTGCTATTGCGGAAACTGCAGCATAGTCAGTGTAATAATCGTACTGTTGCTTTTTCATTTTTTT
TTTTTTTTTTTTTTTTCTCATATAGAAAAACTGTTATTGAATGTTTTGACTAGTCTCATGAAATAAACATT
AATAGTTA

GTGCTACTTTAGTAAACGTTCACATCAAAAGGTAGTATTAATTAAAAAAAAAAAAAAAGCCAATAATTTCGC
GCAAGCTTTGCTCTACATAATAAAAATACAATTAGGATAGAAATGAATATTTTTTTAGCGGCAGTTTTACC
ATAAGTAG

CATCTCTTCAAAATTTCACAGTCATGCTCACCCTTAAGTTCTCAACCTTTTAGTTTTTTTTTTTTTTTTTT
TTTATTTTGTATGGCATACTAACTATACAAATATTTATATGTACATTTATACAGTAACCTATTATTTACCG
ATATTCAC

ACAATGTGTATGATGAGGAGGAATGTACCTAAGCCAAAAAAAAAAAAAAAAAAAAAAAAAAAAAAAAGAAAC
AGCTTTTGCATATTCAATCCAGGCATAGGGCGACTATTTAGCACTCAACGATTTTTAAGCTTGTGTATTGC
TGACATAA

TATTTACATAGAACCGTACACGCTTTTAGCCAAAAAAAAAAAGAAAGGTAGAATATAAAATAAAAACTCGTA
TTTCGTATTAAGACAAATATACAGAAATACAAATGAGATCATAAACGAACTAAAATAGGAAAATGAAAAAA
AAAAAAAA

TGCTTATCCAATTTTAGTTAGGAACTTTGGCATAATTTCAGTATTACATACTACCTAATCGACATAAAATA
ATATTACATATTCATTTTTTTAAAAAATAATCATAATCAATTTATCCTTTTTATTCCTTTTTTCTCCTGTG
CAAGGTTG

ATCATAATTAAATATTTTTATCGTTATTAATTATTATTATTATTACTATTATTATTATTATTATTATTATT
ATTATTATTATTTATTATTTATTAATTATTAATTGTTAATATTATTAATTCTTGTCTATTGTCTTTTGATT
TATCTGCA

AAAAAAAAATTGTATTTAAGGCATCAAATGCGCGACATCATATACCTTTGTCCGCTGTTATCATACCTTGA
ACCTCTACATTTCAGCCTAGCGCGCGCAAAAAGAAAAAAAAATCATAAGCAAGAGTGGTACGGTAACCACA
AGGCAATG

TATTATATTATTTGATACTTAACACAAAAATAAGTACGGATCACGTTTTTATTTTTTTATTTCATTTTTCT
TGTAATGATATATTATTAATAAAAAACTAACAAAAGTTAATTATCTTAACGGGAAGTGGCGGCAACCTTTT
GGAAAGCA

CTATATGTGGCGGCATCTTTTCTTTTTCTTTTCTTTTTTTTTTTTTCACTCTCCCTTTTAATGCCTCCACG
GAGGTTCGAATGGGTTATACTAGAAAGAAAAAAAAAATAGTGATAATTAACAATGAAAAAAAAAAAACAGCA
ACAAAATG

CCGTCCACGAAAAAAATGTTAACATAAAATGCAAGAGAACAATTAATCGAATAATGTTAAATTATTGTAAAA
CAATGTGTATGATGAGGAGGAATGTACCTAAGCCAAAAAAAAAAAAAAAAAAAAAAAAAAAAAAAAGAAACA
GCTTTTGC

CTTCTATTTCTTTTTTTTTTTTTTTTTTTTTTTTTTTTTTTCAGTTTTCGAAGAAACTGCCTTTTTAGAATT
GTAAAAGACGAAGTGAAGTATTCAGGAGTATATTATTACATACATACAAAGCAAGACAAAGAAACATTTTT
AGATCTAA

ATTAAAAAAAAAAAAAAAGCCAATAATTTCGCGCAAGCTTTGCTCTACATAATAAAAATACAATTAGGATAG
AAATGAATATTTTTTTAGCGGCAGTTTTACCATAAGTAGCAAAACCCTAACAATACAATGTGAGACTAAAG
TAAATCCG

AGAAAGAAAAAATATGAAATCAAAATTAAACATTTTTCTGTAATTTCCGTAATACAAAAAACTATTTTCTT
TTCGGAAGGCAAAAACAAAAATTATAATAAAAATAAAAATATGTATAATATAATTTTTATCTTCTAACTTT
TTATTTAT

GGTGAAATCTACGAAACTGGTAAGTTCTTTTAAGTATCACATTACAATAACAAAACTGCAACTACCATAAA
AAAAAATTGAAAAATCATAAATTAAAAAAAAAAAAATCAATTGAATTTTTTTTTTTCATGATTACGTTTTG
ACATTTTT

AGAAAACGATATGATTATCCGATACCGACAAATTCTCATGTTTATTTTTTTTTTTTTTTTTTTTTTTTTTT
AATTTTTCTTGGGCCTTTACATTGAGTACAGGGGCATAAGTGCGAGGAGTTACCAGACGACAACTAGCAGG
CGGATGAA

GAGGAGGGAAAAAAAAAAAATTGTATTTAAGGCATCAAATGCGCGACATCATATACCTTTGTCCGCTGTTAT
CATACCTTGAACCTCTACATTTCAGCCTAGCGCGCGCAAAAAGAAAAAAAAATCATAAGCAAGAGTGGTAC
GGTAACCA

GGAAGAACACTAAAAGTACTTACATTTTCACATGTATGTATACCTATATATATATATATATATACTCTTAT
AGATATATTTACAAATTAAAGGAAAAAATAATAAAATAACCTCCCTGTCACAAGTTAAAACACGGCCCCAT
CACTTATA

TCCCGGTTGTGTGATTGAAAACGGAACTAAGCTTTACTAATCAACAATATTAACTTTATAATAGATTCATA
TATGCGCGTACATATATTATATACAATTTTTCTACACAAGAATGCTTAATAAAGATACGATAATTTTTTGT
TCGCTTTT

GGTAGTGAGATGGCAGTTCGAGGGGTTTTTTATTCAAAATAATAACTTCTGGCTTTTCGCTTTTATATAGC
AGAAAAAAAAGCCGTCGAGGCGCGCGCGTTCATGCAATGGCTCAGTAACCTCGGGATAGAAAAAGGGCAAC
AATGTTGA

TCGACTCCAACAGGAATGTATATTCTACGAAAACGAGATGTATTGAATAGTGATGTTATGACCTTTTTTTT
TACGCCTTTTTTTTTTTTTTTTTTTTTTTTAATTTATCTCACTTTTCCCTTCTATTTTATGTACTAACAGA
TAATTTTA

TTGTATCACGGTCTTTTATATATTATATTATTTGATACTTAACACAAAAATAAGTACGGATCACGTTTTTA
TTTTTTTATTTCATTTTTCTTGTAATGATATATTATTAATAAAAAACTAACAAAAGTTAATTATCTTAACG
GGAAGTGG

AAAATAAAAATATGTATAATATAATTTTTATCTTCTAACTTTTTATTTATTGAATTTTTATATAGTAAATA
TAACACGTGATATATTTGAAATGTGATTATATTATATATATTGATGTTTACATATTTTTTATGTAGTACAA
TTAATTTT

AAGAAAACACAACCTTTTCAGTTGCGGTGCAAAAACATTCTTTTGATCTCGAAGTAGGAAAACTGACATTA
GAAAAAGAAAAAAAAAACAATCTGCGAATTATGCATTTCTTTTTTTCGCTTTTTTATTTTCTTTTTTCTTT
TTTTCGAC

TTATAACTATATTAATAAAAAATTTTATTTTCTATTGAAAACCAATTTTTTACACCAAATATCAGACACGC
TTTGTCTATTTCTATCATATAGCGACTGTGATATAATGCGCGCGTGTTTTGAACGGCATTTTTTTAGCCTG
CGAATATG

AACCTTTTCAGTTGCGGTGCAAAAACATTCTTTTGATCTCGAAGTAGGAAAACTGACATTAGAAAAAGAAA
AAAAAAACAATCTGCGAATTATGCATTTCTTTTTTTCGCTTTTTTATTTTCTTTTTTCTTTTTTTCGACCT
CCAATAGG

AGCGTTCTTTCTTTCTTTCGTGTAATGTGACACAATTTTGCTATTGCGGAAACTGCAGCATAGTCAGTGTA
ATAATCGTACTGTTGCTTTTTCATTTTTTTTTTTTTTTTTTTTTTTCTCATATAGAAAAACTGTTATTGAA
TGTTTTGA

TGCCAAACCCATTCTGCTTCTTTGTTTAATGGTGTAAGCTGCCTATATGTTACTATTGAGTACTCATCTCA
TCGCTTCTTTCAGAACAAAATTTTTCATATTTTTTTTTTTCCTTTTCTTTTTTTTTTTTTTCTTTGACTGT
TACCCGGT

ATACTCATCCTAATCATCTGTTTCATTTTCCTTCTATTTCTTTTTTTTTTTTTTTTTTTTTTTTTTTTTTC
AGTTTTCGAAGAAACTGCCTTTTTAGAATTGTAAAAGACGAAGTGAAGTATTCAGGAGTATATTATTACAT
ACATACAA

CTTTATTTTAAACATTATACAAGTTTTTCATTTTGAGGGGTCTTTAACTGGAAAAAAAAAGCTTTCACGAC
TCAAAGAAAAAAAAAAAATTTTTTTTAGGGTAAAAAAAAACTAAGGGCTAATATGAAAAATACAAAGGTTA
TGTTACTT

AAATCGACATAATGCACCTCAGTATAAATTGTAAAATATAACATAAATCAAAGAAAAACGAGAAAACGATA
TGATTATCCGATACCGACAAATTCTCATGTTTATTTTTTTTTTTTTTTTTTTTTTTTTTTAATTTTTCTTG
GGCCTTTA

TCTTTCTTTATAATGCATAGCAATAAATAGTCATATATATATATATATATATATATATATGTACATATATA
TGAACATATATATGAACAAATAAATGATAATGGTATGTGATAGTCATTATTAACGATTAAGCGTTTTTCTT
CAGTTTCT

TTATTGTAAAACAATGTGTATGATGAGGAGGAATGTACCTAAGCCAAAAAAAAAAAAAAAAAAAAAAAAAA
AAAAAGAAACAGCTTTTGCATATTCAATCCAGGCATAGGGCGACTATTTAGCACTCAACGATTTTTAAGCT
TGTGTATT

TCCAAAAATGTTTCTGACGGCATCGAAGAGCCGAACTCTTATACAATACATGTCATTTTTAACAATGATGC
AAATAACTTTTAAGGCCTTAACACTCTACATAATATGCTAAAAACATTAAAAAAAAAAAAAGGAAAATAAA
AAAAAAAA

AAGCAAAAGAAAAGAAAAAAAAAAAAAAACTGAAATTTCCACCTTACCCGTTTTTTTAATGGAACAGTGATT
GTTACGACATTGAGATTTTAGATTTATATAATCACATACATATTAAATAGTTAATGCACCTTTAATTCTTT
TGCACTTC

ACAATTTGTTATCGGTTATAACTGCCGATACCCGTATCTTTTGTGTTTGATTCAAGGAATACTTATTTAGA
GTAGAAAGAATATATTTACTTAGATAACTTAATGTATAATATATAAAAAAAATCAAAAAAACAGGAAAAAA
AAAAATCA

AGATAATTTTTTAAAAAAAAAAAAAAAATACAAGTGGAACATTATCAAATAAAAAGCATTATAGCGCCACA
TGCGATGAACCTGAGCTTTTATCTTCATTAATATATAGTAATTACAACATGTTATTTGAAATGCACAGCAC
TTATCCAG

AATCTTTTAAATATATGTATACACGTTCATTACCACGTATACATATCCATATATATATATATATATATACA
TACTATTTATGATAAATTTTTAACAAGCAAACCCATTATTAAATGCTATAAATAAAAGACAGCTTCTTTCT
AATTATTA

ATATCACAGTTGACGAAAGAAGACACGTCGCCTAAACGCGTAATGAATCGCAATATACGAAAAGCTAAACG
GGTTTTTCAGAATCCTCTTACCCAGCCTTGGATAATAGTTTTGGCCGAAAAAAAAAAAAAGAAAGAAAAAA
ATTGAAAT

ATTTAAATTTCAATTAAAACAGTAGTAGTATGTGGGGAAGATATGGGATAAAAAAAAGTAAGGGAAAATAA
GGAATTATTATAATTATAATGCGGAAAAAATGAATAAATTATTAGTTGCTGCAACAGCAATACTATTTTCT
CTTGGATG

GGGGGGCGCGGCCATTCGGTTTTTGCAATATGACCTGTGGGCCAAAAATCGAAAAAAAAAAAAAATAAGAGG
CGGCTGCGGAATTTTATAAGACAAGCGCAGGGCCAAAGAAAAAATAATAATTGACGTGGCTGAACAACAGT
CTCTCCCC

TACTTATTGTGACCTTTGGGGTACCGTTACCGTCAGTTTTCTTCAGCTAAGGCGCGCGCGCCAGATAACTA
AAAAAAAATATAGTTGCTGCTTAAAAAACAATACACCCGTACTCTCTTGCCTGTAAAAACCTCGAAGGACC
AAAGATAC

CGCTAATTGAAGTTTTTTTTGATCACTCCAATAGGCAAATCTATAGATATATAAAAAATATAGACAAGACT
TTTTTTTACATTGCCAGTTTTCTTTTTTCCTTTTTAGTATCTATTCAAATGGGCGACCCTATTGTCTAATT
TCATTAGC

CAGCGCGTTTCTTTTTTTTTTTTTTTTTTTTTTTATTTTTCGGTTTTTTTCGCACGTTTCTTTTTGGTGA
GCGAGAAAGCTCGTTGCCTCCTAATATAAAGGATGAGATGAGATGAGCAGTGCCGCAAATGAGGGATGTGT
TTATTTGT

ATGACGTTTTCAACGTAACGCAGATTTCTATTGTATCACGGTCTTTTATATATTATATTATTTGATACTTA
ACACAAAAATAAGTACGGATCACGTTTTTATTTTTTTATTTCATTTTTCTTGTAATGATATATTATTAATA
AAAAACTA

CTTGTTATATATACCAAGCTTTTTGGAAAGCTAAGGATAAAAACAATTCTTTTCATTAGATGTATTTTTTC
ATCGTATTAGGTGTAATATATATATATATGTATATATATATATATATATAATATATATGTAGTATACACAC
GTGGACGA

ACTTTTTTTTCCCTTTTTCTGTCACAGGTTTCCCAATTTTTTTGACGACTAAATAATAACATAACCACTAC
GTTCACTTCCCCGTATATATACATATATACTTAATATTATAAACGCATATACTTTTATAAACTTGGAATAA
GTATTATA

ACTTTGCTGCTTAGGCTTTTAATGAGGTATGCTTTTAAAGATTTATATATTTTTTATACACGCTATATACA
CAATTTTCGCTGATGATTTTGTTTTATTATTGATTTTTAAATTAACCGTAGAAAATACTAAGTGGAAAGAT
CCGATAGT

ACCTCATGGTTTCTCCATCACCCATTTTTCATTTCATTTGCCGGGCGGAAAAAAAAAAGGAAAAAAAAAAA
AAAAAAAAATAAATGAACACATGGAAATAAGTCAAGGATTAGCGGATATGTAGTTCCAGTCCGGGTTATAC
CATCACGT

TTTGATACTTAACACAAAAATAAGTACGGATCACGTTTTTATTTTTTTATTTCATTTTTCTTGTAATGATA
TATTATTAATAAAAAACTAACAAAAGTTAATTATCTTAACGGGAAGTGGCGGCAACCTTTTGGAAAGCACC
CTTAGCTT

ACTAAGACAATCAAAATATGTATTCTTACTATCATAATTAAATATTTTTATCGTTATTAATTATTATTATT
ATTACTATTATTATTATTATTATTATTATTATTATTATTTATTATTTATTAATTATTAATTGTTAATA
TTATTAAT

TGCGTATTCATATTTCCCTTTTGCATTTGTCTTCTTCGGTTTTCCTGGTAGAATTTCCCTTAATGGGAGAG
TAGTAACAATGCAAAAAATGAAGAAAATTGAGAAAAAACAAAAAAGAAAAAAAAAAAAAAAAAAAAAAAGA
GAGCTATG

GATACACACAGATAAACATATATATATTATGTATATATATATATATATATATATATATATATATATGTATA
TATCAACGCCCATACCATTATCATTATCAATAAGTTTGCGAGGCGGGTACCCTCCCTCGTGCTATTATATT
ACTGTTGC

GTTTATAGTCATCTGTGAAATTTCTCAAAAACCTCATGGTTTCTCCATCACCCATTTTTCATTTCATTTGC
CGGGCGGAAAAAAAAAAGGAAAAAAAAAAAAAAAAAAAATAAATGAACACATGGAAATAAGTCAAGGATTA
GCGGATAT

TTTAAGCCTACTGGACTGAAAAAAAGGCAATTCGCGTACAATTTTCGTTGATCGTTCTTTATATAACCTTT
GCATTAAATAAATTTAACAAAAAAAGTTCTTTCTAAAATAATATTATGGTGATACATGAATGTGCTTTAGT
TTTTTCGT

TAATTGTTGTGATATTTGCATCTTCATCTTATGATGATGAGAATATAAATAATTTTTAATCGTATTAAGTG
TATATATGAATAAGCTATTAACCCTACCCATTAATTTCCGGGCGGGTTGTTATTTTTATCAGTATTGGCCT
CTGCCGGG

AAAGAGTATGTAGAAATGTCCGACGTTTTTTTGAGAAGTTCCTAATATAAAATATTAAAAATTAATTTTTC
AGAATATATGTGGAAATTTGTTATTTTTGTATCGTATATATGAATGACTAGAACATCATTATTTTTACCGG
AAACTTGC

GTCATACGCGCTACCGATTGCGCCAACAAGGCTATTTCGTTGAAAAATTCAGGCTTCAAAATTATTATATG
TATCAAAAATATTTTTTTGAGAAATTTCAAATCATTATCTACGCTAGCCTTACTATTTTTTCACCCAGAAG
ATAGTAAC

ATTGTTCGAAATAACCTGTATTGTTCAATACTGGACAATTGTTCTGATATATATATATATATATAATTTTC
AATGAAAAAAAAAAAAAAAAGAAAGAAAATTAGAAGCTAGAAAGAAATAAGGAAAAGGAAAAGCAAACAAC
AATAGGAA

TTGAGGGTTTGTTTTTTTTTTTTTAATGCAGATGTACGTACATATTTTCTTTATCAAAAGGATGTGTAGAG
AACTATGTATATATGTATATGTGTAGGTTTTTTTTTTTTAGGATGACTAGAAAAGGAAATCGAAAAAGAAA
AAATAAGC

TAATGAAGATGGAATAAGCAAAACAAAAACAAAGAAGGAAAAAAAAATAAAAAAATCGTATTTATTTATTT
AAAAAATCATGTTGATGACGACAATGGAAAAAAAAAACCGATTTCACTTTCTCATCCTTATATTTTTCAAA
GGTTGATG

CTTTTAAACTTTTTTCCATTCTGATGGAATAATGGTCTTGATCCCCTAAATATTTCCTTTTTTATTGCAAT
TATGATAGAAATTAATAGTAGTCTATGCTGGATGATATATATATCTCTTTTGAGCTTGCTTCAAATTTTTT
CCCGTTAT

ATTAAGTGAGAATAAACTTTTCGAAAAATTTTTATTTATGATAATTTTACTAATTCTTACTTATTTCTTTT
ATATTCGATAAAAAAAATTCAAAATAAAAAAAGATTAATCCTTTGATAGGAACTAAAGGTACTAGGTATTT
TGGGCGGC

TTGCATTTGTCTTCTTCGGTTTTCCTGGTAGAATTTCCCTTAATGGGAGAGTAGTAACAATGCAAAAAATG
AAGAAAATTGAGAAAAAACAAAAAAGAAAAAAAAAAAAAAAAAAAAAAAGAGAGCTATGTAGGTTGATTGT
GTTGTTCT

TGCGTATGAGGCCGAGGTATTTAGAGCATAAGAAAAAATGTGAACAAAAATAGTGTCATCTCCAGTAAGGC
TTAAATGTATATTGATTTTCTATATATATATATATATATATATATATTTATGAAATAAACCATATAATTAT
AAGTAGTA

GGTAGTATTAATTAAAAAAAAAAAAAAGCCAATAATTTCGCGCAAGCTTTGCTCTACATAATAAAAATACA
ATTAGGATAGAAATGAATATTTTTTTAGCGGCAGTTTTACCATAAGTAGCAAAACCCTAACAATACAATGT
GAGACTAA

CCCATTTTTCATTTCATTTGCCGGGCGGAAAAAAAAAAGGAAAAAAAAAAAAAAAAAAAATAAATGAACAC
ATGGAAATAAGTCAAGGATTAGCGGATATGTAGTTCCAGTCCGGGTTATACCATCACGTGATAATAAATCC
AAATGAGA

GATCTCTTCGGAGGCGCGGGTTCAAACCCCGCGGGTATCAATATTTTTTTGAATCTACGCACTTCAATAAG
AATTAGGCTGTTAGGATAGCATTTTTTTTCTATGTACCAAAAGTATTCATACGTGGCTCCTTGTTACCCCA
CAAAACTT

TAATGAATCGCAATATACGAAAAGCTAAACGGGTTTTTCAGAATCCTCTTACCCAGCCTTGGATAATAGTT
TTGGCCGAAAAAAAAAAAAAGAAAGAAAAAAATTGAAATTTCGCGATCCGAACAAACAATGAACAGAAAAA
CAGAATAT

CTTCTTCGGTTTTCCTGGTAGAATTTCCCTTAATGGGAGAGTAGTAACAATGCAAAAAATGAAGAAAATTG
AGAAAAAACAAAAAAGAAAAAAAAAAAAAAAAAAAAAAAGAGAGCTATGTAGGTTGATTGTGTTGTTCTAT
GTAGTGCC

ATTTTGTTATCTAATTTTTTCCTTCCCACGTCCGCGGGAATCTGTGTATATTACTGCATCTAGATATATGT
TATCTTATCTTGGCGCGTACATTTAATTTTCAACGTATTCTATAAGAAATTGCGGGAGTTTTTTTCATGTA
GATGATAC

GGGCAGCCAGAAAAGGCATACTTGAGGGGTGAGTTTGCCCAGGATTTAATGAAGTGTACATATAAGATATA
AGTTTTTTTATTTTTATTTCTAAAAAAAAGAAAAATAGTAAAACATGGACTGAAGCTAACGCTTTTGGAGT
AATTAGTA

TTGTTTCTGTCATCACTTTTCTATACTTTCTCTTCCCCGCGTGTTTTCCGTACACCAACAATATATGCCAT
AATACACGTAACATTTTTTTATAAAAAGAAAAGGTAAGTGATATATATAAAATAGCGCCATGAGTAGGAAA
CTTTTCTG

ACTATATTGCAATCTTTTAAATATATGTATACACGTTCATTACCACGTATACATATCCATATATATATATA
TATATATACATACTATTTATGATAAATTTTTAACAAGCAAACCCATTATTAAATGCTATAAATAAAAGACA
GCTTCTTT

CGGCGGGAAAAATTATTAAAAAAAGCACTTCCTGAACGCGACGCTGAATAACTTATAATACGTTACATAAT
TGTATACCGTCTAGTAAACTTACATACGCTTGTCTATCTTGTATGCTGATGCATTTTATCAAATAGCGTTA
TTTCCCGC

TTTTATCCCCTGCTTATCCAATTTTAGTTAGGAACTTTGGCATAATTTCAGTATTACATACTACCTAATCG
ACATAAAATAATATTACATATTCATTTTTTTAAAAAATAATCATAATCAATTTATCCTTTTTATTCCTTTT
TTCTCCTG

ATAATACATCTACACTCTTCTCATTTTTAAGTTCATTCTCTTTCATTTTCGGTAGTGAGATGGCAGTTCGA
GGGGTTTTTTATTCAAAATAATAACTTCTGGCTTTTCGCTTTTATATAGCAGAAAAAAAAGCCGTCGAGGC
GCGCGCGT

GTCATCCAAAAGATAGTTATAAATCTATGATTGTATGGTTTCTTTGACATATTTATAGTTAATATATATAT
ATATATGTGTTTAATTTTATAAAAATTCCATCGTTTATATTCGATTATTTTTATATGTCAACCTTTTTTTT
AAGAAATG

TCTTTTTTCCTCTTTATCGCTTTCGTACTATGGTCAGTCATTCATTCATTATATACGCGCTCTCCATAACC
CGTAACTTTTTATTATATATAGACTCGTTTACAATACAACGATAGCGATACCATTCAATTGAAGTTGTGAG
ACCAGGTA

TGCAGACAGCAACAGCTACACTTAAGTACTTTTAATATTGGATTGAAAATTTTCGTTCTTGCGATGAGCTG
CCCTTGCAAATTTTTCACCAAAAAAAAAAAAAAAAAAGAAATGGAGTGAAAAAAGAAACGAAAAAAAAAGG
GCATGGGG

TATTTTTGTGCGTCCTTTTTCAATTATAGCTTTTTTTTTATTTTTTTTTTTTCTCGTACTGTTTCACTGAC
AAAAGTTTTTTTTTCAAGAAAAATTTTCGATGCCGCGTTCTCTGTGTGCAACGGATGGATGGTAGATGGAA
TTTCAATA

TTTTTCCAATACTTGATTAACCTCTTTTTCGTTTCTTGTCTTTATTTTAGATTTGTTTTAATATCGCCTAA
TTTTTCCTTCTTTACTTTATATTTTTTTTATTTTTCGCCTAAAGATTTGTATCAATTAATTAGCCAACAAA
AACAAAAA

GTAGTAACAATGCAAAAAAATGAAGAAAATTGAGAAAAAAACAAAAAAGAAAAAAAAAAAAAAAAAAAAAAG
AGAGCTATGTAGGTTGATTGTGTTGTTCTATGTAGTGCCCTTTATTTTTCTTTGGATTTAAATTTACAGAC
ACATTATA

CTATAAAGTTTAAAATACTTCCCCGCTATTTGTATATATGCATATCTTTATATTATGAAAGATATTTTTTT
ATTTACTTTTAGTCCGTATACTTTTATTTTGTTTTGTTAGACAATCTCGAAGAACAAAAAAAAAAGATAAA
ACAGAAAG

TTACTCTGGAAAGAAAACACAACCTTTTCAGTTGCGGTGCAAAAACATTCTTTTGATCTCGAAGTAGGAAA
ACTGACATTAGAAAAAGAAAAAAAAAACAATCTGCGAATTATGCATTTCTTTTTTTCGCTTTTTTATTTTC
TTTTTTCT

TTCCATTGTAATAACTAATTGTGTGCGTTGCGCGGGGTTGTGTGGCTCAAAAAGATCTGTTTTTGTATTAG
CAATTTTTTATTTCCCTTTTTAAATGAATTTTTTTTCTTTCTGTTTCGAGACAAGGCCTTTTGCAAAAAAA
AAAAAAGT

TTACGCCTTTTTTTTTTTTTTTTTTTTTTTTTAATTTATCTCACTTTTCCCTTCTATTTTATGTACTAACAG
ATAATTTTATAATGATTTCATGTATTTTTGGTTCAGAATTTTGACAAGGCAACTCTTGAATAATTGTCGTA
TGCAAAAA

CACTCACTCCCAAACTTATGCCATTTCTATCATGATAACCAAAAATAGTCTTAACAAAATACATATAAGTA
AATAATGCTAAATAAAAAAGCAAAAAAATAAAATTTATATCACACTTGTAAAACCTTTAAATGATCTTCTT
TTTTACCT

TTCTTTTTGTTTGTAATTGTTTTTTATTTTTTTTTTTGCGCGAAACTTTGCTATATTGGGTAACGCGTAAA
ATACTTTTTATTATTGCAGTAAGGCGGAAGGGTCTTCCCCTTTGCATGTTAAATAGCATACATGGCACCAC
TCAGGTCC

TGGCAGACTTCCATCGTAATGGGATGATCATATGATGGGCGCTATCCTCATCGCGACTCGATAACGACGTG
AGAAACGATTTTTTTTTTCTTTTTCACCGTATTTTTGTGCGTCCTTTTTCAATTATAGCTTTTTTTTTATT
TTTTTTTT

CAGGAATGTATATTCTACGAAAACGAGATGTATTGAATAGTGATGTTATGACCTTTTTTTTTACGCCTTTT
TTTTTTTTTTTTTTTTTTTTAATTTATCTCACTTTTCCCTTCTATTTTATGTACTAACAGATAATTTTATA
ATGATTTC

CCCGTATCTTTTGTGTTTGATTCAAGGAATACTTATTTAGAGTAGAAAGAATATATTTACTTAGATAACTT
AATGTATAATATATAAAAAAAAATCAAAAAAACAGGAAAAAAAAAAAATCATGAGTGAGTGGCACTTTTAACT
GCAAGAAT

AAAAAAAAAAAAAGGAAAATAAAAAAAAAAAAAAAAAAAAAAAAAACGGGAGATTAACCGAATAGCAAACTCTTA
AACTATGAAAAGGCAATCAATCCACTGTTTTCTTTTTCCATTTCTTCTAGCCTATTTGAACTGTCTGCTTC
TGTTTCTT

TTAGTCAAATGCACTAGAACATGGCGCAAGTACTTATTGTGACCTTTGGGGTACCGTTACCGTCAGTTTTC
TTCAGCTAAGGCGCGCGCGCCAGATAACTAAAAAAAAATATAGTTGCTGCTTAAAAAACAATACACCCGTA
CTCTCTTG

AGCAGTAGTTGCAAAAGTGTCCTTTTTTACTTTTTGGGCTACATTATTCTTGAAAAAAGTTCATGTTTTGT
TATTTCTTTCTCACATACTTTATAAAAAAAGATAATTTTTTTAAAAAAAAAAAAAAAATACAAGTGGAACAT
TATCAAAT

AAAAATGTTAACATAAAATGCAAGAGAACAATTAATCGAATAATGTTAAATTATTGTAAAACAATGTGTAT
GATGAGGAGGAATGTACCTAAGCCAAAAAAAAAAAAAAAAAAAAAAAAAAAAAAAAGAAACAGCTTTTGCAT
ATTCAATC

CAAGAAAGACCAAAGGATGCTCCCGGTTGTGTGATTGAAAACGGAACTAAGCTTTACTAATCAACAATATT
AACTTTATAATAGATTCATATATGCGCGTACATATATTATATACAATTTTTCTACACAAGAATGCTTAATA
AAGATACG

ATGCTTTTGTTGTTTGCTTGATATTTTTTGTTTTTAATGAACTGTAAGCACACAGAGCTATGGGATGTGTG
GCAATGACTGTATATATATGTATATATATATATATATATATATATATTTATTTATTTATTTATTTAAAA
TATAGCTA

CTTGAGGGGTGAGTTTGCCCAGGATTTAATGAAGTGTACATATAAGATATAAGTTTTTTTATTTTTATTTC
TAAAAAAAAGAAAAATAGTAAAACATGGACTGAAGCTAACGCTTTTGGAGTAATTAGTAGCATTATATATG
GTAAAAGT

GGTACCTGCAATCTTTATTTATTTTATTTGAAGTAATAGGAATTATTTATATTATTGTGATAAGAAGTAGT
ATTCTATTATTATAACTATATTAATAAAAAATTTTATTTTCTATTGAAAACCAATTTTTTACACCAAATAT
CAGACACG

TGATGAGGAGGAATGTACCTAAGCCAAAAAAAAAAAAAAAAAAAAAAAAAAAAAAAAGAAACAGCTTTTGCA
TATTCAATCCAGGCATAGGGCGACTATTTAGCACTCAACGATTTTTAAGCTTGTGTATTGCTGACATAAAT
TCCGGCTT

CGTCTGTTCATTGTGCAAAGCTTTTTAAAGAGATTTTTGCCTATAAAGTTTAAAATACTTCCCCGCTATTT
GTATATATGCATATCTTTATATTATGAAAGATATTTTTTTATTTACTTTTAGTCCGTATACTTTTATTTTG
TTTTGTTA

AAAATATGAAAAAAAAAAGAAAATTAAAAACTAAACAAATTTTATGACAGGAATAAAAACTATAAAAAAATGA
AAAGCAAAAAAAAAAAAGGCTACTGGATTTTTAACATCTTGTCCTTTTCTTTCCCTTTATCATTTTTTCTCA
TTTACTTT

AAACAATTCTTTTCATTAGATGTATTTTTTTCATCGTATTAGGTGTAATATATATATATATGTATATATATA
TATATATATAATATATATGTAGTATACACACGTGGACGACCATTAACGAATGTTGTATATGCTTATGCGGA
ACCTTTGT

AACACGAAAAATATCACAGTTGACGAAAGAAGACACGTCGCCTAAACGCGTAATGAATCGCAATATACGAA
AAGCTAAACGGGTTTTTCAGAATCCTCTTACCCAGCCTTGGATAATAGTTTTGGCCGAAAAAAAAAAAAAG
AAAGAAAA

ACCTCGCCCCAGTGCATAATAAAAGTAAAAAAAAAAAAAATCAAACTTTATTGGGATAAAATAAAAATCAGA
AAAACCCTTATATATGTATATGTAGTTAAATGAAGCCAGTTTTTTTCGTCTGGTTTGTTTCAGCCTATATG
CCCTCTTT

GTACCGTTACCGTCAGTTTTCTTCAGCTAAGGCGCGCGCGCCAGATAACTAAAAAAAAATATAGTTGCTGC
TTAAAAAACAATACACCCGTACTCTCTTGCCTGTAAAAACCTCGAAGGACCAAAGATACCCTCAAGGTTCT
CATCTGTG

ACTTATTTATTTACCAATCAAATGATTTTTTTAACTTTTCACGATCTATATAATTACGTTAATTTCGAGTA
TATAACAATGAAAATATATATCTACGTTGTTTTAAATTGAAAAAAGAAGAACGGAAGTTCTATTAATGAGC
CGAGACCG

ACTTTCCCCAAAGAATAATAATACGAGTACAATACATATATATATATATATATATAATATACACATGTAAGTG
ATGACTATTCTTAATACAAAAAAATTTCCCTTTTATAATAACTATTCTTAGCATTAAATAACCCGTTACTG
ACAAAAAG

TTTTAATATTTTTTTTTTGTTTTTGGTTGTCATTACTGTCATTACTATCATTATTATTACATTTATCTTCT
AATGTATTGGAAGTAATAGTATTATCATTTATATTTAAATTTTTCATGGCTTTCTCTAGTGAGTTTTGATC
AAGAGTGT

AATTCAACCGAATAAGGAAAACTAAGCCACTTCACGCGGTTCGCGATATTTGTCCAGCCTCTTTTTCCGAA
AAAAAAAAAATTAAATAATAAAATGAAACGGACAGGAATTGAACCTGCAACCCTTCGATTGCAATCTTATT
CCGTGGAA

ATGATTAAGAGTCATACGCGCTACCGATTGCGCCAACAAGGCTATTTCGTTGAAAAATTCAGGCTTCAAAA
TTATTATATGTATCAAAAATATTTTTTTGAGAAATTTCAAATCATTATCTACGCTAGCCTTACTATTTTTT
CACCCAGA

TTGTAATTGTTTTTTATTTTTTTTTTTGCGCGAAACTTTGCTATATTGGGTAACGCGTAAAATACTTTTTA
TTATTGCAGTAAGGCGGAAGGGTCTTCCCCTTTGCATGTTAAATAGCATACATGGCACCACTCAGGTCCAG
AACGTGAC

CATTTACTTTGTTATTAATTTTTTTTTTCAAGTTCTCCACATATACATATGTAAGAATTGAGTCTATCTCT
TTCTAATATTTAAGAAATACATAAAAAAACCAATTTTTTTTTTTTATTACTAGCAAATTTATTTTTGTGAA
GTGAAATT

AAGCCAAAAAAAAAAAAAAAAAAAAAAAAAAAAAAAAGAAACAGCTTTTGCATATTCAATCCAGGCATAGGG
CGACTATTTAGCACTCAACGATTTTTAAGCTTGTGTATTGCTGACATAAATTCCGGCTTTAGAATCCAATA
TTGAAAAA

GGGGGAGTAATATGATATCAAGAAGGCGATCACTTATTGAAGTCCACATAGATACACACAGATAAACATAT
ATATATTATGTATATATATATATATATATATATATATATATATATATGTATATATCAACGCCCATACCATTAT
CATTATCA

TAATCATCTGTTTCATTTTCCTTCTATTTCTTTTTTTTTTTTTTTTTTTTTTTTTTTTTTTCAGTTTTCGAA
GAAACTGCCTTTTTAGAATTGTAAAAGACGAAGTGAAGTATTCAGGAGTATATTATTACATACATACAAAG
CAAGACAA

TAATGTTAAATTATTGTAAAACAATGTGTATGATGAGGAGGAATGTACCTAAGCCAAAAAAAAAAAAAAAA
AAAAAAAAAAAAAAAGAAACAGCTTTTGCATATTCAATCCAGGCATAGGGCGACTATTTAGCACTCAACGA
TTTTTAAG

GCGATGAGATGAGTTTAAAATTTTCATTTTTTAGCCGATCAAGCAAAAGAAAAGAAAAAAAAAAAAAAACTG
AAATTTCCACCTTACCCGTTTTTTTAATGGAACAGTGATTGTTACGACATTGAGATTTTAGATTTATATAA
TCACATAC

ATCTGTGAAATTTCTCAAAAACCTCATGGTTTCTCCATCACCCATTTTTCATTTCATTTGCCGGGCGGAAA
AAAAAAAGGAAAAAAAAAAAAAAAAAAAAAATAAATGAACACATGGAAATAAGTCAAGGATTAGCGGATATGT
AGTTCCAG

CTTAAATGTATATTGATTTTCTATATATATATATATATATATATATATATTTATGAAATAAACCATATAATTA
TAAGTAGTAGCTTGGAAAAACGCCAAAGTTGTACTTAGAATGAGGTTTTAAAAAAAAATTATAAAAAAAAA
CCAAGCGA

GTTATTAATTTTTTTTTTCAAGTTCTCCACATATACATATGTAAGAATTGAGTCTATCTCTTTCTAATATT
TAAGAAATACATAAAAAAACCAATTTTTTTTTTTTATTACTAGCAAATTTATTTTTGTGAAGTGAAATTCA
TACATTGG

TTTCATTTTCCTTCTATTTCTTTTTTTTTTTTTTTTTTTTTTTTTTTTCAGTTTTCGAAGAAACTGCCT
TTTTAGAATTGTAAAAGACGAAGTGAAGTATTCAGGAGTATATTATTACATACATACAAAGCAAGACAAAG
AAACATTT

ATTTTGAAACACTTTGCTGCTTAGGCTTTTAATGAGGTATGCTTTTAAAGATTTATATATTTTTTATACAC
GCTATATACACAATTTTCGCTGATGATTTTGTTTTATTATTGATTTTTAAATTAACCGTAGAAAATACTAA
GTGGAAAG

TTTTTTTTTTGCCATTCTTATACATTTTCTTTCCTTCTGAAATTAACTGTACACCCATACCCTATATACAC
CCATACCCTATTTTTAAATATAAAAAGTAAACTTCATTTTGAAAGACCACTCTGCATCAGCACGCGGGCTC
TGGAAGGA

CAAAACCCTAACAATACAATGTGAGACTAAAGTAAATCCGGGTAATACAAAATACAATTTTTTTTTATTCT
TCACAGCGTTTTTTCGACGGAGGGAAAAAAAAAAAAAATTGTATTTAAGGCATCAAATGCGCGACATCATA
TACCTTTG

ATAAAAAAAAAGTAGCATTAAATGTTATGGATGATTGGGATATATATGCAAATAACCCAATCTTACATATG
TTTCTTTCAAATATATGAGTATATATAACGATTAATTGTTTTTTTTTATTGGGTTTGTTGATTTTTCCAAC
GCACCATA

TGCAAAAAATGAAGAAAATTGAGAAAAAACAAAAAAGAAAAAAAAAAAAAAAAAAAAAAAGAGAGCTATGT
AGGTTGATTGTGTTGTTCTATGTAGTGCCCTTTATTTTTCTTTGGATTTAAATTTACAGACACATTATATC
TTGAGGTT

AAAAGTATTATGCATATATATATATATATATATATATATATATTTATATATTTATACACATAGAATTATTT
TACAGTAAAGGATGCTACACCACCCCGGGCTAACTTTATGGCTTCTACTATTTCAGGTTCCCTTTCGCGTC
TTTTATCT

TTTAAGGTCTCTATATGTGGCGGCATCTTTTCTTTTTCTTTTCTTTTTTTTTTTTTCACTCTCCCTTTTAA
TGCCTCCACGGAGGTTCGAATGGGTTATACTAGAAAGAAAAAAAAATAGTGATAATTAACAATGAAAAAAA
AAAAACAG

TTCTATCTGTTAAGCGTGTTATGGTTATCTGCGATGAGATGAGTTTAAAATTTTCATTTTTTAGCCGATCA
AGCAAAAGAAAAGAAAAAAAAAAAAAACTGAAATTTCCACCTTACCCGTTTTTTTAATGGAACAGTGATTG
TTACGACA

TGGCAAATATTGTCATAATAAATATAAAAAAAAACTGTAGTTTCTTTTTAAATTTTTCTACTTAAGTTTAG
TTATACTGTACCTAGGTATATATAAATATATATAAATAAAAGTGGCCAAGAATAAAAGAACGCACCCCGTC
GTTGACTT

TATTCTATTATTATAACTATATTAATAAAAAAATTTATTTTCTATTGAAAACCAATTTTTTACACCAAATA
TCAGACACGCTTTGTCTATTTCTATCATATAGCGACTGTGATATAATGCGCGCGTGTTTTGAACGGCATTT
TTTTAGCC

TTTTTAAATTAATTCAGGTTAATTCTAAAAACTATATTGCAATCTTTTAAATATATGTATACACGTTCATT
ACCACGTATACATATCCATATATATATATATATATACATACTATTTATGATAAATTTTTAACAAGCAAA
CCCATTAT

ATCATGTCCTTTTTTTTTTTGCCATTCTTATACATTTTCTTTCCTTCTGAAATTAACTGTACACCCATACC
CTATATACACCCATACCCTATTTTTAAATATAAAAAGTAAACTTCATTTTGAAAGACCACTCTGCATCAGC
ACGCGGGC

CGAACTAAGTGAAAACATGGTCTTGCTTCACCCTATTCTCGCCGAGAGCTGTACAAGATATTTTTTACTTT
TGCCATCTTATACTCATCCTAATCATCTGTTTCATTTTCCTTCTATTTCTTTTTTTTTTTTTTTTTTTTTT
TTTTTTTT

TTGTGCGTACAGTATAATGCACTTATTTATTTACCAATCAAATGATTTTTTTAACTTTTCACGATCTATAT
AATTACGTTAATTTCGAGTATATAACAATGAAAATATATATCTACGTTGTTTTAAATTGAAAAAAGAAGAA
CGGAAGTT

TGTTTATTCAAAATGATCATGTACCTTCTTCTCGCGAAAGTTTTAATTAAAATTTACTGATAGTTTTTTAA
TGATGAACTTCTATAACTAAACCATAATAATGAAAATTACAGGTAATATTTATATCACAAGAAACGGCCCC
TCTTTTCT

AATATTTTTATCGTTATTAATTATTATTATTATTACTATTATTATTATTATTATTATTATTATTATTATTA
TTTATTATTTATTAATTATTAATTGTTAATATTATTAATTCTTGTCTATTGTCTTTTGATTTATCTGCACC
GCCAAAAA

ACGTTAGGAATCGACTCCAACAGGAATGTATATTCTACGAAAACGAGATGTATTGAATAGTGATGTTATGA
CCTTTTTTTTTACGCCTTTTTTTTTTTTTTTTTTTTTTTTAATTTATCTCACTTTTCCCTTCTATTTTATG
TACTAACA

ACTAAGCCACTTCACGCGGTTCGCGATATTTGTCCAGCCTCTTTTTCCGAAAAAAAAAAAATTAAATAATA
AAATGAAACGGACAGGAATTGAACCTGCAACCCTTCGATTGCAATCTTATTCCGTGGAATTTCCAAGATTT
AATTGGAG

GGCGCAAATGTTAGAAGTTCAAAATGAAGCCTAAGTATCACGCTAATTGAAGTTTTTTTTGATCACTCCAA
TAGGCAAATCTATAGATATATAAAAAATATAGACAAGACTTTTTTTTACATTGCCAGTTTTCTTTTTTCCT
TTTTAGTA

CGGAAGCTAACAAAATTTTTGAAAAACGAAATAAAGAAGAAAGATTATTATTATTAGTTACTTTTTTTATT
GTACTCCAGTATGGACCTCTTAGGTGAGTGATCTTATTAAAAAAAATAAAAAAAATAAAAAAATATGAAAA
AAAAAGAA

AAAAGGCATACTTGAGGGGTGAGTTTGCCCAGGATTTAATGAAGTGTACATATAAGATATAAGTTTTTTTA
TTTTTATTTCTAAAAAAAAGAAAAATAGTAAAACATGGACTGAAGCTAACGCTTTTGGAGTAATTAGTAGC
ATTATATA

TTGTGCAAAGCTTTTTAAAGAGATTTTTGCCTATAAAGTTTAAAATACTTCCCCGCTATTTGTATATATGC
ATATCTTTATATTATGAAAGATATTTTTTTTATTTACTTTTAGTCCGTATACTTTTATTTGTTTTGTTAGA
CAATCTCG

AAAAAAAAGAAATTTTTTCCAAAAGAGTATATTTATATGTATGTATACATGTAGGGAAAATAAGAAACTTT
ATTAATAGTAAAAAAGCATATATACTTTATTATTAACTCTTTTGTTTTTCTCGAGAAGCTTAATTTTGGGT
CAATTCAA

TTAACTAGCTGGGGTATATTTTTTTTCTTTCTTTTGAATGTGTGTATAAAAGAGAGAAAAAATGGTTATTT
TCCAATTAATTAATTATTAACGATTGTTAAAGAAAAAATTTATCAACGAGGTTGATAGAAAAAAAAAGTTT
TTGTAGAT

TCTATCTATATATATATGTATGTTCTTTATTGTTTGTCTTTACCTTTATATATAAACATATATAGCCATAT
AAGAATATAATATTACCACTCCACTGTATTTTGTATCCTGCCTATTTTTGCTAGATTACAGGACGCGCGAG
CCTTTCAG

TTTTTTTTTTTTTTTTTTTTTAATTTATCTCACTTTTCCCTTCTATTTTATGTACTAACAGATAATTTTAT
AATGATTTCATGTATTTTTGGTTCAGAATTTTGACAAGGCAACTCTTGAATAATTGTCGTATGCAAAAAAA
ATTGTTTA

CTTTTTAAAGAGATTTTTGCCTATAAAGTTTAAAATACTTCCCCGCTATTTGTATATATGCATATCTTTAT
ATTATGAAAGATATTTTTTTATTTACTTTTAGTCCGTATACTTTTATTTTGTTTTGTTAGACAATCTCGAA
GAACAAAA

CCCGTTATTATGATCGCTCGTGACGGATCGTCTTTGCCCTTTTTGGTAAAACGTAAACAAAATAACAATAG
AAAAAATAACAACTTTATCAATGTTTATTTTTATTTATTAAGTATTTGATGTGAAGTAGTTTTTCTAAATG
CTACTTCA

GAAACGAAACAAATCATGTGTTACCCGTCCTGCACAATCATGAGTGCCTGCGGTTTCCATATTGGAACGGC
ACCTTCCTTTTTTGGAAGTCACGTTCCTGCAGCGCGTTTCTTTTTTTTTTTTTTTTTTTTTTTATTTTTC
GGTTTTTT

ACAAAATATATAGGTACCAAACCTCATTATAATTTCTGCCATTTAATAGTTTAAGATGCTATTCCGTTTCG
TATTTTTTCAATTTTTATACCTACTATTGCGCGAAATCAAAATAGCATACTAAGAAAAAGCAATATCATGA
ATTGCTGA

TCGTTCTAACGTTGGTAAAATGAAAAATATGAAAAACAGTTATCATGGCTACTATAATAACAATAATAATA
ATAATAATAATAACAATAATAATAATAACAGTAATGCTACCAACAGCAACAGCGCGGAAAAACAACGTAAA
ATTGAGGA

ATGTCTATTTCAATGCAATTACGTGAAAAATAAAAGAACGCTACCATAAAAATGTTAAAAACTGGTTAAAA
ATGTCAAAAAATGGTCCGAAAACTGAAAAAATATTCGTGATACAGTTTGCATAAAAAAAAACTATTGTGCG
CGATTAAT

GACCTTTGGGGTACCGTTACCGTCAGTTTTCTTCAGCTAAGGCGCGCGCGCCAGATAACTAAAAAAAAATA
TAGTTGCTGCTTAAAAAACAATACACCCGTACTCTCTTGCCTGTAAAAACCTCGAAGGACCAAAGATACCC
TCAAGGTT

CGCGGTTGCGCGATATTTCCCTTGCTTAATGCCGAAGTAATTCTCTTCGTTTACCCATACTCGAATCTATT
GTGTTGTTGAAAAATAAAAAAAAAAAGAACCAGGTTATATAATTGTGAGTTGATCAGTATATAAATGAGAG
AAAAGAAG

TTTTGATCTCGAAGTAGGAAAACTGACATTAGAAAAAGAAAAAAAAAACAATCTGCGAATTATGCATTTCT
TTTTTTCGCTTTTTTATTTTCTTTTTTCTTTTTTTCGACCTCCAATAGGGCTTTGGAAGCCCTATATGTAA
TCTAGGGT

TTCTTTTTTTTTTTTTCACTCTCCCTTTTAATGCCTCCACGGAGGTTCGAATGGGTTATACTAGAAAGAAA
AAAAAATAGTGATAATTAACAATGAAAAAAAAAAAAACAGCAACAAAATGTGGTATTGGGGTCACACCACTC
ATTGACTC

TACAAGAAAATGGCAAATATTGTCATAATAAATATAAAAAAAAACTGTAGTTTCTTTTTAAATTTTTCTAC
TTAAGTTTAGTTATACTGTACCTAGGTATATATAAATATATATAAATAAAAGTGGCCAAGAATAAAAGAAC
GCACCCCG

TAGTAAGAGAGGTGAAATCTACGAAACTGGTAAGTTCTTTTAAGTATCACATTACAATAACAAAACTGCAA
CTACCATAAAAAAAAATTGAAAAATCATAAATTAAAAAAAAAAAAATCAATTGAATTTTTTTTTTTCATGA
TTACGTTT

TCTGCGGAAGACTAAGACAATCAAAATATGTATTCTTACTATCATAATTAAATATTTTTATCGTTATTAAT
TATTATTATTATTACTATTATTATTATTATTATTATTATTATTATTATTATTATTTATTATTTATTAATTATTA
ATTGTTAA

AATTATAATAAAAATAAAAATATGTATAATATAATTTTTATCTTCTAACTTTTTATTTATTGAATTTTTAT
ATAGTAAATATAACACGTGATATATTTGAAATGTGATTATATTATATATATTGATGTTTACATATTTTTTA
TGTAGTAC

GAAGAAAATTGAGAAAAAACAAAAAAGAAAAAAAAAAAAAAAAAAAAAAAAGAGAGCTATGTAGGTTGATTG
TGTTGTTCTATGTAGTGCCCTTTATTTTTCTTTGGATTTAAATTTACAGACACATTATATCTTGAGGTTTT
TAGTCCCT

GATAAGCGCGGGCTTTTTCGCCCTATACGGCTTGGGCCTGACTAAAATGTGATTCTGTGTTTTACGTATAT
GCGCGTATATAGAGCAATTGATTGACTTATTACGCAGTTTATCAAGATAAATTCTTTGTTCTTGCTTACGG
GAGTGTGG

CCCTTTTTCTGTCACAGGTTTCCCAATTTTTTTGACGACTAAATAATAACATAACCACTACGTTCACTTCC
CCGTATATATACATATATACTTAATATTATAAACGCATATACTTTTATAAACTTGGAATAAGTATTATATA
TTTATTTT

GTCCTTTTCTTTCCCTTTATCATTTTTTCTCATTTACTTTGTTATTAATTTTTTTTTTCAAGTTCTCCACA
TATACATATGTAAGAATTGAGTCTATCTCTTTCTAATATTTAAGAAATACATAAAAAAACCAATTTTTTTT
TTTTATTA

AAAATGAAAAAAAAAAAAAAAAGATTAGCGAAGGCAAACCAGAAAGCAGTCCTTTGATATAACAGAGAAAAA
GAAGATTATCAAAGCCTTAATTTAAAGTTAAAAATATTGGGTATCGTTTTTTAAATCAAGATTTTTTTTGT
TTTTTTTT

TATAATTGCATTTTCTTTATACAAAATATATAGGTACCAAACCTCATTATAATTTCTGCCATTTAATAGTT
TAAGATGCTATTCCGTTTCGTATTTTTTCAATTTTTATACCTACTATTGCGCGAAATCAAAATAGCATACT
AAGAAAAA

TTAATAAAGACAAATGCAAAAGTTTTTTTAAAGTATGGATGTATAATAATAATAACGATAATACGAACACT
ATTGGGACTAATAATGATTTATAGTTTTTTTTACACAGGGGATTTAACGTTCCGCGACCCTTTTTCCTTTA
ATTTAACC

ACAACCACTAAACAATCTCGTTCAAAAGCTCTAATTACTTGTATATTATTTTATACGTTTATGTCTATTTC
AATGCAATTACGTGAAAAATAAAAGAACGCTACCATAAAAATGTTAAAAACTGGTTAAAAATGTCAAAAAA
TGGTCCGA

ACATTTTTTTTGTTTATTCAAAATGATCATGTACCTTCTTCTCGCGAAAGTTTTAATTAAAATTTACTGAT
AGTTTTTTAATGATGAACTTCTATAACTAAACCATAATAATGAAAATTACAGGTAATATTTATATCACAAG
AAACGGCC

ATTAATAAAAAATTTTATTTTCTATTGAAAACCAATTTTTTACACCAAATATCAGACACGCTTTGTCTATT
TCTATCATATAGCGACTGTGATATAATGCGCGCGTGTTTTGAACGGCATTTTTTTAGCCTGCGAATATGGA
TAGATGAT

GAAACAACCAAGTCCTGTGTCCTGTGGTAGACCCACTTGTTTTACCGCAGAGGGCCCTTTTTTTGTTTACC
GTCGCAAAAAAAAAAACGCGAAGAATACGCGTTGGTCGTTAAATTTTGTGGTACGCGTAAAACTGCTATTTG
GTATCCTC

CTACCATAAAAATGTTAAAAACTGGTTAAAAATGTCAAAAAATGGTCCGAAAACTGAAAAAATATTCGTGA
TACAGTTTGCATAAAAAAAAACTATTGTGCGCGATTAATATAACCTACTAGACTTAAATGACGACACTTTA
ATATGAAG

TGAAGAAAAAAAAAAAAAAATGGATGAGCCTTCCAGTTCATTGAAATTCTTCAGTTCTTATTTTTTTTTCAA
AAAGAAAAGTTTCAGTACCCTGTTCCAAAAAAAAATGAAAAAAATGAAAATCCAGTATCCTTCCCATAAAAA
TTAAAAGT

TTAGAAGTTCAAAATGAAGCCTAAGTATCACGCTAATTGAAGTTTTTTTTGATCACTCCAATAGGCAAATC
TATAGATATATAAAAAATATAGACAAGACTTTTTTTTACATTGCCAGTTTCTTTTTTCCTTTTTAGTATC
TATTCAAA

TGAAGCCATTCCTGGGTTATCTGCGGGAGTGAACAATGATAATACAAAGGAGAGTAAAGATGTGAAAATGA
ATTAAAAGGAAAAGTATTATGCATATATATATATATATATATATATATATATTTATATATTTATACACATA
GAATTATT

AATAAAAAAATATGGGAGTATAATGATAGAGAAGTGGATGGTAGATTATACCTCGCGAATGAATTATACAG
AAAATAATACTAAATTGAAATATTTAAAAATGATTTAAAAGAAAAATATTCATTGTACATAATAAGTTCGA
GCATAAAT

GAAACTAACTAAAAATATACGCCATTTTTTTGGGGAGGGGGGGCCGCACTGAAACGCGAAATGCCAAAAAA
ACGCGAAACGCCGCAGTAAAAGGTCAGTACCGCGCAAAAAATACAAAAATGACAACAATATTGGCCTTTCT
TTTCCTAA

ATTATTGTGATAAGAAGTAGTATTCTATTATTATAACTATATTAATAAAAAATTTTATTTTCTATTGAAAA
CCAATTTTTTACACCAAATATCAGACACGCTTTGTCTATTTCTATCATATAGCGACTGTGATATAATGCGC
GCGTGTTT

AAAAATATACGCCATTTTTTTGGGGAGGGGGGGCCGCACTGAAACGCGAAATGCCAAAAAAACGCGAAACG
CCGCAGTAAAAGGTCAGTACCGCGCAAAAAATACAAAAATGACAACAATATTGGCCTTTCTTTTCCTAAAG
CAATTAAC

TATTTATTCCTAGCGACGAAAAATGCGAGATCTCGACCAAAAAAAGGGGGTAGGGTAATAAAATTAACCCT
ATTATTTTTTAACTTTAAAACCTATAATGTGCTAATATTTTATTATAAACCTCCTTTTTTTGCGTTCAAAC
CCTGACAC

TTTTCATTTTTTAGCCGATCAAGCAAAAGAAAAGAAAAAAAAAAAAAAACTGAAATTTCCACCTTACCCGTT
TTTTTAATGGAACAGTGATTGTTACGACATTGAGATTTTAGATTTATATAATCACATACATATTAAATAGT
TAATGCAC

CTAAGTATCACGCTAATTGAAGTTTTTTTTGATCACTCCAATAGGCAAATCTATAGATATATATAAAAAATAT
AGACAAGACTTTTTTTTACATTGCCAGTTTTCTTTTTTCCTTTTTAGTATCTATTCAAATGGGCGACCCTA
TTGTCTAA

TTTTTTCGGAATTACCTAAAATGGGTTTACGGCATAGAAGATAGATAGATTAAGAGGTAGGTTGACTTTTC
CTTTGAAATTTGTAATTTTAATAAAAATTCCAAAAAAAAAAAAAAAAAAATTCAAAGTAAAAAGACAAACTT
TGGTGAGA

TTTTAGTTTTGTTGCTTTGTATTGAGCTGTATAAAAATCATTAGATCCACATTACTAATTATTGAATATGG
ACAATAATATCAAATATATTATTATCAAATTGATTATTTTGTGACGCGGCGTCAAATTTTAATCGCGTCTG
ACGAATGA

TACTAGGTATTTTGGGCGGCGAAGCATCATTCCGTTCTTAAACATCTATATATATATATAATTATATTGCAAA
TGTATAAGCATTTAAAAACATAAACATGCACCTATGAAGAAGAATTTAAGACGTTTTATTTTTATGTTCCA
CGCGTTTT

CTATTGCATCCAACCAGGTACCAGTTAAGAGTACATTGTATATGAAAATATATATATGAGTAATATATATA
TATATAGTTTTATTAATAACTATTAGCACATCATAATCGTATCTGGAGACACATACATAGTGGGCCAAACG
GAAACCCC

GGACAATTTCAGTGTTTGTAAAAATATATTTATATTTATATATTTAAATATACTAAATAAAAATAATACTA
TCATCGTTGTGATGCAAAAAAAAAAAAAAAATGAACAGAAAATAATAGTGATGTGAATGGTTTCGTGCCAAG
GAACAAGG

CCTTCGTTTTTTTGTCTTCGCGCGTTACAGGGTAAATGAACCTCTTTATGGGTGAATGGGTTACACAAATG
CACGGATGCATATATAAAATATAGAATTTTGCTTTATATTATTTTGAGCGGGCTAGAATCCACTCAGTGTA
CAAAGTAC

TTAGCCGATCAAGCAAAAGAAAAGAAAAAAAAAAAAAACTGAAATTTCCACCTTACCCGTTTTTTTAATGG
AACAGTGATTGTTACGACATTGAGATTTTAGATTTATATAATCACATACATATTAAATAGTTAATGCACCT
TTAATTCT

CCGCTTTTTTTCCTTTTTTTTTTTTTACTTTGTTTGACCTCCCCTAACTCTTTCTTTTTACCTTCACAAT
TGTTTATTTTAATATATGATTTTTAAATTAATTCAGGTTAATTCTAAAAACTATATTGCAATCTTTTAAAT
ATATGTAT

AAAAAAATTGAAAAAATTTGAAAAAAAAAAAATCTAGAAACATATTTTCCTAAAATGAAAATCGGAAGCTAAC
AAAATTTTTGAAAAACGAAATAAAGAAGAAAGATTATTATTATTAGTTACTTTTTTTATTGTACTCCAGTA
TGGACCTC

TGCTACATTGACGTTAGGAATCGACTCCAACAGGAATGTATATTCTACGAAAACGAGATGTATTGAATAGT
GATGTTATGACCTTTTTTTTTACGCCTTTTTTTTTTTTTTTTTTTTTTTAATTTATCTCACTTTTCCCTT
CTATTTTA

CCTCCCAGTGACAATTTTGTTCTTTTTTCCTCTTTATCGCTTTCGTACTATGGTCAGTCATTCATTCATTA
TATACGCGCTCTCCATAACCCGTAACTTTTTATTATATATAGACTCGTTTACAATACAACGATAGCGATAC
CATTCAAT

TTTTTTTTTTTTCGTTATATAGGTTCTGCGCATGTGAAAAAAAAGAGAAGAAAATAAAGCCAATTATAAGT
CATTACTGACGAAATTATAAGATAAATTAGAGGTACAATATACAAACTAGTGAATAACAGAACTATATAGG
ACTTAGAT

TAAGCGTGTTATGGTTATCTGCGATGAGATGAGTTTAAAATTTCATTTTTTAGCCGATCAAGCAAAAGAA
AAGAAAAAAAAAAAAAACTGAAATTTCCACCTTACCCGTTTTTTTTAATGGAACAGTGATTGTTACGACATT
GAGATTTT

TTTGTTTAATGGTGTAAGCTGCCTATATGTTACTATTGAGTACTCATCTCATCGCTTCTTTCAGAACAAAA
TTTTTCATATTTTTTTTTTTTCCTTTTCTTTTTTTTTTTTCTTTGACTGTTACCCGGTTGTTTATATTTG
TAGGAAAA

TAATATATGATTTTTAAATTAATTCAGGTTAATTCTAAAAACTATATTGCAATCTTTTAAATATATGTATA
CACGTTCATTACCACGTATACATATCCATATATATATATATATATATACATACTATTTATGATAAATTTTT
AACAAGCA

CACGGGTAAAAATTTTGCATCGTGATGATACCAAGAAGTAGTAAAAGCTCATGGCTTCTTATATATTATAT
ATGGAATACATTTATAATAAAATAATAAGAATTATATATTTTATGATTATATTATTACATAAAGTATTCCC
CATTATAA

TTTTTTTTCAAAAAGAAAAGTTTCAGTACCCTGTTCCAAAAAAAAATGAAAAAAAATGAAAATCCAGTATCCT
TCCCATAAAAATTAAAAGTTCCATTATTTATGGGTAAGTAAGTACCACTGATTATATACCATATTTATAAA
GTGCATGA

TAGAGGCTAAATAAAAAAAAAAGTAGCATTAAATGTTATGGATGATTGGGATATATATGCAAATAACCCAAT
CTTACATATGTTTCTTTCAAATATATGAGTATATATAACGATTAATTGTTTTTTTTTATTGGGTTTGTTGA
TTTTTCCA

TATAAGAAATAGCGTTCTTTCTTTCTTTCGTGTAATGTGACACAATTTTGCTATTGCGGAAACTGCAGCAT
AGTCAGTGTAATAATCGTACTGTTGCTTTTTCATTTTTTTTTTTTTTTTTTTTTTTCTCATATAGAAAAAC
TGTTATTG

CTACGGCAAATTAGTCAAATGCACTAGAACATGGCGCAAGTACTTATTGTGACCTTTGGGGTACCGTTACC
GTCAGTTTTCTTCAGCTAAGGCGCGCGCCAGATAACTAAAAAAAAATATAGTTGCTGCTTAAAAAACAA
TACACCCG

TTTCATATATAATGAGTCATTTGTTTCTGTCATCACTTTTCTATACTTTCTCTTCCCCGCGTGTTTTCCGT
ACACCAACAATATATGCCATAATACACGTAACATTTTTTTATAAAAAGAAAAGGTAAGTGATATATATAAA
ATAGCGCC

CTAATTTTTTCCTTCCCACGTCCGCGGGAATCTGTGTATATTACTGCATCTAGATATATGTTATCTTATCT
TGGCGCGTACATTTAATTTTCAACGTATTCTATAAGAAATTGCGGGAGTTTTTTTCATGTAGATGATACTG
ACTGCACG

TAAGAAGTAGTATTCTATTATTATAACTATATTAATAAAAAAATTTATTTTCTATTGAAAACCAATTTTTT
ACACCAAATATCAGACACGCTTTGTCTATTTCTATCATATAGCGACTGTGATATAATGCGCGCGTGTTTTG
AACGGCAT

GAATGTACCTAAGCCAAAAAAAAAAAAAAAAAAAAAAAAAAAAAAAGAAACAGCTTTTGCATATTCAATCC
AGGCATAGGGCGACTATTTAGCACTCAACGATTTTTAAGCTTGTGTATTGCTGACATAAATTCCGGCTTTA
GAATCCAA

GAAAAACGAAATAAAGAAGAAAGATTATTATTATTAGTTACTTTTTTTATTGTACTCCAGTATGGACCTCT
TAGGTGAGTGATCTTATTAAAAAAATAAAAAAAATAAAAAAAATATGAAAAAAAAAGAAAATTAAAAACTA
AACAAATT

TTAACAAAATACATATAAGTAAATAATGCTAAATAAAAAAGCAAAAAAATAAAATTTATATCACACTTGTA
AAACCTTTAAATGATCTTCTTTTTTACCTGTTTTATTCACTTGTTTTTTTTTTTCTGAGTTGTTGCGCCTT
GGAAGAAT

CATTTTTTCTCATTTACTTTGTTATTAATTTTTTTTTTCAAGTTCTCCACATATACATATGTAAGAATTGA
GTCTATCTCTTTCTAATATTTAAGAAATACATAAAAAAACCAATTTTTTTTTTTTATTACTAGCAAATTTA
TTTTTGTG

ATGGTTATCTGCGATGAGATGAGTTTAAAATTTTCATTTTTTAGCCGATCAAGCAAAAGAAAAGAAAAAAA
AAAAAAACTGAAATTTCCACCTTACCCGTTTTTTTAATGGAACAGTGATTGTTACGACATTGAGATTTTAG
ATTTATAT

TTTGACGACTAAATAATAACATAACCACTACGTTCACTTCCCCGTATATATACATATATACTTAATATTAT
AAACGCATATACTTTTATAAACTTGGAATAAGTATTATATATTTATTTTGACCATCCGGAAATGCTTTATT
TTACACTT

AGGATCGAAAAATATCAAAAACATAAATCAGAAAAGGGGATCAAGGGGTTTTTTCATAGAATTTTCGATTA
AGTGGCATAGCAACATTTAGTTCGCAATGCGTTTACTTCAGAATCATTCTTTTTTTTTTTTTTTTTTTTTT
TTTGCCTC

GAAAGTATATATAAATTTGGGGTACCTGCAATCTTTATTTATTTTATTTGAAGTAATAGGAATTATTTATA
TTATTGTGATAAGAAGTAGTATTCTATTATTATAACTATATTAATAAAAAAATTTATTTTCTATTGAAAAC
CAATTTTT

TGTTTTTTTTTTTTAATGCAGATGTACGTACATATTTTCTTTATCAAAAGGATGTGTAGAGAACTATGTAT
ATATGTATATGTGTAGGTTTTTTTTTTTTAGGATGACTAGAAAAGGAAATCGAAAAAGAAAAAATAAGCAT
GAATCATG

GAGTTTAAAATTTTCATTTTTTAGCCGATCAAGCAAAAGAAAAGAAAAAAAAAAAAAACTGAAATTTCCAC
CTTACCCGTTTTTTTAATGGAACAGTGATTGTTACGACATTGAGATTTTAGATTTATATAATCACATACAT
ATTAAATA

AATATCAAAAACATAAATCAGAAAAGGGGATCAAGGGGTTTTTTCATAGAATTTTCGATTAAGTGGCATAG
CAACATTTAGTTCGCAATGCGTTTACTTCAGAATCATTCTTTTTTTTTTTTTTTTTTTTTTTTTGCCTCAC
ATTATATT

TTTAGTCATTCTATCCCTCGGATTATAGACTGTGAATATTGCATATGCAACTTTGACTCAAATTTTTCCAA
AATTTGATATATATATATATATATATATGTTTGTATGTATATATATATATACGTATATATATCATATATAC
GAAAAGTA

TGGACTCTTTTTTATTACCGTTTTTACCATTGGGATTGTTCTTGATGCGATCAAAAATATGATTTTTTTTA
TCATACCTGATACTTCGCGAAAACCTTAGAATAGCAAATAAGGCCATCGCAACATTAATAGACAAAAATTA
GAGTCACT

CTCCCGGTAGCGGGCCGTCGTTTAGTCATTCTATCCCTCGGATTATAGACTGTGAATATTGCATATGCAAC
TTTGACTCAAATTTTTCCAAAATTTGATATATATATATATATATATATGTTTGTATGTATATATATATATA
CGTATATA

TTAGAATGGCTAGTAAGAGAGGTGAAATCTACGAAACTGGTAAGTTCTTTTAAGTATCACATTACAATAAC
AAAACTGCAACTACCATAAAAAAAAATTGAAAAATCATAAATTAAAAAAAAAAAAATCAATTGAATTTTTT
TTTTTCAT

GTGATTGAAAACGGAACTAAGCTTTACTAATCAACAATATTAACTTTATAATAGATTCATATATGCGCGTA
CATATATTATATACAATTTTTCTACACAAGAATGCTTAATAAAGATACGATAATTTTTTGTTCGCTTTTAC
TTTTCCGC

TACTATTGAGTACTCATCTCATCGCTTCTTTCAGAACAAAATTTTTCATATTTTTTTTTTTTCCTTTTCTT
TTTTTTTTTTTCTTTGACTGTTACCCGGTTGTTTATATTTGTAGGAAAACAACAACGACAGAGAAAATATC
CTTGCAGT

ACCCAGTTCTTTGGAAATAACAGGAAAGTGCAGAAAGTTCATTAGAAATCAACGATGATCTGATATACATA
TATATATATATATATATATATATATATAAAGGTTATTAATAGCCTTCGAACAACTCATCTATCTGTGCGAT
GACGTTAA

CAATGCAATTACGTGAAAAATAAAAGAACGCTACCATAAAAATGTTAAAAACTGGTTAAAAATGTCAAAAA
ATGGTCCGAAAACTGAAAAAATATTCGTGATACAGTTTGCATAAAAAAAAACTATTGTGCGCGATTAATAT
AACCTACT

ACGTGAAAAATAAAAGAACGCTACCATAAAAATGTTAAAAACTGGTTAAAAATGTCAAAAAATGGTCCGAA
AACTGAAAAAATATTCGTGATACAGTTTGCATAAAAAAAAACTATTGTGCGCGATTAATATAACCTACTAG
ACTTAAAT

AGTCCTGTGTCCTGTGGTAGACCCACTTGTTTTACCGCAGAGGGCCCTTTTTTTGTTTACCGTCGCAAAAA
AAAAACGCGAAGAATACGCGTTGGTCGTTAAATTTTGTGGTACGCGTAAAACTGCTATTTGGTATCCTCTT
TAGGGACG

ATTTCATTTGCCGGGCGGAAAAAAAAAAAGGAAAAAAAAAAAAAAAAAAAAATAAATGAACACATGGAAATAA
GTCAAGGATTAGCGGATATGTAGTTCCAGTCCGGGTTATACCATCACGTGATAATAAATCCAAATGAGAAT
GAGGGTGT

CTAATCTATCATTTTATTATTTTCTCTCATGAGAAAACAGAATTTCGAAACAGATATAATAAAAAAATTGA
AAAATTTGAAAAAAAAAAATCTAGAAACATATTTTCCTAAAATGAAAATCGGAAGCTAACAAAATTTTTGA
AAAACGAA

AAATCTATGATTGTATGGTTTCTTTGACATATTTATAGTTAATATATATATATATATGTGTTTAATTTTAT
AAAAATTCCATCGTTTATATTCGATTATTTTTATATGTCAACCTTTTTTTTAAGAAATGTTCATATTTAGT
CAAGTTCT

GTGGTCAACTGTGTGGGGCCCGTTCAAGAATGGGAGGTATTGGGACGGGTGGACGTTTAAGCATACATTTA
TGTGCACTGTAAGTAATTATATAGAATTTTTTTTTTTTTTTTTTTTCATATCTGTCAATACTTGAAAAT
GTTCTAGT

AATTGAAATATGATAAAAACCCAAACATATTAAAAAACCCAGACATAAAGAAAAAAACTTTGAAAAAAAAA
GTATTATGCCGCTGAGCGCGCCACGTTAAAAGCTCGAATTCTTCGCATTGGTAGTAGCGCAAAGTCCAATG
AGCGCACC

TTTGACATATATATAGCTCGCAGTAGAAGAATGCAGCGTAACATAATATAATATAATATAGTACGGCGCCG
CCCTCCATTAGAACGCGCAACACAATAAAGACAAATAAAAGAATCAGCTATAAAGCTGGCCATTGCATGGT
TCGCGCTG

ATAAATTTGGGGTACCTGCAATCTTTATTTATTTTATTTGAAGTAATAGGAATTATTTATATTATTGTGAT
AAGAAGTAGTATTCTATTATTATAACTATATTAATAAAAAATTTTATTTTCTATTGAAAACCAATTTTTTA
CACCAAAT

TTTTTTTTTTTTTTTTTTTTTTTTTGCCTCACATTATATTCTTGGCATCTGCCTAGCTTTCGACTTTTTCG
TTCAATTCCTAAAGCGCTAAAAAAATAGTCTCCCCCTGAAAGTGGAGTAGGATAGTAGTATATGAAGTGAG
GCAGAAAA

CGTGATGATACCAAGAAGTAGTAAAAGCTCATGGCTTCTTATATATTATATATGGAATACATTTATAATAA
AATAATAAGAATTATATATTTTATGATTATATTATTACATAAAGTATTCCCCATTATAAATTCTGAGTTTC
GTATTTAA

TTTATAATGTATAATATACCCGAATCTTATTTATTTACCTTTCCTATTTTTTGACGACCAGTAAATACTAA
TACATAATTAGGAACAAAAGTTAAATAAAAAAAAAAATAATAATTTAACGCATCCAATTAACGTGTCCTTT
TTTCATCA

TGGCTTACTGCATCTCTTCAAAATTTCACAGTCATGCTCACCCTTAAGTTCTCAACCTTTTAGTTTTTTTT
TTTTTTTTTTTTTATTTTGTATGGCATACTAACTATACAAATATTTATATGTACATTTATACAGTAACCTA
TTATTTAC

TGTTCTATTTCTTGAGGTGATTTCAAATTGTGGCTCCAACAAGTAATCTCGGCAAGCTATCCCTTTTTAAA
TAATATTTTGCATTCTTTTTCTTTTTTTTTTTTTTTTTTTCCATCTCACAGCCCTTCTTTATTTTTTCAA
TAAGGTAA

CGTCAGTTTTCTTCAGCTAAGGCGCGCGCGCCAGATAACTAAAAAAAAATATAGTTGCTGCTTAAAAAACA
ATACACCCGTACTCTCTTGCCTGTAAAAACCTCGAAGGACCAAAGATACCCTCAAGGTTCTCATCTGTGCG
GTATTCTT

ACAACTTAATTATATAGAATTTAATAAAGACAAATGCAAAAGTTTTTTTAAAGTATGGATGTATAATAATA
ATAACGATAATACGAACACTATTGGGACTAATAATGATTTATAGTTTTTTTTACACAGGGGATTTAACGTT
CCGCGACC

TATTCTCTTCATATTCAACATACTCGAAAAGGAAGAACACTAAAAGTACTTACATTTTCACATGTATGTAT
ACCTATATATATATATATATATATACTCTTATAGATATATTTACAAATTAAAGGAAAAAATAATAAAATAACC
TCCCTGTC

CTGATTTTACTTTTTTCGGAATTACCTAAAATGGGTTTACGGCATAGAAGATAGATAGATTAAGAGGTAGG
TTGACTTTTCCTTTGAAATTTGTAATTTTAATAAAAATTCCAAAAAAAAAAAAAAAAAATTCAAAGTAAAA
AGACAAAC

AAGACACAGGTCGTTCTAACGTTGGTAAAATGAAAAATATGAAAAACAGTTATCATGGCTACTATAATAAC
AATAATAATAATAATAATAACAATAATAATAATAACAGTAATGCTACCAACAGCAACAGCGCGGAAAA
ACAACGTA

ATTTTATTATTTTCTCTCATGAGAAAACAGAATTTCGAAACAGATATAATAAAAAAATTGAAAAATTTGAA
AAAAAAAAATCTAGAAACATATTTTCCTAAAATGAAAATCGGAAGCTAACAAAATTTTTGAAAAACGAAT
AAAGAAGA

ACATATAAGTAAATAATGCTAAATAAAAAAGCAAAAAAATAAAATTTATATCACACTTGTAAAACCTTTAA
ATGATCTTCTTTTTTACCTGTTTTATTCACTTGTTTTTTTTTTTCTGAGTTGTTGCGCCTTGGAAGAATGG
AAAAGCAA

GAAGTAGGAAAACTGACATTAGAAAAAGAAAAAAAAAAACAATCTGCGAATTATGCATTTCTTTTTTTTCGCT
TTTTTATTTTCTTTTTTCTTTTTTTCGACCTCCAATAGGGCTTTGGAAGCCCTATATGTAATCTAGGGTAA
TGACATAT

TGTGTATAAAAGAGAGAAAAAATGGTTATTTTCCAATTAATTAATTATTAACGATTGTTAAAGAAAAAATT
TATCAACGAGGTTGATAGAAAAAAAAAGTTTTTGTAGATATAGAAGAAAAAAAGATTTTCACTATTGGTGT
TATAGGAA

ACTTATTCAGCTTTATTTTAAACATTATACAAGTTTTTCATTTTGAGGGGTCTTTAACTGGAAAAAAAAAG
CTTTCACGACTCAAAGAAAAAAAAAAAAAATTTTTTTAGGGTAAAAAAAAACTAAGGGCTAATATGAAAAAT
ACAAAGGT

AAAAAACGCGAAGAATACGCGTTGGTCGTTAAATTTTGTGGTACGCGTAAAACTGCTATTTGGTATCCTCT
TTAGGGACGCGCCCATTGTCTATACGGTAATATAGGGCTTTCTTCATTACCTGCTTGCGTCAATTAGCTTT
ACTCATGT

TTTTTATTTTTTTTTTTGCGCGAAACTTTGCTATATTGGGTAACGCGTAAAATACTTTTTATTATTGCAGT
AAGGCGGAAGGGTCTTCCCCTTTGCATGTTAAATAGCATACATGGCACCACTCAGGTCCAGAACGTGACAC
ATCTTTGC

CGTTCAAGAATGGGAGGTATTGGGACGGGTGGACGTTTAAGCATACATTTATGTGCACTGTAAGTAATTAT
ATAGAATTTTTTTTTTTTTTTTTTTTTTCATATCTGTCAATACTTGAAAATGTTCTAGTTAGAGATTTACG
TATACTGA

AAATTCTCGGCTTAGGCATCGAACGATGAAGAAACTAACTAAAAATATACGCCATTTTTTTGGGGAGGGGG
GGCCGCACTGAAACGCGAAATGCCAAAAAAAACGCGAAACGCCGCAGTAAAAGGTCAGTACCGCGCAAAAAA
TACAAAAA

AAATGCGAGATCTCGACCAAAAAAAAGGGGGTAGGGTAATAAAATTAACCCTATTATTTTTTAACTTTAAAA
CCTATAATGTGCTAATATTTTATTATAAACCTCCTTTTTTTGCGTTCAAACCCTGACACATTTTAAGCCCT
ATATTTAC

GAGTGAATTTCCGTCCACGAAAAAATGTTAACATAAAATGCAAGAGAACAATTAATCGAATAATGTTAAAT
TATTGTAAAACAATGTGTATGATGAGGAGGAATGTACCTAAGCCAAAAAAAAAAAAAAAAAAAAAAAAAAA
AAAAGAAA

TTGTTCAATACTGGACAATTGTTCTGATATATATATATATATATATAATTTTCAATGAAAAAAAAAAAAAAAA
GAAAGAAAATTAGAAGCTAGAAAGAAATAAGGAAAAGGAAAAGCAAACAACAATAGGAAAAAAAAGCATAG
AAACATAT

TTTTTTTTCAAGTTCTCCACATATACATATGTAAGAATTGAGTCTATCTCTTTCTAATATTTAAGAAATAC
ATAAAAAAACCAATTTTTTTTTTTTTATTACTAGCAAATTTATTTTTGTGAAGTGAAATTCATACATTGGCT
TATTAAAG

AAGAATTTAATAATGAAGATGGGAATAAGCAAAACAAAAACAAAGAAGGGAAAAAAAATAAAAAATCGTAT
TTATTTATTTAAAAAATCATGTTGATGACGACAATGGAAAAAAAAAAACCGATTTCACTTTCTCATCCTTAT
ATTTTTCA

CTATTGCGGAAACTGCAGCATAGTCAGTGTAATAATCGTACTGTTGCTTTTTCATTTTTTTTTTTTTTTTT
TTTTTTCTCATATAGAAAAACTGTTATTGAATGTTTTGACTAGTCTCATGAAATAAACATTAATAGTTACT
AAAGGGTT

CAACAGCCATTCCACAGATAGTGCTACTTTAGTAAACGTTCACATCAAAAGGTAGTATTAATTAAAAAAAA
AAAAAAGCCAATAATTTCGCGCAAGCTTTGCTCTACATAATAAAAATACAATTAGGATAGAAATGAATATT
TTTTTAGC

TTTTTTTCATTTTAGTATTTTATTCTTCGTTATTTATGTATAGAAATTTTCATTTTCATTTAGATTCAGAT
TTGGTTATCTTTTTTCATTATATATCTTTTGCACTAAGTTTCAACTTAAGTTCTATTTTTTATTTTTTTTT
TCTGGGCC

ATATTCAACATACTCGAAAAGGAAGAACACTAAAAGTACTTACATTTTCACATGTATGTATACCTATATAT
ATATATATATATACTCTTATAGATATATTTACAAATTAAAGGAAAAAATAATAAAATAACCTCCCTGTCAC
AAGTTAAA

GTTTTTTTAGTGGTAGAACGTTCTATCTGTTAAGCGTGTTATGGTTATCTGCGATGAGATGAGTTTAAAAT
TTTCATTTTTTAGCCGATCAAGCAAAAGAAAAGAAAAAAAAAAAAAAACTGAAATTTCCACCTTACCCGTTT
TTTTAATG

TGTCATAATAAATATAAAAAAAAAACTGTAGTTTCTTTTTAAATTTTTCTACTTAAGTTTAGTTATACTGTA
CCTAGGTATATATAAATATATATAAATAAAAGTGGCCAAGAATAAAAGAACGCACCCCGTCGTTGACTTAG
AAGTCATC

TTTTCTTTATACAAAATATATAGGTACCAAACCTCATTATAATTTCTGCCATTTAATAGTTTAAGATGCTA
TTCCGTTTCGTATTTTTTCAATTTTTATACCTACTATTGCGCGAAATCAAAATAGCATACTAAGAAAAAGC
AATATCAT

AAAAAGGCAATTCGCGTACAATTTTCGTTGATCGTTCTTTATATAACCTTTGCATTAAATAAATTTAACAA
AAAAAGTTCTTTCTAAAATAATATTATGGTGATACATGAATGTGCTTTAGTTTTTTCGTAGGCTCATCCAT
GTATATAT

ACAATACAATGTGAGACTAAAGTAAATCCGGGTAATACAAAATACAATTTTTTTTTTATTCTTCACAGCGTT
TTTTCGACGGAGGAGGGAAAAAAAAAAAATTGTATTTAAGGCATCAAATGCGCGACATCATATACCTTTGTC
CGCTGTTA

AAAATGAAGCCTAAGTATCACGCTAATTGAAGTTTTTTTTGATCACTCCAATAGGCAAATCTATAGATATA
TAAAAAAATATAGACAAGACTTTTTTTTTACATTGCCAGTTTTCTTTTTTTCCTTTTTAGTATCTATTCAAATG
GGCGACCC

TGTGGCTCAAAAAGATCTGTTTTTGTATTAGCAATTTTTTATTTCCCTTTTTAAATGAATTTTTTTTTCTTT
CTGTTTCGAGACAAGGCCTTTTGCAAAAAAAAAAAAAAGTAAAAAAGTGAAATTTGATAGTGTAAGGGGACA
GAGGTGGG

AACGAGCTAAGAAATAGTACACAGGGCAAGGTCATTAAATAGCGTATATAATCATTTAATATAGTATGTTC
TCGAAGCTGATCGCGTAAGGCGCAGAGCGAACTAAAAAAAAATACCGGCACCCATGCACCTCACACCGCCGC
ACGCGAGT

GGATGCGCTTCACAAGAATAGAAGCAAAAAGTCCGGTTAAAATTGAAATATGATAAAAACCCAAACATATT
AAAAAACCCAGACATAAAGAAAAAAACTTTGAAAAAAAAAGTATTATGCCGCTGAGCGCGCCACGTTAAAA
GCTCGAAT

CCATAAGTAGCAAAACCCTAACAATACAATGTGAGACTAAAGTAAATCCGGGTAATACAAAATACAATTTT
TTTTTATTCTTCACAGCGTTTTTTCGACGGAGGAGGGAAAAAAAAAAAATTGTATTTAAGGCATCAAATGCG
CGACATCA

CTCGCGAAAGTTTTAATTAAAATTTACTGATAGTTTTTTAATGATGAACTTCTATAACTAAACCATAATAA
TGAAAATTACAGGTAATATTTATATCACAAGAAACGGCCCCTCTTTTCTGTTTTTATATAGTAACCATTAA
GAATAGTT

AGAAAAAGAAAAAAAAAAACAATCTGCGAATTATGCATTTCTTTTTTTCGCTTTTTTATTTTCTTTTTTCTT
TTTTTCGACCTCCAATAGGGCTTTGGAAGCCCTATATGTAATCTAGGGTAATGACATATGTCACGTGCTTT
AGGGCTTG

TTAGATCCACATTACTAATTATTGAATATGGACAATAATATCAAATATATTATTATCAAATTGATTATTTT
GTGACGCGGCGTCAAATTTTAATCGCGTCTGACGAATGAGCGGGTAATAAACTGTCTTTTTTTCATGAATG
AATCGAGC

AATGTTAAAAACTGGTTAAAAATGTCAAAAAATGGTCCGAAAACTGAAAAAATATTCGTGATACAGTTTGC
ATAAAAAAAAAACTATTGTGCGCGATTAATATAACCTACTAGACTTAAATGACGACACTTTAATATGAAGTT
CAGTAGTG

ATTTTAGTTAGGAACTTTGGCATAATTTCAGTATTACATACTACCTAATCGACATAAAATAATATTACATA
TTCATTTTTTTAAAAAATAATCATAATCAATTTATCCTTTTTATTCCTTTTTTCTCCTGTGCAAGGTTGAT
GGTGATGT

CTAAACTCGAAAAAAAAAAAATTGAAAAATTGGAAAAAAGCGAGAGGTACTGAAAATTGTGAAGAGATTCCC
GGGTAAATATAAACATAATTAATACAATAATAGCAATATATGTGACGAACTGTGTTCTACTTTTTAGTATT
TACAGGTA

ATAGTTTCACCTTAAAACACATCTCCTTTTAAAACTTATTGTAATTATTCATTAGAATTTTTATATTTAAT
TAATCTATCCTTTTTACTAATGAAAAATTCTTTAAATAAAAAAATCTATACATCCACTAGAAAGTATATAT
AAATTTGG

TCGCGATGGTTAATGCTTTGTATTCGCGCTATCTCGATTTCTACCTATATAGTTAATCTCTGTACAAAAAC
AATCTTTCCAACTATCCATTAATCATAGTATATTATCAGCGTCGGCGATTTTACCACGCTTGACAAAAGCC
GCGGGCGG

CCGTGCGTGAGGGGGGCGCGGCCATTCGGTTTTTGCAATATGACCTGTGGGCCAAAAATCGAAAAAAAAAA
AAATAAGAGGCGGCTGCGGAATTTTATAAGACAAGCGCAGGGCCAAAGAAAAAATAATAATTGACGTGGCT
GAACAACA

AATTTCGAAACAGATATAATAAAAAAATTGAAAAATTTGAAAAAAAAAAATCTAGAAACATATTTTCCTAA
AATGAAAATCGGAAGCTAACAAAATTTTTGAAAAACGAAATAAAGAAGAAAGATTATTATTATTAGTTACT
TTTTTTAT

AATTAAAAACTAAACAAATTTTATGACAGGAATAAAAACTATAAAAAATGAAAAGCAAAAAAAAAAAGGCT
ACTGGATTTTTAACATCTTGTCCTTTTCTTTCCCTTTATCATTTTTTCTCATTTACTTTGTTATTAATTTT
TTTTTTCA

TGATAAAAACCCAAACATATTAAAAAACCCAGACATAAAGAAAAAAACTTTGAAAAAAAAAGTATTATGCC
GCTGAGCGCGCCACGTTAAAAGCTCGAATTCTTCGCATTGGTAGTAGCGCAAAGTCCAATGAGCGCACCTC
AAGTGAGA

AGGGTAGTAGTAAGAGCTTACGGAACAGCCCGAAAGGCTGTAAAAGTTTAATATACTTACTGTTCTTGAAA
TTCTGTCTATATACTTGACTATTTAAAGTTACTATCTTTGGATTTGAACCTTATAAAAAAAAAAAAAAAAG
GTACATGC

AAAGCTAAACGGGTTTTTCAGAATCCTCTTACCCAGCCTTGGATAATAGTTTTGGCCGAAAAAAAAAAAAA
GAAAGAAAAAAAATTGAAATTTCGCGATCCGAACAAACAATGAACAGAAAAACAGAATATATATATAACGCTGC
CAAGCCTA

CAGGAAAGTGCAGAAAGTTCATTAGAAATCAACGATGATCTGATATACATATATATATATATATATATATA
TATATATAAAGGTTATTAATAGCCTTCGAACAACTCATCTATCTGTGCGATGACGTTAATATGAAGTATTT
CTCCTTTT

ATTCTGCTTCTTTGTTTAATGGTGTAAGCTGCCTATATGTTACTATTGAGTACTCATCTCATCGCTTCTTT
CAGAACAAAATTTTTCATATTTTTTTTTTTTCCTTTTCTTTTTTTTTTTTTCTTTGACTGTTACCCGGTTG
TTTATATT

TTATTTCTTTCTCACATACTTTATAAAAAAAGATAATTTTTTTTTAAAAAAAAAAAAATACAAGTGGAACA
TTATCAAATAAAAAGCATTATAGCGCCACATGCGATGAACCTGAGCTTTTATCTTCATTAATATATAGTAA
TTACAACA

TTTTTGATACAGGGCGCTATGATTTTAGTACAAGCAGCTTTAGGTTACGTGTACCTATTTCTAGTTGAATT
TTCTCTCTATGAAATAGTCCTATAAATTTATATAAATCTATTTTTTTTTTTTTTTCGACACAAAATGTCTA
TTTCTTGG

TCCCCATCTATCCTTCGCCTCTCCTTCGCTCTGTAATTTTTTTTACTCGCGCGCTTCCGACTTTTGAAAGA
AGGAGCAATAAAGTTAAATAAATGTAATTAAATTATGCTTTTTTTAGGCAAGTTCGGACTTTGTTGCCACG
TATTGCTC

AATAAACTTTTCGAAAAATTTTTATTTATGATAATTTTACTAATTCTTACTTATTTCTTTTATATTCGATA
AAAAAAATTCAAAATAAAAAAAGATTAATCCTTTGATAGGAACTAAAGGTACTAGGTATTTTGGGCGGCGA
AGCATCAT

CAGTTTTCATTTTTTTTTTTTTCGTTATATAGGTTCTGCGCATGTGAAAAAAAAGAGAAGAAAATAAAGCC
AATTATAAGTCATTACTGACGAAATTATAAGATAAATTAGAGGTACAATATACAAACTAGTGAATAACAGA
ACTATATA

TCCCCAAATATTGAGGGTTTTGTTTTTTTTTTTTAATGCAGATGTACGTACATATTTTCTTTATCAAAAGG
ATGTGTAGAGAACTATGTATATATGTATATGTGTAGGTTTTTTTTTTTTAGGATGACTAGAAAAGGAAATC
GAAAAAGA

TCCCAATTTTTTTGACGACTAAATAATAACATAACCACTACGTTCACTTCCCCGTATATATACATATATAC
TTAATATTATAAACGCATATACTTTTATAAACTTGGAATAAGTATTATATATTTATTTTGACCATCCGGAA
ATGCTTTA

TCCTTTTTTTTTTTTTTACTTTGTTTGACCTCCCCTAACTCTTTCTTTTTACCTTCACAATTGTTTATTTT
AATATATGATTTTTAAATTAATTCAGGTTAATTCTAAAAACTATATTGCAATCTTTTAAATATATGTATAC
ACGTTCAT

TGTGCATACTGATATATGTTCTATTTTACCCATTTGAGGGGCCTAAATTGTCTATTAATTTTTTTACTTTA
ATACAGTTTGTATAGAAACATGTGGCACTAACAGCTTTATCCGCCCGCAGAAAAGATAAAAAAAAGGGTT
TTTACCCG

ATACCAAATATTTTCAAATCCTACTCAATAAAAAATTAATGAATAAATTAGTGTGTGTGCATTATATATAT
TAAAAATTAAGAATTAGACTAAATAAAGTGTTTCTAAAAAAATATTAAAGTTGAAATGTGCGTGTTGTGAA
TTGTGCTC

CGACGTTATATAATTGTTGTGATATTTGCATCTTCATCTTATGATGATGAGAATATAAATAATTTTTAATC
GTATTAAGTGTATATATGAATAAGCTATTAACCCTACCCATTAATTTCCGGGCGGGTTGTTATTTTTATCA
GTATTGGC

CTCATTTTCACTAAACTCGAAAAAAAAAAATTGAAAAATTGGAAAAAAGCGAGAGGTACTGAAAATTGTGA
AGAGATTCCCGGGTAAATATAAACATAATTAATACAATAATAGCAATATATGTGACGAACTGTGTTCTACT
TTTTAGTA

TTTTTACCATTGGGATTGTTCTTGATGCGATCAAAAATATGATTTTTTTTATCATACCTGATACTTCGCGA
AAACCTTAGAATAGCAAATAAGGCCATCGCAACATTAATAGACAAAAATTAGAGTCACTTCACTTCGTTGA
TTTTTGAT

CTTTTGAATGTGTGTATAAAAGAGAGAAAAAAATGGTTATTTTCCAATTAATTAATTATTAACGATTGTTAA
AGAAAAAAATTTATCAACGAGGTTGATAGAAAAAAAAAGTTTTTGTAGATATAGAAGAAAAAAAGATTTTCA
CTATTGGT

GCAACATTTAGTTCGCAATGCGTTTACTTCAGAATCATTCTTTTTTTTTTTTTTTTTTTTTTTTTGCCTCA
CATTATATTCTTGGCATCTGCCTAGCTTTCGACTTTTTCGTTCAATTCCTAAAGCGCTAAAAAAAATAGTCT
CCCCCTGA

GCCATTCGGTTTTTGCAATATGACCTGTGGGCCAAAAATCGAAAAAAAAAAAAAATAAGAGGCGGCTGCGGA
ATTTTATAAGACAAGCGCAGGGCCAAAGAAAAAATAATAATTGACGTGGCTGAACAACAGTCTCTCCCCAC
CCCTTTCC

TTAGTAAAAATAATTACGTGCGCGGTTGCGCGATATTTCCCTTGCTTAATGCCGAAGTAATTCTCTTCGTT
TACCCATACTCGAATCTATTGTGTTGTTGAAAAATAAAAAAAAAAAGAACCAGGTTATATAATTGTGAGTT
GATCAGTA

TATCCGGTTTTTTTTCTTGTTGACCAATAGGAAAAAAAAAAAATTAAAATAAAGTCACAGAAATTCAATGAA
TATTATAAATTTTTCTTGTTTGTTTCCCTAGTTGTTATTTTTATAAAAAAAATTCTTGTAGACAATAAAAT
AAGAAATG

GACCTGGTATTATCCGGTTTTTTTTCTTGTTGACCAATAGGAAAAAAAAAAAATTAAAATAAAGTCACAGAA
ATTCAATGAATATTATAAATTTTTCTTGTTTGTTTCCCTAGTTGTTATTTTTATAAAAAAAATTCTTGTAG
ACAATAAA

GTTCGAAGCTGACCTGGTATTATCCGGTTTTTTTTCTTGTTGACCAATAGGAAAAAAAAAAAATTAAAATAA
AGTCACAGAAATTCAATGAATATTATAAATTTTTCTTGTTTGTTTCCCTAGTTGTTATTTTTATAAAAAAA
ATTCTTGT

ATAATATACCCGAATCTTATTTATTTACCTTTCCTATTTTTTGACGACCAGTAAATACTAATACATAATTA
GGAACAAAAGTTAAATAAAAAAAAAAAATAATAATTTAACGCATCCAATTAACGTGTCCTTTTTTCATCATT
AATTTATC

TTTTTATTTATTTTTTTCATTTTAGTATTTTATTCTTCGTTATTTATGTATAGAAATTTTCATTTTCATTT
AGATTCAGATTTGGTTATCTTTTTTCATTATATATCTTTTGCACTAAGTTTCAACTTAAGTTCTATTTTTT
ATTTTTTT

GTATATTATTTTATACGTTTATGTCTATTTCAATGCAATTACGTGAAAAATAAAAGAACGCTACCATAAAA
ATGTTAAAAACTGGTTAAAAATGTCAAAAAATGGTCCGAAAACTGAAAAAATATTCGTGATACAGTTTGCA
TAAAAAAA

AGTGCATAATAAAAGTAAAAAAAAAAAAAATCAAACTTTATTGGGATAAAATAAAAATCAGAAAAACCCTTA
TATATGTATATGTAGTTAAATGAAGCCAGTTTTTTTCGTCTGGTTTGTTTCAGCCTATATGCCCTCTTTGC
TTTCTTGC

ACAGCAAACAGTACCTCACGTCTTTTTTTTGAATAGTTTTTTTTTTTGTTGAAACAGAAAAAAAACTTTCT
TCCGTATATTACATTGTACATTATTTTTATTGTATTTAGTTTCCAACGTTAGGATTTGAGCCGTCATTAA
TATTATTC

TCGCTCGGCGCCATACCGAAAATCCTCAGGAAAGTGTTAGTGTTATATATTATATTATATTATATCATATT
ATATTAAGTTTATTGTATACATAATGGTCATACATCAATAGTGTACATCCTCTCTTCGCTTGTCTCGCAAA
AAATGTTC

GATATTTGCATCTTCATCTTATGATGATGAGAATATAAATAATTTTTAATCGTATTAAGTGTATATATGAA
TAAGCTATTAACCCTACCCATTAATTTCCGGGCGGGTTGTTATTTTTATCAGTATTGGCCTCTGCCGGGCG
GCTAGCAC

TGGTTTAGCGAAGGCACCATTATGAAGATAGACACATTCTTCTTTTTTTTTTTTTTTTTTTTTTTTTTTTT
TTTTTTTTCATTTACTTTTATTTCTCGCGGTCGGTAAATTTTTCGTGGGTTTCTTTGAATCTATTAGCCGA
CATAAGAA

TCTTTTTTTTTTTTTTTTTTTTTTTTTTTTTTTTTTTTTCATTTACTTTTATTTCTCGCGGTCGGTAAATT
TTTCGTGGGTTTCTTTGAATCTATTAGCCGACATAAGAATAATGCATAAATAATATTTTTAATGTCTTCCT
ATGCCCAA

CTTTTATTAAGGATGCGCGCAATAGAAGACACAGGCAATAATAGAATTTATTAGGGTATTATTGTAAGGAT
AGGCATTACAAAGATAAAATAAGTTTTAAAAAAAAAAAAAAAAAAAAAAAAAAAAAAGAAAAATAAAAATA
AATATGGA

TATGAAGATAGACACATTCTTCTTTTTTTTTTTTTTTTTTTTTTTTTTTTTTTTTTTTTCATTTACTTTTA
TTTCTCGCGGTCGGTAAATTTTTCGTGGGTTTCTTTGAATCTATTAGCCGACATAAGAATAATGCATAAAT
AATATTTT

CTAAGTTTTCCTTTTATTAAGGATGCGCGCAATAGAAGACACAGGCAATAATAGAATTTATTAGGGTATTA
TTGTAAGGATAGGCATTACAAAGATAAAATAAGTTTTAAAAAAAAAAAAAAAAAAAAAAAAAAAAAAGAAA
AATAAAAA

CTATAAAGCGTCAGAAAAGATGGTTTAGCGAAGGCACCATTATGAAGATAGACACATTCTTCTTTTTTTTT
TTTTTTTTTTTTTTTTTTTTTTTTTTTTCATTTACTTTTATTTCTCGCGGTCGGTAAATTTTTCGTGGGTT
TCTTTGAA

GACACATTCTTCTTTTTTTTTTTTTTTTTTTTTTTTTTTTTTTTTTTTTCATTTACTTTTATTTCTCGCGG
TCGGTAAATTTTTCGTGGGTTTCTTTGAATCTATTAGCCGACATAAGAATAATGCATAAATAATATTTTTA
ATGTCTTC

AAGGCACCATTATGAAGATAGACACATTCTTCTTTTTTTTTTTTTTTTTTTTTTTTTTTTTTTTTTTTTCA
TTTACTTTTATTTCTCGCGGTCGGTAAATTTTTCGTGGGTTTCTTTGAATCTATTAGCCGACATAAGAATA
ATGCATAA

GGATGCGCGCAATAGAAGACACAGGCAATAATAGAATTTATTAGGGTATTATTGTAAGGATAGGCATTACA
AAGATAAAATAAGTTTTAAAAAAAAAAAAAAAAAAAAAAAAAAAAAAGAAAAATAAAAATAAATATGGATG
GAAATCAT

TGTGTTCGAACTATAAAGCGTCAGAAAAGATGGTTTAGCGAAGGCACCATTATGAAGATAGACACATTCTT
CTTTTTTTTTTTTTTTTTTTTTTTTTTTTTTTTTTTTTCATTTACTTTTATTTCTCGCGGTCGGTAAATTT
TTCGTGGG

GCATTCGGGTTAGCCCCGTTATAGGGAAAATGAAAAAAAAAAAAATAATAAAAAAAAAAAAGACTAAAAAA
ATAGAAGAGCACGGCAACTTAAAAAGAAAAAAAAAATTTATGATAACTACAAAATAGTAAAGAACCCTGGC
AAAAGAAA

TCAGAAAAGATGGTTTAGCGAAGGCACCATTATGAAGATAGACACATTCTTCTTTTTTTTTTTTTTTTTTT
TTTTTTTTTTTTTTTTTTCATTTACTTTTATTTCTCGCGGTCGGTAAATTTTTCGTGGGTTTCTTTGAATC
TATTAGCC

TTTACGCGGTTGAAGAATGCTGTGTTCGAACTATAAAGCGTCAGAAAAGATGGTTTAGCGAAGGCACCATT
ATGAAGATAGACACATTCTTCTTTTTTTTTTTTTTTTTTTTTTTTTTTTTTTTTTTTTCATTTACTTTTAT
TTCTCGCG

TAATGCATGCGCATTCGGGTTAGCCCCGTTATAGGGAAAATGAAAAAAAAAAAAATAATAAAAAAAAAAAA
GACTAAAAAAATAGAAGAGCACGGCAACTTAAAAAGAAAAAAAAAATTTATGATAACTACAAAATAGTAAA
GAACCCTG

CGCGCAGGCACGCCGCGCATCTCTTTTTCTCGAAGAAAGCGGAAAAATAAAAACGAAAAAATAAAAAAAAA
AAAAAGTATAAATAGTGAAGACTTTTTCCATTTGATATTTGGTAAAAATTTCGGCTGGAAGTTTTTTGCTG
AACATTTA

TTTTTTTTTTTTTTTTTTTTTTTTTTTTTCATTTACTTTTATTTCTCGCGGTCGGTAAATTTTTCGTGGGT
TTCTTTGAATCTATTAGCCGACATAAGAATAATGCATAAATAATATTTTTAATGTCTTCCTATGCCCAAAA
GAAGAAGT

CTTCCGTCTTCTAAGTTTTCCTTTTATTAAGGATGCGCGCAATAGAAGACACAGGCAATAATAGAATTTAT
TAGGGTATTATTGTAAGGATAGGCATTACAAAGATAAAATAAGTTTTTAAAAAAAAAAAAAAAAAAAAAAA
AAAAAAGA

TAGCCCCGTTATAGGGAAAATGAAAAAAAAAAAAATAATAAAAAAAAAAAAGACTAAAAAAATAGAAGAGC
ACGGCAACTTAAAAAGAAAAAAAAAATTTATGATAACTACAAAATAGTAAAGAACCCTGGCAAAAGAAATA
AATCCGGT

TGAAGAATGCTGTGTTCGAACTATAAAGCGTCAGAAAAGATGGTTTAGCGAAGGCACCATTATGAAGATAG
ACACATTCTTCTTTTTTTTTTTTTTTTTTTTTTTTTTTTTTTTTTTTTCATTTACTTTTATTTCTCGCGGT
CGGTAAAT

TCGTAACCGCCGCGCAGGCACGCCGCGCATCTCTTTTTCTCGAAGAAAGCGGAAAAATAAAAACGAAAAAA
TAAAAAAAAAAAAAAGTATAAATAGTGAAGACTTTTTCCATTTGATATTTGGTAAAAATTTCGGCTGGAAG
TTTTTTGC

TACATATTCTTTGACGTAATAATATCGGAATAATGCATGCGCATTCGGGTTAGCCCCGTTATAGGGAAAAT
GAAAAAAAAAAAAATAATAAAAAAAAAAAAGACTAAAAAAATAGAAGAGCACGGCAACTTAAAAAGAAAAA
AAAAATTT

AATATCGGAATAATGCATGCGCATTCGGGTTAGCCCCGTTATAGGGAAAATGAAAAAAAAAAAAATAATAA
AAAAAAAAAAGACTAAAAAAATAGAAGAGCACGGCAACTTAAAAAGAAAAAAAAAATTTATGATAACTACA
AAATAGTA

TTATTCAAAATTATTGGTTTTCGTAACCGCCGCGCAGGCACGCCGCGCATCTCTTTTTCTCGAAGAAAGCG
GAAAAATAAAAACGAAAAAATAAAAAAAAAAAAAAGTATAAATAGTGAAGACTTTTTCCATTTGATATTTG
GTAAAAAT

TGATTTTAACTTTACGCGGTTGAAGAATGCTGTGTTCGAACTATAAAGCGTCAGAAAAGATGGTTTAGCGA
AGGCACCATTATGAAGATAGACACATTCTTCTTTTTTTTTTTTTTTTTTTTTTTTTTTTTTTTTTTTTCAT
TTACTTTT

ATAGGGAAAATGAAAAAAAAAAAAATAATAAAAAAAAAAAAGACTAAAAAAATAGAAGAGCACGGCAACTT
AAAAAGAAAAAAAAAATTTATGATAACTACAAAATAGTAAAGAACCCTGGCAAAAGAAATAAATCCGGTAA
GGGAAGAT

TTGACGTAATAATATCGGAATAATGCATGCGCATTCGGGTTAGCCCCGTTATAGGGAAAATGAAAAAAAAA
AAAATAATAAAAAAAAAAAAGACTAAAAAAATAGAAGAGCACGGCAACTTAAAAAGAAAAAAAAAATTTAT
GATAACTA

ACAGGCAATAATAGAATTTATTAGGGTATTATTGTAAGGATAGGCATTACAAAGATAAAATAAGTTTTTAA
AAAAAAAAAAAAAAAAAAAAAAAAAAAGAAAAATAAAAATAAATATGGATGGAAATCATAGGTTTACTCCA
GATTCCAA

ATAGAATTTATTAGGGTATTATTGTAAGGATAGGCATTACAAAGATAAAATAAGTTTTTAAAAAAAAAAAA
AAAAAAAAAAAAAAAAAGAAAAATAAAAATAAATATGGATGGAAATCATAGGTTTACTCCAGATTCCAAAG
AATTTAAT

TTATTGGTTTTCGTAACCGCCGCGCAGGCACGCCGCGCATCTCTTTTTCTCGAAGAAAGCGGAAAAATAAA
AACGAAAAAATAAAAAAAAAAAAAAAGTATAAATAGTGAAGACTTTTTCCATTTGATATTTGGTAAAAATTT
CGGCTGGA

CGCCGCGCATCTCTTTTTCTCGAAGAAAGCGGAAAAATAAAAACGAAAAAATAAAAAAAAAAAAAAAGTATA
AATAGTGAAGACTTTTTCCATTTGATATTTGGTAAAAATTTCGGCTGGAAGTTTTTTGCTGAACATTTAAC
CGGAGAAT

GTCTTTTCGCGAATTCGCGTCGCGATTCGTGGATTGTGATGATAATAGCGATGTCTACTACCCGGGTACGT
ATTTAATTATCATTTATACATTATATGTTATAGGTTAGCGTATCGTTTTAAGGCTTGTGTCTTTTGGAAGC
ATGGCCTA

TGCAGCATGAATAAGATATAGGTGAGAGCTAGCGATTTGACATTTAGTACATAGATATTTATATATATATA
TAAGGGAGTTTTTTTTTATTTTTTTTTTCAAAATACGAAAATAGCAGTTCACGAGGCACTCACTTACTGGT
TTTTAAAA

TTAGGGTATTATTGTAAGGATAGGCATTACAAAGATAAAATAAGTTTTTAAAAAAAAAAAAAAAAAAAAAA
AAAAAAAGAAAAATAAAAATAAATATGGATGGAAATCATAGGTTTACTCCAGATTCCAAAGAATTTAATAC
TGTTGTAA

CAGAACTTGCGACCAGCTGTTAAATTCACAATCAGACCAATTTTTTACTCGACTTTTATTATATAATAGAT
TATATATATATATATATATATATATCTCTTTTGCAGCTAAATGAAAGAAAAAAAAAGAAATGGCACATATCAA
AAATAATC

GACCAGCTGTTAAATTCACAATCAGACCAATTTTTTACTCGACTTTTATTATATAATAGATTATATATATA
TATATATATATATCTCTTTTGCAGCTAAATGAAAGAAAAAAAAAGAAATGGCACATATCAAAAATAATCAA
TTCTCTCG

TGAAAAAAAAAAAAAATAATAAAAAAAAAAAAAGACTAAAAAAAATAGAAGAGCACGGCAACTTAAAAAGAAAA
AAAAAATTTATGATAACTACAAAATAGTAAAGAACCCTGGCAAAAGAAATAAATCCGGTAAGGGAAGATAG
ACCGGCTT

AATAGAAGACACAGGCAATAATAGAATTTATTAGGGTATTATTGTAAGGATAGGCATTACAAAGATAAAAT
AAGTTTTTAAAAAAAAAAAAAAAAAAAAAAAAAAAAAGAAAAATAAAAATAAATATGGATGGAAATCATAG
GTTTACTC

AACATTCAAGTGATTTTAACTTTACGCGGTTGAAGAATGCTGTGTTCGAACTATAAAGCGTCAGAAAAGAT
GGTTTAGCGAAGGCACCATTATGAAGATAGACACATTCTTCTTTTTTTTTTTTTTTTTTTTTTTTTTTTT
TTTTTTTC

ATTGTAAGGATAGGCATTACAAAGATAAAATAAGTTTTTAAAAAAAAAAAAAAAAAAAAAAAAAAAAAAGAA
AAATAAAAATAAATATGGATGGAAATCATAGGTTTACTCCAGATTCCAAAGAATTTAATACTGTTGTAAAA
AGTAAAGA

ATAAGATATAGGTGAGAGCTAGCGATTTGACATTTAGTACATAGATATTTATATATATATATATAAGGGAGTT
TTTTTTTATTTTTTTTTTCAAAATACGAAAATAGCAGTTCACGAGGCACTCACTTACTGGTTTTTAAAAGT
GCACCCCT

AGGGTTTTTTCAGTTGGTCAACACTCTTTAGAGGTAAAAAAAAAAAAAAAAAAAAAAAAAAAGAGAATTCTT
CATGTAATTTACCATGATTCTACGTTTTTGCAAGCAAAAATGAAGATAATCCGAGCGCATGCGAAGTAGTC
CCTGCCAT

TATTGTTGTTGTCTTTTCGCGAATTCGCGTCGCGATTCGTGGATTGTGATGATAATAGCGATGTCTACTAC
CCGGGTACGTATTTAATTATCATTTATACATTATATGTTATAGGTTAGCGTATCGTTTTAAGGCTTGTGTC
TTTTGGAA

CAATAATTAACAACAAATTCCAGAACTTGCGACCAGCTGTTAAATTCACAATCAGACCAATTTTTTACTCG
ACTTTTATTATATAATAGATTATATATATATATATATATATATCTCTTTTGCAGCTAAATGAAAGAAAAAA
AAAGAAAT

AAAAAAAAAGAAAAATTGTATGTATAATGCTTGTCACTTTTTTTATAATTGATATATGTATATGTATATGT
ATACATACATATATATAGTTGATTTAGAATTTATTTATATTAAACAAAAGACGAAGAGAGGGCAGATAACA
ATATTTAA

TGTGTATACCATGTACAATTGCTAAATGCTCATTGATTAATTTTAAATTTTTCATTTTTATCGCCTTAGAC
GAAATTGAAATTTTTCACTGAAAAAAAAAAAAAAAAAAAAAGTAGACGAAGAAGCTCATCTCATCGCGATAT
TAGTTCGA

TCTTTGTTTTTTTTTTCCTTTATTGTTGTTGTCTTTTCGCGAATTCGCGTCGCGATTCGTGGATTGTGATG
ATAATAGCGATGTCTACTACCCGGGTACGTATTTAATTATCATTTATACATTATATGTTATAGGTTAGCGT
ATCGTTTT

TTTTTTGTCTATTCGTTGTTTTCCTTCTCTATGGAAATCGTCGAAAAATTTCTTTGTTTTTTTTTTCCTTT
ATTGTTGTTGTCTTTTCGCGAATTCGCGTCGCGATTCGTGGATTGTGATGATAATAGCGATGTCTACTACC
CGGGTACG

TTTTTTTTTTTTTTTTTTTCATTTACTTTTATTTCTCGCGGTCGGTAAATTTTTCGTGGGTTTCTTTGAAT
CTATTAGCCGACATAAGAATAATGCATAAATAATATTTTTAATGTCTTCCTATGCCCAAAAGAAGAAGTCT
TGAAGTTG

TACTTTTTTTTTATCATCACTAGCTTCGGCTAAGCCCTTGCATTTTCAATATTCAATTACCAAATAATATA
TATATATATATATATATATGTATGTATGGTATACCTTGTGTTACTGATGTGTTTTTTGTTGACGTCATCCT
TTTGATTT

AGTGCTTCTTTTATTCAAAATTATTGGTTTTCGTAACCGCCGCGCAGGCACGCCGCGCATCTCTTTTTCTC
GAAGAAAGCGGAAAAATAAAAACGAAAAAATAAAAAAAAAAAAAAGTATAAATAGTGAAGACTTTTTCCAT
TTGATATT

AAAGATAAAATAAGTTTTTAAAAAAAAAAAAAAAAAAAAAAAAAAAAAAGAAAAATAAAAATAAATATGGAT
GGAAATCATAGGTTTACTCCAGATTCCAAAGAATTTAATACTGTTGTAAAAAGTAAAGAGTCTTCCACTGG
CAGAAATC

TATGTGGTATTACTTTTTTTTTATCATCACTAGCTTCGGCTAAGCCCTTGCATTTTCAATATTCAATTACC
AAATAATATATATATATATATATATATATATGTATGTATGGTATACCTTGTGTTACTGATGTGTTTTTTGTTG
ACGTCATC

TGATGGCGGGTATGAAGCAGGTTTTTTTTTTTTTGGCTTTTTCTTCTAAGAAAAAAATTTATAGAGAAAAGAT
GTCTTTAATTATAACCTATTCTTTTTTTTTTTGCCAGCTAATACCAGTAAGTTTTGTTTTTTTGCGACACT
AGATTGGA

TTTTTTCCTTTATTGTTGTTGTCTTTTCGCGAATTCGCGTCGCGATTCGTGGATTGTGATGATAATAGCGA
TGTCTACTACCCGGGTACGTATTTAATTATCATTTATACATTATATGTTATAGGTTAGCGTATCGTTTTAA
GGCTTGTG

TAGGCATTACAAAGATAAAATAAGTTTTTAAAAAAAAAAAAAAAAAAAAAAAAAAAAAAGAAAAATAAAAAT
AAATATGGATGGAAATCATAGGTTTACTCCAGATTCCAAAGAATTTAATACTGTTGTAAAAAGTAAAGAGT
CTTCCACT

TTTTACCACTACAGTAAATACTTCTGATAAGAACGGCATGAATAATCAAGTAGGTTTTATAATACTGGTAT
ACATTAATATAATCAACTATTTATACTATCAAAAAAAAAAAGCTTTTTTTTTTTTGTTTTTTTAAGTTTTAT
GGAATTTC

ATCACTCTTTTAGATAGACCTCTACAGCTTTTACATAATGGCACCAGTAATAAATGATGAACGATTTCGTT
ACCCTCGGTAAAACCGGCCAAAAAAAAAAAAAAAAAAAAAAAAAAATGTTCTCAAAGTGTAAACTTAATCA
AAAGCTTA

ACAGTAAATACTTCTGATAAGAACGGCATGAATAATCAAGTAGGTTTTATAATACTGGTATACATTAATAT
AATCAACTATTTATACTATCAAAAAAAAAAAGCTTTTTTTTTTTGTTTTTTTAAGTTTTATGGAATTTCAT
TCGTAAAG

CTATAAGATTTGATGGCGGGTATGAAGCAGGTTTTTTTTTTTTGGCTTTTTCTTCTAAGAAAAAATTTATA
GAGAAAAGATGTCTTTAATTATAACCTATTCTTTTTTTTTTTGCCAGCTAATACCAGTAAGTTTTGTTTTT
TTGCGACA

TAGCGCGGCGTCGCGGATTAAACAAGGTAACAATGGTTAAAGGAAAAATTATTTGTTAAGGTGGGTATATT
ATAGATTGCTAATTGTAAAGTATTAATAGTTTATTAAGTATTAAACTAGAGTTCGCACATGCTTTTATTTT
TTTGTTGC

TAAGTTTTTAAAAAAAAAAAAAAAAAAAAAAAAAAAAAGAAAAATAAAAATAAATATGGATGGAAATCATA
GGTTTACTCCAGATTCCAAAGAATTTAATACTGTTGTAAAAAGTAAAGAGTCTTCCACTGGCAGAAATCCG
TATCAGAC

TAAATTCACAATCAGACCAATTTTTTACTCGACTTTTATTATATAATAGATTATATATATATATATATATA
TATCTCTTTTGCAGCTAAATGAAAGAAAAAAAAAGAAATGGCACATATCAAAAATAATCAATTCTCTCGTC
TTCACACT

TGTTCTCTACAGTGCTTCTTTTATTCAAAATTATTGGTTTTCGTAACCGCCGCGCAGGCACGCCGCGCATC
TCTTTTTCTCGAAGAAAGCGGAAAAATAAAAACGAAAAAATAAAAAAAAAAAAAAGTATAAATAGTGAAGA
CTTTTTCC

ATTGTATGAAATCACTCTTTTAGATAGACCTCTACAGCTTTTACATAATGGCACCAGTAATAAATGATGAA
CGATTTCGTTACCCTCGGTAAAACCGGCCAAAAAAAAAAAAAAAAAAAAAAAAAAATGTTCTCAAAGTGTA
AACTTAAT

AGGCACTATATTTTACCACTACAGTAAATACTTCTGATAAGAACGGCATGAATAATCAAGTAGGTTTTATA
ATACTGGTATACATTAATATAATCAACTATTTATACTATCAAAAAAAAAAAGCTTTTTTTTTTTGTTTTTT
TAAGTTTT

CCATTGAGGTTGCAGCATGAATAAGATATAGGTGAGAGCTAGCGATTTGACATTTAGTACATAGATATTTA
TATATATATATAAGGGAGTTTTTTTTTATTTTTTTTTTCAAAATACGAAAATAGCAGTTCACGAGGCACTC
ACTTACTG

CAATTTAGCGTCTTTTTTTTTTTGCGTTGGTTTTTCCAATTTGTTTGCGTCGGTTATTATTTTTCAATAAA
AATAAATAAGAAAATGAGAAAAAAATGAAAAATGAAAAAAAAAAAATCTGATGGCCTAGATATGTTTGGGT
GTTTTCTT

GTCACAATTCTGTGTATACCATGTACAATTGCTAAATGCTCATTGATTAATTTTAAATTTTTCATTTTTAT
CGCCTTAGACGAAATTGAAATTTTTCACTGAAAAAAAAAAAAAAAAAAAAGTAGACGAAGAAGCTCATCTC
ATCGCGAT

CAACAAATTCCAGAACTTGCGACCAGCTGTTAAATTCACAATCAGACCAATTTTTTACTCGACTTTTATTA
TATAATAGATTATATATATATATATATATATATCTCTTTTGCAGCTAAATGAAAGAAAAAAAAAGAAATGG
CACATATC

TACCGCAATAAAATGGACCCTTGTCGGTCACGTGAGCAAAAATACTATTGCCGCCGACGGCCGTAGATAAT
AACAAAGGAATAAAAAAAAAAAAGCGGTAACTCGCGGAAGCATTGCCGATGCTTACCGAGATTTGCCGCGG
ATAACCGA

TCGAAAAATTTCTTTGTTTTTTTTTTCCTTTATTGTTGTTGTCTTTTCGCGAATTCGCGTCGCGATTCGTG
GATTGTGATGATAATAGCGATGTCTACTACCCGGGTACGTATTTAATTATCATTTATACATTATATGTTAT
AGGTTAGC

CCGCAATTTTGAGAATATTCCCTTTTTTTTACCGGATTAAAAAAAAAAAAAAAAAATTCTGTTATAGATGCA
CATCATATCTCTCTCCTTTTTCCCGCTTTTATATATTCGTTCGTATTAGTCACAACAAAAACCAAAAGAAT
ACTCGAAA

TCTTTTTTTTTTTGCGTTGGTTTTTCCAATTTGTTTGCGTCGGTTATTATTTTTCAATAAAAATAAATAAG
AAAATGAGAAAAAAAATGAAAAATGAAAAAAAAAAAAATCTGATGGCCTAGATATGTTTGGGTGTTTTCTTCA
TAATACTA

CGCTTTCCCTCCGCAATTTTGAGAATATTCCCTTTTTTTTACCGGATTAAAAAAAAAAAAAAAAAATTCTGT
TATAGATGCACATCATATCTCTCTCCTTTTTCCCGCTTTTATATATTCGTTCGTATTAGTCACAACAAAAA
CCAAAAGA

CAGTTGGTCAACACTCTTTAGAGGTAAAAAAAAAAAAAAAAAAAAAAAAAAAGAGAATTCTTCATGTAATTT
ACCATGATTCTACGTTTTTGCAAGCAAAAATGAAGATAATCCGAGCGCATGCGAAGTAGTCCCTGCCATGC
CGCTTCGG

TAGATAGACCTCTACAGCTTTTACATAATGGCACCAGTAATAAATGATGAACGATTTCGTTACCCTCGGTA
AAACCGGCCAAAAAAAAAAAAAAAAAAAAAAAAAAAATGTTCTCAAAGTGTAAACTTAATCAAAAGCTTATT
TGTTTGTT

CGCCCATCATCAACTTATTTATTGTATGAAATCACTCTTTTAGATAGACCTCTACAGCTTTTACATAATGG
CACCAGTAATAAATGATGAACGATTTCGTTACCCTCGGTAAAACCGGCCAAAAAAAAAAAAAAAAAAAAAA
AAAAATGT

TTTCCTCTTCGAGCTGCAAAAAAGTTTATCGCTTCGCGAAAAAAAAAAAAAAAAAAAATTTAAAGAATTTCA
AAACGTGTCAGGGCTACTAAATAATTGAACGAGGTCTATGAAATAAAATGGGCTGAGAAAATTTCAAGAAA
AAGGAAAT

TACTAATATTGGCAATAATGCTAACACATTACTAATAATAGCAATAATATTAATAATAATAATAATAATAA
TAAATTATAATAATAGCAGTAATAAAAATATCAAATAATAATAATAGTAATAGTAGTAGTAGTAGTAGCAG
TTGTAGTA

GCACCAGTAATAAATGATGAACGATTTCGTTACCCTCGGTAAAACCGGCCAAAAAAAAAAAAAAAAAAAAA
AAAAAATGTTCTCAAAGTGTAAACTTAATCAAAAGCTTATTTGTTTGTTATCTGTTTCTCATCGGTTTCGT
TTGTTGCT

TTACATAATGGCACCAGTAATAAATGATGAACGATTTCGTTACCCTCGGTAAAACCGGCCAAAAAAAAAAA
AAAAAAAAAAAAAAAATGTTCTCAAAGTGTAAACTTAATCAAAAGCTTATTTGTTTGTTATCTGTTTCTCA
TCGGTTTC

ACACTCTTTAGAGGTAAAAAAAAAAAAAAAAAAAAAAAAAAAGAGAATTCTTCATGTAATTTACCATGATTC
TACGTTTTTGCAAGCAAAAATGAAGATAATCCGAGCGCATGCGAAGTAGTCCCTGCCATGCCGCTTCGGAA
AAACTTTC

CTCTTGAAGAAGGGTTTTTTCAGTTGGTCAACACTCTTTAGAGGTAAAAAAAAAAAAAAAAAAAAAAAAAA
GAGAATTCTTCATGTAATTTACCATGATTCTACGTTTTTGCAAGCAAAAATGAAGATAATCCGAGCGCATG
CGAAGTAG

GATGTTTATTTACATATTCTTTGACGTAATAATATCGGAATAATGCATGCGCATTCGGGTTAGCCCCGTTA
TAGGGAAAATGAAAAAAAAAAAAAATAATAAAAAAAAAAAAAGACTAAAAAAAATAGAAGAGCACGGCAACTTA
AAAAGAAA

TATGAAGCAGGTTTTTTTTTTTTGGCTTTTTCTTCTAAGAAAAAATTTATAGAGAAAAGATGTCTTTAATT
ATAACCTATTCTTTTTTTTTTTGCCAGCTAATACCAGTAAGTTTTGTTTTTTTGCGACACTAGATTGGAAG
AGTAGAAT

TCTACAGCTTTTACATAATGGCACCAGTAATAAATGATGAACGATTTCGTTACCCTCGGTAAAACCGGCCA
AAAAAAAAAAAAAAAAAAAAAAAAAATGTTCTCAAAGTGTAAACTTAATCAAAAGCTTATTTGTTTGTTAT
CTGTTTCT

TACTGAAAATTGTGGCATTTTTTTTTTTTATTTTTTTGACGTAAATTTTTTATGTATATAGCAGTAAATGT
ATCGATAACGTGTTGGCTAATACTATAGTGAATAACAAAAAGTACTCTTAGTCTCCTTTTAGCATTTGTTT
TCACCAAA

GAGGTAAAAAAAAAAAAAAAAAAAAAAAAAAGAGAATTCTTCATGTAATTTACCATGATTCTACGTTTTTG
CAAGCAAAAATGAAGATAATCCGAGCGCATGCGAAGTAGTCCCTGCCATGCCGCTTCGGAAAAACTTTCGA
AACCAATA

TGTTCGAGATGATGTTTATTTACATATTCTTTGACGTAATAATATCGGAATAATGCATGCGCATTCGGGTT
AGCCCCGTTATAGGGAAAATGAAAAAAAAAAAAAAATAATAAAAAAAAAAGACTAAAAAAATAGAAGAGCA
CGGCAACT

CTTAACCAATTTTCTAAGTTCGGAACACAAAACCCAATGTTTAATCATTTTTAATAAACAATTTAGTATAT
ATATTATTTTAATTTTATTAAATTAATTAAAATTAAAAGAATTATTAGTCATGTGATTACCATCCTACTTA
TTATACTA

TGCGTTTCGAGAAAGTGAAAACCAATTGAATACAAAATAAAAAAAAAAGAAGAAAGAAATAGCAGGTCTAA
GATATATAAGAAAGTTAATATCATTTTTGAACATTTTATTTTAGACGCCTTCAGCCGCGCGACGCCCGGAG
TAATCATA

CTCTCAAATTCACTCTCATTTCTTTTTTCTTTGTTTTTCTTATATTTACTATATATATATATATATATATA
TATATATAGCCAAAAACAATAACAGACACAGTAATTTAGTATGCGTTTATCTCTTATCTTTCATCCACTGC
GACCGAAA

GGCAATAATGCTAACACATTACTAATAATAGCAATAATATTAATAATAATAATAATAATAATAAATTATAA
TAATAGCAGTAATAAAAATATCAAATAATAATAATAGTAATAGTAGTAGTAGTAGTAGCAGTTGTAGTAAT
GATAATAA

GGAATGATATTATGTGGTATTACTTTTTTTTTATCATCACTAGCTTCGGCTAAGCCCTTGCATTTTCAATA
TTCAATTACCAAATAATATATATATATATATATATATATGTATGTATGGTATACCTTGTGTTACTGATGTG
TTTTTTGT

CTTTCGAGTTTTGATTACAAGTCACTCGAAATGTTTTTTTCCTTTTATATATTCTTCCTTTTGTCTATTCT
TATATACACATATTTTAATTTTTGGTAGATTTTTTTTTTTTTTACTTACATTTGTTACAATAGAGAGATTT
ACCAATGG

TTCCTATTTGGGTGAGCAGTTTTCCTCTTCGAGCTGCAAAAAAGTTTATCGCTTCGCGAAAAAAAAAAAAA
AAAAAATTTAAAGAATTTCAAAACGTGTCAGGGCTACTAAATAATTGAACGAGGTCTATGAAATAAAATGG
GCTGAGAA

CAGAAATAACGAAAAATGAACACTTTTATTTTCTTTTGGCTTGTTAACCTAAACTTGTACATATGCCCATA
TATCCTTAAATATATATATATAGCCAACCCGAAGACGCTAAATAAACGCATTTTTTTTTTTTTTTACAGAA
TTATTACT

AGTCCCATCTAGGTCAAAAAGACAAAGATCTACTGAAAATTGTGGCATTTTTTTTTTTTATTTTTTTGACG
TAAATTTTTTATGTATATAGCAGTAAATGTATCGATAACGTGTTGGCTAATACTATAGTGAATAACAAAAA
GTACTCTT

GTAGCACATAGAGAAAACATCGCTTTCCCTCCGCAATTTTGAGAATATTCCCTTTTTTTTACCGGATTAAA
AAAAAAAAAAAAAAATTCTGTTATAGATGCACATCATATCTCTCTCCTTTTTCCCGCTTTTATATATTCGTT
CGTATTAG

ATTTAAGCCTTGCGTTTCGAGAAAGTGAAAACCAATTGAATACAAAATAAAAAAAAAAGAAGAAAGAAATA
GCAGGTCTAAGATATATAAGAAAGTTAATATCATTTTTGAACATTTTATTTTAGACGCCTTCAGCCGCGCG
ACGCCCGG

GAGTGAATACGTAGCACATAGAGAAAACATCGCTTTCCCTCCGCAATTTTGAGAATATTCCCTTTTTTTTA
CCGGATTAAAAAAAAAAAAAAAAAATTCTGTTATAGATGCACATCATATCTCTCTCCTTTTTCCCGCTTTTA
TATATTCG

AAAAATAATAAAAAAAAAAAAAAGACTAAAAAAAATAGAAGAGCACGGCAACTTAAAAAGAAAAAAAAAAATTTA
TGATAACTACAAAATAGTAAAGAACCCTGGCAAAAGAAATAAATCCGGTAAGGGAAGATAGACCGGCTTAA
AAAGTACT

ATGTACAATTGCTAAATGCTCATTGATTAATTTTAAATTTTTCATTTTTATCGCCTTAGACGAAATTGAAA
TTTTTCACTGAAAAAAAAAAAAAAAAAAAAAGTAGACGAAGAAGCTCATCTCATCGCGATATTAGTTCGATA
TCTGGGTT

AGTATATATCCTACCGTGTCTGTTCTCTACAGTGCTTCTTTTATTCAAAATTATTGGTTTTCGTAACCGCC
GCGCAGGCACGCCGCGCATCTCTTTTTCTCGAAGAAAGCGGAAAAATAAAAACGAAAAAATAAAAAAAAAA
AAAAGTAT

TTTTAAATTTTTCATTTTTATCGCCTTAGACGAAATTGAAATTTTTCACTGAAAAAAAAAAAAAAAAAAAA
GTAGACGAAGAAGCTCATCTCATCGCGATATTAGTTCGATATCTGGGTTTGAGTGAGTAGTATGTTTAATA
ATAAAAGA

GAGCTGCAAAAAAGTTTATCGCTTCGCGAAAAAAAAAAAAAAAAAAAAATTTAAAGAATTTCAAAACGTGTCA
GGGCTACTAAATAATTGAACGAGGTCTATGAAATAAAATGGGCTGAGAAAATTTCAAGAAAAAGGAAATAA
GTAAGGGC

CAACTTATTTATTGTATGAAATCACTCTTTTAGATAGACCTCTACAGCTTTTACATAATGGCACCAGTAAT
AAATGATGAACGATTTCGTTACCCTCGGTAAAACCGGCCAAAAAAAAAAAAAAAAAAAAAAAAAAAAAATGTTC
TCAAAGTG

AGGTCAAAAAGACAAAGATCTACTGAAAATTGTGGCATTTTTTTTTTTTTATTTTTTTGACGTAAATTTTTT
ATGTATATAGCAGTAAATGTATCGATAACGTGTTGGCTAATACTATAGTGAATAACAAAAAGTACTCTTAG
TCTCCTTT

ACAGATACGAGTTTAGCGAATAATACCGTTCGCAATCATAACTTTATAAAACTCATCCTATTACTTACTAT
TCGACTGGAAAATTTTCGCAGGCAAAAAAAAAAAAAAAAAAAAAAAGAAAAGAAAAATCGATGAGCCTGCCG
TACACAGA

AGGTAGTGAACCATTGAGGTTGCAGCATGAATAAGATATAGGTGAGAGCTAGCGATTTGACATTTAGTACA
TAGATATTTATATATATATATAAGGGAGTTTTTTTTTATTTTTTTTTTCAAAATACGAAAATAGCAGTTCA
CGAGGCAC

CCTTTATAAACAATTTAGCGTCTTTTTTTTTTTTGCGTTGGTTTTTCCAATTTGTTTGCGTCGGTTATTATT
TTTCAATAAAAATAAATAAGAAAATGAGAAAAAAAATGAAAAATGAAAAAAAAAAAAATCTGATGGCCTAGAT
ATGTTTGG

TATAAGAAAAAAATTAAAAAAAAAAAGAAAGAAAGTATAATACTAATATTGGCAATAATGCTAACACATTA
CTAATAATAGCAATAATATTAATAATAATAATAATAATAATAAATTATAATAATAGCAGTAATAAAAATAT
CAAATAAT

CACTTTTATTTTCTTTTGGCTTGTTAACCTAAACTTGTACATATGCCCATATATCCTTAAATATATATATA
TAGCCAACCCGAAGACGCTAAATAAACGCATTTTTTTTTTTTTTTACAGAATTATTACTTTCGTACTTGGG
TTTATGCT

GAAAAATGAACACTTTTATTTTCTTTTGGCTTGTTAACCTAAACTTGTACATATGCCCATATATCCTTAAA
TATATATATATAGCCAACCCGAAGACGCTAAATAAACGCATTTTTTTTTTTTTTTACAGAATTATTACTTT
CGTACTTG

ATTCGTTGTTTTCCTTCTCTATGGAAATCGTCGAAAAATTTCTTTGTTTTTTTTTTCCTTTATTGTTGTTG
TCTTTTCGCGAATTCGCGTCGCGATTCGTGGATTGTGATGATAATAGCGATGTCTACTACCCGGGTACGTA
TTTAATTA

CTATCAATATATTTCACACTACGCAGCTTAATTAGTAACTGTATAATAGCATTTAAGTTCATTACTTCTTT
GTAATTTATCTTTTTTTTTTTTTTATATAGTTTTCAGCGCGCAGCAGAGTTTCATTGGAACGCTTCGCACG
TTGGAAGA

TTCCTTCTCTATGGAAATCGTCGAAAAATTTCTTTGTTTTTTTTTTCCTTTATTGTTGTTGTCTTTTCGCG
AATTCGCGTCGCGATTCGTGGATTGTGATGATAATAGCGATGTCTACTACCCGGGTACGTATTTAATTATC
ATTTATAC

AAGAGGGTACGTGATTATAAACAGATACGAGTTTAGCGAATAATACCGTTCGCAATCATAACTTTATAAAA
CTCATCCTATTACTTACTATTCGACTGGAAAATTTTCGCAGGCAAAAAAAAAAAAAAAAAAAAAAAGAAAAG
AAAAATCG

GAGAATATTCCCTTTTTTTTACCGGATTAAAAAAAAAAAAAAAAATTCTGTTATAGATGCACATCATATCT
CTCTCCTTTTTCCCGCTTTTATATATTCGTTCGTATTAGTCACAACAAAAACCAAAAGAATACTCGAAATG
TTAGTTTA

GAATTCGCGTCGCGATTCGTGGATTGTGATGATAATAGCGATGTCTACTACCCGGGTACGTATTTAATTAT
CATTTATACATTATATGTTATAGGTTAGCGTATCGTTTTAAGGCTTGTGTCTTTTGGAAGCATGGCCTAAT
TGTTCAGC

ATGGAAATCGTCGAAAAATTTCTTTGTTTTTTTTTTCCTTTATTGTTGTTGTCTTTTCGCGAATTCGCGTC
GCGATTCGTGGATTGTGATGATAATAGCGATGTCTACTACCCGGGTACGTATTTAATTATCATTTATACAT
TATATGTT

GGTGAGCAGTTTTCCTCTTCGAGCTGCAAAAAAGTTTATCGCTTCGCGAAAAAAAAAAAAAAAAAAATTTA
AAGAATTTCAAAACGTGTCAGGGCTACTAAATAATTGAACGAGGTCTATGAAATAAAATGGGCTGAGAAAA
TTTCAAGA

TGATAGATACAAATTCCTCGCCTTTATAAACAATTTAGCGTCTTTTTTTTTTTGCGTTGGTTTTTCCAATT
TGTTTGCGTCGGTTATTATTTTTCAATAAAAATAAATAAGAAAATGAGAAAAAAAATGAAAAATGAAAAAAA
AAAAATCT

TCTATAAGGCCTATAAGATTTGATGGCGGGTATGAAGCAGGTTTTTTTTTTTTGGCTTTTTCTTCTAAGAA
AAAATTTATAGAGAAAAGATGTCTTTAATTATAACCTATTCTTTTTTTTTTTGCCAGCTAATACCAGTAAG
TTTTGTTT

ATCAGACCAATTTTTTACTCGACTTTTATTATATAATAGATTATATATATATATATATATATATCTCTTTT
GCAGCTAAATGAAAGAAAAAAAAAGAAATGGCACATATCAAAAATAATCAATTCTCTCGTCTTCACACTTG
TAGATATT

TTTTTCCTAAAGATATTAGAAAAAAAAATGTTATGTATCAATATTACTAGGTAAAATACAAAAATGCGCTT
TTGAAGTATAAATAATACAGTTTTGTTTTAAGAAATGCTATATATGAATTAATAATAAATGAAACGAAAAA
AAGCACTA

GACAAAGATCTACTGAAAATTGTGGCATTTTTTTTTTTTATTTTTTTGACGTAAATTTTTTATGTATATAG
CAGTAAATGTATCGATAACGTGTTGGCTAATACTATAGTGAATAACAAAAAGTACTCTTAGTCTCCTTTTA
GCATTTGT

GTAAGTAAATAAGAGGGTACGTGATTATAAACAGATACGAGTTTAGCGAATAATACCGTTCGCAATCATAA
CTTTATAAAACTCATCCTATTACTTACTATTCGACTGGAAAATTTTCGCAGGCAAAAAAAAAAAAAAAAAA
AAAAGAAA

GTGATTATAAACAGATACGAGTTTAGCGAATAATACCGTTCGCAATCATAACTTTATAAAACTCATCCTAT
TACTTACTATTCGACTGGAAAATTTTCGCAGGCAAAAAAAAAAAAAAAAAAAAAAGAAAAGAAAAATCGAT
GAGCCTGC

CATTGATTAATTTTAAATTTTTCATTTTTATCGCCTTAGACGAAATTGAAATTTTTCACTGAAAAAAAAA
AAAAAAAAAAGTAGACGAAGAAGCTCATCTCATCGCGATATTAGTTCGATATCTGGGTTTGAGTGAGTAGT
ATGTTTAA

TTGTTGTCCTTTTTTTATTTGTGGAAAATTCCTCAATTTGAAATAAGATTCGTATTTATTTTGTAAAACGA
TTTTAGTTTTATTTTTTTTTTTTTCTTCCCTTTTACCTTATTATTTGTTTTACCCTTTATGGATATTCATC
CACTCAGA

ACAATGCACTCTTCCGTCTTCTAAGTTTTCCTTTTATTAAGGATGCGCGCAATAGAAGACACAGGCAATAA
TAGAATTTATTAGGGTATTATTGTAAGGATAGGCATTACAAAGATAAAATAAGTTTTAAAAAAAAAAAAAA
AAAAAAAA

AAAAAAAAAAAGACTAAAAAAATAGAAGAGCACGGCAACTTAAAAAGAAAAAAAAAATTTATGATAACTAC
AAATAGTAAAGAACCCTGGCAAAAGAAATAAATCCGGTAAGGGAAGATAGACCGGCTTAAAAAGTACTTT
TTCAGATT

CTTTCTGCCGCTCTCAAATTCACTCTCATTTCTTTTTTCTTTGTTTTTCTTATATTTACTATATATATATA
TATATATATATATATATAGCCAAAAACAATAACAGACACAGTAATTTAGTATGCGTTTATCTCTTATCTTT
CATCCACT

TGCTGACAATAGTCCCATCTAGGTCAAAAAGACAAAGATCTACTGAAAATTGTGGCATTTTTTTTTTTTAT
TTTTTTGACGTAAATTTTTTATGTATATAGCAGTAAATGTATCGATAACGTGTTGGCTAATACTATAGTGA
ATAACAAA

ACAATACATACTTTCGAGTTTTGATTACAAGTCACTCGAAATGTTTTTTTCCTTTTATATATTCTTCCTTT
TGTCTATTCTTATATACACATATTTTAATTTTGGTAGATTTTTTTTTTTTTTTACTTACATTTGTTACAAT
AGAGAGAT

AAAACTACAACTATCAATATATTTCACACTACGCAGCTTAATTAGTAACTGTATAATAGCATTTAAGTTCA
TTACTTCTTTGTAATTTTATCTTTTTTTTTTTTTATATAGTTTTCAGCGCGCAGCAGAGTTTCATTGGAAC
GCTTCGCA

TGTCTAATAGAATGGGTGAACTTTTTTTATTTCTTGGCTCATGATTATATTGTAAAATACATAGCATTTTA
TATAATTGTGGTGCTAACCCTAAGTACTTTTTTTTTTCGCCCTTTCCGGGTTTCTTTTTTTCACCTTTTCG
TTTTTCTA

CTGAAAATGTTGAAAAATAAGCTGAATGGGGCATTACATGTTTTCATCCAAACATGCCTTGTCAATAACGA
GATTCGCGACGTATTCTTTTAGTATTCTGTTCCTTTTTTTTTTTTTGCTCCGCGCACTTGAACATCTTGCA
TATTTTTC

TTTCTAAGTTCGGAACACAAAACCCAATGTTTAATCATTTTTAATAAACAATTTAGTATATATATTATTTT
AATTTTATTAAATTAATTAAAATTAAAAGAATTATTAGTCATGTGATTACCATCCTACTTATTATACTAAA
TCGTTTTG

ATCCCCAAATTTTTTTGTCTATTCGTTGTTTTCCTTCTCTATGGAAATCGTCGAAAAATTTCTTTGTTTTT
TTTTTCCTTTATTGTTGTTGTCTTTTCGCGAATTCGCGTCGCGATTCGTGGATTGTGATGATAATAGCGAT
GTCTACTA

TAGAGCAAACAAAAAGCAAGAAAAAAAAGGTAATAAATGTAACAAATCTATTAACGTTAGAAAATAACAAAA
AAAAAAAAAGAAAAAGATAATATAATATAATACCTTTATATATGTGATTTATCTAGCGTCACAGTATGTAA
TATAAATA

CTACCGTGTCTGTTCTCTACAGTGCTTCTTTTATTCAAAATTATTGGTTTTCGTAACCGCCGCGCAGGCAC
GCCGCGCATCTCTTTTTCTCGAAGAAAGCGGAAAAATAAAAACGAAAAAATAAAAAAAAAAAAAAGTATAA
ATAGTGAA

AAATTCCTCGCCTTTATAAACAATTTAGCGTCTTTTTTTTTTTGCGTTGGTTTTTCCAATTTGTTTGCGTC
GGTTATTATTTTTCAATAAAAATAAATAAGAAAATGAGAAAAAAATGAAAAATGAAAAAAAAAAAAATCTGA
TGGCCTAG

TAAGACTAAAAAAAATTTCAAGATTAATTCCCAACCTATATATATATATATATATATATATATACTGTTTAGAT
CAAGTATATCTTCCTGCTAAATAATTAATATGCTCGTATGTAGAAAACAAAAAAGAGGTCTTTTCTCTTTT
TTCTCCCG

AAAAAGCAAGAAAAAAAAGGTAATAAATGTAACAAATCTATTAACGTTAGAAAATAACAAAAAAAAAAAAAG
AAAAAGATAATATAATATAATACCTTTATATATGTGATTTATCTAGCGTCACAGTATGTAATATAAATATC
AATGAGTT

AGATATTAGAAAAAAAAAATGTTATGTATCAATATTACTAGGTAAAATACAAAAATGCGCTTTTGAAGTATA
AATAATACAGTTTTGTTTTAAGAAATGCTATATATGAATTAATAATAAATGAAACGAAAAAAAGCACTACT
TGAGTTTT

GAGAAAACATCGCTTTCCCTCCGCAATTTTGAGAATATTCCCTTTTTTTTACCGGATTAAAAAAAAAAAAA
AAAATTCTGTTATAGATGCACATCATATCTCTCTCCTTTTTCCCGCTTTTATATATTCGTTCGTATTAGTC
ACAACAAA

GCCAACTATTTTGCACAGGAAAGAAAAATCACCAATACAATACTACTATATTTTACCCAAGTTAATACGGG
AAAGTATATTTTCTTTTCCATATTTTTCTTTTTTATTGACGTTTGCATTTTTTAGAGCAACTAGTAAACAA
AAAACCAG

CTCTGCTTATTGTTCGAGATGATGTTTATTTACATATTCTTTGACGTAATAATATCGGAATAATGCATGCG
CATTCGGGTTAGCCCCGTTATAGGGAAAATGAAAAAAAAAAAAAATAATAAAAAAAAAAAAGACTAAAAAAA
TAGAAGAG

AATTTTATTTCTTTCTGCCGCTCTCAAATTCACTCTCATTTCTTTTTTCTTTGTTTTTCTTATATTTACTA
TATATATATATATATATATATATATATATAGCCAAAAACAATAACAGACACAGTAATTTAGTATGCGTTTATC
TCTTATCT

TCGAATTCCCTCTCTTGGCCGTGTTTCGGGCCACGAGGCGAAAATTTTTTTTTTTTAATCGAAAAAAAAAG
GAAAAAAAGATTAACGTTTCTTTTTCGTTCTATCGTCTTCTACGATATCGCATGTAACACAATGCAGCTAT
CTGATAAT

TTCTTTTGGCTTGTTAACCTAAACTTGTACATATGCCCATATATCCTTAAATATATATATATATAGCCAACCC
GAAGACGCTAAATAAACGCATTTTTTTTTTTTTTTACAGAATTATTACTTTCGTACTTGGGTTTATGCTTC
TTATAACA

ACATTTCATGAAAAAAAAAGAAAAATTGTATGTATAATGCTTGTCACTTTTTTTATAATTGATATATGTAT
ATGTATATGTATACATACATATATATAGTTGATTTAGAATTTATTTATATTAAACAAAAGACGAAGAGAGG
GCAGATAA

TAGTTTAACTCTCTTGAAGAAGGGTTTTTTCAGTTGGTCAACACTCTTTAGAGGTAAAAAAAAAAAAAAAA
AAAAAAAAAAGAGAATTCTTCATGTAATTTACCATGATTCTACGTTTTTGCAAGCAAAAATGAAGATAATC
CGAGCGCA

ATAAAAAAAAAAAAAGCGGTAACTCGCGGAAGCATTGCCGATGCTTACCGAGATTTGCCGCGGATAACCGAA
CCATCATTGGGGTAGGAAACATTATAAATATTTGATATATATAAAGGAAAAAGCTTGATTTGGTCTTGTAA
TATCCCAG

GTTTTTTTTTTTTGGCTTTTTCTTCTAAGAAAAAATTTATAGAGAAAAGATGTCTTTAATTATAACCTATT
CTTTTTTTTTTTGCCAGCTAATACCAGTAAGTTTTGTTTTTTTGCGACACTAGATTGGAAGAGTAGAATCA
GAAAATGA

TGTCACTGCTGAAGATAACGTAAGACTAAAAAAATTTCAAGATTAATTCCCAACCTATATATATATATATA
TATATATATACTGTTTAGATCAAGTATATCTTCCTGCTAAATAATTAATATGCTCGTATGTAGAAAACAAA
AAAGAGGT

GGCTACAATTAGCCTCACCGATGTAACGTTATATAACGCTGCGGTCGTAGAATATGATTTTTCTGAATTTT
AATTTTTTTTTTTTTTTTTTCATTTCTTTAACATCTAAAATAGATTTTAGAATCAGAAAAGTGAAGAGTA
TTCCACAT

TGAAAAATAAGCTGAATGGGGCATTACATGTTTTCATCCAAACATGCCTTGTCAATAACGAGATTCGCGAC
GTATTCTTTTAGTATTCTGTTCCTTTTTTTTTTTGCTCCGCGCACTTGAACATCTTGCATATTTTTCTT
TTCTGTTT

GAAAGTGAAAACCAATTGAATACAAAATAAAAAAAAAAGAAGAAAGAAATAGCAGGTCTAAGATATATAAG
AAAGTTAATATCATTTTTGAACATTTTATTTTAGACGCCTTCAGCCGCGCGACGCCCGGAGTAATCATATG
CCCATGAC

AGAGAATTTTGAGATGAAAAAAAAAAAAAATGAATCTGTACTATTTCTTTTTTTAGTGCAAAAGTTTTGTTT
TAATATAGAGATAAATAAAAAAATGAACATATTACTATAATATCAACAAAATATGATGTAATGTCTGCAAT
AAAGATGA

CTAACACATTACTAATAATAGCAATAATATTAATAATAATAATAATAATAAATTATAATAATAGCAGT
AATAAAAATATCAAATAATAATAATAGTAATAGTAGTAGTAGTAGTAGCAGTTGTAGTAATGATAATAATA
ATATAATA

CCTTTTTTTTACCGGATTAAAAAAAAAAAAAAAAAATTCTGTTATAGATGCACATCATATCTCTCTCCTTTT
TCCCGCTTTTATATATTCGTTCGTATTAGTCACAACAAAAACCAAAAGAATACTCGAAATGTTAGTTTATA
TATATATA

TATACAAGACTAGAGCAAACAAAAAGCAAGAAAAAAAAGGTAATAAATGTAACAAATCTATTAACGTTAGAA
AATAACAAAAAAAAAAAAAGAAAAAGATAATATAATATAATACCTTTATATATGTGATTTATCTAGCGTCA
CAGTATGT

ATTAAAAAGATTAGTTTACTTTTTCTGTAGAATGTTAATTAGTTTATTGCATAGAAAATCTTTAATACTGA
ATGACAAAATTTCCACAATTTTTTTTAAAAAAATTATCGACAACTGCAGGACTCGAACCTGCGCGGGCAAA
GCCCAAAA

GGTGAGAGCTAGCGATTTGACATTTAGTACATAGATATTTATATATATATATAAGGGAGTTTTTTTTTATT
TTTTTTTTCAAAATACGAAAATAGCAGTTCACGAGGCACTCACTTACTGGTTTTTAAAAGTGCACCCCTTT
CGAAACGT

ATCATAGAAATATAAGAAAAAAATTAAAAAAAAAAAGAAAGAAAGTATAATACTAATATTGGCAATAATGC
TAACACATTACTAATAATAGCAATAATATTAATAATAATAATAATAATAAATTATAATAATAGCAGTA
ATAAAAAT

TAAATGATGAACGATTTCGTTACCCTCGGTAAAACCGGCCAAAAAAAAAAAAAAAAAAAAAAAAAAAATGTT
CTCAAAGTGTAAACTTAATCAAAAGCTTATTTGTTTGTTATCTGTTTCTCATCGGTTTCGTTTGTTGCTTA
AAGGGTGG

GTTTAGCGAATAATACCGTTCGCAATCATAACTTTATAAAACTCATCCTATTACTTACTATTCGACTGGAA
AATTTTCGCAGGCAAAAAAAAAAAAAAAAAAAAAAAGAAAAGAAAAATCGATGAGCCTGCCGTACACAGAGA
TATAATAA

CGGTTCTTTTCTTTTCTTTTTTTCTTTTTTTACGATGCATGTATATGTATTTTAATAGTAAAATATATTAT
TAAATAGGGGAGTGTGGTTGTGTGATTTAGTAGAGTAGTTAGAATAAAAACGACACAGTGGAAAAAAAAAG
GTATTTTC

GCTACAGTGTGAGTGAATACGTAGCACATAGAGAAAACATCGCTTTCCCTCCGCAATTTTGAGAATATTCC
CTTTTTTTTACCGGATTAAAAAAAAAAAAAAAAAATTCTGTTATAGATGCACATCATATCTCTCTCCTTTTT
CCCGCTTT

TCAGATAATACCTTAAGCGTCGAAACCTCATACCCCCTCATCTGGCTCGGGAGCCAGTATACTATATATAT
ATATATATATATATATATATATATATAAATTCCTAAAATACTCTCACACAGAAGCACAATAATGAGTGACG
CAAATGCA

TCTCTTGGCCGTGTTTCGGGCCACGAGGCGAAAATTTTTTTTTTTAATCGAAAAAAAAAGGAAAAAAAGA
TTAACGTTTCTTTTTCGTTCTATCGTCTTCTACGATATCGCATGTAACACAATGCAGCTATCTGATAATAT
GGTCCCAG

AAAAAAGAAAGAAAGTATAATACTAATATTGGCAATAATGCTAACACATTACTAATAATAGCAATAATATT
AATAATAATAATAATAATAATAAATTATAATAATAGCAGTAATAAAAATATCAAATAATAATAATAGTAAT
AGTAGTAG

TACCAGGTAAGGCTACAATTAGCCTCACCGATGTAACGTTATATAACGCTGCGGTCGTAGAATATGATTTT
TCTGAATTTTAATTTTTTTTTTTTTTTTTTTCATTTCTTTAACATCTAAAATAGATTTTAGAATCAGAAAA
GTGAAGAG

TTTTTTATTTGTGGAAAATTCCTCAATTTGAAATAAGATTCGTATTTATTTTGTAAAACGATTTTAGTTTT
TATTTTTTTTTTTTCTTCCCTTTTACCTTATTATTTGTTTTACCCTTTATGGATATTCATCCACTCAGAAA
CTAATATA

GTTTTTTTAAGGGAAAACGTATATCACTTAGAGATTATATTAATGAAAAAAGGTGTATACAAATGTATAAT
AATAATAGTATATAAGAAAAGTATTCGCTATTTATAGTGATTTTAGTTTGTACAATGTACTGTATTTTTTT
TAAATGTA

ATCGCTTGGCCAGAAATAACGAAAAATGAACACTTTTATTTTCTTTTGGCTTGTTAACCTAAACTTGTACA
TATGCCCATATATCCTTAAATATATATATATAGCCAACCCGAAGACGCTAAATAAACGCATTTTTTTTTT
TTTTACAG

TTCCCGCTTTTATATATTCGTTCGTATTAGTCACAACAAAAACCAAAAGAATACTCGAAATGTTAGTTTAT
ATATATATATATAGTGTGCGTTCTTTGCGGATATGGAAAATCTTGATATCTGCTGCATTCACGACTATGTC
GCGCGGCA

CATTGTACATTACCAGGTAAGGCTACAATTAGCCTCACCGATGTAACGTTATATAACGCTGCGGTCGTAGA
ATATGATTTTTCTGAATTTTAATTTTTTTTTTTTTTTTTTTCATTTCTTTAACATCTAAAATAGATTTTAG
AATCAGAA

TCCGGCGGTATTGAAGGAGAAAAATTTGGGCGGGAGCATATTATTGTAATTAATATATACATATATATATA
TATATATACATCAACCTTACAACTATGCGTTTAGAATCCTAAACTTTGATCTTACCATCAATAACGGTAGT
GGCATGAC

TTATCATCACTAGCTTCGGCTAAGCCCTTGCATTTTCAATATTCAATTACCAAATAATATATATATATATA
TATATATATGTATGTATGGTATACCTTGTGTTACTGATGTGTTTTTTGTTGACGTCATCCTTTTGATTTAC
TCACATGC

TTCATTTTTATCGCCTTAGACGAAATTGAAATTTTTCACTGAAAAAAAAAAAAAAAAAAAAAGTAGACGAAG
AAGCTCATCTCATCGCGATATTAGTTCGATATCTGGGTTTGAGTGAGTAGTATGTTTAATAATAAAAGAGT
ATAATCCA

CCTTAAGCGTCGAAACCTCATACCCCCTCATCTGGCTCGGGAGCCAGTATACTATATATATATATATATAT
ATATATATATATATATAAATTCCTAAAATACTCTCACACAGAAGCACAATAATGAGTGACGCAAATGCAAT
TTATGACT

CAAAAAAAAAAGAATGAAAGTACTATCAGCTTATAAGTCCTGATTAATTAAAAAAAGAATGCAGAATAGCA
CCTTATAGTAAAAAAAGTAAATTTAAGTAATTAAATATAACTATACTGTAACTTTTTTGAATTGTATTTTC
CCTGGCAT

AAAAAAAATGTTATGTATCAATATTACTAGGTAAAATACAAAAATGCGCTTTTGAAGTATAAATAATACAG
TTTTGTTTTAAGAAATGCTATATATGAATTAATAATAAATGAAACGAAAAAAAGCACTACTTGAGTTTTAA
TGTCGTAA

AAAATGTTTGAATTTTATTTCTTTCTGCCGCTCTCAAATTCACTCTCATTTCTTTTTTCTTTGTTTTTCTT
ATATTTACTATATATATATATATATATATATATATATAGCCAAAAACAATAACAGACACAGTAATTTAGTA
TGCGTTTA

AAATGGACCCTTGTCGGTCACGTGAGCAAAAATACTATTGCCGCCGACGGCCGTAGATAATAACAAAGGAA
TAAAAAAAAAAAAGCGGTAACTCGCGGAAGCATTGCCGATGCTTACCGAGATTTGCCGCGGATAACCGAAC
CATCATTG

AAATTAAAAAAAAAAAAGAAAGAAAGTATAATACTAATATTGGCAATAATGCTAACACATTACTAATAATAG
CAATAATATTAATAATAATAATAATAATAATAAATTATAATAATAGCAGTAATAAAAATATCAAATAATAA
TAATAGTA

CGGTAGACTTTCGAATTCCCTCTCTTGGCCGTGTTTCGGGCCACGAGGCGAAAATTTTTTTTTTTTAATCG
AAAAAAAAAGGAAAAAAAGATTAACGTTTCTTTTTCGTTCTATCGTCTTCTACGATATCGCATGTAACACA
ATGCAGCT

AAAATTTCAAGATTAATTCCCAACCTATATATATATATATATATATATATATACTGTTTAGATCAAGTATATC
TTCCTGCTAAATAATTAATATGCTCGTATGTAGAAAACAAAAAAGAGGTCTTTTCTCTTTTTTCTCCCGTT
ACTTTTTT

TCCTAATATAACAATACATACTTTCGAGTTTTGATTACAAGTCACTCGAAATGTTTTTTTCCTTTTATATA
TTCTTCCTTTTGTCTATTCTTATATACACATATTTTAATTTTTGGTAGATTTTTTTTTTTTTACTTACATT
TTGTTACA

AGCCTCACCGATGTAACGTTATATAACGCTGCGGTCGTAGAATATGATTTTTCTGAATTTTAATTTTTTTT
TTTTTTTTTTTCATTTCTTTAACATCTAAAATAGATTTTAGAATCAGAAAAGTGAAGAGTATTCCACATCA
TCGACTTA

GCTGAATGGGGCATTACATGTTTTCATCCAAACATGCCTTGTCAATAACGAGATTCGCGACGTATTCTTTT
AGTATTCTGTTCCTTTTTTTTTTTTGCTCCGCGCGCACTTGAACATCTTGCATATTTTTCTTTTCTGTTTTC
ATCCAGAG

ATCTTGAACAAAAGTAATACTATTTAAGGGTGGGCGCGGGTTGTTATACCGTTTTGAGACGATCTACTATA
ACGTTATATTTAATTATATAAGTGATGAATTTAATGTCCCGTCCACAACACGAAATCGCACCTCATTTCAA
CGCGGCAA

TACAAAATAAAAAAAAAAGAAGAAAGAAATAGCAGGTCTAAGATATATAAGAAAGTTAATATCATTTTTGA
ACATTTTATTTTAGACGCCTTCAGCCGCGCGACGCCCGGAGTAATCATATGCCCATGACTTTACCAAAAGG
CAACAGGG

TTGTAAAACGATTTTAGTTTTTATTTTTTTTTTTTTCTTCCCTTTTACCTTATTATTTGTTTTACCCTTTAT
GGATATTCATCCACTCAGAAACTAATATATCAAAATCAACGATAAGATCACAAGAGGTGAAATAAGAAAAG
TAAAAGAA

ATTGCATTATGGTCAACCAAAAATCAAAAAATTATTAGTAATATCAACATATGTATATTTAATGTATGTAA
ACAATGATTAAATATTAATAAAATATATCTACATATAAGTATACGCAAATGGGGTTTTTCTTCAAAAAAAT
TGTTTGCT

GAACTTTTACTTAATCCGCGGGTGAAATTTCATTTTTATATTTTCCATTTTATAACAGGCCGTCCCCTAAA
AACACCGCGTAAGAACATAGTTTATACACTTATATAAGTAAAAGGTGTAGTAGGTTGGAATGGTAAAGGGC
GTATTGTG

CGAAACCTCATACCCCCTCATCTGGCTCGGGAGCCAGTATACTATATATATATATATATATATATATATAT
ATATATAAATTCCTAAAATACTCTCACACAGAAGCACAATAATGAGTGACGCAAATGCAATTTATGACTAA
TTCATCAC

TTCCTCGATATACCTCTGTCTTACTATCAGTGGTTAAACCTTATGCAAATATAATATATATATATATATAT
ATATATATATCTCATACTTTTGTTGATTCTTGTGTAATTATTGGAAAAGACAAAACAAAGCAAGCGTTTCT
ATTCATAT

GCTAAATGCTCATTGATTAATTTTAAATTTTTCATTTTTATCGCCTTAGACGAAATTGAAATTTTTCACTG
AAAAAAAAAAAAAAAAAAAAGTAGACGAAGAAGCTCATCTCATCGCGATATTAGTTCGATATCTGGGTTTG
AGTGAGTA

GCTTGTTCTCCTCTGCTTATTGTTCGAGATGATGTTTATTTACATATTCTTTGACGTAATAATATCGGAAT
AATGCATGCGCATTCGGGTTAGCCCCGTTATAGGGAAAATGAAAAAAAAAAAAAATAATAAAAAAAAAAAAG
ACTAAAAA

ATATATTGCTTCTAAGATATGTCACAATTCTGTGTATACCATGTACAATTGCTAAATGCTCATTGATTAAT
TTTAAATTTTTCATTTTTATCGCCTTAGACGAAATTGAAATTTTTCACTGAAAAAAAAAAAAAAAAAAAAG
TAGACGAA

ACTTCCCATGGTCCAAGTATCAAAGCTTCAACAAAGCAATCACCTTAAAAGGTTATTTAAGGTCATTATTA
TATTAAAACGTTTGAAAATTAATATTATTATTAAAAAATAAATTTTTTATGCAAAATTTCTCATTTTCAAT
TTAAGCGG

TTGTCTTTTTCGGTTCTTTTCTTTTCTTTTTTTCTTTTTTTACGATGCATGTATATGTATTTTAATAGTAA
AATATATTATTAAATAGGGGAGTGTGGTTGTGTGATTTAGTAGAGTAGTTAGAATAAAAACGACACAGTGG
AAAAAAAA

CTTCTGATAAGAACGGCATGAATAATCAAGTAGGTTTTATAATACTGGTATACATTAATATAATCAACTAT
TTATACTATCAAAAAAAAAAGCTTTTTTTTTTTGTTTTTTTAAGTTTTATGGAATTTCATTCGTAAAGGC
GTTTGTCT

GACCCCGTCTTGTCTAATAGAATGGGTGAACTTTTTTTATTTCTTGGCTCATGATTATATTGTAAAATACA
TAGCATTTTATATAATTGTGGTGCTAACCCTAAGTACTTTTTTTTTTCGCCCTTTCCGGGTTTCTTTTTTT
CACCTTTT

CAATACCGCCTCCGGCGGTATTGAAGGAGAAAAATTTGGGCGGGAGCATATTATTGTAATTAATATATACA
TATATATATATATATATACATCAACCTTACAACTATGCGTTTAGAATCCTAAACTTTGATCTTACCATCAA
TAACGGTA

CTCTTTTTCTCGAAGAAAGCGGAAAAATAAAAACGAAAAAATAAAAAAAAAAAAAAGTATAAATAGTGAAG
ACTTTTTCCATTTGATATTTGGTAAAAATTTCGGCTGGAAGTTTTTTGCTGAACATTTAACCGGAGAATCT
TGGTGGCT

CCCACTCTAGGTAAACACAGTTCCTCGATATACCTCTGTCTTACTATCAGTGGTTAAACCTTATGCAAATA
TAATATATATATATATATATATATATATATCTCATACTTTTGTTGATTCTTGTGTAATTATTGGAAAAGAC
AAAACAAA

GAATCTTGGTATATATTGCTTCTAAGATATGTCACAATTCTGTGTATACCATGTACAATTGCTAAATGCTC
ATTGATTAATTTTAAATTTTTCATTTTTATCGCCTTAGACGAAATTGAAATTTTTCACTGAAAAAAAAAAA
AAAAAAAA

TGTGGCATTTTTTTTTTTTTATTTTTTTGACGTAAATTTTTTATGTATATAGCAGTAAATGTATCGATAACG
TGTTGGCTAATACTATAGTGAATAACAAAAAGTACTCTTAGTCTCCTTTTAGCATTTGTTTTCACCAAAGA
AGATGTTG

TTTTTATTTTTCGCGTTTTTAAGATAATGTCTTAACCAATTTTCTAAGTTCGGAACACAAAACCCAATGTT
TAATCATTTTTAATAAACAATTTAGTATATATATTATTTTAATTTTATTAAATTAATTAAAATTAAAAGAA
TTATTAGT

AATTCTTTATGTTTTTTTAAGGGAAAACGTATATCACTTAGAGATTATATTAATGAAAAAAGGTGTATACA
AATGTATAATAATAATAGTATATAAGAAAAGTATTCGCTATTTATAGTGATTTTAGTTTGTACAATGTACT
GTATTTTT

CACTCTCATTTCTTTTTTCTTTGTTTTTCTTATATTTACTATATATATATATATATATATATATATATAGC
CAAAAACAATAACAGACACAGTAATTTAGTATGCGTTTATCTCTTATCTTTCATCCACTGCGACCGAAACG
TTTGAATC

TTTAGAAATTTCCTTTTTTTTTTTTTTTTTTCCCCTTGACTTTCTTTTCTTTTATGTACCTATAATGAAAGAC
TATTTTATAATTAGAGCTATTTAAAAATTATTAATGAGGTTGATGAGTGGATAGAATATTATATTTTGAAA
GCTAAATT

CTGCCAAAAGCGGTAGACTTTCGAATTCCCTCTCTTGGCCGTGTTTCGGGCCACGAGGCGAAAATTTTTTT
TTTTTAATCGAAAAAAAAAAGGAAAAAAAGATTAACGTTTCTTTTTCGTTCTATCGTCTTCTACGATATCGC
ATGTAACA

AACGTTACTATTATTGTCTTAGGCAACAAAAATCCATGAAAAGAATTTTACCGTTATCGATACCATTGTAT
TTATTTTATTTATTTATTTAATTTTTTTTTTTTTTGGTTTATATCCTGCAAACAACACTTCGAATTCAATT
CGATATTT

CGGAACACAAAACCCAATGTTTAATCATTTTTAATAAACAATTTAGTATATATATTATTTTAATTTTATTA
AATTAATTAAAATTAAAAGAATTATTAGTCATGTGATTACCATCCTACTTATTATACTAAATCGTTTTGTG
GATGTGTA

AAGATAATGTCTTAACCAATTTTCTAAGTTCGGAACACAAAACCCAATGTTTAATCATTTTTAATAAACAA
TTTAGTATATATATTATTTTAATTTTATTAAATTAATTAAAATTAAAAGAATTATTAGTCATGTGATTACC
ATCCTACT

TTTTAAGAGAATTGCATTATGGTCAACCAAAAATCAAAAAATTATTAGTAATATCAACATATGTATATTTA
ATGTATGTAAACAATGATTAAATATTAATAAAATATATCTACATATAAGTATACGCAAATGGGGTTTTTCT
TCAAAAAA

TTGATTACAAGTCACTCGAAATGTTTTTTTCCTTTTATATATTCTTCCTTTTGTCTATTCTTATATACACA
TATTTTAATTTTTGGTAGATTTTTTTTTTTTTTACTTACATTTTGTTACAATAGAGAGATTTACCAATGGTG
TATCTCGA

GAGATGAAAAAAAAAAAAAATGAATCTGTACTATTTCTTTTTTTAGTGCAAAAGTTTTGTTTTAATATAGAG
ATAAATAAAAAAAATGAACATATTACTATAATATCAACAAAATATGATGTAATGTCTGCAATAAAGATGAGT
CAATTGCA

ACCAATTGAATACAAAATAAAAAAAAAAGAAGAAAGAAATAGCAGGTCTAAGATATATAAGAAAGTTAATA
TCATTTTTGAACATTTTATTTTAGACGCCTTCAGCCGCGCGACGCCCGGAGTAATCATATGCCCATGACTT
TACCAAAA

AAGAGAAAGAGAGAGAGAGAGAGAGAAAAAAAAAAAAGAAATATATAGATTTAGGTATTCGTTAAATATATAC
ACATTAAATGGCCTCAGAAATTTTAAATATATAAATAAATAAGCTCTTATATGTACAAATTTGTGCATATA
CTTTTCTT

AGGCAAAAAAAAAAAAAAAAAAAAAAAGAAAAGAAAAATCGATGAGCCTGCCGTACACAGAGATATAATAAA
TTTAGAATGCAAGAATGGTATGGCGGTGATGAAGATAATCATTTCCCTGCTTTTTTATTTTTGTTGCGCTT
CTTCTTGC

AAAAAAGTTAAGAATTGATGAGAGAATTTTGAGATGAAAAAAAAAAAAAATGAATCTGTACTATTTCTTTTT
TTAGTGCAAAAGTTTTGTTTTAATATAGAGATAAATAAAAAAAATGAACATATTACTATAATATCAACAAAA
TATGATGT

ATGAGATATCTGAGATACATGTGTATGTATATATGCACGTTTTCTTTTATTATCTAAAAATCATATTATAT
TAAGTAAGAGAAAAAAAATGTACAACTATATAAATATATATTTATTTAAAATGGTTTTGAATTTTTCCTATT
CTGGTTGA

AGAAAAAAAAAAAAAAAAAAAAAAAGGCTGAATGGATAAAGCGAGAGTCGTAAAGACCGAATGCAATGTCAG
TTTATACATTTTTATTCTATTGGTATTATATTATAAAAAGATATATGATTAGAAAACTAGAATGTAATGCA
ATATTACA

CGACTTTTGAGCTTGTTCTCCTCTGCTTATTGTTCGAGATGATGTTTATTTACATATTCTTTGACGTAATA
ATATCGGAATAATGCATGCGCATTCGGGTTAGCCCCGTTATAGGGAAAATGAAAAAAAAAAAAAATAATAAA
AAAAAAAA

AAAACAATCTTATACAAGACTAGAGCAAACAAAAAGCAAGAAAAAAAAGGTAATAAATGTAACAAATCTATT
AACGTTAGAAAATAACAAAAAAAAAAAAAAGAAAAAGATAATATAATATAATACCTTTATATATGTGATTTA
TCTAGCGT

ATAAAACCTCAAAACTACAACTATCAATATATTTCACACTACGCAGCTTAATTAGTAACTGTATAATAGCA
TTTAAGTTCATTACTTCTTTGTAATTTTATCTTTTTTTTTTTTATATAGTTTTCAGCGCGCAGCAGAGTT
TCATTGGA

CCATTCTCCGAGGTAGTGAACCATTGAGGTTGCAGCATGAATAAGATATAGGTGAGAGCTAGCGATTTGAC
ATTTAGTACATAGATATTTATATATATATATAAGGGAGTTTTTTTTATTTTTTTTTTCAAAATACGAAAA
TAGCAGTT

AAAAAAAAAAAAAAAAAAAAAAAAAAAAAGAAAAATAAAAATAAATATGGATGGAAATCATAGGTTTACTCC
AGATTCCAAAGAATTTAATACTGTTGTAAAAAGTAAAGAGTCTTCCACTGGCAGAAATCCGTATCAGACTC
CGCCCTTA

CTTCTTGGGGAAATCATTGTATTACGTACTTTTTTCCTAAAGATATTAGAAAAAAAAAATGTTATGTATCAA
TATTACTAGGTAAAATACAAAAATGCGCTTTTGAAGTATAAATAATACAGTTTTGTTTTAAGAAATGCTAT
ATATGAAT

TATATATTCGTTCGTATTAGTCACAACAAAAACCAAAAGAATACTCGAAATGTTAGTTTATATATATATAT
ATAGTGTGCGTTCTTTGCGGATATGGAAAATCTTGATATCTGCTGCATTCACGACTATGTCGCGCGGCAAA
GGTATGCT

GTTTTCCTTTGAACTTTTACTTAATCCGCGGGTGAAATTTCATTTTTATATTTTCCATTTTATAACAGGCC
GTCCCCTAAAAACACCGCGTAAGAACATAGTTTATACACTTATATAAGTAAAAGGTGTAGTAGGTTGGAAT
GGTAAAGG

AGGTAGAGGAGACGATGTAATGTAACTGCCGCCTTTCAGTTTGGCCCCATATATATATATATATATAAACAGA
CGGATAATTATATCATATTAAATGCGATAGCATAAATTTCTCGATGCGCATTGGTAAATTGTAGCATGGCG
CGAAGTCT

TTGTCGGTCACGTGAGCAAAAATACTATTGCCGCCGACGGCCGTAGATAATAACAAAGGAATAAAAAAAAA
AAAGCGGTAACTCGCGGAAGCATTGCCGATGCTTACCGAGATTTGCCGCGGATAACCGAACCATCATTGGG
GTAGGAAA

GAAAAATTACGCCAAATAGGCAGGTTTTCAATATTAAAGAGTTAAGTATGCAAAATTTACAAAAAGTTAAT
GGTCAAAGTATAACTATAAAAATATAATAAAACAATAAACAATAACAACTCTCAGAATTGCAAAGCTTGAC
CCTTTTGC

TGGATTATCAAGGCACTATATTTTACCACTACAGTAAATACTTCTGATAAGAACGGCATGAATAATCAAGT
AGGTTTTATAATACTGGTATACATTAATATAATCAACTATTTATACTATCAAAAAAAAAAGCTTTTTTTTT
TTTGTTTT

CTATAAAAGTTGAACAGTGAAGATTAAACCTATGTGTTATTTGCATATCATATATAAATTGTAGTTTTTTG
TATAAAAGTTGGGAAAAAGTTATAGTCGCGTATATTTTAAATTACTCTAAGCAACTAAATGGTCACGGTTT
AGAGCATG

GAGAAAAAAAAAAAAAGAAATATATAGATTTAGGTATTCGTTAAATATATACACATTAAATGGCCTCAGAAA
TTTTAAATATATAAATAAATAAGCTCTTATATGTACAAATTTGTGCATATACTTTTCTTGACCTTTTTACT
CCTCGGCT

ATAACATAAATTTAGAAATTTCCTTTTTTTTTTTTTTTTCCCCTTGACTTTCTTTTCTTTTATGTACCTAT
AATGAAAGACTATTTTATAATTAGAGCTATTTAAAAATTATTAATGAGGTTGATGAGTGGATAGAATATTA
TATTTTGA

ATTTTTCACTGAAAAAAAAAAAAAAAAAAAAAGTAGACGAAGAAGCTCATCTCATCGCGATATTAGTTCGAT
ATCTGGGTTTGAGTGAGTAGTATGTTTAATAATAAAAGAGTATAATCCAGATATAGCAGACAATAAAATTT
CAAGATGG

GAACGGCATGAATAATCAAGTAGGTTTTATAATACTGGTATACATTAATATAATCAACTATTTATACTATC
AAAAAAAAAAGCTTTTTTTTTTTGTTTTTTAAGTTTTATGGAATTTCATTCGTAAAGGCGTTTGTCTCT
AGTTTGTG

AAAAGAAAAAGTGGAGGAAGGTTGGTTGTATTGTCTTTTTCGGTTCTTTTCTTTTCTTTTTTTCTTTTTTT
ACGATGCATGTATATGTATTTTAATAGTAAAATATATTATTAAATAGGGGAGTGTGGTTGTGTGATTTAGT
AGAGTAGT

GAAAAAAAAAAAAAAAAAAAAGTAGACGAAGAAGCTCATCTCATCGCGATATTAGTTCGATATCTGGGTTT
GAGTGAGTAGTATGTTTAATAATAAAAGAGTATAATCCAGATATAGCAGACAATAAAATTTCAAGATGGCC
AAGAGAAG

TTTTTGACAACTATAAAAGTTGAACAGTGAAGATTAAACCTATGTGTTATTTGCATATCATATATAAATTG
TAGTTTTTTGTATAAAAGTTGGGAAAAAGTTATAGTCGCGTATATTTTAAATTACTCTAAGCAACTAAATG
GTCACGGT

GTCCAAGTATCAAAGCTTCAACAAAGCAATCACCTTAAAAGGTTATTTAAGGTCATTATTATATTAAAACG
TTTGAAAATTAATATTATTATTAAAAAATAAATTTTTATGCAAAATTTCTCATTTTCAATTTAAGCGGAG
TCGGAGTT

GAGAGAGCCGTACCGCAATAAAATGGACCCTTGTCGGTCACGTGAGCAAAAATACTATTGCCGCCGACGGC
CGTAGATAATAACAAAGGAATAAAAAAAAAAAAAGCGGTAACTCGCGGAAGCATTGCCGATGCTTACCGAGA
TTTGCCGC

TTGTTAACCTAAACTTGTACATATGCCCATATATCCTTAAATATATATATATAGCCAACCCGAAGACGCTA
AATAAACGCATTTTTTTTTTTTTTTACAGAATTATTACTTTCGTACTTGGGTTTATGCTTCTTATAACAAC
AACTATGA

ACAGGTACAATGTAACAGAATATGCCTTGGATCCCCAAATTTTTTTGTCTATTCGTTGTTTTCCTTCTCTA
TGGAAATCGTCGAAAAATTTCTTTGTTTTTTTTTTCCTTTATTGTTGTTGTCTTTTCGCGAATTCGCGTCG
CGATTCGT

GCAGTTGTTGTGCTGACAATAGTCCCATCTAGGTCAAAAAGACAAAGATCTACTGAAAATTGTGGCATTTT
TTTTTTTTATTTTTTTGACGTAAATTTTTTATGTATATAGCAGTAAATGTATCGATAACGTGTTGGCTAAT
ACTATAGT

TCTATCATTTTGGAAGAGGAATTAATGCAAAACACATTTGTATTTTTTTTTATTTTTGCATATTCTCGATT
ATCTATACATATTTTTAAACTGGATCATAAAAGGTATAAAAAGTAATTGATCCATACAACATTTTTAAATA
TTTACCCC

GAAGATAACGTAAGACTAAAAAAATTTCAAGATTAATTCCCAACCTATATATATATATATATATATATATA
CTGTTTAGATCAAGTATATCTTCCTGCTAAATAATTAATATGCTCGTATGTAGAAAACAAAAAAGAGGTCT
TTTCTCTT

CCTCAATTTGAAATAAGATTCGTATTTATTTTGTAAAACGATTTTAGTTTTTATTTTTTTTTTTTCTTCCC
TTTTACCTTATTATTTGTTTTACCCTTTATGGATATTCATCCACTCAGAAACTAATATATCAAAATCAACG
ATAAGATC

AATAATCATTTTTTTTATTTTTCGCGTTTTTAAGATAATGTCTTAACCAATTTTCTAAGTTCGGAACACAAA
ACCCAATGTTTAATCATTTTTAATAAACAATTTAGTATATATATTATTTTAATTTTATTAAATTAATTAAA
ATTAAAAG

TCGCGTTTTTAAGATAATGTCTTAACCAATTTTCTAAGTTCGGAACACAAAACCCAATGTTTAATCATTTT
TAATAAACAATTTAGTATATATATTATTTTAATTTTATTAAATTAATTAAAATTAAAAGAATTATTAGTCA
TGTGATTA

GAAAGTATAATACTAATATTGGCAATAATGCTAACACATTACTAATAATAGCAATAATATTAATAATAATA
ATAATAATAATAAATTATAATAATAGCAGTAATAAAAATATCAAATAATAATAATAGTAATAGTAGTAGTA
GTAGTAGC

TGAGATACATGTGTATGTATATATGCACGTTTTCTTTTATTATCTAAAAATCATATTATATTAAGTAAGAG
AAAAAAATGTACAACTATATAAATATATATTTATTTAAAATGGTTTTGAATTTTTCCTATTCTGGTTGATA
TTGCCCAA

TACCACACAACGTCTGTAATTTGTTGTCCTTTTTTTATTTGTGGAAAATTCCTCAATTTGAAATAAGATTC
GTATTTATTTTGTAAAACGATTTTAGTTTTTATTTTTTTTTTTTTCTTCCCTTTTACCTTATTATTTGTTTT
ACCCTTTA

GACGATGTAATGTAACTGCCGCCTTTCAGTTTGGCCCCATATATATATATATATAAACAGACGGATAATTA
TATCATATTAAATGCGATAGCATAAATTTCTCGATGCGCATTGGTAAATTGTAGCATGGCGCGAAGTCTCT
CATGGATA

GGCTTGCAAAATCGCTTGGCCAGAAATAACGAAAAATGAACACTTTTATTTTCTTTTGGCTTGTTAACCTA
AACTTGTACATATGCCCATATATCCTTAAATATATATATATAGCCAACCCGAAGACGCTAAATAAACGCAT
TTTTTTTT

ACGATTTCGTTACCCTCGGTAAAACCGGCCAAAAAAAAAAAAAAAAAAAAAAAAAAATGTTCTCAAAGTGT
AAACTTAATCAAAAGCTTATTTGTTTGTTATCTGTTTCTCATCGGTTTCGTTTGTTGCTTAAAGGGTGGAT
CGGTCATT

TAACGTTAGAAAATAACAAAAAAAAAAAAAGAAAAAGATAATATAATATAATACCTTTATATATGTGATTT
ATCTAGCGTCACAGTATGTAATATAAATATCAATGAGTTTCTTAACATGACCTCCAAAACTACATGATCGC
TTAGTATT

AATGGGTGAACTTTTTTTATTTCTTGGCTCATGATTATATTGTAAAATACATAGCATTTTATATAATTGTG
GTGCTAACCCTAAGTACTTTTTTTTTTCGCCCTTTCCGGGTTTCTTTTTTTCACCTTTTCGTTTTTCTAGA
ATTTCGCA

AAATTTTTTTTTTTTTAATCGAAAAAAAAAGGAAAAAAAGATTAACGTTTCTTTTTCGTTCTATCGTCTTC
TACGATATCGCATGTAACACAATGCAGCTATCTGATAATATGGTCCCAGAATCGCAATAGCCGGGCAGGCA
CGCTACTA

TCTAAGATATGTCACAATTCTGTGTATACCATGTACAATTGCTAAATGCTCATTGATTAATTTTAAATTTT
TCATTTTTATCGCCTTAGACGAAATTGAAATTTTTCACTGAAAAAAAAAAAAAAAAAAAAGTAGACGAAGA
AGCTCATC

GAGAGAGAGAGAGAAAAAAAAAAAAGAAATATATAGATTTAGGTATTCGTTAAATATATACACATTAAATG
GCCTCAGAAATTTTAAATATATAAATAAATAAGCTCTTATATGTACAAATTTGTGCATATACTTTTCTTGA
CCTTTTTA

ACACATGCCCTAGCGCGGCGTCGCGGATTAAACAAGGTAACAATGGTTAAAGGAAAAATTATTTGTTAAGG
TGGGTATATTATAGATTGCTAATTGTAAAGTATTAATAGTTTATTAAGTATTAAACTAGAGTTCGCACATG
CTTTTATT

TAAATATTTTCCCTGGCCGCCACTTTTTTGAGAGGTATGACGACTCTATGTTATATATATATATATATATA
TATATATATATAGCACAAAATTTCATGTGATCAATCTACTAGGCGAACACCTAAATTGTTATTTATTTCCA
TGTCTATA

TCCTTTTTTTTTTTTTTTTCCCCTTGACTTTCTTTTCTTTTATGTACCTATAATGAAAGACTATTTTATAA
TTAGAGCTATTTAAAAATTATTAATGAGGTTGATGAGTGGATAGAATATTATATTTTGAAAGCTAAATTAT
TTCATCAA

ATTGTTGTCTAAACTGCAATTGTTAGATGATTCATAATCCGGTATATTTATTGCCCTCTTTTGTATTTTAC
AAATAGTAAATATATATAATATCTTGTGATCTACATAAGCTTTCGCGGGGTCTTCAGAAAAATAAAAAGTT
TTTAAAAG

TTTTCATCCAAACATGCCTTGTCAATAACGAGATTCGCGACGTATTCTTTTAGTATTCTGTTCCTTTTTTT
TTTTGCTCCGCGCGCACTTGAACATCTTGCATATTTTTCTTTTCTGTTTTCATCCAGAGGTTGTGTTCTAG
GACTTTCT

TCGCGGATTAAACAAGGTAACAATGGTTAAAGGAAAAATTATTTGTTAAGGTGGGTATATTATAGATTGCT
AATTGTAAAGTATTAATAGTTTATTAAGTATTAAACTAGAGTTCGCACATGCTTTTATTTTTTTGTTGCTT
TTCTTCAC

TTAATCCGCGGGTGAAATTTCATTTTTATATTTTCCATTTTATAACAGGCCGTCCCCTAAAAACACCGCGT
AAGAACATAGTTTATACACTTATATAAGTAAAAGGTGTAGTAGGTTGGAATGGTAAAGGGCGTATTGTGTG
TCAATTAT

TTGCACAGGAAAGAAAAATCACCAATACAATACTACTATATTTTACCCAAGTTAATACGGGAAAGTATATT
TTCTTTTCCATATTTTTCTTTTTTATTGACGTTTGCATTTTTTAGAGCAACTAGTAAACAAAAAAACCAGTC
AGGCCCAG

GCGTGTTACTCTGAAAATGTTGAAAAATAAGCTGAATGGGGCATTACATGTTTTCATCCAAACATGCCTTG
TCAATAACGAGATTCGCGACGTATTCTTTTAGTATTCTGTTCCTTTTTTTTTTTGCTCCGCGCGCACTTGA
ACATCTTG

CCTTGTCTTTTCGATGAACTTTCCTATTTGGGTGAGCAGTTTTCCTCTTCGAGCTGCAAAAAAGTTTATCG
CTTCGCGAAAAAAAAAAAAAAAAAAAATTTAAAGAATTTCAAAACGTGTCAGGGCTACTAAATAATTGAACG
AGGTCTAT

AAAAATTGTATGTATAATGCTTGTCACTTTTTTTATAATTGATATATGTATATGTATATGTATACATACAT
ATATATAGTTGATTTAGAATTTATTTATATTAAACAAAAGACGAAGAGAGGGCAGATAACAATATTTAAAA
TGTTTACA

GTAATGCTCTGCCAACTATTTTGCACAGGAAAGAAAAATCACCAATACAATACTACTATATTTTACCCAAG
TTAATACGGGAAAGTATATTTTCTTTTCCATATTTTTCTTTTTTATTGACGTTTGCATTTTTTAGAGCAAC
TAGTAAAC

CGTTTTATTACTTATGTAGGAAAAGAGAATGAGGTTTACTAAATTAAAAGAAATATATAAAAAGCTGATTT
TTTTCTTTTTTTAAATATTTCCCAAATGCTAATAAATATTAAAATATGGCAAAAAGAAAATAAGGTGTTGT
TGCGCAGT

TCTTCCCTTAATTAAAAAGATTAGTTTACTTTTTCTGTAGAATGTTAATTAGTTTATTGCATAGAAAATCT
TTAATACTGAATGACAAAATTTCCACAATTTTTTTAAAAAAATTATCGACAACTGCAGGACTCGAACCTG
CGCGGGCA

ATTTTAGTTTTTATTTTTTTTTTTTCTTCCCTTTTACCTTATTATTTGTTTTACCCTTTATGGATATTCAT
CCACTCAGAAACTAATATATCAAAATCAACGATAAGATCACAAGAGGTGAAATAAGAAAAGTAAAAGAAAA
ATATTTAA

CTTTTCTTTTTTTCTTTTTTTTACGATGCATGTATATGTATTTTAATAGTAAAATATATTATTAAATAGGGG
AGTGTGGTTGTGTGATTTAGTAGAGTAGTTAGAATAAAAACGACACAGTGGAAAAAAAAAGGTATTTTCTT
TCTTTCTT

TAATAAAAATATCAAATAATAATAATAGTAATAGTAGTAGTAGTAGTAGCAGTTGTAGTAATGATAATAAT
AATATAATAATAATATAATAATAGTATAATAATAGTATAAGTAGTTGTAGGTAAAAATCTTTGATTTATCT
TATCTGGT

TGTTAGATGATTCATAATCCGGTATATTTATTGCCCTCTTTTGTATTTTACAAATAGTAAATATATATAAT
ATCTTGTGATCTACATAAGCTTTCGCGGGGTCTTCAGAAAAATAAAAAGTTTTTAAAAGAATATACGCTTA
GCAAAGGA

TTGAAGGAGAAAAATTTGGGCGGGAGCATATTATTGTAATTAATATATACATATATATATATATATATACA
TCAACCTTACAACTATGCGTTTAGAATCCTAAACTTTGATCTTACCATCAATAACGGTAGTGGCATGACCT
GCTATATA

TGGTTAGTCTTAGTTTAACTCTCTTGAAGAAGGGTTTTTTCAGTTGGTCAACACTCTTTAGAGGTAAAAAA
AAAAAAAAAAAAAAAAAAAAGAGAATTCTTCATGTAATTTACCATGATTCTACGTTTTTGCAAGCAAAAAT
GAAGATAA

GTGTTTCGGGCCACGAGGCGAAAATTTTTTTTTTTTAATCGAAAAAAAAAAGGAAAAAAAGATTAACGTTTC
TTTTTCGTTCTATCGTCTTCTACGATATCGCATGTAACACAATGCAGCTATCTGATAATATGGTCCCAGAA
TCGCAATA

GTCGTTTGTTTAACTACGGCGGCTTCTGGTATTACTGAATTTCTCTTTTTTTCATTTAAATCAATTTATT
ACAGGTAAATCAATCAAACAAATATATATATATATAGTATACAGAACAAACGGTAATTTCTTTTTTTTTTT
TTCGACGT

ATTACGTACTTTTTTCCTAAAGATATTAGAAAAAAAAAATGTTATGTATCAATATTACTAGGTAAAATACAA
AAATGCGCTTTTGAAGTATAAATAATACAGTTTTGTTTTAAGAAATGCTATATATGAATTAATAATAAATG
AAACGAAA

CGACTGTGATTCAGATAATACCTTAAGCGTCGAAACCTCATACCCCCTCATCTGGCTCGGGAGCCAGTATA
CTATATATATATATATATATATATATATATATATATATAAATTCCTAAAATACTCTCACACAGAAGCACAATA
ATGAGTGA

ATTCCTTTTTTGCGTCACCTTCATTTTTTCTTCTCTGTTCTTTTCTGTATTTGTCGAAAATTTTATATTGA
TGTATCTTAATTTTATTTTTAGTATTATTATACTGTAAATGTTTCTTACTGTGTCAAGATTTCCCGTTGCG
CTATCTTC

TCATAAAGAAAACGTTACTATTATTGTCTTAGGCAACAAAAATCCATGAAAAGAATTTTACCGTTATCGAT
ACCATTGTATTTATTTTATTTATTTATTTAATTTTTTTTTTTTTTGGTTTATATCCTGCAAACAACACTTC
GAATTCAA

AAAAAAAAAAGAGAGAGCCGTACCGCAATAAAATGGACCCTTGTCGGTCACGTGAGCAAAAATACTATTGC
CGCCGACGGCCGTAGATAATAACAAAGGAATAAAAAAAAAAAAAGCGGTAACTCGCGGAAGCATTGCCGATG
CTTACCGA

ATAATAGCAGTAATAAAAATATCAAATAATAATAATAGTAATAGTAGTAGTAGTAGTAGCAGTTGTAGTAA
TGATAATAATAATATAATAATAATATAATAATAGTATAATAATAGTATAAGTAGTTGTAGGTAAAAATCTT
TGATTTAT

GACTAATGAAGGAATGATATTATGTGGTATTACTTTTTTTTTATCATCACTAGCTTCGGCTAAGCCCTTGC
ATTTTCAATATTCAATTACCAAATAATATATATATATATATATATATATATGTATGTATGGTATACCTTGTGT
TACTGATG

CGTCTGTAATTTGTTGTCCTTTTTTTTATTTGTGGAAAATTCCTCAATTTGAAATAAGATTCGTATTTATTT
TGTAAAACGATTTTAGTTTTATTTTTTTTTTTTCTTCCCTTTTACCTTATTATTTGTTTTACCCTTTATG
GATATTCA

AATTGGCCGGATTTCCAATGGAAGCGTTAAAAGAAGCCCGCGAAATATTGGGATAACTTTTGAATACAACT
ATTAATTGTATATAATTTGACATGTAATATAATAAGATGTGGAATCAATTTCCCTGTCTTTTTTTTCAAAA
GCGACTGT

GTCTTCTTCTGCGTGTTACTCTGAAAATGTTGAAAAATAAGCTGAATGGGGCATTACATGTTTTCATCCAA
ACATGCCTTGTCAATAACGAGATTCGCGACGTATTCTTTTAGTATTCTGTTCCTTTTTTTTTTTGCTCCGC
GCGCACTT

CGTCAGTGTAAACATTCAAGTGATTTTAACTTTACGCGGTTGAAGAATGCTGTGTTCGAACTATAAAGCGT
CAGAAAAGATGGTTTAGCGAAGGCACCATTATGAAGATAGACACATTCTTCTTTTTTTTTTTTTTTTTTT
TTTTTTTT

GGTCAACCAAAAATCAAAAAATTATTAGTAATATCAACATATGTATATTTAATGTATGTAAACAATGATTA
AATATTAATAAAATATATCTACATATAAGTATACGCAAATGGGGTTTTTCTTCAAAAAAATTGTTTGCTGG
TGTTTTCA

AAAGTTTATCGCTTCGCGAAAAAAAAAAAAAAAAAAAAATTTAAAGAATTTCAAAACGTGTCAGGGCTACTAA
ATAATTGAACGAGGTCTATGAAATAAAATGGGCTGAGAAAATTTCAAGAAAAAGGAAATAAGTAAGGGCTA
TTTAGGGT

CCCTGGCCGCCACTTTTTTGAGAGGTATGACGACTCTATGTTATATATATATATATATATATATATATATA
TAGCACAAAATTTCATGTGATCAATCTACTAGGCGAACACCTAAATTGTTATTTATTTCCATGTCTATATC
CATTTCAA

CCTTTACAAAAAAGCTTATGGGATAGAATTTTAAGGTTAATTCAGGTATTATTTTTATGGCGGTTTCAGGT
ATTTTACGTAATATTATTATTATATCATTTTTGTAGCAATATATTATTTTTAGCGGTGCTTTATAAAACAA
TGGATACT

AAATCATTGTATTACGTACTTTTTTCCTAAAGATATTAGAAAAAAAAATGTTATGTATCAATATTACTAGG
TAAAATACAAAAATGCGCTTTTGAAGTATAAATAATACAGTTTTGTTTTAAGAAATGCTATATATGAATTA
ATAATAAA

GGCCAAGACGGTTGCTTATACATCTATAATCAATGAGCATTATCTATCGCTTTATTATTTATTATTTATTA
TTATCATTATTATTATTATCATTATTATTATTACTACCAATACCTTCTATATCCCTGTGATCAGCTAAAAT
GATTCAAA

GGGACAAAAATTAATAGTGTTAACCGTACTTTGTAGCACCATTTCTTTTTCTTCTAATTTATATAATTAAT
GAATATATATATTTGGGATAAATAGCCTTTAATGAAATATTAACAGAACTTTTTTGCTTGGGCAGCAACAG
ATTTATAT

ATTTCACACTACGCAGCTTAATTAGTAACTGTATAATAGCATTTAAGTTCATTACTTCTTTGTAATTTTAT
CTTTTTTTTTTTTTATATAGTTTTCAGCGCGCAGCAGAGTTTCATTGGAACGCTTCGCACGTTGGAAGAAT
AGAACTGT

ACAAATCTATTAACGTTAGAAAATAACAAAAAAAAAAAAAGAAAAAGATAATATAATATAATACCTTTATA
TATGTGATTTATCTAGCGTCACAGTATGTAATATAAATATCAATGAGTTTCTTAACATGACCTCCAAAACT
ACATGATC

AGTGACACATAATATATAAATGATCGGTTGAGCGCGATATTATATAAGAACATCCATATGAGTATATATCC
TACCGTGTCTGTTCTCTACAGTGCTTCTTTTATTCAAAATTATTGGTTTTCGTAACCGCCGCGCAGGCACG
CCGCGCAT

AAGGCAGGATGAAAAATTACGCCAAATAGGCAGGTTTTCAATATTAAAGAGTTAAGTATGCAAAATTTACA
AAAAGTTAATGGTCAAAGTATAACTATAAAAATATAATAAAACAATAAACAATAACAACTCTCAGAATTGC
AAAGCTTG

AGAAATATGTGTAATGCTCTGCCAACTATTTTGCACAGGAAAGAAAAATCACCAATACAATACTACTATAT
TTTACCCAAGTTAATACGGGAAAGTATATTTTCTTTTCCATATTTTTCTTTTTTATTGACGTTTGCATTTT
TTAGAGCA

GTTGCTTATACATCTATAATCAATGAGCATTATCTATCGCTTTATTATTTATTATTTATTATTATCATTAT
TATTATTATCATTATTATTATTACTACCAATACCTTCTATATCCCTGTGATCAGCTAAAATGATTCAAATA
ACTGTCAT

TTCGACTGGAAAATTTTCGCAGGCAAAAAAAAAAAAAAAAAAAAAAAGAAAAGAAAAAATCGATGAGCCTGCC
GTACACAGAGATATAATAAATTTAGAATGCAAGAATGGTATGGCGGTGATGAAGATAATCATTTCCCTGCT
TTTTTATT

TATCTACATCAAAATGTTTGAATTTTATTTCTTTCTGCCGCTCTCAAATTCACTCTCATTTCTTTTTTCTT
TGTTTTTCTTATATTTACTATATATATATATATATATATATATATATAGCCAAAAACAATAACAGACACAG
TAATTTAG

GCTTTTTTGTGTTCTATTTCAGGAAAGAAGAATGGGAAAAATAATAAAACAATAAATAACTATATAAAAAA
CAAAAACCTAGAATATACCATTAAAGAATTACTATTCTATTCTGTGCTTCACCGTTGCGTCAATAAATGTG
AAGTTGAT

GTGTATGTATATATGCACGTTTTCTTTTATTATCTAAAAATCATATTATATTAAGTAAGAGAAAAAAATGT
ACAACTATATAAATATATATTTATTTAAAATGGTTTTGAATTTTTCCTATTCTGGTTGATATTGCCCAAAA
GCTATTCA

AATAATAGTAATAGTAGTAGTAGTAGTAGTAGCAGTTGTAGTAATGATAATAATAATATAATAATAATATAATA
ATAGTATAATAATAGTATAAGTAGTTGTAGGTAAAAATCTTTGATTTATCTTATCTGGTTGCAATGCTTCT
CCCCTCTG

AAGGAATGAACTGAAACTATTAAATATTTTCCCTGGCCGCCACTTTTTTGAGAGGTATGACGACTCTATGT
TATATATATATATATATATATATATATATATATAGCACAAAATTTCATGTGATCAATCTACTAGGCGAACACC
TAAATTGT

TCCCTTTTTCTATCACGAGGTTACTGAGCCATTGCATGAACGCGCGCGCCTCGGCGGCTTTTTTTTTCTGC
TGTGCTGTATAAAAGCGAAAAGTCAGAAGTTACTATTTCGAATAAAAAACCCCTCGAACTGCCATCTCACT
ACCGAAAA

TCAAGAAAAAGTAAGTAAATAAGAGGGTACGTGATTATAAACAGATACGAGTTTAGCGAATAATACCGTTC
GCAATCATAACTTTATAAAACTCATCCTATTACTTACTATTCGACTGGAAAATTTTCGCAGGCAAAAAAAA
AAAAAAAA

CTCAATCGCTCCTCTTGCCCTTCCTCTTCTTTTCCTTTTCTTTTTATTTTTTTCTAATTTTTCTATCTTTT
ATTTCTACTTCTATTATTAATTTGTATCTTCTAATCGTTTTTCCCACTTTATCGAAACATTTCTTCTATTT
ATATATGT

AGAGTTGCGGAATTGGCCGGATTTCCAATGGAAGCGTTAAAAGAAGCCCGCGAAATATTGGGATAACTTTT
GAATACAACTATTAATTGTATATAATTTGACATGTAATATAATAAGATGTGGAATCAATTTCCCTGTCTTT
TTTTTCAA

AGAAGAGGAAAAAAAAAATTTTTACCTCATCGCGGATTGTAAATAGTAAATATAAACTGGCAATTTACTTAG
TTTTTGAATAAATAATTGTATAGGAATATTATCAGTCGTATAATACAGACGCTTTAGATCTCCGTAGCATC
ATATCAGA

TATGCCTTGGATCCCCAAATTTTTTTGTCTATTCGTTGTTTTCCTTCTCTATGGAAATCGTCGAAAAATTT
CTTTGTTTTTTTTTTCCTTTATTGTTGTTGTCTTTTCGCGAATTCGCGTCGCGATTCGTGGATTGTGATGA
TAATAGCG

TAATCAGAAGGACCCCGTCTTGTCTAATAGAATGGGTGAACTTTTTTTATTTCTTGGCTCATGATTATATT
GTAAAATACATAGCATTTTATATAATTGTGGTGCTAACCCTAAGTACTTTTTTTTTTCGCCCTTTCCGGGT
TTCTTTTT

GGTATATTTATTGCCCTCTTTTGTATTTTACAAATAGTAAATATATATAATATCTTGTGATCTACATAAGC
TTTCGCGGGGTCTTCAGAAAAATAAAAAGTTTTAAAAGAATATACGCTTAGCAAAGGAATAGACAGATTT
AGTTTGTA

ATAGTAGTAGTAGTAGTAGTAGCAGTTGTAGTAATGATAATAATAATATAATAATAATATAATAATAGTATAAT
AATAGTATAAGTAGTTGTAGGTAAAAATCTTTGATTTATCTTATCTGGTTGCAATGCTTCTCCCCTCTGAT
GCCTAATA

GGTTTTAGCCGCCCACTTTTCACGCGTTAACAGGGTAAGCTATATCAGATGTGACTTTTCTTTTTCACTCA
TCTAATGTTGTTTTTCGCGTCACATTTTTTGCCAATAAATAAATACATCACAATGTTCTATATTGTATATT
CAATGGAA

TCATTTTTTTCTTCGGCGATGAAAAATTTTCAAAAAAAAAAACAGACCTAACAAAAGGGCAAAAGACCTAT
TATACTATATGACCGAAAAAACTTACGCCCTATTAAATAAAGAAGAAACCTAAAGAAGATAAACTAAAAAC
CCTTCTAA

CACTGGTTACACATTTCATGAAAAAAAAAGAAAAATTGTATGTATAATGCTTGTCACTTTTTTTATAATTG
ATATATGTATATGTATATGTATACATACATATATATAGTTGATTTAGAATTTATTTATATTAAACAAAAGA
CGAAGAGA

CTTTTTTTATTTCTTGGCTCATGATTATATTGTAAAATACATAGCATTTTATATAATTGTGGTGCTAACCC
TAAGTACTTTTTTTTTTCGCCCTTTCCGGGTTTCTTTTTTTCACCTTTTCGTTTTTCTAGAATTTCGCAAA
GTATATAA

TCTTTTTTTCTTTTGAATAAGTCGCGTTATAAGGACAAAACATTAAAAAAAAATATGTATTGATTCACTGA
TTTATATATACAGAAGTAAGTACCGGGGTACCTAAATATACGCATAAAAGTCTCTTTCTTTTTTTTTTTTT
TTTTTTTA

TAAATCTCAGCCTAACCCTTTTACACGAAGTTTTCCCAACAAAAAAAAAAAAAAAAAAAAAAAAAAAAAGA
AGGGCGGTTGTCTTTGGCATATCGCGAAAATAATAGATCTTATCAAGGGGTTTTTAAGTCATCCTCTGATT
ATTGGAAG

TGTACACACGTACACGCACAAGCTACAGACGCCACGCGGCCCGGCATTCATATATGTACATACATTTATGC
ACATATATATATATATATATATGTATATGTAAATGTATATATACAGCTTGTGATTTCGCGCGCCGAATGGG
CTGAAAAG

TACACGCACAAGCTACAGACGCCACGCGGCCCGGCATTCATATATGTACATACATTTATGCACATATATAT
ATATATATATATGTATATGTAAATGTATATATACAGCTTGTGATTTCGCGCGCCGAATGGGCTGAAAAGCA
CAGTATAG

CCTAACCCTTTTACACGAAGTTTTCCCAACAAAAAAAAAAAAAAAAAAAAAAAAAAAAAGAAGGGCGGTTG
TCTTTGGCATATCGCGAAAATAATAGATCTTATCAAGGGGTTTTTAAGTCATCCTCTGATTATTGGAAGTC
ATAATCAC

CGTCTCTAGATAAATCTCAGCCTAACCCTTTTACACGAAGTTTTCCCAACAAAAAAAAAAAAAAAAAAAAA
AAAAAAAAGAAGGGCGGTTGTCTTTGGCATATCGCGAAAATAATAGATCTTATCAAGGGGTTTTTAAGTCA
TCCTCTGA

CTGTAAAAAAATATATATACATATGATTCACGTGAAAATTTTTATATTTTTAATTAAATTTTTATAATATTA
TAAATTATTATAATATTGATATTTAAAATTAAAAACAAATTATTAATGGTTTTGTTTTCCGAAATGTTTTT
TATAATTA

TTTTGAATAAGTCGCGTTATAAGGACAAAACATTAAAAAAAAATATGTATTGATTCACTGATTTATATATA
CAGAAGTAAGTACCGGGGTACCTAAATATACGCATAAAAGTCTCTTTCTTTTTTTTTTTTTTTTTTTTAGC
TTCCTACA

TTAAAGAAGAAAAAAAAAAGGTTGGTTTTATCACTTCATTAGTCTATTTGAATAATTCATAAAATATTTCTG
ATTTTTTTTTTTTTTTTTTTTTTTCATATTATTTTAAACGTGACTTTGGCTGCGATTCATGGTTTTTGCCC
TTATATCT

AGCTACAGACGCCACGCGGCCCGGCATTCATATATGTACATACATTTATGCACATATATATATATATATAT
ATGTATATGTAAATGTATATATACAGCTTGTGATTTCGCGCGCCGAATGGGCTGAAAAGCACAGTATAGTG
GAGAAGAA

TTACACGAAGTTTTCCCAACAAAAAAAAAAAAAAAAAAAAAAAAAAAAAGAAGGGCGGTTGTCTTTGGCAT
ATCGCGAAAATAATAGATCTTATCAAGGGGTTTTTAAGTCATCCTCTGATTATTGGAAGTCATAATCACGT
TCAATGGT

GTACCGGGGTACCTAAATATACGCATAAAAGTCTCTTTCTTTTTTTTTTTTTTTTTTTTAGCTTCCTACAT
TTCGTTAATAATATTATATAGATTATATTTATATTTAAGAAAAGTAATATCAGCATATTATGAATAGACAA
AAAAGTCT

CGAAGGCTAGCGTCTCTAGATAAATCTCAGCCTAACCCTTTTACACGAAGTTTTCCCAACAAAAAAAAAAA
AAAAAAAAAAAAAAAAAAGAAGGGCGGTTGTCTTTGGCATATCGCGAAAATAATAGATCTTATCAAGGGGT
TTTTAAGT

TATATATACATATGATTCACGTGAAAATTTTTATATTTTTAATTAAATTTTTATAATATTATAAATTATTA
TAATATTGATATTTAAAATTAAAAACAAATTATTAATGGTTTTGTTTTCCGAAATGTTTTTTATAATTATT
ACTTCAGA

GATACTTTTTCTGTAAAAAATATATATACATATGATTCACGTGAAAATTTTTATATTTTTAATTAAATTTT
TATAATATTATAAATTATTATAATATTGATATTTAAAATTAAAAACAAATTATTAATGGTTTTGTTTTCCG
AAATGTTT

TCAAAGTAAATGGAGAATTTCGAAGGCTAGCGTCTCTAGATAAATCTCAGCCTAACCCTTTTACACGAAGT
TTTCCCAACAAAAAAAAAAAAAAAAAAAAAAAAAAAAAGAAGGGCGGTTGTCTTTGGCATATCGCGAAAAT
AATAGATC

TTATATAACTGTAGAACCAGTTTAGTTTCTTGTTGTTCTTTCCTCTAATTAGAGTCATCTTAAGAATATTT
TTCCACGGGAAAAAAGTGAAAAAAAAAAAAAAAAAAAAAAAAAAGAGTGGGAATTTTCAGTGCAGTTGAAA
AAATCGAG

TGGCAATGCTTCAAAGTAAATGGAGAATTTCGAAGGCTAGCGTCTCTAGATAAATCTCAGCCTAACCCTTT
TACACGAAGTTTTCCCAACAAAAAAAAAAAAAAAAAAAAAAAAAAAAAGAAGGGCGGTTGTCTTTGGCATA
TCGCGAAA

AAAAAAAAAAAAAAAAAAAAAAAAAAAAAGAAGGGCGGTTGTCTTTGGCATATCGCGAAAATAATAGATCT
TATCAAGGGGTTTTTAAGTCATCCTCTGATTATTGGAAGTCATAATCACGTTCAATGGTGGGAGGCCTTCG
GAAAAGAA

GTCGCGTTATAAGGACAAAACATTAAAAAAAAATATGTATTGATTCACTGATTTATATATACAGAAGTAAG
TACCGGGTACCTAAATATACGCATAAAAGTCTCTTTCTTTTTTTTTTTTTTTTTTTTTAGCTTCCTACATT
TCGTTAAT

CAATTTTTTTAAAAAAAAACTCAATACCTACATCACCGCGAAATGCCTGAAGTAAAAGCATATAACAAACG
CGACCCTTAAAATTACGTATATATATGTATATTTCTTTATACTAAGTTTTACTGGCAGAGTATCATACAG
TTCTTCTA

AAAAAAAAGGTTGGTTTTATCACTTCATTAGTCTATTTGAATAATTCATAAAATATTTCTGATTTTTTTTT
TTTTTTTTTTTTTTCATATTATTTAAACGTGACTTTGGCTGCGATTCATGGTTTTTGCCCTTATATCTTC
GTTGTCAT

TGGAGAATTTCGAAGGCTAGCGTCTCTAGATAAATCTCAGCCTAACCCTTTTACACGAAGTTTTCCCAACA
AAAAAAAAAAAAAAAAAAAAAAAAAAAAGAAGGGCGGTTGTCTTTGGCATATCGCGAAAATAATAGATCTT
ATCAAGGG

ACAATATACCTTAAAGAAGAAAAAAAAAGGTTGGTTTTATCACTTCATTAGTCTATTTGAATAATTCATAA
AATATTTCTGATTTTTTTTTTTTTTTTTTTTTTTTCATATTATTTAAACGTGACTTTGGCTGCGATTCATG
GTTTTTGC

TTTTCCCAACAAAAAAAAAAAAAAAAAAAAAAAAAAAAAGAAGGGCGGTTGTCTTTGGCATATCGCGAAAA
TAATAGATCTTATCAAGGGGTTTTTAAGTCATCCTCTGATTATTGGAAGTCATAATCACGTTCAATGGTGG
GAGGCCTT

GCCACGCGGCCCGGCATTCATATATGTACATACATTTATGCACATATATATATATATATATATGTATATGT
AAATGTATATATACAGCTTGTGATTTCGCGCGCCGAATGGGCTGAAAAGCACAGTATAGTGGAGAAGAAGA
GAGAGGAG

ACCTAAATATACGCATAAAAGTCTCTTTCTTTTTTTTTTTTTTTTTTTTAGCTTCCTACATTTCGTTAATA
ATATTATATAGATTATATTTATATTTAAGAAAAGTAATATCAGCATATTATGAATAGACAAAAAAGTCTAA
GGTCAAGA

GTCTCTTTCTTTTTTTTTTTTTTTTTTTTAGCTTCCTACATTTCGTTAATAATATTATATAGATTATATTT
ATATTTAAGAAAAGTAATATCAGCATATTATGAATAGACAAAAAAGTCTAAGGTCAAGATTTATTAAATGT
TAGATTAT

ACGCATAAAAGTCTCTTTCTTTTTTTTTTTTTTTTTTTTAGCTTCCTACATTTCGTTAATAATATTATATA
GATTATATTTATATTTAAGAAAAGTAATATCAGCATATTATGAATAGACAAAAAAGTCTAAGGTCAAGATT
TATTAAAT

GGAGTGGGACGAAAAGATCTCGCTTTTGTCTCACGCGCAATGCTATAAATAATAAATATACATATATATAT
ATTTATATGTAGATAGATGTGTGTATATATATGCTAGGTAAAGAAACGGAAAAATCGAAAAATCGAAGTTG
GAGAGGAA

TAAGAAGTATGATACTTTTTCTGTAAAAAATATATATACATATGATTCACGTGAAAATTTTTATATTTTTA
ATTAAATTTTTATAATATTATAAATTATTATAATATTGATATTTAAAATTAAAAACAAATTATTAATGGTT
TTGTTTTC

TATTTTTACGCGTCACTGCGAACGGATGAATATTGCCAATGACTGCTGCGCACTCTTAAAAAATATAATGA
CAATTATAATAAGAAGTATGATACTTTTTCTGTAAAAAATATATATACATATGATTCACGTGAAAATTTTT
ATATTTTT

ACAATTATAATAAGAAGTATGATACTTTTTCTGTAAAAAATATATATACATATGATTCACGTGAAAATTTT
TATATTTTTAATTAAATTTTTATAATATTATAAATTATTATAATATTGATATTTAAAATTAAAAACAAATT
ATTAATGG

ACAGAAGTAAGTACCGGGGTACCTAAATATACGCATAAAAGTCTCTTTCTTTTTTTTTTTTTTTTTTTTAG
CTTCCTACATTTCGTTAATAATATTATATAGATTATATTTATATTTAAGAAAAGTAATATCAGCATATTAT
GAATAGAC

TATGATTCACGTGAAAATTTTTATATTTTTAATTAAATTTTTATAATATTATAAATTATTATAATATTGAT
ATTTAAAATTAAAAACAAATTATTAATGGTTTTGTTTTCCGAAATGTTTTTTATAATTATTACTTCAGAAT
AGTTATTT

TGATCAATGCGGAGTGGGACGAAAAGATCTCGCTTTTGTCTCACGCGCAATGCTATAAATAATAAATATAC
ATATATATATATTTATATGTAGATAGATGTGTGTATATATATGCTAGGTAAAGAAACGGAAAAATCGAAAA
ATCGAAGT

ATTCATACAATATACTTAAGAAAACAATATACTTTTCTTAGTTTCATAACTTTGGAATCTATAATGTTGTT
TTTTTTTTTTTAAGTATATTTGTATATTATGCATATAAGTCAACGCGTAGCGGTGGTATTACGCGCAAGCT
TTTGATTT

AAAACAATATACTTTTCTTAGTTTCATAACTTTGGAATCTATAATGTTGTTTTTTTTTTTTTAAGTATATT
TGTATATTATGCATATAAGTCAACGCGTAGCGGTGGTATTACGCGCAAGCTTTTGATTTCTCTTACTATTC
TTGGTTGT

TAAGAATATTTTTCCACGGAAAAAAGTGAAAAAAAAAAAAAAAAAAAAAAAAAAAAGAGTGGAATTTTCAG
TGCAGTTGAAAAAATCGAGGTGGAATTTGAGAACGACAATTACTTAGCACTACATGTGTTGTTCTCGTTTG
TTTTTAAT

GCATGGACTTCTTTTTTTTCTTTTGAATAAGTCGCGTTATAAGGACAAAACATTAAAAAAAAATATGTATT
GATTCACTGATTTATATATACAGAAGTAAGTACCGGGGTACCTAAATATACGCATAAAAGTCTCTTTCTTT
TTTTTTTT

GTATCAAGAGAGAAATATGTGAATGACGAGCAAACTAGAGGTGTTATTAATAATAATGGCATAAAAACTAG
ATTTTATTCTATATATATATATATATATTTCTTCGACTTTTTTTTTTTTTACCCGCACACCCTGAATTTTG
TGCTATTT

AGAAATATGTGAATGACGAGCAAACTAGAGGTGTTATTAATAATAATGGCATAAAAACTAGATTTTATTCT
ATATATATATATATATATTTCTTCGACTTTTTTTTTTTTTACCCGCACACCCTGAATTTTGTGCTATTTTG
AAGCAGAG

TGTTGAAGTGTGTACACACGTACACGCACAAGCTACAGACGCCACGCGGCCCGGCATTCATATATGTACAT
ACATTTATGCACATATATATATATATATATATGTATATGTAAATGTATATATACAGCTTGTGATTTCGCGC
GCCGAATG

TATACTTAAGAAAACAATATACTTTTCTTAGTTTCATAACTTTGGAATCTATAATGTTGTTTTTTTTTTTT
TAAGTATATTTGTATATTATGCATATAAGTCAACGCGTAGCGGTGGTATTACGCGCAAGCTTTTGATTTCT
CTTACTAT

TCCTCTAATTAGAGTCATCTTAAGAATATTTTTCCACGGGAAAAAAGTGAAAAAAAAAAAAAAAAAAAAAA
AAAAGAGTGGGAATTTTCAGTGCAGTTGAAAAAATCGAGGTGGAATTTGAGAACGACAATTACTTAGCACT
ACATGTGT

AGAGTCATCTTAAGAATATTTTTCCACGGGAAAAAAGTGAAAAAAAAAAAAAAAAAAAAAAAAAAGAGTGG
GAATTTTCAGTGCAGTTGAAAAAATCGAGGTGGAATTTGAGAACGACAATTACTTAGCACTACATGTGTTG
TTCTCGTT

AAGGACAAAACATTAAAAAAAAATATGTATTGATTCACTGATTTATATATACAGAAGTAAGTACCGGGGTA
CCTAAATATACGCATAAAAGTCTCTTTCTTTTTTTTTTTTTTTTTTTTAGCTTCCTACATTTCGTTAATAA
TATTATAT

CAAACAATGTCAATTTTTTTAAAAAAAAACTCAATACCTACATCACCGCGAAATGCCTGAAGTAAAAGCAT
ATAACAAACGCGACCCTTAAAATTACGTATATATATGTATATTTCTTTATACTAAGTTTTACTGGGCAGAG
TATCATAC

ATTGCGTCGAACAATATACCTTAAAGAAGAAAAAAAAAGGTTGGTTTTATCACTTCATTAGTCTATTTGAA
TAATTCATAAAATATTTCTGATTTTTTTTTTTTTTTTTTTTTTTTCATATTATTTAAACGTGACTTTGGCT
GCGATTCA

TTTAGTTTCTTGTTGTTCTTTCCTCTAATTAGAGTCATCTTAAGAATATTTTTCCACGGGAAAAAAGTGAA
AAAAAAAAAAAAAAAAAAAAAAAAGAGTGGGAATTTTCAGTGCAGTTGAAAAAATCGAGGTGGAATTTGAG
AACGACAA

GTAGAACCAGTTTAGTTTCTTGTTGTTCTTTCCTCTAATTAGAGTCATCTTAAGAATATTTTTCCACGGGA
AAAAAGTGAAAAAAAAAAAAAAAAAAAAAAAAAAGAGTGGGAATTTTCAGTGCAGTTGAAAAAATCGAGGT
GGAATTTG

TTTCCACGGGAAAAAAGTGAAAAAAAAAAAAAAAAAAAAAAAAAAGAGTGGGAATTTTCAGTGCAGTTGAA
AAAATCGAGGTGGAATTTGAGAACGACAATTACTTAGCACTACATGTGTTGTTCTCGTTTGTTTTTAATTA
TATACAGG

AAAAAAAAACTCAATACCTACATCACCGCGAAATGCCTGAAGTAAAAGCATATAACAAACGCGACCCTTAA
AATTACGTATATATATGTATATTTCTTTATACTAAGTTTTACTGGGCAGAGTATCATACAGTTCTTCTATC
CGATATAT

TCTATAGTATGTATCAAGAGAGAAATATGTGAATGACGAGCAAACTAGAGGTGTTATTAATAATAATGGCA
TAAAAACTAGATTTTATTCTATATATATATATATATATTTCTTCGACTTTTTTTTTTTTTACCCGCACACC
CTGAATTT

TAAGATGTCCTTATATAACTGTAGAACCAGTTTAGTTTCTTGTTGTTCTTTCCTCTAATTAGAGTCATCTT
AAGAATATTTTTCCACGGGAAAAAAGTGAAAAAAAAAAAAAAAAAAAAAAAAAAGAGTGGGAATTTTCAGT
GCAGTTGA

AAACAGGAAATATTGCCTATTTTCGTACAAGGTTACTTCCTAGATGCTATATGTCCCTTTACATAATAAAT
TAAAAAATTTTTTTATAAATTATAATAATTTCTTTTTATTTCTAATAGTATCTTGGGATTAAATAAATCA
CTTACAAT

GTGAAAATTTTTATATTTTTAATTAAATTTTTATAATATTATAAATTATTATAATATTGATATTTAAAATT
AAAAACAAATTATTAATGGTTTTGTTTTCCGAAATGTTTTTTATAATTATTACTTCAGAATAGTTATTTTC
ACGAATAC

GAAAAGATCTCGCTTTTGTCTCACGCGCAATGCTATAAATAATAAATATACATATATATATATTTATATGT
AGATAGATGTGTGTATATATATGCTAGGTAAAGAAACGGAAAAATCGAAAAATCGAAGTTGGAGAGGAAAA
ACAAGACA

CATAATATTAATTCATACAATATACTTAAGAAAACAATATACTTTTCTTAGTTTCATAACTTTGGAATCTA
TAATGTTGTTTTTTTTTTTTTAAGTATATTTGTATATTATGCATATAAGTCAACGCGTAGCGGTGGTATTA
CGCGCAAG

ACTTTTCTTAGTTTCATAACTTTGGAATCTATAATGTTGTTTTTTTTTTTTTAAGTATATTTGTATATTAT
GCATATAAGTCAACGCGTAGCGGTGGTATTACGCGCAAGCTTTTGATTTCTCTTACTATTCTTGGTTGTTC
ATTTGTTA

ACACTTCATGATTGCGTCGAACAATATACCTTAAAGAAGAAAAAAAAAAGGTTGGTTTTATCACTTCATTAG
TCTATTTGAATAATTCATAAAATATTTCTGATTTTTTTTTTTTTTTTTTTTTTTCATATTATTTTAAACGT
GACTTTGG

CTAAATCAGTTAAGATGTCCTTATATAACTGTAGAACCAGTTTAGTTTCTTGTTGTTCTTTCCTCTAATTA
GAGTCATCTTAAGAATATTTTTCCACGGGAAAAAAGTGAAAAAAAAAAAAAAAAAAAAAAAAAAAGAGTGGG
AATTTTCA

AAATATAATGACAATTATAATAAGAAGTATGATACTTTTTCTGTAAAAAATATATATACATATGATTCACG
TGAAAATTTTTATATTTTTAATTAAATTTTTATAATATTATAAATTATTATAATATTGATATTTAAAATTA
AAAACAAA

GAATGACGAGCAAACTAGAGGTGTTATTAATAATAATGGCATAAAAACTAGATTTTATTCTATATATATAT
ATATATATTTCTTCGACTTTTTTTTTTTTTTACCCGCACACCCTGAATTTTGTGCTATTTTGAAGCAGAGCG
TACTGAAC

TTTTTTTTTTTTTTTTTTTAGCTTCCTACATTTCGTTAATAATATTATATAGATTATATTTATATTTAAGA
AAAGTAATATCAGCATATTATGAATAGACAAAAAAGTCTAAGGTCAAGATTTATTAAATGTTAGATTATTA
AGATTACA

CTTCATTCTCTTTCATTTGGTAGTGAGATGGCAGTTCGAGGTTTTTTTATTCAAAGTAATAACTTCTTCCT
TTTCGCTTTTATGTAGCAGAAAAAAAAAGCCGCCGAAGCGCGCGCGTTCGTGCAATCTCATGATAGAAAAAG
GGCAACAA

CAAAATTCGTGGCTCAGTGCTCTCAAGACAATGTTGCAACCCTTTGCGTCAATTATATATATATATATATA
TATATATATATCCCGTCCGCTTTCTTTTTTTTGTCAGTTGGGTCGCAACGCAGGGTCTCGAGACCTGAAAA
AAGCTTCA

GTATTCCAAACCAATTTAAGTAAGTATTGATATGTAGTTATTTTTAAAATAAATATATGTAAGTATATTTG
GATGCTTTAATTAGGAATGTTCTATTTTTTTTTTTATTTTTTATATTATGGAAGAAAATGGACGTGACGAAT
CAATAAAA

GCAATAATGACAAGATTTTTGCATGGGACTTCTTTTTTTCTTTTGAATAAGTCGCGTTATAAGGACAAAAC
ATTAAAAAAAAATATGTATTGATTCACTGATTTATATATACAGAAGTAAGTACCGGGGTACCTAAATATAC
GCATAAAA

TCTAGTGCGATGGCAATGCTTCAAAGTAAATGGAGAATTTCGAAGGCTAGCGTCTCTAGATAAATCTCAGC
CTAACCCTTTTACACGAAGTTTTCCCAACAAAAAAAAAAAAAAAAAAAAAAAAAAAAAAGAAGGGCGGTTGT
CTTTGGCA

TCAACCTCGCCAAACAATGTCAATTTTTTTAAAAAAAAAACTCAATACCTACATCACCGCGAAATGCCTGAA
GTAAAAGCATATAACAAACGCGACCCTTAAAATTACGTATATATATGTATATTTCTTTATACTAAGTTTTA
CTGGGCAG

AAGTACTTCAAGGAGACGTTATATAAGCTGGCCAATGAATAAATAGATTAATTTAAACAGTATATGTACAG
TTTTATATATATATATATATATATATACATATATAAAGAAACCTGTGCGTTTTTTGTATTTTCAAATACAT
TTAGTTTT

GTTTCATAACTTTGGAATCTATAATGTTGTTTTTTTTTTTTTAAGTATATTTGTATATTATGCATATAAGT
CAACGCGTAGCGGTGGTATTACGCGCAAGCTTTTGATTTCTCTTACTATTCTTGGTTGTTCATTTGTTATT
TTTCTTTC

CAAGATTTTTGCATGGGACTTCTTTTTTTCTTTTGAATAAGTCGCGTTATAAGGACAAAACATTAAAAAAA
AATATGTATTGATTCACTGATTTATATATACAGAAGTAAGTACCGGGGTACCTAAATATACGCATAAAAGT
CTCTTTCT

AATATAAACAAAAAACCGCAACACTTCATGATTGCGTCGAACAATATACCTTAAAGAAGAAAAAAAAAGGT
TGGTTTTATCACTTCATTAGTCTATTTGAATAATTCATAAAATATTTCTGATTTTTTTTTTTTTTTTTTT
TTTCATAT

TTAGGATATACCTCGAAAATAATAAACCGCCACACTGTCATTATTATAATTAGAAACAGAACGCAAAAATT
ATCCACTATATAATTCAAAGACGCGAAAAAAAAAGAACAACGCGTCATAGAACTTTTGGCAATTCGCGTCA
CAAATAAA

ATGTCCCTTTACATAATAAATTAAAAAATTTTTTTTATAAATTATAATAATTTCTTTTTATTTCTAATAGT
ATCTTGGGATTAAATAAATCACTTACAATATTTATTTTATTATATTGCTTTGTTCAATTAAATTTTAATAC
GAAATTAT

CCTCAGAATAAACAAGGTCATCTAGTGCGATGGCAATGCTTCAAAGTAAATGGAGAATTTCGAAGGCTAGC
GTCTCTAGATAAATCTCAGCCTAACCCTTTTACACGAAGTTTTCCCAACAAAAAAAAAAAAAAAAAAAAAA
AAAAAAAG

ATAAAATGCTTCACGAACACCGTCATTGATCAAATAGGTCTATAATATTAATATACATTTATATAATCTAC
GGTATTTATATCATCAAAAAAAAGTAGTTTTTTATTTATTTTATTTTGTTCGTTAATTTTCAATGTCTATG
GAAGCCCG

GGCTCAGTGCTCTCAAGACAATGTTGCAACCCTTTGCGTCAATTATATATATATATATATATATATATATA
TCCCGTCCGCTTTCTTTTTTTTGTCAGTTGGGTCGCAACGCAGGGTCTCGAGACCTGAAAAAAGCTTCATA
GCGATATT

ACATTTGATAAAGTACTTCAAGGAGACGTTATATAAGCTGGCCAATGAATAAATAGATTAATTTAAACAGT
ATATGTACAGTTTTATATATATATATATATATATATATACATATATAAAGAAACCTGTGCGTTTTTTGTATTT
TCAAATAC

AATCTGCGTCAAACAGGAAATATTGCCTATTTTCGTACAAGGTTACTTCCTAGATGCTATATGTCCCTTTA
CATAATAAATTAAAAAATTTTTTTTATAAATTATAATAATTTCTTTTTATTTCTAATAGTATCTTGGGATT
AAATAAAT

GATTTTTTTTTTTTTTTTTTTTTTTCATATTATTTAAACGTGACTTTGGCTGCGATTCATGGTTTTTGCC
CTTATATCTTCGTTGTCATTATTATCAGCATTATTAGGATAATCCTTTCCATGTAATAGAAAACTTTTATT
TTTCAACA

GATAGTCGCGTATACATAAATAACAAAAAAACAAATTCAATAACATAAAATAAAAATACTTTTACATACAG
AAGAAGAAACTTTATATATTCGACAAAGGAAAATGTAAAAAAAAAAAAACACATTATTTTAAAAGAACTGCG
CTCATTAT

TGTTGTTCTTTCCTCTAATTAGAGTCATCTTAAGAATATTTTTCCACGGGAAAAAAGTGAAAAAAAAAAAA
AAAAAAAAAAAAAAGAGTGGGAATTTTCAGTGCAGTTGAAAAAATCGAGGTGGAATTTGAGAACGACAATT
ACTTAGCA

CGTCACTGCGAACGGATGAATATTGCCAATGACTGCTGCGCACTCTTAAAAAATATAATGACAATTATAAT
AAGAAGTATGATACTTTTTCTGTAAAAAATATATATACATATGATTCACGTGAAAATTTTTATATTTTTAA
TTAAATTT

CACTCTTAAAAAATATAATGACAATTATAATAAGAAGTATGATACTTTTTCTGTAAAAAATATATATACAT
ATGATTCACGTGAAAATTTTTATATTTTTAATTAAATTTTTATAATATTATAAATTATTATAATATTGATA
TTTAAAAT

ATTTATATATACAGAAGTAAGTACCGGGGTACCTAAATATACGCATAAAAGTCTCTTTCTTTTTTTTTTTT
TTTTTTTTAGCTTCCTACATTTCGTTAATAATATTATATAGATTATATTTATATTTAAGAAAAGTAATATC
AGCATATT

CAAACTAGAGGTGTTATTAATAATAATGGCATAAAAACTAGATTTTATTCTATATATATATATATATATTT
CTTCGACTTTTTTTTTTTTTACCCGCACACCCTGAATTTTGTGCTATTTTGAAGCAGAGCGTACTGAACAA
ATTGGCAG

TTTCGTACAAGGTTACTTCCTAGATGCTATATGTCCCTTTACATAATAAATTAAAAAATTTTTTTTATAAA
TTATAATAATTTCTTTTTATTTCTAATAGTATCTTGGGATTAAATAAATCACTTACAATATTTATTTTATT
ATATTGCT

TAGATGCTATATGTCCCTTTACATAATAAATTAAAAAATTTTTTTTATAAATTATAATAATTTCTTTTTAT
TTCTAATAGTATCTTGGGATTAAATAAATCACTTACAATATTTATTTTATTATATTGCTTTGTTCAATTAA
ATTTTAAT

CATTTTTTTTTTGTTCATGATGTTAGCATCTGCTTAGTCTTTTTAAAATTATTTAATTTAACGTTTTATTT
ATGTGAACCTTTTTTTTATTAGTTGTATTTACTATTTATTCAAGAATCATTCTTTACTTTATATTGATATCG
TTTTTTTC

AAAAAAAAAAAAAAAAAAAGAAGGGCGGTTGTCTTTGGCATATCGCGAAAATAATAGATCTTATCAAGGGG
TTTTTAAGTCATCCTCTGATTATTGGAAGTCATAATCACGTTCAATGGTGGGAGGCCTTCGGAAAAGAAAA
GAAAAGAA

ACGGAGCTTTATAGTAATTAGTCAAGGTTTTTTTTTTCTTATTTTTTTTCTATTTCATAACTTAGTCATATC
AAAATATATCTATTAAATTATTTTCCATATATACGTAGATATTCTTCATTTGCATAATTCTTTGGGAAAAA
GGAAAACA

TGGCTACAGCACTTGCTGAACATAAGCTTAAAACGTTTATGTGTGTATTTATATATGATAATAATAATAAT
AATAATAATAATAATAATAATAATAATAATAATAATAATAATGGTGATAAACTGCAATAACAAAAGAA
AATAGTAC

CGCTTTTGTCTCACGCGCAATGCTATAAATAATAAATATACATATATATATATTTATATGTAGATAGATGT
GTGTATATATATGCTAGGTAAAGAAACGGAAAAATCGAAAAATCGAAGTTGGAGAGGAAAAACAAGACATC
AGGCCCGC

GCCAATGAATAAATAGATTAATTTAAACAGTATATGTACAGTTTATATATATATATATATATATATACAT
ATATAAAGAAACCTGTGCGTTTTTTGTATTTTCAAATACATTTAGTTTTGGCGCCACTTCTATAAAAGGTC
TCATGATA

TATTGCCTATTTTCGTACAAGGTTACTTCCTAGATGCTATATGTCCCTTTACATAATAAATTAAAAAATTT
TTTTTATAAATTATAATAATTTCTTTTTATTTCTAATAGTATCTTGGGATTAAATAAATCACTTACAATAT
TTATTTTA

ATGAATAAACTCTATAGTATGTATCAAGAGAGAAATATGTGAATGACGAGCAAACTAGAGGTGTTATTAAT
AATAATGGCATAAAAACTAGATTTTATTCTATATATATATATATATATTTCTTCGACTTTTTTTTTTTTTA
CCCGCACA

TCAAGGAATGCAAAATTCGTGGCTCAGTGCTCTCAAGACAATGTTGCAACCCTTTGCGTCAATTATATATA
TATATATATATATATATATATCCCGTCCGCTTTCTTTTTTTTGTCAGTTGGGTCGCAACGCAGGGTCTCGA
GACCTGAA

GTCAAGGTTTTTTTTTTCTTATTTTTTTCTATTTCATAACTTAGTCATATCAAAATATATCTATTAAATTA
TTTTCCATATATACGTAGATATTCTTCATTTGCATAATTCTTTGGGAAAAAGGAAAACAGTTATAGAAAAA
TATAAACA

AAAAACCGCAACACTTCATGATTGCGTCGAACAATATACCTTAAAGAAGAAAAAAAAAGGTTGGTTTTATC
ACTTCATTAGTCTATTTGAATAATTCATAAAATATTTCTGATTTTTTTTTTTTTTTTTTTTTTCATATTA
TTTTAAAC

TTGGTTTTATCACTTCATTAGTCTATTTGAATAATTCATAAAATATTTCTGATTTTTTTTTTTTTTTTTT
TTTTCATATTATTTTAAACGTGACTTTGGCTGCGATTCATGGTTTTTGCCCTTATATCTTCGTTGTCATTA
TTATCAGC

CATTAAAAAAAAAATATGTATTGATTCACTGATTTATATATACAGAAGTAAGTACCGGGGTACCTAAATATA
CGCATAAAAGTCTCTTTCTTTTTTTTTTTTTTTTTTTAGCTTCCTACATTTCGTTAATAATATTATATAG
ATTATATT

TGATTCACTGATTTATATATACAGAAGTAAGTACCGGGGTACCTAAATATACGCATAAAAGTCTCTTTCTT
TTTTTTTTTTTTTTTTTAGCTTCCTACATTTCGTTAATAATATTATATAGATTATATTTATATTTAAGAA
AAGTAATA

ATATAAGCTGGCCAATGAATAAATAGATTAATTTAAACAGTATATGTACAGTTTTATATATATATATATAT
ATATATACATATATAAAGAAACCTGTGCGTTTTTTGTATTTTCAAATACATTTAGTTTTGGCGCCACTTCT
ATAAAAGG

TAAAATGTAGTGAGTTGGCTTTTGTTCGTGCTGTTTTCGATGAACTATTAATCCCAAATTTATGTAGTAAT
AATCACTAAAAAATGCGAAGAGGAAATATTAACGCGCGGCGCAAAAAGATCGGGTAATTTGGGCGTTTAAG
TACATGAA

TGTAACATATTTAGGATATACCTCGAAAATAATAAACCGCCACACTGTCATTATTATAATTAGAAACAGAA
CGCAAAAATTATCCACTATATAATTCAAAGACGCGAAAAAAAAAGAACAACGCGTCATAGAACTTTTGGCA
ATTCGCGT

TTGGCAGAAGAGGGAACCATTTCATCCAAAAAAAATAAAAAAATAAAAAAAATCCAAATATTAAGCTAAAA
AAATACTTAACTGTTTACACAAAGCGTATATAAAATGCATATTAAGAACCTATCTCGTTTCAGAGGAATAA
ACTTTTAA

AACAAGGTCATCTAGTGCGATGGCAATGCTTCAAAGTAAATGGAGAATTTCGAAGGCTAGCGTCTCTAGAT
AAATCTCAGCCTAACCCTTTTACACGAAGTTTTCCCAACAAAAAAAAAAAAAAAAAAAAAAAAAAAAAAGAA
GGGCGGTT

AAATATTTCTGATTTTTTTTTTTTTTTTTTTTTTTTCATATTATTTTAAACGTGACTTTGGCTGCGATTCAT
GGTTTTTGCCCTTATATCTTCGTTGTCATTATTATCAGCATTATTAGGATAATCCTTTCCATGTAATAGAA
AACTTTTA

AAAAAAGTGAAAAAAAAAAAAAAAAAAAAAAAAAAGAGTGGGAATTTTCAGTGCAGTTGAAAAAATCGAGG
TGGAATTTGAGAACGACAATTACTTAGCACTACATGTGTTGTTCTCGTTTGTTTTTAATTATATACAGGTT
GGTTAGGT

TTCGAGGTACCAAACTGATAGATAGTCGCGTATACATAAATAACAAAAAAACAAATTCAATAACATAAAAT
AAAAATACTTTTACATACAGAAGAAGAAACTTTATATATTCGACAAAGGAAAATGTAAAAAAAAAAAACAC
ATTATTTT

CGGAATCTGCTAAAATGTAGTGAGTTGGCTTTTGTTCGTGCTGTTTTCGATGAACTATTAATCCCAAATTT
ATGTAGTAATAATCACTAAAAAATGCGAAGAGGAAATATTAACGCGCGGCGCAAAAAGATCGGGTAATTTG
GGCGTTTA

ACTTGCTGAACATAAGCTTAAAACGTTTATGTGTGTATTTATATATGATAATAATAATAATAATAATAATA
ATAATAATAATAATAATAATAATAATAATAATAATGGTGATAAACTGCAATAACAAAAGAAAATAGTACCA
ATCCTGGA

ATCTCTGGTGTTCGAGGTACCAAACTGATAGATAGTCGCGTATACATAAATAACAAAAAAACAAATTCAAT
AACATAAAATAAAAATACTTTTACATACAGAAGAAGAAACTTTATATATTCGACAAAGGAAAATGTAAAAA
AAAAAAAC

AAAATAAAATAAAAATGTATGATTTCCCTCCATTTCCGACCAATTGTATAATTTTATATCTGCATGACTTA
ATAATATAATATAATACTTATAAAATACGAATAGAAAAATTTAAACCGATGTAATGCATCCTTTTCTTTGT
CGTCTTCG

CCCTTCTCAAGTATTCCAAACCAATTTAAGTAAGTATTGATATGTAGTTATTTTTAAAATAAATATATGTA
AGTATATTTGGATGCTTTAATTAGGAATGTTCTATTTTTTTTTTTATTTTTATATTATGGAAGAAAATGGA
CGTGACGA

GTGTTATTAATAATAATGGCATAAAAACTAGATTTTATTCTATATATATATATATATATTTCTTCGACTTT
TTTTTTTTTTACCCGCACACCCTGAATTTTGTGCTATTTTGAAGCAGAGCGTACTGAACAAATTGGCAGAT
TTACAAGA

GGACATTATCTCAAGGTACCGCTTACTATGCCTATACATTCCTGCAATTTTTTTTTTTTTTTTTTTGATAA
GTATTACTGGGTAATGGGAAGTATAATCACTAGGCATATTATTTTTGACGCAGAGGAGTGGCAGCCTGTCT
ACTATGTA

TTTGGGCTAAAATCTGCGTCAAACAGGAAATATTGCCTATTTTCGTACAAGGTTACTTCCTAGATGCTATA
TGTCCCTTTACATAATAAATTAAAAAATTTTTTTTATAAATTATAATAATTTCTTTTTATTTCTAATAGTA
TCTTGGGA

TTTGGAATCTATAATGTTGTTTTTTTTTTTTTAAGTATATTTGTATATTATGCATATAAGTCAACGCGTAG
CGGTGGTATTACGCGCAAGCTTTTGATTTCTCTTACTATTCTTGGTTGTTCATTTGTTATTTTTCTTTCCC
TGATATGC

GGGGCCAGTCAGAAGATAAGAAAAAAGGAAAATGTTATCATTGTTAAAAACATAAAGTTGTTGAAAATGTA
TTAACATGTGTATTATATATATAAATTATTTACAGTTAAATTTGACTACAAACTAAGTAACCCCGAGTCGC
GAAATAAA

TTTGGATTTTCTTGTTGCTTCAACGGATTATCTTAAAAAAATCTATCATATTTCAAAATATAAATTCTTA
TTTTTACAAAGAAGATATAGATTATGCATAATATTATTTTGTTACATTTTTTTTCTTTTACTTTTTATTTT
CTTTTCTT

GCAGTTCGAGGTTTTTTTATTCAAAGTAATAACTTCTTCCTTTTCGCTTTTATGTAGCAGAAAAAAAAGCC
GCCGAAGCGCGCGCGTTCGTGCAATCTCATGATAGAAAAAGGGCAACAATGTTGGGCTATTTTAGACAAAG
AAACTCAG

CTCCCCTTCATTGGCAGAAGAGGGAACCATTTCATCCAAAAAAAATAAAAAAATAAAAAAAATCCAAATAT
TAAGCTAAAAAAATACTTAACTGTTTACACAAAGCGTATATAAAATGCATATTAAGAACCTATCTCGTTTC
AGAGGAAT

CGTCATTGATCAAATAGGTCTATAATATTAATATACATTTATATAATCTACGGTATTTATATCATCAAAAA
AAAGTAGTTTTTTATTTATTTTATTTTGTTCGTTAATTTTCAATGTCTATGGAAGCCCGTTCGTAAAATTG
GCGTTTGT
