# Supplementary material for: iNuc-PhysChem: A Sequence-Based Predictor for Identifying Nucleosomes via Physicochemical Properties
Source: PLoS One. 2012 Oct 29;7(10):e47843. doi: 10.1371/journal.pone.0047843 (PMC3483203; doi:10.1371/journal.pone.0047843)
Supplement: Information S2 — The original numerical values for the 12 physicochemical properties of dinucleotide, where the physicochemical property “A-philicity” [22] is denoted by P(1); “base stacking” [23] by P(2); “B-DNA twist” [24] by P(3); “bendability” [25] by P(4); “DNA bending stiffness” [26] by P(5); “DNA denaturation” [27] by P(6); “duplex disrupt energy” [28] by P(7); “duplex free energy” [29] by P(8); “propeller twist” [30] by P(9); “protein deformation” [31] by P(10); “protein-DNA twist” [31] by P(11); and “Z-DNA” [32] by P(12). Their values were taken from the papers cited above, respectively. (PDF) [file pone.0047843.s002.pdf]

Supporting Information S2. The original numerical values for the 12 physicochemical properties of dinucleotide, where the physicochemical property "A-philicity" [22] is denoted by P(1); "base stacking" [23] by P(2); "B-DNA twist" [24] by P(3); "bendability" [25] by P(4); "DNA bending stiffness" [26] by P(5); "DNA denaturation" [27] by P(6); "duplex disrupt energy" [28] by P(7); "duplex free energy" [29] by P(8); "propeller twist" [30] by P(9); "protein deformation" [31] by P(10); "protein-DNA twist" [31] by P(11); and "Z-DNA" [32] by P(12). Their values were taken from the papers cited above, respectively.

Dinuc-leotide	Physicochemical properties [a]											
	P(1)	P(2)	P(3)	P(4)	P(5)	P(6)	P(7)	P(8)	P(9)	P(10)	P(11)	P(12)
AA	0.97	-5.37	35.5	-0.27	35	66.51	1.9	-1.2	-18.66	12.1	35.1	3.9
AC	0.13	-10.51	33.1	-0.21	60	108.8	1.3	-1.5	-13.1	9.8	31.5	4.6
AG	0.33	-6.78	30.6	-0.08	60	85.12	1.6	-1.5	-14.0	6.3	31.9	3.4
AT	0.58	-6.57	43.2	-0.28	20	72.29	0.9	-0.9	-15.01	2.1	29.3	5.9
CA	1.04	-6.57	37.7	-0.01	60	64.92	1.9	-1.7	-9.45	6.1	37.3	1.3
CC	0.19	-8.26	35.3	-0.03	130	99.31	3.1	-2.3	-8.11	2.9	32.9	2.4
CG	0.52	-9.69	31.3	-0.03	85	88.84	3.6	-2.8	-10.03	4.5	36.1	0.7
CT	0.33	-6.78	30.6	-0.18	60	85.12	1.6	-1.5	-14	1.6	31.9	3.4
GA	0.98	-9.81	39.6	0.03	60	80.03	1.6	-1.5	-13.48	2.3	36.3	3.4
GC	0.73	-14.6	38.4	0.02	85	135.83	3.1	-2.3	-11.08	4	33.6	4.0
GG	0.19	-8.26	35.3	-0.06	130	99.31	3.1	-2.3	-8.11	6.1	32.9	2.4
GT	0.13	-10.51	33.1	-0.18	60	108.8	1.3	-1.5	-13.1	2.1	31.5	4.6
TA	0.73	-3.82	31.6	0.18	20	50.11	1.5	-0.9	-11.85	2.3	37.8	2.5
TC	0.98	-9.81	39.6	-0.11	60	80.03	1.6	-1.5	-13.48	4.5	36.3	3.4
TG	1.04	-6.57	37.7	0.13	60	64.92	1.9	-1.7	-9.45	9.8	37.3	1.3
TT	0.97	-5.37	35.5	-0.28	35	66.51	1.9	-1.2	-18.66	2.8	35.1	3.9
